# Metaproteomics-informed stoichiometric modeling reveals the responses of wetland microbial communities to oxygen and sulfate exposure

**DOI:** 10.1038/s41522-024-00525-5

**Published:** 2024-07-03

**Authors:** Dongyu Wang, Pieter Candry, Kristopher A. Hunt, Zachary Flinkstrom, Zheng Shi, Yunlong Liu, Neil Q. Wofford, Michael J. McInerney, Ralph S. Tanner, Kara B. De Leόn, Jizhong Zhou, Mari-Karoliina H. Winkler, David A. Stahl, Chongle Pan

**Affiliations:** 1 School of Biological Sciences, University of Oklahoma, Norman, OK USA; 2 Department of Civil and Environmental Engineering, University of Washington, Seattle, WA USA; 3 Laboratory of Systems and Synthetic Biology, Wageningen University & Research, Wageningen, The Netherlands; 4 Institute for Environmental Genomics, University of Oklahoma, Norman, OK USA; 5 School of Computer Science, University of Oklahoma, Norman, OK USA; 6 School of Civil Engineering and Environmental Sciences, University of Oklahoma, Norman, OK USA; 7 Earth and Environmental Sciences, Lawrence Berkeley National Laboratory, Berkeley, CA USA

**Keywords:** Microbiome, Soil microbiology

## Abstract

Climate changes significantly impact greenhouse gas emissions from wetland soil. Specifically, wetland soil may be exposed to oxygen (O_2_) during droughts, or to sulfate (SO_4_^2-^) as a result of sea level rise. How these stressors – separately and together – impact microbial food webs driving carbon cycling in the wetlands is still not understood. To investigate this, we integrated geochemical analysis, proteogenomics, and stoichiometric modeling to characterize the impact of elevated SO_4_^2-^ and O_2_ levels on microbial methane (CH_4_) and carbon dioxide (CO_2_) emissions. The results uncovered the adaptive responses of this community to changes in SO_4_^2-^ and O_2_ availability and identified altered microbial guilds and metabolic processes driving CH_4_ and CO_2_ emissions. Elevated SO_4_^2-^ reduced CH_4_ emissions, with hydrogenotrophic methanogenesis more suppressed than acetoclastic. Elevated O_2_ shifted the greenhouse gas emissions from CH_4_ to CO_2_. The metabolic effects of combined SO_4_^2-^ and O_2_ exposures on CH_4_ and CO_2_ emissions were similar to those of O_2_ exposure alone. The reduction in CH_4_ emission by increased SO_4_^2-^ and O_2_ was much greater than the concomitant increase in CO_2_ emission. Thus, greater SO_4_^2-^ and O_2_ exposure in wetlands is expected to reduce the aggregate warming effect of CH_4_ and CO_2_. Metaproteomics and stoichiometric modeling revealed a unique subnetwork involving carbon metabolism that converts lactate and SO_4_^2-^ to produce acetate, H_2_S, and CO_2_ when SO_4_^2-^ is elevated under oxic conditions. This study provides greater quantitative resolution of key metabolic processes necessary for the prediction of CH_4_ and CO_2_ emissions from wetlands under future climate scenarios.

## Introduction

Wetlands store approximately one-third of global soil organic carbon (SOC) and play important roles in regulating and stabilizing global climate^1–3^. CH_4_ and CO_2_, the two greatest contributors to the greenhouse effect, are the dominant gaseous end-products from the mineralization of organic carbon in wetland ecosystems^4,5^. Carbon fluxes in a wetland ecosystem are closely linked to its hydrological features^6^. Climate changes may introduce specific hydrology-related stressors. For instance, freshwater wetlands are increasingly vulnerable to drought events, which lower water tables and introduce O_2_ into wetland soils. This leads to more frequent exposure of soil organic matter to O_2_, thereby affecting the organic carbon balance^7^. In addition, freshwater wetlands face perturbations as a result of seawater intrusions. As the sea level rises, the inundation of wetlands by seawater brings a high concentration of sulfate ions (SO_4_^2-^), substantially altering the sediment chemistry^2,8,9^. These stressors can change the production patterns of CH_4_ and CO_2_, resulting in feedback that is poorly characterized qualitatively in terms of the direction of the changes and quantitatively in terms of the magnitudes of the changes. Understanding how environmental disturbances affect the dynamic of metabolic processes and the succession of ecological communities is critical for accurately modeling changes in greenhouse gas emissions from wetland ecosystems in future climate scenarios.

Microbial interactions are vital in modulating organic matter decomposition and greenhouse gas emissions within wetland ecosystems^10,11^. These interactions are driven by fermentation and respiration, coupling the oxidation of organic compounds by electron transfer to electron acceptors^12–14^. In the absence of disturbance by droughts and seawater intrusions, obligatory anaerobic fermenters become dominant^15^. Syntrophic interactions among these diverse anaerobic microorganisms facilitate the stepwise breakdown of complex organic substrates into simpler, chemically stable compounds, ultimately resulting in the production of CH_4_ and CO_2_^16^. However, O_2_ exposure introduced during drought can promote aerobic microbial respiration of carbon stocks, with CO_2_ as the primary product^17^. The introduction of SO_4_^2-^ from seawater inundation as an alternative electronic acceptor leads to sulfate-reducing bacteria (SRB) to producing more H_2_S, utilizing H_2_ and/ or organic acids as electron donors. The competition from SRBs for H_2_ and organic acids may reduce methane production by methanogens^18,19^. Characterizing these metabolic interactions provides insight into biochemical transformations within the community under changing redox conditions, and allows for investigations into how changes in these conditions, induced by climate change, will affect the microbial metabolic networks that control carbon cycling in wetland ecosystems.

Carbon cycling and gas emissions in wetlands have been studied extensively in both field and laboratory conditions^20–23^. Elevated CO_2_ levels, reduced carbon accumulation, and decreased CH_4_ emissions have been observed when introducing O_2_ into previously anoxic wetland soil^22,24^. Previous studies have also demonstrated lower CH_4_ emissions to the atmosphere from lacustrine wetlands following seawater intrusion [21-23]. However, few studies have characterized how the critical syntrophic interactions controlling CH_4_ and CO_2_ productions respond to environmental redox perturbations, such as increased O_2_ from periods of drought or SO_4_^2-^ from seawater intrusion.

**Fig. 1:**
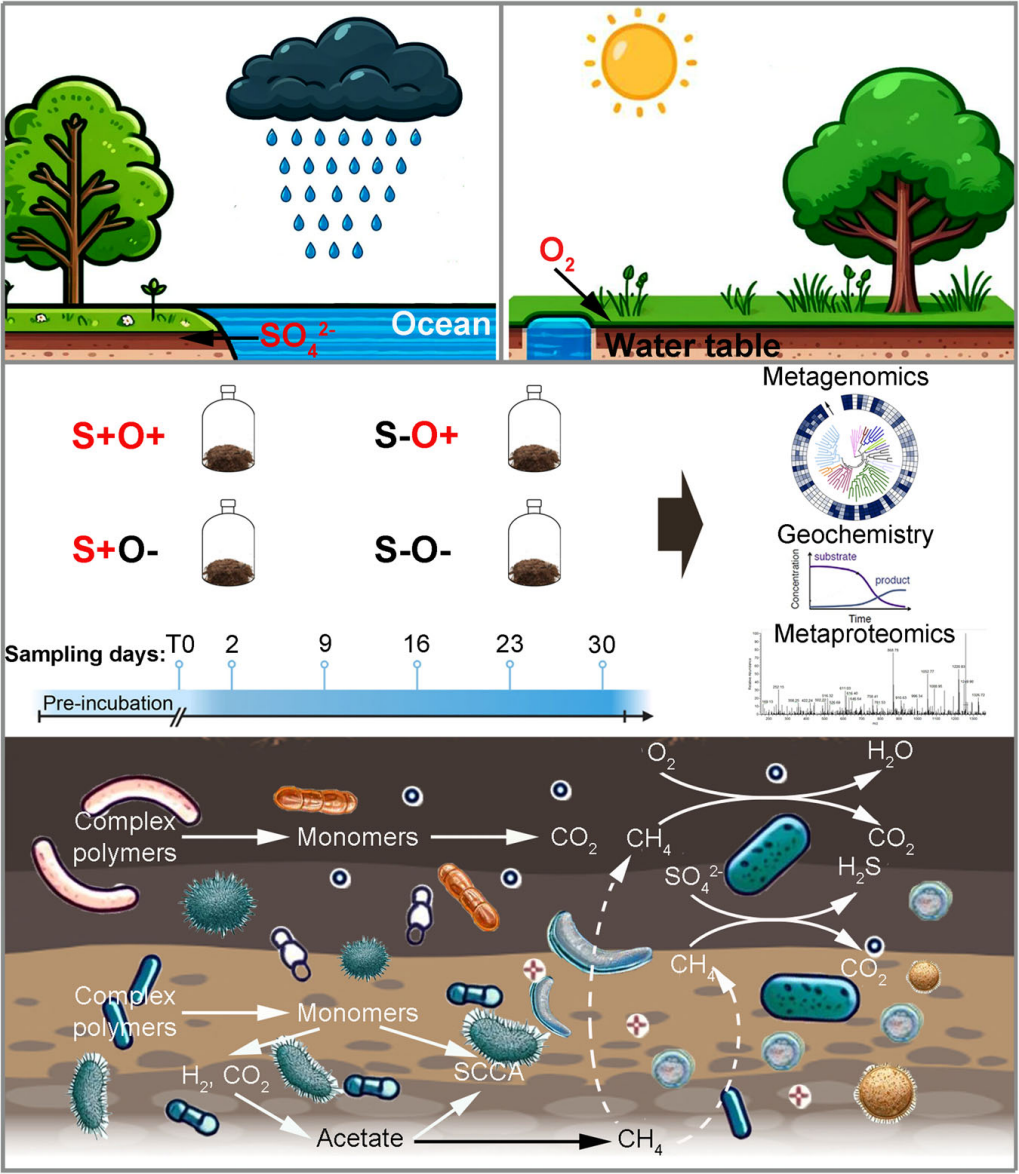
Schematic diagram of study design. Emissions of methane and carbon dioxide from wetland ecosystems can be severely perturbed by climate-change-induced stressors, such as seawater intrusions and droughts. To investigate this, we constructed laboratory microcosms using freshwater wetland soils under four incubation conditions, including anoxic non-sulfate addition (S-O-), anoxic sulfate-addition (S+O-), oxic non-sulfate addition (S-O+) and oxic sulfate-addition (S+O+) conditions. The soil communities were characterized by integrating geochemical analysis, metagenomics, metaproteomics and stoichiometric modeling. The results uncovered the molecular mechanism on how wetland microbial communities modulate greenhouse gas fluxes under future climate scenarios.

Establishing a clear connection between environmental disturbance and microbial adaptations remains a challenge, due to the complex and dynamic nature of microbial communities^25,26^. In this study, we set up laboratory microcosms to investigate the effects of SO_4_^2-^ exposure and O_2_ exposure alone or in combination on microbial activities and interactions, as well as the resulting fate of carbon within wetland soil (Fig. 1). We used proteogenomics to characterize the biochemical and physiological responses of microbial communities to individual perturbations and their combined effects. Stoichiometric models were employed to deconvolute carbon exchanges among the main functional guilds. By integrating geochemical, metaproteomic, and stoichiometric analyses, we aimed to develop a molecular understanding of how climate change-induced stressors modulate wetland microbial communities and greenhouse gas fluxes.

## Results

### SO_4_^2-^ and O_2_ additions changed community structure and activities

Laboratory microcosms were constructed using lacustrine wetland soils and incubated under four conditions: anoxic non-sulfate-addition (S-O-), anoxic sulfate-addition (S+O-), oxic non-sulfate-addition (S-O+ ), and oxic sulfate-addition (S+O+ ) (Fig. 1). Sterile air was introduced under S-O+ and S+O+ conditions. The final concentration of SO_4_^2-^ following addition under the S+O- and S+O+ conditions was approximately 7.2 mM, falling within the range of natural seawater concentration^27^.

**Fig. 2:**
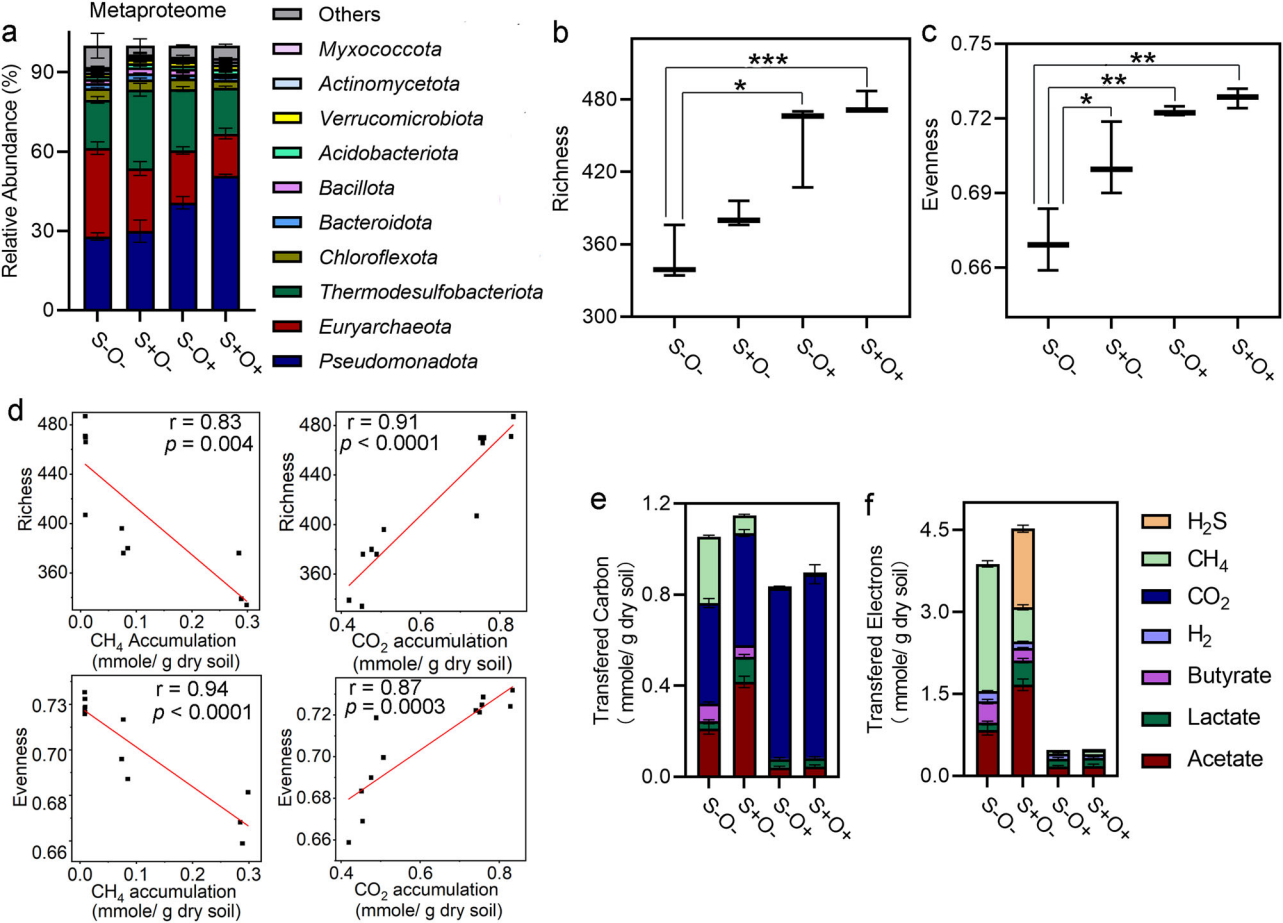
Effects of SO_4_^2-^ and O_2_ exposures on microbial community composition and activities. **a** Relative proteomic abundance of microbial phyla under four treatment scenarios. The relative proteomic abundance is expressed as the proportion of total proteins attributable to each microbial species. **b**, **c** Community species diversity indices, with **b** showing the richness and **c** the evenness across the four experimental conditions. Significant differences with *p*-values determined using Student’s t-test and adjusted for the false discovery rate, are marked by * for *p*-value < 0.05, ** for *p*-value < 0.01 and *** for *p*-value < 0.001. **d** Correlation between microbial species diversity and the accumulations of CH_4_ and CO_2_, where each dot represents an experimental measurement (X-axis for geochemical measurements, Y-axis for metaproteomic measurements) from the four conditions with three replicates, and the red line displays best linear fit. r is the Pearson correlation coefficient, and p values were determined by t-statistic. **e**, **f** Cumulative amounts of fermentation products are measured by carbon molarity **e** and electron molarity **f** within the microcosms. The error bars are defined as standard deviation.

Metaproteomic analysis identified a total of 10598 proteins based on assignments of unique peptide identifications to individual proteins. These proteins originated from 807 microbial species and 50 phyla, with *Pseudomonadota* (~51% of total proteome abundance) and *Euryarchaeota* (~33% of total proteome abundance) being the dominant phyla (Fig. 2a and Supplementary Table 2). All experimental treatments resulted in an increase of *Pseudomonadota* in proteome abundance compared to the S-O- condition. However, the increase under dual exposure (S+O+ , 82%, *p*-value < 0.000, t-test) was more than the additive effect of the increase observed under the S+O- condition (45%, *p*-value = 0.001, t-test) and S-O+ condition (7%, *p*-value = 0.5, t-test). This suggests a synergistic interaction between the effects of SO_4_^2-^ and O_2_ on the proteome abundance of *Pseudomonadota*, where their combined impact on microbial communities is greater than the sum of their individual effects. Conversely, *Euryarchaeota* consistently decreased in proteome abundance upon stress exposure, although this reduction was less under dual exposure (S+ O+ , 52%, *p-*value = 0.0006, t-test) than the sum of the decreases under S+O- condition (29%, *p-*value = 0.008, t-test) and S-O+ condition (41%, *p-*value = 0.001, t-test) (Fig. 2a). This suggests an antagonistic interaction between the effects of SO_4_^2-^ and O_2_ on the proteome abundance of *Euryarchaeota*, where their combined impact on microbial communities is less than the sum of their individual effects.

Compared to the S-O- condition, the metaproteome abundances of active communities showed increased richness under both the S+O- (28%, *p*-value = 0.02, t-test) and S-O+ conditions (36%, *p*-value = 0.0009, t-test). There was also an increase in evenness under S+O- (5%, *p-*value = 0.04, t-test) condition, S-O+ condition (8%, *p-*value < 0.002, t-test), and S+O+ condition (9%, *p*-value = 0.001, t-test) (Fig. 2b, c). This suggests that SO_4_^2-^ and O_2_ exposures contributed to the proliferation of low-abundance microbial species. Moreover, we found a negative correlation between the increased richness and evenness of the active community and the accumulation of CH_4_ and a positive correlation with CO_2_ accumulation (Fig. 2d).

The primary metabolic products in the microcosm system were quantified by carbon (Fig. 2e) and electron molarity (Fig. 2f). Under the S-O- condition, the soil carbon was fermented into 0.32 ± 0.03 mmole/g dry soil of organic acids, 0.44 ± 0.02 mmole/g dry soil of CO_2_, and 0.29 ± 0.01 mmole/g dry soil of CH_4_ over seven days of incubation. Compared to S-O-, the SO_4_^2-^ addition increased the carbon transfer to organic acids by 94% (*p*-value = 0.0003, t-test) and decreased the CH_4_ production by 73% (*p*-value < 0.0001, t-test), and the O_2_ exposure decreased the carbon transfer to organic acids by 76% (*p*-value = 0.0002, t-test), increased that to CO_2_ by 70% (*p*-value < 0.0001, t-test), and eliminated the CH_4_ production. Under the S-O- condition, 1.36 ± 0.13 mmole/g dry soil of electrons was transferred to organic acids, 0.19 ± 0.01 mmole/g dry soil to H_2_, and 2.32 ± 0.63 mmole/g dry soil to CH_4_. In comparison, the SO_4_^2-^ addition diverted 1.44 ± 0.07 mmole/g dry soil of electrons to H_2_S and decreased the electron transfer to H_2_ by 40% (*p*-value = 0.0004, t-test), and the O_2_ exposure decreased the electron transfer to H_2_ by 55% (*p*-value = 0.001, t-test). The effects of the combined exposure to both SO_4_^2-^ and O_2_ on the carbon and electron transfers were similar to the effects of the O_2_ exposure only.

### SO_4_^2-^ and O_2_ additions altered depolymerization of plant polysaccharides

The formation of hydrolysis products derived from plant polysaccharides, such as glucose, xylose, mannose, and galacturonic acid, was affected by exposure to SO_4_^2-^ and O_2_ over time (Supplementary Fig. 1a – 1d). Under both anoxic and oxic conditions, the addition of SO_4_^2-^ slowed down the accumulation of glucose. This resulted in a 12% lower accumulation under anoxic conditions (*p*-value = 0.04, t-test) and a 14% reduced glucose accumulation (*p*-value = 0.02, t-test) under oxic conditions (Supplementary Fig. 1). Xylose reached its peak accumulation later under anoxic conditions compared to oxic conditions. The highest accumulation of xylose was observed under the S-O- condition, while its accumulation was relatively similar under the other three conditions. Mannose and galacturonic acid continuously accumulated under anoxic conditions. Their accumulation peaked between 16 to 23 days and then decreased under oxic conditions.

**Fig. 3:**
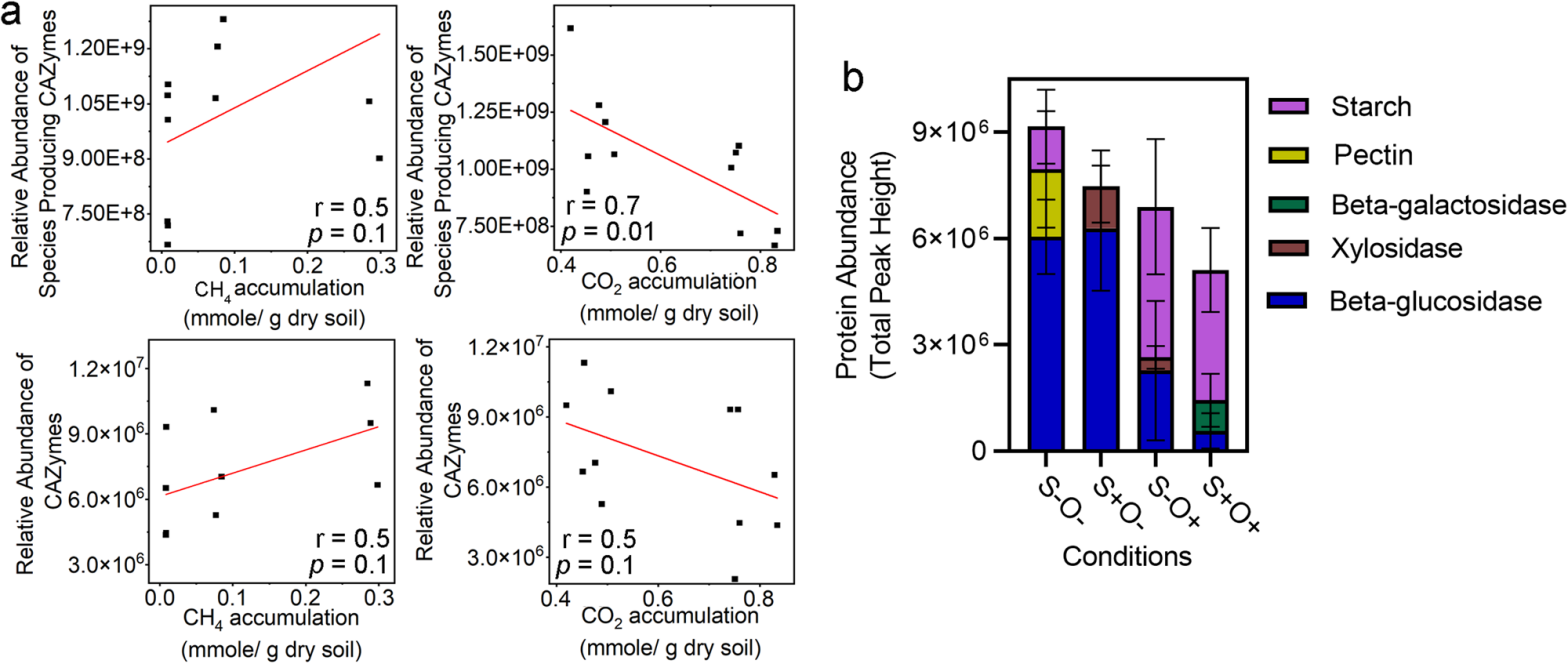
Effects of SO_4_^2-^ and O_2_ exposures on plant polysaccharide degradation. **a** Correlation analysis between the relative abundances of plant polymer degradation species and CAZymes with the accumulation of CH_4_ and CO_2_, where each dot represents experimental measurements (X-axis for geochemical measurements, Y-axis for metaproteomic measurements) from four conditions with three replicates, and the red line is the result of linear fitting. r is the Pearson correlation coefficient, and *p* values were determined by t-test. **b** Protein abundances of CAZyme under the four conditions. The error bars are defined as standard deviation.

The metaproteomic analysis identified five key enzymes from species involved in the degradation of plant polysaccharides. These species had the highest proteome abundance under anoxic conditions. Pairwise comparisons of proteome richness and evenness of these species, between the S-O- condition and the S-O+ condition, as well as between the S+O+ condition and the S+O+ condition, revealed no significant changes under different oxic conditions, which suggests that the actual species composition remained relatively stable across four conditions. A strong negative correlation (r = -0.70, *p-*value = 0.01, t-test) was observed between the total proteome abundance of plant polysaccharide-degrading microbes and the accumulation of CO_2_ (Fig. 3a). Conversely, a weak positive correlation was observed between the total proteome abundance of these microbes and the accumulation of CH_4_ (Fig. 3a).

The identified carbohydrate-active enzymes (CAZymes), including pectate lyase, beta-glucosidase, xylosidase, beta-galactosidase, and starch phosphorylase, showed the highest total abundance under the S-O- condition, and the lowest abundance under the S+O+ condition (Fig. 3b). This indicates that elevated SO_4_^2-^ and O_2_ levels suppressed the activities of plant polysaccharide degradation.

Among the identified CAZymes, beta-glucosidase was detected across all conditions, with the highest abundance observed under the S+O- condition (fold change > 4.0, *q-*value < 0.007). Xylosidase, involved in xylan degradation, showed higher abundance under the S+O- condition (fold change > 3.2, *q-*value < 0.0001) (Supplementary Table 3), indicating enhanced cellulose and xylan degradation capacity under this condition. Beta-galactosidase was only detected under the S+O+ condition (Supplementary Table 3). This enzyme can release galactose from hemicellulose, which enters the glycolysis pathway after conversion into glucose 1-phosphate by glucuronate isomerase (UXA). The highest abundance of UXA proteins was detected under the S+O+ condition (fold change > 1.7, *q-*value < 0.0001) (Supplementary Table 3), suggesting enhanced hemicellulose utilization under this condition. These observations collectively demonstrate that changes in SO_4_^2-^ and O_2_ levels altered the preference of the microbial community in degrading plant polysaccharides. Specifically, the enzymes enriched under oxic conditions use more complex structural substrates (e.g., hemicellulose) compared to those enriched under anoxic conditions (e.g., cellulose).

### SO_4_^2-^ and O_2_ decreased methanogenesis and promoted methane oxidation

**Fig. 4:**
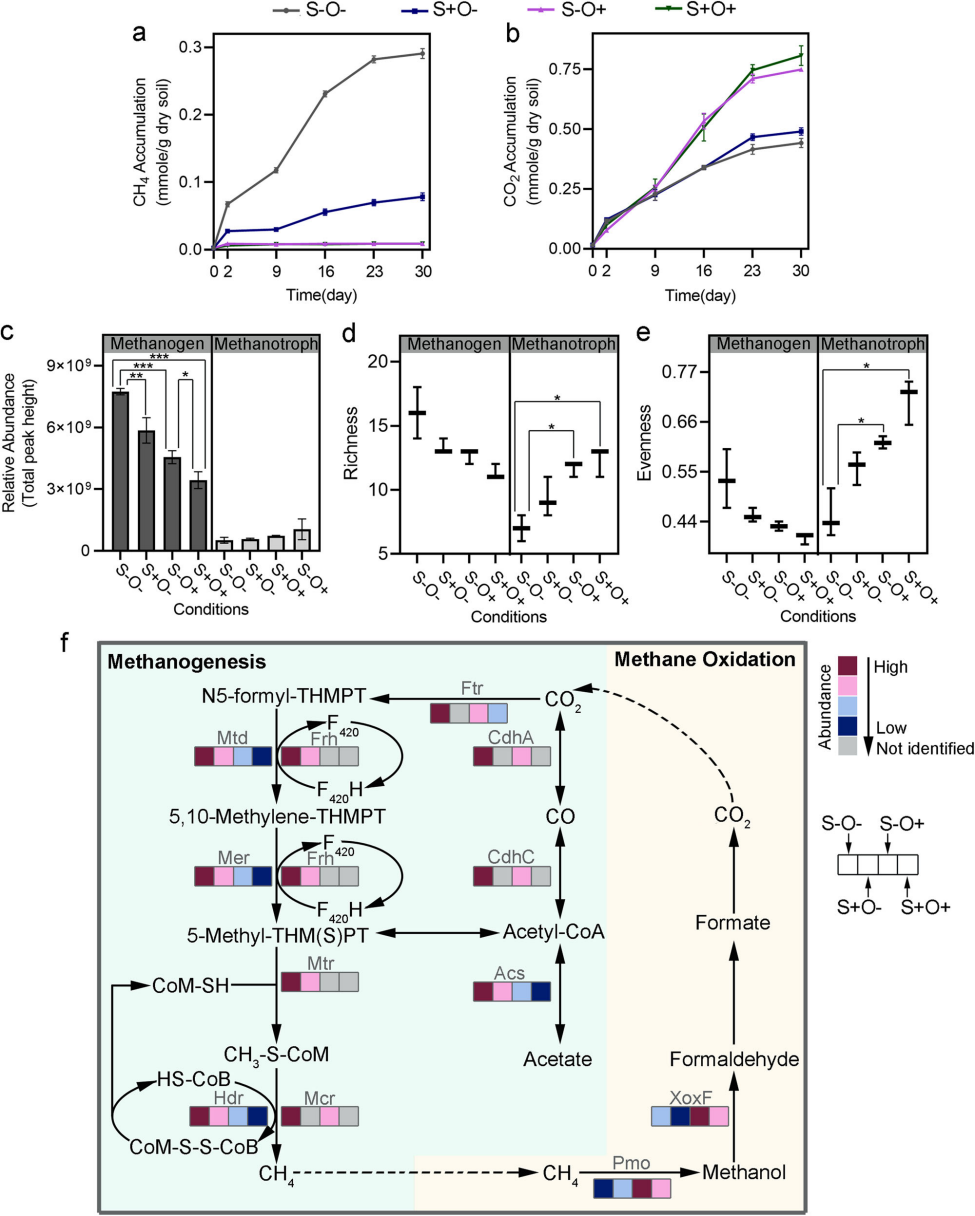
Modulation of methane metabolism by SO_4_^2-^ and O_2_ exposures. **a**, **b** Changes in molar mass of CH_4_ **a** and CO_2_ **b** under the four conditions, over a 30-day incubation. **c**–**e** Comparison of methanogens and methanotrophs in terms of relative abundance **c**, richness **d**, and evenness **e** under the four conditions. Relative abundance is quantified as the aggregate of protein abundances attributable to a specific species. **f** Schematic representation of methane metabolic pathways. The boxes in the pathway map, arranged from left to right, correspond to the S-O-, S+O-, S-O+ and S+O+ conditions. The color gradient from red to blue represents the relative abundance ranking of proteins among the four conditions, with red indicating the highest ranked relative abundance and blue the lowest. The gray color indicates proteins not detected by LC-MS/MS. Significant differences with *p*-values determined using Student’s t-test, and adjusted for the false discovery rate, are marked by * for *p*-value < 0.05, ** for *p*-value < 0.01 and *** for *p*-value < 0.001. The error bars are defined as standard deviation.

Under the S-O- condition, the microbial community continuously produced CH_4_ (0.29 ± 0.007 mmole/g dry soil) and CO_2_ (0.44 ± 0.02 mmole/g dry soil) throughout the experiment (Fig. 4a, b). The two-way ANOVA indicated a significant interaction between SO_4_^2-^ and O_2_ on CH_4_ accumulation (F (1,8) = 1590, *p*-value < 0.0001). CH_4_ release was reduced by 73% (*p*-value < 0.0001, t-test) under the S+O- condition, and by 97% (*p*-value < 0.000, t-test) under the S-O+ condition (Fig. 4a and Table 1), highlighting the higher sensitivity of CH_4_ accumulation to O_2_ than to SO_4_^2-^. However, the same analysis showed no significant interaction between SO_4_^2-^ and O_2_ (F (1, 8) = 0.1, *p*-value = 0.75). The main effects were observed, CO_2_ accumulation increased by 11% higher under the S+O- condition (*p-*value = 0.03, t-test), by 70% higher under the S-O+ condition (*p-*value < 0.0001, t-test), and by 83% higher under the S+O+ condition (*p*-value = 0.0002, t-test), compared to the S-O- condition (Fig. 4b), suggesting an additive effect of SO_4_^2-^ and O_2_ on CO_2_ accumulations.

**Table 1:** Accumulation of metabolic products in the microcosms under the four incubation conditions

Conditions				
Metabolites (mmole/g dry soil)	S-O-	S+O-	S-O+	S+O+
Glucose	0.023 ± 0.003	0.02 ± 0.002	0.02 ± 0.001	0.016 ± 0.001
Xylose	0.033 ± 0.001	0.023 ± 0.004	0.02 ± 0.002	0.02 ± 0.001
Mannose	0.008 ± 0.001	0.008 ± 0.001	0.011 ± 0.001	0.011 ± 0.001
Galacturonic acid	0.015 ± 0.003	0.016 ± 0.002	0.019 ± 0.001	0.015 ± 0.001
H_2_	0.096 ± 0.005	0.058 ± 0.003	0.044 ± 0.009	0.037 ± 0.001
CO_2_	0.44 ± 0.02	0.49 ± 0.02	0.75 ± 0.008	0.81 ± 0.04
CH_4_	0.29 ± 0.007	0.079 ± 0.006	0.009 ± 0.001	0.008 ± 0.001
H_2_S	0	0.18 ± 0.008	0	0.003 ± 0.001
Acetate	0.11 ± 0.012	0.21 ± 0.013	0.021 ± 0.002	0.022 ± 0.004
Lactate	0.01 ± 0.002	0.037 ± 0.004	0.012 ± 0.002	0.013 ± 0.002
Butyrate	0.02 ± 0.002	0.012 ± 0.004	0	0

Metaproteomic analysis identified 634 proteins from methanogens (Supplementary Table 1). These methanogens showed the highest proteome abundance under the S-O- condition, constituting 22% of the total metaproteome (Supplementary Table 1). The addition of SO_4_^2-^ and O_2_, either individually or in combination, led to a reduction in the proteome abundance (*p*-value < 0.0001, one-way ANOVA), richness (*p*-value = 0.005, one-way ANOVA), and evenness (*p*-value = 0.008, one-way ANOVA) of methanogenic populations (Fig. 4c–e). Furthermore, strong positive correlations were observed between the CH_4_ accumulation and the proteome abundance (r = 0.9, *p-*value < 0.0001, t-test), richness (r = 0.9, *p*-value = 0.0001, t-test), as well as evenness (r = 0.8, *p*-value = 0.002, t-test) of methanogens (Supplementary Fig. 2). In summary, the observed relationships among SO_4_^2-^ and O_2_ availability, CH_4_ accumulation, and the composition of methanogen populations could serve as diagnostics for more predictive climate modeling.

Metaproteomics revealed significant suppression of both hydrogenotrophic and acetoclastic methanogenesis by SO_4_^2-^ and O_2_. Many marker proteins involved in methanogenesis, including methyl-coenzyme M reductase (Mcr), 5,10-methylenetetrahydromethanopterin reductase (Mer), tetrahydromethanopterin S-methyltransferase (Mtr), acetyl-CoA decarbonylase/synthetase (CdhA) and methylenetetrahydromethanopterin dehydrogenase (Mtd) were most abundant under S-O- condition (Fig. 4f and Table 2). The addition of SO_4_^2-^ and O_2_, either individually or in combination, led to a significant decrease in the abundance of these proteins (Fig. 4f). This decrease coincided with the decrease in CH_4_ emissions observed when exposed to SO_4_^2-^ and O_2_ stressors (Fig. 4a).

**Table 2:** Key proteins in methane metabolism and sulfate reduction pathways

Processes	Enzymes	Symbol	Comparison				
			S-O- vs. S+O-	S-O+vs. S+O+	S-O- vs. S-O+	S+O- vs. S+O+	S-O- vs. S+O+
			Fold Change (*padj*)	Fold Change (*padj*)	Fold Change (*padj*)	Fold Change (*padj*)	Fold Change (*padj*)
	methyl-coenzyme M reductase	Mcr	—	—	3.1 (*) ▼	—	—
Hydrogenotrophic Methanogenesis	5,10-methylenetetrahydromethanopterin reductase	Mer	3.4 (**) ▼	—	—	9.2 (***) ▼	—
	tetrahydromethanopterin S-methyltransferase	Mtr	5.8 (***) ▼	—	—	5.0 (*) ▼	8.7 (***) ▼
	methylenetetrahydromethanopterin dehydrogenase	Mtd	—	—	7.0 (***) ▼	—	—
Acetoclastic Methanogenesis	acetyl-CoA decarbonylase/synthetase	CdhA	1.2 (***) ▼	—			
	acetyl-CoA synthetase	Acs	—	—	2.6 (*) ▼	1.5 (**) ▼	—
	methyl-coenzyme M reductase	Mcr	—	—	2.1 (***) ▼	-	1.7 (*) ▼
Methane Oxidation	methane/ammonia monooxygenase	Pmo	2.3 (***) ▲	—	3.9 (***) ▲	1.7 (**) ▲	3.9 (**) ▲
	lanthanide-dependent methanol dehydrogenase	XoxF	1.4 (***) ▲	1.7 (**) ▲	2.5 (**) ▲	3.0 (***) ▲	4.2 (***) ▲
Sulfate Reduction	pyruvate ferredoxin oxidoreductase	Por	8.0 (***) ▲	1.8 (**) ▲	1.4 (***) ▼		5.7 (***)
	acetyl-CoA synthetase	Acs	5.3 (***) ▲	2.4 (***) ▲	3.2 (***) ▼	2.0 (***) ▼	—
	sulfate adenylyltransferase	Sat	1.4 (**) ▲	2.8 (***) ▲	2.3 (**) ▼	1.2 (*) ▼	1.2 (***) ▼
	adenylylsulfate reductase	Apr	1.8 (***) ▲	-	1.3 (**) ▼	1.5 (**) ▼	1.2 (**) ▼
	dissimilatory sulfite reductase	Dsr	1.5 (**) ▲	1.6 (***) ▲	1.4 (***) ▼	—	—

▲ significant increase, ▼ significant decrease, - no significant changes were observed.

* for padj < 0.05, ** for padj < 0.01 and *** for padj < 0.001.

The enzymes listed in the table have shown significant changes in at least one of the five comparison groups.

Furthermore, the SO_4_^2-^ and O_2_ exposures suppressed hydrogenotrophic methanogenesis much more than acetoclastic methanogenesis. Specifically, after SO_4_^2-^ addition to the anoxic sediment, the abundance of acetyl-CoA decarbonylase/synthetase essential for acetoclastic methanogenesis, decreased by 1.2-fold (*q-*value = 0.0001). And there was a more pronounced decrease in proteins for hydrogenotrophic methanogenesis (Fig. 4f, Table 2), including a 3.4-fold decrease of the 5,10-methylenetetrahydromethanopterin reductase (*q-*value = 0.003) and a 5.8-fold decrease of the tetrahydromethanopterin S-methyltransferase (*q*-value < 0.0001). This indicated a stronger suppression of hydrogenotrophic methanogenesis by SO_4_^2-^. Under S-O+ condition, we observed a decrease in proteins for both the acetoclastic and hydrogenotrophic pathways of methanogenesis when compared to S-O- condition. In the acetoclastic pathway, there was a 2.6-fold decrease in acetyl-CoA synthetase (Acs) (*q*-value = 0.01) and a 2.1-fold decrease in methyl-coenzyme M reductase (*q*-value < 0.0001) (Fig. 4f, Table 2). In the hydrogenotrophic pathway, there was a 7.0-fold decrease in the methylenetetrahydromethanopterin dehydrogenase (*q-*value < 0.0001) and a 3.1-fold decrease in methyl-coenzyme M reductase (*q-*value = 0.02). Similarly, under S+O+ condition, compared to the S+O- condition, there was a more substantial decrease in key enzymes associated with hydrogenotrophic methanogenesis, including a 9.2-fold decrease in 5,10-methylenetetrahydromethanopterin reductase (*q-*value < 0.0001) and a 5.0-fold decrease in tetrahydromethanopterin S-methyltransferase (*q-*value < 0.01), compared to a 1.5-fold decrease in acetyl-CoA decarbonylase/synthetase (*q*-value = 0.006). These results highlight that O_2_ had a greater suppression of hydrogenotrophic methanogenesis than acetoclastic methanogenesis. Moreover, compared to the S-O- condition, under S+O+ condition, the abundance of methyl-coenzyme M reductase decreased by 1.7-fold (*q-*value = 0.01), and the abundance of tetrahydromethanopterin S-methyltransferase decreased by 8.7-fold (*q-*value < 0.0001). This suggests that the combined addition of SO_4_^2-^ and O_2_ had a more significant impact on hydrogenotrophic methanogenesis compared to acetoclastic methanogenesis.

In addition, metaproteomics identified 208 proteins from methanotrophs (Supplementary Table 1). Under the S+O+ condition, methanotrophs showed the highest richness (*p*-value = 0.0001, one-way ANOVA), and evenness (*p*-value = 0.0004, one-way ANOVA) (Fig. 4c–e). Moreover, significant negative correlations were found among the CH_4_ accumulation and proteome abundance (r = -0.7, *p-*value = 0.02, t-test), richness (r = -0.9, *p*-value < 0.0001, t-test), and evenness (r = -0.8, *p*-value = 0.002, t-test) of active methanotrophs (Supplementary Fig. 2).

Key enzymes involved in methane oxidation, including methane/ammonia monooxygenase (Pmo) and lanthanide-dependent methanol dehydrogenase (XoxF), showed distinct responses to elevated SO_4_^2-^ and O_2_ (Fig. 4f and Table 2). Under the anoxic condition, SO_4_^2-^ addition increased the abundance of methane/ammonia monooxygenase and lanthanide-dependent methanol dehydrogenase by 2.3-fold (*q-*value < 0.0001) and 1.4-fold (*q-*value = 0.0002), respectively. However, under the oxic conditions, SO_4_^2-^ addition decreased the abundance of lanthanide-dependent methanol dehydrogenase by 1.7-fold (*q*-value = 0.005). Oxygen addition led to an increase in the abundance of methane/ammonia monooxygenase and lanthanide-dependent methanol dehydrogenase by 3.9-fold (*q-*value < 0.0001) and 2.5-fold (*q*-value = 0.006), respectively, under the non-sulfate-addition condition. Under the sulfate-addition condition, their abundance increased by 1.7-fold (*q-*value = 0.005) and 3.0-fold (*q-*value < 0.0001), respectively. Furthermore, when compared to S-O- condition, the abundance of methane/ammonia monooxygenase and lanthanide-dependent methanol dehydrogenase increased by 4.2-fold (*q-*value < 0.0001) and by 3.9-fold (*q-*value = 0.001) under S+O+ condition (Fig. 4f, Table 2).

### SO_4_^2-^ and O_2_ additions changed metabolic behaviors of sulfate-reducing bacteria

**Fig. 5:**
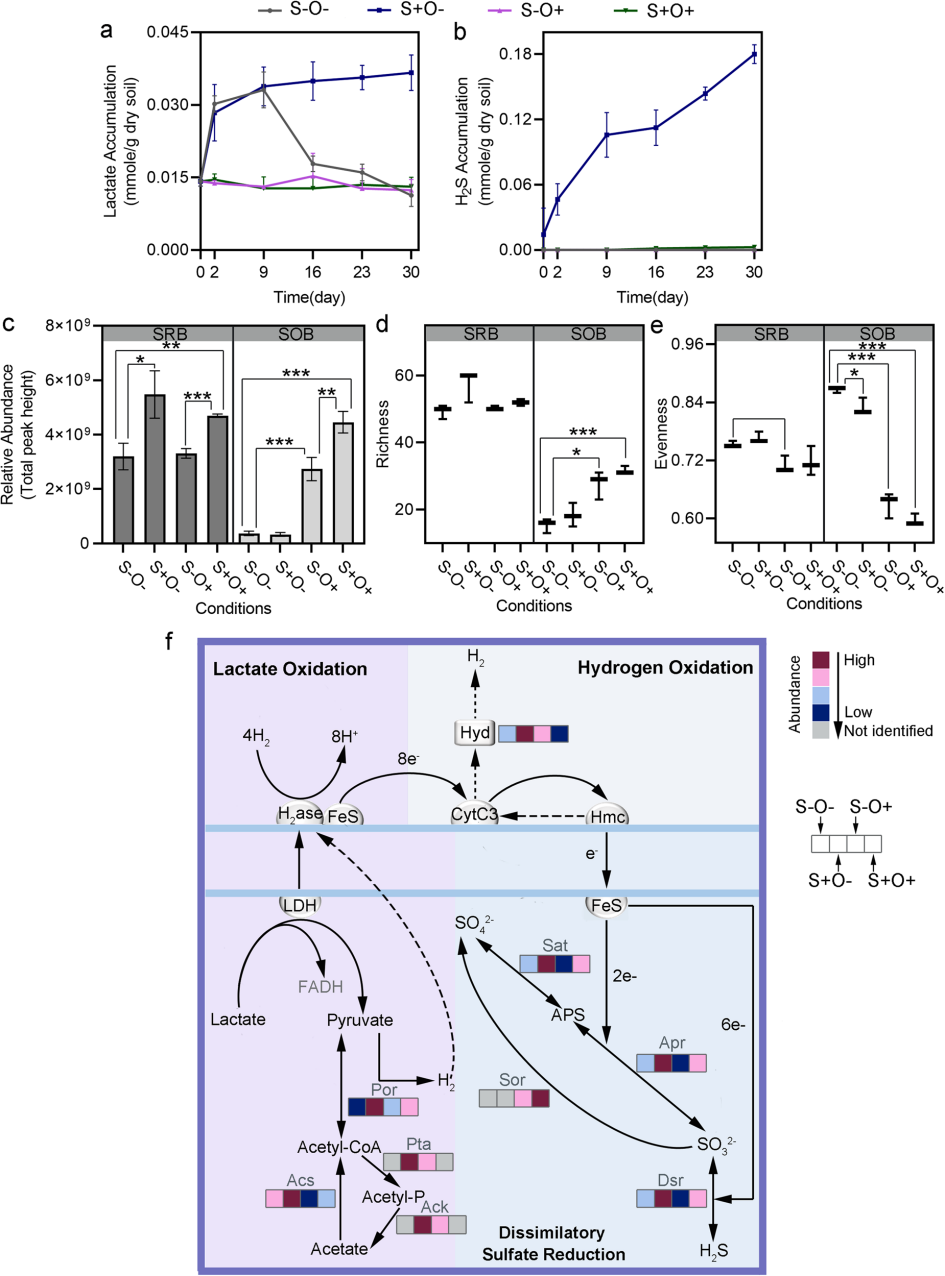
Effects of SO_4_^2-^ and O_2_ exposures on sulfate reduction and sulfite/sulfide oxidation processes. **a**, **b** Changes in molar mass of lactate **a** and H_2_S **b** over the 30 days of incubation. **c**–**e** Relative abundances **c**, richness **d**, and evenness **e** of sulfate-reducing bacteria (SRB) and sulfide/sulfur-oxidizing bacteria (SOB). Significant differences with *p*-values determined using Student’s t-test, and adjusted for the false discovery rate, are marked by * for *p*-value < 0.05, ** for *p*-value < 0.01 and *** for *p*-value < 0.001. **f** Sulfur metabolism pathways. The boxes in the pathway map, arranged from left to right, correspond to the S-O-, S+O-, S-O+ and S+O+ conditions. The color gradient from red to blue represents the relative abundance ranking of proteins among the four conditions, with red indicating the highest ranked relative abundance and blue the lowest. The gray color indicates proteins not detected by LC-MS/MS. The error bars are defined as standard deviation.

Under the sulfate-limited conditions, some sulfate-reducing bacteria (SRB) function as syntrophic oxidizers, metabolizing lactate to produce acetate, H_2,_ and CO_2_. With added SO_4_^2-^, SRB may oxidize lactate to acetate and CO_2_ and concurrently reduce SO_4_^2-^ to H_2_S^28^. Two-way ANOVA indicated a significant interaction between SO_4_^2-^ and O_2_ on lactate accumulation (F (1,8) = 66.51, *p*-value < 0.0001), with significant main effects of SO_4_^2-^ (F (1, 8) = 55.4, *p*-value < 0.0001) and O_2_ (F (1, 8) = 74.76, *p*-value < 0.0001). Under the S-O- condition, lactate substantially accumulated, reaching its peak of 0.033 ± 0.004 mmole/g dry soil on day 9, and then decreased to 0.018 ± 0.002 mmole/g dry soil over the next 7 days. Under the S+O- condition, lactate accumulation continually increased, reaching 0.037 ± 0.004 mmole/g dry soil at the end of incubation. Under oxic conditions, lactate accumulation remained consistently low at less than 0.012 ± 0.004 mmole/g dry soil over 30 days (Fig. 5a). Similarly, the two-way ANOVA for H_2_S accumulation revealed a significant interaction between SO_4_^2-^ and O_2_ (F (1, 8) = 1222, *p*-value < 0.0001), with significant main effects for both SO_4_^2-^ and O_2_. Specifically, a substantial accumulation of H_2_S (0.18 ± 0.008 mmole/g dry soil) was observed under the S+O- condition, while only trace amounts of H_2_S (0.003 ± 0.0002 mmole/g dry soil) were detected under the S+O+ condition (Fig. 5b).

Metaproteomics identified a total of 145 proteins from SRB species, with the highest SRB proteome abundance observed under S+O- condition (fold change > 1.3, *p-*value < 0.006, t-test) (Fig. 5c, Supplementary Table 1). SO_4_^2-^ addition enhanced the activities of both lactate oxidation and dissimilatory sulfate reduction within this community (Fig. 5d–f and Table 2). In the lactate oxidation pathway, SO_4_^2-^ addition resulted in an 8.0-fold increase in the abundance of pyruvate ferredoxin oxidoreductase (Por) (*q-*value < 0.0001) and a 5.3-fold increase in acetyl-CoA synthetase (*q-*value < 0.0001). In the dissimilatory sulfate reduction pathway, the abundance of adenylylsulfate reductase (Apr) increased by 1.8-fold (*q-*value < 0.0001), sulfate adenylyltransferase (Sat) increased by 1.4-fold (*q-*value = 0.002), and dissimilatory sulfate reductase (Dsr) increased by 1.5-fold (*q-*value = 0.005) following SO_4_^2-^ addition (Fig. 5f, Table 2).

Oxygen addition significantly suppressed the activities of lactate oxidation and dissimilatory sulfate reduction. The response of key proteins in both pathways to O_2_ varied with the availability of SO_4_^2-^. In the lactate oxidation pathway, the reduction in the abundance of acetyl-CoA synthetase was more significant when comparing S-O- to S-O+ condition (2.6- fold decrease, *q-*value = 0.01), than when comparing S+O- to the S+O+ condition (1.5-fold decrease, *q-*value = 0.006). A 1.4-fold reduction (*q*-value < 0.0001) in pyruvate ferredoxin oxidoreductase was observed only when comparing S-O- to S-O+ condition, suggesting that O_2_ showed a stronger suppressive effect under the sulfate-sufficient condition. Conversely, in the dissimilatory sulfate reduction pathway, the suppressive effect of O_2_ was intensified under sulfate-limited conditions, as indicated by a larger decrease in the abundance of sulfate adenylyltransferase when comparing S-O- to S-O+ condition (2.3-fold decrease, *q-*value = 0.007), than when comparing S+O- to S+O+ condition (1.2-fold decrease, *q-*value = 0.01). Additionally, a 1.4-fold (*q-*value = 0.0001) decrease in the abundance of dissimilatory sulfate reductase was only observed when comparing S-O- to S-O+ condition (Fig. 5f, Table 2) and no significant change when comparing S+O- to S+O+ condition.

Furthermore, metaproteomics analysis identified a total of 779 proteins from sulfide/sulfur-oxidizing bacteria (SOB), with the highest SOB proteome abundance observed under S+O+ condition (Fig. 5c and Supplementary Table 1). The proteome abundance of SOBs was negatively correlated with the CH_4_ accumulation (r = -0.7, *p-*value = 0.01, t-test), while the evenness of SOBs showed a positive correlation with the CH_4_ accumulation (r = 0.8, *p-*value = 0.0006, t-test) (Supplementary Fig. 2).

### Modeling of carbon and energy flow through the microbial communities

A stoichiometric model was constructed to model the fluxes through the primary carbon metabolism and energy conservation pathways among the predominant microbial guilds in the wetland communities. The overall model comprised nine reactions, including aerobic heterotrophy, lactate fermentation, hydrogenic acetogenesis, sulfidogenic lactate oxidation, sulfidogenic hydrogen oxidation, hydrogenotrophic methanogenesis, acetoclastic methanogenesis, methane oxidation, and butyrate production (Supplementary Fig. 3). The fluxes of these reactions were fitted to the cumulative amounts of acetate, lactate, butyrate, H_2_, CO_2_, CH_4,_ and H_2_S produced or consumed over the 30 days of incubation. The correlation coefficient R^2^, exceeded 0.9 in all four conditions (Supplementary Fig. 4). The consistency between the experimental data and the modeling results indicated a plausible estimation of the carbon and energy flows.

**Fig. 6:**
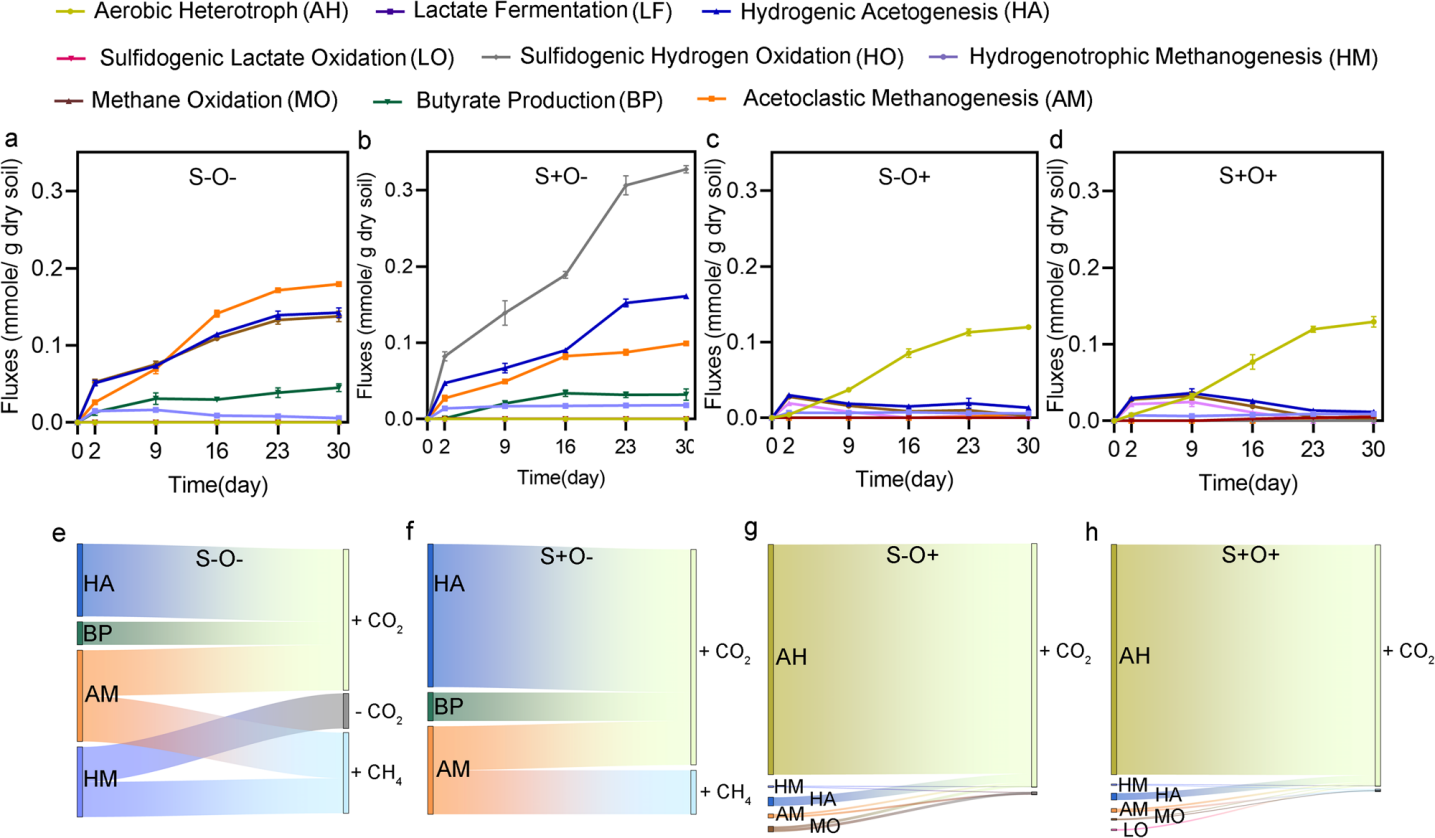
Stoichiometric modeling of the wetland microbial community. **a**–**d** fluxes of the key reactions under four experimental conditions. **e**–**h** Production (+) and consumption (-) of CO_2_ and CH_4_ by these reactions. The error bars are defined as standard deviation.

Under the S-O- condition, the primary reactions involved were hydrogenic acetogenesis, hydrogenotrophic methanogenesis, and acetoclastic methanogenesis. SO_4_^2-^ addition significantly increased the flux from 0 to 0.33 ± 0.005 mmole/g dry soil through sulfidogenic hydrogen oxidation (Fig. 6a, b). This observation was consistent with the increased abundance of functional proteins, including sulfate adenylyltransferase (1.4-fold, *q-*value = 0.002), adenylylsulfate reductase (1.8-fold, *q-*value < 0.001) and dissimilatory sulfate reductase (1.5-fold, *q-*value = 0.001) in the dissimilatory sulfate reduction pathway (Fig. 5c). Furthermore, SO_4_^2-^ addition halted hydrogenotrophic methanogenesis and significantly reduced the flux by 1.7- folds through acetoclastic methanogenesis (*p*-value = 0.0014, Wald type II χ² tests) (Fig. 6b). These reductions were supported by the fact that the abundance of functional proteins for hydrogenotrophic methanogenesis decreased much more than for acetoclastic methanogenesis. Under the two oxic conditions, the dominant reaction shifted to aerobic heterotrophy. The fluxes through dominant reactions observed under anoxic conditions were consistently lower than oxic conditions, such as hydrogenic acetogenesis (fold change > 3.9, *p-*value = 0.000, Wald type II χ² tests), hydrogenotrophic methanogenesis (fold change = 6.0, *p-*value = 0.004, Wald type II χ² tests), and acetoclastic methanogenesis (fold change > 35.4, *p-*value = 0.0003, Wald type II χ² tests). Moreover, O_2_ also eliminated sulfidogenic hydrogen oxidation and butyrate production (Fig. 6c, d). Under S+O+ condition, sulfidogenic lactate oxidation replaced sulfidogenic hydrogen oxidation for sulfate reduction compared to S+O- condition (Fig. 6b, d). These findings indicate that the addition of SO_4_^2-^ and O_2_ restructured the carbon flows across the metabolic network of the wetland community.

The models estimated the contributions of each reaction to the CO_2_ and CH_4_ accumulations. Under S-O- condition – where CO_2_ accumulation was lowest – it was estimated 51% of CO_2_ was derived from hydrogenic acetogenesis (0.28 ± 0.01 mmole/g dry soil), 32% from acetoclastic methanogenesis (0.18 ± 0.002 mmole/g dry soil), and 17% from butyrate production (0.091 ± 0.01 mmole/g dry soil). Additionally, 0.14 ± 0.008 mmole/g dry soil CO_2_ was used for hydrogenotrophic methanogenesis to produce CH_4_. SO_4_^2-^ addition reduced the contributions of butyrate production (0.064 ± 0.014 mmole/g dry soil) and acetoclastic methanogenesis (0.099 ± 0.002 mmole/g dry soil) to CO_2_ accumulation by 31% and 45%, respectively. However, the contribution of CO_2_ accumulation from hydrogenotrophic acetogenesis increased by 13%, and no flux was detected from hydrogenotrophic methanogenesis using CO_2_ to produce CH_4_. These overall changes resulted in a 16% increase in total CO_2_ accumulation. The highest CH_4_ accumulation was observed under S-O- condition, with acetoclastic methanogenesis contributing 56.6% (0.18 ± 0.002 mmole/g dry soil) and hydrogenotrophic methanogenesis contributing 43.4% (0.14 ± 0.008 mmole/g dry soil) (Fig. 6e). Under the S+O- condition, only acetoclastic methanogenesis contributed to CH_4_ accumulation, and the total CH_4_ accumulation decreased by 69% compared to the S-O- condition (Fig. 6f). Under the two oxic conditions, aerobic heterotrophs contributed 94% and 96% of the CO_2_ produced under non-sulfate addition and sulfate addition conditions, respectively (Fig. 6g, h).

## Discussion

**Fig. 7:**
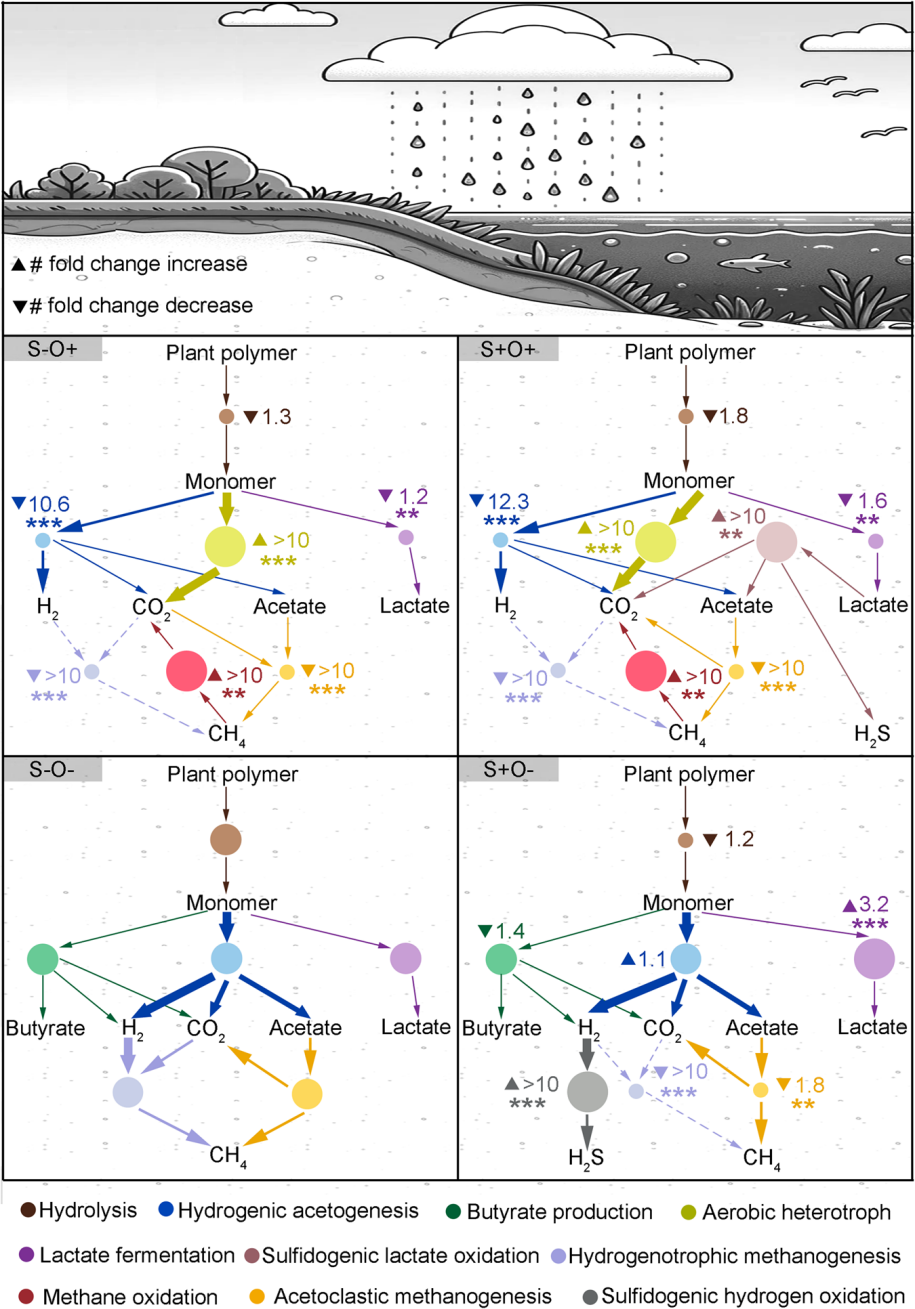
Conceptual food web responding to SO_4_^2-^ and O_2_ perturbations. The S-O- condition serves as the baseline. Circle size corresponds to the relative abundance of marker proteins within the reaction, with larger circles indicating higher abundance, and smaller circles denoting lower abundance. Numbers alongside circles indicate the fold change in metabolic fluxes as inferred from stoichiometric models relative to the control. Edge thickness represents the magnitude of fluxes that consume or produce metabolites. Dashed lines indicate fluxes for nearly eliminated hydrogenotrophic methanogenesis post-treatment. Statistical significance was assessed by Wald type II χ² tests, with *p-*values adjusted for the false discovery rate indicated by: *** *p* < 0.001, ** *p* < 0.01, * *p* < 0.05.

Climate change will result in increased hydrological challenges to wetlands, including seawater intrusion and changing regional regimes of drought and flooding. These stressors lead to recurrent perturbations in wetland microbial communities, primarily due to elevated O_2_ during periods of drought and elevated SO_4_^2-^ from seawater intrusion^22,29^. Our findings demonstrated that the addition of SO_4_^2-^ and O_2_ diminished CH_4_ emissions and increased CO_2_ emissions, which is consistent with observations in natural wetland ecosystems exposed to increased seawater and O_2_^1,30,31^. However, it remains challenging in natural wetland soil to determine how SO_4_^2-^ and O_2_ shape microbial processes that ultimately impact CH_4_ and CO_2_ emissions^32,33^. In this study, we developed a conceptual food web connecting the metabolic activities of key microbial populations to CH_4_ and CO_2_ emissions, under the perturbation of elevated SO_4_^2-^ and O_2_ (Fig. 7). Our findings indicated that elevated availabilities of SO_4_^2-^ and O_2_ changed the compositions of functional guilds and their metabolic activities, including plant polymer breakdown, methane production and oxidation, as well as sulfide/sulfur oxidation. Elevated SO_4_^2-^ reduced CH_4_ emission by 3.7-fold and increased CO_2_ emission by 1.1-fold. Exposure to O_2_ resulted in a 33.3-fold decrease in CH_4_ emission and only a 1.7-fold increase in CO_2_ emission. Because the warming effect of CH_4_ is approximately 28-36 times greater than that of CO_2_^34^, the elevated SO_4_^2-^ and O_2_ exposures may reduce the overall warming effect of gas emissions from wetlands.

The reduction in the CH_4_ production by SO_4_^2-^ exposure was attributed primarily to the sharp decrease in H_2_ available for hydrogenotrophic methanogenesis and acetate needed for acetoclastic methanogenesis. Notably, hydrogenotrophic methanogenesis appeared to be more sensitive to elevated SO_4_^2-^ compared to acetoclastic methanogenesis. This sensitivity is rooted in the thermodynamic basis of competition for H_2_ and carbon by methanogenic consortia and SRB, as hydrogenotrophic sulfate reduction is more energetically favorable than either hydrogenotrophic methanogenesis or acetoclastic methanogenesis^22,35,36^. Even under oxic conditions, SO_4_^2-^ addition facilitated the co-existence of SOB and SRB. Oxygen consumed by sulfide-oxidizing SOB fosters the growth of anaerobic sulfide-generating SRB. In contrast, acetoclastic methanogens do not directly compete with SRB, as most SRBs have an incomplete TCA cycle, which prevents them from completing the oxidation of acetate to CO_2_^37,38^. In addition, our results indicated that a key factor contributing to the increase in CO_2_ emission due to the addition of SO_4_^2-^ is the inhibition of hydrogenotrophic methanogenesis, which consumes CO_2_. SO_4_^2-^ addition reorganized carbon fluxes through the community due to SRB outcompeting hydrogenotrophic methanogens, in turn driving accumulation of short-chain fatty acids and increasing CO_2_ emissions.

O_2_ suppressed both hydrogenotrophic methanogenesis and acetoclastic methanogenesis. In the absence of SO_4_^2-^, elevated O_2_ suppressed reactions that produce H_2_, such as hydrogenic acetogenesis. The decreased H_2_ production constrained the hydrogenotrophic methanogenesis^39^. Under the sulfate addition conditions, the introduction of O_2_ was found to enhance the consumption of H_2_S by SOB. Consequently, this promoted H_2_ uptake by sulfate reduction and intensified the competition between hydrogenotrophic methanogens and SRB^38^. The proteomic abundances of carbohydrate degradation enzymes also indicated that O_2_ altered the preference for the degradation of plant polysaccharides. Exposure to O_2_ favored microbial communities that hydrolyze polysaccharides with more complex structures. Upon recovery from oxic conditions, this may lead to prolonged release of CO_2_^24^.

Approximately 80% of the studies investigating the impact of anthropogenic pressures on ecosystem functions have primarily focused on analyzing the effect of individual stressors in isolation^40,41^. However, climate change may introduce many co-occurring stressors, which need to be investigated in combination. In this study, we found an antagonistic interaction between the effects of sulfate and O_2_ on CH_4,_ and CO_2_ emissions, where the reduction in CH_4_ accumulation and the increase in CO_2_ resulting from the combined treatment of SO_4_^2-^ and O_2_ was less pronounced compared to the sum of individual effects. This non-additive effect can be attributed to the high functional redundancy stemming from extraordinarily diverse functional guilds in soils^41–43^. Furthermore, both SO_4_^2-^ and O_2_ affect certain metabolic processes in a similar manner. For instance, elevated levels of either SO_4_^2-^ or O_2_ nearly completely halted hydrogenotrophic methanogenesis, resulting in a reduction in CH_4_ accumulation. Therefore, when SO_4_^2-^ and O_2_ exposures are combined, the observed methanogenesis decrease is not additive.

In this study, we generated a functional understanding of the decreased CH_4_ and increased CO_2_ emissions in wetland microcosms subjected to SO_4_^2-^ and O_2_ pressure by integrating geochemical analysis, proteogenomics, and stoichiometric modeling. The geochemical analysis quantified the end-products’ accumulation levels and the intermediates’ standing levels in the metabolic network, which were used by the stoichiometric modeling to estimate the flux rates across key metabolic processes under different conditions. Many of the changes in the estimated flux rates were supported by the concordant changes in the protein abundances of enzymes involved in those metabolic processes. The obtained results may enable climate models to better estimate greenhouse gas emissions under changing environmental conditions.

## Methods

### Sampling and soil microcosm set up

Triplicate soil samples were collected from a seasonally flooded urban freshwater wetland connected to Lake Washington in Seattle, WA, USA (Coordinates: 47.642196^o^N, -122.296236^o^W) in May 2022 using a soil core sampler with 2” x 6” plastic liners (AMS, Inc., American Falls, ID). The core sampler was forced approximately 60 cm into the sediment bed with a slide hammer to cut roots and other plant material. The three replicate soil cores were collected approximately one meter apart from each other, avoiding large tree branches or roots. The broader location was selected based on its variably submerged nature, with the water table generally being below the surface during the fall and winter months and above the surface during the spring and summer months. At the time of sampling, the water table was above the soil surface and the soil was completely saturated with water. The cores were sealed air-tight upon removal from the natural habitat, to protect the redox state of samples. Samples were then transported to the lab on ice, for immediate processing.

Microcosms were set up by filling 160 mL bottles with 40 g fresh sediment soil slurry in an anaerobic chamber. The chamber gas contained N_2_ : H_2_ (97:3). All bottles were preincubated for five days at room temperature and then incubated at 30 °C for 30 days. Soil microcosms were divided into four groups, with each group receiving specific treatments every two days: anoxic non-sulfate-addition, anoxic sulfate-addition (resulting in a final SO_4_^2-^ concentration of approximately 7.2 mM), oxic non-sulfate-addition (where half of the gas phase was replaced by sterile air), and oxic sulfate-addition, which was treated with both SO_4_^2-^ and sterile air. Sampling was conducted on days 2, 9, 16, 23, and 30, with each group sacrificing three bottles for sampling at the indicated time points. During sampling, 10 mL of gas phase products were collected in vacuumed Labco exetainer gas vails for later analysis. Then 10 ml of PBS buffer was injected into the bottle, and shaken at 200 rpm for 10 min, after which 10 ml of well-shaken culture liquid was sampled, and stored at -80 °C for subsequent analysis.

### Chemical analysis

Headspace pressure was measured using a pressure gauge (Cole Parmer SK-68900-24) before sampling. pH measurements were taken immediately after sampling. Analysis of H_2_, CO_2_ and CH_4_ was carried out using a gas chromatograph (GC-8A, Shimadzu, Japan). H_2_S in the gas phase was measured by an H_2_S monitor (Forensics, FD-103-H2S, US), the H_2_S dissolved in slurry was calculated based on the pH and the amount of H_2_S in the gas phase. Absolute gas composition was calculated with the ideal gas law. The soluble sugars (glucose, xylose, mannose and galacturonic acid) and fermentation products (acetate, lactate, and butyrate) in the slurry were analyzed by high-performance liquid chromatography (HPLC) as reported previously^44^. The metabolic measurements were standardized using the gram of dry soil at the endpoint, which constitutes 22.9 ± 0.1% of soil slurry.

### DNA extraction and metagenomic sequencing

Only the initial soils from the field were sequenced using metagenomic analysis. The total DNA in the triplicate field soil samples was extracted using the PowerMaxSoil DNA isolation kit as described previously^45^. Metagenomic library preparation and DNA sequencing were performed at the Joint Genome Institute. The metagenomic libraries were prepared for sequencing using 2 × 151-bp lanes on the Illumina NovaSeq S4 platform. A total of 404,050,411 ± 23,970,278 sequence reads were obtained.

### Metagenomic data processing

Metagenomic reads were preprocessed using BBTools for removing adaptors, trimming reads, and sequencing error correction^46^. The pre-processed reads from three replicates were co-assembled into a combined metagenome with SPAdes v3.15.5^47^, A total of 857,101 scaffolds with a length > 1 kbp were retained. The total length and L50 of the assembly were 1,939,450,947 bp and 2420 bp, respectively. The percentage of sequencing reads mapped onto the scaffolds is 87%. Genes were called from these retained scaffolds using the Prodigal algorithm^48^. A total of 26,768,938 genes were identified. Gene functions were annotated using Kofamscan^49^. Taxonomic classification was carried out at the scaffold level using Kaiju, based on reference species in the NCBI RefSeq^50^. Metagenome-assembled genomes (MAGs) were obtained using MetaBAT v2.12.1 with default parameters^51^. The quality of MAGs was estimated using CheckM v1.1.2. The MAGs obtained were classified into high-quality MAGs with completeness > = 50% and contamination < 5%. The taxonomy classifications of high-quality MAGs were inferred using GTDB-Tk v2.3.2^52^.

### Metaproteomics measurement

Soil cultures at the end of the experiment were analyzed by metaproteomics. Proteins were extracted as described previously. Briefly, soil samples were suspended in lysis buffer (containing 10 mM tris-HCl, 4% SDS and 10 mm dithiothreitol), boiled for 5 min and further disrupted by sonication for a 2 min 10% pulse 5 times. The supernatant was collected by centrifugation at 14,000 g for 10 min. Proteins were then precipitated by TCA (trichloroacetic acid) overnight and pelleted by centrifugation. Protein pellets were washed with ice-cold acetone three times and resuspended in guanidine buffer. Protein concentrations were quantified by Bicinchoninic Acid Assay. Twenty mg samples of proteins were processed using FASP (filter-aided sample preparation) and digested by Trypsin/LysC mixture. Each sample was analyzed using 2D-LC-MS/MS (two-dimensional liquid chromatography-tandem mass spectrometry) on an Orbitrap Fusion Tribrid mass spectrometer (Thermo Fisher Scientific, USA) at the IDeA National Resource for Quantitative Proteomics. Tryptic peptides were separated into 46 fractions on a 100 × 1.0 mm Acquity BEH C18 column (Waters) using an UltiMate 3000 UHPLC system (Thermo) with a 50 min gradient from 99:1 to 60:40 buffer A:B ratio under basic pH conditions, then consolidated into 12 super-fractions. Each super-fraction was then separated by reverse phase XSelect CSH C18 2.5 um resin (Waters) on an in-line 120 × 0.075 mm column using an UltiMate 3000 RSLCnano system (Thermo). Peptides were eluted using a 60 min gradient from 98:2 to 65:35 buffer A:B ratio. Eluted peptides were ionized by electrospray (2.4 kV) followed by mass spectrometric analysis on an Orbitrap Fusion Tribrid mass spectrometer (Thermo). MS data were acquired using the FTMS analyzer in profile mode at a resolution of 240,000 over a range of 375 to 1500 m/z. Following HCD activation, MS/MS data were acquired using the ion trap analyzer in centroid mode and normal mass range with normalized collision energy of 28–31% depending on charge state and precursor selection range. Protein identification and quantification followed established procedures. Briefly, mass spectrometry spectra were searched using Sipros Ensemble against a protein database constructed from the metagenome. Raw search results were filtered to achieve 1% FDR (false discovery rate) at the peptide level, which was estimated using the target-decoy approach. Peptide identifications are assigned to protein or protein groups in accordance with the parsimonious rule. To avoid ambiguity in data analysis, protein groups were excluded from biological analysis. Protein quantification was achieved through Intensity-based label-free analysis using ProRata^14^. Protein abundances were quantified by the total peak height of all quantified peptides from a protein, normalized against the average total of all data sets^53–55^.

Following previously described methods^56–58^, the taxonomic annotations were assigned to the identified peptides based on the matched corresponding scaffold’s taxonomic annotation information, which had a length greater than 1000 bp. The relative abundance of a species is expressed as the sum of the abundances of all proteins detected that belong to this species. If a protein was identified in more than one species, its abundance was equally divided among these identified species. The richness and evenness of the communities were calculated using the vegan R package, based on the total protein abundance of species^59^.

### Construction of the stoichiometric model

A set of overall reactions depicting the documented metabolism of major functional populations was fit to the measured amounts of metabolites in the microcosms (Supplementary Fig. 3, Supplementary Fig. 4). The code used in fitting can be found on the Github https://github.com/thepanlab/WetlandSoil. The resulting fits provide a basis for quantifying metabolic contributions. Deviations between the inferred metabolic transformations and measured transformations indicate uncertainty in our understanding of the metabolic transformations or limitations in analytic capacity.

### Statistical analysis

Pairwise comparisons for metabolic measurement data were carried out using Student’s t-test, while metabolic fluxes from modeling were analyzed using Wald type II χ² tests. One-way or two-way ANOVA was used for comparisons among groups of more than two. Differences in protein abundance in the metaproteome were analyzed by Deseq2 R package, which is based on a model using the negative binomial distribution to account for variance and mean linkage through local regression^60–62^. The *p-*values were adjusted to *q-*values using the Benjamini and Hochberg method for multiple comparison correction^63^. Differences with *q-*value < 0.05 were regarded as statistically significant.

### Supplementary information

Supplementary Fig. 1, 2, 3, 4, 5, Supplementary Table 1, 2, 3, 4

## Data Availability

The metagenome data was deposited in NCBI under the accession code PRJNA1112840. Proteomic data are available at the ProteomeXchange Consortium via the PRIDE (Proteomics Identification Database) partner repository with the dataset identifier PXD047453. The rest generated or analyzed data during this study are included in this published article and Supplementary Tables 1-3.
