# Supplementary material for: Metaproteomics-informed stoichiometric modeling reveals the responses of wetland microbial communities to oxygen and sulfate exposure
Source: NPJ Biofilms Microbiomes. 2024 Jul 3;10:55. doi: 10.1038/s41522-024-00525-5 (PMC11222425; doi:10.1038/s41522-024-00525-5)
Supplement: Supplementary file 1 — Supplementary Fig. 1, 2, 3, 4, 5, Supplementary Table 1, 2, 3, 4 [file 41522_2024_525_MOESM1_ESM.pdf]

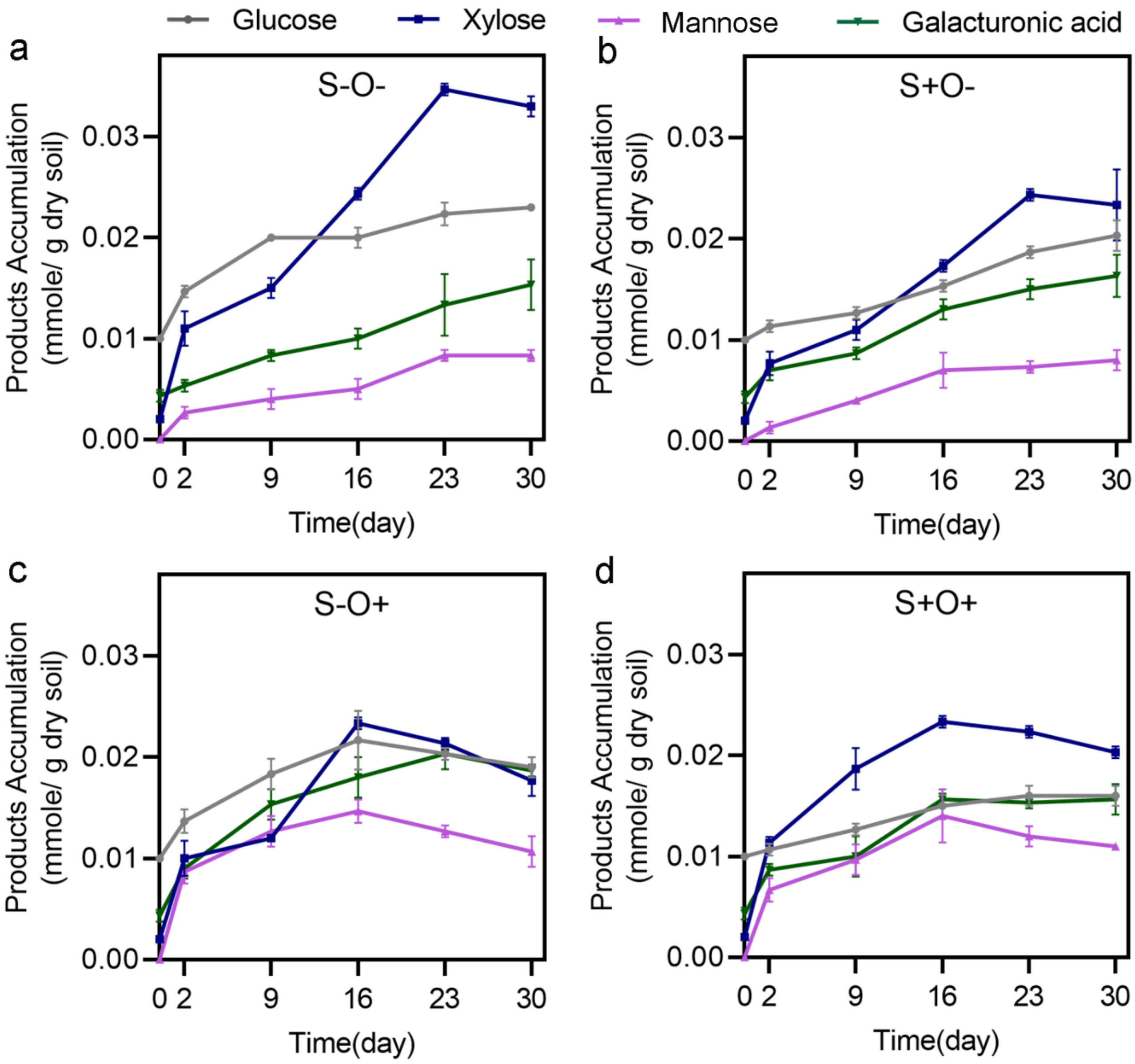


**Supplementary Fig. 1: Accumulation of monosaccharides over a 30-day incubation under the four conditions.** The error bars are defined as standard deviation.

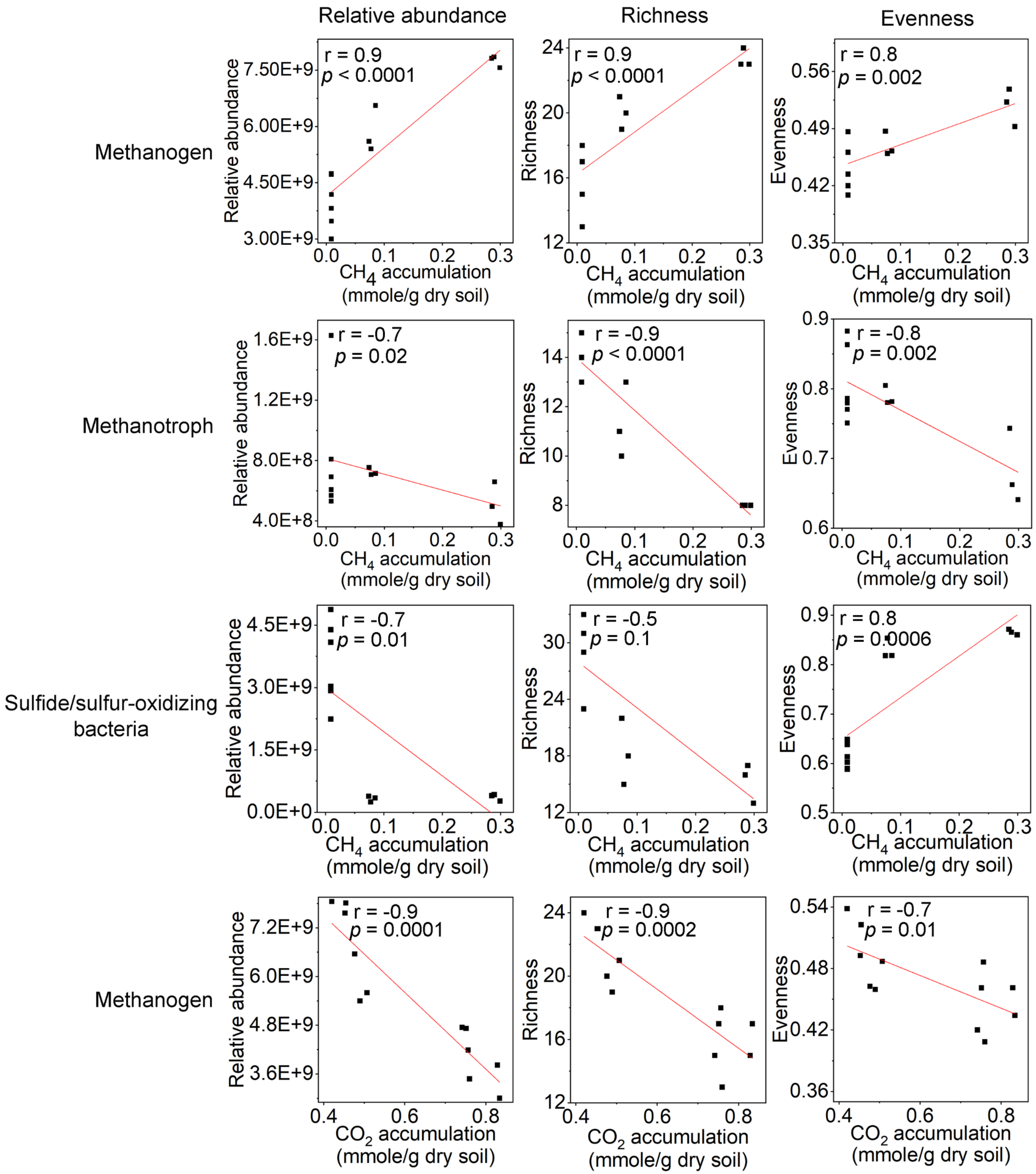


**Supplementary Fig. 2: Correlation between the accumulations of $CH_4$ and $CO_2$ , and the proteome abundance, richness, and evenness of methanogen, methanotroph, and sulfide/sulfur-oxidizing bacterial communities. E**ach dot represents an experimental measurement (X-axis for geochemical measurements, Y-axis for metaproteomic measurements) from the four conditions with three replicates, and the red line displays best linear fit. r is the Pearson correlation coefficient, and *p* values were determined by t-test.

| Reactions | Glucose | Hydrogen | Carbon deoxide | Methane | Sulfide | Lactate | Acetate | Butyrate |
|---|---|---|---|---|---|---|---|---|
| Aerobic heterotroph | -1 | 0 | 6 | 0 | 0 | 0 | 0 | 0 |
| Lactate fermentation | -1 | 0 | 0 | 0 | 0 | 2 | 0 | 0 |
| Hydrogenic acetogenesis | -1 | 4 | 2 | 0 | 0 | 0 | 2 | 0 |
| Sulfidogenic lactate oxidation | 0 | 0 | 1 | 0 | 0.5 | -1 | 1 | 0 |
| Sulfidogenic hydrogen oxidation | 0 | -2 | 0 | 0 | 0.5 | 0 | 0 | 0 |
| Hydrogenic methanogenesis | 0 | -4 | -1 | 1 | 0 | 0 | 0 | 0 |
| Acetoclastic methanogenesis | 0 | 0 | 1 | 1 | 0 | 0 | -1 | 0 |
| Methane oxidation | 0 | 0 | 1 | -1 | 0 | 0 | 0 | 0 |
| Butyrate production | -1 | 2 | 2 | 0 | 0 | 0 | 0 | 1 |

Metabolites

**Supplementary Fig. 3: The stoichiometric matrix in modeling analysis.** Each matrix element is the stoichiometric coefficient of a metabolite in a reaction. Positive values correspond to the amounts of metabolites produced by the reaction. Negative values denote consumption. A value of zero indicates no involvement of metabolites in the reaction.

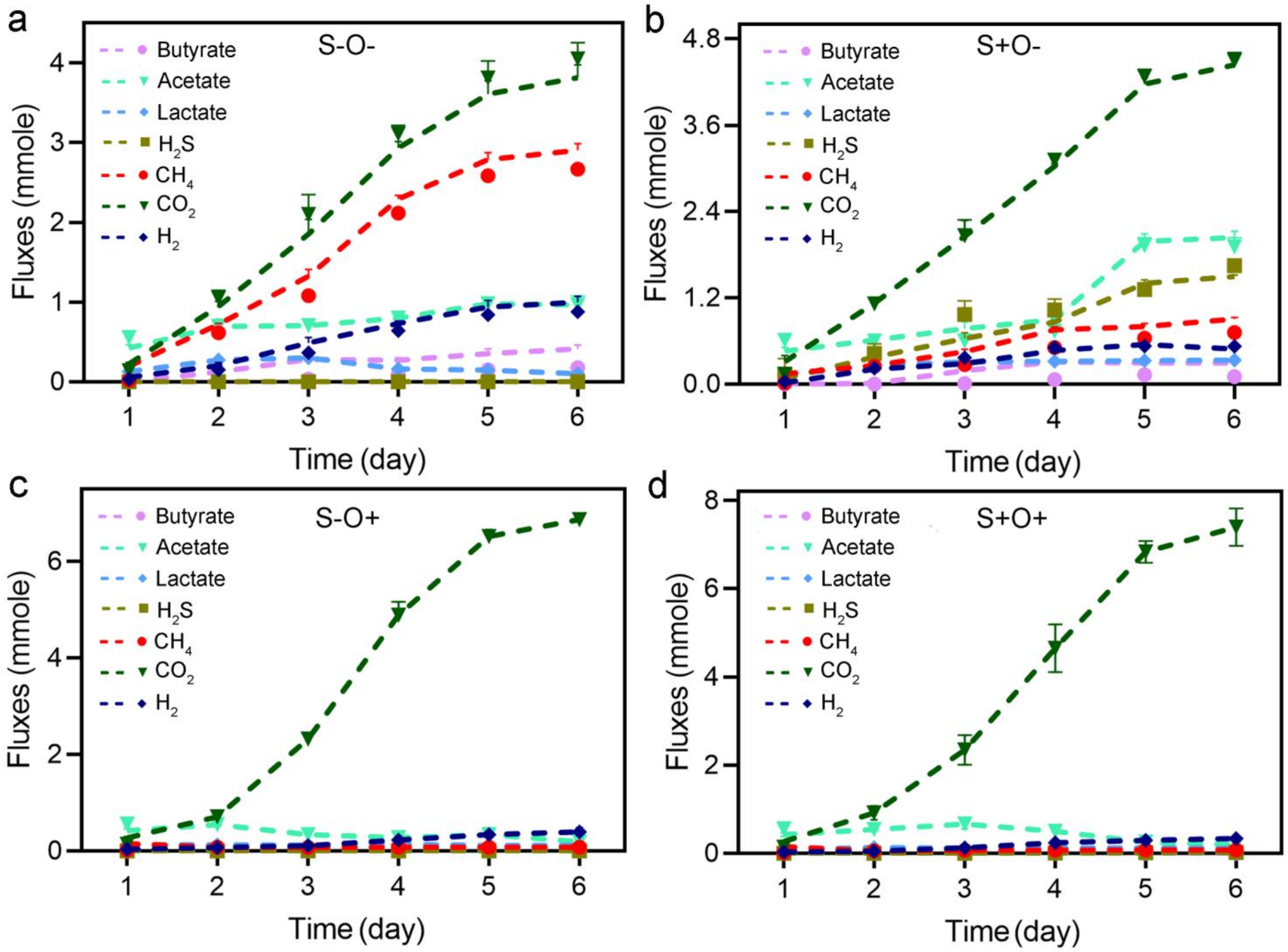


**Supplementary Fig. 4: Model fitting and average fluxes under four conditions.** (a-d) Modeling fitting in the S-O- (A), S+O- (B), S-O+ (C) and S+O+ (D) conditions. The resulting fits indicate the relative contribution of each reaction to the overall observed transformations. Deviations between the inferred metabolic transformations and the measured transformations indicate uncertainty in the understanding of metabolic transformations or limitations in analytical capacity.

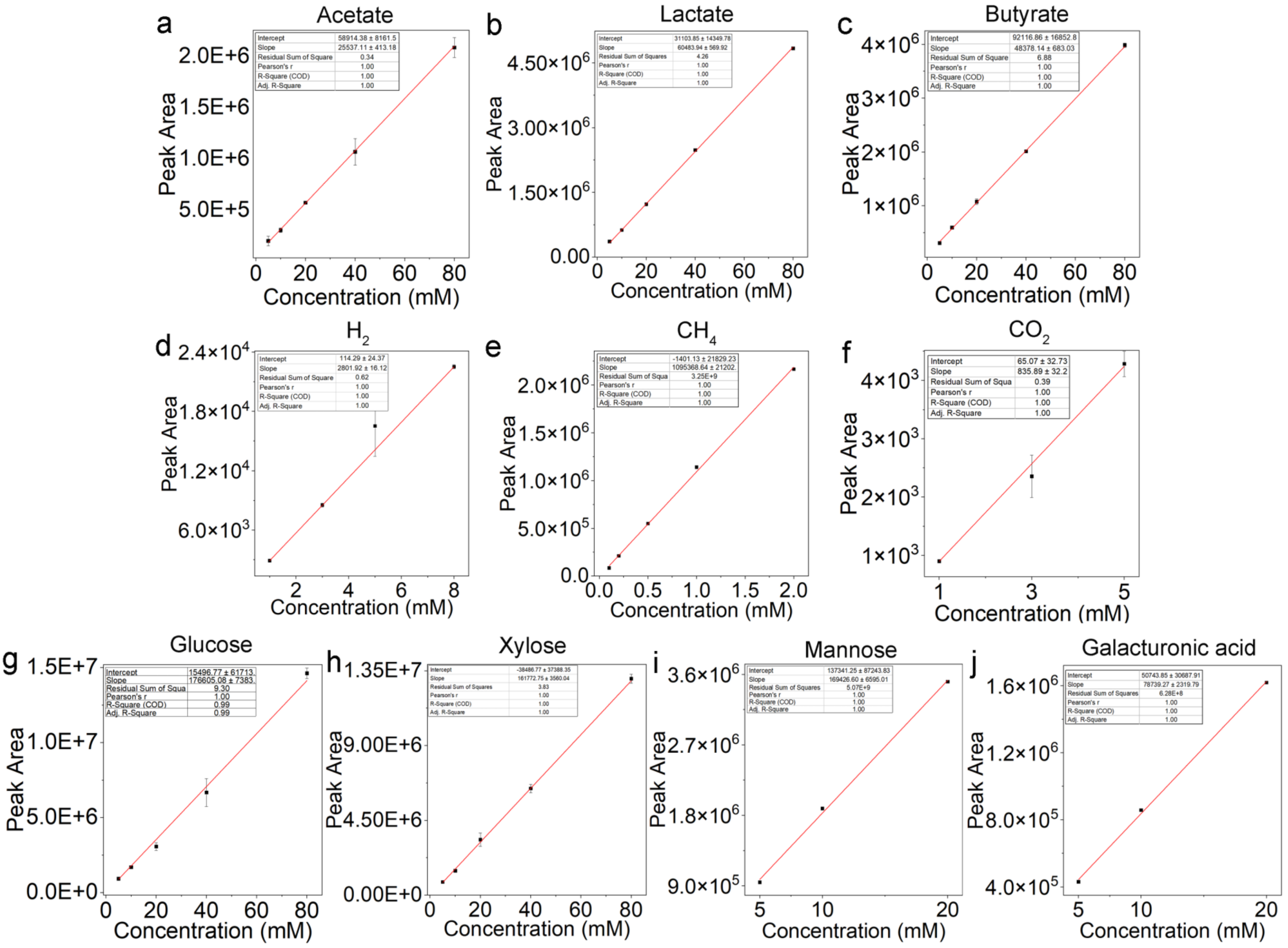


**Supplementary Fig. 5: Calibration curves of known standards.**

Supplementary Table 1. Main functional guilts involved in methane metabolism and sulfate reduction.

| Category | Species | Total proteome abundance | | | | | | | | | | | |
|---|---|---|---|---|---|---|---|---|---|---|---|---|---|
| | | S-O-_1 | S-O-_2 | S-O-_3 | S+O-_1 | S+O-_2 | S+O-_3 | S-O+_1 | S-O+_2 | S-O+_3 | S+O+_1 | S+O+_2 | S+O+_3 |
| Methanogenesis | *Methanocella arvoryzae* | 0.00 | 91120000.00 | 176782000.00 | 0.00 | 29252000.00 | 152051000.00 | 0.00 | 0.00 | 0.00 | 0.00 | 0.00 | 0.00 |
| | *Methanofollis fontis* | 11910000.00 | 46668400.00 | 0.00 | 15105000.00 | 27768800.00 | 20827600.59 | 24829200.00 | 21191400.00 | 24664500.00 | 11627000.00 | 14616800.00 | 13957500.00 |
| | *Methanofollis formosanus* | 84370000.00 | 9860000.00 | 4050717.44 | 43120500.00 | 37904000.00 | 37938709.50 | 32148000.00 | 21697700.00 | 23878800.00 | 19866000.00 | 17248000.00 | 20505000.00 |
| | *Methanomassiliicoccus luminyensis* | 358042738.00 | 331411600.00 | 345792201.60 | 267159000.00 | 224076500.00 | 285866559.30 | 71599054.32 | 65782400.00 | 45167682.51 | 22684200.00 | 31213600.00 | 8719049.25 |
| | *Methanoregula boonei* | 502717086.00 | 479540987.80 | 553420295.50 | 164136561.60 | 335738800.00 | 511337218.80 | 284195383.40 | 175885355.30 | 269216143.30 | 274182212.20 | 196811289.80 | 308598063.00 |
| | *Methanoregula formicica* | 1528983905.00 | 859756668.60 | 795420299.40 | 624551249.60 | 669905968.30 | 645203645.70 | 745692334.90 | 491797813.00 | 611151735.20 | 811158168.60 | 736824593.10 | 780961158.80 |
| | *Methanosarcina barkeri* | 0.00 | 277708.60 | 2616542.50 | 15409000.00 | 19668959.31 | 16232952.85 | 0.00 | 4300500.00 | 5653800.00 | 0.00 | 0.00 | 0.00 |
| | *Methanosarcina horonobensis* | 5210000.00 | 4753200.00 | 0.00 | 6146500.00 | 9918900.00 | 0.00 | 1645685.76 | 1573800.00 | 5337900.00 | 0.00 | 0.00 | 0.00 |
| | *Methanosarcina lacustris* | 0.00 | 0.00 | 0.00 | 6773500.00 | 9774700.00 | 0.00 | 0.00 | 0.00 | 0.00 | 0.00 | 0.00 | 0.00 |
| | *Methanosarcina mazei* | 0.00 | 0.00 | 0.00 | 5424500.00 | 7210000.00 | 0.00 | 0.00 | 0.00 | 0.00 | 0.00 | 0.00 | 0.00 |
| | *Methanosarcina spelaei* | 5640000.00 | 9934800.00 | 15520750.22 | 46879085.70 | 37257696.63 | 84866435.92 | 12790800.00 | 28026737.92 | 77138814.24 | 2895200.00 | 3062400.00 | 1740000.00 |
| | *Methanosarcina thermophila* | 0.00 | 0.00 | 0.00 | 3714500.00 | 3852200.00 | 0.00 | 0.00 | 0.00 | 0.00 | 0.00 | 0.00 | 0.00 |
| | *Methanosarcina vacuolata* | 1002666.00 | 0.00 | 4738635.88 | 0.00 | 6756800.00 | 11487877.84 | 0.00 | 0.00 | 0.00 | 0.00 | 0.00 | 0.00 |
| | *Methanosphaerula palustris* | 293107853.00 | 60805600.00 | 236966700.00 | 643618678.70 | 111652000.00 | 396291486.00 | 153056400.00 | 121935957.30 | 312984000.00 | 152806500.00 | 27024800.00 | 38287500.00 |
| | *Methanospirillum lacunae* | 46830000.00 | 47893381.24 | 15631959.61 | 25194000.00 | 49666600.00 | 6832747.15 | 0.00 | 23676305.15 | 17172000.00 | 24891642.16 | 29103807.04 | 8249915.25 |
| | *Methanospirillum stamsii* | 0.00 | 0.00 | 0.00 | 0.00 | 0.00 | 0.00 | 0.00 | 1836100.00 | 1660500.00 | 1424500.00 | 2103200.00 | 0.00 |
| | *Methanothrix harundinacea* | 62990000.00 | 40560034.80 | 35300100.00 | 34960000.00 | 12555700.00 | 38630902.82 | 7159200.00 | 2409500.00 | 5783400.00 | 2810500.00 | 22668800.00 | 6307500.00 |
| | *Methanothrix soehngenii* | 4523979093.00 | 5583763208.00 | 5220457833.00 | 4443857291.00 | 3826816195.00 | 2957875714.00 | 3370593032.00 | 3094589394.00 | 3218587908.00 | 2393909811.00 | 2365878057.00 | 1763694120.00 |
| | *Methanothrix thermoacetophila* | 391700000.00 | 287927037.50 | 160407612.30 | 214282000.00 | 185358800.00 | 239176326.20 | 45878132.64 | 135639600.00 | 106516178.60 | 102618076.30 | 34126218.72 | 54186612.00 |
| Methanotroph | *Methylobacillus arboreus* | 17109400.00 | 11545600.00 | 5167500.00 | 0.00 | 0.00 | 0.00 | 3008133.96 | 868658.91 | 527842.98 | 0.00 | 0.00 | 0.00 |
| | *Methylobacillus gramineus* | 0.00 | 0.00 | 0.00 | 3344000.00 | 0.00 | 393690.61 | 0.00 | 0.00 | 0.00 | 8720000.00 | 9016800.00 | 5621521.22 |
| | *Methylobacillus rhizosphaerae* | 17440500.00 | 28520800.00 | 47610000.00 | 0.00 | 0.00 | 0.00 | 2599200.00 | 4074800.00 | 10481400.00 | 0.00 | 0.00 | 0.00 |
| | *Methylobacter luteus* | 3680600.00 | 0.00 | 7072500.00 | 0.00 | 0.00 | 0.00 | 0.00 | 5984100.00 | 3888000.00 | 21080000.00 | 12702400.00 | 0.00 |
| | *Methylobacter psychrophilus* | 59197600.00 | 17177600.00 | 17107500.00 | 13985447.80 | 17777800.00 | 2013079.81 | 25057200.00 | 34251500.00 | 13856598.72 | 49430475.00 | 53378213.64 | 173798100.00 |
| | *Methylobacter tundripaludum* | 29905024.38 | 38182041.04 | 28837500.00 | 42417500.00 | 291777.37 | 52233508.35 | 36284193.60 | 17692716.94 | 32634900.00 | 49503075.00 | 71211026.64 | 87256560.63 |
| | *Methylobacterium gnaphalii* | 3984333.43 | 3940948.00 | 7116369.00 | 0.00 | 0.00 | 0.00 | 0.00 | 2635200.00 | 2106000.00 | 14550000.00 | 8812800.00 | 0.00 |
| | *Methyloceanibacter marginalis* | 0.00 | 0.00 | 0.00 | 3496000.00 | 7552992.06 | 0.00 | 0.00 | 0.00 | 0.00 | 0.00 | 0.00 | 0.00 |
| | *Methyloceanibacter methanicus* | 4712400.00 | 0.00 | 6427500.00 | 5155412.50 | 0.00 | 3065495.05 | 0.00 | 1903200.00 | 16969500.00 | 7940000.00 | 4127600.00 | 2516942.17 |
| | *Methyloceanibacter superfactus* | 189103309.80 | 378138387.40 | 73325940.00 | 180509500.00 | 33979700.00 | 57516581.88 | 198656400.00 | 68307800.00 | 247312282.90 | 41750000.00 | 32102800.00 | 158878600.00 |
| | *Methylocella silvestris* | 4650800.00 | 4963200.00 | 0.00 | 0.00 | 0.00 | 0.00 | 0.00 | 0.00 | 0.00 | 4700000.00 | 3536000.00 | 0.00 |
| | *Methylocystis parvus* | 52567900.00 | 45011521.28 | 34500000.00 | 89953704.50 | 122982000.00 | 40494000.00 | 49077727.32 | 56040700.00 | 37389600.00 | 58700000.00 | 77227600.00 | 66888425.45 |
| | *Methyloglobulus morosus* | 0.00 | 0.00 | 0.00 | 0.00 | 0.00 | 0.00 | 21477600.00 | 4648200.00 | 28422900.00 | 0.00 | 0.00 | 0.00 |
| | *Methylomagnum ishizawai* | 109386200.00 | 122390400.00 | 143707500.00 | 376285500.00 | 571722100.00 | 551830000.00 | 266912734.90 | 328966900.00 | 167985380.00 | 529840221.00 | 409133575.60 | 1125740277.00 |
| | *Methylomicrobium lacus* | 0.00 | 0.00 | 0.00 | 0.00 | 0.00 | 0.00 | 0.00 | 1793400.00 | 1279800.00 | 0.00 | 0.00 | 0.00 |
| | *Methylotenera oryzisoli* | 0.00 | 1812800.00 | 1237500.00 | 0.00 | 0.00 | 0.00 | 0.00 | 0.00 | 0.00 | 0.00 | 0.00 | 0.00 |
| | *Methylotenera versatilis* | 5028100.00 | 8395200.00 | 5542500.00 | 0.00 | 0.00 | 0.00 | 4947600.00 | 4825100.00 | 7047000.00 | 23510000.00 | 11968000.00 | 9345800.00 |
| Sulfate-reducing bacteria | *Desulfacinum hydrothermale* | 49596282.00 | 105434000.00 | 72480276.57 | 72036652.25 | 138807582.30 | 171901488.30 | 101403652.10 | 110905729.90 | 83940329.16 | 63217000.00 | 71531935.20 | 49786467.00 |
| | *Desulfacinum infernum* | 18431386.00 | 152421923.20 | 84246965.31 | 53104078.50 | 51448500.00 | 212216159.40 | 84818444.16 | 52719304.90 | 147063600.00 | 49380100.00 | 21183969.84 | 15361371.75 |
| | *Desulfamplus magnetovallimortis* | 3150000.00 | 4046000.00 | 0.00 | 0.00 | 0.00 | 0.00 | 0.00 | 5026400.00 | 3102300.00 | 3757600.00 | 2692800.00 | 0.00 |
| | *Desulfarculus baarsii* | 46546369.00 | 66299235.68 | 101171100.00 | 37935856.95 | 1122700.00 | 21180485.56 | 5310293.28 | 22019787.32 | 88334963.91 | 3426500.00 | 4312000.00 | 67129006.50 |
| | *Desulfatiglans anilini* | 110094313.00 | 349071871.20 | 122611181.60 | 289350996.20 | 309304616.30 | 356785731.00 | 157283070.80 | 146177189.00 | 224151255.00 | 195501911.20 | 269571288.80 | 237360882.00 |
| | *Desulfatirhabdium butyrativorans* | 46990000.00 | 75612090.00 | 106688500.00 | 132296449.00 | 96489187.69 | 181463519.20 | 119663957.80 | 65111067.55 | 84384747.81 | 85413511.26 | 37975220.80 | 13147500.00 |
| | *Desulfatitalea alkaliphila* | 24810000.00 | 82917948.12 | 217949303.20 | 14240779.30 | 338127591.50 | 47035051.40 | 17625004.20 | 180111258.40 | 223736127.20 | 11314969.05 | 29198400.00 | 130353467.30 |
| | *Desulfatitalea tepidiphila* | 13284275.00 | 44823343.76 | 41267900.00 | 20425000.00 | 83378500.00 | 35785456.93 | 68035200.00 | 61742634.74 | 130256100.00 | 48771800.00 | 115711200.00 | 16714495.50 |
| | *Desulfitibacter alkalitolerans* | 0.00 | 0.00 | 0.00 | 0.00 | 0.00 | 0.00 | 0.00 | 5483900.00 | 523354.77 | 0.00 | 0.00 | 0.00 |
| | *Desulfobacca acetoxidans* | 893732402.00 | 992942834.00 | 702364347.20 | 1162852018.00 | 1578252132.00 | 1889985230.00 | 996889689.60 | 931353005.70 | 852188828.10 | 570621014.80 | 383356945.00 | 472086699.80 |
| | *Desulfobacter hydrogenophilus* | 0.00 | 0.00 | 0.00 | 12754825.40 | 10403000.00 | 0.00 | 10123200.00 | 0.00 | 18013197.15 | 0.00 | 0.00 | 0.00 |
| | *Desulfobacter postgatei* | 2250000.00 | 469943.92 | 0.00 | 0.00 | 1971428.24 | 1746919.10 | 0.00 | 568869.53 | 282956.49 | 3287900.00 | 0.00 | 3427500.00 |
| | *Desulfobulbus alkaliphilus* | 25920000.00 | 47321200.00 | 11274497.25 | 116251500.00 | 29633100.00 | 46480887.04 | 54829672.56 | 32403200.00 | 42444000.00 | 34371814.40 | 22764435.76 | 66691560.00 |
| | *Desulfobulbus elongatus* | 12670000.00 | 42432000.00 | 62493000.00 | 60011500.00 | 54445800.00 | 58548686.60 | 56680800.00 | 40327100.00 | 42346800.00 | 29421700.00 | 35173600.00 | 37605000.00 |
| | *Desulfobulbus oligotrophicus* | 2740000.00 | 0.00 | 4144637.10 | 0.00 | 5180900.00 | 9060453.10 | 0.00 | 0.00 | 0.00 | 4304300.00 | 4993134.96 | 2910000.00 |
| | *Desulfobulbus oralis* | 0.00 | 3372800.00 | 1657038.49 | 0.00 | 0.00 | 0.00 | 0.00 | 287476.53 | 307106.64 | 3183170.76 | 307067.20 | 400651.50 |
| | *Desulfobulbus propionicus* | 0.00 | 0.00 | 0.00 | 66338500.00 | 37399300.00 | 35491800.00 | 19357200.00 | 19703000.00 | 19739700.00 | 4150300.00 | 1707200.00 | 6045000.00 |
| | *Desulfobulbus rhabdoformis* | 9460000.00 | 12607200.00 | 0.00 | 28101000.00 | 16716900.00 | 10582225.56 | 4035600.00 | 10692202.00 | 9383406.93 | 2348500.00 | 0.00 | 1500000.00 |
| | *Desulfocapsa sulfexigens* | 4610000.00 | 2094400.00 | 0.00 | 0.00 | 4377500.00 | 19171395.99 | 2781600.00 | 2183800.00 | 4973400.00 | 0.00 | 1469600.00 | 1942500.00 |
| | *Desulfococcus multivorans* | 41980000.00 | 44789020.08 | 179872205.70 | 143440500.00 | 120540900.00 | 237463187.90 | 93320400.00 | 68002800.00 | 267094541.10 | 64385394.92 | 83747270.64 | 89170640.25 |
| | *Desulfocurvibacter africanus* | 1270000.00 | 5956800.00 | 2937829.71 | 10592500.00 | 11546300.00 | 1393700.26 | 6779135.40 | 5770600.00 | 4451879.88 | 5205200.00 | 2756157.36 | 7074987.75 |
| | *Desulfocurvus vexinensis* | 0.00 | 0.00 | 0.00 | 0.00 | 0.00 | 0.00 | 0.00 | 0.00 | 0.00 | 8855000.00 | 0.00 | 15975000.00 |
| | *Desulfofalx alkaliphila* | 0.00 | 0.00 | 0.00 | 13309500.00 | 0.00 | 3084836.89 | 0.00 | 159210000.00 | 2980800.00 | 0.00 | 0.00 | 0.00 |
| | *Desulfoferrobacter suflitae* | 343454841.00 | 343200596.80 | 386479028.80 | 755193297.40 | 656756461.00 | 863539102.00 | 702434445.20 | 456933142.90 | 485661794.20 | 311571889.10 | 299061914.80 | 385897768.10 |
| | *Desulfofundulus thermobenzoicus* | 0.00 | 0.00 | 0.00 | 0.00 | 0.00 | 0.00 | 0.00 | 276820.44 | 20655000.00 | 0.00 | 0.00 | 0.00 |
| | *Desulfofustis glycolicus* | 1500000.00 | 7860800.00 | 1146819.74 | 21897500.00 | 21115000.00 | 4605200.00 | 5084400.00 | 1159000.00 | 2940300.00 | 10395000.00 | 20864800.00 | 0.00 |
| | *Desulfoglaeba alkanexedens* | 115230000.00 | 252497600.00 | 65063356.23 | 117763214.10 | 590352728.70 | 334857881.40 | 256687416.00 | 583130116.70 | 507555980.00 | 423271009.70 | 512670400.00 | 441607500.00 |
| | *Desulfogranum marinum* | 0.00 | 0.00 | 0.00 | 9690000.00 | 10609000.00 | 7900300.00 | 0.00 | 7795800.00 | 4074300.00 | 0.00 | 0.00 | 0.00 |
| | *Desulfogranum mediterraneum* | 5170000.00 | 6766000.00 | 5911500.00 | 4750475.00 | 28469200.00 | 3749597.51 | 2325600.00 | 2204252.08 | 51921000.00 | 0.00 | 19421600.00 | 3978023.25 |
| | *Desulfohalobium retbaense* | 3980000.00 | 1319200.00 | 0.00 | 0.00 | 3543200.00 | 331186.13 | 832154.40 | 0.00 | 464697.00 | 0.00 | 0.00 | 0.00 |
| | *Desulfohalovibrio alkalitolerans* | 0.00 | 0.00 | 0.00 | 0.00 | 0.00 | 0.00 | 0.00 | 0.00 | 0.00 | 7176400.00 | 3977600.00 | 0.00 |
| | *Desulfolithobacter dissulfuricans* | 1030000.00 | 8092000.00 | 188717600.00 | 106400000.00 | 1369900.00 | 237643159.90 | 183084000.00 | 166694700.00 | 0.00 | 179410000.00 | 154000000.00 | 151237500.00 |
| | *Desulfoluna limicola* | 13010000.00 | 0.00 | 7882000.00 | 0.00 | 0.00 | 0.00 | 0.00 | 0.00 | 0.00 | 0.00 | 0.00 | 0.00 |
| | *Desulfomarina profundi* | 0.00 | 0.00 | 0.00 | 0.00 | 0.00 | 0.00 | 3237600.00 | 2220400.00 | 25385400.00 | 0.00 | 0.00 | 0.00 |
| | *Desulfomonile tiedjei* | 81101467.00 | 124239390.50 | 226190761.80 | 249335075.30 | 207327367.20 | 165672733.00 | 205212338.50 | 168477210.90 | 121281546.20 | 65014659.71 | 150129153.70 | 50499714.75 |
| | *Desulfonatronum lacustre* | 0.00 | 0.00 | 0.00 | 0.00 | 0.00 | 0.00 | 18691339.68 | 1134600.00 | 1385100.00 | 2440900.00 | 0.00 | 1740000.00 |
| | *Desulfonatronum thioautotrophicum* | 0.00 | 0.00 | 0.00 | 0.00 | 0.00 | 0.00 | 5677200.00 | 1671400.00 | 2025000.00 | 0.00 | 0.00 | 0.00 |
| | *Desulfonatronum thiosulfatophilum* | 0.00 | 924800.00 | 6136700.00 | 1881000.00 | 0.00 | 8535500.00 | 6377910.12 | 1134600.00 | 0.00 | 0.00 | 4681600.00 | 567057.75 |
| | *Desulfonauticus submarinus* | 0.00 | 0.00 | 0.00 | 9937000.00 | 3203300.00 | 9598459.56 | 5973600.00 | 6421701.80 | 4625100.00 | 6457260.81 | 10919517.84 | 1567500.00 |
| | *Desulfonema ishimotonii* | 1358575.00 | 39168000.00 | 4042424.45 | 3992193.55 | 0.00 | 38945700.00 | 80028000.00 | 85687152.01 | 14890439.79 | 8570100.00 | 1091302.96 | 162517500.00 |

| | | | | | | | | | | | | | |
|---|---|---|---|---|---|---|---|---|---|---|---|---|---|
| | *Desulfonema limicola* | 79002548.00 | 76780863.12 | 131658875.90 | 137611449.20 | 133125887.00 | 136685757.70 | 127260849.40 | 89599119.83 | 64324219.77 | 90830740.00 | 89543803.36 | 98884860.15 |
| | *Desulfonema magnum* | 143957478.00 | 45621754.20 | 6991880.11 | 107699262.80 | 52711565.31 | 44447909.59 | 63589200.00 | 74335910.89 | 76282389.90 | 101521004.20 | 31249994.16 | 29626838.25 |
| | *Desulfonispora thiosulfatigenes* | 0.00 | 0.00 | 0.00 | 0.00 | 0.00 | 0.00 | 0.00 | 370562.19 | 186295.14 | 0.00 | 0.00 | 0.00 |
| | *Desulfopila aestuarii* | 19394874.00 | 28421187.52 | 14902925.28 | 12530500.00 | 2863400.00 | 7694947.78 | 5761751.52 | 10132100.00 | 8002800.00 | 35558600.00 | 38702400.00 | 23055000.00 |
| | *Desulfoprunum benzoelyticum* | 19197825.00 | 11938321.40 | 0.00 | 35836785.40 | 106429900.00 | 0.00 | 10237200.00 | 11260420.05 | 13936156.92 | 0.00 | 2490400.00 | 12735000.00 |
| | *Desulforapulum autotrophicum* | 2680000.00 | 1462000.00 | 1059718.01 | 0.00 | 0.00 | 0.00 | 25627200.00 | 5593700.00 | 1344600.00 | 1232000.00 | 12188000.00 | 0.00 |
| | *Desulforegula conservatrix* | 0.00 | 0.00 | 0.00 | 0.00 | 6205149.51 | 231848000.00 | 0.00 | 0.00 | 0.00 | 7338100.00 | 0.00 | 3165000.00 |
| | *Desulforhopalus singaporensis* | 3350000.00 | 0.00 | 18973100.00 | 14829500.00 | 27305300.00 | 39405648.32 | 6315600.00 | 11681500.00 | 9460800.00 | 4173400.00 | 0.00 | 4327500.00 |
| | *Desulfosalsimonas propionicica* | 27230000.00 | 8636000.00 | 17699655.93 | 29526000.00 | 11731700.00 | 14379984.33 | 13665462.72 | 38692300.00 | 148718038.00 | 9979200.00 | 4573044.08 | 6235604.25 |
| | *Desulfosarcina alkanivorans* | 55976960.00 | 41255500.04 | 32533372.18 | 77662500.00 | 43579300.00 | 58239900.00 | 114834646.40 | 79342700.00 | 37972800.00 | 52945200.00 | 42001941.52 | 41767500.00 |
| | *Desulfosarcina cetonica* | 10170000.00 | 12926976.12 | 8976939.29 | 31065000.00 | 47294869.47 | 22829516.76 | 13058560.92 | 43181900.00 | 25320600.00 | 23677500.00 | 14043839.66 | 14415000.00 |
| | Desulfosarcina ovata | 34020000 | 33830000 | 13259353.75 | 47547500 | 51446176.32 | 24627533.73 | 34154400 | 37252700 | 43772400 | 30723000 | 57780800 | 26107213.5 |
| | Desulfosarcina widdelii | 7362870 | 22249600 | 34734904.3 | 18069000 | 43946298.27 | 18269959.85 | 203250647.9 | 188616530.5 | 147260592.8 | 96237675.38 | 81760800 | 221752500 |
| | Desulfoscipio geothermicus | 0 | 0 | 0 | 0 | 0 | 0 | 0 | 0 | 0 | 1139600 | 861907.2 | 355342.5 |
| | Desulfosediminicola flagellatus | 6540000 | 2475200 | 0 | 3106500 | 1246300 | 0 | 0 | 1878800 | 3248100 | 0 | 0 | 0 |
| | Desulfosediminicola ganghwensis | 607108 | 664547 | 3541754.18 | 0 | 0 | 0 | 0 | 1471805.56 | 560163.6 | 0 | 0 | 0 |
| | Desulfosoma caldarium | 126939428 | 133488050.8 | 75889855.24 | 142720842.7 | 189460842 | 265489758.6 | 317296003.9 | 270196076.1 | 199131441.7 | 161809451.7 | 177790675 | 173036710.5 |
| | Desulfospira joergensenii | 43600000 | 66272800 | 349228900 | 42009000 | 18900500 | 21914400 | 280602908.3 | 97451152.07 | 46251000 | 14129500 | 177980000 | 244800000 |
| | Desulfosudis oleivorans | 9240000 | 21290800 | 22357079.06 | 31442925.2 | 33814900 | 31785912.19 | 13653048.12 | 29440968.02 | 46713462.21 | 188041700 | 110413600 | 41700000 |
| | Desulfotomaculum copahuensis | 31640000 | 5310800 | 31209415.19 | 137949500 | 35576200 | 23823573 | 6349786.32 | 68798864.03 | 7112592.18 | 5182100 | 4979336.56 | 1687500 |
| | Desulfovermiculus halophilus | 0 | 12899533.36 | 1068095.45 | 9690000 | 35514400 | 3978845.16 | 24810358.08 | 21087700 | 27256500 | 0 | 0 | 0 |
| | Desulfovibrio aminophilus | 0 | 0 | 0 | 0 | 0 | 0 | 22549200 | 0 | 12717000 | 0 | 0 | 0 |
| | Desulfovibrio cuneatus | 0 | 0 | 0 | 755834.25 | 644673.91 | 0 | 0 | 0 | 0 | 0 | 0 | 0 |
| | Desulfovibrio inopinatus | 3988584 | 1917600 | 0 | 0 | 0 | 0 | 0 | 0 | 0 | 0 | 0 | 0 |
| | Desulfovibrio litoralis | 0 | 0 | 0 | 7932500 | 8219400 | 0 | 0 | 0 | 0 | 13090000 | 11352000 | 0 |
| | Desulfovibrio psychrotolerans | 41280000 | 0 | 6305600 | 28376500 | 0 | 10599900 | 19174800 | 36500507.78 | 17455500 | 0 | 11744907.68 | 20992281.75 |
| | Desulfovibrio sulfodismutans | 1970000 | 0 | 13286800 | 3756083.4 | 13704139.7 | 94315960.93 | 5767195.25 | 2526473.6 | 6917818.77 | 9548000 | 21665600 | 3562500 |
| | Desulfurella multipotens | 0 | 5766400 | 22013300 | 0 | 0 | 0 | 0 | 0 | 0 | 0 | 0 | 0 |
| | Desulfurispirillum indicum | 393982 | 0 | 646430.97 | 0 | 0 | 0 | 0 | 0 | 0 | 0 | 0 | 0 |
| | Desulfurivibrio alkaliphilus | 0 | 433765.88 | 887749.66 | 0 | 769793.16 | 675169.96 | 0 | 0 | 0 | 877800 | 648177.2 | 665154 |
| | Desulfuromonas acetexigens | 0 | 0 | 0 | 1957000 | 0 | 736502.49 | 1552556.88 | 447973.02 | 0 | 0 | 976800 | 1350000 |
| | Dethiosulfatarculus sandiegensis | 16852251 | 5105819.44 | 4312822.09 | 22573768.9 | 33207230.9 | 3323441.83 | 7272087.36 | 8558300 | 5630623.47 | 4057900 | 13455200 | 6067500 |
| | Dinghuibacter silviterrae | 580459 | 550462.72 | 0 | 1016500 | 1174200 | 0 | 0 | 2275300 | 6235066.53 | 0 | 0 | 0 |
| | Dissulfurimicrobium hydrothermale | 32000000 | 29954000 | 53034600 | 42845000 | 118264600 | 51620671.76 | 116872800 | 37336745.19 | 93222900 | 46508000 | 31468800 | 30082500 |
| | Dissulfurirhabdus thermomarina | 0 | 0 | 0 | 7315000 | 638749.35 | 0 | 0 | 0 | 0 | 0 | 0 | 0 |
| | Dissulfurispira thermophila | 37428444 | 54764437.16 | 43878086.23 | 43871000 | 150915600 | 165207296.5 | 46135635.84 | 130381400 | 88632370.8 | 48433495.11 | 77849293.28 | 57457500 |
| | Lamprocystis purpurea | 6330000 | 21783993.8 | 20476615.71 | 30798928.75 | 24932476.64 | 26293302.06 | 121709573.8 | 28523600 | 38641297.86 | 40002364.71 | 5212368.48 | 39067641.75 |
| | Sulfuricaulis limicola | 67870000 | 41276944.52 | 66965258.06 | 14516000 | 6082936.92 | 17082346.26 | 48361435.68 | 37332562.79 | 48698183.34 | 143367429.6 | 150842653.3 | 59893983.53 |
| | Sulfuricella denitrificans | 0 | 0 | 0 | 0 | 0 | 0 | 22138800 | 123470100 | 12004200 | 5854946.79 | 2728000 | 19833810.75 |
| | Sulfuriferula multivorans | 12325460 | 7548000 | 0 | 9329000 | 7745600 | 25685900 | 141865539.8 | 158095179.9 | 155576700 | 166859000 | 174053807.8 | 142252500 |
| | Sulfuriferula nivalis | 0 | 0 | 0 | 1729000 | 1761300 | 0 | 40584000 | 82833565.91 | 176282394.7 | 128528400 | 178314400 | 152542500 |
| | Sulfuriferula plumbiphila | 99691532 | 84799122.56 | 30057432.74 | 28215000 | 66064200 | 27899186.91 | 147394033.7 | 394587122.4 | 335390822.6 | 849440900 | 928391200 | 817200139.5 |
| | Sulfuriferula thiophila | 0 | 0 | 0 | 0 | 0 | 0 | 3078000 | 7039400 | 14474700 | 30661400 | 22906400 | 16867500 |
| | Sulfuriflexus mobilis | 0 | 0 | 0 | 0 | 0 | 0 | 0 | 4465200 | 3361500 | 4335100 | 4831200 | 6495000 |
| | Sulfurifustis variabilis | 0 | 0 | 0 | 0 | 0 | 0 | 0 | 2177700 | 3005100 | 2902900 | 5623200 | 8137500 |
| | Sulfurimicrobium lacus | 11160000 | 4358800 | 0 | 0 | 0 | 0 | 4833600 | 14963300 | 35996400 | 49665000 | 41333681.84 | 34652503.5 |
| | Sulfurirhabdus autotrophica | 21620000 | 41242000 | 36951531.01 | 13281000 | 9105200 | 34874453.09 | 46101600 | 113622016 | 86005800 | 121991100 | 198214139.2 | 173244867 |
| | Sulfurisoma sediminicola | 11143447 | 7485586.2 | 7151309.89 | 9623500 | 12699900 | 1305640.9 | 10337394.6 | 71735454.05 | 28879627.41 | 29138962.93 | 44121476.08 | 33483972.75 |
| | Sulfuritalea hydrogenivorans | 16385708 | 9752550.48 | 6787516.74 | 0 | 9352400 | 3090541.78 | 2964000 | 18130951.68 | 21495285.9 | 40740700 | 12096692.08 | 25259094.75 |
| | Sulfuritortus calidifontis | 17380000 | 9710400 | 2657528.9 | 0 | 36812200 | 2793911.32 | 104971200 | 133308789.4 | 100614212.4 | 177241526.8 | 186692000 | 129244215.8 |
| | Sulfurivermis fontis | 0 | 0 | 0 | 0 | 0 | 0 | 34678800 | 31469900 | 28609200 | 46169200 | 47819200 | 33480000 |
| | sulfur-oxidizing symbionts | 5800000 | 8296000 | 0 | 18278000 | 7230600 | 5041900 | 0 | 7722600 | 3215700 | 0 | 0 | 0 |
| | Thioalkalivibrio sulfidiphilus | 0 | 0 | 0 | 0 | 0 | 0 | 1145278.2 | 0 | 230806.26 | 218175.65 | 302638.16 | 11413101 |
| | Thiobacillus denitrificans | 44560000 | 65776726.4 | 33689509.01 | 111036000 | 81576000 | 40359055.73 | 588183426.4 | 976470579 | 880418728.1 | 1639208767 | 1908763183 | 1542461190 |
| sulfide/sulfur-oxidizing bacteria | Thiobacillus thioparus | 41304533 | 34224400 | 37786786.55 | 40922961.9 | 46012198.11 | 32301642.98 | 814732376.7 | 679660838.6 | 861720150 | 635549734.8 | 695629565 | 637097069.3 |
| | Thiocapsa imhoffii | 8200000 | 4426800 | 8136808.17 | 0 | 4841000 | 15205100 | 8367600 | 0 | 2583900 | 8600900 | 5033600 | 8977500 |
| | Thiocapsa rosea | 0 | 0 | 0 | 3458000 | 4387800 | 4565500 | 1385790.84 | 2818200 | 5516100 | 4458300 | 0 | 8032500 |
| | Thiocapsa roseopersicina | 0 | 0 | 0 | 0 | 1534700 | 984691.01 | 0 | 11529000 | 1004400 | 15092000 | 9504000 | 6787500 |
| | Thioclava electrotropha | 0 | 0 | 0 | 4740500 | 6571400 | 0 | 42636000 | 34892000 | 7346700 | 89628000 | 89760000 | 81000000 |
| | Thiocystis minor | 0 | 0 | 0 | 4227500 | 23896000 | 0 | 15048000 | 4471300 | 11826000 | 8778000 | 27359200 | 10125000 |
| | Thiocystis violacea | 0 | 0 | 0 | 0 | 0 | 0 | 36229200 | 3737374.23 | 9995400 | 81991671.3 | 60183200 | 52350000 |
| | Thioflavicoccus mobilis | 13100000 | 4311200 | 12389969.15 | 17575000 | 2588333.35 | 0 | 0 | 0 | 0 | 0 | 7594400 | 7447890.75 |
| | Thioflexithrix psekupsensis | 0 | 0 | 0 | 17290000 | 17407000 | 0 | 0 | 23241000 | 4268700 | 21175000 | 0 | 379512 |
| | Thiogranum longum | 0 | 0 | 0 | 0 | 0 | 0 | 0 | 0 | 0 | 0 | 4637600 | 1935000 |
| | Thiohalocapsa halophila | 0 | 0 | 0 | 0 | 0 | 0 | 0 | 0 | 0 | 0 | 2147200 | 14925000 |
| | Thiohalocapsa marina | 0 | 30736000 | 9402100 | 14630000 | 9682000 | 13696500 | 6178800 | 32635000 | 4050000 | 0 | 0 | 0 |
| | Thiohalomonas denitrificans | 0 | 0 | 0 | 0 | 0 | 0 | 0 | 5575400 | 7087500 | 5867400 | 0 | 4777500 |
| | Thiohalorhabdus denitrificans | 0 | 0 | 0 | 5975500 | 7931000 | 0 | 0 | 0 | 0 | 0 | 0 | 0 |
| | Thiomicrospira microaerophila | 0 | 0 | 0 | 0 | 0 | 0 | 0 | 0 | 0 | 2733500 | 3220800 | 3229033.5 |
| | Thiorhodococcus minor | 2800000 | 11043200 | 0 | 2686033.8 | 332542.71 | 0 | 2453337 | 14341100 | 23611500 | 18172621.39 | 4191136.4 | 1065447 |
| | Thiospirillum jenense | 24000000 | 40256000 | 1972262.19 | 0 | 0 | 0 | 0 | 0 | 0 | 0 | 0 | 0 |
| | Thiothrix eikelboomii | 0 | 0 | 0 | 0 | 0 | 0 | 0 | 0 | 0 | 7059718.05 | 13024000 | 0 |
| | Thiothrix fructosivorans | 0 | 0 | 0 | 0 | 0 | 0 | 0 | 5996300 | 4941000 | 862400 | 2877600 | 2842500 |
| | Thiothrix nivea | 0 | 0 | 0 | 0 | 0 | 0 | 0 | 3574600 | 8425293.57 | 18195100 | 43225600 | 6765000 |

Supplementary Table 2. Summary of metaproteomic database searching results.

| ID | Peak Height | | | | | | | | | | | | KO Number | Description | Taxa |
|---|---|---|---|---|---|---|---|---|---|---|---|---|---|---|---|
| | S-O-_1 | S-O-_2 | S-O-_3 | S+O-_1 | S+O-_2 | S+O-_3 | S-O+_1 | S-O+_2 | S-O+_3 | S+O+_1 | S+O+_2 | S+O+_3 | | | |
| Ga0586324_0078845_1967_3262 | 6510000.00 | 0.00 | 0.00 | 0.00 | 0.00 | 0.00 | 1477296.36 | 0.00 | 0.00 | 0.00 | 0.00 | 0.00 | KO:K00003 | homoserine dehydrogenase | Bacteria;Proteobacteria;Deltaproteobacteria;Myxococcales;Myxococcaceae;Corallococcus;Corallococcus sp. H22C18031201;Corallococcus sp. H22C18031201 |
| Ga0586324_0145763_870_2096 | 0.00 | 0.00 | 0.00 | 0.00 | 0.00 | 0.00 | 160742.51 | 0.00 | 0.00 | 0.00 | 0.00 | 0.00 | KO:K00003 | homoserine dehydrogenase | Bacteria;Actinobacteria;Thermoleophilia;Solirubrobacterales;Solirubrobacteraceae;Solirubrobacter;Solirubrobacter pauli;Solirubrobacter pauli DSM 14954 |
| Ga0586324_0260785_1668_2189 | 207568.00 | 0.00 | 0.00 | 0.00 | 0.00 | 0.00 | 0.00 | 0.00 | 0.00 | 0.00 | 0.00 | 0.00 | KO:K00003 | homoserine dehydrogenase | Bacteria;Chloroflexi;Dehalococcoidia;unclassified Dehalococcoidia;unclassified Dehalococcoidia;Dehalogenimonas;Dehalogenimonas formicexedens;Dehalogenimonas formicexedens NSZ-14 |
| Ga0586324_0502813_1_891 | 127031.00 | 0.00 | 0.00 | 0.00 | 0.00 | 0.00 | 0.00 | 1104100.00 | 0.00 | 1771000.00 | 0.00 | 0.00 | KO:K00003 | homoserine dehydrogenase | Bacteria;Proteobacteria;Betaproteobacteria;Nitrosomonadales;Thiobacillaceae;Thiobacillus;Thiobacillus denitrificans;Thiobacillus denitrificans RG |
| Ga0586324_1832549_1_936 | 0.00 | 0.00 | 0.00 | 0.00 | 0.00 | 0.00 | 0.00 | 0.00 | 0.00 | 725802.00 | 0.00 | 0.00 | KO:K00003 | homoserine dehydrogenase | Bacteria;Proteobacteria;Betaproteobacteria;unclassified Betaproteobacteria;unclassified Betaproteobacteria;unclassified Betaproteobacteria;Betaproteobacteria bacterium GR16-43;Betaproteobacteria bacterium GR16-43 |
| Ga0586324_1967702_1_909 | 438574.00 | 0.00 | 0.00 | 0.00 | 0.00 | 0.00 | 0.00 | 0.00 | 0.00 | 0.00 | 0.00 | 0.00 | KO:K00003 | homoserine dehydrogenase | Archaea;Euryarchaeota;Methanococci;Methanococcales;Methanocaldococcaceae;Methanocaldococcus;Methanocaldococcus vulcanius;Methanocaldococcus vulcanius M7, DSM 12094 |
| Ga0586324_2597129_2_715 | 0.00 | 0.00 | 0.00 | 0.00 | 0.00 | 0.00 | 0.00 | 0.00 | 433827.90 | 0.00 | 0.00 | 0.00 | KO:K00003 | homoserine dehydrogenase | Bacteria;Proteobacteria;Alphaproteobacteria;Hyphomicrobiales;unclassified Hyphomicrobiales;Methyloceanibacter;Methyloceanibacter marginalis;Methyloceanibacter marginalis R-67177 |
| Ga0586324_4195391_3_386 | 0.00 | 0.00 | 0.00 | 0.00 | 0.00 | 0.00 | 0.00 | 0.00 | 0.00 | 0.00 | 0.00 | 1365000.00 | KO:K00003 | homoserine dehydrogenase | Bacteria;Firmicutes;Clostridia;Halanaerobiales;Halobacteroidaceae;Candidatus Frackibacter;Candidatus Frackibacter sp. WG12;Candidatus Frackibacter sp. WG12 |
| Ga0586324_7253247_1_528 | 0.00 | 0.00 | 0.00 | 0.00 | 49852000.00 | 0.00 | 0.00 | 0.00 | 0.00 | 0.00 | 0.00 | 0.00 | KO:K00003 | homoserine dehydrogenase | Bacteria;Actinobacteria;Thermoleophilia;Solirubrobacterales;unclassified Solirubrobacterales;unclassified Solirubrobacterales;Solirubrobacterales bacterium URHD0059;Solirubrobacterales bacterium URHD0059 |
| Ga0586324_0025058_2814_3860 | 0.00 | 0.00 | 0.00 | 0.00 | 0.00 | 0.00 | 0.00 | 860100.00 | 0.00 | 0.00 | 0.00 | 0.00 | KO:K00004 | (R,R)-butanediol dehydrogenase/meso-butanediol dehydrogenase/diacetyl reductase | Bacteria;Firmicutes;Clostridia;Eubacteriales;Lachnospiraceae;Anaerostipes;Anaerostipes rhamnosivorans;Anaerostipes rhamnosivorans 1y2 |
| Ga0586324_0382308_3_1019 | 0.00 | 0.00 | 0.00 | 0.00 | 0.00 | 0.00 | 0.00 | 658800.00 | 0.00 | 0.00 | 0.00 | 1170000.00 | KO:K00005 | glycerol dehydrogenase | Bacteria;Firmicutes;Clostridia;Eubacteriales;Peptococcaceae;Thermoanaerosceptrum;Thermoanaerosceptrum fracticalcis;Thermoanaerosceptrum fracticalcis DRI-13 |
| Ga0586324_1655994_2_979 | 6880000.00 | 768400.00 | 0.00 | 0.00 | 0.00 | 0.00 | 0.00 | 0.00 | 0.00 | 0.00 | 0.00 | 741722.25 | KO:K00010 | myo-inositol 2-dehydrogenase/D-chiro-inositol 1-dehydrogenase | Bacteria;Verrucomicrobia;Verrucomicrobiae;Verrucomicrobiales;Verrucomicrobia subdivision 3;Pedosphaera;Pedosphaera parvula;Pedosphaera parvula Ellin514 |
| Ga0586324_6179853_1_564 | 0.00 | 342329.68 | 0.00 | 0.00 | 0.00 | 0.00 | 0.00 | 0.00 | 0.00 | 0.00 | 0.00 | 0.00 | KO:K00010 | myo-inositol 2-dehydrogenase/D-chiro-inositol 1-dehydrogenase | Bacteria;Planctomycetes;Planctomycetia;Pirellulales;Thermoguttaceae;Thermogutta;Thermogutta terrifontis;Thermogutta terrifontis R1 |
| Ga0586324_0000005_55168_56490 | 0.00 | 0.00 | 0.00 | 0.00 | 0.00 | 0.00 | 0.00 | 0.00 | 0.00 | 2849000.00 | 0.00 | 6165000.00 | KO:K00012 | UDPglucose 6-dehydrogenase | Bacteria;Proteobacteria;Betaproteobacteria;Nitrosomonadales;Thiobacillaceae;Annwoodia;Annwoodia aquaesulis;Annwoodia aquaesulis DSM 4255 |
| Ga0586324_0017638_5301_6623 | 0.00 | 0.00 | 0.00 | 0.00 | 0.00 | 0.00 | 0.00 | 0.00 | 0.00 | 0.00 | 2525600.00 | 0.00 | KO:K00012 | UDPglucose 6-dehydrogenase | Bacteria;Proteobacteria;Betaproteobacteria;Nitrosomonadales;Thiobacillaceae;Thiobacillus;Thiobacillus thioparus;Thiobacillus thioparus DSM 505 |
| Ga0586324_0213322_395_1720 | 0.00 | 0.00 | 0.00 | 0.00 | 0.00 | 4367000.00 | 0.00 | 0.00 | 0.00 | 0.00 | 0.00 | 0.00 | KO:K00012 | UDPglucose 6-dehydrogenase | Bacteria;Bacteroidetes;Flavobacteriia;Flavobacteriales;Flavobacteriaceae;Flavobacterium;Flavobacterium succinicans;Flavobacterium succinicans LMG 10402 |
| Ga0586324_0290841_36_1349 | 0.00 | 0.00 | 0.00 | 0.00 | 0.00 | 0.00 | 467317.92 | 0.00 | 0.00 | 0.00 | 0.00 | 0.00 | KO:K00012 | UDPglucose 6-dehydrogenase | Bacteria;Proteobacteria;Alphaproteobacteria;Hyphomicrobiales;Xanthobacteraceae;Pseudolabrys;Pseudolabrys taiwanensis;Pseudolabrys taiwanensis CC-BB4 |
| Ga0586324_1905144_1_711 | 0.00 | 0.00 | 0.00 | 0.00 | 0.00 | 0.00 | 0.00 | 0.00 | 0.00 | 793100.00 | 0.00 | 0.00 | KO:K00012 | UDPglucose 6-dehydrogenase | Bacteria;Proteobacteria;Betaproteobacteria;Nitrosomonadales;Thiobacillaceae;Thiobacillus;Thiobacillus denitrificans;Thiobacillus denitrificans DSM 12475 |
| Ga0586324_7629892_1_519 | 0.00 | 0.00 | 0.00 | 0.00 | 0.00 | 0.00 | 0.00 | 0.00 | 0.00 | 272508.39 | 0.00 | 0.00 | KO:K00012 | UDPglucose 6-dehydrogenase | Bacteria;Proteobacteria;Betaproteobacteria;Nitrosomonadales;Thiobacillaceae;Thiobacillus;Thiobacillus thioparus;Thiobacillus thioparus DSM 505 |
| Ga0586324_0262451_344_1663 | 0.00 | 0.00 | 0.00 | 0.00 | 0.00 | 0.00 | 0.00 | 0.00 | 0.00 | 0.00 | 5702400.00 | 0.00 | KO:K00013 | histidinol dehydrogenase | Bacteria;Proteobacteria;Betaproteobacteria;Neisseriales;Chromobacteriaceae;Laribacter;Laribacter hongkongensis;Laribacter hongkongensis LHGZ1 |
| Ga0586324_0336379_69_1367 | 0.00 | 0.00 | 0.00 | 0.00 | 0.00 | 0.00 | 2827200.00 | 0.00 | 0.00 | 0.00 | 0.00 | 0.00 | KO:K00013 | histidinol dehydrogenase | Bacteria;Proteobacteria;Betaproteobacteria;Nitrosomonadales;Gallionellaceae;Sideroxydans;Sideroxydans lithotrophicus;Sideroxydans lithotrophicus ES-1 |
| Ga0586324_1199669_163_1122 | 0.00 | 0.00 | 0.00 | 0.00 | 0.00 | 0.00 | 0.00 | 0.00 | 0.00 | 0.00 | 0.00 | 5347500.00 | KO:K00013 | histidinol dehydrogenase | Bacteria;Proteobacteria;Betaproteobacteria;Burkholderiales;unclassified Burkholderiales;unclassified Burkholderiales;unclassified Burkholderiales;Burkholderiales A1 |
| Ga0586324_0000007_28526_29476 | 0.00 | 0.00 | 0.00 | 0.00 | 0.00 | 0.00 | 0.00 | 0.00 | 0.00 | 0.00 | 0.00 | 472403.25 | KO:K00018 | glycerate dehydrogenase | Bacteria;Proteobacteria;Betaproteobacteria;Nitrosomonadales;Thiobacillaceae;Annwoodia;Annwoodia aquaesulis;Annwoodia aquaesulis DSM 4255 |
| Ga0586324_0071673_1_627 | 0.00 | 0.00 | 0.00 | 8578500.00 | 0.00 | 0.00 | 0.00 | 0.00 | 0.00 | 0.00 | 0.00 | 0.00 | KO:K00023 | acetoacetyl-CoA reductase | Bacteria;Proteobacteria;Betaproteobacteria;Burkholderiales;Oxalobacteraceae;Herbaspirillum;Herbaspirillum sp. K2R10-39;Herbaspirillum sp. K2R10-39 |
| Ga0586324_2256833_409_858 | 0.00 | 0.00 | 3731974.99 | 0.00 | 0.00 | 6272600.00 | 4332000.00 | 2757200.00 | 2502900.00 | 3164700.00 | 0.00 | 0.00 | KO:K00023 | acetoacetyl-CoA reductase | Bacteria;Proteobacteria;Gammaproteobacteria;unclassified Gammaproteobacteria;Candidatus Competibacteraceae;Candidatus Contendobacter;Candidatus Contendobacter odensis;Candidatus Contendobacter odensis Run_B_J11 |
| Ga0586324_4860956_3_623 | 0.00 | 0.00 | 1144106.08 | 0.00 | 0.00 | 0.00 | 0.00 | 0.00 | 2008800.00 | 0.00 | 0.00 | 2775000.00 | KO:K00023 | acetoacetyl-CoA reductase | Bacteria;Proteobacteria;Gammaproteobacteria;Chromatiales;Ectothiorhodospiraceae;Alkalilimnicola;Alkalilimnicola ehrlichii;Alkalilimnicola ehrlichii MLHE-1 |
| Ga0586324_0000238_38779_39711 | 0.00 | 0.00 | 0.00 | 0.00 | 0.00 | 2228146.62 | 0.00 | 0.00 | 0.00 | 0.00 | 0.00 | 0.00 | KO:K00024 | malate dehydrogenase | Bacteria;Proteobacteria;Gammaproteobacteria;Thiotrichales;Thiotrichaceae;Achromatium;Achromatium sp. WMS3;Achromatium sp. WMS3 |
| Ga0586324_0032997_1606_2535 | 0.00 | 0.00 | 0.00 | 0.00 | 0.00 | 0.00 | 0.00 | 0.00 | 0.00 | 0.00 | 0.00 | 5947500.00 | KO:K00024 | malate dehydrogenase | Bacteria;Proteobacteria;Deltaproteobacteria;Syntrophobacterales;Syntrophaceae;Desulfobacca;Desulfobacca acetoxidans;Desulfobacca acetoxidans ASRB2, DSM 11109 |
| Ga0586324_0287020_1360_2097 | 0.00 | 0.00 | 0.00 | 0.00 | 0.00 | 846666.02 | 0.00 | 0.00 | 0.00 | 0.00 | 0.00 | 0.00 | KO:K00024 | malate dehydrogenase | Bacteria;Proteobacteria;Gammaproteobacteria;unclassified Gammaproteobacteria;unclassified Gammaproteobacteria;unclassified Gammaproteobacteria;endosymbiont of Riftia pachyptila;endosymbiont of Riftia pachyptila (vent Mk28) |
| Ga0586324_0340850_875_1810 | 0.00 | 0.00 | 0.00 | 6840000.00 | 0.00 | 0.00 | 13862400.00 | 6222000.00 | 1263600.00 | 0.00 | 0.00 | 0.00 | KO:K00024 | malate dehydrogenase | Bacteria;Proteobacteria;Deltaproteobacteria;Desulfobacterales;Desulfobacteraceae;Desulfatirhabdium;Desulfatirhabdium butyrativorans;Desulfatirhabdium butyrativorans DSM 18734 |
| Ga0586324_0360500_1_540 | 1510000.00 | 0.00 | 0.00 | 4522000.00 | 7570500.00 | 2703879.66 | 0.00 | 3891800.00 | 2097900.00 | 0.00 | 0.00 | 1365000.00 | KO:K00024 | malate dehydrogenase | Bacteria;Proteobacteria;Deltaproteobacteria;Syntrophobacterales;Syntrophaceae;Desulfobacca;Desulfobacca acetoxidans;Desulfobacca acetoxidans ASRB2, DSM 11109 |
| Ga0586324_0428719_867_1754 | 0.00 | 0.00 | 13399400.00 | 0.00 | 0.00 | 0.00 | 0.00 | 0.00 | 0.00 | 0.00 | 0.00 | 0.00 | KO:K00024 | malate dehydrogenase | Bacteria;Proteobacteria;Betaproteobacteria;unclassified Betaproteobacteria;unclassified Betaproteobacteria;unclassified Betaproteobacteria;uncultured beta proteobacterium;unclassified betaproteobacterium ENR4 |
| Ga0586324_0554577_1_951 | 0.00 | 0.00 | 2831585.98 | 0.00 | 0.00 | 436866.74 | 0.00 | 0.00 | 0.00 | 0.00 | 0.00 | 0.00 | KO:K00024 | malate dehydrogenase | Bacteria;Proteobacteria;Gammaproteobacteria;Chromatiales;Woeseiaceae;Woeseia;Woeseia oceani;Woeseia oceani XK5 |
| Ga0586324_0755426_539_1369 | 0.00 | 0.00 | 0.00 | 0.00 | 0.00 | 0.00 | 0.00 | 0.00 | 4592700.00 | 0.00 | 0.00 | 0.00 | KO:K00024 | malate dehydrogenase | Bacteria;Proteobacteria;Deltaproteobacteria;Desulfobacterales;Desulfobacteraceae;Desulfatirhabdium;Desulfatirhabdium butyrativorans;Desulfatirhabdium butyrativorans DSM 18734 |
| Ga0586324_1196366_706_1125 | 0.00 | 0.00 | 0.00 | 2118500.00 | 0.00 | 0.00 | 0.00 | 4532300.00 | 1701000.00 | 0.00 | 0.00 | 0.00 | KO:K00024 | malate dehydrogenase | Bacteria;Proteobacteria;Deltaproteobacteria;Desulfuromonadales;Geobacteraceae;Geobacter;Geobacter pickeringii;Geobacter pickeringii G13 |
| Ga0586324_1553294_680_1006 | 0.00 | 0.00 | 0.00 | 0.00 | 0.00 | 0.00 | 0.00 | 0.00 | 22275000.00 | 0.00 | 0.00 | 0.00 | KO:K00024 | malate dehydrogenase | Bacteria;Deferribacteres;Deferribacteres;Deferribacterales;Deferribacteraceae;Seleniivibrio;Seleniivibrio woodruffii;Seleniivibrio woodruffii S4 |
| Ga0586324_1845493_97_933 | 0.00 | 0.00 | 1084191.62 | 4978000.00 | 6787700.00 | 1584657.26 | 1316036.52 | 3434300.00 | 1846800.00 | 0.00 | 0.00 | 0.00 | KO:K00024 | malate dehydrogenase | Bacteria;Proteobacteria;Deltaproteobacteria;Syntrophobacterales;Syntrophaceae;Desulfobacca;Desulfobacca acetoxidans;Desulfobacca acetoxidans ASRB2, DSM 11109 |
| Ga0586324_1914176_3_653 | 0.00 | 0.00 | 0.00 | 0.00 | 0.00 | 0.00 | 0.00 | 0.00 | 0.00 | 0.00 | 3995200.00 | 0.00 | KO:K00024 | malate dehydrogenase | Bacteria;Proteobacteria;Deltaproteobacteria;Syntrophobacterales;Syntrophobacteraceae;Desulfacinum;Desulfacinum hydrothermale;Desulfacinum hydrothermale DSM 13146 |
| Ga0586324_2063688_410_892 | 0.00 | 0.00 | 0.00 | 5339000.00 | 0.00 | 0.00 | 0.00 | 0.00 | 0.00 | 0.00 | 0.00 | 0.00 | KO:K00024 | malate dehydrogenase | Bacteria;Proteobacteria;Deltaproteobacteria;Syntrophobacterales;Syntrophobacteraceae;Desulfacinum;Desulfacinum hydrothermale;Desulfacinum hydrothermale DSM 13146 |
| Ga0586324_3352322_3_683 | 0.00 | 0.00 | 0.00 | 0.00 | 0.00 | 0.00 | 0.00 | 811300.00 | 0.00 | 0.00 | 976800.00 | 1005000.00 | KO:K00024 | malate dehydrogenase | Bacteria;Proteobacteria;Deltaproteobacteria;Syntrophobacterales;Syntrophobacteraceae;Desulfosoma;Desulfosoma caldarium;Desulfosoma caldarium DSM 22027 |
| Ga0586324_3442363_1_720 | 0.00 | 0.00 | 0.00 | 0.00 | 0.00 | 13458300.00 | 0.00 | 0.00 | 1304100.00 | 0.00 | 0.00 | 0.00 | KO:K00024 | malate dehydrogenase | Bacteria;Proteobacteria;Deltaproteobacteria;Desulfobacterales;Desulfobacteraceae;Desulfatirhabdium;Desulfatirhabdium butyrativorans;Desulfatirhabdium butyrativorans DSM 18734 |
| Ga0586324_5358720_1_600 | 0.00 | 0.00 | 0.00 | 0.00 | 0.00 | 0.00 | 0.00 | 4148000.00 | 0.00 | 0.00 | 0.00 | 0.00 | KO:K00024 | malate dehydrogenase | Bacteria;Nitrospirae;Thermodesulfovibrionia;Thermodesulfovibrionales;Thermodesulfovibrionaceae;Thermodesulfovibrio;Thermodesulfovibrio hydrogeniphilus;Thermodesulfovibrio hydrogeniphilus DSM 18151 |
| Ga0586324_5849606_1_579 | 0.00 | 0.00 | 0.00 | 0.00 | 0.00 | 26162300.00 | 0.00 | 11956000.00 | 0.00 | 0.00 | 0.00 | 0.00 | KO:K00024 | malate dehydrogenase | Bacteria;Proteobacteria;Alphaproteobacteria;Sphingomonadales;Erythrobacteraceae;Porphyrobacter;Porphyrobacter sp. AAP82;Porphyrobacter sp. AAP82 |
| Ga0586324_6361370_1_558 | 0.00 | 0.00 | 0.00 | 0.00 | 0.00 | 0.00 | 7455600.00 | 5233800.00 | 5572800.00 | 0.00 | 0.00 | 2685000.00 | KO:K00024 | malate dehydrogenase | Bacteria;Proteobacteria;Deltaproteobacteria;Syntrophobacterales;Syntrophobacteraceae;Desulfacinum;Desulfacinum infernum;Desulfacinum infernum DSM 9756 |
| Ga0586324_0729134_3_1391 | 0.00 | 0.00 | 0.00 | 0.00 | 8888900.00 | 0.00 | 0.00 | 0.00 | 0.00 | 0.00 | 0.00 | 0.00 | KO:K00029 | malate dehydrogenase (oxaloacetate-decarboxylating)(NADP+) | Bacteria;Proteobacteria;Deltaproteobacteria;Myxococcales;Anaeromyxobacteraceae;Anaeromyxobacter;Anaeromyxobacter sp. PSR-1;Anaeromyxobacter sp. PSR-1 |
| Ga0586324_0755447_2_1369 | 11300000.00 | 0.00 | 0.00 | 0.00 | 0.00 | 0.00 | 0.00 | 0.00 | 0.00 | 0.00 | 0.00 | 0.00 | KO:K00029 | malate dehydrogenase (oxaloacetate-decarboxylating)(NADP+) | Bacteria;Acidobacteria;Thermoanaerobaculia;Thermoanaerobaculales;Thermoanaerobaculaceae;Thermoanaerobaculum;Thermoanaerobaculum aquaticum;Thermoanaerobaculum aquaticum MP-01 |
| Ga0586324_1007402_2_1210 | 0.00 | 0.00 | 0.00 | 0.00 | 0.00 | 0.00 | 0.00 | 0.00 | 0.00 | 0.00 | 18656000.00 | 0.00 | KO:K00029 | malate dehydrogenase (oxaloacetate-decarboxylating)(NADP+) | Bacteria;Acidobacteria;Acidobacteriia;Bryobacterales;Solibacteraceae;Candidatus Solibacter;Candidatus Solibacter usitatus;Candidatus Solibacter usitatus Ellin6076 |
| Ga0586324_0000606_11127_12380 | 0.00 | 0.00 | 0.00 | 0.00 | 0.00 | 0.00 | 0.00 | 0.00 | 0.00 | 0.00 | 38984000.00 | 0.00 | KO:K00031 | isocitrate dehydrogenase | Bacteria;Firmicutes;Bacilli;Bacillales;Alicyclobacillaceae;Alicyclobacillus;Alicyclobacillus acidocaldarius;Alicyclobacillus acidocaldarius LAA1 |
| Ga0586324_2002003_474_902 | 0.00 | 0.00 | 0.00 | 0.00 | 0.00 | 0.00 | 0.00 | 0.00 | 0.00 | 0.00 | 3616800.00 | 0.00 | KO:K00031 | isocitrate dehydrogenase | Bacteria;Proteobacteria;Deltaproteobacteria;unclassified Deltaproteobacteria;unclassified Deltaproteobacteria;Dissulfuribacter;Dissulfuribacter thermophilus;Dissulfuribacter thermophilus S69 |
| Ga0586324_2734715_76_792 | 0.00 | 0.00 | 0.00 | 0.00 | 0.00 | 0.00 | 0.00 | 0.00 | 923400.00 | 0.00 | 0.00 | 0.00 | KO:K00031 | isocitrate dehydrogenase | Bacteria;Proteobacteria;Gammaproteobacteria;unclassified Gammaproteobacteria;Candidatus Competibacteraceae;Candidatus Competibacter;Candidatus Competibacter denitrificans;Candidatus Competibacter denitrificans Run_A_D11 |
| Ga0586324_2788721_3_695 | 0.00 | 0.00 | 0.00 | 0.00 | 0.00 | 21954100.00 | 0.00 | 0.00 | 0.00 | 0.00 | 0.00 | 0.00 | KO:K00031 | isocitrate dehydrogenase | Bacteria;Verrucomicrobia;Verrucomicrobiae;Verrucomicrobiales;Verrucomicrobia subdivision 3;Pedosphaera;Pedosphaera parvula;Pedosphaera parvula Ellin514 |
| Ga0586324_0031695_3671_5137 | 0.00 | 0.00 | 0.00 | 0.00 | 0.00 | 0.00 | 1501405.08 | 0.00 | 0.00 | 0.00 | 0.00 | 0.00 | KO:K00033 | 6-phosphogluconate dehydrogenase | Bacteria;Bacteroidetes;unclassified Bacteroidetes;Bacteroidetes Order II. Incertae sedis;Rhodothermaceae;unclassified Rhodothermaceae;Rhodothermaceae bacterium RA;Halothermobacillus malaysiensis RA |
| Ga0586324_1018913_1_1203 | 0.00 | 0.00 | 0.00 | 0.00 | 0.00 | 0.00 | 0.00 | 0.00 | 0.00 | 0.00 | 0.00 | 4335000.00 | KO:K00033 | 6-phosphogluconate dehydrogenase | Bacteria;Proteobacteria;Gammaproteobacteria;Methylococcales;Methylococcaceae;Methylomicrobium;Methylomicrobium lacus;Methylosarcina lacus LW14 |
| Ga0586324_1893531_2_925 | 0.00 | 0.00 | 0.00 | 0.00 | 0.00 | 0.00 | 6726000.00 | 0.00 | 0.00 | 0.00 | 0.00 | 0.00 | KO:K00033 | 6-phosphogluconate dehydrogenase | Bacteria;Bacteroidetes;Bacteroidia;Marinilabiliales;Prolixibacteraceae;Sunxiuqinia;Sunxiuqinia elliptica;Sunxiuqinia elliptica 114D |
| Ga0586324_2040485_2_799 | 0.00 | 0.00 | 0.00 | 0.00 | 0.00 | 0.00 | 2827200.00 | 0.00 | 0.00 | 0.00 | 0.00 | 0.00 | KO:K00033 | 6-phosphogluconate dehydrogenase | Bacteria;Acidobacteria;Vicinamibacteria;unclassified Vicinamibacteria;Vicinamibacteraceae;Luteitalea;Luteitalea pratensis;Luteitalea pratensis DSM 100886 |
| Ga0586324_2088782_2_859 | 0.00 | 0.00 | 0.00 | 0.00 | 0.00 | 0.00 | 4810800.00 | 0.00 | 0.00 | 0.00 | 0.00 | 0.00 | KO:K00033 | 6-phosphogluconate dehydrogenase | Bacteria;Verrucomicrobia;Opitutae;Opitutales;Opitutaceae;Lacunisphaera;Lacunisphaera limnophila;Opitutaceae bacterium IG16b |
| Ga0586324_2584116_3_779 | 0.00 | 4984400.00 | 0.00 | 0.00 | 0.00 | 0.00 | 0.00 | 0.00 | 0.00 | 0.00 | 0.00 | 0.00 | KO:K00033 | 6-phosphogluconate dehydrogenase | Bacteria;Verrucomicrobia;Spartobacteria;Chthoniobacterales;Chthoniobacteraceae;Chthoniobacter;Chthoniobacter flavus;Chthoniobacter flavus Ellin428 |
| Ga0586324_0251031_3_1406 | 0.00 | 911200.00 | 0.00 | 0.00 | 0.00 | 0.00 | 0.00 | 0.00 | 0.00 | 0.00 | 0.00 | 0.00 | KO:K00036 | glucose-6-phosphate 1-dehydrogenase | Bacteria;Proteobacteria;Deltaproteobacteria;Myxococcales;Anaeromyxobacteraceae;Anaeromyxobacter;Anaeromyxobacter sp. PSR-1;Anaeromyxobacter sp. PSR-1 |
| Ga0586324_0553196_3_527 | 696883.00 | 0.00 | 0.00 | 4902000.00 | 6499300.00 | 0.00 | 0.00 | 0.00 | 0.00 | 0.00 | 0.00 | 0.00 | KO:K00042 | 2-hydroxy-3-oxopropionate reductase | Bacteria;Firmicutes;Clostridia;Eubacteriales;Peptococcaceae;Syntrophobotulus;Syntrophobotulus glycolicus;Syntrophobotulus glycolicus FlGlyR, DSM 8271 |
| Ga0586324_0667409_273_1196 | 0.00 | 5589600.00 | 0.00 | 0.00 | 0.00 | 0.00 | 0.00 | 7015000.00 | 0.00 | 0.00 | 0.00 | 0.00 | KO:K00042 | 2-hydroxy-3-oxopropionate reductase | Bacteria;Firmicutes;Clostridia;Thermoanaerobacterales;Thermoanaerobacteraceae;Moorella;Moorella thermoacetica;Moorella thermoacetica DSM 11768 |
| Ga0586324_0080700_1374_2486 | 0.00 | 0.00 | 0.00 | 1805000.00 | 0.00 | 0.00 | 0.00 | 0.00 | 0.00 | 0.00 | 0.00 | 0.00 | KO:K00052 | 3-isopropylmalate dehydrogenase | Bacteria;Proteobacteria;Alphaproteobacteria;Rhodospirillales;Acetobacteraceae;Rhodopila;Rhodopila globiformis;Rhodopila globiformis DSM 161 |
| Ga0586324_0108084_1932_2999 | 0.00 | 0.00 | 0.00 | 4949500.00 | 0.00 | 0.00 | 0.00 | 0.00 | 4187700.00 | 0.00 | 0.00 | 0.00 | KO:K00052 | 3-isopropylmalate dehydrogenase | Bacteria;Proteobacteria;Deltaproteobacteria;Syntrophobacterales;Syntrophaceae;Desulfobacca;Desulfobacca acetoxidans;Desulfobacca acetoxidans ASRB2, DSM 11109 |
| Ga0586324_0258097_637_1761 | 0.00 | 0.00 | 0.00 | 0.00 | 0.00 | 0.00 | 0.00 | 0.00 | 0.00 | 0.00 | 0.00 | 454591.50 | KO:K00052 | 3-isopropylmalate dehydrogenase | Bacteria;Proteobacteria;Alphaproteobacteria;Hyphomicrobiales;unclassified Hyphomicrobiales;Methyloceanibacter;Methyloceanibacter marginalis;Methyloceanibacter marginalis R-67177 |
| Ga0586324_1457159_3_1034 | 0.00 | 0.00 | 0.00 | 0.00 | 0.00 | 0.00 | 0.00 | 0.00 | 0.00 | 372270.36 | 0.00 | 0.00 | KO:K00052 | 3-isopropylmalate dehydrogenase | Bacteria;Proteobacteria;Alphaproteobacteria;Hyphomicrobiales;Hyphomicrobiaceae;Rhodoplanes;Rhodoplanes sp. Z2-YC6860;Rhodoplanes sp. Z2-YC6860 |
| Ga0586324_0000037_18910_19926 | 0.00 | 0.00 | 0.00 | 0.00 | 0.00 | 0.00 | 0.00 | 1403000.00 | 8181000.00 | 9471000.00 | 34760000.00 | 7080000.00 | KO:K00053 | ketol-acid reductoisomerase | Bacteria;Proteobacteria;Betaproteobacteria;Nitrosomonadales;Thiobacillaceae;Annwoodia;Annwoodia aquaesulis;Annwoodia aquaesulis DSM 4255 |
| Ga0586324_0011512_4736_5797 | 0.00 | 0.00 | 0.00 | 1795500.00 | 0.00 | 0.00 | 0.00 | 0.00 | 1482300.00 | 0.00 | 0.00 | 0.00 | KO:K00053 | ketol-acid reductoisomerase | Bacteria;Proteobacteria;Deltaproteobacteria;Syntrophobacterales;Syntrophobacteraceae;Desulfoglaeba;Desulfoglaeba alkanexedens;Desulfoglaeba alkanexedens ALDC |
| Ga0586324_0039922_1587_2603 | 0.00 | 0.00 | 0.00 | 0.00 | 0.00 | 0.00 | 0.00 | 3117100.00 | 2438100.00 | 0.00 | 0.00 | 0.00 | KO:K00053 | ketol-acid reductoisomerase | Bacteria;Proteobacteria;Betaproteobacteria;Nitrosomonadales;Thiobacillaceae;Thiobacillus;Thiobacillus thioparus;Thiobacillus thioparus DSM 505 |
| Ga0586324_0044729_388_1377 | 0.00 | 0.00 | 0.00 | 0.00 | 0.00 | 1594276.57 | 0.00 | 0.00 | 0.00 | 0.00 | 0.00 | 0.00 | KO:K00053 | ketol-acid reductoisomerase | Bacteria;Proteobacteria;Deltaproteobacteria;Syntrophobacterales;Syntrophaceae;Desulfobacca;Desulfobacca acetoxidans;Desulfobacca acetoxidans ASRB2, DSM 11109 |
| Ga0586324_0057865_1741_2739 | 0.00 | 0.00 | 0.00 | 0.00 | 1926100.00 | 0.00 | 0.00 | 0.00 | 0.00 | 0.00 | 0.00 | 0.00 | KO:K00053 | ketol-acid reductoisomerase | Bacteria;Cyanobacteria;unclassified Cyanobacteria;Synechococcales;Pseudanabaenaceae;Pseudanabaena;Pseudanabaena sp. 'Roaring Creek';Pseudanabaena sp. Roaring Creek |
| Ga0586324_0164613_547_1563 | 0.00 | 0.00 | 0.00 | 0.00 | 2925200.00 | 0.00 | 0.00 | 0.00 | 0.00 | 0.00 | 0.00 | 0.00 | KO:K00053 | ketol-acid reductoisomerase | Bacteria;Proteobacteria;Gammaproteobacteria;Chromatiales;Ectothiorhodospiraceae;Acidihalobacter;Acidihalobacter ferrooxydans;Acidihalobacter ferrooxydans V8 |
| Ga0586324_0219185_735_1751 | 0.00 | 0.00 | 0.00 | 0.00 | 0.00 | 0.00 | 0.00 | 524068.08 | 0.00 | 3711400.00 | 4144800.00 | 2497500.00 | KO:K00053 | ketol-acid reductoisomerase | Bacteria;Proteobacteria;Betaproteobacteria;Nitrosomonadales;Thiobacillaceae;Annwoodia;Annwoodia aquaesulis;Annwoodia aquaesulis DSM 4255 |
| Ga0586324_0946541_3_728 | 0.00 | 0.00 | 0.00 | 0.00 | 0.00 | 0.00 | 0.00 | 2421700.00 | 0.00 | 0.00 | 0.00 | 0.00 | KO:K00053 | ketol-acid reductoisomerase | Bacteria;Proteobacteria;Deltaproteobacteria;Syntrophorhabdales;Syntrophorhabdaceae;Syntrophorhabdus;Syntrophorhabdus aromaticivorans;Syntrophorhabdus aromaticivorans UI |
| Ga0586324_1501224_3_1004 | 0.00 | 0.00 | 0.00 | 0.00 | 0.00 | 0.00 | 0.00 | 6344000.00 | 6795900.00 | 0.00 | 0.00 | 0.00 | KO:K00053 | ketol-acid reductoisomerase | Bacteria;Proteobacteria;Gammaproteobacteria;Methylococcales;Methylococcaceae;Methylobacter;Methylobacter luteus;Methylobacter luteus IMV-B-3098 |
| Ga0586324_1573152_612_1001 | 0.00 | 0.00 | 0.00 | 1881000.00 | 0.00 | 0.00 | 4833600.00 | 1958100.00 | 0.00 | 2733500.00 | 0.00 | 0.00 | KO:K00053 | ketol-acid reductoisomerase | Bacteria;Proteobacteria;Gammaproteobacteria;Methylococcales;Methylococcaceae;Methyloterricola;Methyloterricola oryzae;Methyloterricola oryzae 73a |
| Ga0586324_1871075_28_930 | 0.00 | 0.00 | 0.00 | 0.00 | 5510500.00 | 0.00 | 0.00 | 0.00 | 3183300.00 | 0.00 | 0.00 | 0.00 | KO:K00053 | ketol-acid reductoisomerase | Bacteria;Proteobacteria;Deltaproteobacteria;Syntrophobacterales;Syntrophaceae;Desulfobacca;Desulfobacca acetoxidans;Desulfobacca acetoxidans ASRB2, DSM 11109 |
| Ga0586324_2061862_1_705 | 0.00 | 0.00 | 0.00 | 0.00 | 0.00 | 0.00 | 0.00 | 0.00 | 1441800.00 | 0.00 | 1390400.00 | 930000.00 | KO:K00053 | ketol-acid reductoisomerase | Bacteria;Proteobacteria;Betaproteobacteria;unclassified Betaproteobacteria;unclassified Betaproteobacteria;unclassified Betaproteobacteria;uncultured beta proteobacterium;unclassified betaproteobacterium ENR4 |
| Ga0586324_2146832_73_876 | 0.00 | 0.00 | 0.00 | 817760.00 | 0.00 | 0.00 | 0.00 | 0.00 | 0.00 | 0.00 | 0.00 | 0.00 | KO:K00053 | ketol-acid reductoisomerase | Bacteria;Proteobacteria;Deltaproteobacteria;Desulfuromonadales;Geobacteraceae;Geobacter;Geobacter sp. DSM 2909;Geobacter sp. DSM 2909 |
| Ga0586324_2420743_3_734 | 0.00 | 2930800.00 | 0.00 | 0.00 | 5788600.00 | 0.00 | 0.00 | 1213900.00 | 0.00 | 0.00 | 0.00 | 0.00 | KO:K00053 | ketol-acid reductoisomerase | Bacteria;Chloroflexi;Dehalococcoidia;unclassified Dehalococcoidia;unclassified Dehalococcoidia;Dehalogenimonas;Dehalogenimonas sp. WBC-2;Dehalogenimonas sp. WBC-2 |
| Ga0586324_2831769_3_779 | 0.00 | 0.00 | 0.00 | 0.00 | 0.00 | 0.00 | 0.00 | 0.00 | 0.00 | 0.00 | 0.00 | 5985000.00 | KO:K00053 | ketol-acid reductoisomerase | Bacteria;Acidobacteria;Acidobacteriia;Acidobacteriales;Acidobacteriaceae;Candidatus Koribacter;Candidatus Koribacter versatilis;Candidatus Koribacter versatilis Ellin345 |
| Ga0586324_2910032_1_603 | 0.00 | 0.00 | 2168265.01 | 0.00 | 0.00 | 0.00 | 3260400.00 | 0.00 | 0.00 | 0.00 | 0.00 | 0.00 | KO:K00053 | ketol-acid reductoisomerase | Bacteria;Proteobacteria;Deltaproteobacteria;Syntrophobacterales;Syntrophobacteraceae;Syntrophobacter;Syntrophobacter fumaroxidans;Syntrophobacter fumaroxidans MPOB |
| Ga0586324_3425754_3_281 | 0.00 | 0.00 | 0.00 | 0.00 | 0.00 | 0.00 | 0.00 | 0.00 | 644915.52 | 0.00 | 0.00 | 0.00 | KO:K00053 | ketol-acid reductoisomerase | Bacteria;Proteobacteria;Betaproteobacteria;Nitrosomonadales;Methylophilaceae;Candidatus Methylopumilus;Candidatus Methylopumilus turicensis;Candidatus Methylopumilus turicensis MMS-10A-171 |
| Ga0586324_3609987_362_706 | 0.00 | 0.00 | 0.00 | 0.00 | 0.00 | 0.00 | 12403200.00 | 0.00 | 0.00 | 0.00 | 0.00 | 0.00 | KO:K00053 | ketol-acid reductoisomerase | Bacteria;Proteobacteria;Betaproteobacteria;Nitrosomonadales;Methylophilaceae;Candidatus Methylopumilus;Candidatus Methylopumilus turicensis;Candidatus Methylopumilus turicensis MMS-10A-171 |
| Ga0586324_7181410_213_530 | 0.00 | 0.00 | 0.00 | 0.00 | 0.00 | 0.00 | 0.00 | 207667.79 | 0.00 | 0.00 | 0.00 | 0.00 | KO:K00053 | ketol-acid reductoisomerase | Bacteria;Chloroflexi;Dehalococcoidia;unclassified Dehalococcoidia;unclassified Dehalococcoidia;Dehalogenimonas;Dehalogenimonas sp. WBC-2;Dehalogenimonas sp. WBC-2 |
| Ga0586324_0001449_20691_22274 | 0.00 | 0.00 | 0.00 | 0.00 | 0.00 | 0.00 | 0.00 | 0.00 | 0.00 | 1609300.00 | 0.00 | 0.00 | KO:K00058 | D-3-phosphoglycerate dehydrogenase | Bacteria;Proteobacteria;Alphaproteobacteria;Hyphomicrobiales;Hyphomicrobiaceae;Rhodoplanes;Rhodoplanes sp. Z2-YC6860;Rhodoplanes sp. Z2-YC6860 |
| Ga0586324_0127315_1_1512 | 2770000.00 | 0.00 | 0.00 | 5415000.00 | 0.00 | 0.00 | 0.00 | 0.00 | 988200.00 | 0.00 | 0.00 | 0.00 | KO:K00058 | D-3-phosphoglycerate dehydrogenase | Bacteria;Proteobacteria;Deltaproteobacteria;Syntrophobacterales;Syntrophaceae;Desulfobacca;Desulfobacca acetoxidans;Desulfobacca acetoxidans ASRB2, DSM 11109 |
| Ga0586324_0441336_3_947 | 0.00 | 0.00 | 0.00 | 16340000.00 | 0.00 | 3799047.83 | 0.00 | 0.00 | 0.00 | 0.00 | 0.00 | 0.00 | KO:K00058 | D-3-phosphoglycerate dehydrogenase | Bacteria;Proteobacteria;Deltaproteobacteria;Syntrophobacterales;Syntrophaceae;Desulfobacca;Desulfobacca acetoxidans;Desulfobacca acetoxidans ASRB2, DSM 11109 |
| Ga0586324_2916780_2_772 | 0.00 | 0.00 | 0.00 | 0.00 | 0.00 | 0.00 | 0.00 | 0.00 | 1514700.00 | 0.00 | 0.00 | 0.00 | KO:K00058 | D-3-phosphoglycerate dehydrogenase | Bacteria;Proteobacteria;Deltaproteobacteria;Syntrophobacterales;Syntrophaceae;Desulfobacca;Desulfobacca acetoxidans;Desulfobacca acetoxidans ASRB2, DSM 11109 |
| Ga0586324_4272258_3_659 | 0.00 | 0.00 | 0.00 | 4997000.00 | 0.00 | 0.00 | 0.00 | 0.00 | 0.00 | 0.00 | 0.00 | 0.00 | KO:K00058 | D-3-phosphoglycerate dehydrogenase | Bacteria;Proteobacteria;Deltaproteobacteria;Syntrophobacterales;Syntrophaceae;Desulfobacca;Desulfobacca acetoxidans;Desulfobacca acetoxidans ASRB2, DSM 11109 |
| Ga0586324_5514232_1_591 | 0.00 | 0.00 | 0.00 | 4636000.00 | 0.00 | 0.00 | 0.00 | 0.00 | 0.00 | 0.00 | 0.00 | 0.00 | KO:K00058 | D-3-phosphoglycerate dehydrogenase | Bacteria;Proteobacteria;Deltaproteobacteria;Syntrophobacterales;Syntrophaceae;Desulfobacca;Desulfobacca acetoxidans;Desulfobacca acetoxidans ASRB2, DSM 11109 |
| Ga0586324_6126046_2_568 | 0.00 | 0.00 | 0.00 | 0.00 | 1596500.00 | 0.00 | 0.00 | 0.00 | 0.00 | 0.00 | 0.00 | 0.00 | KO:K00058 | D-3-phosphoglycerate dehydrogenase | Bacteria;Proteobacteria;Deltaproteobacteria;Desulfobacterales;Desulfobacteraceae;Desulfococcus;Desulfococcus multivorans;Desulfococcus multivorans DSM 2059 |
| Ga0586324_6244574_1_564 | 0.00 | 0.00 | 0.00 | 0.00 | 0.00 | 0.00 | 0.00 | 0.00 | 778265.82 | 0.00 | 0.00 | 0.00 | KO:K00058 | D-3-phosphoglycerate dehydrogenase | Bacteria;Chloroflexi;Anaerolineae;Anaerolineales;Anaerolineaceae;Thermanaerothrix;Thermanaerothrix daxensis;Thermanaerothrix daxensis GNS-1 |
| Ga0586324_6415862_2_556 | 0.00 | 0.00 | 0.00 | 2736000.00 | 0.00 | 0.00 | 0.00 | 0.00 | 0.00 | 0.00 | 0.00 | 0.00 | KO:K00058 | D-3-phosphoglycerate dehydrogenase | Bacteria;Chloroflexi;unclassified Chloroflexi;unclassified Chloroflexi;unclassified Chloroflexi;unclassified Chloroflexi;Chloroflexi sp. G233 |
| Ga0586324_0385632_643_1437 | 7210000.00 | 8024000.00 | 0.00 | 0.00 | 0.00 | 0.00 | 0.00 | 0.00 | 0.00 | 0.00 | 0.00 | 0.00 | KO:K00059 | 3-oxoacyl-[acyl-carrier protein] reductase | Bacteria;Proteobacteria;Gammaproteobacteria;Chromatiales;Chromatiaceae;Thiodictyon;Candidatus Thiodictyon syntrophicum;Candidatus Thiodictyon syntrophicum Cad16 |
| Ga0586324_2635495_410_805 | 0.00 | 0.00 | 0.00 | 0.00 | 2266000.00 | 0.00 | 0.00 | 0.00 | 0.00 | 0.00 | 0.00 | 0.00 | KO:K00059 | 3-oxoacyl-[acyl-carrier protein] reductase | Bacteria;Proteobacteria;Deltaproteobacteria;Desulfobacterales;Desulfobacteraceae;Desulfosarcina;Desulfosarcina variabilis;Desulfosarcina variabilis Montpellier |
| Ga0586324_2973754_2_544 | 0.00 | 0.00 | 0.00 | 0.00 | 0.00 | 0.00 | 0.00 | 549764.33 | 437533.65 | 0.00 | 0.00 | 0.00 | KO:K00059 | 3-oxoacyl-[acyl-carrier protein] reductase | Bacteria;Proteobacteria;Gammaproteobacteria;Methylococcales;Methylococcaceae;Methylobacter;Methylobacter marinus;Methylobacter marinus A45 |
| Ga0586324_3659485_200_700 | 0.00 | 3474800.00 | 0.00 | 0.00 | 0.00 | 0.00 | 0.00 | 0.00 | 0.00 | 0.00 | 0.00 | 0.00 | KO:K00059 | 3-oxoacyl-[acyl-carrier protein] reductase | Bacteria;Proteobacteria;Alphaproteobacteria;Rhodospirillales;Acetobacteraceae;Rhodopila;unclassified Rhodopila;Rhodopila sp. LVNP |
| Ga0586324_0153596_2_757 | 1760000.00 | 0.00 | 0.00 | 0.00 | 0.00 | 0.00 | 0.00 | 0.00 | 0.00 | 0.00 | 0.00 | 0.00 | KO:K00065 | 2-deoxy-D-gluconate 3-dehydrogenase | Bacteria;Acidobacteria;Blastocatellia;Blastocatellales;Pyrinomonadaceae;Pyrinomonas;Pyrinomonas methylaliphatogenes;Pyrinomonas methylaliphatogenes K22 |
| Ga0586324_0026778_2687_3535 | 0.00 | 0.00 | 0.00 | 0.00 | 0.00 | 0.00 | 0.00 | 0.00 | 1976400.00 | 0.00 | 0.00 | 0.00 | KO:K00074 | 3-hydroxybutyryl-CoA dehydrogenase | Bacteria;Proteobacteria;Betaproteobacteria;Rhodocyclales;Rhodocyclaceae;Azospira;unclassified Azospira;Azospira sp. ZAP |
| Ga0586324_0174597_2249_2635 | 0.00 | 5263200.00 | 0.00 | 8274500.00 | 7343900.00 | 10004400.00 | 0.00 | 1165100.00 | 6058800.00 | 0.00 | 0.00 | 6645000.00 | KO:K00074 | 3-hydroxybutyryl-CoA dehydrogenase | Bacteria;Proteobacteria;Deltaproteobacteria;Desulfobacterales;Desulfobacteraceae;Desulfospira;Desulfospira joergensenii;Desulfospira joergensenii DSM 10085 |
| Ga0586324_2091747_2_580 | 0.00 | 183370.16 | 1106120.47 | 0.00 | 638940.93 | 0.00 | 0.00 | 0.00 | 0.00 | 0.00 | 0.00 | 0.00 | KO:K00074 | 3-hydroxybutyryl-CoA dehydrogenase | Bacteria;Proteobacteria;Deltaproteobacteria;Desulfobacterales;Desulfobacteraceae;Desulfococcus;Desulfococcus multivorans;Desulfococcus multivorans DSM 2059 |
| Ga0586324_0000018_73627_75090 | 0.00 | 0.00 | 0.00 | 0.00 | 0.00 | 0.00 | 0.00 | 1098000.00 | 0.00 | 0.00 | 0.00 | 4322503.50 | KO:K00088 | IMP dehydrogenase | Bacteria;Proteobacteria;Betaproteobacteria;Nitrosomonadales;Thiobacillaceae;Annwoodia;Annwoodia aquaesulis;Annwoodia aquaesulis DSM 4255 |
| Ga0586324_0000385_16260_17720 | 0.00 | 0.00 | 0.00 | 0.00 | 0.00 | 0.00 | 16735200.00 | 0.00 | 10611000.00 | 993300.00 | 4963200.00 | 0.00 | KO:K00088 | IMP dehydrogenase | Bacteria;Proteobacteria;Betaproteobacteria;Nitrosomonadales;Thiobacillaceae;Thiobacillus;Thiobacillus denitrificans;Thiobacillus denitrificans ATCC 25259 |
| Ga0586324_0052310_3_791 | 0.00 | 0.00 | 0.00 | 0.00 | 0.00 | 0.00 | 0.00 | 0.00 | 0.00 | 0.00 | 14344000.00 | 0.00 | KO:K00088 | IMP dehydrogenase | Bacteria;Acidobacteria;Blastocatellia;unclassified Blastocatellia;unclassified Blastocatellia;Chloracidobacterium;Chloracidobacterium thermophilum;Chloracidobacterium thermophilum OC1 |
| Ga0586324_0102347_3_872 | 0.00 | 3950800.00 | 0.00 | 0.00 | 0.00 | 0.00 | 0.00 | 0.00 | 0.00 | 0.00 | 0.00 | 0.00 | KO:K00088 | IMP dehydrogenase | Bacteria;Proteobacteria;Betaproteobacteria;Nitrosomonadales;Gallionellaceae;Gallionella;Gallionella capsiferriformans;Gallionella capsiferriformans ES-2 |
| Ga0586324_0118577_1130_2659 | 0.00 | 0.00 | 0.00 | 3496000.00 | 0.00 | 0.00 | 0.00 | 0.00 | 0.00 | 0.00 | 0.00 | 0.00 | KO:K00088 | IMP dehydrogenase | Bacteria;Proteobacteria;Alphaproteobacteria;Hyphomicrobiales;unclassified Hyphomicrobiales;Methyloceanibacter;Methyloceanibacter marginalis;Methyloceanibacter marginalis R-67177 |
| Ga0586324_0452266_632_1714 | 0.00 | 0.00 | 0.00 | 0.00 | 0.00 | 0.00 | 0.00 | 0.00 | 0.00 | 13552000.00 | 1293600.00 | 552920.25 | KO:K00088 | IMP dehydrogenase | Bacteria;Proteobacteria;Betaproteobacteria;Nitrosomonadales;Gallionellaceae;Gallionella;Gallionella capsiferriformans;Gallionella capsiferriformans ES-2 |
| Ga0586324_1922906_1_381 | 0.00 | 0.00 | 0.00 | 0.00 | 2739800.00 | 0.00 | 0.00 | 1762900.00 | 0.00 | 2048200.00 | 0.00 | 0.00 | KO:K00088 | IMP dehydrogenase | Bacteria;Proteobacteria;Betaproteobacteria;Burkholderiales;unclassified Burkholderiales;Aquincola;Aquincola tertiaricarbonis;Aquincola tertiaricarbonis MIMtkpLc11 |
| Ga0586324_1781463_2_901 | 0.00 | 0.00 | 0.00 | 0.00 | 0.00 | 0.00 | 0.00 | 0.00 | 0.00 | 2710400.00 | 2851200.00 | 0.00 | KO:K00104 | glycolate oxidase | Bacteria;Proteobacteria;Deltaproteobacteria;Syntrophorhabdales;Syntrophorhabdaceae;Syntrophorhabdus;Syntrophorhabdus aromaticivorans;Syntrophorhabdus aromaticivorans UI |
| Ga0586324_0561832_69_1559 | 0.00 | 686800.00 | 0.00 | 0.00 | 0.00 | 0.00 | 0.00 | 0.00 | 0.00 | 0.00 | 0.00 | 0.00 | KO:K00108 | choline dehydrogenase | Bacteria;Proteobacteria;Gammaproteobacteria;Methylococcales;Methylococcaceae;Methylomicrobium;Methylomicrobium lacus;Methylosarcina lacus LW14 |
| Ga0586324_0000002_162709_164094 | 0.00 | 0.00 | 0.00 | 0.00 | 0.00 | 0.00 | 0.00 | 0.00 | 0.00 | 5035800.00 | 17529600.00 | 0.00 | KO:K00113 | glycerol-3-phosphate dehydrogenase subunit C | Bacteria;Proteobacteria;Betaproteobacteria;Nitrosomonadales;Thiobacillaceae;Annwoodia;Annwoodia aquaesulis;Annwoodia aquaesulis DSM 4255 |
| Ga0586324_0002371_12216_13559 | 0.00 | 0.00 | 0.00 | 0.00 | 0.00 | 0.00 | 0.00 | 0.00 | 189283.23 | 0.00 | 0.00 | 0.00 | KO:K00113 | glycerol-3-phosphate dehydrogenase subunit C | Bacteria;Proteobacteria;Gammaproteobacteria;Acidiferrobacterales;Acidiferrobacteraceae;Sulfuricaulis;Sulfuricaulis limicola;Sulfuricaulis limicola HA5 |
| Ga0586324_0007845_10314_11345 | 0.00 | 0.00 | 0.00 | 0.00 | 0.00 | 0.00 | 0.00 | 2006900.00 | 3134700.00 | 0.00 | 0.00 | 0.00 | KO:K00113 | glycerol-3-phosphate dehydrogenase subunit C | Bacteria;Proteobacteria;Betaproteobacteria;Nitrosomonadales;Thiobacillaceae;Thiobacillus;Thiobacillus denitrificans;Thiobacillus denitrificans RG |
| Ga0586324_8163619_1_369 | 0.00 | 0.00 | 0.00 | 0.00 | 0.00 | 0.00 | 0.00 | 0.00 | 0.00 | 0.00 | 0.00 | 1050000.00 | KO:K00113 | glycerol-3-phosphate dehydrogenase subunit C | Bacteria;Proteobacteria;Alphaproteobacteria;Hyphomicrobiales;Reyranellaceae;Reyranella;Reyranella massiliensis;Reyranella massiliensis 521, DSM 23428 |
| Ga0586324_0007859_4741_6408 | 2810000.00 | 0.00 | 0.00 | 0.00 | 0.00 | 0.00 | 0.00 | 0.00 | 0.00 | 0.00 | 0.00 | 0.00 | KO:K00114 | alcohol dehydrogenase (cytochrome c) | Bacteria;Proteobacteria;Alphaproteobacteria;Hyphomicrobiales;Bradyrhizobiaceae;Bradyrhizobium;Bradyrhizobium canariense;Bradyrhizobium canariense GAS369 |
| Ga0586324_0029023_1_2133 | 0.00 | 2801600.00 | 0.00 | 0.00 | 0.00 | 0.00 | 0.00 | 0.00 | 5362200.00 | 0.00 | 0.00 | 5520000.00 | KO:K00114 | alcohol dehydrogenase (cytochrome c) | Bacteria;Proteobacteria;Betaproteobacteria;Burkholderiales;unclassified Burkholderiales;unclassified Burkholderiales;Burkholderiales bacterium JOSHI_001;Burkholderiales sp. JOSHI_001 |
| Ga0586324_0076382_1724_3529 | 12250000.00 | 2556800.00 | 528546.09 | 232558.10 | 338422.98 | 6279871.45 | 223118.06 | 383315.46 | 24369497.19 | 3177371.12 | 691326.24 | 1140000.00 | KO:K00114 | alcohol dehydrogenase (cytochrome c) | Bacteria;Proteobacteria;Betaproteobacteria;Nitrosomonadales;Sterolibacteriaceae;Methyloversatilis;Methyloversatilis discipulorum;Methyloversatilis discipulorum FAM1 |
| Ga0586324_0083097_1_1632 | 0.00 | 2312000.00 | 0.00 | 0.00 | 0.00 | 0.00 | 0.00 | 0.00 | 0.00 | 0.00 | 0.00 | 0.00 | KO:K00114 | alcohol dehydrogenase (cytochrome c) | Bacteria;Proteobacteria;Alphaproteobacteria;Hyphomicrobiales;Bradyrhizobiaceae;Bradyrhizobium;Bradyrhizobium erythrophlei;Bradyrhizobium erythrophlei GAS478 |
| Ga0586324_0145572_140_1879 | 0.00 | 0.00 | 0.00 | 0.00 | 0.00 | 3461482.70 | 0.00 | 0.00 | 0.00 | 0.00 | 3000800.00 | 1619916.00 | KO:K00114 | alcohol dehydrogenase (cytochrome c) | Bacteria;Proteobacteria;Betaproteobacteria;Burkholderiales;unclassified Burkholderiales;unclassified Burkholderiales;Burkholderiales bacterium JOSHI_001;Burkholderiales sp. JOSHI_001 |
| Ga0586324_0158644_796_2469 | 0.00 | 0.00 | 0.00 | 0.00 | 0.00 | 0.00 | 0.00 | 0.00 | 0.00 | 0.00 | 2904000.00 | 1815000.00 | KO:K00114 | alcohol dehydrogenase (cytochrome c) | Bacteria;Proteobacteria;Betaproteobacteria;Burkholderiales;Burkholderiaceae;Paraburkholderia;Paraburkholderia susongensis;Paraburkholderia susongensis LMG 29540 |
| Ga0586324_0192193_568_2319 | 14205991.00 | 16708168.48 | 10214608.09 | 452620.85 | 6992300.23 | 386616.07 | 149187.92 | 484269.85 | 292687.02 | 9429314.51 | 403699.12 | 7005489.75 | KO:K00114 | alcohol dehydrogenase (cytochrome c) | Bacteria;Proteobacteria;Betaproteobacteria;Nitrosomonadales;Sterolibacteriaceae;Methyloversatilis;Methyloversatilis discipulorum;Methyloversatilis discipulorum FAM1 |
| Ga0586324_0215684_143_1882 | 7710000.00 | 5290400.00 | 0.00 | 0.00 | 0.00 | 2254416.11 | 2508000.00 | 2604700.00 | 3377700.00 | 0.00 | 1460800.00 | 3285000.00 | KO:K00114 | alcohol dehydrogenase (cytochrome c) | Bacteria;Proteobacteria;Betaproteobacteria;Burkholderiales;unclassified Burkholderiales;unclassified Burkholderiales;Burkholderiales bacterium JOSHI_001;Burkholderiales sp. JOSHI_001 |
| Ga0586324_0567520_157_1551 | 288700.00 | 1417181.88 | 1684518.52 | 423957.45 | 0.00 | 0.00 | 0.00 | 802933.85 | 0.00 | 2086700.00 | 0.00 | 1369850.25 | KO:K00114 | alcohol dehydrogenase (cytochrome c) | Bacteria;Proteobacteria;Betaproteobacteria;Nitrosomonadales;Sterolibacteriaceae;Methyloversatilis;Methyloversatilis universalis;Methyloversatilis universalis EHg5 |
| Ga0586324_0952556_2_859 | 0.00 | 0.00 | 0.00 | 0.00 | 0.00 | 0.00 | 0.00 | 2671800.00 | 0.00 | 5058900.00 | 0.00 | 0.00 | KO:K00114 | alcohol dehydrogenase (cytochrome c) | Bacteria;Proteobacteria;Alphaproteobacteria;Hyphomicrobiales;Bradyrhizobiaceae;Bradyrhizobium;Bradyrhizobium sp. STM 3843;Bradyrhizobium sp. STM 3843 |
| Ga0586324_0953658_1_1167 | 0.00 | 6446400.00 | 15820300.00 | 0.00 | 0.00 | 0.00 | 0.00 | 0.00 | 0.00 | 0.00 | 0.00 | 5962500.00 | KO:K00114 | alcohol dehydrogenase (cytochrome c) | Bacteria;Proteobacteria;Betaproteobacteria;Burkholderiales;Burkholderiaceae;Cupriavidus;Cupriavidus sp. IDO;Cupriavidus sp. IDO |
| Ga0586324_1018899_2_679 | 0.00 | 24684000.00 | 0.00 | 0.00 | 0.00 | 0.00 | 0.00 | 0.00 | 0.00 | 0.00 | 0.00 | 8475000.00 | KO:K00114 | alcohol dehydrogenase (cytochrome c) | Bacteria;Proteobacteria;Betaproteobacteria;Burkholderiales;unclassified Burkholderiales;unclassified Burkholderiales;Burkholderiales bacterium JOSHI_001;Burkholderiales sp. JOSHI_001 |
| Ga0586324_1052426_3_1004 | 0.00 | 0.00 | 27812200.00 | 0.00 | 0.00 | 0.00 | 0.00 | 0.00 | 0.00 | 0.00 | 0.00 | 0.00 | KO:K00114 | alcohol dehydrogenase (cytochrome c) | Bacteria;Proteobacteria;Alphaproteobacteria;Rhodospirillales;Acetobacteraceae;Rhodopila;Rhodopila globiformis;Rhodopila globiformis DSM 161 |
| Ga0586324_1392089_3_1052 | 0.00 | 2516000.00 | 7700196.04 | 1577000.00 | 269120.46 | 9686800.00 | 0.00 | 1488400.00 | 0.00 | 4958800.00 | 8069600.00 | 11445000.00 | KO:K00114 | alcohol dehydrogenase (cytochrome c) | Bacteria;Proteobacteria;Betaproteobacteria;Nitrosomonadales;Sterolibacteriaceae;Methyloversatilis;Methyloversatilis discipulorum;Methyloversatilis discipulorum FAM1 |
| Ga0586324_1460660_1_1032 | 5350000.00 | 5684800.00 | 0.00 | 0.00 | 4902800.00 | 17031300.00 | 1993727.76 | 3879600.00 | 3920400.00 | 4897200.00 | 2921600.00 | 3472500.00 | KO:K00114 | alcohol dehydrogenase (cytochrome c) | Bacteria;Proteobacteria;Betaproteobacteria;Nitrosomonadales;Sterolibacteriaceae;Methyloversatilis;Methyloversatilis discipulorum;Methyloversatilis discipulorum FAM1 |
| Ga0586324_1461177_1_1032 | 0.00 | 2116454.44 | 0.00 | 0.00 | 0.00 | 0.00 | 0.00 | 128211.02 | 0.00 | 1131900.00 | 2340800.00 | 864315.75 | KO:K00114 | alcohol dehydrogenase (cytochrome c) | Bacteria;Proteobacteria;Betaproteobacteria;Nitrosomonadales;Sterolibacteriaceae;Methyloversatilis;Methyloversatilis universalis;Methyloversatilis universalis EHg5 |
| Ga0586324_1808437_3_941 | 3910000.00 | 0.00 | 2841922.66 | 0.00 | 0.00 | 4764000.00 | 2371200.00 | 1409100.00 | 1911600.00 | 1940400.00 | 0.00 | 1522500.00 | KO:K00114 | alcohol dehydrogenase (cytochrome c) | Bacteria;Proteobacteria;Betaproteobacteria;Burkholderiales;unclassified Burkholderiales;unclassified Burkholderiales;Burkholderiales bacterium JOSHI_001;Burkholderiales sp. JOSHI_001 |
| Ga0586324_4028859_29_673 | 0.00 | 0.00 | 0.00 | 8170000.00 | 0.00 | 0.00 | 0.00 | 0.00 | 0.00 | 0.00 | 0.00 | 1890000.00 | KO:K00114 | alcohol dehydrogenase (cytochrome c) | Bacteria;Proteobacteria;Alphaproteobacteria;Rhodospirillales;Acetobacteraceae;Rhodopila;unclassified Rhodopila;Rhodopila sp. LVNP |
| Ga0586324_4800458_3_626 | 0.00 | 573420.20 | 0.00 | 0.00 | 0.00 | 0.00 | 0.00 | 1573800.00 | 0.00 | 434928.34 | 2076800.00 | 990000.00 | KO:K00114 | alcohol dehydrogenase (cytochrome c) | Bacteria;Proteobacteria;Alphaproteobacteria;Hyphomicrobiales;Bradyrhizobiaceae;Bradyrhizobium;Bradyrhizobium elkanii;Bradyrhizobium elkanii WSM2783 |
| Ga0586324_5419857_3_596 | 0.00 | 0.00 | 0.00 | 0.00 | 0.00 | 0.00 | 0.00 | 2873100.00 | 0.00 | 0.00 | 0.00 | 0.00 | KO:K00114 | alcohol dehydrogenase (cytochrome c) | Bacteria;Proteobacteria;Betaproteobacteria;Burkholderiales;unclassified Burkholderiales;unclassified Burkholderiales;Burkholderiales bacterium JOSHI_001;Burkholderiales sp. JOSHI_001 |
| Ga0586324_5724762_3_584 | 6130000.00 | 19856000.00 | 40704900.00 | 0.00 | 0.00 | 26162300.00 | 0.00 | 8784000.00 | 8343000.00 | 25949000.00 | 16632000.00 | 1965000.00 | KO:K00114 | alcohol dehydrogenase (cytochrome c) | Bacteria;Proteobacteria;Alphaproteobacteria;Rhodospirillales;Acetobacteraceae;Rhodopila;Rhodopila globiformis;Rhodopila globiformis DSM 161 |
| Ga0586324_6493512_3_554 | 0.00 | 23936000.00 | 0.00 | 0.00 | 0.00 | 0.00 | 0.00 | 0.00 | 0.00 | 0.00 | 0.00 | 0.00 | KO:K00114 | alcohol dehydrogenase (cytochrome c) | Bacteria;Proteobacteria;Betaproteobacteria;Burkholderiales;unclassified Burkholderiales;Methylibium;Methylibium sp. Pch-M;Methylibium sp. Pch-M |
| Ga0586324_0001544_10986_12095 | 0.00 | 0.00 | 0.00 | 0.00 | 0.00 | 0.00 | 0.00 | 1457900.00 | 1296000.00 | 0.00 | 0.00 | 0.00 | KO:K00121 | S-(hydroxymethyl)glutathione dehydrogenase/alcohol dehydrogenase | Bacteria;Proteobacteria;Betaproteobacteria;Nitrosomonadales;Thiobacillaceae;Thiobacillus;Thiobacillus thioparus;Thiobacillus thioparus DSM 505 |

| | | | | | | | | | | | | | | | |
|---|---|---|---|---|---|---|---|---|---|---|---|---|---|---|---|
| Ga0586324 4844825 1 420 | 0.00 | 0.00 | 0.00 | 1121000.00 | 5984300.00 | 0.00 | 0.00 | 0.00 | 0.00 | 0.00 | 0.00 | 2040000.00 | KO:K00121 | S-(hydroxymethyl)glutathione dehydrogenase/alcohol dehydrogenase | Bacteria;Proteobacteria;Alphaproteobacteria;Rhodospirillales;Acetobacteraceae;Rhodopila;unclassified Rhodopila;Rhodopila sp. LVNP |
| Ga0586324 0068903 1 1809 | 0.00 | 5338000.00 | 0.00 | 0.00 | 0.00 | 0.00 | 0.00 | 0.00 | 0.00 | 0.00 | 0.00 | 0.00 | KO:K00123 | formate dehydrogenase major subunit | Bacteria;Proteobacteria;Betaproteobacteria;Burkholderiales;Burkholderiaceae;unclassified Burkholderiaceae;Burkholderiaceae bacterium PBA;Burkholderiaceae bacterium PBA |
| Ga0586324 0417493 2 1774 | 0.00 | 0.00 | 0.00 | 0.00 | 2111500.00 | 25527100.00 | 0.00 | 0.00 | 0.00 | 0.00 | 0.00 | 0.00 | KO:K00123 | formate dehydrogenase major subunit | Bacteria;Proteobacteria;Deltaproteobacteria;Syntrophobacterales;Syntrophobacteraceae;Desulfacinum;Desulfacinum infernum;Desulfacinum infernum DSM 9756 |
| Ga0586324 0490105 3 1511 | 0.00 | 0.00 | 0.00 | 0.00 | 0.00 | 0.00 | 0.00 | 1451800.00 | 0.00 | 0.00 | 0.00 | 0.00 | KO:K00123 | formate dehydrogenase major subunit | Bacteria;Proteobacteria;Deltaproteobacteria;Syntrophobacterales;Syntrophobacteraceae;Desulfosoma;Desulfosoma caldarium;Desulfosoma caldarium DSM 22027 |
| Ga0586324 1645445 2 982 | 13100000.00 | 0.00 | 0.00 | 0.00 | 0.00 | 0.00 | 0.00 | 0.00 | 0.00 | 0.00 | 0.00 | 0.00 | KO:K00123 | formate dehydrogenase major subunit | Bacteria;Proteobacteria;Deltaproteobacteria;Syntrophobacterales;Syntrophaceae;Desulfomonile;Desulfomonile tiedjei;Desulfomonile tiedjei DCB-1, DSM 6799 |
| Ga0586324 1678795 110 973 | 0.00 | 0.00 | 0.00 | 4674000.00 | 0.00 | 0.00 | 0.00 | 0.00 | 0.00 | 0.00 | 0.00 | 0.00 | KO:K00123 | formate dehydrogenase major subunit | Bacteria;Proteobacteria;Deltaproteobacteria;Syntrophobacterales;Syntrophaceae;Desulfomonile;Desulfomonile tiedjei;Desulfomonile tiedjei DCB-1, DSM 6799 |
| Ga0586324 2189294 3 869 | 0.00 | 0.00 | 5564196.56 | 0.00 | 0.00 | 0.00 | 0.00 | 0.00 | 0.00 | 0.00 | 0.00 | 0.00 | KO:K00123 | formate dehydrogenase major subunit | Bacteria;Proteobacteria;Gammaproteobacteria;Nevskiales;Sinobacteraceae;Hydrocarboniphaga;Hydrocarboniphaga effusa;Hydrocarboniphaga effusa AP103 |
| Ga0586324 3195700 2 742 | 0.00 | 0.00 | 0.00 | 0.00 | 0.00 | 0.00 | 0.00 | 0.00 | 0.00 | 0.00 | 0.00 | 2880000.00 | KO:K00123 | formate dehydrogenase major subunit | Bacteria;Proteobacteria;Betaproteobacteria;Burkholderiales;Comamonadaceae;Ramlibacter;Ramlibacter sp. Leaf400;Ramlibacter sp. Leaf400 |
| Ga0586324 4389209 2 556 | 0.00 | 0.00 | 0.00 | 0.00 | 0.00 | 0.00 | 0.00 | 0.00 | 1125900.00 | 536274.97 | 0.00 | 0.00 | KO:K00123 | formate dehydrogenase major subunit | Bacteria;Proteobacteria;Betaproteobacteria;unclassified Betaproteobacteria;unclassified Betaproteobacteria;unclassified Betaproteobacteria;uncultured beta proteobacterium;unclassified betaproteobacterium ENR4 |
| Ga0586324 2573282 552 812 | 0.00 | 0.00 | 0.00 | 0.00 | 0.00 | 0.00 | 1093442.40 | 0.00 | 0.00 | 0.00 | 0.00 | 0.00 | KO:K00124 | formate dehydrogenase iron-sulfur subunit | Bacteria;Proteobacteria;Betaproteobacteria;Burkholderiales;unclassified Burkholderiales;Leptothrix;Leptothrix cholodnii;Leptothrix cholodnii SP-6 |
| Ga0586324 3257948 3 737 | 0.00 | 0.00 | 0.00 | 0.00 | 0.00 | 0.00 | 0.00 | 0.00 | 1166400.00 | 0.00 | 0.00 | 0.00 | KO:K00124 | formate dehydrogenase iron-sulfur subunit | Bacteria;Firmicutes;Clostridia;Thermoanaerobacterales;Thermoanaerobacteraceae;Calderihabitans;Calderihabitans maritimus;Calderihabitans maritimus KKC1 |
| Ga0586324 3862363 2 685 | 0.00 | 1611600.00 | 0.00 | 2764500.00 | 2132100.00 | 0.00 | 0.00 | 0.00 | 0.00 | 0.00 | 0.00 | 0.00 | KO:K00124 | formate dehydrogenase iron-sulfur subunit | Bacteria;Proteobacteria;Deltaproteobacteria;Desulfovibrionales;Desulfovibrionaceae;Pseudodesulfovibrio;Pseudodesulfovibrio profundus;Pseudodesulfovibrio profundus 500-1 |
| Ga0586324 6131834 265 567 | 0.00 | 0.00 | 0.00 | 1121000.00 | 0.00 | 0.00 | 0.00 | 258113.57 | 0.00 | 0.00 | 0.00 | 0.00 | KO:K00124 | formate dehydrogenase iron-sulfur subunit | Bacteria;Proteobacteria;Deltaproteobacteria;Syntrophobacterales;Syntrophaceae;Desulfomonile;Desulfomonile tiedjei;Desulfomonile tiedjei DCB-1, DSM 6799 |
| Ga0586324 0060940 152 1327 | 0.00 | 0.00 | 0.00 | 0.00 | 0.00 | 1174322.03 | 0.00 | 3397700.00 | 0.00 | 0.00 | 0.00 | 0.00 | KO:K00125 | formate dehydrogenase subunit beta | Archaea;Euryarchaeota;Methanomicrobia;Methanomicrobiales;Methanoregulaceae;Methanoregula;Methanoregula formicica;Methanoregula formicica SMSP, DSM 22288 |
| Ga0586324 0067805 2831 4081 | 1880000.00 | 17000000.00 | 16552200.00 | 18145000.00 | 0.00 | 0.00 | 12540000.00 | 0.00 | 13365000.00 | 0.00 | 0.00 | 10050000.00 | KO:K00125 | formate dehydrogenase subunit beta | Archaea;Euryarchaeota;Methanomicrobia;Methanomicrobiales;Methanoregulaceae;Methanoregula;Methanoregula formicica;Methanoregula formicica SMSP, DSM 22288 |
| Ga0586324 0335549 722 1873 | 0.00 | 0.00 | 0.00 | 0.00 | 0.00 | 0.00 | 0.00 | 0.00 | 0.00 | 0.00 | 15400000.00 | 0.00 | KO:K00125 | formate dehydrogenase subunit beta | Archaea;Euryarchaeota;Methanomicrobia;Methanomicrobiales;Methanoregulaceae;Methanoregula;Methanoregula formicica;Methanoregula formicica SMSP, DSM 22288 |
| Ga0586324 0351754 95 1270 | 0.00 | 1897200.00 | 0.00 | 0.00 | 0.00 | 0.00 | 0.00 | 0.00 | 0.00 | 0.00 | 0.00 | 0.00 | KO:K00125 | formate dehydrogenase subunit beta | Archaea;Euryarchaeota;Methanomicrobia;Methanomicrobiales;Methanomicrobiaceae;Methanoculleus;Methanoculleus bourgensis;Methanoculleus bourgensis MS2 |
| Ga0586324 0514119 1 843 | 0.00 | 0.00 | 0.00 | 1615000.00 | 0.00 | 0.00 | 0.00 | 0.00 | 0.00 | 0.00 | 0.00 | 0.00 | KO:K00125 | formate dehydrogenase subunit beta | Archaea;Euryarchaeota;Methanomicrobia;Methanomicrobiales;Methanomicrobiaceae;Methanoculleus;Methanoculleus sp. MH98A;Methanoculleus sp. MH98A |
| Ga0586324 0538212 2 1057 | 0.00 | 0.00 | 0.00 | 0.00 | 0.00 | 3055431.10 | 0.00 | 1836100.00 | 1660500.00 | 0.00 | 0.00 | 0.00 | KO:K00125 | formate dehydrogenase subunit beta | Archaea;Euryarchaeota;Methanomicrobia;Methanomicrobiales;Methanomicrobiaceae;Methanoculleus;Methanoculleus sp. MH98A;Methanoculleus sp. MH98A |
| Ga0586324 0811947 359 1327 | 0.00 | 0.00 | 0.00 | 0.00 | 0.00 | 0.00 | 0.00 | 1146800.00 | 0.00 | 0.00 | 0.00 | 0.00 | KO:K00125 | formate dehydrogenase subunit beta | Archaea;Euryarchaeota;Methanomicrobia;Methanomicrobiales;Methanoregulaceae;Methanoregula;Methanoregula formicica;Methanoregula formicica SMSP, DSM 22288 |
| Ga0586324 0042298 2 1552 | 0.00 | 0.00 | 0.00 | 0.00 | 0.00 | 0.00 | 0.00 | 0.00 | 2632500.00 | 0.00 | 0.00 | 1477500.00 | KO:K00128 | aldehyde dehydrogenase (NAD+) | Bacteria;Proteobacteria;Alphaproteobacteria;Hyphomicrobiales;Bradyrhizobiaceae;Bradyrhizobium;Bradyrhizobium lablabi;Bradyrhizobium lablabi GAS499 |
| Ga0586324 0000003 78914 80026 | 0.00 | 0.00 | 0.00 | 0.00 | 0.00 | 0.00 | 0.00 | 0.00 | 1174500.00 | 0.00 | 0.00 | 1875000.00 | KO:K00133 | aspartate-semialdehyde dehydrogenase | Bacteria;Proteobacteria;Gammaproteobacteria;Chromatiales;Thioalkalispiraceae;Sulfurivermis;Sulfurivermis fontis;Sulfurivermis fontis JG42 |
| Ga0586324 0143030 976 2013 | 0.00 | 0.00 | 0.00 | 0.00 | 0.00 | 0.00 | 0.00 | 109246.12 | 0.00 | 0.00 | 0.00 | 0.00 | KO:K00133 | aspartate-semialdehyde dehydrogenase | Bacteria;Deferribacteres;Deferribacteres;Deferribacterales;Deferribacteraceae;Calditerrivibrio;Calditerrivibrio nitroreducens;Calditerrivibrio nitroreducens Yu37-1, DSM 19672 |
| Ga0586324 0789210 192 1214 | 0.00 | 0.00 | 0.00 | 0.00 | 0.00 | 1249311.36 | 0.00 | 0.00 | 0.00 | 0.00 | 0.00 | 0.00 | KO:K00133 | aspartate-semialdehyde dehydrogenase | Bacteria;Proteobacteria;Deltaproteobacteria;unclassified Deltaproteobacteria;unclassified Deltaproteobacteria;Deferrisoma;Deferrisoma camini;Deferrisoma camini S3R1 |
| Ga0586324 8143512 2 505 | 0.00 | 0.00 | 0.00 | 0.00 | 0.00 | 0.00 | 0.00 | 0.00 | 0.00 | 0.00 | 0.00 | 1072500.00 | KO:K00133 | aspartate-semialdehyde dehydrogenase | Bacteria;Coprothermobacterota;Coprothermobacteria;Coprothermobacterales;Coprothermobacteraceae;Coprothermobacter;Coprothermobacter platensis;Coprothermobacter platensis DSM 11748 |
| Ga0586324 0000001 222665 223663 | 1060000.00 | 0.00 | 956649.60 | 1976000.00 | 1957000.00 | 2104298.50 | 9735600.00 | 11163000.00 | 11988000.00 | 0.00 | 13481600.00 | 12255000.00 | KO:K00134 | glyceraldehyde 3-phosphate dehydrogenase | Bacteria;Proteobacteria;Betaproteobacteria;Nitrosomonadales;Thiobacillaceae;Thiobacillus;Thiobacillus thiophilus;Thiobacillus thiophilus DSM 19892 |
| Ga0586324 0002733 772 1785 | 0.00 | 0.00 | 0.00 | 0.00 | 0.00 | 0.00 | 0.00 | 0.00 | 1814400.00 | 0.00 | 0.00 | 0.00 | KO:K00134 | glyceraldehyde 3-phosphate dehydrogenase | Bacteria;Proteobacteria;Deltaproteobacteria;Syntrophobacterales;Syntrophobacteraceae;Desulfosoma;Desulfosoma caldarium;Desulfosoma caldarium DSM 22027 |
| Ga0586324 0002802 12818 13888 | 0.00 | 0.00 | 0.00 | 0.00 | 0.00 | 0.00 | 0.00 | 0.00 | 0.00 | 816200.00 | 0.00 | 0.00 | KO:K00134 | glyceraldehyde 3-phosphate dehydrogenase | Bacteria;Firmicutes;unclassified Firmicutes;unclassified Firmicutes;unclassified Firmicutes;Hydrogenispora;Hydrogenispora ethanolica;Hydrogenispora ethanolica DSM 25471 |
| Ga0586324 0003422 2950 3951 | 0.00 | 0.00 | 0.00 | 0.00 | 0.00 | 0.00 | 0.00 | 0.00 | 0.00 | 13321000.00 | 0.00 | 13050000.00 | KO:K00134 | glyceraldehyde 3-phosphate dehydrogenase | Bacteria;Proteobacteria;Betaproteobacteria;Nitrosomonadales;Methylophilaceae;Novimethylophilus;Novimethylophilus kurashikiensis;Novimethylophilus kurashikiensis La2-4 |
| Ga0586324 0013553 7091 8092 | 3180000.00 | 0.00 | 0.00 | 1387000.00 | 1359600.00 | 0.00 | 547366.44 | 719800.00 | 470181.51 | 6691300.00 | 8184000.00 | 0.00 | KO:K00134 | glyceraldehyde 3-phosphate dehydrogenase | Bacteria;Proteobacteria;Gammaproteobacteria;unclassified Gammaproteobacteria;unclassified Gammaproteobacteria;unclassified Gammaproteobacteria;Codakia orbicularis gill symbiont;Codakia orbicularis gill symbiont |
| Ga0586324 0015731 2660 3673 | 0.00 | 2516000.00 | 0.00 | 0.00 | 0.00 | 0.00 | 4651200.00 | 30012000.00 | 7452000.00 | 0.00 | 0.00 | 0.00 | KO:K00134 | glyceraldehyde 3-phosphate dehydrogenase | Bacteria;Proteobacteria;Betaproteobacteria;Burkholderiales;Burkholderiaceae;Pandoraea;Pandoraea thiooxydans;Pandoraea thiooxydans DSM 25325 |
| Ga0586324 0018035 716 1726 | 0.00 | 0.00 | 0.00 | 0.00 | 0.00 | 0.00 | 0.00 | 0.00 | 365884.29 | 0.00 | 0.00 | 0.00 | KO:K00134 | glyceraldehyde 3-phosphate dehydrogenase | Bacteria;Gemmatimonadetes;Longimicrobia;Longimicrobiales;Longimicrobiaceae;Longimicrobium;Longimicrobium terrae;Longimicrobium terrae CECT 8660 |
| Ga0586324 0074606 1714 2739 | 0.00 | 0.00 | 0.00 | 0.00 | 0.00 | 0.00 | 0.00 | 0.00 | 0.00 | 4096400.00 | 3537600.00 | 0.00 | KO:K00134 | glyceraldehyde 3-phosphate dehydrogenase | Bacteria;Actinobacteria;Coriobacteriia;Eggerthellales;Eggerthellaceae;Slackia;Slackia piriformis;Slackia piriformis YIT 12062 |
| Ga0586324 0082036 1334 2392 | 6090000.00 | 0.00 | 5855200.00 | 0.00 | 0.00 | 5835900.00 | 0.00 | 0.00 | 0.00 | 0.00 | 0.00 | 0.00 | KO:K00134 | glyceraldehyde 3-phosphate dehydrogenase | Bacteria;Chloroflexi;Anaerolineae;Anaerolineales;Anaerolineaceae;Pelolinea;Pelolinea submarina;Pelolinea submarina DSM 23923 |
| Ga0586324 0082242 2840 3766 | 0.00 | 0.00 | 0.00 | 1729000.00 | 1761300.00 | 0.00 | 0.00 | 1701900.00 | 805374.09 | 5212900.00 | 4382400.00 | 0.00 | KO:K00134 | glyceraldehyde 3-phosphate dehydrogenase | Bacteria;Proteobacteria;Betaproteobacteria;Nitrosomonadales;Gallionellaceae;Sideroxydans;Sideroxydans lithotrophicus;Sideroxydans lithotrophicus ES-1 |
| Ga0586324 0085611 1568 2707 | 0.00 | 2522800.00 | 0.00 | 0.00 | 0.00 | 0.00 | 0.00 | 0.00 | 0.00 | 0.00 | 0.00 | 0.00 | KO:K00134 | glyceraldehyde 3-phosphate dehydrogenase | Bacteria;Deinococcus-Thermus;Deinococci;Thermales;Thermaceae;Calidithermus;Calidithermus chliarophilus;Meiothermus chliarophilus ALT-8, DSM 9957 |
| Ga0586324 0092083 2587 3567 | 4500000.00 | 0.00 | 0.00 | 6659500.00 | 471816.22 | 0.00 | 0.00 | 4715300.00 | 0.00 | 0.00 | 0.00 | 0.00 | KO:K00134 | glyceraldehyde 3-phosphate dehydrogenase | Bacteria;Proteobacteria;Deltaproteobacteria;Desulfobacterales;Desulfobacteraceae;Desulfosarcina;Desulfosarcina variabilis;Desulfosarcina variabilis Montpellier |
| Ga0586324 0099870 131 1135 | 0.00 | 0.00 | 0.00 | 3790500.00 | 0.00 | 0.00 | 0.00 | 1165100.00 | 972000.00 | 0.00 | 0.00 | 0.00 | KO:K00134 | glyceraldehyde 3-phosphate dehydrogenase | Bacteria;Firmicutes;Clostridia;Thermoanaerobacterales;Thermoanaerobacteraceae;Carboxydothermus;Carboxydothermus islandicus;Carboxydothermus islandicus SET IS-9 |
| Ga0586324 0100418 2488 3423 | 0.00 | 0.00 | 0.00 | 0.00 | 5057300.00 | 0.00 | 0.00 | 0.00 | 0.00 | 0.00 | 0.00 | 0.00 | KO:K00134 | glyceraldehyde 3-phosphate dehydrogenase | Bacteria;Proteobacteria;Betaproteobacteria;Burkholderiales;unclassified Burkholderiales;Rubrivivax;Rubrivivax gelatinosus;Rubrivivax gelatinosus IL144 |
| Ga0586324 0103761 946 1944 | 0.00 | 0.00 | 0.00 | 0.00 | 0.00 | 0.00 | 0.00 | 300231.02 | 365365.89 | 0.00 | 0.00 | 0.00 | KO:K00134 | glyceraldehyde 3-phosphate dehydrogenase | Bacteria;Proteobacteria;Gammaproteobacteria;unclassified Gammaproteobacteria;Candidatus Competibacteraceae;Candidatus Contendobacter;Candidatus Contendobacter odensis;Candidatus Contendobacter odensis Run B J11 |
| Ga0586324 0160522 467 1507 | 0.00 | 0.00 | 0.00 | 0.00 | 0.00 | 10083800.00 | 0.00 | 0.00 | 0.00 | 0.00 | 0.00 | 0.00 | KO:K00134 | glyceraldehyde 3-phosphate dehydrogenase | Bacteria;Planctomycetes;Planctomycetia;Gemmatales;Gemmataceae;Zavarzinella;Zavarzinella formosa;Zavarzinella formosa DSM 19928 |
| Ga0586324 0166635 856 1857 | 0.00 | 0.00 | 0.00 | 0.00 | 513980.30 | 0.00 | 0.00 | 0.00 | 0.00 | 0.00 | 0.00 | 0.00 | KO:K00134 | glyceraldehyde 3-phosphate dehydrogenase | Bacteria;Proteobacteria;Deltaproteobacteria;Desulfobacterales;Desulfobacteraceae;Desulfococcus;Desulfococcus multivorans;Desulfococcus multivorans DSM 2059 |
| Ga0586324 0216117 543 1544 | 3690000.00 | 2951200.00 | 0.00 | 0.00 | 0.00 | 0.00 | 0.00 | 0.00 | 0.00 | 0.00 | 0.00 | 0.00 | KO:K00134 | glyceraldehyde 3-phosphate dehydrogenase | Bacteria;Proteobacteria;Gammaproteobacteria;Chromatiales;Chromatiaceae;Thiobaca;Thiobaca trueperi;Thiobaca trueperi DSM 13587 |
| Ga0586324 0293051 705 1763 | 3160000.00 | 0.00 | 0.00 | 0.00 | 149746.55 | 3946211.76 | 0.00 | 0.00 | 0.00 | 0.00 | 0.00 | 0.00 | KO:K00134 | glyceraldehyde 3-phosphate dehydrogenase | Bacteria;Chloroflexi;Anaerolineae;Anaerolineales;Anaerolineaceae;Thermanaerothrix;Thermanaerothrix daxensis;Thermanaerothrix daxensis GNS-1 |
| Ga0586324 0452081 3 293 | 0.00 | 0.00 | 0.00 | 0.00 | 0.00 | 2456167.54 | 0.00 | 0.00 | 0.00 | 0.00 | 0.00 | 0.00 | KO:K00134 | glyceraldehyde 3-phosphate dehydrogenase | Bacteria;Chloroflexi;Caldilineae;Caldilineales;Caldilineaceae;Caldilinea;Caldilinea aerophila;Caldilinea aerophila STL-6-O1, DSM 14535 |
| Ga0586324 0557522 1 417 | 0.00 | 0.00 | 0.00 | 0.00 | 0.00 | 0.00 | 0.00 | 1073600.00 | 0.00 | 0.00 | 0.00 | 0.00 | KO:K00134 | glyceraldehyde 3-phosphate dehydrogenase | Bacteria;Chloroflexi;Caldilineae;Caldilineales;Caldilineaceae;Caldilinea;Caldilinea aerophila;Caldilinea aerophila STL-6-O1, DSM 14535 |
| Ga0586324 0585333 3 989 | 0.00 | 0.00 | 0.00 | 0.00 | 2111500.00 | 0.00 | 0.00 | 0.00 | 0.00 | 0.00 | 0.00 | 0.00 | KO:K00134 | glyceraldehyde 3-phosphate dehydrogenase | Bacteria;Proteobacteria;Deltaproteobacteria;Desulfuromonadales;Geobacteraceae;Geobacter;Geobacter sp. DSM 2909;Geobacter sp. DSM 2909 |
| Ga0586324 0745820 1 624 | 0.00 | 775200.00 | 0.00 | 0.00 | 0.00 | 0.00 | 0.00 | 0.00 | 0.00 | 0.00 | 0.00 | 0.00 | KO:K00134 | glyceraldehyde 3-phosphate dehydrogenase | Bacteria;Acidobacteria;Acidobacteriia;Bryobacterales;Solibacteraceae;Candidatus Solibacter;Candidatus Solibacter usitatus;Candidatus Solibacter usitatus Ellin6076 |
| Ga0586324 0787902 839 1345 | 887590.00 | 2917200.00 | 0.00 | 0.00 | 0.00 | 0.00 | 0.00 | 0.00 | 1271700.00 | 0.00 | 2622400.00 | 0.00 | KO:K00134 | glyceraldehyde 3-phosphate dehydrogenase | Bacteria;Proteobacteria;Betaproteobacteria;Burkholderiales;Oxalobacteraceae;Janthinobacterium;Janthinobacterium sp. B9-8;Janthinobacterium sp. B9-8 |
| Ga0586324 0823836 1 987 | 16700000.00 | 0.00 | 7206400.00 | 19190000.00 | 0.00 | 4962500.00 | 6817200.00 | 10370000.00 | 10854000.00 | 0.00 | 10912000.00 | 8400000.00 | KO:K00134 | glyceraldehyde 3-phosphate dehydrogenase | Bacteria;Firmicutes;unclassified Firmicutes;unclassified Firmicutes;unclassified Firmicutes;Hydrogenispora;Hydrogenispora ethanolica;Hydrogenispora ethanolica DSM 25471 |
| Ga0586324 1161930 1 507 | 0.00 | 0.00 | 0.00 | 0.00 | 0.00 | 0.00 | 0.00 | 0.00 | 0.00 | 0.00 | 0.00 | 1597500.00 | KO:K00134 | glyceraldehyde 3-phosphate dehydrogenase | Bacteria;Firmicutes;unclassified Firmicutes;unclassified Firmicutes;unclassified Firmicutes;Hydrogenispora;Hydrogenispora ethanolica;Hydrogenispora ethanolica DSM 25471 |
| Ga0586324 1213490 117 1118 | 0.00 | 0.00 | 0.00 | 0.00 | 0.00 | 0.00 | 0.00 | 370562.19 | 186295.14 | 95803.40 | 0.00 | 0.00 | KO:K00134 | glyceraldehyde 3-phosphate dehydrogenase | Bacteria;Firmicutes;Clostridia;Thermoanaerobacterales;Thermoanaerobacteraceae;Carboxydothermus;Carboxydothermus pertinax;Carboxydothermus pertinax Ug1 |
| Ga0586324 1570723 1 393 | 0.00 | 0.00 | 0.00 | 0.00 | 0.00 | 0.00 | 0.00 | 346032.87 | 0.00 | 0.00 | 0.00 | 314784.00 | KO:K00134 | glyceraldehyde 3-phosphate dehydrogenase | Bacteria;Proteobacteria;Betaproteobacteria;Nitrosomonadales;Gallionellaceae;Ferriphaselus;Ferriphaselus amnicola;Ferriphaselus amnicola OYT1 |
| Ga0586324 1805037 471 944 | 1630000.00 | 25024000.00 | 3070686.45 | 2289500.00 | 2873700.00 | 3101772.91 | 2325600.00 | 2415600.00 | 3231900.00 | 45507000.00 | 1786400.00 | 2107500.00 | KO:K00134 | glyceraldehyde 3-phosphate dehydrogenase | Bacteria;Proteobacteria;Gammaproteobacteria;Xanthomonadales;Xanthomonadaceae;Pseudoxanthomonas;Pseudoxanthomonas sp. OG2;Pseudoxanthomonas sp. OG2 |
| Ga0586324 2084087 296 889 | 0.00 | 2074000.00 | 0.00 | 0.00 | 0.00 | 0.00 | 0.00 | 0.00 | 0.00 | 0.00 | 0.00 | 0.00 | KO:K00134 | glyceraldehyde 3-phosphate dehydrogenase | Bacteria;Verrucomicrobia;Verrucomicrobiae;Verrucomicrobiales;Verrucomicrobia subdivision 3;Pedosphaera;Pedosphaera parvula;Pedosphaera parvula Ellin514 |
| Ga0586324 2556392 7 813 | 0.00 | 0.00 | 0.00 | 0.00 | 0.00 | 0.00 | 0.00 | 0.00 | 0.00 | 0.00 | 0.00 | 1927500.00 | KO:K00134 | glyceraldehyde 3-phosphate dehydrogenase | Bacteria;Proteobacteria;Deltaproteobacteria;Syntrophobacterales;Syntrophobacteraceae;Syntrophobacter;Syntrophobacter fumaroxidans;Syntrophobacter fumaroxidans MPOB |
| Ga0586324 2725378 1 645 | 0.00 | 2992000.00 | 0.00 | 2403500.00 | 0.00 | 2014485.19 | 0.00 | 0.00 | 0.00 | 1978900.00 | 0.00 | 0.00 | KO:K00134 | glyceraldehyde 3-phosphate dehydrogenase | Bacteria;Proteobacteria;Deltaproteobacteria;Desulfovibrionales;Desulfovibrionaceae;Desulfovibrio;Desulfovibrio alcoholivorans;Desulfovibrio alcoholivorans DSM 5433 |
| Ga0586324 2992414 271 762 | 0.00 | 0.00 | 0.00 | 0.00 | 0.00 | 0.00 | 0.00 | 0.00 | 0.00 | 6968500.00 | 0.00 | 0.00 | KO:K00134 | glyceraldehyde 3-phosphate dehydrogenase | Bacteria;Actinobacteria;Thermoleophilia;Solirubrobacterales;Solirubrobacteraceae;Solirubrobacter;Solirubrobacter soli;Solirubrobacter soli DSM 22325 |
| Ga0586324 3128612 1 666 | 4140000.00 | 4256800.00 | 0.00 | 0.00 | 0.00 | 4565500.00 | 509096.64 | 3050000.00 | 0.00 | 0.00 | 0.00 | 4042500.00 | KO:K00134 | glyceraldehyde 3-phosphate dehydrogenase | Bacteria;Firmicutes;unclassified Firmicutes;unclassified Firmicutes;unclassified Firmicutes;Hydrogenispora;Hydrogenispora ethanolica;Hydrogenispora ethanolica DSM 25471 |
| Ga0586324 3205221 316 741 | 0.00 | 0.00 | 0.00 | 0.00 | 0.00 | 6113800.00 | 0.00 | 0.00 | 0.00 | 0.00 | 0.00 | 0.00 | KO:K00134 | glyceraldehyde 3-phosphate dehydrogenase | Bacteria;Bacteroidetes;Bacteroidia;Bacteroidales;Odoribacteraceae;Odoribacter;Odoribacter sp. AF15-53;Odoribacter sp. AF15-53 |
| Ga0586324 3239904 3 566 | 0.00 | 3046400.00 | 0.00 | 2118500.00 | 0.00 | 0.00 | 0.00 | 2830400.00 | 1725300.00 | 3249400.00 | 4039200.00 | 1387500.00 | KO:K00134 | glyceraldehyde 3-phosphate dehydrogenase | Bacteria;Proteobacteria;Gammaproteobacteria;unclassified Gammaproteobacteria;Candidatus Competibacteraceae;Candidatus Competibacter;Candidatus Competibacter denitrificans;Candidatus Competibacter denitrificans Run A D11 |
| Ga0586324 3372229 5 724 | 0.00 | 0.00 | 0.00 | 0.00 | 0.00 | 8178200.00 | 0.00 | 0.00 | 0.00 | 0.00 | 0.00 | 0.00 | KO:K00134 | glyceraldehyde 3-phosphate dehydrogenase | Bacteria;Chloroflexi;Caldilineae;Caldilineales;Caldilineaceae;Caldilinea;Caldilinea aerophila;Caldilinea aerophila STL-6-O1, DSM 14535 |
| Ga0586324 3889702 3 683 | 0.00 | 0.00 | 0.00 | 0.00 | 0.00 | 0.00 | 0.00 | 0.00 | 0.00 | 0.00 | 1152800.00 | 0.00 | KO:K00134 | glyceraldehyde 3-phosphate dehydrogenase | Bacteria;Chloroflexi;Anaerolineae;Anaerolineales;Anaerolineaceae;Levilinea;Levilinea saccharolytica;Levilinea saccharolytica DSM 16555 |
| Ga0586324 4041407 138 674 | 0.00 | 0.00 | 0.00 | 0.00 | 6406600.00 | 0.00 | 0.00 | 0.00 | 6277500.00 | 0.00 | 0.00 | 0.00 | KO:K00134 | glyceraldehyde 3-phosphate dehydrogenase | Bacteria;Firmicutes;Clostridia;Eubacteriales;Oscillospiraceae;Acetivibrio;Acetivibrio thermocellus;Acetivibrio thermocellus ATCC 27405 |
| Ga0586324 4234642 1 465 | 0.00 | 0.00 | 0.00 | 15580000.00 | 17201000.00 | 11473300.00 | 2781600.00 | 1903200.00 | 0.00 | 0.00 | 0.00 | 0.00 | KO:K00134 | glyceraldehyde 3-phosphate dehydrogenase | Bacteria;Proteobacteria;Deltaproteobacteria;Syntrophobacterales;Syntrophobacteraceae;Syntrophobacter;Syntrophobacter fumaroxidans;Syntrophobacter fumaroxidans MPOB |
| Ga0586324 4758797 3 629 | 0.00 | 0.00 | 0.00 | 0.00 | 0.00 | 0.00 | 0.00 | 0.00 | 0.00 | 0.00 | 0.00 | 2947500.00 | KO:K00134 | glyceraldehyde 3-phosphate dehydrogenase | Bacteria;Nitrospirae;Nitrospira;Nitrospirales;Nitrospiraceae;Candidatus Magnetobacterium;Candidatus Magnetobacterium casensis;Candidatus Magnetobacterium casensis MYR-1 |
| Ga0586324 4856886 2 532 | 934007.00 | 5331200.00 | 0.00 | 1558000.00 | 1483200.00 | 0.00 | 1328674.56 | 1177300.00 | 1539000.00 | 0.00 | 0.00 | 5100000.00 | KO:K00134 | glyceraldehyde 3-phosphate dehydrogenase | Bacteria;Proteobacteria;Gammaproteobacteria;Chromatiales;Chromatiaceae;Thiocystis;Thiocystis violascens;Thiocystis violascens 611, DSM 198 |
| Ga0586324 5818880 3 578 | 0.00 | 0.00 | 0.00 | 0.00 | 0.00 | 0.00 | 0.00 | 0.00 | 0.00 | 0.00 | 4910400.00 | 0.00 | KO:K00134 | glyceraldehyde 3-phosphate dehydrogenase | Bacteria;Proteobacteria;Betaproteobacteria;Nitrosomonadales;Methylophilaceae;Methylobacillus;Methylobacillus rhizosphaerae;Methylobacillus rhizosphaerae Ca-68 |
| Ga0586324 6081475 2 568 | 0.00 | 0.00 | 1417110.41 | 0.00 | 0.00 | 1741488.14 | 0.00 | 3348900.00 | 5427000.00 | 0.00 | 2736800.00 | 0.00 | KO:K00134 | glyceraldehyde 3-phosphate dehydrogenase | Bacteria;Proteobacteria;Alphaproteobacteria;Sphingomonadales;Sphingomonadaceae;Novosphingobium;Novosphingobium lentum;Novosphingobium lentum NBRC 107847 |
| Ga0586324 6090820 3 383 | 0.00 | 0.00 | 0.00 | 12730000.00 | 0.00 | 0.00 | 0.00 | 0.00 | 0.00 | 0.00 | 0.00 | 0.00 | KO:K00134 | glyceraldehyde 3-phosphate dehydrogenase | Bacteria;Proteobacteria;Deltaproteobacteria;Syntrophobacterales;Syntrophobacteraceae;Syntrophobacter;Syntrophobacter fumaroxidans;Syntrophobacter fumaroxidans MPOB |
| Ga0586324 6151579 3 566 | 0.00 | 0.00 | 0.00 | 0.00 | 9012500.00 | 0.00 | 0.00 | 0.00 | 0.00 | 0.00 | 0.00 | 0.00 | KO:K00134 | glyceraldehyde 3-phosphate dehydrogenase | Bacteria;Proteobacteria;Deltaproteobacteria;Desulfuromonadales;Desulfuromonadaceae;Desulfuromonas;Desulfuromonas sp. TF;Desulfuromonas sp. TF |
| Ga0586324 6309032 116 559 | 0.00 | 10608000.00 | 0.00 | 0.00 | 0.00 | 0.00 | 0.00 | 0.00 | 0.00 | 0.00 | 0.00 | 0.00 | KO:K00134 | glyceraldehyde 3-phosphate dehydrogenase | Bacteria;Proteobacteria;Gammaproteobacteria;Methylococcales;Methylococcaceae;Methylomonas;Methylomonas sp. LW13;Methylomonas sp. LW13 |
| Ga0586324 6704607 2 547 | 0.00 | 0.00 | 0.00 | 0.00 | 0.00 | 0.00 | 0.00 | 0.00 | 0.00 | 0.00 | 1082400.00 | 0.00 | KO:K00134 | glyceraldehyde 3-phosphate dehydrogenase | Bacteria;Proteobacteria;Deltaproteobacteria;Desulfuromonadales;Desulfuromonadaceae;Desulfuromonas;Desulfuromonas sp. TF;Desulfuromonas sp. TF |
| Ga0586324 6820463 2 541 | 0.00 | 0.00 | 0.00 | 0.00 | 0.00 | 0.00 | 3306000.00 | 0.00 | 4714200.00 | 0.00 | 0.00 | 0.00 | KO:K00134 | glyceraldehyde 3-phosphate dehydrogenase | Bacteria;Deinococcus-Thermus;Deinococci;Thermales;Thermaceae;Calidithermus;Calidithermus timidus;Meiothermus timidus DSM 17022 |
| Ga0586324 6882224 23 541 | 0.00 | 0.00 | 1516097.07 | 7714000.00 | 0.00 | 0.00 | 0.00 | 3635600.00 | 3912300.00 | 0.00 | 0.00 | 0.00 | KO:K00134 | glyceraldehyde 3-phosphate dehydrogenase | Bacteria;Chloroflexi;Caldilineae;Caldilineales;Caldilineaceae;Caldilinea;Caldilinea aerophila;Caldilinea aerophila STL-6-O1, DSM 14535 |
| Ga0586324 7216091 3 530 | 0.00 | 0.00 | 0.00 | 0.00 | 0.00 | 0.00 | 0.00 | 0.00 | 2502900.00 | 0.00 | 0.00 | 0.00 | KO:K00134 | glyceraldehyde 3-phosphate dehydrogenase | Bacteria;Proteobacteria;Alphaproteobacteria;Rhodospirillales;Acetobacteraceae;Rhodopila;Rhodopila globiformis;Rhodopila globiformis DSM 161 |
| Ga0586324 7414915 2 523 | 0.00 | 0.00 | 0.00 | 0.00 | 4120000.00 | 0.00 | 0.00 | 0.00 | 0.00 | 0.00 | 0.00 | 0.00 | KO:K00134 | glyceraldehyde 3-phosphate dehydrogenase | Bacteria;Proteobacteria;Alphaproteobacteria;Hyphomicrobiales;unclassified Hyphomicrobiales;Pseudorhodoplanes;Pseudorhodoplanes sinuspersici;Pseudorhodoplanes sinuspersici RIPI110 |
| Ga0586324 7572884 29 520 | 0.00 | 0.00 | 0.00 | 13775000.00 | 0.00 | 17190100.00 | 0.00 | 0.00 | 7508700.00 | 0.00 | 0.00 | 0.00 | KO:K00134 | glyceraldehyde 3-phosphate dehydrogenase | Bacteria;Firmicutes;unclassified Firmicutes;unclassified Firmicutes;unclassified Firmicutes;Hydrogenispora;Hydrogenispora ethanolica;Hydrogenispora ethanolica DSM 25471 |
| Ga0586324 0000437 4149 5702 | 0.00 | 0.00 | 3683540.10 | 0.00 | 0.00 | 4922800.00 | 3237600.00 | 2397300.00 | 0.00 | 0.00 | 0.00 | 0.00 | KO:K00138 | aldehyde dehydrogenase | Bacteria;Gemmatimonadetes;Gemmatimonadetes;Gemmatimonadales;Gemmatimonadaceae;Gemmatimonas;Gemmatimonas aurantiaca;Gemmatimonas aurantiaca T-27T |
| Ga0586324 0945277 167 1243 | 0.00 | 0.00 | 0.00 | 0.00 | 0.00 | 0.00 | 4058400.00 | 0.00 | 0.00 | 0.00 | 0.00 | 0.00 | KO:K00140 | malonate-semialdehyde dehydrogenase (acetylating)/methylmalonate-semialdehyde dehydroger | Bacteria;Proteobacteria;Deltaproteobacteria;Desulfobacterales;Desulfobacteraceae;Desulfobacterium;Desulfobacterium vacuolatum;Desulfobacterium vacuolatum DSM 3385 |
| Ga0586324 0000423 30607 31635 | 0.00 | 0.00 | 0.00 | 0.00 | 0.00 | 0.00 | 0.00 | 0.00 | 6836400.00 | 0.00 | 0.00 | 0.00 | KO:K00145 | N-acetyl-gamma-glutamyl-phosphate reductase | Bacteria;Proteobacteria;Betaproteobacteria;Nitrosomonadales;Thiobacillaceae;Annwoodia;Annwoodia aquaesulis;Annwoodia aquaesulis DSM 4255 |
| Ga0586324 0020730 1674 2732 | 0.00 | 0.00 | 0.00 | 0.00 | 0.00 | 0.00 | 0.00 | 1262700.00 | 0.00 | 0.00 | 0.00 | 0.00 | KO:K00145 | N-acetyl-gamma-glutamyl-phosphate reductase | Bacteria;Proteobacteria;Betaproteobacteria;Nitrosomonadales;Thiobacillaceae;Thiobacillus;Thiobacillus thioparus;Thiobacillus thioparus DSM 505 |
| Ga0586324 0000011 100569 101825 | 0.00 | 0.00 | 0.00 | 0.00 | 0.00 | 0.00 | 1576802.40 | 0.00 | 1603800.00 | 5382300.00 | 5324000.00 | 5347500.00 | KO:K00147 | glutamate-5-semialdehyde dehydrogenase | Bacteria;Proteobacteria;Betaproteobacteria;Nitrosomonadales;Thiobacillaceae;Annwoodia;Annwoodia aquaesulis;Annwoodia aquaesulis DSM 4255 |
| Ga0586324 0012925 8015 9088 | 0.00 | 0.00 | 0.00 | 0.00 | 0.00 | 0.00 | 0.00 | 111630000.00 | 0.00 | 1821877.75 | 0.00 | 0.00 | KO:K00147 | glutamate-5-semialdehyde dehydrogenase | Bacteria;Proteobacteria;Betaproteobacteria;Nitrosomonadales;Sulfuricellaceae;Sulfuricella;Sulfuricella denitrificans;Sulfuricella denitrificans skB26 |
| Ga0586324 3253916 1 735 | 0.00 | 0.00 | 0.00 | 0.00 | 0.00 | 0.00 | 0.00 | 0.00 | 10530000.00 | 0.00 | 0.00 | 0.00 | KO:K00147 | glutamate-5-semialdehyde dehydrogenase | Bacteria;Proteobacteria;Betaproteobacteria;Nitrosomonadales;Nitrosomonadaceae;Nitrosomonas;Nitrosomonas aestuarii;Nitrosomonas aestuarii Nm69 |
| Ga0586324 3565397 3 671 | 0.00 | 0.00 | 0.00 | 0.00 | 0.00 | 0.00 | 0.00 | 0.00 | 0.00 | 0.00 | 0.00 | 1957500.00 | KO:K00147 | glutamate-5-semialdehyde dehydrogenase | Bacteria;Proteobacteria;Deltaproteobacteria;Syntrophobacterales;Syntrophaceae;Desulfobacca;Desulfobacca acetoxidans;Desulfobacca acetoxidans ASRB2, DSM 11109 |
| Ga0586324 0716937 2 1030 | 0.00 | 0.00 | 1477002.35 | 0.00 | 0.00 | 3283200.00 | 0.00 | 0.00 | 0.00 | 0.00 | 0.00 | 0.00 | KO:K00150 | glyceraldehyde-3-phosphate dehydrogenase (NAD(P)) | Archaea;Euryarchaeota;Methanomicrobia;Methanosarcinales;Methanotrichaceae;Methanothrix;Methanothrix soehngenii;Methanothrix soehngenii GP-6 |
| Ga0586324 0758393 858 1367 | 0.00 | 0.00 | 4936186.95 | 0.00 | 6035800.00 | 0.00 | 0.00 | 0.00 | 0.00 | 0.00 | 0.00 | 0.00 | KO:K00150 | glyceraldehyde-3-phosphate dehydrogenase (NAD(P)) | Archaea;Euryarchaeota;Methanomicrobia;Methanosarcinales;Methanotrichaceae;Methanothrix;Methanothrix soehngenii;Methanothrix soehngenii GP-6 |
| Ga0586324 7157457 3 533 | 0.00 | 0.00 | 0.00 | 0.00 | 0.00 | 2532748.84 | 0.00 | 0.00 | 0.00 | 0.00 | 0.00 | 0.00 | KO:K00150 | glyceraldehyde-3-phosphate dehydrogenase (NAD(P)) | Archaea;Euryarchaeota;Methanomicrobia;Methanosarcinales;Methanotrichaceae;Methanothrix;Methanothrix soehngenii;Methanothrix soehngenii GP-6 |
| Ga0586324 0000003 97280 98281 | 0.00 | 0.00 | 0.00 | 0.00 | 0.00 | 0.00 | 0.00 | 0.00 | 0.00 | 6244700.00 | 2824800.00 | 6502500.00 | KO:K00161 | pyruvate dehydrogenase E1 component alpha subunit | Bacteria;Proteobacteria;Betaproteobacteria;Nitrosomonadales;Thiobacillaceae;Annwoodia;Annwoodia aquaesulis;Annwoodia aquaesulis DSM 4255 |
| Ga0586324 0067507 2687 4087 | 0.00 | 0.00 | 0.00 | 0.00 | 0.00 | 0.00 | 0.00 | 0.00 | 0.00 | 0.00 | 0.00 | 1357500.00 | KO:K00162 | pyruvate dehydrogenase E1 component beta subunit | Bacteria;Proteobacteria;Alphaproteobacteria;Hyphomicrobiales;Bradyrhizobiaceae;Bradyrhizobium;Bradyrhizobium sp. URHD0069;Bradyrhizobium sp. URHD0069 |
| Ga0586324 0010511 1327 4011 | 0.00 | 0.00 | 0.00 | 0.00 | 0.00 | 0.00 | 0.00 | 1640900.00 | 0.00 | 0.00 | 0.00 | 0.00 | KO:K00163 | pyruvate dehydrogenase E1 component | Bacteria;Verrucomicrobia;Verrucomicrobiae;Verrucomicrobiales;Verrucomicrobia subdivision 3;Pedosphaera;Pedosphaera parvula;Pedosphaera parvula Ellin514 |
| Ga0586324 0299652 1 2058 | 0.00 | 0.00 | 0.00 | 0.00 | 0.00 | 0.00 | 0.00 | 0.00 | 160975.35 | 0.00 | 0.00 | 0.00 | KO:K00163 | pyruvate dehydrogenase E1 component | Bacteria;Proteobacteria;Betaproteobacteria;Burkholderiales;Burkholderiaceae;Cupriavidus;Cupriavidus gilardii;Cupriavidus gilardii CR3 |
| Ga0586324 1423867 2 1042 | 0.00 | 0.00 | 0.00 | 0.00 | 0.00 | 0.00 | 0.00 | 5264300.00 | 0.00 | 0.00 | 0.00 | 0.00 | KO:K00163 | pyruvate dehydrogenase E1 component | Bacteria;Planctomycetes;Planctomycetia;Pirellulales;Pirellulaceae;Pirellula;Pirellula staleyi;Pirellula staleyi DSM 6068 |
| Ga0586324 2238652 3 860 | 0.00 | 0.00 | 0.00 | 0.00 | 0.00 | 0.00 | 0.00 | 1512800.00 | 0.00 | 0.00 | 0.00 | 0.00 | KO:K00163 | pyruvate dehydrogenase E1 component | Bacteria;Proteobacteria;Betaproteobacteria;Nitrosomonadales;Nitrosomonadaceae;Nitrosomonas;Nitrosomonas eutropha;Nitrosomonas eutropha Nm24 |
| Ga0586324 0551009 546 1571 | 0.00 | 0.00 | 0.00 | 0.00 | 0.00 | 7423900.00 | 0.00 | 3190300.00 | 3831300.00 | 0.00 | 0.00 | 0.00 | KO:K00169 | pyruvate ferredoxin oxidoreductase alpha subunit | Bacteria;Proteobacteria;Deltaproteobacteria;Syntrophobacterales;Syntrophobacteraceae;Syntrophobacter;Syntrophobacter fumaroxidans;Syntrophobacter fumaroxidans MPOB |
| Ga0586324 3467679 1 405 | 0.00 | 1441600.00 | 0.00 | 1320500.00 | 1720100.00 | 422936.01 | 0.00 | 1354200.00 | 939600.00 | 0.00 | 0.00 | 0.00 | KO:K00169 | pyruvate ferredoxin oxidoreductase alpha subunit | Archaea;Euryarchaeota;Methanomicrobia;Methanosarcinales;Methanotrichaceae;Methanothrix;Methanothrix soehngenii;Methanothrix soehngenii GP-6 |
| Ga0586324 0551009 3 545 | 0.00 | 4168400.00 | 0.00 | 0.00 | 0.00 | 0.00 | 0.00 | 0.00 | 2454300.00 | 0.00 | 0.00 | 0.00 | KO:K00170 | pyruvate ferredoxin oxidoreductase beta subunit | Bacteria;Proteobacteria;Deltaproteobacteria;Syntrophobacterales;Syntrophobacteraceae;Syntrophobacter;Syntrophobacter fumaroxidans;Syntrophobacter fumaroxidans MPOB |
| Ga0586324 8095922 3 506 | 0.00 | 0.00 | 0.00 | 0.00 | 0.00 | 0.00 | 0.00 | 0.00 | 0.00 | 0.00 | 0.00 | 1140000.00 | KO:K00170 | pyruvate ferredoxin oxidoreductase beta subunit | Archaea;Euryarchaeota;Methanomicrobia;Methanosarcinales;Methanotrichaceae;Methanothrix;Methanothrix soehngenii;Methanothrix soehngenii GP-6 |
| Ga0586324 0928363 153 524 | 0.00 | 0.00 | 0.00 | 0.00 | 0.00 | 0.00 | 2690400.00 | 0.00 | 0.00 | 0.00 | 0.00 | 0.00 | KO:K00171 | pyruvate ferredoxin oxidoreductase delta subunit | Archaea;Crenarchaeota;Thermoprotei;Thermoproteales;Thermoproteaceae;Pyrobaculum;Pyrobaculum sp. WP30;Pyrobaculum sp. WP30 |
| Ga0586324 0432004 115 651 | 0.00 | 0.00 | 0.00 | 0.00 | 0.00 | 0.00 | 0.00 | 3562400.00 | 0.00 | 0.00 | 0.00 | 0.00 | KO:K00172 | pyruvate ferredoxin oxidoreductase gamma subunit | Archaea;Euryarchaeota;Methanomicrobia;Methanosarcinales;Methanotrichaceae;Methanothrix;Methanothrix soehngenii;Methanothrix soehngenii GP-6 |
| Ga0586324 0836466 862 1311 | 0.00 | 5399200.00 | 0.00 | 0.00 | 0.00 | 0.00 | 0.00 | 0.00 | 0.00 | 0.00 | 0.00 | 0.00 | KO:K00172 | pyruvate ferredoxin oxidoreductase gamma subunit | Bacteria;Proteobacteria;Deltaproteobacteria;Syntrophobacterales;Syntrophobacteraceae;Syntrophobacter;Syntrophobacter fumaroxidans;Syntrophobacter fumaroxidans MPOB |
| Ga0586324 1653619 117 752 | 0.00 | 0.00 | 0.00 | 10450000.00 | 0.00 | 2848482.94 | 0.00 | 0.00 | 33615000.00 | 0.00 | 0.00 | 0.00 | KO:K00172 | pyruvate ferredoxin oxidoreductase gamma subunit | Bacteria;Proteobacteria;Deltaproteobacteria;Syntrophobacterales;Syntrophaceae;Desulfomonile;Desulfomonile tiedjei;Desulfomonile tiedjei DCB-1, DSM 6799 |
| Ga0586324 3749835 293 694 | 0.00 | 0.00 | 0.00 | 0.00 | 0.00 | 0.00 | 0.00 | 0.00 | 0.00 | 0.00 | 7867200.00 | 0.00 | KO:K00172 | pyruvate ferredoxin oxidoreductase gamma subunit | Bacteria;Proteobacteria;Deltaproteobacteria;unclassified Deltaproteobacteria;unclassified Deltaproteobacteria;Dissulfuribacter;Dissulfuribacter thermophilus;Dissulfuribacter thermophilus S69 |
| Ga0586324 5389599 13 564 | 0.00 | 0.00 | 0.00 | 0.00 | 0.00 | 15244800.00 | 0.00 | 0.00 | 0.00 | 0.00 | 0.00 | 0.00 | KO:K00172 | pyruvate ferredoxin oxidoreductase gamma subunit | Bacteria;Proteobacteria;Deltaproteobacteria;Syntrophobacterales;Syntrophobacteraceae;Syntrophobacter;Syntrophobacter fumaroxidans;Syntrophobacter fumaroxidans MPOB |
| Ga0586324 8007698 194 508 | 0.00 | 0.00 | 0.00 | 0.00 | 0.00 | 0.00 | 0.00 | 23485000.00 | 0.00 | 0.00 | 0.00 | 0.00 | KO:K00172 | pyruvate ferredoxin oxidoreductase gamma subunit | Bacteria;Proteobacteria;Deltaproteobacteria;Desulfarculales;Desulfarculaceae;Desulfarculus;Desulfarculus baarsii;Desulfarculus baarsii 2st14, DSM 2075 |
| Ga0586324 1042922 151 1191 | 13900000.00 | 0.00 | 0.00 | 8607000.00 | 13287000.00 | 0.00 | 0.00 | 0.00 | 0.00 | 0.00 | 0.00 | 1417500.00 | KO:K00174 | 2-oxoglutarate ferredoxin oxidoreductase subunit alpha | Bacteria;Proteobacteria;Deltaproteobacteria;Desulfuromonadales;Geobacteraceae;Geobacter;Geobacter sp. OR-1;Geobacter sp. OR-1 |
| Ga0586324 1102614 3 905 | 0.00 | 240985.20 | 0.00 | 0.00 | 0.00 | 0.00 | 0.00 | 0.00 | 0.00 | 0.00 | 0.00 | 0.00 | KO:K00174 | 2-oxoglutarate ferredoxin oxidoreductase subunit alpha | Bacteria;Proteobacteria;Deltaproteobacteria;Desulfobacterales;Desulfobacteraceae;Desulfofaba;Desulfofaba hansenii;Desulfofaba hansenii DSM 12642 |
| Ga0586324 0020602 131 2548 | 0.00 | 0.00 | 0.00 | 0.00 | 0.00 | 0.00 | 0.00 | 4215100.00 | 0.00 | 0.00 | 0.00 | 0.00 | KO:K00192 | acetyl-CoA decarbonylase/synthase complex subunit alpha | Archaea;Euryarchaeota;Methanomicrobia;Methanosarcinales;Methanotrichaceae;Methanothrix;Methanothrix thermoacetophila;Methanothrix thermoacetophila PT |
| Ga0586324 0092445 170 2680 | 13900000.00 | 20168800.00 | 0.00 | 22420000.00 | 0.00 | 25606500.00 | 11103600.00 | 0.00 | 24786000.00 | 1262800.00 | 0.00 | 2257500.00 | KO:K00192 | acetyl-CoA decarbonylase/synthase complex subunit alpha | Archaea;Euryarchaeota;Methanomicrobia;Methanosarcinales;Methanotrichaceae;Methanothrix;Methanothrix thermoacetophila;Methanothrix thermoacetophila PT |
| Ga0586324 0096290 231 2696 | 3349790.00 | 18387200.00 | 8951700.00 | 5092000.00 | 71337800.00 | 9051600.00 | 32170800.00 | 18727000.00 | 5572800.00 | 16100700.00 | 0.00 | 10950000.00 | KO:K00192 | acetyl-CoA decarbonylase/synthase complex subunit alpha | Archaea;Euryarchaeota;Methanomicrobia;Methanosarcinales;Methanotrichaceae;Methanothrix;Methanothrix thermoacetophila;Methanothrix thermoacetophila PT |
| Ga0586324 0108059 610 3075 | 240470.00 | 18033600.00 | 7882000.00 | 15485000.00 | 0.00 | 0.00 | 8344800.00 | 8784000.00 | 0.00 | 1610379.54 | 0.00 | 1471332.00 | KO:K00192 | acetyl-CoA decarbonylase/synthase complex subunit alpha | Archaea;Euryarchaeota;Methanomicrobia;Methanosarcinales;Methanotrichaceae;Methanothrix;Methanothrix soehngenii;Methanothrix soehngenii GP-6 |
| Ga0586324 0148932 1 2172 | 0.00 | 0.00 | 0.00 | 6184500.00 | 4816359.31 | 6592518.48 | 0.00 | 0.00 | 0.00 | 0.00 | 0.00 | 0.00 | KO:K00192 | acetyl-CoA decarbonylase/synthase complex subunit alpha | Archaea;Euryarchaeota;Methanomicrobia;Methanosarcinales;Methanosarcinaceae;Methanosarcina;Methanosarcina barkeri;Methanosarcina barkeri 3 |
| Ga0586324 0698650 1 1416 | 6280000.00 | 12988000.00 | 0.00 | 4883000.00 | 4037600.00 | 0.00 | 1635959.28 | 10461500.00 | 5985900.00 | 5120500.00 | 9653600.00 | 6045000.00 | KO:K00192 | acetyl-CoA decarbonylase/synthase complex subunit alpha | Archaea;Euryarchaeota;Methanomicrobia;Methanosarcinales;Methanotrichaceae;Methanothrix;Methanothrix thermoacetophila;Methanothrix thermoacetophila PT |
| Ga0586324 1141555 1 1146 | 0.00 | 0.00 | 0.00 | 0.00 | 414144.46 | 0.00 | 0.00 | 519113.66 | 173831.67 | 0.00 | 0.00 | 0.00 | KO:K00192 | acetyl-CoA decarbonylase/synthase complex subunit alpha | Archaea;Euryarchaeota;Methanomicrobia;Methanosarcinales;Methanotrichaceae;Methanothrix;Methanothrix thermoacetophila;Methanothrix thermoacetophila PT |
| Ga0586324 2484576 2 823 | 0.00 | 19176000.00 | 0.00 | 0.00 | 0.00 | 0.00 | 0.00 | 0.00 | 0.00 | 0.00 | 0.00 | 0.00 | KO:K00192 | acetyl-CoA decarbonylase/synthase complex subunit alpha | Archaea;Euryarchaeota;Methanomicrobia;Methanosarcinales;Methanotrichaceae;Methanothrix;Methanothrix thermoacetophila;Methanothrix thermoacetophila PT |
| Ga0586324 0047107 1 1116 | 3850000.00 | 150892000.00 | 19029400.00 | 10830000.00 | 13493000.00 | 21676200.00 | 216372000.00 | 19739600.00 | 114566400.00 | 11242000.00 | 77352000.00 | 122700000.00 | KO:K00193 | acetyl-CoA decarbonylase/synthase complex subunit beta | Archaea;Euryarchaeota;Methanomicrobia;Methanosarcinales;Methanotrichaceae;Methanothrix;Methanothrix soehngenii;Methanothrix soehngenii GP-6 |
| Ga0586324 0549039 273 1574 | 132110000.00 | 111044000.00 | 48793160.68 | 58140000.00 | 0.00 | 29060400.00 | 46758766.68 | 45329100.00 | 52594039.53 | 21714000.00 | 22862400.00 | 22822500.00 | KO:K00193 | acetyl-CoA decarbonylase/synthase complex subunit beta | Archaea;Euryarchaeota;Methanomicrobia;Methanosarcinales;Methanotrichaceae;Methanothrix;Methanothrix soehngenii;Methanothrix soehngenii GP-6 |
| Ga0586324 0580456 113 1519 | 4770000.00 | 34068000.00 | 4919060.49 | 12654000.00 | 28840000.00 | 8337000.00 | 1731190.32 | 5111800.00 | 11907000.00 | 5698000.00 | 6820000.00 | 960000.00 | KO:K00193 | acetyl-CoA decarbonylase/synthase complex subunit beta | Archaea;Euryarchaeota;Methanomicrobia;Methanosarcinales;Methanotrichaceae;Methanothrix;Methanothrix soehngenii;Methanothrix soehngenii GP-6 |
| Ga0586324 4003423 3 251 | 0.00 | 0.00 | 144691000.00 | 0.00 | 0.00 | 161182000.00 | 55176000.00 | 89060000.00 | 69660000.00 | 0.00 | 31416000.00 | 0.00 | KO:K00193 | acetyl-CoA decarbonylase/synthase complex subunit beta | Archaea;Euryarchaeota;Methanomicrobia;Methanosarcinales;Methanotrichaceae;Methanothrix;Methanothrix soehngenii;Methanothrix soehngenii GP-6 |
| Ga0586324 4217445 3 662 | 0.00 | 34170000.00 | 0.00 | 0.00 | 14420000.00 | 0.00 | 0.00 | 0.00 | 0.00 | 0.00 | 0.00 | 0.00 | KO:K00193 | acetyl-CoA decarbonylase/synthase complex subunit beta | Archaea;Euryarchaeota;Methanomicrobia;Methanosarcinales;Methanotrichaceae;Methanothrix;Methanothrix soehngenii;Methanothrix soehngenii GP-6 |
| Ga0586324 4288142 1 657 | 0.00 | 0.00 | 2308817.96 | 0.00 | 0.00 | 0.00 | 0.00 | 246652.89 | 0.00 | 0.00 | 0.00 | 0.00 | KO:K00193 | acetyl-CoA decarbonylase/synthase complex subunit beta | Archaea;Euryarchaeota;Methanomicrobia;Methanocellales;Methanocellaceae;Methanocella;Methanocella arvoryzae;Methanocella arvoryzae MRE50 |
| Ga0586324 5210879 3 605 | 8650000.00 | 0.00 | 0.00 | 0.00 | 9074300.00 | 0.00 | 0.00 | 0.00 | 0.00 | 0.00 | 0.00 | 0.00 | KO:K00193 | acetyl-CoA decarbonylase/synthase complex subunit beta | Archaea;Euryarchaeota;Methanomicrobia;Methanosarcinales;Methanotrichaceae;Methanothrix;Methanothrix soehngenii;Methanothrix soehngenii GP-6 |
| Ga0586324 6026791 1 570 | 0.00 | 0.00 | 0.00 | 4408000.00 | 5665000.00 | 0.00 | 0.00 | 3739300.00 | 4317300.00 | 0.00 | 0.00 | 0.00 | KO:K00193 | acetyl-CoA decarbonylase/synthase complex subunit beta | Archaea;Euryarchaeota;Methanomicrobia;Methanosarcinales;Methanosarcinaceae;Methanosarcina;Methanosarcina mazei;Methanosarcina mazei YBB.H.M.1B.1 |
| Ga0586324 7869255 1 345 | 0.00 | 9520000.00 | 0.00 | 0.00 | 0.00 | 0.00 | 0.00 | 0.00 | 5192100.00 | 0.00 | 0.00 | 0.00 | KO:K00193 | acetyl-CoA decarbonylase/synthase complex subunit beta | Archaea;Euryarchaeota;Methanomicrobia;Methanosarcinales;Methanotrichaceae;Methanothrix;Methanothrix soehngenii;Methanothrix soehngenii GP-6 |
| Ga0586324 0047107 2388 3740 | 26800000.00 | 10132000.00 | 11541500.00 | 2679000.00 | 0.00 | 6550500.00 | 0.00 | 3531900.00 | 3507300.00 | 0.00 | 0.00 | 2565000.00 | KO:K00194 | acetyl-CoA decarbonylase/synthase complex subunit delta | Archaea;Euryarchaeota;Methanomicrobia;Methanosarcinales;Methanotrichaceae;Methanothrix;Methanothrix soehngenii;Methanothrix soehngenii GP-6 |
| Ga0586324 0048671 1773 2717 | 3560000.00 | 3944000.00 | 9627300.00 | 7837500.00 | 6684700.00 | 15117823.52 | 11127277.80 | 12497375.00 | 11210400.00 | 10610600.00 | 11655337.76 | 5445000.00 | KO:K00194 | acetyl-CoA decarbonylase/synthase complex subunit delta | Bacteria;Proteobacteria;Deltaproteobacteria;Syntrophobacterales;Syntrophobacteraceae;Syntrophobacter;Syntrophobacter fumaroxidans;Syntrophobacter fumaroxidans MPOB |
| Ga0586324 0059416 3142 4086 | 2430000.00 | 0.00 | 0.00 | 0.00 | 0.00 | 4724300.00 | 0.00 | 0.00 | 0.00 | 0.00 | 0.00 | 0.00 | KO:K00194 | acetyl-CoA decarbonylase/synthase complex subunit delta | Bacteria;Proteobacteria;Deltaproteobacteria;Syntrophobacterales;Syntrophaceae;Desulfobacca;Desulfobacca acetoxidans;Desulfobacca acetoxidans ASRB2, DSM 11109 |
| Ga0586324 0125148 1885 2829 | 17580000.00 | 6242400.00 | 6814005.89 | 8474000.00 | 12988300.00 | 9968340.49 | 7546800.00 | 7027200.00 | 14855400.00 | 2470380.99 | 1660642.72 | 2853537.75 | KO:K00194 | acetyl-CoA decarbonylase/synthase complex subunit delta | Bacteria;Proteobacteria;Deltaproteobacteria;Syntrophobacterales;Syntrophobacteraceae;Syntrophobacter;Syntrophobacter fumaroxidans;Syntrophobacter fumaroxidans MPOB |
| Ga0586324 0147716 298 1245 | 23400000.00 | 0.00 | 0.00 | 0.00 | 0.00 | 0.00 | 0.00 | 0.00 | 0.00 | 0.00 | 0.00 | 0.00 | KO:K00194 | acetyl-CoA decarbonylase/synthase complex subunit delta | Bacteria;Proteobacteria;Deltaproteobacteria;Syntrophobacterales;Syntrophaceae;Desulfobacca;Desulfobacca acetoxidans;Desulfobacca acetoxidans ASRB2, DSM 11109 |
| Ga0586324 0154681 731 2269 | 2030000.00 | 1190000.00 | 1334304.37 | 1415500.00 | 2575000.00 | 0.00 | 1943346.60 | 4404200.00 | 13446000.00 | 1255100.00 | 1575200.00 | 0.00 | KO:K00194 | acetyl-CoA decarbonylase/synthase complex subunit delta | Bacteria;Spirochaetes;Spirochaetia;Spirochaetales;Spirochaetaceae;Alkalispirochaeta;Alkalispirochaeta odontotermitis;Alkalispirochaeta odontotermitis JC202 |
| Ga0586324 0190386 1512 2453 | 0.00 | 0.00 | 1896251.56 | 5928000.00 | 6097600.00 | 1499754.84 | 5084400.00 | 5959700.00 | 4212000.00 | 2171400.00 | 3018400.00 | 3180000.00 | KO:K00194 | acetyl-CoA decarbonylase/synthase complex subunit delta | Bacteria;Proteobacteria;Deltaproteobacteria;Syntrophobacterales;Syntrophaceae;Desulfobacca;Desulfobacca acetoxidans;Desulfobacca acetoxidans ASRB2, DSM 11109 |
| Ga0586324 0251909 1374 2225 | 5240000.00 | 4209200.00 | 0.00 | 13585000.00 | 17355500.00 | 0.00 | 10716000.00 | 1403000.00 | 8002800.00 | 5713400.00 | 0.00 | 6420000.00 | KO:K00194 | acetyl-CoA decarbonylase/synthase complex subunit delta | Bacteria;Proteobacteria;Deltaproteobacteria;Syntrophobacterales;Syntrophaceae;Desulfobacca;Desulfobacca acetoxidans;Desulfobacca acetoxidans ASRB2, DSM 11109 |
| Ga0586324 0277895 1208 2128 | 0.00 | 0.00 | 0.00 | 5149000.00 | 0.00 | 0.00 | 0.00 | 0.00 | 0.00 | 0.00 | 0.00 | 0.00 | KO:K00194 | acetyl-CoA decarbonylase/synthase complex subunit delta | Bacteria;Proteobacteria;Deltaproteobacteria;Syntrophobacterales;Syntrophaceae;Desulfobacca;Desulfobacca acetoxidans;Desulfobacca acetoxidans ASRB2, DSM 11109 |
| Ga0586324 0420772 259 1203 | 1110000.00 | 1285200.00 | 1149302.57 | 1900000.00 | 1596500.00 | 1329398.17 | 2000408.16 | 2098400.00 | 1417500.00 | 1116500.00 | 1478400.00 | 1140000.00 | KO:K00194 | acetyl-CoA decarbonylase/synthase complex subunit delta | Bacteria;Proteobacteria;Deltaproteobacteria;Syntrophobacterales;Syntrophobacteraceae;Syntrophobacter;Syntrophobacter fumaroxidans;Syntrophobacter fumaroxidans MPOB |
| Ga0586324 0624194 491 1489 | 0.00 | 0.00 | 0.00 | 0.00 | 0.00 | 0.00 | 0.00 | 1152900.00 | 0.00 | 0.00 | 0.00 | 0.00 | KO:K00194 | acetyl-CoA decarbonylase/synthase complex subunit delta | Archaea;Euryarchaeota;Methanomicrobia;Methanosarcinales;Methanotrichaceae;Methanothrix;Methanothrix soehngenii;Methanothrix soehngenii GP-6 |
| Ga0586324 0765685 140 1087 | 0.00 | 0.00 | 0.00 | 0.00 | 0.00 | 0.00 | 0.00 | 628300.00 | 793261.35 | 0.00 | 0.00 | 0.00 | KO:K00194 | acetyl-CoA decarbonylase/synthase complex subunit delta | Bacteria;Proteobacteria;Deltaproteobacteria;Syntrophobacterales;Syntrophaceae;Desulfobacca;Desulfobacca acetoxidans;Desulfobacca acetoxidans ASRB2, DSM 11109 |
| Ga0586324 1308214 3 593 | 0.00 | 0.00 | 0.00 | 198968.95 | 176959.15 | 0.00 | 0.00 | 0.00 | 0.00 | 0.00 | 0.00 | 0.00 | KO:K00194 | acetyl-CoA decarbonylase/synthase complex subunit delta | Bacteria;Proteobacteria;Deltaproteobacteria;Syntrophobacterales;Syntrophobacteraceae;Syntrophobacter;Syntrophobacter fumaroxidans;Syntrophobacter fumaroxidans MPOB |
| Ga0586324 2484922 1 573 | 0.00 | 0.00 | 0.00 | 0.00 | 27501000.00 | 0.00 | 0.00 | 0.00 | 0.00 | 0.00 | 0.00 | 0.00 | KO:K00194 | acetyl-CoA decarbonylase/synthase complex subunit delta | Bacteria;Proteobacteria;Deltaproteobacteria;Syntrophobacterales;Syntrophaceae;Desulfobacca;Desulfobacca acetoxidans;Desulfobacca acetoxidans ASRB2, DSM 11109 |

| | | | | | | | | | | | | | | | |
|---|---|---|---|---|---|---|---|---|---|---|---|---|---|---|---|
| Ga0586324 2668118 1 774 | 0.00 | 3712800.00 | 0.00 | 0.00 | 0.00 | 0.00 | 0.00 | 0.00 | 0.00 | 0.00 | 0.00 | 0.00 | KO:K00194 | acetyl-CoA decarbonylase/synthase complex subunit delta | Archaea;Euryarchaeota;Methanomicrobia;Methanosarcinales;Methanotrichaceae;Methanothrix;Methanothrix soehngenii;Methanothrix soehngenii GP-6 |
| Ga0586324 2916911 2 772 | 0.00 | 0.00 | 0.00 | 0.00 | 0.00 | 0.00 | 0.00 | 2379000.00 | 0.00 | 0.00 | 0.00 | 0.00 | KO:K00194 | acetyl-CoA decarbonylase/synthase complex subunit delta | Bacteria;Proteobacteria;Deltaproteobacteria;Desulfobacterales;Desulfobacteraceae;Desulfosarcina;Desulfosarcina sp. BuS5;Desulfosarcina sp. BuS5 |
| Ga0586324 3114661 123 749 | 0.00 | 0.00 | 0.00 | 15105000.00 | 0.00 | 0.00 | 0.00 | 0.00 | 0.00 | 0.00 | 0.00 | 0.00 | KO:K00194 | acetyl-CoA decarbonylase/synthase complex subunit delta | Bacteria;Proteobacteria;Deltaproteobacteria;Syntrophobacterales;Syntrophobacteraceae;Syntrophobacter;Syntrophobacter fumaroxidans;Syntrophobacter fumaroxidans MPOB |
| Ga0586324 3132122 3 749 | 0.00 | 0.00 | 0.00 | 0.00 | 0.00 | 0.00 | 0.00 | 0.00 | 0.00 | 396983.51 | 0.00 | 0.00 | KO:K00194 | acetyl-CoA decarbonylase/synthase complex subunit delta | Bacteria;Proteobacteria;Deltaproteobacteria;Syntrophobacterales;Syntrophaceae;Desulfobacca;Desulfobacca acetoxidans;Desulfobacca acetoxidans ASRB2, DSM 11109 |
| Ga0586324 3773907 29 691 | 9150000.00 | 6800000.00 | 0.00 | 0.00 | 0.00 | 0.00 | 0.00 | 0.00 | 0.00 | 0.00 | 0.00 | 0.00 | KO:K00194 | acetyl-CoA decarbonylase/synthase complex subunit delta | Archaea;Euryarchaeota;Methanomicrobia;Methanosarcinales;Methanotrichaceae;Methanothrix;Methanothrix soehngenii;Methanothrix soehngenii GP-6 |
| Ga0586324 6069467 266 568 | 0.00 | 0.00 | 0.00 | 2137500.00 | 1751000.00 | 526219.53 | 0.00 | 695400.00 | 0.00 | 0.00 | 0.00 | 0.00 | KO:K00194 | acetyl-CoA decarbonylase/synthase complex subunit delta | Bacteria;Proteobacteria;Deltaproteobacteria;Syntrophobacterales;Syntrophobacteraceae;Syntrophobacter;Syntrophobacter fumaroxidans;Syntrophobacter fumaroxidans MPOB |
| Ga0586324 6267432 3 563 | 31230000.00 | 32408800.00 | 36707600.00 | 36033500.00 | 33093900.00 | 29298600.00 | 22389600.00 | 23338600.00 | 15985392.93 | 22645700.00 | 21498400.00 | 21420000.00 | KO:K00194 | acetyl-CoA decarbonylase/synthase complex subunit delta | Archaea;Euryarchaeota;Methanomicrobia;Methanosarcinales;Methanotrichaceae;Methanothrix;Methanothrix soehngenii;Methanothrix soehngenii GP-6 |
| Ga0586324 6478852 3 554 | 0.00 | 0.00 | 0.00 | 14563500.00 | 2914900.00 | 6947500.00 | 0.00 | 3775900.00 | 2057400.00 | 0.00 | 0.00 | 0.00 | KO:K00194 | acetyl-CoA decarbonylase/synthase complex subunit delta | Bacteria;Proteobacteria;Deltaproteobacteria;Syntrophobacterales;Syntrophobacteraceae;Desulfoglaeba;Desulfoglaeba alkanexedens;Desulfoglaeba alkanexedens ALDC |
| Ga0586324 8084193 143 505 | 1680000.00 | 972400.00 | 4449248.25 | 7714000.00 | 12895600.00 | 7265100.00 | 0.00 | 0.00 | 4659414.03 | 0.00 | 0.00 | 0.00 | KO:K00194 | acetyl-CoA decarbonylase/synthase complex subunit delta | Archaea;Euryarchaeota;Methanomicrobia;Methanosarcinales;Methanotrichaceae;Methanothrix;Methanothrix soehngenii;Methanothrix soehngenii GP-6 |
| Ga0586324 0413777 442 954 | 0.00 | 0.00 | 0.00 | 6270000.00 | 6180000.00 | 5716800.00 | 0.00 | 0.00 | 0.00 | 0.00 | 0.00 | 0.00 | KO:K00195 | acetyl-CoA decarbonylase/synthase complex subunit epsilon | Archaea;Euryarchaeota;Methanomicrobia;Methanosarcinales;Methanosarcinaceae;Methanosarcina;Methanosarcina barkeri;Methanosarcina barkeri 3 |
| Ga0586324 1755254 356 868 | 0.00 | 0.00 | 0.00 | 3714500.00 | 3852200.00 | 0.00 | 0.00 | 0.00 | 2988900.00 | 0.00 | 0.00 | 0.00 | KO:K00195 | acetyl-CoA decarbonylase/synthase complex subunit epsilon | Archaea;Euryarchaeota;Methanomicrobia;Methanosarcinales;Methanosarcinaceae;Methanosarcina;Methanosarcina sp. DSM 11855;Methanosarcina sp. DSM 11855 |
| Ga0586324 2280975 180 692 | 0.00 | 0.00 | 0.00 | 0.00 | 0.00 | 7423900.00 | 0.00 | 0.00 | 0.00 | 0.00 | 0.00 | 0.00 | KO:K00195 | acetyl-CoA decarbonylase/synthase complex subunit epsilon | Archaea;Euryarchaeota;Methanomicrobia;Methanosarcinales;Methanosarcinaceae;Methanosarcina;Methanosarcina horonobensis;Methanosarcina horonobensis HB-1 |
| Ga0586324 0008881 6841 8193 | 3140000.00 | 2516000.00 | 0.00 | 5975500.00 | 1998200.00 | 0.00 | 0.00 | 0.00 | 0.00 | 1293600.00 | 3018400.00 | 1102500.00 | KO:K00197 | acetyl-CoA decarbonylase/synthase complex subunit gamma | Bacteria;Proteobacteria;Deltaproteobacteria;Syntrophobacterales;Syntrophobacteraceae;Syntrophobacter;Syntrophobacter fumaroxidans;Syntrophobacter fumaroxidans MPOB |
| Ga0586324 0013713 1337 2677 | 33500000.00 | 0.00 | 0.00 | 0.00 | 0.00 | 0.00 | 0.00 | 0.00 | 0.00 | 29799000.00 | 0.00 | 0.00 | KO:K00197 | acetyl-CoA decarbonylase/synthase complex subunit gamma | Bacteria;Proteobacteria;Deltaproteobacteria;Syntrophobacterales;Syntrophaceae;Desulfobacca;Desulfobacca acetoxidans;Desulfobacca acetoxidans ASRB2, DSM 11109 |
| Ga0586324 0016535 727 2067 | 0.00 | 0.00 | 0.00 | 0.00 | 0.00 | 0.00 | 3192000.00 | 5929200.00 | 7443900.00 | 0.00 | 0.00 | 2677500.00 | KO:K00197 | acetyl-CoA decarbonylase/synthase complex subunit gamma | Bacteria;Spirochaetes;Spirochaetia;Spirochaetales;Spirochaetaceae;Alkalispirochaeta;Alkalispirochaeta odontotermitis;Alkalispirochaeta odontotermitis JC202 |
| Ga0586324 0053197 3504 4640 | 0.00 | 0.00 | 0.00 | 5586000.00 | 5901900.00 | 2307816.58 | 0.00 | 0.00 | 0.00 | 0.00 | 0.00 | 0.00 | KO:K00197 | acetyl-CoA decarbonylase/synthase complex subunit gamma | Bacteria;Proteobacteria;Deltaproteobacteria;Syntrophobacterales;Syntrophaceae;Desulfobacca;Desulfobacca acetoxidans;Desulfobacca acetoxidans ASRB2, DSM 11109 |
| Ga0586324 0076486 2717 3895 | 0.00 | 0.00 | 0.00 | 0.00 | 0.00 | 845435.32 | 0.00 | 0.00 | 0.00 | 1901900.00 | 0.00 | 0.00 | KO:K00197 | acetyl-CoA decarbonylase/synthase complex subunit gamma | Bacteria;Spirochaetes;Spirochaetia;Spirochaetales;Spirochaetaceae;Alkalispirochaeta;Alkalispirochaeta odontotermitis;Alkalispirochaeta odontotermitis JC202 |
| Ga0586324 0091922 3 350 | 0.00 | 0.00 | 0.00 | 5994500.00 | 2163000.00 | 0.00 | 4605600.00 | 1665300.00 | 0.00 | 0.00 | 0.00 | 0.00 | KO:K00197 | acetyl-CoA decarbonylase/synthase complex subunit gamma | Bacteria;Proteobacteria;Deltaproteobacteria;Syntrophobacterales;Syntrophaceae;Desulfobacca;Desulfobacca acetoxidans;Desulfobacca acetoxidans ASRB2, DSM 11109 |
| Ga0586324 0209267 265 1608 | 0.00 | 0.00 | 0.00 | 0.00 | 0.00 | 0.00 | 0.00 | 0.00 | 0.00 | 847000.00 | 0.00 | 0.00 | KO:K00197 | acetyl-CoA decarbonylase/synthase complex subunit gamma | Bacteria;Proteobacteria;Deltaproteobacteria;Syntrophobacterales;Syntrophaceae;Desulfobacca;Desulfobacca acetoxidans;Desulfobacca acetoxidans ASRB2, DSM 11109 |
| Ga0586324 0253929 903 2216 | 0.00 | 19992000.00 | 0.00 | 0.00 | 0.00 | 0.00 | 0.00 | 0.00 | 0.00 | 0.00 | 0.00 | 0.00 | KO:K00197 | acetyl-CoA decarbonylase/synthase complex subunit gamma | Archaea;Euryarchaeota;Methanomicrobia;Methanosarcinales;Methanotrichaceae;Methanothrix;Methanothrix soehngenii;Methanothrix soehngenii GP-6 |
| Ga0586324 0412699 832 1785 | 0.00 | 0.00 | 0.00 | 0.00 | 0.00 | 0.00 | 2217774.24 | 0.00 | 0.00 | 0.00 | 0.00 | 0.00 | KO:K00197 | acetyl-CoA decarbonylase/synthase complex subunit gamma | Bacteria;Proteobacteria;Deltaproteobacteria;Desulfobacterales;Desulfobulbaceae;Desulfoprunum;Desulfoprunum benzoelyticum;Desulfoprunum benzoelyticum DSM 28570 |
| Ga0586324 0483466 2 634 | 0.00 | 0.00 | 0.00 | 0.00 | 0.00 | 0.00 | 0.00 | 6527000.00 | 0.00 | 0.00 | 0.00 | 0.00 | KO:K00197 | acetyl-CoA decarbonylase/synthase complex subunit gamma | Bacteria;Proteobacteria;Deltaproteobacteria;Syntrophobacterales;Syntrophaceae;Desulfobacca;Desulfobacca acetoxidans;Desulfobacca acetoxidans ASRB2, DSM 11109 |
| Ga0586324 1473762 2 1027 | 0.00 | 0.00 | 0.00 | 8265000.00 | 12669000.00 | 26003500.00 | 0.00 | 0.00 | 0.00 | 0.00 | 0.00 | 0.00 | KO:K00197 | acetyl-CoA decarbonylase/synthase complex subunit gamma | Bacteria;Proteobacteria;Deltaproteobacteria;Syntrophobacterales;Syntrophobacteraceae;Syntrophobacter;Syntrophobacter fumaroxidans;Syntrophobacter fumaroxidans MPOB |
| Ga0586324 1503230 551 1018 | 0.00 | 0.00 | 0.00 | 0.00 | 395456.14 | 0.00 | 0.00 | 2061800.00 | 0.00 | 0.00 | 0.00 | 0.00 | KO:K00197 | acetyl-CoA decarbonylase/synthase complex subunit gamma | Bacteria;Proteobacteria;Deltaproteobacteria;Syntrophobacterales;Syntrophobacteraceae;Desulfacinum;Desulfacinum infernum;Desulfacinum infernum DSM 9756 |
| Ga0586324 1583786 3 869 | 0.00 | 0.00 | 0.00 | 8407500.00 | 0.00 | 0.00 | 0.00 | 0.00 | 7063200.00 | 0.00 | 0.00 | 0.00 | KO:K00197 | acetyl-CoA decarbonylase/synthase complex subunit gamma | Bacteria;Proteobacteria;Deltaproteobacteria;Desulfobacterales;Desulfobacteraceae;Desulfosarcina;Desulfosarcina sp. BuS5;Desulfosarcina sp. BuS5 |
| Ga0586324 2775484 1 786 | 0.00 | 0.00 | 0.00 | 0.00 | 1000615.13 | 0.00 | 0.00 | 0.00 | 0.00 | 0.00 | 0.00 | 0.00 | KO:K00197 | acetyl-CoA decarbonylase/synthase complex subunit gamma | Bacteria;Proteobacteria;Deltaproteobacteria;Syntrophobacterales;Syntrophaceae;Desulfobacca;Desulfobacca acetoxidans;Desulfobacca acetoxidans ASRB2, DSM 11109 |
| Ga0586324 2910733 2 772 | 0.00 | 0.00 | 0.00 | 8882500.00 | 7436600.00 | 0.00 | 0.00 | 0.00 | 4592700.00 | 0.00 | 0.00 | 0.00 | KO:K00197 | acetyl-CoA decarbonylase/synthase complex subunit gamma | Bacteria;Proteobacteria;Deltaproteobacteria;Syntrophobacterales;Syntrophaceae;Desulfobacca;Desulfobacca acetoxidans;Desulfobacca acetoxidans ASRB2, DSM 11109 |
| Ga0586324 3638298 1 702 | 0.00 | 0.00 | 0.00 | 0.00 | 16274000.00 | 0.00 | 0.00 | 0.00 | 0.00 | 0.00 | 0.00 | 0.00 | KO:K00197 | acetyl-CoA decarbonylase/synthase complex subunit gamma | Bacteria;Proteobacteria;Deltaproteobacteria;Syntrophobacterales;Syntrophaceae;Desulfobacca;Desulfobacca acetoxidans;Desulfobacca acetoxidans ASRB2, DSM 11109 |
| Ga0586324 4011345 2 676 | 0.00 | 0.00 | 0.00 | 0.00 | 18746000.00 | 0.00 | 0.00 | 0.00 | 0.00 | 0.00 | 0.00 | 0.00 | KO:K00197 | acetyl-CoA decarbonylase/synthase complex subunit gamma | Bacteria;Proteobacteria;Deltaproteobacteria;Syntrophobacterales;Syntrophaceae;Desulfobacca;Desulfobacca acetoxidans;Desulfobacca acetoxidans ASRB2, DSM 11109 |
| Ga0586324 4724276 3 632 | 5330000.00 | 4508400.00 | 6643400.00 | 4227500.00 | 4305400.00 | 12068800.00 | 0.00 | 2928000.00 | 6301800.00 | 2402400.00 | 2939200.00 | 2842500.00 | KO:K00197 | acetyl-CoA decarbonylase/synthase complex subunit gamma | Bacteria;Proteobacteria;Deltaproteobacteria;Syntrophobacterales;Syntrophaceae;Desulfobacca;Desulfobacca acetoxidans;Desulfobacca acetoxidans ASRB2, DSM 11109 |
| Ga0586324 4778256 2 628 | 0.00 | 0.00 | 0.00 | 0.00 | 0.00 | 59153000.00 | 0.00 | 0.00 | 38556000.00 | 0.00 | 0.00 | 0.00 | KO:K00197 | acetyl-CoA decarbonylase/synthase complex subunit gamma | Archaea;Euryarchaeota;Methanomicrobia;Methanosarcinales;Methanosarcinaceae;Methanosarcina;Methanosarcina sp. Kolksee;Methanosarcina sp. Kolksee |
| Ga0586324 5381534 2 598 | 1920000.00 | 12240000.00 | 0.00 | 13395000.00 | 14111000.00 | 8694300.00 | 12175200.00 | 11590000.00 | 9639000.00 | 0.00 | 0.00 | 8775000.00 | KO:K00197 | acetyl-CoA decarbonylase/synthase complex subunit gamma | Bacteria;Proteobacteria;Deltaproteobacteria;Syntrophobacterales;Syntrophobacteraceae;Syntrophobacter;Syntrophobacter fumaroxidans;Syntrophobacter fumaroxidans MPOB |
| Ga0586324 5486413 3 593 | 0.00 | 0.00 | 0.00 | 0.00 | 0.00 | 0.00 | 0.00 | 0.00 | 670300.92 | 0.00 | 0.00 | 0.00 | KO:K00197 | acetyl-CoA decarbonylase/synthase complex subunit gamma | Bacteria;Proteobacteria;Deltaproteobacteria;Syntrophobacterales;Syntrophobacteraceae;Syntrophobacter;Syntrophobacter fumaroxidans;Syntrophobacter fumaroxidans MPOB |
| Ga0586324 5716143 1 585 | 5850000.00 | 0.00 | 0.00 | 0.00 | 0.00 | 0.00 | 0.00 | 0.00 | 0.00 | 0.00 | 0.00 | 0.00 | KO:K00197 | acetyl-CoA decarbonylase/synthase complex subunit gamma | Bacteria;Proteobacteria;Deltaproteobacteria;Syntrophobacterales;Syntrophaceae;Desulfobacca;Desulfobacca acetoxidans;Desulfobacca acetoxidans ASRB2, DSM 11109 |
| Ga0586324 6806504 3 542 | 393252.00 | 0.00 | 0.00 | 0.00 | 0.00 | 0.00 | 0.00 | 0.00 | 0.00 | 0.00 | 0.00 | 0.00 | KO:K00197 | acetyl-CoA decarbonylase/synthase complex subunit gamma | Bacteria;Proteobacteria;Deltaproteobacteria;Syntrophobacterales;Syntrophaceae;Desulfobacca;Desulfobacca acetoxidans;Desulfobacca acetoxidans ASRB2, DSM 11109 |
| Ga0586324 7948469 2 508 | 0.00 | 0.00 | 0.00 | 12730000.00 | 11639000.00 | 0.00 | 0.00 | 7381000.00 | 0.00 | 0.00 | 0.00 | 0.00 | KO:K00197 | acetyl-CoA decarbonylase/synthase complex subunit gamma | Bacteria;Proteobacteria;Deltaproteobacteria;Syntrophobacterales;Syntrophaceae;Desulfobacca;Desulfobacca acetoxidans;Desulfobacca acetoxidans ASRB2, DSM 11109 |
| Ga0586324 0002429 11032 12942 | 0.00 | 0.00 | 0.00 | 0.00 | 0.00 | 0.00 | 2245252.80 | 0.00 | 0.00 | 0.00 | 0.00 | 0.00 | KO:K00198 | carbon-monoxide dehydrogenase catalytic subunit | Bacteria;Proteobacteria;Deltaproteobacteria;Syntrophobacterales;Syntrophobacteraceae;Syntrophobacter;Syntrophobacter fumaroxidans;Syntrophobacter fumaroxidans MPOB |
| Ga0586324 0009689 9758 10336 | 0.00 | 346733.36 | 0.00 | 0.00 | 0.00 | 0.00 | 0.00 | 0.00 | 0.00 | 0.00 | 0.00 | 0.00 | KO:K00198 | carbon-monoxide dehydrogenase catalytic subunit | Bacteria;Proteobacteria;Deltaproteobacteria;Desulfobacterales;Desulfobacteraceae;Desulfatitalea;Desulfatitalea tepidiphila;Desulfatitalea tepidiphila S28bF |
| Ga0586324 0017935 2919 4955 | 0.00 | 0.00 | 0.00 | 3610000.00 | 10186700.00 | 13061300.00 | 6315600.00 | 5856000.00 | 3280500.00 | 0.00 | 0.00 | 0.00 | KO:K00198 | carbon-monoxide dehydrogenase catalytic subunit | Bacteria;Proteobacteria;Deltaproteobacteria;Desulfarculales;Desulfarculaceae;Desulfocarbo;Desulfocarbo indianensis;Desulfocarbo indianensis SCBM |
| Ga0586324 0032078 209 2251 | 0.00 | 0.00 | 0.00 | 0.00 | 0.00 | 0.00 | 3693600.00 | 0.00 | 0.00 | 0.00 | 0.00 | 0.00 | KO:K00198 | carbon-monoxide dehydrogenase catalytic subunit | Bacteria;Proteobacteria;Deltaproteobacteria;Desulfarculales;Desulfarculaceae;Desulfocarbo;Desulfocarbo indianensis;Desulfocarbo indianensis SCBM |
| Ga0586324 0033443 450 2417 | 0.00 | 0.00 | 0.00 | 0.00 | 3306300.00 | 0.00 | 0.00 | 0.00 | 0.00 | 0.00 | 0.00 | 0.00 | KO:K00198 | carbon-monoxide dehydrogenase catalytic subunit | Bacteria;Proteobacteria;Deltaproteobacteria;Syntrophobacterales;Syntrophobacteraceae;Desulfacinum;Desulfacinum infernum;Desulfacinum infernum DSM 9756 |
| Ga0586324 0059416 971 2995 | 0.00 | 0.00 | 0.00 | 0.00 | 0.00 | 13855300.00 | 0.00 | 0.00 | 0.00 | 0.00 | 0.00 | 745284.00 | KO:K00198 | carbon-monoxide dehydrogenase catalytic subunit | Bacteria;Proteobacteria;Deltaproteobacteria;Syntrophobacterales;Syntrophaceae;Desulfobacca;Desulfobacca acetoxidans;Desulfobacca acetoxidans ASRB2, DSM 11109 |
| Ga0586324 0147716 1404 2849 | 0.00 | 0.00 | 0.00 | 0.00 | 0.00 | 2474993.28 | 0.00 | 0.00 | 0.00 | 0.00 | 0.00 | 0.00 | KO:K00198 | carbon-monoxide dehydrogenase catalytic subunit | Bacteria;Proteobacteria;Deltaproteobacteria;Syntrophobacterales;Syntrophaceae;Desulfobacca;Desulfobacca acetoxidans;Desulfobacca acetoxidans ASRB2, DSM 11109 |
| Ga0586324 0172951 1984 2646 | 0.00 | 0.00 | 0.00 | 0.00 | 0.00 | 2793153.05 | 0.00 | 0.00 | 0.00 | 0.00 | 0.00 | 0.00 | KO:K00198 | carbon-monoxide dehydrogenase catalytic subunit | Bacteria;Proteobacteria;Deltaproteobacteria;Desulfobacterales;Desulfobacteraceae;Desulfatitalea;Desulfatitalea tepidiphila;Desulfatitalea tepidiphila S28bF |
| Ga0586324 0185286 357 2333 | 0.00 | 0.00 | 0.00 | 0.00 | 0.00 | 0.00 | 0.00 | 0.00 | 772743.24 | 0.00 | 0.00 | 0.00 | KO:K00198 | carbon-monoxide dehydrogenase catalytic subunit | Bacteria;Proteobacteria;Deltaproteobacteria;Desulfobacterales;Desulfobacteraceae;Desulfatiglans;Desulfatiglans anilini;Desulfatiglans anilini DSM 4660 |
| Ga0586324 0222223 303 2330 | 0.00 | 0.00 | 0.00 | 3002000.00 | 2245400.00 | 0.00 | 0.00 | 0.00 | 0.00 | 0.00 | 0.00 | 0.00 | KO:K00198 | carbon-monoxide dehydrogenase catalytic subunit | Bacteria;Proteobacteria;Deltaproteobacteria;Desulfobacterales;Desulfobacteraceae;Desulfosalsimonas;Desulfosalsimonas propionicica;Desulfosalsimonas propionicica DSM 17721 |
| Ga0586324 0291747 2 1762 | 906787.00 | 0.00 | 0.00 | 0.00 | 0.00 | 0.00 | 0.00 | 2342400.00 | 0.00 | 0.00 | 0.00 | 1567500.00 | KO:K00198 | carbon-monoxide dehydrogenase catalytic subunit | Bacteria;Proteobacteria;Deltaproteobacteria;Desulfobacterales;Desulfobacteraceae;Desulfatiglans;Desulfatiglans anilini;Desulfatiglans anilini DSM 4660 |
| Ga0586324 0325179 3 737 | 4290000.00 | 0.00 | 0.00 | 0.00 | 0.00 | 0.00 | 0.00 | 14030000.00 | 11664000.00 | 14399000.00 | 9152000.00 | 0.00 | KO:K00198 | carbon-monoxide dehydrogenase catalytic subunit | Bacteria;Proteobacteria;Deltaproteobacteria;Syntrophobacterales;Syntrophaceae;Desulfobacca;Desulfobacca acetoxidans;Desulfobacca acetoxidans ASRB2, DSM 11109 |
| Ga0586324 0340151 2 958 | 0.00 | 53584000.00 | 0.00 | 0.00 | 170980000.00 | 0.00 | 0.00 | 90890000.00 | 0.00 | 0.00 | 0.00 | 0.00 | KO:K00198 | carbon-monoxide dehydrogenase catalytic subunit | Bacteria;Proteobacteria;Deltaproteobacteria;Desulfobacterales;Desulfobacteraceae;Desulfosalsimonas;Desulfosalsimonas propionicica;Desulfosalsimonas propionicica DSM 17721 |
| Ga0586324 0342779 3 1787 | 0.00 | 7860800.00 | 0.00 | 13110000.00 | 9651100.00 | 0.00 | 0.00 | 0.00 | 4406400.00 | 0.00 | 0.00 | 0.00 | KO:K00198 | carbon-monoxide dehydrogenase catalytic subunit | Bacteria;Proteobacteria;Deltaproteobacteria;Syntrophobacterales;Syntrophaceae;Desulfobacca;Desulfobacca acetoxidans;Desulfobacca acetoxidans ASRB2, DSM 11109 |
| Ga0586324 0357415 3 1568 | 0.00 | 0.00 | 7150100.00 | 4854500.00 | 0.00 | 0.00 | 33972000.00 | 0.00 | 0.00 | 21560000.00 | 0.00 | 0.00 | KO:K00198 | carbon-monoxide dehydrogenase catalytic subunit | Bacteria;Proteobacteria;Deltaproteobacteria;Desulfobacterales;Desulfobacteraceae;Desulfatirhabdium;Desulfatirhabdium butyrativorans;Desulfatirhabdium butyrativorans DSM 18734 |
| Ga0586324 0360485 3 1784 | 14300000.00 | 0.00 | 9345800.00 | 0.00 | 12875000.00 | 0.00 | 15116400.00 | 7198000.00 | 8343000.00 | 0.00 | 0.00 | 0.00 | KO:K00198 | carbon-monoxide dehydrogenase catalytic subunit | Bacteria;Proteobacteria;Deltaproteobacteria;Syntrophobacterales;Syntrophobacteraceae;Syntrophobacter;Syntrophobacter fumaroxidans;Syntrophobacter fumaroxidans MPOB |
| Ga0586324 0406659 831 1796 | 6540000.00 | 3060000.00 | 0.00 | 10830000.00 | 0.00 | 0.00 | 0.00 | 0.00 | 0.00 | 8162000.00 | 0.00 | 0.00 | KO:K00198 | carbon-monoxide dehydrogenase catalytic subunit | Bacteria;Proteobacteria;Deltaproteobacteria;Syntrophobacterales;Syntrophaceae;Desulfobacca;Desulfobacca acetoxidans;Desulfobacca acetoxidans ASRB2, DSM 11109 |
| Ga0586324 0874128 2 649 | 6540000.00 | 2475200.00 | 0.00 | 3106500.00 | 1246300.00 | 0.00 | 0.00 | 1878800.00 | 3248100.00 | 0.00 | 0.00 | 0.00 | KO:K00198 | carbon-monoxide dehydrogenase catalytic subunit | Bacteria;Proteobacteria;Deltaproteobacteria;Syntrophobacterales;Syntrophaceae;Desulfobacca;Desulfobacca acetoxidans;Desulfobacca acetoxidans ASRB2, DSM 11109 |
| Ga0586324 0914745 1 1260 | 0.00 | 0.00 | 0.00 | 2593500.00 | 4346600.00 | 0.00 | 0.00 | 0.00 | 0.00 | 0.00 | 0.00 | 0.00 | KO:K00198 | carbon-monoxide dehydrogenase catalytic subunit | Bacteria;Proteobacteria;Deltaproteobacteria;Desulfobacterales;Desulfobulbaceae;Desulfopila;Desulfopila aestuarii;Desulfopila aestuarii DSM 18488 |
| Ga0586324 0960244 3 1235 | 0.00 | 11621200.00 | 2936664.30 | 0.00 | 0.00 | 0.00 | 0.00 | 0.00 | 874800.00 | 0.00 | 0.00 | 0.00 | KO:K00198 | carbon-monoxide dehydrogenase catalytic subunit | Bacteria;Proteobacteria;Deltaproteobacteria;Syntrophobacterales;Syntrophaceae;Desulfobacca;Desulfobacca acetoxidans;Desulfobacca acetoxidans ASRB2, DSM 11109 |
| Ga0586324 1069603 2 1141 | 1860000.00 | 5100000.00 | 0.00 | 0.00 | 0.00 | 0.00 | 5768400.00 | 4776300.00 | 5516100.00 | 0.00 | 0.00 | 0.00 | KO:K00198 | carbon-monoxide dehydrogenase catalytic subunit | Bacteria;Proteobacteria;Deltaproteobacteria;Syntrophobacterales;Syntrophaceae;Desulfobacca;Desulfobacca acetoxidans;Desulfobacca acetoxidans ASRB2, DSM 11109 |
| Ga0586324 1165256 1 870 | 0.00 | 0.00 | 0.00 | 1054500.00 | 0.00 | 0.00 | 0.00 | 230228.64 | 0.00 | 0.00 | 0.00 | 0.00 | KO:K00198 | carbon-monoxide dehydrogenase catalytic subunit | Bacteria;Proteobacteria;Deltaproteobacteria;Syntrophobacterales;Syntrophobacteraceae;Desulfacinum;Desulfacinum infernum;Desulfacinum infernum DSM 9756 |
| Ga0586324 1175033 38 1132 | 3330000.00 | 0.00 | 0.00 | 0.00 | 0.00 | 0.00 | 0.00 | 0.00 | 0.00 | 0.00 | 0.00 | 0.00 | KO:K00198 | carbon-monoxide dehydrogenase catalytic subunit | Archaea;Euryarchaeota;Methanomicrobia;Methanosarcinales;Methanotrichaceae;Methanothrix;Methanothrix soehngenii;Methanothrix soehngenii GP-6 |
| Ga0586324 1478217 1 1026 | 0.00 | 462922.24 | 0.00 | 0.00 | 0.00 | 8892800.00 | 0.00 | 0.00 | 9477000.00 | 0.00 | 0.00 | 0.00 | KO:K00198 | carbon-monoxide dehydrogenase catalytic subunit | Bacteria;Proteobacteria;Deltaproteobacteria;Syntrophobacterales;Syntrophaceae;Desulfobacca;Desulfobacca acetoxidans;Desulfobacca acetoxidans ASRB2, DSM 11109 |
| Ga0586324 1640061 2 895 | 0.00 | 2454800.00 | 0.00 | 0.00 | 0.00 | 0.00 | 0.00 | 0.00 | 0.00 | 0.00 | 0.00 | 0.00 | KO:K00198 | carbon-monoxide dehydrogenase catalytic subunit | Bacteria;Proteobacteria;Deltaproteobacteria;Syntrophobacterales;Syntrophaceae;Desulfobacca;Desulfobacca acetoxidans;Desulfobacca acetoxidans ASRB2, DSM 11109 |
| Ga0586324 1891998 311 925 | 0.00 | 0.00 | 0.00 | 1159000.00 | 2369000.00 | 0.00 | 0.00 | 0.00 | 0.00 | 0.00 | 0.00 | 0.00 | KO:K00198 | carbon-monoxide dehydrogenase catalytic subunit | Bacteria;Proteobacteria;Deltaproteobacteria;Syntrophobacterales;Syntrophobacteraceae;Desulfacinum;Desulfacinum infernum;Desulfacinum infernum DSM 9756 |
| Ga0586324 1983577 2 778 | 0.00 | 0.00 | 0.00 | 0.00 | 0.00 | 0.00 | 0.00 | 0.00 | 0.00 | 578112.15 | 0.00 | 0.00 | KO:K00198 | carbon-monoxide dehydrogenase catalytic subunit | Archaea;Euryarchaeota;Methanomicrobia;Methanomicrobiales;Methanoregulaceae;Methanoregula;Methanoregula boonei;Methanoregula boonei 6A8 |
| Ga0586324 2135921 30 878 | 0.00 | 0.00 | 0.00 | 1045000.00 | 0.00 | 0.00 | 0.00 | 0.00 | 0.00 | 0.00 | 0.00 | 0.00 | KO:K00198 | carbon-monoxide dehydrogenase catalytic subunit | Bacteria;Proteobacteria;Deltaproteobacteria;Syntrophobacterales;Syntrophobacteraceae;Syntrophobacter;Syntrophobacter fumaroxidans;Syntrophobacter fumaroxidans MPOB |
| Ga0586324 2244057 1 861 | 4090000.00 | 0.00 | 0.00 | 0.00 | 0.00 | 0.00 | 0.00 | 628300.00 | 0.00 | 0.00 | 0.00 | 0.00 | KO:K00198 | carbon-monoxide dehydrogenase catalytic subunit | Bacteria;Proteobacteria;Deltaproteobacteria;Syntrophobacterales;Syntrophaceae;Desulfobacca;Desulfobacca acetoxidans;Desulfobacca acetoxidans ASRB2, DSM 11109 |
| Ga0586324 2855795 182 778 | 3020000.00 | 3998400.00 | 7825700.00 | 16340000.00 | 10918000.00 | 57104519.70 | 24122400.00 | 7625000.00 | 10602900.00 | 4419800.00 | 11105600.00 | 15075000.00 | KO:K00198 | carbon-monoxide dehydrogenase catalytic subunit | Bacteria;Proteobacteria;Deltaproteobacteria;Syntrophobacterales;Syntrophobacteraceae;Desulfoglaeba;Desulfoglaeba alkanexedens;Desulfoglaeba alkanexedens ALDC |
| Ga0586324 3153100 220 747 | 0.00 | 0.00 | 0.00 | 0.00 | 0.00 | 727494.56 | 1246754.16 | 0.00 | 1134000.00 | 0.00 | 0.00 | 0.00 | KO:K00198 | carbon-monoxide dehydrogenase catalytic subunit | Bacteria;Proteobacteria;Deltaproteobacteria;Syntrophobacterales;Syntrophobacteraceae;Syntrophobacter;Syntrophobacter fumaroxidans;Syntrophobacter fumaroxidans MPOB |
| Ga0586324 3575595 3 707 | 0.00 | 0.00 | 0.00 | 0.00 | 0.00 | 0.00 | 0.00 | 3043900.00 | 0.00 | 0.00 | 0.00 | 0.00 | KO:K00198 | carbon-monoxide dehydrogenase catalytic subunit | Bacteria;Proteobacteria;Deltaproteobacteria;Syntrophobacterales;Syntrophaceae;Desulfobacca;Desulfobacca acetoxidans;Desulfobacca acetoxidans ASRB2, DSM 11109 |
| Ga0586324 3789943 3 692 | 0.00 | 0.00 | 0.00 | 3439000.00 | 0.00 | 0.00 | 0.00 | 0.00 | 0.00 | 0.00 | 0.00 | 0.00 | KO:K00198 | carbon-monoxide dehydrogenase catalytic subunit | Bacteria;Proteobacteria;Deltaproteobacteria;Syntrophobacterales;Syntrophobacteraceae;Desulfoglaeba;Desulfoglaeba alkanexedens;Desulfoglaeba alkanexedens ALDC |
| Ga0586324 4134727 104 667 | 0.00 | 0.00 | 0.00 | 216425.20 | 0.00 | 0.00 | 0.00 | 0.00 | 0.00 | 0.00 | 0.00 | 0.00 | KO:K00198 | carbon-monoxide dehydrogenase catalytic subunit | Bacteria;Proteobacteria;Deltaproteobacteria;Syntrophobacterales;Syntrophobacteraceae;Desulfacinum;Desulfacinum infernum;Desulfacinum infernum DSM 9756 |
| Ga0586324 4510073 1 642 | 0.00 | 13260000.00 | 0.00 | 0.00 | 0.00 | 0.00 | 0.00 | 0.00 | 0.00 | 0.00 | 0.00 | 0.00 | KO:K00198 | carbon-monoxide dehydrogenase catalytic subunit | Bacteria;Proteobacteria;Deltaproteobacteria;Syntrophobacterales;Syntrophaceae;Desulfobacca;Desulfobacca acetoxidans;Desulfobacca acetoxidans ASRB2, DSM 11109 |
| Ga0586324 4788000 3 629 | 0.00 | 0.00 | 0.00 | 0.00 | 0.00 | 0.00 | 96444000.00 | 0.00 | 0.00 | 0.00 | 0.00 | 0.00 | KO:K00198 | carbon-monoxide dehydrogenase catalytic subunit | Bacteria;Proteobacteria;Deltaproteobacteria;Syntrophobacterales;Syntrophobacteraceae;Desulfacinum;Desulfacinum infernum;Desulfacinum infernum DSM 9756 |
| Ga0586324 5091440 1 612 | 0.00 | 0.00 | 0.00 | 4104000.00 | 0.00 | 0.00 | 0.00 | 0.00 | 0.00 | 0.00 | 0.00 | 0.00 | KO:K00198 | carbon-monoxide dehydrogenase catalytic subunit | Bacteria;Proteobacteria;Deltaproteobacteria;Syntrophobacterales;Syntrophaceae;Desulfobacca;Desulfobacca acetoxidans;Desulfobacca acetoxidans ASRB2, DSM 11109 |
| Ga0586324 5265810 3 605 | 0.00 | 0.00 | 0.00 | 0.00 | 2121800.00 | 0.00 | 0.00 | 0.00 | 0.00 | 0.00 | 0.00 | 0.00 | KO:K00198 | carbon-monoxide dehydrogenase catalytic subunit | Bacteria;Proteobacteria;Deltaproteobacteria;Syntrophobacterales;Syntrophaceae;Desulfobacca;Desulfobacca acetoxidans;Desulfobacca acetoxidans ASRB2, DSM 11109 |
| Ga0586324 5577880 3 590 | 0.00 | 0.00 | 0.00 | 0.00 | 19158000.00 | 14847800.00 | 8253600.00 | 12200000.00 | 0.00 | 0.00 | 0.00 | 0.00 | KO:K00198 | carbon-monoxide dehydrogenase catalytic subunit | Bacteria;Proteobacteria;Deltaproteobacteria;Syntrophobacterales;Syntrophaceae;Desulfobacca;Desulfobacca acetoxidans;Desulfobacca acetoxidans ASRB2, DSM 11109 |
| Ga0586324 5719635 1 582 | 0.00 | 0.00 | 0.00 | 0.00 | 0.00 | 0.00 | 0.00 | 0.00 | 473316.21 | 0.00 | 0.00 | 0.00 | KO:K00198 | carbon-monoxide dehydrogenase catalytic subunit | Bacteria;Proteobacteria;Deltaproteobacteria;Syntrophobacterales;Syntrophaceae;Desulfobacca;Desulfobacca acetoxidans;Desulfobacca acetoxidans ASRB2, DSM 11109 |
| Ga0586324 6602596 3 551 | 0.00 | 344543.76 | 0.00 | 0.00 | 0.00 | 0.00 | 0.00 | 0.00 | 0.00 | 0.00 | 0.00 | 0.00 | KO:K00198 | carbon-monoxide dehydrogenase catalytic subunit | Bacteria;Proteobacteria;Deltaproteobacteria;Desulfobacterales;Desulfobacteraceae;Desulfatitalea;Desulfatitalea tepidiphila;Desulfatitalea tepidiphila S28bF |
| Ga0586324 7103589 1 534 | 0.00 | 0.00 | 0.00 | 0.00 | 0.00 | 0.00 | 0.00 | 157764.91 | 0.00 | 0.00 | 0.00 | 0.00 | KO:K00198 | carbon-monoxide dehydrogenase catalytic subunit | Bacteria;Proteobacteria;Deltaproteobacteria;Syntrophobacterales;Syntrophaceae;Desulfobacca;Desulfobacca acetoxidans;Desulfobacca acetoxidans ASRB2, DSM 11109 |
| Ga0586324 7382473 1 525 | 0.00 | 39236000.00 | 0.00 | 98800000.00 | 316807.40 | 0.00 | 0.00 | 52887000.00 | 43254000.00 | 0.00 | 0.00 | 0.00 | KO:K00198 | carbon-monoxide dehydrogenase catalytic subunit | Bacteria;Proteobacteria;Deltaproteobacteria;Syntrophobacterales;Syntrophobacteraceae;Syntrophobacter;Syntrophobacter fumaroxidans;Syntrophobacter fumaroxidans MPOB |
| Ga0586324 7570091 2 520 | 0.00 | 0.00 | 0.00 | 0.00 | 0.00 | 0.00 | 0.00 | 5892600.00 | 0.00 | 0.00 | 0.00 | 0.00 | KO:K00200 | formylmethanofuran dehydrogenase subunit A | Archaea;Euryarchaeota;Methanomicrobia;Methanomicrobiales;Methanoregulaceae;Methanoregula;Methanoregula formicica;Methanoregula formicica SMSP, DSM 22288 |
| Ga0586324 0676120 424 1437 | 6490000.00 | 0.00 | 11823000.00 | 0.00 | 0.00 | 0.00 | 0.00 | 0.00 | 0.00 | 0.00 | 0.00 | 0.00 | KO:K00201 | formylmethanofuran dehydrogenase subunit B | Archaea;Euryarchaeota;Methanomicrobia;Methanomicrobiales;Methanoregulaceae;Methanoregula;Methanoregula formicica;Methanoregula formicica SMSP, DSM 22288 |
| Ga0586324 1148343 1 1143 | 0.00 | 0.00 | 9120600.00 | 0.00 | 0.00 | 0.00 | 0.00 | 0.00 | 0.00 | 0.00 | 0.00 | 0.00 | KO:K00205 | 4Fe-4S ferredoxin | Archaea;Euryarchaeota;Methanomicrobia;Methanomicrobiales;Methanomicrobiaceae;Methanofollis;Methanofollis liminatans;Methanofollis liminatans GKZPZ, DSM 4140 |
| Ga0586324 0037004 4348 5121 | 0.00 | 0.00 | 0.00 | 0.00 | 0.00 | 0.00 | 0.00 | 3721000.00 | 0.00 | 0.00 | 0.00 | 0.00 | KO:K00208 | enoyl-[acyl-carrier protein] reductase I | Bacteria;Nitrospirae;Nitrospira;Nitrospirales;Nitrospiraceae;Nitrospira;Nitrospira defluvii;Nitrospira defluvii |
| Ga0586324 0183631 1523 2305 | 0.00 | 0.00 | 0.00 | 0.00 | 0.00 | 0.00 | 0.00 | 0.00 | 0.00 | 0.00 | 1355200.00 | 0.00 | KO:K00208 | enoyl-[acyl-carrier protein] reductase I | Bacteria;Proteobacteria;Gammaproteobacteria;Pseudomonadales;Pseudomonadaceae;Pseudomonas;Pseudomonas linyingensis;Pseudomonas linyingensis LMG 25967 |
| Ga0586324 0528289 2 655 | 0.00 | 0.00 | 3052405.84 | 0.00 | 0.00 | 0.00 | 0.00 | 0.00 | 0.00 | 0.00 | 0.00 | 0.00 | KO:K00208 | enoyl-[acyl-carrier protein] reductase I | Bacteria;Proteobacteria;Gammaproteobacteria;Methylococcales;Methylococcaceae;Methylobacter;Methylobacter tundripaludum;Methylobacter tundripaludum SV96 |
| Ga0586324 7177985 3 530 | 0.00 | 0.00 | 0.00 | 0.00 | 1668600.00 | 0.00 | 0.00 | 0.00 | 0.00 | 0.00 | 0.00 | 0.00 | KO:K00208 | enoyl-[acyl-carrier protein] reductase I | Bacteria;Proteobacteria;Gammaproteobacteria;Oceanospirillales;Balneatrichaceae;Pokkaliibacter;Pokkaliibacter plantistimulans;Pokkaliibacter plantistimulans L1E11 |
| Ga0586324 7601373 2 517 | 0.00 | 0.00 | 2926761.13 | 0.00 | 0.00 | 0.00 | 0.00 | 0.00 | 0.00 | 0.00 | 0.00 | 0.00 | KO:K00208 | enoyl-[acyl-carrier protein] reductase I | Bacteria;Proteobacteria;Gammaproteobacteria;Methylococcales;Methylococcaceae;Methylobacter;Methylobacter marinus;Methylobacter marinus A45 |
| Ga0586324 0171168 616 1422 | 0.00 | 0.00 | 0.00 | 0.00 | 0.00 | 0.00 | 0.00 | 0.00 | 0.00 | 0.00 | 4919200.00 | 0.00 | KO:K00215 | 4-hydroxy-tetrahydrodipicolinate reductase | Bacteria;Proteobacteria;Betaproteobacteria;Nitrosomonadales;Gallionellaceae;Gallionella;Gallionella capsiferriformans;Gallionella capsiferriformans ES-2 |
| Ga0586324 0438024 1 1434 | 425674.00 | 0.00 | 0.00 | 0.00 | 0.00 | 0.00 | 0.00 | 1439600.00 | 0.00 | 0.00 | 0.00 | 0.00 | KO:K00219 | 2,4-dienoyl-CoA reductase (NADPH2) | Bacteria;Proteobacteria;Deltaproteobacteria;Desulfarculales;Desulfarculaceae;Desulfocarbo;Desulfocarbo indianensis;Desulfocarbo indianensis SCBM |
| Ga0586324 4330780 3 653 | 0.00 | 0.00 | 0.00 | 0.00 | 0.00 | 0.00 | 0.00 | 0.00 | 0.00 | 0.00 | 1064800.00 | 0.00 | KO:K00228 | coproporphyrinogen III oxidase | Bacteria;Proteobacteria;Gammaproteobacteria;Chromatiales;Ectothiorhodospiraceae;Thioalkalivibrio;Thioalkalivibrio sulfidiphilus;Thioalkalivibrio sulfidophilus HL-EbGR7 |
| Ga0586324 0057175 1788 3578 | 0.00 | 0.00 | 0.00 | 0.00 | 0.00 | 1010944.62 | 0.00 | 0.00 | 0.00 | 0.00 | 0.00 | 0.00 | KO:K00239 | succinate dehydrogenase / fumarate reductase flavoprotein subunit | Bacteria;Proteobacteria;Gammaproteobacteria;Chromatiales;Woeseiaceae;unclassified Woeseiaceae;unclassified Woeseiaceae;uncultured Woeseiaceae SAG 1868 B |
| Ga0586324 0058809 3010 4419 | 0.00 | 0.00 | 0.00 | 0.00 | 0.00 | 0.00 | 0.00 | 0.00 | 0.00 | 0.00 | 358526.96 | 0.00 | KO:K00239 | succinate dehydrogenase / fumarate reductase flavoprotein subunit | Bacteria;Proteobacteria;Alphaproteobacteria;Hyphomicrobiales;unclassified Hyphomicrobiales;Methyloceanibacter;Methyloceanibacter superfactus;Methyloceanibacter superfactus R-67175 |
| Ga0586324 0173151 2 619 | 0.00 | 0.00 | 0.00 | 7790000.00 | 0.00 | 0.00 | 0.00 | 0.00 | 0.00 | 0.00 | 0.00 | 0.00 | KO:K00239 | succinate dehydrogenase / fumarate reductase flavoprotein subunit | Bacteria;Bacteroidetes;Cytophagia;Cytophagales;Hymenobacteraceae;Pontibacter;Pontibacter oryzae;Pontibacter oryzae KIRAN |
| Ga0586324 0265555 191 2140 | 0.00 | 0.00 | 0.00 | 959500.00 | 0.00 | 0.00 | 0.00 | 0.00 | 0.00 | 0.00 | 0.00 | 0.00 | KO:K00239 | succinate dehydrogenase / fumarate reductase flavoprotein subunit | Bacteria;Chloroflexi;Chloroflexia;Chloroflexales;Chloroflexaceae;Chloroflexus;Chloroflexus aggregans;Chloroflexus aggregans DSM 9485 |
| Ga0586324 0532104 1 1434 | 0.00 | 0.00 | 0.00 | 57190000.00 | 0.00 | 0.00 | 0.00 | 0.00 | 0.00 | 0.00 | 0.00 | 0.00 | KO:K00239 | succinate dehydrogenase / fumarate reductase flavoprotein subunit | Bacteria;Proteobacteria;Deltaproteobacteria;Desulfuromonadales;Geobacteraceae;Geobacter;Geobacter sp. OR-1;Geobacter sp. OR-1 |
| Ga0586324 0565421 257 1552 | 0.00 | 0.00 | 0.00 | 0.00 | 0.00 | 0.00 | 0.00 | 0.00 | 5661900.00 | 0.00 | 0.00 | 0.00 | KO:K00239 | succinate dehydrogenase / fumarate reductase flavoprotein subunit | Bacteria;Acidobacteria;Acidobacteriia;Bryobacterales;Solibacteraceae;Candidatus Solibacter;Candidatus Solibacter usitatus;Candidatus Solibacter usitatus Ellin6076 |
| Ga0586324 0576188 1 1392 | 3120000.00 | 0.00 | 0.00 | 0.00 | 0.00 | 0.00 | 0.00 | 0.00 | 4301100.00 | 0.00 | 0.00 | 0.00 | KO:K00239 | succinate dehydrogenase / fumarate reductase flavoprotein subunit | Bacteria;Proteobacteria;Gammaproteobacteria;unclassified Gammaproteobacteria;Candidatus Competibacteraceae;Candidatus Competibacter;Candidatus Competibacter denitrificans;Candidatus Competibacter denitrificans Run A D11 |
| Ga0586324 0666186 174 1448 | 0.00 | 0.00 | 0.00 | 0.00 | 0.00 | 0.00 | 0.00 | 817400.00 | 0.00 | 0.00 | 0.00 | 0.00 | KO:K00239 | succinate dehydrogenase / fumarate reductase flavoprotein subunit | Bacteria;Proteobacteria;Alphaproteobacteria;Hyphomicrobiales;Xanthobacteraceae;Pseudolabrys;Pseudolabrys sp. Root1462;Pseudolabrys sp. Root1462 |
| Ga0586324 0668243 314 1444 | 0.00 | 0.00 | 0.00 | 0.00 | 2183600.00 | 0.00 | 0.00 | 0.00 | 0.00 | 0.00 | 0.00 | 0.00 | KO:K00239 | succinate dehydrogenase / fumarate reductase flavoprotein subunit | Bacteria;Bacteroidetes;Cytophagia;Cytophagales;Hymenobacteraceae;Rufibacter;Rufibacter latericius;Rufibacter latericius byd1 |
| Ga0586324 0724072 1 972 | 14300000.00 | 0.00 | 0.00 | 0.00 | 0.00 | 0.00 | 0.00 | 0.00 | 0.00 | 0.00 | 0.00 | 0.00 | KO:K00239 | succinate dehydrogenase / fumarate reductase flavoprotein subunit | Bacteria;Proteobacteria;Deltaproteobacteria;Desulfobacterales;Desulfobacteraceae;Desulfatitalea;Desulfatitalea tepidiphila;Desulfatitalea tepidiphila S28bF |
| Ga0586324 0948251 2 1243 | 0.00 | 0.00 | 0.00 | 0.00 | 0.00 | 0.00 | 0.00 | 0.00 | 0.00 | 0.00 | 1075888.00 | 0.00 | KO:K00239 | succinate dehydrogenase / fumarate reductase flavoprotein subunit | Bacteria;Proteobacteria;Alphaproteobacteria;Hyphomicrobiales;Xanthobacteraceae;Pseudolabrys;Pseudolabrys sp. Root1462;Pseudolabrys sp. Root1462 |
| Ga0586324 1039564 3 1193 | 0.00 | 0.00 | 0.00 | 0.00 | 0.00 | 0.00 | 0.00 | 0.00 | 755011.53 | 0.00 | 0.00 | 0.00 | KO:K00239 | succinate dehydrogenase / fumarate reductase flavoprotein subunit | Bacteria;Bacteroidetes;Bacteroidia;Marinilabiliales;Prolixibacteraceae;Roseimarinus;Roseimarinus sediminis;Roseimarinus sediminis DSM 28824 |
| Ga0586324 1106617 1 1161 | 0.00 | 0.00 | 0.00 | 0.00 | 0.00 | 0.00 | 0.00 | 0.00 | 0.00 | 0.00 | 8976000.00 | 0.00 | KO:K00239 | succinate dehydrogenase / fumarate reductase flavoprotein subunit | Bacteria;Ignavibacteriae;Ignavibacteria;Ignavibacteriales;Ignavibacteriaceae;Ignavibacterium;Ignavibacterium album;Ignavibacterium album Mat9-16, JCM 16511 |
| Ga0586324 1188999 1 1128 | 0.00 | 0.00 | 0.00 | 0.00 | 0.00 | 0.00 | 13406400.00 | 0.00 | 0.00 | 0.00 | 0.00 | 0.00 | KO:K00239 | succinate dehydrogenase / fumarate reductase flavoprotein subunit | Bacteria;Verrucomicrobia;Verrucomicrobiae;Verrucomicrobiales;Verrucomicrobia subdivision 3;Pedosphaera;Pedosphaera parvula;Pedosphaera parvula Ellin514 |
| Ga0586324 1400090 1 936 | 0.00 | 0.00 | 0.00 | 0.00 | 29355000.00 | 0.00 | 0.00 | 0.00 | 2867400.00 | 0.00 | 0.00 | 0.00 | KO:K00239 | succinate dehydrogenase / fumarate reductase flavoprotein subunit | Bacteria;Proteobacteria;Deltaproteobacteria;Desulfobacterales;Desulfobulbaceae;Desulfobulbus;Desulfobulbus elongatus;Desulfobulbus elongatus DSM 2908 |
| Ga0586324 1483764 1 630 | 0.00 | 0.00 | 0.00 | 0.00 | 0.00 | 0.00 | 0.00 | 0.00 | 138629.88 | 0.00 | 0.00 | 0.00 | KO:K00239 | succinate dehydrogenase / fumarate reductase flavoprotein subunit | Bacteria;Acidobacteria;Acidobacteriia;Bryobacterales;Solibacteraceae;Candidatus Solibacter;Candidatus Solibacter usitatus;Candidatus Solibacter usitatus Ellin6076 |
| Ga0586324 1497595 1 1020 | 0.00 | 0.00 | 0.00 | 3439000.00 | 0.00 | 0.00 | 0.00 | 0.00 | 0.00 | 0.00 | 0.00 | 0.00 | KO:K00239 | succinate dehydrogenase / fumarate reductase flavoprotein subunit | Bacteria;Actinobacteria;Actinomycetia;Streptosporangiales;Thermomonosporaceae;Thermomonospora;Thermomonospora curvata;Thermomonospora curvata DSM 43183 |
| Ga0586324 1654603 3 608 | 0.00 | 0.00 | 0.00 | 0.00 | 2678000.00 | 0.00 | 0.00 | 0.00 | 0.00 | 0.00 | 0.00 | 0.00 | KO:K00239 | succinate dehydrogenase / fumarate reductase flavoprotein subunit | Bacteria;Proteobacteria;Betaproteobacteria;Rhodocyclales;Azonexaceae;Dechloromonas;Dechloromonas aromatica;Dechloromonas aromatica RCB |
| Ga0586324 1842949 1 936 | 0.00 | 0.00 | 0.00 | 0.00 | 701105.55 | 0.00 | 0.00 | 0.00 | 0.00 | 0.00 | 0.00 | 0.00 | KO:K00239 | succinate dehydrogenase / fumarate reductase flavoprotein subunit | Bacteria;Chloroflexi;Chloroflexia;Chloroflexales;Roseiflexaceae;Roseiflexus;Roseiflexus castenholzii;Roseiflexus castenholzii HLO8, DSM 13941 |
| Ga0586324 1869805 2 514 | 0.00 | 0.00 | 0.00 | 5747500.00 | 0.00 | 6629900.00 | 0.00 | 0.00 | 3555900.00 | 0.00 | 0.00 | 0.00 | KO:K00239 | succinate dehydrogenase / fumarate reductase flavoprotein subunit | Bacteria;Bacteroidetes;Sphingobacteriia;Sphingobacteriales;Sphingobacteriaceae;Mucilaginibacter;Mucilaginibacter sp. X4EP1;Mucilaginibacter sp. X4EP1 |
| Ga0586324 1929326 1 918 | 0.00 | 0.00 | 0.00 | 0.00 | 0.00 | 0.00 | 0.00 | 0.00 | 1474200.00 | 0.00 | 0.00 | 0.00 | KO:K00239 | succinate dehydrogenase / fumarate reductase flavoprotein subunit | Bacteria;Verrucomicrobia;Verrucomicrobiae;Verrucomicrobiales;Verrucomicrobia subdivision 3;Pedosphaera;Pedosphaera parvula;Pedosphaera parvula Ellin514 |
| Ga0586324 3809141 2 691 | 0.00 | 0.00 | 0.00 | 0.00 | 0.00 | 0.00 | 0.00 | 0.00 | 0.00 | 0.00 | 6934400.00 | 0.00 | KO:K00239 | succinate dehydrogenase / fumarate reductase flavoprotein subunit | Bacteria;Chloroflexi;Chloroflexia;Chloroflexales;Roseiflexaceae;Roseiflexus;Roseiflexus sp. RS-1;Roseiflexus sp. RS-1 |
| Ga0586324 5074373 3 614 | 0.00 | 0.00 | 0.00 | 0.00 | 0.00 | 0.00 | 0.00 | 0.00 | 0.00 | 2156000.00 | 0.00 | 0.00 | KO:K00239 | succinate dehydrogenase / fumarate reductase flavoprotein subunit | Bacteria;Proteobacteria;Deltaproteobacteria;Desulfobacterales;Desulfobulbaceae;Desulfobulbus;Desulfobulbus propionicus;Desulfobulbus propionicus 1pr3, DSM 2032 |
| Ga0586324 6566519 1 552 | 0.00 | 15436000.00 | 0.00 | 0.00 | 0.00 | 0.00 | 0.00 | 0.00 | 0.00 | 0.00 | 0.00 | 0.00 | KO:K00239 | succinate dehydrogenase / fumarate reductase flavoprotein subunit | Bacteria;Ignavibacteriae;Ignavibacteria;Ignavibacteriales;Ignavibacteriaceae;Ignavibacterium;Ignavibacterium album;Ignavibacterium album Mat9-16, JCM 16511 |
| Ga0586324 1363083 2 475 | 0.00 | 0.00 | 0.00 | 0.00 | 0.00 | 0.00 | 0.00 | 0.00 | 0.00 | 1640100.00 | 0.00 | 0.00 | KO:K00240 | succinate dehydrogenase / fumarate reductase iron-sulfur subunit | Bacteria;Proteobacteria;Betaproteobacteria;Rhodocyclales;Zoogloeaceae;unclassified Zoogloeaceae;Zoogloeaceae bacteirum Par-f-2;Zoogloeaceae bacteirum Par-f-2 |
| Ga0586324 0075554 2 1582 | 0.00 | 0.00 | 24884600.00 | 0.00 | 0.00 | 0.00 | 0.00 | 0.00 | 0.00 | 0.00 | 0.00 | 0.00 | KO:K00244 | fumarate reductase flavoprotein subunit | Bacteria;Proteobacteria;Deltaproteobacteria;Syntrophobacterales;Syntrophobacteraceae;Syntrophobacter;Syntrophobacter fumaroxidans;Syntrophobacter fumaroxidans MPOB |
| Ga0586324 0241395 106 2046 | 0.00 | 0.00 | 0.00 | 0.00 | 0.00 | 2995404.70 | 0.00 | 0.00 | 0.00 | 0.00 | 0.00 | 0.00 | KO:K00244 | fumarate reductase flavoprotein subunit | Bacteria;Proteobacteria;Betaproteobacteria;Burkholderiales;Comamonadaceae;Acidovorax;Acidovorax carolinensis;Acidovorax carolinensis P4 |
| Ga0586324 0985021 159 1223 | 0.00 | 0.00 | 0.00 | 0.00 | 0.00 | 0.00 | 0.00 | 0.00 | 0.00 | 0.00 | 0.00 | 670857.00 | KO:K00244 | fumarate reductase flavoprotein subunit | Bacteria;Proteobacteria;Deltaproteobacteria;Desulfuromonadales;Geobacteraceae;Geobacter;Geobacter thiogenes;Geobacter thiogenes ATCC BAA-34 |
| Ga0586324 1029122 260 1198 | 0.00 | 0.00 | 18973100.00 | 0.00 | 0.00 | 0.00 | 0.00 | 0.00 | 0.00 | 0.00 | 0.00 | 0.00 | KO:K00248 | butyryl-CoA dehydrogenase | Bacteria;Proteobacteria;Deltaproteobacteria;Desulfobacterales;Desulfobulbaceae;Desulfobulbus;Desulfobulbus propionicus;Desulfobulbus propionicus 1pr3, DSM 2032 |
| Ga0586324 1218963 32 1114 | 0.00 | 0.00 | 0.00 | 0.00 | 0.00 | 0.00 | 0.00 | 0.00 | 1984500.00 | 0.00 | 0.00 | 0.00 | KO:K00248 | butyryl-CoA dehydrogenase | Bacteria;Firmicutes;Clostridia;Eubacteriales;Clostridiaceae;Caldisalinibacter;Caldisalinibacter kiritimatiensis;Clostridiaceae bacterium L21-TH-D2 |
| Ga0586324 1792850 3 311 | 0.00 | 1509600.00 | 0.00 | 6308000.00 | 6025500.00 | 5835900.00 | 0.00 | 0.00 | 0.00 | 0.00 | 0.00 | 0.00 | KO:K00248 | butyryl-CoA dehydrogenase | Bacteria;Proteobacteria;Deltaproteobacteria;Desulfobacterales;Desulfobulbaceae;Desulfobulbus;Desulfobulbus elongatus;Desulfobulbus elongatus DSM 2908 |
| Ga0586324 0025665 4276 5418 | 0.00 | 1543600.00 | 3174436.09 | 1795500.00 | 2111500.00 | 0.00 | 0.00 | 1683600.00 | 0.00 | 0.00 | 0.00 | 0.00 | KO:K00249 | acyl-CoA dehydrogenase | Bacteria;Proteobacteria;Deltaproteobacteria;Syntrophobacterales;Syntrophaceae;Smithella;Smithella sp. F21;Smithella sp. F21 |
| Ga0586324 0032054 3246 4511 | 0.00 | 0.00 | 0.00 | 0.00 | 0.00 | 0.00 | 0.00 | 0.00 | 0.00 | 0.00 | 1531200.00 | 0.00 | KO:K00249 | acyl-CoA dehydrogenase | Bacteria;Chloroflexi;Chloroflexia;Chloroflexales;Roseiflexaceae;Roseiflexus;Roseiflexus sp. RS-1;Roseiflexus sp. RS-1 |
| Ga0586324 0109383 1137 2423 | 0.00 | 0.00 | 0.00 | 11210000.00 | 0.00 | 0.00 | 0.00 | 199424.86 | 0.00 | 0.00 | 0.00 | 0.00 | KO:K00249 | acyl-CoA dehydrogenase | Bacteria;Proteobacteria;Gammaproteobacteria;Cellvibrionales;Spongiibacteraceae;unclassified Spongiibacteraceae;marine gamma proteobacterium HTCC2143;Marine gamma proteobacterium sp. HTCC2143 |
| Ga0586324 0120710 1722 2864 | 0.00 | 0.00 | 0.00 | 0.00 | 0.00 | 0.00 | 0.00 | 0.00 | 0.00 | 3018400.00 | 0.00 | 0.00 | KO:K00249 | acyl-CoA dehydrogenase | Bacteria;Proteobacteria;Deltaproteobacteria;Syntrophobacterales;Syntrophaceae;Smithella;Smithella sp. F21;Smithella sp. F21 |
| Ga0586324 0294655 1 990 | 0.00 | 0.00 | 0.00 | 0.00 | 1040300.00 | 0.00 | 0.00 | 0.00 | 0.00 | 0.00 | 0.00 | 0.00 | KO:K00249 | acyl-CoA dehydrogenase | Bacteria;Proteobacteria;Deltaproteobacteria;unclassified Deltaproteobacteria;unclassified Deltaproteobacteria;unclassified Deltaproteobacteria;delta proteobacterium NaphS2;delta proteobacterium NaphS2 |
| Ga0586324 0551615 37 1287 | 2050000.00 | 0.00 | 0.00 | 0.00 | 0.00 | 0.00 | 0.00 | 0.00 | 0.00 | 0.00 | 0.00 | 0.00 | KO:K00249 | acyl-CoA dehydrogenase | Bacteria;Chloroflexi;Chloroflexia;Chloroflexales;Roseiflexaceae;Roseiflexus;Roseiflexus sp. RS-1;Roseiflexus sp. RS-1 |
| Ga0586324 3899562 2 682 | 0.00 | 0.00 | 0.00 | 0.00 | 0.00 | 0.00 | 0.00 | 0.00 | 939600.00 | 0.00 | 0.00 | 0.00 | KO:K00249 | acyl-CoA dehydrogenase | Bacteria;Proteobacteria;Alphaproteobacteria;Hyphomicrobiales;unclassified Hyphomicrobiales;unclassified Hyphomicrobiales;Rhizobiales bacterium YIM 77505;Rhizobiales bacterium YIM 77505 |
| Ga0586324 0008898 9260 10444 | 0.00 | 0.00 | 0.00 | 0.00 | 0.00 | 0.00 | 0.00 | 0.00 | 0.00 | 0.00 | 10736000.00 | 0.00 | KO:K00253 | isovaleryl-CoA dehydrogenase | Bacteria;Proteobacteria;Deltaproteobacteria;Syntrophobacterales;Syntrophobacteraceae;Desulfacinum;Desulfacinum infernum;Desulfacinum infernum DSM 9756 |
| Ga0586324 0147090 954 2144 | 0.00 | 16252000.00 | 26517300.00 | 0.00 | 0.00 | 23820000.00 | 0.00 | 0.00 | 0.00 | 0.00 | 0.00 | 0.00 | KO:K00253 | isovaleryl-CoA dehydrogenase | Bacteria;Proteobacteria;Deltaproteobacteria;Syntrophobacterales;Syntrophobacteraceae;Syntrophobacter;Syntrophobacter fumaroxidans;Syntrophobacter fumaroxidans MPOB |
| Ga0586324 2656903 3 800 | 0.00 | 0.00 | 0.00 | 732181.15 | 0.00 | 549809.27 | 0.00 | 0.00 | 0.00 | 0.00 | 0.00 | 0.00 | KO:K00253 | isovaleryl-CoA dehydrogenase | Bacteria;Proteobacteria;Deltaproteobacteria;Syntrophobacterales;Syntrophobacteraceae;Desulfacinum;Desulfacinum hydrothermale;Desulfacinum hydrothermale DSM 13146 |
| Ga0586324 0093698 696 1982 | 0.00 | 0.00 | 0.00 | 3135000.00 | 2791300.00 | 0.00 | 3488400.00 | 0.00 | 0.00 | 0.00 | 0.00 | 0.00 | KO:K00260 | glutamate dehydrogenase | Bacteria;Calditrichaeota;Calditrichae;Calditrichales;Calditrichaceae;Caldithrix;Caldithrix abyssi;Caldithrix abyssi LF13, DSM 13497 |
| Ga0586324 0874120 3 860 | 0.00 | 0.00 | 0.00 | 0.00 | 2008500.00 | 0.00 | 0.00 | 0.00 | 0.00 | 0.00 | 0.00 | 0.00 | KO:K00260 | glutamate dehydrogenase | Bacteria;Chloroflexi;Anaerolineae;Anaerolineales;Anaerolineaceae;Thermanaerothrix;Thermanaerothrix daxensis;Thermanaerothrix daxensis GNS-1 |
| Ga0586324 1019905 1 570 | 0.00 | 0.00 | 0.00 | 0.00 | 0.00 | 0.00 | 0.00 | 1366400.00 | 1652400.00 | 0.00 | 1320000.00 | 0.00 | KO:K00260 | glutamate dehydrogenase | Bacteria;Chloroflexi;Anaerolineae;Anaerolineales;Anaerolineaceae;Levilinea;Levilinea saccharolytica;Levilinea saccharolytica DSM 16555 |
| Ga0586324 0008686 4351 5697 | 0.00 | 0.00 | 0.00 | 31663500.00 | 0.00 | 24931600.00 | 0.00 | 5849900.00 | 0.00 | 0.00 | 0.00 | 0.00 | KO:K00262 | glutamate dehydrogenase (NADP+) | Bacteria;Proteobacteria;Deltaproteobacteria;Desulfuromonadales;Geobacteraceae;Geobacter;Geobacter thiogenes;Geobacter thiogenes ATCC BAA-34 |
| Ga0586324 0011932 972 2309 | 0.00 | 0.00 | 0.00 | 0.00 | 1071200.00 | 0.00 | 0.00 | 0.00 | 0.00 | 10780000.00 | 0.00 | 13275000.00 | KO:K00262 | glutamate dehydrogenase (NADP+) | Bacteria;Bacteroidetes;Bacteroidia;Bacteroidales;Lentimicrobiaceae;Lentimicrobium;Lentimicrobium saccharophilum;Bacteroidales bacterium TBC1 |
| Ga0586324 0014023 3727 5064 | 0.00 | 0.00 | 0.00 | 0.00 | 0.00 | 3970000.00 | 0.00 | 969900.00 | 709378.56 | 0.00 | 0.00 | 0.00 | KO:K00262 | glutamate dehydrogenase (NADP+) | Bacteria;Bacteroidetes;Bacteroidia;Bacteroidales;unclassified Bacteroidales;unclassified Bacteroidales;Bacteroidales bacterium 6E;Bacteroidales bacterium 6E |
| Ga0586324 0028772 4653 5999 | 1390000.00 | 0.00 | 0.00 | 0.00 | 0.00 | 0.00 | 0.00 | 0.00 | 0.00 | 0.00 | 0.00 | 0.00 | KO:K00262 | glutamate dehydrogenase (NADP+) | Bacteria;Bacteroidetes;Bacteroidia;Bacteroidales;Lentimicrobiaceae;Lentimicrobium;Lentimicrobium saccharophilum;Bacteroidales bacterium TBC1 |
| Ga0586324 0030454 3949 5310 | 0.00 | 0.00 | 0.00 | 0.00 | 927896.10 | 0.00 | 6178800.00 | 0.00 | 1028700.00 | 0.00 | 0.00 | 234629.25 | KO:K00262 | glutamate dehydrogenase (NADP+) | Bacteria;Calditrichaeota;Calditrichae;Calditrichales;Calditrichaceae;Caldithrix;Caldithrix abyssi;Caldithrix abyssi LF13, DSM 13497 |

| | | | | | | | | | | | | | | | |
|---|---|---|---|---|---|---|---|---|---|---|---|---|---|---|---|
| Ga0586324 0032905 3921 5417 | 0.00 | 2284800.00 | 0.00 | 0.00 | 0.00 | 0.00 | 0.00 | 0.00 | 0.00 | 0.00 | 0.00 | 0.00 | KO:K00262 | glutamate dehydrogenase (NADP+) | Bacteria;Proteobacteria;Deltaproteobacteria;Syntrophorhabdales;Syntrophorhabdaceae;Syntrophorhabdus;Syntrophorhabdus aromaticivorans;Syntrophorhabdus aromaticivorans UI |
| Ga0586324 0068126 1 1056 | 0.00 | 593742.68 | 34849700.00 | 0.00 | 0.00 | 0.00 | 747356.64 | 0.00 | 0.00 | 0.00 | 0.00 | 0.00 | KO:K00262 | glutamate dehydrogenase (NADP+) | Bacteria;Proteobacteria;Deltaproteobacteria;Desulfobacterales;Desulfobacteraceae;Desulfatirhabdium;Desulfatirhabdium butyrativorans;Desulfatirhabdium butyrativorans DSM 18734 |
| Ga0586324 0092990 1497 2831 | 0.00 | 8704000.00 | 0.00 | 0.00 | 0.00 | 0.00 | 0.00 | 0.00 | 0.00 | 0.00 | 0.00 | 0.00 | KO:K00262 | glutamate dehydrogenase (NADP+) | Bacteria;Bacteroidetes;Bacteroidia;Bacteroidales;Lentimicrobiaceae;Lentimicrobium;Lentimicrobium saccharophilum;Bacteroidales bacterium TBC1 |
| Ga0586324 0111980 1845 3248 | 0.00 | 15096000.00 | 0.00 | 0.00 | 0.00 | 0.00 | 0.00 | 0.00 | 0.00 | 0.00 | 0.00 | 0.00 | KO:K00262 | glutamate dehydrogenase (NADP+) | Bacteria;Bacteroidetes;Bacteroidia;Bacteroidales;Lentimicrobiaceae;Lentimicrobium;Lentimicrobium saccharophilum;Bacteroidales bacterium TBC1 |
| Ga0586324 0179203 1063 2400 | 0.00 | 972400.00 | 0.00 | 0.00 | 0.00 | 0.00 | 0.00 | 0.00 | 28593000.00 | 37653000.00 | 0.00 | 0.00 | KO:K00262 | glutamate dehydrogenase (NADP+) | Bacteria;Verrucomicrobia;Opitutae;Opitutales;Opitutaceae;Opitutus;Opitutus terrae;Opitutus terrae PB90-1 |
| Ga0586324 0186054 1089 2438 | 1790000.00 | 0.00 | 1524784.16 | 0.00 | 440862.66 | 0.00 | 0.00 | 590950.92 | 0.00 | 0.00 | 0.00 | 0.00 | KO:K00262 | glutamate dehydrogenase (NADP+) | Bacteria;Proteobacteria;Deltaproteobacteria;Desulfobacterales;Desulfobacteraceae;Desulfosarcina;Desulfosarcina cetonica;Desulfosarcina cetonica JCM 12296 |
| Ga0586324 0248429 542 1915 | 0.00 | 0.00 | 0.00 | 0.00 | 0.00 | 0.00 | 0.00 | 0.00 | 198219.96 | 0.00 | 0.00 | 0.00 | KO:K00262 | glutamate dehydrogenase (NADP+) | Bacteria;Proteobacteria;Deltaproteobacteria;unclassified Deltaproteobacteria;unclassified Deltaproteobacteria;unclassified Deltaproteobacteria;delta proteobacterium MLMS-1;delta proteobacterium sp. MLMS-1 |
| Ga0586324 0336544 3 1196 | 850950.00 | 0.00 | 0.00 | 0.00 | 1133000.00 | 0.00 | 0.00 | 0.00 | 0.00 | 0.00 | 0.00 | 0.00 | KO:K00262 | glutamate dehydrogenase (NADP+) | Bacteria;Bacteroidetes;Bacteroidia;Bacteroidales;Lentimicrobiaceae;Lentimicrobium;Lentimicrobium saccharophilum;Bacteroidales bacterium TBC1 |
| Ga0586324 0352482 3 1364 | 65760000.00 | 2631600.00 | 2536906.15 | 0.00 | 0.00 | 0.00 | 7136400.00 | 1561600.00 | 2107815.21 | 2825900.00 | 2716736.88 | 0.00 | KO:K00262 | glutamate dehydrogenase (NADP+) | Bacteria;Bacteroidetes;Bacteroidia;Bacteroidales;unclassified Bacteroidales;unclassified Bacteroidales;Bacteroidales bacterium 6E;Bacteroidales bacterium 6E |
| Ga0586324 0465820 3 1052 | 0.00 | 26996000.00 | 0.00 | 0.00 | 0.00 | 0.00 | 0.00 | 0.00 | 0.00 | 0.00 | 0.00 | 0.00 | KO:K00262 | glutamate dehydrogenase (NADP+) | Bacteria;Candidatus Hydrogenedentes;unclassified Candidatus Hydrogenedentes;unclassified Candidatus Hydrogenedentes;unclassified Candidatus Hydrogenedentes;unclassified Candidatus Hydrogenedentes;Hydrogenedentes bacterium JGI 0000077-D07;Hydrogenedentes bacterium JGI 0000077-D07 (contamination screened) |
| Ga0586324 0500303 3 377 | 0.00 | 0.00 | 0.00 | 60135000.00 | 30694000.00 | 0.00 | 0.00 | 0.00 | 0.00 | 0.00 | 0.00 | 0.00 | KO:K00262 | glutamate dehydrogenase (NADP+) | Bacteria;Bacteroidetes;Bacteroidia;Bacteroidales;Lentimicrobiaceae;Lentimicrobium;Lentimicrobium saccharophilum;Bacteroidales bacterium TBC1 |
| Ga0586324 0555854 2 1294 | 845906.00 | 0.00 | 0.00 | 0.00 | 768591.15 | 0.00 | 1787241.84 | 0.00 | 2146500.00 | 0.00 | 1381600.00 | 0.00 | KO:K00262 | glutamate dehydrogenase (NADP+) | Bacteria;Bacteroidetes;Bacteroidia;Bacteroidales;Lentimicrobiaceae;Lentimicrobium;Lentimicrobium saccharophilum;Bacteroidales bacterium TBC1 |
| Ga0586324 0557853 3 1283 | 0.00 | 479123.24 | 0.00 | 978500.00 | 2058178.96 | 0.00 | 0.00 | 0.00 | 0.00 | 793100.00 | 770823.68 | 358455.00 | KO:K00262 | glutamate dehydrogenase (NADP+) | Bacteria;Bacteroidetes;Flavobacteriia;Flavobacteriales;Flavobacteriaceae;Dokdonia;Dokdonia sp. 4H-3-7-5;Dokdonia sp. 4H-3-7-5 |
| Ga0586324 0682672 3 845 | 0.00 | 0.00 | 0.00 | 4085000.00 | 93627000.00 | 8138500.00 | 0.00 | 0.00 | 0.00 | 0.00 | 0.00 | 0.00 | KO:K00262 | glutamate dehydrogenase (NADP+) | Bacteria;Ignavibacteriae;Ignavibacteria;Ignavibacteriales;Ignavibacteriaceae;Ignavibacterium;Ignavibacterium album;Ignavibacterium album Mat9-16, JCM 16511 |
| Ga0586324 0751368 171 1373 | 0.00 | 0.00 | 0.00 | 0.00 | 0.00 | 0.00 | 0.00 | 277658.58 | 0.00 | 0.00 | 0.00 | 0.00 | KO:K00262 | glutamate dehydrogenase (NADP+) | Bacteria;Proteobacteria;Deltaproteobacteria;Desulfuromonadales;Geobacteraceae;Geobacter;Geobacter sp. OR-1;Geobacter sp. OR-1 |
| Ga0586324 0906816 163 1266 | 0.00 | 0.00 | 0.00 | 0.00 | 0.00 | 0.00 | 12220800.00 | 0.00 | 0.00 | 0.00 | 0.00 | 0.00 | KO:K00262 | glutamate dehydrogenase (NADP+) | Bacteria;Proteobacteria;Alphaproteobacteria;Hyphomicrobiales;Reyranellaceae;Reyranella;Reyranella massiliensis;Reyranella massiliensis 521, DSM 23428 |
| Ga0586324 0929384 3 863 | 0.00 | 0.00 | 0.00 | 0.00 | 0.00 | 8972200.00 | 11331600.00 | 8174000.00 | 0.00 | 7530600.00 | 0.00 | 0.00 | KO:K00262 | glutamate dehydrogenase (NADP+) | Bacteria;Bacteroidetes;Bacteroidia;Bacteroidales;unclassified Bacteroidales;unclassified Bacteroidales;Bacteroidales bacterium 6E;Bacteroidales bacterium 6E |
| Ga0586324 0933455 3 1250 | 0.00 | 0.00 | 0.00 | 4702500.00 | 4356900.00 | 0.00 | 0.00 | 1579900.00 | 77193000.00 | 0.00 | 0.00 | 0.00 | KO:K00262 | glutamate dehydrogenase (NADP+) | Bacteria;Proteobacteria;Gammaproteobacteria;unclassified Gammaproteobacteria;Candidatus Competibacteraceae;Candidatus Competibacter;Candidatus Competibacter denitrificans;Candidatus Competibacter denitrificans Run A D11 |
| Ga0586324 1044948 3 1190 | 573375.00 | 0.00 | 0.00 | 0.00 | 0.00 | 0.00 | 0.00 | 0.00 | 0.00 | 0.00 | 189302.08 | 0.00 | KO:K00262 | glutamate dehydrogenase (NADP+) | Bacteria;Bacteroidetes;Bacteroidia;Bacteroidales;unclassified Bacteroidales;unclassified Bacteroidales;Bacteroidales bacterium 6E;Bacteroidales bacterium 6E |
| Ga0586324 1075850 3 845 | 0.00 | 0.00 | 0.00 | 3040000.00 | 0.00 | 0.00 | 0.00 | 0.00 | 0.00 | 0.00 | 0.00 | 0.00 | KO:K00262 | glutamate dehydrogenase (NADP+) | Bacteria;Proteobacteria;Gammaproteobacteria;unclassified Gammaproteobacteria;Candidatus Competibacteraceae;Candidatus Competibacter;Candidatus Competibacter denitrificans;Candidatus Competibacter denitrificans Run A D11 |
| Ga0586324 1369927 3 956 | 0.00 | 15300000.00 | 0.00 | 0.00 | 0.00 | 0.00 | 0.00 | 0.00 | 0.00 | 0.00 | 0.00 | 0.00 | KO:K00262 | glutamate dehydrogenase (NADP+) | Bacteria;Ignavibacteriae;Ignavibacteria;Ignavibacteriales;Ignavibacteriaceae;Ignavibacterium;Ignavibacterium album;Ignavibacterium album Mat9-16, JCM 16511 |
| Ga0586324 1584074 2 757 | 2670000.00 | 0.00 | 0.00 | 0.00 | 0.00 | 0.00 | 0.00 | 0.00 | 0.00 | 0.00 | 0.00 | 0.00 | KO:K00262 | glutamate dehydrogenase (NADP+) | Bacteria;Bacteroidetes;Bacteroidia;Bacteroidales;unclassified Bacteroidales;unclassified Bacteroidales;Bacteroidales bacterium 6E;Bacteroidales bacterium 6E |
| Ga0586324 1588273 3 995 | 1140000.00 | 0.00 | 0.00 | 0.00 | 0.00 | 0.00 | 0.00 | 0.00 | 0.00 | 0.00 | 0.00 | 0.00 | KO:K00262 | glutamate dehydrogenase (NADP+) | Bacteria;Bacteroidetes;Flavobacteriia;Flavobacteriales;Flavobacteriaceae;Tenacibaculum;Tenacibaculum adriaticum;Tenacibaculum adriaticum DSM 18961 |
| Ga0586324 1614471 1 990 | 0.00 | 0.00 | 0.00 | 0.00 | 2636800.00 | 2227368.50 | 0.00 | 0.00 | 0.00 | 0.00 | 0.00 | 0.00 | KO:K00262 | glutamate dehydrogenase (NADP+) | Bacteria;Ignavibacteriae;Ignavibacteria;Ignavibacteriales;Ignavibacteriaceae;Ignavibacterium;Ignavibacterium album;Ignavibacterium album Mat9-16, JCM 16511 |
| Ga0586324 1672889 1 975 | 0.00 | 15368000.00 | 0.00 | 0.00 | 0.00 | 0.00 | 0.00 | 0.00 | 3985200.00 | 0.00 | 0.00 | 0.00 | KO:K00262 | glutamate dehydrogenase (NADP+) | Bacteria;Candidatus Cloacimonetes;unclassified Candidatus Cloacimonetes;unclassified Candidatus Cloacimonetes;unclassified Candidatus Cloacimonetes;unclassified Candidatus Cloacimonetes;Cloacimonetes bacterium SCGC AAA252-M08;Cloacimonetes bacterium SCGC AAA252-M08 (contamination screened) |
| Ga0586324 1711341 3 965 | 0.00 | 0.00 | 335788.40 | 0.00 | 0.00 | 0.00 | 0.00 | 0.00 | 0.00 | 0.00 | 0.00 | 0.00 | KO:K00262 | glutamate dehydrogenase (NADP+) | Bacteria;Bacteroidetes;Flavobacteriia;Flavobacteriales;Flavobacteriaceae;Confluentibacter;Confluentibacter citreus;Confluentibacter citreus XJNY |
| Ga0586324 2062633 1 891 | 0.00 | 0.00 | 0.00 | 0.00 | 0.00 | 0.00 | 0.00 | 0.00 | 0.00 | 0.00 | 0.00 | 8550000.00 | KO:K00262 | glutamate dehydrogenase (NADP+) | Bacteria;Ignavibacteriae;Ignavibacteria;Ignavibacteriales;Melioribacteraceae;Melioribacter;Melioribacter roseus;Melioribacter roseus P3M |
| Ga0586324 2088401 1 888 | 0.00 | 0.00 | 0.00 | 0.00 | 4315700.00 | 0.00 | 0.00 | 0.00 | 0.00 | 0.00 | 0.00 | 0.00 | KO:K00262 | glutamate dehydrogenase (NADP+) | Bacteria;Calditrichaeota;Calditrichae;Calditrichales;Calditrichaceae;Caldithrix;Caldithrix abyssi;Caldithrix abyssi LF13, DSM 13497 |
| Ga0586324 2202616 3 866 | 74400000.00 | 850000.00 | 515893.23 | 950000.00 | 1256600.00 | 75033000.00 | 1652461.92 | 1982500.00 | 1004400.00 | 0.00 | 0.00 | 272833.50 | KO:K00262 | glutamate dehydrogenase (NADP+) | Bacteria;Bacteroidetes;Bacteroidia;Bacteroidales;Lentimicrobiaceae;Lentimicrobium;Lentimicrobium saccharophilum;Bacteroidales bacterium TBC1 |
| Ga0586324 2442559 1 831 | 0.00 | 0.00 | 0.00 | 2346500.00 | 0.00 | 0.00 | 0.00 | 7076000.00 | 0.00 | 7700000.00 | 10718400.00 | 105711.75 | KO:K00262 | glutamate dehydrogenase (NADP+) | Bacteria;Firmicutes;Clostridia;Eubacteriales;Oscillospiraceae;Faecalibacterium;Faecalibacterium prausnitzii;Faecalibacterium prausnitzii 2789STDY5834930 |
| Ga0586324 2699587 262 795 | 2090000.00 | 2094400.00 | 0.00 | 988000.00 | 0.00 | 333073.08 | 653404.68 | 1433500.00 | 947700.00 | 1155000.00 | 1214400.00 | 1042500.00 | KO:K00262 | glutamate dehydrogenase (NADP+) | Bacteria;Bacteroidetes;Bacteroidia;Bacteroidales;Lentimicrobiaceae;Lentimicrobium;Lentimicrobium saccharophilum;Bacteroidales bacterium TBC1 |
| Ga0586324 2895491 3 773 | 0.00 | 0.00 | 0.00 | 0.00 | 12257000.00 | 0.00 | 0.00 | 0.00 | 0.00 | 0.00 | 0.00 | 0.00 | KO:K00262 | glutamate dehydrogenase (NADP+) | Bacteria;Acidobacteria;Acidobacteriia;Acidobacteriales;Acidobacteriaceae;Candidatus Koribacter;Candidatus Koribacter versatilis;Candidatus Koribacter versatilis Ellin345 |
| Ga0586324 3319486 1 729 | 0.00 | 0.00 | 0.00 | 0.00 | 0.00 | 0.00 | 0.00 | 9516000.00 | 0.00 | 0.00 | 0.00 | 0.00 | KO:K00262 | glutamate dehydrogenase (NADP+) | Bacteria;Proteobacteria;Deltaproteobacteria;Syntrophobacterales;Syntrophaceae;Smithella;Smithella sp. SCADC;Smithella sp. SCADC |
| Ga0586324 3364032 3 725 | 0.00 | 0.00 | 0.00 | 6621500.00 | 0.00 | 0.00 | 0.00 | 0.00 | 0.00 | 0.00 | 0.00 | 0.00 | KO:K00262 | glutamate dehydrogenase (NADP+) | Bacteria;Bacteroidetes;Bacteroidia;Bacteroidales;unclassified Bacteroidales;unclassified Bacteroidales;Bacteroidales bacterium 6E;Bacteroidales bacterium 6E |
| Ga0586324 3387266 1 723 | 0.00 | 0.00 | 0.00 | 0.00 | 0.00 | 19135400.00 | 0.00 | 0.00 | 0.00 | 0.00 | 0.00 | 0.00 | KO:K00262 | glutamate dehydrogenase (NADP+) | Bacteria;Ignavibacteriae;Ignavibacteria;Ignavibacteriales;Ignavibacteriaceae;Ignavibacterium;Ignavibacterium album;Ignavibacterium album Mat9-16, JCM 16511 |
| Ga0586324 3634302 1 702 | 0.00 | 0.00 | 0.00 | 0.00 | 0.00 | 0.00 | 5335200.00 | 0.00 | 0.00 | 16170000.00 | 0.00 | 0.00 | KO:K00262 | glutamate dehydrogenase (NADP+) | Bacteria;Proteobacteria;Deltaproteobacteria;Desulfobacterales;Desulfobulbaceae;Desulfopila;Desulfopila aestuarii;Desulfopila aestuarii DSM 18488 |
| Ga0586324 3646517 1 702 | 0.00 | 0.00 | 0.00 | 0.00 | 0.00 | 0.00 | 0.00 | 0.00 | 0.00 | 0.00 | 0.00 | 5137500.00 | KO:K00262 | glutamate dehydrogenase (NADP+) | Bacteria;Proteobacteria;Deltaproteobacteria;Desulfobacterales;Desulfobacteraceae;Desulfosalsimonas;Desulfosalsimonas propionicica;Desulfosalsimonas propionicica DSM 17721 |
| Ga0586324 3767416 2 694 | 0.00 | 7276000.00 | 2842080.30 | 3306000.00 | 0.00 | 0.00 | 10579200.00 | 16280900.00 | 7808400.00 | 63240100.00 | 7972800.00 | 38400000.00 | KO:K00262 | glutamate dehydrogenase (NADP+) | Bacteria;Bacteroidetes;Bacteroidia;Bacteroidales;Lentimicrobiaceae;Lentimicrobium;Lentimicrobium saccharophilum;Bacteroidales bacterium TBC1 |
| Ga0586324 3823422 3 689 | 0.00 | 0.00 | 0.00 | 0.00 | 0.00 | 0.00 | 642358.08 | 0.00 | 0.00 | 0.00 | 0.00 | 0.00 | KO:K00262 | glutamate dehydrogenase (NADP+) | Bacteria;Bacteroidetes;Flavobacteriia;Flavobacteriales;Flavobacteriaceae;Maribacter;Maribacter cobaltidurans;Maribacter cobaltidurans B1 |
| Ga0586324 4145543 176 667 | 0.00 | 0.00 | 0.00 | 6365000.00 | 5943100.00 | 59153000.00 | 0.00 | 0.00 | 0.00 | 0.00 | 0.00 | 0.00 | KO:K00262 | glutamate dehydrogenase (NADP+) | Bacteria;Bacteroidetes;Bacteroidia;Bacteroidales;Lentimicrobiaceae;Lentimicrobium;Lentimicrobium saccharophilum;Bacteroidales bacterium TBC1 |
| Ga0586324 4172883 3 665 | 0.00 | 0.00 | 0.00 | 0.00 | 1421400.00 | 0.00 | 0.00 | 0.00 | 0.00 | 0.00 | 0.00 | 0.00 | KO:K00262 | glutamate dehydrogenase (NADP+) | Bacteria;Verrucomicrobia;Opitutae;Opitutales;Opitutaceae;Opitutus;Opitutus sp. ER46;Opitutus sp. ER46 |
| Ga0586324 4370703 86 652 | 0.00 | 0.00 | 0.00 | 0.00 | 49543000.00 | 0.00 | 0.00 | 0.00 | 0.00 | 0.00 | 0.00 | 0.00 | KO:K00262 | glutamate dehydrogenase (NADP+) | Bacteria;Bacteroidetes;Bacteroidia;Bacteroidales;Lentimicrobiaceae;Lentimicrobium;Lentimicrobium saccharophilum;Bacteroidales bacterium TBC1 |
| Ga0586324 4712227 2 631 | 46500000.00 | 35428000.00 | 52640500.00 | 22705000.00 | 21733000.00 | 56374000.00 | 34200000.00 | 0.00 | 26082000.00 | 0.00 | 16808000.00 | 25650000.00 | KO:K00262 | glutamate dehydrogenase (NADP+) | Bacteria;Bacteroidetes;Bacteroidia;Bacteroidales;Lentimicrobiaceae;Lentimicrobium;Lentimicrobium saccharophilum;Bacteroidales bacterium TBC1 |
| Ga0586324 4728710 1 525 | 0.00 | 0.00 | 0.00 | 0.00 | 0.00 | 834962.46 | 0.00 | 0.00 | 0.00 | 0.00 | 0.00 | 0.00 | KO:K00262 | glutamate dehydrogenase (NADP+) | Bacteria;Calditrichaeota;Calditrichae;Calditrichales;Calditrichaceae;Caldithrix;Caldithrix abyssi;Caldithrix abyssi LF13, DSM 13497 |
| Ga0586324 5345648 2 478 | 0.00 | 0.00 | 0.00 | 0.00 | 668354.64 | 0.00 | 0.00 | 719800.00 | 0.00 | 568025.92 | 58432000.00 | 421349.25 | KO:K00262 | glutamate dehydrogenase (NADP+) | Bacteria;Proteobacteria;Deltaproteobacteria;Desulfuromonadales;Geobacteraceae;Citrifermentans;Citrifermentans pelophilum;Geobacter pelophilus Dfr2 |
| Ga0586324 5752561 3 581 | 0.00 | 0.00 | 0.00 | 825642.15 | 362506.44 | 0.00 | 0.00 | 0.00 | 0.00 | 0.00 | 0.00 | 0.00 | KO:K00262 | glutamate dehydrogenase (NADP+) | Bacteria;Firmicutes;Clostridia;Eubacteriales;Peptococcaceae;unclassified Peptococcaceae;Peptococcaceae bacterium DCMF;Peptococcaceae bacterium DCMF |
| Ga0586324 5812287 2 568 | 498370.00 | 0.00 | 0.00 | 0.00 | 0.00 | 0.00 | 0.00 | 0.00 | 0.00 | 0.00 | 0.00 | 0.00 | KO:K00262 | glutamate dehydrogenase (NADP+) | Bacteria;Ignavibacteriae;Ignavibacteria;Ignavibacteriales;Melioribacteraceae;Melioribacter;Melioribacter roseus;Melioribacter roseus P3M |
| Ga0586324 5967626 2 574 | 0.00 | 2631600.00 | 0.00 | 0.00 | 0.00 | 0.00 | 0.00 | 0.00 | 0.00 | 0.00 | 0.00 | 0.00 | KO:K00262 | glutamate dehydrogenase (NADP+) | Bacteria;Acidobacteria;Acidobacteriia;Acidobacteriales;Acidobacteriaceae;Candidatus Koribacter;Candidatus Koribacter versatilis;Candidatus Koribacter versatilis Ellin345 |
| Ga0586324 6118036 1 567 | 0.00 | 0.00 | 0.00 | 0.00 | 257134.35 | 0.00 | 0.00 | 0.00 | 0.00 | 0.00 | 0.00 | 0.00 | KO:K00262 | glutamate dehydrogenase (NADP+) | Bacteria;Bacteroidetes;Bacteroidia;Bacteroidales;Lentimicrobiaceae;Lentimicrobium;Lentimicrobium saccharophilum;Bacteroidales bacterium TBC1 |
| Ga0586324 6514359 3 554 | 0.00 | 0.00 | 0.00 | 0.00 | 0.00 | 0.00 | 0.00 | 0.00 | 0.00 | 3180100.00 | 0.00 | 0.00 | KO:K00262 | glutamate dehydrogenase (NADP+) | Bacteria;Candidatus Cloacimonetes;unclassified Candidatus Cloacimonetes;unclassified Candidatus Cloacimonetes;unclassified Candidatus Cloacimonetes;unclassified Candidatus Cloacimonetes;Cloacimonetes bacterium JGI 0000014-K11;Cloacimonetes bacterium JGI 0000014-K11 (contamination screened) |
| Ga0586324 6677739 1 546 | 0.00 | 70040000.00 | 0.00 | 0.00 | 0.00 | 0.00 | 0.00 | 0.00 | 0.00 | 0.00 | 0.00 | 0.00 | KO:K00262 | glutamate dehydrogenase (NADP+) | Bacteria;Bacteroidetes;Bacteroidia;Bacteroidales;Lentimicrobiaceae;Lentimicrobium;Lentimicrobium saccharophilum;Bacteroidales bacterium TBC1 |
| Ga0586324 6832207 160 543 | 1130000.00 | 539293.72 | 0.00 | 0.00 | 0.00 | 0.00 | 0.00 | 817400.00 | 0.00 | 0.00 | 0.00 | 0.00 | KO:K00262 | glutamate dehydrogenase (NADP+) | Bacteria;Calditrichaeota;Calditrichae;Calditrichales;Calditrichaceae;Caldithrix;Caldithrix abyssi;Caldithrix abyssi LF13, DSM 13497 |
| Ga0586324 7233843 2 529 | 0.00 | 0.00 | 0.00 | 0.00 | 768163.70 | 0.00 | 0.00 | 0.00 | 0.00 | 0.00 | 0.00 | 0.00 | KO:K00262 | glutamate dehydrogenase (NADP+) | Bacteria;Acidobacteria;Acidobacteriia;Acidobacteriales;Acidobacteriaceae;Candidatus Koribacter;Candidatus Koribacter versatilis;Candidatus Koribacter versatilis Ellin345 |
| Ga0586324 7433660 1 522 | 0.00 | 0.00 | 2009938.15 | 0.00 | 0.00 | 0.00 | 0.00 | 0.00 | 0.00 | 1678600.00 | 0.00 | 0.00 | KO:K00262 | glutamate dehydrogenase (NADP+) | Bacteria;Bacteroidetes;Flavobacteriia;Flavobacteriales;Flavobacteriaceae;Tenacibaculum;Tenacibaculum caenipelagi;Tenacibaculum caenipelagi CECT 8283 |
| Ga0586324 0000001 156184 160662 | 0.00 | 0.00 | 0.00 | 0.00 | 0.00 | 0.00 | 1151393.16 | 2107368.83 | 0.00 | 14614600.00 | 7040000.00 | 17827500.00 | KO:K00265 | glutamate synthase (NADPH/NADH) large chain | Bacteria;Proteobacteria;Betaproteobacteria;Nitrosomonadales;Thiobacillaceae;Annwoodia;Annwoodia aquaesulis;Annwoodia aquaesulis DSM 4255 |
| Ga0586324 0020713 2689 7173 | 0.00 | 0.00 | 0.00 | 0.00 | 0.00 | 0.00 | 183150.58 | 1427400.00 | 6147900.00 | 1393700.00 | 0.00 | 437656.50 | KO:K00265 | glutamate synthase (NADPH/NADH) large chain | Bacteria;Proteobacteria;Betaproteobacteria;Nitrosomonadales;Thiobacillaceae;Thiobacillus;Thiobacillus thioparus;Thiobacillus thioparus DSM 505 |
| Ga0586324 0022846 2653 6981 | 0.00 | 0.00 | 0.00 | 0.00 | 0.00 | 0.00 | 211229.92 | 0.00 | 0.00 | 0.00 | 0.00 | 0.00 | KO:K00265 | glutamate synthase (NADPH/NADH) large chain | Bacteria;Armatimonadetes;Chthonomonadetes;Chthonomonadales;Chthonomonadaceae;Chthonomonas;Chthonomonas calidirosea;Chthonomonas calidirosea T49, DSM 23976 |
| Ga0586324 0067693 3 2561 | 0.00 | 0.00 | 0.00 | 0.00 | 0.00 | 0.00 | 0.00 | 0.00 | 0.00 | 0.00 | 0.00 | 1350000.00 | KO:K00265 | glutamate synthase (NADPH/NADH) large chain | Bacteria;Proteobacteria;Betaproteobacteria;Burkholderiales;Oxalobacteraceae;Herbaspirillum;Herbaspirillum sp. K1R23-30;Herbaspirillum sp. K1R23-30 |
| Ga0586324 0093241 2 3223 | 0.00 | 0.00 | 5911500.00 | 0.00 | 4223000.00 | 0.00 | 0.00 | 0.00 | 0.00 | 0.00 | 0.00 | 0.00 | KO:K00265 | glutamate synthase (NADPH/NADH) large chain | Bacteria;Proteobacteria;Alphaproteobacteria;Hyphomicrobiales;unclassified Hyphomicrobiales;Methyloceanibacter;Methyloceanibacter superfactus;Methyloceanibacter superfactus R-67175 |
| Ga0586324 0673587 1 1440 | 0.00 | 0.00 | 0.00 | 0.00 | 0.00 | 0.00 | 0.00 | 5569300.00 | 0.00 | 0.00 | 0.00 | 0.00 | KO:K00265 | glutamate synthase (NADPH/NADH) large chain | Bacteria;Proteobacteria;Betaproteobacteria;Burkholderiales;unclassified Burkholderiales;Methylibium;Methylibium petroleiphilum;Methylibium petroleiphilum PM1 |
| Ga0586324 1216031 1 1116 | 0.00 | 1244400.00 | 0.00 | 0.00 | 0.00 | 0.00 | 0.00 | 0.00 | 0.00 | 0.00 | 0.00 | 0.00 | KO:K00265 | glutamate synthase (NADPH/NADH) large chain | Bacteria;Proteobacteria;Betaproteobacteria;unclassified Betaproteobacteria;unclassified Betaproteobacteria;unclassified Betaproteobacteria;Betaproteobacteria bacterium UKL13-2;Betaproteobacteria bacterium UKL13-2 |
| Ga0586324 1248798 2 1105 | 0.00 | 0.00 | 0.00 | 0.00 | 0.00 | 0.00 | 0.00 | 0.00 | 0.00 | 0.00 | 0.00 | 389169.00 | KO:K00265 | glutamate synthase (NADPH/NADH) large chain | Bacteria;Proteobacteria;Betaproteobacteria;Rhodocyclales;Azonexaceae;Dechloromonas;Dechloromonas agitata;Dechloromonas agitata is5 |
| Ga0586324 1405554 2 1048 | 0.00 | 0.00 | 0.00 | 0.00 | 0.00 | 0.00 | 0.00 | 0.00 | 1579500.00 | 0.00 | 0.00 | 0.00 | KO:K00265 | glutamate synthase (NADPH/NADH) large chain | Bacteria;Proteobacteria;Betaproteobacteria;Rhodocyclales;Azonexaceae;Dechloromonas;Dechloromonas sp. SG708;Dechloromonas sp. SG708 |
| Ga0586324 2252900 1 858 | 0.00 | 0.00 | 0.00 | 0.00 | 0.00 | 0.00 | 0.00 | 4507900.00 | 0.00 | 0.00 | 0.00 | 0.00 | KO:K00265 | glutamate synthase (NADPH/NADH) large chain | Bacteria;Proteobacteria;Alphaproteobacteria;Hyphomicrobiales;Xanthobacteraceae;Pseudolabrys;Pseudolabrys taiwanensis;Pseudolabrys taiwanensis CC-BB4 |
| Ga0586324 7429040 1 522 | 4070000.00 | 0.00 | 0.00 | 0.00 | 0.00 | 0.00 | 0.00 | 0.00 | 0.00 | 0.00 | 0.00 | 0.00 | KO:K00265 | glutamate synthase (NADPH/NADH) large chain | Bacteria;Proteobacteria;Gammaproteobacteria;Methylococcales;Methylococcaceae;Methylobacter;Methylobacter tundripaludum;Methylobacter tundripaludum OWC-DMM |
| Ga0586324 0167176 1775 2689 | 0.00 | 0.00 | 0.00 | 502386.60 | 0.00 | 0.00 | 0.00 | 0.00 | 0.00 | 0.00 | 0.00 | 0.00 | KO:K00266 | glutamate synthase (NADPH/NADH) small chain | Bacteria;Proteobacteria;Deltaproteobacteria;Desulfuromonadales;Geobacteraceae;Geobacter;Geobacter argillaceus;Geobacter argillaceus ATCC BAA-1139 |
| Ga0586324 0200422 141 1559 | 0.00 | 5827600.00 | 0.00 | 0.00 | 0.00 | 0.00 | 0.00 | 0.00 | 0.00 | 0.00 | 0.00 | 0.00 | KO:K00266 | glutamate synthase (NADPH/NADH) small chain | Bacteria;Proteobacteria;Deltaproteobacteria;Syntrophobacterales;Syntrophaceae;Desulfobacca;Desulfobacca acetoxidans;Desulfobacca acetoxidans ASRB2, DSM 11109 |
| Ga0586324 0751029 214 1374 | 0.00 | 5093200.00 | 0.00 | 3904500.00 | 0.00 | 0.00 | 0.00 | 0.00 | 0.00 | 0.00 | 0.00 | 0.00 | KO:K00266 | glutamate synthase (NADPH/NADH) small chain | Bacteria;Planctomycetes;Planctomycetia;Pirellulales;Pirellulaceae;Pirellula;Pirellula staleyi;Pirellula staleyi DSM 6068 |
| Ga0586324 0756407 2 1090 | 0.00 | 0.00 | 0.00 | 5348500.00 | 6046100.00 | 6431400.00 | 0.00 | 0.00 | 0.00 | 0.00 | 0.00 | 0.00 | KO:K00266 | glutamate synthase (NADPH/NADH) small chain | Bacteria;Proteobacteria;Deltaproteobacteria;Syntrophobacterales;Syntrophaceae;Desulfobacca;Desulfobacca acetoxidans;Desulfobacca acetoxidans ASRB2, DSM 11109 |
| Ga0586324 0848450 763 1302 | 0.00 | 0.00 | 0.00 | 22610000.00 | 0.00 | 0.00 | 0.00 | 13725000.00 | 0.00 | 0.00 | 0.00 | 0.00 | KO:K00266 | glutamate synthase (NADPH/NADH) small chain | Bacteria;Proteobacteria;Deltaproteobacteria;Syntrophobacterales;Syntrophaceae;Desulfobacca;Desulfobacca acetoxidans;Desulfobacca acetoxidans ASRB2, DSM 11109 |
| Ga0586324 2184694 3 869 | 0.00 | 0.00 | 0.00 | 0.00 | 0.00 | 1199535.50 | 0.00 | 0.00 | 0.00 | 0.00 | 0.00 | 0.00 | KO:K00266 | glutamate synthase (NADPH/NADH) small chain | Bacteria;Proteobacteria;Deltaproteobacteria;Desulfobacterales;Desulfobacteraceae;Desulfatirhabdium;Desulfatirhabdium butyrativorans;Desulfatirhabdium butyrativorans DSM 18734 |
| Ga0586324 0000004 109487 110935 | 0.00 | 0.00 | 0.00 | 0.00 | 0.00 | 0.00 | 3670800.00 | 2976800.00 | 1409400.00 | 0.00 | 5438400.00 | 4642500.00 | KO:K00283 | glycine dehydrogenase subunit 2 | Bacteria;Proteobacteria;Betaproteobacteria;Nitrosomonadales;Thiobacillaceae;Annwoodia;Annwoodia aquaesulis;Annwoodia aquaesulis DSM 4255 |
| Ga0586324 0023337 519 2012 | 0.00 | 0.00 | 0.00 | 0.00 | 0.00 | 0.00 | 0.00 | 0.00 | 0.00 | 0.00 | 994400.00 | 0.00 | KO:K00283 | glycine dehydrogenase subunit 2 | Bacteria;Firmicutes;Clostridia;Eubacteriales;Clostridiales Family XVI. Incertae Sedis;Carboxydocella;Carboxydocella sporoproducens;Carboxydocella sporoproducens DSM 16521 |
| Ga0586324 7044560 1 528 | 0.00 | 0.00 | 0.00 | 0.00 | 0.00 | 0.00 | 0.00 | 0.00 | 0.00 | 0.00 | 0.00 | 6150000.00 | KO:K00286 | pyrroline-5-carboxylate reductase | Bacteria;Proteobacteria;Gammaproteobacteria;Methylococcales;Methylococcaceae;Methylobacter;Methylobacter tundripaludum;Methylobacter tundripaludum 31/32 |
| Ga0586324 0010707 6837 8867 | 0.00 | 0.00 | 0.00 | 0.00 | 0.00 | 0.00 | 467751.12 | 0.00 | 0.00 | 0.00 | 0.00 | 0.00 | KO:K00288 | methylenetetrahydrofolate dehydrogenase (NADP+)/methenyltetrahydrofolate cyclohydrolase/f | Bacteria;Chloroflexi;Anaerolineae;Anaerolineales;Anaerolineaceae;Levilinea;Levilinea saccharolytica;Levilinea saccharolytica DSM 16555 |
| Ga0586324 0021358 3 1469 | 11200000.00 | 0.00 | 0.00 | 0.00 | 0.00 | 0.00 | 0.00 | 0.00 | 0.00 | 0.00 | 0.00 | 0.00 | KO:K00288 | methylenetetrahydrofolate dehydrogenase (NADP+)/methenyltetrahydrofolate cyclohydrolase/f | Bacteria;Chloroflexi;Anaerolineae;Anaerolineales;Anaerolineaceae;Anaerolinea;Anaerolinea thermophila;Anaerolinea thermophila UNI-1 |
| Ga0586324 0054348 2 1690 | 7460000.00 | 0.00 | 3484046.68 | 0.00 | 0.00 | 174283000.00 | 152076000.00 | 0.00 | 3620700.00 | 0.00 | 0.00 | 0.00 | KO:K00288 | methylenetetrahydrofolate dehydrogenase (NADP+)/methenyltetrahydrofolate cyclohydrolase/f | Bacteria;Proteobacteria;Deltaproteobacteria;Syntrophobacterales;Syntrophobacteraceae;Thermodesulforhabdus;Thermodesulforhabdus norvegica;Thermodesulforhabdus norvegica DSM 9990 |
| Ga0586324 0072455 1 1383 | 0.00 | 0.00 | 0.00 | 0.00 | 0.00 | 0.00 | 0.00 | 0.00 | 111384.72 | 0.00 | 0.00 | 0.00 | KO:K00288 | methylenetetrahydrofolate dehydrogenase (NADP+)/methenyltetrahydrofolate cyclohydrolase/f | Bacteria;Chloroflexi;Anaerolineae;Anaerolineales;Anaerolineaceae;Thermanaerothrix;Thermanaerothrix daxensis;Thermanaerothrix daxensis GNS-1 |
| Ga0586324 0323763 272 1987 | 0.00 | 0.00 | 0.00 | 0.00 | 0.00 | 0.00 | 0.00 | 0.00 | 0.00 | 4981900.00 | 0.00 | 0.00 | KO:K00288 | methylenetetrahydrofolate dehydrogenase (NADP+)/methenyltetrahydrofolate cyclohydrolase/f | Bacteria;Chloroflexi;Anaerolineae;Anaerolineales;Anaerolineaceae;Anaerolinea;Anaerolinea thermophila;Anaerolinea thermophila UNI-1 |
| Ga0586324 1052693 1 981 | 0.00 | 378590.00 | 0.00 | 1273000.00 | 2049700.00 | 0.00 | 0.00 | 1830000.00 | 0.00 | 0.00 | 0.00 | 0.00 | KO:K00288 | methylenetetrahydrofolate dehydrogenase (NADP+)/methenyltetrahydrofolate cyclohydrolase/f | Bacteria;Proteobacteria;Deltaproteobacteria;Syntrophobacterales;Syntrophaceae;Desulfobacca;Desulfobacca acetoxidans;Desulfobacca acetoxidans ASRB2, DSM 11109 |
| Ga0586324 1465804 1 1029 | 0.00 | 0.00 | 0.00 | 0.00 | 0.00 | 815469.76 | 0.00 | 0.00 | 0.00 | 0.00 | 0.00 | 0.00 | KO:K00288 | methylenetetrahydrofolate dehydrogenase (NADP+)/methenyltetrahydrofolate cyclohydrolase/f | Bacteria;Chloroflexi;Anaerolineae;Anaerolineales;Anaerolineaceae;Thermanaerothrix;Thermanaerothrix daxensis;Thermanaerothrix daxensis GNS-1 |
| Ga0586324 1648706 231 980 | 0.00 | 0.00 | 0.00 | 0.00 | 0.00 | 0.00 | 0.00 | 0.00 | 0.00 | 0.00 | 0.00 | 216776.25 | KO:K00288 | methylenetetrahydrofolate dehydrogenase (NADP+)/methenyltetrahydrofolate cyclohydrolase/f | Bacteria;Chloroflexi;Anaerolineae;Anaerolineales;Anaerolineaceae;Anaerolinea;Anaerolinea thermophila;Anaerolinea thermophila UNI-1 |
| Ga0586324 2298868 2 853 | 55000000.00 | 0.00 | 0.00 | 102600000.00 | 0.00 | 0.00 | 0.00 | 0.00 | 80352000.00 | 0.00 | 0.00 | 0.00 | KO:K00288 | methylenetetrahydrofolate dehydrogenase (NADP+)/methenyltetrahydrofolate cyclohydrolase/f | Bacteria;Proteobacteria;Deltaproteobacteria;Syntrophobacterales;Syntrophobacteraceae;Syntrophobacter;Syntrophobacter fumaroxidans;Syntrophobacter fumaroxidans MPOB |
| Ga0586324 2936669 3 767 | 6300000.00 | 0.00 | 0.00 | 0.00 | 1421400.00 | 8178200.00 | 0.00 | 0.00 | 0.00 | 0.00 | 0.00 | 1380000.00 | KO:K00288 | methylenetetrahydrofolate dehydrogenase (NADP+)/methenyltetrahydrofolate cyclohydrolase/f | Bacteria;Proteobacteria;Deltaproteobacteria;Syntrophobacterales;Syntrophaceae;Desulfobacca;Desulfobacca acetoxidans;Desulfobacca acetoxidans ASRB2, DSM 11109 |
| Ga0586324 3752408 1 693 | 0.00 | 0.00 | 4241276.05 | 0.00 | 0.00 | 0.00 | 3420000.00 | 6893000.00 | 0.00 | 0.00 | 0.00 | 0.00 | KO:K00288 | methylenetetrahydrofolate dehydrogenase (NADP+)/methenyltetrahydrofolate cyclohydrolase/f | Bacteria;Proteobacteria;Deltaproteobacteria;Syntrophobacterales;Syntrophaceae;Desulfobacca;Desulfobacca acetoxidans;Desulfobacca acetoxidans ASRB2, DSM 11109 |
| Ga0586324 4060651 1 672 | 0.00 | 0.00 | 0.00 | 0.00 | 10815000.00 | 0.00 | 0.00 | 0.00 | 0.00 | 0.00 | 0.00 | 0.00 | KO:K00288 | methylenetetrahydrofolate dehydrogenase (NADP+)/methenyltetrahydrofolate cyclohydrolase/f | Bacteria;Proteobacteria;Deltaproteobacteria;Syntrophobacterales;Syntrophobacteraceae;Syntrophobacter;Syntrophobacter fumaroxidans;Syntrophobacter fumaroxidans MPOB |
| Ga0586324 4285021 1 657 | 0.00 | 0.00 | 0.00 | 15010000.00 | 14626000.00 | 0.00 | 0.00 | 0.00 | 4114800.00 | 0.00 | 0.00 | 0.00 | KO:K00288 | methylenetetrahydrofolate dehydrogenase (NADP+)/methenyltetrahydrofolate cyclohydrolase/f | Bacteria;Proteobacteria;Deltaproteobacteria;Desulfobacterales;Desulfobacteraceae;Desulfatirhabdium;Desulfatirhabdium butyrativorans;Desulfatirhabdium butyrativorans DSM 18734 |
| Ga0586324 5898678 2 376 | 0.00 | 0.00 | 0.00 | 0.00 | 0.00 | 756995.63 | 0.00 | 0.00 | 0.00 | 0.00 | 0.00 | 0.00 | KO:K00288 | methylenetetrahydrofolate dehydrogenase (NADP+)/methenyltetrahydrofolate cyclohydrolase/f | Bacteria;Chloroflexi;Anaerolineae;Anaerolineales;Anaerolineaceae;Pelolinea;Pelolinea submarina;Pelolinea submarina DSM 23923 |
| Ga0586324 6204116 93 563 | 0.00 | 0.00 | 0.00 | 0.00 | 0.00 | 1201615.78 | 0.00 | 0.00 | 0.00 | 0.00 | 0.00 | 0.00 | KO:K00288 | methylenetetrahydrofolate dehydrogenase (NADP+)/methenyltetrahydrofolate cyclohydrolase/f | Bacteria;Proteobacteria;Deltaproteobacteria;Syntrophobacterales;Syntrophobacteraceae;Desulfoglaeba;Desulfoglaeba alkanexedens;Desulfoglaeba alkanexedens ALDC |
| Ga0586324 8106829 3 506 | 0.00 | 27744000.00 | 52021200.00 | 0.00 | 2152700.00 | 136568000.00 | 22800000.00 | 0.00 | 50463000.00 | 0.00 | 7559200.00 | 0.00 | KO:K00288 | methylenetetrahydrofolate dehydrogenase (NADP+)/methenyltetrahydrofolate cyclohydrolase/f | Bacteria;Proteobacteria;Deltaproteobacteria;Syntrophobacterales;Syntrophobacteraceae;Syntrophobacter;Syntrophobacter fumaroxidans;Syntrophobacter fumaroxidans MPOB |
| Ga0586324 0246928 1238 2245 | 0.00 | 0.00 | 0.00 | 0.00 | 0.00 | 0.00 | 0.00 | 0.00 | 1312200.00 | 0.00 | 0.00 | 0.00 | KO:K00297 | methylenetetrahydrofolate reductase (NADPH) | Bacteria;Proteobacteria;Deltaproteobacteria;Syntrophobacterales;Syntrophobacteraceae;Syntrophobacter;Syntrophobacter fumaroxidans;Syntrophobacter fumaroxidans MPOB |
| Ga0586324 0889749 359 1276 | 0.00 | 0.00 | 0.00 | 0.00 | 0.00 | 1173865.48 | 1907099.16 | 1268800.00 | 0.00 | 0.00 | 0.00 | 0.00 | KO:K00297 | methylenetetrahydrofolate reductase (NADPH) | Bacteria;Proteobacteria;Deltaproteobacteria;Syntrophobacterales;Syntrophaceae;Desulfobacca;Desulfobacca acetoxidans;Desulfobacca acetoxidans ASRB2, DSM 11109 |
| Ga0586324 2295482 3 851 | 0.00 | 0.00 | 0.00 | 0.00 | 0.00 | 0.00 | 0.00 | 0.00 | 801488.52 | 0.00 | 0.00 | 0.00 | KO:K00297 | methylenetetrahydrofolate reductase (NADPH) | Bacteria;Proteobacteria;Deltaproteobacteria;Syntrophobacterales;Syntrophobacteraceae;Thermodesulforhabdus;Thermodesulforhabdus norvegica;Thermodesulforhabdus norvegica DSM 9990 |
| Ga0586324 2690226 1 798 | 0.00 | 0.00 | 0.00 | 1387000.00 | 0.00 | 0.00 | 0.00 | 0.00 | 0.00 | 0.00 | 0.00 | 0.00 | KO:K00297 | methylenetetrahydrofolate reductase (NADPH) | Bacteria;Proteobacteria;Deltaproteobacteria;Desulfobacterales;Desulfobulbaceae;Desulfoprunum;Desulfoprunum benzoelyticum;Desulfoprunum benzoelyticum DSM 28570 |
| Ga0586324 0007546 3786 4625 | 677922.00 | 0.00 | 0.00 | 0.00 | 0.00 | 0.00 | 0.00 | 0.00 | 0.00 | 0.00 | 0.00 | 0.00 | KO:K00319 | methylenetetrahydromethanopterin dehydrogenase | Archaea;Euryarchaeota;Methanomicrobia;Methanomicrobiales;Methanoregulaceae;Methanoregula;Methanoregula boonei;Methanoregula boonei 6A8 |
| Ga0586324 0010839 6085 6924 | 5930000.00 | 22780000.00 | 0.00 | 0.00 | 0.00 | 0.00 | 0.00 | 0.00 | 0.00 | 8008000.00 | 8245600.00 | 0.00 | KO:K00319 | methylenetetrahydromethanopterin dehydrogenase | Archaea;Euryarchaeota;Methanomicrobia;Methanomicrobiales;Methanoregulaceae;Methanoregula;Methanoregula boonei;Methanoregula boonei 6A8 |
| Ga0586324 0027622 464 1303 | 0.00 | 0.00 | 0.00 | 0.00 | 0.00 | 0.00 | 0.00 | 0.00 | 0.00 | 15092000.00 | 0.00 | 0.00 | KO:K00319 | methylenetetrahydromethanopterin dehydrogenase | Archaea;Euryarchaeota;Methanomicrobia;Methanomicrobiales;Methanoregulaceae;Methanoregula;Methanoregula formicica;Methanoregula formicica SMSP, DSM 22288 |
| Ga0586324 0031012 3875 4714 | 2380000.00 | 8098800.00 | 2440818.94 | 5985000.00 | 0.00 | 527636.82 | 9712800.00 | 4403650.39 | 3645000.00 | 0.00 | 9944000.00 | 2032500.00 | KO:K00319 | methylenetetrahydromethanopterin dehydrogenase | Archaea;Euryarchaeota;Methanomicrobia;Methanomicrobiales;Methanoregulaceae;Methanoregula;Methanoregula boonei;Methanoregula boonei 6A8 |
| Ga0586324 0101723 3297 3401 | 11400000.00 | 0.00 | 0.00 | 0.00 | 0.00 | 0.00 | 0.00 | 0.00 | 0.00 | 0.00 | 0.00 | 0.00 | KO:K00319 | methylenetetrahydromethanopterin dehydrogenase | Archaea;Euryarchaeota;Methanomicrobia;Methanomicrobiales;Methanoregulaceae;Methanoregula;Methanoregula formicica;Methanoregula formicica SMSP, DSM 22288 |
| Ga0586324 0219196 245 1087 | 0.00 | 5630400.00 | 0.00 | 0.00 | 0.00 | 0.00 | 0.00 | 0.00 | 0.00 | 0.00 | 0.00 | 0.00 | KO:K00319 | methylenetetrahydromethanopterin dehydrogenase | Archaea;Euryarchaeota;Methanomicrobia;Methanomicrobiales;Methanocorpusculaceae;Methanocorpusculum;Methanocorpusculum bavaricum;Methanocorpusculum bavaricum DSM 4179 |
| Ga0586324 0223906 797 1639 | 16500000.00 | 631222.92 | 2559600.68 | 15874500.00 | 6365400.00 | 3730215.97 | 0.00 | 2013000.00 | 2081700.00 | 5982900.00 | 1223200.00 | 0.00 | KO:K00319 | methylenetetrahydromethanopterin dehydrogenase | Archaea;Euryarchaeota;Methanomicrobia;Methanomicrobiales;Methanomicrobiaceae;Methanofollis;Methanofollis liminatans;Methanofollis liminatans GKZPZ, DSM 4140 |
| Ga0586324 0317102 257 1096 | 6830000.00 | 4957200.00 | 3218834.27 | 0.00 | 3862500.00 | 3040984.27 | 0.00 | 3586800.00 | 1190700.00 | 870100.00 | 0.00 | 1605000.00 | KO:K00319 | methylenetetrahydromethanopterin dehydrogenase | Archaea;Euryarchaeota;Methanomicrobia;Methanomicrobiales;Methanoregulaceae;Methanoregula;Methanoregula boonei;Methanoregula boonei 6A8 |
| Ga0586324 0342133 1 525 | 30700000.00 | 0.00 | 33273300.00 | 25745000.00 | 21527000.00 | 0.00 | 0.00 | 0.00 | 0.00 | 0.00 | 0.00 | 24075000.00 | KO:K00319 | methylenetetrahydromethanopterin dehydrogenase | Archaea;Euryarchaeota;Methanomicrobia;Methanomicrobiales;Methanoregulaceae;Methanoregula;Methanoregula boonei;Methanoregula boonei 6A8 |
| Ga0586324 0408587 447 1289 | 6550000.00 | 0.00 | 11034800.00 | 10260000.00 | 11227000.00 | 0.00 | 0.00 | 0.00 | 0.00 | 0.00 | 0.00 | 0.00 | KO:K00319 | methylenetetrahydromethanopterin dehydrogenase | Archaea;Euryarchaeota;Methanomicrobia;Methanomicrobiales;Methanomicrobiaceae;Methanofollis;Methanofollis ethanolicus;Methanofollis ethanolicus JCM 15103 |
| Ga0586324 0508055 486 1325 | 0.00 | 2543200.00 | 0.00 | 0.00 | 520163.39 | 0.00 | 0.00 | 1024800.00 | 0.00 | 0.00 | 0.00 | 3480000.00 | KO:K00319 | methylenetetrahydromethanopterin dehydrogenase | Archaea;Euryarchaeota;Methanomicrobia;Methanomicrobiales;Methanoregulaceae;Methanoregula;Methanoregula boonei;Methanoregula boonei 6A8 |
| Ga0586324 1245046 2 787 | 0.00 | 70720000.00 | 0.00 | 0.00 | 0.00 | 0.00 | 0.00 | 0.00 | 0.00 | 0.00 | 19536000.00 | 0.00 | KO:K00319 | methylenetetrahydromethanopterin dehydrogenase | Archaea;Euryarchaeota;Methanomicrobia;Methanomicrobiales;Methanoregulaceae;Methanoregula;Methanoregula boonei;Methanoregula boonei 6A8 |
| Ga0586324 2207042 3 626 | 3330000.00 | 0.00 | 0.00 | 0.00 | 0.00 | 0.00 | 1631385.60 | 1207800.00 | 2899800.00 | 1247400.00 | 0.00 | 0.00 | KO:K00319 | methylenetetrahydromethanopterin dehydrogenase | Archaea;Euryarchaeota;Methanomicrobia;Methanomicrobiales;Methanoregulaceae;Methanoregula;Methanoregula boonei;Methanoregula boonei 6A8 |
| Ga0586324 2629042 1 714 | 0.00 | 2380000.00 | 0.00 | 0.00 | 0.00 | 0.00 | 0.00 | 15189000.00 | 0.00 | 0.00 | 0.00 | 0.00 | KO:K00319 | methylenetetrahydromethanopterin dehydrogenase | Archaea;Euryarchaeota;Methanomicrobia;Methanomicrobiales;Methanoregulaceae;Methanoregula;Methanoregula formicica;Methanoregula formicica SMSP, DSM 22288 |
| Ga0586324 3737181 6 695 | 0.00 | 0.00 | 0.00 | 0.00 | 0.00 | 0.00 | 0.00 | 0.00 | 0.00 | 1901900.00 | 0.00 | 0.00 | KO:K00319 | methylenetetrahydromethanopterin dehydrogenase | Archaea;Euryarchaeota;Methanomicrobia;Methanomicrobiales;Methanoregulaceae;Methanoregula;Methanoregula formicica;Methanoregula formicica SMSP, DSM 22288 |
| Ga0586324 6682139 1 546 | 29000000.00 | 6310400.00 | 33909349.25 | 0.00 | 47895000.00 | 12584900.00 | 0.00 | 306188.89 | 0.00 | 0.00 | 19448000.00 | 0.00 | KO:K00319 | methylenetetrahydromethanopterin dehydrogenase | Archaea;Euryarchaeota;Methanomicrobia;Methanomicrobiales;Methanoregulaceae;Methanoregula;Methanoregula boonei;Methanoregula boonei 6A8 |
| Ga0586324 0000676 22706 23695 | 3650000.00 | 635547.04 | 10895705.22 | 42558914.15 | 42436000.00 | 630432.03 | 29643759.72 | 26535000.00 | 17690400.00 | 14553000.00 | 13552000.00 | 13725000.00 | KO:K00320 | 5,10-methylenetetrahydromethanopterin reductase | Archaea;Euryarchaeota;Methanomicrobia;Methanomicrobiales;Methanospirillaceae;Methanospirillum;Methanospirillum hungatei;Methanospirillum hungatei JF-1 |
| Ga0586324 0003276 653 1642 | 0.00 | 3114400.00 | 0.00 | 0.00 | 0.00 | 0.00 | 0.00 | 0.00 | 0.00 | 13829200.00 | 4672800.00 | 0.00 | KO:K00320 | 5,10-methylenetetrahydromethanopterin reductase | Archaea;Euryarchaeota;Methanomicrobia;Methanomicrobiales;Methanoregulaceae;Methanoregula;Methanoregula boonei;Methanoregula boonei 6A8 |
| Ga0586324 0010839 8033 9022 | 0.00 | 45560000.00 | 202117000.00 | 116850000.00 | 57268000.00 | 66299000.00 | 54264000.00 | 33977000.00 | 47142000.00 | 34111000.00 | 19096000.00 | 33300000.00 | KO:K00320 | 5,10-methylenetetrahydromethanopterin reductase | Archaea;Euryarchaeota;Methanomicrobia;Methanomicrobiales;Methanospirillaceae;Methanospirillum;Methanospirillum hungatei;Methanospirillum hungatei JF-1 |
| Ga0586324 0029955 4003 4989 | 24295315.00 | 9316000.00 | 26775756.41 | 18581592.45 | 14028600.00 | 28158392.18 | 21477600.00 | 21197500.00 | 18281700.00 | 12104400.00 | 5473600.00 | 7305000.00 | KO:K00320 | 5,10-methylenetetrahydromethanopterin reductase | Archaea;Euryarchaeota;Methanomicrobia;Methanomicrobiales;Methanoregulaceae;Methanoregula;Methanoregula formicica;Methanoregula formicica SMSP, DSM 22288 |
| Ga0586324 0074860 1438 2463 | 0.00 | 0.00 | 0.00 | 1273000.00 | 0.00 | 0.00 | 0.00 | 0.00 | 0.00 | 0.00 | 0.00 | 0.00 | KO:K00320 | 5,10-methylenetetrahydromethanopterin reductase | Archaea;Euryarchaeota;Methanomicrobia;Methanosarcinales;Methanotrichaceae;Methanothrix;Methanothrix soehngenii;Methanothrix soehngenii GP-6 |
| Ga0586324 0147711 1796 2782 | 9110000.00 | 0.00 | 16327000.00 | 16245000.00 | 15862000.00 | 12743700.00 | 10419600.00 | 0.00 | 11583000.00 | 0.00 | 0.00 | 0.00 | KO:K00320 | 5,10-methylenetetrahydromethanopterin reductase | Archaea;Euryarchaeota;Methanomicrobia;Methanomicrobiales;Methanoregulaceae;Methanoregula;Methanoregula formicica;Methanoregula formicica SMSP, DSM 22288 |
| Ga0586324 0211396 1 804 | 0.00 | 0.00 | 7825700.00 | 219450000.00 | 0.00 | 0.00 | 7045200.00 | 0.00 | 0.00 | 0.00 | 0.00 | 0.00 | KO:K00320 | 5,10-methylenetetrahydromethanopterin reductase | Archaea;Euryarchaeota;Methanomicrobia;Methanomicrobiales;Methanoregulaceae;Methanoregula;Methanoregula formicica;Methanoregula formicica SMSP, DSM 22288 |
| Ga0586324 0249475 1 861 | 1850000.00 | 0.00 | 3460057.25 | 0.00 | 2461700.00 | 737387.80 | 0.00 | 0.00 | 0.00 | 0.00 | 2508000.00 | 2362500.00 | KO:K00320 | 5,10-methylenetetrahydromethanopterin reductase | Archaea;Euryarchaeota;Methanomicrobia;Methanomicrobiales;Methanoregulaceae;Methanoregula;Methanoregula formicica;Methanoregula formicica SMSP, DSM 22288 |
| Ga0586324 0346381 1021 1929 | 166000000.00 | 0.00 | 0.00 | 203300000.00 | 0.00 | 299338000.00 | 62950800.00 | 60756000.00 | 226314000.00 | 110880000.00 | 0.00 | 0.00 | KO:K00320 | 5,10-methylenetetrahydromethanopterin reductase | Archaea;Euryarchaeota;Methanomicrobia;Methanomicrobiales;Methanoregulaceae;Methanoregula;Methanoregula formicica;Methanoregula formicica SMSP, DSM 22288 |
| Ga0586324 0351136 813 1802 | 9300000.00 | 8500000.00 | 10697000.00 | 20710000.00 | 19776000.00 | 14411100.00 | 9006000.00 | 16165000.00 | 12798000.00 | 5605600.00 | 5702400.00 | 4987500.00 | KO:K00320 | 5,10-methylenetetrahydromethanopterin reductase | Archaea;Euryarchaeota;Methanomicrobia;Methanomicrobiales;Methanospirillaceae;Methanospirillum;Methanospirillum hungatei;Methanospirillum hungatei JF-1 |
| Ga0586324 1053042 506 1186 | 0.00 | 91120000.00 | 176782000.00 | 0.00 | 0.00 | 152051000.00 | 0.00 | 0.00 | 99630000.00 | 0.00 | 0.00 | 0.00 | KO:K00320 | 5,10-methylenetetrahydromethanopterin reductase | Archaea;Euryarchaeota;Methanomicrobia;Methanocellales;Methanocellaceae;Methanocella;Methanocella arvoryzae;Methanocella arvoryzae MRE50 |
| Ga0586324 3200496 2 742 | 0.00 | 0.00 | 0.00 | 8588000.00 | 18746000.00 | 0.00 | 9370800.00 | 10004000.00 | 0.00 | 0.00 | 2226400.00 | 0.00 | KO:K00320 | 5,10-methylenetetrahydromethanopterin reductase | Archaea;Euryarchaeota;Methanomicrobia;Methanomicrobiales;Methanoregulaceae;Methanoregula;Methanoregula formicica;Methanoregula formicica SMSP, DSM 22288 |
| Ga0586324 4031605 1 675 | 20300000.00 | 0.00 | 0.00 | 5833000.00 | 0.00 | 0.00 | 136662.29 | 0.00 | 0.00 | 0.00 | 0.00 | 0.00 | KO:K00320 | 5,10-methylenetetrahydromethanopterin reductase | Archaea;Euryarchaeota;Methanomicrobia;Methanomicrobiales;Methanoregulaceae;Methanoregula;Methanoregula formicica;Methanoregula formicica SMSP, DSM 22288 |
| Ga0586324 4081332 1 669 | 1120000.00 | 1047200.00 | 0.00 | 1358500.00 | 0.00 | 0.00 | 0.00 | 732000.00 | 688198.68 | 0.00 | 263289.84 | 335742.75 | KO:K00320 | 5,10-methylenetetrahydromethanopterin reductase | Archaea;Euryarchaeota;Methanomicrobia;Methanomicrobiales;Methanoregulaceae;Methanoregula;Methanoregula formicica;Methanoregula formicica SMSP, DSM 22288 |
| Ga0586324 4097756 3 668 | 75300000.00 | 0.00 | 0.00 | 0.00 | 0.00 | 0.00 | 0.00 | 0.00 | 0.00 | 0.00 | 0.00 | 0.00 | KO:K00320 | 5,10-methylenetetrahydromethanopterin reductase | Archaea;Euryarchaeota;Methanomicrobia;Methanomicrobiales;Methanoregulaceae;Methanoregula;Methanoregula formicica;Methanoregula formicica SMSP, DSM 22288 |
| Ga0586324 4186676 63 662 | 0.00 | 0.00 | 0.00 | 0.00 | 0.00 | 0.00 | 869368.56 | 0.00 | 0.00 | 0.00 | 0.00 | 0.00 | KO:K00320 | 5,10-methylenetetrahydromethanopterin reductase | Archaea;Euryarchaeota;Methanobacteria;Methanobacteriales;Methanobacteriaceae;Methanobacterium;Methanobacterium sp. SMA-27;Methanobacterium sp. SMA-27 |
| Ga0586324 4841177 3 626 | 0.00 | 1564000.00 | 0.00 | 0.00 | 0.00 | 0.00 | 0.00 | 0.00 | 0.00 | 0.00 | 0.00 | 0.00 | KO:K00320 | 5,10-methylenetetrahydromethanopterin reductase | Archaea;Euryarchaeota;Methanomicrobia;Methanomicrobiales;Methanoregulaceae;Methanoregula;Methanoregula formicica;Methanoregula formicica SMSP, DSM 22288 |
| Ga0586324 4858464 3 611 | 0.00 | 0.00 | 0.00 | 0.00 | 0.00 | 0.00 | 137028000.00 | 0.00 | 0.00 | 0.00 | 0.00 | 0.00 | KO:K00320 | 5,10-methylenetetrahydromethanopterin reductase | Archaea;Euryarchaeota;Methanomicrobia;Methanomicrobiales;Methanoregulaceae;Methanoregula;Methanoregula formicica;Methanoregula formicica SMSP, DSM 22288 |
| Ga0586324 5088018 3 611 | 20700000.00 | 0.00 | 0.00 | 8702000.00 | 0.00 | 0.00 | 0.00 | 0.00 | 0.00 | 0.00 | 0.00 | 0.00 | KO:K00320 | 5,10-methylenetetrahydromethanopterin reductase | Archaea;Euryarchaeota;Methanomicrobia;Methanomicrobiales;Methanoregulaceae;Methanoregula;Methanoregula formicica;Methanoregula formicica SMSP, DSM 22288 |
| Ga0586324 5431567 152 595 | 24700000.00 | 0.00 | 0.00 | 0.00 | 14852600.00 | 0.00 | 0.00 | 0.00 | 3159000.00 | 0.00 | 0.00 | 0.00 | KO:K00320 | 5,10-methylenetetrahydromethanopterin reductase | Archaea;Euryarchaeota;Methanomicrobia;Methanomicrobiales;Methanoregulaceae;Methanosphaerula;Methanosphaerula palustris;Methanosphaerula palustris E1-9c, DSM 19958 |
| Ga0586324 5913206 2 574 | 0.00 | 0.00 | 0.00 | 0.00 | 0.00 | 3818222.93 | 0.00 | 1464000.00 | 980100.00 | 0.00 | 0.00 | 0.00 | KO:K00320 | 5,10-methylenetetrahydromethanopterin reductase | Archaea;Euryarchaeota;Methanomicrobia;Methanomicrobiales;Methanoregulaceae;Methanoregula;Methanoregula formicica;Methanoregula formicica SMSP, DSM 22288 |
| Ga0586324 6216927 3 563 | 3250000.00 | 3631200.00 | 3649045.09 | 3306000.00 | 3594700.00 | 4287600.00 | 1766947.56 | 2250900.00 | 1944000.00 | 4173400.00 | 4540800.00 | 4012500.00 | KO:K00320 | 5,10-methylenetetrahydromethanopterin reductase | Archaea;Euryarchaeota;Methanomicrobia;Methanomicrobiales;Methanoregulaceae;Methanoregula;Methanoregula boonei;Methanoregula boonei 6A8 |
| Ga0586324 0466237 226 1608 | 0.00 | 0.00 | 0.00 | 0.00 | 0.00 | 0.00 | 0.00 | 0.00 | 10044000.00 | 0.00 | 0.00 | 6367500.00 | KO:K00324 | NAD(P) transhydrogenase subunit alpha | Bacteria;Proteobacteria;Betaproteobacteria;Burkholderiales;Oxalobacteraceae;Noviherbaspirillum;Noviherbaspirillum sp. Root189;Noviherbaspirillum sp. Root189 |
| Ga0586324 1269635 82 1095 | 0.00 | 0.00 | 0.00 | 0.00 | 0.00 | 0.00 | 0.00 | 0.00 | 0.00 | 0.00 | 1531200.00 | 240813.75 | KO:K00324 | NAD(P) transhydrogenase subunit alpha | Bacteria;Proteobacteria;Betaproteobacteria;Nitrosomonadales;Gallionellaceae;Sideroxydans;Sideroxydans lithotrophicus;Sideroxydans lithotrophicus ES-1 |
| Ga0586324 8261528 3 239 | 0.00 | 0.00 | 0.00 | 0.00 | 0.00 | 0.00 | 0.00 | 0.00 | 0.00 | 0.00 | 0.00 | 1020000.00 | KO:K00324 | NAD(P) transhydrogenase subunit alpha | Bacteria;Proteobacteria;Betaproteobacteria;unclassified Betaproteobacteria;unclassified Betaproteobacteria;unclassified Betaproteobacteria;beta proteobacterium AAP51;Betaproteobacteria bacterium AAP51 |
| Ga0586324 3137418 3 569 | 0.00 | 2278000.00 | 0.00 | 0.00 | 0.00 | 0.00 | 0.00 | 0.00 | 0.00 | 0.00 | 0.00 | 0.00 | KO:K00331 | NADH-quinone oxidoreductase subunit B | Bacteria;Chloroflexi;Anaerolineae;Anaerolineales;Anaerolineaceae;Anaerolinea;Anaerolinea thermophila;Anaerolinea thermophila UNI-1 |
| Ga0586324 8133884 2 505 | 0.00 | 0.00 | 0.00 | 0.00 | 0.00 | 4406700.00 | 0.00 | 0.00 | 0.00 | 0.00 | 0.00 | 0.00 | KO:K00331 | NADH-quinone oxidoreductase subunit B | Bacteria;Chloroflexi;Caldilineae;Caldilineales;Caldilineaceae;Caldilinea;Caldilinea aerophila;Caldilinea aerophila STL-6-O1, DSM 14535 |
| Ga0586324 0003069 5143 6264 | 0.00 | 0.00 | 0.00 | 0.00 | 0.00 | 0.00 | 0.00 | 2818200.00 | 0.00 | 0.00 | 0.00 | 0.00 | KO:K00333 | NADH-quinone oxidoreductase subunit D | Bacteria;Proteobacteria;Deltaproteobacteria;Syntrophobacterales;Syntrophobacteraceae;Desulfacinum;Desulfacinum infernum;Desulfacinum infernum DSM 9756 |
| Ga0586324 0000030 29090 31450 | 0.00 | 0.00 | 0.00 | 0.00 | 0.00 | 0.00 | 0.00 | 0.00 | 0.00 | 1848000.00 | 1988800.00 | 1117500.00 | KO:K00336 | NADH-quinone oxidoreductase subunit G | Bacteria;Proteobacteria;Betaproteobacteria;Nitrosomonadales;Thiobacillaceae;Annwoodia;Annwoodia aquaesulis;Annwoodia aquaesulis DSM 4255 |

| | | | | | | | | | | | | | | | |
|---|---|---|---|---|---|---|---|---|---|---|---|---|---|---|---|
| Ga0586324_1168874_3_1136 | 0.00 | 0.00 | 0.00 | 1824000.00 | 0.00 | 0.00 | 0.00 | 0.00 | 0.00 | 0.00 | 0.00 | 0.00 | KO:K00336 | NADH-quinone oxidoreductase subunit G | Bacteria;Proteobacteria;Alphaproteobacteria;Hyphomicrobiales;Xanthobacteraceae;Pseudolabrys;Pseudolabrys taiwanensis;Pseudolabrys taiwanensis CC-BB4 |
| Ga0586324_0022751_6184_6996 | 0.00 | 0.00 | 0.00 | 0.00 | 0.00 | 0.00 | 0.00 | 0.00 | 0.00 | 0.00 | 2543200.00 | 0.00 | KO:K00344 | NADPH2:quinone reductase | Bacteria;Proteobacteria;Alphaproteobacteria;Hyphomicrobiales;Bradyrhizobiaceae;Bradyrhizobium;Bradyrhizobium lablabi;Bradyrhizobium lablabi GAS499 |
| Ga0586324_0063496_1338_2729 | 0.00 | 7010800.00 | 0.00 | 0.00 | 0.00 | 0.00 | 0.00 | 0.00 | 0.00 | 4127200.00 | 0.00 | 0.00 | KO:K00346 | Na+-transporting NADH:ubiquinone oxidoreductase subunit A | Bacteria;Proteobacteria;Gammaproteobacteria;Methylococcales;Methylococcaceae;Methylobacter;Methylobacter tundripaludum;Methylobacter tundripaludum SV96 |
| Ga0586324_0087269_2902_3660 | 0.00 | 0.00 | 0.00 | 0.00 | 0.00 | 0.00 | 0.00 | 0.00 | 1174500.00 | 0.00 | 0.00 | 0.00 | KO:K00346 | Na+-transporting NADH:ubiquinone oxidoreductase subunit A | Bacteria;Proteobacteria;Gammaproteobacteria;Enterobacterales;Yersiniaceae;Chania;Chania multitudinisentens;Chania multitudinisentens RB-25 |
| Ga0586324_0154193_1674_2792 | 0.00 | 0.00 | 8219800.00 | 0.00 | 0.00 | 0.00 | 0.00 | 0.00 | 0.00 | 0.00 | 0.00 | 0.00 | KO:K00346 | Na+-transporting NADH:ubiquinone oxidoreductase subunit A | Bacteria;Proteobacteria;Gammaproteobacteria;Methylococcales;Methylococcaceae;Methylobacter;Methylobacter tundripaludum;Methylobacter tundripaludum SV96 |
| Ga0586324_2187877_3_869 | 0.00 | 0.00 | 0.00 | 0.00 | 0.00 | 0.00 | 0.00 | 2549800.00 | 0.00 | 0.00 | 0.00 | 0.00 | KO:K00346 | Na+-transporting NADH:ubiquinone oxidoreductase subunit A | Bacteria;Proteobacteria;Gammaproteobacteria;Methylococcales;Methylococcaceae;Methylobacter;Methylobacter luteus;Methylobacter luteus IMV-B-3098 |
| Ga0586324_2655027_1_801 | 0.00 | 6636800.00 | 0.00 | 0.00 | 0.00 | 0.00 | 0.00 | 872300.00 | 0.00 | 0.00 | 6872800.00 | 0.00 | KO:K00346 | Na+-transporting NADH:ubiquinone oxidoreductase subunit A | Bacteria;Proteobacteria;Gammaproteobacteria;Methylococcales;Methylococcaceae;Methylobacter;Methylobacter luteus;Methylobacter luteus IMV-B-3098 |
| Ga0586324_2802866_3_782 | 6930000.00 | 0.00 | 0.00 | 0.00 | 0.00 | 0.00 | 0.00 | 0.00 | 0.00 | 0.00 | 0.00 | 0.00 | KO:K00346 | Na+-transporting NADH:ubiquinone oxidoreductase subunit A | Bacteria;Proteobacteria;Gammaproteobacteria;Methylococcales;Methylococcaceae;Methylobacter;Methylobacter luteus;Methylobacter luteus IMV-B-3098 |
| Ga0586324_3244751_1_537 | 0.00 | 0.00 | 0.00 | 0.00 | 0.00 | 0.00 | 0.00 | 307716.94 | 0.00 | 2171400.00 | 0.00 | 0.00 | KO:K00346 | Na+-transporting NADH:ubiquinone oxidoreductase subunit A | Bacteria;Proteobacteria;Gammaproteobacteria;Methylococcales;Methylococcaceae;Methylobacter;Methylobacter tundripaludum;Methylobacter tundripaludum SV96 |
| Ga0586324_0226816_1_2334 | 7810000.00 | 0.00 | 0.00 | 0.00 | 598516.52 | 0.00 | 0.00 | 0.00 | 0.00 | 0.00 | 0.00 | 0.00 | KO:K00370 | nitrate reductase alpha subunit | Bacteria;Nitrospirae;Nitrospira;Nitrospirales;Nitrospiraceae;Nitrospira;Nitrospira moscoviensis;Nitrospira moscoviensis NSP M-1 |
| Ga0586324_0702168_1_1413 | 0.00 | 10404000.00 | 17678200.00 | 13015000.00 | 13287000.00 | 0.00 | 0.00 | 9150000.00 | 0.00 | 23100000.00 | 4276800.00 | 8925000.00 | KO:K00370 | nitrate reductase alpha subunit | Bacteria;Nitrospirae;Nitrospira;Nitrospirales;Nitrospiraceae;Nitrospira;Nitrospira lenta;Nitrospira lenta N. lenta BS10 |
| Ga0586324_1546373_1_1008 | 0.00 | 0.00 | 0.00 | 0.00 | 0.00 | 0.00 | 0.00 | 0.00 | 1368900.00 | 0.00 | 0.00 | 0.00 | KO:K00370 | nitrate reductase alpha subunit | Bacteria;Nitrospirae;Nitrospira;Nitrospirales;Nitrospiraceae;Nitrospira;Nitrospira japonica;Nitrospira japonica NJ11 |
| Ga0586324_0017038_6378_7667 | 0.00 | 0.00 | 0.00 | 0.00 | 0.00 | 4764000.00 | 0.00 | 0.00 | 0.00 | 0.00 | 0.00 | 0.00 | KO:K00371 | nitrate reductase beta subunit | Bacteria;Nitrospirae;Nitrospira;Nitrospirales;Nitrospiraceae;Nitrospira;Nitrospira moscoviensis;Nitrospira moscoviensis NSP M-1 |
| Ga0586324_1733195_3_959 | 0.00 | 0.00 | 0.00 | 8854000.00 | 0.00 | 0.00 | 0.00 | 0.00 | 0.00 | 0.00 | 0.00 | 0.00 | KO:K00371 | nitrate reductase beta subunit | Bacteria;Proteobacteria;Alphaproteobacteria;Hyphomicrobiales;Hyphomicrobiaceae;Rhodoplanes;Rhodoplanes sp. Z2-YC6860;Rhodoplanes sp. Z2-YC6860 |
| Ga0586324_1781545_3_950 | 0.00 | 2189600.00 | 2972662.52 | 0.00 | 0.00 | 0.00 | 0.00 | 0.00 | 0.00 | 0.00 | 0.00 | 0.00 | KO:K00371 | nitrate reductase beta subunit | Bacteria;Nitrospirae;Nitrospira;Nitrospirales;Nitrospiraceae;Nitrospira;Nitrospira japonica;Nitrospira japonica NJ11 |
| Ga0586324_0006115_9453_11315 | 0.00 | 2964800.00 | 0.00 | 0.00 | 0.00 | 0.00 | 0.00 | 0.00 | 0.00 | 0.00 | 0.00 | 0.00 | KO:K00376 | nitrous-oxide reductase | Bacteria;Proteobacteria;Deltaproteobacteria;Myxococcales;Anaeromyxobacteraceae;Anaeromyxobacter;Anaeromyxobacter sp. PSR-1;Anaeromyxobacter sp. PSR-1 |
| Ga0586324_0011253_2338_4311 | 0.00 | 741200.00 | 0.00 | 484169.40 | 0.00 | 0.00 | 0.00 | 0.00 | 0.00 | 0.00 | 0.00 | 0.00 | KO:K00376 | nitrous-oxide reductase | Bacteria;Bacteroidetes;unclassified Bacteroidetes;Bacteroidetes Order II. Incertae sedis;Rhodothermaceae;unclassified Rhodothermaceae;Rhodothermaceae bacterium RA;Halothermobacillus malaysiensis RA |
| Ga0586324_0044754_194_2506 | 0.00 | 1217200.00 | 0.00 | 0.00 | 0.00 | 0.00 | 0.00 | 0.00 | 0.00 | 1609300.00 | 0.00 | 0.00 | KO:K00376 | nitrous-oxide reductase | Bacteria;Proteobacteria;Gammaproteobacteria;Chromatiales;Chromatiaceae;Thiocapsa;Thiocapsa marina;Thiocapsa marina 5811, DSM 5653 |
| Ga0586324_0192962_857_2515 | 0.00 | 2652000.00 | 2107202.03 | 0.00 | 0.00 | 0.00 | 0.00 | 2292300.00 | 0.00 | 0.00 | 0.00 | 5745000.00 | KO:K00376 | nitrous-oxide reductase | Bacteria;Proteobacteria;Gammaproteobacteria;Chromatiales;Chromatiaceae;Thiocapsa;Thiocapsa roseopersicina;Thiocapsa roseopersicina DSM 217 |
| Ga0586324_0663683_2_1141 | 0.00 | 5773200.00 | 0.00 | 0.00 | 3254800.00 | 0.00 | 0.00 | 4355400.00 | 4827600.00 | 685185.27 | 0.00 | 0.00 | KO:K00376 | nitrous-oxide reductase | Bacteria;Proteobacteria;Gammaproteobacteria;Chromatiales;Chromatiaceae;Thiocapsa;Thiocapsa marina;Thiocapsa marina 5811, DSM 5653 |
| Ga0586324_1001390_3_1214 | 5800000.00 | 8296000.00 | 0.00 | 6213000.00 | 7230600.00 | 5041900.00 | 0.00 | 6161000.00 | 3215700.00 | 0.00 | 0.00 | 2602500.00 | KO:K00376 | nitrous-oxide reductase | Bacteria;Proteobacteria;Gammaproteobacteria;Chromatiales;Ectothiorhodospiraceae;Thioalbus;Thioalbus denitrificans;Thioalbus denitrificans DSM 26407 |
| Ga0586324_1023610_3_1202 | 0.00 | 0.00 | 0.00 | 0.00 | 0.00 | 0.00 | 0.00 | 0.00 | 0.00 | 0.00 | 1768800.00 | 0.00 | KO:K00376 | nitrous-oxide reductase | Bacteria;Proteobacteria;Betaproteobacteria;Rhodocyclales;Rhodocyclaceae;Azospira;unclassified Azospira;Azospira sp. ZAP |
| Ga0586324_1111901_2_1105 | 0.00 | 0.00 | 0.00 | 0.00 | 0.00 | 0.00 | 0.00 | 1213900.00 | 0.00 | 0.00 | 0.00 | 0.00 | KO:K00376 | nitrous-oxide reductase | Bacteria;Proteobacteria;Deltaproteobacteria;Myxococcales;Anaeromyxobacteraceae;Anaeromyxobacter;Anaeromyxobacter sp. Fw109-5;Anaeromyxobacter sp. Fw109-5 |
| Ga0586324_2034537_3_896 | 0.00 | 0.00 | 0.00 | 0.00 | 0.00 | 0.00 | 7957200.00 | 21008400.00 | 15430500.00 | 0.00 | 0.00 | 4002632.25 | KO:K00376 | nitrous-oxide reductase | Bacteria;Proteobacteria;Betaproteobacteria;Rhodocyclales;Azonexaceae;Dechloromonas;Dechloromonas agitata;Dechloromonas agitata is5 |
| Ga0586324_2035543_2_895 | 0.00 | 3094000.00 | 0.00 | 0.00 | 0.00 | 0.00 | 0.00 | 0.00 | 3094200.00 | 0.00 | 0.00 | 0.00 | KO:K00376 | nitrous-oxide reductase | Bacteria;Proteobacteria;Betaproteobacteria;Rhodocyclales;Azonexaceae;Dechloromonas;Dechloromonas sp. SG708;Dechloromonas sp. SG708 |
| Ga0586324_2639717_3_632 | 0.00 | 0.00 | 0.00 | 0.00 | 0.00 | 0.00 | 0.00 | 0.00 | 0.00 | 954800.00 | 6969600.00 | 4020000.00 | KO:K00376 | nitrous-oxide reductase | Bacteria;Proteobacteria;Gammaproteobacteria;Chromatiales;Chromatiaceae;Thiocapsa;Thiocapsa marina;Thiocapsa marina 5811, DSM 5653 |
| Ga0586324_3433664_84_719 | 0.00 | 0.00 | 0.00 | 0.00 | 2060000.00 | 0.00 | 0.00 | 0.00 | 0.00 | 0.00 | 0.00 | 0.00 | KO:K00376 | nitrous-oxide reductase | Bacteria;Proteobacteria;Gammaproteobacteria;unclassified Gammaproteobacteria;unclassified Gammaproteobacteria;Sedimenticola;Sedimenticola selenatireducens;Sedimenticola selenatireducens DSM 17993 |
| Ga0586324_3522386_1_711 | 0.00 | 0.00 | 0.00 | 0.00 | 10918000.00 | 0.00 | 0.00 | 0.00 | 0.00 | 0.00 | 0.00 | 0.00 | KO:K00376 | nitrous-oxide reductase | Bacteria;Proteobacteria;Betaproteobacteria;Rhodocyclales;Rhodocyclaceae;Dechlorobacter;Dechlorobacter hydrogenophilus;Dechlorobacter hydrogenophilus LT-1 |
| Ga0586324_6375887_3_557 | 0.00 | 0.00 | 0.00 | 0.00 | 0.00 | 0.00 | 8436000.00 | 9943000.00 | 1312200.00 | 0.00 | 9416000.00 | 0.00 | KO:K00376 | nitrous-oxide reductase | Bacteria;Proteobacteria;Betaproteobacteria;Rhodocyclales;Azonexaceae;Dechloromonas;Dechloromonas sp. HYN0024;Dechloromonas sp. HYN0024 |
| Ga0586324_0000003_99423_102416 | 0.00 | 0.00 | 0.00 | 0.00 | 0.00 | 0.00 | 0.00 | 4623800.00 | 0.00 | 5305300.00 | 7418400.00 | 0.00 | KO:K00382 | dihydrolipoamide dehydrogenase | Bacteria;Proteobacteria;Betaproteobacteria;Nitrosomonadales;Thiobacillaceae;Annwoodia;Annwoodia aquaesulis;Annwoodia aquaesulis DSM 4255 |
| Ga0586324_0089864_2539_3609 | 0.00 | 0.00 | 0.00 | 0.00 | 0.00 | 0.00 | 0.00 | 0.00 | 0.00 | 0.00 | 4426400.00 | 0.00 | KO:K00382 | dihydrolipoamide dehydrogenase | Bacteria;Proteobacteria;Betaproteobacteria;Burkholderiales;unclassified Burkholderiales;Rivibacter;Rivibacter subsaxonicus;Rivibacter subsaxonicus DSM 19570 |
| Ga0586324_1405166_200_1045 | 0.00 | 0.00 | 0.00 | 0.00 | 0.00 | 5518300.00 | 0.00 | 0.00 | 0.00 | 0.00 | 0.00 | 0.00 | KO:K00382 | dihydrolipoamide dehydrogenase | Bacteria;Proteobacteria;Betaproteobacteria;Burkholderiales;Comamonadaceae;Ramlibacter;Ramlibacter tataouinensis;Ramlibacter tataouinensis 5-10 |
| Ga0586324_0000002_211845_213866 | 0.00 | 0.00 | 0.00 | 0.00 | 0.00 | 0.00 | 57684000.00 | 6551400.00 | 29848403.85 | 123816000.00 | 150656000.00 | 65400000.00 | KO:K00394 | adenylylsulfate reductase subunit A | Bacteria;Proteobacteria;Betaproteobacteria;Nitrosomonadales;Thiobacillaceae;Annwoodia;Annwoodia aquaesulis;Annwoodia aquaesulis DSM 4255 |
| Ga0586324_0005409_1_1071 | 0.00 | 0.00 | 2451977.60 | 2489000.00 | 1256600.00 | 5076272.26 | 177134499.60 | 160430000.00 | 140053041.90 | 72100148.89 | 60816800.00 | 205920000.00 | KO:K00394 | adenylylsulfate reductase subunit A | Bacteria;Proteobacteria;Deltaproteobacteria;Desulfobacterales;Desulfobacteraceae;Desulfatirhabdium;Desulfatirhabdium butyrativorans;Desulfatirhabdium butyrativorans DSM 18734 |
| Ga0586324_0008738_6807_8732 | 210650000.00 | 16517200.00 | 245749500.00 | 55432500.00 | 57422500.00 | 77637462.92 | 172504800.00 | 81435000.00 | 23911200.00 | 35035000.00 | 207090400.00 | 16642500.00 | KO:K00394 | adenylylsulfate reductase subunit A | Archaea;Euryarchaeota;Archaeoglobi;Archaeoglobales;Archaeoglobaceae;Archaeoglobus;Archaeoglobus sulfaticallidus;Archaeoglobus sulfaticallidus PM70-1, DSM 19444 |
| Ga0586324_0014308_7605_8675 | 0.00 | 0.00 | 2420759.25 | 0.00 | 1761300.00 | 0.00 | 0.00 | 0.00 | 0.00 | 4450600.00 | 0.00 | 0.00 | KO:K00394 | adenylylsulfate reductase subunit A | Bacteria;Proteobacteria;Deltaproteobacteria;Desulfobacterales;Desulfobacteraceae;Desulfosarcina;Desulfosarcina sp. BuS5;Desulfosarcina sp. BuS5 |
| Ga0586324_0032719_268_2280 | 0.00 | 4114000.00 | 0.00 | 0.00 | 0.00 | 0.00 | 10738800.00 | 6222000.00 | 6180300.00 | 14968800.00 | 8395200.00 | 5040000.00 | KO:K00394 | adenylylsulfate reductase subunit A | Bacteria;Proteobacteria;Gammaproteobacteria;Chromatiales;Thioalkalispiraceae;Sulfurivermis;Sulfurivermis fontis;Sulfurivermis fontis JG42 |
| Ga0586324_0037456_2363_4279 | 0.00 | 0.00 | 0.00 | 1605500.00 | 0.00 | 0.00 | 0.00 | 2177700.00 | 0.00 | 0.00 | 2596000.00 | 0.00 | KO:K00394 | adenylylsulfate reductase subunit A | Bacteria;Proteobacteria;Betaproteobacteria;unclassified Betaproteobacteria;unclassified Betaproteobacteria;unclassified Betaproteobacteria;uncultured beta proteobacterium;unclassified betaproteobacterium ENR4 |
| Ga0586324_0041609_1769_3808 | 0.00 | 0.00 | 0.00 | 0.00 | 0.00 | 0.00 | 0.00 | 0.00 | 17577000.00 | 0.00 | 0.00 | 0.00 | KO:K00394 | adenylylsulfate reductase subunit A | Bacteria;Nitrospirae;Thermodesulfovibrionia;Thermodesulfovibrionales;Thermodesulfovibrionaceae;Thermodesulfovibrio;Thermodesulfovibrio yellowstonii;Thermodesulfovibrio yellowstonii DSM 11347 |
| Ga0586324_0046593_893_2932 | 0.00 | 14708425.84 | 0.00 | 1567500.00 | 0.00 | 22390800.00 | 24624000.00 | 0.00 | 593073.90 | 11881100.00 | 44783200.00 | 17550000.00 | KO:K00394 | adenylylsulfate reductase subunit A | Bacteria;Nitrospirae;Thermodesulfovibrionia;Thermodesulfovibrionales;Thermodesulfovibrionaceae;Thermodesulfovibrio;Thermodesulfovibrio yellowstonii;Thermodesulfovibrio yellowstonii DSM 11347 |
| Ga0586324_0057114_1_981 | 0.00 | 256181.16 | 0.00 | 0.00 | 0.00 | 0.00 | 0.00 | 0.00 | 0.00 | 916300.00 | 1029600.00 | 0.00 | KO:K00394 | adenylylsulfate reductase subunit A | Bacteria;Nitrospirae;Thermodesulfovibrionia;Thermodesulfovibrionales;Thermodesulfovibrionaceae;Thermodesulfovibrio;Thermodesulfovibrio sp. N1;Thermodesulfovibrio sp. N1 |
| Ga0586324_0068875_1106_3082 | 28890000.00 | 31878400.00 | 13259353.75 | 47547500.00 | 51446176.32 | 23073004.80 | 26698800.00 | 36478000.00 | 40589100.00 | 22869000.00 | 47256000.00 | 25773726.75 | KO:K00394 | adenylylsulfate reductase subunit A | Bacteria;Proteobacteria;Deltaproteobacteria;Desulfobacterales;Desulfobacteraceae;Desulfatitalea;Desulfatitalea tepidiphila;Desulfatitalea tepidiphila S28bF |
| Ga0586324_0072275_1_1707 | 0.00 | 6534800.00 | 0.00 | 0.00 | 0.00 | 326699.52 | 0.00 | 0.00 | 769468.41 | 0.00 | 0.00 | 0.00 | KO:K00394 | adenylylsulfate reductase subunit A | Bacteria;Nitrospirae;Thermodesulfovibrionia;Thermodesulfovibrionales;Thermodesulfovibrionaceae;Thermodesulfovibrio;Thermodesulfovibrio yellowstonii;Thermodesulfovibrio yellowstonii DSM 11347 |
| Ga0586324_0072572_2313_3995 | 0.00 | 30736000.00 | 0.00 | 0.00 | 9682000.00 | 0.00 | 0.00 | 32635000.00 | 0.00 | 30030000.00 | 0.00 | 0.00 | KO:K00394 | adenylylsulfate reductase subunit A | Bacteria;Proteobacteria;Gammaproteobacteria;Chromatiales;Chromatiaceae;Thiocystis;Thiocystis violascens;Thiocystis violascens 611, DSM 198 |
| Ga0586324_0083535_949_2982 | 0.00 | 0.00 | 0.00 | 43985000.00 | 71585000.00 | 31879100.00 | 0.00 | 33672000.00 | 0.00 | 0.00 | 0.00 | 35400000.00 | KO:K00394 | adenylylsulfate reductase subunit A | Bacteria;Proteobacteria;Gammaproteobacteria;Chromatiales;Chromatiaceae;Thiodictyon;Candidatus Thiodictyon syntrophicum;Candidatus Thiodictyon syntrophicum Cad16 |
| Ga0586324_0095010_3020_3514 | 0.00 | 0.00 | 0.00 | 0.00 | 0.00 | 0.00 | 0.00 | 0.00 | 0.00 | 2864400.00 | 0.00 | 0.00 | KO:K00394 | adenylylsulfate reductase subunit A | Bacteria;Proteobacteria;Betaproteobacteria;Nitrosomonadales;Thiobacillaceae;Thiobacillus;Thiobacillus denitrificans;Thiobacillus denitrificans ATCC 25259 |
| Ga0586324_0113654_2_1273 | 0.00 | 0.00 | 0.00 | 0.00 | 0.00 | 0.00 | 0.00 | 0.00 | 858600.00 | 0.00 | 0.00 | 0.00 | KO:K00394 | adenylylsulfate reductase subunit A | Bacteria;Proteobacteria;Deltaproteobacteria;Desulfobacterales;Desulfobacteraceae;Desulfatiglans;Desulfatiglans anilini;Desulfatiglans anilini DSM 4660 |
| Ga0586324_0124704_72_2075 | 1190000.00 | 3243600.00 | 1887412.46 | 1121000.00 | 0.00 | 0.00 | 815676.84 | 15170700.00 | 1611900.00 | 18552152.85 | 1029600.00 | 0.00 | KO:K00394 | adenylylsulfate reductase subunit A | Bacteria;Nitrospirae;Thermodesulfovibrionia;Thermodesulfovibrionales;Thermodesulfovibrionaceae;Thermodesulfovibrio;Thermodesulfovibrio hydrogeniphilus;Thermodesulfovibrio hydrogeniphilus DSM 18151 |
| Ga0586324_0128575_601_2604 | 0.00 | 7072000.00 | 22463700.00 | 612730.05 | 0.00 | 11513000.00 | 5631600.00 | 3483100.00 | 12069000.00 | 0.00 | 7198400.00 | 7132500.00 | KO:K00394 | adenylylsulfate reductase subunit A | Bacteria;Nitrospirae;Thermodesulfovibrionia;Thermodesulfovibrionales;Thermodesulfovibrionaceae;Thermodesulfovibrio;Thermodesulfovibrio hydrogeniphilus;Thermodesulfovibrio hydrogeniphilus DSM 18151 |
| Ga0586324_0130014_3_1319 | 0.00 | 0.00 | 0.00 | 3410500.00 | 0.00 | 0.00 | 0.00 | 0.00 | 0.00 | 0.00 | 0.00 | 0.00 | KO:K00394 | adenylylsulfate reductase subunit A | Bacteria;Proteobacteria;Gammaproteobacteria;unclassified Gammaproteobacteria;unclassified Gammaproteobacteria;Candidatus Thioglobus;Candidatus Thioglobus singularis;Candidatus Thioglobus singularis PS1 |
| Ga0586324_0140711_2070_2915 | 0.00 | 0.00 | 0.00 | 0.00 | 0.00 | 0.00 | 2090951.52 | 0.00 | 0.00 | 0.00 | 0.00 | 0.00 | KO:K00394 | adenylylsulfate reductase subunit A | Bacteria;Proteobacteria;Deltaproteobacteria;Desulfobacterales;Desulfobulbaceae;Desulfopila;Desulfopila aestuarii;Desulfopila aestuarii DSM 18488 |
| Ga0586324_0142556_2352_2897 | 0.00 | 3080400.00 | 2179457.45 | 8274500.00 | 19153930.47 | 35531500.00 | 8276400.00 | 1811700.00 | 7200900.00 | 0.00 | 0.00 | 0.00 | KO:K00394 | adenylylsulfate reductase subunit A | Bacteria;Proteobacteria;Deltaproteobacteria;Syntrophobacterales;Syntrophaceae;Desulfomonile;Desulfomonile tiedjei;Desulfomonile tiedjei DCB-1, DSM 6799 |
| Ga0586324_0145463_2_1045 | 0.00 | 0.00 | 0.00 | 0.00 | 0.00 | 0.00 | 0.00 | 0.00 | 4754700.00 | 0.00 | 0.00 | 0.00 | KO:K00394 | adenylylsulfate reductase subunit A | Bacteria;Proteobacteria;Deltaproteobacteria;Desulfobacterales;Desulfobacteraceae;Desulfococcus;Desulfococcus oleovorans;Desulfococcus oleovorans Hxd3 |
| Ga0586324_0154655_217_2097 | 7030000.00 | 0.00 | 0.00 | 29640000.00 | 0.00 | 0.00 | 1010445.84 | 0.00 | 0.00 | 0.00 | 0.00 | 0.00 | KO:K00394 | adenylylsulfate reductase subunit A | Bacteria;Proteobacteria;Deltaproteobacteria;Desulfobacterales;Desulfobacteraceae;Desulfatiglans;Desulfatiglans anilini;Desulfatiglans anilini DSM 4660 |
| Ga0586324_0155179_2_1552 | 0.00 | 1448400.00 | 0.00 | 6213000.00 | 0.00 | 0.00 | 0.00 | 4465200.00 | 3361500.00 | 4335100.00 | 4831200.00 | 6495000.00 | KO:K00394 | adenylylsulfate reductase subunit A | Bacteria;Proteobacteria;Betaproteobacteria;Nitrosomonadales;Gallionellaceae;Sideroxydans;Sideroxydans lithotrophicus;Sideroxydans lithotrophicus ES-1 |
| Ga0586324_0180229_197_2161 | 0.00 | 1366800.00 | 0.00 | 3496000.00 | 92494000.00 | 0.00 | 0.00 | 2067900.00 | 0.00 | 28259000.00 | 0.00 | 0.00 | KO:K00394 | adenylylsulfate reductase subunit A | Bacteria;Proteobacteria;Deltaproteobacteria;Syntrophobacterales;Syntrophaceae;Desulfobacca;Desulfobacca acetoxidans;Desulfobacca acetoxidans ASRB2, DSM 11109 |
| Ga0586324_0194934_3_980 | 0.00 | 0.00 | 0.00 | 0.00 | 0.00 | 0.00 | 3374400.00 | 0.00 | 0.00 | 0.00 | 0.00 | 0.00 | KO:K00394 | adenylylsulfate reductase subunit A | Bacteria;Firmicutes;Clostridia;Eubacteriales;Peptococcaceae;Desulfallas;Desulfallas alcoholivorax;Desulfotomaculum alcoholivorax DSM 16058 |
| Ga0586324_0200605_1215_2471 | 0.00 | 0.00 | 0.00 | 0.00 | 0.00 | 0.00 | 3214800.00 | 701500.00 | 0.00 | 0.00 | 2173600.00 | 9975000.00 | KO:K00394 | adenylylsulfate reductase subunit A | Bacteria;Proteobacteria;Betaproteobacteria;Nitrosomonadales;Sulfuricellaceae;Sulfurirhabdus;Sulfurirhabdus autotrophica;Sulfurirhabdus autotrophica DSM 100309 |
| Ga0586324_0271257_1614_2150 | 8160000.00 | 0.00 | 0.00 | 18525000.00 | 0.00 | 0.00 | 8048400.00 | 0.00 | 0.00 | 939400.00 | 2551959.52 | 421699.50 | KO:K00394 | adenylylsulfate reductase subunit A | Bacteria;Proteobacteria;Deltaproteobacteria;unclassified Deltaproteobacteria;unclassified Deltaproteobacteria;unclassified Deltaproteobacteria;delta proteobacterium NaphS2;delta proteobacterium NaphS2 |
| Ga0586324_0282363_1478_2113 | 0.00 | 0.00 | 0.00 | 0.00 | 0.00 | 7622400.00 | 8208000.00 | 0.00 | 3985200.00 | 0.00 | 1663200.00 | 2355000.00 | KO:K00394 | adenylylsulfate reductase subunit A | Archaea;Euryarchaeota;Archaeoglobi;Archaeoglobales;Archaeoglobaceae;Archaeoglobus;Archaeoglobus sulfaticallidus;Archaeoglobus sulfaticallidus PM70-1, DSM 19444 |
| Ga0586324_0306156_2_784 | 0.00 | 0.00 | 0.00 | 0.00 | 65920000.00 | 94883000.00 | 0.00 | 0.00 | 0.00 | 0.00 | 0.00 | 0.00 | KO:K00394 | adenylylsulfate reductase subunit A | Bacteria;Proteobacteria;Deltaproteobacteria;Syntrophobacterales;Syntrophaceae;Desulfobacca;Desulfobacca acetoxidans;Desulfobacca acetoxidans ASRB2, DSM 11109 |
| Ga0586324_0310959_42_2006 | 6960000.00 | 3440800.00 | 81072000.00 | 10070000.00 | 11433000.00 | 193426359.85 | 1419265.80 | 4776300.00 | 5340048.12 | 0.00 | 0.00 | 31350000.00 | KO:K00394 | adenylylsulfate reductase subunit A | Bacteria;Proteobacteria;Deltaproteobacteria;Syntrophobacterales;Syntrophaceae;Desulfobacca;Desulfobacca acetoxidans;Desulfobacca acetoxidans ASRB2, DSM 11109 |
| Ga0586324_0355347_696_1907 | 17500000.00 | 0.00 | 0.00 | 4208500.00 | 0.00 | 0.00 | 0.00 | 159210000.00 | 1417500.00 | 0.00 | 0.00 | 0.00 | KO:K00394 | adenylylsulfate reductase subunit A | Archaea;Euryarchaeota;Archaeoglobi;Archaeoglobales;Archaeoglobaceae;Archaeoglobus;Archaeoglobus sulfaticallidus;Archaeoglobus sulfaticallidus PM70-1, DSM 19444 |
| Ga0586324_0393277_931_1821 | 15600000.00 | 2858152.88 | 3949946.07 | 0.00 | 0.00 | 3090541.78 | 0.00 | 36760.19 | 0.00 | 11349800.00 | 7048800.00 | 1777500.00 | KO:K00394 | adenylylsulfate reductase subunit A | Bacteria;Proteobacteria;Gammaproteobacteria;Chromatiales;Chromatiaceae;Thiodictyon;Candidatus Thiodictyon syntrophicum;Candidatus Thiodictyon syntrophicum Cad16 |
| Ga0586324_0414519_1_420 | 0.00 | 2223600.00 | 3557518.18 | 0.00 | 0.00 | 0.00 | 0.00 | 1878800.00 | 2041200.00 | 4412100.00 | 6230400.00 | 3472500.00 | KO:K00394 | adenylylsulfate reductase subunit A | Bacteria;Proteobacteria;Gammaproteobacteria;Acidiferrobacterales;Acidiferrobacteraceae;Sulfuricaulis;Sulfuricaulis limicola;Sulfuricaulis limicola HA5 |
| Ga0586324_0469781_2_505 | 24000000.00 | 40256000.00 | 1972262.19 | 0.00 | 0.00 | 0.00 | 0.00 | 0.00 | 0.00 | 0.00 | 676091.68 | 0.00 | KO:K00394 | adenylylsulfate reductase subunit A | Bacteria;Proteobacteria;Gammaproteobacteria;Chromatiales;Chromatiaceae;Thiocapsa;Thiocapsa roseopersicina;Thiocapsa roseopersicina DSM 217 |
| Ga0586324_0483263_940_1662 | 0.00 | 0.00 | 0.00 | 0.00 | 0.00 | 0.00 | 1179396.12 | 0.00 | 0.00 | 0.00 | 0.00 | 0.00 | KO:K00394 | adenylylsulfate reductase subunit A | Bacteria;Proteobacteria;Deltaproteobacteria;Desulfobacterales;Desulfobacteraceae;Desulfatiglans;Desulfatiglans anilini;Desulfatiglans anilini DSM 4660 |
| Ga0586324_0501999_70_1635 | 0.00 | 2142000.00 | 0.00 | 0.00 | 0.00 | 0.00 | 0.00 | 0.00 | 2640600.00 | 0.00 | 0.00 | 0.00 | KO:K00394 | adenylylsulfate reductase subunit A | Bacteria;Proteobacteria;Deltaproteobacteria;Syntrophobacterales;Syntrophaceae;Desulfomonile;Desulfomonile tiedjei;Desulfomonile tiedjei DCB-1, DSM 6799 |
| Ga0586324_0506566_2_1369 | 8580000.00 | 12580000.00 | 0.00 | 1976000.00 | 0.00 | 943986.60 | 4468800.00 | 1525000.00 | 891000.00 | 3087700.00 | 1408000.00 | 3187500.00 | KO:K00394 | adenylylsulfate reductase subunit A | Bacteria;Proteobacteria;Deltaproteobacteria;Desulfobacterales;Desulfobulbaceae;Desulfobulbus;Desulfobulbus elongatus;Desulfobulbus elongatus DSM 2908 |
| Ga0586324_0600243_3_527 | 0.00 | 0.00 | 0.00 | 10545000.00 | 10403000.00 | 6709300.00 | 141360000.00 | 47885000.00 | 8699400.00 | 0.00 | 0.00 | 0.00 | KO:K00394 | adenylylsulfate reductase subunit A | Bacteria;Proteobacteria;Deltaproteobacteria;Syntrophobacterales;Syntrophaceae;Desulfomonile;Desulfomonile tiedjei;Desulfomonile tiedjei DCB-1, DSM 6799 |
| Ga0586324_0603787_1_906 | 0.00 | 0.00 | 0.00 | 0.00 | 0.00 | 3232040.52 | 3283200.00 | 4007700.00 | 0.00 | 652526.49 | 1381600.00 | 1320000.00 | KO:K00394 | adenylylsulfate reductase subunit A | Bacteria;Proteobacteria;Deltaproteobacteria;Desulfobacterales;Desulfobacteraceae;Desulfococcus;Desulfococcus oleovorans;Desulfococcus oleovorans Hxd3 |
| Ga0586324_0635473_191_1477 | 0.00 | 0.00 | 0.00 | 0.00 | 0.00 | 0.00 | 3146400.00 | 0.00 | 2259900.00 | 0.00 | 0.00 | 0.00 | KO:K00394 | adenylylsulfate reductase subunit A | Bacteria;Proteobacteria;Gammaproteobacteria;Chromatiales;Chromatiaceae;Thiodictyon;Candidatus Thiodictyon syntrophicum;Candidatus Thiodictyon syntrophicum Cad16 |
| Ga0586324_0645393_2_1468 | 22760000.00 | 5902400.00 | 0.00 | 26077500.00 | 12679300.00 | 0.00 | 10807200.00 | 12187800.00 | 0.00 | 12212200.00 | 4963200.00 | 5467500.00 | KO:K00394 | adenylylsulfate reductase subunit A | Archaea;Euryarchaeota;Archaeoglobi;Archaeoglobales;Archaeoglobaceae;Archaeoglobus;Archaeoglobus sulfaticallidus;Archaeoglobus sulfaticallidus PM70-1, DSM 19444 |
| Ga0586324_0659785_1_1452 | 1390000.00 | 1679600.00 | 0.00 | 675917.40 | 0.00 | 1808021.37 | 0.00 | 2232600.00 | 2470500.00 | 0.00 | 0.00 | 0.00 | KO:K00394 | adenylylsulfate reductase subunit A | Bacteria;Proteobacteria;Deltaproteobacteria;Syntrophobacterales;Syntrophaceae;Desulfobacca;Desulfobacca acetoxidans;Desulfobacca acetoxidans ASRB2, DSM 11109 |
| Ga0586324_0664042_109_1449 | 1140000.00 | 5310800.00 | 5098038.19 | 5643000.00 | 24565500.00 | 6781498.42 | 2063386.32 | 16189400.00 | 2843100.00 | 4381300.00 | 4400000.00 | 1687500.00 | KO:K00394 | adenylylsulfate reductase subunit A | Bacteria;Firmicutes;Clostridia;Eubacteriales;Peptococcaceae;Desulfofundulus;Desulfofundulus thermosubterraneus;Desulfofundulus thermosubterraneus DSM 16057 |
| Ga0586324_0749437_1182_1376 | 0.00 | 0.00 | 0.00 | 0.00 | 0.00 | 0.00 | 0.00 | 398197.63 | 0.00 | 0.00 | 0.00 | 0.00 | KO:K00394 | adenylylsulfate reductase subunit A | Bacteria;Proteobacteria;Betaproteobacteria;Nitrosomonadales;Thiobacillaceae;Thiobacillus;Thiobacillus denitrificans;Thiobacillus denitrificans ATCC 25259 |
| Ga0586324_0751888_609_1373 | 23000000.00 | 0.00 | 0.00 | 0.00 | 0.00 | 0.00 | 0.00 | 0.00 | 0.00 | 0.00 | 0.00 | 0.00 | KO:K00394 | adenylylsulfate reductase subunit A | Bacteria;Proteobacteria;Deltaproteobacteria;Desulfobacterales;Desulfobacteraceae;Desulfatiglans;Desulfatiglans anilini;Desulfatiglans anilini DSM 4660 |
| Ga0586324_0758874_1_693 | 916369.00 | 0.00 | 0.00 | 0.00 | 0.00 | 285714.55 | 0.00 | 0.00 | 0.00 | 0.00 | 0.00 | 319006.50 | KO:K00394 | adenylylsulfate reductase subunit A | Bacteria;Proteobacteria;Deltaproteobacteria;Desulfobacterales;Desulfobacteraceae;Desulfatiglans;Desulfatiglans anilini;Desulfatiglans anilini DSM 4660 |
| Ga0586324_0908185_1_732 | 10660000.00 | 7140000.00 | 0.00 | 7343500.00 | 13287000.00 | 0.00 | 5244000.00 | 0.00 | 0.00 | 0.00 | 7312800.00 | 0.00 | KO:K00394 | adenylylsulfate reductase subunit A | Archaea;Euryarchaeota;Archaeoglobi;Archaeoglobales;Archaeoglobaceae;Archaeoglobus;Archaeoglobus sulfaticallidus;Archaeoglobus sulfaticallidus PM70-1, DSM 19444 |
| Ga0586324_0913754_240_1262 | 0.00 | 0.00 | 0.00 | 6621500.00 | 0.00 | 0.00 | 0.00 | 0.00 | 0.00 | 0.00 | 0.00 | 0.00 | KO:K00394 | adenylylsulfate reductase subunit A | Bacteria;Proteobacteria;Deltaproteobacteria;Syntrophobacterales;Syntrophaceae;Desulfomonile;Desulfomonile tiedjei;Desulfomonile tiedjei DCB-1, DSM 6799 |
| Ga0586324_1049568_565_1188 | 925460.00 | 0.00 | 0.00 | 0.00 | 0.00 | 0.00 | 0.00 | 0.00 | 0.00 | 0.00 | 0.00 | 0.00 | KO:K00394 | adenylylsulfate reductase subunit A | Bacteria;Proteobacteria;Betaproteobacteria;Nitrosomonadales;Thiobacillaceae;Annwoodia;Annwoodia aquaesulis;Annwoodia aquaesulis DSM 4255 |
| Ga0586324_1071246_3_935 | 0.00 | 0.00 | 0.00 | 0.00 | 0.00 | 0.00 | 0.00 | 5575400.00 | 7087500.00 | 5867400.00 | 0.00 | 4777500.00 | KO:K00394 | adenylylsulfate reductase subunit A | Bacteria;Proteobacteria;Betaproteobacteria;Nitrosomonadales;Sterolibacteriaceae;Sulfurisoma;Sulfurisoma sediminicola;Sulfurisoma sediminicola DSM 26916 |
| Ga0586324_1119347_2_1156 | 9970000.00 | 1421200.00 | 0.00 | 206435000.00 | 99395000.00 | 359920200.00 | 50768383.80 | 81130000.00 | 46251000.00 | 1848000.00 | 2059200.00 | 2580000.00 | KO:K00394 | adenylylsulfate reductase subunit A | Bacteria;Proteobacteria;Deltaproteobacteria;Syntrophobacterales;Syntrophobacteraceae;Desulfosoma;Desulfosoma caldarium;Desulfosoma caldarium DSM 22027 |
| Ga0586324_1145541_241_1146 | 0.00 | 239337.56 | 0.00 | 0.00 | 0.00 | 0.00 | 0.00 | 1599631.67 | 0.00 | 11899369.79 | 12279824.48 | 0.00 | KO:K00394 | adenylylsulfate reductase subunit A | Bacteria;Proteobacteria;Betaproteobacteria;Nitrosomonadales;Thiobacillaceae;Thiobacillus;Thiobacillus denitrificans;Thiobacillus denitrificans DSM 12475 |
| Ga0586324_1178762_1_1131 | 10100000.00 | 9248000.00 | 9064300.00 | 13585000.00 | 8682900.00 | 22271700.00 | 14044800.00 | 10248000.00 | 9914400.00 | 0.00 | 0.00 | 2970000.00 | KO:K00394 | adenylylsulfate reductase subunit A | Bacteria;Proteobacteria;Deltaproteobacteria;Syntrophobacterales;Syntrophaceae;Desulfomonile;Desulfomonile tiedjei;Desulfomonile tiedjei DCB-1, DSM 6799 |
| Ga0586324_1281566_304_1092 | 0.00 | 447548.12 | 0.00 | 0.00 | 0.00 | 0.00 | 0.00 | 0.00 | 465178.95 | 0.00 | 0.00 | 0.00 | KO:K00394 | adenylylsulfate reductase subunit A | Bacteria;Proteobacteria;Deltaproteobacteria;Desulfobacterales;Desulfobacteraceae;Desulfosalsimonas;Desulfosalsimonas propionicica;Desulfosalsimonas propionicica DSM 17721 |
| Ga0586324_1307846_3_1034 | 103000000.00 | 0.00 | 0.00 | 0.00 | 0.00 | 0.00 | 0.00 | 0.00 | 11259000.00 | 0.00 | 0.00 | 5887500.00 | KO:K00394 | adenylylsulfate reductase subunit A | Bacteria;Nitrospirae;Thermodesulfovibrionia;Thermodesulfovibrionales;Thermodesulfovibrionaceae;Thermodesulfovibrio;Thermodesulfovibrio islandicus;Thermodesulfovibrio islandicus DSM 12570 |
| Ga0586324_1331044_636_1073 | 9730000.00 | 0.00 | 0.00 | 5244000.00 | 0.00 | 0.00 | 0.00 | 0.00 | 0.00 | 0.00 | 0.00 | 0.00 | KO:K00394 | adenylylsulfate reductase subunit A | Archaea;Euryarchaeota;Archaeoglobi;Archaeoglobales;Archaeoglobaceae;Archaeoglobus;Archaeoglobus sulfaticallidus;Archaeoglobus sulfaticallidus PM70-1, DSM 19444 |
| Ga0586324_1346276_1_615 | 0.00 | 8636000.00 | 0.00 | 0.00 | 0.00 | 0.00 | 0.00 | 0.00 | 0.00 | 1940400.00 | 0.00 | 0.00 | KO:K00394 | adenylylsulfate reductase subunit A | Bacteria;Proteobacteria;Deltaproteobacteria;Syntrophobacterales;Syntrophaceae;Desulfomonile;Desulfomonile tiedjei;Desulfomonile tiedjei DCB-1, DSM 6799 |
| Ga0586324_1397345_7_1053 | 0.00 | 2400400.00 | 0.00 | 4436500.00 | 0.00 | 0.00 | 0.00 | 0.00 | 3466800.00 | 352951.06 | 0.00 | 0.00 | KO:K00394 | adenylylsulfate reductase subunit A | Bacteria;Nitrospirae;Thermodesulfovibrionia;Thermodesulfovibrionales;Thermodesulfovibrionaceae;Thermodesulfovibrio;Thermodesulfovibrio hydrogeniphilus;Thermodesulfovibrio hydrogeniphilus DSM 18151 |
| Ga0586324_1399658_3_1052 | 0.00 | 14531600.00 | 20042800.00 | 7571500.00 | 14265500.00 | 12783400.00 | 61491600.00 | 7564000.00 | 17309700.00 | 21556310.93 | 9603420.64 | 30277500.00 | KO:K00394 | adenylylsulfate reductase subunit A | Bacteria;Proteobacteria;Deltaproteobacteria;Desulfobacterales;Desulfobacteraceae;Desulfatiglans;Desulfatiglans anilini;Desulfatiglans anilini DSM 4660 |
| Ga0586324_1429045_261_1043 | 0.00 | 0.00 | 0.00 | 0.00 | 0.00 | 0.00 | 0.00 | 0.00 | 915300.00 | 0.00 | 0.00 | 0.00 | KO:K00394 | adenylylsulfate reductase subunit A | Bacteria;Proteobacteria;Deltaproteobacteria;Desulfobacterales;Desulfobacteraceae;Desulfosalsimonas;Desulfosalsimonas propionicica;Desulfosalsimonas propionicica DSM 17721 |
| Ga0586324_1433796_2_631 | 16200000.00 | 7412000.00 | 0.00 | 0.00 | 0.00 | 0.00 | 0.00 | 2171600.00 | 0.00 | 0.00 | 0.00 | 504890.25 | KO:K00394 | adenylylsulfate reductase subunit A | Bacteria;Proteobacteria;Betaproteobacteria;Nitrosomonadales;Sulfuricellaceae;Sulfuriferula;Sulfuriferula multivorans;Sulfuriferula multivorans TTN |
| Ga0586324_1536243_1_1011 | 2390000.00 | 9458800.00 | 0.00 | 0.00 | 0.00 | 13220100.00 | 2394000.00 | 9394000.00 | 55080000.00 | 0.00 | 0.00 | 0.00 | KO:K00394 | adenylylsulfate reductase subunit A | Bacteria;Proteobacteria;Deltaproteobacteria;Syntrophobacterales;Syntrophobacteraceae;Syntrophobacter;Syntrophobacter fumaroxidans;Syntrophobacter fumaroxidans MPOB |
| Ga0586324_1555830_3_1004 | 785708.00 | 522797.60 | 0.00 | 0.00 | 0.00 | 0.00 | 2964000.00 | 2452200.00 | 2106000.00 | 1162700.00 | 1020800.00 | 4792500.00 | KO:K00394 | adenylylsulfate reductase subunit A | Bacteria;Proteobacteria;Betaproteobacteria;Nitrosomonadales;Sterolibacteriaceae;Sulfurisoma;Sulfurisoma sediminicola;Sulfurisoma sediminicola DSM 26916 |
| Ga0586324_1638870_3_983 | 0.00 | 0.00 | 0.00 | 0.00 | 0.00 | 0.00 | 0.00 | 0.00 | 0.00 | 552157.76 | 0.00 | 0.00 | KO:K00394 | adenylylsulfate reductase subunit A | Bacteria;Proteobacteria;Gammaproteobacteria;Chromatiales;Chromatiaceae;Thiocystis;Thiocystis violascens;Thiocystis violascens 611, DSM 198 |
| Ga0586324_1651631_3_428 | 0.00 | 5059200.00 | 0.00 | 0.00 | 0.00 | 0.00 | 0.00 | 0.00 | 0.00 | 0.00 | 0.00 | 0.00 | KO:K00394 | adenylylsulfate reductase subunit A | Bacteria;Proteobacteria;Deltaproteobacteria;Desulfobacterales;Desulfobacteraceae;Desulfobacula;Desulfobacula toluolica;Desulfobacula toluolica Tol2 |
| Ga0586324_1657902_1_861 | 0.00 | 0.00 | 0.00 | 0.00 | 0.00 | 0.00 | 1053943.68 | 0.00 | 0.00 | 0.00 | 0.00 | 0.00 | KO:K00394 | adenylylsulfate reductase subunit A | Bacteria;Firmicutes;Clostridia;Eubacteriales;Peptococcaceae;Desulfofundulus;Desulfofundulus thermocisternus;Desulfofundulus thermocisternus DSM 10259 |
| Ga0586324_1685144_395_970 | 6930000.00 | 0.00 | 0.00 | 0.00 | 0.00 | 0.00 | 0.00 | 0.00 | 0.00 | 0.00 | 0.00 | 0.00 | KO:K00394 | adenylylsulfate reductase subunit A | Archaea;Euryarchaeota;Archaeoglobi;Archaeoglobales;Archaeoglobaceae;Archaeoglobus;Archaeoglobus sulfaticallidus;Archaeoglobus sulfaticallidus PM70-1, DSM 19444 |
| Ga0586324_1716769_270_965 | 0.00 | 0.00 | 0.00 | 8160500.00 | 0.00 | 0.00 | 0.00 | 0.00 | 0.00 | 0.00 | 0.00 | 0.00 | KO:K00394 | adenylylsulfate reductase subunit A | Bacteria;Proteobacteria;Deltaproteobacteria;Desulfobacterales;Desulfobulbaceae;Desulfobulbus;Desulfobulbus mediterraneus;Desulfobulbus mediterraneus DSM 13871 |
| Ga0586324_1764877_1_951 | 0.00 | 0.00 | 0.00 | 0.00 | 0.00 | 0.00 | 0.00 | 0.00 | 0.00 | 39424000.00 | 50248000.00 | 0.00 | KO:K00394 | adenylylsulfate reductase subunit A | Bacteria;Proteobacteria;Betaproteobacteria;Nitrosomonadales;Thiobacillaceae;Thiobacillus;Thiobacillus denitrificans;Thiobacillus denitrificans DSM 12475 |
| Ga0586324_1847551_1_933 | 1030000.00 | 648122.96 | 0.00 | 461407.40 | 483546.89 | 444330.34 | 570218.88 | 350438.90 | 0.00 | 0.00 | 0.00 | 0.00 | KO:K00394 | adenylylsulfate reductase subunit A | Bacteria;Proteobacteria;Gammaproteobacteria;Chromatiales;Chromatiaceae;Thiodictyon;Candidatus Thiodictyon syntrophicum;Candidatus Thiodictyon syntrophicum Cad16 |
| Ga0586324_1867848_1_930 | 0.00 | 0.00 | 0.00 | 2394000.00 | 1864300.00 | 0.00 | 0.00 | 0.00 | 0.00 | 0.00 | 0.00 | 0.00 | KO:K00394 | adenylylsulfate reductase subunit A | Bacteria;Proteobacteria;Deltaproteobacteria;unclassified Deltaproteobacteria;unclassified Deltaproteobacteria;unclassified Deltaproteobacteria;delta proteobacterium NaphS2;delta proteobacterium NaphS2 |
| Ga0586324_1959900_2_787 | 1120000.00 | 2067200.00 | 0.00 | 0.00 | 0.00 | 0.00 | 0.00 | 1854400.00 | 8748000.00 | 0.00 | 0.00 | 0.00 | KO:K00394 | adenylylsulfate reductase subunit A | Bacteria;Proteobacteria;Deltaproteobacteria;unclassified Deltaproteobacteria;unclassified Deltaproteobacteria;unclassified Deltaproteobacteria;delta proteobacterium NaphS2;delta proteobacterium NaphS2 |
| Ga0586324_2040812_1_894 | 0.00 | 0.00 | 0.00 | 0.00 | 0.00 | 0.00 | 0.00 | 0.00 | 1676700.00 | 1740200.00 | 1592800.00 | 0.00 | KO:K00394 | adenylylsulfate reductase subunit A | Bacteria;Proteobacteria;Betaproteobacteria;unclassified Betaproteobacteria;unclassified Betaproteobacteria;unclassified Betaproteobacteria;uncultured beta proteobacterium;unclassified betaproteobacterium ENR4 |
| Ga0586324_2071586_468_890 | 0.00 | 0.00 | 0.00 | 0.00 | 6715600.00 | 0.00 | 0.00 | 30622000.00 | 0.00 | 0.00 | 0.00 | 0.00 | KO:K00394 | adenylylsulfate reductase subunit A | Bacteria;Proteobacteria;Deltaproteobacteria;Syntrophobacterales;Syntrophaceae;Desulfobacca;Desulfobacca acetoxidans;Desulfobacca acetoxidans ASRB2, DSM 11109 |
| Ga0586324_2219016_2_865 | 36000000.00 | 9125600.00 | 0.00 | 1472500.00 | 1143300.00 | 59947000.00 | 0.00 | 0.00 | 0.00 | 0.00 | 0.00 | 0.00 | KO:K00394 | adenylylsulfate reductase subunit A | Archaea;Euryarchaeota;Archaeoglobi;Archaeoglobales;Archaeoglobaceae;Archaeoglobus;Archaeoglobus sulfaticallidus;Archaeoglobus sulfaticallidus PM70-1, DSM 19444 |
| Ga0586324_2253579_2_859 | 0.00 | 5691600.00 | 0.00 | 0.00 | 0.00 | 0.00 | 0.00 | 0.00 | 0.00 | 0.00 | 0.00 | 0.00 | KO:K00394 | adenylylsulfate reductase subunit A | Bacteria;Proteobacteria;Deltaproteobacteria;Syntrophobacterales;Syntrophaceae;Desulfobacca;Desulfobacca acetoxidans;Desulfobacca acetoxidans ASRB2, DSM 11109 |
| Ga0586324_2268807_1_855 | 0.00 | 0.00 | 0.00 | 0.00 | 0.00 | 0.00 | 0.00 | 222919.62 | 1425600.00 | 0.00 | 0.00 | 9150000.00 | KO:K00394 | adenylylsulfate reductase subunit A | Bacteria;Proteobacteria;Betaproteobacteria;unclassified Betaproteobacteria;unclassified Betaproteobacteria;unclassified Betaproteobacteria;uncultured beta proteobacterium;unclassified betaproteobacterium ENR4 |
| Ga0586324_2283428_3_671 | 54400000.00 | 31960000.00 | 16552200.00 | 4275000.00 | 325655100.00 | 224212678.05 | 12745200.00 | 0.00 | 13543200.00 | 0.00 | 4382400.00 | 0.00 | KO:K00394 | adenylylsulfate reductase subunit A | Bacteria;Proteobacteria;Deltaproteobacteria;Syntrophobacterales;Syntrophaceae;Desulfomonile;Desulfomonile tiedjei;Desulfomonile tiedjei DCB-1, DSM 6799 |
| Ga0586324_2286594_1_681 | 0.00 | 0.00 | 0.00 | 0.00 | 1905500.00 | 0.00 | 0.00 | 2830400.00 | 2415723.75 | 7923300.00 | 6080800.00 | 2902500.00 | KO:K00394 | adenylylsulfate reductase subunit A | Bacteria;Proteobacteria;Gammaproteobacteria;Thiotrichales;Thiotrichaceae;Thioploca;Thioploca ingrica;Thioploca ingrica Lake Okotanpe |
| Ga0586324_2348371_1_828 | 0.00 | 0.00 | 0.00 | 0.00 | 0.00 | 187018.76 | 2179755.24 | 939400.00 | 0.00 | 1170400.00 | 841886.32 | 772500.00 | KO:K00394 | adenylylsulfate reductase subunit A | Bacteria;Proteobacteria;Deltaproteobacteria;Syntrophobacterales;Syntrophaceae;Desulfobacca;Desulfobacca acetoxidans;Desulfobacca acetoxidans ASRB2, DSM 11109 |
| Ga0586324_2432445_3_833 | 847825.00 | 2584000.00 | 0.00 | 8632866.60 | 8353300.00 | 0.00 | 6178800.00 | 0.00 | 0.00 | 0.00 | 0.00 | 0.00 | KO:K00394 | adenylylsulfate reductase subunit A | Bacteria;Proteobacteria;Deltaproteobacteria;Desulfobacterales;Desulfobulbaceae;Desulfoprunum;Desulfoprunum benzoelyticum;Desulfoprunum benzoelyticum DSM 28570 |
| Ga0586324_2441858_3_683 | 0.00 | 0.00 | 0.00 | 0.00 | 0.00 | 118128.54 | 0.00 | 0.00 | 0.00 | 0.00 | 0.00 | 0.00 | KO:K00394 | adenylylsulfate reductase subunit A | Bacteria;Proteobacteria;Deltaproteobacteria;Desulfobacterales;Desulfobacteraceae;Desulfatitalea;Desulfatitalea tepidiphila;Desulfatitalea tepidiphila S28bF |
| Ga0586324_2582724_3_812 | 28350000.00 | 25704000.00 | 30112612.93 | 45562000.00 | 41818000.00 | 49307400.00 | 97128000.00 | 40961500.00 | 30528900.00 | 24932600.00 | 5183200.00 | 20505000.00 | KO:K00394 | adenylylsulfate reductase subunit A | Bacteria;Proteobacteria;Deltaproteobacteria;Desulfobacterales;Desulfobacteraceae;Desulfatirhabdium;Desulfatirhabdium butyrativorans;Desulfatirhabdium butyrativorans DSM 18734 |
| Ga0586324_2639906_3_803 | 0.00 | 0.00 | 6981200.00 | 6716500.00 | 6252100.00 | 9091300.00 | 5152800.00 | 5014200.00 | 4649400.00 | 3403400.00 | 4954400.00 | 3510000.00 | KO:K00394 | adenylylsulfate reductase subunit A | Bacteria;Proteobacteria;Deltaproteobacteria;Syntrophobacterales;Syntrophaceae;Desulfomonile;Desulfomonile tiedjei;Desulfomonile tiedjei DCB-1, DSM 6799 |
| Ga0586324_2651662_50_802 | 0.00 | 0.00 | 0.00 | 0.00 | 0.00 | 355311.43 | 0.00 | 664900.00 | 0.00 | 1139600.00 | 861907.20 | 355342.50 | KO:K00394 | adenylylsulfate reductase subunit A | Bacteria;Firmicutes;Clostridia;Eubacteriales;Peptococcaceae;Desulfallas;Desulfallas thermosapovorans;Desulfallas thermosapovorans DSM 6562 |
| Ga0586324_2666361_3_383 | 0.00 | 0.00 | 0.00 | 0.00 | 0.00 | 0.00 | 0.00 | 0.00 | 0.00 | 0.00 | 0.00 | 50100000.00 | KO:K00394 | adenylylsulfate reductase subunit A | Bacteria;Proteobacteria;Deltaproteobacteria;Syntrophobacterales;Syntrophaceae;Desulfobacca;Desulfobacca acetoxidans;Desulfobacca acetoxidans ASRB2, DSM 11109 |
| Ga0586324_2768881_64_786 | 1330000.00 | 284313.44 | 0.00 | 0.00 | 1122700.00 | 2376021.18 | 3442800.00 | 2116700.00 | 1085400.00 | 3426500.00 | 3132800.00 | 3052500.00 | KO:K00394 | adenylylsulfate reductase subunit A | Bacteria;Proteobacteria;Deltaproteobacteria;Desulfobacterales;Desulfobacteraceae;Desulfatiglans;Desulfatiglans anilini;Desulfatiglans anilini DSM 4660 |
| Ga0586324_3028006_1_759 | 0.00 | 0.00 | 0.00 | 0.00 | 0.00 | 0.00 | 2964000.00 | 0.00 | 0.00 | 0.00 | 0.00 | 0.00 | KO:K00394 | adenylylsulfate reductase subunit A | Bacteria;Nitrospirae;Thermodesulfovibrionia;Thermodesulfovibrionales;Thermodesulfovibrionaceae;Thermodesulfovibrio;Thermodesulfovibrio islandicus;Thermodesulfovibrio islandicus DSM 12570 |
| Ga0586324_3035968_3_674 | 1290000.00 | 0.00 | 0.00 | 1938000.00 | 0.00 | 13855300.00 | 36252000.00 | 5605900.00 | 4131000.00 | 1832600.00 | 985600.00 | 4170000.00 | KO:K00394 | adenylylsulfate reductase subunit A | Bacteria;Proteobacteria;Deltaproteobacteria;Desulfobacterales;Desulfobulbaceae;Desulfobulbus;Desulfobulbus propionicus;Desulfobulbus propionicus 1pr3, DSM 2032 |
| Ga0586324_3059403_1_756 | 0.00 | 0.00 | 0.00 | 0.00 | 0.00 | 36801900.00 | 0.00 | 0.00 | 299086.83 | 0.00 | 6758400.00 | 10200000.00 | KO:K00394 | adenylylsulfate reductase subunit A | Bacteria;Proteobacteria;Deltaproteobacteria;Desulfobacterales;Desulfobacteraceae;Desulfatiglans;Desulfatiglans anilini;Desulfatiglans anilini DSM 4660 |
| Ga0586324_3102018_1_750 | 30500000.00 | 0.00 | 20774700.00 | 125685000.00 | 11010700.00 | 17042074.58 | 4286400.00 | 52609464.03 | 3410892.18 | 800800.00 | 579336.56 | 0.00 | KO:K00394 | adenylylsulfate reductase subunit A | Bacteria;Firmicutes;Clostridia;Eubacteriales;Peptococcaceae;Desulfofundulus;Desulfofundulus australicus;Desulfofundulus australicus DSM 11792 |
| Ga0586324_3102902_1_714 | 0.00 | 0.00 | 0.00 | 0.00 | 0.00 | 0.00 | 440961.12 | 0.00 | 0.00 | 0.00 | 0.00 | 0.00 | KO:K00394 | adenylylsulfate reductase subunit A | Bacteria;Proteobacteria;Deltaproteobacteria;Desulfobacterales;Desulfobacteraceae;Desulfatiglans;Desulfatiglans anilini;Desulfatiglans anilini DSM 4660 |
| Ga0586324_3161249_1_744 | 0.00 | 0.00 | 0.00 | 0.00 | 0.00 | 9567700.00 | 0.00 | 0.00 | 0.00 | 0.00 | 0.00 | 0.00 | KO:K00394 | adenylylsulfate reductase subunit A | Bacteria;Proteobacteria;Deltaproteobacteria;Syntrophobacterales;Syntrophobacteraceae;Syntrophobacter;Syntrophobacter fumaroxidans;Syntrophobacter fumaroxidans MPOB |
| Ga0586324_3216323_385_741 | 0.00 | 2101200.00 | 0.00 | 0.00 | 46659000.00 | 0.00 | 0.00 | 6893000.00 | 6115500.00 | 2148300.00 | 0.00 | 0.00 | KO:K00394 | adenylylsulfate reductase subunit A | Bacteria;Proteobacteria;Deltaproteobacteria;Syntrophobacterales;Syntrophobacteraceae;Desulfoglaeba;Desulfoglaeba alkanexedens;Desulfoglaeba alkanexedens ALDC |
| Ga0586324_3312272_1_732 | 3900000.00 | 0.00 | 5911500.00 | 21375000.00 | 0.00 | 22430500.00 | 0.00 | 0.00 | 0.00 | 0.00 | 0.00 | 0.00 | KO:K00394 | adenylylsulfate reductase subunit A | Bacteria;Proteobacteria;Deltaproteobacteria;Syntrophobacterales;Syntrophobacteraceae;Desulfacinum;Desulfacinum hydrothermale;Desulfacinum hydrothermale DSM 13146 |
| Ga0586324_3351633_28_726 | 0.00 | 0.00 | 0.00 | 0.00 | 0.00 | 0.00 | 0.00 | 719800.00 | 0.00 | 486717.00 | 0.00 | 0.00 | KO:K00394 | adenylylsulfate reductase subunit A | Bacteria;Proteobacteria;Betaproteobacteria;unclassified Betaproteobacteria;unclassified Betaproteobacteria;unclassified Betaproteobacteria;uncultured beta proteobacterium;unclassified betaproteobacterium ENR4 |
| Ga0586324_3404058_1_723 | 0.00 | 0.00 | 0.00 | 0.00 | 0.00 | 0.00 | 0.00 | 0.00 | 662831.91 | 0.00 | 0.00 | 0.00 | KO:K00394 | adenylylsulfate reductase subunit A | Bacteria;Proteobacteria;Gammaproteobacteria;Chromatiales;Chromatiaceae;Thiodictyon;Candidatus Thiodictyon syntrophicum;Candidatus Thiodictyon syntrophicum Cad16 |
| Ga0586324_3543379_65_712 | 0.00 | 0.00 | 0.00 | 0.00 | 0.00 | 0.00 | 0.00 | 0.00 | 0.00 | 977900.00 | 0.00 | 0.00 | KO:K00394 | adenylylsulfate reductase subunit A | Bacteria;Proteobacteria;Gammaproteobacteria;Thiotrichales;Thiotrichaceae;Beggiatoa;Beggiatoa sp. 'Orange Guaymas';Beggiatoa sp. Orange Guaymas |
| Ga0586324_3552065_2_709 | 1790000.00 | 1788400.00 | 0.00 | 2850000.00 | 3296000.00 | 4883100.00 | 1911659.16 | 1366400.00 | 1887300.00 | 770000.00 | 640607.44 | 0.00 | KO:K00394 | adenylylsulfate reductase subunit A | Bacteria;Proteobacteria;Deltaproteobacteria;Syntrophobacterales;Syntrophaceae;Desulfomonile;Desulfomonile tiedjei;Desulfomonile tiedjei DCB-1, DSM 6799 |
| Ga0586324_3679387_3_701 | 16160000.00 | 15164000.00 | 7656800.00 | 32917500.00 | 29396200.00 | 26357365.95 | 19562400.00 | 17202000.00 | 15552000.00 | 7623000.00 | 8219200.00 | 5955000.00 | KO:K00394 | adenylylsulfate reductase subunit A | Bacteria;Proteobacteria;Deltaproteobacteria;Syntrophobacterales;Syntrophaceae;Desulfobacca;Desulfobacca acetoxidans;Desulfobacca acetoxidans ASRB2, DSM 11109 |
| Ga0586324_3758533_3_695 | 1280000.00 | 93792755.64 | 0.00 | 720062.95 | 1565600.00 | 854558.38 | 0.00 | 994300.00 | 1360800.00 | 0.00 | 0.00 | 0.00 | KO:K00394 | adenylylsulfate reductase subunit A | Bacteria;Proteobacteria;Deltaproteobacteria;Syntrophobacterales;Syntrophaceae;Desulfobacca;Desulfobacca acetoxidans;Desulfobacca acetoxidans ASRB2, DSM 11109 |
| Ga0586324_3832797_2_544 | 19500000.00 | 26071200.00 | 27662684.09 | 31350000.00 | 28438300.00 | 44940400.00 | 12129600.00 | 36539000.00 | 27564300.00 | 8008000.00 | 8685600.00 | 21682500.00 | KO:K00394 | adenylylsulfate reductase subunit A | Bacteria;Proteobacteria;Deltaproteobacteria;Syntrophobacterales;Syntrophaceae;Desulfobacca;Desulfobacca acetoxidans;Desulfobacca acetoxidans ASRB2, DSM 11109 |
| Ga0586324_3972786_3_677 | 0.00 | 0.00 | 0.00 | 0.00 | 0.00 | 0.00 | 0.00 | 0.00 | 0.00 | 257287.80 | 0.00 | 0.00 | KO:K00394 | adenylylsulfate reductase subunit A | Bacteria;Proteobacteria;Betaproteobacteria;Nitrosomonadales;Thiobacillaceae;Thiobacillus;Thiobacillus denitrificans;Thiobacillus denitrificans DSM 12475 |
| Ga0586324_4025496_2_673 | 0.00 | 0.00 | 0.00 | 2470000.00 | 3234200.00 | 3317125.56 | 4035600.00 | 3884602.00 | 2146500.00 | 0.00 | 0.00 | 0.00 | KO:K00394 | adenylylsulfate reductase subunit A | Bacteria;Proteobacteria;Deltaproteobacteria;Desulfobacterales;Desulfobulbaceae;Desulfobulbus;Desulfobulbus propionicus;Desulfobulbus propionicus 1pr3, DSM 2032 |
| Ga0586324_4073583_3_392 | 18400000.00 | 0.00 | 0.00 | 3857000.00 | 0.00 | 3084836.89 | 0.00 | 0.00 | 1563300.00 | 1447600.00 | 0.00 | 0.00 | KO:K00394 | adenylylsulfate reductase subunit A | Archaea;Euryarchaeota;Archaeoglobi;Archaeoglobales;Archaeoglobaceae;Archaeoglobus;Archaeoglobus sulfaticallidus;Archaeoglobus sulfaticallidus PM70-1, DSM 19444 |
| Ga0586324_4200489_3_632 | 30100000.00 | 0.00 | 0.00 | 0.00 | 0.00 | 451385.03 | 1737353.16 | 0.00 | 0.00 | 0.00 | 0.00 | 0.00 | KO:K00394 | adenylylsulfate reductase subunit A | Bacteria;Proteobacteria;Deltaproteobacteria;Syntrophobacterales;Syntrophaceae;Desulfobacca;Desulfobacca acetoxidans;Desulfobacca acetoxidans ASRB2, DSM 11109 |
| Ga0586324_4250192_1_468 | 10400000.00 | 0.00 | 0.00 | 0.00 | 0.00 | 0.00 | 0.00 | 0.00 | 0.00 | 0.00 | 0.00 | 0.00 | KO:K00394 | adenylylsulfate reductase subunit A | Bacteria;Proteobacteria;Deltaproteobacteria;Syntrophobacterales;Syntrophaceae;Desulfobacca;Desulfobacca acetoxidans;Desulfobacca acetoxidans ASRB2, DSM 11109 |

| | | | | | | | | | | | | | | | |
|---|---|---|---|---|---|---|---|---|---|---|---|---|---|---|---|
| Ga0586324 4371959 1 594 | 0.00 | 0.00 | 0.00 | 6469500.00 | 22248000.00 | 0.00 | 30780000.00 | 14579000.00 | 17415000.00 | 0.00 | 25256000.00 | 15525000.00 | KO:K00394 | adenylylsulfate reductase subunit A | Bacteria;Nitrospirae;Thermodesulfovibrionia;Thermodesulfovibrionales;Thermodesulfovibrionaceae;Thermodesulfovibrio;Thermodesulfovibrio thiophilus;Thermodesulfovibrio thiophilus DSM 17215 |
| Ga0586324 4460902 3 647 | 0.00 | 0.00 | 0.00 | 0.00 | 0.00 | 0.00 | 0.00 | 4666500.00 | 0.00 | 0.00 | 0.00 | 0.00 | KO:K00394 | adenylylsulfate reductase subunit A | Bacteria;Nitrospirae;Thermodesulfovibrionia;Thermodesulfovibrionales;Thermodesulfovibrionaceae;Thermodesulfovibrio;Thermodesulfovibrio sp. N1;Thermodesulfovibrio sp. N1 |
| Ga0586324 4637138 3 635 | 10300000.00 | 0.00 | 0.00 | 0.00 | 27913000.00 | 0.00 | 2439600.00 | 0.00 | 0.00 | 0.00 | 0.00 | 0.00 | KO:K00394 | adenylylsulfate reductase subunit A | Archaea;Euryarchaeota;Archaeoglobi;Archaeoglobales;Archaeoglobaceae;Archaeoglobus;Archaeoglobus sulfaticallidus;Archaeoglobus sulfaticallidus PM70-1, DSM 19444 |
| Ga0586324 4669016 114 635 | 22290000.00 | 2325600.00 | 15482500.00 | 18867000.00 | 11082800.00 | 9069687.32 | 5131801.20 | 2714500.00 | 2924100.00 | 4096400.00 | 0.00 | 0.00 | KO:K00394 | adenylylsulfate reductase subunit A | Bacteria;Proteobacteria;Deltaproteobacteria;Syntrophobacterales;Syntrophaceae;Desulfobacca;Desulfobacca acetoxidans;Desulfobacca acetoxidans ASRB2, DSM 11109 |
| Ga0586324 4669363 78 635 | 2360000.00 | 2060400.00 | 3092795.46 | 0.00 | 0.00 | 425921.45 | 0.00 | 652700.00 | 0.00 | 0.00 | 0.00 | 787500.00 | KO:K00394 | adenylylsulfate reductase subunit A | Bacteria;Firmicutes;Clostridia;Thermoanaerobacterales;Thermoanaerobacteraceae;Desulfovirgula;Desulfovirgula thermocuniculi;Desulfovirgula thermocuniculi DSM 16036 |
| Ga0586324 4733930 2 487 | 3071313.00 | 0.00 | 0.00 | 0.00 | 0.00 | 0.00 | 0.00 | 0.00 | 0.00 | 0.00 | 0.00 | 0.00 | KO:K00394 | adenylylsulfate reductase subunit A | Bacteria;Proteobacteria;Deltaproteobacteria;Syntrophobacterales;Syntrophaceae;Desulfomonile;Desulfomonile tiedjei;Desulfomonile tiedjei DCB-1, DSM 6799 |
| Ga0586324 4966543 3 617 | 0.00 | 0.00 | 0.00 | 0.00 | 0.00 | 0.00 | 0.00 | 0.00 | 0.00 | 1963500.00 | 14784000.00 | 0.00 | KO:K00394 | adenylylsulfate reductase subunit A | Bacteria;Proteobacteria;Deltaproteobacteria;Desulfobacterales;Desulfobacteraceae;Desulfatitalea;Desulfatitalea tepidiphila;Desulfatitalea tepidiphila S28bF |
| Ga0586324 5110930 2 610 | 20200000.00 | 8636000.00 | 3816886.65 | 0.00 | 0.00 | 10163200.00 | 3488400.00 | 12566000.00 | 27099329.22 | 6583500.00 | 0.00 | 6652500.00 | KO:K00394 | adenylylsulfate reductase subunit A | Bacteria;Proteobacteria;Deltaproteobacteria;Syntrophobacterales;Syntrophaceae;Desulfobacca;Desulfobacca acetoxidans;Desulfobacca acetoxidans ASRB2, DSM 11109 |
| Ga0586324 5134048 1 609 | 0.00 | 0.00 | 0.00 | 0.00 | 0.00 | 0.00 | 0.00 | 2220400.00 | 5297400.00 | 0.00 | 0.00 | 0.00 | KO:K00394 | adenylylsulfate reductase subunit A | Bacteria;Proteobacteria;Deltaproteobacteria;Desulfobacterales;Desulfobulbaceae;Desulfoprunum;Desulfoprunum benzoelyticum;Desulfoprunum benzoelyticum DSM 28570 |
| Ga0586324 5197660 1 564 | 0.00 | 0.00 | 0.00 | 0.00 | 5098500.00 | 0.00 | 0.00 | 0.00 | 0.00 | 0.00 | 0.00 | 0.00 | KO:K00394 | adenylylsulfate reductase subunit A | Bacteria;Firmicutes;Clostridia;Eubacteriales;Desulfallaceae;Desulfoscipio;Desulfoscipio geothermicus;Desulfallas geothermicus DSM 3669 |
| Ga0586324 5264039 45 605 | 0.00 | 0.00 | 0.00 | 0.00 | 0.00 | 0.00 | 0.00 | 3037800.00 | 0.00 | 0.00 | 0.00 | 0.00 | KO:K00394 | adenylylsulfate reductase subunit A | Bacteria;Proteobacteria;Betaproteobacteria;Nitrosomonadales;Thiobacillaceae;Sulfuritortus;Sulfuritortus calidifontis;Sulfuritortus calidifontis DSM 103923 |
| Ga0586324 5346895 2 601 | 0.00 | 0.00 | 0.00 | 0.00 | 0.00 | 0.00 | 0.00 | 10370000.00 | 0.00 | 0.00 | 0.00 | 0.00 | KO:K00394 | adenylylsulfate reductase subunit A | Archaea;Euryarchaeota;Archaeoglobi;Archaeoglobales;Archaeoglobaceae;Archaeoglobus;Archaeoglobus sulfaticallidus;Archaeoglobus sulfaticallidus PM70-1, DSM 19444 |
| Ga0586324 5476145 1 594 | 2900000.00 | 1339600.00 | 0.00 | 0.00 | 0.00 | 0.00 | 0.00 | 0.00 | 1182600.00 | 0.00 | 0.00 | 0.00 | KO:K00394 | adenylylsulfate reductase subunit A | Bacteria;Proteobacteria;Gammaproteobacteria;Thiotrichales;Thiotrichaceae;Beggiatoa;Beggiatoa sp. 'Orange Guaymas';Beggiatoa sp. Orange Guaymas |
| Ga0586324 5639838 1 471 | 0.00 | 0.00 | 6587100.00 | 0.00 | 4933700.00 | 4724300.00 | 3009600.00 | 2135000.00 | 2389500.00 | 0.00 | 0.00 | 0.00 | KO:K00394 | adenylylsulfate reductase subunit A | Bacteria;Proteobacteria;Deltaproteobacteria;Syntrophobacterales;Syntrophaceae;Desulfobacca;Desulfobacca acetoxidans;Desulfobacca acetoxidans ASRB2, DSM 11109 |
| Ga0586324 5861616 1 579 | 0.00 | 7956000.00 | 0.00 | 5234500.00 | 5860700.00 | 10599900.00 | 0.00 | 5880400.00 | 6253200.00 | 0.00 | 0.00 | 0.00 | KO:K00394 | adenylylsulfate reductase subunit A | Bacteria;Proteobacteria;Deltaproteobacteria;Syntrophobacterales;Syntrophaceae;Desulfobacca;Desulfobacca acetoxidans;Desulfobacca acetoxidans ASRB2, DSM 11109 |
| Ga0586324 5881603 3 578 | 0.00 | 0.00 | 0.00 | 0.00 | 0.00 | 11155700.00 | 0.00 | 3696600.00 | 0.00 | 16786000.00 | 0.00 | 717534.75 | KO:K00394 | adenylylsulfate reductase subunit A | Bacteria;Proteobacteria;Deltaproteobacteria;Syntrophobacterales;Syntrophaceae;Desulfobacca;Desulfobacca acetoxidans;Desulfobacca acetoxidans ASRB2, DSM 11109 |
| Ga0586324 5962425 2 574 | 0.00 | 0.00 | 0.00 | 0.00 | 0.00 | 13021600.00 | 0.00 | 0.00 | 0.00 | 0.00 | 0.00 | 0.00 | KO:K00394 | adenylylsulfate reductase subunit A | Bacteria;Proteobacteria;Deltaproteobacteria;unclassified Deltaproteobacteria;unclassified Deltaproteobacteria;unclassified Deltaproteobacteria;delta proteobacterium NaphS2;delta proteobacterium NaphS2 |
| Ga0586324 6017615 2 571 | 8580000.00 | 8500000.00 | 0.00 | 12065000.00 | 0.00 | 12942200.00 | 0.00 | 0.00 | 7767900.00 | 10857000.00 | 0.00 | 8250000.00 | KO:K00394 | adenylylsulfate reductase subunit A | Bacteria;Proteobacteria;Deltaproteobacteria;Desulfobacterales;Desulfobacteraceae;Desulfospira;Desulfospira joergensenii;Desulfospira joergensenii DSM 10085 |
| Ga0586324 6534953 2 553 | 44400000.00 | 39236000.00 | 17396700.00 | 23750000.00 | 6684700.00 | 5359500.00 | 12836400.00 | 3385500.00 | 5759100.00 | 21252000.00 | 0.00 | 19275000.00 | KO:K00394 | adenylylsulfate reductase subunit A | Bacteria;Proteobacteria;Deltaproteobacteria;Syntrophobacterales;Syntrophaceae;Desulfobacca;Desulfobacca acetoxidans;Desulfobacca acetoxidans ASRB2, DSM 11109 |
| Ga0586324 6690651 1 546 | 0.00 | 0.00 | 0.00 | 0.00 | 0.00 | 200401.23 | 0.00 | 0.00 | 0.00 | 0.00 | 0.00 | 0.00 | KO:K00394 | adenylylsulfate reductase subunit A | Bacteria;Nitrospirae;Thermodesulfovibrionia;Thermodesulfovibrionales;Thermodesulfovibrionaceae;Thermodesulfovibrio;Thermodesulfovibrio hydrogeniphilus;Thermodesulfovibrio hydrogeniphilus DSM 18151 |
| Ga0586324 6866642 1 540 | 0.00 | 0.00 | 0.00 | 0.00 | 0.00 | 0.00 | 8276400.00 | 0.00 | 0.00 | 0.00 | 0.00 | 0.00 | KO:K00394 | adenylylsulfate reductase subunit A | Bacteria;Proteobacteria;Gammaproteobacteria;Acidiferrobacterales;Acidiferrobacteraceae;Sulfuricaulis;Sulfuricaulis limicola;Sulfuricaulis limicola HA5 |
| Ga0586324 6920091 3 467 | 0.00 | 71400000.00 | 3822359.01 | 16055000.00 | 97438000.00 | 199651300.00 | 0.00 | 1653100.00 | 66460500.00 | 35112000.00 | 61195200.00 | 117750000.00 | KO:K00394 | adenylylsulfate reductase subunit A | Bacteria;Proteobacteria;Deltaproteobacteria;Syntrophobacterales;Syntrophaceae;Desulfomonile;Desulfomonile tiedjei;Desulfomonile tiedjei DCB-1, DSM 6799 |
| Ga0586324 7352739 1 525 | 0.00 | 754800.00 | 0.00 | 0.00 | 4377500.00 | 19171395.99 | 2781600.00 | 2183800.00 | 1053000.00 | 0.00 | 1469600.00 | 1942500.00 | KO:K00394 | adenylylsulfate reductase subunit A | Bacteria;Proteobacteria;Deltaproteobacteria;Desulfobacterales;Desulfobulbaceae;Desulfoprunum;Desulfoprunum benzoelyticum;Desulfoprunum benzoelyticum DSM 28570 |
| Ga0586324 7688927 3 515 | 0.00 | 0.00 | 0.00 | 1767000.00 | 0.00 | 0.00 | 0.00 | 0.00 | 0.00 | 1917300.00 | 2543200.00 | 0.00 | KO:K00394 | adenylylsulfate reductase subunit A | Bacteria;Proteobacteria;Betaproteobacteria;Nitrosomonadales;Thiobacillaceae;Thiobacillus;Thiobacillus thioparus;Thiobacillus thioparus DSM 505 |
| Ga0586324 7838490 2 511 | 104000000.00 | 77520000.00 | 176782000.00 | 175750000.00 | 148320000.00 | 0.00 | 21956400.00 | 110410000.00 | 104490000.00 | 69762000.00 | 52096000.00 | 92250000.00 | KO:K00394 | adenylylsulfate reductase subunit A | Archaea;Euryarchaeota;Archaeoglobi;Archaeoglobales;Archaeoglobaceae;Archaeoglobus;Archaeoglobus sulfaticallidus;Archaeoglobus sulfaticallidus PM70-1, DSM 19444 |
| Ga0586324 7976711 3 509 | 0.00 | 0.00 | 0.00 | 0.00 | 0.00 | 0.00 | 0.00 | 0.00 | 196207.92 | 0.00 | 0.00 | 0.00 | KO:K00394 | adenylylsulfate reductase subunit A | Bacteria;Proteobacteria;Deltaproteobacteria;Desulfobacterales;Desulfobacteraceae;Desulfatiglans;Desulfatiglans anilini;Desulfatiglans anilini DSM 4660 |
| Ga0586324 0000002 211332 211817 | 0.00 | 0.00 | 0.00 | 0.00 | 0.00 | 0.00 | 0.00 | 0.00 | 0.00 | 0.00 | 252822.24 | 0.00 | KO:K00395 | adenylylsulfate reductase subunit B | Bacteria;Proteobacteria;Betaproteobacteria;Nitrosomonadales;Thiobacillaceae;Annwoodia;Annwoodia aquaesulis;Annwoodia aquaesulis DSM 4255 |
| Ga0586324 0009502 1883 2362 | 0.00 | 0.00 | 0.00 | 0.00 | 0.00 | 0.00 | 0.00 | 0.00 | 0.00 | 0.00 | 0.00 | 840000.00 | KO:K00395 | adenylylsulfate reductase subunit B | Bacteria;Proteobacteria;Gammaproteobacteria;Chromatiales;Chromatiaceae;Thiodictyon;Candidatus Thiodictyon syntrophicum;Candidatus Thiodictyon syntrophicum Cad16 |
| Ga0586324 0014308 7058 7543 | 1880000.00 | 0.00 | 0.00 | 0.00 | 0.00 | 2265416.98 | 0.00 | 0.00 | 0.00 | 0.00 | 0.00 | 0.00 | KO:K00395 | adenylylsulfate reductase subunit B | Bacteria;Proteobacteria;Deltaproteobacteria;Desulfobacterales;Desulfobacteraceae;Desulfatitalea;Desulfatitalea tepidiphila;Desulfatitalea tepidiphila S28bF |
| Ga0586324 0032719 2335 2802 | 23400000.00 | 0.00 | 25335000.00 | 0.00 | 0.00 | 0.00 | 0.00 | 0.00 | 0.00 | 2340800.00 | 3097600.00 | 0.00 | KO:K00395 | adenylylsulfate reductase subunit B | Bacteria;Proteobacteria;Gammaproteobacteria;Chromatiales;Thioalkalispiraceae;Sulfurivermis;Sulfurivermis fontis;Sulfurivermis fontis JG42 |
| Ga0586324 0046593 2947 3378 | 5420000.00 | 4025600.00 | 3904359.96 | 4740500.00 | 3872800.00 | 3954243.07 | 0.00 | 5740100.00 | 5848200.00 | 5936700.00 | 3960000.00 | 0.00 | KO:K00395 | adenylylsulfate reductase subunit B | Bacteria;Nitrospirae;Nitrospira;Nitrospirales;Nitrospiraceae;Candidatus Magnetobacterium;Candidatus Magnetobacterium casensis;Candidatus Magnetobacterium casensis MYR-1 |
| Ga0586324 0072572 1797 2264 | 0.00 | 2284800.00 | 12949000.00 | 2660000.00 | 0.00 | 4644900.00 | 0.00 | 2501000.00 | 1903500.00 | 800800.00 | 2824800.00 | 3285000.00 | KO:K00395 | adenylylsulfate reductase subunit B | Bacteria;Proteobacteria;Gammaproteobacteria;Chromatiales;Chromatiaceae;Thiocystis;Thiocystis violascens;Thiocystis violascens 611, DSM 198 |
| Ga0586324 0095010 2518 2991 | 0.00 | 0.00 | 0.00 | 0.00 | 0.00 | 0.00 | 0.00 | 0.00 | 0.00 | 0.00 | 0.00 | 32550000.00 | KO:K00395 | adenylylsulfate reductase subunit B | Bacteria;Proteobacteria;Betaproteobacteria;Nitrosomonadales;Thiobacillaceae;Thiobacillus;Thiobacillus thiophilus;Thiobacillus thiophilus DSM 19892 |
| Ga0586324 0154655 2116 2577 | 383584.00 | 1434800.00 | 3318175.62 | 2147000.00 | 0.00 | 2004659.44 | 686444.16 | 1018700.00 | 0.00 | 2410100.00 | 231169.84 | 1928871.75 | KO:K00395 | adenylylsulfate reductase subunit B | Bacteria;Proteobacteria;Deltaproteobacteria;Desulfarculales;Desulfarculaceae;Dethiosulfatarculus;Dethiosulfatarculus sandiegensis;Dethiosulfatarculus sandiegensis SPR |
| Ga0586324 0200605 668 1135 | 0.00 | 0.00 | 0.00 | 0.00 | 0.00 | 0.00 | 23940000.00 | 15677000.00 | 14175000.00 | 23562000.00 | 23760000.00 | 25725000.00 | KO:K00395 | adenylylsulfate reductase subunit B | Bacteria;Proteobacteria;Betaproteobacteria;Nitrosomonadales;Sulfuricellaceae;Sulfurirhabdus;Sulfurirhabdus autotrophica;Sulfurirhabdus autotrophica DSM 100309 |
| Ga0586324 0246733 1369 1830 | 457802.00 | 0.00 | 0.00 | 494052.25 | 0.00 | 0.00 | 0.00 | 0.00 | 0.00 | 0.00 | 1707200.00 | 1425000.00 | KO:K00395 | adenylylsulfate reductase subunit B | Bacteria;Proteobacteria;Deltaproteobacteria;Desulfarculales;Desulfarculaceae;Dethiosulfatarculus;Dethiosulfatarculus sandiegensis;Dethiosulfatarculus sandiegensis SPR |
| Ga0586324 0282363 1019 1465 | 1710000.00 | 0.00 | 0.00 | 0.00 | 0.00 | 0.00 | 0.00 | 0.00 | 0.00 | 0.00 | 0.00 | 0.00 | KO:K00395 | adenylylsulfate reductase subunit B | Bacteria;Firmicutes;Clostridia;Thermoanaerobacterales;Thermoanaerobacteraceae;Ammonifex;Ammonifex thiophilus;Ammonifex thiophilus SR |
| Ga0586324 0355574 1111 1572 | 1210000.00 | 519728.76 | 2338459.91 | 2308500.00 | 1854030.90 | 1537525.42 | 852617.40 | 762500.00 | 0.00 | 1663200.00 | 4408800.00 | 4117500.00 | KO:K00395 | adenylylsulfate reductase subunit B | Bacteria;Proteobacteria;Deltaproteobacteria;Desulfarculales;Desulfarculaceae;Desulfocarbo;Desulfocarbo indianensis;Desulfocarbo indianensis SCBM |
| Ga0586324 0457934 1392 1703 | 0.00 | 1822400.00 | 0.00 | 0.00 | 0.00 | 0.00 | 0.00 | 0.00 | 0.00 | 0.00 | 2217600.00 | 0.00 | KO:K00395 | adenylylsulfate reductase subunit B | Bacteria;Proteobacteria;Deltaproteobacteria;unclassified Deltaproteobacteria;unclassified Deltaproteobacteria;unclassified Deltaproteobacteria;delta proteobacterium NaphS2;delta proteobacterium NaphS2 |
| Ga0586324 0483263 460 921 | 1000000.00 | 30532000.00 | 13030989.69 | 41230000.00 | 32136000.00 | 40494000.00 | 2553600.00 | 25459813.39 | 28998000.00 | 26103000.00 | 10648000.00 | 1507500.00 | KO:K00395 | adenylylsulfate reductase subunit B | Bacteria;Proteobacteria;Deltaproteobacteria;Desulfarculales;Desulfarculaceae;Dethiosulfatarculus;Dethiosulfatarculus sandiegensis;Dethiosulfatarculus sandiegensis SPR |
| Ga0586324 0560875 326 799 | 0.00 | 0.00 | 0.00 | 0.00 | 0.00 | 0.00 | 0.00 | 50203000.00 | 0.00 | 0.00 | 0.00 | 0.00 | KO:K00395 | adenylylsulfate reductase subunit B | Bacteria;Proteobacteria;Betaproteobacteria;Nitrosomonadales;Sterolibacteriaceae;Sulfurisoma;Sulfurisoma sediminicola;Sulfurisoma sediminicola DSM 26916 |
| Ga0586324 0731072 171 638 | 6600000.00 | 0.00 | 0.00 | 3296500.00 | 0.00 | 2506062.50 | 0.00 | 0.00 | 0.00 | 0.00 | 0.00 | 0.00 | KO:K00395 | adenylylsulfate reductase subunit B | Bacteria;Proteobacteria;Gammaproteobacteria;Chromatiales;Chromatiaceae;Thiocystis;Thiocystis violascens;Thiocystis violascens 611, DSM 198 |
| Ga0586324 1285972 2 349 | 0.00 | 0.00 | 0.00 | 0.00 | 0.00 | 0.00 | 0.00 | 0.00 | 0.00 | 0.00 | 1742400.00 | 0.00 | KO:K00395 | adenylylsulfate reductase subunit B | Bacteria;Proteobacteria;Betaproteobacteria;Nitrosomonadales;Sterolibacteriaceae;Sulfurisoma;Sulfurisoma sediminicola;Sulfurisoma sediminicola DSM 26916 |
| Ga0586324 1651631 497 931 | 0.00 | 0.00 | 0.00 | 6336500.00 | 0.00 | 0.00 | 0.00 | 0.00 | 0.00 | 0.00 | 0.00 | 0.00 | KO:K00395 | adenylylsulfate reductase subunit B | Bacteria;Proteobacteria;Deltaproteobacteria;Desulfobacterales;Desulfobacteraceae;Desulfatibacillum;Desulfatibacillum alkenivorans;Desulfatibacillum alkenivorans DSM 16219 |
| Ga0586324 1685144 3 383 | 0.00 | 25296000.00 | 0.00 | 0.00 | 0.00 | 50816000.00 | 0.00 | 0.00 | 20655000.00 | 0.00 | 0.00 | 0.00 | KO:K00395 | adenylylsulfate reductase subunit B | Bacteria;Firmicutes;Clostridia;Thermoanaerobacterales;Thermoanaerobacteraceae;Ammonifex;Ammonifex thiophilus;Ammonifex thiophilus SR |
| Ga0586324 3256386 244 735 | 0.00 | 2794800.00 | 0.00 | 0.00 | 0.00 | 0.00 | 0.00 | 0.00 | 0.00 | 0.00 | 0.00 | 0.00 | KO:K00395 | adenylylsulfate reductase subunit B | Bacteria;Firmicutes;Clostridia;Eubacteriales;Peptococcaceae;Desulfofundulus;Desulfofundulus salinum;Desulfofundulus salinum 435 |
| Ga0586324 4242405 273 659 | 2100000.00 | 0.00 | 0.00 | 9880000.00 | 9177300.00 | 18460500.00 | 0.00 | 0.00 | 0.00 | 0.00 | 0.00 | 0.00 | KO:K00395 | adenylylsulfate reductase subunit B | Bacteria;Proteobacteria;Deltaproteobacteria;Syntrophobacterales;Syntrophobacteraceae;Syntrophobacter;Syntrophobacter fumaroxidans;Syntrophobacter fumaroxidans MPOB |
| Ga0586324 5162953 157 609 | 0.00 | 0.00 | 0.00 | 0.00 | 0.00 | 0.00 | 0.00 | 0.00 | 0.00 | 0.00 | 0.00 | 757500.00 | KO:K00395 | adenylylsulfate reductase subunit B | Bacteria;Proteobacteria;Deltaproteobacteria;Desulfobacterales;Desulfobacteraceae;Desulfatitalea;Desulfatitalea tepidiphila;Desulfatitalea tepidiphila S28bF |
| Ga0586324 7463017 161 523 | 0.00 | 0.00 | 0.00 | 0.00 | 0.00 | 0.00 | 0.00 | 0.00 | 0.00 | 23793000.00 | 0.00 | 0.00 | KO:K00395 | adenylylsulfate reductase subunit B | Bacteria;Proteobacteria;Gammaproteobacteria;Acidiferrobacterales;Acidiferrobacteraceae;Sulfuricaulis;Sulfuricaulis limicola;Sulfuricaulis limicola HA5 |
| Ga0586324 0000267 32259 33962 | 12600000.00 | 0.00 | 0.00 | 0.00 | 0.00 | 0.00 | 0.00 | 0.00 | 0.00 | 0.00 | 0.00 | 0.00 | KO:K00399 | methyl-coenzyme M reductase alpha subunit | Archaea;Euryarchaeota;Methanomicrobia;Methanomicrobiales;Methanomicrobiaceae;Methanofollis;Methanofollis liminatans;Methanofollis liminatans GKZPZ, DSM 4140 |
| Ga0586324 0005577 2364 4028 | 12950000.00 | 1795200.00 | 0.00 | 0.00 | 0.00 | 0.00 | 0.00 | 0.00 | 0.00 | 0.00 | 0.00 | 0.00 | KO:K00399 | methyl-coenzyme M reductase alpha subunit | Archaea;Candidatus Thermoplasmatota;Thermoplasmata;Methanomassiliicoccales;Methanomassiliicoccaceae;Methanomassiliicoccus;Candidatus Methanomassiliicoccus intestinalis;Candidatus Methanomassiliicoccus intestinalis Issoire-Mx1 |
| Ga0586324 0095431 2601 3506 | 2940000.00 | 3488400.00 | 0.00 | 0.00 | 2090900.00 | 0.00 | 0.00 | 3904000.00 | 3240000.00 | 1678600.00 | 0.00 | 2857500.00 | KO:K00399 | methyl-coenzyme M reductase alpha subunit | Archaea;Euryarchaeota;Methanomicrobia;Methanosarcinales;Methanotrichaceae;Methanothrix;Methanothrix thermoacetophila;Methanothrix thermoacetophila PT |
| Ga0586324 0159140 1464 2753 | 0.00 | 7684000.00 | 0.00 | 0.00 | 0.00 | 2168600.59 | 0.00 | 12383000.00 | 11178000.00 | 0.00 | 0.00 | 0.00 | KO:K00399 | methyl-coenzyme M reductase alpha subunit | Archaea;Euryarchaeota;Methanomicrobia;Methanomicrobiales;Methanomicrobiaceae;Methanofollis;Methanofollis ethanolicus;Methanofollis ethanolicus JCM 15103 |
| Ga0586324 0172585 1519 2649 | 0.00 | 0.00 | 0.00 | 0.00 | 4490800.00 | 0.00 | 0.00 | 0.00 | 0.00 | 0.00 | 0.00 | 0.00 | KO:K00399 | methyl-coenzyme M reductase alpha subunit | Archaea;Candidatus Thermoplasmatota;Thermoplasmata;Methanomassiliicoccales;Methanomassiliicoccaceae;Methanomassiliicoccus;Methanomassiliicoccus luminyensis;Methanomassiliicoccus luminyensis B10 |
| Ga0586324 0257221 978 2204 | 27860000.00 | 98804000.00 | 275025500.00 | 16435000.00 | 0.00 | 67410600.00 | 112563600.00 | 195749000.00 | 16807500.00 | 182028000.00 | 65322400.00 | 18030000.00 | KO:K00399 | methyl-coenzyme M reductase alpha subunit | Archaea;Euryarchaeota;Methanomicrobia;Methanosarcinales;Methanotrichaceae;Methanothrix;Methanothrix soehngenii;Methanothrix soehngenii GP-6 |
| Ga0586324 0269169 3 1247 | 0.00 | 0.00 | 0.00 | 0.00 | 7694100.00 | 0.00 | 0.00 | 0.00 | 0.00 | 0.00 | 0.00 | 0.00 | KO:K00399 | methyl-coenzyme M reductase alpha subunit | Archaea;Euryarchaeota;Methanomicrobia;Methanomicrobiales;Methanoregulaceae;Methanoregula;Methanoregula formicica;Methanoregula formicica SMSP, DSM 22288 |
| Ga0586324 0316704 226 1920 | 0.00 | 0.00 | 139229900.00 | 0.00 | 28943000.00 | 24018500.00 | 0.00 | 0.00 | 0.00 | 16940000.00 | 0.00 | 0.00 | KO:K00399 | methyl-coenzyme M reductase alpha subunit | Archaea;Euryarchaeota;Methanomicrobia;Methanomicrobiales;Methanoregulaceae;Methanoregula;Methanoregula boonei;Methanoregula boonei 6A8 |
| Ga0586324 0432841 954 1745 | 0.00 | 0.00 | 0.00 | 4873500.00 | 5489900.00 | 3719635.92 | 0.00 | 2440000.00 | 512814.24 | 0.00 | 0.00 | 0.00 | KO:K00399 | methyl-coenzyme M reductase alpha subunit | Archaea;Euryarchaeota;Methanomicrobia;Methanosarcinales;Methanosarcinaceae;Methanosarcina;Methanosarcina spelaei;Methanosarcina spelaei MC-15 |
| Ga0586324 0460264 1 1161 | 0.00 | 2250800.00 | 0.00 | 0.00 | 0.00 | 2510695.49 | 0.00 | 0.00 | 0.00 | 0.00 | 0.00 | 0.00 | KO:K00399 | methyl-coenzyme M reductase alpha subunit | Archaea;Euryarchaeota;Methanomicrobia;Methanomicrobiales;Methanoregulaceae;Methanoregula;Methanoregula boonei;Methanoregula boonei 6A8 |
| Ga0586324 0623905 376 1488 | 2160000.00 | 0.00 | 0.00 | 0.00 | 0.00 | 0.00 | 0.00 | 0.00 | 0.00 | 0.00 | 0.00 | 0.00 | KO:K00399 | methyl-coenzyme M reductase alpha subunit | Archaea;Candidatus Thermoplasmatota;Thermoplasmata;Methanomassiliicoccales;Methanomassiliicoccaceae;Methanomassiliicoccus;Candidatus Methanomassiliicoccus intestinalis;Candidatus Methanomassiliicoccus intestinalis Issoire-Mx1 |
| Ga0586324 0630844 2 1480 | 6070000.00 | 8024000.00 | 0.00 | 0.00 | 0.00 | 0.00 | 17966400.00 | 1793400.00 | 0.00 | 0.00 | 0.00 | 0.00 | KO:K00399 | methyl-coenzyme M reductase alpha subunit | Archaea;Euryarchaeota;Methanomicrobia;Methanomicrobiales;Methanoregulaceae;Methanoregula;Methanoregula formicica;Methanoregula formicica SMSP, DSM 22288 |
| Ga0586324 0719171 685 1398 | 0.00 | 16456000.00 | 0.00 | 0.00 | 0.00 | 0.00 | 0.00 | 0.00 | 0.00 | 0.00 | 0.00 | 0.00 | KO:K00399 | methyl-coenzyme M reductase alpha subunit | Archaea;Euryarchaeota;Methanobacteria;Methanobacteriales;Methanobacteriaceae;Methanobacterium;Methanobacterium sp. SMA-27;Methanobacterium sp. SMA-27 |
| Ga0586324 0761427 3 1364 | 0.00 | 4610400.00 | 9796200.00 | 0.00 | 0.00 | 0.00 | 0.00 | 0.00 | 0.00 | 0.00 | 0.00 | 0.00 | KO:K00399 | methyl-coenzyme M reductase alpha subunit | Archaea;Euryarchaeota;Methanomicrobia;Methanomicrobiales;Methanoregulaceae;Methanoregula;Methanoregula boonei;Methanoregula boonei 6A8 |
| Ga0586324 0774128 1 915 | 13490000.00 | 11084000.00 | 7713100.00 | 4930500.00 | 0.00 | 0.00 | 0.00 | 3977200.00 | 0.00 | 0.00 | 0.00 | 0.00 | KO:K00399 | methyl-coenzyme M reductase alpha subunit | Archaea;Euryarchaeota;Methanomicrobia;Methanomicrobiales;Methanoregulaceae;Methanoregula;Methanoregula boonei;Methanoregula boonei 6A8 |
| Ga0586324 0800103 3 1292 | 156000000.00 | 0.00 | 0.00 | 0.00 | 0.00 | 0.00 | 0.00 | 0.00 | 0.00 | 0.00 | 0.00 | 0.00 | KO:K00399 | methyl-coenzyme M reductase alpha subunit | Archaea;Euryarchaeota;Methanomicrobia;Methanomicrobiales;Methanoregulaceae;Methanoregula;Methanoregula formicica;Methanoregula formicica SMSP, DSM 22288 |
| Ga0586324 1153740 3 848 | 962738.00 | 0.00 | 0.00 | 0.00 | 0.00 | 0.00 | 0.00 | 0.00 | 0.00 | 0.00 | 0.00 | 0.00 | KO:K00399 | methyl-coenzyme M reductase alpha subunit | Archaea;Candidatus Thermoplasmatota;Thermoplasmata;Methanomassiliicoccales;Methanomassiliicoccaceae;Methanomassiliicoccus;Methanomassiliicoccus luminyensis;Methanomassiliicoccus luminyensis B10 |
| Ga0586324 1274864 1 1095 | 0.00 | 0.00 | 0.00 | 0.00 | 0.00 | 695615.46 | 0.00 | 0.00 | 0.00 | 0.00 | 0.00 | 0.00 | KO:K00399 | methyl-coenzyme M reductase alpha subunit | Archaea;Euryarchaeota;Methanomicrobia;Methanomicrobiales;Methanomicrobiaceae;Methanofollis;Methanofollis liminatans;Methanofollis liminatans GKZPZ, DSM 4140 |
| Ga0586324 1288639 84 1088 | 0.00 | 0.00 | 52753100.00 | 0.00 | 0.00 | 7701800.00 | 0.00 | 0.00 | 0.00 | 0.00 | 0.00 | 0.00 | KO:K00399 | methyl-coenzyme M reductase alpha subunit | Archaea;Euryarchaeota;Methanomicrobia;Methanomicrobiales;Methanoregulaceae;Methanoregula;Methanoregula formicica;Methanoregula formicica SMSP, DSM 22288 |
| Ga0586324 2186377 2 595 | 977853.00 | 0.00 | 0.00 | 0.00 | 0.00 | 1940968.73 | 0.00 | 0.00 | 0.00 | 0.00 | 0.00 | 0.00 | KO:K00399 | methyl-coenzyme M reductase alpha subunit | Archaea;Euryarchaeota;Methanomicrobia;Methanomicrobiales;Methanoregulaceae;Methanosphaerula;Methanosphaerula palustris;Methanosphaerula palustris E1-9c, DSM 19958 |
| Ga0586324 2296551 2 772 | 69960000.00 | 61200000.00 | 27686797.38 | 30305000.00 | 20589700.00 | 25032874.70 | 32148000.00 | 40674800.00 | 69757200.00 | 8855000.00 | 6538400.00 | 13125000.00 | KO:K00399 | methyl-coenzyme M reductase alpha subunit | Archaea;Euryarchaeota;Methanomicrobia;Methanosarcinales;Methanotrichaceae;Methanothrix;Methanothrix soehngenii;Methanothrix soehngenii GP-6 |
| Ga0586324 3134344 2 748 | 913216.00 | 254367600.00 | 0.00 | 0.00 | 0.00 | 0.00 | 0.00 | 0.00 | 5151600.00 | 0.00 | 0.00 | 3075000.00 | KO:K00399 | methyl-coenzyme M reductase alpha subunit | Archaea;Euryarchaeota;Methanomicrobia;Methanosarcinales;Methanotrichaceae;Methanothrix;Methanothrix soehngenii;Methanothrix soehngenii GP-6 |
| Ga0586324 3280632 3 515 | 0.00 | 5433200.00 | 3969352.68 | 0.00 | 0.00 | 0.00 | 0.00 | 0.00 | 0.00 | 0.00 | 0.00 | 0.00 | KO:K00399 | methyl-coenzyme M reductase alpha subunit | Archaea;Euryarchaeota;Methanomicrobia;Methanosarcinales;Methanotrichaceae;Methanothrix;Methanothrix soehngenii;Methanothrix soehngenii GP-6 |
| Ga0586324 3576771 3 674 | 37800000.00 | 76976000.00 | 97455300.00 | 110798500.00 | 88312200.00 | 106435700.00 | 49498800.00 | 13700600.00 | 66760200.00 | 69531000.00 | 103144800.00 | 20497500.00 | KO:K00399 | methyl-coenzyme M reductase alpha subunit | Archaea;Euryarchaeota;Methanomicrobia;Methanosarcinales;Methanotrichaceae;Methanothrix;Methanothrix thermoacetophila;Methanothrix thermoacetophila PT |
| Ga0586324 3682142 1 699 | 1530000.00 | 0.00 | 0.00 | 16055000.00 | 0.00 | 0.00 | 0.00 | 0.00 | 0.00 | 0.00 | 0.00 | 0.00 | KO:K00399 | methyl-coenzyme M reductase alpha subunit | Archaea;Euryarchaeota;Methanomicrobia;Methanosarcinales;Methanotrichaceae;Methanothrix;Methanothrix soehngenii;Methanothrix soehngenii GP-6 |
| Ga0586324 3993454 2 676 | 5100000.00 | 0.00 | 0.00 | 0.00 | 0.00 | 0.00 | 0.00 | 0.00 | 11259000.00 | 0.00 | 0.00 | 0.00 | KO:K00399 | methyl-coenzyme M reductase alpha subunit | Archaea;Euryarchaeota;Methanomicrobia;Methanosarcinales;Methanosarcinaceae;Methanosarcina;Methanosarcina spelaei;Methanosarcina spelaei MC-15 |
| Ga0586324 4220254 3 662 | 0.00 | 21148000.00 | 0.00 | 0.00 | 0.00 | 0.00 | 0.00 | 0.00 | 0.00 | 21098000.00 | 17925600.00 | 0.00 | KO:K00399 | methyl-coenzyme M reductase alpha subunit | Archaea;Euryarchaeota;Methanomicrobia;Methanosarcinales;Methanotrichaceae;Methanothrix;Methanothrix soehngenii;Methanothrix soehngenii GP-6 |
| Ga0586324 4302432 3 656 | 0.00 | 2862800.00 | 1289596.54 | 15105000.00 | 0.00 | 2258529.03 | 4582800.00 | 7076000.00 | 7857000.00 | 0.00 | 0.00 | 0.00 | KO:K00399 | methyl-coenzyme M reductase alpha subunit | Archaea;Euryarchaeota;Methanomicrobia;Methanosarcinales;Methanosarcinaceae;Methanosarcina;unclassified Methanosarcina;Methanosarcina sp. DH2 |
| Ga0586324 5101883 2 598 | 0.00 | 0.00 | 0.00 | 0.00 | 0.00 | 21080700.00 | 13543200.00 | 12810000.00 | 0.00 | 0.00 | 0.00 | 0.00 | KO:K00399 | methyl-coenzyme M reductase alpha subunit | Archaea;Euryarchaeota;Methanomicrobia;Methanosarcinales;Methanosarcinaceae;Methanosarcina;Methanosarcina barkeri;Methanosarcina barkeri 227 |
| Ga0586324 5905733 3 575 | 0.00 | 3991600.00 | 0.00 | 0.00 | 0.00 | 0.00 | 0.00 | 0.00 | 0.00 | 0.00 | 0.00 | 0.00 | KO:K00399 | methyl-coenzyme M reductase alpha subunit | Archaea;Euryarchaeota;Methanomicrobia;Methanomicrobiales;Methanomicrobiaceae;Methanofollis;Methanofollis ethanolicus;Methanofollis ethanolicus JCM 15103 |
| Ga0586324 6074327 3 569 | 0.00 | 28356000.00 | 0.00 | 0.00 | 0.00 | 0.00 | 0.00 | 0.00 | 0.00 | 0.00 | 0.00 | 0.00 | KO:K00399 | methyl-coenzyme M reductase alpha subunit | Archaea;Euryarchaeota;Methanomicrobia;Methanosarcinales;Methanotrichaceae;Methanothrix;Methanothrix soehngenii;Methanothrix soehngenii GP-6 |
| Ga0586324 2323430 2 847 | 810498.00 | 0.00 | 0.00 | 0.00 | 0.00 | 0.00 | 0.00 | 0.00 | 0.00 | 0.00 | 0.00 | 0.00 | KO:K00400 | methyl coenzyme M reductase system subunit A2 | Archaea;Euryarchaeota;Methanomicrobia;Methanosarcinales;Methanotrichaceae;Methanothrix;Methanothrix soehngenii;Methanothrix soehngenii GP-6 |
| Ga0586324 0000267 29055 30368 | 92200000.00 | 84320000.00 | 56863000.00 | 40418698.10 | 53332332.92 | 52646701.98 | 32832000.00 | 67405000.00 | 27297000.00 | 50358000.00 | 22000000.00 | 34965000.00 | KO:K00401 | methyl-coenzyme M reductase beta subunit | Archaea;Euryarchaeota;Methanomicrobia;Methanomicrobiales;Methanoregulaceae;Methanoregula;Methanoregula formicica;Methanoregula formicica SMSP, DSM 22288 |
| Ga0586324 0003112 7231 8535 | 7480000.00 | 0.00 | 0.00 | 0.00 | 0.00 | 0.00 | 0.00 | 0.00 | 0.00 | 0.00 | 4030400.00 | 4177500.00 | KO:K00401 | methyl-coenzyme M reductase beta subunit | Archaea;Euryarchaeota;Methanomicrobia;Methanosarcinales;Candidatus Methanoperedenaceae;Candidatus Methanoperedens;Candidatus Methanoperedens nitroreducens;Candidatus Methanoperedens nitroreducens ANME-2d |
| Ga0586324 0005577 5233 6564 | 3360000.00 | 9452000.00 | 0.00 | 6773500.00 | 5974000.00 | 0.00 | 0.00 | 3690500.00 | 0.00 | 0.00 | 5288800.00 | 0.00 | KO:K00401 | methyl-coenzyme M reductase beta subunit | Archaea;Candidatus Thermoplasmatota;Thermoplasmata;Methanomassiliicoccales;Methanomassiliicoccaceae;Methanomassiliicoccus;Methanomassiliicoccus luminyensis;Methanomassiliicoccus luminyensis B10 |
| Ga0586324 0007977 3 848 | 0.00 | 0.00 | 0.00 | 0.00 | 0.00 | 0.00 | 167580000.00 | 0.00 | 183060000.00 | 274120000.00 | 362560000.00 | 148500000.00 | KO:K00401 | methyl-coenzyme M reductase beta subunit | Archaea;Euryarchaeota;Methanomicrobia;Methanomicrobiales;Methanoregulaceae;Methanoregula;Methanoregula formicica;Methanoregula formicica SMSP, DSM 22288 |
| Ga0586324 0031912 3 1019 | 393870000.00 | 9241200.00 | 58664600.00 | 59755000.00 | 28737000.00 | 20445500.00 | 0.00 | 467522.91 | 17820000.00 | 14907200.00 | 8492000.00 | 10125000.00 | KO:K00401 | methyl-coenzyme M reductase beta subunit | Archaea;Euryarchaeota;Methanomicrobia;Methanomicrobiales;Methanoregulaceae;Methanoregula;Methanoregula boonei;Methanoregula boonei 6A8 |
| Ga0586324 0095431 3 1292 | 785340953.00 | 576878000.00 | ############# | 715606500.00 | 769440900.00 | 346501600.00 | 561564000.00 | 389131200.00 | 473769000.00 | 378301000.00 | 365024000.00 | 375862500.00 | KO:K00401 | methyl-coenzyme M reductase beta subunit | Archaea;Euryarchaeota;Methanomicrobia;Methanosarcinales;Methanotrichaceae;Methanothrix;Methanothrix soehngenii;Methanothrix soehngenii GP-6 |
| Ga0586324 0134678 1663 2976 | 29100000.00 | 33986400.00 | 98468700.00 | 37563000.00 | 52385800.00 | 80114600.00 | 44916000.00 | 33159600.00 | 49839300.00 | 38985100.00 | 21982400.00 | 17137500.00 | KO:K00401 | methyl-coenzyme M reductase beta subunit | Archaea;Euryarchaeota;Methanomicrobia;Methanomicrobiales;Methanoregulaceae;Methanoregula;Methanoregula formicica;Methanoregula formicica SMSP, DSM 22288 |
| Ga0586324 0142898 1773 2894 | 0.00 | 24548000.00 | 0.00 | 0.00 | 0.00 | 0.00 | 0.00 | 0.00 | 0.00 | 0.00 | 0.00 | 0.00 | KO:K00401 | methyl-coenzyme M reductase beta subunit | Archaea;Euryarchaeota;Methanomicrobia;Methanomicrobiales;Methanoregulaceae;Methanoregula;Methanoregula boonei;Methanoregula boonei 6A8 |
| Ga0586324 0152511 1292 2602 | 602075.00 | 29539200.00 | 318798.75 | 0.00 | 0.00 | 0.00 | 0.00 | 382743.89 | 13861961.73 | 0.00 | 23548800.00 | 12166837.50 | KO:K00401 | methyl-coenzyme M reductase beta subunit | Archaea;Euryarchaeota;Methanomicrobia;Methanomicrobiales;Methanoregulaceae;Methanoregula;Methanoregula boonei;Methanoregula boonei 6A8 |
| Ga0586324 0164625 1446 2708 | 50400000.00 | 3148400.00 | 6699700.00 | 17100000.00 | 110210000.00 | 71063000.00 | 79116000.00 | 0.00 | 47628000.00 | 42350000.00 | 0.00 | 1582500.00 | KO:K00401 | methyl-coenzyme M reductase beta subunit | Archaea;Euryarchaeota;Methanomicrobia;Methanomicrobiales;Methanoregulaceae;Methanoregula;Methanoregula formicica;Methanoregula formicica SMSP, DSM 22288 |
| Ga0586324 0191529 1821 2525 | 6530000.00 | 0.00 | 0.00 | 0.00 | 0.00 | 0.00 | 0.00 | 1342000.00 | 0.00 | 0.00 | 0.00 | 0.00 | KO:K00401 | methyl-coenzyme M reductase beta subunit | Archaea;Euryarchaeota;Methanomicrobia;Methanomicrobiales;Methanoregulaceae;Methanosphaerula;Methanosphaerula palustris;Methanosphaerula palustris E1-9c, DSM 19958 |
| Ga0586324 0227838 3 692 | 0.00 | 8527200.00 | 0.00 | 0.00 | 8971300.00 | 0.00 | 0.00 | 0.00 | 3734100.00 | 0.00 | 3229600.00 | 1725000.00 | KO:K00401 | methyl-coenzyme M reductase beta subunit | Archaea;Euryarchaeota;Methanomicrobia;Methanomicrobiales;Methanoregulaceae;Methanoregula;Methanoregula boonei;Methanoregula boonei 6A8 |
| Ga0586324 0310286 3 932 | 0.00 | 0.00 | 0.00 | 1453500.00 | 1472900.00 | 0.00 | 887843.40 | 0.00 | 0.00 | 0.00 | 0.00 | 0.00 | KO:K00401 | methyl-coenzyme M reductase beta subunit | Archaea;Euryarchaeota;Methanomicrobia;Methanosarcinales;Methanosarcinaceae;Methanosarcina;Methanosarcina flavescens;Methanosarcina flavescens E03.2 |
| Ga0586324 0506373 3 605 | 0.00 | 0.00 | 0.00 | 0.00 | 0.00 | 0.00 | 1738664.16 | 1323700.00 | 0.00 | 0.00 | 0.00 | 1665000.00 | KO:K00401 | methyl-coenzyme M reductase beta subunit | Archaea;Euryarchaeota;Methanomicrobia;Methanomicrobiales;Methanoregulaceae;Methanosphaerula;Methanosphaerula palustris;Methanosphaerula palustris E1-9c, DSM 19958 |
| Ga0586324 0669245 540 1445 | 60270000.00 | 0.00 | 91206000.00 | 42465000.00 | 0.00 | 3054240.10 | 0.00 | 4038200.00 | 31071600.00 | 0.00 | 0.00 | 37275000.00 | KO:K00401 | methyl-coenzyme M reductase beta subunit | Archaea;Euryarchaeota;Methanomicrobia;Methanomicrobiales;Methanoregulaceae;Methanoregula;Methanoregula boonei;Methanoregula boonei 6A8 |
| Ga0586324 0844541 224 1306 | 0.00 | 0.00 | 0.00 | 11305000.00 | 0.00 | 7820900.00 | 0.00 | 0.00 | 0.00 | 0.00 | 0.00 | 0.00 | KO:K00401 | methyl-coenzyme M reductase beta subunit | Archaea;Candidatus Thermoplasmatota;Thermoplasmata;Methanomassiliicoccales;Methanomassiliicoccaceae;Methanomassiliicoccus;Methanomassiliicoccus luminyensis;Methanomassiliicoccus luminyensis B10 |
| Ga0586324 0854196 454 1299 | 407410.00 | 1870000.00 | 0.00 | 942854.10 | 0.00 | 0.00 | 0.00 | 0.00 | 0.00 | 0.00 | 0.00 | 0.00 | KO:K00401 | methyl-coenzyme M reductase beta subunit | Archaea;Euryarchaeota;Methanomicrobia;Methanosarcinales;Candidatus Methanoperedenaceae;Candidatus Methanoperedens;Candidatus Methanoperedens nitroreducens;Candidatus Methanoperedens nitroreducens ANME-2d |
| Ga0586324 0889043 1 555 | 5210000.00 | 0.00 | 0.00 | 0.00 | 0.00 | 0.00 | 0.00 | 0.00 | 0.00 | 0.00 | 0.00 | 10575000.00 | KO:K00401 | methyl-coenzyme M reductase beta subunit | Archaea;Euryarchaeota;Methanomicrobia;Methanosarcinales;Candidatus Methanoperedenaceae;Candidatus Methanoperedens;Candidatus Methanoperedens nitroreducens;Candidatus Methanoperedens nitroreducens ANME-2d |
| Ga0586324 0943269 3 623 | 0.00 | 0.00 | 0.00 | 4883000.00 | 0.00 | 0.00 | 0.00 | 0.00 | 0.00 | 0.00 | 0.00 | 0.00 | KO:K00401 | methyl-coenzyme M reductase beta subunit | Archaea;Candidatus Thermoplasmatota;Thermoplasmata;Methanomassiliicoccales;Methanomassiliicoccaceae;Methanomassiliicoccus;Methanomassiliicoccus luminyensis;Methanomassiliicoccus luminyensis B10 |
| Ga0586324 0946083 172 1242 | 12300000.00 | 237320000.00 | 20887300.00 | 0.00 | 0.00 | 16634300.00 | 0.00 | 0.00 | 200070000.00 | 0.00 | 0.00 | 5010000.00 | KO:K00401 | methyl-coenzyme M reductase beta subunit | Archaea;Euryarchaeota;Methanomicrobia;Methanomicrobiales;Methanoregulaceae;Methanoregula;Methanoregula formicica;Methanoregula formicica SMSP, DSM 22288 |
| Ga0586324 1065373 3 1181 | 749790000.00 | ############# | 405198542.86 | 844312500.00 | 606278600.00 | 233447993.37 | 497925105.12 | 522172200.00 | 572491800.00 | 574011900.00 | 498740000.00 | 376222500.00 | KO:K00401 | methyl-coenzyme M reductase beta subunit | Archaea;Euryarchaeota;Methanomicrobia;Methanosarcinales;Methanotrichaceae;Methanothrix;Methanothrix soehngenii;Methanothrix soehngenii GP-6 |
| Ga0586324 1069325 1 1179 | 196820000.00 | 146200000.00 | 191983000.00 | 0.00 | 0.00 | 271825900.00 | 134816400.00 | 42944000.00 | 49572000.00 | 85470000.00 | 35288000.00 | 123195000.00 | KO:K00401 | methyl-coenzyme M reductase beta subunit | Archaea;Euryarchaeota;Methanomicrobia;Methanomicrobiales;Methanoregulaceae;Methanoregula;Methanoregula boonei;Methanoregula boonei 6A8 |
| Ga0586324 1395796 2 1051 | 25600000.00 | 7686737.00 | 0.00 | 11495000.00 | 11124000.00 | 8694300.00 | 6931200.00 | 4459100.00 | 5249016.27 | 0.00 | 0.00 | 3022500.00 | KO:K00401 | methyl-coenzyme M reductase beta subunit | Archaea;Euryarchaeota;Methanomicrobia;Methanosarcinales;Methanotrichaceae;Methanothrix;Methanothrix soehngenii;Methanothrix soehngenii GP-6 |
| Ga0586324 1410674 3 572 | 4720000.00 | 7888000.00 | 0.00 | 3990000.00 | 3337200.00 | 0.00 | 0.00 | 1793400.00 | 0.00 | 0.00 | 0.00 | 3765000.00 | KO:K00401 | methyl-coenzyme M reductase beta subunit | Archaea;Euryarchaeota;Methanomicrobia;Methanomicrobiales;Methanoregulaceae;Methanoregula;Methanoregula boonei;Methanoregula boonei 6A8 |
| Ga0586324 1427021 3 629 | 883652.00 | 5412800.00 | 627626.77 | 4365845.65 | 107830700.00 | 0.00 | 105868259.16 | 185019100.00 | 866700.00 | 192885000.00 | 171512000.00 | 3331557.00 | KO:K00401 | methyl-coenzyme M reductase beta subunit | Archaea;Euryarchaeota;Methanomicrobia;Methanosarcinales;Methanotrichaceae;Methanothrix;Methanothrix soehngenii;Methanothrix soehngenii GP-6 |
| Ga0586324 1465892 3 1031 | 447148.00 | 0.00 | 0.00 | 0.00 | 0.00 | 0.00 | 0.00 | 0.00 | 0.00 | 0.00 | 0.00 | 0.00 | KO:K00401 | methyl-coenzyme M reductase beta subunit | Archaea;Euryarchaeota;Methanomicrobia;Methanomicrobiales;Methanoregulaceae;Methanoregula;Methanoregula boonei;Methanoregula boonei 6A8 |
| Ga0586324 1526255 3 692 | 0.00 | 3638000.00 | 0.00 | 0.00 | 0.00 | 0.00 | 0.00 | 0.00 | 0.00 | 0.00 | 0.00 | 10275000.00 | KO:K00401 | methyl-coenzyme M reductase beta subunit | Archaea;Euryarchaeota;Methanomicrobia;Methanomicrobiales;Methanoregulaceae;Methanoregula;Methanoregula boonei;Methanoregula boonei 6A8 |
| Ga0586324 1591276 2 871 | 0.00 | 38941287.32 | 0.00 | 0.00 | 0.00 | 0.00 | 0.00 | 0.00 | 0.00 | 0.00 | 0.00 | 0.00 | KO:K00401 | methyl-coenzyme M reductase beta subunit | Archaea;Euryarchaeota;Methanomicrobia;Methanomicrobiales;Methanoregulaceae;Methanoregula;Methanoregula boonei;Methanoregula boonei 6A8 |
| Ga0586324 1673222 2 973 | 0.00 | 0.00 | 0.00 | 0.00 | 8868300.00 | 0.00 | 0.00 | 0.00 | 0.00 | 0.00 | 0.00 | 0.00 | KO:K00401 | methyl-coenzyme M reductase beta subunit | Archaea;Euryarchaeota;Methanomicrobia;Methanomicrobiales;Methanoregulaceae;Methanoregula;Methanoregula formicica;Methanoregula formicica SMSP, DSM 22288 |
| Ga0586324 2044502 1 894 | 34810000.00 | 352377964.52 | 168717959.58 | 373245500.00 | 181383000.00 | 161897012.88 | 209532000.00 | 199568871.85 | 278324100.00 | 211241800.00 | 123569600.00 | 21097500.00 | KO:K00401 | methyl-coenzyme M reductase beta subunit | Archaea;Euryarchaeota;Methanomicrobia;Methanosarcinales;Methanotrichaceae;Methanothrix;Methanothrix soehngenii;Methanothrix soehngenii GP-6 |
| Ga0586324 2069110 1 591 | 2970000.00 | 4841600.00 | 15520750.22 | 36575000.00 | 9754100.00 | 21993800.00 | 0.00 | 17873000.00 | 16451100.00 | 2895200.00 | 3062400.00 | 1740000.00 | KO:K00401 | methyl-coenzyme M reductase beta subunit | Archaea;Euryarchaeota;Methanomicrobia;Methanosarcinales;Methanosarcinaceae;Methanosarcina;Methanosarcina spelaei;Methanosarcina spelaei MC-15 |
| Ga0586324 2831159 2 565 | 0.00 | 0.00 | 0.00 | 14155000.00 | 7189400.00 | 1880862.93 | 0.00 | 9394000.00 | 8262000.00 | 0.00 | 0.00 | 0.00 | KO:K00401 | methyl-coenzyme M reductase beta subunit | Archaea;Euryarchaeota;Methanomicrobia;Methanosarcinales;Methanosarcinaceae;Methanosarcina;Methanosarcina lacustris;Methanosarcina lacustris Z-7289 |
| Ga0586324 3312776 1 732 | 0.00 | 0.00 | 0.00 | 0.00 | 0.00 | 0.00 | 0.00 | 0.00 | 0.00 | 0.00 | 0.00 | 17700000.00 | KO:K00401 | methyl-coenzyme M reductase beta subunit | Archaea;Euryarchaeota;Methanomicrobia;Methanomicrobiales;Methanoregulaceae;Methanoregula;Methanoregula boonei;Methanoregula boonei 6A8 |
| Ga0586324 3367902 644 727 | 0.00 | 67728000.00 | 0.00 | 30970000.00 | 0.00 | 0.00 | 0.00 | 0.00 | 0.00 | 0.00 | 0.00 | 0.00 | KO:K00401 | methyl-coenzyme M reductase beta subunit | Archaea;Euryarchaeota;Methanomicrobia;Methanosarcinales;Methanotrichaceae;Methanothrix;Methanothrix soehngenii;Methanothrix soehngenii GP-6 |
| Ga0586324 3893222 3 98 | 0.00 | 2713200.00 | 0.00 | 0.00 | 0.00 | 0.00 | 0.00 | 0.00 | 0.00 | 0.00 | 0.00 | 0.00 | KO:K00401 | methyl-coenzyme M reductase beta subunit | Archaea;Euryarchaeota;Methanomicrobia;Methanosarcinales;Methanotrichaceae;Methanothrix;Methanothrix soehngenii;Methanothrix soehngenii GP-6 |
| Ga0586324 4042508 3 674 | 79300000.00 | 1734000.00 | 54273200.00 | 0.00 | 0.00 | 0.00 | 0.00 | 66490000.00 | 0.00 | 0.00 | 0.00 | 260118.75 | KO:K00401 | methyl-coenzyme M reductase beta subunit | Archaea;Euryarchaeota;Methanomicrobia;Methanomicrobiales;Methanoregulaceae;Methanoregula;Methanoregula formicica;Methanoregula formicica SMSP, DSM 22288 |
| Ga0586324 4450616 61 648 | 0.00 | 1557200.00 | 0.00 | 0.00 | 0.00 | 0.00 | 0.00 | 392605.15 | 0.00 | 0.00 | 0.00 | 442415.25 | KO:K00401 | methyl-coenzyme M reductase beta subunit | Archaea;Euryarchaeota;Methanomicrobia;Methanomicrobiales;Methanoregulaceae;Methanoregula;Methanoregula formicica;Methanoregula formicica SMSP, DSM 22288 |
| Ga0586324 4796160 3 626 | 229178.00 | 92480000.00 | 0.00 | 0.00 | 0.00 | 0.00 | 12859200.00 | 0.00 | 13284000.00 | 8162000.00 | 0.00 | 0.00 | KO:K00401 | methyl-coenzyme M reductase beta subunit | Archaea;Euryarchaeota;Methanomicrobia;Methanomicrobiales;Methanoregulaceae;Methanoregula;Methanoregula formicica;Methanoregula formicica SMSP, DSM 22288 |
| Ga0586324 5084416 2 613 | 0.00 | 0.00 | 0.00 | 2907000.00 | 0.00 | 0.00 | 0.00 | 0.00 | 2543400.00 | 0.00 | 0.00 | 0.00 | KO:K00401 | methyl-coenzyme M reductase beta subunit | Archaea;Euryarchaeota;Methanomicrobia;Methanosarcinales;Methanosarcinaceae;Methanosarcina;Methanosarcina horonobensis;Methanosarcina horonobensis HB-1 |
| Ga0586324 5234100 56 604 | 154800000.00 | 279317228.40 | 279698400.00 | 332120000.00 | 336604000.00 | 81107100.00 | 239400000.00 | 56608000.00 | 42240889.26 | 37499000.00 | 161216000.00 | 45225000.00 | KO:K00401 | methyl-coenzyme M reductase beta subunit | Archaea;Euryarchaeota;Methanomicrobia;Methanosarcinales;Methanotrichaceae;Methanothrix;Methanothrix soehngenii;Methanothrix soehngenii GP-6 |
| Ga0586324 5684930 3 584 | 76700000.00 | 0.00 | 6361900.00 | 0.00 | 0.00 | 0.00 | 6178800.00 | 26901000.00 | 0.00 | 0.00 | 0.00 | 20550000.00 | KO:K00401 | methyl-coenzyme M reductase beta subunit | Archaea;Euryarchaeota;Methanomicrobia;Methanosarcinales;Methanotrichaceae;Methanothrix;Methanothrix soehngenii;Methanothrix soehngenii GP-6 |
| Ga0586324 6009941 3 572 | 0.00 | 40664000.00 | 0.00 | 0.00 | 0.00 | 0.00 | 0.00 | 0.00 | 0.00 | 0.00 | 0.00 | 0.00 | KO:K00401 | methyl-coenzyme M reductase beta subunit | Archaea;Euryarchaeota;Methanomicrobia;Methanomicrobiales;Methanoregulaceae;Methanoregula;Methanoregula boonei;Methanoregula boonei 6A8 |
| Ga0586324 6249662 2 553 | 0.00 | 112880000.00 | 94021000.00 | 822061.60 | 89919000.00 | 64314000.00 | 0.00 | 57462000.00 | 44550000.00 | 82390000.00 | 73480000.00 | 0.00 | KO:K00401 | methyl-coenzyme M reductase beta subunit | Archaea;Euryarchaeota;Methanomicrobia;Methanomicrobiales;Methanoregulaceae;Methanoregula;Methanoregula formicica;Methanoregula formicica SMSP, DSM 22288 |
| Ga0586324 6761995 2 544 | 0.00 | 0.00 | 0.00 | 0.00 | 2667700.00 | 0.00 | 0.00 | 652700.00 | 0.00 | 0.00 | 1126400.00 | 0.00 | KO:K00401 | methyl-coenzyme M reductase beta subunit | Archaea;Euryarchaeota;Methanomicrobia;Methanomicrobiales;Methanoregulaceae;Methanoregula;Methanoregula boonei;Methanoregula boonei 6A8 |
| Ga0586324 6781589 1 543 | 33300000.00 | 0.00 | 0.00 | 0.00 | 0.00 | 0.00 | 0.00 | 0.00 | 0.00 | 0.00 | 0.00 | 0.00 | KO:K00401 | methyl-coenzyme M reductase beta subunit | Archaea;Euryarchaeota;Methanomicrobia;Methanomicrobiales;Methanoregulaceae;Methanoregula;Methanoregula formicica;Methanoregula formicica SMSP, DSM 22288 |
| Ga0586324 7240576 3 374 | 0.00 | 0.00 | 27699600.00 | 14725000.00 | 11845000.00 | 16832800.00 | 0.00 | 0.00 | 17739000.00 | 8239000.00 | 0.00 | 6960000.00 | KO:K00401 | methyl-coenzyme M reductase beta subunit | Archaea;Euryarchaeota;Methanomicrobia;Methanomicrobiales;Methanoregulaceae;Methanoregula;Methanoregula boonei;Methanoregula boonei 6A8 |
| Ga0586324 7256471 1 528 | 3670000.00 | 1366800.00 | 1536387.59 | 0.00 | 7364500.00 | 6788700.00 | 2485200.00 | 4202900.00 | 5151600.00 | 1155000.00 | 0.00 | 322937.25 | KO:K00401 | methyl-coenzyme M reductase beta subunit | Archaea;Euryarchaeota;Methanomicrobia;Methanosarcinales;Methanosarcinaceae;Methanosarcina;Methanosarcina lacustris;Methanosarcina lacustris Z-7289 |
| Ga0586324 0000267 31489 32253 | 84370000.00 | 9860000.00 | 4050717.44 | 43120500.00 | 37904000.00 | 37938709.50 | 32148000.00 | 19721300.00 | 23878800.00 | 19866000.00 | 17248000.00 | 20505000.00 | KO:K00402 | methyl-coenzyme M reductase gamma subunit | Archaea;Euryarchaeota;Methanomicrobia;Methanomicrobiales;Methanomicrobiaceae;Methanoculleus;Methanoculleus bourgensis;Methanoculleus bourgensis MS2 |
| Ga0586324 0005577 4032 4811 | 1080000.00 | 0.00 | 0.00 | 0.00 | 0.00 | 0.00 | 0.00 | 0.00 | 0.00 | 0.00 | 0.00 | 0.00 | KO:K00402 | methyl-coenzyme M reductase gamma subunit | Archaea;Euryarchaeota;Methanomicrobia;Methanomicrobiales;unclassified Methanomicrobiales;unclassified Methanomicrobiales;methanogenic archaeon mixed culture ISO4-G1;Unclassified archaeon ISO4-G1 |
| Ga0586324 0095431 1815 2582 | 142800000.00 | 136292400.00 | 296701000.00 | 214700000.00 | 198378000.00 | 179047000.00 | 93708000.00 | 81313000.00 | 101331000.00 | 32648000.00 | 67496000.00 | 87000000.00 | KO:K00402 | methyl-coenzyme M reductase gamma subunit | Archaea;Euryarchaeota;Methanomicrobia;Methanosarcinales;Methanotrichaceae;Methanothrix;Methanothrix soehngenii;Methanothrix soehngenii GP-6 |
| Ga0586324 0172585 741 1514 | 8220000.00 | 2109164.84 | 16383300.00 | 6802000.00 | 0.00 | 29576500.00 | 11696400.00 | 1677500.00 | 0.00 | 0.00 | 0.00 | 0.00 | KO:K00402 | methyl-coenzyme M reductase gamma subunit | Archaea;Candidatus Thermoplasmatota;Thermoplasmata;Methanomassiliicoccales;Methanomassiliicoccaceae;Methanomassiliicoccus;Methanomassiliicoccus luminyensis;Methanomassiliicoccus luminyensis B10 |
| Ga0586324 0257221 196 963 | 251610000.00 | 202368000.00 | 163551500.00 | 65835000.00 | 33773700.00 | 59351500.00 | 39945600.00 | 8479000.00 | 7767900.00 | 6706700.00 | 5825600.00 | 33750000.00 | KO:K00402 | methyl-coenzyme M reductase gamma subunit | Archaea;Euryarchaeota;Methanomicrobia;Methanosarcinales;Methanotrichaceae;Methanothrix;Methanothrix soehngenii;Methanothrix soehngenii GP-6 |
| Ga0586324 0417828 1 204 | 0.00 | 0.00 | 0.00 | 0.00 | 8466600.00 | 0.00 | 0.00 | 0.00 | 0.00 | 0.00 | 0.00 | 0.00 | KO:K00402 | methyl-coenzyme M reductase gamma subunit | Archaea;Euryarchaeota;Methanomicrobia;Methanosarcinales;Methanosarcinaceae;Methanosarcina;Methanosarcina horonobensis;Methanosarcina horonobensis HB-1 |
| Ga0586324 0432841 197 943 | 1640000.00 | 3658400.00 | 0.00 | 0.00 | 4326000.00 | 19207800.89 | 0.00 | 4672600.00 | 3037500.00 | 0.00 | 0.00 | 0.00 | KO:K00402 | methyl-coenzyme M reductase gamma subunit | Archaea;Euryarchaeota;Methanomicrobia;Methanosarcinales;Methanosarcinaceae;Methanosarcina;Methanosarcina lacustris;Methanosarcina lacustris Z-7289 |
| Ga0586324 0458932 557 1324 | 0.00 | 5310800.00 | 0.00 | 0.00 | 0.00 | 0.00 | 0.00 | 0.00 | 0.00 | 0.00 | 0.00 | 0.00 | KO:K00402 | methyl-coenzyme M reductase gamma subunit | Archaea;Euryarchaeota;Methanomicrobia;Methanomicrobiales;Methanoregulaceae;Methanoregula;Methanoregula boonei;Methanoregula boonei 6A8 |
| Ga0586324 0519656 259 1029 | 3960000.00 | 0.00 | 0.00 | 0.00 | 0.00 | 0.00 | 0.00 | 0.00 | 795882.51 | 0.00 | 0.00 | 0.00 | KO:K00402 | methyl-coenzyme M reductase gamma subunit | Archaea;Candidatus Thermoplasmatota;Thermoplasmata;Methanomassiliicoccales;Methanomassiliicoccaceae;Methanomassiliicoccus;Methanomassiliicoccus luminyensis;Methanomassiliicoccus luminyensis B10 |
| Ga0586324 0663604 1 648 | 20870000.00 | 16490000.00 | 36801475.75 | 16720000.00 | 25595500.00 | 30132300.00 | 16393200.00 | 12450100.00 | 9639000.00 | 8085000.00 | 8720800.00 | 7395000.00 | KO:K00402 | methyl-coenzyme M reductase gamma subunit | Archaea;Euryarchaeota;Methanomicrobia;Methanomicrobiales;Methanoregulaceae;Methanoregula;Methanoregula formicica;Methanoregula formicica SMSP, DSM 22288 |
| Ga0586324 0774128 921 1355 | 8130000.00 | 34544000.00 | 1769447.07 | 16910000.00 | 16377000.00 | 0.00 | 0.00 | 18666000.00 | 0.00 | 0.00 | 0.00 | 13425000.00 | KO:K00402 | methyl-coenzyme M reductase gamma subunit | Archaea;Euryarchaeota;Methanomicrobia;Methanomicrobiales;Methanoregulaceae;Methanosphaerula;Methanosphaerula palustris;Methanosphaerula palustris E1-9c, DSM 19958 |

| | | | | | | | | | | | | | | | |
|---|---|---|---|---|---|---|---|---|---|---|---|---|---|---|---|
| Ga0586324_1898585_2_673 | 0.00 | 0.00 | 0.00 | 0.00 | 0.00 | 0.00 | 0.00 | 0.00 | 0.00 | 15631000.00 | 0.00 | 17700000.00 | KO:K00402 | methyl-coenzyme M reductase gamma subunit | Archaea;Euryarchaeota;Methanomicrobia;Methanomicrobiales;Methanoregulaceae;Methanoregula;Methanoregula boonei;Methanoregula boonei 6A8 |
| Ga0586324_1914067_476_919 | 58730000.00 | 10907200.00 | 0.00 | 8227000.00 | 4387800.00 | 3970000.00 | 0.00 | 0.00 | 1952100.00 | 3488100.00 | 0.00 | 0.00 | KO:K00402 | methyl-coenzyme M reductase gamma subunit | Archaea;Euryarchaeota;Methanomicrobia;Methanomicrobiales;Methanoregulaceae;Methanoregula;Methanoregula formicica;Methanoregula formicica SMSP, DSM 22288 |
| Ga0586324_2168875_183_872 | 36500000.00 | 41480000.00 | 68686000.00 | 3125500.00 | 36771000.00 | 45774100.00 | 24624000.00 | 21777000.00 | 23660100.00 | 0.00 | 19976000.00 | 16687500.00 | KO:K00402 | methyl-coenzyme M reductase gamma subunit | Archaea;Euryarchaeota;Methanomicrobia;Methanomicrobiales;Methanoregulaceae;Methanosphaerula;Methanosphaerula palustris;Methanosphaerula palustris E1-9c, DSM 19958 |
| Ga0586324_2259985_441_857 | 0.00 | 9180000.00 | 0.00 | 0.00 | 0.00 | 0.00 | 0.00 | 0.00 | 0.00 | 0.00 | 0.00 | 0.00 | KO:K00402 | methyl-coenzyme M reductase gamma subunit | Archaea;Euryarchaeota;Methanomicrobia;Methanosarcinales;Methanotrichaceae;Methanothrix;Methanothrix soehngenii;Methanothrix soehngenii GP-6 |
| Ga0586324_2666297_2_790 | 29770000.00 | 197077600.00 | 5028682.22 | 23227500.00 | 15450000.00 | 4009700.00 | 0.00 | 12810000.00 | 50916600.00 | 33572000.00 | 29559200.00 | 9825000.00 | KO:K00402 | methyl-coenzyme M reductase gamma subunit | Archaea;Euryarchaeota;Methanomicrobia;Methanomicrobiales;Methanoregulaceae;Methanoregula;Methanoregula boonei;Methanoregula boonei 6A8 |
| Ga0586324_2763665_3_194 | 0.00 | 3808000.00 | 0.00 | 0.00 | 0.00 | 0.00 | 0.00 | 0.00 | 0.00 | 0.00 | 0.00 | 0.00 | KO:K00402 | methyl-coenzyme M reductase gamma subunit | Archaea;Euryarchaeota;Methanomicrobia;Methanomicrobiales;Methanoregulaceae;Methanoregula;Methanoregula boonei;Methanoregula boonei 6A8 |
| Ga0586324_3432428_127_720 | 47500000.00 | 33088800.00 | 0.00 | 24652500.00 | 43218800.00 | 4724300.00 | 64752000.00 | 0.00 | 20298600.00 | 759677.38 | 5315200.00 | 0.00 | KO:K00402 | methyl-coenzyme M reductase gamma subunit | Archaea;Euryarchaeota;Methanomicrobia;Methanomicrobiales;Methanoregulaceae;Methanoregula;Methanoregula formicica;Methanoregula formicica SMSP, DSM 22288 |
| Ga0586324_3458292_47_718 | 105220000.00 | 12002000.00 | 0.00 | 0.00 | 0.00 | 0.00 | 0.00 | 0.00 | 0.00 | 0.00 | 0.00 | 0.00 | KO:K00402 | methyl-coenzyme M reductase gamma subunit | Archaea;Euryarchaeota;Methanomicrobia;Methanosarcinales;Methanotrichaceae;Methanothrix;Methanothrix soehngenii;Methanothrix soehngenii GP-6 |
| Ga0586324_3689976_3_575 | 0.00 | 5242800.00 | 0.00 | 0.00 | 58195000.00 | 0.00 | 0.00 | 0.00 | 0.00 | 0.00 | 1892000.00 | 0.00 | KO:K00402 | methyl-coenzyme M reductase gamma subunit | Archaea;Euryarchaeota;Methanomicrobia;Methanomicrobiales;Methanoregulaceae;Methanoregula;Methanoregula formicica;Methanoregula formicica SMSP, DSM 22288 |
| Ga0586324_3834023_3_689 | 2670000.00 | 5093200.00 | 0.00 | 1792085.70 | 18645596.63 | 0.00 | 12790800.00 | 6347337.92 | 19140300.00 | 0.00 | 0.00 | 0.00 | KO:K00402 | methyl-coenzyme M reductase gamma subunit | Archaea;Euryarchaeota;Methanomicrobia;Methanosarcinales;Methanosarcinaceae;Methanosarcina;Methanosarcina spelaei;Methanosarcina spelaei MC-15 |
| Ga0586324_4860244_302_625 | 9090000.00 | 0.00 | 0.00 | 1472500.00 | 0.00 | 0.00 | 0.00 | 0.00 | 0.00 | 0.00 | 0.00 | 4147500.00 | KO:K00402 | methyl-coenzyme M reductase gamma subunit | Archaea;Euryarchaeota;Methanomicrobia;Methanomicrobiales;Methanoregulaceae;Methanoregula;Methanoregula boonei;Methanoregula boonei 6A8 |
| Ga0586324_4920357_161_622 | 0.00 | 0.00 | 0.00 | 59945000.00 | 0.00 | 0.00 | 0.00 | 0.00 | 0.00 | 0.00 | 0.00 | 0.00 | KO:K00402 | methyl-coenzyme M reductase gamma subunit | Archaea;Euryarchaeota;Methanomicrobia;Methanomicrobiales;Methanoregulaceae;Methanoregula;Methanoregula formicica;Methanoregula formicica SMSP, DSM 22288 |
| Ga0586324_6706731_7_546 | 0.00 | 3338800.00 | 0.00 | 0.00 | 0.00 | 0.00 | 0.00 | 0.00 | 0.00 | 0.00 | 0.00 | 0.00 | KO:K00402 | methyl-coenzyme M reductase gamma subunit | Archaea;Euryarchaeota;Methanomicrobia;Methanosarcinales;Methanotrichaceae;Methanothrix;Methanothrix thermoacetophila;Methanothrix thermoacetophila PT |
| Ga0586324_7026917_96_536 | 0.00 | 0.00 | 0.00 | 0.00 | 0.00 | 0.00 | 0.00 | 2403400.00 | 0.00 | 0.00 | 0.00 | 0.00 | KO:K00402 | methyl-coenzyme M reductase gamma subunit | Archaea;Euryarchaeota;Methanomicrobia;Methanomicrobiales;Methanoregulaceae;Methanoregula;Methanoregula formicica;Methanoregula formicica SMSP, DSM 22288 |
| Ga0586324_7443777_1_237 | 0.00 | 14824000.00 | 0.00 | 0.00 | 0.00 | 0.00 | 0.00 | 0.00 | 0.00 | 0.00 | 0.00 | 0.00 | KO:K00402 | methyl-coenzyme M reductase gamma subunit | Archaea;Euryarchaeota;Methanomicrobia;Methanomicrobiales;Methanomicrobiaceae;Methanoplanus;Methanoplanus limicola;Methanoplanus limicola M3, DSM 2279 |
| Ga0586324_7980584_1_507 | 0.00 | 3141600.00 | 0.00 | 0.00 | 0.00 | 0.00 | 0.00 | 0.00 | 0.00 | 0.00 | 0.00 | 0.00 | KO:K00402 | methyl-coenzyme M reductase gamma subunit | Archaea;Euryarchaeota;Methanomicrobia;Methanomicrobiales;Methanomicrobiaceae;Methanoculleus;Methanoculleus bourgensis;Methanoculleus bourgensis MS2 |
| Ga0586324_0000407_16454_17410 | 0.00 | 0.00 | 0.00 | 0.00 | 0.00 | 0.00 | 0.00 | 0.00 | 0.00 | 19712000.00 | 20944000.00 | 20400000.00 | KO:K00405 | cytochrome c oxidase cbb3-type subunit 2 | Bacteria;Proteobacteria;Betaproteobacteria;Nitrosomonadales;Gallionellaceae;Sideroxydans;Sideroxydans lithotrophicus;Sideroxydans lithotrophicus ES-1 |
| Ga0586324_0136816_1634_2581 | 0.00 | 0.00 | 0.00 | 0.00 | 0.00 | 0.00 | 0.00 | 3519700.00 | 0.00 | 0.00 | 0.00 | 0.00 | KO:K00405 | cytochrome c oxidase cbb3-type subunit 2 | Bacteria;Proteobacteria;Betaproteobacteria;Nitrosomonadales;Gallionellaceae;Sideroxydans;Sideroxydans lithotrophicus;Sideroxydans lithotrophicus ES-1 |
| Ga0586324_0169027_447_1403 | 0.00 | 0.00 | 0.00 | 0.00 | 0.00 | 0.00 | 0.00 | 0.00 | 0.00 | 6768300.00 | 3943089.04 | 8760000.00 | KO:K00405 | cytochrome c oxidase cbb3-type subunit 2 | Bacteria;Proteobacteria;Betaproteobacteria;Nitrosomonadales;Gallionellaceae;Sideroxydans;Sideroxydans lithotrophicus;Sideroxydans lithotrophicus ES-1 |
| Ga0586324_0222612_1409_2356 | 0.00 | 0.00 | 0.00 | 0.00 | 0.00 | 0.00 | 0.00 | 0.00 | 0.00 | 545163.85 | 851776.64 | 0.00 | KO:K00405 | cytochrome c oxidase cbb3-type subunit 2 | Bacteria;Proteobacteria;Betaproteobacteria;Nitrosomonadales;Gallionellaceae;Sideroxydans;Sideroxydans lithotrophicus;Sideroxydans lithotrophicus ES-1 |
| Ga0586324_0629580_500_1456 | 0.00 | 0.00 | 0.00 | 0.00 | 0.00 | 0.00 | 12654000.00 | 0.00 | 1725300.00 | 7184100.00 | 10736000.00 | 7200000.00 | KO:K00405 | cytochrome c oxidase cbb3-type subunit 2 | Bacteria;Proteobacteria;Betaproteobacteria;Nitrosomonadales;Gallionellaceae;Sideroxydans;Sideroxydans lithotrophicus;Sideroxydans lithotrophicus ES-1 |
| Ga0586324_0000002_10407_11393 | 0.00 | 0.00 | 0.00 | 0.00 | 0.00 | 0.00 | 0.00 | 0.00 | 0.00 | 0.00 | 0.00 | 1320000.00 | KO:K00406 | cytochrome c oxidase cbb3-type subunit 3 | Bacteria;Proteobacteria;Betaproteobacteria;Nitrosomonadales;Thiobacillaceae;Annwoodia;Annwoodia aquaesulis;Annwoodia aquaesulis DSM 4255 |
| Ga0586324_0006396_1783_2382 | 0.00 | 0.00 | 0.00 | 0.00 | 0.00 | 0.00 | 1205230.80 | 0.00 | 0.00 | 0.00 | 0.00 | 0.00 | KO:K00411 | ubiquinol-cytochrome c reductase iron-sulfur subunit | Bacteria;Proteobacteria;Gammaproteobacteria;Methylococcales;Methylococcaceae;Methylobacter;Methylobacter tundripaludum;Methylobacter tundripaludum OWC-G53F |
| Ga0586324_0015399_5457_6086 | 0.00 | 0.00 | 0.00 | 0.00 | 0.00 | 0.00 | 0.00 | 0.00 | 0.00 | 27489000.00 | 0.00 | 0.00 | KO:K00411 | ubiquinol-cytochrome c reductase iron-sulfur subunit | Bacteria;Proteobacteria;Betaproteobacteria;Nitrosomonadales;Thiobacillaceae;Thiobacillus;Thiobacillus denitrificans;Thiobacillus denitrificans ATCC 25259 |
| Ga0586324_0816134_2_580 | 1380000.00 | 0.00 | 0.00 | 0.00 | 0.00 | 0.00 | 0.00 | 0.00 | 0.00 | 0.00 | 0.00 | 0.00 | KO:K00411 | ubiquinol-cytochrome c reductase iron-sulfur subunit | Bacteria;Proteobacteria;Gammaproteobacteria;Methylococcales;Methylococcaceae;Methylobacter;Methylobacter tundripaludum;Methylobacter tundripaludum OWC-G53F |
| Ga0586324_0169086_1_771 | 0.00 | 958800.00 | 0.00 | 1672000.00 | 1648000.00 | 0.00 | 1045313.88 | 0.00 | 1174500.00 | 0.00 | 0.00 | 0.00 | KO:K00413 | ubiquinol-cytochrome c reductase cytochrome c1 subunit | Bacteria;Proteobacteria;Alphaproteobacteria;Rhodospirillales;Acetobacteraceae;Rhodopila;Rhodopila globiformis;Rhodopila globiformis DSM 161 |
| Ga0586324_0003931_9354_10520 | 0.00 | 0.00 | 0.00 | 0.00 | 0.00 | 0.00 | 0.00 | 0.00 | 0.00 | 0.00 | 3080000.00 | 1567500.00 | KO:K00428 | cytochrome c peroxidase | Bacteria;Proteobacteria;Deltaproteobacteria;Desulfuromonadales;Desulfuromonadaceae;Desulfuromonas;Desulfuromonas acetexigens;Desulfuromonas acetexigens DSM 1397 |
| Ga0586324_0009492_600_1604 | 8470000.00 | 0.00 | 0.00 | 0.00 | 0.00 | 10282300.00 | 0.00 | 0.00 | 0.00 | 0.00 | 0.00 | 4477500.00 | KO:K00428 | cytochrome c peroxidase | Bacteria;Proteobacteria;Gammaproteobacteria;Methylococcales;Methylococcaceae;Methylobacter;Methylobacter tundripaludum;Methylobacter tundripaludum OWC-G53F |
| Ga0586324_0078476_507_1595 | 1020000.00 | 0.00 | 1479794.83 | 0.00 | 0.00 | 0.00 | 0.00 | 793000.00 | 1287900.00 | 800800.00 | 771724.80 | 282831.00 | KO:K00428 | cytochrome c peroxidase | Bacteria;Nitrospirae;Nitrospira;Nitrospirales;Nitrospiraceae;Nitrospira;Candidatus Nitrospira nitrificans;Candidatus Nitrospira nitrificans COMA2 |
| Ga0586324_0322406_3_620 | 0.00 | 0.00 | 0.00 | 0.00 | 0.00 | 0.00 | 0.00 | 0.00 | 0.00 | 0.00 | 5825600.00 | 0.00 | KO:K00428 | cytochrome c peroxidase | Bacteria;Proteobacteria;Gammaproteobacteria;Methylococcales;Methylococcaceae;Methylobacter;Methylobacter sp. BBA5.1;Methylobacter sp. BBA5.1 |
| Ga0586324_0445644_358_1449 | 2550000.00 | 0.00 | 0.00 | 0.00 | 0.00 | 0.00 | 0.00 | 0.00 | 0.00 | 0.00 | 0.00 | 0.00 | KO:K00428 | cytochrome c peroxidase | Bacteria;Nitrospirae;Nitrospira;Nitrospirales;Nitrospiraceae;Nitrospira;Candidatus Nitrospira nitrificans;Candidatus Nitrospira nitrificans COMA2 |
| Ga0586324_0864418_1_972 | 1703944.00 | 7684000.00 | 33498500.00 | 0.00 | 0.00 | 0.00 | 0.00 | 658800.00 | 7840800.00 | 9394000.00 | 0.00 | 6435000.00 | KO:K00428 | cytochrome c peroxidase | Bacteria;Proteobacteria;Gammaproteobacteria;Methylococcales;Methylococcaceae;Methylobacter;Methylobacter tundripaludum;Methylobacter tundripaludum OWC-G53F |
| Ga0586324_3178698_8_742 | 0.00 | 0.00 | 0.00 | 0.00 | 0.00 | 0.00 | 0.00 | 0.00 | 0.00 | 0.00 | 15312000.00 | 0.00 | KO:K00428 | cytochrome c peroxidase | Bacteria;Proteobacteria;Gammaproteobacteria;Methylococcales;Methylococcaceae;Methylobacter;Methylobacter tundripaludum;Methylobacter tundripaludum 31/32 |
| Ga0586324_4776736_3_356 | 0.00 | 0.00 | 0.00 | 0.00 | 0.00 | 0.00 | 0.00 | 0.00 | 0.00 | 583137.17 | 0.00 | 0.00 | KO:K00428 | cytochrome c peroxidase | Bacteria;Proteobacteria;Gammaproteobacteria;Methylococcales;Methylococcaceae;Methylocaldum;Methylocaldum szegediense;Methylocaldum szegediense O-12 |
| Ga0586324_5569871_3_590 | 0.00 | 0.00 | 0.00 | 0.00 | 0.00 | 0.00 | 0.00 | 0.00 | 0.00 | 0.00 | 2050400.00 | 0.00 | KO:K00428 | cytochrome c peroxidase | Bacteria;Proteobacteria;Deltaproteobacteria;Syntrophobacterales;Syntrophaceae;Syntrophus;Syntrophus gentianae;Syntrophus gentianae DSM 8423 |
| Ga0586324_6671412_2_493 | 0.00 | 0.00 | 0.00 | 0.00 | 0.00 | 0.00 | 0.00 | 6466000.00 | 0.00 | 0.00 | 0.00 | 0.00 | KO:K00436 | NAD-reducing hydrogenase large subunit | Bacteria;Proteobacteria;Deltaproteobacteria;Syntrophobacterales;Syntrophobacteraceae;Syntrophobacter;Syntrophobacter fumaroxidans;Syntrophobacter fumaroxidans MPOB |
| Ga0586324_0031028_121_1764 | 0.00 | 2998800.00 | 0.00 | 1634000.00 | 1895200.00 | 0.00 | 0.00 | 0.00 | 0.00 | 2102100.00 | 3722400.00 | 0.00 | KO:K00437 | [NiFe] hydrogenase large subunit | Bacteria;Proteobacteria;Deltaproteobacteria;Syntrophobacterales;Syntrophobacteraceae;Desulfacinum;Desulfacinum infernum;Desulfacinum infernum DSM 9756 |
| Ga0586324_0040009_820_2463 | 0.00 | 4420000.00 | 0.00 | 12996000.00 | 21939000.00 | 10996900.00 | 0.00 | 12932000.00 | 6212700.00 | 9394000.00 | 5676000.00 | 4680000.00 | KO:K00437 | [NiFe] hydrogenase large subunit | Bacteria;Proteobacteria;Deltaproteobacteria;Syntrophobacterales;Syntrophobacteraceae;Desulfosoma;Desulfosoma caldarium;Desulfosoma caldarium DSM 22027 |
| Ga0586324_0089541_2_736 | 13400000.00 | 15300000.00 | 0.00 | 18430000.00 | 0.00 | 0.00 | 0.00 | 8845000.00 | 0.00 | 9163000.00 | 13728000.00 | 11475000.00 | KO:K00437 | [NiFe] hydrogenase large subunit | Bacteria;Proteobacteria;Deltaproteobacteria;Desulfobacterales;Desulfobacteraceae;Desulfosarcina;Desulfosarcina variabilis;Desulfosarcina variabilis Montpellier |
| Ga0586324_0363543_456_1883 | 0.00 | 0.00 | 0.00 | 0.00 | 0.00 | 0.00 | 0.00 | 0.00 | 2211300.00 | 0.00 | 0.00 | 0.00 | KO:K00437 | [NiFe] hydrogenase large subunit | Bacteria;Proteobacteria;Deltaproteobacteria;Desulfobacterales;Desulfobacteraceae;Desulfatirhabdium;Desulfatirhabdium butyrativorans;Desulfatirhabdium butyrativorans DSM 18734 |
| Ga0586324_0379005_1168_1851 | 0.00 | 0.00 | 0.00 | 7334000.00 | 0.00 | 0.00 | 0.00 | 23607000.00 | 0.00 | 0.00 | 20328000.00 | 0.00 | KO:K00437 | [NiFe] hydrogenase large subunit | Bacteria;Proteobacteria;Deltaproteobacteria;Desulfobacterales;Desulfobacteraceae;Desulfosarcina;Desulfosarcina variabilis;Desulfosarcina variabilis Montpellier |
| Ga0586324_0860535_133_1296 | 0.00 | 8772000.00 | 0.00 | 0.00 | 0.00 | 0.00 | 0.00 | 0.00 | 0.00 | 0.00 | 0.00 | 0.00 | KO:K00437 | [NiFe] hydrogenase large subunit | Bacteria;Proteobacteria;Deltaproteobacteria;Desulfobacterales;Desulfobacteraceae;Desulfatirhabdium;Desulfatirhabdium butyrativorans;Desulfatirhabdium butyrativorans DSM 18734 |
| Ga0586324_0884872_2_1279 | 0.00 | 671383.04 | 0.00 | 0.00 | 0.00 | 0.00 | 0.00 | 0.00 | 0.00 | 0.00 | 0.00 | 0.00 | KO:K00437 | [NiFe] hydrogenase large subunit | Bacteria;Proteobacteria;Deltaproteobacteria;Syntrophobacterales;Syntrophaceae;Desulfomonile;Desulfomonile tiedjei;Desulfomonile tiedjei DCB-1, DSM 6799 |
| Ga0586324_3053203_1_756 | 0.00 | 0.00 | 0.00 | 2394000.00 | 1987900.00 | 0.00 | 0.00 | 817400.00 | 0.00 | 0.00 | 0.00 | 0.00 | KO:K00437 | [NiFe] hydrogenase large subunit | Bacteria;Proteobacteria;Deltaproteobacteria;Syntrophobacterales;Syntrophobacteraceae;Syntrophobacter;Syntrophobacter fumaroxidans;Syntrophobacter fumaroxidans MPOB |
| Ga0586324_3529764_1_711 | 0.00 | 0.00 | 0.00 | 0.00 | 3460800.00 | 0.00 | 0.00 | 0.00 | 0.00 | 0.00 | 0.00 | 0.00 | KO:K00437 | [NiFe] hydrogenase large subunit | Bacteria;Proteobacteria;Deltaproteobacteria;Syntrophobacterales;Syntrophobacteraceae;Desulfosoma;Desulfosoma caldarium;Desulfosoma caldarium DSM 22027 |
| Ga0586324_3867077_1_642 | 0.00 | 31416000.00 | 0.00 | 54055000.00 | 66126000.00 | 10083800.00 | 44688000.00 | 33428000.00 | 0.00 | 19789000.00 | 0.00 | 0.00 | KO:K00437 | [NiFe] hydrogenase large subunit | Bacteria;Proteobacteria;Deltaproteobacteria;Desulfobacterales;Desulfobacteraceae;Desulfatitalea;Desulfatitalea tepidiphila;Desulfatitalea tepidiphila S28bF |
| Ga0586324_0005418_1075_2442 | 5080000.00 | 0.00 | 0.00 | 0.00 | 0.00 | 0.00 | 0.00 | 0.00 | 0.00 | 0.00 | 0.00 | 0.00 | KO:K00440 | coenzyme F420 hydrogenase subunit alpha | Archaea;Euryarchaeota;Methanomicrobia;Methanomicrobiales;Methanomicrobiaceae;Methanoculleus;Methanoculleus thermophilus;Methanoculleus thermophilus DSM 2373 |
| Ga0586324_0098772_2465_3448 | 0.00 | 0.00 | 0.00 | 0.00 | 0.00 | 0.00 | 0.00 | 0.00 | 0.00 | 2032800.00 | 0.00 | 0.00 | KO:K00440 | coenzyme F420 hydrogenase subunit alpha | Archaea;Euryarchaeota;Methanomicrobia;Methanomicrobiales;Methanoregulaceae;Methanosphaerula;Methanosphaerula palustris;Methanosphaerula palustris E1-9c, DSM 19958 |
| Ga0586324_0110913_1955_3262 | 0.00 | 5684800.00 | 0.00 | 5424500.00 | 7210000.00 | 0.00 | 0.00 | 0.00 | 0.00 | 0.00 | 0.00 | 0.00 | KO:K00440 | coenzyme F420 hydrogenase subunit alpha | Archaea;Euryarchaeota;Methanomicrobia;Methanomicrobiales;Methanomicrobiaceae;Methanoculleus;Methanoculleus bourgensis;Methanoculleus bourgensis MBBA |
| Ga0586324_0198712_1_1140 | 5210000.00 | 4753200.00 | 0.00 | 3239500.00 | 2513200.00 | 0.00 | 1645685.76 | 1573800.00 | 1142100.00 | 1909600.00 | 0.00 | 0.00 | KO:K00440 | coenzyme F420 hydrogenase subunit alpha | Archaea;Euryarchaeota;Methanomicrobia;Methanosarcinales;Methanosarcinaceae;Methanosarcina;Methanosarcina acetivorans;Methanosarcina acetivorans C2A |
| Ga0586324_0713882_3_1181 | 0.00 | 0.00 | 44308100.00 | 0.00 | 0.00 | 0.00 | 0.00 | 0.00 | 27054000.00 | 0.00 | 0.00 | 0.00 | KO:K00440 | coenzyme F420 hydrogenase subunit alpha | Archaea;Euryarchaeota;Methanomicrobia;Methanomicrobiales;Methanoregulaceae;Methanoregula;Methanoregula formicica;Methanoregula formicica SMSP, DSM 22288 |
| Ga0586324_1054547_1_1188 | 0.00 | 594642.32 | 0.00 | 0.00 | 0.00 | 0.00 | 0.00 | 0.00 | 0.00 | 0.00 | 0.00 | 0.00 | KO:K00440 | coenzyme F420 hydrogenase subunit alpha | Archaea;Euryarchaeota;Methanomicrobia;Methanomicrobiales;Methanomicrobiaceae;Methanofollis;Methanofollis ethanolicus;Methanofollis ethanolicus JCM 15103 |
| Ga0586324_2626538_3_806 | 75200000.00 | 4338400.00 | 0.00 | 5310500.00 | 0.00 | 0.00 | 0.00 | 0.00 | 0.00 | 30261000.00 | 0.00 | 0.00 | KO:K00440 | coenzyme F420 hydrogenase subunit alpha | Archaea;Euryarchaeota;Methanomicrobia;Methanomicrobiales;Methanoregulaceae;Methanoregula;Methanoregula formicica;Methanoregula formicica SMSP, DSM 22288 |
| Ga0586324_4147740_3_665 | 0.00 | 12512000.00 | 0.00 | 9158000.00 | 0.00 | 0.00 | 0.00 | 5398500.00 | 0.00 | 0.00 | 0.00 | 6570000.00 | KO:K00440 | coenzyme F420 hydrogenase subunit alpha | Archaea;Euryarchaeota;Methanomicrobia;Methanomicrobiales;Methanoregulaceae;Methanoregula;Methanoregula formicica;Methanoregula formicica SMSP, DSM 22288 |
| Ga0586324_4216383_167_661 | 0.00 | 0.00 | 0.00 | 0.00 | 0.00 | 0.00 | 0.00 | 0.00 | 0.00 | 0.00 | 0.00 | 35325000.00 | KO:K00440 | coenzyme F420 hydrogenase subunit alpha | Archaea;Euryarchaeota;Methanomicrobia;Methanomicrobiales;Methanoregulaceae;Methanoregula;Methanoregula formicica;Methanoregula formicica SMSP, DSM 22288 |
| Ga0586324_7086809_3_533 | 0.00 | 0.00 | 0.00 | 0.00 | 0.00 | 19532400.00 | 0.00 | 0.00 | 0.00 | 0.00 | 10912000.00 | 0.00 | KO:K00440 | coenzyme F420 hydrogenase subunit alpha | Archaea;Euryarchaeota;Methanomicrobia;Methanomicrobiales;Methanoregulaceae;Methanoregula;Methanoregula formicica;Methanoregula formicica SMSP, DSM 22288 |
| Ga0586324_0168802_1918_2676 | 0.00 | 0.00 | 0.00 | 7666500.00 | 12051000.00 | 0.00 | 0.00 | 0.00 | 0.00 | 0.00 | 0.00 | 0.00 | KO:K00441 | coenzyme F420 hydrogenase subunit beta | Archaea;Euryarchaeota;Methanomicrobia;Methanomicrobiales;Methanoregulaceae;Methanoregula;Methanoregula formicica;Methanoregula formicica SMSP, DSM 22288 |
| Ga0586324_0229114_786_1712 | 0.00 | 0.00 | 0.00 | 1719500.00 | 0.00 | 0.00 | 0.00 | 0.00 | 0.00 | 0.00 | 0.00 | 0.00 | KO:K00441 | coenzyme F420 hydrogenase subunit beta | Archaea;Euryarchaeota;Methanomicrobia;Methanomicrobiales;Methanoregulaceae;Methanoregula;Methanoregula formicica;Methanoregula formicica SMSP, DSM 22288 |
| Ga0586324_0274523_1327_2139 | 0.00 | 23460000.00 | 0.00 | 0.00 | 0.00 | 0.00 | 0.00 | 0.00 | 0.00 | 0.00 | 0.00 | 0.00 | KO:K00441 | coenzyme F420 hydrogenase subunit beta | Archaea;Euryarchaeota;Methanomicrobia;Methanomicrobiales;Methanoregulaceae;Methanoregula;Methanoregula formicica;Methanoregula formicica SMSP, DSM 22288 |
| Ga0586324_1584353_264_998 | 58100000.00 | 0.00 | 0.00 | 0.00 | 0.00 | 0.00 | 0.00 | 0.00 | 0.00 | 0.00 | 0.00 | 0.00 | KO:K00441 | coenzyme F420 hydrogenase subunit beta | Archaea;Euryarchaeota;Methanomicrobia;Methanomicrobiales;Methanoregulaceae;Methanoregula;Methanoregula boonei;Methanoregula boonei 6A8 |
| Ga0586324_0030998_2041_2904 | 0.00 | 0.00 | 0.00 | 0.00 | 0.00 | 0.00 | 0.00 | 0.00 | 0.00 | 2610300.00 | 0.00 | 0.00 | KO:K00443 | coenzyme F420 hydrogenase subunit gamma | Archaea;Euryarchaeota;Methanomicrobia;Methanomicrobiales;Methanoregulaceae;Methanoregula;Methanoregula formicica;Methanoregula formicica SMSP, DSM 22288 |
| Ga0586324_1235981_199_1086 | 0.00 | 0.00 | 0.00 | 0.00 | 0.00 | 0.00 | 0.00 | 1128500.00 | 0.00 | 0.00 | 0.00 | 1807500.00 | KO:K00443 | coenzyme F420 hydrogenase subunit gamma | Archaea;Euryarchaeota;Methanomicrobia;Methanomicrobiales;Methanoregulaceae;Methanoregula;Methanoregula formicica;Methanoregula formicica SMSP, DSM 22288 |
| Ga0586324_0776730_1_192 | 5090000.00 | 0.00 | 0.00 | 0.00 | 0.00 | 0.00 | 0.00 | 0.00 | 0.00 | 0.00 | 0.00 | 0.00 | KO:K00518 | nickel superoxide dismutase | Bacteria;Bacteroidetes;Flavobacteriia;Flavobacteriales;Flavobacteriaceae;Psychroflexus;Psychroflexus torquis;Psychroflexus torquis ATCC 700755 |
| Ga0586324_0057586_231_3143 | 0.00 | 0.00 | 0.00 | 0.00 | 0.00 | 0.00 | 0.00 | 5490000.00 | 0.00 | 0.00 | 0.00 | 0.00 | KO:K00525 | ribonucleoside-diphosphate reductase alpha chain | Bacteria;Proteobacteria;Betaproteobacteria;Burkholderiales;unclassified Burkholderiales;Ideonella;Ideonella sp. A 288;Ideonella sp. A 288 |
| Ga0586324_0721191_3_752 | 0.00 | 0.00 | 0.00 | 0.00 | 0.00 | 0.00 | 0.00 | 2891400.00 | 0.00 | 0.00 | 0.00 | 0.00 | KO:K00528 | ferredoxin--NADP+ reductase | Bacteria;Proteobacteria;Deltaproteobacteria;Desulfobacterales;Desulfobacteraceae;Desulfatiglans;Desulfatiglans anilini;Desulfatiglans anilini DSM 4660 |
| Ga0586324_0000004_1795_5466 | 0.00 | 0.00 | 0.00 | 0.00 | 0.00 | 0.00 | 0.00 | 0.00 | 0.00 | 696341.80 | 0.00 | 0.00 | KO:K00548 | 5-methyltetrahydrofolate--homocysteine methyltransferase | Bacteria;Proteobacteria;Betaproteobacteria;Nitrosomonadales;Thiobacillaceae;Annwoodia;Annwoodia aquaesulis;Annwoodia aquaesulis DSM 4255 |
| Ga0586324_0003216_8879_12556 | 0.00 | 0.00 | 0.00 | 0.00 | 0.00 | 0.00 | 354546.84 | 1836100.00 | 0.00 | 0.00 | 0.00 | 0.00 | KO:K00548 | 5-methyltetrahydrofolate--homocysteine methyltransferase | Bacteria;Proteobacteria;Gammaproteobacteria;Methylococcales;Methylococcaceae;Methylobacter;Methylobacter tundripaludum;Methylobacter tundripaludum SV96 |
| Ga0586324_0023800_1943_5578 | 0.00 | 0.00 | 0.00 | 0.00 | 0.00 | 0.00 | 2275316.88 | 0.00 | 0.00 | 0.00 | 0.00 | 0.00 | KO:K00548 | 5-methyltetrahydrofolate--homocysteine methyltransferase | Bacteria;Actinobacteria;Actinomycetia;Streptosporangiales;Thermomonosporaceae;Actinomadura;Actinomadura sp. WAC 06369;Actinomadura sp. WAC 06369 |
| Ga0586324_0056591_2337_4502 | 0.00 | 0.00 | 0.00 | 0.00 | 0.00 | 0.00 | 0.00 | 0.00 | 0.00 | 169118.18 | 0.00 | 0.00 | KO:K00548 | 5-methyltetrahydrofolate--homocysteine methyltransferase | Bacteria;Bacteroidetes;Sphingobacteriia;Sphingobacteriales;Sphingobacteriaceae;Solitalea;Solitalea canadensis;Solitalea canadensis USAM 9D, DSM 3403 |
| Ga0586324_0108686_3_2138 | 0.00 | 0.00 | 0.00 | 0.00 | 0.00 | 0.00 | 642586.08 | 0.00 | 0.00 | 0.00 | 28336000.00 | 0.00 | KO:K00548 | 5-methyltetrahydrofolate--homocysteine methyltransferase | Bacteria;Proteobacteria;Deltaproteobacteria;Myxococcales;Polyangiaceae;Sorangium;Sorangium cellulosum;Sorangium cellulosum So0149 |
| Ga0586324_0190120_1_2535 | 0.00 | 0.00 | 0.00 | 0.00 | 0.00 | 0.00 | 251219.52 | 0.00 | 0.00 | 0.00 | 0.00 | 0.00 | KO:K00548 | 5-methyltetrahydrofolate--homocysteine methyltransferase | Bacteria;Verrucomicrobia;Verrucomicrobiae;Verrucomicrobiales;Verrucomicrobia subdivision 3;Pedosphaera;Pedosphaera parvula;Pedosphaera parvula Ellin514 |
| Ga0586324_0320323_316_951 | 0.00 | 0.00 | 0.00 | 2289500.00 | 4810100.00 | 4565500.00 | 0.00 | 0.00 | 2932200.00 | 0.00 | 0.00 | 0.00 | KO:K00548 | 5-methyltetrahydrofolate--homocysteine methyltransferase | Bacteria;Chloroflexi;Anaerolineae;Anaerolineales;Anaerolineaceae;Pelolinea;Pelolinea submarina;Pelolinea submarina DSM 23923 |
| Ga0586324_0328286_569_1975 | 0.00 | 0.00 | 0.00 | 0.00 | 0.00 | 0.00 | 0.00 | 0.00 | 0.00 | 1701700.00 | 0.00 | 0.00 | KO:K00548 | 5-methyltetrahydrofolate--homocysteine methyltransferase | Bacteria;Proteobacteria;Betaproteobacteria;Rhodocyclales;Azonexaceae;Dechloromonas;Dechloromonas sp. HYN0024;Dechloromonas sp. HYN0024 |
| Ga0586324_0772135_515_1153 | 0.00 | 5385600.00 | 0.00 | 0.00 | 0.00 | 3285782.41 | 0.00 | 0.00 | 0.00 | 0.00 | 0.00 | 0.00 | KO:K00548 | 5-methyltetrahydrofolate--homocysteine methyltransferase | Bacteria;Firmicutes;Clostridia;Thermoanaerobacterales;Thermoanaerobacteraceae;Caldanaerobius;Caldanaerobius fijiensis;Caldanaerobius fijiensis DSM 17918 |
| Ga0586324_3027710_476_760 | 0.00 | 0.00 | 0.00 | 0.00 | 0.00 | 0.00 | 0.00 | 0.00 | 1372090.59 | 0.00 | 0.00 | 0.00 | KO:K00548 | 5-methyltetrahydrofolate--homocysteine methyltransferase | Bacteria;Firmicutes;Clostridia;Thermoanaerobacterales;Thermoanaerobacterales Family IV. Incertae Sedis;Mahella;Mahella australiensis;Mahella australiensis 50-1 BON, DSM 15567 |
| Ga0586324_4389250_2_562 | 0.00 | 0.00 | 0.00 | 0.00 | 0.00 | 0.00 | 0.00 | 0.00 | 637730.01 | 0.00 | 0.00 | 0.00 | KO:K00548 | 5-methyltetrahydrofolate--homocysteine methyltransferase | Bacteria;Verrucomicrobia;Opitutae;Opitutales;Opitutaceae;unclassified Opitutaceae;Opitutaceae bacterium TSB47;Opitutaceae bacterium TSB47 |
| Ga0586324_5723725_2_583 | 0.00 | 0.00 | 0.00 | 0.00 | 0.00 | 0.00 | 0.00 | 0.00 | 0.00 | 1355200.00 | 0.00 | 0.00 | KO:K00548 | 5-methyltetrahydrofolate--homocysteine methyltransferase | Bacteria;Deinococcus-Thermus;Deinococci;Trueperales;Trueperaceae;Truepera;Truepera radiovictrix;Truepera radiovictrix RQ-24, DSM 17093 |
| Ga0586324_7559851_2_520 | 0.00 | 0.00 | 0.00 | 0.00 | 0.00 | 0.00 | 0.00 | 0.00 | 0.00 | 770000.00 | 0.00 | 0.00 | KO:K00548 | 5-methyltetrahydrofolate--homocysteine methyltransferase | Bacteria;Verrucomicrobia;Verrucomicrobiae;Verrucomicrobiales;Verrucomicrobia subdivision 3;Pedosphaera;Pedosphaera parvula;Pedosphaera parvula Ellin514 |
| Ga0586324_7894095_1_510 | 2310000.00 | 1292000.00 | 0.00 | 1567500.00 | 0.00 | 0.00 | 0.00 | 0.00 | 0.00 | 0.00 | 1830400.00 | 1327500.00 | KO:K00548 | 5-methyltetrahydrofolate--homocysteine methyltransferase | Bacteria;Proteobacteria;Alphaproteobacteria;Hyphomicrobiales;Pleomorphomonadaceae;Pleomorphomonas;Pleomorphomonas koreensis;Pleomorphomonas koreensis DSM 23070 |
| Ga0586324_3056202_2_757 | 0.00 | 0.00 | 0.00 | 0.00 | 0.00 | 0.00 | 0.00 | 0.00 | 0.00 | 0.00 | 1804000.00 | 0.00 | KO:K00567 | methylated-DNA-[protein]-cysteine S-methyltransferase | Archaea;Euryarchaeota;Methanomicrobia;Methanosarcinales;Methanosarcinaceae;Methanosarcina;Methanosarcina horonobensis;Methanosarcina horonobensis HB-1 |
| Ga0586324_8236775_2_445 | 0.00 | 0.00 | 0.00 | 0.00 | 0.00 | 4406700.00 | 0.00 | 0.00 | 0.00 | 0.00 | 0.00 | 0.00 | KO:K00567 | methylated-DNA-[protein]-cysteine S-methyltransferase | Bacteria;Proteobacteria;Gammaproteobacteria;Oceanospirillales;Halomonadaceae;Halomonas;Halomonas ilicicola;Halomonas ilicicola DSM 19980 |
| Ga0586324_0345011_3_701 | 9270000.00 | 16728000.00 | 0.00 | 0.00 | 0.00 | 24256700.00 | 0.00 | 3324500.00 | 2713500.00 | 0.00 | 0.00 | 0.00 | KO:K00577 | tetrahydromethanopterin S-methyltransferase subunit A | Archaea;Euryarchaeota;Methanomicrobia;Methanosarcinales;Methanotrichaceae;Methanothrix;Methanothrix thermoacetophila;Methanothrix thermoacetophila PT |
| Ga0586324_0502518_420_1151 | 0.00 | 6752400.00 | 0.00 | 1881000.00 | 4562900.00 | 0.00 | 0.00 | 12200000.00 | 0.00 | 954800.00 | 0.00 | 0.00 | KO:K00577 | tetrahydromethanopterin S-methyltransferase subunit A | Archaea;Euryarchaeota;Methanomicrobia;Methanosarcinales;Methanotrichaceae;Methanothrix;Methanothrix thermoacetophila;Methanothrix thermoacetophila PT |
| Ga0586324_0604409_411_1133 | 0.00 | 1740800.00 | 0.00 | 0.00 | 0.00 | 0.00 | 9804000.00 | 0.00 | 0.00 | 0.00 | 0.00 | 0.00 | KO:K00577 | tetrahydromethanopterin S-methyltransferase subunit A | Archaea;Euryarchaeota;Methanomicrobia;Methanomicrobiales;Methanoregulaceae;Methanoregula;Methanoregula formicica;Methanoregula formicica SMSP, DSM 22288 |
| Ga0586324_2873307_88_774 | 0.00 | 0.00 | 0.00 | 0.00 | 0.00 | 0.00 | 0.00 | 0.00 | 4479300.00 | 0.00 | 0.00 | 0.00 | KO:K00577 | tetrahydromethanopterin S-methyltransferase subunit A | Archaea;Euryarchaeota;Methanomicrobia;Methanosarcinales;Methanotrichaceae;Methanothrix;Methanothrix thermoacetophila;Methanothrix thermoacetophila PT |
| Ga0586324_5430374_3_596 | 0.00 | 0.00 | 0.00 | 0.00 | 0.00 | 2675390.94 | 0.00 | 0.00 | 0.00 | 0.00 | 0.00 | 0.00 | KO:K00577 | tetrahydromethanopterin S-methyltransferase subunit A | Archaea;Euryarchaeota;Methanomicrobia;Methanosarcinales;Methanotrichaceae;Methanothrix;Methanothrix thermoacetophila;Methanothrix thermoacetophila PT |
| Ga0586324_0502518_1153_1512 | 0.00 | 0.00 | 0.00 | 0.00 | 3471100.00 | 0.00 | 0.00 | 0.00 | 0.00 | 0.00 | 0.00 | 0.00 | KO:K00578 | tetrahydromethanopterin S-methyltransferase subunit B | Archaea;Euryarchaeota;Methanomicrobia;Methanosarcinales;Methanotrichaceae;Methanothrix;Methanothrix thermoacetophila;Methanothrix thermoacetophila PT |
| Ga0586324_0000676_342_1379 | 0.00 | 4005200.00 | 0.00 | 0.00 | 5510500.00 | 0.00 | 3192000.00 | 3910100.00 | 4252500.00 | 0.00 | 4945600.00 | 0.00 | KO:K00584 | tetrahydromethanopterin S-methyltransferase subunit H | Archaea;Euryarchaeota;Methanomicrobia;Methanomicrobiales;Methanoregulaceae;Methanoregula;Methanoregula formicica;Methanoregula formicica SMSP, DSM 22288 |
| Ga0586324_0029143_4841_5878 | 7270000.00 | 0.00 | 0.00 | 0.00 | 0.00 | 0.00 | 0.00 | 0.00 | 0.00 | 5297600.00 | 0.00 | 0.00 | KO:K00584 | tetrahydromethanopterin S-methyltransferase subunit H | Archaea;Euryarchaeota;Methanomicrobia;Methanomicrobiales;Methanoregulaceae;Methanoregula;Methanoregula boonei;Methanoregula boonei 6A8 |
| Ga0586324_0133327_566_1603 | 0.00 | 0.00 | 4168271.84 | 4579000.00 | 0.00 | 0.00 | 0.00 | 3312300.00 | 0.00 | 0.00 | 0.00 | 0.00 | KO:K00584 | tetrahydromethanopterin S-methyltransferase subunit H | Archaea;Euryarchaeota;Methanomicrobia;Methanomicrobiales;Methanoregulaceae;Methanoregula;Methanoregula formicica;Methanoregula formicica SMSP, DSM 22288 |
| Ga0586324_0172633_1714_2649 | 0.00 | 0.00 | 0.00 | 0.00 | 0.00 | 0.00 | 0.00 | 0.00 | 0.00 | 954800.00 | 0.00 | 0.00 | KO:K00584 | tetrahydromethanopterin S-methyltransferase subunit H | Archaea;Euryarchaeota;Methanomicrobia;Methanomicrobiales;Methanoregulaceae;Methanoregula;Methanoregula formicica;Methanoregula formicica SMSP, DSM 22288 |
| Ga0586324_0200176_1696_2475 | 6200000.00 | 13328000.00 | 0.00 | 0.00 | 0.00 | 0.00 | 0.00 | 0.00 | 0.00 | 0.00 | 0.00 | 0.00 | KO:K00584 | tetrahydromethanopterin S-methyltransferase subunit H | Archaea;Euryarchaeota;Methanomicrobia;Methanosarcinales;Methanotrichaceae;Methanothrix;Methanothrix soehngenii;Methanothrix soehngenii GP-6 |
| Ga0586324_0235258_494_1531 | 4100000.00 | 4834800.00 | 0.00 | 5757000.00 | 5108800.00 | 0.00 | 6201600.00 | 4953200.00 | 7200900.00 | 7268800.00 | 4637600.00 | 2542500.00 | KO:K00584 | tetrahydromethanopterin S-methyltransferase subunit H | Archaea;Euryarchaeota;Methanomicrobia;Methanomicrobiales;Methanomicrobiaceae;Methanoculleus;Methanoculleus marisnigri;Methanoculleus marisnigri JR1, DSM 1498 |
| Ga0586324_0253111_561_1601 | 6180000.00 | 3689437.92 | 0.00 | 2992500.00 | 1977600.00 | 0.00 | 509324.64 | 1055300.00 | 972000.00 | 1255100.00 | 0.00 | 2475000.00 | KO:K00584 | tetrahydromethanopterin S-methyltransferase subunit H | Archaea;Euryarchaeota;Methanomicrobia;Methanomicrobiales;Methanoregulaceae;Methanoregula;Methanoregula formicica;Methanoregula formicica SMSP, DSM 22288 |
| Ga0586324_0320598_1086_1994 | 10300000.00 | 0.00 | 0.00 | 0.00 | 0.00 | 0.00 | 0.00 | 0.00 | 0.00 | 0.00 | 0.00 | 3900000.00 | KO:K00584 | tetrahydromethanopterin S-methyltransferase subunit H | Archaea;Euryarchaeota;Methanomicrobia;Methanomicrobiales;Methanoregulaceae;Methanoregula;Methanoregula formicica;Methanoregula formicica SMSP, DSM 22288 |
| Ga0586324_0324697_826_1863 | 0.00 | 408703.80 | 0.00 | 0.00 | 0.00 | 0.00 | 0.00 | 0.00 | 0.00 | 0.00 | 0.00 | 0.00 | KO:K00584 | tetrahydromethanopterin S-methyltransferase subunit H | Archaea;Euryarchaeota;Methanomicrobia;Methanomicrobiales;Methanoregulaceae;Methanoregula;Methanoregula formicica;Methanoregula formicica SMSP, DSM 22288 |
| Ga0586324_0331716_1246_1965 | 0.00 | 0.00 | 0.00 | 2954500.00 | 0.00 | 0.00 | 0.00 | 0.00 | 0.00 | 0.00 | 0.00 | 0.00 | KO:K00584 | tetrahydromethanopterin S-methyltransferase subunit H | Archaea;Euryarchaeota;Methanomicrobia;Methanosarcinales;Methanosarcinaceae;Methanosarcina;Methanosarcina barkeri;Methanosarcina barkeri Fusaro, DSM 804 |
| Ga0586324_0481038_334_1374 | 0.00 | 606877.56 | 544403.55 | 0.00 | 0.00 | 0.00 | 0.00 | 0.00 | 0.00 | 0.00 | 0.00 | 0.00 | KO:K00584 | tetrahydromethanopterin S-methyltransferase subunit H | Archaea;Euryarchaeota;Methanomicrobia;Methanomicrobiales;Methanoregulaceae;Methanoregula;Methanoregula formicica;Methanoregula formicica SMSP, DSM 22288 |
| Ga0586324_0785220_187_1098 | 0.00 | 0.00 | 0.00 | 0.00 | 0.00 | 0.00 | 0.00 | 0.00 | 3839400.00 | 0.00 | 0.00 | 0.00 | KO:K00584 | tetrahydromethanopterin S-methyltransferase subunit H | Archaea;Euryarchaeota;Methanomicrobia;Methanosarcinales;Methanosarcinaceae;Methanolobus;Methanolobus tindarius;Methanolobus tindarius DSM 2278 |
| Ga0586324_1169601_1_945 | 8360000.00 | 0.00 | 0.00 | 0.00 | 0.00 | 0.00 | 0.00 | 0.00 | 0.00 | 0.00 | 0.00 | 0.00 | KO:K00584 | tetrahydromethanopterin S-methyltransferase subunit H | Archaea;Euryarchaeota;Methanomicrobia;Methanomicrobiales;Methanoregulaceae;Methanoregula;Methanoregula formicica;Methanoregula formicica SMSP, DSM 22288 |
| Ga0586324_1756787_143_955 | 0.00 | 0.00 | 0.00 | 0.00 | 0.00 | 0.00 | 0.00 | 0.00 | 0.00 | 0.00 | 0.00 | 2850000.00 | KO:K00584 | tetrahydromethanopterin S-methyltransferase subunit H | Archaea;Euryarchaeota;Methanomicrobia;Methanomicrobiales;Methanoregulaceae;Methanoregula;Methanoregula boonei;Methanoregula boonei 6A8 |
| Ga0586324_2293891_10_852 | 0.00 | 8568000.00 | 0.00 | 0.00 | 0.00 | 0.00 | 0.00 | 0.00 | 0.00 | 0.00 | 0.00 | 0.00 | KO:K00584 | tetrahydromethanopterin S-methyltransferase subunit H | Archaea;Euryarchaeota;Methanomicrobia;Methanosarcinales;Methanotrichaceae;Methanothrix;Methanothrix thermoacetophila;Methanothrix thermoacetophila PT |
| Ga0586324_2960723_109_765 | 14700000.00 | 0.00 | 18241200.00 | 0.00 | 0.00 | 1787496.47 | 1938209.76 | 1537200.00 | 0.00 | 687040.20 | 0.00 | 0.00 | KO:K00584 | tetrahydromethanopterin S-methyltransferase subunit H | Archaea;Euryarchaeota;Methanomicrobia;Methanomicrobiales;Methanoregulaceae;Methanoregula;Methanoregula formicica;Methanoregula formicica SMSP, DSM 22288 |
| Ga0586324_5482395_13_594 | 0.00 | 4039200.00 | 17621900.00 | 12730000.00 | 0.00 | 0.00 | 0.00 | 0.00 | 0.00 | 0.00 | 0.00 | 6480000.00 | KO:K00584 | tetrahydromethanopterin S-methyltransferase subunit H | Archaea;Euryarchaeota;Methanomicrobia;Methanomicrobiales;Methanoregulaceae;Methanoregula;Methanoregula formicica;Methanoregula formicica SMSP, DSM 22288 |
| Ga0586324_5958587_3_575 | 30100000.00 | 53108000.00 | 0.00 | 51300000.00 | 47689000.00 | 52801000.00 | 0.00 | 37332000.00 | 32643000.00 | 0.00 | 16192000.00 | 0.00 | KO:K00584 | tetrahydromethanopterin S-methyltransferase subunit H | Archaea;Euryarchaeota;Methanomicrobia;Methanomicrobiales;Methanoregulaceae;Methanoregula;Methanoregula formicica;Methanoregula formicica SMSP, DSM 22288 |
| Ga0586324_7713618_1_516 | 19800000.00 | 0.00 | 55230300.00 | 0.00 | 10506000.00 | 9607400.00 | 0.00 | 0.00 | 3815100.00 | 37422000.00 | 0.00 | 5122500.00 | KO:K00584 | tetrahydromethanopterin S-methyltransferase subunit H | Archaea;Euryarchaeota;Methanomicrobia;Methanosarcinales;Methanotrichaceae;Methanothrix;Methanothrix thermoacetophila;Methanothrix thermoacetophila PT |
| Ga0586324_0000535_14767_16023 | 0.00 | 0.00 | 0.00 | 0.00 | 0.00 | 0.00 | 7259000.00 | 0.00 | 0.00 | 0.00 | 0.00 | 0.00 | KO:K00600 | glycine hydroxymethyltransferase | Bacteria;Proteobacteria;Gammaproteobacteria;Acidiferrobacterales;Acidiferrobacteraceae;Sulfuricaulis;Sulfuricaulis limicola;Sulfuricaulis limicola HA5 |
| Ga0586324_0099684_3_485 | 11400000.00 | 0.00 | 18466400.00 | 0.00 | 0.00 | 0.00 | 0.00 | 0.00 | 0.00 | 0.00 | 0.00 | 543215.25 | KO:K00600 | glycine hydroxymethyltransferase | Archaea;Euryarchaeota;Methanomicrobia;Methanosarcinales;Methanotrichaceae;Methanothrix;Methanothrix soehngenii;Methanothrix soehngenii GP-6 |
| Ga0586324_0188092_1_1206 | 0.00 | 0.00 | 0.00 | 0.00 | 0.00 | 0.00 | 0.00 | 0.00 | 0.00 | 0.00 | 0.00 | 254373.75 | KO:K00600 | glycine hydroxymethyltransferase | Bacteria;Proteobacteria;Deltaproteobacteria;Desulfuromonadales;Geobacteraceae;Geobacter;Geobacter uraniireducens;Geobacter uraniireducens Rf4 |
| Ga0586324_0191219_567_1817 | 0.00 | 0.00 | 0.00 | 0.00 | 0.00 | 0.00 | 0.00 | 0.00 | 0.00 | 8859422.11 | 0.00 | 0.00 | KO:K00600 | glycine hydroxymethyltransferase | Bacteria;Proteobacteria;Betaproteobacteria;Nitrosomonadales;Sulfuricellaceae;Sulfurirhabdus;Sulfurirhabdus autotrophica;Sulfurirhabdus autotrophica DSM 100309 |
| Ga0586324_0404757_255_1535 | 0.00 | 6426000.00 | 0.00 | 0.00 | 0.00 | 0.00 | 0.00 | 0.00 | 0.00 | 0.00 | 0.00 | 0.00 | KO:K00600 | glycine hydroxymethyltransferase | Bacteria;Acidobacteria;Acidobacteriia;Bryobacterales;Solibacteraceae;Candidatus Solibacter;Candidatus Solibacter usitatus;Candidatus Solibacter usitatus Ellin6076 |
| Ga0586324_1528886_351_1013 | 0.00 | 0.00 | 0.00 | 0.00 | 0.00 | 0.00 | 0.00 | 0.00 | 0.00 | 0.00 | 9592000.00 | 0.00 | KO:K00600 | glycine hydroxymethyltransferase | Bacteria;Proteobacteria;Deltaproteobacteria;Syntrophobacterales;Syntrophobacteraceae;Desulfacinum;Desulfacinum hydrothermale;Desulfacinum hydrothermale DSM 13146 |
| Ga0586324_1843878_2_934 | 0.00 | 0.00 | 0.00 | 0.00 | 0.00 | 5240400.00 | 0.00 | 0.00 | 0.00 | 4681600.00 | 0.00 | 0.00 | KO:K00600 | glycine hydroxymethyltransferase | Bacteria;Proteobacteria;Deltaproteobacteria;Syntrophobacterales;Syntrophaceae;Desulfobacca;Desulfobacca acetoxidans;Desulfobacca acetoxidans ASRB2, DSM 11109 |
| Ga0586324_0001379_559_2133 | 0.00 | 0.00 | 0.00 | 0.00 | 0.00 | 0.00 | 0.00 | 4093100.00 | 0.00 | 0.00 | 2490400.00 | 0.00 | KO:K00602 | phosphoribosylaminoimidazolecarboxamide formyltransferase/IMP cyclohydrolase | Bacteria;Proteobacteria;Betaproteobacteria;Nitrosomonadales;Thiobacillaceae;Thiobacillus;Thiobacillus thioparus;Thiobacillus thioparus DSM 505 |
| Ga0586324_0642177_1_1347 | 0.00 | 216692.88 | 0.00 | 0.00 | 0.00 | 0.00 | 0.00 | 0.00 | 0.00 | 0.00 | 0.00 | 0.00 | KO:K00602 | phosphoribosylaminoimidazolecarboxamide formyltransferase/IMP cyclohydrolase | Bacteria;Nitrospirae;Nitrospira;Nitrospirales;Nitrospiraceae;Candidatus Magnetobacterium;Candidatus Magnetobacterium casensis;Candidatus Magnetobacterium casensis MYR-1 |
| Ga0586324_3298425_3_671 | 0.00 | 0.00 | 0.00 | 0.00 | 0.00 | 0.00 | 0.00 | 0.00 | 0.00 | 0.00 | 0.00 | 3037500.00 | KO:K00602 | phosphoribosylaminoimidazolecarboxamide formyltransferase/IMP cyclohydrolase | Bacteria;Proteobacteria;Deltaproteobacteria;Syntrophobacterales;Syntrophobacteraceae;Syntrophobacter;Syntrophobacter fumaroxidans;Syntrophobacter fumaroxidans MPOB |
| Ga0586324_5747152_2_556 | 0.00 | 0.00 | 0.00 | 0.00 | 0.00 | 2312838.63 | 0.00 | 0.00 | 0.00 | 0.00 | 0.00 | 0.00 | KO:K00602 | phosphoribosylaminoimidazolecarboxamide formyltransferase/IMP cyclohydrolase | Bacteria;Proteobacteria;Deltaproteobacteria;Syntrophobacterales;Syntrophobacteraceae;Desulfacinum;Desulfacinum hydrothermale;Desulfacinum hydrothermale DSM 13146 |
| Ga0586324_6567321_2_550 | 0.00 | 0.00 | 0.00 | 0.00 | 0.00 | 0.00 | 0.00 | 0.00 | 6544800.00 | 0.00 | 0.00 | 0.00 | KO:K00602 | phosphoribosylaminoimidazolecarboxamide formyltransferase/IMP cyclohydrolase | Bacteria;Proteobacteria;Betaproteobacteria;Nitrosomonadales;Sulfuricellaceae;Sulfuricella;Sulfuricella denitrificans;Sulfuricella denitrificans skB26 |
| Ga0586324_3629135_3_704 | 0.00 | 0.00 | 0.00 | 0.00 | 297415.59 | 0.00 | 0.00 | 0.00 | 0.00 | 0.00 | 0.00 | 0.00 | KO:K00605 | aminomethyltransferase | Bacteria;Proteobacteria;Deltaproteobacteria;Myxococcales;Polyangiaceae;Sorangium;Sorangium cellulosum;Sorangium cellulosum So0008-312 |
| Ga0586324_0320399_959_1825 | 0.00 | 0.00 | 0.00 | 0.00 | 0.00 | 0.00 | 0.00 | 0.00 | 595161.27 | 0.00 | 0.00 | 0.00 | KO:K00606 | 3-methyl-2-oxobutanoate hydroxymethyltransferase | Bacteria;Proteobacteria;Deltaproteobacteria;Desulfobacterales;Desulfobacteraceae;Desulfococcus;Desulfococcus multivorans;Desulfococcus multivorans DSM 2059 |
| Ga0586324_0000047_47834_48790 | 0.00 | 0.00 | 0.00 | 0.00 | 0.00 | 1019638.92 | 0.00 | 0.00 | 785835.27 | 0.00 | 0.00 | 318096.00 | KO:K00609 | aspartate carbamoyltransferase catalytic subunit | Bacteria;Chloroflexi;Anaerolineae;Anaerolineales;Anaerolineaceae;Pelolinea;Pelolinea submarina;Pelolinea submarina DSM 23923 |
| Ga0586324_0028981_2449_3444 | 0.00 | 1808800.00 | 2238898.99 | 0.00 | 1205100.00 | 2517365.09 | 0.00 | 0.00 | 939600.00 | 0.00 | 0.00 | 0.00 | KO:K00609 | aspartate carbamoyltransferase catalytic subunit | Bacteria;Chloroflexi;Anaerolineae;Anaerolineales;Anaerolineaceae;Levilinea;Levilinea saccharolytica;Levilinea saccharolytica DSM 16555 |
| Ga0586324_0097906_1329_2282 | 0.00 | 0.00 | 16383300.00 | 0.00 | 0.00 | 0.00 | 0.00 | 0.00 | 0.00 | 0.00 | 0.00 | 0.00 | KO:K00609 | aspartate carbamoyltransferase catalytic subunit | Bacteria;Chloroflexi;Anaerolineae;Anaerolineales;Anaerolineaceae;Thermanaerothrix;Thermanaerothrix daxensis;Thermanaerothrix daxensis GNS-1 |
| Ga0586324_0857921_147_1127 | 12400000.00 | 0.00 | 0.00 | 0.00 | 0.00 | 0.00 | 0.00 | 0.00 | 0.00 | 0.00 | 0.00 | 0.00 | KO:K00609 | aspartate carbamoyltransferase catalytic subunit | Bacteria;Chloroflexi;Anaerolineae;Anaerolineales;Anaerolineaceae;Thermanaerothrix;Thermanaerothrix daxensis;Thermanaerothrix daxensis GNS-1 |
| Ga0586324_2365091_2_610 | 0.00 | 0.00 | 2378686.26 | 0.00 | 0.00 | 0.00 | 0.00 | 0.00 | 0.00 | 0.00 | 0.00 | 0.00 | KO:K00609 | aspartate carbamoyltransferase catalytic subunit | Bacteria;Chloroflexi;Anaerolineae;Anaerolineales;Anaerolineaceae;Thermanaerothrix;Thermanaerothrix daxensis;Thermanaerothrix daxensis GNS-1 |
| Ga0586324_5734429_74_583 | 0.00 | 0.00 | 0.00 | 0.00 | 0.00 | 0.00 | 0.00 | 0.00 | 0.00 | 0.00 | 0.00 | 2130000.00 | KO:K00609 | aspartate carbamoyltransferase catalytic subunit | Bacteria;Chloroflexi;Anaerolineae;Anaerolineales;Anaerolineaceae;Levilinea;Levilinea saccharolytica;Levilinea saccharolytica DSM 16555 |
| Ga0586324_0000001_223778_225763 | 0.00 | 0.00 | 0.00 | 0.00 | 0.00 | 0.00 | 7273200.00 | 0.00 | 8505000.00 | 3203200.00 | 2208800.00 | 2017500.00 | KO:K00615 | transketolase | Bacteria;Proteobacteria;Betaproteobacteria;Nitrosomonadales;Thiobacillaceae;Annwoodia;Annwoodia aquaesulis;Annwoodia aquaesulis DSM 4255 |
| Ga0586324_0000401_4914_6935 | 0.00 | 0.00 | 0.00 | 0.00 | 0.00 | 0.00 | 0.00 | 0.00 | 0.00 | 0.00 | 4083200.00 | 0.00 | KO:K00615 | transketolase | Bacteria;Proteobacteria;Gammaproteobacteria;Acidiferrobacterales;Acidiferrobacteraceae;Sulfuricaulis;Sulfuricaulis limicola;Sulfuricaulis limicola HA5 |
| Ga0586324_0066885_2985_4157 | 0.00 | 0.00 | 0.00 | 0.00 | 0.00 | 0.00 | 0.00 | 0.00 | 0.00 | 0.00 | 594132.88 | 0.00 | KO:K00615 | transketolase | Bacteria;Proteobacteria;Gammaproteobacteria;Methylococcales;Methylococcaceae;Methylobacter;Methylobacter luteus;Methylobacter luteus IMV-B-3098 |
| Ga0586324_0144642_1723_2877 | 0.00 | 0.00 | 0.00 | 0.00 | 0.00 | 0.00 | 0.00 | 0.00 | 0.00 | 0.00 | 0.00 | 1837500.00 | KO:K00615 | transketolase | Bacteria;Proteobacteria;Betaproteobacteria;Nitrosomonadales;Sulfuricellaceae;Sulfuriferula;Sulfuriferula multivorans;Sulfuriferula multivorans TTN |
| Ga0586324_0413985_3_983 | 0.00 | 0.00 | 0.00 | 0.00 | 0.00 | 0.00 | 0.00 | 0.00 | 0.00 | 0.00 | 0.00 | 11025000.00 | KO:K00615 | transketolase | Bacteria;Proteobacteria;Betaproteobacteria;Nitrosomonadales;Sulfuricellaceae;Sulfuriferula;unclassified Sulfuriferula;Sulfuriferula sp. AH1 re-analysis |
| Ga0586324_0516489_30_1616 | 0.00 | 46444000.00 | 353564000.00 | 0.00 | 0.00 | 0.00 | 0.00 | 44896000.00 | 0.00 | 0.00 | 0.00 | 0.00 | KO:K00615 | transketolase | Bacteria;Proteobacteria;Gammaproteobacteria;Methylococcales;Methylococcaceae;Methylococcus;Methylococcus capsulatus;Methylococcus capsulatus Texas, ATCC 19069 |
| Ga0586324_0554142_1_903 | 0.00 | 0.00 | 0.00 | 0.00 | 0.00 | 0.00 | 0.00 | 1878800.00 | 0.00 | 0.00 | 0.00 | 0.00 | KO:K00615 | transketolase | Bacteria;Proteobacteria;Betaproteobacteria;Nitrosomonadales;Thiobacillaceae;Thiobacillus;Thiobacillus thioparus;Thiobacillus thioparus DSM 505 |
| Ga0586324_1107133_1_1161 | 13300000.00 | 25364000.00 | 0.00 | 0.00 | 0.00 | 0.00 | 23256000.00 | 17141000.00 | 0.00 | 11165000.00 | 2948000.00 | 31200000.00 | KO:K00615 | transketolase | Bacteria;Proteobacteria;Gammaproteobacteria;Methylococcales;Methylococcaceae;Methyloglobulus;Methyloglobulus morosus;Methyloglobulus morosus KoM1 |
| Ga0586324_1794392_3_947 | 0.00 | 0.00 | 0.00 | 0.00 | 0.00 | 0.00 | 0.00 | 2982900.00 | 0.00 | 0.00 | 0.00 | 0.00 | KO:K00615 | transketolase | Bacteria;Proteobacteria;Betaproteobacteria;Nitrosomonadales;Thiobacillaceae;Thiobacillus;Thiobacillus denitrificans;Thiobacillus denitrificans DSM 12475 |
| Ga0586324_2295782_3_851 | 0.00 | 0.00 | 58552000.00 | 0.00 | 0.00 | 0.00 | 0.00 | 0.00 | 0.00 | 0.00 | 0.00 | 0.00 | KO:K00615 | transketolase | Bacteria;Proteobacteria;Gammaproteobacteria;Methylococcales;Methylococcaceae;Methylobacter;Methylobacter luteus;Methylobacter luteus IMV-B-3098 |
| Ga0586324_3276223_41_733 | 3620000.00 | 7548000.00 | 0.00 | 0.00 | 0.00 | 0.00 | 0.00 | 0.00 | 0.00 | 5651800.00 | 0.00 | 4597500.00 | KO:K00615 | transketolase | Bacteria;Proteobacteria;Gammaproteobacteria;Methylococcales;Methylococcaceae;Methyloglobulus;Methyloglobulus morosus;Methyloglobulus morosus KoM1 |
| Ga0586324_4539050_2_394 | 0.00 | 0.00 | 0.00 | 0.00 | 0.00 | 601724.96 | 0.00 | 0.00 | 0.00 | 0.00 | 0.00 | 0.00 | KO:K00615 | transketolase | Bacteria;Chloroflexi;Anaerolineae;Anaerolineales;Anaerolineaceae;Pelolinea;Pelolinea submarina;Pelolinea submarina DSM 23923 |
| Ga0586324_0006832_7913_8899 | 0.00 | 1944800.00 | 0.00 | 0.00 | 0.00 | 0.00 | 0.00 | 0.00 | 0.00 | 0.00 | 0.00 | 0.00 | KO:K00616 | transaldolase | Bacteria;Proteobacteria;Gammaproteobacteria;Methylococcales;Methylococcaceae;Methylomonas;Methylomonas sp. LW13;Methylomonas sp. LW13 |

| | | | | | | | | | | | | | | | |
|---|---|---|---|---|---|---|---|---|---|---|---|---|---|---|---|
| Ga0586324 0087282 1815 2765 | 0.00 | 0.00 | 0.00 | 0.00 | 0.00 | 0.00 | 0.00 | 11651000.00 | 0.00 | 1963500.00 | 0.00 | 22350000.00 | KO:K00616 | transaldolase | Bacteria;Proteobacteria;Betaproteobacteria;Nitrosomonadales;Methylophilaceae;Candidatus Methylopumilus;Candidatus Methylopumilus planktonicus;Candidatus Methylopumilus planktonicus MMS-2-53 |
| Ga0586324 0676559 198 1181 | 842621.00 | 0.00 | 0.00 | 0.00 | 0.00 | 0.00 | 0.00 | 0.00 | 0.00 | 0.00 | 0.00 | 0.00 | KO:K00616 | transaldolase | Bacteria;Proteobacteria;Gammaproteobacteria;Methylococcales;Methylococcaceae;Methylomarinum;Methylomarinum vadi;Methylomarinum vadi IT-4 |
| Ga0586324 1667332 2 529 | 0.00 | 0.00 | 0.00 | 460913.40 | 0.00 | 0.00 | 895289.88 | 334369.06 | 0.00 | 0.00 | 0.00 | 0.00 | KO:K00616 | transaldolase | Bacteria;Proteobacteria;Alphaproteobacteria;Rhodospirillales;Acetobacteraceae;Acidocella;Acidocella aromatica;Acidocella aromatica DSM 27026 |
| Ga0586324 3389030 161 724 | 0.00 | 6868000.00 | 0.00 | 0.00 | 0.00 | 0.00 | 14706000.00 | 8601000.00 | 8002800.00 | 5028100.00 | 7119200.00 | 4777500.00 | KO:K00616 | transaldolase | Bacteria;Proteobacteria;Gammaproteobacteria;Methylococcales;Methylococcaceae;Methylovulum;Methylovulum miyakonense;Methylovulum miyakonense HT12 |
| Ga0586324 5091533 111 611 | 0.00 | 2006000.00 | 0.00 | 0.00 | 0.00 | 0.00 | 0.00 | 0.00 | 0.00 | 0.00 | 0.00 | 0.00 | KO:K00616 | transaldolase | Bacteria;Proteobacteria;Gammaproteobacteria;Methylococcales;Methylococcaceae;Methyloglobulus;Methyloglobulus morosus;Methyloglobulus morosus KoM1 |
| Ga0586324 5505500 2 592 | 0.00 | 1972000.00 | 0.00 | 0.00 | 0.00 | 0.00 | 0.00 | 0.00 | 0.00 | 0.00 | 0.00 | 0.00 | KO:K00616 | transaldolase | Bacteria;Proteobacteria;Gammaproteobacteria;Methylococcales;Methylococcaceae;Methylosarcina;Methylosarcina fibrata;Methylosarcina fibrata AML-C10 |
| Ga0586324 5881308 3 578 | 1993980.00 | 0.00 | 0.00 | 0.00 | 0.00 | 0.00 | 0.00 | 0.00 | 0.00 | 0.00 | 0.00 | 0.00 | KO:K00616 | transaldolase | Bacteria;Proteobacteria;Gammaproteobacteria;Methylococcales;Methylococcaceae;Methylobacter;Methylobacter tundripaludum;Methylobacter tundripaludum 31/32 |
| Ga0586324 6666006 1 459 | 9200000.00 | 0.00 | 0.00 | 0.00 | 0.00 | 0.00 | 0.00 | 0.00 | 0.00 | 0.00 | 0.00 | 0.00 | KO:K00616 | transaldolase | Bacteria;Proteobacteria;Gammaproteobacteria;Methylococcales;Methylococcaceae;Methyloglobulus;Methyloglobulus morosus;Methyloglobulus morosus KoM1 |
| Ga0586324 0000001 131005 132231 | 0.00 | 0.00 | 7262700.00 | 0.00 | 0.00 | 0.00 | 798369.36 | 496696.16 | 4082400.00 | 1570800.00 | 1548800.00 | 1012500.00 | KO:K00620 | glutamate N-acetyltransferase/amino-acid N-acetyltransferase | Bacteria;Proteobacteria;Betaproteobacteria;Nitrosomonadales;Thiobacillaceae;Annwoodia;Annwoodia aquaesulis;Annwoodia aquaesulis DSM 4255 |
| Ga0586324 0014239 5372 6598 | 0.00 | 0.00 | 0.00 | 0.00 | 0.00 | 0.00 | 739458.72 | 580216.14 | 579751.02 | 893200.00 | 1144000.00 | 787500.00 | KO:K00620 | glutamate N-acetyltransferase/amino-acid N-acetyltransferase | Bacteria;Proteobacteria;Betaproteobacteria;Nitrosomonadales;Thiobacillaceae;Thiobacillus;Thiobacillus thiophilus;Thiobacillus thiophilus DSM 19892 |
| Ga0586324 0057163 1341 2297 | 2530000.00 | 0.00 | 0.00 | 1862000.00 | 1998200.00 | 0.00 | 0.00 | 0.00 | 0.00 | 1617000.00 | 2824800.00 | 0.00 | KO:K00625 | phosphate acetyltransferase | Bacteria;Proteobacteria;Alphaproteobacteria;Hyphomicrobiales;Methylocystaceae;Methylocystis;Methylocystis parvus;Methylocystis parvus OBBP |
| Ga0586324 0121195 412 1362 | 0.00 | 0.00 | 0.00 | 0.00 | 0.00 | 0.00 | 0.00 | 0.00 | 0.00 | 0.00 | 0.00 | 3472500.00 | KO:K00625 | phosphate acetyltransferase | Bacteria;Acidobacteria;Vicinamibacteria;unclassified Vicinamibacteria;Vicinamibacteraceae;Luteitalea;Luteitalea pratensis;Luteitalea pratensis DSM 100886 |
| Ga0586324 4021481 2 673 | 0.00 | 0.00 | 0.00 | 0.00 | 1452300.00 | 1633944.81 | 1028968.56 | 0.00 | 0.00 | 0.00 | 0.00 | 937500.00 | KO:K00625 | phosphate acetyltransferase | Bacteria;Proteobacteria;Alphaproteobacteria;Hyphomicrobiales;Bradyrhizobiaceae;Bradyrhizobium;Bradyrhizobium erythrophlei;Bradyrhizobium erythrophlei GAS242 |
| Ga0586324 0031267 174 1358 | 0.00 | 467827.08 | 0.00 | 783222.75 | 797059.32 | 0.00 | 0.00 | 707600.00 | 458920.08 | 0.00 | 0.00 | 0.00 | KO:K00626 | acetyl-CoA C-acetyltransferase | Bacteria;Proteobacteria;Betaproteobacteria;Burkholderiales;unclassified Burkholderiales;Methylibium;Methylibium sp. NZG;Methylibium sp. NZG |
| Ga0586324 0032785 4029 5219 | 0.00 | 0.00 | 0.00 | 0.00 | 0.00 | 0.00 | 0.00 | 0.00 | 0.00 | 0.00 | 1091200.00 | 276912.00 | KO:K00626 | acetyl-CoA C-acetyltransferase | Bacteria;Proteobacteria;Deltaproteobacteria;Myxococcales;Anaeromyxobacteraceae;Anaeromyxobacter;Anaeromyxobacter sp. PSR-1;Anaeromyxobacter sp. PSR-1 |
| Ga0586324 0132964 2616 2996 | 0.00 | 0.00 | 0.00 | 0.00 | 0.00 | 0.00 | 0.00 | 0.00 | 0.00 | 0.00 | 0.00 | 10575000.00 | KO:K00626 | acetyl-CoA C-acetyltransferase | Bacteria;Proteobacteria;Deltaproteobacteria;Syntrophorhabdales;Syntrophorhabdaceae;Syntrophorhabdus;Syntrophorhabdus aromaticivorans;Syntrophorhabdus aromaticivorans UI |
| Ga0586324 0150139 1171 2616 | 0.00 | 0.00 | 512436.97 | 0.00 | 7961900.00 | 1113660.43 | 0.00 | 0.00 | 12958838.46 | 0.00 | 0.00 | 0.00 | KO:K00626 | acetyl-CoA C-acetyltransferase | Bacteria;Proteobacteria;Deltaproteobacteria;Syntrophobacterales;Syntrophaceae;Smithella;Smithella sp. SCADC;Smithella sp. SCADC |
| Ga0586324 0162834 1297 2724 | 0.00 | 0.00 | 0.00 | 0.00 | 0.00 | 0.00 | 0.00 | 0.00 | 0.00 | 2433200.00 | 0.00 | 0.00 | KO:K00626 | acetyl-CoA C-acetyltransferase | Bacteria;Proteobacteria;Alphaproteobacteria;Hyphomicrobiales;Xanthobacteraceae;Pseudolabrys;Pseudolabrys sp. Root1462;Pseudolabrys sp. Root1462 |
| Ga0586324 0175962 128 1315 | 1450000.00 | 0.00 | 0.00 | 0.00 | 0.00 | 0.00 | 0.00 | 0.00 | 0.00 | 0.00 | 0.00 | 0.00 | KO:K00626 | acetyl-CoA C-acetyltransferase | Bacteria;Proteobacteria;Betaproteobacteria;Burkholderiales;Comamonadaceae;Rhodoferax;Rhodoferax ferrireducens;Rhodoferax ferrireducens T118 |
| Ga0586324 0261347 1 1140 | 0.00 | 0.00 | 0.00 | 0.00 | 336076.64 | 0.00 | 0.00 | 0.00 | 0.00 | 0.00 | 0.00 | 0.00 | KO:K00626 | acetyl-CoA C-acetyltransferase | Bacteria;Proteobacteria;Alphaproteobacteria;Rhodospirillales;Rhodospirillaceae;Indioceanicola;Indioceanicola profundi;Indioceanicola profundi SCSIO 08040 |
| Ga0586324 0526880 335 1603 | 0.00 | 0.00 | 1276073.28 | 0.00 | 0.00 | 0.00 | 0.00 | 0.00 | 529790.22 | 0.00 | 0.00 | 0.00 | KO:K00626 | acetyl-CoA C-acetyltransferase | Bacteria;Proteobacteria;Deltaproteobacteria;Syntrophobacterales;Syntrophaceae;Smithella;Smithella sp. SCADC;Smithella sp. SCADC |
| Ga0586324 0778936 550 1353 | 0.00 | 0.00 | 0.00 | 22325000.00 | 0.00 | 0.00 | 0.00 | 0.00 | 0.00 | 0.00 | 0.00 | 0.00 | KO:K00626 | acetyl-CoA C-acetyltransferase | Bacteria;Proteobacteria;Deltaproteobacteria;Syntrophorhabdales;Syntrophorhabdaceae;Syntrophorhabdus;Syntrophorhabdus aromaticivorans;Syntrophorhabdus aromaticivorans UI |
| Ga0586324 0983266 3 896 | 0.00 | 0.00 | 0.00 | 0.00 | 0.00 | 0.00 | 0.00 | 1104100.00 | 0.00 | 0.00 | 0.00 | 0.00 | KO:K00626 | acetyl-CoA C-acetyltransferase | Bacteria;Proteobacteria;Betaproteobacteria;Rhodocyclales;Rhodocyclaceae;Dechlorobacter;Dechlorobacter hydrogenophilus;Dechlorobacter hydrogenophilus LT-1 |
| Ga0586324 1285149 1 756 | 0.00 | 1740800.00 | 0.00 | 0.00 | 2770700.00 | 0.00 | 0.00 | 0.00 | 0.00 | 0.00 | 0.00 | 0.00 | KO:K00626 | acetyl-CoA C-acetyltransferase | Bacteria;Proteobacteria;Deltaproteobacteria;Syntrophorhabdales;Syntrophorhabdaceae;Syntrophorhabdus;Syntrophorhabdus aromaticivorans;Syntrophorhabdus aromaticivorans UI |
| Ga0586324 1623132 105 986 | 0.00 | 3236800.00 | 0.00 | 0.00 | 6355100.00 | 0.00 | 0.00 | 0.00 | 0.00 | 0.00 | 0.00 | 0.00 | KO:K00626 | acetyl-CoA C-acetyltransferase | Bacteria;Proteobacteria;Deltaproteobacteria;Syntrophobacterales;Syntrophaceae;Smithella;Smithella sp. SCADC;Smithella sp. SCADC |
| Ga0586324 1905346 1 666 | 0.00 | 0.00 | 707251.86 | 0.00 | 0.00 | 0.00 | 0.00 | 0.00 | 0.00 | 0.00 | 0.00 | 0.00 | KO:K00626 | acetyl-CoA C-acetyltransferase | Bacteria;Proteobacteria;Gammaproteobacteria;unclassified Gammaproteobacteria;Candidatus Competibacteraceae;Candidatus Competibacter;Candidatus Competibacter denitrificans;Candidatus Competibacter denitrificans Run A D11 |
| Ga0586324 1948913 77 913 | 11500000.00 | 0.00 | 0.00 | 0.00 | 0.00 | 0.00 | 0.00 | 0.00 | 0.00 | 0.00 | 0.00 | 0.00 | KO:K00626 | acetyl-CoA C-acetyltransferase | Bacteria;Proteobacteria;Alphaproteobacteria;Hyphomicrobiales;Hyphomicrobiaceae;Rhodomicrobium;Rhodomicrobium udaipurense;Rhodomicrobium udaipurense JA643 |
| Ga0586324 2435782 3 470 | 7340000.00 | 0.00 | 0.00 | 0.00 | 0.00 | 0.00 | 0.00 | 0.00 | 0.00 | 0.00 | 0.00 | 0.00 | KO:K00626 | acetyl-CoA C-acetyltransferase | Bacteria;Proteobacteria;Deltaproteobacteria;Syntrophorhabdales;Syntrophorhabdaceae;Syntrophorhabdus;Syntrophorhabdus aromaticivorans;Syntrophorhabdus aromaticivorans UI |
| Ga0586324 3844000 3 686 | 0.00 | 1802000.00 | 0.00 | 0.00 | 1359600.00 | 0.00 | 0.00 | 0.00 | 0.00 | 0.00 | 0.00 | 0.00 | KO:K00626 | acetyl-CoA C-acetyltransferase | Bacteria;Proteobacteria;Deltaproteobacteria;Desulfobacterales;Desulfobacteraceae;Desulfosarcina;Desulfosarcina variabilis;Desulfosarcina variabilis Montpellier |
| Ga0586324 4607209 1 639 | 0.00 | 0.00 | 0.00 | 0.00 | 0.00 | 0.00 | 0.00 | 0.00 | 0.00 | 916300.00 | 0.00 | 0.00 | KO:K00626 | acetyl-CoA C-acetyltransferase | Bacteria;Proteobacteria;Deltaproteobacteria;Syntrophorhabdales;Syntrophorhabdaceae;Syntrophorhabdus;Syntrophorhabdus aromaticivorans;Syntrophorhabdus aromaticivorans UI |
| Ga0586324 5279422 2 604 | 0.00 | 0.00 | 891144.55 | 0.00 | 0.00 | 1012504.83 | 1336209.96 | 0.00 | 0.00 | 0.00 | 0.00 | 0.00 | KO:K00626 | acetyl-CoA C-acetyltransferase | Bacteria;Proteobacteria;Alphaproteobacteria;Hyphomicrobiales;Bradyrhizobiaceae;Bradyrhizobium;Bradyrhizobium lablabi;Bradyrhizobium lablabi GAS522 |
| Ga0586324 5645522 1 588 | 0.00 | 1856400.00 | 0.00 | 0.00 | 0.00 | 0.00 | 0.00 | 0.00 | 0.00 | 0.00 | 0.00 | 0.00 | KO:K00626 | acetyl-CoA C-acetyltransferase | Bacteria;Firmicutes;Bacilli;Bacillales;Thermoactinomycetaceae;Laceyella;Laceyella putida;Laceyella putida DSM 44608 |
| Ga0586324 5826956 3 578 | 0.00 | 0.00 | 0.00 | 1282500.00 | 0.00 | 0.00 | 0.00 | 0.00 | 0.00 | 0.00 | 0.00 | 0.00 | KO:K00626 | acetyl-CoA C-acetyltransferase | Bacteria;Proteobacteria;Deltaproteobacteria;unclassified Deltaproteobacteria;unclassified Deltaproteobacteria;Deferrisoma;Deferrisoma camini;Deferrisoma camini S3R1 |
| Ga0586324 0381192 259 1536 | 0.00 | 0.00 | 0.00 | 0.00 | 0.00 | 0.00 | 0.00 | 0.00 | 0.00 | 0.00 | 4470400.00 | 0.00 | KO:K00627 | pyruvate dehydrogenase E2 component (dihydrolipoamide acetyltransferase) | Bacteria;Proteobacteria;Deltaproteobacteria;Syntrophobacterales;Syntrophaceae;Desulfobacca;Desulfobacca acetoxidans;Desulfobacca acetoxidans ASRB2, DSM 11109 |
| Ga0586324 0211066 301 1500 | 0.00 | 0.00 | 0.00 | 0.00 | 0.00 | 0.00 | 0.00 | 0.00 | 0.00 | 602756.77 | 0.00 | 0.00 | KO:K00632 | acetyl-CoA acyltransferase | Bacteria;Proteobacteria;Betaproteobacteria;Nitrosomonadales;Nitrosomonadaceae;Nitrosospira;Nitrosospira multiformis;Nitrosospira multiformis Nl1 |
| Ga0586324 3173758 1 744 | 0.00 | 0.00 | 0.00 | 0.00 | 0.00 | 1784348.26 | 0.00 | 0.00 | 0.00 | 0.00 | 0.00 | 0.00 | KO:K00639 | glycine C-acetyltransferase | Bacteria;Proteobacteria;Gammaproteobacteria;Nevskiales;Steroidobacteraceae;Povalibacter;Povalibacter uvarum;Povalibacter uvarum DSM 26723 |
| Ga0586324 7972110 2 508 | 3190000.00 | 0.00 | 0.00 | 0.00 | 0.00 | 0.00 | 0.00 | 0.00 | 0.00 | 0.00 | 0.00 | 0.00 | KO:K00639 | glycine C-acetyltransferase | Bacteria;Bacteroidetes;Bacteroidia;Marinilabiliales;Prolixibacteraceae;Prolixibacter;Prolixibacter denitrificans;Prolixibacter denitrificans DSM 27267 |
| Ga0586324 1297463 59 1087 | 0.00 | 0.00 | 0.00 | 6194000.00 | 0.00 | 0.00 | 0.00 | 0.00 | 0.00 | 0.00 | 0.00 | 0.00 | KO:K00658 | 2-oxoglutarate dehydrogenase E2 component (dihydrolipoamide succinyltransferase) | Bacteria;Proteobacteria;Alphaproteobacteria;Sphingomonadales;Sphingomonadaceae;Sphingomonas;Sphingomonas koreensis;Sphingomonas koreensis ABOJV |
| Ga0586324 1413418 1 876 | 2420000.00 | 6133600.00 | 0.00 | 0.00 | 0.00 | 0.00 | 0.00 | 0.00 | 0.00 | 1178100.00 | 0.00 | 0.00 | KO:K00666 | fatty-acyl-CoA synthase | Bacteria;Proteobacteria;Deltaproteobacteria;Syntrophorhabdales;Syntrophorhabdaceae;Syntrophorhabdus;Syntrophorhabdus aromaticivorans;Syntrophorhabdus aromaticivorans UI |
| Ga0586324 6528763 3 194 | 0.00 | 0.00 | 0.00 | 0.00 | 16068000.00 | 0.00 | 0.00 | 0.00 | 0.00 | 0.00 | 0.00 | 0.00 | KO:K00666 | fatty-acyl-CoA synthase | Bacteria;Proteobacteria;Deltaproteobacteria;Desulfobacterales;Desulfobacteraceae;Desulfobacterium;Desulfobacterium autotrophicum;Desulfobacterium autotrophicum HRM2, DSM 3382 |
| Ga0586324 0008680 220 1104 | 0.00 | 0.00 | 1109194.45 | 4151500.00 | 5469300.00 | 1023366.75 | 1884650.28 | 4526200.00 | 5094900.00 | 0.00 | 0.00 | 1552500.00 | KO:K00672 | formylmethanofuran--tetrahydromethanopterin N-formyltransferase | Archaea;Euryarchaeota;Methanomicrobia;Methanomicrobiales;Methanoregulaceae;Methanoregula;Methanoregula formicica;Methanoregula formicica SMSP, DSM 22288 |
| Ga0586324 0036510 2100 2918 | 3250000.00 | 0.00 | 0.00 | 0.00 | 0.00 | 0.00 | 0.00 | 3269600.00 | 0.00 | 0.00 | 2719200.00 | 1815000.00 | KO:K00674 | 2,3,4,5-tetrahydropyridine-2-carboxylate N-succinyltransferase | Bacteria;Proteobacteria;Gammaproteobacteria;Methylococcales;Methylococcaceae;Methylomicrobium;Methylomicrobium album;Methylomicrobium album BG8 |
| Ga0586324 0113557 455 3007 | 0.00 | 0.00 | 0.00 | 0.00 | 0.00 | 0.00 | 0.00 | 2311900.00 | 0.00 | 0.00 | 0.00 | 0.00 | KO:K00688 | starch phosphorylase | Bacteria;Proteobacteria;Deltaproteobacteria;Syntrophobacterales;Syntrophobacteraceae;Syntrophobacter;Syntrophobacter fumaroxidans;Syntrophobacter fumaroxidans MPOB |
| Ga0586324 0450220 3 1319 | 0.00 | 0.00 | 0.00 | 0.00 | 0.00 | 0.00 | 0.00 | 0.00 | 0.00 | 8239000.00 | 0.00 | 0.00 | KO:K00688 | starch phosphorylase | Bacteria;Planctomycetes;Candidatus Brocadiae;Candidatus Brocadiales;Candidatus Brocadiaceae;Candidatus Brocadia;Candidatus Brocadia sinica;Candidatus Brocadia sinica JPN1 |
| Ga0586324 1252744 1 870 | 0.00 | 0.00 | 0.00 | 0.00 | 0.00 | 0.00 | 0.00 | 0.00 | 0.00 | 0.00 | 2472800.00 | 0.00 | KO:K00688 | starch phosphorylase | Bacteria;Planctomycetes;Candidatus Brocadiae;Candidatus Brocadiales;Candidatus Brocadiaceae;Candidatus Brocadia;Candidatus Brocadia sinica;Candidatus Brocadia sinica JPN1 |
| Ga0586324 2004684 2 901 | 0.00 | 0.00 | 0.00 | 0.00 | 0.00 | 0.00 | 0.00 | 0.00 | 0.00 | 0.00 | 0.00 | 2055000.00 | KO:K00688 | starch phosphorylase | Bacteria;Proteobacteria;Betaproteobacteria;Nitrosomonadales;Sterolibacteriaceae;Sulfurisoma;Sulfurisoma sediminicola;Sulfurisoma sediminicola DSM 26916 |
| Ga0586324 2908841 1 771 | 0.00 | 3624400.00 | 0.00 | 0.00 | 0.00 | 0.00 | 0.00 | 0.00 | 0.00 | 0.00 | 0.00 | 0.00 | KO:K00688 | starch phosphorylase | Bacteria;Proteobacteria;Betaproteobacteria;Burkholderiales;Burkholderiaceae;Paraburkholderia;Paraburkholderia sprentiae;Paraburkholderia sprentiae WSM5005 |
| Ga0586324 6933597 3 539 | 0.00 | 0.00 | 0.00 | 0.00 | 0.00 | 0.00 | 8732400.00 | 0.00 | 0.00 | 0.00 | 0.00 | 0.00 | KO:K00688 | starch phosphorylase | Bacteria;Proteobacteria;Deltaproteobacteria;Desulfuromonadales;Geobacteraceae;Geobacter;Geobacter uraniireducens;Geobacter uraniireducens Rf4 |
| Ga0586324 0001390 14517 16952 | 0.00 | 0.00 | 0.00 | 0.00 | 0.00 | 0.00 | 0.00 | 0.00 | 0.00 | 0.00 | 0.00 | 7725000.00 | KO:K00700 | 1,4-alpha-glucan branching enzyme | Bacteria;Proteobacteria;Gammaproteobacteria;Methylococcales;Methylococcaceae;Methylobacter;Methylobacter tundripaludum;Methylobacter tundripaludum OWC-G53F |
| Ga0586324 0076386 3 941 | 0.00 | 0.00 | 0.00 | 3173000.00 | 0.00 | 0.00 | 0.00 | 0.00 | 0.00 | 4504500.00 | 0.00 | 0.00 | KO:K00756 | pyrimidine-nucleoside phosphorylase | Bacteria;Chloroflexi;Anaerolineae;Anaerolineales;Anaerolineaceae;Thermanaerothrix;Thermanaerothrix daxensis;Thermanaerothrix daxensis GNS-1 |
| Ga0586324 0000003 67559 69082 | 0.00 | 0.00 | 0.00 | 0.00 | 0.00 | 0.00 | 0.00 | 0.00 | 0.00 | 2548700.00 | 0.00 | 0.00 | KO:K00764 | amidophosphoribosyltransferase | Bacteria;Proteobacteria;Betaproteobacteria;Nitrosomonadales;Thiobacillaceae;Annwoodia;Annwoodia aquaesulis;Annwoodia aquaesulis DSM 4255 |
| Ga0586324 2609340 3 806 | 0.00 | 0.00 | 0.00 | 0.00 | 0.00 | 0.00 | 0.00 | 0.00 | 0.00 | 6367900.00 | 0.00 | 0.00 | KO:K00764 | amidophosphoribosyltransferase | Bacteria;Proteobacteria;Betaproteobacteria;Nitrosomonadales;Methylophilaceae;Methylobacillus;Methylobacillus sp. MM3;Methylobacillus sp. MM2 |
| Ga0586324 0000011 18843 20006 | 0.00 | 0.00 | 0.00 | 0.00 | 0.00 | 0.00 | 0.00 | 0.00 | 0.00 | 3226300.00 | 0.00 | 0.00 | KO:K00789 | S-adenosylmethionine synthetase | Bacteria;Proteobacteria;Betaproteobacteria;Nitrosomonadales;Thiobacillaceae;Annwoodia;Annwoodia aquaesulis;Annwoodia aquaesulis DSM 4255 |
| Ga0586324 0000464 23162 24337 | 0.00 | 0.00 | 0.00 | 0.00 | 0.00 | 0.00 | 0.00 | 9272000.00 | 12636000.00 | 0.00 | 0.00 | 0.00 | KO:K00789 | S-adenosylmethionine synthetase | Bacteria;Proteobacteria;Alphaproteobacteria;Hyphomicrobiales;unclassified Hyphomicrobiales;Methyloceanibacter;Methyloceanibacter marginalis;Methyloceanibacter marginalis R-67177 |
| Ga0586324 0002541 16011 17150 | 0.00 | 288155.44 | 0.00 | 0.00 | 0.00 | 0.00 | 0.00 | 0.00 | 0.00 | 383348.35 | 0.00 | 0.00 | KO:K00789 | S-adenosylmethionine synthetase | Bacteria;Acidobacteria;Acidobacteriia;Acidobacteriales;Acidobacteriaceae;Candidatus Koribacter;Candidatus Koribacter versatilis;Candidatus Koribacter versatilis Ellin345 |
| Ga0586324 0004912 2 421 | 0.00 | 0.00 | 0.00 | 0.00 | 0.00 | 1214010.12 | 0.00 | 0.00 | 0.00 | 0.00 | 0.00 | 0.00 | KO:K00789 | S-adenosylmethionine synthetase | Bacteria;Nitrospirae;Nitrospira;Nitrospirales;Nitrospiraceae;Nitrospira;Nitrospira japonica;Nitrospira japonica NJ11 |
| Ga0586324 0035298 1435 2598 | 0.00 | 27336000.00 | 0.00 | 0.00 | 0.00 | 0.00 | 0.00 | 0.00 | 0.00 | 0.00 | 0.00 | 0.00 | KO:K00789 | S-adenosylmethionine synthetase | Bacteria;Planctomycetes;Candidatus Brocadiae;Candidatus Brocadiales;Candidatus Brocadiaceae;Candidatus Brocadia;Candidatus Brocadia sapporoensis;Candidatus Brocadia sapporoensis 40 |
| Ga0586324 0053628 42 1211 | 0.00 | 0.00 | 0.00 | 0.00 | 0.00 | 0.00 | 0.00 | 0.00 | 0.00 | 0.00 | 0.00 | 247837.50 | KO:K00789 | S-adenosylmethionine synthetase | Bacteria;Proteobacteria;Deltaproteobacteria;Syntrophobacterales;Syntrophobacteraceae;Desulfacinum;Desulfacinum infernum;Desulfacinum infernum DSM 9756 |
| Ga0586324 0078445 1223 2491 | 0.00 | 0.00 | 0.00 | 0.00 | 0.00 | 0.00 | 0.00 | 530357.18 | 0.00 | 185544.59 | 0.00 | 0.00 | KO:K00789 | S-adenosylmethionine synthetase | Bacteria;Chloroflexi;Anaerolineae;Anaerolineales;Anaerolineaceae;Anaerolinea;Anaerolinea thermophila;Anaerolinea thermophila UNI-1 |
| Ga0586324 0098631 1106 2287 | 0.00 | 0.00 | 0.00 | 6583500.00 | 0.00 | 0.00 | 0.00 | 0.00 | 0.00 | 0.00 | 0.00 | 0.00 | KO:K00789 | S-adenosylmethionine synthetase | Bacteria;Proteobacteria;Gammaproteobacteria;unclassified Gammaproteobacteria;unclassified Gammaproteobacteria;Pseudohongiella;Pseudohongiella spirulinae;Pseudohongiella spirulinae KCTC 32221 |
| Ga0586324 0293004 416 1612 | 0.00 | 0.00 | 0.00 | 0.00 | 0.00 | 294889.62 | 0.00 | 0.00 | 0.00 | 0.00 | 0.00 | 0.00 | KO:K00789 | S-adenosylmethionine synthetase | Bacteria;Actinobacteria;Actinomycetia;Propionibacteriales;Nocardioidaceae;Nocardioides;Nocardioides sp. YIM ART13;Nocardioides sp. YIM ART13 |
| Ga0586324 0306222 543 1763 | 0.00 | 0.00 | 0.00 | 0.00 | 893539.42 | 0.00 | 0.00 | 349834.39 | 0.00 | 1178100.00 | 0.00 | 12150000.00 | KO:K00789 | S-adenosylmethionine synthetase | Bacteria;Chloroflexi;Anaerolineae;Anaerolineales;Anaerolineaceae;Anaerolinea;Anaerolinea thermophila;Anaerolinea thermophila UNI-1 |
| Ga0586324 0368529 454 1722 | 2200000.00 | 0.00 | 0.00 | 0.00 | 0.00 | 0.00 | 0.00 | 0.00 | 0.00 | 0.00 | 0.00 | 0.00 | KO:K00789 | S-adenosylmethionine synthetase | Bacteria;Proteobacteria;Alphaproteobacteria;Hyphomicrobiales;Bradyrhizobiaceae;Bradyrhizobium;Bradyrhizobium erythrophlei;Bradyrhizobium erythrophlei GAS138 |
| Ga0586324 0604130 2 775 | 0.00 | 0.00 | 0.00 | 0.00 | 423347.51 | 0.00 | 0.00 | 341326.72 | 0.00 | 0.00 | 0.00 | 0.00 | KO:K00789 | S-adenosylmethionine synthetase | Bacteria;Proteobacteria;Deltaproteobacteria;Myxococcales;unclassified Myxococcales;Minicystis;Minicystis rosea;Minicystis rosea DSM 24000 |
| Ga0586324 0646488 479 1465 | 0.00 | 0.00 | 0.00 | 2128000.00 | 0.00 | 0.00 | 0.00 | 0.00 | 0.00 | 0.00 | 0.00 | 0.00 | KO:K00789 | S-adenosylmethionine synthetase | Bacteria;Proteobacteria;Alphaproteobacteria;Hyphomicrobiales;Xanthobacteraceae;Pseudolabrys;Pseudolabrys sp. Root1462;Pseudolabrys sp. Root1462 |
| Ga0586324 1125837 646 1155 | 0.00 | 0.00 | 0.00 | 0.00 | 0.00 | 0.00 | 0.00 | 0.00 | 0.00 | 4527600.00 | 0.00 | 0.00 | KO:K00789 | S-adenosylmethionine synthetase | Bacteria;Proteobacteria;Deltaproteobacteria;Myxococcales;Myxococcaceae;Myxococcus;Myxococcus fulvus;Myxococcus fulvus DSM 16525 |
| Ga0586324 1404974 3 614 | 0.00 | 0.00 | 0.00 | 0.00 | 0.00 | 0.00 | 0.00 | 0.00 | 1320300.00 | 0.00 | 0.00 | 0.00 | KO:K00789 | S-adenosylmethionine synthetase | Bacteria;Proteobacteria;Alphaproteobacteria;Hyphomicrobiales;Hyphomicrobiaceae;Hyphomicrobium;Hyphomicrobium facile;Hyphomicrobium facile facile DSM 1565 |
| Ga0586324 1432644 806 1042 | 0.00 | 0.00 | 0.00 | 1653000.00 | 0.00 | 0.00 | 0.00 | 0.00 | 0.00 | 0.00 | 0.00 | 0.00 | KO:K00789 | S-adenosylmethionine synthetase | Bacteria;Proteobacteria;Deltaproteobacteria;Desulfobacterales;Desulfobacteraceae;Desulfatibacillum;Desulfatibacillum alkenivorans;Desulfatibacillum alkenivorans DSM 16219 |
| Ga0586324 1677286 181 972 | 0.00 | 0.00 | 0.00 | 0.00 | 0.00 | 0.00 | 0.00 | 1049200.00 | 0.00 | 0.00 | 0.00 | 0.00 | KO:K00789 | S-adenosylmethionine synthetase | Bacteria;Acidobacteria;Blastocatellia;unclassified Blastocatellia;unclassified Blastocatellia;Chloracidobacterium;Chloracidobacterium thermophilum;Chloracidobacterium thermophilum B |
| Ga0586324 1703159 1 966 | 0.00 | 0.00 | 0.00 | 0.00 | 0.00 | 0.00 | 0.00 | 0.00 | 567437.40 | 0.00 | 0.00 | 0.00 | KO:K00789 | S-adenosylmethionine synthetase | Bacteria;Actinobacteria;Actinomycetia;unclassified Actinomycetia;unclassified Actinomycetia;unclassified Actinomycetia;Actinobacteria bacterium IMCC26256;Actinobacteria bacterium IMCC26256 |
| Ga0586324 1725743 2 883 | 0.00 | 0.00 | 0.00 | 0.00 | 0.00 | 0.00 | 0.00 | 564608.07 | 0.00 | 0.00 | 0.00 | 0.00 | KO:K00789 | S-adenosylmethionine synthetase | Bacteria;Chloroflexi;Anaerolineae;Anaerolineales;Anaerolineaceae;Anaerolinea;Anaerolinea thermophila;Anaerolinea thermophila UNI-1 |
| Ga0586324 2373703 2 790 | 0.00 | 0.00 | 0.00 | 3097000.00 | 0.00 | 0.00 | 0.00 | 0.00 | 0.00 | 0.00 | 0.00 | 0.00 | KO:K00789 | S-adenosylmethionine synthetase | Bacteria;Proteobacteria;Deltaproteobacteria;Desulfobacterales;Desulfobacteraceae;Desulfatirhabdium;Desulfatirhabdium butyrativorans;Desulfatirhabdium butyrativorans DSM 18734 |
| Ga0586324 2504088 3 821 | 0.00 | 0.00 | 0.00 | 0.00 | 0.00 | 0.00 | 0.00 | 0.00 | 0.00 | 159708.01 | 0.00 | 0.00 | KO:K00789 | S-adenosylmethionine synthetase | Bacteria;Planctomycetes;Candidatus Brocadiae;Candidatus Brocadiales;Candidatus Brocadiaceae;Candidatus Brocadia;Candidatus Brocadia sapporoensis;Candidatus Brocadia sapporoensis 40 |
| Ga0586324 2577144 140 811 | 0.00 | 0.00 | 0.00 | 0.00 | 0.00 | 0.00 | 2781600.00 | 0.00 | 0.00 | 0.00 | 0.00 | 0.00 | KO:K00789 | S-adenosylmethionine synthetase | Bacteria;Proteobacteria;Deltaproteobacteria;Syntrophobacterales;Syntrophobacteraceae;Desulfacinum;Desulfacinum hydrothermale;Desulfacinum hydrothermale DSM 13146 |
| Ga0586324 2669935 3 755 | 0.00 | 0.00 | 0.00 | 0.00 | 0.00 | 0.00 | 0.00 | 0.00 | 0.00 | 0.00 | 0.00 | 1072500.00 | KO:K00789 | S-adenosylmethionine synthetase | Bacteria;Acidobacteria;Acidobacteriia;Bryobacterales;Bryobacteraceae;Bryobacter;Bryobacter aggregatus;Bryobacter aggregatus DSM 18758 |
| Ga0586324 4393533 1 651 | 11500000.00 | 0.00 | 0.00 | 0.00 | 0.00 | 0.00 | 0.00 | 0.00 | 0.00 | 0.00 | 0.00 | 0.00 | KO:K00789 | S-adenosylmethionine synthetase | Bacteria;Actinobacteria;Actinomycetia;Propionibacteriales;Nocardioidaceae;Nocardioides;Nocardioides sp. XL1;Nocardioides sp. XL1 |
| Ga0586324 4767419 1 621 | 0.00 | 0.00 | 0.00 | 0.00 | 0.00 | 19254500.00 | 0.00 | 0.00 | 0.00 | 0.00 | 0.00 | 0.00 | KO:K00789 | S-adenosylmethionine synthetase | Bacteria;Bacteroidetes;Bacteroidia;Bacteroidales;Lentimicrobiaceae;Lentimicrobium;Lentimicrobium saccharophilum;Bacteroidales bacterium TBC1 |
| Ga0586324 7943898 3 509 | 0.00 | 0.00 | 0.00 | 0.00 | 0.00 | 0.00 | 0.00 | 0.00 | 0.00 | 0.00 | 0.00 | 273408.75 | KO:K00789 | S-adenosylmethionine synthetase | Bacteria;Acidobacteria;Acidobacteriia;Bryobacterales;Solibacteraceae;Candidatus Solibacter;Candidatus Solibacter usitatus;Candidatus Solibacter usitatus Ellin6076 |
| Ga0586324 0162383 1952 2728 | 0.00 | 0.00 | 14581700.00 | 0.00 | 0.00 | 0.00 | 0.00 | 0.00 | 0.00 | 0.00 | 0.00 | 0.00 | KO:K00791 | tRNA dimethylallyltransferase | Bacteria;Proteobacteria;Alphaproteobacteria;Rhodospirillales;unclassified Rhodospirillales;unclassified Rhodospirillales;Rhodospirillales bacterium URHD0017;Rhodospirillales bacterium URHD0017 |
| Ga0586324 6787824 3 542 | 0.00 | 0.00 | 0.00 | 0.00 | 0.00 | 0.00 | 0.00 | 976000.00 | 0.00 | 0.00 | 0.00 | 0.00 | KO:K00794 | 6,7-dimethyl-8-ribityllumazine synthase | Bacteria;Firmicutes;Clostridia;Thermosediminibacterales;Thermosediminibacteraceae;Thermosediminibacter;Thermosediminibacter oceani;Thermosediminibacter oceani JW/IW-1228P, DSM 16646 |
| Ga0586324 0079055 269 1291 | 0.00 | 1366800.00 | 0.00 | 0.00 | 0.00 | 0.00 | 0.00 | 0.00 | 0.00 | 0.00 | 0.00 | 0.00 | KO:K00809 | deoxyhypusine synthase | Bacteria;Proteobacteria;Deltaproteobacteria;Myxococcales;Anaeromyxobacteraceae;Anaeromyxobacter;Anaeromyxobacter dehalogenans;Anaeromyxobacter dehalogenans 2CP-1 |
| Ga0586324 0799327 3 1136 | 1640000.00 | 0.00 | 0.00 | 0.00 | 0.00 | 0.00 | 0.00 | 0.00 | 0.00 | 0.00 | 0.00 | 0.00 | KO:K00809 | deoxyhypusine synthase | Bacteria;Planctomycetes;Planctomycetia;Pirellulales;Thermoguttaceae;Thermogutta;Thermogutta terrifontis;Thermogutta terrifontis R1 |
| Ga0586324 4801784 393 626 | 0.00 | 0.00 | 903147.71 | 0.00 | 0.00 | 0.00 | 0.00 | 0.00 | 0.00 | 0.00 | 0.00 | 0.00 | KO:K00812 | aspartate aminotransferase | Bacteria;Verrucomicrobia;Verrucomicrobiae;Verrucomicrobiales;Verrucomicrobia subdivision 3;Pedosphaera;Pedosphaera parvula;Pedosphaera parvula Ellin514 |
| Ga0586324 0000001 59872 61701 | 0.00 | 0.00 | 0.00 | 0.00 | 0.00 | 0.00 | 0.00 | 0.00 | 0.00 | 5397700.00 | 0.00 | 4305000.00 | KO:K00820 | glucosamine--fructose-6-phosphate aminotransferase (isomerizing) | Bacteria;Proteobacteria;Betaproteobacteria;Nitrosomonadales;Thiobacillaceae;Annwoodia;Annwoodia aquaesulis;Annwoodia aquaesulis DSM 4255 |
| Ga0586324 0032971 3066 4925 | 0.00 | 0.00 | 0.00 | 0.00 | 0.00 | 258530.77 | 0.00 | 0.00 | 0.00 | 0.00 | 0.00 | 0.00 | KO:K00820 | glucosamine--fructose-6-phosphate aminotransferase (isomerizing) | Bacteria;Acidobacteria;Vicinamibacteria;unclassified Vicinamibacteria;Vicinamibacteraceae;Luteitalea;Luteitalea pratensis;Luteitalea pratensis DSM 100886 |
| Ga0586324 1736314 2 958 | 0.00 | 0.00 | 0.00 | 823137.95 | 0.00 | 0.00 | 0.00 | 0.00 | 0.00 | 0.00 | 0.00 | 0.00 | KO:K00820 | glucosamine--fructose-6-phosphate aminotransferase (isomerizing) | Bacteria;Proteobacteria;Deltaproteobacteria;Desulfuromonadales;Geobacteraceae;Geobacter;Geobacter argillaceus;Geobacter argillaceus ATCC BAA-1139 |
| Ga0586324 3537915 3 710 | 0.00 | 0.00 | 3972392.88 | 0.00 | 0.00 | 0.00 | 0.00 | 0.00 | 0.00 | 0.00 | 0.00 | 0.00 | KO:K00820 | glucosamine--fructose-6-phosphate aminotransferase (isomerizing) | Bacteria;Proteobacteria;Gammaproteobacteria;Acidiferrobacterales;Acidiferrobacteraceae;Sulfuricaulis;Sulfuricaulis limicola;Sulfuricaulis limicola HA5 |
| Ga0586324 0000003 17594 18766 | 0.00 | 0.00 | 0.00 | 0.00 | 0.00 | 0.00 | 0.00 | 0.00 | 0.00 | 0.00 | 0.00 | 528462.75 | KO:K00821 | acetylornithine/N-succinyldiaminopimelate aminotransferase | Bacteria;Proteobacteria;Betaproteobacteria;Nitrosomonadales;Thiobacillaceae;Annwoodia;Annwoodia aquaesulis;Annwoodia aquaesulis DSM 4255 |
| Ga0586324 3646771 83 703 | 0.00 | 0.00 | 729681.78 | 0.00 | 0.00 | 0.00 | 0.00 | 0.00 | 0.00 | 0.00 | 0.00 | 0.00 | KO:K00822 | beta-alanine--pyruvate transaminase | Bacteria;Proteobacteria;Alphaproteobacteria;Rhodospirillales;Rhodospirillaceae;Nitrospirillum;Nitrospirillum amazonense;Nitrospirillum amazonense BR 11140 |
| Ga0586324 0360870 1 588 | 0.00 | 0.00 | 0.00 | 0.00 | 0.00 | 0.00 | 0.00 | 0.00 | 0.00 | 185147.27 | 0.00 | 0.00 | KO:K00826 | branched-chain amino acid aminotransferase | Bacteria;Proteobacteria;Deltaproteobacteria;Desulfobacterales;Desulfobacteraceae;Desulfosarcina;Desulfosarcina cetonica;Desulfosarcina cetonica JCM 12296 |
| Ga0586324 5977157 2 451 | 1290000.00 | 0.00 | 4393781.49 | 0.00 | 0.00 | 0.00 | 0.00 | 0.00 | 0.00 | 0.00 | 0.00 | 0.00 | KO:K00830 | alanine-glyoxylate transaminase/serine-glyoxylate transaminase/serine-pyruvate transaminase | Bacteria;Proteobacteria;Gammaproteobacteria;Methylococcales;Methylococcaceae;Methylobacter;Methylobacter tundripaludum;Methylobacter tundripaludum OWC-G53F |
| Ga0586324 0000005 67335 68417 | 0.00 | 0.00 | 0.00 | 0.00 | 0.00 | 0.00 | 0.00 | 0.00 | 2835000.00 | 0.00 | 3256000.00 | 1912500.00 | KO:K00831 | phosphoserine aminotransferase | Bacteria;Proteobacteria;Betaproteobacteria;Nitrosomonadales;Thiobacillaceae;Annwoodia;Annwoodia aquaesulis;Annwoodia aquaesulis DSM 4255 |
| Ga0586324 0261792 345 1907 | 2080000.00 | 0.00 | 0.00 | 0.00 | 0.00 | 0.00 | 0.00 | 0.00 | 0.00 | 0.00 | 0.00 | 0.00 | KO:K00854 | xylulokinase | Bacteria;Firmicutes;Clostridia;Eubacteriales;Clostridiaceae;Clostridium;Clostridium sp. KNHs216;Clostridium sp. KNHs216 |
| Ga0586324 5982476 2 574 | 1790000.00 | 0.00 | 0.00 | 0.00 | 0.00 | 0.00 | 0.00 | 0.00 | 0.00 | 0.00 | 0.00 | 0.00 | KO:K00854 | xylulokinase | Bacteria;Firmicutes;Clostridia;Eubacteriales;Clostridiaceae;Clostridium;Clostridium sp. KNHs216;Clostridium sp. KNHs216 |
| Ga0586324 0000013 94922 95812 | 0.00 | 0.00 | 0.00 | 0.00 | 0.00 | 0.00 | 0.00 | 3574600.00 | 8425293.57 | 18195100.00 | 43225600.00 | 6765000.00 | KO:K00855 | phosphoribulokinase | Bacteria;Proteobacteria;Betaproteobacteria;Nitrosomonadales;Thiobacillaceae;Annwoodia;Annwoodia aquaesulis;Annwoodia aquaesulis DSM 4255 |
| Ga0586324 0007570 3 563 | 0.00 | 0.00 | 0.00 | 0.00 | 0.00 | 0.00 | 0.00 | 3458700.00 | 0.00 | 0.00 | 4778400.00 | 0.00 | KO:K00855 | phosphoribulokinase | Bacteria;Proteobacteria;Betaproteobacteria;Nitrosomonadales;Thiobacillaceae;Thiobacillus;Thiobacillus thiophilus;Thiobacillus thiophilus DSM 19892 |
| Ga0586324 0007597 4147 5034 | 0.00 | 0.00 | 0.00 | 0.00 | 0.00 | 0.00 | 0.00 | 10065000.00 | 9558000.00 | 705822.81 | 8888000.00 | 10500000.00 | KO:K00855 | phosphoribulokinase | Bacteria;Proteobacteria;Betaproteobacteria;Nitrosomonadales;Thiobacillaceae;Thiobacillus;Thiobacillus thiophilus;Thiobacillus thiophilus DSM 19892 |
| Ga0586324 0011325 1 336 | 0.00 | 0.00 | 7037500.00 | 5472000.00 | 6025500.00 | 3759863.93 | 15504000.00 | 0.00 | 0.00 | 11319000.00 | 0.00 | 0.00 | KO:K00855 | phosphoribulokinase | Bacteria;Proteobacteria;Betaproteobacteria;Nitrosomonadales;Thiobacillaceae;Thiobacillus;Thiobacillus thioparus;Thiobacillus thioparus DSM 505 |
| Ga0586324 0086170 915 1796 | 0.00 | 0.00 | 0.00 | 0.00 | 0.00 | 0.00 | 0.00 | 466332.19 | 0.00 | 0.00 | 301202.00 | 0.00 | KO:K00855 | phosphoribulokinase | Bacteria;Proteobacteria;Betaproteobacteria;Nitrosomonadales;Sulfuricellaceae;Sulfurirhabdus;Sulfurirhabdus autotrophica;Sulfurirhabdus autotrophica DSM 100309 |
| Ga0586324 0435599 2 313 | 0.00 | 0.00 | 0.00 | 0.00 | 0.00 | 0.00 | 0.00 | 793000.00 | 0.00 | 0.00 | 0.00 | 0.00 | KO:K00855 | phosphoribulokinase | Bacteria;Proteobacteria;Gammaproteobacteria;Chromatiales;unclassified Chromatiales;Thiohalobacter;Thiohalobacter thiocyanaticus;Thiohalobacter thiocyanaticus FOKN1 |
| Ga0586324 0580733 489 1376 | 0.00 | 0.00 | 0.00 | 0.00 | 0.00 | 0.00 | 0.00 | 0.00 | 3329100.00 | 0.00 | 0.00 | 0.00 | KO:K00855 | phosphoribulokinase | Bacteria;Proteobacteria;Gammaproteobacteria;Chromatiales;Ectothiorhodospiraceae;Thiogranum;Thiogranum longum;Thiogranum longum DSM 19610 |
| Ga0586324 0768608 3 377 | 0.00 | 0.00 | 0.00 | 0.00 | 0.00 | 0.00 | 0.00 | 0.00 | 0.00 | 0.00 | 0.00 | 2602500.00 | KO:K00855 | phosphoribulokinase | Bacteria;Proteobacteria;Betaproteobacteria;Nitrosomonadales;Sulfuricellaceae;Sulfurirhabdus;Sulfurirhabdus autotrophica;Sulfurirhabdus autotrophica DSM 100309 |
| Ga0586324 0189746 3 830 | 1070000.00 | 1829200.00 | 1643526.49 | 0.00 | 0.00 | 841778.95 | 0.00 | 732000.00 | 1231200.00 | 1555400.00 | 0.00 | 0.00 | KO:K00864 | glycerol kinase | Bacteria;Chloroflexi;Anaerolineae;Anaerolineales;Anaerolineaceae;Pelolinea;Pelolinea submarina;Pelolinea submarina DSM 23923 |
| Ga0586324 0235029 1492 2298 | 0.00 | 0.00 | 15388547.69 | 0.00 | 4645300.00 | 0.00 | 9484800.00 | 9949100.00 | 3742200.00 | 5305300.00 | 4452800.00 | 7499561.25 | KO:K00864 | glycerol kinase | Bacteria;Chloroflexi;Anaerolineae;Anaerolineales;Anaerolineaceae;Pelolinea;Pelolinea submarina;Pelolinea submarina DSM 23923 |
| Ga0586324 0345198 1123 1929 | 0.00 | 0.00 | 0.00 | 0.00 | 0.00 | 0.00 | 4605600.00 | 0.00 | 0.00 | 0.00 | 0.00 | 0.00 | KO:K00864 | glycerol kinase | Bacteria;Chloroflexi;Anaerolineae;Anaerolineales;Anaerolineaceae;Thermanaerothrix;Thermanaerothrix daxensis;Thermanaerothrix daxensis GNS-1 |
| Ga0586324 0495637 108 1604 | 0.00 | 0.00 | 0.00 | 0.00 | 0.00 | 0.00 | 0.00 | 0.00 | 1401300.00 | 0.00 | 0.00 | 0.00 | KO:K00864 | glycerol kinase | Bacteria;Chloroflexi;Anaerolineae;Anaerolineales;Anaerolineaceae;Thermanaerothrix;Thermanaerothrix daxensis;Thermanaerothrix daxensis GNS-1 |
| Ga0586324 0610455 3 1301 | 0.00 | 0.00 | 0.00 | 376563.85 | 2090900.00 | 1771453.70 | 960862.68 | 1354200.00 | 1134000.00 | 0.00 | 941600.00 | 741504.00 | KO:K00864 | glycerol kinase | Bacteria;Cyanobacteria;unclassified Cyanobacteria;Nostocales;Hapalosiphonaceae;Fischerella;Fischerella thermalis;Fischerella thermalis CCMEE 5330 |
| Ga0586324 1215220 3 641 | 0.00 | 26248000.00 | 7262700.00 | 0.00 | 0.00 | 0.00 | 0.00 | 0.00 | 0.00 | 0.00 | 0.00 | 9150000.00 | KO:K00864 | glycerol kinase | Bacteria;Chloroflexi;Anaerolineae;Anaerolineales;Anaerolineaceae;Pelolinea;Pelolinea submarina;Pelolinea submarina DSM 23923 |
| Ga0586324 1710511 389 964 | 0.00 | 0.00 | 40141900.00 | 38760000.00 | 42024000.00 | 50419000.00 | 54264000.00 | 31720000.00 | 33453000.00 | 42273000.00 | 46200000.00 | 34875000.00 | KO:K00864 | glycerol kinase | Bacteria;Chloroflexi;Ardenticatenia;Ardenticatenales;Ardenticatenaceae;Ardenticatena;Ardenticatena maritima;Ardenticatena maritima 110S |
| Ga0586324 1740299 3 959 | 0.00 | 0.00 | 0.00 | 0.00 | 0.00 | 0.00 | 0.00 | 0.00 | 0.00 | 8624000.00 | 0.00 | 0.00 | KO:K00864 | glycerol kinase | Bacteria;Chloroflexi;Anaerolineae;Anaerolineales;Anaerolineaceae;Pelolinea;Pelolinea submarina;Pelolinea submarina DSM 23923 |
| Ga0586324 1856967 299 931 | 0.00 | 0.00 | 0.00 | 0.00 | 0.00 | 0.00 | 0.00 | 793000.00 | 0.00 | 0.00 | 0.00 | 0.00 | KO:K00864 | glycerol kinase | Bacteria;Chloroflexi;Anaerolineae;Anaerolineales;Anaerolineaceae;Pelolinea;Pelolinea submarina;Pelolinea submarina DSM 23923 |
| Ga0586324 2061252 1 729 | 0.00 | 0.00 | 0.00 | 0.00 | 2688300.00 | 0.00 | 0.00 | 0.00 | 0.00 | 0.00 | 0.00 | 0.00 | KO:K00864 | glycerol kinase | Bacteria;Chloroflexi;Anaerolineae;Anaerolineales;Anaerolineaceae;Thermanaerothrix;Thermanaerothrix daxensis;Thermanaerothrix daxensis GNS-1 |
| Ga0586324 2527423 1 414 | 0.00 | 2441200.00 | 0.00 | 0.00 | 0.00 | 0.00 | 0.00 | 0.00 | 0.00 | 0.00 | 0.00 | 0.00 | KO:K00864 | glycerol kinase | Bacteria;Chloroflexi;Anaerolineae;Anaerolineales;Anaerolineaceae;Pelolinea;Pelolinea submarina;Pelolinea submarina DSM 23923 |
| Ga0586324 4695881 2 514 | 30700000.00 | 2924000.00 | 15989200.00 | 0.00 | 0.00 | 0.00 | 0.00 | 963800.00 | 2673000.00 | 0.00 | 697616.48 | 0.00 | KO:K00864 | glycerol kinase | Bacteria;Chloroflexi;Anaerolineae;Anaerolineales;Anaerolineaceae;Pelolinea;Pelolinea submarina;Pelolinea submarina DSM 23923 |
| Ga0586324 5617154 3 587 | 0.00 | 0.00 | 1419508.79 | 1539000.00 | 2224800.00 | 422606.50 | 425591.64 | 283600.59 | 0.00 | 295865.57 | 482430.96 | 377618.25 | KO:K00864 | glycerol kinase | Bacteria;Chloroflexi;Anaerolineae;Anaerolineales;Anaerolineaceae;Pelolinea;Pelolinea submarina;Pelolinea submarina DSM 23923 |
| Ga0586324 7308322 1 528 | 0.00 | 0.00 | 648243.83 | 354258.80 | 0.00 | 0.00 | 411697.32 | 0.00 | 0.00 | 2025100.00 | 0.00 | 670894.50 | KO:K00864 | glycerol kinase | Bacteria;Chloroflexi;Anaerolineae;Anaerolineales;Anaerolineaceae;Pelolinea;Pelolinea submarina;Pelolinea submarina DSM 23923 |
| Ga0586324 0000001 219808 221244 | 0.00 | 0.00 | 0.00 | 0.00 | 0.00 | 0.00 | 0.00 | 1701900.00 | 0.00 | 2333100.00 | 2112000.00 | 0.00 | KO:K00873 | pyruvate kinase | Bacteria;Proteobacteria;Betaproteobacteria;Nitrosomonadales;Thiobacillaceae;Annwoodia;Annwoodia aquaesulis;Annwoodia aquaesulis DSM 4255 |
| Ga0586324 0658499 1 891 | 1190000.00 | 0.00 | 0.00 | 0.00 | 0.00 | 0.00 | 0.00 | 0.00 | 0.00 | 0.00 | 0.00 | 0.00 | KO:K00873 | pyruvate kinase | Bacteria;Proteobacteria;Gammaproteobacteria;Methylococcales;Crenotrichaceae;Crenothrix;Crenothrix polyspora;Crenothrix polyspora ERS1545889 |
| Ga0586324 2022659 85 900 | 0.00 | 660086.88 | 0.00 | 0.00 | 0.00 | 0.00 | 0.00 | 0.00 | 0.00 | 0.00 | 0.00 | 0.00 | KO:K00873 | pyruvate kinase | Bacteria;Verrucomicrobia;Spartobacteria;Chthoniobacterales;Chthoniobacteraceae;Chthoniobacter;Chthoniobacter flavus;Chthoniobacter flavus DSM 22515 |
| Ga0586324 6148799 1 567 | 0.00 | 0.00 | 0.00 | 1216000.00 | 0.00 | 0.00 | 0.00 | 0.00 | 0.00 | 3865400.00 | 0.00 | 0.00 | KO:K00873 | pyruvate kinase | Bacteria;Proteobacteria;Deltaproteobacteria;Syntrophobacterales;Syntrophobacteraceae;Syntrophobacter;Syntrophobacter fumaroxidans;Syntrophobacter fumaroxidans MPOB |
| Ga0586324 0445172 310 1443 | 0.00 | 0.00 | 0.00 | 0.00 | 0.00 | 0.00 | 0.00 | 0.00 | 0.00 | 0.00 | 0.00 | 427081.50 | KO:K00874 | 2-dehydro-3-deoxygluconokinase | Bacteria;Candidatus Latescibacteria;unclassified Candidatus Latescibacteria;unclassified Candidatus Latescibacteria;unclassified Candidatus Latescibacteria;unclassified Candidatus Latescibacteria;Latescibacteria bacterium SCGC AAA252-B13;Latescibacteria bacterium SCGC AAA252-B13 (contamination screened) |
| Ga0586324 4827029 1 627 | 0.00 | 0.00 | 0.00 | 1092500.00 | 529531.24 | 2625126.77 | 0.00 | 0.00 | 0.00 | 0.00 | 0.00 | 0.00 | KO:K00895 | pyrophosphate--fructose-6-phosphate 1-phosphotransferase | Bacteria;Proteobacteria;Deltaproteobacteria;Syntrophobacterales;Syntrophobacteraceae;Syntrophobacter;Syntrophobacter fumaroxidans;Syntrophobacter fumaroxidans MPOB |
| Ga0586324 0048165 3 1106 | 0.00 | 0.00 | 0.00 | 0.00 | 7405700.00 | 0.00 | 0.00 | 0.00 | 1652400.00 | 0.00 | 0.00 | 0.00 | KO:K00925 | acetate kinase | Archaea;Euryarchaeota;Methanomicrobia;Methanosarcinales;Methanosarcinaceae;Methanosarcina;Methanosarcina horonobensis;Methanosarcina horonobensis HB-1 |
| Ga0586324 0000001 221333 222517 | 0.00 | 0.00 | 0.00 | 0.00 | 0.00 | 0.00 | 0.00 | 9570900.00 | 0.00 | 7638400.00 | 0.00 | 0.00 | KO:K00927 | phosphoglycerate kinase | Bacteria;Proteobacteria;Betaproteobacteria;Nitrosomonadales;Sterolibacteriaceae;Sulfurisoma;Sulfurisoma sediminicola;Sulfurisoma sediminicola DSM 26916 |
| Ga0586324 0006147 2 1213 | 0.00 | 0.00 | 0.00 | 0.00 | 0.00 | 11870300.00 | 0.00 | 7259000.00 | 0.00 | 0.00 | 6045600.00 | 5812500.00 | KO:K00927 | phosphoglycerate kinase | Bacteria;Proteobacteria;Betaproteobacteria;Nitrosomonadales;Gallionellaceae;Gallionella;Gallionella capsiferriformans;Gallionella capsiferriformans ES-2 |
| Ga0586324 0013127 2192 3373 | 0.00 | 0.00 | 1417116.04 | 0.00 | 0.00 | 0.00 | 0.00 | 0.00 | 0.00 | 0.00 | 0.00 | 0.00 | KO:K00927 | phosphoglycerate kinase | Bacteria;Proteobacteria;Gammaproteobacteria;Acidiferrobacterales;Acidiferrobacteraceae;Sulfuricaulis;Sulfuricaulis limicola;Sulfuricaulis limicola HA5 |
| Ga0586324 0211041 507 1679 | 0.00 | 1407600.00 | 1213512.72 | 1510500.00 | 1586200.00 | 0.00 | 0.00 | 1848300.00 | 2284200.00 | 2656500.00 | 0.00 | 1035000.00 | KO:K00927 | phosphoglycerate kinase | Bacteria;Proteobacteria;Gammaproteobacteria;Methylococcales;Crenotrichaceae;Crenothrix;Crenothrix polyspora;Crenothrix polyspora ERS1545888 |
| Ga0586324 0528637 3 785 | 0.00 | 1217200.00 | 0.00 | 198307.75 | 0.00 | 0.00 | 0.00 | 1073600.00 | 1806300.00 | 0.00 | 0.00 | 60398.03 | KO:K00927 | phosphoglycerate kinase | Bacteria;Proteobacteria;Gammaproteobacteria;Methylococcales;Methylococcaceae;Methylovulum;Methylovulum miyakonense;Methylovulum miyakonense HT12 |
| Ga0586324 0575363 1 918 | 0.00 | 0.00 | 4366988.32 | 0.00 | 0.00 | 0.00 | 0.00 | 0.00 | 0.00 | 0.00 | 0.00 | 0.00 | KO:K00927 | phosphoglycerate kinase | Bacteria;Proteobacteria;Betaproteobacteria;Nitrosomonadales;Thiobacillaceae;Sulfuritortus;Sulfuritortus calidifontis;Sulfuritortus calidifontis DSM 103923 |
| Ga0586324 0710885 145 1407 | 0.00 | 0.00 | 0.00 | 9880000.00 | 11845000.00 | 0.00 | 0.00 | 0.00 | 0.00 | 0.00 | 0.00 | 0.00 | KO:K00927 | phosphoglycerate kinase | Bacteria;Proteobacteria;Gammaproteobacteria;Chromatiales;Ectothiorhodospiraceae;Ectothiorhodospira;Ectothiorhodospira magna;Ectothiorhodospira magna B7-7 |
| Ga0586324 0947291 3 1010 | 0.00 | 0.00 | 791752.53 | 0.00 | 0.00 | 0.00 | 0.00 | 0.00 | 0.00 | 0.00 | 0.00 | 0.00 | KO:K00927 | phosphoglycerate kinase | Bacteria;Proteobacteria;Gammaproteobacteria;unclassified Gammaproteobacteria;Candidatus Competibacteraceae;Candidatus Competibacter;Candidatus Competibacter denitrificans;Candidatus Competibacter denitrificans Run A D11 |
| Ga0586324 2319952 289 849 | 0.00 | 0.00 | 0.00 | 2118500.00 | 5119100.00 | 0.00 | 0.00 | 2787700.00 | 0.00 | 0.00 | 0.00 | 0.00 | KO:K00927 | phosphoglycerate kinase | Bacteria;Proteobacteria;Gammaproteobacteria;Chromatiales;Chromatiaceae;Thiorhodococcus;Thiorhodococcus drewsii;Thiorhodococcus drewsii AZ1 |
| Ga0586324 2329215 2 664 | 0.00 | 1115200.00 | 0.00 | 0.00 | 0.00 | 0.00 | 0.00 | 0.00 | 0.00 | 0.00 | 0.00 | 0.00 | KO:K00927 | phosphoglycerate kinase | Archaea;Euryarchaeota;Methanomicrobia;Methanosarcinales;Methanotrichaceae;Methanothrix;Methanothrix soehngenii;Methanothrix soehngenii GP-6 |
| Ga0586324 4235855 1 660 | 0.00 | 918000.00 | 0.00 | 0.00 | 3007600.00 | 0.00 | 0.00 | 0.00 | 0.00 | 0.00 | 0.00 | 0.00 | KO:K00927 | phosphoglycerate kinase | Bacteria;Proteobacteria;Betaproteobacteria;Burkholderiales;Alcaligenaceae;Bordetella;Bordetella sp. N;Bordetella sp. N |
| Ga0586324 0018138 758 1645 | 0.00 | 0.00 | 0.00 | 0.00 | 0.00 | 0.00 | 0.00 | 0.00 | 0.00 | 0.00 | 1311200.00 | 0.00 | KO:K00930 | acetylglutamate kinase | Bacteria;Proteobacteria;Betaproteobacteria;Nitrosomonadales;Nitrosomonadaceae;Nitrosospira;Nitrosospira lacus;Nitrosospira lacus APG3 |
| Ga0586324 0583270 117 1001 | 0.00 | 0.00 | 0.00 | 0.00 | 0.00 | 0.00 | 0.00 | 0.00 | 0.00 | 2202200.00 | 0.00 | 0.00 | KO:K00930 | acetylglutamate kinase | Bacteria;Proteobacteria;Betaproteobacteria;Nitrosomonadales;Sterolibacteriaceae;Sulfurisoma;Sulfurisoma sediminicola;Sulfurisoma sediminicola DSM 26916 |
| Ga0586324 0000070 48906 49562 | 0.00 | 0.00 | 0.00 | 0.00 | 0.00 | 0.00 | 18354000.00 | 15067000.00 | 16702200.00 | 3865400.00 | 5086400.00 | 8430000.00 | KO:K00939 | adenylate kinase | Bacteria;Proteobacteria;Betaproteobacteria;Nitrosomonadales;Thiobacillaceae;Thiobacillus;Thiobacillus thioparus;Thiobacillus thioparus DSM 505 |
| Ga0586324 0001048 10750 11694 | 0.00 | 0.00 | 0.00 | 0.00 | 0.00 | 0.00 | 0.00 | 0.00 | 1328400.00 | 1162700.00 | 0.00 | 1680000.00 | KO:K00939 | adenylate kinase | Bacteria;Proteobacteria;Gammaproteobacteria;Acidiferrobacterales;Acidiferrobacteraceae;Sulfurifustis;Sulfurifustis variabilis;Sulfurifustis variabilis skN76 |
| Ga0586324 0020859 3521 4189 | 7390000.00 | 0.00 | 0.00 | 18430000.00 | 7755900.00 | 0.00 | 0.00 | 6832000.00 | 9315000.00 | 0.00 | 0.00 | 0.00 | KO:K00939 | adenylate kinase | Archaea;Euryarchaeota;Methanomicrobia;Methanosarcinales;Methanotrichaceae;Methanothrix;Methanothrix soehngenii;Methanothrix soehngenii GP-6 |

| | | | | | | | | | | | | | | | |
|---|---|---|---|---|---|---|---|---|---|---|---|---|---|---|---|
| Ga0586324 0033408 2929 3621 | 0.00 | 17340000.00 | 0.00 | 0.00 | 0.00 | 0.00 | 0.00 | 0.00 | 0.00 | 0.00 | 0.00 | 0.00 | KO:K00939 | adenylate kinase | Bacteria;Proteobacteria;Deltaproteobacteria;Syntrophobacterales;Syntrophaceae;Desulfobacca;Desulfobacca acetoxidans;Desulfobacca acetoxidans ASRB2, DSM 11109 |
| Ga0586324 0034737 880 1524 | 9390000.00 | 21216000.00 | 19536100.00 | 22116000.00 | 0.00 | 9369200.00 | 0.00 | 0.00 | 3709800.00 | 0.00 | 0.00 | 2160000.00 | KO:K00939 | adenylate kinase | Archaea;Euryarchaeota;Methanomicrobia;Methanosarcinales;Methanotrichaceae;Methanothrix;Methanothrix soehngenii;Methanothrix soehngenii GP-6 |
| Ga0586324 0057467 1360 2007 | 0.00 | 0.00 | 0.00 | 3401000.00 | 0.00 | 0.00 | 2325600.00 | 0.00 | 0.00 | 5374600.00 | 0.00 | 0.00 | KO:K00939 | adenylate kinase | Bacteria;Proteobacteria;Deltaproteobacteria;Syntrophobacterales;Syntrophaceae;Desulfomonile;Desulfomonile tiedjei;Desulfomonile tiedjei DCB-1, DSM 6799 |
| Ga0586324 0145170 2293 2874 | 0.00 | 0.00 | 0.00 | 0.00 | 19261000.00 | 0.00 | 0.00 | 23125100.00 | 12312000.00 | 0.00 | 0.00 | 0.00 | KO:K00939 | adenylate kinase | Bacteria;Proteobacteria;Deltaproteobacteria;Syntrophobacterales;Syntrophaceae;Desulfobacca;Desulfobacca acetoxidans;Desulfobacca acetoxidans ASRB2, DSM 11109 |
| Ga0586324 0186661 173 841 | 0.00 | 1183200.00 | 0.00 | 3030500.00 | 7354200.00 | 0.00 | 0.00 | 799100.00 | 217097.82 | 0.00 | 0.00 | 294338.25 | KO:K00939 | adenylate kinase | Archaea;Euryarchaeota;Methanomicrobia;Methanosarcinales;Methanotrichaceae;Methanothrix;Methanothrix soehngenii;Methanothrix soehngenii GP-6 |
| Ga0586324 0191452 1356 2003 | 0.00 | 16456943.84 | 34906000.00 | 50825000.00 | 45423000.00 | 48259911.53 | 27360000.00 | 32269000.00 | 20688572.88 | 25102000.00 | 29277600.00 | 15675000.00 | KO:K00939 | adenylate kinase | Bacteria;Proteobacteria;Deltaproteobacteria;Syntrophobacterales;Syntrophaceae;Desulfobacca;Desulfobacca acetoxidans;Desulfobacca acetoxidans ASRB2, DSM 11109 |
| Ga0586324 0701932 678 1325 | 0.00 | 0.00 | 0.00 | 0.00 | 3162100.00 | 6907800.00 | 4810800.00 | 0.00 | 0.00 | 0.00 | 5764000.00 | 3832500.00 | KO:K00939 | adenylate kinase | Bacteria;Proteobacteria;Deltaproteobacteria;Syntrophobacterales;Syntrophaceae;Desulfomonile;Desulfomonile tiedjei;Desulfomonile tiedjei DCB-1, DSM 6799 |
| Ga0586324 0887243 445 1092 | 0.00 | 385963.92 | 0.00 | 0.00 | 0.00 | 0.00 | 0.00 | 0.00 | 0.00 | 6006000.00 | 0.00 | 0.00 | KO:K00939 | adenylate kinase | Bacteria;Proteobacteria;Deltaproteobacteria;Syntrophobacterales;Syntrophaceae;Desulfobacca;Desulfobacca acetoxidans;Desulfobacca acetoxidans ASRB2, DSM 11109 |
| Ga0586324 0960057 323 985 | 3310000.00 | 3155200.00 | 12723800.00 | 0.00 | 14317000.00 | 0.00 | 12836400.00 | 6405000.00 | 14904000.00 | 6814500.00 | 8800000.00 | 4177500.00 | KO:K00939 | adenylate kinase | Bacteria;Proteobacteria;Gammaproteobacteria;Chromatiales;Ectothiorhodospiraceae;Inmirania;Inmirania thermothiophila;Inmirania thermothiophila DSM 100275 |
| Ga0586324 2952612 1 672 | 5190000.00 | 0.00 | 0.00 | 0.00 | 0.00 | 0.00 | 0.00 | 0.00 | 0.00 | 0.00 | 0.00 | 0.00 | KO:K00939 | adenylate kinase | Bacteria;Actinobacteria;Acidimicrobiia;Acidimicrobiales;Ilumatobacteraceae;Ilumatobacter;Ilumatobacter fluminis;Ilumatobacter fluminis DSM 18936 |
| Ga0586324 3678679 3 626 | 22600000.00 | 24208000.00 | 0.00 | 25555000.00 | 0.00 | 0.00 | 0.00 | 27694000.00 | 51597000.00 | 27720000.00 | 0.00 | 0.00 | KO:K00939 | adenylate kinase | Bacteria;Proteobacteria;Deltaproteobacteria;Syntrophobacterales;Syntrophaceae;Desulfobacca;Desulfobacca acetoxidans;Desulfobacca acetoxidans ASRB2, DSM 11109 |
| Ga0586324 6460918 1 555 | 3700000.00 | 0.00 | 0.00 | 0.00 | 0.00 | 0.00 | 0.00 | 0.00 | 0.00 | 0.00 | 0.00 | 0.00 | KO:K00939 | adenylate kinase | Bacteria;Proteobacteria;Betaproteobacteria;Nitrosomonadales;Sterolibacteriaceae;Sulfurisoma;Sulfurisoma sediminicola;Sulfurisoma sediminicola DSM 26916 |
| Ga0586324 8114557 3 215 | 0.00 | 0.00 | 0.00 | 0.00 | 0.00 | 1800784.06 | 0.00 | 0.00 | 0.00 | 0.00 | 0.00 | 0.00 | KO:K00939 | adenylate kinase | Bacteria;Nitrospirae;Thermodesulfovibrionia;Thermodesulfovibrionales;Thermodesulfovibrionaceae;Thermodesulfovibrio;Thermodesulfovibrio sp. N1;Thermodesulfovibrio sp. N1 |
| Ga0586324 0000184 28941 29372 | 0.00 | 0.00 | 5379374.92 | 0.00 | 0.00 | 0.00 | 0.00 | 0.00 | 0.00 | 0.00 | 0.00 | 0.00 | KO:K00940 | nucleoside-diphosphate kinase | Bacteria;Proteobacteria;Gammaproteobacteria;Methylococcales;Methylococcaceae;Methylobacter;Methylobacter luteus;Methylobacter luteus IMV-B-3098 |
| Ga0586324 0003751 7165 7590 | 0.00 | 0.00 | 0.00 | 0.00 | 0.00 | 0.00 | 0.00 | 0.00 | 1733400.00 | 0.00 | 0.00 | 0.00 | KO:K00940 | nucleoside-diphosphate kinase | Bacteria;Proteobacteria;Betaproteobacteria;Burkholderiales;Oxalobacteraceae;Noviherbaspirillum;Noviherbaspirillum massiliense;Noviherbaspirillum massiliense JC206 |
| Ga0586324 0093725 1778 2203 | 0.00 | 0.00 | 0.00 | 0.00 | 0.00 | 0.00 | 0.00 | 0.00 | 0.00 | 0.00 | 0.00 | 750000.00 | KO:K00940 | nucleoside-diphosphate kinase | Bacteria;Proteobacteria;Betaproteobacteria;Nitrosomonadales;Gallionellaceae;Sideroxydans;Sideroxydans lithotrophicus;Sideroxydans lithotrophicus ES-1 |
| Ga0586324 1426975 136 558 | 0.00 | 1332800.00 | 0.00 | 0.00 | 0.00 | 0.00 | 0.00 | 0.00 | 0.00 | 0.00 | 0.00 | 0.00 | KO:K00940 | nucleoside-diphosphate kinase | Bacteria;Proteobacteria;Alphaproteobacteria;Hyphomicrobiales;Bradyrhizobiaceae;Bradyrhizobium;Bradyrhizobium elkanii;Bradyrhizobium elkanii WSM1741 |
| Ga0586324 1858645 387 818 | 0.00 | 0.00 | 0.00 | 7628500.00 | 42642000.00 | 0.00 | 6406800.00 | 5063000.00 | 27378000.00 | 0.00 | 33528000.00 | 7875000.00 | KO:K00940 | nucleoside-diphosphate kinase | Bacteria;Proteobacteria;Gammaproteobacteria;Methylococcales;Crenotrichaceae;Crenothrix;Crenothrix polyspora;Crenothrix polyspora ERS1545888 |
| Ga0586324 0000041 19268 20218 | 0.00 | 0.00 | 0.00 | 0.00 | 0.00 | 0.00 | 0.00 | 0.00 | 0.00 | 0.00 | 0.00 | 1822500.00 | KO:K00948 | ribose-phosphate pyrophosphokinase | Bacteria;Proteobacteria;Betaproteobacteria;Nitrosomonadales;Thiobacillaceae;Annwoodia;Annwoodia aquaesulis;Annwoodia aquaesulis DSM 4255 |
| Ga0586324 1399391 287 1051 | 0.00 | 0.00 | 0.00 | 0.00 | 0.00 | 0.00 | 0.00 | 0.00 | 0.00 | 3003000.00 | 0.00 | 0.00 | KO:K00948 | ribose-phosphate pyrophosphokinase | Bacteria;Acidobacteria;Vicinamibacteria;unclassified Vicinamibacteria;Vicinamibacteraceae;Luteitalea;Luteitalea pratensis;Luteitalea pratensis DSM 100886 |
| Ga0586324 0008738 9441 10673 | 0.00 | 20400000.00 | 0.00 | 109345000.00 | 69422000.00 | 0.00 | 0.00 | 0.00 | 666659.16 | 35112000.00 | 38720000.00 | 3660000.00 | KO:K00958 | sulfate adenylyltransferase | Bacteria;Proteobacteria;Deltaproteobacteria;Syntrophobacterales;Syntrophobacteraceae;Syntrophobacter;Syntrophobacter fumaroxidans;Syntrophobacter fumaroxidans MPOB |
| Ga0586324 0009502 2467 3675 | 0.00 | 0.00 | 14018700.00 | 0.00 | 0.00 | 0.00 | 0.00 | 0.00 | 0.00 | 0.00 | 0.00 | 0.00 | KO:K00958 | sulfate adenylyltransferase | Bacteria;Proteobacteria;Betaproteobacteria;Nitrosomonadales;Thiobacillaceae;Thiobacillus;Thiobacillus thioparus;Thiobacillus thioparus DSM 505 |
| Ga0586324 0018169 1791 3047 | 0.00 | 0.00 | 0.00 | 0.00 | 0.00 | 0.00 | 1408866.72 | 0.00 | 0.00 | 0.00 | 0.00 | 0.00 | KO:K00958 | sulfate adenylyltransferase | Bacteria;Proteobacteria;Gammaproteobacteria;Chromatiales;Ectothiorhodospiraceae;Thioalkalivibrio;Thioalkalivibrio sulfidiphilus;Thioalkalivibrio sulfidiphilus HL-EbGR7 |
| Ga0586324 0046593 3503 4744 | 0.00 | 4148000.00 | 0.00 | 4750000.00 | 0.00 | 4287600.00 | 7546800.00 | 0.00 | 687195.09 | 0.00 | 0.00 | 1785000.00 | KO:K00958 | sulfate adenylyltransferase | Bacteria;Nitrospirae;Nitrospira;Nitrospirales;Nitrospiraceae;Candidatus Magnetoovum;Candidatus Magnetoovum chiemensis;Candidatus Magnetoovum chiemensis CS-04 |
| Ga0586324 0057953 2783 4057 | 0.00 | 16592000.00 | 0.00 | 10165000.00 | 6664100.00 | 0.00 | 0.00 | 25986000.00 | 6747300.00 | 0.00 | 0.00 | 0.00 | KO:K00958 | sulfate adenylyltransferase | Bacteria;Proteobacteria;Deltaproteobacteria;Desulfobacterales;Desulfobacteraceae;Desulfatirhabdium;Desulfatirhabdium butyrativorans;Desulfatirhabdium butyrativorans DSM 18734 |
| Ga0586324 0086383 1552 2793 | 0.00 | 0.00 | 0.00 | 0.00 | 0.00 | 0.00 | 0.00 | 0.00 | 1069200.00 | 0.00 | 0.00 | 0.00 | KO:K00958 | sulfate adenylyltransferase | Bacteria;Nitrospirae;Nitrospira;Nitrospirales;Nitrospiraceae;Candidatus Magnetoovum;Candidatus Magnetoovum chiemensis;Candidatus Magnetoovum chiemensis CS-04 |
| Ga0586324 0173887 1466 2641 | 14700000.00 | 0.00 | 36651300.00 | 0.00 | 0.00 | 0.00 | 675901.44 | 0.00 | 0.00 | 0.00 | 0.00 | 0.00 | KO:K00958 | sulfate adenylyltransferase | Bacteria;Proteobacteria;Deltaproteobacteria;Syntrophobacterales;Syntrophobacteraceae;Desulfoglaeba;Desulfoglaeba alkanexedens;Desulfoglaeba alkanexedens ALDC |
| Ga0586324 0200605 2 586 | 0.00 | 0.00 | 0.00 | 0.00 | 0.00 | 0.00 | 0.00 | 0.00 | 0.00 | 7615300.00 | 7656000.00 | 0.00 | KO:K00958 | sulfate adenylyltransferase | Bacteria;Proteobacteria;Betaproteobacteria;Nitrosomonadales;Thiobacillaceae;Thiobacillus;Thiobacillus denitrificans;Thiobacillus denitrificans RG |
| Ga0586324 0299664 1108 2058 | 0.00 | 0.00 | 0.00 | 0.00 | 0.00 | 0.00 | 1602876.48 | 0.00 | 0.00 | 0.00 | 0.00 | 0.00 | KO:K00958 | sulfate adenylyltransferase | Bacteria;Proteobacteria;Deltaproteobacteria;Syntrophobacterales;Syntrophaceae;Desulfobacca;Desulfobacca acetoxidans;Desulfobacca acetoxidans ASRB2, DSM 11109 |
| Ga0586324 0331734 614 1849 | 7170000.00 | 10200000.00 | 10386612.47 | 17765000.00 | 18540000.00 | 7662100.00 | 7296000.00 | 12242700.00 | 10611000.00 | 9471000.00 | 10190400.00 | 6855000.00 | KO:K00958 | sulfate adenylyltransferase | Bacteria;Proteobacteria;Deltaproteobacteria;Syntrophobacterales;Syntrophaceae;Desulfobacca;Desulfobacca acetoxidans;Desulfobacca acetoxidans ASRB2, DSM 11109 |
| Ga0586324 0344379 1 978 | 0.00 | 0.00 | 0.00 | 2954500.00 | 0.00 | 0.00 | 0.00 | 0.00 | 0.00 | 0.00 | 0.00 | 0.00 | KO:K00958 | sulfate adenylyltransferase | Bacteria;Proteobacteria;Betaproteobacteria;Nitrosomonadales;Thiobacillaceae;Thiobacillus;Thiobacillus thioparus;Thiobacillus thioparus DSM 505 |
| Ga0586324 0375356 391 1647 | 1600000.00 | 1924400.00 | 0.00 | 0.00 | 1503800.00 | 0.00 | 0.00 | 0.00 | 0.00 | 2764300.00 | 5394400.00 | 0.00 | KO:K00958 | sulfate adenylyltransferase | Bacteria;Proteobacteria;Deltaproteobacteria;Syntrophobacterales;Syntrophaceae;Desulfomonile;Desulfomonile tiedjei;Desulfomonile tiedjei DCB-1, DSM 6799 |
| Ga0586324 0515793 3 926 | 0.00 | 10676000.00 | 0.00 | 0.00 | 0.00 | 0.00 | 0.00 | 0.00 | 0.00 | 0.00 | 0.00 | 0.00 | KO:K00958 | sulfate adenylyltransferase | Bacteria;Proteobacteria;Deltaproteobacteria;Syntrophobacterales;Syntrophaceae;Desulfobacca;Desulfobacca acetoxidans;Desulfobacca acetoxidans ASRB2, DSM 11109 |
| Ga0586324 0537992 3 1256 | 1710000.00 | 0.00 | 3089760.89 | 2308500.00 | 13390000.00 | 5716800.00 | 5358000.00 | 2641300.00 | 4446900.00 | 3264800.00 | 0.00 | 0.00 | KO:K00958 | sulfate adenylyltransferase | Bacteria;Proteobacteria;Deltaproteobacteria;Desulfobacterales;Desulfobacteraceae;Desulfatirhabdium;Desulfatirhabdium butyrativorans;Desulfatirhabdium butyrativorans DSM 18734 |
| Ga0586324 0549740 537 1571 | 8070000.00 | 10404000.00 | 0.00 | 24320000.00 | 19776000.00 | 20286700.00 | 0.00 | 11651000.00 | 7605900.00 | 11242000.00 | 13376000.00 | 8100000.00 | KO:K00958 | sulfate adenylyltransferase | Bacteria;Nitrospirae;Nitrospira;Nitrospirales;Nitrospiraceae;Candidatus Magnetoovum;Candidatus Magnetoovum chiemensis;Candidatus Magnetoovum chiemensis CS-04 |
| Ga0586324 0671325 119 721 | 0.00 | 0.00 | 0.00 | 0.00 | 0.00 | 0.00 | 0.00 | 19703000.00 | 0.00 | 0.00 | 0.00 | 0.00 | KO:K00958 | sulfate adenylyltransferase | Bacteria;Proteobacteria;Deltaproteobacteria;Desulfobacterales;Desulfobacteraceae;Desulfonema;Desulfonema limicola;Desulfonema limicola Jadebusen, DSM 2076 |
| Ga0586324 0671325 699 1322 | 5700000.00 | 10744000.00 | 0.00 | 0.00 | 0.00 | 0.00 | 0.00 | 0.00 | 0.00 | 0.00 | 0.00 | 0.00 | KO:K00958 | sulfate adenylyltransferase | Bacteria;Proteobacteria;Deltaproteobacteria;Desulfobacterales;Desulfobacteraceae;Desulfatirhabdium;Desulfatirhabdium butyrativorans;Desulfatirhabdium butyrativorans DSM 18734 |
| Ga0586324 0700391 168 1397 | 17100000.00 | 19448000.00 | 41380500.00 | 0.00 | 43548400.00 | 27670900.00 | 0.00 | 29829000.00 | 47790000.00 | 6344800.00 | 0.00 | 0.00 | KO:K00958 | sulfate adenylyltransferase | Bacteria;Proteobacteria;Deltaproteobacteria;Syntrophobacterales;Syntrophaceae;Desulfobacca;Desulfobacca acetoxidans;Desulfobacca acetoxidans ASRB2, DSM 11109 |
| Ga0586324 0780472 3 713 | 0.00 | 0.00 | 0.00 | 2014000.00 | 0.00 | 0.00 | 0.00 | 0.00 | 0.00 | 0.00 | 0.00 | 0.00 | KO:K00958 | sulfate adenylyltransferase | Bacteria;Planctomycetes;Planctomycetia;Pirellulales;Thermoguttaceae;Thermogutta;Thermogutta terrifontis;Thermogutta terrifontis R1 |
| Ga0586324 0875961 1 843 | 0.00 | 0.00 | 0.00 | 0.00 | 0.00 | 0.00 | 0.00 | 0.00 | 0.00 | 0.00 | 508667.28 | 0.00 | KO:K00958 | sulfate adenylyltransferase | Bacteria;Proteobacteria;Deltaproteobacteria;Desulfobacterales;Desulfobacteraceae;Desulfatirhabdium;Desulfatirhabdium butyrativorans;Desulfatirhabdium butyrativorans DSM 18734 |
| Ga0586324 0923162 3 959 | 0.00 | 29920000.00 | 0.00 | 0.00 | 0.00 | 0.00 | 0.00 | 38674000.00 | 35559000.00 | 0.00 | 25960000.00 | 21900000.00 | KO:K00958 | sulfate adenylyltransferase | Bacteria;Proteobacteria;Deltaproteobacteria;Syntrophobacterales;Syntrophobacteraceae;Desulfoglaeba;Desulfoglaeba alkanexedens;Desulfoglaeba alkanexedens ALDC |
| Ga0586324 1182430 676 1131 | 7930000.00 | 0.00 | 0.00 | 35910000.00 | 0.00 | 7622400.00 | 35568000.00 | 9211000.00 | 0.00 | 0.00 | 0.00 | 0.00 | KO:K00958 | sulfate adenylyltransferase | Bacteria;Proteobacteria;Deltaproteobacteria;Desulfobacterales;Desulfobacteraceae;Desulfatirhabdium;Desulfatirhabdium butyrativorans;Desulfatirhabdium butyrativorans DSM 18734 |
| Ga0586324 1539617 44 1009 | 30100000.00 | 377431.28 | 0.00 | 0.00 | 0.00 | 0.00 | 0.00 | 0.00 | 0.00 | 0.00 | 0.00 | 0.00 | KO:K00958 | sulfate adenylyltransferase | Bacteria;Proteobacteria;Deltaproteobacteria;Syntrophobacterales;Syntrophobacteraceae;Syntrophobacter;Syntrophobacter fumaroxidans;Syntrophobacter fumaroxidans MPOB |
| Ga0586324 1602106 3 686 | 14000000.00 | 0.00 | 0.00 | 0.00 | 0.00 | 0.00 | 0.00 | 0.00 | 0.00 | 0.00 | 0.00 | 0.00 | KO:K00958 | sulfate adenylyltransferase | Bacteria;Proteobacteria;Deltaproteobacteria;Syntrophobacterales;Syntrophaceae;Desulfobacca;Desulfobacca acetoxidans;Desulfobacca acetoxidans ASRB2, DSM 11109 |
| Ga0586324 1683363 256 972 | 0.00 | 0.00 | 0.00 | 0.00 | 0.00 | 0.00 | 5973600.00 | 0.00 | 0.00 | 0.00 | 0.00 | 0.00 | KO:K00958 | sulfate adenylyltransferase | Bacteria;Proteobacteria;Betaproteobacteria;Nitrosomonadales;Sulfuricellaceae;Sulfuricella;Sulfuricella sp. T08;Sulfuricella sp. T08 |
| Ga0586324 2391261 2 601 | 0.00 | 0.00 | 0.00 | 0.00 | 0.00 | 0.00 | 0.00 | 0.00 | 6739200.00 | 0.00 | 0.00 | 0.00 | KO:K00958 | sulfate adenylyltransferase | Bacteria;Proteobacteria;Deltaproteobacteria;Syntrophobacterales;Syntrophobacteraceae;Syntrophobacter;Syntrophobacter fumaroxidans;Syntrophobacter fumaroxidans MPOB |
| Ga0586324 2690852 2 796 | 0.00 | 2121600.00 | 0.00 | 15770000.00 | 0.00 | 0.00 | 0.00 | 0.00 | 6682500.00 | 5320700.00 | 0.00 | 0.00 | KO:K00958 | sulfate adenylyltransferase | Bacteria;Proteobacteria;Deltaproteobacteria;Syntrophobacterales;Syntrophaceae;Desulfobacca;Desulfobacca acetoxidans;Desulfobacca acetoxidans ASRB2, DSM 11109 |
| Ga0586324 2858862 3 776 | 0.00 | 0.00 | 0.00 | 0.00 | 0.00 | 1027019.15 | 0.00 | 1842200.00 | 0.00 | 0.00 | 0.00 | 0.00 | KO:K00958 | sulfate adenylyltransferase | Bacteria;Proteobacteria;Deltaproteobacteria;Desulfobacterales;Desulfobacteraceae;Desulfatirhabdium;Desulfatirhabdium butyrativorans;Desulfatirhabdium butyrativorans DSM 18734 |
| Ga0586324 3104600 375 752 | 0.00 | 0.00 | 0.00 | 0.00 | 0.00 | 0.00 | 0.00 | 0.00 | 12474000.00 | 0.00 | 0.00 | 0.00 | KO:K00958 | sulfate adenylyltransferase | Bacteria;Proteobacteria;Deltaproteobacteria;Syntrophobacterales;Syntrophobacteraceae;Syntrophobacter;Syntrophobacter fumaroxidans;Syntrophobacter fumaroxidans MPOB |
| Ga0586324 3520170 222 713 | 0.00 | 1278400.00 | 0.00 | 0.00 | 0.00 | 0.00 | 0.00 | 0.00 | 0.00 | 0.00 | 0.00 | 0.00 | KO:K00958 | sulfate adenylyltransferase | Bacteria;Proteobacteria;Deltaproteobacteria;Desulfobacterales;Desulfobacteraceae;Desulfatirhabdium;Desulfatirhabdium butyrativorans;Desulfatirhabdium butyrativorans DSM 18734 |
| Ga0586324 3888883 3 668 | 0.00 | 0.00 | 0.00 | 0.00 | 0.00 | 8694300.00 | 0.00 | 0.00 | 0.00 | 0.00 | 0.00 | 0.00 | KO:K00958 | sulfate adenylyltransferase | Bacteria;Proteobacteria;Deltaproteobacteria;Syntrophobacterales;Syntrophaceae;Desulfobacca;Desulfobacca acetoxidans;Desulfobacca acetoxidans ASRB2, DSM 11109 |
| Ga0586324 5039500 2 616 | 18800000.00 | 22644000.00 | 0.00 | 7505000.00 | 12257000.00 | 4208200.00 | 4263600.00 | 19581000.00 | 6617700.00 | 14476000.00 | 10208000.00 | 0.00 | KO:K00958 | sulfate adenylyltransferase | Bacteria;Proteobacteria;Deltaproteobacteria;Syntrophobacterales;Syntrophobacteraceae;Syntrophobacter;Syntrophobacter fumaroxidans;Syntrophobacter fumaroxidans MPOB |
| Ga0586324 5573539 2 589 | 0.00 | 0.00 | 0.00 | 0.00 | 0.00 | 0.00 | 0.00 | 0.00 | 9234000.00 | 0.00 | 0.00 | 0.00 | KO:K00958 | sulfate adenylyltransferase | Bacteria;Proteobacteria;Deltaproteobacteria;Desulfobacterales;Desulfobacteraceae;Desulfatirhabdium;Desulfatirhabdium butyrativorans;Desulfatirhabdium butyrativorans DSM 18734 |
| Ga0586324 5667825 3 272 | 3760000.00 | 0.00 | 0.00 | 3353500.00 | 0.00 | 0.00 | 0.00 | 0.00 | 0.00 | 2902900.00 | 4021600.00 | 0.00 | KO:K00958 | sulfate adenylyltransferase | Bacteria;Nitrospirae;Nitrospira;Nitrospirales;Nitrospiraceae;Candidatus Magnetoovum;Candidatus Magnetoovum chiemensis;Candidatus Magnetoovum chiemensis CS-04 |
| Ga0586324 7510884 2 520 | 215037.00 | 5038800.00 | 0.00 | 0.00 | 0.00 | 0.00 | 0.00 | 0.00 | 6706800.00 | 0.00 | 0.00 | 0.00 | KO:K00958 | sulfate adenylyltransferase | Bacteria;Proteobacteria;Deltaproteobacteria;Syntrophobacterales;Syntrophaceae;Desulfobacca;Desulfobacca acetoxidans;Desulfobacca acetoxidans ASRB2, DSM 11109 |
| Ga0586324 0001368 1948 4110 | 0.00 | 0.00 | 0.00 | 0.00 | 4830700.00 | 0.00 | 0.00 | 0.00 | 3288600.00 | 1386000.00 | 0.00 | 0.00 | KO:K00962 | polyribonucleotide nucleotidyltransferase | Bacteria;Proteobacteria;Alphaproteobacteria;Hyphomicrobiales;Xanthobacteraceae;Pseudolabrys;Pseudolabrys taiwanensis;Pseudolabrys taiwanensis CC-BB4 |
| Ga0586324 0001370 13726 15861 | 0.00 | 0.00 | 0.00 | 0.00 | 0.00 | 0.00 | 0.00 | 5837700.00 | 3564000.00 | 12273800.00 | 14044800.00 | 15022500.00 | KO:K00962 | polyribonucleotide nucleotidyltransferase | Bacteria;Proteobacteria;Betaproteobacteria;Nitrosomonadales;Thiobacillaceae;Annwoodia;Annwoodia aquaesulis;Annwoodia aquaesulis DSM 4255 |
| Ga0586324 0002100 12067 14229 | 0.00 | 0.00 | 0.00 | 0.00 | 0.00 | 0.00 | 12950400.00 | 0.00 | 0.00 | 1563100.00 | 34936000.00 | 4567500.00 | KO:K00962 | polyribonucleotide nucleotidyltransferase | Bacteria;Proteobacteria;Betaproteobacteria;Nitrosomonadales;Thiobacillaceae;Thiobacillus;Thiobacillus thioparus;Thiobacillus thioparus DSM 505 |
| Ga0586324 0002329 12843 14942 | 0.00 | 0.00 | 0.00 | 0.00 | 0.00 | 0.00 | 0.00 | 504851.25 | 0.00 | 0.00 | 0.00 | 0.00 | KO:K00962 | polyribonucleotide nucleotidyltransferase | Bacteria;Proteobacteria;Betaproteobacteria;unclassified Betaproteobacteria;unclassified Betaproteobacteria;unclassified Betaproteobacteria;uncultured beta proteobacterium;unclassified betaproteobacterium ENR4 |
| Ga0586324 0004094 2600 4711 | 0.00 | 0.00 | 0.00 | 0.00 | 1369900.00 | 0.00 | 0.00 | 0.00 | 0.00 | 0.00 | 0.00 | 0.00 | KO:K00962 | polyribonucleotide nucleotidyltransferase | Bacteria;Gemmatimonadetes;Gemmatimonadetes;Gemmatimonadales;Gemmatimonadaceae;Gemmatirosa;Gemmatirosa kalamazoonesis;Gemmatimonadetes bacterium KBS708 |
| Ga0586324 0006042 3130 5280 | 0.00 | 0.00 | 0.00 | 0.00 | 0.00 | 0.00 | 0.00 | 707600.00 | 996300.00 | 0.00 | 0.00 | 0.00 | KO:K00962 | polyribonucleotide nucleotidyltransferase | Bacteria;Verrucomicrobia;Verrucomicrobiae;Verrucomicrobiales;Verrucomicrobia subdivision 3;Pedosphaera;Pedosphaera parvula;Pedosphaera parvula Ellin514 |
| Ga0586324 0009301 3 1310 | 0.00 | 3366000.00 | 0.00 | 0.00 | 0.00 | 0.00 | 0.00 | 0.00 | 0.00 | 0.00 | 0.00 | 0.00 | KO:K00962 | polyribonucleotide nucleotidyltransferase | Bacteria;Spirochaetes;Spirochaetia;Spirochaetales;Spirochaetaceae;Spirochaeta;Spirochaeta isovalerica;Spirochaeta isovalerica DSM 2461 |
| Ga0586324 0010745 3311 5548 | 0.00 | 0.00 | 0.00 | 0.00 | 0.00 | 0.00 | 0.00 | 0.00 | 2737800.00 | 0.00 | 0.00 | 0.00 | KO:K00962 | polyribonucleotide nucleotidyltransferase | Bacteria;Chloroflexi;Anaerolineae;Anaerolineales;Anaerolineaceae;Levilinea;Levilinea saccharolytica;Levilinea saccharolytica DSM 16555 |
| Ga0586324 0014811 6478 8535 | 0.00 | 0.00 | 0.00 | 0.00 | 0.00 | 0.00 | 3739200.00 | 0.00 | 0.00 | 0.00 | 0.00 | 0.00 | KO:K00962 | polyribonucleotide nucleotidyltransferase | Bacteria;Proteobacteria;Deltaproteobacteria;Syntrophobacterales;Syntrophobacteraceae;Desulfacinum;Desulfacinum infernum;Desulfacinum infernum DSM 9756 |
| Ga0586324 0017391 1 1248 | 0.00 | 0.00 | 0.00 | 0.00 | 0.00 | 0.00 | 606311.28 | 7838500.00 | 3896100.00 | 2533300.00 | 2930400.00 | 8325000.00 | KO:K00962 | polyribonucleotide nucleotidyltransferase | Bacteria;Proteobacteria;Betaproteobacteria;Nitrosomonadales;Thiobacillaceae;Thiobacillus;Thiobacillus thioparus;Thiobacillus thioparus DSM 505 |
| Ga0586324 0018681 4164 6476 | 0.00 | 0.00 | 0.00 | 0.00 | 0.00 | 0.00 | 2781600.00 | 0.00 | 0.00 | 0.00 | 0.00 | 0.00 | KO:K00962 | polyribonucleotide nucleotidyltransferase | Bacteria;Acidobacteria;Vicinamibacteria;unclassified Vicinamibacteria;Vicinamibacteraceae;Luteitalea;Luteitalea pratensis;Luteitalea pratensis DSM 100886 |
| Ga0586324 0029440 3 1472 | 0.00 | 0.00 | 0.00 | 0.00 | 114550.42 | 0.00 | 0.00 | 0.00 | 0.00 | 0.00 | 0.00 | 0.00 | KO:K00962 | polyribonucleotide nucleotidyltransferase | Bacteria;Actinobacteria;Actinomycetia;Glycomycetales;Glycomycetaceae;Glycomyces;Glycomyces arizonensis;Glycomyces arizonensis DSM 44726 |
| Ga0586324 0038600 4142 5428 | 0.00 | 0.00 | 0.00 | 459599.55 | 0.00 | 0.00 | 0.00 | 1909300.00 | 260581.86 | 0.00 | 0.00 | 0.00 | KO:K00962 | polyribonucleotide nucleotidyltransferase | Bacteria;Proteobacteria;Deltaproteobacteria;Syntrophobacterales;Syntrophobacteraceae;Syntrophobacter;Syntrophobacter fumaroxidans;Syntrophobacter fumaroxidans MPOB |
| Ga0586324 0053148 2 1285 | 0.00 | 0.00 | 0.00 | 0.00 | 0.00 | 0.00 | 0.00 | 3190300.00 | 0.00 | 0.00 | 0.00 | 0.00 | KO:K00962 | polyribonucleotide nucleotidyltransferase | Bacteria;Proteobacteria;Deltaproteobacteria;Myxococcales;Anaeromyxobacteraceae;Anaeromyxobacter;Anaeromyxobacter dehalogenans;Anaeromyxobacter dehalogenans 2CP-C |
| Ga0586324 0073213 1547 3745 | 333422.00 | 1768000.00 | 0.00 | 1624500.00 | 0.00 | 0.00 | 0.00 | 0.00 | 0.00 | 0.00 | 0.00 | 0.00 | KO:K00962 | polyribonucleotide nucleotidyltransferase | Bacteria;Chloroflexi;Thermoflexia;Thermoflexales;Thermoflexaceae;Thermoflexus;Thermoflexus hugenholtzii;Thermoflexus hugenholtzii JAD2 |
| Ga0586324 0104103 3 1781 | 0.00 | 1856400.00 | 0.00 | 0.00 | 0.00 | 0.00 | 0.00 | 0.00 | 0.00 | 703169.39 | 0.00 | 0.00 | KO:K00962 | polyribonucleotide nucleotidyltransferase | Bacteria;Proteobacteria;Gammaproteobacteria;Acidiferrobacterales;Acidiferrobacteraceae;Sulfuricaulis;Sulfuricaulis limicola;Sulfuricaulis limicola HA5 |
| Ga0586324 0121032 2052 3131 | 0.00 | 0.00 | 0.00 | 0.00 | 0.00 | 0.00 | 0.00 | 0.00 | 0.00 | 0.00 | 8703200.00 | 0.00 | KO:K00962 | polyribonucleotide nucleotidyltransferase | Bacteria;Proteobacteria;Alphaproteobacteria;Hyphomicrobiales;Xanthobacteraceae;Pseudolabrys;Pseudolabrys taiwanensis;Pseudolabrys taiwanensis CC-BB4 |
| Ga0586324 0122637 1 1206 | 0.00 | 0.00 | 0.00 | 0.00 | 0.00 | 0.00 | 0.00 | 5703500.00 | 0.00 | 0.00 | 0.00 | 0.00 | KO:K00962 | polyribonucleotide nucleotidyltransferase | Bacteria;Proteobacteria;Betaproteobacteria;Rhodocyclales;Rhodocyclaceae;Azospira;unclassified Azospira;Azospira sp. ZAP |
| Ga0586324 0175914 3 1895 | 3560000.00 | 0.00 | 0.00 | 2945000.00 | 0.00 | 1302727.71 | 1413438.12 | 2202100.00 | 2519100.00 | 0.00 | 0.00 | 495704.25 | KO:K00962 | polyribonucleotide nucleotidyltransferase | Bacteria;Proteobacteria;Deltaproteobacteria;Syntrophobacterales;Syntrophaceae;Desulfobacca;Desulfobacca acetoxidans;Desulfobacca acetoxidans ASRB2, DSM 11109 |
| Ga0586324 0177209 3 620 | 0.00 | 0.00 | 0.00 | 0.00 | 0.00 | 0.00 | 0.00 | 2208200.00 | 0.00 | 0.00 | 0.00 | 0.00 | KO:K00962 | polyribonucleotide nucleotidyltransferase | Bacteria;Proteobacteria;Alphaproteobacteria;Hyphomicrobiales;Methylocystaceae;Methylocystis;Methylocystis parvus;Methylocystis parvus OBBP |
| Ga0586324 0184497 185 2359 | 0.00 | 0.00 | 0.00 | 0.00 | 0.00 | 0.00 | 0.00 | 1146800.00 | 0.00 | 0.00 | 0.00 | 0.00 | KO:K00962 | polyribonucleotide nucleotidyltransferase | Bacteria;Acidobacteria;Vicinamibacteria;unclassified Vicinamibacteria;Vicinamibacteraceae;Luteitalea;Luteitalea pratensis;Luteitalea pratensis DSM 100886 |
| Ga0586324 0221149 2 1105 | 2710000.00 | 0.00 | 0.00 | 0.00 | 0.00 | 0.00 | 0.00 | 0.00 | 0.00 | 0.00 | 0.00 | 0.00 | KO:K00962 | polyribonucleotide nucleotidyltransferase | Bacteria;Proteobacteria;Deltaproteobacteria;Syntrophobacterales;Syntrophaceae;Desulfobacca;Desulfobacca acetoxidans;Desulfobacca acetoxidans ASRB2, DSM 11109 |
| Ga0586324 0228457 1 1938 | 3160000.00 | 6936000.00 | 2305676.42 | 0.00 | 0.00 | 0.00 | 4879200.00 | 4148000.00 | 9882000.00 | 8547000.00 | 13552000.00 | 3270000.00 | KO:K00962 | polyribonucleotide nucleotidyltransferase | Bacteria;Proteobacteria;Gammaproteobacteria;Acidiferrobacterales;Acidiferrobacteraceae;Sulfuricaulis;Sulfuricaulis limicola;Sulfuricaulis limicola HA5 |
| Ga0586324 0266664 1290 2168 | 0.00 | 0.00 | 0.00 | 0.00 | 0.00 | 0.00 | 0.00 | 0.00 | 328119.66 | 0.00 | 0.00 | 0.00 | KO:K00962 | polyribonucleotide nucleotidyltransferase | Bacteria;Proteobacteria;Betaproteobacteria;Rhodocyclales;Azonexaceae;Dechloromonas;Dechloromonas agitata;Dechloromonas agitata is5 |
| Ga0586324 0299496 1079 2056 | 0.00 | 0.00 | 0.00 | 0.00 | 0.00 | 0.00 | 0.00 | 0.00 | 0.00 | 7832000.00 | 0.00 | 0.00 | KO:K00962 | polyribonucleotide nucleotidyltransferase | Bacteria;Verrucomicrobia;Spartobacteria;unclassified Spartobacteria;unclassified Spartobacteria;unclassified Spartobacteria;Spartobacteria bacterium LR76;Spartobacteria bacterium LR76 |
| Ga0586324 0312556 2 1459 | 0.00 | 1428000.00 | 0.00 | 770002.55 | 0.00 | 1324630.20 | 971843.16 | 982100.00 | 1004400.00 | 0.00 | 0.00 | 0.00 | KO:K00962 | polyribonucleotide nucleotidyltransferase | Bacteria;Proteobacteria;Deltaproteobacteria;Syntrophobacterales;Syntrophobacteraceae;Desulfacinum;Desulfacinum infernum;Desulfacinum infernum DSM 9756 |
| Ga0586324 0319417 980 1999 | 0.00 | 0.00 | 0.00 | 0.00 | 0.00 | 0.00 | 0.00 | 0.00 | 1239300.00 | 0.00 | 0.00 | 0.00 | KO:K00962 | polyribonucleotide nucleotidyltransferase | Bacteria;Proteobacteria;Betaproteobacteria;Nitrosomonadales;Thiobacillaceae;Thiobacillus;Thiobacillus denitrificans;Thiobacillus denitrificans DSM 12475 |
| Ga0586324 0326625 3 1721 | 0.00 | 707200.00 | 0.00 | 0.00 | 0.00 | 0.00 | 0.00 | 464332.00 | 0.00 | 0.00 | 0.00 | 0.00 | KO:K00962 | polyribonucleotide nucleotidyltransferase | Bacteria;Proteobacteria;Deltaproteobacteria;Desulfobacterales;Desulfobacteraceae;Desulfosarcina;Desulfosarcina variabilis;Desulfosarcina variabilis Montpellier |
| Ga0586324 0329812 2 1969 | 0.00 | 0.00 | 0.00 | 0.00 | 0.00 | 0.00 | 0.00 | 2476600.00 | 0.00 | 0.00 | 0.00 | 0.00 | KO:K00962 | polyribonucleotide nucleotidyltransferase | Bacteria;Nitrospirae;Nitrospira;Nitrospirales;Nitrospiraceae;Nitrospira;Nitrospira moscoviensis;Nitrospira moscoviensis NSP M-1 |
| Ga0586324 0443178 3 1151 | 2050000.00 | 0.00 | 0.00 | 0.00 | 0.00 | 0.00 | 0.00 | 0.00 | 0.00 | 0.00 | 0.00 | 0.00 | KO:K00962 | polyribonucleotide nucleotidyltransferase | Bacteria;Verrucomicrobia;Verrucomicrobiae;Verrucomicrobiales;Verrucomicrobia subdivision 3;Pedosphaera;Pedosphaera parvula;Pedosphaera parvula Ellin514 |
| Ga0586324 0462311 137 1696 | 877296.00 | 0.00 | 0.00 | 0.00 | 0.00 | 845915.69 | 587907.12 | 596430.55 | 0.00 | 3934700.00 | 6107200.00 | 832500.00 | KO:K00962 | polyribonucleotide nucleotidyltransferase | Bacteria;Verrucomicrobia;Verrucomicrobiae;Verrucomicrobiales;Verrucomicrobia subdivision 3;Pedosphaera;Pedosphaera parvula;Pedosphaera parvula Ellin514 |
| Ga0586324 0519142 1 1611 | 0.00 | 0.00 | 0.00 | 0.00 | 0.00 | 0.00 | 0.00 | 0.00 | 1838700.00 | 0.00 | 0.00 | 0.00 | KO:K00962 | polyribonucleotide nucleotidyltransferase | Bacteria;Acidobacteria;Vicinamibacteria;unclassified Vicinamibacteria;Vicinamibacteraceae;Luteitalea;Luteitalea pratensis;Luteitalea pratensis DSM 100886 |
| Ga0586324 0587382 1 1527 | 0.00 | 0.00 | 0.00 | 0.00 | 0.00 | 0.00 | 0.00 | 1159000.00 | 0.00 | 0.00 | 0.00 | 0.00 | KO:K00962 | polyribonucleotide nucleotidyltransferase | Bacteria;Proteobacteria;Gammaproteobacteria;Nevskiales;Steroidobacteraceae;Povalibacter;Povalibacter uvarum;Povalibacter uvarum DSM 26723 |
| Ga0586324 0598543 1 1515 | 0.00 | 0.00 | 0.00 | 0.00 | 0.00 | 0.00 | 0.00 | 0.00 | 0.00 | 0.00 | 0.00 | 547621.50 | KO:K00962 | polyribonucleotide nucleotidyltransferase | Bacteria;Acidobacteria;Vicinamibacteria;unclassified Vicinamibacteria;Vicinamibacteraceae;Luteitalea;Luteitalea pratensis;Luteitalea pratensis DSM 100886 |
| Ga0586324 0702725 844 1413 | 0.00 | 625166.16 | 0.00 | 0.00 | 0.00 | 1932020.35 | 0.00 | 0.00 | 0.00 | 0.00 | 0.00 | 0.00 | KO:K00962 | polyribonucleotide nucleotidyltransferase | Bacteria;Proteobacteria;Gammaproteobacteria;Chromatiales;Chromatiaceae;Lamprocystis;Lamprocystis purpurea;Lamprocystis purpurea DSM 4197 |
| Ga0586324 0768569 1 1359 | 0.00 | 0.00 | 0.00 | 0.00 | 0.00 | 0.00 | 0.00 | 0.00 | 519551.82 | 4820200.00 | 7136800.00 | 4905000.00 | KO:K00962 | polyribonucleotide nucleotidyltransferase | Bacteria;Proteobacteria;Betaproteobacteria;Nitrosomonadales;Sulfuricellaceae;Sulfuricella;Sulfuricella denitrificans;Sulfuricella denitrificans skB26 |
| Ga0586324 1046793 2 1033 | 0.00 | 0.00 | 0.00 | 367191.15 | 0.00 | 927602.41 | 0.00 | 738100.00 | 0.00 | 0.00 | 0.00 | 0.00 | KO:K00962 | polyribonucleotide nucleotidyltransferase | Bacteria;Proteobacteria;Deltaproteobacteria;Syntrophobacterales;Syntrophaceae;Desulfobacca;Desulfobacca acetoxidans;Desulfobacca acetoxidans ASRB2, DSM 11109 |
| Ga0586324 1599991 3 992 | 0.00 | 0.00 | 0.00 | 0.00 | 3110600.00 | 0.00 | 0.00 | 0.00 | 0.00 | 0.00 | 0.00 | 0.00 | KO:K00962 | polyribonucleotide nucleotidyltransferase | Bacteria;Bacteroidetes;Bacteroidia;Bacteroidales;Lentimicrobiaceae;Lentimicrobium;Lentimicrobium saccharophilum;Bacteroidales bacterium TBC1 |
| Ga0586324 1600664 1 993 | 0.00 | 0.00 | 0.00 | 0.00 | 0.00 | 0.00 | 19448400.00 | 0.00 | 0.00 | 0.00 | 0.00 | 10725000.00 | KO:K00962 | polyribonucleotide nucleotidyltransferase | Bacteria;Proteobacteria;Gammaproteobacteria;Methylococcales;Methylococcaceae;Methylobacter;Methylobacter tundripaludum;Methylobacter tundripaludum 31/32 |
| Ga0586324 2096603 2 886 | 0.00 | 0.00 | 0.00 | 0.00 | 0.00 | 0.00 | 0.00 | 0.00 | 0.00 | 0.00 | 0.00 | 219087.00 | KO:K00962 | polyribonucleotide nucleotidyltransferase | Bacteria;Verrucomicrobia;Verrucomicrobiae;Verrucomicrobiales;Verrucomicrobiaceae;Roseimicrobium;Roseimicrobium gellanilyticum;Roseimicrobium gellanilyticum DSM 25532 |
| Ga0586324 2242229 2 862 | 0.00 | 0.00 | 0.00 | 0.00 | 0.00 | 0.00 | 134702.40 | 0.00 | 0.00 | 0.00 | 0.00 | 0.00 | KO:K00962 | polyribonucleotide nucleotidyltransferase | Bacteria;Actinobacteria;Actinomycetia;Streptosporangiales;Thermomonosporaceae;Thermomonospora;Thermomonospora cellulosilytica;Thermomonospora cellulosilytica DSM 45823 |
| Ga0586324 2382067 2 838 | 0.00 | 0.00 | 3264302.15 | 0.00 | 0.00 | 0.00 | 0.00 | 0.00 | 0.00 | 0.00 | 0.00 | 0.00 | KO:K00962 | polyribonucleotide nucleotidyltransferase | Bacteria;Proteobacteria;Deltaproteobacteria;Myxococcales;Anaeromyxobacteraceae;Anaeromyxobacter;Anaeromyxobacter dehalogenans;Anaeromyxobacter dehalogenans 2CP-C |
| Ga0586324 2876056 2 526 | 0.00 | 0.00 | 0.00 | 0.00 | 0.00 | 0.00 | 0.00 | 0.00 | 0.00 | 0.00 | 0.00 | 710862.00 | KO:K00962 | polyribonucleotide nucleotidyltransferase | Bacteria;Proteobacteria;Betaproteobacteria;Burkholderiales;unclassified Betaproteobacteria;unclassified Betaproteobacteria;beta proteobacterium AAP65;Rubrivivax sp. AAP65 |
| Ga0586324 2876665 3 776 | 0.00 | 0.00 | 0.00 | 0.00 | 1112400.00 | 0.00 | 0.00 | 0.00 | 0.00 | 0.00 | 0.00 | 0.00 | KO:K00962 | polyribonucleotide nucleotidyltransferase | Bacteria;Firmicutes;Clostridia;Eubacteriales;Oscillospiraceae;Faecalibacterium;Faecalibacterium prausnitzii;Faecalibacterium prausnitzii 2789STDY5834930 |
| Ga0586324 2952665 1 768 | 0.00 | 0.00 | 0.00 | 0.00 | 0.00 | 0.00 | 0.00 | 0.00 | 0.00 | 994400.00 | 0.00 | 0.00 | KO:K00962 | polyribonucleotide nucleotidyltransferase | Bacteria;Verrucomicrobia;Verrucomicrobiae;Verrucomicrobiales;Verrucomicrobia subdivision 3;Pedosphaera;Pedosphaera parvula;Pedosphaera parvula Ellin514 |
| Ga0586324 3407941 3 722 | 0.00 | 0.00 | 0.00 | 2356000.00 | 307652.76 | 0.00 | 0.00 | 0.00 | 0.00 | 0.00 | 0.00 | 0.00 | KO:K00962 | polyribonucleotide nucleotidyltransferase | Bacteria;Acidobacteria;Acidobacteriia;Bryobacterales;Solibacteraceae;Candidatus Solibacter;Candidatus Solibacter usitatus;Candidatus Solibacter usitatus Ellin6076 |
| Ga0586324 3655171 3 701 | 507689.00 | 0.00 | 0.00 | 0.00 | 0.00 | 0.00 | 0.00 | 0.00 | 0.00 | 0.00 | 0.00 | 0.00 | KO:K00962 | polyribonucleotide nucleotidyltransferase | Bacteria;Verrucomicrobia;Spartobacteria;unclassified Spartobacteria;unclassified Spartobacteria;unclassified Spartobacteria;Spartobacteria bacterium LR76;Spartobacteria bacterium LR76 |
| Ga0586324 4028824 1 675 | 0.00 | 0.00 | 0.00 | 0.00 | 0.00 | 0.00 | 0.00 | 0.00 | 0.00 | 0.00 | 0.00 | 378465.75 | KO:K00962 | polyribonucleotide nucleotidyltransferase | Bacteria;Ignavibacteriae;Ignavibacteria;Ignavibacteriales;Ignavibacteriaceae;Ignavibacterium;Ignavibacterium album;Ignavibacterium album Mat9-16, JCM 16511 |
| Ga0586324 5008984 1 615 | 10100000.00 | 0.00 | 0.00 | 0.00 | 0.00 | 0.00 | 0.00 | 0.00 | 0.00 | 0.00 | 0.00 | 0.00 | KO:K00962 | polyribonucleotide nucleotidyltransferase | Bacteria;Proteobacteria;Deltaproteobacteria;Desulfobacterales;Desulfobulbaceae;Desulfurivibrio;Desulfurivibrio alkaliphilus;Desulfurivibrio alkaliphilus AHT2 |
| Ga0586324 5304746 3 602 | 0.00 | 0.00 | 0.00 | 0.00 | 0.00 | 0.00 | 2530800.00 | 0.00 | 0.00 | 0.00 | 0.00 | 0.00 | KO:K00962 | polyribonucleotide nucleotidyltransferase | Bacteria;Proteobacteria;Betaproteobacteria;Nitrosomonadales;Nitrosomonadaceae;Nitrosovibrio;Nitrosovibrio sp. Nv4;Nitrosovibrio sp. Nv4 |
| Ga0586324 5319008 158 601 | 0.00 | 0.00 | 0.00 | 0.00 | 0.00 | 0.00 | 0.00 | 0.00 | 0.00 | 871933.92 | 0.00 | 0.00 | KO:K00962 | polyribonucleotide nucleotidyltransferase | Bacteria;Planctomycetes;Candidatus Brocadiae;Candidatus Brocadiales;Candidatus Brocadiaceae;Candidatus Kuenenia;Candidatus Kuenenia stuttgartiensis;Candidatus Kuenenia stuttgartiensis MBR1 |
| Ga0586324 6372709 2 559 | 0.00 | 0.00 | 0.00 | 0.00 | 0.00 | 3016167.80 | 0.00 | 0.00 | 0.00 | 0.00 | 0.00 | 0.00 | KO:K00962 | polyribonucleotide nucleotidyltransferase | Bacteria;Bacteroidetes;Bacteroidia;Bacteroidales;Salinivirgaceae;Salinivirga;Salinivirga cyanobacteriivorans;Salinivirga cyanobacteriivorans L21-Spi-D4 |
| Ga0586324 6437556 3 557 | 0.00 | 6147200.00 | 4402918.98 | 6526500.00 | 0.00 | 0.00 | 3534000.00 | 3525800.00 | 4592700.00 | 0.00 | 0.00 | 12750000.00 | KO:K00962 | polyribonucleotide nucleotidyltransferase | Bacteria;Proteobacteria;Deltaproteobacteria;unclassified Deltaproteobacteria;unclassified Deltaproteobacteria;unclassified Deltaproteobacteria;delta proteobacterium NaphS2;delta proteobacterium NaphS2 |
| Ga0586324 7074902 1 534 | 0.00 | 0.00 | 0.00 | 0.00 | 0.00 | 0.00 | 0.00 | 281802.31 | 0.00 | 0.00 | 0.00 | 0.00 | KO:K00962 | polyribonucleotide nucleotidyltransferase | Bacteria;Proteobacteria;Deltaproteobacteria;Syntrophobacterales;Syntrophaceae;Desulfobacca;Desulfobacca acetoxidans;Desulfobacca acetoxidans ASRB2, DSM 11109 |
| Ga0586324 7620469 1 519 | 0.00 | 1176400.00 | 0.00 | 0.00 | 0.00 | 0.00 | 0.00 | 0.00 | 0.00 | 0.00 | 0.00 | 0.00 | KO:K00962 | polyribonucleotide nucleotidyltransferase | Bacteria;Acidobacteria;Vicinamibacteria;unclassified Vicinamibacteria;Vicinamibacteraceae;Luteitalea;Luteitalea pratensis;Luteitalea pratensis DSM 100886 |
| Ga0586324 8043305 3 506 | 0.00 | 0.00 | 0.00 | 0.00 | 0.00 | 0.00 | 1552379.04 | 0.00 | 0.00 | 0.00 | 0.00 | 0.00 | KO:K00962 | polyribonucleotide nucleotidyltransferase | Bacteria;Proteobacteria;Deltaproteobacteria;Desulfobacterales;Desulfobacteraceae;Desulfatiglans;Desulfatiglans anilini;Desulfatiglans anilini DSM 4660 |
| Ga0586324 0000002 32082 32951 | 0.00 | 0.00 | 0.00 | 0.00 | 0.00 | 0.00 | 0.00 | 1403000.00 | 0.00 | 3826900.00 | 8400000.00 | 0.00 | KO:K00963 | UTP--glucose-1-phosphate uridylyltransferase | Bacteria;Proteobacteria;Betaproteobacteria;Nitrosomonadales;Thiobacillaceae;Annwoodia;Annwoodia aquaesulis;Annwoodia aquaesulis DSM 4255 |
| Ga0586324 0178351 2 136 | 0.00 | 0.00 | 0.00 | 0.00 | 0.00 | 0.00 | 0.00 | 0.00 | 0.00 | 5597900.00 | 0.00 | 0.00 | KO:K00963 | UTP--glucose-1-phosphate uridylyltransferase | Bacteria;Proteobacteria;Alphaproteobacteria;Hyphomicrobiales;Bradyrhizobiaceae;Bradyrhizobium;Bradyrhizobium sp. URHA0013;Bradyrhizobium sp. URHA0013 |
| Ga0586324 0287651 963 1841 | 0.00 | 0.00 | 0.00 | 0.00 | 0.00 | 0.00 | 0.00 | 0.00 | 0.00 | 451273.13 | 0.00 | 0.00 | KO:K00963 | UTP--glucose-1-phosphate uridylyltransferase | Bacteria;Proteobacteria;Betaproteobacteria;Nitrosomonadales;Nitrosomonadaceae;Nitrosospira;Nitrosospira sp. NpAV;Nitrosospira sp. NpAV |
| Ga0586324 1053206 3 245 | 0.00 | 0.00 | 0.00 | 0.00 | 0.00 | 0.00 | 138928.72 | 0.00 | 0.00 | 0.00 | 0.00 | 0.00 | KO:K00963 | UTP--glucose-1-phosphate uridylyltransferase | Bacteria;Proteobacteria;Betaproteobacteria;Burkholderiales;Oxalobacteraceae;Herbaspirillum;Herbaspirillum rubrisubalbicans;Herbaspirillum seropedicae Os45 |
| Ga0586324 0066025 562 2190 | 0.00 | 0.00 | 0.00 | 0.00 | 0.00 | 0.00 | 0.00 | 1134000.00 | 0.00 | 0.00 | 0.00 | 0.00 | KO:K00970 | poly(A) polymerase | Bacteria;Proteobacteria;Betaproteobacteria;Burkholderiales;Comamonadaceae;Caenimonas;Caenimonas koreensis;Caenimonas koreensis DSM 17982 |
| Ga0586324 0676191 1 1077 | 0.00 | 0.00 | 0.00 | 0.00 | 2719200.00 | 0.00 | 0.00 | 0.00 | 0.00 | 0.00 | 0.00 | 0.00 | KO:K00975 | glucose-1-phosphate adenylyltransferase | Bacteria;Proteobacteria;Gammaproteobacteria;unclassified Gammaproteobacteria;Candidatus Competibacteraceae;Candidatus Contendobacter;Candidatus Contendobacter odensis;Candidatus Contendobacter odensis Run B J11 |
| Ga0586324 7445173 2 523 | 0.00 | 1509600.00 | 0.00 | 0.00 | 0.00 | 0.00 | 0.00 | 0.00 | 0.00 | 0.00 | 0.00 | 0.00 | KO:K00989 | ribonuclease PH | Bacteria;Proteobacteria;Alphaproteobacteria;Hyphomicrobiales;Stappiaceae;Stappia;Stappia sp. ARW1T;Stappia sp. ARW1T |
| Ga0586324 0002895 13062 15833 | 0.00 | 0.00 | 0.00 | 3686000.00 | 0.00 | 0.00 | 0.00 | 0.00 | 0.00 | 0.00 | 0.00 | 0.00 | KO:K01006 | pyruvate,orthophosphate dikinase | Bacteria;Ignavibacteriae;Ignavibacteria;Ignavibacteriales;Ignavibacteriaceae;Ignavibacterium;Ignavibacterium album;Ignavibacterium album Mat9-16, JCM 16511 |
| Ga0586324 0012712 1320 4142 | 0.00 | 0.00 | 0.00 | 0.00 | 6025500.00 | 0.00 | 0.00 | 0.00 | 0.00 | 9086000.00 | 0.00 | 3270000.00 | KO:K01006 | pyruvate,orthophosphate dikinase | Bacteria;Proteobacteria;Deltaproteobacteria;Desulfobacterales;Desulfobacteraceae;Desulfatiglans;Desulfatiglans anilini;Desulfatiglans anilini DSM 4660 |
| Ga0586324 0026074 2270 5020 | 0.00 | 0.00 | 0.00 | 0.00 | 0.00 | 0.00 | 0.00 | 9720000.00 | 0.00 | 0.00 | 0.00 | 0.00 | KO:K01006 | pyruvate,orthophosphate dikinase | Bacteria;Chloroflexi;Anaerolineae;Anaerolineales;Anaerolineaceae;Anaerolinea;Anaerolinea thermophila;Anaerolinea thermophila UNI-1 |
| Ga0586324 0039278 1 1764 | 0.00 | 0.00 | 2951144.66 | 0.00 | 0.00 | 1268946.98 | 0.00 | 0.00 | 0.00 | 0.00 | 0.00 | 0.00 | KO:K01006 | pyruvate,orthophosphate dikinase | Bacteria;Verrucomicrobia;Verrucomicrobiae;Verrucomicrobiales;Verrucomicrobia subdivision 3;Pedosphaera;Pedosphaera parvula;Pedosphaera parvula Ellin514 |
| Ga0586324 0053355 3382 4632 | 0.00 | 0.00 | 0.00 | 0.00 | 1452300.00 | 0.00 | 0.00 | 0.00 | 0.00 | 0.00 | 0.00 | 4665000.00 | KO:K01006 | pyruvate,orthophosphate dikinase | Bacteria;Planctomycetes;Planctomycetia;Pirellulales;Thermoguttaceae;Thermogutta;Thermogutta terrifontis;Thermogutta terrifontis R1 |
| Ga0586324 0055648 2419 4539 | 8190000.00 | 10404000.00 | 0.00 | 4892500.00 | 0.00 | 3819068.54 | 0.00 | 0.00 | 0.00 | 0.00 | 0.00 | 0.00 | KO:K01006 | pyruvate,orthophosphate dikinase | Bacteria;Deferribacteres;Deferribacteres;Deferribacterales;Deferribacteraceae;Deferribacter;Deferribacter desulfuricans;Deferribacter desulfuricans SSM1 |
| Ga0586324 0067701 1 2373 | 0.00 | 0.00 | 0.00 | 0.00 | 1823100.00 | 0.00 | 0.00 | 0.00 | 0.00 | 0.00 | 0.00 | 0.00 | KO:K01006 | pyruvate,orthophosphate dikinase | Bacteria;Ignavibacteriae;Ignavibacteria;Ignavibacteriales;Ignavibacteriaceae;Ignavibacterium;Ignavibacterium album;Ignavibacterium album Mat9-16, JCM 16511 |
| Ga0586324 0070688 563 3391 | 0.00 | 0.00 | 0.00 | 0.00 | 0.00 | 0.00 | 0.00 | 0.00 | 1757700.00 | 1809500.00 | 2173600.00 | 2865000.00 | KO:K01006 | pyruvate,orthophosphate dikinase | Bacteria;Proteobacteria;Gammaproteobacteria;Acidiferrobacterales;Acidiferrobacteraceae;Sulfurifustis;Sulfurifustis variabilis;Sulfurifustis variabilis skN76 |
| Ga0586324 0072270 1322 4003 | 0.00 | 0.00 | 0.00 | 16305834.20 | 7694100.00 | 0.00 | 0.00 | 0.00 | 18468000.00 | 0.00 | 0.00 | 0.00 | KO:K01006 | pyruvate,orthophosphate dikinase | Bacteria;Proteobacteria;Gammaproteobacteria;Chromatiales;Chromatiaceae;Thioflavicoccus;Thioflavicoccus mobilis;Thioflavicoccus mobilis 8321 |
| Ga0586324 0074994 1525 3933 | 4550000.00 | 0.00 | 0.00 | 0.00 | 0.00 | 0.00 | 0.00 | 0.00 | 0.00 | 0.00 | 0.00 | 0.00 | KO:K01006 | pyruvate,orthophosphate dikinase | Bacteria;Nitrospirae;Thermodesulfovibrionia;Thermodesulfovibrionales;Thermodesulfovibrionaceae;Thermodesulfovibrio;Thermodesulfovibrio hydrogeniphilus;Thermodesulfovibrio hydrogeniphilus DSM 18151 |
| Ga0586324 0106064 1549 3336 | 1730000.00 | 0.00 | 0.00 | 0.00 | 0.00 | 0.00 | 0.00 | 0.00 | 0.00 | 0.00 | 0.00 | 0.00 | KO:K01006 | pyruvate,orthophosphate dikinase | Bacteria;Deferribacteres;Deferribacteres;Deferribacterales;Deferribacteraceae;Calditerrivibrio;Calditerrivibrio nitroreducens;Calditerrivibrio nitroreducens Yu37-1, DSM 19672 |
| Ga0586324 0107803 1102 3309 | 0.00 | 0.00 | 0.00 | 1586500.00 | 0.00 | 0.00 | 0.00 | 0.00 | 0.00 | 0.00 | 0.00 | 0.00 | KO:K01006 | pyruvate,orthophosphate dikinase | Bacteria;Proteobacteria;Deltaproteobacteria;Desulfuromonadales;Geobacteraceae;Geobacter;Geobacter lovleyi;Geobacter lovleyi SZ |
| Ga0586324 0124958 1 1827 | 0.00 | 0.00 | 1045890.73 | 0.00 | 0.00 | 0.00 | 0.00 | 0.00 | 0.00 | 0.00 | 0.00 | 0.00 | KO:K01006 | pyruvate,orthophosphate dikinase | Bacteria;Candidatus Marinimicrobia;unclassified Candidatus Marinimicrobia;unclassified Candidatus Marinimicrobia;unclassified Candidatus Marinimicrobia;unclassified Candidatus Marinimicrobia;Marinimicrobia bacterium JGI 0000039-D08;Marinimicrobia bacterium JGI 0000039-D08 (contamination screened) |
| Ga0586324 0263241 1 1446 | 0.00 | 0.00 | 0.00 | 1263500.00 | 0.00 | 0.00 | 0.00 | 0.00 | 939600.00 | 0.00 | 0.00 | 0.00 | KO:K01006 | pyruvate,orthophosphate dikinase | Bacteria;Proteobacteria;Deltaproteobacteria;Syntrophobacterales;Syntrophobacteraceae;Syntrophobacter;Syntrophobacter fumaroxidans;Syntrophobacter fumaroxidans MPOB |
| Ga0586324 0263331 270 2180 | 0.00 | 0.00 | 0.00 | 0.00 | 0.00 | 165549000.00 | 0.00 | 0.00 | 0.00 | 0.00 | 0.00 | 0.00 | KO:K01006 | pyruvate,orthophosphate dikinase | Bacteria;Acidobacteria;Thermoanaerobaculia;Thermoanaerobaculales;Thermoanaerobaculaceae;Thermoanaerobaculum;Thermoanaerobaculum aquaticum;Thermoanaerobaculum aquaticum MP-01 |
| Ga0586324 0307839 2 2032 | 0.00 | 0.00 | 0.00 | 0.00 | 0.00 | 0.00 | 0.00 | 0.00 | 145723.05 | 0.00 | 0.00 | 0.00 | KO:K01006 | pyruvate,orthophosphate dikinase | Bacteria;Proteobacteria;Gammaproteobacteria;Thiotrichales;Thiotrichaceae;Thioploca;Thioploca ingrica;Thioploca ingrica Lake Okotanpe |
| Ga0586324 0366005 3 1655 | 0.00 | 5841200.00 | 0.00 | 0.00 | 0.00 | 3463828.97 | 0.00 | 0.00 | 0.00 | 0.00 | 0.00 | 2167500.00 | KO:K01006 | pyruvate,orthophosphate dikinase | Bacteria;Planctomycetes;Planctomycetia;Pirellulales;Thermoguttaceae;Thermogutta;Thermogutta terrifontis;Thermogutta terrifontis R1 |
| Ga0586324 0373156 2 1864 | 8280000.00 | 1516400.00 | 0.00 | 6346000.00 | 1091800.00 | 0.00 | 0.00 | 7088200.00 | 826200.00 | 2340800.00 | 2710400.00 | 2715000.00 | KO:K01006 | pyruvate,orthophosphate dikinase | Bacteria;Proteobacteria;Gammaproteobacteria;unclassified Gammaproteobacteria;Candidatus Competibacteraceae;Candidatus Contendobacter;Candidatus Contendobacter odensis;Candidatus Contendobacter odensis Run B J11 |
| Ga0586324 0407685 1 1794 | 0.00 | 0.00 | 0.00 | 0.00 | 0.00 | 0.00 | 0.00 | 0.00 | 0.00 | 0.00 | 0.00 | 2415000.00 | KO:K01006 | pyruvate,orthophosphate dikinase | Bacteria;Chloroflexi;Anaerolineae;Anaerolineales;Anaerolineaceae;Thermanaerothrix;Thermanaerothrix daxensis;Thermanaerothrix daxensis GNS-1 |
| Ga0586324 0490081 236 1654 | 0.00 | 0.00 | 0.00 | 0.00 | 0.00 | 0.00 | 0.00 | 1842200.00 | 1530900.00 | 0.00 | 0.00 | 0.00 | KO:K01006 | pyruvate,orthophosphate dikinase | Bacteria;Proteobacteria;Gammaproteobacteria;unclassified Gammaproteobacteria;Candidatus Competibacteraceae;Candidatus Competibacter;Candidatus Competibacter denitrificans;Candidatus Competibacter denitrificans Run A D11 |
| Ga0586324 0529416 2 1402 | 949465.00 | 6745600.00 | 0.00 | 1586500.00 | 4511400.00 | 0.00 | 0.00 | 6161000.00 | 4617000.00 | 4697000.00 | 5438400.00 | 2092500.00 | KO:K01006 | pyruvate,orthophosphate dikinase | Bacteria;Proteobacteria;Gammaproteobacteria;Methylococcales;Methylococcaceae;Methyloglobulus;Methyloglobulus morosus;Methyloglobulus morosus KoM1 |

| | | | | | | | | | | | | | | | |
|---|---|---|---|---|---|---|---|---|---|---|---|---|---|---|---|
| Ga0586324 0573186 2 1543 | 0.00 | 0.00 | 0.00 | 0.00 | 0.00 | 0.00 | 4468800.00 | 0.00 | 0.00 | 0.00 | 0.00 | 0.00 | KO:K01006 | pyruvate,orthophosphate dikinase | Bacteria;Verrucomicrobia;Verrucomicrobiae;Verrucomicrobiales;Verrucomicrobiaceae;Prosthecobacter;Prosthecobacter vanneervenii;Prosthecobacter vanneervenii DSM 12252 |
| Ga0586324 0588571 127 1527 | 0.00 | 0.00 | 0.00 | 0.00 | 0.00 | 0.00 | 0.00 | 0.00 | 0.00 | 1894200.00 | 0.00 | 0.00 | KO:K01006 | pyruvate,orthophosphate dikinase | Bacteria;Proteobacteria;Gammaproteobacteria;unclassified Gammaproteobacteria;Candidatus Competibacteraceae;Candidatus Competibacter;Candidatus Competibacter denitrificans;Candidatus Competibacter denitrificans Run A D11 |
| Ga0586324 0645498 3 857 | 0.00 | 0.00 | 0.00 | 2498500.00 | 0.00 | 0.00 | 0.00 | 0.00 | 4001400.00 | 0.00 | 0.00 | 0.00 | KO:K01006 | pyruvate,orthophosphate dikinase | Bacteria;Verrucomicrobia;Verrucomicrobiae;Verrucomicrobiales;Verrucomicrobiaceae;Prosthecobacter;Prosthecobacter vanneervenii;Prosthecobacter vanneervenii DSM 12252 |
| Ga0586324 0681531 664 1431 | 0.00 | 8908000.00 | 0.00 | 0.00 | 288256.83 | 0.00 | 0.00 | 0.00 | 0.00 | 0.00 | 0.00 | 0.00 | KO:K01006 | pyruvate,orthophosphate dikinase | Bacteria;Proteobacteria;Gammaproteobacteria;unclassified Gammaproteobacteria;Candidatus Competibacteraceae;Candidatus Competibacter;Candidatus Competibacter denitrificans;Candidatus Competibacter denitrificans Run A D11 |
| Ga0586324 0708751 2 463 | 0.00 | 1482400.00 | 0.00 | 0.00 | 0.00 | 0.00 | 0.00 | 0.00 | 0.00 | 0.00 | 0.00 | 0.00 | KO:K01006 | pyruvate,orthophosphate dikinase | Bacteria;Proteobacteria;Gammaproteobacteria;unclassified Gammaproteobacteria;Candidatus Competibacteraceae;Candidatus Competibacter;Candidatus Competibacter denitrificans;Candidatus Competibacter denitrificans Run A D11 |
| Ga0586324 0815359 2 1327 | 0.00 | 1720400.00 | 2750632.21 | 0.00 | 0.00 | 0.00 | 0.00 | 732000.00 | 0.00 | 0.00 | 0.00 | 0.00 | KO:K01006 | pyruvate,orthophosphate dikinase | Bacteria;Planctomycetes;Planctomycetia;Pirellulales;Thermoguttaceae;Thermogutta;Thermogutta terrifontis;Thermogutta terrifontis R1 |
| Ga0586324 0842789 2 1306 | 0.00 | 0.00 | 0.00 | 0.00 | 0.00 | 2228067.22 | 0.00 | 0.00 | 0.00 | 0.00 | 0.00 | 0.00 | KO:K01006 | pyruvate,orthophosphate dikinase | Bacteria;Planctomycetes;Planctomycetia;Pirellulales;Thermoguttaceae;Thermogutta;Thermogutta terrifontis;Thermogutta terrifontis R1 |
| Ga0586324 0857313 2 1297 | 2430000.00 | 0.00 | 0.00 | 4598000.00 | 6355100.00 | 0.00 | 0.00 | 3605100.00 | 3539700.00 | 0.00 | 0.00 | 0.00 | KO:K01006 | pyruvate,orthophosphate dikinase | Bacteria;Proteobacteria;Deltaproteobacteria;Syntrophobacterales;Syntrophaceae;Desulfobacca;Desulfobacca acetoxidans;Desulfobacca acetoxidans ASRB2, DSM 11109 |
| Ga0586324 0899167 1 1272 | 0.00 | 2284800.00 | 0.00 | 4845000.00 | 0.00 | 6232900.00 | 5791200.00 | 0.00 | 0.00 | 0.00 | 0.00 | 0.00 | KO:K01006 | pyruvate,orthophosphate dikinase | Bacteria;Proteobacteria;Deltaproteobacteria;Syntrophobacterales;Syntrophaceae;Desulfobacca;Desulfobacca acetoxidans;Desulfobacca acetoxidans ASRB2, DSM 11109 |
| Ga0586324 1006885 2 922 | 0.00 | 0.00 | 0.00 | 0.00 | 0.00 | 0.00 | 0.00 | 0.00 | 0.00 | 0.00 | 1355200.00 | 0.00 | KO:K01006 | pyruvate,orthophosphate dikinase | Bacteria;Proteobacteria;Betaproteobacteria;Nitrosomonadales;Sulfuricellaceae;Sulfuricella;Sulfuricella sp. T08;Sulfuricella sp. T08 |
| Ga0586324 1024913 148 1200 | 0.00 | 0.00 | 0.00 | 0.00 | 3440200.00 | 1530327.81 | 0.00 | 2098400.00 | 0.00 | 0.00 | 4109600.00 | 0.00 | KO:K01006 | pyruvate,orthophosphate dikinase | Bacteria;Verrucomicrobia;Opitutae;Opitutales;Opitutaceae;unclassified Opitutaceae;Opitutaceae bacterium;Opitutaceae bacterium TAV3 |
| Ga0586324 1417947 3 1046 | 0.00 | 0.00 | 0.00 | 0.00 | 0.00 | 0.00 | 0.00 | 454523.81 | 0.00 | 0.00 | 0.00 | 0.00 | KO:K01006 | pyruvate,orthophosphate dikinase | Bacteria;Ignavibacteriae;Ignavibacteria;Ignavibacteriales;Ignavibacteriaceae;Ignavibacterium;Ignavibacterium album;Ignavibacterium album Mat9-16, JCM 16511 |
| Ga0586324 1489919 1 1023 | 0.00 | 0.00 | 0.00 | 0.00 | 0.00 | 2439231.52 | 0.00 | 0.00 | 0.00 | 0.00 | 0.00 | 0.00 | KO:K01006 | pyruvate,orthophosphate dikinase | Bacteria;Proteobacteria;Gammaproteobacteria;unclassified Gammaproteobacteria;Candidatus Competibacteraceae;Candidatus Competibacter;Candidatus Competibacter denitrificans;Candidatus Competibacter denitrificans Run A D11 |
| Ga0586324 1753045 1 954 | 0.00 | 0.00 | 0.00 | 0.00 | 0.00 | 0.00 | 0.00 | 0.00 | 431321.76 | 0.00 | 0.00 | 0.00 | KO:K01006 | pyruvate,orthophosphate dikinase | Bacteria;Ignavibacteriae;Ignavibacteria;Ignavibacteriales;Ignavibacteriaceae;Ignavibacterium;Ignavibacterium album;Ignavibacterium album Mat9-16, JCM 16511 |
| Ga0586324 1757151 2 955 | 0.00 | 0.00 | 0.00 | 0.00 | 0.00 | 0.00 | 0.00 | 817400.00 | 0.00 | 0.00 | 0.00 | 324107.25 | KO:K01006 | pyruvate,orthophosphate dikinase | Bacteria;Verrucomicrobia;Verrucomicrobiae;Verrucomicrobiales;Verrucomicrobia subdivision 3;Pedosphaera;Pedosphaera parvula;Pedosphaera parvula Ellin514 |
| Ga0586324 2128486 1 852 | 0.00 | 0.00 | 436782.16 | 0.00 | 0.00 | 0.00 | 0.00 | 0.00 | 0.00 | 0.00 | 0.00 | 0.00 | KO:K01006 | pyruvate,orthophosphate dikinase | Bacteria;Firmicutes;Clostridia;Eubacteriales;Oscillospiraceae;Thermoclostridium;Thermoclostridium caenicola;Thermoclostridium caenicola DSM 19027 |
| Ga0586324 3099012 1 750 | 0.00 | 0.00 | 0.00 | 2346500.00 | 0.00 | 0.00 | 0.00 | 0.00 | 0.00 | 0.00 | 0.00 | 0.00 | KO:K01006 | pyruvate,orthophosphate dikinase | Bacteria;Proteobacteria;Deltaproteobacteria;Desulfobacterales;Desulfobacteraceae;Desulfobotulus;Desulfobotulus sapovorans;Desulfobotulus sapovorans DSM 2055 |
| Ga0586324 3112058 3 749 | 0.00 | 1373600.00 | 0.00 | 0.00 | 0.00 | 0.00 | 0.00 | 0.00 | 0.00 | 0.00 | 0.00 | 0.00 | KO:K01006 | pyruvate,orthophosphate dikinase | Bacteria;Bacteroidetes;Bacteroidia;Bacteroidales;Lentimicrobiaceae;Lentimicrobium;Lentimicrobium saccharophilum;Bacteroidales bacterium TBC1 |
| Ga0586324 3971727 2 679 | 0.00 | 0.00 | 0.00 | 386348.85 | 0.00 | 4843400.00 | 0.00 | 0.00 | 0.00 | 0.00 | 0.00 | 0.00 | KO:K01006 | pyruvate,orthophosphate dikinase | Bacteria;Planctomycetes;Planctomycetia;Pirellulales;Thermoguttaceae;Thermogutta;Thermogutta terrifontis;Thermogutta terrifontis R1 |
| Ga0586324 3988390 3 677 | 8840000.00 | 0.00 | 0.00 | 0.00 | 0.00 | 0.00 | 0.00 | 0.00 | 0.00 | 0.00 | 0.00 | 0.00 | KO:K01006 | pyruvate,orthophosphate dikinase | Bacteria;Planctomycetes;Planctomycetia;Pirellulales;Thermoguttaceae;Thermogutta;Thermogutta terrifontis;Thermogutta terrifontis R1 |
| Ga0586324 4192866 15 662 | 0.00 | 0.00 | 0.00 | 0.00 | 0.00 | 0.00 | 0.00 | 3977200.00 | 0.00 | 0.00 | 0.00 | 0.00 | KO:K01006 | pyruvate,orthophosphate dikinase | Bacteria;Chloroflexi;Thermoflexia;Thermoflexales;Thermoflexaceae;Thermoflexus;Thermoflexus hugenholtzii;Thermoflexus hugenholtzii JAD2 |
| Ga0586324 4200438 15 662 | 0.00 | 0.00 | 0.00 | 0.00 | 3594700.00 | 0.00 | 0.00 | 0.00 | 0.00 | 0.00 | 0.00 | 0.00 | KO:K01006 | pyruvate,orthophosphate dikinase | Bacteria;Verrucomicrobia;Opitutae;Opitutales;Opitutaceae;unclassified Opitutaceae;Opitutaceae bacterium EW11;Opitutaceae bacterium EW11 |
| Ga0586324 4371104 1 522 | 0.00 | 0.00 | 0.00 | 0.00 | 0.00 | 0.00 | 1854734.40 | 0.00 | 1579500.00 | 0.00 | 0.00 | 0.00 | KO:K01006 | pyruvate,orthophosphate dikinase | Bacteria;Proteobacteria;Deltaproteobacteria;Syntrophobacterales;Syntrophaceae;Desulfobacca;Desulfobacca acetoxidans;Desulfobacca acetoxidans ASRB2, DSM 11109 |
| Ga0586324 5236640 3 605 | 0.00 | 0.00 | 0.00 | 0.00 | 0.00 | 0.00 | 0.00 | 2257000.00 | 0.00 | 0.00 | 0.00 | 0.00 | KO:K01006 | pyruvate,orthophosphate dikinase | Bacteria;Planctomycetes;Planctomycetia;Pirellulales;Thermoguttaceae;Thermogutta;Thermogutta terrifontis;Thermogutta terrifontis R1 |
| Ga0586324 6170799 2 565 | 0.00 | 0.00 | 0.00 | 0.00 | 0.00 | 0.00 | 0.00 | 0.00 | 0.00 | 0.00 | 580466.48 | 0.00 | KO:K01006 | pyruvate,orthophosphate dikinase | Bacteria;Proteobacteria;Deltaproteobacteria;unclassified Deltaproteobacteria;unclassified Deltaproteobacteria;unclassified Deltaproteobacteria;delta proteobacterium NaphS2;delta proteobacterium NaphS2 |
| Ga0586324 6341735 2 559 | 0.00 | 0.00 | 0.00 | 1121000.00 | 0.00 | 0.00 | 0.00 | 0.00 | 0.00 | 0.00 | 0.00 | 0.00 | KO:K01006 | pyruvate,orthophosphate dikinase | Bacteria;Proteobacteria;Deltaproteobacteria;Myxococcales;Anaeromyxobacteraceae;Anaeromyxobacter;Anaeromyxobacter sp. Fw109-5;Anaeromyxobacter sp. Fw109-5 |
| Ga0586324 6344377 1 558 | 0.00 | 0.00 | 0.00 | 0.00 | 0.00 | 0.00 | 0.00 | 0.00 | 330219.99 | 0.00 | 0.00 | 0.00 | KO:K01006 | pyruvate,orthophosphate dikinase | Bacteria;Verrucomicrobia;Opitutae;Opitutales;Opitutaceae;Opitutus;Opitutus sp. ER46;Opitutus sp. ER46 |
| Ga0586324 6430069 2 556 | 0.00 | 215389.32 | 0.00 | 0.00 | 0.00 | 0.00 | 0.00 | 0.00 | 0.00 | 0.00 | 0.00 | 0.00 | KO:K01006 | pyruvate,orthophosphate dikinase | Bacteria;Bacteroidetes;Bacteroidia;Marinilabiliales;Prolixibacteraceae;Draconibacterium;Draconibacterium orientale;Draconibacterium orientale FH5 |
| Ga0586324 6527845 3 551 | 0.00 | 0.00 | 0.00 | 0.00 | 1740700.00 | 0.00 | 0.00 | 0.00 | 0.00 | 0.00 | 0.00 | 0.00 | KO:K01006 | pyruvate,orthophosphate dikinase | Bacteria;Chloroflexi;Thermoflexia;Thermoflexales;Thermoflexaceae;Thermoflexus;Thermoflexus hugenholtzii;Thermoflexus hugenholtzii JAD2 |
| Ga0586324 6647208 1 549 | 0.00 | 9363600.00 | 0.00 | 0.00 | 0.00 | 0.00 | 0.00 | 0.00 | 0.00 | 0.00 | 0.00 | 0.00 | KO:K01006 | pyruvate,orthophosphate dikinase | Bacteria;Acidobacteria;Acidobacteriia;Bryobacterales;Solibacteraceae;Candidatus Solibacter;Candidatus Solibacter usitatus;Candidatus Solibacter usitatus Ellin6076 |
| Ga0586324 7040427 2 535 | 0.00 | 0.00 | 0.00 | 0.00 | 0.00 | 0.00 | 0.00 | 1640900.00 | 0.00 | 0.00 | 0.00 | 0.00 | KO:K01006 | pyruvate,orthophosphate dikinase | Bacteria;Proteobacteria;Deltaproteobacteria;Syntrophobacterales;Syntrophaceae;Desulfobacca;Desulfobacca acetoxidans;Desulfobacca acetoxidans ASRB2, DSM 11109 |
| Ga0586324 7639387 1 516 | 0.00 | 0.00 | 0.00 | 2945000.00 | 0.00 | 0.00 | 0.00 | 0.00 | 0.00 | 0.00 | 0.00 | 0.00 | KO:K01006 | pyruvate,orthophosphate dikinase | Bacteria;Bacteroidetes;Bacteroidia;Bacteroidales;Bacteroidaceae;Bacteroides;Bacteroides acidifaciens;Bacteroides acidifaciens 1e8A |
| Ga0586324 0062816 2755 4281 | 3650000.00 | 3250400.00 | 4672601.61 | 4294000.00 | 4253900.00 | 0.00 | 8048400.00 | 4050400.00 | 3717900.00 | 3842300.00 | 3792800.00 | 3165000.00 | KO:K01007 | pyruvate,water dikinase | Bacteria;Proteobacteria;Deltaproteobacteria;Desulfobacterales;Desulfobacteraceae;Desulfatiglans;Desulfatiglans anilini;Desulfatiglans anilini DSM 4660 |
| Ga0586324 0135424 92 1774 | 0.00 | 0.00 | 0.00 | 4854500.00 | 0.00 | 0.00 | 0.00 | 0.00 | 0.00 | 0.00 | 0.00 | 0.00 | KO:K01007 | pyruvate,water dikinase | Bacteria;Proteobacteria;Deltaproteobacteria;Desulfobacterales;Desulfobacteraceae;Desulfatiglans;Desulfatiglans anilini;Desulfatiglans anilini DSM 4660 |
| Ga0586324 0234527 1038 2300 | 0.00 | 0.00 | 0.00 | 3809500.00 | 0.00 | 0.00 | 0.00 | 0.00 | 0.00 | 0.00 | 0.00 | 0.00 | KO:K01007 | pyruvate,water dikinase | Bacteria;Proteobacteria;Deltaproteobacteria;Desulfobacterales;Desulfobacteraceae;Desulfatiglans;Desulfatiglans anilini;Desulfatiglans anilini DSM 4660 |
| Ga0586324 0455699 654 1706 | 0.00 | 0.00 | 0.00 | 0.00 | 0.00 | 2377692.55 | 0.00 | 0.00 | 0.00 | 0.00 | 0.00 | 0.00 | KO:K01007 | pyruvate,water dikinase | Bacteria;Proteobacteria;Gammaproteobacteria;Chromatiales;Ectothiorhodospiraceae;Inmirania;Inmirania thermothiophila;Inmirania thermothiophila DSM 100275 |
| Ga0586324 0456925 157 1704 | 0.00 | 2148800.00 | 0.00 | 0.00 | 0.00 | 0.00 | 0.00 | 0.00 | 0.00 | 0.00 | 0.00 | 0.00 | KO:K01007 | pyruvate,water dikinase | Bacteria;Proteobacteria;Deltaproteobacteria;Desulfobacterales;Desulfobacteraceae;Desulfatiglans;Desulfatiglans anilini;Desulfatiglans anilini DSM 4660 |
| Ga0586324 0638308 2 1048 | 5360000.00 | 7881200.00 | 4575326.47 | 0.00 | 7642600.00 | 0.00 | 2121259.56 | 1537200.00 | 3604500.00 | 13767600.00 | 16456000.00 | 8025000.00 | KO:K01007 | pyruvate,water dikinase | Bacteria;Proteobacteria;Deltaproteobacteria;Desulfobacterales;Desulfobacteraceae;Desulfatiglans;Desulfatiglans anilini;Desulfatiglans anilini DSM 4660 |
| Ga0586324 0828998 229 1317 | 683854.00 | 1319200.00 | 0.00 | 0.00 | 0.00 | 0.00 | 0.00 | 0.00 | 0.00 | 0.00 | 0.00 | 0.00 | KO:K01007 | pyruvate,water dikinase | Bacteria;Proteobacteria;Deltaproteobacteria;Desulfobacterales;Desulfobacteraceae;Desulfatiglans;Desulfatiglans anilini;Desulfatiglans anilini DSM 4660 |
| Ga0586324 0946464 2 1243 | 6580000.00 | 0.00 | 0.00 | 0.00 | 0.00 | 0.00 | 0.00 | 0.00 | 3207600.00 | 0.00 | 0.00 | 0.00 | KO:K01007 | pyruvate,water dikinase | Bacteria;Proteobacteria;Deltaproteobacteria;Desulfobacterales;Desulfobacteraceae;Desulfatiglans;Desulfatiglans anilini;Desulfatiglans anilini DSM 4660 |
| Ga0586324 1109716 150 1160 | 0.00 | 0.00 | 0.00 | 0.00 | 0.00 | 0.00 | 5335200.00 | 0.00 | 0.00 | 4134900.00 | 0.00 | 0.00 | KO:K01007 | pyruvate,water dikinase | Bacteria;Proteobacteria;Deltaproteobacteria;Desulfobacterales;Desulfobacteraceae;Desulfatiglans;Desulfatiglans anilini;Desulfatiglans anilini DSM 4660 |
| Ga0586324 6068272 3 569 | 0.00 | 0.00 | 0.00 | 0.00 | 0.00 | 0.00 | 0.00 | 0.00 | 0.00 | 0.00 | 0.00 | 2580000.00 | KO:K01007 | pyruvate,water dikinase | Bacteria;Proteobacteria;Deltaproteobacteria;Desulfobacterales;Desulfobacteraceae;Desulfatiglans;Desulfatiglans anilini;Desulfatiglans anilini DSM 4660 |
| Ga0586324 0003104 696 1733 | 0.00 | 7344000.00 | 0.00 | 0.00 | 0.00 | 12942200.00 | 0.00 | 0.00 | 0.00 | 8778000.00 | 9240000.00 | 0.00 | KO:K01039 | glutaconate CoA-transferase subunit A | Bacteria;Proteobacteria;Deltaproteobacteria;Syntrophobacterales;Syntrophobacteraceae;Syntrophobacter;Syntrophobacter fumaroxidans;Syntrophobacter fumaroxidans MPOB |
| Ga0586324 0056515 3902 4504 | 4640000.00 | 7140000.00 | 0.00 | 8987000.00 | 0.00 | 0.00 | 0.00 | 0.00 | 0.00 | 3056900.00 | 3836800.00 | 0.00 | KO:K01039 | glutaconate CoA-transferase subunit A | Bacteria;Proteobacteria;Deltaproteobacteria;Desulfobacterales;Desulfobacteraceae;Desulfobacula;Desulfobacula toluolica;Desulfobacula toluolica Tol2 |
| Ga0586324 2580066 3 812 | 5410000.00 | 5868400.00 | 0.00 | 5196500.00 | 6870100.00 | 1482124.07 | 0.00 | 4331000.00 | 1887300.00 | 0.00 | 0.00 | 0.00 | KO:K01039 | glutaconate CoA-transferase subunit A | Bacteria;Firmicutes;Bacilli;Bacillales;Bacillaceae;Quasibacillus;Quasibacillus thermotolerans;Quasibacillus thermotolerans SGZ-8 |
| Ga0586324 3454815 31 717 | 0.00 | 0.00 | 0.00 | 1453500.00 | 0.00 | 0.00 | 0.00 | 0.00 | 0.00 | 0.00 | 0.00 | 0.00 | KO:K01039 | glutaconate CoA-transferase subunit A | Bacteria;Proteobacteria;Deltaproteobacteria;Syntrophobacterales;Syntrophobacteraceae;Syntrophobacter;Syntrophobacter fumaroxidans;Syntrophobacter fumaroxidans MPOB |
| Ga0586324 7613355 20 517 | 0.00 | 7276000.00 | 0.00 | 0.00 | 0.00 | 0.00 | 0.00 | 0.00 | 0.00 | 0.00 | 0.00 | 0.00 | KO:K01039 | glutaconate CoA-transferase subunit A | Bacteria;Firmicutes;Bacilli;Bacillales;Bacillaceae;Quasibacillus;Quasibacillus thermotolerans;Quasibacillus thermotolerans SGZ-8 |
| Ga0586324 0003104 1736 2503 | 15570000.00 | 19108000.00 | 0.00 | 0.00 | 0.00 | 901809.32 | 5084400.00 | 0.00 | 4414500.00 | 3726800.00 | 2666400.00 | 5017500.00 | KO:K01040 | glutaconate CoA-transferase subunit B | Bacteria;Firmicutes;Bacilli;Bacillales;Alicyclobacillaceae;Effusibacillus;Effusibacillus lacus;Effusibacillus lacus DSM 27172 |
| Ga0586324 0079188 690 1589 | 0.00 | 2686000.00 | 2875105.88 | 0.00 | 0.00 | 2771603.89 | 0.00 | 0.00 | 1409400.00 | 0.00 | 0.00 | 0.00 | KO:K01040 | glutaconate CoA-transferase subunit B | Bacteria;Firmicutes;Clostridia;Eubacteriales;Peptococcaceae;Desulfofarcimen;Desulfofarcimen acetoxidans;Desulfofarcimen acetoxidans DSM 771 |
| Ga0586324 0468029 1326 1688 | 2570000.00 | 0.00 | 0.00 | 0.00 | 0.00 | 0.00 | 0.00 | 0.00 | 0.00 | 0.00 | 0.00 | 0.00 | KO:K01040 | glutaconate CoA-transferase subunit B | Bacteria;Firmicutes;Clostridia;Eubacteriales;Peptococcaceae;Desulfofarcimen;Desulfofarcimen acetoxidans;Desulfofarcimen acetoxidans DSM 771 |
| Ga0586324 1911111 79 846 | 5400000.00 | 7344000.00 | 1856115.29 | 5985000.00 | 1555300.00 | 1594328.18 | 3643672.56 | 4373700.00 | 1020600.00 | 877800.00 | 808435.76 | 3609060.00 | KO:K01040 | glutaconate CoA-transferase subunit B | Bacteria;Firmicutes;Bacilli;Bacillales;Alicyclobacillaceae;Effusibacillus;Effusibacillus lacus;Effusibacillus lacus DSM 27172 |
| Ga0586324 3524985 1 552 | 277392.00 | 0.00 | 0.00 | 0.00 | 5644400.00 | 0.00 | 29001600.00 | 0.00 | 0.00 | 2610300.00 | 0.00 | 0.00 | KO:K01040 | glutaconate CoA-transferase subunit B | Bacteria;Firmicutes;Clostridia;Eubacteriales;Peptococcaceae;Desulfosporosinus;Desulfosporosinus orientis;Desulfosporosinus orientis Singapore I, DSM 765 |
| Ga0586324 7610785 2 334 | 0.00 | 0.00 | 0.00 | 0.00 | 3399000.00 | 0.00 | 0.00 | 0.00 | 0.00 | 0.00 | 0.00 | 0.00 | KO:K01040 | glutaconate CoA-transferase subunit B | Bacteria;Proteobacteria;Deltaproteobacteria;Syntrophobacterales;Syntrophobacteraceae;Syntrophobacter;Syntrophobacter fumaroxidans;Syntrophobacter fumaroxidans MPOB |
| Ga0586324 1483937 255 1025 | 0.00 | 0.00 | 0.00 | 0.00 | 0.00 | 0.00 | 0.00 | 0.00 | 0.00 | 0.00 | 2684000.00 | 0.00 | KO:K01046 | triacylglycerol lipase | Bacteria;Actinobacteria;Actinomycetia;Streptomycetales;Streptomycetaceae;Streptomyces;Streptomyces sp. Mg1;Streptomyces sp. Mg1 |
| Ga0586324 0006638 3229 4302 | 0.00 | 0.00 | 0.00 | 6555000.00 | 0.00 | 0.00 | 0.00 | 0.00 | 0.00 | 0.00 | 0.00 | 0.00 | KO:K01053 | gluconolactonase | Bacteria;Proteobacteria;Betaproteobacteria;Burkholderiales;unclassified Burkholderiales;unclassified Burkholderiales;Burkholderiales bacterium JOSHI 001;Burkholderiales sp. JOSHI 001 |
| Ga0586324 0018497 421 1656 | 0.00 | 0.00 | 0.00 | 0.00 | 0.00 | 0.00 | 0.00 | 0.00 | 0.00 | 0.00 | 0.00 | 885000.00 | KO:K01053 | gluconolactonase | Bacteria;Proteobacteria;Alphaproteobacteria;Hyphomicrobiales;Boseaceae;Bosea;Bosea sp. LC85;Bosea sp. LC85 |
| Ga0586324 0057890 2038 3096 | 7690000.00 | 7140000.00 | 5967800.00 | 7391000.00 | 8559300.00 | 4327300.00 | 0.00 | 0.00 | 4479300.00 | 4858700.00 | 0.00 | 7320000.00 | KO:K01053 | gluconolactonase | Bacteria;Proteobacteria;Betaproteobacteria;Burkholderiales;Burkholderiaceae;Cupriavidus;Cupriavidus sp. ASC-9842;Cupriavidus tamaulipasensis ASC-9842 |
| Ga0586324 0365164 1340 1882 | 0.00 | 0.00 | 0.00 | 0.00 | 0.00 | 0.00 | 0.00 | 0.00 | 0.00 | 0.00 | 0.00 | 400068.00 | KO:K01053 | gluconolactonase | Bacteria;Proteobacteria;Alphaproteobacteria;Hyphomicrobiales;unclassified Hyphomicrobiales;unclassified Hyphomicrobiales;Rhizobiales bacterium YIM 77505;Rhizobiales bacterium YIM 77505 |
| Ga0586324 0669765 544 1443 | 0.00 | 2652000.00 | 4073518.94 | 2593500.00 | 3687400.00 | 3016862.55 | 1483032.84 | 0.00 | 5556600.00 | 4727800.00 | 2666400.00 | 4838445.00 | KO:K01053 | gluconolactonase | Bacteria;Proteobacteria;Alphaproteobacteria;Rhodospirillales;Acetobacteraceae;Rhodopila;Rhodopila globiformis;Rhodopila globiformis DSM 161 |
| Ga0586324 5747177 3 563 | 0.00 | 0.00 | 11597800.00 | 0.00 | 0.00 | 0.00 | 0.00 | 0.00 | 0.00 | 5428500.00 | 0.00 | 0.00 | KO:K01053 | gluconolactonase | Bacteria;Proteobacteria;Alphaproteobacteria;Rhodospirillales;Acetobacteraceae;Rhodopila;Rhodopila globiformis;Rhodopila globiformis DSM 161 |
| Ga0586324 4568759 1 519 | 0.00 | 0.00 | 0.00 | 0.00 | 0.00 | 0.00 | 0.00 | 0.00 | 0.00 | 0.00 | 0.00 | 683942.25 | KO:K01061 | carboxymethylenebutenolidase | Bacteria;Proteobacteria;Alphaproteobacteria;Hyphomicrobiales;Reyranellaceae;Reyranella;Reyranella sp. OAS946;Reyranella sp. OAS946 |
| Ga0586324 2160468 188 874 | 3290000.00 | 0.00 | 0.00 | 0.00 | 0.00 | 0.00 | 0.00 | 0.00 | 0.00 | 0.00 | 0.00 | 0.00 | KO:K01077 | alkaline phosphatase | Bacteria;Firmicutes;Clostridia;Eubacteriales;Desulfallaceae;Desulfoscipio;Desulfoscipio gibsoniae;Desulfallas gibsoniae Groll, DSM 7213 |
| Ga0586324 1113056 3 1160 | 4240000.00 | 0.00 | 0.00 | 0.00 | 0.00 | 0.00 | 0.00 | 0.00 | 0.00 | 0.00 | 0.00 | 0.00 | KO:K01081 | 5'-nucleotidase | Bacteria;Actinobacteria;Actinomycetia;Propionibacteriales;Nocardioidaceae;Nocardioides;Nocardioides sp. Root122;Nocardioides sp. Root122 |
| Ga0586324 4185838 2 664 | 1320000.00 | 0.00 | 0.00 | 0.00 | 0.00 | 0.00 | 0.00 | 0.00 | 0.00 | 0.00 | 0.00 | 0.00 | KO:K01081 | 5'-nucleotidase | Bacteria;Actinobacteria;Actinomycetia;Propionibacteriales;Nocardioidaceae;Nocardioides;Nocardioides soli;Nocardioides soli DSM 105498 |
| Ga0586324 0003161 11133 15230 | 0.00 | 0.00 | 260243.94 | 0.00 | 0.00 | 1418250.74 | 970582.32 | 0.00 | 1085400.00 | 1500923.27 | 463838.32 | 832500.00 | KO:K01119 | 2',3'-cyclic-nucleotide 2'-phosphodiesterase/3'-nucleotidase | Bacteria;Actinobacteria;Actinomycetia;Micrococcales;Beutenbergiaceae;Salana;Salana multivorans;Salana multivorans DSM 13521 |
| Ga0586324 2050843 2 895 | 0.00 | 3706000.00 | 0.00 | 0.00 | 0.00 | 0.00 | 0.00 | 0.00 | 0.00 | 0.00 | 0.00 | 0.00 | KO:K01178 | glucoamylase | Bacteria;Chloroflexi;Ktedonobacteria;Ktedonobacterales;Ktedonobacteraceae;Ktedonobacter;Ktedonobacter racemifer;Ktedonobacter racemifer SOSP1-21, DSM 44963 |
| Ga0586324 0484346 49 1662 | 0.00 | 0.00 | 0.00 | 0.00 | 1328700.00 | 0.00 | 0.00 | 0.00 | 0.00 | 0.00 | 0.00 | 0.00 | KO:K01179 | endoglucanase | Bacteria;Bacteroidetes;Chitinophagia;Chitinophagales;Chitinophagaceae;Niastella;Niastella koreensis;Niastella koreensis GR20-10, DSM 17620 |
| Ga0586324 1257648 1 1101 | 0.00 | 0.00 | 0.00 | 0.00 | 0.00 | 0.00 | 0.00 | 2586400.00 | 0.00 | 0.00 | 0.00 | 0.00 | KO:K01190 | beta-galactosidase | Bacteria;Acidobacteria;Acidobacteriia;Acidobacteriales;Acidobacteriaceae;unclassified Acidobacteriaceae;Acidobacteriaceae bacterium URHE0068;Acidobacteriaceae bacterium URHE0068 |
| Ga0586324 0823852 3 1319 | 0.00 | 0.00 | 0.00 | 0.00 | 0.00 | 0.00 | 0.00 | 0.00 | 0.00 | 0.00 | 0.00 | 1200000.00 | KO:K01214 | isoamylase | Bacteria;Proteobacteria;Deltaproteobacteria;Desulfuromonadales;Desulfuromonadaceae;Pelobacter;Pelobacter propionicus;Pelobacter propionicus DSM 2379 |
| Ga0586324 0000011 20366 21802 | 0.00 | 0.00 | 0.00 | 0.00 | 0.00 | 0.00 | 0.00 | 0.00 | 0.00 | 12012000.00 | 0.00 | 9750000.00 | KO:K01251 | adenosylhomocysteinase | Bacteria;Proteobacteria;Betaproteobacteria;Nitrosomonadales;Thiobacillaceae;Annwoodia;Annwoodia aquaesulis;Annwoodia aquaesulis DSM 4255 |
| Ga0586324 0002917 1643 2935 | 0.00 | 3746800.00 | 0.00 | 0.00 | 0.00 | 0.00 | 21477600.00 | 0.00 | 0.00 | 0.00 | 0.00 | 0.00 | KO:K01251 | adenosylhomocysteinase | Bacteria;Proteobacteria;Gammaproteobacteria;Methylococcales;Methylococcaceae;Methylobacter;Methylobacter tundripaludum;Methylobacter tundripaludum OWC-G53F |
| Ga0586324 0053628 1508 2764 | 0.00 | 0.00 | 0.00 | 0.00 | 2997300.00 | 0.00 | 0.00 | 4202900.00 | 5589000.00 | 0.00 | 3212000.00 | 0.00 | KO:K01251 | adenosylhomocysteinase | Bacteria;Proteobacteria;Deltaproteobacteria;Syntrophobacterales;Syntrophobacteraceae;Syntrophobacter;Syntrophobacter fumaroxidans;Syntrophobacter fumaroxidans MPOB |
| Ga0586324 0161725 2 1048 | 0.00 | 0.00 | 0.00 | 0.00 | 0.00 | 0.00 | 0.00 | 0.00 | 10125000.00 | 0.00 | 0.00 | 0.00 | KO:K01251 | adenosylhomocysteinase | Bacteria;Proteobacteria;Betaproteobacteria;Nitrosomonadales;Thiobacillaceae;Annwoodia;Annwoodia aquaesulis;Annwoodia aquaesulis DSM 4255 |
| Ga0586324 0170397 390 1646 | 0.00 | 0.00 | 0.00 | 0.00 | 0.00 | 0.00 | 0.00 | 0.00 | 2980800.00 | 13090000.00 | 0.00 | 0.00 | KO:K01251 | adenosylhomocysteinase | Bacteria;Proteobacteria;Deltaproteobacteria;unclassified Deltaproteobacteria;unclassified Deltaproteobacteria;Dissulfuribacter;Dissulfuribacter thermophilus;Dissulfuribacter thermophilus S69 |
| Ga0586324 0176469 1815 2624 | 0.00 | 0.00 | 0.00 | 0.00 | 15759000.00 | 0.00 | 0.00 | 0.00 | 0.00 | 0.00 | 7471200.00 | 0.00 | KO:K01251 | adenosylhomocysteinase | Bacteria;Proteobacteria;Gammaproteobacteria;Nevskiales;Steroidobacteraceae;Povalibacter;Povalibacter uvarum;Povalibacter uvarum DSM 26723 |
| Ga0586324 0512168 3 1040 | 0.00 | 4454000.00 | 0.00 | 0.00 | 0.00 | 0.00 | 0.00 | 0.00 | 0.00 | 0.00 | 0.00 | 0.00 | KO:K01251 | adenosylhomocysteinase | Bacteria;Proteobacteria;Betaproteobacteria;Burkholderiales;unclassified Burkholderiales;Aquabacterium;Aquabacterium tepidiphilum;Aquabacterium tepidiphilum YIM 730274 |
| Ga0586324 0616485 554 1495 | 0.00 | 0.00 | 0.00 | 0.00 | 0.00 | 0.00 | 0.00 | 13176000.00 | 0.00 | 0.00 | 0.00 | 0.00 | KO:K01251 | adenosylhomocysteinase | Bacteria;Proteobacteria;Betaproteobacteria;Burkholderiales;Burkholderiaceae;unclassified Burkholderiaceae;Burkholderiaceae bacterium PBA;Burkholderiaceae bacterium PBA |
| Ga0586324 1759418 181 954 | 0.00 | 0.00 | 0.00 | 0.00 | 0.00 | 0.00 | 3237600.00 | 0.00 | 0.00 | 0.00 | 0.00 | 0.00 | KO:K01251 | adenosylhomocysteinase | Bacteria;Proteobacteria;Deltaproteobacteria;Desulfobacterales;Desulfobulbaceae;Desulfocapsa;Desulfocapsa sulfexigens;Desulfocapsa sulfexigens DSM 10523 |
| Ga0586324 2723529 27 794 | 0.00 | 0.00 | 0.00 | 0.00 | 0.00 | 0.00 | 0.00 | 0.00 | 4851900.00 | 0.00 | 0.00 | 0.00 | KO:K01251 | adenosylhomocysteinase | Bacteria;Actinobacteria;Rubrobacteria;Gaiellales;Gaiellaceae;Gaiella;Gaiella occulta;Gaiella occulta F2-233 |
| Ga0586324 3224310 1 738 | 0.00 | 0.00 | 0.00 | 0.00 | 3007600.00 | 0.00 | 0.00 | 0.00 | 0.00 | 0.00 | 0.00 | 0.00 | KO:K01251 | adenosylhomocysteinase | Bacteria;Proteobacteria;Deltaproteobacteria;Desulfarculales;Desulfarculaceae;Desulfocarbo;Desulfocarbo indianensis;Desulfocarbo indianensis SCBM |
| Ga0586324 3281469 1 735 | 0.00 | 0.00 | 5425749.23 | 0.00 | 0.00 | 0.00 | 0.00 | 0.00 | 0.00 | 0.00 | 0.00 | 0.00 | KO:K01251 | adenosylhomocysteinase | Bacteria;Proteobacteria;Deltaproteobacteria;Desulfobacterales;Desulfobacteraceae;Desulfonema;Desulfonema limicola;Desulfonema limicola Jadebusen, DSM 2076 |
| Ga0586324 4150652 1 666 | 17800000.00 | 0.00 | 0.00 | 0.00 | 0.00 | 0.00 | 0.00 | 0.00 | 0.00 | 0.00 | 0.00 | 0.00 | KO:K01251 | adenosylhomocysteinase | Bacteria;Proteobacteria;Alphaproteobacteria;Hyphomicrobiales;Xanthobacteraceae;Xanthobacter;Xanthobacter autotrophicus;Xanthobacter autotrophicus Py2 |
| Ga0586324 4537571 3 641 | 0.00 | 0.00 | 0.00 | 4940000.00 | 0.00 | 0.00 | 6703200.00 | 0.00 | 0.00 | 0.00 | 0.00 | 0.00 | KO:K01251 | adenosylhomocysteinase | Bacteria;Acidobacteria;Acidobacteriia;Acidobacteriales;Acidobacteriaceae;Acidobacterium;Acidobacterium ailaaui;Acidobacterium ailaaui PMMR2 |
| Ga0586324 5897568 2 577 | 0.00 | 0.00 | 0.00 | 0.00 | 0.00 | 0.00 | 0.00 | 0.00 | 7136100.00 | 11550000.00 | 570970.40 | 10875000.00 | KO:K01251 | adenosylhomocysteinase | Bacteria;Proteobacteria;Deltaproteobacteria;Myxococcales;Archangiaceae;Archangium;Archangium sp. Cb G35;Archangium sp. Cb G35 |
| Ga0586324 6556621 2 550 | 0.00 | 0.00 | 0.00 | 4294000.00 | 0.00 | 0.00 | 0.00 | 0.00 | 0.00 | 839300.00 | 0.00 | 5400000.00 | KO:K01251 | adenosylhomocysteinase | Bacteria;Proteobacteria;Gammaproteobacteria;Xanthomonadales;Rhodanobacteraceae;Ahniella;Ahniella affigens;Ahniella affigens D13 |
| Ga0586324 6741891 3 545 | 4030000.00 | 0.00 | 0.00 | 0.00 | 0.00 | 0.00 | 0.00 | 0.00 | 0.00 | 0.00 | 0.00 | 0.00 | KO:K01251 | adenosylhomocysteinase | Bacteria;Proteobacteria;Deltaproteobacteria;unclassified Deltaproteobacteria;unclassified Deltaproteobacteria;Dissulfuribacter;Dissulfuribacter thermophilus;Dissulfuribacter thermophilus S69 |
| Ga0586324 0000037 26947 28452 | 0.00 | 0.00 | 0.00 | 0.00 | 0.00 | 0.00 | 0.00 | 0.00 | 0.00 | 4535300.00 | 4250400.00 | 0.00 | KO:K01255 | leucyl aminopeptidase | Bacteria;Proteobacteria;Betaproteobacteria;Nitrosomonadales;Thiobacillaceae;Annwoodia;Annwoodia aquaesulis;Annwoodia aquaesulis DSM 4255 |
| Ga0586324 0102001 433 1935 | 0.00 | 0.00 | 0.00 | 0.00 | 0.00 | 0.00 | 1230757.68 | 0.00 | 0.00 | 0.00 | 0.00 | 0.00 | KO:K01255 | leucyl aminopeptidase | Bacteria;Proteobacteria;Alphaproteobacteria;Hyphomicrobiales;Xanthobacteraceae;Pseudolabrys;Pseudolabrys taiwanensis;Pseudolabrys taiwanensis CC-BB4 |
| Ga0586324 0147937 844 2331 | 0.00 | 0.00 | 0.00 | 0.00 | 0.00 | 0.00 | 629665.32 | 0.00 | 0.00 | 0.00 | 0.00 | 0.00 | KO:K01255 | leucyl aminopeptidase | Bacteria;Fibrobacteres;Fibrobacteria;unclassified Fibrobacteria;unclassified Fibrobacteria;unclassified Fibrobacteria;Fibrobacteria bacterium AD312;Fibrobacteria bacterium AD312 AD 312 |
| Ga0586324 0851941 3 1091 | 0.00 | 0.00 | 0.00 | 0.00 | 0.00 | 0.00 | 0.00 | 0.00 | 0.00 | 0.00 | 0.00 | 787500.00 | KO:K01255 | leucyl aminopeptidase | Bacteria;Proteobacteria;Alphaproteobacteria;Hyphomicrobiales;Xanthobacteraceae;Pseudolabrys;Pseudolabrys taiwanensis;Pseudolabrys taiwanensis CC-BB4 |
| Ga0586324 4538733 1 642 | 0.00 | 0.00 | 0.00 | 0.00 | 0.00 | 0.00 | 2177685.00 | 0.00 | 0.00 | 0.00 | 0.00 | 0.00 | KO:K01255 | leucyl aminopeptidase | Bacteria;Verrucomicrobia;unclassified Verrucomicrobia;unclassified Verrucomicrobia;unclassified Verrucomicrobia;unclassified Verrucomicrobia;Verrucomicrobia bacterium LW23;Verrucomicrobia bacterium LW23 |
| Ga0586324 1923500 1 804 | 0.00 | 0.00 | 0.00 | 5168000.00 | 0.00 | 0.00 | 0.00 | 0.00 | 0.00 | 0.00 | 0.00 | 0.00 | KO:K01270 | dipeptidase D | Bacteria;Bacteroidetes;Bacteroidia;Marinilabiliales;Marinifilaceae;Ancylomarina;Ancylomarina subtilis;Ancylomarina subtilis DSM 28825 |
| Ga0586324 0000137 32897 35299 | 0.00 | 0.00 | 0.00 | 0.00 | 0.00 | 0.00 | 0.00 | 0.00 | 0.00 | 3357200.00 | 32648000.00 | 4657500.00 | KO:K01338 | ATP-dependent Lon protease | Bacteria;Proteobacteria;Betaproteobacteria;Nitrosomonadales;Thiobacillaceae;Annwoodia;Annwoodia aquaesulis;Annwoodia aquaesulis DSM 4255 |
| Ga0586324 0010606 3967 6381 | 0.00 | 0.00 | 0.00 | 0.00 | 0.00 | 0.00 | 0.00 | 0.00 | 0.00 | 5266800.00 | 0.00 | 4725000.00 | KO:K01338 | ATP-dependent Lon protease | Bacteria;Proteobacteria;Betaproteobacteria;Nitrosomonadales;Thiobacillaceae;Thiobacillus;Thiobacillus thioparus;Thiobacillus thioparus DSM 505 |
| Ga0586324 0014466 5954 8443 | 0.00 | 0.00 | 0.00 | 0.00 | 0.00 | 0.00 | 0.00 | 0.00 | 0.00 | 7700000.00 | 0.00 | 0.00 | KO:K01338 | ATP-dependent Lon protease | Bacteria;Proteobacteria;Alphaproteobacteria;Hyphomicrobiales;Xanthobacteraceae;Pseudolabrys;Pseudolabrys taiwanensis;Pseudolabrys taiwanensis CC-BB4 |
| Ga0586324 0022685 3046 5049 | 0.00 | 1285200.00 | 0.00 | 0.00 | 0.00 | 0.00 | 0.00 | 0.00 | 0.00 | 0.00 | 0.00 | 0.00 | KO:K01338 | ATP-dependent Lon protease | Bacteria;Proteobacteria;Deltaproteobacteria;unclassified Deltaproteobacteria;Candidatus Desulfofervidaceae;Candidatus Desulfofervidus;Candidatus Desulfofervidus auxilii;delta proteobacterium HotSeep1 |
| Ga0586324 0033750 4643 5794 | 0.00 | 0.00 | 0.00 | 0.00 | 0.00 | 0.00 | 0.00 | 0.00 | 0.00 | 0.00 | 3968800.00 | 0.00 | KO:K01338 | ATP-dependent Lon protease | Bacteria;Proteobacteria;Betaproteobacteria;Nitrosomonadales;Nitrosomonadaceae;Nitrosospira;Nitrosospira briensis;Nitrosospira briensis C-128 |
| Ga0586324 0110198 1013 3271 | 0.00 | 0.00 | 0.00 | 3790500.00 | 0.00 | 0.00 | 0.00 | 0.00 | 0.00 | 0.00 | 0.00 | 0.00 | KO:K01338 | ATP-dependent Lon protease | Bacteria;Proteobacteria;Gammaproteobacteria;Nevskiales;Steroidobacteraceae;Povalibacter;Povalibacter uvarum;Povalibacter uvarum DSM 26723 |
| Ga0586324 0128720 1 1245 | 0.00 | 0.00 | 0.00 | 0.00 | 0.00 | 0.00 | 0.00 | 0.00 | 0.00 | 17479000.00 | 15752000.00 | 3945000.00 | KO:K01338 | ATP-dependent Lon protease | Bacteria;Proteobacteria;Betaproteobacteria;Nitrosomonadales;Sulfuricellaceae;Sulfuricella;Sulfuricella sp. T08;Sulfuricella sp. T08 |
| Ga0586324 0136108 3 2129 | 0.00 | 0.00 | 3153143.43 | 0.00 | 0.00 | 0.00 | 0.00 | 0.00 | 0.00 | 0.00 | 0.00 | 0.00 | KO:K01338 | ATP-dependent Lon protease | Bacteria;Proteobacteria;Gammaproteobacteria;unclassified Gammaproteobacteria;Candidatus Competibacteraceae;Candidatus Contendobacter;Candidatus Contendobacter odensis;Candidatus Contendobacter odensis Run B J11 |
| Ga0586324 0157983 209 2650 | 0.00 | 0.00 | 0.00 | 0.00 | 0.00 | 0.00 | 0.00 | 0.00 | 0.00 | 461977.67 | 0.00 | 0.00 | KO:K01338 | ATP-dependent Lon protease | Bacteria;Proteobacteria;Deltaproteobacteria;Syntrophorhabdales;Syntrophorhabdaceae;Syntrophorhabdus;Syntrophorhabdus aromaticivorans;Syntrophorhabdus aromaticivorans UI |
| Ga0586324 0201531 3 1262 | 0.00 | 0.00 | 0.00 | 927759.55 | 0.00 | 0.00 | 0.00 | 0.00 | 0.00 | 0.00 | 0.00 | 0.00 | KO:K01338 | ATP-dependent Lon protease | Bacteria;Proteobacteria;Alphaproteobacteria;Rhodospirillales;Acetobacteraceae;Rhodopila;Rhodopila globiformis;Rhodopila globiformis DSM 161 |
| Ga0586324 0390849 2 1828 | 0.00 | 0.00 | 0.00 | 0.00 | 0.00 | 0.00 | 0.00 | 0.00 | 0.00 | 0.00 | 0.00 | 478444.50 | KO:K01338 | ATP-dependent Lon protease | Bacteria;Proteobacteria;Betaproteobacteria;Nitrosomonadales;Thiobacillaceae;Thiobacillus;Thiobacillus denitrificans;Thiobacillus denitrificans DSM 12475 |
| Ga0586324 0675852 3 1439 | 0.00 | 0.00 | 0.00 | 0.00 | 0.00 | 0.00 | 0.00 | 0.00 | 0.00 | 0.00 | 0.00 | 1425000.00 | KO:K01338 | ATP-dependent Lon protease | Bacteria;Proteobacteria;Betaproteobacteria;Nitrosomonadales;Thiobacillaceae;Thiobacillus;Thiobacillus denitrificans;Thiobacillus denitrificans RG |
| Ga0586324 0681878 272 1432 | 0.00 | 1475600.00 | 0.00 | 2327500.00 | 2997300.00 | 0.00 | 0.00 | 0.00 | 0.00 | 0.00 | 0.00 | 2025000.00 | KO:K01338 | ATP-dependent Lon protease | Bacteria;Proteobacteria;Alphaproteobacteria;Rhodospirillales;Acetobacteraceae;Rhodopila;unclassified Rhodopila;Rhodopila sp. LVNP |
| Ga0586324 0990913 3 1220 | 0.00 | 0.00 | 0.00 | 0.00 | 0.00 | 0.00 | 0.00 | 0.00 | 0.00 | 247740.57 | 0.00 | 0.00 | KO:K01338 | ATP-dependent Lon protease | Bacteria;Proteobacteria;Betaproteobacteria;Nitrosomonadales;Methylophilaceae;Methylotenera;Methylotenera versatilis;Methylotenera versatilis 79 |
| Ga0586324 2829375 3 641 | 0.00 | 0.00 | 12836400.00 | 5586000.00 | 0.00 | 1821594.80 | 0.00 | 0.00 | 2867400.00 | 0.00 | 0.00 | 0.00 | KO:K01338 | ATP-dependent Lon protease | Bacteria;Nitrospirae;unclassified Nitrospirae;unclassified Nitrospirae;unclassified Nitrospirae;unclassified Nitrospirae;Nitrospirae bacterium HCH-1;Nitrospirae bacterium HCH-1 |
| Ga0586324 4128190 3 668 | 0.00 | 0.00 | 0.00 | 0.00 | 0.00 | 0.00 | 0.00 | 0.00 | 0.00 | 439994.94 | 0.00 | 0.00 | KO:K01338 | ATP-dependent Lon protease | Bacteria;Proteobacteria;Deltaproteobacteria;Syntrophobacterales;Syntrophaceae;Desulfobacca;Desulfobacca acetoxidans;Desulfobacca acetoxidans ASRB2, DSM 11109 |
| Ga0586324 5119985 2 610 | 0.00 | 5514800.00 | 0.00 | 0.00 | 0.00 | 0.00 | 0.00 | 0.00 | 6536700.00 | 0.00 | 4100800.00 | 4515000.00 | KO:K01338 | ATP-dependent Lon protease | Bacteria;Acidobacteria;Acidobacteriia;Bryobacterales;Solibacteraceae;Candidatus Solibacter;Candidatus Solibacter usitatus;Candidatus Solibacter usitatus Ellin6076 |
| Ga0586324 5488961 3 593 | 0.00 | 727600.00 | 0.00 | 0.00 | 0.00 | 0.00 | 0.00 | 628300.00 | 305336.79 | 2025100.00 | 2622400.00 | 0.00 | KO:K01338 | ATP-dependent Lon protease | Bacteria;Candidatus Fervidibacteria;unclassified Candidatus Fervidibacteria;unclassified Candidatus Fervidibacteria;unclassified Candidatus Fervidibacteria;unclassified Candidatus Fervidibacteria;Fervidibacteria bacterium JGI 0000001-B9;Fervidibacteria bacterium JGI 0000001-B9 (contamination screened) |
| Ga0586324 0115112 702 2552 | 0.00 | 276321.40 | 0.00 | 2726500.00 | 0.00 | 0.00 | 0.00 | 0.00 | 0.00 | 0.00 | 0.00 | 0.00 | KO:K01342 | subtilisin | Bacteria;Proteobacteria;Gammaproteobacteria;Vibrionales;Vibrionaceae;Vibrio;Vibrio sp. EJY3;Vibrio sp. EJY3 |
| Ga0586324 0392996 143 772 | 0.00 | 0.00 | 0.00 | 0.00 | 0.00 | 0.00 | 563777.88 | 0.00 | 0.00 | 0.00 | 0.00 | 0.00 | KO:K01358 | ATP-dependent Clp protease protease subunit | Bacteria;Proteobacteria;Deltaproteobacteria;Syntrophobacterales;Syntrophaceae;Desulfobacca;Desulfobacca acetoxidans;Desulfobacca acetoxidans ASRB2, DSM 11109 |
| Ga0586324 8280633 1 501 | 0.00 | 0.00 | 0.00 | 0.00 | 0.00 | 0.00 | 0.00 | 0.00 | 0.00 | 1655500.00 | 0.00 | 0.00 | KO:K01358 | ATP-dependent Clp protease protease subunit | Bacteria;Proteobacteria;Deltaproteobacteria;Syntrophobacterales;Syntrophaceae;Desulfobacca;Desulfobacca acetoxidans;Desulfobacca acetoxidans ASRB2, DSM 11109 |
| Ga0586324 0000169 21223 22101 | 0.00 | 0.00 | 0.00 | 0.00 | 0.00 | 0.00 | 0.00 | 0.00 | 0.00 | 0.00 | 4769600.00 | 6495000.00 | KO:K01423 | predicted Zn-dependent protease | Bacteria;Proteobacteria;Betaproteobacteria;Nitrosomonadales;Thiobacillaceae;Annwoodia;Annwoodia aquaesulis;Annwoodia aquaesulis DSM 4255 |
| Ga0586324 0001043 10586 11659 | 0.00 | 0.00 | 1627317.72 | 1463000.00 | 0.00 | 0.00 | 0.00 | 0.00 | 0.00 | 0.00 | 0.00 | 862500.00 | KO:K01424 | L-asparaginase | Bacteria;Proteobacteria;Betaproteobacteria;Burkholderiales;Alcaligenaceae;Achromobacter;Achromobacter xylosoxidans;Achromobacter xylosoxidans IYER ADAF13 |
| Ga0586324 0042502 3286 4374 | 67392.60 | 0.00 | 0.00 | 0.00 | 0.00 | 0.00 | 0.00 | 0.00 | 0.00 | 0.00 | 0.00 | 0.00 | KO:K01424 | L-asparaginase | Bacteria;Proteobacteria;Gammaproteobacteria;Cellvibrionales;Halieaceae;Halioglobus;Halioglobus lutimaris;Halioglobus lutimaris HF004 |
| Ga0586324 0099996 1316 2383 | 0.00 | 294108.16 | 0.00 | 0.00 | 0.00 | 0.00 | 0.00 | 0.00 | 0.00 | 0.00 | 0.00 | 0.00 | KO:K01424 | L-asparaginase | Bacteria;Bacteroidetes;Flavobacteriia;Flavobacteriales;Flavobacteriaceae;Flavobacterium;Flavobacterium sp. URHB0058;Flavobacterium sp. URHB0058 |
| Ga0586324 0385181 401 1465 | 0.00 | 368766.72 | 0.00 | 0.00 | 0.00 | 1048806.51 | 0.00 | 640500.00 | 0.00 | 119502.46 | 0.00 | 0.00 | KO:K01424 | L-asparaginase | Bacteria;Bacteroidetes;Flavobacteriia;Flavobacteriales;Flavobacteriaceae;Flavobacterium;Flavobacterium saccharophilum;Flavobacterium saccharophilum DSM 1811 |
| Ga0586324 6269408 240 563 | 0.00 | 0.00 | 0.00 | 0.00 | 0.00 | 0.00 | 4126800.00 | 0.00 | 1255500.00 | 0.00 | 1795200.00 | 3727500.00 | KO:K01424 | L-asparaginase | Bacteria;Verrucomicrobia;Opitutae;Opitutales;Opitutaceae;Opitutus;Opitutus terrae;Opitutus terrae PB90-1 |
| Ga0586324 0001043 7467 8687 | 0.00 | 0.00 | 2852107.33 | 2128000.00 | 0.00 | 0.00 | 0.00 | 0.00 | 0.00 | 954800.00 | 0.00 | 668823.75 | KO:K01425 | glutaminase | Bacteria;Proteobacteria;Gammaproteobacteria;Cellvibrionales;Spongiibacteraceae;Oceanicoccus;Oceanicoccus sagamiensis;Oceanicoccus sagamiensis NBRC 107125 |
| Ga0586324 0027881 5418 6350 | 0.00 | 0.00 | 0.00 | 0.00 | 5273600.00 | 0.00 | 0.00 | 0.00 | 0.00 | 0.00 | 0.00 | 0.00 | KO:K01425 | glutaminase | Bacteria;Proteobacteria;Gammaproteobacteria;Nevskiales;Steroidobacteraceae;Povalibacter;Povalibacter uvarum;Povalibacter uvarum DSM 26723 |
| Ga0586324 0077702 1242 2270 | 0.00 | 1183200.00 | 1699015.77 | 0.00 | 667065.08 | 682466.82 | 0.00 | 1104100.00 | 834300.00 | 0.00 | 819329.28 | 990000.00 | KO:K01425 | glutaminase | Bacteria;Bacteroidetes;Chitinophagia;Chitinophagales;Chitinophagaceae;Flavisolibacter;Flavisolibacter tropicus;Flavisolibacter tropicus LCS9 |
| Ga0586324 0088508 252 1295 | 0.00 | 0.00 | 0.00 | 0.00 | 966008.16 | 0.00 | 0.00 | 0.00 | 0.00 | 0.00 | 0.00 | 0.00 | KO:K01425 | glutaminase | Bacteria;Acidobacteria;Vicinamibacteria;unclassified Vicinamibacteria;Vicinamibacteraceae;Luteitalea;Luteitalea pratensis;Luteitalea pratensis DSM 100886 |
| Ga0586324 0134455 57 1124 | 0.00 | 0.00 | 0.00 | 0.00 | 0.00 | 2427873.35 | 0.00 | 0.00 | 0.00 | 0.00 | 0.00 | 0.00 | KO:K01425 | glutaminase | Bacteria;Proteobacteria;Gammaproteobacteria;Cellvibrionales;Halieaceae;Halioglobus;Halioglobus sediminis;Halioglobus sediminis U0301 U0301 |
| Ga0586324 0270362 519 1559 | 0.00 | 4576400.00 | 0.00 | 0.00 | 0.00 | 29116976.47 | 0.00 | 0.00 | 0.00 | 0.00 | 0.00 | 0.00 | KO:K01425 | glutaminase | Bacteria;Proteobacteria;Gammaproteobacteria;Alteromonadales;Alteromonadaceae;unclassified Alteromonadaceae;Alteromonadaceae bacterium 1081L.S.0a.03;Alteromonadaceae bacterium 1081L.S.0a.03 |
| Ga0586324 0942500 321 1244 | 0.00 | 0.00 | 12836400.00 | 0.00 | 0.00 | 0.00 | 0.00 | 0.00 | 0.00 | 0.00 | 0.00 | 0.00 | KO:K01425 | glutaminase | Bacteria;Proteobacteria;Gammaproteobacteria;Alteromonadales;Alteromonadaceae;unclassified Alteromonadaceae;Alteromonadaceae bacterium 1081L.S.0a.03;Alteromonadaceae bacterium 1081L.S.0a.03 |
| Ga0586324 2083398 2 889 | 0.00 | 0.00 | 0.00 | 5434000.00 | 0.00 | 0.00 | 0.00 | 0.00 | 0.00 | 0.00 | 0.00 | 0.00 | KO:K01425 | glutaminase | Bacteria;Proteobacteria;Gammaproteobacteria;Alteromonadales;Alteromonadaceae;unclassified Alteromonadaceae;Alteromonadaceae bacterium 1081L.S.0a.03;Alteromonadaceae bacterium 1081L.S.0a.03 |
| Ga0586324 7489879 1 522 | 0.00 | 0.00 | 0.00 | 0.00 | 0.00 | 0.00 | 0.00 | 0.00 | 0.00 | 0.00 | 1108800.00 | 0.00 | KO:K01448 | N-acetylmuramoyl-L-alanine amidase | Bacteria;Proteobacteria;Gammaproteobacteria;Nevskiales;Steroidobacteraceae;Povalibacter;Povalibacter uvarum;Povalibacter uvarum DSM 26723 |
| Ga0586324 1984603 3 905 | 0.00 | 0.00 | 0.00 | 0.00 | 0.00 | 0.00 | 0.00 | 0.00 | 3402000.00 | 0.00 | 0.00 | 0.00 | KO:K01464 | dihydropyrimidinase | Bacteria;Proteobacteria;Alphaproteobacteria;Rhodospirillales;Acetobacteraceae;Rhodopila;unclassified Rhodopila;Rhodopila sp. LVNP |
| Ga0586324 0000007 110868 112097 | 0.00 | 0.00 | 0.00 | 0.00 | 0.00 | 0.00 | 0.00 | 0.00 | 0.00 | 4981900.00 | 2024000.00 | 0.00 | KO:K01478 | arginine deiminase | Bacteria;Proteobacteria;Alphaproteobacteria;Hyphomicrobiales;Rhizobiaceae;Rhizobium;Rhizobium sp. YR295;Rhizobium sp. YR295 |
| Ga0586324 0098969 984 2249 | 0.00 | 0.00 | 0.00 | 0.00 | 2410200.00 | 0.00 | 0.00 | 0.00 | 0.00 | 0.00 | 0.00 | 0.00 | KO:K01478 | arginine deiminase | Bacteria;Proteobacteria;Gammaproteobacteria;Chromatiales;Chromatiaceae;Lamprocystis;Lamprocystis purpurea;Lamprocystis purpurea DSM 4197 |
| Ga0586324 0000241 25343 26230 | 0.00 | 0.00 | 0.00 | 0.00 | 2430800.00 | 0.00 | 0.00 | 0.00 | 4811400.00 | 0.00 | 0.00 | 0.00 | KO:K01491 | methylenetetrahydrofolate dehydrogenase (NADP+)/methenyltetrahydrofolate cyclohydrolase | Bacteria;Proteobacteria;Deltaproteobacteria;Syntrophobacterales;Syntrophaceae;Syntrophus;Syntrophus gentianae;Syntrophus gentianae DSM 8423 |
| Ga0586324 0017644 5900 6820 | 0.00 | 5555600.00 | 15088400.00 | 5105871.90 | 3172400.00 | 9840701.02 | 21158400.00 | 1409100.00 | 2106000.00 | 3803800.00 | 5693600.00 | 5947500.00 | KO:K01491 | methylenetetrahydrofolate dehydrogenase (NADP+)/methenyltetrahydrofolate cyclohydrolase | Bacteria;Proteobacteria;Deltaproteobacteria;Syntrophobacterales;Syntrophobacteraceae;Desulfacinum;Desulfacinum infernum;Desulfacinum infernum DSM 9756 |
| Ga0586324 0069787 2794 3684 | 0.00 | 6752400.00 | 0.00 | 16055000.00 | 40788000.00 | 2903499.20 | 0.00 | 10736000.00 | 18063000.00 | 3896200.00 | 0.00 | 3922500.00 | KO:K01491 | methylenetetrahydrofolate dehydrogenase (NADP+)/methenyltetrahydrofolate cyclohydrolase | Bacteria;Proteobacteria;Deltaproteobacteria;Syntrophobacterales;Syntrophobacteraceae;Desulfoglaeba;Desulfoglaeba alkanexedens;Desulfoglaeba alkanexedens ALDC |
| Ga0586324 0234267 1 495 | 0.00 | 0.00 | 0.00 | 0.00 | 0.00 | 0.00 | 5084400.00 | 0.00 | 0.00 | 0.00 | 0.00 | 0.00 | KO:K01491 | methylenetetrahydrofolate dehydrogenase (NADP+)/methenyltetrahydrofolate cyclohydrolase | Bacteria;Proteobacteria;Deltaproteobacteria;Syntrophobacterales;Syntrophaceae;Desulfobacca;Desulfobacca acetoxidans;Desulfobacca acetoxidans ASRB2, DSM 11109 |
| Ga0586324 0439497 65 964 | 0.00 | 0.00 | 0.00 | 0.00 | 0.00 | 0.00 | 10693200.00 | 0.00 | 0.00 | 0.00 | 0.00 | 0.00 | KO:K01491 | methylenetetrahydrofolate dehydrogenase (NADP+)/methenyltetrahydrofolate cyclohydrolase | Bacteria;Proteobacteria;Deltaproteobacteria;Syntrophobacterales;Syntrophaceae;Desulfobacca;Desulfobacca acetoxidans;Desulfobacca acetoxidans ASRB2, DSM 11109 |
| Ga0586324 0466468 1205 1690 | 0.00 | 4630800.00 | 0.00 | 14535000.00 | 18643000.00 | 7463600.00 | 0.00 | 2659600.00 | 8910000.00 | 0.00 | 0.00 | 4942500.00 | KO:K01491 | methylenetetrahydrofolate dehydrogenase (NADP+)/methenyltetrahydrofolate cyclohydrolase | Bacteria;Proteobacteria;Deltaproteobacteria;Syntrophobacterales;Syntrophaceae;Desulfobacca;Desulfobacca acetoxidans;Desulfobacca acetoxidans ASRB2, DSM 11109 |
| Ga0586324 0842840 2 538 | 0.00 | 0.00 | 0.00 | 0.00 | 0.00 | 10560200.00 | 0.00 | 9333000.00 | 8910000.00 | 0.00 | 0.00 | 0.00 | KO:K01491 | methylenetetrahydrofolate dehydrogenase (NADP+)/methenyltetrahydrofolate cyclohydrolase | Bacteria;Proteobacteria;Deltaproteobacteria;Desulfobacterales;Desulfobacteraceae;Desulfatiglans;Desulfatiglans anilini;Desulfatiglans anilini DSM 4660 |
| Ga0586324 0955091 78 986 | 0.00 | 0.00 | 0.00 | 0.00 | 1359600.00 | 0.00 | 0.00 | 1067500.00 | 0.00 | 0.00 | 0.00 | 0.00 | KO:K01491 | methylenetetrahydrofolate dehydrogenase (NADP+)/methenyltetrahydrofolate cyclohydrolase | Bacteria;Proteobacteria;Deltaproteobacteria;Desulfobacterales;Desulfobacteraceae;Desulfatiglans;Desulfatiglans anilini;Desulfatiglans anilini DSM 4660 |

| | | | | | | | | | | | | | | | |
|---|---|---|---|---|---|---|---|---|---|---|---|---|---|---|---|
| Ga0586324 1124653 1 807 | 0.00 | 727600.00 | 0.00 | 0.00 | 0.00 | 0.00 | 0.00 | 0.00 | 0.00 | 0.00 | 0.00 | 0.00 | KO:K01491 | methylenetetrahydrofolate dehydrogenase (NADP+)/methenyltetrahydrofolate cyclohydrolase | Bacteria;Proteobacteria;Deltaproteobacteria;Syntrophobacterales;Syntrophaceae;Syntrophus;Syntrophus gentianae;Syntrophus gentianae DSM 8423 |
| Ga0586324 2730062 2 535 | 0.00 | 0.00 | 0.00 | 0.00 | 0.00 | 1847173.51 | 0.00 | 0.00 | 0.00 | 0.00 | 0.00 | 0.00 | KO:K01491 | methylenetetrahydrofolate dehydrogenase (NADP+)/methenyltetrahydrofolate cyclohydrolase | Bacteria;Proteobacteria;Deltaproteobacteria;Syntrophobacterales;Syntrophaceae;Desulfobacca;Desulfobacca acetoxidans;Desulfobacca acetoxidans ASRB2, DSM 11109 |
| Ga0586324 3305410 140 730 | 0.00 | 0.00 | 0.00 | 0.00 | 1606800.00 | 0.00 | 0.00 | 1409100.00 | 0.00 | 0.00 | 0.00 | 0.00 | KO:K01491 | methylenetetrahydrofolate dehydrogenase (NADP+)/methenyltetrahydrofolate cyclohydrolase | Bacteria;Proteobacteria;Deltaproteobacteria;Desulfobacterales;Desulfobacteraceae;Desulfococcus;Desulfococcus oleovorans;Desulfococcus oleovorans Hxd3 |
| Ga0586324 3668690 1 702 | 0.00 | 0.00 | 0.00 | 0.00 | 0.00 | 0.00 | 0.00 | 0.00 | 441556.92 | 0.00 | 0.00 | 0.00 | KO:K01491 | methylenetetrahydrofolate dehydrogenase (NADP+)/methenyltetrahydrofolate cyclohydrolase | Bacteria;Proteobacteria;Deltaproteobacteria;Desulfobacterales;Desulfobulbaceae;Desulfoprunum;Desulfoprunum benzoelyticum;Desulfoprunum benzoelyticum DSM 28570 |
| Ga0586324 0689083 7 693 | 0.00 | 0.00 | 0.00 | 0.00 | 0.00 | 0.00 | 1761329.64 | 0.00 | 0.00 | 0.00 | 0.00 | 0.00 | KO:K01507 | inorganic pyrophosphatase | Bacteria;Proteobacteria;Deltaproteobacteria;Desulfovibrionales;Desulfovibrionaceae;Desulfovibrio;Desulfovibrio inopinatus;Desulfovibrio inopinatus DSM 10711 |
| Ga0586324 0004139 1672 3147 | 0.00 | 0.00 | 0.00 | 2546000.00 | 0.00 | 0.00 | 0.00 | 0.00 | 0.00 | 0.00 | 0.00 | 0.00 | KO:K01524 | exopolyphosphatase/guanosine-5'-triphosphate,3'-diphosphate pyrophosphatase | Bacteria;Proteobacteria;Betaproteobacteria;Nitrosomonadales;Gallionellaceae;Gallionella;Gallionella capsiferriformans;Gallionella capsiferriformans ES-2 |
| Ga0586324 0072301 2224 3717 | 0.00 | 0.00 | 0.00 | 0.00 | 0.00 | 0.00 | 0.00 | 0.00 | 0.00 | 1262800.00 | 0.00 | 0.00 | KO:K01524 | exopolyphosphatase/guanosine-5'-triphosphate,3'-diphosphate pyrophosphatase | Bacteria;Proteobacteria;Gammaproteobacteria;Chromatiales;Chromatiaceae;Thiocapsa;Thiocapsa roseopersicina;Thiocapsa roseopersicina DSM 217 |
| Ga0586324 2223888 3 863 | 0.00 | 0.00 | 0.00 | 0.00 | 0.00 | 0.00 | 0.00 | 11529000.00 | 0.00 | 15092000.00 | 9504000.00 | 6787500.00 | KO:K01524 | exopolyphosphatase/guanosine-5'-triphosphate,3'-diphosphate pyrophosphatase | Bacteria;Proteobacteria;Gammaproteobacteria;Chromatiales;Chromatiaceae;Thiocapsa;Thiocapsa roseopersicina;Thiocapsa roseopersicina DSM 217 |
| Ga0586324 3140380 2 748 | 0.00 | 0.00 | 0.00 | 0.00 | 0.00 | 0.00 | 0.00 | 0.00 | 0.00 | 1386000.00 | 0.00 | 0.00 | KO:K01531 | Mg2+-importing ATPase | Bacteria;Proteobacteria;Deltaproteobacteria;Myxococcales;Anaeromyxobacteraceae;Anaeromyxobacter;Anaeromyxobacter sp. PSR-1;Anaeromyxobacter sp. PSR-1 |
| Ga0586324 3680882 3 701 | 0.00 | 0.00 | 0.00 | 0.00 | 0.00 | 17507700.00 | 0.00 | 0.00 | 0.00 | 0.00 | 0.00 | 0.00 | KO:K01537 | Ca2+-transporting ATPase | Archaea;Euryarchaeota;Methanomicrobia;Methanosarcinales;Methanosarcinaceae;Methanosarcina;Methanosarcina mazei;Methanosarcina mazei 3.H.A.2.4 |
| Ga0586324 0140086 1186 2121 | 1630000.00 | 0.00 | 0.00 | 0.00 | 0.00 | 0.00 | 0.00 | 0.00 | 0.00 | 0.00 | 0.00 | 0.00 | KO:K01551 | arsenite-transporting ATPase | Bacteria;Proteobacteria;Deltaproteobacteria;Syntrophobacterales;Syntrophaceae;Desulfomonile;Desulfomonile tiedjei;Desulfomonile tiedjei DCB-1, DSM 6799 |
| Ga0586324 0187333 1068 2054 | 1260000.00 | 0.00 | 0.00 | 0.00 | 0.00 | 0.00 | 0.00 | 0.00 | 0.00 | 0.00 | 0.00 | 0.00 | KO:K01551 | arsenite-transporting ATPase | Bacteria;Proteobacteria;Deltaproteobacteria;Syntrophobacterales;Syntrophaceae;Desulfomonile;Desulfomonile tiedjei;Desulfomonile tiedjei DCB-1, DSM 6799 |
| Ga0586324 1839571 2 808 | 0.00 | 0.00 | 0.00 | 0.00 | 0.00 | 0.00 | 0.00 | 0.00 | 349374.06 | 0.00 | 0.00 | 0.00 | KO:K01551 | arsenite-transporting ATPase | Bacteria;Proteobacteria;Deltaproteobacteria;Syntrophobacterales;Syntrophaceae;Desulfomonile;Desulfomonile tiedjei;Desulfomonile tiedjei DCB-1, DSM 6799 |
| Ga0586324 4168625 1 321 | 0.00 | 0.00 | 0.00 | 0.00 | 0.00 | 0.00 | 0.00 | 0.00 | 0.00 | 0.00 | 0.00 | 2490000.00 | KO:K01551 | arsenite-transporting ATPase | Bacteria;Chloroflexi;Thermoflexia;Thermoflexales;Thermoflexaceae;Thermoflexus;Thermoflexus hugenholtzii;Thermoflexus hugenholtzii JAD2 |
| Ga0586324 5925554 3 575 | 0.00 | 0.00 | 0.00 | 0.00 | 0.00 | 0.00 | 0.00 | 2787700.00 | 0.00 | 0.00 | 0.00 | 0.00 | KO:K01551 | arsenite-transporting ATPase | Bacteria;Proteobacteria;Deltaproteobacteria;Syntrophobacterales;Syntrophaceae;Desulfomonile;Desulfomonile tiedjei;Desulfomonile tiedjei DCB-1, DSM 6799 |
| Ga0586324 0088131 587 2197 | 0.00 | 0.00 | 0.00 | 14630000.00 | 0.00 | 0.00 | 0.00 | 0.00 | 0.00 | 0.00 | 0.00 | 0.00 | KO:K01576 | benzoylformate decarboxylase | Bacteria;Proteobacteria;Alphaproteobacteria;Rhodospirillales;unclassified Rhodospirillales;unclassified Rhodospirillales;Rhodospirillales bacterium URHD0088;Rhodospirillales bacterium URHD0088 |
| Ga0586324 0030710 4622 6064 | 0.00 | 0.00 | 0.00 | 2527000.00 | 0.00 | 0.00 | 0.00 | 0.00 | 0.00 | 0.00 | 0.00 | 0.00 | KO:K01577 | oxalyl-CoA decarboxylase | Bacteria;Proteobacteria;Betaproteobacteria;Burkholderiales;Comamonadaceae;Polaromonas;Polaromonas sp. EUR3 1.2.1;Polaromonas sp. EUR3 1.2.1 |
| Ga0586324 0280958 1 1716 | 0.00 | 0.00 | 0.00 | 0.00 | 0.00 | 0.00 | 0.00 | 0.00 | 0.00 | 0.00 | 0.00 | 2827500.00 | KO:K01577 | oxalyl-CoA decarboxylase | Bacteria;Proteobacteria;Betaproteobacteria;Burkholderiales;Comamonadaceae;Polaromonas;Polaromonas sp. EUR3 1.2.1;Polaromonas sp. EUR3 1.2.1 |
| Ga0586324 7585586 3 518 | 0.00 | 0.00 | 0.00 | 0.00 | 0.00 | 0.00 | 0.00 | 1976400.00 | 0.00 | 0.00 | 0.00 | 0.00 | KO:K01577 | oxalyl-CoA decarboxylase | Bacteria;Proteobacteria;Alphaproteobacteria;Hyphomicrobiales;Bradyrhizobiaceae;Afipia;Afipia birgiae;Afipia birgiae 34632 |
| Ga0586324 0008831 9359 10780 | 0.00 | 0.00 | 0.00 | 0.00 | 0.00 | 0.00 | 0.00 | 156165.49 | 0.00 | 0.00 | 0.00 | 0.00 | KO:K01580 | glutamate decarboxylase | Bacteria;Proteobacteria;Gammaproteobacteria;Alteromonadales;Shewanellaceae;Shewanella;Shewanella sp. cp20;Shewanella sp. cp20 |
| Ga0586324 5873646 69 578 | 0.00 | 0.00 | 7262700.00 | 0.00 | 0.00 | 6868100.00 | 0.00 | 738100.00 | 0.00 | 0.00 | 0.00 | 0.00 | KO:K01580 | glutamate decarboxylase | Bacteria;Proteobacteria;Gammaproteobacteria;Vibrionales;Vibrionaceae;Vibrio;Vibrio breoganii;Vibrio breoganii ZF-55 |
| Ga0586324 0000007 104139 106559 | 0.00 | 0.00 | 0.00 | 0.00 | 0.00 | 0.00 | 0.00 | 0.00 | 0.00 | 0.00 | 4109600.00 | 6720000.00 | KO:K01581 | ornithine decarboxylase | Bacteria;Proteobacteria;Alphaproteobacteria;Hyphomicrobiales;Phyllobacteriaceae;Mesorhizobium;Mesorhizobium sophorae;Mesorhizobium sophorae ICMP 19535 |
| Ga0586324 0007318 6848 9091 | 0.00 | 0.00 | 0.00 | 0.00 | 0.00 | 0.00 | 0.00 | 0.00 | 0.00 | 1170400.00 | 1760000.00 | 1185000.00 | KO:K01584 | arginine decarboxylase | Bacteria;Proteobacteria;Betaproteobacteria;Nitrosomonadales;Thiobacillaceae;Annwoodia;Annwoodia aquaesulis;Annwoodia aquaesulis DSM 4255 |
| Ga0586324 0025661 1467 4226 | 0.00 | 0.00 | 0.00 | 0.00 | 0.00 | 0.00 | 0.00 | 0.00 | 0.00 | 0.00 | 0.00 | 3067500.00 | KO:K01584 | arginine decarboxylase | Bacteria;Proteobacteria;Betaproteobacteria;Burkholderiales;Burkholderiaceae;Paraburkholderia;Paraburkholderia fungorum;Paraburkholderia fungorum GAS106B |
| Ga0586324 0094753 3 1739 | 0.00 | 897600.00 | 0.00 | 0.00 | 0.00 | 0.00 | 0.00 | 0.00 | 0.00 | 0.00 | 1672000.00 | 1515000.00 | KO:K01584 | arginine decarboxylase | Bacteria;Proteobacteria;Betaproteobacteria;Burkholderiales;Burkholderiaceae;Ralstonia;Ralstonia sp. 25mfcol4.1;Ralstonia sp. 25MFCol4.1 |
| Ga0586324 0135668 1 2175 | 0.00 | 7344000.00 | 0.00 | 0.00 | 0.00 | 0.00 | 1580929.20 | 1317600.00 | 0.00 | 0.00 | 0.00 | 16350000.00 | KO:K01584 | arginine decarboxylase | Bacteria;Proteobacteria;Betaproteobacteria;Burkholderiales;Burkholderiaceae;Cupriavidus;Cupriavidus plantarum;Cupriavidus plantarum ASC-64 |
| Ga0586324 1089376 1 897 | 0.00 | 231897.68 | 0.00 | 0.00 | 0.00 | 0.00 | 0.00 | 0.00 | 0.00 | 764123.36 | 0.00 | 0.00 | KO:K01584 | arginine decarboxylase | Bacteria;Proteobacteria;Betaproteobacteria;Burkholderiales;Burkholderiaceae;Cupriavidus;Cupriavidus plantarum;Cupriavidus plantarum ASC-64 |
| Ga0586324 1102099 1 1164 | 0.00 | 0.00 | 0.00 | 0.00 | 0.00 | 0.00 | 0.00 | 0.00 | 0.00 | 0.00 | 2085600.00 | 0.00 | KO:K01584 | arginine decarboxylase | Bacteria;Proteobacteria;Alphaproteobacteria;Hyphomicrobiales;Bradyrhizobiaceae;Afipia;Afipia broomeae;Afipia broomeae GAS525 |
| Ga0586324 1690420 1 969 | 0.00 | 0.00 | 0.00 | 0.00 | 0.00 | 1533341.04 | 0.00 | 0.00 | 0.00 | 0.00 | 0.00 | 0.00 | KO:K01584 | arginine decarboxylase | Bacteria;Proteobacteria;Betaproteobacteria;Burkholderiales;Burkholderiaceae;Cupriavidus;Cupriavidus plantarum;Cupriavidus plantarum MA1-2za |
| Ga0586324 0023837 6022 6528 | 0.00 | 0.00 | 0.00 | 0.00 | 0.00 | 0.00 | 0.00 | 0.00 | 0.00 | 2140600.00 | 0.00 | 0.00 | KO:K01588 | 5-(carboxyamino)imidazole ribonucleotide mutase | Bacteria;Proteobacteria;Betaproteobacteria;Nitrosomonadales;Sulfuricellaceae;Sulfurirhabdus;Sulfurirhabdus autotrophica;Sulfurirhabdus autotrophica DSM 100309 |
| Ga0586324 0000002 19492 22308 | 0.00 | 0.00 | 0.00 | 0.00 | 0.00 | 0.00 | 198004.32 | 0.00 | 0.00 | 0.00 | 0.00 | 0.00 | KO:K01595 | phosphoenolpyruvate carboxylase | Bacteria;Proteobacteria;Betaproteobacteria;Nitrosomonadales;Thiobacillaceae;Annwoodia;Annwoodia aquaesulis;Annwoodia aquaesulis DSM 4255 |
| Ga0586324 0202422 275 2119 | 0.00 | 0.00 | 0.00 | 0.00 | 0.00 | 0.00 | 0.00 | 0.00 | 0.00 | 0.00 | 0.00 | 3052500.00 | KO:K01596 | phosphoenolpyruvate carboxykinase (GTP) | Bacteria;Planctomycetes;Planctomycetia;Gemmatales;Gemmataceae;Gemmata;Gemmata obscuriglobus;Gemmata obscuriglobus DSM 5831 |
| Ga0586324 0927541 183 1253 | 10800000.00 | 0.00 | 0.00 | 0.00 | 0.00 | 0.00 | 0.00 | 0.00 | 0.00 | 0.00 | 0.00 | 0.00 | KO:K01596 | phosphoenolpyruvate carboxykinase (GTP) | Bacteria;Actinobacteria;Actinomycetia;Frankiales;Frankiaceae;Frankia;Frankia inefficax;Frankia inefficax EuI1c |
| Ga0586324 1548495 406 1008 | 0.00 | 1638800.00 | 0.00 | 0.00 | 0.00 | 0.00 | 0.00 | 0.00 | 0.00 | 0.00 | 0.00 | 0.00 | KO:K01596 | phosphoenolpyruvate carboxykinase (GTP) | Bacteria;Proteobacteria;Gammaproteobacteria;unclassified Gammaproteobacteria;Candidatus Competibacteraceae;Candidatus Competibacter;Candidatus Competibacter denitrificans;Candidatus Competibacter denitrificans Run A D11 |
| Ga0586324 6415023 1 555 | 0.00 | 0.00 | 0.00 | 0.00 | 0.00 | 2249802.97 | 0.00 | 0.00 | 0.00 | 0.00 | 0.00 | 0.00 | KO:K01599 | uroporphyrinogen decarboxylase | Bacteria;Verrucomicrobia;Opitutae;Opitutales;Opitutaceae;unclassified Opitutaceae;Opitutaceae bacterium;Opitutaceae bacterium TAV3 |
| Ga0586324 0000011 64953 66374 | 0.00 | 0.00 | 0.00 | 0.00 | 0.00 | 0.00 | 0.00 | 0.00 | 0.00 | 15092000.00 | 2921600.00 | 16725000.00 | KO:K01601 | ribulose-bisphosphate carboxylase large chain | Bacteria;Proteobacteria;Betaproteobacteria;Nitrosomonadales;Thiobacillaceae;Thiobacillus;Thiobacillus denitrificans;Thiobacillus denitrificans DSM 12475 |
| Ga0586324 0000656 9055 10434 | 0.00 | 0.00 | 0.00 | 0.00 | 0.00 | 0.00 | 0.00 | 0.00 | 0.00 | 0.00 | 6767200.00 | 0.00 | KO:K01601 | ribulose-bisphosphate carboxylase large chain | Bacteria;Proteobacteria;Betaproteobacteria;Nitrosomonadales;Thiobacillaceae;Thiobacillus;Thiobacillus denitrificans;Thiobacillus denitrificans DSM 12475 |
| Ga0586324 0037134 4148 5527 | 0.00 | 1339600.00 | 0.00 | 0.00 | 0.00 | 0.00 | 0.00 | 0.00 | 11664000.00 | 0.00 | 0.00 | 5445000.00 | KO:K01601 | ribulose-bisphosphate carboxylase large chain | Bacteria;Proteobacteria;Betaproteobacteria;Burkholderiales;Comamonadaceae;Rhodoferax;Rhodoferax antarcticus;Rhodoferax antarcticus DSM 24876 |
| Ga0586324 0069998 2528 3964 | 52570000.00 | 21222800.00 | 102785902.23 | 93271000.00 | 100394100.00 | 65761827.24 | 0.00 | 33964800.00 | 24957433.26 | 18403000.00 | 12346400.00 | 5685000.00 | KO:K01601 | ribulose-bisphosphate carboxylase large chain | Eukaryota;Streptophyta;Magnoliopsida;Malpighiales;Salicaceae;Populus;Populus trichocarpa;Populus balsamifera trichocarpa |
| Ga0586324 0469282 982 1686 | 0.00 | 0.00 | 0.00 | 0.00 | 0.00 | 0.00 | 49248000.00 | 0.00 | 0.00 | 0.00 | 41536000.00 | 46725000.00 | KO:K01601 | ribulose-bisphosphate carboxylase large chain | Bacteria;Proteobacteria;Acidithiobacillia;Acidithiobacillales;Acidithiobacillaceae;Acidithiobacillus;Acidithiobacillus ferrooxidans;Acidithiobacillus ferrooxidans ATCC 53993 |
| Ga0586324 0731259 3 1049 | 0.00 | 0.00 | 0.00 | 0.00 | 0.00 | 0.00 | 0.00 | 0.00 | 5499900.00 | 0.00 | 0.00 | 0.00 | KO:K01601 | ribulose-bisphosphate carboxylase large chain | Bacteria;Proteobacteria;Betaproteobacteria;Nitrosomonadales;Thiobacillaceae;Thiobacillus;Thiobacillus thioparus;Thiobacillus thioparus DSM 505 |
| Ga0586324 0890895 3 1253 | 0.00 | 0.00 | 0.00 | 0.00 | 0.00 | 0.00 | 0.00 | 11590000.00 | 0.00 | 0.00 | 0.00 | 0.00 | KO:K01601 | ribulose-bisphosphate carboxylase large chain | Bacteria;Proteobacteria;Gammaproteobacteria;Acidiferrobacterales;Acidiferrobacteraceae;Sulfurifustis;Sulfurifustis variabilis;Sulfurifustis variabilis skN76 |
| Ga0586324 1095597 1 1050 | 0.00 | 3413600.00 | 0.00 | 0.00 | 0.00 | 3349346.08 | 0.00 | 0.00 | 0.00 | 0.00 | 0.00 | 0.00 | KO:K01601 | ribulose-bisphosphate carboxylase large chain | Eukaryota;Streptophyta;Magnoliopsida;Malpighiales;Salicaceae;Populus;Populus trichocarpa;Populus balsamifera trichocarpa |
| Ga0586324 1176306 1 945 | 0.00 | 0.00 | 0.00 | 0.00 | 0.00 | 0.00 | 0.00 | 0.00 | 2502900.00 | 2240700.00 | 0.00 | 1762500.00 | KO:K01601 | ribulose-bisphosphate carboxylase large chain | Eukaryota;Bacillariophyta;Coscinodiscophyceae;Thalassiosirales;Thalassiosiraceae;Thalassiosira;Thalassiosira pseudonana;Thalassiosira pseudonana CCMP 1335 |
| Ga0586324 1293263 448 1086 | 0.00 | 0.00 | 0.00 | 0.00 | 0.00 | 0.00 | 0.00 | 0.00 | 3110400.00 | 0.00 | 0.00 | 0.00 | KO:K01601 | ribulose-bisphosphate carboxylase large chain | Bacteria;Proteobacteria;Betaproteobacteria;Nitrosomonadales;Sulfuricellaceae;Sulfuriferula;Sulfuriferula thiophila;Sulfuriferula thiophila mst6 |
| Ga0586324 1870281 1 891 | 0.00 | 0.00 | 0.00 | 0.00 | 0.00 | 0.00 | 0.00 | 0.00 | 2316600.00 | 0.00 | 0.00 | 0.00 | KO:K01601 | ribulose-bisphosphate carboxylase large chain | Bacteria;Proteobacteria;Betaproteobacteria;Burkholderiales;Comamonadaceae;Limnohabitans;Limnohabitans sp. 63ED37-2;Limnohabitans sp. 63ED37-2 |
| Ga0586324 2387207 1 837 | 0.00 | 2176000.00 | 0.00 | 0.00 | 0.00 | 0.00 | 0.00 | 0.00 | 0.00 | 0.00 | 0.00 | 0.00 | KO:K01601 | ribulose-bisphosphate carboxylase large chain | Bacteria;Proteobacteria;Gammaproteobacteria;Chromatiales;Chromatiaceae;Allochromatium;Allochromatium warmingii;Allochromatium warmingii DSM 173 |
| Ga0586324 3945909 3 680 | 0.00 | 0.00 | 0.00 | 0.00 | 0.00 | 0.00 | 0.00 | 0.00 | 0.00 | 3796100.00 | 0.00 | 0.00 | KO:K01601 | ribulose-bisphosphate carboxylase large chain | Bacteria;Proteobacteria;Betaproteobacteria;Nitrosomonadales;Sulfuricellaceae;Sulfuricella;Sulfuricella denitrificans;Sulfuricella denitrificans skB26 |
| Ga0586324 6832111 2 541 | 0.00 | 0.00 | 0.00 | 0.00 | 8167900.00 | 0.00 | 0.00 | 0.00 | 0.00 | 0.00 | 0.00 | 0.00 | KO:K01601 | ribulose-bisphosphate carboxylase large chain | Bacteria;Proteobacteria;Betaproteobacteria;Nitrosomonadales;Sulfuricellaceae;Sulfuriferula;Sulfuriferula thiophila;Sulfuriferula thiophila mst6 |
| Ga0586324 8050146 2 505 | 0.00 | 0.00 | 0.00 | 0.00 | 0.00 | 0.00 | 0.00 | 0.00 | 1927800.00 | 0.00 | 5676000.00 | 0.00 | KO:K01601 | ribulose-bisphosphate carboxylase large chain | Bacteria;Proteobacteria;Betaproteobacteria;Burkholderiales;unclassified Burkholderiales;Sphaerotilus;Sphaerotilus montanus;Sphaerotilus montanus DSM 21226 |
| Ga0586324 0432435 64 1620 | 0.00 | 2679200.00 | 56300000.00 | 0.00 | 0.00 | 0.00 | 0.00 | 0.00 | 0.00 | 0.00 | 0.00 | 0.00 | KO:K01604 | acetyl-CoA carboxylase carboxyltransferase component | Bacteria;Bacteroidetes;Bacteroidia;Marinilabiliales;Marinifilaceae;Ancylomarina;Ancylomarina subtilis;Ancylomarina subtilis DSM 28825 |
| Ga0586324 1080705 3 1175 | 0.00 | 0.00 | 0.00 | 0.00 | 0.00 | 0.00 | 0.00 | 0.00 | 647326.08 | 0.00 | 0.00 | 0.00 | KO:K01604 | acetyl-CoA carboxylase carboxyltransferase component | Bacteria;Proteobacteria;Deltaproteobacteria;Syntrophobacterales;Syntrophaceae;Desulfobacca;Desulfobacca acetoxidans;Desulfobacca acetoxidans ASRB2, DSM 11109 |
| Ga0586324 2288425 2 502 | 0.00 | 0.00 | 0.00 | 0.00 | 0.00 | 0.00 | 0.00 | 31415000.00 | 0.00 | 0.00 | 0.00 | 0.00 | KO:K01604 | acetyl-CoA carboxylase carboxyltransferase component | Bacteria;Bacteroidetes;Cytophagia;Cytophagales;Flammeovirgaceae;Sediminitomix;Sediminitomix flava;Sediminitomix flava DSM 28229 |
| Ga0586324 2679214 48 797 | 0.00 | 0.00 | 0.00 | 0.00 | 0.00 | 8972200.00 | 0.00 | 0.00 | 0.00 | 0.00 | 0.00 | 0.00 | KO:K01604 | acetyl-CoA carboxylase carboxyltransferase component | Bacteria;Proteobacteria;Deltaproteobacteria;Syntrophobacterales;Syntrophobacteraceae;Desulfosoma;Desulfosoma caldarium;Desulfosoma caldarium DSM 22027 |
| Ga0586324 3724333 3 695 | 0.00 | 0.00 | 0.00 | 2061500.00 | 0.00 | 0.00 | 0.00 | 0.00 | 0.00 | 0.00 | 0.00 | 0.00 | KO:K01604 | acetyl-CoA carboxylase carboxyltransferase component | Bacteria;Proteobacteria;Deltaproteobacteria;Syntrophobacterales;Syntrophobacteraceae;Desulfoglaeba;Desulfoglaeba alkanexedens;Desulfoglaeba alkanexedens ALDC |
| Ga0586324 4998944 165 617 | 0.00 | 0.00 | 0.00 | 0.00 | 0.00 | 0.00 | 0.00 | 0.00 | 0.00 | 0.00 | 1117600.00 | 0.00 | KO:K01604 | acetyl-CoA carboxylase carboxyltransferase component | Bacteria;Bacteroidetes;Bacteroidia;Bacteroidales;Salinivirgaceae;Salinivirga;Salinivirga cyanobacteriivorans;Salinivirga cyanobacteriivorans L21-Spi-D4 |
| Ga0586324 3081647 2 370 | 0.00 | 2801600.00 | 0.00 | 0.00 | 0.00 | 0.00 | 0.00 | 368418.04 | 0.00 | 0.00 | 0.00 | 862500.00 | KO:K01607 | 4-carboxymuconolactone decarboxylase | Archaea;Euryarchaeota;Methanomicrobia;Methanomicrobiales;Methanoregulaceae;Methanoregula;Methanoregula formicica;Methanoregula formicica SMSP, DSM 22288 |
| Ga0586324 0051560 2 1189 | 0.00 | 0.00 | 0.00 | 0.00 | 0.00 | 0.00 | 475886.16 | 0.00 | 0.00 | 0.00 | 0.00 | 0.00 | KO:K01610 | phosphoenolpyruvate carboxykinase (ATP) | Bacteria;Ignavibacteriae;Ignavibacteria;Ignavibacteriales;Melioribacteraceae;Melioribacter;Melioribacter roseus;Melioribacter roseus P3M |
| Ga0586324 0047080 2 1321 | 0.00 | 0.00 | 0.00 | 0.00 | 0.00 | 0.00 | 0.00 | 1976400.00 | 0.00 | 0.00 | 0.00 | 0.00 | KO:K01615 | glutaconyl-CoA decarboxylase | Bacteria;Proteobacteria;Betaproteobacteria;Burkholderiales;Comamonadaceae;Ramlibacter;Ramlibacter lithotrophicus;Ramlibacter lithotrophicus RBP-1 |
| Ga0586324 0121115 1 1479 | 0.00 | 0.00 | 0.00 | 0.00 | 1720100.00 | 1541070.63 | 0.00 | 0.00 | 3491100.00 | 0.00 | 0.00 | 0.00 | KO:K01615 | glutaconyl-CoA decarboxylase | Bacteria;Proteobacteria;Deltaproteobacteria;unclassified Deltaproteobacteria;unclassified Deltaproteobacteria;unclassified Deltaproteobacteria;delta proteobacterium NaphS2;delta proteobacterium NaphS2 |
| Ga0586324 1353430 3 1067 | 0.00 | 0.00 | 0.00 | 0.00 | 1833400.00 | 733910.08 | 0.00 | 0.00 | 0.00 | 0.00 | 0.00 | 0.00 | KO:K01615 | glutaconyl-CoA decarboxylase | Bacteria;Proteobacteria;Deltaproteobacteria;unclassified Deltaproteobacteria;unclassified Deltaproteobacteria;unclassified Deltaproteobacteria;delta proteobacterium NaphS2;delta proteobacterium NaphS2 |
| Ga0586324 1426755 1 1041 | 0.00 | 0.00 | 0.00 | 866133.05 | 0.00 | 0.00 | 0.00 | 0.00 | 0.00 | 0.00 | 0.00 | 0.00 | KO:K01615 | glutaconyl-CoA decarboxylase | Bacteria;Proteobacteria;Deltaproteobacteria;Syntrophobacterales;Syntrophaceae;Smithella;Smithella sp. F21;Smithella sp. F21 |
| Ga0586324 1791803 2 946 | 0.00 | 0.00 | 0.00 | 0.00 | 0.00 | 0.00 | 0.00 | 550489.62 | 2527200.00 | 0.00 | 0.00 | 0.00 | KO:K01615 | glutaconyl-CoA decarboxylase | Bacteria;Proteobacteria;Deltaproteobacteria;Syntrophorhabdales;Syntrophorhabdaceae;Syntrophorhabdus;Syntrophorhabdus aromaticivorans;Syntrophorhabdus aromaticivorans UI |
| Ga0586324 3507117 3 713 | 0.00 | 0.00 | 0.00 | 0.00 | 0.00 | 0.00 | 0.00 | 1360300.00 | 0.00 | 0.00 | 0.00 | 0.00 | KO:K01615 | glutaconyl-CoA decarboxylase | Bacteria;Proteobacteria;Deltaproteobacteria;Desulfobacterales;Desulfobacteraceae;Desulfatiglans;Desulfatiglans anilini;Desulfatiglans anilini DSM 4660 |
| Ga0586324 0002350 3357 4397 | 0.00 | 0.00 | 0.00 | 0.00 | 0.00 | 0.00 | 0.00 | 0.00 | 0.00 | 1655500.00 | 0.00 | 3727500.00 | KO:K01619 | deoxyribose-phosphate aldolase | Bacteria;Chloroflexi;Chloroflexia;Kallotenuales;Kallotenuaceae;Kallotenue;Kallotenue papyrolyticum;Kallotenue papyrolyticum JKG1 |
| Ga0586324 0357590 1 1899 | 0.00 | 0.00 | 0.00 | 0.00 | 0.00 | 0.00 | 0.00 | 0.00 | 0.00 | 20713000.00 | 19360000.00 | 0.00 | KO:K01621 | xylulose-5-phosphate/fructose-6-phosphate phosphoketolase | Bacteria;Proteobacteria;Betaproteobacteria;Nitrosomonadales;Nitrosomonadaceae;Nitrosospira;Nitrosospira multiformis;Nitrosospira multiformis Nl1 |
| Ga0586324 0820728 2 1150 | 0.00 | 0.00 | 0.00 | 0.00 | 0.00 | 0.00 | 0.00 | 0.00 | 0.00 | 476397.46 | 0.00 | 0.00 | KO:K01621 | xylulose-5-phosphate/fructose-6-phosphate phosphoketolase | Bacteria;Acidobacteria;Acidobacteriia;Acidobacteriales;Acidobacteriaceae;unclassified Acidobacteriaceae;Acidobacteriaceae bacterium URHE0068;Acidobacteriaceae bacterium URHE0068 |
| Ga0586324 2022343 1 900 | 0.00 | 0.00 | 0.00 | 0.00 | 0.00 | 1733115.41 | 0.00 | 0.00 | 0.00 | 0.00 | 0.00 | 0.00 | KO:K01621 | xylulose-5-phosphate/fructose-6-phosphate phosphoketolase | Bacteria;Acidobacteria;Acidobacteriia;Acidobacteriales;Acidobacteriaceae;unclassified Acidobacteriaceae;Acidobacteriaceae bacterium KBS 89;Acidobacteriaceae bacterium KBS 89, DSM 24296 |
| Ga0586324 3479160 160 717 | 0.00 | 0.00 | 0.00 | 0.00 | 0.00 | 0.00 | 0.00 | 0.00 | 0.00 | 0.00 | 0.00 | 3157500.00 | KO:K01623 | fructose-bisphosphate aldolase class I | Bacteria;Actinobacteria;Rubrobacteria;Gaiellales;Gaiellaceae;Gaiella;Gaiella occulta;Gaiella occulta F2-233 |
| Ga0586324 0000001 218717 219784 | 0.00 | 0.00 | 0.00 | 0.00 | 0.00 | 0.00 | 0.00 | 37197800.00 | 0.00 | 2648800.00 | 2147200.00 | 654325.50 | KO:K01624 | fructose-bisphosphate aldolase class II | Bacteria;Proteobacteria;Betaproteobacteria;Nitrosomonadales;Thiobacillaceae;Sulfuritortus;Sulfuritortus calidifontis;Sulfuritortus calidifontis DSM 103923 |
| Ga0586324 0000401 3 875 | 0.00 | 0.00 | 0.00 | 0.00 | 0.00 | 0.00 | 10396800.00 | 0.00 | 0.00 | 24871000.00 | 26400000.00 | 17025000.00 | KO:K01624 | fructose-bisphosphate aldolase class II | Bacteria;Proteobacteria;Gammaproteobacteria;Acidiferrobacterales;Acidiferrobacteraceae;Sulfurifustis;Sulfurifustis variabilis;Sulfurifustis variabilis skN76 |
| Ga0586324 0001255 3421 4488 | 0.00 | 0.00 | 0.00 | 0.00 | 0.00 | 0.00 | 0.00 | 1207800.00 | 0.00 | 0.00 | 436299.60 | 0.00 | KO:K01624 | fructose-bisphosphate aldolase class II | Bacteria;Proteobacteria;Betaproteobacteria;Nitrosomonadales;Thiobacillaceae;Thiobacillus;Thiobacillus thioparus;Thiobacillus thioparus DSM 505 |
| Ga0586324 0006832 6731 7810 | 595749.00 | 518258.60 | 13005300.00 | 0.00 | 0.00 | 0.00 | 0.00 | 0.00 | 0.00 | 0.00 | 0.00 | 310264.50 | KO:K01624 | fructose-bisphosphate aldolase class II | Bacteria;Proteobacteria;Gammaproteobacteria;Methylococcales;Methylococcaceae;Methylobacter;Methylobacter tundripaludum;Methylobacter tundripaludum SV96 |
| Ga0586324 0008824 3 329 | 0.00 | 0.00 | 0.00 | 0.00 | 5438400.00 | 0.00 | 5061600.00 | 0.00 | 0.00 | 0.00 | 0.00 | 0.00 | KO:K01624 | fructose-bisphosphate aldolase class II | Bacteria;Proteobacteria;Betaproteobacteria;Burkholderiales;unclassified Burkholderiales;Methylibium;Methylibium sp. NZG;Methylibium sp. NZG |
| Ga0586324 0013553 2980 4044 | 0.00 | 0.00 | 0.00 | 0.00 | 0.00 | 0.00 | 0.00 | 0.00 | 1579500.00 | 0.00 | 0.00 | 0.00 | KO:K01624 | fructose-bisphosphate aldolase class II | Bacteria;Proteobacteria;Gammaproteobacteria;Alteromonadales;Alteromonadaceae;unclassified Alteromonadaceae;Alteromonadaceae bacterium 2719K.STB50.0a.01;Alteromonadaceae bacterium 2719K.STB50.0a.01 |
| Ga0586324 0083446 1858 2937 | 0.00 | 1142400.00 | 0.00 | 0.00 | 0.00 | 0.00 | 0.00 | 1305400.00 | 0.00 | 0.00 | 0.00 | 0.00 | KO:K01624 | fructose-bisphosphate aldolase class II | Bacteria;Proteobacteria;Gammaproteobacteria;Methylococcales;Methylococcaceae;Methylobacter;Methylobacter marinus;Methylobacter marinus A45 |
| Ga0586324 0116333 1365 2429 | 0.00 | 0.00 | 3661566.21 | 0.00 | 0.00 | 0.00 | 14637600.00 | 0.00 | 0.00 | 0.00 | 0.00 | 0.00 | KO:K01624 | fructose-bisphosphate aldolase class II | Bacteria;Proteobacteria;Betaproteobacteria;Rhodocyclales;Rhodocyclaceae;Azospira;unclassified Azospira;Azospira sp. ZAP |
| Ga0586324 0137442 120 1184 | 0.00 | 0.00 | 13343100.00 | 0.00 | 0.00 | 0.00 | 0.00 | 0.00 | 0.00 | 0.00 | 0.00 | 0.00 | KO:K01624 | fructose-bisphosphate aldolase class II | Bacteria;Proteobacteria;Betaproteobacteria;unclassified Betaproteobacteria;unclassified Betaproteobacteria;unclassified Betaproteobacteria;uncultured beta proteobacterium;unclassified betaproteobacterium ENR4 |
| Ga0586324 0205310 1650 2444 | 0.00 | 0.00 | 0.00 | 0.00 | 0.00 | 0.00 | 0.00 | 0.00 | 0.00 | 823900.00 | 1540000.00 | 0.00 | KO:K01624 | fructose-bisphosphate aldolase class II | Bacteria;Proteobacteria;Betaproteobacteria;Rhodocyclales;Rhodocyclaceae;Rhodocyclus;Rhodocyclus tenuis;Rhodocyclus tenuis DSM 109 |
| Ga0586324 0237412 556 1635 | 0.00 | 0.00 | 0.00 | 0.00 | 0.00 | 0.00 | 0.00 | 0.00 | 4171500.00 | 0.00 | 0.00 | 0.00 | KO:K01624 | fructose-bisphosphate aldolase class II | Bacteria;Proteobacteria;Gammaproteobacteria;Methylococcales;Methylococcaceae;Methylobacter;Methylobacter tundripaludum;Methylobacter tundripaludum SV96 |
| Ga0586324 0345032 1014 1931 | 12700000.00 | 0.00 | 0.00 | 0.00 | 0.00 | 0.00 | 0.00 | 0.00 | 0.00 | 0.00 | 0.00 | 0.00 | KO:K01624 | fructose-bisphosphate aldolase class II | Bacteria;Proteobacteria;Gammaproteobacteria;Cellvibrionales;Halieaceae;Halioglobus;Halioglobus sediminis;Halioglobus sediminis U0301 U0301 |
| Ga0586324 1195375 367 1125 | 0.00 | 5154400.00 | 0.00 | 0.00 | 0.00 | 0.00 | 0.00 | 7320000.00 | 10530000.00 | 10934000.00 | 12672000.00 | 9900000.00 | KO:K01624 | fructose-bisphosphate aldolase class II | Bacteria;Proteobacteria;Gammaproteobacteria;Chromatiales;unclassified Chromatiales;Thiohalobacter;Thiohalobacter thiocyanaticus;Thiohalobacter thiocyanaticus FOKN1 |
| Ga0586324 2923935 492 770 | 0.00 | 0.00 | 0.00 | 0.00 | 0.00 | 0.00 | 0.00 | 0.00 | 0.00 | 2387000.00 | 0.00 | 0.00 | KO:K01624 | fructose-bisphosphate aldolase class II | Bacteria;Proteobacteria;Gammaproteobacteria;Methylococcales;Methylococcaceae;Methylosarcina;Methylosarcina fibrata;Methylosarcina fibrata AML-C10 |
| Ga0586324 4118822 3 509 | 823075.00 | 181369.60 | 0.00 | 0.00 | 0.00 | 0.00 | 0.00 | 0.00 | 0.00 | 0.00 | 0.00 | 0.00 | KO:K01624 | fructose-bisphosphate aldolase class II | Bacteria;Proteobacteria;Gammaproteobacteria;Methylococcales;Methylococcaceae;Methylobacter;Methylobacter tundripaludum;Methylobacter tundripaludum OWC-G53F |
| Ga0586324 6019010 1 570 | 0.00 | 0.00 | 0.00 | 0.00 | 0.00 | 0.00 | 0.00 | 0.00 | 0.00 | 0.00 | 0.00 | 6937500.00 | KO:K01624 | fructose-bisphosphate aldolase class II | Bacteria;Proteobacteria;Gammaproteobacteria;Chromatiales;Chromatiaceae;Thiobaca;Thiobaca trueperi;Thiobaca trueperi DSM 13587 |
| Ga0586324 7017408 142 537 | 6070000.00 | 5304000.00 | 0.00 | 0.00 | 0.00 | 0.00 | 0.00 | 0.00 | 0.00 | 0.00 | 0.00 | 0.00 | KO:K01624 | fructose-bisphosphate aldolase class II | Bacteria;Proteobacteria;Gammaproteobacteria;Methylococcales;Methylococcaceae;Methylobacter;Methylobacter whittenburyi;Methylobacter whittenburyi ACM 3310 |
| Ga0586324 7674346 1 489 | 0.00 | 0.00 | 0.00 | 0.00 | 0.00 | 3075165.97 | 0.00 | 0.00 | 0.00 | 0.00 | 0.00 | 0.00 | KO:K01624 | fructose-bisphosphate aldolase class II | Bacteria;Spirochaetes;Spirochaetia;Spirochaetales;Treponemataceae;Treponema;Treponema caldarium;Treponema caldarium DSM 7334 |
| Ga0586324 0024967 4697 5542 | 0.00 | 0.00 | 0.00 | 0.00 | 0.00 | 0.00 | 0.00 | 0.00 | 0.00 | 0.00 | 0.00 | 2257500.00 | KO:K01627 | 2-dehydro-3-deoxyphosphooctonate aldolase (KDO 8-P synthase) | Bacteria;Proteobacteria;Gammaproteobacteria;Acidiferrobacterales;Acidiferrobacteraceae;Sulfuricaulis;Sulfuricaulis limicola;Sulfuricaulis limicola HA5 |
| Ga0586324 0027087 1669 2988 | 0.00 | 0.00 | 0.00 | 0.00 | 0.00 | 0.00 | 0.00 | 0.00 | 0.00 | 0.00 | 0.00 | 440930.25 | KO:K01637 | isocitrate lyase | Bacteria;Proteobacteria;Betaproteobacteria;unclassified Betaproteobacteria;unclassified Betaproteobacteria;unclassified Betaproteobacteria;beta proteobacterium AAP99;Methylibium sp. AAP99 |
| Ga0586324 0572149 3 1004 | 0.00 | 15232000.00 | 0.00 | 0.00 | 0.00 | 0.00 | 0.00 | 0.00 | 0.00 | 0.00 | 0.00 | 0.00 | KO:K01637 | isocitrate lyase | Bacteria;Proteobacteria;Gammaproteobacteria;unclassified Gammaproteobacteria;Candidatus Competibacteraceae;Candidatus Competibacter;Candidatus Competibacter denitrificans;Candidatus Competibacter denitrificans Run A D11 |
| Ga0586324 0375934 538 1434 | 0.00 | 0.00 | 6136700.00 | 0.00 | 0.00 | 5994700.00 | 0.00 | 0.00 | 0.00 | 0.00 | 2921600.00 | 595407.75 | KO:K01644 | citrate lyase subunit beta/citryl-CoA lyase | Bacteria;Proteobacteria;Deltaproteobacteria;Desulfobacterales;Desulfobulbaceae;Desulfofustis;Desulfofustis glycolicus;Desulfofustis glycolicus DSM 9705 |
| Ga0586324 0624373 78 974 | 1500000.00 | 5535200.00 | 1146819.74 | 2992500.00 | 0.00 | 4605200.00 | 0.00 | 0.00 | 0.00 | 0.00 | 0.00 | 0.00 | KO:K01644 | citrate lyase subunit beta/citryl-CoA lyase | Bacteria;Proteobacteria;Deltaproteobacteria;Desulfobacterales;Desulfobulbaceae;Desulfofustis;Desulfofustis glycolicus;Desulfofustis glycolicus DSM 9705 |
| Ga0586324 1306750 422 1081 | 3620000.00 | 4658000.00 | 0.00 | 0.00 | 0.00 | 0.00 | 4446000.00 | 0.00 | 1782000.00 | 0.00 | 0.00 | 315144.00 | KO:K01644 | citrate lyase subunit beta/citryl-CoA lyase | Bacteria;Proteobacteria;Deltaproteobacteria;Desulfobacterales;Desulfobulbaceae;Desulfofustis;Desulfofustis glycolicus;Desulfofustis glycolicus DSM 9705 |
| Ga0586324 3864232 17 685 | 0.00 | 140766.12 | 0.00 | 0.00 | 0.00 | 0.00 | 0.00 | 186946.09 | 0.00 | 0.00 | 0.00 | 0.00 | KO:K01644 | citrate lyase subunit beta/citryl-CoA lyase | Bacteria;Proteobacteria;Deltaproteobacteria;Desulfobacterales;Desulfobulbaceae;Desulfofustis;Desulfofustis glycolicus;Desulfofustis glycolicus DSM 9705 |
| Ga0586324 4861346 2 187 | 0.00 | 0.00 | 0.00 | 235814.70 | 0.00 | 0.00 | 0.00 | 0.00 | 0.00 | 0.00 | 0.00 | 0.00 | KO:K01644 | citrate lyase subunit beta/citryl-CoA lyase | Bacteria;Firmicutes;Clostridia;Eubacteriales;Peptococcaceae;unclassified Peptococcaceae;Peptococcaceae bacterium CEB3;Peptococcaceae bacterium CEB3 |
| Ga0586324 0001131 4083 5378 | 0.00 | 0.00 | 0.00 | 0.00 | 0.00 | 0.00 | 1289456.28 | 228266.27 | 1101600.00 | 0.00 | 0.00 | 1500000.00 | KO:K01647 | citrate synthase | Bacteria;Proteobacteria;Betaproteobacteria;Nitrosomonadales;Thiobacillaceae;Thiobacillus;Thiobacillus thioparus;Thiobacillus thioparus DSM 505 |
| Ga0586324 0010533 8008 9339 | 0.00 | 0.00 | 0.00 | 6897000.00 | 13287000.00 | 5518300.00 | 7501200.00 | 0.00 | 6463800.00 | 5189800.00 | 0.00 | 0.00 | KO:K01647 | citrate synthase | Bacteria;Candidatus Aminicenantes;unclassified Candidatus Aminicenantes;unclassified Candidatus Aminicenantes;unclassified Candidatus Aminicenantes;unclassified Candidatus Aminicenantes;Aminicenantes bacterium SCGC AAA255-E05;Aminicenantes bacterium SCGC AAA255-E05 (contamination screened) |
| Ga0586324 0016290 3605 4900 | 0.00 | 0.00 | 0.00 | 0.00 | 0.00 | 0.00 | 4195200.00 | 0.00 | 0.00 | 0.00 | 0.00 | 0.00 | KO:K01647 | citrate synthase | Bacteria;Proteobacteria;Deltaproteobacteria;Myxococcales;Archangiaceae;Stigmatella;Stigmatella aurantiaca;Stigmatella aurantiaca DSM 17044 |
| Ga0586324 0038359 3 923 | 0.00 | 0.00 | 0.00 | 0.00 | 9640800.00 | 0.00 | 0.00 | 0.00 | 0.00 | 0.00 | 0.00 | 0.00 | KO:K01647 | citrate synthase | Bacteria;Proteobacteria;Deltaproteobacteria;Desulfuromonadales;Geobacteraceae;Geobacter;Geobacter argillaceus;Geobacter argillaceus ATCC BAA-1139 |
| Ga0586324 0078366 2 766 | 0.00 | 3202800.00 | 0.00 | 0.00 | 0.00 | 0.00 | 16780800.00 | 4068700.00 | 11583000.00 | 6876100.00 | 0.00 | 0.00 | KO:K01647 | citrate synthase | Bacteria;Proteobacteria;Betaproteobacteria;Burkholderiales;unclassified Burkholderiales;Ideonella;Ideonella sp. A 288;Ideonella sp. A 288 |
| Ga0586324 0150270 1061 2341 | 0.00 | 0.00 | 0.00 | 0.00 | 0.00 | 0.00 | 1931369.76 | 0.00 | 0.00 | 0.00 | 0.00 | 0.00 | KO:K01647 | citrate synthase | Bacteria;Calditrichaeota;Calditrichae;Calditrichales;Calditrichaceae;Caldithrix;Caldithrix abyssi;Caldithrix abyssi LF13, DSM 13497 |
| Ga0586324 0910436 58 1263 | 0.00 | 0.00 | 0.00 | 0.00 | 0.00 | 0.00 | 5586000.00 | 0.00 | 0.00 | 0.00 | 0.00 | 0.00 | KO:K01647 | citrate synthase | Bacteria;Proteobacteria;Deltaproteobacteria;Desulfuromonadales;Geobacteraceae;Geobacter;Geobacter lovleyi;Geobacter lovleyi SZ |
| Ga0586324 1166305 341 1135 | 0.00 | 0.00 | 0.00 | 0.00 | 0.00 | 0.00 | 0.00 | 0.00 | 0.00 | 0.00 | 3150400.00 | 0.00 | KO:K01647 | citrate synthase | Bacteria;Proteobacteria;Betaproteobacteria;Burkholderiales;Alcaligenaceae;Derxia;Derxia gummosa;Derxia gummosa DSM 723 |
| Ga0586324 2700769 1 417 | 0.00 | 0.00 | 0.00 | 0.00 | 0.00 | 0.00 | 0.00 | 0.00 | 0.00 | 0.00 | 3564000.00 | 0.00 | KO:K01647 | citrate synthase | Bacteria;Proteobacteria;Deltaproteobacteria;Desulfuromonadales;Desulfuromonadaceae;Malonomonas;Malonomonas rubra;Malonomonas rubra DSM 5091 |
| Ga0586324 2813544 63 782 | 0.00 | 0.00 | 0.00 | 0.00 | 0.00 | 0.00 | 1521209.16 | 0.00 | 0.00 | 0.00 | 0.00 | 0.00 | KO:K01647 | citrate synthase | Bacteria;Proteobacteria;Deltaproteobacteria;Desulfuromonadales;Geobacteraceae;Geothermobacter;Geothermobacter ehrlichii;Geothermobacter ehrlichii DSM 15274 |
| Ga0586324 3270106 78 734 | 0.00 | 0.00 | 0.00 | 0.00 | 0.00 | 0.00 | 0.00 | 7747000.00 | 0.00 | 0.00 | 0.00 | 0.00 | KO:K01647 | citrate synthase | Bacteria;Proteobacteria;Deltaproteobacteria;Myxococcales;Anaeromyxobacteraceae;Anaeromyxobacter;Anaeromyxobacter dehalogenans;Anaeromyxobacter dehalogenans 2CP-C |
| Ga0586324 3661136 3 542 | 14700000.00 | 0.00 | 0.00 | 0.00 | 0.00 | 0.00 | 1433757.48 | 0.00 | 0.00 | 0.00 | 0.00 | 2235000.00 | KO:K01647 | citrate synthase | Bacteria;Ignavibacteriae;Ignavibacteria;Ignavibacteriales;Ignavibacteriaceae;Ignavibacterium;Ignavibacterium album;Ignavibacterium album Mat9-16, JCM 16511 |
| Ga0586324 6192135 2 565 | 0.00 | 0.00 | 0.00 | 0.00 | 0.00 | 0.00 | 0.00 | 0.00 | 27216000.00 | 0.00 | 0.00 | 0.00 | KO:K01647 | citrate synthase | Bacteria;Proteobacteria;Deltaproteobacteria;Desulfuromonadales;Geobacteraceae;Geoalkalibacter;Geoalkalibacter ferrihydriticus;Geoalkalibacter ferrihydriticus DSM 17813 |
| Ga0586324 7265254 2 301 | 0.00 | 0.00 | 0.00 | 0.00 | 0.00 | 0.00 | 0.00 | 5349700.00 | 0.00 | 0.00 | 0.00 | 0.00 | KO:K01647 | citrate synthase | Bacteria;Proteobacteria;Betaproteobacteria;Burkholderiales;Alcaligenaceae;Azohydromonas;Azohydromonas australica;Azohydromonas australica DSM 1124 |
| Ga0586324 0002719 3080 4630 | 0.00 | 0.00 | 0.00 | 0.00 | 0.00 | 0.00 | 0.00 | 0.00 | 0.00 | 0.00 | 270383.52 | 0.00 | KO:K01649 | 2-isopropylmalate synthase | Bacteria;Proteobacteria;Betaproteobacteria;Burkholderiales;unclassified Burkholderiales;Tepidimonas;Tepidimonas taiwanensis;Tepidimonas taiwanensis VT154-175 |
| Ga0586324 0035173 4010 5557 | 0.00 | 0.00 | 0.00 | 0.00 | 0.00 | 0.00 | 1458645.96 | 0.00 | 0.00 | 660854.04 | 0.00 | 2242500.00 | KO:K01649 | 2-isopropylmalate synthase | Bacteria;Proteobacteria;Betaproteobacteria;Nitrosomonadales;Thiobacillaceae;Thiobacillus;Thiobacillus denitrificans;Thiobacillus denitrificans ATCC 25259 |
| Ga0586324 0047902 1119 2666 | 0.00 | 0.00 | 0.00 | 0.00 | 0.00 | 0.00 | 0.00 | 811300.00 | 0.00 | 0.00 | 0.00 | 0.00 | KO:K01649 | 2-isopropylmalate synthase | Bacteria;Proteobacteria;Betaproteobacteria;Nitrosomonadales;Thiobacillaceae;Thiobacillus;Thiobacillus denitrificans;Thiobacillus denitrificans ATCC 25259 |
| Ga0586324 0372236 517 1866 | 0.00 | 0.00 | 0.00 | 0.00 | 0.00 | 0.00 | 0.00 | 0.00 | 732468.42 | 0.00 | 524899.76 | 0.00 | KO:K01649 | 2-isopropylmalate synthase | Bacteria;Proteobacteria;Betaproteobacteria;Nitrosomonadales;Methylophilaceae;Methylobacillus;Methylobacillus sp. MM3;Methylobacillus sp. MM2 |
| Ga0586324 0862753 2 1294 | 0.00 | 0.00 | 0.00 | 0.00 | 0.00 | 0.00 | 0.00 | 0.00 | 0.00 | 0.00 | 0.00 | 1687500.00 | KO:K01649 | 2-isopropylmalate synthase | Bacteria;Proteobacteria;Betaproteobacteria;Nitrosomonadales;Thiobacillaceae;Sulfuritortus;Sulfuritortus calidifontis;Sulfuritortus calidifontis DSM 103923 |
| Ga0586324 6514484 3 554 | 0.00 | 0.00 | 0.00 | 0.00 | 0.00 | 0.00 | 0.00 | 0.00 | 0.00 | 0.00 | 0.00 | 396637.50 | KO:K01649 | 2-isopropylmalate synthase | Bacteria;Chloroflexi;Dehalococcoidia;Dehalococcoidales;Dehalococcoidaceae;Dehalococcoides;Dehalococcoides mccartyi;Dehalococcoides mccartyi GY50 |
| Ga0586324 0000003 91033 92700 | 0.00 | 0.00 | 0.00 | 0.00 | 0.00 | 0.00 | 0.00 | 0.00 | 0.00 | 0.00 | 184881.84 | 0.00 | KO:K01652 | acetolactate synthase-1/2/3 large subunit | Bacteria;Proteobacteria;Betaproteobacteria;Nitrosomonadales;Thiobacillaceae;Sulfuritortus;Sulfuritortus calidifontis;Sulfuritortus calidifontis DSM 103923 |
| Ga0586324 0000037 20444 22156 | 0.00 | 0.00 | 0.00 | 0.00 | 0.00 | 0.00 | 239128.68 | 2677900.00 | 252072.81 | 5320700.00 | 2332000.00 | 7080000.00 | KO:K01652 | acetolactate synthase-1/2/3 large subunit | Bacteria;Proteobacteria;Betaproteobacteria;Nitrosomonadales;Thiobacillaceae;Annwoodia;Annwoodia aquaesulis;Annwoodia aquaesulis DSM 4255 |
| Ga0586324 0000431 12081 13790 | 0.00 | 1700000.00 | 0.00 | 11590000.00 | 0.00 | 0.00 | 0.00 | 5075200.00 | 0.00 | 6552700.00 | 0.00 | 0.00 | KO:K01652 | acetolactate synthase-1/2/3 large subunit | Bacteria;Proteobacteria;Betaproteobacteria;Nitrosomonadales;Gallionellaceae;Gallionella;Gallionella capsiferriformans;Gallionella capsiferriformans ES-2 |
| Ga0586324 0014235 1101 2777 | 0.00 | 0.00 | 0.00 | 0.00 | 0.00 | 0.00 | 0.00 | 0.00 | 0.00 | 839300.00 | 0.00 | 0.00 | KO:K01652 | acetolactate synthase-1/2/3 large subunit | Archaea;Euryarchaeota;Methanomicrobia;Methanomicrobiales;Methanoregulaceae;Methanoregula;Methanoregula formicica;Methanoregula formicica SMSP, DSM 22288 |
| Ga0586324 0015292 3519 5309 | 0.00 | 0.00 | 0.00 | 0.00 | 0.00 | 0.00 | 0.00 | 0.00 | 133126.74 | 0.00 | 0.00 | 0.00 | KO:K01652 | acetolactate synthase-1/2/3 large subunit | Bacteria;Proteobacteria;Alphaproteobacteria;Hyphomicrobiales;Hyphomicrobiaceae;Rhodoplanes;Rhodoplanes sp. Z2-YC6860;Rhodoplanes sp. Z2-YC6860 |
| Ga0586324 0016207 1152 2822 | 0.00 | 0.00 | 0.00 | 3724000.00 | 0.00 | 9845600.00 | 0.00 | 0.00 | 0.00 | 0.00 | 0.00 | 0.00 | KO:K01652 | acetolactate synthase-1/2/3 large subunit | Archaea;Euryarchaeota;Methanomicrobia;Methanomicrobiales;Methanoregulaceae;Methanoregula;Methanoregula formicica;Methanoregula formicica SMSP, DSM 22288 |
| Ga0586324 0038292 3096 4820 | 0.00 | 0.00 | 0.00 | 648755.00 | 0.00 | 0.00 | 0.00 | 0.00 | 0.00 | 0.00 | 0.00 | 0.00 | KO:K01652 | acetolactate synthase-1/2/3 large subunit | Bacteria;Chloroflexi;Dehalococcoidia;Dehalococcoidales;Dehalococcoidaceae;Dehalococcoides;Dehalococcoides mccartyi;Dehalococcoides mccartyi VS |
| Ga0586324 0039922 3122 4834 | 0.00 | 0.00 | 0.00 | 0.00 | 0.00 | 0.00 | 0.00 | 0.00 | 1190700.00 | 0.00 | 0.00 | 0.00 | KO:K01652 | acetolactate synthase-1/2/3 large subunit | Bacteria;Proteobacteria;Betaproteobacteria;Nitrosomonadales;Thiobacillaceae;Thiobacillus;Thiobacillus denitrificans;Thiobacillus denitrificans ATCC 25259 |
| Ga0586324 0190220 395 2113 | 0.00 | 0.00 | 0.00 | 4018500.00 | 0.00 | 0.00 | 0.00 | 0.00 | 0.00 | 0.00 | 0.00 | 0.00 | KO:K01652 | acetolactate synthase-1/2/3 large subunit | Bacteria;Chloroflexi;Dehalococcoidia;Dehalococcoidales;Dehalococcoidaceae;Dehalococcoides;Dehalococcoides mccartyi;Dehalococcoides mccartyi VS |
| Ga0586324 0467894 2 1432 | 0.00 | 0.00 | 0.00 | 0.00 | 0.00 | 0.00 | 0.00 | 0.00 | 0.00 | 0.00 | 0.00 | 2115000.00 | KO:K01652 | acetolactate synthase-1/2/3 large subunit | Bacteria;Cyanobacteria;unclassified Cyanobacteria;Oscillatoriales;Microcoleaceae;Microcoleus;Microcoleus sp. PCC 7113;Microcoleus sp. PCC 7113 |
| Ga0586324 2583062 1 810 | 3970000.00 | 0.00 | 0.00 | 0.00 | 0.00 | 0.00 | 0.00 | 0.00 | 0.00 | 0.00 | 0.00 | 0.00 | KO:K01652 | acetolactate synthase-1/2/3 large subunit | Bacteria;Chloroflexi;unclassified Chloroflexi;unclassified Chloroflexi;unclassified Chloroflexi;Thermobaculum;Thermobaculum terrenum;Thermobaculum terrenum YNP1, ATCC BAA-798 |
| Ga0586324 2595013 2 577 | 0.00 | 0.00 | 0.00 | 0.00 | 0.00 | 0.00 | 0.00 | 0.00 | 4471200.00 | 0.00 | 0.00 | 0.00 | KO:K01652 | acetolactate synthase-1/2/3 large subunit | Bacteria;Actinobacteria;Rubrobacteria;Gaiellales;Gaiellaceae;Gaiella;Gaiella occulta;Gaiella occulta F2-233 |
| Ga0586324 3727638 1 696 | 0.00 | 0.00 | 0.00 | 0.00 | 0.00 | 5756500.00 | 0.00 | 0.00 | 0.00 | 0.00 | 0.00 | 0.00 | KO:K01652 | acetolactate synthase-1/2/3 large subunit | Bacteria;Proteobacteria;Betaproteobacteria;Nitrosomonadales;Gallionellaceae;Gallionella;Gallionella capsiferriformans;Gallionella capsiferriformans ES-2 |
| Ga0586324 6721905 171 545 | 0.00 | 0.00 | 0.00 | 0.00 | 0.00 | 0.00 | 16438800.00 | 1433500.00 | 0.00 | 0.00 | 0.00 | 0.00 | KO:K01652 | acetolactate synthase-1/2/3 large subunit | Bacteria;Proteobacteria;Betaproteobacteria;Burkholderiales;Comamonadaceae;Variovorax;Variovorax sp. 770b2;Variovorax sp. 770b2 |
| Ga0586324 0108934 1296 1781 | 0.00 | 1298800.00 | 0.00 | 0.00 | 0.00 | 0.00 | 0.00 | 0.00 | 10854000.00 | 0.00 | 0.00 | 0.00 | KO:K01653 | acetolactate synthase-1/3 small subunit | Bacteria;Proteobacteria;Deltaproteobacteria;Syntrophobacterales;Syntrophaceae;Smithella;Smithella sp. SCADC;Smithella sp. SCADC |
| Ga0586324 2929277 2 403 | 0.00 | 1441600.00 | 0.00 | 0.00 | 0.00 | 0.00 | 0.00 | 0.00 | 2251800.00 | 0.00 | 0.00 | 0.00 | KO:K01653 | acetolactate synthase-1/3 small subunit | Bacteria;Verrucomicrobia;Verrucomicrobiae;Verrucomicrobiales;Verrucomicrobia subdivision 3;Pedosphaera;Pedosphaera parvula;Pedosphaera parvula Ellin514 |
| Ga0586324 4252587 1 660 | 0.00 | 0.00 | 0.00 | 2926000.00 | 0.00 | 0.00 | 0.00 | 0.00 | 0.00 | 0.00 | 0.00 | 0.00 | KO:K01667 | tryptophanase | Bacteria;Bacteroidetes;Bacteroidia;Bacteroidales;Salinivirgaceae;Salinivirga;Salinivirga cyanobacteriivorans;Salinivirga cyanobacteriivorans L21-Spi-D4 |
| Ga0586324 3235915 2 739 | 3220000.00 | 0.00 | 0.00 | 0.00 | 0.00 | 0.00 | 0.00 | 0.00 | 0.00 | 0.00 | 0.00 | 0.00 | KO:K01668 | tyrosine phenol-lyase | Bacteria;Ignavibacteriae;Ignavibacteria;Ignavibacteriales;Ignavibacteriaceae;Ignavibacterium;Ignavibacterium album;Ignavibacterium album Mat9-16, JCM 16511 |
| Ga0586324 4544117 3 641 | 0.00 | 0.00 | 0.00 | 0.00 | 0.00 | 0.00 | 0.00 | 2305800.00 | 0.00 | 0.00 | 0.00 | 0.00 | KO:K01668 | tyrosine phenol-lyase | Bacteria;Acidobacteria;Acidobacteriia;Acidobacteriales;Acidobacteriaceae;Candidatus Koribacter;Candidatus Koribacter versatilis;Candidatus Koribacter versatilis Ellin345 |
| Ga0586324 0006505 4456 5031 | 5040000.00 | 0.00 | 0.00 | 0.00 | 0.00 | 0.00 | 0.00 | 0.00 | 0.00 | 0.00 | 0.00 | 0.00 | KO:K01673 | carbonic anhydrase | Bacteria;Proteobacteria;Gammaproteobacteria;Methylococcales;Methylococcaceae;Methylobacter;Methylobacter marinus;Methylobacter marinus A45 |
| Ga0586324 0009019 4665 5249 | 9110000.00 | 10268000.00 | 21337700.00 | 0.00 | 11742000.00 | 15165400.00 | 0.00 | 11285000.00 | 12879000.00 | 0.00 | 18744000.00 | 8625000.00 | KO:K01673 | carbonic anhydrase | Bacteria;Proteobacteria;Gammaproteobacteria;Methylococcales;Methylococcaceae;Methylomicrobium;Methylomicrobium agile;Methylomicrobium agile ATCC 35068 |
| Ga0586324 0025501 2250 2831 | 0.00 | 0.00 | 0.00 | 0.00 | 0.00 | 0.00 | 0.00 | 1134600.00 | 0.00 | 0.00 | 0.00 | 0.00 | KO:K01673 | carbonic anhydrase | Bacteria;Proteobacteria;Gammaproteobacteria;Methylococcales;Methylococcaceae;Methylomicrobium;Methylomicrobium lacus;Methylosarcina lacus LW14 |
| Ga0586324 1143222 317 904 | 0.00 | 0.00 | 0.00 | 0.00 | 0.00 | 0.00 | 69084000.00 | 0.00 | 0.00 | 0.00 | 0.00 | 67425000.00 | KO:K01673 | carbonic anhydrase | Bacteria;Proteobacteria;Alphaproteobacteria;Hyphomicrobiales;Methylocystaceae;Methylocystis;Methylocystis parvus;Methylocystis parvus OBBP |
| Ga0586324 3086010 3 275 | 0.00 | 0.00 | 0.00 | 0.00 | 0.00 | 0.00 | 0.00 | 0.00 | 0.00 | 0.00 | 0.00 | 5617500.00 | KO:K01673 | carbonic anhydrase | Bacteria;Proteobacteria;Gammaproteobacteria;Methylococcales;Methyloprofundus;Methyloprofundus sedimenti;Methyloprofundus sedimenti WF1 |

| | | | | | | | | | | | | | | | |
|---|---|---|---|---|---|---|---|---|---|---|---|---|---|---|---|
| Ga0586324 7134363 3 533 | 0.00 | 0.00 | 0.00 | 0.00 | 0.00 | 0.00 | 0.00 | 0.00 | 626386.77 | 0.00 | 424179.36 | 0.00 | KO:K01673 | carbonic anhydrase | Bacteria;Proteobacteria;Gammaproteobacteria;Methylococcales;Methylococcaceae;Methylobacter;Methylobacter tundripaludum;Methylobacter tundripaludum SV96 |
| Ga0586324 7608941 1 420 | 0.00 | 6772800.00 | 1997343.84 | 1178000.00 | 8930100.00 | 0.00 | 1567107.84 | 5282600.00 | 4738500.00 | 7161000.00 | 7312800.00 | 5572500.00 | KO:K01673 | carbonic anhydrase | Bacteria;Proteobacteria;Gammaproteobacteria;Methylococcales;Methylococcaceae;Methylobacter;Methylobacter tundripaludum;Methylobacter tundripaludum SV96 |
| Ga0586324 0444947 2 1555 | 0.00 | 0.00 | 0.00 | 0.00 | 0.00 | 0.00 | 0.00 | 0.00 | 2535300.00 | 0.00 | 0.00 | 0.00 | KO:K01676 | fumarate hydratase class I | Bacteria;Proteobacteria;Deltaproteobacteria;Syntrophobacterales;Syntrophobacteraceae;Syntrophobacter;Syntrophobacter fumaroxidans;Syntrophobacter fumaroxidans MPOB |
| Ga0586324 1394746 1 1053 | 0.00 | 1931200.00 | 0.00 | 0.00 | 0.00 | 0.00 | 0.00 | 0.00 | 0.00 | 0.00 | 0.00 | 0.00 | KO:K01679 | fumarate hydratase class II | Bacteria;Firmicutes;Clostridia;Eubacteriales;Clostridiales Family XVII. Incertae Sedis;Thermaerobacter;Thermaerobacter subterraneus;Thermaerobacter subterraneus C21, DSM 13965 |
| Ga0586324 0000793 19927 22575 | 0.00 | 0.00 | 0.00 | 0.00 | 0.00 | 0.00 | 0.00 | 2299700.00 | 0.00 | 0.00 | 0.00 | 0.00 | KO:K01681 | aconitate hydratase | Bacteria;Proteobacteria;Betaproteobacteria;Nitrosomonadales;Thiobacillaceae;Thiobacillus;Thiobacillus thioparus;Thiobacillus thioparus DSM 505 |
| Ga0586324 0006682 8298 11117 | 0.00 | 0.00 | 0.00 | 0.00 | 8507800.00 | 0.00 | 0.00 | 0.00 | 0.00 | 0.00 | 0.00 | 0.00 | KO:K01681 | aconitate hydratase | Bacteria;Firmicutes;Bacilli;Bacillales;Alicyclobacillaceae;Alicyclobacillus;Alicyclobacillus macrosporangiidus;Alicyclobacillus macrosporangiidus DSM 17980 |
| Ga0586324 0019988 972 3620 | 0.00 | 0.00 | 0.00 | 0.00 | 0.00 | 0.00 | 0.00 | 0.00 | 0.00 | 0.00 | 3572800.00 | 0.00 | KO:K01681 | aconitate hydratase | Bacteria;Proteobacteria;Betaproteobacteria;Nitrosomonadales;Thiobacillaceae;Thiobacillus;Thiobacillus thioparus;Thiobacillus thioparus DSM 505 |
| Ga0586324 1080172 1 1173 | 0.00 | 0.00 | 0.00 | 0.00 | 0.00 | 0.00 | 0.00 | 0.00 | 0.00 | 1216600.00 | 0.00 | 0.00 | KO:K01681 | aconitate hydratase | Bacteria;Proteobacteria;Alphaproteobacteria;Rhodospirillales;Acetobacteraceae;Rhodopila;Rhodopila globiformis;Rhodopila globiformis DSM 161 |
| Ga0586324 1138273 3 1049 | 0.00 | 0.00 | 0.00 | 0.00 | 0.00 | 0.00 | 0.00 | 1769000.00 | 0.00 | 0.00 | 0.00 | 0.00 | KO:K01681 | aconitate hydratase | Bacteria;Proteobacteria;Betaproteobacteria;Nitrosomonadales;Thiobacillaceae;Thiobacillus;Thiobacillus thioparus;Thiobacillus thioparus DSM 505 |
| Ga0586324 1438707 3 1040 | 0.00 | 0.00 | 0.00 | 0.00 | 1792200.00 | 0.00 | 0.00 | 1213900.00 | 0.00 | 0.00 | 0.00 | 0.00 | KO:K01681 | aconitate hydratase | Bacteria;Proteobacteria;Alphaproteobacteria;Rhodospirillales;Acetobacteraceae;Rhodopila;Rhodopila globiformis;Rhodopila globiformis DSM 161 |
| Ga0586324 3282799 3 734 | 0.00 | 0.00 | 0.00 | 0.00 | 0.00 | 0.00 | 0.00 | 0.00 | 0.00 | 0.00 | 1698400.00 | 0.00 | KO:K01681 | aconitate hydratase | Bacteria;Proteobacteria;Betaproteobacteria;Burkholderiales;unclassified Burkholderiales;Methylibium;Methylibium petroleiphilum;Methylibium petroleiphilum PM1 |
| Ga0586324 4660419 3 635 | 0.00 | 1740800.00 | 0.00 | 0.00 | 0.00 | 0.00 | 0.00 | 0.00 | 0.00 | 0.00 | 0.00 | 0.00 | KO:K01681 | aconitate hydratase | Bacteria;Verrucomicrobia;Opitutae;Opitutales;Opitutaceae;Opitutus;Opitutus sp. GAS368;Opitutus sp. GAS368 |
| Ga0586324 0010177 7747 10116 | 0.00 | 1020000.00 | 0.00 | 2137500.00 | 0.00 | 0.00 | 0.00 | 0.00 | 1198800.00 | 0.00 | 0.00 | 0.00 | KO:K01682 | aconitate hydratase 2/2-methylisocitrate dehydratase | Bacteria;Proteobacteria;Betaproteobacteria;Burkholderiales;unclassified Burkholderiales;unclassified Burkholderiales;[Polyangium] brachysporum;Polyangium brachysporum DSM 7029 |
| Ga0586324 0259536 2 2092 | 0.00 | 0.00 | 0.00 | 0.00 | 0.00 | 0.00 | 0.00 | 0.00 | 1231200.00 | 0.00 | 0.00 | 0.00 | KO:K01682 | aconitate hydratase 2/2-methylisocitrate dehydratase | Bacteria;Proteobacteria;Betaproteobacteria;unclassified Betaproteobacteria;unclassified Betaproteobacteria;unclassified Betaproteobacteria;beta proteobacterium AAP51;Betaproteobacteria bacterium AAP51 |
| Ga0586324 7913473 2 511 | 3940000.00 | 0.00 | 0.00 | 0.00 | 0.00 | 0.00 | 0.00 | 0.00 | 0.00 | 0.00 | 0.00 | 0.00 | KO:K01682 | aconitate hydratase 2/2-methylisocitrate dehydratase | Bacteria;Proteobacteria;Betaproteobacteria;Rhodocyclales;Azonexaceae;Azonexus;Azonexus hydrophilus;Azonexus hydrophilus DSM 23864 |
| Ga0586324 0023853 4338 6833 | 0.00 | 0.00 | 0.00 | 0.00 | 0.00 | 0.00 | 0.00 | 1726300.00 | 1684800.00 | 0.00 | 0.00 | 900000.00 | KO:K01687 | dihydroxy-acid dehydratase | Bacteria;Proteobacteria;Betaproteobacteria;Nitrosomonadales;Thiobacillaceae;Thiobacillus;Thiobacillus thioparus;Thiobacillus thioparus DSM 505 |
| Ga0586324 4080978 3 671 | 0.00 | 0.00 | 0.00 | 0.00 | 0.00 | 0.00 | 0.00 | 0.00 | 0.00 | 0.00 | 384945.44 | 0.00 | KO:K01687 | dihydroxy-acid dehydratase | Bacteria;Verrucomicrobia;Opitutae;Opitutales;Opitutaceae;Opitutus;Opitutus terrae;Opitutus terrae PB90-1 |
| Ga0586324 0000002 36711 37994 | 0.00 | 0.00 | 0.00 | 0.00 | 0.00 | 0.00 | 0.00 | 0.00 | 0.00 | 23100000.00 | 0.00 | 0.00 | KO:K01689 | enolase | Bacteria;Proteobacteria;Betaproteobacteria;Nitrosomonadales;Thiobacillaceae;Annwoodia;Annwoodia aquaesulis;Annwoodia aquaesulis DSM 4255 |
| Ga0586324 0002948 13928 15211 | 0.00 | 0.00 | 701008.19 | 0.00 | 0.00 | 0.00 | 0.00 | 0.00 | 0.00 | 0.00 | 0.00 | 0.00 | KO:K01689 | enolase | Bacteria;Ignavibacteriae;Ignavibacteria;Ignavibacteriales;Melioribacteraceae;Melioribacter;Melioribacter roseus;Melioribacter roseus P3M |
| Ga0586324 0009045 5510 6805 | 0.00 | 0.00 | 0.00 | 0.00 | 0.00 | 0.00 | 0.00 | 0.00 | 0.00 | 0.00 | 0.00 | 515145.00 | KO:K01689 | enolase | Bacteria;Ignavibacteriae;Ignavibacteria;Ignavibacteriales;Ignavibacteriaceae;Ignavibacterium;Ignavibacterium album;Ignavibacterium album Mat9-16, JCM 16511 |
| Ga0586324 0012096 3217 4653 | 0.00 | 0.00 | 0.00 | 0.00 | 0.00 | 0.00 | 0.00 | 0.00 | 0.00 | 0.00 | 1918400.00 | 0.00 | KO:K01689 | enolase | Bacteria;Tenericutes;Mollicutes;Acholeplasmatales;Acholeplasmataceae;Acholeplasma;Acholeplasma brassicae;Acholeplasma brassicae 0502 |
| Ga0586324 0012602 3470 4750 | 0.00 | 0.00 | 0.00 | 14582500.00 | 1957000.00 | 0.00 | 0.00 | 2287500.00 | 2211300.00 | 2564100.00 | 0.00 | 0.00 | KO:K01689 | enolase | Bacteria;Proteobacteria;Deltaproteobacteria;Syntrophobacterales;Syntrophobacteraceae;Syntrophobacter;Syntrophobacter fumaroxidans;Syntrophobacter fumaroxidans MPOB |
| Ga0586324 0012986 1971 3260 | 0.00 | 381137.28 | 0.00 | 0.00 | 0.00 | 0.00 | 0.00 | 0.00 | 0.00 | 0.00 | 0.00 | 0.00 | KO:K01689 | enolase | Bacteria;Ignavibacteriae;Ignavibacteria;Ignavibacteriales;Melioribacteraceae;Melioribacter;Melioribacter roseus;Melioribacter roseus P3M |
| Ga0586324 0014827 2667 3956 | 0.00 | 0.00 | 0.00 | 0.00 | 0.00 | 0.00 | 0.00 | 3336700.00 | 0.00 | 4735500.00 | 279102.56 | 3690000.00 | KO:K01689 | enolase | Bacteria;Proteobacteria;Betaproteobacteria;Nitrosomonadales;Thiobacillaceae;Thiobacillus;Thiobacillus thiophilus;Thiobacillus thiophilus DSM 19892 |
| Ga0586324 0049291 2333 3610 | 0.00 | 0.00 | 0.00 | 0.00 | 0.00 | 0.00 | 0.00 | 0.00 | 0.00 | 0.00 | 0.00 | 2955000.00 | KO:K01689 | enolase | Bacteria;Bacteroidetes;Bacteroidia;Bacteroidales;Rikenellaceae;Alistipes;Alistipes indistinctus;Alistipes indistinctus YIT 12060 |
| Ga0586324 0068805 1422 2717 | 0.00 | 507819.92 | 0.00 | 0.00 | 0.00 | 0.00 | 0.00 | 0.00 | 0.00 | 0.00 | 0.00 | 0.00 | KO:K01689 | enolase | Bacteria;Proteobacteria;Deltaproteobacteria;Desulfuromonadales;Geobacteraceae;Geobacter;Geobacter lovleyi;Geobacter lovleyi SZ |
| Ga0586324 0073340 816 2120 | 0.00 | 0.00 | 0.00 | 0.00 | 0.00 | 0.00 | 0.00 | 0.00 | 0.00 | 0.00 | 0.00 | 1162500.00 | KO:K01689 | enolase | Bacteria;Calditrichaeota;Calditrichae;Calditrichales;Calditrichaceae;Caldithrix;Caldithrix abyssi;Caldithrix abyssi LF13, DSM 13497 |
| Ga0586324 0081131 305 1588 | 0.00 | 0.00 | 0.00 | 0.00 | 0.00 | 0.00 | 0.00 | 0.00 | 0.00 | 0.00 | 215006.00 | 214029.75 | KO:K01689 | enolase | Bacteria;Proteobacteria;Betaproteobacteria;Nitrosomonadales;Gallionellaceae;Sideroxydans;Sideroxydans lithotrophicus;Sideroxydans lithotrophicus ES-1 |
| Ga0586324 0084561 2264 3544 | 0.00 | 0.00 | 0.00 | 0.00 | 0.00 | 0.00 | 0.00 | 2751100.00 | 2778300.00 | 3388000.00 | 3687200.00 | 2790000.00 | KO:K01689 | enolase | Bacteria;Proteobacteria;Gammaproteobacteria;Methylococcales;Methylococcaceae;Methylobacter;Methylobacter tundripaludum;Methylobacter tundripaludum 31/32 |
| Ga0586324 0124146 1 1098 | 0.00 | 0.00 | 0.00 | 0.00 | 0.00 | 0.00 | 0.00 | 0.00 | 0.00 | 0.00 | 932800.00 | 0.00 | KO:K01689 | enolase | Bacteria;Proteobacteria;Deltaproteobacteria;Syntrophobacterales;Syntrophobacteraceae;Syntrophobacter;Syntrophobacter fumaroxidans;Syntrophobacter fumaroxidans MPOB |
| Ga0586324 0125913 1 633 | 0.00 | 0.00 | 0.00 | 0.00 | 0.00 | 0.00 | 0.00 | 0.00 | 980100.00 | 0.00 | 0.00 | 0.00 | KO:K01689 | enolase | Bacteria;Proteobacteria;Betaproteobacteria;Nitrosomonadales;Gallionellaceae;Ferriphaselus;Ferriphaselus amnicola;Ferriphaselus amnicola OYT1 |
| Ga0586324 0129165 745 2022 | 0.00 | 7344000.00 | 0.00 | 0.00 | 0.00 | 0.00 | 0.00 | 0.00 | 0.00 | 1162700.00 | 0.00 | 0.00 | KO:K01689 | enolase | Bacteria;Bacteroidetes;Bacteroidia;Bacteroidales;Rikenellaceae;Alistipes;Alistipes sp. An31A;Alistipes sp. An31A |
| Ga0586324 0133332 231 1511 | 0.00 | 0.00 | 0.00 | 11210000.00 | 0.00 | 0.00 | 0.00 | 0.00 | 0.00 | 8547000.00 | 7656000.00 | 0.00 | KO:K01689 | enolase | Bacteria;Proteobacteria;Deltaproteobacteria;Syntrophobacterales;Syntrophobacteraceae;Desulfacinum;Desulfacinum hydrothermale;Desulfacinum hydrothermale DSM 13146 |
| Ga0586324 0155704 3 1313 | 0.00 | 0.00 | 1651126.99 | 0.00 | 0.00 | 0.00 | 0.00 | 0.00 | 0.00 | 0.00 | 0.00 | 1590000.00 | KO:K01689 | enolase | Bacteria;Proteobacteria;Betaproteobacteria;Nitrosomonadales;Nitrosomonadaceae;Nitrosospira;Nitrosospira sp. NpAV;Nitrosospira sp. NpAV |
| Ga0586324 0218062 518 1801 | 4060000.00 | 0.00 | 0.00 | 0.00 | 0.00 | 0.00 | 0.00 | 4654300.00 | 0.00 | 7345800.00 | 0.00 | 4260000.00 | KO:K01689 | enolase | Archaea;Euryarchaeota;unclassified Euryarchaeota;unclassified Euryarchaeota;unclassified Euryarchaeota;unclassified Euryarchaeota;Euryarchaeota archaeon SCGC AAA252-I15;Euryarchaeota archaeon SCGC AAA252-I15 (contamination screened) |
| Ga0586324 0355736 1 1242 | 1880000.00 | 884000.00 | 0.00 | 0.00 | 4253900.00 | 2039980.53 | 3260400.00 | 0.00 | 1239300.00 | 1370600.00 | 0.00 | 1470000.00 | KO:K01689 | enolase | Bacteria;Proteobacteria;Betaproteobacteria;Burkholderiales;unclassified Burkholderiales;unclassified Burkholderiales;Burkholderiales bacterium JOSHI 001;Burkholderiales sp. JOSHI 001 |
| Ga0586324 0566292 221 1516 | 0.00 | 0.00 | 0.00 | 12350000.00 | 0.00 | 0.00 | 0.00 | 0.00 | 0.00 | 0.00 | 0.00 | 0.00 | KO:K01689 | enolase | Bacteria;Chloroflexi;Anaerolineae;Anaerolineales;Anaerolineaceae;Anaerolinea;Anaerolinea thermophila;Anaerolinea thermophila UNI-1 |
| Ga0586324 0573970 1302 1544 | 0.00 | 0.00 | 0.00 | 0.00 | 0.00 | 957484.60 | 0.00 | 0.00 | 0.00 | 0.00 | 0.00 | 0.00 | KO:K01689 | enolase | Bacteria;Proteobacteria;Betaproteobacteria;Burkholderiales;Comamonadaceae;unclassified Comamonadaceae;Comamonadaceae bacterium;Comamonadaceae sp. OAS658 |
| Ga0586324 0632361 2 1132 | 0.00 | 0.00 | 0.00 | 0.00 | 0.00 | 0.00 | 2508000.00 | 0.00 | 0.00 | 0.00 | 0.00 | 0.00 | KO:K01689 | enolase | Bacteria;Bacteroidetes;Bacteroidia;Bacteroidales;Rikenellaceae;Alistipes;Alistipes sp. An31A;Alistipes sp. An31A |
| Ga0586324 0967476 1 945 | 0.00 | 0.00 | 0.00 | 12445000.00 | 0.00 | 0.00 | 0.00 | 2159400.00 | 0.00 | 0.00 | 0.00 | 0.00 | KO:K01689 | enolase | Bacteria;Proteobacteria;Deltaproteobacteria;Desulfobacterales;Desulfobacteraceae;Desulfatitalea;Desulfatitalea tepidiphila;Desulfatitalea tepidiphila S28bF |
| Ga0586324 0988249 239 1219 | 1050000.00 | 0.00 | 0.00 | 0.00 | 0.00 | 0.00 | 0.00 | 0.00 | 0.00 | 0.00 | 0.00 | 0.00 | KO:K01689 | enolase | Bacteria;Proteobacteria;Betaproteobacteria;Nitrosomonadales;Sterolibacteriaceae;Sulfurisoma;Sulfurisoma sediminicola;Sulfurisoma sediminicola DSM 26916 |
| Ga0586324 1063918 85 777 | 0.00 | 657540.96 | 0.00 | 894610.25 | 1153600.00 | 0.00 | 0.00 | 1146800.00 | 799829.64 | 885500.00 | 719947.36 | 615881.25 | KO:K01689 | enolase | Bacteria;Acidobacteria;Acidobacteriia;Acidobacteriales;Acidobacteriaceae;Edaphobacter;Edaphobacter modestus;Edaphobacter modestus DSM 18101 |
| Ga0586324 1286941 107 1090 | 3150000.00 | 4046000.00 | 0.00 | 0.00 | 4532000.00 | 0.00 | 0.00 | 3928400.00 | 3102300.00 | 3757600.00 | 2692800.00 | 0.00 | KO:K01689 | enolase | Bacteria;Proteobacteria;Deltaproteobacteria;Desulfobacterales;Desulfobacteraceae;Desulfosarcina;Desulfosarcina variabilis;Desulfosarcina variabilis Montpellier |
| Ga0586324 1462201 153 1031 | 0.00 | 300583.80 | 0.00 | 0.00 | 0.00 | 0.00 | 0.00 | 0.00 | 0.00 | 0.00 | 0.00 | 0.00 | KO:K01689 | enolase | Bacteria;Proteobacteria;Gammaproteobacteria;Thiotrichales;Thiotrichaceae;Beggiatoa;Beggiatoa alba;Beggiatoa alba B18LD |
| Ga0586324 1556904 593 1003 | 0.00 | 0.00 | 0.00 | 0.00 | 0.00 | 0.00 | 0.00 | 0.00 | 0.00 | 2779700.00 | 2437600.00 | 3802500.00 | KO:K01689 | enolase | Bacteria;Proteobacteria;Betaproteobacteria;Nitrosomonadales;Nitrosomonadaceae;Nitrosospira;Nitrosospira multiformis;Nitrosospira multiformis NI1 |
| Ga0586324 1627883 3 986 | 0.00 | 0.00 | 0.00 | 17641500.00 | 15645700.00 | 7816793.83 | 0.00 | 0.00 | 625149.09 | 2618000.00 | 2807200.00 | 0.00 | KO:K01689 | enolase | Bacteria;Proteobacteria;Deltaproteobacteria;Syntrophobacterales;Syntrophobacteraceae;Syntrophobacter;Syntrophobacter fumaroxidans;Syntrophobacter fumaroxidans MPOB |
| Ga0586324 1670959 3 887 | 0.00 | 3114400.00 | 0.00 | 0.00 | 4326000.00 | 0.00 | 0.00 | 0.00 | 0.00 | 0.00 | 0.00 | 0.00 | KO:K01689 | enolase | Bacteria;Proteobacteria;Gammaproteobacteria;Methylococcales;Methylococcaceae;Methylobacter;Methylobacter tundripaludum;Methylobacter tundripaludum 31/32 |
| Ga0586324 1907544 2 922 | 0.00 | 0.00 | 0.00 | 1102000.00 | 0.00 | 0.00 | 0.00 | 0.00 | 0.00 | 0.00 | 0.00 | 0.00 | KO:K01689 | enolase | Archaea;Euryarchaeota;Methanomicrobia;Methanosarcinales;Methanotrichaceae;Methanothrix;Methanothrix soehngenii;Methanothrix soehngenii GP-6 |
| Ga0586324 2115540 3 881 | 0.00 | 0.00 | 0.00 | 0.00 | 0.00 | 0.00 | 0.00 | 0.00 | 0.00 | 0.00 | 0.00 | 2505000.00 | KO:K01689 | enolase | Bacteria;Acidobacteria;Vicinamibacteria;unclassified Vicinamibacteria;Vicinamibacteraceae;Luteitalea;Luteitalea pratensis;Luteitalea pratensis DSM 100886 |
| Ga0586324 2286772 1 687 | 0.00 | 0.00 | 0.00 | 3572000.00 | 0.00 | 308021.58 | 0.00 | 0.00 | 0.00 | 0.00 | 0.00 | 0.00 | KO:K01689 | enolase | Bacteria;Proteobacteria;Deltaproteobacteria;Syntrophobacterales;Syntrophobacteraceae;Desulfoglaeba;Desulfoglaeba alkanexedens;Desulfoglaeba alkanexedens ALDC |
| Ga0586324 2388084 1 837 | 0.00 | 0.00 | 0.00 | 0.00 | 2935500.00 | 0.00 | 0.00 | 0.00 | 0.00 | 0.00 | 0.00 | 0.00 | KO:K01689 | enolase | Bacteria;Ignavibacteriae;Ignavibacteria;Ignavibacteriales;Ignavibacteriaceae;Ignavibacterium;Ignavibacterium album;Ignavibacterium album Mat9-16, JCM 16511 |
| Ga0586324 2546528 1 816 | 2730000.00 | 0.00 | 0.00 | 0.00 | 0.00 | 0.00 | 0.00 | 0.00 | 0.00 | 0.00 | 0.00 | 0.00 | KO:K01689 | enolase | Bacteria;Chloroflexi;Anaerolineae;Anaerolineales;Anaerolineaceae;Anaerolinea;Anaerolinea thermophila;Anaerolinea thermophila UNI-1 |
| Ga0586324 2739620 2 790 | 0.00 | 0.00 | 0.00 | 0.00 | 5150000.00 | 0.00 | 0.00 | 2787700.00 | 0.00 | 4242700.00 | 0.00 | 5190000.00 | KO:K01689 | enolase | Bacteria;Proteobacteria;Gammaproteobacteria;unclassified Gammaproteobacteria;Candidatus Competibacteraceae;Candidatus Competibacter;Candidatus Competibacter denitrificans;Candidatus Competibacter denitrificans Run A D11 |
| Ga0586324 3093656 2 751 | 0.00 | 466857.40 | 0.00 | 0.00 | 0.00 | 0.00 | 0.00 | 0.00 | 0.00 | 0.00 | 0.00 | 0.00 | KO:K01689 | enolase | Bacteria;Proteobacteria;Betaproteobacteria;Burkholderiales;Comamonadaceae;Xenophilus;Xenophilus sp. E41;Xenophilus sp. E41 |
| Ga0586324 3771142 1 693 | 3570000.00 | 0.00 | 0.00 | 0.00 | 0.00 | 0.00 | 0.00 | 0.00 | 0.00 | 0.00 | 0.00 | 0.00 | KO:K01689 | enolase | Bacteria;Verrucomicrobia;Verrucomicrobiae;Verrucomicrobiales;Akkermansiaceae;Akkermansia;Akkermansia sp. KLE1798;Akkermansia sp. KLE1798 |
| Ga0586324 3797872 1 690 | 0.00 | 0.00 | 0.00 | 0.00 | 5747400.00 | 0.00 | 0.00 | 0.00 | 0.00 | 0.00 | 0.00 | 0.00 | KO:K01689 | enolase | Bacteria;Proteobacteria;Gammaproteobacteria;unclassified Gammaproteobacteria;Candidatus Competibacteraceae;Candidatus Competibacter;Candidatus Competibacter denitrificans;Candidatus Competibacter denitrificans Run A D11 |
| Ga0586324 3913961 1 681 | 0.00 | 0.00 | 0.00 | 0.00 | 0.00 | 0.00 | 0.00 | 3294000.00 | 3645000.00 | 0.00 | 0.00 | 0.00 | KO:K01689 | enolase | Bacteria;Proteobacteria;Betaproteobacteria;Nitrosomonadales;Thiobacillaceae;Thiobacillus;Thiobacillus denitrificans;Thiobacillus denitrificans DSM 12475 |
| Ga0586324 3991981 124 678 | 1170000.00 | 952000.00 | 0.00 | 0.00 | 0.00 | 0.00 | 0.00 | 0.00 | 0.00 | 0.00 | 0.00 | 0.00 | KO:K01689 | enolase | Bacteria;Proteobacteria;Deltaproteobacteria;Syntrophobacterales;Syntrophobacteraceae;Syntrophobacter;Syntrophobacter fumaroxidans;Syntrophobacter fumaroxidans MPOB |
| Ga0586324 4301279 379 657 | 0.00 | 0.00 | 0.00 | 0.00 | 0.00 | 0.00 | 0.00 | 5239900.00 | 0.00 | 0.00 | 0.00 | 0.00 | KO:K01689 | enolase | Bacteria;Proteobacteria;Deltaproteobacteria;Desulfuromonadales;Desulfuromonadaceae;Desulfuromonas;Desulfuromonas thiophila;Desulfuromonas thiophila DSM 8987 |
| Ga0586324 4428305 3 647 | 0.00 | 0.00 | 0.00 | 0.00 | 0.00 | 0.00 | 0.00 | 0.00 | 0.00 | 0.00 | 8210400.00 | 0.00 | KO:K01689 | enolase | Bacteria;Proteobacteria;Gammaproteobacteria;Chromatiales;Granulosicoccaceae;Sulfuriflexus;Sulfuriflexus mobilis;Sulfuriflexus mobilis aks1 |
| Ga0586324 4701635 3 632 | 0.00 | 0.00 | 0.00 | 0.00 | 0.00 | 0.00 | 0.00 | 0.00 | 0.00 | 0.00 | 0.00 | 1342500.00 | KO:K01689 | enolase | Bacteria;Calditrichaeota;Calditrichae;Calditrichales;Calditrichaceae;Caldithrix;Caldithrix abyssi;Caldithrix abyssi LF13, DSM 13497 |
| Ga0586324 4928770 1 420 | 1970000.00 | 0.00 | 0.00 | 0.00 | 0.00 | 0.00 | 0.00 | 0.00 | 0.00 | 0.00 | 0.00 | 0.00 | KO:K01689 | enolase | Bacteria;Proteobacteria;Betaproteobacteria;Burkholderiales;Sutterellaceae;Sutterella;Sutterella parvirubra;Sutterella parvirubra YIT 11816 |
| Ga0586324 5767279 2 580 | 5050000.00 | 0.00 | 0.00 | 0.00 | 0.00 | 0.00 | 0.00 | 0.00 | 0.00 | 1640100.00 | 0.00 | 1875000.00 | KO:K01689 | enolase | Bacteria;Ignavibacteriae;Ignavibacteria;Ignavibacteriales;Melioribacteraceae;Melioribacter;Melioribacter roseus;Melioribacter roseus P3M |
| Ga0586324 6108104 2 568 | 1200000.00 | 0.00 | 0.00 | 0.00 | 0.00 | 0.00 | 0.00 | 0.00 | 0.00 | 0.00 | 0.00 | 0.00 | KO:K01689 | enolase | Bacteria;Spirochaetes;Spirochaetia;Spirochaetales;Spirochaetaceae;Spirochaeta;Spirochaeta africana;Spirochaeta africana Z-7692, DSM 8902 |
| Ga0586324 6131382 1 567 | 1950000.00 | 0.00 | 0.00 | 0.00 | 0.00 | 0.00 | 0.00 | 0.00 | 0.00 | 0.00 | 0.00 | 0.00 | KO:K01689 | enolase | Bacteria;Proteobacteria;Deltaproteobacteria;Desulfuromonadales;Geobacteraceae;Geobacter;Geobacter argillaceus;Geobacter argillaceus ATCC BAA-1139 |
| Ga0586324 0031138 4230 4817 | 0.00 | 0.00 | 0.00 | 0.00 | 0.00 | 0.00 | 0.00 | 0.00 | 0.00 | 1031800.00 | 1284800.00 | 0.00 | KO:K01693 | imidazoleglycerol-phosphate dehydratase | Bacteria;Proteobacteria;Betaproteobacteria;Nitrosomonadales;Sterolibacteriaceae;Methyloversatilis;Methyloversatilis universalis;Methyloversatilis universalis FAM5 |
| Ga0586324 0269262 1040 1711 | 0.00 | 0.00 | 0.00 | 0.00 | 0.00 | 0.00 | 0.00 | 0.00 | 0.00 | 0.00 | 0.00 | 16650000.00 | KO:K01693 | imidazoleglycerol-phosphate dehydratase | Bacteria;Proteobacteria;Betaproteobacteria;unclassified Betaproteobacteria;unclassified Betaproteobacteria;unclassified Betaproteobacteria;beta proteobacterium AAP51;Betaproteobacteria bacterium AAP51 |
| Ga0586324 0534486 732 1349 | 0.00 | 2930800.00 | 0.00 | 0.00 | 0.00 | 0.00 | 0.00 | 0.00 | 0.00 | 0.00 | 0.00 | 0.00 | KO:K01693 | imidazoleglycerol-phosphate dehydratase | Bacteria;Proteobacteria;Gammaproteobacteria;Chromatiales;Chromatiaceae;Thiocapsa;Thiocapsa marina;Thiocapsa marina 5811, DSM 5653 |
| Ga0586324 0981295 735 1223 | 0.00 | 0.00 | 0.00 | 0.00 | 0.00 | 0.00 | 0.00 | 19459000.00 | 18873000.00 | 0.00 | 0.00 | 0.00 | KO:K01693 | imidazoleglycerol-phosphate dehydratase | Bacteria;Proteobacteria;Betaproteobacteria;Burkholderiales;Alcaligenaceae;Parvibium;Parvibium lacunae;Parvibium lacunae KMB9 |
| Ga0586324 3554928 205 711 | 0.00 | 0.00 | 0.00 | 0.00 | 0.00 | 0.00 | 0.00 | 0.00 | 0.00 | 0.00 | 0.00 | 1470000.00 | KO:K01695 | tryptophan synthase alpha chain | Bacteria;Acidobacteria;Acidobacteriia;Bryobacterales;Solibacteraceae;Candidatus Solibacter;Candidatus Solibacter usitatus;Candidatus Solibacter usitatus Ellin6076 |
| Ga0586324 0114909 1486 2712 | 0.00 | 0.00 | 0.00 | 0.00 | 0.00 | 0.00 | 0.00 | 0.00 | 0.00 | 10241000.00 | 4356000.00 | 0.00 | KO:K01696 | tryptophan synthase beta chain | Bacteria;Proteobacteria;Betaproteobacteria;Burkholderiales;Alcaligenaceae;Pigmentiphaga;Pigmentiphaga sp. NML030171;Pigmentiphaga sp. NML030171 |
| Ga0586324 0710170 415 1407 | 0.00 | 0.00 | 0.00 | 0.00 | 0.00 | 0.00 | 0.00 | 0.00 | 0.00 | 3927000.00 | 0.00 | 0.00 | KO:K01696 | tryptophan synthase beta chain | Bacteria;Proteobacteria;Gammaproteobacteria;Methylococcales;Methylococcaceae;Methylosarcina;Methylosarcina fibrata;Methylosarcina fibrata AML-C10 |
| Ga0586324 1630123 3 761 | 0.00 | 0.00 | 0.00 | 0.00 | 0.00 | 0.00 | 0.00 | 0.00 | 0.00 | 0.00 | 1724800.00 | 0.00 | KO:K01696 | tryptophan synthase beta chain | Bacteria;Proteobacteria;Betaproteobacteria;Burkholderiales;Burkholderiaceae;Pandoraea;Pandoraea pulmonicola;Pandoraea pulmonicola NCTC 13159 |
| Ga0586324 3246915 2 736 | 0.00 | 0.00 | 0.00 | 0.00 | 0.00 | 0.00 | 0.00 | 1640900.00 | 0.00 | 0.00 | 0.00 | 0.00 | KO:K01696 | tryptophan synthase beta chain | Bacteria;Proteobacteria;Betaproteobacteria;Burkholderiales;Oxalobacteraceae;Herminiimonas;Herminiimonas sp. CN;Herminiimonas sp. CN |
| Ga0586324 6767694 2 544 | 0.00 | 0.00 | 0.00 | 0.00 | 0.00 | 0.00 | 0.00 | 0.00 | 2292300.00 | 0.00 | 0.00 | 0.00 | KO:K01696 | tryptophan synthase beta chain | Bacteria;Proteobacteria;Betaproteobacteria;Burkholderiales;Oxalobacteraceae;unclassified Oxalobacteraceae;Oxalobacteraceae bacterium IMCC9480;beta proteobacterium sp. IMCC9480 |
| Ga0586324 0077970 3014 3862 | 0.00 | 0.00 | 0.00 | 0.00 | 0.00 | 0.00 | 0.00 | 1695800.00 | 0.00 | 0.00 | 0.00 | 0.00 | KO:K01698 | porphobilinogen synthase | Bacteria;Proteobacteria;Deltaproteobacteria;Syntrophobacterales;Syntrophaceae;Desulfobacca;Desulfobacca acetoxidans;Desulfobacca acetoxidans ASRB2, DSM 11109 |
| Ga0586324 0273432 344 1279 | 0.00 | 0.00 | 0.00 | 0.00 | 0.00 | 0.00 | 0.00 | 0.00 | 0.00 | 0.00 | 10472000.00 | 0.00 | KO:K01710 | dTDP-glucose 4,6-dehydratase | Bacteria;Proteobacteria;Deltaproteobacteria;Myxococcales;Anaeromyxobacteraceae;Anaeromyxobacter;Anaeromyxobacter sp. Fw109-5;Anaeromyxobacter sp. Fw109-5 |
| Ga0586324 0460038 32 1048 | 0.00 | 0.00 | 0.00 | 0.00 | 0.00 | 0.00 | 0.00 | 0.00 | 0.00 | 0.00 | 0.00 | 2475000.00 | KO:K01711 | GDPmannose 4,6-dehydratase | Bacteria;Actinobacteria;Rubrobacteria;Gaiellales;Gaiellaceae;Gaiella;Gaiella occulta;Gaiella occulta F2-233 |
| Ga0586324 3412142 1 723 | 54300000.00 | 0.00 | 0.00 | 0.00 | 0.00 | 0.00 | 0.00 | 0.00 | 0.00 | 0.00 | 0.00 | 0.00 | KO:K01711 | GDPmannose 4,6-dehydratase | Bacteria;Proteobacteria;Alphaproteobacteria;Rhodospirillales;Rhodospirillaceae;Magnetospirillum;Magnetospirillum sp. WD;Magnetospirillum sp. WD |
| Ga0586324 0022106 6140 7096 | 0.00 | 0.00 | 0.00 | 1463000.00 | 4356900.00 | 0.00 | 268196.40 | 1360300.00 | 0.00 | 0.00 | 0.00 | 0.00 | KO:K01714 | 4-hydroxy-tetrahydrodipicolinate synthase | Archaea;Euryarchaeota;Methanomicrobia;Methanomicrobiales;Methanoregulaceae;Methanoregula;Methanoregula formicica;Methanoregula formicica SMSP, DSM 22288 |
| Ga0586324 0066543 1913 2848 | 8630000.00 | 0.00 | 13512000.00 | 1577000.00 | 1998200.00 | 0.00 | 2302800.00 | 1421300.00 | 0.00 | 4019400.00 | 0.00 | 0.00 | KO:K01714 | 4-hydroxy-tetrahydrodipicolinate synthase | Archaea;Euryarchaeota;Methanomicrobia;Methanomicrobiales;Methanoregulaceae;Methanoregula;Methanoregula formicica;Methanoregula formicica SMSP, DSM 22288 |
| Ga0586324 0067970 3308 4123 | 0.00 | 0.00 | 0.00 | 1168500.00 | 2121800.00 | 0.00 | 0.00 | 1671400.00 | 0.00 | 0.00 | 0.00 | 0.00 | KO:K01714 | 4-hydroxy-tetrahydrodipicolinate synthase | Bacteria;Proteobacteria;Deltaproteobacteria;Syntrophobacterales;Syntrophaceae;Desulfobacca;Desulfobacca acetoxidans;Desulfobacca acetoxidans ASRB2, DSM 11109 |
| Ga0586324 0370288 303 1178 | 0.00 | 2781200.00 | 0.00 | 0.00 | 0.00 | 0.00 | 0.00 | 4129700.00 | 3183300.00 | 2633400.00 | 0.00 | 0.00 | KO:K01714 | 4-hydroxy-tetrahydrodipicolinate synthase | Archaea;Euryarchaeota;Methanomicrobia;Methanosarcinales;Methanotrichaceae;Methanothrix;Methanothrix soehngenii;Methanothrix soehngenii GP-6 |
| Ga0586324 2285925 652 855 | 0.00 | 0.00 | 0.00 | 2983000.00 | 0.00 | 0.00 | 0.00 | 0.00 | 0.00 | 0.00 | 0.00 | 267106.50 | KO:K01714 | 4-hydroxy-tetrahydrodipicolinate synthase | Bacteria;Proteobacteria;Deltaproteobacteria;Syntrophobacterales;Syntrophaceae;Desulfobacca;Desulfobacca acetoxidans;Desulfobacca acetoxidans ASRB2, DSM 11109 |
| Ga0586324 2389852 47 838 | 0.00 | 0.00 | 0.00 | 3610000.00 | 0.00 | 0.00 | 2850000.00 | 0.00 | 732050.46 | 0.00 | 2930400.00 | 0.00 | KO:K01714 | 4-hydroxy-tetrahydrodipicolinate synthase | Bacteria;Proteobacteria;Alphaproteobacteria;Rhodospirillales;Acetobacteraceae;Rhodopila;unclassified Rhodopila;Rhodopila sp. LVNP |
| Ga0586324 7061567 3 536 | 1170000.00 | 0.00 | 0.00 | 0.00 | 0.00 | 0.00 | 0.00 | 0.00 | 1409400.00 | 0.00 | 0.00 | 0.00 | KO:K01714 | 4-hydroxy-tetrahydrodipicolinate synthase | Bacteria;Proteobacteria;Deltaproteobacteria;Syntrophobacterales;Syntrophaceae;Syntrophus;Syntrophus gentianae;Syntrophus gentianae DSM 8423 |
| Ga0586324 1707440 78 872 | 0.00 | 0.00 | 0.00 | 0.00 | 1709800.00 | 0.00 | 0.00 | 0.00 | 0.00 | 0.00 | 0.00 | 0.00 | KO:K01715 | enoyl-CoA hydratase | Bacteria;Proteobacteria;Deltaproteobacteria;Desulfobacterales;Desulfobacteraceae;Desulfatirhabdium;Desulfatirhabdium butyrativorans;Desulfatirhabdium butyrativorans DSM 18734 |
| Ga0586324 2223278 1 441 | 0.00 | 0.00 | 0.00 | 0.00 | 5706200.00 | 0.00 | 0.00 | 0.00 | 0.00 | 0.00 | 0.00 | 0.00 | KO:K01715 | enoyl-CoA hydratase | Bacteria;Proteobacteria;Deltaproteobacteria;Syntrophobacterales;Syntrophobacteraceae;Desulfoglaeba;Desulfoglaeba alkanexedens;Desulfoglaeba alkanexedens ALDC |
| Ga0586324 0031709 3844 5172 | 5700000.00 | 0.00 | 0.00 | 0.00 | 0.00 | 0.00 | 0.00 | 0.00 | 0.00 | 0.00 | 0.00 | 0.00 | KO:K01728 | pectate lyase | Bacteria;Proteobacteria;Gammaproteobacteria;Nevskiales;Steroidobacteraceae;Povalibacter;Povalibacter uvarum;Povalibacter uvarum DSM 26723 |
| Ga0586324 0009601 9857 10375 | 0.00 | 0.00 | 0.00 | 0.00 | 8332700.00 | 0.00 | 0.00 | 0.00 | 0.00 | 0.00 | 0.00 | 0.00 | KO:K01738 | cysteine synthase A | Bacteria;Proteobacteria;Alphaproteobacteria;Hyphomicrobiales;Bradyrhizobiaceae;Bradyrhizobium;Bradyrhizobium erythrophlei;Bradyrhizobium erythrophlei GAS138 |
| Ga0586324 0065815 2887 4191 | 0.00 | 0.00 | 0.00 | 0.00 | 0.00 | 0.00 | 0.00 | 0.00 | 0.00 | 0.00 | 0.00 | 1455000.00 | KO:K01738 | cysteine synthase A | Bacteria;Proteobacteria;Betaproteobacteria;Rhodocyclales;Rhodocyclaceae;Propionivibrio;Propionivibrio dicarboxylicus;Propionivibrio dicarboxylicus DSM 5885 |
| Ga0586324 0076521 1390 2367 | 0.00 | 0.00 | 0.00 | 0.00 | 0.00 | 0.00 | 0.00 | 0.00 | 0.00 | 0.00 | 0.00 | 1215000.00 | KO:K01738 | cysteine synthase A | Bacteria;Proteobacteria;Alphaproteobacteria;Rhodospirillales;Acetobacteraceae;Rhodopila;unclassified Rhodopila;Rhodopila sp. LVNP |
| Ga0586324 0500408 495 1637 | 0.00 | 0.00 | 0.00 | 4227500.00 | 0.00 | 0.00 | 0.00 | 0.00 | 0.00 | 0.00 | 0.00 | 0.00 | KO:K01738 | cysteine synthase A | Bacteria;Chloroflexi;Anaerolineae;Anaerolineales;Anaerolineaceae;Levilinea;Levilinea saccharolytica;Levilinea saccharolytica DSM 16555 |
| Ga0586324 4750224 1 630 | 0.00 | 0.00 | 0.00 | 0.00 | 0.00 | 0.00 | 35340000.00 | 0.00 | 0.00 | 0.00 | 0.00 | 0.00 | KO:K01738 | cysteine synthase A | Bacteria;Chloroflexi;Anaerolineae;Anaerolineales;Anaerolineaceae;Thermanaerothrix;Thermanaerothrix daxensis;Thermanaerothrix daxensis GNS-1 |
| Ga0586324 0032548 1750 3042 | 2250000.00 | 0.00 | 0.00 | 5899500.00 | 4120000.00 | 2833250.05 | 0.00 | 0.00 | 3021300.00 | 3857700.00 | 3502400.00 | 0.00 | KO:K01740 | O-acetylhomoserine (thiol)-lyase | Bacteria;Proteobacteria;Alphaproteobacteria;Rhodospirillales;Acetobacteraceae;Rhodopila;unclassified Rhodopila;Rhodopila sp. LVNP |
| Ga0586324 3678040 2 520 | 0.00 | 0.00 | 0.00 | 0.00 | 0.00 | 0.00 | 0.00 | 0.00 | 0.00 | 7515200.00 | 0.00 | 0.00 | KO:K01740 | O-acetylhomoserine (thiol)-lyase | Archaea;Euryarchaeota;Methanomicrobia;Methanomicrobiales;Methanoregulaceae;Methanoregula;Methanoregula boonei;Methanoregula boonei 6A8 |
| Ga0586324 0211729 806 2410 | 0.00 | 0.00 | 8613900.00 | 0.00 | 0.00 | 0.00 | 0.00 | 0.00 | 0.00 | 0.00 | 0.00 | 0.00 | KO:K01752 | L-serine dehydratase | Bacteria;Acidobacteria;Vicinamibacteria;unclassified Vicinamibacteria;Vicinamibacteraceae;Luteitalea;Luteitalea pratensis;Luteitalea pratensis DSM 100886 |
| Ga0586324 3006702 2 352 | 0.00 | 0.00 | 0.00 | 0.00 | 0.00 | 0.00 | 0.00 | 0.00 | 4009500.00 | 0.00 | 0.00 | 0.00 | KO:K01752 | L-serine dehydratase | Bacteria;Proteobacteria;Alphaproteobacteria;Rhodospirillales;unclassified Rhodospirillales;unclassified Rhodospirillales;Rhodospirillales bacterium URHD0017;Rhodospirillales bacterium URHD0017 |
| Ga0586324 0000013 15062 16582 | 0.00 | 0.00 | 0.00 | 0.00 | 0.00 | 0.00 | 0.00 | 0.00 | 0.00 | 6545000.00 | 4048000.00 | 0.00 | KO:K01754 | threonine dehydratase | Bacteria;Proteobacteria;Betaproteobacteria;Nitrosomonadales;Thiobacillaceae;Annwoodia;Annwoodia aquaesulis;Annwoodia aquaesulis DSM 4255 |
| Ga0586324 0001056 857 2374 | 0.00 | 0.00 | 0.00 | 0.00 | 0.00 | 0.00 | 0.00 | 0.00 | 1093500.00 | 0.00 | 0.00 | 1147500.00 | KO:K01754 | threonine dehydratase | Bacteria;Proteobacteria;Betaproteobacteria;Nitrosomonadales;Thiobacillaceae;Thiobacillus;Thiobacillus thioparus;Thiobacillus thioparus DSM 505 |
| Ga0586324 0001208 12142 13659 | 0.00 | 0.00 | 0.00 | 0.00 | 0.00 | 0.00 | 0.00 | 0.00 | 702052.11 | 862400.00 | 0.00 | 0.00 | KO:K01754 | threonine dehydratase | Bacteria;Proteobacteria;Betaproteobacteria;Nitrosomonadales;Thiobacillaceae;Thiobacillus;Thiobacillus denitrificans;Thiobacillus denitrificans RG |
| Ga0586324 2047594 1 894 | 0.00 | 0.00 | 0.00 | 0.00 | 0.00 | 0.00 | 0.00 | 3830800.00 | 0.00 | 0.00 | 0.00 | 0.00 | KO:K01754 | threonine dehydratase | Bacteria;Proteobacteria;Gammaproteobacteria;Nevskiales;Steroidobacteraceae;Povalibacter;Povalibacter uvarum;Povalibacter uvarum DSM 26723 |
| Ga0586324 0000004 69176 70558 | 0.00 | 0.00 | 0.00 | 0.00 | 0.00 | 0.00 | 0.00 | 0.00 | 694264.77 | 0.00 | 0.00 | 0.00 | KO:K01755 | argininosuccinate lyase | Bacteria;Proteobacteria;Betaproteobacteria;Nitrosomonadales;Thiobacillaceae;Annwoodia;Annwoodia aquaesulis;Annwoodia aquaesulis DSM 4255 |
| Ga0586324 0000090 38706 40076 | 0.00 | 0.00 | 0.00 | 0.00 | 0.00 | 0.00 | 0.00 | 5233800.00 | 0.00 | 6229300.00 | 0.00 | 0.00 | KO:K01756 | adenylosuccinate lyase | Bacteria;Proteobacteria;Betaproteobacteria;Nitrosomonadales;Thiobacillaceae;Annwoodia;Annwoodia aquaesulis;Annwoodia aquaesulis DSM 4255 |
| Ga0586324 0722342 327 1397 | 0.00 | 0.00 | 0.00 | 0.00 | 0.00 | 0.00 | 0.00 | 0.00 | 0.00 | 0.00 | 1390400.00 | 0.00 | KO:K01756 | adenylosuccinate lyase | Bacteria;Proteobacteria;Betaproteobacteria;Nitrosomonadales;Sulfuricellaceae;Sulfuriferula;Sulfuriferula multivorans;Sulfuriferula multivorans TTN |
| Ga0586324 0000169 26450 27253 | 0.00 | 0.00 | 0.00 | 0.00 | 0.00 | 0.00 | 0.00 | 332816.00 | 0.00 | 0.00 | 1698400.00 | 0.00 | KO:K01776 | glutamate racemase | Bacteria;Proteobacteria;Betaproteobacteria;Nitrosomonadales;Thiobacillaceae;Annwoodia;Annwoodia aquaesulis;Annwoodia aquaesulis DSM 4255 |
| Ga0586324 0000363 16477 17166 | 0.00 | 0.00 | 0.00 | 0.00 | 0.00 | 0.00 | 0.00 | 0.00 | 0.00 | 0.00 | 0.00 | 11175000.00 | KO:K01783 | ribulose-phosphate 3-epimerase | Bacteria;Proteobacteria;Gammaproteobacteria;Acidiferrobacterales;Acidiferrobacteraceae;Sulfuricaulis;Sulfuricaulis limicola;Sulfuricaulis limicola HA5 |
| Ga0586324 0308776 952 1623 | 0.00 | 0.00 | 0.00 | 0.00 | 0.00 | 0.00 | 3420000.00 | 2757200.00 | 0.00 | 0.00 | 0.00 | 0.00 | KO:K01783 | ribulose-phosphate 3-epimerase | Bacteria;Proteobacteria;Gammaproteobacteria;Methylococcales;Crenotrichaceae;Crenothrix;Crenothrix polyspora;Crenothrix polyspora ERS1545889 |
| Ga0586324 2319257 2 694 | 0.00 | 6133600.00 | 0.00 | 0.00 | 0.00 | 0.00 | 0.00 | 0.00 | 0.00 | 6267800.00 | 0.00 | 0.00 | KO:K01783 | ribulose-phosphate 3-epimerase | Bacteria;Proteobacteria;Gammaproteobacteria;Methylococcales;Crenotrichaceae;Crenothrix;Crenothrix polyspora;Crenothrix polyspora ERS1545889 |
| Ga0586324 1367307 69 1061 | 1310000.00 | 0.00 | 0.00 | 0.00 | 0.00 | 0.00 | 0.00 | 0.00 | 0.00 | 0.00 | 0.00 | 0.00 | KO:K01785 | aldose 1-epimerase | Bacteria;Actinobacteria;Actinomycetia;Jiangellales;Jiangellaceae;Haloactinopolyspora;Haloactinopolyspora alba;Haloactinopolyspora alba DSM 45211 |
| Ga0586324 6883135 79 540 | 1320000.00 | 0.00 | 0.00 | 0.00 | 0.00 | 0.00 | 0.00 | 0.00 | 0.00 | 0.00 | 0.00 | 0.00 | KO:K01785 | aldose 1-epimerase | Bacteria;Actinobacteria;Actinomycetia;Micromonosporales;Micromonosporaceae;Actinoplanes;Actinoplanes subtropicus;Actinoplanes subtropicus NRRL B-24665 |
| Ga0586324 0000030 22988 23740 | 0.00 | 0.00 | 0.00 | 0.00 | 0.00 | 0.00 | 0.00 | 0.00 | 10449000.00 | 12012000.00 | 0.00 | 0.00 | KO:K01803 | triosephosphate isomerase | Bacteria;Proteobacteria;Betaproteobacteria;Nitrosomonadales;Thiobacillaceae;Annwoodia;Annwoodia aquaesulis;Annwoodia aquaesulis DSM 4255 |
| Ga0586324 0093625 618 1382 | 2050000.00 | 0.00 | 0.00 | 0.00 | 0.00 | 0.00 | 0.00 | 0.00 | 0.00 | 0.00 | 0.00 | 0.00 | KO:K01803 | triosephosphate isomerase | Bacteria;Firmicutes;Clostridia;Thermoanaerobacterales;Thermoanaerobacteraceae;Desulfovirgula;Desulfovirgula thermocuniculi;Desulfovirgula thermocuniculi DSM 16036 |
| Ga0586324 0197056 293 1039 | 0.00 | 1217200.00 | 0.00 | 0.00 | 0.00 | 0.00 | 0.00 | 3287900.00 | 0.00 | 0.00 | 4127200.00 | 204825.00 | KO:K01803 | triosephosphate isomerase | Bacteria;Proteobacteria;Betaproteobacteria;Nitrosomonadales;Thiobacillaceae;Thiobacillus;Thiobacillus denitrificans;Thiobacillus denitrificans RG |
| Ga0586324 0219564 118 879 | 0.00 | 0.00 | 0.00 | 0.00 | 1390500.00 | 0.00 | 0.00 | 0.00 | 0.00 | 0.00 | 0.00 | 0.00 | KO:K01803 | triosephosphate isomerase | Bacteria;Proteobacteria;Gammaproteobacteria;Chromatiales;Chromatiaceae;Thiorhodovibrio;Thiorhodovibrio sp. 970;Thiorhodovibrio sp. 970 |
| Ga0586324 0528455 210 956 | 0.00 | 0.00 | 0.00 | 0.00 | 0.00 | 0.00 | 898593.60 | 2220400.00 | 0.00 | 0.00 | 0.00 | 0.00 | KO:K01803 | triosephosphate isomerase | Bacteria;Proteobacteria;Gammaproteobacteria;Methylococcales;Methylococcaceae;Methylobacter;Methylobacter tundripaludum;Methylobacter tundripaludum OWC-DMM |
| Ga0586324 2700807 1 474 | 0.00 | 0.00 | 0.00 | 0.00 | 0.00 | 0.00 | 0.00 | 0.00 | 7492500.00 | 0.00 | 0.00 | 0.00 | KO:K01803 | triosephosphate isomerase | Bacteria;Proteobacteria;Betaproteobacteria;Nitrosomonadales;Thiobacillaceae;Thiobacillus;Thiobacillus denitrificans;Thiobacillus denitrificans RG |
| Ga0586324 7598167 1 519 | 0.00 | 0.00 | 0.00 | 0.00 | 0.00 | 0.00 | 1934253.96 | 0.00 | 0.00 | 0.00 | 0.00 | 0.00 | KO:K01803 | triosephosphate isomerase | Bacteria;Firmicutes;Clostridia;Eubacteriales;Oscillospiraceae;Pseudoclostridium;Pseudoclostridium thermosuccinogenes;Pseudoclostridium thermosuccinogenes DSM 5806 |
| Ga0586324 4309507 1 600 | 0.00 | 0.00 | 0.00 | 3534000.00 | 0.00 | 0.00 | 0.00 | 0.00 | 0.00 | 0.00 | 0.00 | 0.00 | KO:K01805 | xylose isomerase | Bacteria;Candidatus Latescibacteria;unclassified Candidatus Latescibacteria;unclassified Candidatus Latescibacteria;unclassified Candidatus Latescibacteria;Candidatus Latescibacter;Candidatus Latescibacter anaerobius;Candidatus Latescibacter anaerobius SCGC AAA252-E07 (contamination screened) |
| Ga0586324 6981021 142 537 | 0.00 | 0.00 | 0.00 | 0.00 | 0.00 | 0.00 | 0.00 | 0.00 | 0.00 | 0.00 | 1108800.00 | 0.00 | KO:K01805 | xylose isomerase | Bacteria;Chloroflexi;Ardenticatenia;Ardenticatenales;Ardenticatenaceae;Candidatus Promineofilum;Candidatus Promineofilum breve;Candidatus Promineofilum breve Cfx-K |
| Ga0586324 3694626 1 444 | 0.00 | 0.00 | 0.00 | 0.00 | 0.00 | 0.00 | 0.00 | 0.00 | 0.00 | 0.00 | 0.00 | 687008.25 | KO:K01807 | ribose 5-phosphate isomerase A | Bacteria;Proteobacteria;Alphaproteobacteria;Hyphomicrobiales;Xanthobacteraceae;Pseudolabrys;Pseudolabrys taiwanensis;Pseudolabrys taiwanensis CC-BB4 |
| Ga0586324 4651464 1 510 | 0.00 | 0.00 | 0.00 | 0.00 | 0.00 | 0.00 | 0.00 | 6710000.00 | 0.00 | 0.00 | 0.00 | 0.00 | KO:K01807 | ribose 5-phosphate isomerase A | Bacteria;Proteobacteria;Gammaproteobacteria;unclassified Gammaproteobacteria;Candidatus Competibacteraceae;Plasticicumulans;Plasticicumulans lactativorans;Plasticicumulans lactativorans DSM 25287 |
| Ga0586324 0000943 4956 5711 | 0.00 | 0.00 | 0.00 | 0.00 | 0.00 | 0.00 | 0.00 | 2586400.00 | 0.00 | 0.00 | 0.00 | 3330000.00 | KO:K01814 | phosphoribosylformimino-5-aminoimidazole carboxamide ribotide isomerase | Bacteria;Proteobacteria;Betaproteobacteria;Nitrosomonadales;Thiobacillaceae;Thiobacillus;Thiobacillus denitrificans;Thiobacillus denitrificans DSM 12475 |
| Ga0586324 0001072 12175 12930 | 0.00 | 0.00 | 0.00 | 0.00 | 0.00 | 0.00 | 0.00 | 0.00 | 538040.07 | 0.00 | 0.00 | 0.00 | KO:K01814 | phosphoribosylformimino-5-aminoimidazole carboxamide ribotide isomerase | Bacteria;Proteobacteria;Betaproteobacteria;Nitrosomonadales;Thiobacillaceae;Thiobacillus;Thiobacillus denitrificans;Thiobacillus denitrificans DSM 12475 |
| Ga0586324 1104884 557 1162 | 0.00 | 0.00 | 0.00 | 0.00 | 0.00 | 0.00 | 0.00 | 0.00 | 1611900.00 | 0.00 | 0.00 | 0.00 | KO:K01834 | 2,3-bisphosphoglycerate-dependent phosphoglycerate mutase | Bacteria;Chlorobi;Chlorobia;Chlorobiales;Chlorobiaceae;Chlorobaculum;Chlorobaculum tepidum;Chlorobaculum tepidum TLS |
| Ga0586324 1972730 250 909 | 0.00 | 0.00 | 0.00 | 0.00 | 0.00 | 0.00 | 9598800.00 | 0.00 | 0.00 | 0.00 | 0.00 | 0.00 | KO:K01834 | 2,3-bisphosphoglycerate-dependent phosphoglycerate mutase | Bacteria;Proteobacteria;Deltaproteobacteria;Desulfuromonadales;Geobacteraceae;Geobacter;Geobacter metallireducens;Geobacter metallireducens GS-15 |
| Ga0586324 2887324 39 773 | 0.00 | 0.00 | 5411786.83 | 0.00 | 0.00 | 0.00 | 0.00 | 0.00 | 0.00 | 0.00 | 0.00 | 0.00 | KO:K01834 | 2,3-bisphosphoglycerate-dependent phosphoglycerate mutase | Bacteria;Spirochaetes;Spirochaetia;Spirochaetales;Spirochaetaceae;Alkalispirochaeta;Alkalispirochaeta americana;Alkalispirochaeta americana ASpG1 |
| Ga0586324 0492972 3 1382 | 0.00 | 0.00 | 0.00 | 0.00 | 4202400.00 | 0.00 | 2622000.00 | 0.00 | 0.00 | 0.00 | 0.00 | 0.00 | KO:K01835 | phosphoglucomutase | Bacteria;Proteobacteria;Gammaproteobacteria;Methylococcales;Methylococcaceae;Methylobacter;Methylobacter tundripaludum;Methylobacter tundripaludum 31/32 |
| Ga0586324 6844372 1 516 | 0.00 | 0.00 | 0.00 | 0.00 | 1390500.00 | 0.00 | 0.00 | 0.00 | 0.00 | 0.00 | 0.00 | 0.00 | KO:K01835 | phosphoglucomutase | Bacteria;Proteobacteria;Gammaproteobacteria;Methylococcales;Methylococcaceae;Methylocaldum;Methylocaldum sp. 0917;Methylocaldum sp. 0917 |
| Ga0586324 0528850 243 1598 | 0.00 | 0.00 | 0.00 | 0.00 | 0.00 | 0.00 | 0.00 | 0.00 | 0.00 | 0.00 | 0.00 | 2190000.00 | KO:K01843 | lysine 2,3-aminomutase | Bacteria;Chloroflexi;Anaerolineae;Anaerolineales;Anaerolineaceae;Thermanaerothrix;Thermanaerothrix daxensis;Thermanaerothrix daxensis GNS-1 |
| Ga0586324 0160574 1904 2740 | 0.00 | 0.00 | 0.00 | 0.00 | 0.00 | 0.00 | 0.00 | 1958100.00 | 0.00 | 0.00 | 0.00 | 0.00 | KO:K01845 | glutamate-1-semialdehyde 2,1-aminomutase | Bacteria;Proteobacteria;Deltaproteobacteria;Desulfuromonadales;Geobacteraceae;Geobacter;Geobacter sp. DSM 9736;Geobacter sp. DSM 9736 |
| Ga0586324 0248102 2 1240 | 0.00 | 0.00 | 0.00 | 0.00 | 0.00 | 0.00 | 4582800.00 | 0.00 | 0.00 | 0.00 | 0.00 | 1942500.00 | KO:K01845 | glutamate-1-semialdehyde 2,1-aminomutase | Bacteria;Firmicutes;Clostridia;Thermoanaerobacterales;Thermoanaerobacteraceae;Calderihabitans;Calderihabitans maritimus;Calderihabitans maritimus KKC1 |
| Ga0586324 2402316 10 837 | 0.00 | 0.00 | 0.00 | 0.00 | 0.00 | 3601552.24 | 0.00 | 0.00 | 0.00 | 0.00 | 0.00 | 0.00 | KO:K01845 | glutamate-1-semialdehyde 2,1-aminomutase | Bacteria;Proteobacteria;Deltaproteobacteria;Desulfuromonadales;Geobacteraceae;Geobacter;Geobacter sp. DSM 9736;Geobacter sp. DSM 9736 |
| Ga0586324 0028796 3056 5233 | 0.00 | 0.00 | 0.00 | 0.00 | 0.00 | 0.00 | 0.00 | 0.00 | 0.00 | 0.00 | 4021600.00 | 0.00 | KO:K01847 | methylmalonyl-CoA mutase | Bacteria;Proteobacteria;Betaproteobacteria;Burkholderiales;unclassified Burkholderiales;Aquabacterium;Aquabacterium tepidiphilum;Aquabacterium tepidiphilum YIM 730274 |
| Ga0586324 1079055 585 1175 | 0.00 | 0.00 | 0.00 | 0.00 | 2080600.00 | 0.00 | 0.00 | 0.00 | 0.00 | 0.00 | 0.00 | 0.00 | KO:K01847 | methylmalonyl-CoA mutase | Bacteria;Proteobacteria;Betaproteobacteria;Rhodocyclales;Azonexaceae;Dechloromonas;Dechloromonas sp. SG708;Dechloromonas sp. SG708 |
| Ga0586324 4036731 3 674 | 0.00 | 0.00 | 0.00 | 0.00 | 0.00 | 0.00 | 0.00 | 0.00 | 0.00 | 0.00 | 0.00 | 787500.00 | KO:K01847 | methylmalonyl-CoA mutase | Bacteria;Proteobacteria;Betaproteobacteria;Burkholderiales;Comamonadaceae;Pseudacidovorax;Pseudacidovorax sp. RU35E;Pseudacidovorax sp. RU35E |
| Ga0586324 5207862 2 607 | 0.00 | 0.00 | 3845768.55 | 0.00 | 0.00 | 9647100.00 | 6634800.00 | 4013800.00 | 4309200.00 | 0.00 | 0.00 | 0.00 | KO:K01847 | methylmalonyl-CoA mutase | Bacteria;Proteobacteria;Deltaproteobacteria;Desulfobacterales;Desulfobulbaceae;Desulfobulbus;Desulfobulbus mediterraneus;Desulfobulbus mediterraneus DSM 13871 |
| Ga0586324 0021460 6608 7198 | 0.00 | 4773600.00 | 0.00 | 2080500.00 | 0.00 | 0.00 | 0.00 | 0.00 | 0.00 | 0.00 | 0.00 | 0.00 | KO:K01848 | methylmalonyl-CoA mutase N-terminal domain/subunit | Bacteria;Proteobacteria;Deltaproteobacteria;Desulfobacterales;Desulfobacteraceae;Desulfobotulus;Desulfobotulus alkaliphilus;Desulfobotulus alkaliphilus ASO4-4 |
| Ga0586324 0158128 1771 2760 | 0.00 | 0.00 | 0.00 | 0.00 | 0.00 | 0.00 | 0.00 | 1915400.00 | 0.00 | 0.00 | 0.00 | 0.00 | KO:K01848 | methylmalonyl-CoA mutase N-terminal domain/subunit | Bacteria;Proteobacteria;Deltaproteobacteria;Syntrophobacterales;Syntrophobacteraceae;Syntrophobacter;Syntrophobacter fumaroxidans;Syntrophobacter fumaroxidans MPOB |

| | | | | | | | | | | | | | | | |
|---|---|---|---|---|---|---|---|---|---|---|---|---|---|---|---|
| Ga0586324 0774052 1 1251 | 0.00 | 0.00 | 0.00 | 0.00 | 0.00 | 944649.59 | 0.00 | 0.00 | 0.00 | 0.00 | 0.00 | 0.00 | KO:K01848 | methylmalonyl-CoA mutase N-terminal domain/subunit | Bacteria;Chloroflexi;Chloroflexia;Chloroflexales;Chloroflexaceae;Chloroflexus;Chloroflexus sp. Y-396-1;Chloroflexus sp. Y-396-1 |
| Ga0586324 1720164 354 962 | 0.00 | 2903600.00 | 2760833.77 | 0.00 | 0.00 | 0.00 | 0.00 | 0.00 | 0.00 | 0.00 | 0.00 | 0.00 | KO:K01848 | methylmalonyl-CoA mutase N-terminal domain/subunit | Bacteria;Proteobacteria;Deltaproteobacteria;Desulfobacterales;Desulfobacteraceae;Desulfobotulus;Desulfobotulus alkaliphilus;Desulfobotulus alkaliphilus ASO4-4 |
| Ga0586324 1724375 3 962 | 0.00 | 6392000.00 | 9233200.00 | 0.00 | 0.00 | 0.00 | 3967200.00 | 0.00 | 0.00 | 0.00 | 0.00 | 0.00 | KO:K01848 | methylmalonyl-CoA mutase N-terminal domain/subunit | Bacteria;Proteobacteria;Deltaproteobacteria;Syntrophobacterales;Syntrophobacteraceae;Syntrophobacter;Syntrophobacter fumaroxidans;Syntrophobacter fumaroxidans MPOB |
| Ga0586324 2377158 3 839 | 0.00 | 224221.84 | 0.00 | 0.00 | 0.00 | 0.00 | 0.00 | 0.00 | 438722.73 | 0.00 | 0.00 | 0.00 | KO:K01848 | methylmalonyl-CoA mutase N-terminal domain/subunit | Bacteria;Proteobacteria;Deltaproteobacteria;Syntrophobacterales;Syntrophobacteraceae;Syntrophobacter;Syntrophobacter fumaroxidans;Syntrophobacter fumaroxidans MPOB |
| Ga0586324 0507793 410 1627 | 0.00 | 0.00 | 4600785.33 | 0.00 | 0.00 | 0.00 | 0.00 | 0.00 | 0.00 | 0.00 | 0.00 | 0.00 | KO:K01858 | myo-inositol-1-phosphate synthase | Bacteria;Chloroflexi;unclassified Chloroflexi;unclassified Chloroflexi;unclassified Chloroflexi;unclassified Chloroflexi;unclassified Chloroflexi;Chloroflexi sp. G233 |
| Ga0586324 2995723 3 761 | 0.00 | 0.00 | 0.00 | 0.00 | 0.00 | 0.00 | 0.00 | 0.00 | 1271700.00 | 0.00 | 0.00 | 1215000.00 | KO:K01858 | myo-inositol-1-phosphate synthase | Bacteria;Proteobacteria;Deltaproteobacteria;Myxococcales;Polyangiaceae;Sorangium;Sorangium cellulosum;Sorangium cellulosum So ce836 |
| Ga0586324 3725454 3 695 | 0.00 | 0.00 | 0.00 | 0.00 | 0.00 | 0.00 | 0.00 | 0.00 | 915300.00 | 0.00 | 0.00 | 0.00 | KO:K01858 | myo-inositol-1-phosphate synthase | Bacteria;Proteobacteria;Deltaproteobacteria;Myxococcales;Polyangiaceae;Sorangium;Sorangium cellulosum;Sorangium cellulosum So ce836 |
| Ga0586324 0000423 34769 35959 | 0.00 | 0.00 | 0.00 | 0.00 | 0.00 | 0.00 | 1113837.00 | 0.00 | 0.00 | 0.00 | 0.00 | 0.00 | KO:K01866 | tyrosyl-tRNA synthetase | Bacteria;Proteobacteria;Betaproteobacteria;Nitrosomonadales;Thiobacillaceae;Annwoodia;Annwoodia aquaesulis;Annwoodia aquaesulis DSM 4255 |
| Ga0586324 0000586 13358 14560 | 0.00 | 0.00 | 0.00 | 0.00 | 0.00 | 0.00 | 0.00 | 0.00 | 0.00 | 1478400.00 | 0.00 | 0.00 | KO:K01867 | tryptophanyl-tRNA synthetase | Bacteria;Proteobacteria;Betaproteobacteria;Nitrosomonadales;Thiobacillaceae;Annwoodia;Annwoodia aquaesulis;Annwoodia aquaesulis DSM 4255 |
| Ga0586324 0024769 895 2802 | 0.00 | 0.00 | 0.00 | 0.00 | 0.00 | 0.00 | 0.00 | 0.00 | 0.00 | 0.00 | 0.00 | 802500.00 | KO:K01868 | threonyl-tRNA synthetase | Bacteria;Proteobacteria;Betaproteobacteria;Nitrosomonadales;Thiobacillaceae;Thiobacillus;Thiobacillus denitrificans;Thiobacillus denitrificans DSM 12475 |
| Ga0586324 1311232 1 1005 | 0.00 | 0.00 | 0.00 | 0.00 | 0.00 | 0.00 | 0.00 | 0.00 | 1385100.00 | 0.00 | 0.00 | 0.00 | KO:K01868 | threonyl-tRNA synthetase | Bacteria;Bacteroidetes;Bacteroidia;Bacteroidales;Odoribacteraceae;Butyricimonas;Butyricimonas synergistica;Butyricimonas synergistica DSM 23225 |
| Ga0586324 1893074 2 844 | 0.00 | 240503.76 | 0.00 | 0.00 | 0.00 | 0.00 | 0.00 | 0.00 | 0.00 | 0.00 | 0.00 | 0.00 | KO:K01868 | threonyl-tRNA synthetase | Bacteria;Bacteroidetes;Bacteroidia;Bacteroidales;Odoribacteraceae;Butyricimonas;Butyricimonas sp. Marseille-P3923;Butyricimonas sp. Marseille-P3923 |
| Ga0586324 0303982 3 929 | 0.00 | 0.00 | 0.00 | 0.00 | 0.00 | 0.00 | 0.00 | 0.00 | 1644300.00 | 0.00 | 0.00 | 0.00 | KO:K01869 | leucyl-tRNA synthetase | Bacteria;Proteobacteria;Deltaproteobacteria;unclassified Deltaproteobacteria;unclassified Deltaproteobacteria;Deferrisoma;Deferrisoma camini;Deferrisoma camini S3R1 |
| Ga0586324 0009510 149 2830 | 0.00 | 0.00 | 0.00 | 0.00 | 490795.00 | 0.00 | 0.00 | 0.00 | 0.00 | 0.00 | 0.00 | 0.00 | KO:K01872 | alanyl-tRNA synthetase | Bacteria;Proteobacteria;Alphaproteobacteria;Hyphomicrobiales;Xanthobacteraceae;Pseudolabrys;Pseudolabrys taiwanensis;Pseudolabrys taiwanensis CC-BB4 |
| Ga0586324 0087944 611 3349 | 0.00 | 2135200.00 | 0.00 | 0.00 | 0.00 | 0.00 | 0.00 | 0.00 | 0.00 | 0.00 | 0.00 | 0.00 | KO:K01872 | alanyl-tRNA synthetase | Archaea;Euryarchaeota;Methanomicrobia;Methanosarcinales;Candidatus Methanoperedenaceae;Candidatus Methanoperedens;Candidatus Methanoperedens nitroreducens;Candidatus Methanoperedens nitroreducens ANME-2d |
| Ga0586324 0171329 3 1340 | 0.00 | 0.00 | 0.00 | 0.00 | 0.00 | 0.00 | 3693600.00 | 0.00 | 0.00 | 0.00 | 0.00 | 0.00 | KO:K01872 | alanyl-tRNA synthetase | Bacteria;Proteobacteria;Betaproteobacteria;Burkholderiales;Burkholderiaceae;Quisquiliibacterium;Quisquiliibacterium transsilvanicum;Quisquiliibacterium transsilvanicum DSM 29781 |
| Ga0586324 0257727 2 766 | 0.00 | 0.00 | 0.00 | 0.00 | 0.00 | 0.00 | 0.00 | 0.00 | 0.00 | 1347500.00 | 0.00 | 0.00 | KO:K01872 | alanyl-tRNA synthetase | Bacteria;Proteobacteria;Betaproteobacteria;Nitrosomonadales;Sulfuricellaceae;Sulfurirhabdus;Sulfurirhabdus autotrophica;Sulfurirhabdus autotrophica DSM 100309 |
| Ga0586324 0463865 1 1677 | 0.00 | 0.00 | 0.00 | 0.00 | 0.00 | 0.00 | 0.00 | 0.00 | 0.00 | 0.00 | 1108800.00 | 0.00 | KO:K01872 | alanyl-tRNA synthetase | Bacteria;Proteobacteria;Betaproteobacteria;Burkholderiales;unclassified Burkholderiales;Rubrivivax;Rubrivivax gelatinosus;Rubrivivax gelatinosus DSM 1709 |
| Ga0586324 0719407 3 1400 | 0.00 | 0.00 | 0.00 | 0.00 | 0.00 | 0.00 | 0.00 | 0.00 | 0.00 | 0.00 | 0.00 | 6172500.00 | KO:K01872 | alanyl-tRNA synthetase | Bacteria;Proteobacteria;Betaproteobacteria;Burkholderiales;Burkholderiaceae;Quisquiliibacterium;Quisquiliibacterium transsilvanicum;Quisquiliibacterium transsilvanicum DSM 29781 |
| Ga0586324 4397453 3 605 | 0.00 | 0.00 | 0.00 | 0.00 | 0.00 | 0.00 | 0.00 | 0.00 | 0.00 | 0.00 | 1636800.00 | 0.00 | KO:K01873 | valyl-tRNA synthetase | Bacteria;Firmicutes;Clostridia;Eubacteriales;Peptococcaceae;Thermoanaerosceptrum;Thermoanaerosceptrum fracticalcis;Thermoanaerosceptrum fracticalcis DRI-13 |
| Ga0586324 8195938 1 501 | 0.00 | 0.00 | 0.00 | 0.00 | 0.00 | 0.00 | 0.00 | 0.00 | 0.00 | 15092000.00 | 0.00 | 0.00 | KO:K01873 | valyl-tRNA synthetase | Bacteria;Proteobacteria;Deltaproteobacteria;Desulfuromonadales;Geobacteraceae;Geobacter;Geobacter anodireducens;Geobacter anodireducens SD-1 |
| Ga0586324 0098615 2 1627 | 0.00 | 0.00 | 0.00 | 0.00 | 0.00 | 0.00 | 0.00 | 1415200.00 | 0.00 | 0.00 | 0.00 | 0.00 | KO:K01874 | methionyl-tRNA synthetase | Bacteria;Proteobacteria;Betaproteobacteria;unclassified Betaproteobacteria;unclassified Betaproteobacteria;unclassified Betaproteobacteria;beta proteobacterium AAP51;Betaproteobacteria bacterium AAP51 |
| Ga0586324 0000014 13008 14789 | 0.00 | 0.00 | 0.00 | 0.00 | 0.00 | 0.00 | 0.00 | 0.00 | 11502000.00 | 0.00 | 3388000.00 | 9847500.00 | KO:K01876 | aspartyl-tRNA synthetase | Bacteria;Proteobacteria;Betaproteobacteria;Nitrosomonadales;Thiobacillaceae;Annwoodia;Annwoodia aquaesulis;Annwoodia aquaesulis DSM 4255 |
| Ga0586324 0000100 43706 45502 | 0.00 | 0.00 | 0.00 | 0.00 | 0.00 | 0.00 | 0.00 | 0.00 | 0.00 | 0.00 | 4030400.00 | 0.00 | KO:K01876 | aspartyl-tRNA synthetase | Bacteria;Firmicutes;Bacilli;Bacillales;Alicyclobacillaceae;Effusibacillus;Effusibacillus pohliae;Effusibacillus pohliae DSM 22757 |
| Ga0586324 0005072 2752 4554 | 0.00 | 0.00 | 0.00 | 0.00 | 0.00 | 0.00 | 0.00 | 0.00 | 0.00 | 223333.88 | 0.00 | 552129.75 | KO:K01876 | aspartyl-tRNA synthetase | Bacteria;Proteobacteria;Betaproteobacteria;Nitrosomonadales;Gallionellaceae;Sideroxydans;Sideroxydans lithotrophicus;Sideroxydans lithotrophicus ES-1 |
| Ga0586324 0022446 2838 4679 | 0.00 | 0.00 | 0.00 | 0.00 | 0.00 | 0.00 | 0.00 | 0.00 | 0.00 | 0.00 | 1900800.00 | 0.00 | KO:K01876 | aspartyl-tRNA synthetase | Bacteria;Proteobacteria;Alphaproteobacteria;Hyphomicrobiales;unclassified Hyphomicrobiales;Methyloceanibacter;Methyloceanibacter superfactus;Methyloceanibacter superfactus R-67175 |
| Ga0586324 0161140 3 1163 | 0.00 | 0.00 | 0.00 | 0.00 | 0.00 | 0.00 | 0.00 | 0.00 | 0.00 | 7461300.00 | 0.00 | 0.00 | KO:K01876 | aspartyl-tRNA synthetase | Bacteria;Proteobacteria;Deltaproteobacteria;Myxococcales;Anaeromyxobacteraceae;Anaeromyxobacter;Anaeromyxobacter sp. Fw109-5;Anaeromyxobacter sp. Fw109-5 |
| Ga0586324 0300550 1 1110 | 0.00 | 0.00 | 0.00 | 0.00 | 0.00 | 0.00 | 0.00 | 0.00 | 0.00 | 0.00 | 0.00 | 2002500.00 | KO:K01876 | aspartyl-tRNA synthetase | Bacteria;Proteobacteria;Betaproteobacteria;Nitrosomonadales;Gallionellaceae;Gallionella;Gallionella capsiferriformans;Gallionella capsiferriformans ES-2 |
| Ga0586324 3180476 1 744 | 0.00 | 0.00 | 0.00 | 0.00 | 0.00 | 0.00 | 0.00 | 0.00 | 557953.92 | 0.00 | 0.00 | 0.00 | KO:K01876 | aspartyl-tRNA synthetase | Bacteria;Proteobacteria;Gammaproteobacteria;Nevskiales;Steroidobacteraceae;Povalibacter;Povalibacter uvarum;Povalibacter uvarum DSM 26723 |
| Ga0586324 3511312 1 714 | 0.00 | 0.00 | 0.00 | 0.00 | 0.00 | 0.00 | 0.00 | 0.00 | 0.00 | 6853000.00 | 5359200.00 | 2010000.00 | KO:K01876 | aspartyl-tRNA synthetase | Bacteria;Spirochaetes;Spirochaetia;Spirochaetales;Spirochaetaceae;Sediminispirochaeta;Sediminispirochaeta smaragdinae;Sediminispirochaeta smaragdinae DSM 11293 |
| Ga0586324 3731106 1 696 | 0.00 | 0.00 | 0.00 | 0.00 | 0.00 | 0.00 | 0.00 | 3574600.00 | 0.00 | 0.00 | 0.00 | 0.00 | KO:K01876 | aspartyl-tRNA synthetase | Bacteria;Proteobacteria;Deltaproteobacteria;Desulfobacterales;Desulfobacteraceae;Desulfatibacillum;Desulfatibacillum alkenivorans;Desulfatibacillum alkenivorans DSM 16219 |
| Ga0586324 6519505 2 553 | 0.00 | 0.00 | 0.00 | 0.00 | 0.00 | 0.00 | 0.00 | 0.00 | 1279800.00 | 0.00 | 0.00 | 0.00 | KO:K01876 | aspartyl-tRNA synthetase | Bacteria;Proteobacteria;Gammaproteobacteria;Methylococcales;Methylococcaceae;Methylobacter;Methylobacter tundripaludum;Methylobacter tundripaludum OWC-DMM |
| Ga0586324 0302496 3 1010 | 0.00 | 0.00 | 0.00 | 0.00 | 0.00 | 0.00 | 0.00 | 5727900.00 | 0.00 | 0.00 | 0.00 | 0.00 | KO:K01879 | glycyl-tRNA synthetase beta chain | Bacteria;Proteobacteria;Alphaproteobacteria;Hyphomicrobiales;Xanthobacteraceae;Pseudolabrys;Pseudolabrys taiwanensis;Pseudolabrys taiwanensis CC-BB4 |
| Ga0586324 5070130 1 612 | 0.00 | 0.00 | 0.00 | 0.00 | 0.00 | 0.00 | 0.00 | 0.00 | 1498500.00 | 0.00 | 0.00 | 0.00 | KO:K01880 | glycyl-tRNA synthetase | Bacteria;Bacteroidetes;Bacteroidia;Bacteroidales;unclassified Bacteroidales;Candidatus Symbiothrix;Candidatus Symbiothrix dinenymphae;Candidatus Symbiothrix dinenymphae B4-10h |
| Ga0586324 1402820 24 1049 | 22100000.00 | 0.00 | 0.00 | 0.00 | 0.00 | 0.00 | 0.00 | 0.00 | 0.00 | 0.00 | 0.00 | 0.00 | KO:K01881 | prolyl-tRNA synthetase | Bacteria;Actinobacteria;Rubrobacteria;Gaiellales;Gaiellaceae;Gaiella;Gaiella occulta;Gaiella occulta F2-233 |
| Ga0586324 0239509 3 815 | 0.00 | 0.00 | 0.00 | 0.00 | 0.00 | 0.00 | 0.00 | 0.00 | 0.00 | 0.00 | 2543200.00 | 2535000.00 | KO:K01892 | histidyl-tRNA synthetase | Bacteria;Proteobacteria;Betaproteobacteria;Nitrosomonadales;Sulfuricellaceae;Sulfuricella;Sulfuricella sp. T08;Sulfuricella sp. T08 |
| Ga0586324 4190210 2 664 | 0.00 | 0.00 | 0.00 | 0.00 | 0.00 | 0.00 | 1884116.76 | 0.00 | 0.00 | 0.00 | 0.00 | 0.00 | KO:K01892 | histidyl-tRNA synthetase | Bacteria;Spirochaetes;Spirochaetia;Spirochaetales;Treponemataceae;Treponema;Treponema caldarium;Treponema caldarium DSM 7334 |
| Ga0586324 0125442 1085 2473 | 0.00 | 0.00 | 0.00 | 1216000.00 | 0.00 | 0.00 | 0.00 | 0.00 | 0.00 | 0.00 | 0.00 | 0.00 | KO:K01893 | asparaginyl-tRNA synthetase | Bacteria;Bacteroidetes;Bacteroidia;Bacteroidales;Rikenellaceae;Alistipes;Alistipes indistinctus;Alistipes indistinctus YIT 12060 |
| Ga0586324 0000123 46446 48416 | 0.00 | 0.00 | 0.00 | 0.00 | 0.00 | 0.00 | 0.00 | 2958500.00 | 0.00 | 0.00 | 841577.44 | 0.00 | KO:K01895 | acetyl-CoA synthetase | Bacteria;Proteobacteria;Betaproteobacteria;Nitrosomonadales;Thiobacillaceae;Annwoodia;Annwoodia aquaesulis;Annwoodia aquaesulis DSM 4255 |
| Ga0586324 0011980 414 2393 | 0.00 | 0.00 | 0.00 | 0.00 | 0.00 | 0.00 | 0.00 | 0.00 | 0.00 | 2772000.00 | 0.00 | 0.00 | KO:K01895 | acetyl-CoA synthetase | Bacteria;Firmicutes;Clostridia;Eubacteriales;Desulfallaceae;Desulfoscipio;Desulfoscipio geothermicus;Desulfallas geothermicus DSM 3669 |
| Ga0586324 0020828 1 1560 | 3960000.00 | 0.00 | 0.00 | 0.00 | 0.00 | 0.00 | 0.00 | 0.00 | 0.00 | 0.00 | 0.00 | 0.00 | KO:K01895 | acetyl-CoA synthetase | Bacteria;Proteobacteria;Betaproteobacteria;Burkholderiales;unclassified Burkholderiales;Ideonella;Ideonella sakaiensis;Ideonella sp. 201-F6 |
| Ga0586324 0026882 1 1752 | 111000000.00 | 55216000.00 | 0.00 | 144495000.00 | 0.00 | 0.00 | 0.00 | 38198200.00 | 48276000.00 | 0.00 | 0.00 | 0.00 | KO:K01895 | acetyl-CoA synthetase | Archaea;Euryarchaeota;Methanomicrobia;Methanosarcinales;Methanotrichaceae;Methanothrix;Methanothrix soehngenii;Methanothrix soehngenii GP-6 |
| Ga0586324 0026882 2085 4106 | 3890000.00 | 6120000.00 | 0.00 | 0.00 | 13390000.00 | 8485795.60 | 0.00 | 11907200.00 | 11809800.00 | 0.00 | 0.00 | 3225000.00 | KO:K01895 | acetyl-CoA synthetase | Archaea;Euryarchaeota;Methanomicrobia;Methanosarcinales;Methanotrichaceae;Methanothrix;Methanothrix thermoacetophila;Methanothrix thermoacetophila PT |
| Ga0586324 0026882 4561 6459 | 0.00 | 0.00 | 1106272.48 | 4617000.00 | 5191200.00 | 2844370.02 | 0.00 | 7259000.00 | 7184700.00 | 5443900.00 | 6450400.00 | 6682500.00 | KO:K01895 | acetyl-CoA synthetase | Archaea;Euryarchaeota;Methanomicrobia;Methanosarcinales;Methanotrichaceae;Methanothrix;Methanothrix soehngenii;Methanothrix soehngenii GP-6 |
| Ga0586324 0048598 1389 3383 | 0.00 | 0.00 | 0.00 | 0.00 | 0.00 | 17745900.00 | 0.00 | 10858000.00 | 0.00 | 0.00 | 0.00 | 0.00 | KO:K01895 | acetyl-CoA synthetase | Bacteria;Proteobacteria;Deltaproteobacteria;Syntrophorhabdales;Syntrophorhabdaceae;Syntrophorhabdus;Syntrophorhabdus aromaticivorans;Syntrophorhabdus aromaticivorans UI |
| Ga0586324 0063273 2538 4268 | 0.00 | 0.00 | 0.00 | 0.00 | 0.00 | 0.00 | 0.00 | 0.00 | 68040000.00 | 0.00 | 0.00 | 0.00 | KO:K01895 | acetyl-CoA synthetase | Bacteria;Proteobacteria;Deltaproteobacteria;Desulfobacterales;Desulfobacteraceae;Desulfatitalea;Desulfatitalea tepidiphila;Desulfatitalea tepidiphila S28bF |
| Ga0586324 0071218 2742 4034 | 451788.00 | 39168000.00 | 0.00 | 0.00 | 0.00 | 7940000.00 | 80028000.00 | 82594000.00 | 14256000.00 | 1247400.00 | 808168.24 | 36450000.00 | KO:K01895 | acetyl-CoA synthetase | Bacteria;Proteobacteria;Deltaproteobacteria;Desulfobacterales;Desulfobacteraceae;Desulfatirhabdium;Desulfatirhabdium butyrativorans;Desulfatirhabdium butyrativorans DSM 18734 |
| Ga0586324 0099152 1104 3086 | 0.00 | 0.00 | 5073998.09 | 0.00 | 16377000.00 | 0.00 | 0.00 | 0.00 | 0.00 | 0.00 | 0.00 | 0.00 | KO:K01895 | acetyl-CoA synthetase | Bacteria;Proteobacteria;Gammaproteobacteria;Chromatiales;Ectothiorhodospiraceae;Inmirania;Inmirania thermothiophila;Inmirania thermothiophila DSM 100275 |
| Ga0586324 0123298 2 1615 | 0.00 | 0.00 | 0.00 | 0.00 | 0.00 | 0.00 | 0.00 | 0.00 | 1563300.00 | 0.00 | 0.00 | 0.00 | KO:K01895 | acetyl-CoA synthetase | Bacteria;Proteobacteria;Betaproteobacteria;unclassified Betaproteobacteria;unclassified Betaproteobacteria;unclassified Betaproteobacteria;Betaproteobacteria bacterium GR16-43;Betaproteobacteria bacterium GR16-43 |
| Ga0586324 0156934 1 1689 | 0.00 | 1387200.00 | 0.00 | 0.00 | 0.00 | 0.00 | 0.00 | 0.00 | 0.00 | 1617000.00 | 0.00 | 0.00 | KO:K01895 | acetyl-CoA synthetase | Bacteria;Proteobacteria;Deltaproteobacteria;Syntrophorhabdales;Syntrophorhabdaceae;Syntrophorhabdus;Syntrophorhabdus aromaticivorans;Syntrophorhabdus aromaticivorans UI |
| Ga0586324 0184767 2 1783 | 80900000.00 | 232412723.56 | 329355000.00 | 236740000.00 | 111549000.00 | 226369400.00 | 147766800.00 | 141825000.00 | 154467000.00 | 89782000.00 | 128568000.00 | 118650000.00 | KO:K01895 | acetyl-CoA synthetase | Archaea;Euryarchaeota;Methanomicrobia;Methanosarcinales;Methanotrichaceae;Methanothrix;Methanothrix soehngenii;Methanothrix soehngenii GP-6 |
| Ga0586324 0273610 1251 2144 | 0.00 | 0.00 | 0.00 | 0.00 | 0.00 | 0.00 | 0.00 | 0.00 | 0.00 | 0.00 | 0.00 | 2572500.00 | KO:K01895 | acetyl-CoA synthetase | Bacteria;Firmicutes;Clostridia;Eubacteriales;Desulfallaceae;Desulfoscipio;Desulfoscipio geothermicus;Desulfallas geothermicus DSM 3669 |
| Ga0586324 0281551 156 2114 | 0.00 | 0.00 | 0.00 | 1444000.00 | 0.00 | 0.00 | 0.00 | 0.00 | 0.00 | 0.00 | 0.00 | 0.00 | KO:K01895 | acetyl-CoA synthetase | Bacteria;Actinobacteria;Thermoleophilia;Solirubrobacterales;Solirubrobacteraceae;Solirubrobacter;Solirubrobacter sp. URHD0082;Solirubrobacter sp. URHD0082 |
| Ga0586324 0338197 1414 1947 | 0.00 | 0.00 | 0.00 | 0.00 | 0.00 | 0.00 | 35112000.00 | 20374000.00 | 0.00 | 0.00 | 0.00 | 0.00 | KO:K01895 | acetyl-CoA synthetase | Bacteria;Proteobacteria;Deltaproteobacteria;Desulfobacterales;Desulfobacteraceae;Desulfococcus;Desulfococcus multivorans;Desulfococcus multivorans DSM 2059 |
| Ga0586324 0425730 1 354 | 0.00 | 0.00 | 0.00 | 0.00 | 0.00 | 0.00 | 8800800.00 | 7747000.00 | 0.00 | 0.00 | 0.00 | 0.00 | KO:K01895 | acetyl-CoA synthetase | Bacteria;Proteobacteria;Gammaproteobacteria;Xanthomonadales;Xanthomonadaceae;Pseudoxanthomonas;Pseudoxanthomonas taiwanensis;Pseudoxanthomonas taiwanensis DSM 22914 |
| Ga0586324 0469707 1 1686 | 3102597.00 | 101360800.00 | 0.00 | 0.00 | 0.00 | 732512.64 | 0.00 | 823500.00 | 569059.02 | 0.00 | 0.00 | 0.00 | KO:K01895 | acetyl-CoA synthetase | Archaea;Euryarchaeota;Methanomicrobia;Methanosarcinales;Methanotrichaceae;Methanothrix;Methanothrix thermoacetophila;Methanothrix thermoacetophila PT |
| Ga0586324 0499595 1 1638 | 0.00 | 2386800.00 | 13343100.00 | 10260000.00 | 0.00 | 10044100.00 | 0.00 | 0.00 | 0.00 | 0.00 | 0.00 | 0.00 | KO:K01895 | acetyl-CoA synthetase | Archaea;Euryarchaeota;Methanomicrobia;Methanosarcinales;Methanotrichaceae;Methanothrix;Methanothrix soehngenii;Methanothrix soehngenii GP-6 |
| Ga0586324 0618063 3 1496 | 0.00 | 0.00 | 0.00 | 0.00 | 0.00 | 0.00 | 0.00 | 1104100.00 | 0.00 | 0.00 | 0.00 | 0.00 | KO:K01895 | acetyl-CoA synthetase | Bacteria;Proteobacteria;Alphaproteobacteria;Hyphomicrobiales;Bradyrhizobiaceae;Bradyrhizobium;Bradyrhizobium zhanjiangense;Bradyrhizobium zhanjiangense CCBAU 51778 |
| Ga0586324 0680543 443 1432 | 0.00 | 0.00 | 0.00 | 0.00 | 0.00 | 0.00 | 0.00 | 738100.00 | 0.00 | 0.00 | 0.00 | 0.00 | KO:K01895 | acetyl-CoA synthetase | Archaea;Euryarchaeota;Methanomicrobia;Methanosarcinales;Methanotrichaceae;Methanothrix;Methanothrix thermoacetophila;Methanothrix thermoacetophila PT |
| Ga0586324 0734981 3 1385 | 1670000.00 | 0.00 | 2084180.96 | 0.00 | 0.00 | 0.00 | 0.00 | 0.00 | 0.00 | 0.00 | 0.00 | 0.00 | KO:K01895 | acetyl-CoA synthetase | Archaea;Euryarchaeota;Methanomicrobia;Methanosarcinales;Methanotrichaceae;Methanothrix;Methanothrix soehngenii;Methanothrix soehngenii GP-6 |
| Ga0586324 0827138 2 1318 | 0.00 | 0.00 | 0.00 | 0.00 | 0.00 | 0.00 | 0.00 | 0.00 | 0.00 | 0.00 | 0.00 | 16275000.00 | KO:K01895 | acetyl-CoA synthetase | Bacteria;Thermodesulfobacteria;Thermodesulfobacteria;Thermodesulfobacteriales;Thermodesulfobacteriaceae;Thermodesulfatator;Thermodesulfatator autotrophicus;Thermodesulfatator autotrophicus S606 |
| Ga0586324 0852764 2 1300 | 37800000.00 | 0.00 | 0.00 | 0.00 | 1318400.00 | 0.00 | 2758800.00 | 0.00 | 0.00 | 0.00 | 0.00 | 0.00 | KO:K01895 | acetyl-CoA synthetase | Archaea;Euryarchaeota;Methanomicrobia;Methanosarcinales;Methanotrichaceae;Methanothrix;Methanothrix thermoacetophila;Methanothrix thermoacetophila PT |
| Ga0586324 0896192 84 1274 | 0.00 | 1686400.00 | 0.00 | 25460000.00 | 38728000.00 | 0.00 | 0.00 | 15006000.00 | 26082000.00 | 0.00 | 0.00 | 0.00 | KO:K01895 | acetyl-CoA synthetase | Archaea;Euryarchaeota;Methanomicrobia;Methanosarcinales;Methanotrichaceae;Methanothrix;Methanothrix thermoacetophila;Methanothrix thermoacetophila PT |
| Ga0586324 0927146 1 720 | 0.00 | 6351200.00 | 0.00 | 0.00 | 0.00 | 0.00 | 0.00 | 1586000.00 | 748737.27 | 104720000.00 | 1918400.00 | 142597500.00 | KO:K01895 | acetyl-CoA synthetase | Bacteria;Proteobacteria;Deltaproteobacteria;Syntrophobacterales;Syntrophaceae;Syntrophus;Syntrophus aciditrophicus;Syntrophus aciditrophicus SB |
| Ga0586324 0928591 1 1254 | 9470000.00 | 18218156.08 | 0.00 | 0.00 | 0.00 | 0.00 | 0.00 | 0.00 | 0.00 | 1570800.00 | 0.00 | 0.00 | KO:K01895 | acetyl-CoA synthetase | Archaea;Euryarchaeota;Methanomicrobia;Methanosarcinales;Methanotrichaceae;Methanothrix;Methanothrix thermoacetophila;Methanothrix thermoacetophila PT |
| Ga0586324 0991946 539 1219 | 0.00 | 0.00 | 0.00 | 0.00 | 0.00 | 0.00 | 0.00 | 0.00 | 6407100.00 | 0.00 | 0.00 | 0.00 | KO:K01895 | acetyl-CoA synthetase | Bacteria;Proteobacteria;Deltaproteobacteria;unclassified Deltaproteobacteria;unclassified Deltaproteobacteria;Dissulfuribacter;Dissulfuribacter thermophilus;Dissulfuribacter thermophilus S69 |
| Ga0586324 1124373 1 1035 | 84000000.00 | 21630800.00 | 0.00 | 2422500.00 | 0.00 | 0.00 | 0.00 | 0.00 | 649178.55 | 0.00 | 0.00 | 741612.00 | KO:K01895 | acetyl-CoA synthetase | Archaea;Euryarchaeota;Methanomicrobia;Methanosarcinales;Methanotrichaceae;Methanothrix;Methanothrix thermoacetophila;Methanothrix thermoacetophila PT |
| Ga0586324 1134037 342 1151 | 13000000.00 | 0.00 | 0.00 | 17385000.00 | 8291500.00 | 0.00 | 0.00 | 3074400.00 | 0.00 | 0.00 | 0.00 | 0.00 | KO:K01895 | acetyl-CoA synthetase | Bacteria;Proteobacteria;Deltaproteobacteria;Syntrophobacterales;Syntrophaceae;Smithella;Smithella sp. SCADC;Smithella sp. SCADC |
| Ga0586324 1433018 2 1039 | 0.00 | 0.00 | 0.00 | 0.00 | 0.00 | 0.00 | 0.00 | 0.00 | 0.00 | 0.00 | 0.00 | 3900000.00 | KO:K01895 | acetyl-CoA synthetase | Bacteria;Proteobacteria;Deltaproteobacteria;Syntrophobacterales;Syntrophaceae;Smithella;Smithella sp. SCADC;Smithella sp. SCADC |
| Ga0586324 1555587 3 1004 | 1390000.00 | 0.00 | 0.00 | 0.00 | 0.00 | 0.00 | 0.00 | 0.00 | 0.00 | 0.00 | 0.00 | 0.00 | KO:K01895 | acetyl-CoA synthetase | Bacteria;Proteobacteria;Betaproteobacteria;unclassified Betaproteobacteria;unclassified Betaproteobacteria;unclassified Betaproteobacteria;Betaproteobacteria bacterium GR16-43;Betaproteobacteria bacterium GR16-43 |
| Ga0586324 1634321 416 985 | 0.00 | 0.00 | 0.00 | 0.00 | 0.00 | 0.00 | 0.00 | 10553000.00 | 0.00 | 0.00 | 0.00 | 0.00 | KO:K01895 | acetyl-CoA synthetase | Bacteria;Proteobacteria;Deltaproteobacteria;Desulfobacterales;Desulfobacteraceae;Desulfatiglans;Desulfatiglans anilini;Desulfatiglans anilini DSM 4660 |
| Ga0586324 1742430 252 959 | 0.00 | 0.00 | 0.00 | 0.00 | 0.00 | 0.00 | 0.00 | 384086.50 | 0.00 | 0.00 | 0.00 | 0.00 | KO:K01895 | acetyl-CoA synthetase | Bacteria;Proteobacteria;Deltaproteobacteria;Syntrophorhabdales;Syntrophorhabdaceae;Syntrophorhabdus;Syntrophorhabdus aromaticivorans;Syntrophorhabdus aromaticivorans UI |
| Ga0586324 1838812 1 936 | 2080000.00 | 2169200.00 | 0.00 | 0.00 | 0.00 | 0.00 | 0.00 | 0.00 | 0.00 | 0.00 | 0.00 | 0.00 | KO:K01895 | acetyl-CoA synthetase | Archaea;Euryarchaeota;Methanomicrobia;Methanosarcinales;Methanotrichaceae;Methanothrix;Methanothrix thermoacetophila;Methanothrix thermoacetophila PT |
| Ga0586324 1874112 3 929 | 0.00 | 0.00 | 0.00 | 0.00 | 0.00 | 0.00 | 0.00 | 0.00 | 0.00 | 1270500.00 | 0.00 | 0.00 | KO:K01895 | acetyl-CoA synthetase | Bacteria;Proteobacteria;Gammaproteobacteria;Cellvibrionales;Cellvibrionaceae;Teredinibacter;Teredinibacter waterburyi;Teredinibacter waterburyi Bs02 |
| Ga0586324 1933884 2 796 | 0.00 | 0.00 | 2646150.67 | 0.00 | 0.00 | 0.00 | 2918400.00 | 0.00 | 1717200.00 | 0.00 | 0.00 | 0.00 | KO:K01895 | acetyl-CoA synthetase | Bacteria;Proteobacteria;Betaproteobacteria;Rhodocyclales;Azonexaceae;Dechloromonas;Dechloromonas aromatica;Dechloromonas aromatica RCB |
| Ga0586324 2062576 1 450 | 0.00 | 0.00 | 2671660.20 | 1434500.00 | 14832000.00 | 0.00 | 0.00 | 3623400.00 | 4600800.00 | 1755600.00 | 0.00 | 0.00 | KO:K01895 | acetyl-CoA synthetase | Archaea;Euryarchaeota;Methanomicrobia;Methanosarcinales;Methanotrichaceae;Methanothrix;Methanothrix soehngenii;Methanothrix soehngenii GP-6 |
| Ga0586324 2281009 1 855 | 1950000.00 | 836400.00 | 0.00 | 0.00 | 0.00 | 0.00 | 0.00 | 0.00 | 0.00 | 0.00 | 0.00 | 0.00 | KO:K01895 | acetyl-CoA synthetase | Archaea;Euryarchaeota;Methanomicrobia;Methanosarcinales;Methanotrichaceae;Methanothrix;Methanothrix thermoacetophila;Methanothrix thermoacetophila PT |
| Ga0586324 2329312 3 587 | 0.00 | 0.00 | 0.00 | 0.00 | 0.00 | 0.00 | 0.00 | 0.00 | 6520500.00 | 0.00 | 0.00 | 0.00 | KO:K01895 | acetyl-CoA synthetase | Bacteria;Proteobacteria;Deltaproteobacteria;Syntrophobacterales;Syntrophaceae;Smithella;Smithella sp. SCADC;Smithella sp. SCADC |
| Ga0586324 2689522 2 796 | 0.00 | 0.00 | 0.00 | 0.00 | 0.00 | 0.00 | 0.00 | 1439600.00 | 0.00 | 0.00 | 0.00 | 0.00 | KO:K01895 | acetyl-CoA synthetase | Bacteria;Nitrospirae;Thermodesulfovibrionia;Thermodesulfovibrionales;Thermodesulfovibrionaceae;Thermodesulfovibrio;Thermodesulfovibrio sp. N1;Thermodesulfovibrio sp. N1 |
| Ga0586324 3003435 1 759 | 0.00 | 0.00 | 0.00 | 9975000.00 | 14214000.00 | 0.00 | 0.00 | 0.00 | 0.00 | 0.00 | 0.00 | 0.00 | KO:K01895 | acetyl-CoA synthetase | Bacteria;Proteobacteria;Deltaproteobacteria;Syntrophobacterales;Syntrophobacteraceae;Syntrophobacter;Syntrophobacter fumaroxidans;Syntrophobacter fumaroxidans MPOB |
| Ga0586324 3066518 202 756 | 0.00 | 0.00 | 0.00 | 0.00 | 0.00 | 0.00 | 0.00 | 0.00 | 0.00 | 0.00 | 0.00 | 22725000.00 | KO:K01895 | acetyl-CoA synthetase | Archaea;Euryarchaeota;Methanomicrobia;Methanosarcinales;Methanotrichaceae;Methanothrix;Methanothrix soehngenii;Methanothrix soehngenii GP-6 |
| Ga0586324 3269833 3 734 | 0.00 | 634864.32 | 0.00 | 0.00 | 0.00 | 0.00 | 0.00 | 0.00 | 0.00 | 0.00 | 0.00 | 0.00 | KO:K01895 | acetyl-CoA synthetase | Bacteria;Firmicutes;Clostridia;Eubacteriales;Syntrophomonadaceae;Dethiobacter;Dethiobacter alkaliphilus;Dethiobacter alkaliphilus AHT1 |
| Ga0586324 3296253 3 584 | 0.00 | 0.00 | 0.00 | 0.00 | 0.00 | 0.00 | 0.00 | 0.00 | 0.00 | 0.00 | 0.00 | 1950000.00 | KO:K01895 | acetyl-CoA synthetase | Archaea;Euryarchaeota;Methanomicrobia;Methanosarcinales;Methanotrichaceae;Methanothrix;Methanothrix thermoacetophila;Methanothrix thermoacetophila PT |
| Ga0586324 3357151 1 726 | 0.00 | 2244000.00 | 0.00 | 0.00 | 0.00 | 0.00 | 0.00 | 0.00 | 0.00 | 0.00 | 0.00 | 0.00 | KO:K01895 | acetyl-CoA synthetase | Archaea;Euryarchaeota;Methanomicrobia;Methanosarcinales;Methanotrichaceae;Methanothrix;Methanothrix thermoacetophila;Methanothrix thermoacetophila PT |
| Ga0586324 3388155 2 724 | 0.00 | 0.00 | 0.00 | 0.00 | 8198800.00 | 0.00 | 0.00 | 0.00 | 0.00 | 2394700.00 | 0.00 | 0.00 | KO:K01895 | acetyl-CoA synthetase | Bacteria;Proteobacteria;Deltaproteobacteria;Syntrophobacterales;Syntrophaceae;Syntrophus;Syntrophus aciditrophicus;Syntrophus aciditrophicus SB |
| Ga0586324 3468872 3 716 | 0.00 | 0.00 | 0.00 | 0.00 | 0.00 | 0.00 | 0.00 | 1098000.00 | 589111.38 | 0.00 | 0.00 | 0.00 | KO:K01895 | acetyl-CoA synthetase | Bacteria;Proteobacteria;Deltaproteobacteria;Desulfobacterales;Desulfobacteraceae;Desulfatitalea;Desulfatitalea tepidiphila;Desulfatitalea tepidiphila S28bF |
| Ga0586324 3718501 3 698 | 0.00 | 0.00 | 0.00 | 0.00 | 0.00 | 0.00 | 0.00 | 17812000.00 | 0.00 | 0.00 | 0.00 | 0.00 | KO:K01895 | acetyl-CoA synthetase | Archaea;Euryarchaeota;Methanomicrobia;Methanosarcinales;Methanotrichaceae;Methanothrix;Methanothrix soehngenii;Methanothrix soehngenii GP-6 |
| Ga0586324 4092525 2 670 | 0.00 | 0.00 | 19198300.00 | 0.00 | 5695900.00 | 0.00 | 0.00 | 0.00 | 4422600.00 | 0.00 | 0.00 | 0.00 | KO:K01895 | acetyl-CoA synthetase | Archaea;Euryarchaeota;Methanomicrobia;Methanosarcinales;Methanotrichaceae;Methanothrix;Methanothrix thermoacetophila;Methanothrix thermoacetophila PT |
| Ga0586324 4195005 3 662 | 7720000.00 | 0.00 | 0.00 | 0.00 | 0.00 | 0.00 | 0.00 | 0.00 | 0.00 | 0.00 | 0.00 | 0.00 | KO:K01895 | acetyl-CoA synthetase | Archaea;Euryarchaeota;Methanomicrobia;Methanosarcinales;Methanotrichaceae;Methanothrix;Methanothrix thermoacetophila;Methanothrix thermoacetophila PT |
| Ga0586324 4582793 2 640 | 0.00 | 1591200.00 | 0.00 | 0.00 | 0.00 | 0.00 | 0.00 | 0.00 | 0.00 | 0.00 | 0.00 | 0.00 | KO:K01895 | acetyl-CoA synthetase | Archaea;Euryarchaeota;Methanomicrobia;Methanosarcinales;Methanotrichaceae;Methanothrix;Methanothrix soehngenii;Methanothrix soehngenii GP-6 |
| Ga0586324 5025484 50 616 | 45200000.00 | 38964000.00 | 0.00 | 19190000.00 | 27192000.00 | 18579600.00 | 14272800.00 | 9821000.00 | 20655000.00 | 22360800.00 | 0.00 | 0.00 | KO:K01895 | acetyl-CoA synthetase | Archaea;Euryarchaeota;Methanomicrobia;Methanosarcinales;Methanotrichaceae;Methanothrix;Methanothrix soehngenii;Methanothrix soehngenii GP-6 |
| Ga0586324 5264864 3 605 | 0.00 | 0.00 | 0.00 | 0.00 | 0.00 | 980784.53 | 0.00 | 0.00 | 0.00 | 0.00 | 0.00 | 0.00 | KO:K01895 | acetyl-CoA synthetase | Archaea;Euryarchaeota;Methanomicrobia;Methanosarcinales;Methanotrichaceae;Methanothrix;Methanothrix soehngenii;Methanothrix soehngenii GP-6 |
| Ga0586324 5321395 3 365 | 13740000.00 | 0.00 | 0.00 | 1871500.00 | 0.00 | 5402002.82 | 2576400.00 | 2409500.00 | 2073600.00 | 1239700.00 | 1900800.00 | 0.00 | KO:K01895 | acetyl-CoA synthetase | Archaea;Euryarchaeota;Methanomicrobia;Methanosarcinales;Methanotrichaceae;Methanothrix;Methanothrix thermoacetophila;Methanothrix thermoacetophila PT |
| Ga0586324 5367519 164 598 | 0.00 | 5936400.00 | 0.00 | 7324500.00 | 9064000.00 | 0.00 | 0.00 | 5313100.00 | 7468200.00 | 1863400.00 | 3924800.00 | 0.00 | KO:K01895 | acetyl-CoA synthetase | Archaea;Euryarchaeota;Methanomicrobia;Methanosarcinales;Methanotrichaceae;Methanothrix;Methanothrix soehngenii;Methanothrix soehngenii GP-6 |
| Ga0586324 5546180 3 572 | 0.00 | 0.00 | 0.00 | 0.00 | 0.00 | 0.00 | 0.00 | 0.00 | 0.00 | 60676000.00 | 0.00 | 49725000.00 | KO:K01895 | acetyl-CoA synthetase | Archaea;Euryarchaeota;Methanomicrobia;Methanosarcinales;Methanotrichaceae;Methanothrix;Methanothrix soehngenii;Methanothrix soehngenii GP-6 |
| Ga0586324 5642265 1 588 | 0.00 | 1502800.00 | 0.00 | 0.00 | 0.00 | 0.00 | 0.00 | 0.00 | 2187000.00 | 1439900.00 | 0.00 | 0.00 | KO:K01895 | acetyl-CoA synthetase | Archaea;Euryarchaeota;Methanomicrobia;Methanosarcinales;Methanotrichaceae;Methanothrix;Methanothrix thermoacetophila;Methanothrix thermoacetophila PT |
| Ga0586324 5713005 34 585 | 69900000.00 | 56576000.00 | 49487700.00 | 23655000.00 | 13184000.00 | 37437100.00 | 15982800.00 | 23045800.00 | 19553400.00 | 5197500.00 | 11528000.00 | 15135000.00 | KO:K01895 | acetyl-CoA synthetase | Archaea;Euryarchaeota;Methanomicrobia;Methanosarcinales;Methanotrichaceae;Methanothrix;Methanothrix thermoacetophila;Methanothrix thermoacetophila PT |
| Ga0586324 5820712 3 578 | 1910000.00 | 0.00 | 0.00 | 0.00 | 0.00 | 0.00 | 0.00 | 0.00 | 0.00 | 0.00 | 0.00 | 0.00 | KO:K01895 | acetyl-CoA synthetase | Archaea;Euryarchaeota;Methanomicrobia;Methanosarcinales;Methanotrichaceae;Methanothrix;Methanothrix thermoacetophila;Methanothrix thermoacetophila PT |
| Ga0586324 6888291 125 541 | 0.00 | 0.00 | 0.00 | 0.00 | 0.00 | 2297558.10 | 0.00 | 0.00 | 0.00 | 0.00 | 0.00 | 0.00 | KO:K01895 | acetyl-CoA synthetase | Bacteria;Proteobacteria;Gammaproteobacteria;Xanthomonadales;Xanthomonadaceae;Vulcaniibacterium;Vulcaniibacterium tengchongense;Vulcaniibacterium tengchongense DSM 25623 |
| Ga0586324 7509009 112 522 | 0.00 | 0.00 | 0.00 | 4389000.00 | 0.00 | 0.00 | 0.00 | 0.00 | 0.00 | 0.00 | 0.00 | 0.00 | KO:K01895 | acetyl-CoA synthetase | Bacteria;Proteobacteria;Deltaproteobacteria;Desulfobacterales;Desulfobacteraceae;Desulfatirhabdium;Desulfatirhabdium butyrativorans;Desulfatirhabdium butyrativorans DSM 18734 |
| Ga0586324 4041257 40 672 | 0.00 | 0.00 | 0.00 | 0.00 | 0.00 | 0.00 | 0.00 | 0.00 | 0.00 | 2156000.00 | 0.00 | 0.00 | KO:K01896 | medium-chain acyl-CoA synthetase | Bacteria;Proteobacteria;Deltaproteobacteria;Desulfobacterales;Desulfobacteraceae;Desulfatirhabdium;Desulfatirhabdium butyrativorans;Desulfatirhabdium butyrativorans DSM 18734 |
| Ga0586324 0262442 258 1961 | 0.00 | 0.00 | 0.00 | 1358500.00 | 0.00 | 0.00 | 0.00 | 0.00 | 0.00 | 0.00 | 0.00 | 0.00 | KO:K01897 | long-chain acyl-CoA synthetase | Bacteria;Proteobacteria;Deltaproteobacteria;Syntrophobacterales;Syntrophaceae;Smithella;Smithella sp. F21;Smithella sp. F21 |
| Ga0586324 2031252 3 374 | 0.00 | 0.00 | 0.00 | 443087.60 | 0.00 | 0.00 | 0.00 | 478801.20 | 426867.57 | 0.00 | 0.00 | 0.00 | KO:K01897 | long-chain acyl-CoA synthetase | Bacteria;Proteobacteria;Deltaproteobacteria;Syntrophobacterales;Syntrophaceae;Smithella;Smithella sp. F21;Smithella sp. F21 |
| Ga0586324 5535383 3 590 | 0.00 | 0.00 | 0.00 | 0.00 | 0.00 | 0.00 | 0.00 | 0.00 | 2818800.00 | 0.00 | 0.00 | 0.00 | KO:K01897 | long-chain acyl-CoA synthetase | Bacteria;Proteobacteria;Deltaproteobacteria;Myxococcales;Anaeromyxobacteraceae;Anaeromyxobacter;Anaeromyxobacter sp. Fw109-5;Anaeromyxobacter sp. Fw109-5 |
| Ga0586324 0843547 575 1306 | 0.00 | 0.00 | 0.00 | 0.00 | 0.00 | 0.00 | 0.00 | 0.00 | 0.00 | 0.00 | 0.00 | 2077500.00 | KO:K01902 | succinyl-CoA synthetase alpha subunit | Bacteria;Proteobacteria;Deltaproteobacteria;Desulfobacterales;Desulfobulbaceae;Desulfobulbus;Desulfobulbus propionicus;Desulfobulbus propionicus 1pr3, DSM 2032 |
| Ga0586324 1193910 141 1019 | 0.00 | 0.00 | 0.00 | 1444000.00 | 6901000.00 | 5280100.00 | 0.00 | 0.00 | 0.00 | 0.00 | 0.00 | 0.00 | KO:K01902 | succinyl-CoA synthetase alpha subunit | Bacteria;Proteobacteria;Deltaproteobacteria;Syntrophobacterales;Syntrophobacteraceae;Syntrophobacter;Syntrophobacter fumaroxidans;Syntrophobacter fumaroxidans MPOB |
| Ga0586324 5257265 290 604 | 0.00 | 0.00 | 0.00 | 3382000.00 | 0.00 | 0.00 | 0.00 | 0.00 | 0.00 | 0.00 | 0.00 | 0.00 | KO:K01902 | succinyl-CoA synthetase alpha subunit | Bacteria;Bacteroidetes;Chitinophagia;Chitinophagales;Chitinophagaceae;Chitinophaga;Chitinophaga pinensis;Chitinophaga pinensis 1204 |
| Ga0586324 7497443 133 522 | 0.00 | 0.00 | 0.00 | 0.00 | 0.00 | 0.00 | 0.00 | 0.00 | 1352700.00 | 0.00 | 0.00 | 0.00 | KO:K01902 | succinyl-CoA synthetase alpha subunit | Bacteria;Proteobacteria;Alphaproteobacteria;Hyphomicrobiales;Bradyrhizobiaceae;Bradyrhizobium;Bradyrhizobium erythrophlei;Bradyrhizobium erythrophlei GAS401 |
| Ga0586324 0104753 2390 3355 | 0.00 | 8432000.00 | 0.00 | 1615000.00 | 5829800.00 | 0.00 | 0.00 | 1836100.00 | 3547800.00 | 1355200.00 | 6538400.00 | 8250000.00 | KO:K01903 | succinyl-CoA synthetase beta subunit | Bacteria;Proteobacteria;Gammaproteobacteria;unclassified Gammaproteobacteria;Candidatus Competibacteraceae;Candidatus Competibacter;Candidatus Competibacter denitrificans;Candidatus Competibacter denitrificans Run A D11 |
| Ga0586324 0178732 463 1626 | 0.00 | 0.00 | 7262700.00 | 0.00 | 0.00 | 0.00 | 0.00 | 0.00 | 0.00 | 0.00 | 0.00 | 0.00 | KO:K01903 | succinyl-CoA synthetase beta subunit | Bacteria;Proteobacteria;Gammaproteobacteria;unclassified Gammaproteobacteria;Candidatus Competibacteraceae;Candidatus Competibacter;Candidatus Competibacter denitrificans;Candidatus Competibacter denitrificans Run A D11 |
| Ga0586324 0867594 2 412 | 11400000.00 | 8160000.00 | 16270700.00 | 0.00 | 14626000.00 | 0.00 | 8983200.00 | 8357000.00 | 8748000.00 | 13244000.00 | 0.00 | 7447500.00 | KO:K01903 | succinyl-CoA synthetase beta subunit | Bacteria;Proteobacteria;Gammaproteobacteria;unclassified Gammaproteobacteria;Candidatus Competibacteraceae;Candidatus Contendobacter;Candidatus Contendobacter odensis;Candidatus Contendobacter odensis Run B J11 |
| Ga0586324 0985279 2 934 | 0.00 | 9968800.00 | 0.00 | 0.00 | 0.00 | 0.00 | 0.00 | 0.00 | 0.00 | 1247400.00 | 0.00 | 0.00 | KO:K01903 | succinyl-CoA synthetase beta subunit | Bacteria;Proteobacteria;Deltaproteobacteria;Syntrophobacterales;Syntrophobacteraceae;Syntrophobacter;Syntrophobacter fumaroxidans;Syntrophobacter fumaroxidans MPOB |
| Ga0586324 8285042 2 499 | 0.00 | 0.00 | 0.00 | 0.00 | 0.00 | 0.00 | 0.00 | 0.00 | 5953500.00 | 0.00 | 0.00 | 0.00 | KO:K01903 | succinyl-CoA synthetase beta subunit | Bacteria;Proteobacteria;Betaproteobacteria;Nitrosomonadales;Sterolibacteriaceae;Sulfurisoma;Sulfurisoma sediminicola;Sulfurisoma sediminicola DSM 26916 |
| Ga0586324 0050041 1992 3878 | 0.00 | 0.00 | 1423556.76 | 0.00 | 0.00 | 0.00 | 0.00 | 0.00 | 1571400.00 | 3649800.00 | 0.00 | 458315.25 | KO:K01908 | propionyl-CoA synthetase | Bacteria;Proteobacteria;Gammaproteobacteria;unclassified Gammaproteobacteria;Candidatus Competibacteraceae;Plasticicumulans;Plasticicumulans lactativorans;Plasticicumulans lactativorans DSM 25287 |
| Ga0586324 0071218 677 2581 | 0.00 | 0.00 | 0.00 | 0.00 | 0.00 | 0.00 | 0.00 | 0.00 | 14717700.00 | 0.00 | 0.00 | 0.00 | KO:K01908 | propionyl-CoA synthetase | Bacteria;Proteobacteria;Deltaproteobacteria;Desulfobacterales;Desulfobacteraceae;Desulfatirhabdium;Desulfatirhabdium butyrativorans;Desulfatirhabdium butyrativorans DSM 18734 |
| Ga0586324 0268832 94 2013 | 0.00 | 0.00 | 0.00 | 0.00 | 4181800.00 | 0.00 | 0.00 | 0.00 | 0.00 | 0.00 | 0.00 | 0.00 | KO:K01908 | propionyl-CoA synthetase | Bacteria;Proteobacteria;Alphaproteobacteria;Hyphomicrobiales;Hyphomicrobiaceae;Rhodoplanes;Rhodoplanes sp. Z2-YC6860;Rhodoplanes sp. Z2-YC6860 |
| Ga0586324 1111371 3 1160 | 0.00 | 7643200.00 | 0.00 | 0.00 | 3677100.00 | 0.00 | 5175600.00 | 4196800.00 | 7168500.00 | 8631700.00 | 4945600.00 | 5212500.00 | KO:K01908 | propionyl-CoA synthetase | Bacteria;Proteobacteria;Gammaproteobacteria;unclassified Gammaproteobacteria;Candidatus Competibacteraceae;Candidatus Competibacter;Candidatus Competibacter denitrificans;Candidatus Competibacter denitrificans Run A D11 |
| Ga0586324 1974689 80 907 | 0.00 | 3162000.00 | 6756000.00 | 6726000.00 | 4624700.00 | 4327300.00 | 19585200.00 | 1421300.00 | 3709800.00 | 0.00 | 748454.96 | 6517500.00 | KO:K01908 | propionyl-CoA synthetase | Bacteria;Proteobacteria;Gammaproteobacteria;unclassified Gammaproteobacteria;Candidatus Competibacteraceae;Candidatus Competibacter;Candidatus Competibacter denitrificans;Candidatus Competibacter denitrificans Run A D11 |
| Ga0586324 4472634 1 645 | 0.00 | 0.00 | 0.00 | 0.00 | 0.00 | 0.00 | 0.00 | 1738500.00 | 0.00 | 0.00 | 0.00 | 0.00 | KO:K01908 | propionyl-CoA synthetase | Bacteria;Proteobacteria;Gammaproteobacteria;unclassified Gammaproteobacteria;Candidatus Competibacteraceae;Candidatus Contendobacter;Candidatus Contendobacter odensis;Candidatus Contendobacter odensis Run B J11 |
| Ga0586324 6684701 225 548 | 0.00 | 0.00 | 0.00 | 0.00 | 0.00 | 0.00 | 0.00 | 1006500.00 | 0.00 | 0.00 | 0.00 | 0.00 | KO:K01908 | propionyl-CoA synthetase | Bacteria;Proteobacteria;Betaproteobacteria;Rhodocyclales;Azonexaceae;Azonexus;Azonexus fungiphilus;Azonexus fungiphilus DSM 23841 |
| Ga0586324 7874620 262 510 | 0.00 | 0.00 | 0.00 | 0.00 | 0.00 | 0.00 | 0.00 | 0.00 | 315879.75 | 0.00 | 0.00 | 0.00 | KO:K01912 | phenylacetate-CoA ligase | Bacteria;Proteobacteria;Betaproteobacteria;Burkholderiales;Burkholderiaceae;Cupriavidus;Cupriavidus taiwanensis;Cupriavidus taiwanensis ERS2038951 |
| Ga0586324 0000035 2669 4078 | 1010000.00 | 0.00 | 0.00 | 0.00 | 0.00 | 0.00 | 0.00 | 0.00 | 0.00 | 0.00 | 0.00 | 1305000.00 | KO:K01915 | glutamine synthetase | Bacteria;Proteobacteria;Gammaproteobacteria;Acidiferrobacterales;Acidiferrobacteraceae;Sulfuricaulis;Sulfuricaulis limicola;Sulfuricaulis limicola HA5 |
| Ga0586324 0004232 3423 4757 | 1080000.00 | 2536400.00 | 0.00 | 0.00 | 0.00 | 0.00 | 0.00 | 0.00 | 0.00 | 0.00 | 0.00 | 0.00 | KO:K01915 | glutamine synthetase | Archaea;Euryarchaeota;Methanomicrobia;Methanosarcinales;Methanotrichaceae;Methanothrix;Methanothrix soehngenii;Methanothrix soehngenii GP-6 |
| Ga0586324 0008932 581 1990 | 11600000.00 | 0.00 | 8107200.00 | 0.00 | 0.00 | 0.00 | 0.00 | 0.00 | 0.00 | 0.00 | 0.00 | 0.00 | KO:K01915 | glutamine synthetase | Bacteria;Proteobacteria;Gammaproteobacteria;Methylococcales;Methylococcaceae;Methylobacter;Methylobacter tundripaludum;Methylobacter tundripaludum SV96 |
| Ga0586324 0034848 2 520 | 0.00 | 4515200.00 | 6474500.00 | 4075500.00 | 0.00 | 4525800.00 | 0.00 | 2318000.00 | 1863000.00 | 0.00 | 1716000.00 | 3322500.00 | KO:K01915 | glutamine synthetase | Bacteria;Acidobacteria;Acidobacteriia;Bryobacterales;Solibacteraceae;Candidatus Solibacter;Candidatus Solibacter usitatus;Candidatus Solibacter usitatus Ellin6076 |
| Ga0586324 0044888 3 782 | 0.00 | 0.00 | 0.00 | 0.00 | 0.00 | 1446596.54 | 0.00 | 0.00 | 0.00 | 0.00 | 0.00 | 0.00 | KO:K01915 | glutamine synthetase | Bacteria;Proteobacteria;Alphaproteobacteria;Hyphomicrobiales;Bradyrhizobiaceae;Bradyrhizobium;Bradyrhizobium erythrophlei;Bradyrhizobium erythrophlei GAS478 |
| Ga0586324 0055397 1540 2952 | 1970000.00 | 985086.76 | 0.00 | 0.00 | 0.00 | 0.00 | 0.00 | 0.00 | 0.00 | 469450.52 | 0.00 | 0.00 | KO:K01915 | glutamine synthetase | Bacteria;Proteobacteria;Gammaproteobacteria;Chromatiales;Chromatiaceae;Thiobaca;Thiobaca trueperi;Thiobaca trueperi DSM 13587 |
| Ga0586324 0078508 378 1793 | 0.00 | 567791.16 | 0.00 | 0.00 | 0.00 | 0.00 | 0.00 | 0.00 | 0.00 | 0.00 | 0.00 | 0.00 | KO:K01915 | glutamine synthetase | Bacteria;Proteobacteria;Gammaproteobacteria;Nevskiales;Sinobacteraceae;Solimonas;Solimonas flava;Solimonas flava DSM 18980 |
| Ga0586324 0114939 1844 3211 | 0.00 | 0.00 | 0.00 | 0.00 | 0.00 | 0.00 | 0.00 | 0.00 | 0.00 | 21945000.00 | 0.00 | 0.00 | KO:K01915 | glutamine synthetase | Bacteria;Proteobacteria;Deltaproteobacteria;Syntrophobacterales;Syntrophaceae;Syntrophus;Syntrophus gentianae;Syntrophus gentianae DSM 8423 |
| Ga0586324 0142475 1 1947 | 5680000.00 | 0.00 | 0.00 | 0.00 | 0.00 | 0.00 | 0.00 | 0.00 | 0.00 | 0.00 | 0.00 | 0.00 | KO:K01915 | glutamine synthetase | Bacteria;Bacteroidetes;Chitinophagia;Chitinophagales;Chitinophagaceae;Sediminibacterium;Sediminibacterium goheungense;Sediminibacterium goheungense DSM 28323 |
| Ga0586324 0143141 854 2263 | 0.00 | 5304000.00 | 0.00 | 0.00 | 0.00 | 623508.35 | 7182000.00 | 0.00 | 17439300.00 | 8085000.00 | 9829600.00 | 1702500.00 | KO:K01915 | glutamine synthetase | Bacteria;Proteobacteria;Gammaproteobacteria;Methylococcales;Methylococcaceae;Methylobacter;Methylobacter tundripaludum;Methylobacter tundripaludum OWC-G53F |
| Ga0586324 0158561 1134 2546 | 0.00 | 0.00 | 0.00 | 0.00 | 0.00 | 1098010.69 | 0.00 | 0.00 | 0.00 | 0.00 | 0.00 | 0.00 | KO:K01915 | glutamine synthetase | Bacteria;Proteobacteria;Gammaproteobacteria;Nevskiales;Sinobacteraceae;Panacagrimonas;Panacagrimonas perspica;Panacagrimonas perspica DSM 26377 |
| Ga0586324 0189793 351 1763 | 0.00 | 0.00 | 3590757.70 | 0.00 | 0.00 | 0.00 | 0.00 | 0.00 | 0.00 | 0.00 | 0.00 | 0.00 | KO:K01915 | glutamine synthetase | Bacteria;Proteobacteria;Deltaproteobacteria;Syntrophobacterales;Syntrophobacteraceae;Syntrophobacter;Syntrophobacter fumaroxidans;Syntrophobacter fumaroxidans MPOB |
| Ga0586324 0203462 2 520 | 442906.00 | 0.00 | 0.00 | 0.00 | 0.00 | 0.00 | 0.00 | 0.00 | 0.00 | 0.00 | 0.00 | 0.00 | KO:K01915 | glutamine synthetase | Bacteria;Proteobacteria;Gammaproteobacteria;Chromatiales;Chromatiaceae;Thiodictyon;Candidatus Thiodictyon syntrophicum;Candidatus Thiodictyon syntrophicum Cad16 |
| Ga0586324 0252535 1488 2222 | 13800000.00 | 0.00 | 0.00 | 0.00 | 0.00 | 0.00 | 0.00 | 0.00 | 0.00 | 0.00 | 0.00 | 0.00 | KO:K01915 | glutamine synthetase | Bacteria;Proteobacteria;Gammaproteobacteria;Chromatiales;Ectothiorhodospiraceae;Thiogranum;Thiogranum longum;Thiogranum longum DSM 19610 |
| Ga0586324 0392178 369 1823 | 0.00 | 0.00 | 0.00 | 0.00 | 0.00 | 0.00 | 0.00 | 0.00 | 1150200.00 | 0.00 | 0.00 | 0.00 | KO:K01915 | glutamine synthetase | Archaea;Euryarchaeota;Methanomicrobia;Methanosarcinales;Methanosarcinaceae;Methanosarcina;Methanosarcina barkeri;Methanosarcina barkeri 3 |
| Ga0586324 0674082 1 723 | 0.00 | 669545.00 | 0.00 | 0.00 | 0.00 | 0.00 | 0.00 | 2714500.00 | 679909.14 | 1031800.00 | 0.00 | 1005000.00 | KO:K01915 | glutamine synthetase | Bacteria;Proteobacteria;Alphaproteobacteria;Rhodospirillales;Acetobacteraceae;Acidiphilium;Acidiphilium angustum;Acidiphilium angustum ATCC 35903 |
| Ga0586324 0858374 98 1297 | 0.00 | 0.00 | 14806900.00 | 0.00 | 0.00 | 0.00 | 0.00 | 0.00 | 0.00 | 0.00 | 0.00 | 0.00 | KO:K01915 | glutamine synthetase | Bacteria;Proteobacteria;Gammaproteobacteria;unclassified Gammaproteobacteria;Candidatus Competibacteraceae;Candidatus Contendobacter;Candidatus Contendobacter odensis;Candidatus Contendobacter odensis Run B J11 |
| Ga0586324 0950157 709 1242 | 0.00 | 0.00 | 0.00 | 16245000.00 | 0.00 | 0.00 | 0.00 | 0.00 | 0.00 | 0.00 | 0.00 | 0.00 | KO:K01915 | glutamine synthetase | Bacteria;Proteobacteria;Deltaproteobacteria;unclassified Deltaproteobacteria;unclassified Deltaproteobacteria;Dissulfuribacter;Dissulfuribacter thermophilus;Dissulfuribacter thermophilus S69 |

| | | | | | | | | | | | | | | | |
|---|---|---|---|---|---|---|---|---|---|---|---|---|---|---|---|
| Ga0586324 0954393 3 1127 | 0.00 | 0.00 | 0.00 | 0.00 | 0.00 | 0.00 | 0.00 | 0.00 | 0.00 | 0.00 | 0.00 | 4447500.00 | KO:K01915 | glutamine synthetase | Bacteria;Gemmatimonadetes;Gemmatimonadetes;Gemmatimonadales;Gemmatimonadaceae;Gemmatirosa;Gemmatirosa kalamazoonesis;Gemmatimonadetes bacterium KBS708 |
| Ga0586324 0963047 1 741 | 0.00 | 0.00 | 0.00 | 0.00 | 2987000.00 | 0.00 | 0.00 | 0.00 | 0.00 | 0.00 | 0.00 | 0.00 | KO:K01915 | glutamine synthetase | Bacteria;Acidobacteria;Acidobacteriia;Acidobacteriales;Acidobacteriaceae;unclassified Acidobacteriaceae;Acidobacteriaceae bacterium KBS 96;Acidobacteriaceae bacterium KBS 96, DSM 24297 |
| Ga0586324 1047107 203 1189 | 0.00 | 0.00 | 0.00 | 0.00 | 0.00 | 0.00 | 0.00 | 0.00 | 0.00 | 1093400.00 | 0.00 | 795000.00 | KO:K01915 | glutamine synthetase | Bacteria;Proteobacteria;Gammaproteobacteria;Methylococcales;Methylococcaceae;Methylotuvimicrobium;Methylotuvimicrobium buryatense;Methylotuvimicrobium buryatense 5G |
| Ga0586324 1268046 2 721 | 0.00 | 0.00 | 0.00 | 0.00 | 0.00 | 0.00 | 0.00 | 0.00 | 0.00 | 0.00 | 5236000.00 | 0.00 | KO:K01915 | glutamine synthetase | Bacteria;Proteobacteria;Deltaproteobacteria;Syntrophobacterales;Syntrophobacteraceae;Desulfosoma;Desulfosoma caldarium;Desulfosoma caldarium DSM 22027 |
| Ga0586324 1296513 258 1085 | 0.00 | 0.00 | 0.00 | 0.00 | 0.00 | 0.00 | 0.00 | 0.00 | 0.00 | 0.00 | 0.00 | 4200000.00 | KO:K01915 | glutamine synthetase | Bacteria;Nitrospirae;Nitrospira;Nitrospirales;Nitrospiraceae;Nitrospira;Nitrospira lenta;Nitrospira lenta N. lenta BS10 |
| Ga0586324 1302157 1 285 | 0.00 | 0.00 | 0.00 | 0.00 | 0.00 | 0.00 | 0.00 | 0.00 | 0.00 | 0.00 | 2560800.00 | 0.00 | KO:K01915 | glutamine synthetase | Bacteria;Proteobacteria;Gammaproteobacteria;Acidiferrobacterales;Acidiferrobacteraceae;Sulfurifustis;Sulfurifustis variabilis;Sulfurifustis variabilis skN76 |
| Ga0586324 1964561 1 909 | 0.00 | 0.00 | 0.00 | 6469500.00 | 0.00 | 0.00 | 0.00 | 0.00 | 0.00 | 0.00 | 0.00 | 0.00 | KO:K01915 | glutamine synthetase | Bacteria;Acidobacteria;Vicinamibacteria;unclassified Vicinamibacteria;Vicinamibacteraceae;Luteitalea;Luteitalea pratensis;Luteitalea pratensis DSM 100886 |
| Ga0586324 2247358 3 461 | 0.00 | 0.00 | 0.00 | 0.00 | 2873700.00 | 0.00 | 0.00 | 0.00 | 0.00 | 0.00 | 0.00 | 0.00 | KO:K01915 | glutamine synthetase | Bacteria;Chloroflexi;Chloroflexia;Chloroflexales;Roseiflexaceae;Roseiflexus;Roseiflexus sp. RS-1;Roseiflexus sp. RS-1 |
| Ga0586324 2731795 31 792 | 0.00 | 1863200.00 | 0.00 | 0.00 | 0.00 | 0.00 | 0.00 | 0.00 | 0.00 | 0.00 | 0.00 | 0.00 | KO:K01915 | glutamine synthetase | Bacteria;Proteobacteria;Gammaproteobacteria;Methylococcales;Methylococcaceae;Methylobacter;Methylobacter tundripaludum;Methylobacter tundripaludum OWC-G53F |
| Ga0586324 2964906 2 766 | 0.00 | 30464000.00 | 0.00 | 40755000.00 | 0.00 | 46846000.00 | 70452000.00 | 74420000.00 | 0.00 | 106260000.00 | 476319.36 | 99750000.00 | KO:K01915 | glutamine synthetase | Bacteria;Proteobacteria;Betaproteobacteria;Burkholderiales;Comamonadaceae;Ramlibacter;Ramlibacter tataouinensis;Ramlibacter tataouinensis 5-10 |
| Ga0586324 3211795 2 739 | 0.00 | 0.00 | 0.00 | 0.00 | 0.00 | 0.00 | 0.00 | 0.00 | 64395000.00 | 0.00 | 0.00 | 0.00 | KO:K01915 | glutamine synthetase | Bacteria;Proteobacteria;Betaproteobacteria;Burkholderiales;Comamonadaceae;Hylemonella;Hylemonella gracilis;Hylemonella gracilis Niagara R |
| Ga0586324 3381102 2 724 | 0.00 | 0.00 | 0.00 | 0.00 | 0.00 | 0.00 | 0.00 | 0.00 | 0.00 | 0.00 | 0.00 | 372427.50 | KO:K01915 | glutamine synthetase | Bacteria;Proteobacteria;Gammaproteobacteria;Chromatiales;Ectothiorhodospiraceae;Ectothiorhodospira;Ectothiorhodospira sp. BSL-9;Ectothiorhodospira sp. BSL-9 |
| Ga0586324 3775338 1 693 | 0.00 | 0.00 | 0.00 | 0.00 | 0.00 | 0.00 | 0.00 | 4934900.00 | 0.00 | 0.00 | 6723200.00 | 0.00 | KO:K01915 | glutamine synthetase | Bacteria;Acidobacteria;Vicinamibacteria;unclassified Vicinamibacteria;Vicinamibacteraceae;Luteitalea;Luteitalea pratensis;Luteitalea pratensis DSM 100886 |
| Ga0586324 4573284 3 482 | 0.00 | 0.00 | 0.00 | 0.00 | 0.00 | 0.00 | 0.00 | 0.00 | 0.00 | 0.00 | 1108800.00 | 0.00 | KO:K01915 | glutamine synthetase | Bacteria;Proteobacteria;Gammaproteobacteria;Methylococcales;Methylococcaceae;Methylobacter;Methylobacter tundripaludum;Methylobacter tundripaludum OWC-G53F |
| Ga0586324 4657805 158 634 | 0.00 | 91486.52 | 47235700.00 | 0.00 | 0.00 | 0.00 | 0.00 | 0.00 | 0.00 | 31801000.00 | 0.00 | 34050000.00 | KO:K01915 | glutamine synthetase | Bacteria;Proteobacteria;Gammaproteobacteria;Methylococcales;Methylococcaceae;Methylobacter;Methylobacter tundripaludum;Methylobacter tundripaludum SV96 |
| Ga0586324 5098573 3 611 | 0.00 | 0.00 | 0.00 | 0.00 | 0.00 | 0.00 | 10328400.00 | 0.00 | 0.00 | 0.00 | 0.00 | 0.00 | KO:K01915 | glutamine synthetase | Bacteria;Fibrobacteres;Fibrobacteria;unclassified Fibrobacteria;unclassified Fibrobacteria;unclassified Fibrobacteria;Fibrobacteria bacterium AD312;Fibrobacteria bacterium AD312 AD 312 |
| Ga0586324 5187566 1 606 | 626176.00 | 0.00 | 0.00 | 0.00 | 0.00 | 0.00 | 0.00 | 0.00 | 0.00 | 0.00 | 0.00 | 0.00 | KO:K01915 | glutamine synthetase | Bacteria;Proteobacteria;Alphaproteobacteria;Hyphomicrobiales;Bradyrhizobiaceae;Bradyrhizobium;Bradyrhizobium sp. URHD0069;Bradyrhizobium sp. URHD0069 |
| Ga0586324 5947572 3 575 | 0.00 | 1373600.00 | 0.00 | 0.00 | 0.00 | 0.00 | 0.00 | 0.00 | 263182.77 | 0.00 | 0.00 | 0.00 | KO:K01915 | glutamine synthetase | Bacteria;Proteobacteria;Gammaproteobacteria;Methylococcales;Methylococcaceae;Methylomagnum;Methylomagnum ishizawai;Methylomagnum ishizawai 175 |
| Ga0586324 0164779 1704 2591 | 0.00 | 0.00 | 0.00 | 0.00 | 0.00 | 0.00 | 0.00 | 198967.36 | 13932000.00 | 0.00 | 1452000.00 | 772500.00 | KO:K01923 | phosphoribosylaminoimidazole-succinocarboxamide synthase | Bacteria;Proteobacteria;Betaproteobacteria;Nitrosomonadales;Sulfuricellaceae;Sulfuricella;Sulfuricella denitrificans;Sulfuricella denitrificans skB26 |
| Ga0586324 0000002 38919 40568 | 0.00 | 0.00 | 0.00 | 0.00 | 0.00 | 0.00 | 0.00 | 0.00 | 0.00 | 0.00 | 1355200.00 | 0.00 | KO:K01937 | CTP synthase | Bacteria;Proteobacteria;Betaproteobacteria;Nitrosomonadales;Thiobacillaceae;Annwoodia;Annwoodia aquaesulis;Annwoodia aquaesulis DSM 4255 |
| Ga0586324 0016274 6464 8092 | 0.00 | 0.00 | 0.00 | 0.00 | 0.00 | 0.00 | 0.00 | 0.00 | 0.00 | 0.00 | 0.00 | 404463.75 | KO:K01937 | CTP synthase | Bacteria;Proteobacteria;Alphaproteobacteria;Hyphomicrobiales;Hyphomicrobiaceae;Rhodoplanes;Rhodoplanes sp. Z2-YC6860;Rhodoplanes sp. Z2-YC6860 |
| Ga0586324 0004695 10245 11984 | 0.00 | 0.00 | 0.00 | 0.00 | 0.00 | 0.00 | 0.00 | 0.00 | 0.00 | 0.00 | 0.00 | 277078.50 | KO:K01938 | formate--tetrahydrofolate ligase | Bacteria;Proteobacteria;Alphaproteobacteria;Hyphomicrobiales;unclassified Hyphomicrobiales;Methyloceanibacter;Methyloceanibacter superfactus;Methyloceanibacter superfactus R-67175 |
| Ga0586324 0032718 2118 3794 | 0.00 | 0.00 | 0.00 | 0.00 | 0.00 | 0.00 | 0.00 | 1415200.00 | 0.00 | 0.00 | 0.00 | 0.00 | KO:K01938 | formate--tetrahydrofolate ligase | Bacteria;Proteobacteria;Betaproteobacteria;unclassified Betaproteobacteria;unclassified Betaproteobacteria;unclassified Betaproteobacteria;uncultured beta proteobacterium;unclassified betaproteobacterium ENR4 |
| Ga0586324 0244934 378 2141 | 0.00 | 0.00 | 0.00 | 0.00 | 0.00 | 7543000.00 | 0.00 | 581903.40 | 0.00 | 0.00 | 0.00 | 0.00 | KO:K01938 | formate--tetrahydrofolate ligase | Bacteria;Proteobacteria;Deltaproteobacteria;Desulfobacterales;Desulfobacteraceae;Desulfatiglans;Desulfatiglans anilini;Desulfatiglans anilini DSM 4660 |
| Ga0586324 0376185 606 1859 | 0.00 | 0.00 | 0.00 | 0.00 | 0.00 | 0.00 | 0.00 | 0.00 | 0.00 | 0.00 | 0.00 | 2205000.00 | KO:K01938 | formate--tetrahydrofolate ligase | Bacteria;Proteobacteria;Betaproteobacteria;unclassified Betaproteobacteria;unclassified Betaproteobacteria;unclassified Betaproteobacteria;Betaproteobacteria bacterium GR16-43;Betaproteobacteria bacterium GR16-43 |
| Ga0586324 0610033 3 1130 | 2900000.00 | 0.00 | 3963942.25 | 2118500.00 | 1328700.00 | 0.00 | 4719600.00 | 0.00 | 0.00 | 0.00 | 0.00 | 0.00 | KO:K01938 | formate--tetrahydrofolate ligase | Bacteria;Proteobacteria;Alphaproteobacteria;Rhodospirillales;Acetobacteraceae;Rhodopila;unclassified Rhodopila;Rhodopila sp. LVNP |
| Ga0586324 1217988 1 1089 | 0.00 | 0.00 | 0.00 | 0.00 | 0.00 | 0.00 | 0.00 | 945500.00 | 0.00 | 0.00 | 0.00 | 0.00 | KO:K01938 | formate--tetrahydrofolate ligase | Bacteria;Bacteroidetes;Bacteroidia;Marinilabiliales;Prolixibacteraceae;Prolixibacter;Prolixibacter denitrificans;Prolixibacter denitrificans DSM 27267 |
| Ga0586324 0000002 96468 97760 | 0.00 | 0.00 | 0.00 | 0.00 | 0.00 | 0.00 | 0.00 | 4477400.00 | 6543047.97 | 0.00 | 0.00 | 413851.50 | KO:K01939 | adenylosuccinate synthase | Bacteria;Proteobacteria;Betaproteobacteria;Nitrosomonadales;Thiobacillaceae;Annwoodia;Annwoodia aquaesulis;Annwoodia aquaesulis DSM 4255 |
| Ga0586324 0140213 2 1096 | 0.00 | 0.00 | 0.00 | 0.00 | 0.00 | 0.00 | 0.00 | 0.00 | 0.00 | 0.00 | 994400.00 | 0.00 | KO:K01939 | adenylosuccinate synthase | Bacteria;Proteobacteria;Alphaproteobacteria;Hyphomicrobiales;Xanthobacteraceae;Pseudolabrys;Pseudolabrys taiwanensis;Pseudolabrys taiwanensis CC-BB4 |
| Ga0586324 0269272 2 793 | 0.00 | 0.00 | 0.00 | 0.00 | 0.00 | 0.00 | 0.00 | 0.00 | 1150200.00 | 0.00 | 0.00 | 0.00 | KO:K01939 | adenylosuccinate synthase | Bacteria;Proteobacteria;Betaproteobacteria;Nitrosomonadales;Sulfuricellaceae;Sulfurirhabdus;Sulfurirhabdus autotrophica;Sulfurirhabdus autotrophica DSM 100309 |
| Ga0586324 0434229 456 1745 | 0.00 | 0.00 | 0.00 | 0.00 | 0.00 | 0.00 | 0.00 | 4678700.00 | 5896800.00 | 5405400.00 | 0.00 | 0.00 | KO:K01939 | adenylosuccinate synthase | Bacteria;Proteobacteria;Betaproteobacteria;Nitrosomonadales;Gallionellaceae;Sideroxydans;Sideroxydans lithotrophicus;Sideroxydans lithotrophicus ES-1 |
| Ga0586324 1188691 527 1126 | 0.00 | 0.00 | 0.00 | 0.00 | 0.00 | 0.00 | 0.00 | 0.00 | 7573500.00 | 0.00 | 0.00 | 0.00 | KO:K01939 | adenylosuccinate synthase | Bacteria;Proteobacteria;Betaproteobacteria;Nitrosomonadales;Nitrosomonadaceae;Nitrosospira;Nitrosospira briensis;Nitrosospira briensis Nsp8 |
| Ga0586324 3575327 3 707 | 0.00 | 0.00 | 0.00 | 0.00 | 0.00 | 0.00 | 0.00 | 0.00 | 0.00 | 0.00 | 446810.32 | 0.00 | KO:K01939 | adenylosuccinate synthase | Bacteria;Proteobacteria;Betaproteobacteria;Nitrosomonadales;Gallionellaceae;Gallionella;Gallionella capsiferriformans;Gallionella capsiferriformans ES-2 |
| Ga0586324 5845274 1 579 | 12800000.00 | 0.00 | 0.00 | 0.00 | 0.00 | 0.00 | 0.00 | 0.00 | 0.00 | 0.00 | 0.00 | 0.00 | KO:K01939 | adenylosuccinate synthase | Bacteria;Proteobacteria;Betaproteobacteria;Nitrosomonadales;Gallionellaceae;Gallionella;Gallionella capsiferriformans;Gallionella capsiferriformans ES-2 |
| Ga0586324 0000003 15190 16422 | 0.00 | 0.00 | 0.00 | 0.00 | 0.00 | 0.00 | 0.00 | 5929200.00 | 0.00 | 2340800.00 | 1364000.00 | 5902500.00 | KO:K01940 | argininosuccinate synthase | Bacteria;Proteobacteria;Betaproteobacteria;Nitrosomonadales;Thiobacillaceae;Thiobacillus;Thiobacillus thioparus;Thiobacillus thioparus DSM 505 |
| Ga0586324 0844112 152 1306 | 0.00 | 0.00 | 0.00 | 0.00 | 0.00 | 0.00 | 0.00 | 0.00 | 0.00 | 342422.08 | 0.00 | 0.00 | KO:K01940 | argininosuccinate synthase | Bacteria;Proteobacteria;Betaproteobacteria;Nitrosomonadales;Thiobacillaceae;Thiobacillus;Thiobacillus thioparus;Thiobacillus thioparus DSM 505 |
| Ga0586324 5761231 2 583 | 0.00 | 0.00 | 0.00 | 0.00 | 0.00 | 0.00 | 0.00 | 1689700.00 | 0.00 | 0.00 | 0.00 | 0.00 | KO:K01940 | argininosuccinate synthase | Bacteria;Proteobacteria;Betaproteobacteria;Nitrosomonadales;Nitrosomonadaceae;Nitrosospira;Nitrosospira sp. Nsp13;Nitrosospira sp. Nsp13 |
| Ga0586324 0000018 72011 73573 | 0.00 | 0.00 | 0.00 | 0.00 | 0.00 | 0.00 | 0.00 | 0.00 | 0.00 | 0.00 | 0.00 | 3045000.00 | KO:K01951 | GMP synthase (glutamine-hydrolysing) | Bacteria;Proteobacteria;Betaproteobacteria;Nitrosomonadales;Thiobacillaceae;Annwoodia;Annwoodia aquaesulis;Annwoodia aquaesulis DSM 4255 |
| Ga0586324 0200613 2 1324 | 0.00 | 0.00 | 0.00 | 0.00 | 0.00 | 0.00 | 0.00 | 0.00 | 0.00 | 0.00 | 410499.76 | 0.00 | KO:K01951 | GMP synthase (glutamine-hydrolysing) | Bacteria;Proteobacteria;Betaproteobacteria;Nitrosomonadales;Sulfuricellaceae;Sulfuricella;Sulfuricella sp. T08;Sulfuricella sp. T08 |
| Ga0586324 2132602 1 879 | 0.00 | 0.00 | 0.00 | 0.00 | 0.00 | 0.00 | 710739.84 | 0.00 | 0.00 | 0.00 | 0.00 | 0.00 | KO:K01951 | GMP synthase (glutamine-hydrolysing) | Bacteria;Proteobacteria;Betaproteobacteria;Nitrosomonadales;Sulfuricellaceae;Sulfuriferula;Sulfuriferula multivorans;Sulfuriferula multivorans TTN |
| Ga0586324 0000030 8858 12061 | 0.00 | 0.00 | 0.00 | 0.00 | 0.00 | 0.00 | 5038800.00 | 799100.00 | 3572100.00 | 3434200.00 | 2728000.00 | 19620000.00 | KO:K01955 | carbamoyl-phosphate synthase large subunit | Bacteria;Proteobacteria;Betaproteobacteria;Nitrosomonadales;Thiobacillaceae;Annwoodia;Annwoodia aquaesulis;Annwoodia aquaesulis DSM 4255 |
| Ga0586324 0001926 5182 8409 | 0.00 | 0.00 | 0.00 | 0.00 | 0.00 | 0.00 | 0.00 | 0.00 | 0.00 | 0.00 | 0.00 | 478732.50 | KO:K01955 | carbamoyl-phosphate synthase large subunit | Bacteria;Proteobacteria;Gammaproteobacteria;Acidiferrobacterales;Acidiferrobacteraceae;Sulfuricaulis;Sulfuricaulis limicola;Sulfuricaulis limicola HA5 |
| Ga0586324 0003837 6141 9359 | 0.00 | 0.00 | 0.00 | 0.00 | 0.00 | 0.00 | 0.00 | 0.00 | 915300.00 | 0.00 | 0.00 | 0.00 | KO:K01955 | carbamoyl-phosphate synthase large subunit | Bacteria;Proteobacteria;Gammaproteobacteria;Methylococcales;Methylococcaceae;Methylobacter;Methylobacter tundripaludum;Methylobacter tundripaludum SV96 |
| Ga0586324 1187023 2 1129 | 0.00 | 0.00 | 0.00 | 0.00 | 136564.61 | 0.00 | 0.00 | 0.00 | 0.00 | 0.00 | 0.00 | 0.00 | KO:K01955 | carbamoyl-phosphate synthase large subunit | Bacteria;Proteobacteria;Betaproteobacteria;unclassified Betaproteobacteria;unclassified Betaproteobacteria;unclassified Betaproteobacteria;beta proteobacterium AAP51;Betaproteobacteria bacterium AAP51 |
| Ga0586324 1261158 2 1099 | 0.00 | 0.00 | 0.00 | 3619500.00 | 0.00 | 0.00 | 0.00 | 0.00 | 0.00 | 0.00 | 0.00 | 0.00 | KO:K01955 | carbamoyl-phosphate synthase large subunit | Bacteria;Proteobacteria;Gammaproteobacteria;Methylococcales;Methylococcaceae;Methylobacter;Methylobacter luteus;Methylobacter luteus IMV-B-3098 |
| Ga0586324 2336718 1 846 | 0.00 | 0.00 | 0.00 | 0.00 | 0.00 | 0.00 | 0.00 | 0.00 | 0.00 | 0.00 | 311728.56 | 0.00 | KO:K01955 | carbamoyl-phosphate synthase large subunit | Bacteria;Proteobacteria;Deltaproteobacteria;Desulfuromonadales;Geobacteraceae;Geobacter;Geobacter uraniireducens;Geobacter uraniireducens Rf4 |
| Ga0586324 2402826 1 837 | 0.00 | 0.00 | 0.00 | 0.00 | 0.00 | 0.00 | 0.00 | 229173.95 | 0.00 | 0.00 | 0.00 | 0.00 | KO:K01955 | carbamoyl-phosphate synthase large subunit | Bacteria;Proteobacteria;Gammaproteobacteria;Chromatiales;Ectothiorhodospiraceae;Acidihalobacter;Acidihalobacter prosperus;Acidihalobacter prosperus F5 |
| Ga0586324 2433908 1 831 | 0.00 | 0.00 | 0.00 | 0.00 | 0.00 | 0.00 | 0.00 | 145771.70 | 0.00 | 0.00 | 0.00 | 0.00 | KO:K01955 | carbamoyl-phosphate synthase large subunit | Bacteria;Ignavibacteriae;Ignavibacteria;Ignavibacteriales;Melioribacteraceae;Melioribacter;Melioribacter roseus;Melioribacter roseus P3M |
| Ga0586324 4148247 1 666 | 0.00 | 0.00 | 0.00 | 0.00 | 0.00 | 3544209.56 | 0.00 | 0.00 | 0.00 | 0.00 | 0.00 | 0.00 | KO:K01955 | carbamoyl-phosphate synthase large subunit | Bacteria;Proteobacteria;Betaproteobacteria;unclassified Betaproteobacteria;unclassified Betaproteobacteria;unclassified Betaproteobacteria;Betaproteobacteria bacterium GR16-43;Betaproteobacteria bacterium GR16-43 |
| Ga0586324 8033815 1 507 | 0.00 | 0.00 | 0.00 | 0.00 | 0.00 | 0.00 | 0.00 | 0.00 | 3515400.00 | 0.00 | 0.00 | 0.00 | KO:K01955 | carbamoyl-phosphate synthase large subunit | Bacteria;Firmicutes;Clostridia;Eubacteriales;Peptococcaceae;Desulfofarcimen;Desulfofarcimen intricatum;Desulfofarcimen intricatum NBRC 109411 |
| Ga0586324 0484716 578 1660 | 0.00 | 0.00 | 0.00 | 0.00 | 0.00 | 0.00 | 0.00 | 1238300.00 | 1571400.00 | 0.00 | 0.00 | 0.00 | KO:K01956 | carbamoyl-phosphate synthase small subunit | Bacteria;Proteobacteria;Betaproteobacteria;Nitrosomonadales;Thiobacillaceae;Thiobacillus;Thiobacillus denitrificans;Thiobacillus denitrificans RG |
| Ga0586324 0157350 3 1064 | 0.00 | 0.00 | 0.00 | 0.00 | 0.00 | 0.00 | 0.00 | 0.00 | 0.00 | 0.00 | 2112000.00 | 0.00 | KO:K01959 | pyruvate carboxylase subunit A | Bacteria;Proteobacteria;Gammaproteobacteria;Chromatiales;Chromatiaceae;Thiocapsa;Thiocapsa rosea;Thiocapsa rosea DSM 235 |
| Ga0586324 6492234 3 554 | 0.00 | 0.00 | 0.00 | 0.00 | 0.00 | 0.00 | 0.00 | 1671400.00 | 0.00 | 0.00 | 0.00 | 0.00 | KO:K01959 | pyruvate carboxylase subunit A | Bacteria;Proteobacteria;Betaproteobacteria;Nitrosomonadales;Thiobacillaceae;Thiobacillus;Thiobacillus denitrificans;Thiobacillus denitrificans RG |
| Ga0586324 0037097 2 571 | 0.00 | 0.00 | 3711098.95 | 0.00 | 8559300.00 | 8495800.00 | 0.00 | 5008100.00 | 7411500.00 | 4519900.00 | 4628800.00 | 2505000.00 | KO:K01960 | pyruvate carboxylase subunit B | Bacteria;Proteobacteria;Deltaproteobacteria;Syntrophobacterales;Syntrophobacteraceae;Desulfoglaeba;Desulfoglaeba alkanexedens;Desulfoglaeba alkanexedens ALDC |
| Ga0586324 0223333 1191 2351 | 0.00 | 0.00 | 0.00 | 0.00 | 0.00 | 0.00 | 0.00 | 0.00 | 0.00 | 0.00 | 0.00 | 14850000.00 | KO:K01960 | pyruvate carboxylase subunit B | Bacteria;Proteobacteria;Deltaproteobacteria;Syntrophobacterales;Syntrophobacteraceae;Desulfacinum;Desulfacinum infernum;Desulfacinum infernum DSM 9756 |
| Ga0586324 0242358 1077 2264 | 0.00 | 0.00 | 0.00 | 0.00 | 3491700.00 | 0.00 | 0.00 | 0.00 | 0.00 | 0.00 | 0.00 | 0.00 | KO:K01960 | pyruvate carboxylase subunit B | Bacteria;Proteobacteria;Deltaproteobacteria;Syntrophobacterales;Syntrophobacteraceae;Desulfacinum;Desulfacinum infernum;Desulfacinum infernum DSM 9756 |
| Ga0586324 0253491 3 1652 | 0.00 | 0.00 | 0.00 | 0.00 | 0.00 | 0.00 | 0.00 | 0.00 | 1539000.00 | 0.00 | 0.00 | 0.00 | KO:K01960 | pyruvate carboxylase subunit B | Bacteria;Proteobacteria;Betaproteobacteria;Nitrosomonadales;Thiobacillaceae;Sulfuritortus;Sulfuritortus calidifontis;Sulfuritortus calidifontis DSM 103923 |
| Ga0586324 0704890 255 1412 | 3090000.00 | 13872000.00 | 0.00 | 23085000.00 | 25132000.00 | 0.00 | 0.00 | 0.00 | 0.00 | 0.00 | 0.00 | 0.00 | KO:K01960 | pyruvate carboxylase subunit B | Bacteria;Proteobacteria;Deltaproteobacteria;Syntrophobacterales;Syntrophobacteraceae;Syntrophobacter;Syntrophobacter fumaroxidans;Syntrophobacter fumaroxidans MPOB |
| Ga0586324 0738637 2 1384 | 0.00 | 0.00 | 0.00 | 0.00 | 0.00 | 14649300.00 | 0.00 | 0.00 | 0.00 | 0.00 | 4136000.00 | 0.00 | KO:K01960 | pyruvate carboxylase subunit B | Bacteria;Proteobacteria;Deltaproteobacteria;Desulfobacterales;Desulfobacteraceae;Desulfatirhabdium;Desulfatirhabdium butyrativorans;Desulfatirhabdium butyrativorans DSM 18734 |
| Ga0586324 1307662 1 1008 | 0.00 | 0.00 | 0.00 | 0.00 | 4830700.00 | 0.00 | 0.00 | 0.00 | 0.00 | 0.00 | 0.00 | 0.00 | KO:K01960 | pyruvate carboxylase subunit B | Bacteria;Proteobacteria;Deltaproteobacteria;Desulfobacterales;Desulfobacteraceae;Desulfatitalea;Desulfatitalea tepidiphila;Desulfatitalea tepidiphila S28bF |
| Ga0586324 2223330 3 578 | 0.00 | 0.00 | 0.00 | 8521500.00 | 12669000.00 | 46052000.00 | 0.00 | 0.00 | 9153000.00 | 0.00 | 0.00 | 0.00 | KO:K01960 | pyruvate carboxylase subunit B | Bacteria;Proteobacteria;Deltaproteobacteria;Desulfobacterales;Desulfobacteraceae;Desulfatitalea;Desulfatitalea tepidiphila;Desulfatitalea tepidiphila S28bF |
| Ga0586324 2303162 3 851 | 0.00 | 0.00 | 0.00 | 14725000.00 | 0.00 | 0.00 | 0.00 | 0.00 | 0.00 | 0.00 | 0.00 | 0.00 | KO:K01960 | pyruvate carboxylase subunit B | Bacteria;Proteobacteria;Deltaproteobacteria;Desulfobacterales;Desulfobulbaceae;Desulfobulbus;Desulfobulbus propionicus;Desulfobulbus propionicus 1pr3, DSM 2032 |
| Ga0586324 3009546 3 554 | 0.00 | 0.00 | 0.00 | 0.00 | 0.00 | 15721200.00 | 0.00 | 0.00 | 0.00 | 0.00 | 0.00 | 0.00 | KO:K01960 | pyruvate carboxylase subunit B | Bacteria;Proteobacteria;Deltaproteobacteria;Desulfobacterales;Desulfobulbaceae;Desulfobulbus;Desulfobulbus propionicus;Desulfobulbus propionicus 1pr3, DSM 2032 |
| Ga0586324 3376139 1 726 | 0.00 | 2332400.00 | 0.00 | 0.00 | 3419600.00 | 0.00 | 0.00 | 11468000.00 | 0.00 | 8932000.00 | 0.00 | 3217500.00 | KO:K01960 | pyruvate carboxylase subunit B | Bacteria;Proteobacteria;Deltaproteobacteria;Syntrophobacterales;Syntrophobacteraceae;Syntrophobacter;Syntrophobacter fumaroxidans;Syntrophobacter fumaroxidans MPOB |
| Ga0586324 4649803 3 635 | 0.00 | 307368.84 | 0.00 | 2916500.00 | 962671.99 | 0.00 | 0.00 | 0.00 | 0.00 | 0.00 | 0.00 | 0.00 | KO:K01960 | pyruvate carboxylase subunit B | Bacteria;Proteobacteria;Deltaproteobacteria;Syntrophobacterales;Syntrophobacteraceae;Syntrophobacter;Syntrophobacter fumaroxidans;Syntrophobacter fumaroxidans MPOB |
| Ga0586324 5196215 94 606 | 0.00 | 0.00 | 0.00 | 4978000.00 | 257174.52 | 0.00 | 3488400.00 | 0.00 | 0.00 | 0.00 | 0.00 | 0.00 | KO:K01960 | pyruvate carboxylase subunit B | Bacteria;Proteobacteria;Deltaproteobacteria;Syntrophorhabdales;Syntrophorhabdaceae;Syntrophorhabdus;Syntrophorhabdus aromaticivorans;Syntrophorhabdus aromaticivorans UI |
| Ga0586324 5659920 1 585 | 22990000.00 | 24153600.00 | 21337700.00 | 37525000.00 | 33372000.00 | 26321100.00 | 40287600.00 | 38674000.00 | 36774000.00 | 22099000.00 | 19184000.00 | 9000000.00 | KO:K01960 | pyruvate carboxylase subunit B | Bacteria;Proteobacteria;Deltaproteobacteria;Syntrophobacterales;Syntrophobacteraceae;Syntrophobacter;Syntrophobacter fumaroxidans;Syntrophobacter fumaroxidans MPOB |
| Ga0586324 0000003 71417 72283 | 0.00 | 0.00 | 0.00 | 0.00 | 0.00 | 0.00 | 0.00 | 0.00 | 1782000.00 | 0.00 | 0.00 | 0.00 | KO:K01963 | acetyl-CoA carboxylase carboxyl transferase subunit beta | Bacteria;Proteobacteria;Betaproteobacteria;Nitrosomonadales;Thiobacillaceae;Annwoodia;Annwoodia aquaesulis;Annwoodia aquaesulis DSM 4255 |
| Ga0586324 0385529 134 1669 | 0.00 | 0.00 | 0.00 | 0.00 | 0.00 | 0.00 | 0.00 | 0.00 | 0.00 | 3465000.00 | 0.00 | 0.00 | KO:K01966 | propionyl-CoA carboxylase beta chain | Bacteria;Proteobacteria;Alphaproteobacteria;Hyphomicrobiales;Methylocystaceae;Methylocystis;Methylocystis parvus;Methylocystis parvus OBBP |
| Ga0586324 2153160 1 876 | 0.00 | 0.00 | 0.00 | 0.00 | 0.00 | 0.00 | 0.00 | 0.00 | 0.00 | 3996300.00 | 3889600.00 | 0.00 | KO:K01966 | propionyl-CoA carboxylase beta chain | Bacteria;Proteobacteria;Gammaproteobacteria;unclassified Gammaproteobacteria;Candidatus Competibacteraceae;Candidatus Competibacter;Candidatus Competibacter denitrificans;Candidatus Competibacter denitrificans Run A D11 |
| Ga0586324 3724465 1 696 | 0.00 | 0.00 | 0.00 | 0.00 | 0.00 | 10877800.00 | 0.00 | 0.00 | 0.00 | 0.00 | 0.00 | 0.00 | KO:K01966 | propionyl-CoA carboxylase beta chain | Bacteria;Actinobacteria;Rubrobacteria;Gaiellales;Gaiellaceae;Gaiella;Gaiella occulta;Gaiella occulta F2-233 |
| Ga0586324 6071201 139 570 | 0.00 | 0.00 | 0.00 | 0.00 | 1153600.00 | 0.00 | 0.00 | 0.00 | 0.00 | 0.00 | 0.00 | 0.00 | KO:K01966 | propionyl-CoA carboxylase beta chain | Bacteria;Proteobacteria;Alphaproteobacteria;Hyphomicrobiales;unclassified Hyphomicrobiales;Pseudorhodoplanes;Pseudorhodoplanes sinuspersici;Pseudorhodoplanes sinuspersici RIPI110 |
| Ga0586324 6695013 2 547 | 0.00 | 0.00 | 0.00 | 0.00 | 0.00 | 291855.34 | 0.00 | 0.00 | 0.00 | 0.00 | 0.00 | 0.00 | KO:K01966 | propionyl-CoA carboxylase beta chain | Bacteria;Proteobacteria;Deltaproteobacteria;Myxococcales;Labilitrichaceae;Labilithrix;Labilithrix luteola;Labilithrix luteola DSM 27648 |
| Ga0586324 3568232 1 708 | 0.00 | 0.00 | 25391300.00 | 0.00 | 0.00 | 0.00 | 0.00 | 0.00 | 0.00 | 0.00 | 0.00 | 0.00 | KO:K01968 | 3-methylcrotonyl-CoA carboxylase alpha subunit | Bacteria;Proteobacteria;Betaproteobacteria;Burkholderiales;Alcaligenaceae;Bordetella;Bordetella hinzii;Bordetella hinzii ATCC 51730 |
| Ga0586324 0171315 733 2403 | 0.00 | 17952000.00 | 18330379.20 | 13395000.00 | 13390000.00 | 14530200.00 | 12654000.00 | 10187000.00 | 5054400.00 | 0.00 | 0.00 | 0.00 | KO:K01969 | 3-methylcrotonyl-CoA carboxylase beta subunit | Bacteria;Proteobacteria;Deltaproteobacteria;Syntrophobacterales;Syntrophaceae;Smithella;Smithella sp. F21;Smithella sp. F21 |
| Ga0586324 0814648 196 1326 | 0.00 | 0.00 | 5011769.70 | 0.00 | 0.00 | 3970000.00 | 6657600.00 | 8601000.00 | 8505000.00 | 0.00 | 0.00 | 0.00 | KO:K01969 | 3-methylcrotonyl-CoA carboxylase beta subunit | Bacteria;Proteobacteria;Deltaproteobacteria;Syntrophobacterales;Syntrophaceae;Smithella;Smithella sp. F21;Smithella sp. F21 |
| Ga0586324 0434807 893 1744 | 0.00 | 0.00 | 0.00 | 0.00 | 0.00 | 0.00 | 1098230.40 | 0.00 | 0.00 | 0.00 | 0.00 | 0.00 | KO:K01989 | putative ABC transport system substrate-binding protein | Bacteria;Proteobacteria;Betaproteobacteria;Burkholderiales;Comamonadaceae;Rhodoferax;Rhodoferax antarcticus;Rhodoferax antarcticus DSM 24876 |
| Ga0586324 2755831 1 789 | 1350000.00 | 1652400.00 | 0.00 | 2346500.00 | 0.00 | 0.00 | 0.00 | 0.00 | 0.00 | 0.00 | 0.00 | 0.00 | KO:K01989 | putative ABC transport system substrate-binding protein | Bacteria;Proteobacteria;Betaproteobacteria;Burkholderiales;Comamonadaceae;Rhodoferax;Rhodoferax antarcticus;Rhodoferax antarcticus DSM 24876 |
| Ga0586324 7978686 83 508 | 0.00 | 0.00 | 0.00 | 0.00 | 1462600.00 | 0.00 | 0.00 | 0.00 | 0.00 | 0.00 | 0.00 | 0.00 | KO:K01990 | ABC-2 type transport system ATP-binding protein | Bacteria;Bacteroidetes;Bacteroidia;Marinilabiliales;Marinilabiliaceae;Marinilabilia;Marinilabilia rubra;Marinilabilia rubra WTE16 |
| Ga0586324 4647728 2 634 | 0.00 | 0.00 | 0.00 | 0.00 | 0.00 | 0.00 | 0.00 | 0.00 | 0.00 | 0.00 | 20240000.00 | 0.00 | KO:K01996 | branched-chain amino acid transport system ATP-binding protein | Bacteria;Firmicutes;Clostridia;Eubacteriales;Peptococcaceae;Desulfotomaculum;Desulfotomaculum nigrificans;Desulfotomaculum carboxydivorans CO-1-SRB, DSM 14880 |
| Ga0586324 0000047 61496 62782 | 10400000.00 | 6181200.00 | 0.00 | 0.00 | 0.00 | 0.00 | 0.00 | 0.00 | 0.00 | 0.00 | 0.00 | 3352500.00 | KO:K01999 | branched-chain amino acid transport system substrate-binding protein | Bacteria;Chloroflexi;Chloroflexia;Kallotenuales;Kallotenuaceae;Kallotenue;Kallotenue papyrolyticum;Kallotenue papyrolyticum JKG1 |
| Ga0586324 0001442 3241 4533 | 0.00 | 0.00 | 0.00 | 0.00 | 0.00 | 0.00 | 0.00 | 0.00 | 0.00 | 0.00 | 0.00 | 413034.75 | KO:K01999 | branched-chain amino acid transport system substrate-binding protein | Bacteria;Proteobacteria;Alphaproteobacteria;Hyphomicrobiales;Bradyrhizobiaceae;Bradyrhizobium;Bradyrhizobium elkanii;Bradyrhizobium elkanii WSM2783 |
| Ga0586324 0002026 518 1645 | 2300000.00 | 0.00 | 0.00 | 3163500.00 | 0.00 | 0.00 | 0.00 | 0.00 | 0.00 | 0.00 | 1038400.00 | 0.00 | KO:K01999 | branched-chain amino acid transport system substrate-binding protein | Bacteria;Proteobacteria;Deltaproteobacteria;Syntrophobacterales;Syntrophobacteraceae;Desulfacinum;Desulfacinum infernum;Desulfacinum infernum DSM 9756 |
| Ga0586324 0002141 15111 16361 | 1970000.00 | 0.00 | 1668596.88 | 0.00 | 0.00 | 0.00 | 0.00 | 0.00 | 0.00 | 3187800.00 | 3960000.00 | 1365000.00 | KO:K01999 | branched-chain amino acid transport system substrate-binding protein | Bacteria;Cyanobacteria;unclassified Cyanobacteria;Oscillatoriales;Microcoleaceae;Microcoleus;Microcoleus sp. PCC 7113;Microcoleus sp. PCC 7113 |
| Ga0586324 0002221 5119 6270 | 0.00 | 2543200.00 | 0.00 | 0.00 | 0.00 | 0.00 | 0.00 | 0.00 | 0.00 | 0.00 | 0.00 | 0.00 | KO:K01999 | branched-chain amino acid transport system substrate-binding protein | Bacteria;Proteobacteria;Betaproteobacteria;Nitrosomonadales;Thiobacillaceae;Thiobacillus;Thiobacillus thioparus;Thiobacillus thioparus DSM 505 |
| Ga0586324 0003092 16005 16811 | 5100000.00 | 3672000.00 | 2775905.28 | 0.00 | 1689200.00 | 1807739.50 | 0.00 | 2793800.00 | 4114800.00 | 5582500.00 | 6160000.00 | 7150922.25 | KO:K01999 | branched-chain amino acid transport system substrate-binding protein | Bacteria;Proteobacteria;Betaproteobacteria;Nitrosomonadales;Sulfuricellaceae;Sulfuriferula;Sulfuriferula multivorans;Sulfuriferula multivorans TTN |
| Ga0586324 0003407 9005 10279 | 0.00 | 11288000.00 | 0.00 | 0.00 | 0.00 | 0.00 | 0.00 | 0.00 | 3896100.00 | 0.00 | 0.00 | 0.00 | KO:K01999 | branched-chain amino acid transport system substrate-binding protein | Bacteria;Spirochaetes;Spirochaetia;Spirochaetales;Spirochaetaceae;Alkalispirochaeta;Alkalispirochaeta odontotermitis;Alkalispirochaeta odontotermitis JC202 |
| Ga0586324 0004471 5801 7123 | 0.00 | 0.00 | 0.00 | 0.00 | 0.00 | 0.00 | 0.00 | 0.00 | 0.00 | 0.00 | 725814.32 | 0.00 | KO:K01999 | branched-chain amino acid transport system substrate-binding protein | Bacteria;Proteobacteria;Deltaproteobacteria;Desulfovibrionales;Desulfovibrionaceae;Desulfovibrio;Desulfovibrio carbinolicus;Desulfovibrio carbinolicus DSM 3852 |
| Ga0586324 0006674 2480 3655 | 0.00 | 0.00 | 0.00 | 2774000.00 | 0.00 | 0.00 | 0.00 | 0.00 | 0.00 | 0.00 | 0.00 | 0.00 | KO:K01999 | branched-chain amino acid transport system substrate-binding protein | Bacteria;Proteobacteria;Alphaproteobacteria;Hyphomicrobiales;Xanthobacteraceae;Pseudolabrys;Pseudolabrys taiwanensis;Pseudolabrys taiwanensis CC-BB4 |
| Ga0586324 0010118 2906 4129 | 0.00 | 0.00 | 0.00 | 0.00 | 0.00 | 0.00 | 0.00 | 0.00 | 5815800.00 | 0.00 | 1152800.00 | 11145000.00 | KO:K01999 | branched-chain amino acid transport system substrate-binding protein | Bacteria;Proteobacteria;Betaproteobacteria;Nitrosomonadales;Thiobacillaceae;Thiobacillus;Thiobacillus thioparus;Thiobacillus thioparus DSM 505 |
| Ga0586324 0014268 3574 4749 | 0.00 | 0.00 | 0.00 | 0.00 | 0.00 | 0.00 | 0.00 | 0.00 | 0.00 | 1031800.00 | 0.00 | 0.00 | KO:K01999 | branched-chain amino acid transport system substrate-binding protein | Bacteria;Proteobacteria;Deltaproteobacteria;Desulfobacterales;Desulfobacteraceae;Desulfobacter;Desulfobacter curvatus;Desulfobacter curvatus DSM 3379 |
| Ga0586324 0014791 1880 3037 | 9670000.00 | 4556000.00 | 567943.14 | 4759500.00 | 4058200.00 | 1777551.62 | 4354800.00 | 2562000.00 | 3620700.00 | 1432200.00 | 706398.88 | 1425000.00 | KO:K01999 | branched-chain amino acid transport system substrate-binding protein | Bacteria;Chloroflexi;Anaerolineae;Anaerolineales;Anaerolineaceae;Anaerolinea;Anaerolinea thermophila;Anaerolinea thermophila UNI-1 |
| Ga0586324 0015442 5314 6609 | 3040000.00 | 0.00 | 0.00 | 0.00 | 0.00 | 0.00 | 0.00 | 0.00 | 0.00 | 0.00 | 0.00 | 0.00 | KO:K01999 | branched-chain amino acid transport system substrate-binding protein | Bacteria;Proteobacteria;Deltaproteobacteria;Syntrophobacterales;Syntrophobacteraceae;Syntrophobacter;Syntrophobacter fumaroxidans;Syntrophobacter fumaroxidans MPOB |
| Ga0586324 0017344 691 1932 | 0.00 | 0.00 | 0.00 | 0.00 | 0.00 | 0.00 | 0.00 | 0.00 | 0.00 | 0.00 | 2833600.00 | 2295000.00 | KO:K01999 | branched-chain amino acid transport system substrate-binding protein | Bacteria;Actinobacteria;Rubrobacteria;Gaiellales;Gaiellaceae;Gaiella;Gaiella occulta;Gaiella occulta F2-233 |
| Ga0586324 0017797 1310 2539 | 4300000.00 | 2896800.00 | 14714995.88 | 0.00 | 0.00 | 0.00 | 0.00 | 0.00 | 7565400.00 | 8547000.00 | 17415200.00 | 9000000.00 | KO:K01999 | branched-chain amino acid transport system substrate-binding protein | Bacteria;Spirochaetes;Spirochaetia;Spirochaetales;Spirochaetaceae;Alkalispirochaeta;Alkalispirochaeta odontotermitis;Alkalispirochaeta odontotermitis JC202 |
| Ga0586324 0018728 3237 4388 | 0.00 | 0.00 | 0.00 | 7296000.00 | 0.00 | 0.00 | 0.00 | 5282304.15 | 818100.00 | 0.00 | 0.00 | 0.00 | KO:K01999 | branched-chain amino acid transport system substrate-binding protein | Bacteria;Chloroflexi;Anaerolineae;Anaerolineales;Anaerolineaceae;Thermanaerothrix;Thermanaerothrix daxensis;Thermanaerothrix daxensis GNS-1 |
| Ga0586324 0018801 6733 7650 | 0.00 | 0.00 | 2546696.72 | 0.00 | 2544100.00 | 0.00 | 0.00 | 0.00 | 1903500.00 | 0.00 | 0.00 | 0.00 | KO:K01999 | branched-chain amino acid transport system substrate-binding protein | Bacteria;Proteobacteria;Alphaproteobacteria;Rhodospirillales;Geminicoccaceae;Geminicoccus;Geminicoccus roseus;Geminicoccus roseus DSM 18922 |
| Ga0586324 0021223 4688 5914 | 0.00 | 0.00 | 0.00 | 0.00 | 0.00 | 0.00 | 5517600.00 | 0.00 | 4357800.00 | 0.00 | 0.00 | 0.00 | KO:K01999 | branched-chain amino acid transport system substrate-binding protein | Bacteria;Proteobacteria;Alphaproteobacteria;Hyphomicrobiales;Bradyrhizobiaceae;Bradyrhizobium;Bradyrhizobium erythrophlei;Bradyrhizobium erythrophlei GAS242 |
| Ga0586324 0021538 5419 6585 | 0.00 | 5922800.00 | 0.00 | 0.00 | 2688300.00 | 0.00 | 0.00 | 8637600.00 | 8181000.00 | 7168700.00 | 5200800.00 | 1222500.00 | KO:K01999 | branched-chain amino acid transport system substrate-binding protein | Bacteria;Chloroflexi;Anaerolineae;Anaerolineales;Anaerolineaceae;Anaerolinea;Anaerolinea thermophila;Anaerolinea thermophila UNI-1 |
| Ga0586324 0022068 678 1925 | 0.00 | 918000.00 | 0.00 | 2194500.00 | 1225700.00 | 0.00 | 0.00 | 2104500.00 | 0.00 | 0.00 | 0.00 | 0.00 | KO:K01999 | branched-chain amino acid transport system substrate-binding protein | Bacteria;Proteobacteria;Alphaproteobacteria;Hyphomicrobiales;Xanthobacteraceae;Pseudolabrys;Pseudolabrys sp. Root1462;Pseudolabrys sp. Root1462 |
| Ga0586324 0024067 2795 4129 | 6570000.00 | 0.00 | 0.00 | 0.00 | 0.00 | 0.00 | 4058400.00 | 0.00 | 0.00 | 0.00 | 0.00 | 5947500.00 | KO:K01999 | branched-chain amino acid transport system substrate-binding protein | Bacteria;Proteobacteria;Deltaproteobacteria;Desulfobacterales;Desulfobulbaceae;Desulfoprunum;Desulfoprunum benzoelyticum;Desulfoprunum benzoelyticum DSM 28570 |
| Ga0586324 0026274 3413 4582 | 2940000.00 | 0.00 | 0.00 | 3914000.00 | 2121800.00 | 3126998.29 | 672403.92 | 1543300.00 | 1822500.00 | 0.00 | 0.00 | 1725000.00 | KO:K01999 | branched-chain amino acid transport system substrate-binding protein | Bacteria;Proteobacteria;Deltaproteobacteria;Syntrophobacterales;Syntrophobacteraceae;Syntrophobacter;Syntrophobacter fumaroxidans;Syntrophobacter fumaroxidans MPOB |
| Ga0586324 0027185 3496 4740 | 0.00 | 0.00 | 0.00 | 0.00 | 2863400.00 | 0.00 | 0.00 | 0.00 | 0.00 | 0.00 | 0.00 | 0.00 | KO:K01999 | branched-chain amino acid transport system substrate-binding protein | Bacteria;Proteobacteria;Deltaproteobacteria;unclassified Deltaproteobacteria;unclassified Deltaproteobacteria;Deferrisoma;Deferrisoma camini;Deferrisoma camini S3R1 |
| Ga0586324 0028591 3246 4511 | 0.00 | 0.00 | 0.00 | 0.00 | 0.00 | 0.00 | 0.00 | 3562400.00 | 0.00 | 4781700.00 | 0.00 | 0.00 | KO:K01999 | branched-chain amino acid transport system substrate-binding protein | Bacteria;Proteobacteria;Alphaproteobacteria;Hyphomicrobiales;Bradyrhizobiaceae;Afipia;Afipia sp. 1NLS2;Afipia sp. 1NLS2 |
| Ga0586324 0029283 970 2241 | 0.00 | 10200000.00 | 0.00 | 0.00 | 0.00 | 0.00 | 0.00 | 0.00 | 0.00 | 0.00 | 0.00 | 0.00 | KO:K01999 | branched-chain amino acid transport system substrate-binding protein | Bacteria;Proteobacteria;Deltaproteobacteria;Desulfobacterales;Desulfobacteraceae;Desulfatiglans;Desulfatiglans anilini;Desulfatiglans anilini DSM 4660 |
| Ga0586324 0029862 3658 4890 | 0.00 | 530323.16 | 0.00 | 0.00 | 0.00 | 0.00 | 0.00 | 0.00 | 0.00 | 0.00 | 0.00 | 0.00 | KO:K01999 | branched-chain amino acid transport system substrate-binding protein | Bacteria;Proteobacteria;Deltaproteobacteria;Syntrophobacterales;Syntrophobacteraceae;Desulfacinum;Desulfacinum infernum;Desulfacinum infernum DSM 9756 |
| Ga0586324 0030991 1553 2704 | 4820000.00 | 18958400.00 | 12442300.00 | 14630000.00 | 14626000.00 | 10798400.00 | 14272800.00 | 12555326.83 | 9882000.00 | 7777000.00 | 7128000.00 | 5632500.00 | KO:K01999 | branched-chain amino acid transport system substrate-binding protein | Bacteria;Chloroflexi;Anaerolineae;Anaerolineales;Anaerolineaceae;Thermanaerothrix;Thermanaerothrix daxensis;Thermanaerothrix daxensis GNS-1 |
| Ga0586324 0035148 922 2106 | 0.00 | 0.00 | 0.00 | 0.00 | 0.00 | 0.00 | 0.00 | 0.00 | 0.00 | 0.00 | 2587200.00 | 2977500.00 | KO:K01999 | branched-chain amino acid transport system substrate-binding protein | Bacteria;Proteobacteria;Alphaproteobacteria;Hyphomicrobiales;Xanthobacteraceae;Pseudolabrys;Pseudolabrys taiwanensis;Pseudolabrys taiwanensis CC-BB4 |
| Ga0586324 0036343 4175 5338 | 5260000.00 | 7820000.00 | 0.00 | 0.00 | 0.00 | 0.00 | 0.00 | 0.00 | 0.00 | 0.00 | 0.00 | 0.00 | KO:K01999 | branched-chain amino acid transport system substrate-binding protein | Bacteria;Chloroflexi;Anaerolineae;Anaerolineales;Anaerolineaceae;Thermanaerothrix;Thermanaerothrix daxensis;Thermanaerothrix daxensis GNS-1 |
| Ga0586324 0038515 4447 5433 | 11520000.00 | 15402000.00 | 0.00 | 0.00 | 0.00 | 0.00 | 0.00 | 0.00 | 0.00 | 0.00 | 0.00 | 5017500.00 | KO:K01999 | branched-chain amino acid transport system substrate-binding protein | Bacteria;Spirochaetes;Spirochaetia;Spirochaetales;Spirochaetaceae;Alkalispirochaeta;Alkalispirochaeta odontotermitis;Alkalispirochaeta odontotermitis JC202 |
| Ga0586324 0038693 3550 4797 | 5000000.00 | 5542000.00 | 0.00 | 5301000.00 | 4130300.00 | 0.00 | 0.00 | 4831200.00 | 4819500.00 | 0.00 | 6996000.00 | 4440000.00 | KO:K01999 | branched-chain amino acid transport system substrate-binding protein | Bacteria;Proteobacteria;Alphaproteobacteria;unclassified Alphaproteobacteria;unclassified Alphaproteobacteria;Phreatobacter;Phreatobacter stygius;Phreatobacter stygius KCTC 52518 |
| Ga0586324 0039391 2999 4315 | 3110000.00 | 0.00 | 0.00 | 0.00 | 7601400.00 | 0.00 | 0.00 | 5343600.00 | 5216400.00 | 5428500.00 | 3608000.00 | 3765000.00 | KO:K01999 | branched-chain amino acid transport system substrate-binding protein | Bacteria;Proteobacteria;Deltaproteobacteria;Syntrophobacterales;Syntrophobacteraceae;Syntrophobacter;Syntrophobacter fumaroxidans;Syntrophobacter fumaroxidans MPOB |
| Ga0586324 0042527 3954 5177 | 3290000.00 | 0.00 | 0.00 | 0.00 | 8930100.00 | 14689000.00 | 21409200.00 | 1653100.00 | 0.00 | 0.00 | 19360000.00 | 10950000.00 | KO:K01999 | branched-chain amino acid transport system substrate-binding protein | Bacteria;Proteobacteria;Betaproteobacteria;Burkholderiales;Comamonadaceae;Hydrogenophaga;Hydrogenophaga sp. PML113;Hydrogenophaga sp. PML113 |
| Ga0586324 0043746 3428 4639 | 0.00 | 0.00 | 0.00 | 0.00 | 0.00 | 0.00 | 0.00 | 0.00 | 0.00 | 916300.00 | 0.00 | 0.00 | KO:K01999 | branched-chain amino acid transport system substrate-binding protein | Bacteria;Proteobacteria;Gammaproteobacteria;Alteromonadales;Alteromonadaceae;Mangrovitalea;Mangrovitalea sediminis;Mangrovitalea sediminis M11-4 |
| Ga0586324 0047246 4467 4913 | 0.00 | 0.00 | 2241601.39 | 0.00 | 2616200.00 | 0.00 | 0.00 | 0.00 | 0.00 | 0.00 | 0.00 | 0.00 | KO:K01999 | branched-chain amino acid transport system substrate-binding protein | Bacteria;Chloroflexi;Anaerolineae;Anaerolineales;Anaerolineaceae;Thermanaerothrix;Thermanaerothrix daxensis;Thermanaerothrix daxensis GNS-1 |
| Ga0586324 0047865 2151 3320 | 0.00 | 2788000.00 | 0.00 | 0.00 | 0.00 | 0.00 | 0.00 | 0.00 | 0.00 | 0.00 | 0.00 | 0.00 | KO:K01999 | branched-chain amino acid transport system substrate-binding protein | Bacteria;Proteobacteria;Deltaproteobacteria;Syntrophobacterales;Syntrophobacteraceae;Syntrophobacter;Syntrophobacter fumaroxidans;Syntrophobacter fumaroxidans MPOB |
| Ga0586324 0048995 3 851 | 3920000.00 | 1570800.00 | 0.00 | 0.00 | 0.00 | 1053109.99 | 1680820.56 | 774700.00 | 0.00 | 0.00 | 0.00 | 0.00 | KO:K01999 | branched-chain amino acid transport system substrate-binding protein | Bacteria;Proteobacteria;Alphaproteobacteria;Rhodospirillales;Geminicoccaceae;Geminicoccus;Geminicoccus roseus;Geminicoccus roseus DSM 18922 |
| Ga0586324 0052026 2208 3377 | 0.00 | 0.00 | 0.00 | 0.00 | 0.00 | 2294878.35 | 0.00 | 2989000.00 | 0.00 | 3572800.00 | 19624000.00 | 6067500.00 | KO:K01999 | branched-chain amino acid transport system substrate-binding protein | Bacteria;Proteobacteria;Betaproteobacteria;Burkholderiales;Comamonadaceae;Polaromonas;Polaromonas sp. C04;Polaromonas sp. C04 |
| Ga0586324 0053104 811 1917 | 5170000.00 | 0.00 | 5911500.00 | 2261000.00 | 0.00 | 1237707.05 | 2325600.00 | 2013000.00 | 0.00 | 0.00 | 11017600.00 | 3697500.00 | KO:K01999 | branched-chain amino acid transport system substrate-binding protein | Bacteria;Proteobacteria;Deltaproteobacteria;Desulfobacterales;Desulfobulbaceae;Desulfobulbus;Desulfobulbus mediterraneus;Desulfobulbus mediterraneus DSM 13871 |
| Ga0586324 0055083 546 1748 | 14500000.00 | 14892000.00 | 31415400.00 | 17480000.00 | 15965000.00 | 3873961.73 | 16393200.00 | 16409000.00 | 14499000.00 | 10934000.00 | 850479.52 | 3217500.00 | KO:K01999 | branched-chain amino acid transport system substrate-binding protein | Bacteria;Proteobacteria;Betaproteobacteria;Burkholderiales;unclassified Burkholderiales;Leptothrix;Leptothrix mobilis;Leptothrix mobilis DSM 10617 |
| Ga0586324 0056492 1784 2941 | 2680000.00 | 0.00 | 0.00 | 0.00 | 0.00 | 0.00 | 0.00 | 0.00 | 0.00 | 0.00 | 0.00 | 0.00 | KO:K01999 | branched-chain amino acid transport system substrate-binding protein | Bacteria;Proteobacteria;Deltaproteobacteria;Desulfobacterales;Desulfobacteraceae;Desulfobacterium;Desulfobacterium autotrophicum;Desulfobacterium autotrophicum HRM2, DSM 3382 |
| Ga0586324 0056775 749 1948 | 0.00 | 4889200.00 | 4983732.30 | 0.00 | 0.00 | 0.00 | 2394000.00 | 0.00 | 0.00 | 2902900.00 | 0.00 | 0.00 | KO:K01999 | branched-chain amino acid transport system substrate-binding protein | Bacteria;Chloroflexi;Thermoflexia;Thermoflexales;Thermoflexaceae;Thermoflexus;Thermoflexus hugenholtzii;Thermoflexus hugenholtzii JAD2 |
| Ga0586324 0061443 1117 2349 | 21600000.00 | 0.00 | 4004934.28 | 12445000.00 | 0.00 | 0.00 | 2644800.00 | 0.00 | 0.00 | 2240700.00 | 0.00 | 263370.75 | KO:K01999 | branched-chain amino acid transport system substrate-binding protein | Bacteria;Proteobacteria;Deltaproteobacteria;Syntrophobacterales;Syntrophobacteraceae;Desulfacinum;Desulfacinum infernum;Desulfacinum infernum DSM 9756 |
| Ga0586324 0064439 432 1709 | 0.00 | 0.00 | 0.00 | 0.00 | 0.00 | 0.00 | 0.00 | 0.00 | 1093500.00 | 0.00 | 0.00 | 0.00 | KO:K01999 | branched-chain amino acid transport system substrate-binding protein | Bacteria;Proteobacteria;Alphaproteobacteria;Hyphomicrobiales;Xanthobacteraceae;Pseudolabrys;Pseudolabrys taiwanensis;Pseudolabrys taiwanensis CC-BB4 |
| Ga0586324 0064742 3 626 | 0.00 | 0.00 | 0.00 | 0.00 | 0.00 | 0.00 | 0.00 | 601265.41 | 0.00 | 0.00 | 0.00 | 278047.50 | KO:K01999 | branched-chain amino acid transport system substrate-binding protein | Bacteria;Proteobacteria;Alphaproteobacteria;Hyphomicrobiales;Bradyrhizobiaceae;Bradyrhizobium;Bradyrhizobium erythrophlei;Bradyrhizobium erythrophlei GAS478 |
| Ga0586324 0072321 712 1833 | 0.00 | 2250800.00 | 3186315.39 | 2603000.00 | 0.00 | 0.00 | 2371200.00 | 0.00 | 0.00 | 0.00 | 0.00 | 0.00 | KO:K01999 | branched-chain amino acid transport system substrate-binding protein | Bacteria;Proteobacteria;Alphaproteobacteria;Rhodospirillales;Acetobacteraceae;Rhodopila;unclassified Rhodopila;Rhodopila sp. LVNP |
| Ga0586324 0074902 1713 2993 | 0.00 | 1346400.00 | 0.00 | 0.00 | 2472000.00 | 0.00 | 0.00 | 2232600.00 | 0.00 | 3287900.00 | 3810400.00 | 2790000.00 | KO:K01999 | branched-chain amino acid transport system substrate-binding protein | Bacteria;Proteobacteria;Alphaproteobacteria;Rhodospirillales;Acetobacteraceae;Rhodopila;unclassified Rhodopila;Rhodopila sp. LVNP |
| Ga0586324 0076069 1081 2352 | 691789.00 | 0.00 | 0.00 | 0.00 | 0.00 | 0.00 | 0.00 | 0.00 | 0.00 | 0.00 | 0.00 | 0.00 | KO:K01999 | branched-chain amino acid transport system substrate-binding protein | Bacteria;Proteobacteria;Deltaproteobacteria;Desulfobacterales;Desulfobacteraceae;Desulfatirhabdium;Desulfatirhabdium butyrativorans;Desulfatirhabdium butyrativorans DSM 18734 |
| Ga0586324 0076120 1340 2608 | 0.00 | 0.00 | 3166019.24 | 0.00 | 0.00 | 0.00 | 0.00 | 0.00 | 0.00 | 0.00 | 0.00 | 0.00 | KO:K01999 | branched-chain amino acid transport system substrate-binding protein | Bacteria;Proteobacteria;Alphaproteobacteria;Rhodospirillales;Stellaceae;Stella;Stella humosa;Stella humosa DSM 5900 |
| Ga0586324 0080640 476 1648 | 0.00 | 0.00 | 0.00 | 0.00 | 0.00 | 0.00 | 0.00 | 0.00 | 0.00 | 2310000.00 | 627992.64 | 0.00 | KO:K01999 | branched-chain amino acid transport system substrate-binding protein | Bacteria;Proteobacteria;Alphaproteobacteria;Rhodospirillales;unclassified Rhodospirillales;unclassified Rhodospirillales;Rhodospirillales bacterium URHD0088;Rhodospirillales bacterium URHD0088 |
| Ga0586324 0093812 2418 3536 | 1120000.00 | 0.00 | 0.00 | 0.00 | 0.00 | 0.00 | 0.00 | 0.00 | 0.00 | 0.00 | 0.00 | 0.00 | KO:K01999 | branched-chain amino acid transport system substrate-binding protein | Bacteria;Proteobacteria;Alphaproteobacteria;Hyphomicrobiales;Xanthobacteraceae;Pseudolabrys;Pseudolabrys sp. Root1462;Pseudolabrys sp. Root1462 |
| Ga0586324 0095607 1568 2752 | 900459.00 | 0.00 | 0.00 | 0.00 | 0.00 | 0.00 | 0.00 | 0.00 | 1036800.00 | 0.00 | 0.00 | 0.00 | KO:K01999 | branched-chain amino acid transport system substrate-binding protein | Bacteria;Proteobacteria;Deltaproteobacteria;Desulfobacterales;Desulfobacteraceae;Desulfatiglans;Desulfatiglans anilini;Desulfatiglans anilini DSM 4660 |
| Ga0586324 0097279 1589 2743 | 2320000.00 | 0.00 | 0.00 | 0.00 | 2884000.00 | 0.00 | 0.00 | 4446900.00 | 0.00 | 0.00 | 0.00 | 0.00 | KO:K01999 | branched-chain amino acid transport system substrate-binding protein | Bacteria;Proteobacteria;Deltaproteobacteria;Syntrophobacterales;Syntrophaceae;Desulfomonile;Desulfomonile tiedjei;Desulfomonile tiedjei DCB-1, DSM 6799 |
| Ga0586324 0100237 740 2116 | 0.00 | 0.00 | 0.00 | 0.00 | 0.00 | 0.00 | 0.00 | 0.00 | 0.00 | 597349.06 | 0.00 | 0.00 | KO:K01999 | branched-chain amino acid transport system substrate-binding protein | Bacteria;Spirochaetes;Spirochaetia;Spirochaetales;Spirochaetaceae;Alkalispirochaeta;Alkalispirochaeta odontotermitis;Alkalispirochaeta odontotermitis JC202 |
| Ga0586324 0102892 1 699 | 0.00 | 3910000.00 | 0.00 | 3980500.00 | 3615300.00 | 0.00 | 2010777.60 | 0.00 | 1620000.00 | 0.00 | 0.00 | 0.00 | KO:K01999 | branched-chain amino acid transport system substrate-binding protein | Bacteria;Chloroflexi;Anaerolineae;Anaerolineales;Anaerolineaceae;Thermanaerothrix;Thermanaerothrix daxensis;Thermanaerothrix daxensis GNS-1 |
| Ga0586324 0104193 2056 3219 | 0.00 | 4263600.00 | 0.00 | 0.00 | 0.00 | 0.00 | 0.00 | 0.00 | 0.00 | 0.00 | 0.00 | 3375000.00 | KO:K01999 | branched-chain amino acid transport system substrate-binding protein | Bacteria;Proteobacteria;Alphaproteobacteria;Hyphomicrobiales;Hyphomicrobiaceae;Rhodomicrobium;Rhodomicrobium vannielii;Rhodomicrobium vannielii R RK 2 |
| Ga0586324 0104455 1 843 | 0.00 | 0.00 | 0.00 | 969000.00 | 0.00 | 0.00 | 0.00 | 3092700.00 | 0.00 | 0.00 | 0.00 | 0.00 | KO:K01999 | branched-chain amino acid transport system substrate-binding protein | Bacteria;Proteobacteria;Deltaproteobacteria;Syntrophobacterales;Syntrophobacteraceae;Syntrophobacter;Syntrophobacter fumaroxidans;Syntrophobacter fumaroxidans MPOB |
| Ga0586324 0107085 293 1570 | 0.00 | 7140000.00 | 5033726.70 | 4275000.00 | 2822200.00 | 0.00 | 0.00 | 4428600.00 | 0.00 | 0.00 | 2464000.00 | 2587500.00 | KO:K01999 | branched-chain amino acid transport system substrate-binding protein | Bacteria;Proteobacteria;Deltaproteobacteria;Desulfobacterales;Desulfobacteraceae;Desulfatirhabdium;Desulfatirhabdium butyrativorans;Desulfatirhabdium butyrativorans DSM 18734 |
| Ga0586324 0110514 1039 2274 | 0.00 | 0.00 | 0.00 | 0.00 | 0.00 | 0.00 | 0.00 | 0.00 | 0.00 | 7107100.00 | 6309600.00 | 0.00 | KO:K01999 | branched-chain amino acid transport system substrate-binding protein | Bacteria;Proteobacteria;Betaproteobacteria;Burkholderiales;Comamonadaceae;Rhodoferax;Rhodoferax ferrireducens;Rhodoferax ferrireducens T118 |

| | | | | | | | | | | | | | | | |
|---|---|---|---|---|---|---|---|---|---|---|---|---|---|---|---|
| Ga0586324 0111580 1393 2577 | 9791532.00 | 17955122.56 | 2852968.72 | 0.00 | 13287000.00 | 6232900.00 | 9348000.00 | 17735222.35 | 15262622.64 | 66389400.00 | 76806400.00 | 36568084.50 | KO:K01999 | branched-chain amino acid transport system substrate-binding protein | Bacteria;Proteobacteria;Betaproteobacteria;Nitrosomonadales;Sulfuricellaceae;Sulfurirhabdus;Sulfurirhabdus autotrophica;Sulfurirhabdus autotrophica DSM 100309 |
| Ga0586324 0120267 770 2026 | 0.00 | 0.00 | 0.00 | 0.00 | 0.00 | 0.00 | 0.00 | 0.00 | 24543000.00 | 0.00 | 0.00 | 0.00 | KO:K01999 | branched-chain amino acid transport system substrate-binding protein | Bacteria;Proteobacteria;Deltaproteobacteria;Syntrophobacterales;Syntrophaceae;Desulfomonile;Desulfomonile tiedjei;Desulfomonile tiedjei DCB-1, DSM 6799 |
| Ga0586324 0129944 341 1474 | 0.00 | 3318400.00 | 0.00 | 0.00 | 0.00 | 0.00 | 0.00 | 0.00 | 0.00 | 0.00 | 0.00 | 0.00 | KO:K01999 | branched-chain amino acid transport system substrate-binding protein | Bacteria;Proteobacteria;Deltaproteobacteria;Syntrophobacterales;Syntrophaceae;Desulfobacca;Desulfobacca acetoxidans;Desulfobacca acetoxidans ASRB2, DSM 11109 |
| Ga0586324 0130101 1312 2493 | 3190000.00 | 4807600.00 | 4193342.23 | 0.00 | 3399000.00 | 0.00 | 0.00 | 4416400.00 | 7889400.00 | 0.00 | 12144000.00 | 0.00 | KO:K01999 | branched-chain amino acid transport system substrate-binding protein | Bacteria;Proteobacteria;Alphaproteobacteria;Rhodospirillales;Acetobacteraceae;Dankookia;Dankookia rubra;Dankookia rubra JCM30602 |
| Ga0586324 0138051 1 1374 | 0.00 | 0.00 | 0.00 | 0.00 | 3079700.00 | 0.00 | 3214800.00 | 0.00 | 0.00 | 0.00 | 0.00 | 0.00 | KO:K01999 | branched-chain amino acid transport system substrate-binding protein | Bacteria;Proteobacteria;Alphaproteobacteria;Hyphomicrobiales;Bradyrhizobiaceae;Bradyrhizobium;Bradyrhizobium lablabi;Bradyrhizobium lablabi GAS165 |
| Ga0586324 0139990 412 1587 | 0.00 | 0.00 | 0.00 | 0.00 | 0.00 | 0.00 | 0.00 | 744200.00 | 0.00 | 0.00 | 0.00 | 0.00 | KO:K01999 | branched-chain amino acid transport system substrate-binding protein | Bacteria;Proteobacteria;Betaproteobacteria;Burkholderiales;Comamonadaceae;Variovorax;Variovorax sp. Root411;Variovorax sp. Root411 |
| Ga0586324 0140221 1760 2902 | 9070000.00 | 12321600.00 | 7431600.00 | 1558000.00 | 7004000.00 | 0.00 | 8664000.00 | 13603000.00 | 2300400.00 | 27720000.00 | 26804800.00 | 14287500.00 | KO:K01999 | branched-chain amino acid transport system substrate-binding protein | Bacteria;Proteobacteria;Betaproteobacteria;Burkholderiales;Comamonadaceae;Polaromonas;Polaromonas jejuensis;Polaromonas jejuensis NBRC 106434 |
| Ga0586324 0141244 1671 2885 | 0.00 | 0.00 | 0.00 | 0.00 | 0.00 | 2742745.96 | 0.00 | 3531900.00 | 3790800.00 | 15939000.00 | 5860800.00 | 4776012.75 | KO:K01999 | branched-chain amino acid transport system substrate-binding protein | Bacteria;Proteobacteria;Betaproteobacteria;Burkholderiales;Burkholderiaceae;Pandoraea;Pandoraea thiooxydans;Pandoraea thiooxydans DSM 25325 |
| Ga0586324 0150506 1077 2279 | 0.00 | 0.00 | 0.00 | 0.00 | 0.00 | 0.00 | 0.00 | 902800.00 | 0.00 | 0.00 | 0.00 | 0.00 | KO:K01999 | branched-chain amino acid transport system substrate-binding protein | Bacteria;Chloroflexi;Thermoflexia;Thermoflexales;Thermoflexaceae;Thermoflexus;Thermoflexus hugenholtzii;Thermoflexus hugenholtzii JAD2 |
| Ga0586324 0150727 2 700 | 0.00 | 0.00 | 0.00 | 207233.00 | 0.00 | 0.00 | 0.00 | 0.00 | 0.00 | 0.00 | 0.00 | 0.00 | KO:K01999 | branched-chain amino acid transport system substrate-binding protein | Bacteria;Proteobacteria;Betaproteobacteria;unclassified Betaproteobacteria;unclassified Betaproteobacteria;unclassified Betaproteobacteria;Betaproteobacteria bacterium GR16-43;Betaproteobacteria bacterium GR16-43 |
| Ga0586324 0151613 1771 2814 | 961978.00 | 128921.88 | 0.00 | 0.00 | 0.00 | 0.00 | 0.00 | 0.00 | 0.00 | 0.00 | 4514400.00 | 0.00 | KO:K01999 | branched-chain amino acid transport system substrate-binding protein | Bacteria;Spirochaetes;Spirochaetia;Spirochaetales;Spirochaetaceae;Alkalispirochaeta;Alkalispirochaeta odontotermitis;Alkalispirochaeta odontotermitis JC202 |
| Ga0586324 0157471 278 1531 | 0.00 | 0.00 | 0.00 | 11780000.00 | 10403000.00 | 6510800.00 | 5494800.00 | 0.00 | 0.00 | 0.00 | 0.00 | 0.00 | KO:K01999 | branched-chain amino acid transport system substrate-binding protein | Bacteria;Proteobacteria;Deltaproteobacteria;Syntrophobacterales;Syntrophaceae;Desulfomonile;Desulfomonile tiedjei;Desulfomonile tiedjei DCB-1, DSM 6799 |
| Ga0586324 0159712 850 1992 | 0.00 | 2420800.00 | 0.00 | 0.00 | 0.00 | 0.00 | 0.00 | 0.00 | 0.00 | 0.00 | 0.00 | 0.00 | KO:K01999 | branched-chain amino acid transport system substrate-binding protein | Bacteria;Firmicutes;Clostridia;Thermoanaerobacterales;Thermoanaerobacteraceae;Desulfovirgula;Desulfovirgula thermocuniculi;Desulfovirgula thermocuniculi DSM 16036 |
| Ga0586324 0169318 214 1461 | 0.00 | 0.00 | 0.00 | 0.00 | 0.00 | 0.00 | 0.00 | 2263100.00 | 0.00 | 0.00 | 0.00 | 0.00 | KO:K01999 | branched-chain amino acid transport system substrate-binding protein | Bacteria;Proteobacteria;Alphaproteobacteria;Hyphomicrobiales;Bradyrhizobiaceae;Bradyrhizobium;Bradyrhizobium sp. KBS0725;Bradyrhizobium sp. KBS0725 |
| Ga0586324 0173027 1639 2646 | 0.00 | 0.00 | 0.00 | 4265500.00 | 0.00 | 0.00 | 0.00 | 0.00 | 3515400.00 | 0.00 | 0.00 | 0.00 | KO:K01999 | branched-chain amino acid transport system substrate-binding protein | Bacteria;Proteobacteria;Alphaproteobacteria;Rhodospirillales;unclassified Rhodospirillales;unclassified Rhodospirillales;Rhodospirillales bacterium URHD0088;Rhodospirillales bacterium URHD0088 |
| Ga0586324 0173127 747 1961 | 0.00 | 0.00 | 0.00 | 0.00 | 0.00 | 0.00 | 0.00 | 0.00 | 3993300.00 | 0.00 | 0.00 | 5827500.00 | KO:K01999 | branched-chain amino acid transport system substrate-binding protein | Bacteria;Proteobacteria;Betaproteobacteria;Burkholderiales;Comamonadaceae;Hydrogenophaga;Hydrogenophaga sp. PML113;Hydrogenophaga sp. PML113 |
| Ga0586324 0174010 1104 2495 | 0.00 | 0.00 | 0.00 | 0.00 | 2327800.00 | 0.00 | 0.00 | 2446100.00 | 0.00 | 0.00 | 0.00 | 0.00 | KO:K01999 | branched-chain amino acid transport system substrate-binding protein | Bacteria;Proteobacteria;Deltaproteobacteria;Syntrophobacterales;Syntrophobacteraceae;Syntrophobacter;Syntrophobacter fumaroxidans;Syntrophobacter fumaroxidans MPOB |
| Ga0586324 0185860 1326 2561 | 0.00 | 0.00 | 0.00 | 0.00 | 0.00 | 0.00 | 0.00 | 0.00 | 972000.00 | 0.00 | 0.00 | 0.00 | KO:K01999 | branched-chain amino acid transport system substrate-binding protein | Bacteria;Proteobacteria;Betaproteobacteria;Burkholderiales;Burkholderiaceae;Paraburkholderia;Paraburkholderia terricola;Paraburkholderia terricola LMG 20594 |
| Ga0586324 0190376 1476 2531 | 0.00 | 0.00 | 0.00 | 2802500.00 | 0.00 | 0.00 | 2758800.00 | 0.00 | 2770200.00 | 0.00 | 0.00 | 0.00 | KO:K01999 | branched-chain amino acid transport system substrate-binding protein | Bacteria;Proteobacteria;Deltaproteobacteria;Desulfobacterales;Desulfobacteraceae;Desulfatirhabdium;Desulfatirhabdium butyrativorans;Desulfatirhabdium butyrativorans DSM 18734 |
| Ga0586324 0191621 640 1956 | 0.00 | 2264400.00 | 0.00 | 0.00 | 0.00 | 0.00 | 0.00 | 0.00 | 0.00 | 0.00 | 0.00 | 0.00 | KO:K01999 | branched-chain amino acid transport system substrate-binding protein | Bacteria;Proteobacteria;Deltaproteobacteria;unclassified Deltaproteobacteria;unclassified Deltaproteobacteria;Deferrisoma;Deferrisoma camini;Deferrisoma camini S3R1 |
| Ga0586324 0194603 1037 2284 | 0.00 | 605510.08 | 0.00 | 0.00 | 0.00 | 0.00 | 0.00 | 0.00 | 0.00 | 0.00 | 0.00 | 0.00 | KO:K01999 | branched-chain amino acid transport system substrate-binding protein | Bacteria;Proteobacteria;Deltaproteobacteria;Desulfovibrionales;Desulfovibrionaceae;Desulfovibrio;Desulfovibrio ferrophilus;Desulfovibrio ferrophilus IS5 |
| Ga0586324 0209445 3 1157 | 0.00 | 0.00 | 0.00 | 2555500.00 | 0.00 | 0.00 | 0.00 | 0.00 | 1134000.00 | 0.00 | 0.00 | 0.00 | KO:K01999 | branched-chain amino acid transport system substrate-binding protein | Bacteria;Proteobacteria;Deltaproteobacteria;Syntrophobacterales;Syntrophaceae;Desulfomonile;Desulfomonile tiedjei;Desulfomonile tiedjei DCB-1, DSM 6799 |
| Ga0586324 0219033 286 1416 | 0.00 | 0.00 | 0.00 | 0.00 | 0.00 | 0.00 | 0.00 | 0.00 | 0.00 | 0.00 | 1346400.00 | 0.00 | KO:K01999 | branched-chain amino acid transport system substrate-binding protein | Bacteria;Proteobacteria;Alphaproteobacteria;Hyphomicrobiales;Xanthobacteraceae;Pseudolabrys;Pseudolabrys taiwanensis;Pseudolabrys taiwanensis CC-BB4 |
| Ga0586324 0221128 1123 2364 | 0.00 | 4182000.00 | 0.00 | 0.00 | 0.00 | 0.00 | 0.00 | 0.00 | 2486700.00 | 2263800.00 | 0.00 | 0.00 | KO:K01999 | branched-chain amino acid transport system substrate-binding protein | Bacteria;Actinobacteria;Thermoleophilia;Solirubrobacterales;unclassified Solirubrobacterales;unclassified Solirubrobacterales;Solirubrobacterales bacterium URHD0059;Solirubrobacterales bacterium URHD0059 |
| Ga0586324 0225573 3 1112 | 0.00 | 0.00 | 0.00 | 1149500.00 | 0.00 | 0.00 | 0.00 | 0.00 | 0.00 | 0.00 | 0.00 | 421020.00 | KO:K01999 | branched-chain amino acid transport system substrate-binding protein | Bacteria;Proteobacteria;Deltaproteobacteria;Desulfuromonadales;Geobacteraceae;Geobacter;Geobacter argillaceus;Geobacter argillaceus ATCC BAA-1139 |
| Ga0586324 0225640 3 506 | 0.00 | 0.00 | 0.00 | 0.00 | 5974000.00 | 0.00 | 0.00 | 0.00 | 3029400.00 | 0.00 | 0.00 | 0.00 | KO:K01999 | branched-chain amino acid transport system substrate-binding protein | Bacteria;Proteobacteria;Deltaproteobacteria;Syntrophobacterales;Syntrophobacteraceae;Desulfacinum;Desulfacinum hydrothermale;Desulfacinum hydrothermale DSM 13146 |
| Ga0586324 0233062 1541 2305 | 0.00 | 0.00 | 0.00 | 0.00 | 0.00 | 0.00 | 0.00 | 0.00 | 0.00 | 5205200.00 | 11202400.00 | 3607500.00 | KO:K01999 | branched-chain amino acid transport system substrate-binding protein | Bacteria;Actinobacteria;Rubrobacteria;Gaiellales;Gaiellaceae;Gaiella;Gaiella occulta;Gaiella occulta F2-233 |
| Ga0586324 0234962 334 1509 | 0.00 | 2774400.00 | 0.00 | 3182500.00 | 0.00 | 2361336.15 | 2986800.00 | 0.00 | 0.00 | 0.00 | 0.00 | 0.00 | KO:K01999 | branched-chain amino acid transport system substrate-binding protein | Bacteria;Proteobacteria;Gammaproteobacteria;unclassified Gammaproteobacteria;Candidatus Competibacteraceae;Candidatus Contendobacter;Candidatus Contendobacter odensis;Candidatus Contendobacter odensis Run B J11 |
| Ga0586324 0238988 1050 2279 | 0.00 | 4114000.00 | 0.00 | 0.00 | 0.00 | 0.00 | 0.00 | 0.00 | 0.00 | 0.00 | 0.00 | 0.00 | KO:K01999 | branched-chain amino acid transport system substrate-binding protein | Bacteria;Proteobacteria;Deltaproteobacteria;Desulfobacterales;Desulfobulbaceae;Desulfoprunum;Desulfoprunum benzoelyticum;Desulfoprunum benzoelyticum DSM 28570 |
| Ga0586324 0243009 222 1388 | 5910000.00 | 3991600.00 | 2636759.83 | 3857000.00 | 4027300.00 | 1758801.31 | 1367468.76 | 1964200.00 | 1514700.00 | 0.00 | 0.00 | 0.00 | KO:K01999 | branched-chain amino acid transport system substrate-binding protein | Bacteria;Chloroflexi;Anaerolineae;Anaerolineales;Anaerolineaceae;Thermanaerothrix;Thermanaerothrix daxensis;Thermanaerothrix daxensis GNS-1 |
| Ga0586324 0255222 373 1551 | 0.00 | 0.00 | 0.00 | 1406000.00 | 4078800.00 | 0.00 | 0.00 | 576601.28 | 0.00 | 0.00 | 0.00 | 0.00 | KO:K01999 | branched-chain amino acid transport system substrate-binding protein | Bacteria;Proteobacteria;Deltaproteobacteria;Syntrophobacterales;Syntrophaceae;Desulfobacca;Desulfobacca acetoxidans;Desulfobacca acetoxidans ASRB2, DSM 11109 |
| Ga0586324 0265577 1683 2171 | 0.00 | 0.00 | 0.00 | 0.00 | 0.00 | 0.00 | 0.00 | 0.00 | 0.00 | 0.00 | 72688000.00 | 0.00 | KO:K01999 | branched-chain amino acid transport system substrate-binding protein | Bacteria;Proteobacteria;Deltaproteobacteria;Desulfobacterales;Desulfobacteraceae;Desulfatitalea;Desulfatitalea tepidiphila;Desulfatitalea tepidiphila S28bF |
| Ga0586324 0273414 851 2107 | 0.00 | 4080000.00 | 8276100.00 | 0.00 | 3728600.00 | 5875600.00 | 4856400.00 | 4123600.00 | 4098600.00 | 9471000.00 | 9152000.00 | 9075000.00 | KO:K01999 | branched-chain amino acid transport system substrate-binding protein | Bacteria;Proteobacteria;Betaproteobacteria;Burkholderiales;Oxalobacteraceae;Glaciimonas;Glaciimonas immobilis;Glaciimonas immobilis DSM 23240 |
| Ga0586324 0275499 345 1619 | 0.00 | 0.00 | 0.00 | 0.00 | 1524400.00 | 0.00 | 0.00 | 0.00 | 0.00 | 0.00 | 0.00 | 0.00 | KO:K01999 | branched-chain amino acid transport system substrate-binding protein | Bacteria;Proteobacteria;Deltaproteobacteria;Desulfobacterales;Desulfobacteraceae;Desulfatitalea;Desulfatitalea tepidiphila;Desulfatitalea tepidiphila S28bF |
| Ga0586324 0276570 107 1336 | 0.00 | 0.00 | 0.00 | 0.00 | 0.00 | 0.00 | 0.00 | 957700.00 | 0.00 | 0.00 | 0.00 | 0.00 | KO:K01999 | branched-chain amino acid transport system substrate-binding protein | Bacteria;Proteobacteria;Deltaproteobacteria;Desulfobacterales;Desulfobacteraceae;Desulfatibacillum;Desulfatibacillum aliphaticivorans;Desulfatibacillum aliphaticivorans AK-01 |
| Ga0586324 0280953 3 1271 | 0.00 | 0.00 | 0.00 | 0.00 | 0.00 | 0.00 | 0.00 | 0.00 | 0.00 | 2263800.00 | 2376000.00 | 0.00 | KO:K01999 | branched-chain amino acid transport system substrate-binding protein | Archaea;Euryarchaeota;Halobacteria;Haloferacales;Haloferacaceae;Halobellus;Halobellus limi;Halobellus limi CGMCC 1.10331 |
| Ga0586324 0282627 407 1669 | 0.00 | 0.00 | 0.00 | 0.00 | 0.00 | 9131000.00 | 2371200.00 | 0.00 | 0.00 | 0.00 | 0.00 | 0.00 | KO:K01999 | branched-chain amino acid transport system substrate-binding protein | Bacteria;Proteobacteria;Deltaproteobacteria;Desulfobacterales;Desulfobacteraceae;Desulfatirhabdium;Desulfatirhabdium butyrativorans;Desulfatirhabdium butyrativorans DSM 18734 |
| Ga0586324 0315081 1237 2010 | 0.00 | 0.00 | 0.00 | 0.00 | 0.00 | 0.00 | 0.00 | 0.00 | 0.00 | 0.00 | 0.00 | 1995000.00 | KO:K01999 | branched-chain amino acid transport system substrate-binding protein | Bacteria;Chloroflexi;Thermoflexia;Thermoflexales;Thermoflexaceae;Thermoflexus;Thermoflexus hugenholtzii;Thermoflexus hugenholtzii JAD2 |
| Ga0586324 0321895 2 1198 | 0.00 | 0.00 | 0.00 | 0.00 | 0.00 | 0.00 | 0.00 | 0.00 | 0.00 | 2286900.00 | 0.00 | 0.00 | KO:K01999 | branched-chain amino acid transport system substrate-binding protein | Bacteria;Proteobacteria;Betaproteobacteria;Rhodocyclales;Rhodocyclaceae;Azovibrio;Azovibrio restrictus;Azovibrio restrictus DSM 23866 |
| Ga0586324 0326187 745 1980 | 11100000.00 | 7140000.00 | 0.00 | 0.00 | 3172400.00 | 0.00 | 0.00 | 2507100.00 | 0.00 | 0.00 | 0.00 | 0.00 | KO:K01999 | branched-chain amino acid transport system substrate-binding protein | Bacteria;Spirochaetes;Spirochaetia;Spirochaetales;Spirochaetaceae;Alkalispirochaeta;Alkalispirochaeta odontotermitis;Alkalispirochaeta odontotermitis JC202 |
| Ga0586324 0326441 958 1980 | 0.00 | 0.00 | 0.00 | 0.00 | 0.00 | 3839950.74 | 0.00 | 0.00 | 0.00 | 0.00 | 0.00 | 0.00 | KO:K01999 | branched-chain amino acid transport system substrate-binding protein | Bacteria;Proteobacteria;Betaproteobacteria;Burkholderiales;unclassified Burkholderiales;Rhizobacter;Rhizobacter sp. Root404;Rhizobacter sp. Root404 |
| Ga0586324 0326822 349 1650 | 0.00 | 0.00 | 2365084.18 | 297490.60 | 0.00 | 0.00 | 4491600.00 | 0.00 | 0.00 | 0.00 | 2261600.00 | 0.00 | KO:K01999 | branched-chain amino acid transport system substrate-binding protein | Bacteria;Proteobacteria;Alphaproteobacteria;Hyphomicrobiales;Bradyrhizobiaceae;Bradyrhizobium;Bradyrhizobium sp. OAE829;Bradyrhizobium sp. OAE829 (Illumina draft) |
| Ga0586324 0355022 666 1907 | 4250000.00 | 0.00 | 8219800.00 | 0.00 | 8827100.00 | 12584900.00 | 22412400.00 | 0.00 | 6447600.00 | 3834600.00 | 0.00 | 0.00 | KO:K01999 | branched-chain amino acid transport system substrate-binding protein | Bacteria;Chloroflexi;Anaerolineae;Anaerolineales;Anaerolineaceae;Thermanaerothrix;Thermanaerothrix daxensis;Thermanaerothrix daxensis GNS-1 |
| Ga0586324 0370354 306 1586 | 0.00 | 0.00 | 7600500.00 | 0.00 | 0.00 | 5597700.00 | 4582800.00 | 2690100.00 | 2486700.00 | 0.00 | 2613600.00 | 0.00 | KO:K01999 | branched-chain amino acid transport system substrate-binding protein | Bacteria;Proteobacteria;Alphaproteobacteria;Rhodobacterales;Acuticoccaceae;Amorphus;Amorphus coralli;Amorphus coralli DSM 19760 |
| Ga0586324 0374484 368 1537 | 8030000.00 | 0.00 | 0.00 | 0.00 | 0.00 | 0.00 | 0.00 | 0.00 | 0.00 | 0.00 | 0.00 | 0.00 | KO:K01999 | branched-chain amino acid transport system substrate-binding protein | Bacteria;Chloroflexi;Anaerolineae;Anaerolineales;Anaerolineaceae;Thermanaerothrix;Thermanaerothrix daxensis;Thermanaerothrix daxensis GNS-1 |
| Ga0586324 0381317 474 1718 | 0.00 | 0.00 | 0.00 | 0.00 | 0.00 | 0.00 | 0.00 | 0.00 | 0.00 | 3326400.00 | 0.00 | 0.00 | KO:K01999 | branched-chain amino acid transport system substrate-binding protein | Bacteria;Actinobacteria;Rubrobacteria;Gaiellales;Gaiellaceae;Gaiella;Gaiella occulta;Gaiella occulta F2-233 |
| Ga0586324 0381918 2 1081 | 0.00 | 0.00 | 0.00 | 0.00 | 0.00 | 0.00 | 0.00 | 0.00 | 471377.07 | 0.00 | 0.00 | 0.00 | KO:K01999 | branched-chain amino acid transport system substrate-binding protein | Bacteria;Proteobacteria;Alphaproteobacteria;Hyphomicrobiales;Xanthobacteraceae;Pseudolabrys;Pseudolabrys taiwanensis;Pseudolabrys taiwanensis CC-BB4 |
| Ga0586324 0385410 624 1838 | 118724.00 | 0.00 | 0.00 | 0.00 | 0.00 | 0.00 | 0.00 | 0.00 | 0.00 | 0.00 | 0.00 | 0.00 | KO:K01999 | branched-chain amino acid transport system substrate-binding protein | Bacteria;Proteobacteria;Deltaproteobacteria;Desulfarculales;Desulfarculaceae;Desulfocarbo;Desulfocarbo indianensis;Desulfocarbo indianensis SCBM |
| Ga0586324 0387607 559 1833 | 0.00 | 0.00 | 0.00 | 0.00 | 0.00 | 0.00 | 0.00 | 0.00 | 0.00 | 0.00 | 5878400.00 | 0.00 | KO:K01999 | branched-chain amino acid transport system substrate-binding protein | Bacteria;Proteobacteria;Deltaproteobacteria;Desulfobacterales;Desulfobacteraceae;Desulfatiglans;Desulfatiglans anilini;Desulfatiglans anilini DSM 4660 |
| Ga0586324 0389883 780 1829 | 0.00 | 0.00 | 0.00 | 0.00 | 0.00 | 0.00 | 0.00 | 0.00 | 0.00 | 0.00 | 2367200.00 | 0.00 | KO:K01999 | branched-chain amino acid transport system substrate-binding protein | Bacteria;Proteobacteria;Deltaproteobacteria;Desulfobacterales;Desulfobacteraceae;Desulfatiglans;Desulfatiglans anilini;Desulfatiglans anilini DSM 4660 |
| Ga0586324 0391594 100 1218 | 5900000.00 | 0.00 | 0.00 | 0.00 | 0.00 | 0.00 | 0.00 | 0.00 | 0.00 | 0.00 | 0.00 | 0.00 | KO:K01999 | branched-chain amino acid transport system substrate-binding protein | Bacteria;Proteobacteria;Alphaproteobacteria;Hyphomicrobiales;Bradyrhizobiaceae;Bradyrhizobium;Bradyrhizobium sp. KBS0725;Bradyrhizobium sp. KBS0725 |
| Ga0586324 0391949 92 1336 | 2810000.00 | 0.00 | 0.00 | 0.00 | 0.00 | 1506055.23 | 0.00 | 0.00 | 0.00 | 0.00 | 0.00 | 0.00 | KO:K01999 | branched-chain amino acid transport system substrate-binding protein | Bacteria;Proteobacteria;Betaproteobacteria;Burkholderiales;Oxalobacteraceae;unclassified Oxalobacteraceae;Oxalobacteraceae bacterium IMCC9480;beta proteobacterium sp. IMCC9480 |
| Ga0586324 0395314 477 1736 | 821204.00 | 2522800.00 | 0.00 | 0.00 | 0.00 | 0.00 | 0.00 | 0.00 | 0.00 | 0.00 | 0.00 | 0.00 | KO:K01999 | branched-chain amino acid transport system substrate-binding protein | Bacteria;Spirochaetes;Spirochaetia;Spirochaetales;Spirochaetaceae;Alkalispirochaeta;Alkalispirochaeta odontotermitis;Alkalispirochaeta odontotermitis JC202 |
| Ga0586324 0397175 1 951 | 0.00 | 1013200.00 | 0.00 | 0.00 | 0.00 | 0.00 | 0.00 | 0.00 | 0.00 | 0.00 | 0.00 | 0.00 | KO:K01999 | branched-chain amino acid transport system substrate-binding protein | Bacteria;Proteobacteria;Betaproteobacteria;unclassified Betaproteobacteria;unclassified Betaproteobacteria;unclassified Betaproteobacteria;Betaproteobacteria bacterium GR16-43;Betaproteobacteria bacterium GR16-43 |
| Ga0586324 0430382 122 1390 | 34950000.00 | 12920000.00 | 0.00 | 10206678.40 | 1503800.00 | 0.00 | 0.00 | 0.00 | 0.00 | 11781000.00 | 8333600.00 | 3367500.00 | KO:K01999 | branched-chain amino acid transport system substrate-binding protein | Bacteria;Spirochaetes;Spirochaetia;Spirochaetales;Spirochaetaceae;Alkalispirochaeta;Alkalispirochaeta odontotermitis;Alkalispirochaeta odontotermitis JC202 |
| Ga0586324 0434930 2 844 | 8110000.00 | 8228000.00 | 0.00 | 0.00 | 0.00 | 0.00 | 0.00 | 0.00 | 0.00 | 3380300.00 | 2666400.00 | 0.00 | KO:K01999 | branched-chain amino acid transport system substrate-binding protein | Bacteria;Chloroflexi;Anaerolineae;Anaerolineales;Anaerolineaceae;Anaerolinea;Anaerolinea thermophila;Anaerolinea thermophila UNI-1 |
| Ga0586324 0441081 388 1539 | 0.00 | 0.00 | 0.00 | 0.00 | 0.00 | 0.00 | 0.00 | 0.00 | 0.00 | 831600.00 | 0.00 | 0.00 | KO:K01999 | branched-chain amino acid transport system substrate-binding protein | Bacteria;Chloroflexi;Anaerolineae;Anaerolineales;Anaerolineaceae;Anaerolinea;Anaerolinea thermophila;Anaerolinea thermophila UNI-1 |
| Ga0586324 0475817 115 1449 | 0.00 | 0.00 | 0.00 | 0.00 | 0.00 | 0.00 | 0.00 | 0.00 | 1919700.00 | 0.00 | 0.00 | 0.00 | KO:K01999 | branched-chain amino acid transport system substrate-binding protein | Bacteria;Proteobacteria;Betaproteobacteria;Burkholderiales;unclassified Burkholderiales;Thiomonas;Thiomonas arsenitoxydans;Thiomonas arsenitoxydans 3As |
| Ga0586324 0486535 1 981 | 0.00 | 0.00 | 0.00 | 3201500.00 | 0.00 | 0.00 | 0.00 | 2281400.00 | 0.00 | 0.00 | 0.00 | 0.00 | KO:K01999 | branched-chain amino acid transport system substrate-binding protein | Bacteria;Spirochaetes;Spirochaetia;Spirochaetales;Spirochaetaceae;Alkalispirochaeta;Alkalispirochaeta odontotermitis;Alkalispirochaeta odontotermitis JC202 |
| Ga0586324 0491881 72 1358 | 0.00 | 0.00 | 0.00 | 0.00 | 0.00 | 0.00 | 6885600.00 | 564686.15 | 427910.85 | 719973.10 | 786690.96 | 125596.50 | KO:K01999 | branched-chain amino acid transport system substrate-binding protein | Bacteria;Proteobacteria;Alphaproteobacteria;Rhodospirillales;Acetobacteraceae;Rhodopila;unclassified Rhodopila;Rhodopila sp. LVNP |
| Ga0586324 0500137 815 1639 | 0.00 | 2359600.00 | 0.00 | 0.00 | 0.00 | 0.00 | 0.00 | 0.00 | 0.00 | 0.00 | 0.00 | 0.00 | KO:K01999 | branched-chain amino acid transport system substrate-binding protein | Bacteria;Actinobacteria;Thermoleophilia;Solirubrobacterales;unclassified Solirubrobacterales;unclassified Solirubrobacterales;Solirubrobacterales bacterium URHD0059;Solirubrobacterales bacterium URHD0059 |
| Ga0586324 0510468 66 1322 | 6190000.00 | 9588000.00 | 3729987.60 | 0.00 | 0.00 | 1757340.35 | 1928205.12 | 4026000.00 | 10773000.00 | 4242700.00 | 5878400.00 | 4650000.00 | KO:K01999 | branched-chain amino acid transport system substrate-binding protein | Bacteria;Proteobacteria;Betaproteobacteria;Burkholderiales;Comamonadaceae;Variovorax;Variovorax sp. PDC80;Variovorax sp. PDC80 |
| Ga0586324 0519395 336 1577 | 3630000.00 | 7072000.00 | 5855200.00 | 0.00 | 0.00 | 0.00 | 0.00 | 0.00 | 3960900.00 | 4673900.00 | 5491200.00 | 0.00 | KO:K01999 | branched-chain amino acid transport system substrate-binding protein | Bacteria;Proteobacteria;Deltaproteobacteria;Desulfobacterales;Desulfobacteraceae;Desulfatiglans;Desulfatiglans anilini;Desulfatiglans anilini DSM 4660 |
| Ga0586324 0529584 890 1597 | 0.00 | 0.00 | 0.00 | 0.00 | 0.00 | 0.00 | 0.00 | 0.00 | 996300.00 | 0.00 | 0.00 | 0.00 | KO:K01999 | branched-chain amino acid transport system substrate-binding protein | Bacteria;Proteobacteria;Deltaproteobacteria;Syntrophobacterales;Syntrophobacteraceae;Desulfoglaeba;Desulfoglaeba alkanexedens;Desulfoglaeba alkanexedens ALDC |
| Ga0586324 0552256 548 1570 | 0.00 | 0.00 | 0.00 | 0.00 | 0.00 | 0.00 | 0.00 | 0.00 | 0.00 | 0.00 | 0.00 | 780000.00 | KO:K01999 | branched-chain amino acid transport system substrate-binding protein | Bacteria;Proteobacteria;Deltaproteobacteria;Syntrophobacterales;Syntrophaceae;Desulfomonile;Desulfomonile tiedjei;Desulfomonile tiedjei DCB-1, DSM 6799 |
| Ga0586324 0555484 746 1564 | 0.00 | 0.00 | 0.00 | 6631000.00 | 0.00 | 0.00 | 0.00 | 0.00 | 0.00 | 0.00 | 0.00 | 0.00 | KO:K01999 | branched-chain amino acid transport system substrate-binding protein | Bacteria;Spirochaetes;Spirochaetia;Spirochaetales;Spirochaetaceae;Alkalispirochaeta;Alkalispirochaeta odontotermitis;Alkalispirochaeta odontotermitis JC202 |
| Ga0586324 0617698 192 1496 | 7550000.00 | 6670800.00 | 7206400.00 | 11400000.00 | 11742000.00 | 7622400.00 | 11172000.00 | 9760000.00 | 9072000.00 | 0.00 | 7224800.00 | 6090000.00 | KO:K01999 | branched-chain amino acid transport system substrate-binding protein | Bacteria;Chloroflexi;Thermomicrobia;Thermomicrobiales;Thermomicrobiaceae;Thermomicrobium;Thermomicrobium roseum;Thermomicrobium roseum DSM 5159 |
| Ga0586324 0622428 575 1489 | 0.00 | 8751600.00 | 0.00 | 0.00 | 1771600.00 | 0.00 | 0.00 | 0.00 | 0.00 | 5243700.00 | 4382400.00 | 2040000.00 | KO:K01999 | branched-chain amino acid transport system substrate-binding protein | Bacteria;Proteobacteria;Deltaproteobacteria;Desulfobacterales;Desulfobacteraceae;Desulfatirhabdium;Desulfatirhabdium butyrativorans;Desulfatirhabdium butyrativorans DSM 18734 |
| Ga0586324 0634350 998 1477 | 0.00 | 768400.00 | 0.00 | 0.00 | 0.00 | 0.00 | 0.00 | 0.00 | 0.00 | 0.00 | 0.00 | 0.00 | KO:K01999 | branched-chain amino acid transport system substrate-binding protein | Bacteria;Proteobacteria;Alphaproteobacteria;Hyphomicrobiales;Bradyrhizobiaceae;Bradyrhizobium;Bradyrhizobium sp. OAE819;Bradyrhizobium sp. OAE819 |
| Ga0586324 0637657 214 1473 | 2160000.00 | 0.00 | 0.00 | 0.00 | 0.00 | 0.00 | 0.00 | 0.00 | 0.00 | 0.00 | 0.00 | 0.00 | KO:K01999 | branched-chain amino acid transport system substrate-binding protein | Bacteria;Proteobacteria;Alphaproteobacteria;unclassified Alphaproteobacteria;unclassified Alphaproteobacteria;Phreatobacter;Phreatobacter stygius;Phreatobacter stygius KCTC 52518 |
| Ga0586324 0660989 1 327 | 0.00 | 1013200.00 | 0.00 | 0.00 | 1297800.00 | 0.00 | 0.00 | 0.00 | 0.00 | 0.00 | 0.00 | 0.00 | KO:K01999 | branched-chain amino acid transport system substrate-binding protein | Bacteria;Proteobacteria;Deltaproteobacteria;Syntrophobacterales;Syntrophobacteraceae;Syntrophobacter;Syntrophobacter fumaroxidans;Syntrophobacter fumaroxidans MPOB |
| Ga0586324 0687283 586 1428 | 0.00 | 0.00 | 0.00 | 0.00 | 0.00 | 0.00 | 0.00 | 0.00 | 1109700.00 | 0.00 | 0.00 | 0.00 | KO:K01999 | branched-chain amino acid transport system substrate-binding protein | Bacteria;Proteobacteria;Betaproteobacteria;unclassified Betaproteobacteria;unclassified Betaproteobacteria;unclassified Betaproteobacteria;Betaproteobacteria bacterium GR16-43;Betaproteobacteria bacterium GR16-43 |
| Ga0586324 0695736 174 1391 | 20200000.00 | 20332000.00 | 0.00 | 17765000.00 | 22454000.00 | 13617100.00 | 0.00 | 1140700.00 | 23328000.00 | 970200.00 | 0.00 | 17925000.00 | KO:K01999 | branched-chain amino acid transport system substrate-binding protein | Bacteria;Chloroflexi;Thermomicrobia;Thermomicrobiales;Thermomicrobiaceae;Thermomicrobium;Thermomicrobium roseum;Thermomicrobium roseum DSM 5159 |
| Ga0586324 0701796 3 914 | 0.00 | 0.00 | 0.00 | 0.00 | 0.00 | 0.00 | 0.00 | 0.00 | 0.00 | 0.00 | 0.00 | 2587500.00 | KO:K01999 | branched-chain amino acid transport system substrate-binding protein | Bacteria;Proteobacteria;Alphaproteobacteria;Rhodospirillales;Rhodospirillaceae;Dongia;Dongia sp. URHE0060;Dongia sp. URHE0060 |
| Ga0586324 0715666 313 1401 | 951359.00 | 2516000.00 | 0.00 | 0.00 | 0.00 | 0.00 | 11149200.00 | 0.00 | 0.00 | 0.00 | 0.00 | 0.00 | KO:K01999 | branched-chain amino acid transport system substrate-binding protein | Bacteria;Chloroflexi;Anaerolineae;Anaerolineales;Anaerolineaceae;Levilinea;Levilinea saccharolytica;Levilinea saccharolytica DSM 16555 |
| Ga0586324 0717404 3 746 | 0.00 | 0.00 | 0.00 | 0.00 | 1050600.00 | 0.00 | 0.00 | 0.00 | 0.00 | 0.00 | 0.00 | 0.00 | KO:K01999 | branched-chain amino acid transport system substrate-binding protein | Bacteria;Proteobacteria;Betaproteobacteria;Burkholderiales;Comamonadaceae;Hydrogenophaga;Hydrogenophaga borbori;Hydrogenophaga borbori LA-38 |
| Ga0586324 0744731 1 999 | 0.00 | 0.00 | 0.00 | 0.00 | 0.00 | 0.00 | 0.00 | 0.00 | 0.00 | 0.00 | 0.00 | 1597500.00 | KO:K01999 | branched-chain amino acid transport system substrate-binding protein | Bacteria;Proteobacteria;Deltaproteobacteria;Syntrophobacterales;Syntrophobacteraceae;Syntrophobacter;Syntrophobacter fumaroxidans;Syntrophobacter fumaroxidans MPOB |
| Ga0586324 0761397 3 1175 | 0.00 | 0.00 | 0.00 | 0.00 | 0.00 | 0.00 | 0.00 | 0.00 | 0.00 | 0.00 | 5544000.00 | 3487500.00 | KO:K01999 | branched-chain amino acid transport system substrate-binding protein | Bacteria;Proteobacteria;Alphaproteobacteria;Rhodospirillales;Rhodospirillaceae;Dongia;Dongia sp. URHE0060;Dongia sp. URHE0060 |
| Ga0586324 0767327 93 1262 | 0.00 | 0.00 | 0.00 | 0.00 | 0.00 | 0.00 | 0.00 | 0.00 | 0.00 | 5266800.00 | 0.00 | 0.00 | KO:K01999 | branched-chain amino acid transport system substrate-binding protein | Bacteria;Proteobacteria;Betaproteobacteria;Burkholderiales;Comamonadaceae;Polaromonas;Polaromonas sp. C04;Polaromonas sp. C04 |
| Ga0586324 0769873 464 1360 | 4620000.00 | 4909600.00 | 0.00 | 5491000.00 | 4346600.00 | 3562590.66 | 3944400.00 | 0.00 | 3828250.35 | 0.00 | 0.00 | 0.00 | KO:K01999 | branched-chain amino acid transport system substrate-binding protein | Bacteria;Chloroflexi;Anaerolineae;Anaerolineales;Anaerolineaceae;Anaerolinea;Anaerolinea thermophila;Anaerolinea thermophila UNI-1 |
| Ga0586324 0774711 3 656 | 967277.00 | 2196400.00 | 3356825.57 | 2783500.00 | 0.00 | 2894824.75 | 6224400.00 | 1390800.00 | 4446900.00 | 0.00 | 0.00 | 0.00 | KO:K01999 | branched-chain amino acid transport system substrate-binding protein | Bacteria;Chloroflexi;Anaerolineae;Anaerolineales;Anaerolineaceae;Thermanaerothrix;Thermanaerothrix daxensis;Thermanaerothrix daxensis GNS-1 |
| Ga0586324 0791490 106 1344 | 0.00 | 0.00 | 0.00 | 0.00 | 0.00 | 31363000.00 | 0.00 | 0.00 | 0.00 | 0.00 | 0.00 | 0.00 | KO:K01999 | branched-chain amino acid transport system substrate-binding protein | Bacteria;Proteobacteria;Deltaproteobacteria;Desulfobacterales;Desulfobacteraceae;Desulfatitalea;Desulfatitalea tepidiphila;Desulfatitalea tepidiphila S28bF |
| Ga0586324 0798405 198 1337 | 1180000.00 | 0.00 | 0.00 | 0.00 | 0.00 | 0.00 | 0.00 | 0.00 | 2786400.00 | 0.00 | 0.00 | 0.00 | KO:K01999 | branched-chain amino acid transport system substrate-binding protein | Bacteria;Proteobacteria;Deltaproteobacteria;Desulfobacterales;Desulfobulbaceae;Desulfobulbus;Desulfobulbus elongatus;Desulfobulbus elongatus DSM 2908 |
| Ga0586324 0798484 714 1337 | 3850000.00 | 1230800.00 | 0.00 | 0.00 | 0.00 | 0.00 | 0.00 | 0.00 | 0.00 | 0.00 | 0.00 | 0.00 | KO:K01999 | branched-chain amino acid transport system substrate-binding protein | Bacteria;Proteobacteria;Deltaproteobacteria;Syntrophobacterales;Syntrophaceae;Desulfomonile;Desulfomonile tiedjei;Desulfomonile tiedjei DCB-1, DSM 6799 |
| Ga0586324 0803995 258 1334 | 0.00 | 0.00 | 0.00 | 0.00 | 0.00 | 871129.16 | 0.00 | 0.00 | 0.00 | 0.00 | 0.00 | 0.00 | KO:K01999 | branched-chain amino acid transport system substrate-binding protein | Bacteria;Proteobacteria;Alphaproteobacteria;Rhodospirillales;Stellaceae;Stella;Stella humosa;Stella humosa DSM 5900 |
| Ga0586324 0810320 149 1330 | 0.00 | 0.00 | 0.00 | 0.00 | 0.00 | 0.00 | 0.00 | 945500.00 | 0.00 | 0.00 | 0.00 | 0.00 | KO:K01999 | branched-chain amino acid transport system substrate-binding protein | Bacteria;Proteobacteria;Alphaproteobacteria;Hyphomicrobiales;Bradyrhizobiaceae;Bradyrhizobium;Bradyrhizobium erythrophlei;Bradyrhizobium erythrophlei GAS242 |
| Ga0586324 0833728 209 1312 | 2130000.00 | 0.00 | 0.00 | 0.00 | 0.00 | 0.00 | 0.00 | 0.00 | 0.00 | 0.00 | 0.00 | 0.00 | KO:K01999 | branched-chain amino acid transport system substrate-binding protein | Bacteria;Proteobacteria;Betaproteobacteria;Burkholderiales;Comamonadaceae;Pelomonas;Pelomonas puraquae;Pelomonas puraquae CCUG 52769 |
| Ga0586324 0835679 137 1303 | 0.00 | 0.00 | 0.00 | 0.00 | 0.00 | 1873180.98 | 0.00 | 0.00 | 0.00 | 0.00 | 0.00 | 4297500.00 | KO:K01999 | branched-chain amino acid transport system substrate-binding protein | Bacteria;Proteobacteria;Deltaproteobacteria;Syntrophobacterales;Syntrophobacteraceae;Desulfacinum;Desulfacinum infernum;Desulfacinum infernum DSM 9756 |
| Ga0586324 0837668 402 1310 | 0.00 | 0.00 | 0.00 | 0.00 | 0.00 | 0.00 | 0.00 | 0.00 | 0.00 | 839300.00 | 0.00 | 0.00 | KO:K01999 | branched-chain amino acid transport system substrate-binding protein | Bacteria;Proteobacteria;Alphaproteobacteria;Rhodospirillales;Rhodospirillaceae;Dongia;Dongia sp. URHE0060;Dongia sp. URHE0060 |
| Ga0586324 0838107 755 1309 | 639041.00 | 0.00 | 0.00 | 0.00 | 0.00 | 0.00 | 0.00 | 0.00 | 0.00 | 0.00 | 0.00 | 0.00 | KO:K01999 | branched-chain amino acid transport system substrate-binding protein | Bacteria;Chloroflexi;Anaerolineae;Anaerolineales;Anaerolineaceae;Thermanaerothrix;Thermanaerothrix daxensis;Thermanaerothrix daxensis GNS-1 |
| Ga0586324 0850265 395 1303 | 0.00 | 28356000.00 | 62493000.00 | 42655000.00 | 40479000.00 | 45655000.00 | 41952000.00 | 34953000.00 | 35640000.00 | 26334000.00 | 32560000.00 | 31500000.00 | KO:K01999 | branched-chain amino acid transport system substrate-binding protein | Bacteria;Proteobacteria;Deltaproteobacteria;Desulfobacterales;Desulfobulbaceae;Desulfoprunum;Desulfoprunum benzoelyticum;Desulfoprunum benzoelyticum DSM 28570 |
| Ga0586324 0943069 386 1246 | 0.00 | 0.00 | 0.00 | 0.00 | 0.00 | 2128551.23 | 0.00 | 0.00 | 0.00 | 0.00 | 0.00 | 0.00 | KO:K01999 | branched-chain amino acid transport system substrate-binding protein | Bacteria;Proteobacteria;Betaproteobacteria;unclassified Betaproteobacteria;unclassified Betaproteobacteria;unclassified Betaproteobacteria;beta proteobacterium AAP51;Betaproteobacteria bacterium AAP51 |
| Ga0586324 0976788 2 637 | 0.00 | 0.00 | 0.00 | 0.00 | 0.00 | 0.00 | 0.00 | 0.00 | 0.00 | 585476.43 | 578982.80 | 559779.75 | KO:K01999 | branched-chain amino acid transport system substrate-binding protein | Bacteria;Proteobacteria;Betaproteobacteria;Burkholderiales;Comamonadaceae;Polaromonas;Polaromonas jejuensis;Polaromonas jejuensis NBRC 106434 |
| Ga0586324 0999571 180 1214 | 0.00 | 0.00 | 0.00 | 0.00 | 0.00 | 0.00 | 0.00 | 0.00 | 1223100.00 | 0.00 | 1830400.00 | 0.00 | KO:K01999 | branched-chain amino acid transport system substrate-binding protein | Bacteria;Chloroflexi;Chloroflexia;Kallotenuales;Kallotenuaceae;Kallotenue;Kallotenue papyrolyticum;Kallotenue papyrolyticum JKG1 |
| Ga0586324 1000411 2 1030 | 0.00 | 5208800.00 | 0.00 | 0.00 | 0.00 | 0.00 | 0.00 | 0.00 | 0.00 | 0.00 | 0.00 | 0.00 | KO:K01999 | branched-chain amino acid transport system substrate-binding protein | Bacteria;Chloroflexi;Thermomicrobia;Thermomicrobiales;Thermomicrobiaceae;Thermomicrobium;Thermomicrobium roseum;Thermomicrobium roseum DSM 5159 |
| Ga0586324 1005979 172 1212 | 0.00 | 0.00 | 0.00 | 0.00 | 0.00 | 0.00 | 901313.64 | 0.00 | 0.00 | 2094400.00 | 3704800.00 | 2700000.00 | KO:K01999 | branched-chain amino acid transport system substrate-binding protein | Bacteria;Proteobacteria;Alphaproteobacteria;Hyphomicrobiales;Bradyrhizobiaceae;Afipia;Afipia sp. 1NLS2;Afipia sp. 1NLS2 |
| Ga0586324 1019476 3 1067 | 0.00 | 0.00 | 4042424.45 | 452105.95 | 0.00 | 0.00 | 0.00 | 0.00 | 0.00 | 2240700.00 | 0.00 | 0.00 | KO:K01999 | branched-chain amino acid transport system substrate-binding protein | Bacteria;Proteobacteria;Deltaproteobacteria;Desulfobacterales;Desulfobacteraceae;Desulfatirhabdium;Desulfatirhabdium butyrativorans;Desulfatirhabdium butyrativorans DSM 18734 |
| Ga0586324 1065950 307 1182 | 3430000.00 | 3209600.00 | 5855200.00 | 0.00 | 0.00 | 0.00 | 0.00 | 0.00 | 0.00 | 0.00 | 0.00 | 0.00 | KO:K01999 | branched-chain amino acid transport system substrate-binding protein | Bacteria;Proteobacteria;Deltaproteobacteria;Desulfobacterales;Desulfobacteraceae;Desulfatirhabdium;Desulfatirhabdium butyrativorans;Desulfatirhabdium butyrativorans DSM 18734 |
| Ga0586324 1077015 533 1177 | 0.00 | 782000.00 | 4497576.17 | 0.00 | 9548100.00 | 0.00 | 0.00 | 0.00 | 0.00 | 0.00 | 1927200.00 | 0.00 | KO:K01999 | branched-chain amino acid transport system substrate-binding protein | Bacteria;Chloroflexi;Anaerolineae;Anaerolineales;Anaerolineaceae;Anaerolinea;Anaerolinea thermophila;Anaerolinea thermophila UNI-1 |
| Ga0586324 1195691 82 1125 | 0.00 | 0.00 | 0.00 | 0.00 | 0.00 | 0.00 | 0.00 | 387983.18 | 694434.87 | 0.00 | 0.00 | 0.00 | KO:K01999 | branched-chain amino acid transport system substrate-binding protein | Bacteria;Proteobacteria;Alphaproteobacteria;Hyphomicrobiales;Xanthobacteraceae;Pseudolabrys;Pseudolabrys taiwanensis;Pseudolabrys taiwanensis CC-BB4 |
| Ga0586324 1232760 361 1110 | 0.00 | 0.00 | 0.00 | 0.00 | 0.00 | 0.00 | 0.00 | 0.00 | 693372.96 | 0.00 | 0.00 | 0.00 | KO:K01999 | branched-chain amino acid transport system substrate-binding protein | Bacteria;Proteobacteria;Deltaproteobacteria;Desulfobacterales;Desulfobulbaceae;Desulfopila;Desulfopila sp. IMCC35005;Desulfopila sp. IMCC35005 |
| Ga0586324 1257558 428 1099 | 4860000.00 | 4182000.00 | 0.00 | 0.00 | 0.00 | 3538191.04 | 1937069.76 | 2177700.00 | 2016900.00 | 0.00 | 0.00 | 4102500.00 | KO:K01999 | branched-chain amino acid transport system substrate-binding protein | Bacteria;Proteobacteria;Alphaproteobacteria;Rhodobacterales;Rhodobacteraceae;Paracoccus;Paracoccus pantotrophus;Paracoccus pantotrophus DSM 2944 |
| Ga0586324 1272706 3 731 | 0.00 | 0.00 | 0.00 | 0.00 | 0.00 | 0.00 | 0.00 | 0.00 | 0.00 | 0.00 | 2939200.00 | 2025000.00 | KO:K01999 | branched-chain amino acid transport system substrate-binding protein | Bacteria;Proteobacteria;Betaproteobacteria;Nitrosomonadales;Sulfuricellaceae;Sulfurirhabdus;Sulfurirhabdus autotrophica;Sulfurirhabdus autotrophica BiS0 |
| Ga0586324 1290433 3 974 | 13200000.00 | 38624000.00 | 6080400.00 | 3344000.00 | 0.00 | 0.00 | 7546800.00 | 7442000.00 | 5200200.00 | 28182000.00 | 10736000.00 | 9225000.00 | KO:K01999 | branched-chain amino acid transport system substrate-binding protein | Bacteria;Chloroflexi;Thermomicrobia;Thermomicrobiales;Thermomicrobiaceae;Thermomicrobium;Thermomicrobium roseum;Thermomicrobium roseum DSM 5159 |
| Ga0586324 1302609 406 1083 | 0.00 | 0.00 | 5451467.07 | 0.00 | 0.00 | 0.00 | 0.00 | 677100.00 | 0.00 | 0.00 | 0.00 | 0.00 | KO:K01999 | branched-chain amino acid transport system substrate-binding protein | Bacteria;Proteobacteria;Betaproteobacteria;Burkholderiales;Comamonadaceae;Rhodoferax;Rhodoferax koreense;Rhodoferax koreense DCY110 |
| Ga0586324 1341950 338 1069 | 0.00 | 1462000.00 | 1059718.01 | 0.00 | 0.00 | 0.00 | 4993200.00 | 988200.00 | 0.00 | 1232000.00 | 2508000.00 | 0.00 | KO:K01999 | branched-chain amino acid transport system substrate-binding protein | Bacteria;Proteobacteria;Deltaproteobacteria;Desulfarculales;Desulfarculaceae;Desulfocarbo;Desulfocarbo indianensis;Desulfocarbo indianensis SCBM |
| Ga0586324 1361201 2 973 | 0.00 | 0.00 | 0.00 | 0.00 | 0.00 | 0.00 | 0.00 | 2226500.00 | 1344600.00 | 0.00 | 0.00 | 0.00 | KO:K01999 | branched-chain amino acid transport system substrate-binding protein | Bacteria;Proteobacteria;Deltaproteobacteria;Desulfobacterales;Desulfobacteraceae;Desulfobacterium;Desulfobacterium autotrophicum;Desulfobacterium autotrophicum HRM2, DSM 3382 |
| Ga0586324 1363727 83 1063 | 0.00 | 0.00 | 0.00 | 0.00 | 3563800.00 | 0.00 | 0.00 | 0.00 | 0.00 | 0.00 | 0.00 | 0.00 | KO:K01999 | branched-chain amino acid transport system substrate-binding protein | Bacteria;Firmicutes;Clostridia;Eubacteriales;unclassified Eubacteriales;unclassified Eubacteriales;Clostridiales bacterium PH28 bin88;Clostridiales bacterium PH28 bin88 |
| Ga0586324 1392794 866 1054 | 0.00 | 3617600.00 | 0.00 | 0.00 | 0.00 | 4525800.00 | 0.00 | 3477000.00 | 3920400.00 | 2279200.00 | 0.00 | 0.00 | KO:K01999 | branched-chain amino acid transport system substrate-binding protein | Bacteria;Proteobacteria;Alphaproteobacteria;Hyphomicrobiales;Phyllobacteriaceae;Pseudaminobacter;Pseudaminobacter manganicus;Pseudaminobacter manganicus JH-7 |
| Ga0586324 1483572 225 1025 | 778904.00 | 394489.08 | 0.00 | 0.00 | 0.00 | 7423900.00 | 16712400.00 | 0.00 | 6828300.00 | 0.00 | 0.00 | 0.00 | KO:K01999 | branched-chain amino acid transport system substrate-binding protein | Bacteria;Chloroflexi;Anaerolineae;Anaerolineales;Anaerolineaceae;Thermanaerothrix;Thermanaerothrix daxensis;Thermanaerothrix daxensis GNS-1 |
| Ga0586324 1599366 1 993 | 0.00 | 0.00 | 0.00 | 0.00 | 0.00 | 0.00 | 0.00 | 0.00 | 0.00 | 2571800.00 | 0.00 | 0.00 | KO:K01999 | branched-chain amino acid transport system substrate-binding protein | Bacteria;Proteobacteria;Betaproteobacteria;Burkholderiales;Oxalobacteraceae;Noviherbaspirillum;Noviherbaspirillum denitrificans;Noviherbaspirillum denitrificans TSA40 |
| Ga0586324 1600509 107 994 | 0.00 | 6766000.00 | 0.00 | 0.00 | 0.00 | 0.00 | 0.00 | 0.00 | 0.00 | 0.00 | 0.00 | 0.00 | KO:K01999 | branched-chain amino acid transport system substrate-binding protein | Bacteria;Proteobacteria;Deltaproteobacteria;Desulfobacterales;Desulfobulbaceae;Desulfobulbus;Desulfobulbus mediterraneus;Desulfobulbus mediterraneus DSM 13871 |
| Ga0586324 1600840 3 992 | 639171.00 | 0.00 | 0.00 | 0.00 | 0.00 | 0.00 | 0.00 | 0.00 | 435950.91 | 0.00 | 0.00 | 0.00 | KO:K01999 | branched-chain amino acid transport system substrate-binding protein | Bacteria;Proteobacteria;Deltaproteobacteria;Desulfobacterales;Desulfobacteraceae;Desulfatirhabdium;Desulfatirhabdium butyrativorans;Desulfatirhabdium butyrativorans DSM 18734 |
| Ga0586324 1653280 1 978 | 0.00 | 0.00 | 2394410.85 | 0.00 | 0.00 | 2112444.94 | 1939559.52 | 1427400.00 | 0.00 | 0.00 | 0.00 | 0.00 | KO:K01999 | branched-chain amino acid transport system substrate-binding protein | Bacteria;Proteobacteria;Deltaproteobacteria;Syntrophobacterales;Syntrophaceae;Desulfobacca;Desulfobacca acetoxidans;Desulfobacca acetoxidans ASRB2, DSM 11109 |
| Ga0586324 1720874 3 962 | 0.00 | 0.00 | 0.00 | 0.00 | 0.00 | 0.00 | 0.00 | 677100.00 | 0.00 | 0.00 | 0.00 | 0.00 | KO:K01999 | branched-chain amino acid transport system substrate-binding protein | Bacteria;Chloroflexi;Thermomicrobia;Thermomicrobiales;Thermomicrobiaceae;Thermomicrobium;Thermomicrobium roseum;Thermomicrobium roseum DSM 5159 |
| Ga0586324 1721926 2 361 | 0.00 | 0.00 | 0.00 | 0.00 | 0.00 | 0.00 | 0.00 | 0.00 | 0.00 | 0.00 | 4048000.00 | 7410000.00 | KO:K01999 | branched-chain amino acid transport system substrate-binding protein | Bacteria;Proteobacteria;Alphaproteobacteria;Hyphomicrobiales;Bradyrhizobiaceae;Afipia;Afipia massiliensis;Afipia massiliensis LC387 |
| Ga0586324 1729228 195 962 | 0.00 | 843200.00 | 0.00 | 0.00 | 0.00 | 0.00 | 0.00 | 0.00 | 0.00 | 0.00 | 2499200.00 | 1387500.00 | KO:K01999 | branched-chain amino acid transport system substrate-binding protein | Bacteria;Proteobacteria;Betaproteobacteria;Burkholderiales;unclassified Burkholderiales;Rhizobacter;Rhizobacter sp. Root16D2;Rhizobacter sp. Root16D2 |
| Ga0586324 1808877 122 943 | 0.00 | 1747600.00 | 0.00 | 0.00 | 0.00 | 0.00 | 0.00 | 0.00 | 0.00 | 0.00 | 0.00 | 0.00 | KO:K01999 | branched-chain amino acid transport system substrate-binding protein | Bacteria;Proteobacteria;Deltaproteobacteria;Desulfobacterales;Desulfobacteraceae;Desulfonema;Desulfonema limicola;Desulfonema limicola Jadebusen, DSM 2076 |
| Ga0586324 1814175 1 942 | 0.00 | 0.00 | 0.00 | 0.00 | 0.00 | 0.00 | 0.00 | 0.00 | 0.00 | 0.00 | 0.00 | 997500.00 | KO:K01999 | branched-chain amino acid transport system substrate-binding protein | Bacteria;Spirochaetes;Spirochaetia;Spirochaetales;Spirochaetaceae;Alkalispirochaeta;Alkalispirochaeta odontotermitis;Alkalispirochaeta odontotermitis JC202 |
| Ga0586324 1839227 1 936 | 0.00 | 0.00 | 0.00 | 7201000.00 | 737798.27 | 0.00 | 0.00 | 0.00 | 0.00 | 0.00 | 0.00 | 0.00 | KO:K01999 | branched-chain amino acid transport system substrate-binding protein | Bacteria;Proteobacteria;Deltaproteobacteria;Desulfobacterales;Desulfobacteraceae;Desulfatirhabdium;Desulfatirhabdium butyrativorans;Desulfatirhabdium butyrativorans DSM 18734 |
| Ga0586324 1891133 1 924 | 0.00 | 3270800.00 | 0.00 | 0.00 | 0.00 | 0.00 | 0.00 | 0.00 | 0.00 | 0.00 | 0.00 | 0.00 | KO:K01999 | branched-chain amino acid transport system substrate-binding protein | Bacteria;Spirochaetes;Spirochaetia;Spirochaetales;Spirochaetaceae;Alkalispirochaeta;Alkalispirochaeta odontotermitis;Alkalispirochaeta odontotermitis JC202 |
| Ga0586324 1911993 1 921 | 0.00 | 0.00 | 0.00 | 8027500.00 | 1431700.00 | 0.00 | 0.00 | 0.00 | 0.00 | 0.00 | 0.00 | 0.00 | KO:K01999 | branched-chain amino acid transport system substrate-binding protein | Bacteria;Proteobacteria;Alphaproteobacteria;Hyphomicrobiales;Hyphomicrobiaceae;Rhodoplanes;Rhodoplanes sp. Z2-YC6860;Rhodoplanes sp. Z2-YC6860 |
| Ga0586324 1916237 100 921 | 0.00 | 0.00 | 0.00 | 0.00 | 0.00 | 0.00 | 0.00 | 515840.40 | 0.00 | 763972.44 | 0.00 | 5986373.25 | KO:K01999 | branched-chain amino acid transport system substrate-binding protein | Bacteria;Proteobacteria;Betaproteobacteria;Burkholderiales;Comamonadaceae;Variovorax;Variovorax sp. OV700;Variovorax sp. OV700 |
| Ga0586324 1935117 3 554 | 0.00 | 3794400.00 | 0.00 | 0.00 | 3697700.00 | 0.00 | 0.00 | 0.00 | 0.00 | 0.00 | 8113600.00 | 2017500.00 | KO:K01999 | branched-chain amino acid transport system substrate-binding protein | Bacteria;Spirochaetes;Spirochaetia;Spirochaetales;Spirochaetaceae;Alkalispirochaeta;Alkalispirochaeta odontotermitis;Alkalispirochaeta odontotermitis JC202 |
| Ga0586324 1939908 124 915 | 0.00 | 0.00 | 0.00 | 0.00 | 0.00 | 0.00 | 0.00 | 0.00 | 0.00 | 0.00 | 4752000.00 | 0.00 | KO:K01999 | branched-chain amino acid transport system substrate-binding protein | Bacteria;Proteobacteria;Betaproteobacteria;Rhodocyclales;Azonexaceae;Dechloromonas;Dechloromonas sp. HYN0024;Dechloromonas sp. HYN0024 |
| Ga0586324 1945572 2 448 | 0.00 | 0.00 | 0.00 | 0.00 | 0.00 | 0.00 | 0.00 | 0.00 | 0.00 | 1178100.00 | 1452000.00 | 1110000.00 | KO:K01999 | branched-chain amino acid transport system substrate-binding protein | Bacteria;Proteobacteria;Betaproteobacteria;Burkholderiales;Comamonadaceae;Polaromonas;Polaromonas jejuensis;Polaromonas jejuensis NBRC 106434 |
| Ga0586324 1979620 3 908 | 0.00 | 0.00 | 0.00 | 0.00 | 0.00 | 0.00 | 0.00 | 0.00 | 19197000.00 | 0.00 | 0.00 | 0.00 | KO:K01999 | branched-chain amino acid transport system substrate-binding protein | Bacteria;Proteobacteria;Alphaproteobacteria;Hyphomicrobiales;Xanthobacteraceae;Pseudolabrys;Pseudolabrys taiwanensis;Pseudolabrys taiwanensis CC-BB4 |
| Ga0586324 2059459 1 891 | 0.00 | 0.00 | 0.00 | 0.00 | 0.00 | 0.00 | 0.00 | 515041.30 | 0.00 | 0.00 | 0.00 | 0.00 | KO:K01999 | branched-chain amino acid transport system substrate-binding protein | Bacteria;Actinobacteria;Actinomycetia;Frankiales;Frankiaceae;Frankia;Frankia elaeagni;Frankia elaeagni BMG5.12 |
| Ga0586324 2126576 36 881 | 0.00 | 0.00 | 0.00 | 0.00 | 0.00 | 4327300.00 | 0.00 | 988200.00 | 0.00 | 0.00 | 0.00 | 0.00 | KO:K01999 | branched-chain amino acid transport system substrate-binding protein | Bacteria;Proteobacteria;Deltaproteobacteria;Syntrophobacterales;Syntrophaceae;Desulfobacca;Desulfobacca acetoxidans;Desulfobacca acetoxidans ASRB2, DSM 11109 |
| Ga0586324 2218808 146 865 | 0.00 | 0.00 | 0.00 | 0.00 | 0.00 | 0.00 | 0.00 | 0.00 | 32400000.00 | 0.00 | 0.00 | 0.00 | KO:K01999 | branched-chain amino acid transport system substrate-binding protein | Bacteria;Proteobacteria;Alphaproteobacteria;Rhodospirillales;Rhodospirillaceae;Dongia;Dongia sp. URHE0060;Dongia sp. URHE0060 |
| Ga0586324 2254755 1 858 | 0.00 | 0.00 | 0.00 | 0.00 | 0.00 | 0.00 | 0.00 | 1634800.00 | 0.00 | 2664200.00 | 0.00 | 0.00 | KO:K01999 | branched-chain amino acid transport system substrate-binding protein | Bacteria;Chloroflexi;Chloroflexia;Kallotenuales;Kallotenuaceae;Kallotenue;Kallotenue papyrolyticum;Kallotenue papyrolyticum JKG1 |
| Ga0586324 2265438 2 721 | 3920000.00 | 7677200.00 | 9521014.05 | 411958.95 | 0.00 | 1352368.59 | 3260400.00 | 1177300.00 | 1287900.00 | 0.00 | 0.00 | 3037500.00 | KO:K01999 | branched-chain amino acid transport system substrate-binding protein | Bacteria;Chloroflexi;Anaerolineae;Anaerolineales;Anaerolineaceae;Thermanaerothrix;Thermanaerothrix daxensis;Thermanaerothrix daxensis GNS-1 |
| Ga0586324 2327196 1 846 | 0.00 | 0.00 | 0.00 | 0.00 | 0.00 | 0.00 | 0.00 | 1549400.00 | 0.00 | 0.00 | 0.00 | 0.00 | KO:K01999 | branched-chain amino acid transport system substrate-binding protein | Bacteria;Proteobacteria;Alphaproteobacteria;Rhodospirillales;Rhodospirillaceae;Dongia;Dongia mobilis;Dongia mobilis CGMCC 1.7660 |
| Ga0586324 2333501 2 820 | 0.00 | 0.00 | 0.00 | 0.00 | 3594700.00 | 0.00 | 0.00 | 3489200.00 | 5354100.00 | 8778000.00 | 6996000.00 | 4567500.00 | KO:K01999 | branched-chain amino acid transport system substrate-binding protein | Bacteria;Actinobacteria;Thermoleophilia;Solirubrobacterales;unclassified Solirubrobacterales;unclassified Solirubrobacterales;Solirubrobacterales bacterium URHD0059;Solirubrobacterales bacterium URHD0059 |
| Ga0586324 2420736 2 832 | 0.00 | 3277600.00 | 0.00 | 0.00 | 0.00 | 0.00 | 0.00 | 0.00 | 0.00 | 0.00 | 0.00 | 2865000.00 | KO:K01999 | branched-chain amino acid transport system substrate-binding protein | Bacteria;Cyanobacteria;unclassified Cyanobacteria;Synechococcales;Merismopediaceae;Merismopedia;Merismopedia glauca;Merismopedia glauca CCAP 1448/3 |
| Ga0586324 2497281 3 821 | 6940000.00 | 0.00 | 0.00 | 0.00 | 0.00 | 0.00 | 0.00 | 0.00 | 0.00 | 0.00 | 0.00 | 0.00 | KO:K01999 | branched-chain amino acid transport system substrate-binding protein | Bacteria;Proteobacteria;Betaproteobacteria;Burkholderiales;Burkholderiaceae;Paraburkholderia;Paraburkholderia terricola;Paraburkholderia terricola LMG 20594 |
| Ga0586324 2539154 1 816 | 0.00 | 0.00 | 0.00 | 0.00 | 0.00 | 0.00 | 0.00 | 0.00 | 0.00 | 9240000.00 | 10120000.00 | 0.00 | KO:K01999 | branched-chain amino acid transport system substrate-binding protein | Bacteria;Spirochaetes;Spirochaetia;Spirochaetales;Spirochaetaceae;Alkalispirochaeta;Alkalispirochaeta odontotermitis;Alkalispirochaeta odontotermitis JC202 |
| Ga0586324 2886093 106 774 | 0.00 | 0.00 | 0.00 | 0.00 | 0.00 | 0.00 | 0.00 | 0.00 | 0.00 | 0.00 | 4127200.00 | 0.00 | KO:K01999 | branched-chain amino acid transport system substrate-binding protein | Bacteria;Proteobacteria;Deltaproteobacteria;Syntrophobacterales;Syntrophobacteraceae;Desulfacinum;Desulfacinum infernum;Desulfacinum infernum DSM 9756 |
| Ga0586324 3122252 149 748 | 0.00 | 0.00 | 0.00 | 0.00 | 0.00 | 0.00 | 2827200.00 | 0.00 | 0.00 | 0.00 | 0.00 | 0.00 | KO:K01999 | branched-chain amino acid transport system substrate-binding protein | Bacteria;Proteobacteria;Alphaproteobacteria;Hyphomicrobiales;Bradyrhizobiaceae;Bradyrhizobium;Bradyrhizobium erythrophlei;Bradyrhizobium erythrophlei GAS478 |

| | | | | | | | | | | | | | | | |
|---|---|---|---|---|---|---|---|---|---|---|---|---|---|---|---|
| Ga0586324 3127526 3 455 | 0.00 | 0.00 | 0.00 | 0.00 | 0.00 | 0.00 | 1421575.44 | 0.00 | 0.00 | 2171400.00 | 0.00 | 0.00 | KO:K01999 | branched-chain amino acid transport system substrate-binding protein | Bacteria;Chloroflexi;Chloroflexia;Kallotenuales;Kallotenuaceae;Kallotenue;Kallotenue papyrolyticum;Kallotenue papyrolyticum JKG1 |
| Ga0586324 3193123 120 743 | 9920000.00 | 0.00 | 0.00 | 0.00 | 7302700.00 | 8813400.00 | 0.00 | 6771000.00 | 6682500.00 | 0.00 | 0.00 | 11550000.00 | KO:K01999 | branched-chain amino acid transport system substrate-binding protein | Bacteria;Proteobacteria;Deltaproteobacteria;Desulfobacterales;Desulfobacteraceae;Desulfosarcina;Desulfosarcina variabilis;Desulfosarcina variabilis Montpellier |
| Ga0586324 3194836 1 684 | 0.00 | 0.00 | 0.00 | 0.00 | 0.00 | 0.00 | 0.00 | 0.00 | 6147900.00 | 0.00 | 0.00 | 0.00 | KO:K01999 | branched-chain amino acid transport system substrate-binding protein | Bacteria;Proteobacteria;Betaproteobacteria;Burkholderiales;Oxalobacteraceae;Herbaspirillum;Herbaspirillum robiniae;Herbaspirillum robiniae HZ10 |
| Ga0586324 3297303 1 732 | 8290000.00 | 6453200.00 | 0.00 | 0.00 | 1318400.00 | 0.00 | 0.00 | 0.00 | 0.00 | 0.00 | 5922400.00 | 5610000.00 | KO:K01999 | branched-chain amino acid transport system substrate-binding protein | Bacteria;Proteobacteria;Betaproteobacteria;unclassified Betaproteobacteria;unclassified Betaproteobacteria;unclassified Betaproteobacteria;Betaproteobacteria bacterium GR16-43;Betaproteobacteria bacterium GR16-43 |
| Ga0586324 3347053 3 728 | 1130000.00 | 0.00 | 0.00 | 0.00 | 0.00 | 0.00 | 0.00 | 0.00 | 0.00 | 0.00 | 0.00 | 0.00 | KO:K01999 | branched-chain amino acid transport system substrate-binding protein | Bacteria;Spirochaetes;Spirochaetia;Spirochaetales;Treponemataceae;Treponema;Treponema primitia;Treponema primitia ZAS-2 |
| Ga0586324 3446255 2 718 | 0.00 | 2108000.00 | 0.00 | 0.00 | 0.00 | 0.00 | 0.00 | 0.00 | 0.00 | 0.00 | 0.00 | 440502.00 | KO:K01999 | branched-chain amino acid transport system substrate-binding protein | Bacteria;Proteobacteria;Deltaproteobacteria;Syntrophobacterales;Syntrophobacteraceae;Syntrophobacter;Syntrophobacter fumaroxidans;Syntrophobacter fumaroxidans MPOB |
| Ga0586324 3660172 42 701 | 0.00 | 2067200.00 | 0.00 | 0.00 | 1133000.00 | 0.00 | 0.00 | 0.00 | 0.00 | 0.00 | 0.00 | 0.00 | KO:K01999 | branched-chain amino acid transport system substrate-binding protein | Bacteria;Proteobacteria;Betaproteobacteria;Burkholderiales;unclassified Burkholderiales;Ideonella;Ideonella sp. A 288;Ideonella sp. A 288 |
| Ga0586324 3696861 3 404 | 0.00 | 0.00 | 0.00 | 0.00 | 0.00 | 0.00 | 0.00 | 0.00 | 1854900.00 | 0.00 | 0.00 | 0.00 | KO:K01999 | branched-chain amino acid transport system substrate-binding protein | Bacteria;Proteobacteria;Deltaproteobacteria;Syntrophobacterales;Syntrophobacteraceae;Desulfosoma;Desulfosoma caldarium;Desulfosoma caldarium DSM 22027 |
| Ga0586324 3721274 3 695 | 0.00 | 0.00 | 0.00 | 0.00 | 0.00 | 5637400.00 | 0.00 | 0.00 | 0.00 | 0.00 | 0.00 | 0.00 | KO:K01999 | branched-chain amino acid transport system substrate-binding protein | Bacteria;Proteobacteria;Deltaproteobacteria;Syntrophobacterales;Syntrophobacteraceae;Syntrophobacter;Syntrophobacter fumaroxidans;Syntrophobacter fumaroxidans MPOB |
| Ga0586324 3805167 2 691 | 0.00 | 0.00 | 0.00 | 0.00 | 0.00 | 0.00 | 0.00 | 0.00 | 0.00 | 0.00 | 0.00 | 495897.00 | KO:K01999 | branched-chain amino acid transport system substrate-binding protein | Bacteria;Actinobacteria;Actinomycetia;Streptomycetales;Streptomycetaceae;Streptomyces;Streptomyces sp. QL37;Streptomyces sp. QL37 |
| Ga0586324 3822931 3 689 | 0.00 | 379045.60 | 1874643.62 | 0.00 | 0.00 | 0.00 | 2302800.00 | 1952000.00 | 0.00 | 0.00 | 0.00 | 0.00 | KO:K01999 | branched-chain amino acid transport system substrate-binding protein | Bacteria;Proteobacteria;Deltaproteobacteria;Syntrophobacterales;Syntrophaceae;Smithella;Smithella sp. F21;Smithella sp. F21 |
| Ga0586324 3842650 1 687 | 0.00 | 6228800.00 | 0.00 | 9880000.00 | 9228800.00 | 0.00 | 3944400.00 | 0.00 | 6245100.00 | 0.00 | 7321600.00 | 7072500.00 | KO:K01999 | branched-chain amino acid transport system substrate-binding protein | Bacteria;Proteobacteria;Alphaproteobacteria;Hyphomicrobiales;Roseiarcaceae;Roseiarcus;Roseiarcus fermentans;Roseiarcus fermentans DSM 24875 |
| Ga0586324 3889230 3 683 | 5500000.00 | 0.00 | 0.00 | 0.00 | 0.00 | 0.00 | 0.00 | 0.00 | 0.00 | 0.00 | 0.00 | 0.00 | KO:K01999 | branched-chain amino acid transport system substrate-binding protein | Bacteria;Proteobacteria;Deltaproteobacteria;Syntrophobacterales;Syntrophaceae;Desulfomonile;Desulfomonile tiedjei;Desulfomonile tiedjei DCB-1, DSM 6799 |
| Ga0586324 3970555 1 678 | 14100000.00 | 14293600.00 | 0.00 | 8702000.00 | 0.00 | 5955000.00 | 1608715.56 | 6161000.00 | 6182341.20 | 3472700.00 | 1953600.00 | 2760000.00 | KO:K01999 | branched-chain amino acid transport system substrate-binding protein | Bacteria;Chloroflexi;Anaerolineae;Anaerolineales;Anaerolineaceae;Thermanaerothrix;Thermanaerothrix daxensis;Thermanaerothrix daxensis GNS-1 |
| Ga0586324 3996975 2 676 | 2880000.00 | 3440800.00 | 0.00 | 4987500.00 | 0.00 | 0.00 | 0.00 | 5331400.00 | 4212000.00 | 0.00 | 0.00 | 0.00 | KO:K01999 | branched-chain amino acid transport system substrate-binding protein | Bacteria;Chloroflexi;Anaerolineae;Anaerolineales;Anaerolineaceae;Levilinea;Levilinea saccharolytica;Levilinea saccharolytica DSM 16555 |
| Ga0586324 4106299 3 653 | 0.00 | 1795200.00 | 0.00 | 0.00 | 0.00 | 0.00 | 1889296.92 | 7930000.00 | 1344600.00 | 10703000.00 | 16896000.00 | 2895000.00 | KO:K01999 | branched-chain amino acid transport system substrate-binding protein | Bacteria;Proteobacteria;Alphaproteobacteria;Hyphomicrobiales;Methylobacteriaceae;Methylobacterium;Methylobacterium crusticola;Methylobacterium crusticola MIMD6 |
| Ga0586324 4132245 1 666 | 0.00 | 0.00 | 0.00 | 0.00 | 0.00 | 0.00 | 0.00 | 0.00 | 1992600.00 | 0.00 | 0.00 | 0.00 | KO:K01999 | branched-chain amino acid transport system substrate-binding protein | Bacteria;Proteobacteria;Alphaproteobacteria;Hyphomicrobiales;unclassified Hyphomicrobiales;Pseudorhodoplanes;Pseudorhodoplanes sinuspersici;Pseudorhodoplanes sinuspersici RIPI110 |
| Ga0586324 4142061 2 667 | 0.00 | 0.00 | 0.00 | 0.00 | 2482300.00 | 0.00 | 12859200.00 | 0.00 | 0.00 | 0.00 | 0.00 | 0.00 | KO:K01999 | branched-chain amino acid transport system substrate-binding protein | Bacteria;Proteobacteria;Deltaproteobacteria;Syntrophobacterales;Syntrophobacteraceae;Desulfacinum;Desulfacinum infernum;Desulfacinum infernum DSM 9756 |
| Ga0586324 4157606 1 666 | 0.00 | 0.00 | 0.00 | 0.00 | 11742000.00 | 0.00 | 0.00 | 0.00 | 0.00 | 0.00 | 6890400.00 | 8475000.00 | KO:K01999 | branched-chain amino acid transport system substrate-binding protein | Bacteria;Proteobacteria;Alphaproteobacteria;Hyphomicrobiales;Bradyrhizobiaceae;Bradyrhizobium;Bradyrhizobium erythrophlei;Bradyrhizobium erythrophlei GAS242 |
| Ga0586324 4269433 2 658 | 0.00 | 6555200.00 | 0.00 | 3287000.00 | 0.00 | 0.00 | 2394000.00 | 0.00 | 4608900.00 | 0.00 | 0.00 | 0.00 | KO:K01999 | branched-chain amino acid transport system substrate-binding protein | Bacteria;Chloroflexi;Anaerolineae;Anaerolineales;Anaerolineaceae;Anaerolinea;Anaerolinea thermophila;Anaerolinea thermophila UNI-1 |
| Ga0586324 4535180 33 641 | 0.00 | 0.00 | 0.00 | 0.00 | 0.00 | 0.00 | 3853200.00 | 0.00 | 0.00 | 0.00 | 0.00 | 0.00 | KO:K01999 | branched-chain amino acid transport system substrate-binding protein | Bacteria;Proteobacteria;Alphaproteobacteria;Hyphomicrobiales;Bradyrhizobiaceae;Bradyrhizobium;Bradyrhizobium jicamae;Bradyrhizobium jicamae PAC68 |
| Ga0586324 4537252 1 642 | 0.00 | 0.00 | 0.00 | 0.00 | 0.00 | 0.00 | 0.00 | 0.00 | 0.00 | 0.00 | 0.00 | 1365000.00 | KO:K01999 | branched-chain amino acid transport system substrate-binding protein | Bacteria;Proteobacteria;Betaproteobacteria;Nitrosomonadales;Sulfuricellaceae;Sulfuriferula;Sulfuriferula multivorans;Sulfuriferula multivorans TTN |
| Ga0586324 4647427 1 636 | 0.00 | 0.00 | 0.00 | 2669500.00 | 0.00 | 0.00 | 0.00 | 0.00 | 0.00 | 0.00 | 0.00 | 0.00 | KO:K01999 | branched-chain amino acid transport system substrate-binding protein | Bacteria;Proteobacteria;Deltaproteobacteria;Syntrophobacterales;Syntrophobacteraceae;Desulfacinum;Desulfacinum infernum;Desulfacinum infernum DSM 9756 |
| Ga0586324 4724334 2 631 | 0.00 | 1414400.00 | 0.00 | 0.00 | 0.00 | 0.00 | 0.00 | 0.00 | 0.00 | 0.00 | 0.00 | 0.00 | KO:K01999 | branched-chain amino acid transport system substrate-binding protein | Bacteria;Proteobacteria;Deltaproteobacteria;Syntrophobacterales;Syntrophobacteraceae;Syntrophobacter;Syntrophobacter fumaroxidans;Syntrophobacter fumaroxidans MPOB |
| Ga0586324 4764405 3 629 | 0.00 | 775200.00 | 0.00 | 0.00 | 0.00 | 0.00 | 0.00 | 0.00 | 0.00 | 0.00 | 0.00 | 0.00 | KO:K01999 | branched-chain amino acid transport system substrate-binding protein | Bacteria;Proteobacteria;Gammaproteobacteria;unclassified Gammaproteobacteria;Candidatus Competibacteraceae;Candidatus Contendobacter;Candidatus Contendobacter odensis;Candidatus Contendobacter odensis Run B J11 |
| Ga0586324 4798595 2 370 | 0.00 | 0.00 | 12554900.00 | 0.00 | 0.00 | 0.00 | 0.00 | 8723000.00 | 0.00 | 0.00 | 0.00 | 0.00 | KO:K01999 | branched-chain amino acid transport system substrate-binding protein | Bacteria;Chloroflexi;Anaerolineae;Anaerolineales;Anaerolineaceae;Thermanaerothrix;Thermanaerothrix daxensis;Thermanaerothrix daxensis GNS-1 |
| Ga0586324 4911705 2 622 | 0.00 | 0.00 | 0.00 | 0.00 | 0.00 | 0.00 | 0.00 | 0.00 | 0.00 | 854700.00 | 0.00 | 0.00 | KO:K01999 | branched-chain amino acid transport system substrate-binding protein | Bacteria;Proteobacteria;Alphaproteobacteria;Rhodospirillales;Rhodospirillaceae;Dongia;Dongia sp. URHE0060;Dongia sp. URHE0060 |
| Ga0586324 5421083 1 597 | 0.00 | 0.00 | 0.00 | 0.00 | 0.00 | 0.00 | 0.00 | 0.00 | 3936600.00 | 0.00 | 0.00 | 7200000.00 | KO:K01999 | branched-chain amino acid transport system substrate-binding protein | Bacteria;Cyanobacteria;unclassified Cyanobacteria;Nostocales;Calotrichaceae;Calothrix;Calothrix desertica;Calothrix desertica PCC 7102 |
| Ga0586324 5531583 3 590 | 0.00 | 0.00 | 2249607.25 | 3534000.00 | 0.00 | 5002200.00 | 0.00 | 3678300.00 | 0.00 | 0.00 | 0.00 | 0.00 | KO:K01999 | branched-chain amino acid transport system substrate-binding protein | Bacteria;Chloroflexi;Anaerolineae;Anaerolineales;Anaerolineaceae;Thermanaerothrix;Thermanaerothrix daxensis;Thermanaerothrix daxensis GNS-1 |
| Ga0586324 5598731 1 339 | 0.00 | 0.00 | 0.00 | 0.00 | 0.00 | 0.00 | 1279415.16 | 1891000.00 | 2211300.00 | 1232000.00 | 0.00 | 3165000.00 | KO:K01999 | branched-chain amino acid transport system substrate-binding protein | Bacteria;Proteobacteria;Betaproteobacteria;Burkholderiales;Alcaligenaceae;Candidimonas;Candidimonas bauzanensis;Candidimonas bauzanensis CGMCC 1.10190 |
| Ga0586324 5627504 2 586 | 250476.00 | 1122000.00 | 0.00 | 900312.15 | 0.00 | 0.00 | 1036433.28 | 526968.63 | 13608000.00 | 0.00 | 0.00 | 0.00 | KO:K01999 | branched-chain amino acid transport system substrate-binding protein | Bacteria;Chloroflexi;Anaerolineae;Anaerolineales;Anaerolineaceae;Thermanaerothrix;Thermanaerothrix daxensis;Thermanaerothrix daxensis GNS-1 |
| Ga0586324 5634709 3 587 | 0.00 | 1380400.00 | 0.00 | 0.00 | 0.00 | 0.00 | 0.00 | 1329800.00 | 1628100.00 | 0.00 | 0.00 | 0.00 | KO:K01999 | branched-chain amino acid transport system substrate-binding protein | Bacteria;Spirochaetes;Spirochaetia;Spirochaetales;Spirochaetaceae;Alkalispirochaeta;Alkalispirochaeta odontotermitis;Alkalispirochaeta odontotermitis JC202 |
| Ga0586324 5748569 3 581 | 0.00 | 0.00 | 0.00 | 0.00 | 0.00 | 0.00 | 6156000.00 | 0.00 | 0.00 | 0.00 | 0.00 | 2212500.00 | KO:K01999 | branched-chain amino acid transport system substrate-binding protein | Bacteria;Proteobacteria;Deltaproteobacteria;Syntrophobacterales;Syntrophaceae;Desulfobacca;Desulfobacca acetoxidans;Desulfobacca acetoxidans ASRB2, DSM 11109 |
| Ga0586324 5934463 1 576 | 0.00 | 0.00 | 0.00 | 0.00 | 0.00 | 0.00 | 0.00 | 0.00 | 4001400.00 | 0.00 | 0.00 | 0.00 | KO:K01999 | branched-chain amino acid transport system substrate-binding protein | Bacteria;Proteobacteria;Deltaproteobacteria;Desulfobacterales;Desulfobacteraceae;Desulfosarcina;Desulfosarcina variabilis;Desulfosarcina variabilis Montpellier |
| Ga0586324 6011290 1 573 | 0.00 | 0.00 | 12160800.00 | 12065000.00 | 0.00 | 0.00 | 14774400.00 | 0.00 | 0.00 | 0.00 | 0.00 | 0.00 | KO:K01999 | branched-chain amino acid transport system substrate-binding protein | Bacteria;Proteobacteria;Alphaproteobacteria;Hyphomicrobiales;Roseiarcaceae;Roseiarcus;Roseiarcus fermentans;Roseiarcus fermentans DSM 24875 |
| Ga0586324 6019969 3 572 | 0.00 | 0.00 | 0.00 | 0.00 | 0.00 | 0.00 | 0.00 | 0.00 | 0.00 | 0.00 | 295590.24 | 2655000.00 | KO:K01999 | branched-chain amino acid transport system substrate-binding protein | Bacteria;Proteobacteria;Deltaproteobacteria;Desulfobacterales;Desulfobacteraceae;Desulfatiglans;Desulfatiglans anilini;Desulfatiglans anilini DSM 4660 |
| Ga0586324 6113169 1 567 | 0.00 | 3624400.00 | 0.00 | 0.00 | 0.00 | 0.00 | 0.00 | 0.00 | 0.00 | 0.00 | 0.00 | 0.00 | KO:K01999 | branched-chain amino acid transport system substrate-binding protein | Bacteria;Cyanobacteria;unclassified Cyanobacteria;Oscillatoriales;Microcoleaceae;Microcoleus;Microcoleus sp. PCC 7113;Microcoleus sp. PCC 7113 |
| Ga0586324 6366183 1 558 | 0.00 | 2570400.00 | 0.00 | 0.00 | 0.00 | 0.00 | 0.00 | 0.00 | 0.00 | 0.00 | 1918400.00 | 0.00 | KO:K01999 | branched-chain amino acid transport system substrate-binding protein | Bacteria;Chloroflexi;Chloroflexia;Kallotenuales;Kallotenuaceae;Kallotenue;Kallotenue papyrolyticum;Kallotenue papyrolyticum JKG1 |
| Ga0586324 6572246 1 552 | 0.00 | 553772.96 | 0.00 | 0.00 | 0.00 | 0.00 | 0.00 | 628300.00 | 0.00 | 0.00 | 0.00 | 440794.50 | KO:K01999 | branched-chain amino acid transport system substrate-binding protein | Bacteria;Proteobacteria;Deltaproteobacteria;Desulfuromonadales;Geobacteraceae;Geobacter;Geobacter argillaceus;Geobacter argillaceus ATCC BAA-1139 |
| Ga0586324 6590820 3 551 | 0.00 | 0.00 | 0.00 | 0.00 | 0.00 | 0.00 | 0.00 | 0.00 | 0.00 | 0.00 | 6036800.00 | 0.00 | KO:K01999 | branched-chain amino acid transport system substrate-binding protein | Bacteria;Chloroflexi;Anaerolineae;Anaerolineales;Anaerolineaceae;Thermanaerothrix;Thermanaerothrix daxensis;Thermanaerothrix daxensis GNS-1 |
| Ga0586324 6619265 1 549 | 0.00 | 0.00 | 0.00 | 0.00 | 0.00 | 0.00 | 0.00 | 2446100.00 | 0.00 | 10626000.00 | 0.00 | 11775000.00 | KO:K01999 | branched-chain amino acid transport system substrate-binding protein | Bacteria;Chloroflexi;Anaerolineae;Anaerolineales;Anaerolineaceae;Thermanaerothrix;Thermanaerothrix daxensis;Thermanaerothrix daxensis GNS-1 |
| Ga0586324 6834269 1 348 | 0.00 | 0.00 | 5686300.00 | 0.00 | 0.00 | 0.00 | 0.00 | 0.00 | 0.00 | 0.00 | 0.00 | 0.00 | KO:K01999 | branched-chain amino acid transport system substrate-binding protein | Bacteria;Proteobacteria;Deltaproteobacteria;Syntrophobacterales;Syntrophobacteraceae;Desulfacinum;Desulfacinum infernum;Desulfacinum infernum DSM 9756 |
| Ga0586324 6926063 3 539 | 0.00 | 0.00 | 0.00 | 0.00 | 0.00 | 0.00 | 0.00 | 0.00 | 0.00 | 0.00 | 0.00 | 1087500.00 | KO:K01999 | branched-chain amino acid transport system substrate-binding protein | Bacteria;Actinobacteria;Thermoleophilia;Solirubrobacterales;unclassified Solirubrobacterales;unclassified Solirubrobacterales;Solirubrobacterales bacterium URHD0059;Solirubrobacterales bacterium URHD0059 |
| Ga0586324 6934991 1 540 | 4410000.00 | 0.00 | 0.00 | 0.00 | 30282000.00 | 0.00 | 0.00 | 0.00 | 14661000.00 | 0.00 | 0.00 | 0.00 | KO:K01999 | branched-chain amino acid transport system substrate-binding protein | Bacteria;Chloroflexi;Anaerolineae;Anaerolineales;Anaerolineaceae;Thermanaerothrix;Thermanaerothrix daxensis;Thermanaerothrix daxensis GNS-1 |
| Ga0586324 7270410 2 529 | 0.00 | 0.00 | 0.00 | 0.00 | 0.00 | 3890290.34 | 0.00 | 0.00 | 0.00 | 0.00 | 0.00 | 0.00 | KO:K01999 | branched-chain amino acid transport system substrate-binding protein | Bacteria;Proteobacteria;Deltaproteobacteria;Desulfobacterales;Desulfobacteraceae;Desulfosarcina;Desulfosarcina variabilis;Desulfosarcina variabilis Montpellier |
| Ga0586324 7410547 2 523 | 0.00 | 0.00 | 13286800.00 | 0.00 | 0.00 | 0.00 | 0.00 | 0.00 | 0.00 | 0.00 | 0.00 | 0.00 | KO:K01999 | branched-chain amino acid transport system substrate-binding protein | Bacteria;Proteobacteria;Deltaproteobacteria;Desulfobacterales;Desulfobacteraceae;Desulfosalsimonas;Desulfosalsimonas propionicica;Desulfosalsimonas propionicica DSM 17721 |
| Ga0586324 7658989 1 516 | 2940000.00 | 284199.88 | 0.00 | 492873.30 | 30282000.00 | 17388600.00 | 831224.16 | 847900.00 | 0.00 | 0.00 | 0.00 | 0.00 | KO:K01999 | branched-chain amino acid transport system substrate-binding protein | Bacteria;Chloroflexi;Anaerolineae;Anaerolineales;Anaerolineaceae;Thermanaerothrix;Thermanaerothrix daxensis;Thermanaerothrix daxensis GNS-1 |
| Ga0586324 7847692 1 399 | 0.00 | 0.00 | 0.00 | 0.00 | 0.00 | 0.00 | 0.00 | 1586000.00 | 0.00 | 0.00 | 0.00 | 0.00 | KO:K01999 | branched-chain amino acid transport system substrate-binding protein | Bacteria;Proteobacteria;Alphaproteobacteria;Rhodospirillales;Geminicoccaceae;Geminicoccus;Geminicoccus roseus;Geminicoccus roseus DSM 18922 |
| Ga0586324 8036773 59 505 | 0.00 | 0.00 | 0.00 | 0.00 | 0.00 | 0.00 | 0.00 | 0.00 | 0.00 | 870100.00 | 857258.16 | 0.00 | KO:K01999 | branched-chain amino acid transport system substrate-binding protein | Bacteria;Proteobacteria;Betaproteobacteria;Burkholderiales;Oxalobacteraceae;Massilia;Massilia violacea;Massilia violacea CECT 8897 |
| Ga0586324 8233062 2 502 | 0.00 | 4474400.00 | 0.00 | 0.00 | 0.00 | 0.00 | 0.00 | 0.00 | 2462400.00 | 0.00 | 0.00 | 2475000.00 | KO:K01999 | branched-chain amino acid transport system substrate-binding protein | Bacteria;Proteobacteria;Deltaproteobacteria;Syntrophobacterales;Syntrophobacteraceae;Thermodesulforhabdus;Thermodesulforhabdus norvegica;Thermodesulforhabdus norvegica DSM 9990 |
| Ga0586324 0517839 89 1072 | 0.00 | 0.00 | 0.00 | 0.00 | 0.00 | 0.00 | 0.00 | 0.00 | 0.00 | 12320000.00 | 0.00 | 0.00 | KO:K02002 | glycine betaine/proline transport system substrate-binding protein | Bacteria;Actinobacteria;Rubrobacteria;Gaiellales;Gaiellaceae;Gaiella;Gaiella occulta;Gaiella occulta F2-233 |
| Ga0586324 0013583 2452 3204 | 0.00 | 0.00 | 0.00 | 0.00 | 0.00 | 0.00 | 0.00 | 0.00 | 0.00 | 0.00 | 0.00 | 30975000.00 | KO:K02003 | putative ABC transport system ATP-binding protein | Bacteria;Acidobacteria;Acidobacteriia;Acidobacteriales;Acidobacteriaceae;Silvibacterium;Silvibacterium bohemicum;Acidobacteriaceae bacterium S15 |
| Ga0586324 0059739 226 963 | 0.00 | 0.00 | 0.00 | 0.00 | 0.00 | 0.00 | 0.00 | 0.00 | 747181.26 | 0.00 | 0.00 | 0.00 | KO:K02003 | putative ABC transport system ATP-binding protein | Bacteria;Acidobacteria;Blastocatellia;Blastocatellales;Pyrinomonadaceae;Pyrinomonas;Pyrinomonas methylaliphatogenes;Pyrinomonas methylaliphatogenes K22 |
| Ga0586324 0719113 17 718 | 7300000.00 | 0.00 | 0.00 | 0.00 | 0.00 | 0.00 | 0.00 | 0.00 | 0.00 | 0.00 | 0.00 | 0.00 | KO:K02003 | putative ABC transport system ATP-binding protein | Bacteria;Bacteroidetes;Flavobacteriia;Flavobacteriales;Flavobacteriaceae;Gramella;Gramella fulva;Gramella fulva SH35 |
| Ga0586324 0034208 171 2795 | 0.00 | 0.00 | 0.00 | 0.00 | 0.00 | 1390623.51 | 0.00 | 0.00 | 0.00 | 0.00 | 0.00 | 0.00 | KO:K02004 | putative ABC transport system permease protein | Bacteria;Gemmatimonadetes;Gemmatimonadetes;Gemmatimonadales;Gemmatimonadaceae;Gemmatirosa;Gemmatirosa kalamazoonesis;Gemmatimonadetes bacterium KBS708 |
| Ga0586324 1518127 3 860 | 0.00 | 0.00 | 0.00 | 0.00 | 0.00 | 0.00 | 0.00 | 0.00 | 0.00 | 0.00 | 0.00 | 679045.50 | KO:K02005 | HlyD family secretion protein | Bacteria;Bacteroidetes;Bacteroidia;Marinilabiliales;Prolixibacteraceae;Draconibacterium;Draconibacterium orientale;Draconibacterium orientale FH5 |
| Ga0586324 7594958 2 520 | 0.00 | 0.00 | 0.00 | 0.00 | 0.00 | 6590200.00 | 0.00 | 0.00 | 0.00 | 2841300.00 | 2569600.00 | 0.00 | KO:K02005 | HlyD family secretion protein | Bacteria;Bacteroidetes;Flavobacteriia;Flavobacteriales;Schleiferiaceae;Owenweeksia;Owenweeksia hongkongensis;Owenweeksia hongkongensis DSM 17368 |
| Ga0586324 1756950 30 953 | 0.00 | 8160000.00 | 0.00 | 0.00 | 0.00 | 0.00 | 0.00 | 0.00 | 0.00 | 0.00 | 0.00 | 0.00 | KO:K02010 | iron(III) transport system ATP-binding protein | Bacteria;Proteobacteria;Deltaproteobacteria;Syntrophobacterales;Syntrophobacteraceae;Syntrophobacter;Syntrophobacter fumaroxidans;Syntrophobacter fumaroxidans MPOB |
| Ga0586324 0027290 173 1189 | 0.00 | 0.00 | 0.00 | 0.00 | 0.00 | 0.00 | 0.00 | 0.00 | 1117800.00 | 0.00 | 0.00 | 0.00 | KO:K02012 | iron(III) transport system substrate-binding protein | Bacteria;Firmicutes;Bacilli;Bacillales;Bacillaceae;Salirhabdus;Salirhabdus euzebyi;Salirhabdus euzebyi DSM 19612 |
| Ga0586324 0440390 150 1160 | 0.00 | 0.00 | 0.00 | 0.00 | 2317500.00 | 0.00 | 0.00 | 0.00 | 0.00 | 0.00 | 0.00 | 0.00 | KO:K02012 | iron(III) transport system substrate-binding protein | Bacteria;Proteobacteria;Deltaproteobacteria;Desulfobacterales;Desulfobacteraceae;Desulfobotulus;Desulfobotulus alkaliphilus;Desulfobotulus alkaliphilus ASO4-4 |
| Ga0586324 0002704 3 2534 | 0.00 | 0.00 | 0.00 | 0.00 | 0.00 | 474677.02 | 0.00 | 0.00 | 0.00 | 1054900.00 | 0.00 | 0.00 | KO:K02014 | iron complex outermembrane receptor protein | Bacteria;Proteobacteria;Alphaproteobacteria;Sphingomonadales;Erythrobacteraceae;Erythrobacter;Erythrobacter sp. SG61-1L;Erythrobacter sp. SG61-1L |
| Ga0586324 0002891 137 3106 | 5360000.00 | 1448400.00 | 0.00 | 0.00 | 1946700.00 | 5637400.00 | 0.00 | 2104500.00 | 0.00 | 3967823.86 | 5317324.32 | 3150000.00 | KO:K02014 | iron complex outermembrane receptor protein | Bacteria;Proteobacteria;Alphaproteobacteria;Sphingomonadales;Sphingomonadaceae;unclassified Sphingomonadaceae;unclassified Sphingomonadaceae;Sphingomonadaceae bacterium R-39594 |
| Ga0586324 0003622 10750 13386 | 0.00 | 0.00 | 0.00 | 0.00 | 0.00 | 0.00 | 0.00 | 0.00 | 0.00 | 0.00 | 6327200.00 | 0.00 | KO:K02014 | iron complex outermembrane receptor protein | Bacteria;Proteobacteria;Alphaproteobacteria;Sphingomonadales;Sphingomonadaceae;Sphingobium;Sphingobium sp. SYK-6;Sphingobium sp. SYK-6 |
| Ga0586324 0005583 2386 5343 | 20080645.00 | 12573200.00 | 38903300.00 | 14535000.00 | 15522100.00 | 11314500.00 | 6589200.00 | 15451300.00 | 17139600.00 | 12009807.04 | 20688800.00 | 15135000.00 | KO:K02014 | iron complex outermembrane receptor protein | Bacteria;Proteobacteria;Alphaproteobacteria;Sphingomonadales;Sphingomonadaceae;unclassified Sphingomonadaceae;unclassified Sphingomonadaceae;Sphingomonadaceae bacterium R-39594 |
| Ga0586324 0007783 5976 9671 | 1840000.00 | 0.00 | 0.00 | 0.00 | 0.00 | 0.00 | 0.00 | 0.00 | 0.00 | 0.00 | 0.00 | 0.00 | KO:K02014 | iron complex outermembrane receptor protein | Bacteria;Proteobacteria;Alphaproteobacteria;Sphingomonadales;Sphingomonadaceae;Sphingobium;Sphingobium sp. SYK-6;Sphingobium sp. SYK-6 |
| Ga0586324 0012541 7850 9220 | 0.00 | 0.00 | 0.00 | 0.00 | 20497000.00 | 33506800.00 | 0.00 | 19825000.00 | 0.00 | 0.00 | 0.00 | 0.00 | KO:K02014 | iron complex outermembrane receptor protein | Bacteria;Proteobacteria;Alphaproteobacteria;Caulobacterales;Caulobacteraceae;Phenylobacterium;Phenylobacterium sp. Root700;Phenylobacterium sp. Root700 |
| Ga0586324 0024366 3179 5590 | 0.00 | 0.00 | 0.00 | 0.00 | 0.00 | 0.00 | 0.00 | 0.00 | 0.00 | 0.00 | 671686.40 | 0.00 | KO:K02014 | iron complex outermembrane receptor protein | Bacteria;Proteobacteria;Gammaproteobacteria;Nevskiales;Steroidobacteraceae;Povalibacter;Povalibacter uvarum;Povalibacter uvarum DSM 26723 |
| Ga0586324 0024695 1234 3771 | 16450000.00 | 3032800.00 | 8557600.00 | 8455000.00 | 18952000.00 | 9964700.00 | 0.00 | 12742900.00 | 2988900.00 | 7931000.00 | 17644000.00 | 9315000.00 | KO:K02014 | iron complex outermembrane receptor protein | Bacteria;Proteobacteria;Alphaproteobacteria;Sphingomonadales;Sphingomonadaceae;Novosphingobium;Novosphingobium sp. B 225;Novosphingobium sp. B 225 B 225 |
| Ga0586324 0026501 255 3314 | 0.00 | 150162.36 | 0.00 | 3002000.00 | 0.00 | 0.00 | 0.00 | 0.00 | 0.00 | 0.00 | 0.00 | 0.00 | KO:K02014 | iron complex outermembrane receptor protein | Bacteria;Proteobacteria;Alphaproteobacteria;unclassified Alphaproteobacteria;unclassified Alphaproteobacteria;unclassified Alphaproteobacteria;alpha proteobacterium Q-1;Alpha proteobacterium Q-1 |
| Ga0586324 0029416 144 3044 | 5058848.00 | 7650000.00 | 2303711.55 | 10554500.00 | 14058549.31 | 10877800.00 | 16297918.80 | 13853100.00 | 8910000.00 | 14414400.00 | 10956000.00 | 9643459.50 | KO:K02014 | iron complex outermembrane receptor protein | Bacteria;Proteobacteria;Betaproteobacteria;Burkholderiales;Comamonadaceae;unclassified Comamonadaceae;Comamonadaceae bacterium URHA0028;Comamonadaceae bacterium URHA0028 |
| Ga0586324 0029718 3429 6158 | 0.00 | 904400.00 | 1917983.36 | 2888000.00 | 1720100.00 | 2557037.30 | 2207500.56 | 6222000.00 | 5022000.00 | 0.00 | 4435200.00 | 2175000.00 | KO:K02014 | iron complex outermembrane receptor protein | Bacteria;Proteobacteria;Gammaproteobacteria;Cellvibrionales;Cellvibrionaceae;unclassified Cellvibrionaceae;Cellvibrionaceae bacterium 1162T.S.0a.05;Cellvibrionaceae bacterium 1162T.S.0a.05 |
| Ga0586324 0035918 189 3239 | 477548.00 | 0.00 | 0.00 | 0.00 | 2430800.00 | 0.00 | 0.00 | 0.00 | 802137.33 | 267296.26 | 660277.20 | 0.00 | KO:K02014 | iron complex outermembrane receptor protein | Bacteria;Proteobacteria;Alphaproteobacteria;Hyphomonadales;Hyphomonadaceae;Henriciella;Henriciella barbarensis;Henriciella barbarensis CCUG 66934 |
| Ga0586324 0035918 3309 5624 | 0.00 | 0.00 | 0.00 | 0.00 | 0.00 | 1266088.58 | 0.00 | 0.00 | 0.00 | 1547700.00 | 0.00 | 1252500.00 | KO:K02014 | iron complex outermembrane receptor protein | Bacteria;Proteobacteria;Alphaproteobacteria;Sphingomonadales;Sphingomonadaceae;Sphingomonas;Sphingomonas sp. Root710;Sphingomonas sp. Root710 |
| Ga0586324 0042146 2262 5048 | 0.00 | 342159.00 | 0.00 | 0.00 | 2811900.00 | 0.00 | 0.00 | 7356600.00 | 1231200.00 | 1455300.00 | 0.00 | 0.00 | KO:K02014 | iron complex outermembrane receptor protein | Bacteria;Proteobacteria;Betaproteobacteria;Burkholderiales;Oxalobacteraceae;Collimonas;Collimonas sp. OK412;Collimonas sp. OK412 |
| Ga0586324 0043243 2132 5134 | 4780000.00 | 0.00 | 0.00 | 2204000.00 | 4490800.00 | 0.00 | 0.00 | 3995500.00 | 0.00 | 0.00 | 0.00 | 0.00 | KO:K02014 | iron complex outermembrane receptor protein | Bacteria;Proteobacteria;Alphaproteobacteria;Sphingomonadales;Sphingomonadaceae;Sphingopyxis;Sphingopyxis flava;Sphingopyxis flava R11H |
| Ga0586324 0049329 1372 4410 | 1623861.00 | 1026800.00 | 0.00 | 0.00 | 0.00 | 0.00 | 0.00 | 0.00 | 0.00 | 0.00 | 0.00 | 4185000.00 | KO:K02014 | iron complex outermembrane receptor protein | Bacteria;Proteobacteria;Betaproteobacteria;Burkholderiales;Oxalobacteraceae;Massilia;Massilia namucuonensis;Massilia namucuonensis CGMCC 1.11014 |
| Ga0586324 0052059 849 3599 | 55154884.00 | 95737200.00 | 30675021.22 | 70566000.00 | 106076209.33 | 25737498.09 | 35796000.00 | 121146000.00 | 154280700.00 | 240694300.00 | 227400800.00 | 105615000.00 | KO:K02014 | iron complex outermembrane receptor protein | Bacteria;Proteobacteria;Betaproteobacteria;Burkholderiales;unclassified Burkholderiales;Rhizobacter;Rhizobacter sp. Root16D2;Rhizobacter sp. Root16D2 |
| Ga0586324 0055574 2 2032 | 0.00 | 0.00 | 0.00 | 51775000.00 | 0.00 | 107984000.00 | 0.00 | 0.00 | 0.00 | 90860000.00 | 880000.00 | 97500000.00 | KO:K02014 | iron complex outermembrane receptor protein | Bacteria;Proteobacteria;Gammaproteobacteria;Xanthomonadales;Xanthomonadaceae;Pseudoxanthomonas;Pseudoxanthomonas suwonensis;Pseudoxanthomonas suwonensis J42 |
| Ga0586324 0075194 710 3673 | 0.00 | 0.00 | 0.00 | 0.00 | 0.00 | 0.00 | 0.00 | 0.00 | 0.00 | 1763300.00 | 0.00 | 0.00 | KO:K02014 | iron complex outermembrane receptor protein | Bacteria;Proteobacteria;Alphaproteobacteria;Sphingomonadales;Erythrobacteraceae;Altererythrobacter;Altererythrobacter sp. Root672;Altererythrobacter sp. Root672 |
| Ga0586324 0090931 2709 3590 | 0.00 | 0.00 | 0.00 | 0.00 | 919157.58 | 0.00 | 0.00 | 0.00 | 0.00 | 0.00 | 1645600.00 | 1297500.00 | KO:K02014 | iron complex outermembrane receptor protein | Bacteria;Proteobacteria;Gammaproteobacteria;Cellvibrionales;Spongiibacteraceae;unclassified Spongiibacteraceae;marine gamma proteobacterium HTCC2143;Marine gamma proteobacterium sp. HTCC2143 |
| Ga0586324 0098872 689 3448 | 0.00 | 0.00 | 0.00 | 6621500.00 | 4779200.00 | 5677100.00 | 0.00 | 0.00 | 6484615.38 | 0.00 | 0.00 | 0.00 | KO:K02014 | iron complex outermembrane receptor protein | Bacteria;Proteobacteria;Betaproteobacteria;Burkholderiales;unclassified Burkholderiales;Rhizobacter;Rhizobacter sp. OV335;Rhizobacter sp. OV335 |
| Ga0586324 0113532 1326 3227 | 0.00 | 2951200.00 | 0.00 | 12948500.00 | 9947851.24 | 5875600.00 | 28500000.00 | 9894200.00 | 6326100.00 | 1439900.00 | 0.00 | 1282500.00 | KO:K02014 | iron complex outermembrane receptor protein | Bacteria;Proteobacteria;Betaproteobacteria;Neisseriales;Chromobacteriaceae;Iodobacter;Iodobacter sp. H11R3;Iodobacter sp. H11R3 |
| Ga0586324 0136992 984 2954 | 0.00 | 0.00 | 46391200.00 | 10260000.00 | 0.00 | 16038800.00 | 0.00 | 0.00 | 0.00 | 0.00 | 140503.44 | 0.00 | KO:K02014 | iron complex outermembrane receptor protein | Bacteria;Proteobacteria;Betaproteobacteria;Burkholderiales;Oxalobacteraceae;Duganella;Duganella sp. CF458;Duganella sp. CF458 |
| Ga0586324 0141669 3 1694 | 0.00 | 0.00 | 0.00 | 0.00 | 0.00 | 0.00 | 0.00 | 223751.66 | 0.00 | 0.00 | 0.00 | 0.00 | KO:K02014 | iron complex outermembrane receptor protein | Bacteria;Proteobacteria;Betaproteobacteria;unclassified Betaproteobacteria;unclassified Betaproteobacteria;unclassified Betaproteobacteria;Betaproteobacteria bacterium GR16-43;Betaproteobacteria bacterium GR16-43 |
| Ga0586324 0152736 1802 2806 | 6330000.00 | 0.00 | 0.00 | 0.00 | 0.00 | 0.00 | 0.00 | 0.00 | 558973.71 | 247569.63 | 1249600.00 | 855000.00 | KO:K02014 | iron complex outermembrane receptor protein | Bacteria;Proteobacteria;Betaproteobacteria;Burkholderiales;Oxalobacteraceae;Undibacterium;Undibacterium pigrum;Undibacterium pigrum DSM 19792 |
| Ga0586324 0168909 1 2676 | 1924951.00 | 1924400.00 | 0.00 | 7182000.00 | 13472400.00 | 0.00 | 0.00 | 4758000.00 | 3879900.00 | 3873100.00 | 3132800.00 | 1290000.00 | KO:K02014 | iron complex outermembrane receptor protein | Bacteria;Proteobacteria;Betaproteobacteria;Burkholderiales;Comamonadaceae;Tibeticola;Tibeticola sediminis;Tibeticola sediminis DSM 101684 |
| Ga0586324 0199535 1 2478 | 0.00 | 0.00 | 0.00 | 0.00 | 0.00 | 0.00 | 0.00 | 0.00 | 0.00 | 0.00 | 2384800.00 | 1162500.00 | KO:K02014 | iron complex outermembrane receptor protein | Bacteria;Proteobacteria;Gammaproteobacteria;Cellvibrionales;Halieaceae;Halioglobus;Halioglobus sp. HI00S01;Halioglobus sp. HI00S01 |
| Ga0586324 0209486 1 1797 | 5420000.00 | 0.00 | 0.00 | 0.00 | 0.00 | 0.00 | 5152800.00 | 0.00 | 0.00 | 0.00 | 0.00 | 0.00 | KO:K02014 | iron complex outermembrane receptor protein | Bacteria;Proteobacteria;Gammaproteobacteria;Xanthomonadales;Xanthomonadaceae;Pseudoxanthomonas;Pseudoxanthomonas suwonensis;Pseudoxanthomonas suwonensis J42 |
| Ga0586324 0209895 279 2420 | 0.00 | 0.00 | 0.00 | 0.00 | 831274.89 | 0.00 | 0.00 | 0.00 | 0.00 | 0.00 | 0.00 | 0.00 | KO:K02014 | iron complex outermembrane receptor protein | Bacteria;Proteobacteria;Alphaproteobacteria;Sphingomonadales;Sphingomonadaceae;Novosphingobium;Novosphingobium sp. B 225;Novosphingobium sp. B 225 B 225 |
| Ga0586324 0242681 1089 2264 | 0.00 | 0.00 | 0.00 | 1710000.00 | 0.00 | 0.00 | 7729200.00 | 0.00 | 0.00 | 0.00 | 0.00 | 0.00 | KO:K02014 | iron complex outermembrane receptor protein | Bacteria;Proteobacteria;Betaproteobacteria;Burkholderiales;Oxalobacteraceae;Janthinobacterium;Janthinobacterium sp. CG3;Janthinobacterium sp. CG3 |
| Ga0586324 0264597 65 2176 | 528064.00 | 1523200.00 | 0.00 | 0.00 | 0.00 | 6113800.00 | 0.00 | 3531900.00 | 2430000.00 | 0.00 | 4153600.00 | 0.00 | KO:K02014 | iron complex outermembrane receptor protein | Bacteria;Proteobacteria;Betaproteobacteria;Burkholderiales;Comamonadaceae;unclassified Comamonadaceae;Comamonadaceae bacterium;Comamonadaceae sp. OAS658 |
| Ga0586324 0288755 2 2092 | 0.00 | 0.00 | 0.00 | 5443500.00 | 0.00 | 8614900.00 | 6543600.00 | 0.00 | 5589000.00 | 7522900.00 | 0.00 | 10875000.00 | KO:K02014 | iron complex outermembrane receptor protein | Bacteria;Proteobacteria;Betaproteobacteria;Burkholderiales;Oxalobacteraceae;Massilia;Massilia namucuonensis;Massilia namucuonensis CGMCC 1.11014 |
| Ga0586324 0306867 1 1893 | 9680000.00 | 5276800.00 | 0.00 | 3819000.00 | 1256600.00 | 6907800.00 | 1787980.56 | 6594100.00 | 9801000.00 | 0.00 | 0.00 | 1747500.00 | KO:K02014 | iron complex outermembrane receptor protein | Bacteria;Proteobacteria;Betaproteobacteria;Burkholderiales;Comamonadaceae;unclassified Comamonadaceae;Comamonadaceae bacterium URHA0028;Comamonadaceae bacterium URHA0028 |
| Ga0586324 0316530 1420 2007 | 0.00 | 0.00 | 0.00 | 0.00 | 0.00 | 0.00 | 0.00 | 0.00 | 882900.00 | 0.00 | 0.00 | 0.00 | KO:K02014 | iron complex outermembrane receptor protein | Bacteria;Proteobacteria;Betaproteobacteria;Burkholderiales;Oxalobacteraceae;Janthinobacterium;unclassified Janthinobacterium;Janthinobacterium sp. HH101 |
| Ga0586324 0319519 1 1506 | 0.00 | 0.00 | 0.00 | 1187500.00 | 0.00 | 0.00 | 0.00 | 0.00 | 0.00 | 0.00 | 0.00 | 0.00 | KO:K02014 | iron complex outermembrane receptor protein | Bacteria;Proteobacteria;Betaproteobacteria;Burkholderiales;Oxalobacteraceae;Collimonas;Collimonas sp. OK412;Collimonas sp. OK412 |
| Ga0586324 0338689 1 1014 | 7120000.00 | 5467200.00 | 0.00 | 5890000.00 | 3079700.00 | 4843400.00 | 0.00 | 5483900.00 | 5159700.00 | 8547000.00 | 7673600.00 | 5715000.00 | KO:K02014 | iron complex outermembrane receptor protein | Bacteria;Proteobacteria;Betaproteobacteria;Burkholderiales;Comamonadaceae;Ramlibacter;Ramlibacter tataouinensis;Ramlibacter tataouinensis 5-10 |
| Ga0586324 0493314 3 1649 | 6390000.00 | 9180000.00 | 806869.08 | 0.00 | 931959.45 | 1333304.65 | 0.00 | 1024800.00 | 842400.00 | 1686300.00 | 1531200.00 | 2242500.00 | KO:K02014 | iron complex outermembrane receptor protein | Bacteria;Proteobacteria;Alphaproteobacteria;Sphingomonadales;Erythrobacteraceae;Altererythrobacter;Altererythrobacter atlanticus;Altererythrobacter atlanticus 26DY36 |
| Ga0586324 0536881 3 1589 | 566561.00 | 0.00 | 0.00 | 0.00 | 0.00 | 0.00 | 0.00 | 0.00 | 0.00 | 0.00 | 0.00 | 0.00 | KO:K02014 | iron complex outermembrane receptor protein | Bacteria;Proteobacteria;Gammaproteobacteria;Nevskiales;Steroidobacteraceae;Povalibacter;Povalibacter uvarum;Povalibacter uvarum DSM 26723 |
| Ga0586324 0563977 3 1556 | 0.00 | 2244000.00 | 0.00 | 0.00 | 1215400.00 | 0.00 | 0.00 | 0.00 | 419076.99 | 962500.00 | 0.00 | 922500.00 | KO:K02014 | iron complex outermembrane receptor protein | Bacteria;Proteobacteria;Alphaproteobacteria;Sphingomonadales;Sphingomonadaceae;unclassified Sphingomonadaceae;unclassified Sphingomonadaceae;Sphingomonadaceae bacterium R-39594 |
| Ga0586324 0572483 366 1544 | 0.00 | 6113200.00 | 11654100.00 | 0.00 | 0.00 | 0.00 | 0.00 | 0.00 | 4325400.00 | 0.00 | 0.00 | 0.00 | KO:K02014 | iron complex outermembrane receptor protein | Bacteria;Proteobacteria;Alphaproteobacteria;Sphingomonadales;Sphingomonadaceae;Sphingomonas;Sphingomonas sp. SRS2;Sphingomonas sp. SRS2 |
| Ga0586324 0653422 1 588 | 0.00 | 4746400.00 | 0.00 | 0.00 | 0.00 | 0.00 | 2895600.00 | 0.00 | 1790100.00 | 4242700.00 | 5112800.00 | 0.00 | KO:K02014 | iron complex outermembrane receptor protein | Bacteria;Proteobacteria;Betaproteobacteria;Burkholderiales;Comamonadaceae;unclassified Comamonadaceae;Comamonadaceae bacterium URHA0028;Comamonadaceae bacterium URHA0028 |
| Ga0586324 0654255 320 1459 | 2240000.00 | 0.00 | 2974846.96 | 1596000.00 | 2626500.00 | 0.00 | 2462400.00 | 0.00 | 0.00 | 2002000.00 | 2692800.00 | 2122500.00 | KO:K02014 | iron complex outermembrane receptor protein | Bacteria;Proteobacteria;Betaproteobacteria;Burkholderiales;Oxalobacteraceae;Collimonas;Collimonas sp. OK412;Collimonas sp. OK412 |
| Ga0586324 0669081 1 849 | 1770000.00 | 1380400.00 | 5437921.29 | 12084000.00 | 8847700.00 | 5438900.00 | 0.00 | 8039800.00 | 2446200.00 | 20266400.00 | 14484800.00 | 1912500.00 | KO:K02014 | iron complex outermembrane receptor protein | Bacteria;Proteobacteria;Betaproteobacteria;Rhodocyclales;Rhodocyclaceae;Niveibacterium;Niveibacterium umoris;Niveibacterium umoris DSM 106739 |
| Ga0586324 0686484 1 1428 | 2260000.00 | 14484000.00 | 0.00 | 0.00 | 0.00 | 36682800.00 | 0.00 | 0.00 | 0.00 | 17941000.00 | 0.00 | 0.00 | KO:K02014 | iron complex outermembrane receptor protein | Bacteria;Proteobacteria;Alphaproteobacteria;unclassified Alphaproteobacteria;unclassified Alphaproteobacteria;unclassified Alphaproteobacteria;alpha proteobacterium Q-1;Alpha proteobacterium Q-1 |
| Ga0586324 0783023 1 1350 | 3590000.00 | 4816887.44 | 5289351.22 | 10355000.00 | 16366700.00 | 4922800.00 | 4104000.00 | 10028400.00 | 5540400.00 | 4019400.00 | 2648800.00 | 3247500.00 | KO:K02014 | iron complex outermembrane receptor protein | Bacteria;Proteobacteria;Betaproteobacteria;unclassified Betaproteobacteria;unclassified Betaproteobacteria;unclassified Betaproteobacteria;Betaproteobacteria bacterium GR16-43;Betaproteobacteria bacterium GR16-43 |
| Ga0586324 0811372 589 1329 | 0.00 | 0.00 | 0.00 | 0.00 | 0.00 | 0.00 | 0.00 | 0.00 | 0.00 | 5328400.00 | 2816000.00 | 0.00 | KO:K02014 | iron complex outermembrane receptor protein | Bacteria;Proteobacteria;Betaproteobacteria;Burkholderiales;Oxalobacteraceae;Massilia;Massilia flava;Massilia flava CGMCC 1.10685 |
| Ga0586324 0896310 322 1272 | 2880000.00 | 0.00 | 0.00 | 2251500.00 | 0.00 | 0.00 | 0.00 | 0.00 | 1992600.00 | 0.00 | 0.00 | 0.00 | KO:K02014 | iron complex outermembrane receptor protein | Bacteria;Proteobacteria;Betaproteobacteria;Burkholderiales;unclassified Burkholderiales;Rivibacter;Rivibacter subsaxonicus;Rivibacter subsaxonicus DSM 19570 |
| Ga0586324 0908515 1 993 | 0.00 | 472976.72 | 0.00 | 0.00 | 0.00 | 0.00 | 0.00 | 0.00 | 0.00 | 0.00 | 0.00 | 0.00 | KO:K02014 | iron complex outermembrane receptor protein | Bacteria;Proteobacteria;Alphaproteobacteria;Maricaulales;Maricaulaceae;Maricaulis;Maricaulis salignorans;Maricaulis salignorans DSM 16077 |
| Ga0586324 0956193 1 1239 | 8220000.00 | 0.00 | 0.00 | 0.00 | 0.00 | 0.00 | 0.00 | 0.00 | 1555200.00 | 0.00 | 0.00 | 1290000.00 | KO:K02014 | iron complex outermembrane receptor protein | Bacteria;Proteobacteria;Betaproteobacteria;Burkholderiales;Oxalobacteraceae;Massilia;Massilia niastensis;Massilia niastensis DSM 21313 |
| Ga0586324 1004783 2 1213 | 0.00 | 0.00 | 0.00 | 2574500.00 | 0.00 | 0.00 | 0.00 | 0.00 | 0.00 | 0.00 | 0.00 | 0.00 | KO:K02014 | iron complex outermembrane receptor protein | Bacteria;Proteobacteria;Betaproteobacteria;Burkholderiales;unclassified Burkholderiales;Methylibium;Methylibium sp. CF059;Methylibium sp. CF059 |
| Ga0586324 1082175 409 1173 | 0.00 | 627921.52 | 0.00 | 0.00 | 0.00 | 0.00 | 0.00 | 0.00 | 0.00 | 0.00 | 0.00 | 0.00 | KO:K02014 | iron complex outermembrane receptor protein | Bacteria;Proteobacteria;Alphaproteobacteria;Sphingomonadales;Sphingomonadaceae;Sphingomonas;Sphingomonas leidyi;Sphingomonas leidyi DSM 4733 |
| Ga0586324 1273692 573 1094 | 0.00 | 0.00 | 0.00 | 3809500.00 | 0.00 | 1175123.97 | 0.00 | 0.00 | 0.00 | 0.00 | 0.00 | 0.00 | KO:K02014 | iron complex outermembrane receptor protein | Bacteria;Proteobacteria;Betaproteobacteria;Burkholderiales;Comamonadaceae;unclassified Comamonadaceae;Comamonadaceae bacterium URHA0028;Comamonadaceae bacterium URHA0028 |
| Ga0586324 1274503 545 1093 | 0.00 | 0.00 | 0.00 | 0.00 | 0.00 | 0.00 | 0.00 | 7198000.00 | 5418900.00 | 0.00 | 0.00 | 0.00 | KO:K02014 | iron complex outermembrane receptor protein | Bacteria;Proteobacteria;Betaproteobacteria;Burkholderiales;unclassified Burkholderiales;Paucibacter;Paucibacter sp. KCTC 42545;Paucibacter sp. KCTC 42545 |
| Ga0586324 1348275 1 1068 | 1140000.00 | 0.00 | 0.00 | 0.00 | 0.00 | 0.00 | 0.00 | 0.00 | 0.00 | 0.00 | 0.00 | 0.00 | KO:K02014 | iron complex outermembrane receptor protein | Bacteria;Proteobacteria;Alphaproteobacteria;Sphingomonadales;Sphingomonadaceae;Sphingobium;Sphingobium sp. SYK-6;Sphingobium sp. SYK-6 |
| Ga0586324 1435057 1 1041 | 0.00 | 1496000.00 | 0.00 | 0.00 | 1833400.00 | 0.00 | 0.00 | 2464400.00 | 1782000.00 | 0.00 | 0.00 | 0.00 | KO:K02014 | iron complex outermembrane receptor protein | Bacteria;Proteobacteria;Betaproteobacteria;Burkholderiales;unclassified Burkholderiales;Aquincola;Aquincola tertiaricarbonis;Aquincola tertiaricarbonis MIMtkpLc11 |
| Ga0586324 1534195 2 1012 | 7181625.00 | 3814800.00 | 2951944.12 | 0.00 | 0.00 | 0.00 | 0.00 | 1103994.47 | 0.00 | 0.00 | 1786400.00 | 2475000.00 | KO:K02014 | iron complex outermembrane receptor protein | Bacteria;Proteobacteria;Gammaproteobacteria;Cellvibrionales;Halieaceae;Parahaliea;Parahaliea mediterranea;Parahaliea mediterranea DSM 21924 |
| Ga0586324 1559587 1 840 | 0.00 | 0.00 | 0.00 | 0.00 | 3728600.00 | 0.00 | 0.00 | 0.00 | 0.00 | 0.00 | 0.00 | 0.00 | KO:K02014 | iron complex outermembrane receptor protein | Bacteria;Proteobacteria;Gammaproteobacteria;Nevskiales;Steroidobacteraceae;Povalibacter;Povalibacter uvarum;Povalibacter uvarum DSM 26723 |
| Ga0586324 1746616 3 956 | 0.00 | 0.00 | 0.00 | 0.00 | 0.00 | 0.00 | 3420000.00 | 0.00 | 0.00 | 0.00 | 1003200.00 | 0.00 | KO:K02014 | iron complex outermembrane receptor protein | Bacteria;Proteobacteria;Alphaproteobacteria;Sphingomonadales;Sphingomonadaceae;Sphingomonas;Sphingomonas desiccabilis;Sphingomonas desiccabilis DSM 16792 |
| Ga0586324 1786598 2 817 | 0.00 | 0.00 | 0.00 | 0.00 | 3635900.00 | 0.00 | 0.00 | 0.00 | 0.00 | 5751900.00 | 0.00 | 0.00 | KO:K02014 | iron complex outermembrane receptor protein | Bacteria;Verrucomicrobia;Opitutae;Opitutales;Opitutaceae;Opitutus;Opitutus terrae;Opitutus terrae PB90-1 |
| Ga0586324 1849201 3 935 | 8140000.00 | 0.00 | 0.00 | 3325000.00 | 0.00 | 0.00 | 0.00 | 0.00 | 0.00 | 4004000.00 | 776548.08 | 133110.75 | KO:K02014 | iron complex outermembrane receptor protein | Bacteria;Proteobacteria;Alphaproteobacteria;Sphingomonadales;Sphingomonadaceae;Sphingomonas;Sphingomonas carotinifaciens;Sphingomonas carotinifaciens L9-754 |
| Ga0586324 1914154 3 920 | 0.00 | 0.00 | 0.00 | 0.00 | 7828000.00 | 0.00 | 0.00 | 0.00 | 0.00 | 0.00 | 1496000.00 | 0.00 | KO:K02014 | iron complex outermembrane receptor protein | Bacteria;Proteobacteria;Betaproteobacteria;Burkholderiales;Comamonadaceae;unclassified Comamonadaceae;Comamonadaceae bacterium URHA0028;Comamonadaceae bacterium URHA0028 |
| Ga0586324 2197932 3 869 | 0.00 | 0.00 | 0.00 | 0.00 | 0.00 | 0.00 | 0.00 | 829600.00 | 0.00 | 0.00 | 0.00 | 0.00 | KO:K02014 | iron complex outermembrane receptor protein | Bacteria;Proteobacteria;Alphaproteobacteria;Sphingomonadales;Erythrobacteraceae;Erythrobacter;Erythrobacter sp. SG61-1L;Erythrobacter sp. SG61-1L |
| Ga0586324 2369356 1 840 | 0.00 | 1938000.00 | 0.00 | 4341500.00 | 0.00 | 1025093.70 | 0.00 | 2366800.00 | 0.00 | 0.00 | 3220800.00 | 0.00 | KO:K02014 | iron complex outermembrane receptor protein | Bacteria;Proteobacteria;Betaproteobacteria;Burkholderiales;Comamonadaceae;unclassified Comamonadaceae;Comamonadaceae bacterium;Comamonadaceae sp. OAS658 |
| Ga0586324 2468168 1 825 | 2500000.00 | 2788000.00 | 3186810.83 | 835600.05 | 1236000.00 | 0.00 | 1349889.96 | 805200.00 | 4916700.00 | 18865000.00 | 24843873.12 | 781905.00 | KO:K02014 | iron complex outermembrane receptor protein | Bacteria;Proteobacteria;Betaproteobacteria;Burkholderiales;Oxalobacteraceae;Collimonas;Collimonas sp. OK412;Collimonas sp. OK412 |
| Ga0586324 2570696 3 812 | 0.00 | 0.00 | 0.00 | 3135000.00 | 0.00 | 0.00 | 0.00 | 0.00 | 0.00 | 0.00 | 0.00 | 0.00 | KO:K02014 | iron complex outermembrane receptor protein | Bacteria;Verrucomicrobia;Opitutae;Opitutales;Opitutaceae;Opitutus;Opitutus sp. ER46;Opitutus sp. ER46 |
| Ga0586324 2679148 1 495 | 433152.00 | 0.00 | 0.00 | 0.00 | 0.00 | 0.00 | 0.00 | 0.00 | 0.00 | 0.00 | 210641.20 | 0.00 | KO:K02014 | iron complex outermembrane receptor protein | Bacteria;Proteobacteria;Betaproteobacteria;Burkholderiales;Comamonadaceae;unclassified Comamonadaceae;Comamonadaceae bacterium;Comamonadaceae sp. OAS658 |
| Ga0586324 2717030 2 793 | 0.00 | 0.00 | 0.00 | 0.00 | 900517.67 | 0.00 | 0.00 | 0.00 | 0.00 | 0.00 | 0.00 | 0.00 | KO:K02014 | iron complex outermembrane receptor protein | Bacteria;Proteobacteria;Gammaproteobacteria;Cellvibrionales;Halieaceae;Halioglobus;Halioglobus pacificus;Halioglobus pacificus RR3-57 |
| Ga0586324 2844060 3 779 | 0.00 | 0.00 | 0.00 | 3011500.00 | 3357800.00 | 0.00 | 0.00 | 3074400.00 | 3037500.00 | 3603600.00 | 0.00 | 3337500.00 | KO:K02014 | iron complex outermembrane receptor protein | Bacteria;Proteobacteria;Alphaproteobacteria;Sphingomonadales;Sphingomonadaceae;Sphingomonas;Sphingomonas leidyi;Sphingomonas leidyi DSM 4733 |
| Ga0586324 2941761 1 768 | 1120000.00 | 0.00 | 0.00 | 0.00 | 0.00 | 2183456.33 | 0.00 | 0.00 | 0.00 | 0.00 | 0.00 | 1005000.00 | KO:K02014 | iron complex outermembrane receptor protein | Bacteria;Proteobacteria;Gammaproteobacteria;Xanthomonadales;Xanthomonadaceae;Lysobacter;Lysobacter antibioticus;Lysobacter antibioticus 13-6 |
| Ga0586324 3178487 2 574 | 0.00 | 863600.00 | 0.00 | 0.00 | 2914900.00 | 0.00 | 0.00 | 0.00 | 0.00 | 0.00 | 0.00 | 0.00 | KO:K02014 | iron complex outermembrane receptor protein | Bacteria;Proteobacteria;Gammaproteobacteria;Alteromonadales;Alteromonadaceae;Paraglaciecola;Paraglaciecola sp. MB-3u-78;Paraglaciecola sp. MB-3u-78 |
| Ga0586324 3186393 3 743 | 1510000.00 | 3039600.00 | 0.00 | 0.00 | 0.00 | 0.00 | 0.00 | 0.00 | 0.00 | 0.00 | 0.00 | 0.00 | KO:K02014 | iron complex outermembrane receptor protein | Bacteria;Proteobacteria;Betaproteobacteria;Burkholderiales;Comamonadaceae;unclassified Comamonadaceae;Comamonadaceae bacterium;Comamonadaceae sp. OAS658 |
| Ga0586324 3329483 2 730 | 0.00 | 0.00 | 0.00 | 0.00 | 0.00 | 6034400.00 | 0.00 | 0.00 | 2308500.00 | 0.00 | 0.00 | 3690000.00 | KO:K02014 | iron complex outermembrane receptor protein | Bacteria;Proteobacteria;Alphaproteobacteria;Sphingomonadales;Sphingomonadaceae;Sphingobium;Sphingobium sp. B12D2A;Sphingobium sp. B12D2A |
| Ga0586324 3538615 3 710 | 3770000.00 | 2978400.00 | 0.00 | 0.00 | 0.00 | 0.00 | 0.00 | 0.00 | 0.00 | 0.00 | 0.00 | 0.00 | KO:K02014 | iron complex outermembrane receptor protein | Bacteria;Proteobacteria;Alphaproteobacteria;Sphingomonadales;Erythrobacteraceae;Altererythrobacter;Altererythrobacter atlanticus;Altererythrobacter atlanticus 26DY36 |
| Ga0586324 3669325 3 701 | 6190000.00 | 614557.48 | 0.00 | 0.00 | 0.00 | 0.00 | 0.00 | 0.00 | 0.00 | 0.00 | 0.00 | 0.00 | KO:K02014 | iron complex outermembrane receptor protein | Bacteria;Proteobacteria;Betaproteobacteria;Burkholderiales;Comamonadaceae;Pelomonas;Pelomonas saccharophila;Pelomonas saccharophila DSM 654 |
| Ga0586324 3700876 1 699 | 0.00 | 0.00 | 0.00 | 0.00 | 0.00 | 0.00 | 0.00 | 0.00 | 425893.14 | 0.00 | 0.00 | 0.00 | KO:K02014 | iron complex outermembrane receptor protein | Bacteria;Proteobacteria;Betaproteobacteria;Burkholderiales;Oxalobacteraceae;Massilia;Massilia namucuonensis;Massilia namucuonensis CGMCC 1.11014 |
| Ga0586324 3954688 3 632 | 0.00 | 0.00 | 0.00 | 1406000.00 | 2070300.00 | 0.00 | 0.00 | 0.00 | 0.00 | 0.00 | 0.00 | 0.00 | KO:K02014 | iron complex outermembrane receptor protein | Bacteria;Proteobacteria;Alphaproteobacteria;Sphingomonadales;Sphingomonadaceae;Sphingopyxis;Sphingopyxis sp. MG;Sphingopyxis sp. MG |
| Ga0586324 4118922 2 667 | 3310000.00 | 0.00 | 0.00 | 0.00 | 0.00 | 0.00 | 0.00 | 0.00 | 0.00 | 0.00 | 0.00 | 0.00 | KO:K02014 | iron complex outermembrane receptor protein | Bacteria;Proteobacteria;Gammaproteobacteria;Xanthomonadales;Rhodanobacteraceae;Frateuria;Frateuria sp. Soil773;Frateuria sp. Soil773 |
| Ga0586324 4184351 3 665 | 0.00 | 1312400.00 | 0.00 | 0.00 | 0.00 | 0.00 | 0.00 | 0.00 | 0.00 | 0.00 | 0.00 | 0.00 | KO:K02014 | iron complex outermembrane receptor protein | Bacteria;Proteobacteria;Alphaproteobacteria;Sphingomonadales;Erythrobacteraceae;Erythrobacter;Erythrobacter sp. SG61-1L;Erythrobacter sp. SG61-1L |
| Ga0586324 4248628 150 659 | 0.00 | 0.00 | 0.00 | 0.00 | 0.00 | 2179375.17 | 0.00 | 0.00 | 0.00 | 0.00 | 0.00 | 0.00 | KO:K02014 | iron complex outermembrane receptor protein | Bacteria;Proteobacteria;Betaproteobacteria;Burkholderiales;Comamonadaceae;unclassified Comamonadaceae;Comamonadaceae bacterium URHA0028;Comamonadaceae bacterium URHA0028 |

| | | | | | | | | | | | | | | | |
|---|---|---|---|---|---|---|---|---|---|---|---|---|---|---|---|
| Ga0586324 4435807 2 649 | 0.00 | 0.00 | 0.00 | 0.00 | 0.00 | 10242600.00 | 0.00 | 0.00 | 0.00 | 0.00 | 0.00 | 0.00 | KO:K02014 | iron complex outermembrane receptor protein | Bacteria;Proteobacteria;Betaproteobacteria;Burkholderiales;unclassified Burkholderiales;Paucibacter;Paucibacter toxinivorans;Paucibacter toxinivorans DSM 16998 |
| Ga0586324 4548026 1 642 | 0.00 | 0.00 | 0.00 | 0.00 | 0.00 | 0.00 | 1972505.52 | 0.00 | 0.00 | 0.00 | 0.00 | 0.00 | KO:K02014 | iron complex outermembrane receptor protein | Bacteria;Proteobacteria;Betaproteobacteria;Burkholderiales;unclassified Burkholderiales;Methylibium;Methylibium sp. CF059;Methylibium sp. CF059 |
| Ga0586324 5300730 3 602 | 0.00 | 0.00 | 0.00 | 0.00 | 0.00 | 121474.46 | 0.00 | 218193.95 | 0.00 | 0.00 | 1188000.00 | 0.00 | KO:K02014 | iron complex outermembrane receptor protein | Bacteria;Proteobacteria;Betaproteobacteria;Burkholderiales;Oxalobacteraceae;Massilia;Massilia armeniaca;Massilia armeniaca ZMN-3 |
| Ga0586324 5797868 1 579 | 0.00 | 646722.16 | 0.00 | 0.00 | 8147300.00 | 0.00 | 0.00 | 0.00 | 0.00 | 0.00 | 0.00 | 0.00 | KO:K02014 | iron complex outermembrane receptor protein | Bacteria;Proteobacteria;Betaproteobacteria;Burkholderiales;Comamonadaceae;unclassified Comamonadaceae;Comamonadaceae bacterium URHA0028;Comamonadaceae bacterium URHA0028 |
| Ga0586324 5819553 3 578 | 368788.00 | 734400.00 | 0.00 | 0.00 | 363343.83 | 0.00 | 7957200.00 | 756400.00 | 8910000.00 | 413613.20 | 0.00 | 6720000.00 | KO:K02014 | iron complex outermembrane receptor protein | Bacteria;Proteobacteria;Betaproteobacteria;Burkholderiales;Oxalobacteraceae;Massilia;Massilia sp. 9096;Massilia sp. 9096 |
| Ga0586324 6143111 2 565 | 0.00 | 0.00 | 0.00 | 0.00 | 0.00 | 0.00 | 0.00 | 0.00 | 5418900.00 | 7137900.00 | 1865600.00 | 0.00 | KO:K02014 | iron complex outermembrane receptor protein | Bacteria;Proteobacteria;Betaproteobacteria;Burkholderiales;Comamonadaceae;unclassified Comamonadaceae;Comamonadaceae bacterium URHA0028;Comamonadaceae bacterium URHA0028 |
| Ga0586324 6200431 2 565 | 5750000.00 | 4882400.00 | 0.00 | 9690000.00 | 0.00 | 0.00 | 0.00 | 0.00 | 0.00 | 5775000.00 | 3352800.00 | 4897500.00 | KO:K02014 | iron complex outermembrane receptor protein | Bacteria;Proteobacteria;Betaproteobacteria;Burkholderiales;Oxalobacteraceae;Undibacterium;Undibacterium pigrum;Undibacterium pigrum DSM 19792 |
| Ga0586324 6447792 1 555 | 840122.00 | 0.00 | 0.00 | 0.00 | 0.00 | 0.00 | 0.00 | 0.00 | 0.00 | 0.00 | 0.00 | 0.00 | KO:K02014 | iron complex outermembrane receptor protein | Bacteria;Proteobacteria;Alphaproteobacteria;Caulobacterales;Caulobacteraceae;Brevundimonas;Brevundimonas lutea;Brevundimonas lutea NS26 |
| Ga0586324 6518970 1 552 | 0.00 | 0.00 | 0.00 | 0.00 | 0.00 | 0.00 | 0.00 | 921100.00 | 572241.51 | 0.00 | 0.00 | 0.00 | KO:K02014 | iron complex outermembrane receptor protein | Bacteria;Proteobacteria;Alphaproteobacteria;Sphingomonadales;Sphingomonadaceae;Sphingomonas;Sphingomonas sp. LH128;Sphingomonas sp. LH128 |
| Ga0586324 7163010 3 530 | 0.00 | 429721.92 | 0.00 | 0.00 | 0.00 | 0.00 | 0.00 | 0.00 | 0.00 | 0.00 | 0.00 | 0.00 | KO:K02014 | iron complex outermembrane receptor protein | Bacteria;Proteobacteria;Alphaproteobacteria;Sphingomonadales;Sphingomonadaceae;Sphingobium;Sphingobium xanthum;Sphingobium xanthum DSM 100901 |
| Ga0586324 7344699 3 527 | 1860000.00 | 0.00 | 0.00 | 0.00 | 0.00 | 0.00 | 0.00 | 0.00 | 0.00 | 0.00 | 0.00 | 0.00 | KO:K02014 | iron complex outermembrane receptor protein | Bacteria;Proteobacteria;Alphaproteobacteria;Caulobacterales;Caulobacteraceae;Brevundimonas;Brevundimonas aveniformis;Brevundimonas aveniformis DSM 17977 |
| Ga0586324 7611086 2 517 | 0.00 | 0.00 | 0.00 | 0.00 | 0.00 | 0.00 | 0.00 | 0.00 | 0.00 | 0.00 | 261524.56 | 1072500.00 | KO:K02014 | iron complex outermembrane receptor protein | Bacteria;Proteobacteria;Gammaproteobacteria;Nevskiales;Steroidobacteraceae;Povalibacter;Povalibacter uvarum;Povalibacter uvarum DSM 26723 |
| Ga0586324 7718698 1 516 | 0.00 | 3175600.00 | 0.00 | 0.00 | 0.00 | 0.00 | 0.00 | 0.00 | 3612600.00 | 0.00 | 0.00 | 0.00 | KO:K02014 | iron complex outermembrane receptor protein | Bacteria;Proteobacteria;Betaproteobacteria;Burkholderiales;unclassified Burkholderiales;Ideonella;Ideonella sakaiensis;Ideonella sp. 201-F6 |
| Ga0586324 7848633 2 511 | 0.00 | 0.00 | 0.00 | 0.00 | 1390500.00 | 0.00 | 0.00 | 0.00 | 692982.54 | 0.00 | 0.00 | 0.00 | KO:K02014 | iron complex outermembrane receptor protein | Bacteria;Ignavibacteriae;Ignavibacteria;Ignavibacteriales;Melioribacteraceae;Melioribacter;Melioribacter roseus;Melioribacter roseus P3M |
| Ga0586324 7981204 1 507 | 0.00 | 0.00 | 0.00 | 1757500.00 | 2832500.00 | 0.00 | 0.00 | 0.00 | 0.00 | 0.00 | 0.00 | 0.00 | KO:K02014 | iron complex outermembrane receptor protein | Bacteria;Verrucomicrobia;Opitutae;Opitutales;Opitutaceae;Opitutus;Opitutus sp. GAS368;Opitutus sp. GAS368 |
| Ga0586324 1482272 2 1027 | 81100000.00 | 104046800.00 | 96836000.00 | 77520000.00 | 61903000.00 | 120529200.00 | 94916400.00 | 73242700.00 | 64962000.00 | 84700000.00 | 89680800.00 | 88147500.00 | KO:K02024 | maltoporin | Bacteria;Proteobacteria;Betaproteobacteria;Burkholderiales;unclassified Burkholderiales;unclassified Burkholderiales;[Polyangium] brachysporum;Polyangium brachysporum DSM 7029 |
| Ga0586324 4505996 2 643 | 0.00 | 0.00 | 0.00 | 2337000.00 | 0.00 | 0.00 | 0.00 | 2080100.00 | 1660500.00 | 0.00 | 0.00 | 0.00 | KO:K02024 | maltoporin | Bacteria;Proteobacteria;Deltaproteobacteria;Myxococcales;Anaeromyxobacteraceae;Anaeromyxobacter;Anaeromyxobacter dehalogenans;Anaeromyxobacter dehalogenans 2CP-1 |
| Ga0586324 8113544 234 503 | 5490000.00 | 6432800.00 | 0.00 | 0.00 | 0.00 | 0.00 | 0.00 | 0.00 | 0.00 | 1909600.00 | 0.00 | 787500.00 | KO:K02024 | maltoporin | Bacteria;Proteobacteria;Betaproteobacteria;Burkholderiales;unclassified Burkholderiales;unclassified Burkholderiales;Burkholderiales bacterium JOSHI 001;Burkholderiales sp. JOSHI 001 |
| Ga0586324 0133145 1787 2674 | 0.00 | 0.00 | 0.00 | 0.00 | 0.00 | 0.00 | 0.00 | 0.00 | 1620000.00 | 0.00 | 0.00 | 0.00 | KO:K02026 | multiple sugar transport system permease protein | Bacteria;Actinobacteria;Actinomycetia;Streptosporangiales;Thermomonosporaceae;Actinomadura;Actinomadura chibensis;Actinomadura chibensis NBRC 106107 |
| Ga0586324 0002768 3057 4370 | 0.00 | 0.00 | 0.00 | 0.00 | 0.00 | 0.00 | 0.00 | 0.00 | 1563300.00 | 0.00 | 0.00 | 0.00 | KO:K02027 | multiple sugar transport system substrate-binding protein | Bacteria;Proteobacteria;Alphaproteobacteria;Rhodospirillales;unclassified Rhodospirillales;unclassified Rhodospirillales;Rhodospirillales bacterium URHD0017;Rhodospirillales bacterium URHD0017 |
| Ga0586324 0004543 5116 6426 | 0.00 | 0.00 | 0.00 | 0.00 | 0.00 | 0.00 | 0.00 | 0.00 | 497165.04 | 0.00 | 0.00 | 418787.25 | KO:K02027 | multiple sugar transport system substrate-binding protein | Bacteria;Chloroflexi;Anaerolineae;Anaerolineales;Anaerolineaceae;Levilinea;Levilinea saccharolytica;Levilinea saccharolytica DSM 16555 |
| Ga0586324 0005220 3428 4849 | 995083.00 | 1122000.00 | 0.00 | 2413000.00 | 2534884.59 | 0.00 | 3142147.80 | 2940200.00 | 1709100.00 | 0.00 | 1821600.00 | 1515000.00 | KO:K02027 | multiple sugar transport system substrate-binding protein | Bacteria;Chloroflexi;Caldilineae;Caldilineales;Caldilineaceae;Caldilinea;Caldilinea aerophila;Caldilinea aerophila STL-6-O1, DSM 14535 |
| Ga0586324 0006105 10114 11586 | 6870000.00 | 5848000.00 | 1202601.78 | 3619500.00 | 3502000.00 | 4565500.00 | 518674.92 | 2641300.00 | 1790100.00 | 0.00 | 620312.00 | 0.00 | KO:K02027 | multiple sugar transport system substrate-binding protein | Bacteria;Actinobacteria;Actinomycetia;Glycomycetales;Glycomycetaceae;Glycomyces;Glycomyces tenuis;Glycomyces tenuis DSM 44171 |
| Ga0586324 0008566 6256 7587 | 0.00 | 0.00 | 0.00 | 0.00 | 0.00 | 0.00 | 0.00 | 0.00 | 0.00 | 0.00 | 1733600.00 | 0.00 | KO:K02027 | multiple sugar transport system substrate-binding protein | Bacteria;Thermotogae;Thermotogae;Kosmotogales;Kosmotogaceae;Kosmotoga;Kosmotoga arenicorallina;Kosmotoga arenicorallina S304 |
| Ga0586324 0010779 7276 8706 | 4440000.00 | 8540800.00 | 0.00 | 0.00 | 0.00 | 0.00 | 0.00 | 0.00 | 0.00 | 3195500.00 | 0.00 | 0.00 | KO:K02027 | multiple sugar transport system substrate-binding protein | Bacteria;Chloroflexi;Caldilineae;Caldilineales;Caldilineaceae;Caldilinea;Caldilinea aerophila;Caldilinea aerophila STL-6-O1, DSM 14535 |
| Ga0586324 0010825 130 1665 | 0.00 | 924800.00 | 0.00 | 0.00 | 0.00 | 0.00 | 0.00 | 216137.64 | 0.00 | 0.00 | 0.00 | 1320000.00 | KO:K02027 | multiple sugar transport system substrate-binding protein | Bacteria;Chloroflexi;Ktedonobacteria;Thermogemmatisporales;Thermogemmatisporaceae;Thermogemmatispora;Thermogemmatispora onikobensis;Thermogemmatispora onikobensis NBRC 111776 |
| Ga0586324 0012760 6679 7980 | 0.00 | 2883200.00 | 0.00 | 1966500.00 | 1792200.00 | 0.00 | 0.00 | 1744600.00 | 0.00 | 6421800.00 | 5350400.00 | 2700000.00 | KO:K02027 | multiple sugar transport system substrate-binding protein | Bacteria;Proteobacteria;Alphaproteobacteria;Hyphomicrobiales;Bradyrhizobiaceae;Bradyrhizobium;Bradyrhizobium lablabi;Bradyrhizobium lablabi GAS165 |
| Ga0586324 0017818 2876 4204 | 6840000.00 | 8976000.00 | 0.00 | 0.00 | 0.00 | 0.00 | 0.00 | 0.00 | 0.00 | 0.00 | 0.00 | 3075000.00 | KO:K02027 | multiple sugar transport system substrate-binding protein | Bacteria;Proteobacteria;Deltaproteobacteria;unclassified Deltaproteobacteria;unclassified Deltaproteobacteria;unclassified Deltaproteobacteria;SAR324 cluster bacterium JCVI-SC AAA005;SAR324 cluster bacterium JCVI-SC AAA005 |
| Ga0586324 0019574 3138 4418 | 0.00 | 0.00 | 6868600.00 | 0.00 | 0.00 | 0.00 | 0.00 | 0.00 | 0.00 | 0.00 | 0.00 | 0.00 | KO:K02027 | multiple sugar transport system substrate-binding protein | Bacteria;Proteobacteria;Betaproteobacteria;Burkholderiales;Alcaligenaceae;Bordetella;Bordetella hinzii;Bordetella hinzii ATCC 51730 |
| Ga0586324 0026250 781 2310 | 0.00 | 2219472.40 | 3888854.94 | 0.00 | 0.00 | 0.00 | 2241789.48 | 3013400.00 | 0.00 | 0.00 | 0.00 | 0.00 | KO:K02027 | multiple sugar transport system substrate-binding protein | Bacteria;Chloroflexi;unclassified Chloroflexi;unclassified Chloroflexi;unclassified Chloroflexi;Thermobaculum;Thermobaculum terrenum;Thermobaculum terrenum YNP1, ATCC BAA-798 |
| Ga0586324 0040747 2397 3839 | 1360000.00 | 4413200.00 | 0.00 | 0.00 | 0.00 | 0.00 | 0.00 | 0.00 | 0.00 | 0.00 | 0.00 | 0.00 | KO:K02027 | multiple sugar transport system substrate-binding protein | Bacteria;Proteobacteria;Alphaproteobacteria;Hyphomicrobiales;Aurantimonadaceae;Aurantimonas;Aurantimonas sp. Leaf443;Aurantimonas sp. Leaf443 |
| Ga0586324 0041937 315 1670 | 0.00 | 0.00 | 3780387.36 | 0.00 | 4974900.00 | 0.00 | 0.00 | 0.00 | 0.00 | 0.00 | 0.00 | 0.00 | KO:K02027 | multiple sugar transport system substrate-binding protein | Bacteria;Deinococcus-Thermus;Deinococci;Deinococcales;Deinococcaceae;Deinococcus;Deinococcus humi;Deinococcus humi DSM 27939 |
| Ga0586324 0056785 2829 4241 | 0.00 | 0.00 | 14975800.00 | 3524500.00 | 0.00 | 0.00 | 0.00 | 0.00 | 0.00 | 0.00 | 0.00 | 0.00 | KO:K02027 | multiple sugar transport system substrate-binding protein | Bacteria;Firmicutes;Bacilli;Bacillales;Paenibacillaceae;Paenibacillus;Paenibacillus sp. VKM B-2647;Paenibacillus sp. VKM B-2647 |
| Ga0586324 0072185 186 1727 | 2520000.00 | 1462000.00 | 0.00 | 0.00 | 0.00 | 0.00 | 0.00 | 0.00 | 0.00 | 0.00 | 0.00 | 0.00 | KO:K02027 | multiple sugar transport system substrate-binding protein | Bacteria;Chloroflexi;Chloroflexia;Chloroflexales;Chloroflexaceae;Chloroflexus;Chloroflexus aurantiacus;Chloroflexus aurantiacus J-10-fl |
| Ga0586324 0112384 926 2365 | 0.00 | 8432000.00 | 0.00 | 0.00 | 0.00 | 0.00 | 0.00 | 0.00 | 5062500.00 | 0.00 | 0.00 | 0.00 | KO:K02027 | multiple sugar transport system substrate-binding protein | Bacteria;Chloroflexi;Chloroflexia;Kallotenuales;Kallotenuaceae;Kallotenue;Kallotenue papyrolyticum;Kallotenue papyrolyticum JKG1 |
| Ga0586324 0197573 3 1253 | 0.00 | 0.00 | 8163500.00 | 0.00 | 6159400.00 | 6868100.00 | 0.00 | 3745400.00 | 3823200.00 | 3311000.00 | 0.00 | 0.00 | KO:K02027 | multiple sugar transport system substrate-binding protein | Bacteria;Firmicutes;Bacilli;Bacillales;Paenibacillaceae;Paenibacillus;Paenibacillus elgii;Paenibacillus elgii B69 |
| Ga0586324 0211741 331 1635 | 0.00 | 0.00 | 0.00 | 0.00 | 0.00 | 0.00 | 0.00 | 0.00 | 0.00 | 3249400.00 | 0.00 | 0.00 | KO:K02027 | multiple sugar transport system substrate-binding protein | Bacteria;Firmicutes;Bacilli;Bacillales;Bacillaceae;Bacillus;Bacillus sp. AFS040349;Bacillus sp. AFS040349 |
| Ga0586324 0284540 915 2105 | 3160000.00 | 9588000.00 | 0.00 | 0.00 | 0.00 | 0.00 | 0.00 | 0.00 | 0.00 | 0.00 | 0.00 | 0.00 | KO:K02027 | multiple sugar transport system substrate-binding protein | Bacteria;Proteobacteria;Deltaproteobacteria;unclassified Deltaproteobacteria;unclassified Deltaproteobacteria;unclassified Deltaproteobacteria;SAR324 cluster bacterium JCVI-SC AAA005;SAR324 cluster bacterium JCVI-SC AAA005 |
| Ga0586324 0367272 2 1039 | 2320000.00 | 0.00 | 0.00 | 0.00 | 0.00 | 0.00 | 0.00 | 0.00 | 0.00 | 0.00 | 0.00 | 0.00 | KO:K02027 | multiple sugar transport system substrate-binding protein | Bacteria;Proteobacteria;Deltaproteobacteria;unclassified Deltaproteobacteria;unclassified Deltaproteobacteria;unclassified Deltaproteobacteria;SAR324 cluster bacterium JCVI-SC AAA005;SAR324 cluster bacterium JCVI-SC AAA005 |
| Ga0586324 0380406 1 1380 | 0.00 | 822800.00 | 0.00 | 0.00 | 0.00 | 0.00 | 0.00 | 0.00 | 0.00 | 0.00 | 0.00 | 0.00 | KO:K02027 | multiple sugar transport system substrate-binding protein | Bacteria;Chloroflexi;Chloroflexia;Chloroflexales;Chloroflexaceae;Chloroflexus;Chloroflexus aurantiacus;Chloroflexus aurantiacus J-10-fl |
| Ga0586324 0454741 250 1710 | 0.00 | 563885.92 | 0.00 | 0.00 | 0.00 | 0.00 | 0.00 | 0.00 | 0.00 | 0.00 | 0.00 | 0.00 | KO:K02027 | multiple sugar transport system substrate-binding protein | Bacteria;Spirochaetes;Spirochaetia;Spirochaetales;Spirochaetaceae;Spirochaeta;Spirochaeta thermophila;Spirochaeta thermophila DSM 6192 |
| Ga0586324 0490453 3 1088 | 3780000.00 | 0.00 | 0.00 | 0.00 | 0.00 | 0.00 | 0.00 | 0.00 | 0.00 | 3796100.00 | 0.00 | 2175000.00 | KO:K02027 | multiple sugar transport system substrate-binding protein | Bacteria;Spirochaetes;Spirochaetia;Spirochaetales;Sphaerochaetaceae;Sphaerochaeta;Sphaerochaeta associata;Sphaerochaeta associata GLS2 |
| Ga0586324 0511879 2 988 | 0.00 | 897600.00 | 0.00 | 0.00 | 0.00 | 0.00 | 0.00 | 0.00 | 0.00 | 0.00 | 0.00 | 0.00 | KO:K02027 | multiple sugar transport system substrate-binding protein | Bacteria;Chloroflexi;Caldilineae;Caldilineales;Caldilineaceae;Caldilinea;Caldilinea aerophila;Caldilinea aerophila STL-6-O1, DSM 14535 |
| Ga0586324 0687487 1 939 | 0.00 | 0.00 | 0.00 | 10925000.00 | 0.00 | 0.00 | 0.00 | 0.00 | 0.00 | 0.00 | 0.00 | 0.00 | KO:K02027 | multiple sugar transport system substrate-binding protein | Bacteria;Proteobacteria;Betaproteobacteria;Burkholderiales;Comamonadaceae;Rhodoferax;Rhodoferax ferrireducens;Rhodoferax ferrireducens T118 |
| Ga0586324 0757472 3 1187 | 1530000.00 | 0.00 | 0.00 | 0.00 | 0.00 | 0.00 | 0.00 | 0.00 | 0.00 | 0.00 | 0.00 | 0.00 | KO:K02027 | multiple sugar transport system substrate-binding protein | Bacteria;Thermotogae;Thermotogae;Kosmotogales;Kosmotogaceae;Kosmotoga;Kosmotoga arenicorallina;Kosmotoga arenicorallina S304 |
| Ga0586324 0864730 3 1004 | 0.00 | 0.00 | 5219505.44 | 0.00 | 0.00 | 0.00 | 0.00 | 0.00 | 0.00 | 0.00 | 0.00 | 0.00 | KO:K02027 | multiple sugar transport system substrate-binding protein | Bacteria;Thermotogae;Thermotogae;Petrotogales;Petrotogaceae;Oceanotoga;Oceanotoga teriensis;Oceanotoga teriensis DSM 24906 |
| Ga0586324 0906963 3 1265 | 6040000.00 | 7255600.00 | 4746754.34 | 7163000.00 | 4676200.00 | 0.00 | 0.00 | 0.00 | 0.00 | 0.00 | 0.00 | 0.00 | KO:K02027 | multiple sugar transport system substrate-binding protein | Bacteria;Chloroflexi;Caldilineae;Caldilineales;Caldilineaceae;Caldilinea;Caldilinea aerophila;Caldilinea aerophila STL-6-O1, DSM 14535 |
| Ga0586324 1054469 3 917 | 0.00 | 0.00 | 0.00 | 0.00 | 0.00 | 0.00 | 0.00 | 0.00 | 1215000.00 | 0.00 | 0.00 | 0.00 | KO:K02027 | multiple sugar transport system substrate-binding protein | Bacteria;Chloroflexi;Chloroflexia;Kallotenuales;Kallotenuaceae;Kallotenue;Kallotenue papyrolyticum;Kallotenue papyrolyticum JKG1 |
| Ga0586324 1099724 196 1164 | 5730000.00 | 5827600.00 | 6966077.82 | 8436000.00 | 7227171.13 | 0.00 | 3670800.00 | 0.00 | 3474900.00 | 4874100.00 | 3344000.00 | 6817500.00 | KO:K02027 | multiple sugar transport system substrate-binding protein | Bacteria;Proteobacteria;Alphaproteobacteria;Rhodospirillales;Acetobacteraceae;Rhodopila;unclassified Rhodopila;Rhodopila sp. LVNP |
| Ga0586324 1270454 90 1094 | 0.00 | 0.00 | 0.00 | 0.00 | 609826.95 | 0.00 | 0.00 | 0.00 | 0.00 | 0.00 | 0.00 | 0.00 | KO:K02027 | multiple sugar transport system substrate-binding protein | Bacteria;Proteobacteria;Betaproteobacteria;Burkholderiales;Comamonadaceae;Variovorax;Variovorax sp. Root411;Variovorax sp. Root411 |
| Ga0586324 1385347 1 1056 | 0.00 | 2516000.00 | 0.00 | 0.00 | 0.00 | 0.00 | 3214800.00 | 0.00 | 0.00 | 0.00 | 0.00 | 0.00 | KO:K02027 | multiple sugar transport system substrate-binding protein | Bacteria;Chloroflexi;Caldilineae;Caldilineales;Caldilineaceae;Caldilinea;Caldilinea aerophila;Caldilinea aerophila STL-6-O1, DSM 14535 |
| Ga0586324 1549075 2 1006 | 0.00 | 0.00 | 0.00 | 2622000.00 | 0.00 | 0.00 | 0.00 | 0.00 | 0.00 | 0.00 | 0.00 | 0.00 | KO:K02027 | multiple sugar transport system substrate-binding protein | Bacteria;Atribacterota;unclassified Atribacterota;unclassified Atribacterota;unclassified Atribacterota;Candidatus Caldatribacterium;Candidatus Caldatribacterium saccharofermentans;Candidatus Caldatribacterium saccharofermentans OP9-77CS |
| Ga0586324 2983970 1 612 | 0.00 | 0.00 | 736708.02 | 0.00 | 0.00 | 0.00 | 0.00 | 0.00 | 0.00 | 0.00 | 0.00 | 0.00 | KO:K02027 | multiple sugar transport system substrate-binding protein | Bacteria;Proteobacteria;Betaproteobacteria;Burkholderiales;Comamonadaceae;Variovorax;Variovorax paradoxus;Variovorax paradoxus KB5 |
| Ga0586324 2990319 1 762 | 0.00 | 727600.00 | 0.00 | 0.00 | 0.00 | 0.00 | 0.00 | 0.00 | 0.00 | 0.00 | 0.00 | 0.00 | KO:K02027 | multiple sugar transport system substrate-binding protein | Bacteria;Chloroflexi;Caldilineae;Caldilineales;Caldilineaceae;Caldilinea;Caldilinea aerophila;Caldilinea aerophila STL-6-O1, DSM 14535 |
| Ga0586324 3116083 1 750 | 0.00 | 0.00 | 0.00 | 0.00 | 0.00 | 0.00 | 0.00 | 0.00 | 617875.29 | 0.00 | 0.00 | 0.00 | KO:K02027 | multiple sugar transport system substrate-binding protein | Bacteria;Chloroflexi;Chloroflexia;Kallotenuales;Kallotenuaceae;Kallotenue;Kallotenue papyrolyticum;Kallotenue papyrolyticum JKG1 |
| Ga0586324 3710006 3 347 | 12000000.00 | 0.00 | 0.00 | 0.00 | 0.00 | 0.00 | 0.00 | 0.00 | 0.00 | 0.00 | 0.00 | 0.00 | KO:K02027 | multiple sugar transport system substrate-binding protein | Bacteria;Atribacterota;unclassified Atribacterota;unclassified Atribacterota;unclassified Atribacterota;Candidatus Caldatribacterium;Candidatus Caldatribacterium saccharofermentans;Candidatus Caldatribacterium saccharofermentans OP9-77CS |
| Ga0586324 4828929 3 626 | 1080000.00 | 0.00 | 0.00 | 0.00 | 0.00 | 0.00 | 0.00 | 0.00 | 0.00 | 0.00 | 0.00 | 0.00 | KO:K02027 | multiple sugar transport system substrate-binding protein | Bacteria;Actinobacteria;Nitriliruptoria;Euzebyales;Euzebyaceae;Euzebya;Euzebya rosea;Euzebya rosea DSW09 |
| Ga0586324 5502893 3 593 | 0.00 | 4513588.40 | 0.00 | 4683500.00 | 0.00 | 0.00 | 0.00 | 0.00 | 0.00 | 2048200.00 | 0.00 | 0.00 | KO:K02027 | multiple sugar transport system substrate-binding protein | Bacteria;Chloroflexi;Chloroflexia;Kallotenuales;Kallotenuaceae;Kallotenue;Kallotenue papyrolyticum;Kallotenue papyrolyticum JKG1 |
| Ga0586324 6447106 1 555 | 17200000.00 | 0.00 | 0.00 | 0.00 | 0.00 | 0.00 | 0.00 | 0.00 | 0.00 | 0.00 | 0.00 | 0.00 | KO:K02027 | multiple sugar transport system substrate-binding protein | Bacteria;Chloroflexi;Chloroflexia;Kallotenuales;Kallotenuaceae;Kallotenue;Kallotenue papyrolyticum;Kallotenue papyrolyticum JKG1 |
| Ga0586324 6851984 3 542 | 1020000.00 | 0.00 | 34286700.00 | 0.00 | 0.00 | 0.00 | 0.00 | 0.00 | 0.00 | 0.00 | 0.00 | 0.00 | KO:K02027 | multiple sugar transport system substrate-binding protein | Bacteria;Chloroflexi;Caldilineae;Caldilineales;Caldilineaceae;Caldilinea;Caldilinea aerophila;Caldilinea aerophila STL-6-O1, DSM 14535 |
| Ga0586324 7854389 2 511 | 0.00 | 1006400.00 | 0.00 | 0.00 | 0.00 | 1606063.50 | 0.00 | 0.00 | 858600.00 | 0.00 | 0.00 | 0.00 | KO:K02027 | multiple sugar transport system substrate-binding protein | Bacteria;Proteobacteria;Alphaproteobacteria;Hyphomicrobiales;Aurantimonadaceae;Aureimonas;Aureimonas flava;Aureimonas flava M2BS4Y-1 |
| Ga0586324 0028656 2 907 | 0.00 | 0.00 | 7713100.00 | 0.00 | 0.00 | 0.00 | 0.00 | 0.00 | 0.00 | 0.00 | 0.00 | 0.00 | KO:K02030 | polar amino acid transport system substrate-binding protein | Bacteria;Chloroflexi;Chloroflexia;Kallotenuales;Kallotenuaceae;Kallotenue;Kallotenue papyrolyticum;Kallotenue papyrolyticum JKG1 |
| Ga0586324 0033343 2107 2922 | 0.00 | 1229918.04 | 0.00 | 2812000.00 | 0.00 | 0.00 | 7159200.00 | 0.00 | 0.00 | 6414100.00 | 3106400.00 | 0.00 | KO:K02030 | polar amino acid transport system substrate-binding protein | Bacteria;Proteobacteria;Deltaproteobacteria;Desulfuromonadales;Geobacteraceae;Geobacter;Geobacter sp. M18;Geobacter sp. M18 |
| Ga0586324 0057444 1319 2146 | 0.00 | 0.00 | 0.00 | 0.00 | 0.00 | 0.00 | 0.00 | 0.00 | 0.00 | 4589200.00 | 0.00 | 0.00 | KO:K02030 | polar amino acid transport system substrate-binding protein | Bacteria;Proteobacteria;Gammaproteobacteria;Enterobacterales;Erwiniaceae;Izhakiella;Izhakiella australiensis;Izhakiella australiensis 98 |
| Ga0586324 0063684 898 1713 | 0.00 | 53176000.00 | 0.00 | 0.00 | 0.00 | 0.00 | 0.00 | 0.00 | 0.00 | 0.00 | 0.00 | 0.00 | KO:K02030 | polar amino acid transport system substrate-binding protein | Bacteria;Proteobacteria;Deltaproteobacteria;Desulfuromonadales;Geobacteraceae;Geobacter;Geobacter sp. M18;Geobacter sp. M18 |
| Ga0586324 0141148 997 1818 | 0.00 | 0.00 | 0.00 | 4873500.00 | 0.00 | 0.00 | 0.00 | 0.00 | 0.00 | 0.00 | 0.00 | 0.00 | KO:K02030 | polar amino acid transport system substrate-binding protein | Bacteria;Proteobacteria;Deltaproteobacteria;Desulfuromonadales;Geobacteraceae;Geobacter;Geobacter sp. M18;Geobacter sp. M18 |
| Ga0586324 0148029 2054 2848 | 0.00 | 0.00 | 0.00 | 0.00 | 0.00 | 9647100.00 | 0.00 | 0.00 | 0.00 | 0.00 | 0.00 | 0.00 | KO:K02030 | polar amino acid transport system substrate-binding protein | Bacteria;Proteobacteria;Deltaproteobacteria;Syntrophobacterales;Syntrophobacteraceae;Syntrophobacter;Syntrophobacter fumaroxidans;Syntrophobacter fumaroxidans MPOB |
| Ga0586324 0263309 463 1212 | 0.00 | 1727200.00 | 0.00 | 0.00 | 0.00 | 0.00 | 0.00 | 0.00 | 0.00 | 0.00 | 0.00 | 0.00 | KO:K02030 | polar amino acid transport system substrate-binding protein | Bacteria;Spirochaetes;Spirochaetia;Spirochaetales;Spirochaetaceae;Spirochaeta;Spirochaeta isovalerica;Spirochaeta isovalerica DSM 2461 |
| Ga0586324 0549274 975 1574 | 0.00 | 0.00 | 0.00 | 0.00 | 9218500.00 | 0.00 | 0.00 | 0.00 | 0.00 | 0.00 | 0.00 | 0.00 | KO:K02030 | polar amino acid transport system substrate-binding protein | Bacteria;Proteobacteria;Deltaproteobacteria;Syntrophobacterales;Syntrophobacteraceae;Syntrophobacter;Syntrophobacter fumaroxidans;Syntrophobacter fumaroxidans MPOB |
| Ga0586324 1998008 3 350 | 0.00 | 415484.08 | 0.00 | 1976000.00 | 0.00 | 0.00 | 0.00 | 0.00 | 0.00 | 0.00 | 0.00 | 0.00 | KO:K02030 | polar amino acid transport system substrate-binding protein | Bacteria;Proteobacteria;Deltaproteobacteria;Desulfuromonadales;Geobacteraceae;Geobacter;Geobacter sp. M18;Geobacter sp. M18 |
| Ga0586324 2084228 2 484 | 0.00 | 0.00 | 0.00 | 0.00 | 0.00 | 0.00 | 0.00 | 0.00 | 0.00 | 0.00 | 0.00 | 392770.50 | KO:K02030 | polar amino acid transport system substrate-binding protein | Bacteria;Proteobacteria;Deltaproteobacteria;Desulfovibrionales;Desulfovibrionaceae;Desulfovibrio;Desulfovibrio vulgaris;Desulfovibrio vulgaris Miyazaki F |
| Ga0586324 3365737 248 727 | 0.00 | 0.00 | 0.00 | 0.00 | 0.00 | 0.00 | 0.00 | 0.00 | 1344600.00 | 0.00 | 0.00 | 0.00 | KO:K02030 | polar amino acid transport system substrate-binding protein | Bacteria;Proteobacteria;Deltaproteobacteria;Syntrophobacterales;Syntrophobacteraceae;Desulfacinum;Desulfacinum infernum;Desulfacinum infernum DSM 9756 |
| Ga0586324 5226036 2 604 | 0.00 | 178674.76 | 0.00 | 0.00 | 0.00 | 0.00 | 0.00 | 0.00 | 0.00 | 0.00 | 0.00 | 0.00 | KO:K02030 | polar amino acid transport system substrate-binding protein | Bacteria;Proteobacteria;Deltaproteobacteria;Desulfuromonadales;Geobacteraceae;Geobacter;Geobacter pickeringii;Geobacter pickeringii G13 |
| Ga0586324 0341563 932 1939 | 0.00 | 3019200.00 | 0.00 | 0.00 | 0.00 | 0.00 | 0.00 | 0.00 | 0.00 | 0.00 | 0.00 | 0.00 | KO:K02032 | peptide/nickel transport system ATP-binding protein | Bacteria;Actinobacteria;Actinomycetia;Geodermatophilales;Geodermatophilaceae;Blastococcus;Blastococcus xanthinilyticus;Blastococcus xanthinilyticus DSM 46842 |
| Ga0586324 0000019 21816 24407 | 19000000.00 | 0.00 | 10008845.10 | 3619500.00 | 2564700.00 | 2438985.38 | 7041643.20 | 7557900.00 | 2583900.00 | 6360200.00 | 4796000.00 | 3945000.00 | KO:K02035 | peptide/nickel transport system substrate-binding protein | Archaea;Crenarchaeota;Thermoprotei;Desulfurococcales;Desulfurococcaceae;Ignisphaera;Ignisphaera aggregans;Ignisphaera aggregans AQ1.S1, DSM 17230 |
| Ga0586324 0000272 26498 28426 | 0.00 | 3536000.00 | 0.00 | 0.00 | 0.00 | 0.00 | 0.00 | 0.00 | 0.00 | 0.00 | 0.00 | 0.00 | KO:K02035 | peptide/nickel transport system substrate-binding protein | Bacteria;Chloroflexi;Caldilineae;Caldilineales;Caldilineaceae;Caldilinea;Caldilinea aerophila;Caldilinea aerophila STL-6-O1, DSM 14535 |
| Ga0586324 0000907 6169 7908 | 0.00 | 1360000.00 | 0.00 | 0.00 | 0.00 | 0.00 | 0.00 | 0.00 | 0.00 | 2140600.00 | 1293600.00 | 0.00 | KO:K02035 | peptide/nickel transport system substrate-binding protein | Bacteria;Chloroflexi;Chloroflexia;Chloroflexales;Oscillochloridaceae;Oscillochloris;Oscillochloris trichoides;Oscillochloris trichoides DG6 |
| Ga0586324 0001936 7157 9292 | 0.00 | 2393600.00 | 0.00 | 0.00 | 3780100.00 | 0.00 | 1851663.24 | 5209400.00 | 1871100.00 | 0.00 | 13112000.00 | 429330.75 | KO:K02035 | peptide/nickel transport system substrate-binding protein | Bacteria;Proteobacteria;Alphaproteobacteria;Hyphomicrobiales;Devosiaceae;Devosia;Devosia sp. Root685;Devosia sp. Root685 |
| Ga0586324 0001998 17754 19544 | 0.00 | 4186730.76 | 0.00 | 0.00 | 0.00 | 0.00 | 0.00 | 1250500.00 | 3564000.00 | 0.00 | 5016000.00 | 4575000.00 | KO:K02035 | peptide/nickel transport system substrate-binding protein | Bacteria;Chloroflexi;Anaerolineae;Anaerolineales;Anaerolineaceae;Anaerolinea;Anaerolinea thermophila;Anaerolinea thermophila UNI-1 |
| Ga0586324 0003145 4680 6461 | 0.00 | 856800.00 | 0.00 | 0.00 | 1174200.00 | 0.00 | 0.00 | 0.00 | 0.00 | 4951100.00 | 1812800.00 | 0.00 | KO:K02035 | peptide/nickel transport system substrate-binding protein | Archaea;Crenarchaeota;Thermoprotei;Thermoproteales;Thermoproteaceae;Pyrobaculum;Pyrobaculum arsenaticum;Pyrobaculum arsenaticum PZ6, DSM 13514 |
| Ga0586324 0003635 1485 3230 | 7690000.00 | 3189200.00 | 0.00 | 0.00 | 0.00 | 0.00 | 0.00 | 5526600.00 | 5192100.00 | 0.00 | 0.00 | 1080000.00 | KO:K02035 | peptide/nickel transport system substrate-binding protein | Bacteria;Chloroflexi;Anaerolineae;Anaerolineales;Anaerolineaceae;Anaerolinea;Anaerolinea thermophila;Anaerolinea thermophila UNI-1 |
| Ga0586324 0007399 5215 6909 | 0.00 | 0.00 | 0.00 | 0.00 | 0.00 | 0.00 | 0.00 | 0.00 | 0.00 | 0.00 | 0.00 | 3757500.00 | KO:K02035 | peptide/nickel transport system substrate-binding protein | Bacteria;Actinobacteria;Rubrobacteria;Gaiellales;Gaiellaceae;Gaiella;Gaiella occulta;Gaiella occulta F2-233 |
| Ga0586324 0012773 5921 7636 | 0.00 | 12920000.00 | 0.00 | 0.00 | 0.00 | 0.00 | 0.00 | 4684800.00 | 0.00 | 0.00 | 7224800.00 | 0.00 | KO:K02035 | peptide/nickel transport system substrate-binding protein | Bacteria;Chloroflexi;Anaerolineae;Anaerolineales;Anaerolineaceae;Anaerolinea;Anaerolinea thermophila;Anaerolinea thermophila UNI-1 |
| Ga0586324 0014535 2924 4981 | 32040000.00 | 22504595.92 | 0.00 | 10070000.00 | 0.00 | 21954100.00 | 13793146.37 | 11248400.00 | 10449000.00 | 1492511.79 | 23654400.00 | 64620000.00 | KO:K02035 | peptide/nickel transport system substrate-binding protein | Bacteria;Chloroflexi;Ardenticatenia;Ardenticatenales;Ardenticatenaceae;Candidatus Promineofilum;Candidatus Promineofilum breve;Candidatus Promineofilum breve Cfx-K |
| Ga0586324 0023171 3339 5420 | 0.00 | 0.00 | 0.00 | 0.00 | 0.00 | 0.00 | 0.00 | 3129300.00 | 1368900.00 | 4535300.00 | 2428800.00 | 750000.00 | KO:K02035 | peptide/nickel transport system substrate-binding protein | Bacteria;Chloroflexi;Ardenticatenia;Ardenticatenales;Ardenticatenaceae;Candidatus Promineofilum;Candidatus Promineofilum breve;Candidatus Promineofilum breve Cfx-K |
| Ga0586324 0028684 3881 5830 | 18600000.00 | 2781200.00 | 11316300.00 | 0.00 | 0.00 | 0.00 | 0.00 | 2147200.00 | 0.00 | 0.00 | 0.00 | 0.00 | KO:K02035 | peptide/nickel transport system substrate-binding protein | Bacteria;Chloroflexi;Ardenticatenia;Ardenticatenales;Ardenticatenaceae;Candidatus Promineofilum;Candidatus Promineofilum breve;Candidatus Promineofilum breve Cfx-K |
| Ga0586324 0029403 3 1217 | 0.00 | 0.00 | 0.00 | 0.00 | 0.00 | 0.00 | 0.00 | 0.00 | 5977800.00 | 0.00 | 0.00 | 0.00 | KO:K02035 | peptide/nickel transport system substrate-binding protein | Bacteria;Chloroflexi;Anaerolineae;Anaerolineales;Anaerolineaceae;Pelolinea;Pelolinea submarina;Pelolinea submarina DSM 23923 |
| Ga0586324 0036883 1489 3414 | 0.00 | 1523200.00 | 0.00 | 0.00 | 0.00 | 0.00 | 0.00 | 0.00 | 0.00 | 3041500.00 | 0.00 | 0.00 | KO:K02035 | peptide/nickel transport system substrate-binding protein | Bacteria;Chloroflexi;Caldilineae;Caldilineales;Caldilineaceae;Caldilinea;Caldilinea aerophila;Caldilinea aerophila STL-6-O1, DSM 14535 |
| Ga0586324 0041667 3073 5124 | 0.00 | 0.00 | 0.00 | 0.00 | 0.00 | 0.00 | 2121679.08 | 0.00 | 0.00 | 0.00 | 6573600.00 | 5310000.00 | KO:K02035 | peptide/nickel transport system substrate-binding protein | Bacteria;Chloroflexi;Anaerolineae;Anaerolineales;Anaerolineaceae;Pelolinea;Pelolinea submarina;Pelolinea submarina DSM 23923 |
| Ga0586324 0048601 2145 4196 | 12280000.00 | 9744400.00 | 0.00 | 42332000.00 | 27948550.45 | 4577914.19 | 46554941.52 | 15060900.00 | 8010900.00 | 6583500.00 | 21172800.00 | 4819669.50 | KO:K02035 | peptide/nickel transport system substrate-binding protein | Bacteria;Chloroflexi;Ardenticatenia;Ardenticatenales;Ardenticatenaceae;Candidatus Promineofilum;Candidatus Promineofilum breve;Candidatus Promineofilum breve Cfx-K |
| Ga0586324 0059223 2805 4403 | 0.00 | 4576400.00 | 8107200.00 | 4408000.00 | 5376600.00 | 0.00 | 7957200.00 | 5593700.00 | 6480000.00 | 0.00 | 9064000.00 | 0.00 | KO:K02035 | peptide/nickel transport system substrate-binding protein | Bacteria;Chloroflexi;Thermoflexia;Thermoflexales;Thermoflexaceae;Thermoflexus;Thermoflexus hugenholtzii;Thermoflexus hugenholtzii JAD2 |
| Ga0586324 0062208 77 2233 | 0.00 | 3672000.00 | 0.00 | 0.00 | 0.00 | 0.00 | 0.00 | 3483100.00 | 3474900.00 | 0.00 | 0.00 | 0.00 | KO:K02035 | peptide/nickel transport system substrate-binding protein | Bacteria;Chloroflexi;Chloroflexia;Chloroflexales;Roseiflexaceae;Roseiflexus;Roseiflexus sp. RS-1;Roseiflexus sp. RS-1 |
| Ga0586324 0062557 710 2329 | 0.00 | 9588000.00 | 8670200.00 | 0.00 | 0.00 | 0.00 | 0.00 | 0.00 | 0.00 | 11858000.00 | 11616000.00 | 1530000.00 | KO:K02035 | peptide/nickel transport system substrate-binding protein | Bacteria;Firmicutes;Clostridia;Eubacteriales;Clostridiales Family XVI. Incertae Sedis;Carboxydocella;Carboxydocella thermautotrophica;Carboxydocella thermautotrophica 041 |
| Ga0586324 0068177 509 2257 | 3830000.00 | 0.00 | 0.00 | 793604.35 | 0.00 | 0.00 | 0.00 | 0.00 | 0.00 | 0.00 | 0.00 | 0.00 | KO:K02035 | peptide/nickel transport system substrate-binding protein | Bacteria;Actinobacteria;Actinomycetia;Streptosporangiales;Thermomonosporaceae;Thermostaphylospora;Thermostaphylospora chromogena;Thermostaphylospora chromogena DSM 43794 |
| Ga0586324 0074399 2054 3949 | 0.00 | 6650400.00 | 0.00 | 0.00 | 14729000.00 | 2618389.68 | 0.00 | 2799900.00 | 0.00 | 0.00 | 0.00 | 0.00 | KO:K02035 | peptide/nickel transport system substrate-binding protein | Archaea;unclassified Archaea;unclassified Archaea;unclassified Archaea;Thermofilaceae;Thermofilum;Thermofilum pendens;Thermofilum pendens Hrk 5 |
| Ga0586324 0088374 2277 3638 | 0.00 | 0.00 | 0.00 | 0.00 | 0.00 | 0.00 | 0.00 | 1701900.00 | 0.00 | 0.00 | 0.00 | 0.00 | KO:K02035 | peptide/nickel transport system substrate-binding protein | Bacteria;Chloroflexi;Anaerolineae;Anaerolineales;Anaerolineaceae;Pelolinea;Pelolinea submarina;Pelolinea submarina DSM 23923 |
| Ga0586324 0101802 3 1826 | 0.00 | 0.00 | 0.00 | 541947.45 | 0.00 | 2950015.69 | 0.00 | 0.00 | 0.00 | 0.00 | 0.00 | 0.00 | KO:K02035 | peptide/nickel transport system substrate-binding protein | Archaea;Crenarchaeota;Thermoprotei;Thermoproteales;Thermoproteaceae;Pyrobaculum;Pyrobaculum arsenaticum;Pyrobaculum arsenaticum PZ6, DSM 13514 |
| Ga0586324 0123166 757 3105 | 0.00 | 0.00 | 0.00 | 0.00 | 0.00 | 0.00 | 5768400.00 | 0.00 | 0.00 | 0.00 | 0.00 | 0.00 | KO:K02035 | peptide/nickel transport system substrate-binding protein | Archaea;Crenarchaeota;Thermoprotei;Desulfurococcales;Desulfurococcaceae;Ignisphaera;Ignisphaera aggregans;Ignisphaera aggregans AQ1.S1, DSM 17230 |
| Ga0586324 0162930 1095 2723 | 0.00 | 5168000.00 | 0.00 | 5871000.00 | 1627400.00 | 0.00 | 0.00 | 0.00 | 3053700.00 | 0.00 | 0.00 | 0.00 | KO:K02035 | peptide/nickel transport system substrate-binding protein | Bacteria;Proteobacteria;Alphaproteobacteria;Rhodospirillales;unclassified Rhodospirillales;unclassified Rhodospirillales;Rhodospirillales bacterium URHD0017;Rhodospirillales bacterium URHD0017 |
| Ga0586324 0174077 1311 2639 | 0.00 | 3019200.00 | 0.00 | 0.00 | 0.00 | 0.00 | 0.00 | 0.00 | 0.00 | 0.00 | 0.00 | 0.00 | KO:K02035 | peptide/nickel transport system substrate-binding protein | Bacteria;Chloroflexi;Anaerolineae;Anaerolineales;Anaerolineaceae;Anaerolinea;Anaerolinea thermophila;Anaerolinea thermophila UNI-1 |
| Ga0586324 0178360 2 2335 | 3800000.00 | 3325200.00 | 0.00 | 0.00 | 0.00 | 0.00 | 4012800.00 | 5441200.00 | 2867400.00 | 8316000.00 | 7814400.00 | 6930000.00 | KO:K02035 | peptide/nickel transport system substrate-binding protein | Archaea;Crenarchaeota;Thermoprotei;Desulfurococcales;Desulfurococcaceae;Ignisphaera;Ignisphaera aggregans;Ignisphaera aggregans AQ1.S1, DSM 17230 |
| Ga0586324 0201315 950 2467 | 1230000.00 | 0.00 | 0.00 | 6374500.00 | 8476900.00 | 0.00 | 0.00 | 0.00 | 0.00 | 0.00 | 0.00 | 0.00 | KO:K02035 | peptide/nickel transport system substrate-binding protein | Archaea;Crenarchaeota;Thermoprotei;Desulfurococcales;Desulfurococcaceae;Ignisphaera;Ignisphaera aggregans;Ignisphaera aggregans AQ1.S1, DSM 17230 |
| Ga0586324 0203873 1 1605 | 0.00 | 931600.00 | 0.00 | 14440000.00 | 0.00 | 0.00 | 0.00 | 0.00 | 1539000.00 | 0.00 | 0.00 | 0.00 | KO:K02035 | peptide/nickel transport system substrate-binding protein | Bacteria;Chloroflexi;Ardenticatenia;Ardenticatenales;Ardenticatenaceae;Candidatus Promineofilum;Candidatus Promineofilum breve;Candidatus Promineofilum breve Cfx-K |
| Ga0586324 0206541 2 1750 | 0.00 | 0.00 | 0.00 | 24890000.00 | 0.00 | 0.00 | 2713200.00 | 0.00 | 13608000.00 | 0.00 | 0.00 | 0.00 | KO:K02035 | peptide/nickel transport system substrate-binding protein | Bacteria;Proteobacteria;Alphaproteobacteria;Rhodospirillales;Rhodospirillaceae;Fodinicurvata;Fodinicurvata sediminis;Fodinicurvata sediminis DSM 21159 |
| Ga0586324 0214231 1113 2396 | 0.00 | 1672800.00 | 0.00 | 0.00 | 0.00 | 0.00 | 0.00 | 0.00 | 0.00 | 0.00 | 0.00 | 0.00 | KO:K02035 | peptide/nickel transport system substrate-binding protein | Bacteria;Chloroflexi;Anaerolineae;Anaerolineales;Anaerolineaceae;Anaerolinea;Anaerolinea thermophila;Anaerolinea thermophila UNI-1 |
| Ga0586324 0230572 2 2029 | 2130000.00 | 3950800.00 | 0.00 | 1377500.00 | 1163900.00 | 0.00 | 0.00 | 0.00 | 1142100.00 | 0.00 | 1856800.00 | 0.00 | KO:K02035 | peptide/nickel transport system substrate-binding protein | Archaea;Crenarchaeota;Thermoprotei;Desulfurococcales;Desulfurococcaceae;Ignisphaera;Ignisphaera aggregans;Ignisphaera aggregans AQ1.S1, DSM 17230 |
| Ga0586324 0256811 672 2207 | 0.00 | 0.00 | 0.00 | 0.00 | 0.00 | 0.00 | 0.00 | 1128500.00 | 0.00 | 0.00 | 0.00 | 0.00 | KO:K02035 | peptide/nickel transport system substrate-binding protein | Bacteria;Actinobacteria;Actinomycetia;Kineosporiales;Kineosporiaceae;Kineosporia;Kineosporia sp. A 224;Kineosporia sp. A 224 A 224 |
| Ga0586324 0263496 2 1726 | 0.00 | 0.00 | 0.00 | 0.00 | 0.00 | 0.00 | 0.00 | 152123.02 | 0.00 | 0.00 | 0.00 | 0.00 | KO:K02035 | peptide/nickel transport system substrate-binding protein | Bacteria;Chloroflexi;Ardenticatenia;Ardenticatenales;Ardenticatenaceae;Candidatus Promineofilum;Candidatus Promineofilum breve;Candidatus Promineofilum breve Cfx-K |
| Ga0586324 0278361 57 2126 | 0.00 | 0.00 | 0.00 | 0.00 | 0.00 | 0.00 | 0.00 | 0.00 | 0.00 | 2564100.00 | 2050400.00 | 0.00 | KO:K02035 | peptide/nickel transport system substrate-binding protein | Bacteria;Proteobacteria;Gammaproteobacteria;Oceanospirillales;Halomonadaceae;Halomonas;Halomonas heilongjiangensis;Halomonas heilongjiangensis DSM 26881 |
| Ga0586324 0305797 3 1166 | 0.00 | 0.00 | 0.00 | 0.00 | 0.00 | 0.00 | 0.00 | 0.00 | 562399.20 | 0.00 | 0.00 | 0.00 | KO:K02035 | peptide/nickel transport system substrate-binding protein | Bacteria;Proteobacteria;Deltaproteobacteria;Syntrophobacterales;Syntrophobacteraceae;Desulfacinum;Desulfacinum infernum;Desulfacinum infernum DSM 9756 |
| Ga0586324 0320949 1 1548 | 0.00 | 0.00 | 0.00 | 0.00 | 0.00 | 0.00 | 0.00 | 444002.53 | 0.00 | 0.00 | 0.00 | 0.00 | KO:K02035 | peptide/nickel transport system substrate-binding protein | Bacteria;Chloroflexi;Ardenticatenia;Ardenticatenales;Ardenticatenaceae;Ardenticatena;Ardenticatena maritima;Ardenticatena maritima 110S |
| Ga0586324 0321242 808 1992 | 1730000.00 | 0.00 | 0.00 | 0.00 | 0.00 | 0.00 | 0.00 | 0.00 | 0.00 | 0.00 | 0.00 | 0.00 | KO:K02035 | peptide/nickel transport system substrate-binding protein | Bacteria;Proteobacteria;Deltaproteobacteria;Desulfarculales;Desulfarculaceae;Dethiosulfatarculus;Dethiosulfatarculus sandiegensis;Dethiosulfatarculus sandiegensis SPR |
| Ga0586324 0365080 2 1882 | 13673704.00 | 20323905.96 | 28656700.00 | 5263000.00 | 3584400.00 | 2590492.49 | 3579600.00 | 8576600.00 | 10918800.00 | 11111100.00 | 7893600.00 | 3907500.00 | KO:K02035 | peptide/nickel transport system substrate-binding protein | Bacteria;Chloroflexi;Ardenticatenia;Ardenticatenales;Ardenticatenaceae;Candidatus Promineofilum;Candidatus Promineofilum breve;Candidatus Promineofilum breve Cfx-K |
| Ga0586324 0426076 399 1757 | 0.00 | 8364000.00 | 17790800.00 | 0.00 | 0.00 | 0.00 | 0.00 | 0.00 | 1150200.00 | 0.00 | 0.00 | 12150000.00 | KO:K02035 | peptide/nickel transport system substrate-binding protein | Bacteria;Actinobacteria;Actinomycetia;Streptosporangiales;Streptosporangiaceae;Microtetraspora;Microtetraspora glauca;Microtetraspora glauca NBRC 14761 |
| Ga0586324 0474303 3 1676 | 2360000.00 | 0.00 | 0.00 | 0.00 | 0.00 | 0.00 | 0.00 | 0.00 | 0.00 | 0.00 | 0.00 | 1522500.00 | KO:K02035 | peptide/nickel transport system substrate-binding protein | Archaea;Crenarchaeota;Thermoprotei;Desulfurococcales;Desulfurococcaceae;Ignisphaera;Ignisphaera aggregans;Ignisphaera aggregans AQ1.S1, DSM 17230 |
| Ga0586324 0498076 634 1641 | 0.00 | 0.00 | 0.00 | 0.00 | 0.00 | 0.00 | 0.00 | 0.00 | 587576.43 | 0.00 | 0.00 | 1057500.00 | KO:K02035 | peptide/nickel transport system substrate-binding protein | Bacteria;Firmicutes;Bacilli;Bacillales;Bacillaceae;Bacillus;Bacillus sp. OK085;Bacillus sp. OK085 |
| Ga0586324 0512241 1 1203 | 0.00 | 0.00 | 0.00 | 0.00 | 0.00 | 0.00 | 0.00 | 0.00 | 1077300.00 | 0.00 | 0.00 | 0.00 | KO:K02035 | peptide/nickel transport system substrate-binding protein | Bacteria;Chloroflexi;Ardenticatenia;Ardenticatenales;Ardenticatenaceae;Candidatus Promineofilum;Candidatus Promineofilum breve;Candidatus Promineofilum breve Cfx-K |
| Ga0586324 0520359 816 1610 | 0.00 | 0.00 | 0.00 | 2916500.00 | 0.00 | 0.00 | 1372053.84 | 0.00 | 826200.00 | 0.00 | 0.00 | 0.00 | KO:K02035 | peptide/nickel transport system substrate-binding protein | Bacteria;Chloroflexi;Anaerolineae;Anaerolineales;Anaerolineaceae;Levilinea;Levilinea saccharolytica;Levilinea saccharolytica DSM 16555 |
| Ga0586324 0532211 1 1596 | 0.00 | 0.00 | 0.00 | 0.00 | 2338100.00 | 0.00 | 0.00 | 0.00 | 0.00 | 0.00 | 0.00 | 0.00 | KO:K02035 | peptide/nickel transport system substrate-binding protein | Bacteria;Chloroflexi;Anaerolineae;Anaerolineales;Anaerolineaceae;Thermanaerothrix;Thermanaerothrix daxensis;Thermanaerothrix daxensis GNS-1 |
| Ga0586324 0571536 1 1545 | 13710000.00 | 0.00 | 0.00 | 0.00 | 0.00 | 5796200.00 | 11012400.00 | 0.00 | 0.00 | 0.00 | 2481600.00 | 2812500.00 | KO:K02035 | peptide/nickel transport system substrate-binding protein | Archaea;Thaumarchaeota;unclassified Thaumarchaeota;unclassified Thaumarchaeota;unclassified Thaumarchaeota;unclassified Thaumarchaeota;Aigarchaeota archaeon JGI 0000001-H6;Aigarchaeota archaeon JGI 0000001-H6 (contamination screened) |
| Ga0586324 0588782 2 1525 | 3540000.00 | 0.00 | 952674.82 | 0.00 | 0.00 | 0.00 | 0.00 | 0.00 | 0.00 | 1532300.00 | 3220800.00 | 5752500.00 | KO:K02035 | peptide/nickel transport system substrate-binding protein | Archaea;Thaumarchaeota;unclassified Thaumarchaeota;unclassified Thaumarchaeota;unclassified Thaumarchaeota;unclassified Thaumarchaeota;Aigarchaeota archaeon SCGC AAA471-F17;Aigarchaeota archaeon SCGC AAA471-F17 (contamination screened) |
| Ga0586324 0616346 3 881 | 0.00 | 2597600.00 | 0.00 | 0.00 | 0.00 | 0.00 | 0.00 | 0.00 | 0.00 | 0.00 | 0.00 | 0.00 | KO:K02035 | peptide/nickel transport system substrate-binding protein | Bacteria;Chloroflexi;Chloroflexia;Chloroflexales;Oscillochloridaceae;Oscillochloris;Oscillochloris trichoides;Oscillochloris trichoides DG6 |
| Ga0586324 0848622 3 1304 | 0.00 | 0.00 | 0.00 | 0.00 | 0.00 | 6153500.00 | 6612000.00 | 0.00 | 3523500.00 | 3880800.00 | 0.00 | 5685000.00 | KO:K02035 | peptide/nickel transport system substrate-binding protein | Bacteria;Chloroflexi;Ardenticatenia;Ardenticatenales;Ardenticatenaceae;Candidatus Promineofilum;Candidatus Promineofilum breve;Candidatus Promineofilum breve Cfx-K |
| Ga0586324 0897867 268 1272 | 0.00 | 0.00 | 0.00 | 0.00 | 0.00 | 0.00 | 3100800.00 | 0.00 | 0.00 | 0.00 | 0.00 | 0.00 | KO:K02035 | peptide/nickel transport system substrate-binding protein | Bacteria;Proteobacteria;Deltaproteobacteria;unclassified Deltaproteobacteria;unclassified Deltaproteobacteria;unclassified Deltaproteobacteria;SAR324 cluster bacterium JCVI-SC AAA005;SAR324 cluster bacterium JCVI-SC AAA005 |
| Ga0586324 0904305 1 1008 | 0.00 | 0.00 | 0.00 | 0.00 | 0.00 | 0.00 | 0.00 | 0.00 | 0.00 | 5528600.00 | 5086400.00 | 4687500.00 | KO:K02035 | peptide/nickel transport system substrate-binding protein | Bacteria;Proteobacteria;Betaproteobacteria;Burkholderiales;Comamonadaceae;Polaromonas;Polaromonas sp. EUR3 1.2.1;Polaromonas sp. EUR3 1.2.1 |
| Ga0586324 0933310 3 1250 | 0.00 | 2645200.00 | 3479621.50 | 0.00 | 4037600.00 | 0.00 | 3169200.00 | 3220800.00 | 3256200.00 | 4858700.00 | 5139200.00 | 3592500.00 | KO:K02035 | peptide/nickel transport system substrate-binding protein | Archaea;Crenarchaeota;Thermoprotei;Desulfurococcales;Desulfurococcaceae;Ignisphaera;Ignisphaera aggregans;Ignisphaera aggregans AQ1.S1, DSM 17230 |
| Ga0586324 0941009 159 1247 | 0.00 | 0.00 | 0.00 | 0.00 | 0.00 | 0.00 | 2964000.00 | 0.00 | 0.00 | 0.00 | 0.00 | 0.00 | KO:K02035 | peptide/nickel transport system substrate-binding protein | Bacteria;Proteobacteria;Deltaproteobacteria;Desulfobacterales;Desulfobacteraceae;Desulfospira;Desulfospira joergensenii;Desulfospira joergensenii DSM 10085 |
| Ga0586324 0958491 3 1235 | 608745.00 | 0.00 | 0.00 | 0.00 | 0.00 | 0.00 | 0.00 | 0.00 | 0.00 | 0.00 | 0.00 | 0.00 | KO:K02035 | peptide/nickel transport system substrate-binding protein | Bacteria;Actinobacteria;Actinomycetia;Streptosporangiales;Streptosporangiaceae;Microtetraspora;Microtetraspora malaysiensis;Microtetraspora malaysiensis NBRC 100735 |
| Ga0586324 1005395 2 1210 | 0.00 | 0.00 | 0.00 | 0.00 | 0.00 | 0.00 | 2918400.00 | 2854800.00 | 1903500.00 | 0.00 | 3317600.00 | 0.00 | KO:K02035 | peptide/nickel transport system substrate-binding protein | Archaea;Crenarchaeota;Thermoprotei;Desulfurococcales;Desulfurococcaceae;Ignisphaera;Ignisphaera aggregans;Ignisphaera aggregans AQ1.S1, DSM 17230 |
| Ga0586324 1045598 3 1190 | 0.00 | 0.00 | 3271975.84 | 0.00 | 0.00 | 0.00 | 0.00 | 0.00 | 0.00 | 0.00 | 0.00 | 0.00 | KO:K02035 | peptide/nickel transport system substrate-binding protein | Bacteria;Chloroflexi;Caldilineae;Caldilineales;Caldilineaceae;Caldilinea;Caldilinea aerophila;Caldilinea aerophila STL-6-O1, DSM 14535 |
| Ga0586324 1148658 1 966 | 1430000.00 | 0.00 | 0.00 | 0.00 | 0.00 | 0.00 | 0.00 | 0.00 | 0.00 | 4442900.00 | 0.00 | 0.00 | KO:K02035 | peptide/nickel transport system substrate-binding protein | Bacteria;Proteobacteria;Alphaproteobacteria;Rhodospirillales;unclassified Rhodospirillales;unclassified Rhodospirillales;Rhodospirillales bacterium URHD0017;Rhodospirillales bacterium URHD0017 |
| Ga0586324 1172850 131 1132 | 0.00 | 0.00 | 4676306.15 | 0.00 | 2729500.00 | 0.00 | 0.00 | 3409900.00 | 3515400.00 | 7399700.00 | 7744000.00 | 3030000.00 | KO:K02035 | peptide/nickel transport system substrate-binding protein | Bacteria;Proteobacteria;Alphaproteobacteria;Hyphomicrobiales;Devosiaceae;Devosia;Devosia sp. A16;Devosia sp. DDS-1 A16 |
| Ga0586324 1188274 3 1127 | 0.00 | 843200.00 | 0.00 | 0.00 | 0.00 | 0.00 | 0.00 | 0.00 | 0.00 | 0.00 | 0.00 | 0.00 | KO:K02035 | peptide/nickel transport system substrate-binding protein | Archaea;Crenarchaeota;Thermoprotei;Desulfurococcales;Desulfurococcaceae;Ignisphaera;Ignisphaera aggregans;Ignisphaera aggregans AQ1.S1, DSM 17230 |
| Ga0586324 1331589 1 537 | 911909.00 | 0.00 | 0.00 | 0.00 | 0.00 | 0.00 | 0.00 | 0.00 | 0.00 | 0.00 | 0.00 | 0.00 | KO:K02035 | peptide/nickel transport system substrate-binding protein | Bacteria;Synergistetes;Synergistia;Synergistales;Synergistaceae;Acetomicrobium;Acetomicrobium thermoterrenum;Acetomicrobium thermoterrenum DSM 13490 |
| Ga0586324 1420166 2 1045 | 0.00 | 0.00 | 0.00 | 0.00 | 135248.27 | 0.00 | 141570.22 | 71181.51 | 1231200.00 | 0.00 | 0.00 | 0.00 | KO:K02035 | peptide/nickel transport system substrate-binding protein | Bacteria;Chloroflexi;Ardenticatenia;Ardenticatenales;Ardenticatenaceae;Candidatus Promineofilum;Candidatus Promineofilum breve;Candidatus Promineofilum breve Cfx-K |
| Ga0586324 1503865 2 622 | 0.00 | 0.00 | 0.00 | 0.00 | 0.00 | 0.00 | 0.00 | 0.00 | 0.00 | 0.00 | 0.00 | 1312500.00 | KO:K02035 | peptide/nickel transport system substrate-binding protein | Bacteria;Proteobacteria;Betaproteobacteria;Burkholderiales;Comamonadaceae;Caenimonas;Caenimonas koreensis;Caenimonas koreensis DSM 17982 |
| Ga0586324 1506101 3 803 | 0.00 | 6011200.00 | 2387677.37 | 3543500.00 | 0.00 | 0.00 | 2895600.00 | 4349300.00 | 0.00 | 5382300.00 | 7480000.00 | 5520000.00 | KO:K02035 | peptide/nickel transport system substrate-binding protein | Bacteria;Chloroflexi;Anaerolineae;Anaerolineales;Anaerolineaceae;Levilinea;Levilinea saccharolytica;Levilinea saccharolytica DSM 16555 |
| Ga0586324 1510120 1 1017 | 0.00 | 0.00 | 0.00 | 0.00 | 0.00 | 0.00 | 0.00 | 0.00 | 0.00 | 1909600.00 | 0.00 | 0.00 | KO:K02035 | peptide/nickel transport system substrate-binding protein | Bacteria;Actinobacteria;Actinomycetia;Propionibacteriales;Kribbellaceae;Kribbella;Kribbella soli;Kribbella soli KCTC 29219 |
| Ga0586324 1791127 1 945 | 0.00 | 0.00 | 4533095.84 | 0.00 | 0.00 | 0.00 | 0.00 | 0.00 | 0.00 | 0.00 | 0.00 | 4995000.00 | KO:K02035 | peptide/nickel transport system substrate-binding protein | Bacteria;Chloroflexi;Ardenticatenia;Ardenticatenales;Ardenticatenaceae;Candidatus Promineofilum;Candidatus Promineofilum breve;Candidatus Promineofilum breve Cfx-K |

| | | | | | | | | | | | | | | | |
|---|---|---|---|---|---|---|---|---|---|---|---|---|---|---|---|
| Ga0586324 1802373 1 777 | 0.00 | 172854.64 | 0.00 | 0.00 | 0.00 | 0.00 | 0.00 | 0.00 | 0.00 | 0.00 | 0.00 | 0.00 | KO:K02035 | peptide/nickel transport system substrate-binding protein | Bacteria;Proteobacteria;Alphaproteobacteria;Rhodospirillales;unclassified Rhodospirillales;unclassified Rhodospirillales;Rhodospirillales bacterium URHD0088;Rhodospirillales bacterium URHD0088 |
| Ga0586324 1985302 3 905 | 0.00 | 0.00 | 890643.48 | 0.00 | 1102100.00 | 0.00 | 0.00 | 0.00 | 0.00 | 0.00 | 0.00 | 0.00 | KO:K02035 | peptide/nickel transport system substrate-binding protein | Bacteria;Proteobacteria;Alphaproteobacteria;Rhodospirillales;unclassified Rhodospirillales;unclassified Rhodospirillales;Rhodospirillales bacterium URHD0088;Rhodospirillales bacterium URHD0088 |
| Ga0586324 1998411 3 902 | 0.00 | 504577.68 | 0.00 | 0.00 | 0.00 | 0.00 | 0.00 | 0.00 | 0.00 | 0.00 | 0.00 | 0.00 | KO:K02035 | peptide/nickel transport system substrate-binding protein | Bacteria;Chloroflexi;Ardenticatenia;Ardenticatenales;Ardenticatenaceae;Candidatus Promineofilum;Candidatus Promineofilum breve;Candidatus Promineofilum breve Cfx-K |
| Ga0586324 2461992 1 828 | 0.00 | 0.00 | 0.00 | 0.00 | 1833400.00 | 0.00 | 0.00 | 3043900.00 | 0.00 | 1848000.00 | 0.00 | 0.00 | KO:K02035 | peptide/nickel transport system substrate-binding protein | Bacteria;Actinobacteria;Actinomycetia;Propionibacteriales;Actinopolymorphaceae;Actinopolymorpha;Actinopolymorpha rutila;Actinopolymorpha rutila DSM 18448 |
| Ga0586324 2631910 3 803 | 6360000.00 | 0.00 | 11091100.00 | 0.00 | 7333600.00 | 0.00 | 0.00 | 0.00 | 0.00 | 0.00 | 0.00 | 8025000.00 | KO:K02035 | peptide/nickel transport system substrate-binding protein | Bacteria;Chloroflexi;Anaerolineae;Anaerolineales;Anaerolineaceae;Anaerolinea;Anaerolinea thermophila;Anaerolinea thermophila UNI-1 |
| Ga0586324 2706644 2 796 | 2010000.00 | 0.00 | 0.00 | 0.00 | 0.00 | 0.00 | 0.00 | 0.00 | 0.00 | 0.00 | 0.00 | 0.00 | KO:K02035 | peptide/nickel transport system substrate-binding protein | Bacteria;Chloroflexi;Anaerolineae;Anaerolineales;Anaerolineaceae;Anaerolinea;Anaerolinea thermophila;Anaerolinea thermophila UNI-1 |
| Ga0586324 2726113 3 794 | 0.00 | 0.00 | 0.00 | 0.00 | 0.00 | 0.00 | 0.00 | 0.00 | 2818800.00 | 0.00 | 0.00 | 0.00 | KO:K02035 | peptide/nickel transport system substrate-binding protein | Bacteria;Proteobacteria;Betaproteobacteria;Burkholderiales;Comamonadaceae;unclassified Comamonadaceae;Comamonadaceae bacterium URHA0028;Comamonadaceae bacterium URHA0028 |
| Ga0586324 2778502 329 787 | 0.00 | 3175600.00 | 0.00 | 0.00 | 0.00 | 3726714.43 | 0.00 | 0.00 | 0.00 | 0.00 | 0.00 | 0.00 | KO:K02035 | peptide/nickel transport system substrate-binding protein | Bacteria;Firmicutes;Bacilli;Bacillales;Paenibacillaceae;Paenibacillus;Paenibacillus xerothermodurans;Paenibacillus xerothermodurans ATCC 27380 |
| Ga0586324 2819041 2 781 | 0.00 | 0.00 | 0.00 | 0.00 | 0.00 | 0.00 | 0.00 | 1695800.00 | 0.00 | 0.00 | 0.00 | 0.00 | KO:K02035 | peptide/nickel transport system substrate-binding protein | Bacteria;Chloroflexi;Anaerolineae;Anaerolineales;Anaerolineaceae;Anaerolinea;Anaerolinea thermophila;Anaerolinea thermophila UNI-1 |
| Ga0586324 2820145 3 782 | 0.00 | 0.00 | 0.00 | 0.00 | 0.00 | 0.00 | 1080818.04 | 1323700.00 | 0.00 | 0.00 | 0.00 | 0.00 | KO:K02035 | peptide/nickel transport system substrate-binding protein | Bacteria;Chloroflexi;Ardenticatenia;Ardenticatenales;Ardenticatenaceae;Candidatus Promineofilum;Candidatus Promineofilum breve;Candidatus Promineofilum breve Cfx-K |
| Ga0586324 2837286 13 780 | 0.00 | 0.00 | 0.00 | 1596000.00 | 0.00 | 0.00 | 0.00 | 0.00 | 0.00 | 0.00 | 0.00 | 0.00 | KO:K02035 | peptide/nickel transport system substrate-binding protein | Bacteria;Actinobacteria;Actinomycetia;Streptosporangiales;Streptosporangiaceae;Microtetraspora;Microtetraspora glauca;Microtetraspora glauca NBRC 14761 |
| Ga0586324 3161446 3 746 | 0.00 | 856800.00 | 0.00 | 0.00 | 0.00 | 0.00 | 0.00 | 0.00 | 0.00 | 0.00 | 0.00 | 0.00 | KO:K02035 | peptide/nickel transport system substrate-binding protein | Bacteria;Candidatus Aerophobetes;unclassified Candidatus Aerophobetes;unclassified Candidatus Aerophobetes;unclassified Candidatus Aerophobetes;unclassified Candidatus Aerophobetes;Aerophobetes bacterium SCGC AAA255-F10;Aerophobetes bacterium SCGC AAA255-F10 (contamination screened) |
| Ga0586324 3369313 2 727 | 0.00 | 6738800.00 | 6024100.00 | 7799500.00 | 7642600.00 | 4327300.00 | 4560000.00 | 0.00 | 9234000.00 | 8778000.00 | 10472000.00 | 7575000.00 | KO:K02035 | peptide/nickel transport system substrate-binding protein | Bacteria;Proteobacteria;Alphaproteobacteria;Rhodospirillales;unclassified Rhodospirillales;unclassified Rhodospirillales;Rhodospirillales bacterium URHD0017;Rhodospirillales bacterium URHD0017 |
| Ga0586324 3562698 3 710 | 0.00 | 0.00 | 0.00 | 0.00 | 0.00 | 0.00 | 1202460.60 | 0.00 | 0.00 | 0.00 | 0.00 | 0.00 | KO:K02035 | peptide/nickel transport system substrate-binding protein | Bacteria;Spirochaetes;Spirochaetia;Spirochaetales;Spirochaetaceae;Sediminispirochaeta;Sediminispirochaeta smaragdinae;Sediminispirochaeta smaragdinae DSM 11293 |
| Ga0586324 3770465 2 694 | 0.00 | 0.00 | 0.00 | 0.00 | 0.00 | 0.00 | 0.00 | 72981.01 | 0.00 | 0.00 | 0.00 | 0.00 | KO:K02035 | peptide/nickel transport system substrate-binding protein | Bacteria;Chloroflexi;Ardenticatenia;Ardenticatenales;Ardenticatenaceae;Candidatus Promineofilum;Candidatus Promineofilum breve;Candidatus Promineofilum breve Cfx-K |
| Ga0586324 3890864 2 685 | 0.00 | 3427200.00 | 4490155.83 | 0.00 | 0.00 | 0.00 | 0.00 | 0.00 | 0.00 | 0.00 | 0.00 | 0.00 | KO:K02035 | peptide/nickel transport system substrate-binding protein | Bacteria;Firmicutes;Bacilli;Bacillales;Paenibacillaceae;Paenibacillus;Paenibacillus sp. 597;Paenibacillus sp. 597 |
| Ga0586324 4206806 2 661 | 3210000.00 | 10064000.00 | 0.00 | 3078000.00 | 0.00 | 0.00 | 0.00 | 2726700.00 | 1919700.00 | 6237000.00 | 0.00 | 8325000.00 | KO:K02035 | peptide/nickel transport system substrate-binding protein | Bacteria;Chloroflexi;Ardenticatenia;Ardenticatenales;Ardenticatenaceae;Candidatus Promineofilum;Candidatus Promineofilum breve;Candidatus Promineofilum breve Cfx-K |
| Ga0586324 5014743 3 617 | 0.00 | 4780400.00 | 0.00 | 7020500.00 | 0.00 | 0.00 | 0.00 | 1878800.00 | 0.00 | 0.00 | 0.00 | 0.00 | KO:K02035 | peptide/nickel transport system substrate-binding protein | Bacteria;Chloroflexi;Caldilineae;Caldilineales;Caldilineaceae;Caldilinea;Caldilinea aerophila;Caldilinea aerophila STL-6-O1, DSM 14535 |
| Ga0586324 5871918 1 576 | 0.00 | 0.00 | 0.00 | 0.00 | 0.00 | 578826.00 | 1749687.96 | 0.00 | 0.00 | 0.00 | 0.00 | 0.00 | KO:K02035 | peptide/nickel transport system substrate-binding protein | Bacteria;Actinobacteria;Acidimicrobiia;Acidimicrobiales;Ilumatobacteraceae;Ilumatobacter;Ilumatobacter nonamiensis;Ilumatobacter nonamiensis YM16-303 |
| Ga0586324 5965983 3 572 | 0.00 | 3529200.00 | 0.00 | 2641000.00 | 0.00 | 0.00 | 0.00 | 4733600.00 | 6706800.00 | 6398700.00 | 5112800.00 | 0.00 | KO:K02035 | peptide/nickel transport system substrate-binding protein | Bacteria;Chloroflexi;Caldilineae;Caldilineales;Caldilineaceae;Caldilinea;Caldilinea aerophila;Caldilinea aerophila STL-6-O1, DSM 14535 |
| Ga0586324 6271209 2 562 | 0.00 | 0.00 | 0.00 | 0.00 | 0.00 | 0.00 | 6543600.00 | 0.00 | 0.00 | 0.00 | 0.00 | 0.00 | KO:K02035 | peptide/nickel transport system substrate-binding protein | Bacteria;Chloroflexi;Anaerolineae;Anaerolineales;Anaerolineaceae;Pelolinea;Pelolinea submarina;Pelolinea submarina DSM 23923 |
| Ga0586324 6516357 269 553 | 0.00 | 0.00 | 0.00 | 0.00 | 0.00 | 0.00 | 0.00 | 0.00 | 0.00 | 0.00 | 0.00 | 217121.25 | KO:K02035 | peptide/nickel transport system substrate-binding protein | Bacteria;Chloroflexi;Ardenticatenia;Ardenticatenales;Ardenticatenaceae;Candidatus Promineofilum;Candidatus Promineofilum breve;Candidatus Promineofilum breve Cfx-K |
| Ga0586324 7190220 3 530 | 0.00 | 0.00 | 0.00 | 4740500.00 | 0.00 | 0.00 | 0.00 | 0.00 | 0.00 | 0.00 | 0.00 | 0.00 | KO:K02035 | peptide/nickel transport system substrate-binding protein | Bacteria;Proteobacteria;Alphaproteobacteria;Rhodospirillales;unclassified Rhodospirillales;unclassified Rhodospirillales;Rhodospirillales bacterium URHD0088;Rhodospirillales bacterium URHD0088 |
| Ga0586324 7273037 1 528 | 5630000.00 | 4902800.00 | 0.00 | 0.00 | 0.00 | 0.00 | 0.00 | 0.00 | 0.00 | 0.00 | 0.00 | 1372500.00 | KO:K02035 | peptide/nickel transport system substrate-binding protein | Bacteria;Chloroflexi;Anaerolineae;Anaerolineales;Anaerolineaceae;Anaerolinea;Anaerolinea thermophila;Anaerolinea thermophila UNI-1 |
| Ga0586324 7325205 1 525 | 26100000.00 | 0.00 | 0.00 | 0.00 | 0.00 | 0.00 | 0.00 | 0.00 | 0.00 | 0.00 | 0.00 | 0.00 | KO:K02035 | peptide/nickel transport system substrate-binding protein | Bacteria;Chloroflexi;Thermoflexia;Thermoflexales;Thermoflexaceae;Thermoflexus;Thermoflexus hugenholtzii;Thermoflexus hugenholtzii JAD2 |
| Ga0586324 7913060 3 509 | 0.00 | 2284800.00 | 3926063.61 | 0.00 | 0.00 | 3389454.99 | 0.00 | 0.00 | 0.00 | 0.00 | 0.00 | 0.00 | KO:K02035 | peptide/nickel transport system substrate-binding protein | Bacteria;Chloroflexi;Ardenticatenia;Ardenticatenales;Ardenticatenaceae;Candidatus Promineofilum;Candidatus Promineofilum breve;Candidatus Promineofilum breve Cfx-K |
| Ga0586324 2537977 306 818 | 0.00 | 0.00 | 0.00 | 0.00 | 0.00 | 0.00 | 0.00 | 0.00 | 0.00 | 0.00 | 0.00 | 2692500.00 | KO:K02036 | phosphate transport system ATP-binding protein | Bacteria;Ignavibacteriae;Ignavibacteria;Ignavibacteriales;Melioribacteraceae;Melioribacter;Melioribacter roseus;Melioribacter roseus P3M |
| Ga0586324 0244160 2 331 | 0.00 | 0.00 | 0.00 | 0.00 | 0.00 | 0.00 | 0.00 | 0.00 | 1368900.00 | 0.00 | 0.00 | 0.00 | KO:K02039 | phosphate transport system protein | Bacteria;Proteobacteria;Betaproteobacteria;Nitrosomonadales;Sulfuricellaceae;Sulfurirhabdus;Sulfurirhabdus autotrophica;Sulfurirhabdus autotrophica DSM 100309 |
| Ga0586324 3575851 3 545 | 0.00 | 0.00 | 1920049.57 | 0.00 | 0.00 | 0.00 | 0.00 | 0.00 | 0.00 | 0.00 | 0.00 | 0.00 | KO:K02039 | phosphate transport system protein | Archaea;Thaumarchaeota;Nitrososphaeria;Candidatus Nitrosocaldales;Candidatus Nitrosocaldaceae;Candidatus Nitrosocaldus;Candidatus Nitrosocaldus islandicus;Candidatus Nitrosocaldus islandicus |
| Ga0586324 0000030 18723 19742 | 0.00 | 0.00 | 0.00 | 0.00 | 0.00 | 0.00 | 0.00 | 0.00 | 0.00 | 0.00 | 4065600.00 | 6967500.00 | KO:K02040 | phosphate transport system substrate-binding protein | Bacteria;Proteobacteria;Betaproteobacteria;Nitrosomonadales;Thiobacillaceae;Thiobacillus;Thiobacillus denitrificans;Thiobacillus denitrificans RG |
| Ga0586324 0002092 12647 13666 | 0.00 | 0.00 | 0.00 | 0.00 | 0.00 | 0.00 | 0.00 | 4593300.00 | 0.00 | 0.00 | 0.00 | 0.00 | KO:K02040 | phosphate transport system substrate-binding protein | Bacteria;Proteobacteria;Betaproteobacteria;Nitrosomonadales;Thiobacillaceae;Thiobacillus;Thiobacillus denitrificans;Thiobacillus denitrificans RG |
| Ga0586324 0011195 2807 3823 | 0.00 | 0.00 | 0.00 | 0.00 | 0.00 | 0.00 | 7113600.00 | 1756800.00 | 1773900.00 | 2764300.00 | 2552000.00 | 7455000.00 | KO:K02040 | phosphate transport system substrate-binding protein | Bacteria;Proteobacteria;Betaproteobacteria;Nitrosomonadales;Thiobacillaceae;Thiobacillus;Thiobacillus thioparus;Thiobacillus thioparus DSM 505 |
| Ga0586324 0013673 3562 4611 | 0.00 | 0.00 | 0.00 | 0.00 | 0.00 | 0.00 | 0.00 | 0.00 | 0.00 | 7854000.00 | 12584000.00 | 0.00 | KO:K02040 | phosphate transport system substrate-binding protein | Bacteria;Proteobacteria;Betaproteobacteria;Nitrosomonadales;Gallionellaceae;Gallionella;Gallionella capsiferriformans;Gallionella capsiferriformans ES-2 |
| Ga0586324 0016031 3366 4211 | 0.00 | 2475200.00 | 3699613.75 | 0.00 | 0.00 | 0.00 | 0.00 | 0.00 | 0.00 | 0.00 | 0.00 | 0.00 | KO:K02040 | phosphate transport system substrate-binding protein | Archaea;Euryarchaeota;Methanomicrobia;Methanosarcinales;Methanotrichaceae;Methanothrix;Methanothrix soehngenii;Methanothrix soehngenii GP-6 |
| Ga0586324 0018253 3079 4116 | 0.00 | 0.00 | 0.00 | 0.00 | 0.00 | 0.00 | 0.00 | 2006900.00 | 0.00 | 17224900.00 | 5156800.00 | 0.00 | KO:K02040 | phosphate transport system substrate-binding protein | Bacteria;Proteobacteria;Betaproteobacteria;Nitrosomonadales;Sulfuricellaceae;Sulfuricella;Sulfuricella sp. T08;Sulfuricella sp. T08 |
| Ga0586324 0025083 5299 6348 | 0.00 | 0.00 | 0.00 | 607928.75 | 4871900.00 | 0.00 | 0.00 | 1043100.00 | 3191400.00 | 924000.00 | 0.00 | 1252500.00 | KO:K02040 | phosphate transport system substrate-binding protein | Bacteria;Proteobacteria;Gammaproteobacteria;Chromatiales;Chromatiaceae;Allochromatium;Allochromatium warmingii;Allochromatium warmingii DSM 173 |
| Ga0586324 0028264 5420 6307 | 0.00 | 0.00 | 0.00 | 0.00 | 0.00 | 0.00 | 0.00 | 0.00 | 0.00 | 0.00 | 0.00 | 1620000.00 | KO:K02040 | phosphate transport system substrate-binding protein | Bacteria;Proteobacteria;Gammaproteobacteria;Acidiferrobacterales;Acidiferrobacteraceae;Sulfuricaulis;Sulfuricaulis limicola;Sulfuricaulis limicola HA5 |
| Ga0586324 0871830 195 1007 | 0.00 | 0.00 | 15482500.00 | 0.00 | 0.00 | 0.00 | 0.00 | 0.00 | 0.00 | 0.00 | 0.00 | 0.00 | KO:K02040 | phosphate transport system substrate-binding protein | Bacteria;Nitrospirae;unclassified Nitrospirae;unclassified Nitrospirae;unclassified Nitrospirae;unclassified Nitrospirae;Nitrospirae bacterium HCH-1;Nitrospirae bacterium HCH-1 |
| Ga0586324 0873257 448 1287 | 0.00 | 0.00 | 0.00 | 0.00 | 0.00 | 0.00 | 0.00 | 0.00 | 0.00 | 0.00 | 0.00 | 3697500.00 | KO:K02040 | phosphate transport system substrate-binding protein | Bacteria;Proteobacteria;Betaproteobacteria;Nitrosomonadales;Thiobacillaceae;Thiobacillus;Thiobacillus denitrificans;Thiobacillus denitrificans RG |
| Ga0586324 1457350 1 939 | 1720000.00 | 0.00 | 0.00 | 0.00 | 0.00 | 0.00 | 0.00 | 0.00 | 0.00 | 0.00 | 0.00 | 0.00 | KO:K02040 | phosphate transport system substrate-binding protein | Bacteria;Proteobacteria;Betaproteobacteria;Nitrosomonadales;Thiobacillaceae;Thiobacillus;Thiobacillus denitrificans;Thiobacillus denitrificans RG |
| Ga0586324 0000004 81057 82490 | 0.00 | 0.00 | 0.00 | 0.00 | 0.00 | 0.00 | 0.00 | 0.00 | 0.00 | 0.00 | 0.00 | 5706465.00 | KO:K02051 | NitT/TauT family transport system substrate-binding protein | Bacteria;Proteobacteria;Betaproteobacteria;Nitrosomonadales;Thiobacillaceae;Thiobacillus;Thiobacillus thioparus;Thiobacillus thioparus DSM 505 |
| Ga0586324 0000082 18939 20570 | 0.00 | 1054000.00 | 0.00 | 0.00 | 0.00 | 0.00 | 0.00 | 0.00 | 0.00 | 0.00 | 0.00 | 0.00 | KO:K02051 | NitT/TauT family transport system substrate-binding protein | Bacteria;Firmicutes;Bacilli;Bacillales;Bacillaceae;Salirhabdus;Salirhabdus euzebyi;Salirhabdus euzebyi DSM 19612 |
| Ga0586324 2859778 2 778 | 0.00 | 0.00 | 0.00 | 0.00 | 0.00 | 0.00 | 0.00 | 1348100.00 | 1684800.00 | 0.00 | 0.00 | 0.00 | KO:K02051 | NitT/TauT family transport system substrate-binding protein | Bacteria;Proteobacteria;Betaproteobacteria;Burkholderiales;unclassified Burkholderiales;Methylibium;Methylibium sp. CF059;Methylibium sp. CF059 |
| Ga0586324 0270177 1 939 | 0.00 | 0.00 | 0.00 | 5842500.00 | 0.00 | 0.00 | 0.00 | 0.00 | 0.00 | 0.00 | 0.00 | 0.00 | KO:K02052 | putative spermidine/putrescine transport system ATP-binding protein | Bacteria;Proteobacteria;Alphaproteobacteria;Hyphomicrobiales;Hyphomicrobiaceae;Hyphomicrobium;Hyphomicrobium facile;Hyphomicrobium facile facile DSM 1565 |
| Ga0586324 3560828 124 708 | 0.00 | 0.00 | 0.00 | 0.00 | 0.00 | 0.00 | 0.00 | 3391600.00 | 0.00 | 0.00 | 0.00 | 0.00 | KO:K02052 | putative spermidine/putrescine transport system ATP-binding protein | Bacteria;Actinobacteria;Rubrobacteria;Gaiellales;Gaiellaceae;Gaiella;Gaiella occulta;Gaiella occulta F2-233 |
| Ga0586324 0008056 7618 8946 | 0.00 | 0.00 | 0.00 | 0.00 | 0.00 | 0.00 | 0.00 | 622200.00 | 0.00 | 0.00 | 0.00 | 0.00 | KO:K02055 | putative spermidine/putrescine transport system substrate-binding protein | Bacteria;Proteobacteria;Alphaproteobacteria;Rhodospirillales;Zavarziniaceae;Zavarzinia;Zavarzinia aquatilis;Zavarzinia aquatilis HR-AS |
| Ga0586324 0043327 3 1010 | 0.00 | 0.00 | 12667500.00 | 0.00 | 0.00 | 0.00 | 0.00 | 0.00 | 0.00 | 0.00 | 0.00 | 0.00 | KO:K02055 | putative spermidine/putrescine transport system substrate-binding protein | Bacteria;Firmicutes;Clostridia;Eubacteriales;Vallitaleaceae;Vallitalea;Vallitalea okinawensis;Vallitalea okinawensis S15 |
| Ga0586324 0475222 751 1677 | 0.00 | 2298400.00 | 0.00 | 0.00 | 0.00 | 0.00 | 0.00 | 1091900.00 | 0.00 | 0.00 | 0.00 | 0.00 | KO:K02055 | putative spermidine/putrescine transport system substrate-binding protein | Bacteria;Proteobacteria;Alphaproteobacteria;Hyphomicrobiales;Rhizobiaceae;Neorhizobium;Neorhizobium sp. R1-B;Neorhizobium sp. R1-B |
| Ga0586324 4387707 1 651 | 0.00 | 0.00 | 0.00 | 0.00 | 0.00 | 0.00 | 0.00 | 0.00 | 0.00 | 1963500.00 | 0.00 | 0.00 | KO:K02055 | putative spermidine/putrescine transport system substrate-binding protein | Bacteria;Proteobacteria;Alphaproteobacteria;Hyphomicrobiales;Rhizobiaceae;Pseudorhizobium;Pseudorhizobium pelagicum;Pseudorhizobium pelagicum R1-200B2 |
| Ga0586324 0041529 4274 5236 | 0.00 | 4630800.00 | 5345195.19 | 0.00 | 0.00 | 0.00 | 0.00 | 0.00 | 0.00 | 0.00 | 0.00 | 0.00 | KO:K02058 | simple sugar transport system substrate-binding protein | Bacteria;Atribacterota;unclassified Atribacterota;unclassified Atribacterota;unclassified Atribacterota;unclassified Atribacterota;Atribacteria bacterium JGI 0000079-F20;Atribacteria bacterium JGI 0000079-F20 (contamination screened) |
| Ga0586324 0141170 1697 2692 | 3060000.00 | 1693200.00 | 0.00 | 0.00 | 0.00 | 0.00 | 0.00 | 0.00 | 0.00 | 0.00 | 0.00 | 2295000.00 | KO:K02058 | simple sugar transport system substrate-binding protein | Bacteria;Chloroflexi;Caldilineae;Caldilineales;Caldilineaceae;Caldilinea;Caldilinea aerophila;Caldilinea aerophila STL-6-O1, DSM 14535 |
| Ga0586324 0154201 1688 2794 | 0.00 | 0.00 | 0.00 | 0.00 | 3450500.00 | 0.00 | 0.00 | 0.00 | 0.00 | 0.00 | 0.00 | 0.00 | KO:K02058 | simple sugar transport system substrate-binding protein | Bacteria;Chloroflexi;Anaerolineae;Anaerolineales;Anaerolineaceae;Flexilinea;Flexilinea flocculi;Flexilinea flocculi TC1 |
| Ga0586324 0538390 570 1553 | 1500000.00 | 0.00 | 0.00 | 0.00 | 0.00 | 0.00 | 0.00 | 0.00 | 0.00 | 0.00 | 0.00 | 0.00 | KO:K02058 | simple sugar transport system substrate-binding protein | Bacteria;Proteobacteria;Alphaproteobacteria;Hyphomicrobiales;Aurantimonadaceae;Martelella;Martelella endophytica;Martelella endophytica YC6887 |
| Ga0586324 0903093 110 1198 | 1210000.00 | 0.00 | 0.00 | 0.00 | 0.00 | 0.00 | 0.00 | 0.00 | 0.00 | 0.00 | 0.00 | 0.00 | KO:K02058 | simple sugar transport system substrate-binding protein | Bacteria;Actinobacteria;Actinomycetia;Acidothermales;Acidothermaceae;Acidothermus;Acidothermus cellulolyticus;Acidothermus cellulolyticus 11B |
| Ga0586324 1003170 80 1162 | 0.00 | 0.00 | 0.00 | 0.00 | 0.00 | 0.00 | 0.00 | 0.00 | 2251800.00 | 0.00 | 0.00 | 0.00 | KO:K02058 | simple sugar transport system substrate-binding protein | Bacteria;Chloroflexi;Caldilineae;Caldilineales;Caldilineaceae;Caldilinea;Caldilinea aerophila;Caldilinea aerophila STL-6-O1, DSM 14535 |
| Ga0586324 1633117 320 985 | 0.00 | 0.00 | 0.00 | 0.00 | 0.00 | 2197375.15 | 0.00 | 0.00 | 0.00 | 0.00 | 0.00 | 0.00 | KO:K02058 | simple sugar transport system substrate-binding protein | Bacteria;Proteobacteria;Alphaproteobacteria;Hyphomicrobiales;Bradyrhizobiaceae;Bradyrhizobium;Bradyrhizobium sp. STM 3809;Bradyrhizobium sp. STM 3809 |
| Ga0586324 2067519 144 890 | 0.00 | 0.00 | 1826709.80 | 0.00 | 0.00 | 0.00 | 0.00 | 0.00 | 0.00 | 0.00 | 0.00 | 0.00 | KO:K02058 | simple sugar transport system substrate-binding protein | Bacteria;Chloroflexi;Caldilineae;Caldilineales;Caldilineaceae;Caldilinea;Caldilinea aerophila;Caldilinea aerophila STL-6-O1, DSM 14535 |
| Ga0586324 2607227 1 492 | 0.00 | 2611200.00 | 0.00 | 0.00 | 0.00 | 0.00 | 0.00 | 1726300.00 | 1360800.00 | 0.00 | 0.00 | 0.00 | KO:K02058 | simple sugar transport system substrate-binding protein | Bacteria;Proteobacteria;Alphaproteobacteria;Hyphomicrobiales;unclassified Hyphomicrobiales;Bauldia;Bauldia litoralis;Bauldia litoralis ATCC 35022 |
| Ga0586324 3060140 3 755 | 0.00 | 4243200.00 | 0.00 | 0.00 | 0.00 | 0.00 | 0.00 | 0.00 | 915300.00 | 0.00 | 0.00 | 0.00 | KO:K02058 | simple sugar transport system substrate-binding protein | Bacteria;Proteobacteria;Alphaproteobacteria;Hyphomicrobiales;Kaistiaceae;Kaistia;Kaistia hirudinis;Kaistia hirudinis DSM 25966 |
| Ga0586324 3380632 3 725 | 0.00 | 5827600.00 | 0.00 | 0.00 | 0.00 | 0.00 | 0.00 | 0.00 | 0.00 | 0.00 | 0.00 | 6667500.00 | KO:K02058 | simple sugar transport system substrate-binding protein | Bacteria;Actinobacteria;Actinomycetia;Acidothermales;Acidothermaceae;Acidothermus;Acidothermus cellulolyticus;Acidothermus cellulolyticus 11B |
| Ga0586324 6810456 48 542 | 0.00 | 6936000.00 | 0.00 | 0.00 | 0.00 | 0.00 | 0.00 | 0.00 | 0.00 | 0.00 | 1742400.00 | 0.00 | KO:K02058 | simple sugar transport system substrate-binding protein | Bacteria;Chloroflexi;Caldilineae;Caldilineales;Caldilineaceae;Caldilinea;Caldilinea aerophila;Caldilinea aerophila STL-6-O1, DSM 14535 |
| Ga0586324 7534709 1 519 | 0.00 | 0.00 | 0.00 | 2071000.00 | 0.00 | 0.00 | 0.00 | 0.00 | 0.00 | 0.00 | 0.00 | 0.00 | KO:K02058 | simple sugar transport system substrate-binding protein | Bacteria;Chloroflexi;Anaerolineae;Anaerolineales;Anaerolineaceae;Flexilinea;Flexilinea flocculi;Flexilinea flocculi TC1 |
| Ga0586324 2151783 42 875 | 0.00 | 319780.20 | 0.00 | 0.00 | 0.00 | 0.00 | 0.00 | 0.00 | 0.00 | 0.00 | 0.00 | 0.00 | KO:K02067 | phospholipid/cholesterol/gamma-HCH transport system substrate-binding protein | Bacteria;Proteobacteria;Betaproteobacteria;Burkholderiales;Comamonadaceae;Curvibacter;Curvibacter delicatus;Curvibacter delicatus NBRC 14919 |
| Ga0586324 0899492 737 1063 | 0.00 | 0.00 | 0.00 | 0.00 | 1205100.00 | 0.00 | 0.00 | 0.00 | 0.00 | 0.00 | 0.00 | 0.00 | KO:K02107 | V/A-type H+-transporting ATPase subunit G/H | Archaea;Euryarchaeota;Methanomicrobia;Methanosarcinales;Methanotrichaceae;Methanothrix;Methanothrix soehngenii;Methanothrix soehngenii GP-6 |
| Ga0586324 0000001 71963 72433 | 0.00 | 0.00 | 0.00 | 0.00 | 0.00 | 0.00 | 0.00 | 0.00 | 0.00 | 4319700.00 | 5456000.00 | 4875000.00 | KO:K02109 | F-type H+-transporting ATPase subunit b | Bacteria;Proteobacteria;Betaproteobacteria;Nitrosomonadales;Thiobacillaceae;Annwoodia;Annwoodia aquaesulis;Annwoodia aquaesulis DSM 4255 |
| Ga0586324 0000102 12104 12685 | 0.00 | 0.00 | 0.00 | 0.00 | 0.00 | 0.00 | 0.00 | 480698.91 | 0.00 | 0.00 | 0.00 | 0.00 | KO:K02109 | F-type H+-transporting ATPase subunit b | Bacteria;Aquificae;Aquificae;Desulfurobacteriales;Desulfurobacteriaceae;Thermovibrio;Thermovibrio ammonificans;Thermovibrio ammonificans HB-1 |
| Ga0586324 0000524 24570 25040 | 0.00 | 0.00 | 0.00 | 0.00 | 0.00 | 0.00 | 5768400.00 | 0.00 | 0.00 | 0.00 | 0.00 | 3682500.00 | KO:K02109 | F-type H+-transporting ATPase subunit b | Bacteria;Proteobacteria;Betaproteobacteria;Nitrosomonadales;Thiobacillaceae;Thiobacillus;Thiobacillus thioparus;Thiobacillus thioparus DSM 505 |
| Ga0586324 0190616 1922 2530 | 0.00 | 0.00 | 0.00 | 3401000.00 | 0.00 | 0.00 | 0.00 | 0.00 | 0.00 | 0.00 | 0.00 | 0.00 | KO:K02109 | F-type H+-transporting ATPase subunit b | Bacteria;Proteobacteria;Deltaproteobacteria;Desulfobacterales;Desulfobacteraceae;Desulfatirhabdium;Desulfatirhabdium butyrativorans;Desulfatirhabdium butyrativorans DSM 18734 |
| Ga0586324 1371552 379 987 | 0.00 | 0.00 | 0.00 | 0.00 | 0.00 | 3293111.03 | 1851763.56 | 0.00 | 2559600.00 | 475628.23 | 0.00 | 0.00 | KO:K02109 | F-type H+-transporting ATPase subunit b | Bacteria;Proteobacteria;Deltaproteobacteria;Syntrophobacterales;Syntrophaceae;Desulfobacca;Desulfobacca acetoxidans;Desulfobacca acetoxidans ASRB2, DSM 11109 |
| Ga0586324 3017929 3 521 | 0.00 | 0.00 | 0.00 | 0.00 | 0.00 | 3411143.10 | 0.00 | 0.00 | 0.00 | 0.00 | 0.00 | 0.00 | KO:K02109 | F-type H+-transporting ATPase subunit b | Bacteria;Proteobacteria;Deltaproteobacteria;Desulfobacterales;Desulfobacteraceae;Desulfatirhabdium;Desulfatirhabdium butyrativorans;Desulfatirhabdium butyrativorans DSM 18734 |
| Ga0586324 5941300 170 574 | 0.00 | 0.00 | 0.00 | 0.00 | 0.00 | 0.00 | 0.00 | 0.00 | 5937300.00 | 0.00 | 0.00 | 0.00 | KO:K02109 | F-type H+-transporting ATPase subunit b | Bacteria;Proteobacteria;Gammaproteobacteria;Methylococcales;Methylococcaceae;Methylobacter;Methylobacter luteus;Methylobacter luteus IMV-B-3098 |
| Ga0586324 7437370 43 522 | 0.00 | 0.00 | 0.00 | 978500.00 | 1256600.00 | 302864.55 | 0.00 | 750300.00 | 614310.48 | 0.00 | 0.00 | 0.00 | KO:K02109 | F-type H+-transporting ATPase subunit b | Bacteria;Proteobacteria;Deltaproteobacteria;Syntrophobacterales;Syntrophaceae;Desulfobacca;Desulfobacca acetoxidans;Desulfobacca acetoxidans ASRB2, DSM 11109 |
| Ga0586324 6925671 3 191 | 0.00 | 0.00 | 0.00 | 0.00 | 0.00 | 0.00 | 0.00 | 8357000.00 | 0.00 | 0.00 | 0.00 | 0.00 | KO:K02110 | F-type H+-transporting ATPase subunit c | Bacteria;Candidatus Calescamantes;unclassified Candidatus Calescamantes;unclassified Candidatus Calescamantes;unclassified Candidatus Calescamantes;unclassified Candidatus Calescamantes;Calescamantes bacterium JGI 0000106-I5;Calescamantes bacterium JGI 0000106-I5 (contamination screened) |
| Ga0586324 0000001 69794 71341 | 0.00 | 0.00 | 0.00 | 0.00 | 0.00 | 0.00 | 0.00 | 56693400.00 | 21213900.00 | 80619000.00 | 42240000.00 | 95242500.00 | KO:K02111 | F-type H+-transporting ATPase subunit alpha | Bacteria;Proteobacteria;Betaproteobacteria;Nitrosomonadales;Thiobacillaceae;Annwoodia;Annwoodia aquaesulis;Annwoodia aquaesulis DSM 4255 |
| Ga0586324 0000524 25626 27167 | 0.00 | 0.00 | 0.00 | 0.00 | 0.00 | 0.00 | 47652000.00 | 40522300.00 | 30456000.00 | 25487000.00 | 46024000.00 | 29700000.00 | KO:K02111 | F-type H+-transporting ATPase subunit alpha | Bacteria;Proteobacteria;Betaproteobacteria;Nitrosomonadales;Thiobacillaceae;Thiobacillus;Thiobacillus thioparus;Thiobacillus thioparus DSM 505 |
| Ga0586324 0000808 25068 26609 | 0.00 | 0.00 | 0.00 | 1045000.00 | 0.00 | 0.00 | 0.00 | 0.00 | 0.00 | 0.00 | 0.00 | 0.00 | KO:K02111 | F-type H+-transporting ATPase subunit alpha | Bacteria;Proteobacteria;Betaproteobacteria;Nitrosomonadales;Gallionellaceae;Ferriphaselus;Ferriphaselus amnicola;Ferriphaselus amnicola OYT1 |
| Ga0586324 0002013 6939 8480 | 0.00 | 0.00 | 0.00 | 0.00 | 0.00 | 0.00 | 0.00 | 31346574.47 | 0.00 | 28336000.00 | 32128800.00 | 0.00 | KO:K02111 | F-type H+-transporting ATPase subunit alpha | Bacteria;Proteobacteria;Betaproteobacteria;Nitrosomonadales;Thiobacillaceae;Thiobacillus;Thiobacillus thioparus;Thiobacillus thioparus DSM 505 |
| Ga0586324 0002419 11842 13407 | 0.00 | 0.00 | 15595100.00 | 19380000.00 | 16480000.00 | 18936900.00 | 0.00 | 6710000.00 | 7095600.00 | 7499800.00 | 6274400.00 | 6930000.00 | KO:K02111 | F-type H+-transporting ATPase subunit alpha | Bacteria;Proteobacteria;Deltaproteobacteria;Myxococcales;Kofleriaceae;Haliangium;Haliangium ochraceum;Haliangium ochraceum SMP-2, DSM 14365 |
| Ga0586324 0003391 13966 15507 | 0.00 | 0.00 | 0.00 | 0.00 | 0.00 | 0.00 | 2255996.16 | 0.00 | 0.00 | 0.00 | 0.00 | 0.00 | KO:K02111 | F-type H+-transporting ATPase subunit alpha | Bacteria;Proteobacteria;Betaproteobacteria;Nitrosomonadales;Gallionellaceae;Sideroxydans;Sideroxydans lithotrophicus;Sideroxydans lithotrophicus ES-1 |
| Ga0586324 0005566 7808 9325 | 0.00 | 7004000.00 | 0.00 | 0.00 | 0.00 | 0.00 | 0.00 | 0.00 | 0.00 | 0.00 | 0.00 | 3037500.00 | KO:K02111 | F-type H+-transporting ATPase subunit alpha | Bacteria;Proteobacteria;Deltaproteobacteria;Desulfobacterales;Desulfobacteraceae;Desulfosarcina;Desulfosarcina sp. BuS5;Desulfosarcina sp. BuS5 |
| Ga0586324 0008747 6339 7856 | 42900000.00 | 0.00 | 71501000.00 | 9690000.00 | 39243000.00 | 118306000.00 | 93252000.00 | 0.00 | 739229.49 | 0.00 | 25256000.00 | 63000000.00 | KO:K02111 | F-type H+-transporting ATPase subunit alpha | Bacteria;Proteobacteria;Deltaproteobacteria;Syntrophobacterales;Syntrophobacteraceae;Desulfacinum;Desulfacinum infernum;Desulfacinum infernum DSM 9756 |
| Ga0586324 0026187 5371 6537 | 0.00 | 0.00 | 20831000.00 | 0.00 | 0.00 | 10004400.00 | 0.00 | 0.00 | 0.00 | 0.00 | 0.00 | 0.00 | KO:K02111 | F-type H+-transporting ATPase subunit alpha | Bacteria;Proteobacteria;Gammaproteobacteria;Chromatiales;Woeseiaceae;unclassified Woeseiaceae;unclassified Woeseiaceae;uncultured Woeseiaceae SAG 1868 B |
| Ga0586324 0041167 2442 3983 | 0.00 | 0.00 | 0.00 | 0.00 | 0.00 | 0.00 | 0.00 | 0.00 | 0.00 | 0.00 | 2112000.00 | 2167500.00 | KO:K02111 | F-type H+-transporting ATPase subunit alpha | Bacteria;Proteobacteria;Betaproteobacteria;unclassified Betaproteobacteria;unclassified Betaproteobacteria;unclassified Betaproteobacteria;Betaproteobacteria bacterium GR16-43;Betaproteobacteria bacterium GR16-43 |
| Ga0586324 0042123 4042 5202 | 0.00 | 0.00 | 0.00 | 0.00 | 0.00 | 0.00 | 0.00 | 0.00 | 0.00 | 2402400.00 | 0.00 | 0.00 | KO:K02111 | F-type H+-transporting ATPase subunit alpha | Bacteria;Chloroflexi;Anaerolineae;Anaerolineales;Anaerolineaceae;Thermanaerothrix;Thermanaerothrix daxensis;Thermanaerothrix daxensis GNS-1 |
| Ga0586324 0056416 2 1249 | 0.00 | 0.00 | 3549754.41 | 5358000.00 | 0.00 | 0.00 | 0.00 | 0.00 | 0.00 | 0.00 | 0.00 | 0.00 | KO:K02111 | F-type H+-transporting ATPase subunit alpha | Bacteria;Proteobacteria;Betaproteobacteria;Burkholderiales;unclassified Burkholderiales;Ideonella;Ideonella sp. A 288;Ideonella sp. A 288 |
| Ga0586324 0060913 76 1599 | 34340000.00 | 91392000.00 | 34737100.00 | 104690000.00 | 142984600.00 | 68006100.00 | 47880000.00 | 33489000.00 | 24405300.00 | 83437200.00 | 31926400.00 | 32250000.00 | KO:K02111 | F-type H+-transporting ATPase subunit alpha | Eukaryota;Streptophyta;Magnoliopsida;Malpighiales;Salicaceae;Populus;Populus trichocarpa;Populus balsamifera trichocarpa |
| Ga0586324 0063998 2590 4098 | 0.00 | 3787600.00 | 0.00 | 7058500.00 | 0.00 | 0.00 | 0.00 | 14023900.00 | 13527000.00 | 623556.78 | 6476800.00 | 4665000.00 | KO:K02111 | F-type H+-transporting ATPase subunit alpha | Bacteria;Proteobacteria;Deltaproteobacteria;Desulfuromonadales;Geobacteraceae;Geothermobacter;Geothermobacter ehrlichii;Geothermobacter ehrlichii DSM 15274 |
| Ga0586324 0064613 3127 4224 | 0.00 | 0.00 | 0.00 | 0.00 | 0.00 | 0.00 | 0.00 | 0.00 | 11097000.00 | 0.00 | 0.00 | 0.00 | KO:K02111 | F-type H+-transporting ATPase subunit alpha | Bacteria;Bacteroidetes;Sphingobacteriia;Sphingobacteriales;Sphingobacteriaceae;Parapedobacter;Parapedobacter luteus;Parapedobacter luteus DSM 22899 |
| Ga0586324 0074767 1538 3058 | 23900000.00 | 0.00 | 0.00 | 0.00 | 51294000.00 | 29139800.00 | 0.00 | 22997000.00 | 0.00 | 0.00 | 0.00 | 0.00 | KO:K02111 | F-type H+-transporting ATPase subunit alpha | Bacteria;Proteobacteria;Deltaproteobacteria;Syntrophobacterales;Syntrophaceae;Desulfobacca;Desulfobacca acetoxidans;Desulfobacca acetoxidans ASRB2, DSM 11109 |
| Ga0586324 0081172 1202 2725 | 0.00 | 0.00 | 0.00 | 611508.35 | 0.00 | 0.00 | 0.00 | 0.00 | 0.00 | 0.00 | 0.00 | 0.00 | KO:K02111 | F-type H+-transporting ATPase subunit alpha | Bacteria;Nitrospirae;Thermodesulfovibrionia;Thermodesulfovibrionales;Thermodesulfovibrionaceae;Thermodesulfovibrio;Thermodesulfovibrio sp. N1;Thermodesulfovibrio sp. N1 |
| Ga0586324 0081762 877 2418 | 0.00 | 0.00 | 0.00 | 0.00 | 0.00 | 0.00 | 0.00 | 0.00 | 0.00 | 13475000.00 | 0.00 | 0.00 | KO:K02111 | F-type H+-transporting ATPase subunit alpha | Bacteria;Proteobacteria;Betaproteobacteria;Nitrosomonadales;Thiobacillaceae;Thiobacillus;Thiobacillus thioparus;Thiobacillus thioparus DSM 505 |
| Ga0586324 0084942 3 1235 | 0.00 | 0.00 | 0.00 | 0.00 | 4398100.00 | 0.00 | 0.00 | 1342000.00 | 3871800.00 | 4296600.00 | 8976000.00 | 1350000.00 | KO:K02111 | F-type H+-transporting ATPase subunit alpha | Bacteria;Proteobacteria;Deltaproteobacteria;Desulfuromonadales;Geobacteraceae;Geobacter;Geobacter argillaceus;Geobacter argillaceus ATCC BAA-1139 |
| Ga0586324 0085483 1439 2980 | 0.00 | 0.00 | 0.00 | 0.00 | 0.00 | 0.00 | 0.00 | 0.00 | 0.00 | 7476700.00 | 8016800.00 | 0.00 | KO:K02111 | F-type H+-transporting ATPase subunit alpha | Bacteria;Proteobacteria;Betaproteobacteria;Nitrosomonadales;Gallionellaceae;Sideroxydans;Sideroxydans lithotrophicus;Sideroxydans lithotrophicus ES-1 |
| Ga0586324 0114086 1734 3221 | 0.00 | 0.00 | 0.00 | 0.00 | 0.00 | 0.00 | 0.00 | 0.00 | 0.00 | 0.00 | 0.00 | 3975000.00 | KO:K02111 | F-type H+-transporting ATPase subunit alpha | Bacteria;Proteobacteria;Alphaproteobacteria;Rhodospirillales;Acetobacteraceae;Rhodopila;Rhodopila globiformis;Rhodopila globiformis DSM 161 |
| Ga0586324 0127188 1140 2669 | 0.00 | 0.00 | 0.00 | 0.00 | 0.00 | 0.00 | 0.00 | 0.00 | 7063200.00 | 0.00 | 0.00 | 0.00 | KO:K02111 | F-type H+-transporting ATPase subunit alpha | Bacteria;Proteobacteria;Alphaproteobacteria;Rhodospirillales;Rhodospirillaceae;Dongia;Dongia mobilis;Dongia mobilis CGMCC 1.7660 |
| Ga0586324 0150677 2290 2823 | 0.00 | 0.00 | 0.00 | 7334000.00 | 3656500.00 | 15205100.00 | 12790800.00 | 3977200.00 | 3734100.00 | 0.00 | 0.00 | 0.00 | KO:K02111 | F-type H+-transporting ATPase subunit alpha | Bacteria;Proteobacteria;Deltaproteobacteria;Syntrophobacterales;Syntrophaceae;Desulfobacca;Desulfobacca acetoxidans;Desulfobacca acetoxidans ASRB2, DSM 11109 |
| Ga0586324 0158659 1 1236 | 0.00 | 0.00 | 0.00 | 0.00 | 0.00 | 0.00 | 0.00 | 0.00 | 0.00 | 0.00 | 0.00 | 3397500.00 | KO:K02111 | F-type H+-transporting ATPase subunit alpha | Bacteria;Proteobacteria;Betaproteobacteria;Nitrosomonadales;Methylophilaceae;Methylobacillus;Methylobacillus sp. MM3;Methylobacillus sp. MM2 |
| Ga0586324 0169296 2 1165 | 0.00 | 0.00 | 0.00 | 0.00 | 0.00 | 0.00 | 10966800.00 | 0.00 | 4438800.00 | 0.00 | 0.00 | 0.00 | KO:K02111 | F-type H+-transporting ATPase subunit alpha | Bacteria;Proteobacteria;Gammaproteobacteria;Chromatiales;Chromatiaceae;Thiocystis;Thiocystis violascens;Thiocystis violascens 611, DSM 198 |
| Ga0586324 0172003 1424 2653 | 0.00 | 0.00 | 0.00 | 0.00 | 0.00 | 0.00 | 0.00 | 0.00 | 1393200.00 | 0.00 | 0.00 | 0.00 | KO:K02111 | F-type H+-transporting ATPase subunit alpha | Bacteria;Proteobacteria;Betaproteobacteria;Burkholderiales;unclassified Burkholderiales;Methylibium;Methylibium sp. CF059;Methylibium sp. CF059 |
| Ga0586324 0172380 496 2043 | 0.00 | 0.00 | 0.00 | 0.00 | 0.00 | 0.00 | 0.00 | 0.00 | 0.00 | 0.00 | 0.00 | 2827500.00 | KO:K02111 | F-type H+-transporting ATPase subunit alpha | Bacteria;Proteobacteria;Betaproteobacteria;unclassified Betaproteobacteria;unclassified Betaproteobacteria;unclassified Betaproteobacteria;Betaproteobacteria bacterium GR16-43;Betaproteobacteria bacterium GR16-43 |
| Ga0586324 0195810 1049 2500 | 0.00 | 39916000.00 | 86139000.00 | 29830000.00 | 0.00 | 51610000.00 | 0.00 | 0.00 | 0.00 | 0.00 | 0.00 | 0.00 | KO:K02111 | F-type H+-transporting ATPase subunit alpha | Bacteria;Proteobacteria;Gammaproteobacteria;Methylococcales;Methylococcaceae;Methylobacter;Methylobacter luteus;Methylobacter luteus IMV-B-3098 |
| Ga0586324 0227902 2 1495 | 0.00 | 0.00 | 0.00 | 594093.90 | 0.00 | 0.00 | 0.00 | 0.00 | 0.00 | 0.00 | 0.00 | 0.00 | KO:K02111 | F-type H+-transporting ATPase subunit alpha | Bacteria;Proteobacteria;Deltaproteobacteria;Syntrophobacterales;Syntrophaceae;Desulfomonile;Desulfomonile tiedjei;Desulfomonile tiedjei DCB-1, DSM 6799 |
| Ga0586324 0302525 1 1065 | 8290000.00 | 0.00 | 14300200.00 | 5168000.00 | 4779200.00 | 16912200.00 | 9644400.00 | 5447300.00 | 7419600.00 | 0.00 | 8791200.00 | 13950000.00 | KO:K02111 | F-type H+-transporting ATPase subunit alpha | Bacteria;Thermodesulfobacteria;Thermodesulfobacteria;Thermodesulfobacteriales;Thermodesulfobacteriaceae;Thermosulfuriphilus;Thermosulfuriphilus ammonigenes;Thermosulfuriphilus ammonigenes DSM 102941 |
| Ga0586324 0304569 217 1752 | 0.00 | 0.00 | 0.00 | 0.00 | 0.00 | 0.00 | 0.00 | 2837967.05 | 5945400.00 | 0.00 | 0.00 | 795000.00 | KO:K02111 | F-type H+-transporting ATPase subunit alpha | Bacteria;Proteobacteria;Betaproteobacteria;Nitrosomonadales;Thiobacillaceae;Sulfuritortus;Sulfuritortus calidifontis;Sulfuritortus calidifontis DSM 103923 |
| Ga0586324 0310401 550 2025 | 0.00 | 0.00 | 0.00 | 0.00 | 0.00 | 0.00 | 0.00 | 0.00 | 0.00 | 0.00 | 0.00 | 1650000.00 | KO:K02111 | F-type H+-transporting ATPase subunit alpha | Bacteria;Proteobacteria;Betaproteobacteria;unclassified Betaproteobacteria;unclassified Betaproteobacteria;unclassified Betaproteobacteria;Betaproteobacteria bacterium GR16-43;Betaproteobacteria bacterium GR16-43 |
| Ga0586324 0330161 823 1968 | 0.00 | 0.00 | 0.00 | 0.00 | 0.00 | 0.00 | 0.00 | 0.00 | 1036800.00 | 23408000.00 | 2948000.00 | 0.00 | KO:K02111 | F-type H+-transporting ATPase subunit alpha | Bacteria;Proteobacteria;Betaproteobacteria;Rhodocyclales;Zoogloeaceae;Uliginosibacterium;Uliginosibacterium gangwonense;Uliginosibacterium gangwonense DSM 18521 |
| Ga0586324 0330685 1757 1966 | 0.00 | 0.00 | 0.00 | 0.00 | 0.00 | 0.00 | 0.00 | 2580300.00 | 0.00 | 2063600.00 | 2349600.00 | 0.00 | KO:K02111 | F-type H+-transporting ATPase subunit alpha | Bacteria;Proteobacteria;Betaproteobacteria;Nitrosomonadales;Nitrosomonadaceae;Nitrosospira;Nitrosospira sp. Nsp2;Nitrosospira sp. Nsp2 |
| Ga0586324 0390798 937 1827 | 0.00 | 0.00 | 0.00 | 0.00 | 0.00 | 0.00 | 0.00 | 2147200.00 | 2430000.00 | 3372600.00 | 0.00 | 0.00 | KO:K02111 | F-type H+-transporting ATPase subunit alpha | Bacteria;Proteobacteria;Gammaproteobacteria;Chromatiales;Granulosicoccaceae;Sulfuriflexus;Sulfuriflexus mobilis;Sulfuriflexus mobilis aks1 |
| Ga0586324 0393358 1386 1823 | 0.00 | 0.00 | 0.00 | 0.00 | 0.00 | 0.00 | 0.00 | 36234000.00 | 33372000.00 | 26411000.00 | 0.00 | 0.00 | KO:K02111 | F-type H+-transporting ATPase subunit alpha | Bacteria;Proteobacteria;Gammaproteobacteria;Acidiferrobacterales;Acidiferrobacteraceae;Sulfuricaulis;Sulfuricaulis limicola;Sulfuricaulis limicola HA5 |
| Ga0586324 0480409 3 944 | 0.00 | 0.00 | 0.00 | 0.00 | 0.00 | 0.00 | 0.00 | 0.00 | 0.00 | 0.00 | 9856000.00 | 0.00 | KO:K02111 | F-type H+-transporting ATPase subunit alpha | Bacteria;Proteobacteria;Betaproteobacteria;Nitrosomonadales;Methylophilaceae;Novimethylophilus;Novimethylophilus kurashikiensis;Novimethylophilus kurashikiensis La2-4 |
| Ga0586324 0536721 365 1588 | 8410000.00 | 33252000.00 | 0.00 | 41135000.00 | 96408000.00 | 122276000.00 | 1353079.68 | 0.00 | 9169200.00 | 4196500.00 | 1795200.00 | 8737500.00 | KO:K02111 | F-type H+-transporting ATPase subunit alpha | Bacteria;Proteobacteria;Gammaproteobacteria;unclassified Gammaproteobacteria;Candidatus Competibacteraceae;Candidatus Competibacter;Candidatus Competibacter denitrificans;Candidatus Competibacter denitrificans Run A D11 |
| Ga0586324 0543783 386 1579 | 0.00 | 0.00 | 0.00 | 0.00 | 0.00 | 0.00 | 0.00 | 0.00 | 0.00 | 23485000.00 | 28160000.00 | 0.00 | KO:K02111 | F-type H+-transporting ATPase subunit alpha | Bacteria;Proteobacteria;Deltaproteobacteria;Desulfobacterales;Desulfobacteraceae;Desulfatiglans;Desulfatiglans anilini;Desulfatiglans anilini DSM 4660 |
| Ga0586324 0573113 1 1251 | 0.00 | 0.00 | 0.00 | 0.00 | 0.00 | 0.00 | 0.00 | 18954000.00 | 0.00 | 0.00 | 0.00 | 0.00 | KO:K02111 | F-type H+-transporting ATPase subunit alpha | Bacteria;Proteobacteria;Deltaproteobacteria;Myxococcales;Archangiaceae;Stigmatella;Stigmatella erecta;Stigmatella erecta DSM 16858 |
| Ga0586324 0700345 3 1415 | 0.00 | 618855.08 | 0.00 | 0.00 | 831810.49 | 0.00 | 7660800.00 | 0.00 | 0.00 | 12551000.00 | 0.00 | 3397500.00 | KO:K02111 | F-type H+-transporting ATPase subunit alpha | Bacteria;Proteobacteria;Deltaproteobacteria;Syntrophobacterales;Syntrophaceae;Smithella;Smithella sp. SCADC;Smithella sp. SCADC |
| Ga0586324 0723293 2 1396 | 12600000.00 | 0.00 | 0.00 | 0.00 | 0.00 | 0.00 | 0.00 | 0.00 | 0.00 | 0.00 | 0.00 | 0.00 | KO:K02111 | F-type H+-transporting ATPase subunit alpha | Bacteria;Bacteroidetes;Cytophagia;Cytophagales;Fulvivirgaceae;Ohtaekwangia;Ohtaekwangia koreensis;Ohtaekwangia koreensis DSM 25262 |
| Ga0586324 0723361 629 1396 | 0.00 | 0.00 | 0.00 | 0.00 | 0.00 | 0.00 | 0.00 | 969900.00 | 0.00 | 0.00 | 0.00 | 0.00 | KO:K02111 | F-type H+-transporting ATPase subunit alpha | Bacteria;Proteobacteria;Betaproteobacteria;Nitrosomonadales;Thiobacillaceae;Thiobacillus;Thiobacillus thiophilus;Thiobacillus thiophilus DSM 19892 |
| Ga0586324 0726423 39 1394 | 0.00 | 20060000.00 | 0.00 | 26828000.00 | 3831600.00 | 0.00 | 0.00 | 0.00 | 0.00 | 42504000.00 | 8756000.00 | 0.00 | KO:K02111 | F-type H+-transporting ATPase subunit alpha | Bacteria;Proteobacteria;Deltaproteobacteria;Desulfobacterales;Desulfobacteraceae;Desulfatirhabdium;Desulfatirhabdium butyrativorans;Desulfatirhabdium butyrativorans DSM 18734 |
| Ga0586324 0750617 639 1373 | 0.00 | 31348000.00 | 0.00 | 0.00 | 0.00 | 0.00 | 0.00 | 0.00 | 0.00 | 0.00 | 0.00 | 0.00 | KO:K02111 | F-type H+-transporting ATPase subunit alpha | Bacteria;Proteobacteria;Gammaproteobacteria;Cellvibrionales;Halieaceae;Halioglobus;Halioglobus sediminis;Halioglobus sediminis U0301 U0301 |
| Ga0586324 0797971 332 1339 | 1010000.00 | 2080800.00 | 5524527.58 | 0.00 | 5994600.00 | 0.00 | 4970400.00 | 2860900.00 | 8100000.00 | 2671900.00 | 2288000.00 | 0.00 | KO:K02111 | F-type H+-transporting ATPase subunit alpha | Bacteria;Proteobacteria;Betaproteobacteria;unclassified Betaproteobacteria;unclassified Betaproteobacteria;unclassified Betaproteobacteria;Betaproteobacteria bacterium GR16-43;Betaproteobacteria bacterium GR16-43 |
| Ga0586324 0813683 44 1327 | 3050000.00 | 21624000.00 | 0.00 | 3961500.00 | 0.00 | 0.00 | 1166149.32 | 3946700.00 | 0.00 | 19825726.69 | 8694400.00 | 7800000.00 | KO:K02111 | F-type H+-transporting ATPase subunit alpha | Bacteria;Proteobacteria;Gammaproteobacteria;unclassified Gammaproteobacteria;Candidatus Competibacteraceae;Candidatus Competibacter;Candidatus Competibacter denitrificans;Candidatus Competibacter denitrificans Run A D11 |
| Ga0586324 0854583 3 1298 | 0.00 | 1462000.00 | 0.00 | 0.00 | 0.00 | 0.00 | 0.00 | 2616900.00 | 0.00 | 1309000.00 | 0.00 | 3414229.50 | KO:K02111 | F-type H+-transporting ATPase subunit alpha | Bacteria;Proteobacteria;Betaproteobacteria;unclassified Betaproteobacteria;unclassified Betaproteobacteria;unclassified Betaproteobacteria;Betaproteobacteria bacterium GR16-43;Betaproteobacteria bacterium GR16-43 |
| Ga0586324 0875723 783 1286 | 0.00 | 0.00 | 0.00 | 0.00 | 0.00 | 4247900.00 | 0.00 | 0.00 | 0.00 | 0.00 | 0.00 | 0.00 | KO:K02111 | F-type H+-transporting ATPase subunit alpha | Bacteria;Proteobacteria;Deltaproteobacteria;Syntrophobacterales;Syntrophaceae;Desulfobacca;Desulfobacca acetoxidans;Desulfobacca acetoxidans ASRB2, DSM 11109 |
| Ga0586324 0896203 1 1071 | 0.00 | 0.00 | 0.00 | 0.00 | 0.00 | 659198.65 | 0.00 | 0.00 | 0.00 | 0.00 | 0.00 | 0.00 | KO:K02111 | F-type H+-transporting ATPase subunit alpha | Bacteria;Proteobacteria;Gammaproteobacteria;unclassified Gammaproteobacteria;Candidatus Competibacteraceae;Candidatus Competibacter;Candidatus Competibacter denitrificans;Candidatus Competibacter denitrificans Run A D11 |
| Ga0586324 0925125 1 1254 | 0.00 | 0.00 | 0.00 | 6460000.00 | 0.00 | 0.00 | 0.00 | 0.00 | 0.00 | 0.00 | 0.00 | 0.00 | KO:K02111 | F-type H+-transporting ATPase subunit alpha | Bacteria;Proteobacteria;Deltaproteobacteria;Desulfuromonadales;Desulfuromonadaceae;Desulfuromonas;Desulfuromonas sp. DDH964;Desulfuromonas sp. DDH964 |
| Ga0586324 0926215 392 1255 | 0.00 | 0.00 | 0.00 | 11618500.00 | 72821000.00 | 77172798.24 | 0.00 | 3907437.96 | 1304100.00 | 6976200.00 | 0.00 | 0.00 | KO:K02111 | F-type H+-transporting ATPase subunit alpha | Bacteria;Proteobacteria;Deltaproteobacteria;Syntrophobacterales;Syntrophobacteraceae;Syntrophobacter;Syntrophobacter fumaroxidans;Syntrophobacter fumaroxidans MPOB |
| Ga0586324 0935114 86 1249 | 7140000.00 | 0.00 | 7037500.00 | 5491000.00 | 7374800.00 | 3842531.24 | 79822800.00 | 2610800.00 | 2794500.00 | 3449600.00 | 2420000.00 | 2835000.00 | KO:K02111 | F-type H+-transporting ATPase subunit alpha | Bacteria;Proteobacteria;Deltaproteobacteria;Syntrophobacterales;Syntrophobacteraceae;Desulfacinum;Desulfacinum infernum;Desulfacinum infernum DSM 9756 |
| Ga0586324 0972030 911 1228 | 0.00 | 0.00 | 0.00 | 11875000.00 | 6334500.00 | 11870300.00 | 6999600.00 | 8540000.00 | 6123600.00 | 4643100.00 | 5139200.00 | 2617500.00 | KO:K02111 | F-type H+-transporting ATPase subunit alpha | Bacteria;Proteobacteria;Deltaproteobacteria;Desulfarculales;Desulfarculaceae;Dethiosulfatarculus;Dethiosulfatarculus sandiegensis;Dethiosulfatarculus sandiegensis SPR |
| Ga0586324 0988878 3 1076 | 0.00 | 0.00 | 0.00 | 0.00 | 0.00 | 0.00 | 0.00 | 0.00 | 0.00 | 0.00 | 4725600.00 | 0.00 | KO:K02111 | F-type H+-transporting ATPase subunit alpha | Bacteria;Proteobacteria;Gammaproteobacteria;Methylococcales;Methylococcaceae;Methylobacter;Methylobacter luteus;Methylobacter luteus IMV-B-3098 |
| Ga0586324 1056312 1 1185 | 1200000.00 | 16333600.00 | 0.00 | 0.00 | 12875000.00 | 0.00 | 0.00 | 21716000.00 | 23004000.00 | 26950000.00 | 27896000.00 | 840000.00 | KO:K02111 | F-type H+-transporting ATPase subunit alpha | Bacteria;Proteobacteria;Betaproteobacteria;unclassified Betaproteobacteria;unclassified Betaproteobacteria;unclassified Betaproteobacteria;Betaproteobacteria bacterium GR16-43;Betaproteobacteria bacterium GR16-43 |
| Ga0586324 1304267 2 1084 | 0.00 | 0.00 | 1906120.95 | 3135000.00 | 3059100.00 | 0.00 | 0.00 | 2244800.00 | 1466100.00 | 0.00 | 0.00 | 563016.75 | KO:K02111 | F-type H+-transporting ATPase subunit alpha | Eukaryota;Bacillariophyta;Coscinodiscophyceae;Thalassiosirales;Thalassiosiraceae;Thalassiosira;Thalassiosira pseudonana;Thalassiosira pseudonana CCMP 1335 |
| Ga0586324 1326456 1 1074 | 0.00 | 0.00 | 0.00 | 0.00 | 0.00 | 0.00 | 0.00 | 287558.88 | 0.00 | 0.00 | 0.00 | 0.00 | KO:K02111 | F-type H+-transporting ATPase subunit alpha | Eukaryota;Bacillariophyta;Coscinodiscophyceae;Thalassiosirales;Thalassiosiraceae;Thalassiosira;Thalassiosira pseudonana;Thalassiosira pseudonana CCMP 1335 |
| Ga0586324 1328262 1 1074 | 0.00 | 0.00 | 0.00 | 0.00 | 0.00 | 21636500.00 | 0.00 | 4416400.00 | 0.00 | 0.00 | 0.00 | 0.00 | KO:K02111 | F-type H+-transporting ATPase subunit alpha | Bacteria;Proteobacteria;Deltaproteobacteria;Syntrophobacterales;Syntrophaceae;Desulfomonile;Desulfomonile tiedjei;Desulfomonile tiedjei DCB-1, DSM 6799 |
| Ga0586324 1390830 3 1025 | 5150000.00 | 4209200.00 | 0.00 | 112100000.00 | 12978000.00 | 95478500.00 | 12782336.64 | 36356000.00 | 26568000.00 | 0.00 | 0.00 | 0.00 | KO:K02111 | F-type H+-transporting ATPase subunit alpha | Bacteria;Proteobacteria;Deltaproteobacteria;Syntrophobacterales;Syntrophaceae;Desulfobacca;Desulfobacca acetoxidans;Desulfobacca acetoxidans ASRB2, DSM 11109 |
| Ga0586324 1479217 343 1026 | 0.00 | 0.00 | 0.00 | 0.00 | 0.00 | 0.00 | 0.00 | 0.00 | 28593000.00 | 0.00 | 0.00 | 0.00 | KO:K02111 | F-type H+-transporting ATPase subunit alpha | Bacteria;Proteobacteria;Gammaproteobacteria;Xanthomonadales;Rhodanobacteraceae;Mizugakiibacter;Mizugakiibacter sediminis;Mizugakiibacter sediminis skMP5 |
| Ga0586324 1618290 3 989 | 0.00 | 23936000.00 | 0.00 | 77140000.00 | 85902000.00 | 13259800.00 | 0.00 | 36417000.00 | 0.00 | 30954000.00 | 0.00 | 0.00 | KO:K02111 | F-type H+-transporting ATPase subunit alpha | Bacteria;Proteobacteria;Deltaproteobacteria;Syntrophobacterales;Syntrophobacteraceae;Desulfacinum;Desulfacinum hydrothermale;Desulfacinum hydrothermale DSM 13146 |
| Ga0586324 1796339 3 944 | 0.00 | 0.00 | 0.00 | 2574500.00 | 0.00 | 0.00 | 0.00 | 0.00 | 0.00 | 1309000.00 | 0.00 | 7192500.00 | KO:K02111 | F-type H+-transporting ATPase subunit alpha | Bacteria;Chloroflexi;Anaerolineae;Anaerolineales;Anaerolineaceae;Thermanaerothrix;Thermanaerothrix daxensis;Thermanaerothrix daxensis GNS-1 |
| Ga0586324 1874413 3 929 | 2920000.00 | 1128800.00 | 1242124.38 | 417004.40 | 10403000.00 | 3495342.83 | 4822453.08 | 2385100.00 | 858600.00 | 1339800.00 | 0.00 | 855000.00 | KO:K02111 | F-type H+-transporting ATPase subunit alpha | Eukaryota;Bacillariophyta;Coscinodiscophyceae;Thalassiosirales;Thalassiosiraceae;Thalassiosira;Thalassiosira pseudonana;Thalassiosira pseudonana CCMP 1335 |
| Ga0586324 1914528 601 921 | 0.00 | 0.00 | 0.00 | 0.00 | 0.00 | 0.00 | 0.00 | 0.00 | 0.00 | 0.00 | 1680800.00 | 1305000.00 | KO:K02111 | F-type H+-transporting ATPase subunit alpha | Bacteria;Proteobacteria;Gammaproteobacteria;Chromatiales;Ectothiorhodospiraceae;Thioalkalivibrio;Thioalkalivibrio thiocyanodenitrificans;Thioalkalivibrio thiocyanodenitrificans ARhD 1 |
| Ga0586324 1920505 2 919 | 0.00 | 0.00 | 0.00 | 0.00 | 0.00 | 0.00 | 0.00 | 0.00 | 0.00 | 0.00 | 650337.60 | 0.00 | KO:K02111 | F-type H+-transporting ATPase subunit alpha | Bacteria;Chloroflexi;Dehalococcoidia;Dehalococcoidales;Dehalococcoidaceae;Dehalococcoides;Dehalococcoides mccartyi;Dehalococcoides mccartyi CG1 |

| | | | | | | | | | | | | | | | |
|---|---|---|---|---|---|---|---|---|---|---|---|---|---|---|---|
| Ga0586324_1926553_1_882 | 0.00 | 0.00 | 0.00 | 0.00 | 4346600.00 | 6364271.27 | 0.00 | 0.00 | 0.00 | 0.00 | 0.00 | 0.00 | KO:K02111 | F-type H+-transporting ATPase subunit alpha | Bacteria;Proteobacteria;Deltaproteobacteria;Syntrophobacterales;Syntrophaceae;Desulfobacca;Desulfobacca acetoxidans;Desulfobacca acetoxidans ASRB2, DSM 11109 |
| Ga0586324_1940453_243_914 | 0.00 | 0.00 | 0.00 | 0.00 | 9197900.00 | 0.00 | 0.00 | 0.00 | 0.00 | 2733500.00 | 0.00 | 0.00 | KO:K02111 | F-type H+-transporting ATPase subunit alpha | Bacteria;Proteobacteria;Deltaproteobacteria;Syntrophobacterales;Syntrophobacteraceae;Desulfacinum;Desulfacinum infernum;Desulfacinum infernum DSM 9756 |
| Ga0586324_1981772_1_906 | 0.00 | 0.00 | 1516311.01 | 0.00 | 0.00 | 0.00 | 0.00 | 0.00 | 0.00 | 0.00 | 0.00 | 0.00 | KO:K02111 | F-type H+-transporting ATPase subunit alpha | Bacteria;Proteobacteria;Gammaproteobacteria;Methylococcales;Methylococcaceae;Methylovulum;Methylovulum miyakonense;Methylovulum miyakonense HT12 |
| Ga0586324_2099567_3_884 | 0.00 | 0.00 | 0.00 | 0.00 | 0.00 | 0.00 | 0.00 | 0.00 | 0.00 | 15246000.00 | 10208000.00 | 0.00 | KO:K02111 | F-type H+-transporting ATPase subunit alpha | Bacteria;Acidobacteria;Blastocatellia;Blastocatellales;Pyrinomonadaceae;Pyrinomonas;Pyrinomonas methylaliphatogenes;Pyrinomonas methylaliphatogenes K22 |
| Ga0586324_2163977_3_875 | 2440000.00 | 14416000.00 | 559093.34 | 0.00 | 658873.49 | 0.00 | 0.00 | 14518000.00 | 17010000.00 | 0.00 | 0.00 | 9150000.00 | KO:K02111 | F-type H+-transporting ATPase subunit alpha | Bacteria;Proteobacteria;Gammaproteobacteria;Oceanospirillales;Oceanospirillaceae;Marinobacterium;Marinobacterium lutimaris;Marinobacterium lutimaris DSM 22012 |
| Ga0586324_2188619_3_608 | 0.00 | 611032.36 | 0.00 | 4132500.00 | 0.00 | 0.00 | 0.00 | 0.00 | 0.00 | 0.00 | 0.00 | 0.00 | KO:K02111 | F-type H+-transporting ATPase subunit alpha | Bacteria;Proteobacteria;Deltaproteobacteria;Syntrophobacterales;Syntrophaceae;Smithella;Smithella sp. SCADC;Smithella sp. SCADC |
| Ga0586324_2602352_1_723 | 0.00 | 0.00 | 0.00 | 23560000.00 | 0.00 | 0.00 | 0.00 | 0.00 | 0.00 | 0.00 | 0.00 | 0.00 | KO:K02111 | F-type H+-transporting ATPase subunit alpha | Bacteria;Proteobacteria;Deltaproteobacteria;Desulfobacterales;Desulfobulbaceae;Desulfobulbus;Desulfobulbus mediterraneus;Desulfobulbus mediterraneus DSM 13871 |
| Ga0586324_2752996_3_788 | 0.00 | 0.00 | 0.00 | 0.00 | 7436600.00 | 0.00 | 8572800.00 | 5471700.00 | 3960900.00 | 0.00 | 0.00 | 0.00 | KO:K02111 | F-type H+-transporting ATPase subunit alpha | Bacteria;Proteobacteria;Deltaproteobacteria;Syntrophobacterales;Syntrophaceae;Desulfobacca;Desulfobacca acetoxidans;Desulfobacca acetoxidans ASRB2, DSM 11109 |
| Ga0586324_2828084_3_527 | 0.00 | 1353200.00 | 0.00 | 0.00 | 0.00 | 0.00 | 0.00 | 0.00 | 0.00 | 0.00 | 0.00 | 0.00 | KO:K02111 | F-type H+-transporting ATPase subunit alpha | Bacteria;Proteobacteria;Deltaproteobacteria;Syntrophobacterales;Syntrophaceae;Desulfobacca;Desulfobacca acetoxidans;Desulfobacca acetoxidans ASRB2, DSM 11109 |
| Ga0586324_2866790_1_777 | 0.00 | 5113600.00 | 0.00 | 0.00 | 0.00 | 0.00 | 0.00 | 0.00 | 0.00 | 0.00 | 0.00 | 0.00 | KO:K02111 | F-type H+-transporting ATPase subunit alpha | Bacteria;Proteobacteria;Deltaproteobacteria;Syntrophobacterales;Syntrophaceae;Desulfobacca;Desulfobacca acetoxidans;Desulfobacca acetoxidans ASRB2, DSM 11109 |
| Ga0586324_2993485_1_762 | 0.00 | 0.00 | 0.00 | 897628.40 | 22557000.00 | 0.00 | 1737971.04 | 0.00 | 1053000.00 | 0.00 | 2560800.00 | 1012500.00 | KO:K02111 | F-type H+-transporting ATPase subunit alpha | Bacteria;Proteobacteria;Deltaproteobacteria;Desulfuromonadales;Geobacteraceae;Geobacter;Geobacter argillaceus;Geobacter argillaceus ATCC BAA-1139 |
| Ga0586324_3497667_1_714 | 0.00 | 0.00 | 0.00 | 0.00 | 605091.01 | 0.00 | 0.00 | 0.00 | 0.00 | 0.00 | 0.00 | 0.00 | KO:K02111 | F-type H+-transporting ATPase subunit alpha | Bacteria;Proteobacteria;Deltaproteobacteria;Syntrophobacterales;Syntrophaceae;Desulfobacca;Desulfobacca acetoxidans;Desulfobacca acetoxidans ASRB2, DSM 11109 |
| Ga0586324_3682930_362_700 | 0.00 | 1298800.00 | 0.00 | 0.00 | 0.00 | 0.00 | 0.00 | 0.00 | 0.00 | 0.00 | 0.00 | 0.00 | KO:K02111 | F-type H+-transporting ATPase subunit alpha | Bacteria;Proteobacteria;Alphaproteobacteria;Hyphomicrobiales;Bradyrhizobiaceae;Bradyrhizobium;Bradyrhizobium sp. WSM2254;Bradyrhizobium sp. WSM2254 |
| Ga0586324_4190606_3_662 | 0.00 | 0.00 | 0.00 | 0.00 | 0.00 | 0.00 | 0.00 | 2409500.00 | 0.00 | 0.00 | 0.00 | 0.00 | KO:K02111 | F-type H+-transporting ATPase subunit alpha | Bacteria;Verrucomicrobia;Spartobacteria;Chthoniobacterales;Chthoniobacteraceae;Chthoniobacter;Chthoniobacter flavus;Chthoniobacter flavus Ellin428 |
| Ga0586324_4388021_1_651 | 0.00 | 0.00 | 0.00 | 0.00 | 0.00 | 0.00 | 0.00 | 2684000.00 | 0.00 | 0.00 | 0.00 | 0.00 | KO:K02111 | F-type H+-transporting ATPase subunit alpha | Bacteria;Proteobacteria;Deltaproteobacteria;Syntrophobacterales;Syntrophobacteraceae;Desulfoglaeba;Desulfoglaeba alkanexedens;Desulfoglaeba alkanexedens ALDC |
| Ga0586324_4402512_2_649 | 9060000.00 | 0.00 | 0.00 | 357231.35 | 0.00 | 0.00 | 0.00 | 4111400.00 | 4965300.00 | 0.00 | 11616000.00 | 7020000.00 | KO:K02111 | F-type H+-transporting ATPase subunit alpha | Bacteria;Proteobacteria;Alphaproteobacteria;Hyphomicrobiales;Hyphomicrobiaceae;Rhodoplanes;Rhodoplanes sp. Z2-YC6860;Rhodoplanes sp. Z2-YC6860 |
| Ga0586324_4483113_1_645 | 0.00 | 0.00 | 0.00 | 0.00 | 0.00 | 0.00 | 0.00 | 0.00 | 3653100.00 | 0.00 | 0.00 | 0.00 | KO:K02111 | F-type H+-transporting ATPase subunit alpha | Bacteria;Proteobacteria;Deltaproteobacteria;Syntrophorhabdales;Syntrophorhabdaceae;Syntrophorhabdus;Syntrophorhabdus aromaticivorans;Syntrophorhabdus aromaticivorans UI |
| Ga0586324_4496120_2_643 | 0.00 | 0.00 | 0.00 | 0.00 | 0.00 | 0.00 | 0.00 | 0.00 | 0.00 | 0.00 | 6265600.00 | 0.00 | KO:K02111 | F-type H+-transporting ATPase subunit alpha | Bacteria;Proteobacteria;Deltaproteobacteria;Desulfuromonadales;Geobacteraceae;Geobacter;Geobacter metallireducens;Geobacter metallireducens GS-15 |
| Ga0586324_4503758_62_643 | 4090000.00 | 0.00 | 0.00 | 8417000.00 | 10609000.00 | 11949700.00 | 7774800.00 | 1146800.00 | 0.00 | 0.00 | 1205600.00 | 1230000.00 | KO:K02111 | F-type H+-transporting ATPase subunit alpha | Bacteria;Proteobacteria;Deltaproteobacteria;Desulfobacterales;Desulfobulbaceae;Desulfobulbus;Desulfobulbus elongatus;Desulfobulbus elongatus DSM 2908 |
| Ga0586324_4546583_391_642 | 0.00 | 0.00 | 0.00 | 3771500.00 | 0.00 | 2489590.97 | 0.00 | 1970300.00 | 2276100.00 | 0.00 | 0.00 | 0.00 | KO:K02111 | F-type H+-transporting ATPase subunit alpha | Bacteria;Thermodesulfobacteria;Thermodesulfobacteria;Thermodesulfobacteriales;Thermodesulfobacteriaceae;Thermodesulfatator;Thermodesulfatator autotrophicus;Thermodesulfatator autotrophicus S606 |
| Ga0586324_4614294_1_537 | 0.00 | 0.00 | 0.00 | 0.00 | 0.00 | 0.00 | 0.00 | 0.00 | 1441800.00 | 0.00 | 0.00 | 0.00 | KO:K02111 | F-type H+-transporting ATPase subunit alpha | Bacteria;Proteobacteria;Deltaproteobacteria;Syntrophobacterales;Syntrophaceae;Desulfomonile;Desulfomonile tiedjei;Desulfomonile tiedjei DCB-1, DSM 6799 |
| Ga0586324_4739257_3_512 | 0.00 | 0.00 | 0.00 | 0.00 | 0.00 | 4922800.00 | 0.00 | 0.00 | 0.00 | 0.00 | 0.00 | 0.00 | KO:K02111 | F-type H+-transporting ATPase subunit alpha | Bacteria;Proteobacteria;Deltaproteobacteria;Desulfarculales;Desulfarculaceae;Dethiosulfatarculus;Dethiosulfatarculus sandiegensis;Dethiosulfatarculus sandiegensis SPR |
| Ga0586324_4833078_8_625 | 0.00 | 0.00 | 0.00 | 0.00 | 921500.83 | 0.00 | 0.00 | 0.00 | 0.00 | 0.00 | 0.00 | 0.00 | KO:K02111 | F-type H+-transporting ATPase subunit alpha | Bacteria;Proteobacteria;Deltaproteobacteria;Desulfobacterales;Desulfobacteraceae;Desulfatirhabdium;Desulfatirhabdium butyrativorans;Desulfatirhabdium butyrativorans DSM 18734 |
| Ga0586324_4896602_3_623 | 0.00 | 0.00 | 0.00 | 5329500.00 | 1369900.00 | 0.00 | 0.00 | 0.00 | 0.00 | 0.00 | 0.00 | 0.00 | KO:K02111 | F-type H+-transporting ATPase subunit alpha | Bacteria;Proteobacteria;Betaproteobacteria;Burkholderiales;Burkholderiaceae;Burkholderia;Burkholderia multivorans;Burkholderia multivorans ATCC 17616 |
| Ga0586324_4972470_158_619 | 0.00 | 0.00 | 0.00 | 0.00 | 0.00 | 0.00 | 0.00 | 0.00 | 53055000.00 | 0.00 | 0.00 | 34875000.00 | KO:K02111 | F-type H+-transporting ATPase subunit alpha | Bacteria;Proteobacteria;Deltaproteobacteria;Syntrophobacterales;Syntrophaceae;Desulfobacca;Desulfobacca acetoxidans;Desulfobacca acetoxidans ASRB2, DSM 11109 |
| Ga0586324_5166791_3_407 | 0.00 | 0.00 | 0.00 | 0.00 | 0.00 | 0.00 | 0.00 | 0.00 | 0.00 | 4912600.00 | 0.00 | 0.00 | KO:K02111 | F-type H+-transporting ATPase subunit alpha | Bacteria;Proteobacteria;Deltaproteobacteria;Desulfuromonadales;Geobacteraceae;Citrifermentans;Citrifermentans bremense;Geobacter bremensis R1 |
| Ga0586324_5637166_1_441 | 0.00 | 904400.00 | 0.00 | 0.00 | 0.00 | 0.00 | 0.00 | 0.00 | 0.00 | 0.00 | 0.00 | 0.00 | KO:K02111 | F-type H+-transporting ATPase subunit alpha | Bacteria;Proteobacteria;Deltaproteobacteria;Syntrophobacterales;Syntrophaceae;Smithella;Smithella sp. SCADC;Smithella sp. SCADC |
| Ga0586324_5649367_3_587 | 0.00 | 0.00 | 0.00 | 0.00 | 0.00 | 0.00 | 0.00 | 0.00 | 0.00 | 0.00 | 0.00 | 534474.75 | KO:K02111 | F-type H+-transporting ATPase subunit alpha | Bacteria;Chloroflexi;Anaerolineae;Anaerolineales;Anaerolineaceae;Thermanaerothrix;Thermanaerothrix daxensis;Thermanaerothrix daxensis GNS-1 |
| Ga0586324_5809929_30_581 | 0.00 | 0.00 | 0.00 | 0.00 | 0.00 | 14133200.00 | 0.00 | 0.00 | 0.00 | 0.00 | 0.00 | 0.00 | KO:K02111 | F-type H+-transporting ATPase subunit alpha | Bacteria;Proteobacteria;Deltaproteobacteria;Syntrophobacterales;Syntrophobacteraceae;Desulfacinum;Desulfacinum infernum;Desulfacinum infernum DSM 9756 |
| Ga0586324_5961788_3_572 | 3310000.00 | 0.00 | 0.00 | 0.00 | 0.00 | 0.00 | 0.00 | 0.00 | 0.00 | 0.00 | 0.00 | 757500.00 | KO:K02111 | F-type H+-transporting ATPase subunit alpha | Bacteria;Nitrospirae;Nitrospira;Nitrospirales;Nitrospiraceae;Candidatus Magnetobacterium;Candidatus Magnetobacterium casensis;Candidatus Magnetobacterium casensis MYR-1 |
| Ga0586324_6142220_3_566 | 0.00 | 0.00 | 0.00 | 0.00 | 0.00 | 0.00 | 0.00 | 0.00 | 0.00 | 4666200.00 | 0.00 | 0.00 | KO:K02111 | F-type H+-transporting ATPase subunit alpha | Bacteria;Firmicutes;Clostridia;Eubacteriales;Oscillospiraceae;Acetivibrio;Acetivibrio clariflavus;Hungateiclostridium clariflavum EBR 45, DSM 19732 |
| Ga0586324_6443197_1_555 | 0.00 | 0.00 | 0.00 | 0.00 | 0.00 | 0.00 | 1582972.08 | 860100.00 | 0.00 | 847000.00 | 1328800.00 | 0.00 | KO:K02111 | F-type H+-transporting ATPase subunit alpha | Bacteria;Proteobacteria;Gammaproteobacteria;Alteromonadales;Alteromonadaceae;Lacimicrobium;Lacimicrobium sp. SS2-24;Lacimicrobium sp. SS2-24 |
| Ga0586324_6879627_3_539 | 0.00 | 36992000.00 | 0.00 | 0.00 | 87962000.00 | 0.00 | 0.00 | 3611200.00 | 3604500.00 | 21714000.00 | 21560000.00 | 20475000.00 | KO:K02111 | F-type H+-transporting ATPase subunit alpha | Bacteria;Proteobacteria;Deltaproteobacteria;Syntrophobacterales;Syntrophaceae;Desulfobacca;Desulfobacca acetoxidans;Desulfobacca acetoxidans ASRB2, DSM 11109 |
| Ga0586324_6890196_186_539 | 0.00 | 0.00 | 0.00 | 0.00 | 0.00 | 27154800.00 | 0.00 | 0.00 | 0.00 | 0.00 | 0.00 | 0.00 | KO:K02111 | F-type H+-transporting ATPase subunit alpha | Bacteria;Proteobacteria;Deltaproteobacteria;Desulfuromonadales;Geobacteraceae;Geobacter;Geobacter sp. OR-1;Geobacter sp. OR-1 |
| Ga0586324_7134917_1_531 | 14000000.00 | 0.00 | 0.00 | 0.00 | 0.00 | 0.00 | 0.00 | 0.00 | 0.00 | 0.00 | 0.00 | 7072500.00 | KO:K02111 | F-type H+-transporting ATPase subunit alpha | Bacteria;Proteobacteria;Deltaproteobacteria;Desulfobacterales;Desulfobacteraceae;Desulfatibacillum;Desulfatibacillum alkenivorans;Desulfatibacillum alkenivorans DSM 16219 |
| Ga0586324_7372957_3_524 | 0.00 | 0.00 | 5686300.00 | 0.00 | 0.00 | 0.00 | 0.00 | 3568500.00 | 0.00 | 0.00 | 0.00 | 0.00 | KO:K02111 | F-type H+-transporting ATPase subunit alpha | Bacteria;Firmicutes;Bacilli;Bacillales;Paenibacillaceae;Brevibacillus;Brevibacillus sp. SCSIO 07484;Brevibacillus sp. SCSIO 07484 |
| Ga0586324_7653908_1_516 | 0.00 | 3005600.00 | 0.00 | 0.00 | 0.00 | 0.00 | 0.00 | 0.00 | 0.00 | 0.00 | 0.00 | 0.00 | KO:K02111 | F-type H+-transporting ATPase subunit alpha | Bacteria;Proteobacteria;Gammaproteobacteria;Methylococcales;Methylococcaceae;Methylobacter;Methylobacter tundripaludum;Methylobacter tundripaludum 31/32 |
| Ga0586324_8178600_2_502 | 0.00 | 0.00 | 0.00 | 0.00 | 0.00 | 0.00 | 0.00 | 5465600.00 | 0.00 | 0.00 | 0.00 | 0.00 | KO:K02111 | F-type H+-transporting ATPase subunit alpha | Bacteria;Proteobacteria;Deltaproteobacteria;Syntrophobacterales;Syntrophobacteraceae;Syntrophobacter;Syntrophobacter fumaroxidans;Syntrophobacter fumaroxidans MPOB |
| Ga0586324_8204170_1_384 | 0.00 | 0.00 | 0.00 | 21945000.00 | 23690000.00 | 0.00 | 0.00 | 0.00 | 0.00 | 12551000.00 | 15312000.00 | 2730000.00 | KO:K02111 | F-type H+-transporting ATPase subunit alpha | Bacteria;Proteobacteria;Deltaproteobacteria;Desulfurellales;Desulfurellaceae;Hippea;Hippea sp. KM1;Hippea medeae KM1 |
| Ga0586324_8305578_1_360 | 0.00 | 0.00 | 0.00 | 0.00 | 0.00 | 1550003.13 | 0.00 | 1000400.00 | 1417500.00 | 0.00 | 0.00 | 0.00 | KO:K02111 | F-type H+-transporting ATPase subunit alpha | Bacteria;Thermodesulfobacteria;Thermodesulfobacteria;Thermodesulfobacteriales;Thermodesulfobacteriaceae;Caldimicrobium;Caldimicrobium thiodismutans;Caldimicrobium thiodismutans TF1 |
| Ga0586324_0000001_67484_68863 | 0.00 | 5650800.00 | 0.00 | 0.00 | 0.00 | 0.00 | 12615290.16 | 111047482.33 | 20650912.74 | 146528994.92 | 134237466.88 | 95175000.00 | KO:K02112 | F-type H+-transporting ATPase subunit beta | Bacteria;Proteobacteria;Betaproteobacteria;Nitrosomonadales;Thiobacillaceae;Annwoodia;Annwoodia aquaesulis;Annwoodia aquaesulis DSM 4255 |
| Ga0586324_0000290_24862_26298 | 0.00 | 0.00 | 0.00 | 0.00 | 1720100.00 | 0.00 | 0.00 | 4312700.00 | 4560300.00 | 0.00 | 0.00 | 0.00 | KO:K02112 | F-type H+-transporting ATPase subunit beta | Bacteria;Proteobacteria;Alphaproteobacteria;Hyphomicrobiales;Hyphomicrobiaceae;Rhodoplanes;Rhodoplanes sp. Z2-YC6860;Rhodoplanes sp. Z2-YC6860 |
| Ga0586324_0000524_28100_29479 | 0.00 | 0.00 | 0.00 | 0.00 | 0.00 | 0.00 | 29184000.00 | 13583620.30 | 16694100.00 | 11688600.00 | 11052800.00 | 10200000.00 | KO:K02112 | F-type H+-transporting ATPase subunit beta | Bacteria;Proteobacteria;Betaproteobacteria;Nitrosomonadales;Thiobacillaceae;Thiobacillus;Thiobacillus denitrificans;Thiobacillus denitrificans ATCC 25259 |
| Ga0586324_0001726_9325_10746 | 0.00 | 0.00 | 0.00 | 0.00 | 0.00 | 1881343.30 | 0.00 | 0.00 | 0.00 | 0.00 | 0.00 | 26699.55 | KO:K02112 | F-type H+-transporting ATPase subunit beta | Bacteria;Spirochaetes;Spirochaetia;Leptospirales;Leptospiraceae;Leptospira;Leptospira sp. B5-022;Leptospira sp. B5-022 |
| Ga0586324_0003019_15038_16471 | 0.00 | 0.00 | 0.00 | 0.00 | 204466.33 | 0.00 | 0.00 | 0.00 | 0.00 | 0.00 | 0.00 | 0.00 | KO:K02112 | F-type H+-transporting ATPase subunit beta | Bacteria;Acidobacteria;Vicinamibacteria;unclassified Vicinamibacteria;Vicinamibacteraceae;Luteitalea;Luteitalea pratensis;Luteitalea pratensis DSM 100886 |
| Ga0586324_0005566_10342_11766 | 1694760.00 | 3216400.00 | 0.00 | 2437613.55 | 4223000.00 | 855118.15 | 1482412.68 | 2293600.00 | 1798200.00 | 2471700.00 | 0.00 | 640110.75 | KO:K02112 | F-type H+-transporting ATPase subunit beta | Bacteria;Proteobacteria;Deltaproteobacteria;Desulfobacterales;Desulfobacteraceae;Desulfonema;Desulfonema limicola;Desulfonema limicola Jadebusen, DSM 2076 |
| Ga0586324_0005643_10111_11634 | 0.00 | 0.00 | 0.00 | 0.00 | 0.00 | 0.00 | 0.00 | 1769000.00 | 0.00 | 0.00 | 0.00 | 0.00 | KO:K02112 | F-type H+-transporting ATPase subunit beta | Bacteria;Proteobacteria;Gammaproteobacteria;Acidiferrobacterales;Acidiferrobacteraceae;Sulfuricaulis;Sulfuricaulis limicola;Sulfuricaulis limicola HA5 |
| Ga0586324_0006195_1_1065 | 0.00 | 0.00 | 0.00 | 2863400.00 | 0.00 | 0.00 | 0.00 | 0.00 | 0.00 | 12012000.00 | 0.00 | 0.00 | KO:K02112 | F-type H+-transporting ATPase subunit beta | Bacteria;Verrucomicrobia;Verrucomicrobiae;Verrucomicrobiales;Verrucomicrobia subdivision 3;Pedosphaera;Pedosphaera parvula;Pedosphaera parvula Ellin514 |
| Ga0586324_0008747_3960_5378 | 1688689.00 | 1380400.00 | 1655529.65 | 4398500.00 | 1627400.00 | 2820033.92 | 2221150.92 | 1652177.07 | 2089800.00 | 0.00 | 0.00 | 11512500.00 | KO:K02112 | F-type H+-transporting ATPase subunit beta | Bacteria;Proteobacteria;Deltaproteobacteria;Syntrophobacterales;Syntrophobacteraceae;Desulfacinum;Desulfacinum infernum;Desulfacinum infernum DSM 9756 |
| Ga0586324_0014086_8338_8733 | 0.00 | 0.00 | 0.00 | 0.00 | 0.00 | 0.00 | 0.00 | 0.00 | 0.00 | 0.00 | 0.00 | 1155000.00 | KO:K02112 | F-type H+-transporting ATPase subunit beta | Bacteria;Proteobacteria;Betaproteobacteria;Burkholderiales;Burkholderiaceae;Cupriavidus;Cupriavidus necator;Cupriavidus necator N-1, ATCC 43291 |
| Ga0586324_0015259_2_1414 | 0.00 | 0.00 | 0.00 | 0.00 | 0.00 | 0.00 | 0.00 | 0.00 | 30537000.00 | 0.00 | 0.00 | 0.00 | KO:K02112 | F-type H+-transporting ATPase subunit beta | Bacteria;Proteobacteria;Betaproteobacteria;Nitrosomonadales;Gallionellaceae;Sideroxydans;Sideroxydans lithotrophicus;Sideroxydans lithotrophicus ES-1 |
| Ga0586324_0015593_3462_4883 | 0.00 | 670694.20 | 0.00 | 0.00 | 0.00 | 1767622.65 | 0.00 | 0.00 | 1587600.00 | 0.00 | 0.00 | 0.00 | KO:K02112 | F-type H+-transporting ATPase subunit beta | Bacteria;Proteobacteria;Deltaproteobacteria;Desulfobacterales;Desulfobacteraceae;Desulfosarcina;Desulfosarcina sp. BuS5;Desulfosarcina sp. BuS5 |
| Ga0586324_0022066_5788_7101 | 6860000.00 | 0.00 | 0.00 | 0.00 | 0.00 | 0.00 | 0.00 | 8662000.00 | 0.00 | 0.00 | 5288800.00 | 7725000.00 | KO:K02112 | F-type H+-transporting ATPase subunit beta | Bacteria;Proteobacteria;Betaproteobacteria;unclassified Betaproteobacteria;unclassified Betaproteobacteria;unclassified Betaproteobacteria;Betaproteobacteria bacterium GR16-43;Betaproteobacteria bacterium GR16-43 |
| Ga0586324_0023875_5019_6434 | 0.00 | 0.00 | 0.00 | 1719500.00 | 0.00 | 3145808.15 | 0.00 | 0.00 | 0.00 | 0.00 | 0.00 | 0.00 | KO:K02112 | F-type H+-transporting ATPase subunit beta | Bacteria;Proteobacteria;Deltaproteobacteria;Desulfobacterales;Desulfobacteraceae;Desulfatiglans;Desulfatiglans anilini;Desulfatiglans anilini DSM 4660 |
| Ga0586324_0025745_1478_2857 | 0.00 | 0.00 | 0.00 | 2470000.00 | 0.00 | 0.00 | 0.00 | 0.00 | 0.00 | 0.00 | 0.00 | 0.00 | KO:K02112 | F-type H+-transporting ATPase subunit beta | Bacteria;Proteobacteria;Gammaproteobacteria;Chromatiales;Ectothiorhodospiraceae;Ectothiorhodospira;Ectothiorhodospira mobilis;Ectothiorhodospira marismortui DSM 4180 |
| Ga0586324_0025999_3300_4721 | 0.00 | 0.00 | 0.00 | 0.00 | 0.00 | 0.00 | 0.00 | 0.00 | 0.00 | 0.00 | 0.00 | 72415.43 | KO:K02112 | F-type H+-transporting ATPase subunit beta | Bacteria;Nitrospirae;Nitrospira;Nitrospirales;Nitrospiraceae;Nitrospira;Candidatus Nitrospira inopinata;Candidatus Nitrospira inopinata ENR4 |
| Ga0586324_0029967_5011_6132 | 0.00 | 0.00 | 0.00 | 0.00 | 0.00 | 0.00 | 0.00 | 16409000.00 | 0.00 | 0.00 | 0.00 | 0.00 | KO:K02112 | F-type H+-transporting ATPase subunit beta | Bacteria;Proteobacteria;Betaproteobacteria;Rhodocyclales;Rhodocyclaceae;Rhodocyclus;Rhodocyclus tenuis;Rhodocyclus tenuis DSM 109 |
| Ga0586324_0031467_4794_5993 | 0.00 | 0.00 | 0.00 | 1824000.00 | 0.00 | 0.00 | 0.00 | 0.00 | 0.00 | 0.00 | 0.00 | 0.00 | KO:K02112 | F-type H+-transporting ATPase subunit beta | Bacteria;Proteobacteria;Deltaproteobacteria;Syntrophobacterales;Syntrophobacteraceae;Desulfosoma;Desulfosoma caldarium;Desulfosoma caldarium DSM 22027 |
| Ga0586324_0031564_1264_2676 | 0.00 | 809200.00 | 0.00 | 0.00 | 0.00 | 0.00 | 0.00 | 1177300.00 | 1198800.00 | 0.00 | 0.00 | 0.00 | KO:K02112 | F-type H+-transporting ATPase subunit beta | Bacteria;Proteobacteria;Betaproteobacteria;Burkholderiales;Comamonadaceae;Comamonas;Comamonas granuli;Comamonas granuli NBRC 101663 |
| Ga0586324_0033563_4368_5813 | 0.00 | 0.00 | 0.00 | 0.00 | 1122700.00 | 0.00 | 0.00 | 0.00 | 0.00 | 0.00 | 0.00 | 0.00 | KO:K02112 | F-type H+-transporting ATPase subunit beta | Bacteria;Chloroflexi;Anaerolineae;Anaerolineales;Anaerolineaceae;Thermanaerothrix;Thermanaerothrix daxensis;Thermanaerothrix daxensis GNS-1 |
| Ga0586324_0035361_3255_4673 | 2250000.00 | 8059877.48 | 0.00 | 3838000.00 | 15250465.31 | 19351102.01 | 0.00 | 9371110.36 | 0.00 | 14352800.00 | 368256.24 | 441024.75 | KO:K02112 | F-type H+-transporting ATPase subunit beta | Bacteria;Proteobacteria;Deltaproteobacteria;Desulfobacterales;Desulfobacteraceae;Desulfonema;Desulfonema limicola;Desulfonema limicola Jadebusen, DSM 2076 |
| Ga0586324_0036132_1_1470 | 0.00 | 1455200.00 | 0.00 | 3391500.00 | 0.00 | 5756500.00 | 0.00 | 0.00 | 0.00 | 0.00 | 0.00 | 0.00 | KO:K02112 | F-type H+-transporting ATPase subunit beta | Bacteria;Proteobacteria;Deltaproteobacteria;Syntrophobacterales;Syntrophaceae;Desulfobacca;Desulfobacca acetoxidans;Desulfobacca acetoxidans ASRB2, DSM 11109 |
| Ga0586324_0036372_2904_4313 | 1488821.00 | 2278000.00 | 1711148.42 | 0.00 | 1184500.00 | 5163159.68 | 839664.72 | 3141500.00 | 29670300.00 | 0.00 | 0.00 | 997500.00 | KO:K02112 | F-type H+-transporting ATPase subunit beta | Bacteria;Proteobacteria;Deltaproteobacteria;Syntrophobacterales;Syntrophaceae;Desulfobacca;Desulfobacca acetoxidans;Desulfobacca acetoxidans ASRB2, DSM 11109 |
| Ga0586324_0038418_4132_5442 | 6640000.00 | 1298800.00 | 901785.25 | 0.00 | 0.00 | 543687.53 | 14500800.00 | 2379000.00 | 0.00 | 0.00 | 0.00 | 8400000.00 | KO:K02112 | F-type H+-transporting ATPase subunit beta | Bacteria;Proteobacteria;Gammaproteobacteria;Methylococcales;Methylococcaceae;Methylobacter;Methylobacter tundripaludum;Methylobacter tundripaludum SV96 |
| Ga0586324_0042799_2430_3830 | 0.00 | 0.00 | 0.00 | 0.00 | 0.00 | 0.00 | 0.00 | 0.00 | 0.00 | 0.00 | 941600.00 | 0.00 | KO:K02112 | F-type H+-transporting ATPase subunit beta | Bacteria;Proteobacteria;Betaproteobacteria;Burkholderiales;Comamonadaceae;Caldimonas;Caldimonas manganoxidans;Caldimonas manganoxidans ATCC BAA-369 |
| Ga0586324_0045726_3090_4598 | 1130000.00 | 2482000.00 | 0.00 | 0.00 | 0.00 | 0.00 | 0.00 | 0.00 | 0.00 | 0.00 | 0.00 | 1950000.00 | KO:K02112 | F-type H+-transporting ATPase subunit beta | Bacteria;Proteobacteria;Betaproteobacteria;Rhodocyclales;Rhodocyclaceae;Rhodocyclus;Rhodocyclus tenuis;Rhodocyclus tenuis DSM 109 |
| Ga0586324_0045889_4034_4984 | 49300000.00 | 0.00 | 0.00 | 0.00 | 0.00 | 0.00 | 0.00 | 0.00 | 0.00 | 0.00 | 0.00 | 0.00 | KO:K02112 | F-type H+-transporting ATPase subunit beta | Bacteria;Proteobacteria;Gammaproteobacteria;Methylococcales;Methylococcaceae;Methylobacter;Methylobacter tundripaludum;Methylobacter tundripaludum OWC-G53F |
| Ga0586324_0068363_1889_3307 | 4470000.00 | 8908000.00 | 0.00 | 0.00 | 0.00 | 0.00 | 5563200.00 | 0.00 | 5694300.00 | 0.00 | 5324000.00 | 8475000.00 | KO:K02112 | F-type H+-transporting ATPase subunit beta | Bacteria;Actinobacteria;Thermoleophilia;Thermoleophilales;Thermoleophilaceae;Thermoleophilum;Thermoleophilum album;Thermoleophilum album ATCC 35263 |
| Ga0586324_0069998_223_1719 | 17310000.00 | 28220000.00 | 5279780.22 | 18382500.00 | 40180300.00 | 7895246.19 | 20018400.00 | 5599800.00 | 8464500.00 | 29290800.00 | 9618400.00 | 15108974.25 | KO:K02112 | F-type H+-transporting ATPase subunit beta | Eukaryota;Streptophyta;Magnoliopsida;Malpighiales;Salicaceae;Populus;Populus trichocarpa;Populus balsamifera trichocarpa |
| Ga0586324_0071678_1320_2741 | 0.00 | 1210400.00 | 0.00 | 0.00 | 0.00 | 0.00 | 0.00 | 0.00 | 6561000.00 | 0.00 | 0.00 | 0.00 | KO:K02112 | F-type H+-transporting ATPase subunit beta | Bacteria;Proteobacteria;Deltaproteobacteria;Desulfuromonadales;Desulfuromonadaceae;Desulfuromusa;Desulfuromusa kysingii;Desulfuromusa kysingii DSM 7343 |
| Ga0586324_0079090_913_2289 | 0.00 | 999600.00 | 0.00 | 5272500.00 | 0.00 | 0.00 | 0.00 | 0.00 | 663875.19 | 0.00 | 0.00 | 0.00 | KO:K02112 | F-type H+-transporting ATPase subunit beta | Bacteria;Proteobacteria;Gammaproteobacteria;unclassified Gammaproteobacteria;Candidatus Competibacteraceae;Candidatus Contendobacter;Candidatus Contendobacter odensis;Candidatus Contendobacter odensis Run B J11 |
| Ga0586324_0095662_1422_2834 | 22200000.00 | 3128000.00 | 0.00 | 7229500.00 | 0.00 | 0.00 | 0.00 | 4324900.00 | 4657500.00 | 0.00 | 3669600.00 | 5437500.00 | KO:K02112 | F-type H+-transporting ATPase subunit beta | Bacteria;Proteobacteria;Deltaproteobacteria;Desulfobacterales;Desulfobacteraceae;Desulfatiglans;Desulfatiglans anilini;Desulfatiglans anilini DSM 4660 |
| Ga0586324_0099773_1269_2687 | 5720000.00 | 0.00 | 0.00 | 0.00 | 0.00 | 0.00 | 0.00 | 0.00 | 0.00 | 0.00 | 0.00 | 0.00 | KO:K02112 | F-type H+-transporting ATPase subunit beta | Bacteria;Proteobacteria;Deltaproteobacteria;Desulfobacterales;Desulfobulbaceae;Desulfopila;Desulfopila sp. IMCC35004;Desulfopila sp. IMCC35004 |
| Ga0586324_0103312_2656_3375 | 0.00 | 0.00 | 0.00 | 0.00 | 0.00 | 0.00 | 0.00 | 0.00 | 0.00 | 4774000.00 | 0.00 | 0.00 | KO:K02112 | F-type H+-transporting ATPase subunit beta | Bacteria;Proteobacteria;Betaproteobacteria;Nitrosomonadales;Gallionellaceae;Sideroxydans;Sideroxydans lithotrophicus;Sideroxydans lithotrophicus ES-1 |
| Ga0586324_0107286_2_931 | 19600000.00 | 0.00 | 0.00 | 0.00 | 0.00 | 969986.13 | 37164000.00 | 3117100.00 | 28026000.00 | 0.00 | 0.00 | 4342500.00 | KO:K02112 | F-type H+-transporting ATPase subunit beta | Bacteria;Proteobacteria;Alphaproteobacteria;Hyphomicrobiales;Bradyrhizobiaceae;Bradyrhizobium;Bradyrhizobium canariense;Bradyrhizobium canariense GAS369 |
| Ga0586324_0132779_1462_2856 | 0.00 | 0.00 | 0.00 | 0.00 | 0.00 | 0.00 | 0.00 | 13603000.00 | 0.00 | 0.00 | 0.00 | 0.00 | KO:K02112 | F-type H+-transporting ATPase subunit beta | Bacteria;Chloroflexi;Dehalococcoidia;unclassified Dehalococcoidia;unclassified Dehalococcoidia;Dehalogenimonas;Dehalogenimonas alkenigignens;Dehalogenimonas alkenigignens IP3-3 |
| Ga0586324_0135477_1_1110 | 300090.00 | 0.00 | 2252382.84 | 3429500.00 | 4202400.00 | 2470904.18 | 0.00 | 2305800.00 | 0.00 | 0.00 | 1751200.00 | 132182.25 | KO:K02112 | F-type H+-transporting ATPase subunit beta | Bacteria;Proteobacteria;Alphaproteobacteria;Rhodospirillales;Acetobacteraceae;Rhodopila;Rhodopila globiformis;Rhodopila globiformis DSM 161 |
| Ga0586324_0139246_1_675 | 0.00 | 0.00 | 0.00 | 0.00 | 0.00 | 0.00 | 0.00 | 0.00 | 0.00 | 0.00 | 0.00 | 347278.50 | KO:K02112 | F-type H+-transporting ATPase subunit beta | Bacteria;Proteobacteria;Betaproteobacteria;Burkholderiales;Alcaligenaceae;Alcaligenes;Alcaligenes aquatilis;Alcaligenes aquatilis QD168 |
| Ga0586324_0169296_2314_2673 | 0.00 | 0.00 | 0.00 | 0.00 | 0.00 | 0.00 | 0.00 | 0.00 | 612338.94 | 0.00 | 0.00 | 832500.00 | KO:K02112 | F-type H+-transporting ATPase subunit beta | Bacteria;Proteobacteria;Gammaproteobacteria;Alteromonadales;Alteromonadaceae;unclassified Alteromonadaceae;Alteromonadaceae bacterium 2141T.STBD.0c.01a;Alteromonadaceae bacterium 2141T.STBD.0c.01a |
| Ga0586324_0170721_1954_2664 | 0.00 | 0.00 | 0.00 | 0.00 | 0.00 | 0.00 | 0.00 | 0.00 | 0.00 | 0.00 | 1971200.00 | 0.00 | KO:K02112 | F-type H+-transporting ATPase subunit beta | Bacteria;Proteobacteria;Betaproteobacteria;Nitrosomonadales;Thiobacillaceae;Thiobacillus;Thiobacillus denitrificans;Thiobacillus denitrificans DSM 12475 |
| Ga0586324_0174943_957_2381 | 0.00 | 0.00 | 0.00 | 0.00 | 0.00 | 0.00 | 0.00 | 3836900.00 | 0.00 | 0.00 | 0.00 | 0.00 | KO:K02112 | F-type H+-transporting ATPase subunit beta | Bacteria;Proteobacteria;Betaproteobacteria;Burkholderiales;Burkholderiaceae;Lautropia;Lautropia mirabilis;Lautropia mirabilis ATCC 51599 |
| Ga0586324_0183678_502_2040 | 0.00 | 0.00 | 0.00 | 0.00 | 1025517.44 | 0.00 | 0.00 | 6832000.00 | 0.00 | 0.00 | 0.00 | 0.00 | KO:K02112 | F-type H+-transporting ATPase subunit beta | Bacteria;Actinobacteria;Thermoleophilia;Solirubrobacterales;Conexibacteraceae;Conexibacter;Conexibacter woesei;Conexibacter woesei ID131577, DSM 14684 |
| Ga0586324_0193593_3_920 | 0.00 | 0.00 | 0.00 | 0.00 | 3635900.00 | 2729827.58 | 269083.32 | 0.00 | 0.00 | 0.00 | 0.00 | 0.00 | KO:K02112 | F-type H+-transporting ATPase subunit beta | Bacteria;Proteobacteria;Deltaproteobacteria;unclassified Deltaproteobacteria;unclassified Deltaproteobacteria;unclassified Deltaproteobacteria;delta proteobacterium NaphS2;delta proteobacterium NaphS2 |
| Ga0586324_0199258_559_1962 | 0.00 | 0.00 | 0.00 | 0.00 | 0.00 | 0.00 | 0.00 | 119710.67 | 0.00 | 0.00 | 0.00 | 0.00 | KO:K02112 | F-type H+-transporting ATPase subunit beta | Bacteria;Chloroflexi;Caldilineae;Caldilineales;Caldilineaceae;Caldilinea;Caldilinea aerophila;Caldilinea aerophila STL-6-O1, DSM 14535 |
| Ga0586324_0211149_120_1529 | 1440000.00 | 0.00 | 0.00 | 0.00 | 1823100.00 | 0.00 | 2530800.00 | 994300.00 | 996300.00 | 0.00 | 404868.64 | 1282500.00 | KO:K02112 | F-type H+-transporting ATPase subunit beta | Bacteria;Proteobacteria;Deltaproteobacteria;Syntrophobacterales;Syntrophobacteraceae;Syntrophobacter;Syntrophobacter fumaroxidans;Syntrophobacter fumaroxidans MPOB |
| Ga0586324_0256224_574_1950 | 0.00 | 0.00 | 0.00 | 0.00 | 0.00 | 0.00 | 0.00 | 1030900.00 | 3345300.00 | 649074.58 | 367121.04 | 2662500.00 | KO:K02112 | F-type H+-transporting ATPase subunit beta | Bacteria;Proteobacteria;Gammaproteobacteria;Chromatiales;Ectothiorhodospiraceae;Ectothiorhodospira;Ectothiorhodospira mobilis;Ectothiorhodospira marismortui DSM 4180 |
| Ga0586324_0288794_689_2092 | 28950000.00 | 81600000.00 | 26911400.00 | 188575000.00 | 57165000.00 | 61932000.00 | 29412000.00 | 50813000.00 | 22275000.00 | 62370000.00 | 52712000.00 | 59940000.00 | KO:K02112 | F-type H+-transporting ATPase subunit beta | Bacteria;Proteobacteria;Deltaproteobacteria;Syntrophobacterales;Syntrophobacteraceae;Desulfacinum;Desulfacinum infernum;Desulfacinum infernum DSM 9756 |
| Ga0586324_0318311_394_1872 | 0.00 | 0.00 | 0.00 | 0.00 | 0.00 | 0.00 | 0.00 | 0.00 | 0.00 | 3484800.00 | 0.00 | 0.00 | KO:K02112 | F-type H+-transporting ATPase subunit beta | Bacteria;Verrucomicrobia;Verrucomicrobiae;Verrucomicrobiales;Verrucomicrobia subdivision 3;Pedosphaera;Pedosphaera parvula;Pedosphaera parvula Ellin514 |
| Ga0586324_0330685_1_729 | 0.00 | 0.00 | 0.00 | 0.00 | 0.00 | 0.00 | 0.00 | 121612.04 | 0.00 | 2733500.00 | 3220800.00 | 3229033.50 | KO:K02112 | F-type H+-transporting ATPase subunit beta | Bacteria;Proteobacteria;Betaproteobacteria;Nitrosomonadales;Nitrosomonadaceae;Nitrosospira;Nitrosospira sp. NI5;Nitrosospira sp. NI5 |
| Ga0586324_0331576_105_1532 | 0.00 | 0.00 | 0.00 | 0.00 | 0.00 | 0.00 | 0.00 | 194936.48 | 0.00 | 0.00 | 0.00 | 0.00 | KO:K02112 | F-type H+-transporting ATPase subunit beta | Bacteria;Proteobacteria;Betaproteobacteria;Burkholderiales;Burkholderiaceae;Lautropia;Lautropia mirabilis;Lautropia mirabilis ATCC 51599 |
| Ga0586324_0348588_2_268 | 0.00 | 0.00 | 822937.10 | 0.00 | 21527000.00 | 239330.66 | 0.00 | 0.00 | 0.00 | 0.00 | 0.00 | 0.00 | KO:K02112 | F-type H+-transporting ATPase subunit beta | Bacteria;Proteobacteria;Betaproteobacteria;Burkholderiales;Comamonadaceae;Pelomonas;Pelomonas sp. HMWF004;Pelomonas sp. HMWF004 |
| Ga0586324_0353783_3_1226 | 0.00 | 0.00 | 0.00 | 0.00 | 0.00 | 0.00 | 1560999.72 | 1830000.00 | 6342300.00 | 1532300.00 | 1504800.00 | 8295000.00 | KO:K02112 | F-type H+-transporting ATPase subunit beta | Bacteria;Proteobacteria;Betaproteobacteria;Burkholderiales;unclassified Burkholderiales;Aquabacterium;Aquabacterium tepidiphilum;Aquabacterium tepidiphilum YIM 730274 |
| Ga0586324_0359566_3_926 | 0.00 | 77390800.00 | 169651351.65 | 5814279.30 | 324737591.45 | 1786837.45 | 5586604.20 | 172980358.38 | 216847648.26 | 3996300.00 | 14414400.00 | 128343467.25 | KO:K02112 | F-type H+-transporting ATPase subunit beta | Bacteria;Proteobacteria;Deltaproteobacteria;Desulfobacterales;Desulfobacteraceae;Desulfatibacillum;Desulfatibacillum aliphaticivorans;Desulfatibacillum aliphaticivorans DSM 15576 |
| Ga0586324_0371952_38_1453 | 0.00 | 0.00 | 0.00 | 8654500.00 | 0.00 | 0.00 | 0.00 | 0.00 | 0.00 | 0.00 | 0.00 | 0.00 | KO:K02112 | F-type H+-transporting ATPase subunit beta | Bacteria;Chlamydiae;Chlamydiia;Parachlamydiales;Parachlamydiaceae;Candidatus Protochlamydia;Candidatus Protochlamydia massiliensis;Candidatus Protochlamydia massiliensis Diamant |
| Ga0586324_0423419_398_1762 | 0.00 | 0.00 | 0.00 | 0.00 | 0.00 | 4446400.00 | 0.00 | 0.00 | 0.00 | 0.00 | 0.00 | 0.00 | KO:K02112 | F-type H+-transporting ATPase subunit beta | Bacteria;Proteobacteria;Betaproteobacteria;Nitrosomonadales;Gallionellaceae;Sideroxydans;Sideroxydans lithotrophicus;Sideroxydans lithotrophicus ES-1 |
| Ga0586324_0428488_505_1755 | 0.00 | 0.00 | 0.00 | 0.00 | 1586200.00 | 0.00 | 0.00 | 0.00 | 0.00 | 0.00 | 0.00 | 0.00 | KO:K02112 | F-type H+-transporting ATPase subunit beta | Bacteria;Proteobacteria;Deltaproteobacteria;Syntrophobacterales;Syntrophaceae;Syntrophus;Syntrophus gentianae;Syntrophus gentianae DSM 8423 |
| Ga0586324_0462827_2_400 | 0.00 | 0.00 | 0.00 | 0.00 | 0.00 | 0.00 | 0.00 | 7747000.00 | 0.00 | 0.00 | 0.00 | 0.00 | KO:K02112 | F-type H+-transporting ATPase subunit beta | Bacteria;Proteobacteria;Gammaproteobacteria;Methylococcales;Methylococcaceae;Methylobacter;Methylobacter tundripaludum;Methylobacter tundripaludum SV96 |
| Ga0586324_0500042_829_1638 | 922541.00 | 3110490.68 | 1974362.18 | 3091034.00 | 0.00 | 1785916.41 | 2429469.96 | 3336700.00 | 616983.48 | 2394700.00 | 2613600.00 | 1102500.00 | KO:K02112 | F-type H+-transporting ATPase subunit beta | Bacteria;Proteobacteria;Deltaproteobacteria;Desulfobacterales;Desulfobacteraceae;Desulfococcus;Desulfococcus multivorans;Desulfococcus multivorans DSM 2059 |
| Ga0586324_0524059_501_1604 | 0.00 | 0.00 | 329435.51 | 4854500.00 | 0.00 | 0.00 | 0.00 | 5941400.00 | 0.00 | 0.00 | 0.00 | 0.00 | KO:K02112 | F-type H+-transporting ATPase subunit beta | Bacteria;Proteobacteria;Alphaproteobacteria;Hyphomicrobiales;Methylocystaceae;Methylocystis;Methylocystis parvus;Methylocystis parvus OBBP |
| Ga0586324_0546419_340_1575 | 3656594.00 | 2091242.08 | 22970400.00 | 2647432.45 | 32362600.00 | 4072648.32 | 5745600.00 | 2506486.34 | 3815100.00 | 0.00 | 0.00 | 0.00 | KO:K02112 | F-type H+-transporting ATPase subunit beta | Bacteria;Proteobacteria;Deltaproteobacteria;unclassified Deltaproteobacteria;unclassified Deltaproteobacteria;Dissulfuribacter;Dissulfuribacter thermophilus;Dissulfuribacter thermophilus S69 |
| Ga0586324_0570762_907_1548 | 0.00 | 0.00 | 0.00 | 0.00 | 0.00 | 0.00 | 0.00 | 0.00 | 0.00 | 0.00 | 527805.52 | 0.00 | KO:K02112 | F-type H+-transporting ATPase subunit beta | Bacteria;Proteobacteria;Gammaproteobacteria;Pseudomonadales;Moraxellaceae;Fluviicoccus;Fluviicoccus keumensis;Fluviicoccus keumensis DSM 105135 |
| Ga0586324_0651745_573_1460 | 4790000.00 | 0.00 | 0.00 | 0.00 | 0.00 | 71460000.00 | 0.00 | 3574600.00 | 2899800.00 | 0.00 | 1056000.00 | 0.00 | KO:K02112 | F-type H+-transporting ATPase subunit beta | Bacteria;Proteobacteria;Deltaproteobacteria;Syntrophobacterales;Syntrophobacteraceae;Syntrophobacter;Syntrophobacter fumaroxidans;Syntrophobacter fumaroxidans MPOB |
| Ga0586324_0688878_2_1249 | 0.00 | 0.00 | 0.00 | 0.00 | 14420000.00 | 0.00 | 0.00 | 0.00 | 0.00 | 0.00 | 0.00 | 0.00 | KO:K02112 | F-type H+-transporting ATPase subunit beta | Bacteria;Proteobacteria;Betaproteobacteria;unclassified Betaproteobacteria;unclassified Betaproteobacteria;unclassified Betaproteobacteria;beta proteobacterium AAP65;Rubrivivax sp. AAP65 |
| Ga0586324_0691757_611_1423 | 0.00 | 0.00 | 0.00 | 0.00 | 0.00 | 0.00 | 0.00 | 0.00 | 0.00 | 8239000.00 | 6635200.00 | 0.00 | KO:K02112 | F-type H+-transporting ATPase subunit beta | Bacteria;Proteobacteria;Betaproteobacteria;Nitrosomonadales;Gallionellaceae;Gallionella;Gallionella capsiferriformans;Gallionella capsiferriformans ES-2 |
| Ga0586324_0730066_1_594 | 0.00 | 0.00 | 0.00 | 0.00 | 0.00 | 0.00 | 0.00 | 1000400.00 | 0.00 | 0.00 | 0.00 | 0.00 | KO:K02112 | F-type H+-transporting ATPase subunit beta | Bacteria;Proteobacteria;Betaproteobacteria;Nitrosomonadales;Sterolibacteriaceae;Sterolibacterium;Sterolibacterium denitrificans;Sterolibacterium denitrificans Chol |
| Ga0586324_0792510_12_1343 | 0.00 | 0.00 | 0.00 | 0.00 | 0.00 | 0.00 | 0.00 | 10797000.00 | 0.00 | 0.00 | 0.00 | 0.00 | KO:K02112 | F-type H+-transporting ATPase subunit beta | Bacteria;Proteobacteria;Gammaproteobacteria;unclassified Gammaproteobacteria;Candidatus Competibacteraceae;Candidatus Competibacter;Candidatus Competibacter denitrificans;Candidatus Competibacter denitrificans Run A D11 |
| Ga0586324_0803547_590_1333 | 9242548.00 | 7571841.48 | 4359050.02 | 44935000.00 | 39886682.02 | 22704871.46 | 14658015.12 | 13834465.11 | 22009819.77 | 8393000.00 | 3379200.00 | 14186616.75 | KO:K02112 | F-type H+-transporting ATPase subunit beta | Bacteria;Proteobacteria;Deltaproteobacteria;Desulfobacterales;Desulfobacteraceae;Desulfatirhabdium;Desulfatirhabdium butyrativorans;Desulfatirhabdium butyrativorans DSM 18734 |
| Ga0586324_0819837_2_1294 | 0.00 | 0.00 | 0.00 | 0.00 | 0.00 | 0.00 | 1215759.84 | 0.00 | 0.00 | 0.00 | 0.00 | 0.00 | KO:K02112 | F-type H+-transporting ATPase subunit beta | Bacteria;Acidobacteria;Acidobacteriia;Acidobacteriales;Acidobacteriaceae;Edaphobacter;Edaphobacter modestus;Edaphobacter modestus DSM 18101 |
| Ga0586324_0826206_2_1117 | 0.00 | 0.00 | 0.00 | 0.00 | 0.00 | 0.00 | 0.00 | 0.00 | 13365000.00 | 507563.21 | 0.00 | 0.00 | KO:K02112 | F-type H+-transporting ATPase subunit beta | Bacteria;Proteobacteria;Gammaproteobacteria;Nevskiales;Steroidobacteraceae;Steroidobacter;Steroidobacter denitrificans;Steroidobacter denitrificans DSM 18526 |
| Ga0586324_0830203_2_1294 | 0.00 | 0.00 | 0.00 | 0.00 | 0.00 | 0.00 | 0.00 | 0.00 | 0.00 | 0.00 | 430502.16 | 0.00 | KO:K02112 | F-type H+-transporting ATPase subunit beta | Bacteria;Cyanobacteria;unclassified Cyanobacteria;Oscillatoriales;Microcoleaceae;Microcoleus;Microcoleus vaginatus;Microcoleus vaginatus PCC 9802 |
| Ga0586324_0889285_3_1019 | 0.00 | 459749.36 | 0.00 | 790861.70 | 243725.81 | 0.00 | 0.00 | 0.00 | 0.00 | 1832600.00 | 1645600.00 | 174588.75 | KO:K02112 | F-type H+-transporting ATPase subunit beta | Bacteria;Proteobacteria;Deltaproteobacteria;Syntrophobacterales;Syntrophobacteraceae;Syntrophobacter;Syntrophobacter fumaroxidans;Syntrophobacter fumaroxidans MPOB |
| Ga0586324_0990869_1_996 | 0.00 | 0.00 | 0.00 | 0.00 | 0.00 | 4208200.00 | 210083.99 | 0.00 | 1773900.00 | 0.00 | 0.00 | 0.00 | KO:K02112 | F-type H+-transporting ATPase subunit beta | Bacteria;Proteobacteria;Gammaproteobacteria;Chromatiales;Chromatiaceae;Lamprocystis;Lamprocystis purpurea;Lamprocystis purpurea DSM 4197 |
| Ga0586324_1138994_398_1147 | 3568407.00 | 924800.00 | 0.00 | 10241000.00 | 8178200.00 | 0.00 | 5234861.76 | 5055623.27 | 1036800.00 | 5220600.00 | 6784800.00 | 3862500.00 | KO:K02112 | F-type H+-transporting ATPase subunit beta | Bacteria;Proteobacteria;Deltaproteobacteria;Syntrophobacterales;Syntrophaceae;Desulfomonile;Desulfomonile tiedjei;Desulfomonile tiedjei DCB-1, DSM 6799 |
| Ga0586324_1144032_363_1145 | 0.00 | 0.00 | 0.00 | 0.00 | 0.00 | 0.00 | 46284000.00 | 0.00 | 0.00 | 0.00 | 0.00 | 0.00 | KO:K02112 | F-type H+-transporting ATPase subunit beta | Bacteria;Proteobacteria;Betaproteobacteria;Nitrosomonadales;Thiobacillaceae;Thiobacillus;Thiobacillus denitrificans;Thiobacillus denitrificans RG |
| Ga0586324_1156491_3_1139 | 0.00 | 15368000.00 | 0.00 | 0.00 | 31827000.00 | 0.00 | 0.00 | 0.00 | 0.00 | 0.00 | 0.00 | 0.00 | KO:K02112 | F-type H+-transporting ATPase subunit beta | Bacteria;Proteobacteria;Deltaproteobacteria;Myxococcales;unclassified Myxococcales;Minicystis;Minicystis rosea;Minicystis rosea DSM 24000 |
| Ga0586324_1173504_2_655 | 0.00 | 0.00 | 0.00 | 673244.10 | 27398000.00 | 0.00 | 0.00 | 191252.08 | 51921000.00 | 0.00 | 8404000.00 | 0.00 | KO:K02112 | F-type H+-transporting ATPase subunit beta | Bacteria;Proteobacteria;Deltaproteobacteria;Desulfobacterales;Desulfobulbaceae;Desulfobulbus;Desulfobulbus propionicus;Desulfobulbus propionicus 1pr3, DSM 2032 |
| Ga0586324_1297400_3_536 | 0.00 | 0.00 | 0.00 | 0.00 | 0.00 | 304323.13 | 0.00 | 0.00 | 0.00 | 0.00 | 7911200.00 | 0.00 | KO:K02112 | F-type H+-transporting ATPase subunit beta | Bacteria;Nitrospirae;Thermodesulfovibrionia;Thermodesulfovibrionales;Thermodesulfovibrionaceae;Thermodesulfovibrio;Thermodesulfovibrio sp. N1;Thermodesulfovibrio sp. N1 |
| Ga0586324_1321835_1_1077 | 8360000.00 | 24956000.00 | 6832522.96 | 12872500.00 | 8095800.00 | 14331700.00 | 9416400.00 | 40504000.00 | 9711900.00 | 28259000.00 | 4743200.00 | 4282500.00 | KO:K02112 | F-type H+-transporting ATPase subunit beta | Bacteria;Proteobacteria;Deltaproteobacteria;Syntrophobacterales;Syntrophobacteraceae;Syntrophobacter;Syntrophobacter fumaroxidans;Syntrophobacter fumaroxidans MPOB |
| Ga0586324_1335244_13_1071 | 4280000.00 | 0.00 | 0.00 | 6308000.00 | 12751400.00 | 4987534.82 | 0.00 | 0.00 | 2114100.00 | 0.00 | 0.00 | 0.00 | KO:K02112 | F-type H+-transporting ATPase subunit beta | Bacteria;Proteobacteria;Deltaproteobacteria;Desulfobacterales;Desulfobacteraceae;Desulfatiglans;Desulfatiglans anilini;Desulfatiglans anilini DSM 4660 |
| Ga0586324_1362985_3_623 | 0.00 | 0.00 | 0.00 | 0.00 | 0.00 | 0.00 | 0.00 | 0.00 | 972000.00 | 0.00 | 0.00 | 0.00 | KO:K02112 | F-type H+-transporting ATPase subunit beta | Bacteria;Acidobacteria;Vicinamibacteria;unclassified Vicinamibacteria;Vicinamibacteraceae;Luteitalea;Luteitalea pratensis;Luteitalea pratensis DSM 100886 |
| Ga0586324_1395531_1_1053 | 8940000.00 | 11573600.00 | 6643400.00 | 2593500.00 | 17520300.00 | 49188300.00 | 32193600.00 | 16610300.00 | 9234000.00 | 0.00 | 2235200.00 | 1215000.00 | KO:K02112 | F-type H+-transporting ATPase subunit beta | Bacteria;Proteobacteria;Deltaproteobacteria;Syntrophobacterales;Syntrophobacteraceae;Desulfacinum;Desulfacinum hydrothermale;Desulfacinum hydrothermale DSM 13146 |
| Ga0586324_1408783_2_481 | 0.00 | 382779.48 | 0.00 | 0.00 | 0.00 | 0.00 | 0.00 | 1476200.00 | 1352700.00 | 1370600.00 | 0.00 | 1327500.00 | KO:K02112 | F-type H+-transporting ATPase subunit beta | Bacteria;Proteobacteria;Betaproteobacteria;Rhodocyclales;Zoogloeaceae;Thauera;unclassified Thauera;Thauera sp. SYD-3 |
| Ga0586324_1421456_1_930 | 0.00 | 8296000.00 | 0.00 | 0.00 | 0.00 | 0.00 | 0.00 | 137728.85 | 0.00 | 0.00 | 0.00 | 0.00 | KO:K02112 | F-type H+-transporting ATPase subunit beta | Bacteria;Proteobacteria;Deltaproteobacteria;Myxococcales;unclassified Myxococcales;Minicystis;Minicystis rosea;Minicystis rosea DSM 24000 |
| Ga0586324_1444813_3_1037 | 2720000.00 | 1679600.00 | 22294800.00 | 3315500.00 | 3254800.00 | 4128800.00 | 2394000.00 | 1427400.00 | 0.00 | 1694000.00 | 2279200.00 | 1560000.00 | KO:K02112 | F-type H+-transporting ATPase subunit beta | Bacteria;Proteobacteria;Deltaproteobacteria;Syntrophobacterales;Syntrophaceae;Desulfobacca;Desulfobacca acetoxidans;Desulfobacca acetoxidans ASRB2, DSM 11109 |
| Ga0586324_1478422_1_900 | 0.00 | 0.00 | 0.00 | 334522.55 | 0.00 | 0.00 | 0.00 | 0.00 | 0.00 | 0.00 | 0.00 | 0.00 | KO:K02112 | F-type H+-transporting ATPase subunit beta | Bacteria;Acidobacteria;Acidobacteriia;Bryobacterales;Solibacteraceae;Candidatus Solibacter;Candidatus Solibacter usitatus;Candidatus Solibacter usitatus Ellin6076 |
| Ga0586324_1500255_3_1019 | 0.00 | 0.00 | 0.00 | 0.00 | 0.00 | 0.00 | 0.00 | 0.00 | 0.00 | 1062600.00 | 0.00 | 0.00 | KO:K02112 | F-type H+-transporting ATPase subunit beta | Bacteria;Proteobacteria;Deltaproteobacteria;Myxococcales;Polyangiaceae;Sorangium;Sorangium cellulosum;Sorangium cellulosum So0008-312 |
| Ga0586324_1517077_1_888 | 1270000.00 | 5956800.00 | 2937829.71 | 8949000.00 | 11546300.00 | 1393700.26 | 6180318.48 | 5770600.00 | 3597149.25 | 5205200.00 | 1988800.00 | 6405000.00 | KO:K02112 | F-type H+-transporting ATPase subunit beta | Bacteria;Nitrospirae;Thermodesulfovibrionia;Thermodesulfovibrionales;Thermodesulfovibrionaceae;Thermodesulfovibrio;Thermodesulfovibrio sp. N1;Thermodesulfovibrio sp. N1 |
| Ga0586324_1541859_133_1008 | 0.00 | 468736.92 | 0.00 | 0.00 | 0.00 | 0.00 | 0.00 | 161043.66 | 321815.43 | 0.00 | 0.00 | 0.00 | KO:K02112 | F-type H+-transporting ATPase subunit beta | Bacteria;Proteobacteria;Gammaproteobacteria;Chromatiales;Chromatiaceae;Thiodictyon;Candidatus Thiodictyon syntrophicum;Candidatus Thiodictyon syntrophicum Cad16 |
| Ga0586324_1556055_1_921 | 0.00 | 0.00 | 0.00 | 7240900.00 | 0.00 | 0.00 | 0.00 | 0.00 | 0.00 | 0.00 | 0.00 | 0.00 | KO:K02112 | F-type H+-transporting ATPase subunit beta | Bacteria;Proteobacteria;Deltaproteobacteria;Desulfobacterales;Desulfobulbaceae;Desulfotalea;Desulfotalea psychrophila;Desulfotalea psychrophila LSv54 |
| Ga0586324_1583176_3_977 | 0.00 | 0.00 | 0.00 | 1064000.00 | 1071200.00 | 2511890.46 | 0.00 | 0.00 | 0.00 | 0.00 | 0.00 | 0.00 | KO:K02112 | F-type H+-transporting ATPase subunit beta | Bacteria;Proteobacteria;Deltaproteobacteria;Desulfobacterales;Desulfobulbaceae;Desulfobulbus;Desulfobulbus propionicus;Desulfobulbus propionicus 1pr3, DSM 2032 |
| Ga0586324_1703614_1_762 | 0.00 | 0.00 | 0.00 | 0.00 | 0.00 | 0.00 | 0.00 | 3843000.00 | 0.00 | 0.00 | 0.00 | 179314.50 | KO:K02112 | F-type H+-transporting ATPase subunit beta | Bacteria;Proteobacteria;Betaproteobacteria;Nitrosomonadales;Nitrosomonadaceae;Nitrosomonas;Nitrosomonas sp. Nm143;Nitrosomonas sp. Nm143 (v3) |
| Ga0586324_1707898_3_473 | 0.00 | 0.00 | 0.00 | 49571000.00 | 55105000.00 | 0.00 | 1065459.96 | 5666900.00 | 5143500.00 | 0.00 | 0.00 | 0.00 | KO:K02112 | F-type H+-transporting ATPase subunit beta | Bacteria;Proteobacteria;Deltaproteobacteria;Syntrophobacterales;Syntrophobacteraceae;Syntrophobacter;Syntrophobacter fumaroxidans;Syntrophobacter fumaroxidans MPOB |
| Ga0586324_1713495_180_965 | 0.00 | 3277600.00 | 0.00 | 0.00 | 0.00 | 0.00 | 0.00 | 0.00 | 0.00 | 0.00 | 0.00 | 0.00 | KO:K02112 | F-type H+-transporting ATPase subunit beta | Bacteria;Verrucomicrobia;Opitutae;Opitutales;Opitutaceae;Opitutus;Opitutus sp. GAS368;Opitutus sp. GAS368 |
| Ga0586324_1744520_281_958 | 0.00 | 3182400.00 | 0.00 | 0.00 | 0.00 | 0.00 | 0.00 | 0.00 | 0.00 | 0.00 | 0.00 | 0.00 | KO:K02112 | F-type H+-transporting ATPase subunit beta | Bacteria;Proteobacteria;Gammaproteobacteria;Chromatiales;Ectothiorhodospiraceae;Alkalispirillum;Alkalispirillum mobile;Alkalispirillum mobile DSM 12769 |
| Ga0586324_1747264_2_955 | 0.00 | 0.00 | 0.00 | 0.00 | 0.00 | 0.00 | 0.00 | 854000.00 | 0.00 | 0.00 | 0.00 | 0.00 | KO:K02112 | F-type H+-transporting ATPase subunit beta | Bacteria;Nitrospirae;Thermodesulfovibrionia;Thermodesulfovibrionales;Thermodesulfovibrionaceae;Thermodesulfovibrio;Thermodesulfovibrio yellowstonii;Thermodesulfovibrio yellowstonii DSM 11347 |
| Ga0586324_1771187_3_950 | 0.00 | 0.00 | 0.00 | 0.00 | 619151.54 | 1661055.94 | 0.00 | 0.00 | 0.00 | 0.00 | 0.00 | 0.00 | KO:K02112 | F-type H+-transporting ATPase subunit beta | Bacteria;Proteobacteria;Deltaproteobacteria;Syntrophobacterales;Syntrophaceae;Desulfobacca;Desulfobacca acetoxidans;Desulfobacca acetoxidans ASRB2, DSM 11109 |
| Ga0586324_1825766_2_940 | 0.00 | 0.00 | 0.00 | 0.00 | 0.00 | 0.00 | 0.00 | 0.00 | 0.00 | 601256.04 | 0.00 | 0.00 | KO:K02112 | F-type H+-transporting ATPase subunit beta | Bacteria;Chloroflexi;Anaerolineae;Anaerolineales;Anaerolineaceae;Thermanaerothrix;Thermanaerothrix daxensis;Thermanaerothrix daxensis GNS-1 |
| Ga0586324_1893782_1_924 | 0.00 | 0.00 | 0.00 | 350466.40 | 0.00 | 0.00 | 0.00 | 1207800.00 | 2371341.42 | 0.00 | 0.00 | 0.00 | KO:K02112 | F-type H+-transporting ATPase subunit beta | Bacteria;Nitrospirae;Thermodesulfovibrionia;Thermodesulfovibrionales;Thermodesulfovibrionaceae;Thermodesulfovibrio;Thermodesulfovibrio sp. N1;Thermodesulfovibrio sp. N1 |
| Ga0586324_1897580_309_923 | 0.00 | 0.00 | 0.00 | 0.00 | 0.00 | 0.00 | 0.00 | 0.00 | 0.00 | 1670900.00 | 0.00 | 0.00 | KO:K02112 | F-type H+-transporting ATPase subunit beta | Bacteria;Proteobacteria;Deltaproteobacteria;Syntrophobacterales;Syntrophaceae;Desulfobacca;Desulfobacca acetoxidans;Desulfobacca acetoxidans ASRB2, DSM 11109 |

| | | | | | | | | | | | | | | | |
|---|---|---|---|---|---|---|---|---|---|---|---|---|---|---|---|
| Ga0586324 1945358 3 914 | 0.00 | 0.00 | 0.00 | 0.00 | 0.00 | 1690048.85 | 0.00 | 0.00 | 1377000.00 | 18634000.00 | 0.00 | 0.00 | KO:K02112 | F-type H+-transporting ATPase subunit beta | Bacteria;Proteobacteria;Deltaproteobacteria;unclassified Deltaproteobacteria;unclassified Deltaproteobacteria;Deferrisoma;Deferrisoma camini;Deferrisoma camini S3R1 |
| Ga0586324 1968621 3 908 | 0.00 | 0.00 | 0.00 | 0.00 | 0.00 | 0.00 | 0.00 | 0.00 | 8019000.00 | 0.00 | 0.00 | 0.00 | KO:K02112 | F-type H+-transporting ATPase subunit beta | Bacteria;Proteobacteria;Alphaproteobacteria;Hyphomicrobiales;Phyllobacteriaceae;Mesorhizobium;Mesorhizobium sp. ORS 3324;Mesorhizobium sp. ORS 3324 |
| Ga0586324 2004515 2 901 | 0.00 | 0.00 | 0.00 | 0.00 | 916896.73 | 1246214.76 | 0.00 | 0.00 | 0.00 | 0.00 | 0.00 | 0.00 | KO:K02112 | F-type H+-transporting ATPase subunit beta | Bacteria;Actinobacteria;Rubrobacteria;Gaiellales;Gaiellaceae;Gaiella;Gaiella occulta;Gaiella occulta F2-233 |
| Ga0586324 2095376 1 882 | 0.00 | 0.00 | 0.00 | 0.00 | 0.00 | 14292000.00 | 12562800.00 | 0.00 | 7946100.00 | 8624000.00 | 0.00 | 17040000.00 | KO:K02112 | F-type H+-transporting ATPase subunit beta | Bacteria;Nitrospirae;Thermodesulfovibrionia;Thermodesulfovibrionales;Thermodesulfovibrionaceae;Thermodesulfovibrio;Thermodesulfovibrio sp. N1;Thermodesulfovibrio sp. N1 |
| Ga0586324 2122718 2 880 | 0.00 | 0.00 | 0.00 | 662150.95 | 2698600.00 | 0.00 | 0.00 | 0.00 | 1255500.00 | 4096400.00 | 0.00 | 423564.00 | KO:K02112 | F-type H+-transporting ATPase subunit beta | Bacteria;Bacteroidetes;Cytophagia;Cytophagales;Thermonemataceae;Thermonema;Thermonema lapsum;Thermonema lapsum DSM 5718 |
| Ga0586324 2122784 31 882 | 2677392.00 | 0.00 | 0.00 | 2755000.00 | 47606600.00 | 0.00 | 0.00 | 0.00 | 2567700.00 | 0.00 | 0.00 | 0.00 | KO:K02112 | F-type H+-transporting ATPase subunit beta | Bacteria;Proteobacteria;Deltaproteobacteria;Syntrophobacterales;Syntrophobacteraceae;Syntrophobacter;Syntrophobacter fumaroxidans;Syntrophobacter fumaroxidans MPOB |
| Ga0586324 2186969 58 870 | 4910000.00 | 4120800.00 | 0.00 | 15485000.00 | 2008500.00 | 12346700.00 | 0.00 | 9699000.00 | 14661000.00 | 0.00 | 0.00 | 4777500.00 | KO:K02112 | F-type H+-transporting ATPase subunit beta | Bacteria;Proteobacteria;Deltaproteobacteria;Syntrophobacterales;Syntrophaceae;Desulfobacca;Desulfobacca acetoxidans;Desulfobacca acetoxidans ASRB2, DSM 11109 |
| Ga0586324 2193072 1 447 | 938584.00 | 1917600.00 | 0.00 | 3629000.00 | 0.00 | 0.00 | 0.00 | 3013400.00 | 0.00 | 0.00 | 0.00 | 0.00 | KO:K02112 | F-type H+-transporting ATPase subunit beta | Bacteria;Proteobacteria;Deltaproteobacteria;Syntrophobacterales;Syntrophaceae;Smithella;Smithella sp. SCADC;Smithella sp. SCADC |
| Ga0586324 2292528 314 853 | 0.00 | 0.00 | 0.00 | 0.00 | 0.00 | 0.00 | 0.00 | 0.00 | 0.00 | 0.00 | 0.00 | 1920000.00 | KO:K02112 | F-type H+-transporting ATPase subunit beta | Bacteria;Planctomycetes;Planctomycetia;Pirellulales;Pirellulaceae;Blastopirellula;Blastopirellula marina;Blastopirellula marina DSM 3645 |
| Ga0586324 2301720 1 852 | 0.00 | 0.00 | 0.00 | 0.00 | 0.00 | 0.00 | 0.00 | 0.00 | 0.00 | 0.00 | 0.00 | 234618.75 | KO:K02112 | F-type H+-transporting ATPase subunit beta | Eukaryota;Streptophyta;Bryopsida;Funariales;Funariaceae;Physcomitrium;Physcomitrium patens;Physcomitrella patens patens |
| Ga0586324 2353330 72 842 | 0.00 | 0.00 | 564672.11 | 0.00 | 0.00 | 0.00 | 0.00 | 0.00 | 0.00 | 0.00 | 0.00 | 0.00 | KO:K02112 | F-type H+-transporting ATPase subunit beta | Bacteria;Proteobacteria;Gammaproteobacteria;Nevskiales;Steroidobacteraceae;Steroidobacter;Steroidobacter denitrificans;Steroidobacter denitrificans DSM 18526 |
| Ga0586324 2474440 3 827 | 0.00 | 0.00 | 0.00 | 0.00 | 0.00 | 0.00 | 0.00 | 0.00 | 1044900.00 | 0.00 | 0.00 | 0.00 | KO:K02112 | F-type H+-transporting ATPase subunit beta | Bacteria;Bacteroidetes;Chitinophagia;Chitinophagales;Chitinophagaceae;Cnuella;Cnuella takakiae;Cnuella takakiae DSM 26897 |
| Ga0586324 2482331 3 824 | 0.00 | 0.00 | 0.00 | 0.00 | 0.00 | 0.00 | 0.00 | 0.00 | 0.00 | 258976.41 | 0.00 | 0.00 | KO:K02112 | F-type H+-transporting ATPase subunit beta | Bacteria;Acidobacteria;Acidobacteriia;Acidobacteriales;Acidobacteriaceae;Edaphobacter;Edaphobacter modestus;Edaphobacter modestus DSM 18101 |
| Ga0586324 2531558 3 545 | 0.00 | 0.00 | 0.00 | 0.00 | 0.00 | 0.00 | 0.00 | 392836.95 | 0.00 | 0.00 | 0.00 | 0.00 | KO:K02112 | F-type H+-transporting ATPase subunit beta | Bacteria;Proteobacteria;Deltaproteobacteria;Desulfuromonadales;Geobacteraceae;Geobacter;Geobacter soli;Geobacter soli GSS01 |
| Ga0586324 2560614 1 813 | 0.00 | 0.00 | 0.00 | 0.00 | 0.00 | 0.00 | 3693600.00 | 0.00 | 0.00 | 0.00 | 0.00 | 0.00 | KO:K02112 | F-type H+-transporting ATPase subunit beta | Bacteria;Proteobacteria;Betaproteobacteria;Nitrosomonadales;Methylophilaceae;Methylotenera;Methylotenera mobilis;Methylotenera mobilis #13 |
| Ga0586324 2704697 3 578 | 21460000.00 | 17326400.00 | 0.00 | 38000000.00 | 41292700.00 | 41934931.35 | 284110800.00 | 20252000.00 | 16327994.58 | 214137000.00 | 14784000.00 | 3645000.00 | KO:K02112 | F-type H+-transporting ATPase subunit beta | Bacteria;Proteobacteria;Deltaproteobacteria;Syntrophobacterales;Syntrophaceae;Desulfobacca;Desulfobacca acetoxidans;Desulfobacca acetoxidans ASRB2, DSM 11109 |
| Ga0586324 2769912 1 693 | 0.00 | 1842800.00 | 1931123.78 | 9386000.00 | 0.00 | 0.00 | 0.00 | 0.00 | 0.00 | 1540000.00 | 0.00 | 0.00 | KO:K02112 | F-type H+-transporting ATPase subunit beta | Bacteria;Proteobacteria;Deltaproteobacteria;Desulfobacterales;Desulfobacteraceae;Desulfatirhabdium;Desulfatirhabdium butyrativorans;Desulfatirhabdium butyrativorans DSM 18734 |
| Ga0586324 2865457 544 777 | 0.00 | 0.00 | 0.00 | 0.00 | 0.00 | 0.00 | 0.00 | 0.00 | 0.00 | 1101100.00 | 0.00 | 0.00 | KO:K02112 | F-type H+-transporting ATPase subunit beta | Bacteria;Nitrospirae;Nitrospira;Nitrospirales;Nitrospiraceae;Nitrospira;Candidatus Nitrospira inopinata;Candidatus Nitrospira inopinata ENR4 |
| Ga0586324 3241292 1 738 | 0.00 | 0.00 | 0.00 | 0.00 | 0.00 | 0.00 | 0.00 | 3885700.00 | 0.00 | 0.00 | 0.00 | 0.00 | KO:K02112 | F-type H+-transporting ATPase subunit beta | Bacteria;Proteobacteria;Gammaproteobacteria;Oceanospirillales;Alcanivoracaceae;Alcanivorax;Alcanivorax borkumensis;Alcanivorax borkumensis SK2 |
| Ga0586324 3363165 381 725 | 0.00 | 0.00 | 0.00 | 0.00 | 0.00 | 0.00 | 0.00 | 0.00 | 0.00 | 0.00 | 28512000.00 | 0.00 | KO:K02112 | F-type H+-transporting ATPase subunit beta | Bacteria;Proteobacteria;Betaproteobacteria;Nitrosomonadales;Thiobacillaceae;Thiobacillus;Thiobacillus thioparus;Thiobacillus thioparus DSM 505 |
| Ga0586324 3383490 1 723 | 150700.00 | 0.00 | 0.00 | 0.00 | 0.00 | 0.00 | 0.00 | 0.00 | 0.00 | 0.00 | 0.00 | 0.00 | KO:K02112 | F-type H+-transporting ATPase subunit beta | Bacteria;Proteobacteria;Gammaproteobacteria;Methylococcales;Methylococcaceae;Methylomagnum;Methylomagnum ishizawai;Methylomagnum ishizawai 175 |
| Ga0586324 3729624 3 392 | 3140000.00 | 0.00 | 0.00 | 0.00 | 0.00 | 0.00 | 0.00 | 19337000.00 | 0.00 | 0.00 | 0.00 | 1410000.00 | KO:K02112 | F-type H+-transporting ATPase subunit beta | Bacteria;Proteobacteria;Deltaproteobacteria;Syntrophobacterales;Syntrophaceae;Desulfobacca;Desulfobacca acetoxidans;Desulfobacca acetoxidans ASRB2, DSM 11109 |
| Ga0586324 3831818 1 687 | 0.00 | 0.00 | 0.00 | 0.00 | 0.00 | 0.00 | 0.00 | 0.00 | 0.00 | 0.00 | 1047200.00 | 0.00 | KO:K02112 | F-type H+-transporting ATPase subunit beta | Bacteria;Planctomycetes;Planctomycetia;Pirellulales;Pirellulaceae;Rhodopirellula;Rhodopirellula europaea;Rhodopirellula europaea 6C |
| Ga0586324 3851641 2 688 | 0.00 | 1298800.00 | 2045542.27 | 0.00 | 11639000.00 | 2396974.84 | 0.00 | 0.00 | 3985200.00 | 1509200.00 | 1258400.00 | 0.00 | KO:K02112 | F-type H+-transporting ATPase subunit beta | Bacteria;Proteobacteria;Gammaproteobacteria;Methylococcales;Methylococcaceae;Methylovulum;Methylovulum miyakonense;Methylovulum miyakonense HT12 |
| Ga0586324 3872611 1 684 | 0.00 | 0.00 | 0.00 | 0.00 | 0.00 | 0.00 | 0.00 | 0.00 | 4932900.00 | 0.00 | 0.00 | 0.00 | KO:K02112 | F-type H+-transporting ATPase subunit beta | Bacteria;Proteobacteria;Gammaproteobacteria;Chromatiales;Ectothiorhodospiraceae;Inmirania;Inmirania thermothiophila;Inmirania thermothiophila DSM 100275 |
| Ga0586324 3958217 84 680 | 607108.00 | 664547.00 | 0.00 | 1520000.00 | 0.00 | 0.00 | 0.00 | 1471805.56 | 560163.60 | 0.00 | 0.00 | 710144.25 | KO:K02112 | F-type H+-transporting ATPase subunit beta | Bacteria;Proteobacteria;Deltaproteobacteria;Syntrophobacterales;Syntrophaceae;Smithella;Smithella sp. SCADC;Smithella sp. SCADC |
| Ga0586324 3980352 3 677 | 0.00 | 0.00 | 0.00 | 997500.00 | 0.00 | 0.00 | 0.00 | 0.00 | 0.00 | 0.00 | 0.00 | 0.00 | KO:K02112 | F-type H+-transporting ATPase subunit beta | Bacteria;Proteobacteria;Deltaproteobacteria;Desulfuromonadales;Geobacteraceae;Geobacter;Geobacter sp. DSM 2909;Geobacter sp. DSM 2909 |
| Ga0586324 4038283 1 672 | 0.00 | 498168.00 | 0.00 | 0.00 | 0.00 | 0.00 | 0.00 | 0.00 | 0.00 | 0.00 | 0.00 | 0.00 | KO:K02112 | F-type H+-transporting ATPase subunit beta | Bacteria;Firmicutes;Clostridia;Thermoanaerobacterales;Thermoanaerobacteraceae;Thermanaeromonas;Thermanaeromonas toyohensis;Thermanaeromonas toyohensis ToBE, DSM 14490 |
| Ga0586324 4087231 249 671 | 6100000.00 | 8364000.00 | 0.00 | 46008500.00 | 41097000.00 | 37397400.00 | 10852800.00 | 16531000.00 | 16200000.00 | 4458300.00 | 9592000.00 | 0.00 | KO:K02112 | F-type H+-transporting ATPase subunit beta | Bacteria;Proteobacteria;Deltaproteobacteria;Syntrophobacterales;Syntrophobacteraceae;Syntrophobacter;Syntrophobacter fumaroxidans;Syntrophobacter fumaroxidans MPOB |
| Ga0586324 4124227 2 667 | 0.00 | 5725600.00 | 0.00 | 0.00 | 0.00 | 0.00 | 0.00 | 1933700.00 | 0.00 | 0.00 | 0.00 | 0.00 | KO:K02112 | F-type H+-transporting ATPase subunit beta | Bacteria;Bacteroidetes;Sphingobacteriia;Sphingobacteriales;Sphingobacteriaceae;Pedobacter;unclassified Pedobacter;Pedobacter steynii DX4 |
| Ga0586324 4124370 1 669 | 0.00 | 0.00 | 0.00 | 0.00 | 0.00 | 0.00 | 0.00 | 0.00 | 0.00 | 1994300.00 | 0.00 | 0.00 | KO:K02112 | F-type H+-transporting ATPase subunit beta | Bacteria;Bacteroidetes;Chitinophagia;Chitinophagales;Chitinophagaceae;unclassified Chitinophagaceae;Chitinophagaceae bacterium IBVUCB2;Chitinophagaceae bacterium IBVUCB2 |
| Ga0586324 4171769 2 664 | 478436.00 | 380230.16 | 0.00 | 1700500.00 | 0.00 | 0.00 | 0.00 | 780800.00 | 236179.80 | 383269.04 | 0.00 | 0.00 | KO:K02112 | F-type H+-transporting ATPase subunit beta | Bacteria;Nitrospirae;Thermodesulfovibrionia;Thermodesulfovibrionales;Thermodesulfovibrionaceae;Thermodesulfovibrio;Thermodesulfovibrio sp. N1;Thermodesulfovibrio sp. N1 |
| Ga0586324 4209262 98 661 | 0.00 | 0.00 | 63619000.00 | 0.00 | 0.00 | 0.00 | 0.00 | 0.00 | 0.00 | 0.00 | 0.00 | 0.00 | KO:K02112 | F-type H+-transporting ATPase subunit beta | Bacteria;Proteobacteria;Deltaproteobacteria;Syntrophobacterales;Syntrophaceae;Desulfobacca;Desulfobacca acetoxidans;Desulfobacca acetoxidans ASRB2, DSM 11109 |
| Ga0586324 4332108 2 655 | 0.00 | 0.00 | 0.00 | 0.00 | 0.00 | 0.00 | 803939.40 | 0.00 | 0.00 | 0.00 | 0.00 | 0.00 | KO:K02112 | F-type H+-transporting ATPase subunit beta | Bacteria;Firmicutes;Clostridia;Thermoanaerobacterales;Thermoanaerobacteraceae;Calderihabitans;Calderihabitans maritimus;Calderihabitans maritimus KKC1 |
| Ga0586324 4590874 2 640 | 251663.00 | 0.00 | 0.00 | 0.00 | 0.00 | 0.00 | 0.00 | 0.00 | 0.00 | 0.00 | 0.00 | 0.00 | KO:K02112 | F-type H+-transporting ATPase subunit beta | Bacteria;Acidobacteria;Acidobacteriia;Acidobacteriales;Acidobacteriaceae;unclassified Acidobacteriaceae;Acidobacteriaceae bacterium KBS 96;Acidobacteriaceae bacterium KBS 96, DSM 24297 |
| Ga0586324 4793598 2 628 | 0.00 | 0.00 | 0.00 | 0.00 | 0.00 | 0.00 | 0.00 | 475382.15 | 1854900.00 | 0.00 | 0.00 | 0.00 | KO:K02112 | F-type H+-transporting ATPase subunit beta | Bacteria;Proteobacteria;Betaproteobacteria;unclassified Betaproteobacteria;unclassified Betaproteobacteria;unclassified Betaproteobacteria;Betaproteobacteria bacterium GR16-43;Betaproteobacteria bacterium GR16-43 |
| Ga0586324 4938910 1 621 | 32000000.00 | 29954000.00 | 53034600.00 | 42845000.00 | 42230000.00 | 49571210.87 | 32604000.00 | 25216045.19 | 29970000.00 | 36498000.00 | 24200000.00 | 19800000.00 | KO:K02112 | F-type H+-transporting ATPase subunit beta | Bacteria;Proteobacteria;Deltaproteobacteria;Syntrophobacterales;Syntrophaceae;Desulfobacca;Desulfobacca acetoxidans;Desulfobacca acetoxidans ASRB2, DSM 11109 |
| Ga0586324 5451485 1 594 | 0.00 | 0.00 | 0.00 | 0.00 | 0.00 | 0.00 | 0.00 | 473910.83 | 0.00 | 0.00 | 0.00 | 0.00 | KO:K02112 | F-type H+-transporting ATPase subunit beta | Bacteria;Chloroflexi;Anaerolineae;Anaerolineales;Anaerolineaceae;Thermanaerothrix;Thermanaerothrix daxensis;Thermanaerothrix daxensis GNS-1 |
| Ga0586324 5507692 3 473 | 0.00 | 0.00 | 0.00 | 0.00 | 3306300.00 | 0.00 | 0.00 | 0.00 | 0.00 | 0.00 | 0.00 | 0.00 | KO:K02112 | F-type H+-transporting ATPase subunit beta | Bacteria;Proteobacteria;Deltaproteobacteria;Desulfobacterales;Desulfobulbaceae;Desulfoprunum;Desulfoprunum benzoelyticum;Desulfoprunum benzoelyticum DSM 28570 |
| Ga0586324 5749551 2 583 | 0.00 | 0.00 | 0.00 | 21517500.00 | 0.00 | 0.00 | 0.00 | 0.00 | 0.00 | 0.00 | 0.00 | 0.00 | KO:K02112 | F-type H+-transporting ATPase subunit beta | Bacteria;Actinobacteria;Actinomycetia;Micrococcales;Micrococcaceae;Paenarthrobacter;Paenarthrobacter nicotinovorans;Paenarthrobacter nicotinovorans Hce-1 |
| Ga0586324 5831344 2 580 | 0.00 | 0.00 | 0.00 | 0.00 | 0.00 | 0.00 | 0.00 | 0.00 | 0.00 | 0.00 | 13376000.00 | 0.00 | KO:K02112 | F-type H+-transporting ATPase subunit beta | Bacteria;Bacteroidetes;Cytophagia;Cytophagales;Reichenbachiellaceae;Marinoscillum;Marinoscillum furvescens;Marinoscillum furvescens DSM 4134 |
| Ga0586324 5844838 1 579 | 0.00 | 0.00 | 0.00 | 0.00 | 0.00 | 0.00 | 0.00 | 2238700.00 | 0.00 | 0.00 | 1566400.00 | 0.00 | KO:K02112 | F-type H+-transporting ATPase subunit beta | Bacteria;Proteobacteria;Gammaproteobacteria;Methylococcales;Methylococcaceae;Methylovulum;Methylovulum psychrotolerans;Methylovulum psychrotolerans HV10 M2 |
| Ga0586324 5979342 180 572 | 0.00 | 4930000.00 | 0.00 | 15010000.00 | 0.00 | 0.00 | 0.00 | 0.00 | 0.00 | 4335100.00 | 0.00 | 0.00 | KO:K02112 | F-type H+-transporting ATPase subunit beta | Bacteria;Proteobacteria;Deltaproteobacteria;Desulfobacterales;Desulfobacteraceae;Desulfatirhabdium;Desulfatirhabdium butyrativorans;Desulfatirhabdium butyrativorans DSM 18734 |
| Ga0586324 5989301 3 572 | 0.00 | 0.00 | 0.00 | 0.00 | 749402.25 | 0.00 | 0.00 | 1189500.00 | 0.00 | 0.00 | 817945.04 | 0.00 | KO:K02112 | F-type H+-transporting ATPase subunit beta | Bacteria;Proteobacteria;Alphaproteobacteria;Hyphomicrobiales;Hyphomicrobiaceae;Hyphomicrobium;Hyphomicrobium denitrificans;Hyphomicrobium denitrificans ATCC 51888 |
| Ga0586324 6011243 101 571 | 0.00 | 1944800.00 | 0.00 | 0.00 | 0.00 | 0.00 | 0.00 | 0.00 | 0.00 | 0.00 | 0.00 | 3375000.00 | KO:K02112 | F-type H+-transporting ATPase subunit beta | Bacteria;Chloroflexi;Anaerolineae;Anaerolineales;Anaerolineaceae;Thermanaerothrix;Thermanaerothrix daxensis;Thermanaerothrix daxensis GNS-1 |
| Ga0586324 6087087 274 570 | 0.00 | 0.00 | 0.00 | 6697500.00 | 0.00 | 0.00 | 0.00 | 0.00 | 0.00 | 3634400.00 | 0.00 | 0.00 | KO:K02112 | F-type H+-transporting ATPase subunit beta | Bacteria;Proteobacteria;Alphaproteobacteria;Hyphomicrobiales;Bradyrhizobiaceae;Bradyrhizobium;Bradyrhizobium japonicum;Bradyrhizobium japonicum J5 |
| Ga0586324 6415463 2 544 | 0.00 | 0.00 | 0.00 | 755834.25 | 644673.91 | 0.00 | 0.00 | 0.00 | 0.00 | 0.00 | 0.00 | 0.00 | KO:K02112 | F-type H+-transporting ATPase subunit beta | Bacteria;Proteobacteria;Deltaproteobacteria;Syntrophobacterales;Syntrophobacteraceae;Syntrophobacter;Syntrophobacter fumaroxidans;Syntrophobacter fumaroxidans MPOB |
| Ga0586324 6418987 1 399 | 0.00 | 3338800.00 | 0.00 | 7400500.00 | 10300000.00 | 0.00 | 3055200.00 | 4495700.00 | 4317300.00 | 4034800.00 | 4716800.00 | 2392500.00 | KO:K02112 | F-type H+-transporting ATPase subunit beta | Bacteria;Proteobacteria;Gammaproteobacteria;Chromatiales;Chromatiaceae;Marichromatium;Marichromatium gracile;Marichromatium gracile YL28 |
| Ga0586324 6488391 2 496 | 0.00 | 0.00 | 0.00 | 0.00 | 0.00 | 0.00 | 5175600.00 | 0.00 | 0.00 | 0.00 | 0.00 | 0.00 | KO:K02112 | F-type H+-transporting ATPase subunit beta | Bacteria;Proteobacteria;Alphaproteobacteria;Hyphomicrobiales;Reyranellaceae;Reyranella;Reyranella massiliensis;Reyranella massiliensis 521, DSM 23428 |
| Ga0586324 6644129 3 548 | 0.00 | 2706400.00 | 0.00 | 0.00 | 0.00 | 0.00 | 0.00 | 0.00 | 0.00 | 0.00 | 0.00 | 0.00 | KO:K02112 | F-type H+-transporting ATPase subunit beta | Bacteria;Acidobacteria;Acidobacteriia;Bryobacterales;Solibacteraceae;Candidatus Solibacter;Candidatus Solibacter usitatus;Candidatus Solibacter usitatus Ellin6076 |
| Ga0586324 7123841 1 534 | 0.00 | 0.00 | 0.00 | 0.00 | 0.00 | 0.00 | 0.00 | 0.00 | 0.00 | 0.00 | 0.00 | 2107500.00 | KO:K02112 | F-type H+-transporting ATPase subunit beta | Bacteria;Proteobacteria;Betaproteobacteria;Burkholderiales;unclassified Burkholderiales;Ideonella;Ideonella sp. A 288;Ideonella sp. A 288 |
| Ga0586324 7138225 118 531 | 0.00 | 0.00 | 0.00 | 0.00 | 0.00 | 0.00 | 0.00 | 0.00 | 758315.52 | 0.00 | 0.00 | 0.00 | KO:K02112 | F-type H+-transporting ATPase subunit beta | Bacteria;Proteobacteria;Alphaproteobacteria;Hyphomicrobiales;Bradyrhizobiaceae;Bradyrhizobium;Bradyrhizobium canariense;Bradyrhizobium canariense GAS369 |
| Ga0586324 7290063 2 529 | 0.00 | 0.00 | 0.00 | 0.00 | 0.00 | 0.00 | 0.00 | 0.00 | 6075000.00 | 0.00 | 0.00 | 0.00 | KO:K02112 | F-type H+-transporting ATPase subunit beta | Bacteria;Proteobacteria;Gammaproteobacteria;Chromatiales;Chromatiaceae;Allochromatium;Allochromatium warmingii;Allochromatium warmingii DSM 173 |
| Ga0586324 7454891 3 524 | 1710000.00 | 0.00 | 0.00 | 0.00 | 0.00 | 0.00 | 0.00 | 0.00 | 2737800.00 | 0.00 | 0.00 | 0.00 | KO:K02112 | F-type H+-transporting ATPase subunit beta | Bacteria;Proteobacteria;Deltaproteobacteria;Desulfobacterales;Desulfobulbaceae;Desulfoprunum;Desulfoprunum benzoelyticum;Desulfoprunum benzoelyticum DSM 28570 |
| Ga0586324 7486805 205 522 | 0.00 | 0.00 | 0.00 | 0.00 | 0.00 | 0.00 | 0.00 | 0.00 | 0.00 | 0.00 | 46992000.00 | 0.00 | KO:K02112 | F-type H+-transporting ATPase subunit beta | Bacteria;Proteobacteria;Deltaproteobacteria;Syntrophobacterales;Syntrophaceae;Desulfobacca;Desulfobacca acetoxidans;Desulfobacca acetoxidans ASRB2, DSM 11109 |
| Ga0586324 8061864 1 507 | 0.00 | 221550.80 | 383377.10 | 1045000.00 | 1060900.00 | 305698.34 | 489899.04 | 664900.00 | 371339.64 | 0.00 | 0.00 | 490147.50 | KO:K02112 | F-type H+-transporting ATPase subunit beta | Bacteria;Proteobacteria;Deltaproteobacteria;Syntrophobacterales;Syntrophobacteraceae;Syntrophobacter;Syntrophobacter fumaroxidans;Syntrophobacter fumaroxidans MPOB |
| Ga0586324 8243576 2 502 | 3850000.00 | 0.00 | 0.00 | 0.00 | 0.00 | 0.00 | 0.00 | 0.00 | 0.00 | 0.00 | 0.00 | 0.00 | KO:K02112 | F-type H+-transporting ATPase subunit beta | Bacteria;Proteobacteria;Gammaproteobacteria;Oceanospirillales;Endozoicomonadaceae;Parendozoicomonas;Parendozoicomonas haliclonae;Parendozoicomonas haliclonae ERS1628946 |
| Ga0586324 8257854 1 501 | 0.00 | 0.00 | 0.00 | 490577.15 | 0.00 | 0.00 | 0.00 | 0.00 | 0.00 | 0.00 | 0.00 | 0.00 | KO:K02112 | F-type H+-transporting ATPase subunit beta | Bacteria;Proteobacteria;Alphaproteobacteria;Hyphomicrobiales;Methylocystaceae;Oharaeibacter;Oharaeibacter diazotrophicus;Oharaeibacter diazotrophicus DSM 102969 |
| Ga0586324 8293552 97 501 | 0.00 | 0.00 | 0.00 | 969000.00 | 0.00 | 0.00 | 0.00 | 0.00 | 0.00 | 0.00 | 0.00 | 0.00 | KO:K02112 | F-type H+-transporting ATPase subunit beta | Bacteria;Proteobacteria;Gammaproteobacteria;unclassified Gammaproteobacteria;Candidatus Competibacteraceae;Candidatus Contendobacter;Candidatus Contendobacter odensis;Candidatus Contendobacter odensis Run B J11 |
| Ga0586324 0000001 71430 71960 | 0.00 | 0.00 | 0.00 | 0.00 | 0.00 | 0.00 | 0.00 | 0.00 | 0.00 | 0.00 | 0.00 | 2677500.00 | KO:K02113 | F-type H+-transporting ATPase subunit delta | Bacteria;Proteobacteria;Betaproteobacteria;Nitrosomonadales;Thiobacillaceae;Annwoodia;Annwoodia aquaesulis;Annwoodia aquaesulis DSM 4255 |
| Ga0586324 0926215 3 392 | 0.00 | 0.00 | 0.00 | 16720000.00 | 17613000.00 | 5875600.00 | 0.00 | 0.00 | 1206900.00 | 0.00 | 0.00 | 0.00 | KO:K02113 | F-type H+-transporting ATPase subunit delta | Bacteria;Proteobacteria;Deltaproteobacteria;Syntrophobacterales;Syntrophobacteraceae;Syntrophobacter;Syntrophobacter fumaroxidans;Syntrophobacter fumaroxidans MPOB |
| Ga0586324 1310703 553 1080 | 3360000.00 | 0.00 | 0.00 | 3144500.00 | 2945800.00 | 0.00 | 0.00 | 2031300.00 | 2187000.00 | 2148300.00 | 0.00 | 0.00 | KO:K02113 | F-type H+-transporting ATPase subunit delta | Bacteria;Proteobacteria;Deltaproteobacteria;Syntrophobacterales;Syntrophaceae;Smithella;Smithella sp. SCADC;Smithella sp. SCADC |
| Ga0586324 2023252 112 663 | 0.00 | 0.00 | 0.00 | 0.00 | 0.00 | 0.00 | 0.00 | 0.00 | 0.00 | 0.00 | 1346400.00 | 0.00 | KO:K02113 | F-type H+-transporting ATPase subunit delta | Bacteria;Proteobacteria;Deltaproteobacteria;Syntrophobacterales;Syntrophobacteraceae;Syntrophobacter;Syntrophobacter fumaroxidans;Syntrophobacter fumaroxidans MPOB |
| Ga0586324 4858125 50 625 | 0.00 | 0.00 | 0.00 | 1577000.00 | 0.00 | 0.00 | 0.00 | 0.00 | 0.00 | 0.00 | 0.00 | 0.00 | KO:K02113 | F-type H+-transporting ATPase subunit delta | Bacteria;Proteobacteria;Deltaproteobacteria;Syntrophobacterales;Syntrophaceae;Desulfobacca;Desulfobacca acetoxidans;Desulfobacca acetoxidans ASRB2, DSM 11109 |
| Ga0586324 5599186 45 590 | 0.00 | 0.00 | 0.00 | 1235000.00 | 1174200.00 | 0.00 | 0.00 | 1201700.00 | 0.00 | 0.00 | 0.00 | 0.00 | KO:K02113 | F-type H+-transporting ATPase subunit delta | Bacteria;Proteobacteria;Deltaproteobacteria;Syntrophobacterales;Syntrophaceae;Syntrophus;Syntrophus gentianae;Syntrophus gentianae DSM 8423 |
| Ga0586324 6333523 285 560 | 0.00 | 0.00 | 0.00 | 1016500.00 | 0.00 | 0.00 | 0.00 | 0.00 | 431714.61 | 0.00 | 0.00 | 0.00 | KO:K02113 | F-type H+-transporting ATPase subunit delta | Bacteria;Proteobacteria;Deltaproteobacteria;Syntrophobacterales;Syntrophobacteraceae;Syntrophobacter;Syntrophobacter fumaroxidans;Syntrophobacter fumaroxidans MPOB |
| Ga0586324 6660940 248 547 | 0.00 | 0.00 | 0.00 | 10640000.00 | 11474200.00 | 4168500.00 | 0.00 | 0.00 | 694132.74 | 0.00 | 0.00 | 0.00 | KO:K02113 | F-type H+-transporting ATPase subunit delta | Bacteria;Proteobacteria;Deltaproteobacteria;Syntrophobacterales;Syntrophobacteraceae;Syntrophobacter;Syntrophobacter fumaroxidans;Syntrophobacter fumaroxidans MPOB |
| Ga0586324 0036132 1495 1902 | 0.00 | 0.00 | 0.00 | 0.00 | 0.00 | 12823100.00 | 0.00 | 0.00 | 0.00 | 0.00 | 0.00 | 0.00 | KO:K02114 | F-type H+-transporting ATPase subunit epsilon | Bacteria;Proteobacteria;Deltaproteobacteria;Syntrophobacterales;Syntrophaceae;Desulfobacca;Desulfobacca acetoxidans;Desulfobacca acetoxidans ASRB2, DSM 11109 |
| Ga0586324 0000001 68894 69766 | 0.00 | 0.00 | 0.00 | 0.00 | 0.00 | 0.00 | 0.00 | 1909300.00 | 915300.00 | 0.00 | 0.00 | 0.00 | KO:K02115 | F-type H+-transporting ATPase subunit gamma | Bacteria;Proteobacteria;Betaproteobacteria;Nitrosomonadales;Thiobacillaceae;Annwoodia;Annwoodia aquaesulis;Annwoodia aquaesulis DSM 4255 |
| Ga0586324 1050284 1 699 | 0.00 | 0.00 | 0.00 | 0.00 | 0.00 | 0.00 | 0.00 | 3775900.00 | 0.00 | 0.00 | 0.00 | 0.00 | KO:K02115 | F-type H+-transporting ATPase subunit gamma | Bacteria;Proteobacteria;Deltaproteobacteria;Syntrophobacterales;Syntrophaceae;Desulfobacca;Desulfobacca acetoxidans;Desulfobacca acetoxidans ASRB2, DSM 11109 |
| Ga0586324 1535791 2 826 | 0.00 | 0.00 | 0.00 | 0.00 | 0.00 | 0.00 | 1351770.96 | 0.00 | 0.00 | 0.00 | 0.00 | 0.00 | KO:K02115 | F-type H+-transporting ATPase subunit gamma | Bacteria;Proteobacteria;Alphaproteobacteria;Rhodospirillales;Acetobacteraceae;Rhodopila;Rhodopila globiformis;Rhodopila globiformis DSM 161 |
| Ga0586324 5108147 3 611 | 0.00 | 0.00 | 0.00 | 12350000.00 | 12257000.00 | 7662100.00 | 0.00 | 4355400.00 | 0.00 | 0.00 | 0.00 | 0.00 | KO:K02115 | F-type H+-transporting ATPase subunit gamma | Bacteria;Proteobacteria;Deltaproteobacteria;Desulfobacterales;Desulfobacteraceae;Desulfonema;Desulfonema limicola;Desulfonema limicola Jadebusen, DSM 2076 |
| Ga0586324 0065220 427 2157 | 0.00 | 0.00 | 0.00 | 15295000.00 | 8178200.00 | 0.00 | 0.00 | 0.00 | 0.00 | 0.00 | 0.00 | 0.00 | KO:K02117 | V/A-type H+-transporting ATPase subunit A | Archaea;Euryarchaeota;Methanomicrobia;Methanosarcinales;Methanotrichaceae;Methanothrix;Methanothrix soehngenii;Methanothrix soehngenii GP-6 |
| Ga0586324 0106853 1 1302 | 0.00 | 0.00 | 0.00 | 0.00 | 10506000.00 | 11195400.00 | 0.00 | 0.00 | 0.00 | 6583500.00 | 0.00 | 9075000.00 | KO:K02117 | V/A-type H+-transporting ATPase subunit A | Archaea;Euryarchaeota;Methanomicrobia;Methanosarcinales;Methanotrichaceae;Methanothrix;Methanothrix soehngenii;Methanothrix soehngenii GP-6 |
| Ga0586324 0402888 898 1803 | 0.00 | 1319200.00 | 0.00 | 3876000.00 | 0.00 | 35055100.00 | 0.00 | 0.00 | 0.00 | 0.00 | 0.00 | 0.00 | KO:K02117 | V/A-type H+-transporting ATPase subunit A | Archaea;Euryarchaeota;Methanomicrobia;Methanosarcinales;Methanotrichaceae;Methanothrix;Methanothrix soehngenii;Methanothrix soehngenii GP-6 |
| Ga0586324 0485266 448 1662 | 76800000.00 | 48212000.00 | 27024000.00 | 42493500.00 | 41509000.00 | 26837200.00 | 29868000.00 | 30012000.00 | 22275000.00 | 25117400.00 | 26514400.00 | 13950000.00 | KO:K02117 | V/A-type H+-transporting ATPase subunit A | Archaea;Euryarchaeota;Methanomicrobia;Methanosarcinales;Methanotrichaceae;Methanothrix;Methanothrix soehngenii;Methanothrix soehngenii GP-6 |
| Ga0586324 0893277 3 1274 | 70500000.00 | 47600000.00 | 88954000.00 | 46075000.00 | 39449000.00 | 67887000.00 | 87780000.00 | 52277000.00 | 45441000.00 | 0.00 | 31680000.00 | 7132500.00 | KO:K02117 | V/A-type H+-transporting ATPase subunit A | Archaea;Euryarchaeota;Methanomicrobia;Methanosarcinales;Methanotrichaceae;Methanothrix;Methanothrix soehngenii;Methanothrix soehngenii GP-6 |
| Ga0586324 2389194 1 837 | 0.00 | 1176400.00 | 0.00 | 0.00 | 0.00 | 0.00 | 0.00 | 0.00 | 0.00 | 0.00 | 0.00 | 0.00 | KO:K02117 | V/A-type H+-transporting ATPase subunit A | Archaea;Euryarchaeota;Methanomicrobia;Methanosarcinales;Methanotrichaceae;Methanothrix;Methanothrix soehngenii;Methanothrix soehngenii GP-6 |
| Ga0586324 2521194 3 152 | 0.00 | 0.00 | 0.00 | 0.00 | 0.00 | 12267300.00 | 0.00 | 8418000.00 | 2203200.00 | 0.00 | 0.00 | 0.00 | KO:K02117 | V/A-type H+-transporting ATPase subunit A | Archaea;Euryarchaeota;Methanomicrobia;Methanosarcinales;Methanotrichaceae;Methanothrix;Methanothrix soehngenii;Methanothrix soehngenii GP-6 |
| Ga0586324 5104332 1 612 | 0.00 | 3352400.00 | 0.00 | 0.00 | 0.00 | 0.00 | 0.00 | 0.00 | 0.00 | 0.00 | 0.00 | 0.00 | KO:K02117 | V/A-type H+-transporting ATPase subunit A | Archaea;Euryarchaeota;Methanomicrobia;Methanosarcinales;Methanotrichaceae;Methanothrix;Methanothrix soehngenii;Methanothrix soehngenii GP-6 |
| Ga0586324 5745279 1 582 | 0.00 | 0.00 | 0.00 | 0.00 | 0.00 | 0.00 | 0.00 | 0.00 | 498563.91 | 0.00 | 0.00 | 0.00 | KO:K02117 | V/A-type H+-transporting ATPase subunit A | Bacteria;Proteobacteria;Deltaproteobacteria;Desulfarculales;Desulfarculaceae;Desulfarculus;Desulfarculus baarsii;Desulfarculus baarsii 2st14, DSM 2075 |
| Ga0586324 7772058 2 214 | 0.00 | 0.00 | 0.00 | 9975000.00 | 10918000.00 | 0.00 | 0.00 | 0.00 | 10554300.00 | 0.00 | 0.00 | 0.00 | KO:K02117 | V/A-type H+-transporting ATPase subunit A | Archaea;Euryarchaeota;Methanomicrobia;Methanosarcinales;Methanotrichaceae;Methanothrix;Methanothrix soehngenii;Methanothrix soehngenii GP-6 |
| Ga0586324 0065220 1 414 | 514796.00 | 0.00 | 0.00 | 0.00 | 0.00 | 0.00 | 0.00 | 0.00 | 0.00 | 0.00 | 0.00 | 0.00 | KO:K02118 | V/A-type H+-transporting ATPase subunit B | Archaea;Euryarchaeota;Methanomicrobia;Methanosarcinales;Methanotrichaceae;Methanothrix;Methanothrix soehngenii;Methanothrix soehngenii GP-6 |
| Ga0586324 0106853 1315 2703 | 0.00 | 0.00 | 0.00 | 0.00 | 0.00 | 0.00 | 0.00 | 0.00 | 0.00 | 8085000.00 | 0.00 | 0.00 | KO:K02118 | V/A-type H+-transporting ATPase subunit B | Archaea;Euryarchaeota;Methanomicrobia;Methanosarcinales;Methanotrichaceae;Methanothrix;Methanothrix soehngenii;Methanothrix soehngenii GP-6 |
| Ga0586324 0409224 1355 1789 | 0.00 | 0.00 | 0.00 | 0.00 | 0.00 | 6391700.00 | 0.00 | 0.00 | 0.00 | 0.00 | 0.00 | 0.00 | KO:K02118 | V/A-type H+-transporting ATPase subunit B | Archaea;Euryarchaeota;Methanomicrobia;Methanosarcinales;Methanosarcinaceae;Methanosarcina;Methanosarcina barkeri;Methanosarcina barkeri Fusaro, DSM 804 |
| Ga0586324 0924854 3 1007 | 0.00 | 2352800.00 | 0.00 | 0.00 | 0.00 | 0.00 | 0.00 | 0.00 | 0.00 | 0.00 | 0.00 | 0.00 | KO:K02118 | V/A-type H+-transporting ATPase subunit B | Archaea;Euryarchaeota;Methanomicrobia;Methanosarcinales;Methanotrichaceae;Methanothrix;Methanothrix soehngenii;Methanothrix soehngenii GP-6 |
| Ga0586324 2521194 165 818 | 39500000.00 | 48144000.00 | 83887000.00 | 53390000.00 | 57577000.00 | 52404000.00 | 27360000.00 | 32086000.00 | 36774000.00 | 38885000.00 | 37048000.00 | 9975000.00 | KO:K02118 | V/A-type H+-transporting ATPase subunit B | Archaea;Euryarchaeota;Methanomicrobia;Methanosarcinales;Methanotrichaceae;Methanothrix;Methanothrix soehngenii;Methanothrix soehngenii GP-6 |
| Ga0586324 4425187 1 603 | 0.00 | 0.00 | 0.00 | 0.00 | 6283000.00 | 0.00 | 0.00 | 0.00 | 0.00 | 0.00 | 5253600.00 | 0.00 | KO:K02118 | V/A-type H+-transporting ATPase subunit B | Archaea;Euryarchaeota;Methanomicrobia;Methanosarcinales;Methanotrichaceae;Methanothrix;Methanothrix thermoacetophila;Methanothrix thermoacetophila PT |
| Ga0586324 8071902 2 505 | 0.00 | 0.00 | 0.00 | 0.00 | 0.00 | 0.00 | 0.00 | 567507.40 | 1539000.00 | 0.00 | 0.00 | 4215000.00 | KO:K02118 | V/A-type H+-transporting ATPase subunit B | Archaea;Euryarchaeota;Methanomicrobia;Methanosarcinales;Methanotrichaceae;Methanothrix;Methanothrix soehngenii;Methanothrix soehngenii GP-6 |
| Ga0586324 0217098 254 1303 | 0.00 | 1271600.00 | 0.00 | 1700500.00 | 2132100.00 | 1182134.99 | 12436503.96 | 1628700.00 | 1555200.00 | 0.00 | 0.00 | 675934.50 | KO:K02119 | V/A-type H+-transporting ATPase subunit C | Archaea;Euryarchaeota;Methanomicrobia;Methanosarcinales;Methanotrichaceae;Methanothrix;Methanothrix soehngenii;Methanothrix soehngenii GP-6 |
| Ga0586324 1030406 1 1077 | 10300000.00 | 1720400.00 | 3507332.36 | 3420000.00 | 3574100.00 | 1858059.25 | 16410534.84 | 3172000.00 | 2486700.00 | 1694000.00 | 16368000.00 | 1312500.00 | KO:K02119 | V/A-type H+-transporting ATPase subunit C | Archaea;Euryarchaeota;Methanomicrobia;Methanosarcinales;Methanotrichaceae;Methanothrix;Methanothrix soehngenii;Methanothrix soehngenii GP-6 |
| Ga0586324 1151439 268 1143 | 8120000.00 | 5929600.00 | 4771627.68 | 16720000.00 | 18437000.00 | 0.00 | 0.00 | 20740000.00 | 19116000.00 | 4027100.00 | 4998400.00 | 5737500.00 | KO:K02119 | V/A-type H+-transporting ATPase subunit C | Archaea;Euryarchaeota;Methanomicrobia;Methanosarcinales;Methanotrichaceae;Methanothrix;Methanothrix soehngenii;Methanothrix soehngenii GP-6 |
| Ga0586324 1377708 3 482 | 6600000.00 | 0.00 | 0.00 | 8293500.00 | 8085500.00 | 0.00 | 0.00 | 0.00 | 1474200.00 | 6021400.00 | 3528800.00 | 1590000.00 | KO:K02119 | V/A-type H+-transporting ATPase subunit C | Archaea;Euryarchaeota;Methanomicrobia;Methanosarcinales;Methanotrichaceae;Methanothrix;Methanothrix thermoacetophila;Methanothrix thermoacetophila PT |
| Ga0586324 1433491 2 865 | 0.00 | 0.00 | 0.00 | 2071000.00 | 0.00 | 0.00 | 0.00 | 0.00 | 0.00 | 1663200.00 | 0.00 | 0.00 | KO:K02119 | V/A-type H+-transporting ATPase subunit C | Archaea;Euryarchaeota;Methanomicrobia;Methanosarcinales;Methanotrichaceae;Methanothrix;Methanothrix soehngenii;Methanothrix soehngenii GP-6 |
| Ga0586324 0109379 2754 3284 | 13100000.00 | 59500000.00 | 38461142.32 | 605652.55 | 56856000.00 | 1166290.72 | 0.00 | 0.00 | 42039000.00 | 267904.56 | 21912000.00 | 397263.75 | KO:K02121 | V/A-type H+-transporting ATPase subunit E | Archaea;Euryarchaeota;Methanomicrobia;Methanosarcinales;Methanotrichaceae;Methanothrix;Methanothrix soehngenii;Methanothrix soehngenii GP-6 |
| Ga0586324 0210395 2176 2418 | 0.00 | 4447200.00 | 7713100.00 | 14858000.00 | 3151800.00 | 11830600.00 | 3237600.00 | 17690000.00 | 0.00 | 5613300.00 | 0.00 | 3952500.00 | KO:K02121 | V/A-type H+-transporting ATPase subunit E | Archaea;Euryarchaeota;Methanomicrobia;Methanosarcinales;Methanotrichaceae;Methanothrix;Methanothrix soehngenii;Methanothrix soehngenii GP-6 |
| Ga0586324 1377708 488 1036 | 26700000.00 | 18965200.00 | 29951600.00 | 28699500.00 | 8693200.00 | 11393900.00 | 12038400.00 | 10736000.00 | 9396000.00 | 5259100.00 | 7013600.00 | 9075000.00 | KO:K02121 | V/A-type H+-transporting ATPase subunit E | Archaea;Euryarchaeota;Methanomicrobia;Methanosarcinales;Methanotrichaceae;Methanothrix;Methanothrix soehngenii;Methanothrix soehngenii GP-6 |
| Ga0586324 1394348 291 839 | 6000000.00 | 18360000.00 | 0.00 | 0.00 | 19415500.00 | 17031300.00 | 0.00 | 11285000.00 | 13932000.00 | 13321000.00 | 0.00 | 0.00 | KO:K02121 | V/A-type H+-transporting ATPase subunit E | Archaea;Euryarchaeota;Methanomicrobia;Methanosarcinales;Methanotrichaceae;Methanothrix;Methanothrix soehngenii;Methanothrix soehngenii GP-6 |
| Ga0586324 8283166 3 173 | 622258.00 | 0.00 | 0.00 | 0.00 | 0.00 | 0.00 | 0.00 | 0.00 | 0.00 | 0.00 | 0.00 | 0.00 | KO:K02121 | V/A-type H+-transporting ATPase subunit E | Archaea;Euryarchaeota;Methanomicrobia;Methanosarcinales;Methanotrichaceae;Methanothrix;Methanothrix soehngenii;Methanothrix soehngenii GP-6 |
| Ga0586324 0402888 608 907 | 0.00 | 0.00 | 0.00 | 0.00 | 0.00 | 0.00 | 0.00 | 0.00 | 0.00 | 0.00 | 0.00 | 2287500.00 | KO:K02122 | V/A-type H+-transporting ATPase subunit F | Archaea;Euryarchaeota;Methanomicrobia;Methanosarcinales;Methanotrichaceae;Methanothrix;Methanothrix soehngenii;Methanothrix soehngenii GP-6 |
| Ga0586324 0485266 158 457 | 0.00 | 2482000.00 | 0.00 | 0.00 | 0.00 | 0.00 | 0.00 | 0.00 | 0.00 | 0.00 | 0.00 | 0.00 | KO:K02122 | V/A-type H+-transporting ATPase subunit F | Archaea;Euryarchaeota;Methanomicrobia;Methanosarcinales;Methanotrichaceae;Methanothrix;Methanothrix soehngenii;Methanothrix soehngenii GP-6 |
| Ga0586324 0109379 347 2389 | 0.00 | 4195600.00 | 0.00 | 0.00 | 0.00 | 0.00 | 0.00 | 664900.00 | 0.00 | 0.00 | 0.00 | 0.00 | KO:K02123 | V/A-type H+-transporting ATPase subunit I | Archaea;Euryarchaeota;Methanomicrobia;Methanosarcinales;Methanotrichaceae;Methanothrix;Methanothrix soehngenii;Methanothrix soehngenii GP-6 |
| Ga0586324 0210395 3 1814 | 0.00 | 0.00 | 0.00 | 1434500.00 | 0.00 | 0.00 | 0.00 | 1836100.00 | 0.00 | 0.00 | 0.00 | 0.00 | KO:K02123 | V/A-type H+-transporting ATPase subunit I | Archaea;Euryarchaeota;Methanomicrobia;Methanosarcinales;Methanotrichaceae;Methanothrix;Methanothrix soehngenii;Methanothrix soehngenii GP-6 |
| Ga0586324 1972399 1 909 | 0.00 | 0.00 | 0.00 | 0.00 | 1205100.00 | 0.00 | 0.00 | 0.00 | 0.00 | 0.00 | 0.00 | 0.00 | KO:K02123 | V/A-type H+-transporting ATPase subunit I | Archaea;Euryarchaeota;Methanomicrobia;Methanosarcinales;Methanotrichaceae;Methanothrix;Methanothrix soehngenii;Methanothrix soehngenii GP-6 |
| Ga0586324 2610707 257 808 | 0.00 | 3189200.00 | 0.00 | 0.00 | 0.00 | 0.00 | 0.00 | 0.00 | 0.00 | 0.00 | 0.00 | 0.00 | KO:K02132 | F-type H+-transporting ATPase subunit alpha | Eukaryota;Streptophyta;Magnoliopsida;Malpighiales;Salicaceae;Populus;Populus trichocarpa;Populus balsamifera trichocarpa |
| Ga0586324 0001554 16576 17064 | 0.00 | 0.00 | 0.00 | 0.00 | 0.00 | 0.00 | 5358000.00 | 0.00 | 1206900.00 | 0.00 | 0.00 | 0.00 | KO:K02217 | ferritin | Archaea;Euryarchaeota;Methanomicrobia;Methanomicrobiales;Methanoregulaceae;Methanoregula;Methanoregula formicica;Methanoregula formicica SMSP, DSM 22288 |
| Ga0586324 0032102 4946 5440 | 0.00 | 0.00 | 3389496.46 | 0.00 | 0.00 | 0.00 | 0.00 | 0.00 | 0.00 | 4658500.00 | 0.00 | 3390000.00 | KO:K02217 | ferritin | Bacteria;Chlorobi;Chlorobia;Chlorobiales;Chlorobiaceae;Prosthecochloris;Prosthecochloris sp. CIB 2401;Prosthecochloris sp. CIB 2401 |
| Ga0586324 0595947 774 1334 | 0.00 | 5773200.00 | 0.00 | 0.00 | 0.00 | 1795083.14 | 0.00 | 0.00 | 0.00 | 0.00 | 0.00 | 0.00 | KO:K02217 | ferritin | Bacteria;Proteobacteria;Deltaproteobacteria;unclassified Deltaproteobacteria;unclassified Deltaproteobacteria;unclassified Deltaproteobacteria;delta proteobacterium NaphS2;delta proteobacterium NaphS2 |
| Ga0586324 1519460 362 853 | 5040000.00 | 1468800.00 | 0.00 | 0.00 | 1699500.00 | 3421441.28 | 0.00 | 4843400.00 | 0.00 | 4442900.00 | 3625600.00 | 2025000.00 | KO:K02217 | ferritin | Bacteria;Chlorobi;Chlorobia;Chlorobiales;Chlorobiaceae;Prosthecochloris;Prosthecochloris sp. CIB 2401;Prosthecochloris sp. CIB 2401 |
| Ga0586324 2311656 457 849 | 0.00 | 0.00 | 0.00 | 0.00 | 0.00 | 0.00 | 0.00 | 0.00 | 0.00 | 0.00 | 0.00 | 2152500.00 | KO:K02217 | ferritin | Archaea;Euryarchaeota;Methanomicrobia;Methanomicrobiales;Methanoregulaceae;Methanoregula;Methanoregula formicica;Methanoregula formicica SMSP, DSM 22288 |
| Ga0586324 6389346 83 556 | 0.00 | 1910800.00 | 0.00 | 7495500.00 | 4315700.00 | 0.00 | 0.00 | 0.00 | 0.00 | 0.00 | 0.00 | 2085000.00 | KO:K02217 | ferritin | Bacteria;Chlorobi;Chlorobia;Chlorobiales;Chlorobiaceae;Prosthecochloris;Prosthecochloris sp. CIB 2401;Prosthecochloris sp. CIB 2401 |
| Ga0586324 0000041 53028 54179 | 0.00 | 0.00 | 0.00 | 0.00 | 0.00 | 0.00 | 0.00 | 0.00 | 4795200.00 | 4851000.00 | 6371200.00 | 0.00 | KO:K02275 | cytochrome c oxidase subunit 2 | Bacteria;Proteobacteria;Betaproteobacteria;Nitrosomonadales;Thiobacillaceae;Thiobacillus;Thiobacillus denitrificans;Thiobacillus denitrificans DSM 12475 |
| Ga0586324 0000001 201859 203424 | 0.00 | 0.00 | 0.00 | 0.00 | 0.00 | 0.00 | 0.00 | 0.00 | 0.00 | 81620000.00 | 1716000.00 | 10110000.00 | KO:K02280 | pilus assembly protein CpaC | Bacteria;Proteobacteria;Betaproteobacteria;Rhodocyclales;Rhodocyclaceae;Aromatoleum;Aromatoleum toluclasticum;Aromatoleum toluclasticum ATCC 700605 |
| Ga0586324 2773738 3 788 | 0.00 | 0.00 | 0.00 | 0.00 | 0.00 | 622722.29 | 0.00 | 0.00 | 0.00 | 0.00 | 0.00 | 0.00 | KO:K02280 | pilus assembly protein CpaC | Bacteria;Proteobacteria;Gammaproteobacteria;unclassified Gammaproteobacteria;unclassified Gammaproteobacteria;unclassified Gammaproteobacteria;gamma proteobacterium L18;gamma proteobacterium L18 |
| Ga0586324 4118707 1 669 | 0.00 | 0.00 | 1985002.88 | 0.00 | 0.00 | 0.00 | 0.00 | 2043500.00 | 0.00 | 1355200.00 | 0.00 | 0.00 | KO:K02280 | pilus assembly protein CpaC | Bacteria;Proteobacteria;Deltaproteobacteria;Syntrophorhabdales;Syntrophorhabdaceae;Syntrophorhabdus;Syntrophorhabdus aromaticivorans;Syntrophorhabdus aromaticivorans UI |
| Ga0586324 4604659 3 638 | 0.00 | 3053200.00 | 0.00 | 0.00 | 0.00 | 0.00 | 0.00 | 0.00 | 0.00 | 0.00 | 0.00 | 0.00 | KO:K02280 | pilus assembly protein CpaC | Bacteria;Proteobacteria;Deltaproteobacteria;Syntrophorhabdales;Syntrophorhabdaceae;Syntrophorhabdus;Syntrophorhabdus aromaticivorans;Syntrophorhabdus aromaticivorans UI |
| Ga0586324 4111374 1 345 | 0.00 | 0.00 | 0.00 | 0.00 | 0.00 | 0.00 | 0.00 | 0.00 | 0.00 | 0.00 | 361272.56 | 0.00 | KO:K02282 | pilus assembly protein CpaE | Bacteria;Proteobacteria;Betaproteobacteria;Neisseriales;Chromobacteriaceae;Pseudogulbenkiania;Pseudogulbenkiania subflava;Pseudogulbenkiania subflava DSM 22618 |
| Ga0586324 0000001 196699 198057 | 0.00 | 0.00 | 0.00 | 0.00 | 0.00 | 0.00 | 0.00 | 373729.92 | 0.00 | 2302300.00 | 2164800.00 | 3870000.00 | KO:K02283 | pilus assembly protein CpaF | Bacteria;Proteobacteria;Betaproteobacteria;Neisseriales;Chromobacteriaceae;Pseudogulbenkiania;Pseudogulbenkiania sp. MAI-1;Pseudogulbenkiania sp. MAI-1 |
| Ga0586324 0117733 1320 3038 | 3010000.00 | 0.00 | 0.00 | 3657500.00 | 0.00 | 0.00 | 0.00 | 0.00 | 0.00 | 0.00 | 0.00 | 0.00 | KO:K02292 | beta-carotene ketolase (CrtO type) | Bacteria;Proteobacteria;Deltaproteobacteria;Desulfobacterales;Desulfobacteraceae;Desulfatiglans;Desulfatiglans anilini;Desulfatiglans anilini DSM 4660 |
| Ga0586324 0024351 3609 5033 | 0.00 | 0.00 | 0.00 | 2318000.00 | 0.00 | 0.00 | 0.00 | 0.00 | 0.00 | 0.00 | 0.00 | 0.00 | KO:K02302 | uroporphyrin-III C-methyltransferase/precorrin-2 dehydrogenase/sirohydrochlorin ferrochelatas | Bacteria;Proteobacteria;Alphaproteobacteria;Hyphomicrobiales;unclassified Hyphomicrobiales;Methyloceanibacter;Methyloceanibacter superfactus;Methyloceanibacter superfactus R-67175 |
| Ga0586324 0070403 1439 2902 | 0.00 | 0.00 | 0.00 | 604581.90 | 0.00 | 0.00 | 0.00 | 0.00 | 0.00 | 0.00 | 0.00 | 0.00 | KO:K02302 | uroporphyrin-III C-methyltransferase/precorrin-2 dehydrogenase/sirohydrochlorin ferrochelatas | Bacteria;Proteobacteria;Alphaproteobacteria;Hyphomicrobiales;unclassified Hyphomicrobiales;Methyloceanibacter;Methyloceanibacter superfactus;Methyloceanibacter superfactus R-67175 |
| Ga0586324 0006821 3848 4963 | 0.00 | 0.00 | 0.00 | 0.00 | 0.00 | 0.00 | 10351200.00 | 0.00 | 0.00 | 0.00 | 0.00 | 0.00 | KO:K02338 | DNA polymerase-3 subunit beta | Bacteria;Proteobacteria;Deltaproteobacteria;unclassified Deltaproteobacteria;unclassified Deltaproteobacteria;unclassified Deltaproteobacteria;delta proteobacterium NaphS2;delta proteobacterium NaphS2 |
| Ga0586324 0010698 8078 9184 | 0.00 | 0.00 | 0.00 | 0.00 | 0.00 | 0.00 | 0.00 | 0.00 | 0.00 | 0.00 | 0.00 | 5347500.00 | KO:K02338 | DNA polymerase-3 subunit beta | Bacteria;Proteobacteria;Betaproteobacteria;Nitrosomonadales;Thiobacillaceae;Thiobacillus;Thiobacillus denitrificans;Thiobacillus denitrificans DSM 12475 |
| Ga0586324 0109064 1042 2157 | 0.00 | 0.00 | 0.00 | 0.00 | 0.00 | 0.00 | 0.00 | 0.00 | 1863000.00 | 0.00 | 0.00 | 0.00 | KO:K02338 | DNA polymerase-3 subunit beta | Bacteria;Proteobacteria;Deltaproteobacteria;unclassified Deltaproteobacteria;unclassified Deltaproteobacteria;unclassified Deltaproteobacteria;delta proteobacterium NaphS2;delta proteobacterium NaphS2 |
| Ga0586324 0181727 3 1184 | 0.00 | 0.00 | 0.00 | 0.00 | 0.00 | 0.00 | 101853.76 | 0.00 | 0.00 | 0.00 | 0.00 | 0.00 | KO:K02338 | DNA polymerase-3 subunit beta | Bacteria;Chloroflexi;Anaerolineae;Anaerolineales;Anaerolineaceae;Levilinea;Levilinea saccharolytica;Levilinea saccharolytica DSM 16555 |
| Ga0586324 0290211 3 563 | 0.00 | 0.00 | 0.00 | 0.00 | 0.00 | 0.00 | 0.00 | 0.00 | 0.00 | 0.00 | 60544000.00 | 0.00 | KO:K02338 | DNA polymerase-3 subunit beta | Bacteria;Proteobacteria;Alphaproteobacteria;Hyphomicrobiales;unclassified Hyphomicrobiales;Methyloceanibacter;Methyloceanibacter superfactus;Methyloceanibacter superfactus R-67175 |
| Ga0586324 0756200 1 786 | 0.00 | 0.00 | 0.00 | 3372500.00 | 3162100.00 | 0.00 | 0.00 | 0.00 | 0.00 | 0.00 | 0.00 | 0.00 | KO:K02338 | DNA polymerase-3 subunit beta | Bacteria;Bacteroidetes;Bacteroidia;Marinilabiliales;Prolixibacteraceae;Sunxiuqinia;Sunxiuqinia elliptica;Sunxiuqinia elliptica CGMCC 1.9156 |
| Ga0586324 1311854 192 1079 | 0.00 | 0.00 | 0.00 | 0.00 | 6231500.00 | 0.00 | 0.00 | 0.00 | 0.00 | 0.00 | 0.00 | 0.00 | KO:K02338 | DNA polymerase-3 subunit beta | Bacteria;Proteobacteria;Betaproteobacteria;Nitrosomonadales;Nitrosomonadaceae;Nitrosospira;Nitrosospira sp. Nl5;Nitrosospira sp. Nl5 |
| Ga0586324 0000041 119 2212 | 0.00 | 0.00 | 0.00 | 0.00 | 0.00 | 0.00 | 13634400.00 | 14865700.00 | 12960000.00 | 14168000.00 | 19624000.00 | 28470000.00 | KO:K02355 | elongation factor G | Bacteria;Proteobacteria;Betaproteobacteria;Nitrosomonadales;Thiobacillaceae;Annwoodia;Annwoodia aquaesulis;Annwoodia aquaesulis DSM 4255 |
| Ga0586324 0000999 15353 17425 | 0.00 | 0.00 | 0.00 | 0.00 | 0.00 | 0.00 | 1532230.68 | 1256600.00 | 1409400.00 | 0.00 | 0.00 | 0.00 | KO:K02355 | elongation factor G | Bacteria;Proteobacteria;Alphaproteobacteria;Hyphomicrobiales;Hyphomicrobiaceae;Rhodoplanes;Rhodoplanes sp. Z2-YC6860;Rhodoplanes sp. Z2-YC6860 |
| Ga0586324 0003932 240 2324 | 0.00 | 0.00 | 0.00 | 0.00 | 0.00 | 0.00 | 0.00 | 0.00 | 0.00 | 3234000.00 | 0.00 | 0.00 | KO:K02355 | elongation factor G | Bacteria;Aquificae;Aquificae;Desulfurobacteriales;Desulfurobacteriaceae;Desulfurobacterium;Desulfurobacterium thermolithotrophum;Desulfurobacterium thermolithotrophum BSA, DSM 11699 |
| Ga0586324 0005725 497 2584 | 2600000.00 | 0.00 | 0.00 | 0.00 | 0.00 | 0.00 | 0.00 | 0.00 | 0.00 | 0.00 | 0.00 | 0.00 | KO:K02355 | elongation factor G | Bacteria;Proteobacteria;Gammaproteobacteria;Methylococcales;Methylococcaceae;Methylobacter;Methylobacter tundripaludum;Methylobacter tundripaludum OWC-DMM |
| Ga0586324 0010172 6800 8890 | 6570000.00 | 0.00 | 0.00 | 0.00 | 0.00 | 2449613.07 | 0.00 | 0.00 | 0.00 | 6229300.00 | 0.00 | 7875000.00 | KO:K02355 | elongation factor G | Bacteria;Proteobacteria;Betaproteobacteria;Nitrosomonadales;Thiobacillaceae;Thiobacillus;Thiobacillus denitrificans;Thiobacillus denitrificans ATCC 25259 |
| Ga0586324 0019487 3 1256 | 1990000.00 | 2692800.00 | 0.00 | 5586000.00 | 5489900.00 | 1820570.54 | 935531.88 | 1805600.00 | 834300.00 | 0.00 | 0.00 | 0.00 | KO:K02355 | elongation factor G | Bacteria;Proteobacteria;Deltaproteobacteria;Syntrophobacterales;Syntrophaceae;Desulfobacca;Desulfobacca acetoxidans;Desulfobacca acetoxidans ASRB2, DSM 11109 |
| Ga0586324 0025854 4370 6460 | 0.00 | 0.00 | 0.00 | 0.00 | 0.00 | 0.00 | 1373506.20 | 1720200.00 | 1952100.00 | 2132900.00 | 1619200.00 | 1147500.00 | KO:K02355 | elongation factor G | Bacteria;Proteobacteria;Betaproteobacteria;Nitrosomonadales;Thiobacillaceae;Thiobacillus;Thiobacillus thioparus;Thiobacillus thioparus DSM 505 |
| Ga0586324 0063855 1299 3404 | 0.00 | 0.00 | 0.00 | 0.00 | 0.00 | 0.00 | 0.00 | 3153700.00 | 0.00 | 453040.28 | 3273600.00 | 8887500.00 | KO:K02355 | elongation factor G | Bacteria;Proteobacteria;Gammaproteobacteria;Acidiferrobacterales;Acidiferrobacteraceae;Sulfuricaulis;Sulfuricaulis limicola;Sulfuricaulis limicola HA5 |
| Ga0586324 0076619 3 2057 | 6280000.00 | 0.00 | 0.00 | 0.00 | 0.00 | 0.00 | 0.00 | 0.00 | 0.00 | 0.00 | 0.00 | 0.00 | KO:K02355 | elongation factor G | Bacteria;Proteobacteria;Deltaproteobacteria;Desulfuromonadales;Geobacteraceae;Geobacter;Geobacter sulfurreducens;Geobacter sulfurreducens AM-1 |
| Ga0586324 0106192 161 2251 | 2080000.00 | 0.00 | 0.00 | 0.00 | 0.00 | 0.00 | 0.00 | 0.00 | 0.00 | 0.00 | 0.00 | 0.00 | KO:K02355 | elongation factor G | Bacteria;Proteobacteria;Betaproteobacteria;Nitrosomonadales;Thiobacillaceae;Thiobacillus;Thiobacillus denitrificans;Thiobacillus denitrificans ATCC 25259 |
| Ga0586324 0108476 3 950 | 0.00 | 572815.68 | 0.00 | 0.00 | 120458.50 | 0.00 | 0.00 | 0.00 | 0.00 | 1170400.00 | 0.00 | 0.00 | KO:K02355 | elongation factor G | Bacteria;Proteobacteria;Deltaproteobacteria;Desulfuromonadales;Geobacteraceae;Geobacter;Geobacter sp. DSM 2909;Geobacter sp. DSM 2909 |

| | | | | | | | | | | | | | | | |
|---|---|---|---|---|---|---|---|---|---|---|---|---|---|---|---|
| Ga0586324 0116474 74 2173 | 0.00 | 0.00 | 0.00 | 0.00 | 0.00 | 0.00 | 0.00 | 0.00 | 10368000.00 | 0.00 | 0.00 | 0.00 | KO:K02355 | elongation factor G | Bacteria;Proteobacteria;Gammaproteobacteria;Chromatiales;Ectothiorhodospiraceae;Thioalbus;Thioalbus denitrificans;Thioalbus denitrificans DSM 26407 |
| Ga0586324 0120170 319 2421 | 0.00 | 0.00 | 0.00 | 0.00 | 0.00 | 0.00 | 0.00 | 0.00 | 0.00 | 0.00 | 2895200.00 | 0.00 | KO:K02355 | elongation factor G | Bacteria;Proteobacteria;Betaproteobacteria;Burkholderiales;unclassified Burkholderiales;Methylibium;Methylibium sp. Pch-M;Methylibium sp. Pch-M |
| Ga0586324 0141084 1 285 | 0.00 | 0.00 | 0.00 | 0.00 | 0.00 | 0.00 | 1666228.56 | 0.00 | 0.00 | 0.00 | 0.00 | 0.00 | KO:K02355 | elongation factor G | Bacteria;Proteobacteria;Alphaproteobacteria;Hyphomicrobiales;Hyphomicrobiaceae;Rhodoplanes;Rhodoplanes sp. Z2-YC6860;Rhodoplanes sp. Z2-YC6860 |
| Ga0586324 0156429 523 2613 | 1770000.00 | 0.00 | 0.00 | 2344228.55 | 0.00 | 0.00 | 0.00 | 0.00 | 0.00 | 0.00 | 617607.76 | 0.00 | KO:K02355 | elongation factor G | Bacteria;Proteobacteria;Deltaproteobacteria;Desulfuromonadales;Geobacteraceae;Geobacter;Geobacter sulfurreducens;Geobacter sulfurreducens PCA |
| Ga0586324 0189057 206 2308 | 0.00 | 0.00 | 0.00 | 1786000.00 | 0.00 | 0.00 | 0.00 | 0.00 | 0.00 | 0.00 | 0.00 | 0.00 | KO:K02355 | elongation factor G | Bacteria;Proteobacteria;Betaproteobacteria;Burkholderiales;Comamonadaceae;Rhodoferax;Rhodoferax sp. YR267;Rhodoferax sp. YR267 |
| Ga0586324 0267521 564 2165 | 0.00 | 0.00 | 0.00 | 0.00 | 0.00 | 0.00 | 0.00 | 0.00 | 0.00 | 589220.17 | 0.00 | 0.00 | KO:K02355 | elongation factor G | Bacteria;Acidobacteria;Vicinamibacteria;unclassified Vicinamibacteria;Vicinamibacteraceae;Luteitalea;Luteitalea pratensis;Luteitalea pratensis DSM 100886 |
| Ga0586324 0274046 488 2140 | 0.00 | 0.00 | 0.00 | 0.00 | 0.00 | 0.00 | 0.00 | 0.00 | 0.00 | 0.00 | 14344000.00 | 0.00 | KO:K02355 | elongation factor G | Bacteria;Proteobacteria;Betaproteobacteria;Nitrosomonadales;Sulfuricellaceae;Sulfuricella;Sulfuricella denitrificans;Sulfuricella denitrificans skB26 |
| Ga0586324 0276490 401 2134 | 0.00 | 0.00 | 0.00 | 0.00 | 0.00 | 0.00 | 332736.36 | 0.00 | 14580000.00 | 370443.15 | 2200191.84 | 0.00 | KO:K02355 | elongation factor G | Bacteria;Proteobacteria;Betaproteobacteria;unclassified Betaproteobacteria;unclassified Betaproteobacteria;unclassified Betaproteobacteria;uncultured beta proteobacterium;unclassified betaproteobacterium ENR4 |
| Ga0586324 0315069 223 2010 | 0.00 | 0.00 | 0.00 | 0.00 | 0.00 | 0.00 | 2394000.00 | 1525000.00 | 18249300.00 | 4620000.00 | 4074400.00 | 3600000.00 | KO:K02355 | elongation factor G | Bacteria;Proteobacteria;Betaproteobacteria;Nitrosomonadales;Sulfuricellaceae;Sulfuricella;Sulfuricella sp. T08;Sulfuricella sp. T08 |
| Ga0586324 0342349 378 1937 | 0.00 | 0.00 | 0.00 | 0.00 | 0.00 | 0.00 | 0.00 | 104919.39 | 0.00 | 0.00 | 0.00 | 0.00 | KO:K02355 | elongation factor G | Bacteria;Proteobacteria;Gammaproteobacteria;Acidiferrobacterales;Acidiferrobacteraceae;Sulfuricaulis;Sulfuricaulis limicola;Sulfuricaulis limicola HA5 |
| Ga0586324 0370950 69 1871 | 0.00 | 0.00 | 0.00 | 0.00 | 0.00 | 0.00 | 0.00 | 0.00 | 0.00 | 0.00 | 0.00 | 4650000.00 | KO:K02355 | elongation factor G | Bacteria;Proteobacteria;Betaproteobacteria;Nitrosomonadales;Nitrosomonadaceae;Nitrosospira;Nitrosospira sp. Nsp2;Nitrosospira sp. Nsp2 |
| Ga0586324 0380749 105 1847 | 2250000.00 | 0.00 | 0.00 | 0.00 | 0.00 | 0.00 | 0.00 | 0.00 | 0.00 | 0.00 | 0.00 | 0.00 | KO:K02355 | elongation factor G | Bacteria;Proteobacteria;Alphaproteobacteria;Hyphomicrobiales;Hyphomicrobiaceae;Rhodoplanes;Rhodoplanes sp. Z2-YC6860;Rhodoplanes sp. Z2-YC6860 |
| Ga0586324 0422089 2 1606 | 6420000.00 | 3910000.00 | 0.00 | 3363000.00 | 0.00 | 0.00 | 0.00 | 0.00 | 0.00 | 0.00 | 0.00 | 0.00 | KO:K02355 | elongation factor G | Bacteria;Proteobacteria;Gammaproteobacteria;Methylococcales;Methylococcaceae;Methylobacter;Methylobacter tundripaludum;Methylobacter tundripaludum OWC-G53F |
| Ga0586324 0465687 3 1691 | 0.00 | 0.00 | 0.00 | 0.00 | 1018025.22 | 0.00 | 0.00 | 0.00 | 0.00 | 0.00 | 0.00 | 0.00 | KO:K02355 | elongation factor G | Bacteria;Bacteroidetes;Bacteroidia;Marinilabiliales;Marinilabiliaceae;Anaerophaga;Anaerophaga thermohalophila;Anaerophaga thermohalophila Fru22, DSM 12881 |
| Ga0586324 0617102 196 1497 | 0.00 | 3182400.00 | 0.00 | 0.00 | 0.00 | 0.00 | 0.00 | 0.00 | 0.00 | 0.00 | 0.00 | 0.00 | KO:K02355 | elongation factor G | Bacteria;Proteobacteria;Gammaproteobacteria;Nevskiales;Steroidobacteraceae;Steroidobacter;Steroidobacter denitrificans;Steroidobacter denitrificans DSM 18526 |
| Ga0586324 0802749 2 1336 | 0.00 | 0.00 | 1784214.56 | 0.00 | 894671.39 | 1177112.94 | 0.00 | 0.00 | 0.00 | 0.00 | 1047200.00 | 0.00 | KO:K02355 | elongation factor G | Bacteria;Proteobacteria;Deltaproteobacteria;Desulfuromonadales;Geobacteraceae;Geobacter;Geobacter sp. DSM 2909;Geobacter sp. DSM 2909 |
| Ga0586324 0988683 3 1220 | 0.00 | 0.00 | 0.00 | 0.00 | 0.00 | 704083.47 | 0.00 | 0.00 | 0.00 | 0.00 | 0.00 | 0.00 | KO:K02355 | elongation factor G | Bacteria;Ignavibacteriae;Ignavibacteria;Ignavibacteriales;Ignavibacteriaceae;Ignavibacterium;Ignavibacterium album;Ignavibacterium album Mat9-16, JCM 16511 |
| Ga0586324 1029262 2 1198 | 5610000.00 | 306461.72 | 0.00 | 0.00 | 3594700.00 | 0.00 | 0.00 | 7259000.00 | 2138400.00 | 13013000.00 | 4109600.00 | 454110.75 | KO:K02355 | elongation factor G | Bacteria;Proteobacteria;Betaproteobacteria;Rhodocyclales;Azonexaceae;Dechloromonas;Dechloromonas sp. HYN0024;Dechloromonas sp. HYN0024 |
| Ga0586324 1262453 149 1099 | 0.00 | 0.00 | 0.00 | 1130500.00 | 0.00 | 0.00 | 0.00 | 0.00 | 0.00 | 0.00 | 0.00 | 0.00 | KO:K02355 | elongation factor G | Bacteria;Nitrospirae;Nitrospira;Nitrospirales;Nitrospiraceae;Nitrospira;Candidatus Nitrospira inopinata;Candidatus Nitrospira inopinata ENR4 |
| Ga0586324 1632541 3 983 | 0.00 | 601449.12 | 0.00 | 0.00 | 0.00 | 0.00 | 0.00 | 0.00 | 0.00 | 0.00 | 0.00 | 0.00 | KO:K02355 | elongation factor G | Bacteria;Acidobacteria;Vicinamibacteria;unclassified Vicinamibacteria;Vicinamibacteraceae;Luteitalea;Luteitalea pratensis;Luteitalea pratensis DSM 100886 |
| Ga0586324 1773358 1 951 | 0.00 | 0.00 | 0.00 | 0.00 | 0.00 | 0.00 | 0.00 | 0.00 | 0.00 | 954800.00 | 0.00 | 0.00 | KO:K02355 | elongation factor G | Bacteria;Proteobacteria;Alphaproteobacteria;Hyphomicrobiales;Xanthobacteraceae;Pseudolabrys;Pseudolabrys sp. Root1462;Pseudolabrys sp. Root1462 |
| Ga0586324 1831390 1 936 | 1230000.00 | 0.00 | 0.00 | 0.00 | 0.00 | 0.00 | 0.00 | 0.00 | 0.00 | 0.00 | 0.00 | 0.00 | KO:K02355 | elongation factor G | Bacteria;Proteobacteria;Betaproteobacteria;Nitrosomonadales;Sterolibacteriaceae;Sulfurisoma;Sulfurisoma sediminicola;Sulfurisoma sediminicola DSM 26916 |
| Ga0586324 1945670 1 915 | 0.00 | 0.00 | 0.00 | 0.00 | 0.00 | 0.00 | 0.00 | 5862100.00 | 0.00 | 7931000.00 | 0.00 | 3727500.00 | KO:K02355 | elongation factor G | Bacteria;Proteobacteria;Betaproteobacteria;Nitrosomonadales;Thiobacillaceae;Thiobacillus;Thiobacillus denitrificans;Thiobacillus denitrificans ATCC 25259 |
| Ga0586324 1947165 240 914 | 0.00 | 0.00 | 0.00 | 0.00 | 0.00 | 0.00 | 0.00 | 0.00 | 7816500.00 | 19250000.00 | 0.00 | 0.00 | KO:K02355 | elongation factor G | Bacteria;Proteobacteria;Gammaproteobacteria;Methylococcales;Methylococcaceae;Methylocaldum;Methylocaldum szegediense;Methylocaldum szegediense O-12 |
| Ga0586324 2200466 1 867 | 0.00 | 0.00 | 0.00 | 0.00 | 0.00 | 0.00 | 0.00 | 835700.00 | 0.00 | 0.00 | 0.00 | 0.00 | KO:K02355 | elongation factor G | Bacteria;Ignavibacteriae;Ignavibacteria;Ignavibacteriales;Ignavibacteriaceae;Ignavibacterium;Ignavibacterium album;Ignavibacterium album Mat9-16, JCM 16511 |
| Ga0586324 2480065 2 598 | 0.00 | 0.00 | 0.00 | 0.00 | 0.00 | 0.00 | 0.00 | 0.00 | 0.00 | 0.00 | 0.00 | 1402500.00 | KO:K02355 | elongation factor G | Bacteria;Chloroflexi;Chloroflexia;Chloroflexales;Roseiflexaceae;Roseiflexus;Roseiflexus sp. RS-1;Roseiflexus sp. RS-1 |
| Ga0586324 3086151 1 753 | 0.00 | 0.00 | 0.00 | 0.00 | 0.00 | 0.00 | 0.00 | 0.00 | 0.00 | 0.00 | 5588000.00 | 2302500.00 | KO:K02355 | elongation factor G | Bacteria;Proteobacteria;Betaproteobacteria;Nitrosomonadales;Sulfuricellaceae;Sulfuricella;Sulfuricella sp. T08;Sulfuricella sp. T08 |
| Ga0586324 3113699 2 751 | 3140000.00 | 2006000.00 | 1633049.06 | 0.00 | 0.00 | 0.00 | 0.00 | 0.00 | 0.00 | 0.00 | 0.00 | 0.00 | KO:K02355 | elongation factor G | Bacteria;Proteobacteria;Gammaproteobacteria;Methylococcales;Methylococcaceae;Methylomonas;Methylomonas koyamae;Methylomonas koyamae R-45383 |
| Ga0586324 3483467 1 717 | 0.00 | 0.00 | 0.00 | 0.00 | 1555300.00 | 0.00 | 0.00 | 0.00 | 0.00 | 0.00 | 0.00 | 0.00 | KO:K02355 | elongation factor G | Bacteria;Proteobacteria;Betaproteobacteria;Rhodocyclales;Azonexaceae;Dechloromonas;Dechloromonas sp. SG708;Dechloromonas sp. SG708 |
| Ga0586324 4281919 2 523 | 0.00 | 0.00 | 0.00 | 0.00 | 0.00 | 0.00 | 0.00 | 14274000.00 | 0.00 | 0.00 | 0.00 | 0.00 | KO:K02355 | elongation factor G | Bacteria;Proteobacteria;Betaproteobacteria;Nitrosomonadales;Sulfuricellaceae;Sulfuriferula;Sulfuriferula multivorans;Sulfuriferula multivorans TTN |
| Ga0586324 5848687 3 578 | 0.00 | 0.00 | 0.00 | 0.00 | 0.00 | 0.00 | 0.00 | 0.00 | 0.00 | 0.00 | 0.00 | 11700000.00 | KO:K02355 | elongation factor G | Bacteria;Proteobacteria;Betaproteobacteria;Nitrosomonadales;Methylophilaceae;Novimethylophilus;Novimethylophilus kurashikiensis;Novimethylophilus kurashikiensis La2-4 |
| Ga0586324 6113768 1 567 | 0.00 | 0.00 | 0.00 | 0.00 | 0.00 | 0.00 | 4674000.00 | 0.00 | 4811400.00 | 0.00 | 0.00 | 0.00 | KO:K02355 | elongation factor G | Bacteria;Proteobacteria;Betaproteobacteria;Rhodocyclales;Rhodocyclaceae;Dechlorobacter;Dechlorobacter hydrogenophilus;Dechlorobacter hydrogenophilus LT-1 |
| Ga0586324 6432521 1 555 | 0.00 | 0.00 | 0.00 | 0.00 | 0.00 | 7344500.00 | 0.00 | 0.00 | 0.00 | 0.00 | 0.00 | 0.00 | KO:K02355 | elongation factor G | Bacteria;Proteobacteria;Betaproteobacteria;Nitrosomonadales;Sulfuricellaceae;Sulfuriferula;Sulfuriferula multivorans;Sulfuriferula multivorans TTN |
| Ga0586324 6489451 2 553 | 1560000.00 | 1856400.00 | 0.00 | 0.00 | 0.00 | 0.00 | 1068410.28 | 0.00 | 0.00 | 0.00 | 0.00 | 0.00 | KO:K02355 | elongation factor G | Bacteria;Proteobacteria;Gammaproteobacteria;Methylococcales;Crenotrichaceae;Crenothrix;Crenothrix polyspora;Crenothrix polyspora ERS1545888 |
| Ga0586324 7190281 2 532 | 0.00 | 0.00 | 0.00 | 0.00 | 0.00 | 0.00 | 0.00 | 4141900.00 | 0.00 | 0.00 | 0.00 | 0.00 | KO:K02355 | elongation factor G | Bacteria;Firmicutes;Clostridia;Thermoanaerobacterales;Thermoanaerobacteraceae;Ammonifex;Ammonifex thiophilus;Ammonifex thiophilus SR |
| Ga0586324 8159089 2 502 | 0.00 | 0.00 | 0.00 | 0.00 | 0.00 | 1191746.36 | 0.00 | 0.00 | 356264.73 | 0.00 | 0.00 | 0.00 | KO:K02355 | elongation factor G | Bacteria;Proteobacteria;Deltaproteobacteria;unclassified Deltaproteobacteria;unclassified Deltaproteobacteria;Deferrisoma;Deferrisoma camini;Deferrisoma camini S3R1 |
| Ga0586324 0000002 83549 84430 | 0.00 | 0.00 | 0.00 | 0.00 | 0.00 | 0.00 | 0.00 | 0.00 | 0.00 | 1794100.00 | 1900800.00 | 3540000.00 | KO:K02357 | elongation factor Ts | Bacteria;Proteobacteria;Betaproteobacteria;Nitrosomonadales;Thiobacillaceae;Annwoodia;Annwoodia aquaesulis;Annwoodia aquaesulis DSM 4255 |
| Ga0586324 0157707 373 1272 | 0.00 | 0.00 | 0.00 | 0.00 | 0.00 | 0.00 | 0.00 | 0.00 | 0.00 | 1516900.00 | 223218.16 | 0.00 | KO:K02357 | elongation factor Ts | Bacteria;Proteobacteria;Betaproteobacteria;Nitrosomonadales;Gallionellaceae;Sideroxydans;Sideroxydans lithotrophicus;Sideroxydans lithotrophicus ES-1 |
| Ga0586324 0000041 15170 16360 | 6400000.00 | 8976000.00 | 5713492.90 | 0.00 | 1421400.00 | 0.00 | 103740000.00 | 96136000.00 | 86427000.00 | 140910511.28 | 230612800.00 | 366562500.00 | KO:K02358 | elongation factor Tu | Bacteria;Proteobacteria;Betaproteobacteria;Nitrosomonadales;Thiobacillaceae;Annwoodia;Annwoodia aquaesulis;Annwoodia aquaesulis DSM 4255 |
| Ga0586324 0000456 3956 5158 | 0.00 | 0.00 | 0.00 | 1045000.00 | 369723.65 | 0.00 | 0.00 | 0.00 | 0.00 | 0.00 | 0.00 | 0.00 | KO:K02358 | elongation factor Tu | Bacteria;Fibrobacteres;Fibrobacteria;unclassified Fibrobacteria;unclassified Fibrobacteria;unclassified Fibrobacteria;Fibrobacteria bacterium GUT77;Fibrobacteria bacterium GUT77 MC 77 |
| Ga0586324 0000999 14115 15305 | 0.00 | 0.00 | 0.00 | 0.00 | 0.00 | 0.00 | 1235570.76 | 1250500.00 | 0.00 | 1339800.00 | 695794.88 | 0.00 | KO:K02358 | elongation factor Tu | Bacteria;Proteobacteria;Alphaproteobacteria;Hyphomicrobiales;Hyphomicrobiaceae;Rhodoplanes;Rhodoplanes sp. Z2-YC6860;Rhodoplanes sp. Z2-YC6860 |
| Ga0586324 0008154 10230 11162 | 0.00 | 0.00 | 0.00 | 0.00 | 2090900.00 | 0.00 | 3988212.48 | 5050800.00 | 1125900.00 | 7476700.00 | 10665600.00 | 6015000.00 | KO:K02358 | elongation factor Tu | Bacteria;Proteobacteria;Betaproteobacteria;Nitrosomonadales;Thiobacillaceae;Thiobacillus;Thiobacillus denitrificans;Thiobacillus denitrificans ATCC 25259 |
| Ga0586324 0008218 7620 8807 | 31890000.00 | 20073600.00 | 0.00 | 33250000.00 | 25853000.00 | 1559209.56 | 63156000.00 | 6319600.00 | 28552500.00 | 25656400.00 | 20451200.00 | 25477500.00 | KO:K02358 | elongation factor Tu | Bacteria;Bacteroidetes;Bacteroidia;Bacteroidales;Lentimicrobiaceae;Lentimicrobium;Lentimicrobium saccharophilum;Bacteroidales bacterium TBC1 |
| Ga0586324 0008657 3 1193 | 0.00 | 0.00 | 0.00 | 0.00 | 2245400.00 | 1071602.25 | 4035600.00 | 2494900.00 | 0.00 | 388615.15 | 0.00 | 1455000.00 | KO:K02358 | elongation factor Tu | Bacteria;Acidobacteria;Acidobacteriia;Acidobacteriales;Acidobacteriaceae;Acidobacterium;Acidobacterium ailaaui;Acidobacterium ailaaui PMMR2 |
| Ga0586324 0010172 8933 10120 | 0.00 | 5195200.00 | 1949376.24 | 0.00 | 6169700.00 | 0.00 | 0.00 | 0.00 | 0.00 | 0.00 | 0.00 | 0.00 | KO:K02358 | elongation factor Tu | Bacteria;Proteobacteria;Betaproteobacteria;Nitrosomonadales;Thiobacillaceae;Thiobacillus;Thiobacillus denitrificans;Thiobacillus denitrificans DSM 12475 |
| Ga0586324 0010668 6209 7411 | 0.00 | 1271600.00 | 0.00 | 0.00 | 0.00 | 5637400.00 | 0.00 | 0.00 | 208597.68 | 0.00 | 3810400.00 | 9112500.00 | KO:K02358 | elongation factor Tu | Bacteria;Proteobacteria;Gammaproteobacteria;Legionellales;Coxiellaceae;Rickettsiella;Rickettsiella grylli;Rickettsiella grylli |
| Ga0586324 0011800 2 679 | 0.00 | 0.00 | 0.00 | 0.00 | 0.00 | 117206.31 | 0.00 | 0.00 | 0.00 | 0.00 | 0.00 | 0.00 | KO:K02358 | elongation factor Tu | Bacteria;Proteobacteria;Deltaproteobacteria;Syntrophobacterales;Syntrophaceae;Desulfobacca;Desulfobacca acetoxidans;Desulfobacca acetoxidans ASRB2, DSM 11109 |
| Ga0586324 0014164 1 1161 | 0.00 | 0.00 | 0.00 | 0.00 | 0.00 | 0.00 | 0.00 | 0.00 | 0.00 | 713996.36 | 0.00 | 0.00 | KO:K02358 | elongation factor Tu | Bacteria;Nitrospirae;Nitrospira;Nitrospirales;Nitrospiraceae;Leptospirillum;Leptospirillum sp. Group II 'C75';Leptospirillum sp. Group II 'C75' |
| Ga0586324 0014646 1 1056 | 0.00 | 0.00 | 0.00 | 0.00 | 0.00 | 0.00 | 0.00 | 0.00 | 0.00 | 0.00 | 0.00 | 396654.75 | KO:K02358 | elongation factor Tu | Bacteria;Gemmatimonadetes;Gemmatimonadetes;Gemmatimonadales;Gemmatimonadaceae;Gemmatimonas;Gemmatimonas aurantiaca;Gemmatimonas aurantiaca T-27T |
| Ga0586324 0018236 7661 7759 | 0.00 | 0.00 | 0.00 | 0.00 | 0.00 | 0.00 | 0.00 | 4898300.00 | 0.00 | 0.00 | 0.00 | 0.00 | KO:K02358 | elongation factor Tu | Bacteria;Rhodothermaeota;Rhodothermia;Rhodothermales;Rubricoccaceae;Rubrivirga;Rubrivirga marina;Rubrivirga marina SAORIC-28 |
| Ga0586324 0018881 25 1212 | 0.00 | 0.00 | 0.00 | 0.00 | 0.00 | 0.00 | 0.00 | 1274900.00 | 0.00 | 0.00 | 0.00 | 0.00 | KO:K02358 | elongation factor Tu | Bacteria;Bacteroidetes;Bacteroidia;Marinilabiliales;Marinilabiliaceae;Anaerophaga;Anaerophaga thermohalophila;Anaerophaga thermohalophila Fru22, DSM 12881 |
| Ga0586324 0019141 6255 7457 | 0.00 | 548793.32 | 0.00 | 6517000.00 | 0.00 | 0.00 | 0.00 | 622200.00 | 742060.44 | 3026100.00 | 2138400.00 | 2617500.00 | KO:K02358 | elongation factor Tu | Bacteria;Chloroflexi;Anaerolineae;Anaerolineales;Anaerolineaceae;Anaerolinea;Anaerolinea thermophila;Anaerolinea thermophila UNI-1 |
| Ga0586324 0021799 4714 5904 | 0.00 | 2822000.00 | 0.00 | 5909000.00 | 0.00 | 5081600.00 | 0.00 | 0.00 | 35559000.00 | 0.00 | 0.00 | 21525000.00 | KO:K02358 | elongation factor Tu | Bacteria;Spirochaetes;Spirochaetia;Spirochaetales;Treponemataceae;Treponema;Treponema caldarium;Treponema caldarium DSM 7334 |
| Ga0586324 0047980 3796 4875 | 722043.00 | 0.00 | 0.00 | 0.00 | 0.00 | 0.00 | 0.00 | 0.00 | 0.00 | 0.00 | 0.00 | 0.00 | KO:K02358 | elongation factor Tu | Bacteria;Verrucomicrobia;Verrucomicrobiae;Verrucomicrobiales;Verrucomicrobia subdivision 3;Pedosphaera;Pedosphaera parvula;Pedosphaera parvula Ellin514 |
| Ga0586324 0059148 3 245 | 0.00 | 1128800.00 | 0.00 | 0.00 | 19158000.00 | 0.00 | 0.00 | 0.00 | 0.00 | 0.00 | 0.00 | 0.00 | KO:K02358 | elongation factor Tu | Bacteria;Chloroflexi;Anaerolineae;Anaerolineales;Anaerolineaceae;Anaerolinea;Anaerolinea thermophila;Anaerolinea thermophila UNI-1 |
| Ga0586324 0059428 1 1005 | 0.00 | 0.00 | 0.00 | 3952000.00 | 0.00 | 0.00 | 0.00 | 0.00 | 0.00 | 0.00 | 0.00 | 0.00 | KO:K02358 | elongation factor Tu | Bacteria;Proteobacteria;Alphaproteobacteria;Rhodospirillales;Rhodospirillaceae;Dongia;Dongia mobilis;Dongia mobilis CGMCC 1.7660 |
| Ga0586324 0060081 2771 3955 | 0.00 | 0.00 | 0.00 | 0.00 | 0.00 | 0.00 | 0.00 | 16897000.00 | 0.00 | 0.00 | 867517.20 | 0.00 | KO:K02358 | elongation factor Tu | Bacteria;Verrucomicrobia;Spartobacteria;unclassified Spartobacteria;unclassified Spartobacteria;unclassified Spartobacteria;Spartobacteria bacterium LR76;Spartobacteria bacterium LR76 |
| Ga0586324 0060324 3 788 | 0.00 | 0.00 | 0.00 | 860098.65 | 1390500.00 | 0.00 | 0.00 | 206613.71 | 197067.33 | 292321.26 | 0.00 | 0.00 | KO:K02358 | elongation factor Tu | Bacteria;Proteobacteria;Deltaproteobacteria;unclassified Deltaproteobacteria;unclassified Deltaproteobacteria;unclassified Deltaproteobacteria;delta proteobacterium NaphS2;delta proteobacterium NaphS2 |
| Ga0586324 0063855 3442 4248 | 0.00 | 0.00 | 0.00 | 0.00 | 2616200.00 | 4506283.48 | 1185800.64 | 26437313.38 | 23093100.00 | 2163700.00 | 58185600.00 | 13200000.00 | KO:K02358 | elongation factor Tu | Bacteria;Proteobacteria;Gammaproteobacteria;Acidiferrobacterales;Acidiferrobacteraceae;Acidiferrobacter;Acidiferrobacter sp. SPIII 3;Acidiferrobacter sp. SPIII 3 |
| Ga0586324 0070664 1 507 | 0.00 | 0.00 | 0.00 | 0.00 | 4470200.00 | 0.00 | 0.00 | 0.00 | 3539700.00 | 0.00 | 0.00 | 1215000.00 | KO:K02358 | elongation factor Tu | Bacteria;Proteobacteria;Gammaproteobacteria;unclassified Gammaproteobacteria;Candidatus Competibacteraceae;Candidatus Competibacter;Candidatus Competibacter denitrificans;Candidatus Competibacter denitrificans Run A D11 |
| Ga0586324 0074758 204 1394 | 0.00 | 0.00 | 0.00 | 0.00 | 0.00 | 0.00 | 0.00 | 0.00 | 0.00 | 0.00 | 0.00 | 361403.25 | KO:K02358 | elongation factor Tu | Bacteria;Verrucomicrobia;Opitutae;Opitutales;Opitutaceae;Opitutus;Opitutus sp. ER46;Opitutus sp. ER46 |
| Ga0586324 0086296 824 2017 | 0.00 | 16660000.00 | 44871100.00 | 18525000.00 | 40067000.00 | 18778100.00 | 15636472.56 | 12200000.00 | 16953300.00 | 3888500.00 | 2736800.00 | 16275000.00 | KO:K02358 | elongation factor Tu | Bacteria;Proteobacteria;Deltaproteobacteria;Syntrophobacterales;Syntrophaceae;Smithella;Smithella sp. SCADC;Smithella sp. SCADC |
| Ga0586324 0090903 2260 3450 | 0.00 | 1190000.00 | 0.00 | 0.00 | 0.00 | 0.00 | 9758400.00 | 3208600.00 | 33210000.00 | 13706000.00 | 16720000.00 | 35917500.00 | KO:K02358 | elongation factor Tu | Bacteria;Proteobacteria;Betaproteobacteria;Nitrosomonadales;Thiobacillaceae;Thiobacillus;Thiobacillus denitrificans;Thiobacillus denitrificans ATCC 25259 |
| Ga0586324 0096831 1803 2996 | 0.00 | 1135600.00 | 0.00 | 10640000.00 | 0.00 | 1932596.00 | 3438780.36 | 3855200.00 | 1530900.00 | 0.00 | 0.00 | 0.00 | KO:K02358 | elongation factor Tu | Bacteria;Proteobacteria;Deltaproteobacteria;Syntrophobacterales;Syntrophaceae;Desulfomonile;Desulfomonile tiedjei;Desulfomonile tiedjei DCB-1, DSM 6799 |
| Ga0586324 0099237 2339 3442 | 0.00 | 0.00 | 0.00 | 969000.00 | 0.00 | 0.00 | 0.00 | 0.00 | 0.00 | 0.00 | 0.00 | 0.00 | KO:K02358 | elongation factor Tu | Bacteria;Proteobacteria;Deltaproteobacteria;Syntrophobacterales;Syntrophaceae;Smithella;Smithella sp. SCADC;Smithella sp. SCADC |
| Ga0586324 0100004 2 1030 | 0.00 | 0.00 | 0.00 | 0.00 | 5211800.00 | 0.00 | 0.00 | 0.00 | 0.00 | 6514200.00 | 1249600.00 | 3015000.00 | KO:K02358 | elongation factor Tu | Bacteria;Proteobacteria;Deltaproteobacteria;Desulfarculales;Desulfarculaceae;Dethiosulfatarculus;Dethiosulfatarculus sandiegensis;Dethiosulfatarculus sandiegensis SPR |
| Ga0586324 0107466 2550 3314 | 0.00 | 1013200.00 | 295684.79 | 0.00 | 0.00 | 634624.35 | 0.00 | 0.00 | 0.00 | 0.00 | 0.00 | 129314.25 | KO:K02358 | elongation factor Tu | Bacteria;Proteobacteria;Deltaproteobacteria;Desulfobacterales;Desulfobulbaceae;Desulfobulbus;Desulfobulbus mediterraneus;Desulfobulbus mediterraneus DSM 13871 |
| Ga0586324 0109327 331 1521 | 0.00 | 979200.00 | 0.00 | 0.00 | 0.00 | 0.00 | 0.00 | 90575.85 | 0.00 | 0.00 | 0.00 | 0.00 | KO:K02358 | elongation factor Tu | Bacteria;Proteobacteria;Deltaproteobacteria;Myxococcales;Anaeromyxobacteraceae;Anaeromyxobacter;Anaeromyxobacter sp. Fw109-5;Anaeromyxobacter sp. Fw109-5 |
| Ga0586324 0111284 213 1406 | 3220000.00 | 0.00 | 0.00 | 0.00 | 0.00 | 0.00 | 0.00 | 0.00 | 0.00 | 0.00 | 0.00 | 0.00 | KO:K02358 | elongation factor Tu | Bacteria;Proteobacteria;Deltaproteobacteria;Syntrophobacterales;Syntrophobacteraceae;Desulfacinum;Desulfacinum infernum;Desulfacinum infernum DSM 9756 |
| Ga0586324 0113572 2468 3226 | 0.00 | 0.00 | 0.00 | 15675000.00 | 0.00 | 0.00 | 0.00 | 0.00 | 0.00 | 0.00 | 0.00 | 0.00 | KO:K02358 | elongation factor Tu | Bacteria;Proteobacteria;Deltaproteobacteria;Syntrophobacterales;Syntrophobacteraceae;Desulfacinum;Desulfacinum infernum;Desulfacinum infernum DSM 9756 |
| Ga0586324 0113865 2259 3224 | 0.00 | 0.00 | 0.00 | 0.00 | 1761300.00 | 0.00 | 0.00 | 2543700.00 | 2705400.00 | 2764300.00 | 1267200.00 | 2257500.00 | KO:K02358 | elongation factor Tu | Bacteria;Acidobacteria;Vicinamibacteria;unclassified Vicinamibacteria;Vicinamibacteraceae;Luteitalea;Luteitalea pratensis;Luteitalea pratensis DSM 100886 |
| Ga0586324 0131316 2325 3011 | 0.00 | 0.00 | 0.00 | 0.00 | 0.00 | 0.00 | 0.00 | 0.00 | 416309.22 | 0.00 | 0.00 | 0.00 | KO:K02358 | elongation factor Tu | Bacteria;Acidobacteria;Acidobacteriia;Bryobacterales;Solibacteraceae;Candidatus Solibacter;Candidatus Solibacter usitatus;Candidatus Solibacter usitatus Ellin6076 |
| Ga0586324 0132574 333 1526 | 0.00 | 2509200.00 | 0.00 | 0.00 | 0.00 | 0.00 | 0.00 | 0.00 | 0.00 | 0.00 | 1003200.00 | 0.00 | KO:K02358 | elongation factor Tu | Bacteria;Proteobacteria;Deltaproteobacteria;Myxococcales;Anaeromyxobacteraceae;Anaeromyxobacter;Anaeromyxobacter sp. Fw109-5;Anaeromyxobacter sp. Fw109-5 |
| Ga0586324 0153136 2400 2801 | 0.00 | 0.00 | 0.00 | 0.00 | 0.00 | 0.00 | 3534000.00 | 0.00 | 0.00 | 0.00 | 0.00 | 0.00 | KO:K02358 | elongation factor Tu | Bacteria;Proteobacteria;Betaproteobacteria;Burkholderiales;Comamonadaceae;Limnohabitans;Limnohabitans sp. Rim28;Limnohabitans sp. Rim28 |
| Ga0586324 0165287 683 1876 | 11700000.00 | 909100.84 | 14963256.36 | 2071000.00 | 2529925.14 | 251392.31 | 1762100.28 | 2181939.50 | 2848759.47 | 3028414.62 | 1575200.00 | 2411313.75 | KO:K02358 | elongation factor Tu | Bacteria;Proteobacteria;Gammaproteobacteria;Oceanospirillales;Alcanivoracaceae;Alcanivorax;Alcanivorax sp. EA2;Alcanivorax sp. EA2 |
| Ga0586324 0174598 3 1148 | 0.00 | 93808.04 | 446909.40 | 0.00 | 0.00 | 0.00 | 453341.52 | 182466.86 | 0.00 | 0.00 | 278931.84 | 213942.00 | KO:K02358 | elongation factor Tu | Bacteria;Proteobacteria;Deltaproteobacteria;unclassified Deltaproteobacteria;unclassified Deltaproteobacteria;unclassified Deltaproteobacteria;delta proteobacterium NaphS2;delta proteobacterium NaphS2 |
| Ga0586324 0195300 568 1758 | 0.00 | 0.00 | 0.00 | 0.00 | 0.00 | 0.00 | 592401.00 | 467815.71 | 0.00 | 0.00 | 0.00 | 0.00 | KO:K02358 | elongation factor Tu | Bacteria;Proteobacteria;Betaproteobacteria;unclassified Betaproteobacteria;unclassified Betaproteobacteria;unclassified Betaproteobacteria;Betaproteobacteria bacterium GR16-43;Betaproteobacteria bacterium GR16-43 |
| Ga0586324 0226515 210 1400 | 0.00 | 918000.00 | 0.00 | 0.00 | 0.00 | 0.00 | 0.00 | 0.00 | 238936.23 | 0.00 | 0.00 | 0.00 | KO:K02358 | elongation factor Tu | Bacteria;Proteobacteria;Alphaproteobacteria;Hyphomicrobiales;Xanthobacteraceae;Pseudolabrys;Pseudolabrys sp. Root1462;Pseudolabrys sp. Root1462 |
| Ga0586324 0244091 372 1562 | 0.00 | 0.00 | 0.00 | 0.00 | 0.00 | 0.00 | 9530400.00 | 0.00 | 0.00 | 0.00 | 0.00 | 0.00 | KO:K02358 | elongation factor Tu | Bacteria;Proteobacteria;Deltaproteobacteria;Desulfuromonadales;Geobacteraceae;Geobacter;Geobacter sp. DSM 2909;Geobacter sp. DSM 2909 |
| Ga0586324 0263949 954 2150 | 0.00 | 0.00 | 0.00 | 0.00 | 2266000.00 | 642822.40 | 3009600.00 | 2574200.00 | 2802600.00 | 0.00 | 0.00 | 433044.00 | KO:K02358 | elongation factor Tu | Bacteria;Acidobacteria;Acidobacteriia;Acidobacteriales;Acidobacteriaceae;Candidatus Koribacter;Candidatus Koribacter versatilis;Candidatus Koribacter versatilis Ellin345 |
| Ga0586324 0275321 1 1206 | 0.00 | 843200.00 | 37270600.00 | 861406.80 | 2327800.00 | 0.00 | 1079865.00 | 5923100.00 | 552612.78 | 230254.64 | 959200.00 | 765000.00 | KO:K02358 | elongation factor Tu | Bacteria;Proteobacteria;Deltaproteobacteria;Desulfobacterales;Desulfobacteraceae;Desulfatirhabdium;Desulfatirhabdium butyrativorans;Desulfatirhabdium butyrativorans DSM 18734 |
| Ga0586324 0303876 609 1808 | 0.00 | 0.00 | 0.00 | 0.00 | 4377500.00 | 0.00 | 0.00 | 0.00 | 0.00 | 0.00 | 0.00 | 0.00 | KO:K02358 | elongation factor Tu | Bacteria;Chloroflexi;Caldilineae;Caldilineales;Caldilineaceae;Caldilinea;Caldilinea aerophila;Caldilinea aerophila STL-6-O1, DSM 14535 |
| Ga0586324 0315005 804 1994 | 0.00 | 0.00 | 0.00 | 0.00 | 0.00 | 2700886.28 | 0.00 | 9760000.00 | 3199500.00 | 34804000.00 | 0.00 | 3667500.00 | KO:K02358 | elongation factor Tu | Bacteria;Proteobacteria;Betaproteobacteria;Nitrosomonadales;Nitrosomonadaceae;Nitrosospira;Nitrosospira lacus;Nitrosospira lacus APG3 |
| Ga0586324 0319557 431 1630 | 0.00 | 0.00 | 8050900.00 | 3410500.00 | 0.00 | 3970000.00 | 0.00 | 1653100.00 | 0.00 | 0.00 | 0.00 | 0.00 | KO:K02358 | elongation factor Tu | Bacteria;Proteobacteria;Deltaproteobacteria;Syntrophorhabdales;Syntrophorhabdaceae;Syntrophorhabdus;Syntrophorhabdus aromaticivorans;Syntrophorhabdus aromaticivorans UI |
| Ga0586324 0322626 1102 1989 | 0.00 | 958800.00 | 0.00 | 0.00 | 0.00 | 0.00 | 0.00 | 0.00 | 0.00 | 0.00 | 0.00 | 0.00 | KO:K02358 | elongation factor Tu | Bacteria;Firmicutes;Clostridia;Eubacteriales;Peptococcaceae;Desulfofundulus;Desulfofundulus australicus;Desulfofundulus australicus DSM 11792 |
| Ga0586324 0360215 3 818 | 0.00 | 0.00 | 7319000.00 | 2403500.00 | 2224800.00 | 444933.78 | 1239385.20 | 22643200.00 | 6439500.00 | 18788000.00 | 22352000.00 | 0.00 | KO:K02358 | elongation factor Tu | Bacteria;Proteobacteria;Gammaproteobacteria;Acidiferrobacterales;Acidiferrobacteraceae;Acidiferrobacter;Acidiferrobacter sp. SPIII 3;Acidiferrobacter sp. SPIII 3 |
| Ga0586324 0412238 1 771 | 0.00 | 0.00 | 421161.16 | 0.00 | 0.00 | 0.00 | 17350800.00 | 1500600.00 | 3321000.00 | 26740105.70 | 21278400.00 | 20400000.00 | KO:K02358 | elongation factor Tu | Bacteria;Proteobacteria;Betaproteobacteria;Nitrosomonadales;Sulfuricellaceae;Sulfuriferula;Sulfuriferula multivorans;Sulfuriferula multivorans TTN |
| Ga0586324 0420737 392 1582 | 0.00 | 0.00 | 0.00 | 0.00 | 0.00 | 0.00 | 1597616.52 | 0.00 | 0.00 | 0.00 | 0.00 | 0.00 | KO:K02358 | elongation factor Tu | Bacteria;Proteobacteria;Gammaproteobacteria;unclassified Gammaproteobacteria;Candidatus Competibacteraceae;Candidatus Competibacter;Candidatus Competibacter denitrificans;Candidatus Competibacter denitrificans Run A D11 |
| Ga0586324 0430907 627 1751 | 0.00 | 782000.00 | 0.00 | 0.00 | 0.00 | 0.00 | 0.00 | 0.00 | 1879200.00 | 4912600.00 | 0.00 | 0.00 | KO:K02358 | elongation factor Tu | Bacteria;Proteobacteria;Gammaproteobacteria;Chromatiales;Chromatiaceae;Thiorhodovibrio;Thiorhodovibrio sp. 970;Thiorhodovibrio sp. 970 |
| Ga0586324 0462246 472 1662 | 0.00 | 0.00 | 0.00 | 0.00 | 0.00 | 0.00 | 0.00 | 0.00 | 0.00 | 0.00 | 0.00 | 71590.73 | KO:K02358 | elongation factor Tu | Bacteria;Proteobacteria;Gammaproteobacteria;Nevskiales;Steroidobacteraceae;Povalibacter;Povalibacter uvarum;Povalibacter uvarum DSM 26723 |
| Ga0586324 0474890 1 849 | 0.00 | 0.00 | 0.00 | 0.00 | 0.00 | 0.00 | 848907.84 | 561073.12 | 0.00 | 2387000.00 | 1020800.00 | 1020000.00 | KO:K02358 | elongation factor Tu | Bacteria;Proteobacteria;Betaproteobacteria;Nitrosomonadales;Nitrosomonadaceae;Nitrosovibrio;Nitrosovibrio tenuis;Nitrosovibrio tenuis Nv1 |
| Ga0586324 0553052 76 1299 | 0.00 | 0.00 | 0.00 | 0.00 | 0.00 | 0.00 | 1211687.76 | 0.00 | 6058800.00 | 0.00 | 0.00 | 0.00 | KO:K02358 | elongation factor Tu | Bacteria;Proteobacteria;Gammaproteobacteria;Cellvibrionales;Halieaceae;Halioglobus;Halioglobus pacificus;Halioglobus pacificus RR3-57 |
| Ga0586324 0557875 124 1380 | 9560000.00 | 0.00 | 0.00 | 0.00 | 0.00 | 0.00 | 3716400.00 | 1982500.00 | 0.00 | 3003000.00 | 2103336.40 | 2167500.00 | KO:K02358 | elongation factor Tu | Bacteria;Proteobacteria;Deltaproteobacteria;Myxococcales;Kofleriaceae;Haliangium;Haliangium ochraceum;Haliangium ochraceum SMP-2, DSM 14365 |
| Ga0586324 0571466 676 1545 | 396960.00 | 0.00 | 0.00 | 0.00 | 0.00 | 8932500.00 | 0.00 | 0.00 | 0.00 | 2541000.00 | 2261600.00 | 0.00 | KO:K02358 | elongation factor Tu | Bacteria;Proteobacteria;Deltaproteobacteria;Desulfobacterales;Desulfobacteraceae;Desulfatitalea;Desulfatitalea tepidiphila;Desulfatitalea tepidiphila S28bF |
| Ga0586324 0607632 3 1268 | 0.00 | 0.00 | 0.00 | 0.00 | 0.00 | 0.00 | 0.00 | 0.00 | 0.00 | 1239700.00 | 613202.48 | 522109.50 | KO:K02358 | elongation factor Tu | Bacteria;Proteobacteria;Deltaproteobacteria;Myxococcales;Kofleriaceae;Haliangium;Haliangium ochraceum;Haliangium ochraceum SMP-2, DSM 14365 |
| Ga0586324 0616387 3 431 | 324839.00 | 0.00 | 0.00 | 0.00 | 0.00 | 0.00 | 0.00 | 0.00 | 1360800.00 | 0.00 | 0.00 | 0.00 | KO:K02358 | elongation factor Tu | Bacteria;Acidobacteria;Blastocatellia;unclassified Blastocatellia;unclassified Blastocatellia;Chloracidobacterium;Chloracidobacterium thermophilum;Chloracidobacterium thermophilum OC1 |
| Ga0586324 0621617 730 1491 | 5720000.00 | 6405600.00 | 0.00 | 0.00 | 0.00 | 0.00 | 0.00 | 1884900.00 | 0.00 | 3711400.00 | 2191200.00 | 0.00 | KO:K02358 | elongation factor Tu | Bacteria;Proteobacteria;Deltaproteobacteria;Syntrophorhabdales;Syntrophorhabdaceae;Syntrophorhabdus;Syntrophorhabdus aromaticivorans;Syntrophorhabdus aromaticivorans UI |
| Ga0586324 0641539 1 966 | 0.00 | 0.00 | 0.00 | 0.00 | 0.00 | 0.00 | 0.00 | 388551.09 | 0.00 | 0.00 | 0.00 | 0.00 | KO:K02358 | elongation factor Tu | Bacteria;Proteobacteria;Deltaproteobacteria;Syntrophorhabdales;Syntrophorhabdaceae;Syntrophorhabdus;Syntrophorhabdus aromaticivorans;Syntrophorhabdus aromaticivorans UI |
| Ga0586324 0665990 424 1446 | 38400000.00 | 22848000.00 | 0.00 | 12350000.00 | 3234200.00 | 38231100.00 | 19106400.00 | 2159400.00 | 2721600.00 | 3426500.00 | 5227200.00 | 2625000.00 | KO:K02358 | elongation factor Tu | Bacteria;Acidobacteria;Vicinamibacteria;unclassified Vicinamibacteria;Vicinamibacteraceae;Luteitalea;Luteitalea pratensis;Luteitalea pratensis DSM 100886 |
| Ga0586324 0666247 475 1446 | 1910000.00 | 0.00 | 26573600.00 | 1586500.00 | 0.00 | 13061300.00 | 16416000.00 | 0.00 | 0.00 | 0.00 | 0.00 | 12075000.00 | KO:K02358 | elongation factor Tu | Bacteria;Proteobacteria;Deltaproteobacteria;Syntrophobacterales;Syntrophaceae;Smithella;Smithella sp. SCADC;Smithella sp. SCADC |
| Ga0586324 0684112 844 1431 | 0.00 | 0.00 | 0.00 | 0.00 | 0.00 | 0.00 | 0.00 | 0.00 | 0.00 | 0.00 | 216627.84 | 0.00 | KO:K02358 | elongation factor Tu | Bacteria;Acidobacteria;Acidobacteriia;Bryobacterales;Solibacteraceae;Candidatus Solibacter;Candidatus Solibacter usitatus;Candidatus Solibacter usitatus Ellin6076 |
| Ga0586324 0710007 2 1084 | 1890000.00 | 0.00 | 0.00 | 0.00 | 0.00 | 0.00 | 0.00 | 0.00 | 0.00 | 0.00 | 0.00 | 0.00 | KO:K02358 | elongation factor Tu | Bacteria;Proteobacteria;Gammaproteobacteria;Chromatiales;Chromatiaceae;Thiorhodovibrio;Thiorhodovibrio sp. 970;Thiorhodovibrio sp. 970 |
| Ga0586324 0723957 210 1394 | 35100000.00 | 23800000.00 | 56300000.00 | 32015000.00 | 37492000.00 | 43670000.00 | 45600000.00 | 32513000.00 | 31185000.00 | 36806000.00 | 40040000.00 | 36225000.00 | KO:K02358 | elongation factor Tu | Bacteria;Proteobacteria;Deltaproteobacteria;Desulfobacterales;Desulfobacteraceae;Desulfatirhabdium;Desulfatirhabdium butyrativorans;Desulfatirhabdium butyrativorans DSM 18734 |
| Ga0586324 0734915 1 972 | 0.00 | 0.00 | 0.00 | 0.00 | 0.00 | 0.00 | 0.00 | 0.00 | 0.00 | 0.00 | 2420000.00 | 0.00 | KO:K02358 | elongation factor Tu | Bacteria;Bacteroidetes;Bacteroidia;Marinilabiliales;Prolixibacteraceae;Sunxiuqinia;Sunxiuqinia dokdonensis;Sunxiuqinia dokdonensis SK |
| Ga0586324 0758457 91 1299 | 0.00 | 165992.76 | 0.00 | 151958.20 | 125138.82 | 0.00 | 0.00 | 0.00 | 0.00 | 0.00 | 1126400.00 | 0.00 | KO:K02358 | elongation factor Tu | Bacteria;Proteobacteria;Deltaproteobacteria;Syntrophobacterales;Syntrophaceae;Desulfobacca;Desulfobacca acetoxidans;Desulfobacca acetoxidans ASRB2, DSM 11109 |
| Ga0586324 0768978 3 1124 | 0.00 | 0.00 | 0.00 | 0.00 | 0.00 | 0.00 | 0.00 | 0.00 | 0.00 | 0.00 | 0.00 | 2122500.00 | KO:K02358 | elongation factor Tu | Bacteria;Acidobacteria;Vicinamibacteria;unclassified Vicinamibacteria;Vicinamibacteraceae;Luteitalea;Luteitalea pratensis;Luteitalea pratensis DSM 100886 |
| Ga0586324 0776234 145 1344 | 0.00 | 0.00 | 0.00 | 1757500.00 | 0.00 | 0.00 | 0.00 | 0.00 | 0.00 | 0.00 | 0.00 | 0.00 | KO:K02358 | elongation factor Tu | Bacteria;Nitrospirae;Nitrospira;Nitrospirales;Nitrospiraceae;Candidatus Magnetoovum;Candidatus Magnetoovum chiemensis;Candidatus Magnetoovum chiemensis CS-04 |
| Ga0586324 0793950 2 1117 | 0.00 | 0.00 | 0.00 | 120690.85 | 0.00 | 0.00 | 166850.40 | 69268.55 | 104340.96 | 0.00 | 0.00 | 109029.00 | KO:K02358 | elongation factor Tu | Bacteria;Proteobacteria;Deltaproteobacteria;Syntrophobacterales;Syntrophaceae;Desulfobacca;Desulfobacca acetoxidans;Desulfobacca acetoxidans ASRB2, DSM 11109 |
| Ga0586324 0809346 3 854 | 0.00 | 0.00 | 0.00 | 0.00 | 0.00 | 0.00 | 5198400.00 | 0.00 | 0.00 | 0.00 | 0.00 | 0.00 | KO:K02358 | elongation factor Tu | Bacteria;Bacteroidetes;Chitinophagia;Chitinophagales;Chitinophagaceae;Ferruginibacter;Ferruginibacter sp. BO-59;Ferruginibacter sp. BO-59 |
| Ga0586324 0817487 3 1190 | 0.00 | 0.00 | 0.00 | 0.00 | 0.00 | 0.00 | 0.00 | 0.00 | 915300.00 | 0.00 | 0.00 | 1290000.00 | KO:K02358 | elongation factor Tu | Bacteria;Proteobacteria;Betaproteobacteria;Nitrosomonadales;Nitrosomonadaceae;Nitrosospira;Nitrosospira lacus;Nitrosospira lacus APG3 |
| Ga0586324 0820679 491 1321 | 0.00 | 0.00 | 0.00 | 0.00 | 311062.06 | 0.00 | 0.00 | 0.00 | 0.00 | 8624000.00 | 8976000.00 | 0.00 | KO:K02358 | elongation factor Tu | Bacteria;Chloroflexi;Anaerolineae;Anaerolineales;Anaerolineaceae;Anaerolinea;Anaerolinea thermophila;Anaerolinea thermophila UNI-1 |
| Ga0586324 0829184 118 1317 | 0.00 | 0.00 | 0.00 | 0.00 | 0.00 | 0.00 | 250239.12 | 1128500.00 | 2867400.00 | 0.00 | 0.00 | 10125000.00 | KO:K02358 | elongation factor Tu | Bacteria;Chloroflexi;Anaerolineae;Anaerolineales;Anaerolineaceae;Anaerolinea;Anaerolinea thermophila;Anaerolinea thermophila UNI-1 |
| Ga0586324 0841095 538 1308 | 0.00 | 0.00 | 0.00 | 6773500.00 | 5356000.00 | 0.00 | 0.00 | 1433500.00 | 0.00 | 0.00 | 0.00 | 0.00 | KO:K02358 | elongation factor Tu | Bacteria;Bacteroidetes;Bacteroidia;Marinilabiliales;Marinilabiliaceae;Anaerophaga;Anaerophaga thermohalophila;Anaerophaga thermohalophila Fru22, DSM 12881 |
| Ga0586324 0849243 619 1302 | 0.00 | 0.00 | 0.00 | 0.00 | 0.00 | 0.00 | 0.00 | 2074000.00 | 0.00 | 4173400.00 | 0.00 | 2572500.00 | KO:K02358 | elongation factor Tu | Bacteria;Planctomycetes;unclassified Planctomycetes;unclassified Planctomycetes;unclassified Planctomycetes;unclassified Planctomycetes;unclassified Planctomycetes;Planctomycetes bacterium P3 |
| Ga0586324 0877283 1 873 | 0.00 | 0.00 | 0.00 | 0.00 | 0.00 | 0.00 | 571586.88 | 0.00 | 0.00 | 0.00 | 0.00 | 0.00 | KO:K02358 | elongation factor Tu | Bacteria;Thermodesulfobacteria;Thermodesulfobacteria;Thermodesulfobacteriales;Thermodesulfobacteriaceae;Thermodesulfatator;Thermodesulfatator indicus;Thermodesulfatator indicus CIR29812, DSM 15286 |
| Ga0586324 0946853 37 1227 | 0.00 | 0.00 | 0.00 | 0.00 | 0.00 | 0.00 | 8322000.00 | 0.00 | 0.00 | 0.00 | 0.00 | 0.00 | KO:K02358 | elongation factor Tu | Bacteria;Proteobacteria;Gammaproteobacteria;Methylococcales;Methylococcaceae;Methylovulum;Methylovulum miyakonense;Methylovulum miyakonense HT12 |
| Ga0586324 0958063 22 1212 | 0.00 | 0.00 | 1252928.35 | 0.00 | 0.00 | 0.00 | 0.00 | 0.00 | 0.00 | 0.00 | 0.00 | 219819.75 | KO:K02358 | elongation factor Tu | Bacteria;Proteobacteria;Betaproteobacteria;Nitrosomonadales;Thiobacillaceae;Annwoodia;Annwoodia aquaesulis;Annwoodia aquaesulis DSM 4255 |
| Ga0586324 0966178 3 485 | 4810000.00 | 4032400.00 | 0.00 | 0.00 | 0.00 | 0.00 | 0.00 | 5343600.00 | 2794500.00 | 0.00 | 0.00 | 0.00 | KO:K02358 | elongation factor Tu | Bacteria;Proteobacteria;Betaproteobacteria;Burkholderiales;unclassified Burkholderiales;Aquabacterium;Aquabacterium parvum;Aquabacterium parvum B6 |
| Ga0586324 0973159 2 796 | 0.00 | 0.00 | 0.00 | 0.00 | 0.00 | 0.00 | 4354800.00 | 0.00 | 0.00 | 0.00 | 0.00 | 0.00 | KO:K02358 | elongation factor Tu | Bacteria;Chloroflexi;Anaerolineae;Anaerolineales;Anaerolineaceae;Anaerolinea;Anaerolinea thermophila;Anaerolinea thermophila UNI-1 |
| Ga0586324 0975914 339 1226 | 0.00 | 0.00 | 0.00 | 1928500.00 | 1359600.00 | 0.00 | 0.00 | 0.00 | 0.00 | 0.00 | 0.00 | 0.00 | KO:K02358 | elongation factor Tu | Bacteria;Proteobacteria;Deltaproteobacteria;Syntrophobacterales;Syntrophaceae;Syntrophus;Syntrophus aciditrophicus;Syntrophus aciditrophicus SB |
| Ga0586324 1046966 3 1148 | 1390000.00 | 24888000.00 | 0.00 | 0.00 | 0.00 | 0.00 | 0.00 | 0.00 | 0.00 | 0.00 | 0.00 | 0.00 | KO:K02358 | elongation factor Tu | Bacteria;Proteobacteria;Gammaproteobacteria;Chromatiales;Ectothiorhodospiraceae;Thioalkalivibrio;Thioalkalivibrio nitratireducens;Thioalkalivibrio nitratireducens DSM 14787 |
| Ga0586324 1061670 75 1184 | 25800000.00 | 4865743.40 | 0.00 | 20140000.00 | 0.00 | 0.00 | 226856.81 | 0.00 | 0.00 | 0.00 | 0.00 | 0.00 | KO:K02358 | elongation factor Tu | Bacteria;Nitrospirae;Nitrospira;Nitrospirales;Nitrospiraceae;Nitrospira;Nitrospira sp. ND1;Nitrospira sp. ND1 |
| Ga0586324 1088278 143 1171 | 0.00 | 0.00 | 0.00 | 294231.15 | 0.00 | 0.00 | 0.00 | 0.00 | 2656800.00 | 0.00 | 0.00 | 0.00 | KO:K02358 | elongation factor Tu | Bacteria;Proteobacteria;Deltaproteobacteria;unclassified Deltaproteobacteria;unclassified Deltaproteobacteria;Deferrisoma;Deferrisoma camini;Deferrisoma camini S3R1 |
| Ga0586324 1124674 289 1155 | 0.00 | 1428000.00 | 0.00 | 0.00 | 4768900.00 | 0.00 | 0.00 | 1116300.00 | 1077300.00 | 1740200.00 | 1645600.00 | 1402500.00 | KO:K02358 | elongation factor Tu | Bacteria;Proteobacteria;Gammaproteobacteria;unclassified Gammaproteobacteria;Candidatus Competibacteraceae;Plasticicumulans;Plasticicumulans acidivorans;Plasticicumulans acidivorans DSM 23606 |
| Ga0586324 1173361 264 1133 | 0.00 | 0.00 | 0.00 | 0.00 | 0.00 | 0.00 | 14409600.00 | 0.00 | 0.00 | 0.00 | 0.00 | 0.00 | KO:K02358 | elongation factor Tu | Bacteria;Proteobacteria;Alphaproteobacteria;Hyphomicrobiales;Xanthobacteraceae;Pseudolabrys;Pseudolabrys taiwanensis;Pseudolabrys taiwanensis CC-BB4 |
| Ga0586324 1183476 2 379 | 0.00 | 0.00 | 0.00 | 0.00 | 0.00 | 0.00 | 2394000.00 | 0.00 | 2608200.00 | 0.00 | 2393600.00 | 0.00 | KO:K02358 | elongation factor Tu | Bacteria;Proteobacteria;Alphaproteobacteria;Rhodospirillales;unclassified Rhodospirillales;unclassified Rhodospirillales;Rhodospirillales bacterium URHD0017;Rhodospirillales bacterium URHD0017 |
| Ga0586324 1197804 24 1124 | 2010000.00 | 2196400.00 | 0.00 | 9329000.00 | 9383300.00 | 0.00 | 19516800.00 | 14588071.92 | 168885.81 | 16478000.00 | 29128000.00 | 16125000.00 | KO:K02358 | elongation factor Tu | Bacteria;Proteobacteria;Deltaproteobacteria;Syntrophobacterales;Syntrophobacteraceae;Syntrophobacter;Syntrophobacter fumaroxidans;Syntrophobacter fumaroxidans MPOB |
| Ga0586324 1203313 3 917 | 0.00 | 1652400.00 | 0.00 | 0.00 | 0.00 | 0.00 | 0.00 | 0.00 | 0.00 | 0.00 | 0.00 | 0.00 | KO:K02358 | elongation factor Tu | Bacteria;Nitrospirae;Thermodesulfovibrionia;Thermodesulfovibrionales;Thermodesulfovibrionaceae;Thermodesulfovibrio;Thermodesulfovibrio thiophilus;Thermodesulfovibrio thiophilus DSM 17215 |
| Ga0586324 1220788 464 1114 | 0.00 | 131376.68 | 0.00 | 0.00 | 0.00 | 0.00 | 0.00 | 0.00 | 0.00 | 0.00 | 0.00 | 0.00 | KO:K02358 | elongation factor Tu | Bacteria;Proteobacteria;Deltaproteobacteria;Syntrophobacterales;Syntrophaceae;Smithella;Smithella sp. SCADC;Smithella sp. SCADC |
| Ga0586324 1255136 3 998 | 0.00 | 0.00 | 0.00 | 0.00 | 0.00 | 0.00 | 0.00 | 19764000.00 | 0.00 | 0.00 | 0.00 | 0.00 | KO:K02358 | elongation factor Tu | Bacteria;Proteobacteria;Gammaproteobacteria;Xanthomonadales;Rhodanobacteraceae;Dyella;Dyella sp.;Dyella sp. HyOG |
| Ga0586324 1296494 2 466 | 0.00 | 0.00 | 0.00 | 0.00 | 0.00 | 2579074.77 | 0.00 | 0.00 | 0.00 | 0.00 | 0.00 | 0.00 | KO:K02358 | elongation factor Tu | Bacteria;Proteobacteria;Deltaproteobacteria;Syntrophobacterales;Syntrophobacteraceae;Syntrophobacter;Syntrophobacter fumaroxidans;Syntrophobacter fumaroxidans MPOB |
| Ga0586324 1306920 1 918 | 0.00 | 292447.60 | 0.00 | 0.00 | 0.00 | 0.00 | 387862.20 | 0.00 | 0.00 | 0.00 | 0.00 | 0.00 | KO:K02358 | elongation factor Tu | Bacteria;Planctomycetes;Planctomycetia;Planctomycetales;Planctomycetaceae;Planctomyces;Planctomyces sp. SH-PL62;Planctomyces sp. SH-PL62 |
| Ga0586324 1330242 3 1061 | 0.00 | 788800.00 | 2797271.13 | 917681.00 | 1025405.17 | 782804.60 | 1825356.60 | 1476200.00 | 1482300.00 | 1393700.00 | 1944800.00 | 1425000.00 | KO:K02358 | elongation factor Tu | Bacteria;Proteobacteria;Betaproteobacteria;Burkholderiales;unclassified Burkholderiales;Methylibium;Methylibium petroleiphilum;Methylibium petroleiphilum PM1 |
| Ga0586324 1359412 3 1049 | 0.00 | 0.00 | 0.00 | 1890500.00 | 0.00 | 0.00 | 0.00 | 0.00 | 0.00 | 0.00 | 0.00 | 0.00 | KO:K02358 | elongation factor Tu | Bacteria;Proteobacteria;Alphaproteobacteria;Rhodospirillales;Elioraeaceae;Elioraea;Elioraea tepidiphila;Elioraea tepidiphila DSM 17972 |
| Ga0586324 1361116 161 1063 | 0.00 | 0.00 | 0.00 | 0.00 | 0.00 | 0.00 | 657791.40 | 0.00 | 1385100.00 | 0.00 | 0.00 | 0.00 | KO:K02358 | elongation factor Tu | Bacteria;Proteobacteria;Gammaproteobacteria;Oceanospirillales;Alcanivoracaceae;Ketobacter;Ketobacter alkanivorans;Ketobacter alkanivorans GI5 |
| Ga0586324 1373314 451 1059 | 0.00 | 0.00 | 0.00 | 0.00 | 0.00 | 0.00 | 0.00 | 0.00 | 0.00 | 0.00 | 0.00 | 333486.75 | KO:K02358 | elongation factor Tu | Bacteria;Proteobacteria;Deltaproteobacteria;Desulfobacterales;Desulfobacteraceae;Desulfatitalea;Desulfatitalea tepidiphila;Desulfatitalea tepidiphila S28bF |
| Ga0586324 1377567 203 1057 | 0.00 | 0.00 | 0.00 | 0.00 | 0.00 | 0.00 | 20041200.00 | 0.00 | 0.00 | 0.00 | 0.00 | 0.00 | KO:K02358 | elongation factor Tu | Bacteria;Proteobacteria;Deltaproteobacteria;Desulfuromonadales;Geobacteraceae;Geobacter;Geobacter uraniireducens;Geobacter uraniireducens Rf4 |
| Ga0586324 1379963 3 752 | 8230000.00 | 0.00 | 6418200.00 | 0.00 | 4367200.00 | 0.00 | 6224400.00 | 0.00 | 0.00 | 0.00 | 0.00 | 0.00 | KO:K02358 | elongation factor Tu | Bacteria;Proteobacteria;Deltaproteobacteria;Syntrophobacterales;Syntrophaceae;Smithella;Smithella sp. SCADC;Smithella sp. SCADC |
| Ga0586324 1392737 2 1054 | 7780000.00 | 0.00 | 0.00 | 0.00 | 0.00 | 0.00 | 0.00 | 0.00 | 0.00 | 0.00 | 985600.00 | 0.00 | KO:K02358 | elongation factor Tu | Bacteria;Nitrospirae;Nitrospira;Nitrospirales;Nitrospiraceae;Nitrospira;Nitrospira lenta;Nitrospira lenta N. lenta BS10 |

| | | | | | | | | | | | | | | | |
|---|---|---|---|---|---|---|---|---|---|---|---|---|---|---|---|
| Ga0586324 1409165 164 1048 | 418166.00 | 0.00 | 0.00 | 0.00 | 0.00 | 0.00 | 0.00 | 0.00 | 0.00 | 0.00 | 0.00 | 0.00 | KO:K02358 | elongation factor Tu | Bacteria;Chloroflexi;Ardenticatenia;Ardenticatenales;Ardenticatenaceae;Candidatus Promineofilum;Candidatus Promineofilum breve;Candidatus Promineofilum breve Cfx-K |
| Ga0586324 1413137 1 828 | 0.00 | 0.00 | 0.00 | 12445000.00 | 0.00 | 0.00 | 0.00 | 0.00 | 0.00 | 0.00 | 0.00 | 0.00 | KO:K02358 | elongation factor Tu | Bacteria;Firmicutes;Clostridia;Eubacteriales;Peptococcaceae;Desulfurispora;Desulfurispora thermophila;Desulfurispora thermophila DSM 16022 |
| Ga0586324 1440916 1 1038 | 533325.00 | 20196000.00 | 35469000.00 | 1111500.00 | 52942000.00 | 18341400.00 | 49476000.00 | 38918000.00 | 27297000.00 | 30954000.00 | 25432000.00 | 378866.25 | KO:K02358 | elongation factor Tu | Bacteria;Proteobacteria;Deltaproteobacteria;Desulfobacterales;Desulfobacteraceae;Desulfatirhabdium;Desulfatirhabdium butyrativorans;Desulfatirhabdium butyrativorans DSM 18734 |
| Ga0586324 1475361 35 1027 | 0.00 | 0.00 | 0.00 | 0.00 | 0.00 | 0.00 | 0.00 | 0.00 | 0.00 | 0.00 | 638945.12 | 0.00 | KO:K02358 | elongation factor Tu | Bacteria;Proteobacteria;Betaproteobacteria;Nitrosomonadales;Thiobacillaceae;Thiobacillus;Thiobacillus denitrificans;Thiobacillus denitrificans DSM 12475 |
| Ga0586324 1495606 3 1010 | 5970000.00 | 0.00 | 0.00 | 7932500.00 | 8219400.00 | 0.00 | 5312400.00 | 0.00 | 0.00 | 13090000.00 | 11352000.00 | 0.00 | KO:K02358 | elongation factor Tu | Bacteria;Nitrospirae;Thermodesulfovibrionia;Thermodesulfovibrionales;Thermodesulfovibrionaceae;Thermodesulfovibrio;Thermodesulfovibrio sp. N1;Thermodesulfovibrio sp. N1 |
| Ga0586324 1507400 2 850 | 0.00 | 1475600.00 | 0.00 | 0.00 | 0.00 | 0.00 | 0.00 | 0.00 | 0.00 | 0.00 | 0.00 | 0.00 | KO:K02358 | elongation factor Tu | Bacteria;Proteobacteria;Deltaproteobacteria;Desulfarculales;Desulfarculaceae;Desulfocarbo;Desulfocarbo indianensis;Desulfocarbo indianensis SCBM |
| Ga0586324 1578087 3 515 | 9000000.00 | 4862000.00 | 1940154.30 | 0.00 | 2266000.00 | 499973.86 | 1951151.04 | 1012600.00 | 0.00 | 2579500.00 | 1566400.00 | 2130000.00 | KO:K02358 | elongation factor Tu | Bacteria;Planctomycetes;Phycisphaerae;Sedimentisphaerales;Anaerohalosphaeraceae;Anaerohalosphaera;Anaerohalosphaera lusitana;Phycisphaerae bacterium ST-NAGAB-D1 |
| Ga0586324 1582051 2 997 | 2240000.00 | 2359600.00 | 2143994.08 | 0.00 | 0.00 | 1034252.49 | 0.00 | 582761.06 | 2146500.00 | 1332100.00 | 0.00 | 526178.25 | KO:K02358 | elongation factor Tu | Bacteria;Proteobacteria;Deltaproteobacteria;Myxococcales;Polyangiaceae;Sorangium;Sorangium cellulosum;Sorangium cellulosum So ceGT47 |
| Ga0586324 1700817 227 967 | 0.00 | 252392.88 | 0.00 | 0.00 | 0.00 | 0.00 | 0.00 | 0.00 | 601685.82 | 0.00 | 0.00 | 0.00 | KO:K02358 | elongation factor Tu | Bacteria;Proteobacteria;Acidithiobacillia;Acidithiobacillales;Acidithiobacillaceae;Acidithiobacillus;Acidithiobacillus ferrooxidans;Acidithiobacillus ferrooxidans ATCC 53993 |
| Ga0586324 1745882 1 957 | 0.00 | 0.00 | 0.00 | 0.00 | 0.00 | 0.00 | 0.00 | 0.00 | 4495500.00 | 0.00 | 0.00 | 0.00 | KO:K02358 | elongation factor Tu | Bacteria;Proteobacteria;Deltaproteobacteria;Desulfarculales;Desulfarculaceae;Desulfocarbo;Desulfocarbo indianensis;Desulfocarbo indianensis SCBM |
| Ga0586324 1786871 688 948 | 0.00 | 0.00 | 0.00 | 0.00 | 0.00 | 0.00 | 0.00 | 0.00 | 0.00 | 0.00 | 0.00 | 5535000.00 | KO:K02358 | elongation factor Tu | Bacteria;Firmicutes;Clostridia;Eubacteriales;Peptostreptococcaceae;Tepidibacter;Tepidibacter mesophilus;Tepidibacter mesophilus JCM 16806 |
| Ga0586324 1838165 3 488 | 0.00 | 0.00 | 0.00 | 0.00 | 0.00 | 0.00 | 2918400.00 | 0.00 | 0.00 | 0.00 | 0.00 | 0.00 | KO:K02358 | elongation factor Tu | Bacteria;Chloroflexi;Anaerolineae;Anaerolineales;Anaerolineaceae;Anaerolinea;Anaerolinea thermophila;Anaerolinea thermophila UNI-1 |
| Ga0586324 1881162 3 926 | 0.00 | 0.00 | 0.00 | 0.00 | 0.00 | 0.00 | 0.00 | 192303.72 | 0.00 | 0.00 | 0.00 | 0.00 | KO:K02358 | elongation factor Tu | Bacteria;Proteobacteria;Betaproteobacteria;unclassified Betaproteobacteria;unclassified Betaproteobacteria;unclassified Betaproteobacteria;Betaproteobacteria bacterium GR16-43;Betaproteobacteria bacterium GR16-43 |
| Ga0586324 1901859 182 922 | 0.00 | 0.00 | 0.00 | 0.00 | 0.00 | 0.00 | 0.00 | 0.00 | 0.00 | 0.00 | 5271200.00 | 0.00 | KO:K02358 | elongation factor Tu | Bacteria;Acidobacteria;Acidobacteriia;Bryobacterales;Solibacteraceae;Candidatus Solibacter;Candidatus Solibacter usitatus;Candidatus Solibacter usitatus Ellin6076 |
| Ga0586324 1977198 1 819 | 0.00 | 0.00 | 0.00 | 0.00 | 0.00 | 0.00 | 0.00 | 0.00 | 0.00 | 0.00 | 0.00 | 311955.75 | KO:K02358 | elongation factor Tu | Bacteria;Proteobacteria;Deltaproteobacteria;Syntrophobacterales;Syntrophaceae;Desulfomonile;Desulfomonile tiedjei;Desulfomonile tiedjei DCB-1, DSM 6799 |
| Ga0586324 2015636 2 514 | 2670000.00 | 0.00 | 0.00 | 0.00 | 0.00 | 0.00 | 0.00 | 0.00 | 0.00 | 0.00 | 0.00 | 0.00 | KO:K02358 | elongation factor Tu | Bacteria;Proteobacteria;Deltaproteobacteria;Myxococcales;Sandaracinaceae;Sandaracinus;Sandaracinus amylolyticus;Sandaracinus amylolyticus DSM 53668 |
| Ga0586324 2060391 3 893 | 0.00 | 136202.64 | 168576.28 | 231949.15 | 189005.00 | 153939.53 | 426834.24 | 1531100.00 | 1004400.00 | 124740.00 | 197403.36 | 38243.40 | KO:K02358 | elongation factor Tu | Bacteria;Proteobacteria;Deltaproteobacteria;Desulfobacterales;Desulfobacteraceae;Desulfatirhabdium;Desulfatirhabdium butyrativorans;Desulfatirhabdium butyrativorans DSM 18734 |
| Ga0586324 2166573 1 873 | 0.00 | 42187200.00 | 12821182.11 | 3885500.00 | 12051000.00 | 18215051.18 | 4199153.52 | 2781600.00 | 4511700.00 | 83160000.00 | 18057600.00 | 5670000.00 | KO:K02358 | elongation factor Tu | Bacteria;Proteobacteria;Deltaproteobacteria;Desulfuromonadales;Syntrophotaleaceae;Syntrophotalea;Syntrophotalea carbinolica;Syntrophotalea carbinolica DSM 2380 |
| Ga0586324 2168160 3 755 | 0.00 | 0.00 | 0.00 | 2498500.00 | 0.00 | 0.00 | 775154.40 | 0.00 | 0.00 | 0.00 | 0.00 | 0.00 | KO:K02358 | elongation factor Tu | Bacteria;Proteobacteria;Deltaproteobacteria;Syntrophobacterales;Syntrophobacteraceae;Syntrophobacter;Syntrophobacter fumaroxidans;Syntrophobacter fumaroxidans MPOB |
| Ga0586324 2204829 3 830 | 0.00 | 0.00 | 0.00 | 0.00 | 3512300.00 | 0.00 | 569398.08 | 0.00 | 0.00 | 0.00 | 0.00 | 0.00 | KO:K02358 | elongation factor Tu | Bacteria;Nitrospirae;Thermodesulfovibrionia;Thermodesulfovibrionales;Thermodesulfovibrionaceae;Thermodesulfovibrio;Thermodesulfovibrio sp. N1;Thermodesulfovibrio sp. N1 |
| Ga0586324 2307868 1 849 | 0.00 | 0.00 | 0.00 | 1776500.00 | 1833400.00 | 0.00 | 7273200.00 | 1982500.00 | 1198800.00 | 6260100.00 | 0.00 | 0.00 | KO:K02358 | elongation factor Tu | Bacteria;Proteobacteria;Betaproteobacteria;Rhodocyclales;Azonexaceae;Dechloromonas;Dechloromonas sp. HYN0024;Dechloromonas sp. HYN0024 |
| Ga0586324 2352092 2 703 | 1790000.00 | 0.00 | 0.00 | 0.00 | 0.00 | 0.00 | 0.00 | 1775100.00 | 765284.76 | 3708228.37 | 143484.88 | 765000.00 | KO:K02358 | elongation factor Tu | Bacteria;Proteobacteria;Gammaproteobacteria;Oceanospirillales;Alcanivoracaceae;Ketobacter;Ketobacter alkanivorans;Ketobacter alkanivorans GI5 |
| Ga0586324 2357244 2 724 | 0.00 | 0.00 | 0.00 | 0.00 | 0.00 | 0.00 | 0.00 | 0.00 | 501334.92 | 0.00 | 3106400.00 | 0.00 | KO:K02358 | elongation factor Tu | Bacteria;Ignavibacteriae;Ignavibacteria;Ignavibacteriales;Melioribacteraceae;Melioribacter;Melioribacter roseus;Melioribacter roseus P3M |
| Ga0586324 2378363 64 840 | 0.00 | 0.00 | 0.00 | 0.00 | 812214.74 | 0.00 | 0.00 | 0.00 | 0.00 | 0.00 | 0.00 | 0.00 | KO:K02358 | elongation factor Tu | Bacteria;Actinobacteria;Rubrobacteria;Gaiellales;Gaiellaceae;Gaiella;Gaiella occulta;Gaiella occulta F2-233 |
| Ga0586324 2388044 2 754 | 24900000.00 | 0.00 | 0.00 | 0.00 | 0.00 | 0.00 | 0.00 | 0.00 | 23409000.00 | 0.00 | 0.00 | 0.00 | KO:K02358 | elongation factor Tu | Bacteria;Proteobacteria;Deltaproteobacteria;Desulfobacterales;Desulfobacteraceae;Desulfatirhabdium;Desulfatirhabdium butyrativorans;Desulfatirhabdium butyrativorans DSM 18734 |
| Ga0586324 2394157 3 836 | 0.00 | 311905.80 | 0.00 | 22610000.00 | 368207.49 | 0.00 | 3625200.00 | 4178500.00 | 0.00 | 4365900.00 | 16456000.00 | 0.00 | KO:K02358 | elongation factor Tu | Bacteria;Proteobacteria;Alphaproteobacteria;Hyphomicrobiales;unclassified Hyphomicrobiales;Pseudorhodoplanes;Pseudorhodoplanes sinuspersici;Pseudorhodoplanes sinuspersici RIPI110 |
| Ga0586324 2475114 1 825 | 0.00 | 0.00 | 0.00 | 0.00 | 0.00 | 0.00 | 0.00 | 0.00 | 0.00 | 2017400.00 | 0.00 | 0.00 | KO:K02358 | elongation factor Tu | Bacteria;Proteobacteria;Deltaproteobacteria;Desulfuromonadales;Geobacteraceae;Geobacter;Geobacter uraniireducens;Geobacter uraniireducens Rf4 |
| Ga0586324 2505788 1 822 | 0.00 | 0.00 | 33273300.00 | 0.00 | 0.00 | 3434427.15 | 3990000.00 | 565723.15 | 0.00 | 30338000.00 | 2596000.00 | 2482500.00 | KO:K02358 | elongation factor Tu | Bacteria;Actinobacteria;Actinomycetia;Micrococcales;Cellulomonadaceae;Cellulomonas;Cellulomonas massiliensis;Cellulomonas massiliensis JC225 |
| Ga0586324 2519003 3 818 | 0.00 | 0.00 | 0.00 | 0.00 | 0.00 | 0.00 | 0.00 | 0.00 | 674210.79 | 0.00 | 0.00 | 0.00 | KO:K02358 | elongation factor Tu | Bacteria;Candidatus Aminicenantes;unclassified Candidatus Aminicenantes;unclassified Candidatus Aminicenantes;unclassified Candidatus Aminicenantes;unclassified Candidatus Aminicenantes;Aminicenantes bacterium SCGC AAA252-G04;Aminicenantes bacterium SCGC AAA252-G04 (contamination screened) |
| Ga0586324 2524496 3 818 | 3760000.00 | 4331600.00 | 30042648.36 | 5671500.00 | 20600000.00 | 32831900.00 | 26858400.00 | 37698000.00 | 30067200.00 | 27558300.00 | 0.00 | 17932500.00 | KO:K02358 | elongation factor Tu | Bacteria;Proteobacteria;Gammaproteobacteria;unclassified Gammaproteobacteria;unclassified Gammaproteobacteria;unclassified Gammaproteobacteria;Solemya velum gill symbiont;Solemya velum gill symbiont WH |
| Ga0586324 2537147 2 817 | 3250000.00 | 2733600.00 | 2800440.82 | 0.00 | 0.00 | 0.00 | 0.00 | 0.00 | 0.00 | 0.00 | 0.00 | 0.00 | KO:K02358 | elongation factor Tu | Bacteria;Proteobacteria;Gammaproteobacteria;Xanthomonadales;Xanthomonadaceae;Pseudoxanthomonas;Pseudoxanthomonas taiwanensis;Pseudoxanthomonas taiwanensis DSM 22914 |
| Ga0586324 2635218 2 805 | 139355.00 | 482124.76 | 0.00 | 0.00 | 973183.14 | 0.00 | 0.00 | 0.00 | 0.00 | 0.00 | 0.00 | 0.00 | KO:K02358 | elongation factor Tu | Bacteria;Verrucomicrobia;Verrucomicrobiae;Verrucomicrobiales;Verrucomicrobia subdivision 3;Pedosphaera;Pedosphaera parvula;Pedosphaera parvula Ellin514 |
| Ga0586324 2649834 3 803 | 4330000.00 | 0.00 | 0.00 | 0.00 | 649560.23 | 0.00 | 8116800.00 | 2647400.00 | 2008800.00 | 1593900.00 | 1557600.00 | 1027500.00 | KO:K02358 | elongation factor Tu | Bacteria;Proteobacteria;Betaproteobacteria;Nitrosomonadales;Thiobacillaceae;Thiobacillus;Thiobacillus thiophilus;Thiobacillus thiophilus DSM 19892 |
| Ga0586324 2656034 3 800 | 0.00 | 0.00 | 0.00 | 774929.25 | 0.00 | 0.00 | 0.00 | 0.00 | 0.00 | 0.00 | 0.00 | 0.00 | KO:K02358 | elongation factor Tu | Bacteria;Proteobacteria;Deltaproteobacteria;Desulfobacterales;Desulfobacteraceae;Desulfatirhabdium;Desulfatirhabdium butyrativorans;Desulfatirhabdium butyrativorans DSM 18734 |
| Ga0586324 2671894 2 538 | 0.00 | 0.00 | 0.00 | 0.00 | 0.00 | 0.00 | 3009600.00 | 0.00 | 0.00 | 0.00 | 0.00 | 0.00 | KO:K02358 | elongation factor Tu | Bacteria;Proteobacteria;Deltaproteobacteria;Myxococcales;Sandaracinaceae;Sandaracinus;Sandaracinus amylolyticus;Sandaracinus amylolyticus DSM 53668 |
| Ga0586324 2676317 1 798 | 3350000.00 | 0.00 | 0.00 | 0.00 | 0.00 | 0.00 | 0.00 | 0.00 | 0.00 | 0.00 | 0.00 | 0.00 | KO:K02358 | elongation factor Tu | Bacteria;Proteobacteria;Deltaproteobacteria;Desulfobacterales;Desulfobulbaceae;Desulfopila;Desulfopila sp. IMCC35004;Desulfopila sp. IMCC35004 |
| Ga0586324 2687186 1 798 | 0.00 | 16728000.00 | 31302800.00 | 5177500.00 | 10176400.00 | 27909100.00 | 10693200.00 | 6893000.00 | 9396000.00 | 10626000.00 | 6705600.00 | 10725000.00 | KO:K02358 | elongation factor Tu | Bacteria;Spirochaetes;Spirochaetia;Spirochaetales;Treponemataceae;Treponema;Treponema caldarium;Treponema caldarium DSM 7334 |
| Ga0586324 2707712 3 794 | 0.00 | 499645.64 | 0.00 | 0.00 | 0.00 | 0.00 | 7227600.00 | 0.00 | 255192.93 | 0.00 | 0.00 | 0.00 | KO:K02358 | elongation factor Tu | Bacteria;Chloroflexi;Anaerolineae;Anaerolineales;Anaerolineaceae;Anaerolinea;Anaerolinea thermophila;Anaerolinea thermophila UNI-1 |
| Ga0586324 2709247 3 794 | 0.00 | 335393.68 | 0.00 | 0.00 | 0.00 | 0.00 | 0.00 | 0.00 | 0.00 | 0.00 | 0.00 | 0.00 | KO:K02358 | elongation factor Tu | Bacteria;Acidobacteria;Vicinamibacteria;unclassified Vicinamibacteria;Vicinamibacteraceae;Luteitalea;Luteitalea pratensis;Luteitalea pratensis DSM 100886 |
| Ga0586324 2735735 1 693 | 0.00 | 18904000.00 | 0.00 | 0.00 | 0.00 | 0.00 | 0.00 | 0.00 | 0.00 | 0.00 | 0.00 | 0.00 | KO:K02358 | elongation factor Tu | Bacteria;Proteobacteria;Betaproteobacteria;Neisseriales;Chromobacteriaceae;Chitiniphilus;Chitiniphilus shinanonensis;Chitiniphilus shinanonensis DSM 23277 |
| Ga0586324 2805125 2 784 | 0.00 | 0.00 | 4324633.83 | 0.00 | 0.00 | 0.00 | 5631600.00 | 2427800.00 | 0.00 | 7338100.00 | 7928800.00 | 6667500.00 | KO:K02358 | elongation factor Tu | Bacteria;Proteobacteria;Gammaproteobacteria;unclassified Gammaproteobacteria;Candidatus Competibacteraceae;Candidatus Competibacter;Candidatus Competibacter denitrificans;Candidatus Competibacter denitrificans Run A D11 |
| Ga0586324 2936151 203 769 | 0.00 | 0.00 | 0.00 | 0.00 | 0.00 | 0.00 | 0.00 | 0.00 | 0.00 | 0.00 | 0.00 | 4372500.00 | KO:K02358 | elongation factor Tu | Bacteria;Proteobacteria;Betaproteobacteria;Burkholderiales;unclassified Burkholderiales;Methylibium;Methylibium sp. Pch-M;Methylibium sp. Pch-M |
| Ga0586324 2938005 207 767 | 0.00 | 0.00 | 0.00 | 0.00 | 0.00 | 0.00 | 0.00 | 0.00 | 939600.00 | 0.00 | 0.00 | 0.00 | KO:K02358 | elongation factor Tu | Bacteria;Acidobacteria;Thermoanaerobaculia;Thermoanaerobaculales;Thermoanaerobaculaceae;Thermoanaerobaculum;Thermoanaerobaculum aquaticum;Thermoanaerobaculum aquaticum MP-01 |
| Ga0586324 2951950 142 768 | 0.00 | 884000.00 | 0.00 | 1681500.00 | 0.00 | 0.00 | 0.00 | 5483900.00 | 523354.77 | 0.00 | 0.00 | 0.00 | KO:K02358 | elongation factor Tu | Bacteria;Proteobacteria;Deltaproteobacteria;Desulfuromonadales;Geobacteraceae;Geobacter;Geobacter sp. DSM 2909;Geobacter sp. DSM 2909 |
| Ga0586324 2982324 1 762 | 0.00 | 0.00 | 0.00 | 0.00 | 0.00 | 0.00 | 0.00 | 1030900.00 | 0.00 | 0.00 | 0.00 | 527639.25 | KO:K02358 | elongation factor Tu | Bacteria;Proteobacteria;Deltaproteobacteria;Desulfuromonadales;Desulfuromonadaceae;Pelobacter;Pelobacter propionicus;Pelobacter propionicus DSM 2379 |
| Ga0586324 3000256 3 761 | 0.00 | 0.00 | 3679807.41 | 8407500.00 | 0.00 | 9051600.00 | 6270000.00 | 5880400.00 | 5175900.00 | 0.00 | 0.00 | 0.00 | KO:K02358 | elongation factor Tu | Bacteria;Chloroflexi;Anaerolineae;Anaerolineales;Anaerolineaceae;Anaerolinea;Anaerolinea thermophila;Anaerolinea thermophila UNI-1 |
| Ga0586324 3004817 3 761 | 0.00 | 0.00 | 0.00 | 6013500.00 | 7374800.00 | 15443300.00 | 0.00 | 7198000.00 | 0.00 | 0.00 | 0.00 | 0.00 | KO:K02358 | elongation factor Tu | Bacteria;Bacteroidetes;Bacteroidia;Marinilabiliales;Marinilabiliaceae;Thermophagus;Thermophagus xiamenensis;Thermophagus xiamenensis HS1 |
| Ga0586324 3058439 79 756 | 0.00 | 0.00 | 0.00 | 0.00 | 0.00 | 0.00 | 2031468.60 | 494367.18 | 0.00 | 0.00 | 0.00 | 668584.50 | KO:K02358 | elongation factor Tu | Bacteria;Calditrichaeota;Calditrichae;Calditrichales;Calditrichaceae;Caldithrix;Caldithrix abyssi;Caldithrix abyssi LF13, DSM 13497 |
| Ga0586324 3058645 15 755 | 0.00 | 11288000.00 | 0.00 | 0.00 | 0.00 | 0.00 | 0.00 | 0.00 | 0.00 | 0.00 | 0.00 | 0.00 | KO:K02358 | elongation factor Tu | Bacteria;Bacteroidetes;Bacteroidia;Marinilabiliales;Marinilabiliaceae;Anaerophaga;Anaerophaga thermohalophila;Anaerophaga thermohalophila Fru22, DSM 12881 |
| Ga0586324 3077947 1 753 | 0.00 | 0.00 | 0.00 | 0.00 | 2657400.00 | 0.00 | 2118416.40 | 2507100.00 | 0.00 | 4851000.00 | 0.00 | 1057500.00 | KO:K02358 | elongation factor Tu | Bacteria;Nitrospirae;Nitrospira;Nitrospirales;Nitrospiraceae;Candidatus Magnetobacterium;Candidatus Magnetobacterium casensis;Candidatus Magnetobacterium casensis MYR-1 |
| Ga0586324 3092868 3 752 | 0.00 | 0.00 | 0.00 | 0.00 | 0.00 | 2313350.76 | 0.00 | 2555900.00 | 44064000.00 | 0.00 | 0.00 | 0.00 | KO:K02358 | elongation factor Tu | Bacteria;Proteobacteria;Alphaproteobacteria;Rhodospirillales;Rhodospirillaceae;Dongia;Dongia sp. URHE0060;Dongia sp. URHE0060 |
| Ga0586324 3112455 1 750 | 4800000.00 | 0.00 | 0.00 | 1411502.40 | 21630000.00 | 8575200.00 | 17328000.00 | 11834000.00 | 9072000.00 | 0.00 | 0.00 | 0.00 | KO:K02358 | elongation factor Tu | Bacteria;Proteobacteria;Deltaproteobacteria;Desulfuromonadales;Geobacteraceae;Geobacter;Geobacter sp. DSM 2909;Geobacter sp. DSM 2909 |
| Ga0586324 3126758 1 564 | 0.00 | 0.00 | 0.00 | 4655000.00 | 0.00 | 0.00 | 0.00 | 0.00 | 0.00 | 0.00 | 0.00 | 0.00 | KO:K02358 | elongation factor Tu | Bacteria;Chloroflexi;Caldilineae;Caldilineales;Caldilineaceae;Caldilinea;Caldilinea aerophila;Caldilinea aerophila STL-6-O1, DSM 14535 |
| Ga0586324 3185260 3 743 | 0.00 | 3556400.00 | 0.00 | 0.00 | 0.00 | 0.00 | 0.00 | 0.00 | 0.00 | 0.00 | 0.00 | 0.00 | KO:K02358 | elongation factor Tu | Bacteria;Spirochaetes;Spirochaetia;Spirochaetales;Spirochaetaceae;Spirochaeta;Spirochaeta lutea;Spirochaeta lutea JC230 |
| Ga0586324 3202370 3 740 | 0.00 | 0.00 | 0.00 | 0.00 | 0.00 | 0.00 | 0.00 | 0.00 | 0.00 | 1155000.00 | 0.00 | 687897.00 | KO:K02358 | elongation factor Tu | Bacteria;Proteobacteria;Betaproteobacteria;unclassified Betaproteobacteria;unclassified Betaproteobacteria;unclassified Betaproteobacteria;Betaproteobacteria bacterium GR16-43;Betaproteobacteria bacterium GR16-43 |
| Ga0586324 3234912 2 739 | 0.00 | 0.00 | 0.00 | 3049500.00 | 0.00 | 0.00 | 0.00 | 0.00 | 0.00 | 0.00 | 0.00 | 0.00 | KO:K02358 | elongation factor Tu | Bacteria;Chloroflexi;Anaerolineae;Anaerolineales;Anaerolineaceae;Anaerolinea;Anaerolinea thermophila;Anaerolinea thermophila UNI-1 |
| Ga0586324 3292188 3 734 | 0.00 | 8364000.00 | 14243900.00 | 0.00 | 2595600.00 | 13617100.00 | 0.00 | 0.00 | 0.00 | 0.00 | 8412800.00 | 10125000.00 | KO:K02358 | elongation factor Tu | Bacteria;Verrucomicrobia;Verrucomicrobiae;Verrucomicrobiales;Verrucomicrobia subdivision 3;Pedosphaera;Pedosphaera parvula;Pedosphaera parvula Ellin514 |
| Ga0586324 3417209 2 721 | 0.00 | 5684800.00 | 0.00 | 7201000.00 | 0.00 | 0.00 | 0.00 | 0.00 | 0.00 | 17171000.00 | 1997600.00 | 5707500.00 | KO:K02358 | elongation factor Tu | Bacteria;Proteobacteria;Betaproteobacteria;Burkholderiales;unclassified Burkholderiales;Rhizobacter;Rhizobacter sp. Root404;Rhizobacter sp. Root404 |
| Ga0586324 3486306 1 714 | 0.00 | 2543200.00 | 6868600.00 | 1596000.00 | 0.00 | 0.00 | 0.00 | 0.00 | 2754000.00 | 0.00 | 0.00 | 4305000.00 | KO:K02358 | elongation factor Tu | Bacteria;Proteobacteria;Alphaproteobacteria;Rhodobacterales;Rhodobacteraceae;Rhodovulum;Rhodovulum sp. PH10;Rhodovulum sp. PH10 |
| Ga0586324 3521286 1 618 | 0.00 | 0.00 | 0.00 | 2071000.00 | 0.00 | 0.00 | 0.00 | 0.00 | 3693600.00 | 0.00 | 0.00 | 0.00 | KO:K02358 | elongation factor Tu | Bacteria;Acidobacteria;Acidobacteriia;Bryobacterales;Solibacteraceae;Candidatus Solibacter;Candidatus Solibacter usitatus;Candidatus Solibacter usitatus Ellin6076 |
| Ga0586324 3523156 2 631 | 0.00 | 5324400.00 | 0.00 | 0.00 | 0.00 | 0.00 | 0.00 | 0.00 | 0.00 | 0.00 | 0.00 | 0.00 | KO:K02358 | elongation factor Tu | Bacteria;Proteobacteria;Deltaproteobacteria;Syntrophobacterales;Syntrophobacteraceae;Desulfacinum;Desulfacinum infernum;Desulfacinum infernum DSM 9756 |
| Ga0586324 3529048 1 711 | 0.00 | 1196800.00 | 0.00 | 0.00 | 0.00 | 0.00 | 0.00 | 0.00 | 0.00 | 0.00 | 0.00 | 0.00 | KO:K02358 | elongation factor Tu | Bacteria;Proteobacteria;Betaproteobacteria;Burkholderiales;Comamonadaceae;Polaromonas;Polaromonas sp. JS666;Polaromonas sp. JS666 |
| Ga0586324 3607285 3 704 | 0.00 | 11084000.00 | 0.00 | 13870000.00 | 15244000.00 | 0.00 | 11764800.00 | 0.00 | 0.00 | 0.00 | 0.00 | 0.00 | KO:K02358 | elongation factor Tu | Bacteria;Proteobacteria;Deltaproteobacteria;Syntrophobacterales;Syntrophobacteraceae;Syntrophobacter;Syntrophobacter fumaroxidans;Syntrophobacter fumaroxidans MPOB |
| Ga0586324 3608813 2 706 | 0.00 | 965600.00 | 0.00 | 0.00 | 0.00 | 0.00 | 0.00 | 0.00 | 0.00 | 0.00 | 0.00 | 0.00 | KO:K02358 | elongation factor Tu | Bacteria;Proteobacteria;Alphaproteobacteria;Caulobacterales;Caulobacteraceae;Phenylobacterium;Phenylobacterium parvum;Phenylobacterium parvum HYN0004 |
| Ga0586324 3672984 196 699 | 0.00 | 4630800.00 | 0.00 | 0.00 | 9661400.00 | 0.00 | 0.00 | 0.00 | 101842.92 | 0.00 | 0.00 | 0.00 | KO:K02358 | elongation factor Tu | Bacteria;Candidatus Parcubacteria;unclassified Candidatus Parcubacteria;unclassified Candidatus Parcubacteria;unclassified Candidatus Parcubacteria;unclassified Candidatus Parcubacteria;Parcubacteria bacterium SCGC AAA011-A09;Parcubacteria bacterium SCGC AAA011-A09 (contamination screened) |
| Ga0586324 3692397 137 700 | 0.00 | 61822.40 | 0.00 | 0.00 | 0.00 | 0.00 | 0.00 | 0.00 | 0.00 | 0.00 | 0.00 | 0.00 | KO:K02358 | elongation factor Tu | Bacteria;Planctomycetes;Phycisphaerae;Phycisphaerales;Phycisphaeraceae;Algisphaera;Algisphaera agarilytica;Algisphaera agarilytica DSM 103725 |
| Ga0586324 3719442 399 698 | 0.00 | 409000.28 | 434273.99 | 1026000.00 | 983365.72 | 892289.26 | 399914.28 | 0.00 | 0.00 | 0.00 | 0.00 | 207323.25 | KO:K02358 | elongation factor Tu | Bacteria;Firmicutes;Clostridia;Thermoanaerobacterales;Thermoanaerobacteraceae;Carboxydothermus;Carboxydothermus pertinax;Carboxydothermus pertinax Ug1 |
| Ga0586324 3748766 3 347 | 0.00 | 0.00 | 0.00 | 0.00 | 0.00 | 0.00 | 0.00 | 212955.88 | 0.00 | 0.00 | 0.00 | 0.00 | KO:K02358 | elongation factor Tu | Bacteria;Proteobacteria;Deltaproteobacteria;Desulfobacterales;Desulfobacteraceae;Desulfosarcina;Desulfosarcina variabilis;Desulfosarcina variabilis Montpellier |
| Ga0586324 3751055 3 695 | 2510000.00 | 583795.64 | 0.00 | 6422000.00 | 0.00 | 0.00 | 0.00 | 0.00 | 0.00 | 2125200.00 | 6538400.00 | 0.00 | KO:K02358 | elongation factor Tu | Bacteria;Proteobacteria;Alphaproteobacteria;Hyphomicrobiales;Reyranellaceae;Reyranella;Reyranella sp. OAS946;Reyranella sp. OAS946 |
| Ga0586324 3800375 3 689 | 1930000.00 | 0.00 | 0.00 | 0.00 | 0.00 | 0.00 | 0.00 | 0.00 | 0.00 | 0.00 | 0.00 | 0.00 | KO:K02358 | elongation factor Tu | Bacteria;Chloroflexi;Anaerolineae;Anaerolineales;Anaerolineaceae;Anaerolinea;Anaerolinea thermophila;Anaerolinea thermophila UNI-1 |
| Ga0586324 3812187 1 618 | 13010000.00 | 0.00 | 0.00 | 0.00 | 6684700.00 | 0.00 | 0.00 | 8723000.00 | 0.00 | 0.00 | 0.00 | 0.00 | KO:K02358 | elongation factor Tu | Bacteria;Proteobacteria;Gammaproteobacteria;Methylococcales;Methylococcaceae;Methylovulum;Methylovulum psychrotolerans;Methylovulum psychrotolerans HV10 M2 |
| Ga0586324 3928200 3 680 | 3090000.00 | 1421200.00 | 0.00 | 9253000.00 | 7519000.00 | 1988410.23 | 0.00 | 1457900.00 | 6374700.00 | 0.00 | 0.00 | 0.00 | KO:K02358 | elongation factor Tu | Bacteria;Proteobacteria;Deltaproteobacteria;Syntrophobacterales;Syntrophaceae;Desulfobacca;Desulfobacca acetoxidans;Desulfobacca acetoxidans ASRB2, DSM 11109 |
| Ga0586324 3967737 2 679 | 0.00 | 0.00 | 0.00 | 1985500.00 | 0.00 | 0.00 | 0.00 | 1494500.00 | 0.00 | 0.00 | 0.00 | 0.00 | KO:K02358 | elongation factor Tu | Bacteria;Proteobacteria;Betaproteobacteria;Nitrosomonadales;Nitrosomonadaceae;Nitrosomonas;Nitrosomonas sp. Nm51;Nitrosomonas sp. 51 Nm51 |
| Ga0586324 3971095 2 679 | 0.00 | 0.00 | 0.00 | 0.00 | 0.00 | 0.00 | 0.00 | 1079700.00 | 0.00 | 0.00 | 0.00 | 0.00 | KO:K02358 | elongation factor Tu | Bacteria;Acidobacteria;Vicinamibacteria;unclassified Vicinamibacteria;Vicinamibacteraceae;Luteitalea;Luteitalea pratensis;Luteitalea pratensis DSM 100886 |
| Ga0586324 3990035 1 678 | 0.00 | 5528400.00 | 0.00 | 0.00 | 0.00 | 0.00 | 0.00 | 2482700.00 | 0.00 | 0.00 | 0.00 | 0.00 | KO:K02358 | elongation factor Tu | Bacteria;Proteobacteria;Gammaproteobacteria;Nevskiales;Sinobacteraceae;Solimonas;Solimonas sp. K1W22B-7;Solimonas sp. K1W22B-7 |
| Ga0586324 4044449 54 674 | 0.00 | 1217200.00 | 0.00 | 1396500.00 | 2244829.59 | 0.00 | 1516145.28 | 0.00 | 1555200.00 | 0.00 | 2305600.00 | 0.00 | KO:K02358 | elongation factor Tu | Bacteria;Candidatus Latescibacteria;unclassified Candidatus Latescibacteria;unclassified Candidatus Latescibacteria;unclassified Candidatus Latescibacteria;unclassified Candidatus Latescibacteria;Latescibacteria bacterium SCGC AAA252-D10;Latescibacteria bacterium SCGC AAA252-D10 (contamination screened) |
| Ga0586324 4237593 169 660 | 0.00 | 0.00 | 0.00 | 0.00 | 611602.67 | 0.00 | 0.00 | 0.00 | 0.00 | 0.00 | 0.00 | 0.00 | KO:K02358 | elongation factor Tu | Bacteria;Firmicutes;Clostridia;Eubacteriales;unclassified Eubacteriales;unclassified Eubacteriales;Clostridiales bacterium oral taxon 876;Clostridiales sp. oral taxon 876 F0540 |
| Ga0586324 4397857 89 649 | 0.00 | 0.00 | 0.00 | 0.00 | 0.00 | 0.00 | 0.00 | 0.00 | 0.00 | 0.00 | 0.00 | 7800000.00 | KO:K02358 | elongation factor Tu | Bacteria;Proteobacteria;Deltaproteobacteria;unclassified Deltaproteobacteria;unclassified Deltaproteobacteria;unclassified Deltaproteobacteria;delta proteobacterium NaphS2;delta proteobacterium NaphS2 |
| Ga0586324 4489457 75 644 | 0.00 | 0.00 | 0.00 | 0.00 | 0.00 | 0.00 | 0.00 | 0.00 | 0.00 | 243182.94 | 0.00 | 0.00 | KO:K02358 | elongation factor Tu | Bacteria;Chloroflexi;Caldilineae;Caldilineales;Caldilineaceae;Caldilinea;Caldilinea aerophila;Caldilinea aerophila STL-6-O1, DSM 14535 |
| Ga0586324 4548196 1 555 | 4360000.00 | 2366400.00 | 0.00 | 0.00 | 0.00 | 18539900.00 | 0.00 | 1110200.00 | 0.00 | 0.00 | 0.00 | 0.00 | KO:K02358 | elongation factor Tu | Bacteria;Proteobacteria;Gammaproteobacteria;Nevskiales;Steroidobacteraceae;Povalibacter;Povalibacter uvarum;Povalibacter uvarum DSM 26723 |
| Ga0586324 4564079 1 639 | 0.00 | 0.00 | 0.00 | 0.00 | 0.00 | 0.00 | 0.00 | 1128500.00 | 0.00 | 0.00 | 0.00 | 0.00 | KO:K02358 | elongation factor Tu | Bacteria;Firmicutes;Clostridia;Eubacteriales;Oscillospiraceae;Pseudoclostridium;Pseudoclostridium thermosuccinogenes;Pseudoclostridium thermosuccinogenes DSM 5806 |
| Ga0586324 4634240 1 636 | 0.00 | 0.00 | 0.00 | 0.00 | 0.00 | 0.00 | 0.00 | 0.00 | 0.00 | 0.00 | 0.00 | 595223.25 | KO:K02358 | elongation factor Tu | Bacteria;Calditrichaeota;Calditrichae;Calditrichales;Calditrichaceae;Caldithrix;Caldithrix abyssi;Caldithrix abyssi LF13, DSM 13497 |
| Ga0586324 4701906 1 633 | 0.00 | 0.00 | 0.00 | 2261000.00 | 2760400.00 | 0.00 | 0.00 | 0.00 | 0.00 | 2510200.00 | 0.00 | 0.00 | KO:K02358 | elongation factor Tu | Bacteria;Proteobacteria;Deltaproteobacteria;unclassified Deltaproteobacteria;unclassified Deltaproteobacteria;unclassified Deltaproteobacteria;delta proteobacterium NaphS2;delta proteobacterium NaphS2 |
| Ga0586324 4732038 1 630 | 0.00 | 0.00 | 0.00 | 0.00 | 0.00 | 0.00 | 5289600.00 | 0.00 | 0.00 | 0.00 | 2675200.00 | 0.00 | KO:K02358 | elongation factor Tu | Bacteria;Proteobacteria;Betaproteobacteria;unclassified Betaproteobacteria;unclassified Betaproteobacteria;unclassified Betaproteobacteria;uncultured beta proteobacterium;unclassified betaproteobacterium ENR4 |
| Ga0586324 4865977 2 625 | 0.00 | 433765.88 | 887749.66 | 0.00 | 0.00 | 675169.96 | 0.00 | 719800.00 | 0.00 | 877800.00 | 648177.20 | 665154.00 | KO:K02358 | elongation factor Tu | Bacteria;Proteobacteria;Deltaproteobacteria;unclassified Deltaproteobacteria;unclassified Deltaproteobacteria;unclassified Deltaproteobacteria;delta proteobacterium NaphS2;delta proteobacterium NaphS2 |
| Ga0586324 4885423 1 624 | 24400000.00 | 0.00 | 0.00 | 0.00 | 31930000.00 | 38866300.00 | 1933278.12 | 0.00 | 0.00 | 0.00 | 21736000.00 | 0.00 | KO:K02358 | elongation factor Tu | Bacteria;Proteobacteria;Deltaproteobacteria;Syntrophobacterales;Syntrophobacteraceae;Desulfacinum;Desulfacinum infernum;Desulfacinum infernum DSM 9756 |
| Ga0586324 4919244 3 512 | 0.00 | 0.00 | 0.00 | 0.00 | 0.00 | 0.00 | 0.00 | 0.00 | 0.00 | 619169.32 | 672975.60 | 0.00 | KO:K02358 | elongation factor Tu | Bacteria;Proteobacteria;Deltaproteobacteria;Myxococcales;Vulgatibacteraceae;Vulgatibacter;Vulgatibacter incomptus;Vulgatibacter incomptus DSM 27710 |
| Ga0586324 5026077 3 590 | 0.00 | 0.00 | 0.00 | 0.00 | 0.00 | 0.00 | 0.00 | 0.00 | 1279800.00 | 1054900.00 | 824850.40 | 1425000.00 | KO:K02358 | elongation factor Tu | Bacteria;Proteobacteria;Betaproteobacteria;Burkholderiales;Comamonadaceae;Ramlibacter;Ramlibacter lithotrophicus;Ramlibacter lithotrophicus RBP-1 |
| Ga0586324 5225748 98 607 | 0.00 | 0.00 | 0.00 | 0.00 | 0.00 | 0.00 | 13566000.00 | 0.00 | 8262000.00 | 0.00 | 0.00 | 0.00 | KO:K02358 | elongation factor Tu | Bacteria;Bacteroidetes;Flavobacteriia;Flavobacteriales;Crocinitomicaceae;Crocinitomix;Crocinitomix algicola;Crocinitomix algicola 0182 |
| Ga0586324 5245040 3 605 | 28500000.00 | 16388000.00 | 0.00 | 732946.85 | 0.00 | 0.00 | 22138800.00 | 152205.98 | 285767.19 | 0.00 | 0.00 | 123859.50 | KO:K02358 | elongation factor Tu | Bacteria;Proteobacteria;Deltaproteobacteria;Desulfuromonadales;Desulfuromonadaceae;Pelobacter;Pelobacter propionicus;Pelobacter propionicus DSM 2379 |
| Ga0586324 5263522 2 604 | 0.00 | 0.00 | 0.00 | 0.00 | 0.00 | 0.00 | 5814000.00 | 5142300.00 | 648144.99 | 6760600.00 | 5227200.00 | 0.00 | KO:K02358 | elongation factor Tu | Bacteria;Proteobacteria;Betaproteobacteria;Nitrosomonadales;Thiobacillaceae;Thiobacillus;Thiobacillus denitrificans;Thiobacillus denitrificans DSM 12475 |
| Ga0586324 5284966 3 590 | 0.00 | 0.00 | 0.00 | 0.00 | 237684.86 | 0.00 | 84148.87 | 0.00 | 75202.02 | 116927.58 | 932800.00 | 519401.25 | KO:K02358 | elongation factor Tu | Bacteria;Firmicutes;Clostridia;Thermosediminibacterales;Thermosediminibacteraceae;Caldanaerovirga;Caldanaerovirga acetigignens;Caldanaerovirga acetigignens DSM 18802 |
| Ga0586324 5297820 1 603 | 0.00 | 0.00 | 0.00 | 0.00 | 573445.29 | 0.00 | 0.00 | 0.00 | 0.00 | 0.00 | 0.00 | 0.00 | KO:K02358 | elongation factor Tu | Bacteria;Proteobacteria;Deltaproteobacteria;Syntrophobacterales;Syntrophaceae;Smithella;Smithella sp. SCADC;Smithella sp. SCADC |
| Ga0586324 5428687 1 597 | 0.00 | 0.00 | 0.00 | 0.00 | 0.00 | 0.00 | 0.00 | 0.00 | 1142100.00 | 0.00 | 0.00 | 0.00 | KO:K02358 | elongation factor Tu | Bacteria;Proteobacteria;Deltaproteobacteria;Desulfuromonadales;Geobacteraceae;Geobacter;Geobacter sp. DSM 2909;Geobacter sp. DSM 2909 |
| Ga0586324 5462698 3 596 | 0.00 | 160767.64 | 0.00 | 0.00 | 0.00 | 0.00 | 0.00 | 0.00 | 0.00 | 0.00 | 0.00 | 0.00 | KO:K02358 | elongation factor Tu | Bacteria;Elusimicrobia;Endomicrobia;Endomicrobiales;Endomicrobiaceae;Endomicrobium;Endomicrobium proavitum;Endomicrobium proavitum Rsa215 |
| Ga0586324 5560046 2 589 | 0.00 | 0.00 | 1324902.27 | 0.00 | 0.00 | 0.00 | 0.00 | 0.00 | 0.00 | 0.00 | 0.00 | 0.00 | KO:K02358 | elongation factor Tu | Bacteria;Proteobacteria;Betaproteobacteria;Nitrosomonadales;Gallionellaceae;Sideroxydans;Sideroxydans lithotrophicus;Sideroxydans lithotrophicus ES-1 |
| Ga0586324 5567433 3 590 | 0.00 | 0.00 | 0.00 | 0.00 | 0.00 | 0.00 | 0.00 | 0.00 | 807740.91 | 0.00 | 0.00 | 0.00 | KO:K02358 | elongation factor Tu | Bacteria;Acidobacteria;Acidobacteriia;Bryobacterales;Solibacteraceae;Candidatus Solibacter;Candidatus Solibacter usitatus;Candidatus Solibacter usitatus Ellin6076 |
| Ga0586324 5580137 3 590 | 0.00 | 0.00 | 0.00 | 0.00 | 0.00 | 0.00 | 0.00 | 725900.00 | 694689.21 | 0.00 | 0.00 | 0.00 | KO:K02358 | elongation factor Tu | Bacteria;Chloroflexi;Anaerolineae;Anaerolineales;Anaerolineaceae;Anaerolinea;Anaerolinea thermophila;Anaerolinea thermophila UNI-1 |
| Ga0586324 5813839 3 578 | 0.00 | 0.00 | 0.00 | 0.00 | 0.00 | 0.00 | 0.00 | 2824300.00 | 2632500.00 | 0.00 | 0.00 | 0.00 | KO:K02358 | elongation factor Tu | Bacteria;Proteobacteria;Betaproteobacteria;Rhodocyclales;Azonexaceae;Dechloromonas;Dechloromonas aromatica;Dechloromonas aromatica RCB |
| Ga0586324 5823015 1 579 | 0.00 | 0.00 | 0.00 | 0.00 | 0.00 | 0.00 | 771563.40 | 0.00 | 709835.40 | 1817200.00 | 1073600.00 | 1170000.00 | KO:K02358 | elongation factor Tu | Bacteria;Proteobacteria;Deltaproteobacteria;Myxococcales;Sandaracinaceae;Sandaracinus;Sandaracinus amylolyticus;Sandaracinus amylolyticus DSM 53668 |
| Ga0586324 5839694 2 577 | 0.00 | 0.00 | 0.00 | 906437.75 | 0.00 | 0.00 | 0.00 | 707600.00 | 0.00 | 0.00 | 0.00 | 362720.25 | KO:K02358 | elongation factor Tu | Bacteria;Actinobacteria;Actinomycetia;Propionibacteriales;Nocardioidaceae;Nocardioides;Nocardioides soli;Nocardioides soli DSM 105498 |
| Ga0586324 6016378 2 412 | 0.00 | 0.00 | 0.00 | 0.00 | 0.00 | 0.00 | 425810.52 | 0.00 | 0.00 | 624752.59 | 505721.92 | 0.00 | KO:K02358 | elongation factor Tu | Bacteria;Proteobacteria;Alphaproteobacteria;Hyphomicrobiales;Roseiarcaceae;Roseiarcus;Roseiarcus fermentans;Roseiarcus fermentans DSM 24875 |
| Ga0586324 6023836 1 570 | 0.00 | 0.00 | 0.00 | 5842500.00 | 0.00 | 0.00 | 0.00 | 0.00 | 0.00 | 0.00 | 0.00 | 0.00 | KO:K02358 | elongation factor Tu | Bacteria;Proteobacteria;Deltaproteobacteria;Syntrophobacterales;Syntrophaceae;Desulfobacca;Desulfobacca acetoxidans;Desulfobacca acetoxidans ASRB2, DSM 11109 |
| Ga0586324 6198119 3 563 | 1400000.00 | 856800.00 | 0.00 | 0.00 | 0.00 | 0.00 | 0.00 | 0.00 | 0.00 | 0.00 | 0.00 | 0.00 | KO:K02358 | elongation factor Tu | Bacteria;Chloroflexi;Dehalococcoidia;unclassified Dehalococcoidia;unclassified Dehalococcoidia;Dehalogenimonas;Dehalogenimonas sp. WBC-2;Dehalogenimonas sp. WBC-2 |
| Ga0586324 6201252 3 563 | 0.00 | 0.00 | 0.00 | 0.00 | 0.00 | 0.00 | 9986400.00 | 0.00 | 0.00 | 0.00 | 0.00 | 0.00 | KO:K02358 | elongation factor Tu | Bacteria;Fibrobacteres;Fibrobacteria;unclassified Fibrobacteria;unclassified Fibrobacteria;unclassified Fibrobacteria;Fibrobacteria bacterium GUT77;Fibrobacteria bacterium GUT77 MC 77 |
| Ga0586324 6232728 3 497 | 0.00 | 0.00 | 0.00 | 0.00 | 0.00 | 0.00 | 0.00 | 0.00 | 0.00 | 0.00 | 0.00 | 13200000.00 | KO:K02358 | elongation factor Tu | Bacteria;Proteobacteria;Deltaproteobacteria;Myxococcales;Polyangiaceae;Sorangium;Sorangium cellulosum;Sorangium cellulosum So0011-07 |
| Ga0586324 6240619 88 564 | 0.00 | 0.00 | 0.00 | 0.00 | 0.00 | 0.00 | 0.00 | 0.00 | 0.00 | 0.00 | 0.00 | 2475000.00 | KO:K02358 | elongation factor Tu | Bacteria;Verrucomicrobia;Opitutae;Opitutales;Opitutaceae;Opitutus;Opitutus terrae;Opitutus terrae PB90-1 |
| Ga0586324 6334840 3 560 | 0.00 | 0.00 | 0.00 | 0.00 | 0.00 | 0.00 | 0.00 | 2165500.00 | 0.00 | 0.00 | 0.00 | 0.00 | KO:K02358 | elongation factor Tu | Bacteria;Planctomycetes;Planctomycetia;Planctomycetales;Planctomycetaceae;Rubinisphaera;Rubinisphaera brasiliensis;Rubinisphaera brasiliensis DSM 5305 |
| Ga0586324 6502264 14 553 | 0.00 | 9724000.00 | 0.00 | 18810000.00 | 0.00 | 26519600.00 | 0.00 | 1317600.00 | 13689000.00 | 0.00 | 14344000.00 | 13350000.00 | KO:K02358 | elongation factor Tu | Bacteria;Proteobacteria;Deltaproteobacteria;Syntrophobacterales;Syntrophaceae;Desulfobacca;Desulfobacca acetoxidans;Desulfobacca acetoxidans ASRB2, DSM 11109 |
| Ga0586324 6531105 1 552 | 0.00 | 0.00 | 0.00 | 0.00 | 0.00 | 0.00 | 0.00 | 0.00 | 0.00 | 44814000.00 | 0.00 | 0.00 | KO:K02358 | elongation factor Tu | Bacteria;Proteobacteria;Alphaproteobacteria;Hyphomicrobiales;unclassified Hyphomicrobiales;Nordella;Nordella sp. OAE833;Nordella sp. OAE833 |
| Ga0586324 6751164 304 546 | 0.00 | 0.00 | 0.00 | 0.00 | 0.00 | 0.00 | 0.00 | 0.00 | 636250.14 | 0.00 | 0.00 | 0.00 | KO:K02358 | elongation factor Tu | Bacteria;Proteobacteria;Alphaproteobacteria;Hyphomicrobiales;Xanthobacteraceae;Pseudolabrys;Pseudolabrys sp. Root1462;Pseudolabrys sp. Root1462 |
| Ga0586324 6845388 2 541 | 0.00 | 7004000.00 | 0.00 | 0.00 | 0.00 | 0.00 | 0.00 | 0.00 | 0.00 | 0.00 | 0.00 | 0.00 | KO:K02358 | elongation factor Tu | Bacteria;Proteobacteria;Betaproteobacteria;Nitrosomonadales;Sterolibacteriaceae;Sulfurisoma;Sulfurisoma sediminicola;Sulfurisoma sediminicola DSM 26916 |
| Ga0586324 6989070 1 294 | 0.00 | 0.00 | 0.00 | 0.00 | 303111.49 | 0.00 | 0.00 | 0.00 | 0.00 | 0.00 | 0.00 | 0.00 | KO:K02358 | elongation factor Tu | Bacteria;Actinobacteria;Thermoleophilia;Solirubrobacterales;Patulibacteraceae;Patulibacter;Patulibacter medicamentivorans;Patulibacter medicamentivorans I11 |
| Ga0586324 7145622 3 533 | 4110000.00 | 33252000.00 | 0.00 | 0.00 | 6283000.00 | 0.00 | 0.00 | 0.00 | 0.00 | 0.00 | 18568000.00 | 2535000.00 | KO:K02358 | elongation factor Tu | Bacteria;Proteobacteria;Gammaproteobacteria;Methylococcales;Methylococcaceae;Methylovulum;Methylovulum psychrotolerans;Methylovulum psychrotolerans HV10 M2 |
| Ga0586324 7225469 1 531 | 0.00 | 0.00 | 0.00 | 0.00 | 0.00 | 0.00 | 0.00 | 58430.25 | 0.00 | 0.00 | 0.00 | 0.00 | KO:K02358 | elongation factor Tu | Bacteria;Proteobacteria;Deltaproteobacteria;Desulfobacterales;Desulfobacteraceae;Desulfatirhabdium;Desulfatirhabdium butyrativorans;Desulfatirhabdium butyrativorans DSM 18734 |
| Ga0586324 7420483 3 524 | 0.00 | 2631600.00 | 0.00 | 1216000.00 | 0.00 | 0.00 | 0.00 | 0.00 | 0.00 | 0.00 | 0.00 | 0.00 | KO:K02358 | elongation factor Tu | Bacteria;Firmicutes;Clostridia;Thermoanaerobacterales;Thermoanaerobacterales Family III. Incertae Sedis;Caldicellulosiruptor;Caldicellulosiruptor kristjanssonii;Caldicellulosiruptor kristjanssonii |
| Ga0586324 7453188 3 524 | 125648.00 | 0.00 | 0.00 | 94484.82 | 0.00 | 0.00 | 0.00 | 0.00 | 0.00 | 0.00 | 0.00 | 0.00 | KO:K02358 | elongation factor Tu | Bacteria;Chloroflexi;Anaerolineae;Anaerolineales;Anaerolineaceae;Flexilinea;Flexilinea flocculi;Flexilinea flocculi TC1 |
| Ga0586324 7472115 1 522 | 0.00 | 0.00 | 0.00 | 0.00 | 0.00 | 0.00 | 0.00 | 1110200.00 | 1198800.00 | 0.00 | 0.00 | 0.00 | KO:K02358 | elongation factor Tu | Bacteria;Proteobacteria;Gammaproteobacteria;Methylococcales;Methylococcaceae;Methyloglobulus;Methyloglobulus morosus;Methyloglobulus morosus KoM1 |
| Ga0586324 7605434 3 488 | 0.00 | 0.00 | 0.00 | 0.00 | 0.00 | 0.00 | 0.00 | 0.00 | 0.00 | 885500.00 | 0.00 | 0.00 | KO:K02358 | elongation factor Tu | Bacteria;Proteobacteria;Alphaproteobacteria;Rhodospirillales;unclassified Rhodospirillales;unclassified Rhodospirillales;Rhodospirillales bacterium URHD0088;Rhodospirillales bacterium URHD0088 |
| Ga0586324 7838063 1 513 | 0.00 | 1706800.00 | 0.00 | 0.00 | 0.00 | 0.00 | 0.00 | 0.00 | 0.00 | 0.00 | 0.00 | 0.00 | KO:K02358 | elongation factor Tu | Bacteria;Proteobacteria;Gammaproteobacteria;Methylococcales;Methylococcaceae;Methylovulum;Methylovulum miyakonense;Methylovulum miyakonense HT12 |
| Ga0586324 7846083 1 513 | 0.00 | 0.00 | 0.00 | 0.00 | 0.00 | 0.00 | 1028375.76 | 0.00 | 0.00 | 962500.00 | 1944800.00 | 0.00 | KO:K02358 | elongation factor Tu | Bacteria;Chloroflexi;Anaerolineae;Anaerolineales;Anaerolineaceae;Anaerolinea;Anaerolinea thermophila;Anaerolinea thermophila UNI-1 |
| Ga0586324 8163988 2 502 | 0.00 | 0.00 | 0.00 | 0.00 | 0.00 | 0.00 | 0.00 | 0.00 | 0.00 | 1586200.00 | 0.00 | 0.00 | KO:K02358 | elongation factor Tu | Bacteria;Firmicutes;Bacilli;Bacillales;Thermoactinomycetaceae;Thermoactinomyces;Thermoactinomyces daqus;Thermoactinomyces daqus H-18 |
| Ga0586324 7091064 3 347 | 0.00 | 0.00 | 0.00 | 0.00 | 0.00 | 0.00 | 0.00 | 0.00 | 0.00 | 1455300.00 | 0.00 | 0.00 | KO:K02372 | 3-hydroxyacyl-[acyl-carrier-protein] dehydratase | Bacteria;Proteobacteria;Gammaproteobacteria;Methylococcales;Methylococcaceae;Methylobacter;Methylobacter tundripaludum;Methylobacter tundripaludum OWC-DMM |
| Ga0586324 1696464 3 968 | 0.00 | 0.00 | 0.00 | 0.00 | 0.00 | 0.00 | 8709600.00 | 0.00 | 0.00 | 5505500.00 | 0.00 | 4965000.00 | KO:K02390 | flagellar hook protein FlgE | Bacteria;Proteobacteria;Deltaproteobacteria;Myxococcales;Labilitrichaceae;Labilithrix;Labilithrix luteola;Labilithrix luteola DSM 27648 |
| Ga0586324 0687791 132 848 | 0.00 | 0.00 | 0.00 | 0.00 | 0.00 | 0.00 | 0.00 | 0.00 | 0.00 | 1617000.00 | 1513600.00 | 832500.00 | KO:K02405 | RNA polymerase sigma factor for flagellar operon FliA | Bacteria;Proteobacteria;Betaproteobacteria;Nitrosomonadales;Sulfuricellaceae;Sulfuricella;Sulfuricella denitrificans;Sulfuricella denitrificans skB26 |
| Ga0586324 0000006 79590 81077 | 0.00 | 0.00 | 0.00 | 947358.05 | 717504.18 | 5955000.00 | 0.00 | 1921500.00 | 0.00 | 4258100.00 | 9310400.00 | 6240000.00 | KO:K02406 | flagellin | Bacteria;Proteobacteria;Gammaproteobacteria;Xanthomonadales;Rhodanobacteraceae;Mizugakiibacter;Mizugakiibacter sediminis;Mizugakiibacter sediminis skMP5 |
| Ga0586324 0001502 3563 5032 | 0.00 | 0.00 | 0.00 | 0.00 | 0.00 | 0.00 | 0.00 | 0.00 | 0.00 | 0.00 | 0.00 | 2700000.00 | KO:K02406 | flagellin | Bacteria;Proteobacteria;Betaproteobacteria;Burkholderiales;Comamonadaceae;Rhodoferax;Rhodoferax ferrireducens;Rhodoferax ferrireducens T118 |
| Ga0586324 0006681 7515 8378 | 0.00 | 2250800.00 | 0.00 | 1311000.00 | 1349300.00 | 0.00 | 2416800.00 | 786900.00 | 363367.62 | 0.00 | 0.00 | 0.00 | KO:K02406 | flagellin | Bacteria;Acidobacteria;Acidobacteriia;Acidobacteriales;Acidobacteriaceae;unclassified Acidobacteriaceae;Acidobacteriaceae bacterium KBS 146;Acidobacteriaceae sp. KBS 146 |
| Ga0586324 0010025 3024 3884 | 958885.00 | 0.00 | 3117820.81 | 0.00 | 0.00 | 0.00 | 0.00 | 0.00 | 3442500.00 | 0.00 | 0.00 | 349935.00 | KO:K02406 | flagellin | Bacteria;Spirochaetes;Spirochaetia;Spirochaetales;Treponemataceae;Treponema;Treponema primitia;Treponema primitia ZAS-2 |
| Ga0586324 0010025 4154 5014 | 12391883.70 | 8809830.44 | 27418100.00 | 12825000.00 | 9343290.68 | 18420800.00 | 22517496.60 | 6361539.33 | 7340662.26 | 8940123.50 | 11367983.44 | 14554230.00 | KO:K02406 | flagellin | Bacteria;Spirochaetes;Spirochaetia;Spirochaetales;Treponemataceae;Treponema;Treponema caldarium;Treponema caldarium DSM 7334 |
| Ga0586324 0040593 1276 2133 | 0.00 | 0.00 | 0.00 | 0.00 | 0.00 | 0.00 | 0.00 | 0.00 | 842400.00 | 0.00 | 0.00 | 0.00 | KO:K02406 | flagellin | Bacteria;Proteobacteria;Deltaproteobacteria;Desulfuromonadales;Geobacteraceae;Geobacter;Geobacter sp. DSM 9736;Geobacter sp. DSM 9736 |
| Ga0586324 0048430 392 1912 | 0.00 | 0.00 | 0.00 | 0.00 | 0.00 | 0.00 | 0.00 | 405409.66 | 0.00 | 0.00 | 0.00 | 0.00 | KO:K02406 | flagellin | Bacteria;Proteobacteria;Gammaproteobacteria;Chromatiales;Ectothiorhodospiraceae;Nitrococcus;Nitrococcus mobilis;Nitrococcus mobilis Nb-231 |
| Ga0586324 0068904 2437 4098 | 0.00 | 0.00 | 0.00 | 414266.50 | 0.00 | 0.00 | 0.00 | 336953.63 | 0.00 | 0.00 | 0.00 | 0.00 | KO:K02406 | flagellin | Bacteria;Proteobacteria;Betaproteobacteria;Nitrosomonadales;Thiobacillaceae;Thiobacillus;Thiobacillus denitrificans;Thiobacillus denitrificans ATCC 25259 |
| Ga0586324 0154286 3 959 | 0.00 | 0.00 | 0.00 | 0.00 | 0.00 | 0.00 | 0.00 | 0.00 | 0.00 | 0.00 | 0.00 | 3382500.00 | KO:K02406 | flagellin | Bacteria;Proteobacteria;Betaproteobacteria;Burkholderiales;Comamonadaceae;unclassified Comamonadaceae;Comamonadaceae bacterium;Comamonadaceae sp. OAS658 |
| Ga0586324 0272795 1567 2145 | 0.00 | 0.00 | 0.00 | 0.00 | 0.00 | 0.00 | 0.00 | 0.00 | 5167800.00 | 0.00 | 0.00 | 0.00 | KO:K02406 | flagellin | Bacteria;Proteobacteria;Betaproteobacteria;Nitrosomonadales;Thiobacillaceae;Sulfuritortus;Sulfuritortus calidifontis;Sulfuritortus calidifontis DSM 103923 |
| Ga0586324 0310516 606 1469 | 3380000.00 | 0.00 | 0.00 | 0.00 | 0.00 | 0.00 | 0.00 | 0.00 | 0.00 | 3434200.00 | 0.00 | 0.00 | KO:K02406 | flagellin | Bacteria;Acidobacteria;Acidobacteriia;Acidobacteriales;Acidobacteriaceae;Granulicella;Granulicella arctica;Granulicella arctica X4EP2 |
| Ga0586324 0377892 1 369 | 0.00 | 0.00 | 0.00 | 0.00 | 0.00 | 0.00 | 0.00 | 0.00 | 0.00 | 0.00 | 0.00 | 1252500.00 | KO:K02406 | flagellin | Bacteria;Proteobacteria;Gammaproteobacteria;Xanthomonadales;Rhodanobacteraceae;Mizugakiibacter;Mizugakiibacter sediminis;Mizugakiibacter sediminis skMP5 |
| Ga0586324 0428009 2 460 | 380740.00 | 1306111.36 | 2167364.21 | 1525411.20 | 6973100.00 | 2457461.76 | 6756241.69 | 312748.83 | 1870156.35 | 2117500.00 | 2913973.92 | 3259074.00 | KO:K02406 | flagellin | Bacteria;Spirochaetes;Spirochaetia;Spirochaetales;Spirochaetaceae;Sediminispirochaeta;Sediminispirochaeta smaragdinae;Sediminispirochaeta smaragdinae DSM 11293 |
| Ga0586324 0439072 178 1050 | 0.00 | 2468400.00 | 908203.45 | 1273000.00 | 1308100.00 | 0.00 | 0.00 | 689300.00 | 915300.00 | 0.00 | 0.00 | 0.00 | KO:K02406 | flagellin | Bacteria;Spirochaetes;Spirochaetia;Spirochaetales;Spirochaetaceae;Spirochaeta;Spirochaeta thermophila;Spirochaeta thermophila DSM 6192 |

| | | | | | | | | | | | | | | | | |
|---|---|---|---|---|---|---|---|---|---|---|---|---|---|---|---|---|
| Ga0586324 0472512 358 1230 | 0.00 | 0.00 | 0.00 | 6545500.00 | 0.00 | 0.00 | 4263600.00 | 0.00 | 325730.97 | 0.00 | 2032800.00 | 162277.50 | KO:K02406 | flagellin | Bacteria;Spirochaetes;Spirochaetia;Spirochaetales;Spirochaetaceae;Sediminispirochaeta;Sediminispirochaeta smaragdinae;Sediminispirochaeta smaragdinae DSM 11293 |
| Ga0586324 0506385 2 799 | 0.00 | 0.00 | 0.00 | 0.00 | 1277200.00 | 2063558.36 | 0.00 | 0.00 | 1036800.00 | 1894200.00 | 1724800.00 | 2520000.00 | KO:K02406 | flagellin | Bacteria;Spirochaetes;Spirochaetia;Spirochaetales;Spirochaetaceae;Sediminispirochaeta;Sediminispirochaeta smaragdinae;Sediminispirochaeta smaragdinae DSM 11293 |
| Ga0586324 0683272 2 592 | 0.00 | 0.00 | 0.00 | 0.00 | 0.00 | 0.00 | 0.00 | 0.00 | 0.00 | 0.00 | 0.00 | 210056.25 | KO:K02406 | flagellin | Bacteria;Acidobacteria;Acidobacteriia;Acidobacteriales;Acidobacteriaceae;Candidatus Koribacter;Candidatus Koribacter versatilis;Candidatus Koribacter versatilis Ellin345 |
| Ga0586324 0834516 244 1050 | 0.00 | 0.00 | 0.00 | 0.00 | 0.00 | 0.00 | 0.00 | 0.00 | 0.00 | 0.00 | 2552000.00 | 0.00 | KO:K02406 | flagellin | Bacteria;Proteobacteria;Betaproteobacteria;Burkholderiales;unclassified Burkholderiales;Ideonella;Ideonella sakaiensis;Ideonella sp. 201-F6 |
| Ga0586324 1013116 2 1183 | 0.00 | 0.00 | 0.00 | 0.00 | 0.00 | 0.00 | 0.00 | 0.00 | 0.00 | 0.00 | 99278.96 | 0.00 | KO:K02406 | flagellin | Bacteria;Proteobacteria;Betaproteobacteria;unclassified Betaproteobacteria;unclassified Betaproteobacteria;unclassified Betaproteobacteria;Betaproteobacteria bacterium GR16-43;Betaproteobacteria bacterium GR16-43 |
| Ga0586324 1077790 416 1177 | 648101.00 | 1441172.28 | 629068.05 | 638457.00 | 605402.07 | 0.00 | 2130703.32 | 520400.15 | 566987.04 | 2979900.00 | 2418503.12 | 363504.75 | KO:K02406 | flagellin | Bacteria;Spirochaetes;Spirochaetia;Spirochaetales;Treponemataceae;Treponema;Treponema caldarium;Treponema caldarium DSM 7334 |
| Ga0586324 1082882 401 1174 | 488603.00 | 382404.12 | 0.00 | 1130500.00 | 1627400.00 | 0.00 | 649195.80 | 195433.02 | 0.00 | 0.00 | 0.00 | 176558.25 | KO:K02406 | flagellin | Bacteria;Spirochaetes;Spirochaetia;Spirochaetales;Treponemataceae;Treponema;Treponema primitia;Treponema primitia ZAS-2 |
| Ga0586324 1089907 218 1069 | 0.00 | 707200.00 | 1296589.00 | 894202.70 | 0.00 | 543048.36 | 0.00 | 0.00 | 0.00 | 0.00 | 1452000.00 | 1942500.00 | KO:K02406 | flagellin | Bacteria;Spirochaetes;Spirochaetia;Leptospirales;Leptospiraceae;Leptonema;Leptonema illini;Leptonema illini 3055, DSM 21528 |
| Ga0586324 1152088 1 561 | 1120000.00 | 1489200.00 | 1260607.67 | 1206500.00 | 48616000.00 | 1768956.57 | 2163250.32 | 34709000.00 | 0.00 | 2410100.00 | 2103200.00 | 3236227.50 | KO:K02406 | flagellin | Bacteria;Spirochaetes;Spirochaetia;Leptospirales;Leptospiraceae;Leptonema;Leptonema illini;Leptonema illini 3055, DSM 21528 |
| Ga0586324 1154575 235 1089 | 0.00 | 1346400.00 | 0.00 | 0.00 | 0.00 | 0.00 | 0.00 | 0.00 | 0.00 | 493370.57 | 0.00 | 0.00 | KO:K02406 | flagellin | Bacteria;Spirochaetes;Spirochaetia;Spirochaetales;Treponemataceae;Treponema;Treponema caldarium;Treponema caldarium DSM 7334 |
| Ga0586324 1159152 1 609 | 0.00 | 0.00 | 0.00 | 0.00 | 0.00 | 0.00 | 0.00 | 0.00 | 0.00 | 722344.70 | 2200000.00 | 0.00 | KO:K02406 | flagellin | Bacteria;Spirochaetes;Spirochaetia;Spirochaetales;Treponemataceae;Treponema;Treponema primitia;Treponema primitia ZAS-1 |
| Ga0586324 1160102 3 377 | 4850000.00 | 0.00 | 0.00 | 177244.35 | 462551.37 | 2061247.82 | 8755200.00 | 0.00 | 0.00 | 0.00 | 0.00 | 221116.50 | KO:K02406 | flagellin | Bacteria;Proteobacteria;Gammaproteobacteria;Nevskiales;Steroidobacteraceae;Steroidobacter;Steroidobacter denitrificans;Steroidobacter denitrificans DSM 18526 |
| Ga0586324 1357795 274 1065 | 3210000.00 | 2770878.96 | 1171867.61 | 2907000.00 | 2600582.11 | 2030083.32 | 11754204.84 | 603803.01 | 340193.52 | 0.00 | 683749.44 | 662008.50 | KO:K02406 | flagellin | Bacteria;Spirochaetes;Spirochaetia;Spirochaetales;Treponemataceae;Treponema;Treponema caldarium;Treponema caldarium DSM 7334 |
| Ga0586324 1492833 187 1023 | 0.00 | 1319200.00 | 5238985.24 | 0.00 | 0.00 | 13736200.00 | 0.00 | 0.00 | 0.00 | 0.00 | 10824000.00 | 6600000.00 | KO:K02406 | flagellin | Bacteria;Spirochaetes;Spirochaetia;Spirochaetales;Treponemataceae;Treponema;Treponema primitia;Treponema primitia ZAS-1 |
| Ga0586324 1712498 3 875 | 0.00 | 0.00 | 0.00 | 0.00 | 0.00 | 0.00 | 0.00 | 0.00 | 0.00 | 2926000.00 | 0.00 | 0.00 | KO:K02406 | flagellin | Bacteria;Spirochaetes;Spirochaetia;Spirochaetales;Treponemataceae;Treponema;unclassified Treponema;Treponema sp. RmG30 |
| Ga0586324 1763445 1 708 | 9840000.00 | 24451663.04 | 8276100.00 | 602968.80 | 14382967.38 | 9101522.75 | 12426000.00 | 1554701.51 | 3393900.00 | 2256100.00 | 12707200.00 | 3787500.00 | KO:K02406 | flagellin | Bacteria;Spirochaetes;Spirochaetia;Spirochaetales;Treponemataceae;Treponema;unclassified Treponema;Treponema sp. RmG30 |
| Ga0586324 1821147 530 940 | 0.00 | 0.00 | 0.00 | 0.00 | 1795500.00 | 0.00 | 0.00 | 0.00 | 0.00 | 0.00 | 0.00 | 0.00 | KO:K02406 | flagellin | Bacteria;Proteobacteria;Deltaproteobacteria;Desulfobacterales;Desulfobacteraceae;Desulfatibacillum;Desulfatibacillum aliphaticivorans;Desulfatibacillum aliphaticivorans DSM 15576 |
| Ga0586324 1911584 2 850 | 0.00 | 1938849.32 | 600174.89 | 587698.50 | 1583862.93 | 1128491.16 | 3792365.04 | 547185.25 | 781497.72 | 546900.97 | 473638.88 | 1400841.75 | KO:K02406 | flagellin | Bacteria;Spirochaetes;Spirochaetia;Spirochaetales;Treponemataceae;Treponema;Treponema caldarium;Treponema caldarium DSM 7334 |
| Ga0586324 1977685 1 885 | 869412.00 | 327605.64 | 720172.71 | 1957953.80 | 1631969.08 | 1048306.29 | 6292800.00 | 250658.15 | 1115578.98 | 1108800.00 | 1777600.00 | 2466333.00 | KO:K02406 | flagellin | Bacteria;Spirochaetes;Spirochaetia;Spirochaetales;Treponemataceae;Treponema;Treponema primitia;Treponema primitia ZAS-2 |
| Ga0586324 1980998 190 906 | 0.00 | 0.00 | 0.00 | 0.00 | 0.00 | 0.00 | 0.00 | 0.00 | 0.00 | 1070300.00 | 0.00 | 0.00 | KO:K02406 | flagellin | Bacteria;Proteobacteria;Oligoflexia;Bdellovibrionales;Bdellovibrionaceae;Bdellovibrio;Bdellovibrio bacteriovorus;Bdellovibrio bacteriovorus W |
| Ga0586324 2071280 80 889 | 0.00 | 0.00 | 0.00 | 0.00 | 0.00 | 0.00 | 0.00 | 0.00 | 501214.23 | 0.00 | 0.00 | 0.00 | KO:K02406 | flagellin | Bacteria;Proteobacteria;Betaproteobacteria;unclassified Betaproteobacteria;unclassified Betaproteobacteria;unclassified Betaproteobacteria;Betaproteobacteria bacterium GR16-43;Betaproteobacteria bacterium GR16-43 |
| Ga0586324 2328423 2 844 | 789000.00 | 1798570.08 | 0.00 | 1360841.75 | 1437315.56 | 2150080.54 | 0.00 | 302803.39 | 0.00 | 0.00 | 128861.04 | 527804.25 | KO:K02406 | flagellin | Bacteria;Spirochaetes;Spirochaetia;Spirochaetales;Treponemataceae;Treponema;Treponema primitia;Treponema primitia ZAS-2 |
| Ga0586324 2598338 153 809 | 0.00 | 0.00 | 0.00 | 1111500.00 | 0.00 | 0.00 | 0.00 | 0.00 | 0.00 | 0.00 | 1654400.00 | 1402500.00 | KO:K02406 | flagellin | Bacteria;Spirochaetes;Spirochaetia;Leptospirales;Leptospiraceae;Leptonema;Leptonema illini;Leptonema illini 3055, DSM 21528 |
| Ga0586324 2721266 35 793 | 0.00 | 0.00 | 0.00 | 0.00 | 0.00 | 0.00 | 32832000.00 | 0.00 | 0.00 | 0.00 | 8888000.00 | 2362500.00 | KO:K02406 | flagellin | Bacteria;Spirochaetes;Spirochaetia;Leptospirales;Leptospiraceae;Leptonema;Leptonema illini;Leptonema illini 3055, DSM 21528 |
| Ga0586324 2929914 349 768 | 0.00 | 0.00 | 0.00 | 0.00 | 0.00 | 0.00 | 0.00 | 0.00 | 0.00 | 0.00 | 271412.24 | 0.00 | KO:K02406 | flagellin | Bacteria;Proteobacteria;Gammaproteobacteria;Thiotrichales;Piscirickettsiaceae;Methylophaga;Methylophaga frappieri;Methylophaga frappieri JAM7 |
| Ga0586324 3019156 2 682 | 0.00 | 0.00 | 464853.90 | 0.00 | 0.00 | 1579651.09 | 0.00 | 0.00 | 0.00 | 2494800.00 | 0.00 | 543148.50 | KO:K02406 | flagellin | Bacteria;Spirochaetes;Spirochaetia;Spirochaetales;Treponemataceae;Treponema;Treponema caldarium;Treponema caldarium DSM 7334 |
| Ga0586324 3878996 3 680 | 0.00 | 0.00 | 0.00 | 0.00 | 0.00 | 0.00 | 0.00 | 0.00 | 0.00 | 0.00 | 0.00 | 2655000.00 | KO:K02406 | flagellin | Bacteria;Spirochaetes;Spirochaetia;Leptospirales;Leptospiraceae;Leptonema;Leptonema illini;Leptonema illini 3055, DSM 21528 |
| Ga0586324 4206317 2 391 | 619242.10 | 0.00 | 1169111.73 | 755592.00 | 439017.93 | 446986.27 | 1244618.48 | 433781.98 | 176691.78 | 0.00 | 0.00 | 429729.00 | KO:K02406 | flagellin | Bacteria;Spirochaetes;Spirochaetia;Spirochaetales;Spirochaetaceae;Sediminispirochaeta;Sediminispirochaeta smaragdinae;Sediminispirochaeta smaragdinae DSM 11293 |
| Ga0586324 4455547 2 646 | 0.00 | 0.00 | 0.00 | 0.00 | 0.00 | 0.00 | 0.00 | 0.00 | 0.00 | 0.00 | 0.00 | 1110000.00 | KO:K02406 | flagellin | Bacteria;Proteobacteria;Gammaproteobacteria;Xanthomonadales;Rhodanobacteraceae;Mizugakiibacter;Mizugakiibacter sediminis;Mizugakiibacter sediminis skMP5 |
| Ga0586324 4466875 1 441 | 0.00 | 0.00 | 0.00 | 0.00 | 0.00 | 0.00 | 0.00 | 0.00 | 0.00 | 0.00 | 1557600.00 | 0.00 | KO:K02406 | flagellin | Bacteria;Spirochaetes;Spirochaetia;Spirochaetales;Treponemataceae;Treponema;Treponema caldarium;Treponema caldarium DSM 7334 |
| Ga0586324 4844448 2 625 | 0.00 | 0.00 | 0.00 | 0.00 | 0.00 | 0.00 | 0.00 | 0.00 | 0.00 | 0.00 | 0.00 | 353607.75 | KO:K02406 | flagellin | Bacteria;Acidobacteria;Acidobacteriia;Acidobacteriales;Acidobacteriaceae;unclassified Acidobacteriaceae;Acidobacteriaceae bacterium KBS 146;Acidobacteriaceae sp. KBS 146 |
| Ga0586324 5042307 153 614 | 0.00 | 5895600.00 | 0.00 | 0.00 | 9187600.00 | 0.00 | 0.00 | 0.00 | 0.00 | 1986600.00 | 0.00 | 0.00 | KO:K02406 | flagellin | Bacteria;Spirochaetes;Spirochaetia;Spirochaetales;Treponemataceae;Treponema;Treponema caldarium;Treponema caldarium DSM 7334 |
| Ga0586324 5055430 1 576 | 4570000.00 | 0.00 | 540575.71 | 0.00 | 0.00 | 585237.55 | 812842.80 | 401292.16 | 0.00 | 281758.40 | 429260.48 | 314274.00 | KO:K02406 | flagellin | Bacteria;Spirochaetes;Spirochaetia;Spirochaetales;Spirochaetaceae;Sediminispirochaeta;Sediminispirochaeta bajacaliforniensis;Sediminispirochaeta bajacaliforniensis DSM 16054 |
| Ga0586324 5327804 243 602 | 0.00 | 0.00 | 0.00 | 188606.35 | 0.00 | 0.00 | 0.00 | 0.00 | 0.00 | 0.00 | 0.00 | 0.00 | KO:K02406 | flagellin | Bacteria;Spirochaetes;Spirochaetia;Spirochaetales;Spirochaetaceae;Sediminispirochaeta;Sediminispirochaeta smaragdinae;Sediminispirochaeta smaragdinae DSM 11293 |
| Ga0586324 5617555 3 305 | 2310000.00 | 1713600.00 | 2489377.69 | 2945000.00 | 0.00 | 3021372.47 | 3625200.00 | 969900.00 | 0.00 | 0.00 | 0.00 | 2437500.00 | KO:K02406 | flagellin | Bacteria;Spirochaetes;Spirochaetia;Spirochaetales;Spirochaetaceae;Spirochaeta;Spirochaeta cellobiosiphila;Spirochaeta cellobiosiphila DSM 17781 |
| Ga0586324 5640677 3 494 | 0.00 | 35428000.00 | 9289500.00 | 0.00 | 0.00 | 4049400.00 | 0.00 | 0.00 | 0.00 | 1101100.00 | 7867200.00 | 2017500.00 | KO:K02406 | flagellin | Bacteria;Spirochaetes;Spirochaetia;Spirochaetales;Treponemataceae;Treponema;Treponema primitia;Treponema primitia ZAS-2 |
| Ga0586324 5748241 2 280 | 0.00 | 0.00 | 0.00 | 0.00 | 0.00 | 0.00 | 0.00 | 0.00 | 5985900.00 | 0.00 | 0.00 | 0.00 | KO:K02406 | flagellin | Bacteria;Acidobacteria;Acidobacteriia;Acidobacteriales;Acidobacteriaceae;Candidatus Koribacter;Candidatus Koribacter versatilis;Candidatus Koribacter versatilis Ellin345 |
| Ga0586324 6039014 3 569 | 0.00 | 3420400.00 | 17284100.00 | 42560000.00 | 8858000.00 | 0.00 | 16963200.00 | 9028000.00 | 4025700.00 | 8085000.00 | 11088000.00 | 12225000.00 | KO:K02406 | flagellin | Bacteria;Spirochaetes;Spirochaetia;Spirochaetales;Treponemataceae;Treponema;Treponema azotonutricium;Treponema azotonutricium ZAS-9 |
| Ga0586324 6201053 2 445 | 1570000.00 | 0.00 | 16383300.00 | 418444.60 | 1452300.00 | 20048500.00 | 18126000.00 | 13310200.00 | 0.00 | 11781000.00 | 16984000.00 | 17775000.00 | KO:K02406 | flagellin | Bacteria;Spirochaetes;Spirochaetia;Spirochaetales;Treponemataceae;Treponema;Treponema caldarium;Treponema caldarium DSM 7334 |
| Ga0586324 7545593 2 409 | 851071.00 | 1108400.00 | 1859161.12 | 679441.90 | 872679.86 | 0.00 | 595490.40 | 0.00 | 0.00 | 235372.06 | 369415.20 | 682749.75 | KO:K02406 | flagellin | Bacteria;Spirochaetes;Spirochaetia;Spirochaetales;Spirochaetaceae;Sediminispirochaeta;Sediminispirochaeta smaragdinae;Sediminispirochaeta smaragdinae DSM 11293 |
| Ga0586324 7905929 1 405 | 0.00 | 0.00 | 0.00 | 0.00 | 1514100.00 | 0.00 | 0.00 | 0.00 | 0.00 | 0.00 | 994400.00 | 0.00 | KO:K02406 | flagellin | Bacteria;Proteobacteria;Betaproteobacteria;Rhodocyclales;Zoogloeaceae;Thauera;unclassified Thauera;Thauera sp. SYD-3 |
| Ga0586324 7995422 2 508 | 17200000.00 | 0.00 | 0.00 | 0.00 | 0.00 | 0.00 | 0.00 | 0.00 | 0.00 | 0.00 | 0.00 | 0.00 | KO:K02406 | flagellin | Bacteria;Spirochaetes;Spirochaetia;Spirochaetales;Treponemataceae;Treponema;Treponema azotonutricium;Treponema azotonutricium ZAS-9 |
| Ga0586324 8070674 3 464 | 0.00 | 0.00 | 0.00 | 0.00 | 0.00 | 0.00 | 0.00 | 1769000.00 | 0.00 | 0.00 | 0.00 | 0.00 | KO:K02406 | flagellin | Bacteria;Proteobacteria;Gammaproteobacteria;Xanthomonadales;Rhodanobacteraceae;Mizugakiibacter;Mizugakiibacter sediminis;Mizugakiibacter sediminis skMP5 |
| Ga0586324 7848707 1 267 | 0.00 | 0.00 | 0.00 | 0.00 | 0.00 | 0.00 | 0.00 | 0.00 | 0.00 | 722983.80 | 0.00 | 0.00 | KO:K02433 | aspartyl-tRNA(Asn)/glutamyl-tRNA(Gln) amidotransferase subunit A | Bacteria;Proteobacteria;Alphaproteobacteria;Hyphomicrobiales;Hyphomicrobiaceae;Rhodoplanes;Rhodoplanes sp. Z2-YC6860;Rhodoplanes sp. Z2-YC6860 |
| Ga0586324 0443573 910 1728 | 0.00 | 0.00 | 0.00 | 0.00 | 0.00 | 0.00 | 0.00 | 0.00 | 0.00 | 0.00 | 3960000.00 | 0.00 | KO:K02434 | aspartyl-tRNA(Asn)/glutamyl-tRNA(Gln) amidotransferase subunit B | Archaea;Euryarchaeota;Methanomicrobia;Methanomicrobiales;Methanomicrobiaceae;Methanoculleus;Methanoculleus bourgensis;Methanoculleus bourgensis MS2 |
| Ga0586324 0003864 12117 14750 | 0.00 | 0.00 | 0.00 | 0.00 | 0.00 | 0.00 | 0.00 | 0.00 | 0.00 | 885500.00 | 0.00 | 0.00 | KO:K02453 | general secretion pathway protein D | Bacteria;Acidobacteria;Vicinamibacteria;unclassified Vicinamibacteria;Vicinamibacteraceae;Luteitalea;Luteitalea pratensis;Luteitalea pratensis DSM 100886 |
| Ga0586324 0110436 1990 3270 | 0.00 | 0.00 | 0.00 | 0.00 | 0.00 | 0.00 | 0.00 | 0.00 | 0.00 | 4312000.00 | 4505600.00 | 4215000.00 | KO:K02453 | general secretion pathway protein D | Bacteria;Verrucomicrobia;Verrucomicrobiae;Verrucomicrobiales;Verrucomicrobia subdivision 3;Pedosphaera;Pedosphaera parvula;Pedosphaera parvula Ellin514 |
| Ga0586324 0126024 19 2535 | 0.00 | 0.00 | 0.00 | 0.00 | 449624.87 | 0.00 | 0.00 | 0.00 | 0.00 | 0.00 | 0.00 | 0.00 | KO:K02453 | general secretion pathway protein D | Bacteria;Proteobacteria;Deltaproteobacteria;Syntrophobacterales;Syntrophaceae;Desulfobacca;Desulfobacca acetoxidans;Desulfobacca acetoxidans ASRB2, DSM 11109 |
| Ga0586324 0576884 980 1540 | 4520000.00 | 661289.80 | 0.00 | 6279500.00 | 0.00 | 0.00 | 0.00 | 0.00 | 0.00 | 0.00 | 0.00 | 0.00 | KO:K02453 | general secretion pathway protein D | Bacteria;Verrucomicrobia;Verrucomicrobiae;Verrucomicrobiales;Verrucomicrobia subdivision 3;Pedosphaera;Pedosphaera parvula;Pedosphaera parvula Ellin514 |
| Ga0586324 1417054 2 1045 | 0.00 | 0.00 | 0.00 | 0.00 | 2029100.00 | 0.00 | 0.00 | 0.00 | 0.00 | 0.00 | 0.00 | 0.00 | KO:K02453 | general secretion pathway protein D | Bacteria;Proteobacteria;Deltaproteobacteria;Myxococcales;Polyangiaceae;Sorangium;Sorangium cellulosum;Sorangium cellulosum So ceGT47 |
| Ga0586324 2875339 2 775 | 0.00 | 0.00 | 0.00 | 0.00 | 0.00 | 0.00 | 0.00 | 3007300.00 | 0.00 | 0.00 | 0.00 | 0.00 | KO:K02453 | general secretion pathway protein D | Bacteria;Verrucomicrobia;Verrucomicrobiae;Verrucomicrobiales;Verrucomicrobia subdivision 3;Pedosphaera;Pedosphaera parvula;Pedosphaera parvula Ellin514 |
| Ga0586324 4107999 1 669 | 0.00 | 0.00 | 0.00 | 0.00 | 2224800.00 | 0.00 | 0.00 | 0.00 | 0.00 | 0.00 | 0.00 | 0.00 | KO:K02453 | general secretion pathway protein D | Bacteria;Verrucomicrobia;Spartobacteria;Chthoniobacterales;Chthoniobacteraceae;Chthoniobacter;Chthoniobacter flavus;Chthoniobacter flavus Ellin428 |
| Ga0586324 4121904 3 668 | 0.00 | 0.00 | 0.00 | 0.00 | 0.00 | 7026900.00 | 0.00 | 0.00 | 1668600.00 | 0.00 | 0.00 | 0.00 | KO:K02453 | general secretion pathway protein D | Bacteria;Planctomycetes;Planctomycetia;Pirellulales;Thermoguttaceae;Thermogutta;Thermogutta terrifontis;Thermogutta terrifontis R1 |
| Ga0586324 4574299 1 639 | 0.00 | 1196800.00 | 0.00 | 0.00 | 0.00 | 0.00 | 3534000.00 | 49931.12 | 0.00 | 0.00 | 0.00 | 0.00 | KO:K02453 | general secretion pathway protein D | Bacteria;Verrucomicrobia;Verrucomicrobiae;Verrucomicrobiales;Verrucomicrobia subdivision 3;Pedosphaera;Pedosphaera parvula;Pedosphaera parvula Ellin514 |
| Ga0586324 0000005 69806 72388 | 0.00 | 0.00 | 0.00 | 0.00 | 0.00 | 0.00 | 0.00 | 0.00 | 339332.49 | 4423640.76 | 0.00 | 817500.00 | KO:K02469 | DNA gyrase subunit A | Bacteria;Proteobacteria;Betaproteobacteria;Nitrosomonadales;Thiobacillaceae;Annwoodia;Annwoodia aquaesulis;Annwoodia aquaesulis DSM 4255 |
| Ga0586324 0056296 1 1635 | 0.00 | 0.00 | 0.00 | 0.00 | 0.00 | 0.00 | 0.00 | 0.00 | 0.00 | 0.00 | 2244000.00 | 0.00 | KO:K02469 | DNA gyrase subunit A | Bacteria;Proteobacteria;Betaproteobacteria;Nitrosomonadales;Thiobacillaceae;Thiobacillus;Thiobacillus denitrificans;Thiobacillus denitrificans RG |
| Ga0586324 0075607 1800 3917 | 0.00 | 0.00 | 0.00 | 0.00 | 0.00 | 0.00 | 0.00 | 164458.44 | 0.00 | 0.00 | 0.00 | 0.00 | KO:K02469 | DNA gyrase subunit A | Bacteria;Proteobacteria;Deltaproteobacteria;Syntrophobacterales;Syntrophaceae;Desulfobacca;Desulfobacca acetoxidans;Desulfobacca acetoxidans ASRB2, DSM 11109 |
| Ga0586324 0234429 1 1317 | 0.00 | 0.00 | 0.00 | 0.00 | 0.00 | 0.00 | 0.00 | 0.00 | 1101600.00 | 0.00 | 0.00 | 0.00 | KO:K02469 | DNA gyrase subunit A | Bacteria;Bacteroidetes;Bacteroidia;Bacteroidales;Rikenellaceae;Acetobacteroides;Acetobacteroides hydrogenigenes;Acetobacteroides hydrogenigenes DSM 24657 |
| Ga0586324 7009357 1 537 | 0.00 | 0.00 | 0.00 | 0.00 | 0.00 | 0.00 | 0.00 | 584003.02 | 0.00 | 0.00 | 0.00 | 0.00 | KO:K02469 | DNA gyrase subunit A | Bacteria;Proteobacteria;Betaproteobacteria;Nitrosomonadales;Thiobacillaceae;Thiobacillus;Thiobacillus thioparus;Thiobacillus thioparus DSM 505 |
| Ga0586324 0000524 3 1208 | 0.00 | 0.00 | 0.00 | 0.00 | 0.00 | 0.00 | 1649826.24 | 279327.54 | 8424000.00 | 0.00 | 0.00 | 0.00 | KO:K02470 | DNA gyrase subunit B | Bacteria;Proteobacteria;Betaproteobacteria;Nitrosomonadales;Thiobacillaceae;Thiobacillus;Thiobacillus thioparus;Thiobacillus thioparus DSM 505 |
| Ga0586324 0115811 749 3064 | 0.00 | 0.00 | 2220950.55 | 0.00 | 0.00 | 0.00 | 0.00 | 0.00 | 0.00 | 0.00 | 0.00 | 0.00 | KO:K02470 | DNA gyrase subunit B | Bacteria;Actinobacteria;Rubrobacteria;Gaiellales;Gaiellaceae;Gaiella;Gaiella occulta;Gaiella occulta F2-233 |
| Ga0586324 0184807 1 1689 | 0.00 | 0.00 | 0.00 | 0.00 | 0.00 | 0.00 | 0.00 | 0.00 | 0.00 | 0.00 | 1566400.00 | 0.00 | KO:K02470 | DNA gyrase subunit B | Bacteria;Proteobacteria;Alphaproteobacteria;Hyphomicrobiales;Xanthobacteraceae;Pseudolabrys;Pseudolabrys sp. Root1462;Pseudolabrys sp. Root1462 |
| Ga0586324 3191729 1 741 | 0.00 | 0.00 | 0.00 | 0.00 | 0.00 | 0.00 | 0.00 | 0.00 | 0.00 | 0.00 | 128066.40 | 0.00 | KO:K02470 | DNA gyrase subunit B | Bacteria;Proteobacteria;Gammaproteobacteria;unclassified Gammaproteobacteria;Candidatus Competibacteraceae;Candidatus Contendobacter;Candidatus Contendobacter odensis;Candidatus Contendobacter odensis Run B J11 |
| Ga0586324 0102272 2233 3270 | 0.00 | 0.00 | 0.00 | 0.00 | 0.00 | 0.00 | 0.00 | 0.00 | 0.00 | 598869.04 | 0.00 | 0.00 | KO:K02473 | UDP-N-acetylglucosamine 4-epimerase | Bacteria;Proteobacteria;Betaproteobacteria;Nitrosomonadales;Sulfuricellaceae;Sulfuricella;Sulfuricella sp. T08;Sulfuricella sp. T08 |
| Ga0586324 2019144 1 744 | 0.00 | 1230800.00 | 0.00 | 0.00 | 0.00 | 0.00 | 0.00 | 0.00 | 0.00 | 0.00 | 0.00 | 0.00 | KO:K02473 | UDP-N-acetylglucosamine 4-epimerase | Bacteria;Lentisphaerae;Lentisphaeria;Victivallales;Victivallaceae;Victivallis;Victivallis vadensis;Victivallis vadensis ATCC BAA-548 |
| Ga0586324 0809289 81 1331 | 0.00 | 0.00 | 0.00 | 0.00 | 0.00 | 0.00 | 0.00 | 0.00 | 0.00 | 6860700.00 | 0.00 | 0.00 | KO:K02482 | two-component system NtrC family sensor kinase | Bacteria;Proteobacteria;Deltaproteobacteria;unclassified Deltaproteobacteria;unclassified Deltaproteobacteria;unclassified Deltaproteobacteria;delta proteobacterium NaphS2;delta proteobacterium NaphS2 |
| Ga0586324 3124758 132 749 | 0.00 | 0.00 | 0.00 | 0.00 | 0.00 | 0.00 | 0.00 | 0.00 | 0.00 | 0.00 | 0.00 | 244008.75 | KO:K02482 | two-component system NtrC family sensor kinase | Bacteria;Proteobacteria;Deltaproteobacteria;Syntrophobacterales;Syntrophobacteraceae;Syntrophobacter;Syntrophobacter fumaroxidans;Syntrophobacter fumaroxidans MPOB |
| Ga0586324 4640118 2 637 | 0.00 | 0.00 | 0.00 | 0.00 | 0.00 | 0.00 | 0.00 | 0.00 | 0.00 | 0.00 | 0.00 | 1155000.00 | KO:K02482 | two-component system NtrC family sensor kinase | Bacteria;Proteobacteria;Betaproteobacteria;Burkholderiales;unclassified Burkholderiales;Rivibacter;Rivibacter subsaxonicus;Rivibacter subsaxonicus DSM 19570 |
| Ga0586324 2353385 486 842 | 0.00 | 0.00 | 0.00 | 7191500.00 | 0.00 | 0.00 | 0.00 | 0.00 | 0.00 | 0.00 | 0.00 | 0.00 | KO:K02483 | two-component system OmpR family response regulator | Archaea;Euryarchaeota;Methanomicrobia;Methanomicrobiales;Methanoregulaceae;Methanoregula;Methanoregula formicica;Methanoregula formicica SMSP, DSM 22288 |
| Ga0586324 0079881 1781 2173 | 0.00 | 0.00 | 0.00 | 0.00 | 1648000.00 | 0.00 | 0.00 | 0.00 | 2373300.00 | 0.00 | 0.00 | 0.00 | KO:K02490 | two-component system response regulator (stage 0 sporulation protein F) | Bacteria;Proteobacteria;Deltaproteobacteria;Syntrophobacterales;Syntrophaceae;Desulfobacca;Desulfobacca acetoxidans;Desulfobacca acetoxidans ASRB2, DSM 11109 |
| Ga0586324 0000013 69686 70936 | 0.00 | 0.00 | 0.00 | 0.00 | 0.00 | 0.00 | 0.00 | 0.00 | 0.00 | 0.00 | 0.00 | 915000.00 | KO:K02492 | glutamyl-tRNA reductase | Bacteria;Proteobacteria;Betaproteobacteria;Nitrosomonadales;Thiobacillaceae;Annwoodia;Annwoodia aquaesulis;Annwoodia aquaesulis DSM 4255 |
| Ga0586324 0001370 9202 11841 | 0.00 | 0.00 | 0.00 | 0.00 | 0.00 | 0.00 | 0.00 | 2574200.00 | 1027953.99 | 0.00 | 0.00 | 7462500.00 | KO:K02519 | translation initiation factor IF-2 | Bacteria;Proteobacteria;Betaproteobacteria;Nitrosomonadales;Thiobacillaceae;Annwoodia;Annwoodia aquaesulis;Annwoodia aquaesulis DSM 4255 |
| Ga0586324 0077755 1 1722 | 0.00 | 0.00 | 0.00 | 0.00 | 3337200.00 | 0.00 | 0.00 | 1939800.00 | 1952100.00 | 0.00 | 0.00 | 0.00 | KO:K02519 | translation initiation factor IF-2 | Bacteria;Acidobacteria;Acidobacteriia;Bryobacterales;Solibacteraceae;Candidatus Solibacter;Candidatus Solibacter usitatus;Candidatus Solibacter usitatus Ellin6076 |
| Ga0586324 0124026 1051 3096 | 0.00 | 0.00 | 0.00 | 0.00 | 0.00 | 0.00 | 0.00 | 0.00 | 0.00 | 0.00 | 0.00 | 574986.75 | KO:K02519 | translation initiation factor IF-2 | Bacteria;Proteobacteria;Deltaproteobacteria;Syntrophobacterales;Syntrophaceae;Syntrophus;Syntrophus aciditrophicus;Syntrophus aciditrophicus SB |
| Ga0586324 0165355 1 2250 | 0.00 | 0.00 | 0.00 | 0.00 | 0.00 | 0.00 | 0.00 | 0.00 | 0.00 | 1724800.00 | 0.00 | 0.00 | KO:K02519 | translation initiation factor IF-2 | Bacteria;Firmicutes;Clostridia;Eubacteriales;Peptococcaceae;Desulfotomaculum;Desulfotomaculum hydrothermale;Desulfotomaculum hydrothermale Lam5 |
| Ga0586324 0230444 1 636 | 0.00 | 0.00 | 0.00 | 0.00 | 799461.28 | 0.00 | 3374400.00 | 3501400.00 | 0.00 | 0.00 | 0.00 | 0.00 | KO:K02519 | translation initiation factor IF-2 | Bacteria;Proteobacteria;Betaproteobacteria;Rhodocyclales;Azonexaceae;Azonexus;Azonexus hydrophilus;Azonexus hydrophilus DSM 23864 |
| Ga0586324 0232454 103 2310 | 2900000.00 | 0.00 | 0.00 | 0.00 | 0.00 | 0.00 | 0.00 | 0.00 | 0.00 | 0.00 | 0.00 | 0.00 | KO:K02519 | translation initiation factor IF-2 | Bacteria;Proteobacteria;Gammaproteobacteria;Chromatiales;Ectothiorhodospiraceae;Thioalbus;Thioalbus denitrificans;Thioalbus denitrificans DSM 26407 |
| Ga0586324 0327199 1 1977 | 0.00 | 452868.44 | 0.00 | 0.00 | 0.00 | 0.00 | 0.00 | 0.00 | 0.00 | 0.00 | 0.00 | 0.00 | KO:K02519 | translation initiation factor IF-2 | Bacteria;Bacteroidetes;Bacteroidia;Bacteroidales;unclassified Bacteroidales;unclassified Bacteroidales;Bacteroidales bacterium 6E;Bacteroidales bacterium 6E |
| Ga0586324 0859498 2 1297 | 0.00 | 0.00 | 0.00 | 0.00 | 0.00 | 0.00 | 0.00 | 0.00 | 0.00 | 0.00 | 0.00 | 3292500.00 | KO:K02519 | translation initiation factor IF-2 | Bacteria;Acidobacteria;Acidobacteriia;Acidobacteriales;Acidobacteriaceae;unclassified Acidobacteriaceae;Acidobacteriaceae bacterium KBS 96;Acidobacteriaceae bacterium KBS 96, DSM 24297 |
| Ga0586324 2597510 2 808 | 0.00 | 0.00 | 0.00 | 0.00 | 0.00 | 0.00 | 0.00 | 0.00 | 754695.63 | 0.00 | 0.00 | 0.00 | KO:K02519 | translation initiation factor IF-2 | Bacteria;Proteobacteria;Betaproteobacteria;Nitrosomonadales;Nitrosomonadaceae;Nitrosospira;Nitrosospira lacus;Nitrosospira lacus APG3 |
| Ga0586324 3989823 177 677 | 0.00 | 0.00 | 0.00 | 0.00 | 0.00 | 2715618.95 | 0.00 | 0.00 | 0.00 | 0.00 | 0.00 | 0.00 | KO:K02519 | translation initiation factor IF-2 | Bacteria;Verrucomicrobia;Verrucomicrobiae;Verrucomicrobiales;Verrucomicrobia subdivision 3;Pedosphaera;Pedosphaera parvula;Pedosphaera parvula Ellin514 |
| Ga0586324 6288122 3 560 | 0.00 | 6800000.00 | 0.00 | 0.00 | 0.00 | 0.00 | 0.00 | 0.00 | 0.00 | 0.00 | 0.00 | 0.00 | KO:K02519 | translation initiation factor IF-2 | Bacteria;Proteobacteria;Betaproteobacteria;Burkholderiales;unclassified Burkholderiales;Rhizobacter;Rhizobacter sp. Root404;Rhizobacter sp. Root404 |
| Ga0586324 8226505 2 502 | 0.00 | 863600.00 | 0.00 | 0.00 | 0.00 | 0.00 | 0.00 | 0.00 | 0.00 | 0.00 | 0.00 | 0.00 | KO:K02519 | translation initiation factor IF-2 | Bacteria;Bacteroidetes;Bacteroidia;Marinilabiliales;Marinilabiliaceae;Anaerophaga;Anaerophaga thermohalophila;Anaerophaga thermohalophila Valhall |
| Ga0586324 0000005 138480 139058 | 0.00 | 0.00 | 0.00 | 0.00 | 0.00 | 0.00 | 0.00 | 0.00 | 0.00 | 0.00 | 2068000.00 | 2265000.00 | KO:K02520 | translation initiation factor IF-3 | Bacteria;Proteobacteria;Betaproteobacteria;Nitrosomonadales;Thiobacillaceae;Sulfuritortus;Sulfuritortus calidifontis;Sulfuritortus calidifontis DSM 103923 |
| Ga0586324 0255870 642 1238 | 0.00 | 0.00 | 0.00 | 0.00 | 0.00 | 0.00 | 0.00 | 915000.00 | 0.00 | 0.00 | 0.00 | 0.00 | KO:K02520 | translation initiation factor IF-3 | Bacteria;Proteobacteria;Deltaproteobacteria;Desulfuromonadales;Geobacteraceae;Geobacter;Geobacter argillaceus;Geobacter argillaceus ATCC BAA-1139 |
| Ga0586324 0000002 225294 226262 | 0.00 | 0.00 | 0.00 | 0.00 | 0.00 | 0.00 | 0.00 | 854000.00 | 0.00 | 931700.00 | 2129600.00 | 0.00 | KO:K02523 | octaprenyl-diphosphate synthase | Bacteria;Proteobacteria;Betaproteobacteria;Nitrosomonadales;Thiobacillaceae;Annwoodia;Annwoodia aquaesulis;Annwoodia aquaesulis DSM 4255 |
| Ga0586324 0550323 390 1358 | 0.00 | 0.00 | 0.00 | 0.00 | 0.00 | 0.00 | 2115368.04 | 0.00 | 0.00 | 0.00 | 1496000.00 | 0.00 | KO:K02523 | octaprenyl-diphosphate synthase | Bacteria;Proteobacteria;Betaproteobacteria;Nitrosomonadales;Thiobacillaceae;Thiobacillus;Thiobacillus thioparus;Thiobacillus thioparus DSM 505 |
| Ga0586324 1417828 3 1046 | 0.00 | 0.00 | 0.00 | 0.00 | 0.00 | 0.00 | 0.00 | 0.00 | 4608900.00 | 0.00 | 0.00 | 0.00 | KO:K02527 | 3-deoxy-D-manno-octulosonic-acid transferase | Bacteria;Proteobacteria;Deltaproteobacteria;Myxococcales;Anaeromyxobacteraceae;Anaeromyxobacter;Anaeromyxobacter dehalogenans;Anaeromyxobacter dehalogenans 2CP-C |
| Ga0586324 0124089 1100 1879 | 0.00 | 0.00 | 0.00 | 0.00 | 0.00 | 0.00 | 0.00 | 0.00 | 0.00 | 0.00 | 0.00 | 6457500.00 | KO:K02557 | chemotaxis protein MotB | Bacteria;Proteobacteria;Gammaproteobacteria;Acidiferrobacterales;Acidiferrobacteraceae;Sulfurifustis;Sulfurifustis variabilis;Sulfurifustis variabilis skN76 |
| Ga0586324 1185879 105 773 | 1050000.00 | 0.00 | 0.00 | 0.00 | 0.00 | 0.00 | 0.00 | 0.00 | 0.00 | 0.00 | 0.00 | 0.00 | KO:K02557 | chemotaxis protein MotB | Bacteria;Proteobacteria;Gammaproteobacteria;Acidiferrobacterales;Acidiferrobacteraceae;Sulfurifustis;Sulfurifustis variabilis;Sulfurifustis variabilis skN76 |
| Ga0586324 1250760 543 1103 | 0.00 | 1033600.00 | 0.00 | 0.00 | 0.00 | 0.00 | 0.00 | 1061400.00 | 0.00 | 1355200.00 | 0.00 | 0.00 | KO:K02557 | chemotaxis protein MotB | Bacteria;Bacteroidetes;Chitinophagia;Chitinophagales;Chitinophagaceae;Deminuibacter;Deminuibacter soli;Deminuibacter soli K23C18032701 |
| Ga0586324 3179615 97 744 | 0.00 | 0.00 | 0.00 | 0.00 | 1174200.00 | 0.00 | 0.00 | 0.00 | 1692900.00 | 0.00 | 1364000.00 | 0.00 | KO:K02557 | chemotaxis protein MotB | Bacteria;Bacteroidetes;Chitinophagia;Chitinophagales;Chitinophagaceae;Terrimonas;Terrimonas ferruginea;Terrimonas ferruginea DSM 30193 |
| Ga0586324 4901708 284 622 | 0.00 | 0.00 | 0.00 | 0.00 | 3151800.00 | 0.00 | 0.00 | 0.00 | 0.00 | 0.00 | 0.00 | 0.00 | KO:K02557 | chemotaxis protein MotB | Bacteria;Proteobacteria;Alphaproteobacteria;Rhodospirillales;Acetobacteraceae;Rhodopila;unclassified Rhodopila;Rhodopila sp. LVNP |
| Ga0586324 0087555 318 3086 | 0.00 | 0.00 | 0.00 | 0.00 | 0.00 | 0.00 | 0.00 | 2220400.00 | 3183300.00 | 0.00 | 0.00 | 0.00 | KO:K02567 | nitrate reductase NapA | Bacteria;Proteobacteria;Betaproteobacteria;Nitrosomonadales;Thiobacillaceae;Annwoodia;Annwoodia aquaesulis;Annwoodia aquaesulis DSM 4255 |
| Ga0586324 0784720 3 1349 | 828441.00 | 1190000.00 | 0.00 | 0.00 | 0.00 | 0.00 | 0.00 | 0.00 | 0.00 | 0.00 | 0.00 | 0.00 | KO:K02567 | nitrate reductase NapA | Bacteria;Proteobacteria;Betaproteobacteria;Rhodocyclales;Azonexaceae;Dechloromonas;Dechloromonas sp. HYN0024;Dechloromonas sp. HYN0024 |
| Ga0586324 3389169 106 723 | 0.00 | 0.00 | 0.00 | 0.00 | 0.00 | 0.00 | 0.00 | 0.00 | 270507.60 | 0.00 | 0.00 | 0.00 | KO:K02585 | nitrogen fixation protein NifB | Archaea;Euryarchaeota;Methanomicrobia;Methanomicrobiales;Methanoregulaceae;Methanoregula;Methanoregula boonei;Methanoregula boonei 6A8 |
| Ga0586324 0001370 7693 9168 | 0.00 | 0.00 | 0.00 | 0.00 | 0.00 | 0.00 | 0.00 | 4422500.00 | 0.00 | 5605600.00 | 0.00 | 6127500.00 | KO:K02600 | N utilization substance protein A | Bacteria;Proteobacteria;Betaproteobacteria;Nitrosomonadales;Thiobacillaceae;Annwoodia;Annwoodia aquaesulis;Annwoodia aquaesulis DSM 4255 |
| Ga0586324 0002100 5981 7459 | 0.00 | 0.00 | 0.00 | 0.00 | 0.00 | 0.00 | 0.00 | 0.00 | 0.00 | 0.00 | 1408000.00 | 0.00 | KO:K02600 | N utilization substance protein A | Bacteria;Proteobacteria;Betaproteobacteria;Nitrosomonadales;Thiobacillaceae;Thiobacillus;Thiobacillus thioparus;Thiobacillus thioparus DSM 505 |
| Ga0586324 0078094 2 1483 | 0.00 | 0.00 | 0.00 | 0.00 | 0.00 | 0.00 | 0.00 | 90903.42 | 0.00 | 0.00 | 0.00 | 0.00 | KO:K02600 | N utilization substance protein A | Bacteria;Proteobacteria;Betaproteobacteria;Burkholderiales;Comamonadaceae;Pelomonas;Pelomonas sp. Root1444;Pelomonas sp. Root1444 |
| Ga0586324 0878243 132 1283 | 0.00 | 0.00 | 0.00 | 0.00 | 0.00 | 0.00 | 0.00 | 0.00 | 0.00 | 1208900.00 | 0.00 | 0.00 | KO:K02600 | N utilization substance protein A | Bacteria;Proteobacteria;Gammaproteobacteria;Nevskiales;Steroidobacteraceae;Povalibacter;Povalibacter uvarum;Povalibacter uvarum DSM 26723 |
| Ga0586324 3201847 1 741 | 0.00 | 0.00 | 0.00 | 0.00 | 0.00 | 0.00 | 0.00 | 0.00 | 0.00 | 0.00 | 0.00 | 11400000.00 | KO:K02600 | N utilization substance protein A | Bacteria;Proteobacteria;Alphaproteobacteria;Hyphomicrobiales;Bradyrhizobiaceae;Bradyrhizobium;Bradyrhizobium sp. Tv2a-2;Bradyrhizobium sp. TV2a.2 |
| Ga0586324 3384242 1 723 | 0.00 | 0.00 | 0.00 | 0.00 | 0.00 | 0.00 | 0.00 | 0.00 | 0.00 | 531021.26 | 0.00 | 0.00 | KO:K02600 | N utilization substance protein A | Bacteria;Proteobacteria;Alphaproteobacteria;Hyphomicrobiales;Bradyrhizobiaceae;Bradyrhizobium;Bradyrhizobium erythrophlei;Bradyrhizobium erythrophlei GAS242 |
| Ga0586324 5446681 1 594 | 0.00 | 0.00 | 0.00 | 0.00 | 0.00 | 0.00 | 0.00 | 0.00 | 1984500.00 | 0.00 | 0.00 | 0.00 | KO:K02600 | N utilization substance protein A | Bacteria;Proteobacteria;Betaproteobacteria;Nitrosomonadales;Methylophilaceae;unclassified Methylophilaceae;unclassified Methylophilaceae;Betaproteobacterium LSUCC0135 |
| Ga0586324 0000344 20374 21054 | 3460000.00 | 0.00 | 0.00 | 0.00 | 0.00 | 0.00 | 0.00 | 0.00 | 0.00 | 0.00 | 0.00 | 0.00 | KO:K02601 | transcriptional antiterminator NusG | Bacteria;Chloroflexi;Anaerolineae;Anaerolineales;Anaerolineaceae;Pelolinea;Pelolinea submarina;Pelolinea submarina DSM 23923 |
| Ga0586324 0019172 6643 7170 | 0.00 | 0.00 | 0.00 | 0.00 | 0.00 | 0.00 | 1974434.40 | 3184200.00 | 1287900.00 | 0.00 | 3379200.00 | 0.00 | KO:K02601 | transcriptional antiterminator NusG | Bacteria;Proteobacteria;Betaproteobacteria;Nitrosomonadales;Thiobacillaceae;Thiobacillus;Thiobacillus thioparus;Thiobacillus thioparus DSM 505 |
| Ga0586324 2225656 438 863 | 0.00 | 0.00 | 0.00 | 0.00 | 18849000.00 | 0.00 | 0.00 | 0.00 | 0.00 | 0.00 | 0.00 | 0.00 | KO:K02601 | transcriptional antiterminator NusG | Bacteria;Proteobacteria;Deltaproteobacteria;Desulfuromonadales;Desulfuromonadaceae;Desulfuromonas;Desulfuromonas sp. TF;Desulfuromonas sp. TF |
| Ga0586324 3751254 177 695 | 0.00 | 0.00 | 0.00 | 0.00 | 2647100.00 | 0.00 | 0.00 | 0.00 | 0.00 | 0.00 | 0.00 | 0.00 | KO:K02601 | transcriptional antiterminator NusG | Bacteria;Proteobacteria;Alphaproteobacteria;Hyphomicrobiales;Bradyrhizobiaceae;Afipia;Afipia sp. GAS231;Afipia sp. GAS231 |
| Ga0586324 4380261 383 652 | 0.00 | 0.00 | 0.00 | 1225500.00 | 5829800.00 | 0.00 | 0.00 | 0.00 | 0.00 | 1155000.00 | 1513600.00 | 0.00 | KO:K02601 | transcriptional antiterminator NusG | Bacteria;Proteobacteria;Alphaproteobacteria;Hyphomicrobiales;Bradyrhizobiaceae;Afipia;Afipia clevelandensis;Afipia clevelandensis ATCC 49720 |
| Ga0586324 0696672 280 1242 | 5710000.00 | 14348000.00 | 0.00 | 5225000.00 | 14553900.00 | 5796200.00 | 7136400.00 | 0.00 | 0.00 | 1632400.00 | 2789600.00 | 0.00 | KO:K02634 | apocytochrome f | Eukaryota;Streptophyta;Magnoliopsida;Malpighiales;Salicaceae;Populus;Populus trichocarpa;Populus balsamifera trichocarpa |
| Ga0586324 1135659 2 670 | 0.00 | 0.00 | 0.00 | 0.00 | 0.00 | 483764.35 | 0.00 | 0.00 | 0.00 | 0.00 | 0.00 | 0.00 | KO:K02634 | apocytochrome f | Eukaryota;Streptophyta;Magnoliopsida;Vitales;Vitaceae;Vitis;Vitis vinifera;Vitis vinifera PN40024 |
| Ga0586324 0018604 6467 6895 | 0.00 | 0.00 | 0.00 | 0.00 | 0.00 | 0.00 | 0.00 | 0.00 | 4592700.00 | 0.00 | 0.00 | 0.00 | KO:K02650 | type IV pilus assembly protein PilA | Bacteria;Proteobacteria;Betaproteobacteria;unclassified Betaproteobacteria;unclassified Betaproteobacteria;unclassified Betaproteobacteria;uncultured beta proteobacterium;unclassified betaproteobacterium ENR4 |
| Ga0586324 0242024 335 2050 | 0.00 | 517858.76 | 0.00 | 0.00 | 0.00 | 0.00 | 0.00 | 0.00 | 0.00 | 0.00 | 0.00 | 0.00 | KO:K02652 | type IV pilus assembly protein PilB | Bacteria;Proteobacteria;Betaproteobacteria;unclassified Betaproteobacteria;unclassified Betaproteobacteria;Chitinivorax;Chitinivorax tropicus;Chitinivorax tropicus DSM 27165 |
| Ga0586324 2498643 94 822 | 0.00 | 0.00 | 0.00 | 0.00 | 0.00 | 0.00 | 876060.36 | 0.00 | 0.00 | 0.00 | 0.00 | 0.00 | KO:K02653 | type IV pilus assembly protein PilC | Bacteria;Proteobacteria;Betaproteobacteria;Rhodocyclales;Rhodocyclaceae;Azospira;unclassified Azospira;Azospira sp. ZAP |
| Ga0586324 4696893 3 632 | 0.00 | 0.00 | 0.00 | 0.00 | 0.00 | 0.00 | 0.00 | 0.00 | 0.00 | 0.00 | 3951200.00 | 0.00 | KO:K02653 | type IV pilus assembly protein PilC | Bacteria;Proteobacteria;Deltaproteobacteria;Desulfobacterales;Desulfobacteraceae;Desulfosarcina;Desulfosarcina variabilis;Desulfosarcina variabilis Montpellier |
| Ga0586324 8039304 3 506 | 8360000.00 | 19992000.00 | 0.00 | 2394000.00 | 0.00 | 0.00 | 0.00 | 0.00 | 0.00 | 0.00 | 0.00 | 0.00 | KO:K02653 | type IV pilus assembly protein PilC | Bacteria;Planctomycetes;Planctomycetia;Pirellulales;Pirellulaceae;Rhodopirellula;Rhodopirellula maiorica;Rhodopirellula maiorica SM1 |
| Ga0586324 0116631 1306 2082 | 0.00 | 0.00 | 0.00 | 0.00 | 0.00 | 0.00 | 0.00 | 0.00 | 1490400.00 | 1632400.00 | 1232000.00 | 0.00 | KO:K02656 | type IV pilus assembly protein PilF | Bacteria;Proteobacteria;Betaproteobacteria;Nitrosomonadales;Thiobacillaceae;Thiobacillus;Thiobacillus thioparus;Thiobacillus thioparus DSM 505 |
| Ga0586324 0000004 125073 125438 | 0.00 | 0.00 | 0.00 | 0.00 | 0.00 | 0.00 | 0.00 | 0.00 | 0.00 | 0.00 | 533492.08 | 0.00 | KO:K02658 | twitching motility two-component system response regulator PilH | Bacteria;Proteobacteria;Betaproteobacteria;Nitrosomonadales;Thiobacillaceae;Annwoodia;Annwoodia aquaesulis;Annwoodia aquaesulis DSM 4255 |
| Ga0586324 0000004 125995 128109 | 0.00 | 0.00 | 0.00 | 0.00 | 0.00 | 0.00 | 596213.16 | 6527000.00 | 321730.38 | 9500900.64 | 11792000.00 | 17775000.00 | KO:K02660 | twitching motility protein PilJ | Bacteria;Proteobacteria;Betaproteobacteria;Nitrosomonadales;Thiobacillaceae;Annwoodia;Annwoodia aquaesulis;Annwoodia aquaesulis DSM 4255 |
| Ga0586324 0002716 12344 14464 | 0.00 | 0.00 | 0.00 | 0.00 | 0.00 | 0.00 | 1041486.90 | 0.00 | 258951.33 | 181475.14 | 1636800.00 | 757500.00 | KO:K02660 | twitching motility protein PilJ | Bacteria;Proteobacteria;Betaproteobacteria;Nitrosomonadales;Thiobacillaceae;Thiobacillus;Thiobacillus thioparus;Thiobacillus thioparus DSM 505 |
| Ga0586324 0102567 1 1218 | 0.00 | 0.00 | 0.00 | 0.00 | 0.00 | 0.00 | 0.00 | 695400.00 | 0.00 | 0.00 | 0.00 | 1192500.00 | KO:K02660 | twitching motility protein PilJ | Bacteria;Proteobacteria;Betaproteobacteria;Nitrosomonadales;Sulfuricellaceae;Sulfurirhabdus;Sulfurirhabdus autotrophica;Sulfurirhabdus autotrophica DSM 100309 |
| Ga0586324 1459024 69 1034 | 0.00 | 0.00 | 0.00 | 0.00 | 0.00 | 0.00 | 0.00 | 0.00 | 0.00 | 216102.81 | 271538.08 | 0.00 | KO:K02660 | twitching motility protein PilJ | Bacteria;Proteobacteria;Gammaproteobacteria;Acidiferrobacterales;Acidiferrobacteraceae;Sulfurifustis;Sulfurifustis variabilis;Sulfurifustis variabilis skN76 |
| Ga0586324 2474992 2 826 | 0.00 | 0.00 | 0.00 | 1624500.00 | 0.00 | 0.00 | 0.00 | 0.00 | 0.00 | 0.00 | 0.00 | 0.00 | KO:K02660 | twitching motility protein PilJ | Bacteria;Proteobacteria;Betaproteobacteria;Nitrosomonadales;Sulfuricellaceae;Sulfurirhabdus;Sulfurirhabdus autotrophica;Sulfurirhabdus autotrophica DSM 100309 |
| Ga0586324 0000270 33118 34206 | 0.00 | 0.00 | 0.00 | 0.00 | 0.00 | 0.00 | 0.00 | 0.00 | 5483700.00 | 2310000.00 | 0.00 | 0.00 | KO:K02662 | type IV pilus assembly protein PilM | Bacteria;Proteobacteria;Betaproteobacteria;Nitrosomonadales;Thiobacillaceae;Thiobacillus;Thiobacillus thioparus;Thiobacillus thioparus DSM 505 |
| Ga0586324 0000764 10120 11208 | 0.00 | 0.00 | 0.00 | 0.00 | 0.00 | 0.00 | 0.00 | 1006500.00 | 444981.60 | 0.00 | 1293600.00 | 1425000.00 | KO:K02662 | type IV pilus assembly protein PilM | Bacteria;Proteobacteria;Betaproteobacteria;Nitrosomonadales;Thiobacillaceae;Annwoodia;Annwoodia aquaesulis;Annwoodia aquaesulis DSM 4255 |
| Ga0586324 0017960 1546 2589 | 0.00 | 0.00 | 0.00 | 0.00 | 0.00 | 0.00 | 0.00 | 2379000.00 | 1660500.00 | 0.00 | 1716000.00 | 0.00 | KO:K02662 | type IV pilus assembly protein PilM | Bacteria;Gemmatimonadetes;Gemmatimonadetes;Gemmatimonadales;Gemmatimonadaceae;Gemmatimonas;Gemmatimonas aurantiaca;Gemmatimonas aurantiaca T-27T |
| Ga0586324 0243266 3 665 | 0.00 | 0.00 | 0.00 | 0.00 | 0.00 | 0.00 | 0.00 | 0.00 | 705339.09 | 0.00 | 0.00 | 0.00 | KO:K02662 | type IV pilus assembly protein PilM | Bacteria;Proteobacteria;Betaproteobacteria;unclassified Betaproteobacteria;unclassified Betaproteobacteria;unclassified Betaproteobacteria;Betaproteobacteria bacterium GR16-43;Betaproteobacteria bacterium GR16-43 |
| Ga0586324 0251217 3 1082 | 0.00 | 0.00 | 0.00 | 0.00 | 0.00 | 0.00 | 0.00 | 628300.00 | 0.00 | 0.00 | 0.00 | 0.00 | KO:K02662 | type IV pilus assembly protein PilM | Bacteria;Proteobacteria;Deltaproteobacteria;unclassified Deltaproteobacteria;unclassified Deltaproteobacteria;unclassified Deltaproteobacteria;delta proteobacterium NaphS2;delta proteobacterium NaphS2 |
| Ga0586324 0353963 512 1579 | 0.00 | 0.00 | 0.00 | 0.00 | 0.00 | 0.00 | 0.00 | 0.00 | 0.00 | 0.00 | 0.00 | 2040000.00 | KO:K02662 | type IV pilus assembly protein PilM | Bacteria;Acidobacteria;Thermoanaerobaculia;Thermoanaerobaculales;Thermoanaerobaculaceae;Thermoanaerobaculum;Thermoanaerobaculum aquaticum;Thermoanaerobaculum aquaticum MP-01 |
| Ga0586324 6591882 3 551 | 0.00 | 0.00 | 0.00 | 0.00 | 0.00 | 0.00 | 1191610.08 | 0.00 | 0.00 | 0.00 | 0.00 | 0.00 | KO:K02662 | type IV pilus assembly protein PilM | Bacteria;Proteobacteria;Betaproteobacteria;Nitrosomonadales;Sulfuricellaceae;Sulfurirhabdus;Sulfurirhabdus autotrophica;Sulfurirhabdus autotrophica DSM 100309 |
| Ga0586324 0000270 34203 34781 | 0.00 | 0.00 | 0.00 | 0.00 | 0.00 | 0.00 | 0.00 | 0.00 | 11745000.00 | 0.00 | 0.00 | 0.00 | KO:K02663 | type IV pilus assembly protein PilN | Bacteria;Proteobacteria;Betaproteobacteria;Nitrosomonadales;Thiobacillaceae;Thiobacillus;Thiobacillus thioparus;Thiobacillus thioparus DSM 505 |
| Ga0586324 0000764 11205 11783 | 0.00 | 0.00 | 0.00 | 0.00 | 0.00 | 0.00 | 2530800.00 | 3153700.00 | 14094000.00 | 2918300.00 | 0.00 | 3457500.00 | KO:K02663 | type IV pilus assembly protein PilN | Bacteria;Proteobacteria;Betaproteobacteria;Nitrosomonadales;Thiobacillaceae;Annwoodia;Annwoodia aquaesulis;Annwoodia aquaesulis DSM 4255 |
| Ga0586324 0188911 1 429 | 0.00 | 1176400.00 | 0.00 | 0.00 | 0.00 | 0.00 | 0.00 | 1848300.00 | 2875500.00 | 0.00 | 0.00 | 1185000.00 | KO:K02663 | type IV pilus assembly protein PilN | Bacteria;Proteobacteria;Betaproteobacteria;unclassified Betaproteobacteria;unclassified Betaproteobacteria;unclassified Betaproteobacteria;Betaproteobacteria bacterium GR16-43;Betaproteobacteria bacterium GR16-43 |
| Ga0586324 2135377 2 469 | 0.00 | 0.00 | 0.00 | 0.00 | 0.00 | 0.00 | 0.00 | 0.00 | 0.00 | 0.00 | 818666.64 | 0.00 | KO:K02663 | type IV pilus assembly protein PilN | Bacteria;Proteobacteria;Betaproteobacteria;unclassified Betaproteobacteria;unclassified Betaproteobacteria;unclassified Betaproteobacteria;Betaproteobacteria bacterium GR16-43;Betaproteobacteria bacterium GR16-43 |
| Ga0586324 0000270 34778 35410 | 0.00 | 0.00 | 0.00 | 0.00 | 0.00 | 0.00 | 0.00 | 2263100.00 | 0.00 | 0.00 | 0.00 | 0.00 | KO:K02664 | type IV pilus assembly protein PilO | Bacteria;Proteobacteria;Betaproteobacteria;Nitrosomonadales;Thiobacillaceae;Thiobacillus;Thiobacillus thioparus;Thiobacillus thioparus DSM 505 |
| Ga0586324 0038063 1559 2182 | 0.00 | 0.00 | 0.00 | 0.00 | 0.00 | 0.00 | 0.00 | 1037000.00 | 1474200.00 | 0.00 | 0.00 | 0.00 | KO:K02664 | type IV pilus assembly protein PilO | Bacteria;Proteobacteria;Betaproteobacteria;Nitrosomonadales;Sulfuricellaceae;Sulfurirhabdus;Sulfurirhabdus autotrophica;Sulfurirhabdus autotrophica DSM 100309 |
| Ga0586324 0000270 35935 38181 | 0.00 | 4290800.00 | 3644833.85 | 2156500.00 | 2482300.00 | 15217168.80 | 24670694.40 | 27498800.00 | 27426600.00 | 32632600.00 | 40876000.00 | 24135000.00 | KO:K02666 | type IV pilus assembly protein PilQ | Bacteria;Proteobacteria;Betaproteobacteria;Nitrosomonadales;Thiobacillaceae;Thiobacillus;Thiobacillus thioparus;Thiobacillus thioparus DSM 505 |
| Ga0586324 0000764 12917 15133 | 0.00 | 0.00 | 0.00 | 0.00 | 0.00 | 0.00 | 29936400.00 | 70363500.00 | 81056700.00 | 107105527.76 | 125479200.00 | 102727500.00 | KO:K02666 | type IV pilus assembly protein PilQ | Bacteria;Proteobacteria;Betaproteobacteria;Nitrosomonadales;Thiobacillaceae;Annwoodia;Annwoodia aquaesulis;Annwoodia aquaesulis DSM 4255 |
| Ga0586324 0001026 3536 5200 | 4010000.00 | 0.00 | 0.00 | 0.00 | 0.00 | 0.00 | 0.00 | 0.00 | 0.00 | 0.00 | 0.00 | 0.00 | KO:K02666 | type IV pilus assembly protein PilQ | Bacteria;Nitrospirae;unclassified Nitrospirae;unclassified Nitrospirae;unclassified Nitrospirae;unclassified Nitrospirae;Nitrospirae bacterium HCH-1;Nitrospirae bacterium HCH-1 |
| Ga0586324 0002176 11890 14109 | 0.00 | 741200.00 | 0.00 | 0.00 | 0.00 | 0.00 | 0.00 | 0.00 | 0.00 | 0.00 | 0.00 | 0.00 | KO:K02666 | type IV pilus assembly protein PilQ | Bacteria;Proteobacteria;Gammaproteobacteria;Chromatiales;Woeseiaceae;Woeseia;Woeseia oceani;Woeseia oceani XK5 |

| | | | | | | | | | | | | | | | |
|---|---|---|---|---|---|---|---|---|---|---|---|---|---|---|---|
| Ga0586324 0003249 11808 14147 | 0.00 | 0.00 | 0.00 | 0.00 | 0.00 | 0.00 | 0.00 | 0.00 | 0.00 | 1124200.00 | 0.00 | 0.00 | KO:K02666 | type IV pilus assembly protein PilQ | Bacteria;Acidobacteria;Vicinamibacteria;unclassified Vicinamibacteria;Vicinamibacteraceae;Luteitalea;Luteitalea pratensis;Luteitalea pratensis DSM 100886 |
| Ga0586324 0008939 1796 3274 | 0.00 | 6514400.00 | 0.00 | 5044500.00 | 3862500.00 | 0.00 | 0.00 | 0.00 | 939600.00 | 0.00 | 0.00 | 982500.00 | KO:K02666 | type IV pilus assembly protein PilQ | Bacteria;Planctomycetes;Phycisphaerae;Sedimentisphaerales;Anaerohalosphaeraceae;Anaerohalosphaera;Anaerohalosphaera lusitana;Phycisphaerae bacterium ST-NAGAB-D1 |
| Ga0586324 0015478 1276 3417 | 0.00 | 0.00 | 0.00 | 0.00 | 0.00 | 0.00 | 1470586.32 | 0.00 | 0.00 | 0.00 | 0.00 | 0.00 | KO:K02666 | type IV pilus assembly protein PilQ | Bacteria;Proteobacteria;Betaproteobacteria;Nitrosomonadales;Methylophilaceae;Methylobacillus;Methylobacillus sp. MM3;Methylobacillus sp. MM2 |
| Ga0586324 0016778 6904 8067 | 655054.00 | 1407600.00 | 0.00 | 0.00 | 3275400.00 | 2674942.33 | 0.00 | 3928400.00 | 0.00 | 5505500.00 | 3053600.00 | 802500.00 | KO:K02666 | type IV pilus assembly protein PilQ | Bacteria;Gemmatimonadetes;Gemmatimonadetes;Gemmatimonadales;Gemmatimonadaceae;Gemmatirosa;Gemmatirosa kalamazoonesis;Gemmatimonadetes bacterium KBS708 |
| Ga0586324 0022280 2961 5720 | 0.00 | 0.00 | 0.00 | 0.00 | 0.00 | 0.00 | 0.00 | 646600.00 | 0.00 | 0.00 | 0.00 | 0.00 | KO:K02666 | type IV pilus assembly protein PilQ | Bacteria;Proteobacteria;Deltaproteobacteria;Desulfuromonadales;Geobacteraceae;Geobacter;Geobacter uraniireducens;Geobacter uraniireducens Rf4 |
| Ga0586324 0023027 3196 5898 | 0.00 | 0.00 | 0.00 | 0.00 | 0.00 | 0.00 | 0.00 | 0.00 | 2065500.00 | 19789000.00 | 3713600.00 | 0.00 | KO:K02666 | type IV pilus assembly protein PilQ | Bacteria;Proteobacteria;Deltaproteobacteria;Desulfobacterales;Desulfobacteraceae;Desulfofaba;Desulfofaba hansenii;Desulfofaba hansenii DSM 12642 |
| Ga0586324 0023547 4357 6492 | 0.00 | 795600.00 | 3838922.47 | 2641000.00 | 0.00 | 2854279.14 | 1453627.68 | 933300.00 | 0.00 | 15076600.00 | 13252800.00 | 11167500.00 | KO:K02666 | type IV pilus assembly protein PilQ | Bacteria;Proteobacteria;Betaproteobacteria;Nitrosomonadales;Gallionellaceae;Ferriphaselus;Ferriphaselus amnicola;Ferriphaselus amnicola OYT1 |
| Ga0586324 0033140 3261 5339 | 0.00 | 0.00 | 0.00 | 0.00 | 0.00 | 0.00 | 0.00 | 1146800.00 | 0.00 | 703188.64 | 2376000.00 | 5647500.00 | KO:K02666 | type IV pilus assembly protein PilQ | Bacteria;Proteobacteria;Betaproteobacteria;unclassified Betaproteobacteria;unclassified Betaproteobacteria;unclassified Betaproteobacteria;uncultured beta proteobacterium;unclassified betaproteobacterium ENR4 |
| Ga0586324 0033725 3242 5440 | 0.00 | 0.00 | 0.00 | 0.00 | 5613500.00 | 0.00 | 0.00 | 0.00 | 0.00 | 0.00 | 0.00 | 0.00 | KO:K02666 | type IV pilus assembly protein PilQ | Bacteria;Proteobacteria;Gammaproteobacteria;Alteromonadales;Alteromonadaceae;unclassified Alteromonadaceae;Alteromonadaceae bacterium 2719K.STB50.0a.01;Alteromonadaceae bacterium 2719K.STB50.0a.01 |
| Ga0586324 0034966 365 2491 | 3930000.00 | 0.00 | 6924900.00 | 0.00 | 8579900.00 | 5915300.00 | 0.00 | 6002400.00 | 0.00 | 0.00 | 0.00 | 0.00 | KO:K02666 | type IV pilus assembly protein PilQ | Bacteria;Proteobacteria;Betaproteobacteria;unclassified Betaproteobacteria;unclassified Betaproteobacteria;unclassified Betaproteobacteria;uncultured beta proteobacterium;unclassified betaproteobacterium ENR4 |
| Ga0586324 0034994 1162 3804 | 0.00 | 0.00 | 0.00 | 0.00 | 0.00 | 0.00 | 0.00 | 0.00 | 0.00 | 0.00 | 0.00 | 1042500.00 | KO:K02666 | type IV pilus assembly protein PilQ | Bacteria;Proteobacteria;Gammaproteobacteria;Acidiferrobacterales;Acidiferrobacteraceae;Sulfuricaulis;Sulfuricaulis limicola;Sulfuricaulis limicola HA5 |
| Ga0586324 0041710 1 1551 | 0.00 | 0.00 | 1253739.07 | 0.00 | 0.00 | 0.00 | 1736676.00 | 0.00 | 0.00 | 0.00 | 0.00 | 597207.75 | KO:K02666 | type IV pilus assembly protein PilQ | Bacteria;Candidatus Aminicenantes;unclassified Candidatus Aminicenantes;unclassified Candidatus Aminicenantes;unclassified Candidatus Aminicenantes;unclassified Candidatus Aminicenantes;Aminicenantes bacterium SCGC AAA255-F06;Aminicenantes bacterium SCGC AAA255-F06 (contamination screened) |
| Ga0586324 0049645 2432 4786 | 0.00 | 5310800.00 | 6587100.00 | 6716500.00 | 6962800.00 | 7423900.00 | 5654400.00 | 4800700.00 | 8181000.00 | 3903900.00 | 7858400.00 | 6052500.00 | KO:K02666 | type IV pilus assembly protein PilQ | Bacteria;Proteobacteria;Deltaproteobacteria;Syntrophobacterales;Syntrophobacteraceae;Desulfacinum;Desulfacinum hydrothermale;Desulfacinum hydrothermale DSM 13146 |
| Ga0586324 0051811 473 3052 | 7180000.00 | 5807200.00 | 0.00 | 2764500.00 | 3038500.00 | 0.00 | 1409179.08 | 1500600.00 | 3450600.00 | 2387000.00 | 1997600.00 | 1035000.00 | KO:K02666 | type IV pilus assembly protein PilQ | Bacteria;Proteobacteria;Deltaproteobacteria;Desulfuromonadales;Desulfuromonadaceae;Desulfuromonas;Desulfuromonas sp. DDH964;Desulfuromonas sp. DDH964 |
| Ga0586324 0055790 1 1644 | 1420000.00 | 0.00 | 0.00 | 0.00 | 0.00 | 0.00 | 0.00 | 0.00 | 0.00 | 0.00 | 0.00 | 0.00 | KO:K02666 | type IV pilus assembly protein PilQ | Bacteria;Nitrospirae;Nitrospira;Nitrospirales;Nitrospiraceae;Nitrospira;Nitrospira japonica;Nitrospira japonica NJ11 |
| Ga0586324 0073164 2140 3774 | 0.00 | 0.00 | 0.00 | 0.00 | 0.00 | 0.00 | 5016000.00 | 0.00 | 0.00 | 0.00 | 0.00 | 0.00 | KO:K02666 | type IV pilus assembly protein PilQ | Bacteria;Proteobacteria;Deltaproteobacteria;Desulfovibrionales;Desulfovibrionaceae;Cupidesulfovibrio;Cupidesulfovibrio termitidis;Desulfovibrio termitidis HI1 |
| Ga0586324 0092509 474 2831 | 0.00 | 0.00 | 0.00 | 0.00 | 0.00 | 0.00 | 0.00 | 0.00 | 0.00 | 0.00 | 0.00 | 4627500.00 | KO:K02666 | type IV pilus assembly protein PilQ | Bacteria;Proteobacteria;Deltaproteobacteria;Syntrophobacterales;Syntrophobacteraceae;Desulfosoma;Desulfosoma caldarium;Desulfosoma caldarium DSM 22027 |
| Ga0586324 0092904 1684 3552 | 0.00 | 0.00 | 0.00 | 0.00 | 0.00 | 0.00 | 0.00 | 0.00 | 553673.88 | 4027100.00 | 0.00 | 0.00 | KO:K02666 | type IV pilus assembly protein PilQ | Bacteria;Proteobacteria;Gammaproteobacteria;Acidiferrobacterales;Acidiferrobacteraceae;Sulfuricaulis;Sulfuricaulis limicola;Sulfuricaulis limicola HA5 |
| Ga0586324 0110299 2711 3271 | 1630000.00 | 0.00 | 0.00 | 2327500.00 | 3120900.00 | 0.00 | 0.00 | 4782400.00 | 0.00 | 197354.08 | 383650.96 | 0.00 | KO:K02666 | type IV pilus assembly protein PilQ | Bacteria;Candidatus Aminicenantes;unclassified Candidatus Aminicenantes;unclassified Candidatus Aminicenantes;unclassified Candidatus Aminicenantes;unclassified Candidatus Aminicenantes;Aminicenantes bacterium SCGC AAA255-F06;Aminicenantes bacterium SCGC AAA255-F06 (contamination screened) |
| Ga0586324 0111280 1 1083 | 2250000.00 | 0.00 | 1054420.18 | 0.00 | 0.00 | 658924.72 | 0.00 | 0.00 | 0.00 | 0.00 | 3608000.00 | 817500.00 | KO:K02666 | type IV pilus assembly protein PilQ | Bacteria;Candidatus Aminicenantes;unclassified Candidatus Aminicenantes;unclassified Candidatus Aminicenantes;unclassified Candidatus Aminicenantes;unclassified Candidatus Aminicenantes;Aminicenantes bacterium SCGC AAA255-F06;Aminicenantes bacterium SCGC AAA255-F06 (contamination screened) |
| Ga0586324 0112968 1661 3235 | 0.00 | 0.00 | 0.00 | 0.00 | 0.00 | 0.00 | 0.00 | 0.00 | 0.00 | 0.00 | 0.00 | 469237.50 | KO:K02666 | type IV pilus assembly protein PilQ | Bacteria;Proteobacteria;Betaproteobacteria;Burkholderiales;Oxalobacteraceae;Noviherbaspirillum;Noviherbaspirillum denitrificans;Noviherbaspirillum denitrificans TSA40 |
| Ga0586324 0114907 160 2370 | 0.00 | 0.00 | 0.00 | 0.00 | 0.00 | 0.00 | 0.00 | 3806400.00 | 0.00 | 4004000.00 | 0.00 | 0.00 | KO:K02666 | type IV pilus assembly protein PilQ | Bacteria;Proteobacteria;Betaproteobacteria;unclassified Betaproteobacteria;unclassified Betaproteobacteria;unclassified Betaproteobacteria;Betaproteobacteria bacterium GR16-43;Betaproteobacteria bacterium GR16-43 |
| Ga0586324 0123409 1394 3103 | 0.00 | 0.00 | 5109793.63 | 0.00 | 8157600.00 | 4089100.00 | 0.00 | 356600.51 | 0.00 | 3796100.00 | 4734400.00 | 3480000.00 | KO:K02666 | type IV pilus assembly protein PilQ | Bacteria;Proteobacteria;Betaproteobacteria;unclassified Betaproteobacteria;unclassified Betaproteobacteria;unclassified Betaproteobacteria;uncultured beta proteobacterium;unclassified betaproteobacterium ENR4 |
| Ga0586324 0156759 1110 2771 | 0.00 | 2223600.00 | 0.00 | 3581500.00 | 1812800.00 | 0.00 | 0.00 | 2269200.00 | 2875500.00 | 2541000.00 | 2666400.00 | 2992500.00 | KO:K02666 | type IV pilus assembly protein PilQ | Bacteria;Proteobacteria;Betaproteobacteria;Burkholderiales;Comamonadaceae;unclassified Comamonadaceae;Comamonadaceae bacterium URHA0028;Comamonadaceae bacterium URHA0028 |
| Ga0586324 0162750 1676 2725 | 0.00 | 481633.12 | 0.00 | 0.00 | 0.00 | 0.00 | 0.00 | 0.00 | 0.00 | 0.00 | 0.00 | 0.00 | KO:K02666 | type IV pilus assembly protein PilQ | Bacteria;Candidatus Aminicenantes;unclassified Candidatus Aminicenantes;unclassified Candidatus Aminicenantes;unclassified Candidatus Aminicenantes;unclassified Candidatus Aminicenantes;Aminicenantes bacterium SCGC AAA252-K06;Aminicenantes bacterium SCGC AAA252-K06 (contamination screened) |
| Ga0586324 0166836 1088 2692 | 14300000.00 | 0.00 | 0.00 | 0.00 | 0.00 | 0.00 | 0.00 | 0.00 | 0.00 | 0.00 | 0.00 | 0.00 | KO:K02666 | type IV pilus assembly protein PilQ | Bacteria;Proteobacteria;Betaproteobacteria;unclassified Betaproteobacteria;unclassified Betaproteobacteria;unclassified Betaproteobacteria;Betaproteobacteria bacterium GR16-43;Betaproteobacteria bacterium GR16-43 |
| Ga0586324 0171803 3 1733 | 0.00 | 298117.44 | 0.00 | 0.00 | 0.00 | 0.00 | 0.00 | 0.00 | 1093500.00 | 2887500.00 | 0.00 | 2141523.00 | KO:K02666 | type IV pilus assembly protein PilQ | Bacteria;Proteobacteria;Betaproteobacteria;Nitrosomonadales;Thiobacillaceae;Thiobacillus;Thiobacillus denitrificans;Thiobacillus denitrificans RG |
| Ga0586324 0184509 373 2499 | 0.00 | 0.00 | 0.00 | 2764500.00 | 0.00 | 0.00 | 0.00 | 1134600.00 | 4171500.00 | 0.00 | 1100000.00 | 0.00 | KO:K02666 | type IV pilus assembly protein PilQ | Bacteria;Proteobacteria;Betaproteobacteria;unclassified Betaproteobacteria;unclassified Betaproteobacteria;unclassified Betaproteobacteria;uncultured beta proteobacterium;unclassified betaproteobacterium ENR4 |
| Ga0586324 0187591 462 2549 | 0.00 | 0.00 | 0.00 | 0.00 | 0.00 | 0.00 | 0.00 | 208070.39 | 0.00 | 0.00 | 0.00 | 0.00 | KO:K02666 | type IV pilus assembly protein PilQ | Bacteria;Proteobacteria;Deltaproteobacteria;unclassified Deltaproteobacteria;unclassified Deltaproteobacteria;unclassified Deltaproteobacteria;delta proteobacterium NaphS2;delta proteobacterium NaphS2 |
| Ga0586324 0188911 1588 2541 | 0.00 | 0.00 | 0.00 | 0.00 | 0.00 | 0.00 | 0.00 | 0.00 | 0.00 | 0.00 | 0.00 | 2835000.00 | KO:K02666 | type IV pilus assembly protein PilQ | Bacteria;Proteobacteria;Betaproteobacteria;unclassified Betaproteobacteria;unclassified Betaproteobacteria;unclassified Betaproteobacteria;Betaproteobacteria bacterium GR16-43;Betaproteobacteria bacterium GR16-43 |
| Ga0586324 0198823 1423 2481 | 0.00 | 0.00 | 0.00 | 0.00 | 0.00 | 0.00 | 0.00 | 0.00 | 0.00 | 0.00 | 1056000.00 | 0.00 | KO:K02666 | type IV pilus assembly protein PilQ | Bacteria;Proteobacteria;Gammaproteobacteria;Nevskiales;Steroidobacteraceae;Steroidobacter;Steroidobacter denitrificans;Steroidobacter denitrificans DSM 18526 |
| Ga0586324 0199306 3 692 | 0.00 | 0.00 | 0.00 | 0.00 | 0.00 | 432702.21 | 1079397.60 | 0.00 | 4349700.00 | 0.00 | 0.00 | 0.00 | KO:K02666 | type IV pilus assembly protein PilQ | Bacteria;Candidatus Aminicenantes;unclassified Candidatus Aminicenantes;unclassified Candidatus Aminicenantes;unclassified Candidatus Aminicenantes;unclassified Candidatus Aminicenantes;Aminicenantes bacterium SCGC AAA255-F06;Aminicenantes bacterium SCGC AAA255-F06 (contamination screened) |
| Ga0586324 0213249 1 1947 | 0.00 | 0.00 | 0.00 | 0.00 | 0.00 | 0.00 | 0.00 | 0.00 | 1101600.00 | 0.00 | 0.00 | 0.00 | KO:K02666 | type IV pilus assembly protein PilQ | Bacteria;Proteobacteria;Betaproteobacteria;unclassified Betaproteobacteria;unclassified Betaproteobacteria;unclassified Betaproteobacteria;Betaproteobacteria bacterium GR16-43;Betaproteobacteria bacterium GR16-43 |
| Ga0586324 0224130 58 2298 | 0.00 | 775200.00 | 0.00 | 304989.90 | 0.00 | 0.00 | 0.00 | 0.00 | 0.00 | 12089000.00 | 3044800.00 | 0.00 | KO:K02666 | type IV pilus assembly protein PilQ | Bacteria;Proteobacteria;Betaproteobacteria;unclassified Betaproteobacteria;unclassified Betaproteobacteria;unclassified Betaproteobacteria;Betaproteobacteria bacterium GR16-43;Betaproteobacteria bacterium GR16-43 |
| Ga0586324 0229359 1 1098 | 0.00 | 0.00 | 0.00 | 0.00 | 0.00 | 0.00 | 0.00 | 0.00 | 0.00 | 0.00 | 8060800.00 | 780000.00 | KO:K02666 | type IV pilus assembly protein PilQ | Bacteria;Proteobacteria;Betaproteobacteria;Nitrosomonadales;Sulfuricellaceae;Sulfurirhabdus;Sulfurirhabdus autotrophica;Sulfurirhabdus autotrophica DSM 100309 |
| Ga0586324 0287781 2 2095 | 577271.00 | 0.00 | 0.00 | 0.00 | 0.00 | 0.00 | 0.00 | 0.00 | 0.00 | 0.00 | 0.00 | 0.00 | KO:K02666 | type IV pilus assembly protein PilQ | Bacteria;Proteobacteria;Betaproteobacteria;Burkholderiales;unclassified Burkholderiales;unclassified Burkholderiales;Burkholderiales bacterium JOSHI 001;Burkholderiales sp. JOSHI 001 |
| Ga0586324 0296842 611 2065 | 0.00 | 2414000.00 | 0.00 | 0.00 | 0.00 | 0.00 | 0.00 | 0.00 | 0.00 | 0.00 | 0.00 | 0.00 | KO:K02666 | type IV pilus assembly protein PilQ | Bacteria;Proteobacteria;Deltaproteobacteria;Desulfuromonadales;Desulfuromonadaceae;Desulfuromonas;Desulfuromonas sp. TF;Desulfuromonas sp. TF |
| Ga0586324 0330647 3 404 | 0.00 | 4052800.00 | 6080400.00 | 6602500.00 | 6118200.00 | 4089100.00 | 0.00 | 2690100.00 | 10044000.00 | 2718100.00 | 225844.08 | 2175000.00 | KO:K02666 | type IV pilus assembly protein PilQ | Bacteria;Proteobacteria;Deltaproteobacteria;Desulfobacterales;Desulfobacteraceae;Desulfatitalea;Desulfatitalea tepidiphila;Desulfatitalea tepidiphila S28bF |
| Ga0586324 0374955 3 1622 | 0.00 | 0.00 | 0.00 | 0.00 | 0.00 | 0.00 | 0.00 | 0.00 | 4471200.00 | 0.00 | 0.00 | 0.00 | KO:K02666 | type IV pilus assembly protein PilQ | Bacteria;Proteobacteria;Deltaproteobacteria;Myxococcales;Anaeromyxobacteraceae;Anaeromyxobacter;Anaeromyxobacter sp. Fw109-5;Anaeromyxobacter sp. Fw109-5 |
| Ga0586324 0436619 3 944 | 0.00 | 0.00 | 0.00 | 0.00 | 0.00 | 0.00 | 0.00 | 0.00 | 2389500.00 | 0.00 | 0.00 | 0.00 | KO:K02666 | type IV pilus assembly protein PilQ | Bacteria;Proteobacteria;Betaproteobacteria;Nitrosomonadales;Gallionellaceae;Ferriphaselus;Ferriphaselus amnicola;Ferriphaselus amnicola OYT1 |
| Ga0586324 0443912 489 1727 | 0.00 | 0.00 | 0.00 | 0.00 | 0.00 | 0.00 | 7182000.00 | 7893400.00 | 7816500.00 | 19188400.00 | 18286400.00 | 10650000.00 | KO:K02666 | type IV pilus assembly protein PilQ | Bacteria;Proteobacteria;Betaproteobacteria;Nitrosomonadales;Sulfuricellaceae;Sulfuricella;Sulfuricella sp. T08;Sulfuricella sp. T08 |
| Ga0586324 0455004 2 1651 | 0.00 | 0.00 | 0.00 | 4322500.00 | 0.00 | 0.00 | 0.00 | 0.00 | 0.00 | 0.00 | 0.00 | 0.00 | KO:K02666 | type IV pilus assembly protein PilQ | Bacteria;Proteobacteria;Betaproteobacteria;unclassified Betaproteobacteria;unclassified Betaproteobacteria;unclassified Betaproteobacteria;beta proteobacterium AAP65;Rubrivivax sp. AAP65 |
| Ga0586324 0532439 853 1593 | 0.00 | 0.00 | 90080000.00 | 4275000.00 | 0.00 | 0.00 | 0.00 | 1579900.00 | 0.00 | 0.00 | 0.00 | 0.00 | KO:K02666 | type IV pilus assembly protein PilQ | Bacteria;Candidatus Aminicenantes;unclassified Candidatus Aminicenantes;unclassified Candidatus Aminicenantes;unclassified Candidatus Aminicenantes;unclassified Candidatus Aminicenantes;Aminicenantes bacterium SCGC AAA255-A06;Aminicenantes bacterium SCGC AAA255-A06 (contamination screened) |
| Ga0586324 0776277 2 1354 | 0.00 | 0.00 | 0.00 | 0.00 | 0.00 | 0.00 | 0.00 | 0.00 | 0.00 | 0.00 | 8289600.00 | 0.00 | KO:K02666 | type IV pilus assembly protein PilQ | Bacteria;Proteobacteria;Betaproteobacteria;unclassified Betaproteobacteria;unclassified Betaproteobacteria;unclassified Betaproteobacteria;uncultured beta proteobacterium;unclassified betaproteobacterium ENR4 |
| Ga0586324 0820576 525 1322 | 0.00 | 0.00 | 0.00 | 0.00 | 0.00 | 0.00 | 0.00 | 0.00 | 704676.51 | 0.00 | 0.00 | 0.00 | KO:K02666 | type IV pilus assembly protein PilQ | Bacteria;Proteobacteria;Betaproteobacteria;Rhodocyclales;Azonexaceae;Dechloromonas;Dechloromonas sp. SG708;Dechloromonas sp. SG708 |
| Ga0586324 0900498 1 822 | 0.00 | 0.00 | 0.00 | 0.00 | 0.00 | 0.00 | 0.00 | 0.00 | 0.00 | 0.00 | 664378.88 | 0.00 | KO:K02666 | type IV pilus assembly protein PilQ | Bacteria;Proteobacteria;Betaproteobacteria;Nitrosomonadales;Sulfuricellaceae;Sulfuricella;Sulfuricella sp. T08;Sulfuricella sp. T08 |
| Ga0586324 1061725 2 1183 | 0.00 | 0.00 | 0.00 | 0.00 | 0.00 | 0.00 | 0.00 | 0.00 | 0.00 | 0.00 | 0.00 | 6172500.00 | KO:K02666 | type IV pilus assembly protein PilQ | Bacteria;Proteobacteria;Betaproteobacteria;unclassified Betaproteobacteria;unclassified Betaproteobacteria;unclassified Betaproteobacteria;Betaproteobacteria bacterium GR16-43;Betaproteobacteria bacterium GR16-43 |
| Ga0586324 1172854 322 1134 | 0.00 | 0.00 | 0.00 | 1510500.00 | 0.00 | 0.00 | 0.00 | 0.00 | 0.00 | 0.00 | 0.00 | 0.00 | KO:K02666 | type IV pilus assembly protein PilQ | Bacteria;Spirochaetes;Spirochaetia;Spirochaetales;Spirochaetaceae;Alkalispirochaeta;Alkalispirochaeta odontotermitis;Alkalispirochaeta odontotermitis JC202 |
| Ga0586324 1280324 3 1091 | 0.00 | 0.00 | 0.00 | 0.00 | 0.00 | 0.00 | 0.00 | 0.00 | 0.00 | 696960.11 | 0.00 | 0.00 | KO:K02666 | type IV pilus assembly protein PilQ | Bacteria;Proteobacteria;Betaproteobacteria;Nitrosomonadales;Thiobacillaceae;Thiobacillus;Thiobacillus denitrificans;Thiobacillus denitrificans DSM 12475 |
| Ga0586324 1295531 3 1046 | 5140000.00 | 0.00 | 0.00 | 0.00 | 0.00 | 0.00 | 0.00 | 0.00 | 0.00 | 0.00 | 0.00 | 0.00 | KO:K02666 | type IV pilus assembly protein PilQ | Bacteria;Proteobacteria;Gammaproteobacteria;Acidiferrobacterales;Acidiferrobacteraceae;Sulfuricaulis;Sulfuricaulis limicola;Sulfuricaulis limicola HA5 |
| Ga0586324 1357044 448 1065 | 0.00 | 0.00 | 0.00 | 1168500.00 | 0.00 | 0.00 | 0.00 | 0.00 | 0.00 | 0.00 | 0.00 | 0.00 | KO:K02666 | type IV pilus assembly protein PilQ | Bacteria;Candidatus Aminicenantes;unclassified Candidatus Aminicenantes;unclassified Candidatus Aminicenantes;unclassified Candidatus Aminicenantes;unclassified Candidatus Aminicenantes;Aminicenantes bacterium SCGC AAA255-A06;Aminicenantes bacterium SCGC AAA255-A06 (contamination screened) |
| Ga0586324 1476659 2 1027 | 1150000.00 | 5018400.00 | 0.00 | 0.00 | 0.00 | 12545200.00 | 0.00 | 0.00 | 6998400.00 | 8393000.00 | 6415200.00 | 7575000.00 | KO:K02666 | type IV pilus assembly protein PilQ | Bacteria;Proteobacteria;Betaproteobacteria;unclassified Betaproteobacteria;unclassified Betaproteobacteria;unclassified Betaproteobacteria;uncultured beta proteobacterium;unclassified betaproteobacterium ENR4 |
| Ga0586324 1535831 103 1011 | 0.00 | 0.00 | 0.00 | 0.00 | 0.00 | 0.00 | 0.00 | 0.00 | 0.00 | 0.00 | 789137.36 | 0.00 | KO:K02666 | type IV pilus assembly protein PilQ | Bacteria;Proteobacteria;Betaproteobacteria;Nitrosomonadales;Sulfuricellaceae;Sulfuricella;Sulfuricella sp. T08;Sulfuricella sp. T08 |
| Ga0586324 1560463 501 1004 | 0.00 | 1516400.00 | 0.00 | 0.00 | 0.00 | 0.00 | 0.00 | 2433900.00 | 0.00 | 2849000.00 | 4259200.00 | 0.00 | KO:K02666 | type IV pilus assembly protein PilQ | Bacteria;Proteobacteria;Betaproteobacteria;Nitrosomonadales;Thiobacillaceae;Thiobacillus;Thiobacillus denitrificans;Thiobacillus denitrificans RG |
| Ga0586324 1571215 2 1000 | 2830000.00 | 4712400.00 | 4838388.22 | 7980000.00 | 1812800.00 | 5280100.00 | 6592891.32 | 5788900.00 | 1547100.00 | 0.00 | 9125600.00 | 1387500.00 | KO:K02666 | type IV pilus assembly protein PilQ | Bacteria;Proteobacteria;Gammaproteobacteria;Nevskiales;Steroidobacteraceae;Steroidobacter;Steroidobacter denitrificans;Steroidobacter denitrificans DSM 18526 |
| Ga0586324 1580150 338 997 | 0.00 | 0.00 | 0.00 | 0.00 | 0.00 | 0.00 | 0.00 | 0.00 | 0.00 | 0.00 | 5156800.00 | 0.00 | KO:K02666 | type IV pilus assembly protein PilQ | Bacteria;Proteobacteria;Betaproteobacteria;Nitrosomonadales;Sulfuricellaceae;Sulfuricella;Sulfuricella denitrificans;Sulfuricella denitrificans skB26 |
| Ga0586324 1769505 348 950 | 0.00 | 0.00 | 0.00 | 0.00 | 0.00 | 0.00 | 0.00 | 0.00 | 1757700.00 | 0.00 | 1821600.00 | 0.00 | KO:K02666 | type IV pilus assembly protein PilQ | Bacteria;Proteobacteria;Deltaproteobacteria;Desulfuromonadales;Desulfuromonadaceae;Pelobacter;Pelobacter propionicus;Pelobacter propionicus DSM 2379 |
| Ga0586324 1890902 3 926 | 0.00 | 0.00 | 0.00 | 0.00 | 0.00 | 0.00 | 0.00 | 2354600.00 | 0.00 | 0.00 | 0.00 | 0.00 | KO:K02666 | type IV pilus assembly protein PilQ | Bacteria;Proteobacteria;Gammaproteobacteria;Oceanospirillales;Oceanospirillaceae;Marinobacterium;Marinobacterium mangrovicola;Marinobacterium mangrovicola DSM 27697 |
| Ga0586324 1993157 3 905 | 0.00 | 2434400.00 | 0.00 | 0.00 | 4583500.00 | 0.00 | 0.00 | 0.00 | 0.00 | 1578500.00 | 0.00 | 0.00 | KO:K02666 | type IV pilus assembly protein PilQ | Bacteria;Proteobacteria;Betaproteobacteria;Rhodocyclales;Azonexaceae;Dechloromonas;Dechloromonas denitrificans;Dechloromonas denitrificans ATCC BAA-841 |
| Ga0586324 2032986 179 898 | 0.00 | 1550400.00 | 0.00 | 0.00 | 0.00 | 0.00 | 0.00 | 0.00 | 0.00 | 0.00 | 0.00 | 0.00 | KO:K02666 | type IV pilus assembly protein PilQ | Bacteria;Proteobacteria;Deltaproteobacteria;unclassified Deltaproteobacteria;unclassified Deltaproteobacteria;unclassified Deltaproteobacteria;delta proteobacterium NaphS2;delta proteobacterium NaphS2 |
| Ga0586324 2241801 3 860 | 0.00 | 0.00 | 0.00 | 0.00 | 0.00 | 0.00 | 0.00 | 695400.00 | 0.00 | 0.00 | 0.00 | 0.00 | KO:K02666 | type IV pilus assembly protein PilQ | Bacteria;Gemmatimonadetes;Gemmatimonadetes;Gemmatimonadales;Gemmatimonadaceae;Gemmatimonas;Gemmatimonas phototrophica;Gemmatimonas phototrophica AP64 |
| Ga0586324 2444409 616 831 | 0.00 | 0.00 | 0.00 | 0.00 | 0.00 | 0.00 | 0.00 | 1952000.00 | 1960200.00 | 0.00 | 0.00 | 1642500.00 | KO:K02666 | type IV pilus assembly protein PilQ | Bacteria;Proteobacteria;Gammaproteobacteria;Cellvibrionales;Spongiibacteraceae;Dasania;Dasania marina;Dasania marina DSM 21967 |
| Ga0586324 2659560 3 551 | 0.00 | 0.00 | 0.00 | 0.00 | 0.00 | 0.00 | 0.00 | 0.00 | 0.00 | 0.00 | 1328800.00 | 0.00 | KO:K02666 | type IV pilus assembly protein PilQ | Bacteria;Proteobacteria;Betaproteobacteria;unclassified Betaproteobacteria;unclassified Betaproteobacteria;unclassified Betaproteobacteria;beta proteobacterium AAP65;Rubrivivax sp. AAP65 |
| Ga0586324 2703861 2 625 | 433172.00 | 0.00 | 0.00 | 0.00 | 0.00 | 0.00 | 0.00 | 0.00 | 0.00 | 0.00 | 0.00 | 0.00 | KO:K02666 | type IV pilus assembly protein PilQ | Bacteria;Proteobacteria;Betaproteobacteria;unclassified Betaproteobacteria;unclassified Betaproteobacteria;unclassified Betaproteobacteria;uncultured beta proteobacterium;unclassified betaproteobacterium ENR4 |
| Ga0586324 2798705 2 784 | 0.00 | 0.00 | 0.00 | 0.00 | 0.00 | 0.00 | 0.00 | 0.00 | 13932000.00 | 0.00 | 0.00 | 0.00 | KO:K02666 | type IV pilus assembly protein PilQ | Bacteria;Proteobacteria;Betaproteobacteria;Nitrosomonadales;Thiobacillaceae;Thiobacillus;Thiobacillus denitrificans;Thiobacillus denitrificans DSM 12475 |
| Ga0586324 2865296 3 776 | 935736.00 | 9860000.00 | 0.00 | 5329500.00 | 9682000.00 | 0.00 | 0.00 | 5276500.00 | 8027100.00 | 0.00 | 0.00 | 0.00 | KO:K02666 | type IV pilus assembly protein PilQ | Bacteria;Proteobacteria;Deltaproteobacteria;Myxococcales;Polyangiaceae;Sorangium;Sorangium cellulosum;Sorangium cellulosum So ceGT47 |
| Ga0586324 2977274 1 765 | 0.00 | 1258000.00 | 2208136.67 | 1273000.00 | 0.00 | 0.00 | 0.00 | 0.00 | 0.00 | 0.00 | 0.00 | 0.00 | KO:K02666 | type IV pilus assembly protein PilQ | Bacteria;Proteobacteria;Gammaproteobacteria;Nevskiales;Steroidobacteraceae;Steroidobacter;Steroidobacter denitrificans;Steroidobacter denitrificans DSM 18526 |
| Ga0586324 3231377 1 567 | 0.00 | 0.00 | 0.00 | 0.00 | 0.00 | 0.00 | 0.00 | 0.00 | 0.00 | 0.00 | 0.00 | 1170000.00 | KO:K02666 | type IV pilus assembly protein PilQ | Bacteria;Proteobacteria;Betaproteobacteria;Nitrosomonadales;Thiobacillaceae;Sulfuritortus;Sulfuritortus calidifontis;Sulfuritortus calidifontis DSM 103923 |
| Ga0586324 3545900 3 575 | 0.00 | 0.00 | 26742500.00 | 20235000.00 | 24411000.00 | 23184800.00 | 0.00 | 0.00 | 20574000.00 | 11627000.00 | 0.00 | 9525000.00 | KO:K02666 | type IV pilus assembly protein PilQ | Bacteria;Proteobacteria;Deltaproteobacteria;Desulfuromonadales;Desulfuromonadaceae;Desulfuromonas;Desulfuromonas sp. TF;Desulfuromonas sp. TF |
| Ga0586324 4429383 59 649 | 0.00 | 0.00 | 0.00 | 0.00 | 0.00 | 0.00 | 0.00 | 0.00 | 0.00 | 0.00 | 8456800.00 | 0.00 | KO:K02666 | type IV pilus assembly protein PilQ | Bacteria;Proteobacteria;Deltaproteobacteria;Myxococcales;Labilitrichaceae;Labilithrix;Labilithrix luteola;Labilithrix luteola DSM 27648 |
| Ga0586324 4876774 394 624 | 0.00 | 0.00 | 0.00 | 0.00 | 0.00 | 0.00 | 0.00 | 0.00 | 0.00 | 1347500.00 | 0.00 | 0.00 | KO:K02666 | type IV pilus assembly protein PilQ | Bacteria;Proteobacteria;Deltaproteobacteria;Syntrophobacterales;Syntrophobacteraceae;Syntrophobacter;Syntrophobacter fumaroxidans;Syntrophobacter fumaroxidans MPOB |
| Ga0586324 5211268 3 605 | 0.00 | 0.00 | 0.00 | 0.00 | 0.00 | 0.00 | 0.00 | 963800.00 | 0.00 | 0.00 | 0.00 | 0.00 | KO:K02666 | type IV pilus assembly protein PilQ | Bacteria;Proteobacteria;Gammaproteobacteria;Chromatiales;unclassified Chromatiales;Thiohalobacter;Thiohalobacter thiocyanaticus;Thiohalobacter thiocyanaticus FOKN1 |
| Ga0586324 5353168 1 498 | 0.00 | 0.00 | 0.00 | 0.00 | 0.00 | 0.00 | 0.00 | 0.00 | 325133.19 | 0.00 | 0.00 | 0.00 | KO:K02666 | type IV pilus assembly protein PilQ | Bacteria;Gemmatimonadetes;Gemmatimonadetes;Gemmatimonadales;Gemmatimonadaceae;Gemmatirosa;Gemmatirosa kalamazoonesis;Gemmatimonadetes bacterium KBS708 |
| Ga0586324 6453553 3 554 | 0.00 | 0.00 | 0.00 | 0.00 | 0.00 | 0.00 | 0.00 | 0.00 | 0.00 | 492073.89 | 0.00 | 301725.75 | KO:K02666 | type IV pilus assembly protein PilQ | Bacteria;Proteobacteria;Betaproteobacteria;Burkholderiales;Oxalobacteraceae;Noviherbaspirillum;Noviherbaspirillum denitrificans;Noviherbaspirillum denitrificans TSA40 |
| Ga0586324 6454295 1 555 | 0.00 | 2237200.00 | 0.00 | 0.00 | 0.00 | 0.00 | 0.00 | 0.00 | 0.00 | 0.00 | 0.00 | 0.00 | KO:K02666 | type IV pilus assembly protein PilQ | Bacteria;Proteobacteria;Betaproteobacteria;Rhodocyclales;Rhodocyclaceae;Propionivibrio;Propionivibrio pelophilus;Propionivibrio pelophilus DSM 12018 |
| Ga0586324 6729485 3 545 | 0.00 | 0.00 | 6418200.00 | 0.00 | 0.00 | 0.00 | 0.00 | 0.00 | 0.00 | 0.00 | 0.00 | 0.00 | KO:K02666 | type IV pilus assembly protein PilQ | Bacteria;Proteobacteria;Gammaproteobacteria;unclassified Gammaproteobacteria;Candidatus Competibacteraceae;Candidatus Contendobacter;Candidatus Contendobacter odensis;Candidatus Contendobacter odensis Run B J11 |
| Ga0586324 7774813 1 513 | 0.00 | 0.00 | 0.00 | 0.00 | 0.00 | 0.00 | 0.00 | 1043100.00 | 0.00 | 0.00 | 0.00 | 0.00 | KO:K02666 | type IV pilus assembly protein PilQ | Bacteria;Proteobacteria;Gammaproteobacteria;Alteromonadales;Alteromonadaceae;Marinobacter;Marinobacter daepoensis;Marinobacter daepoensis DSM 16072 |
| Ga0586324 7903537 154 510 | 140741.00 | 0.00 | 0.00 | 0.00 | 0.00 | 0.00 | 0.00 | 0.00 | 0.00 | 0.00 | 0.00 | 0.00 | KO:K02666 | type IV pilus assembly protein PilQ | Bacteria;Proteobacteria;Deltaproteobacteria;Desulfuromonadales;Geobacteraceae;Geobacter;Geobacter uraniireducens;Geobacter uraniireducens Rf4 |
| Ga0586324 8010566 1 507 | 8720000.00 | 6432800.00 | 2187665.99 | 10117500.00 | 3532900.00 | 2419917.47 | 2302800.00 | 0.00 | 6536700.00 | 1778700.00 | 6028000.00 | 765000.00 | KO:K02666 | type IV pilus assembly protein PilQ | Bacteria;Proteobacteria;Betaproteobacteria;Rhodocyclales;Rhodocyclaceae;Dechlorobacter;Dechlorobacter hydrogenophilus;Dechlorobacter hydrogenophilus LT-1 |
| Ga0586324 0057893 2626 3963 | 0.00 | 3039600.00 | 0.00 | 0.00 | 0.00 | 0.00 | 0.00 | 0.00 | 9072000.00 | 0.00 | 0.00 | 0.00 | KO:K02667 | two-component system response regulator PilR (NtrC family) | Bacteria;Proteobacteria;Betaproteobacteria;unclassified Betaproteobacteria;unclassified Betaproteobacteria;unclassified Betaproteobacteria;uncultured beta proteobacterium;unclassified betaproteobacterium ENR4 |
| Ga0586324 0000011 86075 87115 | 0.00 | 0.00 | 0.00 | 0.00 | 0.00 | 0.00 | 0.00 | 14963300.00 | 3661200.00 | 4573800.00 | 8078400.00 | 0.00 | KO:K02669 | twitching motility protein PilT | Bacteria;Proteobacteria;Betaproteobacteria;Nitrosomonadales;Thiobacillaceae;Annwoodia;Annwoodia aquaesulis;Annwoodia aquaesulis DSM 4255 |
| Ga0586324 3298199 51 731 | 0.00 | 0.00 | 0.00 | 0.00 | 0.00 | 0.00 | 0.00 | 0.00 | 557846.19 | 0.00 | 0.00 | 0.00 | KO:K02669 | twitching motility protein PilT | Bacteria;Planctomycetes;Planctomycetia;Gemmatales;Gemmataceae;Gemmata;Gemmata sp. SH-PL17;Gemmata sp. SH-PL17 |
| Ga0586324 3942644 1 681 | 0.00 | 0.00 | 0.00 | 0.00 | 0.00 | 0.00 | 0.00 | 0.00 | 0.00 | 0.00 | 0.00 | 463290.75 | KO:K02669 | twitching motility protein PilT | Bacteria;Proteobacteria;Betaproteobacteria;Nitrosomonadales;Thiobacillaceae;Thiobacillus;Thiobacillus denitrificans;Thiobacillus denitrificans RG |
| Ga0586324 5336686 206 601 | 0.00 | 0.00 | 0.00 | 0.00 | 0.00 | 0.00 | 0.00 | 0.00 | 0.00 | 0.00 | 11176000.00 | 0.00 | KO:K02669 | twitching motility protein PilT | Bacteria;Proteobacteria;Betaproteobacteria;Nitrosomonadales;Thiobacillaceae;Thiobacillus;Thiobacillus thiophilus;Thiobacillus thiophilus DSM 19892 |
| Ga0586324 0432836 447 1607 | 0.00 | 0.00 | 0.00 | 0.00 | 0.00 | 0.00 | 0.00 | 0.00 | 0.00 | 3765300.00 | 0.00 | 2197500.00 | KO:K02670 | twitching motility protein PilU | Bacteria;Proteobacteria;Betaproteobacteria;Nitrosomonadales;Gallionellaceae;Gallionella;Gallionella capsiferriformans;Gallionella capsiferriformans ES-2 |
| Ga0586324 0568190 660 1550 | 0.00 | 0.00 | 0.00 | 0.00 | 0.00 | 0.00 | 0.00 | 0.00 | 0.00 | 0.00 | 11000000.00 | 0.00 | KO:K02670 | twitching motility protein PilU | Bacteria;Proteobacteria;Gammaproteobacteria;Methylococcales;Methylococcaceae;Methylobacter;Methylobacter whittenburyi;Methylobacter whittenburyi ACM 3310 |
| Ga0586324 0789244 506 1345 | 0.00 | 0.00 | 0.00 | 0.00 | 0.00 | 0.00 | 0.00 | 0.00 | 0.00 | 3056900.00 | 3326400.00 | 704330.25 | KO:K02670 | twitching motility protein PilU | Bacteria;Proteobacteria;Betaproteobacteria;Nitrosomonadales;Gallionellaceae;Gallionella;Gallionella capsiferriformans;Gallionella capsiferriformans ES-2 |
| Ga0586324 1337435 71 1072 | 0.00 | 0.00 | 0.00 | 0.00 | 0.00 | 1449371.57 | 0.00 | 0.00 | 0.00 | 0.00 | 0.00 | 0.00 | KO:K02670 | twitching motility protein PilU | Bacteria;Proteobacteria;Gammaproteobacteria;Chromatiales;Chromatiaceae;Thiorhodococcus;Thiorhodococcus drewsii;Thiorhodococcus drewsii AZ1 |
| Ga0586324 0021562 975 3179 | 0.00 | 5147600.00 | 0.00 | 0.00 | 23278000.00 | 0.00 | 13771200.00 | 5551000.00 | 6034500.00 | 0.00 | 0.00 | 0.00 | KO:K02690 | photosystem I P700 chlorophyll a apoprotein A2 | Eukaryota;Streptophyta;Magnoliopsida;Malpighiales;Salicaceae;Populus;Populus trichocarpa;Populus balsamifera trichocarpa |
| Ga0586324 0477246 428 1510 | 0.00 | 0.00 | 0.00 | 6289000.00 | 1596500.00 | 0.00 | 0.00 | 0.00 | 476389.35 | 0.00 | 0.00 | 0.00 | KO:K02703 | photosystem II P680 reaction center D1 protein | Bacteria;Cyanobacteria;unclassified Cyanobacteria;Nostocales;Nostocaceae;Anabaena;Anabaena sp. 4-3;Anabaena sp. 4-3 |
| Ga0586324 1545951 2 1006 | 0.00 | 0.00 | 0.00 | 0.00 | 415712.12 | 0.00 | 0.00 | 0.00 | 0.00 | 0.00 | 0.00 | 0.00 | KO:K02703 | photosystem II P680 reaction center D1 protein | Bacteria;Cyanobacteria;unclassified Cyanobacteria;Gloeoemargaritales;Gloeomargaritaceae;Gloeomargarita;Gloeomargarita lithophora;Candidatus Gloeomargarita lithophora D10 |
| Ga0586324 7650405 2 517 | 0.00 | 0.00 | 0.00 | 0.00 | 0.00 | 0.00 | 0.00 | 0.00 | 272760.21 | 0.00 | 0.00 | 0.00 | KO:K02703 | photosystem II P680 reaction center D1 protein | Bacteria;Cyanobacteria;unclassified Cyanobacteria;Nostocales;Calotrichaceae;Calothrix;Calothrix desertica;Calothrix desertica PCC 7102 |
| Ga0586324 0174294 8 1537 | 0.00 | 0.00 | 0.00 | 5168000.00 | 5150000.00 | 1832274.10 | 0.00 | 8235000.00 | 10449000.00 | 2094400.00 | 0.00 | 0.00 | KO:K02704 | photosystem II CP47 chlorophyll apoprotein | Eukaryota;Bacillariophyta;Coscinodiscophyceae;Thalassiosirales;Thalassiosiraceae;Thalassiosira;Thalassiosira pseudonana;Thalassiosira pseudonana CCMP 1335 |
| Ga0586324 0433790 339 1400 | 0.00 | 5195200.00 | 0.00 | 0.00 | 20394000.00 | 10441100.00 | 14911200.00 | 12261000.00 | 0.00 | 0.00 | 0.00 | 0.00 | KO:K02706 | photosystem II P680 reaction center D2 protein | Eukaryota;Streptophyta;Magnoliopsida;Malpighiales;Salicaceae;Populus;Populus trichocarpa;Populus balsamifera trichocarpa |
| Ga0586324 4822247 3 626 | 0.00 | 0.00 | 0.00 | 858788.60 | 0.00 | 0.00 | 0.00 | 0.00 | 0.00 | 0.00 | 0.00 | 0.00 | KO:K02806 | PTS system nitrogen regulatory IIA component | Bacteria;Planctomycetes;Planctomycetia;Pirellulales;Pirellulaceae;Roseimaritima;Roseimaritima ulvae;Roseimaritima ulvae DSM 25454 |
| Ga0586324 2275194 3 434 | 0.00 | 0.00 | 0.00 | 0.00 | 0.00 | 0.00 | 866755.68 | 0.00 | 0.00 | 0.00 | 0.00 | 0.00 | KO:K02838 | ribosome recycling factor | Bacteria;Spirochaetes;Spirochaetia;Spirochaetales;Spirochaetaceae;Sediminispirochaeta;Sediminispirochaeta smaragdinae;Sediminispirochaeta smaragdinae DSM 11293 |
| Ga0586324 0000041 12866 13561 | 0.00 | 0.00 | 0.00 | 0.00 | 0.00 | 0.00 | 218884.10 | 3062200.00 | 33534000.00 | 3280200.00 | 8412800.00 | 9945000.00 | KO:K02863 | large subunit ribosomal protein L1 | Bacteria;Proteobacteria;Betaproteobacteria;Nitrosomonadales;Thiobacillaceae;Annwoodia;Annwoodia aquaesulis;Annwoodia aquaesulis DSM 4255 |
| Ga0586324 0019172 5371 6066 | 0.00 | 0.00 | 0.00 | 0.00 | 0.00 | 0.00 | 19608000.00 | 0.00 | 0.00 | 0.00 | 12056000.00 | 7725000.00 | KO:K02863 | large subunit ribosomal protein L1 | Bacteria;Proteobacteria;Betaproteobacteria;Nitrosomonadales;Thiobacillaceae;Thiobacillus;Thiobacillus thioparus;Thiobacillus thioparus DSM 505 |
| Ga0586324 0258180 864 1367 | 0.00 | 440958.24 | 0.00 | 1045000.00 | 0.00 | 0.00 | 0.00 | 0.00 | 530146.62 | 0.00 | 0.00 | 0.00 | KO:K02863 | large subunit ribosomal protein L1 | Bacteria;Proteobacteria;Betaproteobacteria;Nitrosomonadales;Sulfuricellaceae;Sulfuriferula;Sulfuriferula multivorans;Sulfuriferula multivorans TTN |
| Ga0586324 0392193 1 711 | 0.00 | 0.00 | 1765900.17 | 0.00 | 0.00 | 0.00 | 0.00 | 0.00 | 0.00 | 0.00 | 0.00 | 0.00 | KO:K02863 | large subunit ribosomal protein L1 | Bacteria;Proteobacteria;Deltaproteobacteria;Syntrophobacterales;Syntrophaceae;Syntrophus;Syntrophus gentianae;Syntrophus gentianae DSM 8423 |
| Ga0586324 0889534 647 1276 | 0.00 | 0.00 | 0.00 | 0.00 | 0.00 | 0.00 | 0.00 | 1281000.00 | 0.00 | 0.00 | 0.00 | 0.00 | KO:K02863 | large subunit ribosomal protein L1 | Bacteria;Proteobacteria;Betaproteobacteria;Nitrosomonadales;Nitrosomonadaceae;Nitrosovibrio;Nitrosovibrio sp. Nv6;Nitrosovibrio sp. Nv6 |
| Ga0586324 1191178 467 1126 | 0.00 | 0.00 | 0.00 | 0.00 | 0.00 | 0.00 | 0.00 | 41358000.00 | 0.00 | 42658000.00 | 0.00 | 0.00 | KO:K02863 | large subunit ribosomal protein L1 | Bacteria;Proteobacteria;Betaproteobacteria;Rhodocyclales;Azonexaceae;Dechloromonas;Dechloromonas denitrificans;Dechloromonas denitrificans ATCC BAA-841 |
| Ga0586324 1369259 393 1061 | 0.00 | 3386400.00 | 0.00 | 0.00 | 0.00 | 0.00 | 0.00 | 0.00 | 0.00 | 0.00 | 0.00 | 0.00 | KO:K02863 | large subunit ribosomal protein L1 | Bacteria;Acidobacteria;Acidobacteriia;Acidobacteriales;Acidobacteriaceae;Acidipila;Acidipila rosea;Acidipila rosea DSM 103428 |
| Ga0586324 1374041 273 1001 | 0.00 | 0.00 | 0.00 | 0.00 | 0.00 | 0.00 | 0.00 | 2415600.00 | 0.00 | 0.00 | 0.00 | 0.00 | KO:K02863 | large subunit ribosomal protein L1 | Bacteria;Proteobacteria;Betaproteobacteria;Nitrosomonadales;Sterolibacteriaceae;Methyloversatilis;Methyloversatilis thermotolerans;Methyloversatilis thermotolerans NVD |
| Ga0586324 1377377 413 1057 | 0.00 | 0.00 | 0.00 | 0.00 | 0.00 | 0.00 | 0.00 | 890600.00 | 0.00 | 0.00 | 0.00 | 0.00 | KO:K02863 | large subunit ribosomal protein L1 | Bacteria;Proteobacteria;Gammaproteobacteria;Oceanospirillales;Oceanospirillaceae;Motiliproteus;Motiliproteus sp. MSK22-1;Motiliproteus sp. MSK22-1 |
| Ga0586324 1417609 532 1044 | 0.00 | 0.00 | 0.00 | 0.00 | 0.00 | 0.00 | 0.00 | 0.00 | 0.00 | 2802800.00 | 0.00 | 0.00 | KO:K02863 | large subunit ribosomal protein L1 | Bacteria;Proteobacteria;Betaproteobacteria;Nitrosomonadales;Methylophilaceae;unclassified Methylophilaceae;Methylophilaceae bacterium 11;Methylophilaceae bacterium 11 |
| Ga0586324 2095370 427 885 | 0.00 | 2067200.00 | 0.00 | 0.00 | 0.00 | 0.00 | 0.00 | 0.00 | 0.00 | 0.00 | 0.00 | 0.00 | KO:K02863 | large subunit ribosomal protein L1 | Bacteria;Proteobacteria;Betaproteobacteria;Burkholderiales;Comamonadaceae;Hydrogenophaga;Hydrogenophaga sp. A37;Hydrogenophaga sp. A37 |
| Ga0586324 2862010 1 567 | 7210000.00 | 4630800.00 | 6136700.00 | 0.00 | 0.00 | 0.00 | 0.00 | 0.00 | 0.00 | 0.00 | 0.00 | 0.00 | KO:K02863 | large subunit ribosomal protein L1 | Bacteria;Proteobacteria;Gammaproteobacteria;Methylococcales;Methylococcaceae;Methylobacter;Methylobacter tundripaludum;Methylobacter tundripaludum OWC-G53F |
| Ga0586324 4233813 2 595 | 0.00 | 0.00 | 0.00 | 0.00 | 1205100.00 | 0.00 | 0.00 | 0.00 | 0.00 | 0.00 | 0.00 | 0.00 | KO:K02863 | large subunit ribosomal protein L1 | Bacteria;Proteobacteria;Gammaproteobacteria;Acidiferrobacterales;Acidiferrobacteraceae;Sulfuricaulis;Sulfuricaulis limicola;Sulfuricaulis limicola HA5 |
| Ga0586324 4461267 6 647 | 0.00 | 0.00 | 0.00 | 6811500.00 | 7034900.00 | 0.00 | 0.00 | 0.00 | 9072000.00 | 0.00 | 1328800.00 | 656365.50 | KO:K02863 | large subunit ribosomal protein L1 | Archaea;Euryarchaeota;Methanomicrobia;Methanosarcinales;Methanotrichaceae;Methanothrix;Methanothrix soehngenii;Methanothrix soehngenii GP-6 |
| Ga0586324 6847907 28 540 | 0.00 | 0.00 | 0.00 | 0.00 | 0.00 | 0.00 | 0.00 | 0.00 | 0.00 | 0.00 | 0.00 | 3465000.00 | KO:K02863 | large subunit ribosomal protein L1 | Bacteria;Proteobacteria;Betaproteobacteria;Nitrosomonadales;Thiobacillaceae;Thiobacillus;Thiobacillus thiophilus;Thiobacillus thiophilus DSM 19892 |
| Ga0586324 0094364 1606 2577 | 10900000.00 | 1339600.00 | 10359200.00 | 0.00 | 7374800.00 | 0.00 | 0.00 | 0.00 | 326733.75 | 0.00 | 0.00 | 0.00 | KO:K02864 | large subunit ribosomal protein L10 | Archaea;Euryarchaeota;Methanomicrobia;Methanosarcinales;Methanotrichaceae;Methanothrix;Methanothrix soehngenii;Methanothrix soehngenii GP-6 |
| Ga0586324 0104993 1200 1736 | 0.00 | 0.00 | 0.00 | 0.00 | 0.00 | 0.00 | 0.00 | 259892.94 | 0.00 | 0.00 | 0.00 | 0.00 | KO:K02864 | large subunit ribosomal protein L10 | Bacteria;Proteobacteria;Alphaproteobacteria;Rhodospirillales;Acetobacteraceae;Rhodopila;unclassified Rhodopila;Rhodopila sp. LVNP |
| Ga0586324 0115715 151 675 | 0.00 | 0.00 | 0.00 | 1491500.00 | 0.00 | 0.00 | 0.00 | 0.00 | 0.00 | 0.00 | 0.00 | 0.00 | KO:K02864 | large subunit ribosomal protein L10 | Bacteria;Proteobacteria;Deltaproteobacteria;Syntrophobacterales;Syntrophaceae;Desulfobacca;Desulfobacca acetoxidans;Desulfobacca acetoxidans ASRB2, DSM 11109 |
| Ga0586324 0497542 946 1476 | 0.00 | 0.00 | 0.00 | 0.00 | 0.00 | 0.00 | 0.00 | 0.00 | 0.00 | 1717100.00 | 0.00 | 0.00 | KO:K02864 | large subunit ribosomal protein L10 | Bacteria;Proteobacteria;Alphaproteobacteria;Rhodospirillales;Azospirillaceae;Azospirillum;Azospirillum halopraeferens;Azospirillum halopraeferens DSM 3675 |
| Ga0586324 1151284 1 234 | 0.00 | 0.00 | 0.00 | 0.00 | 0.00 | 0.00 | 10123200.00 | 0.00 | 0.00 | 0.00 | 6432800.00 | 0.00 | KO:K02864 | large subunit ribosomal protein L10 | Bacteria;Proteobacteria;Gammaproteobacteria;Methylococcales;Methylococcaceae;Methylosarcina;Methylosarcina fibrata;Methylosarcina fibrata AML-C10 |
| Ga0586324 2982738 376 762 | 0.00 | 0.00 | 0.00 | 0.00 | 0.00 | 0.00 | 0.00 | 0.00 | 2413800.00 | 0.00 | 0.00 | 0.00 | KO:K02864 | large subunit ribosomal protein L10 | Bacteria;Proteobacteria;Betaproteobacteria;Ferrovales;Ferrovaceae;Ferrovum;unclassified Ferrovum;Ferrovum sp. Zulauf-31 |
| Ga0586324 3829450 301 687 | 0.00 | 0.00 | 0.00 | 0.00 | 0.00 | 0.00 | 0.00 | 0.00 | 2559600.00 | 0.00 | 0.00 | 0.00 | KO:K02864 | large subunit ribosomal protein L10 | Bacteria;Proteobacteria;Deltaproteobacteria;Desulfuromonadales;Geobacteraceae;Geobacter;Geobacter anodireducens;Geobacter anodireducens SD-1 |
| Ga0586324 6504580 54 554 | 0.00 | 0.00 | 3235189.42 | 0.00 | 0.00 | 0.00 | 0.00 | 0.00 | 0.00 | 0.00 | 0.00 | 0.00 | KO:K02864 | large subunit ribosomal protein L10 | Bacteria;Proteobacteria;Gammaproteobacteria;Methylococcales;Methylococcaceae;Methylobacter;Methylobacter tundripaludum;Methylobacter tundripaludum 31/32 |
| Ga0586324 4958190 198 620 | 14600000.00 | 9248000.00 | 0.00 | 0.00 | 0.00 | 0.00 | 0.00 | 4733600.00 | 3888000.00 | 3680600.00 | 0.00 | 3667500.00 | KO:K02867 | large subunit ribosomal protein L11 | Bacteria;Proteobacteria;Gammaproteobacteria;Methylococcales;Methylococcaceae;Methylobacter;Methylobacter luteus;Methylobacter luteus IMV-B-3098 |
| Ga0586324 0023786 6037 6357 | 0.00 | 0.00 | 0.00 | 0.00 | 0.00 | 0.00 | 0.00 | 0.00 | 1709100.00 | 0.00 | 0.00 | 0.00 | KO:K02869 | large subunit ribosomal protein L12 | Archaea;Euryarchaeota;Methanomicrobia;Methanomicrobiales;Methanoregulaceae;Methanoregula;Methanoregula boonei;Methanoregula boonei 6A8 |
| Ga0586324 3159150 569 745 | 0.00 | 0.00 | 0.00 | 0.00 | 0.00 | 0.00 | 1256508.00 | 0.00 | 0.00 | 0.00 | 0.00 | 0.00 | KO:K02869 | large subunit ribosomal protein L12 | Archaea;Euryarchaeota;Methanomicrobia;Methanosarcinales;Methanotrichaceae;Methanothrix;Methanothrix soehngenii;Methanothrix soehngenii GP-6 |
| Ga0586324 0002657 14719 15147 | 801055.00 | 0.00 | 0.00 | 0.00 | 0.00 | 0.00 | 0.00 | 0.00 | 0.00 | 0.00 | 0.00 | 0.00 | KO:K02871 | large subunit ribosomal protein L13 | Archaea;Euryarchaeota;Methanomicrobia;Methanomicrobiales;Methanoregulaceae;Methanoregula;Methanoregula formicica;Methanoregula formicica SMSP, DSM 22288 |
| Ga0586324 0013015 2569 2937 | 0.00 | 0.00 | 0.00 | 0.00 | 0.00 | 0.00 | 0.00 | 6527000.00 | 3215700.00 | 0.00 | 0.00 | 9825000.00 | KO:K02874 | large subunit ribosomal protein L14 | Bacteria;Proteobacteria;Betaproteobacteria;Burkholderiales;unclassified Burkholderiales;Leptothrix;Leptothrix cholodnii;Leptothrix cholodnii SP-6 |
| Ga0586324 0024822 1729 2100 | 0.00 | 0.00 | 0.00 | 0.00 | 0.00 | 0.00 | 0.00 | 0.00 | 0.00 | 0.00 | 16368000.00 | 0.00 | KO:K02874 | large subunit ribosomal protein L14 | Bacteria;Chloroflexi;Anaerolineae;Anaerolineales;Anaerolineaceae;Thermanaerothrix;Thermanaerothrix daxensis;Thermanaerothrix daxensis GNS-1 |
| Ga0586324 0147496 2123 2491 | 0.00 | 0.00 | 0.00 | 0.00 | 0.00 | 0.00 | 0.00 | 0.00 | 1911600.00 | 0.00 | 3643200.00 | 0.00 | KO:K02874 | large subunit ribosomal protein L14 | Bacteria;Acidobacteria;Vicinamibacteria;unclassified Vicinamibacteria;Vicinamibacteraceae;Luteitalea;Luteitalea pratensis;Luteitalea pratensis DSM 100886 |
| Ga0586324 0487602 874 1242 | 5040000.00 | 1536800.00 | 41999800.00 | 0.00 | 0.00 | 0.00 | 0.00 | 0.00 | 0.00 | 0.00 | 0.00 | 0.00 | KO:K02874 | large subunit ribosomal protein L14 | Bacteria;Acidobacteria;Vicinamibacteria;unclassified Vicinamibacteria;Vicinamibacteraceae;Luteitalea;Luteitalea pratensis;Luteitalea pratensis DSM 100886 |
| Ga0586324 0856609 754 1122 | 0.00 | 0.00 | 5407609.37 | 0.00 | 0.00 | 0.00 | 0.00 | 0.00 | 0.00 | 0.00 | 0.00 | 0.00 | KO:K02874 | large subunit ribosomal protein L14 | Bacteria;Acidobacteria;Vicinamibacteria;unclassified Vicinamibacteria;Vicinamibacteraceae;Luteitalea;Luteitalea pratensis;Luteitalea pratensis DSM 100886 |
| Ga0586324 1587071 329 697 | 0.00 | 0.00 | 0.00 | 0.00 | 0.00 | 0.00 | 492115.20 | 0.00 | 0.00 | 0.00 | 0.00 | 0.00 | KO:K02874 | large subunit ribosomal protein L14 | Bacteria;Acidobacteria;Vicinamibacteria;unclassified Vicinamibacteria;Vicinamibacteraceae;Luteitalea;Luteitalea pratensis;Luteitalea pratensis DSM 100886 |
| Ga0586324 5913373 47 412 | 0.00 | 2543200.00 | 0.00 | 0.00 | 0.00 | 0.00 | 0.00 | 0.00 | 0.00 | 0.00 | 0.00 | 0.00 | KO:K02874 | large subunit ribosomal protein L14 | Bacteria;Verrucomicrobia;Verrucomicrobiae;Verrucomicrobiales;Verrucomicrobia subdivision 3;Pedosphaera;Pedosphaera parvula;Pedosphaera parvula Ellin514 |
| Ga0586324 0172544 1115 1657 | 0.00 | 0.00 | 0.00 | 0.00 | 0.00 | 21835000.00 | 0.00 | 0.00 | 0.00 | 0.00 | 0.00 | 0.00 | KO:K02879 | large subunit ribosomal protein L17 | Bacteria;Bacteroidetes;Bacteroidia;Marinilabiliales;Prolixibacteraceae;Roseimarinus;Roseimarinus sediminis;Roseimarinus sediminis DSM 28824 |
| Ga0586324 3702026 169 597 | 0.00 | 0.00 | 0.00 | 0.00 | 0.00 | 0.00 | 0.00 | 0.00 | 0.00 | 0.00 | 0.00 | 6810000.00 | KO:K02879 | large subunit ribosomal protein L17 | Bacteria;Proteobacteria;Alphaproteobacteria;Hyphomicrobiales;Hyphomicrobiaceae;Hyphomicrobium;Hyphomicrobium sp. CS1GBMeth3;Hyphomicrobium sp. ERS1433774 |
| Ga0586324 0073520 2817 3335 | 2550000.00 | 0.00 | 0.00 | 0.00 | 2039400.00 | 0.00 | 0.00 | 1323700.00 | 980100.00 | 1501500.00 | 2182400.00 | 1072500.00 | KO:K02881 | large subunit ribosomal protein L18 | Archaea;Euryarchaeota;Methanomicrobia;Methanosarcinales;Methanotrichaceae;Methanothrix;Methanothrix soehngenii;Methanothrix soehngenii GP-6 |
| Ga0586324 0001208 241 624 | 0.00 | 0.00 | 0.00 | 0.00 | 0.00 | 0.00 | 0.00 | 1958100.00 | 0.00 | 0.00 | 0.00 | 0.00 | KO:K02884 | large subunit ribosomal protein L19 | Bacteria;Proteobacteria;Betaproteobacteria;Nitrosomonadales;Thiobacillaceae;Thiobacillus;Thiobacillus thioparus;Thiobacillus thioparus DSM 505 |
| Ga0586324 0271509 1153 1536 | 0.00 | 0.00 | 0.00 | 0.00 | 0.00 | 0.00 | 0.00 | 0.00 | 0.00 | 0.00 | 8580000.00 | 0.00 | KO:K02884 | large subunit ribosomal protein L19 | Bacteria;Proteobacteria;Betaproteobacteria;Neisseriales;Chromobacteriaceae;Silvimonas;Silvimonas terrae;Silvimonas terrae DSM 18233 |
| Ga0586324 4801189 236 628 | 0.00 | 0.00 | 0.00 | 0.00 | 0.00 | 0.00 | 0.00 | 658800.00 | 0.00 | 11550000.00 | 0.00 | 14700000.00 | KO:K02884 | large subunit ribosomal protein L19 | Bacteria;Proteobacteria;Betaproteobacteria;Burkholderiales;Comamonadaceae;Caenimonas;Caenimonas koreensis;Caenimonas koreensis DSM 17982 |
| Ga0586324 0084282 1712 2536 | 0.00 | 775200.00 | 0.00 | 0.00 | 0.00 | 0.00 | 0.00 | 0.00 | 0.00 | 0.00 | 0.00 | 0.00 | KO:K02886 | large subunit ribosomal protein L2 | Bacteria;Proteobacteria;Betaproteobacteria;Burkholderiales;unclassified Burkholderiales;Rhizobacter;Rhizobacter sp. Root1221;Rhizobacter sp. Root1221 |
| Ga0586324 0222240 1225 2052 | 0.00 | 0.00 | 0.00 | 0.00 | 0.00 | 0.00 | 0.00 | 0.00 | 0.00 | 0.00 | 1795200.00 | 0.00 | KO:K02886 | large subunit ribosomal protein L2 | Bacteria;Proteobacteria;Betaproteobacteria;Rhodocyclales;Rhodocyclaceae;Azospira;unclassified Azospira;Azospira sp. ZAP |
| Ga0586324 0461204 484 1308 | 0.00 | 101798.72 | 0.00 | 0.00 | 0.00 | 0.00 | 0.00 | 0.00 | 0.00 | 0.00 | 0.00 | 0.00 | KO:K02886 | large subunit ribosomal protein L2 | Bacteria;Proteobacteria;Betaproteobacteria;Nitrosomonadales;Thiobacillaceae;Thiobacillus;Thiobacillus thioparus;Thiobacillus thioparus DSM 505 |

| | | | | | | | | | | | | | | | |
|---|---|---|---|---|---|---|---|---|---|---|---|---|---|---|---|
| Ga0586324 0665822 3 818 | 0.00 | 5922800.00 | 0.00 | 0.00 | 0.00 | 0.00 | 0.00 | 0.00 | 0.00 | 0.00 | 0.00 | 0.00 | KO:K02886 | large subunit ribosomal protein L2 | Bacteria;Proteobacteria;Gammaproteobacteria;Methylococcales;Crenotrichaceae;Crenothrix;Crenothrix polyspora;Crenothrix polyspora ERS1545888 |
| Ga0586324 2502136 225 821 | 0.00 | 0.00 | 0.00 | 0.00 | 0.00 | 0.00 | 7774800.00 | 0.00 | 0.00 | 0.00 | 0.00 | 0.00 | KO:K02886 | large subunit ribosomal protein L2 | Bacteria;Firmicutes;Clostridia;Halanaerobiales;unclassified Halanaerobiales;Anoxybacter;Anoxybacter fermentans;Anoxybacter fermentans DY22613 |
| Ga0586324 7151541 3 470 | 0.00 | 0.00 | 0.00 | 0.00 | 21424000.00 | 0.00 | 13497600.00 | 0.00 | 10206000.00 | 0.00 | 0.00 | 0.00 | KO:K02890 | large subunit ribosomal protein L22 | Bacteria;Actinobacteria;Rubrobacteria;Gaiellales;Gaiellaceae;Gaiella;Gaiella occulta;Gaiella occulta F2-233 |
| Ga0586324 0273355 133 438 | 0.00 | 0.00 | 0.00 | 0.00 | 0.00 | 0.00 | 0.00 | 0.00 | 0.00 | 0.00 | 0.00 | 9600000.00 | KO:K02892 | large subunit ribosomal protein L23 | Bacteria;Proteobacteria;Betaproteobacteria;Nitrosomonadales;Thiobacillaceae;Thiobacillus;Thiobacillus denitrificans;Thiobacillus denitrificans DSM 12475 |
| Ga0586324 4754146 1 345 | 0.00 | 0.00 | 0.00 | 0.00 | 0.00 | 0.00 | 0.00 | 0.00 | 0.00 | 3857700.00 | 0.00 | 0.00 | KO:K02892 | large subunit ribosomal protein L23 | Bacteria;Proteobacteria;Betaproteobacteria;unclassified Betaproteobacteria;unclassified Betaproteobacteria;unclassified Betaproteobacteria;Betaproteobacteria bacterium GR16-43;Betaproteobacteria bacterium GR16-43 |
| Ga0586324 0000041 18556 19176 | 0.00 | 0.00 | 0.00 | 0.00 | 0.00 | 0.00 | 0.00 | 3586800.00 | 3653100.00 | 8239000.00 | 7708800.00 | 9375000.00 | KO:K02897 | large subunit ribosomal protein L25 | Bacteria;Proteobacteria;Betaproteobacteria;Nitrosomonadales;Thiobacillaceae;Annwoodia;Annwoodia aquaesulis;Annwoodia aquaesulis DSM 4255 |
| Ga0586324 1459098 176 841 | 0.00 | 1135600.00 | 0.00 | 0.00 | 0.00 | 0.00 | 0.00 | 0.00 | 0.00 | 0.00 | 0.00 | 0.00 | KO:K02897 | large subunit ribosomal protein L25 | Bacteria;Proteobacteria;Betaproteobacteria;Nitrosomonadales;Gallionellaceae;Sideroxydans;Sideroxydans lithotrophicus;Sideroxydans lithotrophicus ES-1 |
| Ga0586324 1935596 412 915 | 0.00 | 1890400.00 | 0.00 | 0.00 | 0.00 | 0.00 | 0.00 | 0.00 | 792211.59 | 2040500.00 | 0.00 | 1312500.00 | KO:K02897 | large subunit ribosomal protein L25 | Bacteria;Proteobacteria;Gammaproteobacteria;Methylococcales;Methylococcaceae;Methylomicrobium;Methylomicrobium album;Methylomicrobium album BG8 |
| Ga0586324 0004880 465 1109 | 0.00 | 0.00 | 0.00 | 0.00 | 0.00 | 0.00 | 0.00 | 0.00 | 0.00 | 0.00 | 6001600.00 | 0.00 | KO:K02906 | large subunit ribosomal protein L3 | Bacteria;Proteobacteria;Betaproteobacteria;Nitrosomonadales;Thiobacillaceae;Thiobacillus;Thiobacillus thioparus;Thiobacillus thioparus DSM 505 |
| Ga0586324 0412238 1181 1786 | 0.00 | 0.00 | 0.00 | 0.00 | 0.00 | 0.00 | 0.00 | 0.00 | 0.00 | 4458300.00 | 0.00 | 3037500.00 | KO:K02906 | large subunit ribosomal protein L3 | Bacteria;Proteobacteria;Betaproteobacteria;Nitrosomonadales;Sulfuricellaceae;Sulfuricella;Sulfuricella denitrificans;Sulfuricella denitrificans skB26 |
| Ga0586324 4366748 171 623 | 0.00 | 0.00 | 0.00 | 0.00 | 0.00 | 0.00 | 0.00 | 0.00 | 1053000.00 | 0.00 | 0.00 | 0.00 | KO:K02907 | large subunit ribosomal protein L30 | Archaea;Euryarchaeota;Methanomicrobia;Methanosarcinales;Methanotrichaceae;Methanothrix;Methanothrix soehngenii;Methanothrix soehngenii GP-6 |
| Ga0586324 6602247 3 398 | 9690000.00 | 0.00 | 7150100.00 | 0.00 | 0.00 | 7146000.00 | 7911600.00 | 4892200.00 | 5119200.00 | 1593900.00 | 5209600.00 | 4447500.00 | KO:K02907 | large subunit ribosomal protein L30 | Archaea;Euryarchaeota;Methanomicrobia;Methanosarcinales;Methanotrichaceae;Methanothrix;Methanothrix soehngenii;Methanothrix soehngenii GP-6 |
| Ga0586324 0000703 28153 28419 | 0.00 | 0.00 | 0.00 | 1406000.00 | 0.00 | 0.00 | 0.00 | 0.00 | 0.00 | 0.00 | 0.00 | 0.00 | KO:K02910 | large subunit ribosomal protein L31e | Archaea;Euryarchaeota;Methanomicrobia;Methanosarcinales;Methanotrichaceae;Methanothrix;Methanothrix soehngenii;Methanothrix soehngenii GP-6 |
| Ga0586324 0000625 1230 1847 | 0.00 | 0.00 | 0.00 | 0.00 | 0.00 | 0.00 | 0.00 | 0.00 | 0.00 | 0.00 | 1821600.00 | 0.00 | KO:K02926 | large subunit ribosomal protein L4 | Bacteria;Proteobacteria;Betaproteobacteria;Nitrosomonadales;Thiobacillaceae;Annwoodia;Annwoodia aquaesulis;Annwoodia aquaesulis DSM 4255 |
| Ga0586324 1067142 992 1180 | 0.00 | 0.00 | 0.00 | 0.00 | 0.00 | 0.00 | 0.00 | 0.00 | 0.00 | 0.00 | 0.00 | 2085000.00 | KO:K02926 | large subunit ribosomal protein L4 | Bacteria;Spirochaetes;Spirochaetia;Spirochaetales;Spirochaetaceae;Alkalispirochaeta;Alkalispirochaeta odontotermitis;Alkalispirochaeta odontotermitis JC202 |
| Ga0586324 1623249 341 988 | 0.00 | 0.00 | 0.00 | 0.00 | 0.00 | 0.00 | 0.00 | 0.00 | 0.00 | 962500.00 | 0.00 | 1567500.00 | KO:K02926 | large subunit ribosomal protein L4 | Bacteria;Proteobacteria;Betaproteobacteria;Rhodocyclales;Zoogloeaceae;Zoogloea;Zoogloea sp. LCSB751;Zoogloea schifflangensis LCSB751 |
| Ga0586324 8096821 3 473 | 0.00 | 0.00 | 0.00 | 0.00 | 0.00 | 0.00 | 0.00 | 309100.42 | 0.00 | 0.00 | 0.00 | 0.00 | KO:K02926 | large subunit ribosomal protein L4 | Bacteria;Proteobacteria;Betaproteobacteria;Burkholderiales;Comamonadaceae;Pelomonas;Pelomonas sp. Root1444;Pelomonas sp. Root1444 |
| Ga0586324 5846873 38 577 | 0.00 | 0.00 | 0.00 | 0.00 | 0.00 | 0.00 | 889708.44 | 4398100.00 | 0.00 | 0.00 | 377109.04 | 0.00 | KO:K02927 | large subunit ribosomal protein L40e | Bacteria;Firmicutes;Bacilli;Bacillales;Paenibacillaceae;Cohnella;Cohnella sp. OV330;Cohnella sp. OV330 |
| Ga0586324 0022337 3365 3904 | 0.00 | 0.00 | 0.00 | 0.00 | 0.00 | 0.00 | 0.00 | 0.00 | 0.00 | 0.00 | 21472000.00 | 0.00 | KO:K02931 | large subunit ribosomal protein L5 | Bacteria;Proteobacteria;Deltaproteobacteria;Syntrophobacterales;Syntrophobacteraceae;Syntrophobacter;Syntrophobacter fumaroxidans;Syntrophobacter fumaroxidans MPOB |
| Ga0586324 0121247 637 1185 | 0.00 | 19652000.00 | 0.00 | 1615000.00 | 1359600.00 | 0.00 | 0.00 | 0.00 | 0.00 | 0.00 | 0.00 | 0.00 | KO:K02931 | large subunit ribosomal protein L5 | Bacteria;Proteobacteria;Deltaproteobacteria;Syntrophobacterales;Syntrophaceae;Syntrophus;Syntrophus aciditrophicus;Syntrophus aciditrophicus SB |
| Ga0586324 0229206 1040 1582 | 0.00 | 0.00 | 0.00 | 0.00 | 0.00 | 0.00 | 0.00 | 1091900.00 | 0.00 | 0.00 | 0.00 | 0.00 | KO:K02931 | large subunit ribosomal protein L5 | Bacteria;Proteobacteria;Deltaproteobacteria;Syntrophorhabdales;Syntrophorhabdaceae;Syntrophorhabdus;Syntrophorhabdus aromaticivorans;Syntrophorhabdus aromaticivorans UI |
| Ga0586324 0345493 626 1171 | 0.00 | 0.00 | 0.00 | 0.00 | 8270900.00 | 0.00 | 0.00 | 969900.00 | 0.00 | 0.00 | 0.00 | 0.00 | KO:K02931 | large subunit ribosomal protein L5 | Bacteria;Actinobacteria;Rubrobacteria;Gaiellales;Gaiellaceae;Gaiella;Gaiella occulta;Gaiella occulta F2-233 |
| Ga0586324 0517442 3 623 | 0.00 | 0.00 | 0.00 | 0.00 | 0.00 | 0.00 | 0.00 | 0.00 | 534088.89 | 0.00 | 0.00 | 0.00 | KO:K02931 | large subunit ribosomal protein L5 | Bacteria;Proteobacteria;Deltaproteobacteria;Syntrophobacterales;Syntrophaceae;Desulfobacca;Desulfobacca acetoxidans;Desulfobacca acetoxidans ASRB2, DSM 11109 |
| Ga0586324 0614228 1060 1500 | 0.00 | 0.00 | 0.00 | 0.00 | 0.00 | 0.00 | 0.00 | 1421300.00 | 1320300.00 | 0.00 | 0.00 | 0.00 | KO:K02931 | large subunit ribosomal protein L5 | Bacteria;Firmicutes;Clostridia;Eubacteriales;Peptococcaceae;Desulfofarcimen;Desulfofarcimen intricatum;Desulfofarcimen intricatum NBRC 109411 |
| Ga0586324 1353544 203 919 | 0.00 | 0.00 | 0.00 | 0.00 | 5108800.00 | 0.00 | 0.00 | 0.00 | 0.00 | 0.00 | 0.00 | 0.00 | KO:K02931 | large subunit ribosomal protein L5 | Bacteria;Nitrospirae;Nitrospira;Nitrospirales;Nitrospiraceae;Nitrospira;Nitrospira lenta;Nitrospira lenta N. lenta BS10 |
| Ga0586324 1502234 111 803 | 0.00 | 3413600.00 | 36876500.00 | 17195000.00 | 0.00 | 0.00 | 0.00 | 0.00 | 1085400.00 | 0.00 | 0.00 | 1387500.00 | KO:K02931 | large subunit ribosomal protein L5 | Bacteria;Proteobacteria;Betaproteobacteria;unclassified Betaproteobacteria;unclassified Betaproteobacteria;unclassified Betaproteobacteria;Betaproteobacteria bacterium GR16-43;Betaproteobacteria bacterium GR16-43 |
| Ga0586324 1633704 617 985 | 0.00 | 0.00 | 0.00 | 1216000.00 | 0.00 | 15880000.00 | 11126400.00 | 1342000.00 | 27378000.00 | 0.00 | 0.00 | 0.00 | KO:K02931 | large subunit ribosomal protein L5 | Bacteria;Chloroflexi;Anaerolineae;Anaerolineales;Anaerolineaceae;Levilinea;Levilinea saccharolytica;Levilinea saccharolytica DSM 16555 |
| Ga0586324 2189888 245 841 | 0.00 | 0.00 | 0.00 | 0.00 | 0.00 | 4247900.00 | 0.00 | 0.00 | 0.00 | 0.00 | 0.00 | 1597500.00 | KO:K02931 | large subunit ribosomal protein L5 | Bacteria;Proteobacteria;Deltaproteobacteria;Desulfobacterales;Desulfobacteraceae;Desulfatiglans;Desulfatiglans anilini;Desulfatiglans anilini DSM 4660 |
| Ga0586324 3630896 1 642 | 0.00 | 26044000.00 | 0.00 | 0.00 | 0.00 | 0.00 | 0.00 | 0.00 | 0.00 | 0.00 | 0.00 | 0.00 | KO:K02931 | large subunit ribosomal protein L5 | Bacteria;Proteobacteria;Deltaproteobacteria;Myxococcales;Polyangiaceae;Sorangium;Sorangium cellulosum;Sorangium cellulosum So ceGT47 |
| Ga0586324 4068264 192 671 | 0.00 | 0.00 | 0.00 | 0.00 | 0.00 | 0.00 | 0.00 | 0.00 | 0.00 | 0.00 | 2147200.00 | 14925000.00 | KO:K02931 | large subunit ribosomal protein L5 | Bacteria;Proteobacteria;Gammaproteobacteria;unclassified Gammaproteobacteria;Candidatus Competibacteraceae;Candidatus Competibacter;Candidatus Competibacter denitrificans;Candidatus Competibacter denitrificans Run A D11 |
| Ga0586324 0056322 1368 1901 | 0.00 | 0.00 | 0.00 | 0.00 | 0.00 | 0.00 | 0.00 | 3757600.00 | 0.00 | 14553000.00 | 0.00 | 0.00 | KO:K02933 | large subunit ribosomal protein L6 | Bacteria;Proteobacteria;Betaproteobacteria;Burkholderiales;Oxalobacteraceae;Noviherbaspirillum;Noviherbaspirillum denitrificans;Noviherbaspirillum denitrificans TSA40 |
| Ga0586324 0073520 1450 1980 | 15700000.00 | 0.00 | 0.00 | 0.00 | 0.00 | 0.00 | 0.00 | 1012706.14 | 0.00 | 0.00 | 0.00 | 0.00 | KO:K02933 | large subunit ribosomal protein L6 | Archaea;Euryarchaeota;Methanomicrobia;Methanosarcinales;Methanotrichaceae;Methanothrix;Methanothrix soehngenii;Methanothrix soehngenii GP-6 |
| Ga0586324 0189738 233 766 | 0.00 | 0.00 | 0.00 | 0.00 | 0.00 | 0.00 | 0.00 | 1561600.00 | 0.00 | 0.00 | 0.00 | 0.00 | KO:K02933 | large subunit ribosomal protein L6 | Bacteria;Proteobacteria;Gammaproteobacteria;Chromatiales;Chromatiaceae;Allochromatium;Allochromatium vinosum;Allochromatium vinosum DSM 180 |
| Ga0586324 0259631 1570 2103 | 0.00 | 0.00 | 0.00 | 0.00 | 0.00 | 0.00 | 0.00 | 0.00 | 0.00 | 0.00 | 0.00 | 12150000.00 | KO:K02933 | large subunit ribosomal protein L6 | Bacteria;Proteobacteria;Betaproteobacteria;Nitrosomonadales;Gallionellaceae;Sideroxydans;Sideroxydans lithotrophicus;Sideroxydans lithotrophicus ES-1 |
| Ga0586324 0521636 967 1509 | 0.00 | 2148800.00 | 0.00 | 0.00 | 0.00 | 1846435.09 | 1864939.68 | 1134600.00 | 1385100.00 | 2440900.00 | 0.00 | 1740000.00 | KO:K02933 | large subunit ribosomal protein L6 | Bacteria;Proteobacteria;Deltaproteobacteria;unclassified Deltaproteobacteria;unclassified Deltaproteobacteria;unclassified Deltaproteobacteria;delta proteobacterium NaphS2;delta proteobacterium NaphS2 |
| Ga0586324 2436466 71 604 | 0.00 | 0.00 | 7600500.00 | 0.00 | 0.00 | 0.00 | 0.00 | 0.00 | 0.00 | 0.00 | 0.00 | 0.00 | KO:K02933 | large subunit ribosomal protein L6 | Bacteria;Proteobacteria;Betaproteobacteria;unclassified Betaproteobacteria;unclassified Betaproteobacteria;unclassified Betaproteobacteria;Betaproteobacteria bacterium GR16-43;Betaproteobacteria bacterium GR16-43 |
| Ga0586324 0021444 3733 4122 | 0.00 | 0.00 | 0.00 | 0.00 | 0.00 | 0.00 | 0.00 | 0.00 | 0.00 | 0.00 | 0.00 | 4560000.00 | KO:K02935 | large subunit ribosomal protein L7/L12 | Bacteria;Proteobacteria;Deltaproteobacteria;Desulfobacterales;Desulfobulbaceae;Desulfurivibrio;Desulfurivibrio alkaliphilus;Desulfurivibrio alkaliphilus AHT2 |
| Ga0586324 0031827 3300 3680 | 0.00 | 0.00 | 0.00 | 0.00 | 0.00 | 0.00 | 166304.57 | 0.00 | 0.00 | 0.00 | 0.00 | 0.00 | KO:K02935 | large subunit ribosomal protein L7/L12 | Bacteria;Proteobacteria;Betaproteobacteria;Nitrosomonadales;Nitrosomonadaceae;Nitrosospira;Nitrosospira multiformis;Nitrosospira multiformis Nl2 |
| Ga0586324 0599483 948 1331 | 0.00 | 0.00 | 0.00 | 9452500.00 | 0.00 | 0.00 | 0.00 | 0.00 | 0.00 | 0.00 | 0.00 | 0.00 | KO:K02935 | large subunit ribosomal protein L7/L12 | Bacteria;Acidobacteria;Vicinamibacteria;unclassified Vicinamibacteria;Vicinamibacteraceae;Luteitalea;Luteitalea pratensis;Luteitalea pratensis DSM 100886 |
| Ga0586324 5059289 111 614 | 0.00 | 0.00 | 0.00 | 0.00 | 0.00 | 0.00 | 194875.02 | 0.00 | 0.00 | 0.00 | 0.00 | 0.00 | KO:K02935 | large subunit ribosomal protein L7/L12 | Bacteria;Proteobacteria;Betaproteobacteria;unclassified Betaproteobacteria;unclassified Betaproteobacteria;unclassified Betaproteobacteria;uncultured beta proteobacterium;unclassified betaproteobacterium ENR4 |
| Ga0586324 5891342 3 311 | 0.00 | 0.00 | 0.00 | 0.00 | 0.00 | 0.00 | 3078000.00 | 3440400.00 | 0.00 | 0.00 | 0.00 | 0.00 | KO:K02935 | large subunit ribosomal protein L7/L12 | Bacteria;Proteobacteria;Gammaproteobacteria;Methylococcales;Methylococcaceae;Methylovulum;Methylovulum miyakonense;Methylovulum miyakonense HT12 |
| Ga0586324 6639794 1 360 | 0.00 | 0.00 | 0.00 | 0.00 | 0.00 | 0.00 | 0.00 | 0.00 | 0.00 | 0.00 | 1944800.00 | 0.00 | KO:K02935 | large subunit ribosomal protein L7/L12 | Bacteria;Chloroflexi;Anaerolineae;Anaerolineales;Anaerolineaceae;Thermanaerothrix;Thermanaerothrix daxensis;Thermanaerothrix daxensis GNS-1 |
| Ga0586324 1013208 966 1208 | 0.00 | 0.00 | 0.00 | 7438500.00 | 0.00 | 0.00 | 8800800.00 | 0.00 | 4527900.00 | 0.00 | 0.00 | 0.00 | KO:K02936 | large subunit ribosomal protein L7Ae | Archaea;Euryarchaeota;Methanomicrobia;Methanosarcinales;Methanotrichaceae;Methanothrix;Methanothrix soehngenii;Methanothrix soehngenii GP-6 |
| Ga0586324 0017533 2815 3264 | 0.00 | 5093200.00 | 8670200.00 | 0.00 | 0.00 | 0.00 | 12015600.00 | 17019000.00 | 14418000.00 | 25179000.00 | 36080000.00 | 29550000.00 | KO:K02939 | large subunit ribosomal protein L9 | Bacteria;Proteobacteria;Betaproteobacteria;Nitrosomonadales;Thiobacillaceae;Thiobacillus;Thiobacillus thioparus;Thiobacillus thioparus DSM 505 |
| Ga0586324 0062236 1548 2000 | 0.00 | 0.00 | 0.00 | 647629.25 | 0.00 | 0.00 | 0.00 | 0.00 | 0.00 | 0.00 | 0.00 | 0.00 | KO:K02939 | large subunit ribosomal protein L9 | Bacteria;Proteobacteria;Betaproteobacteria;Burkholderiales;unclassified Burkholderiales;Roseateles;Roseateles depolymerans;Roseateles depolymerans KCTC 42856 |
| Ga0586324 7337287 3 455 | 0.00 | 0.00 | 0.00 | 0.00 | 0.00 | 0.00 | 0.00 | 0.00 | 0.00 | 5767300.00 | 0.00 | 0.00 | KO:K02939 | large subunit ribosomal protein L9 | Bacteria;Proteobacteria;Betaproteobacteria;Nitrosomonadales;Thiobacillaceae;Annwoodia;Annwoodia aquaesulis;Annwoodia aquaesulis DSM 4255 |
| Ga0586324 0000005 59155 60876 | 0.00 | 0.00 | 0.00 | 0.00 | 0.00 | 0.00 | 0.00 | 4800700.00 | 18225000.00 | 25579166.69 | 56408000.00 | 40500000.00 | KO:K02945 | small subunit ribosomal protein S1 | Bacteria;Proteobacteria;Betaproteobacteria;Nitrosomonadales;Thiobacillaceae;Annwoodia;Annwoodia aquaesulis;Annwoodia aquaesulis DSM 4255 |
| Ga0586324 0013812 4351 6111 | 0.00 | 0.00 | 0.00 | 0.00 | 0.00 | 0.00 | 0.00 | 0.00 | 0.00 | 0.00 | 4734400.00 | 0.00 | KO:K02945 | small subunit ribosomal protein S1 | Bacteria;Proteobacteria;Betaproteobacteria;unclassified Betaproteobacteria;unclassified Betaproteobacteria;unclassified Betaproteobacteria;uncultured beta proteobacterium;unclassified betaproteobacterium ENR4 |
| Ga0586324 0024907 2186 3925 | 0.00 | 0.00 | 0.00 | 0.00 | 0.00 | 0.00 | 0.00 | 0.00 | 802023.93 | 0.00 | 0.00 | 0.00 | KO:K02945 | small subunit ribosomal protein S1 | Bacteria;Proteobacteria;Alphaproteobacteria;Hyphomicrobiales;Xanthobacteraceae;Pseudolabrys;Pseudolabrys sp. Root1462;Pseudolabrys sp. Root1462 |
| Ga0586324 0033439 2325 4367 | 0.00 | 6868000.00 | 0.00 | 0.00 | 0.00 | 0.00 | 0.00 | 0.00 | 0.00 | 0.00 | 0.00 | 0.00 | KO:K02945 | small subunit ribosomal protein S1 | Bacteria;Bacteroidetes;Bacteroidia;Marinilabiliales;Marinilabiliaceae;Anaerophaga;Anaerophaga thermohalophila;Anaerophaga thermohalophila Valhall |
| Ga0586324 0040568 4504 5292 | 0.00 | 0.00 | 0.00 | 0.00 | 0.00 | 0.00 | 0.00 | 0.00 | 0.00 | 0.00 | 11528000.00 | 5250000.00 | KO:K02945 | small subunit ribosomal protein S1 | Bacteria;Proteobacteria;Betaproteobacteria;Nitrosomonadales;Thiobacillaceae;Thiobacillus;Thiobacillus thioparus;Thiobacillus thioparus DSM 505 |
| Ga0586324 0167923 1 654 | 0.00 | 0.00 | 0.00 | 0.00 | 0.00 | 0.00 | 0.00 | 0.00 | 0.00 | 3711400.00 | 0.00 | 3052500.00 | KO:K02945 | small subunit ribosomal protein S1 | Bacteria;Proteobacteria;Gammaproteobacteria;Chromatiales;Woeseiaceae;Woeseia;Woeseia oceani;Woeseia oceani XK5 |
| Ga0586324 0171902 306 2033 | 0.00 | 0.00 | 0.00 | 0.00 | 0.00 | 0.00 | 0.00 | 0.00 | 0.00 | 0.00 | 0.00 | 2842500.00 | KO:K02945 | small subunit ribosomal protein S1 | Bacteria;Proteobacteria;Betaproteobacteria;Nitrosomonadales;Sulfuricellaceae;Sulfurirhabdus;Sulfurirhabdus autotrophica;Sulfurirhabdus autotrophica DSM 100309 |
| Ga0586324 0264411 153 1832 | 2610000.00 | 0.00 | 0.00 | 0.00 | 0.00 | 0.00 | 0.00 | 3855200.00 | 0.00 | 0.00 | 0.00 | 0.00 | KO:K02945 | small subunit ribosomal protein S1 | Bacteria;Proteobacteria;Gammaproteobacteria;unclassified Gammaproteobacteria;Candidatus Competibacteraceae;Plasticicumulans;Plasticicumulans acidivorans;Plasticicumulans acidivorans DSM 23606 |
| Ga0586324 0358859 3 836 | 0.00 | 0.00 | 0.00 | 0.00 | 0.00 | 0.00 | 0.00 | 6344000.00 | 0.00 | 0.00 | 0.00 | 0.00 | KO:K02945 | small subunit ribosomal protein S1 | Bacteria;Proteobacteria;Betaproteobacteria;Nitrosomonadales;Nitrosomonadaceae;Nitrosospira;Nitrosospira sp. Nsp14;Nitrosospira sp. Nsp14 |
| Ga0586324 0444810 2 1198 | 0.00 | 0.00 | 0.00 | 2907000.00 | 4233300.00 | 0.00 | 0.00 | 108663.57 | 0.00 | 0.00 | 0.00 | 0.00 | KO:K02945 | small subunit ribosomal protein S1 | Bacteria;Proteobacteria;Gammaproteobacteria;Chromatiales;Chromatiaceae;Thiodictyon;Candidatus Thiodictyon syntrophicum;Candidatus Thiodictyon syntrophicum Cad16 |
| Ga0586324 0487175 2 946 | 0.00 | 0.00 | 0.00 | 0.00 | 0.00 | 0.00 | 0.00 | 0.00 | 0.00 | 0.00 | 0.00 | 715978.50 | KO:K02945 | small subunit ribosomal protein S1 | Bacteria;Proteobacteria;Gammaproteobacteria;Chromatiales;unclassified Chromatiales;Thiohalobacter;Thiohalobacter thiocyanaticus;Thiohalobacter thiocyanaticus FOKN1 |
| Ga0586324 0636937 2 550 | 0.00 | 0.00 | 0.00 | 0.00 | 0.00 | 0.00 | 0.00 | 0.00 | 1474200.00 | 0.00 | 0.00 | 0.00 | KO:K02945 | small subunit ribosomal protein S1 | Bacteria;Proteobacteria;Betaproteobacteria;Rhodocyclales;Azonexaceae;Dechloromonas;Dechloromonas sp. HYN0024;Dechloromonas sp. HYN0024 |
| Ga0586324 4342793 2 652 | 0.00 | 0.00 | 0.00 | 0.00 | 0.00 | 0.00 | 0.00 | 0.00 | 1296000.00 | 0.00 | 0.00 | 0.00 | KO:K02945 | small subunit ribosomal protein S1 | Bacteria;Actinobacteria;Acidimicrobiia;Acidimicrobiales;Ilumatobacteraceae;Ilumatobacter;Ilumatobacter fluminis;Ilumatobacter fluminis DSM 18936 |
| Ga0586324 7672759 2 517 | 0.00 | 0.00 | 0.00 | 0.00 | 0.00 | 0.00 | 6247200.00 | 0.00 | 0.00 | 0.00 | 0.00 | 0.00 | KO:K02945 | small subunit ribosomal protein S1 | Bacteria;Proteobacteria;Gammaproteobacteria;Methylococcales;Methylococcaceae;Methylobacter;Methylobacter tundripaludum;Methylobacter tundripaludum SV96 |
| Ga0586324 0000625 102 410 | 0.00 | 8840000.00 | 0.00 | 0.00 | 0.00 | 0.00 | 0.00 | 4959300.00 | 0.00 | 0.00 | 0.00 | 0.00 | KO:K02946 | small subunit ribosomal protein S10 | Bacteria;Proteobacteria;Betaproteobacteria;Nitrosomonadales;Thiobacillaceae;Annwoodia;Annwoodia aquaesulis;Annwoodia aquaesulis DSM 4255 |
| Ga0586324 0284109 618 956 | 0.00 | 979200.00 | 0.00 | 0.00 | 0.00 | 0.00 | 0.00 | 0.00 | 0.00 | 0.00 | 0.00 | 0.00 | KO:K02946 | small subunit ribosomal protein S10 | Bacteria;Acidobacteria;Vicinamibacteria;unclassified Vicinamibacteria;Vicinamibacteraceae;Luteitalea;Luteitalea pratensis;Luteitalea pratensis DSM 100886 |
| Ga0586324 0108714 462 1139 | 0.00 | 0.00 | 0.00 | 0.00 | 0.00 | 0.00 | 0.00 | 0.00 | 2033100.00 | 0.00 | 0.00 | 0.00 | KO:K02948 | small subunit ribosomal protein S11 | Bacteria;Verrucomicrobia;Verrucomicrobiae;Verrucomicrobiales;Verrucomicrobia subdivision 3;Pedosphaera;Pedosphaera parvula;Pedosphaera parvula Ellin514 |
| Ga0586324 0493077 764 1153 | 0.00 | 0.00 | 0.00 | 0.00 | 0.00 | 0.00 | 0.00 | 3355000.00 | 1239300.00 | 0.00 | 0.00 | 0.00 | KO:K02948 | small subunit ribosomal protein S11 | Archaea;Euryarchaeota;Methanomicrobia;Methanosarcinales;Methanotrichaceae;Methanothrix;Methanothrix soehngenii;Methanothrix soehngenii GP-6 |
| Ga0586324 0864425 569 1261 | 0.00 | 2808400.00 | 4069994.56 | 0.00 | 0.00 | 0.00 | 0.00 | 0.00 | 0.00 | 0.00 | 0.00 | 0.00 | KO:K02948 | small subunit ribosomal protein S11 | Bacteria;Verrucomicrobia;Verrucomicrobiae;Verrucomicrobiales;Verrucomicrobia subdivision 3;Pedosphaera;Pedosphaera parvula;Pedosphaera parvula Ellin514 |
| Ga0586324 1302095 704 1084 | 0.00 | 0.00 | 0.00 | 3933000.00 | 0.00 | 0.00 | 0.00 | 0.00 | 0.00 | 0.00 | 0.00 | 0.00 | KO:K02948 | small subunit ribosomal protein S11 | Archaea;Crenarchaeota;Thermoprotei;Desulfurococcales;Pyrodictiaceae;Hyperthermus;Hyperthermus butylicus;Hyperthermus butylicus DSM 5456 |
| Ga0586324 1403350 631 1050 | 0.00 | 0.00 | 0.00 | 0.00 | 0.00 | 0.00 | 2001746.52 | 0.00 | 0.00 | 0.00 | 0.00 | 0.00 | KO:K02948 | small subunit ribosomal protein S11 | Archaea;Crenarchaeota;Thermoprotei;Desulfurococcales;Pyrodictiaceae;Hyperthermus;Hyperthermus butylicus;Hyperthermus butylicus DSM 5456 |
| Ga0586324 1569434 2 415 | 0.00 | 0.00 | 0.00 | 0.00 | 0.00 | 0.00 | 0.00 | 0.00 | 3393900.00 | 0.00 | 0.00 | 0.00 | KO:K02952 | small subunit ribosomal protein S13 | Bacteria;Proteobacteria;Gammaproteobacteria;Nevskiales;Steroidobacteraceae;Povalibacter;Povalibacter uvarum;Povalibacter uvarum DSM 26723 |
| Ga0586324 0105668 1750 2019 | 0.00 | 0.00 | 0.00 | 0.00 | 0.00 | 0.00 | 0.00 | 0.00 | 1676700.00 | 0.00 | 0.00 | 0.00 | KO:K02956 | small subunit ribosomal protein S15 | Bacteria;Proteobacteria;Betaproteobacteria;Burkholderiales;Burkholderiaceae;Lautropia;Lautropia mirabilis;Lautropia mirabilis ATCC 51599 |
| Ga0586324 0230444 2027 2296 | 0.00 | 0.00 | 0.00 | 0.00 | 0.00 | 0.00 | 0.00 | 5136200.00 | 4398300.00 | 0.00 | 719624.40 | 0.00 | KO:K02956 | small subunit ribosomal protein S15 | Bacteria;Proteobacteria;Betaproteobacteria;Rhodocyclales;Azonexaceae;Dechloromonas;Dechloromonas sp. JJ;Dechloromonas sp. JJ |
| Ga0586324 0541334 591 866 | 0.00 | 0.00 | 0.00 | 0.00 | 219268.46 | 0.00 | 0.00 | 0.00 | 0.00 | 0.00 | 0.00 | 310198.50 | KO:K02956 | small subunit ribosomal protein S15 | Bacteria;Proteobacteria;Deltaproteobacteria;Syntrophobacterales;Syntrophaceae;Syntrophus;Syntrophus aciditrophicus;Syntrophus aciditrophicus SB |
| Ga0586324 2119580 488 757 | 0.00 | 0.00 | 0.00 | 0.00 | 0.00 | 0.00 | 0.00 | 0.00 | 0.00 | 0.00 | 0.00 | 1215000.00 | KO:K02956 | small subunit ribosomal protein S15 | Bacteria;Proteobacteria;Deltaproteobacteria;Syntrophorhabdales;Syntrophorhabdaceae;Syntrophorhabdus;Syntrophorhabdus aromaticivorans;Syntrophorhabdus aromaticivorans UI |
| Ga0586324 2129319 469 735 | 0.00 | 0.00 | 0.00 | 0.00 | 201254.79 | 0.00 | 0.00 | 0.00 | 0.00 | 0.00 | 0.00 | 0.00 | KO:K02956 | small subunit ribosomal protein S15 | Bacteria;Proteobacteria;Deltaproteobacteria;Syntrophobacterales;Syntrophaceae;Syntrophus;Syntrophus aciditrophicus;Syntrophus aciditrophicus SB |
| Ga0586324 2006376 3 323 | 0.00 | 0.00 | 0.00 | 0.00 | 0.00 | 0.00 | 0.00 | 33489000.00 | 0.00 | 0.00 | 0.00 | 0.00 | KO:K02965 | small subunit ribosomal protein S19 | Bacteria;Nitrospinae;Nitrospinia;Nitrospinales;Nitrospinaceae;Nitrospina;Nitrospina gracilis;Nitrospina gracilis 3/211 |
| Ga0586324 1604790 356 973 | 0.00 | 0.00 | 0.00 | 0.00 | 1071200.00 | 0.00 | 0.00 | 0.00 | 0.00 | 0.00 | 0.00 | 0.00 | KO:K02966 | small subunit ribosomal protein S19e | Archaea;Euryarchaeota;Methanomicrobia;Methanomicrobiales;Methanoregulaceae;Methanoregula;Methanoregula formicica;Methanoregula formicica SMSP, DSM 22288 |
| Ga0586324 0000002 84505 85251 | 0.00 | 0.00 | 0.00 | 0.00 | 0.00 | 0.00 | 0.00 | 0.00 | 0.00 | 0.00 | 0.00 | 4057500.00 | KO:K02967 | small subunit ribosomal protein S2 | Bacteria;Proteobacteria;Betaproteobacteria;Nitrosomonadales;Thiobacillaceae;Annwoodia;Annwoodia aquaesulis;Annwoodia aquaesulis DSM 4255 |
| Ga0586324 0000532 20643 21521 | 0.00 | 0.00 | 0.00 | 0.00 | 0.00 | 0.00 | 0.00 | 0.00 | 1587600.00 | 0.00 | 994400.00 | 2055000.00 | KO:K02967 | small subunit ribosomal protein S2 | Bacteria;Proteobacteria;Gammaproteobacteria;Acidiferrobacterales;Acidiferrobacteraceae;Sulfurifustis;Sulfurifustis variabilis;Sulfurifustis variabilis skN76 |
| Ga0586324 0021125 2968 3846 | 0.00 | 0.00 | 0.00 | 0.00 | 0.00 | 0.00 | 0.00 | 0.00 | 2033100.00 | 0.00 | 0.00 | 0.00 | KO:K02967 | small subunit ribosomal protein S2 | Bacteria;Proteobacteria;Deltaproteobacteria;Desulfuromonadales;Geobacteraceae;Geothermobacter;Geothermobacter ehrlichii;Geothermobacter ehrlichii DSM 15274 |
| Ga0586324 0024658 3 521 | 0.00 | 0.00 | 0.00 | 0.00 | 8672600.00 | 0.00 | 0.00 | 0.00 | 0.00 | 7353500.00 | 7955200.00 | 5925000.00 | KO:K02967 | small subunit ribosomal protein S2 | Bacteria;Proteobacteria;Alphaproteobacteria;Hyphomicrobiales;Bradyrhizobiaceae;Bradyrhizobium;Bradyrhizobium lablabi;Bradyrhizobium lablabi GAS499 |
| Ga0586324 0028830 3893 4636 | 7040000.00 | 0.00 | 0.00 | 0.00 | 0.00 | 0.00 | 0.00 | 0.00 | 0.00 | 0.00 | 0.00 | 0.00 | KO:K02967 | small subunit ribosomal protein S2 | Bacteria;Proteobacteria;Betaproteobacteria;Nitrosomonadales;Nitrosomonadaceae;Nitrosospira;Nitrosospira sp. Nsp2;Nitrosospira sp. Nsp2 |
| Ga0586324 0041203 845 1585 | 0.00 | 0.00 | 0.00 | 0.00 | 0.00 | 0.00 | 0.00 | 1409100.00 | 5467500.00 | 0.00 | 0.00 | 0.00 | KO:K02967 | small subunit ribosomal protein S2 | Bacteria;Proteobacteria;Gammaproteobacteria;Methylococcales;Methylococcaceae;Methylobacter;Methylobacter tundripaludum;Methylobacter tundripaludum OWC-G53F |
| Ga0586324 0111210 884 1624 | 0.00 | 0.00 | 0.00 | 0.00 | 0.00 | 0.00 | 0.00 | 0.00 | 0.00 | 2810500.00 | 0.00 | 3592500.00 | KO:K02967 | small subunit ribosomal protein S2 | Bacteria;Proteobacteria;Betaproteobacteria;Nitrosomonadales;Nitrosomonadaceae;Nitrosospira;Nitrosospira sp. Nsp44;Nitrosospira sp. Nsp44 |
| Ga0586324 1136853 38 757 | 0.00 | 0.00 | 0.00 | 0.00 | 0.00 | 0.00 | 0.00 | 0.00 | 5661900.00 | 0.00 | 0.00 | 0.00 | KO:K02967 | small subunit ribosomal protein S2 | Bacteria;Proteobacteria;Gammaproteobacteria;Chromatiales;Chromatiaceae;Thiobaca;Thiobaca trueperi;Thiobaca trueperi DSM 13587 |
| Ga0586324 2909193 2 772 | 0.00 | 0.00 | 0.00 | 0.00 | 0.00 | 0.00 | 0.00 | 0.00 | 0.00 | 993300.00 | 0.00 | 0.00 | KO:K02967 | small subunit ribosomal protein S2 | Bacteria;Acidobacteria;Vicinamibacteria;unclassified Vicinamibacteria;Vicinamibacteraceae;Luteitalea;Luteitalea pratensis;Luteitalea pratensis DSM 100886 |
| Ga0586324 5052952 477 614 | 0.00 | 0.00 | 0.00 | 0.00 | 0.00 | 0.00 | 0.00 | 0.00 | 0.00 | 0.00 | 3115200.00 | 0.00 | KO:K02970 | small subunit ribosomal protein S21 | Bacteria;Proteobacteria;Betaproteobacteria;Burkholderiales;unclassified Burkholderiales;Methylibium;Methylibium sp. CF059;Methylibium sp. CF059 |
| Ga0586324 0000625 3621 4325 | 6530000.00 | 0.00 | 0.00 | 0.00 | 0.00 | 0.00 | 11764800.00 | 14213000.00 | 11340000.00 | 5975200.00 | 7594400.00 | 4710000.00 | KO:K02982 | small subunit ribosomal protein S3 | Bacteria;Proteobacteria;Betaproteobacteria;Nitrosomonadales;Thiobacillaceae;Annwoodia;Annwoodia aquaesulis;Annwoodia aquaesulis DSM 4255 |
| Ga0586324 0004880 3511 4215 | 0.00 | 0.00 | 0.00 | 0.00 | 0.00 | 0.00 | 7729200.00 | 6771000.00 | 3207600.00 | 12243000.00 | 0.00 | 10425000.00 | KO:K02982 | small subunit ribosomal protein S3 | Bacteria;Proteobacteria;Betaproteobacteria;Nitrosomonadales;Thiobacillaceae;Thiobacillus;Thiobacillus thioparus;Thiobacillus thioparus DSM 505 |
| Ga0586324 0007546 9543 10241 | 0.00 | 0.00 | 0.00 | 0.00 | 0.00 | 2165710.43 | 0.00 | 0.00 | 0.00 | 0.00 | 0.00 | 0.00 | KO:K02982 | small subunit ribosomal protein S3 | Archaea;Euryarchaeota;Methanomicrobia;Methanomicrobiales;Methanoregulaceae;Methanoregula;Methanoregula formicica;Methanoregula formicica SMSP, DSM 22288 |
| Ga0586324 0038998 772 1419 | 0.00 | 6398800.00 | 0.00 | 0.00 | 0.00 | 0.00 | 0.00 | 0.00 | 0.00 | 1624700.00 | 2032800.00 | 982500.00 | KO:K02982 | small subunit ribosomal protein S3 | Bacteria;Proteobacteria;Deltaproteobacteria;Desulfobacterales;Desulfobacteraceae;Candidatus Magnetomorum;Candidatus Magnetomorum sp. HK-1;Candidatus Magnetomorum sp. HK-1 |
| Ga0586324 0050838 371 1051 | 0.00 | 0.00 | 0.00 | 0.00 | 0.00 | 0.00 | 0.00 | 0.00 | 0.00 | 2633400.00 | 0.00 | 0.00 | KO:K02982 | small subunit ribosomal protein S3 | Bacteria;Proteobacteria;Gammaproteobacteria;Acidiferrobacterales;Acidiferrobacteraceae;Sulfurifustis;Sulfurifustis variabilis;Sulfurifustis variabilis skN76 |
| Ga0586324 0053090 613 1515 | 0.00 | 0.00 | 6812300.00 | 3572000.00 | 2482300.00 | 0.00 | 5244000.00 | 0.00 | 4884300.00 | 3934700.00 | 16984000.00 | 4477500.00 | KO:K02982 | small subunit ribosomal protein S3 | Bacteria;Proteobacteria;Betaproteobacteria;Burkholderiales;Comamonadaceae;Curvibacter;Curvibacter delicatus;Curvibacter delicatus NBRC 14919 |
| Ga0586324 0156257 1158 1985 | 0.00 | 4943600.00 | 0.00 | 0.00 | 0.00 | 0.00 | 0.00 | 0.00 | 0.00 | 0.00 | 0.00 | 0.00 | KO:K02982 | small subunit ribosomal protein S3 | Archaea;Euryarchaeota;Methanomicrobia;Methanosarcinales;Methanotrichaceae;Methanothrix;Methanothrix soehngenii;Methanothrix soehngenii GP-6 |
| Ga0586324 0222240 3 578 | 0.00 | 0.00 | 0.00 | 0.00 | 0.00 | 0.00 | 0.00 | 0.00 | 0.00 | 0.00 | 0.00 | 11100000.00 | KO:K02982 | small subunit ribosomal protein S3 | Bacteria;Proteobacteria;Betaproteobacteria;Nitrosomonadales;Methylophilaceae;Novimethylophilus;Novimethylophilus kurashikiensis;Novimethylophilus kurashikiensis La2-4 |
| Ga0586324 0351197 425 1522 | 0.00 | 0.00 | 0.00 | 0.00 | 0.00 | 0.00 | 0.00 | 0.00 | 0.00 | 0.00 | 6388800.00 | 0.00 | KO:K02982 | small subunit ribosomal protein S3 | Bacteria;Actinobacteria;Rubrobacteria;Gaiellales;Gaiellaceae;Gaiella;Gaiella occulta;Gaiella occulta F2-233 |
| Ga0586324 0832530 360 1070 | 0.00 | 0.00 | 0.00 | 0.00 | 0.00 | 0.00 | 0.00 | 0.00 | 0.00 | 0.00 | 0.00 | 4102500.00 | KO:K02982 | small subunit ribosomal protein S3 | Bacteria;Proteobacteria;Betaproteobacteria;Nitrosomonadales;Gallionellaceae;Gallionella;Gallionella capsiferriformans;Gallionella capsiferriformans ES-2 |
| Ga0586324 1201587 3 908 | 0.00 | 0.00 | 0.00 | 0.00 | 0.00 | 0.00 | 0.00 | 0.00 | 0.00 | 0.00 | 0.00 | 6330000.00 | KO:K02982 | small subunit ribosomal protein S3 | Bacteria;Proteobacteria;Deltaproteobacteria;Desulfobacterales;Desulfobacteraceae;Desulfococcus;Desulfococcus multivorans;Desulfococcus multivorans DSM 2059 |
| Ga0586324 1274316 100 804 | 0.00 | 0.00 | 10246600.00 | 0.00 | 0.00 | 0.00 | 0.00 | 0.00 | 0.00 | 0.00 | 0.00 | 0.00 | KO:K02982 | small subunit ribosomal protein S3 | Bacteria;Proteobacteria;Betaproteobacteria;Nitrosomonadales;Thiobacillaceae;Sulfuritortus;Sulfuritortus calidifontis;Sulfuritortus calidifontis DSM 103923 |
| Ga0586324 2022549 176 892 | 0.00 | 0.00 | 0.00 | 0.00 | 0.00 | 0.00 | 0.00 | 0.00 | 4536000.00 | 0.00 | 0.00 | 0.00 | KO:K02982 | small subunit ribosomal protein S3 | Bacteria;Proteobacteria;Betaproteobacteria;unclassified Betaproteobacteria;unclassified Betaproteobacteria;unclassified Betaproteobacteria;uncultured beta proteobacterium;unclassified betaproteobacterium ENR4 |
| Ga0586324 3249001 75 728 | 0.00 | 0.00 | 0.00 | 0.00 | 0.00 | 0.00 | 0.00 | 3769800.00 | 0.00 | 0.00 | 0.00 | 0.00 | KO:K02982 | small subunit ribosomal protein S3 | Bacteria;Proteobacteria;Deltaproteobacteria;Syntrophobacterales;Syntrophaceae;Desulfobacca;Desulfobacca acetoxidans;Desulfobacca acetoxidans ASRB2, DSM 11109 |
| Ga0586324 7005238 34 537 | 0.00 | 0.00 | 0.00 | 0.00 | 7107000.00 | 0.00 | 0.00 | 0.00 | 0.00 | 0.00 | 0.00 | 0.00 | KO:K02982 | small subunit ribosomal protein S3 | Bacteria;Proteobacteria;Deltaproteobacteria;Desulfuromonadales;Geobacteraceae;Geoalkalibacter;Geoalkalibacter subterraneus;Geoalkalibacter subterraneus Red1 |
| Ga0586324 0217532 1958 2380 | 2140000.00 | 1292000.00 | 1552326.12 | 0.00 | 0.00 | 0.00 | 0.00 | 0.00 | 0.00 | 0.00 | 0.00 | 0.00 | KO:K02984 | small subunit ribosomal protein S3Ae | Archaea;Euryarchaeota;Methanomicrobia;Methanomicrobiales;Methanoregulaceae;Methanoregula;Methanoregula formicica;Methanoregula formicica SMSP, DSM 22288 |
| Ga0586324 0301255 90 722 | 0.00 | 0.00 | 0.00 | 0.00 | 8260600.00 | 0.00 | 0.00 | 0.00 | 0.00 | 0.00 | 0.00 | 0.00 | KO:K02984 | small subunit ribosomal protein S3Ae | Archaea;Euryarchaeota;Methanomicrobia;Methanosarcinales;Methanotrichaceae;Methanothrix;Methanothrix soehngenii;Methanothrix soehngenii GP-6 |
| Ga0586324 0001105 15965 16585 | 0.00 | 0.00 | 0.00 | 5434000.00 | 6921600.00 | 6629900.00 | 0.00 | 0.00 | 12798000.00 | 17941000.00 | 34408000.00 | 10425000.00 | KO:K02986 | small subunit ribosomal protein S4 | Bacteria;Proteobacteria;Gammaproteobacteria;Methylococcales;Methylococcaceae;Methylobacter;Methylobacter luteus;Methylobacter luteus IMV-B-3098 |
| Ga0586324 0004880 11844 12473 | 0.00 | 0.00 | 0.00 | 0.00 | 0.00 | 0.00 | 0.00 | 3117100.00 | 0.00 | 0.00 | 0.00 | 0.00 | KO:K02986 | small subunit ribosomal protein S4 | Bacteria;Proteobacteria;Betaproteobacteria;Nitrosomonadales;Thiobacillaceae;Thiobacillus;Thiobacillus thioparus;Thiobacillus thioparus DSM 505 |
| Ga0586324 0077492 1524 2150 | 0.00 | 3780800.00 | 1955208.92 | 6507500.00 | 5685600.00 | 0.00 | 0.00 | 0.00 | 4519800.00 | 5759600.00 | 0.00 | 0.00 | KO:K02986 | small subunit ribosomal protein S4 | Bacteria;Proteobacteria;Deltaproteobacteria;Syntrophobacterales;Syntrophaceae;Desulfobacca;Desulfobacca acetoxidans;Desulfobacca acetoxidans ASRB2, DSM 11109 |
| Ga0586324 0081877 36 656 | 0.00 | 4311200.00 | 0.00 | 0.00 | 0.00 | 0.00 | 0.00 | 0.00 | 0.00 | 0.00 | 0.00 | 0.00 | KO:K02986 | small subunit ribosomal protein S4 | Bacteria;Proteobacteria;Gammaproteobacteria;Chromatiales;Woeseiaceae;Woeseia;Woeseia oceani;Woeseia oceani XK5 |
| Ga0586324 1158782 454 1074 | 0.00 | 6670800.00 | 0.00 | 0.00 | 0.00 | 0.00 | 0.00 | 0.00 | 0.00 | 0.00 | 0.00 | 0.00 | KO:K02986 | small subunit ribosomal protein S4 | Bacteria;Proteobacteria;Gammaproteobacteria;Methylococcales;Methylococcaceae;Methylobacter;Methylobacter luteus;Methylobacter luteus IMV-B-3098 |
| Ga0586324 1776657 327 950 | 0.00 | 0.00 | 0.00 | 0.00 | 0.00 | 0.00 | 0.00 | 6588000.00 | 0.00 | 0.00 | 0.00 | 0.00 | KO:K02986 | small subunit ribosomal protein S4 | Bacteria;Proteobacteria;Gammaproteobacteria;Nevskiales;Steroidobacteraceae;Steroidobacter;Steroidobacter denitrificans;Steroidobacter denitrificans DSM 18526 |
| Ga0586324 2370142 346 840 | 0.00 | 0.00 | 0.00 | 0.00 | 0.00 | 2149167.44 | 0.00 | 0.00 | 0.00 | 0.00 | 0.00 | 0.00 | KO:K02986 | small subunit ribosomal protein S4 | Bacteria;Proteobacteria;Deltaproteobacteria;Syntrophobacterales;Syntrophobacteraceae;Syntrophobacter;Syntrophobacter fumaroxidans;Syntrophobacter fumaroxidans MPOB |
| Ga0586324 8093762 69 506 | 4690000.00 | 0.00 | 0.00 | 0.00 | 0.00 | 0.00 | 0.00 | 0.00 | 0.00 | 0.00 | 0.00 | 0.00 | KO:K02986 | small subunit ribosomal protein S4 | Bacteria;Proteobacteria;Deltaproteobacteria;Syntrophobacterales;Syntrophobacteraceae;Syntrophobacter;Syntrophobacter fumaroxidans;Syntrophobacter fumaroxidans MPOB |
| Ga0586324 0000625 8227 8736 | 0.00 | 0.00 | 0.00 | 0.00 | 0.00 | 0.00 | 0.00 | 0.00 | 0.00 | 531034.35 | 0.00 | 627732.75 | KO:K02988 | small subunit ribosomal protein S5 | Bacteria;Proteobacteria;Betaproteobacteria;Nitrosomonadales;Thiobacillaceae;Annwoodia;Annwoodia aquaesulis;Annwoodia aquaesulis DSM 4255 |
| Ga0586324 0008357 9631 10158 | 221488.00 | 0.00 | 0.00 | 0.00 | 0.00 | 0.00 | 0.00 | 0.00 | 0.00 | 0.00 | 0.00 | 0.00 | KO:K02988 | small subunit ribosomal protein S5 | Bacteria;Proteobacteria;Betaproteobacteria;Nitrosomonadales;Thiobacillaceae;Thiobacillus;Thiobacillus thioparus;Thiobacillus thioparus DSM 505 |
| Ga0586324 0316962 618 1130 | 0.00 | 0.00 | 0.00 | 3885500.00 | 0.00 | 0.00 | 0.00 | 2379000.00 | 0.00 | 0.00 | 0.00 | 0.00 | KO:K02988 | small subunit ribosomal protein S5 | Bacteria;Proteobacteria;Deltaproteobacteria;Desulfobacterales;Desulfobulbaceae;Desulfobulbus;Desulfobulbus propionicus;Desulfobulbus propionicus 1pr3, DSM 2032 |
| Ga0586324 1215072 554 1075 | 0.00 | 0.00 | 0.00 | 0.00 | 0.00 | 0.00 | 0.00 | 0.00 | 0.00 | 1455300.00 | 0.00 | 0.00 | KO:K02988 | small subunit ribosomal protein S5 | Bacteria;Proteobacteria;Betaproteobacteria;Nitrosomonadales;Nitrosomonadaceae;Nitrosospira;Nitrosospira briensis;Nitrosospira briensis C-128 |
| Ga0586324 1521632 278 790 | 0.00 | 0.00 | 0.00 | 0.00 | 0.00 | 0.00 | 0.00 | 0.00 | 0.00 | 0.00 | 0.00 | 3795000.00 | KO:K02988 | small subunit ribosomal protein S5 | Bacteria;Proteobacteria;Gammaproteobacteria;Chromatiales;Wenzhouxiangellaceae;Wenzhouxiangella;Wenzhouxiangella marina;Wenzhouxiangella marina KCTC 42284 |
| Ga0586324 2160624 2 379 | 0.00 | 0.00 | 0.00 | 0.00 | 0.00 | 0.00 | 0.00 | 0.00 | 0.00 | 509400.43 | 0.00 | 355980.75 | KO:K02988 | small subunit ribosomal protein S5 | Bacteria;Proteobacteria;Betaproteobacteria;Nitrosomonadales;Nitrosomonadaceae;Nitrosospira;Nitrosospira multiformis;Nitrosospira multiformis Nl7 |
| Ga0586324 2264057 1 510 | 0.00 | 0.00 | 0.00 | 0.00 | 0.00 | 0.00 | 0.00 | 0.00 | 0.00 | 1316700.00 | 0.00 | 0.00 | KO:K02988 | small subunit ribosomal protein S5 | Bacteria;Proteobacteria;Betaproteobacteria;Nitrosomonadales;Thiobacillaceae;Thiobacillus;Thiobacillus thioparus;Thiobacillus thioparus DSM 505 |
| Ga0586324 0014756 2928 3398 | 0.00 | 0.00 | 0.00 | 1396500.00 | 0.00 | 0.00 | 0.00 | 0.00 | 0.00 | 0.00 | 0.00 | 0.00 | KO:K02992 | small subunit ribosomal protein S7 | Bacteria;Proteobacteria;Gammaproteobacteria;Methylococcales;Methylococcaceae;Methylobacter;Methylobacter tundripaludum;Methylobacter tundripaludum 31/32 |
| Ga0586324 0032851 2028 2498 | 9720000.00 | 35156000.00 | 0.00 | 6773500.00 | 6530200.00 | 6947500.00 | 0.00 | 0.00 | 31104000.00 | 0.00 | 0.00 | 0.00 | KO:K02992 | small subunit ribosomal protein S7 | Bacteria;Proteobacteria;Deltaproteobacteria;Syntrophobacterales;Syntrophobacteraceae;Desulfacinum;Desulfacinum infernum;Desulfacinum infernum DSM 9756 |
| Ga0586324 0070025 3270 3740 | 0.00 | 4488000.00 | 0.00 | 3021000.00 | 2863400.00 | 5240400.00 | 0.00 | 0.00 | 1749600.00 | 0.00 | 0.00 | 0.00 | KO:K02992 | small subunit ribosomal protein S7 | Bacteria;Proteobacteria;Betaproteobacteria;Burkholderiales;Comamonadaceae;Kinneretia;Kinneretia asaccharophila;Kinneretia asaccharophila DSM 25082 |
| Ga0586324 0076619 2088 2558 | 2420000.00 | 0.00 | 0.00 | 0.00 | 0.00 | 0.00 | 0.00 | 0.00 | 0.00 | 0.00 | 0.00 | 2355000.00 | KO:K02992 | small subunit ribosomal protein S7 | Bacteria;Proteobacteria;Alphaproteobacteria;Magnetococcales;Magnetococcaceae;Magnetofaba;Magnetofaba australis;Magnetofaba australis IT-1 |
| Ga0586324 0106192 2308 2778 | 0.00 | 0.00 | 0.00 | 0.00 | 0.00 | 0.00 | 0.00 | 0.00 | 2130300.00 | 0.00 | 0.00 | 0.00 | KO:K02992 | small subunit ribosomal protein S7 | Bacteria;Proteobacteria;Betaproteobacteria;Nitrosomonadales;Thiobacillaceae;Thiobacillus;Thiobacillus thioparus;Thiobacillus thioparus DSM 505 |
| Ga0586324 0109811 2278 2748 | 0.00 | 0.00 | 640057.81 | 0.00 | 0.00 | 968799.10 | 4810800.00 | 26352000.00 | 0.00 | 0.00 | 0.00 | 0.00 | KO:K02992 | small subunit ribosomal protein S7 | Bacteria;Proteobacteria;Alphaproteobacteria;Hyphomicrobiales;Methylocystaceae;Methylocystis;Methylocystis sp. SC2;Methylocystis sp. SC2 |
| Ga0586324 1462107 163 633 | 0.00 | 0.00 | 0.00 | 0.00 | 0.00 | 0.00 | 0.00 | 0.00 | 0.00 | 35112000.00 | 0.00 | 0.00 | KO:K02992 | small subunit ribosomal protein S7 | Bacteria;Proteobacteria;Deltaproteobacteria;Myxococcales;Polyangiaceae;Chondromyces;Chondromyces crocatus;Chondromyces crocatus Cm c5 |
| Ga0586324 1869360 2 397 | 0.00 | 2373200.00 | 0.00 | 2223000.00 | 0.00 | 0.00 | 0.00 | 2952400.00 | 1263600.00 | 0.00 | 0.00 | 0.00 | KO:K02992 | small subunit ribosomal protein S7 | Bacteria;Proteobacteria;Betaproteobacteria;Nitrosomonadales;Gallionellaceae;Sideroxydans;Sideroxydans lithotrophicus;Sideroxydans lithotrophicus ES-1 |
| Ga0586324 1958213 202 672 | 0.00 | 0.00 | 518972.27 | 0.00 | 0.00 | 0.00 | 0.00 | 0.00 | 0.00 | 0.00 | 0.00 | 0.00 | KO:K02992 | small subunit ribosomal protein S7 | Bacteria;Actinobacteria;Actinomycetia;unclassified Actinomycetia;unclassified Actinomycetia;unclassified Actinomycetia;Actinobacteria bacterium IMCC26256;Actinobacteria bacterium IMCC26256 |
| Ga0586324 1990641 1 366 | 0.00 | 1863200.00 | 0.00 | 0.00 | 0.00 | 0.00 | 0.00 | 5111800.00 | 0.00 | 970200.00 | 0.00 | 713592.75 | KO:K02992 | small subunit ribosomal protein S7 | Bacteria;Proteobacteria;Gammaproteobacteria;Methylococcales;Methylococcaceae;Methylobacter;Methylobacter marinus;Methylobacter marinus A45 |
| Ga0586324 2382597 2 343 | 0.00 | 0.00 | 0.00 | 0.00 | 0.00 | 1424336.75 | 0.00 | 0.00 | 0.00 | 0.00 | 0.00 | 0.00 | KO:K02992 | small subunit ribosomal protein S7 | Bacteria;Proteobacteria;Deltaproteobacteria;unclassified Deltaproteobacteria;unclassified Deltaproteobacteria;Dissulfuribacter;Dissulfuribacter thermophilus;Dissulfuribacter thermophilus S69 |
| Ga0586324 2901822 270 740 | 567712.00 | 0.00 | 0.00 | 0.00 | 0.00 | 0.00 | 4218000.00 | 2257000.00 | 2292300.00 | 1332100.00 | 2763200.00 | 2970000.00 | KO:K02992 | small subunit ribosomal protein S7 | Bacteria;Proteobacteria;Betaproteobacteria;Nitrosomonadales;Thiobacillaceae;Thiobacillus;Thiobacillus denitrificans;Thiobacillus denitrificans RG |
| Ga0586324 6121896 222 566 | 0.00 | 0.00 | 0.00 | 0.00 | 0.00 | 0.00 | 0.00 | 0.00 | 0.00 | 0.00 | 0.00 | 7177500.00 | KO:K02992 | small subunit ribosomal protein S7 | Bacteria;Firmicutes;Clostridia;Eubacteriales;Peptostreptococcaceae;Tepidibacter;Tepidibacter thalassicus;Tepidibacter thalassicus DSM 15285 |
| Ga0586324 6721465 96 545 | 0.00 | 0.00 | 0.00 | 0.00 | 0.00 | 0.00 | 173597.38 | 0.00 | 0.00 | 0.00 | 0.00 | 0.00 | KO:K02992 | small subunit ribosomal protein S7 | Bacteria;Gemmatimonadetes;Gemmatimonadetes;Gemmatimonadales;Gemmatimonadaceae;Gemmatirosa;Gemmatirosa kalamazoonesis;Gemmatimonadetes bacterium KBS708 |
| Ga0586324 0004880 6809 7204 | 0.00 | 0.00 | 0.00 | 0.00 | 0.00 | 0.00 | 0.00 | 6093900.00 | 0.00 | 2541000.00 | 0.00 | 1672500.00 | KO:K02994 | small subunit ribosomal protein S8 | Bacteria;Proteobacteria;Betaproteobacteria;Nitrosomonadales;Thiobacillaceae;Thiobacillus;Thiobacillus thioparus;Thiobacillus thioparus DSM 505 |
| Ga0586324 0593228 119 517 | 0.00 | 0.00 | 0.00 | 0.00 | 0.00 | 1126519.26 | 0.00 | 0.00 | 0.00 | 0.00 | 0.00 | 0.00 | KO:K02994 | small subunit ribosomal protein S8 | Bacteria;Firmicutes;Clostridia;Thermoanaerobacterales;Thermoanaerobacteraceae;Thermodesulfitimonas;Thermodesulfitimonas autotrophica;Thermodesulfitimonas autotrophica DSM 102936 |
| Ga0586324 2642244 533 802 | 0.00 | 0.00 | 0.00 | 0.00 | 0.00 | 0.00 | 0.00 | 0.00 | 0.00 | 0.00 | 0.00 | 365411.25 | KO:K02994 | small subunit ribosomal protein S8 | Bacteria;Proteobacteria;Betaproteobacteria;Burkholderiales;unclassified Burkholderiales;Ideonella;Ideonella sp. B508-1;Ideonella sp. B508-1 |
| Ga0586324 0017457 7199 7591 | 0.00 | 0.00 | 0.00 | 0.00 | 0.00 | 0.00 | 0.00 | 0.00 | 0.00 | 8239000.00 | 9504000.00 | 9075000.00 | KO:K02996 | small subunit ribosomal protein S9 | Bacteria;Proteobacteria;Betaproteobacteria;Nitrosomonadales;Methylophilaceae;unclassified Methylophilaceae;unclassified Methylophilaceae;Betaproteobacterium LSUCC0135 |
| Ga0586324 1212739 170 562 | 0.00 | 0.00 | 0.00 | 0.00 | 0.00 | 0.00 | 0.00 | 0.00 | 2446200.00 | 0.00 | 0.00 | 0.00 | KO:K02996 | small subunit ribosomal protein S9 | Bacteria;Acidobacteria;Blastocatellia;unclassified Blastocatellia;unclassified Blastocatellia;Chloracidobacterium;Chloracidobacterium thermophilum;Chloracidobacterium thermophilum OC1 |
| Ga0586324 2030916 447 839 | 0.00 | 0.00 | 1604392.36 | 0.00 | 0.00 | 0.00 | 0.00 | 0.00 | 0.00 | 0.00 | 0.00 | 0.00 | KO:K02996 | small subunit ribosomal protein S9 | Bacteria;Proteobacteria;Betaproteobacteria;Rhodocyclales;Azonexaceae;Dechloromonas;Dechloromonas sp. JJ;Dechloromonas sp. JJ |
| Ga0586324 0000475 8344 9348 | 0.00 | 0.00 | 0.00 | 0.00 | 0.00 | 0.00 | 0.00 | 0.00 | 0.00 | 0.00 | 0.00 | 1492500.00 | KO:K03040 | DNA-directed RNA polymerase subunit alpha | Bacteria;Proteobacteria;Gammaproteobacteria;Acidiferrobacterales;Acidiferrobacteraceae;Sulfuricaulis;Sulfuricaulis limicola;Sulfuricaulis limicola HA5 |
| Ga0586324 0000625 12594 13577 | 0.00 | 0.00 | 0.00 | 0.00 | 0.00 | 0.00 | 0.00 | 275634.60 | 0.00 | 1801800.00 | 7594400.00 | 10605499.50 | KO:K03040 | DNA-directed RNA polymerase subunit alpha | Bacteria;Proteobacteria;Betaproteobacteria;Nitrosomonadales;Thiobacillaceae;Annwoodia;Annwoodia aquaesulis;Annwoodia aquaesulis DSM 4255 |

| | | | | | | | | | | | | | | | |
|---|---|---|---|---|---|---|---|---|---|---|---|---|---|---|---|
| Ga0586324 0001419 12543 13592 | 0.00 | 1564000.00 | 0.00 | 868613.50 | 2853100.00 | 0.00 | 0.00 | 0.00 | 0.00 | 0.00 | 2288000.00 | 2280000.00 | KO:K03040 | DNA-directed RNA polymerase subunit alpha | Bacteria;Gemmatimonadetes;Gemmatimonadetes;Gemmatimonadales;Gemmatimonadaceae;Gemmatirosa;Gemmatirosa kalamazoonesis;Gemmatimonadetes bacterium KBS708 |
| Ga0586324 0004880 12503 13483 | 0.00 | 0.00 | 0.00 | 0.00 | 0.00 | 0.00 | 0.00 | 0.00 | 0.00 | 19943000.00 | 17424000.00 | 0.00 | KO:K03040 | DNA-directed RNA polymerase subunit alpha | Bacteria;Proteobacteria;Betaproteobacteria;Nitrosomonadales;Thiobacillaceae;Thiobacillus;Thiobacillus thioparus;Thiobacillus thioparus DSM 505 |
| Ga0586324 0005242 3 878 | 0.00 | 0.00 | 0.00 | 0.00 | 0.00 | 0.00 | 0.00 | 0.00 | 1101600.00 | 1609300.00 | 0.00 | 0.00 | KO:K03040 | DNA-directed RNA polymerase subunit alpha | Bacteria;Proteobacteria;Betaproteobacteria;Nitrosomonadales;Gallionellaceae;Gallionella;Gallionella capsiferriformans;Gallionella capsiferriformans ES-2 |
| Ga0586324 0010273 8451 9428 | 0.00 | 0.00 | 0.00 | 3078000.00 | 4892500.00 | 0.00 | 0.00 | 0.00 | 0.00 | 0.00 | 0.00 | 0.00 | KO:K03040 | DNA-directed RNA polymerase subunit alpha | Bacteria;Chloroflexi;Thermoflexia;Thermoflexales;Thermoflexaceae;Thermoflexus;Thermoflexus hugenholtzii;Thermoflexus hugenholtzii JAD2 |
| Ga0586324 0049551 1002 2003 | 0.00 | 4841600.00 | 0.00 | 0.00 | 0.00 | 0.00 | 0.00 | 1403000.00 | 0.00 | 854700.00 | 0.00 | 0.00 | KO:K03040 | DNA-directed RNA polymerase subunit alpha | Bacteria;Proteobacteria;Deltaproteobacteria;Desulfuromonadales;Geobacteraceae;Geobacter;Geobacter sp. DSM 9736;Geobacter sp. DSM 9736 |
| Ga0586324 0062358 566 1555 | 0.00 | 5174800.00 | 0.00 | 0.00 | 0.00 | 0.00 | 0.00 | 0.00 | 0.00 | 0.00 | 0.00 | 0.00 | KO:K03040 | DNA-directed RNA polymerase subunit alpha | Bacteria;Candidatus Latescibacteria;unclassified Candidatus Latescibacteria;unclassified Candidatus Latescibacteria;unclassified Candidatus Latescibacteria;Candidatus Latescibacter;Candidatus Latescibacter anaerobius;Candidatus Latescibacter anaerobius SCGC AAA252-E07 (contamination screened) |
| Ga0586324 0080266 2914 3807 | 0.00 | 0.00 | 0.00 | 2536500.00 | 0.00 | 0.00 | 0.00 | 0.00 | 0.00 | 0.00 | 0.00 | 0.00 | KO:K03040 | DNA-directed RNA polymerase subunit alpha | Bacteria;Proteobacteria;Alphaproteobacteria;Hyphomicrobiales;Bradyrhizobiaceae;Bradyrhizobium;Bradyrhizobium canariense;Bradyrhizobium canariense GAS369 |
| Ga0586324 0089833 614 1666 | 0.00 | 0.00 | 0.00 | 0.00 | 0.00 | 2660666.21 | 1439343.48 | 0.00 | 0.00 | 1601600.00 | 0.00 | 0.00 | KO:K03040 | DNA-directed RNA polymerase subunit alpha | Bacteria;Nitrospirae;Thermodesulfovibrionia;Thermodesulfovibrionales;Thermodesulfovibrionaceae;Thermodesulfovibrio;Thermodesulfovibrio yellowstonii;Thermodesulfovibrio yellowstonii DSM 11347 |
| Ga0586324 0102437 3 344 | 0.00 | 0.00 | 0.00 | 0.00 | 0.00 | 0.00 | 0.00 | 0.00 | 0.00 | 0.00 | 0.00 | 400059.75 | KO:K03040 | DNA-directed RNA polymerase subunit alpha | Bacteria;Chloroflexi;Anaerolineae;Anaerolineales;Anaerolineaceae;Levilinea;Levilinea saccharolytica;Levilinea saccharolytica DSM 16555 |
| Ga0586324 0142810 1201 2244 | 0.00 | 0.00 | 0.00 | 0.00 | 0.00 | 0.00 | 0.00 | 0.00 | 0.00 | 947100.00 | 0.00 | 0.00 | KO:K03040 | DNA-directed RNA polymerase subunit alpha | Bacteria;Gemmatimonadetes;Gemmatimonadetes;Gemmatimonadales;Gemmatimonadaceae;Gemmatimonas;Gemmatimonas phototrophica;Gemmatimonas phototrophica AP64 |
| Ga0586324 0170406 1714 2667 | 0.00 | 0.00 | 0.00 | 0.00 | 0.00 | 0.00 | 0.00 | 0.00 | 359123.22 | 2394700.00 | 21032000.00 | 0.00 | KO:K03040 | DNA-directed RNA polymerase subunit alpha | Bacteria;Proteobacteria;Betaproteobacteria;Nitrosomonadales;Gallionellaceae;Gallionella;Gallionella capsiferriformans;Gallionella capsiferriformans ES-2 |
| Ga0586324 0177767 1510 2613 | 0.00 | 0.00 | 0.00 | 0.00 | 0.00 | 0.00 | 0.00 | 0.00 | 10692000.00 | 0.00 | 0.00 | 0.00 | KO:K03040 | DNA-directed RNA polymerase subunit alpha | Bacteria;Planctomycetes;unclassified Planctomycetes;unclassified Planctomycetes;unclassified Planctomycetes;unclassified Planctomycetes;unclassified Planctomycetes;Planctomycetes bacterium P3 |
| Ga0586324 0268605 259 1290 | 9080000.00 | 0.00 | 0.00 | 0.00 | 0.00 | 0.00 | 0.00 | 0.00 | 7476300.00 | 0.00 | 0.00 | 0.00 | KO:K03040 | DNA-directed RNA polymerase subunit alpha | Bacteria;Acidobacteria;Acidobacteriia;Bryobacterales;Solibacteraceae;Candidatus Solibacter;Candidatus Solibacter usitatus;Candidatus Solibacter usitatus Ellin6076 |
| Ga0586324 0312883 369 1352 | 13600000.00 | 13396000.00 | 0.00 | 0.00 | 0.00 | 0.00 | 0.00 | 10492000.00 | 0.00 | 6922300.00 | 2930400.00 | 3232500.00 | KO:K03040 | DNA-directed RNA polymerase subunit alpha | Bacteria;Proteobacteria;Betaproteobacteria;Burkholderiales;unclassified Burkholderiales;unclassified Burkholderiales;Burkholderiales bacterium GJ-E10;Burkholderiales bacterium GJ-E10 |
| Ga0586324 0687966 426 1409 | 0.00 | 0.00 | 0.00 | 0.00 | 0.00 | 0.00 | 0.00 | 0.00 | 0.00 | 0.00 | 0.00 | 2985000.00 | KO:K03040 | DNA-directed RNA polymerase subunit alpha | Bacteria;Proteobacteria;Betaproteobacteria;Burkholderiales;unclassified Burkholderiales;Aquabacterium;Aquabacterium sp. NJ1;Aquabacterium sp. NJ1 |
| Ga0586324 0751316 64 1077 | 0.00 | 0.00 | 0.00 | 7961000.00 | 0.00 | 0.00 | 0.00 | 0.00 | 891000.00 | 0.00 | 0.00 | 0.00 | KO:K03040 | DNA-directed RNA polymerase subunit alpha | Bacteria;Proteobacteria;Gammaproteobacteria;Acidiferrobacterales;Acidiferrobacteraceae;Sulfuricaulis;Sulfuricaulis limicola;Sulfuricaulis limicola HA5 |
| Ga0586324 0772478 1 759 | 3580000.00 | 0.00 | 0.00 | 0.00 | 0.00 | 0.00 | 0.00 | 0.00 | 0.00 | 0.00 | 0.00 | 0.00 | KO:K03040 | DNA-directed RNA polymerase subunit alpha | Bacteria;Verrucomicrobia;Opitutae;Opitutales;Opitutaceae;Opitutus;Opitutus terrae;Opitutus terrae PB90-1 |
| Ga0586324 0861856 502 1293 | 2790000.00 | 0.00 | 0.00 | 0.00 | 0.00 | 0.00 | 0.00 | 0.00 | 0.00 | 0.00 | 0.00 | 0.00 | KO:K03040 | DNA-directed RNA polymerase subunit alpha | Bacteria;Proteobacteria;Deltaproteobacteria;Desulfuromonadales;Geobacteraceae;Geothermobacter;Geothermobacter sp. HR-1;Geothermobacter sp. HR-1 |
| Ga0586324 1416361 3 836 | 0.00 | 0.00 | 0.00 | 0.00 | 0.00 | 0.00 | 0.00 | 524459.09 | 0.00 | 725191.39 | 782864.72 | 0.00 | KO:K03040 | DNA-directed RNA polymerase subunit alpha | Bacteria;Proteobacteria;Betaproteobacteria;Nitrosomonadales;Nitrosomonadaceae;Nitrosospira;Nitrosospira lacus;Nitrosospira lacus APG3 |
| Ga0586324 1472390 1 975 | 0.00 | 0.00 | 0.00 | 0.00 | 0.00 | 0.00 | 0.00 | 0.00 | 0.00 | 10318000.00 | 8659200.00 | 0.00 | KO:K03040 | DNA-directed RNA polymerase subunit alpha | Bacteria;Proteobacteria;Betaproteobacteria;Nitrosomonadales;Sulfuricellaceae;Sulfuricella;Sulfuricella denitrificans;Sulfuricella denitrificans skB26 |
| Ga0586324 1478632 1 456 | 0.00 | 0.00 | 0.00 | 0.00 | 0.00 | 0.00 | 0.00 | 0.00 | 1838700.00 | 0.00 | 0.00 | 0.00 | KO:K03040 | DNA-directed RNA polymerase subunit alpha | Bacteria;Proteobacteria;Gammaproteobacteria;unclassified Gammaproteobacteria;Candidatus Competibacteraceae;Candidatus Competibacter;Candidatus Competibacter denitrificans;Candidatus Competibacter denitrificans Run A D11 |
| Ga0586324 1773531 3 806 | 0.00 | 0.00 | 0.00 | 0.00 | 0.00 | 0.00 | 0.00 | 0.00 | 0.00 | 5929000.00 | 0.00 | 0.00 | KO:K03040 | DNA-directed RNA polymerase subunit alpha | Bacteria;Acidobacteria;Acidobacteriia;Acidobacteriales;Acidobacteriaceae;unclassified Acidobacteriaceae;Acidobacteriaceae bacterium KBS 96;Acidobacteriaceae bacterium KBS 96, DSM 24297 |
| Ga0586324 2729846 3 416 | 0.00 | 0.00 | 0.00 | 0.00 | 0.00 | 0.00 | 0.00 | 0.00 | 0.00 | 0.00 | 0.00 | 1320000.00 | KO:K03040 | DNA-directed RNA polymerase subunit alpha | Bacteria;Acidobacteria;Acidobacteriia;Acidobacteriales;Acidobacteriaceae;unclassified Acidobacteriaceae;Acidobacteriaceae bacterium KBS 96;Acidobacteriaceae bacterium KBS 96, DSM 24297 |
| Ga0586324 2775532 2 460 | 0.00 | 0.00 | 0.00 | 0.00 | 0.00 | 0.00 | 0.00 | 0.00 | 0.00 | 6414100.00 | 0.00 | 0.00 | KO:K03040 | DNA-directed RNA polymerase subunit alpha | Bacteria;Proteobacteria;Betaproteobacteria;Rhodocyclales;Azonexaceae;Dechloromonas;Dechloromonas sp. SG708;Dechloromonas sp. SG708 |
| Ga0586324 4959282 1 549 | 0.00 | 0.00 | 0.00 | 0.00 | 0.00 | 2211464.68 | 0.00 | 0.00 | 0.00 | 0.00 | 0.00 | 0.00 | KO:K03040 | DNA-directed RNA polymerase subunit alpha | Bacteria;Acidobacteria;Acidobacteriia;Acidobacteriales;Acidobacteriaceae;Granulicella;Granulicella mallensis;Granulicella mallensis MP5ACTX8 |
| Ga0586324 4968328 3 617 | 4260000.00 | 0.00 | 0.00 | 0.00 | 0.00 | 0.00 | 0.00 | 0.00 | 0.00 | 0.00 | 0.00 | 0.00 | KO:K03040 | DNA-directed RNA polymerase subunit alpha | Bacteria;Verrucomicrobia;Verrucomicrobiae;Verrucomicrobiales;Verrucomicrobia subdivision 3;Pedosphaera;Pedosphaera parvula;Pedosphaera parvula Ellin514 |
| Ga0586324 0004064 1 2667 | 773185.00 | 0.00 | 0.00 | 0.00 | 0.00 | 1721280.84 | 0.00 | 0.00 | 0.00 | 0.00 | 0.00 | 0.00 | KO:K03041 | DNA-directed RNA polymerase subunit A' | Archaea;Euryarchaeota;Methanomicrobia;Methanomicrobiales;Methanoregulaceae;Methanoregula;Methanoregula formicica;Methanoregula formicica SMSP, DSM 22288 |
| Ga0586324 0202702 1370 2461 | 0.00 | 0.00 | 0.00 | 0.00 | 0.00 | 0.00 | 2850000.00 | 0.00 | 0.00 | 0.00 | 0.00 | 0.00 | KO:K03042 | DNA-directed RNA polymerase subunit A" | Archaea;Euryarchaeota;Methanomicrobia;Methanosarcinales;Methanotrichaceae;Methanothrix;Methanothrix soehngenii;Methanothrix soehngenii GP-6 |
| Ga0586324 2013075 1 852 | 1240000.00 | 0.00 | 0.00 | 0.00 | 0.00 | 0.00 | 0.00 | 0.00 | 0.00 | 0.00 | 0.00 | 0.00 | KO:K03042 | DNA-directed RNA polymerase subunit A" | Archaea;Euryarchaeota;Methanomicrobia;Methanomicrobiales;Methanoregulaceae;Methanoregula;Methanoregula formicica;Methanoregula formicica SMSP, DSM 22288 |
| Ga0586324 3770164 1 588 | 0.00 | 0.00 | 0.00 | 0.00 | 0.00 | 0.00 | 0.00 | 1177300.00 | 0.00 | 0.00 | 0.00 | 0.00 | KO:K03042 | DNA-directed RNA polymerase subunit A" | Archaea;Euryarchaeota;Methanomicrobia;Methanomicrobiales;Methanoregulaceae;Methanoregula;Methanoregula formicica;Methanoregula formicica SMSP, DSM 22288 |
| Ga0586324 0000041 7465 11538 | 0.00 | 0.00 | 0.00 | 0.00 | 0.00 | 0.00 | 30232800.00 | 18428100.00 | 30990600.00 | 96222577.99 | 72424000.00 | 73012500.00 | KO:K03043 | DNA-directed RNA polymerase subunit beta | Bacteria;Proteobacteria;Betaproteobacteria;Nitrosomonadales;Thiobacillaceae;Annwoodia;Annwoodia aquaesulis;Annwoodia aquaesulis DSM 4255 |
| Ga0586324 0000999 2 3871 | 0.00 | 0.00 | 0.00 | 0.00 | 0.00 | 0.00 | 2049637.92 | 0.00 | 0.00 | 0.00 | 1636800.00 | 583347.00 | KO:K03043 | DNA-directed RNA polymerase subunit beta | Bacteria;Proteobacteria;Alphaproteobacteria;Hyphomicrobiales;Hyphomicrobiaceae;Rhodoplanes;Rhodoplanes sp. Z2-YC6860;Rhodoplanes sp. Z2-YC6860 |
| Ga0586324 0003508 10697 14827 | 0.00 | 0.00 | 5079110.13 | 0.00 | 0.00 | 0.00 | 0.00 | 0.00 | 0.00 | 0.00 | 0.00 | 0.00 | KO:K03043 | DNA-directed RNA polymerase subunit beta | Bacteria;Proteobacteria;Deltaproteobacteria;Syntrophobacterales;Syntrophaceae;Syntrophus;Syntrophus aciditrophicus;Syntrophus aciditrophicus SB |
| Ga0586324 0003932 7424 11326 | 0.00 | 0.00 | 0.00 | 0.00 | 0.00 | 2026089.50 | 0.00 | 0.00 | 0.00 | 0.00 | 0.00 | 0.00 | KO:K03043 | DNA-directed RNA polymerase subunit beta | Bacteria;Proteobacteria;Deltaproteobacteria;Desulfuromonadales;Geobacteraceae;Geobacter;Geobacter soli;Geobacter soli GSS01 |
| Ga0586324 0004831 5256 9554 | 0.00 | 1128800.00 | 4063660.81 | 0.00 | 0.00 | 0.00 | 0.00 | 0.00 | 0.00 | 0.00 | 0.00 | 1305000.00 | KO:K03043 | DNA-directed RNA polymerase subunit beta | Bacteria;Acidobacteria;Acidobacteriia;Bryobacterales;Solibacteraceae;Candidatus Solibacter;Candidatus Solibacter usitatus;Candidatus Solibacter usitatus Ellin6076 |
| Ga0586324 0005554 6819 11324 | 0.00 | 0.00 | 0.00 | 0.00 | 0.00 | 0.00 | 0.00 | 0.00 | 0.00 | 0.00 | 2147200.00 | 0.00 | KO:K03043 | DNA-directed RNA polymerase subunit beta | Bacteria;Proteobacteria;Deltaproteobacteria;Desulfuromonadales;Geobacteraceae;Geobacter;Geobacter lovleyi;Geobacter lovleyi SZ |
| Ga0586324 0005930 4964 9367 | 2600000.00 | 0.00 | 0.00 | 5158500.00 | 0.00 | 0.00 | 0.00 | 3556300.00 | 0.00 | 0.00 | 0.00 | 0.00 | KO:K03043 | DNA-directed RNA polymerase subunit beta | Bacteria;Acidobacteria;Acidobacteriia;Acidobacteriales;Acidobacteriaceae;Acidobacterium;Acidobacterium capsulatum;Acidobacterium capsulatum ATCC 51196 |
| Ga0586324 0009648 4284 8567 | 0.00 | 0.00 | 0.00 | 969000.00 | 4511400.00 | 0.00 | 0.00 | 0.00 | 0.00 | 0.00 | 0.00 | 0.00 | KO:K03043 | DNA-directed RNA polymerase subunit beta | Bacteria;Candidatus Aminicenantes;unclassified Candidatus Aminicenantes;unclassified Candidatus Aminicenantes;unclassified Candidatus Aminicenantes;unclassified Candidatus Aminicenantes;Aminicenantes bacterium SCGC AAA252-L10;Aminicenantes bacterium SCGC AAA252-L10 (contamination screened) |
| Ga0586324 0010304 3 3698 | 0.00 | 0.00 | 0.00 | 0.00 | 0.00 | 0.00 | 0.00 | 0.00 | 0.00 | 612650.50 | 0.00 | 0.00 | KO:K03043 | DNA-directed RNA polymerase subunit beta | Bacteria;Acidobacteria;Acidobacteriia;Bryobacterales;Solibacteraceae;Candidatus Solibacter;Candidatus Solibacter usitatus;Candidatus Solibacter usitatus Ellin6076 |
| Ga0586324 0012298 2681 6967 | 0.00 | 1142400.00 | 0.00 | 68422.71 | 236208.87 | 0.00 | 0.00 | 8052000.00 | 2032991.46 | 1621325.86 | 9011200.00 | 478338.75 | KO:K03043 | DNA-directed RNA polymerase subunit beta | Bacteria;Proteobacteria;Betaproteobacteria;Rhodocyclales;Rhodocyclaceae;Azospira;unclassified Azospira;Azospira sp. ZAP |
| Ga0586324 0012818 2566 6678 | 0.00 | 3882800.00 | 0.00 | 0.00 | 9939500.00 | 0.00 | 0.00 | 294609.87 | 3466800.00 | 0.00 | 0.00 | 1147500.00 | KO:K03043 | DNA-directed RNA polymerase subunit beta | Bacteria;Proteobacteria;Deltaproteobacteria;Syntrophobacterales;Syntrophobacteraceae;Desulfoglaeba;Desulfoglaeba alkanexedens;Desulfoglaeba alkanexedens ALDC |
| Ga0586324 0013269 2142 6431 | 0.00 | 0.00 | 0.00 | 0.00 | 0.00 | 0.00 | 5494800.00 | 0.00 | 0.00 | 0.00 | 0.00 | 634048.50 | KO:K03043 | DNA-directed RNA polymerase subunit beta | Bacteria;Acidobacteria;Vicinamibacteria;unclassified Vicinamibacteria;Vicinamibacteraceae;Luteitalea;Luteitalea pratensis;Luteitalea pratensis DSM 100886 |
| Ga0586324 0015541 2978 7069 | 0.00 | 0.00 | 0.00 | 0.00 | 404792.06 | 0.00 | 0.00 | 0.00 | 0.00 | 0.00 | 0.00 | 3345000.00 | KO:K03043 | DNA-directed RNA polymerase subunit beta | Bacteria;Proteobacteria;Alphaproteobacteria;Hyphomicrobiales;unclassified Hyphomicrobiales;Methyloceanibacter;Methyloceanibacter superfactus;Methyloceanibacter superfactus R-67175 |
| Ga0586324 0018288 3 3830 | 0.00 | 0.00 | 0.00 | 0.00 | 0.00 | 0.00 | 0.00 | 0.00 | 1830600.00 | 0.00 | 0.00 | 0.00 | KO:K03043 | DNA-directed RNA polymerase subunit beta | Bacteria;Proteobacteria;Deltaproteobacteria;Myxococcales;Anaeromyxobacteraceae;Anaeromyxobacter;Anaeromyxobacter sp. K;Anaeromyxobacter sp. K |
| Ga0586324 0020773 908 5389 | 0.00 | 0.00 | 0.00 | 0.00 | 0.00 | 0.00 | 0.00 | 0.00 | 0.00 | 75086.24 | 0.00 | 0.00 | KO:K03043 | DNA-directed RNA polymerase subunit beta | Bacteria;Acidobacteria;Acidobacteriia;Acidobacteriales;Acidobacteriaceae;unclassified Acidobacteriaceae;Acidobacteriaceae bacterium KBS 96;Acidobacteriaceae bacterium KBS 96, DSM 24297 |
| Ga0586324 0021851 4216 7131 | 0.00 | 0.00 | 0.00 | 0.00 | 0.00 | 0.00 | 0.00 | 0.00 | 0.00 | 0.00 | 0.00 | 640457.25 | KO:K03043 | DNA-directed RNA polymerase subunit beta | Bacteria;Proteobacteria;Betaproteobacteria;Nitrosomonadales;Nitrosomonadaceae;Nitrosospira;Nitrosospira sp. Nsp2;Nitrosospira sp. Nsp2 |
| Ga0586324 0026123 2 2638 | 0.00 | 0.00 | 0.00 | 363342.70 | 483208.02 | 0.00 | 0.00 | 0.00 | 0.00 | 0.00 | 0.00 | 0.00 | KO:K03043 | DNA-directed RNA polymerase subunit beta | Bacteria;Proteobacteria;Deltaproteobacteria;Desulfobacterales;Desulfobacteraceae;Desulfatitalea;Desulfatitalea tepidiphila;Desulfatitalea tepidiphila S28bF |
| Ga0586324 0029393 1761 6191 | 0.00 | 5052400.00 | 2497586.23 | 14060000.00 | 3069400.00 | 7384200.00 | 1285267.92 | 0.00 | 0.00 | 0.00 | 0.00 | 1207500.00 | KO:K03043 | DNA-directed RNA polymerase subunit beta | Bacteria;Proteobacteria;Deltaproteobacteria;Desulfuromonadales;Geobacteraceae;Geobacter;Geobacter lovleyi;Geobacter lovleyi SZ |
| Ga0586324 0029753 1021 5010 | 0.00 | 0.00 | 0.00 | 0.00 | 0.00 | 0.00 | 0.00 | 0.00 | 0.00 | 0.00 | 0.00 | 3120000.00 | KO:K03043 | DNA-directed RNA polymerase subunit beta | Bacteria;Nitrospirae;Nitrospira;Nitrospirales;Nitrospiraceae;Nitrospira;Nitrospira japonica;Nitrospira japonica NJ11 |
| Ga0586324 0030633 4710 6071 | 3140000.00 | 0.00 | 0.00 | 0.00 | 0.00 | 0.00 | 0.00 | 203954.72 | 0.00 | 0.00 | 1188000.00 | 0.00 | KO:K03043 | DNA-directed RNA polymerase subunit beta | Bacteria;Proteobacteria;Deltaproteobacteria;Desulfobacterales;Desulfobacteraceae;Desulfatitalea;Desulfatitalea tepidiphila;Desulfatitalea tepidiphila S28bF |
| Ga0586324 0031827 3831 5960 | 0.00 | 0.00 | 0.00 | 0.00 | 0.00 | 0.00 | 5472000.00 | 0.00 | 0.00 | 0.00 | 0.00 | 0.00 | KO:K03043 | DNA-directed RNA polymerase subunit beta | Bacteria;Proteobacteria;Betaproteobacteria;Nitrosomonadales;Nitrosomonadaceae;Nitrosospira;Nitrosospira sp. Nsp2;Nitrosospira sp. Nsp2 |
| Ga0586324 0035080 702 5174 | 0.00 | 0.00 | 0.00 | 0.00 | 0.00 | 626215.89 | 0.00 | 0.00 | 0.00 | 0.00 | 0.00 | 0.00 | KO:K03043 | DNA-directed RNA polymerase subunit beta | Bacteria;Acidobacteria;Acidobacteriia;Acidobacteriales;Acidobacteriaceae;Candidatus Koribacter;Candidatus Koribacter versatilis;Candidatus Koribacter versatilis Ellin345 |
| Ga0586324 0042280 1106 5191 | 0.00 | 0.00 | 0.00 | 0.00 | 0.00 | 0.00 | 0.00 | 0.00 | 0.00 | 0.00 | 3203200.00 | 0.00 | KO:K03043 | DNA-directed RNA polymerase subunit beta | Bacteria;Proteobacteria;Deltaproteobacteria;Desulfuromonadales;Geobacteraceae;Geobacter;Geobacter metallireducens;Geobacter metallireducens GS-15 |
| Ga0586324 0045809 2 1258 | 0.00 | 0.00 | 0.00 | 0.00 | 0.00 | 0.00 | 0.00 | 1860500.00 | 0.00 | 0.00 | 0.00 | 0.00 | KO:K03043 | DNA-directed RNA polymerase subunit beta | Bacteria;Proteobacteria;Gammaproteobacteria;Alteromonadales;Alteromonadaceae;unclassified Alteromonadaceae;Alteromonadaceae bacterium 2719K.STB50.0a.01;Alteromonadaceae bacterium 2719K.STB50.0a.01 |
| Ga0586324 0049905 2 1978 | 0.00 | 0.00 | 0.00 | 0.00 | 0.00 | 867036.09 | 0.00 | 652700.00 | 0.00 | 0.00 | 0.00 | 0.00 | KO:K03043 | DNA-directed RNA polymerase subunit beta | Bacteria;Proteobacteria;Deltaproteobacteria;Desulfobacterales;Desulfobacteraceae;Desulfonema;Desulfonema limicola;Desulfonema limicola Jadebusen, DSM 2076 |
| Ga0586324 0050188 2 4033 | 0.00 | 0.00 | 0.00 | 0.00 | 0.00 | 0.00 | 0.00 | 0.00 | 2948400.00 | 0.00 | 0.00 | 0.00 | KO:K03043 | DNA-directed RNA polymerase subunit beta | Bacteria;Proteobacteria;Deltaproteobacteria;Desulfuromonadales;Geobacteraceae;Geobacter;Geobacter lovleyi;Geobacter lovleyi SZ |
| Ga0586324 0055913 2 4090 | 0.00 | 0.00 | 0.00 | 0.00 | 0.00 | 31005700.00 | 0.00 | 0.00 | 0.00 | 0.00 | 0.00 | 0.00 | KO:K03043 | DNA-directed RNA polymerase subunit beta | Bacteria;Proteobacteria;Deltaproteobacteria;Desulfobacterales;Desulfobacteraceae;Desulfosarcina;Desulfosarcina variabilis;Desulfosarcina variabilis Montpellier |
| Ga0586324 0059637 1094 4390 | 0.00 | 0.00 | 0.00 | 0.00 | 0.00 | 0.00 | 0.00 | 0.00 | 0.00 | 3249400.00 | 0.00 | 0.00 | KO:K03043 | DNA-directed RNA polymerase subunit beta | Bacteria;Verrucomicrobia;Verrucomicrobiae;Verrucomicrobiales;Verrucomicrobia subdivision 3;Pedosphaera;Pedosphaera parvula;Pedosphaera parvula Ellin514 |
| Ga0586324 0065165 1393 4209 | 0.00 | 279744.52 | 0.00 | 0.00 | 0.00 | 0.00 | 0.00 | 0.00 | 0.00 | 0.00 | 0.00 | 0.00 | KO:K03043 | DNA-directed RNA polymerase subunit beta | Bacteria;Proteobacteria;Gammaproteobacteria;Acidiferrobacterales;Acidiferrobacteraceae;Sulfurifustis;Sulfurifustis variabilis;Sulfurifustis variabilis skN76 |
| Ga0586324 0068948 2486 4096 | 11300000.00 | 483683.32 | 0.00 | 1282500.00 | 0.00 | 0.00 | 0.00 | 0.00 | 766831.86 | 0.00 | 0.00 | 0.00 | KO:K03043 | DNA-directed RNA polymerase subunit beta | Bacteria;Proteobacteria;Deltaproteobacteria;Syntrophobacterales;Syntrophaceae;Desulfobacca;Desulfobacca acetoxidans;Desulfobacca acetoxidans ASRB2, DSM 11109 |
| Ga0586324 0087308 442 3660 | 0.00 | 0.00 | 0.00 | 0.00 | 0.00 | 0.00 | 0.00 | 428665.91 | 7969020.57 | 5536300.00 | 1980000.00 | 5332500.00 | KO:K03043 | DNA-directed RNA polymerase subunit beta | Bacteria;Proteobacteria;Betaproteobacteria;Nitrosomonadales;Sulfuricellaceae;Sulfuricella;Sulfuricella sp. T08;Sulfuricella sp. T08 |
| Ga0586324 0093062 1374 3551 | 0.00 | 0.00 | 0.00 | 0.00 | 0.00 | 0.00 | 0.00 | 0.00 | 1061100.00 | 0.00 | 4637600.00 | 1935000.00 | KO:K03043 | DNA-directed RNA polymerase subunit beta | Bacteria;Proteobacteria;Gammaproteobacteria;Acidiferrobacterales;Acidiferrobacteraceae;Sulfurifustis;Sulfurifustis variabilis;Sulfurifustis variabilis skN76 |
| Ga0586324 0094866 2 2008 | 0.00 | 5344800.00 | 0.00 | 7210500.00 | 402277.83 | 0.00 | 0.00 | 243275.93 | 0.00 | 0.00 | 0.00 | 0.00 | KO:K03043 | DNA-directed RNA polymerase subunit beta | Bacteria;Proteobacteria;Deltaproteobacteria;Syntrophobacterales;Syntrophaceae;Syntrophus;Syntrophus aciditrophicus;Syntrophus aciditrophicus SB |
| Ga0586324 0105937 3 3107 | 0.00 | 0.00 | 0.00 | 0.00 | 0.00 | 0.00 | 0.00 | 0.00 | 2308500.00 | 0.00 | 0.00 | 0.00 | KO:K03043 | DNA-directed RNA polymerase subunit beta | Bacteria;Nitrospirae;Nitrospira;Nitrospirales;Nitrospiraceae;Nitrospira;Nitrospira defluvii;Nitrospira defluvii |
| Ga0586324 0121695 3 3125 | 0.00 | 0.00 | 0.00 | 0.00 | 0.00 | 0.00 | 0.00 | 0.00 | 1773900.00 | 839300.00 | 0.00 | 0.00 | KO:K03043 | DNA-directed RNA polymerase subunit beta | Bacteria;Proteobacteria;Betaproteobacteria;unclassified Betaproteobacteria;unclassified Betaproteobacteria;unclassified Betaproteobacteria;uncultured beta proteobacterium;unclassified betaproteobacterium ENR4 |
| Ga0586324 0132650 3 2990 | 13100000.00 | 0.00 | 0.00 | 17575000.00 | 487133.35 | 0.00 | 0.00 | 0.00 | 0.00 | 0.00 | 0.00 | 0.00 | KO:K03043 | DNA-directed RNA polymerase subunit beta | Bacteria;Proteobacteria;Gammaproteobacteria;Chromatiales;Chromatiaceae;Thiocapsa;Thiocapsa marina;Thiocapsa marina 5811, DSM 5653 |
| Ga0586324 0136197 2 2557 | 13100000.00 | 0.00 | 0.00 | 0.00 | 0.00 | 0.00 | 0.00 | 0.00 | 0.00 | 0.00 | 0.00 | 0.00 | KO:K03043 | DNA-directed RNA polymerase subunit beta | Bacteria;Acidobacteria;Acidobacteriia;Bryobacterales;Solibacteraceae;Candidatus Solibacter;Candidatus Solibacter usitatus;Candidatus Solibacter usitatus Ellin6076 |
| Ga0586324 0140329 3 2921 | 0.00 | 0.00 | 0.00 | 0.00 | 0.00 | 0.00 | 0.00 | 0.00 | 0.00 | 0.00 | 0.00 | 2310000.00 | KO:K03043 | DNA-directed RNA polymerase subunit beta | Bacteria;Proteobacteria;Deltaproteobacteria;Desulfuromonadales;Geobacteraceae;Geobacter;Geobacter lovleyi;Geobacter lovleyi SZ |
| Ga0586324 0177995 1 1692 | 2653896.00 | 562683.68 | 0.00 | 2128000.00 | 1040300.00 | 0.00 | 0.00 | 1528901.56 | 623778.57 | 2733500.00 | 2040438.40 | 1575000.00 | KO:K03043 | DNA-directed RNA polymerase subunit beta | Bacteria;Proteobacteria;Betaproteobacteria;Nitrosomonadales;Thiobacillaceae;Annwoodia;Annwoodia aquaesulis;Annwoodia aquaesulis DSM 4255 |
| Ga0586324 0186705 2 1606 | 0.00 | 0.00 | 0.00 | 0.00 | 0.00 | 0.00 | 0.00 | 0.00 | 0.00 | 0.00 | 0.00 | 321555.00 | KO:K03043 | DNA-directed RNA polymerase subunit beta | Bacteria;Proteobacteria;Gammaproteobacteria;unclassified Gammaproteobacteria;Candidatus Competibacteraceae;Candidatus Contendobacter;Candidatus Contendobacter odensis;Candidatus Contendobacter odensis Run B J11 |
| Ga0586324 0188912 2 2227 | 0.00 | 0.00 | 525342.06 | 0.00 | 0.00 | 0.00 | 0.00 | 0.00 | 0.00 | 0.00 | 0.00 | 0.00 | KO:K03043 | DNA-directed RNA polymerase subunit beta | Bacteria;Proteobacteria;Betaproteobacteria;Rhodocyclales;Rhodocyclaceae;Propionivibrio;Propionivibrio dicarboxylicus;Propionivibrio dicarboxylicus DSM 5885 |
| Ga0586324 0191241 1 2526 | 0.00 | 0.00 | 0.00 | 0.00 | 0.00 | 0.00 | 0.00 | 0.00 | 0.00 | 737758.56 | 0.00 | 0.00 | KO:K03043 | DNA-directed RNA polymerase subunit beta | Bacteria;Proteobacteria;Betaproteobacteria;Nitrosomonadales;Thiobacillaceae;Thiobacillus;Thiobacillus denitrificans;Thiobacillus denitrificans DSM 12475 |
| Ga0586324 0225608 1 2340 | 0.00 | 0.00 | 0.00 | 0.00 | 0.00 | 0.00 | 0.00 | 0.00 | 0.00 | 0.00 | 352280.72 | 0.00 | KO:K03043 | DNA-directed RNA polymerase subunit beta | Bacteria;Acidobacteria;Acidobacteriia;Acidobacteriales;Acidobacteriaceae;unclassified Acidobacteriaceae;Acidobacteriaceae bacterium URHE0068;Acidobacteriaceae bacterium URHE0068 |
| Ga0586324 0237184 3 2234 | 0.00 | 0.00 | 0.00 | 700292.50 | 0.00 | 0.00 | 0.00 | 0.00 | 0.00 | 0.00 | 0.00 | 0.00 | KO:K03043 | DNA-directed RNA polymerase subunit beta | Bacteria;Proteobacteria;Deltaproteobacteria;Syntrophobacterales;Syntrophaceae;Desulfomonile;Desulfomonile tiedjei;Desulfomonile tiedjei DCB-1, DSM 6799 |
| Ga0586324 0247920 1 2241 | 0.00 | 0.00 | 0.00 | 0.00 | 1905500.00 | 0.00 | 0.00 | 0.00 | 0.00 | 0.00 | 0.00 | 0.00 | KO:K03043 | DNA-directed RNA polymerase subunit beta | Bacteria;Proteobacteria;Alphaproteobacteria;Hyphomicrobiales;Methylocystaceae;Methylocystis;Methylocystis sp. ATCC 49242;Methylocystis sp. Rockwell, ATCC 49242 |
| Ga0586324 0262898 3 2015 | 0.00 | 0.00 | 0.00 | 0.00 | 2719200.00 | 0.00 | 0.00 | 437313.27 | 3021300.00 | 0.00 | 0.00 | 0.00 | KO:K03043 | DNA-directed RNA polymerase subunit beta | Bacteria;Proteobacteria;Betaproteobacteria;Burkholderiales;Comamonadaceae;Acidovorax;Acidovorax sp. GW101-3H11;Acidovorax sp. GW101-3H11 |
| Ga0586324 0265284 3 1403 | 0.00 | 0.00 | 0.00 | 0.00 | 0.00 | 0.00 | 0.00 | 301573.63 | 0.00 | 0.00 | 0.00 | 236494.50 | KO:K03043 | DNA-directed RNA polymerase subunit beta | Bacteria;Proteobacteria;Gammaproteobacteria;unclassified Gammaproteobacteria;unclassified Gammaproteobacteria;unclassified Gammaproteobacteria;Codakia orbicularis gill symbiont;Codakia orbicularis gill symbiont |
| Ga0586324 0276179 3 2135 | 0.00 | 0.00 | 0.00 | 0.00 | 0.00 | 0.00 | 0.00 | 4123600.00 | 0.00 | 0.00 | 0.00 | 0.00 | KO:K03043 | DNA-directed RNA polymerase subunit beta | Bacteria;Candidatus Aminicenantes;unclassified Candidatus Aminicenantes;unclassified Candidatus Aminicenantes;unclassified Candidatus Aminicenantes;unclassified Candidatus Aminicenantes;Aminicenantes bacterium SCGC AAA252-O11;Aminicenantes bacterium SCGC AAA252-O11 (contamination screened) |
| Ga0586324 0282802 1 2109 | 0.00 | 2427600.00 | 0.00 | 0.00 | 0.00 | 0.00 | 0.00 | 0.00 | 173952.36 | 22022000.00 | 67341355.84 | 434130.00 | KO:K03043 | DNA-directed RNA polymerase subunit beta | Bacteria;Proteobacteria;Gammaproteobacteria;Acidiferrobacterales;Acidiferrobacteraceae;Sulfuricaulis;Sulfuricaulis limicola;Sulfuricaulis limicola HA5 |
| Ga0586324 0290038 3 2087 | 0.00 | 0.00 | 0.00 | 0.00 | 0.00 | 0.00 | 2576400.00 | 3619887.62 | 2409921.72 | 5176576.02 | 4866400.00 | 10875000.00 | KO:K03043 | DNA-directed RNA polymerase subunit beta | Bacteria;Proteobacteria;Betaproteobacteria;Nitrosomonadales;Thiobacillaceae;Annwoodia;Annwoodia aquaesulis;Annwoodia aquaesulis DSM 4255 |
| Ga0586324 0308649 3 2030 | 0.00 | 0.00 | 0.00 | 0.00 | 0.00 | 0.00 | 0.00 | 0.00 | 0.00 | 0.00 | 15488000.00 | 2115000.00 | KO:K03043 | DNA-directed RNA polymerase subunit beta | Bacteria;Proteobacteria;Betaproteobacteria;Nitrosomonadales;Sulfuricellaceae;Sulfuricella;Sulfuricella sp. T08;Sulfuricella sp. T08 |
| Ga0586324 0321205 2 1993 | 3920000.00 | 0.00 | 0.00 | 0.00 | 0.00 | 0.00 | 0.00 | 0.00 | 0.00 | 0.00 | 0.00 | 0.00 | KO:K03043 | DNA-directed RNA polymerase subunit beta | Bacteria;Proteobacteria;Deltaproteobacteria;Myxococcales;Anaeromyxobacteraceae;Anaeromyxobacter;Anaeromyxobacter sp. Fw109-5;Anaeromyxobacter sp. Fw109-5 |
| Ga0586324 0338571 3 1946 | 0.00 | 0.00 | 0.00 | 0.00 | 0.00 | 0.00 | 0.00 | 0.00 | 0.00 | 2748900.00 | 0.00 | 0.00 | KO:K03043 | DNA-directed RNA polymerase subunit beta | Bacteria;Proteobacteria;Betaproteobacteria;Nitrosomonadales;Sulfuricellaceae;Sulfuricella;Sulfuricella denitrificans;Sulfuricella denitrificans skB26 |
| Ga0586324 0339513 1 1944 | 0.00 | 0.00 | 0.00 | 0.00 | 0.00 | 0.00 | 381145.32 | 45750000.00 | 0.00 | 4412100.00 | 0.00 | 0.00 | KO:K03043 | DNA-directed RNA polymerase subunit beta | Bacteria;Proteobacteria;Betaproteobacteria;Nitrosomonadales;Thiobacillaceae;Thiobacillus;Thiobacillus thioparus;Thiobacillus thioparus DSM 505 |
| Ga0586324 0349055 1 1920 | 0.00 | 0.00 | 0.00 | 0.00 | 0.00 | 0.00 | 0.00 | 0.00 | 0.00 | 0.00 | 0.00 | 481170.00 | KO:K03043 | DNA-directed RNA polymerase subunit beta | Bacteria;Proteobacteria;Betaproteobacteria;unclassified Betaproteobacteria;unclassified Betaproteobacteria;unclassified Betaproteobacteria;uncultured beta proteobacterium;unclassified betaproteobacterium ENR4 |
| Ga0586324 0363190 2 1888 | 0.00 | 0.00 | 0.00 | 0.00 | 0.00 | 0.00 | 0.00 | 516690.13 | 0.00 | 0.00 | 0.00 | 0.00 | KO:K03043 | DNA-directed RNA polymerase subunit beta | Bacteria;Proteobacteria;Deltaproteobacteria;Syntrophobacterales;Syntrophaceae;Desulfobacca;Desulfobacca acetoxidans;Desulfobacca acetoxidans ASRB2, DSM 11109 |
| Ga0586324 0371120 3 1868 | 0.00 | 0.00 | 0.00 | 8008500.00 | 0.00 | 0.00 | 0.00 | 0.00 | 2681100.00 | 0.00 | 0.00 | 0.00 | KO:K03043 | DNA-directed RNA polymerase subunit beta | Bacteria;Bacteroidetes;Chitinophagia;Chitinophagales;Chitinophagaceae;unclassified Chitinophagaceae;Chitinophagaceae bacterium PMP191F;Chitinophagaceae bacterium PMP191F |
| Ga0586324 0406136 2 1795 | 0.00 | 0.00 | 0.00 | 0.00 | 0.00 | 0.00 | 0.00 | 0.00 | 0.00 | 0.00 | 0.00 | 18975000.00 | KO:K03043 | DNA-directed RNA polymerase subunit beta | Bacteria;Chloroflexi;Anaerolineae;Anaerolineales;Anaerolineaceae;Thermanaerothrix;Thermanaerothrix daxensis;Thermanaerothrix daxensis GNS-1 |
| Ga0586324 0499644 2 1639 | 0.00 | 0.00 | 0.00 | 0.00 | 0.00 | 0.00 | 0.00 | 0.00 | 345798.72 | 0.00 | 0.00 | 0.00 | KO:K03043 | DNA-directed RNA polymerase subunit beta | Bacteria;Proteobacteria;Gammaproteobacteria;Methylococcales;Methylococcaceae;Methylovulum;Methylovulum miyakonense;Methylovulum miyakonense HT12 |
| Ga0586324 0525378 2 1603 | 0.00 | 1224000.00 | 0.00 | 0.00 | 0.00 | 0.00 | 0.00 | 0.00 | 0.00 | 0.00 | 0.00 | 0.00 | KO:K03043 | DNA-directed RNA polymerase subunit beta | Bacteria;Proteobacteria;Deltaproteobacteria;Syntrophorhabdales;Syntrophorhabdaceae;Syntrophorhabdus;Syntrophorhabdus aromaticivorans;Syntrophorhabdus aromaticivorans UI |
| Ga0586324 0549030 3 1574 | 0.00 | 0.00 | 0.00 | 7847000.00 | 0.00 | 0.00 | 0.00 | 0.00 | 0.00 | 0.00 | 0.00 | 0.00 | KO:K03043 | DNA-directed RNA polymerase subunit beta | Bacteria;Verrucomicrobia;Verrucomicrobiae;Verrucomicrobiales;Verrucomicrobia subdivision 3;Pedosphaera;Pedosphaera parvula;Pedosphaera parvula Ellin514 |
| Ga0586324 0561001 3 1559 | 3480000.00 | 0.00 | 0.00 | 0.00 | 0.00 | 2122663.72 | 1975029.48 | 0.00 | 1344600.00 | 0.00 | 0.00 | 0.00 | KO:K03043 | DNA-directed RNA polymerase subunit beta | Bacteria;Bacteroidetes;Bacteroidia;Marinilabiliales;Marinifilaceae;Labilibaculum;Labilibaculum filiforme;Labilibaculum filiforme 59.16B |
| Ga0586324 0566469 3 1553 | 16000000.00 | 0.00 | 0.00 | 0.00 | 0.00 | 0.00 | 0.00 | 0.00 | 0.00 | 0.00 | 0.00 | 0.00 | KO:K03043 | DNA-directed RNA polymerase subunit beta | Bacteria;Proteobacteria;Alphaproteobacteria;Hyphomicrobiales;Hyphomicrobiaceae;Rhodoplanes;Rhodoplanes sp. Z2-YC6860;Rhodoplanes sp. Z2-YC6860 |
| Ga0586324 0568462 237 1550 | 0.00 | 0.00 | 0.00 | 0.00 | 908450.73 | 0.00 | 0.00 | 0.00 | 0.00 | 0.00 | 0.00 | 0.00 | KO:K03043 | DNA-directed RNA polymerase subunit beta | Bacteria;Verrucomicrobia;Verrucomicrobiae;Verrucomicrobiales;Verrucomicrobia subdivision 3;Pedosphaera;Pedosphaera parvula;Pedosphaera parvula Ellin514 |
| Ga0586324 0572898 3 1544 | 0.00 | 0.00 | 0.00 | 0.00 | 941247.99 | 0.00 | 0.00 | 0.00 | 0.00 | 0.00 | 0.00 | 0.00 | KO:K03043 | DNA-directed RNA polymerase subunit beta | Bacteria;Acidobacteria;Vicinamibacteria;unclassified Vicinamibacteria;Vicinamibacteraceae;Luteitalea;Luteitalea pratensis;Luteitalea pratensis DSM 100886 |
| Ga0586324 0611569 3 1502 | 0.00 | 0.00 | 0.00 | 0.00 | 22351000.00 | 0.00 | 0.00 | 0.00 | 0.00 | 0.00 | 0.00 | 0.00 | KO:K03043 | DNA-directed RNA polymerase subunit beta | Bacteria;Proteobacteria;Betaproteobacteria;Rhodocyclales;Zoogloeaceae;Azoarcus;Azoarcus olearius;Azoarcus olearius DQS4 |
| Ga0586324 0626760 1 1485 | 0.00 | 17000000.00 | 0.00 | 0.00 | 0.00 | 0.00 | 0.00 | 0.00 | 0.00 | 0.00 | 0.00 | 0.00 | KO:K03043 | DNA-directed RNA polymerase subunit beta | Bacteria;Proteobacteria;Gammaproteobacteria;Methylococcales;Methylococcaceae;Methylobacter;Methylobacter luteus;Methylobacter luteus IMV-B-3098 |
| Ga0586324 0735286 2 1387 | 0.00 | 0.00 | 0.00 | 0.00 | 0.00 | 0.00 | 0.00 | 0.00 | 0.00 | 2063600.00 | 0.00 | 146250000.00 | KO:K03043 | DNA-directed RNA polymerase subunit beta | Bacteria;Proteobacteria;Betaproteobacteria;unclassified Betaproteobacteria;unclassified Betaproteobacteria;unclassified Betaproteobacteria;Betaproteobacteria bacterium GR16-43;Betaproteobacteria bacterium GR16-43 |
| Ga0586324 0741365 1 1380 | 0.00 | 0.00 | 0.00 | 0.00 | 0.00 | 0.00 | 0.00 | 0.00 | 160568.73 | 0.00 | 0.00 | 0.00 | KO:K03043 | DNA-directed RNA polymerase subunit beta | Bacteria;Proteobacteria;Betaproteobacteria;Nitrosomonadales;Thiobacillaceae;Annwoodia;Annwoodia aquaesulis;Annwoodia aquaesulis DSM 4255 |
| Ga0586324 0752773 3 1373 | 0.00 | 0.00 | 0.00 | 0.00 | 0.00 | 0.00 | 0.00 | 0.00 | 0.00 | 0.00 | 0.00 | 410998.50 | KO:K03043 | DNA-directed RNA polymerase subunit beta | Bacteria;Acidobacteria;Acidobacteriia;Bryobacterales;Bryobacteraceae;Bryobacter;Bryobacter aggregatus;Bryobacter aggregatus DSM 18758 |
| Ga0586324 0861056 2 1294 | 0.00 | 0.00 | 0.00 | 24415000.00 | 0.00 | 0.00 | 0.00 | 890600.00 | 0.00 | 27258000.00 | 0.00 | 0.00 | KO:K03043 | DNA-directed RNA polymerase subunit beta | Bacteria;Proteobacteria;Betaproteobacteria;Nitrosomonadales;Thiobacillaceae;Thiobacillus;Thiobacillus denitrificans;Thiobacillus denitrificans DSM 12475 |
| Ga0586324 0890709 96 1277 | 0.00 | 0.00 | 0.00 | 0.00 | 0.00 | 0.00 | 119708.44 | 0.00 | 0.00 | 0.00 | 0.00 | 0.00 | KO:K03043 | DNA-directed RNA polymerase subunit beta | Bacteria;Proteobacteria;Deltaproteobacteria;unclassified Deltaproteobacteria;unclassified Deltaproteobacteria;Deferrisoma;Deferrisoma camini;Deferrisoma camini S3R1 |
| Ga0586324 0977322 3 1226 | 0.00 | 0.00 | 0.00 | 0.00 | 0.00 | 0.00 | 0.00 | 0.00 | 0.00 | 0.00 | 5412000.00 | 0.00 | KO:K03043 | DNA-directed RNA polymerase subunit beta | Bacteria;Cyanobacteria;unclassified Cyanobacteria;Synechococcales;Pseudanabaenaceae;Pseudanabaena;Pseudanabaena biceps;Pseudanabaena biceps PCC 7429 |
| Ga0586324 1036849 1 1194 | 0.00 | 3393200.00 | 0.00 | 0.00 | 0.00 | 0.00 | 0.00 | 0.00 | 0.00 | 0.00 | 0.00 | 0.00 | KO:K03043 | DNA-directed RNA polymerase subunit beta | Bacteria;Proteobacteria;Alphaproteobacteria;Hyphomicrobiales;Hyphomicrobiaceae;Rhodoplanes;Rhodoplanes sp. Z2-YC6860;Rhodoplanes sp. Z2-YC6860 |
| Ga0586324 1055973 153 1187 | 0.00 | 0.00 | 0.00 | 0.00 | 4253900.00 | 0.00 | 0.00 | 0.00 | 0.00 | 0.00 | 0.00 | 0.00 | KO:K03043 | DNA-directed RNA polymerase subunit beta | Bacteria;Proteobacteria;Gammaproteobacteria;Chromatiales;Thioalkalispiraceae;Sulfurivermis;Sulfurivermis fontis;Sulfurivermis fontis JG42 |
| Ga0586324 1125115 2 1153 | 0.00 | 6024800.00 | 0.00 | 0.00 | 0.00 | 0.00 | 0.00 | 0.00 | 0.00 | 0.00 | 0.00 | 0.00 | KO:K03043 | DNA-directed RNA polymerase subunit beta | Bacteria;Acidobacteria;Vicinamibacteria;unclassified Vicinamibacteria;Vicinamibacteraceae;Luteitalea;Luteitalea pratensis;Luteitalea pratensis DSM 100886 |
| Ga0586324 1136729 1 1149 | 0.00 | 0.00 | 0.00 | 0.00 | 0.00 | 0.00 | 0.00 | 0.00 | 0.00 | 1147300.00 | 0.00 | 0.00 | KO:K03043 | DNA-directed RNA polymerase subunit beta | Bacteria;Proteobacteria;Betaproteobacteria;Nitrosomonadales;Thiobacillaceae;Annwoodia;Annwoodia aquaesulis;Annwoodia aquaesulis DSM 4255 |
| Ga0586324 1176960 1 1131 | 0.00 | 0.00 | 0.00 | 0.00 | 0.00 | 0.00 | 0.00 | 0.00 | 0.00 | 0.00 | 655303.44 | 0.00 | KO:K03043 | DNA-directed RNA polymerase subunit beta | Bacteria;Proteobacteria;Betaproteobacteria;Nitrosomonadales;Nitrosomonadaceae;Nitrosospira;Nitrosospira sp. Nsp13;Nitrosospira sp. Nsp13 |
| Ga0586324 1284616 3 1091 | 22200000.00 | 0.00 | 0.00 | 0.00 | 0.00 | 0.00 | 0.00 | 0.00 | 0.00 | 0.00 | 0.00 | 0.00 | KO:K03043 | DNA-directed RNA polymerase subunit beta | Bacteria;Proteobacteria;Gammaproteobacteria;Chromatiales;Ectothiorhodospiraceae;Thioalbus;Thioalbus denitrificans;Thioalbus denitrificans DSM 26407 |
| Ga0586324 1325799 3 1076 | 0.00 | 0.00 | 0.00 | 827694.15 | 0.00 | 0.00 | 0.00 | 884500.00 | 0.00 | 1216600.00 | 1425600.00 | 5325000.00 | KO:K03043 | DNA-directed RNA polymerase subunit beta | Bacteria;Proteobacteria;Deltaproteobacteria;Desulfobacterales;Desulfobacteraceae;Desulfatiglans;Desulfatiglans anilini;Desulfatiglans anilini DSM 4660 |
| Ga0586324 1366171 3 1061 | 0.00 | 4148000.00 | 0.00 | 0.00 | 0.00 | 0.00 | 0.00 | 0.00 | 0.00 | 0.00 | 0.00 | 0.00 | KO:K03043 | DNA-directed RNA polymerase subunit beta | Bacteria;Proteobacteria;Betaproteobacteria;Burkholderiales;Burkholderiaceae;Trinickia;Trinickia caryophylli;Trinickia caryophylli Ballard 720 |
| Ga0586324 1384769 181 1056 | 0.00 | 0.00 | 0.00 | 188983.50 | 0.00 | 20485200.00 | 0.00 | 0.00 | 0.00 | 0.00 | 0.00 | 0.00 | KO:K03043 | DNA-directed RNA polymerase subunit beta | Bacteria;Proteobacteria;Deltaproteobacteria;Syntrophobacterales;Syntrophobacteraceae;Syntrophobacter;Syntrophobacter fumaroxidans;Syntrophobacter fumaroxidans MPOB |
| Ga0586324 1444826 3 1037 | 0.00 | 0.00 | 0.00 | 0.00 | 0.00 | 0.00 | 0.00 | 0.00 | 0.00 | 1024100.00 | 0.00 | 0.00 | KO:K03043 | DNA-directed RNA polymerase subunit beta | Bacteria;Acidobacteria;Acidobacteriia;Acidobacteriales;Acidobacteriaceae;Candidatus Koribacter;Candidatus Koribacter versatilis;Candidatus Koribacter versatilis Ellin345 |
| Ga0586324 1455312 2 637 | 0.00 | 0.00 | 0.00 | 0.00 | 0.00 | 564343.44 | 0.00 | 0.00 | 0.00 | 0.00 | 0.00 | 0.00 | KO:K03043 | DNA-directed RNA polymerase subunit beta | Bacteria;Proteobacteria;Gammaproteobacteria;unclassified Gammaproteobacteria;Candidatus Competibacteraceae;Candidatus Contendobacter;Candidatus Contendobacter odensis;Candidatus Contendobacter odensis Run B J11 |
| Ga0586324 1473755 2 1027 | 0.00 | 0.00 | 0.00 | 0.00 | 0.00 | 0.00 | 0.00 | 1134600.00 | 0.00 | 0.00 | 0.00 | 0.00 | KO:K03043 | DNA-directed RNA polymerase subunit beta | Bacteria;Ignavibacteriae;Ignavibacteria;Ignavibacteriales;Ignavibacteriaceae;Ignavibacterium;Ignavibacterium album;Ignavibacterium album Mat9-16, JCM 16511 |
| Ga0586324 1487310 3 1025 | 0.00 | 0.00 | 0.00 | 1463000.00 | 0.00 | 0.00 | 0.00 | 0.00 | 0.00 | 0.00 | 0.00 | 0.00 | KO:K03043 | DNA-directed RNA polymerase subunit beta | Bacteria;Acidobacteria;Acidobacteriia;Bryobacterales;Bryobacteraceae;Bryobacter;Bryobacter aggregatus;Bryobacter aggregatus DSM 18758 |
| Ga0586324 1497240 1 1020 | 0.00 | 5032000.00 | 0.00 | 0.00 | 0.00 | 0.00 | 0.00 | 0.00 | 0.00 | 0.00 | 0.00 | 0.00 | KO:K03043 | DNA-directed RNA polymerase subunit beta | Bacteria;Proteobacteria;Betaproteobacteria;Nitrosomonadales;Nitrosomonadaceae;Nitrosovibrio;Nitrosovibrio tenuis;Nitrosovibrio tenuis Nv1 |
| Ga0586324 1572000 1 999 | 0.00 | 0.00 | 3717719.83 | 0.00 | 0.00 | 0.00 | 0.00 | 0.00 | 0.00 | 0.00 | 0.00 | 0.00 | KO:K03043 | DNA-directed RNA polymerase subunit beta | Bacteria;Acidobacteria;Acidobacteriia;Acidobacteriales;Acidobacteriaceae;unclassified Acidobacteriaceae;Acidobacteriaceae bacterium KBS 96;Acidobacteriaceae bacterium KBS 96, DSM 24297 |
| Ga0586324 1703739 134 967 | 0.00 | 2781200.00 | 0.00 | 0.00 | 0.00 | 0.00 | 0.00 | 0.00 | 0.00 | 0.00 | 0.00 | 0.00 | KO:K03043 | DNA-directed RNA polymerase subunit beta | Bacteria;Acidobacteria;Acidobacteriia;Acidobacteriales;Acidobacteriaceae;unclassified Acidobacteriaceae;Acidobacteriaceae bacterium KBS 96;Acidobacteriaceae bacterium KBS 96, DSM 24297 |
| Ga0586324 2018130 3 899 | 1140000.00 | 0.00 | 0.00 | 0.00 | 0.00 | 0.00 | 0.00 | 0.00 | 0.00 | 0.00 | 0.00 | 0.00 | KO:K03043 | DNA-directed RNA polymerase subunit beta | Bacteria;Proteobacteria;Betaproteobacteria;Nitrosomonadales;Thiobacillaceae;Sulfuritortus;Sulfuritortus calidifontis;Sulfuritortus calidifontis DSM 103923 |
| Ga0586324 2269883 1 855 | 0.00 | 0.00 | 0.00 | 0.00 | 0.00 | 0.00 | 0.00 | 0.00 | 0.00 | 0.00 | 0.00 | 576865.50 | KO:K03043 | DNA-directed RNA polymerase subunit beta | Bacteria;Proteobacteria;Alphaproteobacteria;Hyphomicrobiales;Hyphomicrobiaceae;Rhodoplanes;Rhodoplanes sp. Z2-YC6860;Rhodoplanes sp. Z2-YC6860 |
| Ga0586324 2481688 3 824 | 0.00 | 0.00 | 0.00 | 0.00 | 0.00 | 0.00 | 0.00 | 233894.74 | 0.00 | 0.00 | 0.00 | 0.00 | KO:K03043 | DNA-directed RNA polymerase subunit beta | Bacteria;Planctomycetes;Planctomycetia;Isosphaerales;Isosphaeraceae;Singulisphaera;Singulisphaera sp. GP187;Singulisphaera sp. GP187 |
| Ga0586324 2528978 2 817 | 0.00 | 0.00 | 0.00 | 0.00 | 0.00 | 0.00 | 4149600.00 | 0.00 | 0.00 | 0.00 | 0.00 | 0.00 | KO:K03043 | DNA-directed RNA polymerase subunit beta | Bacteria;Proteobacteria;Alphaproteobacteria;Hyphomicrobiales;Roseiarcaceae;Roseiarcus;Roseiarcus fermentans;Roseiarcus fermentans DSM 24875 |
| Ga0586324 2626439 3 806 | 0.00 | 0.00 | 8445000.00 | 0.00 | 0.00 | 0.00 | 0.00 | 0.00 | 0.00 | 0.00 | 0.00 | 1222500.00 | KO:K03043 | DNA-directed RNA polymerase subunit beta | Bacteria;Proteobacteria;Deltaproteobacteria;Desulfuromonadales;Geobacteraceae;Geoalkalibacter;Geoalkalibacter subterraneus;Geoalkalibacter subterraneus Red1 |
| Ga0586324 2655744 1 801 | 0.00 | 8296000.00 | 35018600.00 | 0.00 | 0.00 | 0.00 | 0.00 | 0.00 | 0.00 | 0.00 | 0.00 | 0.00 | KO:K03043 | DNA-directed RNA polymerase subunit beta | Bacteria;Proteobacteria;Gammaproteobacteria;Nevskiales;Steroidobacteraceae;Povalibacter;Povalibacter uvarum;Povalibacter uvarum DSM 26723 |
| Ga0586324 2699008 2 796 | 0.00 | 0.00 | 0.00 | 0.00 | 0.00 | 0.00 | 4035600.00 | 2568100.00 | 2754000.00 | 0.00 | 0.00 | 0.00 | KO:K03043 | DNA-directed RNA polymerase subunit beta | Bacteria;Proteobacteria;Betaproteobacteria;Nitrosomonadales;Thiobacillaceae;Thiobacillus;Thiobacillus denitrificans;Thiobacillus denitrificans RG |
| Ga0586324 2725374 311 793 | 0.00 | 0.00 | 0.00 | 0.00 | 0.00 | 0.00 | 6247200.00 | 0.00 | 0.00 | 0.00 | 0.00 | 0.00 | KO:K03043 | DNA-directed RNA polymerase subunit beta | Bacteria;Calditrichaeota;Calditrichae;Calditrichales;Calditrichaceae;Caldithrix;Caldithrix abyssi;Caldithrix abyssi LF13, DSM 13497 |
| Ga0586324 2916066 3 677 | 0.00 | 0.00 | 0.00 | 453401.75 | 0.00 | 0.00 | 0.00 | 646600.00 | 0.00 | 0.00 | 0.00 | 0.00 | KO:K03043 | DNA-directed RNA polymerase subunit beta | Bacteria;Proteobacteria;Deltaproteobacteria;Syntrophobacterales;Syntrophobacteraceae;Desulfacinum;Desulfacinum infernum;Desulfacinum infernum DSM 9756 |
| Ga0586324 2988866 255 764 | 0.00 | 0.00 | 0.00 | 736098.95 | 0.00 | 0.00 | 0.00 | 0.00 | 0.00 | 0.00 | 0.00 | 0.00 | KO:K03043 | DNA-directed RNA polymerase subunit beta | Bacteria;Calditrichaeota;Calditrichae;Calditrichales;Calditrichaceae;Caldithrix;Caldithrix abyssi;Caldithrix abyssi LF13, DSM 13497 |
| Ga0586324 3163912 3 746 | 0.00 | 0.00 | 0.00 | 0.00 | 0.00 | 0.00 | 1161174.36 | 1195600.00 | 0.00 | 0.00 | 4276800.00 | 9825000.00 | KO:K03043 | DNA-directed RNA polymerase subunit beta | Bacteria;Proteobacteria;Betaproteobacteria;Nitrosomonadales;Thiobacillaceae;Thiobacillus;Thiobacillus thiophilus;Thiobacillus thiophilus DSM 19892 |
| Ga0586324 3169337 1 744 | 0.00 | 0.00 | 0.00 | 0.00 | 0.00 | 0.00 | 0.00 | 0.00 | 417501.54 | 0.00 | 0.00 | 0.00 | KO:K03043 | DNA-directed RNA polymerase subunit beta | Bacteria;Verrucomicrobia;Verrucomicrobiae;Verrucomicrobiales;Verrucomicrobia subdivision 3;Pedosphaera;Pedosphaera parvula;Pedosphaera parvula Ellin514 |
| Ga0586324 3190288 2 742 | 938454.00 | 0.00 | 0.00 | 0.00 | 0.00 | 0.00 | 0.00 | 0.00 | 0.00 | 0.00 | 0.00 | 0.00 | KO:K03043 | DNA-directed RNA polymerase subunit beta | Bacteria;Proteobacteria;Alphaproteobacteria;Hyphomicrobiales;Xanthobacteraceae;Pseudolabrys;Pseudolabrys taiwanensis;Pseudolabrys taiwanensis CC-BB4 |
| Ga0586324 3271569 2 505 | 0.00 | 0.00 | 0.00 | 0.00 | 0.00 | 0.00 | 0.00 | 5032500.00 | 0.00 | 0.00 | 0.00 | 0.00 | KO:K03043 | DNA-directed RNA polymerase subunit beta | Bacteria;Actinobacteria;Actinomycetia;Micrococcales;Cellulomonadaceae;Cellulomonas;Cellulomonas sp. Root137;Cellulomonas sp. Root137 |
| Ga0586324 3274325 1 735 | 0.00 | 0.00 | 0.00 | 0.00 | 1967300.00 | 0.00 | 0.00 | 0.00 | 12312000.00 | 0.00 | 0.00 | 0.00 | KO:K03043 | DNA-directed RNA polymerase subunit beta | Bacteria;Proteobacteria;Deltaproteobacteria;Syntrophobacterales;Syntrophaceae;Smithella;Smithella sp. SCADC;Smithella sp. SCADC |
| Ga0586324 3274693 1 735 | 0.00 | 0.00 | 0.00 | 0.00 | 0.00 | 0.00 | 0.00 | 0.00 | 0.00 | 0.00 | 2446400.00 | 0.00 | KO:K03043 | DNA-directed RNA polymerase subunit beta | Bacteria;Proteobacteria;Betaproteobacteria;Nitrosomonadales;Methylophilaceae;Novimethylophilus;Novimethylophilus kurashikiensis;Novimethylophilus kurashikiensis La2-4 |
| Ga0586324 3282739 3 734 | 0.00 | 0.00 | 3167516.82 | 0.00 | 0.00 | 0.00 | 0.00 | 0.00 | 0.00 | 0.00 | 0.00 | 0.00 | KO:K03043 | DNA-directed RNA polymerase subunit beta | Bacteria;Planctomycetes;Phycisphaerae;Phycisphaerales;Phycisphaeraceae;Algisphaera;Algisphaera agarilytica;Algisphaera agarilytica DSM 103725 |
| Ga0586324 3449859 2 718 | 0.00 | 0.00 | 0.00 | 0.00 | 0.00 | 0.00 | 372588.48 | 0.00 | 0.00 | 0.00 | 0.00 | 0.00 | KO:K03043 | DNA-directed RNA polymerase subunit beta | Bacteria;Proteobacteria;Deltaproteobacteria;Myxococcales;unclassified Myxococcales;Enhygromyxa;Enhygromyxa salina;Enhygromyxa salina SWB007 |
| Ga0586324 3838485 1 687 | 0.00 | 251500.04 | 0.00 | 0.00 | 0.00 | 0.00 | 0.00 | 0.00 | 0.00 | 0.00 | 501141.52 | 0.00 | KO:K03043 | DNA-directed RNA polymerase subunit beta | Bacteria;Proteobacteria;Deltaproteobacteria;Desulfobacterales;Desulfobacteraceae;Desulfofaba;Desulfofaba hansenii;Desulfofaba hansenii DSM 12642 |
| Ga0586324 3899046 2 682 | 0.00 | 0.00 | 0.00 | 0.00 | 1472900.00 | 0.00 | 0.00 | 1000400.00 | 0.00 | 0.00 | 0.00 | 0.00 | KO:K03043 | DNA-directed RNA polymerase subunit beta | Bacteria;Proteobacteria;Betaproteobacteria;Nitrosomonadales;Sulfuricellaceae;Sulfuricella;Sulfuricella denitrificans;Sulfuricella denitrificans skB26 |

| | | | | | | | | | | | | | | | |
|---|---|---|---|---|---|---|---|---|---|---|---|---|---|---|---|
| Ga0586324 4191247 2 619 | 0.00 | 0.00 | 0.00 | 0.00 | 0.00 | 9567700.00 | 0.00 | 0.00 | 0.00 | 0.00 | 0.00 | 0.00 | KO:K03043 | DNA-directed RNA polymerase subunit beta | Bacteria;Planctomycetes;Candidatus Brocadiae;Candidatus Brocadiales;Candidatus Brocadiaceae;Candidatus Brocadia;Candidatus Brocadia sinica;Candidatus Brocadia sinica JPN1 |
| Ga0586324 5300910 2 601 | 0.00 | 0.00 | 0.00 | 0.00 | 0.00 | 0.00 | 1935332.40 | 0.00 | 0.00 | 0.00 | 0.00 | 0.00 | KO:K03043 | DNA-directed RNA polymerase subunit beta | Bacteria;Acidobacteria;Acidobacteriia;Acidobacteriales;Acidobacteriaceae;Candidatus Koribacter;Candidatus Koribacter versatilis;Candidatus Koribacter versatilis Ellin345 |
| Ga0586324 5435181 1 597 | 0.00 | 0.00 | 0.00 | 0.00 | 0.00 | 0.00 | 12790800.00 | 0.00 | 0.00 | 0.00 | 0.00 | 0.00 | KO:K03043 | DNA-directed RNA polymerase subunit beta | Bacteria;Proteobacteria;Betaproteobacteria;Rhodocyclales;Rhodocyclaceae;Propionivibrio;Propionivibrio dicarboxylicus;Propionivibrio dicarboxylicus DSM 5885 |
| Ga0586324 5648681 3 587 | 0.00 | 0.00 | 0.00 | 0.00 | 0.00 | 0.00 | 0.00 | 0.00 | 1150200.00 | 0.00 | 0.00 | 0.00 | KO:K03043 | DNA-directed RNA polymerase subunit beta | Bacteria;Proteobacteria;Deltaproteobacteria;Syntrophobacterales;Syntrophaceae;Syntrophus;Syntrophus aciditrophicus;Syntrophus aciditrophicus SB |
| Ga0586324 6209471 3 563 | 0.00 | 0.00 | 0.00 | 0.00 | 0.00 | 0.00 | 0.00 | 0.00 | 15147000.00 | 0.00 | 0.00 | 0.00 | KO:K03043 | DNA-directed RNA polymerase subunit beta | Bacteria;Actinobacteria;Rubrobacteria;Gaiellales;Gaiellaceae;Gaiella;Gaiella occulta;Gaiella occulta F2-233 |
| Ga0586324 6413088 1 555 | 2990000.00 | 0.00 | 0.00 | 0.00 | 0.00 | 0.00 | 0.00 | 0.00 | 0.00 | 1717100.00 | 0.00 | 0.00 | KO:K03043 | DNA-directed RNA polymerase subunit beta | Bacteria;Acidobacteria;Acidobacteriia;Acidobacteriales;Acidobacteriaceae;Candidatus Koribacter;Candidatus Koribacter versatilis;Candidatus Koribacter versatilis Ellin345 |
| Ga0586324 6832363 1 543 | 0.00 | 0.00 | 0.00 | 0.00 | 0.00 | 0.00 | 0.00 | 0.00 | 0.00 | 422726.92 | 0.00 | 0.00 | KO:K03043 | DNA-directed RNA polymerase subunit beta | Bacteria;Acidobacteria;Vicinamibacteria;unclassified Vicinamibacteria;Vicinamibacteraceae;Luteitalea;Luteitalea pratensis;Luteitalea pratensis DSM 100886 |
| Ga0586324 6896119 3 539 | 0.00 | 0.00 | 0.00 | 0.00 | 1781900.00 | 0.00 | 0.00 | 1433500.00 | 0.00 | 1871100.00 | 0.00 | 0.00 | KO:K03043 | DNA-directed RNA polymerase subunit beta | Bacteria;Proteobacteria;Betaproteobacteria;Nitrosomonadales;Sulfuricellaceae;Sulfuricella;Sulfuricella denitrificans;Sulfuricella denitrificans skB26 |
| Ga0586324 7096828 2 535 | 0.00 | 0.00 | 0.00 | 0.00 | 0.00 | 0.00 | 0.00 | 4941000.00 | 0.00 | 0.00 | 0.00 | 0.00 | KO:K03043 | DNA-directed RNA polymerase subunit beta | Bacteria;Proteobacteria;Gammaproteobacteria;Methylococcales;Methylococcaceae;Methylobacter;Methylobacter tundripaludum;Methylobacter tundripaludum SV96 |
| Ga0586324 7240534 1 528 | 0.00 | 0.00 | 0.00 | 0.00 | 0.00 | 0.00 | 0.00 | 0.00 | 0.00 | 0.00 | 4963200.00 | 0.00 | KO:K03043 | DNA-directed RNA polymerase subunit beta | Bacteria;Chloroflexi;Anaerolineae;Anaerolineales;Anaerolineaceae;Levilinea;Levilinea saccharolytica;Levilinea saccharolytica DSM 16555 |
| Ga0586324 7737863 3 515 | 0.00 | 0.00 | 646430.97 | 0.00 | 0.00 | 0.00 | 0.00 | 0.00 | 2130300.00 | 0.00 | 0.00 | 0.00 | KO:K03043 | DNA-directed RNA polymerase subunit beta | Bacteria;Calditrichaeota;Calditrichae;Calditrichales;Calditrichaceae;Caldithrix;Caldithrix abyssi;Caldithrix abyssi LF13, DSM 13497 |
| Ga0586324 7947334 1 510 | 6970000.00 | 0.00 | 0.00 | 0.00 | 0.00 | 0.00 | 0.00 | 0.00 | 0.00 | 0.00 | 0.00 | 0.00 | KO:K03043 | DNA-directed RNA polymerase subunit beta | Bacteria;Proteobacteria;Betaproteobacteria;Burkholderiales;unclassified Burkholderiales;Methylibium;Methylibium sp. Root1272;Methylibium sp. Root1272 |
| Ga0586324 7974502 2 508 | 0.00 | 0.00 | 0.00 | 0.00 | 0.00 | 0.00 | 0.00 | 0.00 | 362374.56 | 6013700.00 | 0.00 | 0.00 | KO:K03043 | DNA-directed RNA polymerase subunit beta | Bacteria;Verrucomicrobia;Verrucomicrobiae;Verrucomicrobiales;Verrucomicrobia subdivision 3;Pedosphaera;Pedosphaera parvula;Pedosphaera parvula Ellin514 |
| Ga0586324 0043575 3698 5113 | 0.00 | 0.00 | 3418220.72 | 0.00 | 0.00 | 0.00 | 0.00 | 0.00 | 0.00 | 0.00 | 0.00 | 0.00 | KO:K03045 | DNA-directed RNA polymerase subunit B" | Archaea;Euryarchaeota;Methanomicrobia;Methanosarcinales;Methanotrichaceae;Methanothrix;Methanothrix soehngenii;Methanothrix soehngenii GP-6 |
| Ga0586324 0000041 3247 7443 | 0.00 | 0.00 | 0.00 | 0.00 | 0.00 | 0.00 | 63181066.32 | 36844000.00 | 81793800.00 | 117949617.17 | 212555200.00 | 117454440.00 | KO:K03046 | DNA-directed RNA polymerase subunit beta' | Bacteria;Proteobacteria;Betaproteobacteria;Nitrosomonadales;Thiobacillaceae;Annwoodia;Annwoodia aquaesulis;Annwoodia aquaesulis DSM 4255 |
| Ga0586324 0000999 22808 26107 | 0.00 | 30736000.00 | 0.00 | 0.00 | 0.00 | 555708.69 | 0.00 | 0.00 | 0.00 | 0.00 | 0.00 | 0.00 | KO:K03046 | DNA-directed RNA polymerase subunit beta' | Bacteria;Proteobacteria;Alphaproteobacteria;Hyphomicrobiales;Hyphomicrobiaceae;Rhodoplanes;Rhodoplanes sp. Z2-YC6860;Rhodoplanes sp. Z2-YC6860 |
| Ga0586324 0004540 5735 9976 | 0.00 | 0.00 | 0.00 | 0.00 | 0.00 | 0.00 | 0.00 | 11590000.00 | 0.00 | 1955800.00 | 3599200.00 | 0.00 | KO:K03046 | DNA-directed RNA polymerase subunit beta' | Bacteria;Chloroflexi;Thermoflexia;Thermoflexales;Thermoflexaceae;Thermoflexus;Thermoflexus hugenholtzii;Thermoflexus hugenholtzii JAD2 |
| Ga0586324 0004831 9734 13906 | 0.00 | 0.00 | 14919500.00 | 11495000.00 | 0.00 | 0.00 | 0.00 | 8967000.00 | 0.00 | 0.00 | 0.00 | 0.00 | KO:K03046 | DNA-directed RNA polymerase subunit beta' | Bacteria;Acidobacteria;Acidobacteriia;Bryobacterales;Solibacteraceae;Candidatus Solibacter;Candidatus Solibacter usitatus;Candidatus Solibacter usitatus Ellin6076 |
| Ga0586324 0005412 3 1244 | 0.00 | 0.00 | 0.00 | 0.00 | 0.00 | 0.00 | 0.00 | 0.00 | 6309900.00 | 0.00 | 0.00 | 0.00 | KO:K03046 | DNA-directed RNA polymerase subunit beta' | Bacteria;Ignavibacteriae;Ignavibacteria;Ignavibacteriales;Ignavibacteriaceae;Ignavibacterium;Ignavibacterium album;Ignavibacterium album Mat9-16, JCM 16511 |
| Ga0586324 0005533 4648 8826 | 0.00 | 829600.00 | 0.00 | 0.00 | 0.00 | 0.00 | 0.00 | 0.00 | 0.00 | 0.00 | 0.00 | 0.00 | KO:K03046 | DNA-directed RNA polymerase subunit beta' | Bacteria;Nitrospirae;Nitrospira;Nitrospirales;Nitrospiraceae;Nitrospira;Nitrospira japonica;Nitrospira japonica NJ11 |
| Ga0586324 0005930 718 4947 | 0.00 | 0.00 | 0.00 | 0.00 | 0.00 | 0.00 | 0.00 | 0.00 | 0.00 | 0.00 | 477279.44 | 0.00 | KO:K03046 | DNA-directed RNA polymerase subunit beta' | Bacteria;Acidobacteria;Blastocatellia;Blastocatellales;Pyrinomonadaceae;Pyrinomonas;Pyrinomonas methylaliphatogenes;Pyrinomonas methylaliphatogenes K22 |
| Ga0586324 0011414 442 4626 | 0.00 | 0.00 | 0.00 | 0.00 | 0.00 | 0.00 | 0.00 | 0.00 | 0.00 | 203741.23 | 0.00 | 0.00 | KO:K03046 | DNA-directed RNA polymerase subunit beta' | Bacteria;Proteobacteria;Deltaproteobacteria;Syntrophobacterales;Syntrophaceae;Smithella;Smithella sp. F21;Smithella sp. F21 |
| Ga0586324 0023828 1 1461 | 0.00 | 0.00 | 0.00 | 0.00 | 0.00 | 0.00 | 0.00 | 1061400.00 | 0.00 | 0.00 | 0.00 | 0.00 | KO:K03046 | DNA-directed RNA polymerase subunit beta' | Bacteria;Proteobacteria;Deltaproteobacteria;Desulfuromonadales;Desulfuromonadaceae;Desulfuromonas;Desulfuromonas sp. DDH964;Desulfuromonas sp. DDH964 |
| Ga0586324 0024299 2940 6776 | 1210000.00 | 0.00 | 188967.57 | 0.00 | 0.00 | 0.00 | 0.00 | 2464400.00 | 1142100.00 | 0.00 | 0.00 | 0.00 | KO:K03046 | DNA-directed RNA polymerase subunit beta' | Bacteria;Nitrospirae;Nitrospira;Nitrospirales;Nitrospiraceae;Nitrospira;Nitrospira japonica;Nitrospira japonica NJ11 |
| Ga0586324 0024940 5721 6695 | 0.00 | 0.00 | 0.00 | 0.00 | 0.00 | 0.00 | 0.00 | 445581.82 | 0.00 | 0.00 | 0.00 | 0.00 | KO:K03046 | DNA-directed RNA polymerase subunit beta' | Bacteria;Candidatus Omnitrophica;unclassified Candidatus Omnitrophica;unclassified Candidatus Omnitrophica;unclassified Candidatus Omnitrophica;Candidatus Omnitrophus;Candidatus Omnitrophus fodinae;Candidatus Omnitrophus fodinae SCGC AAA011-A17 (contamination screened) |
| Ga0586324 0025854 3 3323 | 0.00 | 0.00 | 0.00 | 19760000.00 | 0.00 | 0.00 | 0.00 | 0.00 | 0.00 | 2209900.00 | 932800.00 | 8662500.00 | KO:K03046 | DNA-directed RNA polymerase subunit beta' | Bacteria;Proteobacteria;Betaproteobacteria;Nitrosomonadales;Thiobacillaceae;Thiobacillus;Thiobacillus thioparus;Thiobacillus thioparus DSM 505 |
| Ga0586324 0026673 1 2085 | 0.00 | 0.00 | 0.00 | 0.00 | 0.00 | 0.00 | 0.00 | 0.00 | 0.00 | 0.00 | 0.00 | 1747500.00 | KO:K03046 | DNA-directed RNA polymerase subunit beta' | Bacteria;Proteobacteria;Deltaproteobacteria;Myxococcales;Vulgatibacteraceae;Vulgatibacter;Vulgatibacter incomptus;Vulgatibacter incomptus DSM 27710 |
| Ga0586324 0029965 1 1818 | 0.00 | 0.00 | 0.00 | 0.00 | 633461.33 | 0.00 | 0.00 | 0.00 | 0.00 | 0.00 | 0.00 | 0.00 | KO:K03046 | DNA-directed RNA polymerase subunit beta' | Bacteria;Chloroflexi;Anaerolineae;Anaerolineales;Anaerolineaceae;Anaerolinea;Anaerolinea thermophila;Anaerolinea thermophila UNI-1 |
| Ga0586324 0030633 253 4653 | 0.00 | 0.00 | 0.00 | 0.00 | 0.00 | 0.00 | 0.00 | 271950.81 | 207572.22 | 0.00 | 283134.72 | 124500000.00 | KO:K03046 | DNA-directed RNA polymerase subunit beta' | Bacteria;Proteobacteria;Deltaproteobacteria;Desulfobacterales;Desulfobacteraceae;Desulfatitalea;Desulfatitalea tepidiphila;Desulfatitalea tepidiphila S28bF |
| Ga0586324 0034086 1965 4427 | 0.00 | 11560000.00 | 0.00 | 0.00 | 0.00 | 0.00 | 0.00 | 0.00 | 0.00 | 0.00 | 0.00 | 0.00 | KO:K03046 | DNA-directed RNA polymerase subunit beta' | Bacteria;Proteobacteria;Deltaproteobacteria;Desulfarculales;Desulfarculaceae;Desulfocarbo;Desulfocarbo indianensis;Desulfocarbo indianensis SCBM |
| Ga0586324 0041135 299 4357 | 0.00 | 0.00 | 0.00 | 0.00 | 0.00 | 0.00 | 0.00 | 0.00 | 1109700.00 | 0.00 | 0.00 | 0.00 | KO:K03046 | DNA-directed RNA polymerase subunit beta' | Bacteria;Verrucomicrobia;Spartobacteria;unclassified Spartobacteria;unclassified Spartobacteria;unclassified Spartobacteria;Spartobacteria bacterium LR76;Spartobacteria bacterium LR76 |
| Ga0586324 0043882 1 4107 | 0.00 | 0.00 | 0.00 | 0.00 | 1442000.00 | 0.00 | 0.00 | 0.00 | 0.00 | 0.00 | 0.00 | 0.00 | KO:K03046 | DNA-directed RNA polymerase subunit beta' | Bacteria;Proteobacteria;Deltaproteobacteria;Desulfuromonadales;Geobacteraceae;Geobacter;Geobacter pickeringii;Geobacter pickeringii G13 |
| Ga0586324 0047152 1 3006 | 0.00 | 0.00 | 0.00 | 0.00 | 0.00 | 0.00 | 0.00 | 0.00 | 0.00 | 367959.13 | 0.00 | 0.00 | KO:K03046 | DNA-directed RNA polymerase subunit beta' | Bacteria;Proteobacteria;Betaproteobacteria;Nitrosomonadales;Sulfuricellaceae;Sulfuricella;Sulfuricella sp. T08;Sulfuricella sp. T08 |
| Ga0586324 0048057 501 4628 | 0.00 | 0.00 | 0.00 | 0.00 | 30488000.00 | 0.00 | 0.00 | 0.00 | 0.00 | 3403400.00 | 3208289.92 | 1732500.00 | KO:K03046 | DNA-directed RNA polymerase subunit beta' | Bacteria;Proteobacteria;Deltaproteobacteria;Desulfuromonadales;Geobacteraceae;Geobacter;Geobacter pickeringii;Geobacter pickeringii G13 |
| Ga0586324 0061072 176 4342 | 0.00 | 979200.00 | 0.00 | 0.00 | 0.00 | 0.00 | 0.00 | 0.00 | 0.00 | 0.00 | 0.00 | 0.00 | KO:K03046 | DNA-directed RNA polymerase subunit beta' | Bacteria;Proteobacteria;Deltaproteobacteria;Myxococcales;Kofleriaceae;Haliangium;Haliangium ochraceum;Haliangium ochraceum SMP-2, DSM 14365 |
| Ga0586324 0061426 1739 4330 | 0.00 | 0.00 | 0.00 | 0.00 | 0.00 | 0.00 | 0.00 | 0.00 | 0.00 | 0.00 | 3238400.00 | 0.00 | KO:K03046 | DNA-directed RNA polymerase subunit beta' | Bacteria;Proteobacteria;Deltaproteobacteria;Myxococcales;Sandaracinaceae;Sandaracinus;Sandaracinus amylolyticus;Sandaracinus amylolyticus DSM 53668 |
| Ga0586324 0065235 640 4209 | 0.00 | 0.00 | 0.00 | 0.00 | 0.00 | 0.00 | 0.00 | 878400.00 | 0.00 | 0.00 | 0.00 | 0.00 | KO:K03046 | DNA-directed RNA polymerase subunit beta' | Bacteria;Proteobacteria;Deltaproteobacteria;unclassified Deltaproteobacteria;unclassified Deltaproteobacteria;unclassified Deltaproteobacteria;delta proteobacterium MLMS-1;delta proteobacterium sp. MLMS-1 |
| Ga0586324 0070025 1 1944 | 0.00 | 308918.56 | 0.00 | 0.00 | 0.00 | 0.00 | 0.00 | 0.00 | 0.00 | 0.00 | 0.00 | 0.00 | KO:K03046 | DNA-directed RNA polymerase subunit beta' | Bacteria;Proteobacteria;Betaproteobacteria;Burkholderiales;unclassified Burkholderiales;Ideonella;Ideonella sp. A 288;Ideonella sp. A 288 |
| Ga0586324 0072813 588 3992 | 0.00 | 0.00 | 0.00 | 0.00 | 0.00 | 0.00 | 0.00 | 0.00 | 0.00 | 3503500.00 | 0.00 | 0.00 | KO:K03046 | DNA-directed RNA polymerase subunit beta' | Bacteria;Proteobacteria;Deltaproteobacteria;Desulfobacterales;Desulfobacteraceae;Desulfatiglans;Desulfatiglans anilini;Desulfatiglans anilini DSM 4660 |
| Ga0586324 0084505 845 2440 | 0.00 | 1863200.00 | 0.00 | 0.00 | 0.00 | 0.00 | 0.00 | 0.00 | 0.00 | 0.00 | 0.00 | 0.00 | KO:K03046 | DNA-directed RNA polymerase subunit beta' | Bacteria;Proteobacteria;Deltaproteobacteria;Syntrophobacterales;Syntrophaceae;Smithella;Smithella sp. F21;Smithella sp. F21 |
| Ga0586324 0087505 1 2406 | 0.00 | 0.00 | 0.00 | 0.00 | 0.00 | 0.00 | 0.00 | 0.00 | 0.00 | 0.00 | 0.00 | 15127500.00 | KO:K03046 | DNA-directed RNA polymerase subunit beta' | Bacteria;Gemmatimonadetes;Gemmatimonadetes;Gemmatimonadales;Gemmatimonadaceae;Gemmatirosa;Gemmatirosa kalamazoonesis;Gemmatimonadetes bacterium KBS708 |
| Ga0586324 0094866 2111 3517 | 0.00 | 1190000.00 | 0.00 | 0.00 | 0.00 | 0.00 | 0.00 | 0.00 | 0.00 | 0.00 | 0.00 | 0.00 | KO:K03046 | DNA-directed RNA polymerase subunit beta' | Bacteria;Proteobacteria;Deltaproteobacteria;Syntrophobacterales;Syntrophaceae;Syntrophus;Syntrophus aciditrophicus;Syntrophus aciditrophicus SB |
| Ga0586324 0099463 3 3083 | 0.00 | 0.00 | 0.00 | 3040000.00 | 0.00 | 0.00 | 0.00 | 0.00 | 0.00 | 0.00 | 0.00 | 0.00 | KO:K03046 | DNA-directed RNA polymerase subunit beta' | Bacteria;Proteobacteria;Gammaproteobacteria;Nevskiales;Steroidobacteraceae;Povalibacter;Povalibacter uvarum;Povalibacter uvarum DSM 26723 |
| Ga0586324 0101714 1744 3402 | 0.00 | 0.00 | 0.00 | 0.00 | 0.00 | 883932.41 | 0.00 | 0.00 | 0.00 | 0.00 | 0.00 | 0.00 | KO:K03046 | DNA-directed RNA polymerase subunit beta' | Bacteria;Chloroflexi;Anaerolineae;Anaerolineales;Anaerolineaceae;Anaerolinea;Anaerolinea thermophila;Anaerolinea thermophila UNI-1 |
| Ga0586324 0102658 602 3388 | 0.00 | 0.00 | 0.00 | 0.00 | 0.00 | 0.00 | 0.00 | 1140700.00 | 0.00 | 0.00 | 1751200.00 | 0.00 | KO:K03046 | DNA-directed RNA polymerase subunit beta' | Bacteria;Gemmatimonadetes;Longimicrobia;Longimicrobiales;Longimicrobiaceae;Longimicrobium;Longimicrobium terrae;Longimicrobium terrae CECT 8660 |
| Ga0586324 0107460 2 3142 | 21920000.00 | 4073200.00 | 0.00 | 1064000.00 | 1030000.00 | 0.00 | 977727.84 | 0.00 | 4033800.00 | 877800.00 | 0.00 | 2722500.00 | KO:K03046 | DNA-directed RNA polymerase subunit beta' | Bacteria;Proteobacteria;Alphaproteobacteria;Rhodospirillales;Acetobacteraceae;Rhodopila;Rhodopila globiformis;Rhodopila globiformis DSM 161 |
| Ga0586324 0112931 3 3236 | 0.00 | 0.00 | 0.00 | 0.00 | 0.00 | 0.00 | 0.00 | 0.00 | 0.00 | 0.00 | 1188000.00 | 0.00 | KO:K03046 | DNA-directed RNA polymerase subunit beta' | Bacteria;Proteobacteria;Deltaproteobacteria;Desulfuromonadales;Geobacteraceae;Geobacter;Geobacter uraniireducens;Geobacter uraniireducens Rf4 |
| Ga0586324 0112984 580 3234 | 11900000.00 | 0.00 | 0.00 | 0.00 | 0.00 | 0.00 | 0.00 | 0.00 | 0.00 | 0.00 | 0.00 | 0.00 | KO:K03046 | DNA-directed RNA polymerase subunit beta' | Bacteria;Candidatus Aminicenantes;unclassified Candidatus Aminicenantes;unclassified Candidatus Aminicenantes;unclassified Candidatus Aminicenantes;unclassified Candidatus Aminicenantes;Aminicenantes bacterium SCGC AAA252-O11;Aminicenantes bacterium SCGC AAA252-O11 (contamination screened) |
| Ga0586324 0117182 72 3179 | 0.00 | 0.00 | 0.00 | 0.00 | 0.00 | 0.00 | 0.00 | 0.00 | 0.00 | 0.00 | 0.00 | 8775000.00 | KO:K03046 | DNA-directed RNA polymerase subunit beta' | Bacteria;Proteobacteria;Betaproteobacteria;Nitrosomonadales;Methylophilaceae;Methylobacillus;Methylobacillus sp. MM3;Methylobacillus sp. MM2 |
| Ga0586324 0131073 1 594 | 0.00 | 0.00 | 0.00 | 0.00 | 0.00 | 0.00 | 0.00 | 0.00 | 0.00 | 11781000.00 | 0.00 | 0.00 | KO:K03046 | DNA-directed RNA polymerase subunit beta' | Bacteria;Proteobacteria;Betaproteobacteria;Ferrovales;Ferrovaceae;Ferrovum;Ferrovum myxofaciens;Ferrovum myxofaciens PN-J185 |
| Ga0586324 0143001 1 2073 | 0.00 | 0.00 | 0.00 | 0.00 | 558213.65 | 0.00 | 2172087.60 | 0.00 | 0.00 | 0.00 | 0.00 | 0.00 | KO:K03046 | DNA-directed RNA polymerase subunit beta' | Bacteria;Fibrobacteres;Fibrobacteria;unclassified Fibrobacteria;unclassified Fibrobacteria;unclassified Fibrobacteria;Fibrobacteria bacterium GUT77;Fibrobacteria bacterium GUT77 MC 77 |
| Ga0586324 0151548 1 2817 | 0.00 | 0.00 | 0.00 | 0.00 | 0.00 | 0.00 | 0.00 | 0.00 | 0.00 | 0.00 | 0.00 | 2077500.00 | KO:K03046 | DNA-directed RNA polymerase subunit beta' | Bacteria;Proteobacteria;Gammaproteobacteria;Acidiferrobacterales;Acidiferrobacteraceae;Sulfurifustis;Sulfurifustis variabilis;Sulfurifustis variabilis skN76 |
| Ga0586324 0158425 3 2711 | 0.00 | 0.00 | 1045254.54 | 0.00 | 0.00 | 0.00 | 9188400.00 | 1744600.00 | 0.00 | 2445782.57 | 2913761.84 | 0.00 | KO:K03046 | DNA-directed RNA polymerase subunit beta' | Bacteria;Proteobacteria;Gammaproteobacteria;Acidiferrobacterales;Acidiferrobacteraceae;Sulfuricaulis;Sulfuricaulis limicola;Sulfuricaulis limicola HA5 |
| Ga0586324 0162708 1 2724 | 0.00 | 0.00 | 0.00 | 0.00 | 0.00 | 0.00 | 7546800.00 | 0.00 | 0.00 | 0.00 | 0.00 | 0.00 | KO:K03046 | DNA-directed RNA polymerase subunit beta' | Bacteria;Proteobacteria;Gammaproteobacteria;Pseudomonadales;Pseudomonadaceae;Pseudomonas;Pseudomonas panipatensis;Pseudomonas panipatensis LMG 24738 |
| Ga0586324 0166453 3 2138 | 0.00 | 0.00 | 0.00 | 0.00 | 2214500.00 | 0.00 | 0.00 | 0.00 | 0.00 | 0.00 | 0.00 | 0.00 | KO:K03046 | DNA-directed RNA polymerase subunit beta' | Bacteria;Nitrospirae;Nitrospira;Nitrospirales;Nitrospiraceae;Nitrospira;Candidatus Nitrospira inopinata;Candidatus Nitrospira inopinata ENR4 |
| Ga0586324 0179164 1019 2605 | 0.00 | 0.00 | 0.00 | 0.00 | 13081000.00 | 0.00 | 0.00 | 0.00 | 0.00 | 0.00 | 0.00 | 0.00 | KO:K03046 | DNA-directed RNA polymerase subunit beta' | Bacteria;Proteobacteria;Betaproteobacteria;Nitrosomonadales;Nitrosomonadaceae;Nitrosospira;Nitrosospira sp. Nsp2;Nitrosospira sp. Nsp2 |
| Ga0586324 0182722 1 2031 | 0.00 | 0.00 | 12611200.00 | 0.00 | 0.00 | 0.00 | 830391.96 | 0.00 | 0.00 | 0.00 | 0.00 | 0.00 | KO:K03046 | DNA-directed RNA polymerase subunit beta' | Bacteria;Proteobacteria;Deltaproteobacteria;Myxococcales;Polyangiaceae;Sorangium;Sorangium cellulosum;Sorangium cellulosum So0157-25 |
| Ga0586324 0183373 2 2230 | 0.00 | 0.00 | 188605000.00 | 0.00 | 0.00 | 0.00 | 0.00 | 0.00 | 0.00 | 0.00 | 0.00 | 0.00 | KO:K03046 | DNA-directed RNA polymerase subunit beta' | Bacteria;Proteobacteria;Alphaproteobacteria;Hyphomicrobiales;Hyphomicrobiaceae;Hyphomicrobium;Hyphomicrobium denitrificans;Hyphomicrobium denitrificans 1NES1 |
| Ga0586324 0186705 1668 2555 | 0.00 | 0.00 | 0.00 | 0.00 | 0.00 | 0.00 | 163193.05 | 0.00 | 0.00 | 0.00 | 0.00 | 0.00 | KO:K03046 | DNA-directed RNA polymerase subunit beta' | Bacteria;Proteobacteria;Gammaproteobacteria;unclassified Gammaproteobacteria;Candidatus Competibacteraceae;Candidatus Competibacter;Candidatus Competibacter denitrificans;Candidatus Competibacter denitrificans Run A D11 |
| Ga0586324 0200681 2 730 | 0.00 | 0.00 | 0.00 | 0.00 | 0.00 | 0.00 | 0.00 | 0.00 | 0.00 | 0.00 | 0.00 | 1102500.00 | KO:K03046 | DNA-directed RNA polymerase subunit beta' | Bacteria;Proteobacteria;Gammaproteobacteria;Methylococcales;Methylococcaceae;Methylobacter;Methylobacter tundripaludum;Methylobacter tundripaludum OWC-DMM |
| Ga0586324 0236636 3 2291 | 0.00 | 0.00 | 0.00 | 2555500.00 | 0.00 | 0.00 | 0.00 | 0.00 | 0.00 | 0.00 | 0.00 | 0.00 | KO:K03046 | DNA-directed RNA polymerase subunit beta' | Bacteria;Proteobacteria;Alphaproteobacteria;Hyphomicrobiales;Methylocystaceae;Methylocystis;Methylocystis sp. ATCC 49242;Methylocystis sp. Rockwell, ATCC 49242 |
| Ga0586324 0239232 1 1428 | 0.00 | 0.00 | 0.00 | 1957000.00 | 0.00 | 0.00 | 0.00 | 0.00 | 0.00 | 0.00 | 0.00 | 0.00 | KO:K03046 | DNA-directed RNA polymerase subunit beta' | Bacteria;Proteobacteria;Deltaproteobacteria;Syntrophobacterales;Syntrophaceae;Smithella;Smithella sp. F21;Smithella sp. F21 |
| Ga0586324 0255139 2 2212 | 0.00 | 0.00 | 0.00 | 0.00 | 1328700.00 | 0.00 | 0.00 | 0.00 | 0.00 | 0.00 | 0.00 | 0.00 | KO:K03046 | DNA-directed RNA polymerase subunit beta' | Bacteria;Gemmatimonadetes;Gemmatimonadetes;Gemmatimonadales;Gemmatimonadaceae;Gemmatirosa;Gemmatirosa kalamazoonesis;Gemmatimonadetes bacterium KBS708 |
| Ga0586324 0272321 3 2147 | 0.00 | 0.00 | 0.00 | 0.00 | 0.00 | 0.00 | 0.00 | 0.00 | 0.00 | 0.00 | 0.00 | 1335000.00 | KO:K03046 | DNA-directed RNA polymerase subunit beta' | Bacteria;Planctomycetes;Phycisphaerae;Sedimentisphaerales;Anaerohalosphaeraceae;Anaerohalosphaera;Anaerohalosphaera lusitana;Phycisphaerae bacterium ST-NAGAB-D1 |
| Ga0586324 0279911 2 2122 | 0.00 | 0.00 | 0.00 | 0.00 | 0.00 | 0.00 | 0.00 | 11407000.00 | 0.00 | 0.00 | 0.00 | 0.00 | KO:K03046 | DNA-directed RNA polymerase subunit beta' | Bacteria;Proteobacteria;Deltaproteobacteria;unclassified Deltaproteobacteria;unclassified Deltaproteobacteria;Deferrisoma;Deferrisoma camini;Deferrisoma camini S3R1 |
| Ga0586324 0283283 1 2109 | 0.00 | 0.00 | 0.00 | 0.00 | 0.00 | 0.00 | 0.00 | 0.00 | 0.00 | 6029100.00 | 0.00 | 4582500.00 | KO:K03046 | DNA-directed RNA polymerase subunit beta' | Bacteria;Proteobacteria;Betaproteobacteria;Nitrosomonadales;Gallionellaceae;Gallionella;Gallionella capsiferriformans;Gallionella capsiferriformans ES-2 |
| Ga0586324 0284341 775 2106 | 0.00 | 0.00 | 0.00 | 0.00 | 0.00 | 0.00 | 0.00 | 805200.00 | 0.00 | 0.00 | 0.00 | 0.00 | KO:K03046 | DNA-directed RNA polymerase subunit beta' | Bacteria;Firmicutes;Clostridia;Halanaerobiales;unclassified Halanaerobiales;Anoxybacter;Anoxybacter fermentans;Anoxybacter fermentans DY22613 |
| Ga0586324 0313156 2 2017 | 0.00 | 0.00 | 2087069.15 | 0.00 | 0.00 | 0.00 | 0.00 | 509834.95 | 0.00 | 0.00 | 0.00 | 0.00 | KO:K03046 | DNA-directed RNA polymerase subunit beta' | Bacteria;Proteobacteria;Gammaproteobacteria;Chromatiales;Chromatiaceae;Thioflavicoccus;Thioflavicoccus mobilis;Thioflavicoccus mobilis 8321 |
| Ga0586324 0315286 1 2010 | 0.00 | 0.00 | 0.00 | 0.00 | 0.00 | 0.00 | 0.00 | 0.00 | 0.00 | 0.00 | 0.00 | 686191.50 | KO:K03046 | DNA-directed RNA polymerase subunit beta' | Bacteria;Proteobacteria;Betaproteobacteria;Nitrosomonadales;Sulfuricellaceae;Sulfuricella;Sulfuricella denitrificans;Sulfuricella denitrificans skB26 |
| Ga0586324 0322439 3 596 | 20800000.00 | 0.00 | 0.00 | 0.00 | 0.00 | 0.00 | 0.00 | 0.00 | 0.00 | 0.00 | 19976000.00 | 0.00 | KO:K03046 | DNA-directed RNA polymerase subunit beta' | Bacteria;Acidobacteria;Acidobacteriia;Bryobacterales;Solibacteraceae;Candidatus Solibacter;Candidatus Solibacter usitatus;Candidatus Solibacter usitatus Ellin6076 |
| Ga0586324 0333366 1 1959 | 0.00 | 0.00 | 0.00 | 0.00 | 0.00 | 0.00 | 0.00 | 0.00 | 4171500.00 | 0.00 | 0.00 | 0.00 | KO:K03046 | DNA-directed RNA polymerase subunit beta' | Bacteria;Bacteroidetes;Bacteroidia;Marinilabiliales;Marinilabiliaceae;Anaerophaga;Anaerophaga thermohalophila;Anaerophaga thermohalophila Fru22, DSM 12881 |
| Ga0586324 0341079 3 1940 | 0.00 | 0.00 | 0.00 | 0.00 | 0.00 | 0.00 | 0.00 | 3025600.00 | 0.00 | 0.00 | 0.00 | 0.00 | KO:K03046 | DNA-directed RNA polymerase subunit beta' | Bacteria;Verrucomicrobia;Verrucomicrobiae;Verrucomicrobiales;Verrucomicrobia subdivision 3;Pedosphaera;Pedosphaera parvula;Pedosphaera parvula Ellin514 |
| Ga0586324 0350861 3 1916 | 1560000.00 | 0.00 | 0.00 | 0.00 | 0.00 | 0.00 | 0.00 | 2543700.00 | 0.00 | 0.00 | 0.00 | 0.00 | KO:K03046 | DNA-directed RNA polymerase subunit beta' | Bacteria;Acidobacteria;Acidobacteriia;Acidobacteriales;Acidobacteriaceae;Acidobacterium;Acidobacterium ailaaui;Acidobacterium ailaaui PMMR2 |
| Ga0586324 0359799 2 1576 | 1960000.00 | 0.00 | 0.00 | 0.00 | 0.00 | 0.00 | 0.00 | 0.00 | 0.00 | 0.00 | 0.00 | 683124.00 | KO:K03046 | DNA-directed RNA polymerase subunit beta' | Bacteria;Cyanobacteria;unclassified Cyanobacteria;Synechococcales;Leptolyngbyaceae;unclassified Leptolyngbyaceae;Leptolyngbyaceae cyanobacterium JSC-12;Oscillatoriales cyanobacterium JSC-12 |
| Ga0586324 0362543 1 1890 | 1050000.00 | 0.00 | 0.00 | 0.00 | 0.00 | 0.00 | 0.00 | 0.00 | 0.00 | 0.00 | 0.00 | 0.00 | KO:K03046 | DNA-directed RNA polymerase subunit beta' | Bacteria;Proteobacteria;Deltaproteobacteria;Desulfuromonadales;Geobacteraceae;Geobacter;Geobacter sp. DSM 2909;Geobacter sp. DSM 2909 |
| Ga0586324 0374844 3 1862 | 0.00 | 0.00 | 0.00 | 0.00 | 0.00 | 0.00 | 0.00 | 0.00 | 2340900.00 | 0.00 | 0.00 | 0.00 | KO:K03046 | DNA-directed RNA polymerase subunit beta' | Bacteria;Chloroflexi;Caldilineae;Caldilineales;Caldilineaceae;Caldilinea;Caldilinea aerophila;Caldilinea aerophila STL-6-O1, DSM 14535 |
| Ga0586324 0381259 1 1848 | 0.00 | 0.00 | 0.00 | 0.00 | 0.00 | 0.00 | 0.00 | 3190300.00 | 0.00 | 0.00 | 0.00 | 0.00 | KO:K03046 | DNA-directed RNA polymerase subunit beta' | Bacteria;Acidobacteria;Acidobacteriia;Acidobacteriales;Acidobacteriaceae;Candidatus Koribacter;Candidatus Koribacter versatilis;Candidatus Koribacter versatilis Ellin345 |
| Ga0586324 0421618 2 1471 | 0.00 | 0.00 | 0.00 | 0.00 | 0.00 | 0.00 | 0.00 | 0.00 | 1798200.00 | 0.00 | 0.00 | 0.00 | KO:K03046 | DNA-directed RNA polymerase subunit beta' | Bacteria;Proteobacteria;Gammaproteobacteria;unclassified Gammaproteobacteria;unclassified Gammaproteobacteria;Sedimenticola;unclassified Sedimenticola;Sedimenticola thiotaurini SIP-G1 |
| Ga0586324 0425918 330 1760 | 0.00 | 686800.00 | 0.00 | 0.00 | 0.00 | 0.00 | 0.00 | 0.00 | 0.00 | 0.00 | 0.00 | 0.00 | KO:K03046 | DNA-directed RNA polymerase subunit beta' | Bacteria;Proteobacteria;Deltaproteobacteria;Myxococcales;Polyangiaceae;Chondromyces;Chondromyces apiculatus;Chondromyces apiculatus DSM 436 |
| Ga0586324 0438627 1 1737 | 0.00 | 0.00 | 0.00 | 0.00 | 0.00 | 2845811.13 | 0.00 | 0.00 | 0.00 | 0.00 | 0.00 | 0.00 | KO:K03046 | DNA-directed RNA polymerase subunit beta' | Bacteria;Bacteroidetes;Bacteroidia;Bacteroidales;Rikenellaceae;Acetobacteroides;Acetobacteroides hydrogenigenes;Acetobacteroides hydrogenigenes DSM 24657 |
| Ga0586324 0472170 1 1680 | 0.00 | 0.00 | 0.00 | 0.00 | 0.00 | 0.00 | 0.00 | 0.00 | 0.00 | 0.00 | 0.00 | 1635000.00 | KO:K03046 | DNA-directed RNA polymerase subunit beta' | Bacteria;Proteobacteria;Deltaproteobacteria;Desulfobacterales;Desulfobacteraceae;Desulfatiglans;Desulfatiglans anilini;Desulfatiglans anilini DSM 4660 |
| Ga0586324 0474974 2 1648 | 4880000.00 | 0.00 | 0.00 | 0.00 | 0.00 | 0.00 | 0.00 | 0.00 | 0.00 | 0.00 | 0.00 | 0.00 | KO:K03046 | DNA-directed RNA polymerase subunit beta' | Bacteria;Firmicutes;Clostridia;Eubacteriales;Peptococcaceae;Desulfurispora;Desulfurispora thermophila;Desulfurispora thermophila DSM 16022 |
| Ga0586324 0493355 2 1648 | 0.00 | 0.00 | 0.00 | 0.00 | 0.00 | 0.00 | 553944.24 | 0.00 | 0.00 | 0.00 | 0.00 | 0.00 | KO:K03046 | DNA-directed RNA polymerase subunit beta' | Bacteria;Proteobacteria;Deltaproteobacteria;Desulfuromonadales;Geobacteraceae;Geobacter;Geobacter sp. DSM 9736;Geobacter sp. DSM 9736 |
| Ga0586324 0499075 1 1527 | 0.00 | 0.00 | 0.00 | 0.00 | 0.00 | 0.00 | 0.00 | 474899.03 | 0.00 | 0.00 | 0.00 | 0.00 | KO:K03046 | DNA-directed RNA polymerase subunit beta' | Bacteria;Ignavibacteriae;Ignavibacteria;Ignavibacteriales;Melioribacteraceae;Melioribacter;Melioribacter roseus;Melioribacter roseus P3M |
| Ga0586324 0502996 2 1633 | 0.00 | 0.00 | 0.00 | 0.00 | 0.00 | 0.00 | 0.00 | 0.00 | 0.00 | 0.00 | 915200.00 | 0.00 | KO:K03046 | DNA-directed RNA polymerase subunit beta' | Bacteria;Proteobacteria;Betaproteobacteria;unclassified Betaproteobacteria;unclassified Betaproteobacteria;unclassified Betaproteobacteria;Betaproteobacteria bacterium GR16-43;Betaproteobacteria bacterium GR16-43 |
| Ga0586324 0508665 1 1626 | 0.00 | 0.00 | 0.00 | 0.00 | 0.00 | 0.00 | 0.00 | 0.00 | 0.00 | 0.00 | 0.00 | 419985.00 | KO:K03046 | DNA-directed RNA polymerase subunit beta' | Bacteria;Proteobacteria;Betaproteobacteria;Nitrosomonadales;Gallionellaceae;Gallionella;Gallionella capsiferriformans;Gallionella capsiferriformans ES-2 |
| Ga0586324 0632885 3 1478 | 0.00 | 0.00 | 0.00 | 0.00 | 0.00 | 0.00 | 0.00 | 0.00 | 0.00 | 0.00 | 0.00 | 24825000.00 | KO:K03046 | DNA-directed RNA polymerase subunit beta' | Bacteria;Actinobacteria;Actinomycetia;Corynebacteriales;Mycobacteriaceae;Mycobacterium;Mycobacterium sp. M26;Mycobacterium sp. M26 |
| Ga0586324 0638808 2 1474 | 0.00 | 0.00 | 0.00 | 0.00 | 0.00 | 29457400.00 | 0.00 | 0.00 | 0.00 | 0.00 | 0.00 | 0.00 | KO:K03046 | DNA-directed RNA polymerase subunit beta' | Bacteria;Proteobacteria;Deltaproteobacteria;Desulfuromonadales;Geobacteraceae;Geobacter;Geobacter sp. DSM 2909;Geobacter sp. DSM 2909 |
| Ga0586324 0676222 599 1438 | 0.00 | 0.00 | 0.00 | 1855198.00 | 0.00 | 0.00 | 0.00 | 0.00 | 401512.95 | 0.00 | 0.00 | 0.00 | KO:K03046 | DNA-directed RNA polymerase subunit beta' | Bacteria;Bacteroidetes;Bacteroidia;Marinilabiliales;Marinilabiliaceae;Thermophagus;Thermophagus xiamenensis;Thermophagus xiamenensis HS1 |
| Ga0586324 0683597 2 1432 | 0.00 | 0.00 | 0.00 | 0.00 | 0.00 | 0.00 | 0.00 | 0.00 | 8262000.00 | 0.00 | 0.00 | 0.00 | KO:K03046 | DNA-directed RNA polymerase subunit beta' | Bacteria;Proteobacteria;Betaproteobacteria;Nitrosomonadales;Nitrosomonadaceae;Nitrosospira;Nitrosospira sp. Nsp44;Nitrosospira sp. Nsp44 |
| Ga0586324 0696536 3 1418 | 0.00 | 0.00 | 0.00 | 0.00 | 0.00 | 19572100.00 | 0.00 | 0.00 | 0.00 | 0.00 | 0.00 | 0.00 | KO:K03046 | DNA-directed RNA polymerase subunit beta' | Bacteria;Planctomycetes;Candidatus Brocadiae;Candidatus Brocadiales;Candidatus Brocadiaceae;Candidatus Jettenia;Candidatus Jettenia caeni;Planctomycetaceae bacterium KSU-1 |
| Ga0586324 0717797 3 1400 | 0.00 | 6868000.00 | 0.00 | 0.00 | 0.00 | 0.00 | 0.00 | 0.00 | 0.00 | 0.00 | 0.00 | 0.00 | KO:K03046 | DNA-directed RNA polymerase subunit beta' | Bacteria;Chloroflexi;Chloroflexia;Chloroflexales;Chloroflexaceae;Chloroflexus;Chloroflexus sp. Y-396-1;Chloroflexus sp. Y-396-1 |
| Ga0586324 0760132 92 1366 | 0.00 | 0.00 | 0.00 | 0.00 | 0.00 | 0.00 | 17054400.00 | 0.00 | 0.00 | 0.00 | 14520000.00 | 0.00 | KO:K03046 | DNA-directed RNA polymerase subunit beta' | Bacteria;Ignavibacteriae;Ignavibacteria;Ignavibacteriales;Ignavibacteriaceae;Ignavibacterium;Ignavibacterium album;Ignavibacterium album Mat9-16, JCM 16511 |
| Ga0586324 0820050 177 1322 | 0.00 | 0.00 | 0.00 | 0.00 | 0.00 | 0.00 | 0.00 | 259706.89 | 61037.87 | 0.00 | 0.00 | 0.00 | KO:K03046 | DNA-directed RNA polymerase subunit beta' | Bacteria;Proteobacteria;Deltaproteobacteria;unclassified Deltaproteobacteria;unclassified Deltaproteobacteria;unclassified Deltaproteobacteria;delta proteobacterium NaphS2;delta proteobacterium NaphS2 |
| Ga0586324 0827692 1 789 | 0.00 | 0.00 | 0.00 | 0.00 | 0.00 | 795619.76 | 0.00 | 0.00 | 0.00 | 0.00 | 0.00 | 0.00 | KO:K03046 | DNA-directed RNA polymerase subunit beta' | Bacteria;Bacteroidetes;Bacteroidia;Marinilabiliales;Prolixibacteraceae;Prolixibacter;Prolixibacter denitrificans;Prolixibacter denitrificans DSM 27267 |
| Ga0586324 0832007 1 1314 | 0.00 | 0.00 | 0.00 | 0.00 | 0.00 | 0.00 | 0.00 | 0.00 | 0.00 | 527881.97 | 0.00 | 0.00 | KO:K03046 | DNA-directed RNA polymerase subunit beta' | Bacteria;Proteobacteria;Betaproteobacteria;unclassified Betaproteobacteria;unclassified Betaproteobacteria;unclassified Betaproteobacteria;Betaproteobacteria bacterium GR16-43;Betaproteobacteria bacterium GR16-43 |
| Ga0586324 0868050 2 1291 | 0.00 | 0.00 | 0.00 | 6792500.00 | 0.00 | 0.00 | 0.00 | 0.00 | 0.00 | 0.00 | 0.00 | 0.00 | KO:K03046 | DNA-directed RNA polymerase subunit beta' | Bacteria;Proteobacteria;Deltaproteobacteria;Desulfuromonadales;Geobacteraceae;Geobacter;Geobacter pickeringii;Geobacter pickeringii G13 |
| Ga0586324 0876120 1 1275 | 0.00 | 0.00 | 0.00 | 0.00 | 0.00 | 0.00 | 0.00 | 738100.00 | 0.00 | 0.00 | 0.00 | 0.00 | KO:K03046 | DNA-directed RNA polymerase subunit beta' | Bacteria;Proteobacteria;Betaproteobacteria;unclassified Betaproteobacteria;unclassified Betaproteobacteria;unclassified Betaproteobacteria;uncultured beta proteobacterium;unclassified betaproteobacterium ENR4 |
| Ga0586324 0885538 1 1278 | 0.00 | 5392400.00 | 0.00 | 0.00 | 0.00 | 0.00 | 0.00 | 0.00 | 0.00 | 5336100.00 | 0.00 | 0.00 | KO:K03046 | DNA-directed RNA polymerase subunit beta' | Bacteria;Proteobacteria;Gammaproteobacteria;Chromatiales;Chromatiaceae;Thiocapsa;Thiocapsa rosea;Thiocapsa rosea DSM 235 |
| Ga0586324 0908922 1 1266 | 0.00 | 0.00 | 0.00 | 0.00 | 18540000.00 | 0.00 | 0.00 | 0.00 | 0.00 | 0.00 | 0.00 | 0.00 | KO:K03046 | DNA-directed RNA polymerase subunit beta' | Bacteria;Candidatus Omnitrophica;unclassified Candidatus Omnitrophica;unclassified Candidatus Omnitrophica;unclassified Candidatus Omnitrophica;Candidatus Omnitrophus;Candidatus Omnitrophus fodinae;Candidatus Omnitrophus fodinae SCGC AAA011-A17 (contamination screened) |
| Ga0586324 0940523 3 1247 | 0.00 | 0.00 | 0.00 | 0.00 | 0.00 | 0.00 | 0.00 | 0.00 | 0.00 | 0.00 | 0.00 | 46036.95 | KO:K03046 | DNA-directed RNA polymerase subunit beta' | Bacteria;Proteobacteria;Gammaproteobacteria;Cellvibrionales;Halieaceae;Halioglobus;Halioglobus pacificus;Halioglobus pacificus RR3-57 |
| Ga0586324 1058510 2 1186 | 0.00 | 0.00 | 0.00 | 0.00 | 0.00 | 0.00 | 0.00 | 0.00 | 0.00 | 0.00 | 0.00 | 2647500.00 | KO:K03046 | DNA-directed RNA polymerase subunit beta' | Bacteria;Proteobacteria;Betaproteobacteria;Burkholderiales;unclassified Burkholderiales;Rivibacter;Rivibacter subsaxonicus;Rivibacter subsaxonicus DSM 19570 |
| Ga0586324 1066771 1 1182 | 0.00 | 0.00 | 0.00 | 0.00 | 0.00 | 0.00 | 0.00 | 0.00 | 0.00 | 0.00 | 865993.92 | 0.00 | KO:K03046 | DNA-directed RNA polymerase subunit beta' | Bacteria;Proteobacteria;Alphaproteobacteria;Rhodospirillales;Acetobacteraceae;Granulibacter;Granulibacter bethesdensis;Granulibacter bethesdensis CGDNIH2 |
| Ga0586324 1083258 23 1174 | 882875.00 | 0.00 | 0.00 | 0.00 | 0.00 | 0.00 | 0.00 | 0.00 | 0.00 | 0.00 | 0.00 | 0.00 | KO:K03046 | DNA-directed RNA polymerase subunit beta' | Bacteria;Proteobacteria;Deltaproteobacteria;Desulfuromonadales;Geobacteraceae;Geoalkalibacter;Geoalkalibacter subterraneus;Geoalkalibacter subterraneus Red1 |
| Ga0586324 1107008 3 1163 | 0.00 | 0.00 | 0.00 | 0.00 | 0.00 | 0.00 | 0.00 | 3287900.00 | 63828000.00 | 0.00 | 0.00 | 0.00 | KO:K03046 | DNA-directed RNA polymerase subunit beta' | Bacteria;Proteobacteria;Betaproteobacteria;unclassified Betaproteobacteria;unclassified Betaproteobacteria;unclassified Betaproteobacteria;Betaproteobacteria bacterium GR16-43;Betaproteobacteria bacterium GR16-43 |
| Ga0586324 1124602 2 1153 | 0.00 | 0.00 | 0.00 | 0.00 | 0.00 | 9408900.00 | 0.00 | 0.00 | 0.00 | 0.00 | 0.00 | 0.00 | KO:K03046 | DNA-directed RNA polymerase subunit beta' | Bacteria;Proteobacteria;Betaproteobacteria;Burkholderiales;Comamonadaceae;Curvibacter;Curvibacter delicatus;Curvibacter delicatus NBRC 14919 |
| Ga0586324 1215831 3 1115 | 0.00 | 0.00 | 0.00 | 0.00 | 0.00 | 43273000.00 | 746269.08 | 738100.00 | 0.00 | 0.00 | 0.00 | 0.00 | KO:K03046 | DNA-directed RNA polymerase subunit beta' | Bacteria;Proteobacteria;Gammaproteobacteria;Nevskiales;Steroidobacteraceae;Povalibacter;Povalibacter uvarum;Povalibacter uvarum DSM 26723 |
| Ga0586324 1270998 3 1094 | 0.00 | 2951200.00 | 0.00 | 0.00 | 0.00 | 0.00 | 0.00 | 0.00 | 0.00 | 0.00 | 0.00 | 0.00 | KO:K03046 | DNA-directed RNA polymerase subunit beta' | Bacteria;Ignavibacteriae;Ignavibacteria;Ignavibacteriales;Melioribacteraceae;Melioribacter;Melioribacter roseus;Melioribacter roseus P3M |
| Ga0586324 1272497 2 1096 | 0.00 | 0.00 | 0.00 | 0.00 | 0.00 | 0.00 | 0.00 | 0.00 | 0.00 | 0.00 | 0.00 | 2197500.00 | KO:K03046 | DNA-directed RNA polymerase subunit beta' | Bacteria;Proteobacteria;Alphaproteobacteria;Hyphomicrobiales;unclassified Hyphomicrobiales;Pseudorhodoplanes;Pseudorhodoplanes sinuspersici;Pseudorhodoplanes sinuspersici RIPI110 |
| Ga0586324 1389484 2 1054 | 0.00 | 0.00 | 0.00 | 0.00 | 0.00 | 0.00 | 0.00 | 0.00 | 0.00 | 0.00 | 6758400.00 | 0.00 | KO:K03046 | DNA-directed RNA polymerase subunit beta' | Bacteria;Proteobacteria;Gammaproteobacteria;Methylococcales;Methylococcaceae;Methylobacter;Methylobacter tundripaludum;Methylobacter tundripaludum OWC-G53F |
| Ga0586324 1399002 117 1052 | 0.00 | 0.00 | 0.00 | 0.00 | 1843700.00 | 0.00 | 0.00 | 0.00 | 0.00 | 0.00 | 0.00 | 0.00 | KO:K03046 | DNA-directed RNA polymerase subunit beta' | Bacteria;Acidobacteria;Vicinamibacteria;unclassified Vicinamibacteria;Vicinamibacteraceae;Luteitalea;Luteitalea pratensis;Luteitalea pratensis DSM 100886 |
| Ga0586324 1409022 1 1047 | 0.00 | 0.00 | 0.00 | 0.00 | 0.00 | 0.00 | 0.00 | 0.00 | 150335.19 | 0.00 | 0.00 | 0.00 | KO:K03046 | DNA-directed RNA polymerase subunit beta' | Bacteria;Proteobacteria;Betaproteobacteria;unclassified Betaproteobacteria;unclassified Betaproteobacteria;Chitinivorax;Chitinivorax tropicus;Chitinivorax tropicus DSM 27165 |
| Ga0586324 1443986 1 1038 | 0.00 | 0.00 | 0.00 | 0.00 | 0.00 | 0.00 | 0.00 | 0.00 | 1522800.00 | 0.00 | 0.00 | 0.00 | KO:K03046 | DNA-directed RNA polymerase subunit beta' | Bacteria;Acidobacteria;Acidobacteriia;Acidobacteriales;Acidobacteriaceae;Candidatus Koribacter;Candidatus Koribacter versatilis;Candidatus Koribacter versatilis Ellin345 |
| Ga0586324 1460379 3 1031 | 0.00 | 0.00 | 0.00 | 0.00 | 0.00 | 0.00 | 0.00 | 0.00 | 1871100.00 | 0.00 | 0.00 | 0.00 | KO:K03046 | DNA-directed RNA polymerase subunit beta' | Bacteria;Actinobacteria;Actinomycetia;unclassified Actinomycetia;unclassified Actinomycetia;unclassified Actinomycetia;Actinobacteria bacterium IMCC26207;Actinobacteria bacterium IMCC26207 |
| Ga0586324 1477842 3 1028 | 0.00 | 0.00 | 0.00 | 0.00 | 0.00 | 0.00 | 0.00 | 0.00 | 2632500.00 | 0.00 | 0.00 | 0.00 | KO:K03046 | DNA-directed RNA polymerase subunit beta' | Bacteria;Planctomycetes;Phycisphaerae;Sedimentisphaerales;Anaerohalosphaeraceae;Anaerohalosphaera;Anaerohalosphaera lusitana;Phycisphaerae bacterium ST-NAGAB-D1 |
| Ga0586324 1499808 1 1020 | 0.00 | 0.00 | 0.00 | 0.00 | 0.00 | 0.00 | 0.00 | 0.00 | 0.00 | 0.00 | 1469600.00 | 0.00 | KO:K03046 | DNA-directed RNA polymerase subunit beta' | Bacteria;Acidobacteria;Acidobacteriia;Bryobacterales;Bryobacteraceae;Bryobacter;Bryobacter aggregatus;Bryobacter aggregatus DSM 18758 |
| Ga0586324 1561734 1 1002 | 0.00 | 0.00 | 0.00 | 0.00 | 0.00 | 0.00 | 0.00 | 0.00 | 0.00 | 255828.65 | 0.00 | 0.00 | KO:K03046 | DNA-directed RNA polymerase subunit beta' | Bacteria;Proteobacteria;Betaproteobacteria;Nitrosomonadales;Gallionellaceae;Sideroxydans;Sideroxydans lithotrophicus;Sideroxydans lithotrophicus ES-1 |
| Ga0586324 1577031 132 998 | 0.00 | 210802.04 | 0.00 | 0.00 | 0.00 | 0.00 | 0.00 | 0.00 | 0.00 | 0.00 | 0.00 | 0.00 | KO:K03046 | DNA-directed RNA polymerase subunit beta' | Bacteria;Proteobacteria;Gammaproteobacteria;Methylococcales;Methylococcaceae;Methyloterricola;Methyloterricola oryzae;Methyloterricola oryzae 73a |
| Ga0586324 1662363 3 737 | 0.00 | 0.00 | 0.00 | 0.00 | 0.00 | 0.00 | 440888.16 | 0.00 | 0.00 | 0.00 | 0.00 | 0.00 | KO:K03046 | DNA-directed RNA polymerase subunit beta' | Bacteria;Chloroflexi;Caldilineae;Caldilineales;Caldilineaceae;Caldilinea;Caldilinea aerophila;Caldilinea aerophila STL-6-O1, DSM 14535 |
| Ga0586324 1735193 2 958 | 0.00 | 0.00 | 0.00 | 0.00 | 547646.88 | 0.00 | 0.00 | 0.00 | 0.00 | 0.00 | 0.00 | 0.00 | KO:K03046 | DNA-directed RNA polymerase subunit beta' | Bacteria;Proteobacteria;Deltaproteobacteria;Syntrophobacterales;Syntrophobacteraceae;Desulfacinum;Desulfacinum infernum;Desulfacinum infernum DSM 9756 |
| Ga0586324 1827468 3 938 | 0.00 | 9452000.00 | 0.00 | 0.00 | 0.00 | 0.00 | 0.00 | 0.00 | 0.00 | 0.00 | 0.00 | 0.00 | KO:K03046 | DNA-directed RNA polymerase subunit beta' | Bacteria;Actinobacteria;Rubrobacteria;Gaiellales;Gaiellaceae;Gaiella;Gaiella occulta;Gaiella occulta F2-233 |
| Ga0586324 1842474 326 856 | 0.00 | 0.00 | 0.00 | 0.00 | 0.00 | 0.00 | 0.00 | 0.00 | 0.00 | 0.00 | 0.00 | 3210000.00 | KO:K03046 | DNA-directed RNA polymerase subunit beta' | Bacteria;Proteobacteria;Deltaproteobacteria;Syntrophobacterales;Syntrophaceae;Desulfobacca;Desulfobacca acetoxidans;Desulfobacca acetoxidans ASRB2, DSM 11109 |
| Ga0586324 1863417 3 932 | 0.00 | 1400800.00 | 0.00 | 0.00 | 0.00 | 0.00 | 0.00 | 0.00 | 0.00 | 0.00 | 0.00 | 0.00 | KO:K03046 | DNA-directed RNA polymerase subunit beta' | Bacteria;Chloroflexi;Anaerolineae;Anaerolineales;Anaerolineaceae;Levilinea;Levilinea saccharolytica;Levilinea saccharolytica DSM 16555 |
| Ga0586324 1958610 2 910 | 0.00 | 0.00 | 0.00 | 0.00 | 35329000.00 | 0.00 | 0.00 | 0.00 | 0.00 | 0.00 | 0.00 | 0.00 | KO:K03046 | DNA-directed RNA polymerase subunit beta' | Bacteria;Proteobacteria;Betaproteobacteria;Rhodocyclales;Zoogloeaceae;Zoogloea;Zoogloea sp. LCSB751;Zoogloea schifflangensis LCSB751 |
| Ga0586324 1981991 3 908 | 0.00 | 0.00 | 0.00 | 0.00 | 0.00 | 0.00 | 595697.88 | 0.00 | 448554.51 | 0.00 | 0.00 | 0.00 | KO:K03046 | DNA-directed RNA polymerase subunit beta' | Bacteria;Calditrichaeota;Calditrichae;Calditrichales;Calditrichaceae;Caldithrix;Caldithrix abyssi;Caldithrix abyssi LF13, DSM 13497 |
| Ga0586324 2000655 3 902 | 0.00 | 0.00 | 0.00 | 0.00 | 0.00 | 1120536.47 | 0.00 | 0.00 | 0.00 | 0.00 | 0.00 | 0.00 | KO:K03046 | DNA-directed RNA polymerase subunit beta' | Bacteria;Proteobacteria;Alphaproteobacteria;Rhodobacterales;Rhodobacteraceae;Amaricoccus;Amaricoccus macauensis;Amaricoccus macauensis DSM 101730 |
| Ga0586324 2093746 3 887 | 0.00 | 0.00 | 0.00 | 0.00 | 0.00 | 0.00 | 0.00 | 0.00 | 0.00 | 0.00 | 950400.00 | 0.00 | KO:K03046 | DNA-directed RNA polymerase subunit beta' | Bacteria;Proteobacteria;Betaproteobacteria;Burkholderiales;unclassified Burkholderiales;Rubrivivax;Rubrivivax benzoatilyticus;Rubrivivax benzoatilyticus JA2 |
| Ga0586324 2147048 1 801 | 0.00 | 0.00 | 0.00 | 0.00 | 0.00 | 4486100.00 | 0.00 | 0.00 | 0.00 | 0.00 | 0.00 | 0.00 | KO:K03046 | DNA-directed RNA polymerase subunit beta' | Bacteria;Acidobacteria;Acidobacteriia;Acidobacteriales;Acidobacteriaceae;Acidobacterium;Acidobacterium ailaaui;Acidobacterium ailaaui PMMR2 |
| Ga0586324 2247596 2 859 | 0.00 | 0.00 | 0.00 | 0.00 | 0.00 | 0.00 | 0.00 | 0.00 | 0.00 | 2086700.00 | 0.00 | 0.00 | KO:K03046 | DNA-directed RNA polymerase subunit beta' | Bacteria;Proteobacteria;Betaproteobacteria;Nitrosomonadales;Thiobacillaceae;Sulfuritortus;Sulfuritortus calidifontis;Sulfuritortus calidifontis DSM 103923 |
| Ga0586324 2327775 2 847 | 0.00 | 0.00 | 0.00 | 0.00 | 0.00 | 0.00 | 0.00 | 0.00 | 0.00 | 0.00 | 0.00 | 1380000.00 | KO:K03046 | DNA-directed RNA polymerase subunit beta' | Bacteria;Bacteroidetes;Bacteroidia;Marinilabiliales;Marinilabiliaceae;Marinilabilia;Marinilabilia salmonicolor;Marinilabilia salmonicolor MSL42 |
| Ga0586324 2328348 3 848 | 0.00 | 10744000.00 | 0.00 | 5614500.00 | 0.00 | 0.00 | 0.00 | 5734000.00 | 0.00 | 0.00 | 0.00 | 4515000.00 | KO:K03046 | DNA-directed RNA polymerase subunit beta' | Bacteria;Nitrospinae;Nitrospinia;Nitrospinales;Nitrospinaceae;Nitrospina;Nitrospina gracilis;Nitrospina gracilis 3/211 |
| Ga0586324 2422874 3 833 | 0.00 | 0.00 | 0.00 | 0.00 | 0.00 | 0.00 | 0.00 | 0.00 | 271328.94 | 0.00 | 0.00 | 0.00 | KO:K03046 | DNA-directed RNA polymerase subunit beta' | Bacteria;Proteobacteria;Betaproteobacteria;Nitrosomonadales;Sulfuricellaceae;Sulfurirhabdus;Sulfurirhabdus autotrophica;Sulfurirhabdus autotrophica DSM 100309 |
| Ga0586324 2516303 1 819 | 0.00 | 0.00 | 0.00 | 4645500.00 | 0.00 | 0.00 | 0.00 | 0.00 | 0.00 | 0.00 | 0.00 | 6997500.00 | KO:K03046 | DNA-directed RNA polymerase subunit beta' | Bacteria;Proteobacteria;Betaproteobacteria;Rhodocyclales;Azonexaceae;Dechloromonas;Dechloromonas sp. SG708;Dechloromonas sp. SG708 |
| Ga0586324 2562717 3 812 | 203000000.00 | 0.00 | 0.00 | 431564.10 | 947315.72 | 1423201.33 | 775174.92 | 0.00 | 0.00 | 349485.84 | 0.00 | 232799.25 | KO:K03046 | DNA-directed RNA polymerase subunit beta' | Bacteria;Chloroflexi;Caldilineae;Caldilineales;Caldilineaceae;Caldilinea;Caldilinea aerophila;Caldilinea aerophila STL-6-O1, DSM 14535 |
| Ga0586324 2593542 1 741 | 0.00 | 0.00 | 0.00 | 0.00 | 0.00 | 0.00 | 0.00 | 0.00 | 0.00 | 11473000.00 | 0.00 | 0.00 | KO:K03046 | DNA-directed RNA polymerase subunit beta' | Bacteria;Proteobacteria;Gammaproteobacteria;Pseudomonadales;Pseudomonadaceae;Pseudomonas;Pseudomonas sp. R2A2;Pseudomonas sp. R2A2 |
| Ga0586324 2610761 3 806 | 0.00 | 3060000.00 | 0.00 | 0.00 | 0.00 | 0.00 | 0.00 | 0.00 | 0.00 | 0.00 | 0.00 | 0.00 | KO:K03046 | DNA-directed RNA polymerase subunit beta' | Bacteria;Proteobacteria;Deltaproteobacteria;Desulfuromonadales;Geobacteraceae;Geobacter;Geobacter uraniireducens;Geobacter uraniireducens Rf4 |
| Ga0586324 2662797 1 801 | 0.00 | 0.00 | 0.00 | 0.00 | 0.00 | 0.00 | 0.00 | 0.00 | 0.00 | 3372600.00 | 0.00 | 0.00 | KO:K03046 | DNA-directed RNA polymerase subunit beta' | Bacteria;Proteobacteria;Alphaproteobacteria;Hyphomicrobiales;unclassified Hyphomicrobiales;Pseudorhodoplanes;Pseudorhodoplanes sinuspersici;Pseudorhodoplanes sinuspersici RIPI110 |
| Ga0586324 2703181 1 795 | 0.00 | 0.00 | 0.00 | 0.00 | 0.00 | 0.00 | 0.00 | 0.00 | 6058800.00 | 0.00 | 0.00 | 0.00 | KO:K03046 | DNA-directed RNA polymerase subunit beta' | Bacteria;Bacteroidetes;Sphingobacteriia;Sphingobacteriales;Sphingobacteriaceae;Anseongella;Anseongella ginsenosidimutans;Anseongella ginsenosidimutans DSM 21100 |
| Ga0586324 2813892 1 783 | 0.00 | 0.00 | 21002219.56 | 0.00 | 0.00 | 0.00 | 0.00 | 0.00 | 0.00 | 0.00 | 0.00 | 0.00 | KO:K03046 | DNA-directed RNA polymerase subunit beta' | Bacteria;Proteobacteria;Deltaproteobacteria;Syntrophobacterales;Syntrophaceae;Syntrophus;Syntrophus aciditrophicus;Syntrophus aciditrophicus SB |

| | | | | | | | | | | | | | | | |
|---|---|---|---|---|---|---|---|---|---|---|---|---|---|---|---|
| Ga0586324 2820932 2 781 | 0.00 | 0.00 | 0.00 | 0.00 | 0.00 | 0.00 | 1016731.80 | 0.00 | 0.00 | 0.00 | 0.00 | 0.00 | KO:K03046 | DNA-directed RNA polymerase subunit beta' | Bacteria;Bacteroidetes;Cytophagia;Cytophagales;Hymenobacteraceae;Rufibacter;Rufibacter roseus;Rufibacter roseus CCM 8621 |
| Ga0586324 3016108 3 761 | 0.00 | 0.00 | 0.00 | 0.00 | 0.00 | 0.00 | 0.00 | 0.00 | 0.00 | 1116500.00 | 0.00 | 0.00 | KO:K03046 | DNA-directed RNA polymerase subunit beta' | Bacteria;Proteobacteria;Gammaproteobacteria;Nevskiales;Steroidobacteraceae;Povalibacter;Povalibacter uvarum;Povalibacter uvarum DSM 26723 |
| Ga0586324 3225190 2 739 | 0.00 | 0.00 | 0.00 | 0.00 | 0.00 | 0.00 | 0.00 | 0.00 | 3596400.00 | 0.00 | 0.00 | 2242500.00 | KO:K03046 | DNA-directed RNA polymerase subunit beta' | Bacteria;Proteobacteria;Deltaproteobacteria;Desulfobacterales;Desulfobacteraceae;Desulfatiglans;Desulfatiglans anilini;Desulfatiglans anilini DSM 4660 |
| Ga0586324 3228420 2 739 | 0.00 | 0.00 | 0.00 | 0.00 | 0.00 | 0.00 | 0.00 | 640500.00 | 10692000.00 | 0.00 | 0.00 | 0.00 | KO:K03046 | DNA-directed RNA polymerase subunit beta' | Bacteria;Actinobacteria;Thermoleophilia;Solirubrobacterales;Conexibacteraceae;Conexibacter;Conexibacter arvalis;Conexibacter arvalis DSM 23288 |
| Ga0586324 3294827 3 731 | 0.00 | 0.00 | 0.00 | 0.00 | 0.00 | 0.00 | 0.00 | 0.00 | 0.00 | 545988.52 | 0.00 | 0.00 | KO:K03046 | DNA-directed RNA polymerase subunit beta' | Bacteria;Proteobacteria;Deltaproteobacteria;Myxococcales;Anaeromyxobacteraceae;Anaeromyxobacter;Anaeromyxobacter sp. Fw109-5;Anaeromyxobacter sp. Fw109-5 |
| Ga0586324 3544914 1 711 | 0.00 | 128957.92 | 0.00 | 0.00 | 0.00 | 0.00 | 0.00 | 0.00 | 0.00 | 0.00 | 0.00 | 0.00 | KO:K03046 | DNA-directed RNA polymerase subunit beta' | Bacteria;Proteobacteria;Deltaproteobacteria;Desulfuromonadales;Geobacteraceae;Geobacter;Geobacter thiogenes;Geobacter thiogenes ATCC BAA-34 |
| Ga0586324 3579279 1 708 | 0.00 | 0.00 | 0.00 | 126350000.00 | 0.00 | 0.00 | 0.00 | 0.00 | 0.00 | 0.00 | 0.00 | 0.00 | KO:K03046 | DNA-directed RNA polymerase subunit beta' | Bacteria;Bacteroidetes;Bacteroidia;Marinilabiliales;Marinilabiliaceae;Anaerophaga;Anaerophaga thermohalophila;Anaerophaga thermohalophila Fru22, DSM 12881 |
| Ga0586324 3624590 1 705 | 0.00 | 0.00 | 0.00 | 0.00 | 2966400.00 | 1519033.16 | 0.00 | 0.00 | 0.00 | 0.00 | 0.00 | 0.00 | KO:K03046 | DNA-directed RNA polymerase subunit beta' | Bacteria;Proteobacteria;Gammaproteobacteria;Cellvibrionales;Microbulbiferaceae;Microbulbifer;Microbulbifer donghaiensis;Microbulbifer donghaiensis CGMCC 1.7063 |
| Ga0586324 3779671 2 691 | 0.00 | 0.00 | 0.00 | 0.00 | 0.00 | 0.00 | 8344800.00 | 5660800.00 | 6666300.00 | 0.00 | 0.00 | 0.00 | KO:K03046 | DNA-directed RNA polymerase subunit beta' | Bacteria;Proteobacteria;Betaproteobacteria;Nitrosomonadales;Methylophilaceae;Methylotenera;Methylotenera versatilis;Methylotenera versatilis 301, JCM 17579 |
| Ga0586324 3799574 3 689 | 0.00 | 0.00 | 0.00 | 0.00 | 0.00 | 0.00 | 0.00 | 0.00 | 3580200.00 | 9240000.00 | 11000000.00 | 7275000.00 | KO:K03046 | DNA-directed RNA polymerase subunit beta' | Bacteria;Proteobacteria;Betaproteobacteria;Burkholderiales;Oxalobacteraceae;Noviherbaspirillum;Noviherbaspirillum massiliense;Noviherbaspirillum massiliense JC206 |
| Ga0586324 3808298 3 689 | 0.00 | 0.00 | 0.00 | 0.00 | 288858.35 | 0.00 | 0.00 | 0.00 | 0.00 | 0.00 | 0.00 | 0.00 | KO:K03046 | DNA-directed RNA polymerase subunit beta' | Bacteria;Proteobacteria;Gammaproteobacteria;Nevskiales;Steroidobacteraceae;Povalibacter;Povalibacter uvarum;Povalibacter uvarum DSM 26723 |
| Ga0586324 3906820 3 683 | 0.00 | 0.00 | 0.00 | 0.00 | 0.00 | 0.00 | 0.00 | 93171.40 | 0.00 | 0.00 | 0.00 | 0.00 | KO:K03046 | DNA-directed RNA polymerase subunit beta' | Bacteria;Actinobacteria;Coriobacteriia;Eggerthellales;Eggerthellaceae;Slackia;Slackia isoflavoniconvertens;Slackia isoflavoniconvertens OB21 GAM31 |
| Ga0586324 3922992 1 681 | 0.00 | 0.00 | 0.00 | 0.00 | 7385100.00 | 0.00 | 0.00 | 0.00 | 0.00 | 0.00 | 0.00 | 0.00 | KO:K03046 | DNA-directed RNA polymerase subunit beta' | Bacteria;Nitrospirae;Nitrospira;Nitrospirales;Nitrospiraceae;Nitrospira;Candidatus Nitrospira nitrosa;Candidatus Nitrospira nitrosa COMA1 |
| Ga0586324 4010210 119 676 | 0.00 | 0.00 | 0.00 | 0.00 | 0.00 | 385066.97 | 0.00 | 0.00 | 0.00 | 0.00 | 0.00 | 0.00 | KO:K03046 | DNA-directed RNA polymerase subunit beta' | Bacteria;Proteobacteria;Alphaproteobacteria;Hyphomicrobiales;Methylocystaceae;Methylocystis;Methylocystis sp. ATCC 49242;Methylocystis sp. Rockwell, ATCC 49242 |
| Ga0586324 4095715 3 668 | 0.00 | 0.00 | 0.00 | 0.00 | 11948000.00 | 0.00 | 0.00 | 0.00 | 0.00 | 0.00 | 14168000.00 | 0.00 | KO:K03046 | DNA-directed RNA polymerase subunit beta' | Bacteria;Chloroflexi;Anaerolineae;Anaerolineales;Anaerolineaceae;Thermanaerothrix;Thermanaerothrix daxensis;Thermanaerothrix daxensis GNS-1 |
| Ga0586324 4321259 1 654 | 0.00 | 0.00 | 0.00 | 0.00 | 0.00 | 0.00 | 0.00 | 0.00 | 1020600.00 | 0.00 | 0.00 | 0.00 | KO:K03046 | DNA-directed RNA polymerase subunit beta' | Bacteria;Proteobacteria;Alphaproteobacteria;Hyphomicrobiales;Hyphomicrobiaceae;Hyphomicrobium;Hyphomicrobium sp. CS1GBMeth3;Hyphomicrobium sp. ERS1433774 |
| Ga0586324 4338508 3 485 | 0.00 | 0.00 | 0.00 | 0.00 | 0.00 | 0.00 | 0.00 | 0.00 | 0.00 | 0.00 | 6362400.00 | 0.00 | KO:K03046 | DNA-directed RNA polymerase subunit beta' | Bacteria;Acidobacteria;Acidobacteriia;Bryobacterales;Solibacteraceae;Candidatus Solibacter;Candidatus Solibacter usitatus;Candidatus Solibacter usitatus Ellin6076 |
| Ga0586324 4409811 1 648 | 0.00 | 0.00 | 0.00 | 0.00 | 0.00 | 2291940.55 | 0.00 | 0.00 | 0.00 | 0.00 | 0.00 | 0.00 | KO:K03046 | DNA-directed RNA polymerase subunit beta' | Bacteria;Verrucomicrobia;Verrucomicrobiae;Verrucomicrobiales;Verrucomicrobia subdivision 3;Pedosphaera;Pedosphaera parvula;Pedosphaera parvula Ellin514 |
| Ga0586324 4600995 3 638 | 0.00 | 8500000.00 | 0.00 | 0.00 | 0.00 | 0.00 | 0.00 | 0.00 | 0.00 | 0.00 | 994400.00 | 0.00 | KO:K03046 | DNA-directed RNA polymerase subunit beta' | Bacteria;Proteobacteria;Betaproteobacteria;Burkholderiales;Comamonadaceae;Ramlibacter;Ramlibacter tataouinensis;Ramlibacter tataouinensis TTB310 |
| Ga0586324 4763141 2 628 | 0.00 | 0.00 | 0.00 | 0.00 | 0.00 | 0.00 | 0.00 | 0.00 | 0.00 | 0.00 | 1416800.00 | 0.00 | KO:K03046 | DNA-directed RNA polymerase subunit beta' | Bacteria;Proteobacteria;Deltaproteobacteria;Syntrophorhabdales;Syntrophorhabdaceae;Syntrophorhabdus;Syntrophorhabdus aromaticivorans;Syntrophorhabdus aromaticivorans UI |
| Ga0586324 5028611 3 614 | 0.00 | 0.00 | 0.00 | 0.00 | 0.00 | 0.00 | 0.00 | 0.00 | 0.00 | 1216600.00 | 0.00 | 0.00 | KO:K03046 | DNA-directed RNA polymerase subunit beta' | Bacteria;Chloroflexi;Dehalococcoidia;unclassified Dehalococcoidia;unclassified Dehalococcoidia;Dehalogenimonas;Dehalogenimonas alkenigignens;Dehalogenimonas alkenigignens IP3-3 |
| Ga0586324 5059105 1 462 | 0.00 | 637277.64 | 0.00 | 0.00 | 0.00 | 0.00 | 0.00 | 0.00 | 0.00 | 0.00 | 0.00 | 0.00 | KO:K03046 | DNA-directed RNA polymerase subunit beta' | Bacteria;Proteobacteria;Betaproteobacteria;Nitrosomonadales;Sterolibacteriaceae;Sulfurisoma;Sulfurisoma sediminicola;Sulfurisoma sediminicola DSM 26916 |
| Ga0586324 5326224 2 601 | 2990000.00 | 0.00 | 0.00 | 0.00 | 0.00 | 0.00 | 0.00 | 472509.05 | 6415200.00 | 13706000.00 | 18128000.00 | 0.00 | KO:K03046 | DNA-directed RNA polymerase subunit beta' | Bacteria;Firmicutes;Clostridia;Eubacteriales;Clostridiaceae;Clostridium;Clostridium neonatale;Clostridium neonatale LCDC no.99-A-005 |
| Ga0586324 5411395 3 596 | 0.00 | 0.00 | 0.00 | 0.00 | 0.00 | 0.00 | 249951.84 | 0.00 | 0.00 | 0.00 | 0.00 | 0.00 | KO:K03046 | DNA-directed RNA polymerase subunit beta' | Bacteria;Proteobacteria;Deltaproteobacteria;Syntrophorhabdales;Syntrophorhabdaceae;Syntrophorhabdus;Syntrophorhabdus aromaticivorans;Syntrophorhabdus aromaticivorans UI |
| Ga0586324 5414752 1 597 | 1360000.00 | 0.00 | 0.00 | 0.00 | 0.00 | 0.00 | 4742400.00 | 0.00 | 0.00 | 0.00 | 0.00 | 0.00 | KO:K03046 | DNA-directed RNA polymerase subunit beta' | Bacteria;Proteobacteria;Betaproteobacteria;Nitrosomonadales;Sterolibacteriaceae;Sulfurisoma;Sulfurisoma sediminicola;Sulfurisoma sediminicola DSM 26916 |
| Ga0586324 5672553 1 585 | 0.00 | 0.00 | 0.00 | 0.00 | 271368.95 | 0.00 | 0.00 | 0.00 | 0.00 | 0.00 | 0.00 | 0.00 | KO:K03046 | DNA-directed RNA polymerase subunit beta' | Bacteria;Bacteroidetes;Bacteroidia;Marinilabiliales;Prolixibacteraceae;Prolixibacter;Prolixibacter denitrificans;Prolixibacter denitrificans DSM 27267 |
| Ga0586324 5989644 2 571 | 0.00 | 0.00 | 0.00 | 0.00 | 0.00 | 0.00 | 0.00 | 0.00 | 37584000.00 | 0.00 | 0.00 | 0.00 | KO:K03046 | DNA-directed RNA polymerase subunit beta' | Bacteria;Proteobacteria;Betaproteobacteria;Rhodocyclales;Azonexaceae;Dechloromonas;Dechloromonas sp. JJ;Dechloromonas sp. JJ |
| Ga0586324 6538825 3 551 | 0.00 | 0.00 | 14806900.00 | 0.00 | 0.00 | 0.00 | 0.00 | 0.00 | 0.00 | 0.00 | 0.00 | 0.00 | KO:K03046 | DNA-directed RNA polymerase subunit beta' | Bacteria;Proteobacteria;Betaproteobacteria;Rhodocyclales;Zoogloeaceae;Zoogloea;Zoogloea sp. LCSB751;Zoogloea schifflangensis LCSB751 |
| Ga0586324 6757903 3 545 | 0.00 | 8636000.00 | 0.00 | 0.00 | 0.00 | 0.00 | 0.00 | 0.00 | 0.00 | 0.00 | 0.00 | 0.00 | KO:K03046 | DNA-directed RNA polymerase subunit beta' | Bacteria;Proteobacteria;Betaproteobacteria;unclassified Betaproteobacteria;unclassified Betaproteobacteria;Chitinivorax;Chitinivorax tropicus;Chitinivorax tropicus DSM 27165 |
| Ga0586324 6846401 2 541 | 810803.00 | 0.00 | 0.00 | 0.00 | 0.00 | 0.00 | 0.00 | 0.00 | 0.00 | 0.00 | 0.00 | 0.00 | KO:K03046 | DNA-directed RNA polymerase subunit beta' | Bacteria;Acidobacteria;Vicinamibacteria;unclassified Vicinamibacteria;Vicinamibacteraceae;Luteitalea;Luteitalea pratensis;Luteitalea pratensis DSM 100886 |
| Ga0586324 7320526 1 528 | 0.00 | 0.00 | 0.00 | 0.00 | 0.00 | 2791958.08 | 0.00 | 0.00 | 0.00 | 0.00 | 0.00 | 0.00 | KO:K03046 | DNA-directed RNA polymerase subunit beta' | Bacteria;Acidobacteria;Acidobacteriia;Bryobacterales;Solibacteraceae;Candidatus Solibacter;Candidatus Solibacter usitatus;Candidatus Solibacter usitatus Ellin6076 |
| Ga0586324 7498646 1 522 | 0.00 | 0.00 | 0.00 | 0.00 | 0.00 | 0.00 | 1310297.76 | 1360300.00 | 0.00 | 0.00 | 0.00 | 0.00 | KO:K03046 | DNA-directed RNA polymerase subunit beta' | Bacteria;Proteobacteria;Deltaproteobacteria;Desulfuromonadales;Geobacteraceae;Geothermobacter;Geothermobacter ehrlichii;Geothermobacter ehrlichii DSM 15274 |
| Ga0586324 8065653 3 506 | 0.00 | 0.00 | 0.00 | 0.00 | 487496.94 | 0.00 | 0.00 | 0.00 | 0.00 | 0.00 | 0.00 | 0.00 | KO:K03046 | DNA-directed RNA polymerase subunit beta' | Bacteria;Actinobacteria;Rubrobacteria;Gaiellales;Gaiellaceae;Gaiella;Gaiella occulta;Gaiella occulta F2-233 |
| Ga0586324 8096508 3 506 | 0.00 | 0.00 | 0.00 | 0.00 | 0.00 | 0.00 | 0.00 | 210232.23 | 0.00 | 0.00 | 0.00 | 0.00 | KO:K03046 | DNA-directed RNA polymerase subunit beta' | Bacteria;Acidobacteria;Vicinamibacteria;unclassified Vicinamibacteria;Vicinamibacteraceae;Luteitalea;Luteitalea pratensis;Luteitalea pratensis DSM 100886 |
| Ga0586324 2330632 3 680 | 0.00 | 2522800.00 | 0.00 | 0.00 | 0.00 | 0.00 | 0.00 | 2037400.00 | 1879200.00 | 0.00 | 1443200.00 | 0.00 | KO:K03047 | DNA-directed RNA polymerase subunit D | Archaea;Euryarchaeota;Methanomicrobia;Methanosarcinales;Methanotrichaceae;Methanothrix;Methanothrix soehngenii;Methanothrix soehngenii GP-6 |
| Ga0586324 0000004 150384 153125 | 0.00 | 0.00 | 0.00 | 0.00 | 0.00 | 0.00 | 0.00 | 5477800.00 | 4916700.00 | 10857000.00 | 21938400.00 | 12307500.00 | KO:K03070 | preprotein translocase subunit SecA | Bacteria;Proteobacteria;Betaproteobacteria;Nitrosomonadales;Thiobacillaceae;Annwoodia;Annwoodia aquaesulis;Annwoodia aquaesulis DSM 4255 |
| Ga0586324 0001856 14405 17110 | 0.00 | 0.00 | 0.00 | 0.00 | 0.00 | 0.00 | 0.00 | 0.00 | 0.00 | 947100.00 | 3088800.00 | 0.00 | KO:K03070 | preprotein translocase subunit SecA | Bacteria;Proteobacteria;Betaproteobacteria;Nitrosomonadales;Thiobacillaceae;Thiobacillus;Thiobacillus thioparus;Thiobacillus thioparus DSM 505 |
| Ga0586324 0014099 3730 6516 | 0.00 | 0.00 | 0.00 | 15200000.00 | 0.00 | 0.00 | 0.00 | 0.00 | 0.00 | 0.00 | 0.00 | 0.00 | KO:K03070 | preprotein translocase subunit SecA | Bacteria;Proteobacteria;Deltaproteobacteria;Syntrophobacterales;Syntrophaceae;Desulfomonile;Desulfomonile tiedjei;Desulfomonile tiedjei DCB-1, DSM 6799 |
| Ga0586324 0029083 2217 5396 | 0.00 | 0.00 | 0.00 | 0.00 | 0.00 | 0.00 | 0.00 | 0.00 | 0.00 | 0.00 | 0.00 | 1732500.00 | KO:K03070 | preprotein translocase subunit SecA | Bacteria;Proteobacteria;Deltaproteobacteria;Desulfuromonadales;Geobacteraceae;Citrifermentans;Citrifermentans pelophilum;Geobacter pelophilus Dfr2 |
| Ga0586324 0042548 1245 3770 | 0.00 | 0.00 | 2145249.57 | 0.00 | 0.00 | 1879310.66 | 0.00 | 0.00 | 0.00 | 0.00 | 0.00 | 0.00 | KO:K03070 | preprotein translocase subunit SecA | Bacteria;Proteobacteria;Deltaproteobacteria;Desulfobacterales;Desulfobacteraceae;Desulfosarcina;Desulfosarcina variabilis;Desulfosarcina variabilis Montpellier |
| Ga0586324 0048742 1002 3749 | 0.00 | 0.00 | 0.00 | 0.00 | 0.00 | 0.00 | 0.00 | 0.00 | 980100.00 | 0.00 | 0.00 | 0.00 | KO:K03070 | preprotein translocase subunit SecA | Bacteria;Proteobacteria;Gammaproteobacteria;Cellvibrionales;Spongiibacteraceae;Spongiibacter;Spongiibacter marinus;Spongiibacter marinus DSM 17750 |
| Ga0586324 0131759 1048 3006 | 0.00 | 0.00 | 0.00 | 0.00 | 0.00 | 0.00 | 0.00 | 0.00 | 0.00 | 0.00 | 0.00 | 5032500.00 | KO:K03070 | preprotein translocase subunit SecA | Bacteria;Acidobacteria;Acidobacteriia;Acidobacteriales;Acidobacteriaceae;unclassified Acidobacteriaceae;Acidobacteriaceae bacterium URHE0068;Acidobacteriaceae bacterium URHE0068 |
| Ga0586324 0179639 1 2601 | 0.00 | 0.00 | 0.00 | 0.00 | 0.00 | 0.00 | 0.00 | 0.00 | 8991000.00 | 0.00 | 0.00 | 7725000.00 | KO:K03070 | preprotein translocase subunit SecA | Bacteria;Chloroflexi;unclassified Chloroflexi;unclassified Chloroflexi;unclassified Chloroflexi;Thermobaculum;Thermobaculum terrenum;Thermobaculum terrenum YNP1, ATCC BAA-798 |
| Ga0586324 0380759 3 1574 | 0.00 | 0.00 | 0.00 | 0.00 | 0.00 | 0.00 | 0.00 | 0.00 | 477692.64 | 0.00 | 0.00 | 0.00 | KO:K03070 | preprotein translocase subunit SecA | Bacteria;Bacteroidetes;Bacteroidia;Bacteroidales;Lentimicrobiaceae;Lentimicrobium;Lentimicrobium saccharophilum;Bacteroidales bacterium TBC1 |
| Ga0586324 0960870 2 1234 | 0.00 | 0.00 | 0.00 | 0.00 | 0.00 | 0.00 | 0.00 | 0.00 | 0.00 | 557818.03 | 0.00 | 0.00 | KO:K03070 | preprotein translocase subunit SecA | Bacteria;Proteobacteria;Betaproteobacteria;Nitrosomonadales;Sulfuricellaceae;Sulfuricella;Sulfuricella sp. T08;Sulfuricella sp. T08 |
| Ga0586324 1041362 165 1193 | 0.00 | 0.00 | 0.00 | 0.00 | 0.00 | 0.00 | 0.00 | 0.00 | 0.00 | 3187800.00 | 0.00 | 0.00 | KO:K03070 | preprotein translocase subunit SecA | Bacteria;Proteobacteria;Betaproteobacteria;Burkholderiales;Alcaligenaceae;Azohydromonas;Azohydromonas lata;Azohydromonas lata NBRC 102462 |
| Ga0586324 1522958 3 1013 | 0.00 | 0.00 | 0.00 | 0.00 | 0.00 | 0.00 | 0.00 | 683200.00 | 0.00 | 0.00 | 0.00 | 0.00 | KO:K03070 | preprotein translocase subunit SecA | Bacteria;Actinobacteria;Actinomycetia;Corynebacteriales;Mycobacteriaceae;unclassified Mycobacteriaceae;Mycobacteriaceae bacterium 1482268.1;Mycobacterium sp. 1482268.1 |
| Ga0586324 1755049 3 689 | 0.00 | 0.00 | 0.00 | 0.00 | 0.00 | 0.00 | 0.00 | 439311.02 | 0.00 | 1162700.00 | 0.00 | 0.00 | KO:K03070 | preprotein translocase subunit SecA | Bacteria;Proteobacteria;Betaproteobacteria;Rhodocyclales;Rhodocyclaceae;Rhodocyclus;Rhodocyclus tenuis;Rhodocyclus tenuis DSM 109 |
| Ga0586324 2739528 2 790 | 0.00 | 0.00 | 0.00 | 0.00 | 0.00 | 0.00 | 0.00 | 0.00 | 0.00 | 0.00 | 0.00 | 2340000.00 | KO:K03070 | preprotein translocase subunit SecA | Bacteria;Planctomycetes;Phycisphaerae;Phycisphaerales;Phycisphaeraceae;Algisphaera;Algisphaera agarilytica;Algisphaera agarilytica DSM 103725 |
| Ga0586324 3557908 508 708 | 0.00 | 0.00 | 0.00 | 0.00 | 0.00 | 0.00 | 0.00 | 0.00 | 766417.95 | 0.00 | 0.00 | 0.00 | KO:K03070 | preprotein translocase subunit SecA | Bacteria;Proteobacteria;Gammaproteobacteria;Methylococcales;Methylococcaceae;Methylobacter;Methylobacter sp. BBA5.1;Methylobacter sp. BBA5.1 |
| Ga0586324 7292770 3 527 | 0.00 | 0.00 | 0.00 | 0.00 | 0.00 | 0.00 | 0.00 | 430591.07 | 0.00 | 0.00 | 0.00 | 0.00 | KO:K03070 | preprotein translocase subunit SecA | Bacteria;Proteobacteria;Alphaproteobacteria;Hyphomicrobiales;Hyphomicrobiaceae;Rhodoplanes;Rhodoplanes sp. Z2-YC6860;Rhodoplanes sp. Z2-YC6860 |
| Ga0586324 0000184 30477 32324 | 1810000.00 | 0.00 | 0.00 | 0.00 | 0.00 | 0.00 | 0.00 | 0.00 | 0.00 | 0.00 | 0.00 | 0.00 | KO:K03072 | preprotein translocase subunit SecD | Bacteria;Proteobacteria;Gammaproteobacteria;Methylococcales;Methylococcaceae;Methylobacter;Methylobacter tundripaludum;Methylobacter tundripaludum SV96 |
| Ga0586324 0018538 1094 2992 | 0.00 | 0.00 | 0.00 | 0.00 | 0.00 | 0.00 | 0.00 | 0.00 | 0.00 | 0.00 | 3344000.00 | 0.00 | KO:K03072 | preprotein translocase subunit SecD | Bacteria;Proteobacteria;Gammaproteobacteria;Chromatiales;Ectothiorhodospiraceae;Thiogranum;Thiogranum longum;Thiogranum longum DSM 19610 |
| Ga0586324 0148906 204 1808 | 0.00 | 0.00 | 0.00 | 0.00 | 0.00 | 0.00 | 2268960.24 | 0.00 | 0.00 | 0.00 | 0.00 | 0.00 | KO:K03072 | preprotein translocase subunit SecD | Bacteria;Proteobacteria;Deltaproteobacteria;Syntrophobacterales;Syntrophobacteraceae;Desulfacinum;Desulfacinum infernum;Desulfacinum infernum DSM 9756 |
| Ga0586324 0955719 2 1237 | 0.00 | 0.00 | 0.00 | 0.00 | 0.00 | 0.00 | 0.00 | 0.00 | 0.00 | 298524.38 | 0.00 | 0.00 | KO:K03072 | preprotein translocase subunit SecD | Bacteria;Proteobacteria;Gammaproteobacteria;Methylococcales;Methylococcaceae;Methylobacter;Methylobacter tundripaludum;Methylobacter tundripaludum OWC-DMM |
| Ga0586324 0000013 37084 39024 | 0.00 | 0.00 | 0.00 | 0.00 | 0.00 | 0.00 | 0.00 | 0.00 | 5613300.00 | 549083.92 | 1073600.00 | 215876.25 | KO:K03086 | RNA polymerase primary sigma factor | Bacteria;Proteobacteria;Betaproteobacteria;Nitrosomonadales;Thiobacillaceae;Annwoodia;Annwoodia aquaesulis;Annwoodia aquaesulis DSM 4255 |
| Ga0586324 0000896 12618 14537 | 0.00 | 0.00 | 0.00 | 0.00 | 0.00 | 0.00 | 0.00 | 0.00 | 0.00 | 1155000.00 | 4320800.00 | 0.00 | KO:K03086 | RNA polymerase primary sigma factor | Bacteria;Proteobacteria;Betaproteobacteria;Nitrosomonadales;Thiobacillaceae;Thiobacillus;Thiobacillus denitrificans;Thiobacillus denitrificans ATCC 25259 |
| Ga0586324 0004570 6493 8109 | 0.00 | 1795200.00 | 0.00 | 0.00 | 0.00 | 0.00 | 0.00 | 0.00 | 0.00 | 0.00 | 0.00 | 0.00 | KO:K03086 | RNA polymerase primary sigma factor | Bacteria;Candidatus Aminicenantes;unclassified Candidatus Aminicenantes;unclassified Candidatus Aminicenantes;unclassified Candidatus Aminicenantes;unclassified Candidatus Aminicenantes;Aminicenantes bacterium SCGC AAA252-D06;Aminicenantes bacterium SCGC AAA252-D06 (contamination screened) |
| Ga0586324 0017128 6460 7992 | 7570000.00 | 0.00 | 0.00 | 0.00 | 0.00 | 0.00 | 0.00 | 0.00 | 0.00 | 0.00 | 0.00 | 0.00 | KO:K03086 | RNA polymerase primary sigma factor | Bacteria;Proteobacteria;Gammaproteobacteria;Methylococcales;Methylococcaceae;Methylobacter;Methylobacter tundripaludum;Methylobacter tundripaludum 31/32 |
| Ga0586324 0069687 2132 3997 | 0.00 | 0.00 | 0.00 | 0.00 | 0.00 | 7503300.00 | 0.00 | 0.00 | 0.00 | 0.00 | 0.00 | 0.00 | KO:K03086 | RNA polymerase primary sigma factor | Bacteria;Proteobacteria;Gammaproteobacteria;Chromatiales;Ectothiorhodospiraceae;Thiogranum;Thiogranum longum;Thiogranum longum DSM 19610 |
| Ga0586324 0260081 57 1928 | 3270000.00 | 0.00 | 0.00 | 0.00 | 0.00 | 0.00 | 0.00 | 0.00 | 0.00 | 0.00 | 0.00 | 0.00 | KO:K03086 | RNA polymerase primary sigma factor | Bacteria;Proteobacteria;Gammaproteobacteria;unclassified Gammaproteobacteria;Candidatus Competibacteraceae;Plasticicumulans;Plasticicumulans lactativorans;Plasticicumulans lactativorans DSM 25287 |
| Ga0586324 0376135 1 1518 | 0.00 | 0.00 | 0.00 | 0.00 | 1843700.00 | 0.00 | 0.00 | 0.00 | 0.00 | 0.00 | 0.00 | 0.00 | KO:K03086 | RNA polymerase primary sigma factor | Bacteria;Proteobacteria;Alphaproteobacteria;Hyphomicrobiales;unclassified Hyphomicrobiales;Methyloceanibacter;Methyloceanibacter superfactus;Methyloceanibacter superfactus R-67175 |
| Ga0586324 0388704 3 1802 | 0.00 | 0.00 | 0.00 | 0.00 | 0.00 | 0.00 | 0.00 | 0.00 | 0.00 | 0.00 | 0.00 | 211175.25 | KO:K03086 | RNA polymerase primary sigma factor | Bacteria;Acidobacteria;Vicinamibacteria;unclassified Vicinamibacteria;Vicinamibacteraceae;Luteitalea;Luteitalea pratensis;Luteitalea pratensis DSM 100886 |
| Ga0586324 0646466 2 1303 | 0.00 | 0.00 | 0.00 | 0.00 | 0.00 | 0.00 | 0.00 | 0.00 | 0.00 | 0.00 | 0.00 | 1612500.00 | KO:K03086 | RNA polymerase primary sigma factor | Bacteria;Proteobacteria;Deltaproteobacteria;Syntrophorhabdales;Syntrophorhabdaceae;Syntrophorhabdus;Syntrophorhabdus aromaticivorans;Syntrophorhabdus aromaticivorans UI |
| Ga0586324 0713831 3 1403 | 0.00 | 0.00 | 0.00 | 0.00 | 0.00 | 0.00 | 0.00 | 2446100.00 | 0.00 | 0.00 | 0.00 | 0.00 | KO:K03086 | RNA polymerase primary sigma factor | Bacteria;Proteobacteria;Deltaproteobacteria;Desulfuromonadales;Geobacteraceae;Geobacter;Geobacter uraniireducens;Geobacter uraniireducens Rf4 |
| Ga0586324 1443534 125 1036 | 0.00 | 0.00 | 0.00 | 2869000.00 | 0.00 | 0.00 | 0.00 | 1634800.00 | 0.00 | 2502500.00 | 1777600.00 | 0.00 | KO:K03086 | RNA polymerase primary sigma factor | Bacteria;Chloroflexi;Anaerolineae;Anaerolineales;Anaerolineaceae;Anaerolinea;Anaerolinea thermophila;Anaerolinea thermophila UNI-1 |
| Ga0586324 1485074 1 1026 | 0.00 | 0.00 | 0.00 | 0.00 | 0.00 | 0.00 | 0.00 | 0.00 | 0.00 | 0.00 | 6415200.00 | 0.00 | KO:K03086 | RNA polymerase primary sigma factor | Bacteria;Acidobacteria;Acidobacteriia;Acidobacteriales;Acidobacteriaceae;Terracidiphilus;Terracidiphilus gabretensis;Acidobacteriaceae bacterium S55 |
| Ga0586324 3993030 3 677 | 0.00 | 0.00 | 0.00 | 0.00 | 0.00 | 0.00 | 0.00 | 0.00 | 0.00 | 423258.99 | 0.00 | 0.00 | KO:K03086 | RNA polymerase primary sigma factor | Bacteria;Proteobacteria;Gammaproteobacteria;Chromatiales;Chromatiaceae;Thiocapsa;Thiocapsa roseopersicina;Thiocapsa roseopersicina DSM 217 |
| Ga0586324 4351224 2 652 | 0.00 | 0.00 | 0.00 | 0.00 | 0.00 | 0.00 | 0.00 | 0.00 | 175544.01 | 0.00 | 0.00 | 0.00 | KO:K03086 | RNA polymerase primary sigma factor | Bacteria;Acidobacteria;Vicinamibacteria;unclassified Vicinamibacteria;Vicinamibacteraceae;Luteitalea;Luteitalea pratensis;Luteitalea pratensis DSM 100886 |
| Ga0586324 5572127 1 591 | 0.00 | 0.00 | 0.00 | 3933000.00 | 0.00 | 0.00 | 0.00 | 0.00 | 0.00 | 0.00 | 0.00 | 0.00 | KO:K03086 | RNA polymerase primary sigma factor | Bacteria;Proteobacteria;Deltaproteobacteria;Syntrophorhabdales;Syntrophorhabdaceae;Syntrophorhabdus;Syntrophorhabdus aromaticivorans;Syntrophorhabdus aromaticivorans UI |
| Ga0586324 7079971 2 535 | 0.00 | 0.00 | 0.00 | 0.00 | 1792200.00 | 0.00 | 0.00 | 0.00 | 0.00 | 0.00 | 0.00 | 0.00 | KO:K03086 | RNA polymerase primary sigma factor | Bacteria;Proteobacteria;Gammaproteobacteria;Methylococcales;Methylococcaceae;Methyloterricola;Methyloterricola oryzae;Methyloterricola oryzae 73a |
| Ga0586324 0966245 1 339 | 0.00 | 0.00 | 0.00 | 249431.05 | 0.00 | 0.00 | 0.00 | 0.00 | 0.00 | 0.00 | 0.00 | 0.00 | KO:K03088 | RNA polymerase sigma-70 factor (ECF subfamily) | Bacteria;Proteobacteria;Alphaproteobacteria;Hyphomicrobiales;Bradyrhizobiaceae;Bradyrhizobium;Bradyrhizobium erythrophlei;Bradyrhizobium erythrophlei GAS242 |
| Ga0586324 0076874 576 1907 | 0.00 | 0.00 | 0.00 | 0.00 | 0.00 | 0.00 | 0.00 | 0.00 | 0.00 | 0.00 | 0.00 | 1417500.00 | KO:K03106 | signal recognition particle subunit SRP54 | Bacteria;Proteobacteria;Deltaproteobacteria;Syntrophorhabdales;Syntrophorhabdaceae;Syntrophorhabdus;Syntrophorhabdus aromaticivorans;Syntrophorhabdus aromaticivorans UI |
| Ga0586324 0559778 349 840 | 63700000.00 | 0.00 | 0.00 | 0.00 | 0.00 | 0.00 | 0.00 | 0.00 | 0.00 | 0.00 | 0.00 | 0.00 | KO:K03111 | single-strand DNA-binding protein | Bacteria;Proteobacteria;Deltaproteobacteria;Myxococcales;Anaeromyxobacteraceae;Anaeromyxobacter;Anaeromyxobacter sp. K;Anaeromyxobacter sp. K |
| Ga0586324 1600869 534 992 | 0.00 | 0.00 | 0.00 | 0.00 | 0.00 | 0.00 | 0.00 | 3836900.00 | 0.00 | 0.00 | 0.00 | 0.00 | KO:K03111 | single-strand DNA-binding protein | Bacteria;Proteobacteria;Betaproteobacteria;Burkholderiales;unclassified Burkholderiales;Rhizobacter;Rhizobacter sp. Root404;Rhizobacter sp. Root404 |
| Ga0586324 1861409 1 336 | 3110000.00 | 0.00 | 0.00 | 0.00 | 0.00 | 0.00 | 0.00 | 0.00 | 0.00 | 0.00 | 0.00 | 0.00 | KO:K03111 | single-strand DNA-binding protein | Bacteria;Planctomycetes;Planctomycetia;Pirellulales;Pirellulaceae;Blastopirellula;Blastopirellula cremea;Blastopirellula cremea HEX-1 MGV |
| Ga0586324 2543436 13 525 | 0.00 | 0.00 | 0.00 | 0.00 | 0.00 | 0.00 | 0.00 | 0.00 | 0.00 | 0.00 | 0.00 | 3405000.00 | KO:K03111 | single-strand DNA-binding protein | Bacteria;Proteobacteria;Betaproteobacteria;unclassified Betaproteobacteria;unclassified Betaproteobacteria;Chitinivorax;Chitinivorax tropicus;Chitinivorax tropicus DSM 27165 |
| Ga0586324 2950203 218 727 | 18700000.00 | 0.00 | 0.00 | 0.00 | 0.00 | 0.00 | 0.00 | 0.00 | 0.00 | 0.00 | 0.00 | 0.00 | KO:K03111 | single-strand DNA-binding protein | Bacteria;Proteobacteria;Betaproteobacteria;Burkholderiales;Burkholderiaceae;Quisquiliibacterium;Quisquiliibacterium transsilvanicum;Quisquiliibacterium transsilvanicum DSM 29781 |
| Ga0586324 0031419 5468 5773 | 0.00 | 0.00 | 0.00 | 0.00 | 0.00 | 0.00 | 0.00 | 439261.00 | 0.00 | 0.00 | 0.00 | 0.00 | KO:K03113 | translation initiation factor 1 | Archaea;Euryarchaeota;Methanomicrobia;Methanomicrobiales;Methanoregulaceae;Methanoregula;Methanoregula boonei;Methanoregula boonei 6A8 |
| Ga0586324 0112303 464 772 | 0.00 | 0.00 | 0.00 | 0.00 | 0.00 | 0.00 | 0.00 | 1207800.00 | 0.00 | 0.00 | 0.00 | 0.00 | KO:K03113 | translation initiation factor 1 | Archaea;Euryarchaeota;Methanomicrobia;Methanosarcinales;Methanotrichaceae;Methanothrix;Methanothrix soehngenii;Methanothrix soehngenii GP-6 |
| Ga0586324 0006460 8476 9027 | 0.00 | 965600.00 | 0.00 | 7125000.00 | 4810100.00 | 2925310.38 | 0.00 | 1750700.00 | 1895400.00 | 1201200.00 | 0.00 | 0.00 | KO:K03120 | transcription initiation factor TFIID TATA-box-binding protein | Archaea;Euryarchaeota;Methanomicrobia;Methanosarcinales;Methanotrichaceae;Methanothrix;Methanothrix soehngenii;Methanothrix soehngenii GP-6 |
| Ga0586324 0110194 1683 2348 | 0.00 | 0.00 | 0.00 | 0.00 | 8435700.00 | 2745060.47 | 0.00 | 5215500.00 | 5961600.00 | 0.00 | 0.00 | 0.00 | KO:K03120 | transcription initiation factor TFIID TATA-box-binding protein | Archaea;Euryarchaeota;Methanomicrobia;Methanomicrobiales;Methanoregulaceae;Methanoregula;Methanoregula formicica;Methanoregula formicica SMSP, DSM 22288 |
| Ga0586324 4175700 121 663 | 0.00 | 0.00 | 0.00 | 0.00 | 0.00 | 0.00 | 4993200.00 | 0.00 | 0.00 | 0.00 | 0.00 | 0.00 | KO:K03120 | transcription initiation factor TFIID TATA-box-binding protein | Archaea;Euryarchaeota;Methanomicrobia;Methanomicrobiales;Methanoregulaceae;Methanoregula;Methanoregula formicica;Methanoregula formicica SMSP, DSM 22288 |
| Ga0586324 0056120 195 1157 | 0.00 | 0.00 | 0.00 | 0.00 | 0.00 | 0.00 | 0.00 | 0.00 | 0.00 | 0.00 | 1513600.00 | 0.00 | KO:K03124 | transcription initiation factor TFIIB | Archaea;Candidatus Thermoplasmatota;Thermoplasmata;Thermoplasmatales;unclassified Thermoplasmatales;unclassified Thermoplasmatales;Thermoplasmatales archaeon BRNA1;Thermoplasmatales archaeon BRNA1 |
| Ga0586324 0000004 138971 141025 | 0.00 | 0.00 | 0.00 | 0.00 | 0.00 | 0.00 | 0.00 | 0.00 | 0.00 | 4620000.00 | 0.00 | 0.00 | KO:K03147 | phosphomethylpyrimidine synthase | Bacteria;Proteobacteria;Betaproteobacteria;Nitrosomonadales;Thiobacillaceae;Annwoodia;Annwoodia aquaesulis;Annwoodia aquaesulis DSM 4255 |
| Ga0586324 0000001 98751 101249 | 0.00 | 0.00 | 0.00 | 0.00 | 0.00 | 0.00 | 0.00 | 0.00 | 0.00 | 0.00 | 0.00 | 2685000.00 | KO:K03168 | DNA topoisomerase-1 | Bacteria;Proteobacteria;Betaproteobacteria;Nitrosomonadales;Thiobacillaceae;Annwoodia;Annwoodia aquaesulis;Annwoodia aquaesulis DSM 4255 |
| Ga0586324 0000524 4708 6354 | 0.00 | 0.00 | 0.00 | 0.00 | 0.00 | 0.00 | 0.00 | 0.00 | 0.00 | 690662.28 | 0.00 | 0.00 | KO:K03217 | YidC/Oxa1 family membrane protein insertase | Bacteria;Proteobacteria;Betaproteobacteria;Nitrosomonadales;Thiobacillaceae;Thiobacillus;Thiobacillus thioparus;Thiobacillus thioparus DSM 505 |
| Ga0586324 0000672 18076 19347 | 0.00 | 0.00 | 0.00 | 0.00 | 0.00 | 0.00 | 0.00 | 0.00 | 0.00 | 0.00 | 9240000.00 | 0.00 | KO:K03231 | elongation factor 1-alpha | Archaea;Candidatus Thermoplasmatota;Thermoplasmata;Methanomassiliicoccales;Methanomassiliicoccaceae;Methanomassiliicoccus;Methanomassiliicoccus luminyensis;Methanomassiliicoccus luminyensis B10 |
| Ga0586324 0060050 563 1837 | 0.00 | 938400.00 | 0.00 | 0.00 | 0.00 | 0.00 | 0.00 | 0.00 | 0.00 | 0.00 | 0.00 | 0.00 | KO:K03231 | elongation factor 1-alpha | Archaea;Euryarchaeota;Methanomicrobia;Methanosarcinales;Methanotrichaceae;Methanothrix;Methanothrix soehngenii;Methanothrix soehngenii GP-6 |
| Ga0586324 1375115 3 908 | 0.00 | 0.00 | 0.00 | 0.00 | 0.00 | 0.00 | 0.00 | 0.00 | 0.00 | 436631.58 | 0.00 | 0.00 | KO:K03231 | elongation factor 1-alpha | Archaea;Crenarchaeota;unclassified Crenarchaeota;unclassified Crenarchaeota;unclassified Crenarchaeota;unclassified Crenarchaeota;Crenarchaeota archaeon SCGC AAA471-O08;Crenarchaeota archaeon SCGC AAA471-O08 (contamination screened) |
| Ga0586324 1504889 2 1018 | 4990000.00 | 1706800.00 | 3096145.31 | 2793000.00 | 3687400.00 | 3107636.60 | 1574276.16 | 1640900.00 | 0.00 | 1185800.00 | 1531200.00 | 1785000.00 | KO:K03231 | elongation factor 1-alpha | Archaea;Euryarchaeota;Methanomicrobia;Methanosarcinales;Methanotrichaceae;Methanothrix;Methanothrix soehngenii;Methanothrix soehngenii GP-6 |
| Ga0586324 1919221 2 853 | 0.00 | 9248000.00 | 0.00 | 0.00 | 0.00 | 0.00 | 0.00 | 0.00 | 0.00 | 0.00 | 0.00 | 0.00 | KO:K03231 | elongation factor 1-alpha | Archaea;Euryarchaeota;Methanomicrobia;Methanomicrobiales;Methanoregulaceae;Methanoregula;Methanoregula formicica;Methanoregula formicica SMSP, DSM 22288 |
| Ga0586324 2439614 1 648 | 4140000.00 | 4624000.00 | 0.00 | 2897500.00 | 0.00 | 0.00 | 0.00 | 2250900.00 | 0.00 | 4851000.00 | 4857600.00 | 0.00 | KO:K03231 | elongation factor 1-alpha | Archaea;Euryarchaeota;Methanomicrobia;Methanosarcinales;Methanotrichaceae;Methanothrix;Methanothrix soehngenii;Methanothrix soehngenii GP-6 |
| Ga0586324 2958575 1 765 | 3770000.00 | 1944800.00 | 0.00 | 0.00 | 0.00 | 0.00 | 0.00 | 2415600.00 | 2519100.00 | 0.00 | 0.00 | 0.00 | KO:K03231 | elongation factor 1-alpha | Archaea;Euryarchaeota;Methanomicrobia;Methanosarcinales;Methanotrichaceae;Methanothrix;Methanothrix soehngenii;Methanothrix soehngenii GP-6 |
| Ga0586324 3719607 1 681 | 34400000.00 | 26200400.00 | 949234.89 | 1444000.00 | 1843700.00 | 1943549.23 | 1159742.52 | 1494500.00 | 1101600.00 | 713673.73 | 915200.00 | 0.00 | KO:K03231 | elongation factor 1-alpha | Archaea;Euryarchaeota;Methanomicrobia;Methanosarcinales;Methanotrichaceae;Methanothrix;Methanothrix soehngenii;Methanothrix soehngenii GP-6 |
| Ga0586324 5125926 3 611 | 0.00 | 0.00 | 0.00 | 0.00 | 0.00 | 0.00 | 0.00 | 0.00 | 10530000.00 | 0.00 | 0.00 | 0.00 | KO:K03231 | elongation factor 1-alpha | Archaea;Euryarchaeota;Methanomicrobia;Methanosarcinales;Methanotrichaceae;Methanothrix;Methanothrix soehngenii;Methanothrix soehngenii GP-6 |
| Ga0586324 0007595 96 2291 | 7270000.00 | 0.00 | 0.00 | 0.00 | 0.00 | 0.00 | 0.00 | 0.00 | 0.00 | 0.00 | 0.00 | 0.00 | KO:K03234 | elongation factor 2 | Archaea;Euryarchaeota;Methanomicrobia;Methanosarcinales;Methanotrichaceae;Methanothrix;Methanothrix soehngenii;Methanothrix soehngenii GP-6 |
| Ga0586324 0007291 2967 3638 | 0.00 | 0.00 | 0.00 | 0.00 | 1565600.00 | 0.00 | 0.00 | 0.00 | 0.00 | 0.00 | 0.00 | 0.00 | KO:K03264 | translation initiation factor 6 | Archaea;Euryarchaeota;Methanomicrobia;Methanosarcinales;Methanotrichaceae;Methanothrix;Methanothrix soehngenii;Methanothrix soehngenii GP-6 |
| Ga0586324 0000013 2227 2766 | 0.00 | 0.00 | 0.00 | 0.00 | 0.00 | 0.00 | 0.00 | 0.00 | 0.00 | 0.00 | 0.00 | 907500.00 | KO:K03273 | D-glycero-D-manno-heptose 1,7-bisphosphate phosphatase | Bacteria;Proteobacteria;Betaproteobacteria;Nitrosomonadales;Thiobacillaceae;Annwoodia;Annwoodia aquaesulis;Annwoodia aquaesulis DSM 4255 |
| Ga0586324 0000005 53244 54236 | 0.00 | 0.00 | 0.00 | 0.00 | 0.00 | 0.00 | 0.00 | 0.00 | 0.00 | 1455300.00 | 0.00 | 0.00 | KO:K03274 | ADP-L-glycero-D-manno-heptose 6-epimerase | Bacteria;Proteobacteria;Betaproteobacteria;Nitrosomonadales;Thiobacillaceae;Annwoodia;Annwoodia aquaesulis;Annwoodia aquaesulis DSM 4255 |
| Ga0586324 0181661 3 740 | 6580000.00 | 0.00 | 0.00 | 0.00 | 0.00 | 0.00 | 0.00 | 0.00 | 0.00 | 0.00 | 1698400.00 | 2265000.00 | KO:K03285 | GBP family porin | Bacteria;Proteobacteria;Betaproteobacteria;Burkholderiales;Oxalobacteraceae;Collimonas;Collimonas fungivorans;Collimonas fungivorans Ter6 |
| Ga0586324 1695197 70 969 | 0.00 | 5793600.00 | 0.00 | 0.00 | 0.00 | 3613033.48 | 2553600.00 | 3891800.00 | 0.00 | 4704700.00 | 0.00 | 4462500.00 | KO:K03285 | GBP family porin | Bacteria;Proteobacteria;Betaproteobacteria;Nitrosomonadales;Nitrosomonadaceae;Nitrosomonas;Nitrosomonas sp. Is79A3;Nitrosomonas sp. IS79A3 |
| Ga0586324 3185806 162 743 | 0.00 | 0.00 | 0.00 | 2365500.00 | 0.00 | 0.00 | 14364000.00 | 0.00 | 0.00 | 0.00 | 0.00 | 9675000.00 | KO:K03285 | GBP family porin | Bacteria;Proteobacteria;Betaproteobacteria;Burkholderiales;Alcaligenaceae;Pigmentiphaga;Pigmentiphaga humi;Pigmentiphaga sp. dnA1 |
| Ga0586324 3757314 1 693 | 0.00 | 0.00 | 0.00 | 0.00 | 4717400.00 | 0.00 | 0.00 | 0.00 | 0.00 | 0.00 | 0.00 | 0.00 | KO:K03285 | GBP family porin | Bacteria;Proteobacteria;Betaproteobacteria;Burkholderiales;unclassified Burkholderiales;unclassified Burkholderiales;Burkholderiales bacterium JOSHI 001;Burkholderiales sp. JOSHI 001 |
| Ga0586324 3766956 1 693 | 0.00 | 0.00 | 0.00 | 0.00 | 0.00 | 0.00 | 0.00 | 5959700.00 | 5329800.00 | 0.00 | 0.00 | 0.00 | KO:K03285 | GBP family porin | Bacteria;Proteobacteria;Betaproteobacteria;Nitrosomonadales;Nitrosomonadaceae;Nitrosomonas;Nitrosomonas cryotolerans;Nitrosomonas cryotolerans ATCC 49181 |
| Ga0586324 4819245 2 595 | 25300000.00 | 0.00 | 0.00 | 0.00 | 0.00 | 0.00 | 0.00 | 0.00 | 0.00 | 0.00 | 0.00 | 0.00 | KO:K03285 | GBP family porin | Bacteria;Proteobacteria;Gammaproteobacteria;Acidiferrobacterales;Acidiferrobacteraceae;Sulfuricaulis;Sulfuricaulis limicola;Sulfuricaulis limicola HA5 |
| Ga0586324 5775323 53 580 | 4030000.00 | 2781200.00 | 0.00 | 5605000.00 | 8116400.00 | 0.00 | 0.00 | 9522100.00 | 1117800.00 | 9933000.00 | 21049600.00 | 261318.75 | KO:K03285 | GBP family porin | Bacteria;Proteobacteria;Betaproteobacteria;Nitrosomonadales;Thiobacillaceae;Thiobacillus;Thiobacillus denitrificans;Thiobacillus denitrificans ATCC 25259 |
| Ga0586324 7769628 3 329 | 0.00 | 0.00 | 0.00 | 0.00 | 0.00 | 2926255.24 | 6178800.00 | 0.00 | 1611900.00 | 0.00 | 0.00 | 0.00 | KO:K03285 | GBP family porin | Bacteria;Proteobacteria;Betaproteobacteria;Burkholderiales;Alcaligenaceae;Achromobacter;Achromobacter xylosoxidans;Achromobacter xylosoxidans FDAARGOS 88 |
| Ga0586324 0000013 80642 81916 | 0.00 | 0.00 | 0.00 | 0.00 | 0.00 | 567686.18 | 27968294.88 | 27559800.00 | 25725600.00 | 65288300.00 | 75152000.00 | 70575000.00 | KO:K03286 | OOP family OmpA-OmpF porin | Bacteria;Proteobacteria;Betaproteobacteria;Nitrosomonadales;Thiobacillaceae;Thiobacillus;Thiobacillus thioparus;Thiobacillus thioparus DSM 505 |
| Ga0586324 0001111 10242 11330 | 17450000.00 | 13246400.00 | 16552200.00 | 25935000.00 | 25193800.00 | 8495800.00 | 18992400.00 | 5965800.00 | 13851000.00 | 26634300.00 | 15884000.00 | 9022500.00 | KO:K03286 | OOP family OmpA-OmpF porin | Bacteria;Proteobacteria;Gammaproteobacteria;Methylococcales;Methylococcaceae;Methylosarcina;Methylosarcina fibrata;Methylosarcina fibrata AML-C10 |
| Ga0586324 0003059 9306 10331 | 50350000.00 | 2441200.00 | 0.00 | 731623.50 | 15244000.00 | 0.00 | 0.00 | 1958100.00 | 882900.00 | 0.00 | 2411200.00 | 0.00 | KO:K03286 | OOP family OmpA-OmpF porin | Bacteria;Proteobacteria;Deltaproteobacteria;Syntrophobacterales;Syntrophobacteraceae;Desulfosoma;Desulfosoma caldarium;Desulfosoma caldarium DSM 22027 |
| Ga0586324 0005017 13082 13606 | 0.00 | 0.00 | 0.00 | 0.00 | 0.00 | 0.00 | 0.00 | 0.00 | 0.00 | 6360200.00 | 6846400.00 | 9525000.00 | KO:K03286 | OOP family OmpA-OmpF porin | Bacteria;Proteobacteria;Betaproteobacteria;Nitrosomonadales;Gallionellaceae;Sideroxydans;Sideroxydans lithotrophicus;Sideroxydans lithotrophicus ES-1 |
| Ga0586324 0005137 11010 12122 | 0.00 | 0.00 | 3828720.91 | 3458000.00 | 4387800.00 | 4565500.00 | 1385790.84 | 2818200.00 | 5516100.00 | 4458300.00 | 0.00 | 8032500.00 | KO:K03286 | OOP family OmpA-OmpF porin | Bacteria;Proteobacteria;Gammaproteobacteria;Chromatiales;Chromatiaceae;Lamprocystis;Lamprocystis purpurea;Lamprocystis purpurea DSM 4197 |
| Ga0586324 0013780 8050 8826 | 0.00 | 0.00 | 0.00 | 0.00 | 0.00 | 0.00 | 0.00 | 0.00 | 0.00 | 0.00 | 0.00 | 171092.25 | KO:K03286 | OOP family OmpA-OmpF porin | Bacteria;Proteobacteria;Alphaproteobacteria;Rhodospirillales;Acetobacteraceae;Rhodopila;unclassified Rhodopila;Rhodopila sp. LVNP |
| Ga0586324 0016029 2848 3417 | 0.00 | 1128800.00 | 0.00 | 0.00 | 0.00 | 6034400.00 | 0.00 | 0.00 | 0.00 | 0.00 | 0.00 | 0.00 | KO:K03286 | OOP family OmpA-OmpF porin | Bacteria;Proteobacteria;Betaproteobacteria;Neisseriales;Chromobacteriaceae;Pseudogulbenkiania;Pseudogulbenkiania ferrooxidans;Pseudogulbenkiania ferrooxidans 2002 |
| Ga0586324 0035180 5306 5677 | 0.00 | 0.00 | 0.00 | 0.00 | 0.00 | 0.00 | 0.00 | 0.00 | 0.00 | 9394000.00 | 7594400.00 | 0.00 | KO:K03286 | OOP family OmpA-OmpF porin | Bacteria;Proteobacteria;Gammaproteobacteria;Xanthomonadales;Rhodanobacteraceae;Pseudofulvimonas;Pseudofulvimonas gallinarii;Pseudofulvimonas gallinarii DSM 21944 |
| Ga0586324 0037943 4331 5104 | 0.00 | 4107200.00 | 8670200.00 | 0.00 | 5242700.00 | 4208200.00 | 0.00 | 2799900.00 | 2980800.00 | 0.00 | 0.00 | 0.00 | KO:K03286 | OOP family OmpA-OmpF porin | Bacteria;Proteobacteria;Betaproteobacteria;Rhodocyclales;Zoogloeaceae;Azoarcus;Azoarcus sp. KH32C;Azoarcus sp. KH32C |
| Ga0586324 0046924 4111 4704 | 0.00 | 7004000.00 | 0.00 | 0.00 | 0.00 | 0.00 | 15982800.00 | 12261000.00 | 14175000.00 | 12243000.00 | 14432000.00 | 15675000.00 | KO:K03286 | OOP family OmpA-OmpF porin | Bacteria;Proteobacteria;Gammaproteobacteria;Oceanospirillales;Halomonadaceae;Halomonas;Halomonas heilongjiangensis;Halomonas heilongjiangensis DSM 26881 |
| Ga0586324 0058018 482 1684 | 0.00 | 0.00 | 0.00 | 0.00 | 0.00 | 3946481.72 | 0.00 | 0.00 | 0.00 | 0.00 | 0.00 | 0.00 | KO:K03286 | OOP family OmpA-OmpF porin | Bacteria;Ignavibacteriae;Ignavibacteria;Ignavibacteriales;Melioribacteraceae;Melioribacter;Melioribacter roseus;Melioribacter roseus P3M |
| Ga0586324 0064503 3 767 | 0.00 | 0.00 | 1616114.02 | 0.00 | 14729000.00 | 0.00 | 2827200.00 | 6173200.00 | 248930.82 | 0.00 | 0.00 | 1972500.00 | KO:K03286 | OOP family OmpA-OmpF porin | Bacteria;Proteobacteria;Alphaproteobacteria;Rhodospirillales;Rhodospirillaceae;Dongia;Dongia mobilis;Dongia mobilis CGMCC 1.7660 |
| Ga0586324 0068986 1406 2113 | 0.00 | 0.00 | 0.00 | 0.00 | 0.00 | 0.00 | 3670800.00 | 11956000.00 | 13608000.00 | 0.00 | 0.00 | 0.00 | KO:K03286 | OOP family OmpA-OmpF porin | Bacteria;Proteobacteria;Betaproteobacteria;Nitrosomonadales;Sulfuricellaceae;Sulfurirhabdus;Sulfurirhabdus autotrophica;Sulfurirhabdus autotrophica DSM 100309 |
| Ga0586324 0070884 174 749 | 8200000.00 | 4426800.00 | 3818052.06 | 0.00 | 4841000.00 | 15205100.00 | 8367600.00 | 0.00 | 2583900.00 | 8600900.00 | 5033600.00 | 8977500.00 | KO:K03286 | OOP family OmpA-OmpF porin | Bacteria;Proteobacteria;Gammaproteobacteria;Chromatiales;Chromatiaceae;Thiocapsa;Thiocapsa roseopersicina;Thiocapsa roseopersicina DSM 217 |
| Ga0586324 0076904 321 1415 | 104734527.00 | 11900000.00 | 15325265.36 | 5709500.00 | 22557000.00 | 13180400.00 | 8983200.00 | 19611500.00 | 67675500.00 | 27704600.00 | 21648000.00 | 48900000.00 | KO:K03286 | OOP family OmpA-OmpF porin | Bacteria;Proteobacteria;Gammaproteobacteria;Methylococcales;Methylococcaceae;Methylosarcina;Methylosarcina fibrata;Methylosarcina fibrata AML-C10 |
| Ga0586324 0077996 1214 2287 | 4310000.00 | 2087600.00 | 0.00 | 0.00 | 3728600.00 | 2564473.11 | 1871180.04 | 1165100.00 | 1903500.00 | 0.00 | 2164800.00 | 1057500.00 | KO:K03286 | OOP family OmpA-OmpF porin | Bacteria;Proteobacteria;Gammaproteobacteria;Xanthomonadales;Xanthomonadaceae;Lysobacter;Lysobacter sp. URHA0019;Lysobacter sp. URHA0019 |
| Ga0586324 0085121 3187 3702 | 0.00 | 0.00 | 0.00 | 0.00 | 0.00 | 0.00 | 0.00 | 2537600.00 | 3102300.00 | 0.00 | 0.00 | 0.00 | KO:K03286 | OOP family OmpA-OmpF porin | Bacteria;Proteobacteria;Gammaproteobacteria;Alteromonadales;Pseudoalteromonadaceae;Pseudoalteromonas;Pseudoalteromonas sp. T1lg88;Pseudoalteromonas sp. T1lg88 |
| Ga0586324 0100598 1564 2730 | 5430000.00 | 4719200.00 | 2605918.69 | 2109000.00 | 0.00 | 2712701.00 | 3146400.00 | 4361500.00 | 3369600.00 | 4288900.00 | 3942400.00 | 1147500.00 | KO:K03286 | OOP family OmpA-OmpF porin | Bacteria;Proteobacteria;Deltaproteobacteria;Desulfobacterales;Desulfobacteraceae;Desulfospira;Desulfospira joergensenii;Desulfospira joergensenii DSM 10085 |
| Ga0586324 0105595 3 695 | 0.00 | 5168000.00 | 14412800.00 | 6507500.00 | 5582600.00 | 0.00 | 4058400.00 | 0.00 | 5483700.00 | 3026100.00 | 3986400.00 | 3855000.00 | KO:K03286 | OOP family OmpA-OmpF porin | Bacteria;Proteobacteria;Betaproteobacteria;Rhodocyclales;Zoogloeaceae;Azoarcus;Azoarcus sp. KH32C;Azoarcus sp. KH32C |
| Ga0586324 0113525 1749 2777 | 0.00 | 6120000.00 | 0.00 | 0.00 | 0.00 | 0.00 | 267747.24 | 13359000.00 | 0.00 | 0.00 | 0.00 | 0.00 | KO:K03286 | OOP family OmpA-OmpF porin | Bacteria;Proteobacteria;Deltaproteobacteria;Syntrophobacterales;Syntrophobacteraceae;Thermodesulforhabdus;Thermodesulforhabdus norvegica;Thermodesulforhabdus norvegica DSM 9990 |
| Ga0586324 0113751 399 1553 | 61900000.00 | 75072000.00 | 42788000.00 | 7210500.00 | 85314900.00 | 33784700.00 | 0.00 | 19379700.00 | 33645406.59 | 59444000.00 | 72714400.00 | 17257500.00 | KO:K03286 | OOP family OmpA-OmpF porin | Bacteria;Proteobacteria;Alphaproteobacteria;Rhodospirillales;Acetobacteraceae;Rhodopila;Rhodopila globiformis;Rhodopila globiformis DSM 161 |
| Ga0586324 0128495 129 953 | 0.00 | 0.00 | 0.00 | 0.00 | 2121800.00 | 0.00 | 0.00 | 0.00 | 0.00 | 0.00 | 0.00 | 0.00 | KO:K03286 | OOP family OmpA-OmpF porin | Bacteria;Proteobacteria;Gammaproteobacteria;Chromatiales;Chromatiaceae;Nitrosococcus;Nitrosococcus wardiae;Nitrosococcus wardiae D1FHS |
| Ga0586324 0144946 1269 2414 | 0.00 | 0.00 | 0.00 | 0.00 | 0.00 | 0.00 | 1295343.24 | 0.00 | 0.00 | 0.00 | 0.00 | 0.00 | KO:K03286 | OOP family OmpA-OmpF porin | Bacteria;Proteobacteria;Deltaproteobacteria;Desulfobacterales;Desulfobacteraceae;Desulfatiglans;Desulfatiglans anilini;Desulfatiglans anilini DSM 4660 |
| Ga0586324 0147021 1094 2242 | 0.00 | 0.00 | 0.00 | 0.00 | 0.00 | 2726357.80 | 0.00 | 0.00 | 0.00 | 0.00 | 0.00 | 0.00 | KO:K03286 | OOP family OmpA-OmpF porin | Bacteria;Proteobacteria;Deltaproteobacteria;Desulfobacterales;Desulfobacteraceae;Desulfospira;Desulfospira joergensenii;Desulfospira joergensenii DSM 10085 |
| Ga0586324 0204555 25 846 | 0.00 | 1559465.08 | 0.00 | 1330000.00 | 1915800.00 | 1157493.20 | 2872800.00 | 0.00 | 1579500.00 | 823900.00 | 0.00 | 368249.25 | KO:K03286 | OOP family OmpA-OmpF porin | Bacteria;Proteobacteria;Alphaproteobacteria;Rhodospirillales;Rhodospirillaceae;Dongia;Dongia mobilis;Dongia mobilis CGMCC 1.7660 |
| Ga0586324 0338286 1 531 | 0.00 | 0.00 | 9402100.00 | 0.00 | 0.00 | 0.00 | 6178800.00 | 0.00 | 0.00 | 0.00 | 0.00 | 0.00 | KO:K03286 | OOP family OmpA-OmpF porin | Bacteria;Proteobacteria;Gammaproteobacteria;Chromatiales;Chromatiaceae;Thiocapsa;Thiocapsa marina;Thiocapsa marina 5811, DSM 5653 |
| Ga0586324 0347901 1 378 | 13500000.00 | 24344000.00 | 2981546.66 | 0.00 | 27604000.00 | 4446400.00 | 0.00 | 4910500.00 | 0.00 | 9933000.00 | 11088000.00 | 41475000.00 | KO:K03286 | OOP family OmpA-OmpF porin | Bacteria;Proteobacteria;Alphaproteobacteria;Rhodospirillales;Acetobacteraceae;Rhodopila;unclassified Rhodopila;Rhodopila sp. LVNP |
| Ga0586324 0347901 1156 1923 | 52450000.00 | 47396000.00 | 42056100.00 | 47880000.00 | 25956000.00 | 60224900.00 | 22822800.00 | 15433000.00 | 32886000.00 | 34442100.00 | 41272000.00 | 21450000.00 | KO:K03286 | OOP family OmpA-OmpF porin | Bacteria;Proteobacteria;Alphaproteobacteria;Rhodospirillales;Acetobacteraceae;Rhodopila;unclassified Rhodopila;Rhodopila sp. LVNP |
| Ga0586324 0362200 3 1076 | 0.00 | 7616000.00 | 4710711.08 | 7638000.00 | 6602300.00 | 2493961.94 | 0.00 | 4007700.00 | 1895400.00 | 4889500.00 | 2701600.00 | 0.00 | KO:K03286 | OOP family OmpA-OmpF porin | Bacteria;Proteobacteria;Deltaproteobacteria;Desulfuromonadales;Geobacteraceae;Geobacter;Geobacter soli;Geobacter soli GSS01 |
| Ga0586324 0366124 580 1668 | 8960000.00 | 9384000.00 | 14750600.00 | 0.00 | 6715600.00 | 7106300.00 | 0.00 | 707600.00 | 6755400.00 | 9702000.00 | 9944000.00 | 0.00 | KO:K03286 | OOP family OmpA-OmpF porin | Bacteria;Proteobacteria;Deltaproteobacteria;Desulfobacterales;Desulfobulbaceae;Desulfopila;Desulfopila sp. IMCC35005;Desulfopila sp. IMCC35005 |
| Ga0586324 0653976 655 1458 | 0.00 | 10268000.00 | 22182200.00 | 9034500.00 | 8085500.00 | 0.00 | 0.00 | 0.00 | 0.00 | 0.00 | 0.00 | 6517500.00 | KO:K03286 | OOP family OmpA-OmpF porin | Bacteria;Proteobacteria;Deltaproteobacteria;Desulfobacterales;Desulfobulbaceae;Desulfobulbus;Desulfobulbus mediterraneus;Desulfobulbus mediterraneus DSM 13871 |

| | | | | | | | | | | | | | | | |
|---|---|---|---|---|---|---|---|---|---|---|---|---|---|---|---|
| Ga0586324 0823863 193 1320 | 8380000.00 | 1829200.00 | 0.00 | 0.00 | 0.00 | 6550500.00 | 0.00 | 0.00 | 0.00 | 3349500.00 | 0.00 | 0.00 | KO:K03286 | OOP family OmpA-OmpF porin | Bacteria;Proteobacteria;Deltaproteobacteria;Syntrophobacterales;Syntrophobacteraceae;Thermodesulforhabdus;Thermodesulforhabdus norvegica;Thermodesulforhabdus norvegica DSM 9990 |
| Ga0586324 0908926 1 444 | 0.00 | 0.00 | 0.00 | 0.00 | 0.00 | 0.00 | 0.00 | 0.00 | 0.00 | 0.00 | 3546400.00 | 0.00 | KO:K03286 | OOP family OmpA-OmpF porin | Bacteria;Proteobacteria;Betaproteobacteria;Rhodocyclales;Zoogloeaceae;Azoarcus;Azoarcus sp. KH32C;Azoarcus sp. KH32C |
| Ga0586324 0935192 450 1250 | 0.00 | 2611200.00 | 0.00 | 0.00 | 0.00 | 2812534.59 | 3351600.00 | 9821000.00 | 1514700.00 | 0.00 | 12408000.00 | 11550000.00 | KO:K03286 | OOP family OmpA-OmpF porin | Bacteria;Proteobacteria;Gammaproteobacteria;Chromatiales;Chromatiaceae;Lamprocystis;Lamprocystis purpurea;Lamprocystis purpurea DSM 4197 |
| Ga0586324 1129502 414 1136 | 0.00 | 3325200.00 | 7825700.00 | 2508000.00 | 3265100.00 | 0.00 | 2118304.68 | 8723000.00 | 1790100.00 | 3210900.00 | 2675200.00 | 2130000.00 | KO:K03286 | OOP family OmpA-OmpF porin | Bacteria;Proteobacteria;Betaproteobacteria;Rhodocyclales;Zoogloeaceae;Azoarcus;Azoarcus sp. KH32C;Azoarcus sp. KH32C |
| Ga0586324 1131382 2 733 | 0.00 | 0.00 | 0.00 | 0.00 | 0.00 | 0.00 | 142803.92 | 0.00 | 0.00 | 1717100.00 | 0.00 | 0.00 | KO:K03286 | OOP family OmpA-OmpF porin | Bacteria;Proteobacteria;Gammaproteobacteria;Xanthomonadales;Xanthomonadaceae;Lysobacter;Lysobacter sp. URHA0019;Lysobacter sp. URHA0019 |
| Ga0586324 1425363 1 1044 | 0.00 | 0.00 | 0.00 | 0.00 | 0.00 | 0.00 | 0.00 | 0.00 | 3402000.00 | 0.00 | 0.00 | 0.00 | KO:K03286 | OOP family OmpA-OmpF porin | Bacteria;Proteobacteria;Deltaproteobacteria;Desulfobacterales;Desulfobacteraceae;Desulfatiglans;Desulfatiglans anilini;Desulfatiglans anilini DSM 4660 |
| Ga0586324 1744562 2 958 | 32900000.00 | 13056000.00 | 0.00 | 16150000.00 | 20497000.00 | 0.00 | 0.00 | 0.00 | 0.00 | 0.00 | 0.00 | 0.00 | KO:K03286 | OOP family OmpA-OmpF porin | Bacteria;Proteobacteria;Gammaproteobacteria;Methylococcales;Methylococcaceae;Methylobacter;Methylobacter tundripaludum;Methylobacter tundripaludum OWC-DMM |
| Ga0586324 1773197 2 436 | 0.00 | 0.00 | 0.00 | 0.00 | 0.00 | 0.00 | 0.00 | 0.00 | 0.00 | 0.00 | 0.00 | 2947500.00 | KO:K03286 | OOP family OmpA-OmpF porin | Bacteria;Proteobacteria;Alphaproteobacteria;Sphingomonadales;Erythrobacteraceae;Altererythrobacter;Altererythrobacter sp. Root672;Altererythrobacter sp. Root672 |
| Ga0586324 1801935 1 525 | 0.00 | 0.00 | 0.00 | 0.00 | 0.00 | 0.00 | 0.00 | 0.00 | 0.00 | 0.00 | 195021.20 | 0.00 | KO:K03286 | OOP family OmpA-OmpF porin | Bacteria;Proteobacteria;Gammaproteobacteria;Chromatiales;Chromatiaceae;Lamprocystis;Lamprocystis purpurea;Lamprocystis purpurea DSM 4197 |
| Ga0586324 1834686 649 936 | 5170000.00 | 0.00 | 0.00 | 0.00 | 0.00 | 0.00 | 0.00 | 0.00 | 0.00 | 0.00 | 0.00 | 0.00 | KO:K03286 | OOP family OmpA-OmpF porin | Bacteria;Proteobacteria;Gammaproteobacteria;Methylococcales;Methylococcaceae;Methylomonas;Methylomonas denitrificans;Methylomonas denitrificans FJG1 |
| Ga0586324 2160306 36 875 | 0.00 | 0.00 | 39860400.00 | 3030500.00 | 5273600.00 | 35213900.00 | 99636000.00 | 640500.00 | 0.00 | 1378300.00 | 14071200.00 | 0.00 | KO:K03286 | OOP family OmpA-OmpF porin | Bacteria;Proteobacteria;Deltaproteobacteria;Syntrophobacterales;Syntrophobacteraceae;Desulfosoma;Desulfosoma caldarium;Desulfosoma caldarium DSM 22027 |
| Ga0586324 2254824 2 850 | 12700000.00 | 4467600.00 | 0.00 | 0.00 | 0.00 | 60939500.00 | 0.00 | 0.00 | 11210400.00 | 0.00 | 0.00 | 7335000.00 | KO:K03286 | OOP family OmpA-OmpF porin | Bacteria;Proteobacteria;Deltaproteobacteria;Syntrophobacterales;Syntrophobacteraceae;Desulfosoma;Desulfosoma caldarium;Desulfosoma caldarium DSM 22027 |
| Ga0586324 2265959 207 857 | 0.00 | 0.00 | 0.00 | 0.00 | 0.00 | 0.00 | 0.00 | 774700.00 | 0.00 | 0.00 | 0.00 | 0.00 | KO:K03286 | OOP family OmpA-OmpF porin | Bacteria;Bacteroidetes;Sphingobacteriia;Sphingobacteriales;Sphingobacteriaceae;Solitalea;Solitalea canadensis;Solitalea canadensis USAM 9D, DSM 3403 |
| Ga0586324 2307112 1 849 | 135500000.00 | 12308000.00 | 137372000.00 | 59185000.00 | 129471000.00 | 93930200.00 | 1282983.36 | 35190900.00 | 78302700.00 | 14553000.00 | 46816000.00 | 44550000.00 | KO:K03286 | OOP family OmpA-OmpF porin | Bacteria;Proteobacteria;Deltaproteobacteria;Syntrophobacterales;Syntrophobacteraceae;Thermodesulforhabdus;Thermodesulforhabdus norvegica;Thermodesulforhabdus norvegica DSM 9990 |
| Ga0586324 2445312 1 600 | 0.00 | 0.00 | 0.00 | 0.00 | 0.00 | 0.00 | 0.00 | 0.00 | 874800.00 | 0.00 | 0.00 | 0.00 | KO:K03286 | OOP family OmpA-OmpF porin | Bacteria;Proteobacteria;Deltaproteobacteria;Desulfuromonadales;Geobacteraceae;Geobacter;Geobacter uraniireducens;Geobacter uraniireducens Rf4 |
| Ga0586324 2538409 265 546 | 0.00 | 5664400.00 | 0.00 | 0.00 | 2348400.00 | 0.00 | 0.00 | 628300.00 | 0.00 | 5544000.00 | 0.00 | 0.00 | KO:K03286 | OOP family OmpA-OmpF porin | Bacteria;Proteobacteria;Alphaproteobacteria;Rhodospirillales;Acetobacteraceae;Rhodopila;unclassified Rhodopila;Rhodopila sp. LVNP |
| Ga0586324 3044170 2 757 | 139900000.00 | 56358400.00 | 8163500.00 | 111739000.00 | 117008000.00 | 69157400.00 | 71740067.76 | 69906000.00 | 49491000.00 | 62662600.00 | 82280000.00 | 57052500.00 | KO:K03286 | OOP family OmpA-OmpF porin | Bacteria;Proteobacteria;Deltaproteobacteria;Syntrophobacterales;Syntrophobacteraceae;Thermodesulforhabdus;Thermodesulforhabdus norvegica;Thermodesulforhabdus norvegica DSM 9990 |
| Ga0586324 3679954 3 563 | 0.00 | 0.00 | 0.00 | 0.00 | 0.00 | 0.00 | 6452400.00 | 0.00 | 0.00 | 0.00 | 0.00 | 0.00 | KO:K03286 | OOP family OmpA-OmpF porin | Bacteria;Proteobacteria;Alphaproteobacteria;Sphingomonadales;Erythrobacteraceae;Tsuneonella;Tsuneonella troitsensis;Altererythrobacter troitsensis JCM 17037 |
| Ga0586324 3686422 72 698 | 0.00 | 0.00 | 0.00 | 0.00 | 0.00 | 0.00 | 0.00 | 0.00 | 0.00 | 6622000.00 | 0.00 | 0.00 | KO:K03286 | OOP family OmpA-OmpF porin | Bacteria;Proteobacteria;Gammaproteobacteria;Chromatiales;Chromatiaceae;Lamprocystis;Lamprocystis purpurea;Lamprocystis purpurea DSM 4197 |
| Ga0586324 4014842 178 675 | 0.00 | 6800000.00 | 0.00 | 0.00 | 0.00 | 2759666.10 | 4833600.00 | 4373700.00 | 0.00 | 5305300.00 | 0.00 | 0.00 | KO:K03286 | OOP family OmpA-OmpF porin | Bacteria;Proteobacteria;Deltaproteobacteria;Syntrophobacterales;Syntrophobacteraceae;Thermodesulforhabdus;Thermodesulforhabdus norvegica;Thermodesulforhabdus norvegica DSM 9990 |
| Ga0586324 4303462 1 657 | 0.00 | 12008800.00 | 0.00 | 3439000.00 | 0.00 | 0.00 | 0.00 | 3373300.00 | 0.00 | 5074300.00 | 0.00 | 0.00 | KO:K03286 | OOP family OmpA-OmpF porin | Bacteria;Proteobacteria;Deltaproteobacteria;Syntrophobacterales;Syntrophobacteraceae;Desulfacinum;Desulfacinum infernum;Desulfacinum infernum DSM 9756 |
| Ga0586324 4425237 1 618 | 0.00 | 0.00 | 0.00 | 0.00 | 0.00 | 0.00 | 1277826.00 | 0.00 | 0.00 | 0.00 | 0.00 | 0.00 | KO:K03286 | OOP family OmpA-OmpF porin | Bacteria;Proteobacteria;Deltaproteobacteria;Syntrophobacterales;Syntrophobacteraceae;Thermodesulforhabdus;Thermodesulforhabdus norvegica;Thermodesulforhabdus norvegica DSM 9990 |
| Ga0586324 4812962 1 354 | 0.00 | 0.00 | 0.00 | 21185000.00 | 0.00 | 0.00 | 0.00 | 0.00 | 18792000.00 | 24101000.00 | 0.00 | 0.00 | KO:K03286 | OOP family OmpA-OmpF porin | Bacteria;Proteobacteria;Gammaproteobacteria;Chromatiales;Chromatiaceae;Marichromatium;Marichromatium gracile;Marichromatium gracile YL28 |
| Ga0586324 5053675 3 506 | 0.00 | 336406.88 | 0.00 | 0.00 | 0.00 | 0.00 | 0.00 | 0.00 | 0.00 | 0.00 | 0.00 | 0.00 | KO:K03286 | OOP family OmpA-OmpF porin | Bacteria;Proteobacteria;Gammaproteobacteria;Methylococcales;Methylococcaceae;Methylomonas;Methylomonas sp. MK1;Methylomonas sp. MK1 |
| Ga0586324 5267729 3 605 | 0.00 | 3298000.00 | 0.00 | 0.00 | 0.00 | 1996477.27 | 0.00 | 732000.00 | 1393200.00 | 0.00 | 0.00 | 1627500.00 | KO:K03286 | OOP family OmpA-OmpF porin | Bacteria;Proteobacteria;Gammaproteobacteria;Methylococcales;Methylococcaceae;Methylobacter;Methylobacter tundripaludum;Methylobacter tundripaludum SV96 |
| Ga0586324 6107673 1 567 | 0.00 | 6555200.00 | 0.00 | 4892500.00 | 6015200.00 | 12716307.00 | 17841531.24 | 11663200.00 | 5605200.00 | 11234300.00 | 15435200.00 | 13084272.00 | KO:K03286 | OOP family OmpA-OmpF porin | Bacteria;Proteobacteria;Deltaproteobacteria;Syntrophobacterales;Syntrophobacteraceae;Desulfosoma;Desulfosoma caldarium;Desulfosoma caldarium DSM 22027 |
| Ga0586324 6130599 3 566 | 15300000.00 | 0.00 | 0.00 | 0.00 | 0.00 | 0.00 | 0.00 | 0.00 | 0.00 | 0.00 | 0.00 | 0.00 | KO:K03286 | OOP family OmpA-OmpF porin | Bacteria;Proteobacteria;Alphaproteobacteria;Rhodospirillales;Acetobacteraceae;Humitalea;Humitalea rosea;Humitalea rosea DSM 24525 |
| Ga0586324 6314408 2 559 | 0.00 | 0.00 | 0.00 | 0.00 | 0.00 | 0.00 | 0.00 | 18483000.00 | 0.00 | 0.00 | 0.00 | 0.00 | KO:K03286 | OOP family OmpA-OmpF porin | Bacteria;Proteobacteria;Gammaproteobacteria;Xanthomonadales;Xanthomonadaceae;Lysobacter;Lysobacter sp. URHA0019;Lysobacter sp. URHA0019 |
| Ga0586324 6361437 1 558 | 0.00 | 0.00 | 0.00 | 0.00 | 0.00 | 2267322.58 | 2243681.88 | 0.00 | 2324700.00 | 0.00 | 0.00 | 0.00 | KO:K03286 | OOP family OmpA-OmpF porin | Bacteria;Proteobacteria;Gammaproteobacteria;Chromatiales;Chromatiaceae;Lamprocystis;Lamprocystis purpurea;Lamprocystis purpurea DSM 4197 |
| Ga0586324 6818286 1 333 | 0.00 | 0.00 | 0.00 | 0.00 | 11021000.00 | 0.00 | 0.00 | 0.00 | 0.00 | 0.00 | 0.00 | 6900000.00 | KO:K03286 | OOP family OmpA-OmpF porin | Bacteria;Proteobacteria;Betaproteobacteria;Burkholderiales;unclassified Burkholderiales;Aquabacterium;Aquabacterium sp. NJ1;Aquabacterium sp. NJ1 |
| Ga0586324 6835114 3 542 | 32100000.00 | 0.00 | 97962000.00 | 0.00 | 0.00 | 46449000.00 | 21112800.00 | 44225000.00 | 36288000.00 | 0.00 | 0.00 | 0.00 | KO:K03286 | OOP family OmpA-OmpF porin | Bacteria;Proteobacteria;Gammaproteobacteria;Methylococcales;Methylococcaceae;Methylosarcina;Methylosarcina fibrata;Methylosarcina fibrata AML-C10 |
| Ga0586324 6852716 1 231 | 0.00 | 2924000.00 | 0.00 | 0.00 | 0.00 | 0.00 | 0.00 | 1488400.00 | 1417500.00 | 0.00 | 0.00 | 3135000.00 | KO:K03286 | OOP family OmpA-OmpF porin | Bacteria;Proteobacteria;Gammaproteobacteria;Methylococcales;Methylococcaceae;Methylomonas;Methylomonas denitrificans;Methylomonas denitrificans FJG1 |
| Ga0586324 6979936 3 536 | 0.00 | 0.00 | 0.00 | 0.00 | 0.00 | 0.00 | 0.00 | 0.00 | 0.00 | 0.00 | 0.00 | 16800000.00 | KO:K03286 | OOP family OmpA-OmpF porin | Bacteria;Proteobacteria;Deltaproteobacteria;Desulfobacterales;Desulfobacteraceae;Desulfospira;Desulfospira joergensenii;Desulfospira joergensenii DSM 10085 |
| Ga0586324 7375952 164 526 | 0.00 | 0.00 | 0.00 | 0.00 | 0.00 | 0.00 | 0.00 | 5868200.00 | 0.00 | 0.00 | 0.00 | 0.00 | KO:K03286 | OOP family OmpA-OmpF porin | Bacteria;Proteobacteria;Gammaproteobacteria;Methylococcales;Methylococcaceae;Methylomonas;Methylomonas denitrificans;Methylomonas denitrificans FJG1 |
| Ga0586324 8072570 1 504 | 0.00 | 0.00 | 0.00 | 1425000.00 | 0.00 | 0.00 | 0.00 | 0.00 | 3175200.00 | 2271500.00 | 1346400.00 | 0.00 | KO:K03286 | OOP family OmpA-OmpF porin | Bacteria;Proteobacteria;Gammaproteobacteria;Methylococcales;Methylococcaceae;Methylomagnum;Methylomagnum ishizawai;Methylomagnum ishizawai 175 |
| Ga0586324 0722524 340 1398 | 0.00 | 0.00 | 0.00 | 0.00 | 0.00 | 0.00 | 5472000.00 | 0.00 | 0.00 | 0.00 | 0.00 | 0.00 | KO:K03307 | SSS family solute:Na+ symporter | Bacteria;Proteobacteria;Deltaproteobacteria;Desulfobacterales;Desulfobacteraceae;Desulfatitalea;Desulfatitalea tepidiphila;Desulfatitalea tepidiphila S28bF |
| Ga0586324 0000033 70987 72519 | 0.00 | 0.00 | 0.00 | 0.00 | 0.00 | 0.00 | 12123731.28 | 7381000.00 | 6196500.00 | 4912600.00 | 2992000.00 | 0.00 | KO:K03320 | Amt family ammonium transporter | Bacteria;Proteobacteria;Betaproteobacteria;Nitrosomonadales;Thiobacillaceae;Thiobacillus;Thiobacillus thioparus;Thiobacillus thioparus DSM 505 |
| Ga0586324 0121849 1324 2763 | 0.00 | 0.00 | 0.00 | 0.00 | 0.00 | 0.00 | 10168800.00 | 0.00 | 0.00 | 0.00 | 0.00 | 0.00 | KO:K03385 | nitrite reductase (cytochrome c-552) | Bacteria;Proteobacteria;Deltaproteobacteria;Syntrophobacterales;Syntrophobacteraceae;Syntrophobacter;Syntrophobacter fumaroxidans;Syntrophobacter fumaroxidans MPOB |
| Ga0586324 0349273 550 1920 | 0.00 | 0.00 | 0.00 | 2840500.00 | 0.00 | 0.00 | 0.00 | 0.00 | 0.00 | 0.00 | 0.00 | 0.00 | KO:K03385 | nitrite reductase (cytochrome c-552) | Bacteria;Proteobacteria;Deltaproteobacteria;Desulfovibrionales;Desulfovibrionaceae;Desulfovibrio;Desulfovibrio magneticus;Desulfovibrio cf. magneticus IFRC170 |
| Ga0586324 3053575 178 756 | 0.00 | 0.00 | 0.00 | 0.00 | 0.00 | 11910000.00 | 0.00 | 2403400.00 | 0.00 | 0.00 | 0.00 | 0.00 | KO:K03385 | nitrite reductase (cytochrome c-552) | Bacteria;Proteobacteria;Deltaproteobacteria;Desulfovibrionales;Desulfovibrionaceae;Desulfovibrio;Desulfovibrio gigas;Desulfovibrio gigas DSM 1382, ATCC 19364 |
| Ga0586324 5455826 3 596 | 0.00 | 0.00 | 10753300.00 | 0.00 | 0.00 | 0.00 | 0.00 | 0.00 | 0.00 | 0.00 | 0.00 | 0.00 | KO:K03385 | nitrite reductase (cytochrome c-552) | Bacteria;Proteobacteria;Deltaproteobacteria;Desulfobacterales;Desulfobacteraceae;Desulfatirhabdium;Desulfatirhabdium butyrativorans;Desulfatirhabdium butyrativorans DSM 18734 |
| Ga0586324 6436795 1 555 | 0.00 | 0.00 | 0.00 | 0.00 | 0.00 | 0.00 | 0.00 | 2189900.00 | 2097900.00 | 0.00 | 0.00 | 0.00 | KO:K03385 | nitrite reductase (cytochrome c-552) | Bacteria;Proteobacteria;Deltaproteobacteria;Desulfovibrionales;Desulfovibrionaceae;Desulfovibrio;Desulfovibrio magneticus;Desulfovibrio magneticus RS-1 |
| Ga0586324 7922908 2 508 | 0.00 | 0.00 | 0.00 | 0.00 | 0.00 | 3205913.95 | 0.00 | 0.00 | 0.00 | 0.00 | 0.00 | 0.00 | KO:K03385 | nitrite reductase (cytochrome c-552) | Bacteria;Proteobacteria;Deltaproteobacteria;Desulfovibrionales;Desulfovibrionaceae;Desulfovibrio;Desulfovibrio gigas;Desulfovibrio gigas DSM 1382, ATCC 19364 |
| Ga0586324 0003220 5845 6438 | 0.00 | 0.00 | 0.00 | 0.00 | 0.00 | 0.00 | 0.00 | 0.00 | 1522800.00 | 0.00 | 0.00 | 0.00 | KO:K03386 | peroxiredoxin (alkyl hydroperoxide reductase subunit C) | Bacteria;Thermodesulfobacteria;Thermodesulfobacteria;Thermodesulfobacteriales;Thermodesulfobacteriaceae;Thermosulfuriphilus;Thermosulfuriphilus ammonigenes;Thermosulfuriphilus ammonigenes DSM 102941 |
| Ga0586324 0925640 296 901 | 0.00 | 0.00 | 6474500.00 | 0.00 | 0.00 | 0.00 | 0.00 | 1116300.00 | 1992600.00 | 0.00 | 0.00 | 3270000.00 | KO:K03386 | peroxiredoxin (alkyl hydroperoxide reductase subunit C) | Bacteria;Proteobacteria;Gammaproteobacteria;Methylococcales;Crenotrichaceae;Crenothrix;Crenothrix polyspora;Crenothrix polyspora ERS1545889 |
| Ga0586324 1935851 232 828 | 0.00 | 0.00 | 0.00 | 0.00 | 0.00 | 0.00 | 0.00 | 0.00 | 0.00 | 0.00 | 0.00 | 8100000.00 | KO:K03386 | peroxiredoxin (alkyl hydroperoxide reductase subunit C) | Bacteria;Proteobacteria;Deltaproteobacteria;Desulfuromonadales;Geobacteraceae;Geobacter;Geobacter sp. OR-1;Geobacter sp. OR-1 |
| Ga0586324 3641298 302 703 | 20300000.00 | 0.00 | 0.00 | 0.00 | 0.00 | 0.00 | 0.00 | 0.00 | 0.00 | 0.00 | 0.00 | 0.00 | KO:K03386 | peroxiredoxin (alkyl hydroperoxide reductase subunit C) | Bacteria;Proteobacteria;Gammaproteobacteria;Methylococcales;Methylococcaceae;Methylotuvimicrobium;Methylotuvimicrobium alcaliphilum;Methylomicrobium alcaliphilum 20Z |
| Ga0586324 7965539 3 509 | 0.00 | 0.00 | 0.00 | 0.00 | 0.00 | 0.00 | 0.00 | 3233000.00 | 0.00 | 0.00 | 0.00 | 0.00 | KO:K03387 | alkyl hydroperoxide reductase subunit F | Bacteria;Firmicutes;Negativicutes;Selenomonadales;Sporomusaceae;Propionispora;Propionispora sp. 2/2-37;Propionispora sp. Iso2/2 |
| Ga0586324 0000236 34308 37688 | 0.00 | 0.00 | 0.00 | 0.00 | 0.00 | 0.00 | 0.00 | 957700.00 | 0.00 | 0.00 | 0.00 | 0.00 | KO:K03388 | heterodisulfide reductase subunit A | Bacteria;Proteobacteria;Deltaproteobacteria;Syntrophobacterales;Syntrophobacteraceae;Syntrophobacter;Syntrophobacter fumaroxidans;Syntrophobacter fumaroxidans MPOB |
| Ga0586324 0006360 416 3463 | 0.00 | 0.00 | 0.00 | 2033000.00 | 0.00 | 2515634.17 | 0.00 | 0.00 | 0.00 | 0.00 | 0.00 | 0.00 | KO:K03388 | heterodisulfide reductase subunit A | Bacteria;Proteobacteria;Deltaproteobacteria;Syntrophobacterales;Syntrophaceae;Desulfobacca;Desulfobacca acetoxidans;Desulfobacca acetoxidans ASRB2, DSM 11109 |
| Ga0586324 0014051 1 1755 | 0.00 | 0.00 | 0.00 | 0.00 | 0.00 | 0.00 | 0.00 | 250486.13 | 0.00 | 0.00 | 0.00 | 0.00 | KO:K03388 | heterodisulfide reductase subunit A | Bacteria;Proteobacteria;Deltaproteobacteria;Syntrophobacterales;Syntrophobacteraceae;Syntrophobacter;Syntrophobacter fumaroxidans;Syntrophobacter fumaroxidans MPOB |
| Ga0586324 0101759 10 3039 | 0.00 | 0.00 | 0.00 | 0.00 | 0.00 | 0.00 | 0.00 | 0.00 | 1935900.00 | 0.00 | 0.00 | 0.00 | KO:K03388 | heterodisulfide reductase subunit A | Bacteria;Proteobacteria;Deltaproteobacteria;Syntrophobacterales;Syntrophaceae;Desulfobacca;Desulfobacca acetoxidans;Desulfobacca acetoxidans ASRB2, DSM 11109 |
| Ga0586324 0173665 899 2641 | 0.00 | 0.00 | 9683600.00 | 0.00 | 0.00 | 0.00 | 0.00 | 0.00 | 257975.28 | 0.00 | 0.00 | 0.00 | KO:K03388 | heterodisulfide reductase subunit A | Archaea;Euryarchaeota;Methanomicrobia;Methanomicrobiales;Methanoregulaceae;Methanoregula;Methanoregula boonei;Methanoregula boonei 6A8 |
| Ga0586324 0348560 1 1350 | 0.00 | 5263200.00 | 0.00 | 0.00 | 0.00 | 0.00 | 0.00 | 0.00 | 0.00 | 0.00 | 0.00 | 0.00 | KO:K03388 | heterodisulfide reductase subunit A | Bacteria;Chloroflexi;Thermoflexia;Thermoflexales;Thermoflexaceae;Thermoflexus;Thermoflexus hugenholtzii;Thermoflexus hugenholtzii JAD2 |
| Ga0586324 0504282 2 1633 | 0.00 | 0.00 | 0.00 | 0.00 | 0.00 | 0.00 | 0.00 | 187534.74 | 0.00 | 0.00 | 0.00 | 0.00 | KO:K03388 | heterodisulfide reductase subunit A | Bacteria;Proteobacteria;Deltaproteobacteria;Desulfobacterales;Desulfobacteraceae;Desulfatitalea;Desulfatitalea tepidiphila;Desulfatitalea tepidiphila S28bF |
| Ga0586324 0650577 1 1461 | 0.00 | 0.00 | 0.00 | 0.00 | 544330.28 | 0.00 | 0.00 | 0.00 | 0.00 | 0.00 | 0.00 | 0.00 | KO:K03388 | heterodisulfide reductase subunit A | Archaea;Euryarchaeota;Methanomicrobia;Methanomicrobiales;Methanoregulaceae;Methanoregula;Methanoregula boonei;Methanoregula boonei 6A8 |
| Ga0586324 1050805 2 1189 | 0.00 | 0.00 | 0.00 | 1672000.00 | 0.00 | 0.00 | 0.00 | 1628700.00 | 0.00 | 0.00 | 0.00 | 0.00 | KO:K03388 | heterodisulfide reductase subunit A | Bacteria;Proteobacteria;Deltaproteobacteria;Desulfobacterales;Desulfobacteraceae;Desulfatirhabdium;Desulfatirhabdium butyrativorans;Desulfatirhabdium butyrativorans DSM 18734 |
| Ga0586324 1804249 3 944 | 0.00 | 0.00 | 0.00 | 0.00 | 0.00 | 1568614.49 | 0.00 | 0.00 | 0.00 | 0.00 | 0.00 | 1237500.00 | KO:K03388 | heterodisulfide reductase subunit A | Bacteria;Proteobacteria;Deltaproteobacteria;Syntrophobacterales;Syntrophobacteraceae;Syntrophobacter;Syntrophobacter fumaroxidans;Syntrophobacter fumaroxidans MPOB |
| Ga0586324 2754519 3 788 | 0.00 | 0.00 | 0.00 | 0.00 | 0.00 | 0.00 | 0.00 | 0.00 | 0.00 | 585460.26 | 0.00 | 0.00 | KO:K03388 | heterodisulfide reductase subunit A | Bacteria;Firmicutes;Clostridia;Eubacteriales;Peptococcaceae;Desulfocucumis;Desulfocucumis palustris;Desulfocucumis palustris NAW-5 |
| Ga0586324 3416112 1 723 | 0.00 | 0.00 | 0.00 | 0.00 | 0.00 | 3591770.16 | 0.00 | 0.00 | 0.00 | 0.00 | 0.00 | 0.00 | KO:K03388 | heterodisulfide reductase subunit A | Bacteria;Proteobacteria;Deltaproteobacteria;Syntrophobacterales;Syntrophaceae;Desulfobacca;Desulfobacca acetoxidans;Desulfobacca acetoxidans ASRB2, DSM 11109 |
| Ga0586324 0021934 2769 3680 | 0.00 | 0.00 | 0.00 | 0.00 | 0.00 | 0.00 | 3374400.00 | 2482700.00 | 2843100.00 | 1678600.00 | 2411200.00 | 2205000.00 | KO:K03389 | heterodisulfide reductase subunit B | Bacteria;Proteobacteria;Betaproteobacteria;Nitrosomonadales;Thiobacillaceae;Thiobacillus;Thiobacillus denitrificans;Thiobacillus denitrificans RG |
| Ga0586324 0096786 2180 3484 | 0.00 | 0.00 | 0.00 | 0.00 | 2987000.00 | 0.00 | 0.00 | 0.00 | 0.00 | 0.00 | 0.00 | 0.00 | KO:K03405 | magnesium chelatase subunit I | Bacteria;Chloroflexi;unclassified Chloroflexi;unclassified Chloroflexi;unclassified Chloroflexi;Thermobaculum;Thermobaculum terrenum;Thermobaculum terrenum YNP1, ATCC BAA-798 |
| Ga0586324 0001778 12274 13515 | 0.00 | 897600.00 | 0.00 | 0.00 | 0.00 | 0.00 | 0.00 | 0.00 | 0.00 | 0.00 | 0.00 | 0.00 | KO:K03406 | methyl-accepting chemotaxis protein | Bacteria;Firmicutes;Clostridia;Thermoanaerobacterales;Thermoanaerobacteraceae;Moorella;Moorella thermoacetica;Moorella thermoacetica DSM 21394 |
| Ga0586324 0008150 5773 7467 | 0.00 | 0.00 | 0.00 | 0.00 | 0.00 | 0.00 | 0.00 | 0.00 | 0.00 | 808500.00 | 0.00 | 0.00 | KO:K03406 | methyl-accepting chemotaxis protein | Bacteria;Gemmatimonadetes;Longimicrobia;Longimicrobiales;Longimicrobiaceae;Longimicrobium;Longimicrobium terrae;Longimicrobium terrae CECT 8660 |
| Ga0586324 0033242 2102 4516 | 0.00 | 0.00 | 0.00 | 0.00 | 0.00 | 0.00 | 14797200.00 | 2165500.00 | 0.00 | 0.00 | 6529600.00 | 0.00 | KO:K03406 | methyl-accepting chemotaxis protein | Bacteria;Proteobacteria;Deltaproteobacteria;Syntrophobacterales;Syntrophaceae;Desulfomonile;Desulfomonile tiedjei;Desulfomonile tiedjei DCB-1, DSM 6799 |
| Ga0586324 0086484 1927 3555 | 0.00 | 0.00 | 0.00 | 0.00 | 0.00 | 0.00 | 0.00 | 652700.00 | 0.00 | 0.00 | 0.00 | 0.00 | KO:K03406 | methyl-accepting chemotaxis protein | Bacteria;Proteobacteria;Betaproteobacteria;Rhodocyclales;Rhodocyclaceae;Rhodocyclus;Rhodocyclus tenuis;Rhodocyclus tenuis DSM 109 |
| Ga0586324 0360046 3 1895 | 0.00 | 918000.00 | 0.00 | 0.00 | 0.00 | 0.00 | 0.00 | 0.00 | 0.00 | 0.00 | 0.00 | 0.00 | KO:K03406 | methyl-accepting chemotaxis protein | Bacteria;Proteobacteria;Deltaproteobacteria;Myxococcales;Anaeromyxobacteraceae;Anaeromyxobacter;Anaeromyxobacter sp. PSR-1;Anaeromyxobacter sp. PSR-1 |
| Ga0586324 0872212 1 1287 | 0.00 | 0.00 | 0.00 | 0.00 | 0.00 | 0.00 | 0.00 | 28487000.00 | 0.00 | 0.00 | 0.00 | 0.00 | KO:K03406 | methyl-accepting chemotaxis protein | Bacteria;Proteobacteria;Deltaproteobacteria;Syntrophobacterales;Syntrophaceae;Desulfomonile;Desulfomonile tiedjei;Desulfomonile tiedjei DCB-1, DSM 6799 |
| Ga0586324 1218318 396 1115 | 0.00 | 0.00 | 5227702.72 | 0.00 | 0.00 | 6709300.00 | 0.00 | 0.00 | 0.00 | 0.00 | 0.00 | 0.00 | KO:K03406 | methyl-accepting chemotaxis protein | Bacteria;Proteobacteria;Deltaproteobacteria;Desulfobacterales;Desulfobacteraceae;Candidatus Magnetomorum;Candidatus Magnetomorum sp. HK-1;Candidatus Magnetomorum sp. HK-1 |
| Ga0586324 1573718 3 740 | 0.00 | 0.00 | 0.00 | 0.00 | 0.00 | 0.00 | 0.00 | 0.00 | 0.00 | 0.00 | 0.00 | 1575000.00 | KO:K03406 | methyl-accepting chemotaxis protein | Bacteria;Ignavibacteriae;Ignavibacteria;Ignavibacteriales;Ignavibacteriaceae;Ignavibacterium;Ignavibacterium album;Ignavibacterium album Mat9-16, JCM 16511 |
| Ga0586324 2813901 3 782 | 0.00 | 0.00 | 0.00 | 0.00 | 0.00 | 0.00 | 0.00 | 0.00 | 0.00 | 0.00 | 213305.84 | 2850000.00 | KO:K03406 | methyl-accepting chemotaxis protein | Bacteria;Proteobacteria;Deltaproteobacteria;Syntrophobacterales;Syntrophaceae;Desulfomonile;Desulfomonile tiedjei;Desulfomonile tiedjei DCB-1, DSM 6799 |
| Ga0586324 3076864 1 753 | 0.00 | 0.00 | 0.00 | 0.00 | 0.00 | 0.00 | 1626517.80 | 0.00 | 0.00 | 0.00 | 0.00 | 0.00 | KO:K03406 | methyl-accepting chemotaxis protein | Bacteria;Caldiserica;unclassified Caldiserica;unclassified Caldiserica;unclassified Caldiserica;unclassified Caldiserica;Caldiserica bacterium JGI 0000059-M03;Caldiserica bacterium JGI 0000059-M03 (contamination screened) |
| Ga0586324 4077155 2 670 | 0.00 | 0.00 | 0.00 | 0.00 | 0.00 | 0.00 | 0.00 | 0.00 | 0.00 | 0.00 | 5605600.00 | 0.00 | KO:K03406 | methyl-accepting chemotaxis protein | Bacteria;Proteobacteria;Deltaproteobacteria;Desulfuromonadales;Geobacteraceae;Geobacter;Geobacter uraniireducens;Geobacter uraniireducens Rf4 |
| Ga0586324 5935507 2 574 | 0.00 | 0.00 | 0.00 | 0.00 | 0.00 | 0.00 | 949797.84 | 0.00 | 0.00 | 0.00 | 0.00 | 0.00 | KO:K03406 | methyl-accepting chemotaxis protein | Bacteria;Proteobacteria;Deltaproteobacteria;Syntrophobacterales;Syntrophaceae;Desulfomonile;Desulfomonile tiedjei;Desulfomonile tiedjei DCB-1, DSM 6799 |
| Ga0586324 8244583 2 502 | 0.00 | 0.00 | 24096400.00 | 0.00 | 0.00 | 0.00 | 0.00 | 0.00 | 0.00 | 0.00 | 0.00 | 0.00 | KO:K03406 | methyl-accepting chemotaxis protein | Bacteria;Firmicutes;Bacilli;Bacillales;unclassified Bacillales;Exiguobacterium;Exiguobacterium sp. S17;Exiguobacterium sp. S17 |
| Ga0586324 0002033 923 1357 | 0.00 | 0.00 | 0.00 | 0.00 | 1514100.00 | 0.00 | 0.00 | 1464000.00 | 0.00 | 2556400.00 | 2270400.00 | 2340000.00 | KO:K03413 | two-component system chemotaxis response regulator CheY | Bacteria;Nitrospirae;Nitrospira;Nitrospirales;Nitrospiraceae;Nitrospira;Nitrospira lenta;Nitrospira lenta N. lenta BS10 |
| Ga0586324 0011884 4165 4914 | 13900000.00 | 0.00 | 0.00 | 3752500.00 | 0.00 | 0.00 | 0.00 | 0.00 | 0.00 | 0.00 | 0.00 | 0.00 | KO:K03432 | proteasome alpha subunit | Archaea;Euryarchaeota;Methanomicrobia;Methanosarcinales;Methanosarcinaceae;Methanosarcina;Methanosarcina barkeri;Methanosarcina barkeri 3 |
| Ga0586324 0213878 2 541 | 0.00 | 0.00 | 0.00 | 1805000.00 | 0.00 | 0.00 | 0.00 | 0.00 | 0.00 | 9702000.00 | 0.00 | 0.00 | KO:K03432 | proteasome alpha subunit | Archaea;Euryarchaeota;Methanopyri;Methanopyrales;Methanopyraceae;Methanopyrus;Methanopyrus sp. SNP6;Methanopyrus sp. SNP6 |
| Ga0586324 0550376 169 927 | 0.00 | 0.00 | 9458400.00 | 0.00 | 0.00 | 0.00 | 0.00 | 0.00 | 0.00 | 0.00 | 0.00 | 0.00 | KO:K03432 | proteasome alpha subunit | Archaea;Euryarchaeota;Methanomicrobia;Methanosarcinales;Methanotrichaceae;Methanothrix;Methanosaeta harundinacea;Methanothrix harundinacea 6Ac |
| Ga0586324 0604073 129 857 | 0.00 | 14960000.00 | 0.00 | 0.00 | 0.00 | 0.00 | 0.00 | 0.00 | 0.00 | 0.00 | 0.00 | 0.00 | KO:K03432 | proteasome alpha subunit | Archaea;Euryarchaeota;Methanobacteria;Methanobacteriales;Methanobacteriaceae;Methanobacterium;Methanobacterium congolense;Methanobacterium congolense Buetzberg |
| Ga0586324 1598901 1 693 | 0.00 | 0.00 | 0.00 | 0.00 | 0.00 | 0.00 | 0.00 | 0.00 | 0.00 | 0.00 | 0.00 | 267860.25 | KO:K03432 | proteasome alpha subunit | Archaea;Euryarchaeota;Methanopyri;Methanopyrales;Methanopyraceae;Methanopyrus;Methanopyrus sp. KOL6;Methanopyrus sp. KOL6 |
| Ga0586324 1914603 2 697 | 0.00 | 0.00 | 0.00 | 0.00 | 0.00 | 0.00 | 0.00 | 10004000.00 | 0.00 | 0.00 | 0.00 | 538608.00 | KO:K03432 | proteasome alpha subunit | Archaea;Thaumarchaeota;Nitrososphaeria;Nitrososphaerales;Nitrososphaeraceae;Nitrososphaera;Candidatus Nitrososphaera evergladensis;Candidatus Nitrososphaera evergladensis SR1 |
| Ga0586324 7005744 2 322 | 0.00 | 0.00 | 15764000.00 | 0.00 | 0.00 | 0.00 | 0.00 | 0.00 | 0.00 | 0.00 | 0.00 | 0.00 | KO:K03432 | proteasome alpha subunit | Archaea;Euryarchaeota;Methanomicrobia;Methanosarcinales;Methanotrichaceae;Methanothrix;Methanosaeta harundinacea;Methanothrix harundinacea 6Ac |
| Ga0586324 3925402 38 658 | 2220000.00 | 0.00 | 0.00 | 0.00 | 0.00 | 0.00 | 0.00 | 0.00 | 0.00 | 0.00 | 0.00 | 0.00 | KO:K03433 | proteasome beta subunit | Archaea;Euryarchaeota;Methanomicrobia;Methanosarcinales;Methanotrichaceae;Methanothrix;Methanothrix soehngenii;Methanothrix soehngenii GP-6 |
| Ga0586324 0000001 74183 75034 | 0.00 | 0.00 | 0.00 | 0.00 | 0.00 | 0.00 | 0.00 | 0.00 | 0.00 | 0.00 | 1478400.00 | 3105000.00 | KO:K03497 | ParB family chromosome partitioning protein | Bacteria;Proteobacteria;Betaproteobacteria;Nitrosomonadales;Thiobacillaceae;Annwoodia;Annwoodia aquaesulis;Annwoodia aquaesulis DSM 4255 |
| Ga0586324 0093090 2427 3278 | 0.00 | 0.00 | 0.00 | 0.00 | 0.00 | 0.00 | 0.00 | 175071.22 | 0.00 | 0.00 | 0.00 | 0.00 | KO:K03497 | ParB family chromosome partitioning protein | Bacteria;Proteobacteria;Betaproteobacteria;Nitrosomonadales;Thiobacillaceae;Thiobacillus;Thiobacillus thioparus;Thiobacillus thioparus DSM 505 |
| Ga0586324 0645731 726 1466 | 0.00 | 0.00 | 0.00 | 0.00 | 0.00 | 0.00 | 0.00 | 0.00 | 0.00 | 0.00 | 0.00 | 1732500.00 | KO:K03519 | carbon-monoxide dehydrogenase medium subunit | Bacteria;Proteobacteria;Alphaproteobacteria;Rhodospirillales;Acetobacteraceae;Rhodopila;Rhodopila globiformis;Rhodopila globiformis DSM 161 |
| Ga0586324 0680040 600 1400 | 0.00 | 0.00 | 2124587.47 | 0.00 | 0.00 | 0.00 | 0.00 | 0.00 | 0.00 | 0.00 | 0.00 | 0.00 | KO:K03519 | carbon-monoxide dehydrogenase medium subunit | Bacteria;Proteobacteria;Alphaproteobacteria;Hyphomicrobiales;Bradyrhizobiaceae;Bradyrhizobium;Bradyrhizobium lablabi;Bradyrhizobium lablabi GAS165 |
| Ga0586324 5103354 3 560 | 0.00 | 0.00 | 0.00 | 0.00 | 0.00 | 0.00 | 0.00 | 0.00 | 2397600.00 | 0.00 | 0.00 | 0.00 | KO:K03519 | carbon-monoxide dehydrogenase medium subunit | Bacteria;Proteobacteria;Alphaproteobacteria;Hyphomicrobiales;Hyphomicrobiaceae;Rhodoplanes;Rhodoplanes sp. Z2-YC6860;Rhodoplanes sp. Z2-YC6860 |
| Ga0586324 0473985 2 1546 | 0.00 | 0.00 | 0.00 | 0.00 | 0.00 | 0.00 | 0.00 | 0.00 | 0.00 | 0.00 | 1918400.00 | 0.00 | KO:K03520 | carbon-monoxide dehydrogenase large subunit | Bacteria;Proteobacteria;Alphaproteobacteria;Rhodospirillales;Acetobacteraceae;Rhodopila;Rhodopila globiformis;Rhodopila globiformis DSM 161 |
| Ga0586324 0896345 151 1272 | 0.00 | 0.00 | 21168800.00 | 0.00 | 0.00 | 0.00 | 34200000.00 | 24339000.00 | 0.00 | 0.00 | 0.00 | 0.00 | KO:K03520 | carbon-monoxide dehydrogenase large subunit | Bacteria;Proteobacteria;Alphaproteobacteria;Hyphomicrobiales;Hyphomicrobiaceae;Rhodoplanes;Rhodoplanes sp. Z2-YC6860;Rhodoplanes sp. Z2-YC6860 |
| Ga0586324 1454434 2 1033 | 0.00 | 0.00 | 0.00 | 0.00 | 0.00 | 0.00 | 0.00 | 0.00 | 0.00 | 0.00 | 17072000.00 | 0.00 | KO:K03520 | carbon-monoxide dehydrogenase large subunit | Bacteria;Proteobacteria;Alphaproteobacteria;Hyphomicrobiales;Bradyrhizobiaceae;Bradyrhizobium;Bradyrhizobium erythrophlei;Bradyrhizobium erythrophlei GAS242 |
| Ga0586324 7546514 3 521 | 0.00 | 0.00 | 0.00 | 0.00 | 0.00 | 0.00 | 0.00 | 0.00 | 2567700.00 | 0.00 | 0.00 | 0.00 | KO:K03520 | carbon-monoxide dehydrogenase large subunit | Bacteria;Proteobacteria;Alphaproteobacteria;Rhodospirillales;Acetobacteraceae;Rhodopila;Rhodopila globiformis;Rhodopila globiformis DSM 161 |
| Ga0586324 0001147 714 1619 | 0.00 | 0.00 | 0.00 | 0.00 | 0.00 | 0.00 | 740295.48 | 0.00 | 0.00 | 0.00 | 0.00 | 0.00 | KO:K03521 | electron transfer flavoprotein beta subunit | Bacteria;Proteobacteria;Gammaproteobacteria;Chromatiales;Chromatiaceae;Thiodictyon;Candidatus Thiodictyon syntrophicum;Candidatus Thiodictyon syntrophicum Cad16 |
| Ga0586324 0023830 2874 3701 | 0.00 | 0.00 | 0.00 | 6431500.00 | 0.00 | 0.00 | 0.00 | 0.00 | 0.00 | 0.00 | 0.00 | 747931.50 | KO:K03521 | electron transfer flavoprotein beta subunit | Bacteria;Firmicutes;Clostridia;Thermoanaerobacterales;Thermoanaerobacteraceae;Moorella;Moorella thermoacetica;Moorella thermoacetica Y72 |
| Ga0586324 0026463 5506 6300 | 0.00 | 0.00 | 0.00 | 5890000.00 | 0.00 | 8407183.65 | 0.00 | 0.00 | 0.00 | 0.00 | 0.00 | 1897500.00 | KO:K03521 | electron transfer flavoprotein beta subunit | Bacteria;Proteobacteria;Deltaproteobacteria;unclassified Deltaproteobacteria;unclassified Deltaproteobacteria;unclassified Deltaproteobacteria;delta proteobacterium NaphS2;delta proteobacterium NaphS2 |
| Ga0586324 0045920 1042 1791 | 0.00 | 0.00 | 0.00 | 0.00 | 1390500.00 | 0.00 | 0.00 | 0.00 | 0.00 | 0.00 | 0.00 | 0.00 | KO:K03521 | electron transfer flavoprotein beta subunit | Bacteria;Proteobacteria;Alphaproteobacteria;Rhodospirillales;Acetobacteraceae;Rhodopila;Rhodopila globiformis;Rhodopila globiformis DSM 161 |
| Ga0586324 0667550 1 483 | 0.00 | 0.00 | 0.00 | 0.00 | 0.00 | 895000.77 | 0.00 | 0.00 | 0.00 | 0.00 | 0.00 | 0.00 | KO:K03521 | electron transfer flavoprotein beta subunit | Bacteria;Proteobacteria;Deltaproteobacteria;Desulfobacterales;Desulfobacteraceae;Desulfatiglans;Desulfatiglans anilini;Desulfatiglans anilini DSM 4660 |
| Ga0586324 0961768 51 830 | 0.00 | 1645600.00 | 0.00 | 0.00 | 914362.93 | 0.00 | 0.00 | 1043100.00 | 834300.00 | 0.00 | 0.00 | 0.00 | KO:K03521 | electron transfer flavoprotein beta subunit | Bacteria;Proteobacteria;Deltaproteobacteria;Syntrophobacterales;Syntrophaceae;Desulfomonile;Desulfomonile tiedjei;Desulfomonile tiedjei DCB-1, DSM 6799 |
| Ga0586324 1398495 1 615 | 0.00 | 1591200.00 | 0.00 | 1938000.00 | 0.00 | 0.00 | 0.00 | 1689700.00 | 0.00 | 0.00 | 0.00 | 0.00 | KO:K03521 | electron transfer flavoprotein beta subunit | Bacteria;Proteobacteria;Deltaproteobacteria;Syntrophorhabdales;Syntrophorhabdaceae;Syntrophorhabdus;Syntrophorhabdus aromaticivorans;Syntrophorhabdus aromaticivorans UI |
| Ga0586324 1552437 207 1007 | 5470000.00 | 0.00 | 0.00 | 0.00 | 0.00 | 0.00 | 0.00 | 0.00 | 0.00 | 0.00 | 0.00 | 847500.00 | KO:K03521 | electron transfer flavoprotein beta subunit | Bacteria;Proteobacteria;Deltaproteobacteria;Desulfobacterales;Desulfobacteraceae;Desulfatiglans;Desulfatiglans anilini;Desulfatiglans anilini DSM 4660 |
| Ga0586324 2528822 2 811 | 0.00 | 0.00 | 0.00 | 0.00 | 0.00 | 0.00 | 0.00 | 1262700.00 | 0.00 | 0.00 | 0.00 | 0.00 | KO:K03521 | electron transfer flavoprotein beta subunit | Bacteria;Proteobacteria;Alphaproteobacteria;Hyphomicrobiales;Hyphomicrobiaceae;Caenibius;Caenibius tardaugens;Novosphingobium tardaugens NBRC 16725 |
| Ga0586324 2600776 3 632 | 0.00 | 0.00 | 0.00 | 0.00 | 0.00 | 0.00 | 0.00 | 0.00 | 3831300.00 | 0.00 | 0.00 | 0.00 | KO:K03521 | electron transfer flavoprotein beta subunit | Bacteria;Chloroflexi;Thermoflexia;Thermoflexales;Thermoflexaceae;Thermoflexus;Thermoflexus hugenholtzii;Thermoflexus hugenholtzii JAD2 |
| Ga0586324 2653802 511 801 | 2860000.00 | 0.00 | 0.00 | 0.00 | 0.00 | 0.00 | 0.00 | 0.00 | 0.00 | 0.00 | 0.00 | 0.00 | KO:K03521 | electron transfer flavoprotein beta subunit | Bacteria;Proteobacteria;Deltaproteobacteria;Syntrophobacterales;Syntrophaceae;Syntrophus;Syntrophus aciditrophicus;Syntrophus aciditrophicus SB |
| Ga0586324 3351782 3 728 | 0.00 | 0.00 | 0.00 | 0.00 | 0.00 | 0.00 | 0.00 | 0.00 | 1522800.00 | 0.00 | 0.00 | 0.00 | KO:K03521 | electron transfer flavoprotein beta subunit | Bacteria;Proteobacteria;Deltaproteobacteria;unclassified Deltaproteobacteria;unclassified Deltaproteobacteria;unclassified Deltaproteobacteria;delta proteobacterium NaphS2;delta proteobacterium NaphS2 |
| Ga0586324 5261262 6 605 | 0.00 | 0.00 | 0.00 | 0.00 | 0.00 | 0.00 | 0.00 | 0.00 | 433447.20 | 0.00 | 0.00 | 0.00 | KO:K03521 | electron transfer flavoprotein beta subunit | Bacteria;Bacteroidetes;Bacteroidia;Marinilabiliales;Marinilabiliaceae;Marinilabilia;Marinilabilia rubra;Marinilabilia rubra WTE16 |
| Ga0586324 6045284 3 569 | 4260000.00 | 0.00 | 0.00 | 0.00 | 0.00 | 0.00 | 0.00 | 0.00 | 0.00 | 0.00 | 0.00 | 0.00 | KO:K03521 | electron transfer flavoprotein beta subunit | Bacteria;Proteobacteria;Alphaproteobacteria;Hyphomicrobiales;Rhizobiaceae;Shinella;Shinella sp. HZN7;Shinella sp. HZN7 |
| Ga0586324 0138473 1226 2203 | 0.00 | 0.00 | 1881089.97 | 0.00 | 5901900.00 | 3780575.42 | 2462400.00 | 1403000.00 | 0.00 | 0.00 | 0.00 | 0.00 | KO:K03522 | electron transfer flavoprotein alpha subunit | Bacteria;Proteobacteria;Deltaproteobacteria;Syntrophobacterales;Syntrophaceae;Syntrophus;Syntrophus aciditrophicus;Syntrophus aciditrophicus SB |
| Ga0586324 0225496 1206 2186 | 0.00 | 0.00 | 0.00 | 368528.75 | 0.00 | 0.00 | 0.00 | 0.00 | 0.00 | 0.00 | 0.00 | 0.00 | KO:K03522 | electron transfer flavoprotein alpha subunit | Bacteria;Acidobacteria;Blastocatellia;unclassified Blastocatellia;unclassified Blastocatellia;Chloracidobacterium;Chloracidobacterium thermophilum;Chloracidobacterium thermophilum OC1 |
| Ga0586324 0309446 843 1820 | 0.00 | 5650800.00 | 7544200.00 | 0.00 | 12772000.00 | 0.00 | 0.00 | 0.00 | 0.00 | 0.00 | 0.00 | 0.00 | KO:K03522 | electron transfer flavoprotein alpha subunit | Bacteria;Proteobacteria;Deltaproteobacteria;Syntrophobacterales;Syntrophaceae;Syntrophus;Syntrophus aciditrophicus;Syntrophus aciditrophicus SB |
| Ga0586324 0686403 83 1066 | 0.00 | 0.00 | 0.00 | 3285234.90 | 1987900.00 | 0.00 | 0.00 | 0.00 | 518139.99 | 0.00 | 0.00 | 0.00 | KO:K03522 | electron transfer flavoprotein alpha subunit | Bacteria;Proteobacteria;Deltaproteobacteria;Desulfobacterales;Desulfobacteraceae;Desulfatiglans;Desulfatiglans anilini;Desulfatiglans anilini DSM 4660 |
| Ga0586324 1057523 314 1186 | 0.00 | 0.00 | 0.00 | 0.00 | 0.00 | 0.00 | 0.00 | 0.00 | 0.00 | 0.00 | 479638.72 | 0.00 | KO:K03522 | electron transfer flavoprotein alpha subunit | Bacteria;Acidobacteria;Vicinamibacteria;unclassified Vicinamibacteria;Vicinamibacteraceae;Luteitalea;Luteitalea pratensis;Luteitalea pratensis DSM 100886 |
| Ga0586324 1175515 1 852 | 0.00 | 7276000.00 | 0.00 | 0.00 | 0.00 | 0.00 | 0.00 | 0.00 | 0.00 | 0.00 | 0.00 | 0.00 | KO:K03522 | electron transfer flavoprotein alpha subunit | Bacteria;Proteobacteria;Deltaproteobacteria;Myxococcales;Archangiaceae;Hyalangium;Hyalangium minutum;Hyalangium minutum DSM 14724 |
| Ga0586324 2067610 36 890 | 0.00 | 0.00 | 0.00 | 0.00 | 0.00 | 0.00 | 0.00 | 1152900.00 | 0.00 | 0.00 | 0.00 | 0.00 | KO:K03522 | electron transfer flavoprotein alpha subunit | Bacteria;Proteobacteria;Alphaproteobacteria;Hyphomicrobiales;Xanthobacteraceae;Pseudolabrys;Pseudolabrys taiwanensis;Pseudolabrys taiwanensis CC-BB4 |
| Ga0586324 4112453 2 667 | 0.00 | 0.00 | 0.00 | 0.00 | 0.00 | 0.00 | 0.00 | 0.00 | 3831300.00 | 0.00 | 0.00 | 0.00 | KO:K03522 | electron transfer flavoprotein alpha subunit | Bacteria;Proteobacteria;Deltaproteobacteria;Syntrophobacterales;Syntrophaceae;Syntrophus;Syntrophus aciditrophicus;Syntrophus aciditrophicus SB |
| Ga0586324 4127584 115 666 | 2060000.00 | 1972000.00 | 2478894.63 | 0.00 | 2605900.00 | 3546345.42 | 0.00 | 3287900.00 | 0.00 | 0.00 | 0.00 | 0.00 | KO:K03522 | electron transfer flavoprotein alpha subunit | Bacteria;Proteobacteria;Betaproteobacteria;Burkholderiales;Oxalobacteraceae;Duganella;Duganella zoogloeoides;Duganella zoogloeoides ATCC 25935 |
| Ga0586324 4859393 3 623 | 0.00 | 0.00 | 0.00 | 2384500.00 | 2750100.00 | 0.00 | 0.00 | 0.00 | 0.00 | 0.00 | 0.00 | 0.00 | KO:K03522 | electron transfer flavoprotein alpha subunit | Bacteria;Proteobacteria;Deltaproteobacteria;Syntrophorhabdales;Syntrophorhabdaceae;Syntrophorhabdus;Syntrophorhabdus aromaticivorans;Syntrophorhabdus aromaticivorans UI |
| Ga0586324 0000018 56269 59772 | 0.00 | 0.00 | 0.00 | 0.00 | 0.00 | 0.00 | 0.00 | 0.00 | 0.00 | 0.00 | 0.00 | 585042.00 | KO:K03529 | chromosome segregation protein | Bacteria;Proteobacteria;Betaproteobacteria;Nitrosomonadales;Thiobacillaceae;Annwoodia;Annwoodia aquaesulis;Annwoodia aquaesulis DSM 4255 |
| Ga0586324 0002329 2906 6487 | 0.00 | 0.00 | 0.00 | 0.00 | 0.00 | 0.00 | 0.00 | 0.00 | 0.00 | 1771000.00 | 0.00 | 0.00 | KO:K03529 | chromosome segregation protein | Bacteria;Proteobacteria;Betaproteobacteria;Rhodocyclales;Azonexaceae;Dechloromonas;Dechloromonas sp. HYN0024;Dechloromonas sp. HYN0024 |
| Ga0586324 0012675 3 2957 | 0.00 | 0.00 | 0.00 | 0.00 | 0.00 | 0.00 | 0.00 | 0.00 | 0.00 | 0.00 | 0.00 | 509019.00 | KO:K03529 | chromosome segregation protein | Bacteria;Proteobacteria;Betaproteobacteria;Nitrosomonadales;Gallionellaceae;Sideroxydans;Sideroxydans lithotrophicus;Sideroxydans lithotrophicus ES-1 |
| Ga0586324 0032005 2333 5716 | 0.00 | 0.00 | 0.00 | 0.00 | 0.00 | 0.00 | 0.00 | 0.00 | 0.00 | 0.00 | 2411200.00 | 0.00 | KO:K03529 | chromosome segregation protein | Bacteria;Proteobacteria;Betaproteobacteria;Nitrosomonadales;Sulfuricellaceae;Sulfuricella;Sulfuricella denitrificans;Sulfuricella denitrificans skB26 |
| Ga0586324 0032338 2301 5798 | 0.00 | 0.00 | 0.00 | 0.00 | 0.00 | 0.00 | 0.00 | 2830400.00 | 0.00 | 1047200.00 | 1012000.00 | 547470.75 | KO:K03529 | chromosome segregation protein | Bacteria;Proteobacteria;Betaproteobacteria;Nitrosomonadales;Thiobacillaceae;Thiobacillus;Thiobacillus thioparus;Thiobacillus thioparus DSM 505 |
| Ga0586324 6856840 3 542 | 0.00 | 0.00 | 0.00 | 0.00 | 0.00 | 0.00 | 0.00 | 0.00 | 557804.07 | 0.00 | 0.00 | 0.00 | KO:K03529 | chromosome segregation protein | Bacteria;Proteobacteria;Betaproteobacteria;Burkholderiales;Comamonadaceae;Polaromonas;Polaromonas sp. C04;Polaromonas sp. C04 |
| Ga0586324 0000018 232 525 | 0.00 | 0.00 | 0.00 | 0.00 | 0.00 | 0.00 | 17442000.00 | 13176000.00 | 0.00 | 0.00 | 15400000.00 | 12000000.00 | KO:K03530 | DNA-binding protein HU-beta | Bacteria;Proteobacteria;Betaproteobacteria;Nitrosomonadales;Thiobacillaceae;Annwoodia;Annwoodia aquaesulis;Annwoodia aquaesulis DSM 4255 |
| Ga0586324 0000075 45294 45590 | 0.00 | 3760400.00 | 0.00 | 0.00 | 0.00 | 0.00 | 0.00 | 0.00 | 0.00 | 0.00 | 0.00 | 0.00 | KO:K03530 | DNA-binding protein HU-beta | Bacteria;Proteobacteria;Betaproteobacteria;Nitrosomonadales;Thiobacillaceae;Thiobacillus;Thiobacillus thioparus;Thiobacillus thioparus DSM 505 |
| Ga0586324 0000237 29853 30158 | 0.00 | 4841600.00 | 0.00 | 3078000.00 | 3185900.21 | 0.00 | 0.00 | 835700.00 | 0.00 | 0.00 | 0.00 | 3202500.00 | KO:K03530 | DNA-binding protein HU-beta | Bacteria;Acidobacteria;Vicinamibacteria;unclassified Vicinamibacteria;Vicinamibacteraceae;Luteitalea;Luteitalea pratensis;Luteitalea pratensis DSM 100886 |
| Ga0586324 0004434 13490 13789 | 6860000.00 | 4896000.00 | 0.00 | 0.00 | 0.00 | 1829368.06 | 0.00 | 4501800.00 | 1239300.00 | 0.00 | 0.00 | 3532500.00 | KO:K03530 | DNA-binding protein HU-beta | Bacteria;Acidobacteria;Vicinamibacteria;unclassified Vicinamibacteria;Vicinamibacteraceae;Luteitalea;Luteitalea pratensis;Luteitalea pratensis DSM 100886 |
| Ga0586324 0007221 1167 1475 | 0.00 | 0.00 | 17734500.00 | 2356000.00 | 0.00 | 10996900.00 | 29184000.00 | 15921000.00 | 6431400.00 | 11627000.00 | 12672000.00 | 0.00 | KO:K03530 | DNA-binding protein HU-beta | Bacteria;Proteobacteria;Alphaproteobacteria;Hyphomicrobiales;Xanthobacteraceae;Pseudolabrys;Pseudolabrys sp. Root1462;Pseudolabrys sp. Root1462 |
| Ga0586324 0008258 3083 3364 | 1080000.00 | 0.00 | 0.00 | 0.00 | 0.00 | 0.00 | 0.00 | 0.00 | 0.00 | 0.00 | 0.00 | 0.00 | KO:K03530 | DNA-binding protein HU-beta | Bacteria;Acidobacteria;Vicinamibacteria;unclassified Vicinamibacteria;Vicinamibacteraceae;Luteitalea;Luteitalea pratensis;Luteitalea pratensis DSM 100886 |
| Ga0586324 0008489 104 376 | 0.00 | 0.00 | 0.00 | 0.00 | 0.00 | 3171851.35 | 0.00 | 0.00 | 0.00 | 0.00 | 0.00 | 0.00 | KO:K03530 | DNA-binding protein HU-beta | Bacteria;Nitrospirae;Nitrospira;Nitrospirales;Nitrospiraceae;Nitrospira;Nitrospira defluvii;Nitrospira defluvii |
| Ga0586324 0014120 8444 8725 | 0.00 | 0.00 | 0.00 | 0.00 | 0.00 | 0.00 | 0.00 | 0.00 | 3952800.00 | 0.00 | 0.00 | 0.00 | KO:K03530 | DNA-binding protein HU-beta | Bacteria;Proteobacteria;Betaproteobacteria;Nitrosomonadales;Gallionellaceae;Sideroxydans;Sideroxydans lithotrophicus;Sideroxydans lithotrophicus ES-1 |
| Ga0586324 0062170 3172 3474 | 192559.00 | 0.00 | 0.00 | 0.00 | 0.00 | 0.00 | 0.00 | 0.00 | 0.00 | 0.00 | 0.00 | 0.00 | KO:K03530 | DNA-binding protein HU-beta | Bacteria;Acidobacteria;Acidobacteriia;Bryobacterales;Solibacteraceae;Candidatus Solibacter;Candidatus Solibacter usitatus;Candidatus Solibacter usitatus Ellin6076 |
| Ga0586324 0064874 2594 2890 | 0.00 | 0.00 | 0.00 | 0.00 | 0.00 | 0.00 | 0.00 | 10614000.00 | 0.00 | 0.00 | 0.00 | 0.00 | KO:K03530 | DNA-binding protein HU-beta | Bacteria;Proteobacteria;Alphaproteobacteria;Hyphomicrobiales;Hyphomicrobiaceae;Rhodoplanes;Rhodoplanes sp. Z2-YC6860;Rhodoplanes sp. Z2-YC6860 |
| Ga0586324 0117777 729 1007 | 3160000.00 | 0.00 | 2943026.20 | 0.00 | 4995500.00 | 4486100.00 | 2416800.00 | 0.00 | 0.00 | 0.00 | 0.00 | 0.00 | KO:K03530 | DNA-binding protein HU-beta | Bacteria;Ignavibacteriae;Ignavibacteria;Ignavibacteriales;Ignavibacteriaceae;Ignavibacterium;Ignavibacterium album;Ignavibacterium album Mat9-16, JCM 16511 |
| Ga0586324 0123025 873 1163 | 0.00 | 17544000.00 | 0.00 | 0.00 | 0.00 | 0.00 | 0.00 | 0.00 | 0.00 | 0.00 | 0.00 | 0.00 | KO:K03530 | DNA-binding protein HU-beta | Bacteria;Verrucomicrobia;Verrucomicrobiae;Verrucomicrobiales;Verrucomicrobia subdivision 3;Pedosphaera;Pedosphaera parvula;Pedosphaera parvula Ellin514 |
| Ga0586324 0144173 1028 1300 | 12700000.00 | 3393200.00 | 9627300.00 | 0.00 | 0.00 | 7463600.00 | 0.00 | 3495300.00 | 0.00 | 0.00 | 0.00 | 3517500.00 | KO:K03530 | DNA-binding protein HU-beta | Bacteria;Proteobacteria;Deltaproteobacteria;Desulfobacterales;Desulfobacteraceae;Desulfococcus;Desulfococcus oleovorans;Desulfococcus oleovorans Hxd3 |
| Ga0586324 0148884 1658 1966 | 8270000.00 | 1774800.00 | 2377036.67 | 0.00 | 0.00 | 0.00 | 0.00 | 3019500.00 | 2592000.00 | 0.00 | 0.00 | 0.00 | KO:K03530 | DNA-binding protein HU-beta | Bacteria;Proteobacteria;Alphaproteobacteria;Hyphomicrobiales;Xanthobacteraceae;Pseudolabrys;Pseudolabrys taiwanensis;Pseudolabrys taiwanensis CC-BB4 |
| Ga0586324 0245014 972 1274 | 0.00 | 0.00 | 0.00 | 0.00 | 0.00 | 2176056.25 | 0.00 | 0.00 | 1652400.00 | 0.00 | 0.00 | 1290000.00 | KO:K03530 | DNA-binding protein HU-beta | Bacteria;Acidobacteria;Acidobacteriia;Bryobacterales;Solibacteraceae;Candidatus Solibacter;Candidatus Solibacter usitatus;Candidatus Solibacter usitatus Ellin6076 |
| Ga0586324 0285690 132 431 | 1240000.00 | 999600.00 | 235611.56 | 0.00 | 0.00 | 0.00 | 0.00 | 0.00 | 0.00 | 0.00 | 0.00 | 0.00 | KO:K03530 | DNA-binding protein HU-beta | Bacteria;Acidobacteria;Vicinamibacteria;unclassified Vicinamibacteria;Vicinamibacteraceae;Luteitalea;Luteitalea pratensis;Luteitalea pratensis DSM 100886 |

| | | | | | | | | | | | | | | | |
|---|---|---|---|---|---|---|---|---|---|---|---|---|---|---|---|
| Ga0586324 0302658 3 293 | 1390000.00 | 471038.04 | 0.00 | 0.00 | 0.00 | 0.00 | 0.00 | 319804.70 | 191452.41 | 0.00 | 0.00 | 0.00 | KO:K03530 | DNA-binding protein HU-beta | Bacteria;Acidobacteria;Vicinamibacteria;unclassified Vicinamibacteria;Vicinamibacteraceae;Luteitalea;Luteitalea pratensis;Luteitalea pratensis DSM 100886 |
| Ga0586324 0386824 303 590 | 0.00 | 0.00 | 0.00 | 0.00 | 12154000.00 | 0.00 | 0.00 | 0.00 | 11421000.00 | 0.00 | 0.00 | 0.00 | KO:K03530 | DNA-binding protein HU-beta | Bacteria;Verrucomicrobia;Verrucomicrobiae;Verrucomicrobiales;Verrucomicrobia subdivision 3;Pedosphaera;Pedosphaera parvula;Pedosphaera parvula Ellin514 |
| Ga0586324 0410610 1420 1692 | 0.00 | 0.00 | 0.00 | 0.00 | 1318400.00 | 0.00 | 0.00 | 0.00 | 0.00 | 0.00 | 0.00 | 0.00 | KO:K03530 | DNA-binding protein HU-beta | Bacteria;Proteobacteria;Gammaproteobacteria;Methylococcales;Methylococcaceae;Methylobacter;Methylobacter luteus;Methylobacter luteus IMV-B-3098 |
| Ga0586324 0486259 897 1199 | 0.00 | 0.00 | 0.00 | 0.00 | 0.00 | 11195400.00 | 0.00 | 0.00 | 0.00 | 0.00 | 0.00 | 0.00 | KO:K03530 | DNA-binding protein HU-beta | Bacteria;Actinobacteria;Rubrobacteria;Rubrobacterales;Rubrobacteraceae;Rubrobacter;Rubrobacter radiotolerans;Rubrobacter radiotolerans DSM 5868 |
| Ga0586324 0503738 267 566 | 0.00 | 0.00 | 0.00 | 0.00 | 0.00 | 24653700.00 | 0.00 | 0.00 | 0.00 | 0.00 | 0.00 | 0.00 | KO:K03530 | DNA-binding protein HU-beta | Bacteria;Acidobacteria;Vicinamibacteria;unclassified Vicinamibacteria;Vicinamibacteraceae;Luteitalea;Luteitalea pratensis;Luteitalea pratensis DSM 100886 |
| Ga0586324 0516562 311 610 | 0.00 | 0.00 | 0.00 | 7486000.00 | 0.00 | 0.00 | 0.00 | 0.00 | 0.00 | 0.00 | 0.00 | 0.00 | KO:K03530 | DNA-binding protein HU-beta | Bacteria;Proteobacteria;Alphaproteobacteria;Hyphomicrobiales;Bradyrhizobiaceae;Bradyrhizobium;Bradyrhizobium sp. URHD0069;Bradyrhizobium sp. URHD0069 |
| Ga0586324 0588826 713 994 | 0.00 | 0.00 | 0.00 | 0.00 | 423623.55 | 0.00 | 0.00 | 0.00 | 0.00 | 0.00 | 0.00 | 0.00 | KO:K03530 | DNA-binding protein HU-beta | Bacteria;Acidobacteria;Vicinamibacteria;unclassified Vicinamibacteria;Vicinamibacteraceae;Luteitalea;Luteitalea pratensis;Luteitalea pratensis DSM 100886 |
| Ga0586324 0610780 670 978 | 4690000.00 | 0.00 | 0.00 | 0.00 | 0.00 | 0.00 | 0.00 | 0.00 | 0.00 | 0.00 | 0.00 | 0.00 | KO:K03530 | DNA-binding protein HU-beta | Bacteria;Proteobacteria;Alphaproteobacteria;Hyphomicrobiales;Hyphomicrobiaceae;Hyphomicrobium;Hyphomicrobium sp. 99;Hyphomicrobium sp. 99 |
| Ga0586324 0827137 658 945 | 0.00 | 0.00 | 0.00 | 0.00 | 0.00 | 0.00 | 0.00 | 0.00 | 0.00 | 0.00 | 2534400.00 | 0.00 | KO:K03530 | DNA-binding protein HU-beta | Bacteria;Nitrospirae;Nitrospira;Nitrospirales;Nitrospiraceae;Nitrospira;Candidatus Nitrospira inopinata;Candidatus Nitrospira inopinata ENR4 |
| Ga0586324 0876849 331 618 | 0.00 | 0.00 | 0.00 | 0.00 | 0.00 | 0.00 | 0.00 | 4312700.00 | 0.00 | 0.00 | 0.00 | 0.00 | KO:K03530 | DNA-binding protein HU-beta | Bacteria;Proteobacteria;Deltaproteobacteria;Myxococcales;Anaeromyxobacteraceae;Anaeromyxobacter;Anaeromyxobacter dehalogenans;Anaeromyxobacter dehalogenans 2CP-C |
| Ga0586324 0999817 211 519 | 0.00 | 0.00 | 0.00 | 1406000.00 | 0.00 | 973761.60 | 0.00 | 0.00 | 1117800.00 | 0.00 | 0.00 | 6570000.00 | KO:K03530 | DNA-binding protein HU-beta | Bacteria;Proteobacteria;Alphaproteobacteria;Hyphomicrobiales;Xanthobacteraceae;Pseudolabrys;Pseudolabrys taiwanensis;Pseudolabrys taiwanensis CC-BB4 |
| Ga0586324 1130445 737 1024 | 0.00 | 0.00 | 0.00 | 0.00 | 390609.99 | 0.00 | 0.00 | 155113.24 | 0.00 | 0.00 | 0.00 | 0.00 | KO:K03530 | DNA-binding protein HU-beta | Bacteria;Nitrospirae;Nitrospira;Nitrospirales;Nitrospiraceae;Nitrospira;Nitrospira lenta;Nitrospira lenta N. lenta BS10 |
| Ga0586324 1717853 283 582 | 0.00 | 0.00 | 0.00 | 8863500.00 | 0.00 | 0.00 | 0.00 | 0.00 | 0.00 | 0.00 | 0.00 | 0.00 | KO:K03530 | DNA-binding protein HU-beta | Bacteria;Acidobacteria;Acidobacteriia;Acidobacteriales;Acidobacteriaceae;Bryocella;Bryocella elongata;Bryocella elongata DSM 22489 |
| Ga0586324 1776165 367 663 | 2580000.00 | 0.00 | 0.00 | 0.00 | 0.00 | 595424.57 | 0.00 | 0.00 | 0.00 | 0.00 | 0.00 | 0.00 | KO:K03530 | DNA-binding protein HU-beta | Bacteria;Acidobacteria;Vicinamibacteria;unclassified Vicinamibacteria;Vicinamibacteraceae;Luteitalea;Luteitalea pratensis;Luteitalea pratensis DSM 100886 |
| Ga0586324 1909042 373 702 | 0.00 | 0.00 | 0.00 | 0.00 | 0.00 | 7146000.00 | 0.00 | 0.00 | 0.00 | 0.00 | 0.00 | 0.00 | KO:K03530 | DNA-binding protein HU-beta | Bacteria;Nitrospirae;Nitrospira;Nitrospirales;Nitrospiraceae;Nitrospira;Nitrospira marina;Nitrospira marina Nb-295 |
| Ga0586324 2120252 360 656 | 0.00 | 0.00 | 0.00 | 0.00 | 1026412.51 | 0.00 | 0.00 | 0.00 | 0.00 | 0.00 | 0.00 | 0.00 | KO:K03530 | DNA-binding protein HU-beta | Bacteria;Acidobacteria;Acidobacteriia;Acidobacteriales;Acidobacteriaceae;Candidatus Koribacter;Candidatus Koribacter versatilis;Candidatus Koribacter versatilis Ellin345 |
| Ga0586324 2489142 644 823 | 0.00 | 18156000.00 | 0.00 | 0.00 | 0.00 | 0.00 | 0.00 | 0.00 | 0.00 | 0.00 | 0.00 | 0.00 | KO:K03530 | DNA-binding protein HU-beta | Bacteria;Acidobacteria;Vicinamibacteria;unclassified Vicinamibacteria;Vicinamibacteraceae;Luteitalea;Luteitalea pratensis;Luteitalea pratensis DSM 100886 |
| Ga0586324 2509125 117 416 | 0.00 | 0.00 | 0.00 | 0.00 | 0.00 | 0.00 | 0.00 | 0.00 | 0.00 | 0.00 | 780714.00 | 0.00 | KO:K03530 | DNA-binding protein HU-beta | Bacteria;Acidobacteria;Acidobacteriia;Acidobacteriales;Acidobacteriaceae;Candidatus Koribacter;Candidatus Koribacter versatilis;Candidatus Koribacter versatilis Ellin345 |
| Ga0586324 2603374 507 809 | 1910000.00 | 0.00 | 0.00 | 0.00 | 0.00 | 1022711.70 | 0.00 | 0.00 | 0.00 | 0.00 | 0.00 | 0.00 | KO:K03530 | DNA-binding protein HU-beta | Bacteria;Acidobacteria;Vicinamibacteria;unclassified Vicinamibacteria;Vicinamibacteraceae;Luteitalea;Luteitalea pratensis;Luteitalea pratensis DSM 100886 |
| Ga0586324 2842562 120 428 | 0.00 | 0.00 | 0.00 | 0.00 | 7014300.00 | 5161000.00 | 0.00 | 14658300.00 | 0.00 | 0.00 | 0.00 | 0.00 | KO:K03530 | DNA-binding protein HU-beta | Bacteria;Acidobacteria;Blastocatellia;Blastocatellales;Pyrinomonadaceae;Pyrinomonas;Pyrinomonas methylaliphatogenes;Pyrinomonas methylaliphatogenes K22 |
| Ga0586324 3019090 381 695 | 0.00 | 0.00 | 11654100.00 | 0.00 | 0.00 | 0.00 | 0.00 | 0.00 | 0.00 | 0.00 | 0.00 | 0.00 | KO:K03530 | DNA-binding protein HU-beta | Bacteria;Proteobacteria;Deltaproteobacteria;Myxococcales;Labilitrichaceae;Labilithrix;Labilithrix luteola;Labilithrix luteola DSM 27648 |
| Ga0586324 3595290 295 594 | 0.00 | 0.00 | 0.00 | 1928500.00 | 2873700.00 | 0.00 | 0.00 | 1891000.00 | 1255500.00 | 0.00 | 0.00 | 0.00 | KO:K03530 | DNA-binding protein HU-beta | Bacteria;Acidobacteria;Vicinamibacteria;unclassified Vicinamibacteria;Vicinamibacteraceae;Luteitalea;Luteitalea pratensis;Luteitalea pratensis DSM 100886 |
| Ga0586324 4979010 2 268 | 1570000.00 | 0.00 | 0.00 | 8170000.00 | 2678000.00 | 1392668.06 | 0.00 | 1274900.00 | 1701000.00 | 0.00 | 0.00 | 0.00 | KO:K03530 | DNA-binding protein HU-beta | Bacteria;Acidobacteria;Vicinamibacteria;unclassified Vicinamibacteria;Vicinamibacteraceae;Luteitalea;Luteitalea pratensis;Luteitalea pratensis DSM 100886 |
| Ga0586324 7041576 129 428 | 1080000.00 | 0.00 | 0.00 | 0.00 | 516766.45 | 1310223.07 | 10693200.00 | 537544.81 | 700854.12 | 0.00 | 0.00 | 0.00 | KO:K03530 | DNA-binding protein HU-beta | Bacteria;Acidobacteria;Vicinamibacteria;unclassified Vicinamibacteria;Vicinamibacteraceae;Luteitalea;Luteitalea pratensis;Luteitalea pratensis DSM 100886 |
| Ga0586324 7201329 237 530 | 0.00 | 2652000.00 | 0.00 | 0.00 | 0.00 | 0.00 | 0.00 | 0.00 | 0.00 | 0.00 | 0.00 | 0.00 | KO:K03530 | DNA-binding protein HU-beta | Bacteria;Proteobacteria;Deltaproteobacteria;Syntrophorhabdales;Syntrophorhabdaceae;Syntrophorhabdus;Syntrophorhabdus aromaticivorans;Syntrophorhabdus aromaticivorans UI |
| Ga0586324 0002388 13112 14203 | 0.00 | 4311200.00 | 0.00 | 0.00 | 0.00 | 0.00 | 0.00 | 0.00 | 0.00 | 0.00 | 0.00 | 0.00 | KO:K03531 | cell division protein FtsZ | Archaea;Euryarchaeota;Methanomicrobia;Methanomicrobiales;Methanoregulaceae;Methanoregula;Methanoregula boonei;Methanoregula boonei 6A8 |
| Ga0586324 0022020 3 1781 | 0.00 | 0.00 | 0.00 | 0.00 | 0.00 | 0.00 | 0.00 | 0.00 | 0.00 | 0.00 | 959200.00 | 0.00 | KO:K03531 | cell division protein FtsZ | Bacteria;Proteobacteria;Alphaproteobacteria;Hyphomicrobiales;Hyphomicrobiaceae;Rhodoplanes;Rhodoplanes sp. Z2-YC6860;Rhodoplanes sp. Z2-YC6860 |
| Ga0586324 0236545 219 1436 | 0.00 | 0.00 | 0.00 | 0.00 | 1442000.00 | 0.00 | 0.00 | 0.00 | 0.00 | 0.00 | 1900800.00 | 0.00 | KO:K03531 | cell division protein FtsZ | Bacteria;Firmicutes;Clostridia;Eubacteriales;Peptococcaceae;Desulfallas;Desulfallas thermosapovorans;Desulfallas thermosapovorans DSM 6562 |
| Ga0586324 0527912 674 1600 | 0.00 | 0.00 | 0.00 | 0.00 | 0.00 | 0.00 | 0.00 | 0.00 | 1012500.00 | 0.00 | 0.00 | 0.00 | KO:K03531 | cell division protein FtsZ | Archaea;Euryarchaeota;Methanomicrobia;Methanomicrobiales;Methanoregulaceae;Methanoregula;Methanoregula boonei;Methanoregula boonei 6A8 |
| Ga0586324 1072379 402 1178 | 0.00 | 2883200.00 | 0.00 | 0.00 | 0.00 | 0.00 | 0.00 | 0.00 | 0.00 | 0.00 | 0.00 | 0.00 | KO:K03531 | cell division protein FtsZ | Bacteria;Firmicutes;Clostridia;Thermoanaerobacterales;Thermoanaerobacteraceae;Thermodesulfitimonas;Thermodesulfitimonas autotrophica;Thermodesulfitimonas autotrophica DSM 102936 |
| Ga0586324 1958013 115 912 | 0.00 | 0.00 | 0.00 | 0.00 | 0.00 | 0.00 | 0.00 | 0.00 | 0.00 | 2032800.00 | 0.00 | 0.00 | KO:K03531 | cell division protein FtsZ | Bacteria;Chloroflexi;Ktedonobacteria;Ktedonobacterales;Dictyobacteraceae;Dictyobacter;Dictyobacter aurantiacus;Dictyobacter aurantiacus S27 |
| Ga0586324 0000137 35427 36695 | 0.00 | 0.00 | 0.00 | 0.00 | 0.00 | 0.00 | 0.00 | 0.00 | 0.00 | 0.00 | 1399200.00 | 0.00 | KO:K03544 | ATP-dependent Clp protease ATP-binding subunit ClpX | Bacteria;Proteobacteria;Betaproteobacteria;Nitrosomonadales;Thiobacillaceae;Annwoodia;Annwoodia aquaesulis;Annwoodia aquaesulis DSM 4255 |
| Ga0586324 0036515 80 1315 | 0.00 | 0.00 | 0.00 | 0.00 | 0.00 | 0.00 | 0.00 | 0.00 | 16362000.00 | 0.00 | 0.00 | 0.00 | KO:K03544 | ATP-dependent Clp protease ATP-binding subunit ClpX | Bacteria;Nitrospirae;Nitrospira;Nitrospirales;Nitrospiraceae;Nitrospira;Nitrospira defluvii;Nitrospira defluvii |
| Ga0586324 0048196 560 1828 | 0.00 | 10132000.00 | 0.00 | 0.00 | 0.00 | 0.00 | 0.00 | 0.00 | 0.00 | 0.00 | 0.00 | 0.00 | KO:K03544 | ATP-dependent Clp protease ATP-binding subunit ClpX | Bacteria;Proteobacteria;Betaproteobacteria;Nitrosomonadales;Methylophilaceae;Methylobacillus;Methylobacillus sp. MM3;Methylobacillus sp. MM2 |
| Ga0586324 0980937 3 1013 | 0.00 | 0.00 | 0.00 | 0.00 | 0.00 | 0.00 | 0.00 | 0.00 | 0.00 | 0.00 | 537218.00 | 0.00 | KO:K03544 | ATP-dependent Clp protease ATP-binding subunit ClpX | Bacteria;Actinobacteria;Actinomycetia;Streptomycetales;Streptomycetaceae;Streptomyces;Streptomyces thermoautotrophicus;Streptomyces thermoautotrophicus H1 |
| Ga0586324 1034843 45 1196 | 0.00 | 0.00 | 0.00 | 0.00 | 0.00 | 0.00 | 0.00 | 0.00 | 0.00 | 5082000.00 | 0.00 | 0.00 | KO:K03544 | ATP-dependent Clp protease ATP-binding subunit ClpX | Bacteria;Thermodesulfobacteria;Thermodesulfobacteria;Thermodesulfobacteriales;Thermodesulfobacteriaceae;Thermosulfuriphilus;Thermosulfuriphilus ammonigenes;Thermosulfuriphilus ammonigenes DSM 102941 |
| Ga0586324 3062471 3 602 | 0.00 | 0.00 | 0.00 | 0.00 | 0.00 | 0.00 | 3442800.00 | 0.00 | 0.00 | 0.00 | 0.00 | 0.00 | KO:K03544 | ATP-dependent Clp protease ATP-binding subunit ClpX | Bacteria;Proteobacteria;Deltaproteobacteria;Syntrophorhabdales;Syntrophorhabdaceae;Syntrophorhabdus;Syntrophorhabdus aromaticivorans;Syntrophorhabdus aromaticivorans UI |
| Ga0586324 3387301 3 725 | 0.00 | 0.00 | 0.00 | 0.00 | 0.00 | 5002200.00 | 0.00 | 0.00 | 0.00 | 0.00 | 0.00 | 0.00 | KO:K03544 | ATP-dependent Clp protease ATP-binding subunit ClpX | Bacteria;Bacteroidetes;Chitinophagia;Chitinophagales;Chitinophagaceae;Niastella;Niastella sp. CF465;Niastella sp. CF465 |
| Ga0586324 6556995 2 550 | 0.00 | 0.00 | 0.00 | 0.00 | 0.00 | 0.00 | 0.00 | 0.00 | 0.00 | 0.00 | 0.00 | 3037500.00 | KO:K03544 | ATP-dependent Clp protease ATP-binding subunit ClpX | Bacteria;Proteobacteria;Deltaproteobacteria;Desulfuromonadales;Geobacteraceae;Geobacter;Geobacter sp. DSM 9736;Geobacter sp. DSM 9736 |
| Ga0586324 7532543 2 520 | 0.00 | 0.00 | 16439600.00 | 0.00 | 0.00 | 1948702.29 | 0.00 | 0.00 | 0.00 | 0.00 | 0.00 | 0.00 | KO:K03544 | ATP-dependent Clp protease ATP-binding subunit ClpX | Bacteria;Proteobacteria;Deltaproteobacteria;Desulfobacterales;Desulfobulbaceae;Desulfopila;Desulfopila aestuarii;Desulfopila aestuarii DSM 18488 |
| Ga0586324 0010606 8431 9729 | 0.00 | 0.00 | 0.00 | 0.00 | 0.00 | 0.00 | 0.00 | 1104100.00 | 1077300.00 | 0.00 | 0.00 | 2167500.00 | KO:K03545 | trigger factor | Bacteria;Proteobacteria;Betaproteobacteria;Nitrosomonadales;Thiobacillaceae;Thiobacillus;Thiobacillus thioparus;Thiobacillus thioparus DSM 505 |
| Ga0586324 0413225 223 1524 | 0.00 | 0.00 | 0.00 | 0.00 | 0.00 | 0.00 | 0.00 | 0.00 | 0.00 | 2217600.00 | 0.00 | 2505000.00 | KO:K03545 | trigger factor | Bacteria;Proteobacteria;Betaproteobacteria;Nitrosomonadales;Gallionellaceae;Gallionella;Gallionella capsiferriformans;Gallionella capsiferriformans ES-2 |
| Ga0586324 5819185 1 579 | 0.00 | 0.00 | 0.00 | 0.00 | 0.00 | 0.00 | 0.00 | 0.00 | 511677.00 | 0.00 | 0.00 | 0.00 | KO:K03545 | trigger factor | Bacteria;Proteobacteria;Alphaproteobacteria;Hyphomicrobiales;Reyranellaceae;Reyranella;Reyranella massiliensis;Reyranella massiliensis 521, DSM 23428 |
| Ga0586324 0000014 59366 60475 | 0.00 | 0.00 | 0.00 | 0.00 | 0.00 | 0.00 | 0.00 | 0.00 | 2008800.00 | 5859700.00 | 4769600.00 | 0.00 | KO:K03553 | recombination protein RecA | Bacteria;Proteobacteria;Betaproteobacteria;Nitrosomonadales;Thiobacillaceae;Annwoodia;Annwoodia aquaesulis;Annwoodia aquaesulis DSM 4255 |
| Ga0586324 0002949 2468 3535 | 0.00 | 0.00 | 0.00 | 0.00 | 0.00 | 0.00 | 0.00 | 261427.70 | 0.00 | 0.00 | 0.00 | 0.00 | KO:K03553 | recombination protein RecA | Bacteria;Verrucomicrobia;Verrucomicrobiae;Verrucomicrobiales;Verrucomicrobia subdivision 3;Pedosphaera;Pedosphaera parvula;Pedosphaera parvula Ellin514 |
| Ga0586324 0043749 1788 2852 | 0.00 | 0.00 | 0.00 | 4959000.00 | 0.00 | 0.00 | 0.00 | 0.00 | 0.00 | 0.00 | 0.00 | 0.00 | KO:K03553 | recombination protein RecA | Bacteria;Ignavibacteriae;Ignavibacteria;Ignavibacteriales;Ignavibacteriaceae;Ignavibacterium;Ignavibacterium album;Ignavibacterium album Mat9-16, JCM 16511 |
| Ga0586324 0182688 335 1669 | 0.00 | 0.00 | 0.00 | 0.00 | 0.00 | 0.00 | 1817910.12 | 0.00 | 0.00 | 0.00 | 0.00 | 0.00 | KO:K03553 | recombination protein RecA | Bacteria;Proteobacteria;Deltaproteobacteria;Myxococcales;Labilitrichaceae;Labilithrix;Labilithrix luteola;Labilithrix luteola DSM 27648 |
| Ga0586324 0184112 449 1534 | 0.00 | 0.00 | 310820.48 | 0.00 | 0.00 | 0.00 | 0.00 | 0.00 | 0.00 | 0.00 | 0.00 | 0.00 | KO:K03553 | recombination protein RecA | Bacteria;Verrucomicrobia;Verrucomicrobiae;Verrucomicrobiales;Verrucomicrobia subdivision 3;Pedosphaera;Pedosphaera parvula;Pedosphaera parvula Ellin514 |
| Ga0586324 0217791 321 1358 | 0.00 | 474433.96 | 0.00 | 0.00 | 0.00 | 0.00 | 0.00 | 0.00 | 0.00 | 0.00 | 0.00 | 0.00 | KO:K03553 | recombination protein RecA | Bacteria;Chloroflexi;Dehalococcoidia;Dehalococcoidales;Dehalococcoidaceae;Dehalococcoides;Dehalococcoides mccartyi;Dehalococcoides mccartyi CBDB1 |
| Ga0586324 0227339 1231 2331 | 0.00 | 0.00 | 0.00 | 0.00 | 5623800.00 | 0.00 | 0.00 | 0.00 | 0.00 | 0.00 | 0.00 | 0.00 | KO:K03553 | recombination protein RecA | Bacteria;Proteobacteria;Deltaproteobacteria;Desulfuromonadales;Syntrophotaleaceae;Syntrophotalea;Syntrophotalea acetylenica;Pelobacter acetylenicus DSM 3247 |
| Ga0586324 0234758 688 1722 | 1780000.00 | 0.00 | 0.00 | 1776500.00 | 1452300.00 | 0.00 | 2576400.00 | 933300.00 | 1296000.00 | 0.00 | 0.00 | 2415000.00 | KO:K03553 | recombination protein RecA | Bacteria;Proteobacteria;Deltaproteobacteria;Syntrophobacterales;Syntrophaceae;Syntrophus;Syntrophus aciditrophicus;Syntrophus aciditrophicus SB |
| Ga0586324 0245548 85 1110 | 11100000.00 | 0.00 | 0.00 | 0.00 | 0.00 | 0.00 | 0.00 | 0.00 | 0.00 | 0.00 | 0.00 | 0.00 | KO:K03553 | recombination protein RecA | Bacteria;Proteobacteria;Deltaproteobacteria;Desulfuromonadales;Geobacteraceae;Geobacter;Geobacter pickeringii;Geobacter pickeringii G13 |
| Ga0586324 0250913 1 756 | 0.00 | 2216800.00 | 0.00 | 0.00 | 0.00 | 0.00 | 397577.28 | 0.00 | 2567700.00 | 3388000.00 | 3458400.00 | 0.00 | KO:K03553 | recombination protein RecA | Bacteria;Proteobacteria;Betaproteobacteria;Rhodocyclales;Azonexaceae;Azonexus;Azonexus hydrophilus;Azonexus hydrophilus ZS02 |
| Ga0586324 0257114 1076 2104 | 3860000.00 | 0.00 | 1882914.09 | 1909500.00 | 0.00 | 6431400.00 | 1600728.72 | 1958100.00 | 1182600.00 | 3026100.00 | 0.00 | 4957500.00 | KO:K03553 | recombination protein RecA | Bacteria;Proteobacteria;Betaproteobacteria;Rhodocyclales;Azonexaceae;Azonexus;Azonexus hydrophilus;Azonexus hydrophilus ZS02 |
| Ga0586324 0287493 1431 2096 | 393982.00 | 0.00 | 0.00 | 0.00 | 0.00 | 0.00 | 0.00 | 0.00 | 0.00 | 0.00 | 0.00 | 0.00 | KO:K03553 | recombination protein RecA | Bacteria;Proteobacteria;Deltaproteobacteria;Syntrophobacterales;Syntrophaceae;Desulfobacca;Desulfobacca acetoxidans;Desulfobacca acetoxidans ASRB2, DSM 11109 |
| Ga0586324 0337892 3 1100 | 0.00 | 0.00 | 0.00 | 0.00 | 0.00 | 7900300.00 | 0.00 | 0.00 | 0.00 | 0.00 | 0.00 | 0.00 | KO:K03553 | recombination protein RecA | Bacteria;Proteobacteria;Deltaproteobacteria;Desulfuromonadales;Desulfuromonadaceae;Desulfuromonas;Desulfuromonas sp. DDH964;Desulfuromonas sp. DDH964 |
| Ga0586324 0477497 531 1610 | 0.00 | 0.00 | 4831283.16 | 0.00 | 0.00 | 0.00 | 0.00 | 0.00 | 0.00 | 0.00 | 0.00 | 0.00 | KO:K03553 | recombination protein RecA | Bacteria;Firmicutes;Clostridia;Eubacteriales;Clostridiaceae;Alkaliphilus;Alkaliphilus pronyensis;Alkaliphilus sp. LacV |
| Ga0586324 0714473 22 1119 | 0.00 | 0.00 | 0.00 | 0.00 | 0.00 | 0.00 | 0.00 | 0.00 | 0.00 | 0.00 | 10032000.00 | 0.00 | KO:K03553 | recombination protein RecA | Bacteria;Actinobacteria;Actinomycetia;Micrococcales;Intrasporangiaceae;Ornithinibacter;Ornithinibacter aureus;Ornithinibacter aureus DSM 23364 |
| Ga0586324 0743996 619 1380 | 0.00 | 0.00 | 1961773.50 | 0.00 | 0.00 | 0.00 | 0.00 | 0.00 | 0.00 | 0.00 | 0.00 | 0.00 | KO:K03553 | recombination protein RecA | Bacteria;Proteobacteria;Deltaproteobacteria;Myxococcales;Labilitrichaceae;Labilithrix;Labilithrix luteola;Labilithrix luteola DSM 27648 |
| Ga0586324 0815187 752 1327 | 0.00 | 2264400.00 | 0.00 | 0.00 | 0.00 | 0.00 | 0.00 | 0.00 | 0.00 | 0.00 | 0.00 | 0.00 | KO:K03553 | recombination protein RecA | Bacteria;Proteobacteria;Deltaproteobacteria;Syntrophobacterales;Syntrophaceae;Smithella;Smithella sp. SCADC;Smithella sp. SCADC |
| Ga0586324 1113749 2 1045 | 0.00 | 0.00 | 0.00 | 0.00 | 0.00 | 0.00 | 11764800.00 | 0.00 | 0.00 | 0.00 | 0.00 | 0.00 | KO:K03553 | recombination protein RecA | Bacteria;Proteobacteria;Deltaproteobacteria;Desulfuromonadales;Syntrophotaleaceae;Syntrophotalea;Syntrophotalea acetylenica;Pelobacter acetylenicus DSM 3247 |
| Ga0586324 1170839 272 1135 | 0.00 | 0.00 | 21112500.00 | 0.00 | 0.00 | 57168000.00 | 0.00 | 0.00 | 0.00 | 0.00 | 0.00 | 0.00 | KO:K03553 | recombination protein RecA | Bacteria;Firmicutes;Clostridia;Eubacteriales;Thermohalobacteraceae;Thermohalobacter;Thermohalobacter berrensis;Thermohalobacter berrensis CTT3 |
| Ga0586324 1244973 475 1104 | 0.00 | 0.00 | 0.00 | 0.00 | 0.00 | 0.00 | 0.00 | 0.00 | 0.00 | 2464000.00 | 0.00 | 0.00 | KO:K03553 | recombination protein RecA | Bacteria;Firmicutes;Clostridia;Eubacteriales;Clostridiaceae;Clostridium;Clostridium sp. CT4;Clostridium sp. CT4 |
| Ga0586324 1815020 3 512 | 0.00 | 0.00 | 0.00 | 0.00 | 0.00 | 3381137.84 | 0.00 | 0.00 | 0.00 | 0.00 | 0.00 | 0.00 | KO:K03553 | recombination protein RecA | Bacteria;Proteobacteria;Betaproteobacteria;Rhodocyclales;Azonexaceae;Azonexus;Azonexus fungiphilus;Azonexus fungiphilus DSM 23841 |
| Ga0586324 1887944 3 824 | 0.00 | 10404000.00 | 0.00 | 0.00 | 0.00 | 0.00 | 0.00 | 0.00 | 0.00 | 0.00 | 0.00 | 10425000.00 | KO:K03553 | recombination protein RecA | Bacteria;Proteobacteria;Betaproteobacteria;Nitrosomonadales;Gallionellaceae;Sideroxydans;Sideroxydans lithotrophicus;Sideroxydans lithotrophicus ES-1 |
| Ga0586324 2551207 2 319 | 0.00 | 0.00 | 0.00 | 0.00 | 0.00 | 0.00 | 7843200.00 | 0.00 | 0.00 | 0.00 | 0.00 | 0.00 | KO:K03553 | recombination protein RecA | Bacteria;Proteobacteria;Deltaproteobacteria;Syntrophorhabdales;Syntrophorhabdaceae;Syntrophorhabdus;Syntrophorhabdus aromaticivorans;Syntrophorhabdus aromaticivorans UI |
| Ga0586324 3093301 3 518 | 0.00 | 0.00 | 0.00 | 0.00 | 0.00 | 0.00 | 0.00 | 0.00 | 10044000.00 | 0.00 | 0.00 | 0.00 | KO:K03553 | recombination protein RecA | Bacteria;Spirochaetes;Spirochaetia;Spirochaetales;Spirochaetaceae;Alkalispirochaeta;Alkalispirochaeta americana;Alkalispirochaeta americana ASpG1 |
| Ga0586324 3415984 1 723 | 0.00 | 487963.92 | 0.00 | 0.00 | 0.00 | 0.00 | 0.00 | 0.00 | 0.00 | 0.00 | 0.00 | 0.00 | KO:K03553 | recombination protein RecA | Bacteria;Acidobacteria;Acidobacteriia;Acidobacteriales;Acidobacteriaceae;Candidatus Koribacter;Candidatus Koribacter versatilis;Candidatus Koribacter versatilis Ellin345 |
| Ga0586324 4274426 2 658 | 0.00 | 0.00 | 0.00 | 0.00 | 0.00 | 4049400.00 | 0.00 | 0.00 | 0.00 | 0.00 | 0.00 | 0.00 | KO:K03553 | recombination protein RecA | Bacteria;Proteobacteria;Deltaproteobacteria;Desulfuromonadales;Desulfuromonadaceae;Desulfuromonas;Desulfuromonas acetexigens;Desulfuromonas acetexigens DSM 1397 |
| Ga0586324 4526023 3 401 | 0.00 | 0.00 | 0.00 | 0.00 | 0.00 | 0.00 | 0.00 | 596454.34 | 0.00 | 0.00 | 0.00 | 0.00 | KO:K03553 | recombination protein RecA | Bacteria;Proteobacteria;Betaproteobacteria;Burkholderiales;unclassified Burkholderiales;Methylibium;Methylibium sp. YR605;Methylibium sp. YR605 |
| Ga0586324 5266068 43 603 | 0.00 | 0.00 | 0.00 | 0.00 | 0.00 | 0.00 | 0.00 | 0.00 | 0.00 | 0.00 | 0.00 | 3262500.00 | KO:K03553 | recombination protein RecA | Bacteria;Proteobacteria;Deltaproteobacteria;Desulfuromonadales;Syntrophotaleaceae;Syntrophotalea;Syntrophotalea acetylenica;Pelobacter acetylenicus DSM 3247 |
| Ga0586324 6795281 2 544 | 0.00 | 0.00 | 0.00 | 1491500.00 | 0.00 | 0.00 | 0.00 | 0.00 | 0.00 | 0.00 | 0.00 | 0.00 | KO:K03553 | recombination protein RecA | Bacteria;Chloroflexi;Anaerolineae;Anaerolineales;Anaerolineaceae;Anaerolinea;Anaerolinea thermophila;Anaerolinea thermophila UNI-1 |
| Ga0586324 7902177 142 510 | 0.00 | 0.00 | 0.00 | 0.00 | 0.00 | 0.00 | 0.00 | 0.00 | 1490400.00 | 0.00 | 0.00 | 0.00 | KO:K03553 | recombination protein RecA | Bacteria;Proteobacteria;Deltaproteobacteria;Myxococcales;Nannocystaceae;Plesiocystis;Plesiocystis pacifica;Plesiocystis pacifica SIR-1 |
| Ga0586324 0001547 1350 2258 | 0.00 | 0.00 | 13455700.00 | 0.00 | 7539600.00 | 0.00 | 0.00 | 0.00 | 0.00 | 0.00 | 0.00 | 0.00 | KO:K03554 | recombination associated protein RdgC | Bacteria;Proteobacteria;Gammaproteobacteria;Methylococcales;Methylococcaceae;Methylobacter;Methylobacter tundripaludum;Methylobacter tundripaludum SV96 |
| Ga0586324 0066197 3 500 | 0.00 | 0.00 | 10528100.00 | 12635000.00 | 15656000.00 | 8416400.00 | 0.00 | 14213000.00 | 16767000.00 | 0.00 | 0.00 | 26100000.00 | KO:K03561 | biopolymer transport protein ExbB | Bacteria;Acidobacteria;Acidobacteriia;Acidobacteriales;Acidobacteriaceae;unclassified Acidobacteriaceae;Acidobacteriaceae bacterium KBS 96;Acidobacteriaceae bacterium KBS 96, DSM 24297 |
| Ga0586324 0535034 45 707 | 0.00 | 0.00 | 0.00 | 0.00 | 411197.63 | 0.00 | 0.00 | 0.00 | 0.00 | 0.00 | 0.00 | 0.00 | KO:K03561 | biopolymer transport protein ExbB | Bacteria;Acidobacteria;Vicinamibacteria;unclassified Vicinamibacteria;Vicinamibacteraceae;Luteitalea;Luteitalea pratensis;Luteitalea pratensis DSM 100886 |
| Ga0586324 0558049 2 1183 | 0.00 | 0.00 | 0.00 | 0.00 | 0.00 | 0.00 | 0.00 | 0.00 | 0.00 | 2256100.00 | 0.00 | 0.00 | KO:K03561 | biopolymer transport protein ExbB | Bacteria;Proteobacteria;Gammaproteobacteria;Nevskiales;Sinobacteraceae;Hydrocarboniphaga;Hydrocarboniphaga daqingensis;Hydrocarboniphaga daqingensis CGMCC 1.7049 |
| Ga0586324 6331835 155 559 | 0.00 | 0.00 | 0.00 | 19190000.00 | 22660000.00 | 0.00 | 0.00 | 11773000.00 | 0.00 | 15169000.00 | 10472000.00 | 6570000.00 | KO:K03561 | biopolymer transport protein ExbB | Bacteria;Acidobacteria;Acidobacteriia;Bryobacterales;Solibacteraceae;Candidatus Solibacter;Candidatus Solibacter usitatus;Candidatus Solibacter usitatus Ellin6076 |
| Ga0586324 7790552 274 513 | 0.00 | 0.00 | 0.00 | 11590000.00 | 0.00 | 0.00 | 0.00 | 0.00 | 0.00 | 0.00 | 0.00 | 0.00 | KO:K03561 | biopolymer transport protein ExbB | Bacteria;Acidobacteria;Acidobacteriia;Bryobacterales;Bryobacteraceae;Bryobacter;Bryobacter aggregatus;Bryobacter aggregatus DSM 18758 |
| Ga0586324 0244994 1279 1791 | 0.00 | 0.00 | 0.00 | 0.00 | 0.00 | 0.00 | 0.00 | 0.00 | 0.00 | 0.00 | 529061.28 | 0.00 | KO:K03564 | peroxiredoxin Q/BCP | Bacteria;Proteobacteria;Gammaproteobacteria;Methylococcales;Methylococcaceae;Methylovulum;Methylovulum miyakonense;Methylovulum miyakonense HT12 |
| Ga0586324 1313787 219 713 | 0.00 | 2590800.00 | 0.00 | 0.00 | 0.00 | 0.00 | 0.00 | 3281800.00 | 0.00 | 3103100.00 | 691535.68 | 0.00 | KO:K03564 | peroxiredoxin Q/BCP | Bacteria;Proteobacteria;Gammaproteobacteria;Methylococcales;Methylococcaceae;Methylobacter;Methylobacter tundripaludum;Methylobacter tundripaludum OWC-G53F |
| Ga0586324 0000090 49151 50632 | 0.00 | 0.00 | 0.00 | 0.00 | 0.00 | 0.00 | 0.00 | 0.00 | 0.00 | 2864400.00 | 1557600.00 | 0.00 | KO:K03568 | TldD protein | Bacteria;Proteobacteria;Betaproteobacteria;Nitrosomonadales;Thiobacillaceae;Annwoodia;Annwoodia aquaesulis;Annwoodia aquaesulis DSM 4255 |
| Ga0586324 0175289 104 1558 | 0.00 | 0.00 | 0.00 | 0.00 | 0.00 | 0.00 | 0.00 | 0.00 | 0.00 | 0.00 | 2499200.00 | 0.00 | KO:K03568 | TldD protein | Bacteria;Proteobacteria;Gammaproteobacteria;Chromatiales;Chromatiaceae;Imhoffiella;Imhoffiella purpurea;Imhoffiella purpurea AK35 |
| Ga0586324 0000033 52490 53533 | 0.00 | 0.00 | 0.00 | 0.00 | 0.00 | 0.00 | 0.00 | 0.00 | 3418200.00 | 0.00 | 0.00 | 0.00 | KO:K03569 | rod shape-determining protein MreB | Bacteria;Proteobacteria;Betaproteobacteria;Nitrosomonadales;Thiobacillaceae;Annwoodia;Annwoodia aquaesulis;Annwoodia aquaesulis DSM 4255 |
| Ga0586324 0028926 5327 6235 | 0.00 | 0.00 | 0.00 | 0.00 | 0.00 | 0.00 | 0.00 | 0.00 | 0.00 | 0.00 | 0.00 | 2490000.00 | KO:K03569 | rod shape-determining protein MreB | Bacteria;Proteobacteria;Betaproteobacteria;Nitrosomonadales;Gallionellaceae;Gallionella;Gallionella capsiferriformans;Gallionella capsiferriformans ES-2 |
| Ga0586324 0096959 166 1209 | 2230000.00 | 0.00 | 0.00 | 0.00 | 0.00 | 0.00 | 0.00 | 0.00 | 0.00 | 0.00 | 0.00 | 5700000.00 | KO:K03569 | rod shape-determining protein MreB | Bacteria;Proteobacteria;Betaproteobacteria;Burkholderiales;Comamonadaceae;Curvibacter;Curvibacter sp. PAE-UM;Curvibacter sp. PAE-UM |
| Ga0586324 0188058 1327 2364 | 0.00 | 2407200.00 | 0.00 | 0.00 | 0.00 | 0.00 | 0.00 | 0.00 | 0.00 | 0.00 | 0.00 | 0.00 | KO:K03569 | rod shape-determining protein MreB | Bacteria;Proteobacteria;Oligoflexia;Oligoflexales;Oligoflexaceae;Oligoflexus;Oligoflexus tunisiensis;Oligoflexus tunisiensis Shr3 |
| Ga0586324 0414434 1222 1779 | 0.00 | 0.00 | 0.00 | 1083000.00 | 1122700.00 | 1344559.60 | 0.00 | 1409100.00 | 0.00 | 1670900.00 | 1399200.00 | 1342500.00 | KO:K03569 | rod shape-determining protein MreB | Bacteria;Proteobacteria;Betaproteobacteria;Burkholderiales;Comamonadaceae;Caenimonas;Caenimonas koreensis;Caenimonas koreensis DSM 17982 |
| Ga0586324 0441175 1397 1732 | 0.00 | 0.00 | 0.00 | 0.00 | 0.00 | 0.00 | 0.00 | 0.00 | 0.00 | 2702700.00 | 0.00 | 0.00 | KO:K03569 | rod shape-determining protein MreB | Bacteria;Proteobacteria;Betaproteobacteria;unclassified Betaproteobacteria;unclassified Betaproteobacteria;unclassified Betaproteobacteria;uncultured beta proteobacterium;unclassified betaproteobacterium ENR4 |
| Ga0586324 0685171 562 1428 | 0.00 | 0.00 | 0.00 | 0.00 | 0.00 | 0.00 | 0.00 | 0.00 | 996300.00 | 0.00 | 0.00 | 0.00 | KO:K03569 | rod shape-determining protein MreB | Bacteria;Proteobacteria;Betaproteobacteria;Nitrosomonadales;Methylophilaceae;Novimethylophilus;Novimethylophilus kurashikiensis;Novimethylophilus kurashikiensis La2-4 |
| Ga0586324 1260071 745 1098 | 0.00 | 0.00 | 0.00 | 0.00 | 0.00 | 2069191.79 | 0.00 | 0.00 | 0.00 | 0.00 | 0.00 | 0.00 | KO:K03569 | rod shape-determining protein MreB | Bacteria;Proteobacteria;Deltaproteobacteria;Syntrophobacterales;Syntrophaceae;Desulfobacca;Desulfobacca acetoxidans;Desulfobacca acetoxidans ASRB2, DSM 11109 |
| Ga0586324 1732847 274 960 | 0.00 | 0.00 | 0.00 | 0.00 | 0.00 | 0.00 | 0.00 | 0.00 | 0.00 | 0.00 | 62128000.00 | 0.00 | KO:K03569 | rod shape-determining protein MreB | Bacteria;Proteobacteria;Betaproteobacteria;Rhodocyclales;Zoogloeaceae;Uliginosibacterium;Uliginosibacterium gangwonense;Uliginosibacterium gangwonense DSM 18521 |
| Ga0586324 3543644 3 710 | 0.00 | 0.00 | 0.00 | 0.00 | 0.00 | 0.00 | 0.00 | 1464000.00 | 0.00 | 0.00 | 0.00 | 0.00 | KO:K03569 | rod shape-determining protein MreB | Bacteria;Proteobacteria;Betaproteobacteria;Rhodocyclales;Rhodocyclaceae;Azospira;unclassified Azospira;Azospira sp. ZAP |
| Ga0586324 4004922 2 676 | 0.00 | 0.00 | 0.00 | 0.00 | 0.00 | 0.00 | 0.00 | 0.00 | 0.00 | 2202200.00 | 0.00 | 0.00 | KO:K03569 | rod shape-determining protein MreB | Bacteria;Proteobacteria;Oligoflexia;Oligoflexales;Pseudobacteriovoracaceae;Pseudobacteriovorax;Pseudobacteriovorax antillogorgiicola;Pseudobacteriovorax antillogorgiicola RKEM611 |
| Ga0586324 4291508 20 658 | 0.00 | 0.00 | 0.00 | 0.00 | 0.00 | 0.00 | 0.00 | 0.00 | 0.00 | 0.00 | 0.00 | 4042500.00 | KO:K03569 | rod shape-determining protein MreB | Bacteria;Proteobacteria;Deltaproteobacteria;Myxococcales;Anaeromyxobacteraceae;Anaeromyxobacter;Anaeromyxobacter dehalogenans;Anaeromyxobacter dehalogenans 2CP-C |
| Ga0586324 5964828 3 572 | 0.00 | 0.00 | 0.00 | 0.00 | 0.00 | 4367000.00 | 0.00 | 0.00 | 0.00 | 0.00 | 0.00 | 0.00 | KO:K03569 | rod shape-determining protein MreB | Bacteria;Acidobacteria;Blastocatellia;Blastocatellales;Pyrinomonadaceae;Pyrinomonas;Pyrinomonas methylaliphatogenes;Pyrinomonas methylaliphatogenes K22 |
| Ga0586324 1583889 2 772 | 0.00 | 0.00 | 0.00 | 0.00 | 0.00 | 0.00 | 0.00 | 0.00 | 0.00 | 0.00 | 0.00 | 675298.50 | KO:K03584 | DNA repair protein RecO (recombination protein O) | Bacteria;Proteobacteria;Alphaproteobacteria;Hyphomicrobiales;Roseiarcaceae;Roseiarcus;Roseiarcus fermentans;Roseiarcus fermentans DSM 24875 |
| Ga0586324 0075007 179 1321 | 0.00 | 0.00 | 0.00 | 0.00 | 0.00 | 0.00 | 0.00 | 0.00 | 0.00 | 0.00 | 0.00 | 362088.00 | KO:K03585 | membrane fusion protein (multidrug efflux system) | Bacteria;Proteobacteria;Betaproteobacteria;Nitrosomonadales;Sulfuricellaceae;Sulfuriferula;unclassified Sulfuriferula;Sulfuriferula sp. AH1 re-analysis |
| Ga0586324 0000123 10626 11978 | 0.00 | 0.00 | 0.00 | 0.00 | 0.00 | 0.00 | 0.00 | 0.00 | 0.00 | 0.00 | 709935.60 | 0.00 | KO:K03592 | PmbA protein | Bacteria;Proteobacteria;Betaproteobacteria;Nitrosomonadales;Thiobacillaceae;Annwoodia;Annwoodia aquaesulis;Annwoodia aquaesulis DSM 4255 |
| Ga0586324 0007318 9784 10869 | 0.00 | 0.00 | 0.00 | 0.00 | 0.00 | 0.00 | 0.00 | 376097.33 | 789020.19 | 4543000.00 | 8342400.00 | 0.00 | KO:K03593 | ATP-binding protein involved in chromosome partitioning | Bacteria;Proteobacteria;Betaproteobacteria;Nitrosomonadales;Thiobacillaceae;Annwoodia;Annwoodia aquaesulis;Annwoodia aquaesulis DSM 4255 |
| Ga0586324 0074543 1 945 | 0.00 | 0.00 | 0.00 | 0.00 | 9970400.00 | 11870300.00 | 0.00 | 0.00 | 0.00 | 0.00 | 0.00 | 0.00 | KO:K03593 | ATP-binding protein involved in chromosome partitioning | Bacteria;Proteobacteria;Deltaproteobacteria;unclassified Deltaproteobacteria;unclassified Deltaproteobacteria;unclassified Deltaproteobacteria;delta proteobacterium NaphS2;delta proteobacterium NaphS2 |
| Ga0586324 0152023 1230 2474 | 0.00 | 0.00 | 0.00 | 7514500.00 | 9651100.00 | 0.00 | 0.00 | 0.00 | 0.00 | 0.00 | 0.00 | 0.00 | KO:K03593 | ATP-binding protein involved in chromosome partitioning | Bacteria;Proteobacteria;Deltaproteobacteria;Syntrophobacterales;Syntrophobacteraceae;Desulfacinum;Desulfacinum hydrothermale;Desulfacinum hydrothermale DSM 13146 |
| Ga0586324 0173990 1578 2639 | 0.00 | 0.00 | 0.00 | 0.00 | 0.00 | 0.00 | 0.00 | 0.00 | 0.00 | 530477.64 | 0.00 | 0.00 | KO:K03593 | ATP-binding protein involved in chromosome partitioning | Bacteria;Proteobacteria;Betaproteobacteria;Nitrosomonadales;Thiobacillaceae;Thiobacillus;Thiobacillus denitrificans;Thiobacillus denitrificans DSM 12475 |
| Ga0586324 0296777 1 912 | 0.00 | 0.00 | 0.00 | 0.00 | 0.00 | 0.00 | 0.00 | 447538.70 | 519615.00 | 1601600.00 | 1909600.00 | 9225000.00 | KO:K03593 | ATP-binding protein involved in chromosome partitioning | Bacteria;Proteobacteria;Betaproteobacteria;Nitrosomonadales;Thiobacillaceae;Thiobacillus;Thiobacillus thioparus;Thiobacillus thioparus DSM 505 |
| Ga0586324 0478603 3 752 | 0.00 | 0.00 | 0.00 | 0.00 | 15347000.00 | 0.00 | 0.00 | 0.00 | 0.00 | 0.00 | 0.00 | 0.00 | KO:K03593 | ATP-binding protein involved in chromosome partitioning | Bacteria;Proteobacteria;Deltaproteobacteria;Syntrophobacterales;Syntrophobacteraceae;Desulfosoma;Desulfosoma caldarium;Desulfosoma caldarium DSM 22027 |
| Ga0586324 2754929 37 789 | 0.00 | 0.00 | 0.00 | 0.00 | 0.00 | 0.00 | 0.00 | 9943000.00 | 0.00 | 0.00 | 0.00 | 0.00 | KO:K03593 | ATP-binding protein involved in chromosome partitioning | Bacteria;Proteobacteria;Deltaproteobacteria;unclassified Deltaproteobacteria;unclassified Deltaproteobacteria;unclassified Deltaproteobacteria;delta proteobacterium NaphS2;delta proteobacterium NaphS2 |
| Ga0586324 0007498 611 1078 | 0.00 | 8636000.00 | 4049107.26 | 7647500.00 | 11948000.00 | 0.00 | 0.00 | 3159800.00 | 7038900.00 | 9009000.00 | 0.00 | 2370000.00 | KO:K03594 | bacterioferritin | Bacteria;Spirochaetes;Spirochaetia;Leptospirales;Leptospiraceae;Leptospira;Leptospira borgpetersenii;Leptospira borgpetersenii 56670 |
| Ga0586324 1231511 398 841 | 0.00 | 2040000.00 | 0.00 | 0.00 | 0.00 | 0.00 | 0.00 | 0.00 | 0.00 | 0.00 | 0.00 | 0.00 | KO:K03594 | bacterioferritin | Bacteria;Proteobacteria;Gammaproteobacteria;Methylococcales;Methylococcaceae;Methylosarcina;Methylosarcina fibrata;Methylosarcina fibrata AML-C10 |
| Ga0586324 4599065 61 639 | 0.00 | 0.00 | 0.00 | 0.00 | 0.00 | 0.00 | 0.00 | 0.00 | 0.00 | 0.00 | 0.00 | 4012500.00 | KO:K03594 | bacterioferritin | Bacteria;Dictyoglomi;Dictyoglomia;Dictyoglomales;Dictyoglomaceae;Dictyoglomus;Dictyoglomus sp.;Dictyoglomus sp. OB1-4 |
| Ga0586324 0561810 436 1557 | 0.00 | 4420000.00 | 0.00 | 0.00 | 0.00 | 0.00 | 0.00 | 0.00 | 0.00 | 0.00 | 0.00 | 0.00 | KO:K03596 | GTP-binding protein LepA | Bacteria;Ignavibacteriae;Ignavibacteria;Ignavibacteriales;Melioribacteraceae;Melioribacter;Melioribacter roseus;Melioribacter roseus P3M |
| Ga0586324 0000537 25051 26001 | 0.00 | 0.00 | 0.00 | 0.00 | 0.00 | 0.00 | 0.00 | 0.00 | 0.00 | 6221600.00 | 0.00 | 5400000.00 | KO:K03598 | sigma-E factor negative regulatory protein RseB | Bacteria;Proteobacteria;Betaproteobacteria;Nitrosomonadales;Thiobacillaceae;Annwoodia;Annwoodia aquaesulis;Annwoodia aquaesulis DSM 4255 |
| Ga0586324 0045094 4508 5029 | 0.00 | 0.00 | 0.00 | 0.00 | 0.00 | 0.00 | 0.00 | 0.00 | 0.00 | 2464000.00 | 0.00 | 0.00 | KO:K03599 | RNA polymerase-associated protein | Bacteria;Proteobacteria;Betaproteobacteria;Nitrosomonadales;Thiobacillaceae;Annwoodia;Annwoodia aquaesulis;Annwoodia aquaesulis DSM 4255 |
| Ga0586324 0402217 1023 1805 | 0.00 | 0.00 | 0.00 | 0.00 | 0.00 | 0.00 | 0.00 | 0.00 | 0.00 | 0.00 | 0.00 | 6202500.00 | KO:K03609 | septum site-determining protein MinD | Bacteria;Proteobacteria;Gammaproteobacteria;Methylococcales;Methylococcaceae;Methylobacter;Methylobacter tundripaludum;Methylobacter tundripaludum OWC-G53F |
| Ga0586324 0833848 722 1312 | 0.00 | 0.00 | 0.00 | 3391500.00 | 0.00 | 0.00 | 0.00 | 0.00 | 0.00 | 0.00 | 0.00 | 0.00 | KO:K03609 | septum site-determining protein MinD | Bacteria;Proteobacteria;Gammaproteobacteria;Methylococcales;Methylococcaceae;Methylobacter;Methylobacter tundripaludum;Methylobacter tundripaludum OWC-DMM |
| Ga0586324 2393099 2 640 | 0.00 | 0.00 | 0.00 | 0.00 | 0.00 | 0.00 | 0.00 | 0.00 | 1134000.00 | 0.00 | 0.00 | 0.00 | KO:K03609 | septum site-determining protein MinD | Bacteria;Proteobacteria;Gammaproteobacteria;Methylococcales;Methylococcaceae;Methylobacter;Methylobacter tundripaludum;Methylobacter tundripaludum OWC-G53F |
| Ga0586324 0075547 620 910 | 0.00 | 0.00 | 0.00 | 15295000.00 | 0.00 | 0.00 | 0.00 | 0.00 | 0.00 | 0.00 | 0.00 | 0.00 | KO:K03622 | DNA-binding protein | Archaea;Euryarchaeota;Thermococci;Thermococcales;Thermococcaceae;Thermococcus;Thermococcus sp. P6;Thermococcus sp. P6 |
| Ga0586324 1121786 41 349 | 0.00 | 0.00 | 0.00 | 0.00 | 0.00 | 0.00 | 18650400.00 | 0.00 | 0.00 | 0.00 | 0.00 | 0.00 | KO:K03622 | DNA-binding protein | Archaea;Euryarchaeota;Methanobacteria;Methanobacteriales;Methanobacteriaceae;Methanobrevibacter;Methanobrevibacter filiformis;Methanobrevibacter filiformis DSM11501 |
| Ga0586324 0000001 140395 141657 | 0.00 | 0.00 | 0.00 | 0.00 | 0.00 | 0.00 | 0.00 | 2464400.00 | 8424000.00 | 7854000.00 | 0.00 | 0.00 | KO:K03628 | transcription termination factor Rho | Bacteria;Proteobacteria;Betaproteobacteria;Nitrosomonadales;Thiobacillaceae;Annwoodia;Annwoodia aquaesulis;Annwoodia aquaesulis DSM 4255 |
| Ga0586324 0000117 20288 21553 | 0.00 | 0.00 | 0.00 | 0.00 | 0.00 | 0.00 | 0.00 | 0.00 | 0.00 | 12628000.00 | 0.00 | 0.00 | KO:K03628 | transcription termination factor Rho | Bacteria;Proteobacteria;Alphaproteobacteria;Hyphomicrobiales;Hyphomicrobiaceae;Rhodoplanes;Rhodoplanes sp. Z2-YC6860;Rhodoplanes sp. Z2-YC6860 |
| Ga0586324 0001539 7150 8403 | 0.00 | 952000.00 | 0.00 | 0.00 | 0.00 | 0.00 | 0.00 | 0.00 | 0.00 | 0.00 | 0.00 | 0.00 | KO:K03628 | transcription termination factor Rho | Bacteria;Ignavibacteriae;Ignavibacteria;Ignavibacteriales;Ignavibacteriaceae;Ignavibacterium;Ignavibacterium album;Ignavibacterium album Mat9-16, JCM 16511 |
| Ga0586324 0002601 6707 8215 | 0.00 | 3835200.00 | 0.00 | 459350.65 | 0.00 | 6749000.00 | 0.00 | 0.00 | 0.00 | 0.00 | 0.00 | 275490.75 | KO:K03628 | transcription termination factor Rho | Bacteria;Acidobacteria;Blastocatellia;Blastocatellales;Pyrinomonadaceae;Pyrinomonas;Pyrinomonas methylaliphatogenes;Pyrinomonas methylaliphatogenes K22 |
| Ga0586324 0007803 6152 7915 | 0.00 | 0.00 | 0.00 | 0.00 | 0.00 | 0.00 | 0.00 | 9882000.00 | 0.00 | 0.00 | 0.00 | 0.00 | KO:K03628 | transcription termination factor Rho | Bacteria;Spirochaetes;Spirochaetia;Spirochaetales;Spirochaetaceae;Salinispira;Salinispira pacifica;Salinispira pacifica L21-RPul-D2 |
| Ga0586324 0011394 9093 9632 | 0.00 | 0.00 | 0.00 | 7372000.00 | 0.00 | 0.00 | 0.00 | 0.00 | 0.00 | 0.00 | 0.00 | 0.00 | KO:K03628 | transcription termination factor Rho | Bacteria;Candidatus Aminicenantes;unclassified Candidatus Aminicenantes;unclassified Candidatus Aminicenantes;unclassified Candidatus Aminicenantes;unclassified Candidatus Aminicenantes;Aminicenantes bacterium SCGC AAA252-N07;Aminicenantes bacterium SCGC AAA252-N07 (contamination screened) |
| Ga0586324 0012630 7199 8458 | 0.00 | 0.00 | 24715700.00 | 6935000.00 | 0.00 | 0.00 | 0.00 | 0.00 | 0.00 | 0.00 | 25696000.00 | 7725000.00 | KO:K03628 | transcription termination factor Rho | Bacteria;Proteobacteria;Betaproteobacteria;Rhodocyclales;Zoogloeaceae;Azoarcus;Azoarcus sp. PA01;Azoarcus sp. PA01 |
| Ga0586324 0020160 5530 6777 | 0.00 | 0.00 | 0.00 | 0.00 | 3841900.00 | 4287600.00 | 4104000.00 | 2244800.00 | 2081700.00 | 0.00 | 0.00 | 0.00 | KO:K03628 | transcription termination factor Rho | Bacteria;Acidobacteria;Acidobacteriia;Acidobacteriales;Acidobacteriaceae;Acidipila;Acidipila rosea;Acidipila rosea DSM 103428 |
| Ga0586324 0020400 296 1774 | 1610000.00 | 0.00 | 0.00 | 0.00 | 0.00 | 12862800.00 | 0.00 | 0.00 | 11583000.00 | 0.00 | 0.00 | 0.00 | KO:K03628 | transcription termination factor Rho | Bacteria;Proteobacteria;Gammaproteobacteria;Nevskiales;Steroidobacteraceae;Povalibacter;Povalibacter uvarum;Povalibacter uvarum DSM 26723 |
| Ga0586324 0025392 5077 6324 | 5310000.00 | 829600.00 | 0.00 | 4189500.00 | 2863400.00 | 0.00 | 12175200.00 | 664900.00 | 2875500.00 | 0.00 | 1020800.00 | 325813.50 | KO:K03628 | transcription termination factor Rho | Bacteria;Proteobacteria;Deltaproteobacteria;Desulfobacterales;Desulfobacteraceae;Desulfatirhabdium;Desulfatirhabdium butyrativorans;Desulfatirhabdium butyrativorans DSM 18734 |
| Ga0586324 0028240 2221 3468 | 0.00 | 0.00 | 0.00 | 0.00 | 0.00 | 0.00 | 0.00 | 10675000.00 | 0.00 | 0.00 | 0.00 | 0.00 | KO:K03628 | transcription termination factor Rho | Bacteria;Proteobacteria;Deltaproteobacteria;Syntrophobacterales;Syntrophaceae;Syntrophus;Syntrophus aciditrophicus;Syntrophus aciditrophicus SB |
| Ga0586324 0084969 3 794 | 0.00 | 0.00 | 0.00 | 0.00 | 0.00 | 689199.94 | 0.00 | 0.00 | 0.00 | 0.00 | 0.00 | 0.00 | KO:K03628 | transcription termination factor Rho | Bacteria;Verrucomicrobia;Spartobacteria;unclassified Spartobacteria;unclassified Spartobacteria;Candidatus Xiphinematobacter;Candidatus Xiphinematobacter sp. Idaho Grape;Candidatus Xiphinematobacter sp. Idaho Grape |
| Ga0586324 0096018 1303 2619 | 0.00 | 0.00 | 0.00 | 0.00 | 0.00 | 0.00 | 0.00 | 0.00 | 2762100.00 | 7700000.00 | 0.00 | 5212500.00 | KO:K03628 | transcription termination factor Rho | Bacteria;Proteobacteria;Deltaproteobacteria;Syntrophobacterales;Syntrophobacteraceae;Syntrophobacter;Syntrophobacter fumaroxidans;Syntrophobacter fumaroxidans MPOB |
| Ga0586324 0125965 1558 3072 | 0.00 | 0.00 | 0.00 | 0.00 | 0.00 | 0.00 | 0.00 | 0.00 | 0.00 | 0.00 | 17688000.00 | 0.00 | KO:K03628 | transcription termination factor Rho | Bacteria;Acidobacteria;Acidobacteriia;Acidobacteriales;Acidobacteriaceae;unclassified Acidobacteriaceae;Acidobacteriaceae bacterium KBS 96;Acidobacteriaceae bacterium KBS 96, DSM 24297 |
| Ga0586324 0151327 1512 2819 | 0.00 | 0.00 | 0.00 | 0.00 | 0.00 | 0.00 | 0.00 | 2122800.00 | 0.00 | 0.00 | 0.00 | 0.00 | KO:K03628 | transcription termination factor Rho | Bacteria;Candidatus Omnitrophica;unclassified Candidatus Omnitrophica;unclassified Candidatus Omnitrophica;unclassified Candidatus Omnitrophica;unclassified Candidatus Omnitrophica;Omnitrophica bacterium SCGC AAA257-O07;Omnitrophica bacterium SCGC AAA257-O07 (contamination screened) |
| Ga0586324 0229354 616 1875 | 3750000.00 | 0.00 | 0.00 | 0.00 | 0.00 | 0.00 | 0.00 | 0.00 | 0.00 | 0.00 | 0.00 | 0.00 | KO:K03628 | transcription termination factor Rho | Bacteria;Proteobacteria;Betaproteobacteria;Nitrosomonadales;Sulfuricellaceae;Sulfurirhabdus;Sulfurirhabdus autotrophica;Sulfurirhabdus autotrophica DSM 100309 |
| Ga0586324 0271498 980 2149 | 0.00 | 0.00 | 0.00 | 176594.55 | 0.00 | 0.00 | 0.00 | 0.00 | 0.00 | 0.00 | 0.00 | 0.00 | KO:K03628 | transcription termination factor Rho | Bacteria;Proteobacteria;Gammaproteobacteria;Cellvibrionales;Halieaceae;Kineobactrum;Kineobactrum sediminis;Kineobactrum sediminis F02 |
| Ga0586324 0292501 57 1304 | 0.00 | 0.00 | 0.00 | 0.00 | 476940.47 | 0.00 | 0.00 | 0.00 | 0.00 | 0.00 | 0.00 | 952500.00 | KO:K03628 | transcription termination factor Rho | Bacteria;Proteobacteria;Deltaproteobacteria;Syntrophobacterales;Syntrophaceae;Desulfobacca;Desulfobacca acetoxidans;Desulfobacca acetoxidans ASRB2, DSM 11109 |
| Ga0586324 0370656 90 1340 | 0.00 | 0.00 | 3541754.18 | 0.00 | 0.00 | 0.00 | 0.00 | 0.00 | 0.00 | 0.00 | 0.00 | 0.00 | KO:K03628 | transcription termination factor Rho | Bacteria;Proteobacteria;Deltaproteobacteria;Desulfobacterales;Desulfobulbaceae;Desulfopila;Desulfopila sp. IMCC35004;Desulfopila sp. IMCC35004 |
| Ga0586324 0385139 3 1058 | 0.00 | 0.00 | 0.00 | 0.00 | 0.00 | 0.00 | 0.00 | 248002.21 | 0.00 | 0.00 | 0.00 | 632584.50 | KO:K03628 | transcription termination factor Rho | Bacteria;Proteobacteria;Deltaproteobacteria;Desulfobacterales;Desulfobacteraceae;Desulfatiglans;Desulfatiglans anilini;Desulfatiglans anilini DSM 4660 |
| Ga0586324 0404367 82 1629 | 0.00 | 0.00 | 334018.89 | 0.00 | 0.00 | 0.00 | 0.00 | 0.00 | 0.00 | 0.00 | 0.00 | 0.00 | KO:K03628 | transcription termination factor Rho | Bacteria;Verrucomicrobia;Verrucomicrobiae;Verrucomicrobiales;Verrucomicrobia subdivision 3;Pedosphaera;Pedosphaera parvula;Pedosphaera parvula Ellin514 |
| Ga0586324 0407528 1135 1794 | 0.00 | 15708000.00 | 0.00 | 0.00 | 0.00 | 0.00 | 0.00 | 0.00 | 0.00 | 0.00 | 0.00 | 0.00 | KO:K03628 | transcription termination factor Rho | Bacteria;Proteobacteria;Deltaproteobacteria;Syntrophobacterales;Syntrophaceae;Syntrophus;Syntrophus aciditrophicus;Syntrophus aciditrophicus SB |

| | | | | | | | | | | | | | | | |
|---|---|---|---|---|---|---|---|---|---|---|---|---|---|---|---|
| Ga0586324 0498213 3 1439 | 1930000.00 | 0.00 | 0.00 | 1415500.00 | 1720100.00 | 0.00 | 0.00 | 1769000.00 | 2203200.00 | 1755600.00 | 2173600.00 | 1267500.00 | KO:K03628 | transcription termination factor Rho | Bacteria;Spirochaetes;Spirochaetia;Spirochaetales;Treponemataceae;Treponema;Treponema caldarium;Treponema caldarium DSM 7334 |
| Ga0586324 0529161 31 1599 | 0.00 | 0.00 | 0.00 | 2156500.00 | 0.00 | 0.00 | 0.00 | 0.00 | 8210279.88 | 0.00 | 0.00 | 0.00 | KO:K03628 | transcription termination factor Rho | Bacteria;Spirochaetes;Spirochaetia;Spirochaetales;Spirochaetaceae;Sediminispirochaeta;Sediminispirochaeta bajacaliforniensis;Sediminispirochaeta bajacaliforniensis DSM 16054 |
| Ga0586324 0591387 1 1281 | 0.00 | 34612000.00 | 0.00 | 0.00 | 0.00 | 0.00 | 0.00 | 0.00 | 0.00 | 7059718.05 | 13024000.00 | 0.00 | KO:K03628 | transcription termination factor Rho | Bacteria;Proteobacteria;Gammaproteobacteria;Chromatiales;Woeseiaceae;Woeseia;Woeseia oceani;Woeseia oceani XK5 |
| Ga0586324 0623691 3 1151 | 0.00 | 296764.92 | 0.00 | 0.00 | 0.00 | 0.00 | 0.00 | 0.00 | 0.00 | 0.00 | 0.00 | 0.00 | KO:K03628 | transcription termination factor Rho | Bacteria;Bacteroidetes;Bacteroidia;Bacteroidales;Lentimicrobiaceae;Lentimicrobium;Lentimicrobium saccharophilum;Bacteroidales bacterium TBC1 |
| Ga0586324 0663161 937 1449 | 0.00 | 0.00 | 0.00 | 588617.15 | 0.00 | 0.00 | 0.00 | 0.00 | 0.00 | 0.00 | 0.00 | 0.00 | KO:K03628 | transcription termination factor Rho | Bacteria;Chloroflexi;Caldilineae;Caldilineales;Caldilineaceae;Caldilinea;Caldilinea aerophila;Caldilinea aerophila STL-6-O1, DSM 14535 |
| Ga0586324 0668712 2 751 | 0.00 | 0.00 | 0.00 | 0.00 | 0.00 | 0.00 | 0.00 | 0.00 | 104632.56 | 0.00 | 0.00 | 0.00 | KO:K03628 | transcription termination factor Rho | Bacteria;Proteobacteria;Betaproteobacteria;unclassified Betaproteobacteria;unclassified Betaproteobacteria;unclassified Betaproteobacteria;uncultured beta proteobacterium;unclassified betaproteobacterium ENR4 |
| Ga0586324 0699079 2 841 | 0.00 | 2060400.00 | 0.00 | 0.00 | 0.00 | 0.00 | 0.00 | 0.00 | 0.00 | 0.00 | 0.00 | 0.00 | KO:K03628 | transcription termination factor Rho | Bacteria;Acidobacteria;Thermoanaerobaculia;Thermoanaerobaculales;Thermoanaerobaculaceae;Thermoanaerobaculum;Thermoanaerobaculum aquaticum;Thermoanaerobaculum aquaticum MP-01 |
| Ga0586324 0800807 208 1335 | 0.00 | 1686400.00 | 0.00 | 0.00 | 0.00 | 0.00 | 0.00 | 262511.67 | 1701000.00 | 0.00 | 0.00 | 0.00 | KO:K03628 | transcription termination factor Rho | Bacteria;Proteobacteria;Deltaproteobacteria;Desulfobacterales;Desulfobacteraceae;Desulfatiglans;Desulfatiglans anilini;Desulfatiglans anilini DSM 4660 |
| Ga0586324 0812221 527 1327 | 0.00 | 0.00 | 2886123.79 | 5006500.00 | 0.00 | 0.00 | 0.00 | 1762900.00 | 939600.00 | 0.00 | 0.00 | 0.00 | KO:K03628 | transcription termination factor Rho | Bacteria;Proteobacteria;Deltaproteobacteria;Syntrophobacterales;Syntrophaceae;Syntrophus;Syntrophus aciditrophicus;Syntrophus aciditrophicus SB |
| Ga0586324 0874693 1 801 | 0.00 | 4569600.00 | 0.00 | 0.00 | 0.00 | 0.00 | 0.00 | 0.00 | 0.00 | 0.00 | 0.00 | 0.00 | KO:K03628 | transcription termination factor Rho | Bacteria;Planctomycetes;Planctomycetia;Gemmatales;Gemmataceae;Zavarzinella;Zavarzinella formosa;Zavarzinella formosa DSM 19928 |
| Ga0586324 0953964 474 1238 | 0.00 | 0.00 | 0.00 | 0.00 | 0.00 | 0.00 | 0.00 | 0.00 | 0.00 | 0.00 | 1381600.00 | 0.00 | KO:K03628 | transcription termination factor Rho | Bacteria;Acidobacteria;Acidobacteriia;Bryobacterales;Bryobacteraceae;Bryobacter;Bryobacter aggregatus;Bryobacter aggregatus DSM 18758 |
| Ga0586324 1021362 1 1002 | 0.00 | 0.00 | 0.00 | 0.00 | 7931000.00 | 0.00 | 0.00 | 0.00 | 0.00 | 0.00 | 0.00 | 0.00 | KO:K03628 | transcription termination factor Rho | Bacteria;Candidatus Aminicenantes;unclassified Candidatus Aminicenantes;unclassified Candidatus Aminicenantes;unclassified Candidatus Aminicenantes;unclassified Candidatus Aminicenantes;Aminicenantes bacterium SCGC AAA252-N07;Aminicenantes bacterium SCGC AAA252-N07 (contamination screened) |
| Ga0586324 1071377 171 1178 | 0.00 | 0.00 | 0.00 | 0.00 | 0.00 | 0.00 | 3100800.00 | 0.00 | 0.00 | 0.00 | 0.00 | 0.00 | KO:K03628 | transcription termination factor Rho | Bacteria;Chloroflexi;Thermomicrobia;Sphaerobacterales;Sphaerobacteraceae;Sphaerobacter;Sphaerobacter thermophilus;Sphaerobacter thermophilus 4ac11, DSM 20745 |
| Ga0586324 1223574 203 1114 | 0.00 | 0.00 | 0.00 | 0.00 | 0.00 | 0.00 | 0.00 | 0.00 | 0.00 | 0.00 | 2006400.00 | 0.00 | KO:K03628 | transcription termination factor Rho | Bacteria;Proteobacteria;Deltaproteobacteria;Desulfobacterales;Desulfobacteraceae;Desulfococcus;Desulfococcus multivorans;Desulfococcus multivorans DSM 2059 |
| Ga0586324 1333834 2 1042 | 6750000.00 | 0.00 | 0.00 | 0.00 | 0.00 | 0.00 | 0.00 | 0.00 | 0.00 | 0.00 | 0.00 | 0.00 | KO:K03628 | transcription termination factor Rho | Bacteria;Gemmatimonadetes;Longimicrobia;Longimicrobiales;Longimicrobiaceae;Longimicrobium;Longimicrobium terrae;Longimicrobium terrae CECT 8660 |
| Ga0586324 1342636 3 1070 | 13200000.00 | 0.00 | 0.00 | 9690000.00 | 0.00 | 0.00 | 0.00 | 0.00 | 0.00 | 0.00 | 0.00 | 0.00 | KO:K03628 | transcription termination factor Rho | Bacteria;Proteobacteria;Deltaproteobacteria;Syntrophobacterales;Syntrophaceae;Syntrophus;Syntrophus aciditrophicus;Syntrophus aciditrophicus SB |
| Ga0586324 1632540 27 983 | 0.00 | 0.00 | 0.00 | 0.00 | 0.00 | 0.00 | 0.00 | 0.00 | 0.00 | 0.00 | 4505600.00 | 0.00 | KO:K03628 | transcription termination factor Rho | Bacteria;Proteobacteria;Deltaproteobacteria;Syntrophorhabdales;Syntrophorhabdaceae;Syntrophorhabdus;Syntrophorhabdus aromaticivorans;Syntrophorhabdus aromaticivorans UI |
| Ga0586324 1698989 2 967 | 0.00 | 0.00 | 7206400.00 | 0.00 | 0.00 | 5915300.00 | 0.00 | 0.00 | 0.00 | 0.00 | 0.00 | 0.00 | KO:K03628 | transcription termination factor Rho | Bacteria;Proteobacteria;Deltaproteobacteria;Syntrophobacterales;Syntrophaceae;Syntrophus;Syntrophus aciditrophicus;Syntrophus aciditrophicus SB |
| Ga0586324 1731014 3 959 | 0.00 | 0.00 | 0.00 | 0.00 | 0.00 | 0.00 | 0.00 | 0.00 | 2357100.00 | 0.00 | 0.00 | 0.00 | KO:K03628 | transcription termination factor Rho | Bacteria;Verrucomicrobia;Verrucomicrobiae;Verrucomicrobiales;Verrucomicrobia subdivision 3;Pedosphaera;Pedosphaera parvula;Pedosphaera parvula Ellin514 |
| Ga0586324 1853001 2 934 | 2700000.00 | 0.00 | 0.00 | 1643500.00 | 0.00 | 0.00 | 0.00 | 0.00 | 0.00 | 0.00 | 0.00 | 0.00 | KO:K03628 | transcription termination factor Rho | Bacteria;Acidobacteria;Vicinamibacteria;unclassified Vicinamibacteria;Vicinamibacteraceae;Luteitalea;Luteitalea pratensis;Luteitalea pratensis DSM 100886 |
| Ga0586324 1942700 1 915 | 0.00 | 0.00 | 0.00 | 0.00 | 0.00 | 0.00 | 0.00 | 0.00 | 0.00 | 0.00 | 0.00 | 17700000.00 | KO:K03628 | transcription termination factor Rho | Bacteria;Proteobacteria;Deltaproteobacteria;Syntrophobacterales;Syntrophaceae;Desulfobacca;Desulfobacca acetoxidans;Desulfobacca acetoxidans ASRB2, DSM 11109 |
| Ga0586324 2000243 3 902 | 21300000.00 | 18428000.00 | 0.00 | 9053500.00 | 0.00 | 0.00 | 0.00 | 401387.32 | 0.00 | 0.00 | 0.00 | 4635000.00 | KO:K03628 | transcription termination factor Rho | Bacteria;Proteobacteria;Deltaproteobacteria;unclassified Deltaproteobacteria;unclassified Deltaproteobacteria;Dissulfuribacter;Dissulfuribacter thermophilus;Dissulfuribacter thermophilus S69 |
| Ga0586324 2378483 1 759 | 0.00 | 1496000.00 | 1323033.11 | 0.00 | 0.00 | 0.00 | 0.00 | 0.00 | 0.00 | 854700.00 | 1047200.00 | 0.00 | KO:K03628 | transcription termination factor Rho | Bacteria;Proteobacteria;Deltaproteobacteria;Desulfobacterales;Desulfobacteraceae;Desulfonema;Desulfonema limicola;Desulfonema limicola Jadebusen, DSM 2076 |
| Ga0586324 2509953 2 820 | 0.00 | 0.00 | 0.00 | 0.00 | 0.00 | 0.00 | 0.00 | 0.00 | 0.00 | 7238000.00 | 0.00 | 0.00 | KO:K03628 | transcription termination factor Rho | Bacteria;Verrucomicrobia;Verrucomicrobiae;Verrucomicrobiales;Verrucomicrobia subdivision 3;Pedosphaera;Pedosphaera parvula;Pedosphaera parvula Ellin514 |
| Ga0586324 2637317 140 805 | 0.00 | 0.00 | 0.00 | 0.00 | 0.00 | 0.00 | 0.00 | 0.00 | 2859300.00 | 0.00 | 0.00 | 0.00 | KO:K03628 | transcription termination factor Rho | Bacteria;Proteobacteria;Deltaproteobacteria;Desulfuromonadales;Desulfuromonadaceae;Desulfuromonas;Desulfuromonas thiophila;Desulfuromonas thiophila DSM 8987 |
| Ga0586324 2696847 1 795 | 0.00 | 0.00 | 11147400.00 | 0.00 | 0.00 | 0.00 | 0.00 | 0.00 | 0.00 | 0.00 | 0.00 | 0.00 | KO:K03628 | transcription termination factor Rho | Bacteria;Proteobacteria;Betaproteobacteria;Neisseriales;Chromobacteriaceae;Chromobacterium;Chromobacterium haemolyticum;Chromobacterium haemolyticum DSM 19808 |
| Ga0586324 2978979 3 650 | 0.00 | 0.00 | 0.00 | 0.00 | 4665900.00 | 0.00 | 0.00 | 12627000.00 | 0.00 | 0.00 | 0.00 | 0.00 | KO:K03628 | transcription termination factor Rho | Bacteria;Acidobacteria;Acidobacteriia;Acidobacteriales;Acidobacteriaceae;unclassified Acidobacteriaceae;Acidobacteriaceae bacterium KBS 96;Acidobacteriaceae bacterium KBS 96, DSM 24297 |
| Ga0586324 3768232 3 692 | 0.00 | 0.00 | 0.00 | 0.00 | 0.00 | 0.00 | 0.00 | 0.00 | 0.00 | 283586.38 | 0.00 | 0.00 | KO:K03628 | transcription termination factor Rho | Bacteria;Proteobacteria;Deltaproteobacteria;Syntrophobacterales;Syntrophaceae;Desulfobacca;Desulfobacca acetoxidans;Desulfobacca acetoxidans ASRB2, DSM 11109 |
| Ga0586324 3893138 3 683 | 1920000.00 | 0.00 | 0.00 | 0.00 | 0.00 | 0.00 | 0.00 | 0.00 | 0.00 | 0.00 | 0.00 | 0.00 | KO:K03628 | transcription termination factor Rho | Bacteria;Proteobacteria;Deltaproteobacteria;Desulfuromonadales;Geobacteraceae;Geobacter;Geobacter soli;Geobacter soli GSS01 |
| Ga0586324 4003203 1 675 | 0.00 | 0.00 | 0.00 | 0.00 | 0.00 | 0.00 | 0.00 | 0.00 | 0.00 | 0.00 | 11440000.00 | 0.00 | KO:K03628 | transcription termination factor Rho | Bacteria;Proteobacteria;Deltaproteobacteria;Syntrophobacterales;Syntrophobacteraceae;Syntrophobacter;Syntrophobacter fumaroxidans;Syntrophobacter fumaroxidans MPOB |
| Ga0586324 4553477 3 641 | 0.00 | 0.00 | 6756000.00 | 4303500.00 | 1884900.00 | 2923654.89 | 2197491.36 | 0.00 | 1522800.00 | 0.00 | 0.00 | 0.00 | KO:K03628 | transcription termination factor Rho | Bacteria;Acidobacteria;Blastocatellia;Blastocatellales;Pyrinomonadaceae;Pyrinomonas;Pyrinomonas methylaliphatogenes;Pyrinomonas methylaliphatogenes K22 |
| Ga0586324 4638229 21 635 | 0.00 | 3604000.00 | 0.00 | 0.00 | 0.00 | 0.00 | 0.00 | 0.00 | 0.00 | 0.00 | 0.00 | 0.00 | KO:K03628 | transcription termination factor Rho | Bacteria;Spirochaetes;Spirochaetia;Spirochaetales;Spirochaetaceae;Sediminispirochaeta;Sediminispirochaeta bajacaliforniensis;Sediminispirochaeta bajacaliforniensis DSM 16054 |
| Ga0586324 4719614 1 630 | 13400000.00 | 0.00 | 37495800.00 | 0.00 | 0.00 | 50022000.00 | 0.00 | 21777000.00 | 25920000.00 | 0.00 | 32296000.00 | 22575000.00 | KO:K03628 | transcription termination factor Rho | Bacteria;Chloroflexi;Chloroflexia;Chloroflexales;Chloroflexaceae;Chloroflexus;Chloroflexus aurantiacus;Chloroflexus aurantiacus J-10-fl |
| Ga0586324 5073820 3 614 | 8150000.00 | 0.00 | 0.00 | 0.00 | 0.00 | 0.00 | 0.00 | 0.00 | 0.00 | 0.00 | 0.00 | 0.00 | KO:K03628 | transcription termination factor Rho | Bacteria;Proteobacteria;Deltaproteobacteria;Desulfobacterales;Desulfobacteraceae;Desulfococcus;Desulfococcus multivorans;Desulfococcus multivorans DSM 2059 |
| Ga0586324 5296757 1 603 | 0.00 | 0.00 | 0.00 | 0.00 | 0.00 | 0.00 | 15549600.00 | 0.00 | 0.00 | 0.00 | 0.00 | 15300000.00 | KO:K03628 | transcription termination factor Rho | Bacteria;Proteobacteria;Deltaproteobacteria;Syntrophobacterales;Syntrophaceae;Desulfobacca;Desulfobacca acetoxidans;Desulfobacca acetoxidans ASRB2, DSM 11109 |
| Ga0586324 5657434 1 585 | 0.00 | 0.00 | 0.00 | 0.00 | 0.00 | 0.00 | 0.00 | 2446100.00 | 0.00 | 0.00 | 0.00 | 0.00 | KO:K03628 | transcription termination factor Rho | Bacteria;Verrucomicrobia;Verrucomicrobiae;Verrucomicrobiales;Verrucomicrobia subdivision 3;Pedosphaera;Pedosphaera parvula;Pedosphaera parvula Ellin514 |
| Ga0586324 6327739 3 560 | 0.00 | 0.00 | 0.00 | 0.00 | 0.00 | 0.00 | 0.00 | 3428200.00 | 4074300.00 | 0.00 | 0.00 | 0.00 | KO:K03628 | transcription termination factor Rho | Bacteria;Proteobacteria;Betaproteobacteria;Nitrosomonadales;Thiobacillaceae;Thiobacillus;Thiobacillus denitrificans;Thiobacillus denitrificans ATCC 25259 |
| Ga0586324 6353664 96 560 | 0.00 | 0.00 | 0.00 | 0.00 | 0.00 | 0.00 | 13474800.00 | 9394000.00 | 352891.89 | 0.00 | 0.00 | 11100000.00 | KO:K03628 | transcription termination factor Rho | Bacteria;Proteobacteria;Betaproteobacteria;Burkholderiales;Comamonadaceae;Curvibacter;Curvibacter delicatus;Curvibacter delicatus NBRC 14919 |
| Ga0586324 7213725 3 530 | 0.00 | 0.00 | 0.00 | 0.00 | 868723.63 | 956543.71 | 0.00 | 0.00 | 579495.06 | 0.00 | 0.00 | 0.00 | KO:K03628 | transcription termination factor Rho | Bacteria;Proteobacteria;Deltaproteobacteria;Syntrophobacterales;Syntrophaceae;Desulfobacca;Desulfobacca acetoxidans;Desulfobacca acetoxidans ASRB2, DSM 11109 |
| Ga0586324 7265169 35 529 | 0.00 | 0.00 | 0.00 | 0.00 | 0.00 | 0.00 | 7068000.00 | 0.00 | 0.00 | 0.00 | 0.00 | 0.00 | KO:K03628 | transcription termination factor Rho | Bacteria;Candidatus Aminicenantes;unclassified Candidatus Aminicenantes;unclassified Candidatus Aminicenantes;unclassified Candidatus Aminicenantes;unclassified Candidatus Aminicenantes;Aminicenantes bacterium SCGC AAA252-N07;Aminicenantes bacterium SCGC AAA252-N07 (contamination screened) |
| Ga0586324 7450882 1 411 | 0.00 | 0.00 | 0.00 | 0.00 | 0.00 | 0.00 | 0.00 | 0.00 | 0.00 | 0.00 | 3590400.00 | 0.00 | KO:K03628 | transcription termination factor Rho | Bacteria;Proteobacteria;Deltaproteobacteria;Syntrophobacterales;Syntrophaceae;Smithella;Smithella sp. SCADC;Smithella sp. SCADC |
| Ga0586324 0000007 13412 14023 | 0.00 | 0.00 | 0.00 | 0.00 | 0.00 | 0.00 | 0.00 | 0.00 | 0.00 | 0.00 | 0.00 | 680046.75 | KO:K03634 | outer membrane lipoprotein carrier protein | Bacteria;Proteobacteria;Betaproteobacteria;Nitrosomonadales;Thiobacillaceae;Annwoodia;Annwoodia aquaesulis;Annwoodia aquaesulis DSM 4255 |
| Ga0586324 0000007 37429 38478 | 0.00 | 0.00 | 0.00 | 0.00 | 0.00 | 0.00 | 0.00 | 0.00 | 0.00 | 0.00 | 4320800.00 | 3052500.00 | KO:K03640 | peptidoglycan-associated lipoprotein | Bacteria;Proteobacteria;Alphaproteobacteria;Magnetococcales;Magnetococcaceae;Magnetofaba;Magnetofaba australis;Magnetofaba australis IT-1 |
| Ga0586324 0000014 33858 34454 | 0.00 | 0.00 | 0.00 | 0.00 | 0.00 | 0.00 | 0.00 | 0.00 | 0.00 | 6029100.00 | 1293600.00 | 2715000.00 | KO:K03640 | peptidoglycan-associated lipoprotein | Bacteria;Proteobacteria;Betaproteobacteria;Nitrosomonadales;Thiobacillaceae;Annwoodia;Annwoodia aquaesulis;Annwoodia aquaesulis DSM 4255 |
| Ga0586324 0000018 45120 45743 | 0.00 | 0.00 | 0.00 | 0.00 | 0.00 | 0.00 | 1785349.44 | 4788500.00 | 0.00 | 3003000.00 | 3176800.00 | 3090000.00 | KO:K03640 | peptidoglycan-associated lipoprotein | Bacteria;Acidobacteria;Thermoanaerobaculia;Thermoanaerobaculales;Thermoanaerobaculaceae;Thermoanaerobaculum;Thermoanaerobaculum aquaticum;Thermoanaerobaculum aquaticum MP-01 |
| Ga0586324 0003359 133 747 | 0.00 | 0.00 | 0.00 | 0.00 | 0.00 | 0.00 | 0.00 | 2153300.00 | 0.00 | 0.00 | 0.00 | 0.00 | KO:K03640 | peptidoglycan-associated lipoprotein | Bacteria;Proteobacteria;Deltaproteobacteria;Desulfovibrionales;Desulfovibrionaceae;Desulfovibrio;Desulfovibrio aminophilus;Desulfovibrio aminophilus DSM 12254 |
| Ga0586324 0010725 7348 7983 | 0.00 | 0.00 | 6530800.00 | 21356000.00 | 16171000.00 | 7503300.00 | 11924400.00 | 9766100.00 | 3126151.26 | 7376600.00 | 5060000.00 | 4830000.00 | KO:K03640 | peptidoglycan-associated lipoprotein | Bacteria;Candidatus Aminicenantes;unclassified Candidatus Aminicenantes;unclassified Candidatus Aminicenantes;unclassified Candidatus Aminicenantes;unclassified Candidatus Aminicenantes;Aminicenantes bacterium SCGC AAA252-K06;Aminicenantes bacterium SCGC AAA252-K06 (contamination screened) |
| Ga0586324 0042117 4318 4959 | 0.00 | 1271600.00 | 0.00 | 0.00 | 1575900.00 | 0.00 | 0.00 | 0.00 | 0.00 | 0.00 | 0.00 | 0.00 | KO:K03640 | peptidoglycan-associated lipoprotein | Bacteria;Acidobacteria;Thermoanaerobaculia;Thermoanaerobaculales;Thermoanaerobaculaceae;Thermoanaerobaculum;Thermoanaerobaculum aquaticum;Thermoanaerobaculum aquaticum MP-01 |
| Ga0586324 0081632 3262 3777 | 0.00 | 0.00 | 0.00 | 0.00 | 4367200.00 | 0.00 | 0.00 | 0.00 | 0.00 | 0.00 | 0.00 | 0.00 | KO:K03640 | peptidoglycan-associated lipoprotein | Bacteria;Candidatus Aminicenantes;unclassified Candidatus Aminicenantes;unclassified Candidatus Aminicenantes;unclassified Candidatus Aminicenantes;unclassified Candidatus Aminicenantes;Aminicenantes bacterium SCGC AAA252-K06;Aminicenantes bacterium SCGC AAA252-K06 (contamination screened) |
| Ga0586324 0111645 223 723 | 0.00 | 13464000.00 | 0.00 | 22230000.00 | 0.00 | 0.00 | 0.00 | 8235000.00 | 0.00 | 0.00 | 0.00 | 7500000.00 | KO:K03640 | peptidoglycan-associated lipoprotein | Bacteria;Proteobacteria;Betaproteobacteria;Nitrosomonadales;Gallionellaceae;Gallionella;Gallionella capsiferriformans;Gallionella capsiferriformans ES-2 |
| Ga0586324 0128718 1307 1828 | 5690000.00 | 0.00 | 0.00 | 0.00 | 7704400.00 | 0.00 | 0.00 | 5538800.00 | 0.00 | 0.00 | 0.00 | 0.00 | KO:K03640 | peptidoglycan-associated lipoprotein | Bacteria;Proteobacteria;Deltaproteobacteria;Desulfobacterales;Desulfobacteraceae;Desulfatirhabdium;Desulfatirhabdium butyrativorans;Desulfatirhabdium butyrativorans DSM 18734 |
| Ga0586324 0135721 2171 2800 | 0.00 | 0.00 | 0.00 | 0.00 | 9661400.00 | 1212489.61 | 0.00 | 3092700.00 | 3564000.00 | 0.00 | 0.00 | 4027500.00 | KO:K03640 | peptidoglycan-associated lipoprotein | Bacteria;Acidobacteria;Vicinamibacteria;unclassified Vicinamibacteria;Vicinamibacteraceae;Luteitalea;Luteitalea pratensis;Luteitalea pratensis DSM 100886 |
| Ga0586324 0146844 362 910 | 0.00 | 0.00 | 0.00 | 871066.40 | 0.00 | 0.00 | 0.00 | 0.00 | 0.00 | 0.00 | 0.00 | 0.00 | KO:K03640 | peptidoglycan-associated lipoprotein | Bacteria;Candidatus Aminicenantes;unclassified Candidatus Aminicenantes;unclassified Candidatus Aminicenantes;unclassified Candidatus Aminicenantes;unclassified Candidatus Aminicenantes;Aminicenantes bacterium SCGC AAA255-E05;Aminicenantes bacterium SCGC AAA255-E05 (contamination screened) |
| Ga0586324 0218162 1 432 | 0.00 | 2856000.00 | 0.00 | 0.00 | 0.00 | 0.00 | 4674000.00 | 1817800.00 | 0.00 | 2002000.00 | 1346400.00 | 2872500.00 | KO:K03640 | peptidoglycan-associated lipoprotein | Bacteria;Spirochaetes;Spirochaetia;Spirochaetales;Spirochaetaceae;Alkalispirochaeta;Alkalispirochaeta odontotermitis;Alkalispirochaeta odontotermitis JC202 |
| Ga0586324 0263385 1013 1597 | 0.00 | 6630000.00 | 0.00 | 0.00 | 0.00 | 0.00 | 0.00 | 0.00 | 0.00 | 0.00 | 0.00 | 0.00 | KO:K03640 | peptidoglycan-associated lipoprotein | Bacteria;Proteobacteria;Deltaproteobacteria;Desulfuromonadales;Desulfuromonadaceae;Pelobacter;Pelobacter propionicus;Pelobacter propionicus DSM 2379 |
| Ga0586324 0284806 1412 1960 | 0.00 | 0.00 | 0.00 | 0.00 | 0.00 | 2293659.56 | 0.00 | 0.00 | 0.00 | 0.00 | 0.00 | 0.00 | KO:K03640 | peptidoglycan-associated lipoprotein | Bacteria;Candidatus Aminicenantes;unclassified Candidatus Aminicenantes;unclassified Candidatus Aminicenantes;unclassified Candidatus Aminicenantes;unclassified Candidatus Aminicenantes;Aminicenantes bacterium SCGC AAA255-E05;Aminicenantes bacterium SCGC AAA255-E05 (contamination screened) |
| Ga0586324 0491891 1006 1620 | 0.00 | 0.00 | 3617579.02 | 0.00 | 0.00 | 0.00 | 0.00 | 0.00 | 0.00 | 0.00 | 0.00 | 0.00 | KO:K03640 | peptidoglycan-associated lipoprotein | Bacteria;Acidobacteria;Thermoanaerobaculia;Thermoanaerobaculales;Thermoanaerobaculaceae;Thermoanaerobaculum;Thermoanaerobaculum aquaticum;Thermoanaerobaculum aquaticum MP-01 |
| Ga0586324 0942529 29 799 | 0.00 | 1033600.00 | 0.00 | 0.00 | 0.00 | 0.00 | 0.00 | 0.00 | 0.00 | 0.00 | 0.00 | 0.00 | KO:K03640 | peptidoglycan-associated lipoprotein | Bacteria;Acidobacteria;Acidobacteriia;Bryobacterales;Solibacteraceae;Candidatus Solibacter;Candidatus Solibacter usitatus;Candidatus Solibacter usitatus Ellin6076 |
| Ga0586324 2111835 1 579 | 0.00 | 0.00 | 0.00 | 0.00 | 0.00 | 0.00 | 0.00 | 0.00 | 2527200.00 | 0.00 | 0.00 | 0.00 | KO:K03640 | peptidoglycan-associated lipoprotein | Bacteria;Acidobacteria;Thermoanaerobaculia;Thermoanaerobaculales;Thermoanaerobaculaceae;Thermoanaerobaculum;Thermoanaerobaculum aquaticum;Thermoanaerobaculum aquaticum MP-01 |
| Ga0586324 2687251 46 687 | 0.00 | 0.00 | 0.00 | 2821500.00 | 0.00 | 0.00 | 2417251.44 | 1994700.00 | 1150200.00 | 0.00 | 0.00 | 1170000.00 | KO:K03640 | peptidoglycan-associated lipoprotein | Bacteria;Acidobacteria;Thermoanaerobaculia;Thermoanaerobaculales;Thermoanaerobaculaceae;Thermoanaerobaculum;Thermoanaerobaculum aquaticum;Thermoanaerobaculum aquaticum MP-01 |
| Ga0586324 3991914 1 480 | 0.00 | 5453600.00 | 0.00 | 3857000.00 | 0.00 | 0.00 | 0.00 | 0.00 | 0.00 | 0.00 | 0.00 | 0.00 | KO:K03640 | peptidoglycan-associated lipoprotein | Bacteria;Proteobacteria;Deltaproteobacteria;Desulfobacterales;Desulfobulbaceae;Desulfoprunum;Desulfoprunum benzoelyticum;Desulfoprunum benzoelyticum DSM 28570 |
| Ga0586324 5340972 3 377 | 0.00 | 5344800.00 | 0.00 | 0.00 | 0.00 | 0.00 | 0.00 | 0.00 | 0.00 | 0.00 | 0.00 | 0.00 | KO:K03640 | peptidoglycan-associated lipoprotein | Bacteria;Acidobacteria;Vicinamibacteria;unclassified Vicinamibacteria;Vicinamibacteraceae;Luteitalea;Luteitalea pratensis;Luteitalea pratensis DSM 100886 |
| Ga0586324 6658165 50 547 | 696316.00 | 348697.88 | 0.00 | 0.00 | 0.00 | 0.00 | 0.00 | 0.00 | 0.00 | 0.00 | 0.00 | 0.00 | KO:K03640 | peptidoglycan-associated lipoprotein | Bacteria;Acidobacteria;Acidobacteriia;Bryobacterales;Solibacteraceae;Candidatus Solibacter;Candidatus Solibacter usitatus;Candidatus Solibacter usitatus Ellin6076 |
| Ga0586324 7557939 172 519 | 431630.00 | 0.00 | 0.00 | 0.00 | 0.00 | 0.00 | 0.00 | 0.00 | 0.00 | 6136900.00 | 0.00 | 0.00 | KO:K03640 | peptidoglycan-associated lipoprotein | Bacteria;Acidobacteria;Acidobacteriia;Bryobacterales;Solibacteraceae;Candidatus Solibacter;Candidatus Solibacter usitatus;Candidatus Solibacter usitatus Ellin6076 |
| Ga0586324 0012675 7168 8187 | 1220000.00 | 0.00 | 0.00 | 0.00 | 0.00 | 0.00 | 0.00 | 0.00 | 0.00 | 0.00 | 0.00 | 0.00 | KO:K03651 | Icc protein | Bacteria;Proteobacteria;Gammaproteobacteria;Chromatiales;Chromatiaceae;Thiocapsa;Thiocapsa marina;Thiocapsa marina 5811, DSM 5653 |
| Ga0586324 0031225 2796 3815 | 3440000.00 | 0.00 | 0.00 | 0.00 | 0.00 | 0.00 | 0.00 | 0.00 | 0.00 | 0.00 | 0.00 | 0.00 | KO:K03651 | Icc protein | Bacteria;Proteobacteria;Gammaproteobacteria;Chromatiales;Chromatiaceae;Thiocapsa;Thiocapsa marina;Thiocapsa marina 5811, DSM 5653 |
| Ga0586324 2087096 2 886 | 0.00 | 0.00 | 0.00 | 0.00 | 0.00 | 0.00 | 0.00 | 0.00 | 0.00 | 0.00 | 3141600.00 | 0.00 | KO:K03655 | ATP-dependent DNA helicase RecG | Bacteria;Proteobacteria;Deltaproteobacteria;Syntrophorhabdales;Syntrophorhabdaceae;Syntrophorhabdus;Syntrophorhabdus aromaticivorans;Syntrophorhabdus aromaticivorans UI |
| Ga0586324 0000002 102210 102449 | 0.00 | 0.00 | 0.00 | 0.00 | 0.00 | 0.00 | 0.00 | 1842200.00 | 0.00 | 0.00 | 2235200.00 | 1155000.00 | KO:K03666 | host factor-I protein | Bacteria;Proteobacteria;Betaproteobacteria;Nitrosomonadales;Thiobacillaceae;Annwoodia;Annwoodia aquaesulis;Annwoodia aquaesulis DSM 4255 |
| Ga0586324 0161510 1893 2159 | 0.00 | 4508400.00 | 2667640.38 | 0.00 | 0.00 | 0.00 | 0.00 | 4105300.00 | 5135400.00 | 6645100.00 | 0.00 | 14850000.00 | KO:K03666 | host factor-I protein | Bacteria;Proteobacteria;Betaproteobacteria;Burkholderiales;Comamonadaceae;Alicycliphilus;Alicycliphilus denitrificans;Alicycliphilus denitrificans BC |
| Ga0586324 0177936 2027 2266 | 0.00 | 0.00 | 0.00 | 0.00 | 727889.67 | 0.00 | 0.00 | 0.00 | 3248100.00 | 777700.00 | 0.00 | 0.00 | KO:K03666 | host factor-I protein | Bacteria;Proteobacteria;Betaproteobacteria;unclassified Betaproteobacteria;unclassified Betaproteobacteria;unclassified Betaproteobacteria;Betaproteobacteria bacterium GR16-43;Betaproteobacteria bacterium GR16-43 |
| Ga0586324 1428553 745 996 | 0.00 | 0.00 | 0.00 | 0.00 | 0.00 | 0.00 | 0.00 | 0.00 | 175567.50 | 0.00 | 0.00 | 0.00 | KO:K03666 | host factor-I protein | Bacteria;Proteobacteria;Gammaproteobacteria;unclassified Gammaproteobacteria;unclassified Gammaproteobacteria;unclassified Gammaproteobacteria;Codakia orbicularis gill symbiont;Codakia orbicularis gill symbiont |
| Ga0586324 5284408 58 603 | 13200000.00 | 8280419.16 | 4633219.76 | 21793000.00 | 10718323.17 | 14689000.00 | 0.00 | 2433900.00 | 16686000.00 | 33325116.44 | 10864802.96 | 5400000.00 | KO:K03666 | host factor-I protein | Bacteria;Proteobacteria;Betaproteobacteria;Burkholderiales;Comamonadaceae;Acidovorax;Acidovorax sp. 56;Acidovorax sp. 56 |
| Ga0586324 0578699 1029 1538 | 0.00 | 11764000.00 | 0.00 | 0.00 | 0.00 | 0.00 | 0.00 | 0.00 | 0.00 | 0.00 | 0.00 | 0.00 | KO:K03667 | ATP-dependent HslUV protease ATP-binding subunit HslU | Bacteria;Proteobacteria;Deltaproteobacteria;Syntrophobacterales;Syntrophaceae;Syntrophus;Syntrophus gentianae;Syntrophus gentianae DSM 8423 |
| Ga0586324 1237719 613 1107 | 0.00 | 0.00 | 0.00 | 0.00 | 4511400.00 | 0.00 | 0.00 | 0.00 | 0.00 | 0.00 | 0.00 | 0.00 | KO:K03667 | ATP-dependent HslUV protease ATP-binding subunit HslU | Bacteria;Proteobacteria;Deltaproteobacteria;Syntrophobacterales;Syntrophaceae;Syntrophus;Syntrophus aciditrophicus;Syntrophus aciditrophicus SB |
| Ga0586324 1659100 1 891 | 0.00 | 0.00 | 0.00 | 0.00 | 0.00 | 0.00 | 0.00 | 0.00 | 0.00 | 0.00 | 0.00 | 264075.00 | KO:K03667 | ATP-dependent HslUV protease ATP-binding subunit HslU | Bacteria;Proteobacteria;Gammaproteobacteria;Nevskiales;Steroidobacteraceae;Povalibacter;Povalibacter uvarum;Povalibacter uvarum DSM 26723 |
| Ga0586324 2385056 3 482 | 35000000.00 | 0.00 | 0.00 | 0.00 | 0.00 | 0.00 | 0.00 | 0.00 | 0.00 | 0.00 | 0.00 | 0.00 | KO:K03667 | ATP-dependent HslUV protease ATP-binding subunit HslU | Bacteria;Acidobacteria;Acidobacteriia;Acidobacteriales;Acidobacteriaceae;Acidobacterium;Acidobacterium ailaaui;Acidobacterium ailaaui PMMR2 |
| Ga0586324 3273191 3 734 | 0.00 | 0.00 | 0.00 | 0.00 | 107836.88 | 0.00 | 0.00 | 0.00 | 0.00 | 0.00 | 0.00 | 0.00 | KO:K03667 | ATP-dependent HslUV protease ATP-binding subunit HslU | Bacteria;Proteobacteria;Deltaproteobacteria;Syntrophobacterales;Syntrophobacteraceae;Desulfacinum;Desulfacinum hydrothermale;Desulfacinum hydrothermale DSM 13146 |
| Ga0586324 5732731 267 584 | 0.00 | 0.00 | 0.00 | 0.00 | 0.00 | 0.00 | 42636000.00 | 0.00 | 0.00 | 0.00 | 0.00 | 0.00 | KO:K03667 | ATP-dependent HslUV protease ATP-binding subunit HslU | Bacteria;Proteobacteria;Gammaproteobacteria;unclassified Gammaproteobacteria;Candidatus Competibacteraceae;Candidatus Contendobacter;Candidatus Contendobacter odensis;Candidatus Contendobacter odensis Run B J11 |
| Ga0586324 0007893 9231 9563 | 0.00 | 0.00 | 0.00 | 5139500.00 | 3357800.00 | 0.00 | 3648000.00 | 8591980.54 | 3693600.00 | 0.00 | 0.00 | 0.00 | KO:K03671 | thioredoxin 1 | Bacteria;Proteobacteria;Deltaproteobacteria;Syntrophobacterales;Syntrophobacteraceae;Desulfoglaeba;Desulfoglaeba alkanexedens;Desulfoglaeba alkanexedens ALDC |
| Ga0586324 0124379 1559 1891 | 0.00 | 0.00 | 0.00 | 0.00 | 0.00 | 0.00 | 0.00 | 436235.40 | 0.00 | 0.00 | 0.00 | 0.00 | KO:K03671 | thioredoxin 1 | Bacteria;Proteobacteria;Deltaproteobacteria;Syntrophobacterales;Syntrophobacteraceae;Syntrophobacter;Syntrophobacter fumaroxidans;Syntrophobacter fumaroxidans MPOB |
| Ga0586324 2295030 69 401 | 0.00 | 0.00 | 0.00 | 20615000.00 | 0.00 | 0.00 | 2827200.00 | 0.00 | 9801000.00 | 0.00 | 0.00 | 0.00 | KO:K03671 | thioredoxin 1 | Bacteria;Proteobacteria;Deltaproteobacteria;Syntrophobacterales;Syntrophobacteraceae;Desulfoglaeba;Desulfoglaeba alkanexedens;Desulfoglaeba alkanexedens ALDC |
| Ga0586324 2340841 110 442 | 0.00 | 0.00 | 0.00 | 0.00 | 0.00 | 0.00 | 0.00 | 1860500.00 | 0.00 | 0.00 | 0.00 | 0.00 | KO:K03671 | thioredoxin 1 | Bacteria;Proteobacteria;Deltaproteobacteria;Syntrophobacterales;Syntrophobacteraceae;Syntrophobacter;Syntrophobacter fumaroxidans;Syntrophobacter fumaroxidans MPOB |
| Ga0586324 6366672 197 520 | 0.00 | 0.00 | 0.00 | 0.00 | 0.00 | 0.00 | 5973600.00 | 0.00 | 0.00 | 0.00 | 0.00 | 0.00 | KO:K03671 | thioredoxin 1 | Bacteria;Proteobacteria;Deltaproteobacteria;Syntrophobacterales;Syntrophaceae;Desulfobacca;Desulfobacca acetoxidans;Desulfobacca acetoxidans ASRB2, DSM 11109 |
| Ga0586324 0038953 2981 3631 | 0.00 | 0.00 | 0.00 | 0.00 | 0.00 | 0.00 | 2713200.00 | 6344000.00 | 5127300.00 | 6883800.00 | 6424000.00 | 3450000.00 | KO:K03673 | thiol:disulfide interchange protein DsbA | Bacteria;Proteobacteria;Betaproteobacteria;Nitrosomonadales;Thiobacillaceae;Thiobacillus;Thiobacillus thioparus;Thiobacillus thioparus DSM 505 |
| Ga0586324 0042512 161 841 | 0.00 | 0.00 | 0.00 | 0.00 | 0.00 | 0.00 | 0.00 | 0.00 | 3515400.00 | 3180100.00 | 0.00 | 0.00 | KO:K03673 | thiol:disulfide interchange protein DsbA | Bacteria;Proteobacteria;Betaproteobacteria;Nitrosomonadales;Gallionellaceae;Sideroxydans;Sideroxydans lithotrophicus;Sideroxydans lithotrophicus ES-1 |
| Ga0586324 0386472 1277 1837 | 0.00 | 0.00 | 0.00 | 0.00 | 0.00 | 0.00 | 0.00 | 0.00 | 509116.59 | 760097.80 | 667109.52 | 0.00 | KO:K03673 | thiol:disulfide interchange protein DsbA | Bacteria;Proteobacteria;Gammaproteobacteria;Acidiferrobacterales;Acidiferrobacteraceae;Sulfuricaulis;Sulfuricaulis limicola;Sulfuricaulis limicola HA5 |
| Ga0586324 2266647 583 858 | 5150000.00 | 0.00 | 0.00 | 0.00 | 0.00 | 0.00 | 0.00 | 0.00 | 0.00 | 0.00 | 0.00 | 0.00 | KO:K03673 | thiol:disulfide interchange protein DsbA | Bacteria;Proteobacteria;Gammaproteobacteria;Methylococcales;Methylococcaceae;Methylobacter;Methylobacter tundripaludum;Methylobacter tundripaludum 31/32 |
| Ga0586324 3234989 3 626 | 0.00 | 0.00 | 0.00 | 0.00 | 0.00 | 0.00 | 0.00 | 0.00 | 0.00 | 5544000.00 | 6160000.00 | 0.00 | KO:K03673 | thiol:disulfide interchange protein DsbA | Bacteria;Proteobacteria;Betaproteobacteria;Nitrosomonadales;Sulfuricellaceae;Sulfuriferula;Sulfuriferula multivorans;Sulfuriferula multivorans TTN |
| Ga0586324 0408912 1008 1628 | 15400000.00 | 0.00 | 0.00 | 0.00 | 0.00 | 0.00 | 0.00 | 0.00 | 0.00 | 0.00 | 0.00 | 0.00 | KO:K03687 | molecular chaperone GrpE | Bacteria;Proteobacteria;Deltaproteobacteria;Syntrophobacterales;Syntrophaceae;Desulfobacca;Desulfobacca acetoxidans;Desulfobacca acetoxidans ASRB2, DSM 11109 |
| Ga0586324 0845507 522 1055 | 0.00 | 0.00 | 0.00 | 0.00 | 0.00 | 0.00 | 0.00 | 0.00 | 0.00 | 0.00 | 0.00 | 2197500.00 | KO:K03687 | molecular chaperone GrpE | Bacteria;Proteobacteria;Betaproteobacteria;Nitrosomonadales;Methylophilaceae;Methylobacillus;Methylobacillus sp. MM3;Methylobacillus sp. MM2 |
| Ga0586324 0000007 19305 21560 | 0.00 | 0.00 | 0.00 | 0.00 | 0.00 | 0.00 | 4947600.00 | 149461.59 | 0.00 | 4312000.00 | 2983200.00 | 2036251.50 | KO:K03694 | ATP-dependent Clp protease ATP-binding subunit ClpA | Bacteria;Proteobacteria;Betaproteobacteria;Nitrosomonadales;Thiobacillaceae;Annwoodia;Annwoodia aquaesulis;Annwoodia aquaesulis DSM 4255 |
| Ga0586324 0001173 19852 22107 | 0.00 | 0.00 | 0.00 | 0.00 | 0.00 | 0.00 | 0.00 | 341531.07 | 625838.40 | 0.00 | 0.00 | 266929.50 | KO:K03694 | ATP-dependent Clp protease ATP-binding subunit ClpA | Bacteria;Proteobacteria;Betaproteobacteria;Nitrosomonadales;Thiobacillaceae;Thiobacillus;Thiobacillus thioparus;Thiobacillus thioparus DSM 505 |
| Ga0586324 0016630 4538 6817 | 0.00 | 1951600.00 | 0.00 | 0.00 | 0.00 | 0.00 | 0.00 | 0.00 | 0.00 | 0.00 | 0.00 | 0.00 | KO:K03694 | ATP-dependent Clp protease ATP-binding subunit ClpA | Bacteria;Proteobacteria;Betaproteobacteria;unclassified Betaproteobacteria;unclassified Betaproteobacteria;unclassified Betaproteobacteria;Betaproteobacteria bacterium GR16-43;Betaproteobacteria bacterium GR16-43 |
| Ga0586324 1669665 3 626 | 0.00 | 0.00 | 0.00 | 0.00 | 0.00 | 0.00 | 0.00 | 841800.00 | 0.00 | 0.00 | 0.00 | 0.00 | KO:K03694 | ATP-dependent Clp protease ATP-binding subunit ClpA | Bacteria;Proteobacteria;Betaproteobacteria;Rhodocyclales;Azonexaceae;Dechloromonas;Dechloromonas agitata;Dechloromonas agitata CKB |
| Ga0586324 0000169 33633 36227 | 0.00 | 0.00 | 0.00 | 0.00 | 0.00 | 0.00 | 0.00 | 0.00 | 0.00 | 16000600.00 | 0.00 | 5362500.00 | KO:K03695 | ATP-dependent Clp protease ATP-binding subunit ClpB | Bacteria;Proteobacteria;Betaproteobacteria;Nitrosomonadales;Thiobacillaceae;Annwoodia;Annwoodia aquaesulis;Annwoodia aquaesulis DSM 4255 |
| Ga0586324 0003363 10707 13370 | 0.00 | 0.00 | 0.00 | 0.00 | 0.00 | 0.00 | 0.00 | 0.00 | 899100.00 | 0.00 | 0.00 | 0.00 | KO:K03695 | ATP-dependent Clp protease ATP-binding subunit ClpB | Bacteria;Proteobacteria;Alphaproteobacteria;Hyphomicrobiales;Xanthobacteraceae;Pseudolabrys;Pseudolabrys taiwanensis;Pseudolabrys taiwanensis CC-BB4 |
| Ga0586324 0008491 1956 4559 | 0.00 | 8840000.00 | 0.00 | 0.00 | 0.00 | 0.00 | 0.00 | 0.00 | 0.00 | 0.00 | 0.00 | 0.00 | KO:K03695 | ATP-dependent Clp protease ATP-binding subunit ClpB | Bacteria;Proteobacteria;Deltaproteobacteria;unclassified Deltaproteobacteria;unclassified Deltaproteobacteria;Dissulfuribacter;Dissulfuribacter thermophilus;Dissulfuribacter thermophilus S69 |
| Ga0586324 0009695 4635 7229 | 0.00 | 0.00 | 0.00 | 0.00 | 0.00 | 0.00 | 0.00 | 0.00 | 2673000.00 | 0.00 | 0.00 | 0.00 | KO:K03695 | ATP-dependent Clp protease ATP-binding subunit ClpB | Bacteria;Proteobacteria;Deltaproteobacteria;Desulfobacterales;Desulfobacteraceae;Desulfatiglans;Desulfatiglans anilini;Desulfatiglans anilini DSM 4660 |
| Ga0586324 0011557 6407 8986 | 3050000.00 | 0.00 | 0.00 | 0.00 | 0.00 | 0.00 | 0.00 | 0.00 | 0.00 | 0.00 | 0.00 | 0.00 | KO:K03695 | ATP-dependent Clp protease ATP-binding subunit ClpB | Bacteria;Proteobacteria;Betaproteobacteria;Nitrosomonadales;Gallionellaceae;Gallionella;Gallionella capsiferriformans;Gallionella capsiferriformans ES-2 |
| Ga0586324 0017040 2929 5529 | 0.00 | 0.00 | 0.00 | 0.00 | 0.00 | 0.00 | 0.00 | 0.00 | 0.00 | 808500.00 | 0.00 | 0.00 | KO:K03695 | ATP-dependent Clp protease ATP-binding subunit ClpB | Bacteria;Proteobacteria;Alphaproteobacteria;Hyphomicrobiales;Xanthobacteraceae;Pseudolabrys;Pseudolabrys sp. Root1462;Pseudolabrys sp. Root1462 |
| Ga0586324 0031361 432 3026 | 0.00 | 0.00 | 0.00 | 0.00 | 0.00 | 0.00 | 1268883.84 | 11559500.00 | 539964.63 | 9009000.00 | 2332000.00 | 9975000.00 | KO:K03695 | ATP-dependent Clp protease ATP-binding subunit ClpB | Bacteria;Proteobacteria;Betaproteobacteria;Nitrosomonadales;Thiobacillaceae;Thiobacillus;Thiobacillus thioparus;Thiobacillus thioparus DSM 505 |
| Ga0586324 0046152 793 3462 | 0.00 | 0.00 | 0.00 | 0.00 | 0.00 | 0.00 | 0.00 | 0.00 | 0.00 | 0.00 | 0.00 | 499730.25 | KO:K03695 | ATP-dependent Clp protease ATP-binding subunit ClpB | Bacteria;Acidobacteria;Blastocatellia;Blastocatellales;Pyrinomonadaceae;Pyrinomonas;Pyrinomonas methylaliphatogenes;Pyrinomonas methylaliphatogenes K22 |
| Ga0586324 0049255 1013 3595 | 0.00 | 0.00 | 0.00 | 0.00 | 1884900.00 | 0.00 | 0.00 | 0.00 | 0.00 | 0.00 | 0.00 | 0.00 | KO:K03695 | ATP-dependent Clp protease ATP-binding subunit ClpB | Bacteria;Candidatus Aminicenantes;unclassified Candidatus Aminicenantes;unclassified Candidatus Aminicenantes;unclassified Candidatus Aminicenantes;unclassified Candidatus Aminicenantes;Aminicenantes bacterium SCGC AAA255-E05;Aminicenantes bacterium SCGC AAA255-E05 (contamination screened) |
| Ga0586324 0151276 1879 2817 | 0.00 | 0.00 | 0.00 | 0.00 | 0.00 | 0.00 | 0.00 | 0.00 | 526822.38 | 0.00 | 0.00 | 0.00 | KO:K03695 | ATP-dependent Clp protease ATP-binding subunit ClpB | Bacteria;Proteobacteria;Betaproteobacteria;Burkholderiales;Burkholderiaceae;Ralstonia;Ralstonia sp. NFACC01;Ralstonia sp. NFACC01 |
| Ga0586324 0155265 905 2785 | 14700000.00 | 0.00 | 0.00 | 0.00 | 0.00 | 0.00 | 0.00 | 0.00 | 14175000.00 | 0.00 | 0.00 | 0.00 | KO:K03695 | ATP-dependent Clp protease ATP-binding subunit ClpB | Bacteria;Proteobacteria;Deltaproteobacteria;Myxococcales;Anaeromyxobacteraceae;Anaeromyxobacter;Anaeromyxobacter sp. Fw109-5;Anaeromyxobacter sp. Fw109-5 |
| Ga0586324 0189788 2 2506 | 0.00 | 0.00 | 0.00 | 1121000.00 | 0.00 | 0.00 | 0.00 | 0.00 | 0.00 | 0.00 | 0.00 | 0.00 | KO:K03695 | ATP-dependent Clp protease ATP-binding subunit ClpB | Bacteria;Proteobacteria;Gammaproteobacteria;Nevskiales;Steroidobacteraceae;Povalibacter;Povalibacter uvarum;Povalibacter uvarum DSM 26723 |
| Ga0586324 0200434 265 2472 | 0.00 | 0.00 | 0.00 | 0.00 | 0.00 | 0.00 | 0.00 | 0.00 | 19602000.00 | 0.00 | 0.00 | 0.00 | KO:K03695 | ATP-dependent Clp protease ATP-binding subunit ClpB | Bacteria;Proteobacteria;Betaproteobacteria;Nitrosomonadales;Gallionellaceae;Gallionella;Gallionella capsiferriformans;Gallionella capsiferriformans ES-2 |
| Ga0586324 0236933 306 2288 | 2340000.00 | 2522800.00 | 0.00 | 7400500.00 | 0.00 | 0.00 | 1938706.80 | 1970300.00 | 0.00 | 0.00 | 0.00 | 0.00 | KO:K03695 | ATP-dependent Clp protease ATP-binding subunit ClpB | Bacteria;Proteobacteria;Alphaproteobacteria;Rhodospirillales;Acetobacteraceae;Rhodopila;Rhodopila globiformis;Rhodopila globiformis DSM 161 |
| Ga0586324 0244428 2 1411 | 0.00 | 0.00 | 0.00 | 0.00 | 0.00 | 0.00 | 0.00 | 0.00 | 535243.95 | 0.00 | 0.00 | 0.00 | KO:K03695 | ATP-dependent Clp protease ATP-binding subunit ClpB | Bacteria;Proteobacteria;Gammaproteobacteria;Chromatiales;Chromatiaceae;Marichromatium;Marichromatium sp. AB31;Marichromatium sp. AB31 |
| Ga0586324 0286505 267 2099 | 0.00 | 0.00 | 0.00 | 0.00 | 0.00 | 3664230.60 | 0.00 | 0.00 | 0.00 | 0.00 | 0.00 | 0.00 | KO:K03695 | ATP-dependent Clp protease ATP-binding subunit ClpB | Bacteria;Proteobacteria;Deltaproteobacteria;Desulfobacterales;Desulfobacteraceae;Desulfatirhabdium;Desulfatirhabdium butyrativorans;Desulfatirhabdium butyrativorans DSM 18734 |
| Ga0586324 0542323 51 1583 | 3000000.00 | 0.00 | 0.00 | 0.00 | 0.00 | 0.00 | 0.00 | 0.00 | 0.00 | 0.00 | 0.00 | 0.00 | KO:K03695 | ATP-dependent Clp protease ATP-binding subunit ClpB | Bacteria;Proteobacteria;Alphaproteobacteria;Hyphomicrobiales;Xanthobacteraceae;Pseudolabrys;Pseudolabrys taiwanensis;Pseudolabrys taiwanensis CC-BB4 |
| Ga0586324 0544786 3 1079 | 0.00 | 0.00 | 0.00 | 0.00 | 0.00 | 0.00 | 0.00 | 0.00 | 0.00 | 2818200.00 | 0.00 | 558291.75 | KO:K03695 | ATP-dependent Clp protease ATP-binding subunit ClpB | Bacteria;Proteobacteria;Gammaproteobacteria;Methylococcales;Methylococcaceae;Methylobacter;Methylobacter tundripaludum;Methylobacter tundripaludum 31/32 |
| Ga0586324 0755638 566 1369 | 0.00 | 0.00 | 0.00 | 0.00 | 0.00 | 0.00 | 0.00 | 7808000.00 | 0.00 | 0.00 | 10120000.00 | 832500.00 | KO:K03695 | ATP-dependent Clp protease ATP-binding subunit ClpB | Bacteria;Proteobacteria;Betaproteobacteria;Nitrosomonadales;Methylophilaceae;Novimethylophilus;Novimethylophilus kurashikiensis;Novimethylophilus kurashikiensis La2-4 |
| Ga0586324 0824439 1 993 | 0.00 | 0.00 | 0.00 | 0.00 | 0.00 | 0.00 | 0.00 | 0.00 | 0.00 | 1501500.00 | 0.00 | 0.00 | KO:K03695 | ATP-dependent Clp protease ATP-binding subunit ClpB | Bacteria;Proteobacteria;Deltaproteobacteria;Desulfobacterales;Desulfobacteraceae;Desulfatiglans;Desulfatiglans anilini;Desulfatiglans anilini DSM 4660 |
| Ga0586324 1046622 579 1190 | 0.00 | 0.00 | 0.00 | 1320500.00 | 0.00 | 0.00 | 0.00 | 0.00 | 0.00 | 0.00 | 0.00 | 0.00 | KO:K03695 | ATP-dependent Clp protease ATP-binding subunit ClpB | Bacteria;Proteobacteria;Gammaproteobacteria;Chromatiales;Ectothiorhodospiraceae;Thiogranum;Thiogranum longum;Thiogranum longum DSM 19610 |
| Ga0586324 1620016 3 989 | 1380000.00 | 0.00 | 0.00 | 0.00 | 0.00 | 0.00 | 0.00 | 0.00 | 0.00 | 0.00 | 0.00 | 0.00 | KO:K03695 | ATP-dependent Clp protease ATP-binding subunit ClpB | Bacteria;Proteobacteria;Gammaproteobacteria;Nevskiales;Steroidobacteraceae;Povalibacter;Povalibacter uvarum;Povalibacter uvarum DSM 26723 |
| Ga0586324 2026623 1 897 | 12900000.00 | 0.00 | 0.00 | 0.00 | 0.00 | 0.00 | 0.00 | 0.00 | 0.00 | 0.00 | 0.00 | 0.00 | KO:K03695 | ATP-dependent Clp protease ATP-binding subunit ClpB | Bacteria;Proteobacteria;Alphaproteobacteria;Hyphomicrobiales;Rhizobiaceae;Rhizobium;Rhizobium flavum;Rhizobium flavum CCTCC AB 2013042 |
| Ga0586324 2208549 2 865 | 0.00 | 0.00 | 0.00 | 0.00 | 0.00 | 0.00 | 0.00 | 1372500.00 | 0.00 | 0.00 | 0.00 | 0.00 | KO:K03695 | ATP-dependent Clp protease ATP-binding subunit ClpB | Bacteria;Proteobacteria;Betaproteobacteria;Nitrosomonadales;Thiobacillaceae;Thiobacillus;Thiobacillus thiophilus;Thiobacillus thiophilus DSM 19892 |
| Ga0586324 2394147 3 836 | 0.00 | 0.00 | 0.00 | 2954500.00 | 0.00 | 0.00 | 0.00 | 0.00 | 0.00 | 0.00 | 0.00 | 0.00 | KO:K03695 | ATP-dependent Clp protease ATP-binding subunit ClpB | Bacteria;Proteobacteria;Deltaproteobacteria;Syntrophorhabdales;Syntrophorhabdaceae;Syntrophorhabdus;Syntrophorhabdus aromaticivorans;Syntrophorhabdus aromaticivorans UI |
| Ga0586324 2642572 1 804 | 0.00 | 0.00 | 0.00 | 0.00 | 1689200.00 | 0.00 | 0.00 | 0.00 | 0.00 | 0.00 | 0.00 | 0.00 | KO:K03695 | ATP-dependent Clp protease ATP-binding subunit ClpB | Bacteria;Proteobacteria;Deltaproteobacteria;Syntrophobacterales;Syntrophobacteraceae;Desulfacinum;Desulfacinum infernum;Desulfacinum infernum DSM 9756 |
| Ga0586324 4178344 3 545 | 0.00 | 0.00 | 0.00 | 0.00 | 0.00 | 0.00 | 0.00 | 0.00 | 0.00 | 708483.16 | 0.00 | 0.00 | KO:K03695 | ATP-dependent Clp protease ATP-binding subunit ClpB | Bacteria;Proteobacteria;Betaproteobacteria;Nitrosomonadales;Nitrosomonadaceae;Nitrosomonas;Nitrosomonas sp. Nm132;Nitrosomonas sp. 41 Nm132 |
| Ga0586324 4195013 1 663 | 0.00 | 0.00 | 0.00 | 0.00 | 0.00 | 0.00 | 2325600.00 | 0.00 | 0.00 | 0.00 | 0.00 | 0.00 | KO:K03695 | ATP-dependent Clp protease ATP-binding subunit ClpB | Bacteria;Actinobacteria;Actinomycetia;Corynebacteriales;Mycobacteriaceae;Mycobacterium;Mycobacterium genavense;Mycobacterium genavense ATCC 51234 |
| Ga0586324 4747496 1 603 | 0.00 | 0.00 | 0.00 | 0.00 | 1709800.00 | 0.00 | 0.00 | 0.00 | 0.00 | 0.00 | 0.00 | 0.00 | KO:K03695 | ATP-dependent Clp protease ATP-binding subunit ClpB | Bacteria;Acidobacteria;Blastocatellia;unclassified Blastocatellia;unclassified Blastocatellia;Chloracidobacterium;Chloracidobacterium thermophilum;Chloracidobacterium thermophilum B |
| Ga0586324 5415714 1 480 | 0.00 | 0.00 | 0.00 | 0.00 | 0.00 | 0.00 | 0.00 | 2885300.00 | 0.00 | 0.00 | 0.00 | 0.00 | KO:K03695 | ATP-dependent Clp protease ATP-binding subunit ClpB | Bacteria;Proteobacteria;Betaproteobacteria;Burkholderiales;unclassified Burkholderiales;Rubrivivax;Rubrivivax gelatinosus;Rubrivivax gelatinosus IL144 |
| Ga0586324 6390567 1 558 | 0.00 | 0.00 | 0.00 | 0.00 | 0.00 | 0.00 | 0.00 | 5148400.00 | 0.00 | 0.00 | 0.00 | 0.00 | KO:K03695 | ATP-dependent Clp protease ATP-binding subunit ClpB | Bacteria;Actinobacteria;Rubrobacteria;Gaiellales;Gaiellaceae;Gaiella;Gaiella occulta;Gaiella occulta F2-233 |
| Ga0586324 7730467 3 515 | 0.00 | 0.00 | 0.00 | 0.00 | 0.00 | 0.00 | 0.00 | 0.00 | 0.00 | 6006000.00 | 0.00 | 0.00 | KO:K03695 | ATP-dependent Clp protease ATP-binding subunit ClpB | Bacteria;Proteobacteria;Gammaproteobacteria;Nevskiales;Steroidobacteraceae;Povalibacter;Povalibacter uvarum;Povalibacter uvarum DSM 26723 |
| Ga0586324 0000101 53215 54066 | 1550000.00 | 0.00 | 0.00 | 0.00 | 0.00 | 0.00 | 0.00 | 0.00 | 0.00 | 0.00 | 0.00 | 0.00 | KO:K03696 | ATP-dependent Clp protease ATP-binding subunit ClpC | Bacteria;Chloroflexi;Anaerolineae;Anaerolineales;Anaerolineaceae;Anaerolinea;Anaerolinea thermophila;Anaerolinea thermophila UNI-1 |
| Ga0586324 0015696 3045 5513 | 0.00 | 0.00 | 0.00 | 0.00 | 0.00 | 0.00 | 0.00 | 0.00 | 0.00 | 0.00 | 976800.00 | 0.00 | KO:K03696 | ATP-dependent Clp protease ATP-binding subunit ClpC | Bacteria;Chloroflexi;Anaerolineae;Anaerolineales;Anaerolineaceae;Anaerolinea;Anaerolinea thermophila;Anaerolinea thermophila UNI-1 |
| Ga0586324 0072144 474 2939 | 1960000.00 | 0.00 | 0.00 | 0.00 | 0.00 | 0.00 | 0.00 | 0.00 | 0.00 | 0.00 | 0.00 | 0.00 | KO:K03696 | ATP-dependent Clp protease ATP-binding subunit ClpC | Bacteria;Chloroflexi;Anaerolineae;Anaerolineales;Anaerolineaceae;Anaerolinea;Anaerolinea thermophila;Anaerolinea thermophila UNI-1 |
| Ga0586324 0154346 1 1521 | 0.00 | 0.00 | 0.00 | 0.00 | 0.00 | 0.00 | 0.00 | 0.00 | 0.00 | 0.00 | 233965.60 | 0.00 | KO:K03696 | ATP-dependent Clp protease ATP-binding subunit ClpC | Bacteria;Planctomycetes;Phycisphaerae;Sedimentisphaerales;Anaerohalosphaeraceae;Anaerohalosphaera;Anaerohalosphaera lusitana;Phycisphaerae bacterium ST-NAGAB-D1 |
| Ga0586324 0308449 2 2029 | 0.00 | 10744000.00 | 0.00 | 0.00 | 0.00 | 0.00 | 0.00 | 0.00 | 0.00 | 0.00 | 0.00 | 0.00 | KO:K03696 | ATP-dependent Clp protease ATP-binding subunit ClpC | Bacteria;Chloroflexi;Ktedonobacteria;Thermogemmatisporales;Thermogemmatisporaceae;Thermogemmatispora;Thermogemmatispora onikobensis;Thermogemmatispora onikobensis NBRC 111776 |
| Ga0586324 0413371 1 1641 | 0.00 | 0.00 | 0.00 | 0.00 | 0.00 | 0.00 | 0.00 | 0.00 | 0.00 | 0.00 | 0.00 | 170732.25 | KO:K03696 | ATP-dependent Clp protease ATP-binding subunit ClpC | Bacteria;Gemmatimonadetes;Gemmatimonadetes;Gemmatimonadales;Gemmatimonadaceae;Gemmatirosa;Gemmatirosa kalamazoonesis;Gemmatimonadetes bacterium KBS708 |
| Ga0586324 0927202 2 1255 | 0.00 | 0.00 | 0.00 | 0.00 | 0.00 | 0.00 | 0.00 | 0.00 | 0.00 | 106390.13 | 0.00 | 0.00 | KO:K03696 | ATP-dependent Clp protease ATP-binding subunit ClpC | Bacteria;Chloroflexi;Anaerolineae;Anaerolineales;Anaerolineaceae;Anaerolinea;Anaerolinea thermophila;Anaerolinea thermophila UNI-1 |
| Ga0586324 1679267 268 972 | 0.00 | 0.00 | 0.00 | 0.00 | 0.00 | 0.00 | 0.00 | 0.00 | 0.00 | 0.00 | 3036000.00 | 0.00 | KO:K03696 | ATP-dependent Clp protease ATP-binding subunit ClpC | Bacteria;Chloroflexi;Anaerolineae;Anaerolineales;Anaerolineaceae;Thermanaerothrix;Thermanaerothrix daxensis;Thermanaerothrix daxensis GNS-1 |
| Ga0586324 3923812 3 680 | 0.00 | 0.00 | 0.00 | 0.00 | 0.00 | 0.00 | 0.00 | 0.00 | 0.00 | 0.00 | 0.00 | 1110000.00 | KO:K03696 | ATP-dependent Clp protease ATP-binding subunit ClpC | Bacteria;Chloroflexi;Anaerolineae;Anaerolineales;Anaerolineaceae;Anaerolinea;Anaerolinea thermophila;Anaerolinea thermophila UNI-1 |
| Ga0586324 4024114 2 673 | 0.00 | 0.00 | 1185582.29 | 0.00 | 0.00 | 0.00 | 0.00 | 0.00 | 0.00 | 0.00 | 0.00 | 1537500.00 | KO:K03696 | ATP-dependent Clp protease ATP-binding subunit ClpC | Bacteria;Bacteroidetes;Cytophagia;Cytophagales;Fulvivirgaceae;Ohtaekwangia;Ohtaekwangia koreensis;Ohtaekwangia koreensis DSM 25262 |
| Ga0586324 5427859 3 590 | 0.00 | 0.00 | 0.00 | 2631500.00 | 0.00 | 0.00 | 0.00 | 0.00 | 0.00 | 0.00 | 0.00 | 0.00 | KO:K03696 | ATP-dependent Clp protease ATP-binding subunit ClpC | Bacteria;Bacteroidetes;Cytophagia;Cytophagales;Fulvivirgaceae;Chryseolinea;Chryseolinea soli;Chryseolinea soli KIS68-18 |
| Ga0586324 0009406 5405 8200 | 0.00 | 0.00 | 0.00 | 0.00 | 0.00 | 0.00 | 0.00 | 0.00 | 0.00 | 0.00 | 0.00 | 1372500.00 | KO:K03701 | excinuclease ABC subunit A | Bacteria;Ignavibacteriae;Ignavibacteria;Ignavibacteriales;Ignavibacteriaceae;Ignavibacterium;Ignavibacterium album;Ignavibacterium album Mat9-16, JCM 16511 |
| Ga0586324 0080394 468 3341 | 0.00 | 0.00 | 3976457.74 | 0.00 | 0.00 | 0.00 | 0.00 | 0.00 | 0.00 | 0.00 | 0.00 | 0.00 | KO:K03701 | excinuclease ABC subunit A | Archaea;Euryarchaeota;Methanomicrobia;Methanosarcinales;Methanotrichaceae;Methanothrix;Methanothrix soehngenii;Methanothrix soehngenii GP-6 |

| | | | | | | | | | | | | | | | |
|---|---|---|---|---|---|---|---|---|---|---|---|---|---|---|---|
| Ga0586324 0175763 865 2628 | 0.00 | 0.00 | 0.00 | 0.00 | 0.00 | 0.00 | 0.00 | 165158.72 | 0.00 | 0.00 | 0.00 | 0.00 | KO:K03701 | excinuclease ABC subunit A | Bacteria;Proteobacteria;Betaproteobacteria;Burkholderiales;Burkholderiaceae;Burkholderia;Burkholderia sp. MSMB2040;Burkholderia sp. MSMB2040 |
| Ga0586324 0633053 3 1478 | 0.00 | 0.00 | 0.00 | 0.00 | 0.00 | 0.00 | 0.00 | 0.00 | 574255.17 | 0.00 | 0.00 | 0.00 | KO:K03701 | excinuclease ABC subunit A | Bacteria;Planctomycetes;Planctomycetia;Pirellulales;Thermoguttaceae;Thermogutta;Thermogutta terrifontis;Thermogutta terrifontis R1 |
| Ga0586324 0968060 1 1230 | 0.00 | 0.00 | 0.00 | 0.00 | 0.00 | 0.00 | 0.00 | 0.00 | 120946.77 | 0.00 | 0.00 | 0.00 | KO:K03701 | excinuclease ABC subunit A | Bacteria;Chloroflexi;unclassified Chloroflexi;unclassified Chloroflexi;unclassified Chloroflexi;unclassified Chloroflexi;unclassified Chloroflexi;Chloroflexi sp. G233 |
| Ga0586324 1279881 3 1091 | 0.00 | 0.00 | 1953024.48 | 0.00 | 0.00 | 0.00 | 0.00 | 0.00 | 0.00 | 0.00 | 0.00 | 0.00 | KO:K03701 | excinuclease ABC subunit A | Archaea;Euryarchaeota;Methanomicrobia;Methanomicrobiales;Methanomicrobiaceae;Methanoculleus;Methanoculleus bourgensis;Methanoculleus bourgensis MBBA |
| Ga0586324 4442304 3 647 | 0.00 | 0.00 | 0.00 | 0.00 | 0.00 | 0.00 | 0.00 | 0.00 | 1117800.00 | 0.00 | 0.00 | 0.00 | KO:K03701 | excinuclease ABC subunit A | Bacteria;Acidobacteria;Acidobacteriia;Acidobacteriales;Acidobacteriaceae;Candidatus Koribacter;Candidatus Koribacter versatilis;Candidatus Koribacter versatilis Ellin345 |
| Ga0586324 6414830 2 556 | 0.00 | 0.00 | 0.00 | 0.00 | 0.00 | 0.00 | 0.00 | 2623000.00 | 0.00 | 0.00 | 0.00 | 0.00 | KO:K03701 | excinuclease ABC subunit A | Archaea;Euryarchaeota;Methanomicrobia;Methanosarcinales;Methanosarcinaceae;Methanosarcina;Methanosarcina sp. DSM 11855;Methanosarcina sp. DSM 11855 |
| Ga0586324 0000117 24110 24313 | 0.00 | 0.00 | 0.00 | 0.00 | 0.00 | 0.00 | 0.00 | 0.00 | 2357100.00 | 2587200.00 | 0.00 | 2760000.00 | KO:K03704 | CspA family cold shock protein | Bacteria;Proteobacteria;Alphaproteobacteria;Hyphomicrobiales;Bradyrhizobiaceae;Afipia;Afipia sp. P52-10;Afipia sp. P52-10 |
| Ga0586324 0015773 4519 4722 | 0.00 | 0.00 | 0.00 | 0.00 | 0.00 | 0.00 | 0.00 | 0.00 | 0.00 | 0.00 | 0.00 | 637140.00 | KO:K03704 | CspA family cold shock protein | Bacteria;Thermodesulfobacteria;Thermodesulfobacteria;Thermodesulfobacteriales;Thermodesulfobacteriaceae;Thermodesulfatator;Thermodesulfatator indicus;Thermodesulfatator indicus CIR29812, DSM 15286 |
| Ga0586324 0033969 2940 3143 | 0.00 | 0.00 | 0.00 | 2954500.00 | 0.00 | 0.00 | 0.00 | 3721000.00 | 0.00 | 0.00 | 3792800.00 | 3480000.00 | KO:K03704 | CspA family cold shock protein | Bacteria;Proteobacteria;Alphaproteobacteria;Hyphomicrobiales;Xanthobacteraceae;Pseudolabrys;Pseudolabrys sp. Root1462;Pseudolabrys sp. Root1462 |
| Ga0586324 1065815 207 419 | 0.00 | 0.00 | 460973.70 | 0.00 | 0.00 | 0.00 | 0.00 | 0.00 | 0.00 | 0.00 | 0.00 | 0.00 | KO:K03704 | CspA family cold shock protein | Bacteria;Proteobacteria;Gammaproteobacteria;Oceanospirillales;Oceanospirillaceae;Marinomonas;Marinomonas pollencensis;Marinomonas pollencensis CECT 7375 |
| Ga0586324 1076635 46 240 | 0.00 | 5487600.00 | 0.00 | 8341000.00 | 12772000.00 | 0.00 | 0.00 | 0.00 | 0.00 | 0.00 | 0.00 | 5865000.00 | KO:K03704 | CspA family cold shock protein | Archaea;Euryarchaeota;Methanomicrobia;Methanosarcinales;Methanotrichaceae;Methanothrix;Methanothrix soehngenii;Methanothrix soehngenii GP-6 |
| Ga0586324 1393523 160 360 | 0.00 | 0.00 | 0.00 | 0.00 | 0.00 | 0.00 | 0.00 | 478473.63 | 0.00 | 0.00 | 0.00 | 0.00 | KO:K03704 | CspA family cold shock protein | Bacteria;Proteobacteria;Deltaproteobacteria;Desulfuromonadales;Geobacteraceae;Geobacter;Geobacter uraniireducens;Geobacter uraniireducens Rf4 |
| Ga0586324 6292025 113 307 | 0.00 | 0.00 | 0.00 | 454661.45 | 0.00 | 0.00 | 0.00 | 0.00 | 0.00 | 0.00 | 0.00 | 0.00 | KO:K03704 | CspA family cold shock protein | Archaea;Euryarchaeota;Methanomicrobia;Methanosarcinales;Methanotrichaceae;Methanothrix;Methanothrix soehngenii;Methanothrix soehngenii GP-6 |
| Ga0586324 0001326 15141 15560 | 2650000.00 | 0.00 | 0.00 | 2688500.00 | 0.00 | 0.00 | 0.00 | 1250500.00 | 0.00 | 0.00 | 0.00 | 0.00 | KO:K03711 | Fur family ferric uptake transcriptional regulator | Bacteria;Proteobacteria;Gammaproteobacteria;Methylococcales;Methylococcaceae;Methylobacter;Methylobacter luteus;Methylobacter luteus IMV-B-3098 |
| Ga0586324 0014321 4646 5551 | 0.00 | 0.00 | 808118.94 | 0.00 | 0.00 | 0.00 | 0.00 | 0.00 | 0.00 | 0.00 | 0.00 | 0.00 | KO:K03717 | LysR family transcriptional activator of nhaA | Bacteria;Proteobacteria;Deltaproteobacteria;Myxococcales;Myxococcaceae;Corallococcus;Corallococcus sicarius;Corallococcus sicarius CA040B |
| Ga0586324 0020400 2389 3747 | 0.00 | 0.00 | 0.00 | 0.00 | 0.00 | 0.00 | 0.00 | 0.00 | 1077300.00 | 0.00 | 0.00 | 0.00 | KO:K03732 | ATP-dependent RNA helicase RhlB | Bacteria;Proteobacteria;Gammaproteobacteria;Nevskiales;Steroidobacteraceae;Povalibacter;Povalibacter uvarum;Povalibacter uvarum DSM 26723 |
| Ga0586324 0002188 10959 14609 | 0.00 | 0.00 | 0.00 | 0.00 | 0.00 | 0.00 | 0.00 | 0.00 | 3159000.00 | 0.00 | 0.00 | 1777500.00 | KO:K03737 | pyruvate-ferredoxin/flavodoxin oxidoreductase | Bacteria;Proteobacteria;Gammaproteobacteria;Methylococcales;Methylococcaceae;Methylobacter;Methylobacter tundripaludum;Methylobacter tundripaludum SV96 |
| Ga0586324 0002552 243 3764 | 0.00 | 0.00 | 0.00 | 0.00 | 0.00 | 0.00 | 0.00 | 0.00 | 0.00 | 2733500.00 | 0.00 | 0.00 | KO:K03737 | pyruvate-ferredoxin/flavodoxin oxidoreductase | Bacteria;Candidatus Aminicenantes;unclassified Candidatus Aminicenantes;unclassified Candidatus Aminicenantes;unclassified Candidatus Aminicenantes;unclassified Candidatus Aminicenantes;Aminicenantes bacterium SCGC AAA255-A06;Aminicenantes bacterium SCGC AAA255-A06 (contamination screened) |
| Ga0586324 0004169 11826 14822 | 0.00 | 0.00 | 0.00 | 0.00 | 0.00 | 0.00 | 0.00 | 0.00 | 372209.58 | 0.00 | 0.00 | 0.00 | KO:K03737 | pyruvate-ferredoxin/flavodoxin oxidoreductase | Bacteria;Acidobacteria;Acidobacteriia;Acidobacteriales;Acidobacteriaceae;Candidatus Koribacter;Candidatus Koribacter versatilis;Candidatus Koribacter versatilis Ellin345 |
| Ga0586324 0005236 5091 8669 | 0.00 | 6201600.00 | 3309950.19 | 0.00 | 0.00 | 0.00 | 0.00 | 0.00 | 0.00 | 0.00 | 0.00 | 0.00 | KO:K03737 | pyruvate-ferredoxin/flavodoxin oxidoreductase | Bacteria;Acidobacteria;Acidobacteriia;Bryobacterales;Solibacteraceae;Candidatus Solibacter;Candidatus Solibacter usitatus;Candidatus Solibacter usitatus Ellin6076 |
| Ga0586324 0030562 1 3510 | 4730000.00 | 0.00 | 0.00 | 0.00 | 0.00 | 0.00 | 0.00 | 2208200.00 | 469010.25 | 0.00 | 0.00 | 0.00 | KO:K03737 | pyruvate-ferredoxin/flavodoxin oxidoreductase | Bacteria;Proteobacteria;Deltaproteobacteria;Syntrophobacterales;Syntrophaceae;Desulfobacca;Desulfobacca acetoxidans;Desulfobacca acetoxidans ASRB2, DSM 11109 |
| Ga0586324 0038731 566 4138 | 0.00 | 0.00 | 0.00 | 0.00 | 0.00 | 0.00 | 0.00 | 0.00 | 11421000.00 | 0.00 | 0.00 | 0.00 | KO:K03737 | pyruvate-ferredoxin/flavodoxin oxidoreductase | Bacteria;Candidatus Sumerlaeota;unclassified Candidatus Sumerlaeota;unclassified Candidatus Sumerlaeota;unclassified Candidatus Sumerlaeota;unclassified Candidatus Sumerlaeota;BRC1 bacterium SCGC AAA257-C11;BRC1 bacterium SCGC AAA257-C11 (contamination screened) |
| Ga0586324 0048202 838 4416 | 0.00 | 0.00 | 0.00 | 0.00 | 371216.12 | 0.00 | 0.00 | 0.00 | 0.00 | 0.00 | 0.00 | 0.00 | KO:K03737 | pyruvate-ferredoxin/flavodoxin oxidoreductase | Bacteria;Chloroflexi;Anaerolineae;Anaerolineales;Anaerolineaceae;Anaerolinea;Anaerolinea thermophila;Anaerolinea thermophila UNI-1 |
| Ga0586324 0054740 846 4364 | 0.00 | 0.00 | 0.00 | 0.00 | 0.00 | 0.00 | 0.00 | 0.00 | 0.00 | 1039500.00 | 0.00 | 0.00 | KO:K03737 | pyruvate-ferredoxin/flavodoxin oxidoreductase | Bacteria;Acidobacteria;Thermoanaerobaculia;Thermoanaerobaculales;Thermoanaerobaculaceae;Thermoanaerobaculum;Thermoanaerobaculum aquaticum;Thermoanaerobaculum aquaticum MP-01 |
| Ga0586324 0132153 3 2579 | 1150000.00 | 904400.00 | 0.00 | 681346.65 | 787713.10 | 0.00 | 729034.56 | 552211.65 | 484738.83 | 549300.29 | 444708.00 | 702576.00 | KO:K03737 | pyruvate-ferredoxin/flavodoxin oxidoreductase | Bacteria;Proteobacteria;Deltaproteobacteria;unclassified Deltaproteobacteria;unclassified Deltaproteobacteria;unclassified Deltaproteobacteria;delta proteobacterium NaphS2;delta proteobacterium NaphS2 |
| Ga0586324 0171239 1 1362 | 0.00 | 0.00 | 1375544.12 | 0.00 | 0.00 | 0.00 | 0.00 | 0.00 | 0.00 | 0.00 | 0.00 | 0.00 | KO:K03737 | pyruvate-ferredoxin/flavodoxin oxidoreductase | Bacteria;Candidatus Aminicenantes;unclassified Candidatus Aminicenantes;unclassified Candidatus Aminicenantes;unclassified Candidatus Aminicenantes;unclassified Candidatus Aminicenantes;Aminicenantes bacterium SCGC AAA252-K07;Aminicenantes bacterium SCGC AAA252-K07 (contamination screened) |
| Ga0586324 0331790 1 1497 | 9460000.00 | 7187600.00 | 0.00 | 8664000.00 | 7652900.00 | 7265100.00 | 0.00 | 4428600.00 | 972000.00 | 0.00 | 0.00 | 0.00 | KO:K03737 | pyruvate-ferredoxin/flavodoxin oxidoreductase | Bacteria;Proteobacteria;Deltaproteobacteria;Desulfobacterales;Desulfobulbaceae;Desulfobulbus;Desulfobulbus propionicus;Desulfobulbus propionicus 1pr3, DSM 2032 |
| Ga0586324 0347227 1 1926 | 0.00 | 0.00 | 0.00 | 3068500.00 | 2389600.00 | 0.00 | 0.00 | 0.00 | 0.00 | 0.00 | 0.00 | 0.00 | KO:K03737 | pyruvate-ferredoxin/flavodoxin oxidoreductase | Bacteria;Proteobacteria;Deltaproteobacteria;Desulfobacterales;Desulfobulbaceae;Desulfobulbus;Desulfobulbus propionicus;Desulfobulbus propionicus 1pr3, DSM 2032 |
| Ga0586324 0447207 342 1721 | 0.00 | 4066400.00 | 0.00 | 8227000.00 | 3440200.00 | 0.00 | 0.00 | 0.00 | 5613300.00 | 2348500.00 | 0.00 | 1500000.00 | KO:K03737 | pyruvate-ferredoxin/flavodoxin oxidoreductase | Bacteria;Proteobacteria;Deltaproteobacteria;Desulfobacterales;Desulfobulbaceae;Desulfobulbus;Desulfobulbus propionicus;Desulfobulbus propionicus 1pr3, DSM 2032 |
| Ga0586324 0903673 2 1267 | 0.00 | 0.00 | 0.00 | 769389.80 | 0.00 | 0.00 | 0.00 | 0.00 | 0.00 | 0.00 | 0.00 | 0.00 | KO:K03737 | pyruvate-ferredoxin/flavodoxin oxidoreductase | Bacteria;Candidatus Aminicenantes;unclassified Candidatus Aminicenantes;unclassified Candidatus Aminicenantes;unclassified Candidatus Aminicenantes;unclassified Candidatus Aminicenantes;Aminicenantes bacterium SCGC AAA252-K07;Aminicenantes bacterium SCGC AAA252-K07 (contamination screened) |
| Ga0586324 0978715 387 1226 | 0.00 | 0.00 | 0.00 | 0.00 | 0.00 | 0.00 | 0.00 | 0.00 | 378206.82 | 0.00 | 0.00 | 0.00 | KO:K03737 | pyruvate-ferredoxin/flavodoxin oxidoreductase | Bacteria;Proteobacteria;Deltaproteobacteria;Desulfobacterales;Desulfobulbaceae;Desulfobulbus;Desulfobulbus elongatus;Desulfobulbus elongatus DSM 2908 |
| Ga0586324 1203942 2 1120 | 0.00 | 0.00 | 0.00 | 0.00 | 0.00 | 0.00 | 0.00 | 2982900.00 | 0.00 | 0.00 | 0.00 | 0.00 | KO:K03737 | pyruvate-ferredoxin/flavodoxin oxidoreductase | Bacteria;Actinobacteria;Actinomycetia;Micrococcales;Dermatophilaceae;Piscicoccus;Piscicoccus intestinalis;Piscicoccus intestinalis NBRC 104926 |
| Ga0586324 1674169 3 974 | 0.00 | 0.00 | 0.00 | 0.00 | 9094900.00 | 0.00 | 0.00 | 0.00 | 0.00 | 0.00 | 0.00 | 8550000.00 | KO:K03737 | pyruvate-ferredoxin/flavodoxin oxidoreductase | Bacteria;Candidatus Sumerlaeota;unclassified Candidatus Sumerlaeota;unclassified Candidatus Sumerlaeota;unclassified Candidatus Sumerlaeota;unclassified Candidatus Sumerlaeota;BRC1 bacterium SCGC AAA257-C11;BRC1 bacterium SCGC AAA257-C11 (contamination screened) |
| Ga0586324 2288989 1 852 | 0.00 | 0.00 | 0.00 | 0.00 | 10001300.00 | 0.00 | 0.00 | 12322000.00 | 0.00 | 11011000.00 | 0.00 | 0.00 | KO:K03737 | pyruvate-ferredoxin/flavodoxin oxidoreductase | Bacteria;Actinobacteria;Acidimicrobiia;Acidimicrobiales;Ilumatobacteraceae;Ilumatobacter;Ilumatobacter fluminis;Ilumatobacter fluminis DSM 18936 |
| Ga0586324 2373394 1 840 | 0.00 | 0.00 | 0.00 | 0.00 | 0.00 | 0.00 | 0.00 | 0.00 | 0.00 | 0.00 | 0.00 | 6030000.00 | KO:K03737 | pyruvate-ferredoxin/flavodoxin oxidoreductase | Bacteria;Acidobacteria;Thermoanaerobaculia;Thermoanaerobaculales;Thermoanaerobaculaceae;Thermoanaerobaculum;Thermoanaerobaculum aquaticum;Thermoanaerobaculum aquaticum MP-01 |
| Ga0586324 2416078 1 834 | 10200000.00 | 0.00 | 13737200.00 | 0.00 | 0.00 | 0.00 | 0.00 | 0.00 | 0.00 | 0.00 | 0.00 | 0.00 | KO:K03737 | pyruvate-ferredoxin/flavodoxin oxidoreductase | Bacteria;Proteobacteria;Deltaproteobacteria;Desulfuromonadales;Geobacteraceae;Geobacter;Geobacter soli;Geobacter soli GSS01 |
| Ga0586324 3340306 3 728 | 0.00 | 0.00 | 8501300.00 | 0.00 | 0.00 | 0.00 | 0.00 | 0.00 | 0.00 | 0.00 | 0.00 | 0.00 | KO:K03737 | pyruvate-ferredoxin/flavodoxin oxidoreductase | Bacteria;Proteobacteria;Gammaproteobacteria;Methylococcales;Methylococcaceae;Methylobacter;Methylobacter tundripaludum;Methylobacter tundripaludum SV96 |
| Ga0586324 4770104 3 629 | 0.00 | 0.00 | 0.00 | 0.00 | 0.00 | 0.00 | 0.00 | 0.00 | 0.00 | 5636400.00 | 0.00 | 0.00 | KO:K03737 | pyruvate-ferredoxin/flavodoxin oxidoreductase | Bacteria;Proteobacteria;Deltaproteobacteria;unclassified Deltaproteobacteria;unclassified Deltaproteobacteria;Deferrisoma;Deferrisoma camini;Deferrisoma camini S3R1 |
| Ga0586324 5307293 3 602 | 0.00 | 0.00 | 0.00 | 0.00 | 0.00 | 0.00 | 0.00 | 0.00 | 356785.56 | 644956.62 | 0.00 | 0.00 | KO:K03737 | pyruvate-ferredoxin/flavodoxin oxidoreductase | Bacteria;Proteobacteria;Deltaproteobacteria;Desulfobacterales;Desulfobacteraceae;Desulfatirhabdium;Desulfatirhabdium butyrativorans;Desulfatirhabdium butyrativorans DSM 18734 |
| Ga0586324 6737928 1 546 | 0.00 | 0.00 | 0.00 | 3258500.00 | 0.00 | 0.00 | 0.00 | 0.00 | 0.00 | 0.00 | 0.00 | 0.00 | KO:K03737 | pyruvate-ferredoxin/flavodoxin oxidoreductase | Bacteria;Firmicutes;Clostridia;Thermoanaerobacterales;Thermoanaerobacteraceae;Thermoanaerobacter;Thermoanaerobacter mathranii;Thermoanaerobacter mathranii mathranii A3, DSM 11426 |
| Ga0586324 7441022 58 522 | 0.00 | 0.00 | 0.00 | 0.00 | 0.00 | 0.00 | 0.00 | 474577.56 | 0.00 | 0.00 | 0.00 | 0.00 | KO:K03737 | pyruvate-ferredoxin/flavodoxin oxidoreductase | Bacteria;Acidobacteria;Acidobacteriia;Bryobacterales;Solibacteraceae;Candidatus Solibacter;Candidatus Solibacter usitatus;Candidatus Solibacter usitatus Ellin6076 |
| Ga0586324 0002503 50 1876 | 0.00 | 0.00 | 0.00 | 0.00 | 0.00 | 0.00 | 0.00 | 0.00 | 0.00 | 6999300.00 | 0.00 | 0.00 | KO:K03738 | aldehyde:ferredoxin oxidoreductase | Bacteria;Chloroflexi;Chloroflexia;Chloroflexales;Chloroflexaceae;Chloroflexus;Chloroflexus islandicus;Chloroflexus islandicus isl-2 |
| Ga0586324 0002615 13233 15059 | 0.00 | 0.00 | 0.00 | 0.00 | 0.00 | 0.00 | 0.00 | 2464400.00 | 0.00 | 0.00 | 0.00 | 0.00 | KO:K03738 | aldehyde:ferredoxin oxidoreductase | Bacteria;Proteobacteria;Deltaproteobacteria;Desulfobacterales;Desulfobacteraceae;Desulfobacterium;Desulfobacterium autotrophicum;Desulfobacterium autotrophicum HRM2, DSM 3382 |
| Ga0586324 0004973 2278 4014 | 0.00 | 2964800.00 | 0.00 | 0.00 | 0.00 | 0.00 | 0.00 | 0.00 | 0.00 | 0.00 | 0.00 | 0.00 | KO:K03738 | aldehyde:ferredoxin oxidoreductase | Bacteria;Proteobacteria;Deltaproteobacteria;Syntrophorhabdales;Syntrophorhabdaceae;Syntrophorhabdus;Syntrophorhabdus aromaticivorans;Syntrophorhabdus aromaticivorans UI |
| Ga0586324 0015092 3346 5088 | 0.00 | 0.00 | 0.00 | 0.00 | 0.00 | 0.00 | 0.00 | 0.00 | 0.00 | 10549000.00 | 0.00 | 0.00 | KO:K03738 | aldehyde:ferredoxin oxidoreductase | Bacteria;Spirochaetes;Spirochaetia;Spirochaetales;Spirochaetaceae;Alkalispirochaeta;Alkalispirochaeta odontotermitis;Alkalispirochaeta odontotermitis JC202 |
| Ga0586324 0016054 3706 5448 | 4620000.00 | 3638000.00 | 0.00 | 11210000.00 | 0.00 | 0.00 | 0.00 | 1891000.00 | 0.00 | 0.00 | 0.00 | 0.00 | KO:K03738 | aldehyde:ferredoxin oxidoreductase | Bacteria;Proteobacteria;Deltaproteobacteria;Desulfobacterales;Desulfobacteraceae;Desulfosarcina;Desulfosarcina sp. BuS5;Desulfosarcina sp. BuS5 |
| Ga0586324 0025956 3644 5485 | 0.00 | 1944800.00 | 760202.01 | 0.00 | 0.00 | 0.00 | 944106.96 | 0.00 | 343670.04 | 0.00 | 0.00 | 0.00 | KO:K03738 | aldehyde:ferredoxin oxidoreductase | Bacteria;Chloroflexi;Chloroflexia;Chloroflexales;Chloroflexaceae;Chloroflexus;Chloroflexus islandicus;Chloroflexus islandicus isl-2 |
| Ga0586324 0033238 2305 4047 | 3710000.00 | 5004800.00 | 12386000.00 | 2004500.00 | 0.00 | 2808830.58 | 3397200.00 | 2592500.00 | 2956500.00 | 4173400.00 | 2543200.00 | 2340000.00 | KO:K03738 | aldehyde:ferredoxin oxidoreductase | Bacteria;Proteobacteria;Deltaproteobacteria;Syntrophobacterales;Syntrophobacteraceae;Desulfacinum;Desulfacinum hydrothermale;Desulfacinum hydrothermale DSM 13146 |
| Ga0586324 0040817 445 2181 | 9910000.00 | 1101600.00 | 1778595.82 | 2365500.00 | 2018800.00 | 0.00 | 0.00 | 0.00 | 2405700.00 | 0.00 | 0.00 | 0.00 | KO:K03738 | aldehyde:ferredoxin oxidoreductase | Bacteria;Proteobacteria;Deltaproteobacteria;Desulfobacterales;Desulfobacteraceae;Desulfonema;Desulfonema limicola;Desulfonema limicola Jadebusen, DSM 2076 |
| Ga0586324 0043321 3173 5026 | 403357.00 | 0.00 | 0.00 | 0.00 | 0.00 | 0.00 | 0.00 | 0.00 | 0.00 | 0.00 | 0.00 | 0.00 | KO:K03738 | aldehyde:ferredoxin oxidoreductase | Bacteria;Proteobacteria;Betaproteobacteria;Burkholderiales;Comamonadaceae;Rhodoferax;Rhodoferax ferrireducens;Rhodoferax ferrireducens T118 |
| Ga0586324 0067656 286 2118 | 1760000.00 | 1394000.00 | 0.00 | 0.00 | 0.00 | 0.00 | 0.00 | 0.00 | 0.00 | 0.00 | 0.00 | 0.00 | KO:K03738 | aldehyde:ferredoxin oxidoreductase | Bacteria;Chloroflexi;unclassified Chloroflexi;unclassified Chloroflexi;unclassified Chloroflexi;unclassified Chloroflexi;unclassified Chloroflexi;Chloroflexi sp. G233 |
| Ga0586324 0069864 1398 3137 | 0.00 | 0.00 | 0.00 | 0.00 | 0.00 | 0.00 | 3784800.00 | 0.00 | 0.00 | 0.00 | 0.00 | 0.00 | KO:K03738 | aldehyde:ferredoxin oxidoreductase | Bacteria;Proteobacteria;Deltaproteobacteria;Syntrophobacterales;Syntrophobacteraceae;Desulfosoma;Desulfosoma caldarium;Desulfosoma caldarium DSM 22027 |
| Ga0586324 0079436 2282 3826 | 0.00 | 1591200.00 | 0.00 | 0.00 | 0.00 | 0.00 | 0.00 | 0.00 | 0.00 | 0.00 | 0.00 | 0.00 | KO:K03738 | aldehyde:ferredoxin oxidoreductase | Bacteria;Chloroflexi;Chloroflexia;Chloroflexales;Chloroflexaceae;Chloroflexus;Chloroflexus aggregans;Chloroflexus aggregans DSM 9485 |
| Ga0586324 0258389 2 1516 | 0.00 | 0.00 | 0.00 | 0.00 | 1545000.00 | 0.00 | 0.00 | 0.00 | 0.00 | 0.00 | 0.00 | 0.00 | KO:K03738 | aldehyde:ferredoxin oxidoreductase | Bacteria;Proteobacteria;Deltaproteobacteria;Desulfuromonadales;Geobacteraceae;Geobacter;Geobacter sp. DSM 2909;Geobacter sp. DSM 2909 |
| Ga0586324 0286347 1 2046 | 0.00 | 0.00 | 0.00 | 0.00 | 384791.52 | 0.00 | 567083.88 | 0.00 | 0.00 | 0.00 | 0.00 | 0.00 | KO:K03738 | aldehyde:ferredoxin oxidoreductase | Archaea;Euryarchaeota;Thermococci;Thermococcales;Thermococcaceae;Thermococcus;Thermococcus profundus;Thermococcus profundus DT 5432 |
| Ga0586324 0305596 653 2038 | 0.00 | 1931200.00 | 2612578.98 | 0.00 | 0.00 | 0.00 | 0.00 | 0.00 | 0.00 | 1216600.00 | 0.00 | 0.00 | KO:K03738 | aldehyde:ferredoxin oxidoreductase | Bacteria;Firmicutes;Bacilli;Bacillales;Bacillaceae;Bacillus;Bacillus sp. FJAT-29814;Bacillus sp. FJAT-29814 |
| Ga0586324 0317164 690 2003 | 0.00 | 0.00 | 0.00 | 0.00 | 0.00 | 0.00 | 0.00 | 0.00 | 0.00 | 0.00 | 924000.00 | 937500.00 | KO:K03738 | aldehyde:ferredoxin oxidoreductase | Bacteria;Proteobacteria;Betaproteobacteria;Rhodocyclales;Zoogloeaceae;Zoogloea;Zoogloea sp. LCSB751;Zoogloea schifflangensis LCSB751 |
| Ga0586324 0320023 3 626 | 0.00 | 0.00 | 0.00 | 0.00 | 0.00 | 0.00 | 0.00 | 0.00 | 0.00 | 0.00 | 557847.84 | 0.00 | KO:K03738 | aldehyde:ferredoxin oxidoreductase | Bacteria;Proteobacteria;Betaproteobacteria;Burkholderiales;unclassified Burkholderiales;Leptothrix;Leptothrix cholodnii;Leptothrix cholodnii SP-6 |
| Ga0586324 0376333 560 1858 | 0.00 | 0.00 | 0.00 | 0.00 | 0.00 | 0.00 | 0.00 | 0.00 | 2843100.00 | 0.00 | 0.00 | 0.00 | KO:K03738 | aldehyde:ferredoxin oxidoreductase | Bacteria;Chloroflexi;Anaerolineae;Anaerolineales;Anaerolineaceae;Thermanaerothrix;Thermanaerothrix daxensis;Thermanaerothrix daxensis GNS-1 |
| Ga0586324 0386115 1 1407 | 0.00 | 0.00 | 0.00 | 0.00 | 3388700.00 | 11116000.00 | 0.00 | 3324500.00 | 0.00 | 0.00 | 0.00 | 0.00 | KO:K03738 | aldehyde:ferredoxin oxidoreductase | Bacteria;Chloroflexi;Chloroflexia;Chloroflexales;Chloroflexaceae;Chloroflexus;Chloroflexus islandicus;Chloroflexus islandicus isl-2 |
| Ga0586324 0402987 1 1122 | 0.00 | 0.00 | 0.00 | 464782.75 | 0.00 | 0.00 | 0.00 | 0.00 | 0.00 | 0.00 | 0.00 | 0.00 | KO:K03738 | aldehyde:ferredoxin oxidoreductase | Bacteria;Proteobacteria;Deltaproteobacteria;Desulfobacterales;Desulfobacteraceae;Desulfosarcina;Desulfosarcina sp. BuS5;Desulfosarcina sp. BuS5 |
| Ga0586324 0403295 43 1785 | 1890000.00 | 870400.00 | 2699596.26 | 0.00 | 0.00 | 1391580.28 | 0.00 | 768600.00 | 0.00 | 1293600.00 | 0.00 | 0.00 | KO:K03738 | aldehyde:ferredoxin oxidoreductase | Bacteria;Proteobacteria;Deltaproteobacteria;Syntrophobacterales;Syntrophaceae;Desulfomonile;Desulfomonile tiedjei;Desulfomonile tiedjei DCB-1, DSM 6799 |
| Ga0586324 0408835 831 1790 | 3830000.00 | 0.00 | 0.00 | 0.00 | 0.00 | 0.00 | 0.00 | 0.00 | 0.00 | 0.00 | 0.00 | 0.00 | KO:K03738 | aldehyde:ferredoxin oxidoreductase | Bacteria;Firmicutes;Tissierellia;Tissierellales;Tissierellaceae;Soehngenia;Soehngenia saccharolytica;Soehngenia saccharolytica DSM 12858 |
| Ga0586324 0424214 1 1707 | 9770000.00 | 10608000.00 | 1003153.40 | 0.00 | 0.00 | 0.00 | 6156645.24 | 1640900.00 | 4738500.00 | 0.00 | 0.00 | 0.00 | KO:K03738 | aldehyde:ferredoxin oxidoreductase | Bacteria;Chloroflexi;Anaerolineae;Anaerolineales;Anaerolineaceae;Thermanaerothrix;Thermanaerothrix daxensis;Thermanaerothrix daxensis GNS-1 |
| Ga0586324 0442894 630 1730 | 1410000.00 | 0.00 | 0.00 | 0.00 | 0.00 | 0.00 | 0.00 | 0.00 | 0.00 | 0.00 | 0.00 | 0.00 | KO:K03738 | aldehyde:ferredoxin oxidoreductase | Archaea;Euryarchaeota;Halobacteria;Halobacteriales;Halobacteriaceae;Halodesulfurarchaeum;Halodesulfurarchaeum formicicum;Halodesulfurarchaeum formicicum HSR6 |
| Ga0586324 0533063 24 1592 | 354892.00 | 0.00 | 892067.87 | 0.00 | 0.00 | 275488.62 | 0.00 | 205824.98 | 0.00 | 0.00 | 717075.04 | 0.00 | KO:K03738 | aldehyde:ferredoxin oxidoreductase | Bacteria;Proteobacteria;Deltaproteobacteria;Syntrophobacterales;Syntrophobacteraceae;Syntrophobacter;Syntrophobacter fumaroxidans;Syntrophobacter fumaroxidans MPOB |
| Ga0586324 0570795 206 1546 | 0.00 | 0.00 | 0.00 | 0.00 | 0.00 | 3426550.67 | 0.00 | 0.00 | 0.00 | 0.00 | 0.00 | 0.00 | KO:K03738 | aldehyde:ferredoxin oxidoreductase | Bacteria;Proteobacteria;Deltaproteobacteria;Syntrophobacterales;Syntrophobacteraceae;Desulfosoma;Desulfosoma caldarium;Desulfosoma caldarium DSM 22027 |
| Ga0586324 0627982 1 1464 | 0.00 | 0.00 | 0.00 | 0.00 | 0.00 | 0.00 | 0.00 | 0.00 | 0.00 | 0.00 | 694114.08 | 0.00 | KO:K03738 | aldehyde:ferredoxin oxidoreductase | Bacteria;Proteobacteria;Deltaproteobacteria;Desulfobacterales;Desulfobacteraceae;Desulfobacula;Desulfobacula toluolica;Desulfobacula toluolica Tol2 |
| Ga0586324 0644457 1 1467 | 0.00 | 0.00 | 0.00 | 0.00 | 0.00 | 0.00 | 0.00 | 0.00 | 0.00 | 1185800.00 | 0.00 | 0.00 | KO:K03738 | aldehyde:ferredoxin oxidoreductase | Bacteria;Proteobacteria;Deltaproteobacteria;Syntrophobacterales;Syntrophobacteraceae;Desulfacinum;Desulfacinum infernum;Desulfacinum infernum DSM 9756 |
| Ga0586324 0762491 2 1288 | 0.00 | 0.00 | 0.00 | 3771500.00 | 0.00 | 0.00 | 0.00 | 0.00 | 0.00 | 0.00 | 0.00 | 0.00 | KO:K03738 | aldehyde:ferredoxin oxidoreductase | Bacteria;Proteobacteria;Deltaproteobacteria;Syntrophobacterales;Syntrophaceae;Desulfomonile;Desulfomonile tiedjei;Desulfomonile tiedjei DCB-1, DSM 6799 |
| Ga0586324 0797447 1 498 | 0.00 | 1047200.00 | 0.00 | 0.00 | 0.00 | 0.00 | 0.00 | 0.00 | 0.00 | 0.00 | 0.00 | 0.00 | KO:K03738 | aldehyde:ferredoxin oxidoreductase | Bacteria;Proteobacteria;Deltaproteobacteria;Syntrophobacterales;Syntrophaceae;Desulfomonile;Desulfomonile tiedjei;Desulfomonile tiedjei DCB-1, DSM 6799 |
| Ga0586324 0889247 2 1207 | 0.00 | 573174.04 | 0.00 | 0.00 | 0.00 | 0.00 | 0.00 | 0.00 | 0.00 | 0.00 | 0.00 | 0.00 | KO:K03738 | aldehyde:ferredoxin oxidoreductase | Bacteria;Firmicutes;Clostridia;Eubacteriales;unclassified Eubacteriales;unclassified Eubacteriales;Clostridiales bacterium PH28 bin88;Clostridiales bacterium PH28 bin88 |
| Ga0586324 0918255 2 1087 | 0.00 | 992800.00 | 0.00 | 0.00 | 0.00 | 0.00 | 0.00 | 0.00 | 0.00 | 0.00 | 0.00 | 0.00 | KO:K03738 | aldehyde:ferredoxin oxidoreductase | Bacteria;Proteobacteria;Deltaproteobacteria;Syntrophobacterales;Syntrophobacteraceae;Desulfoglaeba;Desulfoglaeba alkanexedens;Desulfoglaeba alkanexedens ALDC |
| Ga0586324 0960772 1 1041 | 0.00 | 0.00 | 0.00 | 0.00 | 3100300.00 | 0.00 | 0.00 | 9089000.00 | 0.00 | 0.00 | 4417600.00 | 0.00 | KO:K03738 | aldehyde:ferredoxin oxidoreductase | Bacteria;Proteobacteria;Deltaproteobacteria;Syntrophobacterales;Syntrophobacteraceae;Desulfacinum;Desulfacinum infernum;Desulfacinum infernum DSM 9756 |
| Ga0586324 0965852 2 790 | 0.00 | 0.00 | 0.00 | 5025500.00 | 0.00 | 0.00 | 0.00 | 1506700.00 | 0.00 | 0.00 | 0.00 | 0.00 | KO:K03738 | aldehyde:ferredoxin oxidoreductase | Bacteria;Proteobacteria;Deltaproteobacteria;Desulfuromonadales;Geobacteraceae;Geobacter;Geobacter sp. DSM 2909;Geobacter sp. DSM 2909 |
| Ga0586324 0967776 3 1232 | 0.00 | 0.00 | 0.00 | 0.00 | 0.00 | 0.00 | 0.00 | 0.00 | 0.00 | 0.00 | 0.00 | 4515000.00 | KO:K03738 | aldehyde:ferredoxin oxidoreductase | Bacteria;Chloroflexi;Chloroflexia;Chloroflexales;Roseiflexaceae;Roseiflexus;Roseiflexus castenholzii;Roseiflexus castenholzii HLO8, DSM 13941 |
| Ga0586324 1121105 1 1155 | 0.00 | 0.00 | 0.00 | 3600500.00 | 0.00 | 0.00 | 0.00 | 0.00 | 0.00 | 0.00 | 0.00 | 0.00 | KO:K03738 | aldehyde:ferredoxin oxidoreductase | Bacteria;Proteobacteria;Deltaproteobacteria;Desulfuromonadales;Geobacteraceae;Geobacter;Geobacter sp. DSM 2909;Geobacter sp. DSM 2909 |
| Ga0586324 1157792 3 971 | 0.00 | 3162000.00 | 10302900.00 | 0.00 | 0.00 | 1690914.31 | 0.00 | 1335900.00 | 0.00 | 2487100.00 | 0.00 | 0.00 | KO:K03738 | aldehyde:ferredoxin oxidoreductase | Bacteria;Proteobacteria;Deltaproteobacteria;unclassified Deltaproteobacteria;unclassified Deltaproteobacteria;Deferrisoma;Deferrisoma camini;Deferrisoma camini S3R1 |
| Ga0586324 1227206 3 458 | 0.00 | 0.00 | 0.00 | 0.00 | 0.00 | 0.00 | 0.00 | 0.00 | 0.00 | 0.00 | 0.00 | 346743.75 | KO:K03738 | aldehyde:ferredoxin oxidoreductase | Bacteria;Proteobacteria;Deltaproteobacteria;Desulfobacterales;Desulfobacteraceae;Desulfatiglans;Desulfatiglans anilini;Desulfatiglans anilini DSM 4660 |
| Ga0586324 1269480 1 1095 | 5290000.00 | 0.00 | 13117900.00 | 0.00 | 0.00 | 5716800.00 | 0.00 | 0.00 | 0.00 | 0.00 | 3528800.00 | 0.00 | KO:K03738 | aldehyde:ferredoxin oxidoreductase | Bacteria;Chloroflexi;Chloroflexia;Chloroflexales;Chloroflexaceae;Chloroflexus;Chloroflexus aggregans;Chloroflexus aggregans DSM 9485 |
| Ga0586324 1603857 61 993 | 0.00 | 401036.12 | 0.00 | 0.00 | 0.00 | 0.00 | 0.00 | 0.00 | 0.00 | 0.00 | 0.00 | 0.00 | KO:K03738 | aldehyde:ferredoxin oxidoreductase | Bacteria;Chloroflexi;Chloroflexia;Chloroflexales;Chloroflexaceae;Chloroflexus;Chloroflexus islandicus;Chloroflexus islandicus isl-2 |
| Ga0586324 1653824 1 978 | 0.00 | 0.00 | 0.00 | 0.00 | 0.00 | 0.00 | 0.00 | 0.00 | 0.00 | 1001000.00 | 0.00 | 0.00 | KO:K03738 | aldehyde:ferredoxin oxidoreductase | Bacteria;Proteobacteria;Deltaproteobacteria;Syntrophobacterales;Syntrophobacteraceae;Desulfoglaeba;Desulfoglaeba alkanexedens;Desulfoglaeba alkanexedens ALDC |
| Ga0586324 1855350 1 933 | 0.00 | 0.00 | 0.00 | 0.00 | 0.00 | 0.00 | 0.00 | 0.00 | 0.00 | 1740200.00 | 0.00 | 0.00 | KO:K03738 | aldehyde:ferredoxin oxidoreductase | Archaea;Euryarchaeota;Methanomicrobia;Methanomicrobiales;Methanomicrobiaceae;Methanoplanus;Methanoplanus limicola;Methanoplanus limicola M3, DSM 2279 |
| Ga0586324 1919066 3 920 | 0.00 | 5378800.00 | 0.00 | 0.00 | 3790400.00 | 0.00 | 0.00 | 0.00 | 3977100.00 | 0.00 | 1355200.00 | 280966.50 | KO:K03738 | aldehyde:ferredoxin oxidoreductase | Bacteria;Proteobacteria;Deltaproteobacteria;Desulfuromonadales;Geobacteraceae;Geobacter;Geobacter sp. DSM 2909;Geobacter sp. DSM 2909 |
| Ga0586324 2008555 3 368 | 0.00 | 0.00 | 0.00 | 0.00 | 0.00 | 0.00 | 0.00 | 0.00 | 0.00 | 0.00 | 0.00 | 625323.75 | KO:K03738 | aldehyde:ferredoxin oxidoreductase | Bacteria;Proteobacteria;Deltaproteobacteria;Syntrophobacterales;Syntrophobacteraceae;Desulfosoma;Desulfosoma caldarium;Desulfosoma caldarium DSM 22027 |
| Ga0586324 2155592 2 874 | 3050000.00 | 0.00 | 0.00 | 0.00 | 0.00 | 0.00 | 0.00 | 0.00 | 0.00 | 0.00 | 0.00 | 0.00 | KO:K03738 | aldehyde:ferredoxin oxidoreductase | Bacteria;Proteobacteria;Deltaproteobacteria;Syntrophobacterales;Syntrophobacteraceae;Syntrophobacter;Syntrophobacter fumaroxidans;Syntrophobacter fumaroxidans MPOB |
| Ga0586324 2304584 174 851 | 9020000.00 | 0.00 | 0.00 | 0.00 | 0.00 | 0.00 | 0.00 | 0.00 | 0.00 | 0.00 | 0.00 | 0.00 | KO:K03738 | aldehyde:ferredoxin oxidoreductase | Bacteria;Proteobacteria;Deltaproteobacteria;Syntrophobacterales;Syntrophobacteraceae;Desulfacinum;Desulfacinum hydrothermale;Desulfacinum hydrothermale DSM 13146 |
| Ga0586324 2544968 224 817 | 778830.00 | 10676000.00 | 457104.77 | 7923000.00 | 11845000.00 | 5835900.00 | 209395.66 | 713700.00 | 10854000.00 | 7130200.00 | 440934.56 | 4462500.00 | KO:K03738 | aldehyde:ferredoxin oxidoreductase | Bacteria;Proteobacteria;Deltaproteobacteria;Syntrophobacterales;Syntrophaceae;Desulfomonile;Desulfomonile tiedjei;Desulfomonile tiedjei DCB-1, DSM 6799 |
| Ga0586324 2961559 322 765 | 6960000.00 | 0.00 | 0.00 | 0.00 | 0.00 | 0.00 | 0.00 | 4489600.00 | 0.00 | 0.00 | 0.00 | 0.00 | KO:K03738 | aldehyde:ferredoxin oxidoreductase | Bacteria;Spirochaetes;Spirochaetia;Spirochaetales;Spirochaetaceae;Alkalispirochaeta;Alkalispirochaeta odontotermitis;Alkalispirochaeta odontotermitis JC202 |
| Ga0586324 3204579 159 740 | 5220000.00 | 3488400.00 | 6756000.00 | 0.00 | 0.00 | 0.00 | 0.00 | 0.00 | 0.00 | 0.00 | 0.00 | 0.00 | KO:K03738 | aldehyde:ferredoxin oxidoreductase | Bacteria;Proteobacteria;Deltaproteobacteria;Desulfobacterales;Desulfobacteraceae;Desulfatirhabdium;Desulfatirhabdium butyrativorans;Desulfatirhabdium butyrativorans DSM 18734 |
| Ga0586324 3412484 3 722 | 1800000.00 | 0.00 | 0.00 | 0.00 | 0.00 | 0.00 | 0.00 | 0.00 | 0.00 | 0.00 | 0.00 | 0.00 | KO:K03738 | aldehyde:ferredoxin oxidoreductase | Bacteria;Chloroflexi;Chloroflexia;Chloroflexales;Roseiflexaceae;Roseiflexus;Roseiflexus castenholzii;Roseiflexus castenholzii HLO8, DSM 13941 |
| Ga0586324 3420085 3 680 | 0.00 | 0.00 | 0.00 | 0.00 | 0.00 | 0.00 | 4149600.00 | 0.00 | 0.00 | 0.00 | 2508000.00 | 0.00 | KO:K03738 | aldehyde:ferredoxin oxidoreductase | Bacteria;Proteobacteria;Deltaproteobacteria;Syntrophobacterales;Syntrophaceae;Desulfomonile;Desulfomonile tiedjei;Desulfomonile tiedjei DCB-1, DSM 6799 |
| Ga0586324 3467340 3 716 | 0.00 | 1081200.00 | 0.00 | 3097000.00 | 0.00 | 0.00 | 0.00 | 0.00 | 0.00 | 0.00 | 0.00 | 0.00 | KO:K03738 | aldehyde:ferredoxin oxidoreductase | Bacteria;Proteobacteria;Deltaproteobacteria;Desulfobacterales;Desulfobacteraceae;Desulfotignum;Desulfotignum phosphitoxidans;Desulfotignum phosphitoxidans FiPS-3, DSM 13687 |
| Ga0586324 3646399 2 703 | 0.00 | 1577600.00 | 0.00 | 0.00 | 0.00 | 0.00 | 0.00 | 0.00 | 0.00 | 0.00 | 0.00 | 0.00 | KO:K03738 | aldehyde:ferredoxin oxidoreductase | Bacteria;Chloroflexi;Anaerolineae;Anaerolineales;Anaerolineaceae;Thermanaerothrix;Thermanaerothrix daxensis;Thermanaerothrix daxensis GNS-1 |
| Ga0586324 3891870 233 685 | 0.00 | 0.00 | 0.00 | 0.00 | 0.00 | 0.00 | 0.00 | 4904400.00 | 5200200.00 | 0.00 | 0.00 | 0.00 | KO:K03738 | aldehyde:ferredoxin oxidoreductase | Bacteria;Proteobacteria;Deltaproteobacteria;Syntrophobacterales;Syntrophaceae;Desulfomonile;Desulfomonile tiedjei;Desulfomonile tiedjei DCB-1, DSM 6799 |
| Ga0586324 4046597 2 673 | 0.00 | 0.00 | 0.00 | 0.00 | 11330000.00 | 0.00 | 0.00 | 0.00 | 0.00 | 0.00 | 0.00 | 0.00 | KO:K03738 | aldehyde:ferredoxin oxidoreductase | Bacteria;Firmicutes;Tissierellia;Tissierellales;Tissierellaceae;Gudongella;Gudongella oleilytica;Soehngenia sp. W6 |
| Ga0586324 4067416 1 672 | 0.00 | 0.00 | 8163500.00 | 0.00 | 0.00 | 0.00 | 0.00 | 0.00 | 0.00 | 0.00 | 0.00 | 0.00 | KO:K03738 | aldehyde:ferredoxin oxidoreductase | Bacteria;Chloroflexi;Chloroflexia;Chloroflexales;Chloroflexaceae;Chloroflexus;Chloroflexus aggregans;Chloroflexus aggregans DSM 9485 |
| Ga0586324 6065322 3 398 | 0.00 | 0.00 | 0.00 | 0.00 | 0.00 | 0.00 | 0.00 | 3080500.00 | 0.00 | 0.00 | 0.00 | 0.00 | KO:K03738 | aldehyde:ferredoxin oxidoreductase | Bacteria;Proteobacteria;Deltaproteobacteria;Syntrophobacterales;Syntrophaceae;Desulfomonile;Desulfomonile tiedjei;Desulfomonile tiedjei DCB-1, DSM 6799 |
| Ga0586324 7204702 1 522 | 5130000.00 | 0.00 | 0.00 | 0.00 | 0.00 | 0.00 | 0.00 | 0.00 | 569739.42 | 0.00 | 0.00 | 0.00 | KO:K03738 | aldehyde:ferredoxin oxidoreductase | Bacteria;Proteobacteria;Deltaproteobacteria;Syntrophobacterales;Syntrophaceae;Desulfomonile;Desulfomonile tiedjei;Desulfomonile tiedjei DCB-1, DSM 6799 |
| Ga0586324 8124957 3 503 | 0.00 | 0.00 | 0.00 | 0.00 | 0.00 | 16197600.00 | 0.00 | 0.00 | 0.00 | 0.00 | 0.00 | 0.00 | KO:K03738 | aldehyde:ferredoxin oxidoreductase | Bacteria;Firmicutes;Clostridia;Eubacteriales;Peptococcaceae;Desulfurispora;Desulfurispora thermophila;Desulfurispora thermophila DSM 16022 |
| Ga0586324 0000018 10707 11357 | 0.00 | 0.00 | 0.00 | 0.00 | 0.00 | 0.00 | 0.00 | 0.00 | 481998.60 | 7777000.00 | 6916800.00 | 6517500.00 | KO:K03744 | LemA protein | Bacteria;Proteobacteria;Betaproteobacteria;Nitrosomonadales;Thiobacillaceae;Annwoodia;Annwoodia aquaesulis;Annwoodia aquaesulis DSM 4255 |
| Ga0586324 0001984 549 1151 | 0.00 | 0.00 | 0.00 | 1263500.00 | 0.00 | 0.00 | 0.00 | 0.00 | 0.00 | 0.00 | 0.00 | 0.00 | KO:K03744 | LemA protein | Bacteria;Acidobacteria;Holophagae;Holophagales;Holophagaceae;Holophaga;Holophaga foetida;Holophaga foetida TMBS4, DSM 6591 |
| Ga0586324 0004183 9572 10174 | 0.00 | 1591200.00 | 0.00 | 0.00 | 0.00 | 0.00 | 0.00 | 0.00 | 0.00 | 0.00 | 0.00 | 672177.00 | KO:K03744 | LemA protein | Bacteria;Spirochaetes;Spirochaetia;Spirochaetales;Spirochaetaceae;Spirochaeta;Spirochaeta thermophila;Spirochaeta thermophila DSM 6192 |
| Ga0586324 0008208 9452 10078 | 0.00 | 1686400.00 | 0.00 | 0.00 | 4861600.00 | 0.00 | 0.00 | 0.00 | 0.00 | 0.00 | 0.00 | 0.00 | KO:K03744 | LemA protein | Bacteria;Acidobacteria;Holophagae;Holophagales;Holophagaceae;Holophaga;Holophaga foetida;Holophaga foetida TMBS4, DSM 6591 |
| Ga0586324 0011087 1165 1791 | 0.00 | 0.00 | 0.00 | 0.00 | 0.00 | 5518300.00 | 0.00 | 0.00 | 0.00 | 0.00 | 2024000.00 | 0.00 | KO:K03744 | LemA protein | Bacteria;Verrucomicrobia;Opitutae;Opitutales;Opitutaceae;unclassified Opitutaceae;Opitutaceae bacterium EW11;Opitutaceae bacterium EW11 |
| Ga0586324 0053378 1605 2318 | 1170000.00 | 0.00 | 0.00 | 0.00 | 0.00 | 0.00 | 0.00 | 0.00 | 2340900.00 | 2579500.00 | 2296800.00 | 1590000.00 | KO:K03744 | LemA protein | Bacteria;Proteobacteria;Gammaproteobacteria;Immundisolibacterales;Immundisolibacteraceae;Immundisolibacter;Immundisolibacter cernigliae;Immundisolibacter cernigliae TR3.2 |
| Ga0586324 0145676 708 1325 | 0.00 | 0.00 | 0.00 | 0.00 | 0.00 | 0.00 | 6657600.00 | 0.00 | 0.00 | 0.00 | 0.00 | 0.00 | KO:K03744 | LemA protein | Bacteria;Proteobacteria;Gammaproteobacteria;Pseudomonadales;Moraxellaceae;Acinetobacter;Acinetobacter bereziniae;Acinetobacter bereziniae XH901 |
| Ga0586324 0181647 1367 2047 | 0.00 | 0.00 | 0.00 | 0.00 | 0.00 | 0.00 | 0.00 | 0.00 | 0.00 | 0.00 | 3757600.00 | 0.00 | KO:K03744 | LemA protein | Bacteria;Acidobacteria;Thermoanaerobaculia;Thermoanaerobaculales;Thermoanaerobaculaceae;Thermoanaerobaculum;Thermoanaerobaculum aquaticum;Thermoanaerobaculum aquaticum MP-01 |
| Ga0586324 0273503 593 1207 | 0.00 | 0.00 | 0.00 | 0.00 | 220114.09 | 0.00 | 0.00 | 0.00 | 0.00 | 0.00 | 0.00 | 0.00 | KO:K03744 | LemA protein | Bacteria;Proteobacteria;Alphaproteobacteria;Hyphomicrobiales;Beijerinckiaceae;Beijerinckia;Beijerinckia mobilis;Beijerinckia mobilis UQM 1969, DSM 2326 |
| Ga0586324 0376151 203 790 | 0.00 | 0.00 | 0.00 | 0.00 | 0.00 | 0.00 | 7022400.00 | 0.00 | 0.00 | 0.00 | 9328000.00 | 0.00 | KO:K03744 | LemA protein | Bacteria;Proteobacteria;Deltaproteobacteria;Desulfobacterales;Desulfobacteraceae;Desulfatirhabdium;Desulfatirhabdium butyrativorans;Desulfatirhabdium butyrativorans DSM 18734 |
| Ga0586324 0454029 695 1267 | 0.00 | 0.00 | 3591388.26 | 0.00 | 0.00 | 0.00 | 0.00 | 0.00 | 0.00 | 0.00 | 0.00 | 0.00 | KO:K03744 | LemA protein | Bacteria;Nitrospinae;Nitrospinia;Nitrospinales;Nitrospinaceae;Nitrospina;Nitrospina gracilis;Nitrospina gracilis 3/211 |
| Ga0586324 0757735 728 1369 | 0.00 | 0.00 | 0.00 | 0.00 | 0.00 | 0.00 | 0.00 | 0.00 | 0.00 | 0.00 | 2745600.00 | 0.00 | KO:K03744 | LemA protein | Bacteria;Proteobacteria;Betaproteobacteria;Nitrosomonadales;Thiobacillaceae;Thiobacillus;Thiobacillus thioparus;Thiobacillus thioparus DSM 505 |
| Ga0586324 0759944 326 934 | 0.00 | 0.00 | 0.00 | 0.00 | 0.00 | 0.00 | 759983.28 | 0.00 | 0.00 | 0.00 | 0.00 | 0.00 | KO:K03744 | LemA protein | Bacteria;Acidobacteria;Holophagae;Holophagales;Holophagaceae;Holophaga;Holophaga foetida;Holophaga foetida TMBS4, DSM 6591 |
| Ga0586324 0775764 60 650 | 0.00 | 0.00 | 0.00 | 0.00 | 4202400.00 | 0.00 | 0.00 | 0.00 | 2089800.00 | 0.00 | 0.00 | 0.00 | KO:K03744 | LemA protein | Bacteria;Bacteroidetes;Sphingobacteriia;Sphingobacteriales;Sphingobacteriaceae;Anseongella;Anseongella ginsenosidimutans;Anseongella ginsenosidimutans DSM 21100 |
| Ga0586324 0820605 18 668 | 0.00 | 0.00 | 0.00 | 2707500.00 | 0.00 | 0.00 | 0.00 | 0.00 | 0.00 | 0.00 | 0.00 | 0.00 | KO:K03744 | LemA protein | Bacteria;Proteobacteria;Betaproteobacteria;Burkholderiales;Burkholderiaceae;Burkholderia;Burkholderia diffusa;Burkholderia diffusa MSMB375WGS |
| Ga0586324 1879726 185 844 | 0.00 | 0.00 | 0.00 | 0.00 | 0.00 | 0.00 | 0.00 | 945500.00 | 0.00 | 0.00 | 0.00 | 0.00 | KO:K03744 | LemA protein | Bacteria;Proteobacteria;Betaproteobacteria;Burkholderiales;Burkholderiaceae;Burkholderia;Burkholderia pseudomallei;Burkholderia pseudomallei MSHR4462 |
| Ga0586324 2868672 2 661 | 0.00 | 0.00 | 0.00 | 0.00 | 0.00 | 0.00 | 4195200.00 | 0.00 | 0.00 | 0.00 | 0.00 | 0.00 | KO:K03744 | LemA protein | Bacteria;Candidatus Aminicenantes;unclassified Candidatus Aminicenantes;unclassified Candidatus Aminicenantes;unclassified Candidatus Aminicenantes;unclassified Candidatus Aminicenantes;Aminicenantes bacterium SCGC AAA255-E05;Aminicenantes bacterium SCGC AAA255-E05 (contamination screened) |
| Ga0586324 3106489 334 750 | 0.00 | 0.00 | 0.00 | 0.00 | 0.00 | 0.00 | 0.00 | 0.00 | 0.00 | 6906900.00 | 0.00 | 0.00 | KO:K03744 | LemA protein | Bacteria;Proteobacteria;Betaproteobacteria;Nitrosomonadales;Sterolibacteriaceae;Methyloversatilis;Methyloversatilis discipulorum;Methyloversatilis discipulorum RZ18-153 |
| Ga0586324 4420883 313 648 | 0.00 | 0.00 | 0.00 | 0.00 | 0.00 | 0.00 | 0.00 | 829600.00 | 0.00 | 0.00 | 0.00 | 0.00 | KO:K03744 | LemA protein | Bacteria;Acidobacteria;Holophagae;Holophagales;Holophagaceae;Holophaga;Holophaga foetida;Holophaga foetida TMBS4, DSM 6591 |
| Ga0586324 5585494 124 588 | 0.00 | 0.00 | 0.00 | 0.00 | 9156700.00 | 0.00 | 0.00 | 0.00 | 0.00 | 0.00 | 0.00 | 0.00 | KO:K03744 | LemA protein | Archaea;Euryarchaeota;Methanomicrobia;Methanomicrobiales;Methanoregulaceae;Methanoregula;Methanoregula formicica;Methanoregula formicica SMSP, DSM 22288 |
| Ga0586324 5771405 246 581 | 0.00 | 0.00 | 0.00 | 0.00 | 0.00 | 0.00 | 0.00 | 945500.00 | 0.00 | 0.00 | 0.00 | 0.00 | KO:K03744 | LemA protein | Bacteria;Chloroflexi;Dehalococcoidia;Dehalococcoidales;Dehalococcoidaceae;Dehalococcoides;Dehalococcoides mccartyi;Dehalococcoides mccartyi VS |
| Ga0586324 6298902 53 562 | 0.00 | 0.00 | 0.00 | 0.00 | 0.00 | 0.00 | 9142800.00 | 0.00 | 0.00 | 0.00 | 0.00 | 0.00 | KO:K03744 | LemA protein | Bacteria;Candidatus Marinimicrobia;unclassified Candidatus Marinimicrobia;unclassified Candidatus Marinimicrobia;unclassified Candidatus Marinimicrobia;unclassified Candidatus Marinimicrobia;Marinimicrobia bacterium JGI 0000077-B04;Marinimicrobia bacterium JGI 0000077-B04 (contamination screened) |
| Ga0586324 7090431 3 533 | 0.00 | 0.00 | 0.00 | 0.00 | 0.00 | 0.00 | 0.00 | 1037000.00 | 0.00 | 0.00 | 0.00 | 0.00 | KO:K03744 | LemA protein | Bacteria;Firmicutes;Clostridia;Eubacteriales;Clostridiaceae;Thermobrachium;Thermobrachium celere;Thermobrachium celere JW/YL-NZ35, DSM 8682 |
| Ga0586324 1177975 792 1130 | 2240000.00 | 0.00 | 0.00 | 5804500.00 | 0.00 | 0.00 | 0.00 | 0.00 | 0.00 | 4543000.00 | 17776000.00 | 0.00 | KO:K03746 | DNA-binding protein H-NS | Bacteria;Proteobacteria;Gammaproteobacteria;Chromatiales;Chromatiaceae;Lamprocystis;Lamprocystis purpurea;Lamprocystis purpurea DSM 4197 |
| Ga0586324 7496189 91 492 | 0.00 | 0.00 | 0.00 | 5757000.00 | 0.00 | 0.00 | 0.00 | 0.00 | 0.00 | 0.00 | 0.00 | 0.00 | KO:K03746 | DNA-binding protein H-NS | Bacteria;Proteobacteria;Gammaproteobacteria;Chromatiales;Chromatiaceae;Lamprocystis;Lamprocystis purpurea;Lamprocystis purpurea DSM 4197 |
| Ga0586324 0000586 9077 9874 | 0.00 | 0.00 | 0.00 | 0.00 | 0.00 | 0.00 | 0.00 | 0.00 | 0.00 | 0.00 | 2261600.00 | 0.00 | KO:K03769 | peptidyl-prolyl cis-trans isomerase C | Bacteria;Proteobacteria;Betaproteobacteria;Nitrosomonadales;Thiobacillaceae;Annwoodia;Annwoodia aquaesulis;Annwoodia aquaesulis DSM 4255 |
| Ga0586324 0001823 5280 6098 | 0.00 | 0.00 | 0.00 | 0.00 | 0.00 | 0.00 | 2895600.00 | 0.00 | 0.00 | 10241000.00 | 0.00 | 3802500.00 | KO:K03769 | peptidyl-prolyl cis-trans isomerase C | Bacteria;Proteobacteria;Betaproteobacteria;Nitrosomonadales;Thiobacillaceae;Thiobacillus;Thiobacillus thioparus;Thiobacillus thioparus DSM 505 |
| Ga0586324 1601968 244 993 | 0.00 | 0.00 | 0.00 | 0.00 | 0.00 | 0.00 | 0.00 | 0.00 | 0.00 | 0.00 | 0.00 | 3142500.00 | KO:K03769 | peptidyl-prolyl cis-trans isomerase C | Bacteria;Proteobacteria;Betaproteobacteria;Neisseriales;Chromobacteriaceae;Chromobacterium;Chromobacterium piscinae;Chromobacterium piscinae ND17 |
| Ga0586324 1634701 1 414 | 0.00 | 0.00 | 0.00 | 0.00 | 0.00 | 0.00 | 0.00 | 0.00 | 0.00 | 0.00 | 4276800.00 | 0.00 | KO:K03769 | peptidyl-prolyl cis-trans isomerase C | Bacteria;Proteobacteria;Betaproteobacteria;Nitrosomonadales;Thiobacillaceae;Thiobacillus;Thiobacillus thioparus;Thiobacillus thioparus DSM 505 |
| Ga0586324 2277036 2 793 | 0.00 | 0.00 | 0.00 | 5491000.00 | 0.00 | 0.00 | 0.00 | 0.00 | 0.00 | 0.00 | 0.00 | 0.00 | KO:K03769 | peptidyl-prolyl cis-trans isomerase C | Bacteria;Proteobacteria;Betaproteobacteria;Rhodocyclales;Azonexaceae;Dechloromonas;Dechloromonas sp. SG708;Dechloromonas sp. SG708 |
| Ga0586324 3195464 1 414 | 2720000.00 | 2019600.00 | 2757534.59 | 0.00 | 0.00 | 2668117.90 | 0.00 | 0.00 | 0.00 | 0.00 | 0.00 | 0.00 | KO:K03769 | peptidyl-prolyl cis-trans isomerase C | Bacteria;Proteobacteria;Betaproteobacteria;Rhodocyclales;Azonexaceae;Dechloromonas;Dechloromonas denitrificans;Dechloromonas denitrificans ATCC BAA-841 |
| Ga0586324 0000137 30238 32133 | 0.00 | 0.00 | 0.00 | 0.00 | 0.00 | 0.00 | 0.00 | 2013000.00 | 939600.00 | 5243700.00 | 5271200.00 | 9997500.00 | KO:K03770 | peptidyl-prolyl cis-trans isomerase D | Bacteria;Proteobacteria;Betaproteobacteria;Nitrosomonadales;Thiobacillaceae;Annwoodia;Annwoodia aquaesulis;Annwoodia aquaesulis DSM 4255 |
| Ga0586324 0026215 2521 4437 | 0.00 | 0.00 | 0.00 | 0.00 | 0.00 | 0.00 | 0.00 | 0.00 | 0.00 | 2494800.00 | 1108800.00 | 0.00 | KO:K03770 | peptidyl-prolyl cis-trans isomerase D | Bacteria;Proteobacteria;Betaproteobacteria;Nitrosomonadales;Sulfuricellaceae;Sulfuricella;Sulfuricella sp. T08;Sulfuricella sp. T08 |
| Ga0586324 8071595 2 505 | 0.00 | 0.00 | 0.00 | 0.00 | 0.00 | 0.00 | 0.00 | 0.00 | 0.00 | 0.00 | 0.00 | 1110000.00 | KO:K03770 | peptidyl-prolyl cis-trans isomerase D | Bacteria;Proteobacteria;Betaproteobacteria;Nitrosomonadales;Thiobacillaceae;Thiobacillus;Thiobacillus denitrificans;Thiobacillus denitrificans DSM 12475 |
| Ga0586324 0000423 1392 2693 | 0.00 | 0.00 | 0.00 | 0.00 | 0.00 | 0.00 | 0.00 | 0.00 | 461091.69 | 0.00 | 3616800.00 | 3622500.00 | KO:K03771 | peptidyl-prolyl cis-trans isomerase SurA | Bacteria;Proteobacteria;Betaproteobacteria;Nitrosomonadales;Thiobacillaceae;Annwoodia;Annwoodia aquaesulis;Annwoodia aquaesulis DSM 4255 |
| Ga0586324 2296311 3 521 | 0.00 | 0.00 | 0.00 | 14820000.00 | 0.00 | 0.00 | 0.00 | 0.00 | 0.00 | 0.00 | 0.00 | 0.00 | KO:K03773 | FKBP-type peptidyl-prolyl cis-trans isomerase FklB | Bacteria;Proteobacteria;Deltaproteobacteria;Desulfuromonadales;Desulfuromonadaceae;Desulfuromonas;Desulfuromonas acetexigens;Desulfuromonas acetexigens DSM 1397 |
| Ga0586324 0165790 249 1940 | 0.00 | 0.00 | 0.00 | 0.00 | 0.00 | 0.00 | 0.00 | 0.00 | 0.00 | 0.00 | 0.00 | 88146.75 | KO:K03776 | aerotaxis receptor | Bacteria;Proteobacteria;Gammaproteobacteria;Oceanospirillales;Oceanospirillaceae;Marinospirillum;Marinospirillum perlucidum;Marinospirillum perlucidum F3212 |
| Ga0586324 0737557 92 1384 | 0.00 | 0.00 | 0.00 | 0.00 | 0.00 | 0.00 | 347202.96 | 0.00 | 0.00 | 0.00 | 0.00 | 0.00 | KO:K03776 | aerotaxis receptor | Bacteria;Proteobacteria;Betaproteobacteria;Burkholderiales;Alcaligenaceae;Parvibium;Parvibium lacunae;Parvibium lacunae KMB9 |
| Ga0586324 0000002 157231 158616 | 0.00 | 0.00 | 0.00 | 0.00 | 0.00 | 0.00 | 0.00 | 0.00 | 0.00 | 0.00 | 0.00 | 3787500.00 | KO:K03777 | D-lactate dehydrogenase | Bacteria;Proteobacteria;Betaproteobacteria;Nitrosomonadales;Thiobacillaceae;Annwoodia;Annwoodia aquaesulis;Annwoodia aquaesulis DSM 4255 |

| | | | | | | | | | | | | | | | |
|---|---|---|---|---|---|---|---|---|---|---|---|---|---|---|---|
| Ga0586324_0009240_1_1878 | 15000000.00 | 0.00 | 0.00 | 0.00 | 0.00 | 0.00 | 0.00 | 0.00 | 0.00 | 0.00 | 0.00 | 0.00 | KO:K03782 | catalase-peroxidase | Archaea;Euryarchaeota;Methanomicrobia;Methanosarcinales;Methanosarcinaceae;Methanococcoides;Methanococcoides methylutens;Methanococcoides methylutens DSM 2657 |
| Ga0586324_0035016_4086_5690 | 0.00 | 0.00 | 0.00 | 0.00 | 6571400.00 | 0.00 | 0.00 | 0.00 | 0.00 | 0.00 | 0.00 | 0.00 | KO:K03782 | catalase-peroxidase | Archaea;Euryarchaeota;Methanomicrobia;Methanosarcinales;Methanosarcinaceae;Methanococcoides;Methanococcoides methylutens;Methanococcoides methylutens MM1 |
| Ga0586324_0052523_1347_3515 | 0.00 | 0.00 | 3145351.51 | 0.00 | 0.00 | 0.00 | 0.00 | 0.00 | 0.00 | 0.00 | 0.00 | 0.00 | KO:K03782 | catalase-peroxidase | Bacteria;Proteobacteria;Gammaproteobacteria;Acidiferrobacterales;Acidiferrobacteraceae;Sulfuricaulis;Sulfuricaulis limicola;Sulfuricaulis limicola HA5 |
| Ga0586324_0073750_1661_3853 | 0.00 | 0.00 | 0.00 | 0.00 | 0.00 | 0.00 | 0.00 | 4093100.00 | 0.00 | 0.00 | 0.00 | 0.00 | KO:K03782 | catalase-peroxidase | Bacteria;Proteobacteria;Deltaproteobacteria;Syntrophobacterales;Syntrophaceae;Desulfomonile;Desulfomonile tiedjei;Desulfomonile tiedjei DCB-1, DSM 6799 |
| Ga0586324_0079363_1593_3785 | 0.00 | 289478.72 | 0.00 | 0.00 | 0.00 | 0.00 | 0.00 | 0.00 | 0.00 | 0.00 | 0.00 | 0.00 | KO:K03782 | catalase-peroxidase | Bacteria;Firmicutes;Negativicutes;Selenomonadales;Sporomusaceae;Dendrosporobacter;Dendrosporobacter quercicolus;Dendrosporobacter quercicolus DSM 1736 |
| Ga0586324_0091325_245_2458 | 0.00 | 0.00 | 0.00 | 8664000.00 | 0.00 | 0.00 | 13315200.00 | 0.00 | 0.00 | 0.00 | 0.00 | 6855000.00 | KO:K03782 | catalase-peroxidase | Bacteria;Proteobacteria;Alphaproteobacteria;Rhodospirillales;unclassified Rhodospirillales;unclassified Rhodospirillales;Rhodospirillales bacterium URHD0088;Rhodospirillales bacterium URHD0088 |
| Ga0586324_0126621_1_1056 | 0.00 | 0.00 | 3364099.53 | 0.00 | 0.00 | 0.00 | 0.00 | 0.00 | 0.00 | 0.00 | 0.00 | 0.00 | KO:K03782 | catalase-peroxidase | Bacteria;Proteobacteria;Betaproteobacteria;Burkholderiales;Comamonadaceae;Polaromonas;Polaromonas sp. JS666;Polaromonas sp. JS666 |
| Ga0586324_0137830_181_2370 | 0.00 | 0.00 | 0.00 | 0.00 | 0.00 | 25884400.00 | 16826400.00 | 0.00 | 0.00 | 0.00 | 0.00 | 0.00 | KO:K03782 | catalase-peroxidase | Bacteria;Proteobacteria;Betaproteobacteria;Neisseriales;Chromobacteriaceae;Chromobacterium;Chromobacterium piscinae;Chromobacterium piscinae ND17 |
| Ga0586324_0163020_2126_2722 | 0.00 | 277708.60 | 0.00 | 0.00 | 0.00 | 0.00 | 0.00 | 0.00 | 0.00 | 0.00 | 0.00 | 0.00 | KO:K03782 | catalase-peroxidase | Archaea;Euryarchaeota;Methanomicrobia;Methanosarcinales;Methanosarcinaceae;Methanosarcina;Methanosarcina barkeri;Methanosarcina barkeri 227 |
| Ga0586324_0257437_3_1097 | 0.00 | 0.00 | 0.00 | 0.00 | 0.00 | 0.00 | 0.00 | 0.00 | 404437.05 | 0.00 | 0.00 | 0.00 | KO:K03782 | catalase-peroxidase | Archaea;Euryarchaeota;Methanomicrobia;Methanosarcinales;Methanosarcinaceae;Methanosarcina;Methanosarcina barkeri;Methanosarcina barkeri MS |
| Ga0586324_0335083_479_1957 | 0.00 | 4807600.00 | 0.00 | 0.00 | 0.00 | 13259800.00 | 0.00 | 0.00 | 0.00 | 0.00 | 0.00 | 0.00 | KO:K03782 | catalase-peroxidase | Archaea;Euryarchaeota;Methanomicrobia;Methanosarcinales;Methanosarcinaceae;Methanosarcina;Methanosarcina sp. WH1;Methanosarcina sp. WH1 |
| Ga0586324_0399200_3_998 | 3250000.00 | 0.00 | 0.00 | 0.00 | 0.00 | 0.00 | 0.00 | 0.00 | 0.00 | 0.00 | 0.00 | 997500.00 | KO:K03782 | catalase-peroxidase | Archaea;Euryarchaeota;Methanomicrobia;Methanosarcinales;Methanosarcinaceae;Methanosarcina;Methanosarcina sp. WH1;Methanosarcina sp. WH1 |
| Ga0586324_0810207_806_1330 | 0.00 | 0.00 | 0.00 | 0.00 | 0.00 | 0.00 | 1697143.08 | 0.00 | 0.00 | 0.00 | 0.00 | 0.00 | KO:K03782 | catalase-peroxidase | Bacteria;Firmicutes;Clostridia;Eubacteriales;Peptococcaceae;unclassified Peptococcaceae;Peptococcaceae bacterium DCMF;Peptococcaceae bacterium DCMF |
| Ga0586324_0826282_1001_1318 | 0.00 | 12172000.00 | 0.00 | 0.00 | 567246.75 | 0.00 | 1165935.00 | 0.00 | 773424.45 | 278073.95 | 0.00 | 469189.50 | KO:K03782 | catalase-peroxidase | Bacteria;Proteobacteria;Betaproteobacteria;Rhodocyclales;Rhodocyclaceae;Rugosibacter;Rugosibacter aromaticivorans;Rugosibacter aromaticivorans PG1-Ca6 |
| Ga0586324_0972235_2_1228 | 0.00 | 761600.00 | 0.00 | 0.00 | 0.00 | 432817.34 | 0.00 | 0.00 | 0.00 | 0.00 | 0.00 | 0.00 | KO:K03782 | catalase-peroxidase | Archaea;Euryarchaeota;Methanomicrobia;Methanomicrobiales;Methanoregulaceae;Methanosphaerula;Methanosphaerula palustris;Methanosphaerula palustris E1-9c, DSM 19958 |
| Ga0586324_0974593_1_1227 | 0.00 | 0.00 | 0.00 | 0.00 | 0.00 | 10004400.00 | 0.00 | 0.00 | 0.00 | 0.00 | 0.00 | 0.00 | KO:K03782 | catalase-peroxidase | Bacteria;Proteobacteria;Betaproteobacteria;Neisseriales;Chromobacteriaceae;Chromobacterium;Chromobacterium sp. MWU13-2610;Chromobacterium sp. MWU13-2610 |
| Ga0586324_1413144_163_1047 | 0.00 | 0.00 | 2430324.62 | 0.00 | 0.00 | 0.00 | 0.00 | 0.00 | 0.00 | 0.00 | 0.00 | 0.00 | KO:K03782 | catalase-peroxidase | Archaea;Euryarchaeota;Methanomicrobia;Methanosarcinales;Methanosarcinaceae;Methanosarcina;Methanosarcina sp. WH1;Methanosarcina sp. WH1 |
| Ga0586324_1501991_637_1020 | 0.00 | 26724000.00 | 0.00 | 0.00 | 0.00 | 0.00 | 0.00 | 0.00 | 0.00 | 0.00 | 0.00 | 0.00 | KO:K03782 | catalase-peroxidase | Bacteria;Proteobacteria;Deltaproteobacteria;Desulfuromonadales;Desulfuromonadaceae;Desulfuromonas;Desulfuromonas sp. TF;Desulfuromonas sp. TF |
| Ga0586324_1699822_526_969 | 0.00 | 0.00 | 0.00 | 0.00 | 0.00 | 0.00 | 0.00 | 0.00 | 3248100.00 | 0.00 | 0.00 | 0.00 | KO:K03782 | catalase-peroxidase | Archaea;Euryarchaeota;Methanomicrobia;Methanomicrobiales;Methanoregulaceae;Methanosphaerula;Methanosphaerula palustris;Methanosphaerula palustris E1-9c, DSM 19958 |
| Ga0586324_2109125_1_882 | 0.00 | 0.00 | 0.00 | 0.00 | 0.00 | 0.00 | 0.00 | 0.00 | 0.00 | 0.00 | 0.00 | 5137500.00 | KO:K03782 | catalase-peroxidase | Archaea;Euryarchaeota;Methanobacteria;Methanobacteriales;Methanobacteriaceae;Methanobacterium;Methanobacterium paludis;Methanobacterium paludis SWAN-1 |
| Ga0586324_2337394_3_845 | 0.00 | 0.00 | 0.00 | 0.00 | 0.00 | 0.00 | 0.00 | 0.00 | 0.00 | 8393000.00 | 0.00 | 0.00 | KO:K03782 | catalase-peroxidase | Bacteria;Proteobacteria;Deltaproteobacteria;Desulfuromonadales;Syntrophotaleaceae;Syntrophotalea;Syntrophotalea acetylenivorans;Pelobacter sp. SFB93 |
| Ga0586324_2460739_3_827 | 0.00 | 0.00 | 0.00 | 0.00 | 0.00 | 532289.66 | 0.00 | 0.00 | 0.00 | 0.00 | 0.00 | 0.00 | KO:K03782 | catalase-peroxidase | Bacteria;Proteobacteria;Betaproteobacteria;Burkholderiales;Comamonadaceae;Polaromonas;Polaromonas sp. JS666;Polaromonas sp. JS666 |
| Ga0586324_2835918_126_779 | 0.00 | 0.00 | 17734500.00 | 3543500.00 | 6046100.00 | 0.00 | 3602400.00 | 1884900.00 | 2033100.00 | 862400.00 | 0.00 | 2377500.00 | KO:K03782 | catalase-peroxidase | Bacteria;Proteobacteria;Betaproteobacteria;Burkholderiales;Burkholderiaceae;Ralstonia;Ralstonia solanacearum;Ralstonia solanacearum bv. 2 SL2312 |
| Ga0586324_2838472_2_778 | 0.00 | 0.00 | 0.00 | 0.00 | 2832500.00 | 0.00 | 3078000.00 | 0.00 | 1636200.00 | 0.00 | 0.00 | 0.00 | KO:K03782 | catalase-peroxidase | Archaea;Euryarchaeota;Methanomicrobia;Methanosarcinales;Methanosarcinaceae;Methanococcoides;Methanococcoides methylutens;Methanococcoides methylutens MM1 |
| Ga0586324_2920269_2_769 | 4940000.00 | 0.00 | 0.00 | 0.00 | 0.00 | 0.00 | 0.00 | 0.00 | 0.00 | 0.00 | 0.00 | 0.00 | KO:K03782 | catalase-peroxidase | Archaea;Euryarchaeota;Methanomicrobia;Methanosarcinales;Methanosarcinaceae;Methanosarcina;Methanosarcina barkeri;Methanosarcina barkeri MS |
| Ga0586324_3183868_1_744 | 0.00 | 0.00 | 0.00 | 0.00 | 0.00 | 0.00 | 0.00 | 0.00 | 0.00 | 0.00 | 0.00 | 915000.00 | KO:K03782 | catalase-peroxidase | Bacteria;Proteobacteria;Alphaproteobacteria;Hyphomicrobiales;Rhizobiaceae;Rhizobium;Rhizobium sp. BK333;Rhizobium sp. BK333 |
| Ga0586324_5717742_2_583 | 0.00 | 0.00 | 0.00 | 0.00 | 0.00 | 0.00 | 0.00 | 0.00 | 619290.36 | 0.00 | 0.00 | 0.00 | KO:K03782 | catalase-peroxidase | Bacteria;Deinococcus-Thermus;Deinococci;Thermales;Thermaceae;Meiothermus;Meiothermus granaticius;Meiothermus granaticius NBRC 107808 |
| Ga0586324_6013406_86_571 | 0.00 | 0.00 | 0.00 | 0.00 | 0.00 | 0.00 | 0.00 | 1268800.00 | 1514700.00 | 0.00 | 0.00 | 3052500.00 | KO:K03782 | catalase-peroxidase | Bacteria;Deinococcus-Thermus;Deinococci;Thermales;Thermaceae;Meiothermus;Meiothermus granaticius;Meiothermus granaticius NBRC 107808 |
| Ga0586324_7726725_99_515 | 0.00 | 0.00 | 0.00 | 0.00 | 0.00 | 0.00 | 0.00 | 0.00 | 0.00 | 0.00 | 0.00 | 9225000.00 | KO:K03782 | catalase-peroxidase | Bacteria;Proteobacteria;Deltaproteobacteria;Desulfovibrionales;Desulfovibrionaceae;Desulfovibrio;Desulfovibrio putealis;Desulfovibrio putealis DSM 16056 |
| Ga0586324_0000004_99744_100274 | 0.00 | 0.00 | 0.00 | 0.00 | 0.00 | 0.00 | 0.00 | 0.00 | 0.00 | 1755600.00 | 0.00 | 2100000.00 | KO:K03786 | 3-dehydroquinate dehydratase-2 | Bacteria;Proteobacteria;Betaproteobacteria;Nitrosomonadales;Thiobacillaceae;Annwoodia;Annwoodia aquaesulis;Annwoodia aquaesulis DSM 4255 |
| Ga0586324_0000290_20935_22278 | 0.00 | 0.00 | 0.00 | 0.00 | 0.00 | 0.00 | 1154434.68 | 646600.00 | 826200.00 | 1878800.00 | 1496000.00 | 1845000.00 | KO:K03797 | carboxyl-terminal processing protease | Bacteria;Proteobacteria;Alphaproteobacteria;Hyphomicrobiales;Hyphomicrobiaceae;Rhodoplanes;Rhodoplanes sp. Z2-YC6860;Rhodoplanes sp. Z2-YC6860 |
| Ga0586324_0000896_9198_10613 | 0.00 | 0.00 | 0.00 | 0.00 | 0.00 | 0.00 | 0.00 | 0.00 | 0.00 | 0.00 | 4347200.00 | 0.00 | KO:K03797 | carboxyl-terminal processing protease | Bacteria;Proteobacteria;Betaproteobacteria;Nitrosomonadales;Thiobacillaceae;Thiobacillus;Thiobacillus thioparus;Thiobacillus thioparus DSM 505 |
| Ga0586324_0001297_9517_11940 | 0.00 | 0.00 | 0.00 | 0.00 | 0.00 | 0.00 | 0.00 | 902800.00 | 0.00 | 0.00 | 0.00 | 0.00 | KO:K03797 | carboxyl-terminal processing protease | Bacteria;Proteobacteria;Deltaproteobacteria;Myxococcales;Labilitrichaceae;Labilithrix;Labilithrix luteola;Labilithrix luteola DSM 27648 |
| Ga0586324_0009890_8501_9868 | 2710000.00 | 0.00 | 0.00 | 0.00 | 0.00 | 0.00 | 0.00 | 0.00 | 0.00 | 0.00 | 0.00 | 0.00 | KO:K03797 | carboxyl-terminal processing protease | Bacteria;Proteobacteria;Deltaproteobacteria;Desulfobacterales;Desulfobacteraceae;Desulfatirhabdium;Desulfatirhabdium butyrativorans;Desulfatirhabdium butyrativorans DSM 18734 |
| Ga0586324_0172381_1306_2640 | 0.00 | 0.00 | 0.00 | 0.00 | 0.00 | 0.00 | 0.00 | 485067.73 | 226813.77 | 0.00 | 0.00 | 0.00 | KO:K03797 | carboxyl-terminal processing protease | Bacteria;Proteobacteria;Alphaproteobacteria;Hyphomicrobiales;Hyphomicrobiaceae;Rhodoplanes;Rhodoplanes sp. Z2-YC6860;Rhodoplanes sp. Z2-YC6860 |
| Ga0586324_0230560_168_1502 | 0.00 | 0.00 | 0.00 | 0.00 | 11742000.00 | 0.00 | 0.00 | 11346000.00 | 0.00 | 0.00 | 0.00 | 0.00 | KO:K03797 | carboxyl-terminal processing protease | Bacteria;Proteobacteria;Deltaproteobacteria;Desulfuromonadales;Geobacteraceae;Geobacter;Geobacter pickeringii;Geobacter pickeringii G13 |
| Ga0586324_0248273_1153_2241 | 0.00 | 0.00 | 0.00 | 0.00 | 0.00 | 0.00 | 0.00 | 0.00 | 0.00 | 606750.76 | 0.00 | 0.00 | KO:K03797 | carboxyl-terminal processing protease | Bacteria;Proteobacteria;Betaproteobacteria;Nitrosomonadales;Gallionellaceae;Gallionella;Gallionella capsiferriformans;Gallionella capsiferriformans ES-2 |
| Ga0586324_0429509_344_1687 | 0.00 | 0.00 | 0.00 | 0.00 | 0.00 | 0.00 | 0.00 | 0.00 | 0.00 | 0.00 | 906400.00 | 0.00 | KO:K03797 | carboxyl-terminal processing protease | Bacteria;Proteobacteria;Deltaproteobacteria;Desulfuromonadales;Geobacteraceae;Geobacter;Geobacter daltonii;Geobacter daltonii FRC-32 |
| Ga0586324_0468282_758_1687 | 0.00 | 0.00 | 0.00 | 0.00 | 0.00 | 0.00 | 0.00 | 0.00 | 0.00 | 0.00 | 0.00 | 351486.00 | KO:K03797 | carboxyl-terminal processing protease | Bacteria;Proteobacteria;Alphaproteobacteria;Rhodobacterales;Rhodobacteraceae;Rhodovulum;Rhodovulum sp. PH10;Rhodovulum sp. PH10 |
| Ga0586324_0591133_1_876 | 0.00 | 0.00 | 0.00 | 0.00 | 0.00 | 0.00 | 0.00 | 0.00 | 0.00 | 0.00 | 0.00 | 4792500.00 | KO:K03797 | carboxyl-terminal processing protease | Bacteria;Proteobacteria;Gammaproteobacteria;Acidiferrobacterales;Acidiferrobacteraceae;Sulfuricaulis;Sulfuricaulis limicola;Sulfuricaulis limicola HA5 |
| Ga0586324_1099797_82_1164 | 0.00 | 0.00 | 0.00 | 0.00 | 0.00 | 0.00 | 0.00 | 0.00 | 2907900.00 | 6252400.00 | 3062400.00 | 0.00 | KO:K03797 | carboxyl-terminal processing protease | Bacteria;Proteobacteria;Alphaproteobacteria;Hyphomicrobiales;Bradyrhizobiaceae;Bradyrhizobium;Bradyrhizobium lablabi;Bradyrhizobium lablabi GAS499 |
| Ga0586324_1289408_3_935 | 2730000.00 | 0.00 | 0.00 | 0.00 | 0.00 | 0.00 | 0.00 | 0.00 | 0.00 | 0.00 | 0.00 | 0.00 | KO:K03797 | carboxyl-terminal processing protease | Bacteria;Proteobacteria;Gammaproteobacteria;Acidiferrobacterales;Acidiferrobacteraceae;Sulfuricaulis;Sulfuricaulis limicola;Sulfuricaulis limicola HA5 |
| Ga0586324_1650723_1_981 | 0.00 | 0.00 | 0.00 | 0.00 | 0.00 | 0.00 | 0.00 | 564348.21 | 0.00 | 0.00 | 1188000.00 | 0.00 | KO:K03797 | carboxyl-terminal processing protease | Bacteria;Proteobacteria;Deltaproteobacteria;Desulfuromonadales;Geobacteraceae;Geobacter;Geobacter uraniireducens;Geobacter uraniireducens Rf4 |
| Ga0586324_1961461_3_629 | 0.00 | 0.00 | 0.00 | 0.00 | 0.00 | 0.00 | 0.00 | 0.00 | 0.00 | 1139600.00 | 0.00 | 0.00 | KO:K03797 | carboxyl-terminal processing protease | Bacteria;Proteobacteria;Alphaproteobacteria;Rhodospirillales;unclassified Rhodospirillales;unclassified Rhodospirillales;Rhodospirillales bacterium URHD0088;Rhodospirillales bacterium URHD0088 |
| Ga0586324_2899178_1_624 | 0.00 | 7480000.00 | 0.00 | 0.00 | 0.00 | 0.00 | 0.00 | 0.00 | 0.00 | 0.00 | 0.00 | 0.00 | KO:K03797 | carboxyl-terminal processing protease | Bacteria;Proteobacteria;Gammaproteobacteria;Methylococcales;Methylococcaceae;Methylobacter;Methylobacter whittenburyi;Methylobacter whittenburyi ACM 3310 |
| Ga0586324_3383039_1_723 | 7200000.00 | 0.00 | 0.00 | 0.00 | 0.00 | 0.00 | 7729200.00 | 0.00 | 0.00 | 0.00 | 0.00 | 0.00 | KO:K03797 | carboxyl-terminal processing protease | Bacteria;Proteobacteria;Gammaproteobacteria;Methylococcales;Methylococcaceae;Methylobacter;Methylobacter tundripaludum;Methylobacter tundripaludum SV96 |
| Ga0586324_5088416_2_613 | 0.00 | 0.00 | 0.00 | 0.00 | 0.00 | 0.00 | 0.00 | 0.00 | 5111100.00 | 0.00 | 0.00 | 0.00 | KO:K03797 | carboxyl-terminal processing protease | Bacteria;Proteobacteria;Gammaproteobacteria;Methylococcales;Methylococcaceae;Methylobacter;Methylobacter tundripaludum;Methylobacter tundripaludum OWC-DMM |
| Ga0586324_6470024_3_554 | 0.00 | 0.00 | 0.00 | 0.00 | 0.00 | 0.00 | 0.00 | 0.00 | 0.00 | 0.00 | 0.00 | 2865000.00 | KO:K03797 | carboxyl-terminal processing protease | Bacteria;Proteobacteria;Betaproteobacteria;Nitrosomonadales;Sulfuricellaceae;Sulfuricella;Sulfuricella sp. T08;Sulfuricella sp. T08 |
| Ga0586324_0316181_3_1832 | 0.00 | 0.00 | 0.00 | 0.00 | 0.00 | 0.00 | 0.00 | 0.00 | 0.00 | 4889500.00 | 0.00 | 0.00 | KO:K03798 | cell division protease FtsH | Bacteria;Proteobacteria;Betaproteobacteria;Burkholderiales;Oxalobacteraceae;Noviherbaspirillum;Noviherbaspirillum humi;Noviherbaspirillum humi U15 |
| Ga0586324_0535252_1_1026 | 0.00 | 0.00 | 0.00 | 0.00 | 0.00 | 0.00 | 0.00 | 0.00 | 0.00 | 0.00 | 0.00 | 1335000.00 | KO:K03798 | cell division protease FtsH | Bacteria;Proteobacteria;Alphaproteobacteria;Hyphomicrobiales;Bradyrhizobiaceae;Bradyrhizobium;Bradyrhizobium sp. Ai1a-2;Bradyrhizobium sp. Ai1a-2 |
| Ga0586324_0774536_512_1354 | 0.00 | 0.00 | 0.00 | 0.00 | 0.00 | 0.00 | 0.00 | 774700.00 | 0.00 | 0.00 | 0.00 | 0.00 | KO:K03798 | cell division protease FtsH | Bacteria;Proteobacteria;Deltaproteobacteria;Desulfobacterales;Desulfobacteraceae;Desulfosarcina;Desulfosarcina variabilis;Desulfosarcina variabilis Montpellier |
| Ga0586324_0941976_3_1247 | 0.00 | 1224000.00 | 0.00 | 0.00 | 0.00 | 0.00 | 0.00 | 0.00 | 0.00 | 0.00 | 0.00 | 0.00 | KO:K03798 | cell division protease FtsH | Bacteria;Proteobacteria;Gammaproteobacteria;Methylococcales;Methylococcaceae;Methylobacter;Methylobacter whittenburyi;Methylobacter whittenburyi ACM 3310 |
| Ga0586324_6208373_136_564 | 0.00 | 0.00 | 0.00 | 790496.90 | 0.00 | 0.00 | 0.00 | 0.00 | 0.00 | 0.00 | 0.00 | 0.00 | KO:K03832 | protein TonB | Bacteria;Proteobacteria;Gammaproteobacteria;Xanthomonadales;Rhodanobacteraceae;Rhodanobacter;Rhodanobacter sp. K2T2;Rhodanobacter sp. K2T2 |
| Ga0586324_0033085_5231_5854 | 2490000.00 | 1978800.00 | 0.00 | 0.00 | 0.00 | 0.00 | 0.00 | 0.00 | 0.00 | 0.00 | 0.00 | 0.00 | KO:K03841 | fructose-1,6-bisphosphatase I | Archaea;Euryarchaeota;Methanomicrobia;Methanosarcinales;Methanotrichaceae;Methanothrix;Methanothrix soehngenii;Methanothrix soehngenii GP-6 |
| Ga0586324_0185464_642_1661 | 0.00 | 0.00 | 0.00 | 0.00 | 0.00 | 997323.55 | 0.00 | 0.00 | 0.00 | 0.00 | 0.00 | 0.00 | KO:K03841 | fructose-1,6-bisphosphatase I | Bacteria;Proteobacteria;Gammaproteobacteria;unclassified Gammaproteobacteria;Candidatus Competibacteraceae;Candidatus Competibacter;Candidatus Competibacter denitrificans;Candidatus Competibacter denitrificans Run_A_D11 |
| Ga0586324_1897278_1_612 | 0.00 | 720800.00 | 0.00 | 0.00 | 0.00 | 0.00 | 0.00 | 0.00 | 0.00 | 0.00 | 0.00 | 0.00 | KO:K03841 | fructose-1,6-bisphosphatase I | Bacteria;Proteobacteria;Gammaproteobacteria;unclassified Gammaproteobacteria;Candidatus Competibacteraceae;Candidatus Contendobacter;Candidatus Contendobacter odensis;Candidatus Contendobacter odensis Run_B_J11 |
| Ga0586324_2229137_1_756 | 0.00 | 0.00 | 0.00 | 0.00 | 0.00 | 0.00 | 0.00 | 0.00 | 0.00 | 1316700.00 | 0.00 | 0.00 | KO:K03841 | fructose-1,6-bisphosphatase I | Bacteria;Gemmatimonadetes;Gemmatimonadetes;Gemmatimonadales;Gemmatimonadaceae;Gemmatirosa;Gemmatirosa kalamazoonesis;Gemmatimonadetes bacterium KBS708 |
| Ga0586324_0303894_1034_2044 | 0.00 | 0.00 | 0.00 | 0.00 | 0.00 | 0.00 | 0.00 | 902800.00 | 0.00 | 0.00 | 0.00 | 1252500.00 | KO:K03856 | 3-deoxy-7-phosphoheptulonate synthase | Bacteria;Proteobacteria;Betaproteobacteria;Nitrosomonadales;Sulfuricellaceae;Sulfurirhabdus;Sulfurirhabdus autotrophica;Sulfurirhabdus autotrophica DSM 100309 |
| Ga0586324_1838141_24_935 | 0.00 | 0.00 | 0.00 | 0.00 | 0.00 | 0.00 | 0.00 | 0.00 | 2478600.00 | 0.00 | 0.00 | 0.00 | KO:K03856 | 3-deoxy-7-phosphoheptulonate synthase | Bacteria;Proteobacteria;Deltaproteobacteria;Syntrophobacterales;Syntrophobacteraceae;Syntrophobacter;Syntrophobacter fumaroxidans;Syntrophobacter fumaroxidans MPOB |
| Ga0586324_0000075_37944_38981 | 0.00 | 0.00 | 0.00 | 0.00 | 0.00 | 0.00 | 0.00 | 0.00 | 0.00 | 1894200.00 | 0.00 | 0.00 | KO:K03924 | MoxR-like ATPase | Bacteria;Proteobacteria;Betaproteobacteria;Nitrosomonadales;Thiobacillaceae;Thiobacillus;Thiobacillus thioparus;Thiobacillus thioparus DSM 505 |
| Ga0586324_0001512_6702_7700 | 0.00 | 0.00 | 0.00 | 0.00 | 0.00 | 0.00 | 0.00 | 0.00 | 0.00 | 0.00 | 0.00 | 4860000.00 | KO:K03924 | MoxR-like ATPase | Bacteria;Gemmatimonadetes;Gemmatimonadetes;Gemmatimonadales;Gemmatimonadaceae;Gemmatirosa;Gemmatirosa kalamazoonesis;Gemmatimonadetes bacterium KBS708 |
| Ga0586324_0010432_6412_7401 | 0.00 | 0.00 | 0.00 | 0.00 | 0.00 | 0.00 | 0.00 | 6405000.00 | 0.00 | 0.00 | 0.00 | 0.00 | KO:K03924 | MoxR-like ATPase | Bacteria;Ignavibacteriae;Ignavibacteria;Ignavibacteriales;Ignavibacteriaceae;Ignavibacterium;Ignavibacterium album;Ignavibacterium album Mat9-16, JCM 16511 |
| Ga0586324_0710031_2_1042 | 0.00 | 0.00 | 0.00 | 0.00 | 0.00 | 0.00 | 0.00 | 0.00 | 0.00 | 0.00 | 0.00 | 10875000.00 | KO:K03924 | MoxR-like ATPase | Bacteria;Planctomycetes;Planctomycetia;Pirellulales;Pirellulaceae;Blastopirellula;Blastopirellula cremea;Blastopirellula cremea HEX PRIS-MGV |
| Ga0586324_1221701_3_455 | 0.00 | 6364800.00 | 0.00 | 0.00 | 0.00 | 0.00 | 0.00 | 0.00 | 9639000.00 | 0.00 | 0.00 | 0.00 | KO:K03924 | MoxR-like ATPase | Bacteria;Bacteroidetes;Bacteroidia;Marinilabiliales;Marinifilaceae;Labilibaculum;Labilibaculum antarcticum;Labilibaculum antarcticum SPP2 |
| Ga0586324_1267123_3_437 | 0.00 | 0.00 | 0.00 | 0.00 | 0.00 | 0.00 | 0.00 | 7808000.00 | 0.00 | 0.00 | 0.00 | 0.00 | KO:K03924 | MoxR-like ATPase | Bacteria;Actinobacteria;Actinomycetia;Micrococcales;Intrasporangiaceae;Phycicoccus;Phycicoccus sp. Soil802;Phycicoccus sp. Soil802 |
| Ga0586324_2795672_76_783 | 0.00 | 0.00 | 0.00 | 0.00 | 0.00 | 0.00 | 0.00 | 0.00 | 0.00 | 0.00 | 1724800.00 | 0.00 | KO:K03924 | MoxR-like ATPase | Bacteria;Bacteroidetes;Bacteroidia;Marinilabiliales;Marinifilaceae;Labilibaculum;Labilibaculum filiforme;Labilibaculum filiforme 59.16B |
| Ga0586324_7914247_2_511 | 14000000.00 | 0.00 | 0.00 | 0.00 | 0.00 | 0.00 | 0.00 | 0.00 | 0.00 | 0.00 | 0.00 | 0.00 | KO:K03924 | MoxR-like ATPase | Bacteria;Candidatus Hydrogenedentes;unclassified Candidatus Hydrogenedentes;unclassified Candidatus Hydrogenedentes;unclassified Candidatus Hydrogenedentes;unclassified Candidatus Hydrogenedentes;Hydrogenedentes bacterium JGI 0000077-D07;Hydrogenedentes bacterium JGI 0000077-D07 (contamination screened) |
| Ga0586324_0113176_3_1700 | 0.00 | 0.00 | 0.00 | 0.00 | 0.00 | 0.00 | 0.00 | 0.00 | 0.00 | 1347500.00 | 0.00 | 0.00 | KO:K03929 | para-nitrobenzyl esterase | Bacteria;Proteobacteria;Gammaproteobacteria;Pseudomonadales;Pseudomonadaceae;Pseudomonas;Pseudomonas pelagia;Pseudomonas pelagia CL-AP6 |
| Ga0586324_0269651_2_1615 | 0.00 | 0.00 | 0.00 | 1121000.00 | 0.00 | 0.00 | 0.00 | 0.00 | 0.00 | 0.00 | 0.00 | 0.00 | KO:K03929 | para-nitrobenzyl esterase | Bacteria;Proteobacteria;Alphaproteobacteria;Hyphomicrobiales;Rhizobiaceae;Neorhizobium;Neorhizobium galegae;Neorhizobium galegae bv. orientalis HAMBI 2605 |
| Ga0586324_0284597_140_1768 | 0.00 | 0.00 | 0.00 | 0.00 | 0.00 | 0.00 | 0.00 | 0.00 | 0.00 | 1486100.00 | 0.00 | 0.00 | KO:K03929 | para-nitrobenzyl esterase | Bacteria;Proteobacteria;Alphaproteobacteria;Hyphomicrobiales;Bradyrhizobiaceae;Bradyrhizobium;Bradyrhizobium lablabi;Bradyrhizobium lablabi GAS499 |
| Ga0586324_1379880_1_1056 | 0.00 | 782000.00 | 0.00 | 0.00 | 0.00 | 0.00 | 0.00 | 0.00 | 0.00 | 0.00 | 0.00 | 0.00 | KO:K03929 | para-nitrobenzyl esterase | Bacteria;Proteobacteria;Gammaproteobacteria;Pseudomonadales;Pseudomonadaceae;Pseudomonas;Pseudomonas formosensis;Pseudomonas formosensis JCM 18415 |
| Ga0586324_0005739_3132_3812 | 1540000.00 | 0.00 | 0.00 | 0.00 | 0.00 | 0.00 | 0.00 | 0.00 | 0.00 | 0.00 | 1179200.00 | 0.00 | KO:K03969 | phage shock protein A | Bacteria;Chloroflexi;Caldilineae;Caldilineales;Caldilineaceae;Caldilinea;Caldilinea aerophila;Caldilinea aerophila STL-6-O1, DSM 14535 |
| Ga0586324_0048011_3442_4146 | 1400000.00 | 1115200.00 | 1489039.29 | 2308500.00 | 2276300.00 | 2145816.76 | 1590160.92 | 0.00 | 939600.00 | 2163700.00 | 0.00 | 0.00 | KO:K03969 | phage shock protein A | Bacteria;Proteobacteria;Deltaproteobacteria;Syntrophobacterales;Syntrophobacteraceae;Desulfacinum;Desulfacinum hydrothermale;Desulfacinum hydrothermale DSM 13146 |
| Ga0586324_0052820_3732_4436 | 0.00 | 0.00 | 0.00 | 0.00 | 0.00 | 0.00 | 0.00 | 0.00 | 0.00 | 0.00 | 31833.74 | 0.00 | KO:K03969 | phage shock protein A | Bacteria;Proteobacteria;Deltaproteobacteria;Syntrophobacterales;Syntrophobacteraceae;Desulfacinum;Desulfacinum hydrothermale;Desulfacinum hydrothermale DSM 13146 |
| Ga0586324_0121675_2_667 | 0.00 | 0.00 | 0.00 | 0.00 | 887317.19 | 0.00 | 0.00 | 0.00 | 371018.07 | 0.00 | 0.00 | 0.00 | KO:K03969 | phage shock protein A | Bacteria;Chloroflexi;Caldilineae;Caldilineales;Caldilineaceae;Caldilinea;Caldilinea aerophila;Caldilinea aerophila STL-6-O1, DSM 14535 |
| Ga0586324_0159344_3_686 | 0.00 | 0.00 | 0.00 | 0.00 | 0.00 | 0.00 | 0.00 | 1061400.00 | 0.00 | 0.00 | 0.00 | 0.00 | KO:K03969 | phage shock protein A | Bacteria;Proteobacteria;Deltaproteobacteria;Syntrophobacterales;Syntrophobacteraceae;Desulfacinum;Desulfacinum hydrothermale;Desulfacinum hydrothermale DSM 13146 |
| Ga0586324_1678869_192_974 | 0.00 | 0.00 | 1394370.84 | 0.00 | 0.00 | 0.00 | 0.00 | 0.00 | 0.00 | 0.00 | 0.00 | 0.00 | KO:K03969 | phage shock protein A | Bacteria;Actinobacteria;Actinomycetia;Micrococcales;Microbacteriaceae;Compostimonas;Compostimonas suwonensis;Compostimonas suwonensis DSM 25625 |
| Ga0586324_0000002_223334_224398 | 0.00 | 0.00 | 0.00 | 0.00 | 0.00 | 0.00 | 0.00 | 1323700.00 | 1749600.00 | 0.00 | 0.00 | 0.00 | KO:K03979 | GTP-binding protein | Bacteria;Proteobacteria;Betaproteobacteria;Nitrosomonadales;Thiobacillaceae;Annwoodia;Annwoodia aquaesulis;Annwoodia aquaesulis DSM 4255 |
| Ga0586324_2264078_227_505 | 15900000.00 | 0.00 | 0.00 | 0.00 | 0.00 | 0.00 | 0.00 | 0.00 | 0.00 | 0.00 | 0.00 | 0.00 | KO:K04027 | ethanolamine utilization protein EutM | Bacteria;Chloroflexi;Anaerolineae;Anaerolineales;Anaerolineaceae;Anaerolinea;Anaerolinea thermophila;Anaerolinea thermophila UNI-1 |
| Ga0586324_2592728_3_278 | 0.00 | 0.00 | 0.00 | 0.00 | 0.00 | 0.00 | 0.00 | 0.00 | 0.00 | 10087000.00 | 0.00 | 0.00 | KO:K04027 | ethanolamine utilization protein EutM | Bacteria;Firmicutes;Bacilli;Bacillales;Paenibacillaceae;Paenibacillus;Paenibacillus sp. GM2;Paenibacillus sp. GM2 |
| Ga0586324_5203960_500_607 | 0.00 | 0.00 | 0.00 | 0.00 | 0.00 | 514055.45 | 0.00 | 0.00 | 0.00 | 0.00 | 0.00 | 0.00 | KO:K04027 | ethanolamine utilization protein EutM | Bacteria;Proteobacteria;Alphaproteobacteria;Hyphomicrobiales;Afifellaceae;Afifella;Afifella marina;Afifella marina DSM 2698 |
| Ga0586324_7444438_403_522 | 0.00 | 18836000.00 | 0.00 | 0.00 | 0.00 | 0.00 | 0.00 | 0.00 | 0.00 | 0.00 | 0.00 | 0.00 | KO:K04027 | ethanolamine utilization protein EutM | Bacteria;Proteobacteria;Deltaproteobacteria;Desulfobacterales;Desulfobacteraceae;Desulfobotulus;Desulfobotulus sapovorans;Desulfobotulus sapovorans DSM 2055 |
| Ga0586324_8105263_1_390 | 0.00 | 2502400.00 | 5265316.75 | 3714500.00 | 0.00 | 2472075.33 | 0.00 | 0.00 | 0.00 | 0.00 | 0.00 | 0.00 | KO:K04027 | ethanolamine utilization protein EutM | Bacteria;Proteobacteria;Alphaproteobacteria;Hyphomicrobiales;Afifellaceae;Afifella;Afifella marina;Afifella marina DSM 2698 |
| Ga0586324_0000109_2_1171 | 0.00 | 0.00 | 0.00 | 0.00 | 0.00 | 0.00 | 0.00 | 1256600.00 | 0.00 | 0.00 | 0.00 | 0.00 | KO:K04043 | molecular chaperone DnaK | Bacteria;Gemmatimonadetes;Gemmatimonadetes;Gemmatimonadales;Gemmatimonadaceae;Gemmatirosa;Gemmatirosa kalamazoonesis;Gemmatimonadetes bacterium KBS708 |
| Ga0586324_0000586_3_1079 | 0.00 | 0.00 | 0.00 | 0.00 | 0.00 | 0.00 | 6156000.00 | 1964200.00 | 6909300.00 | 11473000.00 | 12276000.00 | 9082500.00 | KO:K04043 | molecular chaperone DnaK | Bacteria;Proteobacteria;Betaproteobacteria;Nitrosomonadales;Thiobacillaceae;Thiobacillus;Thiobacillus denitrificans;Thiobacillus denitrificans ATCC 25259 |
| Ga0586324_0001064_7375_9291 | 0.00 | 3638071.40 | 0.00 | 0.00 | 0.00 | 0.00 | 2690400.00 | 10754300.00 | 17115300.00 | 47639900.00 | 40928800.00 | 13530000.00 | KO:K04043 | molecular chaperone DnaK | Bacteria;Proteobacteria;Betaproteobacteria;Nitrosomonadales;Thiobacillaceae;Thiobacillus;Thiobacillus thioparus;Thiobacillus thioparus DSM 505 |
| Ga0586324_0001380_21539_23017 | 0.00 | 0.00 | 0.00 | 0.00 | 0.00 | 0.00 | 0.00 | 0.00 | 988200.00 | 0.00 | 0.00 | 0.00 | KO:K04043 | molecular chaperone DnaK | Bacteria;Candidatus Aminicenantes;unclassified Candidatus Aminicenantes;unclassified Candidatus Aminicenantes;unclassified Candidatus Aminicenantes;unclassified Candidatus Aminicenantes;Aminicenantes bacterium SCGC AAA252-D06;Aminicenantes bacterium SCGC AAA252-D06 (contamination screened) |
| Ga0586324_0002008_6208_8127 | 0.00 | 0.00 | 0.00 | 0.00 | 0.00 | 0.00 | 1870692.12 | 0.00 | 0.00 | 0.00 | 0.00 | 0.00 | KO:K04043 | molecular chaperone DnaK | Bacteria;Nitrospirae;Nitrospira;Nitrospirales;Nitrospiraceae;Nitrospira;Nitrospira moscoviensis;Nitrospira moscoviensis NSP M-1 |
| Ga0586324_0002409_15404_17335 | 0.00 | 0.00 | 276996000.00 | 0.00 | 0.00 | 0.00 | 246240000.00 | 95160000.00 | 15795000.00 | 0.00 | 162800000.00 | 180750000.00 | KO:K04043 | molecular chaperone DnaK | Bacteria;Proteobacteria;Deltaproteobacteria;Desulfobacterales;Desulfobacteraceae;Desulfatibacillum;Desulfatibacillum alkenivorans;Desulfatibacillum alkenivorans DSM 16219 |
| Ga0586324_0002807_15076_17001 | 0.00 | 2978400.00 | 0.00 | 0.00 | 0.00 | 0.00 | 0.00 | 0.00 | 0.00 | 0.00 | 0.00 | 0.00 | KO:K04043 | molecular chaperone DnaK | Bacteria;Proteobacteria;Deltaproteobacteria;Myxococcales;Kofleriaceae;Haliangium;Haliangium ochraceum;Haliangium ochraceum SMP-2, DSM 14365 |
| Ga0586324_0003346_2573_4522 | 0.00 | 0.00 | 0.00 | 0.00 | 769058.77 | 0.00 | 0.00 | 0.00 | 538706.70 | 0.00 | 0.00 | 0.00 | KO:K04043 | molecular chaperone DnaK | Bacteria;Candidatus Aminicenantes;unclassified Candidatus Aminicenantes;unclassified Candidatus Aminicenantes;unclassified Candidatus Aminicenantes;unclassified Candidatus Aminicenantes;Aminicenantes bacterium SCGC AAA255-F06;Aminicenantes bacterium SCGC AAA255-F06 (contamination screened) |
| Ga0586324_0004268_13910_14686 | 0.00 | 81321.88 | 0.00 | 0.00 | 0.00 | 0.00 | 0.00 | 0.00 | 0.00 | 0.00 | 0.00 | 0.00 | KO:K04043 | molecular chaperone DnaK | Bacteria;Gemmatimonadetes;Longimicrobia;Longimicrobiales;Longimicrobiaceae;Longimicrobium;Longimicrobium terrae;Longimicrobium terrae CECT 8660 |
| Ga0586324_0004407_6617_8548 | 0.00 | 0.00 | 0.00 | 0.00 | 0.00 | 0.00 | 1705581.36 | 0.00 | 0.00 | 0.00 | 906400.00 | 0.00 | KO:K04043 | molecular chaperone DnaK | Bacteria;Proteobacteria;Betaproteobacteria;Nitrosomonadales;Gallionellaceae;Gallionella;Gallionella capsiferriformans;Gallionella capsiferriformans ES-2 |
| Ga0586324_0010215_453_2378 | 0.00 | 0.00 | 0.00 | 0.00 | 0.00 | 0.00 | 0.00 | 0.00 | 0.00 | 11858000.00 | 0.00 | 0.00 | KO:K04043 | molecular chaperone DnaK | Bacteria;Proteobacteria;Deltaproteobacteria;Syntrophobacterales;Syntrophaceae;Desulfomonile;Desulfomonile tiedjei;Desulfomonile tiedjei DCB-1, DSM 6799 |
| Ga0586324_0014894_6739_8511 | 0.00 | 0.00 | 0.00 | 0.00 | 0.00 | 0.00 | 0.00 | 0.00 | 0.00 | 2387000.00 | 0.00 | 0.00 | KO:K04043 | molecular chaperone DnaK | Bacteria;Bacteroidetes;Bacteroidia;Bacteroidales;unclassified Bacteroidales;unclassified Bacteroidales;Bacteroidales bacterium 6E;Bacteroidales bacterium 6E |
| Ga0586324_0022008_1777_3702 | 0.00 | 12240000.00 | 27249200.00 | 0.00 | 0.00 | 9488300.00 | 5244000.00 | 9211000.00 | 0.00 | 14476000.00 | 15752000.00 | 11925000.00 | KO:K04043 | molecular chaperone DnaK | Bacteria;Calditrichaeota;Calditrichae;Calditrichales;Calditrichaceae;Caldithrix;Caldithrix abyssi;Caldithrix abyssi LF13, DSM 13497 |
| Ga0586324_0024493_2358_4292 | 5810000.00 | 0.00 | 0.00 | 0.00 | 0.00 | 0.00 | 0.00 | 0.00 | 0.00 | 0.00 | 0.00 | 0.00 | KO:K04043 | molecular chaperone DnaK | Bacteria;Proteobacteria;Gammaproteobacteria;Methylococcales;Methylococcaceae;Methylobacter;Methylobacter tundripaludum;Methylobacter tundripaludum OWC-DMM |
| Ga0586324_0024506_4302_6266 | 18400000.00 | 0.00 | 0.00 | 0.00 | 0.00 | 0.00 | 0.00 | 0.00 | 1158300.00 | 0.00 | 0.00 | 0.00 | KO:K04043 | molecular chaperone DnaK | Bacteria;Planctomycetes;Phycisphaerae;Sedimentisphaerales;Anaerohalosphaeraceae;Anaerohalosphaera;Anaerohalosphaera lusitana;Phycisphaerae bacterium ST-NAGAB-D1 |
| Ga0586324_0027461_3848_5794 | 0.00 | 2699600.00 | 0.00 | 0.00 | 0.00 | 0.00 | 0.00 | 4751900.00 | 5022000.00 | 5728800.00 | 407604.56 | 534674.25 | KO:K04043 | molecular chaperone DnaK | Bacteria;Proteobacteria;Betaproteobacteria;unclassified Betaproteobacteria;unclassified Betaproteobacteria;unclassified Betaproteobacteria;Betaproteobacteria bacterium GR16-43;Betaproteobacteria bacterium GR16-43 |
| Ga0586324_0027585_1_783 | 0.00 | 0.00 | 0.00 | 0.00 | 0.00 | 231848000.00 | 0.00 | 0.00 | 0.00 | 0.00 | 0.00 | 0.00 | KO:K04043 | molecular chaperone DnaK | Bacteria;Proteobacteria;Deltaproteobacteria;Syntrophobacterales;Syntrophaceae;Desulfobacca;Desulfobacca acetoxidans;Desulfobacca acetoxidans ASRB2, DSM 11109 |
| Ga0586324_0028874_1769_3637 | 3800000.00 | 7908400.00 | 0.00 | 13081500.00 | 17448200.00 | 5121300.00 | 0.00 | 0.00 | 499940.10 | 0.00 | 22264000.00 | 1657500.00 | KO:K04043 | molecular chaperone DnaK | Archaea;Euryarchaeota;Methanomicrobia;Methanosarcinales;Methanotrichaceae;Methanothrix;Methanothrix soehngenii;Methanothrix soehngenii GP-6 |
| Ga0586324_0029318_3_1442 | 0.00 | 0.00 | 0.00 | 0.00 | 0.00 | 0.00 | 0.00 | 0.00 | 0.00 | 8662500.00 | 1645600.00 | 2122523.25 | KO:K04043 | molecular chaperone DnaK | Bacteria;Proteobacteria;Betaproteobacteria;Nitrosomonadales;Gallionellaceae;Sideroxydans;Sideroxydans lithotrophicus;Sideroxydans lithotrophicus ES-1 |
| Ga0586324_0032346_837_2762 | 6570000.00 | 0.00 | 0.00 | 0.00 | 0.00 | 0.00 | 0.00 | 0.00 | 0.00 | 0.00 | 0.00 | 0.00 | KO:K04043 | molecular chaperone DnaK | Bacteria;Nitrospirae;Nitrospira;Nitrospirales;Nitrospiraceae;Nitrospira;Nitrospira moscoviensis;Nitrospira moscoviensis NSP M-1 |
| Ga0586324_0044840_4465_5043 | 0.00 | 0.00 | 0.00 | 0.00 | 0.00 | 0.00 | 0.00 | 0.00 | 0.00 | 0.00 | 0.00 | 14700000.00 | KO:K04043 | molecular chaperone DnaK | Bacteria;Acidobacteria;Blastocatellia;Blastocatellales;Pyrinomonadaceae;Pyrinomonas;Pyrinomonas methylaliphatogenes;Pyrinomonas methylaliphatogenes K22 |
| Ga0586324_0051143_3233_4729 | 0.00 | 5283600.00 | 0.00 | 0.00 | 1936400.00 | 8575200.00 | 10830000.00 | 7503000.00 | 7865100.00 | 5636400.00 | 6820000.00 | 5160000.00 | KO:K04043 | molecular chaperone DnaK | Bacteria;Proteobacteria;Betaproteobacteria;Burkholderiales;unclassified Burkholderiales;Ideonella;Ideonella sp. A 288;Ideonella sp. A 288 |
| Ga0586324_0052192_1_1089 | 0.00 | 0.00 | 0.00 | 0.00 | 0.00 | 0.00 | 0.00 | 2311900.00 | 0.00 | 0.00 | 0.00 | 0.00 | KO:K04043 | molecular chaperone DnaK | Bacteria;Ignavibacteriae;Ignavibacteria;Ignavibacteriales;Ignavibacteriaceae;Ignavibacterium;Ignavibacterium album;Ignavibacterium album Mat9-16, JCM 16511 |
| Ga0586324_0061325_1_1701 | 0.00 | 0.00 | 0.00 | 0.00 | 0.00 | 0.00 | 0.00 | 1915400.00 | 2187000.00 | 2810500.00 | 857470.24 | 2670000.00 | KO:K04043 | molecular chaperone DnaK | Bacteria;Proteobacteria;Betaproteobacteria;Nitrosomonadales;Gallionellaceae;Sideroxydans;Sideroxydans lithotrophicus;Sideroxydans lithotrophicus ES-1 |
| Ga0586324_0066386_3_1148 | 0.00 | 0.00 | 0.00 | 0.00 | 0.00 | 0.00 | 0.00 | 0.00 | 0.00 | 0.00 | 0.00 | 9375000.00 | KO:K04043 | molecular chaperone DnaK | Bacteria;Proteobacteria;Deltaproteobacteria;Desulfobacterales;Desulfobacteraceae;Desulfosarcina;Desulfosarcina variabilis;Desulfosarcina variabilis Montpellier |
| Ga0586324_0070531_1_1416 | 0.00 | 0.00 | 0.00 | 0.00 | 0.00 | 0.00 | 0.00 | 0.00 | 0.00 | 0.00 | 10296000.00 | 2992500.00 | KO:K04043 | molecular chaperone DnaK | Bacteria;Proteobacteria;Gammaproteobacteria;Chromatiales;Ectothiorhodospiraceae;Thiohalomonas;Thiohalomonas denitrificans;Thiohalomonas denitrificans HLD2 |
| Ga0586324_0087210_1260_3182 | 0.00 | 0.00 | 1306537.21 | 0.00 | 0.00 | 0.00 | 0.00 | 0.00 | 0.00 | 0.00 | 0.00 | 0.00 | KO:K04043 | molecular chaperone DnaK | Bacteria;Nitrospirae;Nitrospira;Nitrospirales;Nitrospiraceae;Candidatus Magnetobacterium;Candidatus Magnetobacterium casensis;Candidatus Magnetobacterium casensis MYR-1 |
| Ga0586324_0087454_1_1461 | 0.00 | 0.00 | 0.00 | 0.00 | 0.00 | 0.00 | 0.00 | 5837700.00 | 0.00 | 0.00 | 0.00 | 0.00 | KO:K04043 | molecular chaperone DnaK | Bacteria;Proteobacteria;Betaproteobacteria;Burkholderiales;unclassified Burkholderiales;Ideonella;Ideonella sp. A 288;Ideonella sp. A 288 |
| Ga0586324_0103309_373_2307 | 0.00 | 0.00 | 0.00 | 13490000.00 | 11021000.00 | 12346700.00 | 0.00 | 0.00 | 0.00 | 0.00 | 0.00 | 0.00 | KO:K04043 | molecular chaperone DnaK | Bacteria;Acidobacteria;Acidobacteriia;Acidobacteriales;Acidobacteriaceae;unclassified Acidobacteriaceae;Acidobacteriaceae bacterium KBS 96;Acidobacteriaceae bacterium KBS 96, DSM 24297 |
| Ga0586324_0110594_2666_3268 | 0.00 | 0.00 | 1457432.47 | 0.00 | 0.00 | 0.00 | 0.00 | 0.00 | 0.00 | 0.00 | 0.00 | 0.00 | KO:K04043 | molecular chaperone DnaK | Archaea;Euryarchaeota;Methanomicrobia;Methanosarcinales;Methanotrichaceae;Methanothrix;Methanothrix soehngenii;Methanothrix soehngenii GP-6 |
| Ga0586324_0111567_932_2869 | 0.00 | 0.00 | 0.00 | 0.00 | 0.00 | 2013079.81 | 0.00 | 0.00 | 0.00 | 0.00 | 0.00 | 0.00 | KO:K04043 | molecular chaperone DnaK | Bacteria;Proteobacteria;Gammaproteobacteria;Methylococcales;Methylococcaceae;Methylobacter;Methylobacter tundripaludum;Methylobacter tundripaludum SV96 |
| Ga0586324_0113939_1837_3222 | 0.00 | 0.00 | 0.00 | 752230.90 | 0.00 | 0.00 | 0.00 | 0.00 | 0.00 | 0.00 | 0.00 | 280523.25 | KO:K04043 | molecular chaperone DnaK | Bacteria;Proteobacteria;Deltaproteobacteria;Desulfobacterales;Desulfobulbaceae;Desulfopila;Desulfopila sp. IMCC35004;Desulfopila sp. IMCC35004 |
| Ga0586324_0120733_134_2113 | 0.00 | 6133600.00 | 0.00 | 0.00 | 0.00 | 0.00 | 4400400.00 | 0.00 | 0.00 | 0.00 | 0.00 | 0.00 | KO:K04043 | molecular chaperone DnaK | Bacteria;Nitrospirae;Nitrospira;Nitrospirales;Nitrospiraceae;Nitrospira;Candidatus Nitrospira inopinata;Candidatus Nitrospira inopinata ENR4 |
| Ga0586324_0121563_733_2703 | 0.00 | 0.00 | 0.00 | 0.00 | 0.00 | 0.00 | 0.00 | 0.00 | 0.00 | 1262800.00 | 1452000.00 | 0.00 | KO:K04043 | molecular chaperone DnaK | Bacteria;Proteobacteria;Betaproteobacteria;unclassified Betaproteobacteria;unclassified Betaproteobacteria;unclassified Betaproteobacteria;Betaproteobacteria bacterium UKL13-2;Betaproteobacteria bacterium UKL13-2 |
| Ga0586324_0122252_556_2484 | 0.00 | 0.00 | 0.00 | 0.00 | 8837400.00 | 0.00 | 38076000.00 | 0.00 | 0.00 | 0.00 | 0.00 | 0.00 | KO:K04043 | molecular chaperone DnaK | Bacteria;Proteobacteria;Deltaproteobacteria;Syntrophobacterales;Syntrophaceae;Desulfomonile;Desulfomonile tiedjei;Desulfomonile tiedjei DCB-1, DSM 6799 |
| Ga0586324_0131268_828_2753 | 0.00 | 0.00 | 0.00 | 0.00 | 0.00 | 0.00 | 0.00 | 0.00 | 0.00 | 0.00 | 0.00 | 892500.00 | KO:K04043 | molecular chaperone DnaK | Bacteria;Proteobacteria;Gammaproteobacteria;Oceanospirillales;Oleiphilaceae;Oleiphilus;Oleiphilus messinensis;Oleiphilus messinensis ME102 |
| Ga0586324_0134934_137_2050 | 0.00 | 0.00 | 0.00 | 764472.60 | 0.00 | 0.00 | 0.00 | 0.00 | 0.00 | 0.00 | 0.00 | 0.00 | KO:K04043 | molecular chaperone DnaK | Bacteria;Actinobacteria;Rubrobacteria;Gaiellales;Gaiellaceae;Gaiella;Gaiella occulta;Gaiella occulta F2-233 |
| Ga0586324_0143703_1095_2888 | 0.00 | 0.00 | 0.00 | 0.00 | 0.00 | 0.00 | 0.00 | 0.00 | 0.00 | 0.00 | 3907200.00 | 0.00 | KO:K04043 | molecular chaperone DnaK | Bacteria;Bacteroidetes;Bacteroidia;Marinilabiliales;Marinilabiliaceae;Geofilum;Geofilum rhodophaeum;Geofilum rhodophaeum HF401 |
| Ga0586324_0164355_2_1954 | 0.00 | 0.00 | 0.00 | 0.00 | 0.00 | 3200657.67 | 0.00 | 0.00 | 3701700.00 | 0.00 | 0.00 | 0.00 | KO:K04043 | molecular chaperone DnaK | Bacteria;Acidobacteria;Acidobacteriia;Acidobacteriales;Acidobacteriaceae;unclassified Acidobacteriaceae;Acidobacteriaceae bacterium KBS 96;Acidobacteriaceae bacterium KBS 96, DSM 24297 |
| Ga0586324_0170750_874_2664 | 8150000.00 | 0.00 | 0.00 | 0.00 | 0.00 | 0.00 | 0.00 | 0.00 | 0.00 | 0.00 | 0.00 | 0.00 | KO:K04043 | molecular chaperone DnaK | Bacteria;Ignavibacteriae;Ignavibacteria;Ignavibacteriales;Melioribacteraceae;Melioribacter;Melioribacter roseus;Melioribacter roseus P3M |
| Ga0586324_0174987_1751_2632 | 0.00 | 0.00 | 0.00 | 0.00 | 0.00 | 0.00 | 0.00 | 0.00 | 0.00 | 0.00 | 3572800.00 | 0.00 | KO:K04043 | molecular chaperone DnaK | Bacteria;Proteobacteria;Gammaproteobacteria;Nevskiales;Steroidobacteraceae;Steroidobacter;Steroidobacter denitrificans;Steroidobacter denitrificans DSM 18526 |
| Ga0586324_0183471_831_2576 | 0.00 | 0.00 | 0.00 | 9405000.00 | 2554400.00 | 0.00 | 0.00 | 1116300.00 | 1304100.00 | 0.00 | 0.00 | 0.00 | KO:K04043 | molecular chaperone DnaK | Bacteria;Nitrospirae;Nitrospira;Nitrospirales;Nitrospiraceae;Nitrospira;Nitrospira moscoviensis;Nitrospira moscoviensis NSP M-1 |
| Ga0586324_0192770_2_1771 | 0.00 | 0.00 | 0.00 | 0.00 | 1277200.00 | 0.00 | 0.00 | 0.00 | 0.00 | 0.00 | 0.00 | 0.00 | KO:K04043 | molecular chaperone DnaK | Bacteria;Proteobacteria;Deltaproteobacteria;Syntrophorhabdales;Syntrophorhabdaceae;Syntrophorhabdus;Syntrophorhabdus aromaticivorans;Syntrophorhabdus aromaticivorans UI |
| Ga0586324_0228728_661_2325 | 0.00 | 0.00 | 0.00 | 0.00 | 0.00 | 0.00 | 0.00 | 2135000.00 | 0.00 | 0.00 | 0.00 | 3442500.00 | KO:K04043 | molecular chaperone DnaK | Bacteria;Verrucomicrobia;Opitutae;Opitutales;Opitutaceae;Opitutus;Opitutus sp. GAS368;Opitutus sp. GAS368 |
| Ga0586324_0235488_1_1467 | 0.00 | 11900000.00 | 0.00 | 0.00 | 0.00 | 0.00 | 0.00 | 0.00 | 0.00 | 0.00 | 0.00 | 0.00 | KO:K04043 | molecular chaperone DnaK | Bacteria;Proteobacteria;Deltaproteobacteria;Syntrophobacterales;Syntrophaceae;Desulfomonile;Desulfomonile tiedjei;Desulfomonile tiedjei DCB-1, DSM 6799 |
| Ga0586324_0241281_701_2269 | 0.00 | 0.00 | 6305600.00 | 0.00 | 0.00 | 0.00 | 0.00 | 199890.29 | 0.00 | 0.00 | 0.00 | 9525000.00 | KO:K04043 | molecular chaperone DnaK | Bacteria;Proteobacteria;Deltaproteobacteria;Syntrophobacterales;Syntrophaceae;Syntrophus;Syntrophus aciditrophicus;Syntrophus aciditrophicus SB |
| Ga0586324_0244037_3_1259 | 0.00 | 0.00 | 0.00 | 0.00 | 1771600.00 | 0.00 | 0.00 | 0.00 | 0.00 | 0.00 | 0.00 | 0.00 | KO:K04043 | molecular chaperone DnaK | Bacteria;Proteobacteria;Deltaproteobacteria;Desulfobacterales;Desulfobacteraceae;Desulfatiglans;Desulfatiglans anilini;Desulfatiglans anilini DSM 4660 |
| Ga0586324_0258990_3_1268 | 9820000.00 | 11550139.32 | 12149872.17 | 6270000.00 | 5129400.00 | 1545441.60 | 14881767.48 | 6222082.35 | 8618400.00 | 19057500.00 | 4021600.00 | 7492500.00 | KO:K04043 | molecular chaperone DnaK | Bacteria;Proteobacteria;Alphaproteobacteria;Rhodospirillales;Acetobacteraceae;Rhodopila;unclassified Rhodopila;Rhodopila sp. LVNP |
| Ga0586324_0271723_105_2063 | 0.00 | 0.00 | 0.00 | 0.00 | 0.00 | 0.00 | 0.00 | 5374100.00 | 0.00 | 0.00 | 0.00 | 0.00 | KO:K04043 | molecular chaperone DnaK | Bacteria;Proteobacteria;Deltaproteobacteria;Syntrophobacterales;Syntrophaceae;Desulfomonile;Desulfomonile tiedjei;Desulfomonile tiedjei DCB-1, DSM 6799 |
| Ga0586324_0273692_2_781 | 0.00 | 0.00 | 0.00 | 0.00 | 0.00 | 0.00 | 0.00 | 0.00 | 0.00 | 560388.29 | 3159200.00 | 0.00 | KO:K04043 | molecular chaperone DnaK | Bacteria;Proteobacteria;Gammaproteobacteria;Chromatiales;Chromatiaceae;Thioflavicoccus;Thioflavicoccus mobilis;Thioflavicoccus mobilis 8321 |
| Ga0586324_0279701_2_1489 | 0.00 | 0.00 | 0.00 | 12445000.00 | 0.00 | 0.00 | 0.00 | 0.00 | 0.00 | 0.00 | 0.00 | 0.00 | KO:K04043 | molecular chaperone DnaK | Archaea;Euryarchaeota;Methanomicrobia;Methanomicrobiales;Methanoregulaceae;Methanoregula;Methanoregula formicica;Methanoregula formicica SMSP, DSM 22288 |
| Ga0586324_0284744_127_2022 | 2160000.00 | 0.00 | 0.00 | 0.00 | 0.00 | 0.00 | 4377600.00 | 0.00 | 0.00 | 0.00 | 0.00 | 0.00 | KO:K04043 | molecular chaperone DnaK | Bacteria;Acidobacteria;Acidobacteriia;Acidobacteriales;Acidobacteriaceae;unclassified Acidobacteriaceae;Acidobacteriaceae bacterium KBS 96;Acidobacteriaceae bacterium KBS 96, DSM 24297 |
| Ga0586324_0311447_564_2021 | 980653.00 | 1060800.00 | 0.00 | 842143.65 | 962492.77 | 0.00 | 0.00 | 1079700.00 | 0.00 | 0.00 | 0.00 | 0.00 | KO:K04043 | molecular chaperone DnaK | Bacteria;Proteobacteria;Alphaproteobacteria;Rhodospirillales;Rhodospirillaceae;Dongia;Dongia sp. URHE0060;Dongia sp. URHE0060 |
| Ga0586324_0312778_2_937 | 0.00 | 0.00 | 0.00 | 0.00 | 0.00 | 0.00 | 0.00 | 0.00 | 16281000.00 | 0.00 | 0.00 | 2242500.00 | KO:K04043 | molecular chaperone DnaK | Bacteria;Proteobacteria;Deltaproteobacteria;Desulfobacterales;Desulfobacteraceae;Desulfatiglans;Desulfatiglans anilini;Desulfatiglans anilini DSM 4660 |
| Ga0586324_0315277_254_2008 | 2640000.00 | 0.00 | 0.00 | 0.00 | 0.00 | 0.00 | 0.00 | 0.00 | 0.00 | 0.00 | 0.00 | 0.00 | KO:K04043 | molecular chaperone DnaK | Archaea;Euryarchaeota;Methanomicrobia;Methanomicrobiales;Methanoregulaceae;Methanoregula;Methanoregula boonei;Methanoregula boonei 6A8 |
| Ga0586324_0345733_6_1928 | 0.00 | 4746400.00 | 0.00 | 0.00 | 0.00 | 0.00 | 0.00 | 0.00 | 0.00 | 0.00 | 0.00 | 0.00 | KO:K04043 | molecular chaperone DnaK | Bacteria;Proteobacteria;Deltaproteobacteria;Syntrophorhabdales;Syntrophorhabdaceae;Syntrophorhabdus;Syntrophorhabdus aromaticivorans;Syntrophorhabdus aromaticivorans UI |

| | | | | | | | | | | | | | | | |
|---|---|---|---|---|---|---|---|---|---|---|---|---|---|---|---|
| Ga0586324 0357604 3 1484 | 0.00 | 0.00 | 0.00 | 1719500.00 | 0.00 | 0.00 | 0.00 | 0.00 | 0.00 | 0.00 | 0.00 | 0.00 | KO:K04043 | molecular chaperone DnaK | Bacteria;Proteobacteria;Deltaproteobacteria;Myxococcales;Archangiaceae;Vitiosangium;Vitiosangium sp. GDMCC 1.1324;Vitiosangium sp. GDMCC 1.1324 |
| Ga0586324 0357786 3 1205 | 0.00 | 0.00 | 0.00 | 0.00 | 1287500.00 | 0.00 | 0.00 | 0.00 | 358931.25 | 0.00 | 0.00 | 0.00 | KO:K04043 | molecular chaperone DnaK | Bacteria;Proteobacteria;Deltaproteobacteria;Syntrophobacterales;Syntrophobacteraceae;Syntrophobacter;Syntrophobacter fumaroxidans;Syntrophobacter fumaroxidans MPOB |
| Ga0586324 0372044 920 1867 | 0.00 | 0.00 | 0.00 | 0.00 | 0.00 | 1554528.93 | 0.00 | 0.00 | 0.00 | 0.00 | 0.00 | 0.00 | KO:K04043 | molecular chaperone DnaK | Bacteria;Proteobacteria;Deltaproteobacteria;Desulfobacterales;Desulfobacteraceae;Desulfosarcina;Desulfosarcina cetonica;Desulfosarcina cetonica JCM 12296 |
| Ga0586324 0388513 186 1832 | 0.00 | 0.00 | 0.00 | 0.00 | 0.00 | 0.00 | 0.00 | 0.00 | 0.00 | 186309.20 | 0.00 | 0.00 | KO:K04043 | molecular chaperone DnaK | Bacteria;Proteobacteria;Gammaproteobacteria;Nevskiales;Steroidobacteraceae;Steroidobacter;Steroidobacter denitrificans;Steroidobacter denitrificans DSM 18526 |
| Ga0586324 0389708 1 1023 | 0.00 | 0.00 | 0.00 | 0.00 | 0.00 | 0.00 | 0.00 | 9943000.00 | 0.00 | 0.00 | 0.00 | 0.00 | KO:K04043 | molecular chaperone DnaK | Archaea;Candidatus Thermoplasmatota;Thermoplasmata;Methanomassiliicoccales;Methanomassiliicoccaceae;Methanomassiliicoccus;Methanomassiliicoccus luminyensis;Methanomassiliicoccus luminyensis B10 |
| Ga0586324 0464587 339 1694 | 0.00 | 0.00 | 0.00 | 0.00 | 0.00 | 0.00 | 0.00 | 0.00 | 0.00 | 0.00 | 0.00 | 1552500.00 | KO:K04043 | molecular chaperone DnaK | Bacteria;Proteobacteria;Deltaproteobacteria;Desulfobacterales;Desulfobacteraceae;Desulfococcus;Desulfococcus oleovorans;Desulfococcus oleovorans Hxd3 |
| Ga0586324 0466820 3 1148 | 0.00 | 625349.08 | 0.00 | 1168500.00 | 0.00 | 0.00 | 0.00 | 0.00 | 0.00 | 2679600.00 | 4444000.00 | 5640000.00 | KO:K04043 | molecular chaperone DnaK | Bacteria;Proteobacteria;Deltaproteobacteria;Myxococcales;Anaeromyxobacteraceae;Anaeromyxobacter;Anaeromyxobacter dehalogenans;Anaeromyxobacter dehalogenans 2CP-1 |
| Ga0586324 0489999 1 1530 | 0.00 | 0.00 | 0.00 | 0.00 | 0.00 | 0.00 | 0.00 | 0.00 | 0.00 | 0.00 | 0.00 | 231522.00 | KO:K04043 | molecular chaperone DnaK | Bacteria;Proteobacteria;Deltaproteobacteria;Desulfobacterales;Desulfobacteraceae;Desulfatiglans;Desulfatiglans anilini;Desulfatiglans anilini DSM 4660 |
| Ga0586324 0501856 153 1637 | 0.00 | 0.00 | 0.00 | 0.00 | 0.00 | 38826600.00 | 0.00 | 0.00 | 0.00 | 0.00 | 0.00 | 26850000.00 | KO:K04043 | molecular chaperone DnaK | Bacteria;Chloroflexi;unclassified Chloroflexi;unclassified Chloroflexi;unclassified Chloroflexi;unclassified Chloroflexi;unclassified Chloroflexi;Chloroflexi sp. G233 |
| Ga0586324 0520638 3 1610 | 8750000.00 | 3230000.00 | 20549500.00 | 6165500.00 | 0.00 | 0.00 | 0.00 | 0.00 | 0.00 | 5397700.00 | 8078400.00 | 10950000.00 | KO:K04043 | molecular chaperone DnaK | Bacteria;Proteobacteria;Gammaproteobacteria;Methylococcales;Methylococcaceae;Methylobacter;Methylobacter tundripaludum;Methylobacter tundripaludum SV96 |
| Ga0586324 0544565 504 1580 | 0.00 | 0.00 | 0.00 | 0.00 | 0.00 | 0.00 | 0.00 | 0.00 | 4544100.00 | 0.00 | 0.00 | 0.00 | KO:K04043 | molecular chaperone DnaK | Bacteria;Chloroflexi;Anaerolineae;Anaerolineales;Anaerolineaceae;Levilinea;Levilinea saccharolytica;Levilinea saccharolytica DSM 16555 |
| Ga0586324 0564369 892 1554 | 0.00 | 0.00 | 2103334.22 | 6783000.00 | 5047000.00 | 0.00 | 0.00 | 0.00 | 1393200.00 | 0.00 | 0.00 | 0.00 | KO:K04043 | molecular chaperone DnaK | Bacteria;Proteobacteria;Deltaproteobacteria;Desulfarculales;Desulfarculaceae;Desulfocarbo;Desulfocarbo indianensis;Desulfocarbo indianensis SCBM |
| Ga0586324 0592008 524 1522 | 0.00 | 0.00 | 0.00 | 0.00 | 0.00 | 0.00 | 0.00 | 0.00 | 1036800.00 | 0.00 | 0.00 | 0.00 | KO:K04043 | molecular chaperone DnaK | Bacteria;Proteobacteria;Gammaproteobacteria;unclassified Gammaproteobacteria;Candidatus Competibacteraceae;Candidatus Competibacter;Candidatus Competibacter denitrificans;Candidatus Competibacter denitrificans Run A D11 |
| Ga0586324 0604577 2 1252 | 0.00 | 0.00 | 18353800.00 | 0.00 | 0.00 | 0.00 | 0.00 | 13298000.00 | 2438100.00 | 23177000.00 | 6177600.00 | 18375000.00 | KO:K04043 | molecular chaperone DnaK | Bacteria;Acidobacteria;Blastocatellia;Blastocatellales;Pyrinomonadaceae;Pyrinomonas;Pyrinomonas methylaliphatogenes;Pyrinomonas methylaliphatogenes K22 |
| Ga0586324 0650832 1 1056 | 0.00 | 0.00 | 0.00 | 0.00 | 0.00 | 0.00 | 0.00 | 0.00 | 0.00 | 292500.67 | 0.00 | 0.00 | KO:K04043 | molecular chaperone DnaK | Bacteria;Proteobacteria;Gammaproteobacteria;Oceanospirillales;Halomonadaceae;Halomonas;Halomonas huangheensis;Halomonas huangheensis BJGMM-B45 |
| Ga0586324 0690175 738 1424 | 0.00 | 0.00 | 11823000.00 | 0.00 | 0.00 | 0.00 | 0.00 | 6161000.00 | 0.00 | 0.00 | 0.00 | 0.00 | KO:K04043 | molecular chaperone DnaK | Bacteria;Chloroflexi;Ktedonobacteria;Thermogemmatisporales;Thermogemmatisporaceae;Thermogemmatispora;Thermogemmatispora carboxidivorans;Thermogemmatispora carboxidivorans PM5 |
| Ga0586324 0788616 344 1345 | 0.00 | 0.00 | 0.00 | 0.00 | 28767900.00 | 0.00 | 4719600.00 | 0.00 | 0.00 | 0.00 | 0.00 | 0.00 | KO:K04043 | molecular chaperone DnaK | Archaea;Euryarchaeota;Methanomicrobia;Methanosarcinales;Methanotrichaceae;Methanothrix;Methanothrix thermoacetophila;Methanothrix thermoacetophila PT |
| Ga0586324 0803187 3 1334 | 0.00 | 2046800.00 | 0.00 | 0.00 | 0.00 | 0.00 | 0.00 | 0.00 | 0.00 | 1031800.00 | 0.00 | 0.00 | KO:K04043 | molecular chaperone DnaK | Bacteria;Proteobacteria;Betaproteobacteria;Rhodocyclales;Zoogloeaceae;Azoarcus;Azoarcus sp. KH32C;Azoarcus sp. KH32C |
| Ga0586324 0827555 1 435 | 0.00 | 0.00 | 0.00 | 5339000.00 | 0.00 | 9250100.00 | 0.00 | 0.00 | 0.00 | 0.00 | 0.00 | 0.00 | KO:K04043 | molecular chaperone DnaK | Bacteria;Proteobacteria;Deltaproteobacteria;Syntrophobacterales;Syntrophaceae;Desulfobacca;Desulfobacca acetoxidans;Desulfobacca acetoxidans ASRB2, DSM 11109 |
| Ga0586324 0851005 2 1285 | 0.00 | 3699200.00 | 0.00 | 0.00 | 0.00 | 0.00 | 0.00 | 0.00 | 0.00 | 0.00 | 0.00 | 0.00 | KO:K04043 | molecular chaperone DnaK | Bacteria;Chloroflexi;Anaerolineae;Anaerolineales;Anaerolineaceae;Anaerolinea;Anaerolinea thermophila;Anaerolinea thermophila UNI-1 |
| Ga0586324 0873210 2 1288 | 0.00 | 0.00 | 0.00 | 0.00 | 0.00 | 0.00 | 0.00 | 0.00 | 0.00 | 0.00 | 0.00 | 1335000.00 | KO:K04043 | molecular chaperone DnaK | Bacteria;Nitrospinae;Nitrospinia;Nitrospinales;Nitrospinaceae;Nitrospina;Nitrospina gracilis;Nitrospina gracilis 3/211 |
| Ga0586324 0877683 1 1239 | 0.00 | 0.00 | 0.00 | 0.00 | 0.00 | 0.00 | 0.00 | 1262700.00 | 0.00 | 0.00 | 0.00 | 0.00 | KO:K04043 | molecular chaperone DnaK | Bacteria;Proteobacteria;Betaproteobacteria;Nitrosomonadales;Sulfuricellaceae;Sulfuricella;Sulfuricella denitrificans;Sulfuricella denitrificans skB26 |
| Ga0586324 0892667 591 1274 | 0.00 | 0.00 | 0.00 | 0.00 | 1761300.00 | 0.00 | 0.00 | 0.00 | 0.00 | 0.00 | 0.00 | 0.00 | KO:K04043 | molecular chaperone DnaK | Bacteria;Proteobacteria;Deltaproteobacteria;Syntrophobacterales;Syntrophaceae;Desulfobacca;Desulfobacca acetoxidans;Desulfobacca acetoxidans ASRB2, DSM 11109 |
| Ga0586324 0929413 764 1252 | 0.00 | 0.00 | 0.00 | 0.00 | 0.00 | 2911848.11 | 0.00 | 0.00 | 0.00 | 0.00 | 0.00 | 0.00 | KO:K04043 | molecular chaperone DnaK | Bacteria;Proteobacteria;Deltaproteobacteria;Syntrophobacterales;Syntrophaceae;Desulfobacca;Desulfobacca acetoxidans;Desulfobacca acetoxidans ASRB2, DSM 11109 |
| Ga0586324 0931821 1 735 | 0.00 | 0.00 | 0.00 | 0.00 | 0.00 | 3397871.39 | 0.00 | 0.00 | 0.00 | 0.00 | 0.00 | 0.00 | KO:K04043 | molecular chaperone DnaK | Bacteria;Proteobacteria;Deltaproteobacteria;Syntrophobacterales;Syntrophaceae;Desulfobacca;Desulfobacca acetoxidans;Desulfobacca acetoxidans ASRB2, DSM 11109 |
| Ga0586324 0990497 1 1218 | 0.00 | 0.00 | 0.00 | 0.00 | 0.00 | 0.00 | 0.00 | 0.00 | 0.00 | 5135900.00 | 0.00 | 1147500.00 | KO:K04043 | molecular chaperone DnaK | Bacteria;Proteobacteria;Deltaproteobacteria;Syntrophobacterales;Syntrophaceae;Desulfobacca;Desulfobacca acetoxidans;Desulfobacca acetoxidans ASRB2, DSM 11109 |
| Ga0586324 1049109 3 1190 | 0.00 | 0.00 | 0.00 | 0.00 | 0.00 | 2809926.30 | 0.00 | 0.00 | 0.00 | 0.00 | 0.00 | 0.00 | KO:K04043 | molecular chaperone DnaK | Bacteria;Proteobacteria;Deltaproteobacteria;Syntrophobacterales;Syntrophaceae;Desulfomonile;Desulfomonile tiedjei;Desulfomonile tiedjei DCB-1, DSM 6799 |
| Ga0586324 1063667 2 1183 | 0.00 | 0.00 | 647697.72 | 0.00 | 0.00 | 0.00 | 0.00 | 0.00 | 0.00 | 0.00 | 538884.72 | 650748.75 | KO:K04043 | molecular chaperone DnaK | Bacteria;Proteobacteria;Deltaproteobacteria;Syntrophobacterales;Syntrophaceae;Syntrophus;Syntrophus aciditrophicus;Syntrophus aciditrophicus SB |
| Ga0586324 1159132 2 1138 | 0.00 | 0.00 | 0.00 | 0.00 | 968784.01 | 3111142.11 | 0.00 | 0.00 | 0.00 | 0.00 | 0.00 | 0.00 | KO:K04043 | molecular chaperone DnaK | Bacteria;Proteobacteria;Deltaproteobacteria;Syntrophobacterales;Syntrophaceae;Desulfobacca;Desulfobacca acetoxidans;Desulfobacca acetoxidans ASRB2, DSM 11109 |
| Ga0586324 1239016 2 1108 | 0.00 | 0.00 | 0.00 | 0.00 | 0.00 | 0.00 | 6042000.00 | 0.00 | 0.00 | 0.00 | 0.00 | 0.00 | KO:K04043 | molecular chaperone DnaK | Bacteria;Proteobacteria;Alphaproteobacteria;Hyphomicrobiales;Hyphomicrobiaceae;Rhodoplanes;Rhodoplanes sp. Z2-YC6860;Rhodoplanes sp. Z2-YC6860 |
| Ga0586324 1297123 2 1087 | 0.00 | 0.00 | 0.00 | 0.00 | 0.00 | 8614900.00 | 0.00 | 0.00 | 0.00 | 0.00 | 0.00 | 0.00 | KO:K04043 | molecular chaperone DnaK | Bacteria;Calditrichaeota;Calditrichae;Calditrichales;Calditrichaceae;Caldithrix;Caldithrix abyssi;Caldithrix abyssi LF13, DSM 13497 |
| Ga0586324 1364225 2 1063 | 0.00 | 0.00 | 0.00 | 0.00 | 0.00 | 0.00 | 6840000.00 | 0.00 | 0.00 | 0.00 | 0.00 | 0.00 | KO:K04043 | molecular chaperone DnaK | Bacteria;Proteobacteria;Deltaproteobacteria;Desulfuromonadales;Desulfuromonadaceae;Desulfuromonas;Desulfuromonas thiophila;Desulfuromonas thiophila DSM 8987 |
| Ga0586324 1382540 3 1058 | 0.00 | 6099600.00 | 1289016.65 | 0.00 | 0.00 | 4128800.00 | 1129527.96 | 0.00 | 0.00 | 0.00 | 438171.36 | 462672.00 | KO:K04043 | molecular chaperone DnaK | Bacteria;Proteobacteria;Deltaproteobacteria;Syntrophobacterales;Syntrophaceae;Desulfobacca;Desulfobacca acetoxidans;Desulfobacca acetoxidans ASRB2, DSM 11109 |
| Ga0586324 1389786 1 1053 | 0.00 | 0.00 | 0.00 | 0.00 | 0.00 | 0.00 | 0.00 | 0.00 | 0.00 | 0.00 | 1830400.00 | 0.00 | KO:K04043 | molecular chaperone DnaK | Bacteria;Proteobacteria;Deltaproteobacteria;Desulfuromonadales;Geobacteraceae;Geothermobacter;Geothermobacter ehrlichii;Geothermobacter ehrlichii DSM 15274 |
| Ga0586324 1510683 1 1017 | 7020000.00 | 2298400.00 | 6996125.13 | 969000.00 | 4902800.00 | 3951571.26 | 0.00 | 0.00 | 0.00 | 3819200.00 | 4171200.00 | 4327500.00 | KO:K04043 | molecular chaperone DnaK | Bacteria;Proteobacteria;Deltaproteobacteria;Syntrophobacterales;Syntrophaceae;Desulfomonile;Desulfomonile tiedjei;Desulfomonile tiedjei DCB-1, DSM 6799 |
| Ga0586324 1584969 88 996 | 0.00 | 0.00 | 0.00 | 2109000.00 | 0.00 | 0.00 | 2237035.68 | 2793800.00 | 580756.23 | 2294600.00 | 4048000.00 | 15427500.00 | KO:K04043 | molecular chaperone DnaK | Bacteria;Proteobacteria;Gammaproteobacteria;Acidiferrobacterales;Acidiferrobacteraceae;Sulfuricaulis;Sulfuricaulis limicola;Sulfuricaulis limicola HA5 |
| Ga0586324 1620689 2 988 | 0.00 | 0.00 | 0.00 | 0.00 | 0.00 | 0.00 | 0.00 | 523847.87 | 381731.13 | 0.00 | 0.00 | 0.00 | KO:K04043 | molecular chaperone DnaK | Bacteria;Proteobacteria;Deltaproteobacteria;Syntrophobacterales;Syntrophobacteraceae;Syntrophobacter;Syntrophobacter fumaroxidans;Syntrophobacter fumaroxidans MPOB |
| Ga0586324 1630116 1 984 | 0.00 | 9316000.00 | 0.00 | 0.00 | 0.00 | 0.00 | 0.00 | 8662000.00 | 10044000.00 | 22407000.00 | 21296000.00 | 750000.00 | KO:K04043 | molecular chaperone DnaK | Bacteria;Proteobacteria;Betaproteobacteria;Rhodocyclales;Zoogloeaceae;Azoarcus;Azoarcus sp. KH32C;Azoarcus sp. KH32C |
| Ga0586324 1692915 501 968 | 0.00 | 0.00 | 0.00 | 0.00 | 0.00 | 0.00 | 0.00 | 0.00 | 231206.40 | 0.00 | 0.00 | 0.00 | KO:K04043 | molecular chaperone DnaK | Bacteria;Chloroflexi;Dehalococcoidia;unclassified Dehalococcoidia;unclassified Dehalococcoidia;Dehalogenimonas;Dehalogenimonas formicexedens;Dehalogenimonas formicexedens NSZ-14 |
| Ga0586324 1762837 3 953 | 0.00 | 0.00 | 0.00 | 0.00 | 10300000.00 | 0.00 | 0.00 | 0.00 | 0.00 | 9009000.00 | 0.00 | 0.00 | KO:K04043 | molecular chaperone DnaK | Bacteria;Proteobacteria;Deltaproteobacteria;Syntrophobacterales;Syntrophaceae;Smithella;Smithella sp. F21;Smithella sp. F21 |
| Ga0586324 1868420 2 931 | 1160000.00 | 0.00 | 0.00 | 0.00 | 0.00 | 0.00 | 0.00 | 0.00 | 0.00 | 0.00 | 0.00 | 0.00 | KO:K04043 | molecular chaperone DnaK | Bacteria;Planctomycetes;Planctomycetia;Isosphaerales;Isosphaeraceae;Singulisphaera;Singulisphaera sp. GP187;Singulisphaera sp. GP187 |
| Ga0586324 2143314 1 876 | 4650000.00 | 0.00 | 0.00 | 0.00 | 0.00 | 0.00 | 0.00 | 0.00 | 0.00 | 0.00 | 0.00 | 0.00 | KO:K04043 | molecular chaperone DnaK | Bacteria;Gemmatimonadetes;Gemmatimonadetes;Gemmatimonadales;Gemmatimonadaceae;Gemmatirosa;Gemmatirosa kalamazoonesis;Gemmatimonadetes bacterium KBS708 |
| Ga0586324 2187565 3 644 | 0.00 | 8772000.00 | 0.00 | 0.00 | 1781900.00 | 0.00 | 0.00 | 0.00 | 53541000.00 | 0.00 | 105336000.00 | 73200000.00 | KO:K04043 | molecular chaperone DnaK | Bacteria;Proteobacteria;Deltaproteobacteria;Myxococcales;Labilitrichaceae;Labilithrix;Labilithrix luteola;Labilithrix luteola DSM 27648 |
| Ga0586324 2193286 1 870 | 0.00 | 1496000.00 | 0.00 | 2688500.00 | 3357800.00 | 0.00 | 2485200.00 | 2702300.00 | 2867400.00 | 0.00 | 0.00 | 1687500.00 | KO:K04043 | molecular chaperone DnaK | Bacteria;Proteobacteria;Deltaproteobacteria;Desulfobacterales;Desulfobulbaceae;Desulfobulbus;Desulfobulbus elongatus;Desulfobulbus elongatus DSM 2908 |
| Ga0586324 2320017 2 847 | 0.00 | 0.00 | 0.00 | 0.00 | 0.00 | 0.00 | 0.00 | 5978000.00 | 0.00 | 0.00 | 0.00 | 0.00 | KO:K04043 | molecular chaperone DnaK | Bacteria;Proteobacteria;Alphaproteobacteria;Hyphomicrobiales;Bradyrhizobiaceae;Afipia;Afipia carboxidovorans;Oligotropha carboxidovorans OM4 |
| Ga0586324 2383137 1 840 | 0.00 | 0.00 | 0.00 | 0.00 | 0.00 | 0.00 | 0.00 | 0.00 | 140349.51 | 0.00 | 0.00 | 0.00 | KO:K04043 | molecular chaperone DnaK | Bacteria;Candidatus Latescibacteria;unclassified Candidatus Latescibacteria;unclassified Candidatus Latescibacteria;unclassified Candidatus Latescibacteria;unclassified Candidatus Latescibacteria;Latescibacteria bacterium SCGC AAA252-B13;Latescibacteria bacterium SCGC AAA252-B13 (contamination screened) |
| Ga0586324 2444977 2 829 | 0.00 | 0.00 | 0.00 | 0.00 | 0.00 | 0.00 | 0.00 | 0.00 | 0.00 | 0.00 | 0.00 | 825000.00 | KO:K04043 | molecular chaperone DnaK | Bacteria;Proteobacteria;Deltaproteobacteria;Desulfuromonadales;Desulfuromonadaceae;Desulfuromonas;Desulfuromonas thiophila;Desulfuromonas thiophila DSM 8987 |
| Ga0586324 2514667 401 820 | 0.00 | 0.00 | 0.00 | 0.00 | 0.00 | 0.00 | 141321.92 | 0.00 | 0.00 | 0.00 | 0.00 | 0.00 | KO:K04043 | molecular chaperone DnaK | Bacteria;Proteobacteria;Betaproteobacteria;unclassified Betaproteobacteria;unclassified Betaproteobacteria;unclassified Betaproteobacteria;Betaproteobacteria bacterium GR16-43;Betaproteobacteria bacterium GR16-43 |
| Ga0586324 2658627 3 800 | 0.00 | 0.00 | 0.00 | 0.00 | 0.00 | 0.00 | 0.00 | 0.00 | 0.00 | 3241700.00 | 7682400.00 | 0.00 | KO:K04043 | molecular chaperone DnaK | Bacteria;Proteobacteria;Betaproteobacteria;Nitrosomonadales;Gallionellaceae;Sideroxydans;Sideroxydans lithotrophicus;Sideroxydans lithotrophicus ES-1 |
| Ga0586324 2711771 1 795 | 0.00 | 0.00 | 0.00 | 0.00 | 0.00 | 0.00 | 0.00 | 0.00 | 2907900.00 | 0.00 | 0.00 | 0.00 | KO:K04043 | molecular chaperone DnaK | Bacteria;Acidobacteria;Blastocatellia;Blastocatellales;Pyrinomonadaceae;Pyrinomonas;Pyrinomonas methylaliphatogenes;Pyrinomonas methylaliphatogenes K22 |
| Ga0586324 2744255 2 790 | 0.00 | 0.00 | 0.00 | 0.00 | 0.00 | 0.00 | 0.00 | 0.00 | 0.00 | 0.00 | 0.00 | 4815000.00 | KO:K04043 | molecular chaperone DnaK | Bacteria;Proteobacteria;Deltaproteobacteria;Syntrophobacterales;Syntrophaceae;Syntrophus;Syntrophus aciditrophicus;Syntrophus aciditrophicus SB |
| Ga0586324 2758411 2 790 | 0.00 | 0.00 | 0.00 | 0.00 | 0.00 | 0.00 | 0.00 | 8052000.00 | 0.00 | 0.00 | 0.00 | 0.00 | KO:K04043 | molecular chaperone DnaK | Bacteria;Nitrospirae;Nitrospira;Nitrospirales;Nitrospiraceae;Nitrospira;Nitrospira japonica;Nitrospira japonica NJ11 |
| Ga0586324 2787856 214 786 | 0.00 | 0.00 | 0.00 | 22040000.00 | 11948000.00 | 0.00 | 0.00 | 0.00 | 0.00 | 0.00 | 0.00 | 0.00 | KO:K04043 | molecular chaperone DnaK | Bacteria;Proteobacteria;Deltaproteobacteria;Desulfobacterales;Desulfobacteraceae;Desulfatiglans;Desulfatiglans anilini;Desulfatiglans anilini DSM 4660 |
| Ga0586324 2817946 1 750 | 0.00 | 0.00 | 0.00 | 0.00 | 0.00 | 0.00 | 0.00 | 0.00 | 1701000.00 | 6737500.00 | 7832000.00 | 8175000.00 | KO:K04043 | molecular chaperone DnaK | Bacteria;Proteobacteria;Betaproteobacteria;Nitrosomonadales;Nitrosomonadaceae;Nitrosomonas;Nitrosomonas eutropha;Nitrosomonas eutropha C91 |
| Ga0586324 2823731 3 782 | 0.00 | 0.00 | 0.00 | 0.00 | 6293300.00 | 0.00 | 0.00 | 0.00 | 0.00 | 0.00 | 0.00 | 0.00 | KO:K04043 | molecular chaperone DnaK | Bacteria;Proteobacteria;Deltaproteobacteria;Syntrophobacterales;Syntrophaceae;Desulfobacca;Desulfobacca acetoxidans;Desulfobacca acetoxidans ASRB2, DSM 11109 |
| Ga0586324 2880462 3 776 | 0.00 | 0.00 | 0.00 | 0.00 | 0.00 | 0.00 | 666343.68 | 0.00 | 0.00 | 0.00 | 0.00 | 0.00 | KO:K04043 | molecular chaperone DnaK | Bacteria;Proteobacteria;Deltaproteobacteria;Syntrophobacterales;Syntrophobacteraceae;Syntrophobacter;Syntrophobacter fumaroxidans;Syntrophobacter fumaroxidans MPOB |
| Ga0586324 2887459 1 774 | 0.00 | 0.00 | 0.00 | 2090000.00 | 0.00 | 0.00 | 0.00 | 0.00 | 0.00 | 0.00 | 0.00 | 0.00 | KO:K04043 | molecular chaperone DnaK | Bacteria;Proteobacteria;Deltaproteobacteria;Syntrophobacterales;Syntrophaceae;Desulfobacca;Desulfobacca acetoxidans;Desulfobacca acetoxidans ASRB2, DSM 11109 |
| Ga0586324 2898878 1 774 | 0.00 | 0.00 | 0.00 | 0.00 | 1308100.00 | 0.00 | 0.00 | 0.00 | 3742200.00 | 0.00 | 4162400.00 | 3105000.00 | KO:K04043 | molecular chaperone DnaK | Bacteria;Proteobacteria;Betaproteobacteria;Nitrosomonadales;Thiobacillaceae;Annwoodia;Annwoodia aquaesulis;Annwoodia aquaesulis DSM 4255 |
| Ga0586324 2935005 3 770 | 0.00 | 0.00 | 0.00 | 0.00 | 0.00 | 0.00 | 0.00 | 0.00 | 0.00 | 52899000.00 | 0.00 | 0.00 | KO:K04043 | molecular chaperone DnaK | Archaea;Thaumarchaeota;unclassified Thaumarchaeota;unclassified Thaumarchaeota;unclassified Thaumarchaeota;unclassified Thaumarchaeota;unclassified Thaumarchaeota;Thaumarchaeota archaeon sp. BS1 |
| Ga0586324 3252826 1 495 | 0.00 | 0.00 | 0.00 | 903458.55 | 0.00 | 0.00 | 0.00 | 0.00 | 0.00 | 0.00 | 0.00 | 1822500.00 | KO:K04043 | molecular chaperone DnaK | Bacteria;Proteobacteria;Deltaproteobacteria;Syntrophobacterales;Syntrophaceae;Syntrophus;Syntrophus aciditrophicus;Syntrophus aciditrophicus SB |
| Ga0586324 3380985 2 724 | 0.00 | 0.00 | 0.00 | 0.00 | 0.00 | 0.00 | 0.00 | 0.00 | 0.00 | 0.00 | 1188000.00 | 0.00 | KO:K04043 | molecular chaperone DnaK | Bacteria;Proteobacteria;Alphaproteobacteria;Hyphomicrobiales;Rhizobiaceae;Rhizobium;Rhizobium vallis;Rhizobium vallis LMG 25295 |
| Ga0586324 3383724 265 723 | 0.00 | 210136.32 | 0.00 | 0.00 | 0.00 | 0.00 | 0.00 | 0.00 | 0.00 | 0.00 | 0.00 | 0.00 | KO:K04043 | molecular chaperone DnaK | Archaea;Euryarchaeota;Methanomicrobia;Methanosarcinales;Methanotrichaceae;Methanothrix;Methanothrix soehngenii;Methanothrix soehngenii GP-6 |
| Ga0586324 3433946 3 719 | 0.00 | 0.00 | 0.00 | 0.00 | 0.00 | 0.00 | 2485200.00 | 0.00 | 603415.98 | 0.00 | 0.00 | 0.00 | KO:K04043 | molecular chaperone DnaK | Bacteria;Proteobacteria;Deltaproteobacteria;Syntrophobacterales;Syntrophaceae;Syntrophus;Syntrophus aciditrophicus;Syntrophus aciditrophicus SB |
| Ga0586324 3603920 1 705 | 0.00 | 2774400.00 | 0.00 | 0.00 | 3574100.00 | 0.00 | 3876000.00 | 7686000.00 | 0.00 | 5405400.00 | 4725600.00 | 4132500.00 | KO:K04043 | molecular chaperone DnaK | Bacteria;Proteobacteria;Betaproteobacteria;Rhodocyclales;Rhodocyclaceae;Dechlorobacter;Dechlorobacter hydrogenophilus;Dechlorobacter hydrogenophilus LT-1 |
| Ga0586324 3622014 168 704 | 0.00 | 0.00 | 0.00 | 0.00 | 0.00 | 0.00 | 0.00 | 0.00 | 0.00 | 816200.00 | 0.00 | 0.00 | KO:K04043 | molecular chaperone DnaK | Bacteria;Proteobacteria;Betaproteobacteria;Burkholderiales;unclassified Burkholderiales;Leptothrix;Leptothrix mobilis;Leptothrix mobilis DSM 10617 |
| Ga0586324 3689066 2 700 | 0.00 | 0.00 | 0.00 | 2935500.00 | 2533800.00 | 2061569.39 | 1493363.52 | 0.00 | 581313.51 | 511695.80 | 4558400.00 | 0.00 | KO:K04043 | molecular chaperone DnaK | Bacteria;Proteobacteria;Deltaproteobacteria;Desulfobacterales;Desulfobacteraceae;Desulfatirhabdium;Desulfatirhabdium butyrativorans;Desulfatirhabdium butyrativorans DSM 18734 |
| Ga0586324 3879465 1 684 | 0.00 | 0.00 | 0.00 | 0.00 | 0.00 | 0.00 | 2804400.00 | 0.00 | 1539000.00 | 0.00 | 1443200.00 | 0.00 | KO:K04043 | molecular chaperone DnaK | Bacteria;Proteobacteria;Betaproteobacteria;Rhodocyclales;Rhodocyclaceae;Dechlorobacter;Dechlorobacter hydrogenophilus;Dechlorobacter hydrogenophilus LT-1 |
| Ga0586324 4007574 3 674 | 0.00 | 3148400.00 | 0.00 | 0.00 | 0.00 | 0.00 | 0.00 | 0.00 | 0.00 | 550369.05 | 0.00 | 0.00 | KO:K04043 | molecular chaperone DnaK | Bacteria;Proteobacteria;Deltaproteobacteria;Desulfarculales;Desulfarculaceae;Desulfocarbo;Desulfocarbo indianensis;Desulfocarbo indianensis SCBM |
| Ga0586324 4074325 1 492 | 430684.00 | 0.00 | 0.00 | 0.00 | 0.00 | 0.00 | 0.00 | 0.00 | 0.00 | 0.00 | 0.00 | 0.00 | KO:K04043 | molecular chaperone DnaK | Bacteria;Proteobacteria;Deltaproteobacteria;Desulfobacterales;Desulfobacteraceae;Desulfatibacillum;Desulfatibacillum alkenivorans;Desulfatibacillum alkenivorans DSM 16219 |
| Ga0586324 4143192 3 665 | 0.00 | 0.00 | 0.00 | 0.00 | 0.00 | 0.00 | 0.00 | 0.00 | 0.00 | 0.00 | 0.00 | 5235000.00 | KO:K04043 | molecular chaperone DnaK | Bacteria;Proteobacteria;Betaproteobacteria;unclassified Betaproteobacteria;unclassified Betaproteobacteria;unclassified Betaproteobacteria;Betaproteobacteria bacterium GR16-43;Betaproteobacteria bacterium GR16-43 |
| Ga0586324 4224241 39 662 | 4270000.00 | 0.00 | 0.00 | 0.00 | 0.00 | 0.00 | 0.00 | 0.00 | 0.00 | 0.00 | 0.00 | 0.00 | KO:K04043 | molecular chaperone DnaK | Bacteria;Proteobacteria;Alphaproteobacteria;Hyphomicrobiales;Reyranellaceae;Reyranella;Reyranella sp. OAS946;Reyranella sp. OAS946 |
| Ga0586324 4373246 3 653 | 0.00 | 829600.00 | 42562800.00 | 0.00 | 0.00 | 0.00 | 0.00 | 0.00 | 0.00 | 0.00 | 73920000.00 | 0.00 | KO:K04043 | molecular chaperone DnaK | Bacteria;Proteobacteria;Deltaproteobacteria;Desulfobacterales;Desulfobacteraceae;Desulfatiglans;Desulfatiglans anilini;Desulfatiglans anilini DSM 4660 |
| Ga0586324 4564431 2 640 | 0.00 | 0.00 | 0.00 | 0.00 | 0.00 | 0.00 | 0.00 | 0.00 | 0.00 | 14322000.00 | 0.00 | 0.00 | KO:K04043 | molecular chaperone DnaK | Bacteria;Chloroflexi;Anaerolineae;Anaerolineales;Anaerolineaceae;Levilinea;Levilinea saccharolytica;Levilinea saccharolytica DSM 16555 |
| Ga0586324 4681333 1 633 | 0.00 | 1700000.00 | 0.00 | 0.00 | 0.00 | 0.00 | 0.00 | 0.00 | 0.00 | 0.00 | 1812800.00 | 1447500.00 | KO:K04043 | molecular chaperone DnaK | Bacteria;Proteobacteria;Deltaproteobacteria;Syntrophobacterales;Syntrophaceae;Syntrophus;Syntrophus gentianae;Syntrophus gentianae DSM 8423 |
| Ga0586324 4964823 3 617 | 2000000.00 | 0.00 | 0.00 | 4503000.00 | 0.00 | 0.00 | 0.00 | 0.00 | 0.00 | 0.00 | 0.00 | 0.00 | KO:K04043 | molecular chaperone DnaK | Bacteria;Verrucomicrobia;Opitutae;Opitutales;Opitutaceae;Opitutus;Opitutus sp. GAS368;Opitutus sp. GAS368 |
| Ga0586324 5125027 1 363 | 0.00 | 1122000.00 | 0.00 | 0.00 | 0.00 | 0.00 | 0.00 | 0.00 | 0.00 | 0.00 | 0.00 | 0.00 | KO:K04043 | molecular chaperone DnaK | Bacteria;Proteobacteria;Deltaproteobacteria;Desulfobacterales;Desulfobacteraceae;Desulfatibacillum;Desulfatibacillum alkenivorans;Desulfatibacillum alkenivorans DSM 16219 |
| Ga0586324 5214014 206 607 | 0.00 | 3862400.00 | 0.00 | 0.00 | 0.00 | 0.00 | 0.00 | 0.00 | 542118.42 | 234193.96 | 0.00 | 0.00 | KO:K04043 | molecular chaperone DnaK | Bacteria;Proteobacteria;Betaproteobacteria;Burkholderiales;unclassified Burkholderiales;unclassified Burkholderiales;Burkholderiales bacterium JOSHI 001;Burkholderiales sp. JOSHI 001 |
| Ga0586324 5552180 50 592 | 0.00 | 0.00 | 0.00 | 0.00 | 0.00 | 0.00 | 0.00 | 0.00 | 0.00 | 0.00 | 416721.36 | 0.00 | KO:K04043 | molecular chaperone DnaK | Bacteria;Acidobacteria;Blastocatellia;Blastocatellales;Pyrinomonadaceae;Pyrinomonas;Pyrinomonas methylaliphatogenes;Pyrinomonas methylaliphatogenes K22 |
| Ga0586324 5789712 1 579 | 0.00 | 0.00 | 0.00 | 0.00 | 0.00 | 0.00 | 0.00 | 0.00 | 0.00 | 4327400.00 | 4012800.00 | 0.00 | KO:K04043 | molecular chaperone DnaK | Bacteria;Proteobacteria;Deltaproteobacteria;Syntrophobacterales;Syntrophaceae;Syntrophus;Syntrophus gentianae;Syntrophus gentianae DSM 8423 |
| Ga0586324 5790276 3 581 | 0.00 | 0.00 | 0.00 | 0.00 | 0.00 | 0.00 | 0.00 | 0.00 | 0.00 | 1139600.00 | 0.00 | 0.00 | KO:K04043 | molecular chaperone DnaK | Bacteria;Proteobacteria;Gammaproteobacteria;Methylococcales;Methylococcaceae;Methylovulum;Methylovulum psychrotolerans;Methylovulum psychrotolerans HV10 M2 |
| Ga0586324 6245619 2 562 | 0.00 | 0.00 | 0.00 | 0.00 | 0.00 | 0.00 | 0.00 | 2812100.00 | 0.00 | 0.00 | 0.00 | 0.00 | KO:K04043 | molecular chaperone DnaK | Bacteria;Proteobacteria;Alphaproteobacteria;Rhodospirillales;unclassified Rhodospirillales;Enhydrobacter;Enhydrobacter aerosaccus;Enhydrobacter aerosaccus ATCC 27094 |
| Ga0586324 6795648 2 544 | 0.00 | 7004000.00 | 0.00 | 0.00 | 0.00 | 0.00 | 0.00 | 0.00 | 0.00 | 0.00 | 7295200.00 | 0.00 | KO:K04043 | molecular chaperone DnaK | Bacteria;Proteobacteria;Deltaproteobacteria;Myxococcales;Sandaracinaceae;Sandaracinus;Sandaracinus amylolyticus;Sandaracinus amylolyticus DSM 53668 |
| Ga0586324 6886162 1 540 | 0.00 | 0.00 | 0.00 | 0.00 | 0.00 | 0.00 | 0.00 | 0.00 | 485571.51 | 0.00 | 0.00 | 0.00 | KO:K04043 | molecular chaperone DnaK | Bacteria;Nitrospirae;Nitrospira;Nitrospirales;Nitrospiraceae;Nitrospira;Nitrospira marina;Nitrospira marina Nb-295 |
| Ga0586324 7042569 3 536 | 0.00 | 0.00 | 0.00 | 0.00 | 0.00 | 0.00 | 0.00 | 4782400.00 | 0.00 | 0.00 | 0.00 | 0.00 | KO:K04043 | molecular chaperone DnaK | Bacteria;Gemmatimonadetes;Gemmatimonadetes;Gemmatimonadales;Gemmatimonadaceae;Gemmatimonas;Gemmatimonas aurantiaca;Gemmatimonas aurantiaca T-27T |
| Ga0586324 7073317 3 533 | 0.00 | 0.00 | 0.00 | 0.00 | 0.00 | 5438900.00 | 0.00 | 0.00 | 3458700.00 | 0.00 | 0.00 | 3262500.00 | KO:K04043 | molecular chaperone DnaK | Archaea;Euryarchaeota;Methanomicrobia;Methanomicrobiales;Methanoregulaceae;Methanoregula;Methanoregula boonei;Methanoregula boonei 6A8 |
| Ga0586324 7190110 3 530 | 0.00 | 0.00 | 0.00 | 6137000.00 | 6664100.00 | 0.00 | 0.00 | 0.00 | 0.00 | 0.00 | 0.00 | 0.00 | KO:K04043 | molecular chaperone DnaK | Bacteria;Bacteroidetes;Bacteroidia;Marinilabiliales;Marinilabiliaceae;Alkaliflexus;Alkaliflexus imshenetskii;Alkaliflexus imshenetskii Z-7010, DSM 15055 |
| Ga0586324 7234384 1 528 | 0.00 | 0.00 | 0.00 | 944364.60 | 0.00 | 0.00 | 0.00 | 0.00 | 0.00 | 0.00 | 0.00 | 0.00 | KO:K04043 | molecular chaperone DnaK | Bacteria;Nitrospirae;Thermodesulfovibrionia;Thermodesulfovibrionales;Thermodesulfovibrionaceae;Thermodesulfovibrio;Thermodesulfovibrio sp. N1;Thermodesulfovibrio sp. N1 |
| Ga0586324 7334695 3 527 | 0.00 | 0.00 | 0.00 | 0.00 | 0.00 | 0.00 | 0.00 | 1756800.00 | 1684800.00 | 823900.00 | 906400.00 | 900000.00 | KO:K04043 | molecular chaperone DnaK | Bacteria;Proteobacteria;Gammaproteobacteria;Acidiferrobacterales;Acidiferrobacteraceae;Sulfuricaulis;Sulfuricaulis limicola;Sulfuricaulis limicola HA5 |
| Ga0586324 7377955 1 525 | 228961.00 | 0.00 | 0.00 | 0.00 | 0.00 | 0.00 | 0.00 | 0.00 | 0.00 | 0.00 | 0.00 | 0.00 | KO:K04043 | molecular chaperone DnaK | Bacteria;Candidatus Parcubacteria;unclassified Candidatus Parcubacteria;unclassified Candidatus Parcubacteria;unclassified Candidatus Parcubacteria;unclassified Candidatus Parcubacteria;Parcubacteria bacterium SCGC AAA036-E14;Parcubacteria bacterium SCGC AAA036-E14 (contamination screened) |
| Ga0586324 7465131 1 522 | 0.00 | 2781200.00 | 0.00 | 0.00 | 0.00 | 0.00 | 0.00 | 0.00 | 0.00 | 0.00 | 0.00 | 0.00 | KO:K04043 | molecular chaperone DnaK | Bacteria;Proteobacteria;Deltaproteobacteria;Desulfuromonadales;Geobacteraceae;Geoalkalibacter;Geoalkalibacter subterraneus;Geoalkalibacter subterraneus Red1 |
| Ga0586324 7644862 3 518 | 0.00 | 0.00 | 0.00 | 0.00 | 349036.10 | 0.00 | 0.00 | 0.00 | 0.00 | 0.00 | 0.00 | 0.00 | KO:K04043 | molecular chaperone DnaK | Bacteria;Proteobacteria;Deltaproteobacteria;Syntrophobacterales;Syntrophaceae;Desulfobacca;Desulfobacca acetoxidans;Desulfobacca acetoxidans ASRB2, DSM 11109 |
| Ga0586324 7802945 3 512 | 0.00 | 0.00 | 0.00 | 0.00 | 0.00 | 0.00 | 3055200.00 | 0.00 | 0.00 | 0.00 | 0.00 | 0.00 | KO:K04043 | molecular chaperone DnaK | Bacteria;Proteobacteria;Betaproteobacteria;Burkholderiales;Alcaligenaceae;Bordetella;Bordetella avium;Bordetella avium 197N |
| Ga0586324 8013073 3 506 | 0.00 | 0.00 | 0.00 | 7144000.00 | 6705300.00 | 0.00 | 0.00 | 5636400.00 | 0.00 | 0.00 | 0.00 | 4515000.00 | KO:K04043 | molecular chaperone DnaK | Bacteria;Proteobacteria;Deltaproteobacteria;Syntrophobacterales;Syntrophobacteraceae;Syntrophobacter;Syntrophobacter fumaroxidans;Syntrophobacter fumaroxidans MPOB |
| Ga0586324 0006543 8978 9466 | 0.00 | 0.00 | 0.00 | 0.00 | 0.00 | 0.00 | 0.00 | 0.00 | 0.00 | 0.00 | 1909600.00 | 0.00 | KO:K04047 | starvation-inducible DNA-binding protein | Bacteria;Proteobacteria;Betaproteobacteria;Burkholderiales;Oxalobacteraceae;Herbaspirillum;Herbaspirillum sp. RV1423;Herbaspirillum sp. RV1423 |
| Ga0586324 0037486 3978 4484 | 0.00 | 0.00 | 0.00 | 0.00 | 2523500.00 | 0.00 | 0.00 | 0.00 | 0.00 | 0.00 | 0.00 | 0.00 | KO:K04047 | starvation-inducible DNA-binding protein | Bacteria;Proteobacteria;Betaproteobacteria;Burkholderiales;Oxalobacteraceae;Herbaspirillum;Herbaspirillum sp. meg3;Herbaspirillum sp. meg3 |
| Ga0586324 0048842 2723 3217 | 953163.00 | 0.00 | 0.00 | 0.00 | 0.00 | 0.00 | 0.00 | 0.00 | 0.00 | 0.00 | 1821600.00 | 0.00 | KO:K04047 | starvation-inducible DNA-binding protein | Bacteria;Proteobacteria;Gammaproteobacteria;Pseudomonadales;Pseudomonadaceae;Pseudomonas;Pseudomonas linyingensis;Pseudomonas linyingensis LMG 25967 |
| Ga0586324 1342520 132 1070 | 0.00 | 0.00 | 0.00 | 1282500.00 | 0.00 | 0.00 | 0.00 | 0.00 | 0.00 | 0.00 | 0.00 | 0.00 | KO:K04069 | pyruvate formate lyase activating enzyme | Bacteria;Cyanobacteria;unclassified Cyanobacteria;Synechococcales;Oculatellaceae;Elainella;Elainella saxicola;Elainella saxicola E1 |
| Ga0586324 4844071 260 625 | 0.00 | 0.00 | 0.00 | 2061500.00 | 0.00 | 0.00 | 0.00 | 0.00 | 0.00 | 0.00 | 0.00 | 0.00 | KO:K04069 | pyruvate formate lyase activating enzyme | Bacteria;Cyanobacteria;unclassified Cyanobacteria;Synechococcales;Oculatellaceae;Elainella;Elainella saxicola;Elainella saxicola E1 |
| Ga0586324 1761345 1 891 | 0.00 | 13124000.00 | 0.00 | 0.00 | 17613000.00 | 0.00 | 0.00 | 0.00 | 17010000.00 | 0.00 | 0.00 | 0.00 | KO:K04072 | acetaldehyde dehydrogenase/alcohol dehydrogenase | Bacteria;Proteobacteria;Deltaproteobacteria;Desulfuromonadales;Syntrophotaleaceae;Syntrophotalea;Syntrophotalea carbinolica;Syntrophotalea carbinolica DSM 2380 |
| Ga0586324 2444130 80 829 | 0.00 | 8636000.00 | 0.00 | 0.00 | 0.00 | 0.00 | 0.00 | 0.00 | 0.00 | 0.00 | 0.00 | 0.00 | KO:K04072 | acetaldehyde dehydrogenase/alcohol dehydrogenase | Bacteria;Proteobacteria;Deltaproteobacteria;Desulfuromonadales;Syntrophotaleaceae;Syntrophotalea;Syntrophotalea carbinolica;Syntrophotalea carbinolica DSM 2380 |
| Ga0586324 6021344 3 572 | 7580000.00 | 0.00 | 0.00 | 0.00 | 0.00 | 7940000.00 | 0.00 | 6283000.00 | 0.00 | 0.00 | 0.00 | 2857500.00 | KO:K04072 | acetaldehyde dehydrogenase/alcohol dehydrogenase | Bacteria;Proteobacteria;Deltaproteobacteria;Desulfuromonadales;Syntrophotaleaceae;Syntrophotalea;Syntrophotalea carbinolica;Syntrophotalea carbinolica DSM 2380 |
| Ga0586324 0000001 8899 10542 | 0.00 | 2352800.00 | 2619019.70 | 0.00 | 0.00 | 0.00 | 52212000.00 | 71967800.00 | 44331300.00 | 113343355.51 | 104720000.00 | 153111114.00 | KO:K04077 | chaperonin GroEL | Bacteria;Proteobacteria;Betaproteobacteria;Nitrosomonadales;Thiobacillaceae;Annwoodia;Annwoodia aquaesulis;Annwoodia aquaesulis DSM 4255 |
| Ga0586324 0000075 51485 53128 | 0.00 | 0.00 | 0.00 | 408959.80 | 0.00 | 0.00 | 20428800.00 | 25735900.00 | 23157900.00 | 1008700.00 | 10771200.00 | 13117500.00 | KO:K04077 | chaperonin GroEL | Bacteria;Proteobacteria;Betaproteobacteria;Nitrosomonadales;Thiobacillaceae;Thiobacillus;Thiobacillus thioparus;Thiobacillus thioparus DSM 505 |
| Ga0586324 0000224 26536 28191 | 0.00 | 81600000.00 | 0.00 | 0.00 | 0.00 | 0.00 | 806565.96 | 799100.00 | 1360800.00 | 0.00 | 0.00 | 8325000.00 | KO:K04077 | chaperonin GroEL | Bacteria;Proteobacteria;Gammaproteobacteria;Acidiferrobacterales;Acidiferrobacteraceae;Sulfuricaulis;Sulfuricaulis limicola;Sulfuricaulis limicola HA5 |
| Ga0586324 0000339 2083 3723 | 1370000.00 | 1040400.00 | 0.00 | 2479500.00 | 2544100.00 | 0.00 | 0.00 | 6553718.61 | 3231900.00 | 0.00 | 5126990.88 | 3065607.00 | KO:K04077 | chaperonin GroEL | Bacteria;Proteobacteria;Deltaproteobacteria;Desulfobacterales;Desulfobacteraceae;Desulfatitalea;Desulfatitalea tepidiphila;Desulfatitalea tepidiphila S28bF |
| Ga0586324 0000346 4613 6256 | 4380000.00 | 0.00 | 1395952.87 | 1187500.00 | 0.00 | 0.00 | 0.00 | 0.00 | 0.00 | 3780700.00 | 0.00 | 0.00 | KO:K04077 | chaperonin GroEL | Bacteria;Chloroflexi;Anaerolineae;Anaerolineales;Anaerolineaceae;Anaerolinea;Anaerolinea thermophila;Anaerolinea thermophila UNI-1 |
| Ga0586324 0000364 2032 3672 | 0.00 | 0.00 | 0.00 | 0.00 | 0.00 | 0.00 | 0.00 | 0.00 | 314454.96 | 0.00 | 346538.72 | 0.00 | KO:K04077 | chaperonin GroEL | Bacteria;Proteobacteria;Alphaproteobacteria;Hyphomicrobiales;unclassified Hyphomicrobiales;Methyloceanibacter;Methyloceanibacter superfactus;Methyloceanibacter superfactus R-67175 |
| Ga0586324 0000454 17862 19496 | 0.00 | 5569200.00 | 14356500.00 | 0.00 | 0.00 | 5637400.00 | 347932.56 | 0.00 | 0.00 | 0.00 | 0.00 | 0.00 | KO:K04077 | chaperonin GroEL | Bacteria;Proteobacteria;Alphaproteobacteria;Hyphomicrobiales;Hyphomicrobiaceae;Rhodoplanes;Rhodoplanes sp. Z2-YC6860;Rhodoplanes sp. Z2-YC6860 |
| Ga0586324 0000709 27971 29527 | 0.00 | 0.00 | 0.00 | 0.00 | 0.00 | 0.00 | 0.00 | 1939800.00 | 0.00 | 0.00 | 114270.64 | 0.00 | KO:K04077 | chaperonin GroEL | Bacteria;Acidobacteria;Acidobacteriia;Acidobacteriales;Acidobacteriaceae;Candidatus Koribacter;Candidatus Koribacter versatilis;Candidatus Koribacter versatilis Ellin345 |
| Ga0586324 0000792 4379 5998 | 0.00 | 0.00 | 0.00 | 0.00 | 275802.07 | 0.00 | 0.00 | 0.00 | 0.00 | 0.00 | 465842.08 | 496172.25 | KO:K04077 | chaperonin GroEL | Bacteria;Proteobacteria;Deltaproteobacteria;Desulfobacterales;Desulfobacteraceae;Desulfatiglans;Desulfatiglans anilini;Desulfatiglans anilini DSM 4660 |
| Ga0586324 0001014 22891 24522 | 8190000.00 | 14642335.28 | 48349426.60 | 16625000.00 | 34768742.83 | 9964700.00 | 152828400.00 | 8052000.00 | 9415316.88 | 13729100.00 | 12232000.00 | 6352500.00 | KO:K04077 | chaperonin GroEL | Bacteria;Acidobacteria;Vicinamibacteria;unclassified Vicinamibacteria;Vicinamibacteraceae;Luteitalea;Luteitalea pratensis;Luteitalea pratensis DSM 100886 |
| Ga0586324 0001224 5111 6751 | 0.00 | 0.00 | 0.00 | 0.00 | 0.00 | 0.00 | 0.00 | 125660000.00 | 2583900.00 | 10425800.00 | 27473600.00 | 21300000.00 | KO:K04077 | chaperonin GroEL | Bacteria;Proteobacteria;Alphaproteobacteria;Hyphomicrobiales;Hyphomicrobiaceae;Rhodoplanes;Rhodoplanes sp. Z2-YC6860;Rhodoplanes sp. Z2-YC6860 |
| Ga0586324 0001384 1285 2904 | 0.00 | 686800.00 | 0.00 | 346426.05 | 886100.76 | 0.00 | 0.00 | 259379.93 | 20169000.00 | 0.00 | 0.00 | 997500.00 | KO:K04077 | chaperonin GroEL | Bacteria;Acidobacteria;Vicinamibacteria;unclassified Vicinamibacteria;Vicinamibacteraceae;Luteitalea;Luteitalea pratensis;Luteitalea pratensis DSM 100886 |
| Ga0586324 0001592 20039 21658 | 0.00 | 0.00 | 0.00 | 0.00 | 2811900.00 | 0.00 | 3009600.00 | 0.00 | 0.00 | 0.00 | 0.00 | 0.00 | KO:K04077 | chaperonin GroEL | Bacteria;Proteobacteria;Hydrogenophilalia;Hydrogenophilales;Hydrogenophilaceae;Hydrogenophilus;Hydrogenophilus thermoluteolus;Hydrogenophilus thermoluteolus TH-1 |
| Ga0586324 0001629 7357 8988 | 0.00 | 2169200.00 | 0.00 | 2793000.00 | 0.00 | 0.00 | 0.00 | 6161000.00 | 0.00 | 486380.51 | 390836.16 | 0.00 | KO:K04077 | chaperonin GroEL | Bacteria;Gemmatimonadetes;Gemmatimonadetes;Gemmatimonadales;Gemmatimonadaceae;Gemmatimonas;Gemmatimonas aurantiaca;Gemmatimonas aurantiaca T-27T |
| Ga0586324 0001729 2151 3794 | 698524.00 | 0.00 | 3685854.03 | 0.00 | 1421400.00 | 0.00 | 1098184.80 | 2055700.00 | 0.00 | 2571800.00 | 0.00 | 4334862.75 | KO:K04077 | chaperonin GroEL | Bacteria;Acidobacteria;Vicinamibacteria;unclassified Vicinamibacteria;Vicinamibacteraceae;Luteitalea;Luteitalea pratensis;Luteitalea pratensis DSM 100886 |
| Ga0586324 0001941 6288 7916 | 0.00 | 0.00 | 0.00 | 0.00 | 0.00 | 0.00 | 0.00 | 0.00 | 0.00 | 0.00 | 0.00 | 1230000.00 | KO:K04077 | chaperonin GroEL | Bacteria;Candidatus Omnitrophica;unclassified Candidatus Omnitrophica;unclassified Candidatus Omnitrophica;unclassified Candidatus Omnitrophica;unclassified Candidatus Omnitrophica;Omnitrophica bacterium SCGC AAA257-O07;Omnitrophica bacterium SCGC AAA257-O07 (contamination screened) |
| Ga0586324 0002538 7270 8934 | 8770000.00 | 2862800.00 | 7487900.00 | 15076500.00 | 24102000.00 | 13021600.00 | 11468400.00 | 17269100.00 | 12150000.00 | 16270100.00 | 9152000.00 | 12375000.00 | KO:K04077 | chaperonin GroEL | Bacteria;Proteobacteria;Deltaproteobacteria;Desulfobacterales;Desulfobacteraceae;Desulfosarcina;Desulfosarcina cetonica;Desulfosarcina cetonica JCM 12296 |
| Ga0586324 0002867 6230 7852 | 0.00 | 2509200.00 | 2339259.37 | 1061158.55 | 0.00 | 27591500.00 | 0.00 | 0.00 | 0.00 | 0.00 | 0.00 | 0.00 | KO:K04077 | chaperonin GroEL | Bacteria;Proteobacteria;Deltaproteobacteria;Syntrophobacterales;Syntrophobacteraceae;Desulfosoma;Desulfosoma caldarium;Desulfosoma caldarium DSM 22027 |
| Ga0586324 0003140 11827 13458 | 0.00 | 0.00 | 0.00 | 0.00 | 5819500.00 | 5747547.65 | 0.00 | 0.00 | 4633200.00 | 0.00 | 0.00 | 403244.25 | KO:K04077 | chaperonin GroEL | Bacteria;Chloroflexi;Anaerolineae;Anaerolineales;Anaerolineaceae;Levilinea;Levilinea saccharolytica;Levilinea saccharolytica DSM 16555 |
| Ga0586324 0003621 13233 14876 | 0.00 | 462376.20 | 0.00 | 1130500.00 | 776575.71 | 213427.99 | 9056734.56 | 7801900.00 | 7346700.00 | 0.00 | 6952000.00 | 5557500.00 | KO:K04077 | chaperonin GroEL | Bacteria;Proteobacteria;Betaproteobacteria;Nitrosomonadales;Gallionellaceae;Sideroxydans;Sideroxydans lithotrophicus;Sideroxydans lithotrophicus ES-1 |
| Ga0586324 0004061 7920 9560 | 18260000.00 | 1965200.00 | 1756779.57 | 5206000.00 | 9094900.00 | 1013739.50 | 0.00 | 2860900.00 | 4876200.00 | 0.00 | 4435200.00 | 3127500.00 | KO:K04077 | chaperonin GroEL | Bacteria;Nitrospirae;Nitrospira;Nitrospirales;Nitrospiraceae;Nitrospira;Nitrospira lenta;Nitrospira lenta N. lenta BS10 |
| Ga0586324 0004557 8984 10612 | 0.00 | 1080576.44 | 463350.69 | 0.00 | 0.00 | 0.00 | 2016817.32 | 3976693.70 | 7298100.00 | 4593430.38 | 385745.36 | 4905000.00 | KO:K04077 | chaperonin GroEL | Bacteria;Proteobacteria;Deltaproteobacteria;Myxococcales;Sandaracinaceae;Sandaracinus;Sandaracinus amylolyticus;Sandaracinus amylolyticus DSM 53668 |
| Ga0586324 0005132 4750 6402 | 750947.00 | 9724000.00 | 0.00 | 0.00 | 0.00 | 0.00 | 0.00 | 0.00 | 0.00 | 0.00 | 0.00 | 0.00 | KO:K04077 | chaperonin GroEL | Bacteria;Verrucomicrobia;Verrucomicrobiae;Verrucomicrobiales;Verrucomicrobia subdivision 3;Pedosphaera;Pedosphaera parvula;Pedosphaera parvula Ellin514 |
| Ga0586324 0005954 2432 4078 | 3630000.00 | 2522800.00 | 4071103.67 | 5519500.00 | 4676200.00 | 4168500.00 | 3662788.08 | 4995900.00 | 5832000.00 | 6853000.00 | 5953083.84 | 5323107.00 | KO:K04077 | chaperonin GroEL | Bacteria;Proteobacteria;Deltaproteobacteria;Syntrophorhabdales;Syntrophorhabdaceae;Syntrophorhabdus;Syntrophorhabdus aromaticivorans;Syntrophorhabdus aromaticivorans UI |
| Ga0586324 0006423 11437 12336 | 0.00 | 1162800.00 | 15313600.00 | 0.00 | 0.00 | 0.00 | 0.00 | 13054000.00 | 5321700.00 | 10549000.00 | 11528000.00 | 13275000.00 | KO:K04077 | chaperonin GroEL | Bacteria;Candidatus Calescamantes;unclassified Candidatus Calescamantes;unclassified Candidatus Calescamantes;unclassified Candidatus Calescamantes;unclassified Candidatus Calescamantes;Calescamantes bacterium JGI 0000106-H18;Calescamantes bacterium JGI 0000106-H18 (contamination screened) |
| Ga0586324 0006499 7270 8898 | 19025531.00 | 0.00 | 893013.71 | 0.00 | 5713800.37 | 462651.89 | 16644000.00 | 29945621.02 | 38855700.00 | 0.00 | 2032800.00 | 0.00 | KO:K04077 | chaperonin GroEL | Bacteria;Candidatus Omnitrophica;unclassified Candidatus Omnitrophica;unclassified Candidatus Omnitrophica;unclassified Candidatus Omnitrophica;unclassified Candidatus Omnitrophica;Omnitrophica bacterium SCGC AAA011-L16;Omnitrophica bacterium SCGC AAA011-L16 (contamination screened) |
| Ga0586324 0006560 2165 3805 | 0.00 | 0.00 | 0.00 | 0.00 | 0.00 | 0.00 | 0.00 | 3818600.00 | 0.00 | 0.00 | 0.00 | 0.00 | KO:K04077 | chaperonin GroEL | Bacteria;Proteobacteria;Betaproteobacteria;Nitrosomonadales;Gallionellaceae;Gallionella;Gallionella capsiferriformans;Gallionella capsiferriformans ES-2 |
| Ga0586324 0006676 3 1190 | 10700000.00 | 15041600.00 | 17396700.00 | 15200000.00 | 12447212.16 | 12704000.00 | 27702000.00 | 27633000.00 | 24381000.00 | 17017000.00 | 29392000.00 | 42750000.00 | KO:K04077 | chaperonin GroEL | Bacteria;Proteobacteria;Gammaproteobacteria;Chromatiales;Woeseiaceae;Woeseia;Woeseia oceani;Woeseia oceani XK5 |
| Ga0586324 0007804 5367 7007 | 0.00 | 0.00 | 131742000.00 | 146300000.00 | 0.00 | 5200700.00 | 133380000.00 | 4105300.00 | 0.00 | 0.00 | 255200000.00 | 4305000.00 | KO:K04077 | chaperonin GroEL | Bacteria;Proteobacteria;Alphaproteobacteria;Hyphomicrobiales;unclassified Hyphomicrobiales;Methyloceanibacter;Methyloceanibacter superfactus;Methyloceanibacter superfactus R-67175 |
| Ga0586324 0008370 3743 5380 | 0.00 | 0.00 | 10528100.00 | 0.00 | 16552100.00 | 0.00 | 0.00 | 0.00 | 0.00 | 0.00 | 0.00 | 0.00 | KO:K04077 | chaperonin GroEL | Bacteria;Chloroflexi;Thermoflexia;Thermoflexales;Thermoflexaceae;Thermoflexus;Thermoflexus hugenholtzii;Thermoflexus hugenholtzii JAD2 |
| Ga0586324 0008451 9206 10846 | 0.00 | 0.00 | 0.00 | 209919.60 | 3986100.00 | 0.00 | 5244606.25 | 683200.00 | 8060702.93 | 633494.40 | 6969600.00 | 9766310.25 | KO:K04077 | chaperonin GroEL | Bacteria;Proteobacteria;Alphaproteobacteria;Hyphomicrobiales;Hyphomicrobiaceae;Rhodoplanes;Rhodoplanes sp. Z2-YC6860;Rhodoplanes sp. Z2-YC6860 |
| Ga0586324 0008827 7169 8791 | 0.00 | 3372800.00 | 1657038.49 | 0.00 | 0.00 | 1876225.97 | 0.00 | 287476.53 | 307106.64 | 3183170.76 | 307067.20 | 400651.50 | KO:K04077 | chaperonin GroEL | Bacteria;Acidobacteria;Acidobacteriia;Acidobacteriales;Acidobacteriaceae;Candidatus Koribacter;Candidatus Koribacter versatilis;Candidatus Koribacter versatilis Ellin345 |
| Ga0586324 0009215 3159 4790 | 2340000.00 | 899254.44 | 1052066.84 | 10811000.00 | 9960100.00 | 6153500.00 | 20360400.00 | 10554410.93 | 43416000.00 | 4496800.00 | 16209600.00 | 3136265.25 | KO:K04077 | chaperonin GroEL | Bacteria;Ignavibacteriae;Ignavibacteria;Ignavibacteriales;Ignavibacteriaceae;Ignavibacterium;Ignavibacterium album;Ignavibacterium album Mat9-16, JCM 16511 |
| Ga0586324 0009617 52 1695 | 1020000.00 | 1319200.00 | 0.00 | 0.00 | 0.00 | 0.00 | 1886401.32 | 1213900.00 | 2349000.00 | 16646937.23 | 14068127.04 | 9525000.00 | KO:K04077 | chaperonin GroEL | Bacteria;Verrucomicrobia;Verrucomicrobiae;Verrucomicrobiales;Verrucomicrobia subdivision 3;Pedosphaera;Pedosphaera parvula;Pedosphaera parvula Ellin514 |
| Ga0586324 0009733 8665 10305 | 4650000.00 | 1856400.00 | 0.00 | 0.00 | 493772.73 | 0.00 | 26791224.36 | 27755000.00 | 631024.02 | 1278200.00 | 0.00 | 4837500.00 | KO:K04077 | chaperonin GroEL | Bacteria;Candidatus Calescamantes;unclassified Candidatus Calescamantes;unclassified Candidatus Calescamantes;unclassified Candidatus Calescamantes;unclassified Candidatus Calescamantes;Calescamantes bacterium JGI 0000106-H18;Calescamantes bacterium JGI 0000106-H18 (contamination screened) |
| Ga0586324 0010529 2339 3994 | 3240000.00 | 4515200.00 | 5111820.43 | 3133274.80 | 18525318.38 | 13162983.61 | 43460764.92 | 37210000.00 | 49167000.00 | 30119880.56 | 39820000.00 | 66838795.50 | KO:K04077 | chaperonin GroEL | Bacteria;Proteobacteria;Betaproteobacteria;Nitrosomonadales;Sterolibacteriaceae;Sulfurisoma;Sulfurisoma sediminicola;Sulfurisoma sediminicola DSM 26916 |
| Ga0586324 0011142 6562 8202 | 0.00 | 0.00 | 0.00 | 0.00 | 618435.69 | 0.00 | 0.00 | 10370000.00 | 11664000.00 | 16093000.00 | 16896000.00 | 0.00 | KO:K04077 | chaperonin GroEL | Bacteria;Proteobacteria;Gammaproteobacteria;Chromatiales;Woeseiaceae;Woeseia;Woeseia oceani;Woeseia oceani XK5 |
| Ga0586324 0011604 1604 3205 | 0.00 | 0.00 | 0.00 | 6811500.00 | 3450500.00 | 0.00 | 0.00 | 0.00 | 0.00 | 0.00 | 0.00 | 0.00 | KO:K04077 | chaperonin GroEL | Bacteria;Proteobacteria;Deltaproteobacteria;Myxococcales;Kofleriaceae;Haliangium;Haliangium ochraceum;Haliangium ochraceum SMP-2, DSM 14365 |

| | | | | | | | | | | | | | | | |
|---|---|---|---|---|---|---|---|---|---|---|---|---|---|---|---|
| Ga0586324 0011956 6215 7846 | 0.00 | 610455.72 | 0.00 | 0.00 | 0.00 | 0.00 | 0.00 | 0.00 | 730804.68 | 0.00 | 0.00 | 0.00 | KO:K04077 | chaperonin GroEL | Bacteria;Candidatus Omnitrophica;unclassified Candidatus Omnitrophica;unclassified Candidatus Omnitrophica;unclassified Candidatus Omnitrophica;unclassified Candidatus Omnitrophica;Omnitrophica bacterium SCGC AAA011-L16;Omnitrophica bacterium SCGC AAA011-L16 (contamination screened) |
| Ga0586324 0014375 4921 6552 | 0.00 | 1060800.00 | 0.00 | 0.00 | 0.00 | 0.00 | 0.00 | 0.00 | 0.00 | 0.00 | 0.00 | 0.00 | KO:K04077 | chaperonin GroEL | Bacteria;Acidobacteria;Vicinamibacteria;unclassified Vicinamibacteria;Vicinamibacteraceae;Luteitalea;Luteitalea pratensis;Luteitalea pratensis DSM 100886 |
| Ga0586324 0015151 2916 4538 | 0.00 | 0.00 | 0.00 | 2061500.00 | 3481400.00 | 0.00 | 0.00 | 61000000.00 | 64314000.00 | 71456000.00 | 75328000.00 | 55950000.00 | KO:K04077 | chaperonin GroEL | Bacteria;Proteobacteria;Deltaproteobacteria;Syntrophobacterales;Syntrophobacteraceae;Desulfosoma;Desulfosoma caldarium;Desulfosoma caldarium DSM 22027 |
| Ga0586324 0016019 1 1248 | 0.00 | 0.00 | 0.00 | 0.00 | 0.00 | 0.00 | 0.00 | 0.00 | 0.00 | 124269309.78 | 872521.76 | 358861.50 | KO:K04077 | chaperonin GroEL | Bacteria;Proteobacteria;Alphaproteobacteria;Hyphomicrobiales;unclassified Hyphomicrobiales;Methyloceanibacter;Methyloceanibacter superfactus;Methyloceanibacter superfactus R-67175 |
| Ga0586324 0017301 1331 2965 | 0.00 | 0.00 | 0.00 | 0.00 | 0.00 | 0.00 | 0.00 | 0.00 | 324220.32 | 0.00 | 0.00 | 0.00 | KO:K04077 | chaperonin GroEL | Bacteria;Acidobacteria;Blastocatellia;Blastocatellales;Pyrinomonadaceae;Pyrinomonas;Pyrinomonas methylaliphatogenes;Pyrinomonas methylaliphatogenes K22 |
| Ga0586324 0017747 5165 6823 | 2270000.00 | 10064000.00 | 0.00 | 0.00 | 18231000.00 | 0.00 | 0.00 | 0.00 | 12960000.00 | 10010000.00 | 13200000.00 | 0.00 | KO:K04077 | chaperonin GroEL | Bacteria;Proteobacteria;Deltaproteobacteria;Myxococcales;Anaeromyxobacteraceae;Anaeromyxobacter;Anaeromyxobacter dehalogenans;Anaeromyxobacter dehalogenans 2CP-C |
| Ga0586324 0018000 3579 5201 | 0.00 | 0.00 | 0.00 | 0.00 | 0.00 | 0.00 | 0.00 | 5178900.00 | 0.00 | 0.00 | 0.00 | 0.00 | KO:K04077 | chaperonin GroEL | Bacteria;Proteobacteria;Deltaproteobacteria;Myxococcales;Polyangiaceae;Sorangium;Sorangium cellulosum;Sorangium cellulosum So0157-25 |
| Ga0586324 0018662 1203 2864 | 0.00 | 0.00 | 0.00 | 4512732.75 | 0.00 | 0.00 | 2895600.00 | 3135297.52 | 2269274.13 | 4693609.69 | 8107640.64 | 1116534.00 | KO:K04077 | chaperonin GroEL | Bacteria;Proteobacteria;Deltaproteobacteria;Myxococcales;Sandaracinaceae;Sandaracinus;Sandaracinus amylolyticus;Sandaracinus amylolyticus DSM 53668 |
| Ga0586324 0018690 4476 6143 | 0.00 | 0.00 | 0.00 | 0.00 | 0.00 | 37675300.00 | 0.00 | 0.00 | 0.00 | 0.00 | 0.00 | 0.00 | KO:K04077 | chaperonin GroEL | Bacteria;Proteobacteria;Gammaproteobacteria;Legionellales;Coxiellaceae;Rickettsiella;Rickettsiella grylli;Rickettsiella grylli |
| Ga0586324 0018967 1127 2761 | 0.00 | 0.00 | 0.00 | 104557000.00 | 0.00 | 0.00 | 0.00 | 0.00 | 0.00 | 0.00 | 0.00 | 51750000.00 | KO:K04077 | chaperonin GroEL | Bacteria;Proteobacteria;Deltaproteobacteria;Syntrophobacterales;Syntrophaceae;Smithella;Smithella sp. F21;Smithella sp. F21 |
| Ga0586324 0019147 4693 6351 | 0.00 | 0.00 | 39691500.00 | 0.00 | 1823100.00 | 0.00 | 0.00 | 9680700.00 | 5564700.00 | 10271800.00 | 10560000.00 | 0.00 | KO:K04077 | chaperonin GroEL | Bacteria;Proteobacteria;Deltaproteobacteria;Syntrophobacterales;Syntrophobacteraceae;Desulfacinum;Desulfacinum infernum;Desulfacinum infernum DSM 9756 |
| Ga0586324 0019973 6190 7440 | 607400.00 | 10360347.20 | 1073089.26 | 11020210.90 | 1388982.81 | 528867.52 | 5118141.26 | 15743058.73 | 2217839.13 | 6506500.00 | 15202665.28 | 16996899.75 | KO:K04077 | chaperonin GroEL | Bacteria;Proteobacteria;Betaproteobacteria;unclassified Betaproteobacteria;unclassified Betaproteobacteria;unclassified Betaproteobacteria;beta proteobacterium AAP51;Betaproteobacteria bacterium AAP51 |
| Ga0586324 0020129 1177 2847 | 0.00 | 2590800.00 | 0.00 | 1120949.65 | 183279.23 | 0.00 | 0.00 | 0.00 | 2956500.00 | 496715.45 | 1170400.00 | 757500.00 | KO:K04077 | chaperonin GroEL | Bacteria;Spirochaetes;Spirochaetia;Spirochaetales;Spirochaetaceae;Sediminispirochaeta;Sediminispirochaeta bajacaliforniensis;Sediminispirochaeta bajacaliforniensis DSM 16054 |
| Ga0586324 0020832 4280 5917 | 0.00 | 0.00 | 6024100.00 | 0.00 | 0.00 | 3282368.21 | 4446000.00 | 0.00 | 0.00 | 5459300.00 | 4787200.00 | 0.00 | KO:K04077 | chaperonin GroEL | Bacteria;Actinobacteria;Actinomycetia;unclassified Actinomycetia;unclassified Actinomycetia;unclassified Actinomycetia;Actinobacteria bacterium IMCC26256;Actinobacteria bacterium IMCC26256 |
| Ga0586324 0021034 1 1116 | 16200000.00 | 3424505.84 | 74203400.00 | 0.00 | 33711900.00 | 0.00 | 8857328.04 | 39888230.01 | 6018300.00 | 99476300.00 | 122427470.88 | 116865000.00 | KO:K04077 | chaperonin GroEL | Bacteria;Proteobacteria;Gammaproteobacteria;Methylococcales;Crenotrichaceae;Crenothrix;Crenothrix polyspora;Crenothrix polyspora ERS1545888 |
| Ga0586324 0021252 339 1973 | 0.00 | 0.00 | 0.00 | 997500.00 | 0.00 | 0.00 | 16963200.00 | 0.00 | 22599000.00 | 0.00 | 0.00 | 1695000.00 | KO:K04077 | chaperonin GroEL | Bacteria;Chloroflexi;Thermoflexia;Thermoflexales;Thermoflexaceae;Thermoflexus;Thermoflexus hugenholtzii;Thermoflexus hugenholtzii JAD2 |
| Ga0586324 0021327 2 1243 | 0.00 | 0.00 | 0.00 | 6507500.00 | 6386000.00 | 0.00 | 0.00 | 0.00 | 0.00 | 6806800.00 | 0.00 | 0.00 | KO:K04077 | chaperonin GroEL | Bacteria;Proteobacteria;Deltaproteobacteria;Desulfobacterales;Desulfobacteraceae;Desulfatiglans;Desulfatiglans anilini;Desulfatiglans anilini DSM 4660 |
| Ga0586324 0022153 3214 4857 | 0.00 | 0.00 | 0.00 | 0.00 | 0.00 | 0.00 | 0.00 | 0.00 | 0.00 | 0.00 | 0.00 | 1875000.00 | KO:K04077 | chaperonin GroEL | Bacteria;Proteobacteria;Alphaproteobacteria;Hyphomicrobiales;unclassified Hyphomicrobiales;Methyloceanibacter;Methyloceanibacter caenitepidi;Methyloceanibacter caenitepidi Gela4 |
| Ga0586324 0025186 4403 6061 | 28200000.00 | 38692000.00 | 131404200.00 | 62320000.00 | 71791000.00 | 0.00 | 67032000.00 | 30317000.00 | 76140000.00 | 0.00 | 0.00 | 0.00 | KO:K04077 | chaperonin GroEL | Bacteria;Proteobacteria;Deltaproteobacteria;Myxococcales;Myxococcaceae;Corallococcus;Corallococcus sp. H22C18031201;Corallococcus sp. H22C18031201 |
| Ga0586324 0025299 5639 6652 | 0.00 | 7412000.00 | 0.00 | 10355000.00 | 8930100.00 | 0.00 | 0.00 | 0.00 | 4017600.00 | 0.00 | 4488000.00 | 4815000.00 | KO:K04077 | chaperonin GroEL | Bacteria;Gemmatimonadetes;Gemmatimonadetes;Gemmatimonadales;Gemmatimonadaceae;Gemmatimonas;Gemmatimonas phototrophica;Gemmatimonas phototrophica AP64 |
| Ga0586324 0025519 532 2172 | 0.00 | 0.00 | 0.00 | 0.00 | 0.00 | 23780300.00 | 2553600.00 | 0.00 | 947700.00 | 7777000.00 | 0.00 | 0.00 | KO:K04077 | chaperonin GroEL | Bacteria;Chloroflexi;Anaerolineae;Anaerolineales;Anaerolineaceae;Levilinea;Levilinea saccharolytica;Levilinea saccharolytica DSM 16555 |
| Ga0586324 0026287 959 2599 | 0.00 | 0.00 | 0.00 | 0.00 | 0.00 | 0.00 | 0.00 | 0.00 | 0.00 | 0.00 | 11528000.00 | 0.00 | KO:K04077 | chaperonin GroEL | Bacteria;Proteobacteria;Betaproteobacteria;Nitrosomonadales;Gallionellaceae;Gallionella;Gallionella capsiferriformans;Gallionella capsiferriformans ES-2 |
| Ga0586324 0026425 1541 3172 | 0.00 | 943132.12 | 0.00 | 190000000.00 | 256715.14 | 0.00 | 0.00 | 221253.10 | 49161.01 | 0.00 | 1196800.00 | 7140000.00 | KO:K04077 | chaperonin GroEL | Bacteria;Proteobacteria;Deltaproteobacteria;Myxococcales;Kofleriaceae;Haliangium;Haliangium ochraceum;Haliangium ochraceum SMP-2, DSM 14365 |
| Ga0586324 0026669 3259 4893 | 0.00 | 0.00 | 0.00 | 0.00 | 0.00 | 0.00 | 0.00 | 0.00 | 280141.74 | 0.00 | 0.00 | 0.00 | KO:K04077 | chaperonin GroEL | Bacteria;Proteobacteria;Deltaproteobacteria;Syntrophobacterales;Syntrophaceae;Desulfobacca;Desulfobacca acetoxidans;Desulfobacca acetoxidans ASRB2, DSM 11109 |
| Ga0586324 0026724 4734 6350 | 0.00 | 9996000.00 | 5964821.73 | 15637000.00 | 22072900.00 | 3534784.78 | 12813600.00 | 8314300.00 | 12328200.00 | 16085300.00 | 19711068.08 | 11895000.00 | KO:K04077 | chaperonin GroEL | Bacteria;Proteobacteria;Betaproteobacteria;Nitrosomonadales;Nitrosomonadaceae;Nitrosospira;Nitrosospira sp. Nsp1;Nitrosospira sp. 1 Nsp1 |
| Ga0586324 0027024 1013 2626 | 0.00 | 0.00 | 0.00 | 0.00 | 0.00 | 0.00 | 0.00 | 0.00 | 0.00 | 0.00 | 4989600.00 | 0.00 | KO:K04077 | chaperonin GroEL | Bacteria;Proteobacteria;Hydrogenophilalia;Hydrogenophilales;Hydrogenophilaceae;Tepidiphilus;Tepidiphilus thermophilus;Tepidiphilus thermophilus JCM 19170 |
| Ga0586324 0027423 1255 2865 | 0.00 | 0.00 | 3932577.52 | 5633500.00 | 5335400.00 | 1506063.17 | 1293323.16 | 2342400.00 | 1628100.00 | 0.00 | 0.00 | 630438.75 | KO:K04077 | chaperonin GroEL | Bacteria;Proteobacteria;Deltaproteobacteria;Syntrophobacterales;Syntrophobacteraceae;Desulfosoma;Desulfosoma caldarium;Desulfosoma caldarium DSM 22027 |
| Ga0586324 0027819 3539 5164 | 0.00 | 1285200.00 | 0.00 | 1463000.00 | 0.00 | 0.00 | 0.00 | 0.00 | 0.00 | 0.00 | 0.00 | 1942500.00 | KO:K04077 | chaperonin GroEL | Bacteria;Proteobacteria;Deltaproteobacteria;Desulfobacterales;Desulfobacteraceae;Desulfosarcina;Desulfosarcina cetonica;Desulfosarcina cetonica JCM 12296 |
| Ga0586324 0028147 3702 5330 | 0.00 | 2502400.00 | 0.00 | 0.00 | 0.00 | 0.00 | 0.00 | 0.00 | 3248100.00 | 0.00 | 0.00 | 0.00 | KO:K04077 | chaperonin GroEL | Bacteria;Chloroflexi;Anaerolineae;Anaerolineales;Anaerolineaceae;Thermanaerothrix;Thermanaerothrix daxensis;Thermanaerothrix daxensis GNS-1 |
| Ga0586324 0028606 533 2161 | 0.00 | 505446.04 | 0.00 | 0.00 | 0.00 | 0.00 | 6815649.60 | 1079700.00 | 219878.55 | 1326635.31 | 1091200.00 | 795000.00 | KO:K04077 | chaperonin GroEL | Bacteria;Acidobacteria;Acidobacteriia;Bryobacterales;Solibacteraceae;Candidatus Solibacter;Candidatus Solibacter usitatus;Candidatus Solibacter usitatus Ellin6076 |
| Ga0586324 0028829 2 1129 | 0.00 | 0.00 | 0.00 | 2935500.00 | 0.00 | 0.00 | 15526800.00 | 1794422.97 | 0.00 | 1432200.00 | 0.00 | 945000.00 | KO:K04077 | chaperonin GroEL | Bacteria;Bacteroidetes;Bacteroidia;Bacteroidales;Rikenellaceae;Acetobacteroides;Acetobacteroides hydrogenigenes;Acetobacteroides hydrogenigenes DSM 24657 |
| Ga0586324 0029790 1643 3292 | 17045926.00 | 135164333.72 | 89507184.66 | 5760268.00 | 132849400.00 | 8416400.00 | 85028759.57 | 72038660.04 | 85817597.31 | 109671100.00 | 81566549.68 | 73065000.00 | KO:K04077 | chaperonin GroEL | Bacteria;Proteobacteria;Betaproteobacteria;unclassified Betaproteobacteria;unclassified Betaproteobacteria;unclassified Betaproteobacteria;Betaproteobacteria bacterium GR16-43;Betaproteobacteria bacterium GR16-43 |
| Ga0586324 0029959 792 2420 | 0.00 | 0.00 | 0.00 | 6013500.00 | 8404800.00 | 25646200.00 | 0.00 | 1421300.00 | 6115500.00 | 0.00 | 0.00 | 0.00 | KO:K04077 | chaperonin GroEL | Bacteria;Chloroflexi;Anaerolineae;Anaerolineales;Anaerolineaceae;Thermanaerothrix;Thermanaerothrix daxensis;Thermanaerothrix daxensis GNS-1 |
| Ga0586324 0030182 128 1768 | 0.00 | 466260.36 | 784399.75 | 350578.50 | 549601.82 | 271968.03 | 1114803.72 | 884500.00 | 1385100.00 | 12543300.00 | 3616800.00 | 3345000.00 | KO:K04077 | chaperonin GroEL | Bacteria;Proteobacteria;Betaproteobacteria;Nitrosomonadales;Gallionellaceae;Gallionella;Gallionella capsiferriformans;Gallionella capsiferriformans ES-2 |
| Ga0586324 0031737 3 1337 | 0.00 | 0.00 | 0.00 | 0.00 | 0.00 | 0.00 | 4286400.00 | 4117500.00 | 0.00 | 4573800.00 | 5051200.00 | 0.00 | KO:K04077 | chaperonin GroEL | Bacteria;Proteobacteria;Betaproteobacteria;Burkholderiales;Comamonadaceae;Hydrogenophaga;Hydrogenophaga taeniospiralis;Hydrogenophaga taeniospiralis NBRC 102512 |
| Ga0586324 0033594 486 2135 | 0.00 | 0.00 | 0.00 | 0.00 | 0.00 | 0.00 | 0.00 | 0.00 | 145455.75 | 0.00 | 1038400.00 | 1365000.00 | KO:K04077 | chaperonin GroEL | Bacteria;Proteobacteria;Alphaproteobacteria;Hyphomicrobiales;Bradyrhizobiaceae;Bradyrhizobium;Bradyrhizobium erythrophlei;Bradyrhizobium erythrophlei GAS138 |
| Ga0586324 0033740 3853 4671 | 0.00 | 0.00 | 0.00 | 0.00 | 5428100.00 | 0.00 | 5950800.00 | 0.00 | 0.00 | 0.00 | 0.00 | 0.00 | KO:K04077 | chaperonin GroEL | Bacteria;Bacteroidetes;Bacteroidia;Bacteroidales;Lentimicrobiaceae;Lentimicrobium;Lentimicrobium saccharophilum;Bacteroidales bacterium TBC1 |
| Ga0586324 0033897 1 1062 | 0.00 | 0.00 | 0.00 | 3619500.00 | 4521700.00 | 3407383.51 | 20041200.00 | 1329800.00 | 0.00 | 0.00 | 1487200.00 | 0.00 | KO:K04077 | chaperonin GroEL | Bacteria;Proteobacteria;Deltaproteobacteria;Syntrophobacterales;Syntrophobacteraceae;Desulfosoma;Desulfosoma caldarium;Desulfosoma caldarium DSM 22027 |
| Ga0586324 0034450 4134 5735 | 0.00 | 2930800.00 | 39310202.62 | 303981000.00 | 8315751.35 | 129480061.25 | 14638847.16 | 11865692.55 | 206080200.00 | 41210400.00 | 0.00 | 102300000.00 | KO:K04077 | chaperonin GroEL | Bacteria;Proteobacteria;Deltaproteobacteria;Desulfuromonadales;Geobacteraceae;Geoalkalibacter;Geoalkalibacter ferrihydriticus;Geoalkalibacter ferrihydriticus DSM 17813 |
| Ga0586324 0034806 3170 4843 | 530008.00 | 1876800.00 | 0.00 | 0.00 | 28016000.00 | 3784188.12 | 0.00 | 2543700.00 | 2673000.00 | 0.00 | 541293.28 | 0.00 | KO:K04077 | chaperonin GroEL | Bacteria;Nitrospirae;Nitrospira;Nitrospirales;Nitrospiraceae;Nitrospira;Nitrospira moscoviensis;Nitrospira moscoviensis NSP M-1 |
| Ga0586324 0034930 3448 5076 | 278326.00 | 0.00 | 0.00 | 0.00 | 0.00 | 0.00 | 0.00 | 165904.75 | 0.00 | 0.00 | 0.00 | 0.00 | KO:K04077 | chaperonin GroEL | Bacteria;Chloroflexi;Anaerolineae;Anaerolineales;Anaerolineaceae;Thermanaerothrix;Thermanaerothrix daxensis;Thermanaerothrix daxensis GNS-1 |
| Ga0586324 0035645 1330 2973 | 8380000.00 | 0.00 | 0.00 | 2698000.00 | 0.00 | 0.00 | 0.00 | 0.00 | 1223100.00 | 0.00 | 0.00 | 0.00 | KO:K04077 | chaperonin GroEL | Bacteria;Proteobacteria;Deltaproteobacteria;Syntrophobacterales;Syntrophaceae;Syntrophus;Syntrophus aciditrophicus;Syntrophus aciditrophicus SB |
| Ga0586324 0035654 3 1145 | 0.00 | 945200.00 | 0.00 | 0.00 | 0.00 | 0.00 | 0.00 | 0.00 | 1223100.00 | 0.00 | 0.00 | 0.00 | KO:K04077 | chaperonin GroEL | Bacteria;Proteobacteria;Deltaproteobacteria;unclassified Deltaproteobacteria;unclassified Deltaproteobacteria;Dissulfuribacter;Dissulfuribacter thermophilus;Dissulfuribacter thermophilus S69 |
| Ga0586324 0036009 30 1658 | 0.00 | 0.00 | 0.00 | 173062.45 | 0.00 | 0.00 | 0.00 | 805200.00 | 674716.23 | 314399.47 | 0.00 | 0.00 | KO:K04077 | chaperonin GroEL | Bacteria;Acidobacteria;Acidobacteriia;Acidobacteriales;Acidobacteriaceae;Candidatus Koribacter;Candidatus Koribacter versatilis;Candidatus Koribacter versatilis Ellin345 |
| Ga0586324 0036137 4874 5605 | 2370000.00 | 3311600.00 | 844556.30 | 1605500.00 | 0.00 | 1818232.21 | 3693600.00 | 4370176.64 | 955800.00 | 4802664.02 | 11714929.60 | 6286932.00 | KO:K04077 | chaperonin GroEL | Bacteria;Proteobacteria;Betaproteobacteria;Burkholderiales;unclassified Burkholderiales;Methylibium;Methylibium sp. NZG;Methylibium sp. NZG |
| Ga0586324 0039106 3035 4663 | 0.00 | 0.00 | 0.00 | 0.00 | 0.00 | 0.00 | 3169200.00 | 1531100.00 | 1692900.00 | 0.00 | 1883200.00 | 26775000.00 | KO:K04077 | chaperonin GroEL | Bacteria;Acidobacteria;Acidobacteriia;Acidobacteriales;Acidobacteriaceae;Candidatus Koribacter;Candidatus Koribacter versatilis;Candidatus Koribacter versatilis Ellin345 |
| Ga0586324 0039504 4532 5365 | 0.00 | 0.00 | 0.00 | 0.00 | 0.00 | 0.00 | 0.00 | 0.00 | 939600.00 | 0.00 | 0.00 | 0.00 | KO:K04077 | chaperonin GroEL | Bacteria;Candidatus Aminicenantes;unclassified Candidatus Aminicenantes;unclassified Candidatus Aminicenantes;unclassified Candidatus Aminicenantes;unclassified Candidatus Aminicenantes;Aminicenantes bacterium SCGC AAA255-E10;Aminicenantes bacterium SCGC AAA255-E10 (contamination screened) |
| Ga0586324 0041652 1584 3218 | 2640000.00 | 0.00 | 0.00 | 0.00 | 0.00 | 1252058.60 | 7273200.00 | 0.00 | 10854000.00 | 0.00 | 7726400.00 | 718068.00 | KO:K04077 | chaperonin GroEL | Bacteria;Chloroflexi;Anaerolineae;Anaerolineales;Anaerolineaceae;Thermanaerothrix;Thermanaerothrix daxensis;Thermanaerothrix daxensis GNS-1 |
| Ga0586324 0042533 4440 5174 | 0.00 | 0.00 | 0.00 | 2907000.00 | 1400800.00 | 2878781.98 | 3169200.00 | 1692551.75 | 1798200.00 | 0.00 | 869031.68 | 336498.00 | KO:K04077 | chaperonin GroEL | Bacteria;Acidobacteria;Blastocatellia;Blastocatellales;Pyrinomonadaceae;Pyrinomonas;Pyrinomonas methylaliphatogenes;Pyrinomonas methylaliphatogenes K22 |
| Ga0586324 0043595 472 2103 | 0.00 | 0.00 | 0.00 | 0.00 | 15429400.00 | 1591120.42 | 0.00 | 0.00 | 2664900.00 | 378757.61 | 0.00 | 269497.50 | KO:K04077 | chaperonin GroEL | Bacteria;Chloroflexi;Anaerolineae;Anaerolineales;Anaerolineaceae;Levilinea;Levilinea saccharolytica;Levilinea saccharolytica DSM 16555 |
| Ga0586324 0044217 3 1592 | 0.00 | 0.00 | 0.00 | 3914000.00 | 0.00 | 0.00 | 0.00 | 461702.29 | 216232.74 | 421139.95 | 0.00 | 0.00 | KO:K04077 | chaperonin GroEL | Bacteria;Proteobacteria;Betaproteobacteria;Nitrosomonadales;Nitrosomonadaceae;Nitrosovibrio;Nitrosovibrio sp. Nv4;Nitrosovibrio sp. Nv4 |
| Ga0586324 0044644 724 2352 | 260000000.00 | 0.00 | 0.00 | 93195000.00 | 0.00 | 0.00 | 0.00 | 0.00 | 442123.92 | 0.00 | 11352000.00 | 0.00 | KO:K04077 | chaperonin GroEL | Bacteria;Actinobacteria;Thermoleophilia;Solirubrobacterales;Solirubrobacteraceae;Solirubrobacter;Solirubrobacter pauli;Solirubrobacter pauli DSM 14954 |
| Ga0586324 0047047 910 2565 | 0.00 | 4766800.00 | 8276100.00 | 0.00 | 1421400.00 | 100441000.00 | 0.00 | 0.00 | 0.00 | 0.00 | 0.00 | 301485.00 | KO:K04077 | chaperonin GroEL | Bacteria;Proteobacteria;Deltaproteobacteria;Myxococcales;Kofleriaceae;Haliangium;Haliangium ochraceum;Haliangium ochraceum SMP-2, DSM 14365 |
| Ga0586324 0049017 1534 3177 | 961440.00 | 580653.36 | 1774891.28 | 4275000.00 | 2863400.00 | 0.00 | 0.00 | 2000800.00 | 1917270.81 | 1901900.00 | 3696000.00 | 1627500.00 | KO:K04077 | chaperonin GroEL | Bacteria;Proteobacteria;Betaproteobacteria;Nitrosomonadales;Nitrosomonadaceae;Nitrosospira;Nitrosospira lacus;Nitrosospira lacus APG3 |
| Ga0586324 0052118 1669 3309 | 11000000.00 | 0.00 | 0.00 | 0.00 | 0.00 | 0.00 | 0.00 | 0.00 | 0.00 | 0.00 | 0.00 | 0.00 | KO:K04077 | chaperonin GroEL | Bacteria;Proteobacteria;Alphaproteobacteria;Hyphomicrobiales;Hyphomicrobiaceae;Rhodoplanes;Rhodoplanes sp. Z2-YC6860;Rhodoplanes sp. Z2-YC6860 |
| Ga0586324 0054127 839 2476 | 0.00 | 7004000.00 | 0.00 | 0.00 | 0.00 | 0.00 | 0.00 | 0.00 | 0.00 | 0.00 | 0.00 | 0.00 | KO:K04077 | chaperonin GroEL | Bacteria;Chloroflexi;Thermomicrobia;Sphaerobacterales;Sphaerobacteraceae;Sphaerobacter;Sphaerobacter thermophilus;Sphaerobacter thermophilus 4ac11, DSM 20745 |
| Ga0586324 0056786 1822 3465 | 0.00 | 0.00 | 0.00 | 2916500.00 | 11608100.00 | 0.00 | 0.00 | 1720200.00 | 7200900.00 | 0.00 | 4532000.00 | 5880000.00 | KO:K04077 | chaperonin GroEL | Bacteria;Bacteroidetes;Bacteroidia;Bacteroidales;Lentimicrobiaceae;Lentimicrobium;Lentimicrobium saccharophilum;Bacteroidales bacterium TBC1 |
| Ga0586324 0058586 3532 4428 | 107000000.00 | 700400.00 | 1877813.31 | 1235000.00 | 2533800.00 | 38310500.00 | 0.00 | 2915800.00 | 2875500.00 | 3203200.00 | 1777600.00 | 1140000.00 | KO:K04077 | chaperonin GroEL | Bacteria;Proteobacteria;Alphaproteobacteria;Hyphomicrobiales;Hyphomicrobiaceae;Rhodoplanes;Rhodoplanes sp. Z2-YC6860;Rhodoplanes sp. Z2-YC6860 |
| Ga0586324 0060597 507 2153 | 32641702.00 | 10737200.00 | 2159054.33 | 10800084.50 | 15934100.00 | 11195018.88 | 14250000.00 | 11459441.09 | 15860653.74 | 34973400.00 | 21832800.00 | 22845000.00 | KO:K04077 | chaperonin GroEL | Bacteria;Proteobacteria;Betaproteobacteria;unclassified Betaproteobacteria;unclassified Betaproteobacteria;unclassified Betaproteobacteria;Betaproteobacteria bacterium GR16-43;Betaproteobacteria bacterium GR16-43 |
| Ga0586324 0060706 2 1198 | 186477.00 | 399245.00 | 10584400.00 | 2913118.95 | 0.00 | 0.00 | 4537200.00 | 969900.00 | 9420300.00 | 0.00 | 4540800.00 | 13035000.00 | KO:K04077 | chaperonin GroEL | Bacteria;Candidatus Calescamantes;unclassified Candidatus Calescamantes;unclassified Candidatus Calescamantes;unclassified Candidatus Calescamantes;unclassified Candidatus Calescamantes;Calescamantes bacterium JGI 0000106-H18;Calescamantes bacterium JGI 0000106-H18 (contamination screened) |
| Ga0586324 0064131 3163 4239 | 0.00 | 0.00 | 0.00 | 0.00 | 0.00 | 0.00 | 0.00 | 0.00 | 0.00 | 0.00 | 0.00 | 8025000.00 | KO:K04077 | chaperonin GroEL | Bacteria;Proteobacteria;Betaproteobacteria;Nitrosomonadales;Nitrosomonadaceae;Nitrosovibrio;Nitrosovibrio sp. Nv17;Nitrosovibrio sp. Nv17 |
| Ga0586324 0065559 853 2487 | 0.00 | 0.00 | 0.00 | 0.00 | 336883.13 | 7979700.00 | 0.00 | 0.00 | 0.00 | 947100.00 | 0.00 | 982500.00 | KO:K04077 | chaperonin GroEL | Bacteria;Proteobacteria;Deltaproteobacteria;Desulfobacterales;Desulfobacteraceae;Desulfosarcina;Desulfosarcina variabilis;Desulfosarcina variabilis Montpellier |
| Ga0586324 0070909 3 761 | 0.00 | 0.00 | 0.00 | 0.00 | 0.00 | 0.00 | 0.00 | 0.00 | 29727000.00 | 0.00 | 166672.00 | 40125000.00 | KO:K04077 | chaperonin GroEL | Bacteria;Chloroflexi;Ardenticatenia;Ardenticatenales;Ardenticatenaceae;Candidatus Promineofilum;Candidatus Promineofilum breve;Candidatus Promineofilum breve Cfx-K |
| Ga0586324 0071657 1720 3360 | 4000000.00 | 5372000.00 | 6361900.00 | 10865513.85 | 9213685.78 | 5298516.83 | 3602400.00 | 5794024.61 | 6659982.81 | 5197500.00 | 1751200.00 | 1777500.00 | KO:K04077 | chaperonin GroEL | Bacteria;Proteobacteria;Deltaproteobacteria;Syntrophorhabdales;Syntrophorhabdaceae;Syntrophorhabdus;Syntrophorhabdus aromaticivorans;Syntrophorhabdus aromaticivorans UI |
| Ga0586324 0072295 1 1194 | 7360000.00 | 11036578.16 | 150940300.00 | 105811000.00 | 79134900.00 | 185720367.53 | 53557200.00 | 61494100.00 | 164909179.80 | 10703000.00 | 7118741.52 | 19075640.25 | KO:K04077 | chaperonin GroEL | Bacteria;Proteobacteria;Deltaproteobacteria;Desulfobacterales;Desulfobacteraceae;Desulfatirhabdium;Desulfatirhabdium butyrativorans;Desulfatirhabdium butyrativorans DSM 18734 |
| Ga0586324 0075324 2475 3926 | 0.00 | 0.00 | 0.00 | 0.00 | 30900000.00 | 0.00 | 0.00 | 0.00 | 0.00 | 0.00 | 0.00 | 0.00 | KO:K04077 | chaperonin GroEL | Bacteria;Chloroflexi;Caldilineae;Caldilineales;Caldilineaceae;Caldilinea;Caldilinea aerophila;Caldilinea aerophila STL-6-O1, DSM 14535 |
| Ga0586324 0075963 1126 2769 | 0.00 | 0.00 | 0.00 | 0.00 | 0.00 | 0.00 | 0.00 | 0.00 | 0.00 | 0.00 | 3194400.00 | 0.00 | KO:K04077 | chaperonin GroEL | Bacteria;Proteobacteria;Deltaproteobacteria;Desulfobacterales;Desulfobulbaceae;Desulfoprunum;Desulfoprunum benzoelyticum;Desulfoprunum benzoelyticum DSM 28570 |
| Ga0586324 0076592 2598 3893 | 0.00 | 0.00 | 0.00 | 0.00 | 0.00 | 0.00 | 116052000.00 | 54595000.00 | 329097.33 | 182958.93 | 415569.44 | 4620000.00 | KO:K04077 | chaperonin GroEL | Bacteria;Proteobacteria;Alphaproteobacteria;Hyphomicrobiales;Xanthobacteraceae;Pseudolabrys;Pseudolabrys sp. Root1462;Pseudolabrys sp. Root1462 |
| Ga0586324 0077424 137 1762 | 4840000.00 | 5242800.00 | 5352699.98 | 0.00 | 2307200.00 | 4089100.00 | 1965617.64 | 0.00 | 3677400.00 | 2941400.00 | 1768800.00 | 1477500.00 | KO:K04077 | chaperonin GroEL | Bacteria;Acidobacteria;Acidobacteriia;Acidobacteriales;Acidobacteriaceae;Candidatus Koribacter;Candidatus Koribacter versatilis;Candidatus Koribacter versatilis Ellin345 |
| Ga0586324 0077725 2197 3843 | 0.00 | 0.00 | 0.00 | 0.00 | 0.00 | 6193200.00 | 0.00 | 0.00 | 0.00 | 0.00 | 3660800.00 | 17887500.00 | KO:K04077 | chaperonin GroEL | Bacteria;Proteobacteria;Gammaproteobacteria;Nevskiales;Steroidobacteraceae;Steroidobacter;Steroidobacter denitrificans;Steroidobacter denitrificans DSM 18526 |
| Ga0586324 0077955 2075 3703 | 0.00 | 0.00 | 8050900.00 | 0.00 | 0.00 | 0.00 | 0.00 | 0.00 | 0.00 | 0.00 | 0.00 | 787500.00 | KO:K04077 | chaperonin GroEL | Bacteria;Acidobacteria;Vicinamibacteria;unclassified Vicinamibacteria;Vicinamibacteraceae;Luteitalea;Luteitalea pratensis;Luteitalea pratensis DSM 100886 |
| Ga0586324 0084587 2866 3714 | 0.00 | 0.00 | 0.00 | 0.00 | 0.00 | 0.00 | 0.00 | 0.00 | 0.00 | 0.00 | 0.00 | 3127500.00 | KO:K04077 | chaperonin GroEL | Bacteria;Chloroflexi;Dehalococcoidia;unclassified Dehalococcoidia;unclassified Dehalococcoidia;Dehalogenimonas;Dehalogenimonas sp. WBC-2;Dehalogenimonas sp. WBC-2 |
| Ga0586324 0085943 2151 3686 | 3540000.00 | 112514.84 | 0.00 | 723980.75 | 0.00 | 0.00 | 0.00 | 218119.53 | 3175200.00 | 1509200.00 | 0.00 | 129069.75 | KO:K04077 | chaperonin GroEL | Bacteria;Proteobacteria;Gammaproteobacteria;Chromatiales;Woeseiaceae;Woeseia;Woeseia oceani;Woeseia oceani XK5 |
| Ga0586324 0087456 2910 3656 | 0.00 | 741200.00 | 0.00 | 0.00 | 0.00 | 0.00 | 0.00 | 0.00 | 373802.04 | 0.00 | 0.00 | 0.00 | KO:K04077 | chaperonin GroEL | Bacteria;Proteobacteria;Betaproteobacteria;Nitrosomonadales;Sterolibacteriaceae;Sulfurisoma;Sulfurisoma sediminicola;Sulfurisoma sediminicola DSM 26916 |
| Ga0586324 0089538 701 2317 | 6730000.00 | 1550400.00 | 10217794.11 | 1102000.00 | 7900100.00 | 31659034.96 | 4012800.00 | 26516700.00 | 6270907.41 | 342700.82 | 8712000.00 | 18344241.75 | KO:K04077 | chaperonin GroEL | Bacteria;Candidatus Aminicenantes;unclassified Candidatus Aminicenantes;unclassified Candidatus Aminicenantes;unclassified Candidatus Aminicenantes;unclassified Candidatus Aminicenantes;Aminicenantes bacterium SCGC AAA255-E10;Aminicenantes bacterium SCGC AAA255-E10 (contamination screened) |
| Ga0586324 0089599 1971 3617 | 0.00 | 0.00 | 9458400.00 | 0.00 | 0.00 | 0.00 | 0.00 | 0.00 | 0.00 | 0.00 | 0.00 | 0.00 | KO:K04077 | chaperonin GroEL | Bacteria;Proteobacteria;Deltaproteobacteria;Desulfuromonadales;Geobacteraceae;Geobacter;Geobacter daltonii;Geobacter daltonii FRC-32 |
| Ga0586324 0092320 1 1035 | 1750000.00 | 1706800.00 | 1486196.14 | 3249000.00 | 3429900.00 | 0.00 | 2599200.00 | 3434300.00 | 2486700.00 | 0.00 | 0.00 | 0.00 | KO:K04077 | chaperonin GroEL | Bacteria;Verrucomicrobia;Verrucomicrobiae;Verrucomicrobiales;Verrucomicrobia subdivision 3;Pedosphaera;Pedosphaera parvula;Pedosphaera parvula Ellin514 |
| Ga0586324 0092707 3 1625 | 0.00 | 14280000.00 | 0.00 | 10735000.00 | 0.00 | 0.00 | 0.00 | 12139000.00 | 11097000.00 | 5775000.00 | 13288000.00 | 16125000.00 | KO:K04077 | chaperonin GroEL | Bacteria;Acidobacteria;Vicinamibacteria;unclassified Vicinamibacteria;Vicinamibacteraceae;Luteitalea;Luteitalea pratensis;Luteitalea pratensis DSM 100886 |
| Ga0586324 0095374 1 918 | 14300000.00 | 1331638.56 | 0.00 | 2717000.00 | 1483200.00 | 0.00 | 5768400.00 | 7039400.00 | 209725.20 | 2009700.00 | 1452000.00 | 5790000.00 | KO:K04077 | chaperonin GroEL | Bacteria;Verrucomicrobia;Verrucomicrobiae;Verrucomicrobiales;Verrucomicrobia subdivision 3;Pedosphaera;Pedosphaera parvula;Pedosphaera parvula Ellin514 |
| Ga0586324 0097081 2756 3478 | 0.00 | 897600.00 | 0.00 | 913892.40 | 1205100.00 | 0.00 | 9924630.24 | 2708400.00 | 31517100.00 | 1031800.00 | 1196800.00 | 952500.00 | KO:K04077 | chaperonin GroEL | Bacteria;Verrucomicrobia;Verrucomicrobiae;Verrucomicrobiales;Verrucomicrobia subdivision 3;Pedosphaera;Pedosphaera parvula;Pedosphaera parvula Ellin514 |
| Ga0586324 0097390 535 2190 | 0.00 | 0.00 | 1308822.99 | 0.00 | 4356900.00 | 4843400.00 | 4856400.00 | 3117100.00 | 8424000.00 | 4104100.00 | 2736800.00 | 2557500.00 | KO:K04077 | chaperonin GroEL | Bacteria;Chloroflexi;Dehalococcoidia;Dehalococcoidales;Dehalococcoidaceae;Dehalococcoides;Dehalococcoides mccartyi;Dehalococcoides mccartyi 195 |
| Ga0586324 0097718 163 1806 | 0.00 | 0.00 | 6587100.00 | 3885500.00 | 0.00 | 0.00 | 0.00 | 0.00 | 0.00 | 0.00 | 0.00 | 0.00 | KO:K04077 | chaperonin GroEL | Bacteria;Proteobacteria;Deltaproteobacteria;Desulfobacterales;Desulfobacteraceae;Desulfatiglans;Desulfatiglans anilini;Desulfatiglans anilini DSM 4660 |
| Ga0586324 0098155 2398 3459 | 55900000.00 | 0.00 | 0.00 | 147250000.00 | 149350000.00 | 0.00 | 83904000.00 | 97600000.00 | 87480000.00 | 76769000.00 | 58256000.00 | 677741.25 | KO:K04077 | chaperonin GroEL | Bacteria;Verrucomicrobia;Spartobacteria;unclassified Spartobacteria;unclassified Spartobacteria;unclassified Spartobacteria;Spartobacteria bacterium LR76;Spartobacteria bacterium LR76 |
| Ga0586324 0098293 1560 3185 | 1860000.00 | 3243225.32 | 72025147.37 | 0.00 | 459463.43 | 0.00 | 0.00 | 153856.64 | 1099851.21 | 839300.00 | 3572800.00 | 2745000.00 | KO:K04077 | chaperonin GroEL | Bacteria;Acidobacteria;Acidobacteriia;Bryobacterales;Solibacteraceae;Candidatus Solibacter;Candidatus Solibacter usitatus;Candidatus Solibacter usitatus Ellin6076 |
| Ga0586324 0098904 2327 3448 | 15578462.00 | 6249200.00 | 0.00 | 0.00 | 0.00 | 0.00 | 0.00 | 7747000.00 | 7249500.00 | 9933000.00 | 13640000.00 | 12975000.00 | KO:K04077 | chaperonin GroEL | Bacteria;Actinobacteria;Rubrobacteria;Gaiellales;Gaiellaceae;Gaiella;Gaiella occulta;Gaiella occulta F2-233 |
| Ga0586324 0100766 2481 3416 | 0.00 | 0.00 | 0.00 | 0.00 | 0.00 | 0.00 | 0.00 | 0.00 | 0.00 | 157880.03 | 0.00 | 165712.50 | KO:K04077 | chaperonin GroEL | Bacteria;Proteobacteria;Deltaproteobacteria;Syntrophobacterales;Syntrophaceae;Smithella;Smithella sp. SCADC;Smithella sp. SCADC |
| Ga0586324 0101000 2553 3413 | 0.00 | 0.00 | 0.00 | 0.00 | 0.00 | 0.00 | 0.00 | 0.00 | 0.00 | 0.00 | 0.00 | 1965000.00 | KO:K04077 | chaperonin GroEL | Bacteria;Bacteroidetes;Bacteroidia;Bacteroidales;Porphyromonadaceae;Macellibacteroides;Macellibacteroides fermentans;Macellibacteroides fermentans DSM 23697 |
| Ga0586324 0101299 1305 2924 | 0.00 | 0.00 | 0.00 | 4645500.00 | 0.00 | 0.00 | 4240800.00 | 0.00 | 0.00 | 3395700.00 | 0.00 | 3285000.00 | KO:K04077 | chaperonin GroEL | Bacteria;Proteobacteria;Deltaproteobacteria;Myxococcales;Archangiaceae;Cystobacter;Cystobacter fuscus;Cystobacter fuscus DSM 2262 |
| Ga0586324 0102219 2051 3394 | 0.00 | 0.00 | 0.00 | 0.00 | 0.00 | 918272.91 | 0.00 | 5502200.00 | 2203200.00 | 0.00 | 0.00 | 0.00 | KO:K04077 | chaperonin GroEL | Bacteria;Proteobacteria;Deltaproteobacteria;Myxococcales;Sandaracinaceae;Sandaracinus;Sandaracinus amylolyticus;Sandaracinus amylolyticus DSM 53668 |
| Ga0586324 0103488 1062 2687 | 0.00 | 0.00 | 0.00 | 0.00 | 2461700.00 | 0.00 | 0.00 | 0.00 | 0.00 | 0.00 | 0.00 | 0.00 | KO:K04077 | chaperonin GroEL | Bacteria;Proteobacteria;Alphaproteobacteria;Rhodospirillales;Rhodospirillaceae;Magnetospirillum;Magnetospirillum moscoviense;Magnetospirillum moscoviense BB-1 |
| Ga0586324 0105201 2239 3348 | 0.00 | 0.00 | 5230517.72 | 0.00 | 453840.66 | 0.00 | 0.00 | 4690900.00 | 0.00 | 0.00 | 0.00 | 0.00 | KO:K04077 | chaperonin GroEL | Bacteria;Proteobacteria;Deltaproteobacteria;Syntrophobacterales;Syntrophaceae;Desulfobacca;Desulfobacca acetoxidans;Desulfobacca acetoxidans ASRB2, DSM 11109 |
| Ga0586324 0105447 1437 3071 | 62900000.00 | 0.00 | 0.00 | 0.00 | 0.00 | 0.00 | 0.00 | 0.00 | 0.00 | 0.00 | 0.00 | 0.00 | KO:K04077 | chaperonin GroEL | Bacteria;Proteobacteria;Alphaproteobacteria;Hyphomicrobiales;unclassified Hyphomicrobiales;Methyloceanibacter;Methyloceanibacter superfactus;Methyloceanibacter superfactus R-67175 |
| Ga0586324 0106676 2309 3325 | 0.00 | 5970400.00 | 5520586.58 | 15836500.00 | 6313900.00 | 1376680.87 | 4788000.00 | 7484700.00 | 8650800.00 | 7915600.00 | 10120000.00 | 8430000.00 | KO:K04077 | chaperonin GroEL | Bacteria;Proteobacteria;Gammaproteobacteria;Nevskiales;Steroidobacteraceae;Steroidobacter;Steroidobacter denitrificans;Steroidobacter denitrificans DSM 18526 |
| Ga0586324 0107096 3 1094 | 0.00 | 0.00 | 0.00 | 0.00 | 0.00 | 2968781.88 | 0.00 | 4300500.00 | 0.00 | 0.00 | 0.00 | 0.00 | KO:K04077 | chaperonin GroEL | Bacteria;Proteobacteria;Alphaproteobacteria;Hyphomicrobiales;unclassified Hyphomicrobiales;Methyloceanibacter;Methyloceanibacter caenitepidi;Methyloceanibacter caenitepidi Gela4 |
| Ga0586324 0107517 2065 3312 | 0.00 | 0.00 | 0.00 | 347964.10 | 551180.81 | 732254.59 | 0.00 | 266734.09 | 681293.43 | 2048200.00 | 0.00 | 2312514.00 | KO:K04077 | chaperonin GroEL | Bacteria;Proteobacteria;Betaproteobacteria;Burkholderiales;Comamonadaceae;Polaromonas;Polaromonas naphthalenivorans;Polaromonas naphthalenivorans CJ2 |
| Ga0586324 0109045 1 1518 | 0.00 | 0.00 | 0.00 | 0.00 | 320772.90 | 0.00 | 373163.04 | 634400.00 | 520895.61 | 8778000.00 | 26741148.72 | 275302.50 | KO:K04077 | chaperonin GroEL | Bacteria;Chloroflexi;Anaerolineae;Anaerolineales;Anaerolineaceae;Levilinea;Levilinea saccharolytica;Levilinea saccharolytica DSM 16555 |
| Ga0586324 0109421 1 765 | 0.00 | 1966437.60 | 3030274.31 | 2821500.00 | 3478974.35 | 16157900.00 | 39501392.16 | 40610080.22 | 4711813.74 | 40780859.35 | 15199547.44 | 36771920.25 | KO:K04077 | chaperonin GroEL | Bacteria;Proteobacteria;Gammaproteobacteria;unclassified Gammaproteobacteria;Candidatus Competibacteraceae;Candidatus Competibacter;Candidatus Competibacter denitrificans;Candidatus Competibacter denitrificans Run A D11 |
| Ga0586324 0110415 3 1034 | 0.00 | 0.00 | 0.00 | 0.00 | 0.00 | 0.00 | 17054400.00 | 927200.00 | 0.00 | 0.00 | 0.00 | 0.00 | KO:K04077 | chaperonin GroEL | Bacteria;Chloroflexi;Anaerolineae;Anaerolineales;Anaerolineaceae;Anaerolinea;Anaerolinea thermophila;Anaerolinea thermophila UNI-1 |
| Ga0586324 0112020 2 1261 | 0.00 | 1326000.00 | 0.00 | 4284500.00 | 6262400.00 | 0.00 | 0.00 | 0.00 | 0.00 | 4543000.00 | 0.00 | 0.00 | KO:K04077 | chaperonin GroEL | Bacteria;Chloroflexi;Anaerolineae;Anaerolineales;Anaerolineaceae;Levilinea;Levilinea saccharolytica;Levilinea saccharolytica DSM 16555 |
| Ga0586324 0113053 125 1807 | 0.00 | 1428000.00 | 0.00 | 1178000.00 | 1028155.27 | 0.00 | 0.00 | 890600.00 | 0.00 | 0.00 | 0.00 | 0.00 | KO:K04077 | chaperonin GroEL | Bacteria;Proteobacteria;Deltaproteobacteria;Myxococcales;Anaeromyxobacteraceae;Anaeromyxobacter;Anaeromyxobacter dehalogenans;Anaeromyxobacter dehalogenans 2CP-C |
| Ga0586324 0113057 2097 3233 | 0.00 | 0.00 | 0.00 | 0.00 | 0.00 | 0.00 | 0.00 | 2372900.00 | 0.00 | 0.00 | 0.00 | 0.00 | KO:K04077 | chaperonin GroEL | Bacteria;Proteobacteria;Betaproteobacteria;Nitrosomonadales;Gallionellaceae;Sideroxydans;Sideroxydans lithotrophicus;Sideroxydans lithotrophicus ES-1 |
| Ga0586324 0113280 1 1044 | 0.00 | 0.00 | 0.00 | 0.00 | 0.00 | 0.00 | 0.00 | 0.00 | 0.00 | 625904.51 | 0.00 | 0.00 | KO:K04077 | chaperonin GroEL | Bacteria;Proteobacteria;Alphaproteobacteria;Hyphomicrobiales;Xanthobacteraceae;Pseudolabrys;Pseudolabrys taiwanensis;Pseudolabrys taiwanensis CC-BB4 |
| Ga0586324 0114224 192 1835 | 0.00 | 2686000.00 | 4965507.99 | 1035500.00 | 0.00 | 3105075.95 | 0.00 | 3294000.00 | 1498500.00 | 1232000.00 | 3335200.00 | 4372500.00 | KO:K04077 | chaperonin GroEL | Bacteria;Proteobacteria;Betaproteobacteria;Burkholderiales;unclassified Burkholderiales;Rubrivivax;Rubrivivax gelatinosus;Rubrivivax gelatinosus IL144 |
| Ga0586324 0114456 2177 3217 | 0.00 | 0.00 | 0.00 | 0.00 | 0.00 | 0.00 | 0.00 | 0.00 | 0.00 | 3087700.00 | 0.00 | 1740000.00 | KO:K04077 | chaperonin GroEL | Bacteria;Acidobacteria;Acidobacteriia;Acidobacteriales;Acidobacteriaceae;unclassified Acidobacteriaceae;Acidobacteriaceae bacterium URHE0068;Acidobacteriaceae bacterium URHE0068 |
| Ga0586324 0116850 3 1097 | 0.00 | 0.00 | 4729143.70 | 2451000.00 | 0.00 | 6034400.00 | 1601360.28 | 2360700.00 | 2859300.00 | 0.00 | 1399200.00 | 2400000.00 | KO:K04077 | chaperonin GroEL | Bacteria;Proteobacteria;Deltaproteobacteria;unclassified Deltaproteobacteria;unclassified Deltaproteobacteria;Dissulfuribacter;Dissulfuribacter thermophilus;Dissulfuribacter thermophilus S69 |
| Ga0586324 0119427 1012 2658 | 1280000.00 | 911200.00 | 0.00 | 881033.80 | 332542.71 | 0.00 | 2453337.00 | 12242700.00 | 1417500.00 | 3080621.39 | 4191136.40 | 1065447.00 | KO:K04077 | chaperonin GroEL | Bacteria;Proteobacteria;Gammaproteobacteria;Chromatiales;Chromatiaceae;Thioflavicoccus;Thioflavicoccus mobilis;Thioflavicoccus mobilis 8321 |
| Ga0586324 0122587 2 922 | 0.00 | 0.00 | 0.00 | 0.00 | 644109.47 | 1775225.20 | 0.00 | 0.00 | 268413.75 | 0.00 | 0.00 | 0.00 | KO:K04077 | chaperonin GroEL | Bacteria;Actinobacteria;Actinomycetia;Streptomycetales;Streptomycetaceae;Streptomyces;Streptomyces sp. RFCAC02;Streptomyces sp. RFCAC02 |
| Ga0586324 0123816 337 2001 | 0.00 | 0.00 | 0.00 | 0.00 | 0.00 | 1629053.77 | 0.00 | 5569300.00 | 565410.78 | 1147300.00 | 2622400.00 | 937500.00 | KO:K04077 | chaperonin GroEL | Bacteria;Acidobacteria;Acidobacteriia;Acidobacteriales;Acidobacteriaceae;Candidatus Koribacter;Candidatus Koribacter versatilis;Candidatus Koribacter versatilis Ellin345 |
| Ga0586324 0124030 2 1354 | 144000000.00 | 22712000.00 | 17340400.00 | 4307198.35 | 0.00 | 0.00 | 25992000.00 | 793000.00 | 9477000.00 | 0.00 | 8800000.00 | 14775000.00 | KO:K04077 | chaperonin GroEL | Bacteria;Proteobacteria;Deltaproteobacteria;Syntrophobacterales;Syntrophaceae;Smithella;Smithella sp. SCADC;Smithella sp. SCADC |
| Ga0586324 0132769 1578 2996 | 0.00 | 0.00 | 0.00 | 0.00 | 0.00 | 0.00 | 0.00 | 0.00 | 0.00 | 0.00 | 2606084.80 | 0.00 | KO:K04077 | chaperonin GroEL | Bacteria;Acidobacteria;Vicinamibacteria;unclassified Vicinamibacteria;Vicinamibacteraceae;Luteitalea;Luteitalea pratensis;Luteitalea pratensis DSM 100886 |
| Ga0586324 0136650 1 1206 | 0.00 | 0.00 | 0.00 | 382542.20 | 235623.83 | 0.00 | 0.00 | 549850.34 | 0.00 | 0.00 | 0.00 | 560718.00 | KO:K04077 | chaperonin GroEL | Bacteria;Proteobacteria;Alphaproteobacteria;Rhodospirillales;unclassified Rhodospirillales;unclassified Rhodospirillales;Rhodospirillales bacterium URHD0088;Rhodospirillales bacterium URHD0088 |
| Ga0586324 0136798 1461 2954 | 0.00 | 0.00 | 0.00 | 0.00 | 13802000.00 | 0.00 | 0.00 | 1866600.00 | 1044900.00 | 487297.58 | 6045600.00 | 2530493.25 | KO:K04077 | chaperonin GroEL | Bacteria;Proteobacteria;Deltaproteobacteria;Desulfuromonadales;Desulfuromonadaceae;Pelobacter;Pelobacter propionicus;Pelobacter propionicus DSM 2379 |
| Ga0586324 0138016 2147 2941 | 0.00 | 8704000.00 | 780588.24 | 1301500.00 | 0.00 | 0.00 | 0.00 | 186777.73 | 101171.43 | 159089700.00 | 80836800.00 | 2587500.00 | KO:K04077 | chaperonin GroEL | Bacteria;Acidobacteria;Vicinamibacteria;unclassified Vicinamibacteria;Vicinamibacteraceae;Luteitalea;Luteitalea pratensis;Luteitalea pratensis DSM 100886 |
| Ga0586324 0140392 646 2295 | 0.00 | 381389.56 | 0.00 | 0.00 | 80958000.00 | 0.00 | 740327.40 | 2176842.34 | 15106500.00 | 17688538.56 | 42372000.00 | 17642499.75 | KO:K04077 | chaperonin GroEL | Bacteria;Proteobacteria;Alphaproteobacteria;Hyphomicrobiales;Xanthobacteraceae;Pseudolabrys;Pseudolabrys sp. Root1462;Pseudolabrys sp. Root1462 |
| Ga0586324 0141658 1900 2907 | 0.00 | 0.00 | 0.00 | 12065000.00 | 0.00 | 2342851.83 | 0.00 | 0.00 | 9477000.00 | 0.00 | 0.00 | 0.00 | KO:K04077 | chaperonin GroEL | Bacteria;Acidobacteria;Vicinamibacteria;unclassified Vicinamibacteria;Vicinamibacteraceae;Luteitalea;Luteitalea pratensis;Luteitalea pratensis DSM 100886 |
| Ga0586324 0141765 754 2376 | 20600000.00 | 0.00 | 0.00 | 0.00 | 0.00 | 0.00 | 0.00 | 0.00 | 0.00 | 0.00 | 0.00 | 0.00 | KO:K04077 | chaperonin GroEL | Bacteria;Proteobacteria;Deltaproteobacteria;Desulfobacterales;Desulfobacteraceae;Desulfatiglans;Desulfatiglans anilini;Desulfatiglans anilini DSM 4660 |
| Ga0586324 0141859 1220 2878 | 0.00 | 1489200.00 | 0.00 | 0.00 | 0.00 | 0.00 | 0.00 | 3928400.00 | 0.00 | 0.00 | 0.00 | 0.00 | KO:K04077 | chaperonin GroEL | Bacteria;Nitrospirae;Nitrospira;Nitrospirales;Nitrospiraceae;Nitrospira;Nitrospira marina;Nitrospira marina Nb-295 |
| Ga0586324 0141999 1 1536 | 2880000.00 | 0.00 | 0.00 | 0.00 | 0.00 | 0.00 | 0.00 | 2110600.00 | 0.00 | 2163700.00 | 36784000.00 | 35932500.00 | KO:K04077 | chaperonin GroEL | Bacteria;Proteobacteria;Deltaproteobacteria;Syntrophorhabdales;Syntrophorhabdaceae;Syntrophorhabdus;Syntrophorhabdus aromaticivorans;Syntrophorhabdus aromaticivorans UI |
| Ga0586324 0143946 208 1836 | 16300000.00 | 0.00 | 0.00 | 0.00 | 0.00 | 0.00 | 0.00 | 0.00 | 0.00 | 0.00 | 0.00 | 0.00 | KO:K04077 | chaperonin GroEL | Bacteria;Actinobacteria;Thermoleophilia;Solirubrobacterales;Solirubrobacteraceae;Solirubrobacter;Solirubrobacter pauli;Solirubrobacter pauli DSM 14954 |
| Ga0586324 0144401 1616 2881 | 0.00 | 2638400.00 | 0.00 | 490783.30 | 493752.13 | 0.00 | 0.00 | 0.00 | 0.00 | 0.00 | 0.00 | 0.00 | KO:K04077 | chaperonin GroEL | Bacteria;Proteobacteria;Betaproteobacteria;unclassified Betaproteobacteria;unclassified Betaproteobacteria;unclassified Betaproteobacteria;Betaproteobacteria bacterium UKL13-2;Betaproteobacteria bacterium UKL13-2 |
| Ga0586324 0149695 380 2005 | 0.00 | 0.00 | 0.00 | 0.00 | 0.00 | 0.00 | 298123.68 | 0.00 | 0.00 | 0.00 | 0.00 | 0.00 | KO:K04077 | chaperonin GroEL | Bacteria;Acidobacteria;Acidobacteriia;Acidobacteriales;Acidobacteriaceae;Candidatus Koribacter;Candidatus Koribacter versatilis;Candidatus Koribacter versatilis Ellin345 |
| Ga0586324 0150064 715 2352 | 0.00 | 0.00 | 0.00 | 0.00 | 0.00 | 0.00 | 0.00 | 0.00 | 0.00 | 0.00 | 0.00 | 1327500.00 | KO:K04077 | chaperonin GroEL | Bacteria;Proteobacteria;Alphaproteobacteria;Hyphomicrobiales;Xanthobacteraceae;Pseudolabrys;Pseudolabrys sp. Root1462;Pseudolabrys sp. Root1462 |
| Ga0586324 0150273 381 2018 | 26400000.00 | 0.00 | 0.00 | 0.00 | 0.00 | 55580000.00 | 937754.88 | 1146800.00 | 874800.00 | 758319.10 | 651006.40 | 1042500.00 | KO:K04077 | chaperonin GroEL | Bacteria;Proteobacteria;Alphaproteobacteria;Rhodospirillales;Rhodospirillaceae;Dongia;Dongia sp. URHE0060;Dongia sp. URHE0060 |
| Ga0586324 0150513 1343 2824 | 2120000.00 | 0.00 | 0.00 | 625552.20 | 593076.06 | 0.00 | 0.00 | 408197.97 | 283274.82 | 0.00 | 0.00 | 0.00 | KO:K04077 | chaperonin GroEL | Bacteria;Firmicutes;Negativicutes;Veillonellales;Veillonellaceae;Megasphaera;Megasphaera elsdenii;Megasphaera elsdenii 24-50 |
| Ga0586324 0151589 124 1695 | 0.00 | 0.00 | 0.00 | 0.00 | 0.00 | 0.00 | 0.00 | 1067500.00 | 0.00 | 0.00 | 0.00 | 0.00 | KO:K04077 | chaperonin GroEL | Bacteria;Proteobacteria;Alphaproteobacteria;Hyphomicrobiales;Xanthobacteraceae;Pseudolabrys;Pseudolabrys taiwanensis;Pseudolabrys taiwanensis CC-BB4 |
| Ga0586324 0151934 505 2136 | 0.00 | 0.00 | 0.00 | 3201500.00 | 0.00 | 2557156.40 | 773870.76 | 0.00 | 1312200.00 | 0.00 | 0.00 | 0.00 | KO:K04077 | chaperonin GroEL | Bacteria;Chloroflexi;Anaerolineae;Anaerolineales;Anaerolineaceae;Anaerolinea;Anaerolinea thermophila;Anaerolinea thermophila UNI-1 |
| Ga0586324 0158769 1 1083 | 0.00 | 918000.00 | 4629898.06 | 0.00 | 0.00 | 0.00 | 0.00 | 0.00 | 1263600.00 | 1170400.00 | 1689600.00 | 0.00 | KO:K04077 | chaperonin GroEL | Bacteria;Acidobacteria;Vicinamibacteria;unclassified Vicinamibacteria;Vicinamibacteraceae;Luteitalea;Luteitalea pratensis;Luteitalea pratensis DSM 100886 |
| Ga0586324 0158936 3 1598 | 1260000.00 | 0.00 | 0.00 | 0.00 | 0.00 | 0.00 | 0.00 | 9455000.00 | 3167100.00 | 0.00 | 0.00 | 0.00 | KO:K04077 | chaperonin GroEL | Bacteria;Chloroflexi;Anaerolineae;Anaerolineales;Anaerolineaceae;Anaerolinea;Anaerolinea thermophila;Anaerolinea thermophila UNI-1 |
| Ga0586324 0160095 1516 2745 | 0.00 | 149634.00 | 0.00 | 0.00 | 2904600.00 | 0.00 | 0.00 | 0.00 | 0.00 | 0.00 | 0.00 | 0.00 | KO:K04077 | chaperonin GroEL | Bacteria;Acidobacteria;Acidobacteriia;Acidobacteriales;Acidobacteriaceae;unclassified Acidobacteriaceae;Acidobacteriaceae bacterium KBS 96;Acidobacteriaceae bacterium KBS 96, DSM 24297 |
| Ga0586324 0160442 2 1519 | 2160000.00 | 0.00 | 3868226.62 | 938922.05 | 5335400.00 | 4406700.00 | 0.00 | 3446500.00 | 4001400.00 | 0.00 | 0.00 | 0.00 | KO:K04077 | chaperonin GroEL | Bacteria;Proteobacteria;Alphaproteobacteria;Rhodospirillales;Rhodospirillaceae;Inquilinus;Inquilinus limosus;Inquilinus limosus DSM 16000 |
| Ga0586324 0161052 1630 2736 | 0.00 | 0.00 | 0.00 | 523136.50 | 0.00 | 0.00 | 0.00 | 2067900.00 | 2762100.00 | 0.00 | 0.00 | 440788.50 | KO:K04077 | chaperonin GroEL | Bacteria;Proteobacteria;Alphaproteobacteria;Rhodospirillales;Stellaceae;Stella;Stella humosa;Stella humosa DSM 5900 |
| Ga0586324 0163352 3 1076 | 0.00 | 0.00 | 0.00 | 3771500.00 | 0.00 | 0.00 | 0.00 | 1555500.00 | 2924100.00 | 5228300.00 | 0.00 | 0.00 | KO:K04077 | chaperonin GroEL | Bacteria;Verrucomicrobia;Opitutae;Opitutales;Opitutaceae;Opitutus;Opitutus terrae;Opitutus terrae PB90-1 |
| Ga0586324 0165800 3 1079 | 4640000.00 | 236713.44 | 0.00 | 0.00 | 0.00 | 0.00 | 0.00 | 0.00 | 0.00 | 0.00 | 577284.40 | 0.00 | KO:K04077 | chaperonin GroEL | Bacteria;Proteobacteria;Deltaproteobacteria;Desulfobacterales;Desulfobacteraceae;Desulfatiglans;Desulfatiglans anilini;Desulfatiglans anilini DSM 4660 |
| Ga0586324 0174345 3 860 | 1290392.00 | 3044505.52 | 2575321.33 | 3570558.85 | 3191732.07 | 2476886.57 | 4605112.99 | 4759109.59 | 4837426.92 | 7220341.59 | 6378387.84 | 7210063.50 | KO:K04077 | chaperonin GroEL | Bacteria;Proteobacteria;Betaproteobacteria;Burkholderiales;unclassified Burkholderiales;Methylibium;Methylibium sp. NZG;Methylibium sp. NZG |
| Ga0586324 0176867 952 2586 | 0.00 | 0.00 | 0.00 | 0.00 | 0.00 | 0.00 | 0.00 | 756400.00 | 0.00 | 0.00 | 0.00 | 0.00 | KO:K04077 | chaperonin GroEL | Bacteria;Bacteroidetes;Bacteroidia;Marinilabiliales;Prolixibacteraceae;Draconibacterium;Draconibacterium sediminis;Draconibacterium sediminis JN14CK-3 |
| Ga0586324 0177021 3 860 | 0.00 | 0.00 | 0.00 | 0.00 | 0.00 | 2589071.23 | 0.00 | 0.00 | 0.00 | 0.00 | 0.00 | 0.00 | KO:K04077 | chaperonin GroEL | Bacteria;Chloroflexi;Caldilineae;Caldilineales;Caldilineaceae;Caldilinea;Caldilinea aerophila;Caldilinea aerophila STL-6-O1, DSM 14535 |
| Ga0586324 0180366 2 1396 | 0.00 | 0.00 | 0.00 | 0.00 | 0.00 | 0.00 | 195249.17 | 0.00 | 0.00 | 207601.24 | 0.00 | 297033.75 | KO:K04077 | chaperonin GroEL | Bacteria;Chloroflexi;Anaerolineae;Anaerolineales;Anaerolineaceae;Levilinea;Levilinea saccharolytica;Levilinea saccharolytica DSM 16555 |
| Ga0586324 0183082 923 2578 | 9940000.00 | 12240000.00 | 0.00 | 5519500.00 | 0.00 | 0.00 | 0.00 | 0.00 | 0.00 | 0.00 | 0.00 | 0.00 | KO:K04077 | chaperonin GroEL | Bacteria;Planctomycetes;unclassified Planctomycetes;unclassified Planctomycetes;unclassified Planctomycetes;unclassified Planctomycetes;unclassified Planctomycetes;Planctomycetes bacterium P3 |
| Ga0586324 0186662 2048 2554 | 0.00 | 215223.40 | 0.00 | 0.00 | 804375.41 | 0.00 | 0.00 | 652700.00 | 1012500.00 | 411185.39 | 509788.40 | 0.00 | KO:K04077 | chaperonin GroEL | Bacteria;Proteobacteria;Deltaproteobacteria;Desulfobacterales;Desulfobacteraceae;Desulfobacterium;Desulfobacterium autotrophicum;Desulfobacterium autotrophicum HRM2, DSM 3382 |

| | | | | | | | | | | | | | | | |
|---|---|---|---|---|---|---|---|---|---|---|---|---|---|---|---|
| Ga0586324 0186985 315 1973 | 0.00 | 0.00 | 0.00 | 247894.90 | 0.00 | 0.00 | 344391.72 | 0.00 | 405292.41 | 0.00 | 0.00 | 0.00 | KO:K04077 | chaperonin GroEL | Bacteria;Proteobacteria;Betaproteobacteria;Burkholderiales;unclassified Burkholderiales;unclassified Burkholderiales;Burkholderiales bacterium GJ-E10;Burkholderiales bacterium GJ-E10 |
| Ga0586324 0188679 3 560 | 0.00 | 16184000.00 | 0.00 | 0.00 | 0.00 | 0.00 | 0.00 | 0.00 | 0.00 | 31185000.00 | 1777600.00 | 0.00 | KO:K04077 | chaperonin GroEL | Bacteria;Proteobacteria;Gammaproteobacteria;Chromatiales;Chromatiaceae;Lamprocystis;Lamprocystis purpurea;Lamprocystis purpurea DSM 4197 |
| Ga0586324 0190138 730 2355 | 0.00 | 0.00 | 0.00 | 0.00 | 0.00 | 97265000.00 | 2488688.40 | 37232370.53 | 219012.66 | 0.00 | 777209.84 | 0.00 | KO:K04077 | chaperonin GroEL | Bacteria;Proteobacteria;Deltaproteobacteria;Desulfobacterales;Desulfobacteraceae;Desulfosarcina;Desulfosarcina cetonica;Desulfosarcina cetonica JCM 12296 |
| Ga0586324 0191407 1 1530 | 1870000.00 | 1149200.00 | 0.00 | 0.00 | 0.00 | 0.00 | 0.00 | 18727000.00 | 0.00 | 0.00 | 0.00 | 2040000.00 | KO:K04077 | chaperonin GroEL | Bacteria;Nitrospirae;Nitrospira;Nitrospirales;Nitrospiraceae;Candidatus Magnetobacterium;Candidatus Magnetobacterium casensis;Candidatus Magnetobacterium casensis MYR-1 |
| Ga0586324 0193010 1 1098 | 0.00 | 0.00 | 0.00 | 0.00 | 0.00 | 0.00 | 261144.36 | 0.00 | 0.00 | 0.00 | 0.00 | 0.00 | KO:K04077 | chaperonin GroEL | Bacteria;Firmicutes;Negativicutes;Selenomonadales;Sporomusaceae;Sporomusa;Sporomusa acidovorans;Sporomusa acidovorans DSM 3132 |
| Ga0586324 0195824 1 984 | 0.00 | 0.00 | 0.00 | 0.00 | 0.00 | 0.00 | 0.00 | 4788500.00 | 0.00 | 3780700.00 | 4628800.00 | 4027500.00 | KO:K04077 | chaperonin GroEL | Bacteria;Proteobacteria;Deltaproteobacteria;Desulfobacterales;Desulfobacteraceae;Desulfatiglans;Desulfatiglans anilini;Desulfatiglans anilini DSM 4660 |
| Ga0586324 0196005 456 2096 | 0.00 | 0.00 | 0.00 | 0.00 | 0.00 | 0.00 | 0.00 | 0.00 | 75735000.00 | 0.00 | 0.00 | 0.00 | KO:K04077 | chaperonin GroEL | Bacteria;Verrucomicrobia;Methylacidiphilae;Methylacidiphilales;Methylacidiphilaceae;Methylacidiphilum;Methylacidiphilum infernorum;Methylacidiphilum infernorum V4 |
| Ga0586324 0197204 528 2144 | 0.00 | 608948.84 | 0.00 | 1263500.00 | 0.00 | 0.00 | 8732400.00 | 1836100.00 | 774853.29 | 0.00 | 2692800.00 | 0.00 | KO:K04077 | chaperonin GroEL | Bacteria;Candidatus Aminicenantes;unclassified Candidatus Aminicenantes;unclassified Candidatus Aminicenantes;unclassified Candidatus Aminicenantes;unclassified Candidatus Aminicenantes;Aminicenantes bacterium SCGC AAA255-E10;Aminicenantes bacterium SCGC AAA255-E10 (contamination screened) |
| Ga0586324 0197688 838 2469 | 0.00 | 0.00 | 0.00 | 26362967.40 | 8425400.00 | 0.00 | 0.00 | 15121900.00 | 7209000.00 | 3503500.00 | 11176000.00 | 12690000.00 | KO:K04077 | chaperonin GroEL | Bacteria;Proteobacteria;Deltaproteobacteria;Syntrophobacterales;Syntrophobacteraceae;Desulfosoma;Desulfosoma caldarium;Desulfosoma caldarium DSM 22027 |
| Ga0586324 0201697 566 2212 | 0.00 | 0.00 | 0.00 | 0.00 | 0.00 | 0.00 | 0.00 | 235219.05 | 0.00 | 396231.99 | 0.00 | 509060.25 | KO:K04077 | chaperonin GroEL | Bacteria;Proteobacteria;Betaproteobacteria;Burkholderiales;unclassified Burkholderiales;unclassified Burkholderiales;Burkholderiales bacterium GJ-E10;Burkholderiales bacterium GJ-E10 |
| Ga0586324 0203051 942 2459 | 0.00 | 6704800.00 | 1175572.15 | 593202.80 | 0.00 | 0.00 | 0.00 | 4770200.00 | 977414.85 | 877800.00 | 215966.96 | 8662500.00 | KO:K04077 | chaperonin GroEL | Bacteria;Proteobacteria;Deltaproteobacteria;Myxococcales;Anaeromyxobacteraceae;Anaeromyxobacter;Anaeromyxobacter sp. Fw109-5;Anaeromyxobacter sp. Fw109-5 |
| Ga0586324 0203282 2 1087 | 0.00 | 0.00 | 0.00 | 0.00 | 422908.73 | 0.00 | 5791200.00 | 1305400.00 | 1020600.00 | 469877.87 | 0.00 | 835026.00 | KO:K04077 | chaperonin GroEL | Bacteria;Proteobacteria;Deltaproteobacteria;unclassified Deltaproteobacteria;unclassified Deltaproteobacteria;unclassified Deltaproteobacteria;delta proteobacterium NaphS2;delta proteobacterium NaphS2 |
| Ga0586324 0206080 1253 2440 | 5290000.00 | 0.00 | 0.00 | 0.00 | 0.00 | 0.00 | 0.00 | 0.00 | 0.00 | 0.00 | 0.00 | 0.00 | KO:K04077 | chaperonin GroEL | Bacteria;Acidobacteria;Vicinamibacteria;unclassified Vicinamibacteria;Vicinamibacteraceae;Luteitalea;Luteitalea pratensis;Luteitalea pratensis DSM 100886 |
| Ga0586324 0207531 3 953 | 0.00 | 0.00 | 11260000.00 | 4113500.00 | 3986100.00 | 0.00 | 0.00 | 5758400.00 | 0.00 | 0.00 | 0.00 | 2775000.00 | KO:K04077 | chaperonin GroEL | Bacteria;Proteobacteria;Deltaproteobacteria;Syntrophorhabdales;Syntrophorhabdaceae;Syntrophorhabdus;Syntrophorhabdus aromaticivorans;Syntrophorhabdus aromaticivorans UI |
| Ga0586324 0210491 144 1775 | 4480000.00 | 3658400.00 | 0.00 | 0.00 | 1184500.00 | 0.00 | 0.00 | 0.00 | 0.00 | 0.00 | 0.00 | 0.00 | KO:K04077 | chaperonin GroEL | Bacteria;Proteobacteria;Deltaproteobacteria;Myxococcales;Archangiaceae;Vitiosangium;Vitiosangium sp. GDMCC 1.1324;Vitiosangium sp. GDMCC 1.1324 |
| Ga0586324 0216558 2 1639 | 0.00 | 0.00 | 0.00 | 0.00 | 0.00 | 0.00 | 0.00 | 197454.56 | 763908.57 | 367179.12 | 549236.16 | 242507.25 | KO:K04077 | chaperonin GroEL | Bacteria;Proteobacteria;Deltaproteobacteria;Myxococcales;Anaeromyxobacteraceae;Anaeromyxobacter;Anaeromyxobacter dehalogenans;Anaeromyxobacter dehalogenans 2CP-C |
| Ga0586324 0216662 3 1439 | 0.00 | 0.00 | 0.00 | 0.00 | 0.00 | 0.00 | 1239079.68 | 0.00 | 0.00 | 0.00 | 0.00 | 0.00 | KO:K04077 | chaperonin GroEL | Bacteria;Proteobacteria;Alphaproteobacteria;Hyphomicrobiales;Xanthobacteraceae;Pseudolabrys;Pseudolabrys sp. Root1462;Pseudolabrys sp. Root1462 |
| Ga0586324 0221696 989 2359 | 0.00 | 0.00 | 0.00 | 2736000.00 | 0.00 | 0.00 | 0.00 | 0.00 | 0.00 | 0.00 | 0.00 | 765000.00 | KO:K04077 | chaperonin GroEL | Bacteria;Proteobacteria;Deltaproteobacteria;Syntrophobacterales;Syntrophaceae;Smithella;Smithella sp. F21;Smithella sp. F21 |
| Ga0586324 0224332 866 2347 | 2640000.00 | 7848092.16 | 3305115.71 | 3249000.00 | 4418700.00 | 22630099.69 | 113638373.76 | 2769400.00 | 5653800.00 | 2326264.71 | 318147.28 | 28020141.75 | KO:K04077 | chaperonin GroEL | Bacteria;Proteobacteria;Gammaproteobacteria;Chromatiales;Chromatiaceae;Thiocystis;Thiocystis violascens;Thiocystis violascens 611, DSM 198 |
| Ga0586324 0234920 881 2299 | 0.00 | 0.00 | 0.00 | 0.00 | 0.00 | 0.00 | 0.00 | 0.00 | 0.00 | 0.00 | 1689600.00 | 0.00 | KO:K04077 | chaperonin GroEL | Bacteria;Acidobacteria;Blastocatellia;Blastocatellales;Pyrinomonadaceae;Pyrinomonas;Pyrinomonas methylaliphatogenes;Pyrinomonas methylaliphatogenes K22 |
| Ga0586324 0238716 564 2192 | 14110000.00 | 16639600.00 | 5506849.38 | 4180000.00 | 8971300.00 | 2104564.89 | 14353714.92 | 13877500.00 | 9009209.61 | 17509800.00 | 11017600.00 | 24915000.00 | KO:K04077 | chaperonin GroEL | Bacteria;Acidobacteria;Vicinamibacteria;unclassified Vicinamibacteria;Vicinamibacteraceae;Luteitalea;Luteitalea pratensis;Luteitalea pratensis DSM 100886 |
| Ga0586324 0242391 1209 2264 | 0.00 | 0.00 | 0.00 | 0.00 | 0.00 | 0.00 | 777039.96 | 4001600.00 | 17901000.00 | 37191000.00 | 3238400.00 | 877500.00 | KO:K04077 | chaperonin GroEL | Bacteria;Proteobacteria;Deltaproteobacteria;Syntrophobacterales;Syntrophaceae;Syntrophus;Syntrophus aciditrophicus;Syntrophus aciditrophicus SB |
| Ga0586324 0244757 942 2255 | 0.00 | 652537.52 | 10865900.00 | 0.00 | 0.00 | 0.00 | 667342.32 | 2092221.92 | 0.00 | 2012908.59 | 3320697.60 | 5002500.00 | KO:K04077 | chaperonin GroEL | Bacteria;Verrucomicrobia;Spartobacteria;unclassified Spartobacteria;unclassified Spartobacteria;unclassified Spartobacteria;Spartobacteria bacterium LR76;Spartobacteria bacterium LR76 |
| Ga0586324 0244974 1220 2254 | 0.00 | 0.00 | 0.00 | 0.00 | 0.00 | 0.00 | 0.00 | 0.00 | 0.00 | 0.00 | 0.00 | 6405000.00 | KO:K04077 | chaperonin GroEL | Bacteria;Verrucomicrobia;Verrucomicrobiae;unclassified Verrucomicrobiae;unclassified Verrucomicrobiae;unclassified Verrucomicrobiae;Verrucomicrobiae bacterium DG1235;Verrucomicrobiales sp. DG1235 |
| Ga0586324 0247739 3 1142 | 0.00 | 0.00 | 0.00 | 0.00 | 0.00 | 0.00 | 0.00 | 6093900.00 | 1401300.00 | 0.00 | 2200000.00 | 0.00 | KO:K04077 | chaperonin GroEL | Bacteria;Acidobacteria;Vicinamibacteria;unclassified Vicinamibacteria;Vicinamibacteraceae;Luteitalea;Luteitalea pratensis;Luteitalea pratensis DSM 100886 |
| Ga0586324 0250220 3 1472 | 0.00 | 0.00 | 209133.67 | 1197000.00 | 0.00 | 0.00 | 1145278.20 | 0.00 | 230806.26 | 218175.65 | 302638.16 | 238101.00 | KO:K04077 | chaperonin GroEL | Bacteria;Proteobacteria;Gammaproteobacteria;Chromatiales;Woeseiaceae;Woeseia;Woeseia oceani;Woeseia oceani XK5 |
| Ga0586324 0251325 1083 2228 | 0.00 | 2339200.00 | 0.00 | 0.00 | 0.00 | 0.00 | 0.00 | 0.00 | 891000.00 | 1948100.00 | 0.00 | 0.00 | KO:K04077 | chaperonin GroEL | Bacteria;Acidobacteria;Vicinamibacteria;unclassified Vicinamibacteria;Vicinamibacteraceae;Luteitalea;Luteitalea pratensis;Luteitalea pratensis DSM 100886 |
| Ga0586324 0252067 1231 2226 | 451644.00 | 339205.76 | 0.00 | 2831000.00 | 520961.64 | 0.00 | 0.00 | 289534.06 | 1327130.73 | 1239700.00 | 0.00 | 900000.00 | KO:K04077 | chaperonin GroEL | Bacteria;Proteobacteria;Gammaproteobacteria;unclassified Gammaproteobacteria;Candidatus Competibacteraceae;Candidatus Competibacter;Candidatus Competibacter denitrificans;Candidatus Competibacter denitrificans Run A D11 |
| Ga0586324 0253786 757 2217 | 0.00 | 197376.12 | 0.00 | 0.00 | 0.00 | 0.00 | 0.00 | 20496000.00 | 12231000.00 | 0.00 | 0.00 | 0.00 | KO:K04077 | chaperonin GroEL | Bacteria;Proteobacteria;Deltaproteobacteria;Desulfobacterales;Desulfobacteraceae;Desulfosarcina;Desulfosarcina cetonica;Desulfosarcina cetonica JCM 12296 |
| Ga0586324 0255038 3 1109 | 1260000.00 | 668926.88 | 568061.37 | 4579000.00 | 0.00 | 1253059.04 | 2280000.00 | 1085800.00 | 0.00 | 2471700.00 | 3845600.00 | 4575000.00 | KO:K04077 | chaperonin GroEL | Bacteria;Proteobacteria;Deltaproteobacteria;Myxococcales;Archangiaceae;Archangium;Archangium sp. Cb G35;Archangium sp. Cb G35 |
| Ga0586324 0255394 513 2177 | 0.00 | 0.00 | 0.00 | 915583.40 | 623139.70 | 0.00 | 5767195.25 | 336573.60 | 4819918.77 | 9548000.00 | 15224000.00 | 3562500.00 | KO:K04077 | chaperonin GroEL | Bacteria;Proteobacteria;Deltaproteobacteria;Syntrophobacterales;Syntrophobacteraceae;Syntrophobacter;Syntrophobacter fumaroxidans;Syntrophobacter fumaroxidans MPOB |
| Ga0586324 0255474 1 1278 | 0.00 | 0.00 | 0.00 | 43985000.00 | 1318400.00 | 2881426.00 | 0.00 | 0.00 | 0.00 | 0.00 | 0.00 | 0.00 | KO:K04077 | chaperonin GroEL | Bacteria;Proteobacteria;Deltaproteobacteria;Desulfarculales;Desulfarculaceae;Desulfocarbo;Desulfocarbo indianensis;Desulfocarbo indianensis SCBM |
| Ga0586324 0256513 355 2007 | 7560000.00 | 5365200.00 | 10077700.00 | 2555500.00 | 2080600.00 | 26876900.00 | 45600000.00 | 4306600.00 | 4268700.00 | 49357000.00 | 66088000.00 | 28500000.00 | KO:K04077 | chaperonin GroEL | Bacteria;Proteobacteria;Gammaproteobacteria;Methylococcales;Crenotrichaceae;Crenothrix;Crenothrix polyspora;Crenothrix polyspora ERS1545888 |
| Ga0586324 0268138 1251 2162 | 0.00 | 0.00 | 0.00 | 14155000.00 | 4336300.00 | 0.00 | 0.00 | 0.00 | 383317.11 | 8085000.00 | 4802156.48 | 0.00 | KO:K04077 | chaperonin GroEL | Bacteria;Chloroflexi;Ardenticatenia;Ardenticatenales;Ardenticatenaceae;Candidatus Promineofilum;Candidatus Promineofilum breve;Candidatus Promineofilum breve Cfx-K |
| Ga0586324 0268820 2 1663 | 30620000.00 | 58076151.40 | 53354158.80 | 15571341.70 | 8933698.82 | 26732963.68 | 15526800.00 | 136926700.00 | 65634633.72 | 177089973.06 | 113152044.72 | 106404489.75 | KO:K04077 | chaperonin GroEL | Bacteria;Proteobacteria;Alphaproteobacteria;Rhodospirillales;Acetobacteraceae;Rhodopila;unclassified Rhodopila;Rhodopila sp. LVNP |
| Ga0586324 0271979 325 1989 | 0.00 | 4773600.00 | 0.00 | 0.00 | 0.00 | 6629900.00 | 0.00 | 0.00 | 13680900.00 | 0.00 | 580247.36 | 0.00 | KO:K04077 | chaperonin GroEL | Bacteria;Proteobacteria;Deltaproteobacteria;Myxococcales;Archangiaceae;Vitiosangium;Vitiosangium sp. GDMCC 1.1324;Vitiosangium sp. GDMCC 1.1324 |
| Ga0586324 0273975 860 2140 | 0.00 | 0.00 | 0.00 | 0.00 | 0.00 | 0.00 | 0.00 | 0.00 | 486205.74 | 0.00 | 767357.36 | 669987.75 | KO:K04077 | chaperonin GroEL | Bacteria;Proteobacteria;Deltaproteobacteria;Syntrophobacterales;Syntrophaceae;Syntrophus;Syntrophus aciditrophicus;Syntrophus aciditrophicus SB |
| Ga0586324 0278594 34 1668 | 0.00 | 0.00 | 5798900.00 | 0.00 | 0.00 | 0.00 | 2690400.00 | 2989000.00 | 0.00 | 0.00 | 5324000.00 | 3465000.00 | KO:K04077 | chaperonin GroEL | Bacteria;Bacteroidetes;Bacteroidia;Marinilabiliales;Prolixibacteraceae;Draconibacterium;Draconibacterium sediminis;Draconibacterium sediminis JN14CK-3 |
| Ga0586324 0281552 1 585 | 5080000.00 | 0.00 | 0.00 | 4158378.95 | 3429900.00 | 12267300.00 | 2280000.00 | 4270000.00 | 6147900.00 | 2140600.00 | 0.00 | 0.00 | KO:K04077 | chaperonin GroEL | Bacteria;Chloroflexi;Caldilineae;Caldilineales;Caldilineaceae;Caldilinea;Caldilinea aerophila;Caldilinea aerophila STL-6-O1, DSM 14535 |
| Ga0586324 0285424 1 963 | 851544.00 | 5888800.00 | 1121580.45 | 6042000.00 | 5850400.00 | 0.00 | 1283961.48 | 7143100.00 | 5880520.62 | 3734500.00 | 4840000.00 | 5632500.00 | KO:K04077 | chaperonin GroEL | Bacteria;Proteobacteria;Deltaproteobacteria;Desulfobacterales;Desulfobacteraceae;Desulfococcus;Desulfococcus multivorans;Desulfococcus multivorans DSM 2059 |
| Ga0586324 0288273 527 2092 | 0.00 | 0.00 | 0.00 | 0.00 | 172901.98 | 0.00 | 0.00 | 292132.05 | 244124.28 | 6152300.00 | 0.00 | 0.00 | KO:K04077 | chaperonin GroEL | Bacteria;Proteobacteria;Betaproteobacteria;unclassified Betaproteobacteria;unclassified Betaproteobacteria;unclassified Betaproteobacteria;Betaproteobacteria bacterium GR16-43;Betaproteobacteria bacterium GR16-43 |
| Ga0586324 0289271 925 2088 | 1610000.00 | 4259690.00 | 0.00 | 15760500.00 | 22243882.06 | 83092100.00 | 867692.76 | 10246935.55 | 0.00 | 5074300.00 | 752953.52 | 1545000.00 | KO:K04077 | chaperonin GroEL | Bacteria;Proteobacteria;Deltaproteobacteria;Desulfobacterales;Desulfobacteraceae;Desulfatirhabdium;Desulfatirhabdium butyrativorans;Desulfatirhabdium butyrativorans DSM 18734 |
| Ga0586324 0294719 1 876 | 0.00 | 178652.32 | 0.00 | 4370000.00 | 0.00 | 14530200.00 | 10225533.24 | 9760000.00 | 0.00 | 12326370.21 | 19289600.00 | 6517500.00 | KO:K04077 | chaperonin GroEL | Bacteria;Proteobacteria;Deltaproteobacteria;Desulfobacterales;Desulfobacteraceae;Desulfatiglans;Desulfatiglans anilini;Desulfatiglans anilini DSM 4660 |
| Ga0586324 0298000 908 2062 | 0.00 | 0.00 | 0.00 | 0.00 | 0.00 | 0.00 | 0.00 | 11224000.00 | 0.00 | 0.00 | 0.00 | 0.00 | KO:K04077 | chaperonin GroEL | Bacteria;Proteobacteria;Betaproteobacteria;Nitrosomonadales;Nitrosomonadaceae;Nitrosomonas;Nitrosomonas oligotropha;Nitrosomonas oligotropha Nm75 |
| Ga0586324 0299974 849 2057 | 0.00 | 836400.00 | 0.00 | 8426500.00 | 3203300.00 | 7304800.00 | 0.00 | 6421701.80 | 4625100.00 | 6457260.81 | 10919517.84 | 1567500.00 | KO:K04077 | chaperonin GroEL | Bacteria;Proteobacteria;Deltaproteobacteria;Myxococcales;Anaeromyxobacteraceae;Anaeromyxobacter;Anaeromyxobacter dehalogenans;Anaeromyxobacter dehalogenans 2CP-C |
| Ga0586324 0300965 375 2051 | 0.00 | 0.00 | 0.00 | 2118500.00 | 0.00 | 0.00 | 0.00 | 0.00 | 0.00 | 0.00 | 0.00 | 0.00 | KO:K04077 | chaperonin GroEL | Bacteria;Proteobacteria;Betaproteobacteria;Burkholderiales;Oxalobacteraceae;Noviherbaspirillum;Noviherbaspirillum sp. K1S02-23;Noviherbaspirillum sp. K1S02-23 |
| Ga0586324 0301154 3 1535 | 6010000.00 | 6181200.00 | 0.00 | 0.00 | 0.00 | 0.00 | 0.00 | 1354200.00 | 1182600.00 | 14476000.00 | 0.00 | 0.00 | KO:K04077 | chaperonin GroEL | Bacteria;Proteobacteria;Deltaproteobacteria;Syntrophobacterales;Syntrophaceae;Syntrophus;Syntrophus aciditrophicus;Syntrophus aciditrophicus SB |
| Ga0586324 0301327 140 1774 | 0.00 | 0.00 | 0.00 | 0.00 | 0.00 | 5558000.00 | 1716912.96 | 0.00 | 2697300.00 | 0.00 | 5412000.00 | 0.00 | KO:K04077 | chaperonin GroEL | Bacteria;Bacteroidetes;Bacteroidia;Bacteroidales;Rikenellaceae;Acetobacteroides;Acetobacteroides hydrogenigenes;Acetobacteroides hydrogenigenes DSM 24657 |
| Ga0586324 0303950 1137 2042 | 1680000.00 | 1530680.68 | 6892595.06 | 5089334.30 | 4933700.00 | 20615431.88 | 5694746.88 | 24778605.65 | 10824652.89 | 14814800.00 | 17864000.00 | 29392500.00 | KO:K04077 | chaperonin GroEL | Bacteria;Proteobacteria;Gammaproteobacteria;Chromatiales;Chromatiaceae;Thiodictyon;Candidatus Thiodictyon syntrophicum;Candidatus Thiodictyon syntrophicum Cad16 |
| Ga0586324 0304269 2 598 | 0.00 | 0.00 | 0.00 | 0.00 | 132870000.00 | 18381100.00 | 0.00 | 0.00 | 0.00 | 0.00 | 0.00 | 0.00 | KO:K04077 | chaperonin GroEL | Bacteria;Bacteroidetes;unclassified Bacteroidetes;Bacteroidetes Order II. Incertae sedis;Rhodothermaceae;unclassified Rhodothermaceae;Rhodothermaceae bacterium RA;Halothermobacillus malaysiensis RA |
| Ga0586324 0304465 2 1222 | 0.00 | 0.00 | 0.00 | 0.00 | 0.00 | 0.00 | 0.00 | 0.00 | 0.00 | 0.00 | 2921600.00 | 0.00 | KO:K04077 | chaperonin GroEL | Bacteria;Planctomycetes;Candidatus Brocadiae;Candidatus Brocadiales;Candidatus Brocadiaceae;Candidatus Kuenenia;Candidatus Kuenenia stuttgartiensis;Candidatus Kuenenia stuttgartiensis MBR1 |
| Ga0586324 0306695 812 2035 | 0.00 | 0.00 | 2521890.94 | 0.00 | 0.00 | 1271420.29 | 0.00 | 0.00 | 0.00 | 0.00 | 0.00 | 0.00 | KO:K04077 | chaperonin GroEL | Bacteria;Bacteroidetes;Bacteroidia;Marinilabiliales;Marinilabiliaceae;Alkalitalea;Alkalitalea saponilacus;Alkalitalea saponilacus DSM 24412 |
| Ga0586324 0307071 3 1106 | 11800000.00 | 199144.12 | 0.00 | 0.00 | 0.00 | 0.00 | 0.00 | 297592.77 | 589637.88 | 0.00 | 0.00 | 0.00 | KO:K04077 | chaperonin GroEL | Bacteria;Proteobacteria;Deltaproteobacteria;Syntrophorhabdales;Syntrophorhabdaceae;Syntrophorhabdus;Syntrophorhabdus aromaticivorans;Syntrophorhabdus aromaticivorans UI |
| Ga0586324 0307572 3 791 | 0.00 | 0.00 | 0.00 | 0.00 | 15038000.00 | 10361700.00 | 0.00 | 0.00 | 0.00 | 0.00 | 3819200.00 | 6067500.00 | KO:K04077 | chaperonin GroEL | Bacteria;Proteobacteria;Deltaproteobacteria;Desulfuromonadales;Geobacteraceae;Geobacter;Geobacter pickeringii;Geobacter pickeringii G13 |
| Ga0586324 0308522 3 1625 | 0.00 | 0.00 | 0.00 | 0.00 | 0.00 | 0.00 | 0.00 | 0.00 | 1539000.00 | 121194.92 | 5359200.00 | 0.00 | KO:K04077 | chaperonin GroEL | Bacteria;Proteobacteria;Deltaproteobacteria;Myxococcales;Anaeromyxobacteraceae;Anaeromyxobacter;Anaeromyxobacter sp. Fw109-5;Anaeromyxobacter sp. Fw109-5 |
| Ga0586324 0309156 416 2026 | 0.00 | 0.00 | 0.00 | 0.00 | 0.00 | 0.00 | 0.00 | 106475.50 | 0.00 | 0.00 | 0.00 | 0.00 | KO:K04077 | chaperonin GroEL | Bacteria;Chloroflexi;Anaerolineae;Anaerolineales;Anaerolineaceae;Anaerolinea;Anaerolinea thermophila;Anaerolinea thermophila UNI-1 |
| Ga0586324 0319856 1711 1998 | 0.00 | 0.00 | 0.00 | 0.00 | 0.00 | 0.00 | 0.00 | 0.00 | 9477000.00 | 0.00 | 0.00 | 0.00 | KO:K04077 | chaperonin GroEL | Bacteria;Chloroflexi;Ardenticatenia;Ardenticatenales;Ardenticatenaceae;Candidatus Promineofilum;Candidatus Promineofilum breve;Candidatus Promineofilum breve Cfx-K |
| Ga0586324 0328660 1217 1972 | 0.00 | 0.00 | 0.00 | 0.00 | 0.00 | 0.00 | 0.00 | 1085800.00 | 0.00 | 0.00 | 0.00 | 5820000.00 | KO:K04077 | chaperonin GroEL | Bacteria;Proteobacteria;Betaproteobacteria;Burkholderiales;unclassified Burkholderiales;unclassified Burkholderiales;Burkholderiales bacterium GJ-E10;Burkholderiales bacterium GJ-E10 |
| Ga0586324 0329337 1 852 | 0.00 | 1475600.00 | 77131000.00 | 0.00 | 0.00 | 0.00 | 21888000.00 | 0.00 | 12393000.00 | 12243000.00 | 17512000.00 | 0.00 | KO:K04077 | chaperonin GroEL | Bacteria;Verrucomicrobia;Verrucomicrobiae;Verrucomicrobiales;Verrucomicrobia subdivision 3;Pedosphaera;Pedosphaera parvula;Pedosphaera parvula Ellin514 |
| Ga0586324 0336642 267 1901 | 0.00 | 0.00 | 0.00 | 26980000.00 | 0.00 | 0.00 | 0.00 | 0.00 | 0.00 | 0.00 | 0.00 | 0.00 | KO:K04077 | chaperonin GroEL | Bacteria;Proteobacteria;Alphaproteobacteria;Hyphomicrobiales;Xanthobacteraceae;Pseudolabrys;Pseudolabrys taiwanensis;Pseudolabrys taiwanensis CC-BB4 |
| Ga0586324 0337707 137 1783 | 5280000.00 | 1774800.00 | 261832.16 | 2356000.00 | 0.00 | 0.00 | 0.00 | 2831882.91 | 510483.06 | 0.00 | 0.00 | 0.00 | KO:K04077 | chaperonin GroEL | Bacteria;Proteobacteria;Betaproteobacteria;Nitrosomonadales;Methylophilaceae;unclassified Methylophilaceae;unclassified Methylophilaceae;Betaproteobacterium LSUCC0135 |
| Ga0586324 0337906 1 852 | 0.00 | 0.00 | 0.00 | 0.00 | 0.00 | 0.00 | 0.00 | 1390800.00 | 0.00 | 0.00 | 9240000.00 | 2092500.00 | KO:K04077 | chaperonin GroEL | Bacteria;Acidobacteria;Acidobacteriia;Acidobacteriales;Acidobacteriaceae;Candidatus Koribacter;Candidatus Koribacter versatilis;Candidatus Koribacter versatilis Ellin345 |
| Ga0586324 0338879 627 1946 | 0.00 | 0.00 | 0.00 | 0.00 | 0.00 | 0.00 | 10602000.00 | 149858.09 | 3904200.00 | 1116500.00 | 4400000.00 | 1927500.00 | KO:K04077 | chaperonin GroEL | Bacteria;Gemmatimonadetes;Gemmatimonadetes;Gemmatimonadales;Gemmatimonadaceae;Gemmatimonas;Gemmatimonas phototrophica;Gemmatimonas phototrophica AP64 |
| Ga0586324 0340705 160 1815 | 0.00 | 0.00 | 0.00 | 0.00 | 7601400.00 | 0.00 | 1451001.12 | 23234900.00 | 2162700.00 | 1062600.00 | 5112800.00 | 1207500.00 | KO:K04077 | chaperonin GroEL | Bacteria;Proteobacteria;Betaproteobacteria;unclassified Betaproteobacteria;unclassified Betaproteobacteria;unclassified Betaproteobacteria;Betaproteobacteria bacterium GR16-43;Betaproteobacteria bacterium GR16-43 |
| Ga0586324 0341213 230 1861 | 0.00 | 152651.16 | 0.00 | 186200000.00 | 0.00 | 0.00 | 0.00 | 573872.14 | 122310000.00 | 0.00 | 8157600.00 | 7950000.00 | KO:K04077 | chaperonin GroEL | Bacteria;Calditrichaeota;Calditrichae;Calditrichales;Calditrichaceae;Caldithrix;Caldithrix abyssi;Caldithrix abyssi LF13, DSM 13497 |
| Ga0586324 0342624 1 1392 | 4050000.00 | 0.00 | 0.00 | 17423000.00 | 0.00 | 0.00 | 0.00 | 0.00 | 0.00 | 0.00 | 0.00 | 610888.50 | KO:K04077 | chaperonin GroEL | Bacteria;Proteobacteria;Deltaproteobacteria;Myxococcales;Vulgatibacteraceae;Vulgatibacter;Vulgatibacter incomptus;Vulgatibacter incomptus DSM 27710 |
| Ga0586324 0342965 1 1509 | 0.00 | 0.00 | 0.00 | 438310.05 | 0.00 | 0.00 | 0.00 | 0.00 | 0.00 | 0.00 | 0.00 | 0.00 | KO:K04077 | chaperonin GroEL | Bacteria;Actinobacteria;Actinomycetia;Frankiales;unclassified Frankiales;unclassified Frankiales;Frankineae bacterium MT45;Frankineae sp. MT45 |
| Ga0586324 0346422 3 1283 | 0.00 | 0.00 | 0.00 | 0.00 | 0.00 | 0.00 | 0.00 | 933300.00 | 1806300.00 | 1763300.00 | 0.00 | 0.00 | KO:K04077 | chaperonin GroEL | Bacteria;Proteobacteria;Deltaproteobacteria;Myxococcales;Anaeromyxobacteraceae;Anaeromyxobacter;Anaeromyxobacter dehalogenans;Anaeromyxobacter dehalogenans 2CP-C |
| Ga0586324 0347510 2 1060 | 0.00 | 0.00 | 0.00 | 0.00 | 0.00 | 0.00 | 0.00 | 361388.40 | 0.00 | 3957800.00 | 0.00 | 0.00 | KO:K04077 | chaperonin GroEL | Bacteria;Acidobacteria;Blastocatellia;Blastocatellales;Pyrinomonadaceae;Pyrinomonas;Pyrinomonas methylaliphatogenes;Pyrinomonas methylaliphatogenes K22 |
| Ga0586324 0347958 166 1800 | 0.00 | 1006400.00 | 0.00 | 0.00 | 0.00 | 0.00 | 0.00 | 634400.00 | 0.00 | 468413.33 | 0.00 | 0.00 | KO:K04077 | chaperonin GroEL | Bacteria;Verrucomicrobia;Methylacidiphilae;Methylacidiphilales;Methylacidiphilaceae;Methylacidiphilum;Methylacidiphilum kamchatkense;Methylacidiphilum kamchatkense Kam1 |
| Ga0586324 0348744 2 1600 | 5370000.00 | 656669.20 | 36876500.00 | 6517000.00 | 7498400.00 | 7536139.84 | 40128000.00 | 0.00 | 6439500.00 | 4342800.00 | 0.00 | 6315000.00 | KO:K04077 | chaperonin GroEL | Bacteria;Proteobacteria;Deltaproteobacteria;Syntrophorhabdales;Syntrophorhabdaceae;Syntrophorhabdus;Syntrophorhabdus aromaticivorans;Syntrophorhabdus aromaticivorans UI |
| Ga0586324 0350606 3 1484 | 13767478.00 | 22459076.72 | 834990.93 | 3553000.00 | 3203300.00 | 5994700.00 | 0.00 | 19276000.00 | 2026683.18 | 17463600.00 | 12311914.56 | 14025000.00 | KO:K04077 | chaperonin GroEL | Bacteria;Proteobacteria;Deltaproteobacteria;Desulfobacterales;Desulfobacteraceae;Desulfatitalea;Desulfatitalea tepidiphila;Desulfatitalea tepidiphila S28bF |
| Ga0586324 0351186 114 1766 | 0.00 | 0.00 | 2043740.67 | 879629.70 | 0.00 | 0.00 | 1138907.88 | 683200.00 | 0.00 | 434125.23 | 1091200.00 | 409173.75 | KO:K04077 | chaperonin GroEL | Bacteria;Planctomycetes;Planctomycetia;Isosphaerales;Isosphaeraceae;Paludisphaera;Paludisphaera borealis;Paludisphaera borealis PX4 |
| Ga0586324 0352498 775 1914 | 9400000.00 | 0.00 | 0.00 | 0.00 | 299680.56 | 0.00 | 0.00 | 0.00 | 0.00 | 2625700.00 | 0.00 | 0.00 | KO:K04077 | chaperonin GroEL | Bacteria;Proteobacteria;Deltaproteobacteria;Myxococcales;Polyangiaceae;Chondromyces;Chondromyces apiculatus;Chondromyces apiculatus DSM 436 |
| Ga0586324 0353656 1 855 | 0.00 | 0.00 | 0.00 | 0.00 | 0.00 | 0.00 | 17578800.00 | 0.00 | 2413800.00 | 0.00 | 0.00 | 6285000.00 | KO:K04077 | chaperonin GroEL | Bacteria;Chloroflexi;Anaerolineae;Anaerolineales;Anaerolineaceae;Anaerolinea;Anaerolinea thermophila;Anaerolinea thermophila UNI-1 |
| Ga0586324 0355604 459 1904 | 262770.00 | 0.00 | 0.00 | 0.00 | 0.00 | 0.00 | 0.00 | 5514400.00 | 0.00 | 0.00 | 5632000.00 | 0.00 | KO:K04077 | chaperonin GroEL | Bacteria;Acidobacteria;Blastocatellia;Blastocatellales;Pyrinomonadaceae;Pyrinomonas;Pyrinomonas methylaliphatogenes;Pyrinomonas methylaliphatogenes K22 |
| Ga0586324 0355713 300 1904 | 0.00 | 0.00 | 0.00 | 0.00 | 0.00 | 0.00 | 0.00 | 2647400.00 | 0.00 | 2302300.00 | 369908.00 | 0.00 | KO:K04077 | chaperonin GroEL | Bacteria;Proteobacteria;Gammaproteobacteria;Oceanospirillales;Hahellaceae;Hahella;Hahella sp. KA22;Hahella sp. KA22 |
| Ga0586324 0364283 323 1885 | 1910000.00 | 0.00 | 3797975.48 | 2802500.00 | 0.00 | 542290.09 | 0.00 | 915000.00 | 826200.00 | 0.00 | 1257857.92 | 1072710.75 | KO:K04077 | chaperonin GroEL | Bacteria;Candidatus Aminicenantes;unclassified Candidatus Aminicenantes;unclassified Candidatus Aminicenantes;unclassified Candidatus Aminicenantes;unclassified Candidatus Aminicenantes;Aminicenantes bacterium SCGC AAA252-K07;Aminicenantes bacterium SCGC AAA252-K07 (contamination screened) |
| Ga0586324 0372390 685 1866 | 0.00 | 0.00 | 0.00 | 0.00 | 0.00 | 0.00 | 0.00 | 0.00 | 0.00 | 0.00 | 0.00 | 1012500.00 | KO:K04077 | chaperonin GroEL | Bacteria;Proteobacteria;Betaproteobacteria;Nitrosomonadales;Thiobacillaceae;Annwoodia;Annwoodia aquaesulis;Annwoodia aquaesulis DSM 4255 |
| Ga0586324 0378516 264 1853 | 0.00 | 0.00 | 0.00 | 2280000.00 | 0.00 | 0.00 | 13087200.00 | 194607.08 | 1466100.00 | 0.00 | 7333156.16 | 7998274.50 | KO:K04077 | chaperonin GroEL | Bacteria;Proteobacteria;Deltaproteobacteria;Myxococcales;Sandaracinaceae;Sandaracinus;Sandaracinus amylolyticus;Sandaracinus amylolyticus DSM 53668 |
| Ga0586324 0385463 116 1735 | 0.00 | 0.00 | 0.00 | 0.00 | 0.00 | 0.00 | 0.00 | 3269600.00 | 0.00 | 0.00 | 0.00 | 0.00 | KO:K04077 | chaperonin GroEL | Bacteria;Planctomycetes;Planctomycetia;Pirellulales;Pirellulaceae;Rhodopirellula;Rhodopirellula maiorica;Rhodopirellula maiorica SM1 |
| Ga0586324 0395284 836 1819 | 0.00 | 0.00 | 0.00 | 0.00 | 0.00 | 0.00 | 0.00 | 412419.17 | 0.00 | 0.00 | 0.00 | 0.00 | KO:K04077 | chaperonin GroEL | Bacteria;Proteobacteria;Deltaproteobacteria;Syntrophorhabdales;Syntrophorhabdaceae;Syntrophorhabdus;Syntrophorhabdus aromaticivorans;Syntrophorhabdus aromaticivorans UI |
| Ga0586324 0400484 64 1716 | 1100000.00 | 5105353.64 | 2833407.29 | 8825098.15 | 12658471.34 | 1610756.83 | 16223415.24 | 18284632.88 | 5514218.37 | 13531582.68 | 4363324.24 | 13917145.50 | KO:K04077 | chaperonin GroEL | Bacteria;Proteobacteria;Betaproteobacteria;Burkholderiales;unclassified Burkholderiales;Xylophilus;Xylophilus ampelinus;Xylophilus ampelinus CECT 7646 |
| Ga0586324 0404562 727 1800 | 46600000.00 | 9248000.00 | 153136000.00 | 9595000.00 | 42436000.00 | 0.00 | 140028413.88 | 79300000.00 | 48276000.00 | 64834000.00 | 11704000.00 | 69750000.00 | KO:K04077 | chaperonin GroEL | Bacteria;Thermodesulfobacteria;Thermodesulfobacteria;Thermodesulfobacteriales;Thermodesulfobacteriaceae;Thermodesulfobacterium;Thermodesulfobacterium commune;Thermodesulfobacterium commune DSM 2178 |
| Ga0586324 0407587 2 1036 | 0.00 | 4862000.00 | 4501297.60 | 14630000.00 | 14770200.00 | 17031300.00 | 0.00 | 4672600.00 | 6990300.00 | 6822200.00 | 10560000.00 | 0.00 | KO:K04077 | chaperonin GroEL | Bacteria;Proteobacteria;Deltaproteobacteria;Desulfobacterales;Desulfobacteraceae;Desulfococcus;Desulfococcus multivorans;Desulfococcus multivorans DSM 2059 |
| Ga0586324 0412002 663 1784 | 0.00 | 1407600.00 | 0.00 | 445119.65 | 77662000.00 | 117954.66 | 1114017.12 | 787872.34 | 2349920.97 | 0.00 | 0.00 | 0.00 | KO:K04077 | chaperonin GroEL | Bacteria;Verrucomicrobia;Opitutae;Opitutales;Opitutaceae;unclassified Opitutaceae;Opitutaceae bacterium EW11;Opitutaceae bacterium EW11 |
| Ga0586324 0416418 447 1778 | 0.00 | 666720.96 | 0.00 | 1463000.00 | 0.00 | 0.00 | 1327238.16 | 475434.00 | 1182600.00 | 0.00 | 0.00 | 0.00 | KO:K04077 | chaperonin GroEL | Bacteria;Proteobacteria;Alphaproteobacteria;Hyphomicrobiales;Hyphomicrobiaceae;Hyphomicrobium;Hyphomicrobium sp. NDB2Meth4;Hyphomicrobium sp. ERS1433775 |
| Ga0586324 0424165 566 1762 | 0.00 | 0.00 | 0.00 | 7913500.00 | 10815000.00 | 21438000.00 | 15891600.00 | 8662000.00 | 16038000.00 | 0.00 | 0.00 | 0.00 | KO:K04077 | chaperonin GroEL | Bacteria;Proteobacteria;Deltaproteobacteria;Syntrophobacterales;Syntrophaceae;Desulfobacca;Desulfobacca acetoxidans;Desulfobacca acetoxidans ASRB2, DSM 11109 |
| Ga0586324 0425704 844 1758 | 0.00 | 6208400.00 | 0.00 | 0.00 | 7951600.00 | 0.00 | 0.00 | 8174000.00 | 0.00 | 14784000.00 | 1188000.00 | 0.00 | KO:K04077 | chaperonin GroEL | Bacteria;Acidobacteria;Acidobacteriia;Acidobacteriales;Acidobacteriaceae;Candidatus Koribacter;Candidatus Koribacter versatilis;Candidatus Koribacter versatilis Ellin345 |
| Ga0586324 0426053 57 1685 | 0.00 | 5616800.00 | 3404753.76 | 0.00 | 0.00 | 0.00 | 0.00 | 0.00 | 0.00 | 0.00 | 1654400.00 | 0.00 | KO:K04077 | chaperonin GroEL | Bacteria;Acidobacteria;Acidobacteriia;Acidobacteriales;Acidobacteriaceae;Candidatus Koribacter;Candidatus Koribacter versatilis;Candidatus Koribacter versatilis Ellin345 |
| Ga0586324 0430003 1025 1750 | 0.00 | 641807.12 | 0.00 | 0.00 | 0.00 | 0.00 | 0.00 | 258162.37 | 0.00 | 0.00 | 0.00 | 0.00 | KO:K04077 | chaperonin GroEL | Bacteria;Proteobacteria;Deltaproteobacteria;Desulfobacterales;Desulfobacteraceae;Desulfatiglans;Desulfatiglans anilini;Desulfatiglans anilini DSM 4660 |
| Ga0586324 0432587 76 1707 | 0.00 | 0.00 | 0.00 | 1159000.00 | 1308100.00 | 5822267.02 | 0.00 | 53741000.00 | 94915800.00 | 0.00 | 0.00 | 0.00 | KO:K04077 | chaperonin GroEL | Bacteria;Actinobacteria;Thermoleophilia;Solirubrobacterales;Solirubrobacteraceae;Solirubrobacter;Solirubrobacter sp. URHD0082;Solirubrobacter sp. URHD0082 |
| Ga0586324 0438249 183 1736 | 660720.00 | 0.00 | 0.00 | 0.00 | 0.00 | 0.00 | 0.00 | 0.00 | 0.00 | 0.00 | 0.00 | 0.00 | KO:K04077 | chaperonin GroEL | Bacteria;Proteobacteria;Gammaproteobacteria;Nevskiales;Steroidobacteraceae;Povalibacter;Povalibacter uvarum;Povalibacter uvarum DSM 26723 |
| Ga0586324 0439190 3 569 | 260932.00 | 0.00 | 0.00 | 0.00 | 0.00 | 0.00 | 0.00 | 957700.00 | 1935900.00 | 1386000.00 | 0.00 | 0.00 | KO:K04077 | chaperonin GroEL | Bacteria;Verrucomicrobia;Verrucomicrobiae;Verrucomicrobiales;Verrucomicrobia subdivision 3;Pedosphaera;Pedosphaera parvula;Pedosphaera parvula Ellin514 |
| Ga0586324 0439964 393 1733 | 3260000.00 | 0.00 | 0.00 | 0.00 | 0.00 | 1694002.97 | 0.00 | 0.00 | 3321000.00 | 0.00 | 0.00 | 847500.00 | KO:K04077 | chaperonin GroEL | Bacteria;Proteobacteria;Deltaproteobacteria;Desulfobacterales;Desulfobacteraceae;Desulfatiglans;Desulfatiglans anilini;Desulfatiglans anilini DSM 4660 |
| Ga0586324 0441680 54 1718 | 65500000.00 | 0.00 | 0.00 | 25170102.75 | 0.00 | 47399440.23 | 1345190.88 | 16755445.84 | 14232103.38 | 562286.34 | 0.00 | 67441.88 | KO:K04077 | chaperonin GroEL | Bacteria;Proteobacteria;Deltaproteobacteria;Syntrophobacterales;Syntrophobacteraceae;Syntrophobacter;Syntrophobacter fumaroxidans;Syntrophobacter fumaroxidans MPOB |
| Ga0586324 0446195 2 1081 | 0.00 | 0.00 | 0.00 | 576570.20 | 0.00 | 0.00 | 0.00 | 89469.31 | 0.00 | 0.00 | 0.00 | 0.00 | KO:K04077 | chaperonin GroEL | Bacteria;Acidobacteria;Thermoanaerobaculia;Thermoanaerobaculales;Thermoanaerobaculaceae;Thermoanaerobaculum;Thermoanaerobaculum aquaticum;Thermoanaerobaculum aquaticum MP-01 |
| Ga0586324 0452459 1280 1711 | 0.00 | 0.00 | 0.00 | 0.00 | 0.00 | 0.00 | 0.00 | 0.00 | 5678100.00 | 0.00 | 0.00 | 0.00 | KO:K04077 | chaperonin GroEL | Bacteria;Proteobacteria;Gammaproteobacteria;Chromatiales;Chromatiaceae;Thiodictyon;Candidatus Thiodictyon syntrophicum;Candidatus Thiodictyon syntrophicum Cad16 |
| Ga0586324 0459293 3 1532 | 349790.00 | 0.00 | 1249809.33 | 1355002.10 | 439098.27 | 610887.72 | 0.00 | 0.00 | 0.00 | 2502500.00 | 0.00 | 0.00 | KO:K04077 | chaperonin GroEL | Bacteria;Proteobacteria;Betaproteobacteria;Nitrosomonadales;Thiobacillaceae;Thiobacillus;Thiobacillus denitrificans;Thiobacillus denitrificans RG |
| Ga0586324 0459484 765 1700 | 1430000.00 | 965600.00 | 0.00 | 1434500.00 | 1287500.00 | 10719000.00 | 0.00 | 3592900.00 | 10287000.00 | 9533233.71 | 48752000.00 | 23640000.00 | KO:K04077 | chaperonin GroEL | Bacteria;Proteobacteria;Deltaproteobacteria;Desulfuromonadales;Geobacteraceae;Geobacter;Geobacter sp. OR-1;Geobacter sp. OR-1 |
| Ga0586324 0459569 927 1700 | 0.00 | 673429.84 | 0.00 | 0.00 | 0.00 | 0.00 | 0.00 | 0.00 | 169183.08 | 1655500.00 | 976800.00 | 0.00 | KO:K04077 | chaperonin GroEL | Bacteria;Planctomycetes;Planctomycetia;Pirellulales;Pirellulaceae;Pirellula;Pirellula sp. SH-Sr6A;Pirellula sp. SH-Sr6A |
| Ga0586324 0464055 1 1659 | 0.00 | 0.00 | 929186.46 | 0.00 | 0.00 | 0.00 | 6087600.00 | 3702500.53 | 51309681.66 | 67452000.00 | 16720000.00 | 2137500.00 | KO:K04077 | chaperonin GroEL | Bacteria;Proteobacteria;Deltaproteobacteria;Desulfobacterales;Desulfobacteraceae;Desulfatiglans;Desulfatiglans anilini;Desulfatiglans anilini DSM 4660 |
| Ga0586324 0466728 2 1603 | 0.00 | 3277600.00 | 0.00 | 380456.00 | 366269.03 | 0.00 | 0.00 | 0.00 | 289488.33 | 241613.68 | 358555.12 | 4287651.75 | KO:K04077 | chaperonin GroEL | Bacteria;Candidatus Omnitrophica;unclassified Candidatus Omnitrophica;unclassified Candidatus Omnitrophica;unclassified Candidatus Omnitrophica;unclassified Candidatus Omnitrophica;Omnitrophica bacterium SCGC AAA011-L16;Omnitrophica bacterium SCGC AAA011-L16 (contamination screened) |
| Ga0586324 0467318 1 759 | 0.00 | 0.00 | 0.00 | 0.00 | 1013801.19 | 0.00 | 1458527.40 | 5575400.00 | 0.00 | 0.00 | 0.00 | 0.00 | KO:K04077 | chaperonin GroEL | Bacteria;Chloroflexi;Anaerolineae;Anaerolineales;Anaerolineaceae;Anaerolinea;Anaerolinea thermophila;Anaerolinea thermophila UNI-1 |
| Ga0586324 0469785 1 684 | 0.00 | 0.00 | 0.00 | 0.00 | 0.00 | 0.00 | 0.00 | 2738900.00 | 0.00 | 0.00 | 0.00 | 0.00 | KO:K04077 | chaperonin GroEL | Bacteria;Proteobacteria;Deltaproteobacteria;Syntrophobacterales;Syntrophaceae;Desulfobacca;Desulfobacca acetoxidans;Desulfobacca acetoxidans ASRB2, DSM 11109 |
| Ga0586324 0471233 708 1682 | 0.00 | 0.00 | 0.00 | 1311000.00 | 0.00 | 0.00 | 0.00 | 0.00 | 0.00 | 0.00 | 0.00 | 0.00 | KO:K04077 | chaperonin GroEL | Bacteria;Proteobacteria;Deltaproteobacteria;Syntrophobacterales;Syntrophaceae;Desulfobacca;Desulfobacca acetoxidans;Desulfobacca acetoxidans ASRB2, DSM 11109 |
| Ga0586324 0475701 30 1676 | 0.00 | 0.00 | 0.00 | 0.00 | 0.00 | 5915300.00 | 287663.04 | 11285000.00 | 261079.20 | 0.00 | 0.00 | 0.00 | KO:K04077 | chaperonin GroEL | Bacteria;Verrucomicrobia;Spartobacteria;unclassified Spartobacteria;unclassified Spartobacteria;unclassified Spartobacteria;Spartobacteria bacterium LR76;Spartobacteria bacterium LR76 |
| Ga0586324 0478369 477 1670 | 0.00 | 0.00 | 0.00 | 0.00 | 0.00 | 0.00 | 0.00 | 0.00 | 0.00 | 375301.85 | 0.00 | 0.00 | KO:K04077 | chaperonin GroEL | Bacteria;Actinobacteria;Acidimicrobiia;Acidimicrobiales;Ilumatobacteraceae;Desertimonas;Desertimonas flava;Acidimicrobiales bacterium SYSU D60003 |
| Ga0586324 0493473 1 1467 | 305430.00 | 433055.96 | 0.00 | 0.00 | 0.00 | 0.00 | 0.00 | 692520.80 | 0.00 | 785400.00 | 0.00 | 840000.00 | KO:K04077 | chaperonin GroEL | Bacteria;Proteobacteria;Deltaproteobacteria;Myxococcales;Anaeromyxobacteraceae;Anaeromyxobacter;Anaeromyxobacter sp. Fw109-5;Anaeromyxobacter sp. Fw109-5 |
| Ga0586324 0500874 3 1475 | 0.00 | 0.00 | 0.00 | 0.00 | 0.00 | 0.00 | 0.00 | 2842600.00 | 0.00 | 0.00 | 0.00 | 0.00 | KO:K04077 | chaperonin GroEL | Bacteria;Nitrospirae;Nitrospira;Nitrospirales;Nitrospiraceae;Nitrospira;Nitrospira lenta;Nitrospira lenta N. lenta BS10 |
| Ga0586324 0503240 3 806 | 0.00 | 3461200.00 | 0.00 | 0.00 | 537834.07 | 0.00 | 12631200.00 | 0.00 | 9954900.00 | 9779000.00 | 0.00 | 0.00 | KO:K04077 | chaperonin GroEL | Bacteria;Proteobacteria;Gammaproteobacteria;Acidiferrobacterales;Acidiferrobacteraceae;Sulfuricaulis;Sulfuricaulis limicola;Sulfuricaulis limicola HA5 |
| Ga0586324 0506188 796 1629 | 0.00 | 419257.40 | 0.00 | 0.00 | 566188.94 | 0.00 | 905570.40 | 780800.00 | 915300.00 | 1431346.07 | 1381600.00 | 1192500.00 | KO:K04077 | chaperonin GroEL | Bacteria;Proteobacteria;Betaproteobacteria;Rhodocyclales;Zoogloeaceae;Thauera;Thauera selenatis;Thauera selenatis AX, ATCC 55363 |
| Ga0586324 0507848 479 1627 | 1170000.00 | 3318400.00 | 0.00 | 4284500.00 | 1730400.00 | 1130004.92 | 5147284.68 | 622200.00 | 3086100.00 | 7761600.00 | 4206400.00 | 2604849.75 | KO:K04077 | chaperonin GroEL | Bacteria;Proteobacteria;Alphaproteobacteria;Hyphomicrobiales;Hyphomicrobiaceae;Hyphomicrobium;Hyphomicrobium sp. NDB2Meth4;Hyphomicrobium sp. ERS1433775 |
| Ga0586324 0508048 718 1626 | 0.00 | 0.00 | 0.00 | 0.00 | 272798.59 | 224545.58 | 0.00 | 0.00 | 0.00 | 0.00 | 0.00 | 355897.50 | KO:K04077 | chaperonin GroEL | Bacteria;Proteobacteria;Deltaproteobacteria;Syntrophobacterales;Syntrophaceae;Syntrophus;Syntrophus aciditrophicus;Syntrophus aciditrophicus SB |
| Ga0586324 0511925 462 1622 | 0.00 | 6194800.00 | 0.00 | 0.00 | 0.00 | 0.00 | 0.00 | 0.00 | 0.00 | 0.00 | 3872000.00 | 8850000.00 | KO:K04077 | chaperonin GroEL | Bacteria;Proteobacteria;Alphaproteobacteria;Hyphomicrobiales;Methylocystaceae;Methylocystis;Methylocystis sp. ATCC 49242;Methylocystis sp. Rockwell, ATCC 49242 |
| Ga0586324 0512096 3 485 | 0.00 | 0.00 | 0.00 | 0.00 | 844205.51 | 0.00 | 2202163.08 | 1226100.00 | 798459.12 | 1285900.00 | 1135200.00 | 397914.75 | KO:K04077 | chaperonin GroEL | Bacteria;Proteobacteria;Deltaproteobacteria;Syntrophobacterales;Syntrophaceae;Desulfobacca;Desulfobacca acetoxidans;Desulfobacca acetoxidans ASRB2, DSM 11109 |
| Ga0586324 0512868 1158 1622 | 0.00 | 0.00 | 0.00 | 3325000.00 | 0.00 | 0.00 | 0.00 | 0.00 | 0.00 | 0.00 | 0.00 | 0.00 | KO:K04077 | chaperonin GroEL | Bacteria;Nitrospirae;Nitrospira;Nitrospirales;Nitrospiraceae;Nitrospira;Nitrospira moscoviensis;Nitrospira moscoviensis NSP M-1 |
| Ga0586324 0514331 822 1619 | 5840000.00 | 4209200.00 | 3218209.34 | 6070500.00 | 5737100.00 | 3098291.22 | 0.00 | 7381000.00 | 21384000.00 | 10549000.00 | 11176000.00 | 9375000.00 | KO:K04077 | chaperonin GroEL | Bacteria;Proteobacteria;Betaproteobacteria;Burkholderiales;Comamonadaceae;Ramlibacter;Ramlibacter tataouinensis;Ramlibacter tataouinensis 5-10 |
| Ga0586324 0514337 3 1016 | 0.00 | 0.00 | 0.00 | 0.00 | 4552600.00 | 0.00 | 835221.00 | 0.00 | 8343000.00 | 2348500.00 | 2156000.00 | 0.00 | KO:K04077 | chaperonin GroEL | Bacteria;Bacteroidetes;Bacteroidia;Marinilabiliales;Prolixibacteraceae;Draconibacterium;Draconibacterium sediminis;Draconibacterium sediminis JN14CK-3 |
| Ga0586324 0522750 1 966 | 0.00 | 0.00 | 0.00 | 0.00 | 0.00 | 0.00 | 0.00 | 0.00 | 1690288.56 | 0.00 | 0.00 | 0.00 | KO:K04077 | chaperonin GroEL | Bacteria;Chloroflexi;Chloroflexia;Chloroflexales;Roseiflexaceae;Roseiflexus;Roseiflexus sp. RS-1;Roseiflexus sp. RS-1 |
| Ga0586324 0523664 1 1482 | 0.00 | 0.00 | 0.00 | 11590000.00 | 0.00 | 0.00 | 0.00 | 0.00 | 0.00 | 0.00 | 0.00 | 0.00 | KO:K04077 | chaperonin GroEL | Bacteria;Proteobacteria;Alphaproteobacteria;Hyphomicrobiales;Xanthobacteraceae;Pseudolabrys;Pseudolabrys taiwanensis;Pseudolabrys taiwanensis CC-BB4 |
| Ga0586324 0532102 2 1594 | 0.00 | 0.00 | 0.00 | 855390.45 | 0.00 | 0.00 | 0.00 | 0.00 | 0.00 | 0.00 | 310649.68 | 0.00 | KO:K04077 | chaperonin GroEL | Bacteria;Proteobacteria;Betaproteobacteria;Nitrosomonadales;Nitrosomonadaceae;Nitrosovibrio;Nitrosovibrio tenuis;Nitrosovibrio tenuis Nv1 |
| Ga0586324 0535799 1 1230 | 0.00 | 249503.56 | 0.00 | 0.00 | 0.00 | 0.00 | 0.00 | 0.00 | 0.00 | 1001000.00 | 0.00 | 570230.25 | KO:K04077 | chaperonin GroEL | Bacteria;Verrucomicrobia;Verrucomicrobiae;Verrucomicrobiales;Verrucomicrobia subdivision 3;Pedosphaera;Pedosphaera parvula;Pedosphaera parvula Ellin514 |
| Ga0586324 0538230 80 1588 | 908602.00 | 0.00 | 1141651.40 | 0.00 | 0.00 | 0.00 | 630374.40 | 0.00 | 468472.41 | 0.00 | 0.00 | 649242.75 | KO:K04077 | chaperonin GroEL | Bacteria;Bacteroidetes;Bacteroidia;Marinilabiliales;Prolixibacteraceae;Draconibacterium;Draconibacterium sediminis;Draconibacterium sediminis JN14CK-3 |
| Ga0586324 0539513 888 1586 | 0.00 | 1686400.00 | 0.00 | 0.00 | 0.00 | 0.00 | 0.00 | 0.00 | 0.00 | 0.00 | 0.00 | 276556.50 | KO:K04077 | chaperonin GroEL | Bacteria;Proteobacteria;Deltaproteobacteria;Myxococcales;Sandaracinaceae;Sandaracinus;Sandaracinus amylolyticus;Sandaracinus amylolyticus DSM 53668 |
| Ga0586324 0545029 1 597 | 0.00 | 0.00 | 0.00 | 0.00 | 0.00 | 0.00 | 0.00 | 44678.72 | 88723.35 | 6745200.00 | 0.00 | 0.00 | KO:K04077 | chaperonin GroEL | Bacteria;Proteobacteria;Deltaproteobacteria;Desulfovibrionales;Desulfovibrionaceae;Desulfocurvibacter;Desulfocurvibacter africanus;Desulfocurvibacter africanus Walvis Bay |
| Ga0586324 0548489 402 1574 | 0.00 | 0.00 | 0.00 | 0.00 | 0.00 | 0.00 | 0.00 | 0.00 | 0.00 | 0.00 | 0.00 | 4875000.00 | KO:K04077 | chaperonin GroEL | Bacteria;Proteobacteria;Deltaproteobacteria;Syntrophorhabdales;Syntrophorhabdaceae;Syntrophorhabdus;Syntrophorhabdus aromaticivorans;Syntrophorhabdus aromaticivorans UI |
| Ga0586324 0553330 217 1569 | 0.00 | 0.00 | 0.00 | 0.00 | 0.00 | 0.00 | 0.00 | 0.00 | 0.00 | 10780000.00 | 0.00 | 0.00 | KO:K04077 | chaperonin GroEL | Bacteria;Acidobacteria;Acidobacteriia;Acidobacteriales;Acidobacteriaceae;Candidatus Koribacter;Candidatus Koribacter versatilis;Candidatus Koribacter versatilis Ellin345 |
| Ga0586324 0553701 2 799 | 0.00 | 1142340.84 | 0.00 | 2926000.00 | 0.00 | 0.00 | 1076761.92 | 2940200.00 | 159056.46 | 0.00 | 244646.16 | 2175000.00 | KO:K04077 | chaperonin GroEL | Bacteria;Acidobacteria;Vicinamibacteria;unclassified Vicinamibacteria;Vicinamibacteraceae;Luteitalea;Luteitalea pratensis;Luteitalea pratensis DSM 100886 |
| Ga0586324 0556668 2 883 | 0.00 | 802400.00 | 0.00 | 0.00 | 0.00 | 0.00 | 0.00 | 0.00 | 0.00 | 525710.57 | 0.00 | 0.00 | KO:K04077 | chaperonin GroEL | Bacteria;Proteobacteria;Betaproteobacteria;Nitrosomonadales;Nitrosomonadaceae;Nitrosospira;Nitrosospira briensis;Nitrosospira briensis C-128 |
| Ga0586324 0558459 113 1561 | 0.00 | 0.00 | 0.00 | 0.00 | 2049700.00 | 0.00 | 0.00 | 272574.23 | 0.00 | 0.00 | 0.00 | 0.00 | KO:K04077 | chaperonin GroEL | Bacteria;Verrucomicrobia;Methylacidiphilae;Methylacidiphilales;Methylacidiphilaceae;Methylacidiphilum;Methylacidiphilum kamchatkense;Methylacidiphilum kamchatkense Kam1 |
| Ga0586324 0566822 1 513 | 0.00 | 13532000.00 | 0.00 | 1035500.00 | 44599000.00 | 0.00 | 501923.76 | 562410.24 | 0.00 | 22561000.00 | 0.00 | 23700000.00 | KO:K04077 | chaperonin GroEL | Bacteria;Verrucomicrobia;Spartobacteria;Chthoniobacterales;Chthoniobacteraceae;Chthoniobacter;Chthoniobacter flavus;Chthoniobacter flavus Ellin428 |
| Ga0586324 0569681 458 1549 | 16740000.00 | 3563200.00 | 0.00 | 11305000.00 | 7148200.00 | 1089554.59 | 5312400.00 | 23826600.00 | 0.00 | 4881800.00 | 11695200.00 | 2024838.00 | KO:K04077 | chaperonin GroEL | Bacteria;Proteobacteria;Betaproteobacteria;unclassified Betaproteobacteria;unclassified Betaproteobacteria;unclassified Betaproteobacteria;Betaproteobacteria bacterium UKL13-2;Betaproteobacteria bacterium UKL13-2 |
| Ga0586324 0570800 618 1547 | 0.00 | 0.00 | 0.00 | 0.00 | 0.00 | 0.00 | 0.00 | 0.00 | 0.00 | 0.00 | 2296800.00 | 0.00 | KO:K04077 | chaperonin GroEL | Bacteria;Acidobacteria;Acidobacteriia;Acidobacteriales;Acidobacteriaceae;unclassified Acidobacteriaceae;Acidobacteriaceae bacterium KBS 96;Acidobacteriaceae bacterium KBS 96, DSM 24297 |
| Ga0586324 0570850 3 602 | 4980000.00 | 1285200.00 | 2438673.91 | 1615000.00 | 0.00 | 7304800.00 | 0.00 | 0.00 | 0.00 | 0.00 | 0.00 | 0.00 | KO:K04077 | chaperonin GroEL | Bacteria;Chloroflexi;Anaerolineae;Anaerolineales;Anaerolineaceae;Anaerolinea;Anaerolinea thermophila;Anaerolinea thermophila UNI-1 |
| Ga0586324 0578463 1 1467 | 9140000.00 | 20196000.00 | 15062383.77 | 14297500.00 | 24493400.00 | 13656800.00 | 25106929.08 | 11022700.00 | 17941500.00 | 3257100.00 | 13020362.96 | 5061810.00 | KO:K04077 | chaperonin GroEL | Bacteria;Proteobacteria;Deltaproteobacteria;Syntrophobacterales;Syntrophaceae;Desulfomonile;Desulfomonile tiedjei;Desulfomonile tiedjei DCB-1, DSM 6799 |
| Ga0586324 0578694 3 1298 | 0.00 | 0.00 | 2409640.00 | 0.00 | 0.00 | 0.00 | 0.00 | 0.00 | 0.00 | 0.00 | 0.00 | 0.00 | KO:K04077 | chaperonin GroEL | Bacteria;Proteobacteria;Deltaproteobacteria;unclassified Deltaproteobacteria;unclassified Deltaproteobacteria;Dissulfuribacter;Dissulfuribacter thermophilus;Dissulfuribacter thermophilus S69 |
| Ga0586324 0584170 682 1530 | 0.00 | 0.00 | 0.00 | 0.00 | 0.00 | 0.00 | 2827200.00 | 0.00 | 0.00 | 0.00 | 0.00 | 0.00 | KO:K04077 | chaperonin GroEL | Bacteria;Chloroflexi;Caldilineae;Caldilineales;Caldilineaceae;Caldilinea;Caldilinea aerophila;Caldilinea aerophila STL-6-O1, DSM 14535 |
| Ga0586324 0590025 122 1525 | 7086307.00 | 8840000.00 | 4282769.15 | 6602500.00 | 10083700.00 | 1351391.97 | 1801626.36 | 1274900.00 | 1360800.00 | 1108800.00 | 0.00 | 1245000.00 | KO:K04077 | chaperonin GroEL | Bacteria;Candidatus Calescamantes;unclassified Candidatus Calescamantes;unclassified Candidatus Calescamantes;unclassified Candidatus Calescamantes;unclassified Candidatus Calescamantes;Calescamantes bacterium JGI 0000106-H18;Calescamantes bacterium JGI 0000106-H18 (contamination screened) |

| | | | | | | | | | | | | | | | |
|---|---|---|---|---|---|---|---|---|---|---|---|---|---|---|---|
| Ga0586324 0590606 2 1177 | 0.00 | 5827600.00 | 6136700.00 | 6317500.00 | 1545000.00 | 0.00 | 0.00 | 6466000.00 | 4827600.00 | 3880800.00 | 6644000.00 | 0.00 | KO:K04077 | chaperonin GroEL | Bacteria;Proteobacteria;Deltaproteobacteria;Syntrophobacterales;Syntrophaceae;Syntrophus;Syntrophus gentianae;Syntrophus gentianae DSM 8423 |
| Ga0586324 0590866 3 209 | 0.00 | 1455200.00 | 0.00 | 0.00 | 0.00 | 0.00 | 0.00 | 0.00 | 0.00 | 364746.69 | 0.00 | 0.00 | KO:K04077 | chaperonin GroEL | Bacteria;Proteobacteria;Deltaproteobacteria;Syntrophobacterales;Syntrophaceae;Syntrophus;Syntrophus gentianae;Syntrophus gentianae DSM 8423 |
| Ga0586324 0592122 1 480 | 13320000.00 | 3338800.00 | 5614331.71 | 4493500.00 | 4274500.00 | 0.00 | 0.00 | 0.00 | 7030800.00 | 5058900.00 | 0.00 | 5730000.00 | KO:K04077 | chaperonin GroEL | Bacteria;Proteobacteria;Alphaproteobacteria;Hyphomicrobiales;Methylocystaceae;Methylocystis;Methylocystis parvus;Methylocystis parvus OBBP |
| Ga0586324 0593990 3 1520 | 0.00 | 0.00 | 0.00 | 0.00 | 0.00 | 0.00 | 0.00 | 0.00 | 0.00 | 0.00 | 22176000.00 | 0.00 | KO:K04077 | chaperonin GroEL | Bacteria;Chloroflexi;unclassified Chloroflexi;unclassified Chloroflexi;unclassified Chloroflexi;unclassified Chloroflexi;Chloroflexi bacterium T81;Chloroflexi bacterium T81 |
| Ga0586324 0594172 1 1521 | 16900000.00 | 16728000.00 | 0.00 | 34295000.00 | 19055000.00 | 0.00 | 7090800.00 | 25010000.00 | 12186702.72 | 21406000.00 | 23144000.00 | 11325000.00 | KO:K04077 | chaperonin GroEL | Bacteria;Proteobacteria;Deltaproteobacteria;Syntrophorhabdales;Syntrophorhabdaceae;Syntrophorhabdus;Syntrophorhabdus aromaticivorans;Syntrophorhabdus aromaticivorans UI |
| Ga0586324 0594969 390 1520 | 0.00 | 433130.76 | 0.00 | 639070.70 | 0.00 | 0.00 | 838910.04 | 0.00 | 0.00 | 0.00 | 906400.00 | 723330.75 | KO:K04077 | chaperonin GroEL | Bacteria;Proteobacteria;Deltaproteobacteria;Myxococcales;Kofleriaceae;Haliangium;Haliangium ochraceum;Haliangium ochraceum SMP-2, DSM 14365 |
| Ga0586324 0595215 143 1519 | 0.00 | 0.00 | 0.00 | 0.00 | 336634.90 | 0.00 | 0.00 | 768600.00 | 7727400.00 | 17248000.00 | 665041.52 | 14325000.00 | KO:K04077 | chaperonin GroEL | Bacteria;Nitrospirae;Nitrospira;Nitrospirales;Nitrospiraceae;Nitrospira;Nitrospira marina;Nitrospira marina Nb-295 |
| Ga0586324 0595394 42 1520 | 0.00 | 0.00 | 0.00 | 0.00 | 0.00 | 0.00 | 0.00 | 0.00 | 0.00 | 4373600.00 | 0.00 | 0.00 | KO:K04077 | chaperonin GroEL | Bacteria;Proteobacteria;Deltaproteobacteria;Myxococcales;Anaeromyxobacteraceae;Anaeromyxobacter;Anaeromyxobacter dehalogenans;Anaeromyxobacter dehalogenans 2CP-C |
| Ga0586324 0599560 376 1515 | 3450000.00 | 22678000.00 | 744618.17 | 0.00 | 389971.39 | 0.00 | 9963600.00 | 2257000.00 | 7371687.69 | 17871700.00 | 14064417.84 | 13389162.00 | KO:K04077 | chaperonin GroEL | Bacteria;Acidobacteria;Acidobacteriia;Acidobacteriales;Acidobacteriaceae;Candidatus Koribacter;Candidatus Koribacter versatilis;Candidatus Koribacter versatilis Ellin345 |
| Ga0586324 0600642 3 1292 | 898158.00 | 0.00 | 0.00 | 30020000.00 | 0.00 | 0.00 | 480391.44 | 0.00 | 3442500.00 | 3349500.00 | 3212000.00 | 3607500.00 | KO:K04077 | chaperonin GroEL | Bacteria;Chloroflexi;Caldilineae;Caldilineales;Caldilineaceae;Caldilinea;Caldilinea aerophila;Caldilinea aerophila STL-6-O1, DSM 14535 |
| Ga0586324 0608322 391 1506 | 0.00 | 0.00 | 0.00 | 0.00 | 456917.27 | 0.00 | 0.00 | 0.00 | 0.00 | 793100.00 | 0.00 | 0.00 | KO:K04077 | chaperonin GroEL | Bacteria;Proteobacteria;Deltaproteobacteria;unclassified Deltaproteobacteria;unclassified Deltaproteobacteria;Dissulfuribacter;Dissulfuribacter thermophilus;Dissulfuribacter thermophilus S69 |
| Ga0586324 0608632 526 1506 | 0.00 | 829600.00 | 0.00 | 0.00 | 0.00 | 0.00 | 7455600.00 | 0.00 | 3183300.00 | 7854000.00 | 6661600.00 | 0.00 | KO:K04077 | chaperonin GroEL | Bacteria;Proteobacteria;Deltaproteobacteria;Desulfobacterales;Desulfobacteraceae;Desulfatiglans;Desulfatiglans anilini;Desulfatiglans anilini DSM 4660 |
| Ga0586324 0611607 1 825 | 0.00 | 0.00 | 0.00 | 1168500.00 | 538757.98 | 508032.96 | 1093975.92 | 224265.89 | 626140.53 | 0.00 | 0.00 | 0.00 | KO:K04077 | chaperonin GroEL | Bacteria;Chloroflexi;Anaerolineae;Anaerolineales;Anaerolineaceae;Thermanaerothrix;Thermanaerothrix daxensis;Thermanaerothrix daxensis GNS-1 |
| Ga0586324 0614064 386 1498 | 0.00 | 0.00 | 514472.22 | 0.00 | 0.00 | 0.00 | 0.00 | 0.00 | 0.00 | 0.00 | 0.00 | 0.00 | KO:K04077 | chaperonin GroEL | Bacteria;Proteobacteria;Alphaproteobacteria;unclassified Alphaproteobacteria;unclassified Alphaproteobacteria;unclassified Alphaproteobacteria;alpha proteobacterium BAL199;Nisaea sp BAL199 |
| Ga0586324 0615025 1 1497 | 0.00 | 0.00 | 0.00 | 10640000.00 | 0.00 | 2611831.24 | 3237600.00 | 2763300.00 | 4576500.00 | 2502500.00 | 3238400.00 | 2797500.00 | KO:K04077 | chaperonin GroEL | Bacteria;Acidobacteria;Acidobacteriia;Acidobacteriales;Acidobacteriaceae;Candidatus Koribacter;Candidatus Koribacter versatilis;Candidatus Koribacter versatilis Ellin345 |
| Ga0586324 0617535 2 1066 | 0.00 | 0.00 | 0.00 | 22610000.00 | 0.00 | 0.00 | 0.00 | 16457800.00 | 0.00 | 0.00 | 1179200.00 | 0.00 | KO:K04077 | chaperonin GroEL | Bacteria;Nitrospirae;Nitrospira;Nitrospirales;Nitrospiraceae;Nitrospira;Candidatus Nitrospira nitrosa;Candidatus Nitrospira nitrosa COMA1 |
| Ga0586324 0618976 803 1495 | 0.00 | 3141600.00 | 0.00 | 0.00 | 613599.84 | 0.00 | 11445600.00 | 10400500.00 | 8181000.00 | 8470000.00 | 13200000.00 | 7800000.00 | KO:K04077 | chaperonin GroEL | Bacteria;Proteobacteria;Betaproteobacteria;Nitrosomonadales;Thiobacillaceae;Thiobacillus;Thiobacillus thioparus;Thiobacillus thioparus DSM 505 |
| Ga0586324 0619927 590 1492 | 3620000.00 | 0.00 | 2067088.28 | 7058500.00 | 5139700.00 | 10599900.00 | 4400400.00 | 9272000.00 | 0.00 | 3264800.00 | 1540000.00 | 802500.00 | KO:K04077 | chaperonin GroEL | Bacteria;Chloroflexi;Anaerolineae;Anaerolineales;Anaerolineaceae;Anaerolinea;Anaerolinea thermophila;Anaerolinea thermophila UNI-1 |
| Ga0586324 0627245 188 1486 | 0.00 | 0.00 | 0.00 | 0.00 | 0.00 | 0.00 | 0.00 | 0.00 | 0.00 | 0.00 | 9240000.00 | 2085000.00 | KO:K04077 | chaperonin GroEL | Bacteria;Acidobacteria;Blastocatellia;Blastocatellales;Pyrinomonadaceae;Pyrinomonas;Pyrinomonas methylaliphatogenes;Pyrinomonas methylaliphatogenes K22 |
| Ga0586324 0647813 3 818 | 379256.00 | 0.00 | 0.00 | 626183.00 | 0.00 | 0.00 | 1984783.32 | 647784.01 | 3967025.22 | 1940400.00 | 3869624.88 | 3702549.75 | KO:K04077 | chaperonin GroEL | Bacteria;Proteobacteria;Alphaproteobacteria;Hyphomicrobiales;Bradyrhizobiaceae;Bradyrhizobium;Bradyrhizobium macuxiense;Bradyrhizobium macuxiense BR 10303 |
| Ga0586324 0654544 3 1457 | 0.00 | 0.00 | 0.00 | 0.00 | 0.00 | 0.00 | 0.00 | 0.00 | 0.00 | 0.00 | 3256000.00 | 0.00 | KO:K04077 | chaperonin GroEL | Bacteria;Proteobacteria;Hydrogenophilalia;Hydrogenophilales;Hydrogenophilaceae;Tepidiphilus;Tepidiphilus margaritifer;Tepidiphilus margaritifer DSM 15129 |
| Ga0586324 0658756 145 1455 | 0.00 | 0.00 | 0.00 | 0.00 | 0.00 | 0.00 | 0.00 | 0.00 | 0.00 | 0.00 | 310336.40 | 0.00 | KO:K04077 | chaperonin GroEL | Bacteria;Acidobacteria;Vicinamibacteria;unclassified Vicinamibacteria;Vicinamibacteraceae;Luteitalea;Luteitalea pratensis;Luteitalea pratensis DSM 100886 |
| Ga0586324 0671001 3 752 | 0.00 | 0.00 | 0.00 | 0.00 | 0.00 | 0.00 | 0.00 | 0.00 | 0.00 | 0.00 | 0.00 | 213810.75 | KO:K04077 | chaperonin GroEL | Bacteria;Proteobacteria;Betaproteobacteria;Nitrosomonadales;Methylophilaceae;Novimethylophilus;Novimethylophilus kurashikiensis;Novimethylophilus kurashikiensis La2-4 |
| Ga0586324 0671889 265 1440 | 30800000.00 | 15388400.00 | 20211700.00 | 47405000.00 | 14729000.00 | 0.00 | 12448800.00 | 11699800.00 | 11421000.00 | 16454900.00 | 14872000.00 | 12750000.00 | KO:K04077 | chaperonin GroEL | Bacteria;Proteobacteria;Gammaproteobacteria;Methylococcales;Crenotrichaceae;Crenothrix;Crenothrix polyspora;Crenothrix polyspora ERS1545888 |
| Ga0586324 0675008 100 1437 | 0.00 | 0.00 | 0.00 | 3876000.00 | 0.00 | 30814473.04 | 1180759.56 | 17019000.00 | 8675100.00 | 5959800.00 | 20011200.00 | 17175000.00 | KO:K04077 | chaperonin GroEL | Bacteria;Proteobacteria;Betaproteobacteria;unclassified Betaproteobacteria;unclassified Betaproteobacteria;unclassified Betaproteobacteria;Betaproteobacteria bacterium UKL13-2;Betaproteobacteria bacterium UKL13-2 |
| Ga0586324 0675796 1 1437 | 17700000.00 | 0.00 | 1684507.26 | 0.00 | 0.00 | 19214800.00 | 0.00 | 0.00 | 0.00 | 0.00 | 0.00 | 0.00 | KO:K04077 | chaperonin GroEL | Bacteria;Proteobacteria;Deltaproteobacteria;Myxococcales;Myxococcaceae;Myxococcus;Myxococcus xanthus;Myxococcus xanthus KF3.2.8c11 |
| Ga0586324 0680857 121 1434 | 1380000.00 | 2951200.00 | 30876198.01 | 4474500.00 | 1740700.00 | 782887.97 | 7660800.00 | 2324100.00 | 26632800.00 | 2539200.51 | 26274167.04 | 3187500.00 | KO:K04077 | chaperonin GroEL | Bacteria;Candidatus Calescamantes;unclassified Candidatus Calescamantes;unclassified Candidatus Calescamantes;unclassified Candidatus Calescamantes;unclassified Candidatus Calescamantes;Calescamantes bacterium JGI 0000106-H18;Calescamantes bacterium JGI 0000106-H18 (contamination screened) |
| Ga0586324 0686531 421 1428 | 608459.00 | 1747844.80 | 277774.63 | 15038500.00 | 0.00 | 0.00 | 528966.84 | 658764.62 | 0.00 | 0.00 | 0.00 | 410624.25 | KO:K04077 | chaperonin GroEL | Bacteria;Verrucomicrobia;Verrucomicrobiae;Verrucomicrobiales;Verrucomicrobia subdivision 3;Pedosphaera;Pedosphaera parvula;Pedosphaera parvula Ellin514 |
| Ga0586324 0687455 1 894 | 0.00 | 0.00 | 0.00 | 0.00 | 1266900.00 | 0.00 | 0.00 | 0.00 | 0.00 | 0.00 | 0.00 | 0.00 | KO:K04077 | chaperonin GroEL | Bacteria;Chloroflexi;Anaerolineae;Anaerolineales;Anaerolineaceae;Levilinea;Levilinea saccharolytica;Levilinea saccharolytica DSM 16555 |
| Ga0586324 0690043 3 512 | 0.00 | 0.00 | 0.00 | 0.00 | 0.00 | 0.00 | 0.00 | 0.00 | 668852.64 | 0.00 | 0.00 | 0.00 | KO:K04077 | chaperonin GroEL | Bacteria;Proteobacteria;Gammaproteobacteria;Acidiferrobacterales;Acidiferrobacteraceae;Sulfuricaulis;Sulfuricaulis limicola;Sulfuricaulis limicola HA5 |
| Ga0586324 0690260 240 1424 | 1780000.00 | 0.00 | 0.00 | 0.00 | 0.00 | 0.00 | 0.00 | 0.00 | 0.00 | 0.00 | 0.00 | 0.00 | KO:K04077 | chaperonin GroEL | Bacteria;Chloroflexi;Dehalococcoidia;Dehalococcoidales;Dehalococcoidaceae;Dehalococcoides;Dehalococcoides mccartyi;Dehalococcoides mccartyi VS |
| Ga0586324 0692247 1 1422 | 0.00 | 30117200.00 | 0.00 | 8892000.00 | 13493000.00 | 33847795.21 | 1331007.00 | 44411434.30 | 0.00 | 53284000.00 | 7568000.00 | 1560000.00 | KO:K04077 | chaperonin GroEL | Bacteria;Acidobacteria;Acidobacteriia;Acidobacteriales;Acidobacteriaceae;Candidatus Koribacter;Candidatus Koribacter versatilis;Candidatus Koribacter versatilis Ellin345 |
| Ga0586324 0693023 763 1422 | 0.00 | 4619753.40 | 0.00 | 0.00 | 0.00 | 0.00 | 0.00 | 0.00 | 0.00 | 0.00 | 11704000.00 | 0.00 | KO:K04077 | chaperonin GroEL | Bacteria;Proteobacteria;Gammaproteobacteria;Methylococcales;Methylococcaceae;Methylocaldum;Methylocaldum szegediense;Methylocaldum szegediense O-12 |
| Ga0586324 0695674 3 1304 | 0.00 | 521773.52 | 0.00 | 0.00 | 0.00 | 0.00 | 0.00 | 0.00 | 0.00 | 0.00 | 0.00 | 0.00 | KO:K04077 | chaperonin GroEL | Bacteria;Nitrospirae;Thermodesulfovibrionia;Thermodesulfovibrionales;Thermodesulfovibrionaceae;Thermodesulfovibrio;Thermodesulfovibrio sp. N1;Thermodesulfovibrio sp. N1 |
| Ga0586324 0703513 634 1413 | 0.00 | 0.00 | 0.00 | 0.00 | 0.00 | 0.00 | 0.00 | 0.00 | 0.00 | 0.00 | 1012000.00 | 0.00 | KO:K04077 | chaperonin GroEL | Bacteria;Proteobacteria;Alphaproteobacteria;Rhodospirillales;Acetobacteraceae;Oleomonas;Oleomonas sp. K1W22B-8;Oleomonas sp. K1W22B-8 |
| Ga0586324 0703705 2 1381 | 0.00 | 754800.00 | 0.00 | 0.00 | 0.00 | 0.00 | 14174301.72 | 2122800.00 | 19148400.00 | 1478400.00 | 2646029.76 | 514486.50 | KO:K04077 | chaperonin GroEL | Bacteria;Acidobacteria;Blastocatellia;Blastocatellales;Pyrinomonadaceae;Pyrinomonas;Pyrinomonas methylaliphatogenes;Pyrinomonas methylaliphatogenes K22 |
| Ga0586324 0703887 3 1346 | 0.00 | 0.00 | 0.00 | 1463000.00 | 8044300.00 | 236215000.00 | 0.00 | 1018700.00 | 0.00 | 0.00 | 0.00 | 0.00 | KO:K04077 | chaperonin GroEL | Bacteria;Proteobacteria;Deltaproteobacteria;Syntrophobacterales;Syntrophobacteraceae;Desulfacinum;Desulfacinum infernum;Desulfacinum infernum DSM 9756 |
| Ga0586324 0706076 3 1412 | 0.00 | 0.00 | 0.00 | 0.00 | 0.00 | 0.00 | 0.00 | 0.00 | 0.00 | 0.00 | 0.00 | 15525000.00 | KO:K04077 | chaperonin GroEL | Bacteria;Thermodesulfobacteria;Thermodesulfobacteria;Thermodesulfobacteriales;Thermodesulfobacteriaceae;Thermosulfurimonas;Thermosulfurimonas dismutans;Thermosulfurimonas dismutans S95 |
| Ga0586324 0712577 3 1235 | 3404781.00 | 9356800.00 | 0.00 | 537196.50 | 5339972.17 | 1211258.91 | 2781600.00 | 5207404.08 | 6601500.00 | 0.00 | 0.00 | 960000.00 | KO:K04077 | chaperonin GroEL | Bacteria;Chloroflexi;Thermoflexia;Thermoflexales;Thermoflexaceae;Thermoflexus;Thermoflexus hugenholtzii;Thermoflexus hugenholtzii JAD2 |
| Ga0586324 0712784 3 1406 | 0.00 | 0.00 | 0.00 | 0.00 | 0.00 | 0.00 | 0.00 | 0.00 | 0.00 | 459375.84 | 607992.88 | 0.00 | KO:K04077 | chaperonin GroEL | Bacteria;Proteobacteria;Deltaproteobacteria;Syntrophobacterales;Syntrophaceae;Syntrophus;Syntrophus aciditrophicus;Syntrophus aciditrophicus SB |
| Ga0586324 0719302 3 734 | 0.00 | 3005600.00 | 0.00 | 0.00 | 0.00 | 0.00 | 5973600.00 | 0.00 | 0.00 | 0.00 | 0.00 | 616787.25 | KO:K04077 | chaperonin GroEL | Bacteria;Acidobacteria;Acidobacteriia;Acidobacteriales;Acidobacteriaceae;Candidatus Koribacter;Candidatus Koribacter versatilis;Candidatus Koribacter versatilis Ellin345 |
| Ga0586324 0725488 1 1395 | 6817704.00 | 0.00 | 0.00 | 4921000.00 | 0.00 | 0.00 | 0.00 | 25131.39 | 0.00 | 0.00 | 0.00 | 692658.75 | KO:K04077 | chaperonin GroEL | Bacteria;Proteobacteria;Deltaproteobacteria;Syntrophobacterales;Syntrophobacteraceae;Thermodesulforhabdus;Thermodesulforhabdus norvegica;Thermodesulforhabdus norvegica DSM 9990 |
| Ga0586324 0729127 51 1391 | 0.00 | 0.00 | 0.00 | 3097000.00 | 0.00 | 9131000.00 | 0.00 | 0.00 | 1571400.00 | 0.00 | 0.00 | 0.00 | KO:K04077 | chaperonin GroEL | Bacteria;Bacteroidetes;Bacteroidia;Marinilabiliales;Prolixibacteraceae;Draconibacterium;Draconibacterium sediminis;Draconibacterium sediminis JN14CK-3 |
| Ga0586324 0730369 19 1389 | 0.00 | 0.00 | 0.00 | 0.00 | 0.00 | 0.00 | 499178.64 | 0.00 | 0.00 | 0.00 | 0.00 | 0.00 | KO:K04077 | chaperonin GroEL | Bacteria;Actinobacteria;Coriobacteriia;Eggerthellales;Eggerthellaceae;Slackia;Slackia isoflavoniconvertens;Slackia isoflavoniconvertens DSM 22006 |
| Ga0586324 0734697 2 1387 | 0.00 | 4644400.00 | 0.00 | 0.00 | 813600.09 | 0.00 | 3853200.00 | 16958000.00 | 256094.46 | 5944400.00 | 5500000.00 | 4957500.00 | KO:K04077 | chaperonin GroEL | Bacteria;Nitrospirae;Nitrospira;Nitrospirales;Nitrospiraceae;Nitrospira;Nitrospira marina;Nitrospira marina Nb-295 |
| Ga0586324 0742266 3 1361 | 0.00 | 0.00 | 0.00 | 732153.60 | 0.00 | 0.00 | 0.00 | 0.00 | 0.00 | 0.00 | 0.00 | 0.00 | KO:K04077 | chaperonin GroEL | Bacteria;Proteobacteria;Deltaproteobacteria;Myxococcales;Anaeromyxobacteraceae;Anaeromyxobacter;Anaeromyxobacter sp. Fw109-5;Anaeromyxobacter sp. Fw109-5 |
| Ga0586324 0756070 529 1368 | 0.00 | 0.00 | 0.00 | 0.00 | 0.00 | 0.00 | 0.00 | 0.00 | 2276100.00 | 0.00 | 4628800.00 | 5272500.00 | KO:K04077 | chaperonin GroEL | Bacteria;Proteobacteria;Betaproteobacteria;Burkholderiales;Comamonadaceae;Ramlibacter;Ramlibacter tataouinensis;Ramlibacter tataouinensis 5-10 |
| Ga0586324 0763000 2 1363 | 0.00 | 294189.76 | 0.00 | 2470000.00 | 0.00 | 7026900.00 | 15367200.00 | 12566000.00 | 0.00 | 0.00 | 0.00 | 0.00 | KO:K04077 | chaperonin GroEL | Bacteria;Proteobacteria;Alphaproteobacteria;Hyphomicrobiales;Bradyrhizobiaceae;Bradyrhizobium;Bradyrhizobium erythrophlei;Bradyrhizobium erythrophlei GAS478 |
| Ga0586324 0770063 3 1223 | 0.00 | 0.00 | 0.00 | 0.00 | 53148000.00 | 67093000.00 | 1507567.92 | 38186000.00 | 193517.10 | 723930.13 | 0.00 | 0.00 | KO:K04077 | chaperonin GroEL | Bacteria;Nitrospirae;Nitrospira;Nitrospirales;Nitrospiraceae;Leptospirillum;Leptospirillum sp. Group II 'C75';Leptospirillum sp. Group II 'C75' |
| Ga0586324 0777149 2 565 | 22130000.00 | 0.00 | 0.00 | 49305000.00 | 259033.67 | 49625000.00 | 0.00 | 0.00 | 0.00 | 572128.48 | 0.00 | 6712500.00 | KO:K04077 | chaperonin GroEL | Bacteria;Chloroflexi;Anaerolineae;Anaerolineales;Anaerolineaceae;Pelolinea;Pelolinea submarina;Pelolinea submarina DSM 23923 |
| Ga0586324 0779977 357 1352 | 0.00 | 167241.92 | 1851605.66 | 0.00 | 0.00 | 0.00 | 0.00 | 1525000.00 | 0.00 | 2579500.00 | 2490400.00 | 1147500.00 | KO:K04077 | chaperonin GroEL | Bacteria;Proteobacteria;Deltaproteobacteria;Desulfobacterales;Desulfobacteraceae;Desulfatirhabdium;Desulfatirhabdium butyrativorans;Desulfatirhabdium butyrativorans DSM 18734 |
| Ga0586324 0795904 3 707 | 0.00 | 0.00 | 0.00 | 0.00 | 0.00 | 0.00 | 0.00 | 233570.22 | 0.00 | 0.00 | 0.00 | 0.00 | KO:K04077 | chaperonin GroEL | Bacteria;Proteobacteria;Deltaproteobacteria;Desulfobacterales;Desulfobacteraceae;Desulfatiglans;Desulfatiglans anilini;Desulfatiglans anilini DSM 4660 |
| Ga0586324 0797336 376 1338 | 1840000.00 | 986000.00 | 0.00 | 5538500.00 | 16397600.00 | 39303000.00 | 0.00 | 10695463.06 | 8879602.32 | 2033067.19 | 0.00 | 1762500.00 | KO:K04077 | chaperonin GroEL | Bacteria;Ignavibacteriae;Ignavibacteria;Ignavibacteriales;Ignavibacteriaceae;Ignavibacterium;Ignavibacterium album;Ignavibacterium album Mat9-16, JCM 16511 |
| Ga0586324 0802506 604 1335 | 0.00 | 0.00 | 0.00 | 0.00 | 22969000.00 | 0.00 | 0.00 | 0.00 | 0.00 | 0.00 | 0.00 | 0.00 | KO:K04077 | chaperonin GroEL | Bacteria;Proteobacteria;Deltaproteobacteria;Syntrophorhabdales;Syntrophorhabdaceae;Syntrophorhabdus;Syntrophorhabdus aromaticivorans;Syntrophorhabdus aromaticivorans UI |
| Ga0586324 0803740 385 1335 | 0.00 | 2556800.00 | 1854189.83 | 3154000.00 | 3162100.00 | 0.00 | 3420000.00 | 1762900.00 | 14836291.29 | 727914.11 | 1478400.00 | 1417500.00 | KO:K04077 | chaperonin GroEL | Bacteria;Firmicutes;Negativicutes;Selenomonadales;Selenomonadaceae;Selenomonas;Selenomonas sp. oral taxon 149;Selenomonas sp. oral taxon 149 67H29BP |
| Ga0586324 0812340 3 1025 | 0.00 | 0.00 | 0.00 | 7552500.00 | 6911300.00 | 6272600.00 | 0.00 | 0.00 | 5386500.00 | 0.00 | 0.00 | 0.00 | KO:K04077 | chaperonin GroEL | Bacteria;Proteobacteria;Deltaproteobacteria;Desulfobacterales;Desulfobulbaceae;Desulfoprunum;Desulfoprunum benzoelyticum;Desulfoprunum benzoelyticum DSM 28570 |
| Ga0586324 0813017 1 858 | 14270000.00 | 2721194.76 | 6129409.15 | 45163000.00 | 44454800.00 | 10912132.56 | 13520400.00 | 23180000.00 | 20047500.00 | 7977200.00 | 8395200.00 | 14272500.00 | KO:K04077 | chaperonin GroEL | Bacteria;Chloroflexi;Anaerolineae;Anaerolineales;Anaerolineaceae;Levilinea;Levilinea saccharolytica;Levilinea saccharolytica DSM 16555 |
| Ga0586324 0814458 3 890 | 0.00 | 0.00 | 0.00 | 0.00 | 0.00 | 0.00 | 0.00 | 0.00 | 0.00 | 0.00 | 0.00 | 722877.75 | KO:K04077 | chaperonin GroEL | Bacteria;Proteobacteria;Betaproteobacteria;Burkholderiales;unclassified Burkholderiales;Ideonella;Ideonella sakaiensis;Ideonella sp. 201-F6 |
| Ga0586324 0822566 1 1320 | 0.00 | 7752000.00 | 0.00 | 0.00 | 19776000.00 | 201297111.14 | 23028000.00 | 6588000.00 | 193249800.00 | 8470000.00 | 10032000.00 | 0.00 | KO:K04077 | chaperonin GroEL | Bacteria;Chloroflexi;Anaerolineae;Anaerolineales;Anaerolineaceae;Anaerolinea;Anaerolinea thermophila;Anaerolinea thermophila UNI-1 |
| Ga0586324 0823359 297 1319 | 0.00 | 0.00 | 3756482.38 | 0.00 | 0.00 | 0.00 | 0.00 | 0.00 | 0.00 | 0.00 | 516715.76 | 862500.00 | KO:K04077 | chaperonin GroEL | Bacteria;Ignavibacteriae;Ignavibacteria;Ignavibacteriales;Melioribacteraceae;Melioribacter;Melioribacter roseus;Melioribacter roseus P3M |
| Ga0586324 0828992 586 1317 | 0.00 | 0.00 | 0.00 | 0.00 | 0.00 | 0.00 | 644902.56 | 489307.84 | 0.00 | 0.00 | 606650.88 | 0.00 | KO:K04077 | chaperonin GroEL | Bacteria;Proteobacteria;Deltaproteobacteria;Myxococcales;Archangiaceae;Stigmatella;Stigmatella erecta;Stigmatella erecta DSM 16858 |
| Ga0586324 0829534 2 1243 | 0.00 | 0.00 | 0.00 | 0.00 | 0.00 | 0.00 | 0.00 | 161208.36 | 0.00 | 0.00 | 0.00 | 190152.00 | KO:K04077 | chaperonin GroEL | Bacteria;Proteobacteria;Betaproteobacteria;Rhodocyclales;Zoogloeaceae;Thauera;unclassified Thauera;Thauera sp. SYD-3 |
| Ga0586324 0850166 1 1302 | 0.00 | 0.00 | 0.00 | 0.00 | 7972200.00 | 0.00 | 0.00 | 3580700.00 | 0.00 | 0.00 | 0.00 | 0.00 | KO:K04077 | chaperonin GroEL | Bacteria;Proteobacteria;Deltaproteobacteria;Syntrophobacterales;Syntrophaceae;Smithella;Smithella sp. F21;Smithella sp. F21 |
| Ga0586324 0857779 3 1235 | 0.00 | 0.00 | 0.00 | 0.00 | 0.00 | 1961529.36 | 0.00 | 0.00 | 0.00 | 0.00 | 0.00 | 700482.00 | KO:K04077 | chaperonin GroEL | Bacteria;Proteobacteria;Deltaproteobacteria;unclassified Deltaproteobacteria;unclassified Deltaproteobacteria;Dissulfuribacter;Dissulfuribacter thermophilus;Dissulfuribacter thermophilus S69 |
| Ga0586324 0860205 1 924 | 0.00 | 0.00 | 0.00 | 0.00 | 0.00 | 0.00 | 0.00 | 0.00 | 2365200.00 | 0.00 | 0.00 | 0.00 | KO:K04077 | chaperonin GroEL | Bacteria;Chloroflexi;Anaerolineae;Anaerolineales;Anaerolineaceae;Anaerolinea;Anaerolinea thermophila;Anaerolinea thermophila UNI-1 |
| Ga0586324 0862477 1 1278 | 0.00 | 7820000.00 | 6080400.00 | 5244000.00 | 4944000.00 | 3590793.54 | 82422000.00 | 5032500.00 | 8553600.00 | 73319400.00 | 9680000.00 | 7800000.00 | KO:K04077 | chaperonin GroEL | Bacteria;Proteobacteria;Deltaproteobacteria;Syntrophorhabdales;Syntrophorhabdaceae;Syntrophorhabdus;Syntrophorhabdus aromaticivorans;Syntrophorhabdus aromaticivorans UI |
| Ga0586324 0864860 465 1292 | 0.00 | 0.00 | 0.00 | 0.00 | 0.00 | 0.00 | 0.00 | 0.00 | 0.00 | 0.00 | 12232000.00 | 0.00 | KO:K04077 | chaperonin GroEL | Bacteria;Proteobacteria;Deltaproteobacteria;Syntrophobacterales;Syntrophaceae;Smithella;Smithella sp. F21;Smithella sp. F21 |
| Ga0586324 0868392 3 365 | 0.00 | 0.00 | 0.00 | 0.00 | 0.00 | 0.00 | 0.00 | 0.00 | 0.00 | 2125200.00 | 0.00 | 0.00 | KO:K04077 | chaperonin GroEL | Bacteria;Candidatus Omnitrophica;unclassified Candidatus Omnitrophica;unclassified Candidatus Omnitrophica;unclassified Candidatus Omnitrophica;unclassified Candidatus Omnitrophica;Omnitrophica bacterium SCGC AAA257-O07;Omnitrophica bacterium SCGC AAA257-O07 (contamination screened) |
| Ga0586324 0869957 1 1083 | 0.00 | 0.00 | 1574716.63 | 1567500.00 | 443201.79 | 0.00 | 1037760.24 | 1939454.74 | 133702.65 | 0.00 | 0.00 | 0.00 | KO:K04077 | chaperonin GroEL | Bacteria;Proteobacteria;Deltaproteobacteria;Desulfobacterales;Desulfobacteraceae;Desulfosarcina;Desulfosarcina variabilis;Desulfosarcina variabilis Montpellier |
| Ga0586324 0870498 2 1288 | 0.00 | 0.00 | 0.00 | 455956.30 | 0.00 | 395010.24 | 0.00 | 0.00 | 424388.16 | 0.00 | 0.00 | 476955.00 | KO:K04077 | chaperonin GroEL | Bacteria;Proteobacteria;Deltaproteobacteria;Myxococcales;Anaeromyxobacteraceae;Anaeromyxobacter;Anaeromyxobacter dehalogenans;Anaeromyxobacter dehalogenans 2CP-C |
| Ga0586324 0876795 1 1149 | 0.00 | 0.00 | 0.00 | 842549.30 | 0.00 | 0.00 | 0.00 | 0.00 | 156674.25 | 2879800.00 | 0.00 | 3382500.00 | KO:K04077 | chaperonin GroEL | Bacteria;Bacteroidetes;Chitinophagia;Chitinophagales;Chitinophagaceae;Flavihumibacter;Flavihumibacter sp. ZG627;Flavihumibacter sp. ZG627 CCTCC AB2013368 |
| Ga0586324 0879792 3 1283 | 0.00 | 3978000.00 | 1381720.23 | 7913500.00 | 1596500.00 | 7582700.00 | 1388953.20 | 1335900.00 | 681632.82 | 777700.00 | 1496000.00 | 1185000.00 | KO:K04077 | chaperonin GroEL | Bacteria;Proteobacteria;Deltaproteobacteria;Syntrophobacterales;Syntrophobacteraceae;Syntrophobacter;Syntrophobacter fumaroxidans;Syntrophobacter fumaroxidans MPOB |
| Ga0586324 0883946 279 1280 | 0.00 | 5875200.00 | 540281.26 | 0.00 | 5133455.11 | 0.00 | 0.00 | 13395600.00 | 1927800.00 | 0.00 | 2965600.00 | 0.00 | KO:K04077 | chaperonin GroEL | Bacteria;Proteobacteria;Betaproteobacteria;Burkholderiales;unclassified Burkholderiales;unclassified Burkholderiales;Burkholderiales bacterium GJ-E10;Burkholderiales bacterium GJ-E10 |
| Ga0586324 0888711 2 784 | 4600000.00 | 965600.00 | 0.00 | 1330000.00 | 0.00 | 0.00 | 0.00 | 2879200.00 | 0.00 | 1232000.00 | 0.00 | 0.00 | KO:K04077 | chaperonin GroEL | Bacteria;Proteobacteria;Betaproteobacteria;Nitrosomonadales;Nitrosomonadaceae;Nitrosospira;Nitrosospira briensis;Nitrosospira briensis C-128 |
| Ga0586324 0890796 3 485 | 0.00 | 0.00 | 0.00 | 0.00 | 0.00 | 475209.00 | 0.00 | 0.00 | 253601.28 | 0.00 | 0.00 | 0.00 | KO:K04077 | chaperonin GroEL | Bacteria;Acidobacteria;Vicinamibacteria;unclassified Vicinamibacteria;Vicinamibacteraceae;Luteitalea;Luteitalea pratensis;Luteitalea pratensis DSM 100886 |
| Ga0586324 0891336 2 691 | 1000000.00 | 3474800.00 | 0.00 | 0.00 | 863138.97 | 0.00 | 0.00 | 622200.00 | 0.00 | 4535300.00 | 0.00 | 298197.75 | KO:K04077 | chaperonin GroEL | Bacteria;Proteobacteria;Gammaproteobacteria;Chromatiales;Chromatiaceae;Thiodictyon;Candidatus Thiodictyon syntrophicum;Candidatus Thiodictyon syntrophicum Cad16 |
| Ga0586324 0905080 619 1266 | 14400000.00 | 20808000.00 | 3195242.88 | 734541.90 | 3481689.43 | 438581.78 | 64888366.80 | 413243.28 | 7188541.02 | 6360200.00 | 2235200.00 | 51975000.00 | KO:K04077 | chaperonin GroEL | Bacteria;Proteobacteria;Alphaproteobacteria;Rhodospirillales;Acetobacteraceae;Acidiphilium;Acidiphilium cryptum;Acidiphilium cryptum JF-5 |
| Ga0586324 0905420 3 791 | 0.00 | 1040400.00 | 1973247.44 | 0.00 | 0.00 | 0.00 | 0.00 | 0.00 | 0.00 | 0.00 | 0.00 | 0.00 | KO:K04077 | chaperonin GroEL | Bacteria;Nitrospirae;Thermodesulfovibrionia;Thermodesulfovibrionales;Thermodesulfovibrionaceae;Thermodesulfovibrio;Thermodesulfovibrio thiophilus;Thermodesulfovibrio thiophilus DSM 17215 |
| Ga0586324 0910614 586 1263 | 0.00 | 3556400.00 | 0.00 | 0.00 | 645533.96 | 2513954.86 | 0.00 | 2189900.00 | 2508996.06 | 993300.00 | 0.00 | 0.00 | KO:K04077 | chaperonin GroEL | Bacteria;Proteobacteria;Deltaproteobacteria;unclassified Deltaproteobacteria;unclassified Deltaproteobacteria;Dissulfuribacter;Dissulfuribacter thermophilus;Dissulfuribacter thermophilus S69 |
| Ga0586324 0911088 1 1074 | 514536.00 | 0.00 | 0.00 | 6764000.00 | 10712000.00 | 0.00 | 6885600.00 | 0.00 | 6788841.66 | 2733500.00 | 2120800.00 | 1935000.00 | KO:K04077 | chaperonin GroEL | Bacteria;Proteobacteria;Deltaproteobacteria;Syntrophobacterales;Syntrophobacteraceae;Desulfosoma;Desulfosoma caldarium;Desulfosoma caldarium DSM 22027 |
| Ga0586324 0918487 269 1258 | 0.00 | 0.00 | 0.00 | 0.00 | 0.00 | 0.00 | 0.00 | 0.00 | 0.00 | 0.00 | 343546.72 | 0.00 | KO:K04077 | chaperonin GroEL | Bacteria;Acidobacteria;Vicinamibacteria;unclassified Vicinamibacteria;Vicinamibacteraceae;Luteitalea;Luteitalea pratensis;Luteitalea pratensis DSM 100886 |
| Ga0586324 0936649 315 1250 | 532833.00 | 2570400.00 | 0.00 | 4816500.00 | 6674400.00 | 7543000.00 | 0.00 | 1732400.00 | 6706800.00 | 521742.76 | 0.00 | 0.00 | KO:K04077 | chaperonin GroEL | Bacteria;Proteobacteria;Deltaproteobacteria;Desulfuromonadales;Geobacteraceae;Geobacter;Geobacter sp. DSM 2909;Geobacter sp. DSM 2909 |
| Ga0586324 0943410 3 1244 | 0.00 | 0.00 | 0.00 | 2147000.00 | 300793.99 | 0.00 | 8071200.00 | 5971900.00 | 0.00 | 0.00 | 0.00 | 1117500.00 | KO:K04077 | chaperonin GroEL | Bacteria;Proteobacteria;Deltaproteobacteria;Desulfobacterales;Desulfobacteraceae;Desulfatiglans;Desulfatiglans anilini;Desulfatiglans anilini DSM 4660 |
| Ga0586324 0946649 3 791 | 0.00 | 0.00 | 0.00 | 589030.40 | 0.00 | 0.00 | 0.00 | 548183.21 | 0.00 | 0.00 | 0.00 | 0.00 | KO:K04077 | chaperonin GroEL | Bacteria;Proteobacteria;Betaproteobacteria;Nitrosomonadales;Nitrosomonadaceae;Nitrosospira;Nitrosospira sp. Nsp44;Nitrosospira sp. Nsp44 |
| Ga0586324 0947946 2 742 | 3520000.00 | 7275033.72 | 6057041.13 | 4733976.35 | 4289274.32 | 3407899.61 | 12175200.00 | 5495123.39 | 8035200.00 | 8508028.76 | 7348000.00 | 7852975.50 | KO:K04077 | chaperonin GroEL | Bacteria;Proteobacteria;Gammaproteobacteria;unclassified Gammaproteobacteria;Candidatus Competibacteraceae;Candidatus Contendobacter;Candidatus Contendobacter odensis;Candidatus Contendobacter odensis Run B J11 |
| Ga0586324 0948775 1 555 | 0.00 | 0.00 | 0.00 | 0.00 | 0.00 | 0.00 | 995112.84 | 0.00 | 0.00 | 0.00 | 0.00 | 0.00 | KO:K04077 | chaperonin GroEL | Bacteria;Chloroflexi;Anaerolineae;Anaerolineales;Anaerolineaceae;Pelolinea;Pelolinea submarina;Pelolinea submarina DSM 23923 |
| Ga0586324 0949791 199 1242 | 0.00 | 0.00 | 0.00 | 0.00 | 0.00 | 15125700.00 | 0.00 | 0.00 | 0.00 | 0.00 | 0.00 | 0.00 | KO:K04077 | chaperonin GroEL | Bacteria;Proteobacteria;Deltaproteobacteria;Syntrophobacterales;Syntrophobacteraceae;Thermodesulforhabdus;Thermodesulforhabdus norvegica;Thermodesulforhabdus norvegica DSM 9990 |
| Ga0586324 0953307 1 1041 | 0.00 | 652536.16 | 0.00 | 0.00 | 0.00 | 0.00 | 620670.72 | 0.00 | 0.00 | 3287900.00 | 1152800.00 | 0.00 | KO:K04077 | chaperonin GroEL | Bacteria;Bacteroidetes;Bacteroidia;Marinilabiliales;Prolixibacteraceae;Prolixibacter;Prolixibacter bellariivorans;Prolixibacter bellariivorans ATCC BAA-1284 |
| Ga0586324 0953998 3 1211 | 534494.00 | 0.00 | 0.00 | 0.00 | 0.00 | 0.00 | 0.00 | 0.00 | 2940300.00 | 2017400.00 | 2208800.00 | 2677500.00 | KO:K04077 | chaperonin GroEL | Bacteria;Verrucomicrobia;Opitutae;Opitutales;Opitutaceae;Opitutus;Opitutus sp. GAS368;Opitutus sp. GAS368 |
| Ga0586324 0960197 351 1235 | 0.00 | 0.00 | 0.00 | 0.00 | 0.00 | 0.00 | 0.00 | 8845000.00 | 26163000.00 | 15708000.00 | 16456000.00 | 41625000.00 | KO:K04077 | chaperonin GroEL | Bacteria;Proteobacteria;Betaproteobacteria;Nitrosomonadales;Sulfuricellaceae;Sulfurirhabdus;Sulfurirhabdus autotrophica;Sulfurirhabdus autotrophica DSM 100309 |
| Ga0586324 0965405 1 1065 | 0.00 | 0.00 | 0.00 | 0.00 | 0.00 | 0.00 | 0.00 | 0.00 | 0.00 | 46015200.00 | 23496000.00 | 0.00 | KO:K04077 | chaperonin GroEL | Bacteria;Proteobacteria;Alphaproteobacteria;Hyphomicrobiales;Xanthobacteraceae;Pseudolabrys;Pseudolabrys sp. Root1462;Pseudolabrys sp. Root1462 |
| Ga0586324 0974234 1 786 | 0.00 | 543126.20 | 0.00 | 0.00 | 0.00 | 0.00 | 0.00 | 0.00 | 0.00 | 0.00 | 0.00 | 0.00 | KO:K04077 | chaperonin GroEL | Bacteria;Proteobacteria;Deltaproteobacteria;Syntrophobacterales;Syntrophobacteraceae;Desulfosoma;Desulfosoma caldarium;Desulfosoma caldarium DSM 22027 |
| Ga0586324 0984220 60 1223 | 0.00 | 0.00 | 0.00 | 0.00 | 0.00 | 0.00 | 0.00 | 0.00 | 0.00 | 0.00 | 0.00 | 12600000.00 | KO:K04077 | chaperonin GroEL | Bacteria;Proteobacteria;Deltaproteobacteria;unclassified Deltaproteobacteria;unclassified Deltaproteobacteria;Dissulfuribacter;Dissulfuribacter thermophilus;Dissulfuribacter thermophilus S69 |
| Ga0586324 0992435 3 611 | 230731.00 | 856800.00 | 631708.52 | 915877.90 | 889912.79 | 585146.24 | 1245652.92 | 628300.00 | 842400.00 | 900900.00 | 959200.00 | 945000.00 | KO:K04077 | chaperonin GroEL | Bacteria;Verrucomicrobia;Spartobacteria;unclassified Spartobacteria;unclassified Spartobacteria;Spartobacteria;Spartobacteria bacterium LR76;Spartobacteria bacterium LR76 |
| Ga0586324 1002935 2 1213 | 0.00 | 0.00 | 0.00 | 0.00 | 0.00 | 0.00 | 0.00 | 0.00 | 0.00 | 0.00 | 0.00 | 795000.00 | KO:K04077 | chaperonin GroEL | Bacteria;Proteobacteria;Deltaproteobacteria;Desulfobacterales;Desulfobacteraceae;Desulfosarcina;Desulfosarcina cetonica;Desulfosarcina cetonica JCM 12296 |
| Ga0586324 1003738 559 1212 | 0.00 | 0.00 | 0.00 | 0.00 | 0.00 | 737427.50 | 0.00 | 482056.77 | 0.00 | 11319000.00 | 0.00 | 0.00 | KO:K04077 | chaperonin GroEL | Bacteria;Bacteroidetes;Chitinophagia;Chitinophagales;Chitinophagaceae;Niastella;Niastella koreensis;Niastella koreensis GR20-10, DSM 17620 |
| Ga0586324 1011347 3 1208 | 0.00 | 0.00 | 0.00 | 0.00 | 0.00 | 0.00 | 0.00 | 0.00 | 113101.11 | 0.00 | 0.00 | 0.00 | KO:K04077 | chaperonin GroEL | Bacteria;Proteobacteria;Deltaproteobacteria;Desulfobacterales;Desulfobacteraceae;Desulfatitalea;Desulfatitalea tepidiphila;Desulfatitalea tepidiphila S28bF |
| Ga0586324 1015321 2 739 | 0.00 | 0.00 | 0.00 | 2907000.00 | 0.00 | 0.00 | 0.00 | 0.00 | 0.00 | 0.00 | 0.00 | 0.00 | KO:K04077 | chaperonin GroEL | Bacteria;Chlorobi;Chlorobia;Chlorobiales;Chlorobiaceae;Prosthecochloris;Prosthecochloris sp. GSB1;Prosthecochloris sp. GSB1 |
| Ga0586324 1016059 2 1207 | 0.00 | 338011.68 | 2150547.40 | 0.00 | 2214500.00 | 3970000.00 | 1108768.56 | 744200.00 | 1223100.00 | 0.00 | 624674.16 | 1065000.00 | KO:K04077 | chaperonin GroEL | Bacteria;Proteobacteria;Deltaproteobacteria;Syntrophobacterales;Syntrophaceae;Syntrophus;Syntrophus gentianae;Syntrophus gentianae DSM 8423 |
| Ga0586324 1016526 3 794 | 0.00 | 0.00 | 0.00 | 0.00 | 0.00 | 0.00 | 0.00 | 0.00 | 567503.01 | 0.00 | 0.00 | 0.00 | KO:K04077 | chaperonin GroEL | Bacteria;Chloroflexi;Anaerolineae;Anaerolineales;Anaerolineaceae;Levilinea;Levilinea saccharolytica;Levilinea saccharolytica DSM 16555 |
| Ga0586324 1021385 3 488 | 1340000.00 | 1244400.00 | 1549685.65 | 1586500.00 | 1575900.00 | 1286891.38 | 2879959.20 | 8216700.00 | 4284900.00 | 12258400.00 | 3187582.64 | 817500.00 | KO:K04077 | chaperonin GroEL | Bacteria;Proteobacteria;Gammaproteobacteria;Nevskiales;Steroidobacteraceae;Steroidobacter;Steroidobacter denitrificans;Steroidobacter denitrificans DSM 18526 |
| Ga0586324 1031142 1 1122 | 0.00 | 1910800.00 | 0.00 | 0.00 | 0.00 | 0.00 | 0.00 | 0.00 | 0.00 | 0.00 | 0.00 | 0.00 | KO:K04077 | chaperonin GroEL | Bacteria;Spirochaetes;Spirochaetia;Spirochaetales;Treponemataceae;Treponema;Treponema caldarium;Treponema caldarium DSM 7334 |
| Ga0586324 1046845 1 1191 | 1265477.00 | 4114000.00 | 22312303.67 | 23132500.00 | 22103800.00 | 1677833.16 | 39672000.00 | 10956363.11 | 2795052.42 | 15315300.00 | 17104552.08 | 6668186.25 | KO:K04077 | chaperonin GroEL | Bacteria;Proteobacteria;Betaproteobacteria;Rhodocyclales;Azonexaceae;Dechloromonas;Dechloromonas sp. HYN0024;Dechloromonas sp. HYN0024 |
| Ga0586324 1049617 2 1189 | 0.00 | 0.00 | 1138436.67 | 0.00 | 0.00 | 0.00 | 0.00 | 0.00 | 0.00 | 0.00 | 0.00 | 0.00 | KO:K04077 | chaperonin GroEL | Bacteria;Planctomycetes;Phycisphaerae;Sedimentisphaerales;Anaerohalosphaeraceae;Anaerohalosphaera;Anaerohalosphaera lusitana;Phycisphaerae bacterium ST-NAGAB-D1 |
| Ga0586324 1051209 3 1109 | 0.00 | 0.00 | 578899.12 | 893864.50 | 96637455.16 | 998501.05 | 1428404.04 | 1024800.00 | 2178173.43 | 539411.95 | 0.00 | 0.00 | KO:K04077 | chaperonin GroEL | Bacteria;Chloroflexi;Chloroflexia;Herpetosiphonales;Herpetosiphonaceae;Herpetosiphon;Herpetosiphon aurantiacus;Herpetosiphon aurantiacus DSM 785 |
| Ga0586324 1051741 1 1188 | 1910000.00 | 1067600.00 | 0.00 | 0.00 | 4985200.00 | 0.00 | 0.00 | 0.00 | 373011.48 | 1439900.00 | 692998.24 | 1305000.00 | KO:K04077 | chaperonin GroEL | Bacteria;Proteobacteria;Deltaproteobacteria;Myxococcales;Kofleriaceae;Haliangium;Haliangium ochraceum;Haliangium ochraceum SMP-2, DSM 14365 |
| Ga0586324 1056201 3 1187 | 0.00 | 0.00 | 0.00 | 0.00 | 0.00 | 0.00 | 0.00 | 0.00 | 0.00 | 24871000.00 | 0.00 | 0.00 | KO:K04077 | chaperonin GroEL | Bacteria;Proteobacteria;Alphaproteobacteria;Hyphomicrobiales;Phyllobacteriaceae;Mesorhizobium;Mesorhizobium sp. WSM2561;Mesorhizobium sp. WSM2561 |
| Ga0586324 1072060 1 531 | 0.00 | 0.00 | 0.00 | 0.00 | 0.00 | 0.00 | 0.00 | 0.00 | 2268000.00 | 0.00 | 4074400.00 | 0.00 | KO:K04077 | chaperonin GroEL | Bacteria;Nitrospirae;Nitrospira;Nitrospirales;Nitrospiraceae;Nitrospira;Nitrospira lenta;Nitrospira lenta N. lenta BS10 |
| Ga0586324 1073707 163 1179 | 8760000.00 | 2930800.00 | 9514700.00 | 17198027.65 | 8818588.08 | 12108500.00 | 14136000.00 | 8637600.00 | 7402909.95 | 8239000.00 | 1240800.00 | 7800000.00 | KO:K04077 | chaperonin GroEL | Bacteria;Chloroflexi;Anaerolineae;Anaerolineales;Anaerolineaceae;Thermanaerothrix;Thermanaerothrix daxensis;Thermanaerothrix daxensis GNS-1 |
| Ga0586324 1074696 3 1178 | 0.00 | 0.00 | 0.00 | 0.00 | 0.00 | 0.00 | 0.00 | 0.00 | 0.00 | 0.00 | 359894.48 | 0.00 | KO:K04077 | chaperonin GroEL | Bacteria;Proteobacteria;Deltaproteobacteria;unclassified Deltaproteobacteria;unclassified Deltaproteobacteria;Dissulfuribacter;Dissulfuribacter thermophilus;Dissulfuribacter thermophilus S69 |
| Ga0586324 1075127 66 1178 | 0.00 | 8092000.00 | 0.00 | 0.00 | 0.00 | 0.00 | 0.00 | 14993800.00 | 0.00 | 8855000.00 | 0.00 | 0.00 | KO:K04077 | chaperonin GroEL | Bacteria;Proteobacteria;Deltaproteobacteria;Myxococcales;Sandaracinaceae;Sandaracinus;Sandaracinus amylolyticus;Sandaracinus amylolyticus DSM 53668 |
| Ga0586324 1076140 1 1176 | 0.00 | 80920000.00 | 0.00 | 0.00 | 0.00 | 0.00 | 4195200.00 | 4331000.00 | 0.00 | 2864400.00 | 3678400.00 | 3697500.00 | KO:K04077 | chaperonin GroEL | Bacteria;Acidobacteria;Vicinamibacteria;unclassified Vicinamibacteria;Vicinamibacteraceae;Luteitalea;Luteitalea pratensis;Luteitalea pratensis DSM 100886 |
| Ga0586324 1080154 2 1174 | 1040000.00 | 1190000.00 | 0.00 | 0.00 | 0.00 | 0.00 | 0.00 | 0.00 | 10692000.00 | 0.00 | 0.00 | 0.00 | KO:K04077 | chaperonin GroEL | Bacteria;Actinobacteria;Acidimicrobiia;Acidimicrobiales;Acidimicrobiaceae;Ferrithrix;Ferrithrix thermotolerans;Ferrithrix thermotolerans DSM 19514 |
| Ga0586324 1083406 3 782 | 0.00 | 387689.76 | 0.00 | 0.00 | 0.00 | 0.00 | 0.00 | 286591.42 | 0.00 | 0.00 | 1540000.00 | 0.00 | KO:K04077 | chaperonin GroEL | Bacteria;Proteobacteria;Alphaproteobacteria;Hyphomicrobiales;Hyphomicrobiaceae;Hyphomicrobium;Hyphomicrobium sp. CS1BSMeth3;Hyphomicrobium sp. CS1BSMeth3 |
| Ga0586324 1091643 1 1170 | 0.00 | 0.00 | 0.00 | 10839500.00 | 12895600.00 | 0.00 | 0.00 | 744200.00 | 1069200.00 | 0.00 | 0.00 | 0.00 | KO:K04077 | chaperonin GroEL | Bacteria;Chloroflexi;Anaerolineae;Anaerolineales;Anaerolineaceae;Thermanaerothrix;Thermanaerothrix daxensis;Thermanaerothrix daxensis GNS-1 |
| Ga0586324 1097003 40 1167 | 0.00 | 0.00 | 0.00 | 941963.95 | 0.00 | 0.00 | 0.00 | 0.00 | 0.00 | 0.00 | 0.00 | 0.00 | KO:K04077 | chaperonin GroEL | Bacteria;Proteobacteria;Betaproteobacteria;Nitrosomonadales;Nitrosomonadaceae;Nitrosomonas;Nitrosomonas oligotropha;Nitrosomonas oligotropha Nm75 |
| Ga0586324 1097836 1 1167 | 0.00 | 0.00 | 0.00 | 0.00 | 467778.62 | 0.00 | 922273.68 | 0.00 | 0.00 | 0.00 | 0.00 | 0.00 | KO:K04077 | chaperonin GroEL | Bacteria;Acidobacteria;Acidobacteriia;Acidobacteriales;Acidobacteriaceae;unclassified Acidobacteriaceae;Acidobacteriaceae bacterium KBS 96;Acidobacteriaceae bacterium KBS 96, DSM 24297 |
| Ga0586324 1098593 3 1166 | 0.00 | 0.00 | 0.00 | 0.00 | 0.00 | 0.00 | 0.00 | 0.00 | 493094.79 | 0.00 | 0.00 | 0.00 | KO:K04077 | chaperonin GroEL | Bacteria;Chloroflexi;Anaerolineae;Anaerolineales;Anaerolineaceae;Anaerolinea;Anaerolinea thermophila;Anaerolinea thermophila UNI-1 |
| Ga0586324 1102392 713 1165 | 0.00 | 0.00 | 0.00 | 0.00 | 3151800.00 | 0.00 | 0.00 | 0.00 | 0.00 | 0.00 | 0.00 | 0.00 | KO:K04077 | chaperonin GroEL | Bacteria;Acidobacteria;Vicinamibacteria;unclassified Vicinamibacteria;Vicinamibacteraceae;Luteitalea;Luteitalea pratensis;Luteitalea pratensis DSM 100886 |
| Ga0586324 1106535 1 1161 | 0.00 | 0.00 | 0.00 | 0.00 | 3244500.00 | 0.00 | 2530800.00 | 0.00 | 2025000.00 | 310660.35 | 1944800.00 | 1305000.00 | KO:K04077 | chaperonin GroEL | Bacteria;Proteobacteria;Deltaproteobacteria;Myxococcales;Sandaracinaceae;Sandaracinus;Sandaracinus amylolyticus;Sandaracinus amylolyticus DSM 53668 |
| Ga0586324 1126604 1 1152 | 0.00 | 0.00 | 0.00 | 0.00 | 481125.36 | 0.00 | 0.00 | 315439.54 | 2178900.00 | 0.00 | 0.00 | 0.00 | KO:K04077 | chaperonin GroEL | Bacteria;Proteobacteria;Deltaproteobacteria;Myxococcales;Anaeromyxobacteraceae;Anaeromyxobacter;Anaeromyxobacter dehalogenans;Anaeromyxobacter dehalogenans 2CP-C |
| Ga0586324 1127807 3 713 | 5800000.00 | 5793600.00 | 5014719.82 | 4275000.00 | 5304500.00 | 4287600.00 | 2196643.20 | 3934500.00 | 3928500.00 | 3503500.00 | 3009600.00 | 3682500.00 | KO:K04077 | chaperonin GroEL | Bacteria;Proteobacteria;Deltaproteobacteria;Syntrophorhabdales;Syntrophorhabdaceae;Syntrophorhabdus;Syntrophorhabdus aromaticivorans;Syntrophorhabdus aromaticivorans UI |
| Ga0586324 1137060 1 648 | 585851.00 | 0.00 | 0.00 | 0.00 | 0.00 | 0.00 | 0.00 | 0.00 | 0.00 | 0.00 | 0.00 | 0.00 | KO:K04077 | chaperonin GroEL | Bacteria;Gemmatimonadetes;Gemmatimonadetes;Gemmatimonadales;Gemmatimonadaceae;Gemmatimonas;Gemmatimonas aurantiaca;Gemmatimonas aurantiaca T-27T |
| Ga0586324 1141343 3 1148 | 0.00 | 0.00 | 0.00 | 0.00 | 0.00 | 0.00 | 0.00 | 0.00 | 0.00 | 1909600.00 | 0.00 | 0.00 | KO:K04077 | chaperonin GroEL | Bacteria;Acidobacteria;Acidobacteriia;Acidobacteriales;Acidobacteriaceae;unclassified Acidobacteriaceae;Acidobacteriaceae bacterium KBS 96;Acidobacteriaceae bacterium KBS 96, DSM 24297 |
| Ga0586324 1145097 1 738 | 0.00 | 0.00 | 0.00 | 1026000.00 | 559003.66 | 0.00 | 0.00 | 0.00 | 0.00 | 0.00 | 0.00 | 0.00 | KO:K04077 | chaperonin GroEL | Bacteria;Chloroflexi;Anaerolineae;Anaerolineales;Anaerolineaceae;Pelolinea;Pelolinea submarina;Pelolinea submarina DSM 23923 |
| Ga0586324 1158100 2 1141 | 0.00 | 0.00 | 1782092.05 | 291882.75 | 3955200.00 | 2935783.24 | 12357600.00 | 11285000.00 | 3288600.00 | 885500.00 | 9416000.00 | 11677500.00 | KO:K04077 | chaperonin GroEL | Bacteria;Proteobacteria;Gammaproteobacteria;Cellvibrionales;Halieaceae;unclassified Halieaceae;gamma proteobacterium IMCC3088;gamma proteobacterium IMCC3088 |
| Ga0586324 1160565 3 512 | 0.00 | 0.00 | 0.00 | 0.00 | 0.00 | 0.00 | 0.00 | 0.00 | 0.00 | 1139600.00 | 0.00 | 0.00 | KO:K04077 | chaperonin GroEL | Bacteria;Candidatus Calescamantes;unclassified Candidatus Calescamantes;unclassified Candidatus Calescamantes;unclassified Candidatus Calescamantes;unclassified Candidatus Calescamantes;Calescamantes bacterium JGI 0000106-H18;Calescamantes bacterium JGI 0000106-H18 (contamination screened) |
| Ga0586324 1163241 778 1137 | 0.00 | 0.00 | 0.00 | 0.00 | 0.00 | 0.00 | 0.00 | 0.00 | 0.00 | 0.00 | 0.00 | 847500.00 | KO:K04077 | chaperonin GroEL | Bacteria;Acidobacteria;Acidobacteriia;Acidobacteriales;Acidobacteriaceae;unclassified Acidobacteriaceae;Acidobacteriaceae bacterium URHE0068;Acidobacteriaceae bacterium URHE0068 |
| Ga0586324 1168812 6 1136 | 0.00 | 0.00 | 0.00 | 0.00 | 0.00 | 0.00 | 0.00 | 0.00 | 0.00 | 6121500.00 | 0.00 | 14550000.00 | KO:K04077 | chaperonin GroEL | Bacteria;Proteobacteria;Deltaproteobacteria;Myxococcales;Sandaracinaceae;Sandaracinus;Sandaracinus amylolyticus;Sandaracinus amylolyticus DSM 53668 |
| Ga0586324 1184606 433 1128 | 1110000.00 | 0.00 | 0.00 | 0.00 | 0.00 | 10599900.00 | 0.00 | 0.00 | 0.00 | 0.00 | 0.00 | 3652500.00 | KO:K04077 | chaperonin GroEL | Bacteria;Proteobacteria;Betaproteobacteria;Nitrosomonadales;Nitrosomonadaceae;Nitrosospira;Nitrosospira sp. Nl5;Nitrosospira sp. Nl5 |
| Ga0586324 1189234 5 1126 | 0.00 | 0.00 | 0.00 | 0.00 | 0.00 | 0.00 | 0.00 | 69540000.00 | 0.00 | 0.00 | 0.00 | 0.00 | KO:K04077 | chaperonin GroEL | Bacteria;Actinobacteria;Actinomycetia;Micrococcales;Intrasporangiaceae;Oryzihumus;Oryzihumus leptocrescens;Oryzihumus leptocrescens DSM 18082 |
| Ga0586324 1193034 1 1125 | 0.00 | 0.00 | 0.00 | 3315500.00 | 2493535.24 | 108303.98 | 17599707.60 | 4209000.00 | 17010000.00 | 16786000.00 | 5183200.00 | 5272500.00 | KO:K04077 | chaperonin GroEL | Bacteria;Proteobacteria;Betaproteobacteria;Nitrosomonadales;Nitrosomonadaceae;Nitrosospira;Nitrosospira sp. Nl5;Nitrosospira sp. Nl5 |
| Ga0586324 1203919 31 1122 | 0.00 | 0.00 | 0.00 | 0.00 | 0.00 | 0.00 | 0.00 | 0.00 | 0.00 | 0.00 | 897600.00 | 0.00 | KO:K04077 | chaperonin GroEL | Bacteria;Actinobacteria;Actinomycetia;unclassified Actinomycetia;unclassified Actinomycetia;unclassified Actinomycetia;Actinobacteria bacterium IMCC26256;Actinobacteria bacterium IMCC26256 |
| Ga0586324 1205378 2 1120 | 0.00 | 1391763.48 | 10077700.00 | 4018500.00 | 4810256.56 | 2852111.52 | 10221021.12 | 4080900.00 | 2118460.23 | 2695759.99 | 3330642.48 | 1567500.00 | KO:K04077 | chaperonin GroEL | Bacteria;Candidatus Calescamantes;unclassified Candidatus Calescamantes;unclassified Candidatus Calescamantes;unclassified Candidatus Calescamantes;unclassified Candidatus Calescamantes;Calescamantes bacterium JGI 0000106-H18;Calescamantes bacterium JGI 0000106-H18 (contamination screened) |
| Ga0586324 1210661 149 1117 | 1380000.00 | 0.00 | 4305199.07 | 7457500.00 | 0.00 | 1568900.33 | 1736648.64 | 2421700.00 | 1053000.00 | 1817200.00 | 932800.00 | 1353057.75 | KO:K04077 | chaperonin GroEL | Bacteria;Proteobacteria;Betaproteobacteria;unclassified Betaproteobacteria;unclassified Betaproteobacteria;unclassified Betaproteobacteria;Betaproteobacteria bacterium UKL13-2;Betaproteobacteria bacterium UKL13-2 |
| Ga0586324 1224138 1 1113 | 0.00 | 2366400.00 | 0.00 | 0.00 | 0.00 | 0.00 | 0.00 | 0.00 | 0.00 | 0.00 | 0.00 | 0.00 | KO:K04077 | chaperonin GroEL | Bacteria;Proteobacteria;Deltaproteobacteria;Desulfovibrionales;Desulfohalobiaceae;Desulfonauticus;Desulfonauticus submarinus;Desulfonauticus submarinus DSM 15269 |
| Ga0586324 1227436 736 1113 | 1600000.00 | 0.00 | 0.00 | 0.00 | 0.00 | 0.00 | 0.00 | 0.00 | 0.00 | 0.00 | 0.00 | 0.00 | KO:K04077 | chaperonin GroEL | Bacteria;Proteobacteria;Deltaproteobacteria;Myxococcales;Sandaracinaceae;Sandaracinus;Sandaracinus amylolyticus;Sandaracinus amylolyticus DSM 53668 |
| Ga0586324 1233132 266 1111 | 0.00 | 4263600.00 | 0.00 | 0.00 | 1012212.93 | 1337266.71 | 0.00 | 664900.00 | 1830600.00 | 250022.85 | 0.00 | 0.00 | KO:K04077 | chaperonin GroEL | Bacteria;Proteobacteria;Deltaproteobacteria;Desulfobacterales;Desulfobacteraceae;Desulfatirhabdium;Desulfatirhabdium butyrativorans;Desulfatirhabdium butyrativorans DSM 18734 |
| Ga0586324 1237227 1 1107 | 20800000.00 | 21284000.00 | 10809600.00 | 14155000.00 | 20188000.00 | 12584900.00 | 5768400.00 | 12932000.00 | 61560000.00 | 12545950.34 | 12144000.00 | 32355000.00 | KO:K04077 | chaperonin GroEL | Bacteria;Proteobacteria;Betaproteobacteria;unclassified Betaproteobacteria;unclassified Betaproteobacteria;unclassified Betaproteobacteria;Betaproteobacteria bacterium UKL13-2;Betaproteobacteria bacterium UKL13-2 |

| | | | | | | | | | | | | | | | |
|---|---|---|---|---|---|---|---|---|---|---|---|---|---|---|---|
| Ga0586324 1237710 1 969 | 0.00 | 0.00 | 0.00 | 0.00 | 0.00 | 0.00 | 0.00 | 354390.48 | 603245.88 | 0.00 | 0.00 | 642671.25 | KO:K04077 | chaperonin GroEL | Bacteria;Proteobacteria;Deltaproteobacteria;unclassified Deltaproteobacteria;unclassified Deltaproteobacteria;Dissulfuribacter;Dissulfuribacter thermophilus;Dissulfuribacter thermophilus S69 |
| Ga0586324 1244784 3 524 | 0.00 | 0.00 | 0.00 | 0.00 | 0.00 | 9210400.00 | 0.00 | 0.00 | 0.00 | 0.00 | 0.00 | 0.00 | KO:K04077 | chaperonin GroEL | Bacteria;Bacteroidetes;Sphingobacteriia;Sphingobacteriales;Sphingobacteriaceae;Solitalea;Solitalea canadensis;Solitalea canadensis USAM 9D, DSM 3403 |
| Ga0586324 1244792 2 898 | 4820000.00 | 0.00 | 0.00 | 1388124.80 | 2274918.77 | 0.00 | 2872800.00 | 29896100.00 | 1801783.44 | 24101000.00 | 26928000.00 | 19425000.00 | KO:K04077 | chaperonin GroEL | Bacteria;Chloroflexi;unclassified Chloroflexi;unclassified Chloroflexi;unclassified Chloroflexi;unclassified Chloroflexi;Chloroflexi bacterium T81;Chloroflexi bacterium T81 |
| Ga0586324 1250252 3 692 | 0.00 | 659776.12 | 0.00 | 0.00 | 0.00 | 0.00 | 647928.12 | 0.00 | 0.00 | 0.00 | 0.00 | 817500.00 | KO:K04077 | chaperonin GroEL | Bacteria;Planctomycetes;Planctomycetia;Pirellulales;Thermoguttaceae;Thermogutta;Thermogutta terrifontis;Thermogutta terrifontis R1 |
| Ga0586324 1255417 2 733 | 0.00 | 0.00 | 0.00 | 0.00 | 0.00 | 0.00 | 0.00 | 595988.30 | 450357.57 | 0.00 | 0.00 | 0.00 | KO:K04077 | chaperonin GroEL | Bacteria;Proteobacteria;Deltaproteobacteria;Syntrophobacterales;Syntrophobacteraceae;Syntrophobacter;Syntrophobacter fumaroxidans;Syntrophobacter fumaroxidans MPOB |
| Ga0586324 1268121 1 1095 | 0.00 | 0.00 | 0.00 | 0.00 | 0.00 | 0.00 | 0.00 | 0.00 | 6269400.00 | 3141600.00 | 0.00 | 0.00 | KO:K04077 | chaperonin GroEL | Bacteria;Proteobacteria;Deltaproteobacteria;Myxococcales;Sandaracinaceae;Sandaracinus;Sandaracinus amylolyticus;Sandaracinus amylolyticus DSM 53668 |
| Ga0586324 1271641 621 1094 | 0.00 | 0.00 | 0.00 | 0.00 | 0.00 | 0.00 | 0.00 | 0.00 | 0.00 | 465783.01 | 0.00 | 0.00 | KO:K04077 | chaperonin GroEL | Bacteria;Proteobacteria;Gammaproteobacteria;unclassified Gammaproteobacteria;unclassified Gammaproteobacteria;unclassified Gammaproteobacteria;endosymbiont of Riftia pachyptila;endosymbiont of Riftia pachyptila (vent Mk28) |
| Ga0586324 1277245 3 713 | 0.00 | 0.00 | 0.00 | 0.00 | 54075000.00 | 0.00 | 0.00 | 0.00 | 0.00 | 0.00 | 0.00 | 0.00 | KO:K04077 | chaperonin GroEL | Bacteria;Proteobacteria;Alphaproteobacteria;Hyphomicrobiales;Bradyrhizobiaceae;Bradyrhizobium;unclassified Bradyrhizobium;Bradyrhizobium sp. HAMBI 2127 |
| Ga0586324 1279677 3 755 | 0.00 | 54377.90 | 0.00 | 0.00 | 0.00 | 0.00 | 0.00 | 0.00 | 0.00 | 0.00 | 0.00 | 0.00 | KO:K04077 | chaperonin GroEL | Bacteria;Planctomycetes;Planctomycetia;Pirellulales;Thermoguttaceae;Thermogutta;Thermogutta terrifontis;Thermogutta terrifontis R1 |
| Ga0586324 1284848 1 1089 | 0.00 | 775200.00 | 0.00 | 0.00 | 0.00 | 0.00 | 0.00 | 0.00 | 0.00 | 0.00 | 0.00 | 0.00 | KO:K04077 | chaperonin GroEL | Bacteria;Planctomycetes;Planctomycetia;Pirellulales;Pirellulaceae;Pirellula;Pirellula staleyi;Pirellula staleyi DSM 6068 |
| Ga0586324 1285596 3 1091 | 0.00 | 0.00 | 1268985.11 | 0.00 | 0.00 | 0.00 | 2064722.40 | 0.00 | 3661200.00 | 2548700.00 | 0.00 | 3735000.00 | KO:K04077 | chaperonin GroEL | Bacteria;Bacteroidetes;Chitinophagia;Chitinophagales;Chitinophagaceae;Pseudobacter;Pseudobacter ginsenosidimutans;Pseudobacter ginsenosidimutans DSM 18116 |
| Ga0586324 1294808 515 1087 | 1590000.00 | 0.00 | 0.00 | 6031585.15 | 3573995.97 | 0.00 | 0.00 | 2698477.74 | 2795457.42 | 0.00 | 0.00 | 0.00 | KO:K04077 | chaperonin GroEL | Bacteria;Proteobacteria;Deltaproteobacteria;Syntrophobacterales;Syntrophaceae;Desulfobacca;Desulfobacca acetoxidans;Desulfobacca acetoxidans ASRB2, DSM 11109 |
| Ga0586324 1316497 3 1079 | 0.00 | 0.00 | 0.00 | 0.00 | 0.00 | 0.00 | 0.00 | 0.00 | 0.00 | 2130947.28 | 1638945.44 | 5814103.50 | KO:K04077 | chaperonin GroEL | Bacteria;Proteobacteria;Betaproteobacteria;Burkholderiales;Burkholderiaceae;Trinickia;Trinickia caryophylli;Trinickia caryophylli Ballard 720 |
| Ga0586324 1318742 1 1077 | 1390000.00 | 0.00 | 3291680.84 | 2147000.00 | 0.00 | 2810363.00 | 2986800.00 | 1482300.00 | 0.00 | 0.00 | 1689600.00 | 4515000.00 | KO:K04077 | chaperonin GroEL | Bacteria;Acidobacteria;Vicinamibacteria;unclassified Vicinamibacteria;Vicinamibacteraceae;Luteitalea;Luteitalea pratensis;Luteitalea pratensis DSM 100886 |
| Ga0586324 1324643 2 886 | 0.00 | 4467600.00 | 0.00 | 0.00 | 0.00 | 0.00 | 0.00 | 0.00 | 0.00 | 0.00 | 0.00 | 0.00 | KO:K04077 | chaperonin GroEL | Bacteria;Chloroflexi;Dehalococcoidia;unclassified Dehalococcoidia;unclassified Dehalococcoidia;Dehalogenimonas;Dehalogenimonas sp. WBC-2;Dehalogenimonas sp. WBC-2 |
| Ga0586324 1326564 3 1076 | 4270000.00 | 0.00 | 11823000.00 | 0.00 | 0.00 | 0.00 | 0.00 | 9089000.00 | 7865100.00 | 0.00 | 0.00 | 0.00 | KO:K04077 | chaperonin GroEL | Bacteria;Planctomycetes;Planctomycetia;Pirellulales;Pirellulaceae;Pirellula;Pirellula sp. SH-Sr6A;Pirellula sp. SH-Sr6A |
| Ga0586324 1332878 3 1073 | 0.00 | 0.00 | 0.00 | 0.00 | 0.00 | 0.00 | 379093.32 | 0.00 | 0.00 | 0.00 | 0.00 | 0.00 | KO:K04077 | chaperonin GroEL | Bacteria;Chloroflexi;Dehalococcoidia;unclassified Dehalococcoidia;unclassified Dehalococcoidia;Dehalogenimonas;Dehalogenimonas lykanthroporepellens;Dehalogenimonas lykanthroporepellens BL-DC-9 |
| Ga0586324 1333931 609 1073 | 0.00 | 0.00 | 0.00 | 0.00 | 0.00 | 0.00 | 0.00 | 3269600.00 | 2924100.00 | 0.00 | 0.00 | 0.00 | KO:K04077 | chaperonin GroEL | Bacteria;Chloroflexi;Anaerolineae;Anaerolineales;Anaerolineaceae;Thermanaerothrix;Thermanaerothrix daxensis;Thermanaerothrix daxensis GNS-1 |
| Ga0586324 1339613 3 1070 | 0.00 | 0.00 | 0.00 | 0.00 | 0.00 | 0.00 | 0.00 | 0.00 | 0.00 | 0.00 | 350375.52 | 0.00 | KO:K04077 | chaperonin GroEL | Bacteria;Candidatus Omnitrophica;unclassified Candidatus Omnitrophica;unclassified Candidatus Omnitrophica;unclassified Candidatus Omnitrophica;unclassified Candidatus Omnitrophica;Omnitrophica bacterium SCGC AAA257-O07;Omnitrophica bacterium SCGC AAA257-O07 (contamination screened) |
| Ga0586324 1357176 3 1064 | 0.00 | 0.00 | 0.00 | 0.00 | 0.00 | 0.00 | 0.00 | 0.00 | 0.00 | 0.00 | 738474.88 | 0.00 | KO:K04077 | chaperonin GroEL | Bacteria;Proteobacteria;Deltaproteobacteria;Desulfobacterales;Desulfobacteraceae;Desulfatiglans;Desulfatiglans anilini;Desulfatiglans anilini DSM 4660 |
| Ga0586324 1358918 2 1048 | 0.00 | 4458774.96 | 0.00 | 0.00 | 41251500.00 | 0.00 | 0.00 | 0.00 | 665598.06 | 2394700.00 | 2420000.00 | 1305000.00 | KO:K04077 | chaperonin GroEL | Bacteria;Proteobacteria;Deltaproteobacteria;Myxococcales;Labilitrichaceae;Labilithrix;Labilithrix luteola;Labilithrix luteola DSM 27648 |
| Ga0586324 1359163 3 863 | 0.00 | 0.00 | 0.00 | 0.00 | 0.00 | 0.00 | 0.00 | 0.00 | 0.00 | 0.00 | 1355200.00 | 1072500.00 | KO:K04077 | chaperonin GroEL | Bacteria;Thermodesulfobacteria;Thermodesulfobacteria;Thermodesulfobacteriales;Thermodesulfobacteriaceae;Thermodesulfatator;Thermodesulfatator autotrophicus;Thermodesulfatator autotrophicus S606 |
| Ga0586324 1365126 1 1062 | 0.00 | 312200.24 | 966136.15 | 12445000.00 | 0.00 | 7185700.00 | 0.00 | 24156000.00 | 2883600.00 | 0.00 | 0.00 | 14925000.00 | KO:K04077 | chaperonin GroEL | Bacteria;Planctomycetes;Planctomycetia;Pirellulales;Thermoguttaceae;Thermogutta;Thermogutta terrifontis;Thermogutta terrifontis R1 |
| Ga0586324 1369567 1 1062 | 0.00 | 0.00 | 0.00 | 0.00 | 0.00 | 3267099.59 | 1936066.56 | 6649000.00 | 5151600.00 | 0.00 | 1592800.00 | 0.00 | KO:K04077 | chaperonin GroEL | Bacteria;Acidobacteria;Thermoanaerobaculia;Thermoanaerobaculales;Thermoanaerobaculaceae;Thermoanaerobaculum;Thermoanaerobaculum aquaticum;Thermoanaerobaculum aquaticum MP-01 |
| Ga0586324 1369892 1 1062 | 0.00 | 0.00 | 0.00 | 11127653.05 | 491449.05 | 5637400.00 | 14250000.00 | 5166700.00 | 0.00 | 0.00 | 0.00 | 0.00 | KO:K04077 | chaperonin GroEL | Bacteria;Proteobacteria;Deltaproteobacteria;Syntrophobacterales;Syntrophaceae;Desulfomonile;Desulfomonile tiedjei;Desulfomonile tiedjei DCB-1, DSM 6799 |
| Ga0586324 1371484 3 1061 | 5030000.00 | 17061200.00 | 14578682.32 | 4807000.00 | 29355000.00 | 5121300.00 | 5677200.00 | 5544900.00 | 26073900.00 | 30030000.00 | 26144800.00 | 6075927.00 | KO:K04077 | chaperonin GroEL | Bacteria;Proteobacteria;Gammaproteobacteria;unclassified Gammaproteobacteria;Candidatus Competibacteraceae;Candidatus Competibacter;Candidatus Competibacter denitrificans;Candidatus Competibacter denitrificans Run A D11 |
| Ga0586324 1382410 3 848 | 0.00 | 5807200.00 | 0.00 | 2128000.00 | 0.00 | 0.00 | 0.00 | 2696200.00 | 0.00 | 2541000.00 | 4144800.00 | 0.00 | KO:K04077 | chaperonin GroEL | Bacteria;Proteobacteria;Gammaproteobacteria;Methylococcales;Crenotrichaceae;Crenothrix;Crenothrix polyspora;Crenothrix polyspora ERS1545888 |
| Ga0586324 1407468 3 650 | 10800000.00 | 500537.12 | 2134608.87 | 1843000.00 | 2070300.00 | 8337000.00 | 2895600.00 | 2379000.00 | 2268000.00 | 763521.22 | 1152800.00 | 1290000.00 | KO:K04077 | chaperonin GroEL | Bacteria;Ignavibacteriae;Ignavibacteria;Ignavibacteriales;Ignavibacteriaceae;Ignavibacterium;Ignavibacterium album;Ignavibacterium album Mat9-16, JCM 16511 |
| Ga0586324 1421778 1 1026 | 0.00 | 0.00 | 1053046.46 | 9690000.00 | 5644400.00 | 709716.90 | 0.00 | 2281400.00 | 516242.16 | 4959144.11 | 22149600.00 | 4317594.75 | KO:K04077 | chaperonin GroEL | Bacteria;Proteobacteria;Betaproteobacteria;Nitrosomonadales;Nitrosomonadaceae;Nitrosospira;Nitrosospira sp. Nsp44;Nitrosospira sp. Nsp44 |
| Ga0586324 1421787 1 1044 | 0.00 | 123711.72 | 658710000.00 | 0.00 | 0.00 | 25098089.89 | 0.00 | 26162900.00 | 20574000.00 | 17171000.00 | 23760000.00 | 3412500.00 | KO:K04077 | chaperonin GroEL | Bacteria;Proteobacteria;Deltaproteobacteria;Desulfobacterales;Desulfobacteraceae;Desulfatirhabdium;Desulfatirhabdium butyrativorans;Desulfatirhabdium butyrativorans DSM 18734 |
| Ga0586324 1446228 3 1037 | 0.00 | 132402800.00 | 5742600.00 | 3543500.00 | 0.00 | 0.00 | 1910475.84 | 385202800.00 | 383826600.00 | 368060000.00 | 446652800.00 | 403995000.00 | KO:K04077 | chaperonin GroEL | Bacteria;Proteobacteria;Deltaproteobacteria;Syntrophobacterales;Syntrophobacteraceae;Desulfosoma;Desulfosoma caldarium;Desulfosoma caldarium DSM 22027 |
| Ga0586324 1446626 3 1037 | 0.00 | 0.00 | 0.00 | 0.00 | 0.00 | 0.00 | 1770586.44 | 0.00 | 0.00 | 0.00 | 0.00 | 426674.25 | KO:K04077 | chaperonin GroEL | Bacteria;Proteobacteria;Deltaproteobacteria;Syntrophorhabdales;Syntrophorhabdaceae;Syntrophorhabdus;Syntrophorhabdus aromaticivorans;Syntrophorhabdus aromaticivorans UI |
| Ga0586324 1460185 3 992 | 0.00 | 0.00 | 0.00 | 0.00 | 0.00 | 0.00 | 0.00 | 4629900.00 | 273204.09 | 0.00 | 0.00 | 68260.95 | KO:K04077 | chaperonin GroEL | Bacteria;Acidobacteria;Vicinamibacteria;unclassified Vicinamibacteria;Vicinamibacteraceae;Luteitalea;Luteitalea pratensis;Luteitalea pratensis DSM 100886 |
| Ga0586324 1461475 2 613 | 0.00 | 0.00 | 0.00 | 0.00 | 0.00 | 0.00 | 459315.12 | 0.00 | 0.00 | 0.00 | 0.00 | 0.00 | KO:K04077 | chaperonin GroEL | Bacteria;Chloroflexi;Anaerolineae;Anaerolineales;Anaerolineaceae;Anaerolinea;Anaerolinea thermophila;Anaerolinea thermophila UNI-1 |
| Ga0586324 1468437 3 1031 | 1570000.00 | 0.00 | 0.00 | 0.00 | 907740.03 | 0.00 | 0.00 | 0.00 | 0.00 | 307116.81 | 0.00 | 0.00 | KO:K04077 | chaperonin GroEL | Bacteria;Candidatus Aminicenantes;unclassified Candidatus Aminicenantes;unclassified Candidatus Aminicenantes;unclassified Candidatus Aminicenantes;unclassified Candidatus Aminicenantes;Aminicenantes bacterium SCGC AAA252-P13;Aminicenantes bacterium SCGC AAA252-P13 (contamination screened) |
| Ga0586324 1474592 3 1028 | 0.00 | 112850.76 | 0.00 | 0.00 | 0.00 | 0.00 | 0.00 | 0.00 | 0.00 | 0.00 | 0.00 | 0.00 | KO:K04077 | chaperonin GroEL | Bacteria;Proteobacteria;Deltaproteobacteria;Syntrophobacterales;Syntrophobacteraceae;Desulfosoma;Desulfosoma caldarium;Desulfosoma caldarium DSM 22027 |
| Ga0586324 1474646 413 1027 | 0.00 | 0.00 | 0.00 | 0.00 | 0.00 | 1026233.09 | 0.00 | 0.00 | 0.00 | 0.00 | 0.00 | 0.00 | KO:K04077 | chaperonin GroEL | Bacteria;Proteobacteria;Betaproteobacteria;Burkholderiales;Burkholderiaceae;Pandoraea;Pandoraea thiooxydans;Pandoraea thiooxydans DSM 25325 |
| Ga0586324 1474991 1 1029 | 0.00 | 0.00 | 0.00 | 0.00 | 0.00 | 961224.34 | 0.00 | 0.00 | 0.00 | 0.00 | 0.00 | 0.00 | KO:K04077 | chaperonin GroEL | Bacteria;Chloroflexi;Thermomicrobia;Sphaerobacterales;Sphaerobacteraceae;Sphaerobacter;Sphaerobacter thermophilus;Sphaerobacter thermophilus 4ac11, DSM 20745 |
| Ga0586324 1486277 166 1023 | 0.00 | 0.00 | 0.00 | 0.00 | 0.00 | 0.00 | 0.00 | 0.00 | 300161.70 | 0.00 | 0.00 | 0.00 | KO:K04077 | chaperonin GroEL | Bacteria;Proteobacteria;Alphaproteobacteria;Rhodospirillales;Rhodospirillaceae;Dongia;Dongia sp. URHE0060;Dongia sp. URHE0060 |
| Ga0586324 1503966 3 1019 | 0.00 | 0.00 | 0.00 | 0.00 | 0.00 | 727994.78 | 866003.28 | 4593300.00 | 2247511.05 | 0.00 | 0.00 | 601467.00 | KO:K04077 | chaperonin GroEL | Bacteria;Proteobacteria;Deltaproteobacteria;Syntrophobacterales;Syntrophobacteraceae;Syntrophobacter;Syntrophobacter fumaroxidans;Syntrophobacter fumaroxidans MPOB |
| Ga0586324 1506514 430 1017 | 0.00 | 0.00 | 0.00 | 0.00 | 0.00 | 0.00 | 0.00 | 0.00 | 0.00 | 1416800.00 | 1144000.00 | 0.00 | KO:K04077 | chaperonin GroEL | Bacteria;Chloroflexi;Caldilineae;Caldilineales;Caldilineaceae;Caldilinea;Caldilinea aerophila;Caldilinea aerophila STL-6-O1, DSM 14535 |
| Ga0586324 1513980 3 1016 | 51620000.00 | 14422800.00 | 27812200.00 | 23180000.00 | 26213500.00 | 29497100.00 | 31830503.16 | 25376000.00 | 27945000.00 | 29304902.55 | 23584000.00 | 25995000.00 | KO:K04077 | chaperonin GroEL | Bacteria;Chloroflexi;Chloroflexia;Chloroflexales;Roseiflexaceae;Roseiflexus;Roseiflexus sp. RS-1;Roseiflexus sp. RS-1 |
| Ga0586324 1514848 250 1017 | 0.00 | 0.00 | 0.00 | 443882.75 | 0.00 | 0.00 | 0.00 | 0.00 | 0.00 | 0.00 | 0.00 | 0.00 | KO:K04077 | chaperonin GroEL | Bacteria;Proteobacteria;Deltaproteobacteria;Syntrophobacterales;Syntrophobacteraceae;Desulfacinum;Desulfacinum infernum;Desulfacinum infernum DSM 9756 |
| Ga0586324 1522268 2 586 | 0.00 | 0.00 | 0.00 | 0.00 | 0.00 | 0.00 | 0.00 | 0.00 | 0.00 | 0.00 | 4197600.00 | 0.00 | KO:K04077 | chaperonin GroEL | Bacteria;Proteobacteria;Deltaproteobacteria;Desulfobacterales;Desulfobulbaceae;Desulfobulbus;Desulfobulbus elongatus;Desulfobulbus elongatus DSM 2908 |
| Ga0586324 1525582 340 1014 | 0.00 | 0.00 | 0.00 | 0.00 | 83430000.00 | 0.00 | 0.00 | 0.00 | 0.00 | 0.00 | 0.00 | 0.00 | KO:K04077 | chaperonin GroEL | Bacteria;Proteobacteria;Deltaproteobacteria;Desulfobacterales;Desulfobulbaceae;Desulfoprunum;Desulfoprunum benzoelyticum;Desulfoprunum benzoelyticum DSM 28570 |
| Ga0586324 1528050 2 715 | 0.00 | 0.00 | 0.00 | 0.00 | 0.00 | 0.00 | 1055195.40 | 0.00 | 1296000.00 | 0.00 | 0.00 | 0.00 | KO:K04077 | chaperonin GroEL | Bacteria;Gemmatimonadetes;Gemmatimonadetes;Gemmatimonadales;Gemmatimonadaceae;Gemmatimonas;Gemmatimonas aurantiaca;Gemmatimonas aurantiaca T-27T |
| Ga0586324 1541617 127 1008 | 0.00 | 0.00 | 0.00 | 0.00 | 0.00 | 0.00 | 0.00 | 0.00 | 1611900.00 | 0.00 | 1592800.00 | 0.00 | KO:K04077 | chaperonin GroEL | Bacteria;Firmicutes;Negativicutes;Selenomonadales;Selenomonadaceae;Selenomonas;Selenomonas ruminantium;Selenomonas ruminantium ATCC 12561 |
| Ga0586324 1542576 2 880 | 0.00 | 1101600.00 | 0.00 | 0.00 | 0.00 | 0.00 | 0.00 | 10699400.00 | 0.00 | 24178000.00 | 10208000.00 | 0.00 | KO:K04077 | chaperonin GroEL | Bacteria;Proteobacteria;Gammaproteobacteria;Methylococcales;Crenotrichaceae;Crenothrix;Crenothrix polyspora;Crenothrix polyspora ERS1545888 |
| Ga0586324 1553802 3 1004 | 8230000.00 | 0.00 | 0.00 | 0.00 | 4109700.00 | 0.00 | 0.00 | 0.00 | 2972700.00 | 0.00 | 0.00 | 0.00 | KO:K04077 | chaperonin GroEL | Bacteria;Proteobacteria;Alphaproteobacteria;Hyphomicrobiales;Phyllobacteriaceae;Mesorhizobium;Mesorhizobium amorphae;Mesorhizobium amorphae CCNWGS0123 |
| Ga0586324 1557960 1 1005 | 0.00 | 0.00 | 0.00 | 0.00 | 0.00 | 0.00 | 0.00 | 713700.00 | 0.00 | 0.00 | 0.00 | 0.00 | KO:K04077 | chaperonin GroEL | Bacteria;Proteobacteria;Betaproteobacteria;unclassified Betaproteobacteria;unclassified Betaproteobacteria;unclassified Betaproteobacteria;uncultured beta proteobacterium;unclassified betaproteobacterium ENR4 |
| Ga0586324 1562043 2 664 | 0.00 | 0.00 | 0.00 | 0.00 | 0.00 | 0.00 | 0.00 | 847900.00 | 0.00 | 0.00 | 0.00 | 0.00 | KO:K04077 | chaperonin GroEL | Bacteria;Candidatus Calescamantes;unclassified Candidatus Calescamantes;unclassified Candidatus Calescamantes;unclassified Candidatus Calescamantes;unclassified Candidatus Calescamantes;Calescamantes bacterium JGI 0000106-H18;Calescamantes bacterium JGI 0000106-H18 (contamination screened) |
| Ga0586324 1563383 71 1003 | 0.00 | 0.00 | 0.00 | 3714500.00 | 0.00 | 0.00 | 0.00 | 3446500.00 | 0.00 | 3988600.00 | 2939200.00 | 2272500.00 | KO:K04077 | chaperonin GroEL | Bacteria;Proteobacteria;Deltaproteobacteria;Myxococcales;Archangiaceae;Vitiosangium;Vitiosangium sp. GDMCC 1.1324;Vitiosangium sp. GDMCC 1.1324 |
| Ga0586324 1566228 3 1001 | 0.00 | 0.00 | 0.00 | 0.00 | 2060000.00 | 0.00 | 0.00 | 0.00 | 0.00 | 0.00 | 0.00 | 0.00 | KO:K04077 | chaperonin GroEL | Bacteria;Actinobacteria;Nitriliruptoria;Nitriliruptorales;Nitriliruptoraceae;Nitriliruptor;Nitriliruptor alkaliphilus;Nitriliruptor alkaliphilus DSM 45188 |
| Ga0586324 1569823 11 1000 | 0.00 | 0.00 | 0.00 | 8284000.00 | 0.00 | 0.00 | 0.00 | 0.00 | 0.00 | 0.00 | 0.00 | 0.00 | KO:K04077 | chaperonin GroEL | Bacteria;Chloroflexi;Anaerolineae;Anaerolineales;Anaerolineaceae;Anaerolinea;Anaerolinea thermophila;Anaerolinea thermophila UNI-1 |
| Ga0586324 1573005 1 999 | 0.00 | 2271200.00 | 21450300.00 | 0.00 | 7931000.00 | 2868003.43 | 1526811.12 | 9146507.75 | 10368000.00 | 636251.77 | 473366.96 | 14100000.00 | KO:K04077 | chaperonin GroEL | Bacteria;Proteobacteria;Deltaproteobacteria;Syntrophobacterales;Syntrophaceae;Desulfobacca;Desulfobacca acetoxidans;Desulfobacca acetoxidans ASRB2, DSM 11109 |
| Ga0586324 1574824 240 1001 | 0.00 | 0.00 | 0.00 | 0.00 | 0.00 | 1203648.42 | 0.00 | 572912.61 | 0.00 | 0.00 | 449738.08 | 477342.75 | KO:K04077 | chaperonin GroEL | Bacteria;Proteobacteria;Deltaproteobacteria;Desulfobacterales;Desulfobacteraceae;Desulfatiglans;Desulfatiglans anilini;Desulfatiglans anilini DSM 4660 |
| Ga0586324 1576138 1 999 | 1830000.00 | 0.00 | 0.00 | 0.00 | 0.00 | 0.00 | 4377600.00 | 0.00 | 0.00 | 0.00 | 0.00 | 5047500.00 | KO:K04077 | chaperonin GroEL | Bacteria;Spirochaetes;Spirochaetia;Leptospirales;Leptospiraceae;Leptospira;Leptospira santarosai;Leptospira santarosai U160 |
| Ga0586324 1582416 268 999 | 0.00 | 0.00 | 0.00 | 0.00 | 0.00 | 0.00 | 0.00 | 0.00 | 0.00 | 0.00 | 959200.00 | 1252500.00 | KO:K04077 | chaperonin GroEL | Bacteria;Proteobacteria;Alphaproteobacteria;Hyphomicrobiales;unclassified Hyphomicrobiales;Methyloceanibacter;Methyloceanibacter caenitepidi;Methyloceanibacter caenitepidi Gela4 |
| Ga0586324 1584219 2 997 | 0.00 | 0.00 | 0.00 | 0.00 | 3213600.00 | 0.00 | 0.00 | 0.00 | 0.00 | 0.00 | 0.00 | 0.00 | KO:K04077 | chaperonin GroEL | Bacteria;Proteobacteria;Deltaproteobacteria;unclassified Deltaproteobacteria;unclassified Deltaproteobacteria;Dissulfuribacter;Dissulfuribacter thermophilus;Dissulfuribacter thermophilus S69 |
| Ga0586324 1584744 3 890 | 887797.00 | 1686400.00 | 0.00 | 4123000.00 | 7083333.69 | 0.00 | 1557985.56 | 1030900.00 | 2405700.00 | 1309000.00 | 550693.44 | 370681.50 | KO:K04077 | chaperonin GroEL | Bacteria;Proteobacteria;Betaproteobacteria;Rhodocyclales;Azonexaceae;Dechloromonas;Dechloromonas aromatica;Dechloromonas aromatica RCB |
| Ga0586324 1586373 3 644 | 0.00 | 0.00 | 0.00 | 0.00 | 47895000.00 | 491934.61 | 0.00 | 171114.15 | 127170000.00 | 0.00 | 0.00 | 300623.25 | KO:K04077 | chaperonin GroEL | Bacteria;Proteobacteria;Alphaproteobacteria;Hyphomicrobiales;Bradyrhizobiaceae;Bradyrhizobium;Bradyrhizobium elkanii;Bradyrhizobium elkanii WSM1741 |
| Ga0586324 1586944 155 997 | 0.00 | 0.00 | 0.00 | 0.00 | 0.00 | 0.00 | 0.00 | 1378600.00 | 0.00 | 2279200.00 | 0.00 | 1507500.00 | KO:K04077 | chaperonin GroEL | Bacteria;Verrucomicrobia;Verrucomicrobiae;Verrucomicrobiales;Verrucomicrobia subdivision 3;Pedosphaera;Pedosphaera parvula;Pedosphaera parvula Ellin514 |
| Ga0586324 1587390 2 772 | 9340000.00 | 0.00 | 0.00 | 3334500.00 | 0.00 | 3001585.99 | 0.00 | 0.00 | 4203900.00 | 6183100.00 | 437552.72 | 5385000.00 | KO:K04077 | chaperonin GroEL | Bacteria;Verrucomicrobia;Verrucomicrobiae;Verrucomicrobiales;Verrucomicrobia subdivision 3;Pedosphaera;Pedosphaera parvula;Pedosphaera parvula Ellin514 |
| Ga0586324 1587845 3 995 | 0.00 | 0.00 | 0.00 | 0.00 | 0.00 | 0.00 | 0.00 | 0.00 | 0.00 | 0.00 | 0.00 | 3742500.00 | KO:K04077 | chaperonin GroEL | Bacteria;Actinobacteria;Thermoleophilia;Solirubrobacterales;unclassified Solirubrobacterales;unclassified Solirubrobacterales;Solirubrobacterales bacterium URHD0059;Solirubrobacterales bacterium URHD0059 |
| Ga0586324 1588119 3 995 | 1020000.00 | 0.00 | 0.00 | 0.00 | 0.00 | 0.00 | 0.00 | 0.00 | 0.00 | 0.00 | 0.00 | 0.00 | KO:K04077 | chaperonin GroEL | Bacteria;Proteobacteria;Betaproteobacteria;Nitrosomonadales;Nitrosomonadaceae;Nitrosovibrio;Nitrosovibrio tenuis;Nitrosovibrio tenuis Nv1 |
| Ga0586324 1616324 3 794 | 0.00 | 0.00 | 0.00 | 0.00 | 0.00 | 0.00 | 0.00 | 0.00 | 0.00 | 0.00 | 0.00 | 2490000.00 | KO:K04077 | chaperonin GroEL | Bacteria;Acidobacteria;Blastocatellia;Blastocatellales;Pyrinomonadaceae;Pyrinomonas;Pyrinomonas methylaliphatogenes;Pyrinomonas methylaliphatogenes K22 |
| Ga0586324 1618843 304 987 | 0.00 | 0.00 | 0.00 | 0.00 | 0.00 | 0.00 | 0.00 | 0.00 | 0.00 | 741179.67 | 0.00 | 0.00 | KO:K04077 | chaperonin GroEL | Bacteria;Acidobacteria;Acidobacteriia;Bryobacterales;Bryobacteraceae;Bryobacter;Bryobacter aggregatus;Bryobacter aggregatus DSM 18758 |
| Ga0586324 1623071 69 986 | 755385.00 | 0.00 | 15595100.00 | 0.00 | 0.00 | 0.00 | 0.00 | 0.00 | 0.00 | 0.00 | 0.00 | 2520000.00 | KO:K04077 | chaperonin GroEL | Bacteria;Verrucomicrobia;Verrucomicrobiae;Verrucomicrobiales;Verrucomicrobia subdivision 3;Pedosphaera;Pedosphaera parvula;Pedosphaera parvula Ellin514 |
| Ga0586324 1633705 1 984 | 0.00 | 0.00 | 0.00 | 73438.33 | 0.00 | 0.00 | 109275.38 | 0.00 | 0.00 | 0.00 | 0.00 | 4335000.00 | KO:K04077 | chaperonin GroEL | Bacteria;Chloroflexi;Thermomicrobia;Sphaerobacterales;Sphaerobacteraceae;Sphaerobacter;Sphaerobacter thermophilus;Sphaerobacter thermophilus 4ac11, DSM 20745 |
| Ga0586324 1644817 1 981 | 0.00 | 383282.00 | 0.00 | 0.00 | 0.00 | 0.00 | 0.00 | 0.00 | 0.00 | 0.00 | 0.00 | 0.00 | KO:K04077 | chaperonin GroEL | Bacteria;Proteobacteria;Deltaproteobacteria;Myxococcales;Anaeromyxobacteraceae;Anaeromyxobacter;Anaeromyxobacter sp. Fw109-5;Anaeromyxobacter sp. Fw109-5 |
| Ga0586324 1647720 2 982 | 0.00 | 0.00 | 0.00 | 0.00 | 4418700.00 | 0.00 | 0.00 | 3751500.00 | 0.00 | 4750900.00 | 3564000.00 | 0.00 | KO:K04077 | chaperonin GroEL | Bacteria;Proteobacteria;Deltaproteobacteria;Syntrophobacterales;Syntrophobacteraceae;Desulfacinum;Desulfacinum infernum;Desulfacinum infernum DSM 9756 |
| Ga0586324 1653429 1 978 | 0.00 | 0.00 | 0.00 | 0.00 | 0.00 | 0.00 | 6247200.00 | 0.00 | 0.00 | 1355200.00 | 0.00 | 0.00 | KO:K04077 | chaperonin GroEL | Bacteria;Candidatus Calescamantes;unclassified Candidatus Calescamantes;unclassified Candidatus Calescamantes;unclassified Candidatus Calescamantes;unclassified Candidatus Calescamantes;Calescamantes bacterium JGI 0000106-H18;Calescamantes bacterium JGI 0000106-H18 (contamination screened) |
| Ga0586324 1667162 121 975 | 0.00 | 0.00 | 0.00 | 0.00 | 0.00 | 0.00 | 0.00 | 0.00 | 0.00 | 194188.61 | 0.00 | 0.00 | KO:K04077 | chaperonin GroEL | Bacteria;Bacteroidetes;Bacteroidia;Marinilabiliales;Prolixibacteraceae;Draconibacterium;Draconibacterium sediminis;Draconibacterium sediminis JN14CK-3 |
| Ga0586324 1671387 3 974 | 0.00 | 0.00 | 0.00 | 0.00 | 0.00 | 0.00 | 0.00 | 0.00 | 615634.83 | 0.00 | 0.00 | 0.00 | KO:K04077 | chaperonin GroEL | Bacteria;Verrucomicrobia;Verrucomicrobiae;Verrucomicrobiales;Verrucomicrobia subdivision 3;Pedosphaera;Pedosphaera parvula;Pedosphaera parvula Ellin514 |
| Ga0586324 1689956 368 970 | 0.00 | 0.00 | 0.00 | 0.00 | 0.00 | 0.00 | 0.00 | 0.00 | 0.00 | 0.00 | 3960000.00 | 0.00 | KO:K04077 | chaperonin GroEL | Bacteria;Acidobacteria;Acidobacteriia;Acidobacteriales;Acidobacteriaceae;unclassified Acidobacteriaceae;Acidobacteriaceae bacterium KBS 96;Acidobacteriaceae bacterium KBS 96, DSM 24297 |
| Ga0586324 1697695 2 967 | 339383.00 | 8432000.00 | 0.00 | 0.00 | 0.00 | 0.00 | 0.00 | 0.00 | 327652.29 | 550019.47 | 2955704.40 | 565038.00 | KO:K04077 | chaperonin GroEL | Bacteria;Chloroflexi;Thermomicrobia;Sphaerobacterales;Sphaerobacteraceae;Sphaerobacter;Sphaerobacter thermophilus;Sphaerobacter thermophilus 4ac11, DSM 20745 |
| Ga0586324 1698941 195 968 | 0.00 | 357763.64 | 0.00 | 0.00 | 0.00 | 0.00 | 2122511.28 | 5166700.00 | 3086100.00 | 0.00 | 7436000.00 | 6365376.00 | KO:K04077 | chaperonin GroEL | Bacteria;Ignavibacteriae;Ignavibacteria;Ignavibacteriales;Melioribacteraceae;Melioribacter;Melioribacter roseus;Melioribacter roseus P3M |
| Ga0586324 1701721 126 968 | 0.00 | 0.00 | 0.00 | 0.00 | 0.00 | 0.00 | 0.00 | 0.00 | 0.00 | 0.00 | 207911.44 | 0.00 | KO:K04077 | chaperonin GroEL | Bacteria;Acidobacteria;Blastocatellia;unclassified Blastocatellia;unclassified Blastocatellia;Chloracidobacterium;Chloracidobacterium thermophilum;Chloracidobacterium thermophilum B |
| Ga0586324 1702852 1 966 | 318875.00 | 0.00 | 0.00 | 0.00 | 0.00 | 0.00 | 0.00 | 436650.20 | 398814.03 | 514719.59 | 670362.00 | 428205.00 | KO:K04077 | chaperonin GroEL | Bacteria;Candidatus Calescamantes;unclassified Candidatus Calescamantes;unclassified Candidatus Calescamantes;unclassified Candidatus Calescamantes;unclassified Candidatus Calescamantes;Calescamantes bacterium JGI 0000106-H18;Calescamantes bacterium JGI 0000106-H18 (contamination screened) |
| Ga0586324 1707104 3 965 | 10900000.00 | 11158800.00 | 4778344.27 | 7714000.00 | 742530.09 | 9925000.00 | 18787200.00 | 9375700.00 | 10829700.00 | 16493400.00 | 17855200.00 | 19200000.00 | KO:K04077 | chaperonin GroEL | Bacteria;Proteobacteria;Alphaproteobacteria;Rhodospirillales;Rhodospirillaceae;Dongia;Dongia mobilis;Dongia mobilis CGMCC 1.7660 |
| Ga0586324 1711725 2 964 | 2140000.00 | 0.00 | 0.00 | 0.00 | 0.00 | 0.00 | 3762000.00 | 0.00 | 0.00 | 9317000.00 | 0.00 | 0.00 | KO:K04077 | chaperonin GroEL | Bacteria;Proteobacteria;Deltaproteobacteria;Syntrophorhabdales;Syntrophorhabdaceae;Syntrophorhabdus;Syntrophorhabdus aromaticivorans;Syntrophorhabdus aromaticivorans UI |
| Ga0586324 1735580 135 959 | 2190000.00 | 0.00 | 0.00 | 0.00 | 0.00 | 0.00 | 1558113.24 | 0.00 | 0.00 | 0.00 | 0.00 | 0.00 | KO:K04077 | chaperonin GroEL | Bacteria;Acidobacteria;Vicinamibacteria;unclassified Vicinamibacteria;Vicinamibacteraceae;Luteitalea;Luteitalea pratensis;Luteitalea pratensis DSM 100886 |
| Ga0586324 1745407 2 958 | 0.00 | 0.00 | 0.00 | 0.00 | 12051000.00 | 2420608.25 | 0.00 | 2440000.00 | 2170800.00 | 1493800.00 | 0.00 | 1680000.00 | KO:K04077 | chaperonin GroEL | Bacteria;Acidobacteria;Vicinamibacteria;unclassified Vicinamibacteria;Vicinamibacteraceae;Luteitalea;Luteitalea pratensis;Luteitalea pratensis DSM 100886 |
| Ga0586324 1752923 1 444 | 0.00 | 0.00 | 0.00 | 0.00 | 0.00 | 0.00 | 0.00 | 0.00 | 0.00 | 4219600.00 | 0.00 | 0.00 | KO:K04077 | chaperonin GroEL | Bacteria;Proteobacteria;Betaproteobacteria;unclassified Betaproteobacteria;unclassified Betaproteobacteria;unclassified Betaproteobacteria;beta proteobacterium AAP51;Betaproteobacteria bacterium AAP51 |
| Ga0586324 1753820 1 771 | 0.00 | 0.00 | 0.00 | 2888000.00 | 3388700.00 | 0.00 | 0.00 | 54534000.00 | 0.00 | 0.00 | 0.00 | 67500000.00 | KO:K04077 | chaperonin GroEL | Bacteria;Proteobacteria;Alphaproteobacteria;Hyphomicrobiales;Bradyrhizobiaceae;Bradyrhizobium;Bradyrhizobium arachidis;Bradyrhizobium arachidis USDA 3384 |
| Ga0586324 1756898 1 954 | 1168567.00 | 1550400.00 | 152974368.33 | 2187036.80 | 1785226.90 | 1288483.35 | 459244.44 | 1730809.12 | 2939563.71 | 2911779.64 | 83419677.44 | 2452349.25 | KO:K04077 | chaperonin GroEL | Bacteria;Proteobacteria;Deltaproteobacteria;Syntrophobacterales;Syntrophobacteraceae;Desulfacinum;Desulfacinum infernum;Desulfacinum infernum DSM 9756 |
| Ga0586324 1759098 1 954 | 0.00 | 0.00 | 0.00 | 573770.55 | 0.00 | 2725436.76 | 0.00 | 0.00 | 1287900.00 | 350704.20 | 0.00 | 0.00 | KO:K04077 | chaperonin GroEL | Bacteria;Proteobacteria;Deltaproteobacteria;Desulfobacterales;Desulfobacteraceae;Desulfosarcina;Desulfosarcina variabilis;Desulfosarcina variabilis Montpellier |
| Ga0586324 1760263 3 626 | 0.00 | 0.00 | 0.00 | 0.00 | 2647100.00 | 0.00 | 0.00 | 0.00 | 0.00 | 0.00 | 0.00 | 0.00 | KO:K04077 | chaperonin GroEL | Bacteria;Proteobacteria;Deltaproteobacteria;Syntrophorhabdales;Syntrophorhabdaceae;Syntrophorhabdus;Syntrophorhabdus aromaticivorans;Syntrophorhabdus aromaticivorans UI |
| Ga0586324 1774016 41 949 | 0.00 | 0.00 | 0.00 | 0.00 | 0.00 | 0.00 | 0.00 | 0.00 | 602078.67 | 0.00 | 0.00 | 0.00 | KO:K04077 | chaperonin GroEL | Bacteria;Proteobacteria;Alphaproteobacteria;Hyphomicrobiales;Hyphomicrobiaceae;Rhodoplanes;Rhodoplanes sp. Z2-YC6860;Rhodoplanes sp. Z2-YC6860 |
| Ga0586324 1774180 3 455 | 0.00 | 0.00 | 0.00 | 0.00 | 2492600.00 | 0.00 | 0.00 | 0.00 | 0.00 | 0.00 | 378578.64 | 0.00 | KO:K04077 | chaperonin GroEL | Bacteria;Acidobacteria;Vicinamibacteria;unclassified Vicinamibacteria;Vicinamibacteraceae;Luteitalea;Luteitalea pratensis;Luteitalea pratensis DSM 100886 |
| Ga0586324 1776279 1 951 | 12500000.00 | 0.00 | 0.00 | 26885000.00 | 24514000.00 | 15086000.00 | 0.00 | 15494000.00 | 19917900.00 | 22330000.00 | 0.00 | 427048.50 | KO:K04077 | chaperonin GroEL | Bacteria;Proteobacteria;Deltaproteobacteria;Syntrophorhabdales;Syntrophorhabdaceae;Syntrophorhabdus;Syntrophorhabdus aromaticivorans;Syntrophorhabdus aromaticivorans UI |
| Ga0586324 1779026 1 819 | 0.00 | 0.00 | 0.00 | 0.00 | 0.00 | 0.00 | 0.00 | 0.00 | 923400.00 | 0.00 | 0.00 | 0.00 | KO:K04077 | chaperonin GroEL | Bacteria;Chloroflexi;Anaerolineae;Anaerolineales;Anaerolineaceae;Anaerolinea;Anaerolinea thermophila;Anaerolinea thermophila UNI-1 |
| Ga0586324 1779391 2 880 | 0.00 | 0.00 | 41549400.00 | 31825000.00 | 1359600.00 | 0.00 | 6429600.00 | 4068700.00 | 4552200.00 | 4820200.00 | 4048000.00 | 4942500.00 | KO:K04077 | chaperonin GroEL | Bacteria;Proteobacteria;Gammaproteobacteria;Cellvibrionales;Halieaceae;Haliea;Haliea salexigens;Haliea salexigens DSM 19537 |
| Ga0586324 1782170 1 948 | 0.00 | 8704000.00 | 0.00 | 0.00 | 0.00 | 0.00 | 0.00 | 0.00 | 0.00 | 137901.61 | 0.00 | 0.00 | KO:K04077 | chaperonin GroEL | Bacteria;Acidobacteria;Blastocatellia;Blastocatellales;Pyrinomonadaceae;Pyrinomonas;Pyrinomonas methylaliphatogenes;Pyrinomonas methylaliphatogenes K22 |
| Ga0586324 1784511 3 860 | 0.00 | 3270800.00 | 0.00 | 0.00 | 0.00 | 0.00 | 0.00 | 0.00 | 0.00 | 0.00 | 0.00 | 0.00 | KO:K04077 | chaperonin GroEL | Bacteria;Proteobacteria;Betaproteobacteria;Burkholderiales;Burkholderiaceae;Pandoraea;Candidatus Pandoraea novymonadis;Pandoraea novymonadis E262 |
| Ga0586324 1789579 2 946 | 0.00 | 0.00 | 0.00 | 0.00 | 0.00 | 0.00 | 0.00 | 0.00 | 571232.25 | 0.00 | 0.00 | 0.00 | KO:K04077 | chaperonin GroEL | Bacteria;Proteobacteria;Alphaproteobacteria;Micropepsales;Micropepsaceae;Rhizomicrobium;Rhizomicrobium electricum;Rhizomicrobium electricum DSM 21034 |
| Ga0586324 1794479 177 947 | 0.00 | 0.00 | 0.00 | 0.00 | 0.00 | 0.00 | 0.00 | 0.00 | 0.00 | 2079000.00 | 0.00 | 0.00 | KO:K04077 | chaperonin GroEL | Bacteria;Chloroflexi;Dehalococcoidia;unclassified Dehalococcoidia;unclassified Dehalococcoidia;Dehalogenimonas;Dehalogenimonas formicexedens;Dehalogenimonas formicexedens NSZ-14 |
| Ga0586324 1795689 3 944 | 4780000.00 | 0.00 | 0.00 | 4474500.00 | 2348400.00 | 0.00 | 0.00 | 15677000.00 | 0.00 | 0.00 | 0.00 | 0.00 | KO:K04077 | chaperonin GroEL | Bacteria;Proteobacteria;Deltaproteobacteria;unclassified Deltaproteobacteria;unclassified Deltaproteobacteria;Dissulfuribacter;Dissulfuribacter thermophilus;Dissulfuribacter thermophilus S69 |
| Ga0586324 1796526 3 902 | 0.00 | 428592.44 | 108377.50 | 0.00 | 513203.68 | 210269.07 | 0.00 | 98621.14 | 0.00 | 0.00 | 0.00 | 92524.50 | KO:K04077 | chaperonin GroEL | Bacteria;Proteobacteria;Deltaproteobacteria;Syntrophobacterales;Syntrophaceae;Syntrophus;Syntrophus aciditrophicus;Syntrophus aciditrophicus SB |
| Ga0586324 1798862 1 678 | 0.00 | 0.00 | 0.00 | 0.00 | 5644400.00 | 0.00 | 0.00 | 3812500.00 | 0.00 | 2795100.00 | 0.00 | 1995000.00 | KO:K04077 | chaperonin GroEL | Bacteria;Bacteroidetes;Bacteroidia;Bacteroidales;Porphyromonadaceae;Massilibacteroides;Massilibacteroides vaginae;Massilibacteroides vaginae MV12 |
| Ga0586324 1799381 156 944 | 0.00 | 1870000.00 | 0.00 | 0.00 | 1194800.00 | 0.00 | 0.00 | 0.00 | 2413800.00 | 0.00 | 0.00 | 0.00 | KO:K04077 | chaperonin GroEL | Bacteria;Gemmatimonadetes;Gemmatimonadetes;Gemmatimonadales;Gemmatimonadaceae;Gemmatimonas;Gemmatimonas phototrophica;Gemmatimonas phototrophica AP64 |
| Ga0586324 1820088 3 941 | 0.00 | 0.00 | 0.00 | 15105000.00 | 197605.50 | 0.00 | 9485764.44 | 15054800.00 | 11880043.20 | 3734887.31 | 4901600.00 | 3382985.25 | KO:K04077 | chaperonin GroEL | Bacteria;Proteobacteria;Deltaproteobacteria;Desulfobacterales;Desulfobacteraceae;Desulfosarcina;Desulfosarcina cetonica;Desulfosarcina cetonica JCM 12296 |
| Ga0586324 1833001 109 936 | 0.00 | 0.00 | 0.00 | 0.00 | 0.00 | 0.00 | 0.00 | 0.00 | 0.00 | 839300.00 | 1416800.00 | 0.00 | KO:K04077 | chaperonin GroEL | Bacteria;Gemmatimonadetes;Gemmatimonadetes;Gemmatimonadales;Gemmatimonadaceae;Gemmatirosa;Gemmatirosa kalamazoonesis;Gemmatimonadetes bacterium KBS708 |
| Ga0586324 1838732 112 936 | 3250000.00 | 2061076.60 | 6981200.00 | 21470000.00 | 1903223.70 | 9885300.00 | 5403600.00 | 26846100.00 | 8461903.14 | 8778000.00 | 1816283.92 | 6690000.00 | KO:K04077 | chaperonin GroEL | Bacteria;Acidobacteria;Vicinamibacteria;unclassified Vicinamibacteria;Vicinamibacteraceae;Luteitalea;Luteitalea pratensis;Luteitalea pratensis DSM 100886 |
| Ga0586324 1843088 2 934 | 0.00 | 0.00 | 0.00 | 0.00 | 0.00 | 0.00 | 0.00 | 0.00 | 0.00 | 0.00 | 0.00 | 3127500.00 | KO:K04077 | chaperonin GroEL | Bacteria;Proteobacteria;Alphaproteobacteria;Hyphomicrobiales;Methylobacteriaceae;Microvirga;Microvirga subterranea;Microvirga subterranea DSM 14364 |
| Ga0586324 1846139 3 935 | 710125.00 | 4031559.52 | 192072.52 | 3339069.50 | 3990511.49 | 12281730.95 | 533079.73 | 4426623.60 | 3466312.38 | 1354553.20 | 1642975.84 | 1285359.00 | KO:K04077 | chaperonin GroEL | Bacteria;Proteobacteria;Betaproteobacteria;Rhodocyclales;Azonexaceae;Dechloromonas;Dechloromonas aromatica;Dechloromonas aromatica RCB |
| Ga0586324 1860623 3 932 | 0.00 | 0.00 | 0.00 | 0.00 | 0.00 | 0.00 | 0.00 | 443341.29 | 0.00 | 0.00 | 0.00 | 0.00 | KO:K04077 | chaperonin GroEL | Bacteria;Proteobacteria;Deltaproteobacteria;Syntrophobacterales;Syntrophobacteraceae;Thermodesulforhabdus;Thermodesulforhabdus norvegica;Thermodesulforhabdus norvegica DSM 9990 |
| Ga0586324 1863702 1 930 | 0.00 | 0.00 | 0.00 | 0.00 | 0.00 | 0.00 | 0.00 | 0.00 | 0.00 | 449940.26 | 0.00 | 0.00 | KO:K04077 | chaperonin GroEL | Bacteria;Proteobacteria;Alphaproteobacteria;Rhodospirillales;Rhodospirillaceae;Inquilinus;Inquilinus limosus;Inquilinus limosus DSM 16000 |
| Ga0586324 1881778 3 926 | 0.00 | 0.00 | 0.00 | 0.00 | 0.00 | 0.00 | 0.00 | 0.00 | 23733000.00 | 0.00 | 0.00 | 0.00 | KO:K04077 | chaperonin GroEL | Bacteria;Actinobacteria;Actinomycetia;Acidothermales;Acidothermaceae;Acidothermus;Acidothermus cellulolyticus;Acidothermus cellulolyticus 11B |
| Ga0586324 1887040 1 408 | 0.00 | 0.00 | 0.00 | 0.00 | 0.00 | 0.00 | 0.00 | 0.00 | 0.00 | 3295600.00 | 0.00 | 0.00 | KO:K04077 | chaperonin GroEL | Bacteria;Proteobacteria;Betaproteobacteria;Nitrosomonadales;Gallionellaceae;Gallionella;Gallionella capsiferriformans;Gallionella capsiferriformans ES-2 |
| Ga0586324 1897776 557 922 | 0.00 | 0.00 | 0.00 | 1843000.00 | 1400800.00 | 0.00 | 917107.20 | 1098000.00 | 0.00 | 0.00 | 0.00 | 0.00 | KO:K04077 | chaperonin GroEL | Bacteria;Gemmatimonadetes;Gemmatimonadetes;Gemmatimonadales;Gemmatimonadaceae;Gemmatimonas;Gemmatimonas phototrophica;Gemmatimonas phototrophica AP64 |
| Ga0586324 1904710 2 922 | 6213447.00 | 481586.20 | 7151309.89 | 0.00 | 3656500.00 | 291520.28 | 9085847.88 | 8246654.05 | 3754795.50 | 7771462.93 | 7865476.08 | 1631472.75 | KO:K04077 | chaperonin GroEL | Bacteria;Proteobacteria;Betaproteobacteria;Nitrosomonadales;Sterolibacteriaceae;Sulfurisoma;Sulfurisoma sediminicola;Sulfurisoma sediminicola DSM 26916 |
| Ga0586324 1913151 3 920 | 0.00 | 0.00 | 0.00 | 0.00 | 0.00 | 31442400.00 | 0.00 | 0.00 | 99792000.00 | 0.00 | 67848000.00 | 74250000.00 | KO:K04077 | chaperonin GroEL | Bacteria;Proteobacteria;Deltaproteobacteria;Desulfobacterales;Desulfobacteraceae;Desulfatiglans;Desulfatiglans anilini;Desulfatiglans anilini DSM 4660 |
| Ga0586324 1938466 3 914 | 0.00 | 0.00 | 0.00 | 17480000.00 | 0.00 | 0.00 | 0.00 | 4629900.00 | 0.00 | 0.00 | 924000.00 | 6337500.00 | KO:K04077 | chaperonin GroEL | Bacteria;Proteobacteria;Betaproteobacteria;Nitrosomonadales;Nitrosomonadaceae;Nitrosospira;Nitrosospira multiformis;Nitrosospira multiformis Nl12 |
| Ga0586324 1963886 3 911 | 0.00 | 0.00 | 0.00 | 451861.80 | 0.00 | 5280100.00 | 20132400.00 | 0.00 | 561784.41 | 1216600.00 | 0.00 | 3720000.00 | KO:K04077 | chaperonin GroEL | Bacteria;Proteobacteria;Deltaproteobacteria;Syntrophobacterales;Syntrophaceae;Desulfomonile;Desulfomonile tiedjei;Desulfomonile tiedjei DCB-1, DSM 6799 |
| Ga0586324 1978383 1 906 | 0.00 | 0.00 | 0.00 | 0.00 | 0.00 | 0.00 | 71592000.00 | 0.00 | 0.00 | 0.00 | 0.00 | 0.00 | KO:K04077 | chaperonin GroEL | Bacteria;Planctomycetes;Candidatus Brocadiae;Candidatus Brocadiales;Candidatus Brocadiaceae;Candidatus Brocadia;Candidatus Brocadia sinica;Candidatus Brocadia sinica JPN1 |
| Ga0586324 1981808 3 722 | 0.00 | 4233457.64 | 0.00 | 0.00 | 566889.34 | 0.00 | 0.00 | 1944349.38 | 4258227.51 | 1878800.00 | 0.00 | 1710000.00 | KO:K04077 | chaperonin GroEL | Bacteria;Proteobacteria;Deltaproteobacteria;Myxococcales;Sandaracinaceae;Sandaracinus;Sandaracinus amylolyticus;Sandaracinus amylolyticus DSM 53668 |
| Ga0586324 1992256 1 621 | 0.00 | 0.00 | 0.00 | 0.00 | 0.00 | 0.00 | 444745.92 | 2104500.00 | 1198800.00 | 3041500.00 | 3326400.00 | 2850000.00 | KO:K04077 | chaperonin GroEL | Bacteria;Proteobacteria;Betaproteobacteria;Burkholderiales;Comamonadaceae;Ramlibacter;Ramlibacter tataouinensis;Ramlibacter tataouinensis TTB310 |
| Ga0586324 2002424 3 902 | 0.00 | 0.00 | 499356.23 | 0.00 | 0.00 | 0.00 | 0.00 | 0.00 | 0.00 | 0.00 | 0.00 | 0.00 | KO:K04077 | chaperonin GroEL | Bacteria;Proteobacteria;Deltaproteobacteria;Myxococcales;Sandaracinaceae;Sandaracinus;Sandaracinus amylolyticus;Sandaracinus amylolyticus DSM 53668 |
| Ga0586324 2008342 2 901 | 0.00 | 0.00 | 0.00 | 0.00 | 0.00 | 0.00 | 0.00 | 0.00 | 0.00 | 0.00 | 578929.12 | 0.00 | KO:K04077 | chaperonin GroEL | Bacteria;Acidobacteria;Vicinamibacteria;unclassified Vicinamibacteria;Vicinamibacteraceae;Luteitalea;Luteitalea pratensis;Luteitalea pratensis DSM 100886 |
| Ga0586324 2008638 1 903 | 0.00 | 0.00 | 0.00 | 0.00 | 0.00 | 0.00 | 0.00 | 0.00 | 0.00 | 513048.69 | 950400.00 | 0.00 | KO:K04077 | chaperonin GroEL | Bacteria;Chloroflexi;Dehalococcoidia;unclassified Dehalococcoidia;unclassified Dehalococcoidia;Dehalogenimonas;Dehalogenimonas formicexedens;Dehalogenimonas formicexedens NSZ-14 |
| Ga0586324 2010929 1 513 | 0.00 | 0.00 | 0.00 | 0.00 | 0.00 | 0.00 | 0.00 | 301268.02 | 0.00 | 0.00 | 0.00 | 0.00 | KO:K04077 | chaperonin GroEL | Bacteria;Firmicutes;Clostridia;Thermosediminibacterales;Thermosediminibacteraceae;Caldanaerovirga;Caldanaerovirga acetigignens;Caldanaerovirga acetigignens DSM 18802 |
| Ga0586324 2014554 87 899 | 0.00 | 0.00 | 0.00 | 0.00 | 0.00 | 0.00 | 1171931.40 | 0.00 | 0.00 | 0.00 | 0.00 | 296109.00 | KO:K04077 | chaperonin GroEL | Bacteria;Spirochaetes;Spirochaetia;Spirochaetales;Treponemataceae;Treponema;Treponema caldarium;Treponema caldarium DSM 7334 |
| Ga0586324 2022589 1 900 | 0.00 | 1523200.00 | 0.00 | 0.00 | 0.00 | 0.00 | 5677200.00 | 1671400.00 | 2025000.00 | 4373600.00 | 0.00 | 0.00 | KO:K04077 | chaperonin GroEL | Bacteria;Proteobacteria;Deltaproteobacteria;Desulfobacterales;Desulfobacteraceae;Desulfosarcina;Desulfosarcina cetonica;Desulfosarcina cetonica JCM 12296 |
| Ga0586324 2031217 94 897 | 0.00 | 0.00 | 0.00 | 0.00 | 0.00 | 0.00 | 0.00 | 9760000.00 | 0.00 | 0.00 | 0.00 | 0.00 | KO:K04077 | chaperonin GroEL | Bacteria;Proteobacteria;Deltaproteobacteria;Desulfobacterales;Desulfobacteraceae;Desulfosalsimonas;Desulfosalsimonas propionicica;Desulfosalsimonas propionicica DSM 17721 |
| Ga0586324 2036195 272 895 | 0.00 | 0.00 | 927615.69 | 0.00 | 0.00 | 0.00 | 827220.48 | 3239100.00 | 0.00 | 3010700.00 | 0.00 | 593418.00 | KO:K04077 | chaperonin GroEL | Bacteria;Nitrospirae;Nitrospira;Nitrospirales;Nitrospiraceae;Nitrospira;Nitrospira sp. ND1;Nitrospira sp. ND1 |
| Ga0586324 2037211 1 579 | 0.00 | 0.00 | 0.00 | 0.00 | 0.00 | 0.00 | 0.00 | 2257000.00 | 0.00 | 565526.50 | 0.00 | 0.00 | KO:K04077 | chaperonin GroEL | Bacteria;Planctomycetes;Planctomycetia;Pirellulales;Pirellulaceae;Rhodopirellula;Rhodopirellula rubra;Rhodopirellula rubra CECT 8075 |
| Ga0586324 2051323 2 895 | 0.00 | 0.00 | 0.00 | 0.00 | 0.00 | 0.00 | 0.00 | 0.00 | 1012500.00 | 0.00 | 0.00 | 0.00 | KO:K04077 | chaperonin GroEL | Bacteria;Actinobacteria;Actinomycetia;unclassified Actinomycetia;unclassified Actinomycetia;unclassified Actinomycetia;Actinobacteria bacterium IMCC26256;Actinobacteria bacterium IMCC26256 |
| Ga0586324 2051404 1 894 | 0.00 | 962699.80 | 3914240.61 | 0.00 | 0.00 | 0.00 | 0.00 | 0.00 | 0.00 | 0.00 | 281285.84 | 0.00 | KO:K04077 | chaperonin GroEL | Bacteria;Calditrichaeota;Calditrichae;Calditrichales;Calditrichaceae;Caldithrix;Caldithrix abyssi;Caldithrix abyssi LF13, DSM 13497 |
| Ga0586324 2053567 1 894 | 0.00 | 0.00 | 0.00 | 0.00 | 0.00 | 0.00 | 0.00 | 10126000.00 | 6795900.00 | 0.00 | 9328000.00 | 0.00 | KO:K04077 | chaperonin GroEL | Bacteria;Proteobacteria;Betaproteobacteria;unclassified Betaproteobacteria;unclassified Betaproteobacteria;unclassified Betaproteobacteria;uncultured beta proteobacterium;unclassified betaproteobacterium ENR4 |
| Ga0586324 2059126 1 831 | 0.00 | 0.00 | 0.00 | 1092500.00 | 11453600.00 | 0.00 | 0.00 | 4532300.00 | 19278000.00 | 0.00 | 591367.04 | 3022500.00 | KO:K04077 | chaperonin GroEL | Bacteria;Acidobacteria;Acidobacteriia;Acidobacteriales;Acidobacteriaceae;Candidatus Koribacter;Candidatus Koribacter versatilis;Candidatus Koribacter versatilis Ellin345 |
| Ga0586324 2065411 1 891 | 0.00 | 0.00 | 0.00 | 0.00 | 0.00 | 0.00 | 0.00 | 0.00 | 0.00 | 939400.00 | 436324.24 | 0.00 | KO:K04077 | chaperonin GroEL | Bacteria;Proteobacteria;Deltaproteobacteria;Myxococcales;Anaeromyxobacteraceae;Anaeromyxobacter;Anaeromyxobacter dehalogenans;Anaeromyxobacter dehalogenans 2CP-C |
| Ga0586324 2070496 3 890 | 0.00 | 0.00 | 0.00 | 0.00 | 1709800.00 | 9766200.00 | 0.00 | 0.00 | 0.00 | 0.00 | 0.00 | 0.00 | KO:K04077 | chaperonin GroEL | Bacteria;Chloroflexi;Anaerolineae;Anaerolineales;Anaerolineaceae;Anaerolinea;Anaerolinea thermophila;Anaerolinea thermophila UNI-1 |
| Ga0586324 2072967 48 890 | 3370000.00 | 14280000.00 | 0.00 | 0.00 | 0.00 | 0.00 | 0.00 | 0.00 | 0.00 | 3773000.00 | 5306400.00 | 12825000.00 | KO:K04077 | chaperonin GroEL | Bacteria;Planctomycetes;Planctomycetia;Pirellulales;Pirellulaceae;Blastopirellula;Blastopirellula marina;Blastopirellula marina DSM 3645 |
| Ga0586324 2074483 1 609 | 0.00 | 0.00 | 0.00 | 3458000.00 | 4882200.00 | 0.00 | 2184080.40 | 981902.36 | 0.00 | 908600.00 | 0.00 | 0.00 | KO:K04077 | chaperonin GroEL | Bacteria;Proteobacteria;Deltaproteobacteria;Desulfuromonadales;Geobacteraceae;Geothermobacter;Geothermobacter sp. HR-1;Geothermobacter sp. HR-1 |
| Ga0586324 2086683 3 635 | 0.00 | 0.00 | 0.00 | 36385000.00 | 0.00 | 0.00 | 0.00 | 0.00 | 0.00 | 0.00 | 26312000.00 | 0.00 | KO:K04077 | chaperonin GroEL | Bacteria;Bacteroidetes;Bacteroidia;Bacteroidales;Tannerellaceae;Parabacteroides;Parabacteroides sp. AM58-2XD;Parabacteroides sp. AM58-2XD |
| Ga0586324 2108286 1 882 | 0.00 | 0.00 | 0.00 | 0.00 | 2060000.00 | 0.00 | 468503.52 | 0.00 | 2049300.00 | 9933000.00 | 2930400.00 | 1905000.00 | KO:K04077 | chaperonin GroEL | Bacteria;Verrucomicrobia;Spartobacteria;unclassified Spartobacteria;unclassified Spartobacteria;unclassified Spartobacteria;Spartobacteria bacterium LR76;Spartobacteria bacterium LR76 |
| Ga0586324 2124389 3 881 | 0.00 | 0.00 | 0.00 | 0.00 | 0.00 | 0.00 | 282323.28 | 466975.13 | 1189642.95 | 0.00 | 327301.04 | 323934.75 | KO:K04077 | chaperonin GroEL | Bacteria;Proteobacteria;Alphaproteobacteria;Rhodospirillales;Rhodospirillaceae;Dongia;Dongia mobilis;Dongia mobilis CGMCC 1.7660 |
| Ga0586324 2125141 316 879 | 0.00 | 258486.36 | 0.00 | 0.00 | 0.00 | 0.00 | 0.00 | 0.00 | 0.00 | 118978.86 | 0.00 | 0.00 | KO:K04077 | chaperonin GroEL | Bacteria;Acidobacteria;Thermoanaerobaculia;Thermoanaerobaculales;Thermoanaerobaculaceae;Thermoanaerobaculum;Thermoanaerobaculum aquaticum;Thermoanaerobaculum aquaticum MP-01 |

| | | | | | | | | | | | | | | | |
|---|---|---|---|---|---|---|---|---|---|---|---|---|---|---|---|
| Ga0586324 2129619 1 879 | 0.00 | 0.00 | 0.00 | 0.00 | 0.00 | 0.00 | 0.00 | 0.00 | 0.00 | 0.00 | 1082400.00 | 0.00 | KO:K04077 | chaperonin GroEL | Bacteria;Gemmatimonadetes;Gemmatimonadetes;Gemmatimonadales;Gemmatimonadaceae;Gemmatirosa;Gemmatirosa kalamazoonesis;Gemmatimonadetes bacterium KBS708 |
| Ga0586324 2141489 3 545 | 0.00 | 0.00 | 6305600.00 | 0.00 | 17510000.00 | 0.00 | 0.00 | 0.00 | 0.00 | 0.00 | 0.00 | 0.00 | KO:K04077 | chaperonin GroEL | Bacteria;Proteobacteria;Deltaproteobacteria;Syntrophobacterales;Syntrophaceae;Syntrophus;Syntrophus aciditrophicus;Syntrophus aciditrophicus SB |
| Ga0586324 2144220 2 778 | 0.00 | 0.00 | 0.00 | 0.00 | 0.00 | 0.00 | 0.00 | 2037400.00 | 0.00 | 0.00 | 0.00 | 0.00 | KO:K04077 | chaperonin GroEL | Bacteria;Proteobacteria;Deltaproteobacteria;Syntrophorhabdales;Syntrophorhabdaceae;Syntrophorhabdus;Syntrophorhabdus aromaticivorans;Syntrophorhabdus aromaticivorans UI |
| Ga0586324 2146879 142 876 | 0.00 | 0.00 | 0.00 | 0.00 | 0.00 | 0.00 | 0.00 | 0.00 | 0.00 | 847000.00 | 0.00 | 0.00 | KO:K04077 | chaperonin GroEL | Bacteria;Planctomycetes;Candidatus Brocadiae;Candidatus Brocadiales;Candidatus Brocadiaceae;Candidatus Brocadia;Candidatus Brocadia sinica;Candidatus Brocadia sinica JPN1 |
| Ga0586324 2182224 2 871 | 0.00 | 6269600.00 | 0.00 | 387559.15 | 0.00 | 0.00 | 0.00 | 0.00 | 0.00 | 0.00 | 0.00 | 0.00 | KO:K04077 | chaperonin GroEL | Bacteria;Proteobacteria;Gammaproteobacteria;Nevskiales;Steroidobacteraceae;Povalibacter;Povalibacter uvarum;Povalibacter uvarum DSM 26723 |
| Ga0586324 2187491 2 742 | 0.00 | 6371600.00 | 2837570.67 | 0.00 | 1225700.00 | 0.00 | 0.00 | 1367991.49 | 19389285.90 | 26719000.00 | 3493600.00 | 18525000.00 | KO:K04077 | chaperonin GroEL | Bacteria;Proteobacteria;Betaproteobacteria;Nitrosomonadales;Nitrosomonadaceae;Nitrosospira;Nitrosospira sp. Nl5;Nitrosospira sp. Nl5 |
| Ga0586324 2190598 208 870 | 0.00 | 0.00 | 0.00 | 0.00 | 0.00 | 5319800.00 | 0.00 | 0.00 | 0.00 | 0.00 | 0.00 | 0.00 | KO:K04077 | chaperonin GroEL | Bacteria;Proteobacteria;Gammaproteobacteria;Thiotrichales;Thiotrichaceae;Beggiatoa;Beggiatoa leptomitoformis;Beggiatoa leptomitoformis D-402 |
| Ga0586324 2195520 1 744 | 0.00 | 0.00 | 0.00 | 175380136.50 | 0.00 | 222488863.95 | 5494800.00 | 1756800.00 | 4479300.00 | 8778000.00 | 133654400.00 | 108750000.00 | KO:K04077 | chaperonin GroEL | Bacteria;Proteobacteria;Deltaproteobacteria;Desulfobacterales;Desulfobacteraceae;Desulfatiglans;Desulfatiglans anilini;Desulfatiglans anilini DSM 4660 |
| Ga0586324 2197386 1 867 | 0.00 | 0.00 | 0.00 | 0.00 | 0.00 | 0.00 | 0.00 | 744200.00 | 0.00 | 0.00 | 0.00 | 0.00 | KO:K04077 | chaperonin GroEL | Bacteria;Proteobacteria;Betaproteobacteria;Nitrosomonadales;Nitrosomonadaceae;Nitrosovibrio;Nitrosovibrio tenuis;Nitrosovibrio tenuis Nv1 |
| Ga0586324 2202714 1 867 | 0.00 | 0.00 | 0.00 | 0.00 | 0.00 | 0.00 | 0.00 | 2257000.00 | 1142100.00 | 2787400.00 | 0.00 | 0.00 | KO:K04077 | chaperonin GroEL | Bacteria;Proteobacteria;Gammaproteobacteria;Methylococcales;Crenotrichaceae;Crenothrix;Crenothrix polyspora;Crenothrix polyspora ERS1545888 |
| Ga0586324 2204249 1 867 | 0.00 | 3264000.00 | 0.00 | 0.00 | 0.00 | 0.00 | 0.00 | 0.00 | 6002100.00 | 0.00 | 0.00 | 0.00 | KO:K04077 | chaperonin GroEL | Bacteria;Proteobacteria;Deltaproteobacteria;Desulfobacterales;Desulfobacteraceae;Desulfatiglans;Desulfatiglans anilini;Desulfatiglans anilini DSM 4660 |
| Ga0586324 2205265 3 866 | 0.00 | 0.00 | 0.00 | 0.00 | 0.00 | 0.00 | 0.00 | 4459100.00 | 0.00 | 0.00 | 1724800.00 | 0.00 | KO:K04077 | chaperonin GroEL | Bacteria;Proteobacteria;Deltaproteobacteria;unclassified Deltaproteobacteria;unclassified Deltaproteobacteria;unclassified Deltaproteobacteria;delta proteobacterium NaphS2;delta proteobacterium NaphS2 |
| Ga0586324 2208977 1 489 | 0.00 | 0.00 | 0.00 | 0.00 | 1730400.00 | 0.00 | 0.00 | 0.00 | 0.00 | 0.00 | 0.00 | 0.00 | KO:K04077 | chaperonin GroEL | Bacteria;Chloroflexi;Anaerolineae;Anaerolineales;Anaerolineaceae;Anaerolinea;Anaerolinea thermophila;Anaerolinea thermophila UNI-1 |
| Ga0586324 2214622 1 864 | 0.00 | 0.00 | 0.00 | 0.00 | 0.00 | 0.00 | 0.00 | 0.00 | 0.00 | 7931000.00 | 8060800.00 | 0.00 | KO:K04077 | chaperonin GroEL | Bacteria;Nitrospirae;Thermodesulfovibrionia;Thermodesulfovibrionales;Thermodesulfovibrionaceae;Thermodesulfovibrio;Thermodesulfovibrio thiophilus;Thermodesulfovibrio thiophilus DSM 17215 |
| Ga0586324 2217974 115 864 | 0.00 | 0.00 | 0.00 | 0.00 | 0.00 | 0.00 | 0.00 | 3794200.00 | 0.00 | 0.00 | 2490400.00 | 0.00 | KO:K04077 | chaperonin GroEL | Bacteria;Bacteroidetes;Bacteroidia;Bacteroidales;Dysgonomonadaceae;Dysgonomonas;Dysgonomonas sp. HGC4;Dysgonomonas sp. HGC4 |
| Ga0586324 2230193 337 864 | 0.00 | 0.00 | 0.00 | 0.00 | 0.00 | 0.00 | 0.00 | 0.00 | 0.00 | 0.00 | 0.00 | 1762500.00 | KO:K04077 | chaperonin GroEL | Bacteria;Nitrospirae;Nitrospira;Nitrospirales;Nitrospiraceae;Candidatus Magnetoovum;Candidatus Magnetoovum chiemensis;Candidatus Magnetoovum chiemensis CS-04 |
| Ga0586324 2230913 3 863 | 11700000.00 | 2699600.00 | 8332400.00 | 4208500.00 | 5304500.00 | 16658092.21 | 6680400.00 | 1195600.00 | 4681800.00 | 9209200.00 | 6749600.00 | 4012500.00 | KO:K04077 | chaperonin GroEL | Bacteria;Acidobacteria;Vicinamibacteria;unclassified Vicinamibacteria;Vicinamibacteraceae;Luteitalea;Luteitalea pratensis;Luteitalea pratensis DSM 100886 |
| Ga0586324 2237233 1 831 | 0.00 | 0.00 | 0.00 | 0.00 | 0.00 | 0.00 | 0.00 | 0.00 | 0.00 | 4250400.00 | 0.00 | 0.00 | KO:K04077 | chaperonin GroEL | Bacteria;Acidobacteria;Acidobacteriia;Acidobacteriales;Acidobacteriaceae;unclassified Acidobacteriaceae;Acidobacteriaceae bacterium KBS 96;Acidobacteriaceae bacterium KBS 96, DSM 24297 |
| Ga0586324 2250524 1 858 | 0.00 | 1700000.00 | 2244714.78 | 3239500.00 | 5500200.00 | 893111.05 | 3304039.20 | 3001200.00 | 5710500.00 | 2979900.00 | 2798400.00 | 19087500.00 | KO:K04077 | chaperonin GroEL | Bacteria;Proteobacteria;Deltaproteobacteria;Myxococcales;Anaeromyxobacteraceae;Anaeromyxobacter;Anaeromyxobacter sp. K;Anaeromyxobacter sp. K |
| Ga0586324 2252934 1 858 | 0.00 | 0.00 | 813315.43 | 0.00 | 0.00 | 0.00 | 454476.96 | 0.00 | 0.00 | 0.00 | 0.00 | 0.00 | KO:K04077 | chaperonin GroEL | Bacteria;Chloroflexi;unclassified Chloroflexi;unclassified Chloroflexi;unclassified Chloroflexi;unclassified Chloroflexi;unclassified Chloroflexi;Chloroflexi sp. G233 |
| Ga0586324 2270428 3 857 | 0.00 | 0.00 | 0.00 | 0.00 | 0.00 | 0.00 | 0.00 | 1262700.00 | 0.00 | 0.00 | 0.00 | 0.00 | KO:K04077 | chaperonin GroEL | Bacteria;Proteobacteria;Deltaproteobacteria;Desulfobacterales;Desulfobacteraceae;Desulfatitalea;Desulfatitalea tepidiphila;Desulfatitalea tepidiphila S28bF |
| Ga0586324 2279052 3 854 | 0.00 | 0.00 | 0.00 | 0.00 | 0.00 | 0.00 | 0.00 | 0.00 | 160344.36 | 0.00 | 0.00 | 0.00 | KO:K04077 | chaperonin GroEL | Bacteria;Actinobacteria;Actinomycetia;Motilibacterales;Motilibacteraceae;Motilibacter;Motilibacter peucedani;Motilibacter peucedani DSM 45328 |
| Ga0586324 2284182 221 853 | 0.00 | 0.00 | 0.00 | 0.00 | 0.00 | 0.00 | 0.00 | 8052000.00 | 0.00 | 0.00 | 0.00 | 0.00 | KO:K04077 | chaperonin GroEL | Bacteria;Verrucomicrobia;Verrucomicrobiae;Verrucomicrobiales;Verrucomicrobia subdivision 3;Pedosphaera;Pedosphaera parvula;Pedosphaera parvula Ellin514 |
| Ga0586324 2294525 2 853 | 0.00 | 0.00 | 0.00 | 0.00 | 0.00 | 0.00 | 0.00 | 872300.00 | 0.00 | 0.00 | 0.00 | 0.00 | KO:K04077 | chaperonin GroEL | Bacteria;Calditrichaeota;Calditrichae;Calditrichales;Calditrichaceae;Caldithrix;Caldithrix abyssi;Caldithrix abyssi LF13, DSM 13497 |
| Ga0586324 2297552 3 851 | 0.00 | 0.00 | 0.00 | 0.00 | 0.00 | 0.00 | 0.00 | 0.00 | 0.00 | 6645100.00 | 0.00 | 0.00 | KO:K04077 | chaperonin GroEL | Bacteria;Chloroflexi;Dehalococcoidia;Dehalococcoidales;Dehalococcoidaceae;Dehalococcoides;Dehalococcoides mccartyi;Dehalococcoides mccartyi VS |
| Ga0586324 2306912 1 849 | 0.00 | 5698400.00 | 0.00 | 0.00 | 0.00 | 825291.54 | 0.00 | 505639.37 | 20898000.00 | 28105000.00 | 1240800.00 | 1972773.00 | KO:K04077 | chaperonin GroEL | Bacteria;Proteobacteria;Betaproteobacteria;Burkholderiales;Alcaligenaceae;Pigmentiphaga;Pigmentiphaga sp. NML080357;Pigmentiphaga sp. NML080357 |
| Ga0586324 2320362 66 848 | 0.00 | 0.00 | 0.00 | 0.00 | 0.00 | 0.00 | 0.00 | 0.00 | 1498500.00 | 0.00 | 1214400.00 | 0.00 | KO:K04077 | chaperonin GroEL | Bacteria;Acidobacteria;Vicinamibacteria;unclassified Vicinamibacteria;Vicinamibacteraceae;Luteitalea;Luteitalea pratensis;Luteitalea pratensis DSM 100886 |
| Ga0586324 2332701 3 845 | 11250000.00 | 2951200.00 | 774840.01 | 23778500.00 | 57401900.00 | 1431101.63 | 42499200.00 | 54210700.00 | 13365000.00 | 7422800.00 | 18392000.00 | 12204199.50 | KO:K04077 | chaperonin GroEL | Bacteria;Proteobacteria;Deltaproteobacteria;Syntrophobacterales;Syntrophobacteraceae;Desulfosoma;Desulfosoma caldarium;Desulfosoma caldarium DSM 22027 |
| Ga0586324 2340090 3 845 | 0.00 | 0.00 | 1120505.12 | 0.00 | 0.00 | 0.00 | 0.00 | 0.00 | 0.00 | 0.00 | 0.00 | 333864.00 | KO:K04077 | chaperonin GroEL | Bacteria;Proteobacteria;Deltaproteobacteria;Myxococcales;Anaeromyxobacteraceae;Anaeromyxobacter;Anaeromyxobacter sp. K;Anaeromyxobacter sp. K |
| Ga0586324 2342918 132 845 | 0.00 | 0.00 | 0.00 | 0.00 | 15367600.00 | 959473.57 | 0.00 | 19239400.00 | 3410100.00 | 0.00 | 0.00 | 0.00 | KO:K04077 | chaperonin GroEL | Bacteria;Chloroflexi;Anaerolineae;Anaerolineales;Anaerolineaceae;Levilinea;Levilinea saccharolytica;Levilinea saccharolytica DSM 16555 |
| Ga0586324 2343943 3 845 | 0.00 | 320208.60 | 0.00 | 0.00 | 0.00 | 0.00 | 0.00 | 0.00 | 0.00 | 0.00 | 0.00 | 0.00 | KO:K04077 | chaperonin GroEL | Bacteria;Verrucomicrobia;Opitutae;Opitutales;Opitutaceae;Opitutus;Opitutus terrae;Opitutus terrae PB90-1 |
| Ga0586324 2356242 1 843 | 0.00 | 0.00 | 0.00 | 0.00 | 0.00 | 0.00 | 0.00 | 382169.27 | 0.00 | 0.00 | 0.00 | 195524.25 | KO:K04077 | chaperonin GroEL | Bacteria;Acidobacteria;Vicinamibacteria;unclassified Vicinamibacteria;Vicinamibacteraceae;Luteitalea;Luteitalea pratensis;Luteitalea pratensis DSM 100886 |
| Ga0586324 2367919 3 821 | 0.00 | 0.00 | 0.00 | 0.00 | 0.00 | 0.00 | 2095037.28 | 0.00 | 0.00 | 0.00 | 0.00 | 0.00 | KO:K04077 | chaperonin GroEL | Bacteria;Proteobacteria;Betaproteobacteria;Nitrosomonadales;Thiobacillaceae;Sulfuritortus;Sulfuritortus calidifontis;Sulfuritortus calidifontis DSM 103923 |
| Ga0586324 2368979 1 408 | 0.00 | 0.00 | 0.00 | 0.00 | 0.00 | 0.00 | 0.00 | 4611600.00 | 0.00 | 0.00 | 0.00 | 0.00 | KO:K04077 | chaperonin GroEL | Bacteria;Thermodesulfobacteria;Thermodesulfobacteria;Thermodesulfobacteriales;Thermodesulfobacteriaceae;Thermodesulfobacterium;Thermodesulfobacterium commune;Thermodesulfobacterium commune DSM 2178 |
| Ga0586324 2376545 1 840 | 0.00 | 83082.40 | 110548.43 | 0.00 | 0.00 | 0.00 | 0.00 | 0.00 | 0.00 | 0.00 | 0.00 | 0.00 | KO:K04077 | chaperonin GroEL | Bacteria;Chloroflexi;Thermomicrobia;Thermomicrobiales;Thermomicrobiaceae;Thermomicrobium;Thermomicrobium roseum;Thermomicrobium roseum DSM 5159 |
| Ga0586324 2379196 1 840 | 1030000.00 | 8092000.00 | 188717600.00 | 106400000.00 | 0.00 | 237643159.86 | 183084000.00 | 165816300.00 | 0.00 | 179410000.00 | 154000000.00 | 151237500.00 | KO:K04077 | chaperonin GroEL | Bacteria;Acidobacteria;Vicinamibacteria;unclassified Vicinamibacteria;Vicinamibacteraceae;Luteitalea;Luteitalea pratensis;Luteitalea pratensis DSM 100886 |
| Ga0586324 2385775 1 837 | 0.00 | 0.00 | 0.00 | 0.00 | 0.00 | 0.00 | 0.00 | 0.00 | 2778300.00 | 0.00 | 0.00 | 0.00 | KO:K04077 | chaperonin GroEL | Bacteria;Ignavibacteriae;Ignavibacteria;Ignavibacteriales;Ignavibacteriaceae;Ignavibacterium;Ignavibacterium album;Ignavibacterium album Mat9-16, JCM 16511 |
| Ga0586324 2396465 3 836 | 0.00 | 0.00 | 0.00 | 0.00 | 0.00 | 0.00 | 0.00 | 0.00 | 3167100.00 | 0.00 | 0.00 | 0.00 | KO:K04077 | chaperonin GroEL | Bacteria;Proteobacteria;Alphaproteobacteria;Hyphomicrobiales;Xanthobacteraceae;Pseudolabrys;Pseudolabrys taiwanensis;Pseudolabrys taiwanensis CC-BB4 |
| Ga0586324 2409865 1 834 | 0.00 | 3107600.00 | 0.00 | 6327000.00 | 0.00 | 6153500.00 | 8344800.00 | 0.00 | 0.00 | 9009000.00 | 10736000.00 | 0.00 | KO:K04077 | chaperonin GroEL | Bacteria;Acidobacteria;Vicinamibacteria;unclassified Vicinamibacteria;Vicinamibacteraceae;Luteitalea;Luteitalea pratensis;Luteitalea pratensis DSM 100886 |
| Ga0586324 2415240 3 605 | 0.00 | 0.00 | 1959572.17 | 0.00 | 0.00 | 24534600.00 | 1375856.88 | 17995000.00 | 626102.46 | 0.00 | 0.00 | 1095000.00 | KO:K04077 | chaperonin GroEL | Bacteria;Bacteroidetes;Bacteroidia;Marinilabiliales;Prolixibacteraceae;Prolixibacter;Prolixibacter denitrificans;Prolixibacter denitrificans DSM 27267 |
| Ga0586324 2419001 3 833 | 0.00 | 0.00 | 0.00 | 928317.20 | 0.00 | 0.00 | 5580160.92 | 0.00 | 5378400.00 | 3226300.00 | 2235200.00 | 3555000.00 | KO:K04077 | chaperonin GroEL | Bacteria;Bacteroidetes;Bacteroidia;Bacteroidales;Lentimicrobiaceae;Lentimicrobium;Lentimicrobium saccharophilum;Bacteroidales bacterium TBC1 |
| Ga0586324 2421592 452 832 | 0.00 | 0.00 | 0.00 | 3562500.00 | 3296000.00 | 2911923.54 | 1050142.92 | 2982900.00 | 1441800.00 | 0.00 | 0.00 | 0.00 | KO:K04077 | chaperonin GroEL | Bacteria;Ignavibacteriae;Ignavibacteria;Ignavibacteriales;Ignavibacteriaceae;Ignavibacterium;Ignavibacterium album;Ignavibacterium album Mat9-16, JCM 16511 |
| Ga0586324 2437204 1 396 | 0.00 | 0.00 | 0.00 | 819051.05 | 0.00 | 0.00 | 0.00 | 11102000.00 | 0.00 | 0.00 | 0.00 | 0.00 | KO:K04077 | chaperonin GroEL | Bacteria;Proteobacteria;Deltaproteobacteria;Syntrophobacterales;Syntrophobacteraceae;Desulfosoma;Desulfosoma caldarium;Desulfosoma caldarium DSM 22027 |
| Ga0586324 2437365 3 728 | 5450000.00 | 0.00 | 34624500.00 | 27170000.00 | 0.00 | 8138500.00 | 1416484.20 | 36600000.00 | 456281.10 | 0.00 | 0.00 | 0.00 | KO:K04077 | chaperonin GroEL | Bacteria;Proteobacteria;Alphaproteobacteria;Hyphomicrobiales;unclassified Hyphomicrobiales;Methyloceanibacter;Methyloceanibacter caenitepidi;Methyloceanibacter caenitepidi Gela4 |
| Ga0586324 2437845 84 830 | 0.00 | 0.00 | 0.00 | 6707000.00 | 0.00 | 0.00 | 0.00 | 6649000.00 | 5427000.00 | 15323000.00 | 932800.00 | 0.00 | KO:K04077 | chaperonin GroEL | Bacteria;Proteobacteria;Alphaproteobacteria;Hyphomicrobiales;Xanthobacteraceae;Pseudolabrys;Pseudolabrys sp. Root1462;Pseudolabrys sp. Root1462 |
| Ga0586324 2445890 1 744 | 0.00 | 0.00 | 0.00 | 0.00 | 0.00 | 0.00 | 0.00 | 1445700.00 | 0.00 | 0.00 | 0.00 | 0.00 | KO:K04077 | chaperonin GroEL | Bacteria;Chloroflexi;Anaerolineae;Anaerolineales;Anaerolineaceae;Levilinea;Levilinea saccharolytica;Levilinea saccharolytica DSM 16555 |
| Ga0586324 2448631 313 828 | 0.00 | 0.00 | 0.00 | 0.00 | 0.00 | 0.00 | 0.00 | 0.00 | 0.00 | 0.00 | 1240800.00 | 0.00 | KO:K04077 | chaperonin GroEL | Bacteria;Proteobacteria;Betaproteobacteria;unclassified Betaproteobacteria;unclassified Betaproteobacteria;unclassified Betaproteobacteria;Betaproteobacteria bacterium GR16-43;Betaproteobacteria bacterium GR16-43 |
| Ga0586324 2454760 1 828 | 3020000.00 | 0.00 | 0.00 | 0.00 | 0.00 | 14212600.00 | 8299200.00 | 11468000.00 | 11421000.00 | 0.00 | 0.00 | 6427500.00 | KO:K04077 | chaperonin GroEL | Bacteria;Proteobacteria;Deltaproteobacteria;Syntrophobacterales;Syntrophaceae;Desulfobacca;Desulfobacca acetoxidans;Desulfobacca acetoxidans ASRB2, DSM 11109 |
| Ga0586324 2462045 2 826 | 0.00 | 0.00 | 0.00 | 0.00 | 0.00 | 0.00 | 7774800.00 | 0.00 | 0.00 | 0.00 | 0.00 | 0.00 | KO:K04077 | chaperonin GroEL | Bacteria;Chloroflexi;Anaerolineae;Anaerolineales;Anaerolineaceae;Thermanaerothrix;Thermanaerothrix daxensis;Thermanaerothrix daxensis GNS-1 |
| Ga0586324 2462393 1 828 | 3660000.00 | 2917200.00 | 5572613.41 | 3771500.00 | 2327800.00 | 6267943.19 | 3214800.00 | 2793800.00 | 5645700.00 | 6075300.00 | 0.00 | 2145000.00 | KO:K04077 | chaperonin GroEL | Bacteria;Proteobacteria;Deltaproteobacteria;Desulfobacterales;Desulfobulbaceae;Desulfobulbus;Desulfobulbus elongatus;Desulfobulbus elongatus DSM 2908 |
| Ga0586324 2464544 3 827 | 0.00 | 304579.48 | 0.00 | 0.00 | 0.00 | 0.00 | 0.00 | 0.00 | 0.00 | 862400.00 | 0.00 | 0.00 | KO:K04077 | chaperonin GroEL | Bacteria;Proteobacteria;Alphaproteobacteria;Rhodospirillales;Acetobacteraceae;Roseomonas;Roseomonas lacus;Roseomonas lacus DSM 19439 |
| Ga0586324 2476185 2 826 | 0.00 | 604983.08 | 0.00 | 0.00 | 159887.93 | 0.00 | 0.00 | 169425.06 | 1077300.00 | 0.00 | 0.00 | 0.00 | KO:K04077 | chaperonin GroEL | Bacteria;Nitrospirae;unclassified Nitrospirae;unclassified Nitrospirae;unclassified Nitrospirae;unclassified Nitrospirae;Nitrospirae bacterium HCH-1;Nitrospirae bacterium HCH-1 |
| Ga0586324 2482722 25 825 | 0.00 | 0.00 | 0.00 | 0.00 | 17922000.00 | 0.00 | 0.00 | 0.00 | 0.00 | 0.00 | 2736800.00 | 5542500.00 | KO:K04077 | chaperonin GroEL | Bacteria;Proteobacteria;Deltaproteobacteria;Syntrophobacterales;Syntrophaceae;Desulfobacca;Desulfobacca acetoxidans;Desulfobacca acetoxidans ASRB2, DSM 11109 |
| Ga0586324 2490866 2 823 | 731086.00 | 6800000.00 | 0.00 | 0.00 | 1668600.00 | 0.00 | 3488400.00 | 0.00 | 2762100.00 | 5374600.00 | 10208000.00 | 4755000.00 | KO:K04077 | chaperonin GroEL | Bacteria;Proteobacteria;Betaproteobacteria;Nitrosomonadales;Nitrosomonadaceae;Nitrosomonas;Nitrosomonas sp. AL212;Nitrosomonas sp. AL212 |
| Ga0586324 2500628 3 821 | 0.00 | 727600.00 | 6812300.00 | 4351000.00 | 0.00 | 0.00 | 0.00 | 0.00 | 0.00 | 0.00 | 3511200.00 | 5257500.00 | KO:K04077 | chaperonin GroEL | Bacteria;Proteobacteria;Deltaproteobacteria;Myxococcales;Archangiaceae;Vitiosangium;Vitiosangium sp. GDMCC 1.1324;Vitiosangium sp. GDMCC 1.1324 |
| Ga0586324 2506557 338 820 | 19700000.00 | 8092000.00 | 0.00 | 5485647.70 | 10437970.56 | 0.00 | 0.00 | 4556700.00 | 5375842.83 | 3834600.00 | 14344000.00 | 37650000.00 | KO:K04077 | chaperonin GroEL | Bacteria;Proteobacteria;Deltaproteobacteria;Syntrophobacterales;Syntrophaceae;Desulfobacca;Desulfobacca acetoxidans;Desulfobacca acetoxidans ASRB2, DSM 11109 |
| Ga0586324 2529825 3 818 | 0.00 | 2985200.00 | 0.00 | 0.00 | 0.00 | 0.00 | 0.00 | 0.00 | 0.00 | 0.00 | 7348000.00 | 0.00 | KO:K04077 | chaperonin GroEL | Bacteria;Proteobacteria;Deltaproteobacteria;Myxococcales;Sandaracinaceae;Sandaracinus;Sandaracinus amylolyticus;Sandaracinus amylolyticus DSM 53668 |
| Ga0586324 2532019 3 710 | 0.00 | 0.00 | 0.00 | 10355000.00 | 0.00 | 0.00 | 2508000.00 | 0.00 | 1490400.00 | 3126200.00 | 1865600.00 | 0.00 | KO:K04077 | chaperonin GroEL | Bacteria;Chloroflexi;Dehalococcoidia;Dehalococcoidales;Dehalococcoidaceae;Dehalococcoides;Dehalococcoides mccartyi;Dehalococcoides mccartyi VS |
| Ga0586324 2534706 3 818 | 2250000.00 | 469943.92 | 0.00 | 0.00 | 529901.01 | 1746919.10 | 0.00 | 0.00 | 282956.49 | 3287900.00 | 0.00 | 3427500.00 | KO:K04077 | chaperonin GroEL | Bacteria;Chloroflexi;Thermomicrobia;Sphaerobacterales;Sphaerobacteraceae;Sphaerobacter;Sphaerobacter thermophilus;Sphaerobacter thermophilus 4ac11, DSM 20745 |
| Ga0586324 2535689 1 816 | 0.00 | 0.00 | 0.00 | 0.00 | 0.00 | 0.00 | 0.00 | 0.00 | 0.00 | 10241000.00 | 7964000.00 | 0.00 | KO:K04077 | chaperonin GroEL | Bacteria;Acidobacteria;Acidobacteriia;Bryobacterales;Bryobacteraceae;Bryobacter;Bryobacter aggregatus;Bryobacter aggregatus DSM 18758 |
| Ga0586324 2538623 267 818 | 0.00 | 0.00 | 0.00 | 5073000.00 | 0.00 | 5915300.00 | 4286400.00 | 1281000.00 | 0.00 | 1008700.00 | 1689600.00 | 0.00 | KO:K04077 | chaperonin GroEL | Bacteria;Chloroflexi;Anaerolineae;Anaerolineales;Anaerolineaceae;Anaerolinea;Anaerolinea thermophila;Anaerolinea thermophila UNI-1 |
| Ga0586324 2547692 116 814 | 0.00 | 0.00 | 0.00 | 0.00 | 552581.61 | 2386021.61 | 1482166.44 | 0.00 | 0.00 | 0.00 | 0.00 | 0.00 | KO:K04077 | chaperonin GroEL | Bacteria;Chloroflexi;Anaerolineae;Anaerolineales;Anaerolineaceae;Levilinea;Levilinea saccharolytica;Levilinea saccharolytica DSM 16555 |
| Ga0586324 2551134 150 815 | 23900000.00 | 6759200.00 | 1443284.28 | 1387000.00 | 8435700.00 | 1120341.94 | 8504400.00 | 18605000.00 | 24057000.00 | 11627000.00 | 6468000.00 | 5340000.00 | KO:K04077 | chaperonin GroEL | Bacteria;Proteobacteria;Gammaproteobacteria;Pseudomonadales;Pseudomonadaceae;Pseudomonas;Pseudomonas putida;Pseudomonas putida PC2 |
| Ga0586324 2555285 3 566 | 0.00 | 1054000.00 | 2833229.94 | 0.00 | 0.00 | 0.00 | 0.00 | 0.00 | 0.00 | 0.00 | 0.00 | 0.00 | KO:K04077 | chaperonin GroEL | Bacteria;Proteobacteria;Gammaproteobacteria;Nevskiales;Steroidobacteraceae;Steroidobacter;Steroidobacter denitrificans;Steroidobacter denitrificans DSM 18526 |
| Ga0586324 2558652 1 813 | 0.00 | 0.00 | 0.00 | 9690000.00 | 8549000.00 | 0.00 | 10009200.00 | 0.00 | 7921800.00 | 6221600.00 | 6230400.00 | 5580000.00 | KO:K04077 | chaperonin GroEL | Bacteria;Proteobacteria;Deltaproteobacteria;Syntrophobacterales;Syntrophobacteraceae;Desulfacinum;Desulfacinum infernum;Desulfacinum infernum DSM 9756 |
| Ga0586324 2561104 240 815 | 0.00 | 979200.00 | 2005912.70 | 0.00 | 0.00 | 0.00 | 456243.96 | 957488.94 | 413410.23 | 535074.54 | 543292.64 | 1642500.00 | KO:K04077 | chaperonin GroEL | Bacteria;Proteobacteria;Gammaproteobacteria;Cellvibrionales;Spongiibacteraceae;Dasania;Dasania marina;Dasania marina DSM 21967 |
| Ga0586324 2568076 3 812 | 0.00 | 0.00 | 0.00 | 771547.25 | 0.00 | 0.00 | 386546.64 | 1052623.32 | 354386.34 | 0.00 | 0.00 | 0.00 | KO:K04077 | chaperonin GroEL | Bacteria;Chloroflexi;Anaerolineae;Anaerolineales;Anaerolineaceae;Anaerolinea;Anaerolinea thermophila;Anaerolinea thermophila UNI-1 |
| Ga0586324 2577060 3 812 | 0.00 | 2000207.76 | 0.00 | 8160500.00 | 0.00 | 0.00 | 5722800.00 | 0.00 | 2069440.65 | 0.00 | 1513600.00 | 2925000.00 | KO:K04077 | chaperonin GroEL | Bacteria;Chloroflexi;Anaerolineae;Anaerolineales;Anaerolineaceae;Thermanaerothrix;Thermanaerothrix daxensis;Thermanaerothrix daxensis GNS-1 |
| Ga0586324 2585345 3 755 | 0.00 | 7752000.00 | 0.00 | 6640500.00 | 3625600.00 | 0.00 | 0.00 | 0.00 | 4714200.00 | 0.00 | 0.00 | 0.00 | KO:K04077 | chaperonin GroEL | Bacteria;Proteobacteria;Deltaproteobacteria;Syntrophobacterales;Syntrophaceae;Syntrophus;Syntrophus aciditrophicus;Syntrophus aciditrophicus SB |
| Ga0586324 2592073 2 811 | 0.00 | 0.00 | 0.00 | 7324500.00 | 0.00 | 0.00 | 0.00 | 0.00 | 0.00 | 0.00 | 0.00 | 5122500.00 | KO:K04077 | chaperonin GroEL | Bacteria;Proteobacteria;Deltaproteobacteria;Desulfobacterales;Desulfobacteraceae;Desulfatiglans;Desulfatiglans anilini;Desulfatiglans anilini DSM 4660 |
| Ga0586324 2594150 1 810 | 0.00 | 0.00 | 0.00 | 0.00 | 1524400.00 | 0.00 | 0.00 | 0.00 | 0.00 | 0.00 | 0.00 | 0.00 | KO:K04077 | chaperonin GroEL | Bacteria;Proteobacteria;Deltaproteobacteria;Syntrophobacterales;Syntrophaceae;Desulfomonile;Desulfomonile tiedjei;Desulfomonile tiedjei DCB-1, DSM 6799 |
| Ga0586324 2598219 50 808 | 0.00 | 850000.00 | 0.00 | 0.00 | 0.00 | 0.00 | 2274559.92 | 0.00 | 0.00 | 0.00 | 0.00 | 1702500.00 | KO:K04077 | chaperonin GroEL | Bacteria;Nitrospirae;Nitrospira;Nitrospirales;Nitrospiraceae;Nitrospira;Nitrospira moscoviensis;Nitrospira moscoviensis NSP M-1 |
| Ga0586324 2608977 3 806 | 0.00 | 3060000.00 | 0.00 | 0.00 | 0.00 | 0.00 | 0.00 | 0.00 | 323808.84 | 576326.52 | 2252800.00 | 3180000.00 | KO:K04077 | chaperonin GroEL | Bacteria;Spirochaetes;Spirochaetia;Spirochaetales;Treponemataceae;Treponema;Treponema caldarium;Treponema caldarium DSM 7334 |
| Ga0586324 2611224 1 807 | 0.00 | 0.00 | 0.00 | 0.00 | 0.00 | 0.00 | 0.00 | 0.00 | 0.00 | 0.00 | 2543200.00 | 0.00 | KO:K04077 | chaperonin GroEL | Bacteria;Verrucomicrobia;Opitutae;Opitutales;Opitutaceae;Opitutus;Opitutus terrae;Opitutus terrae PB90-1 |
| Ga0586324 2614839 3 806 | 13600000.00 | 2468400.00 | 0.00 | 11495000.00 | 0.00 | 0.00 | 17556000.00 | 0.00 | 7759800.00 | 2094400.00 | 0.00 | 7950000.00 | KO:K04077 | chaperonin GroEL | Bacteria;Proteobacteria;Deltaproteobacteria;Desulfuromonadales;Geobacteraceae;Geobacter;Geobacter daltonii;Geobacter daltonii FRC-32 |
| Ga0586324 2626164 1 804 | 0.00 | 0.00 | 0.00 | 0.00 | 188773.25 | 0.00 | 0.00 | 0.00 | 144689.49 | 0.00 | 0.00 | 0.00 | KO:K04077 | chaperonin GroEL | Bacteria;Calditrichaeota;Calditrichae;Calditrichales;Calditrichaceae;Caldithrix;Caldithrix abyssi;Caldithrix abyssi LF13, DSM 13497 |
| Ga0586324 2627420 1 354 | 0.00 | 0.00 | 0.00 | 657967.15 | 867278.54 | 0.00 | 0.00 | 921100.00 | 0.00 | 0.00 | 0.00 | 0.00 | KO:K04077 | chaperonin GroEL | Bacteria;Verrucomicrobia;Verrucomicrobiae;Verrucomicrobiales;Verrucomicrobia subdivision 3;Pedosphaera;Pedosphaera parvula;Pedosphaera parvula Ellin514 |
| Ga0586324 2644344 2 802 | 0.00 | 0.00 | 0.00 | 1320500.00 | 510262.00 | 1611033.94 | 0.00 | 0.00 | 3312900.00 | 0.00 | 0.00 | 174421.50 | KO:K04077 | chaperonin GroEL | Bacteria;Actinobacteria;Thermoleophilia;Solirubrobacterales;Conexibacteraceae;Conexibacter;Conexibacter woesei;Conexibacter woesei Iso977N |
| Ga0586324 2644627 1 804 | 0.00 | 0.00 | 0.00 | 14440000.00 | 3780100.00 | 0.00 | 0.00 | 0.00 | 0.00 | 3141600.00 | 3238400.00 | 0.00 | KO:K04077 | chaperonin GroEL | Bacteria;Planctomycetes;Planctomycetia;Planctomycetales;Planctomycetaceae;Planctomyces;Planctomyces sp. SH-PL62;Planctomyces sp. SH-PL62 |
| Ga0586324 2648884 3 803 | 0.00 | 0.00 | 0.00 | 0.00 | 0.00 | 0.00 | 0.00 | 0.00 | 0.00 | 0.00 | 0.00 | 346531.50 | KO:K04077 | chaperonin GroEL | Bacteria;Proteobacteria;Deltaproteobacteria;Syntrophobacterales;Syntrophaceae;Syntrophus;Syntrophus aciditrophicus;Syntrophus aciditrophicus SB |
| Ga0586324 2657261 3 476 | 0.00 | 0.00 | 0.00 | 0.00 | 0.00 | 0.00 | 0.00 | 0.00 | 0.00 | 0.00 | 0.00 | 2602500.00 | KO:K04077 | chaperonin GroEL | Bacteria;Proteobacteria;Alphaproteobacteria;Hyphomicrobiales;unclassified Hyphomicrobiales;Methyloceanibacter;Methyloceanibacter stevinii;Methyloceanibacter stevinii R-67176 |
| Ga0586324 2667495 1 732 | 0.00 | 0.00 | 0.00 | 216885.00 | 1594343.18 | 0.00 | 0.00 | 0.00 | 0.00 | 0.00 | 0.00 | 0.00 | KO:K04077 | chaperonin GroEL | Bacteria;Proteobacteria;Deltaproteobacteria;Desulfobacterales;Desulfobacteraceae;Desulfatibacillum;Desulfatibacillum aliphaticivorans;Desulfatibacillum aliphaticivorans AK-01 |
| Ga0586324 2687556 2 568 | 0.00 | 142549.08 | 0.00 | 0.00 | 0.00 | 0.00 | 0.00 | 0.00 | 0.00 | 0.00 | 0.00 | 0.00 | KO:K04077 | chaperonin GroEL | Bacteria;Acidobacteria;Acidobacteriia;Acidobacteriales;Acidobacteriaceae;Candidatus Koribacter;Candidatus Koribacter versatilis;Candidatus Koribacter versatilis Ellin345 |
| Ga0586324 2692003 514 798 | 0.00 | 0.00 | 0.00 | 0.00 | 2667700.00 | 0.00 | 35340000.00 | 738100.00 | 858600.00 | 0.00 | 26048000.00 | 20550000.00 | KO:K04077 | chaperonin GroEL | Bacteria;Chloroflexi;Anaerolineae;Anaerolineales;Anaerolineaceae;Anaerolinea;Anaerolinea thermophila;Anaerolinea thermophila UNI-1 |
| Ga0586324 2698702 32 796 | 0.00 | 6793200.00 | 0.00 | 0.00 | 0.00 | 0.00 | 0.00 | 0.00 | 0.00 | 939400.00 | 1223200.00 | 0.00 | KO:K04077 | chaperonin GroEL | Bacteria;Acidobacteria;Acidobacteriia;Acidobacteriales;Acidobacteriaceae;Candidatus Koribacter;Candidatus Koribacter versatilis;Candidatus Koribacter versatilis Ellin345 |
| Ga0586324 2701817 1 795 | 0.00 | 638917.12 | 0.00 | 0.00 | 0.00 | 0.00 | 0.00 | 0.00 | 0.00 | 0.00 | 0.00 | 0.00 | KO:K04077 | chaperonin GroEL | Bacteria;Chloroflexi;Caldilineae;Caldilineales;Caldilineaceae;Caldilinea;Caldilinea aerophila;Caldilinea aerophila STL-6-O1, DSM 14535 |
| Ga0586324 2705442 3 794 | 0.00 | 74800000.00 | 0.00 | 0.00 | 0.00 | 0.00 | 3237600.00 | 76250000.00 | 988200.00 | 2310000.00 | 0.00 | 0.00 | KO:K04077 | chaperonin GroEL | Bacteria;Proteobacteria;Gammaproteobacteria;Nevskiales;Steroidobacteraceae;Povalibacter;Povalibacter uvarum;Povalibacter uvarum DSM 26723 |
| Ga0586324 2707456 1 795 | 0.00 | 0.00 | 0.00 | 0.00 | 0.00 | 0.00 | 0.00 | 0.00 | 0.00 | 0.00 | 0.00 | 8025000.00 | KO:K04077 | chaperonin GroEL | Bacteria;Proteobacteria;Deltaproteobacteria;Syntrophobacterales;Syntrophaceae;Syntrophus;Syntrophus aciditrophicus;Syntrophus aciditrophicus SB |
| Ga0586324 2713584 1 795 | 0.00 | 0.00 | 1104718.60 | 0.00 | 44145800.00 | 0.00 | 729796.08 | 3214700.00 | 0.00 | 839300.00 | 861022.80 | 3810000.00 | KO:K04077 | chaperonin GroEL | Bacteria;Bacteroidetes;Bacteroidia;Bacteroidales;Porphyromonadaceae;Gabonia;Gabonia massiliensis;Porphyromonadaceae bacterium GM3 |
| Ga0586324 2723383 282 794 | 0.00 | 0.00 | 0.00 | 2603000.00 | 0.00 | 0.00 | 0.00 | 2189900.00 | 3353400.00 | 0.00 | 0.00 | 2257500.00 | KO:K04077 | chaperonin GroEL | Bacteria;Proteobacteria;Deltaproteobacteria;Syntrophobacterales;Syntrophaceae;Desulfobacca;Desulfobacca acetoxidans;Desulfobacca acetoxidans ASRB2, DSM 11109 |
| Ga0586324 2723763 1 792 | 0.00 | 0.00 | 3709466.25 | 0.00 | 0.00 | 0.00 | 2048853.60 | 0.00 | 0.00 | 0.00 | 0.00 | 0.00 | KO:K04077 | chaperonin GroEL | Bacteria;Proteobacteria;Betaproteobacteria;Burkholderiales;Oxalobacteraceae;Herbaspirillum;Herbaspirillum sp. RV1423;Herbaspirillum sp. RV1423 |
| Ga0586324 2725782 2 793 | 0.00 | 0.00 | 0.00 | 0.00 | 0.00 | 0.00 | 0.00 | 16043000.00 | 0.00 | 0.00 | 0.00 | 0.00 | KO:K04077 | chaperonin GroEL | Bacteria;Proteobacteria;Betaproteobacteria;unclassified Betaproteobacteria;unclassified Betaproteobacteria;unclassified Betaproteobacteria;uncultured beta proteobacterium;unclassified betaproteobacterium ENR4 |
| Ga0586324 2733167 1 792 | 988706.00 | 0.00 | 7150100.00 | 0.00 | 0.00 | 0.00 | 0.00 | 658800000.00 | 0.00 | 0.00 | 0.00 | 588599.25 | KO:K04077 | chaperonin GroEL | Bacteria;Calditrichaeota;Calditrichae;Calditrichales;Calditrichaceae;Caldithrix;Caldithrix abyssi;Caldithrix abyssi LF13, DSM 13497 |
| Ga0586324 2733734 3 743 | 10100000.00 | 4420000.00 | 9233200.00 | 11552000.00 | 12360000.00 | 8217900.00 | 21067200.00 | 18147500.00 | 0.00 | 13706000.00 | 15752000.00 | 10875000.00 | KO:K04077 | chaperonin GroEL | Bacteria;Proteobacteria;Alphaproteobacteria;Rhodospirillales;Acetobacteraceae;Rhodopila;Rhodopila globiformis;Rhodopila globiformis DSM 161 |
| Ga0586324 2736241 2 790 | 0.00 | 0.00 | 0.00 | 5662000.00 | 5654700.00 | 2466680.10 | 3100800.00 | 4782400.00 | 0.00 | 0.00 | 0.00 | 140882.25 | KO:K04077 | chaperonin GroEL | Bacteria;Chloroflexi;Anaerolineae;Anaerolineales;Anaerolineaceae;Anaerolinea;Anaerolinea thermophila;Anaerolinea thermophila UNI-1 |
| Ga0586324 2737342 2 631 | 0.00 | 0.00 | 1482913.85 | 0.00 | 0.00 | 0.00 | 0.00 | 589696.15 | 1433700.00 | 970200.00 | 872361.60 | 2228530.50 | KO:K04077 | chaperonin GroEL | Bacteria;Proteobacteria;Betaproteobacteria;unclassified Betaproteobacteria;unclassified Betaproteobacteria;unclassified Betaproteobacteria;Betaproteobacteria bacterium GR16-43;Betaproteobacteria bacterium GR16-43 |
| Ga0586324 2745910 3 791 | 0.00 | 0.00 | 0.00 | 0.00 | 0.00 | 0.00 | 0.00 | 5917000.00 | 0.00 | 186280.71 | 0.00 | 1462500.00 | KO:K04077 | chaperonin GroEL | Bacteria;Acidobacteria;Vicinamibacteria;unclassified Vicinamibacteria;Vicinamibacteraceae;Luteitalea;Luteitalea pratensis;Luteitalea pratensis DSM 100886 |
| Ga0586324 2758823 3 788 | 0.00 | 0.00 | 0.00 | 0.00 | 0.00 | 0.00 | 0.00 | 1159000.00 | 0.00 | 1078000.00 | 5464800.00 | 0.00 | KO:K04077 | chaperonin GroEL | Bacteria;Proteobacteria;Deltaproteobacteria;Myxococcales;Anaeromyxobacteraceae;Anaeromyxobacter;Anaeromyxobacter dehalogenans;Anaeromyxobacter dehalogenans 2CP-C |
| Ga0586324 2769121 3 788 | 0.00 | 0.00 | 0.00 | 48355000.00 | 42642000.00 | 23700900.00 | 0.00 | 39284000.00 | 37584000.00 | 1955800.00 | 0.00 | 0.00 | KO:K04077 | chaperonin GroEL | Bacteria;Chloroflexi;Anaerolineae;Anaerolineales;Anaerolineaceae;Levilinea;Levilinea saccharolytica;Levilinea saccharolytica DSM 16555 |
| Ga0586324 2781482 1 786 | 0.00 | 0.00 | 0.00 | 0.00 | 0.00 | 18262000.00 | 0.00 | 12932000.00 | 12393000.00 | 0.00 | 0.00 | 0.00 | KO:K04077 | chaperonin GroEL | Bacteria;Chloroflexi;Anaerolineae;Anaerolineales;Anaerolineaceae;Anaerolinea;Anaerolinea thermophila;Anaerolinea thermophila UNI-1 |
| Ga0586324 2784698 1 786 | 0.00 | 0.00 | 0.00 | 0.00 | 0.00 | 0.00 | 0.00 | 0.00 | 0.00 | 51995.71 | 0.00 | 0.00 | KO:K04077 | chaperonin GroEL | Bacteria;Proteobacteria;Deltaproteobacteria;Desulfobacterales;Desulfobacteraceae;Desulfosarcina;Desulfosarcina variabilis;Desulfosarcina variabilis Montpellier |
| Ga0586324 2788525 67 786 | 0.00 | 484476.88 | 0.00 | 0.00 | 0.00 | 0.00 | 0.00 | 0.00 | 0.00 | 0.00 | 0.00 | 0.00 | KO:K04077 | chaperonin GroEL | Bacteria;Ignavibacteriae;Ignavibacteria;Ignavibacteriales;Ignavibacteriaceae;Ignavibacterium;Ignavibacterium album;Ignavibacterium album Mat9-16, JCM 16511 |
| Ga0586324 2803288 1 783 | 5070000.00 | 4005200.00 | 6193000.00 | 0.00 | 0.00 | 4287600.00 | 0.00 | 4282200.00 | 0.00 | 5659500.00 | 5869600.00 | 4995000.00 | KO:K04077 | chaperonin GroEL | Bacteria;Proteobacteria;Gammaproteobacteria;Chromatiales;Ectothiorhodospiraceae;Acidihalobacter;Acidihalobacter ferrooxydans;Acidihalobacter ferrooxydans V8 |
| Ga0586324 2803376 3 782 | 0.00 | 8908000.00 | 0.00 | 22990000.00 | 14523000.00 | 0.00 | 0.00 | 6588000.00 | 291037.05 | 1455300.00 | 0.00 | 10425000.00 | KO:K04077 | chaperonin GroEL | Bacteria;Acidobacteria;Acidobacteriia;Acidobacteriales;Acidobacteriaceae;Candidatus Koribacter;Candidatus Koribacter versatilis;Candidatus Koribacter versatilis Ellin345 |
| Ga0586324 2807804 2 784 | 0.00 | 1441600.00 | 0.00 | 3296500.00 | 0.00 | 0.00 | 0.00 | 622200.00 | 164559.60 | 92007300.00 | 35358400.00 | 746622.00 | KO:K04077 | chaperonin GroEL | Bacteria;Nitrospirae;Thermodesulfovibrionia;Thermodesulfovibrionales;Thermodesulfovibrionaceae;Thermodesulfovibrio;Thermodesulfovibrio thiophilus;Thermodesulfovibrio thiophilus DSM 17215 |
| Ga0586324 2810731 3 782 | 0.00 | 0.00 | 0.00 | 0.00 | 1740700.00 | 0.00 | 0.00 | 0.00 | 0.00 | 0.00 | 0.00 | 0.00 | KO:K04077 | chaperonin GroEL | Bacteria;Actinobacteria;Rubrobacteria;Gaiellales;Gaiellaceae;Gaiella;Gaiella occulta;Gaiella occulta F2-233 |
| Ga0586324 2817838 53 781 | 0.00 | 0.00 | 0.00 | 5187000.00 | 0.00 | 0.00 | 12403200.00 | 0.00 | 11809800.00 | 0.00 | 3960000.00 | 0.00 | KO:K04077 | chaperonin GroEL | Bacteria;Proteobacteria;Betaproteobacteria;unclassified Betaproteobacteria;unclassified Betaproteobacteria;unclassified Betaproteobacteria;Betaproteobacteria bacterium GR16-43;Betaproteobacteria bacterium GR16-43 |
| Ga0586324 2819239 1 783 | 0.00 | 0.00 | 0.00 | 0.00 | 6355100.00 | 0.00 | 0.00 | 0.00 | 0.00 | 0.00 | 0.00 | 0.00 | KO:K04077 | chaperonin GroEL | Bacteria;Planctomycetes;Planctomycetia;Gemmatales;Gemmataceae;Zavarzinella;Zavarzinella formosa;Zavarzinella formosa DSM 19928 |
| Ga0586324 2831100 47 781 | 0.00 | 0.00 | 0.00 | 0.00 | 0.00 | 0.00 | 0.00 | 0.00 | 0.00 | 3149300.00 | 0.00 | 0.00 | KO:K04077 | chaperonin GroEL | Bacteria;Proteobacteria;Betaproteobacteria;Nitrosomonadales;Gallionellaceae;Gallionella;Gallionella capsiferriformans;Gallionella capsiferriformans ES-2 |
| Ga0586324 2832599 2 781 | 0.00 | 0.00 | 0.00 | 0.00 | 0.00 | 0.00 | 0.00 | 0.00 | 0.00 | 0.00 | 15224000.00 | 0.00 | KO:K04077 | chaperonin GroEL | Bacteria;Proteobacteria;Betaproteobacteria;unclassified Betaproteobacteria;unclassified Betaproteobacteria;unclassified Betaproteobacteria;uncultured beta proteobacterium;unclassified betaproteobacterium ENR4 |
| Ga0586324 2863907 2 571 | 1570000.00 | 0.00 | 0.00 | 0.00 | 0.00 | 0.00 | 0.00 | 0.00 | 0.00 | 0.00 | 0.00 | 170462.25 | KO:K04077 | chaperonin GroEL | Bacteria;Candidatus Aminicenantes;unclassified Candidatus Aminicenantes;unclassified Candidatus Aminicenantes;unclassified Candidatus Aminicenantes;unclassified Candidatus Aminicenantes;Aminicenantes bacterium SCGC AAA255-A06;Aminicenantes bacterium SCGC AAA255-A06 (contamination screened) |
| Ga0586324 2871695 376 777 | 13100000.00 | 3740000.00 | 0.00 | 0.00 | 0.00 | 0.00 | 0.00 | 0.00 | 0.00 | 0.00 | 0.00 | 0.00 | KO:K04077 | chaperonin GroEL | Bacteria;Verrucomicrobia;Opitutae;Opitutales;Opitutaceae;unclassified Opitutaceae;Opitutaceae bacterium TSB47;Opitutaceae bacterium TSB47 |
| Ga0586324 2873722 1 774 | 0.00 | 4893379.96 | 1403975.62 | 0.00 | 0.00 | 0.00 | 1434254.52 | 677100.00 | 1880373.69 | 0.00 | 340636.56 | 2137856.25 | KO:K04077 | chaperonin GroEL | Bacteria;Acidobacteria;Acidobacteriia;Bryobacterales;Solibacteraceae;Candidatus Solibacter;Candidatus Solibacter usitatus;Candidatus Solibacter usitatus Ellin6076 |
| Ga0586324 2879480 1 774 | 0.00 | 7072000.00 | 9120600.00 | 10830000.00 | 9618505.65 | 0.00 | 0.00 | 2562000.00 | 7286452.20 | 1932700.00 | 3872000.00 | 0.00 | KO:K04077 | chaperonin GroEL | Bacteria;Proteobacteria;Deltaproteobacteria;Syntrophobacterales;Syntrophaceae;Syntrophus;Syntrophus aciditrophicus;Syntrophus aciditrophicus SB |
| Ga0586324 2892663 3 773 | 0.00 | 1285200.00 | 0.00 | 0.00 | 0.00 | 0.00 | 0.00 | 0.00 | 0.00 | 0.00 | 0.00 | 285052.50 | KO:K04077 | chaperonin GroEL | Bacteria;Proteobacteria;Deltaproteobacteria;Myxococcales;Anaeromyxobacteraceae;Anaeromyxobacter;Anaeromyxobacter sp. K;Anaeromyxobacter sp. K |
| Ga0586324 2906220 2 772 | 0.00 | 4154800.00 | 0.00 | 0.00 | 10712000.00 | 0.00 | 0.00 | 689300.00 | 0.00 | 7492100.00 | 0.00 | 0.00 | KO:K04077 | chaperonin GroEL | Bacteria;Chloroflexi;Caldilineae;Caldilineales;Caldilineaceae;Caldilinea;Caldilinea aerophila;Caldilinea aerophila STL-6-O1, DSM 14535 |
| Ga0586324 2935050 1 768 | 0.00 | 0.00 | 0.00 | 0.00 | 0.00 | 0.00 | 0.00 | 276820.44 | 0.00 | 0.00 | 228969.84 | 0.00 | KO:K04077 | chaperonin GroEL | Bacteria;Verrucomicrobia;Verrucomicrobiae;Verrucomicrobiales;Verrucomicrobia subdivision 3;Pedosphaera;Pedosphaera parvula;Pedosphaera parvula Ellin514 |
| Ga0586324 2939664 3 749 | 0.00 | 0.00 | 0.00 | 0.00 | 1586200.00 | 0.00 | 0.00 | 0.00 | 0.00 | 0.00 | 0.00 | 0.00 | KO:K04077 | chaperonin GroEL | Bacteria;Proteobacteria;Deltaproteobacteria;Desulfobacterales;Desulfobulbaceae;Desulfopila;Desulfopila aestuarii;Desulfopila aestuarii DSM 18488 |
| Ga0586324 2956322 1 765 | 0.00 | 0.00 | 0.00 | 700161.40 | 5644400.00 | 0.00 | 0.00 | 2580300.00 | 3304800.00 | 0.00 | 0.00 | 0.00 | KO:K04077 | chaperonin GroEL | Bacteria;Candidatus Aminicenantes;unclassified Candidatus Aminicenantes;unclassified Candidatus Aminicenantes;unclassified Candidatus Aminicenantes;unclassified Candidatus Aminicenantes;Aminicenantes bacterium SCGC AAA252-P13;Aminicenantes bacterium SCGC AAA252-P13 (contamination screened) |
| Ga0586324 2963603 1 765 | 0.00 | 0.00 | 0.00 | 1786000.00 | 0.00 | 0.00 | 0.00 | 0.00 | 273400.11 | 0.00 | 0.00 | 0.00 | KO:K04077 | chaperonin GroEL | Bacteria;Proteobacteria;Deltaproteobacteria;Desulfobacterales;Desulfobulbaceae;Desulfobulbus;Desulfobulbus elongatus;Desulfobulbus elongatus DSM 2908 |
| Ga0586324 2975408 3 764 | 821703.00 | 9316000.00 | 20475949.68 | 12742712.90 | 15491200.00 | 19214800.00 | 0.00 | 854000.00 | 1077300.00 | 15400000.00 | 932225.36 | 12392838.75 | KO:K04077 | chaperonin GroEL | Bacteria;Proteobacteria;Deltaproteobacteria;Syntrophobacterales;Syntrophobacteraceae;Desulfacinum;Desulfacinum infernum;Desulfacinum infernum DSM 9756 |
| Ga0586324 2987624 1 762 | 1570000.00 | 0.00 | 0.00 | 1710000.00 | 1328700.00 | 0.00 | 0.00 | 433233.59 | 826200.00 | 208861.73 | 0.00 | 542913.75 | KO:K04077 | chaperonin GroEL | Bacteria;Proteobacteria;Deltaproteobacteria;Syntrophobacterales;Syntrophobacteraceae;Desulfoglaeba;Desulfoglaeba alkanexedens;Desulfoglaeba alkanexedens ALDC |
| Ga0586324 2993530 1 762 | 0.00 | 0.00 | 0.00 | 0.00 | 0.00 | 0.00 | 0.00 | 0.00 | 4349700.00 | 0.00 | 0.00 | 0.00 | KO:K04077 | chaperonin GroEL | Bacteria;Proteobacteria;Deltaproteobacteria;Syntrophobacterales;Syntrophobacteraceae;Desulfoglaeba;Desulfoglaeba alkanexedens;Desulfoglaeba alkanexedens ALDC |
| Ga0586324 2997951 2 763 | 0.00 | 0.00 | 0.00 | 0.00 | 0.00 | 0.00 | 0.00 | 3348900.00 | 4868100.00 | 0.00 | 0.00 | 0.00 | KO:K04077 | chaperonin GroEL | Bacteria;Bacteroidetes;Bacteroidia;Bacteroidales;Salinivirgaceae;Salinivirga;Salinivirga cyanobacteriivorans;Salinivirga cyanobacteriivorans L21-Spi-D4 |
| Ga0586324 3000693 1 762 | 191979.00 | 0.00 | 0.00 | 0.00 | 0.00 | 0.00 | 0.00 | 2189900.00 | 232380.09 | 0.00 | 0.00 | 0.00 | KO:K04077 | chaperonin GroEL | Bacteria;Proteobacteria;Betaproteobacteria;Nitrosomonadales;Nitrosomonadaceae;Nitrosospira;Nitrosospira sp. Nsp11;Nitrosospira sp. Nsp11 |
| Ga0586324 3014904 3 761 | 0.00 | 0.00 | 0.00 | 0.00 | 0.00 | 0.00 | 0.00 | 50630000.00 | 0.00 | 0.00 | 65560000.00 | 0.00 | KO:K04077 | chaperonin GroEL | Bacteria;Proteobacteria;Deltaproteobacteria;Syntrophobacterales;Syntrophaceae;Syntrophus;Syntrophus gentianae;Syntrophus gentianae DSM 8423 |
| Ga0586324 3022736 3 758 | 0.00 | 3597200.00 | 0.00 | 0.00 | 0.00 | 0.00 | 0.00 | 0.00 | 0.00 | 0.00 | 0.00 | 0.00 | KO:K04077 | chaperonin GroEL | Bacteria;Nitrospinae;Nitrospinia;Nitrospinales;Nitrospinaceae;Nitrospina;Nitrospina gracilis;Nitrospina gracilis 3/211 |
| Ga0586324 3031846 3 758 | 9880000.00 | 1094800.00 | 0.00 | 0.00 | 0.00 | 0.00 | 0.00 | 3019500.00 | 2016900.00 | 4088700.00 | 0.00 | 2490000.00 | KO:K04077 | chaperonin GroEL | Bacteria;Proteobacteria;Gammaproteobacteria;Methylococcales;Crenotrichaceae;Crenothrix;Crenothrix polyspora;Crenothrix polyspora ERS1545888 |
| Ga0586324 3034876 3 629 | 0.00 | 565090.88 | 0.00 | 351264.40 | 0.00 | 0.00 | 0.00 | 0.00 | 0.00 | 0.00 | 379888.08 | 0.00 | KO:K04077 | chaperonin GroEL | Bacteria;Chloroflexi;Anaerolineae;Anaerolineales;Anaerolineaceae;Thermanaerothrix;Thermanaerothrix daxensis;Thermanaerothrix daxensis GNS-1 |
| Ga0586324 3042103 1 669 | 0.00 | 0.00 | 0.00 | 0.00 | 0.00 | 0.00 | 0.00 | 0.00 | 0.00 | 0.00 | 1073600.00 | 0.00 | KO:K04077 | chaperonin GroEL | Bacteria;Verrucomicrobia;Verrucomicrobiae;Verrucomicrobiales;Verrucomicrobiaceae;Roseimicrobium;Roseimicrobium gellanilyticum;Roseimicrobium gellanilyticum DSM 25532 |
| Ga0586324 3044528 3 758 | 564651.00 | 0.00 | 0.00 | 0.00 | 0.00 | 0.00 | 0.00 | 0.00 | 0.00 | 0.00 | 0.00 | 0.00 | KO:K04077 | chaperonin GroEL | Bacteria;Proteobacteria;Betaproteobacteria;unclassified Betaproteobacteria;unclassified Betaproteobacteria;unclassified Betaproteobacteria;uncultured beta proteobacterium;unclassified betaproteobacterium ENR4 |
| Ga0586324 3048020 2 757 | 0.00 | 0.00 | 0.00 | 158881.80 | 109799.03 | 0.00 | 0.00 | 284890.13 | 166838.13 | 225094.87 | 0.00 | 161649.00 | KO:K04077 | chaperonin GroEL | Bacteria;Proteobacteria;Deltaproteobacteria;Syntrophobacterales;Syntrophaceae;Smithella;Smithella sp. SCADC;Smithella sp. SCADC |
| Ga0586324 3057500 3 755 | 0.00 | 0.00 | 0.00 | 0.00 | 14523000.00 | 9805900.00 | 4788000.00 | 5404600.00 | 4422600.00 | 0.00 | 3995200.00 | 6420000.00 | KO:K04077 | chaperonin GroEL | Bacteria;Proteobacteria;Deltaproteobacteria;Desulfobacterales;Desulfobacteraceae;Desulfococcus;Desulfococcus multivorans;Desulfococcus multivorans DSM 2059 |
| Ga0586324 3061170 1 756 | 0.00 | 0.00 | 0.00 | 0.00 | 0.00 | 0.00 | 0.00 | 0.00 | 1806300.00 | 0.00 | 0.00 | 2047500.00 | KO:K04077 | chaperonin GroEL | Bacteria;Proteobacteria;Betaproteobacteria;unclassified Betaproteobacteria;unclassified Betaproteobacteria;unclassified Betaproteobacteria;uncultured beta proteobacterium;unclassified betaproteobacterium ENR4 |
| Ga0586324 3065152 3 755 | 0.00 | 514673.64 | 0.00 | 0.00 | 0.00 | 0.00 | 0.00 | 0.00 | 0.00 | 0.00 | 0.00 | 0.00 | KO:K04077 | chaperonin GroEL | Bacteria;Verrucomicrobia;Verrucomicrobiae;Verrucomicrobiales;Verrucomicrobia subdivision 3;Pedosphaera;Pedosphaera parvula;Pedosphaera parvula Ellin514 |
| Ga0586324 3079895 2 754 | 0.00 | 0.00 | 0.00 | 0.00 | 0.00 | 0.00 | 0.00 | 4947100.00 | 1239300.00 | 1178100.00 | 0.00 | 0.00 | KO:K04077 | chaperonin GroEL | Bacteria;Acidobacteria;Vicinamibacteria;unclassified Vicinamibacteria;Vicinamibacteraceae;Luteitalea;Luteitalea pratensis;Luteitalea pratensis DSM 100886 |
| Ga0586324 3086126 3 752 | 0.00 | 0.00 | 1671130.38 | 3809500.00 | 0.00 | 8456100.00 | 1724411.88 | 536939.69 | 0.00 | 0.00 | 950400.00 | 3997500.00 | KO:K04077 | chaperonin GroEL | Bacteria;Acidobacteria;Acidobacteriia;Acidobacteriales;Acidobacteriaceae;Candidatus Koribacter;Candidatus Koribacter versatilis;Candidatus Koribacter versatilis Ellin345 |
| Ga0586324 3094691 2 751 | 0.00 | 0.00 | 0.00 | 0.00 | 0.00 | 0.00 | 0.00 | 1647000.00 | 0.00 | 0.00 | 0.00 | 0.00 | KO:K04077 | chaperonin GroEL | Bacteria;Bacteroidetes;Bacteroidia;Marinilabiliales;Marinilabiliaceae;Natronoflexus;Natronoflexus pectinivorans;Natronoflexus pectinivorans DSM 24179 |
| Ga0586324 3095509 1 753 | 228586.00 | 0.00 | 462624.42 | 7647500.00 | 0.00 | 0.00 | 0.00 | 0.00 | 529000.47 | 0.00 | 0.00 | 0.00 | KO:K04077 | chaperonin GroEL | Bacteria;Proteobacteria;Gammaproteobacteria;Cellvibrionales;Halieaceae;Congregibacter;Congregibacter litoralis;Congregibacter litoralis KT71 |

| | | | | | | | | | | | | | | | |
|---|---|---|---|---|---|---|---|---|---|---|---|---|---|---|---|
| Ga0586324 3111926 3 749 | 0.00 | 0.00 | 0.00 | 0.00 | 0.00 | 0.00 | 0.00 | 0.00 | 0.00 | 0.00 | 779134.40 | 0.00 | KO:K04077 | chaperonin GroEL | Bacteria;Proteobacteria;Betaproteobacteria;Rhodocyclales;Rhodocyclaceae;Propionivibrio;Propionivibrio pelophilus;Propionivibrio pelophilus DSM 12018 |
| Ga0586324 3117329 278 748 | 0.00 | 1020008.16 | 1462116.63 | 1125646.45 | 1165451.18 | 0.00 | 0.00 | 0.00 | 1624370.76 | 2849000.00 | 0.00 | 0.00 | KO:K04077 | chaperonin GroEL | Bacteria;Proteobacteria;Deltaproteobacteria;Desulfovibrionales;Desulfovibrionaceae;Desulfovibrio;Desulfovibrio sp. DMSS-1;Desulfovibrio sp. DMSS-1 |
| Ga0586324 3120268 1 750 | 0.00 | 0.00 | 0.00 | 0.00 | 0.00 | 0.00 | 0.00 | 2226500.00 | 0.00 | 0.00 | 0.00 | 0.00 | KO:K04077 | chaperonin GroEL | Bacteria;Proteobacteria;Betaproteobacteria;unclassified Betaproteobacteria;unclassified Betaproteobacteria;unclassified Betaproteobacteria;uncultured beta proteobacterium;unclassified betaproteobacterium ENR4 |
| Ga0586324 3121543 3 749 | 0.00 | 0.00 | 0.00 | 9690000.00 | 10609000.00 | 7900300.00 | 0.00 | 5429000.00 | 4074300.00 | 0.00 | 0.00 | 2227500.00 | KO:K04077 | chaperonin GroEL | Bacteria;Proteobacteria;Deltaproteobacteria;Desulfobacterales;Desulfobulbaceae;Desulfobulbus;Desulfobulbus elongatus;Desulfobulbus elongatus DSM 2908 |
| Ga0586324 3130054 1 747 | 0.00 | 0.00 | 4216211.29 | 0.00 | 0.00 | 393808.12 | 0.00 | 0.00 | 0.00 | 0.00 | 0.00 | 0.00 | KO:K04077 | chaperonin GroEL | Bacteria;Chloroflexi;unclassified Chloroflexi;unclassified Chloroflexi;unclassified Chloroflexi;unclassified Chloroflexi;unclassified Chloroflexi;Chloroflexi sp. G233 |
| Ga0586324 3131039 471 749 | 0.00 | 0.00 | 0.00 | 0.00 | 0.00 | 0.00 | 0.00 | 415677.79 | 0.00 | 0.00 | 0.00 | 0.00 | KO:K04077 | chaperonin GroEL | Bacteria;Verrucomicrobia;Methylacidiphilae;Methylacidiphilales;Methylacidiphilaceae;Methylacidiphilum;Methylacidiphilum fumariolicum;Methylacidiphilum fumariolicum SolV |
| Ga0586324 3137428 2 748 | 0.00 | 0.00 | 0.00 | 0.00 | 0.00 | 0.00 | 0.00 | 0.00 | 0.00 | 1409100.00 | 0.00 | 0.00 | KO:K04077 | chaperonin GroEL | Bacteria;Planctomycetes;Planctomycetia;Planctomycetales;Planctomycetaceae;Planctopirus;Planctopirus limnophila;Planctopirus limnophila DSM 3776 |
| Ga0586324 3140418 19 747 | 5470000.00 | 13219200.00 | 15622062.07 | 23710770.70 | 5026400.00 | 4724300.00 | 4651200.00 | 8506054.11 | 27564300.00 | 6135480.12 | 12034360.80 | 6804111.00 | KO:K04077 | chaperonin GroEL | Bacteria;Verrucomicrobia;Verrucomicrobiae;Verrucomicrobiales;Verrucomicrobia subdivision 3;Pedosphaera;Pedosphaera parvula;Pedosphaera parvula Ellin514 |
| Ga0586324 3164199 1 744 | 0.00 | 0.00 | 0.00 | 0.00 | 592174.81 | 0.00 | 0.00 | 513171.65 | 0.00 | 8162000.00 | 6080800.00 | 0.00 | KO:K04077 | chaperonin GroEL | Bacteria;Candidatus Calescamantes;unclassified Candidatus Calescamantes;unclassified Candidatus Calescamantes;unclassified Candidatus Calescamantes;unclassified Candidatus Calescamantes;Calescamantes bacterium JGI 0000106-H18;Calescamantes bacterium JGI 0000106-H18 (contamination screened) |
| Ga0586324 3178178 2 742 | 0.00 | 0.00 | 0.00 | 5339000.00 | 3110600.00 | 0.00 | 0.00 | 1775100.00 | 1919700.00 | 0.00 | 0.00 | 937500.00 | KO:K04077 | chaperonin GroEL | Bacteria;Proteobacteria;Deltaproteobacteria;Desulfobacterales;Desulfobacteraceae;Desulfatitalea;Desulfatitalea tepidiphila;Desulfatitalea tepidiphila S28bF |
| Ga0586324 3186407 3 743 | 0.00 | 0.00 | 0.00 | 0.00 | 72718000.00 | 0.00 | 0.00 | 158517.65 | 0.00 | 0.00 | 0.00 | 0.00 | KO:K04077 | chaperonin GroEL | Bacteria;Proteobacteria;Betaproteobacteria;Nitrosomonadales;Nitrosomonadaceae;Nitrosospira;Nitrosospira sp. Nl5;Nitrosospira sp. Nl5 |
| Ga0586324 3189150 1 741 | 0.00 | 0.00 | 0.00 | 0.00 | 0.00 | 0.00 | 0.00 | 0.00 | 0.00 | 571809.70 | 0.00 | 0.00 | KO:K04077 | chaperonin GroEL | Bacteria;Proteobacteria;Deltaproteobacteria;Syntrophobacterales;Syntrophobacteraceae;Syntrophobacter;Syntrophobacter fumaroxidans;Syntrophobacter fumaroxidans MPOB |
| Ga0586324 3208261 2 742 | 0.00 | 157107.20 | 876838.72 | 0.00 | 0.00 | 0.00 | 0.00 | 666853.22 | 0.00 | 0.00 | 0.00 | 410690.25 | KO:K04077 | chaperonin GroEL | Bacteria;Candidatus Calescamantes;unclassified Candidatus Calescamantes;unclassified Candidatus Calescamantes;unclassified Candidatus Calescamantes;unclassified Candidatus Calescamantes;Calescamantes bacterium JGI 0000106-H18;Calescamantes bacterium JGI 0000106-H18 (contamination screened) |
| Ga0586324 3211431 3 740 | 0.00 | 0.00 | 0.00 | 0.00 | 0.00 | 6907800.00 | 0.00 | 3586800.00 | 4827600.00 | 0.00 | 0.00 | 0.00 | KO:K04077 | chaperonin GroEL | Bacteria;Candidatus Calescamantes;unclassified Candidatus Calescamantes;unclassified Candidatus Calescamantes;unclassified Candidatus Calescamantes;unclassified Candidatus Calescamantes;Calescamantes bacterium JGI 0000106-H18;Calescamantes bacterium JGI 0000106-H18 (contamination screened) |
| Ga0586324 3225670 3 740 | 0.00 | 0.00 | 0.00 | 13870000.00 | 6656581.00 | 9611830.52 | 0.00 | 6478200.00 | 7970400.00 | 0.00 | 0.00 | 0.00 | KO:K04077 | chaperonin GroEL | Bacteria;Chloroflexi;Anaerolineae;Anaerolineales;Anaerolineaceae;Thermanaerothrix;Thermanaerothrix daxensis;Thermanaerothrix daxensis GNS-1 |
| Ga0586324 3232492 1 738 | 42700000.00 | 49000800.00 | 1632942.09 | 36670000.00 | 0.00 | 0.00 | 72276000.00 | 559166.26 | 8594835.48 | 52062692.99 | 62840800.00 | 3863491.50 | KO:K04077 | chaperonin GroEL | Bacteria;Verrucomicrobia;Verrucomicrobiae;Verrucomicrobiales;Verrucomicrobia subdivision 3;Pedosphaera;Pedosphaera parvula;Pedosphaera parvula Ellin514 |
| Ga0586324 3232943 117 737 | 0.00 | 77342.52 | 0.00 | 0.00 | 0.00 | 0.00 | 0.00 | 0.00 | 0.00 | 0.00 | 0.00 | 1927500.00 | KO:K04077 | chaperonin GroEL | Bacteria;Firmicutes;Limnochordia;Limnochordales;Limnochordaceae;Limnochorda;Limnochorda pilosa;Limnochorda pilosa HC45 |
| Ga0586324 3235615 3 737 | 0.00 | 0.00 | 0.00 | 0.00 | 0.00 | 0.00 | 0.00 | 0.00 | 0.00 | 0.00 | 1056000.00 | 0.00 | KO:K04077 | chaperonin GroEL | Bacteria;Gemmatimonadetes;Longimicrobia;Longimicrobiales;Longimicrobiaceae;Longimicrobium;Longimicrobium terrae;Longimicrobium terrae CECT 8660 |
| Ga0586324 3239191 75 737 | 26600000.00 | 0.00 | 0.00 | 0.00 | 0.00 | 0.00 | 0.00 | 0.00 | 0.00 | 0.00 | 0.00 | 0.00 | KO:K04077 | chaperonin GroEL | Bacteria;Proteobacteria;Betaproteobacteria;Burkholderiales;Comamonadaceae;Hydrogenophaga;Hydrogenophaga sp. Root209;Hydrogenophaga sp. Root209 |
| Ga0586324 3262186 141 734 | 0.00 | 0.00 | 0.00 | 0.00 | 0.00 | 0.00 | 0.00 | 0.00 | 0.00 | 839300.00 | 0.00 | 2647500.00 | KO:K04077 | chaperonin GroEL | Bacteria;Proteobacteria;Deltaproteobacteria;Syntrophobacterales;Syntrophaceae;Desulfobacca;Desulfobacca acetoxidans;Desulfobacca acetoxidans ASRB2, DSM 11109 |
| Ga0586324 3262600 3 464 | 0.00 | 0.00 | 0.00 | 1168500.00 | 0.00 | 0.00 | 0.00 | 494744.16 | 1441800.00 | 0.00 | 0.00 | 0.00 | KO:K04077 | chaperonin GroEL | Bacteria;Proteobacteria;Deltaproteobacteria;Syntrophobacterales;Syntrophaceae;Desulfobacca;Desulfobacca acetoxidans;Desulfobacca acetoxidans ASRB2, DSM 11109 |
| Ga0586324 3264575 3 734 | 0.00 | 0.00 | 22294800.00 | 0.00 | 0.00 | 11790900.00 | 0.00 | 0.00 | 0.00 | 0.00 | 0.00 | 24542985.75 | KO:K04077 | chaperonin GroEL | Bacteria;Proteobacteria;Deltaproteobacteria;Syntrophobacterales;Syntrophaceae;Smithella;Smithella sp. SCADC;Smithella sp. SCADC |
| Ga0586324 3282396 1 732 | 64000000.00 | 144368.76 | 0.00 | 0.00 | 7189400.00 | 0.00 | 0.00 | 0.00 | 0.00 | 0.00 | 0.00 | 891054.75 | KO:K04077 | chaperonin GroEL | Bacteria;Acidobacteria;Vicinamibacteria;unclassified Vicinamibacteria;Vicinamibacteraceae;Luteitalea;Luteitalea pratensis;Luteitalea pratensis DSM 100886 |
| Ga0586324 3285694 3 734 | 0.00 | 32912000.00 | 0.00 | 0.00 | 0.00 | 0.00 | 966891.00 | 2531500.00 | 2081700.00 | 4073300.00 | 3176800.00 | 3195000.00 | KO:K04077 | chaperonin GroEL | Bacteria;Proteobacteria;Alphaproteobacteria;Hyphomicrobiales;Hyphomicrobiaceae;Hyphomicrobium;Hyphomicrobium denitrificans;Hyphomicrobium denitrificans 1NES1 |
| Ga0586324 3285726 1 732 | 0.00 | 0.00 | 0.00 | 0.00 | 0.00 | 0.00 | 0.00 | 683200.00 | 0.00 | 0.00 | 0.00 | 0.00 | KO:K04077 | chaperonin GroEL | Bacteria;Proteobacteria;Deltaproteobacteria;Syntrophobacterales;Syntrophobacteraceae;Desulfoglaeba;Desulfoglaeba alkanexedens;Desulfoglaeba alkanexedens ALDC |
| Ga0586324 3287112 3 734 | 0.00 | 0.00 | 0.00 | 0.00 | 0.00 | 0.00 | 587765.76 | 0.00 | 0.00 | 0.00 | 0.00 | 0.00 | KO:K04077 | chaperonin GroEL | Bacteria;Chloroflexi;Anaerolineae;Anaerolineales;Anaerolineaceae;Pelolinea;Pelolinea submarina;Pelolinea submarina DSM 23923 |
| Ga0586324 3297900 239 733 | 0.00 | 0.00 | 0.00 | 0.00 | 0.00 | 0.00 | 0.00 | 606982.33 | 0.00 | 0.00 | 0.00 | 209139.00 | KO:K04077 | chaperonin GroEL | Bacteria;Verrucomicrobia;Verrucomicrobiae;Verrucomicrobiales;Verrucomicrobia subdivision 3;Pedosphaera;Pedosphaera parvula;Pedosphaera parvula Ellin514 |
| Ga0586324 3305414 2 730 | 0.00 | 0.00 | 0.00 | 6745000.00 | 7210159.65 | 0.00 | 0.00 | 3757600.00 | 4374000.00 | 1809500.00 | 2393600.00 | 1252500.00 | KO:K04077 | chaperonin GroEL | Bacteria;Proteobacteria;Deltaproteobacteria;Syntrophobacterales;Syntrophaceae;Smithella;Smithella sp. F21;Smithella sp. F21 |
| Ga0586324 3314464 3 731 | 0.00 | 795600.00 | 0.00 | 0.00 | 0.00 | 0.00 | 0.00 | 0.00 | 0.00 | 0.00 | 0.00 | 0.00 | KO:K04077 | chaperonin GroEL | Bacteria;Proteobacteria;Deltaproteobacteria;Desulfuromonadales;Geobacteraceae;Geobacter;Geobacter argillaceus;Geobacter argillaceus ATCC BAA-1139 |
| Ga0586324 3321163 3 731 | 0.00 | 0.00 | 0.00 | 0.00 | 3162100.00 | 345022.38 | 0.00 | 0.00 | 0.00 | 631580.95 | 275813.12 | 0.00 | KO:K04077 | chaperonin GroEL | Bacteria;Actinobacteria;Actinomycetia;Micrococcales;Intrasporangiaceae;Tetrasphaera;Tetrasphaera japonica;Tetrasphaera japonica T1-X7 |
| Ga0586324 3323387 115 729 | 0.00 | 4807600.00 | 4349265.08 | 0.00 | 0.00 | 4406700.00 | 1968214.56 | 5331400.00 | 3766500.00 | 5135900.00 | 2226400.00 | 6907500.00 | KO:K04077 | chaperonin GroEL | Bacteria;Actinobacteria;Acidimicrobiia;Acidimicrobiales;Acidimicrobiaceae;Acidithrix;Acidithrix ferrooxidans;Acidithrix ferrooxidans Py-F3 |
| Ga0586324 3323437 1 729 | 0.00 | 10540000.00 | 0.00 | 0.00 | 0.00 | 0.00 | 0.00 | 0.00 | 0.00 | 594512.38 | 0.00 | 0.00 | KO:K04077 | chaperonin GroEL | Bacteria;Proteobacteria;Deltaproteobacteria;Myxococcales;Labilitrichaceae;Labilithrix;Labilithrix luteola;Labilithrix luteola DSM 27648 |
| Ga0586324 3331374 1 729 | 10600000.00 | 22712000.00 | 0.00 | 19095000.00 | 14420000.00 | 18976600.00 | 0.00 | 27633000.00 | 0.00 | 33340445.60 | 0.00 | 19260000.00 | KO:K04077 | chaperonin GroEL | Bacteria;Deferribacteres;Deferribacteres;Deferribacterales;Deferribacteraceae;Deferribacter;Deferribacter desulfuricans;Deferribacter desulfuricans SSM1 |
| Ga0586324 3335780 166 729 | 0.00 | 0.00 | 0.00 | 0.00 | 0.00 | 0.00 | 0.00 | 292726.80 | 0.00 | 0.00 | 5227200.00 | 0.00 | KO:K04077 | chaperonin GroEL | Bacteria;Proteobacteria;Deltaproteobacteria;Myxococcales;Labilitrichaceae;Labilithrix;Labilithrix luteola;Labilithrix luteola DSM 27648 |
| Ga0586324 3342009 1 720 | 0.00 | 843200.00 | 214171.39 | 851439.40 | 51244110.92 | 0.00 | 3214800.00 | 0.00 | 107730000.00 | 426879.53 | 816800.16 | 1129702.50 | KO:K04077 | chaperonin GroEL | Bacteria;Deferribacteres;Deferribacteres;Deferribacterales;Deferribacteraceae;Deferribacter;Deferribacter desulfuricans;Deferribacter desulfuricans SSM1 |
| Ga0586324 3344261 1 729 | 0.00 | 0.00 | 399081.42 | 0.00 | 0.00 | 0.00 | 0.00 | 64070.74 | 0.00 | 0.00 | 100342.88 | 0.00 | KO:K04077 | chaperonin GroEL | Bacteria;Proteobacteria;Deltaproteobacteria;unclassified Deltaproteobacteria;unclassified Deltaproteobacteria;Dissulfuribacter;Dissulfuribacter thermophilus;Dissulfuribacter thermophilus S69 |
| Ga0586324 3344582 1 729 | 0.00 | 0.00 | 0.00 | 0.00 | 927546.93 | 0.00 | 0.00 | 0.00 | 0.00 | 0.00 | 0.00 | 0.00 | KO:K04077 | chaperonin GroEL | Bacteria;Proteobacteria;Deltaproteobacteria;Desulfobacterales;Desulfobacteraceae;Desulfatiglans;Desulfatiglans anilini;Desulfatiglans anilini DSM 4660 |
| Ga0586324 3355633 2 727 | 0.00 | 0.00 | 0.00 | 0.00 | 0.00 | 0.00 | 0.00 | 0.00 | 0.00 | 0.00 | 2657600.00 | 0.00 | KO:K04077 | chaperonin GroEL | Bacteria;Proteobacteria;Betaproteobacteria;unclassified Betaproteobacteria;unclassified Betaproteobacteria;unclassified Betaproteobacteria;beta proteobacterium AAP51;Betaproteobacteria bacterium AAP51 |
| Ga0586324 3361432 3 350 | 0.00 | 0.00 | 0.00 | 0.00 | 0.00 | 0.00 | 0.00 | 0.00 | 0.00 | 0.00 | 0.00 | 3727500.00 | KO:K04077 | chaperonin GroEL | Bacteria;Acidobacteria;Acidobacteriia;Bryobacterales;Solibacteraceae;Candidatus Solibacter;Candidatus Solibacter usitatus;Candidatus Solibacter usitatus Ellin6076 |
| Ga0586324 3371734 147 725 | 0.00 | 924800.00 | 6136700.00 | 1881000.00 | 0.00 | 8535500.00 | 4560000.00 | 1134600.00 | 0.00 | 0.00 | 4681600.00 | 567057.75 | KO:K04077 | chaperonin GroEL | Bacteria;Proteobacteria;Deltaproteobacteria;Syntrophobacterales;Syntrophaceae;Smithella;Smithella sp. F21;Smithella sp. F21 |
| Ga0586324 3377017 1 726 | 0.00 | 0.00 | 0.00 | 0.00 | 0.00 | 0.00 | 0.00 | 1884900.00 | 0.00 | 0.00 | 0.00 | 0.00 | KO:K04077 | chaperonin GroEL | Bacteria;Acidobacteria;Acidobacteriia;Acidobacteriales;Acidobacteriaceae;Candidatus Koribacter;Candidatus Koribacter versatilis;Candidatus Koribacter versatilis Ellin345 |
| Ga0586324 3398575 2 724 | 1870000.00 | 2808400.00 | 6193000.00 | 4978000.00 | 6715600.00 | 5983639.58 | 1133305.92 | 4922700.00 | 3863700.00 | 5905900.00 | 5746400.00 | 5317500.00 | KO:K04077 | chaperonin GroEL | Bacteria;Spirochaetes;Spirochaetia;Spirochaetales;Treponemataceae;Treponema;Treponema caldarium;Treponema caldarium DSM 7334 |
| Ga0586324 3400072 2 724 | 24081504.00 | 27472000.00 | 14697887.44 | 10317000.00 | 18231000.00 | 5597700.00 | 20100327.24 | 43693258.12 | 36155151.90 | 3938409.09 | 1570754.24 | 6855000.00 | KO:K04077 | chaperonin GroEL | Bacteria;Proteobacteria;Betaproteobacteria;Rhodocyclales;Azonexaceae;Dechloromonas;Dechloromonas sp. HYN0024;Dechloromonas sp. HYN0024 |
| Ga0586324 3400951 1 699 | 0.00 | 0.00 | 0.00 | 0.00 | 520371.45 | 0.00 | 0.00 | 0.00 | 0.00 | 0.00 | 0.00 | 0.00 | KO:K04077 | chaperonin GroEL | Bacteria;Proteobacteria;Alphaproteobacteria;Rhodospirillales;Acetobacteraceae;Rhodopila;unclassified Rhodopila;Rhodopila sp. LVNP |
| Ga0586324 3414527 214 723 | 395330.00 | 0.00 | 0.00 | 0.00 | 0.00 | 0.00 | 21386400.00 | 0.00 | 0.00 | 682144.54 | 0.00 | 0.00 | KO:K04077 | chaperonin GroEL | Bacteria;Proteobacteria;Deltaproteobacteria;Desulfuromonadales;Geobacteraceae;Citrifermentans;Citrifermentans bemidjiense;Geobacter bemidjiensis Bem, DSM 16622 |
| Ga0586324 3419818 3 692 | 7800000.00 | 50743478.84 | 0.00 | 75620000.00 | 22763000.00 | 0.00 | 0.00 | 1250500.00 | 826200.00 | 35805000.00 | 416334.16 | 7080000.00 | KO:K04077 | chaperonin GroEL | Bacteria;Proteobacteria;Deltaproteobacteria;Desulfobacterales;Desulfobacteraceae;Desulfatiglans;Desulfatiglans anilini;Desulfatiglans anilini DSM 4660 |
| Ga0586324 3420012 1 720 | 0.00 | 0.00 | 0.00 | 0.00 | 0.00 | 1422538.34 | 1299260.28 | 0.00 | 1992600.00 | 1771000.00 | 2543200.00 | 604921.50 | KO:K04077 | chaperonin GroEL | Bacteria;Proteobacteria;Alphaproteobacteria;Hyphomicrobiales;Bradyrhizobiaceae;Bradyrhizobium;Bradyrhizobium canariense;Bradyrhizobium canariense GAS369 |
| Ga0586324 3425726 3 722 | 0.00 | 0.00 | 0.00 | 0.00 | 0.00 | 0.00 | 0.00 | 0.00 | 0.00 | 0.00 | 251043.76 | 0.00 | KO:K04077 | chaperonin GroEL | Bacteria;Proteobacteria;Deltaproteobacteria;Syntrophorhabdales;Syntrophorhabdaceae;Syntrophorhabdus;Syntrophorhabdus aromaticivorans;Syntrophorhabdus aromaticivorans UI |
| Ga0586324 3430765 2 721 | 0.00 | 0.00 | 0.00 | 0.00 | 715983.90 | 1883082.16 | 2394000.00 | 3233000.00 | 0.00 | 0.00 | 0.00 | 0.00 | KO:K04077 | chaperonin GroEL | Bacteria;Acidobacteria;Blastocatellia;Blastocatellales;Pyrinomonadaceae;Pyrinomonas;Pyrinomonas methylaliphatogenes;Pyrinomonas methylaliphatogenes K22 |
| Ga0586324 3452042 1 717 | 0.00 | 0.00 | 0.00 | 11666000.00 | 16768400.00 | 0.00 | 9758400.00 | 12407400.00 | 14450400.00 | 0.00 | 0.00 | 7575000.00 | KO:K04077 | chaperonin GroEL | Bacteria;Proteobacteria;Deltaproteobacteria;unclassified Deltaproteobacteria;unclassified Deltaproteobacteria;unclassified Deltaproteobacteria;delta proteobacterium NaphS2;delta proteobacterium NaphS2 |
| Ga0586324 3452105 3 719 | 7520000.00 | 795600.00 | 1691201.33 | 4047260.30 | 2348400.00 | 392783.07 | 5571262.08 | 2366800.00 | 4479300.00 | 1355200.00 | 1664084.40 | 3352500.00 | KO:K04077 | chaperonin GroEL | Bacteria;Candidatus Aminicenantes;unclassified Candidatus Aminicenantes;unclassified Candidatus Aminicenantes;unclassified Candidatus Aminicenantes;unclassified Candidatus Aminicenantes;Aminicenantes bacterium SCGC AAA252-K07;Aminicenantes bacterium SCGC AAA252-K07 (contamination screened) |
| Ga0586324 3455018 2 661 | 0.00 | 1686400.00 | 0.00 | 0.00 | 0.00 | 0.00 | 0.00 | 0.00 | 1223100.00 | 0.00 | 0.00 | 0.00 | KO:K04077 | chaperonin GroEL | Bacteria;Verrucomicrobia;Verrucomicrobiae;Verrucomicrobiales;Verrucomicrobia subdivision 3;Pedosphaera;Pedosphaera parvula;Pedosphaera parvula Ellin514 |
| Ga0586324 3473796 1 717 | 0.00 | 0.00 | 0.00 | 0.00 | 0.00 | 0.00 | 0.00 | 0.00 | 0.00 | 0.00 | 0.00 | 4567500.00 | KO:K04077 | chaperonin GroEL | Bacteria;Proteobacteria;Gammaproteobacteria;Chromatiales;Ectothiorhodospiraceae;Inmirania;Inmirania thermothiophila;Inmirania thermothiophila DSM 100275 |
| Ga0586324 3482647 2 715 | 41280000.00 | 0.00 | 6305600.00 | 28376500.00 | 0.00 | 10599900.00 | 19174800.00 | 36500507.78 | 17455500.00 | 0.00 | 11744907.68 | 20992281.75 | KO:K04077 | chaperonin GroEL | Bacteria;Proteobacteria;Deltaproteobacteria;unclassified Deltaproteobacteria;unclassified Deltaproteobacteria;Dissulfuribacter;Dissulfuribacter thermophilus;Dissulfuribacter thermophilus S69 |
| Ga0586324 3486370 1 714 | 0.00 | 0.00 | 0.00 | 0.00 | 0.00 | 0.00 | 0.00 | 0.00 | 0.00 | 0.00 | 0.00 | 577387.50 | KO:K04077 | chaperonin GroEL | Bacteria;Verrucomicrobia;Spartobacteria;unclassified Spartobacteria;unclassified Spartobacteria;unclassified Spartobacteria;Spartobacteria bacterium LR76;Spartobacteria bacterium LR76 |
| Ga0586324 3487625 3 716 | 0.00 | 0.00 | 0.00 | 337749.70 | 0.00 | 0.00 | 0.00 | 0.00 | 0.00 | 0.00 | 0.00 | 0.00 | KO:K04077 | chaperonin GroEL | Bacteria;Proteobacteria;Deltaproteobacteria;Myxococcales;Sandaracinaceae;Sandaracinus;Sandaracinus amylolyticus;Sandaracinus amylolyticus DSM 53668 |
| Ga0586324 3498205 2 715 | 0.00 | 0.00 | 0.00 | 0.00 | 0.00 | 0.00 | 2462400.00 | 884500.00 | 0.00 | 0.00 | 0.00 | 0.00 | KO:K04077 | chaperonin GroEL | Bacteria;Chloroflexi;Ktedonobacteria;Thermogemmatisporales;Thermogemmatisporaceae;Thermogemmatispora;Thermogemmatispora carboxidivorans;Thermogemmatispora carboxidivorans PM5 |
| Ga0586324 3529693 3 713 | 0.00 | 0.00 | 0.00 | 0.00 | 0.00 | 0.00 | 0.00 | 0.00 | 0.00 | 3095400.00 | 2305600.00 | 2872500.00 | KO:K04077 | chaperonin GroEL | Bacteria;Proteobacteria;Alphaproteobacteria;Hyphomicrobiales;Bradyrhizobiaceae;Bradyrhizobium;Bradyrhizobium oligotrophicum;Bradyrhizobium oligotrophicum S58 |
| Ga0586324 3531000 1 678 | 0.00 | 5582800.00 | 0.00 | 0.00 | 0.00 | 0.00 | 0.00 | 4202900.00 | 0.00 | 0.00 | 437552.72 | 0.00 | KO:K04077 | chaperonin GroEL | Bacteria;Verrucomicrobia;Verrucomicrobiae;Verrucomicrobiales;Verrucomicrobia subdivision 3;Pedosphaera;Pedosphaera parvula;Pedosphaera parvula Ellin514 |
| Ga0586324 3533483 3 713 | 0.00 | 0.00 | 0.00 | 0.00 | 0.00 | 0.00 | 469132.80 | 0.00 | 0.00 | 0.00 | 0.00 | 0.00 | KO:K04077 | chaperonin GroEL | Bacteria;Proteobacteria;Betaproteobacteria;unclassified Betaproteobacteria;unclassified Betaproteobacteria;unclassified Betaproteobacteria;uncultured beta proteobacterium;unclassified betaproteobacterium ENR4 |
| Ga0586324 3535917 216 710 | 0.00 | 214574000.00 | 251817221.24 | 4788000.00 | 5757700.00 | 2346871.46 | 237781840.68 | 2623000.00 | 2907900.00 | 3542000.00 | 2851200.00 | 757500.00 | KO:K04077 | chaperonin GroEL | Bacteria;Proteobacteria;Gammaproteobacteria;Methylococcales;Methylococcaceae;Methylocaldum;Methylocaldum szegediense;Methylocaldum szegediense O-12 |
| Ga0586324 3544692 347 712 | 0.00 | 0.00 | 0.00 | 0.00 | 0.00 | 6490024.99 | 0.00 | 0.00 | 0.00 | 0.00 | 0.00 | 0.00 | KO:K04077 | chaperonin GroEL | Bacteria;Bacteroidetes;Bacteroidia;Marinilabiliales;Marinilabiliaceae;Anaerophaga;Anaerophaga thermohalophila;Anaerophaga thermohalophila Fru22, DSM 12881 |
| Ga0586324 3552823 3 710 | 0.00 | 321051.80 | 0.00 | 0.00 | 0.00 | 0.00 | 0.00 | 0.00 | 0.00 | 0.00 | 0.00 | 0.00 | KO:K04077 | chaperonin GroEL | Bacteria;Proteobacteria;Deltaproteobacteria;Myxococcales;Labilitrichaceae;Labilithrix;Labilithrix luteola;Labilithrix luteola DSM 27648 |
| Ga0586324 3565782 40 708 | 0.00 | 0.00 | 0.00 | 0.00 | 0.00 | 0.00 | 0.00 | 756400.00 | 0.00 | 0.00 | 0.00 | 0.00 | KO:K04077 | chaperonin GroEL | Bacteria;Proteobacteria;Betaproteobacteria;Burkholderiales;Comamonadaceae;Ramlibacter;Ramlibacter tataouinensis;Ramlibacter tataouinensis 5-10 |
| Ga0586324 3566643 3 710 | 4900000.00 | 6596000.00 | 0.00 | 6821000.00 | 23690000.00 | 0.00 | 7729200.00 | 1201700.00 | 10287000.00 | 18641833.98 | 17424000.00 | 8100000.00 | KO:K04077 | chaperonin GroEL | Bacteria;Verrucomicrobia;Verrucomicrobiae;Verrucomicrobiales;Verrucomicrobia subdivision 3;Pedosphaera;Pedosphaera parvula;Pedosphaera parvula Ellin514 |
| Ga0586324 3581984 3 677 | 0.00 | 0.00 | 0.00 | 0.00 | 0.00 | 0.00 | 6270000.00 | 5172800.00 | 0.00 | 0.00 | 0.00 | 4492500.00 | KO:K04077 | chaperonin GroEL | Bacteria;Planctomycetes;Planctomycetia;Isosphaerales;Isosphaeraceae;Paludisphaera;Paludisphaera borealis;Paludisphaera borealis PX4 |
| Ga0586324 3588271 269 706 | 0.00 | 0.00 | 0.00 | 0.00 | 0.00 | 0.00 | 0.00 | 0.00 | 0.00 | 0.00 | 0.00 | 615008.25 | KO:K04077 | chaperonin GroEL | Bacteria;Proteobacteria;Gammaproteobacteria;Oceanospirillales;Alcanivoracaceae;Ketobacter;Ketobacter alkanivorans;Ketobacter alkanivorans GI5 |
| Ga0586324 3589490 244 708 | 3510000.00 | 170325.72 | 293886000.00 | 0.00 | 0.00 | 329510000.00 | 339720000.00 | 267790000.00 | 722485.17 | 0.00 | 0.00 | 281250000.00 | KO:K04077 | chaperonin GroEL | Bacteria;Proteobacteria;Betaproteobacteria;unclassified Betaproteobacteria;unclassified Betaproteobacteria;unclassified Betaproteobacteria;Betaproteobacteria bacterium UKL13-2;Betaproteobacteria bacterium UKL13-2 |
| Ga0586324 3606452 3 707 | 11000000.00 | 0.00 | 0.00 | 0.00 | 0.00 | 0.00 | 3602400.00 | 29463000.00 | 30500136.09 | 34881000.00 | 94705600.00 | 4240307.25 | KO:K04077 | chaperonin GroEL | Bacteria;Proteobacteria;Gammaproteobacteria;Chromatiales;Ectothiorhodospiraceae;Thioalkalivibrio;Thioalkalivibrio sulfidiphilus;Thioalkalivibrio sulfidiphilus ALJ17 |
| Ga0586324 3610909 3 704 | 0.00 | 0.00 | 0.00 | 0.00 | 0.00 | 214685.69 | 0.00 | 0.00 | 72425.91 | 358619.80 | 0.00 | 0.00 | KO:K04077 | chaperonin GroEL | Bacteria;Proteobacteria;Deltaproteobacteria;Syntrophobacterales;Syntrophaceae;Desulfomonile;Desulfomonile tiedjei;Desulfomonile tiedjei DCB-1, DSM 6799 |
| Ga0586324 3620236 3 704 | 0.00 | 0.00 | 0.00 | 0.00 | 0.00 | 0.00 | 0.00 | 293748.55 | 0.00 | 0.00 | 0.00 | 0.00 | KO:K04077 | chaperonin GroEL | Bacteria;Chloroflexi;Thermomicrobia;Sphaerobacterales;Sphaerobacteraceae;Sphaerobacter;Sphaerobacter thermophilus;Sphaerobacter thermophilus 4ac11, DSM 20745 |
| Ga0586324 3621639 2 703 | 0.00 | 337231.04 | 0.00 | 0.00 | 0.00 | 0.00 | 0.00 | 329081.58 | 0.00 | 0.00 | 0.00 | 641472.00 | KO:K04077 | chaperonin GroEL | Bacteria;Thermodesulfobacteria;Thermodesulfobacteria;Thermodesulfobacteriales;Thermodesulfobacteriaceae;Thermosulfuriphilus;Thermosulfuriphilus ammonigenes;Thermosulfuriphilus ammonigenes DSM 102941 |
| Ga0586324 3632849 2 703 | 0.00 | 2101200.00 | 0.00 | 4759500.00 | 0.00 | 856618.81 | 9325200.00 | 0.00 | 990826.02 | 0.00 | 0.00 | 409087.50 | KO:K04077 | chaperonin GroEL | Bacteria;Chloroflexi;Anaerolineae;Anaerolineales;Anaerolineaceae;Anaerolinea;Anaerolinea thermophila;Anaerolinea thermophila UNI-1 |
| Ga0586324 3645516 1 702 | 0.00 | 0.00 | 0.00 | 0.00 | 0.00 | 0.00 | 0.00 | 11956000.00 | 0.00 | 0.00 | 0.00 | 0.00 | KO:K04077 | chaperonin GroEL | Bacteria;Gemmatimonadetes;Gemmatimonadetes;Gemmatimonadales;Gemmatimonadaceae;Gemmatimonas;Gemmatimonas aurantiaca;Gemmatimonas aurantiaca T-27T |
| Ga0586324 3654207 2 703 | 0.00 | 0.00 | 0.00 | 0.00 | 638749.35 | 0.00 | 0.00 | 0.00 | 342587.88 | 0.00 | 1856800.00 | 0.00 | KO:K04077 | chaperonin GroEL | Bacteria;Proteobacteria;Deltaproteobacteria;Syntrophorhabdales;Syntrophorhabdaceae;Syntrophorhabdus;Syntrophorhabdus aromaticivorans;Syntrophorhabdus aromaticivorans UI |
| Ga0586324 3657405 2 700 | 0.00 | 1122000.00 | 0.00 | 0.00 | 0.00 | 0.00 | 0.00 | 0.00 | 0.00 | 0.00 | 0.00 | 0.00 | KO:K04077 | chaperonin GroEL | Bacteria;Verrucomicrobia;Verrucomicrobiae;Verrucomicrobiales;Verrucomicrobia subdivision 3;Pedosphaera;Pedosphaera parvula;Pedosphaera parvula Ellin514 |
| Ga0586324 3665444 3 701 | 0.00 | 0.00 | 0.00 | 0.00 | 0.00 | 0.00 | 3990000.00 | 0.00 | 0.00 | 0.00 | 968000.00 | 0.00 | KO:K04077 | chaperonin GroEL | Bacteria;Proteobacteria;Deltaproteobacteria;unclassified Deltaproteobacteria;unclassified Deltaproteobacteria;Dissulfuribacter;Dissulfuribacter thermophilus;Dissulfuribacter thermophilus S69 |
| Ga0586324 3678384 2 418 | 0.00 | 1795200.00 | 0.00 | 0.00 | 8013400.00 | 0.00 | 0.00 | 9455000.00 | 0.00 | 6106100.00 | 0.00 | 0.00 | KO:K04077 | chaperonin GroEL | Bacteria;Verrucomicrobia;Verrucomicrobiae;Verrucomicrobiales;Verrucomicrobia subdivision 3;Pedosphaera;Pedosphaera parvula;Pedosphaera parvula Ellin514 |
| Ga0586324 3681474 1 699 | 0.00 | 0.00 | 0.00 | 0.00 | 0.00 | 0.00 | 0.00 | 0.00 | 0.00 | 0.00 | 932800.00 | 0.00 | KO:K04077 | chaperonin GroEL | Bacteria;Bacteroidetes;Bacteroidia;Bacteroidales;Lentimicrobiaceae;Lentimicrobium;Lentimicrobium saccharophilum;Bacteroidales bacterium TBC1 |
| Ga0586324 3689031 1 699 | 1389641.00 | 84772.88 | 0.00 | 373443.10 | 721896.10 | 858397.37 | 0.00 | 598402.07 | 617547.24 | 0.00 | 0.00 | 0.00 | KO:K04077 | chaperonin GroEL | Bacteria;Chloroflexi;Anaerolineae;Anaerolineales;Anaerolineaceae;Levilinea;Levilinea saccharolytica;Levilinea saccharolytica DSM 16555 |
| Ga0586324 3690820 1 699 | 522593.00 | 1025207.44 | 0.00 | 0.00 | 0.00 | 6590200.00 | 0.00 | 1166482.87 | 780452.82 | 2664200.00 | 0.00 | 0.00 | KO:K04077 | chaperonin GroEL | Bacteria;Proteobacteria;Deltaproteobacteria;unclassified Deltaproteobacteria;unclassified Deltaproteobacteria;Dissulfuribacter;Dissulfuribacter thermophilus;Dissulfuribacter thermophilus S69 |
| Ga0586324 3694437 1 699 | 0.00 | 191360.84 | 0.00 | 9281500.00 | 2224800.00 | 0.00 | 0.00 | 0.00 | 0.00 | 0.00 | 0.00 | 0.00 | KO:K04077 | chaperonin GroEL | Bacteria;Nitrospirae;Nitrospira;Nitrospirales;Nitrospiraceae;Nitrospira;Candidatus Nitrospira inopinata;Candidatus Nitrospira inopinata ENR4 |
| Ga0586324 3699339 3 698 | 0.00 | 0.00 | 0.00 | 0.00 | 294644.89 | 0.00 | 0.00 | 0.00 | 0.00 | 0.00 | 1496000.00 | 0.00 | KO:K04077 | chaperonin GroEL | Bacteria;Bacteroidetes;Bacteroidia;Bacteroidales;Rikenellaceae;Acetobacteroides;Acetobacteroides hydrogenigenes;Acetobacteroides hydrogenigenes DSM 24657 |
| Ga0586324 3715468 3 698 | 0.00 | 0.00 | 0.00 | 0.00 | 0.00 | 0.00 | 0.00 | 0.00 | 0.00 | 389389.77 | 15048000.00 | 243264.00 | KO:K04077 | chaperonin GroEL | Bacteria;Chloroflexi;Dehalococcoidia;Dehalococcoidales;Dehalococcoidaceae;Dehalococcoides;Dehalococcoides mccartyi;Dehalococcoides mccartyi VS |
| Ga0586324 3715859 3 626 | 0.00 | 0.00 | 0.00 | 0.00 | 0.00 | 0.00 | 0.00 | 6771000.00 | 0.00 | 0.00 | 0.00 | 0.00 | KO:K04077 | chaperonin GroEL | Bacteria;Verrucomicrobia;Methylacidiphilae;Methylacidiphilales;Methylacidiphilaceae;Methylacidiphilum;Methylacidiphilum kamchatkense;Methylacidiphilum kamchatkense Kam1 |
| Ga0586324 3722854 2 592 | 0.00 | 0.00 | 0.00 | 0.00 | 0.00 | 0.00 | 0.00 | 0.00 | 744965.91 | 0.00 | 0.00 | 84717.75 | KO:K04077 | chaperonin GroEL | Bacteria;Acidobacteria;Acidobacteriia;Acidobacteriales;Acidobacteriaceae;Acidobacterium;Acidobacterium ailaaui;Acidobacterium ailaaui PMMR2 |
| Ga0586324 3728517 3 629 | 2880000.00 | 0.00 | 2942694.03 | 9785000.00 | 8240000.00 | 0.00 | 10191600.00 | 12871000.00 | 1360800.00 | 5174400.00 | 6494400.00 | 0.00 | KO:K04077 | chaperonin GroEL | Bacteria;Proteobacteria;Alphaproteobacteria;Micropepsales;Micropepsaceae;Rhizomicrobium;Rhizomicrobium sp. SG733;Rhizomicrobium sp. SG733 |
| Ga0586324 3744952 1 696 | 0.00 | 0.00 | 0.00 | 0.00 | 0.00 | 6669600.00 | 0.00 | 1104100.00 | 0.00 | 0.00 | 0.00 | 0.00 | KO:K04077 | chaperonin GroEL | Bacteria;Actinobacteria;Actinomycetia;Acidothermales;Acidothermaceae;Acidothermus;Acidothermus cellulolyticus;Acidothermus cellulolyticus 11B |
| Ga0586324 3759701 3 692 | 0.00 | 166862.48 | 0.00 | 0.00 | 0.00 | 0.00 | 108303.88 | 264101.33 | 323249.94 | 818325.74 | 0.00 | 176892.00 | KO:K04077 | chaperonin GroEL | Bacteria;Proteobacteria;Betaproteobacteria;Nitrosomonadales;Methylophilaceae;Methylotenera;Methylotenera versatilis;Methylotenera versatilis #7 |
| Ga0586324 3762337 1 693 | 0.00 | 506081.16 | 0.00 | 0.00 | 0.00 | 0.00 | 0.00 | 0.00 | 0.00 | 0.00 | 6239200.00 | 0.00 | KO:K04077 | chaperonin GroEL | Bacteria;Nitrospirae;unclassified Nitrospirae;unclassified Nitrospirae;unclassified Nitrospirae;unclassified Nitrospirae;Nitrospirae bacterium HCH-1;Nitrospirae bacterium HCH-1 |
| Ga0586324 3767650 62 694 | 0.00 | 0.00 | 0.00 | 0.00 | 0.00 | 0.00 | 0.00 | 1732400.00 | 159499.53 | 5798100.00 | 7145600.00 | 5272500.00 | KO:K04077 | chaperonin GroEL | Bacteria;Proteobacteria;Alphaproteobacteria;Rhodospirillales;unclassified Rhodospirillales;unclassified Rhodospirillales;Rhodospirillales bacterium URHD0017;Rhodospirillales bacterium URHD0017 |
| Ga0586324 3784240 2 427 | 0.00 | 0.00 | 0.00 | 0.00 | 0.00 | 0.00 | 0.00 | 744200.00 | 0.00 | 0.00 | 0.00 | 0.00 | KO:K04077 | chaperonin GroEL | Bacteria;Proteobacteria;Deltaproteobacteria;Myxococcales;Sandaracinaceae;Sandaracinus;Sandaracinus amylolyticus;Sandaracinus amylolyticus DSM 53668 |
| Ga0586324 3792664 3 683 | 0.00 | 8908000.00 | 8839100.00 | 5633500.00 | 4501100.00 | 2827052.88 | 8048400.00 | 5581500.00 | 6439500.00 | 10241000.00 | 8720800.00 | 7020000.00 | KO:K04077 | chaperonin GroEL | Bacteria;Spirochaetes;Spirochaetia;Spirochaetales;Treponemataceae;Treponema;Treponema caldarium;Treponema caldarium DSM 7334 |
| Ga0586324 3806884 50 691 | 0.00 | 0.00 | 2447513.01 | 0.00 | 0.00 | 0.00 | 0.00 | 0.00 | 1445124.24 | 0.00 | 0.00 | 855000.00 | KO:K04077 | chaperonin GroEL | Bacteria;Proteobacteria;Deltaproteobacteria;Syntrophobacterales;Syntrophaceae;Desulfobacca;Desulfobacca acetoxidans;Desulfobacca acetoxidans ASRB2, DSM 11109 |
| Ga0586324 3808968 2 691 | 0.00 | 1312400.00 | 0.00 | 1263500.00 | 0.00 | 0.00 | 0.00 | 0.00 | 0.00 | 0.00 | 3423200.00 | 1207500.00 | KO:K04077 | chaperonin GroEL | Bacteria;Proteobacteria;Deltaproteobacteria;Syntrophobacterales;Syntrophobacteraceae;Desulfacinum;Desulfacinum infernum;Desulfacinum infernum DSM 9756 |
| Ga0586324 3833054 2 460 | 0.00 | 3243600.00 | 0.00 | 0.00 | 701500.04 | 0.00 | 0.00 | 0.00 | 0.00 | 0.00 | 0.00 | 0.00 | KO:K04077 | chaperonin GroEL | Bacteria;Proteobacteria;Deltaproteobacteria;Desulfuromonadales;Geobacteraceae;Geobacter;Geobacter sp. OR-1;Geobacter sp. OR-1 |
| Ga0586324 3872857 3 686 | 0.00 | 0.00 | 0.00 | 0.00 | 1277200.00 | 0.00 | 0.00 | 0.00 | 764951.85 | 0.00 | 0.00 | 0.00 | KO:K04077 | chaperonin GroEL | Bacteria;Acidobacteria;Blastocatellia;Blastocatellales;Pyrinomonadaceae;Pyrinomonas;Pyrinomonas methylaliphatogenes;Pyrinomonas methylaliphatogenes K22 |
| Ga0586324 3875326 3 686 | 0.00 | 0.00 | 0.00 | 0.00 | 0.00 | 0.00 | 0.00 | 0.00 | 0.00 | 305356.59 | 0.00 | 0.00 | KO:K04077 | chaperonin GroEL | Bacteria;Actinobacteria;Thermoleophilia;Solirubrobacterales;Solirubrobacteraceae;Solirubrobacter;Solirubrobacter soli;Solirubrobacter soli DSM 22325 |
| Ga0586324 3888743 3 683 | 0.00 | 0.00 | 0.00 | 0.00 | 0.00 | 0.00 | 1211972.76 | 884500.00 | 0.00 | 0.00 | 2666400.00 | 1500000.00 | KO:K04077 | chaperonin GroEL | Bacteria;Proteobacteria;Deltaproteobacteria;unclassified Deltaproteobacteria;unclassified Deltaproteobacteria;Dissulfuribacter;Dissulfuribacter thermophilus;Dissulfuribacter thermophilus S69 |
| Ga0586324 3896244 2 682 | 499304.00 | 0.00 | 2382458.36 | 1995000.00 | 1915800.00 | 649690.50 | 2644800.00 | 3836900.00 | 3272400.00 | 2132900.00 | 1619200.00 | 2872500.00 | KO:K04077 | chaperonin GroEL | Bacteria;Proteobacteria;Gammaproteobacteria;Nevskiales;Steroidobacteraceae;Povalibacter;Povalibacter uvarum;Povalibacter uvarum DSM 26723 |
| Ga0586324 3911890 43 681 | 0.00 | 0.00 | 0.00 | 0.00 | 0.00 | 81782000.00 | 25080000.00 | 0.00 | 169290000.00 | 0.00 | 243830.40 | 303252.75 | KO:K04077 | chaperonin GroEL | Bacteria;Proteobacteria;Deltaproteobacteria;Syntrophobacterales;Syntrophaceae;Syntrophus;Syntrophus aciditrophicus;Syntrophus aciditrophicus SB |
| Ga0586324 3916883 1 324 | 410891.00 | 0.00 | 0.00 | 0.00 | 0.00 | 0.00 | 0.00 | 0.00 | 0.00 | 0.00 | 5368000.00 | 0.00 | KO:K04077 | chaperonin GroEL | Bacteria;Proteobacteria;Deltaproteobacteria;Desulfobacterales;Desulfobacteraceae;Desulfococcus;Desulfococcus multivorans;Desulfococcus multivorans DSM 2059 |
| Ga0586324 3939947 2 679 | 0.00 | 0.00 | 0.00 | 0.00 | 0.00 | 0.00 | 0.00 | 0.00 | 0.00 | 0.00 | 0.00 | 241899.00 | KO:K04077 | chaperonin GroEL | Bacteria;Verrucomicrobia;Verrucomicrobiae;Verrucomicrobiales;Verrucomicrobia subdivision 3;Pedosphaera;Pedosphaera parvula;Pedosphaera parvula Ellin514 |
| Ga0586324 3946789 1 681 | 178143106.00 | 34340000.00 | 742045.26 | 0.00 | 175285400.00 | 186647406.20 | 0.00 | 22021000.00 | 140940000.00 | 0.00 | 0.00 | 198000000.00 | KO:K04077 | chaperonin GroEL | Bacteria;Chloroflexi;unclassified Chloroflexi;unclassified Chloroflexi;unclassified Chloroflexi;unclassified Chloroflexi;unclassified Chloroflexi;Chloroflexi sp. G233 |
| Ga0586324 3972848 2 679 | 0.00 | 0.00 | 0.00 | 0.00 | 0.00 | 0.00 | 0.00 | 0.00 | 0.00 | 159266.03 | 0.00 | 0.00 | KO:K04077 | chaperonin GroEL | Bacteria;Candidatus Calescamantes;unclassified Candidatus Calescamantes;unclassified Candidatus Calescamantes;unclassified Candidatus Calescamantes;unclassified Candidatus Calescamantes;Calescamantes bacterium JGI 0000106-H18;Calescamantes bacterium JGI 0000106-H18 (contamination screened) |
| Ga0586324 3976179 1 678 | 6190000.00 | 9112000.00 | 13230500.00 | 0.00 | 0.00 | 0.00 | 11719200.00 | 10309000.00 | 13203000.00 | 19327000.00 | 32648000.00 | 19125000.00 | KO:K04077 | chaperonin GroEL | Bacteria;Bacteroidetes;Bacteroidia;Marinilabiliales;Prolixibacteraceae;Tangfeifania;Tangfeifania diversioriginum;Tangfeifania diversioriginum DSM 27063 |
| Ga0586324 3992756 2 676 | 6460000.00 | 1584400.00 | 0.00 | 5073000.00 | 3399000.00 | 0.00 | 0.00 | 4343200.00 | 54723600.00 | 1486100.00 | 3124000.00 | 3022500.00 | KO:K04077 | chaperonin GroEL | Bacteria;Proteobacteria;Alphaproteobacteria;Caulobacterales;Caulobacteraceae;Phenylobacterium;Phenylobacterium soli;Phenylobacterium soli LX32 |
| Ga0586324 3998077 3 677 | 0.00 | 530893.68 | 0.00 | 0.00 | 0.00 | 0.00 | 0.00 | 3557670.67 | 0.00 | 0.00 | 0.00 | 0.00 | KO:K04077 | chaperonin GroEL | Bacteria;Verrucomicrobia;Verrucomicrobiae;Verrucomicrobiales;Verrucomicrobia subdivision 3;Pedosphaera;Pedosphaera parvula;Pedosphaera parvula Ellin514 |
| Ga0586324 3998592 1 675 | 0.00 | 0.00 | 0.00 | 3439000.00 | 0.00 | 0.00 | 0.00 | 0.00 | 0.00 | 0.00 | 0.00 | 0.00 | KO:K04077 | chaperonin GroEL | Bacteria;Bacteroidetes;Bacteroidia;Bacteroidales;Tannerellaceae;Parabacteroides;Parabacteroides distasonis;Parabacteroides distasonis ATCC 8503 |
| Ga0586324 4030186 3 674 | 0.00 | 0.00 | 0.00 | 0.00 | 0.00 | 0.00 | 0.00 | 0.00 | 0.00 | 0.00 | 10912000.00 | 0.00 | KO:K04077 | chaperonin GroEL | Bacteria;Chloroflexi;Dehalococcoidia;Dehalococcoidales;Dehalococcoidaceae;Dehalococcoides;Dehalococcoides mccartyi;Dehalococcoides mccartyi 195 |
| Ga0586324 4054753 3 671 | 0.00 | 0.00 | 0.00 | 0.00 | 0.00 | 0.00 | 0.00 | 317347.62 | 0.00 | 0.00 | 0.00 | 0.00 | KO:K04077 | chaperonin GroEL | Bacteria;Proteobacteria;Deltaproteobacteria;Desulfobacterales;Desulfobacteraceae;Desulfatiglans;Desulfatiglans anilini;Desulfatiglans anilini DSM 4660 |
| Ga0586324 4055028 2 673 | 0.00 | 0.00 | 0.00 | 331158.60 | 0.00 | 0.00 | 0.00 | 358338.40 | 0.00 | 0.00 | 0.00 | 0.00 | KO:K04077 | chaperonin GroEL | Bacteria;Proteobacteria;Deltaproteobacteria;Syntrophobacterales;Syntrophobacteraceae;Syntrophobacter;Syntrophobacter fumaroxidans;Syntrophobacter fumaroxidans MPOB |
| Ga0586324 4055335 52 672 | 0.00 | 0.00 | 0.00 | 1092500.00 | 0.00 | 0.00 | 0.00 | 0.00 | 0.00 | 0.00 | 0.00 | 0.00 | KO:K04077 | chaperonin GroEL | Bacteria;Planctomycetes;Phycisphaerae;Sedimentisphaerales;Anaerohalosphaeraceae;Anaerohalosphaera;Anaerohalosphaera lusitana;Phycisphaerae bacterium ST-NAGAB-D1 |
| Ga0586324 4062422 1 633 | 0.00 | 0.00 | 979828.31 | 1871500.00 | 556147.47 | 545263.62 | 0.00 | 0.00 | 0.00 | 0.00 | 0.00 | 0.00 | KO:K04077 | chaperonin GroEL | Bacteria;Firmicutes;Negativicutes;Selenomonadales;Sporomusaceae;Propionispora;Propionispora sp. 2/2-37;Propionispora sp. Iso2/2 |
| Ga0586324 4067182 2 670 | 1910000.00 | 0.00 | 0.00 | 719412.20 | 261649.87 | 0.00 | 0.00 | 370642.10 | 1077300.00 | 0.00 | 0.00 | 0.00 | KO:K04077 | chaperonin GroEL | Bacteria;Chloroflexi;Thermomicrobia;Thermomicrobiales;Thermomicrobiaceae;Thermomicrobium;Thermomicrobium roseum;Thermomicrobium roseum DSM 5159 |
| Ga0586324 4080571 1 669 | 0.00 | 0.00 | 13455700.00 | 0.00 | 6664100.00 | 0.00 | 0.00 | 16592000.00 | 17739000.00 | 34881000.00 | 39600000.00 | 30150000.00 | KO:K04077 | chaperonin GroEL | Bacteria;Proteobacteria;Betaproteobacteria;Rhodocyclales;Azonexaceae;Dechloromonas;Dechloromonas sp. HYN0024;Dechloromonas sp. HYN0024 |
| Ga0586324 4085307 3 671 | 0.00 | 0.00 | 0.00 | 0.00 | 3646200.00 | 0.00 | 0.00 | 0.00 | 0.00 | 0.00 | 0.00 | 0.00 | KO:K04077 | chaperonin GroEL | Bacteria;Chloroflexi;Anaerolineae;Anaerolineales;Anaerolineaceae;Anaerolinea;Anaerolinea thermophila;Anaerolinea thermophila UNI-1 |
| Ga0586324 4088480 1 603 | 0.00 | 0.00 | 0.00 | 0.00 | 0.00 | 0.00 | 0.00 | 622200.00 | 0.00 | 0.00 | 0.00 | 0.00 | KO:K04077 | chaperonin GroEL | Bacteria;Proteobacteria;Gammaproteobacteria;Acidiferrobacterales;Acidiferrobacteraceae;Sulfuricaulis;Sulfuricaulis limicola;Sulfuricaulis limicola HA5 |
| Ga0586324 4095950 2 670 | 0.00 | 0.00 | 0.00 | 589741.95 | 0.00 | 0.00 | 0.00 | 29768000.00 | 0.00 | 1116500.00 | 0.00 | 687918.00 | KO:K04077 | chaperonin GroEL | Bacteria;Proteobacteria;Betaproteobacteria;unclassified Betaproteobacteria;unclassified Betaproteobacteria;unclassified Betaproteobacteria;Betaproteobacteria bacterium UKL13-2;Betaproteobacteria bacterium UKL13-2 |
| Ga0586324 4106101 1 669 | 0.00 | 0.00 | 0.00 | 0.00 | 0.00 | 0.00 | 2576400.00 | 0.00 | 0.00 | 0.00 | 0.00 | 298496.25 | KO:K04077 | chaperonin GroEL | Bacteria;Proteobacteria;Deltaproteobacteria;Syntrophobacterales;Syntrophobacteraceae;Desulfosoma;Desulfosoma caldarium;Desulfosoma caldarium DSM 22027 |
| Ga0586324 4128614 1 666 | 0.00 | 5304000.00 | 0.00 | 0.00 | 0.00 | 0.00 | 0.00 | 0.00 | 0.00 | 23485000.00 | 20064000.00 | 0.00 | KO:K04077 | chaperonin GroEL | Bacteria;Proteobacteria;Alphaproteobacteria;Hyphomicrobiales;Bradyrhizobiaceae;Bradyrhizobium;Bradyrhizobium erythrophlei;Bradyrhizobium erythrophlei GAS401 |
| Ga0586324 4131541 3 668 | 0.00 | 0.00 | 0.00 | 1833500.00 | 0.00 | 0.00 | 0.00 | 0.00 | 0.00 | 0.00 | 0.00 | 0.00 | KO:K04077 | chaperonin GroEL | Bacteria;Verrucomicrobia;Spartobacteria;unclassified Spartobacteria;unclassified Spartobacteria;unclassified Spartobacteria;Spartobacteria bacterium LR76;Spartobacteria bacterium LR76 |
| Ga0586324 4131588 2 667 | 0.00 | 17816000.00 | 498480.76 | 278996.95 | 0.00 | 0.00 | 247646.76 | 252859.64 | 707149.44 | 1786400.00 | 1276000.00 | 997500.00 | KO:K04077 | chaperonin GroEL | Bacteria;Proteobacteria;Alphaproteobacteria;Hyphomicrobiales;Hyphomicrobiaceae;Hyphomicrobium;Hyphomicrobium sp. CS1BSMeth3;Hyphomicrobium sp. CS1BSMeth3 |
| Ga0586324 4136767 1 666 | 0.00 | 4467600.00 | 0.00 | 0.00 | 0.00 | 0.00 | 0.00 | 0.00 | 0.00 | 0.00 | 0.00 | 0.00 | KO:K04077 | chaperonin GroEL | Bacteria;Chloroflexi;Chloroflexia;Kallotenuales;Kallotenuaceae;Kallotenue;Kallotenue papyrolyticum;Kallotenue papyrolyticum JKG1 |
| Ga0586324 4148840 3 665 | 0.00 | 0.00 | 0.00 | 0.00 | 0.00 | 0.00 | 270962.04 | 336608.98 | 0.00 | 0.00 | 0.00 | 507580.50 | KO:K04077 | chaperonin GroEL | Bacteria;Proteobacteria;Deltaproteobacteria;Syntrophorhabdales;Syntrophorhabdaceae;Syntrophorhabdus;Syntrophorhabdus aromaticivorans;Syntrophorhabdus aromaticivorans UI |
| Ga0586324 4152771 1 666 | 1220000.00 | 1815600.00 | 1810579.85 | 0.00 | 2029100.00 | 0.00 | 3556800.00 | 2745000.00 | 2227500.00 | 5782700.00 | 6248000.00 | 4440000.00 | KO:K04077 | chaperonin GroEL | Bacteria;Proteobacteria;Betaproteobacteria;unclassified Betaproteobacteria;unclassified Betaproteobacteria;unclassified Betaproteobacteria;Betaproteobacteria bacterium GR16-43;Betaproteobacteria bacterium GR16-43 |
| Ga0586324 4159954 2 664 | 0.00 | 0.00 | 0.00 | 0.00 | 0.00 | 0.00 | 1825545.84 | 0.00 | 404610.39 | 0.00 | 0.00 | 0.00 | KO:K04077 | chaperonin GroEL | Bacteria;Proteobacteria;Betaproteobacteria;Burkholderiales;Oxalobacteraceae;Noviherbaspirillum;Noviherbaspirillum sp. K1S02-23;Noviherbaspirillum sp. K1S02-23 |
| Ga0586324 4167927 2 664 | 0.00 | 0.00 | 0.00 | 0.00 | 0.00 | 0.00 | 0.00 | 0.00 | 0.00 | 0.00 | 7136800.00 | 0.00 | KO:K04077 | chaperonin GroEL | Bacteria;Proteobacteria;Deltaproteobacteria;Desulfobacterales;Desulfobacteraceae;Desulfosarcina;Desulfosarcina variabilis;Desulfosarcina variabilis Montpellier |
| Ga0586324 4175505 2 664 | 0.00 | 0.00 | 0.00 | 0.00 | 361521.76 | 0.00 | 0.00 | 0.00 | 0.00 | 0.00 | 0.00 | 0.00 | KO:K04077 | chaperonin GroEL | Bacteria;Proteobacteria;Deltaproteobacteria;Myxococcales;Vulgatibacteraceae;Vulgatibacter;Vulgatibacter incomptus;Vulgatibacter incomptus DSM 27710 |
| Ga0586324 4179888 2 664 | 2280000.00 | 534507.20 | 828420.72 | 832570.50 | 0.00 | 539618.28 | 3876000.00 | 1790113.93 | 1401300.00 | 2979900.00 | 2728000.00 | 2635155.00 | KO:K04077 | chaperonin GroEL | Bacteria;Proteobacteria;Betaproteobacteria;unclassified Betaproteobacteria;unclassified Betaproteobacteria;unclassified Betaproteobacteria;uncultured beta proteobacterium;unclassified betaproteobacterium ENR4 |
| Ga0586324 4185962 3 662 | 3790000.00 | 13396000.00 | 10302900.00 | 0.00 | 0.00 | 0.00 | 0.00 | 9821000.00 | 12879000.00 | 5094487.09 | 17487631.04 | 1790064.00 | KO:K04077 | chaperonin GroEL | Bacteria;Planctomycetes;Planctomycetia;Pirellulales;Thermoguttaceae;Thermogutta;Thermogutta terrifontis;Thermogutta terrifontis R1 |
| Ga0586324 4194392 2 664 | 0.00 | 386836.36 | 360768.15 | 862015.75 | 0.00 | 0.00 | 633739.68 | 701500.00 | 159311.61 | 0.00 | 0.00 | 0.00 | KO:K04077 | chaperonin GroEL | Bacteria;Proteobacteria;Deltaproteobacteria;Desulfuromonadales;Geobacteraceae;Geobacter;Geobacter sp. DSM 2909;Geobacter sp. DSM 2909 |
| Ga0586324 4206717 1 663 | 0.00 | 87626.16 | 0.00 | 9690000.00 | 0.00 | 11671800.00 | 3351600.00 | 10839700.00 | 699170.94 | 191358.86 | 416909.68 | 2655000.00 | KO:K04077 | chaperonin GroEL | Bacteria;Planctomycetes;Planctomycetia;Pirellulales;Pirellulaceae;Blastopirellula;Blastopirellula cremea;Blastopirellula cremea HEX PRIS-MGV |
| Ga0586324 4211459 2 661 | 0.00 | 0.00 | 0.00 | 0.00 | 0.00 | 0.00 | 0.00 | 0.00 | 2081700.00 | 0.00 | 0.00 | 0.00 | KO:K04077 | chaperonin GroEL | Bacteria;Verrucomicrobia;Opitutae;Opitutales;Opitutaceae;Opitutus;Opitutus terrae;Opitutus terrae PB90-1 |
| Ga0586324 4212312 2 661 | 0.00 | 0.00 | 0.00 | 988000.00 | 106090000.00 | 0.00 | 0.00 | 9247600.00 | 42181763.31 | 0.00 | 0.00 | 0.00 | KO:K04077 | chaperonin GroEL | Bacteria;Proteobacteria;Deltaproteobacteria;unclassified Deltaproteobacteria;Candidatus Desulfofervidaceae;Candidatus Desulfofervidus;Candidatus Desulfofervidus auxilii;delta proteobacterium HotSeep1 |
| Ga0586324 4215379 3 662 | 0.00 | 0.00 | 0.00 | 3904500.00 | 0.00 | 0.00 | 0.00 | 1787300.00 | 0.00 | 0.00 | 0.00 | 0.00 | KO:K04077 | chaperonin GroEL | Bacteria;Proteobacteria;Deltaproteobacteria;Syntrophobacterales;Syntrophaceae;Desulfomonile;Desulfomonile tiedjei;Desulfomonile tiedjei DCB-1, DSM 6799 |
| Ga0586324 4227828 1 660 | 0.00 | 0.00 | 0.00 | 0.00 | 0.00 | 0.00 | 0.00 | 0.00 | 762337.17 | 0.00 | 0.00 | 0.00 | KO:K04077 | chaperonin GroEL | Bacteria;Proteobacteria;Gammaproteobacteria;Methylococcales;Methylococcaceae;Methylovulum;Methylovulum psychrotolerans;Methylovulum psychrotolerans HV10 M2 |
| Ga0586324 4254889 1 660 | 0.00 | 0.00 | 0.00 | 0.00 | 0.00 | 0.00 | 0.00 | 0.00 | 245012.04 | 0.00 | 0.00 | 0.00 | KO:K04077 | chaperonin GroEL | Bacteria;Proteobacteria;Deltaproteobacteria;Syntrophobacterales;Syntrophaceae;Desulfobacca;Desulfobacca acetoxidans;Desulfobacca acetoxidans ASRB2, DSM 11109 |
| Ga0586324 4256017 2 658 | 0.00 | 0.00 | 0.00 | 0.00 | 0.00 | 0.00 | 0.00 | 3318400.00 | 0.00 | 0.00 | 0.00 | 0.00 | KO:K04077 | chaperonin GroEL | Bacteria;Proteobacteria;Gammaproteobacteria;Cellvibrionales;Cellvibrionaceae;Saccharophagus;Saccharophagus degradans;Saccharophagus degradans 2-40 |

| | | | | | | | | | | | | | | | |
|---|---|---|---|---|---|---|---|---|---|---|---|---|---|---|---|
| Ga0586324 4259349 3 554 | 0.00 | 0.00 | 0.00 | 0.00 | 68598000.00 | 2049460.89 | 75696000.00 | 6649000.00 | 59292000.00 | 10010000.00 | 7268800.00 | 7072500.00 | KO:K04077 | chaperonin GroEL | Bacteria;Acidobacteria;Acidobacteriia;Acidobacteriales;Acidobacteriaceae;Granulicella;Granulicella tundricola;Granulicella tundricola MP5ACTX9 |
| Ga0586324 4259535 112 660 | 0.00 | 0.00 | 0.00 | 0.00 | 0.00 | 0.00 | 0.00 | 0.00 | 0.00 | 2987600.00 | 0.00 | 3435000.00 | KO:K04077 | chaperonin GroEL | Bacteria;Proteobacteria;Deltaproteobacteria;Syntrophobacterales;Syntrophobacteraceae;Desulfacinum;Desulfacinum hydrothermale;Desulfacinum hydrothermale DSM 13146 |
| Ga0586324 4278833 1 324 | 0.00 | 0.00 | 4173654.12 | 0.00 | 0.00 | 0.00 | 0.00 | 0.00 | 0.00 | 0.00 | 0.00 | 0.00 | KO:K04077 | chaperonin GroEL | Bacteria;Verrucomicrobia;Spartobacteria;unclassified Spartobacteria;unclassified Spartobacteria;unclassified Spartobacteria;Spartobacteria bacterium LR76;Spartobacteria bacterium LR76 |
| Ga0586324 4286601 3 656 | 0.00 | 0.00 | 0.00 | 0.00 | 0.00 | 0.00 | 0.00 | 0.00 | 0.00 | 254527.35 | 0.00 | 0.00 | KO:K04077 | chaperonin GroEL | Bacteria;Actinobacteria;Thermoleophilia;Solirubrobacterales;Solirubrobacteraceae;Solirubrobacter;Solirubrobacter soli;Solirubrobacter soli DSM 22325 |
| Ga0586324 4301111 56 655 | 0.00 | 0.00 | 0.00 | 0.00 | 846718.71 | 0.00 | 0.00 | 0.00 | 0.00 | 0.00 | 0.00 | 0.00 | KO:K04077 | chaperonin GroEL | Bacteria;Proteobacteria;Deltaproteobacteria;Syntrophobacterales;Syntrophaceae;Desulfobacca;Desulfobacca acetoxidans;Desulfobacca acetoxidans ASRB2, DSM 11109 |
| Ga0586324 4308896 3 656 | 0.00 | 0.00 | 0.00 | 0.00 | 0.00 | 0.00 | 0.00 | 0.00 | 0.00 | 0.00 | 850407.36 | 0.00 | KO:K04077 | chaperonin GroEL | Bacteria;Proteobacteria;Deltaproteobacteria;Desulfobacterales;Desulfobacteraceae;Desulfatiglans;Desulfatiglans anilini;Desulfatiglans anilini DSM 4660 |
| Ga0586324 4308974 1 657 | 3880000.00 | 5086400.00 | 7769400.00 | 10715647.55 | 10650200.00 | 9766200.00 | 10129558.92 | 9636049.83 | 10068300.00 | 2156000.00 | 1082400.00 | 7312500.00 | KO:K04077 | chaperonin GroEL | Bacteria;Chloroflexi;Caldilineae;Caldilineales;Caldilineaceae;Caldilinea;Caldilinea aerophila;Caldilinea aerophila STL-6-O1, DSM 14535 |
| Ga0586324 4323996 114 656 | 56700000.00 | 10812000.00 | 0.00 | 676506.40 | 5057300.00 | 3802473.94 | 8572800.00 | 1903200.00 | 6868800.00 | 1208900.00 | 0.00 | 5722500.00 | KO:K04077 | chaperonin GroEL | Bacteria;Proteobacteria;Deltaproteobacteria;Syntrophorhabdales;Syntrophorhabdaceae;Syntrophorhabdus;Syntrophorhabdus aromaticivorans;Syntrophorhabdus aromaticivorans UI |
| Ga0586324 4334130 2 655 | 0.00 | 451908.28 | 0.00 | 0.00 | 0.00 | 0.00 | 1527043.68 | 0.00 | 1059032.88 | 0.00 | 69327.72 | 0.00 | KO:K04077 | chaperonin GroEL | Bacteria;Proteobacteria;Deltaproteobacteria;Myxococcales;Labilitrichaceae;Labilithrix;Labilithrix luteola;Labilithrix luteola DSM 27648 |
| Ga0586324 4342412 22 654 | 0.00 | 0.00 | 0.00 | 0.00 | 0.00 | 0.00 | 0.00 | 0.00 | 0.00 | 0.00 | 406300.40 | 0.00 | KO:K04077 | chaperonin GroEL | Bacteria;Proteobacteria;Alphaproteobacteria;Hyphomicrobiales;Phyllobacteriaceae;Aquamicrobium;Aquamicrobium sp. LC103;Aquamicrobium sp. LC103 |
| Ga0586324 4355537 1 654 | 0.00 | 0.00 | 0.00 | 551211.85 | 0.00 | 0.00 | 8185200.00 | 0.00 | 0.00 | 0.00 | 0.00 | 0.00 | KO:K04077 | chaperonin GroEL | Bacteria;Chloroflexi;Dehalococcoidia;Dehalococcoidales;Dehalococcoidaceae;Dehalococcoides;Dehalococcoides mccartyi;Dehalococcoides mccartyi VS |
| Ga0586324 4359722 3 521 | 0.00 | 0.00 | 0.00 | 0.00 | 0.00 | 0.00 | 0.00 | 0.00 | 0.00 | 0.00 | 777388.48 | 0.00 | KO:K04077 | chaperonin GroEL | Bacteria;Acidobacteria;Vicinamibacteria;unclassified Vicinamibacteria;Vicinamibacteraceae;Luteitalea;Luteitalea pratensis;Luteitalea pratensis DSM 100886 |
| Ga0586324 4372848 3 653 | 0.00 | 0.00 | 0.00 | 0.00 | 0.00 | 0.00 | 0.00 | 0.00 | 0.00 | 0.00 | 0.00 | 334632.75 | KO:K04077 | chaperonin GroEL | Bacteria;Acidobacteria;Acidobacteriia;Acidobacteriales;Acidobacteriaceae;unclassified Acidobacteriaceae;Acidobacteriaceae bacterium KBS 96;Acidobacteriaceae bacterium KBS 96, DSM 24297 |
| Ga0586324 4383847 2 589 | 0.00 | 124104.08 | 0.00 | 0.00 | 0.00 | 0.00 | 0.00 | 0.00 | 0.00 | 0.00 | 0.00 | 0.00 | KO:K04077 | chaperonin GroEL | Bacteria;Proteobacteria;Deltaproteobacteria;Myxococcales;Polyangiaceae;Sorangium;Sorangium cellulosum;Sorangium cellulosum So ceGT47 |
| Ga0586324 4391855 2 649 | 0.00 | 0.00 | 0.00 | 0.00 | 0.00 | 0.00 | 0.00 | 0.00 | 0.00 | 0.00 | 0.00 | 241465.50 | KO:K04077 | chaperonin GroEL | Bacteria;Proteobacteria;Deltaproteobacteria;Desulfobacterales;Desulfobacteraceae;Desulfatiglans;Desulfatiglans anilini;Desulfatiglans anilini DSM 4660 |
| Ga0586324 4394556 3 650 | 0.00 | 19094400.00 | 0.00 | 0.00 | 0.00 | 0.00 | 3830400.00 | 0.00 | 12636000.00 | 0.00 | 0.00 | 15975000.00 | KO:K04077 | chaperonin GroEL | Bacteria;Chloroflexi;Chloroflexia;Chloroflexales;Roseiflexaceae;Roseiflexus;Roseiflexus sp. RS-1;Roseiflexus sp. RS-1 |
| Ga0586324 4395021 16 651 | 0.00 | 0.00 | 0.00 | 0.00 | 0.00 | 0.00 | 0.00 | 553091.27 | 0.00 | 0.00 | 0.00 | 0.00 | KO:K04077 | chaperonin GroEL | Bacteria;Actinobacteria;Thermoleophilia;Solirubrobacterales;Solirubrobacteraceae;Solirubrobacter;Solirubrobacter pauli;Solirubrobacter pauli DSM 14954 |
| Ga0586324 4402008 3 650 | 30500000.00 | 28696000.00 | 0.00 | 9500000.00 | 7838300.00 | 0.00 | 35112000.00 | 16104000.00 | 15228000.00 | 14014000.00 | 2164800.00 | 13200000.00 | KO:K04077 | chaperonin GroEL | Bacteria;Proteobacteria;Deltaproteobacteria;Syntrophobacterales;Syntrophobacteraceae;Desulfacinum;Desulfacinum hydrothermale;Desulfacinum hydrothermale DSM 13146 |
| Ga0586324 4436809 1 648 | 0.00 | 0.00 | 0.00 | 0.00 | 0.00 | 2615662.29 | 0.00 | 780800.00 | 1432903.77 | 367646.51 | 0.00 | 165000.00 | KO:K04077 | chaperonin GroEL | Bacteria;Chloroflexi;Thermoflexia;Thermoflexales;Thermoflexaceae;Thermoflexus;Thermoflexus hugenholtzii;Thermoflexus hugenholtzii JAD2 |
| Ga0586324 4441838 3 482 | 0.00 | 0.00 | 0.00 | 0.00 | 1678900.00 | 0.00 | 2185284.24 | 0.00 | 0.00 | 0.00 | 2384800.00 | 0.00 | KO:K04077 | chaperonin GroEL | Bacteria;Calditrichaeota;Calditrichae;Calditrichales;Calditrichaceae;Caldithrix;Caldithrix abyssi;Caldithrix abyssi LF13, DSM 13497 |
| Ga0586324 4469378 1 645 | 220452.00 | 0.00 | 151578.18 | 0.00 | 5445936.51 | 255260.68 | 153855.54 | 0.00 | 0.00 | 0.00 | 0.00 | 0.00 | KO:K04077 | chaperonin GroEL | Bacteria;Actinobacteria;Thermoleophilia;Solirubrobacterales;Solirubrobacteraceae;Solirubrobacter;Solirubrobacter sp. URHD0082;Solirubrobacter sp. URHD0082 |
| Ga0586324 4474121 3 644 | 0.00 | 0.00 | 0.00 | 0.00 | 0.00 | 0.00 | 0.00 | 0.00 | 0.00 | 1532300.00 | 0.00 | 0.00 | KO:K04077 | chaperonin GroEL | Bacteria;Proteobacteria;Deltaproteobacteria;Myxococcales;Myxococcaceae;Myxococcus;Myxococcus stipitatus;Myxococcus stipitatus DSM 14675 |
| Ga0586324 4485548 2 646 | 0.00 | 42175.64 | 0.00 | 259580.85 | 1442000.00 | 0.00 | 248668.20 | 240782.25 | 1344600.00 | 1416800.00 | 205018.88 | 136044.00 | KO:K04077 | chaperonin GroEL | Bacteria;Proteobacteria;Deltaproteobacteria;Syntrophobacterales;Syntrophobacteraceae;Desulfosoma;Desulfosoma caldarium;Desulfosoma caldarium DSM 22027 |
| Ga0586324 4498965 1 645 | 0.00 | 0.00 | 0.00 | 0.00 | 0.00 | 0.00 | 0.00 | 568869.53 | 0.00 | 0.00 | 0.00 | 0.00 | KO:K04077 | chaperonin GroEL | Bacteria;Chloroflexi;Thermomicrobia;Sphaerobacterales;Sphaerobacteraceae;Sphaerobacter;Sphaerobacter thermophilus;Sphaerobacter thermophilus 4ac11, DSM 20745 |
| Ga0586324 4500219 1 498 | 0.00 | 0.00 | 0.00 | 0.00 | 2523500.00 | 5081600.00 | 0.00 | 1848300.00 | 0.00 | 0.00 | 0.00 | 0.00 | KO:K04077 | chaperonin GroEL | Bacteria;Chloroflexi;Anaerolineae;Anaerolineales;Anaerolineaceae;Anaerolinea;Anaerolinea thermophila;Anaerolinea thermophila UNI-1 |
| Ga0586324 4514096 3 644 | 0.00 | 0.00 | 0.00 | 0.00 | 0.00 | 0.00 | 0.00 | 0.00 | 0.00 | 0.00 | 0.00 | 3172500.00 | KO:K04077 | chaperonin GroEL | Bacteria;Acidobacteria;Vicinamibacteria;unclassified Vicinamibacteria;Vicinamibacteraceae;Luteitalea;Luteitalea pratensis;Luteitalea pratensis DSM 100886 |
| Ga0586324 4517468 3 644 | 0.00 | 0.00 | 0.00 | 0.00 | 0.00 | 852664.69 | 0.00 | 2074000.00 | 0.00 | 0.00 | 3132800.00 | 2287500.00 | KO:K04077 | chaperonin GroEL | Bacteria;Chloroflexi;Thermomicrobia;Thermomicrobiales;Thermomicrobiaceae;Thermomicrobium;Thermomicrobium roseum;Thermomicrobium roseum DSM 5159 |
| Ga0586324 4518090 3 644 | 0.00 | 467619.00 | 0.00 | 0.00 | 0.00 | 0.00 | 0.00 | 2616900.00 | 0.00 | 0.00 | 0.00 | 0.00 | KO:K04077 | chaperonin GroEL | Bacteria;Proteobacteria;Deltaproteobacteria;Desulfobacterales;Desulfobacteraceae;Desulfosarcina;Desulfosarcina cetonica;Desulfosarcina cetonica JCM 12296 |
| Ga0586324 4523952 1 642 | 0.00 | 0.00 | 0.00 | 6944500.00 | 0.00 | 0.00 | 0.00 | 0.00 | 0.00 | 0.00 | 0.00 | 0.00 | KO:K04077 | chaperonin GroEL | Bacteria;Acidobacteria;Acidobacteriia;Acidobacteriales;Acidobacteriaceae;Candidatus Koribacter;Candidatus Koribacter versatilis;Candidatus Koribacter versatilis Ellin345 |
| Ga0586324 4547100 1 642 | 0.00 | 2121600.00 | 0.00 | 0.00 | 2544100.00 | 0.00 | 0.00 | 2690100.00 | 13851000.00 | 0.00 | 0.00 | 0.00 | KO:K04077 | chaperonin GroEL | Bacteria;Proteobacteria;Deltaproteobacteria;Desulfuromonadales;Geobacteraceae;Geobacter;Geobacter sp. DSM 2909;Geobacter sp. DSM 2909 |
| Ga0586324 4549495 2 640 | 0.00 | 1878540.80 | 0.00 | 0.00 | 5150000.00 | 1054842.10 | 10168800.00 | 0.00 | 0.00 | 8547000.00 | 5850471.44 | 6909438.00 | KO:K04077 | chaperonin GroEL | Bacteria;Proteobacteria;Deltaproteobacteria;Syntrophobacterales;Syntrophobacteraceae;Desulfacinum;Desulfacinum infernum;Desulfacinum infernum DSM 9756 |
| Ga0586324 4567459 1 639 | 0.00 | 0.00 | 0.00 | 0.00 | 0.00 | 0.00 | 0.00 | 0.00 | 0.00 | 480523.89 | 0.00 | 0.00 | KO:K04077 | chaperonin GroEL | Bacteria;Proteobacteria;Betaproteobacteria;unclassified Betaproteobacteria;unclassified Betaproteobacteria;unclassified Betaproteobacteria;uncultured beta proteobacterium;unclassified betaproteobacterium ENR4 |
| Ga0586324 4573927 2 490 | 0.00 | 132283.12 | 0.00 | 0.00 | 0.00 | 0.00 | 0.00 | 0.00 | 0.00 | 0.00 | 0.00 | 0.00 | KO:K04077 | chaperonin GroEL | Bacteria;Ignavibacteriae;Ignavibacteria;Ignavibacteriales;Ignavibacteriaceae;Ignavibacterium;Ignavibacterium album;Ignavibacterium album Mat9-16, JCM 16511 |
| Ga0586324 4581071 3 587 | 0.00 | 334615.76 | 0.00 | 307096.05 | 0.00 | 0.00 | 0.00 | 1713181.34 | 0.00 | 29087537.71 | 681183.36 | 19335823.50 | KO:K04077 | chaperonin GroEL | Bacteria;Verrucomicrobia;Verrucomicrobiae;Verrucomicrobiales;Verrucomicrobia subdivision 3;Pedosphaera;Pedosphaera parvula;Pedosphaera parvula Ellin514 |
| Ga0586324 4585246 1 639 | 0.00 | 0.00 | 0.00 | 0.00 | 0.00 | 0.00 | 2104113.96 | 9845400.00 | 0.00 | 0.00 | 0.00 | 1627500.00 | KO:K04077 | chaperonin GroEL | Bacteria;Proteobacteria;Deltaproteobacteria;Myxococcales;Anaeromyxobacteraceae;Anaeromyxobacter;Anaeromyxobacter dehalogenans;Anaeromyxobacter dehalogenans 2CP-C |
| Ga0586324 4600629 2 637 | 0.00 | 16796000.00 | 0.00 | 17860000.00 | 16583000.00 | 12862800.00 | 0.00 | 12566000.00 | 9072000.00 | 0.00 | 0.00 | 10500000.00 | KO:K04077 | chaperonin GroEL | Bacteria;Proteobacteria;Deltaproteobacteria;Syntrophorhabdales;Syntrophorhabdaceae;Syntrophorhabdus;Syntrophorhabdus aromaticivorans;Syntrophorhabdus aromaticivorans UI |
| Ga0586324 4602367 2 637 | 506025.00 | 0.00 | 0.00 | 1919000.00 | 0.00 | 0.00 | 788353.32 | 0.00 | 0.00 | 0.00 | 1328800.00 | 1282500.00 | KO:K04077 | chaperonin GroEL | Bacteria;Proteobacteria;Deltaproteobacteria;unclassified Deltaproteobacteria;unclassified Deltaproteobacteria;Dissulfuribacter;Dissulfuribacter thermophilus;Dissulfuribacter thermophilus S69 |
| Ga0586324 4603950 1 603 | 0.00 | 0.00 | 0.00 | 219808245.00 | 0.00 | 0.00 | 0.00 | 0.00 | 0.00 | 0.00 | 0.00 | 0.00 | KO:K04077 | chaperonin GroEL | Bacteria;Actinobacteria;Thermoleophilia;Solirubrobacterales;Solirubrobacteraceae;Solirubrobacter;Solirubrobacter pauli;Solirubrobacter pauli DSM 14954 |
| Ga0586324 4607548 2 637 | 1340000.00 | 0.00 | 2751904.59 | 0.00 | 0.00 | 0.00 | 1245851.28 | 591061.94 | 0.00 | 0.00 | 0.00 | 0.00 | KO:K04077 | chaperonin GroEL | Bacteria;Acidobacteria;Blastocatellia;Blastocatellales;Pyrinomonadaceae;Pyrinomonas;Pyrinomonas methylaliphatogenes;Pyrinomonas methylaliphatogenes K22 |
| Ga0586324 4611682 2 637 | 0.00 | 0.00 | 0.00 | 0.00 | 0.00 | 0.00 | 0.00 | 0.00 | 4673700.00 | 0.00 | 0.00 | 0.00 | KO:K04077 | chaperonin GroEL | Bacteria;Proteobacteria;Deltaproteobacteria;Desulfobacterales;Desulfobacteraceae;Desulfatitalea;Desulfatitalea tepidiphila;Desulfatitalea tepidiphila S28bF |
| Ga0586324 4629872 1 636 | 0.00 | 0.00 | 0.00 | 424421.05 | 0.00 | 4009700.00 | 0.00 | 10614000.00 | 0.00 | 0.00 | 0.00 | 0.00 | KO:K04077 | chaperonin GroEL | Bacteria;Chloroflexi;Anaerolineae;Anaerolineales;Anaerolineaceae;Anaerolinea;Anaerolinea thermophila;Anaerolinea thermophila UNI-1 |
| Ga0586324 4677406 3 434 | 0.00 | 5671200.00 | 0.00 | 0.00 | 0.00 | 0.00 | 0.00 | 0.00 | 0.00 | 0.00 | 0.00 | 0.00 | KO:K04077 | chaperonin GroEL | Bacteria;Proteobacteria;Alphaproteobacteria;Hyphomicrobiales;Phyllobacteriaceae;Mesorhizobium;Mesorhizobium metallidurans;Mesorhizobium metallidurans STM 2683 |
| Ga0586324 4716647 3 632 | 0.00 | 8092000.00 | 2825460.54 | 25935000.00 | 4253900.00 | 1216546.95 | 936893.04 | 6527000.00 | 4463100.00 | 1393700.00 | 4963200.00 | 4732500.00 | KO:K04077 | chaperonin GroEL | Bacteria;Acidobacteria;Vicinamibacteria;unclassified Vicinamibacteria;Vicinamibacteraceae;Luteitalea;Luteitalea pratensis;Luteitalea pratensis DSM 100886 |
| Ga0586324 4716648 3 632 | 0.00 | 0.00 | 0.00 | 0.00 | 0.00 | 0.00 | 0.00 | 0.00 | 0.00 | 1963500.00 | 5139200.00 | 0.00 | KO:K04077 | chaperonin GroEL | Bacteria;Verrucomicrobia;Spartobacteria;Chthoniobacterales;Chthoniobacteraceae;Chthoniobacter;Chthoniobacter flavus;Chthoniobacter flavus Ellin428 |
| Ga0586324 4729509 3 632 | 518021.00 | 439524.12 | 0.00 | 1301613.05 | 831371.71 | 0.00 | 250473.96 | 0.00 | 616475.61 | 291502.75 | 296303.92 | 2254713.75 | KO:K04077 | chaperonin GroEL | Bacteria;Proteobacteria;Deltaproteobacteria;Syntrophobacterales;Syntrophobacteraceae;Syntrophobacter;Syntrophobacter fumaroxidans;Syntrophobacter fumaroxidans MPOB |
| Ga0586324 4730002 3 632 | 0.00 | 0.00 | 0.00 | 0.00 | 0.00 | 0.00 | 0.00 | 0.00 | 2924100.00 | 0.00 | 0.00 | 795000.00 | KO:K04077 | chaperonin GroEL | Bacteria;Bacteroidetes;Chitinophagia;Chitinophagales;Chitinophagaceae;Asinibacterium;Asinibacterium sp. OR53;Asinibacterium sp. OR53 |
| Ga0586324 4749751 117 629 | 0.00 | 0.00 | 0.00 | 0.00 | 0.00 | 0.00 | 0.00 | 0.00 | 1061100.00 | 0.00 | 0.00 | 6787500.00 | KO:K04077 | chaperonin GroEL | Bacteria;Cyanobacteria;unclassified Cyanobacteria;Oscillatoriales;Microcoleaceae;Microcoleus;Microcoleus vaginatus;Microcoleus vaginatus PCC 9802 |
| Ga0586324 4776489 1 627 | 1200000.00 | 4624000.00 | 6924900.00 | 14706000.00 | 8415100.00 | 16396100.00 | 6311149.44 | 10040600.00 | 4568400.00 | 7623000.00 | 0.00 | 3217500.00 | KO:K04077 | chaperonin GroEL | Bacteria;Planctomycetes;Planctomycetia;Pirellulales;Pirellulaceae;Rhodopirellula;Rhodopirellula baltica;Rhodopirellula baltica BR-MGV |
| Ga0586324 4786800 1 627 | 0.00 | 2325600.00 | 0.00 | 0.00 | 10609000.00 | 0.00 | 0.00 | 0.00 | 0.00 | 9317000.00 | 15400000.00 | 0.00 | KO:K04077 | chaperonin GroEL | Bacteria;Acidobacteria;Vicinamibacteria;unclassified Vicinamibacteria;Vicinamibacteraceae;Luteitalea;Luteitalea pratensis;Luteitalea pratensis DSM 100886 |
| Ga0586324 4797338 3 626 | 685468.00 | 0.00 | 0.00 | 0.00 | 0.00 | 0.00 | 0.00 | 0.00 | 0.00 | 0.00 | 0.00 | 0.00 | KO:K04077 | chaperonin GroEL | Bacteria;Chloroflexi;Anaerolineae;Anaerolineales;Anaerolineaceae;Pelolinea;Pelolinea submarina;Pelolinea submarina DSM 23923 |
| Ga0586324 4815664 3 626 | 0.00 | 0.00 | 0.00 | 0.00 | 0.00 | 0.00 | 0.00 | 0.00 | 0.00 | 7753900.00 | 2244000.00 | 0.00 | KO:K04077 | chaperonin GroEL | Bacteria;Actinobacteria;Acidimicrobiia;Acidimicrobiales;Ilumatobacteraceae;Ilumatobacter;Ilumatobacter coccineus;Ilumatobacter coccineum YM16-304 |
| Ga0586324 4817938 2 238 | 0.00 | 0.00 | 0.00 | 32585000.00 | 0.00 | 0.00 | 0.00 | 0.00 | 0.00 | 0.00 | 0.00 | 0.00 | KO:K04077 | chaperonin GroEL | Bacteria;Acidobacteria;Acidobacteriia;Acidobacteriales;Acidobacteriaceae;unclassified Acidobacteriaceae;Acidobacteriaceae bacterium KBS 83;Acidobacteriaceae bacterium KBS 83 |
| Ga0586324 4821613 1 627 | 0.00 | 0.00 | 0.00 | 14345000.00 | 0.00 | 6550500.00 | 32832000.00 | 7686000.00 | 13527000.00 | 0.00 | 0.00 | 41025000.00 | KO:K04077 | chaperonin GroEL | Bacteria;Proteobacteria;Deltaproteobacteria;Myxococcales;Anaeromyxobacteraceae;Anaeromyxobacter;Anaeromyxobacter dehalogenans;Anaeromyxobacter dehalogenans 2CP-C |
| Ga0586324 4869981 3 614 | 2931662.00 | 18645600.00 | 1396493.35 | 8711500.00 | 1627400.00 | 6812889.21 | 154564373.76 | 1116300.00 | 6034500.00 | 1747900.00 | 3335200.00 | 6142500.00 | KO:K04077 | chaperonin GroEL | Bacteria;Chloroflexi;Ardenticatenia;Ardenticatenales;Ardenticatenaceae;Candidatus Promineofilum;Candidatus Promineofilum breve;Candidatus Promineofilum breve Cfx-K |
| Ga0586324 4876188 3 623 | 0.00 | 0.00 | 0.00 | 5177500.00 | 0.00 | 0.00 | 0.00 | 1110200.00 | 4390200.00 | 2048200.00 | 1584000.00 | 0.00 | KO:K04077 | chaperonin GroEL | Bacteria;Calditrichaeota;Calditrichae;Calditrichales;Calditrichaceae;Caldithrix;Caldithrix abyssi;Caldithrix abyssi LF13, DSM 13497 |
| Ga0586324 4877856 1 624 | 0.00 | 918000.00 | 543259.53 | 2849128.85 | 902200.69 | 630332.78 | 928511.76 | 0.00 | 312974.28 | 487236.75 | 4918021.68 | 0.00 | KO:K04077 | chaperonin GroEL | Bacteria;Chloroflexi;Thermoflexia;Thermoflexales;Thermoflexaceae;Thermoflexus;Thermoflexus hugenholtzii;Thermoflexus hugenholtzii JAD2 |
| Ga0586324 4900110 3 623 | 0.00 | 0.00 | 0.00 | 374012.15 | 0.00 | 0.00 | 0.00 | 0.00 | 0.00 | 0.00 | 0.00 | 0.00 | KO:K04077 | chaperonin GroEL | Bacteria;Chloroflexi;Anaerolineae;Anaerolineales;Anaerolineaceae;Anaerolinea;Anaerolinea thermophila;Anaerolinea thermophila UNI-1 |
| Ga0586324 4923818 2 622 | 0.00 | 463816.44 | 0.00 | 0.00 | 0.00 | 0.00 | 0.00 | 0.00 | 0.00 | 0.00 | 0.00 | 0.00 | KO:K04077 | chaperonin GroEL | Bacteria;Proteobacteria;Gammaproteobacteria;Pseudomonadales;Pseudomonadaceae;Permianibacter;Permianibacter aggregans;Permianibacter aggregans DSM 103792 |
| Ga0586324 4929362 3 620 | 0.00 | 2080800.00 | 0.00 | 0.00 | 798835.04 | 0.00 | 764848.80 | 501829.92 | 718896.06 | 0.00 | 3499135.20 | 0.00 | KO:K04077 | chaperonin GroEL | Bacteria;Proteobacteria;Deltaproteobacteria;Syntrophobacterales;Syntrophobacteraceae;Syntrophobacter;Syntrophobacter fumaroxidans;Syntrophobacter fumaroxidans MPOB |
| Ga0586324 4931654 1 621 | 0.00 | 0.00 | 0.00 | 1140000.00 | 0.00 | 0.00 | 0.00 | 2049420.05 | 0.00 | 0.00 | 2490400.00 | 0.00 | KO:K04077 | chaperonin GroEL | Bacteria;Proteobacteria;Deltaproteobacteria;Desulfobacterales;Desulfobulbaceae;Desulfopila;Desulfopila sp. IMCC35005;Desulfopila sp. IMCC35005 |
| Ga0586324 4961026 1 618 | 0.00 | 0.00 | 0.00 | 0.00 | 0.00 | 0.00 | 0.00 | 0.00 | 0.00 | 0.00 | 0.00 | 274354.50 | KO:K04077 | chaperonin GroEL | Bacteria;Candidatus Aminicenantes;unclassified Candidatus Aminicenantes;unclassified Candidatus Aminicenantes;unclassified Candidatus Aminicenantes;unclassified Candidatus Aminicenantes;Aminicenantes bacterium SCGC AAA252-P13;Aminicenantes bacterium SCGC AAA252-P13 (contamination screened) |
| Ga0586324 4976400 3 617 | 9160000.00 | 0.00 | 0.00 | 0.00 | 0.00 | 0.00 | 0.00 | 0.00 | 0.00 | 0.00 | 0.00 | 12750000.00 | KO:K04077 | chaperonin GroEL | Bacteria;Chloroflexi;Thermomicrobia;Sphaerobacterales;Sphaerobacteraceae;Sphaerobacter;Sphaerobacter thermophilus;Sphaerobacter thermophilus 4ac11, DSM 20745 |
| Ga0586324 4977351 1 618 | 0.00 | 0.00 | 0.00 | 0.00 | 0.00 | 0.00 | 206268.64 | 0.00 | 0.00 | 0.00 | 0.00 | 0.00 | KO:K04077 | chaperonin GroEL | Bacteria;Proteobacteria;Deltaproteobacteria;Myxococcales;Anaeromyxobacteraceae;Anaeromyxobacter;Anaeromyxobacter sp. K;Anaeromyxobacter sp. K |
| Ga0586324 4995475 23 616 | 0.00 | 0.00 | 0.00 | 0.00 | 22351000.00 | 0.00 | 0.00 | 0.00 | 0.00 | 0.00 | 0.00 | 0.00 | KO:K04077 | chaperonin GroEL | Bacteria;Chloroflexi;Dehalococcoidia;unclassified Dehalococcoidia;unclassified Dehalococcoidia;Dehalogenimonas;Dehalogenimonas formicexedens;Dehalogenimonas formicexedens NSZ-14 |
| Ga0586324 5001488 1 618 | 0.00 | 0.00 | 0.00 | 15675000.00 | 20394000.00 | 5994700.00 | 8572800.00 | 22509000.00 | 33939000.00 | 0.00 | 0.00 | 8400000.00 | KO:K04077 | chaperonin GroEL | Bacteria;Chloroflexi;Anaerolineae;Anaerolineales;Anaerolineaceae;Anaerolinea;Anaerolinea thermophila;Anaerolinea thermophila UNI-1 |
| Ga0586324 5012340 3 617 | 0.00 | 564519.00 | 0.00 | 1624500.00 | 0.00 | 0.00 | 0.00 | 6319600.00 | 4365900.00 | 0.00 | 9768000.00 | 915000.00 | KO:K04077 | chaperonin GroEL | Bacteria;Proteobacteria;Deltaproteobacteria;Syntrophobacterales;Syntrophobacteraceae;Desulfacinum;Desulfacinum infernum;Desulfacinum infernum DSM 9756 |
| Ga0586324 5020227 3 617 | 0.00 | 0.00 | 0.00 | 22800000.00 | 0.00 | 10639600.00 | 0.00 | 0.00 | 0.00 | 2772000.00 | 0.00 | 11775000.00 | KO:K04077 | chaperonin GroEL | Bacteria;Proteobacteria;Deltaproteobacteria;Myxococcales;Sandaracinaceae;Sandaracinus;Sandaracinus amylolyticus;Sandaracinus amylolyticus DSM 53668 |
| Ga0586324 5051766 3 614 | 5110000.00 | 4569600.00 | 5742600.00 | 4978000.00 | 4902800.00 | 11116000.00 | 20235392.16 | 16592000.00 | 17496000.00 | 10502800.00 | 17327576.64 | 42292500.00 | KO:K04077 | chaperonin GroEL | Bacteria;Proteobacteria;Betaproteobacteria;Burkholderiales;unclassified Burkholderiales;Rubrivivax;Rubrivivax benzoatilyticus;Rubrivivax benzoatilyticus ATCC BAA-35 |
| Ga0586324 5082657 1 612 | 0.00 | 113560000.00 | 123297000.00 | 4835500.00 | 455290.90 | 10401400.00 | 1162859.28 | 2732800.00 | 3993300.00 | 80749900.00 | 1240800.00 | 1300705.50 | KO:K04077 | chaperonin GroEL | Bacteria;Proteobacteria;Deltaproteobacteria;Myxococcales;Kofleriaceae;Haliangium;Haliangium ochraceum;Haliangium ochraceum SMP-2, DSM 14365 |
| Ga0586324 5087721 2 565 | 0.00 | 0.00 | 0.00 | 347593.60 | 292683.77 | 0.00 | 0.00 | 0.00 | 0.00 | 0.00 | 0.00 | 0.00 | KO:K04077 | chaperonin GroEL | Bacteria;Acidobacteria;Blastocatellia;Blastocatellales;Pyrinomonadaceae;Pyrinomonas;Pyrinomonas methylaliphatogenes;Pyrinomonas methylaliphatogenes K22 |
| Ga0586324 5100356 3 611 | 0.00 | 0.00 | 0.00 | 0.00 | 0.00 | 0.00 | 0.00 | 0.00 | 0.00 | 1155000.00 | 16456000.00 | 0.00 | KO:K04077 | chaperonin GroEL | Bacteria;Acidobacteria;Acidobacteriia;Bryobacterales;Solibacteraceae;Candidatus Solibacter;Candidatus Solibacter usitatus;Candidatus Solibacter usitatus Ellin6076 |
| Ga0586324 5106740 2 610 | 0.00 | 5274607.00 | 8235445.77 | 0.00 | 0.00 | 0.00 | 4719600.00 | 0.00 | 0.00 | 0.00 | 9847200.00 | 0.00 | KO:K04077 | chaperonin GroEL | Bacteria;Calditrichaeota;Calditrichae;Calditrichales;Calditrichaceae;Caldithrix;Caldithrix abyssi;Caldithrix abyssi LF13, DSM 13497 |
| Ga0586324 5123223 2 610 | 0.00 | 1373600.00 | 28938200.00 | 4522000.00 | 14584800.00 | 4930432.33 | 2021028.48 | 8418000.00 | 1709100.00 | 7045500.00 | 20592000.00 | 3435653.25 | KO:K04077 | chaperonin GroEL | Bacteria;Verrucomicrobia;Verrucomicrobiae;Verrucomicrobiales;Verrucomicrobia subdivision 3;Pedosphaera;Pedosphaera parvula;Pedosphaera parvula Ellin514 |
| Ga0586324 5144876 1 609 | 0.00 | 130576.32 | 0.00 | 0.00 | 0.00 | 0.00 | 0.00 | 1415200.00 | 3977100.00 | 2933700.00 | 5916340.32 | 23302500.00 | KO:K04077 | chaperonin GroEL | Bacteria;Proteobacteria;Betaproteobacteria;Nitrosomonadales;Nitrosomonadaceae;Nitrosospira;Nitrosospira lacus;Nitrosospira lacus APG3 |
| Ga0586324 5147362 3 608 | 0.00 | 149946.80 | 0.00 | 0.00 | 0.00 | 0.00 | 0.00 | 115874.99 | 0.00 | 0.00 | 0.00 | 0.00 | KO:K04077 | chaperonin GroEL | Bacteria;Proteobacteria;Betaproteobacteria;Nitrosomonadales;Nitrosomonadaceae;Nitrosospira;Nitrosospira lacus;Nitrosospira lacus APG3 |
| Ga0586324 5179038 28 609 | 0.00 | 0.00 | 0.00 | 0.00 | 0.00 | 0.00 | 0.00 | 171453.31 | 0.00 | 0.00 | 0.00 | 0.00 | KO:K04077 | chaperonin GroEL | Bacteria;Proteobacteria;Gammaproteobacteria;Chromatiales;Woeseiaceae;Woeseia;Woeseia oceani;Woeseia oceani XK5 |
| Ga0586324 5180716 2 607 | 0.00 | 0.00 | 0.00 | 0.00 | 0.00 | 0.00 | 0.00 | 0.00 | 0.00 | 0.00 | 0.00 | 152547.75 | KO:K04077 | chaperonin GroEL | Bacteria;Actinobacteria;Actinomycetia;Micromonosporales;Micromonosporaceae;unclassified Micromonosporaceae;Micromonosporaceae bacterium URHE0070;Micromonosporaceae bacterium URHE0070 |
| Ga0586324 5184255 2 607 | 6720000.00 | 0.00 | 0.00 | 0.00 | 0.00 | 0.00 | 0.00 | 0.00 | 0.00 | 0.00 | 0.00 | 0.00 | KO:K04077 | chaperonin GroEL | Bacteria;Proteobacteria;Gammaproteobacteria;Methylococcales;Methylococcaceae;Methylocaldum;Methylocaldum szegediense;Methylocaldum szegediense O-12 |
| Ga0586324 5213937 3 605 | 0.00 | 0.00 | 0.00 | 0.00 | 0.00 | 0.00 | 0.00 | 0.00 | 0.00 | 0.00 | 29392000.00 | 0.00 | KO:K04077 | chaperonin GroEL | Bacteria;Acidobacteria;Vicinamibacteria;unclassified Vicinamibacteria;Vicinamibacteraceae;Luteitalea;Luteitalea pratensis;Luteitalea pratensis DSM 100886 |
| Ga0586324 5219370 3 605 | 6290000.00 | 1047200.00 | 0.00 | 10735000.00 | 7436600.00 | 7106300.00 | 7227600.00 | 1756800.00 | 10611000.00 | 2618000.00 | 5174400.00 | 13320000.00 | KO:K04077 | chaperonin GroEL | Bacteria;Proteobacteria;Deltaproteobacteria;Desulfobacterales;Desulfobacteraceae;Desulfosarcina;Desulfosarcina variabilis;Desulfosarcina variabilis Montpellier |
| Ga0586324 5226828 2 604 | 0.00 | 0.00 | 0.00 | 9785000.00 | 14832000.00 | 0.00 | 15640800.00 | 3031700.00 | 14839200.00 | 0.00 | 0.00 | 0.00 | KO:K04077 | chaperonin GroEL | Bacteria;Proteobacteria;Deltaproteobacteria;Syntrophobacterales;Syntrophaceae;Desulfobacca;Desulfobacca acetoxidans;Desulfobacca acetoxidans ASRB2, DSM 11109 |
| Ga0586324 5254236 1 603 | 0.00 | 0.00 | 2460225.55 | 0.00 | 0.00 | 2002670.47 | 0.00 | 0.00 | 0.00 | 0.00 | 0.00 | 0.00 | KO:K04077 | chaperonin GroEL | Bacteria;Acidobacteria;Blastocatellia;Blastocatellales;Pyrinomonadaceae;Pyrinomonas;Pyrinomonas methylaliphatogenes;Pyrinomonas methylaliphatogenes K22 |
| Ga0586324 5262326 3 584 | 0.00 | 0.00 | 0.00 | 0.00 | 0.00 | 0.00 | 0.00 | 957700.00 | 0.00 | 0.00 | 0.00 | 0.00 | KO:K04077 | chaperonin GroEL | Bacteria;Chloroflexi;Anaerolineae;Anaerolineales;Anaerolineaceae;Pelolinea;Pelolinea submarina;Pelolinea submarina DSM 23923 |
| Ga0586324 5263940 2 604 | 826216.00 | 0.00 | 0.00 | 0.00 | 0.00 | 0.00 | 0.00 | 0.00 | 0.00 | 785400.00 | 0.00 | 0.00 | KO:K04077 | chaperonin GroEL | Bacteria;Planctomycetes;Planctomycetia;Gemmatales;Gemmataceae;Zavarzinella;Zavarzinella formosa;Zavarzinella formosa DSM 19928 |
| Ga0586324 5276430 62 604 | 0.00 | 6868000.00 | 5911500.00 | 4930500.00 | 4758600.00 | 3224084.64 | 4081200.00 | 1183400.00 | 3863700.00 | 6483400.00 | 976800.00 | 3780000.00 | KO:K04077 | chaperonin GroEL | Bacteria;Verrucomicrobia;Verrucomicrobiae;Verrucomicrobiales;Verrucomicrobia subdivision 3;Pedosphaera;Pedosphaera parvula;Pedosphaera parvula Ellin514 |
| Ga0586324 5279792 2 604 | 1520000.00 | 0.00 | 17058900.00 | 0.00 | 0.00 | 0.00 | 8588497.80 | 780800.00 | 0.00 | 186872.07 | 1108800.00 | 741042.00 | KO:K04077 | chaperonin GroEL | Bacteria;Chloroflexi;Anaerolineae;Anaerolineales;Anaerolineaceae;Anaerolinea;Anaerolinea thermophila;Anaerolinea thermophila UNI-1 |
| Ga0586324 5344322 3 599 | 0.00 | 0.00 | 0.00 | 0.00 | 0.00 | 0.00 | 0.00 | 0.00 | 185114.97 | 0.00 | 0.00 | 0.00 | KO:K04077 | chaperonin GroEL | Bacteria;Actinobacteria;Acidimicrobiia;Acidimicrobiales;Ilumatobacteraceae;Desertimonas;Desertimonas flava;Acidimicrobiales bacterium SYSU D60003 |
| Ga0586324 5363038 2 598 | 0.00 | 61852800.00 | 0.00 | 0.00 | 0.00 | 601824.21 | 52896000.00 | 500160.96 | 59616000.00 | 75845000.00 | 55247160.32 | 0.00 | KO:K04077 | chaperonin GroEL | Bacteria;Actinobacteria;Acidimicrobiia;Acidimicrobiales;Ilumatobacteraceae;Desertimonas;Desertimonas flava;Acidimicrobiales bacterium SYSU D60003 |
| Ga0586324 5369256 3 599 | 0.00 | 14076000.00 | 0.00 | 0.00 | 0.00 | 0.00 | 0.00 | 0.00 | 0.00 | 0.00 | 0.00 | 0.00 | KO:K04077 | chaperonin GroEL | Bacteria;Verrucomicrobia;Verrucomicrobiae;Verrucomicrobiales;Verrucomicrobia subdivision 3;Pedosphaera;Pedosphaera parvula;Pedosphaera parvula Ellin514 |
| Ga0586324 5401138 1 597 | 0.00 | 2890000.00 | 0.00 | 1368000.00 | 0.00 | 0.00 | 1920393.84 | 1049200.00 | 1077300.00 | 924000.00 | 5295359.52 | 3982500.00 | KO:K04077 | chaperonin GroEL | Bacteria;Chloroflexi;Chloroflexia;Chloroflexales;Roseiflexaceae;Roseiflexus;Roseiflexus sp. RS-1;Roseiflexus sp. RS-1 |
| Ga0586324 5445367 1 594 | 0.00 | 0.00 | 0.00 | 0.00 | 0.00 | 0.00 | 0.00 | 689300.00 | 0.00 | 5228300.00 | 0.00 | 0.00 | KO:K04077 | chaperonin GroEL | Bacteria;Chloroflexi;Anaerolineae;Anaerolineales;Anaerolineaceae;Levilinea;Levilinea saccharolytica;Levilinea saccharolytica DSM 16555 |
| Ga0586324 5451972 3 596 | 0.00 | 0.00 | 0.00 | 6099000.00 | 0.00 | 0.00 | 0.00 | 0.00 | 0.00 | 0.00 | 0.00 | 0.00 | KO:K04077 | chaperonin GroEL | Bacteria;Bacteroidetes;Bacteroidia;Bacteroidales;Tannerellaceae;Parabacteroides;Parabacteroides sp. ASF519;Parabacteroides sp. ASF519 |
| Ga0586324 5462390 3 596 | 0.00 | 0.00 | 0.00 | 18620000.00 | 0.00 | 0.00 | 0.00 | 2409500.00 | 0.00 | 0.00 | 0.00 | 0.00 | KO:K04077 | chaperonin GroEL | Bacteria;Spirochaetes;Spirochaetia;Spirochaetales;Spirochaetaceae;Spirochaeta;Spirochaeta lutea;Spirochaeta lutea JC230 |
| Ga0586324 5462898 1 594 | 5590000.00 | 0.00 | 0.00 | 0.00 | 0.00 | 0.00 | 0.00 | 0.00 | 4220100.00 | 0.00 | 4197600.00 | 0.00 | KO:K04077 | chaperonin GroEL | Bacteria;Spirochaetes;Spirochaetia;Spirochaetales;Treponemataceae;Treponema;Treponema primitia;Treponema primitia ZAS-2 |
| Ga0586324 5482007 3 593 | 0.00 | 0.00 | 0.00 | 0.00 | 0.00 | 0.00 | 0.00 | 0.00 | 0.00 | 0.00 | 251008.56 | 0.00 | KO:K04077 | chaperonin GroEL | Bacteria;Proteobacteria;Alphaproteobacteria;Hyphomicrobiales;Hyphomicrobiaceae;Hyphomicrobium;Hyphomicrobium zavarzinii;Hyphomicrobium zavarzinii ATCC 27496 |
| Ga0586324 5491203 1 594 | 610092.00 | 0.00 | 220469.67 | 13300000.00 | 0.00 | 0.00 | 13725600.00 | 0.00 | 0.00 | 0.00 | 0.00 | 128693.25 | KO:K04077 | chaperonin GroEL | Bacteria;Proteobacteria;Gammaproteobacteria;Nevskiales;Steroidobacteraceae;Steroidobacter;Steroidobacter denitrificans;Steroidobacter denitrificans DSM 18526 |
| Ga0586324 5509060 1 579 | 0.00 | 0.00 | 0.00 | 0.00 | 0.00 | 0.00 | 0.00 | 0.00 | 0.00 | 1070300.00 | 0.00 | 0.00 | KO:K04077 | chaperonin GroEL | Bacteria;Actinobacteria;Acidimicrobiia;Acidimicrobiales;Ilumatobacteraceae;Desertimonas;Desertimonas flava;Acidimicrobiales bacterium SYSU D60003 |
| Ga0586324 5513556 3 593 | 0.00 | 0.00 | 991217.80 | 0.00 | 0.00 | 0.00 | 0.00 | 0.00 | 1134000.00 | 0.00 | 0.00 | 0.00 | KO:K04077 | chaperonin GroEL | Bacteria;Chloroflexi;Thermoflexia;Thermoflexales;Thermoflexaceae;Thermoflexus;Thermoflexus hugenholtzii;Thermoflexus hugenholtzii JAD2 |
| Ga0586324 5516322 1 591 | 0.00 | 0.00 | 0.00 | 0.00 | 1565600.00 | 0.00 | 1812036.84 | 0.00 | 611230.86 | 970200.00 | 2552000.00 | 2827500.00 | KO:K04077 | chaperonin GroEL | Bacteria;Nitrospinae;Nitrospinia;Nitrospinales;Nitrospinaceae;Nitrospina;Nitrospina gracilis;Nitrospina gracilis 3/211 |
| Ga0586324 5527372 2 592 | 12000000.00 | 0.00 | 0.00 | 0.00 | 0.00 | 0.00 | 0.00 | 0.00 | 0.00 | 0.00 | 0.00 | 0.00 | KO:K04077 | chaperonin GroEL | Bacteria;Verrucomicrobia;Verrucomicrobiae;Verrucomicrobiales;Verrucomicrobia subdivision 3;Pedosphaera;Pedosphaera parvula;Pedosphaera parvula Ellin514 |
| Ga0586324 5528329 1 591 | 702490.00 | 6405600.00 | 707910.57 | 0.00 | 1936400.00 | 564661.04 | 1087302.36 | 3421020.91 | 6153871.32 | 1732500.00 | 2208800.00 | 960000.00 | KO:K04077 | chaperonin GroEL | Bacteria;Proteobacteria;Deltaproteobacteria;Myxococcales;Labilitrichaceae;Labilithrix;Labilithrix luteola;Labilithrix luteola DSM 27648 |
| Ga0586324 5531528 3 590 | 0.00 | 1115200.00 | 6226886.97 | 16340000.00 | 1565600.00 | 7106300.00 | 1473785.16 | 327925.63 | 1490400.00 | 1332100.00 | 677858.72 | 1267500.00 | KO:K04077 | chaperonin GroEL | Bacteria;Proteobacteria;Deltaproteobacteria;Desulfuromonadales;Geobacteraceae;Geobacter;Geobacter pickeringii;Geobacter pickeringii G13 |
| Ga0586324 5533111 3 590 | 0.00 | 0.00 | 0.00 | 4861915.70 | 3780100.00 | 0.00 | 0.00 | 3294000.00 | 4463100.00 | 0.00 | 2191200.00 | 359748.75 | KO:K04077 | chaperonin GroEL | Bacteria;Ignavibacteriae;Ignavibacteria;Ignavibacteriales;Ignavibacteriaceae;Ignavibacterium;Ignavibacterium album;Ignavibacterium album Mat9-16, JCM 16511 |
| Ga0586324 5537026 1 591 | 423256.00 | 7323600.00 | 0.00 | 272922.65 | 15244000.00 | 0.00 | 0.00 | 0.00 | 0.00 | 893200.00 | 0.00 | 0.00 | KO:K04077 | chaperonin GroEL | Bacteria;Chloroflexi;Anaerolineae;Anaerolineales;Anaerolineaceae;Anaerolinea;Anaerolinea thermophila;Anaerolinea thermophila UNI-1 |
| Ga0586324 5545720 2 592 | 0.00 | 118613.76 | 0.00 | 0.00 | 116168.55 | 0.00 | 0.00 | 260430.35 | 228526.92 | 0.00 | 0.00 | 394303.50 | KO:K04077 | chaperonin GroEL | Bacteria;Planctomycetes;Planctomycetia;Pirellulales;Thermoguttaceae;Thermogutta;Thermogutta terrifontis;Thermogutta terrifontis R1 |
| Ga0586324 5548207 3 590 | 0.00 | 0.00 | 0.00 | 0.00 | 0.00 | 0.00 | 0.00 | 0.00 | 0.00 | 309791.79 | 0.00 | 674802.75 | KO:K04077 | chaperonin GroEL | Bacteria;Proteobacteria;Betaproteobacteria;unclassified Betaproteobacteria;unclassified Betaproteobacteria;unclassified Betaproteobacteria;uncultured beta proteobacterium;unclassified betaproteobacterium ENR4 |
| Ga0586324 5548872 2 592 | 0.00 | 0.00 | 0.00 | 0.00 | 4017000.00 | 0.00 | 0.00 | 0.00 | 1911600.00 | 210287.00 | 0.00 | 0.00 | KO:K04077 | chaperonin GroEL | Bacteria;Planctomycetes;Planctomycetia;Pirellulales;Pirellulaceae;Roseimaritima;Roseimaritima ulvae;Roseimaritima ulvae DSM 25454 |
| Ga0586324 5555393 1 591 | 0.00 | 0.00 | 0.00 | 0.00 | 0.00 | 0.00 | 6840000.00 | 0.00 | 0.00 | 0.00 | 0.00 | 0.00 | KO:K04077 | chaperonin GroEL | Bacteria;Acidobacteria;Acidobacteriia;Acidobacteriales;Acidobacteriaceae;Candidatus Koribacter;Candidatus Koribacter versatilis;Candidatus Koribacter versatilis Ellin345 |
| Ga0586324 5564265 2 589 | 0.00 | 3461200.00 | 0.00 | 0.00 | 7652900.00 | 0.00 | 0.00 | 2165500.00 | 2583900.00 | 0.00 | 0.00 | 0.00 | KO:K04077 | chaperonin GroEL | Bacteria;Proteobacteria;Deltaproteobacteria;Syntrophorhabdales;Syntrophorhabdaceae;Syntrophorhabdus;Syntrophorhabdus aromaticivorans;Syntrophorhabdus aromaticivorans UI |
| Ga0586324 5573730 2 589 | 4140000.00 | 0.00 | 3568654.88 | 652135.10 | 3769800.00 | 1752762.94 | 608228.76 | 0.00 | 1409400.00 | 1339642.92 | 1196800.00 | 1421443.50 | KO:K04077 | chaperonin GroEL | Bacteria;Proteobacteria;Deltaproteobacteria;Desulfuromonadales;Geobacteraceae;Geobacter;Geobacter sp. M18;Geobacter sp. M18 |
| Ga0586324 5587110 3 590 | 0.00 | 0.00 | 2567854.26 | 0.00 | 22866000.00 | 10163200.00 | 12426000.00 | 12169500.00 | 8262000.00 | 0.00 | 1751200.00 | 1320000.00 | KO:K04077 | chaperonin GroEL | Bacteria;Chloroflexi;Anaerolineae;Anaerolineales;Anaerolineaceae;Thermanaerothrix;Thermanaerothrix daxensis;Thermanaerothrix daxensis GNS-1 |
| Ga0586324 5593874 3 590 | 0.00 | 0.00 | 0.00 | 0.00 | 0.00 | 0.00 | 0.00 | 0.00 | 0.00 | 0.00 | 3220800.00 | 0.00 | KO:K04077 | chaperonin GroEL | Bacteria;Chloroflexi;Anaerolineae;Anaerolineales;Anaerolineaceae;Levilinea;Levilinea saccharolytica;Levilinea saccharolytica DSM 16555 |
| Ga0586324 5598005 1 588 | 0.00 | 14824000.00 | 0.00 | 8388500.00 | 9651100.00 | 0.00 | 0.00 | 11163000.00 | 12474000.00 | 0.00 | 5966400.00 | 8175000.00 | KO:K04077 | chaperonin GroEL | Bacteria;Proteobacteria;Alphaproteobacteria;Rhodospirillales;Acetobacteraceae;Acidibrevibacterium;Acidibrevibacterium fodinaquatile;Acidibrevibacterium fodinaquatile G45-3 |
| Ga0586324 5600474 1 552 | 4670000.00 | 0.00 | 0.00 | 0.00 | 0.00 | 0.00 | 0.00 | 0.00 | 0.00 | 0.00 | 0.00 | 0.00 | KO:K04077 | chaperonin GroEL | Bacteria;Chloroflexi;Dehalococcoidia;unclassified Dehalococcoidia;unclassified Dehalococcoidia;Dehalogenimonas;Dehalogenimonas lykanthroporepellens;Dehalogenimonas lykanthroporepellens BL-DC-9 |
| Ga0586324 5635795 3 587 | 0.00 | 0.00 | 0.00 | 0.00 | 1874600.00 | 0.00 | 0.00 | 0.00 | 0.00 | 0.00 | 0.00 | 0.00 | KO:K04077 | chaperonin GroEL | Bacteria;Proteobacteria;Deltaproteobacteria;Syntrophobacterales;Syntrophobacteraceae;Desulfacinum;Desulfacinum infernum;Desulfacinum infernum DSM 9756 |
| Ga0586324 5652481 3 587 | 0.00 | 0.00 | 0.00 | 0.00 | 0.00 | 0.00 | 0.00 | 0.00 | 0.00 | 0.00 | 3115200.00 | 0.00 | KO:K04077 | chaperonin GroEL | Bacteria;Chloroflexi;Anaerolineae;Anaerolineales;Anaerolineaceae;Anaerolinea;Anaerolinea thermophila;Anaerolinea thermophila UNI-1 |
| Ga0586324 5670379 18 584 | 0.00 | 0.00 | 0.00 | 0.00 | 0.00 | 0.00 | 0.00 | 0.00 | 0.00 | 0.00 | 262070.16 | 0.00 | KO:K04077 | chaperonin GroEL | Bacteria;Chloroflexi;Anaerolineae;Anaerolineales;Anaerolineaceae;Thermanaerothrix;Thermanaerothrix daxensis;Thermanaerothrix daxensis GNS-1 |
| Ga0586324 5685767 3 584 | 0.00 | 0.00 | 0.00 | 0.00 | 1833400.00 | 1883626.05 | 2508000.00 | 1250500.00 | 0.00 | 0.00 | 0.00 | 0.00 | KO:K04077 | chaperonin GroEL | Bacteria;Proteobacteria;Deltaproteobacteria;Desulfarculales;Desulfarculaceae;Desulfocarbo;Desulfocarbo indianensis;Desulfocarbo indianensis SCBM |
| Ga0586324 5693828 1 585 | 0.00 | 0.00 | 0.00 | 0.00 | 0.00 | 0.00 | 22549200.00 | 0.00 | 12717000.00 | 0.00 | 0.00 | 0.00 | KO:K04077 | chaperonin GroEL | Bacteria;Chloroflexi;Thermomicrobia;Sphaerobacterales;Sphaerobacteraceae;Sphaerobacter;Sphaerobacter thermophilus;Sphaerobacter thermophilus 4ac11, DSM 20745 |
| Ga0586324 5729791 1 582 | 0.00 | 0.00 | 0.00 | 0.00 | 413095.92 | 0.00 | 0.00 | 356673.10 | 291310.83 | 1532300.00 | 0.00 | 0.00 | KO:K04077 | chaperonin GroEL | Bacteria;Proteobacteria;Gammaproteobacteria;Cellvibrionales;Halieaceae;Halioglobus;Halioglobus lutimaris;Halioglobus lutimaris HF004 |
| Ga0586324 5787823 2 580 | 0.00 | 0.00 | 0.00 | 0.00 | 0.00 | 0.00 | 0.00 | 0.00 | 0.00 | 5159000.00 | 509974.08 | 0.00 | KO:K04077 | chaperonin GroEL | Bacteria;Acidobacteria;Acidobacteriia;Acidobacteriales;Acidobacteriaceae;Acidobacterium;Acidobacterium ailaaui;Acidobacterium ailaaui PMMR2 |
| Ga0586324 5804281 1 534 | 0.00 | 0.00 | 0.00 | 606939.80 | 0.00 | 0.00 | 1370466.96 | 112850000.00 | 566208.63 | 477246.00 | 859233.76 | 575882.25 | KO:K04077 | chaperonin GroEL | Bacteria;Proteobacteria;Alphaproteobacteria;Rhodospirillales;unclassified Rhodospirillales;unclassified Rhodospirillales;Rhodospirillales bacterium URHD0017;Rhodospirillales bacterium URHD0017 |
| Ga0586324 5808095 1 579 | 2380000.00 | 0.00 | 3236495.58 | 3885500.00 | 0.00 | 2712149.17 | 3990000.00 | 4172400.00 | 5427000.00 | 0.00 | 3986400.00 | 2850000.00 | KO:K04077 | chaperonin GroEL | Bacteria;Proteobacteria;Deltaproteobacteria;Myxococcales;Vulgatibacteraceae;Vulgatibacter;Vulgatibacter incomptus;Vulgatibacter incomptus DSM 27710 |
| Ga0586324 5828789 3 578 | 0.00 | 0.00 | 0.00 | 0.00 | 39140000.00 | 0.00 | 0.00 | 0.00 | 0.00 | 0.00 | 53944000.00 | 0.00 | KO:K04077 | chaperonin GroEL | Bacteria;Acidobacteria;Vicinamibacteria;unclassified Vicinamibacteria;Vicinamibacteraceae;Luteitalea;Luteitalea pratensis;Luteitalea pratensis DSM 100886 |
| Ga0586324 5839649 1 483 | 0.00 | 0.00 | 0.00 | 0.00 | 0.00 | 0.00 | 0.00 | 0.00 | 0.00 | 0.00 | 0.00 | 937500.00 | KO:K04077 | chaperonin GroEL | Bacteria;Spirochaetes;Spirochaetia;Spirochaetales;Spirochaetaceae;Sediminispirochaeta;Sediminispirochaeta bajacaliforniensis;Sediminispirochaeta bajacaliforniensis DSM 16054 |
| Ga0586324 5843233 1 579 | 0.00 | 0.00 | 1082519.51 | 0.00 | 0.00 | 0.00 | 601272.48 | 504310.79 | 0.00 | 616533.61 | 888800.00 | 0.00 | KO:K04077 | chaperonin GroEL | Bacteria;Verrucomicrobia;Verrucomicrobiae;Verrucomicrobiales;Verrucomicrobiaceae;Roseimicrobium;Roseimicrobium gellanilyticum;Roseimicrobium gellanilyticum DSM 25532 |
| Ga0586324 5851923 2 577 | 0.00 | 0.00 | 0.00 | 0.00 | 576979.22 | 0.00 | 0.00 | 0.00 | 0.00 | 0.00 | 0.00 | 0.00 | KO:K04077 | chaperonin GroEL | Bacteria;Proteobacteria;Deltaproteobacteria;Myxococcales;Anaeromyxobacteraceae;Anaeromyxobacter;Anaeromyxobacter sp. Fw109-5;Anaeromyxobacter sp. Fw109-5 |
| Ga0586324 5858538 2 493 | 0.00 | 2012800.00 | 0.00 | 5757000.00 | 0.00 | 0.00 | 3534000.00 | 3446500.00 | 5427000.00 | 4312000.00 | 0.00 | 0.00 | KO:K04077 | chaperonin GroEL | Bacteria;Proteobacteria;Alphaproteobacteria;Hyphomicrobiales;Hyphomicrobiaceae;Hyphomicrobium;Hyphomicrobium sp. NDB2Meth4;Hyphomicrobium sp. ERS1433775 |
| Ga0586324 5872607 240 578 | 0.00 | 829600.00 | 2465027.94 | 1852500.00 | 2111500.00 | 0.00 | 0.00 | 0.00 | 805742.64 | 1039500.00 | 0.00 | 0.00 | KO:K04077 | chaperonin GroEL | Bacteria;Fibrobacteres;Fibrobacteria;Fibrobacterales;Fibrobacteraceae;Fibrobacter;Fibrobacter sp. UWP2;Fibrobacter sp. UWP2 |
| Ga0586324 5899787 2 577 | 0.00 | 0.00 | 0.00 | 0.00 | 0.00 | 0.00 | 0.00 | 0.00 | 0.00 | 0.00 | 7295200.00 | 0.00 | KO:K04077 | chaperonin GroEL | Bacteria;Proteobacteria;Deltaproteobacteria;Desulfuromonadales;Desulfuromonadaceae;Pelobacter;Pelobacter propionicus;Pelobacter propionicus DSM 2379 |
| Ga0586324 5917309 2 574 | 0.00 | 0.00 | 0.00 | 15200000.00 | 11948000.00 | 9289800.00 | 6498000.00 | 0.00 | 0.00 | 5744200.00 | 5825600.00 | 0.00 | KO:K04077 | chaperonin GroEL | Bacteria;Proteobacteria;Deltaproteobacteria;Syntrophobacterales;Syntrophaceae;Desulfobacca;Desulfobacca acetoxidans;Desulfobacca acetoxidans ASRB2, DSM 11109 |
| Ga0586324 5951524 3 575 | 274591.00 | 0.00 | 0.00 | 0.00 | 0.00 | 0.00 | 0.00 | 0.00 | 0.00 | 0.00 | 0.00 | 0.00 | KO:K04077 | chaperonin GroEL | Bacteria;Elusimicrobia;unclassified Elusimicrobia;unclassified Elusimicrobia;unclassified Elusimicrobia;unclassified Elusimicrobia;uncultured Termite group 1 bacterium;uncultured Termite group 1 bacterium Rs-D17 |
| Ga0586324 5960914 2 556 | 0.00 | 0.00 | 0.00 | 7400500.00 | 0.00 | 0.00 | 0.00 | 0.00 | 0.00 | 0.00 | 0.00 | 0.00 | KO:K04077 | chaperonin GroEL | Bacteria;Proteobacteria;Gammaproteobacteria;Legionellales;Legionellaceae;Legionella;Legionella wadsworthii;Legionella wadsworthii ATCC 33877 |
| Ga0586324 5966871 2 574 | 8380000.00 | 0.00 | 0.00 | 0.00 | 1225700.00 | 0.00 | 0.00 | 0.00 | 0.00 | 0.00 | 0.00 | 0.00 | KO:K04077 | chaperonin GroEL | Bacteria;Proteobacteria;Gammaproteobacteria;Methylococcales;Methylococcaceae;Methylomagnum;Methylomagnum ishizawai;Methylomagnum ishizawai 175 |
| Ga0586324 5979568 3 572 | 0.00 | 0.00 | 0.00 | 0.00 | 0.00 | 0.00 | 0.00 | 0.00 | 2997000.00 | 2533300.00 | 0.00 | 0.00 | KO:K04077 | chaperonin GroEL | Bacteria;Chloroflexi;Caldilineae;Caldilineales;Caldilineaceae;Caldilinea;Caldilinea aerophila;Caldilinea aerophila STL-6-O1, DSM 14535 |
| Ga0586324 5987763 2 571 | 3470000.00 | 3128000.00 | 12442300.00 | 6393500.00 | 11229376.21 | 11552700.00 | 7387200.00 | 15189000.00 | 2875500.00 | 7139051.15 | 544460.40 | 4710000.00 | KO:K04077 | chaperonin GroEL | Bacteria;Chloroflexi;Anaerolineae;Anaerolineales;Anaerolineaceae;Thermanaerothrix;Thermanaerothrix daxensis;Thermanaerothrix daxensis GNS-1 |
| Ga0586324 5996079 3 572 | 0.00 | 0.00 | 0.00 | 0.00 | 0.00 | 0.00 | 0.00 | 0.00 | 0.00 | 0.00 | 1003200.00 | 0.00 | KO:K04077 | chaperonin GroEL | Bacteria;Proteobacteria;Deltaproteobacteria;Desulfobacterales;Desulfobacteraceae;Desulfatiglans;Desulfatiglans anilini;Desulfatiglans anilini DSM 4660 |
| Ga0586324 6010697 1 573 | 0.00 | 0.00 | 713957.19 | 0.00 | 0.00 | 867770.54 | 0.00 | 0.00 | 0.00 | 2094400.00 | 0.00 | 0.00 | KO:K04077 | chaperonin GroEL | Bacteria;Verrucomicrobia;Verrucomicrobiae;Verrucomicrobiales;Verrucomicrobiaceae;Roseimicrobium;Roseimicrobium gellanilyticum;Roseimicrobium gellanilyticum DSM 25532 |
| Ga0586324 6049958 11 571 | 0.00 | 1836000.00 | 0.00 | 0.00 | 0.00 | 0.00 | 0.00 | 0.00 | 0.00 | 0.00 | 0.00 | 877500.00 | KO:K04077 | chaperonin GroEL | Bacteria;Proteobacteria;Betaproteobacteria;Ferrovales;Ferrovaceae;Ferrovum;Ferrovum myxofaciens;Ferrovum myxofaciens P3G |

| | | | | | | | | | | | | | | | |
|---|---|---|---|---|---|---|---|---|---|---|---|---|---|---|---|
| Ga0586324 6068063 3 569 | 0.00 | 0.00 | 1404240.23 | 0.00 | 0.00 | 0.00 | 0.00 | 0.00 | 0.00 | 0.00 | 0.00 | 1650000.00 | KO:K04077 | chaperonin GroEL | Bacteria;Proteobacteria;Betaproteobacteria;Nitrosomonadales;Nitrosomonadaceae;Nitrosospira;Nitrosospira sp. Nsp1;Nitrosospira sp. 1 Nsp1 |
| Ga0586324 6069053 2 568 | 0.00 | 0.00 | 0.00 | 3990000.00 | 0.00 | 0.00 | 0.00 | 0.00 | 0.00 | 0.00 | 0.00 | 0.00 | KO:K04077 | chaperonin GroEL | Bacteria;Chloroflexi;Anaerolineae;Anaerolineales;Anaerolineaceae;Anaerolinea;Anaerolinea thermophila;Anaerolinea thermophila UNI-1 |
| Ga0586324 6077464 2 568 | 0.00 | 0.00 | 0.00 | 0.00 | 0.00 | 0.00 | 3351600.00 | 0.00 | 1020600.00 | 0.00 | 0.00 | 168988.50 | KO:K04077 | chaperonin GroEL | Bacteria;Acidobacteria;Blastocatellia;Blastocatellales;Pyrinomonadaceae;Pyrinomonas;Pyrinomonas methylaliphatogenes;Pyrinomonas methylaliphatogenes K22 |
| Ga0586324 6080026 2 460 | 0.00 | 416703.32 | 409008.24 | 0.00 | 278682.98 | 0.00 | 1736587.08 | 0.00 | 0.00 | 137850.79 | 412532.56 | 429447.00 | KO:K04077 | chaperonin GroEL | Bacteria;Proteobacteria;Deltaproteobacteria;Desulfarculales;Desulfarculaceae;Desulfarculus;Desulfarculus baarsii;Desulfarculus baarsii 2st14, DSM 2075 |
| Ga0586324 6104940 1 567 | 0.00 | 620642.80 | 0.00 | 0.00 | 0.00 | 0.00 | 0.00 | 0.00 | 0.00 | 0.00 | 0.00 | 0.00 | KO:K04077 | chaperonin GroEL | Bacteria;Verrucomicrobia;Verrucomicrobiae;Verrucomicrobiales;Verrucomicrobia subdivision 3;Pedosphaera;Pedosphaera parvula;Pedosphaera parvula Ellin514 |
| Ga0586324 6110030 2 568 | 0.00 | 31076000.00 | 0.00 | 0.00 | 0.00 | 0.00 | 0.00 | 0.00 | 0.00 | 0.00 | 0.00 | 0.00 | KO:K04077 | chaperonin GroEL | Bacteria;Proteobacteria;Deltaproteobacteria;Myxococcales;Sandaracinaceae;Sandaracinus;Sandaracinus amylolyticus;Sandaracinus amylolyticus DSM 53668 |
| Ga0586324 6157797 1 567 | 0.00 | 473645.84 | 0.00 | 378426.80 | 0.00 | 0.00 | 0.00 | 250415.37 | 0.00 | 0.00 | 11968000.00 | 459647.25 | KO:K04077 | chaperonin GroEL | Bacteria;Candidatus Bipolaricaulota;unclassified Candidatus Bipolaricaulota;unclassified Candidatus Bipolaricaulota;unclassified Candidatus Bipolaricaulota;unclassified Candidatus Bipolaricaulota;Acetothermia bacterium SCGC AAA255-C06;Acetothermia bacterium SCGC AAA255-C06 (contamination screened) |
| Ga0586324 6160721 50 565 | 0.00 | 0.00 | 0.00 | 0.00 | 0.00 | 0.00 | 0.00 | 394542.51 | 0.00 | 0.00 | 0.00 | 0.00 | KO:K04077 | chaperonin GroEL | Bacteria;Proteobacteria;Deltaproteobacteria;unclassified Deltaproteobacteria;unclassified Deltaproteobacteria;Dissulfuribacter;Dissulfuribacter thermophilus;Dissulfuribacter thermophilus S69 |
| Ga0586324 6163184 1 567 | 1030000.00 | 0.00 | 19705000.00 | 13110000.00 | 0.00 | 0.00 | 0.00 | 0.00 | 0.00 | 0.00 | 0.00 | 0.00 | KO:K04077 | chaperonin GroEL | Bacteria;Proteobacteria;Deltaproteobacteria;Myxococcales;Kofleriaceae;Haliangium;Haliangium ochraceum;Haliangium ochraceum SMP-2, DSM 14365 |
| Ga0586324 6165917 309 566 | 0.00 | 0.00 | 0.00 | 6802000.00 | 0.00 | 0.00 | 0.00 | 0.00 | 0.00 | 0.00 | 0.00 | 0.00 | KO:K04077 | chaperonin GroEL | Bacteria;Chloroflexi;Anaerolineae;Anaerolineales;Anaerolineaceae;Thermanaerothrix;Thermanaerothrix daxensis;Thermanaerothrix daxensis GNS-1 |
| Ga0586324 6169713 1 564 | 28200000.00 | 335890.76 | 0.00 | 0.00 | 3821300.00 | 42885.53 | 631888.32 | 915000.00 | 834300.00 | 0.00 | 723562.40 | 549723.00 | KO:K04077 | chaperonin GroEL | Bacteria;Proteobacteria;Deltaproteobacteria;Syntrophobacterales;Syntrophaceae;Desulfobacca;Desulfobacca acetoxidans;Desulfobacca acetoxidans ASRB2, DSM 11109 |
| Ga0586324 6169737 47 565 | 0.00 | 0.00 | 0.00 | 0.00 | 0.00 | 0.00 | 0.00 | 1488400.00 | 1587600.00 | 0.00 | 0.00 | 0.00 | KO:K04077 | chaperonin GroEL | Bacteria;Proteobacteria;Deltaproteobacteria;Syntrophobacterales;Syntrophobacteraceae;Syntrophobacter;Syntrophobacter fumaroxidans;Syntrophobacter fumaroxidans MPOB |
| Ga0586324 6186299 2 565 | 1320000.00 | 1341155.16 | 916890.54 | 12969268.90 | 763297.98 | 389144.56 | 6657600.00 | 5715700.00 | 19311710.58 | 12080728.66 | 1874400.00 | 2632500.00 | KO:K04077 | chaperonin GroEL | Bacteria;Verrucomicrobia;Verrucomicrobiae;Verrucomicrobiales;Verrucomicrobia subdivision 3;Pedosphaera;Pedosphaera parvula;Pedosphaera parvula Ellin514 |
| Ga0586324 6188187 1 564 | 308111.00 | 0.00 | 0.00 | 0.00 | 165220.24 | 0.00 | 0.00 | 0.00 | 320109.57 | 1001000.00 | 915200.00 | 584372.25 | KO:K04077 | chaperonin GroEL | Bacteria;Acidobacteria;Vicinamibacteria;unclassified Vicinamibacteria;Vicinamibacteraceae;Luteitalea;Luteitalea pratensis;Luteitalea pratensis DSM 100886 |
| Ga0586324 6194576 2 565 | 0.00 | 0.00 | 0.00 | 0.00 | 0.00 | 0.00 | 0.00 | 1421300.00 | 0.00 | 0.00 | 0.00 | 832500.00 | KO:K04077 | chaperonin GroEL | Bacteria;Proteobacteria;Alphaproteobacteria;Hyphomicrobiales;Bradyrhizobiaceae;Bradyrhizobium;Bradyrhizobium erythrophlei;Bradyrhizobium erythrophlei GAS478 |
| Ga0586324 6199609 1 564 | 7530000.00 | 355965.04 | 0.00 | 0.00 | 0.00 | 0.00 | 256903.56 | 2067900.00 | 0.00 | 0.00 | 0.00 | 2062500.00 | KO:K04077 | chaperonin GroEL | Bacteria;Chloroflexi;Dehalococcoidia;Dehalococcoidales;Dehalococcoidaceae;Dehalococcoides;Dehalococcoides mccartyi;Dehalococcoides mccartyi VS |
| Ga0586324 6225363 3 563 | 0.00 | 0.00 | 0.00 | 0.00 | 0.00 | 0.00 | 0.00 | 0.00 | 3944700.00 | 0.00 | 0.00 | 0.00 | KO:K04077 | chaperonin GroEL | Bacteria;Actinobacteria;Actinomycetia;Kineosporiales;Kineosporiaceae;Quadrisphaera;Quadrisphaera granulorum;Quadrisphaera granulorum DSM 44889 |
| Ga0586324 6232871 3 563 | 0.00 | 0.00 | 10809600.00 | 0.00 | 4635000.00 | 0.00 | 0.00 | 0.00 | 5621400.00 | 3826900.00 | 0.00 | 0.00 | KO:K04077 | chaperonin GroEL | Bacteria;Proteobacteria;Betaproteobacteria;Burkholderiales;Comamonadaceae;Rhodoferax;Rhodoferax ferrireducens;Rhodoferax ferrireducens T118 |
| Ga0586324 6237511 1 564 | 3080000.00 | 1989810.56 | 5092351.89 | 5272412.60 | 2407553.93 | 2289967.46 | 3671830.56 | 2726700.00 | 26885531.34 | 6375600.00 | 0.00 | 5805000.00 | KO:K04077 | chaperonin GroEL | Bacteria;Acidobacteria;Acidobacteriia;Acidobacteriales;Acidobacteriaceae;unclassified Acidobacteriaceae;Acidobacteriaceae bacterium KBS 96;Acidobacteriaceae bacterium KBS 96, DSM 24297 |
| Ga0586324 6241248 3 563 | 0.00 | 0.00 | 0.00 | 23560000.00 | 26368000.00 | 0.00 | 0.00 | 0.00 | 24786000.00 | 0.00 | 0.00 | 0.00 | KO:K04077 | chaperonin GroEL | Bacteria;Acidobacteria;Thermoanaerobaculia;Thermoanaerobaculales;Thermoanaerobaculaceae;Thermoanaerobaculum;Thermoanaerobaculum aquaticum;Thermoanaerobaculum aquaticum MP-01 |
| Ga0586324 6251764 1 561 | 0.00 | 0.00 | 0.00 | 0.00 | 0.00 | 0.00 | 0.00 | 0.00 | 0.00 | 126623.42 | 0.00 | 0.00 | KO:K04077 | chaperonin GroEL | Bacteria;Chloroflexi;Dehalococcoidia;unclassified Dehalococcoidia;unclassified Dehalococcoidia;Dehalogenimonas;Dehalogenimonas formicexedens;Dehalogenimonas formicexedens NSZ-14 |
| Ga0586324 6255740 3 563 | 0.00 | 272127.84 | 0.00 | 0.00 | 0.00 | 0.00 | 20998800.00 | 0.00 | 0.00 | 0.00 | 0.00 | 0.00 | KO:K04077 | chaperonin GroEL | Bacteria;Proteobacteria;Alphaproteobacteria;Hyphomicrobiales;Bradyrhizobiaceae;Bradyrhizobium;Bradyrhizobium erythrophlei;Bradyrhizobium erythrophlei GAS138 |
| Ga0586324 6262026 1 561 | 21000000.00 | 0.00 | 0.00 | 0.00 | 0.00 | 0.00 | 0.00 | 0.00 | 0.00 | 0.00 | 0.00 | 0.00 | KO:K04077 | chaperonin GroEL | Bacteria;Proteobacteria;Betaproteobacteria;Burkholderiales;Burkholderiaceae;Trinickia;Burkholderia dabaoshanensis;Burkholderia dabaoshanensis GIMN1.004 |
| Ga0586324 6263746 3 563 | 0.00 | 0.00 | 447184.14 | 0.00 | 0.00 | 0.00 | 0.00 | 0.00 | 0.00 | 0.00 | 0.00 | 0.00 | KO:K04077 | chaperonin GroEL | Bacteria;Proteobacteria;Alphaproteobacteria;Hyphomicrobiales;Hyphomicrobiaceae;Hyphomicrobium;Hyphomicrobium sulfonivorans;Hyphomicrobium sulfonivorans S1 |
| Ga0586324 6270258 2 562 | 120420.00 | 0.00 | 0.00 | 1140000.00 | 0.00 | 0.00 | 1987523.88 | 0.00 | 0.00 | 174106.24 | 0.00 | 0.00 | KO:K04077 | chaperonin GroEL | Bacteria;Chloroflexi;Anaerolineae;Anaerolineales;Anaerolineaceae;Levilinea;Levilinea saccharolytica;Levilinea saccharolytica DSM 16555 |
| Ga0586324 6274565 25 561 | 0.00 | 0.00 | 1862494.08 | 0.00 | 0.00 | 736502.49 | 1552556.88 | 447973.02 | 0.00 | 0.00 | 976800.00 | 1350000.00 | KO:K04077 | chaperonin GroEL | Bacteria;Acidobacteria;Blastocatellia;Blastocatellales;Pyrinomonadaceae;Pyrinomonas;Pyrinomonas methylaliphatogenes;Pyrinomonas methylaliphatogenes K22 |
| Ga0586324 6292103 2 562 | 0.00 | 0.00 | 0.00 | 1064000.00 | 853716.53 | 719546.62 | 0.00 | 1192963.58 | 0.00 | 0.00 | 0.00 | 0.00 | KO:K04077 | chaperonin GroEL | Bacteria;Proteobacteria;Deltaproteobacteria;Syntrophobacterales;Syntrophobacteraceae;Desulfoglaeba;Desulfoglaeba alkanexedens;Desulfoglaeba alkanexedens ALDC |
| Ga0586324 6347514 2 559 | 0.00 | 958800.00 | 0.00 | 82175000.00 | 2389600.00 | 0.00 | 1373175.60 | 9064600.00 | 1449900.00 | 3056900.00 | 1760000.00 | 0.00 | KO:K04077 | chaperonin GroEL | Bacteria;Proteobacteria;Deltaproteobacteria;Syntrophorhabdales;Syntrophorhabdaceae;Syntrophorhabdus;Syntrophorhabdus aromaticivorans;Syntrophorhabdus aromaticivorans UI |
| Ga0586324 6405313 3 557 | 8774003.00 | 8228000.00 | 9685960.66 | 541735.60 | 0.00 | 0.00 | 7476512.16 | 7191900.00 | 5653800.00 | 971730.76 | 775795.68 | 840000.00 | KO:K04077 | chaperonin GroEL | Bacteria;Proteobacteria;Betaproteobacteria;Nitrosomonadales;Sterolibacteriaceae;Sulfurisoma;Sulfurisoma sediminicola;Sulfurisoma sediminicola DSM 26916 |
| Ga0586324 6427977 1 555 | 0.00 | 0.00 | 0.00 | 0.00 | 0.00 | 0.00 | 0.00 | 0.00 | 0.00 | 1316700.00 | 0.00 | 0.00 | KO:K04077 | chaperonin GroEL | Bacteria;Proteobacteria;Deltaproteobacteria;Myxococcales;Sandaracinaceae;Sandaracinus;Sandaracinus amylolyticus;Sandaracinus amylolyticus DSM 53668 |
| Ga0586324 6430205 1 555 | 0.00 | 0.00 | 0.00 | 0.00 | 0.00 | 0.00 | 0.00 | 0.00 | 0.00 | 0.00 | 3625600.00 | 0.00 | KO:K04077 | chaperonin GroEL | Bacteria;Verrucomicrobia;Verrucomicrobiae;Verrucomicrobiales;Verrucomicrobia subdivision 3;Pedosphaera;Pedosphaera parvula;Pedosphaera parvula Ellin514 |
| Ga0586324 6431274 175 555 | 0.00 | 0.00 | 0.00 | 0.00 | 0.00 | 43273000.00 | 0.00 | 0.00 | 0.00 | 0.00 | 0.00 | 0.00 | KO:K04077 | chaperonin GroEL | Bacteria;Acidobacteria;Acidobacteriia;Acidobacteriales;Acidobacteriaceae;Acidobacterium;Acidobacterium ailaaui;Acidobacterium ailaaui PMMR2 |
| Ga0586324 6433814 3 557 | 0.00 | 0.00 | 0.00 | 80479.16 | 0.00 | 0.00 | 0.00 | 0.00 | 0.00 | 0.00 | 0.00 | 0.00 | KO:K04077 | chaperonin GroEL | Bacteria;Acidobacteria;Acidobacteriia;Acidobacteriales;Acidobacteriaceae;Candidatus Koribacter;Candidatus Koribacter versatilis;Candidatus Koribacter versatilis Ellin345 |
| Ga0586324 6456891 3 554 | 0.00 | 0.00 | 0.00 | 0.00 | 0.00 | 0.00 | 0.00 | 1891000.00 | 0.00 | 0.00 | 0.00 | 0.00 | KO:K04077 | chaperonin GroEL | Bacteria;Chloroflexi;Anaerolineae;Anaerolineales;Anaerolineaceae;Thermanaerothrix;Thermanaerothrix daxensis;Thermanaerothrix daxensis GNS-1 |
| Ga0586324 6468426 2 553 | 0.00 | 0.00 | 283686.69 | 0.00 | 0.00 | 0.00 | 0.00 | 0.00 | 0.00 | 0.00 | 0.00 | 0.00 | KO:K04077 | chaperonin GroEL | Bacteria;Acidobacteria;Acidobacteriia;Acidobacteriales;Acidobacteriaceae;Candidatus Koribacter;Candidatus Koribacter versatilis;Candidatus Koribacter versatilis Ellin345 |
| Ga0586324 6482771 3 554 | 0.00 | 0.00 | 0.00 | 3477000.00 | 0.00 | 0.00 | 0.00 | 3580700.00 | 3661200.00 | 0.00 | 0.00 | 5512500.00 | KO:K04077 | chaperonin GroEL | Bacteria;Acidobacteria;Vicinamibacteria;unclassified Vicinamibacteria;Vicinamibacteraceae;Luteitalea;Luteitalea pratensis;Luteitalea pratensis DSM 100886 |
| Ga0586324 6515392 1 552 | 0.00 | 0.00 | 0.00 | 0.00 | 0.00 | 0.00 | 0.00 | 0.00 | 127816.38 | 0.00 | 445542.24 | 559033.50 | KO:K04077 | chaperonin GroEL | Bacteria;Proteobacteria;Deltaproteobacteria;Syntrophobacterales;Syntrophobacteraceae;Desulfacinum;Desulfacinum hydrothermale;Desulfacinum hydrothermale DSM 13146 |
| Ga0586324 6528097 2 553 | 0.00 | 0.00 | 0.00 | 0.00 | 0.00 | 0.00 | 0.00 | 0.00 | 0.00 | 670432.07 | 0.00 | 0.00 | KO:K04077 | chaperonin GroEL | Bacteria;Spirochaetes;Spirochaetia;Leptospirales;Leptospiraceae;Leptospira;Leptospira santarosai;Leptospira santarosai U160 |
| Ga0586324 6529464 1 552 | 0.00 | 0.00 | 0.00 | 0.00 | 1802500.00 | 0.00 | 0.00 | 114983.17 | 0.00 | 0.00 | 0.00 | 0.00 | KO:K04077 | chaperonin GroEL | Bacteria;Proteobacteria;Deltaproteobacteria;Syntrophobacterales;Syntrophaceae;Syntrophus;Syntrophus aciditrophicus;Syntrophus aciditrophicus SB |
| Ga0586324 6534835 1 552 | 0.00 | 0.00 | 0.00 | 0.00 | 0.00 | 0.00 | 0.00 | 0.00 | 17415000.00 | 0.00 | 0.00 | 364927.50 | KO:K04077 | chaperonin GroEL | Bacteria;Proteobacteria;Betaproteobacteria;unclassified Betaproteobacteria;unclassified Betaproteobacteria;unclassified Betaproteobacteria;Betaproteobacteria bacterium UKL13-2;Betaproteobacteria bacterium UKL13-2 |
| Ga0586324 6580831 2 550 | 0.00 | 8903659.56 | 0.00 | 0.00 | 0.00 | 0.00 | 22800000.00 | 6466000.00 | 1524904.38 | 498793.68 | 0.00 | 453739.50 | KO:K04077 | chaperonin GroEL | Bacteria;Candidatus Omnitrophica;unclassified Candidatus Omnitrophica;unclassified Candidatus Omnitrophica;unclassified Candidatus Omnitrophica;unclassified Candidatus Omnitrophica;Omnitrophica bacterium SCGC AAA011-L16;Omnitrophica bacterium SCGC AAA011-L16 (contamination screened) |
| Ga0586324 6589958 2 550 | 0.00 | 0.00 | 0.00 | 0.00 | 0.00 | 0.00 | 0.00 | 0.00 | 0.00 | 0.00 | 0.00 | 477627.00 | KO:K04077 | chaperonin GroEL | Bacteria;Verrucomicrobia;Verrucomicrobiae;Verrucomicrobiales;Verrucomicrobia subdivision 3;Pedosphaera;Pedosphaera parvula;Pedosphaera parvula Ellin514 |
| Ga0586324 6606580 3 551 | 0.00 | 0.00 | 3430617.98 | 0.00 | 0.00 | 0.00 | 0.00 | 1598597.72 | 2162700.00 | 1617000.00 | 707469.84 | 2055000.00 | KO:K04077 | chaperonin GroEL | Bacteria;Proteobacteria;Betaproteobacteria;unclassified Betaproteobacteria;unclassified Betaproteobacteria;unclassified Betaproteobacteria;uncultured beta proteobacterium;unclassified betaproteobacterium ENR4 |
| Ga0586324 6609767 2 550 | 1050000.00 | 0.00 | 10809600.00 | 8132000.00 | 0.00 | 378508.53 | 1123130.28 | 829600.00 | 0.00 | 600569.20 | 985600.00 | 3217500.00 | KO:K04077 | chaperonin GroEL | Bacteria;Chloroflexi;Chloroflexia;Chloroflexales;Roseiflexaceae;Roseiflexus;Roseiflexus sp. RS-1;Roseiflexus sp. RS-1 |
| Ga0586324 6649781 1 549 | 0.00 | 0.00 | 1153896.65 | 1605500.00 | 0.00 | 963999.37 | 0.00 | 902490.73 | 1125900.00 | 0.00 | 0.00 | 0.00 | KO:K04077 | chaperonin GroEL | Bacteria;Chloroflexi;Anaerolineae;Anaerolineales;Anaerolineaceae;Anaerolinea;Anaerolinea thermophila;Anaerolinea thermophila UNI-1 |
| Ga0586324 6662179 1 549 | 3340000.00 | 0.00 | 0.00 | 178600000.00 | 12360000.00 | 0.00 | 0.00 | 124086200.00 | 71358.08 | 0.00 | 0.00 | 113250000.00 | KO:K04077 | chaperonin GroEL | Bacteria;Proteobacteria;Gammaproteobacteria;Nevskiales;Steroidobacteraceae;Steroidobacter;Steroidobacter denitrificans;Steroidobacter denitrificans DSM 18526 |
| Ga0586324 6683545 3 548 | 0.00 | 3740000.00 | 0.00 | 0.00 | 0.00 | 0.00 | 0.00 | 0.00 | 0.00 | 3395700.00 | 0.00 | 0.00 | KO:K04077 | chaperonin GroEL | Bacteria;Acidobacteria;Vicinamibacteria;unclassified Vicinamibacteria;Vicinamibacteraceae;Luteitalea;Luteitalea pratensis;Luteitalea pratensis DSM 100886 |
| Ga0586324 6691141 3 548 | 0.00 | 0.00 | 361015.31 | 0.00 | 15450000.00 | 0.00 | 0.00 | 0.00 | 0.00 | 0.00 | 0.00 | 0.00 | KO:K04077 | chaperonin GroEL | Bacteria;Bacteroidetes;Bacteroidia;Marinilabiliales;Prolixibacteraceae;Prolixibacter;Prolixibacter denitrificans;Prolixibacter denitrificans DSM 27267 |
| Ga0586324 6694713 3 548 | 0.00 | 0.00 | 0.00 | 0.00 | 0.00 | 31442400.00 | 0.00 | 13054000.00 | 0.00 | 0.00 | 0.00 | 13350000.00 | KO:K04077 | chaperonin GroEL | Bacteria;Verrucomicrobia;Verrucomicrobiae;Verrucomicrobiales;Verrucomicrobia subdivision 3;Pedosphaera;Pedosphaera parvula;Pedosphaera parvula Ellin514 |
| Ga0586324 6704468 3 545 | 0.00 | 0.00 | 0.00 | 0.00 | 0.00 | 416703.11 | 0.00 | 0.00 | 0.00 | 0.00 | 0.00 | 840000.00 | KO:K04077 | chaperonin GroEL | Bacteria;Proteobacteria;Deltaproteobacteria;Syntrophobacterales;Syntrophobacteraceae;Syntrophobacter;Syntrophobacter fumaroxidans;Syntrophobacter fumaroxidans MPOB |
| Ga0586324 6722068 3 545 | 320015.00 | 0.00 | 0.00 | 0.00 | 0.00 | 0.00 | 0.00 | 0.00 | 0.00 | 0.00 | 0.00 | 0.00 | KO:K04077 | chaperonin GroEL | Bacteria;Proteobacteria;Deltaproteobacteria;Desulfobacterales;Desulfobacteraceae;Desulfobacter;Desulfobacter postgatei;Desulfobacter postgatei 2ac9 |
| Ga0586324 6723171 2 547 | 0.00 | 0.00 | 0.00 | 0.00 | 8126700.00 | 0.00 | 0.00 | 0.00 | 0.00 | 1509200.00 | 0.00 | 164094.75 | KO:K04077 | chaperonin GroEL | Bacteria;Proteobacteria;Betaproteobacteria;unclassified Betaproteobacteria;unclassified Betaproteobacteria;unclassified Betaproteobacteria;uncultured beta proteobacterium;unclassified betaproteobacterium ENR4 |
| Ga0586324 6731884 3 545 | 0.00 | 292791.00 | 0.00 | 0.00 | 0.00 | 0.00 | 0.00 | 0.00 | 0.00 | 0.00 | 0.00 | 0.00 | KO:K04077 | chaperonin GroEL | Bacteria;Verrucomicrobia;Verrucomicrobiae;Verrucomicrobiales;Verrucomicrobia subdivision 3;Pedosphaera;Pedosphaera parvula;Pedosphaera parvula Ellin514 |
| Ga0586324 6764480 1 543 | 0.00 | 0.00 | 0.00 | 0.00 | 38728000.00 | 0.00 | 0.00 | 0.00 | 3045600.00 | 691801.88 | 675114.00 | 0.00 | KO:K04077 | chaperonin GroEL | Bacteria;Proteobacteria;Alphaproteobacteria;Hyphomicrobiales;Xanthobacteraceae;Pseudolabrys;Pseudolabrys sp. Root1462;Pseudolabrys sp. Root1462 |
| Ga0586324 6782897 3 545 | 0.00 | 0.00 | 0.00 | 0.00 | 1246300.00 | 0.00 | 0.00 | 0.00 | 0.00 | 0.00 | 0.00 | 0.00 | KO:K04077 | chaperonin GroEL | Bacteria;Deferribacteres;Deferribacteres;Deferribacterales;Deferribacteraceae;Deferribacter;Deferribacter desulfuricans;Deferribacter desulfuricans SSM1 |
| Ga0586324 6827283 1 543 | 0.00 | 0.00 | 0.00 | 0.00 | 0.00 | 0.00 | 0.00 | 0.00 | 0.00 | 0.00 | 3229600.00 | 0.00 | KO:K04077 | chaperonin GroEL | Bacteria;Proteobacteria;Alphaproteobacteria;Hyphomicrobiales;Bradyrhizobiaceae;Bradyrhizobium;Bradyrhizobium valentinum;Bradyrhizobium valentinum LmjM6 |
| Ga0586324 6898969 3 539 | 0.00 | 0.00 | 0.00 | 0.00 | 0.00 | 0.00 | 0.00 | 18361000.00 | 0.00 | 0.00 | 0.00 | 0.00 | KO:K04077 | chaperonin GroEL | Bacteria;Bacteroidetes;Bacteroidia;Bacteroidales;Bacteroidaceae;Bacteroides;Bacteroides graminisolvens;Bacteroides graminisolvens DSM 19988 |
| Ga0586324 6902509 1 540 | 0.00 | 0.00 | 0.00 | 0.00 | 0.00 | 0.00 | 0.00 | 0.00 | 0.00 | 0.00 | 3775200.00 | 0.00 | KO:K04077 | chaperonin GroEL | Bacteria;Proteobacteria;Deltaproteobacteria;Syntrophobacterales;Syntrophobacteraceae;Syntrophobacter;Syntrophobacter fumaroxidans;Syntrophobacter fumaroxidans MPOB |
| Ga0586324 6926734 2 538 | 0.00 | 0.00 | 0.00 | 0.00 | 0.00 | 1904405.03 | 0.00 | 0.00 | 0.00 | 0.00 | 0.00 | 922500.00 | KO:K04077 | chaperonin GroEL | Bacteria;Firmicutes;Clostridia;Thermosediminibacterales;Thermosediminibacteraceae;Caldanaerovirga;Caldanaerovirga acetigignens;Caldanaerovirga acetigignens DSM 18802 |
| Ga0586324 6960467 3 329 | 0.00 | 0.00 | 1245249.03 | 0.00 | 0.00 | 0.00 | 0.00 | 0.00 | 1080420.12 | 0.00 | 0.00 | 952500.00 | KO:K04077 | chaperonin GroEL | Bacteria;Verrucomicrobia;Verrucomicrobiae;Verrucomicrobiales;Verrucomicrobia subdivision 3;Pedosphaera;Pedosphaera parvula;Pedosphaera parvula Ellin514 |
| Ga0586324 6963065 2 538 | 0.00 | 0.00 | 0.00 | 0.00 | 0.00 | 0.00 | 0.00 | 199719.49 | 0.00 | 140721.35 | 0.00 | 0.00 | KO:K04077 | chaperonin GroEL | Bacteria;Actinobacteria;Actinomycetia;Propionibacteriales;Nocardioidaceae;Propionicimonas;Propionicimonas paludicola;Propionicimonas paludicola DSM 15597 |
| Ga0586324 6980858 1 537 | 0.00 | 0.00 | 0.00 | 0.00 | 325195.72 | 0.00 | 0.00 | 0.00 | 0.00 | 0.00 | 0.00 | 0.00 | KO:K04077 | chaperonin GroEL | Bacteria;Proteobacteria;Deltaproteobacteria;Syntrophobacterales;Syntrophobacteraceae;Desulfacinum;Desulfacinum hydrothermale;Desulfacinum hydrothermale DSM 13146 |
| Ga0586324 6993014 1 537 | 0.00 | 0.00 | 35975700.00 | 0.00 | 2472000.00 | 0.00 | 36936000.00 | 17690000.00 | 0.00 | 3873100.00 | 4408800.00 | 47250000.00 | KO:K04077 | chaperonin GroEL | Bacteria;Proteobacteria;Alphaproteobacteria;Hyphomicrobiales;Hyphomicrobiaceae;Prosthecomicrobium;Prosthecomicrobium hirschii;Prosthecomicrobium hirschii ATCC 27832 |
| Ga0586324 7029147 3 536 | 0.00 | 1428000.00 | 0.00 | 0.00 | 0.00 | 0.00 | 0.00 | 0.00 | 0.00 | 0.00 | 155865.60 | 0.00 | KO:K04077 | chaperonin GroEL | Bacteria;Proteobacteria;Betaproteobacteria;unclassified Betaproteobacteria;unclassified Betaproteobacteria;unclassified Betaproteobacteria;Betaproteobacteria bacterium UKL13-2;Betaproteobacteria bacterium UKL13-2 |
| Ga0586324 7047159 2 535 | 62000000.00 | 584231.52 | 54048000.00 | 111055000.00 | 64313200.00 | 44148857.43 | 95463600.00 | 232562500.00 | 42006600.00 | 118903400.00 | 119539200.00 | 4995000.00 | KO:K04077 | chaperonin GroEL | Bacteria;Proteobacteria;Deltaproteobacteria;Myxococcales;Labilitrichaceae;Labilithrix;Labilithrix luteola;Labilithrix luteola DSM 27648 |
| Ga0586324 7087088 2 469 | 0.00 | 0.00 | 0.00 | 0.00 | 0.00 | 6709300.00 | 0.00 | 0.00 | 0.00 | 0.00 | 0.00 | 0.00 | KO:K04077 | chaperonin GroEL | Bacteria;Spirochaetes;Spirochaetia;Spirochaetales;Spirochaetaceae;Sediminispirochaeta;Sediminispirochaeta bajacaliforniensis;Sediminispirochaeta bajacaliforniensis DSM 16054 |
| Ga0586324 7087981 20 535 | 0.00 | 0.00 | 0.00 | 627854.05 | 0.00 | 0.00 | 0.00 | 0.00 | 0.00 | 0.00 | 0.00 | 0.00 | KO:K04077 | chaperonin GroEL | Bacteria;Proteobacteria;Deltaproteobacteria;Syntrophobacterales;Syntrophaceae;Syntrophus;Syntrophus gentianae;Syntrophus gentianae DSM 8423 |
| Ga0586324 7135665 3 476 | 0.00 | 0.00 | 30739800.00 | 0.00 | 260994.79 | 1433428.05 | 307396.44 | 835700.00 | 4884300.00 | 7107100.00 | 7436000.00 | 0.00 | KO:K04077 | chaperonin GroEL | Bacteria;Candidatus Aminicenantes;unclassified Candidatus Aminicenantes;unclassified Candidatus Aminicenantes;unclassified Candidatus Aminicenantes;unclassified Candidatus Aminicenantes;Aminicenantes bacterium SCGC AAA255-E10;Aminicenantes bacterium SCGC AAA255-E10 (contamination screened) |
| Ga0586324 7138747 3 533 | 0.00 | 0.00 | 0.00 | 0.00 | 0.00 | 0.00 | 0.00 | 0.00 | 9801000.00 | 0.00 | 0.00 | 0.00 | KO:K04077 | chaperonin GroEL | Bacteria;Proteobacteria;Alphaproteobacteria;Hyphomicrobiales;Hyphomicrobiaceae;Hyphomicrobium;Hyphomicrobium sulfonivorans;Hyphomicrobium sulfonivorans S1 |
| Ga0586324 7156591 1 531 | 0.00 | 0.00 | 0.00 | 0.00 | 0.00 | 0.00 | 0.00 | 1207800.00 | 0.00 | 0.00 | 0.00 | 0.00 | KO:K04077 | chaperonin GroEL | Bacteria;Proteobacteria;Deltaproteobacteria;Desulfuromonadales;Geobacteraceae;Geobacter;Geobacter lovleyi;Geobacter lovleyi SZ |
| Ga0586324 7212010 2 529 | 0.00 | 516074.44 | 0.00 | 0.00 | 0.00 | 0.00 | 0.00 | 0.00 | 0.00 | 0.00 | 0.00 | 0.00 | KO:K04077 | chaperonin GroEL | Bacteria;Proteobacteria;Deltaproteobacteria;unclassified Deltaproteobacteria;unclassified Deltaproteobacteria;Dissulfuribacter;Dissulfuribacter thermophilus;Dissulfuribacter thermophilus S69 |
| Ga0586324 7234226 2 529 | 0.00 | 0.00 | 0.00 | 0.00 | 0.00 | 0.00 | 0.00 | 282720.97 | 0.00 | 0.00 | 0.00 | 0.00 | KO:K04077 | chaperonin GroEL | Bacteria;Verrucomicrobia;Verrucomicrobiae;Verrucomicrobiales;Verrucomicrobia subdivision 3;Pedosphaera;Pedosphaera parvula;Pedosphaera parvula Ellin514 |
| Ga0586324 7284117 2 529 | 0.00 | 514882.40 | 0.00 | 0.00 | 0.00 | 0.00 | 0.00 | 0.00 | 0.00 | 0.00 | 0.00 | 0.00 | KO:K04077 | chaperonin GroEL | Bacteria;Actinobacteria;Acidimicrobiia;Acidimicrobiales;Ilumatobacteraceae;Desertimonas;Desertimonas flava;Acidimicrobiales bacterium SYSU D60003 |
| Ga0586324 7300269 2 526 | 0.00 | 0.00 | 0.00 | 0.00 | 0.00 | 0.00 | 0.00 | 0.00 | 874800.00 | 3711400.00 | 5112800.00 | 3915000.00 | KO:K04077 | chaperonin GroEL | Bacteria;Verrucomicrobia;Verrucomicrobiae;Verrucomicrobiales;Verrucomicrobia subdivision 3;Pedosphaera;Pedosphaera parvula;Pedosphaera parvula Ellin514 |
| Ga0586324 7329156 3 527 | 0.00 | 0.00 | 0.00 | 0.00 | 0.00 | 0.00 | 0.00 | 0.00 | 0.00 | 1670900.00 | 0.00 | 0.00 | KO:K04077 | chaperonin GroEL | Bacteria;Proteobacteria;Gammaproteobacteria;Cellvibrionales;Porticoccaceae;Porticoccus;Porticoccus hydrocarbonoclasticus;Porticoccus hydrocarbonoclasticus MCTG13d |
| Ga0586324 7354239 1 525 | 110690.00 | 0.00 | 0.00 | 0.00 | 0.00 | 0.00 | 0.00 | 0.00 | 0.00 | 0.00 | 0.00 | 0.00 | KO:K04077 | chaperonin GroEL | Bacteria;Actinobacteria;Actinomycetia;unclassified Actinomycetia;unclassified Actinomycetia;unclassified Actinomycetia;Actinobacteria bacterium IMCC26256;Actinobacteria bacterium IMCC26256 |
| Ga0586324 7362969 3 524 | 0.00 | 0.00 | 0.00 | 0.00 | 0.00 | 0.00 | 0.00 | 0.00 | 0.00 | 699338.64 | 0.00 | 0.00 | KO:K04077 | chaperonin GroEL | Bacteria;Actinobacteria;Actinomycetia;Propionibacteriales;Propionibacteriaceae;Microlunatus;Microlunatus phosphovorus;Microlunatus phosphovorus NM-1 |
| Ga0586324 7369589 3 524 | 3730000.00 | 2923475.72 | 6699700.00 | 0.00 | 3605000.00 | 3518670.55 | 7341600.00 | 29456900.00 | 4673700.00 | 14606900.00 | 3748800.00 | 25605000.00 | KO:K04077 | chaperonin GroEL | Bacteria;Proteobacteria;Gammaproteobacteria;unclassified Gammaproteobacteria;Candidatus Competibacteraceae;Candidatus Competibacter;Candidatus Competibacter denitrificans;Candidatus Competibacter denitrificans Run A D11 |
| Ga0586324 7383262 2 526 | 0.00 | 0.00 | 0.00 | 911141.20 | 0.00 | 0.00 | 0.00 | 0.00 | 0.00 | 0.00 | 0.00 | 0.00 | KO:K04077 | chaperonin GroEL | Bacteria;Bacteroidetes;Chitinophagia;Chitinophagales;Chitinophagaceae;unclassified Chitinophagaceae;Chitinophagaceae bacterium PMP191F;Chitinophagaceae bacterium PMP191F |
| Ga0586324 7387731 1 525 | 0.00 | 4882400.00 | 7037500.00 | 0.00 | 0.00 | 0.00 | 8709600.00 | 0.00 | 0.00 | 5397700.00 | 0.00 | 0.00 | KO:K04077 | chaperonin GroEL | Bacteria;Proteobacteria;Betaproteobacteria;Burkholderiales;Sutterellaceae;Sutterella;Sutterella wadsworthensis;Sutterella wadsworthensis 2 1 59BFAA |
| Ga0586324 7390304 1 525 | 0.00 | 0.00 | 0.00 | 0.00 | 0.00 | 28822200.00 | 0.00 | 0.00 | 0.00 | 0.00 | 0.00 | 0.00 | KO:K04077 | chaperonin GroEL | Bacteria;Proteobacteria;Alphaproteobacteria;Hyphomicrobiales;Hyphomicrobiaceae;Hyphomicrobium;Hyphomicrobium zavarzinii;Hyphomicrobium zavarzinii ATCC 27496 |
| Ga0586324 7390395 3 524 | 0.00 | 0.00 | 2982942.90 | 0.00 | 0.00 | 0.00 | 0.00 | 0.00 | 0.00 | 0.00 | 0.00 | 10800000.00 | KO:K04077 | chaperonin GroEL | Bacteria;Candidatus Calescamantes;unclassified Candidatus Calescamantes;unclassified Candidatus Calescamantes;unclassified Candidatus Calescamantes;unclassified Candidatus Calescamantes;Calescamantes bacterium JGI 0000106-H18;Calescamantes bacterium JGI 0000106-H18 (contamination screened) |
| Ga0586324 7453023 1 522 | 0.00 | 0.00 | 0.00 | 0.00 | 0.00 | 0.00 | 15481200.00 | 0.00 | 0.00 | 0.00 | 0.00 | 0.00 | KO:K04077 | chaperonin GroEL | Bacteria;Proteobacteria;Deltaproteobacteria;Desulfobacterales;Desulfobacteraceae;Desulfosalsimonas;Desulfosalsimonas propionicica;Desulfosalsimonas propionicica DSM 17721 |
| Ga0586324 7460490 3 521 | 0.00 | 264831.44 | 0.00 | 0.00 | 0.00 | 0.00 | 0.00 | 0.00 | 0.00 | 0.00 | 0.00 | 0.00 | KO:K04077 | chaperonin GroEL | Bacteria;Chloroflexi;Thermomicrobia;Sphaerobacterales;Sphaerobacteraceae;Sphaerobacter;Sphaerobacter thermophilus;Sphaerobacter thermophilus 4ac11, DSM 20745 |
| Ga0586324 7486040 3 521 | 0.00 | 0.00 | 0.00 | 0.00 | 0.00 | 0.00 | 0.00 | 0.00 | 0.00 | 0.00 | 0.00 | 2242500.00 | KO:K04077 | chaperonin GroEL | Bacteria;Proteobacteria;Alphaproteobacteria;Rhodospirillales;Acetobacteraceae;Roseomonas;Roseomonas gilardii;Roseomonas gilardii rosea ATCC BAA-691 |
| Ga0586324 7504413 1 522 | 0.00 | 195178.36 | 15369900.00 | 0.00 | 0.00 | 0.00 | 2622000.00 | 1464000.00 | 0.00 | 1162700.00 | 1826153.12 | 1425000.00 | KO:K04077 | chaperonin GroEL | Bacteria;Proteobacteria;Deltaproteobacteria;Syntrophobacterales;Syntrophaceae;Syntrophus;Syntrophus gentianae;Syntrophus gentianae DSM 8423 |
| Ga0586324 7531220 2 520 | 0.00 | 1353200.00 | 0.00 | 2289500.00 | 0.00 | 0.00 | 9943091.40 | 2305800.00 | 2203200.00 | 0.00 | 5852000.00 | 5971766.25 | KO:K04077 | chaperonin GroEL | Bacteria;Spirochaetes;Spirochaetia;Leptospirales;Leptospiraceae;Leptospira;Leptospira santarosai;Leptospira santarosai CBC379 |
| Ga0586324 7539811 3 521 | 0.00 | 0.00 | 0.00 | 0.00 | 0.00 | 0.00 | 0.00 | 0.00 | 0.00 | 0.00 | 0.00 | 802500.00 | KO:K04077 | chaperonin GroEL | Bacteria;Proteobacteria;Alphaproteobacteria;Micropepsales;Micropepsaceae;Rhizomicrobium;Rhizomicrobium sp. SG733;Rhizomicrobium sp. SG733 |
| Ga0586324 7547027 3 521 | 0.00 | 0.00 | 0.00 | 0.00 | 0.00 | 0.00 | 0.00 | 0.00 | 0.00 | 429302.72 | 0.00 | 0.00 | KO:K04077 | chaperonin GroEL | Bacteria;Proteobacteria;Betaproteobacteria;Nitrosomonadales;Nitrosomonadaceae;Nitrosovibrio;Nitrosovibrio sp. Nv17;Nitrosovibrio sp. Nv17 |
| Ga0586324 7552295 2 520 | 0.00 | 0.00 | 0.00 | 0.00 | 0.00 | 0.00 | 0.00 | 242704.97 | 609689.43 | 0.00 | 0.00 | 0.00 | KO:K04077 | chaperonin GroEL | Bacteria;Planctomycetes;Candidatus Brocadiae;Candidatus Brocadiales;Candidatus Brocadiaceae;Candidatus Brocadia;Candidatus Brocadia sinica;Candidatus Brocadia sinica JPN1 |
| Ga0586324 7556382 3 521 | 0.00 | 2495600.00 | 2314639.38 | 4436500.00 | 4489526.92 | 0.00 | 4793412.72 | 1464000.00 | 955706.04 | 0.00 | 2912800.00 | 10950000.00 | KO:K04077 | chaperonin GroEL | Bacteria;Acidobacteria;Blastocatellia;Blastocatellales;Pyrinomonadaceae;Pyrinomonas;Pyrinomonas methylaliphatogenes;Pyrinomonas methylaliphatogenes K22 |
| Ga0586324 7574551 1 519 | 4048046.00 | 5542000.00 | 2698976.96 | 4968500.00 | 13052879.97 | 0.00 | 0.00 | 1061400.00 | 641042.91 | 5459300.00 | 0.00 | 2512500.00 | KO:K04077 | chaperonin GroEL | Bacteria;Proteobacteria;Deltaproteobacteria;Myxococcales;Labilitrichaceae;Labilithrix;Labilithrix luteola;Labilithrix luteola DSM 27648 |
| Ga0586324 7588146 2 520 | 0.00 | 0.00 | 0.00 | 0.00 | 0.00 | 0.00 | 0.00 | 0.00 | 260086.14 | 0.00 | 418093.28 | 0.00 | KO:K04077 | chaperonin GroEL | Bacteria;Chloroflexi;Anaerolineae;Anaerolineales;Anaerolineaceae;Anaerolinea;Anaerolinea thermophila;Anaerolinea thermophila UNI-1 |
| Ga0586324 7590119 1 519 | 0.00 | 0.00 | 0.00 | 0.00 | 0.00 | 0.00 | 0.00 | 0.00 | 0.00 | 2194500.00 | 1073600.00 | 885000.00 | KO:K04077 | chaperonin GroEL | Bacteria;Nitrospinae;Nitrospinia;Nitrospinales;Nitrospinaceae;Candidatus Nitromaritima;Candidatus Nitromaritima sp. SCGC AAA799-A02;Candidatus Nitromaritima sp. SCGC AAA799 A02 |
| Ga0586324 7591954 2 520 | 0.00 | 0.00 | 0.00 | 0.00 | 0.00 | 0.00 | 0.00 | 3836900.00 | 0.00 | 0.00 | 0.00 | 0.00 | KO:K04077 | chaperonin GroEL | Bacteria;Candidatus Omnitrophica;unclassified Candidatus Omnitrophica;unclassified Candidatus Omnitrophica;unclassified Candidatus Omnitrophica;unclassified Candidatus Omnitrophica;Omnitrophica bacterium SCGC AAA257-O07;Omnitrophica bacterium SCGC AAA257-O07 (contamination screened) |
| Ga0586324 7595243 1 339 | 0.00 | 0.00 | 0.00 | 0.00 | 7951600.00 | 6312300.00 | 0.00 | 0.00 | 2802600.00 | 0.00 | 0.00 | 0.00 | KO:K04077 | chaperonin GroEL | Bacteria;Bacteroidetes;Bacteroidia;Marinilabiliales;Prolixibacteraceae;Roseimarinus;Roseimarinus sediminis;Roseimarinus sediminis DSM 28824 |
| Ga0586324 7602981 1 519 | 0.00 | 0.00 | 0.00 | 0.00 | 426849.51 | 0.00 | 0.00 | 0.00 | 0.00 | 0.00 | 0.00 | 0.00 | KO:K04077 | chaperonin GroEL | Bacteria;Proteobacteria;Deltaproteobacteria;Syntrophorhabdales;Syntrophorhabdaceae;Syntrophorhabdus;Syntrophorhabdus aromaticivorans;Syntrophorhabdus aromaticivorans UI |
| Ga0586324 7669693 3 515 | 0.00 | 1319200.00 | 352358.62 | 0.00 | 0.00 | 0.00 | 0.00 | 11102000.00 | 899100.00 | 1193500.00 | 0.00 | 555947.25 | KO:K04077 | chaperonin GroEL | Bacteria;Verrucomicrobia;Opitutae;Opitutales;Opitutaceae;Opitutus;Opitutus sp. ER46;Opitutus sp. ER46 |
| Ga0586324 7683023 28 516 | 0.00 | 375146.48 | 0.00 | 0.00 | 0.00 | 0.00 | 0.00 | 0.00 | 0.00 | 0.00 | 0.00 | 475023.00 | KO:K04077 | chaperonin GroEL | Bacteria;Proteobacteria;Deltaproteobacteria;Syntrophobacterales;Syntrophaceae;Desulfobacca;Desulfobacca acetoxidans;Desulfobacca acetoxidans ASRB2, DSM 11109 |
| Ga0586324 7683358 84 515 | 0.00 | 0.00 | 0.00 | 0.00 | 0.00 | 0.00 | 2416800.00 | 2372900.00 | 2567700.00 | 0.00 | 3722400.00 | 4680000.00 | KO:K04077 | chaperonin GroEL | Bacteria;Acidobacteria;Vicinamibacteria;unclassified Vicinamibacteria;Vicinamibacteraceae;Luteitalea;Luteitalea pratensis;Luteitalea pratensis DSM 100886 |
| Ga0586324 7714952 3 374 | 0.00 | 0.00 | 0.00 | 2099500.00 | 0.00 | 0.00 | 0.00 | 0.00 | 0.00 | 0.00 | 0.00 | 0.00 | KO:K04077 | chaperonin GroEL | Bacteria;Acidobacteria;Thermoanaerobaculia;Thermoanaerobaculales;Thermoanaerobaculaceae;Thermoanaerobaculum;Thermoanaerobaculum aquaticum;Thermoanaerobaculum aquaticum MP-01 |
| Ga0586324 7732817 2 478 | 0.00 | 1862195.64 | 1351042.36 | 2473039.05 | 0.00 | 0.00 | 0.00 | 1390800.00 | 689530.32 | 2040500.00 | 2340800.00 | 1290000.00 | KO:K04077 | chaperonin GroEL | Bacteria;Proteobacteria;Deltaproteobacteria;Myxococcales;Labilitrichaceae;Labilithrix;Labilithrix luteola;Labilithrix luteola DSM 27648 |
| Ga0586324 7766463 1 513 | 0.00 | 0.00 | 0.00 | 0.00 | 0.00 | 0.00 | 1536763.32 | 0.00 | 0.00 | 0.00 | 0.00 | 0.00 | KO:K04077 | chaperonin GroEL | Bacteria;Chloroflexi;Anaerolineae;Anaerolineales;Anaerolineaceae;Anaerolinea;Anaerolinea thermophila;Anaerolinea thermophila UNI-1 |
| Ga0586324 7770370 3 515 | 0.00 | 123711.72 | 0.00 | 0.00 | 0.00 | 0.00 | 0.00 | 0.00 | 0.00 | 0.00 | 0.00 | 0.00 | KO:K04077 | chaperonin GroEL | Bacteria;Acidobacteria;Acidobacteriia;Bryobacterales;Solibacteraceae;Candidatus Solibacter;Candidatus Solibacter usitatus;Candidatus Solibacter usitatus Ellin6076 |
| Ga0586324 7774271 1 513 | 0.00 | 100357.80 | 0.00 | 0.00 | 0.00 | 0.00 | 0.00 | 179472.37 | 0.00 | 0.00 | 0.00 | 0.00 | KO:K04077 | chaperonin GroEL | Bacteria;Proteobacteria;Deltaproteobacteria;Syntrophorhabdales;Syntrophorhabdaceae;Syntrophorhabdus;Syntrophorhabdus aromaticivorans;Syntrophorhabdus aromaticivorans UI |
| Ga0586324 7793056 3 512 | 11430000.00 | 0.00 | 0.00 | 0.00 | 2369000.00 | 0.00 | 2065376.76 | 463731.15 | 0.00 | 1543523.52 | 1020800.00 | 588787.50 | KO:K04077 | chaperonin GroEL | Bacteria;Proteobacteria;Deltaproteobacteria;Myxococcales;Sandaracinaceae;Sandaracinus;Sandaracinus amylolyticus;Sandaracinus amylolyticus DSM 53668 |
| Ga0586324 7795577 1 513 | 0.00 | 1115200.00 | 0.00 | 2878500.00 | 4253900.00 | 0.00 | 0.00 | 3342800.00 | 3920400.00 | 2279200.00 | 2816000.00 | 0.00 | KO:K04077 | chaperonin GroEL | Bacteria;Chloroflexi;Anaerolineae;Anaerolineales;Anaerolineaceae;Levilinea;Levilinea saccharolytica;Levilinea saccharolytica DSM 16555 |
| Ga0586324 7809805 3 512 | 0.00 | 0.00 | 0.00 | 7343500.00 | 0.00 | 7185700.00 | 7501200.00 | 5923100.00 | 6439500.00 | 3757600.00 | 4188800.00 | 3427500.00 | KO:K04077 | chaperonin GroEL | Bacteria;Proteobacteria;Deltaproteobacteria;Desulfobacterales;Desulfobacteraceae;Desulfatirhabdium;Desulfatirhabdium butyrativorans;Desulfatirhabdium butyrativorans DSM 18734 |
| Ga0586324 7822267 3 512 | 0.00 | 1142400.00 | 3399394.00 | 0.00 | 0.00 | 0.00 | 0.00 | 0.00 | 0.00 | 0.00 | 0.00 | 0.00 | KO:K04077 | chaperonin GroEL | Bacteria;Proteobacteria;Deltaproteobacteria;Myxococcales;Sandaracinaceae;Sandaracinus;Sandaracinus amylolyticus;Sandaracinus amylolyticus DSM 53668 |
| Ga0586324 7859188 1 510 | 0.00 | 0.00 | 0.00 | 0.00 | 0.00 | 0.00 | 0.00 | 563545.45 | 0.00 | 0.00 | 257664.00 | 0.00 | KO:K04077 | chaperonin GroEL | Bacteria;Nitrospirae;Nitrospira;Nitrospirales;Nitrospiraceae;Candidatus Magnetoovum;Candidatus Magnetoovum chiemensis;Candidatus Magnetoovum chiemensis CS-04 |
| Ga0586324 7868429 3 512 | 0.00 | 0.00 | 0.00 | 297992.20 | 0.00 | 0.00 | 0.00 | 0.00 | 217425.06 | 0.00 | 829023.36 | 0.00 | KO:K04077 | chaperonin GroEL | Bacteria;Proteobacteria;Deltaproteobacteria;Desulfobacterales;Desulfobulbaceae;Desulfobulbus;Desulfobulbus propionicus;Desulfobulbus propionicus 1pr3, DSM 2032 |
| Ga0586324 7868862 3 512 | 0.00 | 0.00 | 0.00 | 0.00 | 0.00 | 463426.04 | 0.00 | 0.00 | 0.00 | 0.00 | 0.00 | 0.00 | KO:K04077 | chaperonin GroEL | Bacteria;Nitrospirae;Nitrospira;Nitrospirales;Nitrospiraceae;Nitrospira;Nitrospira lenta;Nitrospira lenta N. lenta BS10 |
| Ga0586324 7890952 2 511 | 0.00 | 0.00 | 0.00 | 0.00 | 0.00 | 0.00 | 0.00 | 0.00 | 0.00 | 0.00 | 0.00 | 608166.75 | KO:K04077 | chaperonin GroEL | Bacteria;Candidatus Omnitrophica;unclassified Candidatus Omnitrophica;unclassified Candidatus Omnitrophica;unclassified Candidatus Omnitrophica;unclassified Candidatus Omnitrophica;Omnitrophica bacterium SCGC AAA011-L16;Omnitrophica bacterium SCGC AAA011-L16 (contamination screened) |
| Ga0586324 7909277 2 511 | 0.00 | 357470.56 | 0.00 | 0.00 | 996550.75 | 0.00 | 0.00 | 407423.88 | 644950.35 | 808500.00 | 0.00 | 0.00 | KO:K04077 | chaperonin GroEL | Bacteria;Proteobacteria;Deltaproteobacteria;Syntrophobacterales;Syntrophaceae;Desulfobacca;Desulfobacca acetoxidans;Desulfobacca acetoxidans ASRB2, DSM 11109 |
| Ga0586324 7915693 25 510 | 0.00 | 0.00 | 0.00 | 0.00 | 0.00 | 0.00 | 786846.24 | 0.00 | 0.00 | 0.00 | 568785.36 | 0.00 | KO:K04077 | chaperonin GroEL | Bacteria;Acidobacteria;Acidobacteriia;Bryobacterales;Solibacteraceae;Candidatus Solibacter;Candidatus Solibacter usitatus;Candidatus Solibacter usitatus Ellin6076 |
| Ga0586324 7960933 2 508 | 0.00 | 0.00 | 0.00 | 0.00 | 0.00 | 0.00 | 22754400.00 | 505003.14 | 471121.11 | 540338.26 | 24552000.00 | 29025000.00 | KO:K04077 | chaperonin GroEL | Bacteria;Proteobacteria;Betaproteobacteria;Nitrosomonadales;Nitrosomonadaceae;Nitrosomonas;Nitrosomonas europaea;Nitrosomonas europaea ATCC 19718 |
| Ga0586324 7982695 153 509 | 0.00 | 0.00 | 0.00 | 0.00 | 720062.70 | 0.00 | 13360800.00 | 439555.63 | 4430700.00 | 5844300.00 | 0.00 | 0.00 | KO:K04077 | chaperonin GroEL | Bacteria;Chloroflexi;Thermomicrobia;Sphaerobacterales;Sphaerobacteraceae;Sphaerobacter;Sphaerobacter thermophilus;Sphaerobacter thermophilus 4ac11, DSM 20745 |
| Ga0586324 7993154 3 509 | 0.00 | 0.00 | 0.00 | 0.00 | 0.00 | 0.00 | 7364400.00 | 0.00 | 0.00 | 0.00 | 0.00 | 0.00 | KO:K04077 | chaperonin GroEL | Bacteria;Bacteroidetes;unclassified Bacteroidetes;Bacteroidetes Order II. Incertae sedis;Rhodothermaceae;Rhodothermus;Rhodothermus marinus;Rhodothermus marinus R-10, DSM 4252 |
| Ga0586324 8019664 1 507 | 0.00 | 0.00 | 0.00 | 0.00 | 0.00 | 0.00 | 0.00 | 0.00 | 0.00 | 0.00 | 300768.16 | 0.00 | KO:K04077 | chaperonin GroEL | Bacteria;Acidobacteria;Acidobacteriia;Bryobacterales;Solibacteraceae;Candidatus Solibacter;Candidatus Solibacter usitatus;Candidatus Solibacter usitatus Ellin6076 |
| Ga0586324 8039779 3 506 | 0.00 | 0.00 | 0.00 | 0.00 | 857600.66 | 0.00 | 0.00 | 0.00 | 0.00 | 0.00 | 0.00 | 0.00 | KO:K04077 | chaperonin GroEL | Bacteria;Proteobacteria;Gammaproteobacteria;Chromatiales;Ectothiorhodospiraceae;Acidihalobacter;Acidihalobacter prosperus;Acidihalobacter prosperus V6 |
| Ga0586324 8061802 1 468 | 0.00 | 0.00 | 0.00 | 0.00 | 0.00 | 0.00 | 395196.96 | 0.00 | 0.00 | 0.00 | 0.00 | 0.00 | KO:K04077 | chaperonin GroEL | Bacteria;Gemmatimonadetes;Gemmatimonadetes;Gemmatimonadales;Gemmatimonadaceae;Gemmatirosa;Gemmatirosa kalamazoonesis;Gemmatimonadetes bacterium KBS708 |
| Ga0586324 8073518 1 504 | 0.00 | 172282.76 | 0.00 | 0.00 | 0.00 | 0.00 | 0.00 | 0.00 | 0.00 | 0.00 | 0.00 | 0.00 | KO:K04077 | chaperonin GroEL | Bacteria;Nitrospirae;Nitrospira;Nitrospirales;Nitrospiraceae;Nitrospira;Nitrospira moscoviensis;Nitrospira moscoviensis NSP M-1 |
| Ga0586324 8075159 3 506 | 0.00 | 0.00 | 0.00 | 0.00 | 3409300.00 | 0.00 | 0.00 | 0.00 | 2146500.00 | 0.00 | 0.00 | 0.00 | KO:K04077 | chaperonin GroEL | Bacteria;Verrucomicrobia;Methylacidiphilae;Methylacidiphilales;Methylacidiphilaceae;Methylacidiphilum;Methylacidiphilum kamchatkense;Methylacidiphilum kamchatkense Kam1 |
| Ga0586324 8095789 3 506 | 0.00 | 0.00 | 0.00 | 0.00 | 0.00 | 0.00 | 0.00 | 988200.00 | 0.00 | 3110800.00 | 7867200.00 | 0.00 | KO:K04077 | chaperonin GroEL | Bacteria;Proteobacteria;Alphaproteobacteria;Hyphomicrobiales;Reyranellaceae;Reyranella;Reyranella sp. OAS946;Reyranella sp. OAS946 |
| Ga0586324 8129441 2 505 | 0.00 | 0.00 | 0.00 | 0.00 | 0.00 | 0.00 | 0.00 | 0.00 | 0.00 | 3303300.00 | 1487200.00 | 1380000.00 | KO:K04077 | chaperonin GroEL | Bacteria;Acidobacteria;Vicinamibacteria;unclassified Vicinamibacteria;Vicinamibacteraceae;Luteitalea;Luteitalea pratensis;Luteitalea pratensis DSM 100886 |
| Ga0586324 8162787 3 503 | 0.00 | 0.00 | 0.00 | 2175500.00 | 0.00 | 0.00 | 0.00 | 0.00 | 0.00 | 0.00 | 0.00 | 0.00 | KO:K04077 | chaperonin GroEL | Bacteria;Proteobacteria;Gammaproteobacteria;Chromatiales;Chromatiaceae;Thiorhodovibrio;Thiorhodovibrio sp. 970;Thiorhodovibrio sp. 970 |
| Ga0586324 8173622 2 502 | 0.00 | 0.00 | 0.00 | 0.00 | 0.00 | 0.00 | 8094000.00 | 0.00 | 0.00 | 0.00 | 37224000.00 | 0.00 | KO:K04077 | chaperonin GroEL | Bacteria;Proteobacteria;Alphaproteobacteria;Hyphomicrobiales;Hyphomicrobiaceae;Hyphomicrobium;Hyphomicrobium sp. CS1BSMeth3;Hyphomicrobium sp. CS1BSMeth3 |
| Ga0586324 8188665 1 501 | 0.00 | 678983.40 | 269071.78 | 53095500.00 | 56979600.00 | 83924041.29 | 79959600.00 | 12126800.00 | 13810500.00 | 66458700.00 | 403287.28 | 20910868.50 | KO:K04077 | chaperonin GroEL | Bacteria;Proteobacteria;Deltaproteobacteria;Syntrophobacterales;Syntrophaceae;Desulfobacca;Desulfobacca acetoxidans;Desulfobacca acetoxidans ASRB2, DSM 11109 |
| Ga0586324 8206217 3 503 | 0.00 | 516889.08 | 0.00 | 0.00 | 0.00 | 0.00 | 0.00 | 0.00 | 0.00 | 0.00 | 2393600.00 | 0.00 | KO:K04077 | chaperonin GroEL | Bacteria;Proteobacteria;Betaproteobacteria;unclassified Betaproteobacteria;unclassified Betaproteobacteria;unclassified Betaproteobacteria;uncultured beta proteobacterium;unclassified betaproteobacterium ENR4 |
| Ga0586324 8209974 3 503 | 494750.00 | 0.00 | 0.00 | 0.00 | 0.00 | 0.00 | 0.00 | 0.00 | 0.00 | 0.00 | 0.00 | 0.00 | KO:K04077 | chaperonin GroEL | Bacteria;Verrucomicrobia;Verrucomicrobiae;Verrucomicrobiales;Verrucomicrobia subdivision 3;Pedosphaera;Pedosphaera parvula;Pedosphaera parvula Ellin514 |
| Ga0586324 8217539 3 503 | 0.00 | 0.00 | 0.00 | 0.00 | 0.00 | 0.00 | 0.00 | 0.00 | 0.00 | 0.00 | 0.00 | 274063.50 | KO:K04077 | chaperonin GroEL | Bacteria;Acidobacteria;Acidobacteriia;Acidobacteriales;Acidobacteriaceae;Candidatus Koribacter;Candidatus Koribacter versatilis;Candidatus Koribacter versatilis Ellin345 |
| Ga0586324 8221951 3 503 | 0.00 | 0.00 | 0.00 | 0.00 | 0.00 | 0.00 | 0.00 | 0.00 | 0.00 | 1747900.00 | 0.00 | 0.00 | KO:K04077 | chaperonin GroEL | Bacteria;Proteobacteria;Deltaproteobacteria;Syntrophobacterales;Syntrophaceae;Desulfobacca;Desulfobacca acetoxidans;Desulfobacca acetoxidans ASRB2, DSM 11109 |
| Ga0586324 8233794 2 499 | 0.00 | 0.00 | 0.00 | 579883.80 | 0.00 | 793825.32 | 0.00 | 0.00 | 0.00 | 0.00 | 0.00 | 0.00 | KO:K04077 | chaperonin GroEL | Bacteria;Proteobacteria;Deltaproteobacteria;Desulfuromonadales;Geobacteraceae;Geobacter;Geobacter sp. OR-1;Geobacter sp. OR-1 |
| Ga0586324 8234695 2 502 | 0.00 | 0.00 | 4094394.98 | 0.00 | 0.00 | 0.00 | 0.00 | 9455000.00 | 0.00 | 8767930.71 | 0.00 | 8700000.00 | KO:K04077 | chaperonin GroEL | Bacteria;Candidatus Aminicenantes;unclassified Candidatus Aminicenantes;unclassified Candidatus Aminicenantes;unclassified Candidatus Aminicenantes;unclassified Candidatus Aminicenantes;Aminicenantes bacterium SCGC AAA255-E10;Aminicenantes bacterium SCGC AAA255-E10 (contamination screened) |
| Ga0586324 8235524 3 500 | 0.00 | 0.00 | 0.00 | 0.00 | 0.00 | 0.00 | 0.00 | 37405.63 | 0.00 | 0.00 | 0.00 | 15975000.00 | KO:K04077 | chaperonin GroEL | Bacteria;Proteobacteria;Deltaproteobacteria;Desulfobacterales;Desulfobacteraceae;Desulfatiglans;Desulfatiglans anilini;Desulfatiglans anilini DSM 4660 |
| Ga0586324 8263120 3 500 | 0.00 | 0.00 | 0.00 | 0.00 | 0.00 | 0.00 | 0.00 | 0.00 | 0.00 | 707968.80 | 0.00 | 0.00 | KO:K04077 | chaperonin GroEL | Bacteria;Proteobacteria;Deltaproteobacteria;Desulfuromonadales;Desulfuromonadaceae;Desulfuromonas;Desulfuromonas sp. TF;Desulfuromonas sp. TF |
| Ga0586324 8301710 3 500 | 0.00 | 0.00 | 0.00 | 1225500.00 | 0.00 | 865515.58 | 6612000.00 | 0.00 | 0.00 | 4150300.00 | 0.00 | 5002500.00 | KO:K04077 | chaperonin GroEL | Bacteria;Proteobacteria;Alphaproteobacteria;Rhodospirillales;Elioraeaceae;Elioraea;Elioraea tepidiphila;Elioraea tepidiphila DSM 17972 |
| Ga0586324 8311849 1 498 | 0.00 | 4678400.00 | 542169.00 | 0.00 | 0.00 | 393690.61 | 0.00 | 0.00 | 0.00 | 0.00 | 0.00 | 0.00 | KO:K04077 | chaperonin GroEL | Bacteria;Planctomycetes;Planctomycetia;Planctomycetales;Planctomycetaceae;Planctomyces;Planctomyces sp. SH-PL14;Planctomyces sp. SH-PL14 |
| Ga0586324 8318259 3 500 | 0.00 | 0.00 | 0.00 | 0.00 | 0.00 | 0.00 | 1781804.04 | 0.00 | 0.00 | 0.00 | 0.00 | 1987500.00 | KO:K04077 | chaperonin GroEL | Bacteria;Spirochaetes;Spirochaetia;Spirochaetales;Treponemataceae;Treponema;Treponema caldarium;Treponema caldarium DSM 7334 |

| | | | | | | | | | | | | | | | |
|---|---|---|---|---|---|---|---|---|---|---|---|---|---|---|---|
| Ga0586324 8318449 1 498 | 0.00 | 0.00 | 0.00 | 1282500.00 | 0.00 | 0.00 | 0.00 | 0.00 | 0.00 | 0.00 | 0.00 | 0.00 | KO:K04077 | chaperonin GroEL | Bacteria;Proteobacteria;Deltaproteobacteria;Desulfobacterales;Desulfobacteraceae;Desulfatiglans;Desulfatiglans anilini;Desulfatiglans anilini DSM 4660 |
| Ga0586324 8327149 2 499 | 0.00 | 0.00 | 0.00 | 1330000.00 | 6887969.47 | 0.00 | 0.00 | 0.00 | 0.00 | 0.00 | 477605.92 | 0.00 | KO:K04077 | chaperonin GroEL | Bacteria;Proteobacteria;Deltaproteobacteria;Desulfobacterales;Desulfobacteraceae;Desulfatitalea;Desulfatitalea tepidiphila;Desulfatitalea tepidiphila S28bF |
| Ga0586324 8336586 3 500 | 0.00 | 6086000.00 | 969547.93 | 23541000.00 | 18004400.00 | 1368971.13 | 5979644.28 | 20618000.00 | 20646900.00 | 22214500.00 | 20354400.00 | 20077500.00 | KO:K04077 | chaperonin GroEL | Bacteria;Proteobacteria;Deltaproteobacteria;Desulfuromonadales;Geobacteraceae;Geoalkalibacter;Geoalkalibacter subterraneus;Geoalkalibacter subterraneus Red1 |
| Ga0586324 8343122 1 498 | 0.00 | 0.00 | 0.00 | 0.00 | 0.00 | 0.00 | 0.00 | 2098400.00 | 1093500.00 | 0.00 | 0.00 | 714640.50 | KO:K04077 | chaperonin GroEL | Bacteria;Chloroflexi;unclassified Chloroflexi;unclassified Chloroflexi;unclassified Chloroflexi;unclassified Chloroflexi;Chloroflexi bacterium T81;Chloroflexi bacterium T81 |
| Ga0586324 0000709 27603 27890 | 0.00 | 0.00 | 0.00 | 0.00 | 0.00 | 0.00 | 76836000.00 | 0.00 | 0.00 | 0.00 | 0.00 | 0.00 | KO:K04078 | chaperonin GroES | Bacteria;Acidobacteria;Acidobacteriia;Bryobacterales;Solibacteraceae;Candidatus Solibacter;Candidatus Solibacter usitatus;Candidatus Solibacter usitatus Ellin6076 |
| Ga0586324 0028606 161 517 | 2520000.00 | 972400.00 | 0.00 | 0.00 | 0.00 | 0.00 | 0.00 | 1976400.00 | 1514700.00 | 2032800.00 | 3713600.00 | 0.00 | KO:K04078 | chaperonin GroES | Bacteria;Acidobacteria;Acidobacteriia;Bryobacterales;Solibacteraceae;Candidatus Solibacter;Candidatus Solibacter usitatus;Candidatus Solibacter usitatus Ellin6076 |
| Ga0586324 0030188 2979 3287 | 0.00 | 0.00 | 0.00 | 0.00 | 0.00 | 0.00 | 0.00 | 0.00 | 5175900.00 | 0.00 | 0.00 | 7470000.00 | KO:K04078 | chaperonin GroES | Bacteria;Nitrospirae;Nitrospira;Nitrospirales;Nitrospiraceae;Nitrospira;Nitrospira moscoviensis;Nitrospira moscoviensis NSP M-1 |
| Ga0586324 0035645 3001 3294 | 0.00 | 0.00 | 0.00 | 12635000.00 | 0.00 | 0.00 | 0.00 | 0.00 | 0.00 | 0.00 | 0.00 | 0.00 | KO:K04078 | chaperonin GroES | Bacteria;Proteobacteria;Deltaproteobacteria;Syntrophobacterales;Syntrophaceae;Syntrophus;Syntrophus gentianae;Syntrophus gentianae DSM 8423 |
| Ga0586324 0150273 2075 2392 | 0.00 | 340431.12 | 0.00 | 0.00 | 0.00 | 245166.16 | 0.00 | 0.00 | 232723.53 | 0.00 | 0.00 | 0.00 | KO:K04078 | chaperonin GroES | Bacteria;Proteobacteria;Alphaproteobacteria;Hyphomicrobiales;Boseaceae;Bosea;Bosea sp. LC85;Bosea sp. LC85 |
| Ga0586324 0216662 1474 1791 | 3060000.00 | 0.00 | 0.00 | 0.00 | 0.00 | 0.00 | 0.00 | 0.00 | 0.00 | 0.00 | 0.00 | 0.00 | KO:K04078 | chaperonin GroES | Bacteria;Proteobacteria;Alphaproteobacteria;Hyphomicrobiales;Bradyrhizobiaceae;Variibacter;Variibacter gotjawalensis;Variibacter gotjawalensis GJW-30 |
| Ga0586324 0268820 1804 2124 | 10200000.00 | 12920000.00 | 0.00 | 0.00 | 0.00 | 0.00 | 0.00 | 0.00 | 0.00 | 0.00 | 0.00 | 0.00 | KO:K04078 | chaperonin GroES | Bacteria;Proteobacteria;Alphaproteobacteria;Rhodospirillales;Acetobacteraceae;Rhodopila;unclassified Rhodopila;Rhodopila sp. LVNP |
| Ga0586324 0355604 95 409 | 0.00 | 0.00 | 655574.09 | 0.00 | 0.00 | 932529.18 | 0.00 | 0.00 | 0.00 | 916300.00 | 0.00 | 0.00 | KO:K04078 | chaperonin GroES | Bacteria;Proteobacteria;Deltaproteobacteria;unclassified Deltaproteobacteria;unclassified Deltaproteobacteria;Dissulfuribacter;Dissulfuribacter thermophilus;Dissulfuribacter thermophilus S69 |
| Ga0586324 0445115 1065 1427 | 0.00 | 0.00 | 0.00 | 0.00 | 0.00 | 0.00 | 0.00 | 0.00 | 0.00 | 555519.58 | 0.00 | 0.00 | KO:K04078 | chaperonin GroES | Bacteria;Proteobacteria;Alphaproteobacteria;Hyphomicrobiales;Hyphomicrobiaceae;Rhodoplanes;Rhodoplanes sp. Z2-YC6860;Rhodoplanes sp. Z2-YC6860 |
| Ga0586324 0566822 551 844 | 0.00 | 0.00 | 0.00 | 0.00 | 0.00 | 4327300.00 | 0.00 | 0.00 | 0.00 | 0.00 | 0.00 | 0.00 | KO:K04078 | chaperonin GroES | Bacteria;Verrucomicrobia;Spartobacteria;unclassified Spartobacteria;unclassified Spartobacteria;unclassified Spartobacteria;Spartobacteria bacterium LR76;Spartobacteria bacterium LR76 |
| Ga0586324 0660567 421 735 | 830820.00 | 0.00 | 0.00 | 88160000.00 | 109180000.00 | 1049374.22 | 1663200.72 | 459296.45 | 533846.70 | 41888000.00 | 2270400.00 | 1815000.00 | KO:K04078 | chaperonin GroES | Bacteria;Proteobacteria;Alphaproteobacteria;Hyphomicrobiales;Bradyrhizobiaceae;Bradyrhizobium;Bradyrhizobium japonicum;Bradyrhizobium japonicum USDA 6 |
| Ga0586324 0775198 719 1006 | 0.00 | 0.00 | 0.00 | 0.00 | 323274.77 | 0.00 | 0.00 | 182390000.00 | 0.00 | 0.00 | 0.00 | 0.00 | KO:K04078 | chaperonin GroES | Bacteria;Thermodesulfobacteria;Thermodesulfobacteria;Thermodesulfobacteriales;Thermodesulfobacteriaceae;Thermosulfuriphilus;Thermosulfuriphilus ammonigenes;Thermosulfuriphilus ammonigenes DSM 102941 |
| Ga0586324 0783844 214 528 | 0.00 | 0.00 | 0.00 | 0.00 | 0.00 | 0.00 | 0.00 | 0.00 | 1312200.00 | 0.00 | 0.00 | 0.00 | KO:K04078 | chaperonin GroES | Bacteria;Proteobacteria;Alphaproteobacteria;Hyphomicrobiales;unclassified Hyphomicrobiales;unclassified Hyphomicrobiales;Rhizobiales bacterium GAS113;Rhizobiales sp. GAS113 |
| Ga0586324 0846075 700 1035 | 0.00 | 0.00 | 0.00 | 4189500.00 | 4089100.00 | 0.00 | 0.00 | 0.00 | 0.00 | 0.00 | 1214400.00 | 0.00 | KO:K04078 | chaperonin GroES | Bacteria;Chloroflexi;Anaerolineae;Anaerolineales;Anaerolineaceae;Levilinea;Levilinea saccharolytica;Levilinea saccharolytica DSM 16555 |
| Ga0586324 1476271 583 879 | 0.00 | 0.00 | 1594562.38 | 0.00 | 0.00 | 0.00 | 0.00 | 0.00 | 0.00 | 0.00 | 0.00 | 0.00 | KO:K04078 | chaperonin GroES | Bacteria;Acidobacteria;Thermoanaerobaculia;Thermoanaerobaculales;Thermoanaerobaculaceae;Thermoanaerobaculum;Thermoanaerobaculum aquaticum;Thermoanaerobaculum aquaticum MP-01 |
| Ga0586324 1665999 55 372 | 895955.00 | 952000.00 | 0.00 | 0.00 | 0.00 | 813496.67 | 864550.92 | 539672.49 | 0.00 | 0.00 | 860474.56 | 1230000.00 | KO:K04078 | chaperonin GroES | Bacteria;Proteobacteria;Alphaproteobacteria;Hyphomicrobiales;Bradyrhizobiaceae;Bradyrhizobium;Bradyrhizobium erythrophlei;Bradyrhizobium erythrophlei GAS401 |
| Ga0586324 2370034 571 840 | 0.00 | 40596000.00 | 0.00 | 0.00 | 0.00 | 0.00 | 0.00 | 41724000.00 | 0.00 | 0.00 | 0.00 | 34350000.00 | KO:K04078 | chaperonin GroES | Bacteria;Verrucomicrobia;Verrucomicrobiae;Verrucomicrobiales;Verrucomicrobia subdivision 3;Pedosphaera;Pedosphaera parvula;Pedosphaera parvula Ellin514 |
| Ga0586324 2437204 422 709 | 0.00 | 15436000.00 | 0.00 | 0.00 | 38419000.00 | 48434000.00 | 0.00 | 0.00 | 0.00 | 0.00 | 0.00 | 0.00 | KO:K04078 | chaperonin GroES | Bacteria;Proteobacteria;Deltaproteobacteria;Syntrophobacterales;Syntrophobacteraceae;Desulfacinum;Desulfacinum infernum;Desulfacinum infernum DSM 9756 |
| Ga0586324 2622444 348 665 | 0.00 | 0.00 | 0.00 | 0.00 | 0.00 | 3712799.58 | 0.00 | 0.00 | 0.00 | 0.00 | 0.00 | 0.00 | KO:K04078 | chaperonin GroES | Bacteria;Proteobacteria;Alphaproteobacteria;Hyphomicrobiales;Bradyrhizobiaceae;unclassified Bradyrhizobiaceae;Bradyrhizobiaceae sp. OAS957;Bradyrhizobiaceae sp. OAS957 |
| Ga0586324 2626729 108 440 | 0.00 | 0.00 | 0.00 | 4512500.00 | 4243600.00 | 4803700.00 | 0.00 | 933300.00 | 0.00 | 0.00 | 0.00 | 1050000.00 | KO:K04078 | chaperonin GroES | Bacteria;Proteobacteria;Deltaproteobacteria;Syntrophobacterales;Syntrophobacteraceae;Syntrophobacter;Syntrophobacter fumaroxidans;Syntrophobacter fumaroxidans MPOB |
| Ga0586324 3097086 267 584 | 0.00 | 0.00 | 0.00 | 0.00 | 0.00 | 0.00 | 0.00 | 0.00 | 0.00 | 0.00 | 0.00 | 3547500.00 | KO:K04078 | chaperonin GroES | Bacteria;Proteobacteria;Alphaproteobacteria;Hyphomicrobiales;Xanthobacteraceae;Pseudolabrys;Pseudolabrys sp. Root1462;Pseudolabrys sp. Root1462 |
| Ga0586324 3310491 474 731 | 0.00 | 0.00 | 0.00 | 0.00 | 12566000.00 | 5121300.00 | 0.00 | 6466000.00 | 0.00 | 0.00 | 0.00 | 0.00 | KO:K04078 | chaperonin GroES | Bacteria;Proteobacteria;Alphaproteobacteria;Hyphomicrobiales;Hyphomicrobiaceae;Hyphomicrobium;Hyphomicrobium sp. CS1BSMeth3;Hyphomicrobium sp. CS1BSMeth3 |
| Ga0586324 3548584 280 591 | 0.00 | 123760000.00 | 502916.64 | 0.00 | 46762000.00 | 252083.88 | 0.00 | 0.00 | 0.00 | 0.00 | 0.00 | 0.00 | KO:K04078 | chaperonin GroES | Bacteria;Proteobacteria;Deltaproteobacteria;Myxococcales;Polyangiaceae;Chondromyces;Chondromyces apiculatus;Chondromyces apiculatus DSM 436 |
| Ga0586324 3645227 3 227 | 0.00 | 0.00 | 0.00 | 0.00 | 0.00 | 0.00 | 0.00 | 0.00 | 0.00 | 0.00 | 1980000.00 | 0.00 | KO:K04078 | chaperonin GroES | Bacteria;Acidobacteria;Thermoanaerobaculia;Thermoanaerobaculales;Thermoanaerobaculaceae;Thermoanaerobaculum;Thermoanaerobaculum aquaticum;Thermoanaerobaculum aquaticum MP-01 |
| Ga0586324 3664227 209 526 | 0.00 | 0.00 | 0.00 | 226100000.00 | 0.00 | 0.00 | 0.00 | 0.00 | 0.00 | 0.00 | 0.00 | 0.00 | KO:K04078 | chaperonin GroES | Bacteria;Proteobacteria;Alphaproteobacteria;Rhodospirillales;unclassified Rhodospirillales;Enhydrobacter;Enhydrobacter aerosaccus;Enhydrobacter aerosaccus ATCC 27094 |
| Ga0586324 3953440 190 480 | 0.00 | 0.00 | 0.00 | 0.00 | 0.00 | 3234001.70 | 0.00 | 0.00 | 0.00 | 0.00 | 0.00 | 0.00 | KO:K04078 | chaperonin GroES | Bacteria;Proteobacteria;Deltaproteobacteria;Syntrophobacterales;Syntrophobacteraceae;Desulfoglaeba;Desulfoglaeba alkanexedens;Desulfoglaeba alkanexedens ALDC |
| Ga0586324 4630004 187 474 | 3980000.00 | 0.00 | 0.00 | 0.00 | 0.00 | 0.00 | 0.00 | 0.00 | 0.00 | 0.00 | 0.00 | 0.00 | KO:K04078 | chaperonin GroES | Bacteria;Proteobacteria;Deltaproteobacteria;unclassified Deltaproteobacteria;unclassified Deltaproteobacteria;Dissulfuribacter;Dissulfuribacter thermophilus;Dissulfuribacter thermophilus S69 |
| Ga0586324 5406666 267 557 | 0.00 | 0.00 | 0.00 | 0.00 | 734696.94 | 0.00 | 0.00 | 0.00 | 0.00 | 0.00 | 0.00 | 0.00 | KO:K04078 | chaperonin GroES | Bacteria;Proteobacteria;Deltaproteobacteria;Myxococcales;Anaeromyxobacteraceae;Anaeromyxobacter;Anaeromyxobacter dehalogenans;Anaeromyxobacter dehalogenans 2CP-C |
| Ga0586324 5554863 84 485 | 0.00 | 0.00 | 0.00 | 0.00 | 0.00 | 0.00 | 0.00 | 0.00 | 0.00 | 0.00 | 10824000.00 | 0.00 | KO:K04078 | chaperonin GroES | Bacteria;Acidobacteria;Vicinamibacteria;unclassified Vicinamibacteria;Vicinamibacteraceae;Luteitalea;Luteitalea pratensis;Luteitalea pratensis DSM 100886 |
| Ga0586324 5762313 263 583 | 0.00 | 0.00 | 0.00 | 0.00 | 0.00 | 0.00 | 0.00 | 5557100.00 | 0.00 | 1193500.00 | 1425600.00 | 0.00 | KO:K04078 | chaperonin GroES | Bacteria;Proteobacteria;Alphaproteobacteria;Hyphomicrobiales;Phyllobacteriaceae;Mesorhizobium;Mesorhizobium sp. YR577;Mesorhizobium sp. YR577 |
| Ga0586324 6843315 171 461 | 0.00 | 0.00 | 0.00 | 0.00 | 0.00 | 22629000.00 | 0.00 | 0.00 | 0.00 | 0.00 | 0.00 | 0.00 | KO:K04078 | chaperonin GroES | Bacteria;Proteobacteria;Deltaproteobacteria;Syntrophobacterales;Syntrophaceae;Desulfobacca;Desulfobacca acetoxidans;Desulfobacca acetoxidans ASRB2, DSM 11109 |
| Ga0586324 7867390 2 376 | 0.00 | 0.00 | 0.00 | 0.00 | 0.00 | 8892800.00 | 0.00 | 0.00 | 0.00 | 0.00 | 0.00 | 0.00 | KO:K04078 | chaperonin GroES | Bacteria;Proteobacteria;Deltaproteobacteria;Desulfobacterales;Desulfobacteraceae;Desulfococcus;Desulfococcus oleovorans;Desulfococcus oleovorans Hxd3 |
| Ga0586324 0007904 755 2692 | 0.00 | 0.00 | 0.00 | 0.00 | 0.00 | 0.00 | 8595600.00 | 0.00 | 1668600.00 | 6352500.00 | 0.00 | 3007500.00 | KO:K04079 | molecular chaperone HtpG | Bacteria;Proteobacteria;Betaproteobacteria;Burkholderiales;Oxalobacteraceae;Noviherbaspirillum;Noviherbaspirillum humi;Noviherbaspirillum humi U15 |
| Ga0586324 0008820 3598 5643 | 0.00 | 0.00 | 0.00 | 3743000.00 | 0.00 | 0.00 | 0.00 | 0.00 | 0.00 | 0.00 | 0.00 | 0.00 | KO:K04079 | molecular chaperone HtpG | Bacteria;Bacteroidetes;Bacteroidia;Marinilabiliales;Marinilabiliaceae;Alkalitalea;Alkalitalea saponilacus;Alkalitalea saponilacus DSM 24412 |
| Ga0586324 0067629 2557 4134 | 3020000.00 | 0.00 | 0.00 | 0.00 | 0.00 | 0.00 | 0.00 | 0.00 | 0.00 | 0.00 | 0.00 | 0.00 | KO:K04079 | molecular chaperone HtpG | Bacteria;Proteobacteria;Betaproteobacteria;Nitrosomonadales;Thiobacillaceae;Thiobacillus;Thiobacillus denitrificans;Thiobacillus denitrificans ATCC 25259 |
| Ga0586324 0084717 2 925 | 1450000.00 | 3175600.00 | 11541500.00 | 0.00 | 0.00 | 0.00 | 23592468.72 | 16226000.00 | 7857000.00 | 13244000.00 | 14344000.00 | 5850000.00 | KO:K04079 | molecular chaperone HtpG | Bacteria;Proteobacteria;Gammaproteobacteria;Methylococcales;Methylococcaceae;Methylobacter;Methylobacter tundripaludum;Methylobacter tundripaludum 31/32 |
| Ga0586324 0750919 339 1373 | 0.00 | 0.00 | 0.00 | 0.00 | 0.00 | 0.00 | 0.00 | 0.00 | 0.00 | 0.00 | 0.00 | 8850000.00 | KO:K04079 | molecular chaperone HtpG | Bacteria;Proteobacteria;Betaproteobacteria;Nitrosomonadales;Thiobacillaceae;Thiobacillus;Thiobacillus denitrificans;Thiobacillus denitrificans ATCC 25259 |
| Ga0586324 0787744 230 1345 | 0.00 | 0.00 | 450517.10 | 0.00 | 0.00 | 0.00 | 0.00 | 0.00 | 0.00 | 0.00 | 0.00 | 0.00 | KO:K04079 | molecular chaperone HtpG | Bacteria;Proteobacteria;Gammaproteobacteria;Methylococcales;Methylococcaceae;Methylobacter;Methylobacter tundripaludum;Methylobacter tundripaludum SV96 |
| Ga0586324 1410941 421 1047 | 0.00 | 0.00 | 0.00 | 14725000.00 | 0.00 | 0.00 | 0.00 | 6771000.00 | 0.00 | 0.00 | 0.00 | 0.00 | KO:K04079 | molecular chaperone HtpG | Bacteria;Proteobacteria;Deltaproteobacteria;Desulfobacterales;Desulfobacteraceae;Desulfatirhabdium;Desulfatirhabdium butyrativorans;Desulfatirhabdium butyrativorans DSM 18734 |
| Ga0586324 1496025 3 1016 | 0.00 | 0.00 | 0.00 | 0.00 | 0.00 | 0.00 | 0.00 | 2702300.00 | 0.00 | 0.00 | 0.00 | 0.00 | KO:K04079 | molecular chaperone HtpG | Bacteria;Proteobacteria;Betaproteobacteria;Nitrosomonadales;Sulfuricellaceae;Sulfuricella;Sulfuricella sp. T08;Sulfuricella sp. T08 |
| Ga0586324 1542583 422 1009 | 2040000.00 | 2305200.00 | 27530700.00 | 0.00 | 0.00 | 0.00 | 0.00 | 0.00 | 9801000.00 | 0.00 | 0.00 | 0.00 | KO:K04079 | molecular chaperone HtpG | Bacteria;Proteobacteria;Gammaproteobacteria;Methylococcales;Methylococcaceae;Methylobacter;Methylobacter tundripaludum;Methylobacter tundripaludum OWC-G53F |
| Ga0586324 3630403 2 703 | 0.00 | 0.00 | 0.00 | 10260000.00 | 0.00 | 0.00 | 0.00 | 0.00 | 0.00 | 0.00 | 0.00 | 0.00 | KO:K04079 | molecular chaperone HtpG | Bacteria;Proteobacteria;Gammaproteobacteria;Nevskiales;Steroidobacteraceae;Povalibacter;Povalibacter uvarum;Povalibacter uvarum DSM 26723 |
| Ga0586324 4239470 2 661 | 0.00 | 2420800.00 | 0.00 | 0.00 | 8034000.00 | 0.00 | 2325600.00 | 3885700.00 | 0.00 | 3003000.00 | 1795200.00 | 0.00 | KO:K04079 | molecular chaperone HtpG | Bacteria;Proteobacteria;Deltaproteobacteria;Desulfuromonadales;Geobacteraceae;Geobacter;Geobacter sp. DSM 2909;Geobacter sp. DSM 2909 |
| Ga0586324 4361929 2 652 | 0.00 | 0.00 | 0.00 | 0.00 | 0.00 | 0.00 | 0.00 | 0.00 | 0.00 | 0.00 | 0.00 | 421080.75 | KO:K04079 | molecular chaperone HtpG | Bacteria;Acidobacteria;Holophagae;Holophagales;Holophagaceae;Holophaga;Holophaga foetida;Holophaga foetida TMBS4, DSM 6591 |
| Ga0586324 5219447 45 605 | 0.00 | 7888000.00 | 0.00 | 0.00 | 0.00 | 0.00 | 0.00 | 0.00 | 0.00 | 0.00 | 0.00 | 0.00 | KO:K04079 | molecular chaperone HtpG | Bacteria;Proteobacteria;Deltaproteobacteria;Syntrophorhabdales;Syntrophorhabdaceae;Syntrophorhabdus;Syntrophorhabdus aromaticivorans;Syntrophorhabdus aromaticivorans UI |
| Ga0586324 6832212 3 542 | 0.00 | 0.00 | 0.00 | 0.00 | 0.00 | 5121300.00 | 0.00 | 0.00 | 0.00 | 0.00 | 0.00 | 0.00 | KO:K04079 | molecular chaperone HtpG | Bacteria;Proteobacteria;Alphaproteobacteria;Hyphomicrobiales;Bradyrhizobiaceae;Bradyrhizobium;Bradyrhizobium sp. ARR65;Bradyrhizobium sp. ARR65 |
| Ga0586324 7214041 2 529 | 2630000.00 | 2692800.00 | 1117560.63 | 4313000.00 | 0.00 | 0.00 | 5836800.00 | 2610800.00 | 5653800.00 | 7438200.00 | 9627200.00 | 8467500.00 | KO:K04079 | molecular chaperone HtpG | Bacteria;Proteobacteria;Gammaproteobacteria;Methylococcales;Methylococcaceae;Methylosarcina;Methylosarcina fibrata;Methylosarcina fibrata AML-C10 |
| Ga0586324 7467836 3 521 | 0.00 | 0.00 | 15369900.00 | 0.00 | 0.00 | 0.00 | 0.00 | 0.00 | 0.00 | 0.00 | 0.00 | 6247500.00 | KO:K04079 | molecular chaperone HtpG | Bacteria;Proteobacteria;Gammaproteobacteria;Methylococcales;Methylococcaceae;Methylomicrobium;Methylomicrobium lacus;Methylosarcina lacus LW14 |
| Ga0586324 7586862 2 520 | 2600000.00 | 0.00 | 0.00 | 0.00 | 0.00 | 0.00 | 0.00 | 0.00 | 0.00 | 0.00 | 0.00 | 0.00 | KO:K04079 | molecular chaperone HtpG | Bacteria;Proteobacteria;Gammaproteobacteria;Methylococcales;Methylococcaceae;Methylobacter;Methylobacter sp. BBA5.1;Methylobacter sp. BBA5.1 |
| Ga0586324 2734746 3 419 | 0.00 | 578677.28 | 0.00 | 0.00 | 624035.80 | 0.00 | 3260400.00 | 0.00 | 256851.00 | 2102100.00 | 0.00 | 1537528.50 | KO:K04080 | molecular chaperone IbpA | Bacteria;Proteobacteria;Gammaproteobacteria;Chromatiales;Chromatiaceae;Thiocystis;Thiocystis violascens;Thiocystis violascens 611, DSM 198 |
| Ga0586324 6573062 299 550 | 0.00 | 0.00 | 0.00 | 0.00 | 0.00 | 0.00 | 0.00 | 0.00 | 0.00 | 0.00 | 16632000.00 | 0.00 | KO:K04080 | molecular chaperone IbpA | Bacteria;Proteobacteria;Gammaproteobacteria;Chromatiales;Chromatiaceae;Lamprocystis;Lamprocystis purpurea;Lamprocystis purpurea DSM 4197 |
| Ga0586324 0003450 2051 2278 | 0.00 | 0.00 | 0.00 | 0.00 | 0.00 | 0.00 | 0.00 | 7259000.00 | 4981500.00 | 0.00 | 0.00 | 0.00 | KO:K04085 | tRNA 2-thiouridine synthesizing protein A | Bacteria;Proteobacteria;Betaproteobacteria;Nitrosomonadales;Thiobacillaceae;Thiobacillus;Thiobacillus thioparus;Thiobacillus thioparus DSM 505 |
| Ga0586324 0125566 1997 2224 | 0.00 | 0.00 | 0.00 | 0.00 | 0.00 | 0.00 | 0.00 | 0.00 | 767019.78 | 0.00 | 0.00 | 0.00 | KO:K04085 | tRNA 2-thiouridine synthesizing protein A | Bacteria;Proteobacteria;Betaproteobacteria;Nitrosomonadales;Thiobacillaceae;Thiobacillus;Thiobacillus thiophilus;Thiobacillus thiophilus DSM 19892 |
| Ga0586324 0000002 98969 99847 | 0.00 | 0.00 | 0.00 | 0.00 | 0.00 | 0.00 | 6361200.00 | 4483500.00 | 0.00 | 0.00 | 8008000.00 | 4500000.00 | KO:K04087 | membrane protease subunit HflC | Bacteria;Proteobacteria;Betaproteobacteria;Nitrosomonadales;Thiobacillaceae;Annwoodia;Annwoodia aquaesulis;Annwoodia aquaesulis DSM 4255 |
| Ga0586324 0002538 3951 4895 | 0.00 | 2250800.00 | 0.00 | 2061500.00 | 0.00 | 0.00 | 0.00 | 0.00 | 0.00 | 0.00 | 0.00 | 0.00 | KO:K04087 | membrane protease subunit HflC | Bacteria;Proteobacteria;Deltaproteobacteria;Desulfobacterales;Desulfobacteraceae;Desulfatirhabdium;Desulfatirhabdium butyrativorans;Desulfatirhabdium butyrativorans DSM 18734 |
| Ga0586324 0017662 5620 6474 | 0.00 | 0.00 | 0.00 | 0.00 | 0.00 | 0.00 | 0.00 | 1287100.00 | 0.00 | 0.00 | 0.00 | 772500.00 | KO:K04087 | membrane protease subunit HflC | Bacteria;Proteobacteria;Gammaproteobacteria;Methylococcales;Methylococcaceae;Methylobacter;Methylobacter tundripaludum;Methylobacter tundripaludum SV96 |
| Ga0586324 0045143 3141 4052 | 0.00 | 0.00 | 0.00 | 0.00 | 0.00 | 0.00 | 0.00 | 0.00 | 1757700.00 | 0.00 | 0.00 | 0.00 | KO:K04087 | membrane protease subunit HflC | Bacteria;Proteobacteria;Alphaproteobacteria;Hyphomicrobiales;Bradyrhizobiaceae;Bradyrhizobium;Bradyrhizobium lablabi;Bradyrhizobium lablabi GAS499 |
| Ga0586324 0982345 208 1128 | 0.00 | 0.00 | 0.00 | 0.00 | 0.00 | 2468021.96 | 0.00 | 0.00 | 0.00 | 0.00 | 0.00 | 0.00 | KO:K04087 | membrane protease subunit HflC | Bacteria;Proteobacteria;Alphaproteobacteria;Hyphomicrobiales;Xanthobacteraceae;Pseudolabrys;Pseudolabrys sp. Root1462;Pseudolabrys sp. Root1462 |
| Ga0586324 0000002 99844 101025 | 0.00 | 0.00 | 0.00 | 0.00 | 0.00 | 0.00 | 0.00 | 0.00 | 1077300.00 | 0.00 | 0.00 | 4005000.00 | KO:K04088 | membrane protease subunit HflK | Bacteria;Proteobacteria;Betaproteobacteria;Nitrosomonadales;Thiobacillaceae;Annwoodia;Annwoodia aquaesulis;Annwoodia aquaesulis DSM 4255 |
| Ga0586324 0022042 4231 5424 | 0.00 | 0.00 | 0.00 | 0.00 | 0.00 | 0.00 | 0.00 | 969900.00 | 1466100.00 | 0.00 | 0.00 | 0.00 | KO:K04088 | membrane protease subunit HflK | Bacteria;Proteobacteria;Betaproteobacteria;Nitrosomonadales;Thiobacillaceae;Thiobacillus;Thiobacillus thioparus;Thiobacillus thioparus DSM 505 |
| Ga0586324 0043334 812 2011 | 0.00 | 0.00 | 0.00 | 0.00 | 0.00 | 0.00 | 0.00 | 0.00 | 0.00 | 0.00 | 0.00 | 1260000.00 | KO:K04088 | membrane protease subunit HflK | Bacteria;Proteobacteria;Betaproteobacteria;Nitrosomonadales;Gallionellaceae;Gallionella;Gallionella capsiferriformans;Gallionella capsiferriformans ES-2 |
| Ga0586324 0049929 2440 3669 | 0.00 | 0.00 | 0.00 | 0.00 | 0.00 | 0.00 | 0.00 | 0.00 | 0.00 | 0.00 | 0.00 | 1267500.00 | KO:K04088 | membrane protease subunit HflK | Bacteria;Proteobacteria;Betaproteobacteria;Nitrosomonadales;Thiobacillaceae;Sulfuritortus;Sulfuritortus calidifontis;Sulfuritortus calidifontis DSM 103923 |
| Ga0586324 0191051 218 1432 | 0.00 | 0.00 | 0.00 | 0.00 | 0.00 | 0.00 | 0.00 | 1311500.00 | 0.00 | 0.00 | 0.00 | 0.00 | KO:K04088 | membrane protease subunit HflK | Bacteria;Proteobacteria;Betaproteobacteria;Nitrosomonadales;Nitrosomonadaceae;Nitrosovibrio;Nitrosovibrio sp. Nv17;Nitrosovibrio sp. Nv17 |
| Ga0586324 0197417 1103 2398 | 0.00 | 0.00 | 0.00 | 0.00 | 0.00 | 0.00 | 3420000.00 | 0.00 | 508440.24 | 0.00 | 0.00 | 0.00 | KO:K04088 | membrane protease subunit HflK | Bacteria;Proteobacteria;Betaproteobacteria;Rhodocyclales;Azonexaceae;Dechloromonas;Dechloromonas denitrificans;Dechloromonas denitrificans ATCC BAA-841 |
| Ga0586324 3589602 3 572 | 0.00 | 0.00 | 0.00 | 0.00 | 0.00 | 0.00 | 0.00 | 0.00 | 0.00 | 0.00 | 0.00 | 1860000.00 | KO:K04088 | membrane protease subunit HflK | Bacteria;Proteobacteria;Betaproteobacteria;Nitrosomonadales;Thiobacillaceae;Annwoodia;Annwoodia aquaesulis;Annwoodia aquaesulis DSM 4255 |
| Ga0586324 3898795 1 684 | 0.00 | 0.00 | 0.00 | 1130500.00 | 1328700.00 | 0.00 | 0.00 | 0.00 | 0.00 | 0.00 | 0.00 | 0.00 | KO:K04088 | membrane protease subunit HflK | Bacteria;Spirochaetes;Spirochaetia;Spirochaetales;Spirochaetaceae;Spirochaeta;Spirochaeta thermophila;Spirochaeta thermophila DSM 6192 |
| Ga0586324 0069164 1 315 | 35600000.00 | 28696000.00 | 92895000.00 | 106400000.00 | 0.00 | 0.00 | 0.00 | 0.00 | 0.00 | 0.00 | 0.00 | 0.00 | KO:K04480 | methanol--5-hydroxybenzimidazolylcobamide Co-methyltransferase | Archaea;Candidatus Thermoplasmatota;Thermoplasmata;Methanomassiliicoccales;Methanomassiliicoccaceae;Methanomassiliicoccus;Methanomassiliicoccus luminyensis;Methanomassiliicoccus luminyensis B10 |
| Ga0586324 0127643 196 1572 | 43600000.00 | 62689200.00 | 72232900.00 | 36366000.00 | 9434800.00 | 21914400.00 | 29252400.00 | 125652.07 | 30456000.00 | 12012000.00 | 15180000.00 | 39750000.00 | KO:K04480 | methanol--5-hydroxybenzimidazolylcobamide Co-methyltransferase | Bacteria;Proteobacteria;Deltaproteobacteria;Desulfobacterales;Desulfobacteraceae;Desulfospira;Desulfospira joergensenii;Desulfospira joergensenii DSM 10085 |
| Ga0586324 0285324 2 1186 | 194870000.00 | 85965600.00 | 69136400.00 | 55375500.00 | 89630600.00 | 176939803.40 | 10191600.00 | 15616000.00 | 0.00 | 0.00 | 0.00 | 0.00 | KO:K04480 | methanol--5-hydroxybenzimidazolylcobamide Co-methyltransferase | Archaea;Candidatus Thermoplasmatota;Thermoplasmata;Methanomassiliicoccales;Methanomassiliicoccaceae;Methanomassiliicoccus;Methanomassiliicoccus luminyensis;Methanomassiliicoccus luminyensis B10 |
| Ga0586324 0511464 430 1623 | 7650000.00 | 26560800.00 | 7818296.55 | 0.00 | 2132100.00 | 2990652.61 | 3579600.00 | 6649000.00 | 3304800.00 | 6167700.00 | 0.00 | 0.00 | KO:K04480 | methanol--5-hydroxybenzimidazolylcobamide Co-methyltransferase | Bacteria;Proteobacteria;Deltaproteobacteria;Syntrophobacterales;Syntrophaceae;Desulfomonile;Desulfomonile tiedjei;Desulfomonile tiedjei DCB-1, DSM 6799 |
| Ga0586324 0605793 554 1507 | 0.00 | 0.00 | 0.00 | 0.00 | 1215400.00 | 0.00 | 0.00 | 0.00 | 0.00 | 0.00 | 0.00 | 0.00 | KO:K04480 | methanol--5-hydroxybenzimidazolylcobamide Co-methyltransferase | Archaea;Candidatus Thermoplasmatota;Thermoplasmata;Methanomassiliicoccales;Methanomassiliicoccaceae;Methanomassiliicoccus;Methanomassiliicoccus luminyensis;Methanomassiliicoccus luminyensis B10 |
| Ga0586324 0955463 2 760 | 0.00 | 0.00 | 1983026.75 | 1225500.00 | 0.00 | 0.00 | 0.00 | 0.00 | 0.00 | 0.00 | 0.00 | 0.00 | KO:K04480 | methanol--5-hydroxybenzimidazolylcobamide Co-methyltransferase | Archaea;Candidatus Thermoplasmatota;Thermoplasmata;Methanomassiliicoccales;Methanomassiliicoccaceae;Methanomassiliicoccus;Candidatus Methanomassiliicoccus intestinalis;Candidatus Methanomassiliicoccus intestinalis Issoire-Mx1 |
| Ga0586324 1926063 2 805 | 0.00 | 0.00 | 0.00 | 3638500.00 | 3368100.00 | 0.00 | 0.00 | 1366400.00 | 2478600.00 | 0.00 | 0.00 | 0.00 | KO:K04480 | methanol--5-hydroxybenzimidazolylcobamide Co-methyltransferase | Archaea;Euryarchaeota;Methanomicrobia;Methanosarcinales;Methanosarcinaceae;Methanosarcina;Methanosarcina sp. Kolksee;Methanosarcina sp. Kolksee |
| Ga0586324 1951058 1 669 | 5660000.00 | 0.00 | 0.00 | 0.00 | 8219400.00 | 0.00 | 0.00 | 0.00 | 3807000.00 | 0.00 | 0.00 | 0.00 | KO:K04480 | methanol--5-hydroxybenzimidazolylcobamide Co-methyltransferase | Archaea;Candidatus Thermoplasmatota;Thermoplasmata;Methanomassiliicoccales;Methanomassiliicoccaceae;Methanomassiliicoccus;Candidatus Methanomassiliicoccus intestinalis;Candidatus Methanomassiliicoccus intestinalis Issoire-Mx1 |
| Ga0586324 1983712 2 907 | 12500000.00 | 0.00 | 0.00 | 0.00 | 0.00 | 0.00 | 3488400.00 | 13359000.00 | 0.00 | 0.00 | 1592800.00 | 0.00 | KO:K04480 | methanol--5-hydroxybenzimidazolylcobamide Co-methyltransferase | Bacteria;Proteobacteria;Deltaproteobacteria;Desulfobacterales;Desulfobacteraceae;Desulfosalsimonas;Desulfosalsimonas propionicica;Desulfosalsimonas propionicica DSM 17721 |
| Ga0586324 2185705 1 870 | 15600000.00 | 8908000.00 | 16214400.00 | 13585000.00 | 4892500.00 | 3621017.15 | 0.00 | 0.00 | 0.00 | 0.00 | 0.00 | 0.00 | KO:K04480 | methanol--5-hydroxybenzimidazolylcobamide Co-methyltransferase | Archaea;Candidatus Thermoplasmatota;Thermoplasmata;Methanomassiliicoccales;Methanomassiliicoccaceae;Methanomassiliicoccus;Methanomassiliicoccus luminyensis;Methanomassiliicoccus luminyensis B10 |
| Ga0586324 2605692 1 807 | 0.00 | 0.00 | 3078714.83 | 0.00 | 0.00 | 0.00 | 0.00 | 4812900.00 | 2964600.00 | 1293600.00 | 0.00 | 1687500.00 | KO:K04480 | methanol--5-hydroxybenzimidazolylcobamide Co-methyltransferase | Archaea;Candidatus Thermoplasmatota;Thermoplasmata;Methanomassiliicoccales;Methanomassiliicoccaceae;Methanomassiliicoccus;Candidatus Methanomassiliicoccus intestinalis;Candidatus Methanomassiliicoccus intestinalis Issoire-Mx1 |
| Ga0586324 3630952 240 704 | 0.00 | 48416000.00 | 68686000.00 | 34580000.00 | 35020000.00 | 0.00 | 0.00 | 0.00 | 0.00 | 0.00 | 0.00 | 0.00 | KO:K04480 | methanol--5-hydroxybenzimidazolylcobamide Co-methyltransferase | Archaea;Candidatus Thermoplasmatota;Thermoplasmata;Methanomassiliicoccales;Methanomassiliicoccaceae;Methanomassiliicoccus;Methanomassiliicoccus luminyensis;Methanomassiliicoccus luminyensis B10 |
| Ga0586324 6129391 1 567 | 0.00 | 4698800.00 | 0.00 | 9595000.00 | 12360000.00 | 0.00 | 0.00 | 0.00 | 2640600.00 | 3095400.00 | 0.00 | 0.00 | KO:K04480 | methanol--5-hydroxybenzimidazolylcobamide Co-methyltransferase | Bacteria;Proteobacteria;Deltaproteobacteria;Syntrophobacterales;Syntrophaceae;Desulfomonile;Desulfomonile tiedjei;Desulfomonile tiedjei DCB-1, DSM 6799 |
| Ga0586324 7049904 2 535 | 0.00 | 2352800.00 | 0.00 | 0.00 | 0.00 | 4208200.00 | 0.00 | 0.00 | 0.00 | 0.00 | 0.00 | 0.00 | KO:K04480 | methanol--5-hydroxybenzimidazolylcobamide Co-methyltransferase | Archaea;Candidatus Thermoplasmatota;Thermoplasmata;Methanomassiliicoccales;Methanomassiliicoccaceae;Methanomassiliicoccus;Candidatus Methanomassiliicoccus intestinalis;Candidatus Methanomassiliicoccus intestinalis Issoire-Mx1 |
| Ga0586324 0000030 53246 54457 | 0.00 | 0.00 | 0.00 | 0.00 | 0.00 | 0.00 | 0.00 | 0.00 | 0.00 | 0.00 | 915200.00 | 0.00 | KO:K04487 | cysteine desulfurase | Bacteria;Proteobacteria;Betaproteobacteria;Nitrosomonadales;Thiobacillaceae;Annwoodia;Annwoodia aquaesulis;Annwoodia aquaesulis DSM 4255 |
| Ga0586324 0003386 3375 4583 | 0.00 | 0.00 | 0.00 | 0.00 | 0.00 | 0.00 | 0.00 | 994300.00 | 584700.93 | 0.00 | 754728.48 | 1807500.00 | KO:K04487 | cysteine desulfurase | Bacteria;Proteobacteria;Betaproteobacteria;Nitrosomonadales;Thiobacillaceae;Thiobacillus;Thiobacillus thioparus;Thiobacillus thioparus DSM 505 |
| Ga0586324 0814861 413 1327 | 0.00 | 0.00 | 0.00 | 0.00 | 0.00 | 0.00 | 0.00 | 268001.06 | 153354.06 | 0.00 | 0.00 | 0.00 | KO:K04487 | cysteine desulfurase | Bacteria;Proteobacteria;Betaproteobacteria;Nitrosomonadales;Thiobacillaceae;Thiobacillus;Thiobacillus thioparus;Thiobacillus thioparus DSM 505 |
| Ga0586324 0000926 12114 12689 | 5570000.00 | 0.00 | 0.00 | 0.00 | 0.00 | 0.00 | 0.00 | 0.00 | 0.00 | 0.00 | 0.00 | 0.00 | KO:K04564 | Fe-Mn family superoxide dismutase | Bacteria;Planctomycetes;Candidatus Brocadiae;Candidatus Brocadiales;Candidatus Brocadiaceae;Candidatus Brocadia;Candidatus Brocadia sapporoensis;Candidatus Brocadia sapporoensis 40 |
| Ga0586324 0002103 7660 8346 | 0.00 | 0.00 | 0.00 | 0.00 | 2956100.00 | 0.00 | 0.00 | 0.00 | 0.00 | 0.00 | 0.00 | 0.00 | KO:K04564 | Fe-Mn family superoxide dismutase | Bacteria;Proteobacteria;Deltaproteobacteria;Syntrophobacterales;Syntrophobacteraceae;Desulfosoma;Desulfosoma caldarium;Desulfosoma caldarium DSM 22027 |
| Ga0586324 0003786 12855 13439 | 0.00 | 1183200.00 | 0.00 | 0.00 | 0.00 | 0.00 | 0.00 | 0.00 | 0.00 | 0.00 | 0.00 | 0.00 | KO:K04564 | Fe-Mn family superoxide dismutase | Bacteria;Proteobacteria;Betaproteobacteria;Nitrosomonadales;Sterolibacteriaceae;Sulfurisoma;Sulfurisoma sediminicola;Sulfurisoma sediminicola DSM 26916 |
| Ga0586324 0015624 4159 4755 | 0.00 | 0.00 | 0.00 | 0.00 | 0.00 | 0.00 | 0.00 | 0.00 | 2000700.00 | 0.00 | 0.00 | 0.00 | KO:K04564 | Fe-Mn family superoxide dismutase | Bacteria;Proteobacteria;Betaproteobacteria;Nitrosomonadales;Nitrosomonadaceae;Nitrosospira;Nitrosospira multiformis;Nitrosospira multiformis NI14 |
| Ga0586324 0017719 673 1251 | 4200000.00 | 0.00 | 0.00 | 0.00 | 0.00 | 0.00 | 0.00 | 0.00 | 0.00 | 0.00 | 0.00 | 0.00 | KO:K04564 | Fe-Mn family superoxide dismutase | Bacteria;Planctomycetes;Candidatus Brocadiae;Candidatus Brocadiales;Candidatus Brocadiaceae;Candidatus Jettenia;Candidatus Jettenia caeni;Planctomycetaceae bacterium KSU-1 |
| Ga0586324 0021899 2192 2821 | 0.00 | 6133600.00 | 1632058.18 | 0.00 | 0.00 | 0.00 | 10123200.00 | 0.00 | 0.00 | 0.00 | 0.00 | 0.00 | KO:K04564 | Fe-Mn family superoxide dismutase | Bacteria;Planctomycetes;Candidatus Brocadiae;Candidatus Brocadiales;Candidatus Brocadiaceae;Candidatus Jettenia;Candidatus Jettenia caeni;Planctomycetaceae bacterium KSU-1 |
| Ga0586324 0062294 2545 3123 | 0.00 | 0.00 | 0.00 | 0.00 | 0.00 | 0.00 | 0.00 | 473786.39 | 0.00 | 0.00 | 0.00 | 0.00 | KO:K04564 | Fe-Mn family superoxide dismutase | Bacteria;Planctomycetes;Candidatus Brocadiae;Candidatus Brocadiales;Candidatus Brocadiaceae;Candidatus Brocadia;Candidatus Brocadia sapporoensis;Candidatus Brocadia sapporoensis 40 |
| Ga0586324 0108426 1884 2570 | 0.00 | 0.00 | 0.00 | 0.00 | 0.00 | 0.00 | 5517600.00 | 0.00 | 0.00 | 0.00 | 0.00 | 0.00 | KO:K04564 | Fe-Mn family superoxide dismutase | Bacteria;Planctomycetes;Candidatus Brocadiae;Candidatus Brocadiales;Candidatus Brocadiaceae;Candidatus Jettenia;Candidatus Jettenia caeni;Planctomycetaceae bacterium KSU-1 |
| Ga0586324 0117810 251 865 | 1400000.00 | 624063.88 | 0.00 | 0.00 | 0.00 | 0.00 | 0.00 | 2647400.00 | 1684800.00 | 0.00 | 0.00 | 2370000.00 | KO:K04564 | Fe-Mn family superoxide dismutase | Bacteria;Planctomycetes;Candidatus Brocadiae;Candidatus Brocadiales;Candidatus Brocadiaceae;Candidatus Jettenia;Candidatus Jettenia caeni;Planctomycetaceae bacterium KSU-1 |
| Ga0586324 0247355 1022 1615 | 0.00 | 2284800.00 | 0.00 | 4332000.00 | 0.00 | 0.00 | 0.00 | 0.00 | 3013200.00 | 0.00 | 0.00 | 0.00 | KO:K04564 | Fe-Mn family superoxide dismutase | Bacteria;Proteobacteria;Alphaproteobacteria;Rhodospirillales;Acetobacteraceae;Rhodopila;Rhodopila globiformis;Rhodopila globiformis DSM 161 |
| Ga0586324 0277190 1264 1845 | 0.00 | 707200.00 | 0.00 | 0.00 | 0.00 | 0.00 | 0.00 | 0.00 | 0.00 | 0.00 | 0.00 | 0.00 | KO:K04564 | Fe-Mn family superoxide dismutase | Bacteria;Proteobacteria;Gammaproteobacteria;Chromatiales;Chromatiaceae;Thiocapsa;Thiocapsa marina;Thiocapsa marina 5811, DSM 5653 |
| Ga0586324 0307858 190 774 | 0.00 | 0.00 | 0.00 | 0.00 | 0.00 | 0.00 | 0.00 | 3147600.00 | 0.00 | 0.00 | 0.00 | 0.00 | KO:K04564 | Fe-Mn family superoxide dismutase | Bacteria;Proteobacteria;Betaproteobacteria;Rhodocyclales;Azonexaceae;Dechloromonas;Dechloromonas denitrificans;Dechloromonas denitrificans ATCC BAA-841 |
| Ga0586324 0392287 1 594 | 1580000.00 | 0.00 | 0.00 | 0.00 | 0.00 | 0.00 | 0.00 | 0.00 | 0.00 | 0.00 | 0.00 | 0.00 | KO:K04564 | Fe-Mn family superoxide dismutase | Bacteria;Proteobacteria;Deltaproteobacteria;Desulfovibrionales;Desulfovibrionaceae;Desulfovibrio;Desulfovibrio cuneatus;Desulfovibrio cuneatus DSM 11391 |
| Ga0586324 0473570 836 1507 | 0.00 | 0.00 | 0.00 | 0.00 | 4171500.00 | 0.00 | 0.00 | 0.00 | 0.00 | 0.00 | 3115200.00 | 0.00 | KO:K04564 | Fe-Mn family superoxide dismutase | Bacteria;Planctomycetes;Candidatus Brocadiae;Candidatus Brocadiales;Candidatus Brocadiaceae;Candidatus Jettenia;Candidatus Jettenia caeni;Planctomycetaceae bacterium KSU-1 |
| Ga0586324 0511390 896 1474 | 0.00 | 0.00 | 0.00 | 0.00 | 0.00 | 0.00 | 0.00 | 0.00 | 4131000.00 | 0.00 | 0.00 | 0.00 | KO:K04564 | Fe-Mn family superoxide dismutase | Bacteria;Planctomycetes;Candidatus Brocadiae;Candidatus Brocadiales;Candidatus Brocadiaceae;Candidatus Brocadia;Candidatus Brocadia sapporoensis;Candidatus Brocadia sapporoensis 40 |
| Ga0586324 0690008 2 214 | 0.00 | 0.00 | 0.00 | 0.00 | 0.00 | 0.00 | 5882400.00 | 0.00 | 0.00 | 3665200.00 | 0.00 | 4110000.00 | KO:K04564 | Fe-Mn family superoxide dismutase | Bacteria;Firmicutes;Bacilli;Lactobacillales;Enterococcaceae;Enterococcus;Enterococcus timonensis;Enterococcus timonensis Marseille-P2817 |
| Ga0586324 0695808 494 1075 | 0.00 | 2148800.00 | 0.00 | 0.00 | 0.00 | 0.00 | 0.00 | 0.00 | 0.00 | 0.00 | 0.00 | 0.00 | KO:K04564 | Fe-Mn family superoxide dismutase | Bacteria;Planctomycetes;Candidatus Brocadiae;Candidatus Brocadiales;Candidatus Brocadiaceae;Candidatus Brocadia;Candidatus Brocadia sinica;Candidatus Brocadia sinica JPN1 |
| Ga0586324 0732507 347 952 | 7920000.00 | 0.00 | 0.00 | 0.00 | 0.00 | 0.00 | 0.00 | 0.00 | 0.00 | 0.00 | 0.00 | 0.00 | KO:K04564 | Fe-Mn family superoxide dismutase | Bacteria;Cyanobacteria;unclassified Cyanobacteria;Oscillatoriales;Oscillatoriaceae;Oscillatoria;Oscillatoria sp. PCC 10802;Oscillatoria sp. PCC 10802 |
| Ga0586324 0738933 504 1094 | 0.00 | 0.00 | 0.00 | 0.00 | 0.00 | 0.00 | 0.00 | 0.00 | 0.00 | 0.00 | 0.00 | 3135000.00 | KO:K04564 | Fe-Mn family superoxide dismutase | Bacteria;Planctomycetes;Candidatus Brocadiae;Candidatus Brocadiales;Candidatus Brocadiaceae;Candidatus Jettenia;Candidatus Jettenia caeni;Planctomycetaceae bacterium KSU-1 |
| Ga0586324 0740791 108 701 | 0.00 | 0.00 | 0.00 | 0.00 | 0.00 | 0.00 | 0.00 | 0.00 | 0.00 | 0.00 | 0.00 | 2707500.00 | KO:K04564 | Fe-Mn family superoxide dismutase | Archaea;Euryarchaeota;Methanomicrobia;Methanosarcinales;Candidatus Methanoperedenaceae;Candidatus Methanoperedens;Candidatus Methanoperedens nitroreducens;Candidatus Methanoperedens nitroreducens ANME-2d |
| Ga0586324 0827718 299 1024 | 0.00 | 0.00 | 0.00 | 0.00 | 1802500.00 | 0.00 | 0.00 | 0.00 | 1482300.00 | 0.00 | 0.00 | 0.00 | KO:K04564 | Fe-Mn family superoxide dismutase | Bacteria;Firmicutes;Bacilli;Bacillales;Paenibacillaceae;Aneurinibacillus;Aneurinibacillus soli;Aneurinibacillus soli CECT 8566 |
| Ga0586324 0878502 718 1284 | 3110000.00 | 0.00 | 0.00 | 0.00 | 0.00 | 0.00 | 0.00 | 6466000.00 | 0.00 | 4558400.00 | 0.00 | 4567500.00 | KO:K04564 | Fe-Mn family superoxide dismutase | Bacteria;Planctomycetes;Candidatus Brocadiae;Candidatus Brocadiales;Candidatus Brocadiaceae;Candidatus Jettenia;Candidatus Jettenia caeni;Planctomycetaceae bacterium KSU-1 |
| Ga0586324 1032084 1 645 | 0.00 | 321056.56 | 0.00 | 0.00 | 0.00 | 0.00 | 0.00 | 0.00 | 0.00 | 0.00 | 0.00 | 0.00 | KO:K04564 | Fe-Mn family superoxide dismutase | Bacteria;Planctomycetes;Candidatus Brocadiae;Candidatus Brocadiales;Candidatus Brocadiaceae;Candidatus Brocadia;Candidatus Brocadia sinica;Candidatus Brocadia sinica JPN1 |
| Ga0586324 1039281 133 801 | 0.00 | 2985200.00 | 665432.22 | 0.00 | 0.00 | 0.00 | 0.00 | 0.00 | 0.00 | 0.00 | 670133.20 | 620918.25 | KO:K04564 | Fe-Mn family superoxide dismutase | Bacteria;Planctomycetes;Candidatus Brocadiae;Candidatus Brocadiales;Candidatus Brocadiaceae;Candidatus Brocadia;Candidatus Brocadia sinica;Candidatus Brocadia sinica JPN1 |
| Ga0586324 1254421 306 1031 | 6960000.00 | 3250400.00 | 0.00 | 0.00 | 0.00 | 0.00 | 0.00 | 12566000.00 | 3507300.00 | 19866000.00 | 0.00 | 0.00 | KO:K04564 | Fe-Mn family superoxide dismutase | Bacteria;Planctomycetes;Candidatus Brocadiae;Candidatus Brocadiales;Candidatus Brocadiaceae;Candidatus Brocadia;Candidatus Brocadia sapporoensis;Candidatus Brocadia sapporoensis 40 |
| Ga0586324 1765481 154 849 | 0.00 | 14484000.00 | 3724143.66 | 0.00 | 0.00 | 0.00 | 0.00 | 0.00 | 3661200.00 | 0.00 | 0.00 | 22575000.00 | KO:K04564 | Fe-Mn family superoxide dismutase | Bacteria;Planctomycetes;Candidatus Brocadiae;Candidatus Brocadiales;Candidatus Brocadiaceae;Candidatus Jettenia;Candidatus Jettenia caeni;Planctomycetaceae bacterium KSU-1 |
| Ga0586324 2250701 297 860 | 9280000.00 | 0.00 | 631421.39 | 4436500.00 | 4923400.00 | 0.00 | 0.00 | 2604700.00 | 0.00 | 3773000.00 | 0.00 | 0.00 | KO:K04564 | Fe-Mn family superoxide dismutase | Bacteria;Planctomycetes;Candidatus Brocadiae;Candidatus Brocadiales;Candidatus Brocadiaceae;Candidatus Brocadia;Candidatus Brocadia sinica;Candidatus Brocadia sinica JPN1 |
| Ga0586324 2424199 1 735 | 0.00 | 0.00 | 0.00 | 0.00 | 0.00 | 0.00 | 0.00 | 0.00 | 0.00 | 3372600.00 | 0.00 | 0.00 | KO:K04564 | Fe-Mn family superoxide dismutase | Bacteria;Proteobacteria;Betaproteobacteria;Nitrosomonadales;Sterolibacteriaceae;Sulfurisoma;Sulfurisoma sediminicola;Sulfurisoma sediminicola DSM 26916 |
| Ga0586324 2510031 146 820 | 5190000.00 | 0.00 | 0.00 | 0.00 | 0.00 | 0.00 | 0.00 | 0.00 | 0.00 | 0.00 | 0.00 | 0.00 | KO:K04564 | Fe-Mn family superoxide dismutase | Bacteria;Planctomycetes;Candidatus Brocadiae;Candidatus Brocadiales;Candidatus Brocadiaceae;Candidatus Brocadia;Candidatus Brocadia sapporoensis;Candidatus Brocadia sapporoensis 40 |
| Ga0586324 3136304 356 748 | 0.00 | 473280000.00 | 0.00 | 0.00 | 0.00 | 1205863.68 | 0.00 | 0.00 | 0.00 | 0.00 | 0.00 | 0.00 | KO:K04564 | Fe-Mn family superoxide dismutase | Bacteria;Planctomycetes;Candidatus Brocadiae;Candidatus Brocadiales;Candidatus Brocadiaceae;Candidatus Brocadia;Candidatus Brocadia sinica;Candidatus Brocadia sinica JPN1 |
| Ga0586324 4707759 1 582 | 0.00 | 952000.00 | 0.00 | 0.00 | 0.00 | 0.00 | 0.00 | 0.00 | 0.00 | 0.00 | 0.00 | 0.00 | KO:K04564 | Fe-Mn family superoxide dismutase | Bacteria;Planctomycetes;Candidatus Brocadiae;Candidatus Brocadiales;Candidatus Brocadiaceae;Candidatus Brocadia;Candidatus Brocadia sinica;Candidatus Brocadia sinica JPN1 |
| Ga0586324 5280031 2 577 | 0.00 | 0.00 | 0.00 | 4693000.00 | 0.00 | 0.00 | 0.00 | 0.00 | 0.00 | 0.00 | 0.00 | 0.00 | KO:K04564 | Fe-Mn family superoxide dismutase | Bacteria;Planctomycetes;Candidatus Brocadiae;Candidatus Brocadiales;Candidatus Brocadiaceae;Candidatus Jettenia;Candidatus Jettenia caeni;Planctomycetaceae bacterium KSU-1 |
| Ga0586324 6068061 1 570 | 0.00 | 0.00 | 0.00 | 0.00 | 0.00 | 0.00 | 0.00 | 480984.39 | 0.00 | 0.00 | 0.00 | 0.00 | KO:K04564 | Fe-Mn family superoxide dismutase | Bacteria;Planctomycetes;Candidatus Brocadiae;Candidatus Brocadiales;Candidatus Brocadiaceae;Candidatus Jettenia;Candidatus Jettenia caeni;Planctomycetaceae bacterium KSU-1 |
| Ga0586324 7161986 2 289 | 0.00 | 0.00 | 0.00 | 0.00 | 0.00 | 0.00 | 0.00 | 0.00 | 0.00 | 8162000.00 | 0.00 | 0.00 | KO:K04564 | Fe-Mn family superoxide dismutase | Bacteria;Proteobacteria;Deltaproteobacteria;Syntrophobacterales;Syntrophaceae;Smithella;Smithella sp. SCADC;Smithella sp. SCADC |
| Ga0586324 8294672 1 501 | 0.00 | 0.00 | 9008000.00 | 0.00 | 0.00 | 0.00 | 0.00 | 3867400.00 | 0.00 | 0.00 | 0.00 | 5512500.00 | KO:K04564 | Fe-Mn family superoxide dismutase | Bacteria;Planctomycetes;Candidatus Brocadiae;Candidatus Brocadiales;Candidatus Brocadiaceae;Candidatus Brocadia;Candidatus Brocadia sapporoensis;Candidatus Brocadia sapporoensis 40 |
| Ga0586324 0008978 10343 10693 | 0.00 | 13056000.00 | 0.00 | 4579000.00 | 0.00 | 0.00 | 0.00 | 0.00 | 0.00 | 0.00 | 15400000.00 | 0.00 | KO:K04749 | anti-sigma B factor antagonist | Bacteria;Acidobacteria;Vicinamibacteria;unclassified Vicinamibacteria;Vicinamibacteraceae;Luteitalea;Luteitalea pratensis;Luteitalea pratensis DSM 100886 |
| Ga0586324 0010127 240 581 | 0.00 | 12376000.00 | 0.00 | 8939500.00 | 6303600.00 | 0.00 | 0.00 | 7869000.00 | 0.00 | 2718100.00 | 0.00 | 3037500.00 | KO:K04749 | anti-sigma B factor antagonist | Bacteria;Acidobacteria;Vicinamibacteria;unclassified Vicinamibacteria;Vicinamibacteraceae;Luteitalea;Luteitalea pratensis;Luteitalea pratensis DSM 100886 |
| Ga0586324 0092334 691 1032 | 0.00 | 0.00 | 0.00 | 0.00 | 0.00 | 0.00 | 29868000.00 | 0.00 | 0.00 | 0.00 | 0.00 | 0.00 | KO:K04749 | anti-sigma B factor antagonist | Bacteria;Acidobacteria;Vicinamibacteria;unclassified Vicinamibacteria;Vicinamibacteraceae;Luteitalea;Luteitalea pratensis;Luteitalea pratensis DSM 100886 |
| Ga0586324 0545101 1165 1509 | 0.00 | 0.00 | 0.00 | 0.00 | 0.00 | 0.00 | 0.00 | 7625000.00 | 0.00 | 0.00 | 0.00 | 0.00 | KO:K04749 | anti-sigma B factor antagonist | Bacteria;Acidobacteria;Acidobacteriia;Bryobacterales;Solibacteraceae;Candidatus Solibacter;Candidatus Solibacter usitatus;Candidatus Solibacter usitatus Ellin6076 |
| Ga0586324 2471958 1 231 | 0.00 | 0.00 | 0.00 | 0.00 | 0.00 | 0.00 | 0.00 | 0.00 | 0.00 | 2687300.00 | 0.00 | 0.00 | KO:K04749 | anti-sigma B factor antagonist | Bacteria;Verrucomicrobia;Verrucomicrobiae;Verrucomicrobiales;Verrucomicrobia subdivision 3;Pedosphaera;Pedosphaera parvula;Pedosphaera parvula Ellin514 |
| Ga0586324 3375376 305 724 | 0.00 | 0.00 | 0.00 | 0.00 | 0.00 | 4009700.00 | 0.00 | 0.00 | 0.00 | 0.00 | 0.00 | 0.00 | KO:K04749 | anti-sigma B factor antagonist | Bacteria;Proteobacteria;Deltaproteobacteria;Myxococcales;Myxococcaceae;Myxococcus;Myxococcus xanthus;Myxococcus xanthus KF4.3.9c1 |
| Ga0586324 6404909 87 428 | 0.00 | 0.00 | 0.00 | 4759500.00 | 4851300.00 | 0.00 | 0.00 | 2738900.00 | 3288600.00 | 0.00 | 0.00 | 0.00 | KO:K04749 | anti-sigma B factor antagonist | Bacteria;Acidobacteria;Vicinamibacteria;unclassified Vicinamibacteria;Vicinamibacteraceae;Luteitalea;Luteitalea pratensis;Luteitalea pratensis DSM 100886 |
| Ga0586324 0242053 1640 1987 | 0.00 | 0.00 | 1453063.59 | 1396500.00 | 2255700.00 | 1796937.13 | 1953077.64 | 1342000.00 | 1846800.00 | 977900.00 | 0.00 | 0.00 | KO:K04751 | nitrogen regulatory protein P-II 1 | Bacteria;Proteobacteria;Deltaproteobacteria;Desulfuromonadales;Geobacteraceae;Geobacter;Geobacter sp. DSM 2909;Geobacter sp. DSM 2909 |
| Ga0586324 1130224 534 872 | 0.00 | 0.00 | 0.00 | 0.00 | 0.00 | 0.00 | 0.00 | 13176000.00 | 4949100.00 | 0.00 | 0.00 | 0.00 | KO:K04751 | nitrogen regulatory protein P-II 1 | Bacteria;Proteobacteria;Betaproteobacteria;Nitrosomonadales;Gallionellaceae;Ferriphaselus;Ferriphaselus amnicola;Ferriphaselus amnicola OYT1 |
| Ga0586324 2603731 3 398 | 58600000.00 | 0.00 | 0.00 | 107777500.00 | 119480000.00 | 119100000.00 | 0.00 | 0.00 | 0.00 | 0.00 | 95040000.00 | 0.00 | KO:K04751 | nitrogen regulatory protein P-II 1 | Bacteria;Proteobacteria;Deltaproteobacteria;Desulfuromonadales;Geobacteraceae;Geobacter;Geobacter sp. OR-1;Geobacter sp. OR-1 |
| Ga0586324 2694778 451 798 | 0.00 | 0.00 | 0.00 | 0.00 | 0.00 | 0.00 | 0.00 | 0.00 | 568215.00 | 0.00 | 0.00 | 0.00 | KO:K04751 | nitrogen regulatory protein P-II 1 | Bacteria;Proteobacteria;Alphaproteobacteria;Sphingomonadales;Erythrobacteraceae;Paraurantiacibacter;Paraurantiacibacter namhicola;Paraurantiacibacter namhicola JCM 16345 |
| Ga0586324 3731483 2 373 | 0.00 | 1054000.00 | 0.00 | 0.00 | 432781.28 | 0.00 | 0.00 | 0.00 | 0.00 | 0.00 | 0.00 | 0.00 | KO:K04751 | nitrogen regulatory protein P-II 1 | Bacteria;Proteobacteria;Deltaproteobacteria;Myxococcales;Labilitrichaceae;Labilithrix;Labilithrix luteola;Labilithrix luteola DSM 27648 |
| Ga0586324 5009764 137 475 | 0.00 | 0.00 | 0.00 | 0.00 | 0.00 | 0.00 | 0.00 | 1061400.00 | 12312000.00 | 338184.00 | 1592800.00 | 6236211.00 | KO:K04751 | nitrogen regulatory protein P-II 1 | Bacteria;Proteobacteria;Deltaproteobacteria;Myxococcales;Labilitrichaceae;Labilithrix;Labilithrix luteola;Labilithrix luteola DSM 27648 |
| Ga0586324 7665633 149 517 | 0.00 | 0.00 | 0.00 | 0.00 | 11639000.00 | 8376700.00 | 0.00 | 0.00 | 0.00 | 0.00 | 0.00 | 6930000.00 | KO:K04751 | nitrogen regulatory protein P-II 1 | Bacteria;Proteobacteria;Alphaproteobacteria;Rhodospirillales;Azospirillaceae;Azospirillum;Azospirillum sp. B4;Azospirillum sp. B4 |
| Ga0586324 0001095 6369 6707 | 6930000.00 | 18156000.00 | 1253001.54 | 6232000.00 | 6107900.00 | 6113800.00 | 222055.58 | 5172800.00 | 8181000.00 | 1994300.00 | 192307.28 | 144408.00 | KO:K04752 | nitrogen regulatory protein P-II 2 | Bacteria;Proteobacteria;Betaproteobacteria;Burkholderiales;Comamonadaceae;unclassified Comamonadaceae;Comamonadaceae bacterium URHA0028;Comamonadaceae bacterium URHA0028 |
| Ga0586324 0008440 10532 10999 | 0.00 | 0.00 | 0.00 | 0.00 | 0.00 | 0.00 | 0.00 | 582705.55 | 0.00 | 0.00 | 1390400.00 | 1297500.00 | KO:K04752 | nitrogen regulatory protein P-II 2 | Bacteria;Proteobacteria;Alphaproteobacteria;Hyphomicrobiales;Xanthobacteraceae;Pseudolabrys;Pseudolabrys taiwanensis;Pseudolabrys taiwanensis CC-BB4 |
| Ga0586324 0039226 1511 1849 | 0.00 | 0.00 | 0.00 | 0.00 | 0.00 | 0.00 | 0.00 | 0.00 | 0.00 | 0.00 | 0.00 | 1702500.00 | KO:K04752 | nitrogen regulatory protein P-II 2 | Bacteria;Proteobacteria;Alphaproteobacteria;Hyphomicrobiales;Hyphomicrobiaceae;Rhodoplanes;Rhodoplanes sp. Z2-YC6860;Rhodoplanes sp. Z2-YC6860 |

| | | | | | | | | | | | | | | |
|---|---|---|---|---|---|---|---|---|---|---|---|---|---|---|
| Ga0586324 0052710 1386 1724 | 0.00 | 0.00 | 0.00 | 0.00 | 0.00 | 0.00 | 0.00 | 1598200.00 | 0.00 | 0.00 | 0.00 | 0.00 | KO:K04752 | nitrogen regulatory protein P-II 2 | Bacteria;Proteobacteria;Alphaproteobacteria;Hyphomicrobiales;Bradyrhizobiaceae;Nitrobacter;Nitrobacter winogradskyi;Nitrobacter winogradskyi Nb-255 |
| Ga0586324 0078374 577 915 | 0.00 | 1040400.00 | 0.00 | 0.00 | 2842800.00 | 0.00 | 6840000.00 | 8723000.00 | 5540400.00 | 8932000.00 | 11704000.00 | 7327500.00 | KO:K04752 | nitrogen regulatory protein P-II 2 | Bacteria;Proteobacteria;Betaproteobacteria;Nitrosomonadales;Sulfuricellaceae;Sulfurirhabdus;Sulfurirhabdus autotrophica;Sulfurirhabdus autotrophica DSM 100309 |
| Ga0586324 0389007 153 491 | 0.00 | 0.00 | 0.00 | 0.00 | 0.00 | 4922800.00 | 4970400.00 | 3861300.00 | 0.00 | 0.00 | 7823200.00 | 0.00 | KO:K04752 | nitrogen regulatory protein P-II 2 | Bacteria;Proteobacteria;Alphaproteobacteria;Hyphomicrobiales;Bradyrhizobiaceae;Bradyrhizobium;Bradyrhizobium sp. YR681;Bradyrhizobium sp. YR681 |
| Ga0586324 0571820 976 1314 | 0.00 | 0.00 | 0.00 | 0.00 | 8229700.00 | 0.00 | 0.00 | 0.00 | 0.00 | 0.00 | 0.00 | 0.00 | KO:K04752 | nitrogen regulatory protein P-II 2 | Bacteria;Proteobacteria;Betaproteobacteria;Rhodocyclales;Azonexaceae;Dechloromonas;Dechloromonas aromatica;Dechloromonas aromatica RCB |
| Ga0586324 0647447 882 1238 | 0.00 | 297759.76 | 0.00 | 0.00 | 0.00 | 0.00 | 0.00 | 0.00 | 0.00 | 0.00 | 0.00 | 0.00 | KO:K04752 | nitrogen regulatory protein P-II 2 | Bacteria;Proteobacteria;Alphaproteobacteria;Hyphomicrobiales;Xanthobacteraceae;Pseudolabrys;Pseudolabrys taiwanensis;Pseudolabrys taiwanensis CC-BB4 |
| Ga0586324 0832507 386 724 | 6940000.00 | 8500000.00 | 0.00 | 47120000.00 | 11536000.00 | 0.00 | 0.00 | 0.00 | 4195800.00 | 0.00 | 0.00 | 8625000.00 | KO:K04752 | nitrogen regulatory protein P-II 2 | Bacteria;Proteobacteria;Alphaproteobacteria;Hyphomicrobiales;Bradyrhizobiaceae;Bradyrhizobium;Bradyrhizobium elkanii;Bradyrhizobium elkanii WSM1741 |
| Ga0586324 1432914 244 582 | 0.00 | 0.00 | 0.00 | 0.00 | 0.00 | 4049400.00 | 0.00 | 0.00 | 0.00 | 0.00 | 0.00 | 877500.00 | KO:K04752 | nitrogen regulatory protein P-II 2 | Bacteria;Proteobacteria;Betaproteobacteria;Nitrosomonadales;Gallionellaceae;Sideroxydans;Sideroxydans lithotrophicus;Sideroxydans lithotrophicus ES-1 |
| Ga0586324 2073087 317 655 | 0.00 | 0.00 | 0.00 | 0.00 | 0.00 | 0.00 | 4605600.00 | 13481000.00 | 11745000.00 | 0.00 | 0.00 | 5805000.00 | KO:K04752 | nitrogen regulatory protein P-II 2 | Bacteria;Proteobacteria;Betaproteobacteria;Ferrovales;Ferrovaceae;Ferrovum;Ferrovum myxofaciens;Ferrovum myxofaciens PN-J185 |
| Ga0586324 2715460 377 715 | 0.00 | 0.00 | 0.00 | 0.00 | 0.00 | 0.00 | 0.00 | 3629500.00 | 0.00 | 0.00 | 0.00 | 7875000.00 | KO:K04752 | nitrogen regulatory protein P-II 2 | Bacteria;Proteobacteria;Alphaproteobacteria;Hyphomicrobiales;Xanthobacteraceae;Pseudolabrys;Pseudolabrys sp. Root1462;Pseudolabrys sp. Root1462 |
| Ga0586324 3002953 2 250 | 0.00 | 7208000.00 | 0.00 | 7058500.00 | 7755900.00 | 0.00 | 0.00 | 0.00 | 0.00 | 5582500.00 | 6617600.00 | 0.00 | KO:K04752 | nitrogen regulatory protein P-II 2 | Bacteria;Proteobacteria;Gammaproteobacteria;Methylococcales;Methylococcaceae;Methylosarcina;Methylosarcina fibrata;Methylosarcina fibrata AML-C10 |
| Ga0586324 3882447 219 557 | 8670000.00 | 0.00 | 0.00 | 0.00 | 0.00 | 0.00 | 377967.00 | 0.00 | 0.00 | 0.00 | 0.00 | 0.00 | KO:K04752 | nitrogen regulatory protein P-II 2 | Bacteria;Proteobacteria;Alphaproteobacteria;Hyphomicrobiales;Hyphomicrobiaceae;Hyphomicrobium;Hyphomicrobium sp. 99;Hyphomicrobium sp. 99 |
| Ga0586324 4745892 132 470 | 0.00 | 0.00 | 630498.07 | 0.00 | 0.00 | 0.00 | 867961.80 | 0.00 | 0.00 | 0.00 | 515870.96 | 1185000.00 | KO:K04752 | nitrogen regulatory protein P-II 2 | Bacteria;Proteobacteria;Betaproteobacteria;Burkholderiales;Comamonadaceae;Polaromonas;Polaromonas sp. AET17H-212;Polaromonas sp. AET17H-212 |
| Ga0586324 0366923 1489 1764 | 0.00 | 0.00 | 0.00 | 0.00 | 0.00 | 8257600.00 | 0.00 | 0.00 | 0.00 | 0.00 | 0.00 | 0.00 | KO:K04764 | integration host factor subunit alpha | Bacteria;Proteobacteria;Deltaproteobacteria;Syntrophobacterales;Syntrophaceae;Syntrophus;Syntrophus aciditrophicus;Syntrophus aciditrophicus SB |
| Ga0586324 0598872 24 308 | 0.00 | 0.00 | 0.00 | 0.00 | 8404800.00 | 0.00 | 0.00 | 0.00 | 0.00 | 0.00 | 0.00 | 0.00 | KO:K04764 | integration host factor subunit alpha | Bacteria;Proteobacteria;Deltaproteobacteria;Myxococcales;Vulgatibacteraceae;Vulgatibacter;Vulgatibacter incomptus;Vulgatibacter incomptus DSM 27710 |
| Ga0586324 3255206 293 592 | 0.00 | 0.00 | 0.00 | 0.00 | 0.00 | 0.00 | 0.00 | 0.00 | 1628100.00 | 0.00 | 0.00 | 0.00 | KO:K04764 | integration host factor subunit alpha | Bacteria;Proteobacteria;Gammaproteobacteria;unclassified Gammaproteobacteria;Candidatus Competibacteraceae;Candidatus Competibacter;Candidatus Competibacter denitrificans;Candidatus Competibacter denitrificans Run A D11 |
| Ga0586324 2285703 245 721 | 5750000.00 | 2454800.00 | 0.00 | 0.00 | 7374800.00 | 0.00 | 0.00 | 0.00 | 0.00 | 0.00 | 0.00 | 0.00 | KO:K04767 | acetoin utilization protein AcuB | Bacteria;Proteobacteria;Gammaproteobacteria;Methylococcales;Methylococcaceae;Methylobacter;Methylobacter tundripaludum;Methylobacter tundripaludum SV96 |
| Ga0586324 0000537 26465 27862 | 0.00 | 0.00 | 0.00 | 0.00 | 0.00 | 0.00 | 0.00 | 0.00 | 5556600.00 | 77169400.00 | 57024000.00 | 44835000.00 | KO:K04771 | serine protease Do | Bacteria;Proteobacteria;Betaproteobacteria;Nitrosomonadales;Thiobacillaceae;Annwoodia;Annwoodia aquaesulis;Annwoodia aquaesulis DSM 4255 |
| Ga0586324 0000875 1654 3114 | 9350000.00 | 0.00 | 0.00 | 0.00 | 0.00 | 0.00 | 0.00 | 0.00 | 0.00 | 0.00 | 0.00 | 0.00 | KO:K04771 | serine protease Do | Bacteria;Proteobacteria;Gammaproteobacteria;Methylococcales;Methylococcaceae;Methylobacter;Methylobacter tundripaludum;Methylobacter tundripaludum OWC-G53F |
| Ga0586324 0003945 12599 14131 | 0.00 | 0.00 | 0.00 | 0.00 | 0.00 | 0.00 | 0.00 | 0.00 | 0.00 | 1655500.00 | 1760000.00 | 2595000.00 | KO:K04771 | serine protease Do | Bacteria;Proteobacteria;Alphaproteobacteria;Hyphomicrobiales;Hyphomicrobiaceae;Rhodoplanes;Rhodoplanes sp. Z2-YC6860;Rhodoplanes sp. Z2-YC6860 |
| Ga0586324 0006917 4589 6058 | 0.00 | 0.00 | 0.00 | 0.00 | 0.00 | 0.00 | 0.00 | 0.00 | 0.00 | 0.00 | 2842400.00 | 0.00 | KO:K04771 | serine protease Do | Bacteria;Proteobacteria;Deltaproteobacteria;Desulfuromonadales;Geobacteraceae;Geobacter;Geobacter argillaceus;Geobacter argillaceus ATCC BAA-1139 |
| Ga0586324 0017806 1768 3159 | 0.00 | 0.00 | 0.00 | 0.00 | 0.00 | 0.00 | 0.00 | 0.00 | 0.00 | 10314447.22 | 14213823.36 | 8362500.00 | KO:K04771 | serine protease Do | Bacteria;Proteobacteria;Betaproteobacteria;Nitrosomonadales;Thiobacillaceae;Thiobacillus;Thiobacillus thioparus;Thiobacillus thioparus DSM 505 |
| Ga0586324 0020619 4878 6272 | 0.00 | 0.00 | 0.00 | 0.00 | 0.00 | 0.00 | 0.00 | 0.00 | 0.00 | 0.00 | 7427200.00 | 0.00 | KO:K04771 | serine protease Do | Bacteria;Proteobacteria;Betaproteobacteria;Nitrosomonadales;Thiobacillaceae;Thiobacillus;Thiobacillus denitrificans;Thiobacillus denitrificans DSM 12475 |
| Ga0586324 0033345 1391 2875 | 0.00 | 0.00 | 0.00 | 0.00 | 0.00 | 0.00 | 2599200.00 | 4074800.00 | 10481400.00 | 17440500.00 | 28520800.00 | 47610000.00 | KO:K04771 | serine protease Do | Bacteria;Proteobacteria;Betaproteobacteria;Nitrosomonadales;Sulfuricellaceae;Sulfurirhabdus;Sulfurirhabdus autotrophica;Sulfurirhabdus autotrophica DSM 100309 |
| Ga0586324 0034773 4882 5709 | 0.00 | 0.00 | 0.00 | 0.00 | 0.00 | 0.00 | 0.00 | 0.00 | 0.00 | 0.00 | 367392.08 | 0.00 | KO:K04771 | serine protease Do | Bacteria;Acidobacteria;Acidobacteriia;Acidobacteriales;Acidobacteriaceae;Candidatus Koribacter;Candidatus Koribacter versatilis;Candidatus Koribacter versatilis Ellin345 |
| Ga0586324 0036983 2056 3486 | 0.00 | 0.00 | 0.00 | 0.00 | 0.00 | 0.00 | 0.00 | 0.00 | 0.00 | 1801800.00 | 0.00 | 0.00 | KO:K04771 | serine protease Do | Bacteria;Proteobacteria;Betaproteobacteria;Burkholderiales;Comamonadaceae;Acidovorax;Acidovorax citrulli;Acidovorax avenae citrulli AAC00-1 |
| Ga0586324 0130854 588 2024 | 0.00 | 0.00 | 657651.56 | 0.00 | 1050600.00 | 0.00 | 2804400.00 | 4068700.00 | 378456.30 | 3472700.00 | 1179200.00 | 543104.25 | KO:K04771 | serine protease Do | Bacteria;Proteobacteria;Deltaproteobacteria;Desulfobacterales;Desulfobacteraceae;Desulfosarcina;Desulfosarcina variabilis;Desulfosarcina variabilis Montpellier |
| Ga0586324 0151163 3 1496 | 0.00 | 0.00 | 0.00 | 0.00 | 0.00 | 0.00 | 0.00 | 0.00 | 0.00 | 0.00 | 0.00 | 621093.00 | KO:K04771 | serine protease Do | Bacteria;Proteobacteria;Deltaproteobacteria;Desulfobacterales;Desulfobacteraceae;Desulfatibacillum;Desulfatibacillum aliphaticivorans;Desulfatibacillum aliphaticivorans DSM 15576 |
| Ga0586324 0190409 170 2533 | 0.00 | 0.00 | 0.00 | 1235000.00 | 0.00 | 0.00 | 0.00 | 0.00 | 0.00 | 3934700.00 | 0.00 | 0.00 | KO:K04771 | serine protease Do | Bacteria;Chloroflexi;Anaerolineae;Anaerolineales;Anaerolineaceae;Thermanaerothrix;Thermanaerothrix daxensis;Thermanaerothrix daxensis GNS-1 |
| Ga0586324 0219915 223 1740 | 0.00 | 2121600.00 | 0.00 | 0.00 | 0.00 | 0.00 | 0.00 | 0.00 | 2681100.00 | 0.00 | 0.00 | 0.00 | KO:K04771 | serine protease Do | Bacteria;Proteobacteria;Deltaproteobacteria;Syntrophobacterales;Syntrophaceae;Desulfobacca;Desulfobacca acetoxidans;Desulfobacca acetoxidans ASRB2, DSM 11109 |
| Ga0586324 0250355 520 2001 | 0.00 | 0.00 | 0.00 | 0.00 | 0.00 | 0.00 | 0.00 | 0.00 | 0.00 | 0.00 | 1100000.00 | 0.00 | KO:K04771 | serine protease Do | Bacteria;Proteobacteria;Gammaproteobacteria;Acidiferrobacterales;Acidiferrobacteraceae;Sulfuricaulis;Sulfuricaulis limicola;Sulfuricaulis limicola HA5 |
| Ga0586324 0326660 2 1189 | 0.00 | 0.00 | 0.00 | 0.00 | 0.00 | 0.00 | 0.00 | 0.00 | 0.00 | 1832600.00 | 0.00 | 0.00 | KO:K04771 | serine protease Do | Bacteria;Proteobacteria;Alphaproteobacteria;Hyphomicrobiales;Bradyrhizobiaceae;Afipia;Afipia sp. GAS231;Afipia sp. GAS231 |
| Ga0586324 0498233 1021 1641 | 0.00 | 0.00 | 0.00 | 0.00 | 0.00 | 0.00 | 0.00 | 0.00 | 0.00 | 1393700.00 | 4197600.00 | 0.00 | KO:K04771 | serine protease Do | Bacteria;Proteobacteria;Deltaproteobacteria;Syntrophobacterales;Syntrophobacteraceae;Desulfacinum;Desulfacinum infernum;Desulfacinum infernum DSM 9756 |
| Ga0586324 0512796 1 1323 | 0.00 | 0.00 | 0.00 | 0.00 | 0.00 | 0.00 | 0.00 | 0.00 | 0.00 | 0.00 | 0.00 | 1080000.00 | KO:K04771 | serine protease Do | Bacteria;Nitrospirae;unclassified Nitrospirae;unclassified Nitrospirae;unclassified Nitrospirae;unclassified Nitrospirae;Nitrospirae bacterium HCH-1;Nitrospirae bacterium HCH-1 |
| Ga0586324 0514813 59 1543 | 0.00 | 0.00 | 0.00 | 0.00 | 0.00 | 0.00 | 3008133.96 | 868658.91 | 527842.98 | 5020400.00 | 5156800.00 | 5167500.00 | KO:K04771 | serine protease Do | Bacteria;Proteobacteria;Betaproteobacteria;Nitrosomonadales;Sulfuricellaceae;Sulfurirhabdus;Sulfurirhabdus autotrophica;Sulfurirhabdus autotrophica DSM 100309 |
| Ga0586324 0555492 293 1564 | 0.00 | 0.00 | 0.00 | 0.00 | 0.00 | 4962500.00 | 0.00 | 0.00 | 0.00 | 0.00 | 0.00 | 0.00 | KO:K04771 | serine protease Do | Bacteria;Proteobacteria;Deltaproteobacteria;Desulfobacterales;Desulfobacteraceae;Desulfatibacillum;Desulfatibacillum aliphaticivorans;Desulfatibacillum aliphaticivorans DSM 15576 |
| Ga0586324 0561483 1 1302 | 0.00 | 0.00 | 0.00 | 0.00 | 0.00 | 0.00 | 0.00 | 0.00 | 0.00 | 0.00 | 0.00 | 1845000.00 | KO:K04771 | serine protease Do | Bacteria;Proteobacteria;Alphaproteobacteria;Hyphomicrobiales;Xanthobacteraceae;Pseudolabrys;Pseudolabrys taiwanensis;Pseudolabrys taiwanensis CC-BB4 |
| Ga0586324 0595199 3 1376 | 0.00 | 0.00 | 0.00 | 0.00 | 0.00 | 0.00 | 0.00 | 1183400.00 | 0.00 | 1478400.00 | 0.00 | 0.00 | KO:K04771 | serine protease Do | Bacteria;Proteobacteria;Betaproteobacteria;Nitrosomonadales;Thiobacillaceae;Thiobacillus;Thiobacillus denitrificans;Thiobacillus denitrificans DSM 12475 |
| Ga0586324 0635925 58 1476 | 0.00 | 0.00 | 0.00 | 0.00 | 0.00 | 0.00 | 0.00 | 4495700.00 | 0.00 | 0.00 | 0.00 | 0.00 | KO:K04771 | serine protease Do | Bacteria;Proteobacteria;Deltaproteobacteria;Syntrophobacterales;Syntrophaceae;Desulfobacca;Desulfobacca acetoxidans;Desulfobacca acetoxidans ASRB2, DSM 11109 |
| Ga0586324 0724316 912 1394 | 0.00 | 0.00 | 0.00 | 0.00 | 0.00 | 0.00 | 0.00 | 0.00 | 0.00 | 0.00 | 2235200.00 | 0.00 | KO:K04771 | serine protease Do | Bacteria;Proteobacteria;Betaproteobacteria;Burkholderiales;unclassified Burkholderiales;Methylibium;Methylibium sp. YR605;Methylibium sp. YR605 |
| Ga0586324 0727710 1 1368 | 0.00 | 0.00 | 0.00 | 0.00 | 0.00 | 0.00 | 0.00 | 0.00 | 0.00 | 0.00 | 0.00 | 1117500.00 | KO:K04771 | serine protease Do | Bacteria;Proteobacteria;Deltaproteobacteria;Syntrophorhabdales;Syntrophorhabdaceae;Syntrophorhabdus;Syntrophorhabdus aromaticivorans;Syntrophorhabdus aromaticivorans UI |
| Ga0586324 0815363 159 1325 | 0.00 | 0.00 | 0.00 | 0.00 | 416494.92 | 591438.69 | 0.00 | 0.00 | 0.00 | 0.00 | 0.00 | 0.00 | KO:K04771 | serine protease Do | Bacteria;Deferribacteres;Deferribacteres;Deferribacterales;Deferribacteraceae;Flexistipes;Flexistipes sinusarabici;Flexistipes sinusarabici MAS10, DSM 4947 |
| Ga0586324 0854317 3 938 | 0.00 | 0.00 | 0.00 | 0.00 | 0.00 | 0.00 | 0.00 | 0.00 | 0.00 | 0.00 | 440806.96 | 0.00 | KO:K04771 | serine protease Do | Bacteria;Proteobacteria;Alphaproteobacteria;Hyphomicrobiales;Bradyrhizobiaceae;Bradyrhizobium;Bradyrhizobium erythrophlei;Bradyrhizobium erythrophlei GAS401 |
| Ga0586324 0854562 56 1300 | 0.00 | 0.00 | 0.00 | 0.00 | 0.00 | 0.00 | 0.00 | 0.00 | 0.00 | 0.00 | 0.00 | 746511.75 | KO:K04771 | serine protease Do | Bacteria;Proteobacteria;Betaproteobacteria;Nitrosomonadales;Thiobacillaceae;Thiobacillus;Thiobacillus denitrificans;Thiobacillus denitrificans DSM 12475 |
| Ga0586324 0938867 2 889 | 0.00 | 0.00 | 0.00 | 0.00 | 0.00 | 0.00 | 0.00 | 0.00 | 0.00 | 0.00 | 0.00 | 1177500.00 | KO:K04771 | serine protease Do | Bacteria;Proteobacteria;Deltaproteobacteria;Desulfuromonadales;Geobacteraceae;Geobacter;Geobacter sp. OR-1;Geobacter sp. OR-1 |
| Ga0586324 1389803 2 1054 | 0.00 | 0.00 | 0.00 | 0.00 | 0.00 | 0.00 | 0.00 | 0.00 | 0.00 | 0.00 | 0.00 | 5040000.00 | KO:K04771 | serine protease Do | Bacteria;Planctomycetes;Planctomycetia;Planctomycetales;Planctomycetaceae;Fuerstia;Fuerstia marisgermanicae;Fuerstia marisgermanicae NH11 |
| Ga0586324 3634411 3 704 | 0.00 | 0.00 | 0.00 | 0.00 | 0.00 | 0.00 | 0.00 | 0.00 | 0.00 | 1894200.00 | 0.00 | 0.00 | KO:K04771 | serine protease Do | Bacteria;Proteobacteria;Betaproteobacteria;Rhodocyclales;Zoogloeaceae;Azoarcus;Azoarcus sp. BH72;Azoarcus sp. BH72 |
| Ga0586324 4373263 261 653 | 0.00 | 0.00 | 0.00 | 0.00 | 0.00 | 0.00 | 15504000.00 | 0.00 | 0.00 | 0.00 | 0.00 | 0.00 | KO:K04771 | serine protease Do | Bacteria;Proteobacteria;Gammaproteobacteria;Methylococcales;Methylococcaceae;Methylobacter;Methylobacter tundripaludum;Methylobacter tundripaludum OWC-DMM |
| Ga0586324 4701283 3 632 | 0.00 | 0.00 | 0.00 | 0.00 | 0.00 | 0.00 | 0.00 | 0.00 | 0.00 | 0.00 | 0.00 | 2257500.00 | KO:K04771 | serine protease Do | Bacteria;Proteobacteria;Betaproteobacteria;Neisseriales;Chromobacteriaceae;Silvimonas;Silvimonas terrae;Silvimonas terrae DSM 18233 |
| Ga0586324 5106437 3 491 | 0.00 | 0.00 | 2832678.20 | 0.00 | 0.00 | 0.00 | 0.00 | 0.00 | 0.00 | 0.00 | 0.00 | 0.00 | KO:K04771 | serine protease Do | Bacteria;Proteobacteria;Deltaproteobacteria;Syntrophobacterales;Syntrophaceae;Desulfobacca;Desulfobacca acetoxidans;Desulfobacca acetoxidans ASRB2, DSM 11109 |
| Ga0586324 0005837 2814 3998 | 0.00 | 0.00 | 0.00 | 0.00 | 0.00 | 0.00 | 0.00 | 0.00 | 0.00 | 3842300.00 | 0.00 | 0.00 | KO:K04772 | serine protease DegQ | Bacteria;Proteobacteria;Betaproteobacteria;Nitrosomonadales;Thiobacillaceae;Thiobacillus;Thiobacillus thioparus;Thiobacillus thioparus DSM 505 |
| Ga0586324 0000007 51755 52693 | 0.00 | 0.00 | 0.00 | 0.00 | 0.00 | 0.00 | 0.00 | 866200.00 | 760790.88 | 0.00 | 1161600.00 | 653935.50 | KO:K04773 | protease-4 | Bacteria;Proteobacteria;Betaproteobacteria;Nitrosomonadales;Thiobacillaceae;Annwoodia;Annwoodia aquaesulis;Annwoodia aquaesulis DSM 4255 |
| Ga0586324 0073773 2587 3582 | 0.00 | 0.00 | 0.00 | 15675000.00 | 0.00 | 0.00 | 0.00 | 0.00 | 0.00 | 0.00 | 0.00 | 0.00 | KO:K04801 | replication factor C small subunit | Archaea;Euryarchaeota;Methanomicrobia;Methanosarcinales;Methanotrichaceae;Methanothrix;Methanothrix soehngenii;Methanothrix soehngenii GP-6 |
| Ga0586324 1987579 2 424 | 3530000.00 | 1346400.00 | 2724756.73 | 1814500.00 | 1792200.00 | 2232021.34 | 2348400.00 | 1647000.00 | 1717200.00 | 0.00 | 1944800.00 | 960000.00 | KO:K04802 | proliferating cell nuclear antigen | Archaea;Euryarchaeota;Methanomicrobia;Methanosarcinales;Methanotrichaceae;Methanothrix;Methanothrix soehngenii;Methanothrix soehngenii GP-6 |
| Ga0586324 2184007 238 870 | 0.00 | 0.00 | 0.00 | 0.00 | 0.00 | 0.00 | 0.00 | 0.00 | 0.00 | 0.00 | 0.00 | 169592.25 | KO:K04802 | proliferating cell nuclear antigen | Archaea;Thaumarchaeota;Nitrososphaeria;Nitrososphaerales;Nitrososphaeraceae;Candidatus Nitrosocosmicus;Candidatus Nitrosocosmicus hydrocola;Candidatus Nitrosocosmicus exaquare G61 |
| Ga0586324 0560753 1 1257 | 0.00 | 0.00 | 0.00 | 0.00 | 0.00 | 0.00 | 0.00 | 0.00 | 0.00 | 1501500.00 | 0.00 | 0.00 | KO:K05301 | sulfite dehydrogenase | Bacteria;Proteobacteria;Betaproteobacteria;Burkholderiales;Comamonadaceae;Limnohabitans;Limnohabitans curvus;Limnohabitans curvus MWH-C5 |
| Ga0586324 1630631 1 984 | 0.00 | 0.00 | 0.00 | 0.00 | 0.00 | 0.00 | 0.00 | 0.00 | 0.00 | 0.00 | 994400.00 | 0.00 | KO:K05301 | sulfite dehydrogenase | Bacteria;Proteobacteria;Gammaproteobacteria;Xanthomonadales;Rhodanobacteraceae;Rudaea;Rudaea cellulosilytica;Rudaea cellulosilytica DSM 22992 |
| Ga0586324 1900128 1 279 | 0.00 | 0.00 | 0.00 | 0.00 | 0.00 | 0.00 | 0.00 | 0.00 | 0.00 | 5151300.00 | 0.00 | 0.00 | KO:K05301 | sulfite dehydrogenase | Bacteria;Proteobacteria;Alphaproteobacteria;Hyphomicrobiales;Methylobacteriaceae;Methylobacterium;Methylobacterium nodulans;Methylobacterium nodulans ORS 2060 |
| Ga0586324 6091202 3 569 | 2850000.00 | 0.00 | 0.00 | 0.00 | 0.00 | 0.00 | 0.00 | 0.00 | 0.00 | 0.00 | 0.00 | 0.00 | KO:K05301 | sulfite dehydrogenase | Bacteria;Proteobacteria;Betaproteobacteria;Burkholderiales;Comamonadaceae;Limnohabitans;Limnohabitans curvus;Limnohabitans curvus MWH-C5 |
| Ga0586324 0023761 707 4210 | 0.00 | 4834800.00 | 8951700.00 | 8996500.00 | 7571785.44 | 0.00 | 0.00 | 0.00 | 0.00 | 6814500.00 | 0.00 | 0.00 | KO:K05349 | beta-glucosidase | Bacteria;Actinobacteria;Thermoleophilia;Solirubrobacterales;Solirubrobacteraceae;Solirubrobacter;Solirubrobacter soli;Solirubrobacter soli DSM 22325 |
| Ga0586324 0032920 1 2448 | 0.00 | 0.00 | 0.00 | 351309.05 | 0.00 | 0.00 | 0.00 | 0.00 | 0.00 | 0.00 | 0.00 | 0.00 | KO:K05349 | beta-glucosidase | Bacteria;Bacteroidetes;Bacteroidia;Bacteroidales;Bacteroidaceae;Bacteroides;Bacteroides ihuae;Bacteroides ihuae Marseille-P2824 |
| Ga0586324 0164974 179 2707 | 0.00 | 0.00 | 0.00 | 0.00 | 0.00 | 0.00 | 1715323.80 | 0.00 | 0.00 | 0.00 | 0.00 | 0.00 | KO:K05349 | beta-glucosidase | Bacteria;Acidobacteria;Acidobacteriia;Acidobacteriales;Acidobacteriaceae;Edaphobacter;Edaphobacter aggregans;Edaphobacter aggregans EB153 |
| Ga0586324 0172930 2 1852 | 0.00 | 0.00 | 0.00 | 0.00 | 759676.50 | 0.00 | 0.00 | 0.00 | 0.00 | 0.00 | 0.00 | 0.00 | KO:K05349 | beta-glucosidase | Bacteria;Proteobacteria;Alphaproteobacteria;Sphingomonadales;Sphingomonadaceae;Sphingomonas;Sphingomonas hengshuiensis;Sphingomonas hengshuiensis WHSC-8 |
| Ga0586324 0492407 1 1650 | 0.00 | 0.00 | 4362242.23 | 0.00 | 0.00 | 0.00 | 0.00 | 0.00 | 0.00 | 0.00 | 0.00 | 0.00 | KO:K05349 | beta-glucosidase | Bacteria;Proteobacteria;Gammaproteobacteria;Nevskiales;Steroidobacteraceae;Povalibacter;Povalibacter uvarum;Povalibacter uvarum DSM 26723 |
| Ga0586324 0691323 3 1424 | 0.00 | 0.00 | 0.00 | 720110.45 | 0.00 | 0.00 | 0.00 | 0.00 | 0.00 | 0.00 | 0.00 | 0.00 | KO:K05349 | beta-glucosidase | Bacteria;Proteobacteria;Alphaproteobacteria;Micropepsales;Micropepsaceae;Rhizomicrobium;Rhizomicrobium electricum;Rhizomicrobium electricum DSM 21034 |
| Ga0586324 1623366 3 986 | 0.00 | 0.00 | 0.00 | 0.00 | 0.00 | 476709.66 | 0.00 | 0.00 | 0.00 | 0.00 | 0.00 | 0.00 | KO:K05349 | beta-glucosidase | Bacteria;Proteobacteria;Alphaproteobacteria;Micropepsales;Micropepsaceae;Rhizomicrobium;Rhizomicrobium electricum;Rhizomicrobium electricum DSM 21034 |
| Ga0586324 0485554 57 1019 | 0.00 | 0.00 | 0.00 | 0.00 | 0.00 | 0.00 | 0.00 | 0.00 | 0.00 | 164368.82 | 0.00 | 0.00 | KO:K05516 | curved DNA-binding protein | Bacteria;Proteobacteria;Deltaproteobacteria;Myxococcales;Anaeromyxobacteraceae;Anaeromyxobacter;Anaeromyxobacter sp. Fw109-5;Anaeromyxobacter sp. Fw109-5 |
| Ga0586324 0020341 2534 4174 | 0.00 | 14076000.00 | 0.00 | 19285000.00 | 0.00 | 0.00 | 0.00 | 0.00 | 29808000.00 | 0.00 | 0.00 | 0.00 | KO:K05601 | hydroxylamine reductase | Bacteria;Proteobacteria;Deltaproteobacteria;Syntrophobacterales;Syntrophobacteraceae;Syntrophobacter;Syntrophobacter fumaroxidans;Syntrophobacter fumaroxidans MPOB |
| Ga0586324 0024257 2 1561 | 0.00 | 0.00 | 0.00 | 0.00 | 5778300.00 | 0.00 | 10282800.00 | 4776300.00 | 0.00 | 0.00 | 0.00 | 0.00 | KO:K05601 | hydroxylamine reductase | Bacteria;Proteobacteria;Deltaproteobacteria;Syntrophobacterales;Syntrophaceae;Desulfomonile;Desulfomonile tiedjei;Desulfomonile tiedjei DCB-1, DSM 6799 |
| Ga0586324 0536571 1 1590 | 0.00 | 0.00 | 0.00 | 0.00 | 0.00 | 0.00 | 0.00 | 0.00 | 2681100.00 | 0.00 | 0.00 | 0.00 | KO:K05601 | hydroxylamine reductase | Bacteria;Proteobacteria;Deltaproteobacteria;Syntrophobacterales;Syntrophobacteraceae;Syntrophobacter;Syntrophobacter fumaroxidans;Syntrophobacter fumaroxidans MPOB |
| Ga0586324 0939143 315 1247 | 0.00 | 3026000.00 | 0.00 | 0.00 | 0.00 | 0.00 | 0.00 | 0.00 | 0.00 | 0.00 | 0.00 | 0.00 | KO:K05601 | hydroxylamine reductase | Bacteria;Proteobacteria;Deltaproteobacteria;Syntrophobacterales;Syntrophaceae;Desulfomonile;Desulfomonile tiedjei;Desulfomonile tiedjei DCB-1, DSM 6799 |
| Ga0586324 1617287 1 987 | 0.00 | 0.00 | 0.00 | 0.00 | 0.00 | 0.00 | 0.00 | 0.00 | 5799600.00 | 0.00 | 0.00 | 0.00 | KO:K05601 | hydroxylamine reductase | Bacteria;Proteobacteria;Deltaproteobacteria;Syntrophobacterales;Syntrophaceae;Desulfobacca;Desulfobacca acetoxidans;Desulfobacca acetoxidans ASRB2, DSM 11109 |
| Ga0586324 3711326 129 698 | 6030000.00 | 0.00 | 0.00 | 0.00 | 0.00 | 0.00 | 0.00 | 0.00 | 0.00 | 0.00 | 0.00 | 0.00 | KO:K05601 | hydroxylamine reductase | Bacteria;Proteobacteria;Deltaproteobacteria;Syntrophobacterales;Syntrophobacteraceae;Desulfosoma;Desulfosoma caldarium;Desulfosoma caldarium DSM 22027 |
| Ga0586324 0025459 5201 6007 | 0.00 | 0.00 | 0.00 | 0.00 | 0.00 | 0.00 | 0.00 | 0.00 | 0.00 | 0.00 | 0.00 | 2422500.00 | KO:K05772 | tungstate transport system substrate-binding protein | Bacteria;Proteobacteria;Betaproteobacteria;Burkholderiales;Oxalobacteraceae;Massilia;Massilia sp. YMA4;Massilia sp. YMA4 |
| Ga0586324 3946121 2 679 | 0.00 | 0.00 | 0.00 | 0.00 | 0.00 | 0.00 | 0.00 | 0.00 | 0.00 | 0.00 | 0.00 | 2017500.00 | KO:K05772 | tungstate transport system substrate-binding protein | Bacteria;Nitrospirae;Thermodesulfovibrionia;Thermodesulfovibrionales;Thermodesulfovibrionaceae;Thermodesulfovibrio;Thermodesulfovibrio islandicus;Thermodesulfovibrio islandicus DSM 12570 |
| Ga0586324 6902250 18 539 | 0.00 | 0.00 | 0.00 | 0.00 | 0.00 | 611987.41 | 0.00 | 0.00 | 0.00 | 0.00 | 0.00 | 0.00 | KO:K05772 | tungstate transport system substrate-binding protein | Bacteria;Proteobacteria;Deltaproteobacteria;Syntrophobacterales;Syntrophaceae;Syntrophus;Syntrophus aciditrophicus;Syntrophus aciditrophicus SB |
| Ga0586324 0011056 4857 5432 | 0.00 | 1968831.34 | 0.00 | 0.00 | 0.00 | 0.00 | 0.00 | 0.00 | 0.00 | 0.00 | 0.00 | 0.00 | KO:K05795 | tellurium resistance protein TerD | Bacteria;Proteobacteria;Gammaproteobacteria;Methylococcales;Methylococcaceae;Methylobacter;Methylobacter tundripaludum;Methylobacter tundripaludum SV96 |
| Ga0586324 4580906 2 403 | 4310000.00 | 2958000.00 | 0.00 | 1292000.00 | 2276300.00 | 0.00 | 0.00 | 0.00 | 1935900.00 | 2117500.00 | 3256000.00 | 2490000.00 | KO:K05795 | tellurium resistance protein TerD | Bacteria;Proteobacteria;Gammaproteobacteria;Methylococcales;Methylococcaceae;Methylobacter;Methylobacter tundripaludum;Methylobacter tundripaludum SV96 |
| Ga0586324 7044410 199 534 | 0.00 | 134644.08 | 0.00 | 0.00 | 0.00 | 0.00 | 0.00 | 0.00 | 0.00 | 0.00 | 0.00 | 0.00 | KO:K05795 | tellurium resistance protein TerD | Bacteria;Proteobacteria;Gammaproteobacteria;Methylococcales;Methylococcaceae;Methylomicrobium;Methylomicrobium lacus;Methylosarcina lacus LW14 |
| Ga0586324 1027441 269 796 | 0.00 | 822800.00 | 0.00 | 0.00 | 0.00 | 0.00 | 0.00 | 0.00 | 0.00 | 0.00 | 0.00 | 0.00 | KO:K05808 | putative sigma-54 modulation protein | Bacteria;Firmicutes;Clostridia;Thermoanaerobacterales;Thermoanaerobacteraceae;Moorella;Moorella sp. Hama-1;Moorella sp. Hama-1 |
| Ga0586324 3463375 1 717 | 0.00 | 0.00 | 0.00 | 0.00 | 0.00 | 0.00 | 0.00 | 256669.09 | 0.00 | 0.00 | 0.00 | 0.00 | KO:K05813 | sn-glycerol 3-phosphate transport system substrate-binding protein | Bacteria;Firmicutes;Negativicutes;Selenomonadales;Selenomonadaceae;Pectinatus;Pectinatus cerevisiiphilus;Pectinatus cerevisiiphilus DSM 20467 |
| Ga0586324 0402861 900 1802 | 0.00 | 1081200.00 | 816423.19 | 0.00 | 1514100.00 | 0.00 | 0.00 | 0.00 | 0.00 | 1208900.00 | 0.00 | 0.00 | KO:K05816 | sn-glycerol 3-phosphate transport system ATP-binding protein | Bacteria;Proteobacteria;Alphaproteobacteria;Hyphomicrobiales;Phyllobacteriaceae;Mesorhizobium;Mesorhizobium japonicum;Mesorhizobium loti R7A |
| Ga0586324 0760720 58 1155 | 0.00 | 0.00 | 0.00 | 0.00 | 0.00 | 0.00 | 0.00 | 0.00 | 0.00 | 0.00 | 0.00 | 3487500.00 | KO:K05816 | sn-glycerol 3-phosphate transport system ATP-binding protein | Bacteria;Proteobacteria;Alphaproteobacteria;Hyphomicrobiales;Bradyrhizobiaceae;Bradyrhizobium;Bradyrhizobium valentinum;Bradyrhizobium valentinum LmjM3 |
| Ga0586324 0000923 23642 24637 | 0.00 | 0.00 | 0.00 | 0.00 | 0.00 | 0.00 | 0.00 | 0.00 | 0.00 | 2587200.00 | 0.00 | 0.00 | KO:K05878 | dihydroxyacetone kinase-like protein | Bacteria;Bacteroidetes;Flavobacteriia;Flavobacteriales;Flavobacteriaceae;Muricauda;Muricauda pacifica;Muricauda pacifica DSM 25027 |
| Ga0586324 0191842 163 1158 | 0.00 | 0.00 | 0.00 | 0.00 | 0.00 | 0.00 | 0.00 | 0.00 | 0.00 | 0.00 | 30096000.00 | 0.00 | KO:K05878 | dihydroxyacetone kinase-like protein | Bacteria;Actinobacteria;Actinomycetia;Micrococcales;Cellulomonadaceae;Cellulomonas;Cellulomonas sp. HZM;Cellulomonas sp. HZM |
| Ga0586324 0452721 2 1432 | 0.00 | 0.00 | 0.00 | 0.00 | 0.00 | 0.00 | 0.00 | 866200.00 | 0.00 | 0.00 | 0.00 | 0.00 | KO:K05898 | 3-oxosteroid 1-dehydrogenase | Bacteria;Proteobacteria;Betaproteobacteria;Burkholderiales;Comamonadaceae;Alicycliphilus;Alicycliphilus denitrificans;Alicycliphilus denitrificans K601 |
| Ga0586324 0079868 2227 2604 | 2320000.00 | 0.00 | 0.00 | 0.00 | 0.00 | 0.00 | 0.00 | 0.00 | 0.00 | 0.00 | 0.00 | 0.00 | KO:K05919 | superoxide reductase | Bacteria;Proteobacteria;Deltaproteobacteria;Syntrophobacterales;Syntrophobacteraceae;Thermodesulforhabdus;Thermodesulforhabdus norvegica;Thermodesulforhabdus norvegica DSM 9990 |
| Ga0586324 0086671 1 369 | 5840000.00 | 7956000.00 | 11485200.00 | 0.00 | 0.00 | 18698700.00 | 0.00 | 5233800.00 | 9315000.00 | 9702000.00 | 8342400.00 | 8925000.00 | KO:K05919 | superoxide reductase | Bacteria;Proteobacteria;Deltaproteobacteria;Desulfovibrionales;Desulfovibrionaceae;Desulfovibrio;Desulfovibrio vietnamensis;Desulfovibrio vietnamensis DSM 10520 |
| Ga0586324 0099274 1906 2277 | 0.00 | 2550000.00 | 11091100.00 | 6992000.00 | 0.00 | 0.00 | 0.00 | 0.00 | 0.00 | 0.00 | 4444000.00 | 0.00 | KO:K05919 | superoxide reductase | Bacteria;Proteobacteria;Deltaproteobacteria;Syntrophobacterales;Syntrophaceae;Desulfomonile;Desulfomonile tiedjei;Desulfomonile tiedjei DCB-1, DSM 6799 |
| Ga0586324 0313830 158 532 | 0.00 | 0.00 | 2846195.83 | 9500000.00 | 0.00 | 6312300.00 | 14272800.00 | 4507900.00 | 0.00 | 0.00 | 0.00 | 1455000.00 | KO:K05919 | superoxide reductase | Bacteria;Proteobacteria;Deltaproteobacteria;Desulfobacterales;Desulfobacteraceae;Desulfatirhabdium;Desulfatirhabdium butyrativorans;Desulfatirhabdium butyrativorans DSM 18734 |
| Ga0586324 1040023 94 468 | 0.00 | 0.00 | 0.00 | 0.00 | 29252000.00 | 0.00 | 0.00 | 0.00 | 8910000.00 | 0.00 | 6820000.00 | 0.00 | KO:K05919 | superoxide reductase | Bacteria;Proteobacteria;Deltaproteobacteria;Syntrophobacterales;Syntrophaceae;Desulfobacca;Desulfobacca acetoxidans;Desulfobacca acetoxidans ASRB2, DSM 11109 |
| Ga0586324 2019036 168 542 | 2570000.00 | 0.00 | 0.00 | 0.00 | 0.00 | 0.00 | 0.00 | 0.00 | 0.00 | 0.00 | 0.00 | 0.00 | KO:K05919 | superoxide reductase | Bacteria;Proteobacteria;Deltaproteobacteria;Syntrophobacterales;Syntrophaceae;Desulfobacca;Desulfobacca acetoxidans;Desulfobacca acetoxidans ASRB2, DSM 11109 |
| Ga0586324 7003198 90 497 | 0.00 | 0.00 | 5336677.00 | 0.00 | 0.00 | 0.00 | 0.00 | 0.00 | 0.00 | 0.00 | 0.00 | 0.00 | KO:K05919 | superoxide reductase | Bacteria;Proteobacteria;Deltaproteobacteria;Desulfovibrionales;Desulfovibrionaceae;Desulfovibrio;Desulfovibrio vietnamensis;Desulfovibrio vietnamensis DSM 10520 |
| Ga0586324 8002908 49 423 | 0.00 | 0.00 | 0.00 | 0.00 | 0.00 | 0.00 | 11308800.00 | 0.00 | 0.00 | 0.00 | 0.00 | 0.00 | KO:K05919 | superoxide reductase | Bacteria;Proteobacteria;Deltaproteobacteria;Desulfovibrionales;Desulfovibrionaceae;Desulfovibrio;Desulfovibrio fructosivorans;Desulfovibrio fructosovorans JJ |
| Ga0586324 8099205 175 504 | 0.00 | 1203600.00 | 0.00 | 0.00 | 0.00 | 0.00 | 0.00 | 0.00 | 4576500.00 | 0.00 | 0.00 | 0.00 | KO:K05919 | superoxide reductase | Bacteria;Proteobacteria;Deltaproteobacteria;Desulfobacterales;Desulfobacteraceae;Desulfatirhabdium;Desulfatirhabdium butyrativorans;Desulfatirhabdium butyrativorans DSM 18734 |
| Ga0586324 8166719 3 488 | 0.00 | 0.00 | 0.00 | 2992500.00 | 0.00 | 0.00 | 0.00 | 0.00 | 0.00 | 0.00 | 0.00 | 0.00 | KO:K05919 | superoxide reductase | Bacteria;Proteobacteria;Deltaproteobacteria;Desulfovibrionales;Desulfovibrionaceae;Desulfovibrio;Desulfovibrio vietnamensis;Desulfovibrio vietnamensis DSM 10520 |
| Ga0586324 4793142 3 626 | 0.00 | 0.00 | 0.00 | 0.00 | 0.00 | 0.00 | 0.00 | 1403000.00 | 0.00 | 0.00 | 0.00 | 0.00 | KO:K05942 | isopropylmalate/homocitrate/citramalate synthase | Bacteria;Proteobacteria;Deltaproteobacteria;Syntrophobacterales;Syntrophaceae;Syntrophus;Syntrophus aciditrophicus;Syntrophus aciditrophicus SB |
| Ga0586324 2732471 65 793 | 0.00 | 0.00 | 0.00 | 0.00 | 0.00 | 0.00 | 0.00 | 0.00 | 0.00 | 0.00 | 0.00 | 1132500.00 | KO:K05970 | sialate O-acetylesterase | Bacteria;Planctomycetes;Planctomycetia;Planctomycetales;Planctomycetaceae;Planctomyces;Planctomyces sp. SH-PL62;Planctomyces sp. SH-PL62 |
| Ga0586324 0363582 1403 1885 | 10100000.00 | 0.00 | 0.00 | 0.00 | 12360000.00 | 0.00 | 0.00 | 8418000.00 | 8910000.00 | 6021400.00 | 0.00 | 3982500.00 | KO:K06001 | tryptophan synthase beta chain | Bacteria;Proteobacteria;Deltaproteobacteria;Desulfobacterales;Desulfobacteraceae;Desulfatiglans;Desulfatiglans anilini;Desulfatiglans anilini DSM 4660 |
| Ga0586324 0659431 3 1226 | 0.00 | 0.00 | 0.00 | 0.00 | 0.00 | 0.00 | 0.00 | 0.00 | 0.00 | 0.00 | 0.00 | 2070000.00 | KO:K06001 | tryptophan synthase beta chain | Bacteria;Proteobacteria;Deltaproteobacteria;Syntrophobacterales;Syntrophaceae;Desulfomonile;Desulfomonile tiedjei;Desulfomonile tiedjei DCB-1, DSM 6799 |
| Ga0586324 0178887 764 2422 | 0.00 | 0.00 | 0.00 | 0.00 | 0.00 | 0.00 | 0.00 | 0.00 | 0.00 | 1925000.00 | 0.00 | 0.00 | KO:K06020 | sulfate-transporting ATPase | Bacteria;Proteobacteria;Alphaproteobacteria;Hyphomicrobiales;unclassified Hyphomicrobiales;Methyloceanibacter;Methyloceanibacter caenitepidi;Methyloceanibacter caenitepidi Gela4 |
| Ga0586324 5903187 3 575 | 0.00 | 0.00 | 0.00 | 0.00 | 0.00 | 0.00 | 0.00 | 0.00 | 0.00 | 0.00 | 0.00 | 293129.25 | KO:K06020 | sulfate-transporting ATPase | Bacteria;Chloroflexi;Chloroflexia;Chloroflexales;Chloroflexaceae;Chloroflexus;Chloroflexus aggregans;Chloroflexus aggregans DSM 9485 |
| Ga0586324 7056382 3 536 | 0.00 | 0.00 | 0.00 | 0.00 | 0.00 | 0.00 | 0.00 | 0.00 | 0.00 | 885500.00 | 0.00 | 0.00 | KO:K06020 | sulfate-transporting ATPase | Bacteria;Proteobacteria;Alphaproteobacteria;Rhodospirillales;Acetobacteraceae;Rhodopila;Rhodopila globiformis;Rhodopila globiformis DSM 161 |
| Ga0586324 2430858 195 713 | 0.00 | 0.00 | 0.00 | 0.00 | 28531000.00 | 0.00 | 0.00 | 0.00 | 0.00 | 0.00 | 0.00 | 0.00 | KO:K06034 | sulfopyruvate decarboxylase subunit alpha | Bacteria;Proteobacteria;Alphaproteobacteria;Hyphomicrobiales;Bradyrhizobiaceae;Bradyrhizobium;Bradyrhizobium guangzhouense;Bradyrhizobium guangzhouense CCBAU 51670 |
| Ga0586324 0000037 33152 34453 | 17800000.00 | 17884000.00 | 38340300.00 | 26125000.00 | 20703000.00 | 29497100.00 | 33537377.28 | 107756500.00 | 114598800.00 | 276553200.00 | 283712000.00 | 230662500.00 | KO:K06076 | long-chain fatty acid transport protein | Bacteria;Proteobacteria;Betaproteobacteria;Nitrosomonadales;Thiobacillaceae;Annwoodia;Annwoodia aquaesulis;Annwoodia aquaesulis DSM 4255 |
| Ga0586324 0000197 11275 12498 | 0.00 | 7820000.00 | 0.00 | 10165000.00 | 0.00 | 0.00 | 0.00 | 10309000.00 | 9865800.00 | 0.00 | 0.00 | 0.00 | KO:K06076 | long-chain fatty acid transport protein | Bacteria;Aquificae;Aquificae;Aquificales;Aquificaceae;Hydrogenobacter;Hydrogenobacter hydrogenophilus;Hydrogenobacter hydrogenophilus DSM 2913 |
| Ga0586324 0001023 590 1795 | 0.00 | 0.00 | 0.00 | 0.00 | 0.00 | 0.00 | 0.00 | 1494500.00 | 0.00 | 0.00 | 0.00 | 0.00 | KO:K06076 | long-chain fatty acid transport protein | Bacteria;Acidobacteria;Thermoanaerobaculia;Thermoanaerobaculales;Thermoanaerobaculaceae;Thermoanaerobaculum;Thermoanaerobaculum aquaticum;Thermoanaerobaculum aquaticum MP-01 |
| Ga0586324 0003450 3064 4317 | 17100000.00 | 10560400.00 | 3800779.22 | 41610000.00 | 39716800.00 | 0.00 | 21751200.00 | 128466000.00 | 168966000.00 | 70224000.00 | 253440000.00 | 189450000.00 | KO:K06076 | long-chain fatty acid transport protein | Bacteria;Proteobacteria;Betaproteobacteria;Nitrosomonadales;Thiobacillaceae;Thiobacillus;Thiobacillus denitrificans;Thiobacillus denitrificans DSM 12475 |
| Ga0586324 0004429 9130 10401 | 6550000.00 | 10682800.00 | 9289500.00 | 5766500.00 | 1472900.00 | 6590200.00 | 13543200.00 | 4337100.00 | 8505000.00 | 1817200.00 | 9240000.00 | 10905000.00 | KO:K06076 | long-chain fatty acid transport protein | Bacteria;Proteobacteria;Betaproteobacteria;Nitrosomonadales;Gallionellaceae;Sideroxydans;Sideroxydans lithotrophicus;Sideroxydans lithotrophicus ES-1 |
| Ga0586324 0010546 3641 4984 | 11400000.00 | 7548000.00 | 0.00 | 9329000.00 | 7745600.00 | 25685900.00 | 0.00 | 13359000.00 | 9315000.00 | 5343800.00 | 5244800.00 | 6982500.00 | KO:K06076 | long-chain fatty acid transport protein | Bacteria;Proteobacteria;Betaproteobacteria;unclassified Betaproteobacteria;unclassified Betaproteobacteria;unclassified Betaproteobacteria;uncultured beta proteobacterium;unclassified betaproteobacterium ENR4 |
| Ga0586324 0019603 6177 7487 | 0.00 | 5712000.00 | 0.00 | 0.00 | 0.00 | 0.00 | 0.00 | 0.00 | 0.00 | 0.00 | 0.00 | 0.00 | KO:K06076 | long-chain fatty acid transport protein | Bacteria;Proteobacteria;Deltaproteobacteria;Desulfobacterales;Desulfobacteraceae;Desulfosarcina;Desulfosarcina variabilis;Desulfosarcina variabilis Montpellier |
| Ga0586324 0045209 2205 3512 | 0.00 | 0.00 | 0.00 | 0.00 | 0.00 | 0.00 | 0.00 | 2269200.00 | 0.00 | 0.00 | 0.00 | 0.00 | KO:K06076 | long-chain fatty acid transport protein | Bacteria;Proteobacteria;Deltaproteobacteria;Syntrophobacterales;Syntrophobacteraceae;Syntrophobacter;Syntrophobacter fumaroxidans;Syntrophobacter fumaroxidans MPOB |
| Ga0586324 0060955 1 1344 | 8130000.00 | 1577600.00 | 0.00 | 0.00 | 0.00 | 0.00 | 0.00 | 4239500.00 | 5904900.00 | 0.00 | 0.00 | 0.00 | KO:K06076 | long-chain fatty acid transport protein | Bacteria;Proteobacteria;Betaproteobacteria;Nitrosomonadales;Sulfuricellaceae;Sulfuricella;Sulfuricella denitrificans;Sulfuricella denitrificans skB26 |
| Ga0586324 0067902 3108 4127 | 0.00 | 0.00 | 0.00 | 1273000.00 | 0.00 | 0.00 | 0.00 | 0.00 | 0.00 | 0.00 | 0.00 | 0.00 | KO:K06076 | long-chain fatty acid transport protein | Bacteria;Proteobacteria;Deltaproteobacteria;Desulfovibrionales;Desulfovibrionaceae;Desulfovibrio;unclassified Desulfovibrio;Desulfovibrio sp. DF1 |
| Ga0586324 0069193 864 2192 | 0.00 | 3121200.00 | 0.00 | 0.00 | 1431700.00 | 0.00 | 3192000.00 | 5325300.00 | 3604500.00 | 6822200.00 | 0.00 | 6315000.00 | KO:K06076 | long-chain fatty acid transport protein | Bacteria;Proteobacteria;Betaproteobacteria;Nitrosomonadales;Sulfuricellaceae;Sulfuricella;Sulfuricella sp. T08;Sulfuricella sp. T08 |
| Ga0586324 0077972 1603 2874 | 0.00 | 0.00 | 0.00 | 0.00 | 0.00 | 0.00 | 0.00 | 0.00 | 0.00 | 0.00 | 0.00 | 1567500.00 | KO:K06076 | long-chain fatty acid transport protein | Bacteria;Proteobacteria;Betaproteobacteria;Nitrosomonadales;Methylophilaceae;Methylotenera;Methylotenera versatilis;Methylotenera versatilis 79 |
| Ga0586324 0080592 430 1728 | 2870000.00 | 0.00 | 0.00 | 3135000.00 | 4047900.00 | 0.00 | 0.00 | 0.00 | 13786200.00 | 8162000.00 | 8368800.00 | 0.00 | KO:K06076 | long-chain fatty acid transport protein | Bacteria;Proteobacteria;Betaproteobacteria;Nitrosomonadales;Gallionellaceae;Sideroxydans;Sideroxydans lithotrophicus;Sideroxydans lithotrophicus ES-1 |
| Ga0586324 0094538 466 1953 | 0.00 | 0.00 | 0.00 | 0.00 | 0.00 | 0.00 | 0.00 | 2580300.00 | 0.00 | 0.00 | 0.00 | 0.00 | KO:K06076 | long-chain fatty acid transport protein | Bacteria;Proteobacteria;Betaproteobacteria;Nitrosomonadales;Thiobacillaceae;Thiobacillus;Thiobacillus thioparus;Thiobacillus thioparus DSM 505 |
| Ga0586324 0098011 1125 2462 | 31500000.00 | 20332000.00 | 19986500.00 | 0.00 | 8095800.00 | 9327669.83 | 137745158.04 | 114021200.00 | 168868800.00 | 313975200.00 | 210900800.00 | 247260000.00 | KO:K06076 | long-chain fatty acid transport protein | Bacteria;Proteobacteria;Betaproteobacteria;Nitrosomonadales;Sulfuricellaceae;Sulfuricella;Sulfuricella denitrificans;Sulfuricella denitrificans skB26 |
| Ga0586324 0101852 1462 2775 | 0.00 | 3590400.00 | 0.00 | 3268000.00 | 3409300.00 | 0.00 | 3693600.00 | 2513200.00 | 1215000.00 | 5143600.00 | 6283200.00 | 6855000.00 | KO:K06076 | long-chain fatty acid transport protein | Bacteria;Proteobacteria;Betaproteobacteria;Burkholderiales;Comamonadaceae;Curvibacter;Curvibacter sp. PAE-UM;Curvibacter sp. PAE-UM |
| Ga0586324 0125359 2383 3081 | 0.00 | 0.00 | 0.00 | 0.00 | 0.00 | 0.00 | 0.00 | 0.00 | 0.00 | 0.00 | 0.00 | 11400000.00 | KO:K06076 | long-chain fatty acid transport protein | Bacteria;Proteobacteria;Gammaproteobacteria;Acidiferrobacterales;Acidiferrobacteraceae;Sulfuricaulis;Sulfuricaulis limicola;Sulfuricaulis limicola HA5 |
| Ga0586324 0135218 1078 2397 | 12740000.00 | 15096000.00 | 0.00 | 16910000.00 | 0.00 | 4446400.00 | 0.00 | 11956000.00 | 12968100.00 | 13244000.00 | 9944000.00 | 8700000.00 | KO:K06076 | long-chain fatty acid transport protein | Bacteria;Proteobacteria;Betaproteobacteria;Burkholderiales;Comamonadaceae;Limnohabitans;Limnohabitans sp. 63ED37-2;Limnohabitans sp. 63ED37-2 |
| Ga0586324 0146497 954 2432 | 0.00 | 1292000.00 | 0.00 | 0.00 | 0.00 | 757845.21 | 0.00 | 0.00 | 0.00 | 0.00 | 0.00 | 0.00 | KO:K06076 | long-chain fatty acid transport protein | Bacteria;Proteobacteria;Betaproteobacteria;Burkholderiales;unclassified Burkholderiales;Ideonella;Ideonella sp. A 288;Ideonella sp. A 288 |
| Ga0586324 0177026 820 2040 | 0.00 | 0.00 | 0.00 | 0.00 | 16480000.00 | 0.00 | 0.00 | 0.00 | 20088000.00 | 11088000.00 | 0.00 | 0.00 | KO:K06076 | long-chain fatty acid transport protein | Bacteria;Proteobacteria;Deltaproteobacteria;Desulfobacterales;Desulfobulbaceae;Desulfoprunum;Desulfoprunum benzoelyticum;Desulfoprunum benzoelyticum DSM 28570 |
| Ga0586324 0186317 339 1763 | 0.00 | 0.00 | 0.00 | 0.00 | 0.00 | 0.00 | 0.00 | 0.00 | 2697300.00 | 0.00 | 0.00 | 0.00 | KO:K06076 | long-chain fatty acid transport protein | Bacteria;Proteobacteria;Betaproteobacteria;Burkholderiales;unclassified Burkholderiales;Ideonella;Ideonella sp. A 288;Ideonella sp. A 288 |
| Ga0586324 0314543 2 1276 | 0.00 | 2781200.00 | 0.00 | 2042500.00 | 0.00 | 0.00 | 0.00 | 0.00 | 0.00 | 0.00 | 0.00 | 1365000.00 | KO:K06076 | long-chain fatty acid transport protein | Bacteria;Proteobacteria;Betaproteobacteria;unclassified Betaproteobacteria;unclassified Betaproteobacteria;unclassified Betaproteobacteria;uncultured beta proteobacterium;unclassified betaproteobacterium ENR4 |
| Ga0586324 0328305 482 1894 | 0.00 | 0.00 | 0.00 | 0.00 | 0.00 | 1883407.70 | 0.00 | 0.00 | 0.00 | 5343800.00 | 0.00 | 0.00 | KO:K06076 | long-chain fatty acid transport protein | Bacteria;Proteobacteria;Gammaproteobacteria;Chromatiales;Chromatiaceae;Lamprocystis;Lamprocystis purpurea;Lamprocystis purpurea DSM 4197 |
| Ga0586324 0347685 200 1489 | 0.00 | 5535200.00 | 4196528.81 | 1729000.00 | 4532000.00 | 5806287.77 | 12540000.00 | 13426100.00 | 3588300.00 | 12774371.61 | 14168000.00 | 19177500.00 | KO:K06076 | long-chain fatty acid transport protein | Bacteria;Proteobacteria;Betaproteobacteria;Nitrosomonadales;Thiobacillaceae;Thiobacillus;Thiobacillus thioparus;Thiobacillus thioparus DSM 505 |
| Ga0586324 0359940 2 1195 | 0.00 | 4950400.00 | 0.00 | 0.00 | 0.00 | 0.00 | 0.00 | 3660000.00 | 0.00 | 0.00 | 0.00 | 0.00 | KO:K06076 | long-chain fatty acid transport protein | Bacteria;Proteobacteria;Deltaproteobacteria;Syntrophobacterales;Syntrophobacteraceae;Syntrophobacter;Syntrophobacter fumaroxidans;Syntrophobacter fumaroxidans MPOB |
| Ga0586324 0384668 651 1841 | 3650000.00 | 6324000.00 | 5253583.83 | 3458000.00 | 2781000.00 | 0.00 | 3670800.00 | 9150000.00 | 7257600.00 | 3880800.00 | 0.00 | 3742500.00 | KO:K06076 | long-chain fatty acid transport protein | Bacteria;Proteobacteria;Betaproteobacteria;Burkholderiales;Comamonadaceae;Tibeticola;Tibeticola sediminis;Tibeticola sediminis DSM 101684 |
| Ga0586324 0411595 1 1155 | 0.00 | 0.00 | 0.00 | 0.00 | 0.00 | 0.00 | 0.00 | 1726300.00 | 1352700.00 | 0.00 | 3115200.00 | 1162500.00 | KO:K06076 | long-chain fatty acid transport protein | Bacteria;Proteobacteria;Betaproteobacteria;Nitrosomonadales;Thiobacillaceae;Thiobacillus;Thiobacillus thioparus;Thiobacillus thioparus DSM 505 |
| Ga0586324 0415582 152 1609 | 0.00 | 0.00 | 0.00 | 0.00 | 0.00 | 0.00 | 0.00 | 0.00 | 0.00 | 0.00 | 0.00 | 4290000.00 | KO:K06076 | long-chain fatty acid transport protein | Bacteria;Proteobacteria;Deltaproteobacteria;Myxococcales;Anaeromyxobacteraceae;Anaeromyxobacter;Anaeromyxobacter sp. Fw109-5;Anaeromyxobacter sp. Fw109-5 |
| Ga0586324 0426324 669 1757 | 0.00 | 0.00 | 0.00 | 0.00 | 1689200.00 | 6312300.00 | 0.00 | 0.00 | 0.00 | 0.00 | 0.00 | 0.00 | KO:K06076 | long-chain fatty acid transport protein | Bacteria;Proteobacteria;Betaproteobacteria;Rhodocyclales;Azonexaceae;Dechloromonas;Dechloromonas sp. SG708;Dechloromonas sp. SG708 |
| Ga0586324 0428834 1 1131 | 0.00 | 0.00 | 0.00 | 0.00 | 0.00 | 0.00 | 0.00 | 5734000.00 | 4446900.00 | 2887500.00 | 2921600.00 | 13147500.00 | KO:K06076 | long-chain fatty acid transport protein | Bacteria;Proteobacteria;Betaproteobacteria;Nitrosomonadales;Thiobacillaceae;Thiobacillus;Thiobacillus thioparus;Thiobacillus thioparus DSM 505 |
| Ga0586324 0602813 2 1246 | 8460000.00 | 12104000.00 | 18072300.00 | 16340000.00 | 17819000.00 | 16912200.00 | 0.00 | 15372000.00 | 17957700.00 | 9856000.00 | 0.00 | 12675000.00 | KO:K06076 | long-chain fatty acid transport protein | Bacteria;Proteobacteria;Betaproteobacteria;Rhodocyclales;Azonexaceae;Dechloromonas;Dechloromonas sp. JJ;Dechloromonas sp. JJ |
| Ga0586324 0676206 572 1438 | 0.00 | 0.00 | 0.00 | 0.00 | 0.00 | 0.00 | 3328800.00 | 0.00 | 0.00 | 0.00 | 0.00 | 4282500.00 | KO:K06076 | long-chain fatty acid transport protein | Bacteria;Proteobacteria;Betaproteobacteria;Rhodocyclales;Azonexaceae;Dechloromonas;Dechloromonas sp. JJ;Dechloromonas sp. JJ |
| Ga0586324 0824200 3 1172 | 2610000.00 | 3196000.00 | 0.00 | 4455500.00 | 10197000.00 | 0.00 | 7366823.64 | 4349300.00 | 0.00 | 9856000.00 | 5561600.00 | 7650000.00 | KO:K06076 | long-chain fatty acid transport protein | Bacteria;Proteobacteria;Betaproteobacteria;Nitrosomonadales;Gallionellaceae;Sideroxydans;Sideroxydans lithotrophicus;Sideroxydans lithotrophicus ES-1 |
| Ga0586324 0844721 415 1305 | 0.00 | 0.00 | 0.00 | 0.00 | 0.00 | 5796200.00 | 0.00 | 11716338.93 | 2251800.00 | 0.00 | 2314400.00 | 1942500.00 | KO:K06076 | long-chain fatty acid transport protein | Bacteria;Proteobacteria;Gammaproteobacteria;Acidiferrobacterales;Acidiferrobacteraceae;Sulfuricaulis;Sulfuricaulis limicola;Sulfuricaulis limicola HA5 |
| Ga0586324 0950317 2 997 | 442277.00 | 5576000.00 | 0.00 | 0.00 | 0.00 | 0.00 | 0.00 | 6264700.00 | 0.00 | 7700000.00 | 11070400.00 | 8625000.00 | KO:K06076 | long-chain fatty acid transport protein | Bacteria;Proteobacteria;Betaproteobacteria;Nitrosomonadales;Gallionellaceae;Sideroxydans;Sideroxydans lithotrophicus;Sideroxydans lithotrophicus ES-1 |
| Ga0586324 1071248 3 1133 | 0.00 | 0.00 | 0.00 | 0.00 | 0.00 | 0.00 | 0.00 | 0.00 | 3564000.00 | 0.00 | 0.00 | 0.00 | KO:K06076 | long-chain fatty acid transport protein | Bacteria;Proteobacteria;Betaproteobacteria;Nitrosomonadales;Sulfuricellaceae;Sulfuricella;Sulfuricella sp. T08;Sulfuricella sp. T08 |

| | | | | | | | | | | | | | | | |
|---|---|---|---|---|---|---|---|---|---|---|---|---|---|---|---|
| Ga0586324_1153130_3_887 | 0.00 | 2468400.00 | 0.00 | 0.00 | 0.00 | 0.00 | 4856400.00 | 16116200.00 | 11161800.00 | 62631800.00 | 62383200.00 | 11572500.00 | KO:K06076 | long-chain fatty acid transport protein | Bacteria;Proteobacteria;Betaproteobacteria;Nitrosomonadales;Thiobacillaceae;Thiobacillus;Thiobacillus thioparus;Thiobacillus thioparus DSM 505 |
| Ga0586324_1153303_2_1141 | 0.00 | 0.00 | 0.00 | 28310000.00 | 0.00 | 0.00 | 0.00 | 1140700.00 | 0.00 | 0.00 | 0.00 | 2025000.00 | KO:K06076 | long-chain fatty acid transport protein | Bacteria;Proteobacteria;Betaproteobacteria;Rhodocyclales;Azonexaceae;Dechloromonas;Dechloromonas sp. SG708;Dechloromonas sp. SG708 |
| Ga0586324_1303287_2_1060 | 415049.00 | 301605.84 | 0.00 | 0.00 | 0.00 | 0.00 | 0.00 | 403455.83 | 0.00 | 1540000.00 | 0.00 | 423813.75 | KO:K06076 | long-chain fatty acid transport protein | Bacteria;Proteobacteria;Betaproteobacteria;Burkholderiales;Comamonadaceae;Curvibacter;Curvibacter sp. PAE-UM;Curvibacter sp. PAE-UM |
| Ga0586324_1532909_2_1012 | 1480000.00 | 0.00 | 0.00 | 0.00 | 0.00 | 0.00 | 40584000.00 | 80703000.00 | 167508000.00 | 104458200.00 | 171952000.00 | 132900000.00 | KO:K06076 | long-chain fatty acid transport protein | Bacteria;Proteobacteria;Betaproteobacteria;Nitrosomonadales;Thiobacillaceae;Thiobacillus;Thiobacillus thiophilus;Thiobacillus thiophilus DSM 19892 |
| Ga0586324_1735776_1_960 | 0.00 | 12240000.00 | 0.00 | 0.00 | 0.00 | 0.00 | 0.00 | 0.00 | 2786400.00 | 12782000.00 | 0.00 | 0.00 | KO:K06076 | long-chain fatty acid transport protein | Bacteria;Proteobacteria;Deltaproteobacteria;Desulfobacterales;Desulfobacteraceae;Desulfosarcina;Desulfosarcina variabilis;Desulfosarcina variabilis Montpellier |
| Ga0586324_1799772_3_800 | 0.00 | 0.00 | 0.00 | 0.00 | 0.00 | 0.00 | 0.00 | 1238300.00 | 0.00 | 0.00 | 0.00 | 0.00 | KO:K06076 | long-chain fatty acid transport protein | Bacteria;Proteobacteria;Gammaproteobacteria;Chromatiales;Chromatiaceae;Lamprocystis;Lamprocystis purpurea;Lamprocystis purpurea DSM 4197 |
| Ga0586324_2112755_3_881 | 0.00 | 234338.20 | 0.00 | 465690.95 | 0.00 | 0.00 | 0.00 | 572544.78 | 0.00 | 1339800.00 | 817203.20 | 1042500.00 | KO:K06076 | long-chain fatty acid transport protein | Bacteria;Proteobacteria;Betaproteobacteria;Burkholderiales;Comamonadaceae;Ramlibacter;Ramlibacter lithotrophicus;Ramlibacter lithotrophicus RBP-1 |
| Ga0586324_2307312_1_849 | 0.00 | 0.00 | 0.00 | 0.00 | 0.00 | 0.00 | 0.00 | 0.00 | 0.00 | 0.00 | 3528800.00 | 0.00 | KO:K06076 | long-chain fatty acid transport protein | Bacteria;Proteobacteria;Betaproteobacteria;Nitrosomonadales;Sterolibacteriaceae;Sterolibacterium;Sterolibacterium denitrificans;Sterolibacterium denitrificans Chol |
| Ga0586324_2575249_1_813 | 0.00 | 0.00 | 0.00 | 0.00 | 0.00 | 0.00 | 0.00 | 2181103.80 | 3102300.00 | 8932000.00 | 12320000.00 | 12187500.00 | KO:K06076 | long-chain fatty acid transport protein | Bacteria;Proteobacteria;Betaproteobacteria;Burkholderiales;Comamonadaceae;Ramlibacter;Ramlibacter lithotrophicus;Ramlibacter lithotrophicus RBP-1 |
| Ga0586324_2754818_187_789 | 1620000.00 | 2298400.00 | 2663018.15 | 2698000.00 | 4717400.00 | 3575763.12 | 6361200.00 | 2732800.00 | 4155300.00 | 2148300.00 | 2833600.00 | 2520000.00 | KO:K06076 | long-chain fatty acid transport protein | Bacteria;Proteobacteria;Betaproteobacteria;Rhodocyclales;Azonexaceae;Dechloromonas;Dechloromonas sp. JJ;Dechloromonas sp. JJ |
| Ga0586324_3091409_1_753 | 0.00 | 0.00 | 0.00 | 0.00 | 5459000.00 | 0.00 | 0.00 | 0.00 | 0.00 | 0.00 | 0.00 | 0.00 | KO:K06076 | long-chain fatty acid transport protein | Bacteria;Proteobacteria;Betaproteobacteria;Rhodocyclales;Azonexaceae;Dechloromonas;Dechloromonas sp. JJ;Dechloromonas sp. JJ |
| Ga0586324_3243650_3_737 | 423326.00 | 1298800.00 | 0.00 | 3961500.00 | 4305400.00 | 0.00 | 0.00 | 1683600.00 | 0.00 | 0.00 | 2868800.00 | 2820000.00 | KO:K06076 | long-chain fatty acid transport protein | Bacteria;Proteobacteria;Betaproteobacteria;Burkholderiales;Comamonadaceae;Curvibacter;Curvibacter sp. PAE-UM;Curvibacter sp. PAE-UM |
| Ga0586324_3380788_2_724 | 0.00 | 9778400.00 | 0.00 | 6241500.00 | 9846800.00 | 0.00 | 2644800.00 | 3702700.00 | 4082400.00 | 6067600.00 | 5156800.00 | 3030000.00 | KO:K06076 | long-chain fatty acid transport protein | Bacteria;Proteobacteria;Betaproteobacteria;Rhodocyclales;Azonexaceae;Dechloromonas;Dechloromonas sp. SG708;Dechloromonas sp. SG708 |
| Ga0586324_3965962_2_679 | 0.00 | 0.00 | 0.00 | 0.00 | 0.00 | 4009700.00 | 0.00 | 6588000.00 | 6107400.00 | 8162000.00 | 0.00 | 9525000.00 | KO:K06076 | long-chain fatty acid transport protein | Bacteria;Proteobacteria;Betaproteobacteria;Nitrosomonadales;Thiobacillaceae;Thiobacillus;Thiobacillus thioparus;Thiobacillus thioparus DSM 505 |
| Ga0586324_4382573_12_650 | 0.00 | 0.00 | 0.00 | 0.00 | 0.00 | 0.00 | 0.00 | 0.00 | 462185.19 | 0.00 | 3194400.00 | 0.00 | KO:K06076 | long-chain fatty acid transport protein | Bacteria;Proteobacteria;Betaproteobacteria;Nitrosomonadales;Thiobacillaceae;Thiobacillus;Thiobacillus denitrificans;Thiobacillus denitrificans DSM 12475 |
| Ga0586324_4507868_3_644 | 0.00 | 0.00 | 0.00 | 0.00 | 0.00 | 0.00 | 562033.68 | 0.00 | 0.00 | 924000.00 | 0.00 | 0.00 | KO:K06076 | long-chain fatty acid transport protein | Bacteria;Proteobacteria;Betaproteobacteria;Nitrosomonadales;Sulfuricellaceae;Sulfuricella;Sulfuricella sp. T08;Sulfuricella sp. T08 |
| Ga0586324_6399458_3_557 | 0.00 | 0.00 | 955112.61 | 4740500.00 | 6571400.00 | 0.00 | 42636000.00 | 34892000.00 | 7346700.00 | 89628000.00 | 89760000.00 | 81000000.00 | KO:K06076 | long-chain fatty acid transport protein | Bacteria;Proteobacteria;Betaproteobacteria;Nitrosomonadales;Sulfuricellaceae;Sulfuriferula;Sulfuriferula multivorans;Sulfuriferula multivorans TTN |
| Ga0586324_6913809_3_539 | 10800000.00 | 11220000.00 | 14412800.00 | 14155000.00 | 10258800.00 | 5081600.00 | 0.00 | 33428000.00 | 10773000.00 | 12320000.00 | 0.00 | 68100000.00 | KO:K06076 | long-chain fatty acid transport protein | Bacteria;Proteobacteria;Betaproteobacteria;Nitrosomonadales;Thiobacillaceae;Thiobacillus;Thiobacillus thioparus;Thiobacillus thioparus DSM 505 |
| Ga0586324_2016603_3_464 | 0.00 | 0.00 | 0.00 | 0.00 | 0.00 | 0.00 | 491980.68 | 0.00 | 0.00 | 0.00 | 0.00 | 0.00 | KO:K06077 | outer membrane lipoprotein SlyB | Bacteria;Proteobacteria;Betaproteobacteria;Nitrosomonadales;Sulfuricellaceae;Sulfurirhabdus;Sulfurirhabdus autotrophica;Sulfurirhabdus autotrophica DSM 100309 |
| Ga0586324_0005055_10774_11310 | 0.00 | 1822400.00 | 0.00 | 0.00 | 0.00 | 0.00 | 0.00 | 0.00 | 0.00 | 0.00 | 0.00 | 0.00 | KO:K06142 | outer membrane protein | Bacteria;Proteobacteria;Deltaproteobacteria;Desulfuromonadales;Desulfuromonadaceae;Desulfuromonas;Desulfuromonas sp. DDH964;Desulfuromonas sp. DDH964 |
| Ga0586324_0009356_7608_8213 | 0.00 | 0.00 | 0.00 | 0.00 | 1339000.00 | 0.00 | 0.00 | 0.00 | 0.00 | 0.00 | 0.00 | 0.00 | KO:K06142 | outer membrane protein | Bacteria;Deferribacteres;Deferribacteres;Deferribacterales;Deferribacteraceae;Geovibrio;Geovibrio sp. L21-Ace-BES;Geovibrio sp. L21-Ace-BES, DSM 26904 |
| Ga0586324_0522433_873_1394 | 0.00 | 0.00 | 0.00 | 0.00 | 0.00 | 0.00 | 0.00 | 0.00 | 0.00 | 0.00 | 0.00 | 4582500.00 | KO:K06142 | outer membrane protein | Bacteria;Proteobacteria;Gammaproteobacteria;unclassified Gammaproteobacteria;Candidatus Competibacteraceae;Candidatus Contendobacter;Candidatus Contendobacter odensis;Candidatus Contendobacter odensis Run_B_J11 |
| Ga0586324_1919512_542_919 | 0.00 | 0.00 | 0.00 | 0.00 | 0.00 | 1061423.17 | 0.00 | 0.00 | 0.00 | 0.00 | 0.00 | 0.00 | KO:K06142 | outer membrane protein | Bacteria;Bacteroidetes;Bacteroidia;Marinilabiliales;Prolixibacteraceae;Draconibacterium;Draconibacterium orientale;Draconibacterium orientale FH5 |
| Ga0586324_0088330_115_561 | 0.00 | 2305200.00 | 0.00 | 9880000.00 | 0.00 | 0.00 | 3328800.00 | 0.00 | 1377000.00 | 1516900.00 | 0.00 | 0.00 | KO:K06149 | universal stress protein A | Bacteria;Proteobacteria;Deltaproteobacteria;Syntrophorhabdales;Syntrophorhabdaceae;Syntrophorhabdus;Syntrophorhabdus aromaticivorans;Syntrophorhabdus aromaticivorans UI |
| Ga0586324_0202854_1817_2263 | 0.00 | 0.00 | 0.00 | 0.00 | 0.00 | 0.00 | 0.00 | 0.00 | 0.00 | 0.00 | 0.00 | 1635000.00 | KO:K06149 | universal stress protein A | Bacteria;Proteobacteria;Deltaproteobacteria;Syntrophorhabdales;Syntrophorhabdaceae;Syntrophorhabdus;Syntrophorhabdus aromaticivorans;Syntrophorhabdus aromaticivorans UI |
| Ga0586324_1777363_1_438 | 0.00 | 3910000.00 | 0.00 | 0.00 | 4645300.00 | 0.00 | 0.00 | 0.00 | 0.00 | 3488100.00 | 0.00 | 0.00 | KO:K06149 | universal stress protein A | Bacteria;Proteobacteria;Deltaproteobacteria;Syntrophobacterales;Syntrophaceae;Smithella;Smithella sp. SCADC;Smithella sp. SCADC |
| Ga0586324_0008005_7386_9134 | 0.00 | 0.00 | 0.00 | 0.00 | 0.00 | 0.00 | 0.00 | 0.00 | 0.00 | 0.00 | 1469600.00 | 0.00 | KO:K06178 | 23S rRNA pseudouridine2605 synthase | Bacteria;Proteobacteria;Betaproteobacteria;Burkholderiales;Oxalobacteraceae;Massilia;Massilia sp. GV016;Massilia sp. GV016 |
| Ga0586324_0000030_5333_6529 | 0.00 | 0.00 | 0.00 | 0.00 | 0.00 | 0.00 | 0.00 | 0.00 | 0.00 | 0.00 | 1751200.00 | 0.00 | KO:K06186 | outer membrane protein assembly factor BamE | Bacteria;Proteobacteria;Betaproteobacteria;Nitrosomonadales;Thiobacillaceae;Annwoodia;Annwoodia aquaesulis;Annwoodia aquaesulis DSM 4255 |
| Ga0586324_0025592_1267_3117 | 0.00 | 0.00 | 0.00 | 0.00 | 0.00 | 0.00 | 0.00 | 0.00 | 13041000.00 | 0.00 | 0.00 | 0.00 | KO:K06207 | GTP-binding protein | Bacteria;Chloroflexi;Chloroflexia;Kallotenuales;Kallotenuaceae;Kallotenue;Kallotenue papyrolyticum;Kallotenue papyrolyticum JKG1 |
| Ga0586324_0037253_1477_3306 | 0.00 | 0.00 | 11541500.00 | 0.00 | 0.00 | 0.00 | 0.00 | 0.00 | 0.00 | 0.00 | 0.00 | 0.00 | KO:K06207 | GTP-binding protein | Bacteria;Proteobacteria;Alphaproteobacteria;Hyphomicrobiales;Xanthobacteraceae;Pseudolabrys;Pseudolabrys sp. Root1462;Pseudolabrys sp. Root1462 |
| Ga0586324_0347668_617_1924 | 0.00 | 0.00 | 0.00 | 0.00 | 0.00 | 0.00 | 27360000.00 | 0.00 | 0.00 | 0.00 | 0.00 | 0.00 | KO:K06207 | GTP-binding protein | Bacteria;Nitrospirae;Nitrospira;Nitrospirales;Nitrospiraceae;Nitrospira;Candidatus Nitrospira nitrosa;Candidatus Nitrospira nitrosa COMA1 |
| Ga0586324_0396342_1_1791 | 0.00 | 0.00 | 0.00 | 0.00 | 17716000.00 | 0.00 | 0.00 | 0.00 | 0.00 | 0.00 | 0.00 | 0.00 | KO:K06207 | GTP-binding protein | Bacteria;Acidobacteria;Acidobacteriia;Acidobacteriales;Acidobacteriaceae;Acidobacterium;Acidobacterium ailaaui;Acidobacterium ailaaui PMMR2 |
| Ga0586324_0470691_1_1299 | 0.00 | 8976000.00 | 0.00 | 0.00 | 0.00 | 0.00 | 0.00 | 0.00 | 0.00 | 0.00 | 0.00 | 0.00 | KO:K06207 | GTP-binding protein | Bacteria;Proteobacteria;Deltaproteobacteria;Syntrophobacterales;Syntrophaceae;Desulfobacca;Desulfobacca acetoxidans;Desulfobacca acetoxidans ASRB2, DSM 11109 |
| Ga0586324_0667395_2_1447 | 0.00 | 0.00 | 0.00 | 0.00 | 0.00 | 0.00 | 0.00 | 10919000.00 | 0.00 | 0.00 | 0.00 | 0.00 | KO:K06207 | GTP-binding protein | Bacteria;Nitrospirae;unclassified Nitrospirae;unclassified Nitrospirae;unclassified Nitrospirae;unclassified Nitrospirae;Nitrospirae bacterium HCH-1;Nitrospirae bacterium HCH-1 |
| Ga0586324_5148578_1_609 | 1900000.00 | 0.00 | 0.00 | 0.00 | 0.00 | 0.00 | 0.00 | 0.00 | 0.00 | 0.00 | 0.00 | 0.00 | KO:K06207 | GTP-binding protein | Bacteria;Bacteroidetes;Flavobacteriia;Flavobacteriales;Weeksellaceae;Apibacter;Apibacter sp. wkB309;Apibacter sp. wkB309 |
| Ga0586324_0004482_4503_5399 | 0.00 | 0.00 | 0.00 | 0.00 | 0.00 | 0.00 | 0.00 | 10675000.00 | 0.00 | 0.00 | 0.00 | 0.00 | KO:K06215 | pyridoxal 5'-phosphate synthase pdxS subunit | Archaea;Euryarchaeota;Methanomicrobia;Methanomicrobiales;Methanoregulaceae;Methanoregula;Methanoregula formicica;Methanoregula formicica SMSP, DSM 22288 |
| Ga0586324_0461921_836_1696 | 0.00 | 0.00 | 0.00 | 19095000.00 | 0.00 | 0.00 | 0.00 | 0.00 | 0.00 | 0.00 | 0.00 | 0.00 | KO:K06215 | pyridoxal 5'-phosphate synthase pdxS subunit | Bacteria;Proteobacteria;Gammaproteobacteria;unclassified Gammaproteobacteria;Candidatus Competibacteraceae;Candidatus Contendobacter;Candidatus Contendobacter odensis;Candidatus Contendobacter odensis Run_B_J11 |
| Ga0586324_0582334_160_1041 | 0.00 | 8092000.00 | 0.00 | 0.00 | 0.00 | 0.00 | 0.00 | 0.00 | 0.00 | 0.00 | 0.00 | 0.00 | KO:K06215 | pyridoxal 5'-phosphate synthase pdxS subunit | Bacteria;Proteobacteria;Deltaproteobacteria;Syntrophobacterales;Syntrophaceae;Desulfomonile;Desulfomonile tiedjei;Desulfomonile tiedjei DCB-1, DSM 6799 |
| Ga0586324_2166691_2_619 | 0.00 | 0.00 | 0.00 | 0.00 | 8569600.00 | 0.00 | 0.00 | 0.00 | 0.00 | 0.00 | 0.00 | 0.00 | KO:K06215 | pyridoxal 5'-phosphate synthase pdxS subunit | Bacteria;Acidobacteria;Acidobacteriia;Acidobacteriales;Acidobacteriaceae;unclassified Acidobacteriaceae;Acidobacteriaceae bacterium KBS 96;Acidobacteriaceae bacterium KBS 96, DSM 24297 |
| Ga0586324_4082108_25_669 | 0.00 | 0.00 | 0.00 | 0.00 | 0.00 | 0.00 | 0.00 | 1543300.00 | 0.00 | 0.00 | 0.00 | 0.00 | KO:K06215 | pyridoxal 5'-phosphate synthase pdxS subunit | Bacteria;Chloroflexi;Anaerolineae;Anaerolineales;Anaerolineaceae;Anaerolinea;Anaerolinea thermophila;Anaerolinea thermophila UNI-1 |
| Ga0586324_6208543_3_491 | 0.00 | 0.00 | 0.00 | 0.00 | 5778300.00 | 0.00 | 0.00 | 0.00 | 0.00 | 0.00 | 0.00 | 0.00 | KO:K06215 | pyridoxal 5'-phosphate synthase pdxS subunit | Bacteria;Actinobacteria;Acidimicrobiia;Acidimicrobiales;Ilumatobacteraceae;Desertimonas;Desertimonas flava;Acidimicrobiales bacterium SYSU D60003 |
| Ga0586324_1090001_369_1169 | 0.00 | 11832000.00 | 0.00 | 0.00 | 0.00 | 0.00 | 0.00 | 0.00 | 0.00 | 0.00 | 0.00 | 0.00 | KO:K06217 | phosphate starvation-inducible PhoH-like protein | Bacteria;Proteobacteria;Deltaproteobacteria;Myxococcales;Anaeromyxobacteraceae;Anaeromyxobacter;Anaeromyxobacter dehalogenans;Anaeromyxobacter dehalogenans 2CP-1 |
| Ga0586324_0000005_11964_13721 | 0.00 | 0.00 | 0.00 | 0.00 | 0.00 | 0.00 | 0.00 | 1000400.00 | 0.00 | 6229300.00 | 14484800.00 | 6945000.00 | KO:K06281 | hydrogenase large subunit | Bacteria;Proteobacteria;Betaproteobacteria;Nitrosomonadales;Thiobacillaceae;Annwoodia;Annwoodia aquaesulis;Annwoodia aquaesulis DSM 4255 |
| Ga0586324_0000597_2013_3470 | 0.00 | 0.00 | 0.00 | 0.00 | 0.00 | 0.00 | 9393600.00 | 0.00 | 0.00 | 0.00 | 0.00 | 0.00 | KO:K06281 | hydrogenase large subunit | Bacteria;Proteobacteria;Deltaproteobacteria;Desulfobacterales;Desulfobulbaceae;Desulfobulbus;Desulfobulbus propionicus;Desulfobulbus propionicus 1pr3, DSM 2032 |
| Ga0586324_0025429_3523_5223 | 0.00 | 0.00 | 0.00 | 5301000.00 | 0.00 | 0.00 | 0.00 | 3739300.00 | 0.00 | 3888500.00 | 0.00 | 5002500.00 | KO:K06281 | hydrogenase large subunit | Bacteria;Proteobacteria;Gammaproteobacteria;Chromatiales;Ectothiorhodospiraceae;Thioalbus;Thioalbus denitrificans;Thioalbus denitrificans DSM 26407 |
| Ga0586324_0112811_176_1939 | 0.00 | 0.00 | 0.00 | 0.00 | 0.00 | 0.00 | 364428.36 | 6405000.00 | 0.00 | 0.00 | 7400800.00 | 2902500.00 | KO:K06281 | hydrogenase large subunit | Bacteria;Proteobacteria;Betaproteobacteria;Nitrosomonadales;Thiobacillaceae;Thiobacillus;Thiobacillus thioparus;Thiobacillus thioparus DSM 505 |
| Ga0586324_0113263_2550_3230 | 0.00 | 0.00 | 0.00 | 0.00 | 0.00 | 0.00 | 0.00 | 0.00 | 1036800.00 | 0.00 | 0.00 | 0.00 | KO:K06281 | hydrogenase large subunit | Bacteria;Proteobacteria;Betaproteobacteria;Nitrosomonadales;Sterolibacteriaceae;Sulfurisoma;Sulfurisoma sediminicola;Sulfurisoma sediminicola DSM 26916 |
| Ga0586324_0403733_3_1328 | 0.00 | 0.00 | 0.00 | 0.00 | 0.00 | 0.00 | 0.00 | 0.00 | 0.00 | 0.00 | 0.00 | 4762500.00 | KO:K06281 | hydrogenase large subunit | Bacteria;Proteobacteria;Alphaproteobacteria;Hyphomicrobiales;Bradyrhizobiaceae;Rhodopseudomonas;Rhodopseudomonas palustris;Rhodopseudomonas palustris O.U.11, DSM 7375 |
| Ga0586324_0660996_171_1451 | 0.00 | 0.00 | 0.00 | 0.00 | 0.00 | 0.00 | 4696800.00 | 0.00 | 0.00 | 0.00 | 0.00 | 0.00 | KO:K06281 | hydrogenase large subunit | Bacteria;Proteobacteria;Betaproteobacteria;Rhodocyclales;Zoogloeaceae;Azoarcus;Azoarcus indigens;Azoarcus indigens DSM 12121 |
| Ga0586324_1065884_1_1179 | 0.00 | 0.00 | 0.00 | 0.00 | 0.00 | 0.00 | 357656.76 | 0.00 | 0.00 | 0.00 | 0.00 | 0.00 | KO:K06281 | hydrogenase large subunit | Bacteria;Proteobacteria;Betaproteobacteria;Neisseriales;Chromobacteriaceae;Aquaspirillum;Aquaspirillum sp. LM1;Aquaspirillum sp. LM1 |
| Ga0586324_2376205_3_839 | 0.00 | 0.00 | 0.00 | 0.00 | 0.00 | 0.00 | 0.00 | 0.00 | 1304100.00 | 0.00 | 0.00 | 0.00 | KO:K06281 | hydrogenase large subunit | Bacteria;Proteobacteria;Deltaproteobacteria;Desulfobacterales;Desulfobulbaceae;Desulfobulbus;Desulfobulbus propionicus;Desulfobulbus propionicus 1pr3, DSM 2032 |
| Ga0586324_2573607_3_593 | 0.00 | 0.00 | 0.00 | 0.00 | 0.00 | 0.00 | 344008.68 | 0.00 | 0.00 | 0.00 | 0.00 | 0.00 | KO:K06281 | hydrogenase large subunit | Bacteria;Bacteroidetes;Sphingobacteriia;Sphingobacteriales;Sphingobacteriaceae;Mucilaginibacter;Mucilaginibacter gotjawali;Mucilaginibacter gotjawali SA3-7 |
| Ga0586324_3358479_288_728 | 0.00 | 0.00 | 0.00 | 0.00 | 0.00 | 0.00 | 0.00 | 0.00 | 2300400.00 | 0.00 | 0.00 | 0.00 | KO:K06281 | hydrogenase large subunit | Bacteria;Firmicutes;Clostridia;Eubacteriales;Heliobacteriaceae;Heliomicrobium;Heliomicrobium modesticaldum;Heliomicrobium modesticaldum Ice1 |
| Ga0586324_4059606_45_671 | 0.00 | 0.00 | 0.00 | 0.00 | 0.00 | 0.00 | 0.00 | 0.00 | 0.00 | 0.00 | 0.00 | 1957500.00 | KO:K06281 | hydrogenase large subunit | Bacteria;Proteobacteria;Gammaproteobacteria;Chromatiales;Chromatiaceae;Thiocystis;Thiocystis violascens;Thiocystis violascens 611, DSM 198 |
| Ga0586324_0003737_1_1398 | 0.00 | 0.00 | 0.00 | 0.00 | 0.00 | 19135400.00 | 0.00 | 0.00 | 10044000.00 | 0.00 | 0.00 | 0.00 | KO:K06324 | spore coat protein A | Bacteria;Proteobacteria;Deltaproteobacteria;Desulfuromonadales;Geobacteraceae;Geobacter;Geobacter pickeringii;Geobacter pickeringii G13 |
| Ga0586324_0010092_6220_8124 | 24020000.00 | 5780000.00 | 26010600.00 | 22230000.00 | 7992800.00 | 12981900.00 | 0.00 | 6588000.00 | 10837800.00 | 17702300.00 | 0.00 | 7290000.00 | KO:K06324 | spore coat protein A | Bacteria;Firmicutes;Clostridia;Eubacteriales;Clostridiaceae;Abyssisolibacter;Abyssisolibacter fermentans;Abyssisolibacter fermentans MCWD3 |
| Ga0586324_0013881_5782_7956 | 1780000.00 | 0.00 | 468215.57 | 0.00 | 0.00 | 0.00 | 0.00 | 0.00 | 0.00 | 0.00 | 0.00 | 0.00 | KO:K06324 | spore coat protein A | Bacteria;Proteobacteria;Deltaproteobacteria;Desulfuromonadales;Geobacteraceae;Geobacter;Geobacter pickeringii;Geobacter pickeringii G13 |
| Ga0586324_0013978_1720_5040 | 2420000.00 | 0.00 | 0.00 | 25175000.00 | 26697600.00 | 19587638.58 | 4696800.00 | 9570900.00 | 14102100.00 | 0.00 | 0.00 | 0.00 | KO:K06324 | spore coat protein A | Bacteria;Proteobacteria;Gammaproteobacteria;Chromatiales;Chromatiaceae;Nitrosococcus;Nitrosococcus halophilus;Nitrosococcus halophilus Nc4 |
| Ga0586324_0022131_1_2715 | 0.00 | 4399600.00 | 0.00 | 0.00 | 0.00 | 0.00 | 0.00 | 0.00 | 0.00 | 0.00 | 0.00 | 0.00 | KO:K06324 | spore coat protein A | Bacteria;Chloroflexi;Ktedonobacteria;Ktedonobacterales;Dictyobacteraceae;Dictyobacter;Dictyobacter aurantiacus;Dictyobacter aurantiacus S27 |
| Ga0586324_0045294_2_1810 | 0.00 | 0.00 | 0.00 | 0.00 | 4223000.00 | 0.00 | 0.00 | 4007700.00 | 1458000.00 | 4188800.00 | 0.00 | 705665.25 | KO:K06324 | spore coat protein A | Bacteria;Proteobacteria;Deltaproteobacteria;Desulfuromonadales;Geobacteraceae;Geobacter;Geobacter pickeringii;Geobacter pickeringii G13 |
| Ga0586324_0105611_1159_3342 | 0.00 | 2788000.00 | 0.00 | 0.00 | 0.00 | 0.00 | 0.00 | 0.00 | 0.00 | 0.00 | 0.00 | 0.00 | KO:K06324 | spore coat protein A | Bacteria;Proteobacteria;Deltaproteobacteria;Desulfuromonadales;Geobacteraceae;Geobacter;Geobacter pickeringii;Geobacter pickeringii G13 |
| Ga0586324_0113510_3_2096 | 0.00 | 1645600.00 | 2185712.38 | 2394000.00 | 2121800.00 | 0.00 | 0.00 | 475032.62 | 0.00 | 0.00 | 0.00 | 0.00 | KO:K06324 | spore coat protein A | Bacteria;Proteobacteria;Deltaproteobacteria;Desulfuromonadales;Geobacteraceae;Geobacter;Geobacter pickeringii;Geobacter pickeringii G13 |
| Ga0586324_0138117_1_1083 | 0.00 | 2162400.00 | 0.00 | 0.00 | 0.00 | 0.00 | 0.00 | 0.00 | 0.00 | 0.00 | 0.00 | 0.00 | KO:K06324 | spore coat protein A | Bacteria;Proteobacteria;Deltaproteobacteria;Desulfuromonadales;Geobacteraceae;Geobacter;Geobacter pickeringii;Geobacter pickeringii G13 |
| Ga0586324_0160252_1536_2744 | 0.00 | 0.00 | 0.00 | 0.00 | 0.00 | 0.00 | 0.00 | 0.00 | 0.00 | 0.00 | 0.00 | 1717500.00 | KO:K06324 | spore coat protein A | Bacteria;Proteobacteria;Deltaproteobacteria;Desulfuromonadales;Geobacteraceae;Geobacter;Geobacter pickeringii;Geobacter pickeringii G13 |
| Ga0586324_0163708_1_1782 | 12059055.00 | 14229585.48 | 4094625.81 | 15922000.00 | 14646600.00 | 6074560.52 | 6467327.16 | 9872746.30 | 9360234.45 | 9409400.00 | 7840800.00 | 8730000.00 | KO:K06324 | spore coat protein A | Bacteria;Proteobacteria;Deltaproteobacteria;Desulfuromonadales;Geobacteraceae;Geobacter;Geobacter pickeringii;Geobacter pickeringii G13 |
| Ga0586324_0177590_3_2153 | 5910000.00 | 0.00 | 0.00 | 0.00 | 0.00 | 0.00 | 0.00 | 0.00 | 0.00 | 0.00 | 0.00 | 0.00 | KO:K06324 | spore coat protein A | Bacteria;Proteobacteria;Deltaproteobacteria;Desulfuromonadales;Geobacteraceae;Geobacter;Geobacter pickeringii;Geobacter pickeringii G13 |
| Ga0586324_0196986_2_1327 | 12500000.00 | 0.00 | 0.00 | 0.00 | 0.00 | 0.00 | 0.00 | 0.00 | 0.00 | 0.00 | 0.00 | 0.00 | KO:K06324 | spore coat protein A | Bacteria;Proteobacteria;Deltaproteobacteria;Desulfuromonadales;Geobacteraceae;Geobacter;Geobacter pickeringii;Geobacter pickeringii G13 |
| Ga0586324_0325493_201_1982 | 0.00 | 0.00 | 0.00 | 2394000.00 | 0.00 | 0.00 | 0.00 | 0.00 | 0.00 | 0.00 | 0.00 | 0.00 | KO:K06324 | spore coat protein A | Bacteria;Proteobacteria;Deltaproteobacteria;Desulfuromonadales;Geobacteraceae;Geobacter;Geobacter pickeringii;Geobacter pickeringii G13 |
| Ga0586324_0393204_2_1564 | 0.00 | 0.00 | 0.00 | 0.00 | 0.00 | 2397117.76 | 0.00 | 0.00 | 0.00 | 0.00 | 0.00 | 0.00 | KO:K06324 | spore coat protein A | Bacteria;Proteobacteria;Deltaproteobacteria;Myxococcales;unclassified Myxococcales;Minicystis;Minicystis rosea;Minicystis rosea DSM 24000 |
| Ga0586324_0701904_3_1415 | 0.00 | 0.00 | 0.00 | 0.00 | 0.00 | 0.00 | 0.00 | 0.00 | 0.00 | 793100.00 | 624014.16 | 0.00 | KO:K06324 | spore coat protein A | Bacteria;Proteobacteria;Deltaproteobacteria;Desulfuromonadales;Geobacteraceae;Geobacter;Geobacter pickeringii;Geobacter pickeringii G13 |
| Ga0586324_0706127_1_1410 | 30380000.00 | 9717200.00 | 8170210.96 | 7581000.00 | 3874565.42 | 4768093.07 | 6026931.48 | 3020816.99 | 4767327.09 | 8916600.00 | 6221600.00 | 0.00 | KO:K06324 | spore coat protein A | Bacteria;Proteobacteria;Deltaproteobacteria;Desulfuromonadales;Geobacteraceae;Geobacter;Geobacter pickeringii;Geobacter pickeringii G13 |
| Ga0586324_0791572_1_1344 | 3670000.00 | 0.00 | 9120600.00 | 0.00 | 0.00 | 0.00 | 0.00 | 0.00 | 0.00 | 9702000.00 | 0.00 | 0.00 | KO:K06324 | spore coat protein A | Bacteria;Proteobacteria;Betaproteobacteria;Burkholderiales;Burkholderiaceae;Burkholderia;Burkholderia ubonensis;Burkholderia ubonensis MSMB2120WGS |
| Ga0586324_0883833_1_1281 | 0.00 | 0.00 | 0.00 | 0.00 | 0.00 | 1690386.30 | 0.00 | 0.00 | 0.00 | 0.00 | 0.00 | 0.00 | KO:K06324 | spore coat protein A | Bacteria;Proteobacteria;Deltaproteobacteria;Desulfuromonadales;Geobacteraceae;Geobacter;Geobacter argillaceus;Geobacter argillaceus ATCC BAA-1139 |
| Ga0586324_1181769_3_1130 | 0.00 | 2475200.00 | 0.00 | 5111000.00 | 0.00 | 0.00 | 9963600.00 | 0.00 | 4681800.00 | 7053200.00 | 6573600.00 | 12900000.00 | KO:K06324 | spore coat protein A | Bacteria;Proteobacteria;Deltaproteobacteria;Desulfuromonadales;Geobacteraceae;Geobacter;Geobacter pickeringii;Geobacter pickeringii G13 |
| Ga0586324_1512860_1_1017 | 0.00 | 686800.00 | 0.00 | 0.00 | 0.00 | 0.00 | 0.00 | 0.00 | 1069200.00 | 1771000.00 | 0.00 | 1957500.00 | KO:K06324 | spore coat protein A | Bacteria;Proteobacteria;Deltaproteobacteria;Desulfuromonadales;Geobacteraceae;Geobacter;Geobacter pickeringii;Geobacter pickeringii G13 |
| Ga0586324_1664191_3_977 | 0.00 | 0.00 | 1189140.45 | 0.00 | 0.00 | 0.00 | 0.00 | 0.00 | 0.00 | 0.00 | 0.00 | 0.00 | KO:K06324 | spore coat protein A | Bacteria;Proteobacteria;Betaproteobacteria;Neisseriales;Chromobacteriaceae;Pseudogulbenkiania;Pseudogulbenkiania sp. MAI-1;Pseudogulbenkiania sp. MAI-1 |
| Ga0586324_2156411_53_874 | 0.00 | 0.00 | 3434840.48 | 0.00 | 0.00 | 0.00 | 0.00 | 0.00 | 0.00 | 0.00 | 0.00 | 930000.00 | KO:K06324 | spore coat protein A | Bacteria;Proteobacteria;Deltaproteobacteria;Desulfuromonadales;Geobacteraceae;Geobacter;Geobacter pickeringii;Geobacter pickeringii G13 |
| Ga0586324_3383546_2_724 | 896713.00 | 2284800.00 | 0.00 | 1957000.00 | 0.00 | 0.00 | 0.00 | 0.00 | 0.00 | 0.00 | 0.00 | 1470000.00 | KO:K06324 | spore coat protein A | Bacteria;Proteobacteria;Deltaproteobacteria;Desulfuromonadales;Geobacteraceae;Geobacter;Geobacter sp. M18;Geobacter sp. M18 |
| Ga0586324_3463910_1_717 | 13900000.00 | 0.00 | 0.00 | 11020000.00 | 11330000.00 | 0.00 | 0.00 | 0.00 | 0.00 | 11704000.00 | 0.00 | 12225000.00 | KO:K06324 | spore coat protein A | Bacteria;Firmicutes;Clostridia;Eubacteriales;Syntrophomonadaceae;Syntrophomonas;Syntrophomonas zehnderi;Syntrophomonas zehnderi OL-4 |
| Ga0586324_3478110_2_715 | 4320000.00 | 1536800.00 | 0.00 | 0.00 | 0.00 | 0.00 | 0.00 | 0.00 | 0.00 | 0.00 | 0.00 | 0.00 | KO:K06324 | spore coat protein A | Bacteria;Proteobacteria;Deltaproteobacteria;Desulfuromonadales;Geobacteraceae;Geobacter;Geobacter pickeringii;Geobacter pickeringii G13 |
| Ga0586324_4016060_2_652 | 0.00 | 0.00 | 0.00 | 0.00 | 0.00 | 0.00 | 0.00 | 0.00 | 0.00 | 2810500.00 | 0.00 | 0.00 | KO:K06324 | spore coat protein A | Bacteria;Proteobacteria;Deltaproteobacteria;Desulfuromonadales;Geobacteraceae;Geobacter;Geobacter pickeringii;Geobacter pickeringii G13 |
| Ga0586324_4124556_2_667 | 0.00 | 0.00 | 0.00 | 0.00 | 0.00 | 0.00 | 0.00 | 0.00 | 0.00 | 669474.19 | 0.00 | 0.00 | KO:K06324 | spore coat protein A | Bacteria;Proteobacteria;Betaproteobacteria;Burkholderiales;Burkholderiaceae;Burkholderia;Burkholderia ubonensis;Burkholderia ubonensis MSMB782WGS |
| Ga0586324_4834132_62_625 | 0.00 | 0.00 | 0.00 | 0.00 | 0.00 | 0.00 | 0.00 | 0.00 | 0.00 | 3903900.00 | 2261600.00 | 0.00 | KO:K06324 | spore coat protein A | Bacteria;Proteobacteria;Deltaproteobacteria;Desulfuromonadales;Geobacteraceae;Geobacter;Geobacter pickeringii;Geobacter pickeringii G13 |
| Ga0586324_0000004_128140_133716 | 0.00 | 0.00 | 0.00 | 0.00 | 0.00 | 0.00 | 0.00 | 0.00 | 0.00 | 5836600.00 | 3282400.00 | 1597500.00 | KO:K06596 | chemosensory pili system protein ChpA (sensor histidine kinase/response regulator) | Bacteria;Proteobacteria;Betaproteobacteria;Nitrosomonadales;Thiobacillaceae;Annwoodia;Annwoodia aquaesulis;Annwoodia aquaesulis DSM 4255 |
| Ga0586324_0155214_3_2783 | 0.00 | 0.00 | 0.00 | 0.00 | 0.00 | 0.00 | 0.00 | 0.00 | 0.00 | 0.00 | 523479.44 | 0.00 | KO:K06596 | chemosensory pili system protein ChpA (sensor histidine kinase/response regulator) | Bacteria;Proteobacteria;Betaproteobacteria;Nitrosomonadales;Gallionellaceae;Sideroxydans;Sideroxydans lithotrophicus;Sideroxydans lithotrophicus ES-1 |
| Ga0586324_0817481_3_1022 | 0.00 | 0.00 | 0.00 | 2622000.00 | 0.00 | 0.00 | 0.00 | 0.00 | 0.00 | 0.00 | 0.00 | 0.00 | KO:K06871 | uncharacterized protein | Bacteria;Proteobacteria;Deltaproteobacteria;Desulfobacterales;Desulfobacteraceae;Desulfosarcina;Desulfosarcina cetonica;Desulfosarcina cetonica JCM 12296 |
| Ga0586324_0000293_24344_26005 | 11900000.00 | 11628000.00 | 0.00 | 0.00 | 12566000.00 | 0.00 | 0.00 | 0.00 | 0.00 | 0.00 | 5526400.00 | 3240000.00 | KO:K06907 | phage tail sheath protein FI | Bacteria;Proteobacteria;Deltaproteobacteria;Desulfobacterales;Desulfobacteraceae;Desulfatitalea;Desulfatitalea tepidiphila;Desulfatitalea tepidiphila S28bF |
| Ga0586324_0001201_17266_18738 | 0.00 | 0.00 | 0.00 | 31350000.00 | 39449000.00 | 917077.94 | 0.00 | 0.00 | 0.00 | 0.00 | 0.00 | 28950000.00 | KO:K06907 | phage tail sheath protein FI | Bacteria;Proteobacteria;Deltaproteobacteria;Myxococcales;Myxococcaceae;Myxococcus;Myxococcus stipitatus;Myxococcus stipitatus DSM 14675 |
| Ga0586324_0004873_10685_12250 | 3910000.00 | 0.00 | 0.00 | 0.00 | 0.00 | 0.00 | 0.00 | 0.00 | 0.00 | 0.00 | 0.00 | 0.00 | KO:K06907 | phage tail sheath protein FI | Bacteria;Proteobacteria;Betaproteobacteria;Burkholderiales;Comamonadaceae;Variovorax;Variovorax sp. JS1663;Variovorax sp. JS1663 |
| Ga0586324_0005654_6522_8540 | 0.00 | 3107600.00 | 40479700.00 | 29260000.00 | 0.00 | 0.00 | 53352000.00 | 0.00 | 19521000.00 | 31108000.00 | 40040000.00 | 32025000.00 | KO:K06907 | phage tail sheath protein FI | Bacteria;Firmicutes;Bacilli;Bacillales;Paenibacillaceae;Paenibacillus;Paenibacillus sp. A3;Paenibacillus sp. A3 |
| Ga0586324_0006885_7583_8803 | 6780000.00 | 2536400.00 | 0.00 | 4427000.00 | 3646200.00 | 0.00 | 5702512.56 | 3050000.00 | 4633200.00 | 2918300.00 | 0.00 | 2947500.00 | KO:K06907 | phage tail sheath protein FI | Bacteria;Proteobacteria;Deltaproteobacteria;Myxococcales;Kofleriaceae;Haliangium;Haliangium ochraceum;Haliangium ochraceum SMP-2, DSM 14365 |
| Ga0586324_0009957_711_2663 | 0.00 | 0.00 | 0.00 | 0.00 | 1617100.00 | 0.00 | 0.00 | 0.00 | 0.00 | 605556.49 | 0.00 | 0.00 | KO:K06907 | phage tail sheath protein FI | Bacteria;Planctomycetes;Candidatus Brocadiae;Candidatus Brocadiales;Candidatus Brocadiaceae;Candidatus Brocadia;Candidatus Brocadia sinica;Candidatus Brocadia sinica JPN1 |
| Ga0586324_0014495_239_1879 | 0.00 | 1054000.00 | 0.00 | 0.00 | 0.00 | 0.00 | 0.00 | 0.00 | 0.00 | 3210900.00 | 0.00 | 0.00 | KO:K06907 | phage tail sheath protein FI | Bacteria;Actinobacteria;Actinomycetia;Micrococcales;Microbacteriaceae;Microbacterium;Microbacterium trichothecenolyticum;Microbacterium trichothecenolyticum ZKA46 |
| Ga0586324_0014748_2161_4245 | 0.00 | 30328000.00 | 0.00 | 0.00 | 0.00 | 0.00 | 0.00 | 0.00 | 0.00 | 0.00 | 0.00 | 0.00 | KO:K06907 | phage tail sheath protein FI | Bacteria;Proteobacteria;Deltaproteobacteria;Desulfobacterales;Desulfobacteraceae;Desulfobacterium;Desulfobacterium autotrophicum;Desulfobacterium autotrophicum HRM2, DSM 3382 |
| Ga0586324_0035758_1025_2845 | 9150000.00 | 0.00 | 7262700.00 | 6821000.00 | 0.00 | 9051600.00 | 0.00 | 0.00 | 0.00 | 0.00 | 0.00 | 0.00 | KO:K06907 | phage tail sheath protein FI | Bacteria;Proteobacteria;Alphaproteobacteria;Sphingomonadales;Sphingosinicellaceae;Polymorphobacter;Polymorphobacter fuscus;Polymorphobacter fuscus DSM 105347 |
| Ga0586324_0057493_2122_3726 | 0.00 | 318169.28 | 0.00 | 0.00 | 0.00 | 0.00 | 0.00 | 0.00 | 0.00 | 0.00 | 0.00 | 0.00 | KO:K06907 | phage tail sheath protein FI | Bacteria;Actinobacteria;Actinomycetia;Pseudonocardiales;Pseudonocardiaceae;Labedaea;Labedaea rhizosphaerae;Labedaea rhizosphaerae DSM 45361 |
| Ga0586324_0057605_1202_2614 | 0.00 | 1067600.00 | 0.00 | 0.00 | 0.00 | 0.00 | 0.00 | 0.00 | 0.00 | 0.00 | 0.00 | 2220000.00 | KO:K06907 | phage tail sheath protein FI | Bacteria;Proteobacteria;Deltaproteobacteria;Desulfobacterales;Desulfobulbaceae;Desulfobulbus;Desulfobulbus elongatus;Desulfobulbus elongatus DSM 2908 |
| Ga0586324_0070247_3_1763 | 0.00 | 2686000.00 | 0.00 | 0.00 | 0.00 | 0.00 | 0.00 | 0.00 | 0.00 | 0.00 | 2613600.00 | 0.00 | KO:K06907 | phage tail sheath protein FI | Bacteria;Proteobacteria;Gammaproteobacteria;Nevskiales;Sinobacteraceae;Solimonas;Solimonas soli;Solimonas soli DSM 21787 |
| Ga0586324_0094369_1836_3362 | 0.00 | 1543600.00 | 0.00 | 0.00 | 2348400.00 | 0.00 | 1653656.64 | 0.00 | 0.00 | 0.00 | 0.00 | 0.00 | KO:K06907 | phage tail sheath protein FI | Archaea;Euryarchaeota;Methanomicrobia;Methanosarcinales;unclassified Methanosarcinales;unclassified Methanosarcinales;unclassified Methanosarcinales;Methanosarcinales sp. ANME-2a |
| Ga0586324_0136039_1113_2564 | 0.00 | 0.00 | 0.00 | 0.00 | 0.00 | 0.00 | 2824977.00 | 0.00 | 0.00 | 0.00 | 0.00 | 32850000.00 | KO:K06907 | phage tail sheath protein FI | Bacteria;Bacteroidetes;Chitinophagia;Chitinophagales;Chitinophagaceae;Chitinophaga;Chitinophaga pinensis;Chitinophaga pinensis 1204 |
| Ga0586324_0176186_1032_2414 | 0.00 | 938400.00 | 0.00 | 0.00 | 0.00 | 0.00 | 1637641.92 | 0.00 | 0.00 | 14707000.00 | 0.00 | 990000.00 | KO:K06907 | phage tail sheath protein FI | Bacteria;Proteobacteria;Betaproteobacteria;Burkholderiales;Comamonadaceae;Variovorax;Variovorax paradoxus;Variovorax paradoxus MEDvA23 |
| Ga0586324_0239835_200_2275 | 0.00 | 0.00 | 0.00 | 0.00 | 0.00 | 942966.31 | 74100000.00 | 0.00 | 0.00 | 0.00 | 0.00 | 0.00 | KO:K06907 | phage tail sheath protein FI | Bacteria;Cyanobacteria;unclassified Cyanobacteria;Nostocales;Hapalosiphonaceae;Fischerella;Fischerella thermalis;Fischerella thermalis CCMEE 5201 |
| Ga0586324_0244771_2_1261 | 0.00 | 0.00 | 0.00 | 0.00 | 0.00 | 3801981.66 | 0.00 | 0.00 | 0.00 | 0.00 | 0.00 | 0.00 | KO:K06907 | phage tail sheath protein FI | Bacteria;Proteobacteria;Betaproteobacteria;Burkholderiales;Burkholderiaceae;Burkholderia;Burkholderia sp. yr281;Burkholderia sp. YR281 |
| Ga0586324_0365218_1226_1882 | 0.00 | 0.00 | 0.00 | 0.00 | 541763.52 | 0.00 | 0.00 | 0.00 | 0.00 | 0.00 | 0.00 | 0.00 | KO:K06907 | hypothetical protein | Bacteria;Proteobacteria;Betaproteobacteria;Nitrosomonadales;Sulfuricellaceae;Sulfurirhabdus;Sulfurirhabdus autotrophica;Sulfurirhabdus autotrophica DSM 100309 |
| Ga0586324_0439736_672_1733 | 0.00 | 0.00 | 0.00 | 0.00 | 6622900.00 | 0.00 | 0.00 | 3470900.00 | 0.00 | 0.00 | 0.00 | 0.00 | KO:K06907 | phage tail sheath protein FI | Bacteria;Proteobacteria;Gammaproteobacteria;unclassified Gammaproteobacteria;Candidatus Competibacteraceae;Plasticicumulans;Plasticicumulans lactativorans;Plasticicumulans lactativorans DSM 25287 |
| Ga0586324_0445910_2_1078 | 0.00 | 1455200.00 | 0.00 | 0.00 | 0.00 | 0.00 | 0.00 | 0.00 | 0.00 | 0.00 | 0.00 | 0.00 | KO:K06907 | phage tail sheath protein FI | Bacteria;Actinobacteria;Actinomycetia;Micrococcales;Cellulomonadaceae;Cellulomonas;Cellulomonas flavigena;Cellulomonas flavigena 134, DSM 20109 |
| Ga0586324_0465789_3_1478 | 0.00 | 0.00 | 0.00 | 0.00 | 0.00 | 0.00 | 0.00 | 0.00 | 0.00 | 3711400.00 | 0.00 | 0.00 | KO:K06907 | phage tail sheath protein FI | Bacteria;Cyanobacteria;unclassified Cyanobacteria;Nostocales;Calotrichaceae;Calothrix;Calothrix sp. NIES-4101;Calothrix sp. NIES-4101 |
| Ga0586324_1209055_3_473 | 0.00 | 0.00 | 0.00 | 0.00 | 0.00 | 3073474.75 | 1857155.76 | 1244400.00 | 0.00 | 1578500.00 | 0.00 | 0.00 | KO:K06907 | phage tail sheath protein FI | Bacteria;Proteobacteria;Deltaproteobacteria;Myxococcales;Myxococcaceae;Myxococcus;Myxococcus fulvus;Myxococcus fulvus DSM 16525 |
| Ga0586324_1633434_2_550 | 0.00 | 0.00 | 0.00 | 0.00 | 5047000.00 | 0.00 | 0.00 | 0.00 | 0.00 | 0.00 | 0.00 | 0.00 | KO:K06907 | phage tail sheath protein FI | Bacteria;Proteobacteria;Betaproteobacteria;Rhodocyclales;Zoogloeaceae;Azoarcus;Azoarcus communis;Azoarcus communis DSM 12120 |
| Ga0586324_2747143_2_316 | 1650000.00 | 661898.40 | 2726952.43 | 0.00 | 2451400.00 | 0.00 | 2416800.00 | 744200.00 | 0.00 | 1732500.00 | 0.00 | 673827.00 | KO:K06907 | phage tail sheath protein FI | Bacteria;Bacteroidetes;Cytophagia;Cytophagales;Cyclobacteriaceae;Echinicola;Echinicola vietnamensis;Echinicola vietnamensis KMM 6221, DSM 17526 |
| Ga0586324_2869482_3_632 | 0.00 | 0.00 | 0.00 | 0.00 | 0.00 | 0.00 | 0.00 | 1610400.00 | 0.00 | 0.00 | 4752000.00 | 3202500.00 | KO:K06907 | phage tail sheath protein FI | Bacteria;Proteobacteria;Gammaproteobacteria;Alteromonadales;Alteromonadaceae;Marinobacter;Marinobacter sp. X15-166B;Marinobacter sp. X15-166B |
| Ga0586324_2893791_1_774 | 986028.00 | 0.00 | 0.00 | 0.00 | 0.00 | 0.00 | 0.00 | 0.00 | 0.00 | 0.00 | 0.00 | 0.00 | KO:K06907 | phage tail sheath protein FI | Bacteria;Proteobacteria;Gammaproteobacteria;Chromatiales;unclassified Chromatiales;unclassified Chromatiales;unclassified Chromatiales;Chromatiales bacterium |
| Ga0586324_2896216_2_772 | 0.00 | 2808400.00 | 0.00 | 0.00 | 0.00 | 0.00 | 3488400.00 | 0.00 | 2600100.00 | 0.00 | 0.00 | 442683.75 | KO:K06907 | phage tail sheath protein FI | Bacteria;Cyanobacteria;unclassified Cyanobacteria;Nostocales;Calotrichaceae;Calothrix;Calothrix sp. NIES-2100;Calothrix sp. NIES-2100 |
| Ga0586324_3128598_3_602 | 0.00 | 4549200.00 | 0.00 | 0.00 | 0.00 | 0.00 | 0.00 | 2342400.00 | 0.00 | 0.00 | 0.00 | 0.00 | KO:K06907 | phage tail sheath protein FI | Bacteria;Proteobacteria;Gammaproteobacteria;Methylococcales;Methylococcaceae;Methylocaldum;Methylocaldum marinum;Methylocaldum marinum S8 |
| Ga0586324_3445804_2_571 | 0.00 | 0.00 | 0.00 | 0.00 | 0.00 | 1539470.72 | 0.00 | 0.00 | 0.00 | 0.00 | 0.00 | 0.00 | KO:K06907 | phage tail sheath protein FI | Bacteria;Bacteroidetes;Chitinophagia;Chitinophagales;Chitinophagaceae;Chitinophaga;unclassified Chitinophaga;Chitinophaga sp. CF418 |
| Ga0586324_4068948_381_671 | 0.00 | 1196800.00 | 0.00 | 747068.60 | 0.00 | 0.00 | 0.00 | 0.00 | 0.00 | 0.00 | 0.00 | 0.00 | KO:K06907 | phage tail sheath protein FI | Bacteria;Nitrospirae;Nitrospira;Nitrospirales;Nitrospiraceae;Nitrospira;Nitrospira defluvii;Nitrospira defluvii |
| Ga0586324_6827079_3_542 | 0.00 | 0.00 | 0.00 | 6992000.00 | 0.00 | 0.00 | 0.00 | 0.00 | 0.00 | 0.00 | 0.00 | 0.00 | KO:K06907 | phage tail sheath protein FI | Bacteria;Actinobacteria;Actinomycetia;Pseudonocardiales;Pseudonocardiaceae;Kutzneria;Kutzneria sp. 744;Kutzneria sp. 744 |
| Ga0586324_0059848_319_1812 | 0.00 | 0.00 | 0.00 | 0.00 | 0.00 | 0.00 | 0.00 | 0.00 | 2964600.00 | 0.00 | 0.00 | 0.00 | KO:K06915 | DNA helicase HerA-like ATPase | Bacteria;Proteobacteria;Betaproteobacteria;Burkholderiales;unclassified Burkholderiales;Thiomonas;Thiomonas bhubaneswarensis;Thiomonas bhubaneswarensis DSM 18181 |
| Ga0586324_0124759_1059_2525 | 0.00 | 0.00 | 0.00 | 0.00 | 0.00 | 0.00 | 0.00 | 0.00 | 0.00 | 531393.94 | 0.00 | 0.00 | KO:K06915 | DNA helicase HerA-like ATPase | Bacteria;Proteobacteria;Deltaproteobacteria;Desulfuromonadales;Geobacteraceae;Geobacter;Geobacter sp. DSM 2909;Geobacter sp. DSM 2909 |
| Ga0586324_0478266_280_1671 | 0.00 | 0.00 | 624637.24 | 0.00 | 0.00 | 0.00 | 0.00 | 0.00 | 0.00 | 0.00 | 0.00 | 0.00 | KO:K06915 | DNA helicase HerA-like ATPase | Bacteria;Gemmatimonadetes;Longimicrobia;Longimicrobiales;Longimicrobiaceae;Longimicrobium;Longimicrobium terrae;Longimicrobium terrae CECT 8660 |
| Ga0586324_0495317_3_677 | 0.00 | 0.00 | 0.00 | 0.00 | 0.00 | 0.00 | 11924400.00 | 0.00 | 0.00 | 0.00 | 0.00 | 0.00 | KO:K06915 | DNA helicase HerA-like ATPase | Bacteria;Proteobacteria;Betaproteobacteria;Rhodocyclales;Zoogloeaceae;Azoarcus;Azoarcus sp. KH32C;Azoarcus sp. KH32C |
| Ga0586324_1357947_3_1064 | 0.00 | 0.00 | 711626.37 | 0.00 | 0.00 | 0.00 | 0.00 | 0.00 | 0.00 | 0.00 | 0.00 | 0.00 | KO:K06915 | DNA helicase HerA-like ATPase | Bacteria;Acidobacteria;Holophagae;Holophagales;Holophagaceae;Geothrix;Geothrix fermentans;Geothrix fermentans DSM 14018 |
| Ga0586324_1395934_402_1052 | 0.00 | 0.00 | 0.00 | 0.00 | 0.00 | 0.00 | 0.00 | 0.00 | 0.00 | 4134900.00 | 0.00 | 0.00 | KO:K06915 | DNA helicase HerA-like ATPase | Bacteria;Proteobacteria;Alphaproteobacteria;Rhodospirillales;unclassified Rhodospirillales;Enhydrobacter;Enhydrobacter aerosaccus;Enhydrobacter aerosaccus ATCC 27094 |
| Ga0586324_2573583_323_811 | 0.00 | 0.00 | 0.00 | 0.00 | 0.00 | 0.00 | 1408027.68 | 0.00 | 0.00 | 0.00 | 0.00 | 0.00 | KO:K06915 | DNA helicase HerA-like ATPase | Bacteria;Proteobacteria;Betaproteobacteria;Nitrosomonadales;Nitrosomonadaceae;Nitrosospira;Nitrosospira multiformis;Nitrosospira multiformis NI1 |
| Ga0586324_3946224_3_446 | 0.00 | 958800.00 | 0.00 | 0.00 | 0.00 | 6034400.00 | 0.00 | 0.00 | 0.00 | 0.00 | 0.00 | 0.00 | KO:K06915 | DNA helicase HerA-like ATPase | Bacteria;Bacteroidetes;Bacteroidia;Marinilabiliales;Marinilabiliaceae;Breznakibacter;Breznakibacter xylanolyticus;Cytophaga xylanolytica DSM 6779 |
| Ga0586324_4979884_3_563 | 2880000.00 | 0.00 | 0.00 | 2308500.00 | 0.00 | 0.00 | 0.00 | 0.00 | 0.00 | 0.00 | 0.00 | 0.00 | KO:K06915 | DNA helicase HerA-like ATPase | Bacteria;Deinococcus-Thermus;Deinococci;Thermales;Thermaceae;Meiothermus;Meiothermus cerbereus;Meiothermus cerbereus DSM 11376 |
| Ga0586324_0000071_33412_34518 | 0.00 | 0.00 | 0.00 | 0.00 | 0.00 | 1680774.93 | 0.00 | 0.00 | 0.00 | 0.00 | 0.00 | 0.00 | KO:K06942 | GTP-binding protein YchF | Bacteria;Proteobacteria;Alphaproteobacteria;Hyphomicrobiales;Hyphomicrobiaceae;Rhodoplanes;Rhodoplanes sp. Z2-YC6860;Rhodoplanes sp. Z2-YC6860 |
| Ga0586324_0005622_10951_11202 | 0.00 | 0.00 | 0.00 | 0.00 | 0.00 | 0.00 | 0.00 | 0.00 | 0.00 | 0.00 | 0.00 | 1800000.00 | KO:K06960 | predicted RNA-binding protein YlqC (UPF0109 family) | Bacteria;Acidobacteria;Acidobacteriia;Bryobacterales;Solibacteraceae;Candidatus Solibacter;Candidatus Solibacter usitatus;Candidatus Solibacter usitatus Ellin6076 |
| Ga0586324_0000007_87388_89592 | 0.00 | 0.00 | 0.00 | 0.00 | 0.00 | 0.00 | 0.00 | 0.00 | 0.00 | 0.00 | 684603.04 | 0.00 | KO:K07001 | NTE family protein | Bacteria;Proteobacteria;Betaproteobacteria;Burkholderiales;Alcaligenaceae;Achromobacter;Achromobacter sp. B4U2B;Achromobacter sp. B4U2B |
| Ga0586324_0007057_9834_11855 | 0.00 | 0.00 | 0.00 | 0.00 | 0.00 | 0.00 | 0.00 | 0.00 | 0.00 | 0.00 | 2455200.00 | 0.00 | KO:K07001 | NTE family protein | Bacteria;Proteobacteria;Betaproteobacteria;Burkholderiales;Alcaligenaceae;Achromobacter;Achromobacter sp. B4U2B;Achromobacter sp. B4U2B |
| Ga0586324_0262483_3_2183 | 0.00 | 0.00 | 0.00 | 0.00 | 0.00 | 2954045.24 | 0.00 | 0.00 | 0.00 | 0.00 | 0.00 | 0.00 | KO:K07004 | predicted extracellular nuclease | Bacteria;Chloroflexi;Ardenticatenia;Ardenticatenales;Ardenticatenaceae;Candidatus Promineofilum;Candidatus Promineofilum breve;Candidatus Promineofilum breve Cfx-K |
| Ga0586324_0058991_2208_2612 | 4520000.00 | 0.00 | 3075922.35 | 0.00 | 0.00 | 0.00 | 0.00 | 0.00 | 0.00 | 0.00 | 0.00 | 0.00 | KO:K07006 | predicted pyridoxine 5'-phosphate oxidase superfamily flavin-nucleotide-binding protein | Archaea;Euryarchaeota;Methanomicrobia;Methanosarcinales;Methanotrichaceae;Methanothrix;Methanothrix soehngenii;Methanothrix soehngenii GP-6 |
| Ga0586324_0792324_3_725 | 0.00 | 0.00 | 13455700.00 | 0.00 | 11124000.00 | 0.00 | 13087200.00 | 4324900.00 | 5613300.00 | 4404400.00 | 8298400.00 | 6382500.00 | KO:K07045 | hypothetical protein | Bacteria;Proteobacteria;Alphaproteobacteria;Rhodospirillales;Acetobacteraceae;Rhodopila;Rhodopila globiformis;Rhodopila globiformis DSM 161 |
| Ga0586324_1485337_83_940 | 1670000.00 | 0.00 | 0.00 | 0.00 | 0.00 | 0.00 | 0.00 | 0.00 | 0.00 | 0.00 | 0.00 | 0.00 | KO:K07045 | predicted TIM-barrel fold metal-dependent hydrolase | Bacteria;Actinobacteria;Actinomycetia;Streptosporangiales;Streptosporangiaceae;Sinosporangium;Sinosporangium album;Sinosporangium album CPCC 201354 |
| Ga0586324_7566096_71_520 | 0.00 | 0.00 | 5442537.89 | 828967.15 | 0.00 | 1556291.61 | 0.00 | 0.00 | 2025000.00 | 1239700.00 | 0.00 | 0.00 | KO:K07045 | predicted TIM-barrel fold metal-dependent hydrolase | Bacteria;Proteobacteria;Alphaproteobacteria;Rhodospirillales;unclassified Rhodospirillales;unclassified Rhodospirillales;Rhodospirillales bacterium URHD0088;Rhodospirillales bacterium URHD0088 |
| Ga0586324_0000194_6651_7601 | 0.00 | 593263.28 | 0.00 | 0.00 | 0.00 | 0.00 | 0.00 | 0.00 | 0.00 | 0.00 | 0.00 | 0.00 | KO:K07080 | TRAP transporter TAXI family solute receptor | Bacteria;Proteobacteria;Deltaproteobacteria;Syntrophobacterales;Syntrophobacteraceae;Syntrophobacter;Syntrophobacter fumaroxidans;Syntrophobacter fumaroxidans MPOB |
| Ga0586324_0006328_8838_9788 | 208696.00 | 12083600.00 | 0.00 | 10735000.00 | 11021000.00 | 7503300.00 | 7843200.00 | 13975100.00 | 6188400.00 | 1008700.00 | 1522400.00 | 10935030.00 | KO:K07080 | TRAP transporter TAXI family solute receptor | Bacteria;Proteobacteria;Deltaproteobacteria;Syntrophobacterales;Syntrophobacteraceae;Syntrophobacter;Syntrophobacter fumaroxidans;Syntrophobacter fumaroxidans MPOB |
| Ga0586324_0011796_579_1439 | 0.00 | 1734000.00 | 0.00 | 0.00 | 0.00 | 0.00 | 0.00 | 0.00 | 0.00 | 0.00 | 0.00 | 0.00 | KO:K07080 | TRAP transporter TAXI family solute receptor | Bacteria;Proteobacteria;Betaproteobacteria;Burkholderiales;Comamonadaceae;Ramlibacter;Ramlibacter lithotrophicus;Ramlibacter lithotrophicus RBP-1 |
| Ga0586324_0013179_7267_8268 | 0.00 | 0.00 | 0.00 | 0.00 | 0.00 | 0.00 | 0.00 | 0.00 | 3693600.00 | 0.00 | 0.00 | 0.00 | KO:K07080 | TRAP transporter TAXI family solute receptor | Archaea;Candidatus Korarchaeota;unclassified Candidatus Korarchaeota;unclassified Candidatus Korarchaeota;unclassified Candidatus Korarchaeota;Candidatus Korarchaeum;Candidatus Korarchaeum cryptofilum;Candidatus Korarchaeum cryptofilum OPF8 |

| | | | | | | | | | | | | | | | |
|---|---|---|---|---|---|---|---|---|---|---|---|---|---|---|---|
| Ga0586324 0085036 2 748 | 0.00 | 3828400.00 | 63056000.00 | 0.00 | 0.00 | 0.00 | 0.00 | 0.00 | 0.00 | 0.00 | 0.00 | 0.00 | KO:K07080 | TRAP transporter TAXI family solute receptor | Bacteria;Proteobacteria;Deltaproteobacteria;Syntrophobacterales;Syntrophobacteraceae;Syntrophobacter;Syntrophobacter fumaroxidans;Syntrophobacter fumaroxidans MPOB |
| Ga0586324 0107071 1745 2692 | 0.00 | 15436000.00 | 0.00 | 0.00 | 0.00 | 0.00 | 0.00 | 0.00 | 0.00 | 10918600.00 | 8219200.00 | 0.00 | KO:K07080 | TRAP transporter TAXI family solute receptor | Bacteria;Proteobacteria;Deltaproteobacteria;Syntrophobacterales;Syntrophobacteraceae;Syntrophobacter;Syntrophobacter fumaroxidans;Syntrophobacter fumaroxidans MPOB |
| Ga0586324 0206822 1771 2436 | 0.00 | 0.00 | 0.00 | 0.00 | 0.00 | 0.00 | 0.00 | 0.00 | 0.00 | 0.00 | 0.00 | 1470000.00 | KO:K07080 | TRAP transporter TAXI family solute receptor | Bacteria;Proteobacteria;Betaproteobacteria;unclassified Betaproteobacteria;unclassified Betaproteobacteria;unclassified Betaproteobacteria;Betaproteobacteria bacterium GR16-43;Betaproteobacteria bacterium GR16-43 |
| Ga0586324 0259640 1 927 | 0.00 | 0.00 | 0.00 | 0.00 | 2286600.00 | 0.00 | 0.00 | 0.00 | 2357100.00 | 0.00 | 1258400.00 | 0.00 | KO:K07080 | TRAP transporter TAXI family solute receptor | Bacteria;Proteobacteria;Deltaproteobacteria;Syntrophobacterales;Syntrophobacteraceae;Syntrophobacter;Syntrophobacter fumaroxidans;Syntrophobacter fumaroxidans MPOB |
| Ga0586324 0275988 3 443 | 0.00 | 0.00 | 0.00 | 0.00 | 0.00 | 0.00 | 0.00 | 0.00 | 0.00 | 7777000.00 | 0.00 | 1560000.00 | KO:K07080 | TRAP transporter TAXI family solute receptor | Bacteria;Proteobacteria;Deltaproteobacteria;Syntrophobacterales;Syntrophobacteraceae;Syntrophobacter;Syntrophobacter fumaroxidans;Syntrophobacter fumaroxidans MPOB |
| Ga0586324 0335257 713 1750 | 0.00 | 0.00 | 2856774.60 | 0.00 | 0.00 | 0.00 | 0.00 | 0.00 | 0.00 | 0.00 | 0.00 | 0.00 | KO:K07080 | TRAP transporter TAXI family solute receptor | Bacteria;Proteobacteria;Deltaproteobacteria;Desulfobacterales;Desulfobacteraceae;Desulfatirhabdium;Desulfatirhabdium butyrativorans;Desulfatirhabdium butyrativorans DSM 18734 |
| Ga0586324 0400275 1020 1808 | 0.00 | 0.00 | 0.00 | 0.00 | 2193900.00 | 0.00 | 0.00 | 0.00 | 0.00 | 0.00 | 0.00 | 0.00 | KO:K07080 | TRAP transporter TAXI family solute receptor | Bacteria;Proteobacteria;Alphaproteobacteria;Hyphomicrobiales;Hyphomicrobiaceae;Prosthecomicrobium;Prosthecomicrobium hirschii;Prosthecomicrobium hirschii ATCC 27832 |
| Ga0586324 0465987 891 1691 | 0.00 | 0.00 | 0.00 | 0.00 | 1015184.48 | 0.00 | 0.00 | 0.00 | 0.00 | 0.00 | 0.00 | 0.00 | KO:K07080 | TRAP transporter TAXI family solute receptor | Bacteria;Proteobacteria;Deltaproteobacteria;Desulfobacterales;Desulfobacteraceae;Desulfobacter;Desulfobacter postgatei;Desulfobacter postgatei 2ac9 |
| Ga0586324 1081415 2 1054 | 0.00 | 0.00 | 0.00 | 0.00 | 0.00 | 0.00 | 0.00 | 0.00 | 0.00 | 0.00 | 2332000.00 | 0.00 | KO:K07080 | TRAP transporter TAXI family solute receptor | Bacteria;Proteobacteria;Deltaproteobacteria;Desulfarculales;Desulfarculaceae;Dethiosulfatarculus;Dethiosulfatarculus sandiegensis;Dethiosulfatarculus sandiegensis SPR |
| Ga0586324 1219603 82 1041 | 0.00 | 0.00 | 0.00 | 0.00 | 0.00 | 3200125.69 | 0.00 | 5160600.00 | 3426300.00 | 5790400.00 | 8729600.00 | 5985000.00 | KO:K07080 | TRAP transporter TAXI family solute receptor | Bacteria;Proteobacteria;Betaproteobacteria;Rhodocyclales;Rhodocyclaceae;Dechlorobacter;Dechlorobacter hydrogenophilus;Dechlorobacter hydrogenophilus LT-1 |
| Ga0586324 1274246 1 930 | 0.00 | 0.00 | 0.00 | 0.00 | 0.00 | 0.00 | 0.00 | 0.00 | 0.00 | 0.00 | 0.00 | 29400000.00 | KO:K07080 | TRAP transporter TAXI family solute receptor | Bacteria;Proteobacteria;Betaproteobacteria;Burkholderiales;Oxalobacteraceae;Noviherbaspirillum;Noviherbaspirillum sp. Root189;Noviherbaspirillum sp. Root189 |
| Ga0586324 2148589 3 875 | 7910000.00 | 6310400.00 | 0.00 | 540000.90 | 335294.87 | 0.00 | 0.00 | 7564000.00 | 4949100.00 | 8855000.00 | 9152000.00 | 5587500.00 | KO:K07080 | TRAP transporter TAXI family solute receptor | Bacteria;Proteobacteria;Deltaproteobacteria;Syntrophobacterales;Syntrophobacteraceae;Syntrophobacter;Syntrophobacter fumaroxidans;Syntrophobacter fumaroxidans MPOB |
| Ga0586324 2211166 216 866 | 0.00 | 0.00 | 0.00 | 0.00 | 0.00 | 0.00 | 0.00 | 0.00 | 0.00 | 0.00 | 1628000.00 | 0.00 | KO:K07080 | TRAP transporter TAXI family solute receptor | Bacteria;Proteobacteria;Betaproteobacteria;Burkholderiales;Comamonadaceae;Ramlibacter;Ramlibacter tataouinensis;Ramlibacter tataouinensis TTB310 |
| Ga0586324 4940485 3 620 | 0.00 | 0.00 | 0.00 | 0.00 | 0.00 | 0.00 | 0.00 | 0.00 | 104124.69 | 0.00 | 0.00 | 0.00 | KO:K07080 | TRAP transporter TAXI family solute receptor | Bacteria;Proteobacteria;Deltaproteobacteria;Syntrophobacterales;Syntrophaceae;Desulfomonile;Desulfomonile tiedjei;Desulfomonile tiedjei DCB-1, DSM 6799 |
| Ga0586324 5567493 3 590 | 0.00 | 0.00 | 0.00 | 0.00 | 0.00 | 0.00 | 0.00 | 2366800.00 | 0.00 | 0.00 | 0.00 | 3247500.00 | KO:K07080 | TRAP transporter TAXI family solute receptor | Bacteria;Proteobacteria;Deltaproteobacteria;Desulfobacterales;Desulfobacteraceae;Desulfobacter;Desulfobacter curvatus;Desulfobacter curvatus DSM 3379 |
| Ga0586324 6784031 148 543 | 0.00 | 0.00 | 0.00 | 0.00 | 0.00 | 0.00 | 0.00 | 0.00 | 850500.00 | 0.00 | 0.00 | 0.00 | KO:K07092 | predicted peroxiredoxin | Bacteria;Proteobacteria;Deltaproteobacteria;Syntrophobacterales;Syntrophobacteraceae;Desulfacinum;Desulfacinum infernum;Desulfacinum infernum DSM 9756 |
| Ga0586324 0390910 2 1696 | 0.00 | 0.00 | 0.00 | 0.00 | 0.00 | 0.00 | 0.00 | 192484.89 | 0.00 | 0.00 | 0.00 | 0.00 | KO:K07141 | molybdenum cofactor cytidylyltransferase | Bacteria;Proteobacteria;Alphaproteobacteria;Rhodospirillales;Acetobacteraceae;Rhodopila;Rhodopila globiformis;Rhodopila globiformis DSM 161 |
| Ga0586324 0021710 2664 4718 | 0.00 | 0.00 | 0.00 | 0.00 | 0.00 | 0.00 | 0.00 | 0.00 | 1263600.00 | 0.00 | 0.00 | 0.00 | KO:K07180 | serine protein kinase | Bacteria;Planctomycetes;Planctomycetia;Isosphaerales;Isosphaeraceae;Paludisphaera;Paludisphaera borealis;Paludisphaera borealis PX4 |
| Ga0586324 0025633 3 1637 | 647041.00 | 506784.28 | 0.00 | 3914000.00 | 3543200.00 | 0.00 | 0.00 | 2592500.00 | 2681100.00 | 0.00 | 0.00 | 4140000.00 | KO:K07180 | serine protein kinase | Bacteria;Proteobacteria;Betaproteobacteria;Burkholderiales;unclassified Burkholderiales;Rubrivivax;Rubrivivax benzoatilyticus;Rubrivivax benzoatilyticus JA2 |
| Ga0586324 0028345 3591 5666 | 0.00 | 0.00 | 0.00 | 0.00 | 0.00 | 0.00 | 0.00 | 1470100.00 | 2300400.00 | 0.00 | 0.00 | 0.00 | KO:K07180 | serine protein kinase | Bacteria;Proteobacteria;Deltaproteobacteria;Myxococcales;Sandaracinaceae;Sandaracinus;Sandaracinus amylolyticus;Sandaracinus amylolyticus DSM 53668 |
| Ga0586324 0078599 3 1523 | 0.00 | 0.00 | 0.00 | 0.00 | 0.00 | 0.00 | 0.00 | 4507900.00 | 0.00 | 0.00 | 0.00 | 0.00 | KO:K07180 | serine protein kinase | Bacteria;Proteobacteria;Alphaproteobacteria;Hyphomicrobiales;Bradyrhizobiaceae;Bradyrhizobium;Bradyrhizobium erythrophlei;Bradyrhizobium erythrophlei GAS401 |
| Ga0586324 0098158 447 2513 | 0.00 | 734400.00 | 0.00 | 0.00 | 0.00 | 0.00 | 0.00 | 0.00 | 0.00 | 0.00 | 0.00 | 0.00 | KO:K07180 | serine protein kinase | Bacteria;Proteobacteria;Deltaproteobacteria;Myxococcales;Anaeromyxobacteraceae;Anaeromyxobacter;Anaeromyxobacter sp. K;Anaeromyxobacter sp. K |
| Ga0586324 0171911 750 2654 | 0.00 | 0.00 | 0.00 | 0.00 | 0.00 | 0.00 | 0.00 | 0.00 | 1287900.00 | 0.00 | 0.00 | 0.00 | KO:K07180 | serine protein kinase | Bacteria;Planctomycetes;Planctomycetia;Planctomycetales;Planctomycetaceae;Planctomyces;Planctomyces sp. SH-PL62;Planctomyces sp. SH-PL62 |
| Ga0586324 0179755 3 1628 | 0.00 | 0.00 | 0.00 | 0.00 | 0.00 | 0.00 | 0.00 | 0.00 | 0.00 | 0.00 | 0.00 | 4807500.00 | KO:K07180 | serine protein kinase | Bacteria;Proteobacteria;Alphaproteobacteria;Rhodospirillales;Acetobacteraceae;Rhodopila;unclassified Rhodopila;Rhodopila sp. LVNP |
| Ga0586324 0843379 3 203 | 1820000.00 | 0.00 | 0.00 | 0.00 | 0.00 | 0.00 | 0.00 | 0.00 | 0.00 | 0.00 | 0.00 | 0.00 | KO:K07180 | serine protein kinase | Bacteria;Proteobacteria;Deltaproteobacteria;Syntrophobacterales;Syntrophobacteraceae;Desulfacinum;Desulfacinum hydrothermale;Desulfacinum hydrothermale DSM 13146 |
| Ga0586324 1654125 2 979 | 0.00 | 0.00 | 0.00 | 0.00 | 0.00 | 0.00 | 0.00 | 0.00 | 0.00 | 0.00 | 0.00 | 4245000.00 | KO:K07180 | serine protein kinase | Bacteria;Planctomycetes;Planctomycetia;Gemmatales;Gemmataceae;Gemmata;Gemmata massiliana;Gemmata massiliana IIL30 |
| Ga0586324 2355363 3 842 | 0.00 | 0.00 | 0.00 | 0.00 | 0.00 | 0.00 | 0.00 | 0.00 | 0.00 | 0.00 | 5121600.00 | 0.00 | KO:K07180 | serine protein kinase | Bacteria;Proteobacteria;Betaproteobacteria;Burkholderiales;Comamonadaceae;Ramlibacter;Ramlibacter tataouinensis;Ramlibacter tataouinensis TTB310 |
| Ga0586324 4058102 3 671 | 0.00 | 0.00 | 0.00 | 0.00 | 0.00 | 0.00 | 0.00 | 0.00 | 0.00 | 0.00 | 6151200.00 | 0.00 | KO:K07180 | serine protein kinase | Bacteria;Proteobacteria;Gammaproteobacteria;Chromatiales;Chromatiaceae;Thioflavicoccus;Thioflavicoccus mobilis;Thioflavicoccus mobilis 8321 |
| Ga0586324 4797539 2 628 | 0.00 | 484015.84 | 0.00 | 0.00 | 0.00 | 0.00 | 0.00 | 0.00 | 0.00 | 0.00 | 0.00 | 0.00 | KO:K07180 | serine protein kinase | Bacteria;Proteobacteria;Deltaproteobacteria;Myxococcales;Polyangiaceae;Sorangium;Sorangium cellulosum;Sorangium cellulosum So0157-25 |
| Ga0586324 0094425 798 2783 | 0.00 | 0.00 | 0.00 | 0.00 | 2214500.00 | 0.00 | 0.00 | 0.00 | 0.00 | 0.00 | 0.00 | 0.00 | KO:K07182 | CBS domain-containing protein | Bacteria;Proteobacteria;Deltaproteobacteria;Syntrophobacterales;Syntrophaceae;Desulfomonile;Desulfomonile tiedjei;Desulfomonile tiedjei DCB-1, DSM 6799 |
| Ga0586324 0998838 3 1214 | 0.00 | 1985600.00 | 0.00 | 0.00 | 2667700.00 | 0.00 | 0.00 | 0.00 | 1741500.00 | 0.00 | 0.00 | 0.00 | KO:K07221 | phosphate-selective porin OprO/OprP | Bacteria;Proteobacteria;Gammaproteobacteria;Methylococcales;Methylococcaceae;Methylosarcina;Methylosarcina fibrata;Methylosarcina fibrata AML-C10 |
| Ga0586324 4613245 3 638 | 0.00 | 0.00 | 0.00 | 899508.45 | 0.00 | 0.00 | 0.00 | 0.00 | 0.00 | 0.00 | 0.00 | 0.00 | KO:K07221 | phosphate-selective porin OprO/OprP | Bacteria;Proteobacteria;Gammaproteobacteria;Methylococcales;Crenotrichaceae;Crenothrix;Crenothrix polyspora;Crenothrix polyspora ERS1545888 |
| Ga0586324 0000049 63473 63832 | 0.00 | 0.00 | 0.00 | 0.00 | 0.00 | 0.00 | 0.00 | 0.00 | 2818800.00 | 0.00 | 0.00 | 0.00 | KO:K07235 | tRNA 2-thiouridine synthesizing protein D | Bacteria;Proteobacteria;Betaproteobacteria;Nitrosomonadales;Sulfuricellaceae;Sulfuriferula;Sulfuriferula multivorans;Sulfuriferula multivorans TTN |
| Ga0586324 0051016 1616 1975 | 0.00 | 0.00 | 0.00 | 0.00 | 0.00 | 0.00 | 0.00 | 0.00 | 0.00 | 10395000.00 | 10736000.00 | 11175000.00 | KO:K07235 | tRNA 2-thiouridine synthesizing protein D | Bacteria;Proteobacteria;Betaproteobacteria;Nitrosomonadales;Gallionellaceae;Sideroxydans;Sideroxydans lithotrophicus;Sideroxydans lithotrophicus ES-1 |
| Ga0586324 0283980 189 551 | 0.00 | 1434800.00 | 0.00 | 0.00 | 2657400.00 | 0.00 | 0.00 | 0.00 | 0.00 | 3457300.00 | 0.00 | 2902500.00 | KO:K07235 | tRNA 2-thiouridine synthesizing protein D | Bacteria;Proteobacteria;Gammaproteobacteria;Acidiferrobacterales;Acidiferrobacteraceae;Sulfuricaulis;Sulfuricaulis limicola;Sulfuricaulis limicola HA5 |
| Ga0586324 0590777 695 1054 | 0.00 | 0.00 | 0.00 | 0.00 | 0.00 | 0.00 | 0.00 | 8418000.00 | 0.00 | 0.00 | 0.00 | 0.00 | KO:K07235 | tRNA 2-thiouridine synthesizing protein D | Bacteria;Proteobacteria;Betaproteobacteria;Nitrosomonadales;Gallionellaceae;Sideroxydans;Sideroxydans lithotrophicus;Sideroxydans lithotrophicus ES-1 |
| Ga0586324 0000003 177176 177472 | 0.00 | 0.00 | 0.00 | 0.00 | 0.00 | 0.00 | 0.00 | 0.00 | 0.00 | 0.00 | 1557600.00 | 0.00 | KO:K07237 | tRNA 2-thiouridine synthesizing protein B | Bacteria;Proteobacteria;Betaproteobacteria;Nitrosomonadales;Thiobacillaceae;Annwoodia;Annwoodia aquaesulis;Annwoodia aquaesulis DSM 4255 |
| Ga0586324 0051016 2408 2713 | 0.00 | 0.00 | 0.00 | 0.00 | 0.00 | 0.00 | 0.00 | 0.00 | 0.00 | 0.00 | 450508.08 | 0.00 | KO:K07237 | tRNA 2-thiouridine synthesizing protein B | Bacteria;Proteobacteria;Betaproteobacteria;Nitrosomonadales;Sulfuricellaceae;Sulfuriferula;Sulfuriferula multivorans;Sulfuriferula multivorans TTN |
| Ga0586324 0000041 32626 33960 | 0.00 | 0.00 | 0.00 | 0.00 | 0.00 | 0.00 | 0.00 | 0.00 | 0.00 | 0.00 | 5412000.00 | 0.00 | KO:K07263 | zinc protease | Bacteria;Proteobacteria;Betaproteobacteria;Nitrosomonadales;Thiobacillaceae;Annwoodia;Annwoodia aquaesulis;Annwoodia aquaesulis DSM 4255 |
| Ga0586324 0162431 236 2725 | 0.00 | 0.00 | 0.00 | 0.00 | 0.00 | 19135400.00 | 0.00 | 0.00 | 0.00 | 0.00 | 0.00 | 0.00 | KO:K07263 | zinc protease | Bacteria;Bacteroidetes;Bacteroidia;Marinilabiliales;Prolixibacteraceae;Draconibacterium;Draconibacterium sediminis;Draconibacterium sediminis JN14CK-3 |
| Ga0586324 0025664 116 946 | 0.00 | 0.00 | 0.00 | 5966000.00 | 0.00 | 7423900.00 | 6771600.00 | 6283000.00 | 5370300.00 | 4158000.00 | 6670400.00 | 4905000.00 | KO:K07274 | outer membrane protein | Bacteria;Proteobacteria;Betaproteobacteria;Burkholderiales;Oxalobacteraceae;Massilia;Massilia timonae;Massilia timonae CCUG 45783 |
| Ga0586324 0000002 75603 77930 | 0.00 | 0.00 | 0.00 | 0.00 | 0.00 | 0.00 | 0.00 | 0.00 | 0.00 | 4211900.00 | 9724000.00 | 0.00 | KO:K07277 | outer membrane protein insertion porin family | Bacteria;Proteobacteria;Betaproteobacteria;Nitrosomonadales;Thiobacillaceae;Annwoodia;Annwoodia aquaesulis;Annwoodia aquaesulis DSM 4255 |
| Ga0586324 0015454 4236 6536 | 0.00 | 0.00 | 0.00 | 0.00 | 0.00 | 0.00 | 0.00 | 0.00 | 0.00 | 0.00 | 0.00 | 5272500.00 | KO:K07277 | outer membrane protein insertion porin family | Bacteria;Proteobacteria;Betaproteobacteria;Nitrosomonadales;Thiobacillaceae;Thiobacillus;Thiobacillus thioparus;Thiobacillus thioparus DSM 505 |
| Ga0586324 0030649 2002 4533 | 0.00 | 0.00 | 0.00 | 0.00 | 0.00 | 8138500.00 | 8937600.00 | 0.00 | 0.00 | 0.00 | 3150400.00 | 0.00 | KO:K07277 | outer membrane protein insertion porin family | Bacteria;Proteobacteria;Alphaproteobacteria;Hyphomicrobiales;Hyphomicrobiaceae;Rhodoplanes;Rhodoplanes sp. Z2-YC6860;Rhodoplanes sp. Z2-YC6860 |
| Ga0586324 0061388 3 2135 | 7390000.00 | 1523200.00 | 0.00 | 1263500.00 | 933062.58 | 2804237.29 | 0.00 | 0.00 | 0.00 | 2233000.00 | 3344000.00 | 4050000.00 | KO:K07277 | outer membrane protein insertion porin family | Bacteria;Proteobacteria;Alphaproteobacteria;Hyphomicrobiales;Bradyrhizobiaceae;Bradyrhizobium;Bradyrhizobium erythrophlei;Bradyrhizobium erythrophlei GAS401 |
| Ga0586324 0126751 869 3064 | 0.00 | 0.00 | 0.00 | 0.00 | 0.00 | 0.00 | 0.00 | 0.00 | 0.00 | 0.00 | 1936000.00 | 1312500.00 | KO:K07277 | outer membrane protein insertion porin family | Bacteria;Proteobacteria;Betaproteobacteria;Nitrosomonadales;Sulfuricellaceae;Sulfurirhabdus;Sulfurirhabdus autotrophica;Sulfurirhabdus autotrophica DSM 100309 |
| Ga0586324 0169362 725 2674 | 0.00 | 0.00 | 0.00 | 4940000.00 | 4253900.00 | 0.00 | 0.00 | 0.00 | 0.00 | 0.00 | 0.00 | 0.00 | KO:K07277 | outer membrane protein insertion porin family | Bacteria;Proteobacteria;Alphaproteobacteria;Hyphomicrobiales;Methylocystaceae;Methylocystis;Methylocystis parvus;Methylocystis parvus OBBP |
| Ga0586324 0175395 705 2630 | 0.00 | 0.00 | 0.00 | 0.00 | 0.00 | 0.00 | 0.00 | 0.00 | 0.00 | 0.00 | 0.00 | 1185000.00 | KO:K07277 | outer membrane protein insertion porin family | Bacteria;Proteobacteria;Alphaproteobacteria;Hyphomicrobiales;Xanthobacteraceae;Pseudolabrys;Pseudolabrys sp. Root1462;Pseudolabrys sp. Root1462 |
| Ga0586324 1900291 3 923 | 0.00 | 0.00 | 0.00 | 0.00 | 0.00 | 0.00 | 0.00 | 0.00 | 0.00 | 0.00 | 0.00 | 2452500.00 | KO:K07277 | outer membrane protein insertion porin family | Bacteria;Proteobacteria;Alphaproteobacteria;Hyphomicrobiales;Xanthobacteraceae;Pseudolabrys;Pseudolabrys sp. Root1462;Pseudolabrys sp. Root1462 |
| Ga0586324 2178266 1 870 | 0.00 | 0.00 | 0.00 | 0.00 | 0.00 | 0.00 | 0.00 | 0.00 | 0.00 | 0.00 | 0.00 | 4147500.00 | KO:K07277 | outer membrane protein insertion porin family | Bacteria;Proteobacteria;Alphaproteobacteria;Hyphomicrobiales;Bradyrhizobiaceae;Bradyrhizobium;Bradyrhizobium sp. STM 3843;Bradyrhizobium sp. STM 3843 |
| Ga0586324 0838444 3 656 | 873271.00 | 0.00 | 0.00 | 0.00 | 0.00 | 0.00 | 0.00 | 0.00 | 0.00 | 0.00 | 0.00 | 0.00 | KO:K07283 | putative salt-induced outer membrane protein | Bacteria;Proteobacteria;Deltaproteobacteria;Myxococcales;Labilitrichaceae;Labilithrix;Labilithrix luteola;Labilithrix luteola DSM 27648 |
| Ga0586324 0000002 169139 170242 | 0.00 | 0.00 | 0.00 | 0.00 | 0.00 | 0.00 | 0.00 | 0.00 | 0.00 | 0.00 | 0.00 | 4267500.00 | KO:K07287 | outer membrane protein assembly factor BamC | Bacteria;Proteobacteria;Betaproteobacteria;Nitrosomonadales;Thiobacillaceae;Annwoodia;Annwoodia aquaesulis;Annwoodia aquaesulis DSM 4255 |
| Ga0586324 0808886 2 1324 | 0.00 | 0.00 | 0.00 | 0.00 | 0.00 | 0.00 | 0.00 | 0.00 | 303501.33 | 0.00 | 0.00 | 0.00 | KO:K07289 | AsmA protein | Bacteria;Proteobacteria;Deltaproteobacteria;Desulfobacterales;Desulfobacteraceae;Desulfobacula;Desulfobacula phenolica;Desulfobacula phenolica DSM 3384 |
| Ga0586324 0010089 1327 3486 | 0.00 | 3100800.00 | 0.00 | 0.00 | 0.00 | 0.00 | 0.00 | 0.00 | 0.00 | 0.00 | 0.00 | 5407500.00 | KO:K07303 | isoquinoline 1-oxidoreductase beta subunit | Bacteria;Proteobacteria;Alphaproteobacteria;Hyphomicrobiales;Bradyrhizobiaceae;Bradyrhizobium;Bradyrhizobium viridifuturi;Bradyrhizobium viridifuturi SEMIA 690 |
| Ga0586324 0041788 2263 4470 | 0.00 | 0.00 | 0.00 | 0.00 | 0.00 | 0.00 | 0.00 | 0.00 | 0.00 | 1609300.00 | 0.00 | 0.00 | KO:K07303 | isoquinoline 1-oxidoreductase beta subunit | Bacteria;Proteobacteria;Betaproteobacteria;Burkholderiales;Oxalobacteraceae;Collimonas;Collimonas arenae;Collimonas arenae Cal35 |
| Ga0586324 0044225 1910 4234 | 6820000.00 | 0.00 | 0.00 | 2992500.00 | 0.00 | 5677100.00 | 0.00 | 0.00 | 0.00 | 0.00 | 0.00 | 0.00 | KO:K07303 | isoquinoline 1-oxidoreductase beta subunit | Bacteria;Proteobacteria;Alphaproteobacteria;Rhodospirillales;unclassified Rhodospirillales;unclassified Rhodospirillales;Rhodospirillales bacterium URHD0088;Rhodospirillales bacterium URHD0088 |
| Ga0586324 0097230 1015 3228 | 0.00 | 0.00 | 0.00 | 0.00 | 0.00 | 0.00 | 0.00 | 0.00 | 0.00 | 0.00 | 880000.00 | 0.00 | KO:K07303 | isoquinoline 1-oxidoreductase beta subunit | Bacteria;Proteobacteria;Betaproteobacteria;unclassified Betaproteobacteria;unclassified Betaproteobacteria;unclassified Betaproteobacteria;Betaproteobacteria bacterium GR16-43;Betaproteobacteria bacterium GR16-43 |
| Ga0586324 0180579 588 2594 | 0.00 | 0.00 | 0.00 | 0.00 | 0.00 | 0.00 | 0.00 | 0.00 | 0.00 | 0.00 | 4417600.00 | 0.00 | KO:K07303 | isoquinoline 1-oxidoreductase beta subunit | Bacteria;Proteobacteria;Alphaproteobacteria;Hyphomicrobiales;Bradyrhizobiaceae;Bradyrhizobium;Bradyrhizobium lablabi;Bradyrhizobium lablabi GAS499 |
| Ga0586324 0252780 3 1742 | 0.00 | 2291600.00 | 4981621.05 | 0.00 | 0.00 | 0.00 | 0.00 | 0.00 | 0.00 | 6131978.93 | 4206400.00 | 6240000.00 | KO:K07303 | isoquinoline 1-oxidoreductase beta subunit | Bacteria;Proteobacteria;Betaproteobacteria;Burkholderiales;Oxalobacteraceae;Collimonas;Collimonas arenae;Collimonas arenae Cal35 |
| Ga0586324 0267465 3 2000 | 0.00 | 0.00 | 0.00 | 0.00 | 0.00 | 0.00 | 0.00 | 471514.14 | 0.00 | 0.00 | 0.00 | 0.00 | KO:K07303 | isoquinoline 1-oxidoreductase beta subunit | Bacteria;Proteobacteria;Betaproteobacteria;Burkholderiales;Oxalobacteraceae;Collimonas;Collimonas arenae;Collimonas arenae Cal35 |
| Ga0586324 0309052 726 2027 | 1570000.00 | 0.00 | 0.00 | 0.00 | 0.00 | 0.00 | 0.00 | 0.00 | 0.00 | 0.00 | 0.00 | 0.00 | KO:K07303 | isoquinoline 1-oxidoreductase beta subunit | Bacteria;Proteobacteria;Betaproteobacteria;Burkholderiales;Comamonadaceae;Rhodoferax;Rhodoferax saidenbachensis;Rhodoferax saidenbachensis ED16 |
| Ga0586324 0337560 1 1950 | 0.00 | 0.00 | 0.00 | 0.00 | 0.00 | 0.00 | 0.00 | 0.00 | 0.00 | 638891.33 | 0.00 | 0.00 | KO:K07303 | isoquinoline 1-oxidoreductase beta subunit | Bacteria;Proteobacteria;Alphaproteobacteria;Rhodospirillales;unclassified Rhodospirillales;unclassified Rhodospirillales;Rhodospirillales bacterium URHD0088;Rhodospirillales bacterium URHD0088 |
| Ga0586324 0464292 2 1456 | 0.00 | 0.00 | 0.00 | 0.00 | 0.00 | 0.00 | 0.00 | 0.00 | 0.00 | 0.00 | 0.00 | 3832500.00 | KO:K07303 | isoquinoline 1-oxidoreductase beta subunit | Bacteria;Proteobacteria;Betaproteobacteria;Burkholderiales;Comamonadaceae;Variovorax;Variovorax soli;Variovorax soli NBRC 106424 |
| Ga0586324 3182481 1 744 | 0.00 | 0.00 | 0.00 | 0.00 | 34505000.00 | 0.00 | 0.00 | 0.00 | 0.00 | 0.00 | 0.00 | 0.00 | KO:K07303 | isoquinoline 1-oxidoreductase beta subunit | Bacteria;Proteobacteria;Betaproteobacteria;Burkholderiales;Comamonadaceae;Variovorax;Variovorax sp. BK119;Variovorax sp. BK119 |
| Ga0586324 3926626 168 680 | 0.00 | 0.00 | 3639727.44 | 0.00 | 0.00 | 0.00 | 0.00 | 0.00 | 0.00 | 0.00 | 0.00 | 1732500.00 | KO:K07304 | peptide-methionine (S)-S-oxide reductase | Bacteria;Deinococcus-Thermus;Deinococci;Thermales;Thermaceae;Calidithermus;Calidithermus timidus;Meiothermus timidus DSM 17022 |
| Ga0586324 0005259 9651 11696 | 0.00 | 5902400.00 | 0.00 | 0.00 | 0.00 | 0.00 | 0.00 | 6057300.00 | 0.00 | 0.00 | 0.00 | 0.00 | KO:K07335 | basic membrane protein A | Bacteria;Chloroflexi;Chloroflexia;Chloroflexales;Chloroflexaceae;Chloroflexus;Chloroflexus aggregans;Chloroflexus aggregans DSM 9485 |
| Ga0586324 0007772 7846 8919 | 0.00 | 0.00 | 0.00 | 0.00 | 0.00 | 1635008.77 | 0.00 | 0.00 | 0.00 | 0.00 | 0.00 | 0.00 | KO:K07335 | basic membrane protein A | Bacteria;Chloroflexi;Anaerolineae;Anaerolineales;Anaerolineaceae;Thermanaerothrix;Thermanaerothrix daxensis;Thermanaerothrix daxensis GNS-1 |
| Ga0586324 0028479 61 1137 | 2920000.00 | 1285200.00 | 0.00 | 0.00 | 0.00 | 0.00 | 3739200.00 | 0.00 | 0.00 | 5035800.00 | 0.00 | 0.00 | KO:K07335 | basic membrane protein A | Bacteria;Chloroflexi;Caldilineae;Caldilineales;Caldilineaceae;Caldilinea;Caldilinea aerophila;Caldilinea aerophila STL-6-O1, DSM 14535 |
| Ga0586324 0034050 2873 4954 | 0.00 | 0.00 | 17058900.00 | 0.00 | 0.00 | 0.00 | 0.00 | 0.00 | 14661000.00 | 0.00 | 0.00 | 0.00 | KO:K07335 | basic membrane protein A | Bacteria;Chloroflexi;Anaerolineae;Anaerolineales;Anaerolineaceae;Thermanaerothrix;Thermanaerothrix daxensis;Thermanaerothrix daxensis GNS-1 |
| Ga0586324 0041345 988 3102 | 17630000.00 | 11689200.00 | 1270555.88 | 2764500.00 | 11845000.00 | 1643818.20 | 1648718.16 | 9723400.00 | 2041200.00 | 2456300.00 | 2323200.00 | 2655000.00 | KO:K07335 | basic membrane protein A | Bacteria;Chloroflexi;Chloroflexia;Chloroflexales;Chloroflexaceae;Chloroflexus;Chloroflexus islandicus;Chloroflexus islandicus isl-2 |
| Ga0586324 0045154 3235 4257 | 0.00 | 0.00 | 7544200.00 | 0.00 | 0.00 | 0.00 | 0.00 | 0.00 | 0.00 | 4797100.00 | 30509600.00 | 0.00 | KO:K07335 | basic membrane protein A | Bacteria;Actinobacteria;Rubrobacteria;Gaiellales;Gaiellaceae;Gaiella;Gaiella occulta;Gaiella occulta F2-233 |
| Ga0586324 0061263 3 920 | 8360000.00 | 3563200.00 | 0.00 | 0.00 | 0.00 | 0.00 | 0.00 | 0.00 | 0.00 | 0.00 | 0.00 | 0.00 | KO:K07335 | basic membrane protein A | Bacteria;Cyanobacteria;unclassified Cyanobacteria;Synechococcales;Leptolyngbyaceae;Leptolyngbya;Leptolyngbya sp. 2LT21S03;Leptolyngbya sp. 2LT21S03 |
| Ga0586324 0062946 2 1876 | 0.00 | 0.00 | 0.00 | 0.00 | 0.00 | 0.00 | 2177003.28 | 486717.78 | 0.00 | 1863400.00 | 0.00 | 0.00 | KO:K07335 | basic membrane protein A | Bacteria;Chloroflexi;Anaerolineae;Anaerolineales;Anaerolineaceae;Thermanaerothrix;Thermanaerothrix daxensis;Thermanaerothrix daxensis GNS-1 |
| Ga0586324 0129895 1566 2612 | 0.00 | 0.00 | 0.00 | 0.00 | 0.00 | 0.00 | 0.00 | 1470100.00 | 1701000.00 | 0.00 | 0.00 | 0.00 | KO:K07335 | basic membrane protein A | Bacteria;Actinobacteria;Actinomycetia;Kineosporiales;Kineosporiaceae;Kineosporia;Kineosporia sp. R H 3;Kineosporia sp. R H 3R H 3 |
| Ga0586324 0137725 1519 2637 | 0.00 | 1720400.00 | 0.00 | 0.00 | 0.00 | 7543000.00 | 0.00 | 0.00 | 0.00 | 0.00 | 0.00 | 0.00 | KO:K07335 | basic membrane protein A | Bacteria;Coprothermobacterota;Coprothermobacteria;Coprothermobacterales;Coprothermobacteraceae;Coprothermobacter;Coprothermobacter proteolyticus;Coprothermobacter proteolyticus DSM 5265 |
| Ga0586324 0139974 1774 2925 | 0.00 | 3182400.00 | 0.00 | 0.00 | 0.00 | 0.00 | 0.00 | 0.00 | 2810700.00 | 0.00 | 3194400.00 | 2640000.00 | KO:K07335 | basic membrane protein A | Bacteria;Firmicutes;Limnochordia;Limnochordales;Limnochordaceae;Limnochorda;Limnochorda pilosa;Limnochorda pilosa HC45 |
| Ga0586324 0170651 743 1741 | 4010000.00 | 0.00 | 0.00 | 0.00 | 5067600.00 | 0.00 | 0.00 | 0.00 | 0.00 | 0.00 | 0.00 | 0.00 | KO:K07335 | basic membrane protein A | Bacteria;Actinobacteria;Rubrobacteria;Gaiellales;Gaiellaceae;Gaiella;Gaiella occulta;Gaiella occulta F2-233 |
| Ga0586324 0201364 2 262 | 0.00 | 0.00 | 0.00 | 0.00 | 0.00 | 0.00 | 0.00 | 1250500.00 | 1514700.00 | 0.00 | 0.00 | 0.00 | KO:K07335 | basic membrane protein A | Bacteria;Chloroflexi;Chloroflexia;Chloroflexales;Roseiflexaceae;Roseiflexus;Roseiflexus castenholzii;Roseiflexus castenholzii HLO8, DSM 13941 |
| Ga0586324 0220593 773 1906 | 0.00 | 0.00 | 0.00 | 0.00 | 0.00 | 0.00 | 0.00 | 0.00 | 0.00 | 0.00 | 2138400.00 | 0.00 | KO:K07335 | basic membrane protein A | Bacteria;Proteobacteria;Deltaproteobacteria;Myxococcales;Anaeromyxobacteraceae;Anaeromyxobacter;Anaeromyxobacter sp. Fw109-5;Anaeromyxobacter sp. Fw109-5 |
| Ga0586324 0221825 1 690 | 0.00 | 0.00 | 0.00 | 0.00 | 0.00 | 0.00 | 0.00 | 2318000.00 | 0.00 | 0.00 | 0.00 | 0.00 | KO:K07335 | basic membrane protein A | Bacteria;Cyanobacteria;unclassified Cyanobacteria;Synechococcales;Leptolyngbyaceae;Leptolyngbya;Leptolyngbya sp. 2LT21S03;Leptolyngbya sp. 2LT21S03 |
| Ga0586324 0330108 703 1968 | 0.00 | 2475200.00 | 0.00 | 0.00 | 2698600.00 | 0.00 | 0.00 | 0.00 | 2624400.00 | 0.00 | 0.00 | 0.00 | KO:K07335 | basic membrane protein A | Bacteria;Proteobacteria;Deltaproteobacteria;unclassified Deltaproteobacteria;unclassified Deltaproteobacteria;unclassified Deltaproteobacteria;SAR324 cluster bacterium JCVI-SC AAA005;SAR324 cluster bacterium JCVI-SC AAA005 |
| Ga0586324 0363820 2 1417 | 0.00 | 0.00 | 0.00 | 0.00 | 0.00 | 0.00 | 0.00 | 473321.57 | 0.00 | 0.00 | 0.00 | 0.00 | KO:K07335 | basic membrane protein A | Bacteria;Chloroflexi;Anaerolineae;Anaerolineales;Anaerolineaceae;Thermanaerothrix;Thermanaerothrix daxensis;Thermanaerothrix daxensis GNS-1 |
| Ga0586324 0363862 454 1521 | 0.00 | 0.00 | 0.00 | 0.00 | 0.00 | 0.00 | 0.00 | 0.00 | 0.00 | 16401000.00 | 0.00 | 0.00 | KO:K07335 | basic membrane protein A | Bacteria;Cyanobacteria;unclassified Cyanobacteria;Synechococcales;Leptolyngbyaceae;Leptolyngbya;Leptolyngbya sp. 2LT21S03;Leptolyngbya sp. 2LT21S03 |
| Ga0586324 0438842 3 1241 | 3670000.00 | 0.00 | 2828033.45 | 0.00 | 0.00 | 1234662.06 | 0.00 | 1769000.00 | 0.00 | 1532300.00 | 0.00 | 0.00 | KO:K07335 | basic membrane protein A | Bacteria;Chloroflexi;Anaerolineae;Anaerolineales;Anaerolineaceae;Levilinea;Levilinea saccharolytica;Levilinea saccharolytica DSM 16555 |
| Ga0586324 0448765 1 1116 | 0.00 | 0.00 | 0.00 | 0.00 | 0.00 | 0.00 | 0.00 | 4562800.00 | 0.00 | 0.00 | 0.00 | 0.00 | KO:K07335 | basic membrane protein A | Bacteria;Cyanobacteria;unclassified Cyanobacteria;Synechococcales;Leptolyngbyaceae;Leptolyngbya;Leptolyngbya sp. 2LT21S03;Leptolyngbya sp. 2LT21S03 |
| Ga0586324 0461035 82 1146 | 4280000.00 | 0.00 | 0.00 | 0.00 | 0.00 | 0.00 | 0.00 | 0.00 | 0.00 | 3311000.00 | 0.00 | 0.00 | KO:K07335 | basic membrane protein A | Bacteria;Chloroflexi;Anaerolineae;Anaerolineales;Anaerolineaceae;Thermanaerothrix;Thermanaerothrix daxensis;Thermanaerothrix daxensis GNS-1 |
| Ga0586324 0487445 937 1656 | 0.00 | 0.00 | 0.00 | 0.00 | 269096.77 | 0.00 | 0.00 | 0.00 | 0.00 | 0.00 | 0.00 | 0.00 | KO:K07335 | basic membrane protein A | Bacteria;Proteobacteria;Betaproteobacteria;Burkholderiales;unclassified Burkholderiales;unclassified Burkholderiales;[Polyangium] brachysporum;Polyangium brachysporum DSM 7029 |
| Ga0586324 0490131 59 1054 | 741299.00 | 0.00 | 0.00 | 0.00 | 0.00 | 0.00 | 0.00 | 0.00 | 0.00 | 0.00 | 0.00 | 0.00 | KO:K07335 | basic membrane protein A | Bacteria;Firmicutes;Clostridia;Eubacteriales;unclassified Eubacteriales;unclassified Eubacteriales;Clostridiales bacterium PH28 bin88;Clostridiales bacterium PH28 bin88 |
| Ga0586324 0491708 2 958 | 8360000.00 | 0.00 | 0.00 | 0.00 | 0.00 | 0.00 | 0.00 | 8418000.00 | 0.00 | 0.00 | 0.00 | 0.00 | KO:K07335 | basic membrane protein A | Bacteria;Chloroflexi;Anaerolineae;Anaerolineales;Anaerolineaceae;Anaerolinea;Anaerolinea thermophila;Anaerolinea thermophila UNI-1 |
| Ga0586324 0522058 86 1120 | 0.00 | 0.00 | 0.00 | 0.00 | 0.00 | 0.00 | 3967200.00 | 2958500.00 | 0.00 | 0.00 | 0.00 | 0.00 | KO:K07335 | basic membrane protein A | Bacteria;Chloroflexi;Anaerolineae;Anaerolineales;Anaerolineaceae;Levilinea;Levilinea saccharolytica;Levilinea saccharolytica DSM 16555 |
| Ga0586324 0591542 25 1200 | 0.00 | 0.00 | 0.00 | 0.00 | 0.00 | 9369200.00 | 13246800.00 | 0.00 | 0.00 | 0.00 | 4567200.00 | 0.00 | KO:K07335 | basic membrane protein A | Bacteria;Chloroflexi;Chloroflexia;Chloroflexales;Oscillochloridaceae;Oscillochloris;Oscillochloris trichoides;Oscillochloris trichoides DG6 |
| Ga0586324 0719726 2 1156 | 0.00 | 0.00 | 0.00 | 0.00 | 0.00 | 0.00 | 0.00 | 2635200.00 | 0.00 | 0.00 | 0.00 | 0.00 | KO:K07335 | basic membrane protein A | Bacteria;Chloroflexi;Anaerolineae;Anaerolineales;Anaerolineaceae;Anaerolinea;Anaerolinea thermophila;Anaerolinea thermophila UNI-1 |
| Ga0586324 0752410 3 1373 | 3230000.00 | 707200.00 | 7431600.00 | 13328500.00 | 7107000.00 | 6352000.00 | 15618000.00 | 3373300.00 | 6204600.00 | 4820200.00 | 8791200.00 | 0.00 | KO:K07335 | basic membrane protein A | Bacteria;Chloroflexi;Chloroflexia;Chloroflexales;Chloroflexaceae;Chloroflexus;Chloroflexus islandicus;Chloroflexus islandicus isl-2 |
| Ga0586324 0763715 3 662 | 0.00 | 1496000.00 | 0.00 | 0.00 | 0.00 | 0.00 | 0.00 | 570514.70 | 0.00 | 0.00 | 0.00 | 1612500.00 | KO:K07335 | basic membrane protein A | Bacteria;Chloroflexi;Anaerolineae;Anaerolineales;Anaerolineaceae;Levilinea;Levilinea saccharolytica;Levilinea saccharolytica DSM 16555 |
| Ga0586324 0784694 2 1048 | 0.00 | 0.00 | 0.00 | 5054000.00 | 3100300.00 | 0.00 | 0.00 | 0.00 | 0.00 | 0.00 | 0.00 | 0.00 | KO:K07335 | basic membrane protein A | Bacteria;Chloroflexi;Ardenticatenia;Ardenticatenales;Ardenticatenaceae;Candidatus Promineofilum;Candidatus Promineofilum breve;Candidatus Promineofilum breve Cfx-K |
| Ga0586324 0817277 93 1172 | 0.00 | 0.00 | 0.00 | 0.00 | 0.00 | 4168500.00 | 8846400.00 | 6649000.00 | 0.00 | 2294600.00 | 0.00 | 4305000.00 | KO:K07335 | basic membrane protein A | Bacteria;Chloroflexi;Caldilineae;Caldilineales;Caldilineaceae;Caldilinea;Caldilinea aerophila;Caldilinea aerophila STL-6-O1, DSM 14535 |
| Ga0586324 0907826 1 675 | 0.00 | 0.00 | 0.00 | 0.00 | 10403000.00 | 0.00 | 0.00 | 0.00 | 0.00 | 0.00 | 5834400.00 | 0.00 | KO:K07335 | basic membrane protein A | Bacteria;Chloroflexi;Anaerolineae;Anaerolineales;Anaerolineaceae;Anaerolinea;Anaerolinea thermophila;Anaerolinea thermophila UNI-1 |
| Ga0586324 1069784 1 891 | 3930000.00 | 0.00 | 0.00 | 0.00 | 0.00 | 0.00 | 0.00 | 0.00 | 0.00 | 0.00 | 0.00 | 0.00 | KO:K07335 | basic membrane protein A | Bacteria;Coprothermobacterota;Coprothermobacteria;Coprothermobacterales;Coprothermobacteraceae;Coprothermobacter;Coprothermobacter proteolyticus;Coprothermobacter proteolyticus DSM 5265 |
| Ga0586324 1251084 70 1104 | 0.00 | 0.00 | 4527296.94 | 0.00 | 0.00 | 0.00 | 0.00 | 0.00 | 0.00 | 2209900.00 | 0.00 | 0.00 | KO:K07335 | basic membrane protein A | Bacteria;Actinobacteria;Rubrobacteria;Gaiellales;Gaiellaceae;Gaiella;Gaiella occulta;Gaiella occulta F2-233 |
| Ga0586324 1397295 1 1053 | 0.00 | 1278400.00 | 0.00 | 0.00 | 0.00 | 0.00 | 0.00 | 0.00 | 2502900.00 | 0.00 | 0.00 | 0.00 | KO:K07335 | basic membrane protein A | Bacteria;Cyanobacteria;unclassified Cyanobacteria;Synechococcales;Leptolyngbyaceae;Leptolyngbya;Leptolyngbya sp. 2LT21S03;Leptolyngbya sp. 2LT21S03 |
| Ga0586324 1432183 3 455 | 0.00 | 552203.52 | 0.00 | 0.00 | 0.00 | 0.00 | 0.00 | 0.00 | 2195100.00 | 0.00 | 0.00 | 0.00 | KO:K07335 | basic membrane protein A | Bacteria;Firmicutes;Limnochordia;Limnochordales;Limnochordaceae;Limnochorda;Limnochorda pilosa;Limnochorda pilosa HC45 |
| Ga0586324 1882355 3 926 | 0.00 | 0.00 | 0.00 | 0.00 | 0.00 | 0.00 | 0.00 | 0.00 | 0.00 | 701216.67 | 0.00 | 0.00 | KO:K07335 | basic membrane protein A | Bacteria;Chloroflexi;Anaerolineae;Anaerolineales;Anaerolineaceae;Thermanaerothrix;Thermanaerothrix daxensis;Thermanaerothrix daxensis GNS-1 |
| Ga0586324 2710134 3 794 | 0.00 | 0.00 | 0.00 | 0.00 | 0.00 | 0.00 | 8230800.00 | 3836900.00 | 34749000.00 | 17579100.00 | 0.00 | 0.00 | KO:K07335 | basic membrane protein A | Bacteria;Chloroflexi;Anaerolineae;Anaerolineales;Anaerolineaceae;Pelolinea;Pelolinea submarina;Pelolinea submarina DSM 23923 |
| Ga0586324 3400019 3 722 | 0.00 | 0.00 | 0.00 | 0.00 | 0.00 | 3673226.62 | 0.00 | 0.00 | 0.00 | 0.00 | 0.00 | 0.00 | KO:K07335 | basic membrane protein A | Bacteria;Cyanobacteria;unclassified Cyanobacteria;Synechococcales;Leptolyngbyaceae;Leptolyngbya;Leptolyngbya sp. 2LT21S03;Leptolyngbya sp. 2LT21S03 |
| Ga0586324 4478623 67 645 | 0.00 | 0.00 | 0.00 | 0.00 | 0.00 | 0.00 | 0.00 | 1915400.00 | 0.00 | 0.00 | 0.00 | 0.00 | KO:K07335 | basic membrane protein A | Bacteria;Chloroflexi;Anaerolineae;Anaerolineales;Anaerolineaceae;Anaerolinea;Anaerolinea thermophila;Anaerolinea thermophila UNI-1 |
| Ga0586324 5134402 3 611 | 0.00 | 0.00 | 0.00 | 0.00 | 0.00 | 4605200.00 | 0.00 | 0.00 | 0.00 | 0.00 | 0.00 | 0.00 | KO:K07335 | basic membrane protein A | Bacteria;Chloroflexi;Anaerolineae;Anaerolineales;Anaerolineaceae;Levilinea;Levilinea saccharolytica;Levilinea saccharolytica DSM 16555 |
| Ga0586324 5251408 1 603 | 0.00 | 0.00 | 0.00 | 0.00 | 0.00 | 2950174.49 | 0.00 | 0.00 | 0.00 | 0.00 | 0.00 | 0.00 | KO:K07335 | basic membrane protein A | Bacteria;Synergistetes;Synergistia;Synergistales;Synergistaceae;Aminivibrio;Aminivibrio pyruvatiphilus;Aminivibrio pyruvatiphilus DSM 25964 |
| Ga0586324 5799317 62 580 | 0.00 | 0.00 | 0.00 | 0.00 | 0.00 | 0.00 | 0.00 | 0.00 | 5499900.00 | 0.00 | 0.00 | 0.00 | KO:K07335 | basic membrane protein A | Bacteria;Chloroflexi;Anaerolineae;Anaerolineales;Anaerolineaceae;Anaerolinea;Anaerolinea thermophila;Anaerolinea thermophila UNI-1 |
| Ga0586324 6011712 1 573 | 7090000.00 | 6092800.00 | 0.00 | 0.00 | 0.00 | 0.00 | 7296000.00 | 0.00 | 8100000.00 | 0.00 | 0.00 | 0.00 | KO:K07335 | basic membrane protein A | Bacteria;Chloroflexi;Ardenticatenia;Ardenticatenales;Ardenticatenaceae;Ardenticatena;Ardenticatena maritima;Ardenticatena maritima 110S |
| Ga0586324 6302974 8 559 | 0.00 | 0.00 | 0.00 | 0.00 | 0.00 | 0.00 | 0.00 | 0.00 | 0.00 | 0.00 | 2481600.00 | 0.00 | KO:K07335 | basic membrane protein A | Bacteria;Chloroflexi;Anaerolineae;Anaerolineales;Anaerolineaceae;Pelolinea;Pelolinea submarina;Pelolinea submarina DSM 23923 |
| Ga0586324 6580007 3 308 | 0.00 | 0.00 | 0.00 | 0.00 | 0.00 | 0.00 | 8846400.00 | 0.00 | 0.00 | 0.00 | 0.00 | 0.00 | KO:K07335 | basic membrane protein A | Bacteria;Chloroflexi;Caldilineae;Caldilineales;Caldilineaceae;Caldilinea;Caldilinea aerophila;Caldilinea aerophila STL-6-O1, DSM 14535 |
| Ga0586324 7237484 2 475 | 0.00 | 0.00 | 0.00 | 0.00 | 0.00 | 0.00 | 0.00 | 0.00 | 1206900.00 | 0.00 | 0.00 | 0.00 | KO:K07335 | basic membrane protein A | Bacteria;Chloroflexi;Anaerolineae;Anaerolineales;Anaerolineaceae;Thermanaerothrix;Thermanaerothrix daxensis;Thermanaerothrix daxensis GNS-1 |
| Ga0586324 0005323 2031 2444 | 0.00 | 1931200.00 | 0.00 | 0.00 | 0.00 | 0.00 | 0.00 | 0.00 | 0.00 | 0.00 | 0.00 | 0.00 | KO:K07397 | putative redox protein | Bacteria;Proteobacteria;Deltaproteobacteria;Desulfobacterales;Desulfobacteraceae;Desulfatitalea;Desulfatitalea tepidiphila;Desulfatitalea tepidiphila S28bF |
| Ga0586324 0370071 3 1742 | 504639.00 | 0.00 | 0.00 | 0.00 | 0.00 | 0.00 | 0.00 | 0.00 | 0.00 | 0.00 | 0.00 | 0.00 | KO:K07407 | alpha-galactosidase | Bacteria;Chloroflexi;Anaerolineae;Anaerolineales;Anaerolineaceae;Thermanaerothrix;Thermanaerothrix daxensis;Thermanaerothrix daxensis GNS-1 |
| Ga0586324 0000182 5669 7510 | 0.00 | 0.00 | 0.00 | 4474500.00 | 7580800.00 | 0.00 | 0.00 | 0.00 | 0.00 | 0.00 | 0.00 | 0.00 | KO:K07463 | RecJ-like exonuclease | Archaea;Euryarchaeota;Methanomicrobia;Methanomicrobiales;Methanoregulaceae;Methanoregula;Methanoregula formicica;Methanoregula formicica SMSP, DSM 22288 |
| Ga0586324 0005330 10500 12881 | 0.00 | 0.00 | 0.00 | 0.00 | 0.00 | 0.00 | 0.00 | 0.00 | 0.00 | 14245000.00 | 0.00 | 0.00 | KO:K07516 | 3-hydroxyacyl-CoA dehydrogenase | Bacteria;Chloroflexi;Ardenticatenia;Ardenticatenales;Ardenticatenaceae;Candidatus Promineofilum;Candidatus Promineofilum breve;Candidatus Promineofilum breve Cfx-K |
| Ga0586324 0238143 1 2130 | 0.00 | 0.00 | 0.00 | 0.00 | 0.00 | 0.00 | 0.00 | 0.00 | 462433.05 | 0.00 | 0.00 | 0.00 | KO:K07516 | 3-hydroxyacyl-CoA dehydrogenase | Bacteria;Proteobacteria;Deltaproteobacteria;Desulfobacterales;Desulfobacteraceae;Desulfatirhabdium;Desulfatirhabdium butyrativorans;Desulfatirhabdium butyrativorans DSM 18734 |
| Ga0586324 0387438 1158 1835 | 0.00 | 0.00 | 0.00 | 514455.40 | 851701.85 | 0.00 | 0.00 | 30012000.00 | 0.00 | 0.00 | 0.00 | 0.00 | KO:K07516 | 3-hydroxyacyl-CoA dehydrogenase | Bacteria;Proteobacteria;Deltaproteobacteria;Desulfobacterales;Desulfobacteraceae;Desulfatiglans;Desulfatiglans anilini;Desulfatiglans anilini DSM 4660 |
| Ga0586324 0790768 2 1342 | 2150000.00 | 0.00 | 0.00 | 1624500.00 | 2605900.00 | 1138306.19 | 0.00 | 0.00 | 0.00 | 0.00 | 0.00 | 0.00 | KO:K07516 | 3-hydroxyacyl-CoA dehydrogenase | Bacteria;Proteobacteria;Deltaproteobacteria;Desulfobacterales;Desulfobacteraceae;Desulfatiglans;Desulfatiglans anilini;Desulfatiglans anilini DSM 4660 |
| Ga0586324 6097495 69 569 | 0.00 | 0.00 | 0.00 | 3239500.00 | 0.00 | 5478600.00 | 0.00 | 0.00 | 0.00 | 0.00 | 0.00 | 0.00 | KO:K07516 | 3-hydroxyacyl-CoA dehydrogenase | Bacteria;Proteobacteria;Deltaproteobacteria;Desulfobacterales;Desulfobacteraceae;Desulfatirhabdium;Desulfatirhabdium butyrativorans;Desulfatirhabdium butyrativorans DSM 18734 |
| Ga0586324 4092280 168 671 | 341910.00 | 0.00 | 0.00 | 1301500.00 | 0.00 | 473347.07 | 488948.28 | 224130.47 | 0.00 | 0.00 | 0.00 | 0.00 | KO:K07537 | cyclohexa-1,5-dienecarbonyl-CoA hydratase | Bacteria;Proteobacteria;Deltaproteobacteria;Desulfobacterales;Desulfobacteraceae;Desulfococcus;Desulfococcus multivorans;Desulfococcus multivorans DSM 2059 |
| Ga0586324 0021186 1888 3000 | 2180000.00 | 3298000.00 | 0.00 | 0.00 | 1781900.00 | 0.00 | 0.00 | 1329800.00 | 750840.03 | 0.00 | 0.00 | 0.00 | KO:K07538 | 6-hydroxycyclohex-1-ene-1-carbonyl-CoA dehydrogenase | Bacteria;Proteobacteria;Deltaproteobacteria;Syntrophorhabdales;Syntrophorhabdaceae;Syntrophorhabdus;Syntrophorhabdus aromaticivorans;Syntrophorhabdus aromaticivorans UI |
| Ga0586324 0004542 2 1024 | 3140000.00 | 0.00 | 0.00 | 29830000.00 | 9960100.00 | 0.00 | 0.00 | 0.00 | 0.00 | 0.00 | 0.00 | 0.00 | KO:K07539 | 6-oxo-cyclohex-1-ene-carbonyl-CoA hydrolase | Bacteria;Proteobacteria;Deltaproteobacteria;unclassified Deltaproteobacteria;unclassified Deltaproteobacteria;unclassified Deltaproteobacteria;delta proteobacterium NaphS2;delta proteobacterium NaphS2 |
| Ga0586324 0021186 705 1853 | 27100000.00 | 1788400.00 | 38452900.00 | 75620000.00 | 70040000.00 | 67847300.00 | 38355170.04 | 35892400.00 | 26568000.00 | 16940000.00 | 0.00 | 16500000.00 | KO:K07539 | 6-oxo-cyclohex-1-ene-carbonyl-CoA hydrolase | Bacteria;Proteobacteria;Deltaproteobacteria;Syntrophorhabdales;Syntrophorhabdaceae;Syntrophorhabdus;Syntrophorhabdus aromaticivorans;Syntrophorhabdus aromaticivorans UI |
| Ga0586324 0064950 3305 4216 | 6620000.00 | 0.00 | 0.00 | 0.00 | 0.00 | 0.00 | 0.00 | 0.00 | 0.00 | 0.00 | 0.00 | 0.00 | KO:K07539 | 6-oxo-cyclohex-1-ene-carbonyl-CoA hydrolase | Bacteria;Proteobacteria;Deltaproteobacteria;Syntrophobacterales;Syntrophaceae;Syntrophus;Syntrophus aciditrophicus;Syntrophus aciditrophicus SB |
| Ga0586324 0141527 3 620 | 6510000.00 | 0.00 | 12329700.00 | 8303000.00 | 0.00 | 12108500.00 | 11445600.00 | 0.00 | 0.00 | 0.00 | 0.00 | 0.00 | KO:K07539 | 6-oxo-cyclohex-1-ene-carbonyl-CoA hydrolase | Bacteria;Proteobacteria;Deltaproteobacteria;Syntrophobacterales;Syntrophaceae;Desulfomonile;Desulfomonile tiedjei;Desulfomonile tiedjei DCB-1, DSM 6799 |
| Ga0586324 1375992 2 886 | 0.00 | 0.00 | 0.00 | 0.00 | 0.00 | 0.00 | 874637.64 | 0.00 | 0.00 | 0.00 | 0.00 | 0.00 | KO:K07539 | 6-oxo-cyclohex-1-ene-carbonyl-CoA hydrolase | Bacteria;Proteobacteria;Deltaproteobacteria;Syntrophorhabdales;Syntrophorhabdaceae;Syntrophorhabdus;Syntrophorhabdus aromaticivorans;Syntrophorhabdus aromaticivorans UI |
| Ga0586324 2116187 1 852 | 0.00 | 13532000.00 | 0.00 | 0.00 | 23690000.00 | 0.00 | 20360400.00 | 0.00 | 12960000.00 | 12397000.00 | 13464000.00 | 2970000.00 | KO:K07539 | 6-oxo-cyclohex-1-ene-carbonyl-CoA hydrolase | Bacteria;Proteobacteria;Deltaproteobacteria;Syntrophorhabdales;Syntrophorhabdaceae;Syntrophorhabdus;Syntrophorhabdus aromaticivorans;Syntrophorhabdus aromaticivorans UI |
| Ga0586324 2128400 2 880 | 1480000.00 | 7208000.00 | 0.00 | 3087500.00 | 1133000.00 | 0.00 | 0.00 | 0.00 | 6755400.00 | 0.00 | 0.00 | 0.00 | KO:K07539 | 6-oxo-cyclohex-1-ene-carbonyl-CoA hydrolase | Bacteria;Proteobacteria;Deltaproteobacteria;Desulfobacterales;Desulfobacteraceae;Desulfatiglans;Desulfatiglans anilini;Desulfatiglans anilini DSM 4660 |
| Ga0586324 2224347 29 865 | 0.00 | 0.00 | 0.00 | 0.00 | 0.00 | 0.00 | 0.00 | 0.00 | 0.00 | 947100.00 | 0.00 | 0.00 | KO:K07539 | 6-oxo-cyclohex-1-ene-carbonyl-CoA hydrolase | Bacteria;Proteobacteria;Deltaproteobacteria;Syntrophobacterales;Syntrophaceae;Syntrophus;Syntrophus aciditrophicus;Syntrophus aciditrophicus SB |
| Ga0586324 2934599 1 768 | 0.00 | 0.00 | 0.00 | 8122500.00 | 0.00 | 0.00 | 0.00 | 0.00 | 0.00 | 0.00 | 0.00 | 0.00 | KO:K07539 | 6-oxo-cyclohex-1-ene-carbonyl-CoA hydrolase | Bacteria;Proteobacteria;Deltaproteobacteria;Syntrophobacterales;Syntrophaceae;Syntrophus;Syntrophus aciditrophicus;Syntrophus aciditrophicus SB |
| Ga0586324 3154272 1 747 | 0.00 | 1387200.00 | 0.00 | 0.00 | 2513200.00 | 0.00 | 995438.88 | 0.00 | 907200.00 | 0.00 | 0.00 | 0.00 | KO:K07539 | 6-oxo-cyclohex-1-ene-carbonyl-CoA hydrolase | Bacteria;Proteobacteria;Deltaproteobacteria;Syntrophobacterales;Syntrophaceae;Syntrophus;Syntrophus aciditrophicus;Syntrophus aciditrophicus SB |
| Ga0586324 5082594 3 386 | 20000000.00 | 23732000.00 | 28150000.00 | 23465000.00 | 24514000.00 | 18301700.00 | 13862400.00 | 13359000.00 | 13203000.00 | 15708000.00 | 0.00 | 12150000.00 | KO:K07539 | 6-oxo-cyclohex-1-ene-carbonyl-CoA hydrolase | Bacteria;Proteobacteria;Deltaproteobacteria;Syntrophorhabdales;Syntrophorhabdaceae;Syntrophorhabdus;Syntrophorhabdus aromaticivorans;Syntrophorhabdus aromaticivorans UI |
| Ga0586324 0209238 535 1098 | 0.00 | 1863200.00 | 0.00 | 0.00 | 0.00 | 0.00 | 0.00 | 0.00 | 0.00 | 0.00 | 0.00 | 0.00 | KO:K07572 | putative nucleotide binding protein | Archaea;Euryarchaeota;Methanomicrobia;Methanomicrobiales;Methanoregulaceae;Methanoregula;Methanoregula formicica;Methanoregula formicica SMSP, DSM 22288 |
| Ga0586324 0002706 2572 3879 | 0.00 | 0.00 | 0.00 | 0.00 | 1545000.00 | 0.00 | 0.00 | 0.00 | 0.00 | 0.00 | 0.00 | 0.00 | KO:K07662 | two-component system response regulator CpxR | Bacteria;Proteobacteria;Deltaproteobacteria;Desulfobacterales;Desulfobacteraceae;Desulfococcus;Desulfococcus multivorans;Desulfococcus multivorans DSM 2059 |
| Ga0586324 0009977 2 739 | 0.00 | 0.00 | 0.00 | 0.00 | 0.00 | 0.00 | 0.00 | 0.00 | 1441800.00 | 0.00 | 0.00 | 1200000.00 | KO:K07712 | two-component system nitrogen regulation response regulator GlnG | Bacteria;Proteobacteria;Betaproteobacteria;Nitrosomonadales;Thiobacillaceae;Thiobacillus;Thiobacillus thioparus;Thiobacillus thioparus DSM 505 |
| Ga0586324 0235423 545 1987 | 0.00 | 0.00 | 0.00 | 0.00 | 0.00 | 0.00 | 0.00 | 0.00 | 0.00 | 0.00 | 2780800.00 | 0.00 | KO:K07712 | two-component system nitrogen regulation response regulator GlnG | Bacteria;Proteobacteria;Alphaproteobacteria;Hyphomicrobiales;Bradyrhizobiaceae;Bradyrhizobium;Bradyrhizobium lablabi;Bradyrhizobium lablabi GAS499 |
| Ga0586324 0362938 871 1632 | 0.00 | 0.00 | 0.00 | 0.00 | 0.00 | 0.00 | 0.00 | 0.00 | 0.00 | 0.00 | 959200.00 | 0.00 | KO:K07736 | CarD family transcriptional regulator | Bacteria;Proteobacteria;Alphaproteobacteria;Hyphomicrobiales;Xanthobacteraceae;Pseudolabrys;Pseudolabrys taiwanensis;Pseudolabrys taiwanensis CC-BB4 |
| Ga0586324 0382876 309 1304 | 0.00 | 0.00 | 0.00 | 0.00 | 0.00 | 0.00 | 2895600.00 | 0.00 | 0.00 | 0.00 | 0.00 | 0.00 | KO:K07736 | CarD family transcriptional regulator | Bacteria;Proteobacteria;Alphaproteobacteria;Hyphomicrobiales;Bradyrhizobiaceae;Bradyrhizobium;Bradyrhizobium elkanii;Bradyrhizobium elkanii USDA 94 |

| | | | | | | | | | | | | | | | |
|---|---|---|---|---|---|---|---|---|---|---|---|---|---|---|---|
| Ga0586324 1032819 332 1198 | 0.00 | 0.00 | 0.00 | 0.00 | 0.00 | 0.00 | 0.00 | 0.00 | 262029.33 | 0.00 | 0.00 | 0.00 | KO:K07736 | CarD family transcriptional regulator | Bacteria;Proteobacteria;Alphaproteobacteria;Hyphomicrobiales;Bradyrhizobiaceae;Bradyrhizobium;Bradyrhizobium elkanii;Bradyrhizobium elkanii WSM1741 |
| Ga0586324 5240671 209 604 | 0.00 | 0.00 | 0.00 | 8597500.00 | 0.00 | 0.00 | 0.00 | 0.00 | 2559600.00 | 0.00 | 2340800.00 | 0.00 | KO:K07736 | CarD family transcriptional regulator | Bacteria;Proteobacteria;Deltaproteobacteria;Syntrophobacterales;Syntrophobacteraceae;Syntrophobacter;Syntrophobacter fumaroxidans;Syntrophobacter fumaroxidans MPOB |
| Ga0586324 0034737 2423 3310 | 0.00 | 2278000.00 | 0.00 | 2242000.00 | 0.00 | 0.00 | 0.00 | 1677500.00 | 0.00 | 0.00 | 0.00 | 0.00 | KO:K07744 | predicted transcriptional regulator | Archaea;Euryarchaeota;Methanomicrobia;Methanosarcinales;Methanotrichaceae;Methanothrix;Methanothrix soehngenii;Methanothrix soehngenii GP-6 |
| Ga0586324 1627179 3 986 | 0.00 | 0.00 | 0.00 | 0.00 | 0.00 | 0.00 | 900643.32 | 0.00 | 0.00 | 0.00 | 0.00 | 0.00 | KO:K07749 | formyl-CoA transferase | Bacteria;Proteobacteria;Alphaproteobacteria;Hyphomicrobiales;Bradyrhizobiaceae;Bradyrhizobium;Bradyrhizobium icense;Bradyrhizobium icense LMTR 13 |
| Ga0586324 5747967 2 583 | 0.00 | 0.00 | 2135380.18 | 0.00 | 0.00 | 0.00 | 0.00 | 0.00 | 0.00 | 0.00 | 0.00 | 0.00 | KO:K07749 | formyl-CoA transferase | Bacteria;Proteobacteria;Alphaproteobacteria;Hyphomicrobiales;Hyphomicrobiaceae;Rhodoplanes;Rhodoplanes sp. Z2-YC6860;Rhodoplanes sp. Z2-YC6860 |
| Ga0586324 0000626 24085 25089 | 0.00 | 0.00 | 0.00 | 0.00 | 0.00 | 0.00 | 0.00 | 0.00 | 0.00 | 8855000.00 | 0.00 | 0.00 | KO:K07795 | putative tricarboxylic transport membrane protein | Bacteria;Proteobacteria;Alphaproteobacteria;Hyphomicrobiales;Xanthobacteraceae;Pseudolabrys;Pseudolabrys sp. Root1462;Pseudolabrys sp. Root1462 |
| Ga0586324 0008220 8971 9987 | 0.00 | 1074400.00 | 0.00 | 0.00 | 0.00 | 0.00 | 0.00 | 0.00 | 0.00 | 1886500.00 | 0.00 | 0.00 | KO:K07795 | putative tricarboxylic transport membrane protein | Bacteria;Proteobacteria;Betaproteobacteria;Burkholderiales;Comamonadaceae;Polaromonas;Polaromonas sp. EUR3 1.2.1;Polaromonas sp. EUR3 1.2.1 |
| Ga0586324 0020385 2949 3956 | 0.00 | 0.00 | 0.00 | 0.00 | 0.00 | 0.00 | 0.00 | 3373300.00 | 0.00 | 5828900.00 | 6265600.00 | 5295000.00 | KO:K07795 | putative tricarboxylic transport membrane protein | Bacteria;Proteobacteria;Betaproteobacteria;Burkholderiales;Comamonadaceae;Hylemonella;Hylemonella gracilis;Hylemonella gracilis Niagara R |
| Ga0586324 0025491 4548 5561 | 0.00 | 0.00 | 0.00 | 0.00 | 0.00 | 14689000.00 | 0.00 | 0.00 | 0.00 | 0.00 | 0.00 | 0.00 | KO:K07795 | putative tricarboxylic transport membrane protein | Bacteria;Proteobacteria;Betaproteobacteria;unclassified Betaproteobacteria;unclassified Betaproteobacteria;unclassified Betaproteobacteria;Betaproteobacteria bacterium GR16-43;Betaproteobacteria bacterium GR16-43 |
| Ga0586324 0028653 2726 3712 | 2600000.00 | 3060000.00 | 0.00 | 0.00 | 0.00 | 0.00 | 0.00 | 0.00 | 2527200.00 | 4034800.00 | 3916000.00 | 4665000.00 | KO:K07795 | putative tricarboxylic transport membrane protein | Bacteria;Proteobacteria;Betaproteobacteria;unclassified Betaproteobacteria;unclassified Betaproteobacteria;unclassified Betaproteobacteria;uncultured beta proteobacterium;unclassified betaproteobacterium ENR4 |
| Ga0586324 0030312 3287 4288 | 0.00 | 0.00 | 0.00 | 0.00 | 0.00 | 0.00 | 0.00 | 0.00 | 0.00 | 0.00 | 0.00 | 855000.00 | KO:K07795 | putative tricarboxylic transport membrane protein | Bacteria;Proteobacteria;Betaproteobacteria;unclassified Betaproteobacteria;unclassified Betaproteobacteria;unclassified Betaproteobacteria;Betaproteobacteria bacterium GR16-43;Betaproteobacteria bacterium GR16-43 |
| Ga0586324 0062838 236 1213 | 0.00 | 0.00 | 0.00 | 0.00 | 0.00 | 0.00 | 6976800.00 | 3788100.00 | 6407100.00 | 4412100.00 | 0.00 | 8992500.00 | KO:K07795 | putative tricarboxylic transport membrane protein | Bacteria;Proteobacteria;Betaproteobacteria;Burkholderiales;Comamonadaceae;Polaromonas;Polaromonas sp. YR568;Polaromonas sp. YR568 |
| Ga0586324 0133754 1133 2125 | 4400000.00 | 0.00 | 0.00 | 0.00 | 3934600.00 | 30052900.00 | 12950400.00 | 21289000.00 | 0.00 | 0.00 | 0.00 | 23625000.00 | KO:K07795 | putative tricarboxylic transport membrane protein | Bacteria;Proteobacteria;Betaproteobacteria;unclassified Betaproteobacteria;unclassified Betaproteobacteria;unclassified Betaproteobacteria;uncultured beta proteobacterium;unclassified betaproteobacterium ENR4 |
| Ga0586324 0686205 3 902 | 0.00 | 0.00 | 0.00 | 0.00 | 0.00 | 0.00 | 781570.32 | 0.00 | 383328.45 | 1932700.00 | 1812800.00 | 0.00 | KO:K07795 | putative tricarboxylic transport membrane protein | Bacteria;Proteobacteria;Betaproteobacteria;Burkholderiales;Comamonadaceae;Polaromonas;Polaromonas sp. YR568;Polaromonas sp. YR568 |
| Ga0586324 0691410 381 1403 | 0.00 | 0.00 | 0.00 | 0.00 | 0.00 | 0.00 | 5987074.80 | 4721400.00 | 4576500.00 | 5166700.00 | 6168800.00 | 7605000.00 | KO:K07795 | putative tricarboxylic transport membrane protein | Bacteria;Proteobacteria;Betaproteobacteria;unclassified Betaproteobacteria;unclassified Betaproteobacteria;unclassified Betaproteobacteria;uncultured beta proteobacterium;unclassified betaproteobacterium ENR4 |
| Ga0586324 1083090 1 489 | 0.00 | 0.00 | 0.00 | 0.00 | 0.00 | 0.00 | 0.00 | 11956000.00 | 10206000.00 | 0.00 | 0.00 | 0.00 | KO:K07795 | putative tricarboxylic transport membrane protein | Bacteria;Proteobacteria;Betaproteobacteria;Burkholderiales;Oxalobacteraceae;unclassified Oxalobacteraceae;Oxalobacteraceae bacterium;Oxalobacteraceae sp. OAS666 |
| Ga0586324 1721227 2 907 | 2680000.00 | 0.00 | 0.00 | 0.00 | 0.00 | 0.00 | 0.00 | 0.00 | 0.00 | 0.00 | 0.00 | 0.00 | KO:K07795 | putative tricarboxylic transport membrane protein | Bacteria;Proteobacteria;Betaproteobacteria;Burkholderiales;Burkholderiaceae;Polynucleobacter;Polynucleobacter rarus;Polynucleobacter rarus MT-CBb6A5 |
| Ga0586324 2919013 1 771 | 0.00 | 0.00 | 0.00 | 0.00 | 0.00 | 0.00 | 0.00 | 0.00 | 0.00 | 5713400.00 | 0.00 | 0.00 | KO:K07795 | putative tricarboxylic transport membrane protein | Bacteria;Proteobacteria;Betaproteobacteria;Burkholderiales;Burkholderiaceae;Polynucleobacter;Polynucleobacter sp. UB-Domo-W1;Polynucleobacter sp. UB-Domo-W1 |
| Ga0586324 5636256 1 585 | 0.00 | 0.00 | 0.00 | 0.00 | 0.00 | 0.00 | 0.00 | 0.00 | 0.00 | 0.00 | 4760800.00 | 0.00 | KO:K07795 | putative tricarboxylic transport membrane protein | Bacteria;Proteobacteria;Betaproteobacteria;unclassified Betaproteobacteria;unclassified Betaproteobacteria;unclassified Betaproteobacteria;Betaproteobacteria bacterium GR16-43;Betaproteobacteria bacterium GR16-43 |
| Ga0586324 0261288 3 2189 | 0.00 | 0.00 | 0.00 | 0.00 | 0.00 | 0.00 | 0.00 | 0.00 | 1887300.00 | 0.00 | 0.00 | 0.00 | KO:K08086 | pilus assembly protein FimV | Bacteria;Proteobacteria;Betaproteobacteria;Nitrosomonadales;Sulfuricellaceae;Sulfurirhabdus;Sulfurirhabdus autotrophica;Sulfurirhabdus autotrophica DSM 100309 |
| Ga0586324 0475403 352 1677 | 0.00 | 0.00 | 0.00 | 0.00 | 0.00 | 0.00 | 0.00 | 0.00 | 0.00 | 0.00 | 4188800.00 | 0.00 | KO:K08086 | pilus assembly protein FimV | Bacteria;Proteobacteria;Gammaproteobacteria;Nevskiales;Steroidobacteraceae;Povalibacter;Povalibacter uvarum;Povalibacter uvarum DSM 26723 |
| Ga0586324 2612510 145 807 | 0.00 | 0.00 | 0.00 | 0.00 | 774718.62 | 0.00 | 0.00 | 0.00 | 0.00 | 0.00 | 0.00 | 0.00 | KO:K08086 | pilus assembly protein FimV | Bacteria;Proteobacteria;Betaproteobacteria;Rhodocyclales;Azonexaceae;Dechloromonas;Dechloromonas denitrificans;Dechloromonas denitrificans ATCC BAA-841 |
| Ga0586324 0019659 1823 2464 | 0.00 | 0.00 | 103029000.00 | 0.00 | 0.00 | 0.00 | 0.00 | 0.00 | 0.00 | 38192000.00 | 0.00 | 0.00 | KO:K08093 | 3-hexulose-6-phosphate synthase | Bacteria;Proteobacteria;Gammaproteobacteria;Methylococcales;Crenotrichaceae;Crenothrix;Crenothrix polyspora;Crenothrix polyspora ERS1545888 |
| Ga0586324 0112735 1290 1985 | 8720000.00 | 4338400.00 | 5079352.22 | 3344000.00 | 0.00 | 0.00 | 0.00 | 1939800.00 | 0.00 | 0.00 | 0.00 | 0.00 | KO:K08093 | 3-hexulose-6-phosphate synthase | Bacteria;Proteobacteria;Betaproteobacteria;Nitrosomonadales;Methylophilaceae;Methylovorus;Methylovorus glucosotrophus;Methylovorus glucosotrophus DSM 6874 |
| Ga0586324 1623353 1 561 | 15300000.00 | 4624000.00 | 9176900.00 | 0.00 | 0.00 | 0.00 | 0.00 | 4453000.00 | 0.00 | 8239000.00 | 15840000.00 | 9375000.00 | KO:K08093 | 3-hexulose-6-phosphate synthase | Bacteria;Proteobacteria;Betaproteobacteria;Nitrosomonadales;Methylophilaceae;Methylobacillus;Methylobacillus sp. MM3;Methylobacillus sp. MM2 |
| Ga0586324 1625264 2 496 | 7010000.00 | 0.00 | 0.00 | 0.00 | 0.00 | 0.00 | 0.00 | 0.00 | 0.00 | 0.00 | 7110400.00 | 0.00 | KO:K08093 | 3-hexulose-6-phosphate synthase | Bacteria;Proteobacteria;Betaproteobacteria;Nitrosomonadales;Methylophilaceae;Methylotenera;Methylotenera versatilis;Methylotenera versatilis 301, JCM 17579 |
| Ga0586324 1798020 1 480 | 0.00 | 172856000.00 | 184326200.00 | 0.00 | 0.00 | 0.00 | 105792000.00 | 100040000.00 | 140130000.00 | 66759000.00 | 117040000.00 | 113850000.00 | KO:K08093 | 3-hexulose-6-phosphate synthase | Bacteria;Proteobacteria;Gammaproteobacteria;Methylococcales;Methylococcaceae;Methylobacter;Methylobacter tundripaludum;Methylobacter tundripaludum OWC-G53F |
| Ga0586324 3021936 1 387 | 603590.00 | 678090.56 | 924035.01 | 0.00 | 0.00 | 0.00 | 0.00 | 774700.00 | 5224500.00 | 0.00 | 0.00 | 0.00 | KO:K08093 | 3-hexulose-6-phosphate synthase | Bacteria;Proteobacteria;Betaproteobacteria;Nitrosomonadales;Methylophilaceae;Methylobacillus;Methylobacillus sp. MM3;Methylobacillus sp. MM2 |
| Ga0586324 0066885 2307 2852 | 0.00 | 0.00 | 0.00 | 0.00 | 0.00 | 0.00 | 0.00 | 0.00 | 23814000.00 | 0.00 | 0.00 | 0.00 | KO:K08094 | 6-phospho-3-hexuloisomerase | Bacteria;Proteobacteria;Gammaproteobacteria;Methylococcales;Methylococcaceae;Methylobacter;Methylobacter tundripaludum;Methylobacter tundripaludum OWC-G53F |
| Ga0586324 1284577 440 973 | 0.00 | 2087600.00 | 0.00 | 0.00 | 0.00 | 0.00 | 0.00 | 0.00 | 0.00 | 0.00 | 0.00 | 0.00 | KO:K08094 | 6-phospho-3-hexuloisomerase | Bacteria;Proteobacteria;Gammaproteobacteria;Methylococcales;Methylococcaceae;Methylobacter;Methylobacter tundripaludum;Methylobacter tundripaludum OWC-G53F |
| Ga0586324 3783405 102 647 | 0.00 | 952000.00 | 0.00 | 0.00 | 0.00 | 0.00 | 0.00 | 0.00 | 0.00 | 0.00 | 0.00 | 0.00 | KO:K08094 | 6-phospho-3-hexuloisomerase | Bacteria;Proteobacteria;Gammaproteobacteria;Methylococcales;Methylococcaceae;Methylomarinum;Methylomarinum vadi;Methylomarinum vadi IT-4 |
| Ga0586324 0001625 1000 2637 | 17567035.00 | 5752800.00 | 13655441.14 | 0.00 | 2142400.00 | 9616538.94 | 0.00 | 6081700.00 | 5378400.00 | 25625600.00 | 6476800.00 | 11842500.00 | KO:K08295 | 2-aminobenzoate-CoA ligase | Bacteria;Proteobacteria;Deltaproteobacteria;Syntrophobacterales;Syntrophobacteraceae;Desulfacinum;Desulfacinum infernum;Desulfacinum infernum DSM 9756 |
| Ga0586324 0038228 91 1731 | 638057.00 | 0.00 | 0.00 | 0.00 | 0.00 | 0.00 | 0.00 | 0.00 | 0.00 | 0.00 | 0.00 | 0.00 | KO:K08295 | 2-aminobenzoate-CoA ligase | Bacteria;Proteobacteria;Deltaproteobacteria;Syntrophobacterales;Syntrophobacteraceae;Desulfacinum;Desulfacinum infernum;Desulfacinum infernum DSM 9756 |
| Ga0586324 0060271 800 2479 | 27800000.00 | 23364800.00 | 18701970.46 | 21261000.00 | 22927800.00 | 24949965.22 | 57729926.04 | 12627000.00 | 52277400.00 | 25025000.00 | 25432000.00 | 21675000.00 | KO:K08295 | 2-aminobenzoate-CoA ligase | Bacteria;Proteobacteria;Deltaproteobacteria;Syntrophobacterales;Syntrophaceae;Desulfobacca;Desulfobacca acetoxidans;Desulfobacca acetoxidans ASRB2, DSM 11109 |
| Ga0586324 0079167 934 2613 | 54520000.00 | 31320800.00 | 27431060.26 | 91307389.90 | 88784463.24 | 61644987.26 | 62877110.40 | 35422700.00 | 58975537.05 | 12335400.00 | 9820800.00 | 14617500.00 | KO:K08295 | 2-aminobenzoate-CoA ligase | Bacteria;Proteobacteria;Deltaproteobacteria;Syntrophobacterales;Syntrophaceae;Desulfobacca;Desulfobacca acetoxidans;Desulfobacca acetoxidans ASRB2, DSM 11109 |
| Ga0586324 0213130 1047 2402 | 46600000.00 | 0.00 | 0.00 | 0.00 | 0.00 | 0.00 | 0.00 | 14945000.00 | 0.00 | 0.00 | 0.00 | 20175000.00 | KO:K08295 | 2-aminobenzoate-CoA ligase | Bacteria;Proteobacteria;Deltaproteobacteria;Syntrophobacterales;Syntrophaceae;Desulfobacca;Desulfobacca acetoxidans;Desulfobacca acetoxidans ASRB2, DSM 11109 |
| Ga0586324 0319551 218 1903 | 13243369.00 | 10147531.20 | 10068962.24 | 0.00 | 0.00 | 506012.23 | 11514000.00 | 5612000.00 | 8748000.00 | 0.00 | 4338400.00 | 13644832.50 | KO:K08295 | 2-aminobenzoate-CoA ligase | Bacteria;Proteobacteria;Deltaproteobacteria;Syntrophobacterales;Syntrophaceae;Desulfobacca;Desulfobacca acetoxidans;Desulfobacca acetoxidans ASRB2, DSM 11109 |
| Ga0586324 0342351 1 684 | 12700000.00 | 12240000.00 | 0.00 | 0.00 | 18025000.00 | 0.00 | 0.00 | 0.00 | 0.00 | 0.00 | 0.00 | 0.00 | KO:K08295 | 2-aminobenzoate-CoA ligase | Bacteria;Proteobacteria;Deltaproteobacteria;Syntrophobacterales;Syntrophaceae;Desulfobacca;Desulfobacca acetoxidans;Desulfobacca acetoxidans ASRB2, DSM 11109 |
| Ga0586324 0462546 3 233 | 0.00 | 10948000.00 | 15876600.00 | 14440000.00 | 13596000.00 | 31442400.00 | 20816400.00 | 11224000.00 | 12231000.00 | 8162000.00 | 0.00 | 0.00 | KO:K08295 | 2-aminobenzoate-CoA ligase | Bacteria;Proteobacteria;Deltaproteobacteria;Syntrophobacterales;Syntrophaceae;Desulfobacca;Desulfobacca acetoxidans;Desulfobacca acetoxidans ASRB2, DSM 11109 |
| Ga0586324 0491014 1 687 | 0.00 | 0.00 | 0.00 | 0.00 | 0.00 | 0.00 | 0.00 | 200577.15 | 0.00 | 0.00 | 0.00 | 0.00 | KO:K08295 | 2-aminobenzoate-CoA ligase | Bacteria;Proteobacteria;Deltaproteobacteria;Syntrophobacterales;Syntrophaceae;Desulfobacca;Desulfobacca acetoxidans;Desulfobacca acetoxidans ASRB2, DSM 11109 |
| Ga0586324 0492076 3 578 | 0.00 | 4134400.00 | 0.00 | 0.00 | 0.00 | 1658197.54 | 0.00 | 0.00 | 1158300.00 | 0.00 | 0.00 | 0.00 | KO:K08295 | 2-aminobenzoate-CoA ligase | Bacteria;Proteobacteria;Deltaproteobacteria;Syntrophobacterales;Syntrophaceae;Desulfobacca;Desulfobacca acetoxidans;Desulfobacca acetoxidans ASRB2, DSM 11109 |
| Ga0586324 0533033 2 1594 | 3210000.00 | 16592000.00 | 34214241.90 | 23389000.00 | 9712900.00 | 112998657.86 | 56042400.00 | 17446000.00 | 0.00 | 30415000.00 | 0.00 | 3967500.00 | KO:K08295 | 2-aminobenzoate-CoA ligase | Bacteria;Proteobacteria;Deltaproteobacteria;Syntrophobacterales;Syntrophaceae;Desulfobacca;Desulfobacca acetoxidans;Desulfobacca acetoxidans ASRB2, DSM 11109 |
| Ga0586324 0668512 2 703 | 742397.00 | 0.00 | 0.00 | 0.00 | 0.00 | 0.00 | 0.00 | 0.00 | 0.00 | 0.00 | 0.00 | 0.00 | KO:K08295 | 2-aminobenzoate-CoA ligase | Bacteria;Proteobacteria;Deltaproteobacteria;Syntrophobacterales;Syntrophobacteraceae;Desulfacinum;Desulfacinum infernum;Desulfacinum infernum DSM 9756 |
| Ga0586324 0993661 156 1217 | 1770000.00 | 33755200.00 | 0.00 | 16568000.00 | 0.00 | 0.00 | 14158800.00 | 8747400.00 | 8035729.74 | 0.00 | 0.00 | 0.00 | KO:K08295 | 2-aminobenzoate-CoA ligase | Bacteria;Proteobacteria;Deltaproteobacteria;Syntrophobacterales;Syntrophaceae;Desulfobacca;Desulfobacca acetoxidans;Desulfobacca acetoxidans ASRB2, DSM 11109 |
| Ga0586324 1055809 3 1187 | 0.00 | 0.00 | 0.00 | 0.00 | 0.00 | 0.00 | 0.00 | 0.00 | 0.00 | 0.00 | 0.00 | 4305000.00 | KO:K08295 | 2-aminobenzoate-CoA ligase | Bacteria;Proteobacteria;Deltaproteobacteria;Syntrophobacterales;Syntrophaceae;Desulfobacca;Desulfobacca acetoxidans;Desulfobacca acetoxidans ASRB2, DSM 11109 |
| Ga0586324 1353343 1 1065 | 73600000.00 | 44880000.00 | 0.00 | 54910000.00 | 40685000.00 | 84561000.00 | 0.00 | 38613000.00 | 45603000.00 | 0.00 | 0.00 | 2160000.00 | KO:K08295 | 2-aminobenzoate-CoA ligase | Bacteria;Proteobacteria;Deltaproteobacteria;Syntrophobacterales;Syntrophaceae;Desulfobacca;Desulfobacca acetoxidans;Desulfobacca acetoxidans ASRB2, DSM 11109 |
| Ga0586324 1660071 3 977 | 23700000.00 | 24024400.00 | 35469000.00 | 40204000.00 | 135033000.00 | 135518697.24 | 12403200.00 | 81270300.00 | 24364800.00 | 23716000.00 | 22352000.00 | 12300000.00 | KO:K08295 | 2-aminobenzoate-CoA ligase | Bacteria;Proteobacteria;Deltaproteobacteria;Syntrophobacterales;Syntrophaceae;Desulfobacca;Desulfobacca acetoxidans;Desulfobacca acetoxidans ASRB2, DSM 11109 |
| Ga0586324 2053716 149 892 | 0.00 | 0.00 | 0.00 | 0.00 | 0.00 | 0.00 | 42180000.00 | 0.00 | 13932000.00 | 0.00 | 0.00 | 0.00 | KO:K08295 | 2-aminobenzoate-CoA ligase | Bacteria;Proteobacteria;Deltaproteobacteria;Syntrophobacterales;Syntrophaceae;Desulfobacca;Desulfobacca acetoxidans;Desulfobacca acetoxidans ASRB2, DSM 11109 |
| Ga0586324 2073383 3 551 | 0.00 | 0.00 | 0.00 | 9975000.00 | 11433000.00 | 22629000.00 | 5403600.00 | 18422000.00 | 7200900.00 | 0.00 | 0.00 | 0.00 | KO:K08295 | 2-aminobenzoate-CoA ligase | Bacteria;Proteobacteria;Deltaproteobacteria;Syntrophobacterales;Syntrophaceae;Desulfobacca;Desulfobacca acetoxidans;Desulfobacca acetoxidans ASRB2, DSM 11109 |
| Ga0586324 2120516 168 881 | 0.00 | 3869200.00 | 0.00 | 0.00 | 4717400.00 | 0.00 | 0.00 | 2745000.00 | 0.00 | 0.00 | 0.00 | 2992500.00 | KO:K08295 | 2-aminobenzoate-CoA ligase | Bacteria;Proteobacteria;Deltaproteobacteria;Syntrophobacterales;Syntrophaceae;Desulfobacca;Desulfobacca acetoxidans;Desulfobacca acetoxidans ASRB2, DSM 11109 |
| Ga0586324 2135944 159 878 | 0.00 | 0.00 | 0.00 | 0.00 | 0.00 | 0.00 | 0.00 | 2482700.00 | 0.00 | 1270500.00 | 0.00 | 0.00 | KO:K08295 | 2-aminobenzoate-CoA ligase | Bacteria;Proteobacteria;Deltaproteobacteria;Syntrophobacterales;Syntrophobacteraceae;Desulfacinum;Desulfacinum infernum;Desulfacinum infernum DSM 9756 |
| Ga0586324 2243045 1 153 | 0.00 | 0.00 | 0.00 | 0.00 | 0.00 | 153122.50 | 0.00 | 0.00 | 0.00 | 0.00 | 0.00 | 0.00 | KO:K08295 | 2-aminobenzoate-CoA ligase | Bacteria;Proteobacteria;Deltaproteobacteria;Syntrophobacterales;Syntrophaceae;Desulfobacca;Desulfobacca acetoxidans;Desulfobacca acetoxidans ASRB2, DSM 11109 |
| Ga0586324 3097798 1 384 | 0.00 | 0.00 | 0.00 | 40660000.00 | 53457000.00 | 32315800.00 | 53808000.00 | 52216000.00 | 56538000.00 | 0.00 | 0.00 | 0.00 | KO:K08295 | 2-aminobenzoate-CoA ligase | Bacteria;Proteobacteria;Deltaproteobacteria;Syntrophobacterales;Syntrophobacteraceae;Desulfacinum;Desulfacinum hydrothermale;Desulfacinum hydrothermale DSM 13146 |
| Ga0586324 4205099 2 367 | 0.00 | 0.00 | 0.00 | 0.00 | 1668600.00 | 0.00 | 0.00 | 0.00 | 0.00 | 0.00 | 0.00 | 0.00 | KO:K08295 | 2-aminobenzoate-CoA ligase | Bacteria;Proteobacteria;Deltaproteobacteria;Syntrophobacterales;Syntrophaceae;Desulfobacca;Desulfobacca acetoxidans;Desulfobacca acetoxidans ASRB2, DSM 11109 |
| Ga0586324 4384196 2 517 | 2090000.00 | 2794800.00 | 0.00 | 0.00 | 0.00 | 0.00 | 5677200.00 | 2208200.00 | 2284200.00 | 0.00 | 0.00 | 2490000.00 | KO:K08295 | 2-aminobenzoate-CoA ligase | Bacteria;Proteobacteria;Deltaproteobacteria;Syntrophobacterales;Syntrophobacteraceae;Desulfacinum;Desulfacinum infernum;Desulfacinum infernum DSM 9756 |
| Ga0586324 4977739 3 617 | 0.00 | 0.00 | 0.00 | 1900000.00 | 0.00 | 0.00 | 0.00 | 0.00 | 0.00 | 0.00 | 0.00 | 0.00 | KO:K08295 | 2-aminobenzoate-CoA ligase | Bacteria;Proteobacteria;Deltaproteobacteria;Syntrophobacterales;Syntrophaceae;Desulfobacca;Desulfobacca acetoxidans;Desulfobacca acetoxidans ASRB2, DSM 11109 |
| Ga0586324 5410732 114 596 | 33100000.00 | 30464000.00 | 37608400.00 | 78660000.00 | 85284000.00 | 72811376.09 | 46740000.00 | 40748000.00 | 35478000.00 | 27181000.00 | 23584000.00 | 22500000.00 | KO:K08295 | 2-aminobenzoate-CoA ligase | Bacteria;Proteobacteria;Deltaproteobacteria;Syntrophobacterales;Syntrophaceae;Desulfobacca;Desulfobacca acetoxidans;Desulfobacca acetoxidans ASRB2, DSM 11109 |
| Ga0586324 5764971 1 582 | 468986.00 | 1978800.00 | 0.00 | 850692.70 | 0.00 | 0.00 | 0.00 | 0.00 | 0.00 | 0.00 | 0.00 | 0.00 | KO:K08295 | 2-aminobenzoate-CoA ligase | Bacteria;Proteobacteria;Deltaproteobacteria;Syntrophobacterales;Syntrophaceae;Desulfobacca;Desulfobacca acetoxidans;Desulfobacca acetoxidans ASRB2, DSM 11109 |
| Ga0586324 5765977 2 580 | 3160000.00 | 3774000.00 | 0.00 | 0.00 | 0.00 | 0.00 | 0.00 | 1195600.00 | 582922.98 | 0.00 | 0.00 | 0.00 | KO:K08295 | 2-aminobenzoate-CoA ligase | Bacteria;Proteobacteria;Deltaproteobacteria;Syntrophobacterales;Syntrophaceae;Desulfobacca;Desulfobacca acetoxidans;Desulfobacca acetoxidans ASRB2, DSM 11109 |
| Ga0586324 5826454 101 580 | 0.00 | 0.00 | 0.00 | 0.00 | 32445000.00 | 22946600.00 | 0.00 | 13420000.00 | 16443000.00 | 0.00 | 0.00 | 0.00 | KO:K08295 | 2-aminobenzoate-CoA ligase | Bacteria;Proteobacteria;Deltaproteobacteria;Syntrophobacterales;Syntrophaceae;Desulfobacca;Desulfobacca acetoxidans;Desulfobacca acetoxidans ASRB2, DSM 11109 |
| Ga0586324 5874540 203 577 | 0.00 | 1448400.00 | 0.00 | 0.00 | 2029100.00 | 0.00 | 0.00 | 1945900.00 | 1927800.00 | 0.00 | 0.00 | 0.00 | KO:K08295 | 2-aminobenzoate-CoA ligase | Bacteria;Proteobacteria;Deltaproteobacteria;Syntrophobacterales;Syntrophobacteraceae;Desulfosoma;Desulfosoma caldarium;Desulfosoma caldarium DSM 22027 |
| Ga0586324 7794566 2 514 | 0.00 | 0.00 | 0.00 | 0.00 | 0.00 | 1610251.85 | 0.00 | 0.00 | 0.00 | 0.00 | 0.00 | 0.00 | KO:K08295 | 2-aminobenzoate-CoA ligase | Bacteria;Proteobacteria;Deltaproteobacteria;Syntrophobacterales;Syntrophaceae;Desulfobacca;Desulfobacca acetoxidans;Desulfobacca acetoxidans ASRB2, DSM 11109 |
| Ga0586324 8261402 3 500 | 0.00 | 0.00 | 0.00 | 0.00 | 0.00 | 0.00 | 0.00 | 1958100.00 | 0.00 | 0.00 | 0.00 | 0.00 | KO:K08295 | 2-aminobenzoate-CoA ligase | Bacteria;Proteobacteria;Deltaproteobacteria;Syntrophobacterales;Syntrophaceae;Desulfobacca;Desulfobacca acetoxidans;Desulfobacca acetoxidans ASRB2, DSM 11109 |
| Ga0586324 0000007 46820 49429 | 0.00 | 0.00 | 0.00 | 0.00 | 0.00 | 0.00 | 0.00 | 0.00 | 0.00 | 2949100.00 | 0.00 | 0.00 | KO:K08300 | ribonuclease E | Bacteria;Proteobacteria;Betaproteobacteria;Nitrosomonadales;Thiobacillaceae;Annwoodia;Annwoodia aquaesulis;Annwoodia aquaesulis DSM 4255 |
| Ga0586324 0012438 2311 5049 | 0.00 | 328780.00 | 0.00 | 0.00 | 0.00 | 0.00 | 0.00 | 0.00 | 0.00 | 0.00 | 565196.72 | 0.00 | KO:K08300 | ribonuclease E | Bacteria;Proteobacteria;Alphaproteobacteria;Hyphomicrobiales;unclassified Hyphomicrobiales;Methyloceanibacter;Methyloceanibacter caenitepidi;Methyloceanibacter caenitepidi Gela4 |
| Ga0586324 0014901 2418 5192 | 0.00 | 0.00 | 0.00 | 0.00 | 0.00 | 0.00 | 0.00 | 0.00 | 0.00 | 0.00 | 1848000.00 | 0.00 | KO:K08300 | ribonuclease E | Bacteria;Proteobacteria;Betaproteobacteria;Burkholderiales;Burkholderiaceae;Chitinimonas;Chitinimonas koreensis;Chitinimonas koreensis DSM 17726 |
| Ga0586324 0033251 2724 5363 | 0.00 | 0.00 | 0.00 | 1102000.00 | 0.00 | 0.00 | 0.00 | 2928000.00 | 3102300.00 | 0.00 | 3027200.00 | 0.00 | KO:K08300 | ribonuclease E | Bacteria;Proteobacteria;Betaproteobacteria;Nitrosomonadales;Sterolibacteriaceae;Methyloversatilis;Methyloversatilis discipulorum;Methyloversatilis discipulorum FAM1 |
| Ga0586324 0494937 1 1647 | 0.00 | 0.00 | 0.00 | 0.00 | 0.00 | 0.00 | 0.00 | 0.00 | 4365900.00 | 0.00 | 0.00 | 0.00 | KO:K08300 | ribonuclease E | Bacteria;Proteobacteria;Gammaproteobacteria;unclassified Gammaproteobacteria;Candidatus Competibacteraceae;Candidatus Contendobacter;Candidatus Contendobacter odensis;Candidatus Contendobacter odensis Run B J11 |
| Ga0586324 1851278 2 478 | 0.00 | 0.00 | 0.00 | 0.00 | 648568.34 | 0.00 | 0.00 | 0.00 | 0.00 | 0.00 | 0.00 | 0.00 | KO:K08300 | ribonuclease E | Bacteria;Proteobacteria;Alphaproteobacteria;Hyphomicrobiales;Xanthobacteraceae;Pseudolabrys;Pseudolabrys sp. Root1462;Pseudolabrys sp. Root1462 |
| Ga0586324 0000011 46490 47197 | 0.00 | 0.00 | 0.00 | 0.00 | 0.00 | 0.00 | 0.00 | 0.00 | 0.00 | 1463000.00 | 0.00 | 0.00 | KO:K08355 | arsenite oxidase small subunit | Bacteria;Proteobacteria;Betaproteobacteria;Rhodocyclales;Zoogloeaceae;Azoarcus;Azoarcus pumilus;Azoarcus pumilus SY39 |
| Ga0586324 0076339 1603 3900 | 0.00 | 0.00 | 0.00 | 0.00 | 0.00 | 0.00 | 0.00 | 0.00 | 0.00 | 1455300.00 | 0.00 | 0.00 | KO:K08356 | arsenite oxidase large subunit | Bacteria;Proteobacteria;Betaproteobacteria;Burkholderiales;Burkholderiaceae;Paraburkholderia;Paraburkholderia bryophila;Paraburkholderia bryophila H2C3B |
| Ga0586324 0181765 630 1403 | 0.00 | 0.00 | 0.00 | 0.00 | 0.00 | 0.00 | 0.00 | 0.00 | 7079400.00 | 0.00 | 15664000.00 | 0.00 | KO:K08358 | tetrathionate reductase subunit B | Bacteria;Proteobacteria;Betaproteobacteria;Nitrosomonadales;Sulfuricellaceae;Sulfurirhabdus;Sulfurirhabdus autotrophica;Sulfurirhabdus autotrophica DSM 100309 |
| Ga0586324 0000013 58661 60382 | 0.00 | 0.00 | 0.00 | 0.00 | 0.00 | 0.00 | 0.00 | 0.00 | 0.00 | 1047200.00 | 0.00 | 0.00 | KO:K08483 | phosphotransferase system enzyme I (PtsI) | Bacteria;Proteobacteria;Betaproteobacteria;Nitrosomonadales;Thiobacillaceae;Annwoodia;Annwoodia aquaesulis;Annwoodia aquaesulis DSM 4255 |
| Ga0586324 0000406 6350 8785 | 2040000.00 | 12031192.40 | 15482500.00 | 0.00 | 0.00 | 0.00 | 0.00 | 0.00 | 0.00 | 0.00 | 0.00 | 0.00 | KO:K08651 | thermitase | Bacteria;Chloroflexi;Anaerolineae;Anaerolineales;Anaerolineaceae;Levilinea;Levilinea saccharolytica;Levilinea saccharolytica DSM 16555 |
| Ga0586324 0015122 4299 5252 | 11900000.00 | 0.00 | 0.00 | 0.00 | 0.00 | 3307081.46 | 0.00 | 0.00 | 0.00 | 1755600.00 | 627670.56 | 0.00 | KO:K08678 | UDP-glucuronate decarboxylase | Bacteria;Proteobacteria;Deltaproteobacteria;Syntrophobacterales;Syntrophobacteraceae;Syntrophobacter;Syntrophobacter fumaroxidans;Syntrophobacter fumaroxidans MPOB |
| Ga0586324 0504955 3 725 | 0.00 | 0.00 | 0.00 | 0.00 | 0.00 | 0.00 | 0.00 | 0.00 | 0.00 | 0.00 | 2173600.00 | 0.00 | KO:K08678 | UDP-glucuronate decarboxylase | Bacteria;Proteobacteria;Betaproteobacteria;Burkholderiales;Oxalobacteraceae;Janthinobacterium;Janthinobacterium sp. B9-8;Janthinobacterium sp. B9-8 |
| Ga0586324 7757495 1 513 | 0.00 | 0.00 | 0.00 | 2907000.00 | 0.00 | 0.00 | 0.00 | 0.00 | 0.00 | 0.00 | 0.00 | 0.00 | KO:K08678 | UDP-glucuronate decarboxylase | Bacteria;Fibrobacteres;Fibrobacteria;Fibrobacterales;Fibrobacteraceae;Fibrobacter;Fibrobacter sp. UWB7;Fibrobacter sp. UWB7 |
| Ga0586324 3152468 2 745 | 0.00 | 0.00 | 0.00 | 0.00 | 0.00 | 0.00 | 0.00 | 0.00 | 0.00 | 4142600.00 | 0.00 | 0.00 | KO:K08720 | outer membrane protein OmpU | Bacteria;Proteobacteria;Alphaproteobacteria;Rhodospirillales;Rhodospirillaceae;Dongia;Dongia sp. URHE0060;Dongia sp. URHE0060 |
| Ga0586324 0187273 1084 1539 | 0.00 | 2475200.00 | 0.00 | 0.00 | 0.00 | 0.00 | 0.00 | 0.00 | 0.00 | 0.00 | 0.00 | 0.00 | KO:K08738 | cytochrome c | Bacteria;Proteobacteria;Betaproteobacteria;Burkholderiales;Oxalobacteraceae;Noviherbaspirillum;Noviherbaspirillum autotrophicum;Noviherbaspirillum autotrophicum TSA66 |
| Ga0586324 0929066 136 705 | 0.00 | 0.00 | 0.00 | 4028000.00 | 0.00 | 0.00 | 0.00 | 3391600.00 | 0.00 | 4004000.00 | 0.00 | 0.00 | KO:K08738 | cytochrome c | Bacteria;Proteobacteria;Gammaproteobacteria;unclassified Gammaproteobacteria;Candidatus Competibacteraceae;Candidatus Contendobacter;Candidatus Contendobacter odensis;Candidatus Contendobacter odensis Run B J11 |
| Ga0586324 1223168 1 288 | 0.00 | 0.00 | 0.00 | 0.00 | 0.00 | 0.00 | 0.00 | 0.00 | 1603800.00 | 0.00 | 0.00 | 0.00 | KO:K08738 | cytochrome c | Bacteria;Proteobacteria;Betaproteobacteria;Burkholderiales;Oxalobacteraceae;Rugamonas;Rugamonas sp. SG757;Rugamonas sp. SG757 |
| Ga0586324 2191023 21 476 | 0.00 | 0.00 | 0.00 | 0.00 | 0.00 | 16872500.00 | 0.00 | 7808000.00 | 97200000.00 | 0.00 | 0.00 | 0.00 | KO:K08738 | cytochrome c | Bacteria;Proteobacteria;Betaproteobacteria;Burkholderiales;Oxalobacteraceae;Duganella;Duganella sp. CF517;Duganella sp. CF517 |
| Ga0586324 0079051 1941 2381 | 0.00 | 0.00 | 0.00 | 0.00 | 0.00 | 0.00 | 0.00 | 2220400.00 | 0.00 | 0.00 | 0.00 | 0.00 | KO:K08972 | putative membrane protein | Bacteria;Firmicutes;Clostridia;Eubacteriales;Peptococcaceae;Desulfotomaculum;Desulfotomaculum ruminis;Desulfotomaculum ruminis DL, DSM 2154 |
| Ga0586324 0000007 8901 9329 | 0.00 | 0.00 | 0.00 | 0.00 | 0.00 | 0.00 | 0.00 | 11712000.00 | 18630000.00 | 16203726.77 | 35112000.00 | 0.00 | KO:K09004 | intracellular sulfur oxidation DsrE/DsrF family protein | Bacteria;Proteobacteria;Betaproteobacteria;Nitrosomonadales;Thiobacillaceae;Annwoodia;Annwoodia aquaesulis;Annwoodia aquaesulis DSM 4255 |
| Ga0586324 0015399 437 886 | 0.00 | 0.00 | 0.00 | 0.00 | 0.00 | 0.00 | 29868000.00 | 57157000.00 | 44639100.00 | 42627200.00 | 29480000.00 | 44850000.00 | KO:K09004 | intracellular sulfur oxidation DsrE/DsrF family protein | Bacteria;Proteobacteria;Betaproteobacteria;Nitrosomonadales;Thiobacillaceae;Thiobacillus;Thiobacillus denitrificans;Thiobacillus denitrificans ATCC 25259 |
| Ga0586324 0064339 1247 1669 | 0.00 | 0.00 | 0.00 | 0.00 | 0.00 | 0.00 | 0.00 | 0.00 | 0.00 | 0.00 | 9328000.00 | 2295000.00 | KO:K09004 | intracellular sulfur oxidation DsrE/DsrF family protein | Bacteria;Proteobacteria;Betaproteobacteria;Burkholderiales;Comamonadaceae;Polaromonas;Polaromonas sp. CF318;Polaromonas sp. CF318 |
| Ga0586324 0498954 934 1464 | 0.00 | 0.00 | 0.00 | 0.00 | 0.00 | 0.00 | 0.00 | 0.00 | 2016900.00 | 7777000.00 | 7576800.00 | 8512500.00 | KO:K09004 | intracellular sulfur oxidation DsrE/DsrF family protein | Bacteria;Proteobacteria;Gammaproteobacteria;Acidiferrobacterales;Acidiferrobacteraceae;Sulfurifustis;Sulfurifustis variabilis;Sulfurifustis variabilis skN76 |
| Ga0586324 0113018 2085 2867 | 0.00 | 0.00 | 0.00 | 0.00 | 2049700.00 | 0.00 | 0.00 | 0.00 | 0.00 | 0.00 | 0.00 | 0.00 | KO:K09013 | Fe-S cluster assembly ATP-binding protein | Bacteria;Actinobacteria;Rubrobacteria;Gaiellales;Gaiellaceae;Gaiella;Gaiella occulta;Gaiella occulta F2-233 |
| Ga0586324 1896004 72 839 | 0.00 | 0.00 | 0.00 | 0.00 | 0.00 | 0.00 | 7911600.00 | 0.00 | 0.00 | 0.00 | 0.00 | 0.00 | KO:K09013 | Fe-S cluster assembly ATP-binding protein | Bacteria;Actinobacteria;Thermoleophilia;Thermoleophilales;Thermoleophilaceae;Thermoleophilum;Thermoleophilum album;Thermoleophilum album ATCC 35263 |
| Ga0586324 2067982 220 891 | 0.00 | 0.00 | 0.00 | 0.00 | 0.00 | 0.00 | 0.00 | 0.00 | 0.00 | 3665200.00 | 0.00 | 0.00 | KO:K09013 | Fe-S cluster assembly ATP-binding protein | Bacteria;Actinobacteria;Actinomycetia;Propionibacteriales;Nocardioidaceae;Nocardioides;Nocardioides ferulae;Nocardioides ferulae EGI 63112 |
| Ga0586324 3854844 1 456 | 0.00 | 1516400.00 | 0.00 | 0.00 | 0.00 | 0.00 | 0.00 | 0.00 | 0.00 | 0.00 | 0.00 | 0.00 | KO:K09013 | Fe-S cluster assembly ATP-binding protein | Bacteria;Bacteroidetes;Cytophagia;Cytophagales;Fulvivirgaceae;Ohtaekwangia;Ohtaekwangia kribbensis;Ohtaekwangia kribbensis DSM 25221 |
| Ga0586324 6223837 70 564 | 0.00 | 0.00 | 0.00 | 0.00 | 0.00 | 0.00 | 0.00 | 0.00 | 1603800.00 | 0.00 | 0.00 | 0.00 | KO:K09013 | Fe-S cluster assembly ATP-binding protein | Bacteria;Verrucomicrobia;Verrucomicrobiae;Verrucomicrobiales;Verrucomicrobia subdivision 3;Pedosphaera;Pedosphaera parvula;Pedosphaera parvula Ellin514 |
| Ga0586324 8095891 3 506 | 0.00 | 0.00 | 0.00 | 0.00 | 3429900.00 | 0.00 | 0.00 | 0.00 | 0.00 | 0.00 | 0.00 | 0.00 | KO:K09013 | Fe-S cluster assembly ATP-binding protein | Bacteria;Proteobacteria;Deltaproteobacteria;Syntrophobacterales;Syntrophobacteraceae;Desulfacinum;Desulfacinum infernum;Desulfacinum infernum DSM 9756 |
| Ga0586324 0071657 1365 1580 | 0.00 | 0.00 | 0.00 | 0.00 | 0.00 | 18539900.00 | 0.00 | 0.00 | 0.00 | 0.00 | 0.00 | 0.00 | KO:K09165 | flavin-binding protein dodecin | Bacteria;Proteobacteria;Deltaproteobacteria;Syntrophorhabdales;Syntrophorhabdaceae;Syntrophorhabdus;Syntrophorhabdus aromaticivorans;Syntrophorhabdus aromaticivorans UI |
| Ga0586324 0078057 1964 2179 | 0.00 | 0.00 | 3337368.29 | 0.00 | 0.00 | 0.00 | 0.00 | 0.00 | 0.00 | 0.00 | 0.00 | 0.00 | KO:K09165 | flavin-binding protein dodecin | Bacteria;Proteobacteria;Deltaproteobacteria;Syntrophorhabdales;Syntrophorhabdaceae;Syntrophorhabdus;Syntrophorhabdus aromaticivorans;Syntrophorhabdus aromaticivorans UI |
| Ga0586324 0117694 2237 2446 | 8960000.00 | 9724000.00 | 0.00 | 16245000.00 | 16377000.00 | 0.00 | 14956800.00 | 0.00 | 11907000.00 | 12166000.00 | 11176000.00 | 13950000.00 | KO:K09165 | flavin-binding protein dodecin | Bacteria;Proteobacteria;Deltaproteobacteria;unclassified Deltaproteobacteria;unclassified Deltaproteobacteria;unclassified Deltaproteobacteria;delta proteobacterium NaphS2;delta proteobacterium NaphS2 |
| Ga0586324 0205356 2 178 | 1060000.00 | 0.00 | 0.00 | 0.00 | 0.00 | 0.00 | 0.00 | 1518900.00 | 1490400.00 | 1424500.00 | 0.00 | 1297500.00 | KO:K09165 | flavin-binding protein dodecin | Bacteria;Proteobacteria;Gammaproteobacteria;Acidiferrobacterales;Acidiferrobacteraceae;Sulfuricaulis;Sulfuricaulis limicola;Sulfuricaulis limicola HA5 |
| Ga0586324 0585860 222 431 | 0.00 | 0.00 | 8163500.00 | 0.00 | 0.00 | 0.00 | 0.00 | 0.00 | 0.00 | 0.00 | 0.00 | 0.00 | KO:K09165 | flavin-binding protein dodecin | Bacteria;Proteobacteria;Gammaproteobacteria;Methylococcales;Methylococcaceae;Methylomicrobium;Methylomicrobium album;Methylomicrobium album BG8 |
| Ga0586324 2026683 216 431 | 910003.00 | 0.00 | 0.00 | 0.00 | 3883100.00 | 0.00 | 12540000.00 | 3983300.00 | 4033800.00 | 5728800.00 | 2314400.00 | 2385000.00 | KO:K09165 | flavin-binding protein dodecin | Bacteria;Proteobacteria;Deltaproteobacteria;Desulfarculales;Desulfarculaceae;Desulfocarbo;Desulfocarbo indianensis;Desulfocarbo indianensis SCBM |
| Ga0586324 4517172 203 424 | 0.00 | 3366000.00 | 0.00 | 3477000.00 | 990804.38 | 0.00 | 0.00 | 0.00 | 0.00 | 0.00 | 0.00 | 0.00 | KO:K09165 | flavin-binding protein dodecin | Archaea;Euryarchaeota;Methanomicrobia;Methanosarcinales;Methanotrichaceae;Methanothrix;Methanothrix soehngenii;Methanothrix soehngenii GP-6 |
| Ga0586324 4977341 2 196 | 12000000.00 | 0.00 | 0.00 | 0.00 | 0.00 | 0.00 | 0.00 | 0.00 | 0.00 | 0.00 | 0.00 | 0.00 | KO:K09165 | flavin-binding protein dodecin | Bacteria;Proteobacteria;Deltaproteobacteria;Syntrophobacterales;Syntrophaceae;Desulfobacca;Desulfobacca acetoxidans;Desulfobacca acetoxidans ASRB2, DSM 11109 |
| Ga0586324 8040479 3 506 | 0.00 | 0.00 | 0.00 | 0.00 | 0.00 | 0.00 | 0.00 | 0.00 | 0.00 | 1224300.00 | 0.00 | 0.00 | KO:K09458 | 3-oxoacyl-[acyl-carrier-protein] synthase II | Bacteria;Proteobacteria;Deltaproteobacteria;Myxococcales;Labilitrichaceae;Labilithrix;Labilithrix luteola;Labilithrix luteola DSM 27648 |
| Ga0586324 0002060 15894 19115 | 4320000.00 | 2108000.00 | 0.00 | 0.00 | 4140600.00 | 0.00 | 0.00 | 0.00 | 2454300.00 | 0.00 | 3784000.00 | 0.00 | KO:K09607 | immune inhibitor A | Bacteria;Actinobacteria;Actinomycetia;Streptosporangiales;Streptosporangiaceae;Microbispora;Microbispora sp. ATCC PTA-5024;Microbispora sp. ATCC PTA-5024 |
| Ga0586324 0029060 174 3341 | 0.00 | 795600.00 | 1147838.77 | 0.00 | 1266900.00 | 0.00 | 0.00 | 0.00 | 0.00 | 908600.00 | 343938.32 | 703676.25 | KO:K09607 | immune inhibitor A | Bacteria;Actinobacteria;Actinomycetia;Micromonosporales;Micromonosporaceae;Micromonospora;Micromonospora sp. RP3T;Micromonospora sp. RP3T |
| Ga0586324 0034726 3158 5455 | 8250000.00 | 2033200.00 | 0.00 | 0.00 | 0.00 | 0.00 | 0.00 | 0.00 | 0.00 | 0.00 | 0.00 | 0.00 | KO:K09607 | immune inhibitor A | Bacteria;Actinobacteria;Actinomycetia;Corynebacteriales;Corynebacteriaceae;Corynebacterium;Corynebacterium variabile;Corynebacterium variabile NRRL B-4201 |
| Ga0586324 0047970 1980 4274 | 10883992.00 | 9248000.00 | 10697000.00 | 0.00 | 0.00 | 5081600.00 | 0.00 | 0.00 | 0.00 | 0.00 | 0.00 | 12135195.00 | KO:K09607 | immune inhibitor A | Bacteria;Actinobacteria;Actinomycetia;Corynebacteriales;Corynebacteriaceae;Corynebacterium;Corynebacterium variabile;Corynebacterium variabile NRRL B-4201 |
| Ga0586324 0125070 349 3054 | 0.00 | 3182400.00 | 0.00 | 0.00 | 4686500.00 | 0.00 | 0.00 | 0.00 | 0.00 | 0.00 | 0.00 | 0.00 | KO:K09607 | immune inhibitor A | Bacteria;Actinobacteria;Actinomycetia;Micromonosporales;Micromonosporaceae;Micromonospora;Micromonospora echinospora;Micromonospora echinospora DSM 1040 |
| Ga0586324 0184621 221 2407 | 0.00 | 0.00 | 20493200.00 | 0.00 | 0.00 | 14808100.00 | 0.00 | 0.00 | 0.00 | 0.00 | 0.00 | 9750000.00 | KO:K09607 | immune inhibitor A | Bacteria;Actinobacteria;Actinomycetia;Micrococcales;Intrasporangiaceae;Intrasporangium;Intrasporangium calvum;Intrasporangium calvum 7KIP, DSM 43043 |
| Ga0586324 0208452 2 1669 | 1100000.00 | 2380000.00 | 0.00 | 0.00 | 0.00 | 0.00 | 0.00 | 1311500.00 | 1174500.00 | 1640100.00 | 2508000.00 | 0.00 | KO:K09607 | immune inhibitor A | Bacteria;Proteobacteria;Deltaproteobacteria;Myxococcales;Anaeromyxobacteraceae;Anaeromyxobacter;Anaeromyxobacter dehalogenans;Anaeromyxobacter dehalogenans 2CP-C |
| Ga0586324 1129301 3 1151 | 0.00 | 0.00 | 0.00 | 0.00 | 0.00 | 0.00 | 0.00 | 1531100.00 | 0.00 | 2317700.00 | 2376000.00 | 0.00 | KO:K09607 | immune inhibitor A | Bacteria;Actinobacteria;Actinomycetia;Streptosporangiales;Streptosporangiaceae;Thermobispora;Thermobispora bispora;Thermobispora bispora R51, DSM 43833 |
| Ga0586324 1566146 1 1002 | 0.00 | 0.00 | 0.00 | 0.00 | 0.00 | 0.00 | 0.00 | 0.00 | 0.00 | 3826900.00 | 0.00 | 0.00 | KO:K09607 | immune inhibitor A | Bacteria;Actinobacteria;Actinomycetia;Micromonosporales;Micromonosporaceae;Micromonospora;Micromonospora echinospora;Micromonospora echinospora DSM 1040 |
| Ga0586324 2126330 1 879 | 0.00 | 0.00 | 0.00 | 0.00 | 0.00 | 0.00 | 0.00 | 0.00 | 0.00 | 0.00 | 681224.72 | 0.00 | KO:K09607 | immune inhibitor A | Bacteria;Actinobacteria;Actinomycetia;Micrococcales;Intrasporangiaceae;Intrasporangium;Intrasporangium calvum;Intrasporangium calvum 7KIP, DSM 43043 |
| Ga0586324 2242801 3 860 | 0.00 | 0.00 | 0.00 | 0.00 | 0.00 | 0.00 | 0.00 | 3300100.00 | 0.00 | 5821200.00 | 4364800.00 | 3855000.00 | KO:K09607 | immune inhibitor A | Bacteria;Actinobacteria;Actinomycetia;Streptosporangiales;Streptosporangiaceae;Thermobispora;Thermobispora bispora;Thermobispora bispora R51, DSM 43833 |
| Ga0586324 2283599 1 855 | 7190000.00 | 0.00 | 0.00 | 0.00 | 0.00 | 0.00 | 0.00 | 2964600.00 | 0.00 | 8624000.00 | 5878400.00 | 4162500.00 | KO:K09607 | immune inhibitor A | Bacteria;Actinobacteria;Actinomycetia;Streptosporangiales;Streptosporangiaceae;Thermobispora;Thermobispora bispora;Thermobispora bispora R51, DSM 43833 |
| Ga0586324 3697467 3 698 | 0.00 | 0.00 | 0.00 | 0.00 | 0.00 | 0.00 | 0.00 | 0.00 | 0.00 | 0.00 | 2604800.00 | 0.00 | KO:K09607 | immune inhibitor A | Bacteria;Proteobacteria;Deltaproteobacteria;Myxococcales;Anaeromyxobacteraceae;Anaeromyxobacter;Anaeromyxobacter dehalogenans;Anaeromyxobacter dehalogenans 2CP-C |
| Ga0586324 6644121 2 547 | 0.00 | 8228000.00 | 13906100.00 | 0.00 | 11021000.00 | 9766200.00 | 0.00 | 0.00 | 0.00 | 0.00 | 0.00 | 0.00 | KO:K09607 | immune inhibitor A | Bacteria;Proteobacteria;Deltaproteobacteria;Myxococcales;Anaeromyxobacteraceae;Anaeromyxobacter;Anaeromyxobacter dehalogenans;Anaeromyxobacter dehalogenans 2CP-C |
| Ga0586324 0001043 11838 13523 | 0.00 | 0.00 | 0.00 | 1586500.00 | 0.00 | 0.00 | 0.00 | 0.00 | 0.00 | 0.00 | 0.00 | 0.00 | KO:K09758 | aspartate 4-decarboxylase | Bacteria;Proteobacteria;Alphaproteobacteria;Hyphomicrobiales;Xanthobacteraceae;Pseudolabrys;Pseudolabrys taiwanensis;Pseudolabrys taiwanensis CC-BB4 |
| Ga0586324 0536504 772 1590 | 0.00 | 0.00 | 0.00 | 0.00 | 0.00 | 0.00 | 0.00 | 457603.09 | 0.00 | 0.00 | 0.00 | 0.00 | KO:K09758 | aspartate 4-decarboxylase | Bacteria;Actinobacteria;Actinomycetia;Micromonosporales;Micromonosporaceae;Hamadaea;Hamadaea flava;Hamadaea flava DSM 100517 |
| Ga0586324 5650084 1 585 | 0.00 | 0.00 | 0.00 | 77805000.00 | 0.00 | 0.00 | 0.00 | 0.00 | 0.00 | 0.00 | 0.00 | 0.00 | KO:K09759 | nondiscriminating aspartyl-tRNA synthetase | Archaea;Euryarchaeota;Methanomicrobia;Methanosarcinales;Methanotrichaceae;Methanothrix;Methanothrix soehngenii;Methanothrix soehngenii GP-6 |
| Ga0586324 0023621 2422 3216 | 0.00 | 0.00 | 0.00 | 0.00 | 1153600.00 | 0.00 | 0.00 | 0.00 | 0.00 | 0.00 | 0.00 | 0.00 | KO:K09774 | lipopolysaccharide export system protein LptA | Bacteria;Proteobacteria;Betaproteobacteria;Burkholderiales;unclassified Burkholderiales;Rubrivivax;Rubrivivax benzoatilyticus;Rubrivivax benzoatilyticus JA2 |
| Ga0586324 3190665 2 574 | 0.00 | 0.00 | 0.00 | 0.00 | 0.00 | 0.00 | 0.00 | 860100.00 | 0.00 | 0.00 | 0.00 | 0.00 | KO:K09774 | lipopolysaccharide export system protein LptA | Bacteria;Proteobacteria;Betaproteobacteria;Nitrosomonadales;Nitrosomonadaceae;Nitrosospira;Nitrosospira multiformis;Nitrosospira multiformis ATCC 25196 |
| Ga0586324 1775156 547 951 | 0.00 | 741200.00 | 0.00 | 0.00 | 0.00 | 0.00 | 0.00 | 0.00 | 0.00 | 0.00 | 0.00 | 0.00 | KO:K09807 | uncharacterized protein YggE | Archaea;Euryarchaeota;Halobacteria;Halobacteriales;Haloarculaceae;Halorhabdus;Halorhabdus utahensis;Halorhabdus utahensis AX-2, DSM 12940 |
| Ga0586324 0273873 995 1573 | 0.00 | 624426.32 | 0.00 | 813316.85 | 0.00 | 0.00 | 0.00 | 133659.54 | 0.00 | 0.00 | 0.00 | 0.00 | KO:K09924 | hypothetical protein | Bacteria;Proteobacteria;Alphaproteobacteria;Hyphomicrobiales;Bradyrhizobiaceae;Bradyrhizobium;Bradyrhizobium jicamae;Bradyrhizobium jicamae PAC68 |
| Ga0586324 0723732 158 1132 | 0.00 | 0.00 | 0.00 | 0.00 | 0.00 | 0.00 | 0.00 | 0.00 | 0.00 | 0.00 | 0.00 | 664337.25 | KO:K09942 | hypothetical protein | Bacteria;Firmicutes;Clostridia;Eubacteriales;Peptococcaceae;Desulfofundulus;Desulfofundulus kuznetsovii;Desulfofundulus kuznetsovii DSM 6115 |
| Ga0586324 0043768 57 1070 | 19200000.00 | 13124000.00 | 712408.94 | 5139500.00 | 0.00 | 0.00 | 13634400.00 | 1683600.00 | 727222.05 | 8778000.00 | 39336000.00 | 15900000.00 | KO:K09969 | general L-amino acid transport system substrate-binding protein | Bacteria;Proteobacteria;Deltaproteobacteria;Syntrophobacterales;Syntrophobacteraceae;Desulfacinum;Desulfacinum infernum;Desulfacinum infernum DSM 9756 |
| Ga0586324 0071083 2701 3714 | 0.00 | 0.00 | 0.00 | 0.00 | 0.00 | 0.00 | 16416000.00 | 0.00 | 0.00 | 0.00 | 0.00 | 0.00 | KO:K09969 | general L-amino acid transport system substrate-binding protein | Bacteria;Proteobacteria;Deltaproteobacteria;Syntrophobacterales;Syntrophobacteraceae;Desulfacinum;Desulfacinum infernum;Desulfacinum infernum DSM 9756 |
| Ga0586324 0205720 423 1445 | 0.00 | 0.00 | 0.00 | 0.00 | 0.00 | 0.00 | 0.00 | 17202000.00 | 0.00 | 0.00 | 0.00 | 0.00 | KO:K09969 | general L-amino acid transport system substrate-binding protein | Bacteria;Proteobacteria;Alphaproteobacteria;Rhodospirillales;Rhodospirillaceae;Dongia;Dongia mobilis;Dongia mobilis CGMCC 1.7660 |
| Ga0586324 0255949 635 1654 | 0.00 | 2597600.00 | 0.00 | 0.00 | 0.00 | 0.00 | 0.00 | 5038600.00 | 0.00 | 8008000.00 | 9856000.00 | 5032500.00 | KO:K09969 | general L-amino acid transport system substrate-binding protein | Bacteria;Proteobacteria;Betaproteobacteria;Burkholderiales;Burkholderiaceae;Cupriavidus;Cupriavidus necator;Cupriavidus necator NH9 |
| Ga0586324 0298700 741 1883 | 8850000.00 | 5100000.00 | 10640700.00 | 5548000.00 | 0.00 | 2464063.87 | 0.00 | 0.00 | 3385800.00 | 0.00 | 0.00 | 4230000.00 | KO:K09969 | general L-amino acid transport system substrate-binding protein | Bacteria;Proteobacteria;Deltaproteobacteria;Syntrophobacterales;Syntrophobacteraceae;Thermodesulforhabdus;Thermodesulforhabdus norvegica;Thermodesulforhabdus norvegica DSM 9990 |
| Ga0586324 0400516 1 861 | 0.00 | 0.00 | 0.00 | 0.00 | 2842800.00 | 0.00 | 0.00 | 2165500.00 | 0.00 | 0.00 | 0.00 | 24300000.00 | KO:K09969 | general L-amino acid transport system substrate-binding protein | Bacteria;Proteobacteria;Deltaproteobacteria;Desulfobacterales;Desulfobacteraceae;Desulfatirhabdium;Desulfatirhabdium butyrativorans;Desulfatirhabdium butyrativorans DSM 18734 |
| Ga0586324 0454136 2 943 | 0.00 | 0.00 | 1267290.48 | 1026000.00 | 0.00 | 17825300.00 | 0.00 | 580267.99 | 442159.56 | 0.00 | 0.00 | 0.00 | KO:K09969 | general L-amino acid transport system substrate-binding protein | Bacteria;Proteobacteria;Alphaproteobacteria;Hyphomicrobiales;Xanthobacteraceae;Pseudolabrys;Pseudolabrys taiwanensis;Pseudolabrys taiwanensis CC-BB4 |
| Ga0586324 0658878 416 1426 | 0.00 | 0.00 | 0.00 | 0.00 | 1442000.00 | 0.00 | 0.00 | 0.00 | 0.00 | 0.00 | 0.00 | 0.00 | KO:K09969 | general L-amino acid transport system substrate-binding protein | Bacteria;Proteobacteria;Alphaproteobacteria;Hyphomicrobiales;Xanthobacteraceae;Pseudolabrys;Pseudolabrys taiwanensis;Pseudolabrys taiwanensis CC-BB4 |
| Ga0586324 0863799 195 1208 | 0.00 | 0.00 | 0.00 | 0.00 | 0.00 | 0.00 | 0.00 | 0.00 | 0.00 | 11858000.00 | 0.00 | 3210000.00 | KO:K09969 | general L-amino acid transport system substrate-binding protein | Bacteria;Proteobacteria;Deltaproteobacteria;Syntrophobacterales;Syntrophobacteraceae;Desulfacinum;Desulfacinum hydrothermale;Desulfacinum hydrothermale DSM 13146 |
| Ga0586324 0867884 158 1291 | 2490000.00 | 1958400.00 | 0.00 | 929744.10 | 2235100.00 | 0.00 | 0.00 | 0.00 | 0.00 | 0.00 | 0.00 | 0.00 | KO:K09969 | general L-amino acid transport system substrate-binding protein | Bacteria;Chloroflexi;Caldilineae;Caldilineales;Caldilineaceae;Caldilinea;Caldilinea aerophila;Caldilinea aerophila STL-6-O1, DSM 14535 |
| Ga0586324 2257506 301 858 | 0.00 | 0.00 | 5219325.28 | 0.00 | 0.00 | 0.00 | 0.00 | 0.00 | 0.00 | 0.00 | 0.00 | 0.00 | KO:K09969 | general L-amino acid transport system substrate-binding protein | Bacteria;Proteobacteria;Deltaproteobacteria;Desulfobacterales;Desulfobulbaceae;Desulfopila;Desulfopila aestuarii;Desulfopila aestuarii DSM 18488 |
| Ga0586324 2507493 54 821 | 0.00 | 0.00 | 0.00 | 0.00 | 0.00 | 0.00 | 0.00 | 0.00 | 1271700.00 | 0.00 | 0.00 | 0.00 | KO:K09969 | general L-amino acid transport system substrate-binding protein | Bacteria;Proteobacteria;Gammaproteobacteria;Pseudomonadales;Pseudomonadaceae;Pseudomonas;Pseudomonas sagittaria;Pseudomonas sagittaria JCM 18195 |
| Ga0586324 5374907 3 599 | 0.00 | 0.00 | 0.00 | 0.00 | 0.00 | 0.00 | 1108246.44 | 0.00 | 0.00 | 0.00 | 0.00 | 0.00 | KO:K09969 | general L-amino acid transport system substrate-binding protein | Bacteria;Proteobacteria;Deltaproteobacteria;Desulfobacterales;Desulfobulbaceae;Desulfopila;Desulfopila aestuarii;Desulfopila aestuarii DSM 18488 |
| Ga0586324 6862729 1 447 | 0.00 | 0.00 | 0.00 | 0.00 | 0.00 | 8773700.00 | 0.00 | 0.00 | 0.00 | 0.00 | 0.00 | 0.00 | KO:K09969 | general L-amino acid transport system substrate-binding protein | Bacteria;Proteobacteria;Deltaproteobacteria;Desulfobacterales;Desulfobulbaceae;Desulfopila;Desulfopila aestuarii;Desulfopila aestuarii DSM 18488 |
| Ga0586324 0180760 370 1269 | 0.00 | 0.00 | 3944439.93 | 0.00 | 0.00 | 0.00 | 0.00 | 0.00 | 0.00 | 0.00 | 0.00 | 0.00 | KO:K10001 | glutamate/aspartate transport system substrate-binding protein | Bacteria;Proteobacteria;Betaproteobacteria;Burkholderiales;Oxalobacteraceae;Noviherbaspirillum;Noviherbaspirillum massiliense;Noviherbaspirillum massiliense JC206 |

| | | | | | | | | | | | | | | | |
|---|---|---|---|---|---|---|---|---|---|---|---|---|---|---|---|
| Ga0586324_0410950_488_1387 | 0.00 | 0.00 | 0.00 | 0.00 | 0.00 | 0.00 | 0.00 | 0.00 | 0.00 | 2417800.00 | 0.00 | 0.00 | KO:K10001 | glutamate/aspartate transport system substrate-binding protein | Bacteria;Proteobacteria;Betaproteobacteria;Burkholderiales;unclassified Burkholderiales;Rhizobacter;Rhizobacter sp. SG703;Rhizobacter sp. SG703 |
| Ga0586324_0100752_935_2041 | 0.00 | 0.00 | 0.00 | 0.00 | 0.00 | 0.00 | 0.00 | 0.00 | 0.00 | 0.00 | 0.00 | 177605.25 | KO:K10111 | multiple sugar transport system ATP-binding protein | Bacteria;Proteobacteria;Alphaproteobacteria;Hyphomicrobiales;Devosiaceae;Maritalea;Maritalea mobilis;Maritalea mobilis CGMCC 1.7002 |
| Ga0586324_0004979_12021_13118 | 10900000.00 | 4692000.00 | 0.00 | 3752500.00 | 0.00 | 2150203.61 | 111720000.00 | 3983300.00 | 3345300.00 | 5844300.00 | 0.00 | 0.00 | KO:K10112 | multiple sugar transport system ATP-binding protein | Bacteria;Spirochaetes;Spirochaetia;Spirochaetales;Spirochaetaceae;Salinispira;Salinispira pacifica;Salinispira pacifica L21-RPul-D2 |
| Ga0586324_0018862_3212_4411 | 0.00 | 6174400.00 | 606519.90 | 0.00 | 0.00 | 0.00 | 2964000.00 | 4892200.00 | 0.00 | 0.00 | 0.00 | 0.00 | KO:K10112 | multiple sugar transport system ATP-binding protein | Bacteria;Chloroflexi;Caldilineae;Caldilineales;Caldilineaceae;Caldilinea;Caldilinea aerophila;Caldilinea aerophila STL-6-O1, DSM 14535 |
| Ga0586324_0028932_1846_2916 | 0.00 | 0.00 | 0.00 | 0.00 | 5912200.00 | 0.00 | 0.00 | 0.00 | 0.00 | 0.00 | 0.00 | 0.00 | KO:K10112 | multiple sugar transport system ATP-binding protein | Bacteria;Proteobacteria;Betaproteobacteria;Burkholderiales;unclassified Burkholderiales;Methylibium;Methylibium sp. NZG;Methylibium sp. NZG |
| Ga0586324_0050128_3611_4711 | 0.00 | 877200.00 | 0.00 | 884335.05 | 0.00 | 0.00 | 0.00 | 1274900.00 | 0.00 | 0.00 | 737073.92 | 277938.00 | KO:K10112 | multiple sugar transport system ATP-binding protein | Bacteria;Chloroflexi;Anaerolineae;Anaerolineales;Anaerolineaceae;Thermanaerothrix;Thermanaerothrix daxensis;Thermanaerothrix daxensis GNS-1 |
| Ga0586324_0058874_3795_4415 | 0.00 | 0.00 | 0.00 | 0.00 | 0.00 | 0.00 | 0.00 | 0.00 | 0.00 | 624570.87 | 671997.92 | 817500.00 | KO:K10112 | multiple sugar transport system ATP-binding protein | Bacteria;Proteobacteria;Alphaproteobacteria;Rhodospirillales;Stellaceae;Stella;Stella humosa;Stella humosa DSM 5900 |
| Ga0586324_0060506_1_1110 | 0.00 | 0.00 | 0.00 | 0.00 | 940935.90 | 0.00 | 0.00 | 415822.36 | 0.00 | 341388.74 | 65824000.00 | 373577.25 | KO:K10112 | multiple sugar transport system ATP-binding protein | Bacteria;Chloroflexi;Anaerolineae;Anaerolineales;Anaerolineaceae;Thermanaerothrix;Thermanaerothrix daxensis;Thermanaerothrix daxensis GNS-1 |
| Ga0586324_0078451_2156_3256 | 0.00 | 0.00 | 1196825.40 | 0.00 | 0.00 | 0.00 | 0.00 | 561017.00 | 402548.94 | 0.00 | 1135200.00 | 0.00 | KO:K10112 | multiple sugar transport system ATP-binding protein | Bacteria;Chloroflexi;Anaerolineae;Anaerolineales;Anaerolineaceae;Anaerolinea;Anaerolinea thermophila;Anaerolinea thermophila UNI-1 |
| Ga0586324_0135906_1137_2237 | 0.00 | 0.00 | 0.00 | 0.00 | 0.00 | 218499.27 | 0.00 | 0.00 | 0.00 | 0.00 | 773116.96 | 0.00 | KO:K10112 | multiple sugar transport system ATP-binding protein | Bacteria;Chloroflexi;Anaerolineae;Anaerolineales;Anaerolineaceae;Anaerolinea;Anaerolinea thermophila;Anaerolinea thermophila UNI-1 |
| Ga0586324_0169913_1342_2442 | 0.00 | 0.00 | 0.00 | 0.00 | 0.00 | 0.00 | 0.00 | 0.00 | 0.00 | 0.00 | 1293600.00 | 0.00 | KO:K10112 | multiple sugar transport system ATP-binding protein | Bacteria;Chloroflexi;Anaerolineae;Anaerolineales;Anaerolineaceae;Thermanaerothrix;Thermanaerothrix daxensis;Thermanaerothrix daxensis GNS-1 |
| Ga0586324_0174299_1276_2433 | 6430000.00 | 0.00 | 0.00 | 0.00 | 0.00 | 0.00 | 0.00 | 0.00 | 0.00 | 0.00 | 0.00 | 0.00 | KO:K10112 | multiple sugar transport system ATP-binding protein | Bacteria;Chloroflexi;unclassified Chloroflexi;unclassified Chloroflexi;unclassified Chloroflexi;Thermobaculum;Thermobaculum terrenum;Thermobaculum terrenum YNP1, ATCC BAA-798 |
| Ga0586324_0200559_3_950 | 423176.00 | 0.00 | 0.00 | 0.00 | 0.00 | 149193.00 | 1628727.12 | 0.00 | 0.00 | 0.00 | 514304.56 | 0.00 | KO:K10112 | multiple sugar transport system ATP-binding protein | Bacteria;Chloroflexi;Anaerolineae;Anaerolineales;Anaerolineaceae;Anaerolinea;Anaerolinea thermophila;Anaerolinea thermophila UNI-1 |
| Ga0586324_0273290_351_1523 | 1670000.00 | 0.00 | 0.00 | 0.00 | 0.00 | 0.00 | 0.00 | 286094.88 | 0.00 | 0.00 | 0.00 | 0.00 | KO:K10112 | multiple sugar transport system ATP-binding protein | Bacteria;Chloroflexi;Anaerolineae;Anaerolineales;Anaerolineaceae;Thermanaerothrix;Thermanaerothrix daxensis;Thermanaerothrix daxensis GNS-1 |
| Ga0586324_0376035_845_1858 | 0.00 | 0.00 | 13975388.41 | 3439000.00 | 9228800.00 | 10373502.81 | 0.00 | 5782800.00 | 12822300.00 | 0.00 | 0.00 | 5610000.00 | KO:K10112 | multiple sugar transport system ATP-binding protein | Bacteria;Proteobacteria;Alphaproteobacteria;Rhodospirillales;Acetobacteraceae;Rhodopila;Rhodopila globiformis;Rhodopila globiformis DSM 161 |
| Ga0586324_0462852_339_1436 | 0.00 | 0.00 | 0.00 | 0.00 | 0.00 | 0.00 | 0.00 | 0.00 | 0.00 | 0.00 | 426904.72 | 0.00 | KO:K10112 | multiple sugar transport system ATP-binding protein | Bacteria;Proteobacteria;Betaproteobacteria;Burkholderiales;Comamonadaceae;Rhodoferax;Rhodoferax ferrireducens;Rhodoferax ferrireducens T118 |
| Ga0586324_0498261_524_1642 | 0.00 | 0.00 | 0.00 | 0.00 | 0.00 | 0.00 | 0.00 | 555875.92 | 0.00 | 0.00 | 0.00 | 594847.50 | KO:K10112 | multiple sugar transport system ATP-binding protein | Bacteria;Chloroflexi;Anaerolineae;Anaerolineales;Anaerolineaceae;Anaerolinea;Anaerolinea thermophila;Anaerolinea thermophila UNI-1 |
| Ga0586324_0511540_638_1621 | 0.00 | 447557.64 | 0.00 | 9129500.00 | 1472900.00 | 0.00 | 1193910.60 | 768600.00 | 1134000.00 | 0.00 | 1029600.00 | 0.00 | KO:K10112 | multiple sugar transport system ATP-binding protein | Bacteria;Proteobacteria;Betaproteobacteria;Burkholderiales;Comamonadaceae;Rhodoferax;Rhodoferax ferrireducens;Rhodoferax ferrireducens T118 |
| Ga0586324_0556972_1_1563 | 0.00 | 5236000.00 | 0.00 | 0.00 | 0.00 | 0.00 | 0.00 | 0.00 | 0.00 | 0.00 | 0.00 | 0.00 | KO:K10112 | multiple sugar transport system ATP-binding protein | Bacteria;Chloroflexi;Anaerolineae;Anaerolineales;Anaerolineaceae;Thermanaerothrix;Thermanaerothrix daxensis;Thermanaerothrix daxensis GNS-1 |
| Ga0586324_0823989_256_1320 | 0.00 | 775200.00 | 0.00 | 0.00 | 0.00 | 430856.16 | 0.00 | 0.00 | 0.00 | 0.00 | 0.00 | 0.00 | KO:K10112 | multiple sugar transport system ATP-binding protein | Bacteria;Chloroflexi;Anaerolineae;Anaerolineales;Anaerolineaceae;Anaerolinea;Anaerolinea thermophila;Anaerolinea thermophila UNI-1 |
| Ga0586324_0904532_1_816 | 0.00 | 0.00 | 0.00 | 0.00 | 0.00 | 2256254.22 | 0.00 | 0.00 | 0.00 | 0.00 | 0.00 | 0.00 | KO:K10112 | multiple sugar transport system ATP-binding protein | Bacteria;Thermotogae;Thermotogae;Petrotogales;Petrotogaceae;Marinitoga;Marinitoga piezophila;Marinitoga piezophila KA3 |
| Ga0586324_0914960_3_1145 | 0.00 | 0.00 | 0.00 | 868942.20 | 0.00 | 0.00 | 0.00 | 0.00 | 0.00 | 0.00 | 693067.76 | 0.00 | KO:K10112 | multiple sugar transport system ATP-binding protein | Bacteria;Chloroflexi;Anaerolineae;Anaerolineales;Anaerolineaceae;Thermanaerothrix;Thermanaerothrix daxensis;Thermanaerothrix daxensis GNS-1 |
| Ga0586324_1108834_18_1091 | 0.00 | 0.00 | 0.00 | 0.00 | 0.00 | 0.00 | 0.00 | 0.00 | 0.00 | 0.00 | 387056.56 | 0.00 | KO:K10112 | multiple sugar transport system ATP-binding protein | Bacteria;Proteobacteria;Betaproteobacteria;Burkholderiales;Comamonadaceae;Variovorax;Variovorax sp. OK202;Variovorax sp. OK202 |
| Ga0586324_1133738_29_1135 | 9960000.00 | 16150000.00 | 17847100.00 | 16758000.00 | 17242200.00 | 0.00 | 6680400.00 | 8954800.00 | 1328400.00 | 0.00 | 8140000.00 | 6802500.00 | KO:K10112 | multiple sugar transport system ATP-binding protein | Bacteria;Chloroflexi;Caldilineae;Caldilineales;Caldilineaceae;Caldilinea;Caldilinea aerophila;Caldilinea aerophila STL-6-O1, DSM 14535 |
| Ga0586324_1292523_3_866 | 0.00 | 0.00 | 0.00 | 0.00 | 1658300.00 | 0.00 | 0.00 | 0.00 | 0.00 | 0.00 | 0.00 | 0.00 | KO:K10112 | multiple sugar transport system ATP-binding protein | Bacteria;Proteobacteria;Alphaproteobacteria;Rhodospirillales;Azospirillaceae;Azospirillum;Azospirillum palustre;Azospirillum palustre B2 |
| Ga0586324_1296405_3_1085 | 0.00 | 0.00 | 0.00 | 0.00 | 0.00 | 0.00 | 0.00 | 0.00 | 0.00 | 3803800.00 | 0.00 | 0.00 | KO:K10112 | multiple sugar transport system ATP-binding protein | Bacteria;Firmicutes;Clostridia;Halanaerobiales;Halanaerobiaceae;Halanaerobium;Halanaerobium saccharolyticum;Halanaerobium saccharolyticum WC1 |
| Ga0586324_1298080_498_1085 | 3700000.00 | 0.00 | 0.00 | 0.00 | 0.00 | 2805340.95 | 0.00 | 0.00 | 0.00 | 3064600.00 | 2393600.00 | 0.00 | KO:K10112 | multiple sugar transport system ATP-binding protein | Bacteria;Chloroflexi;Anaerolineae;Anaerolineales;Anaerolineaceae;Anaerolinea;Anaerolinea thermophila;Anaerolinea thermophila UNI-1 |
| Ga0586324_1413896_177_1046 | 2930000.00 | 13260000.00 | 0.00 | 9082000.00 | 7735300.00 | 0.00 | 3328800.00 | 4263900.00 | 4212000.00 | 15554000.00 | 13552000.00 | 6262500.00 | KO:K10112 | multiple sugar transport system ATP-binding protein | Bacteria;Spirochaetes;Spirochaetia;Spirochaetales;Spirochaetaceae;Spirochaeta;Spirochaeta thermophila;Spirochaeta thermophila DSM 6192 |
| Ga0586324_1570224_751_1002 | 0.00 | 1516400.00 | 0.00 | 0.00 | 0.00 | 0.00 | 0.00 | 0.00 | 0.00 | 0.00 | 0.00 | 0.00 | KO:K10112 | multiple sugar transport system ATP-binding protein | Bacteria;Chloroflexi;Anaerolineae;Anaerolineales;Anaerolineaceae;Anaerolinea;Anaerolinea thermophila;Anaerolinea thermophila UNI-1 |
| Ga0586324_1651078_1_729 | 1660000.00 | 2699600.00 | 6924900.00 | 2052000.00 | 10557500.00 | 4499018.38 | 0.00 | 1561600.00 | 1587600.00 | 1624700.00 | 1636800.00 | 1792500.00 | KO:K10112 | multiple sugar transport system ATP-binding protein | Bacteria;Spirochaetes;Spirochaetia;Spirochaetales;Spirochaetaceae;Sediminispirochaeta;Sediminispirochaeta bajacaliforniensis;Sediminispirochaeta bajacaliforniensis DSM 16054 |
| Ga0586324_2577840_1_810 | 0.00 | 1584400.00 | 0.00 | 0.00 | 0.00 | 0.00 | 0.00 | 0.00 | 0.00 | 0.00 | 0.00 | 0.00 | KO:K10112 | multiple sugar transport system ATP-binding protein | Bacteria;Proteobacteria;Alphaproteobacteria;Hyphomicrobiales;Chelatococcaceae;Chelatococcus;Chelatococcus sambhunathii;Chelatococcus sambhunathii DSM 18167 |
| Ga0586324_2596184_32_808 | 0.00 | 0.00 | 0.00 | 0.00 | 0.00 | 0.00 | 0.00 | 982100.00 | 0.00 | 0.00 | 0.00 | 0.00 | KO:K10112 | multiple sugar transport system ATP-binding protein | Bacteria;Proteobacteria;Betaproteobacteria;Burkholderiales;Comamonadaceae;Variovorax;Variovorax sp. OV700;Variovorax sp. OV700 |
| Ga0586324_2812491_3_782 | 0.00 | 0.00 | 0.00 | 0.00 | 0.00 | 0.00 | 0.00 | 0.00 | 0.00 | 0.00 | 0.00 | 16831.35 | KO:K10112 | multiple sugar transport system ATP-binding protein | Bacteria;Proteobacteria;Alphaproteobacteria;Rhodospirillales;unclassified Rhodospirillales;unclassified Rhodospirillales;Rhodospirillales bacterium URHD0088;Rhodospirillales bacterium URHD0088 |
| Ga0586324_3175725_2_745 | 0.00 | 0.00 | 0.00 | 0.00 | 0.00 | 0.00 | 0.00 | 0.00 | 0.00 | 576899.40 | 0.00 | 0.00 | KO:K10112 | multiple sugar transport system ATP-binding protein | Bacteria;Chloroflexi;Anaerolineae;Anaerolineales;Anaerolineaceae;Thermanaerothrix;Thermanaerothrix daxensis;Thermanaerothrix daxensis GNS-1 |
| Ga0586324_3285766_1_732 | 0.00 | 0.00 | 0.00 | 0.00 | 0.00 | 1001027.56 | 0.00 | 1085800.00 | 0.00 | 0.00 | 0.00 | 1552500.00 | KO:K10112 | multiple sugar transport system ATP-binding protein | Bacteria;Verrucomicrobia;Opitutae;Opitutales;Opitutaceae;Opitutus;Opitutus terrae;Opitutus terrae PB90-1 |
| Ga0586324_3555070_2_709 | 0.00 | 583123.12 | 0.00 | 0.00 | 0.00 | 0.00 | 0.00 | 0.00 | 0.00 | 446797.12 | 420727.12 | 0.00 | KO:K10112 | multiple sugar transport system ATP-binding protein | Bacteria;Spirochaetes;Spirochaetia;Spirochaetales;Treponemataceae;Treponema;Treponema lecithinolyticum;Treponema lecithinolyticum ATCC 700332 |
| Ga0586324_4070193_41_670 | 4110000.00 | 0.00 | 6924900.00 | 4968500.00 | 4284800.00 | 0.00 | 0.00 | 969900.00 | 3572100.00 | 266552.44 | 0.00 | 1050000.00 | KO:K10112 | multiple sugar transport system ATP-binding protein | Bacteria;Chloroflexi;Anaerolineae;Anaerolineales;Anaerolineaceae;Anaerolinea;Anaerolinea thermophila;Anaerolinea thermophila UNI-1 |
| Ga0586324_4086459_3_671 | 0.00 | 0.00 | 0.00 | 0.00 | 2245400.00 | 1105577.51 | 0.00 | 0.00 | 0.00 | 0.00 | 0.00 | 0.00 | KO:K10112 | multiple sugar transport system ATP-binding protein | Bacteria;Chloroflexi;Caldilineae;Caldilineales;Caldilineaceae;Caldilinea;Caldilinea aerophila;Caldilinea aerophila STL-6-O1, DSM 14535 |
| Ga0586324_4776225_3_629 | 391415.00 | 0.00 | 0.00 | 0.00 | 0.00 | 0.00 | 0.00 | 0.00 | 184440.24 | 0.00 | 0.00 | 0.00 | KO:K10112 | multiple sugar transport system ATP-binding protein | Bacteria;Chloroflexi;Anaerolineae;Anaerolineales;Anaerolineaceae;Anaerolinea;Anaerolinea thermophila;Anaerolinea thermophila UNI-1 |
| Ga0586324_5021867_2_616 | 0.00 | 0.00 | 0.00 | 0.00 | 0.00 | 0.00 | 0.00 | 0.00 | 0.00 | 21560000.00 | 0.00 | 0.00 | KO:K10112 | multiple sugar transport system ATP-binding protein | Bacteria;Proteobacteria;Betaproteobacteria;Burkholderiales;Comamonadaceae;Variovorax;Variovorax sp. EL159;Variovorax sp. EL159 |
| Ga0586324_5355639_1_600 | 0.00 | 0.00 | 0.00 | 0.00 | 0.00 | 0.00 | 191537.78 | 0.00 | 0.00 | 0.00 | 0.00 | 0.00 | KO:K10112 | multiple sugar transport system ATP-binding protein | Bacteria;Proteobacteria;Alphaproteobacteria;Hyphomicrobiales;Bradyrhizobiaceae;Bradyrhizobium;Bradyrhizobium sp. URHD0069;Bradyrhizobium sp. URHD0069 |
| Ga0586324_6444782_1_555 | 9740000.00 | 0.00 | 3685825.88 | 6099000.00 | 8899200.00 | 1764704.70 | 3534000.00 | 6100000.00 | 4746600.00 | 4881800.00 | 0.00 | 0.00 | KO:K10112 | multiple sugar transport system ATP-binding protein | Bacteria;Chloroflexi;Caldilineae;Caldilineales;Caldilineaceae;Caldilinea;Caldilinea aerophila;Caldilinea aerophila STL-6-O1, DSM 14535 |
| Ga0586324_7235249_3_530 | 0.00 | 1455200.00 | 0.00 | 0.00 | 0.00 | 0.00 | 0.00 | 0.00 | 0.00 | 0.00 | 0.00 | 862500.00 | KO:K10112 | multiple sugar transport system ATP-binding protein | Bacteria;Spirochaetes;Spirochaetia;Spirochaetales;Sphaerochaetaceae;Sphaerochaeta;Sphaerochaeta globosa;Sphaerochaeta globosa Buddy |
| Ga0586324_7358589_3_527 | 2410000.00 | 3359200.00 | 296382.34 | 0.00 | 2760400.00 | 685329.19 | 2936601.24 | 680193.92 | 2625999.75 | 2841300.00 | 2411309.12 | 3945000.00 | KO:K10112 | multiple sugar transport system ATP-binding protein | Bacteria;Chloroflexi;Anaerolineae;Anaerolineales;Anaerolineaceae;Anaerolinea;Anaerolinea thermophila;Anaerolinea thermophila UNI-1 |
| Ga0586324_7570789_1_519 | 0.00 | 37060000.00 | 0.00 | 0.00 | 0.00 | 0.00 | 0.00 | 0.00 | 0.00 | 0.00 | 0.00 | 0.00 | KO:K10112 | multiple sugar transport system ATP-binding protein | Bacteria;Spirochaetes;Spirochaetia;Spirochaetales;Treponemataceae;Treponema;Treponema maltophilum;Treponema maltophilum ATCC 51939 |
| Ga0586324_0004496_9834_11186 | 15050000.00 | 22188400.00 | 11597800.00 | 21660000.00 | 0.00 | 0.00 | 10602000.00 | 0.00 | 0.00 | 0.00 | 0.00 | 0.00 | KO:K10117 | raffinose/stachyose/melibiose transport system substrate-binding protein | Bacteria;Chloroflexi;Anaerolineae;Anaerolineales;Anaerolineaceae;Anaerolinea;Anaerolinea thermophila;Anaerolinea thermophila UNI-1 |
| Ga0586324_0004673_4863_6272 | 9790000.00 | 2577200.00 | 0.00 | 4702500.00 | 6777400.00 | 0.00 | 4035600.00 | 2769400.00 | 899100.00 | 2417800.00 | 4074400.00 | 4800000.00 | KO:K10117 | raffinose/stachyose/melibiose transport system substrate-binding protein | Bacteria;Chloroflexi;Anaerolineae;Anaerolineales;Anaerolineaceae;Pelolinea;Pelolinea submarina;Pelolinea submarina DSM 23923 |
| Ga0586324_0015488_7595_8359 | 0.00 | 0.00 | 0.00 | 3116000.00 | 0.00 | 0.00 | 0.00 | 72590000.00 | 0.00 | 0.00 | 0.00 | 0.00 | KO:K10117 | raffinose/stachyose/melibiose transport system substrate-binding protein | Bacteria;Chloroflexi;Anaerolineae;Anaerolineales;Anaerolineaceae;Thermanaerothrix;Thermanaerothrix daxensis;Thermanaerothrix daxensis GNS-1 |
| Ga0586324_0035652_1655_3067 | 10140000.00 | 17183600.00 | 7544200.00 | 2204000.00 | 0.00 | 6510800.00 | 0.00 | 2464400.00 | 0.00 | 3126200.00 | 0.00 | 1650000.00 | KO:K10117 | raffinose/stachyose/melibiose transport system substrate-binding protein | Bacteria;Chloroflexi;Anaerolineae;Anaerolineales;Anaerolineaceae;Anaerolinea;Anaerolinea thermophila;Anaerolinea thermophila UNI-1 |
| Ga0586324_0040551_2187_3545 | 0.00 | 0.00 | 0.00 | 0.00 | 0.00 | 0.00 | 0.00 | 1000400.00 | 0.00 | 0.00 | 1443200.00 | 0.00 | KO:K10117 | raffinose/stachyose/melibiose transport system substrate-binding protein | Bacteria;Chloroflexi;Anaerolineae;Anaerolineales;Anaerolineaceae;Thermanaerothrix;Thermanaerothrix daxensis;Thermanaerothrix daxensis GNS-1 |
| Ga0586324_0073132_587_2020 | 0.00 | 0.00 | 0.00 | 2783500.00 | 0.00 | 0.00 | 0.00 | 0.00 | 0.00 | 0.00 | 0.00 | 0.00 | KO:K10117 | raffinose/stachyose/melibiose transport system substrate-binding protein | Bacteria;Chloroflexi;Anaerolineae;Anaerolineales;Anaerolineaceae;Pelolinea;Pelolinea submarina;Pelolinea submarina DSM 23923 |
| Ga0586324_0081531_116_1501 | 0.00 | 0.00 | 0.00 | 0.00 | 0.00 | 0.00 | 0.00 | 0.00 | 1782000.00 | 0.00 | 0.00 | 0.00 | KO:K10117 | raffinose/stachyose/melibiose transport system substrate-binding protein | Bacteria;Firmicutes;Clostridia;Eubacteriales;Oscillospiraceae;Ruminiclostridium;Ruminiclostridium sufflavum;Ruminiclostridium sufflavum DSM 19573 |
| Ga0586324_0148421_55_1470 | 2330000.00 | 0.00 | 3839603.70 | 0.00 | 0.00 | 0.00 | 0.00 | 0.00 | 0.00 | 0.00 | 0.00 | 0.00 | KO:K10117 | raffinose/stachyose/melibiose transport system substrate-binding protein | Bacteria;Chloroflexi;Anaerolineae;Anaerolineales;Anaerolineaceae;Pelolinea;Pelolinea submarina;Pelolinea submarina DSM 23923 |
| Ga0586324_0210012_940_2334 | 1200000.00 | 0.00 | 0.00 | 0.00 | 0.00 | 0.00 | 0.00 | 0.00 | 0.00 | 0.00 | 0.00 | 0.00 | KO:K10117 | raffinose/stachyose/melibiose transport system substrate-binding protein | Bacteria;Chloroflexi;Anaerolineae;Anaerolineales;Anaerolineaceae;Pelolinea;Pelolinea submarina;Pelolinea submarina DSM 23923 |
| Ga0586324_0400723_936_1808 | 0.00 | 0.00 | 976658.62 | 0.00 | 0.00 | 0.00 | 0.00 | 0.00 | 0.00 | 0.00 | 0.00 | 0.00 | KO:K10117 | raffinose/stachyose/melibiose transport system substrate-binding protein | Bacteria;Proteobacteria;Alphaproteobacteria;Hyphomicrobiales;Phyllobacteriaceae;Mesorhizobium;Mesorhizobium sp. WSM3882;Mesorhizobium sp. WSM3882 |
| Ga0586324_0406488_3_1139 | 0.00 | 0.00 | 0.00 | 0.00 | 0.00 | 0.00 | 0.00 | 0.00 | 0.00 | 0.00 | 0.00 | 2782500.00 | KO:K10117 | raffinose/stachyose/melibiose transport system substrate-binding protein | Bacteria;Chloroflexi;Anaerolineae;Anaerolineales;Anaerolineaceae;Pelolinea;Pelolinea submarina;Pelolinea submarina DSM 23923 |
| Ga0586324_1356416_3_1064 | 8360000.00 | 0.00 | 0.00 | 0.00 | 0.00 | 0.00 | 0.00 | 0.00 | 0.00 | 0.00 | 0.00 | 0.00 | KO:K10117 | raffinose/stachyose/melibiose transport system substrate-binding protein | Bacteria;Chloroflexi;Anaerolineae;Anaerolineales;Anaerolineaceae;Anaerolinea;Anaerolinea thermophila;Anaerolinea thermophila UNI-1 |
| Ga0586324_1361864_3_1064 | 5800000.00 | 0.00 | 0.00 | 0.00 | 0.00 | 0.00 | 0.00 | 0.00 | 0.00 | 0.00 | 0.00 | 0.00 | KO:K10117 | raffinose/stachyose/melibiose transport system substrate-binding protein | Bacteria;Chloroflexi;Anaerolineae;Anaerolineales;Anaerolineaceae;Pelolinea;Pelolinea submarina;Pelolinea submarina DSM 23923 |
| Ga0586324_1603425_2_991 | 0.00 | 2427600.00 | 0.00 | 0.00 | 0.00 | 0.00 | 0.00 | 0.00 | 0.00 | 0.00 | 0.00 | 0.00 | KO:K10117 | raffinose/stachyose/melibiose transport system substrate-binding protein | Bacteria;Chloroflexi;Anaerolineae;Anaerolineales;Anaerolineaceae;Pelolinea;Pelolinea submarina;Pelolinea submarina DSM 23923 |
| Ga0586324_1686914_1_951 | 0.00 | 35142400.00 | 0.00 | 0.00 | 0.00 | 0.00 | 0.00 | 0.00 | 0.00 | 0.00 | 0.00 | 0.00 | KO:K10117 | raffinose/stachyose/melibiose transport system substrate-binding protein | Bacteria;Firmicutes;unclassified Firmicutes;unclassified Firmicutes;unclassified Firmicutes;Hydrogenispora;Hydrogenispora ethanolica;Hydrogenispora ethanolica DSM 25471 |
| Ga0586324_1752563_369_956 | 0.00 | 2271200.00 | 0.00 | 0.00 | 0.00 | 0.00 | 0.00 | 0.00 | 0.00 | 0.00 | 0.00 | 0.00 | KO:K10117 | raffinose/stachyose/melibiose transport system substrate-binding protein | Bacteria;Chloroflexi;Anaerolineae;Anaerolineales;Anaerolineaceae;Levilinea;Levilinea saccharolytica;Levilinea saccharolytica DSM 16555 |
| Ga0586324_1869002_1_648 | 0.00 | 0.00 | 0.00 | 0.00 | 0.00 | 0.00 | 0.00 | 2690100.00 | 0.00 | 0.00 | 0.00 | 0.00 | KO:K10117 | raffinose/stachyose/melibiose transport system substrate-binding protein | Bacteria;Chloroflexi;Anaerolineae;Anaerolineales;Anaerolineaceae;Anaerolinea;Anaerolinea thermophila;Anaerolinea thermophila UNI-1 |
| Ga0586324_2279545_279_854 | 0.00 | 0.00 | 0.00 | 2783500.00 | 0.00 | 0.00 | 0.00 | 0.00 | 0.00 | 0.00 | 0.00 | 0.00 | KO:K10117 | raffinose/stachyose/melibiose transport system substrate-binding protein | Bacteria;Chloroflexi;Anaerolineae;Anaerolineales;Anaerolineaceae;Anaerolinea;Anaerolinea thermophila;Anaerolinea thermophila UNI-1 |
| Ga0586324_3014340_84_761 | 9270000.00 | 5882000.00 | 10584400.00 | 8407500.00 | 0.00 | 9210400.00 | 0.00 | 5801100.00 | 0.00 | 5220600.00 | 5544000.00 | 5295000.00 | KO:K10117 | raffinose/stachyose/melibiose transport system substrate-binding protein | Bacteria;Chloroflexi;Anaerolineae;Anaerolineales;Anaerolineaceae;Anaerolinea;Anaerolinea thermophila;Anaerolinea thermophila UNI-1 |
| Ga0586324_3258692_45_737 | 0.00 | 0.00 | 3078422.07 | 0.00 | 0.00 | 0.00 | 0.00 | 0.00 | 1101600.00 | 4704700.00 | 0.00 | 0.00 | KO:K10117 | raffinose/stachyose/melibiose transport system substrate-binding protein | Bacteria;Chloroflexi;Chloroflexia;Kallotenuales;Kallotenuaceae;Kallotenue;Kallotenue papyrolyticum;Kallotenue papyrolyticum JKG1 |
| Ga0586324_3673999_1_591 | 0.00 | 2917200.00 | 0.00 | 0.00 | 0.00 | 0.00 | 0.00 | 0.00 | 0.00 | 0.00 | 0.00 | 0.00 | KO:K10117 | raffinose/stachyose/melibiose transport system substrate-binding protein | Bacteria;Chloroflexi;Anaerolineae;Anaerolineales;Anaerolineaceae;Thermanaerothrix;Thermanaerothrix daxensis;Thermanaerothrix daxensis GNS-1 |
| Ga0586324_3288341_3_734 | 2610000.00 | 0.00 | 0.00 | 0.00 | 0.00 | 0.00 | 0.00 | 0.00 | 0.00 | 0.00 | 0.00 | 0.00 | KO:K10188 | lactose/L-arabinose transport system substrate-binding protein | Bacteria;Spirochaetes;Spirochaetia;Spirochaetales;Treponemataceae;Treponema;Treponema caldarium;Treponema caldarium DSM 7334 |
| Ga0586324_4583003_3_614 | 0.00 | 0.00 | 9627300.00 | 0.00 | 0.00 | 0.00 | 0.00 | 0.00 | 0.00 | 0.00 | 0.00 | 0.00 | KO:K10206 | LL-diaminopimelate aminotransferase | Archaea;Candidatus Thermoplasmatota;Thermoplasmata;Methanomassiliicoccales;Methanomassiliicoccaceae;Methanomassiliicoccus;Candidatus Methanomassiliicoccus intestinalis;Candidatus Methanomassiliicoccus intestinalis Issoire-Mx1 |
| Ga0586324_0008110_2662_5109 | 1280000.00 | 0.00 | 0.00 | 0.00 | 0.00 | 599819.36 | 31236000.00 | 13054000.00 | 16524000.00 | 0.00 | 0.00 | 12750000.00 | KO:K10232 | alpha-glucoside transport system substrate-binding protein | Bacteria;Chloroflexi;Ardenticatenia;Ardenticatenales;Ardenticatenaceae;Ardenticatena;Ardenticatena maritima;Ardenticatena maritima 110S |
| Ga0586324_0015750_314_2989 | 0.00 | 0.00 | 0.00 | 0.00 | 0.00 | 0.00 | 10396800.00 | 7198000.00 | 8343000.00 | 0.00 | 0.00 | 8925000.00 | KO:K10232 | alpha-glucoside transport system substrate-binding protein | Bacteria;Chloroflexi;Ardenticatenia;Ardenticatenales;Ardenticatenaceae;Candidatus Promineofilum;Candidatus Promineofilum breve;Candidatus Promineofilum breve Cfx-K |
| Ga0586324_0085770_2783_3691 | 0.00 | 3638000.00 | 0.00 | 0.00 | 0.00 | 0.00 | 0.00 | 0.00 | 0.00 | 0.00 | 0.00 | 0.00 | KO:K10232 | alpha-glucoside transport system substrate-binding protein | Bacteria;Chloroflexi;Ardenticatenia;Ardenticatenales;Ardenticatenaceae;Ardenticatena;Ardenticatena maritima;Ardenticatena maritima 110S |
| Ga0586324_0124223_1532_2953 | 0.00 | 0.00 | 0.00 | 0.00 | 0.00 | 0.00 | 0.00 | 0.00 | 0.00 | 0.00 | 0.00 | 110499.75 | KO:K10232 | alpha-glucoside transport system substrate-binding protein | Bacteria;Chloroflexi;Ardenticatenia;Ardenticatenales;Ardenticatenaceae;Ardenticatena;Ardenticatena maritima;Ardenticatena maritima 110S |
| Ga0586324_0595481_1_1485 | 0.00 | 3175600.00 | 7487900.00 | 0.00 | 0.00 | 0.00 | 0.00 | 0.00 | 5629500.00 | 0.00 | 0.00 | 0.00 | KO:K10232 | alpha-glucoside transport system substrate-binding protein | Bacteria;Chloroflexi;Ardenticatenia;Ardenticatenales;Ardenticatenaceae;Ardenticatena;Ardenticatena maritima;Ardenticatena maritima 110S |
| Ga0586324_1385288_1_1056 | 2350000.00 | 1468800.00 | 0.00 | 0.00 | 0.00 | 2628231.31 | 0.00 | 0.00 | 1968300.00 | 0.00 | 0.00 | 665346.00 | KO:K10232 | alpha-glucoside transport system substrate-binding protein | Bacteria;Chloroflexi;Ardenticatenia;Ardenticatenales;Ardenticatenaceae;Ardenticatena;Ardenticatena maritima;Ardenticatena maritima 110S |
| Ga0586324_1784092_56_949 | 0.00 | 0.00 | 0.00 | 0.00 | 0.00 | 0.00 | 24168000.00 | 0.00 | 0.00 | 0.00 | 0.00 | 0.00 | KO:K10232 | alpha-glucoside transport system substrate-binding protein | Bacteria;Chloroflexi;Ardenticatenia;Ardenticatenales;Ardenticatenaceae;Ardenticatena;Ardenticatena maritima;Ardenticatena maritima 110S |
| Ga0586324_1870987_2_928 | 0.00 | 0.00 | 0.00 | 0.00 | 0.00 | 0.00 | 2462400.00 | 2555900.00 | 2924100.00 | 0.00 | 0.00 | 1440000.00 | KO:K10232 | alpha-glucoside transport system substrate-binding protein | Bacteria;Chloroflexi;Ardenticatenia;Ardenticatenales;Ardenticatenaceae;Candidatus Promineofilum;Candidatus Promineofilum breve;Candidatus Promineofilum breve Cfx-K |
| Ga0586324_2547094_3_740 | 0.00 | 0.00 | 0.00 | 0.00 | 0.00 | 0.00 | 0.00 | 0.00 | 1409400.00 | 0.00 | 0.00 | 645597.75 | KO:K10232 | alpha-glucoside transport system substrate-binding protein | Bacteria;Chloroflexi;Ardenticatenia;Ardenticatenales;Ardenticatenaceae;Ardenticatena;Ardenticatena maritima;Ardenticatena maritima 110S |
| Ga0586324_3102044_1_750 | 0.00 | 0.00 | 0.00 | 0.00 | 3254800.00 | 0.00 | 0.00 | 0.00 | 0.00 | 0.00 | 0.00 | 0.00 | KO:K10232 | alpha-glucoside transport system substrate-binding protein | Bacteria;Chloroflexi;Chloroflexia;Chloroflexales;Oscillochloridaceae;Candidatus Viridilinea;Candidatus Viridilinea mediisalina;Viridilinea mediisalina Kir15-3F |
| Ga0586324_5895392_3_575 | 0.00 | 0.00 | 0.00 | 0.00 | 0.00 | 0.00 | 16210800.00 | 0.00 | 0.00 | 0.00 | 0.00 | 0.00 | KO:K10232 | alpha-glucoside transport system substrate-binding protein | Bacteria;Firmicutes;Bacilli;Bacillales;Paenibacillaceae;Paenibacillus;Paenibacillus sp. MBLB1234;Paenibacillus sp. MBLB1234 |
| Ga0586324_3509841_1_714 | 0.00 | 2087600.00 | 0.00 | 0.00 | 0.00 | 0.00 | 0.00 | 0.00 | 2446200.00 | 0.00 | 0.00 | 0.00 | KO:K10236 | trehalose/maltose transport system substrate-binding protein | Bacteria;Firmicutes;Clostridia;Halanaerobiales;unclassified Halanaerobiales;Anoxybacter;Anoxybacter fermentans;Anoxybacter fermentans DY22613 |
| Ga0586324_0044188_4110_5078 | 3080000.00 | 2713200.00 | 0.00 | 0.00 | 2822200.00 | 2164189.92 | 4240800.00 | 0.00 | 0.00 | 0.00 | 0.00 | 2332500.00 | KO:K10355 | actin | Eukaryota;Arthropoda;Insecta;Diptera;Culicidae;Culex;Culex quinquefasciatus;Culex pipiens quinquefasciatus JHB |
| Ga0586324_0001903_9463_10401 | 0.00 | 0.00 | 0.00 | 0.00 | 0.00 | 0.00 | 0.00 | 0.00 | 826200.00 | 0.00 | 0.00 | 0.00 | KO:K10439 | ribose transport system substrate-binding protein | Bacteria;Proteobacteria;Alphaproteobacteria;Rhodospirillales;Rhodospirillaceae;Rhodospirillum;Rhodospirillum rubrum;Rhodospirillum rubrum S1, ATCC 11170 |
| Ga0586324_0007436_5518_7677 | 4909005.00 | 0.00 | 0.00 | 0.00 | 0.00 | 0.00 | 0.00 | 2177700.00 | 437830.92 | 0.00 | 0.00 | 0.00 | KO:K10439 | ribose transport system substrate-binding protein | Bacteria;Chloroflexi;Anaerolineae;Anaerolineales;Anaerolineaceae;Thermanaerothrix;Thermanaerothrix daxensis;Thermanaerothrix daxensis GNS-1 |
| Ga0586324_0022403_5794_6882 | 2390000.00 | 0.00 | 0.00 | 0.00 | 0.00 | 0.00 | 0.00 | 0.00 | 0.00 | 2032800.00 | 0.00 | 0.00 | KO:K10439 | ribose transport system substrate-binding protein | Bacteria;Proteobacteria;Alphaproteobacteria;Hyphomicrobiales;Tepidamorphaceae;Tepidamorphus;Tepidamorphus gemmatus;Tepidamorphus gemmatus DSM 19345 |
| Ga0586324_0025068_2448_3584 | 0.00 | 1162800.00 | 0.00 | 0.00 | 0.00 | 0.00 | 0.00 | 0.00 | 0.00 | 0.00 | 0.00 | 0.00 | KO:K10439 | ribose transport system substrate-binding protein | Bacteria;Proteobacteria;Betaproteobacteria;Burkholderiales;Comamonadaceae;Variovorax;Variovorax sp. OV329;Variovorax sp. OV329 |
| Ga0586324_0025068_874_2022 | 0.00 | 0.00 | 0.00 | 0.00 | 0.00 | 0.00 | 0.00 | 1903200.00 | 0.00 | 3280200.00 | 3053600.00 | 1942500.00 | KO:K10439 | ribose transport system substrate-binding protein | Bacteria;Proteobacteria;Alphaproteobacteria;Hyphomicrobiales;Phyllobacteriaceae;Hoeflea;Hoeflea marina;Hoeflea marina DSM 16791 |
| Ga0586324_0028755_3537_5711 | 0.00 | 0.00 | 0.00 | 0.00 | 5150000.00 | 0.00 | 0.00 | 0.00 | 0.00 | 536124.82 | 0.00 | 0.00 | KO:K10439 | ribose transport system substrate-binding protein | Bacteria;Proteobacteria;Alphaproteobacteria;Hyphomicrobiales;Devosiaceae;Devosia;Devosia sp. Root105;Devosia sp. Root105 |
| Ga0586324_0030539_3604_4551 | 0.00 | 0.00 | 1789174.59 | 0.00 | 0.00 | 0.00 | 2394000.00 | 0.00 | 0.00 | 6006000.00 | 0.00 | 0.00 | KO:K10439 | ribose transport system substrate-binding protein | Bacteria;Proteobacteria;Betaproteobacteria;Burkholderiales;Comamonadaceae;Rhodoferax;Albidiferax sp. OV413;Albidiferax sp. OV413 |
| Ga0586324_0041586_3497_4426 | 0.00 | 4569600.00 | 0.00 | 0.00 | 0.00 | 1621792.64 | 0.00 | 0.00 | 947700.00 | 0.00 | 0.00 | 1557622.50 | KO:K10439 | ribose transport system substrate-binding protein | Bacteria;Firmicutes;Clostridia;Thermoanaerobacterales;Thermoanaerobacteraceae;Thermoanaerobacter;Thermoanaerobacter ethanolicus;Thermoanaerobacter ethanolicus JW 200 |
| Ga0586324_0057470_3242_4468 | 0.00 | 0.00 | 1455844.81 | 0.00 | 0.00 | 6312300.00 | 0.00 | 0.00 | 0.00 | 0.00 | 0.00 | 8475000.00 | KO:K10439 | ribose transport system substrate-binding protein | Bacteria;Proteobacteria;Alphaproteobacteria;Hyphomicrobiales;Phyllobacteriaceae;Mesorhizobium;Mesorhizobium loti;Mesorhizobium loti UFLA 01-765 |
| Ga0586324_0058284_2595_3569 | 375420.00 | 0.00 | 4393314.20 | 499719.95 | 711849.48 | 0.00 | 754349.40 | 177686.90 | 1733400.00 | 527671.76 | 320876.16 | 0.00 | KO:K10439 | ribose transport system substrate-binding protein | Bacteria;Firmicutes;Clostridia;Thermoanaerobacterales;Thermoanaerobacteraceae;Thermanaeromonas;Thermanaeromonas toyohensis;Thermanaeromonas toyohensis ToBE, DSM 14490 |
| Ga0586324_0059902_165_1217 | 6220000.00 | 0.00 | 10779603.36 | 5282000.00 | 0.00 | 2203322.21 | 0.00 | 1671400.00 | 2673000.00 | 695833.60 | 4030400.00 | 825000.00 | KO:K10439 | ribose transport system substrate-binding protein | Bacteria;Spirochaetes;Spirochaetia;Spirochaetales;Spirochaetaceae;Sediminispirochaeta;Sediminispirochaeta smaragdinae;Sediminispirochaeta smaragdinae DSM 11293 |
| Ga0586324_0108219_1_486 | 5240000.00 | 4039200.00 | 0.00 | 3401000.00 | 3399000.00 | 0.00 | 0.00 | 3568500.00 | 2292300.00 | 1316700.00 | 5482400.00 | 1530000.00 | KO:K10439 | ribose transport system substrate-binding protein | Bacteria;Firmicutes;Clostridia;Eubacteriales;Oscillospiraceae;Petroclostridium;Petroclostridium xylanilyticum;Petroclostridium xylanilyticum SK-Y3 |
| Ga0586324_0129328_1748_2734 | 0.00 | 0.00 | 0.00 | 0.00 | 6334500.00 | 0.00 | 0.00 | 2202100.00 | 0.00 | 0.00 | 0.00 | 0.00 | KO:K10439 | ribose transport system substrate-binding protein | Bacteria;Verrucomicrobia;unclassified Verrucomicrobia;unclassified Verrucomicrobia;unclassified Verrucomicrobia;unclassified Verrucomicrobia;Verrucomicrobia bacterium LW23;Verrucomicrobia bacterium LW23 |
| Ga0586324_0196859_622_1629 | 13200000.00 | 8160000.00 | 15595100.00 | 6897000.00 | 7539600.00 | 5955000.00 | 8755200.00 | 12846600.00 | 12425400.00 | 9625000.00 | 10296000.00 | 9900000.00 | KO:K10439 | ribose transport system substrate-binding protein | Bacteria;Firmicutes;Clostridia;Thermoanaerobacterales;Thermoanaerobacteraceae;Thermanaeromonas;Thermanaeromonas toyohensis;Thermanaeromonas toyohensis ToBE, DSM 14490 |
| Ga0586324_0227014_107_1135 | 0.00 | 1910800.00 | 0.00 | 0.00 | 0.00 | 0.00 | 0.00 | 0.00 | 0.00 | 0.00 | 0.00 | 0.00 | KO:K10439 | ribose transport system substrate-binding protein | Bacteria;Chloroflexi;Anaerolineae;Anaerolineales;Anaerolineaceae;Thermanaerothrix;Thermanaerothrix daxensis;Thermanaerothrix daxensis GNS-1 |
| Ga0586324_0254225_1_1176 | 1800000.00 | 1992400.00 | 0.00 | 0.00 | 0.00 | 0.00 | 0.00 | 616100.00 | 0.00 | 854700.00 | 0.00 | 0.00 | KO:K10439 | ribose transport system substrate-binding protein | Bacteria;Firmicutes;Bacilli;Bacillales;Paenibacillaceae;Paenibacillus;Paenibacillus sp. 1_12;Paenibacillus sp. 1_12 |
| Ga0586324_0258796_673_2199 | 0.00 | 754800.00 | 0.00 | 0.00 | 0.00 | 0.00 | 0.00 | 0.00 | 0.00 | 0.00 | 0.00 | 0.00 | KO:K10439 | ribose transport system substrate-binding protein | Bacteria;Firmicutes;Clostridia;Eubacteriales;Oscillospiraceae;Anaerobacterium;Anaerobacterium chartisolvens;Anaerobacterium chartisolvens DSM 27016 |
| Ga0586324_0324990_970_1881 | 0.00 | 0.00 | 0.00 | 0.00 | 0.00 | 2578487.21 | 0.00 | 0.00 | 0.00 | 0.00 | 0.00 | 0.00 | KO:K10439 | ribose transport system substrate-binding protein | Bacteria;Candidatus Aminicenantes;unclassified Candidatus Aminicenantes;unclassified Candidatus Aminicenantes;unclassified Candidatus Aminicenantes;unclassified Candidatus Aminicenantes;Aminicenantes bacterium SCGC AAA252-K07;Aminicenantes bacterium SCGC AAA252-K07 (contamination screened) |
| Ga0586324_0354957_587_1594 | 0.00 | 0.00 | 0.00 | 0.00 | 0.00 | 0.00 | 2872800.00 | 0.00 | 923400.00 | 0.00 | 6679200.00 | 0.00 | KO:K10439 | ribose transport system substrate-binding protein | Bacteria;Firmicutes;Clostridia;Eubacteriales;Oscillospiraceae;Petroclostridium;Petroclostridium xylanilyticum;Petroclostridium xylanilyticum SK-Y3 |
| Ga0586324_0385703_942_1838 | 6700000.00 | 7752000.00 | 9739900.00 | 0.00 | 0.00 | 0.00 | 4833600.00 | 6527000.00 | 7241400.00 | 5544000.00 | 8395200.00 | 5985000.00 | KO:K10439 | ribose transport system substrate-binding protein | Bacteria;Proteobacteria;Alphaproteobacteria;Hyphomicrobiales;Phyllobacteriaceae;Aquamicrobium;Aquamicrobium aerolatum;Aquamicrobium aerolatum DSM 21857 |
| Ga0586324_0470382_13_996 | 2410000.00 | 0.00 | 0.00 | 0.00 | 0.00 | 0.00 | 0.00 | 0.00 | 0.00 | 0.00 | 0.00 | 0.00 | KO:K10439 | ribose transport system substrate-binding protein | Bacteria;Thermotogae;Thermotogae;Kosmotogales;Kosmotogaceae;Kosmotoga;Kosmotoga arenicorallina;Kosmotoga arenicorallina S304 |
| Ga0586324_0536845_336_1589 | 0.00 | 0.00 | 0.00 | 0.00 | 0.00 | 0.00 | 29868000.00 | 0.00 | 0.00 | 34419000.00 | 27720000.00 | 2287500.00 | KO:K10439 | ribose transport system substrate-binding protein | Bacteria;Proteobacteria;Alphaproteobacteria;Hyphomicrobiales;Devosiaceae;Devosia;Devosia geojensis;Devosia geojensis BD-c194 |
| Ga0586324_0605420_1_1101 | 0.00 | 0.00 | 0.00 | 0.00 | 0.00 | 0.00 | 0.00 | 0.00 | 12960000.00 | 0.00 | 0.00 | 0.00 | KO:K10439 | ribose transport system substrate-binding protein | Bacteria;Firmicutes;Clostridia;Thermoanaerobacterales;Thermoanaerobacteraceae;Thermanaeromonas;Thermanaeromonas toyohensis;Thermanaeromonas toyohensis ToBE, DSM 14490 |
| Ga0586324_0614357_1_600 | 0.00 | 0.00 | 0.00 | 0.00 | 0.00 | 0.00 | 8755200.00 | 0.00 | 0.00 | 0.00 | 0.00 | 0.00 | KO:K10439 | ribose transport system substrate-binding protein | Bacteria;Firmicutes;Clostridia;Eubacteriales;Oscillospiraceae;Petroclostridium;Petroclostridium xylanilyticum;Petroclostridium xylanilyticum SK-Y3 |
| Ga0586324_0692329_491_1423 | 0.00 | 0.00 | 0.00 | 0.00 | 0.00 | 0.00 | 0.00 | 0.00 | 0.00 | 2140600.00 | 3106400.00 | 0.00 | KO:K10439 | ribose transport system substrate-binding protein | Bacteria;Proteobacteria;Betaproteobacteria;Burkholderiales;Burkholderiaceae;Paraburkholderia;Paraburkholderia ginsengiterrae;Paraburkholderia ginsengiterrae DCY85-1 |
| Ga0586324_0869601_532_1290 | 0.00 | 0.00 | 441149.35 | 0.00 | 0.00 | 330823.67 | 0.00 | 0.00 | 0.00 | 0.00 | 0.00 | 0.00 | KO:K10439 | ribose transport system substrate-binding protein | Bacteria;Proteobacteria;Betaproteobacteria;Burkholderiales;unclassified Burkholderiales;Rhizobacter;Rhizobacter gummiphilus;Rhizobacter gummiphilus NBRC 109400 |
| Ga0586324_0885194_3_701 | 1370000.00 | 0.00 | 0.00 | 0.00 | 0.00 | 0.00 | 0.00 | 0.00 | 0.00 | 1085700.00 | 0.00 | 0.00 | KO:K10439 | ribose transport system substrate-binding protein | Bacteria;Proteobacteria;Alphaproteobacteria;Hyphomicrobiales;Phyllobacteriaceae;Mesorhizobium;Mesorhizobium sp. DCY119;Mesorhizobium sp. DCY119 |
| Ga0586324_0945418_3_722 | 0.00 | 3073600.00 | 3967915.34 | 0.00 | 8147300.00 | 486348.82 | 0.00 | 0.00 | 5872500.00 | 0.00 | 0.00 | 0.00 | KO:K10439 | ribose transport system substrate-binding protein | Bacteria;Firmicutes;Clostridia;Thermoanaerobacterales;Thermoanaerobacteraceae;Thermoanaerobacter;Thermoanaerobacter ethanolicus;Thermoanaerobacter ethanolicus JW 200 |
| Ga0586324_1025919_102_1202 | 12000000.00 | 2985200.00 | 0.00 | 0.00 | 0.00 | 0.00 | 0.00 | 0.00 | 0.00 | 0.00 | 0.00 | 0.00 | KO:K10439 | ribose transport system substrate-binding protein | Bacteria;Actinobacteria;Nitriliruptoria;Nitriliruptorales;Nitriliruptoraceae;Nitriliruptor;Nitriliruptor alkaliphilus;Nitriliruptor alkaliphilus DSM 45188 |
| Ga0586324_1115434_35_1084 | 0.00 | 607557.56 | 0.00 | 0.00 | 0.00 | 0.00 | 0.00 | 0.00 | 0.00 | 0.00 | 0.00 | 0.00 | KO:K10439 | ribose transport system substrate-binding protein | Bacteria;Chloroflexi;Anaerolineae;Anaerolineales;Anaerolineaceae;Flexilinea;Flexilinea flocculi;Flexilinea flocculi TC1 |
| Ga0586324_1131061_1_567 | 0.00 | 4760000.00 | 8219800.00 | 4978000.00 | 4789500.00 | 4803700.00 | 8253600.00 | 0.00 | 4941000.00 | 8239000.00 | 8888000.00 | 8400000.00 | KO:K10439 | ribose transport system substrate-binding protein | Bacteria;Firmicutes;Clostridia;Thermoanaerobacterales;Thermoanaerobacteraceae;Thermanaeromonas;Thermanaeromonas toyohensis;Thermanaeromonas toyohensis ToBE, DSM 14490 |
| Ga0586324_1256415_2_688 | 0.00 | 4338400.00 | 0.00 | 347200.30 | 0.00 | 5915300.00 | 608730.36 | 304865.80 | 365536.80 | 0.00 | 287276.88 | 0.00 | KO:K10439 | ribose transport system substrate-binding protein | Bacteria;Firmicutes;Clostridia;Thermoanaerobacterales;Thermoanaerobacteraceae;Thermanaeromonas;Thermanaeromonas toyohensis;Thermanaeromonas toyohensis ToBE, DSM 14490 |
| Ga0586324_1321578_157_1077 | 0.00 | 2033200.00 | 0.00 | 0.00 | 0.00 | 0.00 | 0.00 | 0.00 | 0.00 | 0.00 | 0.00 | 0.00 | KO:K10439 | ribose transport system substrate-binding protein | Bacteria;Firmicutes;Clostridia;Thermoanaerobacterales;Thermoanaerobacteraceae;Moorella;Moorella thermoacetica;Moorella thermoacetica Y72 |
| Ga0586324_1577248_18_998 | 1650000.00 | 0.00 | 1570550.43 | 0.00 | 0.00 | 0.00 | 0.00 | 0.00 | 0.00 | 3511200.00 | 0.00 | 0.00 | KO:K10439 | ribose transport system substrate-binding protein | Bacteria;Firmicutes;Bacilli;Bacillales;Paenibacillaceae;Paenibacillus;Paenibacillus alginolyticus;Paenibacillus alginolyticus DSM 5050 |
| Ga0586324_1617947_1_831 | 0.00 | 21692000.00 | 0.00 | 4417500.00 | 1163900.00 | 0.00 | 0.00 | 0.00 | 712250.01 | 0.00 | 0.00 | 0.00 | KO:K10439 | ribose transport system substrate-binding protein | Bacteria;Proteobacteria;Alphaproteobacteria;Hyphomicrobiales;Phyllobacteriaceae;Nitratireductor;Nitratireductor sp. StC3;Nitratireductor sp. StC3 |
| Ga0586324_2014648_3_899 | 0.00 | 0.00 | 27192900.00 | 0.00 | 0.00 | 0.00 | 0.00 | 0.00 | 25434000.00 | 0.00 | 0.00 | 0.00 | KO:K10439 | ribose transport system substrate-binding protein | Bacteria;Proteobacteria;Alphaproteobacteria;Hyphomicrobiales;Rhizobiaceae;Rhizobium;Rhizobium azooxidifex;Rhizobium azooxidifex DSM 100211 |
| Ga0586324_2144317_3_878 | 0.00 | 0.00 | 0.00 | 0.00 | 2255700.00 | 0.00 | 0.00 | 0.00 | 0.00 | 0.00 | 0.00 | 0.00 | KO:K10439 | ribose transport system substrate-binding protein | Bacteria;Chloroflexi;Anaerolineae;Anaerolineales;Anaerolineaceae;Pelolinea;Pelolinea submarina;Pelolinea submarina DSM 23923 |
| Ga0586324_2207592_3_866 | 0.00 | 0.00 | 4560322.52 | 0.00 | 7817700.00 | 8575200.00 | 0.00 | 2348500.00 | 2259900.00 | 2748900.00 | 0.00 | 0.00 | KO:K10439 | ribose transport system substrate-binding protein | Bacteria;Chloroflexi;Caldilineae;Caldilineales;Caldilineaceae;Caldilinea;Caldilinea aerophila;Caldilinea aerophila STL-6-O1, DSM 14535 |
| Ga0586324_2521427_265_819 | 5100000.00 | 4073200.00 | 3100716.87 | 0.00 | 0.00 | 0.00 | 8709600.00 | 0.00 | 0.00 | 14637700.00 | 0.00 | 9030000.00 | KO:K10439 | ribose transport system substrate-binding protein | Bacteria;Firmicutes;Clostridia;Thermoanaerobacterales;Thermoanaerobacteraceae;Thermanaeromonas;Thermanaeromonas toyohensis;Thermanaeromonas toyohensis ToBE, DSM 14490 |
| Ga0586324_2580631_18_812 | 0.00 | 2148800.00 | 0.00 | 0.00 | 0.00 | 0.00 | 0.00 | 0.00 | 0.00 | 0.00 | 0.00 | 0.00 | KO:K10439 | ribose transport system substrate-binding protein | Bacteria;Atribacterota;unclassified Atribacterota;unclassified Atribacterota;unclassified Atribacterota;Candidatus Caldatribacterium;Candidatus Caldatribacterium saccharofermentans;Candidatus Caldatribacterium saccharofermentans OP9-77CS |
| Ga0586324_2599157_3_809 | 1270000.00 | 5582800.00 | 1634591.68 | 0.00 | 2492600.00 | 0.00 | 5266800.00 | 1628700.00 | 3734100.00 | 2695000.00 | 2728000.00 | 5700000.00 | KO:K10439 | ribose transport system substrate-binding protein | Bacteria;Proteobacteria;Alphaproteobacteria;Hyphomicrobiales;Phyllobacteriaceae;Aquamicrobium;Aquamicrobium aerolatum;Aquamicrobium aerolatum DSM 21857 |
| Ga0586324_2672974_2_799 | 2190000.00 | 0.00 | 0.00 | 0.00 | 0.00 | 0.00 | 0.00 | 0.00 | 0.00 | 0.00 | 0.00 | 0.00 | KO:K10439 | ribose transport system substrate-binding protein | Bacteria;Chloroflexi;Chloroflexia;Chloroflexales;Roseiflexaceae;Roseiflexus;Roseiflexus sp. RS-1;Roseiflexus sp. RS-1 |
| Ga0586324_2742681_538_792 | 0.00 | 0.00 | 0.00 | 0.00 | 0.00 | 0.00 | 0.00 | 0.00 | 5491800.00 | 0.00 | 0.00 | 0.00 | KO:K10439 | ribose transport system substrate-binding protein | Bacteria;Proteobacteria;Betaproteobacteria;Burkholderiales;Burkholderiaceae;Burkholderia;Burkholderia vietnamiensis;Burkholderia vietnamiensis FL-2-3-10-S1-D7 |
| Ga0586324_3333182_270_728 | 0.00 | 0.00 | 8276100.00 | 0.00 | 0.00 | 568285.65 | 2713200.00 | 0.00 | 0.00 | 4235000.00 | 0.00 | 0.00 | KO:K10439 | ribose transport system substrate-binding protein | Bacteria;Firmicutes;Bacilli;Bacillales;Paenibacillaceae;Paenibacillus;Paenibacillus alginolyticus;Paenibacillus alginolyticus DSM 5050 |
| Ga0586324_3709890_144_698 | 0.00 | 0.00 | 0.00 | 0.00 | 0.00 | 0.00 | 0.00 | 0.00 | 0.00 | 530593.91 | 0.00 | 0.00 | KO:K10439 | ribose transport system substrate-binding protein | Bacteria;Spirochaetes;Spirochaetia;Spirochaetales;Sphaerochaetaceae;Sphaerochaeta;Sphaerochaeta halotolerans;Sphaerochaeta halotolerans 4-11 |
| Ga0586324_4238327_115_660 | 6160000.00 | 0.00 | 0.00 | 0.00 | 0.00 | 0.00 | 6178800.00 | 1579900.00 | 0.00 | 0.00 | 0.00 | 1627500.00 | KO:K10439 | ribose transport system substrate-binding protein | Bacteria;Firmicutes;Bacilli;Bacillales;Paenibacillaceae;Paenibacillus;Paenibacillus chondroitinus;Paenibacillus chondroitinus OK414 |
| Ga0586324_4624777_261_638 | 0.00 | 8840000.00 | 0.00 | 0.00 | 14214000.00 | 0.00 | 0.00 | 7869000.00 | 0.00 | 10703000.00 | 0.00 | 0.00 | KO:K10439 | ribose transport system substrate-binding protein | Bacteria;Chloroflexi;Anaerolineae;Anaerolineales;Anaerolineaceae;Pelolinea;Pelolinea submarina;Pelolinea submarina DSM 23923 |
| Ga0586324_4975379_2_619 | 985219.00 | 0.00 | 0.00 | 0.00 | 0.00 | 0.00 | 0.00 | 0.00 | 0.00 | 0.00 | 0.00 | 0.00 | KO:K10439 | ribose transport system substrate-binding protein | Bacteria;Proteobacteria;Alphaproteobacteria;Rhodospirillales;Geminicoccaceae;Geminicoccus;Geminicoccus roseus;Geminicoccus roseus DSM 18922 |
| Ga0586324_5011108_3_512 | 0.00 | 1285200.00 | 0.00 | 0.00 | 0.00 | 0.00 | 0.00 | 0.00 | 0.00 | 0.00 | 0.00 | 0.00 | KO:K10439 | ribose transport system substrate-binding protein | Bacteria;Proteobacteria;Betaproteobacteria;Burkholderiales;Burkholderiaceae;Paraburkholderia;Paraburkholderia tropica;Paraburkholderia tropica LMG 22274 |
| Ga0586324_5184316_3_608 | 0.00 | 0.00 | 0.00 | 0.00 | 0.00 | 0.00 | 0.00 | 0.00 | 0.00 | 1324400.00 | 0.00 | 0.00 | KO:K10439 | ribose transport system substrate-binding protein | Bacteria;Firmicutes;Clostridia;Eubacteriales;Clostridiaceae;Clostridium;Clostridium sp. D5;Clostridium sp. D5 |
| Ga0586324_5309649_1_603 | 0.00 | 0.00 | 0.00 | 0.00 | 0.00 | 1572052.51 | 0.00 | 762500.00 | 0.00 | 0.00 | 0.00 | 0.00 | KO:K10439 | ribose transport system substrate-binding protein | Bacteria;Armatimonadetes;unclassified Armatimonadetes;unclassified Armatimonadetes;unclassified Armatimonadetes;unclassified Armatimonadetes;Armatimonadetes bacterium JGI 0000077-K19;Armatimonadetes bacterium JGI 0000077-K19 (contamination screened) |
| Ga0586324_5741125_2_454 | 237074.00 | 0.00 | 0.00 | 0.00 | 0.00 | 0.00 | 0.00 | 0.00 | 0.00 | 0.00 | 0.00 | 0.00 | KO:K10439 | ribose transport system substrate-binding protein | Bacteria;Firmicutes;Clostridia;Thermoanaerobacterales;Thermoanaerobacteraceae;Thermoanaerobacter;Thermoanaerobacter ethanolicus;Thermoanaerobacter ethanolicus JW 200 |
| Ga0586324_5766202_3_581 | 1850000.00 | 3134800.00 | 5176565.43 | 0.00 | 0.00 | 0.00 | 0.00 | 0.00 | 0.00 | 900900.00 | 0.00 | 0.00 | KO:K10439 | ribose transport system substrate-binding protein | Bacteria;Candidatus Fervidibacteria;unclassified Candidatus Fervidibacteria;unclassified Candidatus Fervidibacteria;unclassified Candidatus Fervidibacteria;unclassified Candidatus Fervidibacteria;Fervidibacteria bacterium JGI 0000001-G10;Fervidibacteria bacterium JGI 0000001-G10 (contamination screened) |
| Ga0586324_6853075_2_541 | 0.00 | 0.00 | 0.00 | 0.00 | 14729000.00 | 0.00 | 0.00 | 0.00 | 0.00 | 0.00 | 0.00 | 0.00 | KO:K10439 | ribose transport system substrate-binding protein | Bacteria;Proteobacteria;Alphaproteobacteria;Hyphomicrobiales;Rhizobiaceae;Rhizobium;Rhizobium leguminosarum;Rhizobium leguminosarum bv. trifolii WSM2297 |
| Ga0586324_7610324_1_399 | 503204.00 | 1475600.00 | 0.00 | 0.00 | 0.00 | 0.00 | 0.00 | 0.00 | 0.00 | 0.00 | 1073600.00 | 2190000.00 | KO:K10439 | ribose transport system substrate-binding protein | Bacteria;Firmicutes;Clostridia;Eubacteriales;Oscillospiraceae;Petroclostridium;Petroclostridium xylanilyticum;Petroclostridium xylanilyticum SK-Y3 |
| Ga0586324_7653423_3_518 | 609480.00 | 0.00 | 2105062.63 | 0.00 | 0.00 | 0.00 | 0.00 | 0.00 | 0.00 | 2849000.00 | 0.00 | 2602500.00 | KO:K10439 | ribose transport system substrate-binding protein | Bacteria;Thermotogae;Thermotogae;Kosmotogales;Kosmotogaceae;Kosmotoga;Kosmotoga pacifica;Kosmotoga pacifica SLHLJ1 |
| Ga0586324_8313507_2_499 | 0.00 | 0.00 | 0.00 | 0.00 | 0.00 | 0.00 | 0.00 | 0.00 | 0.00 | 0.00 | 0.00 | 729859.50 | KO:K10439 | ribose transport system substrate-binding protein | Bacteria;Proteobacteria;Alphaproteobacteria;Hyphomicrobiales;Methylobacteriaceae;Microvirga;Microvirga massiliensis;Microvirga massiliensis JC119 |
| Ga0586324_0015365_3513_4988 | 0.00 | 0.00 | 0.00 | 0.00 | 0.00 | 0.00 | 20178000.00 | 3464800.00 | 0.00 | 3796100.00 | 0.00 | 3277500.00 | KO:K10535 | hydroxylamine dehydrogenase | Bacteria;Proteobacteria;Betaproteobacteria;Burkholderiales;Comamonadaceae;Rhodoferax;Rhodoferax ferrireducens;Rhodoferax ferrireducens T118 |

| | | | | | | | | | | | | | | | |
|---|---|---|---|---|---|---|---|---|---|---|---|---|---|---|---|
| Ga0586324 0183225 770 2275 | 5360000.00 | 0.00 | 0.00 | 0.00 | 3594700.00 | 0.00 | 0.00 | 0.00 | 0.00 | 0.00 | 0.00 | 0.00 | KO:K10535 | hydroxylamine dehydrogenase | Bacteria;Proteobacteria;Gammaproteobacteria;Chromatiales;Chromatiaceae;Lamprocystis;Lamprocystis purpurea;Lamprocystis purpurea DSM 4197 |
| Ga0586324 2917349 1 771 | 0.00 | 0.00 | 2094213.62 | 0.00 | 0.00 | 0.00 | 0.00 | 0.00 | 0.00 | 0.00 | 0.00 | 0.00 | KO:K10535 | hydroxylamine dehydrogenase | Bacteria;Proteobacteria;Gammaproteobacteria;Chromatiales;Chromatiaceae;Lamprocystis;Lamprocystis purpurea;Lamprocystis purpurea DSM 4197 |
| Ga0586324 2612995 1 807 | 0.00 | 2964800.00 | 0.00 | 0.00 | 0.00 | 0.00 | 0.00 | 0.00 | 0.00 | 0.00 | 0.00 | 0.00 | KO:K10537 | L-arabinose transport system substrate-binding protein | Bacteria;Chloroflexi;Anaerolineae;Anaerolineales;Anaerolineaceae;Flexilinea;Flexilinea flocculi;Flexilinea flocculi TC1 |
| Ga0586324 3124119 3 749 | 2430000.00 | 0.00 | 0.00 | 0.00 | 0.00 | 0.00 | 0.00 | 0.00 | 0.00 | 0.00 | 0.00 | 0.00 | KO:K10537 | L-arabinose transport system substrate-binding protein | Bacteria;Chloroflexi;Anaerolineae;Anaerolineales;Anaerolineaceae;Flexilinea;Flexilinea flocculi;Flexilinea flocculi TC1 |
| Ga0586324 3713389 1 696 | 0.00 | 0.00 | 17171500.00 | 0.00 | 0.00 | 4962500.00 | 0.00 | 0.00 | 0.00 | 0.00 | 0.00 | 0.00 | KO:K10537 | L-arabinose transport system substrate-binding protein | Bacteria;Proteobacteria;Alphaproteobacteria;Hyphomicrobiales;Phyllobacteriaceae;Mesorhizobium;Mesorhizobium sp. OAE845;Mesorhizobium sp. OAE845 |
| Ga0586324 0381175 3 911 | 0.00 | 616770.88 | 0.00 | 0.00 | 0.00 | 762708.46 | 0.00 | 0.00 | 0.00 | 0.00 | 0.00 | 0.00 | KO:K10540 | methyl-galactoside transport system substrate-binding protein | Bacteria;Firmicutes;unclassified Firmicutes;unclassified Firmicutes;unclassified Firmicutes;Hydrogenispora;Hydrogenispora ethanolica;Hydrogenispora ethanolica DSM 25471 |
| Ga0586324 0001947 5230 6453 | 3400000.00 | 19312000.00 | 18635300.00 | 5073000.00 | 0.00 | 0.00 | 2690400.00 | 8479000.00 | 5062500.00 | 15053500.00 | 9240000.00 | 12075000.00 | KO:K10543 | D-xylose transport system substrate-binding protein | Bacteria;Chloroflexi;Anaerolineae;Anaerolineales;Anaerolineaceae;Levilinea;Levilinea saccharolytica;Levilinea saccharolytica DSM 16555 |
| Ga0586324 0004025 482 1582 | 38930000.00 | 30668000.00 | 29562486.49 | 2099500.00 | 0.00 | 2263801.19 | 13771200.00 | 1122400.00 | 0.00 | 1692003.39 | 14828000.00 | 15037500.00 | KO:K10543 | D-xylose transport system substrate-binding protein | Bacteria;Chloroflexi;Anaerolineae;Anaerolineales;Anaerolineaceae;Anaerolinea;Anaerolinea thermophila;Anaerolinea thermophila UNI-1 |
| Ga0586324 0018461 4815 5852 | 1250000.00 | 0.00 | 0.00 | 0.00 | 0.00 | 0.00 | 0.00 | 0.00 | 0.00 | 0.00 | 0.00 | 0.00 | KO:K10543 | D-xylose transport system substrate-binding protein | Bacteria;Proteobacteria;Betaproteobacteria;Burkholderiales;Comamonadaceae;Hydrogenophaga;Hydrogenophaga taeniospiralis;Hydrogenophaga taeniospiralis NBRC 102512 |
| Ga0586324 0033302 2842 3891 | 13010000.00 | 20196000.00 | 9683600.00 | 12530500.00 | 2863400.00 | 6749000.00 | 0.00 | 10132100.00 | 8002800.00 | 31231200.00 | 30483200.00 | 23055000.00 | KO:K10543 | D-xylose transport system substrate-binding protein | Bacteria;Proteobacteria;Deltaproteobacteria;Desulfobacterales;Desulfobulbaceae;Desulfopila;Desulfopila aestuarii;Desulfopila aestuarii DSM 18488 |
| Ga0586324 0050497 2 1018 | 771802.00 | 0.00 | 0.00 | 0.00 | 0.00 | 0.00 | 0.00 | 0.00 | 0.00 | 0.00 | 0.00 | 0.00 | KO:K10543 | D-xylose transport system substrate-binding protein | Bacteria;Chloroflexi;Anaerolineae;Anaerolineales;Anaerolineaceae;Levilinea;Levilinea saccharolytica;Levilinea saccharolytica DSM 16555 |
| Ga0586324 0142194 885 1988 | 0.00 | 1774800.00 | 0.00 | 1273000.00 | 0.00 | 0.00 | 0.00 | 0.00 | 0.00 | 0.00 | 0.00 | 0.00 | KO:K10543 | D-xylose transport system substrate-binding protein | Bacteria;Proteobacteria;Alphaproteobacteria;Hyphomicrobiales;Phyllobacteriaceae;Mesorhizobium;Mesorhizobium sp. WSM3876;Mesorhizobium sp. WSM3876 |
| Ga0586324 0213301 1590 2402 | 5700000.00 | 596078.48 | 0.00 | 0.00 | 0.00 | 0.00 | 0.00 | 0.00 | 0.00 | 0.00 | 0.00 | 0.00 | KO:K10543 | D-xylose transport system substrate-binding protein | Bacteria;Chloroflexi;Anaerolineae;Anaerolineales;Anaerolineaceae;Levilinea;Levilinea saccharolytica;Levilinea saccharolytica DSM 16555 |
| Ga0586324 0214536 886 2001 | 0.00 | 0.00 | 0.00 | 0.00 | 0.00 | 0.00 | 0.00 | 4568900.00 | 16637400.00 | 4496800.00 | 2921600.00 | 6742500.00 | KO:K10543 | D-xylose transport system substrate-binding protein | Bacteria;Actinobacteria;Thermoleophilia;Solirubrobacterales;Solirubrobacteraceae;Solirubrobacter;Solirubrobacter soli;Solirubrobacter soli DSM 22325 |
| Ga0586324 0227733 1123 2211 | 0.00 | 0.00 | 0.00 | 0.00 | 0.00 | 0.00 | 0.00 | 1500600.00 | 0.00 | 0.00 | 0.00 | 0.00 | KO:K10543 | D-xylose transport system substrate-binding protein | Bacteria;Actinobacteria;Nitriliruptoria;Nitriliruptorales;Nitriliruptoraceae;Nitriliruptor;Nitriliruptor alkaliphilus;Nitriliruptor alkaliphilus DSM 45188 |
| Ga0586324 0228145 1147 2217 | 32500000.00 | 19679200.00 | 20831000.00 | 13585000.00 | 11433000.00 | 8019400.00 | 6384000.00 | 15768500.00 | 14256000.00 | 50119300.00 | 17424000.00 | 25425000.00 | KO:K10543 | D-xylose transport system substrate-binding protein | Bacteria;Actinobacteria;Rubrobacteria;Gaiellales;Gaiellaceae;Gaiella;Gaiella occulta;Gaiella occulta F2-233 |
| Ga0586324 0268256 1654 2163 | 0.00 | 0.00 | 0.00 | 0.00 | 0.00 | 0.00 | 0.00 | 0.00 | 0.00 | 0.00 | 0.00 | 1065000.00 | KO:K10543 | D-xylose transport system substrate-binding protein | Bacteria;Chloroflexi;Anaerolineae;Anaerolineales;Anaerolineaceae;Anaerolinea;Anaerolinea thermophila;Anaerolinea thermophila UNI-1 |
| Ga0586324 0305616 1058 2038 | 7640000.00 | 2509200.00 | 864587.84 | 0.00 | 0.00 | 0.00 | 0.00 | 3202500.00 | 0.00 | 0.00 | 0.00 | 0.00 | KO:K10543 | D-xylose transport system substrate-binding protein | Bacteria;Chloroflexi;Anaerolineae;Anaerolineales;Anaerolineaceae;Anaerolinea;Anaerolinea thermophila;Anaerolinea thermophila UNI-1 |
| Ga0586324 0391902 125 1252 | 3010000.00 | 2407200.00 | 2661498.05 | 0.00 | 0.00 | 0.00 | 3146400.00 | 0.00 | 2114100.00 | 2294600.00 | 3440800.00 | 1770000.00 | KO:K10543 | D-xylose transport system substrate-binding protein | Bacteria;Chloroflexi;Anaerolineae;Anaerolineales;Anaerolineaceae;Levilinea;Levilinea saccharolytica;Levilinea saccharolytica DSM 16555 |
| Ga0586324 0399781 666 1808 | 1760000.00 | 2767600.00 | 4124419.77 | 0.00 | 625714.70 | 2902740.93 | 2667600.00 | 0.00 | 0.00 | 1409100.00 | 2525600.00 | 2617500.00 | KO:K10543 | D-xylose transport system substrate-binding protein | Bacteria;Chloroflexi;Anaerolineae;Anaerolineales;Anaerolineaceae;Anaerolinea;Anaerolinea thermophila;Anaerolinea thermophila UNI-1 |
| Ga0586324 0429683 674 1741 | 0.00 | 2298400.00 | 4160744.53 | 0.00 | 0.00 | 0.00 | 0.00 | 0.00 | 0.00 | 0.00 | 0.00 | 0.00 | KO:K10543 | D-xylose transport system substrate-binding protein | Bacteria;Proteobacteria;Deltaproteobacteria;Desulfobacterales;Desulfobulbaceae;Desulfopila;Desulfopila aestuarii;Desulfopila aestuarii DSM 18488 |
| Ga0586324 0497451 1366 1641 | 1150000.00 | 0.00 | 0.00 | 0.00 | 0.00 | 0.00 | 0.00 | 0.00 | 0.00 | 0.00 | 0.00 | 0.00 | KO:K10543 | D-xylose transport system substrate-binding protein | Bacteria;Chloroflexi;Anaerolineae;Anaerolineales;Anaerolineaceae;Anaerolinea;Anaerolinea thermophila;Anaerolinea thermophila UNI-1 |
| Ga0586324 0512077 553 1623 | 0.00 | 1849600.00 | 0.00 | 0.00 | 0.00 | 0.00 | 0.00 | 0.00 | 0.00 | 1316700.00 | 0.00 | 647589.75 | KO:K10543 | D-xylose transport system substrate-binding protein | Bacteria;Actinobacteria;Rubrobacteria;Gaiellales;Gaiellaceae;Gaiella;Gaiella occulta;Gaiella occulta F2-233 |
| Ga0586324 0645915 112 1122 | 5690000.00 | 0.00 | 3997395.71 | 0.00 | 0.00 | 0.00 | 0.00 | 3550200.00 | 0.00 | 0.00 | 3836800.00 | 4222500.00 | KO:K10543 | D-xylose transport system substrate-binding protein | Bacteria;Firmicutes;Clostridia;Eubacteriales;Oscillospiraceae;Petroclostridium;Petroclostridium xylanilyticum;Petroclostridium xylanilyticum SK-Y3 |
| Ga0586324 0680703 310 1434 | 0.00 | 1033600.00 | 0.00 | 0.00 | 0.00 | 0.00 | 0.00 | 0.00 | 0.00 | 0.00 | 0.00 | 1770000.00 | KO:K10543 | D-xylose transport system substrate-binding protein | Bacteria;Chloroflexi;Anaerolineae;Anaerolineales;Anaerolineaceae;Levilinea;Levilinea saccharolytica;Levilinea saccharolytica DSM 16555 |
| Ga0586324 0701540 412 1416 | 0.00 | 0.00 | 1580690.06 | 0.00 | 0.00 | 0.00 | 0.00 | 0.00 | 0.00 | 0.00 | 0.00 | 0.00 | KO:K10543 | D-xylose transport system substrate-binding protein | Bacteria;Proteobacteria;Alphaproteobacteria;Hyphomicrobiales;Phyllobacteriaceae;Mesorhizobium;Mesorhizobium sp. WSM3876;Mesorhizobium sp. WSM3876 |
| Ga0586324 0751990 3 581 | 0.00 | 0.00 | 0.00 | 0.00 | 0.00 | 0.00 | 0.00 | 0.00 | 0.00 | 777700.00 | 0.00 | 1965000.00 | KO:K10543 | D-xylose transport system substrate-binding protein | Bacteria;Chloroflexi;Anaerolineae;Anaerolineales;Anaerolineaceae;Anaerolinea;Anaerolinea thermophila;Anaerolinea thermophila UNI-1 |
| Ga0586324 0765999 81 1259 | 0.00 | 0.00 | 11203700.00 | 0.00 | 0.00 | 0.00 | 0.00 | 0.00 | 0.00 | 0.00 | 0.00 | 0.00 | KO:K10543 | D-xylose transport system substrate-binding protein | Bacteria;Chloroflexi;Anaerolineae;Anaerolineales;Anaerolineaceae;Levilinea;Levilinea saccharolytica;Levilinea saccharolytica DSM 16555 |
| Ga0586324 0940872 158 1246 | 4620000.00 | 1713600.00 | 1674654.76 | 0.00 | 0.00 | 2057801.86 | 2021058.12 | 0.00 | 818100.00 | 649390.28 | 485801.36 | 2887500.00 | KO:K10543 | D-xylose transport system substrate-binding protein | Bacteria;Chloroflexi;Anaerolineae;Anaerolineales;Anaerolineaceae;Anaerolinea;Anaerolinea thermophila;Anaerolinea thermophila UNI-1 |
| Ga0586324 1434946 3 1040 | 1290000.00 | 5773200.00 | 0.00 | 0.00 | 0.00 | 0.00 | 17419200.00 | 0.00 | 0.00 | 0.00 | 0.00 | 4620000.00 | KO:K10543 | D-xylose transport system substrate-binding protein | Bacteria;Chloroflexi;Anaerolineae;Anaerolineales;Anaerolineaceae;Levilinea;Levilinea saccharolytica;Levilinea saccharolytica DSM 16555 |
| Ga0586324 1500679 1 1020 | 0.00 | 0.00 | 0.00 | 0.00 | 0.00 | 0.00 | 0.00 | 0.00 | 0.00 | 0.00 | 0.00 | 1177500.00 | KO:K10543 | D-xylose transport system substrate-binding protein | Bacteria;Chloroflexi;Anaerolineae;Anaerolineales;Anaerolineaceae;Anaerolinea;Anaerolinea thermophila;Anaerolinea thermophila UNI-1 |
| Ga0586324 1527816 3 860 | 10300000.00 | 13464000.00 | 9120600.00 | 16473000.00 | 0.00 | 7344500.00 | 9444864.66 | 5105700.00 | 2729700.00 | 9308904.99 | 8152513.60 | 11325000.00 | KO:K10543 | D-xylose transport system substrate-binding protein | Bacteria;Chloroflexi;Anaerolineae;Anaerolineales;Anaerolineaceae;Levilinea;Levilinea saccharolytica;Levilinea saccharolytica DSM 16555 |
| Ga0586324 1554725 3 1004 | 0.00 | 2142000.00 | 0.00 | 0.00 | 0.00 | 2630363.20 | 0.00 | 0.00 | 0.00 | 0.00 | 0.00 | 0.00 | KO:K10543 | D-xylose transport system substrate-binding protein | Bacteria;Chloroflexi;Anaerolineae;Anaerolineales;Anaerolineaceae;Anaerolinea;Anaerolinea thermophila;Anaerolinea thermophila UNI-1 |
| Ga0586324 1661960 2 871 | 0.00 | 0.00 | 1371107.68 | 0.00 | 0.00 | 0.00 | 0.00 | 0.00 | 0.00 | 0.00 | 0.00 | 0.00 | KO:K10543 | D-xylose transport system substrate-binding protein | Bacteria;Chloroflexi;Anaerolineae;Anaerolineales;Anaerolineaceae;Anaerolinea;Anaerolinea thermophila;Anaerolinea thermophila UNI-1 |
| Ga0586324 1929304 86 916 | 0.00 | 3842000.00 | 0.00 | 0.00 | 0.00 | 0.00 | 0.00 | 0.00 | 0.00 | 0.00 | 0.00 | 0.00 | KO:K10543 | D-xylose transport system substrate-binding protein | Bacteria;Actinobacteria;Rubrobacteria;Gaiellales;Gaiellaceae;Gaiella;Gaiella occulta;Gaiella occulta F2-233 |
| Ga0586324 2142474 397 876 | 0.00 | 0.00 | 1265674.67 | 1501000.00 | 0.00 | 0.00 | 3146400.00 | 0.00 | 0.00 | 0.00 | 0.00 | 0.00 | KO:K10543 | D-xylose transport system substrate-binding protein | Bacteria;Chloroflexi;Anaerolineae;Anaerolineales;Anaerolineaceae;Anaerolinea;Anaerolinea thermophila;Anaerolinea thermophila UNI-1 |
| Ga0586324 2153717 3 692 | 0.00 | 0.00 | 0.00 | 0.00 | 0.00 | 1910753.06 | 0.00 | 3123200.00 | 3159000.00 | 0.00 | 5024800.00 | 9112500.00 | KO:K10543 | D-xylose transport system substrate-binding protein | Bacteria;Proteobacteria;Deltaproteobacteria;Desulfobacterales;Desulfobulbaceae;Desulfopila;Desulfopila aestuarii;Desulfopila aestuarii DSM 18488 |
| Ga0586324 2539350 400 816 | 4940000.00 | 0.00 | 0.00 | 0.00 | 0.00 | 0.00 | 0.00 | 0.00 | 0.00 | 0.00 | 0.00 | 0.00 | KO:K10543 | D-xylose transport system substrate-binding protein | Bacteria;Chloroflexi;Anaerolineae;Anaerolineales;Anaerolineaceae;Anaerolinea;Anaerolinea thermophila;Anaerolinea thermophila UNI-1 |
| Ga0586324 2567512 84 812 | 2550000.00 | 4182000.00 | 13680900.00 | 0.00 | 0.00 | 0.00 | 0.00 | 0.00 | 0.00 | 10703000.00 | 0.00 | 0.00 | KO:K10543 | D-xylose transport system substrate-binding protein | Bacteria;Proteobacteria;Deltaproteobacteria;Desulfobacterales;Desulfobulbaceae;Desulfopila;Desulfopila sp. IMCC35005;Desulfopila sp. IMCC35005 |
| Ga0586324 2608882 199 807 | 7120000.00 | 2893257.88 | 12667500.00 | 8151000.00 | 4768900.00 | 1723710.48 | 6019200.00 | 1238300.00 | 1304100.00 | 3187800.00 | 3810400.00 | 3915000.00 | KO:K10543 | D-xylose transport system substrate-binding protein | Bacteria;Chloroflexi;Anaerolineae;Anaerolineales;Anaerolineaceae;Anaerolinea;Anaerolinea thermophila;Anaerolinea thermophila UNI-1 |
| Ga0586324 2730394 1 792 | 5650000.00 | 0.00 | 0.00 | 0.00 | 0.00 | 0.00 | 3990000.00 | 0.00 | 0.00 | 0.00 | 5535200.00 | 0.00 | KO:K10543 | D-xylose transport system substrate-binding protein | Bacteria;Chloroflexi;Anaerolineae;Anaerolineales;Anaerolineaceae;Anaerolinea;Anaerolinea thermophila;Anaerolinea thermophila UNI-1 |
| Ga0586324 2769154 1 786 | 6200000.00 | 0.00 | 0.00 | 0.00 | 0.00 | 0.00 | 0.00 | 0.00 | 2741622.39 | 2279200.00 | 287344.64 | 3765000.00 | KO:K10543 | D-xylose transport system substrate-binding protein | Bacteria;Chloroflexi;Anaerolineae;Anaerolineales;Anaerolineaceae;Levilinea;Levilinea saccharolytica;Levilinea saccharolytica DSM 16555 |
| Ga0586324 2783461 2 787 | 948485.00 | 354770.96 | 0.00 | 0.00 | 0.00 | 0.00 | 0.00 | 0.00 | 0.00 | 0.00 | 0.00 | 0.00 | KO:K10543 | D-xylose transport system substrate-binding protein | Bacteria;Chloroflexi;Anaerolineae;Anaerolineales;Anaerolineaceae;Anaerolinea;Anaerolinea thermophila;Anaerolinea thermophila UNI-1 |
| Ga0586324 3037131 1 747 | 0.00 | 1985600.00 | 0.00 | 0.00 | 0.00 | 0.00 | 0.00 | 0.00 | 0.00 | 0.00 | 0.00 | 0.00 | KO:K10543 | D-xylose transport system substrate-binding protein | Bacteria;Proteobacteria;Deltaproteobacteria;Desulfobacterales;Desulfobulbaceae;Desulfopila;Desulfopila sp. IMCC35005;Desulfopila sp. IMCC35005 |
| Ga0586324 3198227 2 730 | 8530000.00 | 0.00 | 0.00 | 0.00 | 0.00 | 7741500.00 | 0.00 | 1201700.00 | 0.00 | 0.00 | 0.00 | 4230000.00 | KO:K10543 | D-xylose transport system substrate-binding protein | Bacteria;Actinobacteria;Nitriliruptoria;Nitriliruptorales;Nitriliruptoraceae;Nitriliruptor;Nitriliruptor alkaliphilus;Nitriliruptor alkaliphilus DSM 45188 |
| Ga0586324 3210933 3 626 | 0.00 | 1101600.00 | 0.00 | 0.00 | 0.00 | 0.00 | 0.00 | 0.00 | 0.00 | 0.00 | 0.00 | 0.00 | KO:K10543 | D-xylose transport system substrate-binding protein | Bacteria;Chloroflexi;Anaerolineae;Anaerolineales;Anaerolineaceae;Levilinea;Levilinea saccharolytica;Levilinea saccharolytica DSM 16555 |
| Ga0586324 3423933 3 629 | 9720000.00 | 4658000.00 | 2092845.53 | 1529500.00 | 1720100.00 | 0.00 | 603707.52 | 1360300.00 | 717258.24 | 2926000.00 | 6168800.00 | 2991564.75 | KO:K10543 | D-xylose transport system substrate-binding protein | Bacteria;Chloroflexi;Anaerolineae;Anaerolineales;Anaerolineaceae;Anaerolinea;Anaerolinea thermophila;Anaerolinea thermophila UNI-1 |
| Ga0586324 3812976 2 388 | 1060000.00 | 0.00 | 0.00 | 0.00 | 0.00 | 0.00 | 0.00 | 0.00 | 0.00 | 0.00 | 0.00 | 0.00 | KO:K10543 | D-xylose transport system substrate-binding protein | Bacteria;Chloroflexi;Anaerolineae;Anaerolineales;Anaerolineaceae;Anaerolinea;Anaerolinea thermophila;Anaerolinea thermophila UNI-1 |
| Ga0586324 3818530 1 690 | 3560000.00 | 0.00 | 0.00 | 0.00 | 0.00 | 0.00 | 0.00 | 0.00 | 0.00 | 0.00 | 0.00 | 1312500.00 | KO:K10543 | D-xylose transport system substrate-binding protein | Bacteria;Chloroflexi;Anaerolineae;Anaerolineales;Anaerolineaceae;Anaerolinea;Anaerolinea thermophila;Anaerolinea thermophila UNI-1 |
| Ga0586324 3822971 2 688 | 0.00 | 1584400.00 | 0.00 | 0.00 | 0.00 | 0.00 | 0.00 | 0.00 | 0.00 | 0.00 | 0.00 | 0.00 | KO:K10543 | D-xylose transport system substrate-binding protein | Bacteria;Chloroflexi;Anaerolineae;Anaerolineales;Anaerolineaceae;Levilinea;Levilinea saccharolytica;Levilinea saccharolytica DSM 16555 |
| Ga0586324 4186681 2 664 | 36200000.00 | 22372000.00 | 14806900.00 | 6640500.00 | 6890700.00 | 12942200.00 | 7501200.00 | 7625000.00 | 8262000.00 | 10087000.00 | 8518400.00 | 14775000.00 | KO:K10543 | D-xylose transport system substrate-binding protein | Bacteria;Chloroflexi;Anaerolineae;Anaerolineales;Anaerolineaceae;Anaerolinea;Anaerolinea thermophila;Anaerolinea thermophila UNI-1 |
| Ga0586324 4749571 3 629 | 0.00 | 2393600.00 | 0.00 | 0.00 | 0.00 | 2578959.64 | 0.00 | 1171200.00 | 972000.00 | 0.00 | 0.00 | 1560000.00 | KO:K10543 | D-xylose transport system substrate-binding protein | Bacteria;Chloroflexi;Anaerolineae;Anaerolineales;Anaerolineaceae;Levilinea;Levilinea saccharolytica;Levilinea saccharolytica DSM 16555 |
| Ga0586324 4831903 2 445 | 0.00 | 0.00 | 0.00 | 0.00 | 0.00 | 0.00 | 0.00 | 0.00 | 0.00 | 6067600.00 | 7075200.00 | 0.00 | KO:K10543 | D-xylose transport system substrate-binding protein | Bacteria;Chloroflexi;Anaerolineae;Anaerolineales;Anaerolineaceae;Anaerolinea;Anaerolinea thermophila;Anaerolinea thermophila UNI-1 |
| Ga0586324 4833877 2 559 | 11400000.00 | 12648000.00 | 16439600.00 | 0.00 | 0.00 | 0.00 | 0.00 | 0.00 | 0.00 | 0.00 | 0.00 | 0.00 | KO:K10543 | D-xylose transport system substrate-binding protein | Bacteria;Chloroflexi;Anaerolineae;Anaerolineales;Anaerolineaceae;Anaerolinea;Anaerolinea thermophila;Anaerolinea thermophila UNI-1 |
| Ga0586324 5064141 2 613 | 0.00 | 0.00 | 4035296.87 | 0.00 | 0.00 | 0.00 | 0.00 | 0.00 | 0.00 | 0.00 | 0.00 | 0.00 | KO:K10543 | D-xylose transport system substrate-binding protein | Bacteria;Chloroflexi;Anaerolineae;Anaerolineales;Anaerolineaceae;Levilinea;Levilinea saccharolytica;Levilinea saccharolytica DSM 16555 |
| Ga0586324 5856859 2 577 | 274365.00 | 0.00 | 0.00 | 0.00 | 0.00 | 0.00 | 0.00 | 0.00 | 0.00 | 0.00 | 0.00 | 0.00 | KO:K10543 | D-xylose transport system substrate-binding protein | Bacteria;Chloroflexi;Anaerolineae;Anaerolineales;Anaerolineaceae;Levilinea;Levilinea saccharolytica;Levilinea saccharolytica DSM 16555 |
| Ga0586324 5911029 1 576 | 4090000.00 | 3121200.00 | 0.00 | 0.00 | 0.00 | 342275.14 | 1202198.40 | 563709.54 | 488313.36 | 0.00 | 1372800.00 | 1170000.00 | KO:K10543 | D-xylose transport system substrate-binding protein | Bacteria;Chloroflexi;Anaerolineae;Anaerolineales;Anaerolineaceae;Anaerolinea;Anaerolinea thermophila;Anaerolinea thermophila UNI-1 |
| Ga0586324 5955861 3 575 | 5890000.00 | 5236000.00 | 0.00 | 0.00 | 0.00 | 0.00 | 0.00 | 0.00 | 0.00 | 3511200.00 | 4646400.00 | 5010000.00 | KO:K10543 | D-xylose transport system substrate-binding protein | Bacteria;Chloroflexi;Anaerolineae;Anaerolineales;Anaerolineaceae;Anaerolinea;Anaerolinea thermophila;Anaerolinea thermophila UNI-1 |
| Ga0586324 6276086 1 561 | 0.00 | 4630800.00 | 0.00 | 0.00 | 0.00 | 0.00 | 0.00 | 0.00 | 0.00 | 0.00 | 0.00 | 0.00 | KO:K10543 | D-xylose transport system substrate-binding protein | Bacteria;Chloroflexi;Anaerolineae;Anaerolineales;Anaerolineaceae;Anaerolinea;Anaerolinea thermophila;Anaerolinea thermophila UNI-1 |
| Ga0586324 6808737 130 543 | 0.00 | 748000.00 | 0.00 | 0.00 | 0.00 | 0.00 | 0.00 | 0.00 | 0.00 | 0.00 | 0.00 | 0.00 | KO:K10543 | D-xylose transport system substrate-binding protein | Bacteria;Chloroflexi;Anaerolineae;Anaerolineales;Anaerolineaceae;Anaerolinea;Anaerolinea thermophila;Anaerolinea thermophila UNI-1 |
| Ga0586324 6922786 2 538 | 664874.00 | 4111187.52 | 0.00 | 0.00 | 0.00 | 0.00 | 0.00 | 0.00 | 0.00 | 0.00 | 1012000.00 | 0.00 | KO:K10543 | D-xylose transport system substrate-binding protein | Bacteria;Proteobacteria;Deltaproteobacteria;Desulfobacterales;Desulfobulbaceae;Desulfopila;Desulfopila aestuarii;Desulfopila aestuarii DSM 18488 |
| Ga0586324 7097857 2 532 | 9460000.00 | 5059200.00 | 0.00 | 0.00 | 0.00 | 0.00 | 0.00 | 0.00 | 0.00 | 0.00 | 0.00 | 0.00 | KO:K10543 | D-xylose transport system substrate-binding protein | Bacteria;Chloroflexi;Anaerolineae;Anaerolineales;Anaerolineaceae;Levilinea;Levilinea saccharolytica;Levilinea saccharolytica DSM 16555 |
| Ga0586324 0076166 989 3148 | 0.00 | 1625200.00 | 0.00 | 0.00 | 0.00 | 0.00 | 0.00 | 0.00 | 0.00 | 0.00 | 0.00 | 0.00 | KO:K10546 | putative multiple sugar transport system substrate-binding protein | Bacteria;Chloroflexi;Ardenticatenia;Ardenticatenales;Ardenticatenaceae;Candidatus Promineofilum;Candidatus Promineofilum breve;Candidatus Promineofilum breve Cfx-K |
| Ga0586324 0092070 724 2733 | 7490000.00 | 3413600.00 | 29670100.00 | 0.00 | 0.00 | 0.00 | 13953600.00 | 201331.72 | 1717200.00 | 0.00 | 7207200.00 | 9450000.00 | KO:K10546 | putative multiple sugar transport system substrate-binding protein | Bacteria;Chloroflexi;Anaerolineae;Anaerolineales;Anaerolineaceae;Pelolinea;Pelolinea submarina;Pelolinea submarina DSM 23923 |
| Ga0586324 0104828 2 892 | 0.00 | 0.00 | 0.00 | 0.00 | 0.00 | 0.00 | 0.00 | 0.00 | 0.00 | 0.00 | 10032000.00 | 0.00 | KO:K10546 | putative multiple sugar transport system substrate-binding protein | Bacteria;Atribacterota;unclassified Atribacterota;unclassified Atribacterota;unclassified Atribacterota;unclassified Atribacterota;Atribacteria bacterium SCGC AB-164-P05;Atribacteria bacterium SCGC AB-164-P05 (contamination screened) |
| Ga0586324 0160636 2179 2739 | 0.00 | 0.00 | 6080400.00 | 0.00 | 0.00 | 0.00 | 0.00 | 2128900.00 | 0.00 | 0.00 | 3555200.00 | 2797500.00 | KO:K10546 | putative multiple sugar transport system substrate-binding protein | Bacteria;Chloroflexi;Anaerolineae;Anaerolineales;Anaerolineaceae;Pelolinea;Pelolinea submarina;Pelolinea submarina DSM 23923 |
| Ga0586324 0218068 1571 2377 | 0.00 | 0.00 | 0.00 | 0.00 | 0.00 | 0.00 | 0.00 | 0.00 | 0.00 | 1347500.00 | 0.00 | 0.00 | KO:K10546 | putative multiple sugar transport system substrate-binding protein | Bacteria;Chloroflexi;Anaerolineae;Anaerolineales;Anaerolineaceae;Pelolinea;Pelolinea submarina;Pelolinea submarina DSM 23923 |
| Ga0586324 0229262 1 570 | 748988.00 | 4488000.00 | 0.00 | 0.00 | 0.00 | 0.00 | 0.00 | 0.00 | 0.00 | 0.00 | 5359200.00 | 698736.75 | KO:K10546 | putative multiple sugar transport system substrate-binding protein | Bacteria;Chloroflexi;Anaerolineae;Anaerolineales;Anaerolineaceae;Pelolinea;Pelolinea submarina;Pelolinea submarina DSM 23923 |
| Ga0586324 0408164 1 1224 | 0.00 | 0.00 | 20999900.00 | 0.00 | 0.00 | 0.00 | 0.00 | 0.00 | 0.00 | 0.00 | 0.00 | 0.00 | KO:K10546 | putative multiple sugar transport system substrate-binding protein | Bacteria;Chloroflexi;Chloroflexia;Chloroflexales;Chloroflexaceae;Chloroflexus;Chloroflexus aggregans;Chloroflexus aggregans DSM 9485 |
| Ga0586324 0461461 1 870 | 525133.00 | 0.00 | 1785683.99 | 0.00 | 18849000.00 | 1551900.79 | 1598268.60 | 324845.74 | 1790100.00 | 823900.00 | 1716000.00 | 27676114.50 | KO:K10546 | putative multiple sugar transport system substrate-binding protein | Bacteria;Chloroflexi;Chloroflexia;Chloroflexales;Chloroflexaceae;Chloroflexus;Chloroflexus aggregans;Chloroflexus aggregans DSM 9485 |
| Ga0586324 0839688 3 1310 | 0.00 | 26248000.00 | 0.00 | 0.00 | 18746000.00 | 0.00 | 0.00 | 0.00 | 0.00 | 0.00 | 0.00 | 0.00 | KO:K10546 | putative multiple sugar transport system substrate-binding protein | Bacteria;Chloroflexi;Ardenticatenia;Ardenticatenales;Ardenticatenaceae;Candidatus Promineofilum;Candidatus Promineofilum breve;Candidatus Promineofilum breve Cfx-K |
| Ga0586324 0857332 3 1220 | 0.00 | 7752000.00 | 0.00 | 0.00 | 0.00 | 0.00 | 0.00 | 0.00 | 0.00 | 690324.25 | 0.00 | 0.00 | KO:K10546 | putative multiple sugar transport system substrate-binding protein | Bacteria;Chloroflexi;Ardenticatenia;Ardenticatenales;Ardenticatenaceae;Candidatus Promineofilum;Candidatus Promineofilum breve;Candidatus Promineofilum breve Cfx-K |
| Ga0586324 1365953 1 492 | 0.00 | 0.00 | 39072200.00 | 0.00 | 0.00 | 0.00 | 0.00 | 2177700.00 | 0.00 | 0.00 | 0.00 | 0.00 | KO:K10546 | putative multiple sugar transport system substrate-binding protein | Bacteria;Atribacterota;unclassified Atribacterota;unclassified Atribacterota;unclassified Atribacterota;unclassified Atribacterota;Atribacteria bacterium SCGC AB-164-P05;Atribacteria bacterium SCGC AB-164-P05 (contamination screened) |
| Ga0586324 1374342 3 1058 | 0.00 | 0.00 | 0.00 | 0.00 | 0.00 | 0.00 | 16712400.00 | 0.00 | 0.00 | 0.00 | 18629600.00 | 0.00 | KO:K10546 | putative multiple sugar transport system substrate-binding protein | Bacteria;Chloroflexi;Chloroflexia;Chloroflexales;Chloroflexaceae;Chloroflexus;Chloroflexus aurantiacus;Chloroflexus aurantiacus J-10-fl |
| Ga0586324 1453070 2 1033 | 3920000.00 | 3787600.00 | 0.00 | 4066000.00 | 4161200.00 | 11790900.00 | 0.00 | 2366800.00 | 0.00 | 0.00 | 0.00 | 0.00 | KO:K10546 | putative multiple sugar transport system substrate-binding protein | Bacteria;Chloroflexi;Ardenticatenia;Ardenticatenales;Ardenticatenaceae;Candidatus Promineofilum;Candidatus Promineofilum breve;Candidatus Promineofilum breve Cfx-K |
| Ga0586324 1823287 1 714 | 0.00 | 0.00 | 0.00 | 1016500.00 | 5376600.00 | 3583758.70 | 4651200.00 | 0.00 | 0.00 | 2502500.00 | 0.00 | 3637500.00 | KO:K10546 | putative multiple sugar transport system substrate-binding protein | Bacteria;Chloroflexi;Chloroflexia;Kallotenuales;Kallotenuaceae;Kallotenue;Kallotenue papyrolyticum;Kallotenue papyrolyticum JKG1 |
| Ga0586324 2427553 3 833 | 16600000.00 | 0.00 | 0.00 | 0.00 | 0.00 | 0.00 | 0.00 | 0.00 | 0.00 | 0.00 | 0.00 | 0.00 | KO:K10546 | putative multiple sugar transport system substrate-binding protein | Bacteria;Chloroflexi;Chloroflexia;Chloroflexales;Chloroflexaceae;Chloroflexus;Chloroflexus aurantiacus;Chloroflexus aurantiacus J-10-fl |
| Ga0586324 3242611 1 738 | 0.00 | 2053600.00 | 0.00 | 0.00 | 4212700.00 | 0.00 | 0.00 | 0.00 | 0.00 | 0.00 | 0.00 | 0.00 | KO:K10546 | putative multiple sugar transport system substrate-binding protein | Bacteria;Atribacterota;unclassified Atribacterota;unclassified Atribacterota;unclassified Atribacterota;unclassified Atribacterota;Atribacteria bacterium SCGC AB-164-P05;Atribacteria bacterium SCGC AB-164-P05 (contamination screened) |
| Ga0586324 3396380 3 701 | 0.00 | 0.00 | 0.00 | 0.00 | 0.00 | 0.00 | 0.00 | 15494000.00 | 13446000.00 | 0.00 | 0.00 | 0.00 | KO:K10546 | putative multiple sugar transport system substrate-binding protein | Bacteria;Chloroflexi;Chloroflexia;Kallotenuales;Kallotenuaceae;Kallotenue;Kallotenue papyrolyticum;Kallotenue papyrolyticum JKG1 |
| Ga0586324 4008606 97 675 | 0.00 | 0.00 | 0.00 | 7001500.00 | 0.00 | 0.00 | 0.00 | 0.00 | 0.00 | 0.00 | 0.00 | 0.00 | KO:K10546 | putative multiple sugar transport system substrate-binding protein | Bacteria;Chloroflexi;Chloroflexia;Chloroflexales;Oscillochloridaceae;Candidatus Chloroploca;Candidatus Chloroploca asiatica;Candidatus Chloroploca asiatica B7-9 |
| Ga0586324 4069835 2 670 | 0.00 | 1754400.00 | 3998307.77 | 0.00 | 1462600.00 | 3612390.34 | 2690400.00 | 1683600.00 | 0.00 | 2356200.00 | 3828000.00 | 3022500.00 | KO:K10546 | putative multiple sugar transport system substrate-binding protein | Bacteria;Chloroflexi;Chloroflexia;Chloroflexales;Chloroflexaceae;Chloroflexus;Chloroflexus aurantiacus;Chloroflexus aurantiacus J-10-fl |
| Ga0586324 4143497 1 666 | 0.00 | 0.00 | 0.00 | 0.00 | 0.00 | 0.00 | 0.00 | 0.00 | 0.00 | 0.00 | 3220800.00 | 339323.25 | KO:K10546 | putative multiple sugar transport system substrate-binding protein | Bacteria;Chloroflexi;Chloroflexia;Kallotenuales;Kallotenuaceae;Kallotenue;Kallotenue papyrolyticum;Kallotenue papyrolyticum JKG1 |
| Ga0586324 4156032 3 665 | 9690000.00 | 2046800.00 | 17790800.00 | 0.00 | 0.00 | 4486100.00 | 0.00 | 1628700.00 | 608367.51 | 4943400.00 | 9416000.00 | 0.00 | KO:K10546 | putative multiple sugar transport system substrate-binding protein | Bacteria;Atribacterota;unclassified Atribacterota;unclassified Atribacterota;unclassified Atribacterota;unclassified Atribacterota;Atribacteria bacterium SCGC AB-164-P05;Atribacteria bacterium SCGC AB-164-P05 (contamination screened) |
| Ga0586324 4313806 1 654 | 376778.00 | 0.00 | 920679.53 | 0.00 | 0.00 | 0.00 | 0.00 | 0.00 | 0.00 | 0.00 | 0.00 | 0.00 | KO:K10546 | putative multiple sugar transport system substrate-binding protein | Bacteria;Chloroflexi;Chloroflexia;Chloroflexales;Chloroflexaceae;Chloroflexus;Chloroflexus aggregans;Chloroflexus aggregans DSM 9485 |
| Ga0586324 4536808 3 641 | 0.00 | 0.00 | 0.00 | 0.00 | 0.00 | 0.00 | 0.00 | 0.00 | 0.00 | 0.00 | 19096000.00 | 23100000.00 | KO:K10546 | putative multiple sugar transport system substrate-binding protein | Bacteria;Atribacterota;unclassified Atribacterota;unclassified Atribacterota;unclassified Atribacterota;unclassified Atribacterota;Atribacteria bacterium SCGC AB-164-P05;Atribacteria bacterium SCGC AB-164-P05 (contamination screened) |
| Ga0586324 5071077 144 614 | 22100000.00 | 0.00 | 5798900.00 | 0.00 | 0.00 | 0.00 | 0.00 | 0.00 | 0.00 | 7361200.00 | 0.00 | 1455000.00 | KO:K10546 | putative multiple sugar transport system substrate-binding protein | Bacteria;Chloroflexi;Chloroflexia;Chloroflexales;Chloroflexaceae;Chloroflexus;Chloroflexus aggregans;Chloroflexus aggregans DSM 9485 |
| Ga0586324 5511200 2 592 | 0.00 | 0.00 | 0.00 | 0.00 | 0.00 | 0.00 | 0.00 | 0.00 | 555849.54 | 0.00 | 0.00 | 0.00 | KO:K10546 | putative multiple sugar transport system substrate-binding protein | Bacteria;Chloroflexi;Chloroflexia;Kallotenuales;Kallotenuaceae;Kallotenue;Kallotenue papyrolyticum;Kallotenue papyrolyticum JKG1 |
| Ga0586324 5579393 1 588 | 0.00 | 0.00 | 0.00 | 0.00 | 0.00 | 0.00 | 0.00 | 0.00 | 713701.53 | 0.00 | 0.00 | 0.00 | KO:K10546 | putative multiple sugar transport system substrate-binding protein | Bacteria;Chloroflexi;Chloroflexia;Chloroflexales;Chloroflexaceae;Chloroflexus;Chloroflexus aggregans;Chloroflexus aggregans DSM 9485 |
| Ga0586324 5618460 1 588 | 0.00 | 0.00 | 0.00 | 0.00 | 0.00 | 0.00 | 21386400.00 | 0.00 | 0.00 | 0.00 | 0.00 | 1282500.00 | KO:K10546 | putative multiple sugar transport system substrate-binding protein | Bacteria;Chloroflexi;Chloroflexia;Chloroflexales;Chloroflexaceae;Chloroflexus;Chloroflexus aggregans;Chloroflexus aggregans DSM 9485 |
| Ga0586324 5632112 2 586 | 0.00 | 0.00 | 0.00 | 0.00 | 0.00 | 0.00 | 0.00 | 0.00 | 0.00 | 0.00 | 0.00 | 699767.25 | KO:K10546 | putative multiple sugar transport system substrate-binding protein | Bacteria;Chloroflexi;Chloroflexia;Chloroflexales;Chloroflexaceae;Chloroflexus;Chloroflexus aggregans;Chloroflexus aggregans DSM 9485 |
| Ga0586324 6032880 1 570 | 0.00 | 0.00 | 0.00 | 0.00 | 0.00 | 0.00 | 17100000.00 | 0.00 | 0.00 | 0.00 | 0.00 | 11850000.00 | KO:K10546 | putative multiple sugar transport system substrate-binding protein | Bacteria;Chloroflexi;Chloroflexia;Chloroflexales;Chloroflexaceae;Chloroflexus;Chloroflexus aggregans;Chloroflexus aggregans DSM 9485 |
| Ga0586324 6069768 2 568 | 0.00 | 0.00 | 0.00 | 0.00 | 0.00 | 0.00 | 0.00 | 0.00 | 5589000.00 | 0.00 | 0.00 | 0.00 | KO:K10546 | putative multiple sugar transport system substrate-binding protein | Bacteria;Chloroflexi;Anaerolineae;Anaerolineales;Anaerolineaceae;Pelolinea;Pelolinea submarina;Pelolinea submarina DSM 23923 |
| Ga0586324 6276107 2 562 | 0.00 | 5501200.00 | 979344.13 | 0.00 | 0.00 | 0.00 | 0.00 | 0.00 | 0.00 | 0.00 | 0.00 | 15300000.00 | KO:K10546 | putative multiple sugar transport system substrate-binding protein | Bacteria;Chloroflexi;Chloroflexia;Chloroflexales;Chloroflexaceae;Chloroflexus;Chloroflexus aurantiacus;Chloroflexus aurantiacus J-10-fl |
| Ga0586324 6472738 3 554 | 0.00 | 0.00 | 0.00 | 0.00 | 0.00 | 0.00 | 0.00 | 0.00 | 14094000.00 | 0.00 | 0.00 | 0.00 | KO:K10546 | putative multiple sugar transport system substrate-binding protein | Bacteria;Chloroflexi;Chloroflexia;Chloroflexales;Chloroflexaceae;Chloroflexus;Chloroflexus islandicus;Chloroflexus islandicus isl-2 |
| Ga0586324 7105679 2 532 | 0.00 | 1883600.00 | 230830000.00 | 132050000.00 | 2049700.00 | 2913269.37 | 2667600.00 | 117120000.00 | 1287900.00 | 2987600.00 | 2895200.00 | 2122500.00 | KO:K10546 | putative multiple sugar transport system substrate-binding protein | Bacteria;Chloroflexi;Chloroflexia;Kallotenuales;Kallotenuaceae;Kallotenue;Kallotenue papyrolyticum;Kallotenue papyrolyticum JKG1 |
| Ga0586324 7378278 3 524 | 5240000.00 | 0.00 | 7037500.00 | 0.00 | 0.00 | 0.00 | 0.00 | 0.00 | 0.00 | 0.00 | 0.00 | 0.00 | KO:K10546 | putative multiple sugar transport system substrate-binding protein | Bacteria;Chloroflexi;Chloroflexia;Chloroflexales;Chloroflexaceae;Chloroflexus;Chloroflexus aggregans;Chloroflexus aggregans DSM 9485 |
| Ga0586324 7747234 2 514 | 0.00 | 0.00 | 45490400.00 | 0.00 | 0.00 | 0.00 | 0.00 | 0.00 | 0.00 | 0.00 | 0.00 | 0.00 | KO:K10546 | putative multiple sugar transport system substrate-binding protein | Bacteria;Atribacterota;unclassified Atribacterota;unclassified Atribacterota;unclassified Atribacterota;unclassified Atribacterota;Atribacteria bacterium SCGC AB-164-P05;Atribacteria bacterium SCGC AB-164-P05 (contamination screened) |
| Ga0586324 0549977 728 1573 | 0.00 | 0.00 | 0.00 | 0.00 | 0.00 | 0.00 | 0.00 | 0.00 | 842400.00 | 0.00 | 0.00 | 0.00 | KO:K10552 | fructose transport system substrate-binding protein | Bacteria;Proteobacteria;Betaproteobacteria;Burkholderiales;Burkholderiaceae;Paraburkholderia;Paraburkholderia megapolitana;Paraburkholderia megapolitana LMG 23650 |
| Ga0586324 7115346 2 532 | 4330000.00 | 1740800.00 | 0.00 | 0.00 | 0.00 | 0.00 | 0.00 | 0.00 | 0.00 | 0.00 | 0.00 | 4252500.00 | KO:K10552 | fructose transport system substrate-binding protein | Bacteria;Proteobacteria;Alphaproteobacteria;Hyphomicrobiales;unclassified Hyphomicrobiales;unclassified Hyphomicrobiales;Rhizobiales bacterium GAS188;Rhizobiales sp. GAS188 |
| Ga0586324 0011339 3804 4901 | 0.00 | 10404000.00 | 0.00 | 0.00 | 0.00 | 0.00 | 0.00 | 0.00 | 0.00 | 8008000.00 | 10208000.00 | 9600000.00 | KO:K10559 | rhamnose transport system substrate-binding protein | Bacteria;Chloroflexi;Chloroflexia;Kallotenuales;Kallotenuaceae;Kallotenue;Kallotenue papyrolyticum;Kallotenue papyrolyticum JKG1 |
| Ga0586324 0464142 3 1271 | 1120000.00 | 0.00 | 0.00 | 0.00 | 0.00 | 0.00 | 0.00 | 0.00 | 2057400.00 | 0.00 | 0.00 | 1342500.00 | KO:K10559 | rhamnose transport system substrate-binding protein | Bacteria;Chloroflexi;Chloroflexia;Chloroflexales;Chloroflexaceae;Chloroflexus;Chloroflexus aurantiacus;Chloroflexus aurantiacus J-10-fl |
| Ga0586324 0670318 280 1443 | 0.00 | 1237600.00 | 0.00 | 0.00 | 0.00 | 0.00 | 0.00 | 0.00 | 0.00 | 0.00 | 0.00 | 0.00 | KO:K10559 | rhamnose transport system substrate-binding protein | Bacteria;Actinobacteria;Coriobacteriia;Coriobacteriales;Atopobiaceae;Olsenella;Olsenella sp. An285;Olsenella sp. An285 |
| Ga0586324 0826414 68 1147 | 0.00 | 0.00 | 0.00 | 0.00 | 0.00 | 0.00 | 1311503.88 | 0.00 | 0.00 | 0.00 | 0.00 | 1267500.00 | KO:K10559 | rhamnose transport system substrate-binding protein | Bacteria;Chloroflexi;Chloroflexia;Kallotenuales;Kallotenuaceae;Kallotenue;Kallotenue papyrolyticum;Kallotenue papyrolyticum JKG1 |
| Ga0586324 2874828 1 774 | 0.00 | 0.00 | 0.00 | 0.00 | 0.00 | 0.00 | 0.00 | 866200.00 | 0.00 | 1262800.00 | 0.00 | 975000.00 | KO:K10559 | rhamnose transport system substrate-binding protein | Bacteria;Proteobacteria;Deltaproteobacteria;unclassified Deltaproteobacteria;unclassified Deltaproteobacteria;unclassified Deltaproteobacteria;SAR324 cluster bacterium JCVI-SC AAA005;SAR324 cluster bacterium JCVI-SC AAA005 |
| Ga0586324 2905479 2 715 | 11100000.00 | 6215200.00 | 0.00 | 2223000.00 | 0.00 | 5319800.00 | 3876000.00 | 2629100.00 | 5483700.00 | 5536300.00 | 4180000.00 | 0.00 | KO:K10559 | rhamnose transport system substrate-binding protein | Bacteria;Chloroflexi;Chloroflexia;Kallotenuales;Kallotenuaceae;Kallotenue;Kallotenue papyrolyticum;Kallotenue papyrolyticum JKG1 |
| Ga0586324 4916960 309 620 | 2740000.00 | 5963600.00 | 4269735.70 | 2831000.00 | 0.00 | 2886178.09 | 4628400.00 | 0.00 | 1425600.00 | 0.00 | 0.00 | 4530000.00 | KO:K10559 | rhamnose transport system substrate-binding protein | Bacteria;Firmicutes;Clostridia;Eubacteriales;Christensenellaceae;Christensenella;Christensenella minuta;Christensenella minuta DSM 22607 |
| Ga0586324 5836699 3 578 | 0.00 | 0.00 | 0.00 | 0.00 | 0.00 | 0.00 | 1620945.48 | 0.00 | 0.00 | 1378300.00 | 0.00 | 1815000.00 | KO:K10559 | rhamnose transport system substrate-binding protein | Bacteria;Spirochaetes;Spirochaetia;Spirochaetales;Spirochaetaceae;Spirochaeta;Spirochaeta thermophila;Spirochaeta thermophila DSM 6192 |
| Ga0586324 0045441 4342 4851 | 8440000.00 | 437820.04 | 6672169.30 | 0.00 | 10063100.00 | 8694300.00 | 13429200.00 | 9345200.00 | 67716000.00 | 6429500.00 | 0.00 | 0.00 | KO:K10713 | 5,6,7,8-tetrahydromethanopterin hydro-lyase | Bacteria;Proteobacteria;Betaproteobacteria;Burkholderiales;unclassified Burkholderiales;Ideonella;Ideonella sp. A 288;Ideonella sp. A 288 |
| Ga0586324 0190629 115 624 | 8850000.00 | 5854800.00 | 4561797.58 | 5852000.00 | 0.00 | 5161000.00 | 0.00 | 2763300.00 | 0.00 | 8085000.00 | 5816800.00 | 0.00 | KO:K10713 | 5,6,7,8-tetrahydromethanopterin hydro-lyase | Bacteria;Proteobacteria;Alphaproteobacteria;Hyphomicrobiales;Methylocystaceae;Methylocystis;Methylocystis parvus;Methylocystis parvus OBBP |
| Ga0586324 0256468 1363 1881 | 0.00 | 57868000.00 | 0.00 | 0.00 | 38522000.00 | 0.00 | 0.00 | 0.00 | 49329000.00 | 0.00 | 29040000.00 | 37200000.00 | KO:K10713 | 5,6,7,8-tetrahydromethanopterin hydro-lyase | Bacteria;Proteobacteria;Gammaproteobacteria;Methylococcales;Methylococcaceae;Methylovulum;Methylovulum miyakonense;Methylovulum miyakonense HT12 |
| Ga0586324 0475964 772 1272 | 0.00 | 0.00 | 0.00 | 0.00 | 0.00 | 0.00 | 2736000.00 | 0.00 | 1571400.00 | 0.00 | 0.00 | 0.00 | KO:K10713 | 5,6,7,8-tetrahydromethanopterin hydro-lyase | Bacteria;Proteobacteria;Betaproteobacteria;Burkholderiales;Burkholderiaceae;Trinickia;Trinickia symbiotica;Trinickia symbiotica JPY347 |
| Ga0586324 0529332 37 618 | 3190000.00 | 0.00 | 0.00 | 0.00 | 0.00 | 0.00 | 0.00 | 0.00 | 0.00 | 0.00 | 0.00 | 0.00 | KO:K10713 | 5,6,7,8-tetrahydromethanopterin hydro-lyase | Bacteria;Proteobacteria;Alphaproteobacteria;Hyphomicrobiales;Methylocystaceae;Methylocystis;Methylocystis parvus;Methylocystis parvus OBBP |
| Ga0586324 0854593 328 843 | 0.00 | 0.00 | 0.00 | 0.00 | 0.00 | 0.00 | 10898400.00 | 29524000.00 | 27864000.00 | 0.00 | 0.00 | 0.00 | KO:K10713 | 5,6,7,8-tetrahydromethanopterin hydro-lyase | Bacteria;Proteobacteria;Gammaproteobacteria;Methylococcales;Methylococcaceae;Methylovulum;Methylovulum miyakonense;Methylovulum miyakonense HT12 |
| Ga0586324 1948731 192 761 | 0.00 | 0.00 | 0.00 | 0.00 | 0.00 | 0.00 | 0.00 | 0.00 | 2559600.00 | 0.00 | 0.00 | 0.00 | KO:K10713 | 5,6,7,8-tetrahydromethanopterin hydro-lyase | Bacteria;Proteobacteria;Alphaproteobacteria;Hyphomicrobiales;Methylocystaceae;Methylocystis;Methylocystis sp. SC2;Methylocystis sp. SC2 |
| Ga0586324 2694260 3 386 | 0.00 | 14756000.00 | 0.00 | 0.00 | 0.00 | 0.00 | 0.00 | 0.00 | 0.00 | 0.00 | 0.00 | 0.00 | KO:K10713 | 5,6,7,8-tetrahydromethanopterin hydro-lyase | Bacteria;Proteobacteria;Gammaproteobacteria;Methylococcales;Methylococcaceae;Methylomonas;Methylomonas koyamae;Methylomonas koyamae R-45383 |
| Ga0586324 3176463 3 419 | 0.00 | 149011.12 | 0.00 | 0.00 | 0.00 | 12227600.00 | 0.00 | 0.00 | 0.00 | 0.00 | 0.00 | 0.00 | KO:K10713 | 5,6,7,8-tetrahydromethanopterin hydro-lyase | Bacteria;Proteobacteria;Betaproteobacteria;Nitrosomonadales;Methylophilaceae;Methylovorus;Methylovorus glucosotrophus;Methylovorus glucosetrophus SIP3-4 |
| Ga0586324 4700569 1 486 | 0.00 | 0.00 | 0.00 | 0.00 | 0.00 | 5240400.00 | 0.00 | 4227300.00 | 0.00 | 0.00 | 0.00 | 0.00 | KO:K10713 | 5,6,7,8-tetrahydromethanopterin hydro-lyase | Bacteria;Proteobacteria;Betaproteobacteria;Burkholderiales;Burkholderiaceae;Burkholderia;Burkholderia sp. YI23;Burkholderia sp. YI23 |
| Ga0586324 4721494 426 632 | 4700000.00 | 3536000.00 | 0.00 | 0.00 | 6550800.00 | 0.00 | 0.00 | 0.00 | 0.00 | 4650800.00 | 4963200.00 | 0.00 | KO:K10713 | 5,6,7,8-tetrahydromethanopterin hydro-lyase | Bacteria;Proteobacteria;Betaproteobacteria;Nitrosomonadales;Sterolibacteriaceae;Methyloversatilis;Methyloversatilis sp. RAC08;Methyloversatilis sp. RAC08 |
| Ga0586324 5820654 1 420 | 116000000.00 | 96573600.00 | 115809100.00 | 22990000.00 | 24617000.00 | 57962000.00 | 0.00 | 1384700.00 | 0.00 | 0.00 | 0.00 | 0.00 | KO:K10713 | 5,6,7,8-tetrahydromethanopterin hydro-lyase | Bacteria;Proteobacteria;Gammaproteobacteria;Methylococcales;Methylococcaceae;Methylovulum;Methylovulum miyakonense;Methylovulum miyakonense HT12 |
| Ga0586324 6249212 1 279 | 0.00 | 0.00 | 0.00 | 0.00 | 0.00 | 0.00 | 4651200.00 | 774700.00 | 0.00 | 0.00 | 0.00 | 0.00 | KO:K10713 | 5,6,7,8-tetrahydromethanopterin hydro-lyase | Bacteria;Proteobacteria;Alphaproteobacteria;Hyphomicrobiales;Hyphomicrobiaceae;Hyphomicrobium;Hyphomicrobium facile;Hyphomicrobium facile facile DSM 1565 |
| Ga0586324 6042799 2 571 | 0.00 | 0.00 | 0.00 | 0.00 | 0.00 | 0.00 | 0.00 | 0.00 | 1814400.00 | 0.00 | 0.00 | 0.00 | KO:K10823 | oligopeptide transport system ATP-binding protein | Bacteria;Proteobacteria;Alphaproteobacteria;Rhodospirillales;Acetobacteraceae;Crenalkalicoccus;Crenalkalicoccus roseus;Crenalkalicoccus roseus YIM 78023 |
| Ga0586324 0145051 280 990 | 0.00 | 0.00 | 0.00 | 0.00 | 0.00 | 0.00 | 0.00 | 0.00 | 3126600.00 | 0.00 | 0.00 | 0.00 | KO:K10914 | CRP/FNR family cyclic AMP-dependent transcriptional regulator | Bacteria;Proteobacteria;Betaproteobacteria;Burkholderiales;Alcaligenaceae;Parvibium;Parvibium lacunae;Parvibium lacunae KMB9 |
| Ga0586324 0009226 9489 10382 | 0.00 | 0.00 | 0.00 | 0.00 | 0.00 | 0.00 | 433941.00 | 0.00 | 0.00 | 0.00 | 0.00 | 0.00 | KO:K10944 | methane/ammonia monooxygenase subunit A | Bacteria;Nitrospirae;Nitrospira;Nitrospirales;Nitrospiraceae;Nitrospira;Candidatus Nitrospira nitrosa;Candidatus Nitrospira nitrosa COMA1 |
| Ga0586324 0009226 8227 9492 | 6800000.00 | 6786400.00 | 0.00 | 0.00 | 1133000.00 | 9595116.82 | 13611600.00 | 0.00 | 1053000.00 | 0.00 | 3317600.00 | 0.00 | KO:K10945 | methane/ammonia monooxygenase subunit B | Bacteria;Nitrospirae;Nitrospira;Nitrospirales;Nitrospiraceae;Nitrospira;Candidatus Nitrospira inopinata;Candidatus Nitrospira inopinata ENR4 |
| Ga0586324 0133958 1864 2985 | ############# | ############# | 200596900.00 | 572185000.00 | 342887000.00 | ############# | ############# | 629825000.00 | 702027000.00 | 229136600.00 | 349096000.00 | 775725000.00 | KO:K10945 | methane/ammonia monooxygenase subunit B | Bacteria;Proteobacteria;Gammaproteobacteria;Methylococcales;Methylococcaceae;Methylovulum;Methylovulum psychrotolerans;Methylovulum psychrotolerans Sph1 |
| Ga0586324 0169447 852 2117 | 0.00 | 2964800.00 | 0.00 | 0.00 | 1668600.00 | 0.00 | 0.00 | 0.00 | 0.00 | 0.00 | 0.00 | 0.00 | KO:K10945 | methane/ammonia monooxygenase subunit B | Bacteria;Nitrospirae;Nitrospira;Nitrospirales;Nitrospiraceae;Nitrospira;Candidatus Nitrospira inopinata;Candidatus Nitrospira inopinata ENR4 |
| Ga0586324 0181757 1198 2517 | 50490000.00 | 77227600.00 | 66888425.45 | 89953704.50 | 122982000.00 | 40494000.00 | 46159327.32 | 56040700.00 | 37389600.00 | 52567900.00 | 45011521.28 | 34500000.00 | KO:K10945 | methane/ammonia monooxygenase subunit B | Bacteria;Proteobacteria;Alphaproteobacteria;Hyphomicrobiales;Methylocystaceae;Methylocystis;Methylocystis parvus;Methylocystis parvus OBBP |
| Ga0586324 0496635 152 1417 | 0.00 | 0.00 | 0.00 | 0.00 | 0.00 | 3430218.95 | 0.00 | 0.00 | 0.00 | 0.00 | 0.00 | 0.00 | KO:K10945 | methane/ammonia monooxygenase subunit B | Bacteria;Nitrospirae;Nitrospira;Nitrospirales;Nitrospiraceae;Nitrospira;Candidatus Nitrospira inopinata;Candidatus Nitrospira inopinata ENR4 |
| Ga0586324 0752065 78 1322 | 0.00 | 30668000.00 | ############# | 420850000.00 | 377701000.00 | 536942500.00 | 28864800.00 | 371008304.96 | 322785000.00 | 18218200.00 | 751147.76 | 0.00 | KO:K10945 | methane/ammonia monooxygenase subunit B | Bacteria;Proteobacteria;Gammaproteobacteria;Methylococcales;Methylococcaceae;Methylovulum;Methylovulum miyakonense;Methylovulum miyakonense HT12 |
| Ga0586324 1967331 1 453 | 206495.00 | 3779013.64 | 43069500.00 | 210447.80 | 1102100.00 | 0.00 | 0.00 | 18586700.00 | 0.00 | 13860000.00 | 1047200.00 | 0.00 | KO:K10945 | methane/ammonia monooxygenase subunit B | Bacteria;Proteobacteria;Gammaproteobacteria;Methylococcales;Methylococcaceae;Methylovulum;Methylovulum psychrotolerans;Methylovulum psychrotolerans Sph1 |
| Ga0586324 2363358 1 810 | 1870000.00 | 0.00 | 5342256.33 | 6137000.00 | 9311200.00 | 22906900.00 | 35568000.00 | 4032100.00 | 4617000.00 | 1101100.00 | 0.00 | 0.00 | KO:K10945 | methane/ammonia monooxygenase subunit B | Bacteria;Proteobacteria;Alphaproteobacteria;Hyphomicrobiales;Methylocystaceae;Methylocystis;Methylocystis rosea;Methylocystis rosea SV97T |
| Ga0586324 2834562 3 779 | 0.00 | 3488400.00 | 2187356.34 | 6146500.00 | 5129400.00 | 3340095.98 | 1470761.88 | 2513200.00 | 2276100.00 | 5243700.00 | 0.00 | 0.00 | KO:K10945 | methane/ammonia monooxygenase subunit B | Bacteria;Proteobacteria;Gammaproteobacteria;Methylococcales;Methylococcaceae;Methylovulum;Methylovulum psychrotolerans;Methylovulum psychrotolerans Sph1 |
| Ga0586324 2879264 1 774 | 39290000.00 | 0.00 | 73079483.10 | 75715000.00 | 97602800.00 | 63123000.00 | 122892000.00 | 63287500.00 | 106677000.00 | 38808000.00 | 0.00 | 42397500.00 | KO:K10945 | methane/ammonia monooxygenase subunit B | Bacteria;Proteobacteria;Gammaproteobacteria;Methylococcales;Methylococcaceae;Methylobacter;Methylobacter whittenburyi;Methylobacter whittenburyi ACM 3310 |
| Ga0586324 3371191 3 554 | 181800000.00 | 111316000.00 | 0.00 | 0.00 | 71276000.00 | 0.00 | 33060000.00 | 47885000.00 | 67934700.00 | 0.00 | 0.00 | 85350000.00 | KO:K10945 | methane/ammonia monooxygenase subunit B | Bacteria;Proteobacteria;Gammaproteobacteria;Methylococcales;Methylococcaceae;Methyloterricola;Methyloterricola oryzae;Methyloterricola oryzae 73a |
| Ga0586324 3676533 3 701 | 0.00 | 10608000.00 | 0.00 | 0.00 | 0.00 | 59947000.00 | 0.00 | 0.00 | 0.00 | 0.00 | 0.00 | 0.00 | KO:K10945 | methane/ammonia monooxygenase subunit B | Bacteria;Proteobacteria;Gammaproteobacteria;Methylococcales;Methylococcaceae;Methylovulum;Methylovulum miyakonense;Methylovulum miyakonense HT12 |
| Ga0586324 4462712 1 573 | 528700000.00 | 362689575.64 | 755004776.84 | 376285500.00 | 571722100.00 | 551830000.00 | 265164000.00 | 282613000.00 | 167670000.00 | 107415000.00 | 117480000.00 | 141450000.00 | KO:K10945 | methane/ammonia monooxygenase subunit B | Bacteria;Proteobacteria;Gammaproteobacteria;Methylococcales;Methylococcaceae;Methylomagnum;Methylomagnum ishizawai;Methylomagnum ishizawai 175 |
| Ga0586324 4734155 28 630 | 0.00 | 4263600.00 | 0.00 | 41705000.00 | 50058000.00 | 58359000.00 | 0.00 | 26779000.00 | 0.00 | 30184000.00 | 0.00 | 0.00 | KO:K10945 | methane/ammonia monooxygenase subunit B | Bacteria;Proteobacteria;Alphaproteobacteria;Hyphomicrobiales;Methylocystaceae;Methylocystis;Methylocystis sp. ATCC 49242;Methylocystis sp. Rockwell, ATCC 49242 |
| Ga0586324 5033266 2 325 | 17466969.00 | 25976000.00 | 0.00 | 0.00 | 0.00 | 0.00 | 0.00 | 0.00 | 0.00 | 1054900.00 | 0.00 | 503290.50 | KO:K10945 | methane/ammonia monooxygenase subunit B | Bacteria;Proteobacteria;Gammaproteobacteria;Methylococcales;Methylococcaceae;Methylomonas;Methylomonas methanica;Methylomonas methanica MC09 |
| Ga0586324 0091155 2731 3246 | 0.00 | 0.00 | 0.00 | 0.00 | 0.00 | 0.00 | 309359.52 | 0.00 | 0.00 | 0.00 | 0.00 | 0.00 | KO:K11065 | thiol peroxidase | Bacteria;Proteobacteria;Deltaproteobacteria;Syntrophobacterales;Syntrophobacteraceae;Desulfoglaeba;Desulfoglaeba alkanexedens;Desulfoglaeba alkanexedens ALDC |

| | | | | | | | | | | | | | | | |
|---|---|---|---|---|---|---|---|---|---|---|---|---|---|---|---|
| Ga0586324_1083067_181_672 | 0.00 | 0.00 | 0.00 | 0.00 | 0.00 | 0.00 | 0.00 | 0.00 | 0.00 | 2271500.00 | 3141600.00 | 0.00 | KO:K11065 | thiol peroxidase | Bacteria;Proteobacteria;Betaproteobacteria;Nitrosomonadales;Thiobacillaceae;Thiobacillus;Thiobacillus thioparus;Thiobacillus thioparus DSM 505 |
| Ga0586324_2806457_279_782 | 0.00 | 0.00 | 0.00 | 0.00 | 276172.87 | 0.00 | 449449.56 | 562082.06 | 850500.00 | 525592.76 | 0.00 | 290614.50 | KO:K11065 | thiol peroxidase | Bacteria;Proteobacteria;Deltaproteobacteria;Syntrophobacterales;Syntrophobacteraceae;Desulfoglaeba;Desulfoglaeba alkanexedens;Desulfoglaeba alkanexedens ALDC |
| Ga0586324_3087077_1_519 | 0.00 | 0.00 | 0.00 | 0.00 | 0.00 | 0.00 | 0.00 | 0.00 | 302893.83 | 0.00 | 0.00 | 0.00 | KO:K11085 | subfamily B ATP-binding cassette protein MsbA | Bacteria;Firmicutes;Negativicutes;Selenomonadales;Sporomusaceae;Sporolituus;Sporolituus thermophilus;Sporolituus thermophilus DSM 23256 |
| Ga0586324_0000003_179518_180810 | 0.00 | 0.00 | 0.00 | 0.00 | 0.00 | 0.00 | 0.00 | 6222000.00 | 11064600.00 | 23331000.00 | 24129600.00 | 23677500.00 | KO:K11180 | sulfite reductase alpha subunit | Bacteria;Proteobacteria;Betaproteobacteria;Nitrosomonadales;Thiobacillaceae;Annwoodia;Annwoodia aquaesulis;Annwoodia aquaesulis DSM 4255 |
| Ga0586324_0000049_60952_62244 | 0.00 | 0.00 | 0.00 | 0.00 | 0.00 | 0.00 | 0.00 | 0.00 | 0.00 | 1886500.00 | 0.00 | 1320000.00 | KO:K11180 | sulfite reductase alpha subunit | Bacteria;Proteobacteria;Gammaproteobacteria;Acidiferrobacterales;Acidiferrobacteraceae;Sulfurifustis;Sulfurifustis variabilis;Sulfurifustis variabilis skN76 |
| Ga0586324_0001021_13892_15193 | 1450000.00 | 6936000.00 | 0.00 | 0.00 | 0.00 | 3215600.75 | 43456800.00 | 77653000.00 | 120406500.00 | 184376500.00 | 273072800.00 | 105232500.00 | KO:K11180 | sulfite reductase alpha subunit | Bacteria;Proteobacteria;Betaproteobacteria;Nitrosomonadales;Thiobacillaceae;Thiobacillus;Thiobacillus denitrificans;Thiobacillus denitrificans ATCC 25259 |
| Ga0586324_0004429_4006_5301 | 0.00 | 0.00 | 0.00 | 0.00 | 0.00 | 0.00 | 2094608.64 | 0.00 | 0.00 | 0.00 | 0.00 | 0.00 | KO:K11180 | sulfite reductase alpha subunit | Bacteria;Proteobacteria;Betaproteobacteria;Nitrosomonadales;Gallionellaceae;Ferriphaselus;Ferriphaselus amnicola;Ferriphaselus amnicola OYT1 |
| Ga0586324_0019654_7270_7497 | 0.00 | 0.00 | 0.00 | 0.00 | 0.00 | 0.00 | 0.00 | 0.00 | 0.00 | 9086000.00 | 0.00 | 0.00 | KO:K11180 | sulfite reductase alpha subunit | Bacteria;Proteobacteria;Betaproteobacteria;Burkholderiales;Comamonadaceae;Curvibacter;Curvibacter sp. PAE-UM;Curvibacter sp. PAE-UM |
| Ga0586324_0028735_2326_3567 | 0.00 | 0.00 | 0.00 | 0.00 | 1277200.00 | 0.00 | 0.00 | 0.00 | 1571400.00 | 1193500.00 | 0.00 | 0.00 | KO:K11180 | sulfite reductase alpha subunit | Bacteria;Proteobacteria;Deltaproteobacteria;Syntrophobacterales;Syntrophaceae;Desulfobacca;Desulfobacca acetoxidans;Desulfobacca acetoxidans ASRB2, DSM 11109 |
| Ga0586324_0028923_1542_2795 | 0.00 | 4875600.00 | 9064300.00 | 0.00 | 0.00 | 0.00 | 0.00 | 5660800.00 | 0.00 | 0.00 | 0.00 | 3555000.00 | KO:K11180 | sulfite reductase alpha subunit | Bacteria;Proteobacteria;Gammaproteobacteria;Chromatiales;Chromatiaceae;Lamprocystis;Lamprocystis purpurea;Lamprocystis purpurea DSM 4197 |
| Ga0586324_0041780_2638_3918 | 8640000.00 | 0.00 | 0.00 | 0.00 | 0.00 | 0.00 | 0.00 | 0.00 | 0.00 | 0.00 | 0.00 | 0.00 | KO:K11180 | sulfite reductase alpha subunit | Bacteria;Proteobacteria;Betaproteobacteria;Burkholderiales;Comamonadaceae;Curvibacter;Curvibacter sp. PAE-UM;Curvibacter sp. PAE-UM |
| Ga0586324_0047188_571_1818 | 0.00 | 0.00 | 0.00 | 0.00 | 0.00 | 8853100.00 | 4286400.00 | 1110200.00 | 2794500.00 | 0.00 | 0.00 | 0.00 | KO:K11180 | sulfite reductase alpha subunit | Bacteria;Proteobacteria;Deltaproteobacteria;Syntrophobacterales;Syntrophaceae;Desulfobacca;Desulfobacca acetoxidans;Desulfobacca acetoxidans ASRB2, DSM 11109 |
| Ga0586324_0069840_2542_3720 | 0.00 | 0.00 | 0.00 | 0.00 | 0.00 | 0.00 | 0.00 | 0.00 | 0.00 | 5567100.00 | 0.00 | 0.00 | KO:K11180 | sulfite reductase alpha subunit | Bacteria;Nitrospirae;Nitrospira;Nitrospirales;Nitrospiraceae;Candidatus Magnetobacterium;Candidatus Magnetobacterium casensis;Candidatus Magnetobacterium casensis MYR-1 |
| Ga0586324_0070982_1658_2959 | 1290000.00 | 0.00 | 0.00 | 0.00 | 0.00 | 0.00 | 6465090.48 | 5648600.00 | 8910000.00 | 8393000.00 | 0.00 | 4245000.00 | KO:K11180 | sulfite reductase alpha subunit | Bacteria;Proteobacteria;Betaproteobacteria;Nitrosomonadales;Thiobacillaceae;Thiobacillus;Thiobacillus denitrificans;Thiobacillus denitrificans ATCC 25259 |
| Ga0586324_0087561_598_1845 | 0.00 | 0.00 | 11710400.00 | 0.00 | 0.00 | 0.00 | 0.00 | 6026800.00 | 5815800.00 | 0.00 | 0.00 | 0.00 | KO:K11180 | sulfite reductase alpha subunit | Bacteria;Proteobacteria;Deltaproteobacteria;Syntrophobacterales;Syntrophaceae;Desulfobacca;Desulfobacca acetoxidans;Desulfobacca acetoxidans ASRB2, DSM 11109 |
| Ga0586324_0144035_1990_2883 | 0.00 | 0.00 | 0.00 | 0.00 | 0.00 | 2709155.79 | 0.00 | 0.00 | 7055100.00 | 2533300.00 | 0.00 | 0.00 | KO:K11180 | sulfite reductase alpha subunit | Bacteria;Proteobacteria;Deltaproteobacteria;Desulfobacterales;Desulfobacteraceae;Desulfococcus;Desulfococcus oleovorans;Desulfococcus oleovorans Hxd3 |
| Ga0586324_0153499_1_282 | 0.00 | 0.00 | 0.00 | 0.00 | 1483200.00 | 0.00 | 0.00 | 0.00 | 714308.22 | 537543.16 | 0.00 | 0.00 | KO:K11180 | sulfite reductase alpha subunit | Bacteria;Proteobacteria;Deltaproteobacteria;Syntrophobacterales;Syntrophaceae;Desulfobacca;Desulfobacca acetoxidans;Desulfobacca acetoxidans ASRB2, DSM 11109 |
| Ga0586324_0156778_602_1900 | 0.00 | 0.00 | 0.00 | 0.00 | 1287500.00 | 0.00 | 0.00 | 0.00 | 0.00 | 0.00 | 0.00 | 0.00 | KO:K11180 | sulfite reductase alpha subunit | Bacteria;Proteobacteria;Betaproteobacteria;Nitrosomonadales;Gallionellaceae;Ferriphaselus;Ferriphaselus amnicola;Ferriphaselus amnicola OYT1 |
| Ga0586324_0187036_755_1933 | 0.00 | 0.00 | 0.00 | 0.00 | 0.00 | 0.00 | 0.00 | 0.00 | 3588300.00 | 0.00 | 0.00 | 0.00 | KO:K11180 | sulfite reductase alpha subunit | Bacteria;Nitrospirae;unclassified Nitrospirae;unclassified Nitrospirae;unclassified Nitrospirae;unclassified Nitrospirae;Nitrospirae bacterium HCH-1;Nitrospirae bacterium HCH-1 |
| Ga0586324_0195715_1299_2498 | 0.00 | 0.00 | 0.00 | 0.00 | 0.00 | 0.00 | 0.00 | 0.00 | 0.00 | 352312.73 | 325403.76 | 0.00 | KO:K11180 | sulfite reductase alpha subunit | Bacteria;Firmicutes;Clostridia;Thermoanaerobacterales;Thermoanaerobacteraceae;Calderihabitans;Calderihabitans maritimus;Calderihabitans maritimus KKC1 |
| Ga0586324_0219864_1_129 | 2460000.00 | 0.00 | 0.00 | 0.00 | 0.00 | 0.00 | 0.00 | 634400.00 | 0.00 | 0.00 | 0.00 | 0.00 | KO:K11180 | sulfite reductase alpha subunit | Bacteria;Proteobacteria;Deltaproteobacteria;Syntrophobacterales;Syntrophobacteraceae;Syntrophobacter;Syntrophobacter fumaroxidans;Syntrophobacter fumaroxidans MPOB |
| Ga0586324_0271695_3_818 | 0.00 | 0.00 | 0.00 | 0.00 | 0.00 | 0.00 | 0.00 | 5483900.00 | 0.00 | 0.00 | 0.00 | 0.00 | KO:K11180 | sulfite reductase alpha subunit | Bacteria;Proteobacteria;Deltaproteobacteria;Desulfobacterales;Desulfobacteraceae;Desulfococcus;Desulfococcus multivorans;Desulfococcus multivorans DSM 2059 |
| Ga0586324_0349685_3_1079 | 11800000.00 | 2094400.00 | 0.00 | 4816500.00 | 705123.58 | 39700000.00 | 42567600.00 | 6527000.00 | 2268000.00 | 3234000.00 | 8395200.00 | 6435000.00 | KO:K11180 | sulfite reductase alpha subunit | Bacteria;Proteobacteria;Deltaproteobacteria;Syntrophobacterales;Syntrophobacteraceae;Syntrophobacter;Syntrophobacter fumaroxidans;Syntrophobacter fumaroxidans MPOB |
| Ga0586324_0354113_156_1469 | 7660000.00 | 0.00 | 0.00 | 0.00 | 0.00 | 0.00 | 840729.48 | 0.00 | 0.00 | 0.00 | 0.00 | 2925000.00 | KO:K11180 | sulfite reductase alpha subunit | Bacteria;Proteobacteria;Deltaproteobacteria;Desulfobacterales;Desulfobacteraceae;Desulfatirhabdium;Desulfatirhabdium butyrativorans;Desulfatirhabdium butyrativorans DSM 18734 |
| Ga0586324_0374606_2_583 | 0.00 | 0.00 | 0.00 | 0.00 | 0.00 | 0.00 | 0.00 | 16958000.00 | 0.00 | 0.00 | 0.00 | 0.00 | KO:K11180 | sulfite reductase alpha subunit | Bacteria;Proteobacteria;Betaproteobacteria;Burkholderiales;unclassified Burkholderiales;unclassified Burkholderiales;Burkholderiales bacterium GJ-E10;Burkholderiales bacterium GJ-E10 |
| Ga0586324_0453980_2_1147 | 0.00 | 0.00 | 0.00 | 0.00 | 0.00 | 0.00 | 0.00 | 2763300.00 | 3418200.00 | 2918300.00 | 0.00 | 2212500.00 | KO:K11180 | sulfite reductase alpha subunit | Bacteria;Proteobacteria;Betaproteobacteria;Nitrosomonadales;Thiobacillaceae;Sulfuritortus;Sulfuritortus calidifontis;Sulfuritortus calidifontis DSM 103923 |
| Ga0586324_0483529_397_1662 | 0.00 | 7684000.00 | 0.00 | 0.00 | 4140600.00 | 0.00 | 5722800.00 | 6527000.00 | 6933600.00 | 2394700.00 | 3194400.00 | 0.00 | KO:K11180 | sulfite reductase alpha subunit | Bacteria;Proteobacteria;Deltaproteobacteria;Syntrophobacterales;Syntrophobacteraceae;Syntrophobacter;Syntrophobacter fumaroxidans;Syntrophobacter fumaroxidans MPOB |
| Ga0586324_0602559_413_1510 | 0.00 | 0.00 | 0.00 | 0.00 | 0.00 | 0.00 | 0.00 | 0.00 | 0.00 | 351874.60 | 0.00 | 0.00 | KO:K11180 | sulfite reductase alpha subunit | Bacteria;Proteobacteria;Betaproteobacteria;Burkholderiales;Comamonadaceae;Ramlibacter;Ramlibacter lithotrophicus;Ramlibacter lithotrophicus RBP-1 |
| Ga0586324_0634589_183_1478 | 20620000.00 | 33585200.00 | 27080300.00 | 35815000.00 | 39861000.00 | 46131400.00 | 39763200.00 | 4983700.00 | 46996200.00 | 50981700.00 | 68200000.00 | 27000000.00 | KO:K11180 | sulfite reductase alpha subunit | Bacteria;Proteobacteria;Deltaproteobacteria;Desulfobacterales;Desulfobacteraceae;Desulfonema;Desulfonema limicola;Desulfonema limicola Jadebusen, DSM 2076 |
| Ga0586324_0635338_3_1100 | 0.00 | 1822400.00 | 0.00 | 3866500.00 | 0.00 | 0.00 | 0.00 | 0.00 | 0.00 | 2525600.00 | 0.00 | 2062500.00 | KO:K11180 | sulfite reductase alpha subunit | Bacteria;Proteobacteria;Deltaproteobacteria;Syntrophobacterales;Syntrophaceae;Desulfobacca;Desulfobacca acetoxidans;Desulfobacca acetoxidans ASRB2, DSM 11109 |
| Ga0586324_0663948_1_945 | 0.00 | 0.00 | 8219800.00 | 2565000.00 | 1011519.74 | 0.00 | 0.00 | 0.00 | 2494800.00 | 0.00 | 0.00 | 0.00 | KO:K11180 | sulfite reductase alpha subunit | Bacteria;Proteobacteria;Deltaproteobacteria;Syntrophobacterales;Syntrophaceae;Desulfobacca;Desulfobacca acetoxidans;Desulfobacca acetoxidans ASRB2, DSM 11109 |
| Ga0586324_0752000_3_1046 | 0.00 | 0.00 | 0.00 | 3458000.00 | 0.00 | 1914135.50 | 0.00 | 896700.00 | 1385100.00 | 0.00 | 0.00 | 0.00 | KO:K11180 | sulfite reductase alpha subunit | Bacteria;Proteobacteria;Deltaproteobacteria;Syntrophobacterales;Syntrophaceae;Desulfobacca;Desulfobacca acetoxidans;Desulfobacca acetoxidans ASRB2, DSM 11109 |
| Ga0586324_1277912_1_621 | 0.00 | 0.00 | 0.00 | 0.00 | 0.00 | 0.00 | 0.00 | 0.00 | 21141000.00 | 0.00 | 0.00 | 0.00 | KO:K11180 | sulfite reductase alpha subunit | Bacteria;Proteobacteria;Betaproteobacteria;Nitrosomonadales;Gallionellaceae;Sideroxydans;Sideroxydans lithotrophicus;Sideroxydans lithotrophicus ES-1 |
| Ga0586324_1336663_3_944 | 577404.00 | 24548000.00 | 0.00 | 1016500.00 | 0.00 | 0.00 | 0.00 | 0.00 | 0.00 | 401149.98 | 0.00 | 0.00 | KO:K11180 | sulfite reductase alpha subunit | Bacteria;Proteobacteria;Deltaproteobacteria;Syntrophobacterales;Syntrophaceae;Desulfobacca;Desulfobacca acetoxidans;Desulfobacca acetoxidans ASRB2, DSM 11109 |
| Ga0586324_1589143_3_926 | 1730000.00 | 2380000.00 | 0.00 | 0.00 | 9795300.00 | 2330461.46 | 440899.56 | 530172.35 | 582846.03 | 1409100.00 | 1918400.00 | 2197500.00 | KO:K11180 | sulfite reductase alpha subunit | Bacteria;Proteobacteria;Deltaproteobacteria;Syntrophobacterales;Syntrophaceae;Desulfobacca;Desulfobacca acetoxidans;Desulfobacca acetoxidans ASRB2, DSM 11109 |
| Ga0586324_1608571_1_990 | 0.00 | 1611600.00 | 0.00 | 0.00 | 0.00 | 0.00 | 11058000.00 | 1610400.00 | 1611900.00 | 2748900.00 | 0.00 | 0.00 | KO:K11180 | sulfite reductase alpha subunit | Bacteria;Proteobacteria;Betaproteobacteria;Nitrosomonadales;Thiobacillaceae;Thiobacillus;Thiobacillus denitrificans;Thiobacillus denitrificans ATCC 25259 |
| Ga0586324_1701750_3_653 | 0.00 | 4229600.00 | 0.00 | 0.00 | 0.00 | 0.00 | 0.00 | 0.00 | 19359000.00 | 0.00 | 0.00 | 0.00 | KO:K11180 | sulfite reductase alpha subunit | Bacteria;Proteobacteria;Deltaproteobacteria;Syntrophobacterales;Syntrophaceae;Desulfobacca;Desulfobacca acetoxidans;Desulfobacca acetoxidans ASRB2, DSM 11109 |
| Ga0586324_1713397_472_963 | 0.00 | 0.00 | 0.00 | 0.00 | 0.00 | 0.00 | 2986800.00 | 0.00 | 0.00 | 0.00 | 0.00 | 0.00 | KO:K11180 | sulfite reductase alpha subunit | Bacteria;Proteobacteria;Deltaproteobacteria;Desulfobacterales;Desulfobacteraceae;Desulfatiglans;Desulfatiglans anilini;Desulfatiglans anilini DSM 4660 |
| Ga0586324_1793868_3_947 | 3270000.00 | 2686000.00 | 0.00 | 0.00 | 0.00 | 0.00 | 0.00 | 1683600.00 | 0.00 | 0.00 | 0.00 | 0.00 | KO:K11180 | sulfite reductase alpha subunit | Bacteria;Proteobacteria;Deltaproteobacteria;Desulfobacterales;Desulfobacteraceae;Desulfatiglans;Desulfatiglans anilini;Desulfatiglans anilini DSM 4660 |
| Ga0586324_1812708_189_941 | 0.00 | 10608000.00 | 0.00 | 0.00 | 25750000.00 | 1272206.35 | 0.00 | 15616000.00 | 22923000.00 | 12782000.00 | 9064000.00 | 0.00 | KO:K11180 | sulfite reductase alpha subunit | Bacteria;Proteobacteria;Deltaproteobacteria;Desulfobacterales;Desulfobulbaceae;Desulfobulbus;Desulfobulbus propionicus;Desulfobulbus propionicus 1pr3, DSM 2032 |
| Ga0586324_1956730_573_911 | 0.00 | 39508000.00 | 0.00 | 0.00 | 33887000.00 | 0.00 | 0.00 | 19215000.00 | 10287000.00 | 23408000.00 | 0.00 | 0.00 | KO:K11180 | sulfite reductase alpha subunit | Bacteria;Proteobacteria;Deltaproteobacteria;Syntrophobacterales;Syntrophaceae;Desulfobacca;Desulfobacca acetoxidans;Desulfobacca acetoxidans ASRB2, DSM 11109 |
| Ga0586324_2094332_336_887 | 4100000.00 | 0.00 | 0.00 | 1786000.00 | 0.00 | 0.00 | 0.00 | 0.00 | 0.00 | 0.00 | 0.00 | 0.00 | KO:K11180 | sulfite reductase alpha subunit | Bacteria;Proteobacteria;Deltaproteobacteria;Syntrophobacterales;Syntrophaceae;Desulfobacca;Desulfobacca acetoxidans;Desulfobacca acetoxidans ASRB2, DSM 11109 |
| Ga0586324_2230265_3_863 | 6690000.00 | 7820000.00 | 0.00 | 0.00 | 0.00 | 0.00 | 5631600.00 | 7869000.00 | 7200900.00 | 0.00 | 0.00 | 0.00 | KO:K11180 | sulfite reductase alpha subunit | Bacteria;Proteobacteria;Deltaproteobacteria;Desulfobacterales;Desulfobacteraceae;Desulfatiglans;Desulfatiglans anilini;Desulfatiglans anilini DSM 4660 |
| Ga0586324_2364420_3_581 | 0.00 | 0.00 | 0.00 | 0.00 | 0.00 | 0.00 | 0.00 | 4587200.00 | 0.00 | 0.00 | 0.00 | 0.00 | KO:K11180 | sulfite reductase alpha subunit | Bacteria;Proteobacteria;Gammaproteobacteria;Thiotrichales;Thiotrichaceae;Thioploca;Thioploca ingrica;Thioploca ingrica Lake Okotanpe |
| Ga0586324_2676346_553_798 | 0.00 | 0.00 | 0.00 | 0.00 | 0.00 | 0.00 | 0.00 | 0.00 | 24462000.00 | 2117500.00 | 26224000.00 | 18825000.00 | KO:K11180 | sulfite reductase alpha subunit | Bacteria;Proteobacteria;Betaproteobacteria;Nitrosomonadales;Gallionellaceae;Sideroxydans;Sideroxydans lithotrophicus;Sideroxydans lithotrophicus ES-1 |
| Ga0586324_2762683_1_789 | 0.00 | 0.00 | 0.00 | 0.00 | 0.00 | 0.00 | 0.00 | 0.00 | 0.00 | 0.00 | 0.00 | 5107500.00 | KO:K11180 | sulfite reductase alpha subunit | Bacteria;Proteobacteria;Betaproteobacteria;Nitrosomonadales;Thiobacillaceae;Thiobacillus;Thiobacillus thioparus;Thiobacillus thioparus DSM 505 |
| Ga0586324_3094512_1_702 | 13500000.00 | 15232000.00 | 0.00 | 7191500.00 | 7148200.00 | 0.00 | 0.00 | 9028000.00 | 6804000.00 | 0.00 | 0.00 | 0.00 | KO:K11180 | sulfite reductase alpha subunit | Bacteria;Proteobacteria;Deltaproteobacteria;Syntrophobacterales;Syntrophaceae;Desulfobacca;Desulfobacca acetoxidans;Desulfobacca acetoxidans ASRB2, DSM 11109 |
| Ga0586324_3152047_3_506 | 0.00 | 9452000.00 | 0.00 | 0.00 | 13287000.00 | 7701800.00 | 1821218.40 | 0.00 | 0.00 | 14091000.00 | 14960000.00 | 15300000.00 | KO:K11180 | sulfite reductase alpha subunit | Bacteria;Proteobacteria;Betaproteobacteria;Nitrosomonadales;Gallionellaceae;Sideroxydans;Sideroxydans lithotrophicus;Sideroxydans lithotrophicus ES-1 |
| Ga0586324_3190264_2_742 | 0.00 | 0.00 | 29332300.00 | 0.00 | 0.00 | 0.00 | 0.00 | 0.00 | 0.00 | 0.00 | 0.00 | 0.00 | KO:K11180 | sulfite reductase alpha subunit | Bacteria;Proteobacteria;Gammaproteobacteria;Acidiferrobacterales;Acidiferrobacteraceae;Sulfuricaulis;Sulfuricaulis limicola;Sulfuricaulis limicola HA5 |
| Ga0586324_3372915_1_726 | 0.00 | 0.00 | 0.00 | 0.00 | 0.00 | 0.00 | 21112800.00 | 17202000.00 | 10773000.00 | 12243000.00 | 15752000.00 | 15750000.00 | KO:K11180 | sulfite reductase alpha subunit | Bacteria;Proteobacteria;Betaproteobacteria;Nitrosomonadales;Thiobacillaceae;Thiobacillus;Thiobacillus thioparus;Thiobacillus thioparus DSM 505 |
| Ga0586324_3639461_2_703 | 0.00 | 727600.00 | 0.00 | 93886.51 | 192759.35 | 0.00 | 0.00 | 446953.71 | 200700.99 | 1439900.00 | 0.00 | 0.00 | KO:K11180 | sulfite reductase alpha subunit | Bacteria;Proteobacteria;Deltaproteobacteria;Syntrophobacterales;Syntrophobacteraceae;Desulfosoma;Desulfosoma caldarium;Desulfosoma caldarium DSM 22027 |
| Ga0586324_3639574_3_704 | 0.00 | 0.00 | 0.00 | 0.00 | 2018800.00 | 6709300.00 | 7866000.00 | 9028000.00 | 3175200.00 | 2002000.00 | 1707200.00 | 1515000.00 | KO:K11180 | sulfite reductase alpha subunit | Bacteria;Proteobacteria;Deltaproteobacteria;Desulfobacterales;Desulfobulbaceae;Desulfobulbus;Desulfobulbus elongatus;Desulfobulbus elongatus DSM 2908 |
| Ga0586324_3936839_1_681 | 0.00 | 0.00 | 0.00 | 0.00 | 0.00 | 0.00 | 0.00 | 0.00 | 1935900.00 | 0.00 | 0.00 | 3247500.00 | KO:K11180 | sulfite reductase alpha subunit | Bacteria;Proteobacteria;Betaproteobacteria;Nitrosomonadales;Thiobacillaceae;Annwoodia;Annwoodia aquaesulis;Annwoodia aquaesulis DSM 4255 |
| Ga0586324_4494296_3_644 | 0.00 | 0.00 | 0.00 | 37335000.00 | 0.00 | 0.00 | 0.00 | 0.00 | 5670000.00 | 0.00 | 0.00 | 0.00 | KO:K11180 | sulfite reductase alpha subunit | Bacteria;Proteobacteria;Deltaproteobacteria;Syntrophobacterales;Syntrophobacteraceae;Syntrophobacter;Syntrophobacter fumaroxidans;Syntrophobacter fumaroxidans MPOB |
| Ga0586324_5040590_238_615 | 0.00 | 2760800.00 | 10190300.00 | 0.00 | 0.00 | 19929400.00 | 11422800.00 | 2714500.00 | 4519800.00 | 6937700.00 | 0.00 | 10500000.00 | KO:K11180 | sulfite reductase alpha subunit | Bacteria;Proteobacteria;Deltaproteobacteria;Syntrophobacterales;Syntrophobacteraceae;Desulfosoma;Desulfosoma caldarium;Desulfosoma caldarium DSM 22027 |
| Ga0586324_5599400_3_590 | 0.00 | 157738.92 | 0.00 | 0.00 | 0.00 | 0.00 | 322339.56 | 121704.15 | 389167.74 | 0.00 | 0.00 | 0.00 | KO:K11180 | sulfite reductase alpha subunit | Bacteria;Proteobacteria;Deltaproteobacteria;Syntrophobacterales;Syntrophaceae;Desulfobacca;Desulfobacca acetoxidans;Desulfobacca acetoxidans ASRB2, DSM 11109 |
| Ga0586324_6546222_2_553 | 2610000.00 | 2638400.00 | 0.00 | 0.00 | 0.00 | 0.00 | 0.00 | 0.00 | 0.00 | 0.00 | 0.00 | 0.00 | KO:K11180 | sulfite reductase alpha subunit | Bacteria;Proteobacteria;Gammaproteobacteria;Thiotrichales;Thiotrichaceae;Thioploca;Thioploca ingrica;Thioploca ingrica Lake Okotanpe |
| Ga0586324_6987753_1_537 | 0.00 | 2869600.00 | 0.00 | 0.00 | 0.00 | 0.00 | 0.00 | 0.00 | 0.00 | 0.00 | 0.00 | 0.00 | KO:K11180 | sulfite reductase alpha subunit | Bacteria;Proteobacteria;Deltaproteobacteria;Desulfobacterales;Desulfobacteraceae;Desulfatirhabdium;Desulfatirhabdium butyrativorans;Desulfatirhabdium butyrativorans DSM 18734 |
| Ga0586324_7430166_185_523 | 552355.00 | 0.00 | 0.00 | 0.00 | 0.00 | 195490.74 | 0.00 | 0.00 | 0.00 | 0.00 | 0.00 | 0.00 | KO:K11180 | sulfite reductase alpha subunit | Bacteria;Proteobacteria;Deltaproteobacteria;Syntrophobacterales;Syntrophaceae;Desulfobacca;Desulfobacca acetoxidans;Desulfobacca acetoxidans ASRB2, DSM 11109 |
| Ga0586324_0000003_178378_179448 | 0.00 | 0.00 | 0.00 | 0.00 | 0.00 | 0.00 | 0.00 | 52539300.00 | 1644300.00 | 60137000.00 | 670865.36 | 6510000.00 | KO:K11181 | sulfite reductase beta subunit | Bacteria;Proteobacteria;Betaproteobacteria;Nitrosomonadales;Thiobacillaceae;Annwoodia;Annwoodia aquaesulis;Annwoodia aquaesulis DSM 4255 |
| Ga0586324_0015809_333_1406 | 0.00 | 5814000.00 | 0.00 | 1330000.00 | 0.00 | 0.00 | 0.00 | 0.00 | 0.00 | 0.00 | 0.00 | 0.00 | KO:K11181 | sulfite reductase beta subunit | Bacteria;Nitrospirae;unclassified Nitrospirae;unclassified Nitrospirae;unclassified Nitrospirae;unclassified Nitrospirae;Nitrospirae bacterium HCH-1;Nitrospirae bacterium HCH-1 |
| Ga0586324_0017488_2749_3849 | 0.00 | 20604000.00 | 44871100.00 | 0.00 | 0.00 | 0.00 | 0.00 | 0.00 | 42606000.00 | 0.00 | 0.00 | 24900000.00 | KO:K11181 | sulfite reductase beta subunit | Bacteria;Proteobacteria;Deltaproteobacteria;Syntrophobacterales;Syntrophobacteraceae;Desulfosoma;Desulfosoma caldarium;Desulfosoma caldarium DSM 22027 |
| Ga0586324_0028146_2701_3840 | 0.00 | 0.00 | 1068095.45 | 0.00 | 0.00 | 1185692.11 | 24168000.00 | 0.00 | 0.00 | 0.00 | 0.00 | 0.00 | KO:K11181 | sulfite reductase beta subunit | Bacteria;Proteobacteria;Deltaproteobacteria;Desulfobacterales;Desulfobacteraceae;Desulfosalsimonas;Desulfosalsimonas propionicica;Desulfosalsimonas propionicica DSM 17721 |
| Ga0586324_0028735_1192_2262 | 0.00 | 0.00 | 0.00 | 4978000.00 | 6447800.00 | 0.00 | 14272800.00 | 0.00 | 0.00 | 0.00 | 0.00 | 0.00 | KO:K11181 | sulfite reductase beta subunit | Bacteria;Proteobacteria;Deltaproteobacteria;Syntrophobacterales;Syntrophaceae;Desulfobacca;Desulfobacca acetoxidans;Desulfobacca acetoxidans ASRB2, DSM 11109 |
| Ga0586324_0051016_465_1535 | 2670000.00 | 0.00 | 0.00 | 0.00 | 0.00 | 0.00 | 8322000.00 | 19520000.00 | 13446000.00 | 11935000.00 | 0.00 | 3505029.00 | KO:K11181 | sulfite reductase beta subunit | Bacteria;Proteobacteria;Betaproteobacteria;Nitrosomonadales;Sulfuricellaceae;Sulfuriferula;Sulfuriferula multivorans;Sulfuriferula multivorans TTN |
| Ga0586324_0070982_3025_4038 | 0.00 | 0.00 | 4579250.58 | 0.00 | 0.00 | 0.00 | 22458000.00 | 8174000.00 | 0.00 | 8701000.00 | 7066400.00 | 2310000.00 | KO:K11181 | sulfite reductase beta subunit | Bacteria;Proteobacteria;Betaproteobacteria;Nitrosomonadales;Thiobacillaceae;Thiobacillus;Thiobacillus thioparus;Thiobacillus thioparus DSM 505 |
| Ga0586324_0163121_3_527 | 31000000.00 | 0.00 | 29557500.00 | 0.00 | 0.00 | 46846000.00 | 52440000.00 | 0.00 | 24138000.00 | 0.00 | 0.00 | 0.00 | KO:K11181 | sulfite reductase beta subunit | Bacteria;Proteobacteria;Deltaproteobacteria;Desulfobacterales;Desulfobacteraceae;Desulfatirhabdium;Desulfatirhabdium butyrativorans;Desulfatirhabdium butyrativorans DSM 18734 |
| Ga0586324_0179166_1421_2494 | 15100000.00 | 13396000.00 | 22632600.00 | 0.00 | 0.00 | 0.00 | 15640800.00 | 18422000.00 | 0.00 | 13706000.00 | 17160000.00 | 15600000.00 | KO:K11181 | sulfite reductase beta subunit | Bacteria;Proteobacteria;Betaproteobacteria;Nitrosomonadales;Thiobacillaceae;Sulfuritortus;Sulfuritortus calidifontis;Sulfuritortus calidifontis DSM 103923 |
| Ga0586324_0187036_1979_2551 | 0.00 | 0.00 | 0.00 | 271418.80 | 0.00 | 0.00 | 0.00 | 0.00 | 0.00 | 0.00 | 0.00 | 0.00 | KO:K11181 | sulfite reductase beta subunit | Bacteria;Nitrospirae;Nitrospira;Nitrospirales;Nitrospiraceae;Candidatus Magnetobacterium;Candidatus Magnetobacterium casensis;Candidatus Magnetobacterium casensis MYR-1 |
| Ga0586324_0268276_204_1346 | 0.00 | 0.00 | 2924267.04 | 4569500.00 | 0.00 | 1097812.19 | 1295448.12 | 2653500.00 | 466590.78 | 0.00 | 1944800.00 | 1620000.00 | KO:K11181 | sulfite reductase beta subunit | Bacteria;Proteobacteria;Deltaproteobacteria;Desulfobacterales;Desulfobacteraceae;Desulfococcus;Desulfococcus oleovorans;Desulfococcus oleovorans Hxd3 |
| Ga0586324_0327527_930_1976 | 0.00 | 1319200.00 | 0.00 | 0.00 | 3543200.00 | 331186.13 | 832154.40 | 0.00 | 464697.00 | 0.00 | 0.00 | 0.00 | KO:K11181 | sulfite reductase beta subunit | Bacteria;Proteobacteria;Deltaproteobacteria;Desulfobacterales;Desulfobacteraceae;Desulfococcus;Desulfococcus oleovorans;Desulfococcus oleovorans Hxd3 |
| Ga0586324_0381402_342_1415 | 3380000.00 | 931600.00 | 0.00 | 5434000.00 | 1349300.00 | 0.00 | 0.00 | 0.00 | 9558000.00 | 0.00 | 924000.00 | 0.00 | KO:K11181 | sulfite reductase beta subunit | Bacteria;Proteobacteria;Gammaproteobacteria;Thiotrichales;Thiolinaceae;Thiolinea;Thiolinea disciformis;Thiolinea disciformis DSM 14473 |
| Ga0586324_0445522_279_1418 | 0.00 | 0.00 | 0.00 | 0.00 | 0.00 | 0.00 | 0.00 | 0.00 | 344481.66 | 0.00 | 0.00 | 0.00 | KO:K11181 | sulfite reductase beta subunit | Bacteria;Proteobacteria;Deltaproteobacteria;Desulfobacterales;Desulfobacteraceae;Desulfococcus;Desulfococcus oleovorans;Desulfococcus oleovorans Hxd3 |
| Ga0586324_0453867_3_1106 | 0.00 | 0.00 | 0.00 | 0.00 | 13081000.00 | 94315960.93 | 0.00 | 0.00 | 0.00 | 0.00 | 3467200.00 | 0.00 | KO:K11181 | sulfite reductase beta subunit | Bacteria;Proteobacteria;Deltaproteobacteria;unclassified Deltaproteobacteria;Candidatus Desulfofervidaceae;Candidatus Desulfofervidus;Candidatus Desulfofervidus auxilii;delta proteobacterium HotSeep1 |
| Ga0586324_0538190_68_1147 | 0.00 | 0.00 | 0.00 | 0.00 | 0.00 | 0.00 | 0.00 | 0.00 | 0.00 | 0.00 | 33792000.00 | 0.00 | KO:K11181 | sulfite reductase beta subunit | Bacteria;Proteobacteria;Deltaproteobacteria;Syntrophobacterales;Syntrophaceae;Desulfobacca;Desulfobacca acetoxidans;Desulfobacca acetoxidans ASRB2, DSM 11109 |
| Ga0586324_0596412_480_1517 | 0.00 | 5453600.00 | 0.00 | 0.00 | 0.00 | 0.00 | 0.00 | 0.00 | 0.00 | 28567000.00 | 0.00 | 0.00 | KO:K11181 | sulfite reductase beta subunit | Bacteria;Proteobacteria;Deltaproteobacteria;Desulfobacterales;Desulfobacteraceae;Desulfosalsimonas;Desulfosalsimonas propionicica;Desulfosalsimonas propionicica DSM 17721 |
| Ga0586324_0671027_1_525 | 26800000.00 | 0.00 | 0.00 | 0.00 | 42230000.00 | 0.00 | 0.00 | 23729000.00 | 0.00 | 0.00 | 0.00 | 0.00 | KO:K11181 | sulfite reductase beta subunit | Bacteria;Proteobacteria;Deltaproteobacteria;Syntrophobacterales;Syntrophaceae;Desulfobacca;Desulfobacca acetoxidans;Desulfobacca acetoxidans ASRB2, DSM 11109 |
| Ga0586324_0747168_150_1376 | 0.00 | 0.00 | 0.00 | 0.00 | 0.00 | 0.00 | 0.00 | 0.00 | 0.00 | 0.00 | 0.00 | 631731.75 | KO:K11181 | sulfite reductase beta subunit | Bacteria;Proteobacteria;Deltaproteobacteria;unclassified Deltaproteobacteria;Candidatus Desulfofervidaceae;Candidatus Desulfofervidus;Candidatus Desulfofervidus auxilii;delta proteobacterium HotSeep1 |
| Ga0586324_1013140_760_1206 | 2190000.00 | 2019600.00 | 6924900.00 | 0.00 | 3708000.00 | 4605200.00 | 0.00 | 2464400.00 | 2462400.00 | 2841300.00 | 0.00 | 2182500.00 | KO:K11181 | sulfite reductase beta subunit | Bacteria;Proteobacteria;Deltaproteobacteria;Syntrophobacterales;Syntrophobacteraceae;Desulfosoma;Desulfosoma caldarium;Desulfosoma caldarium DSM 22027 |
| Ga0586324_1246515_252_1103 | 3320000.00 | 4875600.00 | 0.00 | 3325000.00 | 0.00 | 0.00 | 0.00 | 0.00 | 0.00 | 0.00 | 0.00 | 0.00 | KO:K11181 | sulfite reductase beta subunit | Bacteria;Proteobacteria;Deltaproteobacteria;Desulfobacterales;Desulfobacteraceae;Desulfatirhabdium;Desulfatirhabdium butyrativorans;Desulfatirhabdium butyrativorans DSM 18734 |
| Ga0586324_1267535_3_710 | 11900000.00 | 69360000.00 | 0.00 | 0.00 | 0.00 | 0.00 | 0.00 | 0.00 | 0.00 | 0.00 | 0.00 | 0.00 | KO:K11181 | sulfite reductase beta subunit | Bacteria;Proteobacteria;Deltaproteobacteria;Syntrophobacterales;Syntrophobacteraceae;Desulfacinum;Desulfacinum infernum;Desulfacinum infernum DSM 9756 |
| Ga0586324_1409280_1_1047 | 0.00 | 26520000.00 | 56300000.00 | 0.00 | 0.00 | 1218766.18 | 0.00 | 0.00 | 34668000.00 | 0.00 | 0.00 | 32325000.00 | KO:K11181 | sulfite reductase beta subunit | Bacteria;Proteobacteria;Deltaproteobacteria;Syntrophobacterales;Syntrophobacteraceae;Desulfosoma;Desulfosoma caldarium;Desulfosoma caldarium DSM 22027 |
| Ga0586324_1655678_1_978 | 0.00 | 0.00 | 0.00 | 0.00 | 0.00 | 0.00 | 0.00 | 0.00 | 0.00 | 0.00 | 2974400.00 | 0.00 | KO:K11181 | sulfite reductase beta subunit | Bacteria;Proteobacteria;Deltaproteobacteria;unclassified Deltaproteobacteria;Candidatus Desulfofervidaceae;Candidatus Desulfofervidus;Candidatus Desulfofervidus auxilii;delta proteobacterium HotSeep1 |
| Ga0586324_1973769_56_907 | 21530000.00 | 6167600.00 | 26742500.00 | 46559500.00 | 40170000.00 | 146872218.37 | 45144000.00 | 42645100.00 | 32205600.00 | 21868000.00 | 27825600.00 | 18757500.00 | KO:K11181 | sulfite reductase beta subunit | Bacteria;Proteobacteria;Deltaproteobacteria;Syntrophobacterales;Syntrophobacteraceae;Syntrophobacter;Syntrophobacter fumaroxidans;Syntrophobacter fumaroxidans MPOB |
| Ga0586324_2751395_3_602 | 492567.00 | 3570000.00 | 8163500.00 | 4617000.00 | 6004900.00 | 0.00 | 3762000.00 | 2641300.00 | 2924100.00 | 2879800.00 | 2886400.00 | 2902500.00 | KO:K11181 | sulfite reductase beta subunit | Bacteria;Proteobacteria;Deltaproteobacteria;Syntrophobacterales;Syntrophobacteraceae;Desulfosoma;Desulfosoma caldarium;Desulfosoma caldarium DSM 22027 |
| Ga0586324_2953455_437_766 | 0.00 | 1679600.00 | 0.00 | 0.00 | 0.00 | 0.00 | 0.00 | 0.00 | 0.00 | 0.00 | 0.00 | 0.00 | KO:K11181 | sulfite reductase beta subunit | Bacteria;Proteobacteria;Deltaproteobacteria;Syntrophobacterales;Syntrophobacteraceae;Syntrophobacter;Syntrophobacter fumaroxidans;Syntrophobacter fumaroxidans MPOB |
| Ga0586324_3676637_1_375 | 10600000.00 | 0.00 | 0.00 | 16720000.00 | 9754100.00 | 0.00 | 0.00 | 9211000.00 | 0.00 | 0.00 | 0.00 | 0.00 | KO:K11181 | sulfite reductase beta subunit | Bacteria;Proteobacteria;Deltaproteobacteria;Desulfobacterales;Desulfobulbaceae;Desulfoprunum;Desulfoprunum benzoelyticum;Desulfoprunum benzoelyticum DSM 28570 |
| Ga0586324_3819141_2_688 | 0.00 | 4311200.00 | 10302900.00 | 0.00 | 2101200.00 | 0.00 | 0.00 | 0.00 | 0.00 | 0.00 | 1443200.00 | 0.00 | KO:K11181 | sulfite reductase beta subunit | Bacteria;Proteobacteria;Gammaproteobacteria;Alteromonadales;Alteromonadaceae;unclassified Alteromonadaceae;Alteromonadaceae bacterium 2719K.STB50.0a.01;Alteromonadaceae bacterium 2719K.STB50.0a.01 |
| Ga0586324_4117179_1_489 | 0.00 | 0.00 | 0.00 | 9690000.00 | 0.00 | 0.00 | 0.00 | 0.00 | 0.00 | 0.00 | 0.00 | 0.00 | KO:K11181 | sulfite reductase beta subunit | Bacteria;Proteobacteria;Deltaproteobacteria;Syntrophobacterales;Syntrophobacteraceae;Syntrophobacter;Syntrophobacter fumaroxidans;Syntrophobacter fumaroxidans MPOB |
| Ga0586324_4632641_1_627 | 0.00 | 0.00 | 0.00 | 0.00 | 0.00 | 0.00 | 56088000.00 | 0.00 | 0.00 | 0.00 | 0.00 | 0.00 | KO:K11181 | sulfite reductase beta subunit | Bacteria;Proteobacteria;Deltaproteobacteria;Syntrophobacterales;Syntrophaceae;Desulfobacca;Desulfobacca acetoxidans;Desulfobacca acetoxidans ASRB2, DSM 11109 |
| Ga0586324_5163960_1_480 | 0.00 | 0.00 | 0.00 | 3135000.00 | 0.00 | 10202900.00 | 0.00 | 0.00 | 0.00 | 0.00 | 0.00 | 0.00 | KO:K11181 | sulfite reductase beta subunit | Bacteria;Proteobacteria;Deltaproteobacteria;Desulfobacterales;Desulfobulbaceae;Desulfopila;Desulfopila aestuarii;Desulfopila aestuarii DSM 18488 |
| Ga0586324_5453960_3_596 | 0.00 | 6432800.00 | 0.00 | 0.00 | 0.00 | 0.00 | 0.00 | 0.00 | 0.00 | 0.00 | 6336000.00 | 0.00 | KO:K11181 | sulfite reductase beta subunit | Bacteria;Proteobacteria;Deltaproteobacteria;Syntrophobacterales;Syntrophobacteraceae;Syntrophobacter;Syntrophobacter fumaroxidans;Syntrophobacter fumaroxidans MPOB |
| Ga0586324_5511193_3_593 | 0.00 | 3182400.00 | 0.00 | 0.00 | 1936400.00 | 0.00 | 0.00 | 0.00 | 0.00 | 0.00 | 0.00 | 0.00 | KO:K11181 | sulfite reductase beta subunit | Bacteria;Proteobacteria;Deltaproteobacteria;unclassified Deltaproteobacteria;Candidatus Desulfofervidaceae;Candidatus Desulfofervidus;Candidatus Desulfofervidus auxilii;delta proteobacterium HotSeep1 |
| Ga0586324_6573522_3_374 | 0.00 | 0.00 | 0.00 | 0.00 | 0.00 | 1284322.79 | 0.00 | 0.00 | 0.00 | 0.00 | 0.00 | 0.00 | KO:K11181 | sulfite reductase beta subunit | Bacteria;Proteobacteria;Deltaproteobacteria;Syntrophobacterales;Syntrophaceae;Desulfomonile;Desulfomonile tiedjei;Desulfomonile tiedjei DCB-1, DSM 6799 |
| Ga0586324_6878634_16_540 | 0.00 | 1203600.00 | 0.00 | 0.00 | 0.00 | 0.00 | 0.00 | 0.00 | 0.00 | 0.00 | 0.00 | 0.00 | KO:K11181 | sulfite reductase beta subunit | Bacteria;Proteobacteria;Deltaproteobacteria;Syntrophobacterales;Syntrophobacteraceae;Desulfacinum;Desulfacinum infernum;Desulfacinum infernum DSM 9756 |
| Ga0586324_6946917_3_539 | 0.00 | 3882800.00 | 5798900.00 | 0.00 | 8878600.00 | 0.00 | 11559600.00 | 9516000.00 | 9801000.00 | 8701000.00 | 0.00 | 6982500.00 | KO:K11181 | sulfite reductase beta subunit | Bacteria;Proteobacteria;Deltaproteobacteria;Desulfobacterales;Desulfobacteraceae;Desulfatitalea;Desulfatitalea tepidiphila;Desulfatitalea tepidiphila S28bF |
| Ga0586324_0000090_50629_51573 | 0.00 | 0.00 | 0.00 | 0.00 | 0.00 | 0.00 | 0.00 | 0.00 | 0.00 | 1717100.00 | 2464000.00 | 1957500.00 | KO:K11206 | predicted amidohydrolase | Bacteria;Proteobacteria;Betaproteobacteria;Nitrosomonadales;Thiobacillaceae;Annwoodia;Annwoodia aquaesulis;Annwoodia aquaesulis DSM 4255 |
| Ga0586324_0581071_938_1249 | 154300000.00 | 57596000.00 | 3781896.20 | 0.00 | 0.00 | 55580000.00 | 88008000.00 | 42517000.00 | 5418900.00 | 0.00 | 65560000.00 | 83850000.00 | KO:K11254 | histone H4 | Eukaryota;Zoopagomycota;Kickxellomycetes;Kickxellales;Kickxellaceae;Linderina;Linderina pennispora;Linderina pennispora ATCC 12442 |
| Ga0586324_0604164_1_1509 | 0.00 | 0.00 | 0.00 | 0.00 | 0.00 | 0.00 | 0.00 | 0.00 | 0.00 | 0.00 | 375939.52 | 0.00 | KO:K11381 | 2-oxoisovalerate dehydrogenase E1 component | Bacteria;Proteobacteria;Gammaproteobacteria;Oceanospirillales;Balneatrichaceae;Pokkaliibacter;Pokkaliibacter plantistimulans;Pokkaliibacter plantistimulans L1E11 |
| Ga0586324_1330270_481_1017 | 0.00 | 0.00 | 0.00 | 0.00 | 0.00 | 0.00 | 0.00 | 0.00 | 0.00 | 0.00 | 5429600.00 | 3885000.00 | KO:K11477 | glc operon protein GlcG | Bacteria;Proteobacteria;Alphaproteobacteria;Hyphomicrobiales;Hyphomicrobiaceae;Rhodoplanes;Rhodoplanes sp. Z2-YC6860;Rhodoplanes sp. Z2-YC6860 |
| Ga0586324_0006349_1097_2161 | 0.00 | 0.00 | 0.00 | 0.00 | 0.00 | 0.00 | 0.00 | 0.00 | 1595700.00 | 0.00 | 0.00 | 0.00 | KO:K11645 | class I fructose-bisphosphate aldolase | Bacteria;Verrucomicrobia;Spartobacteria;unclassified Spartobacteria;unclassified Spartobacteria;unclassified Spartobacteria;Spartobacteria bacterium LR76;Spartobacteria bacterium LR76 |
| Ga0586324_0039613_3264_4361 | 0.00 | 0.00 | 0.00 | 0.00 | 0.00 | 0.00 | 22252800.00 | 0.00 | 158254.56 | 0.00 | 0.00 | 0.00 | KO:K11645 | class I fructose-bisphosphate aldolase | Bacteria;Proteobacteria;Betaproteobacteria;unclassified Betaproteobacteria;unclassified Betaproteobacteria;unclassified Betaproteobacteria;uncultured beta proteobacterium;unclassified betaproteobacterium ENR4 |
| Ga0586324_0180889_2_1021 | 0.00 | 0.00 | 0.00 | 0.00 | 1936400.00 | 0.00 | 0.00 | 0.00 | 0.00 | 0.00 | 0.00 | 0.00 | KO:K11645 | class I fructose-bisphosphate aldolase | Bacteria;Proteobacteria;Deltaproteobacteria;Syntrophobacterales;Syntrophaceae;Desulfobacca;Desulfobacca acetoxidans;Desulfobacca acetoxidans ASRB2, DSM 11109 |
| Ga0586324_0309138_963_2027 | 0.00 | 3298000.00 | 0.00 | 0.00 | 0.00 | 0.00 | 0.00 | 0.00 | 0.00 | 0.00 | 0.00 | 0.00 | KO:K11645 | class I fructose-bisphosphate aldolase | Bacteria;Proteobacteria;Gammaproteobacteria;unclassified Gammaproteobacteria;Candidatus Competibacteraceae;Candidatus Competibacter;Candidatus Competibacter denitrificans;Candidatus Competibacter denitrificans Run_A_D11 |
| Ga0586324_0758588_264_1316 | 2640000.00 | 0.00 | 0.00 | 0.00 | 0.00 | 0.00 | 0.00 | 0.00 | 0.00 | 0.00 | 0.00 | 0.00 | KO:K11645 | class I fructose-bisphosphate aldolase | Bacteria;Proteobacteria;Betaproteobacteria;unclassified Betaproteobacteria;unclassified Betaproteobacteria;unclassified Betaproteobacteria;uncultured beta proteobacterium;unclassified betaproteobacterium ENR4 |
| Ga0586324_0766782_566_1360 | 0.00 | 0.00 | 0.00 | 17290000.00 | 17407000.00 | 0.00 | 0.00 | 23241000.00 | 4268700.00 | 21175000.00 | 0.00 | 379512.00 | KO:K11645 | class I fructose-bisphosphate aldolase | Bacteria;Proteobacteria;Gammaproteobacteria;unclassified Gammaproteobacteria;Candidatus Competibacteraceae;Candidatus Competibacter;Candidatus Competibacter denitrificans;Candidatus Competibacter denitrificans Run_A_D11 |
| Ga0586324_1620178_219_989 | 0.00 | 0.00 | 0.00 | 0.00 | 0.00 | 0.00 | 0.00 | 0.00 | 0.00 | 699447.98 | 0.00 | 0.00 | KO:K11645 | class I fructose-bisphosphate aldolase | Bacteria;Chloroflexi;Ktedonobacteria;Ktedonobacterales;Ktedonobacteraceae;Ktedonobacter;Ktedonobacter racemifer;Ktedonobacter racemifer SOSP1-21, DSM 44963 |
| Ga0586324_1648092_3_965 | 0.00 | 0.00 | 0.00 | 0.00 | 2976700.00 | 0.00 | 0.00 | 6100000.00 | 0.00 | 0.00 | 0.00 | 0.00 | KO:K11645 | class I fructose-bisphosphate aldolase | Bacteria;Armatimonadetes;Fimbriimonadia;Fimbriimonadales;Fimbriimonadaceae;Fimbriimonas;Fimbriimonas ginsengisoli;Fimbriimonas ginsengisoli Gsoil 348 |
| Ga0586324_0006346_10821_11840 | 0.00 | 0.00 | 0.00 | 0.00 | 0.00 | 0.00 | 0.00 | 0.00 | 1312200.00 | 0.00 | 0.00 | 0.00 | KO:K11688 | C4-dicarboxylate-binding protein DctP | Bacteria;Proteobacteria;Betaproteobacteria;Nitrosomonadales;Gallionellaceae;Gallionella;Gallionella capsiferriformans;Gallionella capsiferriformans ES-2 |
| Ga0586324_0063675_3406_4254 | 0.00 | 0.00 | 7037500.00 | 0.00 | 0.00 | 7423900.00 | 0.00 | 0.00 | 0.00 | 0.00 | 0.00 | 0.00 | KO:K11688 | C4-dicarboxylate-binding protein DctP | Bacteria;Proteobacteria;Alphaproteobacteria;Hyphomicrobiales;Bradyrhizobiaceae;Bradyrhizobium;Bradyrhizobium lablabi;Bradyrhizobium lablabi GAS499 |
| Ga0586324_0073168_1389_2396 | 2980000.00 | 0.00 | 0.00 | 0.00 | 0.00 | 0.00 | 0.00 | 0.00 | 0.00 | 0.00 | 0.00 | 0.00 | KO:K11688 | C4-dicarboxylate-binding protein DctP | Bacteria;Proteobacteria;Betaproteobacteria;Rhodocyclales;Rhodocyclaceae;Aromatoleum;Aromatoleum toluclasticum;Aromatoleum toluclasticum ATCC 700605 |
| Ga0586324_0367147_1170_1877 | 0.00 | 0.00 | 0.00 | 0.00 | 0.00 | 510355.41 | 0.00 | 0.00 | 0.00 | 0.00 | 0.00 | 0.00 | KO:K11688 | C4-dicarboxylate-binding protein DctP | Bacteria;Proteobacteria;Alphaproteobacteria;Hyphomicrobiales;Bradyrhizobiaceae;Bradyrhizobium;Bradyrhizobium sp. cf659;Bradyrhizobium sp. CF659 |
| Ga0586324_0411088_796_1788 | 0.00 | 0.00 | 0.00 | 0.00 | 0.00 | 0.00 | 0.00 | 1457900.00 | 0.00 | 0.00 | 0.00 | 0.00 | KO:K11688 | C4-dicarboxylate-binding protein DctP | Bacteria;Proteobacteria;Betaproteobacteria;Burkholderiales;Burkholderiaceae;Paraburkholderia;Paraburkholderia mimosarum;Paraburkholderia mimosarum STM3621 |
| Ga0586324_1713552_2_334 | 0.00 | 0.00 | 0.00 | 0.00 | 0.00 | 0.00 | 0.00 | 0.00 | 0.00 | 0.00 | 0.00 | 2925000.00 | KO:K11688 | C4-dicarboxylate-binding protein DctP | Bacteria;Proteobacteria;Alphaproteobacteria;Hyphomicrobiales;Bradyrhizobiaceae;Bradyrhizobium;Bradyrhizobium lablabi;Bradyrhizobium lablabi CCBAU 23086 |
| Ga0586324_6849801_3_359 | 0.00 | 0.00 | 0.00 | 0.00 | 0.00 | 0.00 | 0.00 | 0.00 | 0.00 | 0.00 | 0.00 | 1627500.00 | KO:K11711 | two-component system sensor histidine kinase DctS | Bacteria;Proteobacteria;Betaproteobacteria;Neisseriales;Neisseriaceae;Vitreoscilla;Vitreoscilla filiformis;Vitreoscilla filiformis ATCC 15551 |
| Ga0586324_0118670_1_2709 | 0.00 | 816000.00 | 0.00 | 0.00 | 0.00 | 429362.76 | 0.00 | 0.00 | 3094200.00 | 0.00 | 0.00 | 0.00 | KO:K11751 | 5'-nucleotidase/UDP-sugar diphosphatase | Bacteria;Firmicutes;Clostridia;Halanaerobiales;unclassified Halanaerobiales;Anoxybacter;Anoxybacter fermentans;Anoxybacter fermentans DY22613 |
| Ga0586324_2570386_101_811 | 0.00 | 0.00 | 0.00 | 0.00 | 0.00 | 0.00 | 0.00 | 0.00 | 0.00 | 0.00 | 1029600.00 | 0.00 | KO:K11751 | 5'-nucleotidase/UDP-sugar diphosphatase | Bacteria;Actinobacteria;Actinomycetia;Propionibacteriales;Nocardioidaceae;Nocardioides;Nocardioides soli;Nocardioides soli DSM 105498 |
| Ga0586324_0000398_13718_15205 | 0.00 | 3583600.00 | 0.00 | 5643000.00 | 6622900.00 | 0.00 | 0.00 | 0.00 | 0.00 | 2117500.00 | 0.00 | 0.00 | KO:K11900 | type VI secretion system protein ImpC | Bacteria;Proteobacteria;Deltaproteobacteria;Desulfobacterales;Desulfobacteraceae;Desulfospira;Desulfospira joergensenii;Desulfospira joergensenii DSM 10085 |
| Ga0586324_0003221_10478_11965 | 0.00 | 0.00 | 0.00 | 0.00 | 991572.76 | 0.00 | 0.00 | 1897100.00 | 4600800.00 | 0.00 | 11176000.00 | 6772500.00 | KO:K11900 | type VI secretion system protein ImpC | Bacteria;Acidobacteria;Acidobacteriia;Bryobacterales;Solibacteraceae;Candidatus Solibacter;Candidatus Solibacter usitatus;Candidatus Solibacter usitatus Ellin6076 |
| Ga0586324_0032354_937_2418 | 0.00 | 0.00 | 0.00 | 0.00 | 0.00 | 0.00 | 5084400.00 | 0.00 | 0.00 | 0.00 | 0.00 | 4237500.00 | KO:K11900 | type VI secretion system protein ImpC | Bacteria;Proteobacteria;Gammaproteobacteria;Chromatiales;Ectothiorhodospiraceae;Thioalbus;Thioalbus denitrificans;Thioalbus denitrificans DSM 26407 |
| Ga0586324_0055244_699_2192 | 0.00 | 0.00 | 0.00 | 0.00 | 0.00 | 0.00 | 0.00 | 0.00 | 0.00 | 395836.98 | 0.00 | 1612500.00 | KO:K11900 | type VI secretion system protein ImpC | Bacteria;Proteobacteria;Gammaproteobacteria;Pseudomonadales;Pseudomonadaceae;Pseudomonas;Pseudomonas sp. RIT-PI-r;Pseudomonas sp. RIT-PI-r |
| Ga0586324_0064448_1308_2810 | 5130000.00 | 0.00 | 0.00 | 0.00 | 0.00 | 0.00 | 0.00 | 0.00 | 0.00 | 0.00 | 3863200.00 | 0.00 | KO:K11900 | type VI secretion system protein ImpC | Bacteria;Proteobacteria;Deltaproteobacteria;Desulfuromonadales;Geobacteraceae;Geobacter;Geobacter sp. DSM 2909;Geobacter sp. DSM 2909 |
| Ga0586324_0132873_1_924 | 0.00 | 0.00 | 0.00 | 0.00 | 0.00 | 0.00 | 0.00 | 0.00 | 0.00 | 0.00 | 1707200.00 | 0.00 | KO:K11900 | type VI secretion system protein ImpC | Bacteria;Acidobacteria;Acidobacteriia;Acidobacteriales;Acidobacteriaceae;Granulicella;Granulicella mallensis;Granulicella mallensis X5P3 |
| Ga0586324_0158948_653_2140 | 0.00 | 2067200.00 | 3118226.17 | 1111500.00 | 0.00 | 0.00 | 0.00 | 0.00 | 0.00 | 0.00 | 0.00 | 0.00 | KO:K11900 | type VI secretion system protein ImpC | Bacteria;Acidobacteria;Acidobacteriia;Acidobacteriales;Acidobacteriaceae;unclassified Acidobacteriaceae;Acidobacteriaceae bacterium URHE0068;Acidobacteriaceae bacterium URHE0068 |
| Ga0586324_0903985_2_1267 | 0.00 | 0.00 | 7206400.00 | 0.00 | 0.00 | 0.00 | 0.00 | 0.00 | 0.00 | 0.00 | 0.00 | 0.00 | KO:K11900 | type VI secretion system protein ImpC | Bacteria;Acidobacteria;Acidobacteriia;Acidobacteriales;Acidobacteriaceae;Granulicella;Granulicella mallensis;Granulicella mallensis X5P3 |
| Ga0586324_0027616_4196_4711 | 0.00 | 1693200.00 | 0.00 | 0.00 | 0.00 | 0.00 | 0.00 | 0.00 | 0.00 | 0.00 | 0.00 | 0.00 | KO:K11901 | type VI secretion system protein ImpB | Bacteria;Proteobacteria;Gammaproteobacteria;Chromatiales;Chromatiaceae;Thiocapsa;Thiocapsa rosea;Thiocapsa rosea DSM 235 |
| Ga0586324_1121837_456_965 | 0.00 | 0.00 | 0.00 | 0.00 | 0.00 | 1390452.80 | 0.00 | 0.00 | 0.00 | 0.00 | 0.00 | 0.00 | KO:K11901 | type VI secretion system protein ImpB | Bacteria;Acidobacteria;Acidobacteriia;Acidobacteriales;Acidobacteriaceae;unclassified Acidobacteriaceae;Acidobacteriaceae bacterium URHE0068;Acidobacteriaceae bacterium URHE0068 |
| Ga0586324_3938457_3_476 | 0.00 | 0.00 | 0.00 | 0.00 | 0.00 | 0.00 | 0.00 | 0.00 | 0.00 | 0.00 | 1416800.00 | 0.00 | KO:K11901 | type VI secretion system protein ImpB | Bacteria;Proteobacteria;Gammaproteobacteria;Chromatiales;unclassified Chromatiales;unclassified Chromatiales;unclassified Chromatiales;Chromatiales bacterium |
| Ga0586324_0000924_17015_17512 | 0.00 | 0.00 | 0.00 | 0.00 | 0.00 | 7503300.00 | 0.00 | 0.00 | 0.00 | 0.00 | 0.00 | 0.00 | KO:K11903 | type VI secretion system secreted protein Hcp | Bacteria;Proteobacteria;Deltaproteobacteria;Desulfuromonadales;Geobacteraceae;Geobacter;Geobacter sp. DSM 2909;Geobacter sp. DSM 2909 |
| Ga0586324_0388181_1278_1832 | 0.00 | 0.00 | 0.00 | 0.00 | 0.00 | 0.00 | 0.00 | 0.00 | 0.00 | 0.00 | 0.00 | 355005.00 | KO:K11903 | type VI secretion system secreted protein Hcp | Bacteria;Proteobacteria;Betaproteobacteria;Burkholderiales;Comamonadaceae;Schlegelella;Schlegelella thermodepolymerans;Schlegelella thermodepolymerans DSM 15344 |
| Ga0586324_0286849_3_1916 | 0.00 | 0.00 | 0.00 | 1966500.00 | 0.00 | 0.00 | 0.00 | 0.00 | 0.00 | 0.00 | 0.00 | 0.00 | KO:K11904 | type VI secretion system secreted protein VgrG | Bacteria;Proteobacteria;Gammaproteobacteria;Chromatiales;Chromatiaceae;Thiocystis;Thiocystis violascens;Thiocystis violascens 611, DSM 198 |
| Ga0586324_0000314_28291_31014 | 0.00 | 0.00 | 0.00 | 0.00 | 0.00 | 0.00 | 0.00 | 0.00 | 0.00 | 0.00 | 6793600.00 | 0.00 | KO:K11907 | type VI secretion system protein VasG | Bacteria;Proteobacteria;Gammaproteobacteria;Methylococcales;Methylococcaceae;Methylocaldum;Methylocaldum szegediense;Methylocaldum szegediense O-12 |
| Ga0586324_0000402_34942_36768 | 0.00 | 0.00 | 0.00 | 0.00 | 0.00 | 0.00 | 0.00 | 0.00 | 0.00 | 0.00 | 0.00 | 8025000.00 | KO:K11907 | type VI secretion system protein VasG | Bacteria;Acidobacteria;Vicinamibacteria;unclassified Vicinamibacteria;Vicinamibacteraceae;Luteitalea;Luteitalea pratensis;Luteitalea pratensis DSM 100886 |
| Ga0586324_0003221_1276_3942 | 0.00 | 0.00 | 3742080.84 | 0.00 | 0.00 | 0.00 | 0.00 | 0.00 | 0.00 | 0.00 | 0.00 | 0.00 | KO:K11907 | type VI secretion system protein VasG | Bacteria;Acidobacteria;Acidobacteriia;Bryobacterales;Solibacteraceae;Candidatus Solibacter;Candidatus Solibacter usitatus;Candidatus Solibacter usitatus Ellin6076 |
| Ga0586324_0602564_3_884 | 0.00 | 0.00 | 0.00 | 0.00 | 0.00 | 0.00 | 0.00 | 5764500.00 | 0.00 | 0.00 | 0.00 | 0.00 | KO:K11907 | type VI secretion system protein VasG | Bacteria;Proteobacteria;Alphaproteobacteria;Hyphomicrobiales;Bradyrhizobiaceae;Bradyrhizobium;Bradyrhizobium sp. WSM1417;Bradyrhizobium sp. WSM1417 |
| Ga0586324_2256493_1_858 | 0.00 | 0.00 | 0.00 | 0.00 | 0.00 | 0.00 | 0.00 | 0.00 | 899100.00 | 0.00 | 0.00 | 0.00 | KO:K11907 | type VI secretion system protein VasG | Bacteria;Proteobacteria;Alphaproteobacteria;Rhodospirillales;Elioraeaceae;Elioraea;Elioraea tepidiphila;Elioraea tepidiphila DSM 17972 |
| Ga0586324_0009712_9258_10328 | 0.00 | 0.00 | 0.00 | 0.00 | 0.00 | 0.00 | 0.00 | 1744600.00 | 0.00 | 0.00 | 0.00 | 990000.00 | KO:K11959 | urea transport system substrate-binding protein | Bacteria;Proteobacteria;Betaproteobacteria;Burkholderiales;Burkholderiaceae;Caballeronia;Caballeronia udeis;Caballeronia udeis ES_FE-B12 |
| Ga0586324_0017341_3155_4474 | 242471.00 | 4161600.00 | 4767996.33 | 0.00 | 0.00 | 4724300.00 | 5107200.00 | 559657.31 | 3920400.00 | 5674900.00 | 0.00 | 4005000.00 | KO:K11959 | urea transport system substrate-binding protein | Bacteria;Proteobacteria;Alphaproteobacteria;Hyphomicrobiales;Bradyrhizobiaceae;Bradyrhizobium;Bradyrhizobium lablabi;Bradyrhizobium lablabi GAS499 |
| Ga0586324_0029578_1982_3415 | 0.00 | 0.00 | 0.00 | 0.00 | 0.00 | 0.00 | 0.00 | 59147.86 | 0.00 | 0.00 | 0.00 | 0.00 | KO:K11959 | urea transport system substrate-binding protein | Bacteria;Proteobacteria;Alphaproteobacteria;Hyphomicrobiales;Xanthobacteraceae;Pseudolabrys;Pseudolabrys taiwanensis;Pseudolabrys taiwanensis CC-BB4 |

| | | | | | | | | | | | | | | | |
|---|---|---|---|---|---|---|---|---|---|---|---|---|---|---|---|
| Ga0586324 2186811 3 869 | 496231.00 | 0.00 | 0.00 | 0.00 | 0.00 | 0.00 | 0.00 | 0.00 | 0.00 | 0.00 | 0.00 | 0.00 | KO:K11959 | urea transport system substrate-binding protein | Bacteria;Proteobacteria;Alphaproteobacteria;Hyphomicrobiales;Reyranellaceae;Reyranella;Reyranella massiliensis;Reyranella massiliensis 521, DSM 23428 |
| Ga0586324 4659583 2 634 | 0.00 | 0.00 | 0.00 | 0.00 | 0.00 | 0.00 | 0.00 | 0.00 | 0.00 | 0.00 | 0.00 | 825000.00 | KO:K11959 | urea transport system substrate-binding protein | Bacteria;Proteobacteria;Alphaproteobacteria;Hyphomicrobiales;Phyllobacteriaceae;Zhengella;Zhengella mangrovi;Zhengella mangrovi X9-2-2 |
| Ga0586324 1343858 29 721 | 0.00 | 2924000.00 | 0.00 | 0.00 | 0.00 | 0.00 | 0.00 | 0.00 | 0.00 | 0.00 | 0.00 | 0.00 | KO:K11963 | urea transport system ATP-binding protein | Bacteria;Proteobacteria;Betaproteobacteria;Rhodocyclales;Rhodocyclaceae;Niveibacterium;Niveibacterium umoris;Niveibacterium umoris DSM 106739 |
| Ga0586324 0000078 41484 43568 | 0.00 | 0.00 | 0.00 | 0.00 | 0.00 | 0.00 | 365415.60 | 0.00 | 0.00 | 0.00 | 0.00 | 0.00 | KO:K12132 | HAMP domain-containing protein | Bacteria;Proteobacteria;Gammaproteobacteria;Chromatiales;Wenzhouxiangellaceae;Wenzhouxiangella;Wenzhouxiangella sediminis;Wenzhouxiangella sediminis XDB06 |
| Ga0586324 1918243 3 920 | 0.00 | 0.00 | 0.00 | 0.00 | 0.00 | 0.00 | 0.00 | 0.00 | 3142800.00 | 3572800.00 | 0.00 | 0.00 | KO:K12132 | hypothetical protein | Bacteria;Planctomycetes;Planctomycetia;Isosphaerales;Isosphaeraceae;Singulisphaera;Singulisphaera sp. GP187;Singulisphaera sp. GP187 |
| Ga0586324 4716551 3 533 | 0.00 | 2257600.00 | 0.00 | 0.00 | 0.00 | 0.00 | 0.00 | 0.00 | 0.00 | 0.00 | 0.00 | 0.00 | KO:K12261 | 2-hydroxyacyl-CoA lyase 1 | Bacteria;Proteobacteria;Alphaproteobacteria;Rhodospirillales;Acetobacteraceae;Rhodopila;Rhodopila globiformis;Rhodopila globiformis DSM 161 |
| Ga0586324 0380764 1 1692 | 0.00 | 0.00 | 0.00 | 0.00 | 0.00 | 0.00 | 0.00 | 0.00 | 0.00 | 0.00 | 0.00 | 1260000.00 | KO:K12282 | MSHA biogenesis protein MshL | Bacteria;Proteobacteria;Betaproteobacteria;Nitrosomonadales;Gallionellaceae;Ferriphaselus;Ferriphaselus amnicola;Ferriphaselus amnicola OYT1 |
| Ga0586324 0000003 54335 55699 | 0.00 | 0.00 | 0.00 | 0.00 | 0.00 | 0.00 | 2242535.04 | 16372400.00 | 5961600.00 | 32832800.00 | 31416000.00 | 33892500.00 | KO:K12340 | outer membrane protein | Bacteria;Proteobacteria;Betaproteobacteria;Nitrosomonadales;Thiobacillaceae;Annwoodia;Annwoodia aquaesulis;Annwoodia aquaesulis DSM 4255 |
| Ga0586324 0000004 142184 143491 | 0.00 | 0.00 | 0.00 | 0.00 | 0.00 | 0.00 | 0.00 | 0.00 | 0.00 | 1362900.00 | 1372800.00 | 1942500.00 | KO:K12340 | outer membrane protein | Bacteria;Proteobacteria;Betaproteobacteria;Nitrosomonadales;Thiobacillaceae;Annwoodia;Annwoodia aquaesulis;Annwoodia aquaesulis DSM 4255 |
| Ga0586324 0023208 2962 4410 | 0.00 | 0.00 | 0.00 | 0.00 | 0.00 | 0.00 | 0.00 | 0.00 | 0.00 | 6491100.00 | 0.00 | 0.00 | KO:K12340 | outer membrane protein | Bacteria;Proteobacteria;Alphaproteobacteria;Hyphomicrobiales;Bradyrhizobiaceae;Bradyrhizobium;Bradyrhizobium erythrophlei;Bradyrhizobium erythrophlei GAS242 |
| Ga0586324 0108778 222 1562 | 0.00 | 0.00 | 0.00 | 0.00 | 0.00 | 0.00 | 0.00 | 0.00 | 0.00 | 0.00 | 0.00 | 1432500.00 | KO:K12340 | outer membrane protein | Bacteria;Proteobacteria;Betaproteobacteria;Nitrosomonadales;Sulfuricellaceae;Sulfuricella;Sulfuricella sp. T08;Sulfuricella sp. T08 |
| Ga0586324 0515485 1 1110 | 0.00 | 0.00 | 0.00 | 0.00 | 0.00 | 0.00 | 0.00 | 0.00 | 0.00 | 0.00 | 0.00 | 363114.00 | KO:K12340 | outer membrane protein | Bacteria;Acidobacteria;Acidobacteriia;Bryobacterales;Solibacteraceae;Candidatus Solibacter;Candidatus Solibacter usitatus;Candidatus Solibacter usitatus Ellin6076 |
| Ga0586324 0626403 148 1485 | 0.00 | 0.00 | 0.00 | 0.00 | 0.00 | 0.00 | 0.00 | 3068300.00 | 0.00 | 2387000.00 | 0.00 | 0.00 | KO:K12340 | outer membrane protein | Bacteria;Proteobacteria;Alphaproteobacteria;Hyphomicrobiales;Bradyrhizobiaceae;Bradyrhizobium;Bradyrhizobium sp. KBS0725;Bradyrhizobium sp. KBS0725 |
| Ga0586324 0671718 3 1277 | 0.00 | 0.00 | 0.00 | 0.00 | 0.00 | 0.00 | 0.00 | 0.00 | 0.00 | 0.00 | 0.00 | 7222500.00 | KO:K12340 | outer membrane protein | Bacteria;Proteobacteria;Betaproteobacteria;Nitrosomonadales;Sulfuricellaceae;Sulfuricella;Sulfuricella sp. T08;Sulfuricella sp. T08 |
| Ga0586324 3287222 1 507 | 0.00 | 0.00 | 0.00 | 0.00 | 0.00 | 0.00 | 0.00 | 0.00 | 0.00 | 0.00 | 0.00 | 2400000.00 | KO:K12340 | outer membrane protein | Bacteria;Proteobacteria;Betaproteobacteria;Nitrosomonadales;Thiobacillaceae;Thiobacillus;Thiobacillus denitrificans;Thiobacillus denitrificans DSM 12475 |
| Ga0586324 4075130 3 671 | 0.00 | 0.00 | 0.00 | 0.00 | 0.00 | 4009700.00 | 0.00 | 0.00 | 0.00 | 0.00 | 0.00 | 0.00 | KO:K12340 | outer membrane protein | Bacteria;Proteobacteria;Deltaproteobacteria;Syntrophobacterales;Syntrophaceae;Desulfobacca;Desulfobacca acetoxidans;Desulfobacca acetoxidans ASRB2, DSM 11109 |
| Ga0586324 0110526 1307 3079 | 0.00 | 0.00 | 0.00 | 0.00 | 0.00 | 0.00 | 0.00 | 0.00 | 0.00 | 0.00 | 0.00 | 281208.75 | KO:K12507 | acyl-CoA synthetase | Bacteria;Proteobacteria;Alphaproteobacteria;Hyphomicrobiales;Hyphomicrobiaceae;Rhodoplanes;Rhodoplanes sp. Z2-YC6860;Rhodoplanes sp. Z2-YC6860 |
| Ga0586324 0000011 3246 5447 | 0.00 | 0.00 | 0.00 | 0.00 | 0.00 | 0.00 | 0.00 | 89527.26 | 0.00 | 0.00 | 0.00 | 0.00 | KO:K12541 | ATP-binding cassette subfamily C protein LapB | Bacteria;Proteobacteria;Betaproteobacteria;Nitrosomonadales;Thiobacillaceae;Annwoodia;Annwoodia aquaesulis;Annwoodia aquaesulis DSM 4255 |
| Ga0586324 0000652 22780 24699 | 0.00 | 0.00 | 0.00 | 0.00 | 0.00 | 0.00 | 0.00 | 19373600.00 | 8215791.12 | 40748400.00 | 25810400.00 | 48735651.00 | KO:K12543 | adhesin transport system outer membrane protein | Bacteria;Proteobacteria;Betaproteobacteria;Nitrosomonadales;Thiobacillaceae;Annwoodia;Annwoodia aquaesulis;Annwoodia aquaesulis DSM 4255 |
| Ga0586324 2331687 2 502 | 0.00 | 0.00 | 0.00 | 0.00 | 1112400.00 | 0.00 | 0.00 | 0.00 | 0.00 | 0.00 | 0.00 | 0.00 | KO:K12543 | adhesin transport system outer membrane protein | Bacteria;Proteobacteria;Alphaproteobacteria;Hyphomicrobiales;Phyllobacteriaceae;Mesorhizobium;Mesorhizobium sp. Pch-S;Mesorhizobium sp. Pch-S |
| Ga0586324 0221029 3 1010 | 205948.00 | 0.00 | 0.00 | 550053.80 | 0.00 | 0.00 | 0.00 | 239283.48 | 0.00 | 0.00 | 0.00 | 0.00 | KO:K12574 | ribonuclease J | Archaea;Euryarchaeota;Methanomicrobia;Methanosarcinales;Methanotrichaceae;Methanothrix;Methanothrix soehngenii;Methanothrix soehngenii GP-6 |
| Ga0586324 0253183 1 438 | 0.00 | 0.00 | 0.00 | 0.00 | 0.00 | 0.00 | 0.00 | 234721.90 | 0.00 | 0.00 | 0.00 | 0.00 | KO:K12574 | ribonuclease J | Archaea;Euryarchaeota;Methanomicrobia;Methanosarcinales;Methanotrichaceae;Methanothrix;Methanothrix soehngenii;Methanothrix soehngenii GP-6 |
| Ga0586324 1886349 42 926 | 0.00 | 0.00 | 0.00 | 98577.70 | 0.00 | 0.00 | 0.00 | 0.00 | 0.00 | 0.00 | 0.00 | 0.00 | KO:K12574 | ribonuclease J | Archaea;Euryarchaeota;Methanomicrobia;Methanosarcinales;Methanotrichaceae;Methanothrix;Methanothrix soehngenii;Methanothrix soehngenii GP-6 |
| Ga0586324 0065518 2378 3349 | 0.00 | 1183200.00 | 3148712.62 | 2489000.00 | 1503800.00 | 0.00 | 0.00 | 671000.00 | 1960200.00 | 0.00 | 0.00 | 0.00 | KO:K12686 | outer membrane lipase/esterase | Bacteria;Proteobacteria;Betaproteobacteria;Burkholderiales;unclassified Burkholderiales;Rhizobacter;Rhizobacter sp. Root404;Rhizobacter sp. Root404 |
| Ga0586324 0835308 110 1312 | 0.00 | 0.00 | 0.00 | 0.00 | 0.00 | 0.00 | 0.00 | 0.00 | 0.00 | 0.00 | 0.00 | 1162500.00 | KO:K13015 | UDP-N-acetyl-D-glucosamine dehydrogenase | Bacteria;Proteobacteria;Deltaproteobacteria;Syntrophorhabdales;Syntrophorhabdaceae;Syntrophorhabdus;Syntrophorhabdus aromaticivorans;Syntrophorhabdus aromaticivorans UI |
| Ga0586324 0034865 2658 3533 | 5500000.00 | 0.00 | 7600500.00 | 5671500.00 | 0.00 | 0.00 | 0.00 | 0.00 | 0.00 | 0.00 | 0.00 | 4777500.00 | KO:K13075 | N-acyl homoserine lactone hydrolase | Bacteria;Proteobacteria;Alphaproteobacteria;Hyphomicrobiales;Reyranellaceae;Reyranella;Reyranella sp. OAS946;Reyranella sp. OAS946 |
| Ga0586324 0434970 525 1397 | 599554.00 | 0.00 | 0.00 | 0.00 | 0.00 | 0.00 | 0.00 | 0.00 | 0.00 | 0.00 | 1812800.00 | 0.00 | KO:K13075 | N-acyl homoserine lactone hydrolase | Bacteria;Proteobacteria;Betaproteobacteria;Burkholderiales;Alcaligenaceae;Achromobacter;Achromobacter sp. MYb9;Achromobacter sp. MYb9 |
| Ga0586324 0000519 7515 7823 | 0.00 | 0.00 | 0.00 | 2755000.00 | 0.00 | 0.00 | 0.00 | 0.00 | 0.00 | 0.00 | 0.00 | 0.00 | KO:K13195 | cold-inducible RNA-binding protein | Bacteria;Proteobacteria;Deltaproteobacteria;Myxococcales;Sandaracinaceae;Sandaracinus;Sandaracinus amylolyticus;Sandaracinus amylolyticus DSM 53668 |
| Ga0586324 0002885 7498 7887 | 0.00 | 7480000.00 | 0.00 | 0.00 | 7848600.00 | 13021600.00 | 0.00 | 902800.00 | 0.00 | 0.00 | 0.00 | 0.00 | KO:K13195 | cold-inducible RNA-binding protein | Bacteria;Armatimonadetes;Armatimonadia;Capsulimonadales;Capsulimonadaceae;Capsulimonas;Capsulimonas corticalis;Capsulimonas corticalis AX-7 |
| Ga0586324 0258146 731 1093 | 0.00 | 0.00 | 0.00 | 0.00 | 0.00 | 0.00 | 0.00 | 132853.73 | 0.00 | 0.00 | 0.00 | 0.00 | KO:K13195 | cold-inducible RNA-binding protein | Bacteria;Proteobacteria;Deltaproteobacteria;Syntrophobacterales;Syntrophobacteraceae;Syntrophobacter;Syntrophobacter fumaroxidans;Syntrophobacter fumaroxidans MPOB |
| Ga0586324 1060368 691 1020 | 1710000.00 | 0.00 | 0.00 | 0.00 | 0.00 | 3262093.42 | 0.00 | 3226900.00 | 0.00 | 0.00 | 0.00 | 0.00 | KO:K13195 | cold-inducible RNA-binding protein | Bacteria;Proteobacteria;Deltaproteobacteria;Myxococcales;Sandaracinaceae;Sandaracinus;Sandaracinus amylolyticus;Sandaracinus amylolyticus DSM 53668 |
| Ga0586324 1255818 440 805 | 0.00 | 1196800.00 | 0.00 | 0.00 | 0.00 | 0.00 | 0.00 | 0.00 | 0.00 | 0.00 | 0.00 | 0.00 | KO:K13195 | cold-inducible RNA-binding protein | Bacteria;Planctomycetes;Planctomycetia;Planctomycetales;Planctomycetaceae;Planctomyces;Planctomyces sp. SH-PL14;Planctomyces sp. SH-PL14 |
| Ga0586324 1639625 187 558 | 0.00 | 5222400.00 | 0.00 | 0.00 | 0.00 | 0.00 | 0.00 | 0.00 | 0.00 | 0.00 | 0.00 | 0.00 | KO:K13195 | cold-inducible RNA-binding protein | Bacteria;Proteobacteria;Gammaproteobacteria;Chromatiales;Chromatiaceae;Lamprocystis;Lamprocystis purpurea;Lamprocystis purpurea DSM 4197 |
| Ga0586324 2504379 260 580 | 0.00 | 0.00 | 0.00 | 0.00 | 0.00 | 0.00 | 0.00 | 0.00 | 0.00 | 0.00 | 0.00 | 5332500.00 | KO:K13195 | cold-inducible RNA-binding protein | Bacteria;Proteobacteria;Deltaproteobacteria;Myxococcales;Sandaracinaceae;Sandaracinus;Sandaracinus amylolyticus;Sandaracinus amylolyticus DSM 53668 |
| Ga0586324 4003122 233 559 | 0.00 | 4569600.00 | 4808667.45 | 3743000.00 | 4006700.00 | 2497717.56 | 0.00 | 2110600.00 | 0.00 | 1301300.00 | 1513600.00 | 0.00 | KO:K13195 | cold-inducible RNA-binding protein | Bacteria;Proteobacteria;Deltaproteobacteria;Myxococcales;Sandaracinaceae;Sandaracinus;Sandaracinus amylolyticus;Sandaracinus amylolyticus DSM 53668 |
| Ga0586324 6620345 23 382 | 0.00 | 2985200.00 | 0.00 | 0.00 | 0.00 | 0.00 | 0.00 | 0.00 | 0.00 | 0.00 | 0.00 | 0.00 | KO:K13195 | cold-inducible RNA-binding protein | Bacteria;Proteobacteria;Gammaproteobacteria;Nevskiales;Steroidobacteraceae;Povalibacter;Povalibacter uvarum;Povalibacter uvarum DSM 26723 |
| Ga0586324 0047409 2338 3483 | 0.00 | 0.00 | 0.00 | 0.00 | 0.00 | 0.00 | 340269.48 | 0.00 | 210310.02 | 0.00 | 0.00 | 0.00 | KO:K13290 | aspartate aminotransferase-like enzyme | Bacteria;Proteobacteria;Deltaproteobacteria;Syntrophobacterales;Syntrophaceae;Desulfobacca;Desulfobacca acetoxidans;Desulfobacca acetoxidans ASRB2, DSM 11109 |
| Ga0586324 0441336 944 1732 | 0.00 | 0.00 | 0.00 | 1643500.00 | 0.00 | 0.00 | 258547.44 | 0.00 | 158214.87 | 0.00 | 0.00 | 0.00 | KO:K13290 | aspartate aminotransferase-like enzyme | Bacteria;Proteobacteria;Deltaproteobacteria;Syntrophobacterales;Syntrophaceae;Desulfobacca;Desulfobacca acetoxidans;Desulfobacca acetoxidans ASRB2, DSM 11109 |
| Ga0586324 0105833 1829 2782 | 0.00 | 0.00 | 0.00 | 0.00 | 0.00 | 0.00 | 0.00 | 3220800.00 | 0.00 | 0.00 | 0.00 | 0.00 | KO:K13444 | sulfatase modifying factor 1 | Bacteria;Proteobacteria;Gammaproteobacteria;Thiotrichales;Thiotrichaceae;Achromatium;uncultured Achromatium sp.;Achromatium sp. Cell8 |
| Ga0586324 3623233 2 703 | 0.00 | 1917600.00 | 0.00 | 0.00 | 0.00 | 0.00 | 0.00 | 0.00 | 0.00 | 0.00 | 0.00 | 0.00 | KO:K13444 | sulfatase modifying factor 1 | Bacteria;Planctomycetes;Planctomycetia;Pirellulales;Pirellulaceae;Blastopirellula;Blastopirellula cremea;Blastopirellula cremea HEX-1 MGV |
| Ga0586324 0013466 1 1764 | 6110000.00 | 5256400.00 | 0.00 | 3990000.00 | 0.00 | 0.00 | 0.00 | 5337500.00 | 931500.00 | 6290900.00 | 7031200.00 | 1777500.00 | KO:K13525 | transitional endoplasmic reticulum ATPase | Archaea;Euryarchaeota;Methanomicrobia;Methanosarcinales;Methanotrichaceae;Methanothrix;Methanothrix soehngenii;Methanothrix soehngenii GP-6 |
| Ga0586324 0317319 189 2003 | 0.00 | 795600.00 | 0.00 | 0.00 | 0.00 | 0.00 | 0.00 | 399032.72 | 0.00 | 0.00 | 0.00 | 0.00 | KO:K13525 | transitional endoplasmic reticulum ATPase | Archaea;Euryarchaeota;Methanomicrobia;Methanosarcinales;Methanotrichaceae;Methanothrix;Methanothrix soehngenii;Methanothrix soehngenii GP-6 |
| Ga0586324 0593164 1 576 | 0.00 | 0.00 | 0.00 | 0.00 | 2729500.00 | 0.00 | 0.00 | 0.00 | 0.00 | 0.00 | 0.00 | 0.00 | KO:K13525 | transitional endoplasmic reticulum ATPase | Archaea;Euryarchaeota;Methanomicrobia;Methanosarcinales;Methanotrichaceae;Methanothrix;Methanothrix soehngenii;Methanothrix soehngenii GP-6 |
| Ga0586324 0834648 3 1268 | 0.00 | 78758.28 | 0.00 | 1140000.00 | 0.00 | 0.00 | 0.00 | 0.00 | 1206900.00 | 0.00 | 0.00 | 0.00 | KO:K13525 | transitional endoplasmic reticulum ATPase | Archaea;Euryarchaeota;Methanomicrobia;Methanosarcinales;Methanotrichaceae;Methanothrix;Methanothrix soehngenii;Methanothrix soehngenii GP-6 |
| Ga0586324 1255573 1 1101 | 0.00 | 0.00 | 0.00 | 0.00 | 0.00 | 0.00 | 0.00 | 0.00 | 0.00 | 8855000.00 | 0.00 | 0.00 | KO:K13525 | transitional endoplasmic reticulum ATPase | Archaea;Euryarchaeota;Methanomicrobia;Methanosarcinales;Methanosarcinaceae;Methanosarcina;Methanosarcina siciliae;Methanosarcina siciliae HI350 |
| Ga0586324 1460358 1 1032 | 9180000.00 | 0.00 | 0.00 | 0.00 | 0.00 | 0.00 | 0.00 | 0.00 | 192756.51 | 0.00 | 0.00 | 0.00 | KO:K13525 | transitional endoplasmic reticulum ATPase | Archaea;Euryarchaeota;Methanomicrobia;Methanosarcinales;Methanotrichaceae;Methanothrix;Methanothrix soehngenii;Methanothrix soehngenii GP-6 |
| Ga0586324 1996757 1 903 | 0.00 | 0.00 | 2087361.91 | 0.00 | 0.00 | 0.00 | 0.00 | 0.00 | 0.00 | 0.00 | 0.00 | 0.00 | KO:K13525 | transitional endoplasmic reticulum ATPase | Archaea;unclassified Archaea;unclassified Archaea;unclassified Archaea;Thermofilaceae;Thermofilum;Thermofilum pendens;Thermofilum pendens Hrk 5 |
| Ga0586324 2346188 1 843 | 0.00 | 11628000.00 | 0.00 | 0.00 | 0.00 | 0.00 | 0.00 | 0.00 | 0.00 | 0.00 | 0.00 | 0.00 | KO:K13525 | transitional endoplasmic reticulum ATPase | Archaea;Euryarchaeota;Methanomicrobia;Methanosarcinales;Methanotrichaceae;Methanothrix;Methanothrix soehngenii;Methanothrix soehngenii GP-6 |
| Ga0586324 4485491 2 646 | 0.00 | 0.00 | 0.00 | 0.00 | 0.00 | 0.00 | 0.00 | 2696200.00 | 0.00 | 0.00 | 0.00 | 0.00 | KO:K13525 | transitional endoplasmic reticulum ATPase | Archaea;Euryarchaeota;Methanomicrobia;Methanomicrobiales;Methanoregulaceae;Methanoregula;Methanoregula formicica;Methanoregula formicica SMSP, DSM 22288 |
| Ga0586324 7016145 1 537 | 0.00 | 609117.48 | 0.00 | 0.00 | 0.00 | 0.00 | 1552281.00 | 2793800.00 | 1296000.00 | 0.00 | 0.00 | 354678.75 | KO:K13525 | transitional endoplasmic reticulum ATPase | Archaea;Euryarchaeota;Methanomicrobia;Methanosarcinales;Methanotrichaceae;Methanothrix;Methanothrix soehngenii;Methanothrix soehngenii GP-6 |
| Ga0586324 1083327 3 1172 | 0.00 | 0.00 | 0.00 | 279655.30 | 0.00 | 0.00 | 0.00 | 0.00 | 0.00 | 0.00 | 0.00 | 0.00 | KO:K13542 | uroporphyrinogen III methyltransferase/synthase | Bacteria;Proteobacteria;Deltaproteobacteria;Syntrophobacterales;Syntrophaceae;Syntrophus;Syntrophus aciditrophicus;Syntrophus aciditrophicus SB |
| Ga0586324 0320210 3 1193 | 0.00 | 0.00 | 0.00 | 0.00 | 0.00 | 0.00 | 0.00 | 0.00 | 0.00 | 0.00 | 0.00 | 354812.25 | KO:K13587 | two-component system cell cycle sensor histidine kinase/response regulator CckA | Bacteria;Proteobacteria;Deltaproteobacteria;Syntrophobacterales;Syntrophaceae;Desulfomonile;Desulfomonile tiedjei;Desulfomonile tiedjei DCB-1, DSM 6799 |
| Ga0586324 0599285 2 1447 | 0.00 | 0.00 | 0.00 | 0.00 | 0.00 | 0.00 | 0.00 | 0.00 | 0.00 | 0.00 | 0.00 | 645546.75 | KO:K13587 | two-component system cell cycle sensor histidine kinase/response regulator CckA | Bacteria;Verrucomicrobia;Opitutae;Opitutales;Opitutaceae;Opitutus;Opitutus terrae;Opitutus terrae PB90-1 |
| Ga0586324 1616880 2 988 | 0.00 | 0.00 | 0.00 | 0.00 | 0.00 | 0.00 | 0.00 | 0.00 | 0.00 | 1355200.00 | 0.00 | 0.00 | KO:K13598 | two-component system nitrogen regulation sensor histidine kinase NtrY | Bacteria;Acidobacteria;Vicinamibacteria;unclassified Vicinamibacteria;Vicinamibacteraceae;Luteitalea;Luteitalea pratensis;Luteitalea pratensis DSM 100886 |
| Ga0586324 3018128 3 761 | 0.00 | 0.00 | 0.00 | 0.00 | 0.00 | 0.00 | 0.00 | 0.00 | 0.00 | 299314.40 | 0.00 | 0.00 | KO:K13598 | two-component system nitrogen regulation sensor histidine kinase NtrY | Bacteria;Proteobacteria;Deltaproteobacteria;Desulfobacterales;Desulfobulbaceae;Desulfobulbus;Desulfobulbus propionicus;Desulfobulbus propionicus 1pr3, DSM 2032 |
| Ga0586324 0000001 110890 112167 | 0.00 | 0.00 | 0.00 | 0.00 | 0.00 | 0.00 | 0.00 | 0.00 | 0.00 | 0.00 | 1487200.00 | 0.00 | KO:K13599 | two-component system nitrogen regulation response regulator NtrX | Bacteria;Proteobacteria;Betaproteobacteria;Nitrosomonadales;Thiobacillaceae;Annwoodia;Annwoodia aquaesulis;Annwoodia aquaesulis DSM 4255 |
| Ga0586324 0045136 3 5027 | 0.00 | 0.00 | 0.00 | 3515000.00 | 0.00 | 0.00 | 0.00 | 0.00 | 0.00 | 0.00 | 0.00 | 0.00 | KO:K13688 | cyclic beta-1,2-glucan synthetase | Bacteria;Proteobacteria;Gammaproteobacteria;Acidiferrobacterales;Acidiferrobacteraceae;Sulfuricaulis;Sulfuricaulis limicola;Sulfuricaulis limicola HA5 |
| Ga0586324 0003519 9788 11329 | 0.00 | 0.00 | 2341241.13 | 0.00 | 0.00 | 0.00 | 0.00 | 0.00 | 0.00 | 0.00 | 0.00 | 0.00 | KO:K13889 | glutathione transport system substrate-binding protein | Bacteria;Firmicutes;Bacilli;Bacillales;Alicyclobacillaceae;Effusibacillus;Effusibacillus pohliae;Effusibacillus pohliae DSM 22757 |
| Ga0586324 1670554 365 976 | 0.00 | 0.00 | 0.00 | 0.00 | 0.00 | 0.00 | 0.00 | 1043100.00 | 923400.00 | 0.00 | 0.00 | 0.00 | KO:K13953 | propanol-preferring alcohol dehydrogenase | Bacteria;Proteobacteria;Alphaproteobacteria;Rhodospirillales;Acetobacteraceae;Rhodopila;unclassified Rhodopila;Rhodopila sp. LVNP |
| Ga0586324 0136868 2 1009 | 23450000.00 | 12920000.00 | 0.00 | 10925000.00 | 38357200.00 | 27948800.00 | 36936000.00 | 2549800.00 | 0.00 | 0.00 | 0.00 | 0.00 | KO:K13954 | alcohol dehydrogenase | Bacteria;Proteobacteria;Deltaproteobacteria;Desulfuromonadales;Geobacteraceae;Geobacter;Geobacter sp. DSM 2909;Geobacter sp. DSM 2909 |
| Ga0586324 0881029 727 1281 | 0.00 | 0.00 | 0.00 | 0.00 | 0.00 | 23343600.00 | 20634000.00 | 0.00 | 0.00 | 0.00 | 9680000.00 | 0.00 | KO:K13954 | alcohol dehydrogenase | Bacteria;Proteobacteria;Deltaproteobacteria;Desulfobacterales;Desulfobacteraceae;Desulfococcus;Desulfococcus multivorans;Desulfococcus multivorans DSM 2059 |
| Ga0586324 3425811 2 721 | 0.00 | 3991600.00 | 0.00 | 13585000.00 | 0.00 | 7225400.00 | 11491200.00 | 10675000.00 | 12393000.00 | 2148300.00 | 0.00 | 2452500.00 | KO:K13954 | alcohol dehydrogenase | Bacteria;Proteobacteria;Deltaproteobacteria;Desulfobacterales;Desulfobulbaceae;Desulfobulbus;Desulfobulbus propionicus;Desulfobulbus propionicus 1pr3, DSM 2032 |
| Ga0586324 7725075 2 307 | 0.00 | 0.00 | 0.00 | 4104000.00 | 0.00 | 0.00 | 0.00 | 0.00 | 0.00 | 0.00 | 0.00 | 0.00 | KO:K13954 | alcohol dehydrogenase | Bacteria;Proteobacteria;Deltaproteobacteria;Desulfobacterales;Desulfobacteraceae;Desulfosarcina;Desulfosarcina variabilis;Desulfosarcina variabilis Montpellier |
| Ga0586324 0007287 3047 3502 | 0.00 | 7371200.00 | 0.00 | 0.00 | 0.00 | 0.00 | 0.00 | 0.00 | 0.00 | 0.00 | 0.00 | 0.00 | KO:K13993 | HSP20 family protein | Bacteria;Balneolaeota;Balneolia;Balneolales;Balneolaceae;Aliifodinibius;Aliifodinibius sediminis;Aliifodinibius sediminis DSM 21194 |
| Ga0586324 0009435 1401 1853 | 0.00 | 0.00 | 0.00 | 0.00 | 4707100.00 | 0.00 | 0.00 | 0.00 | 0.00 | 0.00 | 0.00 | 0.00 | KO:K13993 | HSP20 family protein | Bacteria;Proteobacteria;Gammaproteobacteria;Chromatiales;Chromatiaceae;Marichromatium;Marichromatium purpuratum;Marichromatium purpuratum 984 |
| Ga0586324 0018138 1750 2175 | 0.00 | 0.00 | 0.00 | 0.00 | 0.00 | 0.00 | 0.00 | 0.00 | 1198800.00 | 0.00 | 2552000.00 | 2197500.00 | KO:K13993 | HSP20 family protein | Bacteria;Proteobacteria;Betaproteobacteria;Nitrosomonadales;Gallionellaceae;Sideroxydans;Sideroxydans lithotrophicus;Sideroxydans lithotrophicus ES-1 |
| Ga0586324 0048515 91 534 | 0.00 | 0.00 | 0.00 | 0.00 | 0.00 | 0.00 | 0.00 | 0.00 | 0.00 | 0.00 | 0.00 | 772500.00 | KO:K13993 | HSP20 family protein | Bacteria;Proteobacteria;Betaproteobacteria;Nitrosomonadales;Thiobacillaceae;Thiobacillus;Thiobacillus thioparus;Thiobacillus thioparus DSM 505 |
| Ga0586324 0073272 3313 3777 | 0.00 | 0.00 | 8163500.00 | 0.00 | 0.00 | 0.00 | 0.00 | 0.00 | 0.00 | 0.00 | 0.00 | 0.00 | KO:K13993 | HSP20 family protein | Archaea;Euryarchaeota;Methanomicrobia;Methanosarcinales;Methanotrichaceae;Methanothrix;Methanothrix soehngenii;Methanothrix soehngenii GP-6 |
| Ga0586324 0190226 834 1175 | 9480000.00 | 7480000.00 | 0.00 | 0.00 | 0.00 | 0.00 | 0.00 | 0.00 | 0.00 | 0.00 | 0.00 | 0.00 | KO:K13993 | HSP20 family protein | Bacteria;Planctomycetes;Planctomycetia;Gemmatales;Gemmataceae;Fimbriiglobus;Fimbriiglobus ruber;Fimbriiglobus ruber SP5 |
| Ga0586324 0215373 197 640 | 0.00 | 0.00 | 0.00 | 0.00 | 0.00 | 0.00 | 0.00 | 0.00 | 0.00 | 0.00 | 1909600.00 | 982500.00 | KO:K13993 | HSP20 family protein | Bacteria;Proteobacteria;Betaproteobacteria;Nitrosomonadales;Thiobacillaceae;Thiobacillus;Thiobacillus thioparus;Thiobacillus thioparus DSM 505 |
| Ga0586324 0236188 1040 1486 | 0.00 | 0.00 | 0.00 | 0.00 | 739090.92 | 0.00 | 0.00 | 0.00 | 0.00 | 0.00 | 0.00 | 0.00 | KO:K13993 | HSP20 family protein | Bacteria;Proteobacteria;Deltaproteobacteria;Desulfobacterales;Desulfobacteraceae;Desulfatitalea;Desulfatitalea tepidiphila;Desulfatitalea tepidiphila S28bF |
| Ga0586324 0266745 272 649 | 0.00 | 0.00 | 0.00 | 0.00 | 0.00 | 0.00 | 0.00 | 0.00 | 0.00 | 0.00 | 0.00 | 6885000.00 | KO:K13993 | HSP20 family protein | Bacteria;Planctomycetes;Planctomycetia;Gemmatales;Gemmataceae;Fimbriiglobus;Fimbriiglobus ruber;Fimbriiglobus ruber SP5 |
| Ga0586324 0521173 666 1064 | 0.00 | 0.00 | 0.00 | 0.00 | 0.00 | 0.00 | 0.00 | 0.00 | 0.00 | 0.00 | 0.00 | 1612500.00 | KO:K13993 | HSP20 family protein | Bacteria;Proteobacteria;Deltaproteobacteria;Syntrophobacterales;Syntrophobacteraceae;Syntrophobacter;Syntrophobacter fumaroxidans;Syntrophobacter fumaroxidans MPOB |
| Ga0586324 0756214 783 1235 | 0.00 | 0.00 | 653226.38 | 0.00 | 0.00 | 0.00 | 0.00 | 2125324.18 | 0.00 | 717635.38 | 15664000.00 | 1117500.00 | KO:K13993 | HSP20 family protein | Bacteria;Proteobacteria;Betaproteobacteria;Burkholderiales;unclassified Burkholderiales;Ideonella;Ideonella sp. A 288;Ideonella sp. A 288 |
| Ga0586324 0779124 834 1202 | 0.00 | 0.00 | 0.00 | 0.00 | 0.00 | 0.00 | 0.00 | 0.00 | 0.00 | 0.00 | 0.00 | 2370000.00 | KO:K13993 | HSP20 family protein | Bacteria;Planctomycetes;Planctomycetia;Gemmatales;Gemmataceae;Fimbriiglobus;Fimbriiglobus ruber;Fimbriiglobus ruber SP5 |
| Ga0586324 0878275 224 685 | 0.00 | 1965200.00 | 0.00 | 0.00 | 0.00 | 0.00 | 0.00 | 0.00 | 0.00 | 0.00 | 0.00 | 0.00 | KO:K13993 | HSP20 family protein | Bacteria;Nitrospirae;Nitrospira;Nitrospirales;Nitrospiraceae;Nitrospira;Candidatus Nitrospira nitrificans;Candidatus Nitrospira nitrificans COMA2 |
| Ga0586324 1018637 816 1205 | 0.00 | 0.00 | 0.00 | 0.00 | 0.00 | 0.00 | 0.00 | 0.00 | 0.00 | 1270500.00 | 783049.52 | 795000.00 | KO:K13993 | HSP20 family protein | Bacteria;Proteobacteria;Betaproteobacteria;Nitrosomonadales;Nitrosomonadaceae;Nitrosospira;Nitrosospira multiformis;Nitrosospira multiformis NI12 |
| Ga0586324 1317879 440 886 | 0.00 | 0.00 | 0.00 | 5491000.00 | 0.00 | 0.00 | 0.00 | 4971500.00 | 6180300.00 | 4173400.00 | 0.00 | 4327500.00 | KO:K13993 | HSP20 family protein | Bacteria;Proteobacteria;Deltaproteobacteria;Desulfobacterales;Desulfobulbaceae;Desulfopila;Desulfopila aestuarii;Desulfopila aestuarii DSM 18488 |
| Ga0586324 1724519 527 961 | 0.00 | 3107600.00 | 0.00 | 0.00 | 0.00 | 0.00 | 0.00 | 0.00 | 1433700.00 | 0.00 | 0.00 | 0.00 | KO:K13993 | HSP20 family protein | Bacteria;Acidobacteria;Acidobacteriia;Acidobacteriales;Acidobacteriaceae;unclassified Acidobacteriaceae;Acidobacteriaceae bacterium KBS 96;Acidobacteriaceae bacterium KBS 96, DSM 24297 |
| Ga0586324 1740885 379 876 | 0.00 | 12920000.00 | 20718400.00 | 8521500.00 | 0.00 | 0.00 | 0.00 | 0.00 | 0.00 | 0.00 | 0.00 | 0.00 | KO:K13993 | HSP20 family protein | Bacteria;Proteobacteria;Alphaproteobacteria;Rhodospirillales;Acetobacteraceae;Rhodopila;Rhodopila globiformis;Rhodopila globiformis DSM 161 |
| Ga0586324 1759398 3 368 | 0.00 | 0.00 | 0.00 | 1719500.00 | 2533800.00 | 0.00 | 0.00 | 0.00 | 0.00 | 0.00 | 0.00 | 0.00 | KO:K13993 | HSP20 family protein | Archaea;Euryarchaeota;Methanomicrobia;Methanosarcinales;Methanotrichaceae;Methanothrix;Methanothrix soehngenii;Methanothrix soehngenii GP-6 |
| Ga0586324 2077913 263 685 | 0.00 | 0.00 | 0.00 | 0.00 | 0.00 | 0.00 | 0.00 | 0.00 | 0.00 | 3033800.00 | 0.00 | 0.00 | KO:K13993 | HSP20 family protein | Bacteria;Proteobacteria;Betaproteobacteria;Burkholderiales;Oxalobacteraceae;Noviherbaspirillum;Noviherbaspirillum denitrificans;Noviherbaspirillum denitrificans TSA40 |
| Ga0586324 2155781 146 679 | 0.00 | 0.00 | 0.00 | 0.00 | 0.00 | 2730272.22 | 0.00 | 0.00 | 0.00 | 0.00 | 0.00 | 0.00 | KO:K13993 | HSP20 family protein | Bacteria;Proteobacteria;Alphaproteobacteria;Hyphomicrobiales;Phyllobacteriaceae;Chelativorans;Chelativorans sp. BNC1;Chelativorans sp. BNC1 |
| Ga0586324 2496980 211 657 | 0.00 | 0.00 | 0.00 | 0.00 | 12772000.00 | 16141448.32 | 0.00 | 854000.00 | 0.00 | 0.00 | 0.00 | 0.00 | KO:K13993 | HSP20 family protein | Bacteria;Proteobacteria;Deltaproteobacteria;Desulfobacterales;Desulfobulbaceae;Desulfopila;Desulfopila aestuarii;Desulfopila aestuarii DSM 18488 |
| Ga0586324 2547406 136 597 | 0.00 | 2958000.00 | 0.00 | 0.00 | 0.00 | 0.00 | 0.00 | 0.00 | 0.00 | 0.00 | 0.00 | 0.00 | KO:K13993 | HSP20 family protein | Bacteria;Acidobacteria;Holophagae;Holophagales;Holophagaceae;Geothrix;Geothrix fermentans;Geothrix fermentans DSM 14018 |
| Ga0586324 2812697 1 480 | 11000000.00 | 5616800.00 | 5406460.85 | 0.00 | 0.00 | 0.00 | 0.00 | 5422900.00 | 0.00 | 4566100.00 | 4628800.00 | 4230000.00 | KO:K13993 | HSP20 family protein | Bacteria;Proteobacteria;Betaproteobacteria;Rhodocyclales;Azonexaceae;Dechloromonas;Dechloromonas sp. SG708;Dechloromonas sp. SG708 |
| Ga0586324 3239821 2 415 | 0.00 | 0.00 | 0.00 | 0.00 | 0.00 | 0.00 | 0.00 | 0.00 | 0.00 | 0.00 | 1434400.00 | 0.00 | KO:K13993 | HSP20 family protein | Bacteria;Proteobacteria;Gammaproteobacteria;Methylococcales;Methylococcaceae;Methylomicrobium;Methylomicrobium lacus;Methylosarcina lacus LW14 |
| Ga0586324 3475159 108 554 | 0.00 | 0.00 | 0.00 | 0.00 | 0.00 | 0.00 | 10738800.00 | 0.00 | 0.00 | 0.00 | 0.00 | 0.00 | KO:K13993 | HSP20 family protein | Bacteria;Proteobacteria;Deltaproteobacteria;Desulfobacterales;Desulfobacteraceae;Desulfatirhabdium;Desulfatirhabdium butyrativorans;Desulfatirhabdium butyrativorans DSM 18734 |
| Ga0586324 4378537 2 241 | 14600000.00 | 0.00 | 0.00 | 0.00 | 0.00 | 0.00 | 0.00 | 0.00 | 0.00 | 0.00 | 0.00 | 0.00 | KO:K13993 | HSP20 family protein | Bacteria;Firmicutes;Clostridia;Thermoanaerobacterales;Thermodesulfobiaceae;Thermodesulfobium;Thermodesulfobium narugense;Thermodesulfobium narugense Na82, DSM 14796 |
| Ga0586324 6693943 3 416 | 0.00 | 0.00 | 0.00 | 0.00 | 0.00 | 0.00 | 0.00 | 1964200.00 | 0.00 | 0.00 | 0.00 | 0.00 | KO:K13993 | HSP20 family protein | Bacteria;Proteobacteria;Deltaproteobacteria;Desulfobacterales;Desulfobacteraceae;Desulfatitalea;Desulfatitalea tepidiphila;Desulfatitalea tepidiphila S28bF |
| Ga0586324 0259178 840 2198 | 0.00 | 0.00 | 0.00 | 0.00 | 0.00 | 0.00 | 0.00 | 841800.00 | 0.00 | 0.00 | 0.00 | 0.00 | KO:K14068 | F420-nonreducing hydrogenase I large subunit | Archaea;Euryarchaeota;Methanomicrobia;Methanosarcinales;Methanosarcinaceae;Methanosarcina;Methanosarcina barkeri;Methanosarcina barkeri 3 |
| Ga0586324 0285324 1199 2017 | 104700000.00 | 138060400.00 | 105900300.00 | 53827000.00 | 55939300.00 | 94692297.08 | 48039600.00 | 38844800.00 | 27507600.00 | 17448200.00 | 10454400.00 | 5315170.50 | KO:K14081 | methanol corrinoid protein | Archaea;Candidatus Thermoplasmatota;Thermoplasmata;Methanomassiliicoccales;Methanomassiliicoccaceae;Methanomassiliicoccus;Methanomassiliicoccus luminyensis;Methanomassiliicoccus luminyensis B10 |
| Ga0586324 0346200 1 351 | 0.00 | 0.00 | 0.00 | 15485000.00 | 0.00 | 0.00 | 0.00 | 0.00 | 0.00 | 0.00 | 0.00 | 0.00 | KO:K14081 | methanol corrinoid protein | Archaea;Euryarchaeota;Methanomicrobia;Methanosarcinales;Methanosarcinaceae;Methanosarcina;Methanosarcina barkeri;Methanosarcina barkeri 3 |
| Ga0586324 0353579 41 916 | 14500000.00 | 8228000.00 | 19648700.00 | 0.00 | 0.00 | 18857500.00 | 5061600.00 | 3989400.00 | 0.00 | 0.00 | 0.00 | 0.00 | KO:K14081 | methanol corrinoid protein | Archaea;Candidatus Thermoplasmatota;Thermoplasmata;Methanomassiliicoccales;Methanomassiliicoccaceae;Methanomassiliicoccus;Methanomassiliicoccus luminyensis;Methanomassiliicoccus luminyensis B10 |
| Ga0586324 0426406 285 1067 | 0.00 | 0.00 | 2616542.50 | 0.00 | 8672600.00 | 3923634.37 | 0.00 | 3458700.00 | 4503600.00 | 0.00 | 0.00 | 0.00 | KO:K14081 | methanol corrinoid protein | Archaea;Euryarchaeota;Methanomicrobia;Methanosarcinales;Methanosarcinaceae;Methanosarcina;Methanosarcina barkeri;Methanosarcina barkeri Wiesmoor |
| Ga0586324 0605793 3 539 | 2110000.00 | 1849600.00 | 4393477.47 | 5320000.00 | 5150000.00 | 1262277.38 | 2530800.00 | 2818200.00 | 2867400.00 | 3942400.00 | 6230400.00 | 658878.75 | KO:K14081 | methanol corrinoid protein | Archaea;Candidatus Thermoplasmatota;Thermoplasmata;Methanomassiliicoccales;Methanomassiliicoccaceae;Methanomassiliicoccus;Methanomassiliicoccus luminyensis;Methanomassiliicoccus luminyensis B10 |
| Ga0586324 4852330 87 623 | 0.00 | 0.00 | 6924900.00 | 0.00 | 0.00 | 0.00 | 0.00 | 0.00 | 0.00 | 0.00 | 0.00 | 0.00 | KO:K14081 | methanol corrinoid protein | Archaea;Candidatus Thermoplasmatota;Thermoplasmata;Methanomassiliicoccales;Methanomassiliicoccaceae;Methanomassiliicoccus;Methanomassiliicoccus luminyensis;Methanomassiliicoccus luminyensis B10 |
| Ga0586324 5041498 1 579 | 0.00 | 0.00 | 0.00 | 0.00 | 0.00 | 0.00 | 9370800.00 | 0.00 | 0.00 | 0.00 | 0.00 | 0.00 | KO:K14081 | methanol corrinoid protein | Archaea;Candidatus Thermoplasmatota;Thermoplasmata;Methanomassiliicoccales;Methanomassiliicoccaceae;Methanomassiliicoccus;Methanomassiliicoccus luminyensis;Methanomassiliicoccus luminyensis B10 |
| Ga0586324 6494203 272 553 | 11400000.00 | 7480000.00 | 0.00 | 0.00 | 9712900.00 | 8654600.00 | 0.00 | 0.00 | 0.00 | 0.00 | 0.00 | 0.00 | KO:K14081 | methanol corrinoid protein | Archaea;Candidatus Thermoplasmatota;Thermoplasmata;Methanomassiliicoccales;Methanomassiliicoccaceae;Methanomassiliicoccus;Methanomassiliicoccus luminyensis;Methanomassiliicoccus luminyensis B10 |
| Ga0586324 0195978 779 2125 | 0.00 | 8772000.00 | 0.00 | 0.00 | 0.00 | 0.00 | 0.00 | 8296000.00 | 6893100.00 | 0.00 | 0.00 | 7215000.00 | KO:K14126 | F420-non-reducing hydrogenase large subunit | Bacteria;Chloroflexi;Dehalococcoidia;unclassified Dehalococcoidia;unclassified Dehalococcoidia;Dehalogenimonas;Dehalogenimonas lykanthroporepellens;Dehalogenimonas lykanthroporepellens BL-DC-9 |
| Ga0586324 1453580 628 1035 | 0.00 | 0.00 | 0.00 | 5529000.00 | 0.00 | 0.00 | 0.00 | 0.00 | 0.00 | 0.00 | 1636800.00 | 0.00 | KO:K14126 | F420-non-reducing hydrogenase large subunit | Bacteria;Firmicutes;Clostridia;Thermoanaerobacterales;Thermoanaerobacteraceae;Ammonifex;Ammonifex degensii;Ammonifex degensii KC4 |
| Ga0586324 6981726 1 537 | 0.00 | 0.00 | 0.00 | 0.00 | 0.00 | 0.00 | 0.00 | 0.00 | 1628100.00 | 0.00 | 0.00 | 0.00 | KO:K14126 | F420-non-reducing hydrogenase large subunit | Bacteria;Chloroflexi;Dehalococcoidia;unclassified Dehalococcoidia;unclassified Dehalococcoidia;Dehalogenimonas;Dehalogenimonas sp. GP;Dehalogenimonas sp. GP |
| Ga0586324 1421440 612 1037 | 5140000.00 | 0.00 | 0.00 | 0.00 | 0.00 | 0.00 | 0.00 | 0.00 | 0.00 | 0.00 | 0.00 | 0.00 | KO:K14127 | F420-non-reducing hydrogenase iron-sulfur subunit | Archaea;Euryarchaeota;Methanomicrobia;Methanomicrobiales;Methanomicrobiaceae;Methanoculleus;Methanoculleus sp. MH98A;Methanoculleus sp. MH98A |
| Ga0586324 0003570 11991 14207 | 0.00 | 0.00 | 0.00 | 0.00 | 9342100.00 | 0.00 | 0.00 | 7625000.00 | 0.00 | 0.00 | 0.00 | 4095000.00 | KO:K14138 | acetyl-CoA synthase | Bacteria;Spirochaetes;Spirochaetia;Spirochaetales;Spirochaetaceae;Alkalispirochaeta;Alkalispirochaeta odontotermitis;Alkalispirochaeta odontotermitis JC202 |
| Ga0586324 0008881 8268 10481 | 8910000.00 | 0.00 | 0.00 | 0.00 | 0.00 | 15006600.00 | 19174800.00 | 8174000.00 | 21465000.00 | 0.00 | 8976000.00 | 5962500.00 | KO:K14138 | acetyl-CoA synthase | Bacteria;Proteobacteria;Deltaproteobacteria;Syntrophobacterales;Syntrophobacteraceae;Desulfosoma;Desulfosoma caldarium;Desulfosoma caldarium DSM 22027 |
| Ga0586324 0013713 3 1217 | 44310000.00 | 0.00 | 866532.44 | 0.00 | 0.00 | 1390198.72 | 385671.12 | 11346000.00 | 32481000.00 | 18095000.00 | 15400000.00 | 0.00 | KO:K14138 | acetyl-CoA synthase | Bacteria;Proteobacteria;Deltaproteobacteria;Syntrophobacterales;Syntrophobacteraceae;Desulfosoma;Desulfosoma caldarium;Desulfosoma caldarium DSM 22027 |
| Ga0586324 0053197 1161 3392 | 1630000.00 | 0.00 | 5522421.96 | 1757500.00 | 5809200.00 | 0.00 | 18855600.00 | 464755.95 | 4819500.00 | 0.00 | 0.00 | 0.00 | KO:K14138 | acetyl-CoA synthase | Bacteria;Proteobacteria;Deltaproteobacteria;Syntrophobacterales;Syntrophaceae;Desulfobacca;Desulfobacca acetoxidans;Desulfobacca acetoxidans ASRB2, DSM 11109 |
| Ga0586324 0063474 1858 4074 | 0.00 | 0.00 | 0.00 | 3258500.00 | 3563800.00 | 4724300.00 | 0.00 | 3672200.00 | 3296700.00 | 2148300.00 | 2085600.00 | 1612500.00 | KO:K14138 | acetyl-CoA synthase | Bacteria;Proteobacteria;Deltaproteobacteria;Desulfobacterales;Desulfobacteraceae;Desulfatiglans;Desulfatiglans anilini;Desulfatiglans anilini DSM 4660 |
| Ga0586324 0072078 286 2499 | 23490000.00 | 38093600.00 | 51175562.74 | 39520000.00 | 32651000.00 | 21776061.38 | 37164000.00 | 28487000.00 | 37989000.00 | 32501700.00 | 41254400.00 | 46117500.00 | KO:K14138 | acetyl-CoA synthase | Bacteria;Proteobacteria;Deltaproteobacteria;Syntrophobacterales;Syntrophobacteraceae;Desulfacinum;Desulfacinum infernum;Desulfacinum infernum DSM 9756 |
| Ga0586324 0252085 1567 2226 | 0.00 | 0.00 | 0.00 | 0.00 | 0.00 | 1861878.39 | 0.00 | 0.00 | 0.00 | 648451.65 | 0.00 | 0.00 | KO:K14138 | acetyl-CoA synthase | Bacteria;Proteobacteria;Deltaproteobacteria;Syntrophobacterales;Syntrophobacteraceae;Desulfosoma;Desulfosoma caldarium;Desulfosoma caldarium DSM 22027 |
| Ga0586324 0294731 964 2070 | 0.00 | 442975.80 | 0.00 | 0.00 | 0.00 | 0.00 | 0.00 | 0.00 | 0.00 | 0.00 | 0.00 | 0.00 | KO:K14138 | acetyl-CoA synthase | Bacteria;Proteobacteria;Deltaproteobacteria;Syntrophobacterales;Syntrophaceae;Desulfobacca;Desulfobacca acetoxidans;Desulfobacca acetoxidans ASRB2, DSM 11109 |
| Ga0586324 0389801 647 1828 | 0.00 | 1251200.00 | 0.00 | 0.00 | 1017861.45 | 0.00 | 0.00 | 0.00 | 0.00 | 0.00 | 0.00 | 0.00 | KO:K14138 | acetyl-CoA synthase | Bacteria;Proteobacteria;Deltaproteobacteria;Desulfobacterales;Desulfobacteraceae;Desulfatirhabdium;Desulfatirhabdium butyrativorans;Desulfatirhabdium butyrativorans DSM 18734 |
| Ga0586324 0392540 220 1824 | 0.00 | 0.00 | 0.00 | 18905000.00 | 10506000.00 | 0.00 | 0.00 | 0.00 | 2940300.00 | 0.00 | 0.00 | 0.00 | KO:K14138 | acetyl-CoA synthase | Bacteria;Proteobacteria;Deltaproteobacteria;Desulfobacterales;Desulfobulbaceae;Desulfopila;Desulfopila aestuarii;Desulfopila aestuarii DSM 18488 |
| Ga0586324 0439861 3 1706 | 143208.00 | 0.00 | 0.00 | 1947500.00 | 2704815.02 | 38628100.00 | 43456359.96 | 1016726.65 | 4221962.19 | 0.00 | 0.00 | 0.00 | KO:K14138 | acetyl-CoA synthase | Bacteria;Proteobacteria;Deltaproteobacteria;Syntrophobacterales;Syntrophaceae;Desulfobacca;Desulfobacca acetoxidans;Desulfobacca acetoxidans ASRB2, DSM 11109 |
| Ga0586324 0535760 1415 1591 | 0.00 | 0.00 | 0.00 | 0.00 | 0.00 | 8535500.00 | 0.00 | 0.00 | 0.00 | 0.00 | 0.00 | 0.00 | KO:K14138 | acetyl-CoA synthase | Bacteria;Spirochaetes;Spirochaetia;Spirochaetales;Spirochaetaceae;Alkalispirochaeta;Alkalispirochaeta odontotermitis;Alkalispirochaeta odontotermitis JC202 |
| Ga0586324 0689373 3 1424 | 3350000.00 | 0.00 | 0.00 | 0.00 | 0.00 | 0.00 | 0.00 | 0.00 | 0.00 | 0.00 | 0.00 | 0.00 | KO:K14138 | acetyl-CoA synthase | Bacteria;Chloroflexi;Dehalococcoidia;unclassified Dehalococcoidia;unclassified Dehalococcoidia;Dehalogenimonas;Dehalogenimonas sp. WBC-2;Dehalogenimonas sp. WBC-2 |
| Ga0586324 0697252 1 1419 | 1120000.00 | 18421200.00 | 350808.12 | 28234000.00 | 45423000.00 | 1779878.04 | 14400163.08 | 27504900.00 | 21938722.83 | 3237486.56 | 25185600.00 | 21952500.00 | KO:K14138 | acetyl-CoA synthase | Bacteria;Proteobacteria;Deltaproteobacteria;Syntrophobacterales;Syntrophobacteraceae;Syntrophobacter;Syntrophobacter fumaroxidans;Syntrophobacter fumaroxidans MPOB |
| Ga0586324 0718780 1 1401 | 0.00 | 0.00 | 0.00 | 0.00 | 5263300.00 | 0.00 | 0.00 | 0.00 | 0.00 | 0.00 | 0.00 | 0.00 | KO:K14138 | acetyl-CoA synthase | Bacteria;Proteobacteria;Deltaproteobacteria;Syntrophobacterales;Syntrophaceae;Desulfomonile;Desulfomonile tiedjei;Desulfomonile tiedjei DCB-1, DSM 6799 |
| Ga0586324 0917986 3 1259 | 0.00 | 40256000.00 | 39241100.00 | 1719500.00 | 12998600.00 | 65584400.00 | 53352000.00 | 2086200.00 | 0.00 | 0.00 | 0.00 | 19537500.00 | KO:K14138 | acetyl-CoA synthase | Bacteria;Proteobacteria;Deltaproteobacteria;Syntrophobacterales;Syntrophaceae;Desulfobacca;Desulfobacca acetoxidans;Desulfobacca acetoxidans ASRB2, DSM 11109 |
| Ga0586324 0921343 3 1259 | 66570000.00 | 28356000.00 | 18579000.00 | 36100000.00 | 39531400.00 | 10459842.37 | 28932607.20 | 17220300.00 | 24493362.39 | 23716000.00 | 16077600.00 | 0.00 | KO:K14138 | acetyl-CoA synthase | Bacteria;Proteobacteria;Deltaproteobacteria;Syntrophobacterales;Syntrophaceae;Desulfobacca;Desulfobacca acetoxidans;Desulfobacca acetoxidans ASRB2, DSM 11109 |
| Ga0586324 0979259 3 1226 | 4400000.00 | 8432000.00 | 6305600.00 | 9110500.00 | 10506000.00 | 15880000.00 | 7045200.00 | 7076000.00 | 6091200.00 | 0.00 | 4056800.00 | 0.00 | KO:K14138 | acetyl-CoA synthase | Bacteria;Proteobacteria;Deltaproteobacteria;Syntrophobacterales;Syntrophaceae;Desulfobacca;Desulfobacca acetoxidans;Desulfobacca acetoxidans ASRB2, DSM 11109 |
| Ga0586324 1028914 3 611 | 0.00 | 0.00 | 0.00 | 0.00 | 0.00 | 2795364.34 | 4218000.00 | 0.00 | 0.00 | 839300.00 | 0.00 | 0.00 | KO:K14138 | acetyl-CoA synthase | Bacteria;Proteobacteria;Deltaproteobacteria;Syntrophobacterales;Syntrophaceae;Desulfobacca;Desulfobacca acetoxidans;Desulfobacca acetoxidans ASRB2, DSM 11109 |
| Ga0586324 1361676 3 1064 | 0.00 | 0.00 | 0.00 | 0.00 | 0.00 | 0.00 | 0.00 | 0.00 | 1255500.00 | 0.00 | 0.00 | 0.00 | KO:K14138 | acetyl-CoA synthase | Bacteria;Proteobacteria;Deltaproteobacteria;Syntrophobacterales;Syntrophobacteraceae;Desulfosoma;Desulfosoma caldarium;Desulfosoma caldarium DSM 22027 |
| Ga0586324 1514031 288 1016 | 0.00 | 0.00 | 0.00 | 0.00 | 25647000.00 | 0.00 | 0.00 | 9028000.00 | 18306000.00 | 0.00 | 0.00 | 0.00 | KO:K14138 | acetyl-CoA synthase | Bacteria;Proteobacteria;Deltaproteobacteria;Syntrophobacterales;Syntrophobacteraceae;Desulfacinum;Desulfacinum infernum;Desulfacinum infernum DSM 9756 |
| Ga0586324 1572901 3 956 | 0.00 | 0.00 | 0.00 | 0.00 | 0.00 | 0.00 | 2146508.28 | 0.00 | 0.00 | 0.00 | 0.00 | 0.00 | KO:K14138 | acetyl-CoA synthase | Bacteria;Proteobacteria;Deltaproteobacteria;Syntrophobacterales;Syntrophaceae;Desulfomonile;Desulfomonile tiedjei;Desulfomonile tiedjei DCB-1, DSM 6799 |
| Ga0586324 1860850 1 930 | 0.00 | 0.00 | 0.00 | 168614.55 | 105828.38 | 0.00 | 15937200.00 | 224171.34 | 0.00 | 0.00 | 0.00 | 0.00 | KO:K14138 | acetyl-CoA synthase | Bacteria;Proteobacteria;Deltaproteobacteria;Syntrophobacterales;Syntrophobacteraceae;Desulfosoma;Desulfosoma caldarium;Desulfosoma caldarium DSM 22027 |
| Ga0586324 2247774 3 860 | 0.00 | 0.00 | 11091100.00 | 0.00 | 0.00 | 0.00 | 0.00 | 0.00 | 0.00 | 0.00 | 0.00 | 0.00 | KO:K14138 | acetyl-CoA synthase | Bacteria;Proteobacteria;Deltaproteobacteria;Syntrophobacterales;Syntrophobacteraceae;Desulfacinum;Desulfacinum hydrothermale;Desulfacinum hydrothermale DSM 13146 |
| Ga0586324 3578320 1 708 | 1720000.00 | 0.00 | 0.00 | 0.00 | 0.00 | 0.00 | 0.00 | 0.00 | 0.00 | 0.00 | 0.00 | 0.00 | KO:K14138 | acetyl-CoA synthase | Bacteria;Proteobacteria;Deltaproteobacteria;Desulfobacterales;Desulfobacteraceae;Desulfatiglans;Desulfatiglans anilini;Desulfatiglans anilini DSM 4660 |
| Ga0586324 4104197 2 670 | 4850000.00 | 3971200.00 | 890249.38 | 49115000.00 | 25853000.00 | 1636334.75 | 24852000.00 | 2263100.00 | 34942581.09 | 885500.00 | 1372800.00 | 772500.00 | KO:K14138 | acetyl-CoA synthase | Bacteria;Proteobacteria;Deltaproteobacteria;Syntrophobacterales;Syntrophobacteraceae;Desulfacinum;Desulfacinum infernum;Desulfacinum infernum DSM 9756 |
| Ga0586324 4127401 1 666 | 0.00 | 0.00 | 0.00 | 2641000.00 | 0.00 | 0.00 | 0.00 | 0.00 | 0.00 | 0.00 | 0.00 | 0.00 | KO:K14138 | acetyl-CoA synthase | Bacteria;Proteobacteria;Deltaproteobacteria;Syntrophobacterales;Syntrophaceae;Desulfobacca;Desulfobacca acetoxidans;Desulfobacca acetoxidans ASRB2, DSM 11109 |
| Ga0586324 4838020 2 625 | 0.00 | 0.00 | 0.00 | 7077500.00 | 0.00 | 0.00 | 0.00 | 3830800.00 | 0.00 | 0.00 | 0.00 | 0.00 | KO:K14138 | acetyl-CoA synthase | Bacteria;Proteobacteria;Deltaproteobacteria;Syntrophobacterales;Syntrophobacteraceae;Syntrophobacter;Syntrophobacter fumaroxidans;Syntrophobacter fumaroxidans MPOB |
| Ga0586324 5188758 1 606 | 0.00 | 897600.00 | 0.00 | 2099500.00 | 0.00 | 0.00 | 0.00 | 0.00 | 0.00 | 0.00 | 0.00 | 0.00 | KO:K14138 | acetyl-CoA synthase | Bacteria;Proteobacteria;Deltaproteobacteria;Syntrophobacterales;Syntrophobacteraceae;Desulfosoma;Desulfosoma caldarium;Desulfosoma caldarium DSM 22027 |
| Ga0586324 5743070 3 581 | 1530000.00 | 0.00 | 0.00 | 0.00 | 0.00 | 0.00 | 0.00 | 0.00 | 0.00 | 0.00 | 0.00 | 0.00 | KO:K14138 | acetyl-CoA synthase | Bacteria;Proteobacteria;Deltaproteobacteria;Syntrophobacterales;Syntrophobacteraceae;Desulfacinum;Desulfacinum hydrothermale;Desulfacinum hydrothermale DSM 13146 |
| Ga0586324 5796893 3 443 | 980986.00 | 0.00 | 0.00 | 0.00 | 0.00 | 0.00 | 0.00 | 0.00 | 0.00 | 0.00 | 2376000.00 | 0.00 | KO:K14138 | acetyl-CoA synthase | Bacteria;Proteobacteria;Deltaproteobacteria;Desulfobacterales;Desulfobacteraceae;Desulfatiglans;Desulfatiglans anilini;Desulfatiglans anilini DSM 4660 |
| Ga0586324 6206620 1 564 | 0.00 | 4440400.00 | 0.00 | 12445000.00 | 6921600.00 | 0.00 | 0.00 | 3458700.00 | 2762100.00 | 0.00 | 0.00 | 0.00 | KO:K14138 | acetyl-CoA synthase | Bacteria;Proteobacteria;Deltaproteobacteria;Desulfobacterales;Desulfobacteraceae;Desulfatirhabdium;Desulfatirhabdium butyrativorans;Desulfatirhabdium butyrativorans DSM 18734 |
| Ga0586324 6675125 3 548 | 7180000.00 | 5222400.00 | 0.00 | 0.00 | 0.00 | 10838100.00 | 8458800.00 | 9028000.00 | 0.00 | 0.00 | 0.00 | 0.00 | KO:K14138 | acetyl-CoA synthase | Bacteria;Proteobacteria;Deltaproteobacteria;Syntrophobacterales;Syntrophobacteraceae;Desulfosoma;Desulfosoma caldarium;Desulfosoma caldarium DSM 22027 |
| Ga0586324 7009754 2 535 | 0.00 | 0.00 | 0.00 | 0.00 | 0.00 | 3067055.26 | 0.00 | 0.00 | 0.00 | 0.00 | 0.00 | 0.00 | KO:K14138 | acetyl-CoA synthase | Bacteria;Proteobacteria;Deltaproteobacteria;Syntrophobacterales;Syntrophaceae;Desulfomonile;Desulfomonile tiedjei;Desulfomonile tiedjei DCB-1, DSM 6799 |
| Ga0586324 7450588 1 522 | 0.00 | 8024000.00 | 0.00 | 9690000.00 | 9867400.00 | 0.00 | 0.00 | 7564000.00 | 6269400.00 | 0.00 | 0.00 | 0.00 | KO:K14138 | acetyl-CoA synthase | Bacteria;Proteobacteria;Deltaproteobacteria;Syntrophobacterales;Syntrophobacteraceae;Desulfacinum;Desulfacinum infernum;Desulfacinum infernum DSM 9756 |
| Ga0586324 0000001 19104 21206 | 0.00 | 0.00 | 0.00 | 0.00 | 0.00 | 0.00 | 40036800.00 | 17202000.00 | 10020829.14 | 31824100.00 | 10120000.00 | 6075000.00 | KO:K14393 | cation/acetate symporter | Bacteria;Proteobacteria;Betaproteobacteria;Nitrosomonadales;Thiobacillaceae;Annwoodia;Annwoodia aquaesulis;Annwoodia aquaesulis DSM 4255 |

| | | | | | | | | | | | | | | | |
|---|---|---|---|---|---|---|---|---|---|---|---|---|---|---|---|
| Ga0586324 0000075 48573 50588 | 0.00 | 0.00 | 0.00 | 0.00 | 0.00 | 0.00 | 5836800.00 | 7039400.00 | 7500600.00 | 9702000.00 | 0.00 | 0.00 | KO:K14393 | cation/acetate symporter | Bacteria;Proteobacteria;Betaproteobacteria;Nitrosomonadales;Thiobacillaceae;Thiobacillus;Thiobacillus thioparus;Thiobacillus thioparus DSM 505 |
| Ga0586324 1839099 2 937 | 1410000.00 | 0.00 | 0.00 | 0.00 | 0.00 | 0.00 | 0.00 | 0.00 | 0.00 | 0.00 | 0.00 | 0.00 | KO:K14447 | (2R)-ethylmalonyl-CoA mutase | Bacteria;Proteobacteria;Gammaproteobacteria;unclassified Gammaproteobacteria;Candidatus Competibacteraceae;Candidatus Competibacter;Candidatus Competibacter denitrificans;Candidatus Competibacter denitrificans Run A D11 |
| Ga0586324 0050460 2519 3973 | 1870000.00 | 0.00 | 0.00 | 0.00 | 0.00 | 0.00 | 0.00 | 0.00 | 0.00 | 0.00 | 0.00 | 0.00 | KO:K14534 | 4-hydroxybutyryl-CoA dehydratase/vinylacetyl-CoA-Delta-isomerase | Bacteria;Proteobacteria;Deltaproteobacteria;Syntrophobacterales;Syntrophaceae;Desulfomonile;Desulfomonile tiedjei;Desulfomonile tiedjei DCB-1, DSM 6799 |
| Ga0586324 0071715 1 1410 | 1090000.00 | 0.00 | 0.00 | 1643500.00 | 20600000.00 | 0.00 | 0.00 | 0.00 | 0.00 | 0.00 | 0.00 | 0.00 | KO:K14534 | 4-hydroxybutyryl-CoA dehydratase/vinylacetyl-CoA-Delta-isomerase | Bacteria;Proteobacteria;Deltaproteobacteria;Syntrophobacterales;Syntrophaceae;Desulfomonile;Desulfomonile tiedjei;Desulfomonile tiedjei DCB-1, DSM 6799 |
| Ga0586324 0120464 670 2166 | 0.00 | 0.00 | 0.00 | 0.00 | 0.00 | 1534162.83 | 0.00 | 0.00 | 0.00 | 0.00 | 0.00 | 0.00 | KO:K14534 | 4-hydroxybutyryl-CoA dehydratase/vinylacetyl-CoA-Delta-isomerase | Bacteria;Proteobacteria;Deltaproteobacteria;Desulfobacterales;Desulfobacteraceae;Desulfatitalea;Desulfatitalea tepidiphila;Desulfatitalea tepidiphila S28bF |
| Ga0586324 0199858 1 1482 | 0.00 | 0.00 | 0.00 | 0.00 | 0.00 | 0.00 | 0.00 | 393090.10 | 0.00 | 579485.06 | 0.00 | 0.00 | KO:K14534 | 4-hydroxybutyryl-CoA dehydratase/vinylacetyl-CoA-Delta-isomerase | Bacteria;Firmicutes;Clostridia;Eubacteriales;unclassified Eubacteriales;unclassified Eubacteriales;Clostridiales bacterium PH28 bin88;Clostridiales bacterium PH28 bin88 |
| Ga0586324 4110938 1 669 | 865886.00 | 0.00 | 0.00 | 0.00 | 0.00 | 0.00 | 0.00 | 0.00 | 0.00 | 0.00 | 0.00 | 0.00 | KO:K14534 | 4-hydroxybutyryl-CoA dehydratase/vinylacetyl-CoA-Delta-isomerase | Bacteria;Proteobacteria;Deltaproteobacteria;Syntrophobacterales;Syntrophaceae;Desulfomonile;Desulfomonile tiedjei;Desulfomonile tiedjei DCB-1, DSM 6799 |
| Ga0586324 0025359 642 2858 | 0.00 | 3549600.00 | 7994600.00 | 0.00 | 0.00 | 8734000.00 | 0.00 | 5471700.00 | 4981500.00 | 6352500.00 | 5764000.00 | 5355000.00 | KO:K14647 | minor extracellular serine protease Vpr | Bacteria;Proteobacteria;Betaproteobacteria;Rhodocyclales;Rhodocyclaceae;Niveibacterium;Niveibacterium umoris;Niveibacterium umoris DSM 106739 |
| Ga0586324 0647837 3 1463 | 0.00 | 0.00 | 0.00 | 0.00 | 0.00 | 0.00 | 0.00 | 0.00 | 0.00 | 1932700.00 | 0.00 | 0.00 | KO:K14647 | minor extracellular serine protease Vpr | Bacteria;Chloroflexi;Chloroflexia;Chloroflexales;Oscillochloridaceae;Candidatus Chloroploca;Candidatus Chloroploca asiatica;Candidatus Chloroploca asiatica B7-9 |
| Ga0586324 5972308 3 572 | 0.00 | 0.00 | 0.00 | 3410500.00 | 0.00 | 0.00 | 0.00 | 0.00 | 0.00 | 0.00 | 0.00 | 0.00 | KO:K14647 | minor extracellular serine protease Vpr | Bacteria;Actinobacteria;Actinomycetia;Micrococcales;Ornithinimicrobiaceae;Ornithinimicrobium;Ornithinimicrobium cerasi;Ornithinimicrobium cerasi CPCC 203383 |
| Ga0586324 0001392 4583 5668 | 0.00 | 0.00 | 0.00 | 0.00 | 0.00 | 0.00 | 0.00 | 841800.00 | 0.00 | 0.00 | 0.00 | 0.00 | KO:K14652 | 3,4-dihydroxy 2-butanone 4-phosphate synthase/GTP cyclohydrolase II | Bacteria;Proteobacteria;Betaproteobacteria;Nitrosomonadales;Thiobacillaceae;Thiobacillus;Thiobacillus thioparus;Thiobacillus thioparus DSM 505 |
| Ga0586324 0620536 1192 1491 | 0.00 | 0.00 | 0.00 | 0.00 | 0.00 | 0.00 | 0.00 | 0.00 | 0.00 | 0.00 | 2138400.00 | 0.00 | KO:K14742 | tRNA threonylcarbamoyladenosine biosynthesis protein TsaB | Bacteria;Proteobacteria;Alphaproteobacteria;Hyphomicrobiales;Xanthobacteraceae;Pseudolabrys;Pseudolabrys taiwanensis;Pseudolabrys taiwanensis CC-BB4 |
| Ga0586324 0317933 1363 2001 | 0.00 | 0.00 | 0.00 | 0.00 | 0.00 | 0.00 | 0.00 | 0.00 | 0.00 | 0.00 | 5350400.00 | 0.00 | KO:K14981 | two-component system response regulator ChvI | Bacteria;Proteobacteria;Alphaproteobacteria;Hyphomicrobiales;unclassified Hyphomicrobiales;Pseudorhodoplanes;Pseudorhodoplanes sinuspersici;Pseudorhodoplanes sinuspersici RIPI110 |
| Ga0586324 0420574 1212 1769 | 0.00 | 0.00 | 0.00 | 0.00 | 0.00 | 8892800.00 | 0.00 | 0.00 | 0.00 | 0.00 | 0.00 | 0.00 | KO:K14981 | two-component system response regulator ChvI | Bacteria;Proteobacteria;Alphaproteobacteria;Hyphomicrobiales;Xanthobacteraceae;Pseudolabrys;Pseudolabrys sp. Root1462;Pseudolabrys sp. Root1462 |
| Ga0586324 0059579 744 2648 | 0.00 | 0.00 | 0.00 | 0.00 | 0.00 | 0.00 | 0.00 | 0.00 | 2308500.00 | 0.00 | 0.00 | 0.00 | KO:K15022 | formate dehydrogenase beta subunit | Bacteria;Proteobacteria;Deltaproteobacteria;Desulfobacterales;Desulfobacteraceae;Desulfosarcina;Desulfosarcina cetonica;Desulfosarcina cetonica JCM 12296 |
| Ga0586324 3564581 3 710 | 0.00 | 0.00 | 0.00 | 0.00 | 0.00 | 0.00 | 3898800.00 | 0.00 | 0.00 | 0.00 | 0.00 | 0.00 | KO:K15022 | formate dehydrogenase beta subunit | Bacteria;Proteobacteria;Gammaproteobacteria;unclassified Gammaproteobacteria;Candidatus Competibacteraceae;Candidatus Contendobacter;Candidatus Contendobacter odensis;Candidatus Contendobacter odensis Run B J11 |
| Ga0586324 0000241 17021 17923 | 2800000.00 | 0.00 | 0.00 | 0.00 | 0.00 | 0.00 | 0.00 | 0.00 | 0.00 | 0.00 | 0.00 | 0.00 | KO:K15023 | 5-methyltetrahydrofolate corrinoid/iron sulfur protein methyltransferase | Bacteria;Proteobacteria;Deltaproteobacteria;Syntrophobacterales;Syntrophaceae;Desulfobacca;Desulfobacca acetoxidans;Desulfobacca acetoxidans ASRB2, DSM 11109 |
| Ga0586324 0013713 2766 3656 | 38000000.00 | 4562800.00 | 4269887.71 | 0.00 | 0.00 | 0.00 | 34428000.00 | 0.00 | 2843100.00 | 14707000.00 | 30448000.00 | 0.00 | KO:K15023 | 5-methyltetrahydrofolate corrinoid/iron sulfur protein methyltransferase | Bacteria;Proteobacteria;Deltaproteobacteria;Syntrophobacterales;Syntrophaceae;Desulfobacca;Desulfobacca acetoxidans;Desulfobacca acetoxidans ASRB2, DSM 11109 |
| Ga0586324 0091922 436 1326 | 11680000.00 | 3094000.00 | 0.00 | 0.00 | 8476900.00 | 100838000.00 | 66120000.00 | 0.00 | 9153000.00 | 0.00 | 0.00 | 0.00 | KO:K15023 | 5-methyltetrahydrofolate corrinoid/iron sulfur protein methyltransferase | Bacteria;Proteobacteria;Deltaproteobacteria;Syntrophobacterales;Syntrophaceae;Desulfobacca;Desulfobacca acetoxidans;Desulfobacca acetoxidans ASRB2, DSM 11109 |
| Ga0586324 0168591 873 1751 | 0.00 | 0.00 | 0.00 | 0.00 | 0.00 | 14569900.00 | 0.00 | 0.00 | 0.00 | 0.00 | 0.00 | 0.00 | KO:K15023 | 5-methyltetrahydrofolate corrinoid/iron sulfur protein methyltransferase | Bacteria;Proteobacteria;Deltaproteobacteria;Desulfobacterales;Desulfobacteraceae;Desulfatiglans;Desulfatiglans anilini;Desulfatiglans anilini DSM 4660 |
| Ga0586324 0380864 1123 1848 | 277641.00 | 0.00 | 0.00 | 0.00 | 68907000.00 | 0.00 | 0.00 | 45323000.00 | 44307000.00 | 0.00 | 0.00 | 0.00 | KO:K15023 | 5-methyltetrahydrofolate corrinoid/iron sulfur protein methyltransferase | Bacteria;Proteobacteria;Deltaproteobacteria;Syntrophobacterales;Syntrophaceae;Desulfobacca;Desulfobacca acetoxidans;Desulfobacca acetoxidans ASRB2, DSM 11109 |
| Ga0586324 0525890 43 933 | 0.00 | 0.00 | 0.00 | 11419000.00 | 0.00 | 0.00 | 0.00 | 0.00 | 2316600.00 | 0.00 | 0.00 | 0.00 | KO:K15023 | 5-methyltetrahydrofolate corrinoid/iron sulfur protein methyltransferase | Bacteria;Proteobacteria;Deltaproteobacteria;Syntrophobacterales;Syntrophaceae;Desulfobacca;Desulfobacca acetoxidans;Desulfobacca acetoxidans ASRB2, DSM 11109 |
| Ga0586324 1117361 25 903 | 0.00 | 0.00 | 0.00 | 0.00 | 0.00 | 0.00 | 0.00 | 0.00 | 0.00 | 0.00 | 9416000.00 | 0.00 | KO:K15023 | 5-methyltetrahydrofolate corrinoid/iron sulfur protein methyltransferase | Bacteria;Proteobacteria;Deltaproteobacteria;Desulfobacterales;Desulfobacteraceae;Desulfatiglans;Desulfatiglans anilini;Desulfatiglans anilini DSM 4660 |
| Ga0586324 2672514 1 546 | 0.00 | 0.00 | 7656800.00 | 0.00 | 0.00 | 6391700.00 | 5380800.00 | 4812900.00 | 0.00 | 0.00 | 0.00 | 0.00 | KO:K15023 | 5-methyltetrahydrofolate corrinoid/iron sulfur protein methyltransferase | Bacteria;Proteobacteria;Deltaproteobacteria;Syntrophobacterales;Syntrophobacteraceae;Syntrophobacter;Syntrophobacter fumaroxidans;Syntrophobacter fumaroxidans MPOB |
| Ga0586324 1163959 3 995 | 0.00 | 0.00 | 2697169.73 | 0.00 | 0.00 | 0.00 | 0.00 | 0.00 | 0.00 | 0.00 | 0.00 | 0.00 | KO:K15230 | ATP-citrate lyase alpha-subunit | Archaea;Euryarchaeota;Methanomicrobia;Methanosarcinales;Methanotrichaceae;Methanothrix;Methanosaeta harundinacea;Methanothrix harundinacea 6Ac |
| Ga0586324 1468077 1 1029 | 4600000.00 | 0.00 | 0.00 | 0.00 | 0.00 | 0.00 | 0.00 | 0.00 | 0.00 | 0.00 | 0.00 | 0.00 | KO:K15230 | ATP-citrate lyase alpha-subunit | Archaea;Euryarchaeota;Methanomicrobia;Methanosarcinales;Methanotrichaceae;Methanothrix;Methanosaeta harundinacea;Methanothrix harundinacea 6Ac |
| Ga0586324 0135044 1759 2973 | 0.00 | 0.00 | 0.00 | 0.00 | 0.00 | 0.00 | 2093621.40 | 0.00 | 0.00 | 0.00 | 0.00 | 997500.00 | KO:K15515 | sulfoacetaldehyde dehydrogenase | Bacteria;Chloroflexi;Anaerolineae;Anaerolineales;Anaerolineaceae;Pelolinea;Pelolinea submarina;Pelolinea submarina DSM 23923 |
| Ga0586324 0003545 6777 7781 | 0.00 | 0.00 | 0.00 | 0.00 | 0.00 | 0.00 | 0.00 | 0.00 | 0.00 | 0.00 | 1082400.00 | 0.00 | KO:K15553 | sulfonate transport system substrate-binding protein | Bacteria;Proteobacteria;Alphaproteobacteria;Hyphomicrobiales;Xanthobacteraceae;Pseudolabrys;Pseudolabrys taiwanensis;Pseudolabrys taiwanensis CC-BB4 |
| Ga0586324 0575113 1 1107 | 0.00 | 0.00 | 0.00 | 0.00 | 0.00 | 0.00 | 0.00 | 0.00 | 0.00 | 0.00 | 0.00 | 1140000.00 | KO:K15553 | sulfonate transport system substrate-binding protein | Bacteria;Proteobacteria;Alphaproteobacteria;Hyphomicrobiales;Xanthobacteraceae;Pseudolabrys;Pseudolabrys taiwanensis;Pseudolabrys taiwanensis CC-BB4 |
| Ga0586324 2425818 2 832 | 0.00 | 558212.68 | 0.00 | 0.00 | 0.00 | 0.00 | 3055200.00 | 1116300.00 | 907200.00 | 1809500.00 | 1364000.00 | 1702500.00 | KO:K15553 | sulfonate transport system substrate-binding protein | Bacteria;Proteobacteria;Alphaproteobacteria;Hyphomicrobiales;Xanthobacteraceae;Pseudolabrys;Pseudolabrys taiwanensis;Pseudolabrys taiwanensis CC-BB4 |
| Ga0586324 0000004 18266 19660 | 0.00 | 0.00 | 0.00 | 0.00 | 0.00 | 0.00 | 0.00 | 2208200.00 | 0.00 | 0.00 | 0.00 | 3187500.00 | KO:K15576 | nitrate/nitrite transport system substrate-binding protein | Bacteria;Proteobacteria;Betaproteobacteria;Nitrosomonadales;Sulfuricellaceae;Sulfuriferula;Sulfuriferula multivorans;Sulfuriferula multivorans TTN |
| Ga0586324 0019358 1822 3687 | 0.00 | 0.00 | 64745000.00 | 28595000.00 | 2430800.00 | 0.00 | 1850596.20 | 0.00 | 4187700.00 | 0.00 | 0.00 | 0.00 | KO:K15580 | oligopeptide transport system substrate-binding protein | Bacteria;Chloroflexi;Anaerolineae;Anaerolineales;Anaerolineaceae;Thermanaerothrix;Thermanaerothrix daxensis;Thermanaerothrix daxensis GNS-1 |
| Ga0586324 0039565 2559 4217 | 0.00 | 0.00 | 0.00 | 0.00 | 0.00 | 5597700.00 | 0.00 | 0.00 | 0.00 | 0.00 | 0.00 | 0.00 | KO:K15580 | oligopeptide transport system substrate-binding protein | Bacteria;Firmicutes;Clostridia;Halanaerobiales;unclassified Halanaerobiales;Anoxybacter;Anoxybacter fermentans;Anoxybacter fermentans DY22613 |
| Ga0586324 0049724 1146 2999 | 0.00 | 0.00 | 5060688.77 | 2270500.00 | 5871000.00 | 0.00 | 6292800.00 | 3818600.00 | 1879200.00 | 0.00 | 1108800.00 | 0.00 | KO:K15580 | oligopeptide transport system substrate-binding protein | Bacteria;Chloroflexi;Anaerolineae;Anaerolineales;Anaerolineaceae;Levilinea;Levilinea saccharolytica;Levilinea saccharolytica DSM 16555 |
| Ga0586324 0080424 2295 3806 | 0.00 | 0.00 | 0.00 | 0.00 | 0.00 | 0.00 | 0.00 | 0.00 | 515624.94 | 0.00 | 0.00 | 0.00 | KO:K15580 | oligopeptide transport system substrate-binding protein | Bacteria;Chloroflexi;Anaerolineae;Anaerolineales;Anaerolineaceae;Thermanaerothrix;Thermanaerothrix daxensis;Thermanaerothrix daxensis GNS-1 |
| Ga0586324 0138962 1624 2934 | 0.00 | 3270800.00 | 0.00 | 0.00 | 0.00 | 0.00 | 0.00 | 0.00 | 0.00 | 0.00 | 0.00 | 0.00 | KO:K15580 | oligopeptide transport system substrate-binding protein | Bacteria;Chloroflexi;Anaerolineae;Anaerolineales;Anaerolineaceae;Thermanaerothrix;Thermanaerothrix daxensis;Thermanaerothrix daxensis GNS-1 |
| Ga0586324 0168878 1012 2676 | 6790000.00 | 14232400.00 | 0.00 | 6032500.00 | 6705300.00 | 0.00 | 8595600.00 | 10296800.00 | 5111100.00 | 10853409.49 | 16984000.00 | 6217500.00 | KO:K15580 | oligopeptide transport system substrate-binding protein | Bacteria;Chloroflexi;Anaerolineae;Anaerolineales;Anaerolineaceae;Levilinea;Levilinea saccharolytica;Levilinea saccharolytica DSM 16555 |
| Ga0586324 0239191 1 1656 | 3560000.00 | 0.00 | 0.00 | 0.00 | 2029100.00 | 0.00 | 0.00 | 0.00 | 0.00 | 0.00 | 0.00 | 0.00 | KO:K15580 | oligopeptide transport system substrate-binding protein | Bacteria;Chloroflexi;Anaerolineae;Anaerolineales;Anaerolineaceae;Levilinea;Levilinea saccharolytica;Levilinea saccharolytica DSM 16555 |
| Ga0586324 0350424 3 1229 | 6460000.00 | 2516000.00 | 0.00 | 0.00 | 4140600.00 | 0.00 | 2021767.20 | 0.00 | 1831951.89 | 0.00 | 0.00 | 0.00 | KO:K15580 | oligopeptide transport system substrate-binding protein | Bacteria;Chloroflexi;Anaerolineae;Anaerolineales;Anaerolineaceae;Levilinea;Levilinea saccharolytica;Levilinea saccharolytica DSM 16555 |
| Ga0586324 1121999 3 965 | 0.00 | 248508.04 | 0.00 | 0.00 | 0.00 | 0.00 | 0.00 | 0.00 | 4430700.00 | 5628700.00 | 0.00 | 5542500.00 | KO:K15580 | oligopeptide transport system substrate-binding protein | Bacteria;Chloroflexi;Anaerolineae;Anaerolineales;Anaerolineaceae;Levilinea;Levilinea saccharolytica;Levilinea saccharolytica DSM 16555 |
| Ga0586324 1327023 3 1076 | 1250000.00 | 0.00 | 0.00 | 0.00 | 0.00 | 0.00 | 0.00 | 0.00 | 0.00 | 0.00 | 0.00 | 0.00 | KO:K15580 | oligopeptide transport system substrate-binding protein | Bacteria;Spirochaetes;Spirochaetia;Spirochaetales;Spirochaetaceae;Alkalispirochaeta;Alkalispirochaeta sphaeroplastigenens;Alkalispirochaeta sphaeroplastigenens JC133 |
| Ga0586324 1381225 1 1056 | 10300000.00 | 6038400.00 | 0.00 | 0.00 | 0.00 | 2596622.17 | 3420000.00 | 12139000.00 | 1539000.00 | 0.00 | 0.00 | 16950000.00 | KO:K15580 | oligopeptide transport system substrate-binding protein | Bacteria;Chloroflexi;Anaerolineae;Anaerolineales;Anaerolineaceae;Levilinea;Levilinea saccharolytica;Levilinea saccharolytica DSM 16555 |
| Ga0586324 2000255 1 900 | 897474.00 | 0.00 | 0.00 | 0.00 | 0.00 | 0.00 | 0.00 | 0.00 | 0.00 | 0.00 | 0.00 | 0.00 | KO:K15580 | oligopeptide transport system substrate-binding protein | Bacteria;Chloroflexi;Thermomicrobia;Thermomicrobiales;Thermomicrobiaceae;Thermomicrobium;Thermomicrobium roseum;Thermomicrobium roseum DSM 5159 |
| Ga0586324 2949084 9 767 | 0.00 | 0.00 | 0.00 | 0.00 | 0.00 | 0.00 | 0.00 | 0.00 | 0.00 | 0.00 | 0.00 | 772500.00 | KO:K15580 | oligopeptide transport system substrate-binding protein | Bacteria;Chloroflexi;Anaerolineae;Anaerolineales;Anaerolineaceae;Thermanaerothrix;Thermanaerothrix daxensis;Thermanaerothrix daxensis GNS-1 |
| Ga0586324 3447989 2 703 | 0.00 | 0.00 | 0.00 | 0.00 | 0.00 | 0.00 | 0.00 | 0.00 | 0.00 | 1332100.00 | 0.00 | 458932.50 | KO:K15580 | oligopeptide transport system substrate-binding protein | Bacteria;Chloroflexi;Anaerolineae;Anaerolineales;Anaerolineaceae;Levilinea;Levilinea saccharolytica;Levilinea saccharolytica DSM 16555 |
| Ga0586324 4441022 3 647 | 0.00 | 12580000.00 | 0.00 | 4028000.00 | 0.00 | 0.00 | 0.00 | 0.00 | 0.00 | 0.00 | 0.00 | 0.00 | KO:K15580 | oligopeptide transport system substrate-binding protein | Bacteria;Chloroflexi;Anaerolineae;Anaerolineales;Anaerolineaceae;Thermanaerothrix;Thermanaerothrix daxensis;Thermanaerothrix daxensis GNS-1 |
| Ga0586324 5788662 1 579 | 6670000.00 | 0.00 | 8501300.00 | 7229500.00 | 0.00 | 0.00 | 0.00 | 0.00 | 0.00 | 0.00 | 0.00 | 0.00 | KO:K15580 | oligopeptide transport system substrate-binding protein | Bacteria;Chloroflexi;Anaerolineae;Anaerolineales;Anaerolineaceae;Thermanaerothrix;Thermanaerothrix daxensis;Thermanaerothrix daxensis GNS-1 |
| Ga0586324 6933049 3 539 | 0.00 | 6643600.00 | 0.00 | 0.00 | 0.00 | 0.00 | 0.00 | 0.00 | 0.00 | 0.00 | 0.00 | 0.00 | KO:K15580 | oligopeptide transport system substrate-binding protein | Bacteria;Chloroflexi;Anaerolineae;Anaerolineales;Anaerolineaceae;Thermanaerothrix;Thermanaerothrix daxensis;Thermanaerothrix daxensis GNS-1 |
| Ga0586324 7495251 3 521 | 0.00 | 0.00 | 9233200.00 | 988000.00 | 0.00 | 0.00 | 0.00 | 0.00 | 0.00 | 0.00 | 0.00 | 0.00 | KO:K15580 | oligopeptide transport system substrate-binding protein | Bacteria;Chloroflexi;Anaerolineae;Anaerolineales;Anaerolineaceae;Levilinea;Levilinea saccharolytica;Levilinea saccharolytica DSM 16555 |
| Ga0586324 7550333 3 521 | 0.00 | 4984400.00 | 0.00 | 0.00 | 0.00 | 0.00 | 0.00 | 0.00 | 0.00 | 0.00 | 0.00 | 0.00 | KO:K15580 | oligopeptide transport system substrate-binding protein | Bacteria;Chloroflexi;Anaerolineae;Anaerolineales;Anaerolineaceae;Levilinea;Levilinea saccharolytica;Levilinea saccharolytica DSM 16555 |
| Ga0586324 1655780 3 980 | 0.00 | 0.00 | 0.00 | 0.00 | 0.00 | 0.00 | 3648000.00 | 0.00 | 0.00 | 0.00 | 0.00 | 0.00 | KO:K15633 | 2,3-bisphosphoglycerate-independent phosphoglycerate mutase | Bacteria;Proteobacteria;Betaproteobacteria;Nitrosomonadales;Thiobacillaceae;Thiobacillus;Thiobacillus thioparus;Thiobacillus thioparus DSM 505 |
| Ga0586324 4365797 1 651 | 1410000.00 | 0.00 | 0.00 | 0.00 | 0.00 | 0.00 | 0.00 | 0.00 | 0.00 | 0.00 | 0.00 | 885000.00 | KO:K15635 | 2,3-bisphosphoglycerate-independent phosphoglycerate mutase | Bacteria;Verrucomicrobia;Verrucomicrobiae;Verrucomicrobiales;Verrucomicrobia subdivision 3;Pedosphaera;Pedosphaera parvula;Pedosphaera parvula Ellin514 |
| Ga0586324 0083219 2243 3511 | 1490000.00 | 3298000.00 | 0.00 | 1653000.00 | 1957000.00 | 6629900.00 | 0.00 | 1823900.00 | 4122900.00 | 8446900.00 | 5156800.00 | 6420000.00 | KO:K15770 | arabinogalactan oligomer/maltooligosaccharide transport system substrate-binding protein | Bacteria;Firmicutes;Clostridia;Eubacteriales;Clostridiales Family XVI. Incertae Sedis;Carboxydocella;Carboxydocella sporoproducens;Carboxydocella sporoproducens DSM 16521 |
| Ga0586324 0136239 1696 2961 | 2240000.00 | 0.00 | 0.00 | 0.00 | 0.00 | 0.00 | 0.00 | 0.00 | 0.00 | 0.00 | 0.00 | 0.00 | KO:K15770 | arabinogalactan oligomer/maltooligosaccharide transport system substrate-binding protein | Bacteria;Chloroflexi;Chloroflexia;Chloroflexales;Chloroflexaceae;Chloroflexus;Chloroflexus aurantiacus;Chloroflexus aurantiacus J-10-fl |
| Ga0586324 0560066 393 1559 | 1940000.00 | 0.00 | 0.00 | 0.00 | 0.00 | 0.00 | 0.00 | 0.00 | 0.00 | 1147300.00 | 0.00 | 0.00 | KO:K15770 | arabinogalactan oligomer/maltooligosaccharide transport system substrate-binding protein | Bacteria;Chloroflexi;Chloroflexia;Chloroflexales;Chloroflexaceae;Chloroflexus;Chloroflexus aurantiacus;Chloroflexus aurantiacus J-10-fl |
| Ga0586324 0805642 24 1334 | 0.00 | 4678400.00 | 0.00 | 0.00 | 0.00 | 0.00 | 0.00 | 0.00 | 0.00 | 0.00 | 0.00 | 0.00 | KO:K15770 | arabinogalactan oligomer/maltooligosaccharide transport system substrate-binding protein | Bacteria;Chloroflexi;Anaerolineae;Anaerolineales;Anaerolineaceae;Levilinea;Levilinea saccharolytica;Levilinea saccharolytica DSM 16555 |
| Ga0586324 2211455 3 866 | 4280000.00 | 0.00 | 0.00 | 0.00 | 0.00 | 0.00 | 0.00 | 0.00 | 0.00 | 0.00 | 0.00 | 0.00 | KO:K15770 | arabinogalactan oligomer/maltooligosaccharide transport system substrate-binding protein | Bacteria;Chloroflexi;Chloroflexia;Chloroflexales;Chloroflexaceae;Chloroflexus;Chloroflexus aurantiacus;Chloroflexus aurantiacus J-10-fl |
| Ga0586324 3371951 3 725 | 0.00 | 0.00 | 0.00 | 0.00 | 1812800.00 | 0.00 | 0.00 | 0.00 | 0.00 | 0.00 | 0.00 | 0.00 | KO:K15770 | arabinogalactan oligomer/maltooligosaccharide transport system substrate-binding protein | Bacteria;Caldiserica;Caldisericia;Caldisericales;Caldisericaceae;Caldisericum;Caldisericum exile;Caldisericum exile AZM16c01, NBRC 104410 |
| Ga0586324 0000123 34440 35822 | 0.00 | 0.00 | 0.00 | 0.00 | 0.00 | 0.00 | 0.00 | 0.00 | 0.00 | 2571800.00 | 0.00 | 0.00 | KO:K15778 | phosphomannomutase/phosphoglucomutase | Bacteria;Proteobacteria;Betaproteobacteria;Nitrosomonadales;Thiobacillaceae;Annwoodia;Annwoodia aquaesulis;Annwoodia aquaesulis DSM 4255 |
| Ga0586324 0001357 4563 5936 | 0.00 | 0.00 | 0.00 | 0.00 | 0.00 | 0.00 | 0.00 | 0.00 | 0.00 | 1031800.00 | 0.00 | 0.00 | KO:K15778 | phosphomannomutase/phosphoglucomutase | Bacteria;Proteobacteria;Betaproteobacteria;Nitrosomonadales;Thiobacillaceae;Thiobacillus;Thiobacillus denitrificans;Thiobacillus denitrificans RG |
| Ga0586324 0611666 709 1500 | 0.00 | 0.00 | 7487900.00 | 11580500.00 | 2286600.00 | 0.00 | 0.00 | 872300.00 | 0.00 | 0.00 | 0.00 | 3435000.00 | KO:K15778 | phosphomannomutase/phosphoglucomutase | Bacteria;Candidatus Aminicenantes;unclassified Candidatus Aminicenantes;unclassified Candidatus Aminicenantes;unclassified Candidatus Aminicenantes;unclassified Candidatus Aminicenantes;Aminicenantes bacterium SCGC AAA255-A06;Aminicenantes bacterium SCGC AAA255-A06 (contamination screened) |
| Ga0586324 1061337 456 1184 | 0.00 | 0.00 | 0.00 | 0.00 | 0.00 | 0.00 | 0.00 | 0.00 | 0.00 | 2648800.00 | 1082400.00 | 0.00 | KO:K15778 | phosphomannomutase/phosphoglucomutase | Bacteria;Proteobacteria;Betaproteobacteria;Nitrosomonadales;Thiobacillaceae;Thiobacillus;Thiobacillus thiophilus;Thiobacillus thiophilus DSM 19892 |
| Ga0586324 0013864 5664 7262 | 3070000.00 | 0.00 | 0.00 | 0.00 | 0.00 | 0.00 | 0.00 | 0.00 | 0.00 | 0.00 | 4884000.00 | 0.00 | KO:K15864 | nitrite reductase (NO-forming)/hydroxylamine reductase | Bacteria;Proteobacteria;Gammaproteobacteria;Methylococcales;Methylococcaceae;Methylobacter;Methylobacter tundripaludum;Methylobacter tundripaludum OWC-G53F |
| Ga0586324 0027612 1 1539 | 8790000.00 | 9057600.00 | 0.00 | 2261000.00 | 0.00 | 0.00 | 0.00 | 5105700.00 | 5605200.00 | 0.00 | 0.00 | 0.00 | KO:K15864 | nitrite reductase (NO-forming)/hydroxylamine reductase | Bacteria;Proteobacteria;Gammaproteobacteria;Methylococcales;Methylococcaceae;Methylobacter;Methylobacter tundripaludum;Methylobacter tundripaludum 31/32 |
| Ga0586324 0168076 1042 2682 | 0.00 | 0.00 | 14187600.00 | 0.00 | 0.00 | 6352000.00 | 0.00 | 2342400.00 | 0.00 | 4835600.00 | 0.00 | 4792500.00 | KO:K15864 | nitrite reductase (NO-forming)/hydroxylamine reductase | Bacteria;Proteobacteria;Gammaproteobacteria;Chromatiales;Ectothiorhodospiraceae;Thiogranum;Thiogranum longum;Thiogranum longum DSM 19610 |
| Ga0586324 0870388 2 1288 | 0.00 | 5372000.00 | 0.00 | 0.00 | 0.00 | 0.00 | 0.00 | 0.00 | 0.00 | 0.00 | 0.00 | 0.00 | KO:K15864 | nitrite reductase (NO-forming)/hydroxylamine reductase | Bacteria;Proteobacteria;Betaproteobacteria;Burkholderiales;unclassified Burkholderiales;Inhella;Inhella inkyongensis;Inhella inkyongensis IMCC1713 |
| Ga0586324 0962956 2 835 | 0.00 | 4753200.00 | 0.00 | 0.00 | 0.00 | 0.00 | 0.00 | 0.00 | 0.00 | 0.00 | 0.00 | 0.00 | KO:K15864 | nitrite reductase (NO-forming)/hydroxylamine reductase | Bacteria;Proteobacteria;Gammaproteobacteria;Methylococcales;Methylococcaceae;Methylobacter;Methylobacter tundripaludum;Methylobacter tundripaludum 31/32 |
| Ga0586324 1223024 1 789 | 6140000.00 | 0.00 | 0.00 | 0.00 | 0.00 | 0.00 | 0.00 | 0.00 | 0.00 | 0.00 | 0.00 | 0.00 | KO:K15864 | nitrite reductase (NO-forming)/hydroxylamine reductase | Bacteria;Proteobacteria;Gammaproteobacteria;Methylococcales;Methylococcaceae;Methylobacter;Methylobacter tundripaludum;Methylobacter tundripaludum 31/32 |
| Ga0586324 1623591 1 987 | 0.00 | 0.00 | 0.00 | 0.00 | 0.00 | 0.00 | 11126400.00 | 11041000.00 | 6650100.00 | 0.00 | 0.00 | 0.00 | KO:K15864 | nitrite reductase (NO-forming)/hydroxylamine reductase | Bacteria;Proteobacteria;Betaproteobacteria;Nitrosomonadales;Sulfuricellaceae;Sulfuricella;Sulfuricella sp. T08;Sulfuricella sp. T08 |
| Ga0586324 3190517 1 741 | 0.00 | 0.00 | 4350374.19 | 0.00 | 0.00 | 0.00 | 0.00 | 0.00 | 0.00 | 0.00 | 0.00 | 0.00 | KO:K15864 | nitrite reductase (NO-forming)/hydroxylamine reductase | Bacteria;Proteobacteria;Betaproteobacteria;Rhodocyclales;Azonexaceae;Dechloromonas;Dechloromonas denitrificans;Dechloromonas denitrificans ATCC BAA-841 |
| Ga0586324 0010664 3365 4309 | 77320000.00 | 49483600.00 | 45434100.00 | 10070000.00 | 11433000.00 | 24693400.00 | 24168000.00 | 0.00 | 14580000.00 | 15092000.00 | 27984000.00 | 21427500.00 | KO:K15986 | manganese-dependent inorganic pyrophosphatase | Archaea;Euryarchaeota;Methanomicrobia;Methanosarcinales;Methanotrichaceae;Methanothrix;Methanothrix soehngenii;Methanothrix soehngenii GP-6 |
| Ga0586324 0030240 4799 5728 | 0.00 | 0.00 | 0.00 | 0.00 | 0.00 | 0.00 | 3670800.00 | 0.00 | 0.00 | 0.00 | 0.00 | 0.00 | KO:K15986 | manganese-dependent inorganic pyrophosphatase | Bacteria;Proteobacteria;Deltaproteobacteria;Desulfobacterales;Desulfobulbaceae;Desulfopila;Desulfopila aestuarii;Desulfopila aestuarii DSM 18488 |
| Ga0586324 0187049 1409 2407 | 0.00 | 6670800.00 | 0.00 | 0.00 | 0.00 | 0.00 | 0.00 | 732000.00 | 2664900.00 | 0.00 | 0.00 | 0.00 | KO:K15986 | manganese-dependent inorganic pyrophosphatase | Archaea;Euryarchaeota;Methanomicrobia;Methanosarcinales;Methanotrichaceae;Methanothrix;Methanothrix soehngenii;Methanothrix soehngenii GP-6 |
| Ga0586324 0375032 1 558 | 0.00 | 1692284.72 | 0.00 | 0.00 | 0.00 | 0.00 | 0.00 | 0.00 | 0.00 | 0.00 | 0.00 | 0.00 | KO:K15986 | manganese-dependent inorganic pyrophosphatase | Archaea;Euryarchaeota;Methanomicrobia;Methanosarcinales;Methanotrichaceae;Methanothrix;Methanothrix soehngenii;Methanothrix soehngenii GP-6 |
| Ga0586324 1214846 2 475 | 8720000.00 | 0.00 | 14581700.00 | 0.00 | 0.00 | 13339200.00 | 11445600.00 | 0.00 | 0.00 | 0.00 | 0.00 | 0.00 | KO:K15986 | manganese-dependent inorganic pyrophosphatase | Archaea;Euryarchaeota;Methanomicrobia;Methanosarcinales;Methanotrichaceae;Methanothrix;Methanothrix soehngenii;Methanothrix soehngenii GP-6 |
| Ga0586324 1833263 2 808 | 40900000.00 | 9180000.00 | 35131200.00 | 2935500.00 | 0.00 | 0.00 | 0.00 | 1067500.00 | 0.00 | 2833600.00 | 0.00 | 12525000.00 | KO:K15986 | manganese-dependent inorganic pyrophosphatase | Archaea;Euryarchaeota;Methanomicrobia;Methanosarcinales;Methanotrichaceae;Methanothrix;Methanothrix soehngenii;Methanothrix soehngenii GP-6 |
| Ga0586324 2146638 2 877 | 12149099.00 | 3128888.08 | 0.00 | 1691000.00 | 0.00 | 0.00 | 0.00 | 1372500.00 | 1142100.00 | 1786400.00 | 2780800.00 | 438200.25 | KO:K15986 | manganese-dependent inorganic pyrophosphatase | Archaea;Euryarchaeota;Methanomicrobia;Methanosarcinales;Methanotrichaceae;Methanothrix;Methanothrix soehngenii;Methanothrix soehngenii GP-6 |
| Ga0586324 2809471 2 784 | 0.00 | 7622800.00 | 0.00 | 0.00 | 5726800.00 | 0.00 | 3648000.00 | 4196800.00 | 0.00 | 0.00 | 0.00 | 1665000.00 | KO:K15986 | manganese-dependent inorganic pyrophosphatase | Archaea;Euryarchaeota;Methanomicrobia;Methanosarcinales;Methanotrichaceae;Methanothrix;Methanothrix soehngenii;Methanothrix soehngenii GP-6 |
| Ga0586324 3276020 2 406 | 1550000.00 | 2726800.00 | 0.00 | 0.00 | 0.00 | 0.00 | 0.00 | 0.00 | 0.00 | 0.00 | 0.00 | 0.00 | KO:K15986 | manganese-dependent inorganic pyrophosphatase | Archaea;Euryarchaeota;Methanomicrobia;Methanosarcinales;Methanotrichaceae;Methanothrix;Methanothrix soehngenii;Methanothrix soehngenii GP-6 |
| Ga0586324 6003170 1 534 | 0.00 | 635245.12 | 0.00 | 0.00 | 0.00 | 0.00 | 0.00 | 0.00 | 0.00 | 0.00 | 0.00 | 0.00 | KO:K15986 | manganese-dependent inorganic pyrophosphatase | Archaea;Euryarchaeota;Methanomicrobia;Methanosarcinales;Methanotrichaceae;Methanothrix;Methanothrix soehngenii;Methanothrix soehngenii GP-6 |
| Ga0586324 0002303 14722 16767 | 0.00 | 0.00 | 0.00 | 0.00 | 3120900.00 | 0.00 | 0.00 | 57480300.00 | 3685500.00 | 0.00 | 0.00 | 0.00 | KO:K15987 | K(+)-stimulated pyrophosphate-energized sodium pump | Bacteria;Proteobacteria;Deltaproteobacteria;Syntrophobacterales;Syntrophobacteraceae;Syntrophobacter;Syntrophobacter fumaroxidans;Syntrophobacter fumaroxidans MPOB |
| Ga0586324 0065565 1414 3468 | 0.00 | 0.00 | 0.00 | 0.00 | 0.00 | 0.00 | 0.00 | 0.00 | 18144000.00 | 0.00 | 0.00 | 0.00 | KO:K15987 | K(+)-stimulated pyrophosphate-energized sodium pump | Bacteria;Nitrospirae;Nitrospira;Nitrospirales;Nitrospiraceae;Nitrospira;Nitrospira lenta;Nitrospira lenta N. lenta BS10 |
| Ga0586324 0124297 1 1938 | 0.00 | 0.00 | 0.00 | 0.00 | 0.00 | 0.00 | 0.00 | 103700000.00 | 79137000.00 | 0.00 | 0.00 | 0.00 | KO:K15987 | K(+)-stimulated pyrophosphate-energized sodium pump | Archaea;Euryarchaeota;Methanomicrobia;Methanomicrobiales;Methanoregulaceae;Methanosphaerula;Methanosphaerula palustris;Methanosphaerula palustris E1-9c, DSM 19958 |
| Ga0586324 0148642 1 2061 | 0.00 | 12444000.00 | 0.00 | 0.00 | 75293000.00 | 0.00 | 1663196.16 | 0.00 | 0.00 | 0.00 | 0.00 | 0.00 | KO:K15987 | K(+)-stimulated pyrophosphate-energized sodium pump | Bacteria;Proteobacteria;Deltaproteobacteria;Syntrophobacterales;Syntrophaceae;Desulfobacca;Desulfobacca acetoxidans;Desulfobacca acetoxidans ASRB2, DSM 11109 |
| Ga0586324 0187438 3 2033 | 0.00 | 0.00 | 0.00 | 0.00 | 0.00 | 0.00 | 7045200.00 | 0.00 | 0.00 | 0.00 | 0.00 | 0.00 | KO:K15987 | K(+)-stimulated pyrophosphate-energized sodium pump | Bacteria;Proteobacteria;Deltaproteobacteria;Desulfobacterales;Desulfobacteraceae;Desulfatiglans;Desulfatiglans anilini;Desulfatiglans anilini DSM 4660 |
| Ga0586324 0226948 525 2333 | 0.00 | 0.00 | 0.00 | 1216000.00 | 17819000.00 | 0.00 | 5358000.00 | 0.00 | 0.00 | 0.00 | 0.00 | 0.00 | KO:K15987 | K(+)-stimulated pyrophosphate-energized sodium pump | Bacteria;Proteobacteria;Deltaproteobacteria;Syntrophobacterales;Syntrophaceae;Desulfobacca;Desulfobacca acetoxidans;Desulfobacca acetoxidans ASRB2, DSM 11109 |
| Ga0586324 0231778 253 2313 | 0.00 | 0.00 | 0.00 | 0.00 | 0.00 | 0.00 | 291840000.00 | 0.00 | 0.00 | 0.00 | 0.00 | 0.00 | KO:K15987 | K(+)-stimulated pyrophosphate-energized sodium pump | Bacteria;Firmicutes;Clostridia;Thermoanaerobacterales;Thermoanaerobacteraceae;Thermoanaerobacter;Thermoanaerobacter uzonensis;Thermoanaerobacter uzonensis DSM 18761 |
| Ga0586324 0368554 536 1876 | 0.00 | 0.00 | 0.00 | 0.00 | 0.00 | 0.00 | 74100000.00 | 24217000.00 | 19602000.00 | 0.00 | 0.00 | 0.00 | KO:K15987 | K(+)-stimulated pyrophosphate-energized sodium pump | Bacteria;Actinobacteria;Rubrobacteria;Gaiellales;Gaiellaceae;Gaiella;Gaiella occulta;Gaiella occulta F2-233 |
| Ga0586324 1040422 544 1194 | 0.00 | 0.00 | 0.00 | 49495000.00 | 0.00 | 0.00 | 0.00 | 786900.00 | 0.00 | 0.00 | 0.00 | 0.00 | KO:K15987 | K(+)-stimulated pyrophosphate-energized sodium pump | Archaea;Euryarchaeota;Methanomicrobia;Methanomicrobiales;Methanoregulaceae;Methanosphaerula;Methanosphaerula palustris;Methanosphaerula palustris E1-9c, DSM 19958 |
| Ga0586324 1045194 129 1190 | 0.00 | 9248000.00 | 0.00 | 0.00 | 0.00 | 0.00 | 0.00 | 0.00 | 0.00 | 0.00 | 0.00 | 0.00 | KO:K15987 | K(+)-stimulated pyrophosphate-energized sodium pump | Bacteria;Proteobacteria;Betaproteobacteria;Burkholderiales;unclassified Burkholderiales;Ideonella;Ideonella sp. A 288;Ideonella sp. A 288 |
| Ga0586324 1257060 455 1099 | 0.00 | 0.00 | 0.00 | 0.00 | 0.00 | 0.00 | 0.00 | 5898700.00 | 0.00 | 0.00 | 0.00 | 0.00 | KO:K15987 | K(+)-stimulated pyrophosphate-energized sodium pump | Bacteria;Proteobacteria;Betaproteobacteria;Nitrosomonadales;Sterolibacteriaceae;Sterolibacterium;Sterolibacterium denitrificans;Sterolibacterium denitrificans DSM 13999 |
| Ga0586324 2735081 3 689 | 0.00 | 0.00 | 0.00 | 0.00 | 0.00 | 0.00 | 10191600.00 | 0.00 | 0.00 | 0.00 | 0.00 | 0.00 | KO:K15987 | K(+)-stimulated pyrophosphate-energized sodium pump | Bacteria;Nitrospirae;Nitrospira;Nitrospirales;Nitrospiraceae;Candidatus Magnetobacterium;Candidatus Magnetobacterium casensis;Candidatus Magnetobacterium casensis MYR-1 |
| Ga0586324 2828280 3 581 | 0.00 | 0.00 | 0.00 | 0.00 | 0.00 | 0.00 | 6156000.00 | 0.00 | 0.00 | 0.00 | 0.00 | 0.00 | KO:K15987 | K(+)-stimulated pyrophosphate-energized sodium pump | Bacteria;Proteobacteria;Deltaproteobacteria;Syntrophobacterales;Syntrophobacteraceae;Syntrophobacter;Syntrophobacter fumaroxidans;Syntrophobacter fumaroxidans MPOB |
| Ga0586324 5533072 285 590 | 0.00 | 0.00 | 0.00 | 0.00 | 0.00 | 0.00 | 0.00 | 0.00 | 3782700.00 | 0.00 | 0.00 | 0.00 | KO:K15987 | K(+)-stimulated pyrophosphate-energized sodium pump | Bacteria;Proteobacteria;Deltaproteobacteria;Syntrophobacterales;Syntrophobacteraceae;Syntrophobacter;Syntrophobacter fumaroxidans;Syntrophobacter fumaroxidans MPOB |
| Ga0586324 6996178 1 504 | 0.00 | 0.00 | 0.00 | 0.00 | 4274500.00 | 0.00 | 0.00 | 0.00 | 0.00 | 0.00 | 0.00 | 0.00 | KO:K15987 | K(+)-stimulated pyrophosphate-energized sodium pump | Bacteria;Firmicutes;Clostridia;Thermoanaerobacterales;Thermoanaerobacteraceae;Caldanaerobacter;Caldanaerobacter subterraneus;Thermoanaerobacter tengcongensis MB4 |
| Ga0586324 7399756 1 525 | 0.00 | 0.00 | 0.00 | 4911500.00 | 8178200.00 | 0.00 | 12106800.00 | 0.00 | 0.00 | 0.00 | 0.00 | 1927500.00 | KO:K15987 | K(+)-stimulated pyrophosphate-energized sodium pump | Bacteria;Proteobacteria;Betaproteobacteria;unclassified Betaproteobacteria;unclassified Betaproteobacteria;unclassified Betaproteobacteria;Betaproteobacteria bacterium GR16-43;Betaproteobacteria bacterium GR16-43 |
| Ga0586324 0000244 38796 39410 | 7880000.00 | 0.00 | 0.00 | 0.00 | 0.00 | 0.00 | 11058000.00 | 2714500.00 | 27912600.00 | 1586200.00 | 35472800.00 | 42375000.00 | KO:K16079 | outer membrane immunogenic protein | Bacteria;Proteobacteria;Alphaproteobacteria;Hyphomicrobiales;Methylocystaceae;Methylosinus;Methylosinus sp. R-45379;Methylosinus sp. R-45379 |
| Ga0586324 0002577 1398 2234 | 0.00 | 0.00 | 0.00 | 0.00 | 0.00 | 0.00 | 0.00 | 0.00 | 0.00 | 1270500.00 | 0.00 | 0.00 | KO:K16079 | outer membrane immunogenic protein | Bacteria;Proteobacteria;Alphaproteobacteria;Hyphomicrobiales;Bradyrhizobiaceae;Bradyrhizobium;Bradyrhizobium erythrophlei;Bradyrhizobium erythrophlei GAS138 |
| Ga0586324 0003003 11032 11772 | 0.00 | 0.00 | 0.00 | 0.00 | 0.00 | 10322000.00 | 0.00 | 11773000.00 | 0.00 | 0.00 | 0.00 | 0.00 | KO:K16079 | outer membrane immunogenic protein | Bacteria;Proteobacteria;Alphaproteobacteria;Hyphomicrobiales;Bradyrhizobiaceae;Rhodopseudomonas;Rhodopseudomonas palustris;Rhodopseudomonas palustris 2.1.18 |
| Ga0586324 0006227 11928 12482 | 32200000.00 | 30872000.00 | 0.00 | 48640000.00 | 34814000.00 | 0.00 | 0.00 | 19276000.00 | 22275000.00 | 24486000.00 | 18392000.00 | 18225000.00 | KO:K16079 | outer membrane immunogenic protein | Bacteria;Proteobacteria;Alphaproteobacteria;Hyphomicrobiales;Bradyrhizobiaceae;Bradyrhizobium;Bradyrhizobium sp. Leaf396;Bradyrhizobium sp. Leaf396 |
| Ga0586324 0022259 2310 3026 | 6630000.00 | 0.00 | 0.00 | 0.00 | 0.00 | 0.00 | 0.00 | 0.00 | 0.00 | 0.00 | 0.00 | 0.00 | KO:K16079 | outer membrane immunogenic protein | Bacteria;Proteobacteria;Alphaproteobacteria;Hyphomicrobiales;Boseaceae;Bosea;Bosea psychrotolerans;Bosea psychrolimnosis 1131 |
| Ga0586324 0061464 2705 3613 | 0.00 | 0.00 | 0.00 | 0.00 | 0.00 | 0.00 | 0.00 | 0.00 | 0.00 | 0.00 | 9768000.00 | 0.00 | KO:K16079 | outer membrane immunogenic protein | Bacteria;Proteobacteria;Alphaproteobacteria;Hyphomicrobiales;Bradyrhizobiaceae;Bradyrhizobium;Bradyrhizobium sp. Ec3.3;Bradyrhizobium sp. EC3.3 |
| Ga0586324 0099833 2426 3193 | 0.00 | 0.00 | 0.00 | 0.00 | 468020.67 | 0.00 | 0.00 | 0.00 | 0.00 | 0.00 | 0.00 | 0.00 | KO:K16079 | outer membrane immunogenic protein | Bacteria;Proteobacteria;Alphaproteobacteria;Hyphomicrobiales;Bradyrhizobiaceae;Afipia;Afipia broomeae;Afipia broomeae GAS525 |
| Ga0586324 0148851 1949 2719 | 0.00 | 0.00 | 0.00 | 2517500.00 | 0.00 | 0.00 | 0.00 | 0.00 | 0.00 | 0.00 | 0.00 | 0.00 | KO:K16079 | outer membrane immunogenic protein | Bacteria;Proteobacteria;Alphaproteobacteria;Hyphomicrobiales;Bradyrhizobiaceae;Bradyrhizobium;Bradyrhizobium betae;Bradyrhizobium betae CECT 5829 |
| Ga0586324 0192008 318 1112 | 3070000.00 | 3733200.00 | 0.00 | 4702500.00 | 3811000.00 | 0.00 | 0.00 | 3178100.00 | 3636900.00 | 0.00 | 0.00 | 0.00 | KO:K16079 | outer membrane immunogenic protein | Bacteria;Proteobacteria;Alphaproteobacteria;Hyphomicrobiales;Bradyrhizobiaceae;Bradyrhizobium;Bradyrhizobium lablabi;Bradyrhizobium lablabi GAS165 |
| Ga0586324 0278907 946 1617 | 814372.00 | 0.00 | 0.00 | 14725000.00 | 0.00 | 0.00 | 0.00 | 10736000.00 | 0.00 | 0.00 | 0.00 | 6202500.00 | KO:K16079 | outer membrane immunogenic protein | Bacteria;Acidobacteria;Acidobacteriia;Acidobacteriales;Acidobacteriaceae;Granulicella;Granulicella mallensis;Granulicella mallensis MP5ACTX8 |
| Ga0586324 0329348 604 1251 | 0.00 | 0.00 | 0.00 | 0.00 | 0.00 | 0.00 | 9918000.00 | 0.00 | 0.00 | 0.00 | 0.00 | 0.00 | KO:K16079 | outer membrane immunogenic protein | Bacteria;Proteobacteria;Alphaproteobacteria;Hyphomicrobiales;Bradyrhizobiaceae;Bradyrhizobium;Bradyrhizobium jicamae;Bradyrhizobium jicamae PAC68 |
| Ga0586324 0384432 890 1603 | 0.00 | 0.00 | 0.00 | 1130500.00 | 0.00 | 0.00 | 0.00 | 0.00 | 0.00 | 0.00 | 0.00 | 0.00 | KO:K16079 | outer membrane immunogenic protein | Bacteria;Proteobacteria;Alphaproteobacteria;Hyphomicrobiales;Bradyrhizobiaceae;Bradyrhizobium;Bradyrhizobium canariense;Bradyrhizobium canariense GAS369 |
| Ga0586324 5575844 21 590 | 0.00 | 0.00 | 0.00 | 0.00 | 0.00 | 0.00 | 15139200.00 | 0.00 | 0.00 | 0.00 | 0.00 | 0.00 | KO:K16079 | outer membrane immunogenic protein | Bacteria;Proteobacteria;Alphaproteobacteria;Hyphomicrobiales;Bradyrhizobiaceae;Bradyrhizobium;Bradyrhizobium sp. OAE829;Bradyrhizobium sp. OAE829 (Illumina draft) |
| Ga0586324 6846245 1 543 | 0.00 | 140372.40 | 0.00 | 8483500.00 | 0.00 | 0.00 | 831657.36 | 0.00 | 5459400.00 | 0.00 | 1566400.00 | 1365000.00 | KO:K16079 | outer membrane immunogenic protein | Bacteria;Proteobacteria;Alphaproteobacteria;Hyphomicrobiales;Bradyrhizobiaceae;Bradyrhizobium;Bradyrhizobium erythrophlei;Bradyrhizobium erythrophlei GAS401 |
| Ga0586324 0396875 676 1725 | 0.00 | 0.00 | 2363873.73 | 0.00 | 0.00 | 0.00 | 0.00 | 0.00 | 0.00 | 0.00 | 0.00 | 0.00 | KO:K16167 | alkylresorcinol/alkylpyrone synthase | Bacteria;Acidobacteria;Acidobacteriia;Acidobacteriales;Acidobacteriaceae;Candidatus Koribacter;Candidatus Koribacter versatilis;Candidatus Koribacter versatilis Ellin345 |
| Ga0586324 0027042 5029 6192 | 0.00 | 0.00 | 0.00 | 0.00 | 11021000.00 | 0.00 | 0.00 | 0.00 | 515424.87 | 0.00 | 0.00 | 0.00 | KO:K16173 | glutaryl-CoA dehydrogenase (non-decarboxylating) | Bacteria;Proteobacteria;Deltaproteobacteria;Desulfobacterales;Desulfobacteraceae;Desulfatirhabdium;Desulfatirhabdium butyrativorans;Desulfatirhabdium butyrativorans DSM 18734 |
| Ga0586324 0230104 3 350 | 0.00 | 225654.60 | 0.00 | 0.00 | 0.00 | 0.00 | 0.00 | 0.00 | 0.00 | 0.00 | 0.00 | 0.00 | KO:K16173 | glutaryl-CoA dehydrogenase (non-decarboxylating) | Bacteria;Proteobacteria;Deltaproteobacteria;Syntrophorhabdales;Syntrophorhabdaceae;Syntrophorhabdus;Syntrophorhabdus aromaticivorans;Syntrophorhabdus aromaticivorans UI |
| Ga0586324 0513750 3 992 | 0.00 | 0.00 | 0.00 | 0.00 | 0.00 | 0.00 | 0.00 | 0.00 | 470673.18 | 0.00 | 0.00 | 0.00 | KO:K16173 | glutaryl-CoA dehydrogenase (non-decarboxylating) | Bacteria;Proteobacteria;Deltaproteobacteria;Desulfobacterales;Desulfobacteraceae;Desulfatirhabdium;Desulfatirhabdium butyrativorans;Desulfatirhabdium butyrativorans DSM 18734 |
| Ga0586324 0567595 1034 1552 | 0.00 | 225641.68 | 0.00 | 0.00 | 0.00 | 0.00 | 0.00 | 0.00 | 0.00 | 0.00 | 0.00 | 0.00 | KO:K16173 | glutaryl-CoA dehydrogenase (non-decarboxylating) | Bacteria;Proteobacteria;Deltaproteobacteria;Syntrophorhabdales;Syntrophorhabdaceae;Syntrophorhabdus;Syntrophorhabdus aromaticivorans;Syntrophorhabdus aromaticivorans UI |
| Ga0586324 0650534 917 1462 | 0.00 | 0.00 | 0.00 | 0.00 | 0.00 | 0.00 | 0.00 | 0.00 | 233697.15 | 0.00 | 0.00 | 0.00 | KO:K16173 | glutaryl-CoA dehydrogenase (non-decarboxylating) | Bacteria;Proteobacteria;Deltaproteobacteria;Desulfobacterales;Desulfobacteraceae;Desulfatiglans;Desulfatiglans anilini;Desulfatiglans anilini DSM 4660 |
| Ga0586324 0167625 923 1561 | 0.00 | 0.00 | 0.00 | 0.00 | 4387800.00 | 0.00 | 0.00 | 0.00 | 7654500.00 | 0.00 | 0.00 | 0.00 | KO:K16179 | dimethylamine corrinoid protein | Archaea;Candidatus Thermoplasmatota;Thermoplasmata;Methanomassiliicoccales;Methanomassiliicoccaceae;Methanomassiliicoccus;Methanomassiliicoccus luminyensis;Methanomassiliicoccus luminyensis B10 |
| Ga0586324 0502047 1 1368 | 0.00 | 0.00 | 0.00 | 0.00 | 0.00 | 0.00 | 0.00 | 0.00 | 132396.12 | 0.00 | 0.00 | 0.00 | KO:K16692 | tyrosine-protein kinase Etk/Wzc | Bacteria;Bacteroidetes;Bacteroidia;Bacteroidales;Lentimicrobiaceae;Lentimicrobium;Lentimicrobium saccharophilum;Bacteroidales bacterium TBC1 |
| Ga0586324 1305675 1 1083 | 0.00 | 0.00 | 0.00 | 0.00 | 0.00 | 0.00 | 280036.44 | 0.00 | 0.00 | 0.00 | 0.00 | 0.00 | KO:K16881 | mannose-1-phosphate guanylyltransferase/phosphomannomutase | Bacteria;Actinobacteria;Actinomycetia;unclassified Actinomycetia;unclassified Actinomycetia;unclassified Actinomycetia;Actinobacteria bacterium IMCC26207;Actinobacteria bacterium IMCC26207 |
| Ga0586324 0000070 39790 41085 | 0.00 | 0.00 | 0.00 | 0.00 | 0.00 | 0.00 | 0.00 | 0.00 | 0.00 | 22484000.00 | 28952000.00 | 25882500.00 | KO:K16885 | quinone-modifying oxidoreductase subunit QmoA | Bacteria;Proteobacteria;Betaproteobacteria;Nitrosomonadales;Thiobacillaceae;Annwoodia;Annwoodia aquaesulis;Annwoodia aquaesulis DSM 4255 |
| Ga0586324 0035720 3907 5175 | 0.00 | 0.00 | 0.00 | 2118500.00 | 0.00 | 0.00 | 6247200.00 | 4337100.00 | 0.00 | 0.00 | 0.00 | 0.00 | KO:K16885 | quinone-modifying oxidoreductase subunit QmoA | Bacteria;Proteobacteria;Deltaproteobacteria;Syntrophobacterales;Syntrophobacteraceae;Desulfacinum;Desulfacinum hydrothermale;Desulfacinum hydrothermale DSM 13146 |
| Ga0586324 0109371 3 1262 | 0.00 | 1666000.00 | 3086985.30 | 4056500.00 | 4542300.00 | 3837560.80 | 5677200.00 | 2964600.00 | 2089800.00 | 2225300.00 | 2059200.00 | 0.00 | KO:K16885 | quinone-modifying oxidoreductase subunit QmoA | Bacteria;Proteobacteria;Deltaproteobacteria;Syntrophobacterales;Syntrophobacteraceae;Desulfacinum;Desulfacinum hydrothermale;Desulfacinum hydrothermale DSM 13146 |
| Ga0586324 0179143 1131 2417 | 0.00 | 0.00 | 0.00 | 0.00 | 0.00 | 0.00 | 9484800.00 | 1634800.00 | 1757700.00 | 7499800.00 | 14264800.00 | 18517500.00 | KO:K16885 | quinone-modifying oxidoreductase subunit QmoA | Bacteria;Proteobacteria;Betaproteobacteria;Nitrosomonadales;Sulfuricellaceae;Sulfurirhabdus;Sulfurirhabdus autotrophica;Sulfurirhabdus autotrophica DSM 100309 |
| Ga0586324 0388698 1 741 | 0.00 | 0.00 | 0.00 | 2337000.00 | 0.00 | 0.00 | 0.00 | 0.00 | 0.00 | 0.00 | 0.00 | 0.00 | KO:K16885 | quinone-modifying oxidoreductase subunit QmoA | Bacteria;Proteobacteria;Deltaproteobacteria;Syntrophobacterales;Syntrophobacteraceae;Desulfacinum;Desulfacinum hydrothermale;Desulfacinum hydrothermale DSM 13146 |
| Ga0586324 0557942 675 1562 | 0.00 | 0.00 | 0.00 | 4883000.00 | 0.00 | 0.00 | 0.00 | 0.00 | 0.00 | 0.00 | 0.00 | 0.00 | KO:K16885 | quinone-modifying oxidoreductase subunit QmoA | Bacteria;Proteobacteria;Deltaproteobacteria;Syntrophobacterales;Syntrophobacteraceae;Syntrophobacter;Syntrophobacter fumaroxidans;Syntrophobacter fumaroxidans MPOB |
| Ga0586324 0896937 1 1182 | 0.00 | 5304000.00 | 0.00 | 0.00 | 0.00 | 0.00 | 0.00 | 0.00 | 0.00 | 0.00 | 3713600.00 | 0.00 | KO:K16885 | quinone-modifying oxidoreductase subunit QmoA | Bacteria;Proteobacteria;Deltaproteobacteria;Syntrophobacterales;Syntrophaceae;Desulfobacca;Desulfobacca acetoxidans;Desulfobacca acetoxidans ASRB2, DSM 11109 |
| Ga0586324 2885863 1 774 | 1840000.00 | 0.00 | 0.00 | 3163500.00 | 0.00 | 1842913.70 | 0.00 | 0.00 | 0.00 | 0.00 | 0.00 | 0.00 | KO:K16885 | quinone-modifying oxidoreductase subunit QmoA | Bacteria;Proteobacteria;Deltaproteobacteria;Syntrophobacterales;Syntrophaceae;Desulfobacca;Desulfobacca acetoxidans;Desulfobacca acetoxidans ASRB2, DSM 11109 |
| Ga0586324 3080630 3 752 | 0.00 | 0.00 | 0.00 | 0.00 | 0.00 | 3847874.86 | 0.00 | 0.00 | 0.00 | 0.00 | 0.00 | 0.00 | KO:K16885 | quinone-modifying oxidoreductase subunit QmoA | Bacteria;Proteobacteria;Deltaproteobacteria;Syntrophobacterales;Syntrophaceae;Desulfobacca;Desulfobacca acetoxidans;Desulfobacca acetoxidans ASRB2, DSM 11109 |
| Ga0586324 3615294 3 704 | 0.00 | 0.00 | 0.00 | 0.00 | 5778300.00 | 0.00 | 0.00 | 0.00 | 0.00 | 0.00 | 0.00 | 0.00 | KO:K16885 | quinone-modifying oxidoreductase subunit QmoA | Bacteria;Proteobacteria;Deltaproteobacteria;Desulfobacterales;Desulfobacteraceae;Desulfosarcina;Desulfosarcina sp. BuS5;Desulfosarcina sp. BuS5 |
| Ga0586324 4536821 3 641 | 0.00 | 0.00 | 0.00 | 0.00 | 1339000.00 | 0.00 | 0.00 | 0.00 | 0.00 | 0.00 | 0.00 | 0.00 | KO:K16885 | quinone-modifying oxidoreductase subunit QmoA | Bacteria;Proteobacteria;Deltaproteobacteria;Syntrophobacterales;Syntrophobacteraceae;Desulfoglaeba;Desulfoglaeba alkanexedens;Desulfoglaeba alkanexedens ALDC |
| Ga0586324 5341181 49 600 | 0.00 | 0.00 | 0.00 | 11970000.00 | 0.00 | 0.00 | 0.00 | 0.00 | 0.00 | 0.00 | 0.00 | 0.00 | KO:K16885 | quinone-modifying oxidoreductase subunit QmoA | Bacteria;Proteobacteria;Deltaproteobacteria;Syntrophobacterales;Syntrophaceae;Desulfobacca;Desulfobacca acetoxidans;Desulfobacca acetoxidans ASRB2, DSM 11109 |
| Ga0586324 5713958 165 584 | 0.00 | 0.00 | 0.00 | 12350000.00 | 5335400.00 | 12187900.00 | 0.00 | 6527000.00 | 0.00 | 2294600.00 | 0.00 | 0.00 | KO:K16885 | quinone-modifying oxidoreductase subunit QmoA | Bacteria;Proteobacteria;Deltaproteobacteria;Syntrophobacterales;Syntrophaceae;Desulfobacca;Desulfobacca acetoxidans;Desulfobacca acetoxidans ASRB2, DSM 11109 |
| Ga0586324 7123680 2 532 | 0.00 | 0.00 | 0.00 | 0.00 | 0.00 | 25606500.00 | 0.00 | 0.00 | 30618000.00 | 0.00 | 0.00 | 0.00 | KO:K16885 | quinone-modifying oxidoreductase subunit QmoA | Bacteria;Proteobacteria;Deltaproteobacteria;Desulfobacterales;Desulfobacteraceae;Desulfosarcina;Desulfosarcina sp. BuS5;Desulfosarcina sp. BuS5 |
| Ga0586324 0000070 37495 39771 | 0.00 | 0.00 | 0.00 | 0.00 | 0.00 | 0.00 | 43693498.20 | 33428000.00 | 19877400.00 | 67991000.00 | 59312000.00 | 66757500.00 | KO:K16886 | quinone-modifying oxidoreductase subunit QmoB | Bacteria;Proteobacteria;Betaproteobacteria;Nitrosomonadales;Thiobacillaceae;Annwoodia;Annwoodia aquaesulis;Annwoodia aquaesulis DSM 4255 |
| Ga0586324 0000747 8761 10974 | 0.00 | 0.00 | 0.00 | 0.00 | 0.00 | 0.00 | 0.00 | 6100000.00 | 2421900.00 | 571277.63 | 0.00 | 308294.25 | KO:K16886 | quinone-modifying oxidoreductase subunit QmoB | Bacteria;Proteobacteria;Gammaproteobacteria;Acidiferrobacterales;Acidiferrobacteraceae;Sulfuricaulis;Sulfuricaulis limicola;Sulfuricaulis limicola HA5 |
| Ga0586324 0015020 5882 8092 | 0.00 | 0.00 | 0.00 | 0.00 | 0.00 | 0.00 | 0.00 | 0.00 | 0.00 | 0.00 | 3352800.00 | 0.00 | KO:K16886 | quinone-modifying oxidoreductase subunit QmoB | Bacteria;Proteobacteria;Gammaproteobacteria;Acidiferrobacterales;Acidiferrobacteraceae;Sulfuricaulis;Sulfuricaulis limicola;Sulfuricaulis limicola HA5 |
| Ga0586324 0021934 2 2116 | 0.00 | 0.00 | 0.00 | 0.00 | 0.00 | 0.00 | 0.00 | 0.00 | 0.00 | 2618000.00 | 2895200.00 | 1860000.00 | KO:K16886 | quinone-modifying oxidoreductase subunit QmoB | Bacteria;Proteobacteria;Betaproteobacteria;Nitrosomonadales;Thiobacillaceae;Thiobacillus;Thiobacillus denitrificans;Thiobacillus denitrificans DSM 12475 |
| Ga0586324 0033731 3020 5254 | 0.00 | 0.00 | 0.00 | 0.00 | 1545000.00 | 0.00 | 0.00 | 0.00 | 0.00 | 0.00 | 0.00 | 19958.70 | KO:K16886 | quinone-modifying oxidoreductase subunit QmoB | Bacteria;Nitrospirae;Thermodesulfovibrionia;Thermodesulfovibrionales;Thermodesulfovibrionaceae;Thermodesulfovibrio;Thermodesulfovibrio hydrogeniphilus;Thermodesulfovibrio hydrogeniphilus DSM 18151 |
| Ga0586324 0035720 1683 3902 | 11720000.00 | 13858400.00 | 0.00 | 11875000.00 | 46556000.00 | 13696500.00 | 0.00 | 3891800.00 | 37057500.00 | 3973200.00 | 2270400.00 | 0.00 | KO:K16886 | quinone-modifying oxidoreductase subunit QmoB | Bacteria;Proteobacteria;Deltaproteobacteria;Syntrophobacterales;Syntrophobacteraceae;Desulfacinum;Desulfacinum hydrothermale;Desulfacinum hydrothermale DSM 13146 |

| | | | | | | | | | | | | | | | |
|---|---|---|---|---|---|---|---|---|---|---|---|---|---|---|---|
| Ga0586324_0055559_2432_4540 | 0.00 | 0.00 | 0.00 | 3667000.00 | 0.00 | 0.00 | 0.00 | 0.00 | 0.00 | 4473700.00 | 0.00 | 0.00 | KO:K16886 | quinone-modifying oxidoreductase subunit QmoB | Bacteria;Spirochaetes;Spirochaetia;Spirochaetales;Spirochaetaceae;Alkalispirochaeta;Alkalispirochaeta odontotermitis;Alkalispirochaeta odontotermitis JC202 |
| Ga0586324_0122357_866_3085 | 0.00 | 3522400.00 | 0.00 | 1406000.00 | 10083700.00 | 9131000.00 | 0.00 | 0.00 | 5734800.00 | 0.00 | 1865600.00 | 0.00 | KO:K16886 | quinone-modifying oxidoreductase subunit QmoB | Bacteria;Proteobacteria;Deltaproteobacteria;Syntrophobacterales;Syntrophobacteraceae;Desulfacinum;Desulfacinum hydrothermale;Desulfacinum hydrothermale DSM 13146 |
| Ga0586324_0159580_2_1669 | 4170000.00 | 0.00 | 7882000.00 | 0.00 | 0.00 | 6828400.00 | 0.00 | 0.00 | 0.00 | 0.00 | 0.00 | 0.00 | KO:K16886 | quinone-modifying oxidoreductase subunit QmoB | Bacteria;Spirochaetes;Spirochaetia;Spirochaetales;Spirochaetaceae;Alkalispirochaeta;Alkalispirochaeta odontotermitis;Alkalispirochaeta odontotermitis JC202 |
| Ga0586324_0319884_1_1986 | 0.00 | 0.00 | 0.00 | 0.00 | 0.00 | 2251490.22 | 3556800.00 | 780800.00 | 1134000.00 | 0.00 | 0.00 | 0.00 | KO:K16886 | quinone-modifying oxidoreductase subunit QmoB | Bacteria;Proteobacteria;Deltaproteobacteria;Syntrophobacterales;Syntrophobacteraceae;Desulfacinum;Desulfacinum infernum;Desulfacinum infernum DSM 9756 |
| Ga0586324_0355433_1_699 | 0.00 | 0.00 | 0.00 | 0.00 | 0.00 | 0.00 | 0.00 | 0.00 | 0.00 | 0.00 | 0.00 | 2182500.00 | KO:K16886 | quinone-modifying oxidoreductase subunit QmoB | Bacteria;Proteobacteria;Gammaproteobacteria;Acidiferrobacterales;Acidiferrobacteraceae;Sulfuricaulis;Sulfuricaulis limicola;Sulfuricaulis limicola HA5 |
| Ga0586324_0368236_147_1877 | 0.00 | 0.00 | 0.00 | 5130000.00 | 4284800.00 | 0.00 | 0.00 | 2891400.00 | 0.00 | 0.00 | 0.00 | 0.00 | KO:K16886 | quinone-modifying oxidoreductase subunit QmoB | Bacteria;Proteobacteria;Deltaproteobacteria;Syntrophobacterales;Syntrophobacteraceae;Desulfacinum;Desulfacinum hydrothermale;Desulfacinum hydrothermale DSM 13146 |
| Ga0586324_0372832_2_1495 | 0.00 | 0.00 | 0.00 | 3258500.00 | 0.00 | 0.00 | 0.00 | 0.00 | 0.00 | 0.00 | 0.00 | 0.00 | KO:K16886 | quinone-modifying oxidoreductase subunit QmoB | Bacteria;Proteobacteria;Deltaproteobacteria;Syntrophobacterales;Syntrophobacteraceae;Desulfacinum;Desulfacinum infernum;Desulfacinum infernum DSM 9756 |
| Ga0586324_0421817_3_1733 | 0.00 | 0.00 | 0.00 | 382356.95 | 0.00 | 0.00 | 0.00 | 0.00 | 0.00 | 0.00 | 0.00 | 0.00 | KO:K16886 | quinone-modifying oxidoreductase subunit QmoB | Bacteria;Proteobacteria;Deltaproteobacteria;unclassified Deltaproteobacteria;unclassified Deltaproteobacteria;unclassified Deltaproteobacteria;delta proteobacterium NaphS2;delta proteobacterium NaphS2 |
| Ga0586324_0643275_1_1341 | 0.00 | 4182000.00 | 6868600.00 | 26505000.00 | 12051000.00 | 8257600.00 | 0.00 | 0.00 | 3750300.00 | 0.00 | 0.00 | 0.00 | KO:K16886 | quinone-modifying oxidoreductase subunit QmoB | Bacteria;Proteobacteria;Deltaproteobacteria;Desulfobacterales;Desulfobacteraceae;Desulfatirhabdium;Desulfatirhabdium butyrativorans;Desulfatirhabdium butyrativorans DSM 18734 |
| Ga0586324_0766622_1_723 | 0.00 | 0.00 | 0.00 | 0.00 | 0.00 | 0.00 | 0.00 | 841800.00 | 0.00 | 0.00 | 0.00 | 0.00 | KO:K16886 | quinone-modifying oxidoreductase subunit QmoB | Bacteria;Firmicutes;Clostridia;Eubacteriales;Peptococcaceae;Desulfofundulus;Desulfofundulus australicus;Desulfofundulus australicus DSM 11792 |
| Ga0586324_0810901_3_1328 | 0.00 | 0.00 | 0.00 | 1814500.00 | 0.00 | 1482620.32 | 0.00 | 0.00 | 0.00 | 0.00 | 0.00 | 0.00 | KO:K16886 | quinone-modifying oxidoreductase subunit QmoB | Bacteria;Spirochaetes;Spirochaetia;Spirochaetales;Spirochaetaceae;Alkalispirochaeta;Alkalispirochaeta odontotermitis;Alkalispirochaeta odontotermitis JC202 |
| Ga0586324_0848156_1_1305 | 0.00 | 0.00 | 0.00 | 3353500.00 | 0.00 | 0.00 | 0.00 | 0.00 | 0.00 | 0.00 | 0.00 | 0.00 | KO:K16886 | quinone-modifying oxidoreductase subunit QmoB | Bacteria;Spirochaetes;Spirochaetia;Spirochaetales;Spirochaetaceae;Alkalispirochaeta;Alkalispirochaeta odontotermitis;Alkalispirochaeta odontotermitis JC202 |
| Ga0586324_0913840_45_1262 | 0.00 | 0.00 | 0.00 | 0.00 | 0.00 | 0.00 | 0.00 | 0.00 | 0.00 | 6991600.00 | 0.00 | 4605000.00 | KO:K16886 | quinone-modifying oxidoreductase subunit QmoB | Bacteria;Proteobacteria;Betaproteobacteria;unclassified Betaproteobacteria;unclassified Betaproteobacteria;unclassified Betaproteobacteria;uncultured beta proteobacterium;unclassified betaproteobacterium ENR4 |
| Ga0586324_0971510_65_1228 | 0.00 | 0.00 | 0.00 | 2137500.00 | 0.00 | 0.00 | 0.00 | 0.00 | 2948400.00 | 0.00 | 0.00 | 0.00 | KO:K16886 | quinone-modifying oxidoreductase subunit QmoB | Bacteria;Proteobacteria;Deltaproteobacteria;Desulfobacterales;Desulfobulbaceae;Desulfobulbus;Desulfobulbus elongatus;Desulfobulbus elongatus DSM 2908 |
| Ga0586324_1124266_3_1154 | 0.00 | 0.00 | 0.00 | 0.00 | 0.00 | 0.00 | 0.00 | 0.00 | 0.00 | 0.00 | 9944000.00 | 4972500.00 | KO:K16886 | quinone-modifying oxidoreductase subunit QmoB | Bacteria;Proteobacteria;Betaproteobacteria;Nitrosomonadales;Thiobacillaceae;Thiobacillus;Thiobacillus denitrificans;Thiobacillus denitrificans RG |
| Ga0586324_1500001_3_1019 | 0.00 | 0.00 | 0.00 | 0.00 | 0.00 | 0.00 | 0.00 | 0.00 | 0.00 | 1478400.00 | 0.00 | 0.00 | KO:K16886 | quinone-modifying oxidoreductase subunit QmoB | Bacteria;Proteobacteria;Deltaproteobacteria;Syntrophobacterales;Syntrophobacteraceae;Syntrophobacter;Syntrophobacter fumaroxidans;Syntrophobacter fumaroxidans MPOB |
| Ga0586324_1506275_343_1020 | 0.00 | 0.00 | 0.00 | 0.00 | 0.00 | 0.00 | 0.00 | 0.00 | 6941700.00 | 0.00 | 0.00 | 0.00 | KO:K16886 | quinone-modifying oxidoreductase subunit QmoB | Bacteria;Proteobacteria;Deltaproteobacteria;Syntrophobacterales;Syntrophobacteraceae;Desulfacinum;Desulfacinum hydrothermale;Desulfacinum hydrothermale DSM 13146 |
| Ga0586324_1815953_2_940 | 0.00 | 0.00 | 0.00 | 20653000.00 | 6643500.00 | 0.00 | 0.00 | 0.00 | 0.00 | 0.00 | 0.00 | 0.00 | KO:K16886 | quinone-modifying oxidoreductase subunit QmoB | Bacteria;Proteobacteria;Deltaproteobacteria;Syntrophobacterales;Syntrophobacteraceae;Syntrophobacter;Syntrophobacter fumaroxidans;Syntrophobacter fumaroxidans MPOB |
| Ga0586324_1957032_3_911 | 2090000.00 | 0.00 | 0.00 | 2223000.00 | 1843700.00 | 0.00 | 0.00 | 2409500.00 | 1255500.00 | 0.00 | 0.00 | 0.00 | KO:K16886 | quinone-modifying oxidoreductase subunit QmoB | Bacteria;Proteobacteria;Deltaproteobacteria;Syntrophobacterales;Syntrophaceae;Desulfobacca;Desulfobacca acetoxidans;Desulfobacca acetoxidans ASRB2, DSM 11109 |
| Ga0586324_2064329_3_890 | 0.00 | 0.00 | 0.00 | 0.00 | 0.00 | 0.00 | 0.00 | 0.00 | 7079400.00 | 0.00 | 0.00 | 0.00 | KO:K16886 | quinone-modifying oxidoreductase subunit QmoB | Bacteria;Proteobacteria;Betaproteobacteria;Nitrosomonadales;Thiobacillaceae;Thiobacillus;Thiobacillus thiophilus;Thiobacillus thiophilus DSM 19892 |
| Ga0586324_2126129_3_881 | 0.00 | 0.00 | 0.00 | 0.00 | 0.00 | 0.00 | 0.00 | 0.00 | 1020600.00 | 1054900.00 | 0.00 | 907500.00 | KO:K16886 | quinone-modifying oxidoreductase subunit QmoB | Bacteria;Proteobacteria;Gammaproteobacteria;Acidiferrobacterales;Acidiferrobacteraceae;Sulfuricaulis;Sulfuricaulis limicola;Sulfuricaulis limicola HA5 |
| Ga0586324_2170353_2_874 | 0.00 | 0.00 | 0.00 | 0.00 | 0.00 | 0.00 | 0.00 | 0.00 | 0.00 | 0.00 | 871309.12 | 0.00 | KO:K16886 | quinone-modifying oxidoreductase subunit QmoB | Bacteria;Proteobacteria;Betaproteobacteria;Nitrosomonadales;Thiobacillaceae;Thiobacillus;Thiobacillus denitrificans;Thiobacillus denitrificans ATCC 25259 |
| Ga0586324_2384226_2_838 | 0.00 | 1720400.00 | 0.00 | 0.00 | 0.00 | 0.00 | 0.00 | 0.00 | 0.00 | 1078000.00 | 0.00 | 634423.50 | KO:K16886 | quinone-modifying oxidoreductase subunit QmoB | Bacteria;Proteobacteria;Gammaproteobacteria;Thiotrichales;Thiotrichaceae;Achromatium;uncultured Achromatium sp.;Achromatium sp. Cell8 |
| Ga0586324_2631318_2_805 | 0.00 | 0.00 | 0.00 | 0.00 | 5860700.00 | 0.00 | 0.00 | 0.00 | 0.00 | 0.00 | 0.00 | 0.00 | KO:K16886 | quinone-modifying oxidoreductase subunit QmoB | Bacteria;Spirochaetes;Spirochaetia;Spirochaetales;Spirochaetaceae;Alkalispirochaeta;Alkalispirochaeta odontotermitis;Alkalispirochaeta odontotermitis JC202 |
| Ga0586324_3275622_44_733 | 0.00 | 4630800.00 | 0.00 | 0.00 | 0.00 | 0.00 | 0.00 | 0.00 | 0.00 | 0.00 | 0.00 | 0.00 | KO:K16886 | quinone-modifying oxidoreductase subunit QmoB | Bacteria;Proteobacteria;Deltaproteobacteria;Syntrophobacterales;Syntrophaceae;Desulfobacca;Desulfobacca acetoxidans;Desulfobacca acetoxidans ASRB2, DSM 11109 |
| Ga0586324_4065758_2_670 | 0.00 | 4018800.00 | 0.00 | 0.00 | 0.00 | 0.00 | 0.00 | 8906000.00 | 2025000.00 | 0.00 | 0.00 | 0.00 | KO:K16886 | quinone-modifying oxidoreductase subunit QmoB | Bacteria;Proteobacteria;Gammaproteobacteria;Chromatiales;Chromatiaceae;Lamprocystis;Lamprocystis purpurea;Lamprocystis purpurea DSM 4197 |
| Ga0586324_4652899_2_634 | 0.00 | 3787600.00 | 9233200.00 | 3876000.00 | 5984300.00 | 4883100.00 | 6224400.00 | 2928000.00 | 3645000.00 | 0.00 | 0.00 | 3105000.00 | KO:K16886 | quinone-modifying oxidoreductase subunit QmoB | Bacteria;Proteobacteria;Gammaproteobacteria;Chromatiales;Chromatiaceae;Lamprocystis;Lamprocystis purpurea;Lamprocystis purpurea DSM 4197 |
| Ga0586324_4663063_2_634 | 0.00 | 0.00 | 0.00 | 0.00 | 0.00 | 0.00 | 0.00 | 1970300.00 | 0.00 | 0.00 | 0.00 | 0.00 | KO:K16886 | quinone-modifying oxidoreductase subunit QmoB | Bacteria;Proteobacteria;Deltaproteobacteria;Syntrophobacterales;Syntrophaceae;Desulfobacca;Desulfobacca acetoxidans;Desulfobacca acetoxidans ASRB2, DSM 11109 |
| Ga0586324_2592783_1_810 | 0.00 | 0.00 | 0.00 | 0.00 | 0.00 | 2235078.24 | 0.00 | 0.00 | 0.00 | 0.00 | 0.00 | 0.00 | KO:K17080 | prohibitin 1 | Bacteria;Acidobacteria;Thermoanaerobaculia;Thermoanaerobaculales;Thermoanaerobaculaceae;Thermoanaerobaculum;Thermoanaerobaculum aquaticum;Thermoanaerobaculum aquaticum MP-01 |
| Ga0586324_0129816_373_2292 | 0.00 | 0.00 | 0.00 | 0.00 | 7076100.00 | 0.00 | 0.00 | 0.00 | 0.00 | 0.00 | 0.00 | 0.00 | KO:K17202 | erythritol transport system substrate-binding protein | Bacteria;Chloroflexi;Anaerolineae;Anaerolineales;Anaerolineaceae;Levilinea;Levilinea saccharolytica;Levilinea saccharolytica DSM 16555 |
| Ga0586324_3826561_3_650 | 0.00 | 6752400.00 | 0.00 | 0.00 | 0.00 | 0.00 | 0.00 | 0.00 | 0.00 | 0.00 | 0.00 | 0.00 | KO:K17213 | inositol transport system substrate-binding protein | Bacteria;Proteobacteria;Alphaproteobacteria;Rhodospirillales;Azospirillaceae;Azospirillum;Azospirillum sp. TSH100;Azospirillum sp. TSH100 |
| Ga0586324_0000423_19925_21193 | 0.00 | 0.00 | 0.00 | 0.00 | 0.00 | 0.00 | 0.00 | 0.00 | 0.00 | 4396700.00 | 11431200.00 | 3187500.00 | KO:K17218 | sulfide:quinone oxidoreductase | Bacteria;Proteobacteria;Betaproteobacteria;Nitrosomonadales;Thiobacillaceae;Annwoodia;Annwoodia aquaesulis;Annwoodia aquaesulis DSM 4255 |
| Ga0586324_0003206_1730_2998 | 0.00 | 0.00 | 0.00 | 0.00 | 0.00 | 0.00 | 0.00 | 16409000.00 | 0.00 | 0.00 | 0.00 | 7455000.00 | KO:K17218 | sulfide:quinone oxidoreductase | Bacteria;Proteobacteria;Betaproteobacteria;Nitrosomonadales;Thiobacillaceae;Thiobacillus;Thiobacillus thioparus;Thiobacillus thioparus DSM 505 |
| Ga0586324_0022884_3208_4341 | 0.00 | 1625200.00 | 0.00 | 0.00 | 0.00 | 0.00 | 0.00 | 0.00 | 0.00 | 3465000.00 | 2631200.00 | 0.00 | KO:K17218 | sulfide:quinone oxidoreductase | Bacteria;Proteobacteria;Betaproteobacteria;Ferrovales;Ferrovaceae;Ferrovum;unclassified Ferrovum;Ferrovum sp. Zulauf-31 |
| Ga0586324_0109810_1744_3012 | 0.00 | 0.00 | 0.00 | 0.00 | 0.00 | 0.00 | 0.00 | 13847000.00 | 13041000.00 | 0.00 | 0.00 | 0.00 | KO:K17218 | sulfide:quinone oxidoreductase | Bacteria;Proteobacteria;Betaproteobacteria;Nitrosomonadales;Thiobacillaceae;Thiobacillus;Thiobacillus denitrificans;Thiobacillus denitrificans RG |
| Ga0586324_5895249_113_577 | 0.00 | 0.00 | 0.00 | 0.00 | 0.00 | 0.00 | 0.00 | 0.00 | 0.00 | 0.00 | 0.00 | 2482500.00 | KO:K17218 | sulfide:quinone oxidoreductase | Bacteria;Proteobacteria;Betaproteobacteria;Burkholderiales;Comamonadaceae;Curvibacter;Curvibacter sp. PAE-UM;Curvibacter sp. PAE-UM |
| Ga0586324_8181678_2_502 | 0.00 | 0.00 | 0.00 | 0.00 | 0.00 | 0.00 | 0.00 | 0.00 | 0.00 | 0.00 | 0.00 | 3067500.00 | KO:K17218 | sulfide:quinone oxidoreductase | Bacteria;Proteobacteria;Betaproteobacteria;Burkholderiales;Burkholderiaceae;Chitinimonas;Chitinimonas koreensis;Chitinimonas koreensis DSM 17726 |
| Ga0586324_0000037_45798_46628 | 0.00 | 0.00 | 0.00 | 0.00 | 0.00 | 0.00 | 85044000.00 | 314943000.00 | 271269000.00 | 426410600.00 | 447145600.00 | 428017500.00 | KO:K17222 | sulfur-oxidizing protein SoxA | Bacteria;Proteobacteria;Betaproteobacteria;Nitrosomonadales;Thiobacillaceae;Annwoodia;Annwoodia aquaesulis;Annwoodia aquaesulis DSM 4255 |
| Ga0586324_0004144_4156_4998 | 0.00 | 0.00 | 0.00 | 0.00 | 0.00 | 0.00 | 0.00 | 0.00 | 1093500.00 | 0.00 | 0.00 | 847500.00 | KO:K17222 | sulfur-oxidizing protein SoxA | Bacteria;Proteobacteria;Alphaproteobacteria;Hyphomicrobiales;Bradyrhizobiaceae;Afipia;Afipia sp. 1NLS2;Afipia sp. 1NLS2 |
| Ga0586324_0015713_1918_2763 | 0.00 | 0.00 | 0.00 | 0.00 | 0.00 | 0.00 | 0.00 | 25571200.00 | 67246200.00 | 13113100.00 | 15224000.00 | 0.00 | KO:K17222 | sulfur-oxidizing protein SoxA | Bacteria;Proteobacteria;Betaproteobacteria;Nitrosomonadales;Thiobacillaceae;Thiobacillus;Thiobacillus thioparus;Thiobacillus thioparus DSM 505 |
| Ga0586324_0043326_2379_3224 | 0.00 | 0.00 | 0.00 | 0.00 | 0.00 | 0.00 | 0.00 | 0.00 | 0.00 | 7291900.00 | 0.00 | 4680000.00 | KO:K17222 | sulfur-oxidizing protein SoxA | Bacteria;Proteobacteria;Betaproteobacteria;Nitrosomonadales;Thiobacillaceae;Thiobacillus;Thiobacillus thiophilus;Thiobacillus thiophilus DSM 19892 |
| Ga0586324_0408773_771_1601 | 0.00 | 0.00 | 0.00 | 0.00 | 0.00 | 0.00 | 138624000.00 | 139690000.00 | 134306100.00 | 110880000.00 | 132880000.00 | 101250000.00 | KO:K17222 | sulfur-oxidizing protein SoxA | Bacteria;Proteobacteria;Betaproteobacteria;Nitrosomonadales;Sulfuricellaceae;Sulfuriferula;Sulfuriferula multivorans;Sulfuriferula multivorans TTN |
| Ga0586324_1930433_138_917 | 0.00 | 0.00 | 0.00 | 0.00 | 0.00 | 0.00 | 0.00 | 4880000.00 | 5532300.00 | 1609300.00 | 0.00 | 0.00 | KO:K17222 | sulfur-oxidizing protein SoxA | Bacteria;Proteobacteria;Betaproteobacteria;Nitrosomonadales;Thiobacillaceae;Thiobacillus;Thiobacillus thioparus;Thiobacillus thioparus DSM 505 |
| Ga0586324_0000037_44492_44857 | 0.00 | 0.00 | 0.00 | 0.00 | 0.00 | 0.00 | 0.00 | 0.00 | 0.00 | 5682600.00 | 0.00 | 0.00 | KO:K17223 | sulfur-oxidizing protein SoxX | Bacteria;Proteobacteria;Betaproteobacteria;Nitrosomonadales;Thiobacillaceae;Annwoodia;Annwoodia aquaesulis;Annwoodia aquaesulis DSM 4255 |
| Ga0586324_0015713_497_952 | 0.00 | 0.00 | 0.00 | 0.00 | 0.00 | 0.00 | 17464800.00 | 21655000.00 | 17901000.00 | 0.00 | 0.00 | 13200000.00 | KO:K17223 | sulfur-oxidizing protein SoxX | Bacteria;Proteobacteria;Betaproteobacteria;Nitrosomonadales;Thiobacillaceae;Thiobacillus;Thiobacillus thioparus;Thiobacillus thioparus DSM 505 |
| Ga0586324_0000037_46777_48486 | 0.00 | 0.00 | 0.00 | 0.00 | 0.00 | 0.00 | 0.00 | 6136600.00 | 8594100.00 | 30661400.00 | 21736000.00 | 13522500.00 | KO:K17224 | sulfur-oxidizing protein SoxB | Bacteria;Proteobacteria;Betaproteobacteria;Nitrosomonadales;Thiobacillaceae;Annwoodia;Annwoodia aquaesulis;Annwoodia aquaesulis DSM 4255 |
| Ga0586324_0015713_2913_4655 | 0.00 | 0.00 | 0.00 | 0.00 | 0.00 | 0.00 | 0.00 | 2793800.00 | 0.00 | 11935000.00 | 20504000.00 | 15300000.00 | KO:K17224 | sulfur-oxidizing protein SoxB | Bacteria;Proteobacteria;Betaproteobacteria;Nitrosomonadales;Thiobacillaceae;Thiobacillus;Thiobacillus thioparus;Thiobacillus thioparus DSM 505 |
| Ga0586324_0043326_480_2222 | 0.00 | 0.00 | 0.00 | 0.00 | 0.00 | 0.00 | 0.00 | 23161700.00 | 25020900.00 | 5043500.00 | 3634400.00 | 4005000.00 | KO:K17224 | sulfur-oxidizing protein SoxB | Bacteria;Proteobacteria;Betaproteobacteria;Nitrosomonadales;Thiobacillaceae;Thiobacillus;Thiobacillus thioparus;Thiobacillus thioparus DSM 505 |
| Ga0586324_0062093_1802_3886 | 0.00 | 0.00 | 0.00 | 0.00 | 0.00 | 0.00 | 0.00 | 0.00 | 0.00 | 8470000.00 | 0.00 | 11250000.00 | KO:K17224 | sulfur-oxidizing protein SoxB | Bacteria;Proteobacteria;Gammaproteobacteria;Acidiferrobacterales;Acidiferrobacteraceae;Sulfuricaulis;Sulfuricaulis limicola;Sulfuricaulis limicola HA5 |
| Ga0586324_0099464_2_1759 | 0.00 | 0.00 | 4454844.47 | 0.00 | 0.00 | 0.00 | 0.00 | 0.00 | 0.00 | 0.00 | 0.00 | 0.00 | KO:K17224 | sulfur-oxidizing protein SoxB | Bacteria;Firmicutes;Bacilli;Lactobacillales;Carnobacteriaceae;Alkalibacterium;Alkalibacterium putridalgicola;Alkalibacterium putridalgicola DSM 19182 |
| Ga0586324_0117721_1977_3173 | 0.00 | 0.00 | 0.00 | 0.00 | 0.00 | 0.00 | 0.00 | 3763700.00 | 3539700.00 | 0.00 | 0.00 | 4155000.00 | KO:K17224 | sulfur-oxidizing protein SoxB | Bacteria;Proteobacteria;Betaproteobacteria;Nitrosomonadales;Sulfuricellaceae;Sulfurirhabdus;Sulfurirhabdus autotrophica;Sulfurirhabdus autotrophica DSM 100309 |
| Ga0586324_0697354_184_1419 | 0.00 | 0.00 | 0.00 | 0.00 | 0.00 | 0.00 | 0.00 | 0.00 | 0.00 | 0.00 | 0.00 | 311079.00 | KO:K17224 | sulfur-oxidizing protein SoxB | Bacteria;Proteobacteria;Betaproteobacteria;Nitrosomonadales;Sulfuricellaceae;Sulfurirhabdus;Sulfurirhabdus autotrophica;Sulfurirhabdus autotrophica DSM 100309 |
| Ga0586324_0936315_2_1249 | 9180000.00 | 4923200.00 | 0.00 | 3781000.00 | 4748300.00 | 5637400.00 | 0.00 | 0.00 | 0.00 | 0.00 | 0.00 | 0.00 | KO:K17224 | sulfur-oxidizing protein SoxB | Bacteria;Proteobacteria;Betaproteobacteria;Nitrosomonadales;Thiobacillaceae;Thiobacillus;Thiobacillus denitrificans;Thiobacillus denitrificans ATCC 25259 |
| Ga0586324_1287660_3_1088 | 3040000.00 | 6800000.00 | 0.00 | 3106500.00 | 2636800.00 | 0.00 | 0.00 | 0.00 | 1628100.00 | 10626000.00 | 7629600.00 | 0.00 | KO:K17224 | sulfur-oxidizing protein SoxB | Bacteria;Proteobacteria;Gammaproteobacteria;Chromatiales;Ectothiorhodospiraceae;Thiogranum;Thiogranum longum;Thiogranum longum DSM 19610 |
| Ga0586324_1612763_2_988 | 0.00 | 0.00 | 0.00 | 0.00 | 0.00 | 0.00 | 0.00 | 4434700.00 | 0.00 | 0.00 | 0.00 | 0.00 | KO:K17224 | sulfur-oxidizing protein SoxB | Bacteria;Proteobacteria;Betaproteobacteria;Nitrosomonadales;Thiobacillaceae;Thiobacillus;Thiobacillus denitrificans;Thiobacillus denitrificans DSM 12475 |
| Ga0586324_3241436_2_571 | 0.00 | 0.00 | 0.00 | 0.00 | 0.00 | 0.00 | 0.00 | 0.00 | 0.00 | 0.00 | 1584000.00 | 0.00 | KO:K17224 | sulfur-oxidizing protein SoxB | Bacteria;Proteobacteria;Alphaproteobacteria;Hyphomicrobiales;Xanthobacteraceae;Pseudolabrys;Pseudolabrys sp. Root1462;Pseudolabrys sp. Root1462 |
| Ga0586324_0045066_210_680 | 0.00 | 0.00 | 0.00 | 0.00 | 0.00 | 0.00 | 0.00 | 11638800.00 | 1482300.00 | 15392300.00 | 47288201.84 | 19890000.00 | KO:K17226 | sulfur-oxidizing protein SoxY | Bacteria;Proteobacteria;Betaproteobacteria;Nitrosomonadales;Sulfuricellaceae;Sulfurirhabdus;Sulfurirhabdus autotrophica;Sulfurirhabdus autotrophica DSM 100309 |
| Ga0586324_1259963_98_571 | 0.00 | 0.00 | 0.00 | 0.00 | 0.00 | 2420628.10 | 0.00 | 0.00 | 0.00 | 2841300.00 | 0.00 | 0.00 | KO:K17226 | sulfur-oxidizing protein SoxY | Bacteria;Proteobacteria;Gammaproteobacteria;Chromatiales;Chromatiaceae;Thiocapsa;Thiocapsa marina;Thiocapsa marina 5811, DSM 5653 |
| Ga0586324_0000037_45401_45715 | 0.00 | 0.00 | 0.00 | 0.00 | 0.00 | 0.00 | 6133200.00 | 6222000.00 | 18630000.00 | 0.00 | 2481600.00 | 4132500.00 | KO:K17227 | sulfur-oxidizing protein SoxZ | Bacteria;Proteobacteria;Betaproteobacteria;Nitrosomonadales;Thiobacillaceae;Annwoodia;Annwoodia aquaesulis;Annwoodia aquaesulis DSM 4255 |
| Ga0586324_0524969_1001_1315 | 0.00 | 0.00 | 0.00 | 0.00 | 0.00 | 0.00 | 3078000.00 | 0.00 | 5880600.00 | 0.00 | 1170400.00 | 2092500.00 | KO:K17227 | sulfur-oxidizing protein SoxZ | Bacteria;Proteobacteria;Betaproteobacteria;Nitrosomonadales;Sulfuricellaceae;Sulfuriferula;unclassified Sulfuriferula;Sulfuriferula sp. AH1 re-analysis |
| Ga0586324_0000002_52602_53903 | 0.00 | 0.00 | 0.00 | 0.00 | 0.00 | 0.00 | 0.00 | 0.00 | 0.00 | 7299600.00 | 7515200.00 | 0.00 | KO:K17229 | sulfide dehydrogenase [flavocytochrome c] flavoprotein subunit | Bacteria;Proteobacteria;Gammaproteobacteria;Thiotrichales;Thiotrichaceae;Thiothrix;Thiothrix nivea;Thiothrix nivea JP2, DSM 5205 |
| Ga0586324_0000011_47276_48637 | 0.00 | 0.00 | 0.00 | 0.00 | 0.00 | 0.00 | 10054800.00 | 18531800.00 | 15754500.00 | 78917300.00 | 80115200.00 | 51375000.00 | KO:K17229 | sulfide dehydrogenase [flavocytochrome c] flavoprotein subunit | Bacteria;Proteobacteria;Betaproteobacteria;Nitrosomonadales;Thiobacillaceae;Thiobacillus;Thiobacillus thiophilus;Thiobacillus thiophilus DSM 19892 |
| Ga0586324_0001373_3254_4573 | 0.00 | 0.00 | 0.00 | 0.00 | 0.00 | 0.00 | 0.00 | 0.00 | 0.00 | 3888500.00 | 0.00 | 0.00 | KO:K17229 | sulfide dehydrogenase [flavocytochrome c] flavoprotein subunit | Bacteria;Proteobacteria;Gammaproteobacteria;Chromatiales;Ectothiorhodospiraceae;Thiohalospira;Thiohalospira halophila;Thiohalospira halophila HL3 |
| Ga0586324_0009452_6633_7997 | 3150000.00 | 3515600.00 | 0.00 | 0.00 | 0.00 | 0.00 | 4674000.00 | 0.00 | 10044000.00 | 13444200.00 | 0.00 | 13747500.00 | KO:K17229 | sulfide dehydrogenase [flavocytochrome c] flavoprotein subunit | Bacteria;Proteobacteria;Betaproteobacteria;Nitrosomonadales;Thiobacillaceae;Thiobacillus;Thiobacillus thiophilus;Thiobacillus thiophilus DSM 19892 |
| Ga0586324_0040499_3841_5199 | 0.00 | 1645600.00 | 0.00 | 0.00 | 0.00 | 0.00 | 0.00 | 1903200.00 | 5921100.00 | 5020400.00 | 0.00 | 7440000.00 | KO:K17229 | sulfide dehydrogenase [flavocytochrome c] flavoprotein subunit | Bacteria;Proteobacteria;Betaproteobacteria;Nitrosomonadales;Thiobacillaceae;Thiobacillus;Thiobacillus thiophilus;Thiobacillus thiophilus DSM 19892 |
| Ga0586324_0048721_3044_4411 | 0.00 | 0.00 | 0.00 | 0.00 | 0.00 | 0.00 | 2107508.88 | 5160600.00 | 0.00 | 3703700.00 | 2173600.00 | 2918680.50 | KO:K17229 | sulfide dehydrogenase [flavocytochrome c] flavoprotein subunit | Bacteria;Proteobacteria;Betaproteobacteria;Nitrosomonadales;Thiobacillaceae;Thiobacillus;Thiobacillus thiophilus;Thiobacillus thiophilus DSM 19892 |
| Ga0586324_0491732_2_970 | 0.00 | 0.00 | 0.00 | 0.00 | 0.00 | 0.00 | 0.00 | 0.00 | 4706100.00 | 0.00 | 0.00 | 0.00 | KO:K17229 | sulfide dehydrogenase [flavocytochrome c] flavoprotein subunit | Bacteria;Proteobacteria;Gammaproteobacteria;Chromatiales;Ectothiorhodospiraceae;Thiohalomonas;Thiohalomonas denitrificans;Thiohalomonas denitrificans HLD2 |
| Ga0586324_0537497_192_1484 | 0.00 | 614701.64 | 0.00 | 0.00 | 13184000.00 | 0.00 | 3534000.00 | 9028000.00 | 5629500.00 | 4181100.00 | 0.00 | 2520000.00 | KO:K17229 | sulfide dehydrogenase [flavocytochrome c] flavoprotein subunit | Bacteria;Proteobacteria;Gammaproteobacteria;Chromatiales;Chromatiaceae;Lamprocystis;Lamprocystis purpurea;Lamprocystis purpurea DSM 4197 |
| Ga0586324_1179157_2_1132 | 10400000.00 | 1013200.00 | 0.00 | 0.00 | 0.00 | 0.00 | 0.00 | 0.00 | 0.00 | 0.00 | 0.00 | 0.00 | KO:K17229 | sulfide dehydrogenase [flavocytochrome c] flavoprotein subunit | Bacteria;Proteobacteria;Gammaproteobacteria;Chromatiales;Chromatiaceae;Lamprocystis;Lamprocystis purpurea;Lamprocystis purpurea DSM 4197 |
| Ga0586324_1532353_3_1010 | 0.00 | 1570800.00 | 4833248.03 | 0.00 | 0.00 | 0.00 | 0.00 | 0.00 | 0.00 | 0.00 | 0.00 | 0.00 | KO:K17229 | sulfide dehydrogenase [flavocytochrome c] flavoprotein subunit | Bacteria;Proteobacteria;Gammaproteobacteria;Chromatiales;Chromatiaceae;Lamprocystis;Lamprocystis purpurea;Lamprocystis purpurea DSM 4197 |
| Ga0586324_1573603_2_697 | 0.00 | 0.00 | 0.00 | 0.00 | 0.00 | 0.00 | 0.00 | 945500.00 | 12069000.00 | 900900.00 | 0.00 | 0.00 | KO:K17229 | sulfide dehydrogenase [flavocytochrome c] flavoprotein subunit | Bacteria;Proteobacteria;Betaproteobacteria;Nitrosomonadales;Thiobacillaceae;Thiobacillus;Thiobacillus thiophilus;Thiobacillus thiophilus DSM 19892 |
| Ga0586324_3183976_1_603 | 31400000.00 | 0.00 | 0.00 | 0.00 | 0.00 | 0.00 | 0.00 | 0.00 | 0.00 | 0.00 | 0.00 | 0.00 | KO:K17229 | sulfide dehydrogenase [flavocytochrome c] flavoprotein subunit | Bacteria;Proteobacteria;Gammaproteobacteria;Chromatiales;Chromatiaceae;Thiocapsa;Thiocapsa roseopersicina;Thiocapsa roseopersicina DSM 217 |
| Ga0586324_5213702_2_607 | 0.00 | 0.00 | 0.00 | 0.00 | 0.00 | 0.00 | 11012400.00 | 11407000.00 | 10854000.00 | 0.00 | 12672000.00 | 9525000.00 | KO:K17229 | sulfide dehydrogenase [flavocytochrome c] flavoprotein subunit | Bacteria;Proteobacteria;Alphaproteobacteria;Magnetococcales;Magnetococcaceae;Magnetococcus;Magnetococcus marinus;Magnetococcus marinus MC-1 |
| Ga0586324_5497243_2_592 | 0.00 | 7276000.00 | 0.00 | 0.00 | 0.00 | 0.00 | 0.00 | 0.00 | 0.00 | 0.00 | 0.00 | 0.00 | KO:K17229 | sulfide dehydrogenase [flavocytochrome c] flavoprotein subunit | Bacteria;Proteobacteria;Gammaproteobacteria;Chromatiales;Chromatiaceae;Lamprocystis;Lamprocystis purpurea;Lamprocystis purpurea DSM 4197 |
| Ga0586324_0003813_798_2120 | 0.00 | 0.00 | 0.00 | 0.00 | 0.00 | 0.00 | 0.00 | 17446000.00 | 0.00 | 0.00 | 0.00 | 12450000.00 | KO:K17315 | glucose/mannose transport system substrate-binding protein | Bacteria;Chloroflexi;Anaerolineae;Anaerolineales;Anaerolineaceae;Anaerolinea;Anaerolinea thermophila;Anaerolinea thermophila UNI-1 |
| Ga0586324_0006948_8201_9622 | 0.00 | 0.00 | 0.00 | 2622000.00 | 0.00 | 0.00 | 3169200.00 | 0.00 | 3523500.00 | 0.00 | 0.00 | 0.00 | KO:K17315 | glucose/mannose transport system substrate-binding protein | Bacteria;Firmicutes;Bacilli;Bacillales;Paenibacillaceae;Paenibacillus;Paenibacillus sp. 597;Paenibacillus sp. 597 |
| Ga0586324_0009258_3923_5353 | 0.00 | 0.00 | 0.00 | 0.00 | 0.00 | 0.00 | 5289600.00 | 0.00 | 1449900.00 | 0.00 | 0.00 | 0.00 | KO:K17315 | glucose/mannose transport system substrate-binding protein | Archaea;Euryarchaeota;Thermococci;Thermococcales;Thermococcaceae;Thermococcus;Thermococcus barophilus;Thermococcus barophilus MP, DSM 11836 |
| Ga0586324_0015731_1228_2478 | 0.00 | 1237600.00 | 0.00 | 0.00 | 0.00 | 0.00 | 0.00 | 0.00 | 0.00 | 0.00 | 0.00 | 0.00 | KO:K17315 | glucose/mannose transport system substrate-binding protein | Bacteria;Proteobacteria;Betaproteobacteria;Burkholderiales;Burkholderiaceae;Chitinimonas;Chitinimonas koreensis;Chitinimonas koreensis DSM 17726 |
| Ga0586324_0082993_1687_3111 | 0.00 | 4386000.00 | 0.00 | 0.00 | 0.00 | 4605200.00 | 0.00 | 0.00 | 1190700.00 | 0.00 | 0.00 | 0.00 | KO:K17315 | glucose/mannose transport system substrate-binding protein | Archaea;Crenarchaeota;Thermoprotei;Desulfurococcales;Desulfurococcaceae;Staphylothermus;Staphylothermus hellenicus;Staphylothermus hellenicus P8, DSM 12710 |
| Ga0586324_0166473_2_1966 | 13570000.00 | 7888000.00 | 0.00 | 0.00 | 0.00 | 0.00 | 0.00 | 0.00 | 0.00 | 13706000.00 | 7840800.00 | 0.00 | KO:K17315 | glucose/mannose transport system substrate-binding protein | Bacteria;Chloroflexi;Anaerolineae;Anaerolineales;Anaerolineaceae;Anaerolinea;Anaerolinea thermophila;Anaerolinea thermophila UNI-1 |
| Ga0586324_0364701_1_1191 | 0.00 | 0.00 | 0.00 | 0.00 | 0.00 | 0.00 | 0.00 | 0.00 | 0.00 | 0.00 | 0.00 | 2632500.00 | KO:K17315 | glucose/mannose transport system substrate-binding protein | Bacteria;Proteobacteria;Betaproteobacteria;Neisseriales;Chromobacteriaceae;Leeia;Leeia oryzae;Leeia oryzae DSM 17879 |
| Ga0586324_2238901_2_862 | 0.00 | 3243600.00 | 0.00 | 0.00 | 0.00 | 0.00 | 0.00 | 0.00 | 0.00 | 0.00 | 0.00 | 0.00 | KO:K17315 | glucose/mannose transport system substrate-binding protein | Bacteria;Chloroflexi;Anaerolineae;Anaerolineales;Anaerolineaceae;Anaerolinea;Anaerolinea thermophila;Anaerolinea thermophila UNI-1 |
| Ga0586324_3153191_1_531 | 0.00 | 6262800.00 | 0.00 | 0.00 | 0.00 | 0.00 | 0.00 | 0.00 | 0.00 | 0.00 | 0.00 | 0.00 | KO:K17315 | glucose/mannose transport system substrate-binding protein | Bacteria;Chloroflexi;Anaerolineae;Anaerolineales;Anaerolineaceae;Thermanaerothrix;Thermanaerothrix daxensis;Thermanaerothrix daxensis GNS-1 |
| Ga0586324_3186792_3_635 | 0.00 | 0.00 | 0.00 | 0.00 | 0.00 | 0.00 | 355454.28 | 0.00 | 0.00 | 0.00 | 0.00 | 0.00 | KO:K17315 | glucose/mannose transport system substrate-binding protein | Bacteria;Deinococcus-Thermus;Deinococci;Thermales;Thermaceae;Marinithermus;Marinithermus hydrothermalis;Marinithermus hydrothermalis T1, DSM 14884 |
| Ga0586324_3612034_1_537 | 0.00 | 0.00 | 0.00 | 0.00 | 0.00 | 3056344.20 | 0.00 | 0.00 | 0.00 | 0.00 | 0.00 | 0.00 | KO:K17315 | glucose/mannose transport system substrate-binding protein | Bacteria;Atribacterota;unclassified Atribacterota;unclassified Atribacterota;unclassified Atribacterota;unclassified Atribacterota;Atribacteria bacterium JGI 0000014-F07;Atribacteria bacterium JGI 0000014-F07 |
| Ga0586324_4093592_3_653 | 0.00 | 720800.00 | 0.00 | 0.00 | 0.00 | 0.00 | 0.00 | 0.00 | 0.00 | 0.00 | 0.00 | 0.00 | KO:K17315 | glucose/mannose transport system substrate-binding protein | Bacteria;Proteobacteria;Alphaproteobacteria;Hyphomicrobiales;Bradyrhizobiaceae;Bradyrhizobium;Bradyrhizobium manausense;Bradyrhizobium manausense BR3351 |
| Ga0586324_6967120_3_539 | 32700000.00 | 0.00 | 0.00 | 0.00 | 0.00 | 0.00 | 0.00 | 0.00 | 0.00 | 0.00 | 0.00 | 3367500.00 | KO:K17315 | glucose/mannose transport system substrate-binding protein | Bacteria;Deinococcus-Thermus;Deinococci;Thermales;Thermaceae;Marinithermus;Marinithermus hydrothermalis;Marinithermus hydrothermalis T1, DSM 14884 |
| Ga0586324_2377869_1_840 | 0.00 | 0.00 | 8895400.00 | 0.00 | 0.00 | 0.00 | 0.00 | 0.00 | 0.00 | 0.00 | 0.00 | 0.00 | KO:K17318 | putative aldouronate transport system substrate-binding protein | Bacteria;Firmicutes;Clostridia;Eubacteriales;Oscillospiraceae;Thermoclostridium;Thermoclostridium caenicola;Thermoclostridium caenicola DSM 19027 |
| Ga0586324_0019435_5279_7024 | 0.00 | 0.00 | 0.00 | 0.00 | 0.00 | 0.00 | 6680400.00 | 0.00 | 0.00 | 0.00 | 0.00 | 0.00 | KO:K17321 | glycerol transport system substrate-binding protein | Bacteria;Proteobacteria;Deltaproteobacteria;Desulfobacterales;Desulfobacteraceae;Desulfatitalea;Desulfatitalea tepidiphila;Desulfatitalea tepidiphila S28bF |
| Ga0586324_0046981_4466_4924 | 0.00 | 0.00 | 15482500.00 | 0.00 | 0.00 | 0.00 | 0.00 | 0.00 | 0.00 | 0.00 | 0.00 | 0.00 | KO:K17321 | glycerol transport system substrate-binding protein | Bacteria;Proteobacteria;Betaproteobacteria;Burkholderiales;Comamonadaceae;Polaromonas;Polaromonas sp. CG9_12;Polaromonas sp. CG9_12 |
| Ga0586324_0395831_3_1544 | 0.00 | 4909600.00 | 0.00 | 0.00 | 0.00 | 0.00 | 0.00 | 0.00 | 3717900.00 | 0.00 | 0.00 | 0.00 | KO:K17321 | glycerol transport system substrate-binding protein | Bacteria;Proteobacteria;Betaproteobacteria;Burkholderiales;unclassified Burkholderiales;Rhizobacter;Rhizobacter sp. Root16D2;Rhizobacter sp. Root16D2 |
| Ga0586324_0694356_3_986 | 0.00 | 0.00 | 0.00 | 2546000.00 | 0.00 | 0.00 | 0.00 | 0.00 | 0.00 | 0.00 | 0.00 | 0.00 | KO:K17321 | glycerol transport system substrate-binding protein | Bacteria;Proteobacteria;Alphaproteobacteria;Hyphomicrobiales;Tepidamorphaceae;Tepidamorphus;Tepidamorphus gemmatus;Tepidamorphus gemmatus DSM 19345 |
| Ga0586324_1835662_203_937 | 0.00 | 5018400.00 | 0.00 | 0.00 | 0.00 | 2139143.19 | 0.00 | 1579900.00 | 0.00 | 0.00 | 0.00 | 0.00 | KO:K17321 | glycerol transport system substrate-binding protein | Bacteria;Proteobacteria;Betaproteobacteria;Burkholderiales;unclassified Burkholderiales;Methylibium;Methylibium sp. NZG;Methylibium sp. NZG |
| Ga0586324_2291474_217_852 | 572577.00 | 63852000.00 | 0.00 | 0.00 | 0.00 | 107984000.00 | 0.00 | 0.00 | 0.00 | 0.00 | 0.00 | 0.00 | KO:K17321 | glycerol transport system substrate-binding protein | Bacteria;Proteobacteria;Alphaproteobacteria;Hyphomicrobiales;Bradyrhizobiaceae;Bradyrhizobium;Bradyrhizobium lablabi;Bradyrhizobium lablabi CCBAU 23086 |
| Ga0586324_0000002_110635_111819 | 0.00 | 0.00 | 0.00 | 0.00 | 0.00 | 0.00 | 0.00 | 0.00 | 0.00 | 0.00 | 5746400.00 | 7185000.00 | KO:K17713 | outer membrane protein assembly factor BamB | Bacteria;Proteobacteria;Betaproteobacteria;Nitrosomonadales;Thiobacillaceae;Annwoodia;Annwoodia aquaesulis;Annwoodia aquaesulis DSM 4255 |
| Ga0586324_5445728_3_596 | 0.00 | 0.00 | 0.00 | 0.00 | 0.00 | 0.00 | 0.00 | 0.00 | 0.00 | 0.00 | 0.00 | 2032500.00 | KO:K17713 | outer membrane protein assembly factor BamB | Bacteria;Proteobacteria;Betaproteobacteria;Rhodocyclales;Rhodocyclaceae;Rugosibacter;Rugosibacter aromaticivorans;Rugosibacter aromaticivorans PG1-Ca6 |
| Ga0586324_6084979_3_569 | 0.00 | 0.00 | 0.00 | 0.00 | 0.00 | 875539.83 | 0.00 | 0.00 | 0.00 | 0.00 | 0.00 | 0.00 | KO:K17713 | outer membrane protein assembly factor BamB | Bacteria;Planctomycetes;Planctomycetia;Pirellulales;Pirellulaceae;Pirellula;Pirellula sp. SH-Sr6A;Pirellula sp. SH-Sr6A |
| Ga0586324_0012428_543_2795 | 0.00 | 0.00 | 0.00 | 0.00 | 0.00 | 0.00 | 0.00 | 0.00 | 0.00 | 916300.00 | 0.00 | 0.00 | KO:K17734 | serine protease AprX | Bacteria;Acidobacteria;Vicinamibacteria;unclassified Vicinamibacteria;Vicinamibacteraceae;Luteitalea;Luteitalea pratensis;Luteitalea pratensis DSM 100886 |
| Ga0586324_0425789_959_1759 | 0.00 | 0.00 | 0.00 | 0.00 | 0.00 | 0.00 | 0.00 | 0.00 | 0.00 | 0.00 | 0.00 | 8550000.00 | KO:K17995 | sulfhydrogenase subunit gamma (sulfur reductase) | Bacteria;Proteobacteria;Deltaproteobacteria;unclassified Deltaproteobacteria;unclassified Deltaproteobacteria;unclassified Deltaproteobacteria;delta proteobacterium NaphS2;delta proteobacterium NaphS2 |
| Ga0586324_3409297_158_721 | 0.00 | 911200.00 | 0.00 | 0.00 | 0.00 | 0.00 | 0.00 | 0.00 | 0.00 | 0.00 | 0.00 | 0.00 | KO:K18007 | NAD-reducing hydrogenase small subunit | Bacteria;Proteobacteria;Deltaproteobacteria;Syntrophobacterales;Syntrophobacteraceae;Syntrophobacter;Syntrophobacter fumaroxidans;Syntrophobacter fumaroxidans MPOB |
| Ga0586324_2006947_584_901 | 0.00 | 0.00 | 0.00 | 0.00 | 0.00 | 0.00 | 1340174.88 | 0.00 | 0.00 | 0.00 | 0.00 | 0.00 | KO:K18008 | [NiFe] hydrogenase small subunit | Bacteria;Proteobacteria;Deltaproteobacteria;Syntrophobacterales;Syntrophaceae;Desulfomonile;Desulfomonile tiedjei;Desulfomonile tiedjei DCB-1, DSM 6799 |
| Ga0586324_4251299_2_658 | 0.00 | 0.00 | 0.00 | 0.00 | 0.00 | 0.00 | 0.00 | 0.00 | 0.00 | 977900.00 | 0.00 | 0.00 | KO:K18030 | nicotinate dehydrogenase subunit B | Bacteria;Candidatus Aminicenantes;unclassified Candidatus Aminicenantes;unclassified Candidatus Aminicenantes;unclassified Candidatus Aminicenantes;unclassified Candidatus Aminicenantes;Aminicenantes bacterium SCGC AAA255-A06;Aminicenantes bacterium SCGC AAA255-A06 (contamination screened) |
| Ga0586324_0456501_663_1706 | 0.00 | 0.00 | 0.00 | 0.00 | 0.00 | 0.00 | 0.00 | 0.00 | 0.00 | 785400.00 | 1188000.00 | 330094.50 | KO:K18118 | succinyl-CoA:acetate CoA-transferase | Bacteria;Proteobacteria;Deltaproteobacteria;Myxococcales;Anaeromyxobacteraceae;Anaeromyxobacter;Anaeromyxobacter sp. Fw109-5;Anaeromyxobacter sp. Fw109-5 |
| Ga0586324_0955239_3_740 | 0.00 | 0.00 | 0.00 | 0.00 | 0.00 | 0.00 | 0.00 | 0.00 | 0.00 | 2132900.00 | 0.00 | 0.00 | KO:K18118 | succinyl-CoA:acetate CoA-transferase | Bacteria;Proteobacteria;Deltaproteobacteria;Myxococcales;Anaeromyxobacteraceae;Anaeromyxobacter;Anaeromyxobacter sp. Fw109-5;Anaeromyxobacter sp. Fw109-5 |
| Ga0586324_4693918_1_633 | 0.00 | 9520000.00 | 0.00 | 0.00 | 0.00 | 0.00 | 0.00 | 0.00 | 0.00 | 0.00 | 0.00 | 0.00 | KO:K18118 | succinyl-CoA:acetate CoA-transferase | Bacteria;Proteobacteria;Deltaproteobacteria;Myxococcales;Anaeromyxobacteraceae;Anaeromyxobacter;Anaeromyxobacter sp. PSR-1;Anaeromyxobacter sp. PSR-1 |
| Ga0586324_0091552_1081_2622 | 0.00 | 9588000.00 | 0.00 | 0.00 | 0.00 | 0.00 | 0.00 | 3025600.00 | 0.00 | 0.00 | 0.00 | 0.00 | KO:K18122 | 4-hydroxybutyrate CoA-transferase | Bacteria;Proteobacteria;Deltaproteobacteria;Desulfurellales;Desulfurellaceae;Desulfurella;Desulfurella multipotens;Desulfurella multipotens DSM 8415 |
| Ga0586324_0116857_716_2263 | 0.00 | 5766400.00 | 22013300.00 | 0.00 | 0.00 | 0.00 | 0.00 | 0.00 | 0.00 | 0.00 | 0.00 | 0.00 | KO:K18122 | 4-hydroxybutyrate CoA-transferase | Bacteria;Proteobacteria;Deltaproteobacteria;Desulfurellales;Desulfurellaceae;Desulfurella;Desulfurella multipotens;Desulfurella multipotens DSM 8415 |
| Ga0586324_1002882_3_1139 | 0.00 | 0.00 | 0.00 | 0.00 | 7210000.00 | 0.00 | 0.00 | 0.00 | 0.00 | 0.00 | 0.00 | 0.00 | KO:K18122 | 4-hydroxybutyrate CoA-transferase | Bacteria;Chloroflexi;Anaerolineae;Anaerolineales;Anaerolineaceae;Levilinea;Levilinea saccharolytica;Levilinea saccharolytica DSM 16555 |
| Ga0586324_2293992_1_738 | 0.00 | 0.00 | 0.00 | 0.00 | 0.00 | 0.00 | 0.00 | 0.00 | 0.00 | 5913600.00 | 0.00 | 0.00 | KO:K18139 | multidrug efflux system outer membrane protein | Bacteria;Acidobacteria;Acidobacteriia;Acidobacteriales;Acidobacteriaceae;Candidatus Koribacter;Candidatus Koribacter versatilis;Candidatus Koribacter versatilis Ellin345 |
| Ga0586324_0062540_3346_4293 | 0.00 | 0.00 | 0.00 | 0.00 | 0.00 | 0.00 | 0.00 | 260593.83 | 0.00 | 0.00 | 0.00 | 0.00 | KO:K18208 | renalase | Bacteria;Proteobacteria;Betaproteobacteria;Nitrosomonadales;Thiobacillaceae;Thiobacillus;Thiobacillus thioparus;Thiobacillus thioparus DSM 505 |
| Ga0586324_0055510_2_1756 | 0.00 | 0.00 | 0.00 | 0.00 | 0.00 | 1914913.62 | 0.00 | 0.00 | 0.00 | 0.00 | 0.00 | 0.00 | KO:K18331 | NADP-reducing hydrogenase subunit HndC | Bacteria;Proteobacteria;Deltaproteobacteria;Syntrophobacterales;Syntrophaceae;Desulfomonile;Desulfomonile tiedjei;Desulfomonile tiedjei DCB-1, DSM 6799 |
| Ga0586324_0338605_140_1606 | 1950000.00 | 0.00 | 1776574.65 | 2213500.00 | 3543200.00 | 0.00 | 1201192.92 | 1329800.00 | 1320300.00 | 0.00 | 0.00 | 0.00 | KO:K18331 | NADP-reducing hydrogenase subunit HndC | Bacteria;Proteobacteria;Deltaproteobacteria;Desulfuromonadales;Geobacteraceae;Geobacter;Geobacter sp. DSM 2909;Geobacter sp. DSM 2909 |
| Ga0586324_0379245_1_1851 | 0.00 | 0.00 | 0.00 | 0.00 | 0.00 | 0.00 | 0.00 | 0.00 | 589298.49 | 0.00 | 0.00 | 0.00 | KO:K18930 | D-lactate dehydrogenase | Bacteria;Acidobacteria;Holophagae;Holophagales;Holophagaceae;Geothrix;Geothrix fermentans;Geothrix fermentans DSM 14018 |
| Ga0586324_0795981_480_1340 | 0.00 | 0.00 | 0.00 | 0.00 | 0.00 | 0.00 | 0.00 | 719800.00 | 1263600.00 | 0.00 | 0.00 | 0.00 | KO:K19231 | fibronectin-binding autotransporter adhesin | Bacteria;Cyanobacteria;unclassified Cyanobacteria;Gloeoemargaritales;Gloeomargaritaceae;Gloeomargarita;Gloeomargarita lithophora;Candidatus Gloeomargarita lithophora D10 |
| Ga0586324_2497344_51_821 | 0.00 | 0.00 | 2166069.31 | 0.00 | 0.00 | 0.00 | 0.00 | 0.00 | 0.00 | 0.00 | 0.00 | 0.00 | KO:K19231 | fibronectin-binding autotransporter adhesin | Bacteria;Proteobacteria;Betaproteobacteria;Burkholderiales;Burkholderiaceae;Paraburkholderia;Paraburkholderia caballeronis;Paraburkholderia caballeronis LMG 26416 |
| Ga0586324_0235364_279_1286 | 0.00 | 0.00 | 0.00 | 0.00 | 0.00 | 4128800.00 | 0.00 | 0.00 | 0.00 | 0.00 | 0.00 | 0.00 | KO:K19244 | alanine dehydrogenase | Bacteria;Proteobacteria;Deltaproteobacteria;Desulfobacterales;Desulfobacteraceae;Desulfococcus;Desulfococcus multivorans;Desulfococcus multivorans DSM 2059 |
| Ga0586324_0068621_1891_3444 | 0.00 | 0.00 | 0.00 | 0.00 | 5860700.00 | 0.00 | 0.00 | 0.00 | 0.00 | 0.00 | 0.00 | 0.00 | KO:K19519 | transforming growth factor-beta-induced protein | Bacteria;Chloroflexi;Caldilineae;Caldilineales;Caldilineaceae;Caldilinea;Caldilinea aerophila;Caldilinea aerophila STL-6-O1, DSM 14535 |
| Ga0586324_0482736_613_1122 | 0.00 | 0.00 | 0.00 | 0.00 | 0.00 | 0.00 | 0.00 | 0.00 | 0.00 | 0.00 | 0.00 | 3225000.00 | KO:K19519 | transforming growth factor-beta-induced protein | Bacteria;Deinococcus-Thermus;Deinococci;Deinococcales;Deinococcaceae;Deinococcus;Deinococcus soli Cha et al. 2016;Deinococcus soli N5 |
| Ga0586324_0721541_2_1177 | 0.00 | 2509200.00 | 0.00 | 0.00 | 0.00 | 0.00 | 0.00 | 0.00 | 0.00 | 2348500.00 | 0.00 | 0.00 | KO:K19519 | transforming growth factor-beta-induced protein | Bacteria;Planctomycetes;Planctomycetia;Pirellulales;Pirellulaceae;Rhodopirellula;Rhodopirellula sp. SWK7;Rhodopirellula sp. SWK7 |
| Ga0586324_1788408_448_948 | 0.00 | 0.00 | 0.00 | 0.00 | 0.00 | 0.00 | 0.00 | 0.00 | 1458000.00 | 0.00 | 0.00 | 0.00 | KO:K19519 | transforming growth factor-beta-induced protein | Bacteria;Chloroflexi;Anaerolineae;Anaerolineales;Anaerolineaceae;Levilinea;Levilinea saccharolytica;Levilinea saccharolytica DSM 16555 |
| Ga0586324_2089821_13_888 | 0.00 | 0.00 | 0.00 | 2042500.00 | 0.00 | 0.00 | 0.00 | 0.00 | 0.00 | 0.00 | 0.00 | 0.00 | KO:K19715 | 8-amino-3,8-dideoxy-alpha-D-manno-octulosonate transaminase | Bacteria;Planctomycetes;Planctomycetia;Pirellulales;Thermoguttaceae;Thermogutta;Thermogutta terrifontis;Thermogutta terrifontis R1 |
| Ga0586324_0668500_855_1445 | 0.00 | 0.00 | 0.00 | 0.00 | 0.00 | 0.00 | 0.00 | 0.00 | 0.00 | 0.00 | 0.00 | 2317500.00 | KO:K19784 | chromate reductase | Bacteria;Proteobacteria;Betaproteobacteria;Burkholderiales;Oxalobacteraceae;Noviherbaspirillum;Noviherbaspirillum massiliense;Noviherbaspirillum massiliense JC206 |
| Ga0586324_0007462_2520_6041 | 0.00 | 0.00 | 7713100.00 | 3629000.00 | 2513200.00 | 5002200.00 | 3009600.00 | 3409900.00 | 2762100.00 | 0.00 | 3845600.00 | 3502500.00 | KO:K20276 | PKD repeat protein | Archaea;Euryarchaeota;Methanomicrobia;Methanomicrobiales;Methanoregulaceae;Methanosphaerula;Methanosphaerula palustris;Methanosphaerula palustris E1-9c, DSM 19958 |
| Ga0586324_0012740_2_3145 | 1440000.00 | 6364800.00 | 9739900.00 | 2004500.00 | 0.00 | 224399.88 | 0.00 | 0.00 | 0.00 | 1978900.00 | 0.00 | 688734.75 | KO:K20276 | hypothetical protein | Archaea;Euryarchaeota;Methanomicrobia;Methanosarcinales;Methanotrichaceae;Methanothrix;Methanothrix soehngenii;Methanothrix soehngenii GP-6 |
| Ga0586324_0083542_121_2580 | 0.00 | 0.00 | 0.00 | 0.00 | 0.00 | 0.00 | 0.00 | 0.00 | 0.00 | 0.00 | 0.00 | 4057500.00 | KO:K20276 | hypothetical protein | Bacteria;Proteobacteria;Deltaproteobacteria;Desulfuromonadales;Geobacteraceae;Geobacter;Geobacter sp. OR-1;Geobacter sp. OR-1 |
| Ga0586324_0104518_1_2325 | 3230000.00 | 3576800.00 | 0.00 | 0.00 | 0.00 | 0.00 | 0.00 | 0.00 | 0.00 | 2071300.00 | 0.00 | 2205000.00 | KO:K20276 | hypothetical protein | Bacteria;Proteobacteria;Betaproteobacteria;Burkholderiales;Oxalobacteraceae;Massilia;Massilia sp. WF1;Massilia sp. WF1 |
| Ga0586324_0153398_1_1128 | 1820000.00 | 0.00 | 0.00 | 0.00 | 0.00 | 0.00 | 5973600.00 | 2165500.00 | 0.00 | 0.00 | 0.00 | 0.00 | KO:K20276 | subtilase family serine protease | Bacteria;Proteobacteria;Deltaproteobacteria;Myxococcales;Anaeromyxobacteraceae;Anaeromyxobacter;Anaeromyxobacter sp. Fw109-5;Anaeromyxobacter sp. Fw109-5 |
| Ga0586324_1851782_3_932 | 0.00 | 3855600.00 | 0.00 | 1881000.00 | 0.00 | 0.00 | 0.00 | 786900.00 | 0.00 | 1578500.00 | 0.00 | 1290000.00 | KO:K20276 | hypothetical protein | Archaea;Euryarchaeota;Methanomicrobia;Methanosarcinales;Methanotrichaceae;Methanothrix;Methanothrix soehngenii;Methanothrix soehngenii GP-6 |
| Ga0586324_0792630_1_885 | 0.00 | 0.00 | 0.00 | 0.00 | 0.00 | 0.00 | 0.00 | 4587200.00 | 0.00 | 0.00 | 0.00 | 0.00 | KO:K20370 | hypothetical protein | Bacteria;Proteobacteria;Betaproteobacteria;Rhodocyclales;Rhodocyclaceae;Propionivibrio;Propionivibrio dicarboxylicus;Propionivibrio dicarboxylicus DSM 5885 |
| Ga0586324_0033214_1444_2472 | 0.00 | 0.00 | 5089024.56 | 0.00 | 0.00 | 5438900.00 | 0.00 | 3001200.00 | 0.00 | 2641100.00 | 0.00 | 0.00 | KO:K20523 | lipid-binding SYLF domain-containing protein | Bacteria;Proteobacteria;Deltaproteobacteria;Syntrophorhabdales;Syntrophorhabdaceae;Syntrophorhabdus;Syntrophorhabdus aromaticivorans;Syntrophorhabdus aromaticivorans UI |
| Ga0586324_0083877_530_1225 | 0.00 | 0.00 | 0.00 | 0.00 | 0.00 | 0.00 | 0.00 | 0.00 | 10125000.00 | 0.00 | 0.00 | 11400000.00 | KO:K20523 | lipid-binding SYLF domain-containing protein | Bacteria;Proteobacteria;Gammaproteobacteria;Methylococcales;Methylococcaceae;Methylococcus;Methylococcus capsulatus;Methylococcus capsulatus Texas, ATCC 19069 |
| Ga0586324_0933423_543_1232 | 0.00 | 43520000.00 | 0.00 | 0.00 | 0.00 | 0.00 | 0.00 | 0.00 | 0.00 | 0.00 | 0.00 | 0.00 | KO:K20523 | lipid-binding SYLF domain-containing protein | Bacteria;Proteobacteria;Betaproteobacteria;Burkholderiales;Comamonadaceae;Variovorax;Variovorax sp. YR216;Variovorax sp. YR216 |
| Ga0586324_6501281_88_552 | 0.00 | 0.00 | 0.00 | 0.00 | 0.00 | 0.00 | 0.00 | 0.00 | 3831300.00 | 0.00 | 0.00 | 0.00 | KO:K20523 | lipid-binding SYLF domain-containing protein | Bacteria;Acidobacteria;Acidobacteriia;Bryobacterales;Solibacteraceae;Candidatus Solibacter;Candidatus Solibacter usitatus;Candidatus Solibacter usitatus Ellin6076 |
| Ga0586324_0002210_16960_18216 | 0.00 | 0.00 | 0.00 | 0.00 | 0.00 | 0.00 | 0.00 | 27389000.00 | 36855000.00 | 0.00 | 0.00 | 0.00 | KO:K21071 | 6-phosphofructokinase | Bacteria;Proteobacteria;Betaproteobacteria;Nitrosomonadales;Thiobacillaceae;Thiobacillus;Thiobacillus thioparus;Thiobacillus thioparus DSM 505 |
| Ga0586324_0110466_3_1070 | 0.00 | 0.00 | 0.00 | 0.00 | 0.00 | 0.00 | 0.00 | 0.00 | 0.00 | 0.00 | 13728000.00 | 16050000.00 | KO:K21071 | 6-phosphofructokinase | Bacteria;Proteobacteria;Betaproteobacteria;Nitrosomonadales;Gallionellaceae;Sideroxydans;Sideroxydans lithotrophicus;Sideroxydans lithotrophicus ES-1 |
| Ga0586324_0278976_975_2123 | 0.00 | 0.00 | 0.00 | 0.00 | 0.00 | 0.00 | 0.00 | 923400.00 | 0.00 | 0.00 | 0.00 | 0.00 | KO:K21071 | 6-phosphofructokinase | Bacteria;Proteobacteria;Betaproteobacteria;Nitrosomonadales;Thiobacillaceae;Thiobacillus;Thiobacillus denitrificans;Thiobacillus denitrificans ATCC 25259 |
| Ga0586324_0441071_3_1127 | 0.00 | 0.00 | 0.00 | 0.00 | 0.00 | 0.00 | 0.00 | 0.00 | 2956800.00 | 0.00 | 0.00 | 0.00 | KO:K21071 | 6-phosphofructokinase | Bacteria;Proteobacteria;Deltaproteobacteria;Syntrophorhabdales;Syntrophorhabdaceae;Syntrophorhabdus;Syntrophorhabdus aromaticivorans;Syntrophorhabdus aromaticivorans UI |
| Ga0586324_1907649_1_702 | 0.00 | 1067600.00 | 0.00 | 0.00 | 0.00 | 0.00 | 0.00 | 5996300.00 | 4941000.00 | 862400.00 | 2877600.00 | 2842500.00 | KO:K21071 | 6-phosphofructokinase | Bacteria;Proteobacteria;Gammaproteobacteria;Chromatiales;Chromatiaceae;Thiodictyon;Candidatus Thiodictyon syntrophicum;Candidatus Thiodictyon syntrophicum Cad16 |
| Ga0586324_3278208_1_720 | 0.00 | 775200.00 | 0.00 | 0.00 | 0.00 | 0.00 | 0.00 | 0.00 | 0.00 | 0.00 | 0.00 | 0.00 | KO:K21071 | 6-phosphofructokinase | Bacteria;Chloroflexi;Caldilineae;Caldilineales;Caldilineaceae;Caldilinea;Caldilinea aerophila;Caldilinea aerophila STL-6-O1, DSM 14535 |

| | | | | | | | | | | | | | | | |
|---|---|---|---|---|---|---|---|---|---|---|---|---|---|---|---|
| Ga0586324 5757852 251 583 | 0.00 | 0.00 | 0.00 | 642406.15 | 0.00 | 0.00 | 0.00 | 0.00 | 0.00 | 0.00 | 0.00 | 0.00 | KO:K21071 | 6-phosphofructokinase | Bacteria;Proteobacteria;Betaproteobacteria;Nitrosomonadales;Thiobacillaceae;Thiobacillus;Thiobacillus denitrificans;Thiobacillus denitrificans ATCC 25259 |
| Ga0586324 0000025 45736 46770 | 0.00 | 0.00 | 0.00 | 0.00 | 0.00 | 0.00 | 0.00 | 0.00 | 1320300.00 | 0.00 | 0.00 | 0.00 | KO:K21395 | TRAP-type C4-dicarboxylate transport system substrate-binding protein | Bacteria;Proteobacteria;Alphaproteobacteria;Hyphomicrobiales;Hyphomicrobiaceae;Rhodoplanes;Rhodoplanes sp. Z2-YC6860;Rhodoplanes sp. Z2-YC6860 |
| Ga0586324 0000819 19128 20105 | 0.00 | 0.00 | 0.00 | 0.00 | 0.00 | 0.00 | 0.00 | 432960.31 | 382954.23 | 0.00 | 0.00 | 0.00 | KO:K21395 | tripartite ATP-independent transporter DctP family solute receptor | Bacteria;Proteobacteria;Betaproteobacteria;unclassified Betaproteobacteria;unclassified Betaproteobacteria;unclassified Betaproteobacteria;uncultured beta proteobacterium;unclassified betaproteobacterium ENR4 |
| Ga0586324 0006747 2973 4010 | 0.00 | 0.00 | 0.00 | 0.00 | 0.00 | 0.00 | 0.00 | 0.00 | 0.00 | 3680600.00 | 0.00 | 0.00 | KO:K21395 | TRAP-type C4-dicarboxylate transport system substrate-binding protein | Bacteria;Proteobacteria;Deltaproteobacteria;Desulfobacterales;Desulfobacteraceae;Desulfatiglans;Desulfatiglans anilini;Desulfatiglans anilini DSM 4660 |
| Ga0586324 0007732 2934 3959 | 2730000.00 | 0.00 | 0.00 | 0.00 | 0.00 | 0.00 | 0.00 | 0.00 | 0.00 | 0.00 | 0.00 | 0.00 | KO:K21395 | TRAP-type C4-dicarboxylate transport system substrate-binding protein | Bacteria;Proteobacteria;Deltaproteobacteria;Syntrophobacterales;Syntrophaceae;Desulfomonile;Desulfomonile tiedjei;Desulfomonile tiedjei DCB-1, DSM 6799 |
| Ga0586324 0008404 5067 6074 | 1100000.00 | 0.00 | 0.00 | 0.00 | 0.00 | 0.00 | 0.00 | 0.00 | 0.00 | 0.00 | 0.00 | 0.00 | KO:K21395 | tripartite ATP-independent transporter DctP family solute receptor | Bacteria;Proteobacteria;Alphaproteobacteria;Hyphomicrobiales;unclassified Hyphomicrobiales;unclassified Hyphomicrobiales;Rhizobiales bacterium YIM 77505;Rhizobiales bacterium YIM 77505 |
| Ga0586324 0009654 4421 5380 | 0.00 | 1985600.00 | 0.00 | 0.00 | 0.00 | 0.00 | 0.00 | 1860500.00 | 0.00 | 0.00 | 2156000.00 | 1695000.00 | KO:K21395 | TRAP-type C4-dicarboxylate transport system substrate-binding protein | Bacteria;Proteobacteria;Deltaproteobacteria;Desulfuromonadales;Desulfuromonadaceae;Pelobacter;Pelobacter seleniigenes;Pelobacter seleniigenes DSM 18267 |
| Ga0586324 0018383 603 1649 | 0.00 | 1373600.00 | 0.00 | 0.00 | 0.00 | 0.00 | 0.00 | 0.00 | 1247400.00 | 1493800.00 | 0.00 | 0.00 | KO:K21395 | TRAP-type C4-dicarboxylate transport system substrate-binding protein | Bacteria;Proteobacteria;Alphaproteobacteria;Rhodospirillales;Rhodospirillaceae;Tistlia;Tistlia consotensis;Tistlia consotensis USBA 355 |
| Ga0586324 0027822 168 1211 | 19900000.00 | 25840000.00 | 37383200.00 | 0.00 | 0.00 | 9528000.00 | 0.00 | 0.00 | 11761200.00 | 6514200.00 | 0.00 | 6750000.00 | KO:K21395 | tripartite ATP-independent transporter DctP family solute receptor | Bacteria;Proteobacteria;Deltaproteobacteria;Syntrophobacterales;Syntrophobacteraceae;Desulfacinum;Desulfacinum infernum;Desulfacinum infernum DSM 9756 |
| Ga0586324 0040515 3661 4710 | 0.00 | 0.00 | 0.00 | 0.00 | 0.00 | 0.00 | 0.00 | 0.00 | 2932200.00 | 0.00 | 0.00 | 0.00 | KO:K21395 | TRAP-type C4-dicarboxylate transport system substrate-binding protein | Bacteria;Proteobacteria;Deltaproteobacteria;Desulfuromonadales;Geobacteraceae;Geobacter;Geobacter daltonii;Geobacter daltonii FRC-32 |
| Ga0586324 0058812 1 1044 | 5420000.00 | 0.00 | 9627300.00 | 0.00 | 0.00 | 0.00 | 0.00 | 1701900.00 | 0.00 | 0.00 | 3220800.00 | 4545000.00 | KO:K21395 | tripartite ATP-independent transporter DctP family solute receptor | Bacteria;Proteobacteria;Deltaproteobacteria;Syntrophobacterales;Syntrophobacteraceae;Syntrophobacter;Syntrophobacter fumaroxidans;Syntrophobacter fumaroxidans MPOB |
| Ga0586324 0115607 435 1460 | 0.00 | 0.00 | 0.00 | 0.00 | 0.00 | 0.00 | 0.00 | 0.00 | 0.00 | 12012000.00 | 9944000.00 | 0.00 | KO:K21395 | TRAP-type C4-dicarboxylate transport system substrate-binding protein | Bacteria;Proteobacteria;Betaproteobacteria;Rhodocyclales;Azonexaceae;Azonexus;Azonexus hydrophilus;Azonexus hydrophilus ZS02 |
| Ga0586324 0116108 92 1087 | 0.00 | 1054000.00 | 0.00 | 0.00 | 0.00 | 0.00 | 0.00 | 0.00 | 0.00 | 0.00 | 0.00 | 0.00 | KO:K21395 | tripartite ATP-independent transporter DctP family solute receptor | Bacteria;Firmicutes;Clostridia;Eubacteriales;Desulfallaceae;Desulfoscipio;Desulfoscipio geothermicus;Desulfallas geothermicus DSM 3669 |
| Ga0586324 0163265 1495 2487 | 0.00 | 0.00 | 0.00 | 0.00 | 0.00 | 0.00 | 0.00 | 0.00 | 0.00 | 0.00 | 0.00 | 230999.25 | KO:K21395 | tripartite ATP-independent transporter DctP family solute receptor | Bacteria;Atribacterota;unclassified Atribacterota;unclassified Atribacterota;unclassified Atribacterota;unclassified Atribacterota;Atribacteria bacterium JGI 0000079-L04;Atribacteria bacterium JGI 0000079-L04 (contamination screened) |
| Ga0586324 0208045 1459 2430 | 0.00 | 2033200.00 | 0.00 | 0.00 | 0.00 | 0.00 | 0.00 | 0.00 | 0.00 | 0.00 | 0.00 | 0.00 | KO:K21395 | tripartite ATP-independent transporter DctP family solute receptor | Bacteria;Proteobacteria;Alphaproteobacteria;Rhodobacterales;Acuticoccaceae;Acuticoccus;Acuticoccus kandeliae;Acuticoccus kandeliae J103 |
| Ga0586324 0283010 704 1732 | 0.00 | 3454400.00 | 0.00 | 0.00 | 0.00 | 0.00 | 0.00 | 0.00 | 0.00 | 0.00 | 0.00 | 0.00 | KO:K21395 | TRAP-type C4-dicarboxylate transport system substrate-binding protein | Bacteria;Proteobacteria;Deltaproteobacteria;Desulfuromonadales;Geobacteraceae;Geobacter;Geobacter sp. DSM 9736;Geobacter sp. DSM 9736 |
| Ga0586324 0328233 1 537 | 0.00 | 0.00 | 0.00 | 0.00 | 536299.37 | 0.00 | 0.00 | 0.00 | 0.00 | 0.00 | 0.00 | 0.00 | KO:K21395 | TRAP-type C4-dicarboxylate transport system substrate-binding protein | Bacteria;Proteobacteria;Deltaproteobacteria;Syntrophobacterales;Syntrophaceae;Desulfomonile;Desulfomonile tiedjei;Desulfomonile tiedjei DCB-1, DSM 6799 |
| Ga0586324 0341830 781 1788 | 0.00 | 0.00 | 0.00 | 0.00 | 0.00 | 1959520.54 | 0.00 | 0.00 | 0.00 | 0.00 | 1566400.00 | 0.00 | KO:K21395 | tripartite ATP-independent transporter DctP family solute receptor | Bacteria;Thermotogae;Thermotogae;Kosmotogales;Kosmotogaceae;Kosmotoga;Kosmotoga pacifica;Kosmotoga pacifica SLHLJ1 |
| Ga0586324 0350809 677 1732 | 5490000.00 | 5297200.00 | 0.00 | 4113500.00 | 1050600.00 | 0.00 | 1895131.44 | 0.00 | 1692900.00 | 9471000.00 | 7788000.00 | 0.00 | KO:K21395 | TRAP-type C4-dicarboxylate transport system substrate-binding protein | Bacteria;Proteobacteria;Deltaproteobacteria;Syntrophorhabdales;Syntrophorhabdaceae;Syntrophorhabdus;Syntrophorhabdus aromaticivorans;Syntrophorhabdus aromaticivorans UI |
| Ga0586324 0402675 454 1488 | 18000000.00 | 7820000.00 | 0.00 | 0.00 | 2585300.00 | 0.00 | 0.00 | 0.00 | 2292300.00 | 0.00 | 29216000.00 | 1762500.00 | KO:K21395 | tripartite ATP-independent transporter DctP family solute receptor | Bacteria;Proteobacteria;Deltaproteobacteria;Syntrophobacterales;Syntrophobacteraceae;Desulfacinum;Desulfacinum infernum;Desulfacinum infernum DSM 9756 |
| Ga0586324 0422039 758 1765 | 3480000.00 | 3794400.00 | 2178826.89 | 0.00 | 0.00 | 0.00 | 0.00 | 0.00 | 0.00 | 0.00 | 0.00 | 0.00 | KO:K21395 | TRAP-type C4-dicarboxylate transport system substrate-binding protein | Bacteria;Proteobacteria;Deltaproteobacteria;Syntrophobacterales;Syntrophaceae;Desulfomonile;Desulfomonile tiedjei;Desulfomonile tiedjei DCB-1, DSM 6799 |
| Ga0586324 1411352 46 1047 | 0.00 | 1094800.00 | 5742600.00 | 0.00 | 0.00 | 0.00 | 0.00 | 0.00 | 0.00 | 0.00 | 0.00 | 0.00 | KO:K21395 | TRAP-type C4-dicarboxylate transport system substrate-binding protein | Bacteria;Proteobacteria;Deltaproteobacteria;Syntrophobacterales;Syntrophaceae;Desulfomonile;Desulfomonile tiedjei;Desulfomonile tiedjei DCB-1, DSM 6799 |
| Ga0586324 1422360 3 1043 | 0.00 | 1659200.00 | 0.00 | 0.00 | 0.00 | 0.00 | 0.00 | 0.00 | 0.00 | 0.00 | 0.00 | 0.00 | KO:K21395 | TRAP-type C4-dicarboxylate transport system substrate-binding protein | Bacteria;Proteobacteria;Deltaproteobacteria;Syntrophorhabdales;Syntrophorhabdaceae;Syntrophorhabdus;Syntrophorhabdus aromaticivorans;Syntrophorhabdus aromaticivorans UI |
| Ga0586324 1484076 176 1024 | 0.00 | 0.00 | 0.00 | 0.00 | 0.00 | 0.00 | 0.00 | 0.00 | 0.00 | 0.00 | 0.00 | 825000.00 | KO:K21395 | tripartite ATP-independent transporter DctP family solute receptor | Bacteria;Proteobacteria;Betaproteobacteria;Rhodocyclales;Rhodocyclaceae;Propionivibrio;Propionivibrio pelophilus;Propionivibrio pelophilus DSM 12018 |
| Ga0586324 1718969 3 428 | 0.00 | 1591200.00 | 0.00 | 0.00 | 0.00 | 0.00 | 0.00 | 0.00 | 0.00 | 0.00 | 0.00 | 0.00 | KO:K21395 | TRAP-type C4-dicarboxylate transport system substrate-binding protein | Bacteria;Proteobacteria;Deltaproteobacteria;Syntrophobacterales;Syntrophaceae;Desulfomonile;Desulfomonile tiedjei;Desulfomonile tiedjei DCB-1, DSM 6799 |
| Ga0586324 1836198 1 693 | 0.00 | 0.00 | 0.00 | 0.00 | 602119.46 | 0.00 | 0.00 | 0.00 | 0.00 | 0.00 | 0.00 | 0.00 | KO:K21395 | TRAP-type C4-dicarboxylate transport system substrate-binding protein | Bacteria;Proteobacteria;Deltaproteobacteria;Syntrophobacterales;Syntrophaceae;Desulfomonile;Desulfomonile tiedjei;Desulfomonile tiedjei DCB-1, DSM 6799 |
| Ga0586324 2033040 2 181 | 0.00 | 0.00 | 6981200.00 | 0.00 | 0.00 | 0.00 | 0.00 | 0.00 | 0.00 | 0.00 | 0.00 | 0.00 | KO:K21395 | TRAP-type C4-dicarboxylate transport system substrate-binding protein | Bacteria;Proteobacteria;Deltaproteobacteria;Syntrophobacterales;Syntrophobacteraceae;Desulfacinum;Desulfacinum infernum;Desulfacinum infernum DSM 9756 |
| Ga0586324 2043117 240 896 | 0.00 | 0.00 | 0.00 | 0.00 | 0.00 | 4168500.00 | 0.00 | 0.00 | 0.00 | 0.00 | 0.00 | 1207500.00 | KO:K21395 | tripartite ATP-independent transporter DctP family solute receptor | Bacteria;Proteobacteria;Deltaproteobacteria;Syntrophobacterales;Syntrophobacteraceae;Desulfacinum;Desulfacinum infernum;Desulfacinum infernum DSM 9756 |
| Ga0586324 2221236 3 671 | 0.00 | 0.00 | 0.00 | 0.00 | 0.00 | 0.00 | 0.00 | 0.00 | 0.00 | 0.00 | 2068000.00 | 0.00 | KO:K21395 | TRAP-type C4-dicarboxylate transport system substrate-binding protein | Bacteria;Proteobacteria;Deltaproteobacteria;Desulfobacterales;Desulfobacteraceae;Desulfatiglans;Desulfatiglans anilini;Desulfatiglans anilini DSM 4660 |
| Ga0586324 2475573 44 826 | 970571.00 | 0.00 | 0.00 | 0.00 | 0.00 | 0.00 | 0.00 | 0.00 | 0.00 | 0.00 | 0.00 | 1012500.00 | KO:K21395 | TRAP-type C4-dicarboxylate transport system substrate-binding protein | Bacteria;Proteobacteria;Betaproteobacteria;Rhodocyclales;Zoogloeaceae;Azoarcus;Azoarcus indigens;Azoarcus indigens DSM 12121 |
| Ga0586324 2639656 1 762 | 0.00 | 0.00 | 1507657.70 | 0.00 | 0.00 | 916093.38 | 0.00 | 0.00 | 826200.00 | 0.00 | 0.00 | 0.00 | KO:K21395 | tripartite ATP-independent transporter DctP family solute receptor | Bacteria;Proteobacteria;Alphaproteobacteria;Rhodobacterales;Rhodobacteraceae;Pseudooceanicola;Pseudooceanicola batsensis;Pseudooceanicola batsensis HTCC2597 |
| Ga0586324 2884979 3 773 | 0.00 | 4930000.00 | 0.00 | 0.00 | 0.00 | 0.00 | 0.00 | 0.00 | 0.00 | 0.00 | 0.00 | 0.00 | KO:K21395 | tripartite ATP-independent transporter DctP family solute receptor | Bacteria;Proteobacteria;Deltaproteobacteria;Syntrophobacterales;Syntrophobacteraceae;Desulfacinum;Desulfacinum infernum;Desulfacinum infernum DSM 9756 |
| Ga0586324 3960362 1 678 | 0.00 | 0.00 | 0.00 | 0.00 | 0.00 | 0.00 | 0.00 | 0.00 | 0.00 | 3634400.00 | 0.00 | 0.00 | KO:K21395 | TRAP-type C4-dicarboxylate transport system substrate-binding protein | Bacteria;Proteobacteria;Deltaproteobacteria;Syntrophobacterales;Syntrophaceae;Desulfomonile;Desulfomonile tiedjei;Desulfomonile tiedjei DCB-1, DSM 6799 |
| Ga0586324 4225322 2 661 | 0.00 | 0.00 | 3358328.78 | 0.00 | 0.00 | 0.00 | 0.00 | 0.00 | 0.00 | 0.00 | 0.00 | 0.00 | KO:K21395 | TRAP-type C4-dicarboxylate transport system substrate-binding protein | Bacteria;Proteobacteria;Deltaproteobacteria;Desulfuromonadales;Geobacteraceae;Geobacter;Geobacter daltonii;Geobacter daltonii FRC-32 |
| Ga0586324 4396908 2 649 | 0.00 | 0.00 | 0.00 | 0.00 | 0.00 | 0.00 | 0.00 | 0.00 | 0.00 | 0.00 | 0.00 | 2610000.00 | KO:K21395 | TRAP-type C4-dicarboxylate transport system substrate-binding protein | Bacteria;Proteobacteria;Alphaproteobacteria;Rhodospirillales;Rhodospirillaceae;Magnetospirillum;Magnetospirillum moscoviense;Magnetospirillum moscoviense BB-1 |
| Ga0586324 0024968 2860 4005 | 0.00 | 0.00 | 0.00 | 0.00 | 0.00 | 0.00 | 0.00 | 0.00 | 0.00 | 0.00 | 3766400.00 | 0.00 | KO:K21430 | glucose/arabinose dehydrogenase | Bacteria;Proteobacteria;Alphaproteobacteria;Hyphomicrobiales;Bradyrhizobiaceae;Tardiphaga;Tardiphaga sp. OK245;Tardiphaga sp. OK245 |
| Ga0586324 0000652 11010 13133 | 0.00 | 0.00 | 0.00 | 0.00 | 0.00 | 0.00 | 220612800.00 | 93488600.00 | 97159500.00 | 585230800.00 | 322264800.00 | 592680000.00 | KO:K21449 | hypothetical protein | Bacteria;Proteobacteria;Betaproteobacteria;Burkholderiales;Comamonadaceae;Polaromonas;Polaromonas jejuensis;Polaromonas jejuensis NBRC 106434 |
| Ga0586324 0000676 28385 30187 | 27700000.00 | 33864000.00 | 1050259.61 | 20615000.00 | 32537700.00 | 0.00 | 0.00 | 11468000.00 | 14733900.00 | 10792942.16 | 13851200.00 | 3660000.00 | KO:K21449 | PGF-CTERM protein | Archaea;Euryarchaeota;Methanomicrobia;Methanomicrobiales;Methanospirillaceae;Methanospirillum;Methanospirillum hungatei;Methanospirillum hungatei JF-1 |
| Ga0586324 0002518 15696 18209 | 621340000.00 | 91377069.24 | 132420324.92 | 105060500.00 | 90650300.00 | 291314741.16 | 243732000.00 | 152835500.00 | 160355700.00 | 304935400.00 | 236337885.52 | 351945000.00 | KO:K21449 | hypothetical protein | Archaea;Euryarchaeota;Methanomicrobia;Methanomicrobiales;Methanoregulaceae;Methanoregula;Methanoregula formicica;Methanoregula formicica SMSP, DSM 22288 |
| Ga0586324 0014171 2 8365 | 2740000.00 | 0.00 | 0.00 | 0.00 | 0.00 | 0.00 | 0.00 | 0.00 | 0.00 | 0.00 | 0.00 | 0.00 | KO:K21449 | Ca2+-binding RTX toxin-like protein | Bacteria;Cyanobacteria;unclassified Cyanobacteria;Chroococcales;Chroococcaceae;Geminocystis;Geminocystis sp. NIES-3709;Geminocystis sp. NIES-3709 |
| Ga0586324 0019129 1 1884 | 80390000.00 | 58969600.00 | 166085000.00 | 43605000.00 | 96099000.00 | 53584308.79 | 115596000.00 | 47580000.00 | 36612000.00 | 50589000.00 | 51392000.00 | 84825000.00 | KO:K21449 | PGF-CTERM protein | Archaea;Euryarchaeota;Methanomicrobia;Methanomicrobiales;Methanoregulaceae;Methanoregula;Methanoregula formicica;Methanoregula formicica SMSP, DSM 22288 |
| Ga0586324 0025527 2041 4698 | 0.00 | 7412000.00 | 0.00 | 0.00 | 0.00 | 0.00 | 0.00 | 0.00 | 0.00 | 0.00 | 0.00 | 0.00 | KO:K21449 | PGF-CTERM protein | Archaea;Euryarchaeota;Methanomicrobia;Methanomicrobiales;Methanomicrobiaceae;Methanofollis;Methanofollis liminatans;Methanofollis liminatans GKZPZ, DSM 4140 |
| Ga0586324 0029787 408 3920 | 1840000.00 | 0.00 | 0.00 | 0.00 | 0.00 | 0.00 | 0.00 | 0.00 | 0.00 | 0.00 | 0.00 | 0.00 | KO:K21449 | hypothetical protein | Bacteria;Proteobacteria;Gammaproteobacteria;Legionellales;Legionellaceae;Legionella;Legionella gratiana;Legionella gratiana Lyon 8420412 |
| Ga0586324 0031362 1 1965 | 15600000.00 | 20586670.88 | 12780100.00 | 13300000.00 | 0.00 | 0.00 | 0.00 | 5947500.00 | 7055100.00 | 2333100.00 | 1469600.00 | 3150000.00 | KO:K21449 | PGF-CTERM protein | Archaea;Euryarchaeota;Methanomicrobia;Methanomicrobiales;Methanomicrobiaceae;Methanofollis;Methanofollis liminatans;Methanofollis liminatans GKZPZ, DSM 4140 |
| Ga0586324 0033523 4565 5815 | 0.00 | 1727200.00 | 3120337.42 | 0.00 | 0.00 | 2694173.01 | 0.00 | 0.00 | 0.00 | 0.00 | 0.00 | 0.00 | KO:K21449 | ELWxxDGT repeat protein | Bacteria;Bacteroidetes;Chitinophagia;Chitinophagales;Chitinophagaceae;Flavisolibacter;Flavisolibacter ginsengiterrae;Flavisolibacter ginsengiterrae DSM 18136 |
| Ga0586324 0056749 2708 4498 | 1860000.00 | 40913895.24 | 66828100.00 | 8141500.00 | 13596000.00 | 10163200.00 | 0.00 | 0.00 | 9558000.00 | 15253700.00 | 12021943.12 | 22514790.75 | KO:K21449 | PGF-CTERM protein | Archaea;Euryarchaeota;Methanomicrobia;Methanomicrobiales;Methanospirillaceae;Methanospirillum;Methanospirillum hungatei;Methanospirillum hungatei JF-1 |
| Ga0586324 0058422 3 1430 | 64600000.00 | 62689200.00 | 25503900.00 | 5462500.00 | 0.00 | 17261845.84 | 0.00 | 28029500.00 | 6212700.00 | 43551200.00 | 49772800.00 | 43687500.00 | KO:K21449 | hypothetical protein | Archaea;Euryarchaeota;Methanomicrobia;Methanomicrobiales;Methanospirillaceae;Methanospirillum;Methanospirillum hungatei;Methanospirillum hungatei JF-1 |
| Ga0586324 0059539 284 3385 | 28000000.00 | 0.00 | 0.00 | 0.00 | 6427200.00 | 6232900.00 | 9576000.00 | 2543700.00 | 0.00 | 7584500.00 | 5940000.00 | 6577500.00 | KO:K21449 | PGF-CTERM protein | Archaea;Euryarchaeota;Methanomicrobia;Methanomicrobiales;Methanoregulaceae;Methanoregula;Methanoregula formicica;Methanoregula formicica SMSP, DSM 22288 |
| Ga0586324 0083763 1778 3730 | 0.00 | 15170800.00 | 16890000.00 | 1377500.00 | 2348400.00 | 0.00 | 5654400.00 | 0.00 | 658025.37 | 3133900.00 | 0.00 | 0.00 | KO:K21449 | PGF-CTERM protein | Archaea;Euryarchaeota;Methanomicrobia;Methanomicrobiales;Methanospirillaceae;Methanospirillum;Methanospirillum hungatei;Methanospirillum hungatei JF-1 |
| Ga0586324 0105012 2 2152 | 7450000.00 | 15245600.00 | 0.00 | 0.00 | 3625600.00 | 0.00 | 0.00 | 0.00 | 0.00 | 0.00 | 3731200.00 | 1380000.00 | KO:K21449 | hypothetical protein | Archaea;Euryarchaeota;Methanomicrobia;Methanomicrobiales;Methanomicrobiaceae;Methanofollis;Methanofollis liminatans;Methanofollis liminatans GKZPZ, DSM 4140 |
| Ga0586324 0143442 928 2889 | 5920000.00 | 318108.76 | 23420800.00 | 46075000.00 | 34391700.00 | 37476800.00 | 9781200.00 | 3028862.28 | 26568000.00 | 28020300.00 | 23259838.80 | 26775000.00 | KO:K21449 | hypothetical protein | Archaea;Euryarchaeota;Methanomicrobia;Methanomicrobiales;Methanoregulaceae;Methanoregula;Methanoregula formicica;Methanoregula formicica SMSP, DSM 22288 |
| Ga0586324 0144796 3 2375 | 50700000.00 | 46484800.00 | 0.00 | 14535000.00 | 4027300.00 | 3208696.92 | 0.00 | 0.00 | 9963000.00 | 13937000.00 | 13024000.00 | 13260000.00 | KO:K21449 | hypothetical protein | Archaea;Euryarchaeota;Methanomicrobia;Methanomicrobiales;Methanomicrobiaceae;Methanofollis;Methanofollis liminatans;Methanofollis liminatans GKZPZ, DSM 4140 |
| Ga0586324 0218664 2 1198 | 13700000.00 | 11900000.00 | 0.00 | 11970000.00 | 0.00 | 0.00 | 0.00 | 0.00 | 0.00 | 15939000.00 | 13710400.00 | 870000.00 | KO:K21449 | PGF-CTERM protein | Archaea;Euryarchaeota;Methanomicrobia;Methanomicrobiales;Methanoregulaceae;Methanoregula;Methanoregula formicica;Methanoregula formicica SMSP, DSM 22288 |
| Ga0586324 0269586 206 2158 | 0.00 | 0.00 | 0.00 | 0.00 | 0.00 | 0.00 | 0.00 | 0.00 | 0.00 | 0.00 | 0.00 | 1515000.00 | KO:K21449 | hypothetical protein | Bacteria;Chloroflexi;Dehalococcoidia;unclassified Dehalococcoidia;unclassified Dehalococcoidia;Dehalogenimonas;Dehalogenimonas sp. GP;Dehalogenimonas sp. GP |
| Ga0586324 0277913 3 902 | 28900000.00 | 24616000.00 | 34511900.00 | 23465000.00 | 24720000.00 | 22073200.00 | 11628000.00 | 11102000.00 | 8910000.00 | 27566000.00 | 27632000.00 | 21225000.00 | KO:K21449 | hypothetical protein | Archaea;Euryarchaeota;Methanomicrobia;Methanomicrobiales;Methanospirillaceae;Methanospirillum;Methanospirillum hungatei;Methanospirillum hungatei JF-1 |
| Ga0586324 0318397 125 1288 | 8600000.00 | 9805600.00 | 12323991.18 | 4446000.00 | 4264200.00 | 0.00 | 0.00 | 762500.00 | 63722700.00 | 33251405.11 | 40075200.00 | 8745000.00 | KO:K21449 | PGF-CTERM protein | Archaea;Euryarchaeota;Methanomicrobia;Methanomicrobiales;Methanoregulaceae;Methanoregula;Methanoregula formicica;Methanoregula formicica SMSP, DSM 22288 |
| Ga0586324 0346649 3 1001 | 0.00 | 4440400.00 | 0.00 | 0.00 | 0.00 | 0.00 | 0.00 | 0.00 | 0.00 | 3928705.55 | 1108800.00 | 877500.00 | KO:K21449 | PGF-CTERM protein | Archaea;Euryarchaeota;Methanomicrobia;Methanomicrobiales;Methanoregulaceae;Methanoregula;Methanoregula formicica;Methanoregula formicica SMSP, DSM 22288 |
| Ga0586324 0463827 3 1583 | 0.00 | 158097.28 | 0.00 | 0.00 | 0.00 | 0.00 | 0.00 | 0.00 | 0.00 | 146027.42 | 0.00 | 0.00 | KO:K21449 | hypothetical protein | Archaea;Euryarchaeota;Methanomicrobia;Methanomicrobiales;Methanoregulaceae;Methanoregula;Methanoregula formicica;Methanoregula formicica SMSP, DSM 22288 |
| Ga0586324 0516223 3 1616 | 0.00 | 0.00 | 0.00 | 0.00 | 0.00 | 0.00 | 0.00 | 0.00 | 0.00 | 0.00 | 0.00 | 2917500.00 | KO:K21449 | hypothetical protein | Bacteria;Acidobacteria;Acidobacteriia;Acidobacteriales;Acidobacteriaceae;Edaphobacter;Edaphobacter dinghuensis;Edaphobacter dinghuensis EB95 |
| Ga0586324 0541910 3 1583 | 0.00 | 50870800.00 | 0.00 | 0.00 | 0.00 | 0.00 | 0.00 | 14945000.00 | 0.00 | 566793.15 | 0.00 | 4852500.00 | KO:K21449 | hypothetical protein | Archaea;Euryarchaeota;Methanomicrobia;Methanomicrobiales;Methanoregulaceae;Methanoregula;Methanoregula formicica;Methanoregula formicica SMSP, DSM 22288 |
| Ga0586324 0594544 3 1520 | 7630000.00 | 7419781.24 | 14581700.00 | 4579000.00 | 3769800.00 | 5438900.00 | 0.00 | 3355000.00 | 2438100.00 | 2702700.00 | 2349600.00 | 0.00 | KO:K21449 | hypothetical protein | Archaea;Euryarchaeota;Methanomicrobia;Methanomicrobiales;Methanospirillaceae;Methanospirillum;Methanospirillum hungatei;Methanospirillum hungatei JF-1 |
| Ga0586324 0653594 2 1459 | 0.00 | 1108400.00 | 0.00 | 0.00 | 0.00 | 0.00 | 0.00 | 0.00 | 0.00 | 0.00 | 0.00 | 0.00 | KO:K21449 | hypothetical protein | Archaea;Euryarchaeota;Methanomicrobia;Methanomicrobiales;Methanoregulaceae;Methanoregula;Methanoregula formicica;Methanoregula formicica SMSP, DSM 22288 |
| Ga0586324 0839148 2 1309 | 0.00 | 5508000.00 | 0.00 | 0.00 | 0.00 | 0.00 | 0.00 | 0.00 | 0.00 | 4889500.00 | 6001600.00 | 0.00 | KO:K21449 | Ca2+-binding RTX toxin-like protein | Bacteria;Firmicutes;Negativicutes;Selenomonadales;Sporomusaceae;Sporomusa;Sporomusa ovata;Sporomusa ovata DSM 2662 |
| Ga0586324 1205374 3 1121 | 46870000.00 | 0.00 | 0.00 | 0.00 | 51706000.00 | 0.00 | 0.00 | 28487000.00 | 0.00 | 36729000.00 | 31152000.00 | 26625000.00 | KO:K21449 | hypothetical protein | Archaea;Euryarchaeota;Methanomicrobia;Methanomicrobiales;Methanoregulaceae;Methanoregula;Methanoregula formicica;Methanoregula formicica SMSP, DSM 22288 |
| Ga0586324 1233459 3 1109 | 0.00 | 0.00 | 0.00 | 0.00 | 0.00 | 0.00 | 0.00 | 0.00 | 0.00 | 0.00 | 0.00 | 19950000.00 | KO:K21449 | hypothetical protein | Archaea;Euryarchaeota;Methanomicrobia;Methanomicrobiales;Methanomicrobiaceae;Methanofollis;Methanofollis ethanolicus;Methanofollis ethanolicus JCM 15103 |
| Ga0586324 1235181 1 1110 | 0.00 | 349622.00 | 0.00 | 0.00 | 0.00 | 0.00 | 0.00 | 0.00 | 0.00 | 0.00 | 0.00 | 0.00 | KO:K21449 | hypothetical protein | Archaea;Euryarchaeota;Methanomicrobia;Methanomicrobiales;Methanospirillaceae;Methanospirillum;Methanospirillum hungatei;Methanospirillum hungatei JF-1 |
| Ga0586324 1281273 2 1090 | 0.00 | 0.00 | 0.00 | 0.00 | 0.00 | 0.00 | 0.00 | 0.00 | 0.00 | 0.00 | 3493600.00 | 0.00 | KO:K21449 | hypothetical protein | Archaea;Euryarchaeota;Methanomicrobia;Methanomicrobiales;Methanoregulaceae;Methanoregula;Methanoregula boonei;Methanoregula boonei 6A8 |
| Ga0586324 1329291 3 1073 | 0.00 | 6840800.00 | 0.00 | 0.00 | 8631400.00 | 0.00 | 0.00 | 2049600.00 | 2008800.00 | 0.00 | 1628000.00 | 722982.75 | KO:K21449 | hypothetical protein | Archaea;Euryarchaeota;Methanomicrobia;Methanomicrobiales;Methanoregulaceae;Methanoregula;Methanoregula formicica;Methanoregula formicica SMSP, DSM 22288 |
| Ga0586324 1518839 1 1014 | 5960941.00 | 938400.00 | 0.00 | 1083000.00 | 1411100.00 | 0.00 | 0.00 | 805200.00 | 511965.36 | 0.00 | 0.00 | 0.00 | KO:K21449 | hypothetical protein | Archaea;Euryarchaeota;Methanomicrobia;Methanomicrobiales;Methanoregulaceae;Methanoregula;Methanoregula formicica;Methanoregula formicica SMSP, DSM 22288 |
| Ga0586324 1727135 1 960 | 3540000.00 | 401411.48 | 0.00 | 0.00 | 1936400.00 | 0.00 | 0.00 | 0.00 | 963900.00 | 0.00 | 0.00 | 0.00 | KO:K21449 | hypothetical protein | Archaea;Euryarchaeota;Methanomicrobia;Methanomicrobiales;Methanospirillaceae;Methanospirillum;Methanospirillum hungatei;Methanospirillum hungatei JF-1 |
| Ga0586324 1727374 1 960 | 3650000.00 | 12240000.00 | 6193000.00 | 26410000.00 | 32960000.00 | 0.00 | 1642580.40 | 10675000.00 | 10189800.00 | 35550900.00 | 5352877.20 | 21367500.00 | KO:K21449 | hypothetical protein | Archaea;Euryarchaeota;Methanomicrobia;Methanomicrobiales;Methanoregulaceae;Methanoregula;Methanoregula formicica;Methanoregula formicica SMSP, DSM 22288 |
| Ga0586324 1808577 2 943 | 0.00 | 2903600.00 | 0.00 | 0.00 | 0.00 | 0.00 | 0.00 | 0.00 | 0.00 | 0.00 | 0.00 | 1185000.00 | KO:K21449 | hypothetical protein | Archaea;Euryarchaeota;Methanomicrobia;Methanomicrobiales;Methanoregulaceae;Methanoregula;Methanoregula formicica;Methanoregula formicica SMSP, DSM 22288 |
| Ga0586324 1934036 1 915 | 0.00 | 2563600.00 | 0.00 | 0.00 | 0.00 | 0.00 | 0.00 | 0.00 | 0.00 | 25025000.00 | 10648000.00 | 0.00 | KO:K21449 | hypothetical protein | Archaea;Euryarchaeota;Methanomicrobia;Methanomicrobiales;Methanoregulaceae;Methanoregula;Methanoregula formicica;Methanoregula formicica SMSP, DSM 22288 |
| Ga0586324 2565376 1 813 | 0.00 | 1366800.00 | 0.00 | 2859500.00 | 0.00 | 2885979.59 | 0.00 | 0.00 | 0.00 | 0.00 | 2142588.80 | 1905000.00 | KO:K21449 | hypothetical protein | Archaea;Euryarchaeota;Methanomicrobia;Methanomicrobiales;Methanoregulaceae;Methanoregula;Methanoregula formicica;Methanoregula formicica SMSP, DSM 22288 |
| Ga0586324 2569870 3 812 | 0.00 | 0.00 | 0.00 | 0.00 | 0.00 | 1393847.15 | 0.00 | 0.00 | 0.00 | 0.00 | 1355200.00 | 0.00 | KO:K21449 | hypothetical protein | Archaea;Euryarchaeota;Methanomicrobia;Methanomicrobiales;Methanospirillaceae;Methanospirillum;Methanospirillum hungatei;Methanospirillum hungatei JF-1 |
| Ga0586324 2577579 1 810 | 0.00 | 0.00 | 6249300.00 | 0.00 | 0.00 | 4446400.00 | 4674000.00 | 0.00 | 0.00 | 0.00 | 0.00 | 0.00 | KO:K21449 | hypothetical protein | Archaea;Euryarchaeota;Methanomicrobia;Methanomicrobiales;Methanoregulaceae;Methanoregula;Methanoregula formicica;Methanoregula formicica SMSP, DSM 22288 |
| Ga0586324 2685695 2 799 | 623503.00 | 0.00 | 3477583.44 | 13965000.00 | 0.00 | 0.00 | 0.00 | 0.00 | 0.00 | 4635400.00 | 10736000.00 | 5835000.00 | KO:K21449 | hypothetical protein | Archaea;Euryarchaeota;Methanomicrobia;Methanomicrobiales;Methanoregulaceae;Methanoregula;Methanoregula formicica;Methanoregula formicica SMSP, DSM 22288 |
| Ga0586324 2897085 3 773 | 5650000.00 | 1740800.00 | 5106066.57 | 1064000.00 | 1946700.00 | 1758158.17 | 0.00 | 0.00 | 0.00 | 0.00 | 2631200.00 | 1065000.00 | KO:K21449 | hypothetical protein | Archaea;Euryarchaeota;Methanomicrobia;Methanomicrobiales;Methanoregulaceae;Methanoregula;Methanoregula formicica;Methanoregula formicica SMSP, DSM 22288 |
| Ga0586324 2949870 2 571 | 0.00 | 0.00 | 834000.05 | 0.00 | 596727.41 | 0.00 | 0.00 | 0.00 | 0.00 | 0.00 | 0.00 | 226080.00 | KO:K21449 | PGF-CTERM protein | Archaea;Euryarchaeota;Methanomicrobia;Methanomicrobiales;Methanoregulaceae;Methanoregula;Methanoregula formicica;Methanoregula formicica SMSP, DSM 22288 |
| Ga0586324 3063412 2 754 | 4780000.00 | 19482000.00 | 14356500.00 | 5804500.00 | 9208200.00 | 8257600.00 | 4902000.00 | 3519700.00 | 3329100.00 | 7022400.00 | 13112000.00 | 4755000.00 | KO:K21449 | hypothetical protein | Archaea;Euryarchaeota;Methanomicrobia;Methanomicrobiales;Methanoregulaceae;Methanoregula;Methanoregula formicica;Methanoregula formicica SMSP, DSM 22288 |
| Ga0586324 3067693 3 755 | 0.00 | 0.00 | 0.00 | 495066.85 | 1143300.00 | 0.00 | 0.00 | 640500.00 | 297512.19 | 1208900.00 | 1108800.00 | 2910000.00 | KO:K21449 | hypothetical protein | Archaea;Euryarchaeota;Methanomicrobia;Methanomicrobiales;Methanoregulaceae;Methanoregula;Methanoregula formicica;Methanoregula formicica SMSP, DSM 22288 |
| Ga0586324 3090664 2 751 | 6640000.00 | 5127200.00 | 333530.21 | 7296000.00 | 6375700.00 | 0.00 | 63384000.00 | 51728000.00 | 4252500.00 | 83160000.00 | 108240000.00 | 86250000.00 | KO:K21449 | hypothetical protein | Archaea;Euryarchaeota;Methanomicrobia;Methanomicrobiales;Methanoregulaceae;Methanoregula;Methanoregula formicica;Methanoregula formicica SMSP, DSM 22288 |
| Ga0586324 3209122 2 739 | 9610000.00 | 7888000.00 | 15369900.00 | 6479000.00 | 5479600.00 | 7900300.00 | 3146400.00 | 1256600.00 | 2689200.00 | 8855000.00 | 9768000.00 | 7725000.00 | KO:K21449 | hypothetical protein | Archaea;Euryarchaeota;Methanomicrobia;Methanomicrobiales;Methanospirillaceae;Methanospirillum;Methanospirillum hungatei;Methanospirillum hungatei JF-1 |
| Ga0586324 3212859 1 741 | 0.00 | 5052400.00 | 0.00 | 0.00 | 3059100.00 | 0.00 | 0.00 | 2677900.00 | 0.00 | 0.00 | 1434400.00 | 4147500.00 | KO:K21449 | hypothetical protein | Archaea;Euryarchaeota;Methanomicrobia;Methanomicrobiales;Methanoregulaceae;Methanoregula;Methanoregula formicica;Methanoregula formicica SMSP, DSM 22288 |
| Ga0586324 3378461 2 724 | 11500000.00 | 0.00 | 0.00 | 0.00 | 10300000.00 | 0.00 | 0.00 | 5782800.00 | 0.00 | 11396000.00 | 10113407.04 | 0.00 | KO:K21449 | hypothetical protein | Archaea;Euryarchaeota;Methanomicrobia;Methanomicrobiales;Methanospirillaceae;Methanospirillum;Methanospirillum hungatei;Methanospirillum hungatei JF-1 |
| Ga0586324 3602286 1 705 | 109331.00 | 3645591.52 | 1925640.16 | 571740.40 | 3876378.22 | 0.00 | 0.00 | 0.00 | 0.00 | 1393700.00 | 4088202.80 | 1342500.00 | KO:K21449 | hypothetical protein | Archaea;Euryarchaeota;Methanomicrobia;Methanomicrobiales;Methanoregulaceae;Methanoregula;Methanoregula boonei;Methanoregula boonei 6A8 |
| Ga0586324 4144328 3 665 | 32200000.00 | 2563600.00 | 0.00 | 0.00 | 0.00 | 0.00 | 0.00 | 0.00 | 0.00 | 0.00 | 0.00 | 0.00 | KO:K21449 | hypothetical protein | Archaea;Euryarchaeota;Methanomicrobia;Methanomicrobiales;Methanoregulaceae;Methanoregula;Methanoregula formicica;Methanoregula formicica SMSP, DSM 22288 |
| Ga0586324 4145484 1 666 | 4210000.00 | 5814000.00 | 3161701.03 | 5776000.00 | 6828900.00 | 2362531.12 | 0.00 | 2757200.00 | 0.00 | 0.00 | 12232000.00 | 7320000.00 | KO:K21449 | hypothetical protein | Archaea;Euryarchaeota;Methanomicrobia;Methanomicrobiales;Methanoregulaceae;Methanoregula;Methanoregula formicica;Methanoregula formicica SMSP, DSM 22288 |
| Ga0586324 4149929 2 667 | 0.00 | 1564000.00 | 0.00 | 0.00 | 0.00 | 0.00 | 0.00 | 0.00 | 0.00 | 2132900.00 | 0.00 | 0.00 | KO:K21449 | hypothetical protein | Archaea;Euryarchaeota;Methanomicrobia;Methanomicrobiales;Methanoregulaceae;Methanoregula;Methanoregula formicica;Methanoregula formicica SMSP, DSM 22288 |
| Ga0586324 4274118 3 602 | 0.00 | 0.00 | 0.00 | 0.00 | 1823100.00 | 0.00 | 0.00 | 0.00 | 0.00 | 2710400.00 | 1592800.00 | 0.00 | KO:K21449 | hypothetical protein | Archaea;Euryarchaeota;Methanomicrobia;Methanomicrobiales;Methanospirillaceae;Methanospirillum;Methanospirillum hungatei;Methanospirillum hungatei JF-1 |
| Ga0586324 4381451 1 651 | 0.00 | 788800.00 | 4061223.02 | 1311000.00 | 3584400.00 | 2391222.31 | 995477.64 | 1421300.00 | 750269.79 | 4327400.00 | 5667200.00 | 3810000.00 | KO:K21449 | hypothetical protein | Archaea;Euryarchaeota;Methanomicrobia;Methanomicrobiales;Methanoregulaceae;Methanoregula;Methanoregula formicica;Methanoregula formicica SMSP, DSM 22288 |
| Ga0586324 4639590 3 635 | 9040000.00 | 7140000.00 | 0.00 | 0.00 | 0.00 | 0.00 | 0.00 | 0.00 | 0.00 | 7168700.00 | 9240000.00 | 5955000.00 | KO:K21449 | hypothetical protein | Archaea;Euryarchaeota;Methanomicrobia;Methanomicrobiales;Methanoregulaceae;Methanoregula;Methanoregula formicica;Methanoregula formicica SMSP, DSM 22288 |
| Ga0586324 5055669 1 615 | 0.00 | 0.00 | 0.00 | 0.00 | 0.00 | 0.00 | 0.00 | 0.00 | 0.00 | 2564100.00 | 0.00 | 0.00 | KO:K21449 | hypothetical protein | Archaea;Euryarchaeota;Methanomicrobia;Methanomicrobiales;Methanospirillaceae;Methanospirillum;Methanospirillum hungatei;Methanospirillum hungatei JF-1 |
| Ga0586324 6082017 1 570 | 0.00 | 0.00 | 0.00 | 0.00 | 0.00 | 0.00 | 0.00 | 0.00 | 0.00 | 0.00 | 0.00 | 4095000.00 | KO:K21449 | hypothetical protein | Archaea;Euryarchaeota;Methanomicrobia;Methanomicrobiales;Methanoregulaceae;Methanoregula;Methanoregula formicica;Methanoregula formicica SMSP, DSM 22288 |
| Ga0586324 6082566 1 570 | 540802.00 | 5134000.00 | 0.00 | 3030500.00 | 418499.30 | 0.00 | 0.00 | 1403000.00 | 0.00 | 0.00 | 1980000.00 | 1695000.00 | KO:K21449 | hypothetical protein | Archaea;Euryarchaeota;Methanomicrobia;Methanomicrobiales;Methanoregulaceae;Methanoregula;Methanoregula formicica;Methanoregula formicica SMSP, DSM 22288 |
| Ga0586324 6390858 2 556 | 3150000.00 | 0.00 | 2735251.05 | 0.00 | 0.00 | 0.00 | 0.00 | 0.00 | 0.00 | 0.00 | 1786400.00 | 0.00 | KO:K21449 | hypothetical protein | Archaea;Euryarchaeota;Methanomicrobia;Methanomicrobiales;Methanoregulaceae;Methanoregula;Methanoregula formicica;Methanoregula formicica SMSP, DSM 22288 |
| Ga0586324 6662427 1 549 | 3840000.00 | 7004000.00 | 4263756.64 | 5899500.00 | 4820400.00 | 3804292.20 | 134996.52 | 719800.00 | 1976400.00 | 3157000.00 | 3704800.00 | 1635000.00 | KO:K21449 | hypothetical protein | Archaea;Euryarchaeota;Methanomicrobia;Methanomicrobiales;Methanoregulaceae;Methanoregula;Methanoregula formicica;Methanoregula formicica SMSP, DSM 22288 |
| Ga0586324 6774640 3 545 | 23840000.00 | 13532000.00 | 38194809.54 | 17575000.00 | 0.00 | 0.00 | 0.00 | 4831200.00 | 2673000.00 | 9086000.00 | 5632000.00 | 4260000.00 | KO:K21449 | hypothetical protein | Archaea;Euryarchaeota;Methanomicrobia;Methanomicrobiales;Methanoregulaceae;Methanoregula;Methanoregula formicica;Methanoregula formicica SMSP, DSM 22288 |
| Ga0586324 6910977 2 538 | 505354.00 | 0.00 | 0.00 | 0.00 | 0.00 | 0.00 | 0.00 | 0.00 | 0.00 | 0.00 | 0.00 | 0.00 | KO:K21449 | hypothetical protein | Archaea;Euryarchaeota;Methanomicrobia;Methanomicrobiales;Methanoregulaceae;Methanoregula;Methanoregula formicica;Methanoregula formicica SMSP, DSM 22288 |
| Ga0586324 6990621 1 537 | 11590000.00 | 6201600.00 | 2402444.86 | 6127500.00 | 7446900.00 | 0.00 | 0.00 | 2818200.00 | 0.00 | 10980200.00 | 4444000.00 | 7200000.00 | KO:K21449 | hypothetical protein | Archaea;Euryarchaeota;Methanomicrobia;Methanomicrobiales;Methanoregulaceae;Methanoregula;Methanoregula formicica;Methanoregula formicica SMSP, DSM 22288 |
| Ga0586324 7056128 3 536 | 54700000.00 | 43246297.96 | 108076863.63 | 43738916.75 | 41148500.00 | 52840700.00 | 26904000.00 | 9873832.71 | 16856100.00 | 36146825.33 | 43032000.00 | 34875000.00 | KO:K21449 | hypothetical protein | Archaea;Euryarchaeota;Methanomicrobia;Methanomicrobiales;Methanoregulaceae;Methanoregula;Methanoregula formicica;Methanoregula formicica SMSP, DSM 22288 |
| Ga0586324 7121002 2 532 | 0.00 | 3794400.00 | 0.00 | 0.00 | 0.00 | 0.00 | 0.00 | 0.00 | 0.00 | 0.00 | 0.00 | 0.00 | KO:K21449 | hypothetical protein | Archaea;Euryarchaeota;Methanomicrobia;Methanomicrobiales;Methanomicrobiaceae;Methanofollis;Methanofollis liminatans;Methanofollis liminatans GKZPZ, DSM 4140 |
| Ga0586324 7673039 2 517 | 0.00 | 0.00 | 2983702.95 | 0.00 | 0.00 | 0.00 | 0.00 | 0.00 | 0.00 | 2487100.00 | 2323200.00 | 2280000.00 | KO:K21449 | hypothetical protein | Archaea;Euryarchaeota;Methanomicrobia;Methanomicrobiales;Methanoregulaceae;Methanoregula;Methanoregula formicica;Methanoregula formicica SMSP, DSM 22288 |
| Ga0586324 7687869 2 517 | 3760000.00 | 0.00 | 0.00 | 0.00 | 0.00 | 0.00 | 0.00 | 0.00 | 0.00 | 0.00 | 0.00 | 0.00 | KO:K21449 | hypothetical protein | Archaea;Euryarchaeota;Methanomicrobia;Methanomicrobiales;Methanospirillaceae;Methanospirillum;Methanospirillum hungatei;Methanospirillum hungatei JF-1 |
| Ga0586324 7900933 2 478 | 2220000.00 | 2686000.00 | 0.00 | 3087500.00 | 0.00 | 0.00 | 0.00 | 1610400.00 | 322367.85 | 2286900.00 | 2349600.00 | 723747.75 | KO:K21449 | autotransporter adhesin | Bacteria;Proteobacteria;Gammaproteobacteria;Pseudomonadales;Moraxellaceae;Agitococcus;Agitococcus lubricus;Agitococcus lubricus DSM 5822 |
| Ga0586324 8133525 2 505 | 0.00 | 0.00 | 0.00 | 0.00 | 0.00 | 0.00 | 0.00 | 0.00 | 0.00 | 0.00 | 0.00 | 2737500.00 | KO:K21449 | hypothetical protein | Archaea;Euryarchaeota;Methanomicrobia;Methanomicrobiales;Methanoregulaceae;Methanoregula;Methanoregula formicica;Methanoregula formicica SMSP, DSM 22288 |
| Ga0586324 4088529 1 195 | 0.00 | 11220000.00 | 0.00 | 0.00 | 0.00 | 0.00 | 0.00 | 0.00 | 0.00 | 0.00 | 0.00 | 7110000.00 | KO:K21481 | heme-degrading monooxygenase HmoA | Bacteria;Proteobacteria;Deltaproteobacteria;Syntrophobacterales;Syntrophobacteraceae;Desulfoglaeba;Desulfoglaeba alkanexedens;Desulfoglaeba alkanexedens ALDC |
| Ga0586324 0004310 2875 4500 | 0.00 | 0.00 | 0.00 | 9690000.00 | 5139700.00 | 0.00 | 0.00 | 2318000.00 | 0.00 | 0.00 | 0.00 | 0.00 | KO:K21572 | hypothetical protein | Bacteria;Bacteroidetes;Bacteroidia;Bacteroidales;Porphyromonadaceae;unclassified Porphyromonadaceae;Porphyromonadaceae bacterium KHP3R9;Porphyromonadaceae bacterium KHP3R9 |
| Ga0586324 0011002 1983 3764 | 4760000.00 | 3202800.00 | 10190300.00 | 7096500.00 | 5150000.00 | 7423900.00 | 9572217.48 | 4233400.00 | 5872500.00 | 4211900.00 | 4092000.00 | 6067500.00 | KO:K21572 | hypothetical protein | Bacteria;Bacteroidetes;Bacteroidia;Marinilabiliales;Prolixibacteraceae;Prolixibacter;Prolixibacter bellariivorans;Prolixibacter bellariivorans ATCC BAA-1284 |
| Ga0586324 0023977 36 1511 | 0.00 | 1407600.00 | 8557600.00 | 0.00 | 0.00 | 0.00 | 0.00 | 2476600.00 | 2640600.00 | 0.00 | 4127200.00 | 4762500.00 | KO:K21572 | hypothetical protein | Bacteria;Bacteroidetes;Cytophagia;Cytophagales;Cytophagaceae;Arsenicibacter;Arsenicibacter rosenii;Arsenicibacter rosenii SM-1 |
| Ga0586324 0090250 1 1164 | 1160000.00 | 0.00 | 0.00 | 0.00 | 0.00 | 0.00 | 0.00 | 0.00 | 0.00 | 0.00 | 0.00 | 0.00 | KO:K21572 | hypothetical protein | Bacteria;Bacteroidetes;Chitinophagia;Chitinophagales;Chitinophagaceae;Niabella;Niabella drilacis;Niabella drilacis DSM 25811 |
| Ga0586324 0174281 3 1634 | 0.00 | 1060800.00 | 0.00 | 0.00 | 0.00 | 0.00 | 1201703.64 | 1299300.00 | 597805.11 | 0.00 | 0.00 | 0.00 | KO:K21572 | hypothetical protein | Bacteria;Bacteroidetes;Bacteroidia;Marinilabiliales;Prolixibacteraceae;Draconibacterium;Draconibacterium sediminis;Draconibacterium sediminis JN14CK-3 |
| Ga0586324 0181243 3 1706 | 0.00 | 775200.00 | 0.00 | 1434500.00 | 0.00 | 0.00 | 2485200.00 | 1256600.00 | 0.00 | 0.00 | 0.00 | 0.00 | KO:K21572 | hypothetical protein | Bacteria;Bacteroidetes;Cytophagia;Cytophagales;Cytophagaceae;Anditalea;Anditalea andensis;Anditalea andensis LY1 |
| Ga0586324 0195139 2 973 | 0.00 | 0.00 | 0.00 | 0.00 | 0.00 | 0.00 | 0.00 | 0.00 | 0.00 | 847000.00 | 577698.88 | 0.00 | KO:K21572 | hypothetical protein | Bacteria;Bacteroidetes;Cytophagia;Cytophagales;Cyclobacteriaceae;Cyclobacterium;Cyclobacterium lianum;Cyclobacterium lianum CGMCC 1.6102 |
| Ga0586324 0197297 2 1750 | 0.00 | 0.00 | 0.00 | 0.00 | 0.00 | 0.00 | 0.00 | 0.00 | 0.00 | 0.00 | 0.00 | 930000.00 | KO:K21572 | hypothetical protein | Bacteria;Bacteroidetes;Bacteroidia;Marinilabiliales;Prolixibacteraceae;Mariniphaga;Mariniphaga anaerophila;Mariniphaga anaerophila DSM 26910 |
| Ga0586324 0235742 814 2295 | 4710000.00 | 1332800.00 | 1932700.18 | 3515000.00 | 2585734.66 | 821595.47 | 1221147.48 | 0.00 | 0.00 | 2017400.00 | 4056800.00 | 2295000.00 | KO:K21572 | hypothetical protein | Bacteria;Bacteroidetes;Cytophagia;Cytophagales;Cytophagaceae;Anditalea;Anditalea andensis;Anditalea andensis LY1 |
| Ga0586324 0293720 43 1764 | 21580000.00 | 8608800.00 | 6015987.17 | 16026500.00 | 15320383.77 | 9071342.81 | 7835343.12 | 3294000.00 | 8213400.00 | 3280200.00 | 3264800.00 | 11013291.00 | KO:K21572 | hypothetical protein | Bacteria;Bacteroidetes;Saprospiria;Saprospirales;Haliscomenobacteraceae;Haliscomenobacter;Haliscomenobacter hydrossis;Haliscomenobacter hydrossis O, DSM 1100 |
| Ga0586324 0876984 263 1285 | 3510000.00 | 2339200.00 | 4002569.68 | 2299000.00 | 2739800.00 | 3964203.80 | 2530800.00 | 1598200.00 | 1482300.00 | 0.00 | 2279200.00 | 1462500.00 | KO:K21572 | hypothetical protein | Bacteria;Bacteroidetes;Cytophagia;Cytophagales;Cytophagaceae;Anditalea;Anditalea andensis;Anditalea andensis LY1 |
| Ga0586324 1099888 758 1165 | 0.00 | 0.00 | 0.00 | 5481500.00 | 0.00 | 0.00 | 0.00 | 0.00 | 0.00 | 0.00 | 0.00 | 5385000.00 | KO:K21572 | hypothetical protein | Bacteria;Bacteroidetes;Cytophagia;Cytophagales;Cytophagaceae;Arsenicibacter;Arsenicibacter rosenii;Arsenicibacter rosenii SM-1 |
| Ga0586324 1256110 3 419 | 0.00 | 5038800.00 | 0.00 | 0.00 | 0.00 | 0.00 | 0.00 | 0.00 | 0.00 | 0.00 | 0.00 | 0.00 | KO:K21572 | hypothetical protein | Bacteria;Bacteroidetes;Sphingobacteriia;Sphingobacteriales;Sphingobacteriaceae;Pedobacter;Pedobacter yulinensis;Pedobacter yulinensis YL28-9 |
| Ga0586324 1294909 3 1088 | 0.00 | 0.00 | 0.00 | 0.00 | 0.00 | 0.00 | 0.00 | 0.00 | 2883600.00 | 0.00 | 0.00 | 0.00 | KO:K21572 | hypothetical protein | Bacteria;Bacteroidetes;Sphingobacteriia;Sphingobacteriales;Sphingobacteriaceae;Sphingobacterium;Sphingobacterium gobiense;Sphingobacterium gobiense ACCC 05757 |
| Ga0586324 1326101 2 1075 | 0.00 | 1421200.00 | 0.00 | 0.00 | 0.00 | 2546655.75 | 0.00 | 1421300.00 | 0.00 | 0.00 | 2930400.00 | 0.00 | KO:K21572 | hypothetical protein | Bacteria;Bacteroidetes;Chitinophagia;Chitinophagales;Chitinophagaceae;Thermoflavifilum;Thermoflavifilum aggregans;Thermoflavifilum aggregans DSM 27268 |
| Ga0586324 1329594 306 1073 | 0.00 | 0.00 | 0.00 | 0.00 | 0.00 | 0.00 | 11308800.00 | 0.00 | 0.00 | 0.00 | 0.00 | 0.00 | KO:K21572 | hypothetical protein | Bacteria;Bacteroidetes;Chitinophagia;Chitinophagales;Chitinophagaceae;Hydrobacter;Hydrobacter penzbergensis;Hydrobacter penzbergensis DSM 25353 |
| Ga0586324 1756245 3 953 | 12200000.00 | 0.00 | 0.00 | 14250000.00 | 13699000.00 | 0.00 | 5358000.00 | 8967000.00 | 9882000.00 | 11550000.00 | 0.00 | 6097500.00 | KO:K21572 | hypothetical protein | Bacteria;Bacteroidetes;Chitinophagia;Chitinophagales;Chitinophagaceae;Chitinophaga;Chitinophaga rupis;Chitinophaga rupis DSM 21039 |
| Ga0586324 1885660 2 925 | 0.00 | 0.00 | 0.00 | 0.00 | 0.00 | 0.00 | 0.00 | 0.00 | 0.00 | 1547700.00 | 0.00 | 0.00 | KO:K21572 | hypothetical protein | Bacteria;Bacteroidetes;Chitinophagia;Chitinophagales;Chitinophagaceae;Hydrotalea;Hydrotalea sandarakina;Hydrotalea sandarakina DSM 23241 |
| Ga0586324 2132107 93 878 | 0.00 | 0.00 | 0.00 | 0.00 | 3728600.00 | 0.00 | 0.00 | 0.00 | 0.00 | 0.00 | 0.00 | 0.00 | KO:K21572 | hypothetical protein | Bacteria;Bacteroidetes;Chitinophagia;Chitinophagales;Chitinophagaceae;Filimonas;Filimonas endophytica;Filimonas endophytica 3197 |
| Ga0586324 2141040 187 879 | 0.00 | 2794800.00 | 0.00 | 0.00 | 0.00 | 0.00 | 0.00 | 0.00 | 0.00 | 0.00 | 0.00 | 0.00 | KO:K21572 | hypothetical protein | Bacteria;Bacteroidetes;Cytophagia;Cytophagales;Cytophagaceae;Anditalea;Anditalea andensis;Anditalea andensis LY1 |
| Ga0586324 3181252 3 623 | 0.00 | 0.00 | 625340.99 | 0.00 | 0.00 | 0.00 | 0.00 | 0.00 | 0.00 | 0.00 | 0.00 | 0.00 | KO:K21572 | hypothetical protein | Bacteria;Bacteroidetes;Flavobacteriia;Flavobacteriales;Flavobacteriaceae;Polaribacter;Polaribacter filamentus;Polaribacter filamentus ATCC 700397 |
| Ga0586324 6045906 3 569 | 0.00 | 0.00 | 0.00 | 0.00 | 4768900.00 | 0.00 | 0.00 | 0.00 | 1701000.00 | 3457300.00 | 0.00 | 1950000.00 | KO:K21572 | hypothetical protein | Bacteria;Bacteroidetes;Bacteroidia;Bacteroidales;Dysgonomonadaceae;Petrimonas;Petrimonas mucosa;Petrimonas mucosa ING2-E5A |
| Ga0586324 6716912 3 545 | 0.00 | 0.00 | 0.00 | 0.00 | 0.00 | 0.00 | 1524430.80 | 0.00 | 0.00 | 0.00 | 0.00 | 0.00 | KO:K21572 | hypothetical protein | Bacteria;Bacteroidetes;Bacteroidia;Marinilabiliales;Prolixibacteraceae;Draconibacterium;Draconibacterium orientale;Draconibacterium orientale DSM 25947 |
| Ga0586324 0002554 4451 7936 | 12800000.00 | 2522800.00 | 0.00 | 0.00 | 1349300.00 | 0.00 | 0.00 | 0.00 | 0.00 | 0.00 | 0.00 | 0.00 | KO:K21573 | TonB-linked SusC/RagA family outer membrane protein | Bacteria;Bacteroidetes;Cytophagia;Cytophagales;Reichenbachiellaceae;Reichenbachiella;Reichenbachiella sp. MSK19-1;Reichenbachiella sp. MSK19-1 |
| Ga0586324 0003640 11712 14888 | 0.00 | 3060000.00 | 0.00 | 0.00 | 0.00 | 0.00 | 0.00 | 2269200.00 | 0.00 | 0.00 | 0.00 | 0.00 | KO:K21573 | TonB-linked SusC/RagA family outer membrane protein | Bacteria;Bacteroidetes;Cytophagia;Cytophagales;Cytophagaceae;Larkinella;Larkinella sp. BK230;Larkinella sp. BK230 |
| Ga0586324 0004310 4520 7777 | 4350000.00 | 0.00 | 10697000.00 | 4113500.00 | 0.00 | 0.00 | 9712800.00 | 0.00 | 0.00 | 2525600.00 | 0.00 | 0.00 | KO:K21573 | TonB-linked SusC/RagA family outer membrane protein | Bacteria;Bacteroidetes;Bacteroidia;Marinilabiliales;Prolixibacteraceae;Tangfeifania;Tangfeifania diversioriginum;Tangfeifania diversioriginum DSM 27063 |
| Ga0586324 0011002 3801 6971 | 28330000.00 | 40378400.00 | 13859832.66 | 29849000.00 | 34453500.00 | 11724180.18 | 14655703.20 | 23338600.00 | 25288200.00 | 4812500.00 | 10331200.00 | 18870000.00 | KO:K21573 | TonB-linked SusC/RagA family outer membrane protein | Bacteria;Bacteroidetes;Bacteroidia;Marinilabiliales;Prolixibacteraceae;Prolixibacter;Prolixibacter denitrificans;Prolixibacter denitrificans DSM 27267 |
| Ga0586324 0011871 3214 6147 | 1881138.00 | 3529200.00 | 9289500.00 | 0.00 | 6087300.00 | 5200700.00 | 833312.64 | 908900.00 | 49410000.00 | 1101100.00 | 3238400.00 | 5126031.00 | KO:K21573 | TonB-linked SusC/RagA family outer membrane protein | Bacteria;Bacteroidetes;Chitinophagia;Chitinophagales;Chitinophagaceae;Chitinophaga;Chitinophaga niabensis;Chitinophaga niabensis DSM 24787 |
| Ga0586324 0016572 3712 6807 | 0.00 | 0.00 | 5275191.77 | 0.00 | 5562000.00 | 0.00 | 0.00 | 0.00 | 0.00 | 2648800.00 | 0.00 | 1762500.00 | KO:K21573 | TonB-linked SusC/RagA family outer membrane protein | Bacteria;Bacteroidetes;Bacteroidia;Bacteroidales;unclassified Bacteroidales;unclassified Bacteroidales;Bacteroidales bacterium 6E;Bacteroidales bacterium 6E |
| Ga0586324 0025706 2391 5900 | 0.00 | 0.00 | 0.00 | 0.00 | 0.00 | 0.00 | 0.00 | 0.00 | 4147200.00 | 0.00 | 0.00 | 0.00 | KO:K21573 | TonB-linked SusC/RagA family outer membrane protein | Bacteria;Bacteroidetes;Chitinophagia;Chitinophagales;Chitinophagaceae;Chitinophaga;Chitinophaga barathri;Chitinophaga barathri YLT18 |
| Ga0586324 0054246 2960 4594 | 19480000.00 | 16292800.00 | 7938300.00 | 3454473.60 | 13822600.00 | 27790000.00 | 0.00 | 10559100.00 | 1198800.00 | 33425700.00 | 25608000.00 | 10050000.00 | KO:K21573 | TonB-linked SusC/RagA family outer membrane protein | Bacteria;Bacteroidetes;Bacteroidia;Marinilabiliales;Marinilabiliaceae;Thermophagus;Thermophagus xiamenensis;Thermophagus xiamenensis HS1 |
| Ga0586324 0114237 1357 3219 | 0.00 | 0.00 | 0.00 | 3429500.00 | 0.00 | 0.00 | 0.00 | 0.00 | 0.00 | 0.00 | 0.00 | 0.00 | KO:K21573 | TonB-linked SusC/RagA family outer membrane protein | Bacteria;Bacteroidetes;Cytophagia;Cytophagales;Reichenbachiellaceae;Reichenbachiella;Reichenbachiella sp. MSK19-1;Reichenbachiella sp. MSK19-1 |

| | | | | | | | | | | | | | | | |
|---|---|---|---|---|---|---|---|---|---|---|---|---|---|---|---|
| Ga0586324 0148439 64 2844 | 0.00 | 0.00 | 0.00 | 0.00 | 1194800.00 | 534207.17 | 0.00 | 17751000.00 | 0.00 | 0.00 | 0.00 | 0.00 | KO:K21573 | TonB-linked SusC/RagA family outer membrane protein | Bacteria;Bacteroidetes;Flavobacteriia;Flavobacteriales;Flavobacteriaceae;Flavobacterium;Flavobacterium denitrificans;Flavobacterium denitrificans DSM 15936 |
| Ga0586324 0149519 59 2833 | 0.00 | 431894.52 | 0.00 | 0.00 | 0.00 | 0.00 | 0.00 | 0.00 | 0.00 | 0.00 | 0.00 | 0.00 | KO:K21573 | TonB-linked SusC/RagA family outer membrane protein | Bacteria;Bacteroidetes;Bacteroidia;Marinilabiliales;Prolixibacteraceae;Sunxiuqinia;Sunxiuqinia dokdonensis;Sunxiuqinia dokdonensis SK |
| Ga0586324 0183553 3 1919 | 0.00 | 2101200.00 | 5104310.01 | 0.00 | 0.00 | 4247900.00 | 0.00 | 2726700.00 | 0.00 | 2594900.00 | 3388000.00 | 3862500.00 | KO:K21573 | hypothetical protein | Bacteria;Bacteroidetes;Flavobacteriia;Flavobacteriales;Flavobacteriaceae;Arenibacter;Arenibacter sp. ARW7G5Y1;Arenibacter sp. ARW7G5Y1 |
| Ga0586324 0193365 2 2425 | 0.00 | 0.00 | 0.00 | 11400000.00 | 0.00 | 0.00 | 0.00 | 9699000.00 | 0.00 | 0.00 | 0.00 | 0.00 | KO:K21573 | TonB-linked SusC/RagA family outer membrane protein | Bacteria;Bacteroidetes;Cytophagia;Cytophagales;Cyclobacteriaceae;Algoriphagus;Algoriphagus halophilus;Algoriphagus halophilus DSM 15292 |
| Ga0586324 0263775 3 2180 | 567823.00 | 0.00 | 0.00 | 0.00 | 0.00 | 0.00 | 0.00 | 0.00 | 0.00 | 0.00 | 0.00 | 0.00 | KO:K21573 | TonB-linked SusC/RagA family outer membrane protein | Bacteria;Bacteroidetes;Chitinophagia;Chitinophagales;Chitinophagaceae;unclassified Chitinophagaceae;Chitinophagaceae bacterium;Chitinophagaceae sp. OAS944 |
| Ga0586324 0303717 3 2045 | 0.00 | 924800.00 | 0.00 | 433076.50 | 1030000.00 | 0.00 | 0.00 | 497300.06 | 0.00 | 35820400.00 | 976800.00 | 1035000.00 | KO:K21573 | TonB-linked SusC/RagA family outer membrane protein | Bacteria;Bacteroidetes;Cytophagia;Cytophagales;Cytophagaceae;Anditalea;Anditalea andensis;Anditalea andensis LY1 |
| Ga0586324 0312915 3 2015 | 0.00 | 0.00 | 0.00 | 0.00 | 0.00 | 0.00 | 0.00 | 1897100.00 | 0.00 | 0.00 | 0.00 | 0.00 | KO:K21573 | TonB-linked SusC/RagA family outer membrane protein | Bacteria;Bacteroidetes;Bacteroidia;Marinilabiliales;Prolixibacteraceae;Tangfeifania;Tangfeifania diversioriginum;Tangfeifania diversioriginum DSM 27063 |
| Ga0586324 0332722 2 1552 | 0.00 | 0.00 | 0.00 | 0.00 | 0.00 | 0.00 | 0.00 | 0.00 | 0.00 | 0.00 | 1812800.00 | 1327500.00 | KO:K21573 | hypothetical protein | Bacteria;Gemmatimonadetes;Gemmatimonadetes;Gemmatimonadales;Gemmatimonadaceae;Gemmatirosa;Gemmatirosa kalamazoonesis;Gemmatimonadetes bacterium KBS708 |
| Ga0586324 0335483 1 1956 | 19780000.00 | 12172000.00 | 3456966.38 | 50169500.00 | 59379500.00 | 42876000.00 | 46056000.00 | 0.00 | 22456810.17 | 75306000.00 | 43208000.00 | 47475000.00 | KO:K21573 | TonB-linked SusC/RagA family outer membrane protein | Bacteria;Bacteroidetes;Cytophagia;Cytophagales;Cytophagaceae;Anditalea;Anditalea andensis;Anditalea andensis LY1 |
| Ga0586324 0361129 2 1891 | 96600000.00 | 0.00 | 0.00 | 0.00 | 0.00 | 0.00 | 0.00 | 0.00 | 0.00 | 0.00 | 0.00 | 0.00 | KO:K21573 | TonB-linked SusC/RagA family outer membrane protein | Bacteria;Bacteroidetes;Sphingobacteriia;Sphingobacteriales;Sphingobacteriaceae;Mucilaginibacter;Mucilaginibacter sp. OK268;Mucilaginibacter sp. OK268 |
| Ga0586324 0388538 2 1831 | 9090000.00 | 3121200.00 | 19367200.00 | 1064000.00 | 0.00 | 0.00 | 0.00 | 1274900.00 | 740103.48 | 8008000.00 | 7612000.00 | 5137500.00 | KO:K21573 | TonB-linked SusC/RagA family outer membrane protein | Bacteria;Balneolaeota;Balneolia;Balneolales;Balneolaceae;Aliifodinibius;Aliifodinibius roseus;Aliifodinibius roseus DSM 21986 |
| Ga0586324 0439102 3 1736 | 0.00 | 2985200.00 | 3112342.82 | 0.00 | 0.00 | 4605200.00 | 6429600.00 | 2616900.00 | 3879900.00 | 7022400.00 | 1399200.00 | 2662500.00 | KO:K21573 | TonB-linked SusC/RagA family outer membrane protein | Bacteria;Bacteroidetes;Cytophagia;Cytophagales;Cytophagaceae;Anditalea;Anditalea andensis;Anditalea andensis LY1 |
| Ga0586324 0488716 2 1483 | 0.00 | 0.00 | 0.00 | 0.00 | 0.00 | 0.00 | 0.00 | 0.00 | 0.00 | 0.00 | 0.00 | 296803.50 | KO:K21573 | TonB-linked SusC/RagA family outer membrane protein | Bacteria;Bacteroidetes;Flavobacteriia;Flavobacteriales;Flavobacteriaceae;Urechidicola;Urechidicola croceus;Urechidicola croceus LPB0138 |
| Ga0586324 0505509 2 1630 | 1150000.00 | 666797.12 | 390703.98 | 650365.25 | 0.00 | 1035864.31 | 817348.08 | 0.00 | 0.00 | 1316700.00 | 1082400.00 | 1222500.00 | KO:K21573 | TonB-linked SusC/RagA family outer membrane protein | Bacteria;Bacteroidetes;Cytophagia;Cytophagales;Cyclobacteriaceae;Cecembia;Cecembia calidifontis;Cecembia calidifontis DSM 21411 |
| Ga0586324 0571340 2 1546 | 9660000.00 | 10948000.00 | 5323142.48 | 5291500.00 | 3584400.00 | 3862520.19 | 0.00 | 1653100.00 | 6269400.00 | 7430500.00 | 9750400.00 | 21292500.00 | KO:K21573 | TonB-linked SusC/RagA family outer membrane protein | Bacteria;Bacteroidetes;Cytophagia;Cytophagales;Cytophagaceae;Anditalea;Anditalea andensis;Anditalea andensis LY1 |
| Ga0586324 0601043 3 1514 | 0.00 | 0.00 | 0.00 | 805421.40 | 0.00 | 0.00 | 0.00 | 0.00 | 0.00 | 0.00 | 0.00 | 1860000.00 | KO:K21573 | hypothetical protein | Bacteria;Bacteroidetes;Bacteroidia;Marinilabiliales;Prolixibacteraceae;Tangfeifania;Tangfeifania diversioriginum;Tangfeifania diversioriginum DSM 27063 |
| Ga0586324 0720555 181 1398 | 0.00 | 16116000.00 | 0.00 | 0.00 | 0.00 | 0.00 | 0.00 | 0.00 | 0.00 | 0.00 | 0.00 | 0.00 | KO:K21573 | TonB-linked SusC/RagA family outer membrane protein | Bacteria;Bacteroidetes;Cytophagia;Cytophagales;Cytophagaceae;Larkinella;Larkinella sp. BK230;Larkinella sp. BK230 |
| Ga0586324 0780848 525 1352 | 0.00 | 748000.00 | 0.00 | 0.00 | 0.00 | 0.00 | 0.00 | 0.00 | 0.00 | 0.00 | 0.00 | 0.00 | KO:K21573 | hypothetical protein | Bacteria;Bacteroidetes;Chitinophagia;Chitinophagales;Chitinophagaceae;Flavihumibacter;Flavihumibacter sp. ZG627;Flavihumibacter sp. ZG627 CCTCC AB2013368 |
| Ga0586324 0813014 3 1106 | 0.00 | 0.00 | 0.00 | 0.00 | 4274500.00 | 0.00 | 0.00 | 0.00 | 0.00 | 0.00 | 0.00 | 0.00 | KO:K21573 | hypothetical protein | Bacteria;Bacteroidetes;Bacteroidia;Marinilabiliales;Prolixibacteraceae;Prolixibacter;Prolixibacter bellariivorans;Prolixibacter bellariivorans ATCC BAA-1284 |
| Ga0586324 0895708 3 1274 | 0.00 | 0.00 | 0.00 | 4094500.00 | 0.00 | 0.00 | 0.00 | 0.00 | 0.00 | 0.00 | 0.00 | 0.00 | KO:K21573 | TonB-dependent SusC/RagA subfamily outer membrane receptor | Bacteria;Bacteroidetes;Bacteroidia;Bacteroidales;Bacteroidaceae;Bacteroides;Bacteroides luti;Bacteroides luti DSM 26991 |
| Ga0586324 0957835 1 1236 | 0.00 | 0.00 | 17959700.00 | 0.00 | 0.00 | 0.00 | 0.00 | 0.00 | 678623.67 | 0.00 | 0.00 | 7950000.00 | KO:K21573 | hypothetical protein | Bacteria;Bacteroidetes;Cytophagia;Cytophagales;Cytophagaceae;Anditalea;Anditalea andensis;Anditalea andensis LY1 |
| Ga0586324 1133100 2 1150 | 0.00 | 0.00 | 0.00 | 1653000.00 | 1678900.00 | 0.00 | 0.00 | 0.00 | 0.00 | 0.00 | 67672000.00 | 92250000.00 | KO:K21573 | TonB-dependent SusC/RagA subfamily outer membrane receptor | Bacteria;Bacteroidetes;Cytophagia;Cytophagales;Reichenbachiellaceae;Reichenbachiella;Reichenbachiella sp. MSK19-1;Reichenbachiella sp. MSK19-1 |
| Ga0586324 1138020 3 1148 | 0.00 | 0.00 | 0.00 | 0.00 | 0.00 | 0.00 | 0.00 | 0.00 | 0.00 | 1031800.00 | 651065.36 | 598620.00 | KO:K21573 | hypothetical protein | Bacteria;Bacteroidetes;Chitinophagia;Chitinophagales;Chitinophagaceae;Thermoflavifilum;Thermoflavifilum aggregans;Thermoflavifilum aggregans DSM 27268 |
| Ga0586324 1151592 3 1142 | 0.00 | 2189600.00 | 0.00 | 0.00 | 0.00 | 0.00 | 0.00 | 0.00 | 0.00 | 0.00 | 0.00 | 0.00 | KO:K21573 | outer membrane receptor protein involved in Fe transport | Bacteria;Bacteroidetes;Bacteroidia;Bacteroidales;Bacteroidaceae;Bacteroides;Bacteroides stercorirosoris;Bacteroides stercorirosoris JCM 17103 |
| Ga0586324 1242431 2 1105 | 5150000.00 | 2638400.00 | 5030298.03 | 4237000.00 | 12432100.00 | 12624600.00 | 5403600.00 | 3666100.00 | 1733400.00 | 2263800.00 | 2164800.00 | 1942500.00 | KO:K21573 | hypothetical protein | Bacteria;Bacteroidetes;Bacteroidia;Marinilabiliales;Prolixibacteraceae;Draconibacterium;Draconibacterium sediminis;Draconibacterium sediminis JN14CK-3 |
| Ga0586324 1247230 3 1103 | 1440000.00 | 2890000.00 | 0.00 | 5434000.00 | 0.00 | 0.00 | 0.00 | 2928000.00 | 2656800.00 | 0.00 | 0.00 | 0.00 | KO:K21573 | hypothetical protein | Bacteria;Bacteroidetes;Cytophagia;Cytophagales;Cytophagaceae;Anditalea;Anditalea andensis;Anditalea andensis LY1 |
| Ga0586324 1263572 3 722 | 0.00 | 0.00 | 0.00 | 0.00 | 0.00 | 0.00 | 745961.28 | 1122400.00 | 2997000.00 | 0.00 | 0.00 | 0.00 | KO:K21573 | hypothetical protein | Bacteria;Bacteroidetes;Chitinophagia;Chitinophagales;Chitinophagaceae;Segetibacter;Segetibacter koreensis;Segetibacter koreensis DSM 18137 |
| Ga0586324 1276920 3 1094 | 264020.00 | 0.00 | 0.00 | 0.00 | 0.00 | 0.00 | 0.00 | 0.00 | 0.00 | 0.00 | 0.00 | 0.00 | KO:K21573 | hypothetical protein | Bacteria;Bacteroidetes;Bacteroidia;Marinilabiliales;Prolixibacteraceae;Draconibacterium;Draconibacterium sediminis;Draconibacterium sediminis JN14CK-3 |
| Ga0586324 1326501 2 1075 | 3080000.00 | 0.00 | 0.00 | 0.00 | 0.00 | 0.00 | 0.00 | 0.00 | 0.00 | 0.00 | 0.00 | 0.00 | KO:K21573 | hypothetical protein | Bacteria;Bacteroidetes;Chitinophagia;Chitinophagales;Chitinophagaceae;Chitinophaga;Chitinophaga rupis;Chitinophaga rupis DSM 21039 |
| Ga0586324 1329594 2 286 | 580459.00 | 550462.72 | 0.00 | 1016500.00 | 1174200.00 | 0.00 | 0.00 | 2275300.00 | 6235066.53 | 0.00 | 0.00 | 1252500.00 | KO:K21573 | hypothetical protein | Bacteria;Bacteroidetes;Cytophagia;Cytophagales;Cytophagaceae;Anditalea;Anditalea andensis;Anditalea andensis LY1 |
| Ga0586324 1350901 1 1068 | 0.00 | 0.00 | 0.00 | 0.00 | 3934600.00 | 0.00 | 0.00 | 0.00 | 0.00 | 0.00 | 0.00 | 0.00 | KO:K21573 | hypothetical protein | Bacteria;Bacteroidetes;Cytophagia;Cytophagales;Cytophagaceae;Larkinella;Larkinella sp. BK230;Larkinella sp. BK230 |
| Ga0586324 1372640 2 1060 | 0.00 | 7616000.00 | 0.00 | 95000000.00 | 0.00 | 0.00 | 0.00 | 0.00 | 0.00 | 0.00 | 0.00 | 0.00 | KO:K21573 | outer membrane receptor for ferrienterochelin and colicin | Bacteria;Bacteroidetes;Chitinophagia;Chitinophagales;Chitinophagaceae;Hydrotalea;Hydrotalea sandarakina;Hydrotalea sandarakina DSM 23241 |
| Ga0586324 1452202 3 1034 | 12500000.00 | 10744000.00 | 5544953.22 | 3192000.00 | 15347000.00 | 9250100.00 | 9462000.00 | 2738900.00 | 10287000.00 | 4904900.00 | 6318400.00 | 8700000.00 | KO:K21573 | TonB-dependent SusC/RagA subfamily outer membrane receptor | Bacteria;Bacteroidetes;Cytophagia;Cytophagales;Cytophagaceae;Anditalea;Anditalea andensis;Anditalea andensis LY1 |
| Ga0586324 1538271 266 1009 | 0.00 | 0.00 | 0.00 | 2242000.00 | 0.00 | 0.00 | 0.00 | 113803.43 | 7468200.00 | 0.00 | 0.00 | 2085000.00 | KO:K21573 | TonB-dependent SusC/RagA subfamily outer membrane receptor | Bacteria;Bacteroidetes;Chitinophagia;Chitinophagales;Chitinophagaceae;Parafilimonas;Parafilimonas terrae;Parafilimonas terrae DSM 28286 |
| Ga0586324 1600171 2 994 | 0.00 | 0.00 | 0.00 | 1776500.00 | 0.00 | 0.00 | 0.00 | 0.00 | 0.00 | 0.00 | 0.00 | 0.00 | KO:K21573 | hypothetical protein | Bacteria;Bacteroidetes;Sphingobacteriia;Sphingobacteriales;Sphingobacteriaceae;Daejeonella;Daejeonella rubra;Pedobacter ruber DSM 24536 |
| Ga0586324 1752904 97 954 | 0.00 | 216462.36 | 0.00 | 0.00 | 0.00 | 0.00 | 0.00 | 0.00 | 0.00 | 0.00 | 0.00 | 0.00 | KO:K21573 | hypothetical protein | Bacteria;Bacteroidetes;Bacteroidia;Marinilabiliales;Prolixibacteraceae;Draconibacterium;Draconibacterium orientale;Draconibacterium orientale FH5 |
| Ga0586324 1859721 1 930 | 0.00 | 662997.96 | 0.00 | 0.00 | 1483200.00 | 0.00 | 0.00 | 951600.00 | 0.00 | 900900.00 | 6424000.00 | 772500.00 | KO:K21573 | TonB-dependent SusC/RagA subfamily outer membrane receptor | Bacteria;Bacteroidetes;Chitinophagia;Chitinophagales;Chitinophagaceae;Segetibacter;Segetibacter koreensis;Segetibacter koreensis DSM 18137 |
| Ga0586324 1884188 2 925 | 18700000.00 | 13736000.00 | 0.00 | 0.00 | 1380200.00 | 0.00 | 0.00 | 0.00 | 0.00 | 0.00 | 0.00 | 0.00 | KO:K21573 | TonB-dependent SusC/RagA subfamily outer membrane receptor | Bacteria;Balneolaeota;Balneolia;Balneolales;Balneolaceae;Aliifodinibius;Aliifodinibius roseus;Aliifodinibius roseus DSM 21986 |
| Ga0586324 2242612 1 861 | 0.00 | 0.00 | 0.00 | 0.00 | 0.00 | 0.00 | 0.00 | 27145000.00 | 0.00 | 0.00 | 0.00 | 0.00 | KO:K21573 | TonB-dependent SusC/RagA subfamily outer membrane receptor | Bacteria;Bacteroidetes;Bacteroidia;Bacteroidales;Dysgonomonadaceae;Petrimonas;Petrimonas sulfuriphila;Petrimonas sulfuriphila DSM 16547 |
| Ga0586324 2278366 2 856 | 0.00 | 0.00 | 0.00 | 0.00 | 0.00 | 4009700.00 | 0.00 | 0.00 | 0.00 | 0.00 | 0.00 | 0.00 | KO:K21573 | TonB-dependent SusC/RagA subfamily outer membrane receptor | Bacteria;Bacteroidetes;Bacteroidia;Marinilabiliales;Prolixibacteraceae;Mangrovibacterium;Mangrovibacterium marinum;Mangrovibacterium marinum DSM 28823 |
| Ga0586324 2378974 306 839 | 2910000.00 | 2509200.00 | 0.00 | 0.00 | 6313900.00 | 3629294.60 | 0.00 | 2684000.00 | 2762100.00 | 0.00 | 0.00 | 0.00 | KO:K21573 | hypothetical protein | Bacteria;Bacteroidetes;Cytophagia;Cytophagales;Hymenobacteraceae;Pontibacter;Pontibacter korlensis;Pontibacter korlensis X14-1T |
| Ga0586324 2424493 58 834 | 0.00 | 0.00 | 0.00 | 0.00 | 0.00 | 0.00 | 0.00 | 0.00 | 0.00 | 0.00 | 0.00 | 1567500.00 | KO:K21573 | TonB-dependent SusC/RagA subfamily outer membrane receptor | Bacteria;Bacteroidetes;Bacteroidia;Marinilabiliales;Marinilabiliaceae;Alkaliflexus;Alkaliflexus imshenetskii;Alkaliflexus imshenetskii Z-7010, DSM 15055 |
| Ga0586324 2619419 2 805 | 0.00 | 0.00 | 0.00 | 0.00 | 0.00 | 0.00 | 1221883.92 | 0.00 | 0.00 | 970200.00 | 0.00 | 0.00 | KO:K21573 | hypothetical protein | Bacteria;Bacteroidetes;Cytophagia;Cytophagales;Cytophagaceae;Flectobacillus;Flectobacillus major;Flectobacillus major VKMB-859, DSM 103 |
| Ga0586324 2645814 3 803 | 0.00 | 0.00 | 0.00 | 0.00 | 0.00 | 0.00 | 0.00 | 0.00 | 155894.22 | 0.00 | 0.00 | 0.00 | KO:K21573 | hypothetical protein | Bacteria;Bacteroidetes;Cytophagia;Cytophagales;Cytophagaceae;Anditalea;Anditalea andensis;Anditalea andensis LY1 |
| Ga0586324 2685823 1 798 | 0.00 | 0.00 | 0.00 | 0.00 | 0.00 | 0.00 | 0.00 | 126784.23 | 0.00 | 0.00 | 0.00 | 0.00 | KO:K21573 | TonB-dependent SusC/RagA subfamily outer membrane receptor | Bacteria;Bacteroidetes;Bacteroidia;Marinilabiliales;Prolixibacteraceae;Sunxiuqinia;Sunxiuqinia dokdonensis;Sunxiuqinia dokdonensis SK |
| Ga0586324 2719099 1 795 | 959878.00 | 0.00 | 0.00 | 0.00 | 0.00 | 0.00 | 0.00 | 0.00 | 0.00 | 0.00 | 0.00 | 0.00 | KO:K21573 | TonB-dependent SusC/RagA subfamily outer membrane receptor | Bacteria;Bacteroidetes;Bacteroidia;Marinilabiliales;Prolixibacteraceae;Draconibacterium;Draconibacterium orientale;Draconibacterium orientale FH5 |
| Ga0586324 2773839 3 788 | 0.00 | 0.00 | 0.00 | 342994.65 | 0.00 | 0.00 | 0.00 | 0.00 | 0.00 | 0.00 | 0.00 | 0.00 | KO:K21573 | TonB-dependent SusC/RagA subfamily outer membrane receptor | Bacteria;Bacteroidetes;Bacteroidia;Bacteroidales;Bacteroidaceae;Bacteroides;Bacteroides luti;Bacteroides luti DSM 26991 |
| Ga0586324 2828714 56 781 | 735257.00 | 1149200.00 | 0.00 | 0.00 | 6293300.00 | 0.00 | 0.00 | 0.00 | 0.00 | 0.00 | 0.00 | 0.00 | KO:K21573 | hypothetical protein | Bacteria;Bacteroidetes;Chitinophagia;Chitinophagales;Chitinophagaceae;Segetibacter;Segetibacter koreensis;Segetibacter koreensis DSM 18137 |
| Ga0586324 2908078 144 773 | 1100000.00 | 0.00 | 0.00 | 0.00 | 0.00 | 0.00 | 0.00 | 0.00 | 0.00 | 0.00 | 0.00 | 0.00 | KO:K21573 | outer membrane receptor protein involved in Fe transport | Bacteria;Bacteroidetes;Cytophagia;Cytophagales;Cytophagaceae;Leadbetterella;Leadbetterella byssophila;Leadbetterella byssophila 4M15, DSM 17132 |
| Ga0586324 2918769 1 771 | 0.00 | 0.00 | 4265181.03 | 0.00 | 0.00 | 0.00 | 0.00 | 0.00 | 0.00 | 0.00 | 0.00 | 0.00 | KO:K21573 | hypothetical protein | Bacteria;Bacteroidetes;Bacteroidia;Marinilabiliales;Prolixibacteraceae;Draconibacterium;Draconibacterium sediminis;Draconibacterium sediminis JN14CK-3 |
| Ga0586324 3083463 3 752 | 0.00 | 0.00 | 0.00 | 696306.30 | 0.00 | 0.00 | 0.00 | 0.00 | 0.00 | 0.00 | 0.00 | 0.00 | KO:K21573 | hypothetical protein | Bacteria;Bacteroidetes;Bacteroidia;Marinilabiliales;Prolixibacteraceae;Sunxiuqinia;Sunxiuqinia dokdonensis;Sunxiuqinia dokdonensis SK |
| Ga0586324 3117094 3 749 | 0.00 | 0.00 | 0.00 | 0.00 | 5438400.00 | 0.00 | 0.00 | 0.00 | 0.00 | 0.00 | 0.00 | 0.00 | KO:K21573 | hypothetical protein | Bacteria;Bacteroidetes;Cytophagia;Cytophagales;Mooreiaceae;Mooreia;Mooreia alkaloidigena;Mooreia alkaloidigena DSM 25186 |
| Ga0586324 3174560 3 503 | 0.00 | 0.00 | 2790346.23 | 1216000.00 | 1442000.00 | 890284.41 | 895540.68 | 817400.00 | 1028700.00 | 1840300.00 | 2578400.00 | 1830000.00 | KO:K21573 | hypothetical protein | Bacteria;Bacteroidetes;Cytophagia;Cytophagales;Cytophagaceae;Rudanella;Rudanella lutea;Rudanella lutea DSM 19387 |
| Ga0586324 3241194 369 737 | 0.00 | 0.00 | 0.00 | 3429500.00 | 1452300.00 | 1937157.53 | 0.00 | 0.00 | 0.00 | 0.00 | 0.00 | 0.00 | KO:K21573 | hypothetical protein | Bacteria;Bacteroidetes;Bacteroidia;Marinilabiliales;Marinifilaceae;Labilibaculum;Labilibaculum filiforme;Labilibaculum filiforme 59.16B |
| Ga0586324 3323001 2 730 | 6550000.00 | 15436000.00 | 0.00 | 0.00 | 0.00 | 25249200.00 | 6384000.00 | 5923100.00 | 0.00 | 0.00 | 0.00 | 0.00 | KO:K21573 | hypothetical protein | Bacteria;Bacteroidetes;Chitinophagia;Chitinophagales;Chitinophagaceae;Hydrotalea;Hydrotalea sandarakina;Hydrotalea sandarakina DSM 23241 |
| Ga0586324 3444396 2 718 | 0.00 | 0.00 | 0.00 | 0.00 | 0.00 | 0.00 | 0.00 | 0.00 | 0.00 | 1771000.00 | 0.00 | 0.00 | KO:K21573 | hypothetical protein | Bacteria;Bacteroidetes;Chitinophagia;Chitinophagales;Chitinophagaceae;Thermoflavifilum;Thermoflavifilum aggregans;Thermoflavifilum aggregans DSM 27268 |
| Ga0586324 3582599 3 707 | 4410000.00 | 3998400.00 | 0.00 | 440113.15 | 604558.50 | 0.00 | 0.00 | 249719.36 | 0.00 | 0.00 | 0.00 | 0.00 | KO:K21573 | TonB-dependent SusC/RagA subfamily outer membrane receptor | Bacteria;Bacteroidetes;Cytophagia;Cytophagales;Cytophagaceae;Siphonobacter;Siphonobacter aquaeclarae;Siphonobacter aquaeclarae DSM 21668 |
| Ga0586324 3676512 2 700 | 0.00 | 0.00 | 0.00 | 0.00 | 0.00 | 0.00 | 12357600.00 | 0.00 | 0.00 | 0.00 | 0.00 | 0.00 | KO:K21573 | hypothetical protein | Bacteria;Bacteroidetes;Cytophagia;Cytophagales;Cytophagaceae;Anditalea;Anditalea andensis;Anditalea andensis LY1 |
| Ga0586324 3762489 3 692 | 8330000.00 | 6555200.00 | 0.00 | 0.00 | 3048800.00 | 0.00 | 4788000.00 | 4038200.00 | 907200.00 | 5798100.00 | 7189600.00 | 6180000.00 | KO:K21573 | hypothetical protein | Bacteria;Bacteroidetes;Cytophagia;Cytophagales;Cytophagaceae;Anditalea;Anditalea andensis;Anditalea andensis LY1 |
| Ga0586324 3863937 252 686 | 1350000.00 | 0.00 | 85688600.00 | 0.00 | 0.00 | 0.00 | 84132000.00 | 0.00 | 0.00 | 0.00 | 0.00 | 0.00 | KO:K21573 | TonB-dependent SusC/RagA subfamily outer membrane receptor | Bacteria;Bacteroidetes;Chitinophagia;Chitinophagales;Chitinophagaceae;Paracnuella;Paracnuella aquatica;Paracnuella aquatica N24 |
| Ga0586324 3980669 3 677 | 0.00 | 0.00 | 0.00 | 6317500.00 | 3697700.00 | 8773700.00 | 5221200.00 | 2530090.29 | 2494800.00 | 4050200.00 | 6230400.00 | 0.00 | KO:K21573 | TonB-dependent SusC/RagA subfamily outer membrane receptor | Bacteria;Bacteroidetes;Cytophagia;Cytophagales;Cytophagaceae;Anditalea;Anditalea andensis;Anditalea andensis LY1 |
| Ga0586324 4028446 2 673 | 0.00 | 0.00 | 0.00 | 0.00 | 0.00 | 0.00 | 0.00 | 0.00 | 1263600.00 | 0.00 | 0.00 | 0.00 | KO:K21573 | outer membrane receptor protein involved in Fe transport | Bacteria;Bacteroidetes;Saprospiria;Saprospirales;Haliscomenobacteraceae;Haliscomenobacter;Haliscomenobacter hydrossis;Haliscomenobacter hydrossis O, DSM 1100 |
| Ga0586324 4304586 1 342 | 1810000.00 | 0.00 | 0.00 | 0.00 | 0.00 | 0.00 | 0.00 | 0.00 | 0.00 | 0.00 | 0.00 | 0.00 | KO:K21573 | hypothetical protein | Bacteria;Bacteroidetes;Cytophagia;Cytophagales;Cytophagaceae;Anditalea;Anditalea andensis;Anditalea andensis LY1 |
| Ga0586324 4599927 3 638 | 12800000.00 | 0.00 | 0.00 | 0.00 | 0.00 | 0.00 | 0.00 | 0.00 | 0.00 | 0.00 | 0.00 | 0.00 | KO:K21573 | TonB-dependent SusC/RagA subfamily outer membrane receptor | Bacteria;Bacteroidetes;Bacteroidia;Bacteroidales;Dysgonomonadaceae;Petrimonas;Petrimonas mucosa;Petrimonas mucosa ING2-E5A |
| Ga0586324 4789608 2 628 | 0.00 | 1203600.00 | 0.00 | 0.00 | 0.00 | 0.00 | 0.00 | 0.00 | 0.00 | 0.00 | 0.00 | 0.00 | KO:K21573 | TonB-dependent SusC/RagA subfamily outer membrane receptor | Bacteria;Bacteroidetes;Cytophagia;Cytophagales;Cyclobacteriaceae;Algoriphagus;Algoriphagus halophilus;Algoriphagus halophilus DSM 15292 |
| Ga0586324 4949045 1 618 | 3670000.00 | 3624400.00 | 0.00 | 2869000.00 | 1339000.00 | 0.00 | 0.00 | 0.00 | 1749600.00 | 2548700.00 | 897600.00 | 2940000.00 | KO:K21573 | TonB-dependent SusC/RagA subfamily outer membrane receptor | Bacteria;Bacteroidetes;Chitinophagia;Chitinophagales;Chitinophagaceae;Hydrotalea;Hydrotalea flava;Hydrotalea flava CCUG 51397 |
| Ga0586324 5916719 2 574 | 1130000.00 | 1455200.00 | 0.00 | 1168500.00 | 1648000.00 | 0.00 | 0.00 | 0.00 | 802726.20 | 0.00 | 0.00 | 0.00 | KO:K21573 | TonB-dependent SusC/RagA subfamily outer membrane receptor | Bacteria;Bacteroidetes;Cytophagia;Cytophagales;Cytophagaceae;Anditalea;Anditalea andensis;Anditalea andensis LY1 |
| Ga0586324 7178487 2 532 | 0.00 | 13872000.00 | 0.00 | 0.00 | 0.00 | 0.00 | 0.00 | 0.00 | 0.00 | 453233.55 | 0.00 | 0.00 | KO:K21573 | TonB-dependent SusC/RagA subfamily outer membrane receptor | Bacteria;Bacteroidetes;Chitinophagia;Chitinophagales;Chitinophagaceae;Chitinophaga;Chitinophaga pinensis;Chitinophaga pinensis UQM 2034, DSM 2588 |
| Ga0586324 7757229 1 513 | 0.00 | 0.00 | 0.00 | 0.00 | 1854000.00 | 0.00 | 0.00 | 417160.09 | 0.00 | 2949100.00 | 0.00 | 0.00 | KO:K21573 | hypothetical protein | Bacteria;Bacteroidetes;Chitinophagia;Chitinophagales;Chitinophagaceae;Thermoflavifilum;Thermoflavifilum aggregans;Thermoflavifilum aggregans DSM 27268 |
| Ga0586324 8009471 1 507 | 0.00 | 0.00 | 0.00 | 0.00 | 0.00 | 0.00 | 0.00 | 0.00 | 588591.36 | 0.00 | 0.00 | 0.00 | KO:K21573 | hypothetical protein | Bacteria;Bacteroidetes;Flavobacteriia;Flavobacteriales;Flavobacteriaceae;Euzebyella;Euzebyella marina;Euzebyella marina RN62 |
| Ga0586324 8274931 1 501 | 1190000.00 | 0.00 | 0.00 | 0.00 | 6684700.00 | 0.00 | 0.00 | 0.00 | 0.00 | 0.00 | 0.00 | 0.00 | KO:K21573 | hypothetical protein | Bacteria;Bacteroidetes;Chitinophagia;Chitinophagales;Chitinophagaceae;Segetibacter;Segetibacter koreensis;Segetibacter koreensis DSM 18137 |
| Ga0586324 0203970 1219 2382 | 0.00 | 0.00 | 0.00 | 7562000.00 | 0.00 | 0.00 | 0.00 | 0.00 | 0.00 | 0.00 | 0.00 | 0.00 | KO:K21619 | hypothetical protein | Bacteria;Chloroflexi;Ktedonobacteria;Ktedonobacterales;Dictyobacteraceae;Dictyobacter;Dictyobacter aurantiacus;Dictyobacter aurantiacus S27 |
| Ga0586324 0094179 1246 2583 | 1560000.00 | 0.00 | 0.00 | 0.00 | 0.00 | 0.00 | 0.00 | 0.00 | 0.00 | 0.00 | 0.00 | 0.00 | KO:K21677 | squalene-associated FAD-dependent desaturase | Bacteria;Acidobacteria;Blastocatellia;unclassified Blastocatellia;unclassified Blastocatellia;Chloracidobacterium;Chloracidobacterium thermophilum;Chloracidobacterium thermophilum B |
| Ga0586324 1959000 323 910 | 0.00 | 0.00 | 0.00 | 0.00 | 0.00 | 0.00 | 0.00 | 4593300.00 | 0.00 | 0.00 | 0.00 | 0.00 | KO:K21900 | DNA-binding transcriptional LysR family regulator | Bacteria;Verrucomicrobia;Verrucomicrobiae;Verrucomicrobiales;Verrucomicrobia subdivision 3;Pedosphaera;Pedosphaera parvula;Pedosphaera parvula Ellin514 |
| Ga0586324 6803415 199 543 | 1430000.00 | 0.00 | 0.00 | 0.00 | 0.00 | 0.00 | 0.00 | 0.00 | 0.00 | 0.00 | 0.00 | 0.00 | KO:K22010 | AmiR/NasT family two-component response regulator | Bacteria;Chloroflexi;Caldilineae;Caldilineales;Caldilineaceae;Caldilinea;Caldilinea aerophila;Caldilinea aerophila STL-6-O1, DSM 14535 |
| Ga0586324 0008867 4171 5667 | 0.00 | 0.00 | 0.00 | 0.00 | 0.00 | 0.00 | 2781600.00 | 0.00 | 0.00 | 0.00 | 0.00 | 0.00 | KO:K22169 | proton-translocating NADH-quinone oxidoreductase chain N | Archaea;Euryarchaeota;Methanomicrobia;Methanosarcinales;Methanotrichaceae;Methanothrix;Methanothrix soehngenii;Methanothrix soehngenii GP-6 |
| Ga0586324 0024598 6491 6739 | 0.00 | 0.00 | 0.00 | 0.00 | 0.00 | 0.00 | 0.00 | 1006500.00 | 0.00 | 0.00 | 0.00 | 0.00 | KO:K22231 | threonine dehydrogenase-like Zn-dependent dehydrogenase | Bacteria;Chloroflexi;Thermoflexia;Thermoflexales;Thermoflexaceae;Thermoflexus;Thermoflexus hugenholtzii;Thermoflexus hugenholtzii JAD2 |
| Ga0586324 0000126 2018 6964 | 0.00 | 2556800.00 | 0.00 | 0.00 | 0.00 | 0.00 | 0.00 | 0.00 | 0.00 | 0.00 | 0.00 | 0.00 | KO:K22349 | FtsP/CotA-like multicopper oxidase with cupredoxin domain | Bacteria;Nitrospirae;Nitrospira;Nitrospirales;Nitrospiraceae;Nitrospira;Nitrospira sp. ND1;Nitrospira sp. ND1 |
| Ga0586324 0002261 14138 15784 | 0.00 | 0.00 | 0.00 | 0.00 | 0.00 | 2890965.91 | 0.00 | 0.00 | 0.00 | 0.00 | 0.00 | 0.00 | KO:K22447 | thermosome | Archaea;Candidatus Thermoplasmatota;Thermoplasmata;Methanomassiliicoccales;Methanomassiliicoccaceae;Methanomassiliicoccus;Methanomassiliicoccus luminyensis;Methanomassiliicoccus luminyensis B10 |
| Ga0586324 0010156 8147 9793 | 2330000.00 | 3882800.00 | 0.00 | 2251500.00 | 45824700.00 | 0.00 | 0.00 | 7661600.00 | 8569800.00 | 5713400.00 | 0.00 | 2460000.00 | KO:K22447 | thermosome | Archaea;Euryarchaeota;Methanomicrobia;Methanosarcinales;Methanotrichaceae;Methanothrix;Methanothrix soehngenii;Methanothrix soehngenii GP-6 |
| Ga0586324 0018433 2863 4506 | 0.00 | 224729.12 | 500717.56 | 0.00 | 0.00 | 6669600.00 | 638146.92 | 591371.21 | 478846.89 | 0.00 | 950400.00 | 0.00 | KO:K22447 | thermosome | Archaea;Euryarchaeota;Methanomicrobia;Methanomicrobiales;Methanoregulaceae;Methanoregula;Methanoregula formicica;Methanoregula formicica SMSP, DSM 22288 |
| Ga0586324 0028874 4290 5930 | 0.00 | 0.00 | 0.00 | 0.00 | 0.00 | 0.00 | 0.00 | 0.00 | 0.00 | 0.00 | 1804000.00 | 0.00 | KO:K22447 | thermosome | Archaea;Euryarchaeota;Methanomicrobia;Methanosarcinales;Methanotrichaceae;Methanothrix;Methanothrix soehngenii;Methanothrix soehngenii GP-6 |
| Ga0586324 0079760 106 1755 | 0.00 | 0.00 | 0.00 | 0.00 | 623373.51 | 0.00 | 6801654.96 | 0.00 | 102796.29 | 0.00 | 0.00 | 930000.00 | KO:K22447 | thermosome | Archaea;Euryarchaeota;Methanomicrobia;Methanosarcinales;Methanotrichaceae;Methanothrix;Methanothrix soehngenii;Methanothrix soehngenii GP-6 |
| Ga0586324 0091267 2098 3585 | 1400000.00 | 3236800.00 | 0.00 | 4180000.00 | 7786800.00 | 0.00 | 13702800.00 | 0.00 | 7954200.00 | 0.00 | 4936800.00 | 5160000.00 | KO:K22447 | thermosome | Archaea;Euryarchaeota;Methanomicrobia;Methanomicrobiales;Methanoregulaceae;Methanoregula;Methanoregula formicica;Methanoregula formicica SMSP, DSM 22288 |
| Ga0586324 0115263 2 586 | 0.00 | 1904000.00 | 0.00 | 0.00 | 0.00 | 0.00 | 0.00 | 0.00 | 0.00 | 0.00 | 0.00 | 0.00 | KO:K22447 | chaperonin GroEL (HSP60 family) | Archaea;Euryarchaeota;Methanomicrobia;Methanomicrobiales;Methanoregulaceae;Methanoregula;Methanoregula boonei;Methanoregula boonei 6A8 |
| Ga0586324 0140914 646 2322 | 0.00 | 0.00 | 0.00 | 0.00 | 0.00 | 0.00 | 1097190.72 | 0.00 | 0.00 | 0.00 | 0.00 | 0.00 | KO:K22447 | thermosome | Archaea;Euryarchaeota;Thermococci;Thermococcales;Thermococcaceae;Thermococcus;Thermococcus gorgonarius;Thermococcus gorgonarius W-12 |
| Ga0586324 0200638 723 2381 | 6590000.00 | 33054800.00 | 220536.11 | 0.00 | 5119100.00 | 3314715.77 | 1321084.44 | 1407482.28 | 785332.26 | 0.00 | 0.00 | 1200000.00 | KO:K22447 | thermosome | Archaea;Euryarchaeota;Methanomicrobia;Methanosarcinales;Methanotrichaceae;Methanothrix;Methanothrix soehngenii;Methanothrix soehngenii GP-6 |
| Ga0586324 0229908 757 2322 | 0.00 | 7072000.00 | 0.00 | 0.00 | 0.00 | 0.00 | 0.00 | 0.00 | 0.00 | 0.00 | 0.00 | 0.00 | KO:K22447 | thermosome | Archaea;Euryarchaeota;Methanomicrobia;Methanosarcinales;Methanotrichaceae;Methanothrix;Methanothrix soehngenii;Methanothrix soehngenii GP-6 |
| Ga0586324 0255809 2 1588 | 0.00 | 0.00 | 0.00 | 0.00 | 0.00 | 0.00 | 2872800.00 | 0.00 | 0.00 | 0.00 | 0.00 | 0.00 | KO:K22447 | thermosome | Archaea;Euryarchaeota;Methanomicrobia;Methanomicrobiales;Methanoregulaceae;Methanoregula;Methanoregula formicica;Methanoregula formicica SMSP, DSM 22288 |
| Ga0586324 0276332 1392 2132 | 0.00 | 0.00 | 0.00 | 0.00 | 0.00 | 0.00 | 0.00 | 0.00 | 0.00 | 33187000.00 | 0.00 | 0.00 | KO:K22447 | chaperonin GroEL (HSP60 family) | Archaea;Euryarchaeota;Methanomicrobia;Methanomicrobiales;Methanoregulaceae;Methanoregula;Methanoregula formicica;Methanoregula formicica SMSP, DSM 22288 |
| Ga0586324 0289604 20 1678 | 0.00 | 5997600.00 | 0.00 | 278678.70 | 0.00 | 0.00 | 0.00 | 247057.32 | 0.00 | 1370600.00 | 0.00 | 0.00 | KO:K22447 | thermosome | Archaea;Euryarchaeota;Methanomicrobia;Methanomicrobiales;Methanoregulaceae;Methanosphaerula;Methanosphaerula palustris;Methanosphaerula palustris E1-9c, DSM 19958 |
| Ga0586324 0290543 325 1974 | 0.00 | 0.00 | 0.00 | 1824000.00 | 0.00 | 0.00 | 0.00 | 0.00 | 0.00 | 0.00 | 0.00 | 0.00 | KO:K22447 | thermosome | Archaea;Candidatus Thermoplasmatota;Thermoplasmata;Methanomassiliicoccales;Methanomassiliicoccaceae;Methanomassiliicoccus;Candidatus Methanomassiliicoccus intestinalis;Candidatus Methanomassiliicoccus intestinalis Issoire-Mx1 |
| Ga0586324 0307550 65 1720 | 0.00 | 863600.00 | 0.00 | 0.00 | 0.00 | 0.00 | 0.00 | 0.00 | 0.00 | 0.00 | 0.00 | 0.00 | KO:K22447 | thermosome | Archaea;Euryarchaeota;Methanomicrobia;Methanomicrobiales;Methanoregulaceae;Methanosphaerula;Methanosphaerula palustris;Methanosphaerula palustris E1-9c, DSM 19958 |
| Ga0586324 0383934 1 552 | 0.00 | 0.00 | 3442542.32 | 0.00 | 694507.37 | 3659486.45 | 0.00 | 1134600.00 | 2300400.00 | 0.00 | 0.00 | 0.00 | KO:K22447 | chaperonin GroEL (HSP60 family) | Archaea;Euryarchaeota;Methanomicrobia;Methanomicrobiales;Methanoregulaceae;Methanosphaerula;Methanosphaerula palustris;Methanosphaerula palustris E1-9c, DSM 19958 |
| Ga0586324 0447106 1 1017 | 0.00 | 0.00 | 0.00 | 0.00 | 0.00 | 0.00 | 0.00 | 0.00 | 1458000.00 | 0.00 | 0.00 | 0.00 | KO:K22447 | chaperonin GroEL (HSP60 family) | Archaea;Euryarchaeota;Methanomicrobia;Methanosarcinales;Methanotrichaceae;Methanothrix;Methanothrix soehngenii;Methanothrix soehngenii GP-6 |
| Ga0586324 0539427 3 1514 | 0.00 | 0.00 | 0.00 | 0.00 | 0.00 | 0.00 | 0.00 | 0.00 | 0.00 | 0.00 | 20768000.00 | 0.00 | KO:K22447 | thermosome | Archaea;Euryarchaeota;Methanomicrobia;Methanosarcinales;Methanotrichaceae;Methanothrix;Methanothrix soehngenii;Methanothrix soehngenii GP-6 |
| Ga0586324 0615246 147 1499 | 0.00 | 0.00 | 4638838.50 | 0.00 | 0.00 | 0.00 | 0.00 | 0.00 | 0.00 | 0.00 | 0.00 | 0.00 | KO:K22447 | thermosome | Archaea;Euryarchaeota;Methanomicrobia;Methanomicrobiales;Methanoregulaceae;Methanoregula;Methanoregula formicica;Methanoregula formicica SMSP, DSM 22288 |
| Ga0586324 0769596 3 812 | 4080000.00 | 0.00 | 0.00 | 0.00 | 0.00 | 0.00 | 0.00 | 3416000.00 | 3288600.00 | 0.00 | 0.00 | 0.00 | KO:K22447 | chaperonin GroEL (HSP60 family) | Archaea;Euryarchaeota;Methanomicrobia;Methanosarcinales;Methanotrichaceae;Methanothrix;Methanothrix soehngenii;Methanothrix soehngenii GP-6 |
| Ga0586324 0948820 3 527 | 8150000.00 | 0.00 | 0.00 | 9500000.00 | 9084600.00 | 0.00 | 4582800.00 | 0.00 | 0.00 | 0.00 | 0.00 | 0.00 | KO:K22447 | chaperonin GroEL (HSP60 family) | Archaea;Euryarchaeota;Methanomicrobia;Methanosarcinales;Methanotrichaceae;Methanothrix;Methanothrix soehngenii;Methanothrix soehngenii GP-6 |
| Ga0586324 1132215 1 1152 | 0.00 | 0.00 | 1702309.32 | 0.00 | 0.00 | 0.00 | 1466254.32 | 0.00 | 0.00 | 0.00 | 0.00 | 1057500.00 | KO:K22447 | thermosome | Archaea;Candidatus Thermoplasmatota;Thermoplasmata;Methanomassiliicoccales;Methanomassiliicoccaceae;Methanomassiliicoccus;Methanomassiliicoccus luminyensis;Methanomassiliicoccus luminyensis B10 |
| Ga0586324 1166234 2 1135 | 0.00 | 0.00 | 4710164.97 | 0.00 | 0.00 | 0.00 | 0.00 | 4873900.00 | 0.00 | 0.00 | 0.00 | 0.00 | KO:K22447 | chaperonin GroEL (HSP60 family) | Archaea;Candidatus Thermoplasmatota;Thermoplasmata;Methanomassiliicoccales;Methanomassiliicoccaceae;Methanomassiliicoccus;Candidatus Methanomassiliicoccus intestinalis;Candidatus Methanomassiliicoccus intestinalis Issoire-Mx1 |
| Ga0586324 1298294 1 1086 | 6570000.00 | 31756000.00 | 0.00 | 10925000.00 | 19982000.00 | 18659000.00 | 8253600.00 | 7320000.00 | 5532300.00 | 11627000.00 | 9680000.00 | 7372500.00 | KO:K22447 | chaperonin GroEL (HSP60 family) | Archaea;Euryarchaeota;Methanomicrobia;Methanomicrobiales;Methanoregulaceae;Methanoregula;Methanoregula formicica;Methanoregula formicica SMSP, DSM 22288 |
| Ga0586324 1313300 1 774 | 0.00 | 0.00 | 0.00 | 0.00 | 0.00 | 0.00 | 0.00 | 3318400.00 | 0.00 | 0.00 | 0.00 | 0.00 | KO:K22447 | chaperonin GroEL (HSP60 family) | Archaea;Euryarchaeota;Methanomicrobia;Methanosarcinales;Methanotrichaceae;Methanothrix;Methanothrix soehngenii;Methanothrix soehngenii GP-6 |
| Ga0586324 1339443 2 1057 | 3940000.00 | 0.00 | 0.00 | 0.00 | 0.00 | 0.00 | 0.00 | 1488400.00 | 0.00 | 0.00 | 0.00 | 1425000.00 | KO:K22447 | chaperonin GroEL (HSP60 family) | Archaea;Euryarchaeota;Methanomicrobia;Methanomicrobiales;Methanoregulaceae;Methanoregula;Methanoregula formicica;Methanoregula formicica SMSP, DSM 22288 |
| Ga0586324 1682390 3 971 | 0.00 | 0.00 | 0.00 | 0.00 | 0.00 | 0.00 | 0.00 | 2976800.00 | 0.00 | 0.00 | 0.00 | 0.00 | KO:K22447 | chaperonin GroEL (HSP60 family) | Archaea;Euryarchaeota;Methanomicrobia;Methanosarcinales;Methanotrichaceae;Methanothrix;Methanothrix soehngenii;Methanothrix soehngenii GP-6 |
| Ga0586324 2852108 1 777 | 0.00 | 0.00 | 0.00 | 0.00 | 0.00 | 0.00 | 1461719.40 | 17031200.00 | 16524000.00 | 13013000.00 | 0.00 | 0.00 | KO:K22447 | chaperonin GroEL (HSP60 family) | Archaea;Euryarchaeota;Methanomicrobia;Methanomicrobiales;Methanoregulaceae;Methanosphaerula;Methanosphaerula palustris;Methanosphaerula palustris E1-9c, DSM 19958 |
| Ga0586324 3309779 1 732 | 27300000.00 | 0.00 | 0.00 | 0.00 | 0.00 | 0.00 | 0.00 | 0.00 | 0.00 | 0.00 | 0.00 | 0.00 | KO:K22447 | chaperonin GroEL (HSP60 family) | Archaea;Euryarchaeota;Methanomicrobia;Methanomicrobiales;Methanoregulaceae;Methanoregula;Methanoregula formicica;Methanoregula formicica SMSP, DSM 22288 |
| Ga0586324 3363105 1 726 | 0.00 | 0.00 | 0.00 | 0.00 | 0.00 | 8257600.00 | 0.00 | 0.00 | 0.00 | 0.00 | 0.00 | 0.00 | KO:K22447 | chaperonin GroEL (HSP60 family) | Archaea;Euryarchaeota;Methanomicrobia;Methanosarcinales;Methanotrichaceae;Methanothrix;Methanothrix soehngenii;Methanothrix soehngenii GP-6 |
| Ga0586324 4000403 16 675 | 0.00 | 0.00 | 0.00 | 0.00 | 0.00 | 22787800.00 | 3693600.00 | 0.00 | 0.00 | 0.00 | 0.00 | 0.00 | KO:K22447 | chaperonin GroEL (HSP60 family) | Archaea;Euryarchaeota;Methanomicrobia;Methanosarcinales;Methanotrichaceae;Methanothrix;Methanothrix soehngenii;Methanothrix soehngenii GP-6 |
| Ga0586324 4319057 1 654 | 0.00 | 0.00 | 0.00 | 5215500.00 | 0.00 | 0.00 | 0.00 | 0.00 | 0.00 | 0.00 | 0.00 | 0.00 | KO:K22447 | chaperonin GroEL (HSP60 family) | Archaea;Euryarchaeota;Methanomicrobia;Methanosarcinales;Methanotrichaceae;Methanothrix;Methanothrix soehngenii;Methanothrix soehngenii GP-6 |
| Ga0586324 4600698 3 638 | 0.00 | 0.00 | 0.00 | 0.00 | 0.00 | 0.00 | 5244000.00 | 10553000.00 | 0.00 | 0.00 | 0.00 | 0.00 | KO:K22447 | chaperonin GroEL (HSP60 family) | Archaea;Euryarchaeota;Methanomicrobia;Methanosarcinales;Methanotrichaceae;Methanothrix;Methanothrix soehngenii;Methanothrix soehngenii GP-6 |
| Ga0586324 4974467 1 618 | 0.00 | 0.00 | 0.00 | 0.00 | 0.00 | 0.00 | 0.00 | 0.00 | 0.00 | 1978900.00 | 0.00 | 3045000.00 | KO:K22447 | chaperonin GroEL (HSP60 family) | Archaea;Candidatus Thermoplasmatota;unclassified Candidatus Thermoplasmatota;unclassified Candidatus Thermoplasmatota;unclassified Candidatus Thermoplasmatota;Aciduliprofundum;Aciduliprofundum sp. MAR08-339;Aciduliprofundum sp. MAR08-339 |
| Ga0586324 5249717 1 603 | 1560000.00 | 0.00 | 0.00 | 0.00 | 0.00 | 0.00 | 0.00 | 0.00 | 0.00 | 0.00 | 0.00 | 0.00 | KO:K22447 | chaperonin GroEL (HSP60 family) | Archaea;unclassified Archaea;unclassified Archaea;unclassified Archaea;Thermofilaceae;Thermofilum;Thermofilum pendens;Thermofilum pendens Hrk 5 |
| Ga0586324 5362442 2 598 | 0.00 | 0.00 | 0.00 | 0.00 | 0.00 | 5835900.00 | 0.00 | 0.00 | 0.00 | 0.00 | 0.00 | 0.00 | KO:K22447 | chaperonin GroEL (HSP60 family) | Archaea;Euryarchaeota;Methanomicrobia;Methanosarcinales;Methanotrichaceae;Methanothrix;Methanothrix soehngenii;Methanothrix soehngenii GP-6 |
| Ga0586324 6040384 1 570 | 907863.00 | 0.00 | 0.00 | 0.00 | 0.00 | 0.00 | 0.00 | 0.00 | 0.00 | 0.00 | 0.00 | 0.00 | KO:K22447 | chaperonin GroEL (HSP60 family) | Archaea;Euryarchaeota;Methanomicrobia;Methanomicrobiales;Methanoregulaceae;Methanoregula;Methanoregula formicica;Methanoregula formicica SMSP, DSM 22288 |
| Ga0586324 6684862 1 546 | 0.00 | 0.00 | 0.00 | 0.00 | 6458100.00 | 0.00 | 0.00 | 0.00 | 0.00 | 0.00 | 0.00 | 0.00 | KO:K22447 | chaperonin GroEL (HSP60 family) | Archaea;Euryarchaeota;Methanomicrobia;Methanosarcinales;Methanotrichaceae;Methanothrix;Methanothrix soehngenii;Methanothrix soehngenii GP-6 |
| Ga0586324 6957484 3 539 | 0.00 | 0.00 | 0.00 | 0.00 | 0.00 | 0.00 | 0.00 | 0.00 | 0.00 | 1062600.00 | 208126.16 | 0.00 | KO:K22447 | chaperonin GroEL (HSP60 family) | Archaea;Euryarchaeota;Methanomicrobia;Methanosarcinales;Methanotrichaceae;Methanothrix;Methanothrix soehngenii;Methanothrix soehngenii GP-6 |
| Ga0586324 7542085 1 519 | 0.00 | 0.00 | 0.00 | 0.00 | 0.00 | 13061300.00 | 0.00 | 0.00 | 0.00 | 0.00 | 0.00 | 0.00 | KO:K22447 | chaperonin GroEL (HSP60 family) | Archaea;Euryarchaeota;Methanomicrobia;Methanocellales;Methanocellaceae;Methanocella;Methanocella conradii;Methanocella conradii HZ254 |
| Ga0586324 7778650 3 512 | 0.00 | 0.00 | 0.00 | 0.00 | 0.00 | 0.00 | 0.00 | 5093500.00 | 0.00 | 0.00 | 0.00 | 0.00 | KO:K22447 | chaperonin GroEL (HSP60 family) | Archaea;Euryarchaeota;Methanomicrobia;Methanomicrobiales;Methanoregulaceae;Methanoregula;Methanoregula boonei;Methanoregula boonei 6A8 |
| Ga0586324 0474663 149 997 | 0.00 | 0.00 | 0.00 | 0.00 | 0.00 | 0.00 | 0.00 | 1421300.00 | 0.00 | 0.00 | 0.00 | 0.00 | KO:K22468 | polyphosphate kinase 2 | Bacteria;Proteobacteria;Gammaproteobacteria;Chromatiales;Chromatiaceae;Thiobaca;Thiobaca trueperi;Thiobaca trueperi DSM 13587 |
| Ga0586324 4063764 37 672 | 0.00 | 0.00 | 0.00 | 0.00 | 2513200.00 | 0.00 | 0.00 | 0.00 | 0.00 | 0.00 | 0.00 | 0.00 | KO:K22468 | polyphosphate kinase 2 | Bacteria;Proteobacteria;Alphaproteobacteria;Hyphomicrobiales;Bradyrhizobiaceae;Rhodopseudomonas;Rhodopseudomonas palustris;Rhodopseudomonas palustris BisA53 |
| Ga0586324 0001475 11189 13255 | 0.00 | 623929.92 | 0.00 | 0.00 | 0.00 | 0.00 | 0.00 | 0.00 | 0.00 | 0.00 | 0.00 | 0.00 | KO:K22516 | formate dehydrogenase alpha subunit | Archaea;Euryarchaeota;Methanomicrobia;Methanomicrobiales;Methanoregulaceae;Methanosphaerula;Methanosphaerula palustris;Methanosphaerula palustris E1-9c, DSM 19958 |
| Ga0586324 0018309 1 759 | 2140000.00 | 0.00 | 0.00 | 0.00 | 0.00 | 0.00 | 0.00 | 0.00 | 0.00 | 0.00 | 0.00 | 0.00 | KO:K22516 | predicted molibdopterin-dependent oxidoreductase YjgC | Archaea;Euryarchaeota;Methanomicrobia;Methanomicrobiales;Methanomicrobiaceae;Methanoculleus;Methanoculleus marisnigri;Methanoculleus marisnigri JR1, DSM 1498 |
| Ga0586324 0098128 1584 3461 | 0.00 | 0.00 | 0.00 | 0.00 | 0.00 | 0.00 | 0.00 | 0.00 | 0.00 | 0.00 | 9152000.00 | 0.00 | KO:K22516 | formate dehydrogenase alpha subunit | Archaea;Euryarchaeota;Methanomicrobia;Methanomicrobiales;Methanoregulaceae;Methanoregula;Methanoregula formicica;Methanoregula formicica SMSP, DSM 22288 |
| Ga0586324 0331340 1 768 | 0.00 | 1278400.00 | 0.00 | 0.00 | 0.00 | 0.00 | 0.00 | 0.00 | 0.00 | 0.00 | 0.00 | 0.00 | KO:K22516 | predicted molibdopterin-dependent oxidoreductase YjgC | Archaea;Euryarchaeota;Methanomicrobia;Methanomicrobiales;Methanoregulaceae;Methanoregula;Methanoregula formicica;Methanoregula formicica SMSP, DSM 22288 |
| Ga0586324 0641545 1 1470 | 960230.00 | 700400.00 | 0.00 | 0.00 | 0.00 | 0.00 | 0.00 | 0.00 | 0.00 | 0.00 | 0.00 | 1365000.00 | KO:K22516 | predicted molibdopterin-dependent oxidoreductase YjgC | Archaea;Euryarchaeota;Methanomicrobia;Methanomicrobiales;Methanomicrobiaceae;Methanofollis;Methanofollis liminatans;Methanofollis liminatans GKZPZ, DSM 4140 |
| Ga0586324 1592866 2 994 | 4170000.00 | 2346000.00 | 0.00 | 0.00 | 0.00 | 0.00 | 0.00 | 0.00 | 0.00 | 0.00 | 0.00 | 0.00 | KO:K22516 | predicted molibdopterin-dependent oxidoreductase YjgC | Archaea;Euryarchaeota;Methanomicrobia;Methanomicrobiales;Methanoregulaceae;Methanoregula;Methanoregula formicica;Methanoregula formicica SMSP, DSM 22288 |
| Ga0586324 1968842 45 908 | 1530000.00 | 748000.00 | 0.00 | 0.00 | 0.00 | 1558717.28 | 0.00 | 0.00 | 0.00 | 0.00 | 0.00 | 0.00 | KO:K22516 | predicted molibdopterin-dependent oxidoreductase YjgC | Archaea;Euryarchaeota;Methanomicrobia;Methanomicrobiales;Methanoregulaceae;Methanoregula;Methanoregula formicica;Methanoregula formicica SMSP, DSM 22288 |
| Ga0586324 2861521 3 776 | 0.00 | 0.00 | 0.00 | 0.00 | 0.00 | 0.00 | 0.00 | 1976400.00 | 0.00 | 0.00 | 0.00 | 0.00 | KO:K22516 | predicted molibdopterin-dependent oxidoreductase YjgC | Archaea;Euryarchaeota;Methanomicrobia;Methanomicrobiales;Methanomicrobiaceae;Methanoculleus;Methanoculleus sp. MH98A;Methanoculleus sp. MH98A |
| Ga0586324 3277311 2 733 | 0.00 | 0.00 | 0.00 | 0.00 | 0.00 | 0.00 | 0.00 | 0.00 | 15147000.00 | 0.00 | 0.00 | 0.00 | KO:K22516 | predicted molibdopterin-dependent oxidoreductase YjgC | Archaea;Euryarchaeota;Methanomicrobia;Methanomicrobiales;Methanoregulaceae;Methanoregula;Methanoregula formicica;Methanoregula formicica SMSP, DSM 22288 |
| Ga0586324 6776635 1 543 | 9860000.00 | 8568000.00 | 0.00 | 0.00 | 0.00 | 0.00 | 0.00 | 0.00 | 0.00 | 0.00 | 0.00 | 3757500.00 | KO:K22516 | predicted molibdopterin-dependent oxidoreductase YjgC | Archaea;Euryarchaeota;Methanomicrobia;Methanomicrobiales;Methanoregulaceae;Methanoregula;Methanoregula formicica;Methanoregula formicica SMSP, DSM 22288 |
| Ga0586324 0348708 2 313 | 0.00 | 0.00 | 0.00 | 2527000.00 | 2832500.00 | 0.00 | 0.00 | 0.00 | 411582.87 | 0.00 | 0.00 | 0.00 | KO:K22684 | hypothetical protein | Bacteria;Chlorobi;Chlorobia;Chlorobiales;Chlorobiaceae;Pelodictyon;Pelodictyon phaeoclathratiforme;Pelodictyon phaeoclathratiforme BU-1 |
| Ga0586324 1662523 1 828 | 0.00 | 0.00 | 0.00 | 0.00 | 0.00 | 0.00 | 0.00 | 0.00 | 0.00 | 0.00 | 0.00 | 100809.00 | KO:K22684 | hypothetical protein | Bacteria;Proteobacteria;Betaproteobacteria;Burkholderiales;unclassified Burkholderiales;Rubrivivax;Rubrivivax gelatinosus;Rubrivivax gelatinosus CBS |
| Ga0586324 4437739 316 648 | 0.00 | 0.00 | 0.00 | 0.00 | 0.00 | 0.00 | 0.00 | 0.00 | 1085400.00 | 0.00 | 0.00 | 0.00 | KO:K22684 | hypothetical protein | Bacteria;Proteobacteria;Betaproteobacteria;Burkholderiales;unclassified Burkholderiales;Rubrivivax;Rubrivivax benzoatilyticus;Rubrivivax benzoatilyticus JA2 |
| Ga0586324 1519671 304 1014 | 0.00 | 0.00 | 0.00 | 0.00 | 0.00 | 0.00 | 0.00 | 502103.81 | 0.00 | 0.00 | 0.00 | 0.00 | KO:K22719 | septal ring factor EnvC (AmiA/AmiB activator) | Bacteria;Proteobacteria;Alphaproteobacteria;Hyphomicrobiales;Bradyrhizobiaceae;Rhodopseudomonas;Rhodopseudomonas rhenobacensis;Rhodopseudomonas rhenobacensis DSM 12706 |
| Ga0586324 0466462 3 1517 | 0.00 | 0.00 | 0.00 | 0.00 | 0.00 | 0.00 | 0.00 | 0.00 | 0.00 | 0.00 | 2006400.00 | 1455000.00 | KO:K22958 | UbiD family decarboxylase | Bacteria;Proteobacteria;Alphaproteobacteria;Hyphomicrobiales;Hyphomicrobiaceae;Rhodoplanes;Rhodoplanes sp. JGI PP 4-B12;Rhodoplanes sp. JGI PP 4-B12 (contamination screened) |
| Ga0586324 0004429 1479 1814 | 0.00 | 0.00 | 0.00 | 0.00 | 0.00 | 0.00 | 0.00 | 5849900.00 | 0.00 | 2656500.00 | 15400000.00 | 2145000.00 | KO:K23077 | TusE/DsrC/DsvC family sulfur relay protein | Bacteria;Proteobacteria;Betaproteobacteria;Nitrosomonadales;Gallionellaceae;Sideroxydans;Sideroxydans lithotrophicus;Sideroxydans lithotrophicus ES-1 |
| Ga0586324 0184783 741 1058 | 0.00 | 0.00 | 4274836.48 | 0.00 | 0.00 | 6352000.00 | 0.00 | 0.00 | 0.00 | 0.00 | 0.00 | 3285000.00 | KO:K23077 | TusE/DsrC/DsvC family sulfur relay protein | Bacteria;Proteobacteria;Deltaproteobacteria;Syntrophobacterales;Syntrophaceae;Desulfobacca;Desulfobacca acetoxidans;Desulfobacca acetoxidans ASRB2, DSM 11109 |
| Ga0586324 0283980 1320 1652 | 0.00 | 0.00 | 0.00 | 4085000.00 | 0.00 | 0.00 | 0.00 | 3891800.00 | 0.00 | 0.00 | 0.00 | 1155000.00 | KO:K23077 | TusE/DsrC/DsvC family sulfur relay protein | Bacteria;Proteobacteria;Gammaproteobacteria;Acidiferrobacterales;Acidiferrobacteraceae;Sulfuricaulis;Sulfuricaulis limicola;Sulfuricaulis limicola HA5 |

| | | | | | | | | | | | | | | | |
|---|---|---|---|---|---|---|---|---|---|---|---|---|---|---|---|
| Ga0586324_0575289_952_1269 | 0.00 | 0.00 | 0.00 | 2299000.00 | 0.00 | 0.00 | 0.00 | 805200.00 | 1077300.00 | 0.00 | 0.00 | 0.00 | KO:K23077 | TusE/DsrC/DsvC family sulfur relay protein | Bacteria;Proteobacteria;Deltaproteobacteria;Syntrophobacterales;Syntrophobacteraceae;Syntrophobacter;Syntrophobacter fumaroxidans;Syntrophobacter fumaroxidans MPOB |
| Ga0586324_0630249_144_461 | 553659.00 | 19584000.00 | 0.00 | 0.00 | 0.00 | 0.00 | 324252.48 | 522564.43 | 227819.79 | 0.00 | 0.00 | 80508.00 | KO:K23077 | TusE/DsrC/DsvC family sulfur relay protein | Bacteria;Proteobacteria;Deltaproteobacteria;Syntrophobacterales;Syntrophaceae;Desulfobacca;Desulfobacca acetoxidans;Desulfobacca acetoxidans ASRB2, DSM 11109 |
| Ga0586324_0770903_123_440 | 0.00 | 9656000.00 | 10978500.00 | 25840000.00 | 28119000.00 | 21755600.00 | 19995600.00 | 19154000.00 | 22680000.00 | 17325000.00 | 20592000.00 | 14325000.00 | KO:K23077 | TusE/DsrC/DsvC family sulfur relay protein | Bacteria;Proteobacteria;Deltaproteobacteria;Desulfobacterales;Desulfobacteraceae;Desulfatiglans;Desulfatiglans anilini;Desulfatiglans anilini DSM 4660 |
| Ga0586324_1139818_797_1147 | 22200000.00 | 1224000.00 | 0.00 | 10545000.00 | 0.00 | 0.00 | 26676000.00 | 16409000.00 | 16119000.00 | 14861000.00 | 5790400.00 | 14625000.00 | KO:K23077 | TusE/DsrC/DsvC family sulfur relay protein | Bacteria;Proteobacteria;Deltaproteobacteria;Syntrophobacterales;Syntrophaceae;Desulfobacca;Desulfobacca acetoxidans;Desulfobacca acetoxidans ASRB2, DSM 11109 |
| Ga0586324_3372719_43_360 | 0.00 | 0.00 | 0.00 | 3629000.00 | 0.00 | 0.00 | 0.00 | 0.00 | 4706100.00 | 0.00 | 0.00 | 0.00 | KO:K23077 | TusE/DsrC/DsvC family sulfur relay protein | Bacteria;Proteobacteria;Deltaproteobacteria;Desulfobacterales;Desulfobulbaceae;Desulfoprunum;Desulfoprunum benzoelyticum;Desulfoprunum benzoelyticum DSM 28570 |
| Ga0586324_6108798_65_382 | 218670.00 | 587536.32 | 0.00 | 988000.00 | 0.00 | 0.00 | 2667600.00 | 526135.37 | 223732.53 | 0.00 | 0.00 | 840000.00 | KO:K23077 | TusE/DsrC/DsvC family sulfur relay protein | Bacteria;Proteobacteria;Deltaproteobacteria;Syntrophobacterales;Syntrophaceae;Desulfobacca;Desulfobacca acetoxidans;Desulfobacca acetoxidans ASRB2, DSM 11109 |
| Ga0586324_0100146_1_1878 | 0.00 | 0.00 | 0.00 | 0.00 | 0.00 | 0.00 | 0.00 | 0.00 | 327538.89 | 0.00 | 0.00 | 0.00 | KO:K23269 | phosphoribosylformylglycinamidine synthase II | Archaea;Euryarchaeota;Methanomicrobia;Methanosarcinales;Methanotrichaceae;Methanothrix;Methanothrix soehngenii;Methanothrix soehngenii GP-6 |
| Ga0586324_0028555_1941_4424 | 0.00 | 0.00 | 0.00 | 0.00 | 946967.58 | 0.00 | 0.00 | 0.00 | 0.00 | 0.00 | 0.00 | 0.00 | KO:K23302 | anaerobic selenocysteine-containing dehydrogenase | Bacteria;Proteobacteria;Deltaproteobacteria;Syntrophobacterales;Syntrophobacteraceae;Desulfoglaeba;Desulfoglaeba alkanexedens;Desulfoglaeba alkanexedens ALDC |
| Ga0586324_3150718_3_746 | 0.00 | 0.00 | 0.00 | 0.00 | 0.00 | 0.00 | 0.00 | 0.00 | 0.00 | 0.00 | 0.00 | 1357500.00 | KO:K23356 | sugar-specific transcriptional regulator TrmB | Archaea;Nanoarchaeota;unclassified Nanoarchaeota;Nanoarchaeales;Nanopusillaceae;Candidatus Nanopusillus;Candidatus Nanopusillus acidilobi;Nanoarchaeota archaeon 7A |
| Ga0586324_0017284_6656_7687 | 0.00 | 0.00 | 0.00 | 0.00 | 0.00 | 0.00 | 0.00 | 69540000.00 | 1336500.00 | 862400.00 | 0.00 | 0.00 | KO:K23508 | ABC-type sugar transport system substrate-binding protein | Bacteria;Chloroflexi;Anaerolineae;Anaerolineales;Anaerolineaceae;Pelolinea;Pelolinea submarina;Pelolinea submarina DSM 23923 |
| Ga0586324_0080231_2_1234 | 0.00 | 0.00 | 0.00 | 0.00 | 0.00 | 0.00 | 0.00 | 0.00 | 1741500.00 | 0.00 | 0.00 | 0.00 | KO:K23508 | ABC-type sugar transport system substrate-binding protein | Bacteria;Firmicutes;Clostridia;Eubacteriales;Lachnospiraceae;Cellulosilyticum;Cellulosilyticum sp. I15G10I2;Cellulosilyticum sp. I15G10I2 |
| Ga0586324_0083255_1532_2590 | 0.00 | 0.00 | 0.00 | 0.00 | 0.00 | 0.00 | 0.00 | 0.00 | 0.00 | 1193500.00 | 0.00 | 0.00 | KO:K23508 | ABC-type sugar transport system substrate-binding protein | Bacteria;Firmicutes;Negativicutes;Selenomonadales;Sporomusaceae;Propionispora;Propionispora vibrioides;Propionispora vibrioides DSM 13305 |
| Ga0586324_0122508_3_944 | 0.00 | 0.00 | 0.00 | 0.00 | 15965000.00 | 0.00 | 0.00 | 0.00 | 0.00 | 0.00 | 0.00 | 0.00 | KO:K23508 | ABC-type sugar transport system substrate-binding protein | Bacteria;Firmicutes;Clostridia;Eubacteriales;Lachnospiraceae;Cellulosilyticum;Cellulosilyticum sp. I15G10I2;Cellulosilyticum sp. I15G10I2 |
| Ga0586324_0143917_1245_2276 | 38700000.00 | 46110800.00 | 46785300.00 | 24225000.00 | 0.00 | 0.00 | 0.00 | 24461000.00 | 25029000.00 | 0.00 | 22176000.00 | 0.00 | KO:K23508 | ABC-type sugar transport system substrate-binding protein | Bacteria;Chloroflexi;Anaerolineae;Anaerolineales;Anaerolineaceae;Pelolinea;Pelolinea submarina;Pelolinea submarina DSM 23923 |
| Ga0586324_0309029_355_1347 | 0.00 | 0.00 | 0.00 | 0.00 | 0.00 | 0.00 | 0.00 | 0.00 | 453087.27 | 0.00 | 0.00 | 0.00 | KO:K23508 | ABC-type sugar transport system substrate-binding protein | Bacteria;Chloroflexi;Anaerolineae;Anaerolineales;Anaerolineaceae;Pelolinea;Pelolinea submarina;Pelolinea submarina DSM 23923 |
| Ga0586324_0882461_81_1214 | 0.00 | 0.00 | 0.00 | 0.00 | 0.00 | 0.00 | 0.00 | 0.00 | 0.00 | 0.00 | 3502400.00 | 0.00 | KO:K23508 | ABC-type sugar transport system substrate-binding protein | Bacteria;Proteobacteria;Deltaproteobacteria;Myxococcales;Polyangiaceae;Sorangium;Sorangium cellulosum;Sorangium cellulosum So ce836 |
| Ga0586324_1760665_3_842 | 2990000.00 | 4270400.00 | 0.00 | 1691000.00 | 0.00 | 0.00 | 534302.04 | 2430046.63 | 0.00 | 0.00 | 0.00 | 0.00 | KO:K23508 | ABC-type sugar transport system substrate-binding protein | Bacteria;Spirochaetes;Spirochaetia;Spirochaetales;Sphaerochaetaceae;Sphaerochaeta;Sphaerochaeta pleomorpha;Sphaerochaeta pleomorpha Grapes |
| Ga0586324_1856941_146_931 | 2310000.00 | 10336000.00 | 0.00 | 0.00 | 0.00 | 0.00 | 0.00 | 488015.25 | 0.00 | 0.00 | 0.00 | 0.00 | KO:K23508 | ABC-type sugar transport system substrate-binding protein | Bacteria;Proteobacteria;Betaproteobacteria;Burkholderiales;Oxalobacteraceae;Janthinobacterium;Janthinobacterium sp. PC23-8;Janthinobacterium sp. PC23-8 |
| Ga0586324_2471015_1_825 | 0.00 | 1428000.00 | 0.00 | 0.00 | 0.00 | 0.00 | 0.00 | 0.00 | 0.00 | 0.00 | 0.00 | 0.00 | KO:K23508 | ABC-type sugar transport system substrate-binding protein | Bacteria;Firmicutes;Clostridia;Eubacteriales;Lachnospiraceae;Cellulosilyticum;Cellulosilyticum sp. I15G10I2;Cellulosilyticum sp. I15G10I2 |
| Ga0586324_2920810_1_624 | 0.00 | 0.00 | 10528100.00 | 1168500.00 | 0.00 | 1184354.22 | 16142400.00 | 671000.00 | 0.00 | 0.00 | 0.00 | 23100000.00 | KO:K23508 | ABC-type sugar transport system substrate-binding protein | Bacteria;Spirochaetes;Spirochaetia;Spirochaetales;Spirochaetaceae;Spirochaeta;Spirochaeta isovalerica;Spirochaeta isovalerica DSM 2461 |
| Ga0586324_4884351_3_563 | 0.00 | 8098800.00 | 0.00 | 0.00 | 0.00 | 0.00 | 1113096.00 | 0.00 | 0.00 | 0.00 | 0.00 | 0.00 | KO:K23508 | ABC-type sugar transport system substrate-binding protein | Bacteria;Firmicutes;Clostridia;Halanaerobiales;Halanaerobiaceae;Halocella;Halocella sp. SP3-1;Halocella sp. SP3-1 |
| Ga0586324_5166098_3_608 | 38000000.00 | 7276000.00 | 0.00 | 7125000.00 | 9156700.00 | 24296400.00 | 0.00 | 8784000.00 | 0.00 | 0.00 | 0.00 | 0.00 | KO:K23508 | ABC-type sugar transport system substrate-binding protein | Bacteria;Chloroflexi;Anaerolineae;Anaerolineales;Anaerolineaceae;Pelolinea;Pelolinea submarina;Pelolinea submarina DSM 23923 |
| Ga0586324_5510705_2_592 | 0.00 | 1774800.00 | 0.00 | 0.00 | 0.00 | 0.00 | 0.00 | 0.00 | 0.00 | 0.00 | 0.00 | 1852500.00 | KO:K23508 | ABC-type sugar transport system substrate-binding protein | Bacteria;Spirochaetes;Spirochaetia;Spirochaetales;Treponemataceae;Treponema;Treponema caldarium;Treponema caldarium DSM 7334 |
| Ga0586324_6029324_3_572 | 8710000.00 | 9248000.00 | 0.00 | 0.00 | 0.00 | 0.00 | 0.00 | 6032900.00 | 0.00 | 0.00 | 0.00 | 0.00 | KO:K23508 | ABC-type sugar transport system substrate-binding protein | Bacteria;Chloroflexi;Anaerolineae;Anaerolineales;Anaerolineaceae;Pelolinea;Pelolinea submarina;Pelolinea submarina DSM 23923 |
| Ga0586324_6453867_3_554 | 1590000.00 | 0.00 | 0.00 | 0.00 | 0.00 | 0.00 | 0.00 | 0.00 | 0.00 | 0.00 | 0.00 | 0.00 | KO:K23508 | ABC-type sugar transport system substrate-binding protein | Bacteria;Firmicutes;Clostridia;Halanaerobiales;Halanaerobiaceae;Halocella;Halocella sp. SP3-1;Halocella sp. SP3-1 |
| Ga0586324_7608985_3_518 | 0.00 | 351234.96 | 0.00 | 393859.55 | 369466.15 | 0.00 | 0.00 | 364457.92 | 750937.23 | 0.00 | 0.00 | 0.00 | KO:K23508 | ABC-type sugar transport system substrate-binding protein | Bacteria;Chloroflexi;Anaerolineae;Anaerolineales;Anaerolineaceae;Pelolinea;Pelolinea submarina;Pelolinea submarina DSM 23923 |
| Ga0586324_0520671_20_1609 | 0.00 | 1951600.00 | 5128625.98 | 0.00 | 0.00 | 0.00 | 0.00 | 0.00 | 0.00 | 0.00 | 0.00 | 0.00 | KO:K23545 | ABC-type sugar transport system substrate-binding protein | Bacteria;Firmicutes;Clostridia;Eubacteriales;Clostridiaceae;Clostridium;Clostridium estertheticum;Clostridium estertheticum FP3 |
| Ga0586324_5403159_3_596 | 2950000.00 | 0.00 | 0.00 | 0.00 | 0.00 | 0.00 | 0.00 | 0.00 | 0.00 | 0.00 | 0.00 | 0.00 | KO:K23545 | ABC-type sugar transport system substrate-binding protein | Bacteria;Proteobacteria;Alphaproteobacteria;Hyphomicrobiales;Rhizobiaceae;Rhizobium;Rhizobium sp. BK327;Rhizobium sp. BK327 |
| Ga0586324_0000970_23294_25180 | 7940000.00 | 1632000.00 | 0.00 | 148912.50 | 0.00 | 0.00 | 0.00 | 0.00 | 4033800.00 | 4712400.00 | 0.00 | 2962500.00 | KO:K23995 | PQQ-dependent dehydrogenase (methanol/ethanol family) | Bacteria;Proteobacteria;Alphaproteobacteria;Hyphomicrobiales;unclassified Hyphomicrobiales;Methyloceanibacter;Methyloceanibacter superfactus;Methyloceanibacter superfactus R-67175 |
| Ga0586324_0004617_3968_5809 | 81970000.00 | 33688026.88 | 116355339.49 | 4396015.75 | 51217229.98 | 52404000.00 | 12077771.04 | 30811100.00 | 33768900.00 | 61677000.00 | 43806400.00 | 84307500.00 | KO:K23995 | PQQ-dependent dehydrogenase (methanol/ethanol family) | Bacteria;Proteobacteria;Alphaproteobacteria;Hyphomicrobiales;Beijerinckiaceae;Methylocella;Methylocella tundrae;Methylocella tundrae ERS3224381 |
| Ga0586324_0015996_2105_4030 | 0.00 | 0.00 | 0.00 | 0.00 | 0.00 | 5716800.00 | 0.00 | 0.00 | 14337000.00 | 13629000.00 | 2472800.00 | 0.00 | KO:K23995 | PQQ-dependent dehydrogenase (methanol/ethanol family) | Bacteria;Proteobacteria;Gammaproteobacteria;Methylococcales;Crenotrichaceae;Crenothrix;Crenothrix polyspora;Crenothrix polyspora ERS1545889 |
| Ga0586324_0023874_3647_5452 | 0.00 | 0.00 | 0.00 | 0.00 | 0.00 | 1052502.58 | 0.00 | 0.00 | 0.00 | 0.00 | 0.00 | 0.00 | KO:K23995 | PQQ-dependent dehydrogenase (methanol/ethanol family) | Bacteria;Proteobacteria;Betaproteobacteria;Burkholderiales;unclassified Burkholderiales;Rubrivivax;Rubrivivax gelatinosus;Rubrivivax gelatinosus CBS |
| Ga0586324_0035130_1453_3249 | 3510000.00 | 7140000.00 | 5273052.37 | 0.00 | 0.00 | 2272650.32 | 0.00 | 2220400.00 | 0.00 | 8393000.00 | 0.00 | 0.00 | KO:K23995 | PQQ-dependent dehydrogenase (methanol/ethanol family) | Bacteria;Proteobacteria;Gammaproteobacteria;Alteromonadales;Alteromonadaceae;unclassified Alteromonadaceae;Alteromonadaceae bacterium 2719K.STB50.0a.01;Alteromonadaceae bacterium 2719K.STB50.0a.01 |
| Ga0586324_0066014_1141_2961 | 0.00 | 3978000.00 | 4427910.55 | 0.00 | 0.00 | 2106160.43 | 2348400.00 | 2604700.00 | 3790800.00 | 3780700.00 | 4831200.00 | 4072500.00 | KO:K23995 | PQQ-dependent dehydrogenase (methanol/ethanol family) | Bacteria;Proteobacteria;Betaproteobacteria;Burkholderiales;unclassified Burkholderiales;Rhizobacter;Rhizobacter sp. Root16D2;Rhizobacter sp. Root16D2 |
| Ga0586324_0092928_964_2805 | 14550000.00 | 8812800.00 | 0.00 | 5944338.10 | 0.00 | 0.00 | 0.00 | 2635200.00 | 2106000.00 | 3984333.43 | 3940948.00 | 7116369.00 | KO:K23995 | PQQ-dependent dehydrogenase (methanol/ethanol family) | Bacteria;Proteobacteria;Alphaproteobacteria;Hyphomicrobiales;Bradyrhizobiaceae;Bradyrhizobium;Bradyrhizobium elkanii;Bradyrhizobium elkanii USDA 94 |
| Ga0586324_0100545_400_2220 | 0.00 | 0.00 | 0.00 | 706737.30 | 0.00 | 0.00 | 0.00 | 0.00 | 0.00 | 0.00 | 0.00 | 0.00 | KO:K23995 | PQQ-dependent dehydrogenase (methanol/ethanol family) | Bacteria;Proteobacteria;Betaproteobacteria;Burkholderiales;unclassified Burkholderiales;Methylibium;Methylibium sp. CF468;Methylibium sp. CF468 |
| Ga0586324_0104842_1590_3353 | 0.00 | 5732400.00 | 0.00 | 0.00 | 0.00 | 0.00 | 0.00 | 499874.87 | 0.00 | 3041500.00 | 0.00 | 2722500.00 | KO:K23995 | PQQ-dependent dehydrogenase (methanol/ethanol family) | Bacteria;Proteobacteria;Gammaproteobacteria;Alteromonadales;Alteromonadaceae;unclassified Alteromonadaceae;Alteromonadaceae bacterium 2719K.STB50.0a.01;Alteromonadaceae bacterium 2719K.STB50.0a.01 |
| Ga0586324_0177367_1810_2616 | 0.00 | 0.00 | 0.00 | 0.00 | 0.00 | 3519238.26 | 0.00 | 0.00 | 0.00 | 0.00 | 0.00 | 0.00 | KO:K23995 | PQQ-dependent dehydrogenase (methanol/ethanol family) | Bacteria;Proteobacteria;Betaproteobacteria;Burkholderiales;unclassified Burkholderiales;Sphaerotilus;Sphaerotilus montanus;Sphaerotilus montanus DSM 21226 |
| Ga0586324_0222319_3_1508 | 0.00 | 0.00 | 0.00 | 3595870.65 | 0.00 | 0.00 | 0.00 | 0.00 | 0.00 | 0.00 | 0.00 | 0.00 | KO:K23995 | PQQ-dependent dehydrogenase (methanol/ethanol family) | Bacteria;Proteobacteria;Alphaproteobacteria;Hyphomicrobiales;Bradyrhizobiaceae;Bradyrhizobium;Bradyrhizobium erythrophlei;Bradyrhizobium erythrophlei GAS478 |
| Ga0586324_0230246_1_1434 | 31300000.00 | 9513200.00 | 12160800.00 | 0.00 | 0.00 | 0.00 | 0.00 | 0.00 | 0.00 | 0.00 | 0.00 | 0.00 | KO:K23995 | PQQ-dependent dehydrogenase (methanol/ethanol family) | Bacteria;Proteobacteria;Gammaproteobacteria;Methylococcales;Crenotrichaceae;Crenothrix;Crenothrix polyspora;Crenothrix polyspora ERS1545889 |
| Ga0586324_0247183_264_2084 | 0.00 | 11689200.00 | 32993303.21 | 13984000.00 | 9527500.00 | 13299500.00 | 3283200.00 | 4038200.00 | 4722300.00 | 14984200.00 | 9741600.00 | 4914394.50 | KO:K23995 | PQQ-dependent dehydrogenase (methanol/ethanol family) | Bacteria;Proteobacteria;Alphaproteobacteria;Rhodospirillales;Rhodospirillaceae;Inquilinus;Inquilinus limosus;Inquilinus limosus DSM 16000 |
| Ga0586324_0254785_1317_2213 | 0.00 | 0.00 | 0.00 | 0.00 | 0.00 | 3484052.15 | 0.00 | 0.00 | 0.00 | 2772000.00 | 0.00 | 0.00 | KO:K23995 | glucose dehydrogenase | Bacteria;Proteobacteria;Alphaproteobacteria;Hyphomicrobiales;unclassified Hyphomicrobiales;Methyloceanibacter;Methyloceanibacter superfactus;Methyloceanibacter superfactus R-67175 |
| Ga0586324_0294134_2_1000 | 16500000.00 | 11968000.00 | 9345800.00 | 0.00 | 0.00 | 0.00 | 4947600.00 | 3788100.00 | 5572800.00 | 5028100.00 | 1284800.00 | 5542500.00 | KO:K23995 | PQQ-dependent dehydrogenase (methanol/ethanol family) | Bacteria;Proteobacteria;Betaproteobacteria;Nitrosomonadales;Methylophilaceae;Methylotenera;Methylotenera versatilis;Methylotenera versatilis 301, JCM 17579 |
| Ga0586324_0301899_2_1597 | 0.00 | 2495600.00 | 2516942.17 | 5006500.00 | 0.00 | 3065495.05 | 0.00 | 1903200.00 | 12935700.00 | 0.00 | 0.00 | 3465000.00 | KO:K23995 | PQQ-dependent dehydrogenase (methanol/ethanol family) | Bacteria;Proteobacteria;Alphaproteobacteria;Hyphomicrobiales;unclassified Hyphomicrobiales;Methyloceanibacter;Methyloceanibacter superfactus;Methyloceanibacter superfactus R-67175 |
| Ga0586324_0320822_515_1993 | 482018.00 | 0.00 | 0.00 | 1567500.00 | 0.00 | 0.00 | 0.00 | 0.00 | 0.00 | 0.00 | 0.00 | 0.00 | KO:K23995 | PQQ-dependent dehydrogenase (methanol/ethanol family) | Bacteria;Proteobacteria;Betaproteobacteria;Burkholderiales;unclassified Burkholderiales;Rubrivivax;Rubrivivax gelatinosus;Rubrivivax gelatinosus CBS |
| Ga0586324_0335245_1_1449 | 13190000.00 | 34748000.00 | 27755900.00 | 0.00 | 0.00 | 0.00 | 3716400.00 | 0.00 | 0.00 | 1632400.00 | 7128000.00 | 1507500.00 | KO:K23995 | glucose dehydrogenase | Bacteria;Proteobacteria;Betaproteobacteria;Nitrosomonadales;Methylophilaceae;Methylophilus;Methylophilus rhizosphaerae;Methylophilus rhizosphaerae CBMB127 |
| Ga0586324_0340939_65_1942 | 18900000.00 | 0.00 | 0.00 | 0.00 | 11639000.00 | 0.00 | 0.00 | 0.00 | 13284000.00 | 0.00 | 0.00 | 64500000.00 | KO:K23995 | PQQ-dependent dehydrogenase (methanol/ethanol family) | Bacteria;Proteobacteria;Gammaproteobacteria;Methylococcales;Crenotrichaceae;Crenothrix;Crenothrix polyspora;Crenothrix polyspora ERS1545889 |
| Ga0586324_0365898_2_613 | 0.00 | 0.00 | 29107100.00 | 486001.95 | 0.00 | 22311400.00 | 0.00 | 0.00 | 0.00 | 10934000.00 | 11616000.00 | 17925000.00 | KO:K23995 | PQQ-dependent dehydrogenase (methanol/ethanol family) | Bacteria;Proteobacteria;Betaproteobacteria;Burkholderiales;unclassified Burkholderiales;Rubrivivax;Rubrivivax gelatinosus;Rubrivivax gelatinosus CBS |
| Ga0586324_0374591_17_1861 | 14300000.00 | 10880000.00 | 0.00 | 0.00 | 0.00 | 0.00 | 0.00 | 8601000.00 | 0.00 | 0.00 | 0.00 | 3667500.00 | KO:K23995 | PQQ-dependent dehydrogenase (methanol/ethanol family) | Bacteria;Proteobacteria;Gammaproteobacteria;Methylococcales;Crenotrichaceae;Crenothrix;Crenothrix polyspora;Crenothrix polyspora ERS1545889 |
| Ga0586324_0443931_3_905 | 0.00 | 0.00 | 0.00 | 0.00 | 0.00 | 0.00 | 0.00 | 485679.56 | 0.00 | 0.00 | 0.00 | 0.00 | KO:K23995 | PQQ-dependent dehydrogenase (methanol/ethanol family) | Bacteria;Proteobacteria;Betaproteobacteria;Burkholderiales;unclassified Burkholderiales;Ideonella;Ideonella sakaiensis;Ideonella sp. 201-F6 |
| Ga0586324_0540198_366_1583 | 0.00 | 3270800.00 | 0.00 | 2916500.00 | 0.00 | 0.00 | 0.00 | 0.00 | 0.00 | 0.00 | 0.00 | 0.00 | KO:K23995 | PQQ-dependent dehydrogenase (methanol/ethanol family) | Bacteria;Proteobacteria;Alphaproteobacteria;Hyphomicrobiales;unclassified Hyphomicrobiales;Methyloceanibacter;Methyloceanibacter superfactus;Methyloceanibacter superfactus R-67175 |
| Ga0586324_0739858_108_1382 | 0.00 | 0.00 | 0.00 | 0.00 | 0.00 | 0.00 | 0.00 | 0.00 | 0.00 | 0.00 | 0.00 | 2767500.00 | KO:K23995 | PQQ-dependent dehydrogenase (methanol/ethanol family) | Bacteria;Proteobacteria;Alphaproteobacteria;Hyphomicrobiales;Bradyrhizobiaceae;Bradyrhizobium;Bradyrhizobium elkanii;Bradyrhizobium elkanii USDA 94 |
| Ga0586324_1023369_3_1046 | 0.00 | 0.00 | 0.00 | 0.00 | 0.00 | 0.00 | 8094000.00 | 12566000.00 | 0.00 | 14168000.00 | 4769600.00 | 18600000.00 | KO:K23995 | PQQ-dependent dehydrogenase (methanol/ethanol family) | Bacteria;Proteobacteria;Alphaproteobacteria;Hyphomicrobiales;unclassified Hyphomicrobiales;Methyloceanibacter;Methyloceanibacter superfactus;Methyloceanibacter superfactus R-67175 |
| Ga0586324_1160425_1_1041 | 0.00 | 0.00 | 0.00 | 28975000.00 | 0.00 | 22906900.00 | 16621200.00 | 20923000.00 | 22275000.00 | 36806000.00 | 33792000.00 | 32625000.00 | KO:K23995 | PQQ-dependent dehydrogenase (methanol/ethanol family) | Bacteria;Proteobacteria;Alphaproteobacteria;Hyphomicrobiales;unclassified Hyphomicrobiales;Methyloceanibacter;Methyloceanibacter methanicus;Methyloceanibacter methanicus R-67174 |
| Ga0586324_1242512_1_1107 | 0.00 | 0.00 | 0.00 | 783870.65 | 0.00 | 0.00 | 0.00 | 0.00 | 0.00 | 0.00 | 0.00 | 0.00 | KO:K23995 | PQQ-dependent dehydrogenase (methanol/ethanol family) | Bacteria;Proteobacteria;Alphaproteobacteria;Hyphomicrobiales;Bradyrhizobiaceae;Bradyrhizobium;Bradyrhizobium erythrophlei;Bradyrhizobium erythrophlei GAS478 |
| Ga0586324_1408073_3_1049 | 2850000.00 | 3692400.00 | 5284284.22 | 2147000.00 | 0.00 | 6342527.58 | 0.00 | 1860500.00 | 3418200.00 | 6999300.00 | 8676800.00 | 6705000.00 | KO:K23995 | PQQ-dependent dehydrogenase (methanol/ethanol family) | Bacteria;Proteobacteria;Alphaproteobacteria;Rhodospirillales;Rhodospirillaceae;Inquilinus;Inquilinus limosus;Inquilinus limosus DSM 16000 |
| Ga0586324_1583581_1_996 | 6960000.00 | 0.00 | 17340400.00 | 0.00 | 11639000.00 | 16316700.00 | 6680400.00 | 6283000.00 | 0.00 | 0.00 | 9064000.00 | 6607500.00 | KO:K23995 | glucose dehydrogenase | Bacteria;Proteobacteria;Alphaproteobacteria;Hyphomicrobiales;unclassified Hyphomicrobiales;Methyloceanibacter;Methyloceanibacter superfactus;Methyloceanibacter superfactus R-67175 |
| Ga0586324_2060581_73_891 | 13800000.00 | 0.00 | 0.00 | 0.00 | 0.00 | 0.00 | 0.00 | 0.00 | 0.00 | 0.00 | 0.00 | 0.00 | KO:K23995 | PQQ-dependent dehydrogenase (methanol/ethanol family) | Bacteria;Proteobacteria;Gammaproteobacteria;Methylococcales;Methylococcaceae;Methylobacter;Methylobacter whittenburyi;Methylobacter whittenburyi ACM 3310 |
| Ga0586324_2172787_251_871 | 60700000.00 | 112308800.00 | 57426000.00 | 0.00 | 0.00 | 0.00 | 9074400.00 | 35014000.00 | 29079000.00 | 31339000.00 | 0.00 | 37800000.00 | KO:K23995 | glucose dehydrogenase | Bacteria;Proteobacteria;Gammaproteobacteria;Methylococcales;Methylococcaceae;Methylobacter;Methylobacter luteus;Methylobacter luteus IMV-B-3098 |
| Ga0586324_2219072_3_863 | 0.00 | 0.00 | 0.00 | 0.00 | 0.00 | 0.00 | 0.00 | 0.00 | 0.00 | 0.00 | 2587200.00 | 1657500.00 | KO:K23995 | PQQ-dependent dehydrogenase (methanol/ethanol family) | Bacteria;Proteobacteria;Betaproteobacteria;Nitrosomonadales;Methylophilaceae;Methylobacillus;Methylobacillus sp. MM3;Methylobacillus sp. MM2 |
| Ga0586324_2247724_22_861 | 0.00 | 0.00 | 0.00 | 0.00 | 0.00 | 0.00 | 0.00 | 0.00 | 0.00 | 0.00 | 11528000.00 | 13350000.00 | KO:K23995 | PQQ-dependent dehydrogenase (methanol/ethanol family) | Bacteria;Proteobacteria;Alphaproteobacteria;Hyphomicrobiales;Bradyrhizobiaceae;Bradyrhizobium;Bradyrhizobium sp. ARR65;Bradyrhizobium sp. ARR65 |
| Ga0586324_2299633_2_853 | 0.00 | 0.00 | 0.00 | 0.00 | 2420500.00 | 0.00 | 0.00 | 0.00 | 0.00 | 0.00 | 0.00 | 0.00 | KO:K23995 | PQQ-dependent dehydrogenase (methanol/ethanol family) | Bacteria;Proteobacteria;Alphaproteobacteria;Hyphomicrobiales;Hyphomicrobiaceae;Hyphomicrobium;Hyphomicrobium sp. 99;Hyphomicrobium sp. 99 |
| Ga0586324_2756972_1_789 | 75628.90 | 0.00 | 0.00 | 0.00 | 0.00 | 0.00 | 0.00 | 0.00 | 0.00 | 0.00 | 0.00 | 0.00 | KO:K23995 | PQQ-dependent dehydrogenase (methanol/ethanol family) | Bacteria;Proteobacteria;Gammaproteobacteria;Methylococcales;Crenotrichaceae;Crenothrix;Crenothrix polyspora;Crenothrix polyspora ERS1545889 |
| Ga0586324_2962098_1_765 | 0.00 | 0.00 | 0.00 | 0.00 | 0.00 | 0.00 | 0.00 | 0.00 | 0.00 | 0.00 | 10912000.00 | 0.00 | KO:K23995 | PQQ-dependent dehydrogenase (methanol/ethanol family) | Bacteria;Proteobacteria;Alphaproteobacteria;Hyphomicrobiales;unclassified Hyphomicrobiales;Methyloceanibacter;Methyloceanibacter methanicus;Methyloceanibacter methanicus R-67174 |
| Ga0586324_2971396_1_765 | 401997.00 | 0.00 | 0.00 | 0.00 | 0.00 | 0.00 | 0.00 | 0.00 | 0.00 | 0.00 | 0.00 | 0.00 | KO:K23995 | PQQ-dependent dehydrogenase (methanol/ethanol family) | Bacteria;Proteobacteria;Betaproteobacteria;Burkholderiales;unclassified Burkholderiales;Rubrivivax;Rubrivivax gelatinosus;Rubrivivax gelatinosus IL144 |
| Ga0586324_3020901_1_411 | 0.00 | 0.00 | 0.00 | 0.00 | 0.00 | 590061.10 | 0.00 | 0.00 | 0.00 | 0.00 | 0.00 | 0.00 | KO:K23995 | glucose dehydrogenase | Bacteria;Proteobacteria;Alphaproteobacteria;Hyphomicrobiales;Hyphomicrobiaceae;Hyphomicrobium;Hyphomicrobium sp. 99;Hyphomicrobium sp. 99 |
| Ga0586324_3027420_2_511 | 29070000.00 | 47940000.00 | 0.00 | 0.00 | 0.00 | 0.00 | 0.00 | 0.00 | 0.00 | 7368900.00 | 34056000.00 | 0.00 | KO:K23995 | glucose dehydrogenase | Bacteria;Proteobacteria;Gammaproteobacteria;Methylococcales;Methylococcaceae;Methylobacter;Methylobacter marinus;Methylobacter marinus A45 |
| Ga0586324_3162230_3_686 | 79910000.00 | 1910800.00 | 91114625.10 | 2565000.00 | 2801600.00 | 0.00 | 3625200.00 | 4038200.00 | 3936600.00 | 4019400.00 | 5244800.00 | 5640000.00 | KO:K23995 | glucose dehydrogenase | Bacteria;Proteobacteria;Betaproteobacteria;Nitrosomonadales;Methylophilaceae;Methylotenera;Methylotenera versatilis;Methylotenera versatilis 301, JCM 17579 |
| Ga0586324_3270709_1_561 | 0.00 | 0.00 | 0.00 | 0.00 | 0.00 | 0.00 | 0.00 | 640500.00 | 0.00 | 0.00 | 0.00 | 0.00 | KO:K23995 | glucose dehydrogenase | Bacteria;Proteobacteria;Gammaproteobacteria;Methylococcales;Crenotrichaceae;Crenothrix;Crenothrix polyspora;Crenothrix polyspora ERS1545889 |
| Ga0586324_3480127_3_716 | 70900000.00 | 70040000.00 | 85576000.00 | 0.00 | 3677100.00 | 6868100.00 | 20770800.00 | 17690000.00 | 13527000.00 | 9625000.00 | 17072000.00 | 11625000.00 | KO:K23995 | PQQ-dependent dehydrogenase (methanol/ethanol family) | Bacteria;Proteobacteria;Gammaproteobacteria;Chromatiales;Ectothiorhodospiraceae;Thioalkalivibrio;Thioalkalivibrio sp. HK1;Thioalkalivibrio sp. HK1 |
| Ga0586324_3840239_3_227 | 9440000.00 | 9792000.00 | 9796200.00 | 0.00 | 8641700.00 | 10123500.00 | 0.00 | 6832000.00 | 15309000.00 | 13860000.00 | 12232000.00 | 9300000.00 | KO:K23995 | hypothetical protein | Bacteria;Proteobacteria;Alphaproteobacteria;Hyphomicrobiales;unclassified Hyphomicrobiales;Methyloceanibacter;Methyloceanibacter superfactus;Methyloceanibacter superfactus R-67175 |
| Ga0586324_5422384_3_596 | 0.00 | 2801600.00 | 1412775.31 | 0.00 | 0.00 | 0.00 | 0.00 | 0.00 | 0.00 | 0.00 | 0.00 | 0.00 | KO:K23995 | glucose dehydrogenase | Bacteria;Proteobacteria;Betaproteobacteria;Nitrosomonadales;Methylophilaceae;Methylobacillus;Methylobacillus sp. MM3;Methylobacillus sp. MM2 |
| Ga0586324_5446189_3_596 | 0.00 | 0.00 | 2247214.50 | 0.00 | 0.00 | 0.00 | 0.00 | 0.00 | 0.00 | 0.00 | 0.00 | 0.00 | KO:K23995 | PQQ-dependent dehydrogenase (methanol/ethanol family) | Bacteria;Proteobacteria;Alphaproteobacteria;Hyphomicrobiales;Methylocystaceae;Methylocystis;Methylocystis sp. SC2;Methylocystis sp. SC2 |
| Ga0586324_6098200_1_567 | 6430000.00 | 1781600.00 | 20268000.00 | 7106000.00 | 0.00 | 6572239.72 | 0.00 | 11144700.00 | 3134700.00 | 0.00 | 2508000.00 | 5287500.00 | KO:K23995 | glucose dehydrogenase | Bacteria;Proteobacteria;Alphaproteobacteria;Hyphomicrobiales;Hyphomicrobiaceae;Hyphomicrobium;Hyphomicrobium zavarzinii;Hyphomicrobium zavarzinii ATCC 27496 |
| Ga0586324_7039868_1_534 | 1540000.00 | 0.00 | 0.00 | 0.00 | 0.00 | 0.00 | 0.00 | 0.00 | 0.00 | 0.00 | 0.00 | 0.00 | KO:K23995 | glucose dehydrogenase | Bacteria;Proteobacteria;Alphaproteobacteria;Hyphomicrobiales;Hyphomicrobiaceae;Hyphomicrobium;Hyphomicrobium sp. CS1GBMeth3;Hyphomicrobium sp. ERS1433774 |
| Ga0586324_7084956_2_535 | 7880000.00 | 0.00 | 0.00 | 0.00 | 0.00 | 0.00 | 0.00 | 0.00 | 0.00 | 0.00 | 0.00 | 0.00 | KO:K23995 | glucose dehydrogenase | Bacteria;Proteobacteria;Gammaproteobacteria;Methylococcales;Methylococcaceae;Methyloglobulus;Methyloglobulus morosus;Methyloglobulus morosus KoM1 |
| Ga0586324_7699039_3_515 | 989521.00 | 0.00 | 17171500.00 | 0.00 | 0.00 | 0.00 | 1748734.92 | 1457900.00 | 315379.98 | 1971200.00 | 4910400.00 | 2257500.00 | KO:K23995 | glucose dehydrogenase | Bacteria;Proteobacteria;Gammaproteobacteria;Methylococcales;Methylococcaceae;Methylomagnum;Methylomagnum ishizawai;Methylomagnum ishizawai 175 |
| Ga0586324_8189017_2_502 | 0.00 | 0.00 | 94584000.00 | 0.00 | 0.00 | 0.00 | 0.00 | 0.00 | 0.00 | 0.00 | 0.00 | 0.00 | KO:K23995 | PQQ-dependent dehydrogenase (methanol/ethanol family) | Bacteria;Proteobacteria;Gammaproteobacteria;Methylococcales;Methylococcaceae;Methylomicrobium;Methylomicrobium lacus;Methylosarcina lacus LW14 |
| Ga0586324_0089292_1837_3423 | 0.00 | 0.00 | 0.00 | 0.00 | 9640800.00 | 0.00 | 0.00 | 0.00 | 0.00 | 0.00 | 0.00 | 0.00 | KO:K23997 | hydroxyethylthiazole kinase-like uncharacterized protein yjeF | Bacteria;Firmicutes;Clostridia;Thermoanaerobacterales;Thermoanaerobacteraceae;Thermodesulfitimonas;Thermodesulfitimonas autotrophica;Thermodesulfitimonas autotrophica DSM 102936 |
| Ga0586324_0614558_1_1341 | 17800000.00 | 0.00 | 0.00 | 0.00 | 0.00 | 0.00 | 0.00 | 0.00 | 0.00 | 0.00 | 0.00 | 0.00 | KO:K23997 | hydroxyethylthiazole kinase-like uncharacterized protein yjeF | Bacteria;Proteobacteria;Deltaproteobacteria;Desulfuromonadales;Desulfuromonadaceae;Desulfuromonas;Desulfuromonas sp. DDH964;Desulfuromonas sp. DDH964 |
| Ga0586324_0154022_2_1771 | 0.00 | 0.00 | 0.00 | 0.00 | 0.00 | 0.00 | 0.00 | 1098000.00 | 0.00 | 0.00 | 0.00 | 0.00 | KO:K24012 | acetyl coenzyme A synthetase (ADP forming)-like protein | Bacteria;Proteobacteria;Deltaproteobacteria;Desulfobacterales;Desulfobacteraceae;Desulfosalsimonas;Desulfosalsimonas propionicica;Desulfosalsimonas propionicica DSM 17721 |
| Ga0586324_1765093_2_475 | 0.00 | 0.00 | 0.00 | 0.00 | 0.00 | 0.00 | 2302800.00 | 0.00 | 0.00 | 2325400.00 | 0.00 | 0.00 | KO:K24119 | peroxiredoxin | Bacteria;Proteobacteria;Gammaproteobacteria;Nevskiales;Steroidobacteraceae;Povalibacter;Povalibacter uvarum;Povalibacter uvarum DSM 26723 |
| Ga0586324_1805259_200_763 | 0.00 | 5807200.00 | 0.00 | 0.00 | 20291000.00 | 0.00 | 553073.28 | 0.00 | 0.00 | 5921300.00 | 17512000.00 | 4507500.00 | KO:K24119 | peroxiredoxin | Bacteria;Proteobacteria;Gammaproteobacteria;Pseudomonadales;Pseudomonadaceae;Azomonas;Azomonas agilis;Azomonas agilis DSM 375 |
| Ga0586324_0645579_987_1460 | 0.00 | 0.00 | 0.00 | 3410500.00 | 2039400.00 | 0.00 | 0.00 | 0.00 | 0.00 | 4435200.00 | 4250400.00 | 5137500.00 | KO:K24129 | peroxiredoxin | Bacteria;Proteobacteria;Deltaproteobacteria;Myxococcales;Nannocystaceae;Plesiocystis;Plesiocystis pacifica;Plesiocystis pacifica SIR-1 |
| Ga0586324_4324050_7_654 | 0.00 | 5650800.00 | 0.00 | 0.00 | 0.00 | 0.00 | 0.00 | 0.00 | 0.00 | 0.00 | 0.00 | 0.00 | KO:K24129 | peroxiredoxin | Bacteria;Proteobacteria;Deltaproteobacteria;Myxococcales;Nannocystaceae;Nannocystis;Nannocystis exedens;Nannocystis exedens ATCC 25963 |
| Ga0586324_0125028_1434_1916 | 0.00 | 3746800.00 | 0.00 | 0.00 | 0.00 | 3466254.64 | 0.00 | 0.00 | 0.00 | 0.00 | 0.00 | 0.00 | KO:K24138 | peroxiredoxin | Bacteria;Proteobacteria;Alphaproteobacteria;Rhodospirillales;Acetobacteraceae;Rhodopila;unclassified Rhodopila;Rhodopila sp. LVNP |
| Ga0586324_0000001_103210_104415 | 0.00 | 0.00 | 0.00 | 0.00 | 0.00 | 0.00 | 0.00 | 8558300.00 | 6504300.00 | 24070200.00 | 8116592.00 | 24952500.00 | | hypothetical protein | Bacteria;Proteobacteria;Betaproteobacteria;Nitrosomonadales;Thiobacillaceae;Annwoodia;Annwoodia aquaesulis;Annwoodia aquaesulis DSM 4255 |
| Ga0586324_0000002_111816_112457 | 0.00 | 0.00 | 0.00 | 0.00 | 0.00 | 0.00 | 0.00 | 0.00 | 0.00 | 0.00 | 2622400.00 | 1792500.00 | | predicted negative regulator of RcsB-dependent stress response | Bacteria;Proteobacteria;Betaproteobacteria;Nitrosomonadales;Thiobacillaceae;Annwoodia;Annwoodia aquaesulis;Annwoodia aquaesulis DSM 4255 |
| Ga0586324_0000002_189616_190569 | 0.00 | 0.00 | 0.00 | 0.00 | 0.00 | 0.00 | 0.00 | 0.00 | 0.00 | 0.00 | 0.00 | 4087500.00 | | glyoxylase-like metal-dependent hydrolase (beta-lactamase superfamily II) | Bacteria;Proteobacteria;Betaproteobacteria;Nitrosomonadales;Thiobacillaceae;Annwoodia;Annwoodia aquaesulis;Annwoodia aquaesulis DSM 4255 |
| Ga0586324_0000002_56313_58445 | 0.00 | 0.00 | 0.00 | 0.00 | 0.00 | 0.00 | 0.00 | 0.00 | 0.00 | 0.00 | 888800.00 | 236435.25 | | MtrB/PioB family decaheme-associated outer membrane protein | Bacteria;Proteobacteria;Betaproteobacteria;Rhodocyclales;Rhodocyclaceae;Aromatoleum;Aromatoleum toluclasticum;Aromatoleum toluclasticum ATCC 700605 |
| Ga0586324_0000003_12721_13170 | 0.00 | 0.00 | 0.00 | 0.00 | 0.00 | 0.00 | 0.00 | 2488800.00 | 0.00 | 3026100.00 | 1760000.00 | 7935000.00 | | CBS domain-containing protein | Bacteria;Proteobacteria;Betaproteobacteria;Nitrosomonadales;Thiobacillaceae;Annwoodia;Annwoodia aquaesulis;Annwoodia aquaesulis DSM 4255 |
| Ga0586324_0000003_172392_174353 | 6360000.00 | 0.00 | 0.00 | 0.00 | 0.00 | 0.00 | 38554800.00 | 38545900.00 | 26065800.00 | 79387000.00 | 71535200.00 | 70807500.00 | | NADPH-dependent glutamate synthase beta subunit-like oxidoreductase/Pyruvate/2-oxoacid:fer | Bacteria;Proteobacteria;Betaproteobacteria;Nitrosomonadales;Thiobacillaceae;Annwoodia;Annwoodia aquaesulis;Annwoodia aquaesulis DSM 4255 |
| Ga0586324_0000004_122259_122657 | 0.00 | 0.00 | 0.00 | 0.00 | 0.00 | 0.00 | 0.00 | 0.00 | 0.00 | 1208900.00 | 0.00 | 0.00 | | DUF971 family protein | Bacteria;Proteobacteria;Betaproteobacteria;Nitrosomonadales;Thiobacillaceae;Annwoodia;Annwoodia aquaesulis;Annwoodia aquaesulis DSM 4255 |
| Ga0586324_0000004_123664_124560 | 0.00 | 0.00 | 0.00 | 0.00 | 0.00 | 0.00 | 0.00 | 5313100.00 | 7767900.00 | 17109400.00 | 16579200.00 | 10155000.00 | | hypothetical protein | Bacteria;Proteobacteria;Betaproteobacteria;Nitrosomonadales;Thiobacillaceae;Annwoodia;Annwoodia aquaesulis;Annwoodia aquaesulis DSM 4255 |
| Ga0586324_0000005_158962_159300 | 0.00 | 0.00 | 0.00 | 0.00 | 0.00 | 0.00 | 0.00 | 5874300.00 | 0.00 | 0.00 | 0.00 | 0.00 | | nitrogen regulatory protein PII | Bacteria;Proteobacteria;Betaproteobacteria;Nitrosomonadales;Thiobacillaceae;Annwoodia;Annwoodia aquaesulis;Annwoodia aquaesulis DSM 4255 |
| Ga0586324_0000005_36313_37335 | 0.00 | 0.00 | 0.00 | 0.00 | 0.00 | 0.00 | 0.00 | 0.00 | 0.00 | 5667200.00 | 1760000.00 | 34477500.00 | | FtsP/CotA-like multicopper oxidase with cupredoxin domain | Bacteria;Proteobacteria;Betaproteobacteria;Nitrosomonadales;Thiobacillaceae;Thiobacillus;Thiobacillus denitrificans;Thiobacillus denitrificans RG |
| Ga0586324_0000007_112166_113494 | 0.00 | 0.00 | 0.00 | 0.00 | 0.00 | 0.00 | 0.00 | 0.00 | 0.00 | 0.00 | 515973.92 | 1027500.00 | | hypothetical protein | Bacteria;Proteobacteria;Alphaproteobacteria;Hyphomonadales;Hyphomonadaceae;Hyphomonas;Hyphomonas oceanitis;Hyphomonas oceanitis SCH89 |
| Ga0586324_0000007_33285_34436 | 0.00 | 2298400.00 | 2657528.90 | 0.00 | 1483200.00 | 2793911.32 | 47492400.00 | 27138289.39 | 25879500.00 | 69238400.00 | 52368800.00 | 40395000.00 | | TRAP-type mannitol/chloroaromatic compound transport system substrate-binding protein | Bacteria;Proteobacteria;Betaproteobacteria;Nitrosomonadales;Thiobacillaceae;Thiobacillus;Thiobacillus denitrificans;Thiobacillus denitrificans RG |
| Ga0586324_0000007_78078_78644 | 0.00 | 0.00 | 0.00 | 0.00 | 0.00 | 0.00 | 0.00 | 0.00 | 0.00 | 0.00 | 0.00 | 13125000.00 | | lipid-binding SYLF domain-containing protein | Bacteria;Proteobacteria;Betaproteobacteria;Rhodocyclales;Zoogloeaceae;Thauera;Thauera selenatis;Thauera selenatis AX, ATCC 55363 |
| Ga0586324_0000007_83821_84792 | 0.00 | 0.00 | 0.00 | 0.00 | 0.00 | 0.00 | 0.00 | 0.00 | 0.00 | 2517900.00 | 6556000.00 | 1395000.00 | | hypothetical protein | Bacteria;Proteobacteria;Betaproteobacteria;Nitrosomonadales;Thiobacillaceae;Thiobacillus;Thiobacillus thiophilus;Thiobacillus thiophilus DSM 19892 |
| Ga0586324_0000011_31458_32867 | 0.00 | 0.00 | 0.00 | 0.00 | 0.00 | 0.00 | 0.00 | 28609000.00 | 27864000.00 | 62447000.00 | 544759.60 | 50925000.00 | | glutamine synthetase type I | Bacteria;Proteobacteria;Betaproteobacteria;Nitrosomonadales;Thiobacillaceae;Annwoodia;Annwoodia aquaesulis;Annwoodia aquaesulis DSM 4255 |
| Ga0586324_0000011_6456_8294 | 0.00 | 0.00 | 0.00 | 0.00 | 0.00 | 0.00 | 0.00 | 0.00 | 9639000.00 | 7153300.00 | 12716000.00 | 21082500.00 | | hypothetical protein | Bacteria;Proteobacteria;Betaproteobacteria;Nitrosomonadales;Thiobacillaceae;Annwoodia;Annwoodia aquaesulis;Annwoodia aquaesulis DSM 4255 |
| Ga0586324_0000013_45844_46875 | 0.00 | 0.00 | 0.00 | 0.00 | 0.00 | 0.00 | 0.00 | 0.00 | 0.00 | 1047200.00 | 0.00 | 1245000.00 | | RND family efflux transporter MFP subunit | Bacteria;Proteobacteria;Betaproteobacteria;Nitrosomonadales;Thiobacillaceae;Annwoodia;Annwoodia aquaesulis;Annwoodia aquaesulis DSM 4255 |
| Ga0586324_0000013_61899_62333 | 0.00 | 0.00 | 0.00 | 0.00 | 0.00 | 0.00 | 0.00 | 0.00 | 0.00 | 2910600.00 | 0.00 | 0.00 | | quercetin dioxygenase-like cupin family protein | Bacteria;Bacteroidetes;Flavobacteriia;Flavobacteriales;Flavobacteriaceae;Flavobacterium;Flavobacterium denitrificans;Flavobacterium denitrificans DSM 15936 |
| Ga0586324_0000013_71030_74239 | 0.00 | 0.00 | 0.00 | 0.00 | 0.00 | 0.00 | 0.00 | 0.00 | 0.00 | 1085700.00 | 0.00 | 0.00 | | membrane peptidoglycan carboxypeptidase | Bacteria;Proteobacteria;Betaproteobacteria;Nitrosomonadales;Thiobacillaceae;Annwoodia;Annwoodia aquaesulis;Annwoodia aquaesulis DSM 4255 |
| Ga0586324_0000018_16215_17159 | 0.00 | 0.00 | 0.00 | 0.00 | 0.00 | 0.00 | 0.00 | 0.00 | 0.00 | 0.00 | 9416000.00 | 3930000.00 | | DNA-binding transcriptional LysR family regulator | Bacteria;Proteobacteria;Betaproteobacteria;Nitrosomonadales;Thiobacillaceae;Annwoodia;Annwoodia aquaesulis;Annwoodia aquaesulis DSM 4255 |
| Ga0586324_0000018_42136_42597 | 0.00 | 0.00 | 0.00 | 0.00 | 0.00 | 0.00 | 0.00 | 0.00 | 0.00 | 0.00 | 0.00 | 1260000.00 | | uncharacterized protein (DUF302 family) | Bacteria;Proteobacteria;Betaproteobacteria;Nitrosomonadales;Thiobacillaceae;Annwoodia;Annwoodia aquaesulis;Annwoodia aquaesulis DSM 4255 |
| Ga0586324_0000032_29918_30175 | 0.00 | 0.00 | 568630.00 | 648284.75 | 1205100.00 | 840222.71 | 0.00 | 0.00 | 0.00 | 0.00 | 0.00 | 0.00 | | hypothetical protein | |
| Ga0586324_0000033_62152_62676 | 0.00 | 0.00 | 0.00 | 0.00 | 0.00 | 0.00 | 2235843.24 | 9638000.00 | 0.00 | 0.00 | 7092800.00 | 4665000.00 | | hypothetical protein | |
| Ga0586324_0000033_65074_65499 | 0.00 | 0.00 | 0.00 | 0.00 | 0.00 | 0.00 | 2986800.00 | 3806400.00 | 0.00 | 10010000.00 | 10560000.00 | 7027500.00 | | hypothetical protein | Bacteria;Proteobacteria;Betaproteobacteria;Nitrosomonadales;Thiobacillaceae;Annwoodia;Annwoodia aquaesulis;Annwoodia aquaesulis DSM 4255 |
| Ga0586324_0000035_53685_55538 | 0.00 | 0.00 | 0.00 | 0.00 | 0.00 | 0.00 | 0.00 | 0.00 | 0.00 | 0.00 | 0.00 | 2257500.00 | | phosphate/phosphite/phosphonate ABC transporter binding protein | Bacteria;Proteobacteria;Gammaproteobacteria;Oceanospirillales;Oceanospirillaceae;Oceanospirillum;Oceanospirillum multiglobuliferum;Oceanospirillum multiglobuliferum ATCC 33336 |
| Ga0586324_0000037_32177_33070 | 0.00 | 0.00 | 0.00 | 0.00 | 0.00 | 0.00 | 0.00 | 599279.86 | 0.00 | 0.00 | 0.00 | 0.00 | | hypothetical protein | Bacteria;Proteobacteria;Betaproteobacteria;Nitrosomonadales;Sulfuricellaceae;Sulfuriferula;Sulfuriferula multivorans;Sulfuriferula multivorans TTN |
| Ga0586324_0000037_34663_35172 | 0.00 | 0.00 | 0.00 | 0.00 | 0.00 | 0.00 | 2530800.00 | 0.00 | 0.00 | 2587200.00 | 0.00 | 0.00 | | peroxiredoxin family protein | Bacteria;Proteobacteria;Betaproteobacteria;Nitrosomonadales;Thiobacillaceae;Annwoodia;Annwoodia aquaesulis;Annwoodia aquaesulis DSM 4255 |
| Ga0586324_0000037_68802_69647 | 0.00 | 0.00 | 0.00 | 0.00 | 0.00 | 0.00 | 0.00 | 0.00 | 0.00 | 4596900.00 | 3361600.00 | 3607500.00 | | dienelactone hydrolase | Bacteria;Proteobacteria;Betaproteobacteria;Nitrosomonadales;Thiobacillaceae;Annwoodia;Annwoodia aquaesulis;Annwoodia aquaesulis DSM 4255 |
| Ga0586324_0000041_23064_24878 | 0.00 | 0.00 | 0.00 | 0.00 | 0.00 | 0.00 | 0.00 | 0.00 | 0.00 | 0.00 | 5165600.00 | 0.00 | | tetratricopeptide (TPR) repeat protein | Bacteria;Proteobacteria;Betaproteobacteria;Nitrosomonadales;Thiobacillaceae;Annwoodia;Annwoodia aquaesulis;Annwoodia aquaesulis DSM 4255 |
| Ga0586324_0000066_30538_35268 | 0.00 | 0.00 | 0.00 | 0.00 | 0.00 | 0.00 | 0.00 | 0.00 | 0.00 | 1817200.00 | 0.00 | 0.00 | | hypothetical protein | Bacteria;Proteobacteria;Alphaproteobacteria;Hyphomicrobiales;Hyphomicrobiaceae;Rhodoplanes;Rhodoplanes sp. Z2-YC6860;Rhodoplanes sp. Z2-YC6860 |
| Ga0586324_0000070_43313_44773 | 0.00 | 0.00 | 0.00 | 0.00 | 0.00 | 0.00 | 0.00 | 1348100.00 | 1733400.00 | 0.00 | 5271200.00 | 4845000.00 | | uncharacterized repeat protein (TIGR01451 family) | Bacteria;Verrucomicrobia;Opitutae;Puniceicoccales;Puniceicoccaceae;Coraliomargarita;Coraliomargarita sinensis;Coraliomargarita sinensis WN38 |
| Ga0586324_0000080_6188_7249 | 6790000.00 | 7877857.80 | 12386000.00 | 6631000.00 | 14450900.00 | 7026900.00 | 14865600.00 | 24766000.00 | 25312500.00 | 8670200.00 | 11422400.00 | 1642500.00 | | predicted porin | Bacteria;Proteobacteria;Betaproteobacteria;Nitrosomonadales;Sulfuricellaceae;Sulfuriferula;Sulfuriferula multivorans;Sulfuriferula multivorans TTN |
| Ga0586324_0000092_30838_31752 | 0.00 | 0.00 | 0.00 | 0.00 | 0.00 | 0.00 | 0.00 | 0.00 | 0.00 | 1355200.00 | 0.00 | 0.00 | | hypothetical protein | Bacteria;Atribacterota;unclassified Atribacterota;unclassified Atribacterota;unclassified Atribacterota;unclassified Atribacterota;Atribacteria bacterium JGI 0000059-I14;Atribacteria bacterium JGI 0000059-I14 (contamination screened) |
| Ga0586324_0000103_24180_25175 | 0.00 | 0.00 | 0.00 | 0.00 | 0.00 | 0.00 | 0.00 | 0.00 | 306342.81 | 0.00 | 0.00 | 0.00 | | ABC-type nitrate/sulfonate/bicarbonate transport system substrate-binding protein | Bacteria;Proteobacteria;Alphaproteobacteria;Hyphomicrobiales;Bradyrhizobiaceae;Bradyrhizobium;Bradyrhizobium erythrophlei;Bradyrhizobium erythrophlei GAS401 |
| Ga0586324_0000105_22067_23551 | 813917.00 | 1224000.00 | 2257005.07 | 3306000.00 | 3812777.78 | 1278812.43 | 0.00 | 1049200.00 | 2260165.68 | 0.00 | 0.00 | 662704.50 | | hypothetical protein | Archaea;Euryarchaeota;Methanomicrobia;Methanosarcinales;Methanosarcinaceae;Methanosarcina;Methanosarcina horonobensis;Methanosarcina horonobensis HB-1 |
| Ga0586324_0000147_1184_2944 | 0.00 | 0.00 | 0.00 | 0.00 | 0.00 | 0.00 | 0.00 | 0.00 | 980100.00 | 0.00 | 0.00 | 0.00 | | hypothetical protein | Bacteria;Verrucomicrobia;Opitutae;Opitutales;Opitutaceae;Opitutus;Opitutus sp. ER46;Opitutus sp. ER46 |
| Ga0586324_0000151_2249_3661 | 0.00 | 0.00 | 0.00 | 0.00 | 0.00 | 0.00 | 2918400.00 | 2696200.00 | 0.00 | 0.00 | 0.00 | 0.00 | | cytochrome c peroxidase | Bacteria;Proteobacteria;Gammaproteobacteria;Methylococcales;Methylococcaceae;Methylobacter;Methylobacter tundripaludum;Methylobacter tundripaludum SV96 |
| Ga0586324_0000169_6878_8317 | 0.00 | 0.00 | 0.00 | 0.00 | 0.00 | 0.00 | 0.00 | 0.00 | 0.00 | 0.00 | 5605600.00 | 3937500.00 | | hypothetical protein | Bacteria;Proteobacteria;Betaproteobacteria;Nitrosomonadales;Thiobacillaceae;Annwoodia;Annwoodia aquaesulis;Annwoodia aquaesulis DSM 4255 |
| Ga0586324_0000181_14108_17032 | 8340000.00 | 12056400.00 | 0.00 | 3695500.00 | 0.00 | 7580377.55 | 1233258.84 | 2458300.00 | 0.00 | 2589554.66 | 4954400.00 | 5700000.00 | | hypothetical protein | Bacteria;Ignavibacteriae;Ignavibacteria;Ignavibacteriales;Ignavibacteriaceae;Ignavibacterium;Ignavibacterium album;Ignavibacterium album Mat9-16, JCM 16511 |
| Ga0586324_0000181_17052_20468 | 3920000.00 | 10764400.00 | 0.00 | 0.00 | 3069400.00 | 15125700.00 | 0.00 | 0.00 | 788164.83 | 0.00 | 0.00 | 2412843.75 | | hypothetical protein | Bacteria;Ignavibacteriae;Ignavibacteria;Ignavibacteriales;Melioribacteraceae;Melioribacter;Melioribacter roseus;Melioribacter roseus P3M |
| Ga0586324_0000181_20552_21601 | 0.00 | 0.00 | 0.00 | 0.00 | 0.00 | 0.00 | 0.00 | 0.00 | 0.00 | 0.00 | 0.00 | 362399.25 | | hypothetical protein | Bacteria;Ignavibacteriae;Ignavibacteria;Ignavibacteriales;Melioribacteraceae;Melioribacter;Melioribacter roseus;Melioribacter roseus P3M |
| Ga0586324_0000192_5334_8912 | 0.00 | 1135600.00 | 0.00 | 0.00 | 0.00 | 0.00 | 0.00 | 0.00 | 0.00 | 3819200.00 | 0.00 | 0.00 | | 2',3'-cyclic-nucleotide 2'-phosphodiesterase (5'-nucleotidase family) | Bacteria;Firmicutes;Bacilli;Bacillales;Bacillaceae;Bacillus;Bacillus sp. OV166;Bacillus sp. OV166 |
| Ga0586324_0000211_21187_24735 | 0.00 | 945200.00 | 0.00 | 0.00 | 0.00 | 0.00 | 0.00 | 0.00 | 0.00 | 0.00 | 0.00 | 0.00 | | hypothetical protein | Bacteria;Acidobacteria;Vicinamibacteria;unclassified Vicinamibacteria;Vicinamibacteraceae;Luteitalea;Luteitalea pratensis;Luteitalea pratensis DSM 100886 |
| Ga0586324_0000217_1659_4838 | 78410000.00 | 54196000.00 | 98919100.00 | 42655000.00 | 63508962.61 | 35412400.00 | 46489200.00 | 32811900.00 | 26090100.00 | 84469000.00 | 81699200.00 | 79627500.00 | | hypothetical protein | Bacteria;Candidatus Aminicenantes;unclassified Candidatus Aminicenantes;unclassified Candidatus Aminicenantes;unclassified Candidatus Aminicenantes;unclassified Candidatus Aminicenantes;Aminicenantes bacterium SCGC AAA252-D06;Aminicenantes bacterium SCGC AAA252-D06 (contamination screened) |
| Ga0586324_0000219_3914_4954 | 27700000.00 | 904400.00 | 29670100.00 | 16150000.00 | 0.00 | 0.00 | 1551872.88 | 13908000.00 | 11907000.00 | 8778000.00 | 1971200.00 | 2152500.00 | | excisionase family DNA binding protein | Bacteria;Chloroflexi;Anaerolineae;Anaerolineales;Anaerolineaceae;Levilinea;Levilinea saccharolytica;Levilinea saccharolytica DSM 16555 |
| Ga0586324_0000244_83_667 | 0.00 | 0.00 | 0.00 | 0.00 | 0.00 | 0.00 | 0.00 | 2488800.00 | 972000.00 | 15631000.00 | 17952000.00 | 3330000.00 | | intracellular sulfur oxidation DsrE/DsrF family protein | Bacteria;Proteobacteria;Gammaproteobacteria;Acidiferrobacterales;Acidiferrobacteraceae;Sulfurifustis;Sulfurifustis variabilis;Sulfurifustis variabilis skN76 |
| Ga0586324_0000270_22082_24850 | 0.00 | 0.00 | 0.00 | 0.00 | 0.00 | 0.00 | 769205.88 | 0.00 | 545424.03 | 0.00 | 0.00 | 0.00 | | putative PEP-CTERM system TPR-repeat lipoprotein | Bacteria;Proteobacteria;Betaproteobacteria;Nitrosomonadales;Sulfuricellaceae;Sulfuriferula;Sulfuriferula multivorans;Sulfuriferula multivorans TTN |
| Ga0586324_0000291_28342_31605 | 193059.00 | 0.00 | 0.00 | 3106500.00 | 2482300.00 | 3216990.25 | 0.00 | 0.00 | 0.00 | 0.00 | 3344000.00 | 3682500.00 | | hypothetical protein | Bacteria;Acidobacteria;Vicinamibacteria;unclassified Vicinamibacteria;Vicinamibacteraceae;Luteitalea;Luteitalea pratensis;Luteitalea pratensis DSM 100886 |
| Ga0586324_0000309_16191_18146 | 0.00 | 0.00 | 0.00 | 0.00 | 0.00 | 0.00 | 0.00 | 0.00 | 680502.87 | 0.00 | 0.00 | 0.00 | | hypothetical protein | Bacteria;Firmicutes;Clostridia;Eubacteriales;Oscillospiraceae;Papillibacter;Papillibacter cinnamivorans;Papillibacter cinnamivorans DSM 12816 |
| Ga0586324_0000329_2673_5540 | 8740000.00 | 1176400.00 | 10162256.97 | 3220500.00 | 2492600.00 | 0.00 | 866060.28 | 1579900.00 | 1215000.00 | 0.00 | 2129600.00 | 1530000.00 | | hypothetical protein | Bacteria;Acidobacteria;Vicinamibacteria;unclassified Vicinamibacteria;Vicinamibacteraceae;Luteitalea;Luteitalea pratensis;Luteitalea pratensis DSM 100886 |
| Ga0586324_0000337_27529_29658 | 19880000.00 | 19584000.00 | 30739800.00 | 29849000.00 | 47235800.00 | 36682800.00 | 41427600.00 | 15433000.00 | 13713300.00 | 23461900.00 | 11844800.00 | 18450000.00 | | outer membrane protein OmpA-like peptidoglycan-associated protein | Bacteria;Acidobacteria;Acidobacteriia;Acidobacteriales;Acidobacteriaceae;Candidatus Koribacter;Candidatus Koribacter versatilis;Candidatus Koribacter versatilis Ellin345 |
| Ga0586324_0000361_12533_14101 | 0.00 | 0.00 | 0.00 | 0.00 | 0.00 | 0.00 | 0.00 | 2513200.00 | 0.00 | 0.00 | 1205600.00 | 870000.00 | | uncharacterized protein (PEP-CTERM system associated) | Bacteria;Proteobacteria;Betaproteobacteria;Nitrosomonadales;Thiobacillaceae;Thiobacillus;Thiobacillus denitrificans;Thiobacillus denitrificans RG |
| Ga0586324_0000363_631_1803 | 352370000.00 | 108052000.00 | 18090967.39 | 307230000.00 | 341156600.00 | 47946972.31 | 352885904.00 | 74295402.01 | 170518328.55 | 81364744.23 | 112340800.00 | 158557500.00 | | predicted porin | Bacteria;Proteobacteria;Gammaproteobacteria;Acidiferrobacterales;Acidiferrobacteraceae;Sulfurifustis;Sulfurifustis variabilis;Sulfurifustis variabilis skN76 |

| | | | | | | | | | | | | | | |
|---|---|---|---|---|---|---|---|---|---|---|---|---|---|---|
| Ga0586324 0000365 20454 23741 | 0.00 | 1137255.80 | 5855200.00 | 1396500.00 | 1102100.00 | 0.00 | 0.00 | 933300.00 | 328626.72 | 862400.00 | 888800.00 | 381415.50 | hypothetical protein | Bacteria;Acidobacteria;Vicinamibacteria;unclassified Vicinamibacteria;Vicinamibacteraceae;Luteitalea;Luteitalea pratensis;Luteitalea pratensis DSM 100886 |
| Ga0586324 0000365 636 4013 | 13590000.00 | 13933200.00 | 12723800.00 | 16055000.00 | 12586600.00 | 10574916.79 | 13292400.00 | 12718500.00 | 8221500.00 | 10017700.00 | 7708800.00 | 10425000.00 | outer membrane receptor protein involved in Fe transport | Bacteria;Acidobacteria;Acidobacteriia;Acidobacteriales;Acidobacteriaceae;Acidipila;Acidipila rosea;Acidipila rosea DSM 103428 |
| Ga0586324 0000423 24008 25159 | 936392.00 | 37196000.00 | 25756546.25 | 2346500.00 | 5489900.00 | 17150400.00 | 174672537.36 | 356270802.56 | 115077997.62 | 291837700.00 | 364362276.08 | 453452394.00 | predicted porin | Bacteria;Proteobacteria;Betaproteobacteria;Nitrosomonadales;Thiobacillaceae;Sulfuritortus;Sulfuritortus calidifontis;Sulfuritortus calidifontis DSM 103923 |
| Ga0586324 0000429 10739 12514 | 0.00 | 0.00 | 0.00 | 0.00 | 0.00 | 0.00 | 0.00 | 0.00 | 1231200.00 | 0.00 | 0.00 | 0.00 | MSHA type pilus biogenesis protein MshL | Bacteria;Proteobacteria;Betaproteobacteria;Nitrosomonadales;Thiobacillaceae;Thiobacillus;Thiobacillus thioparus;Thiobacillus thioparus DSM 505 |
| Ga0586324 0000431 19186 19905 | 0.00 | 0.00 | 0.00 | 0.00 | 463150.83 | 0.00 | 0.00 | 0.00 | 1101600.00 | 0.00 | 950400.00 | 0.00 | hypothetical protein | Bacteria;Proteobacteria;Betaproteobacteria;Nitrosomonadales;Gallionellaceae;Sideroxydans;Sideroxydans lithotrophicus;Sideroxydans lithotrophicus ES-1 |
| Ga0586324 0000434 6653 9970 | 3950000.00 | 23881600.00 | 12632329.39 | 32110000.00 | 1359600.00 | 15789678.53 | 5859600.00 | 6703900.00 | 11372400.00 | 11357500.00 | 11070400.00 | 8647500.00 | hypothetical protein | Bacteria;Acidobacteria;Vicinamibacteria;unclassified Vicinamibacteria;Vicinamibacteraceae;Luteitalea;Luteitalea pratensis;Luteitalea pratensis DSM 100886 |
| Ga0586324 0000470 22417 22821 | 12400000.00 | 10030000.00 | 0.00 | 10260000.00 | 4964600.00 | 0.00 | 8390400.00 | 15231700.00 | 6131700.00 | 5574800.00 | 7040000.00 | 5730000.00 | hypothetical protein | |
| Ga0586324 0000475 15100 16647 | 0.00 | 1360000.00 | 0.00 | 0.00 | 0.00 | 0.00 | 0.00 | 1750700.00 | 0.00 | 5428500.00 | 4822400.00 | 4012500.00 | hypothetical protein | Bacteria;Proteobacteria;Betaproteobacteria;Nitrosomonadales;Sulfuricellaceae;Sulfurirhabdus;Sulfurirhabdus autotrophica;Sulfurirhabdus autotrophica DSM 100309 |
| Ga0586324 0000491 6575 8887 | 677390.00 | 1813961.20 | 1444911.35 | 1320500.00 | 0.00 | 336977.57 | 0.00 | 0.00 | 0.00 | 0.00 | 0.00 | 423196.50 | hypothetical protein | Bacteria;Proteobacteria;Betaproteobacteria;unclassified Betaproteobacteria;unclassified Betaproteobacteria;unclassified Betaproteobacteria;Betaproteobacteria bacterium GR16-43;Betaproteobacteria bacterium GR16-43 |
| Ga0586324 0000518 24887 28231 | 1320000.00 | 0.00 | 0.00 | 1586500.00 | 0.00 | 0.00 | 0.00 | 0.00 | 0.00 | 0.00 | 941600.00 | 1492500.00 | hypothetical protein | Bacteria;Acidobacteria;Vicinamibacteria;unclassified Vicinamibacteria;Vicinamibacteraceae;Luteitalea;Luteitalea pratensis;Luteitalea pratensis DSM 100886 |
| Ga0586324 0000524 30320 31144 | 0.00 | 0.00 | 0.00 | 0.00 | 0.00 | 0.00 | 0.00 | 0.00 | 2170800.00 | 5667200.00 | 7330400.00 | 1267500.00 | outer membrane protein OmpA-like peptidoglycan-associated protein | Bacteria;Proteobacteria;Betaproteobacteria;Nitrosomonadales;Thiobacillaceae;Annwoodia;Annwoodia aquaesulis;Annwoodia aquaesulis DSM 4255 |
| Ga0586324 0000537 19185 20219 | 0.00 | 0.00 | 0.00 | 0.00 | 0.00 | 0.00 | 0.00 | 0.00 | 0.00 | 0.00 | 0.00 | 1297500.00 | hypothetical protein | Bacteria;Proteobacteria;Betaproteobacteria;Nitrosomonadales;Thiobacillaceae;Annwoodia;Annwoodia aquaesulis;Annwoodia aquaesulis DSM 4255 |
| Ga0586324 0000542 20285 21433 | 0.00 | 2128400.00 | 0.00 | 0.00 | 0.00 | 0.00 | 0.00 | 0.00 | 0.00 | 9317000.00 | 0.00 | 10125000.00 | hypothetical protein | Bacteria;Proteobacteria;Betaproteobacteria;Nitrosomonadales;Thiobacillaceae;Thiobacillus;Thiobacillus thioparus;Thiobacillus thioparus DSM 505 |
| Ga0586324 0000542 21592 24315 | 0.00 | 0.00 | 2231326.64 | 0.00 | 0.00 | 0.00 | 6066799.56 | 8930400.00 | 6658200.00 | 14483700.00 | 17107200.00 | 10260000.00 | hypothetical protein | Bacteria;Proteobacteria;Betaproteobacteria;Nitrosomonadales;Thiobacillaceae;Thiobacillus;Thiobacillus thioparus;Thiobacillus thioparus DSM 505 |
| Ga0586324 0000543 15169 16437 | 0.00 | 10812000.00 | 0.00 | 0.00 | 0.00 | 0.00 | 0.00 | 0.00 | 0.00 | 0.00 | 0.00 | 0.00 | hypothetical protein | Bacteria;Proteobacteria;Deltaproteobacteria;Desulfuromonadales;Geobacteraceae;Geobacter;Geobacter sp. DSM 9736;Geobacter sp. DSM 9736 |
| Ga0586324 0000576 17339 18628 | 0.00 | 0.00 | 0.00 | 0.00 | 0.00 | 0.00 | 0.00 | 567964.29 | 447932.43 | 491637.30 | 530063.60 | 0.00 | hypothetical protein | Bacteria;Proteobacteria;Alphaproteobacteria;Hyphomicrobiales;Hyphomicrobiaceae;Rhodoplanes;Rhodoplanes sp. Z2-YC6860;Rhodoplanes sp. Z2-YC6860 |
| Ga0586324 0000620 19859 23254 | 0.00 | 0.00 | 0.00 | 8787500.00 | 9609900.00 | 0.00 | 0.00 | 0.00 | 6083100.00 | 0.00 | 0.00 | 4777500.00 | hypothetical protein | Bacteria;Acidobacteria;Vicinamibacteria;unclassified Vicinamibacteria;Vicinamibacteraceae;Luteitalea;Luteitalea pratensis;Luteitalea pratensis DSM 100886 |
| Ga0586324 0000630 16007 16582 | 0.00 | 0.00 | 0.00 | 0.00 | 0.00 | 0.00 | 0.00 | 0.00 | 0.00 | 3672900.00 | 4716800.00 | 1057500.00 | polyisoprenoid-binding protein YceI | Bacteria;Proteobacteria;Betaproteobacteria;Nitrosomonadales;Thiobacillaceae;Thiobacillus;Thiobacillus thioparus;Thiobacillus thioparus DSM 505 |
| Ga0586324 0000652 28176 30539 | 0.00 | 0.00 | 0.00 | 0.00 | 0.00 | 0.00 | 0.00 | 0.00 | 0.00 | 0.00 | 1812800.00 | 1237500.00 | Ca2+-binding RTX toxin-like protein | Bacteria;Proteobacteria;Betaproteobacteria;Nitrosomonadales;Thiobacillaceae;Annwoodia;Annwoodia aquaesulis;Annwoodia aquaesulis DSM 4255 |
| Ga0586324 0000679 1574 3100 | 0.00 | 931600.00 | 0.00 | 0.00 | 0.00 | 942755.90 | 0.00 | 0.00 | 0.00 | 0.00 | 0.00 | 0.00 | hypothetical protein | |
| Ga0586324 0000686 24489 27053 | 10800000.00 | 0.00 | 0.00 | 6156000.00 | 0.00 | 0.00 | 0.00 | 0.00 | 3353400.00 | 0.00 | 4760800.00 | 0.00 | hypothetical protein | Bacteria;Bacteroidetes;Bacteroidia;Bacteroidales;Lentimicrobiaceae;Lentimicrobium;Lentimicrobium saccharophilum;Bacteroidales bacterium TBC1 |
| Ga0586324 0000732 5275 7188 | 3340000.00 | 2869600.00 | 0.00 | 5120500.00 | 0.00 | 0.00 | 0.00 | 0.00 | 0.00 | 0.00 | 0.00 | 0.00 | transketolase | Bacteria;Proteobacteria;Gammaproteobacteria;Methylococcales;Methylococcaceae;Methylomonas;Methylomonas lenta;Methylomonas lenta R-45370 |
| Ga0586324 0000741 23484 25919 | 0.00 | 0.00 | 0.00 | 0.00 | 0.00 | 0.00 | 0.00 | 0.00 | 0.00 | 0.00 | 0.00 | 2835000.00 | formate dehydrogenase-N alpha subunit | Bacteria;Proteobacteria;Betaproteobacteria;Nitrosomonadales;Thiobacillaceae;Thiobacillus;Thiobacillus thioparus;Thiobacillus thioparus DSM 505 |
| Ga0586324 0000753 5636 6421 | 0.00 | 0.00 | 0.00 | 0.00 | 0.00 | 0.00 | 0.00 | 0.00 | 0.00 | 1940400.00 | 0.00 | 0.00 | short-subunit dehydrogenase | Bacteria;Proteobacteria;Betaproteobacteria;Rhodocyclales;Rhodocyclaceae;Propionivibrio;Propionivibrio pelophilus;Propionivibrio pelophilus DSM 12018 |
| Ga0586324 0000755 13164 14306 | 0.00 | 4107200.00 | 0.00 | 0.00 | 1740700.00 | 0.00 | 0.00 | 0.00 | 0.00 | 0.00 | 0.00 | 0.00 | alkylation response protein AidB-like acyl-CoA dehydrogenase | Bacteria;Proteobacteria;Deltaproteobacteria;Syntrophobacterales;Syntrophobacteraceae;Desulfacinum;Desulfacinum infernum;Desulfacinum infernum DSM 9756 |
| Ga0586324 0000764 15803 16765 | 0.00 | 0.00 | 0.00 | 0.00 | 0.00 | 0.00 | 0.00 | 0.00 | 0.00 | 1216600.00 | 0.00 | 0.00 | single-stranded DNA-specific DHH superfamily exonuclease | Bacteria;Proteobacteria;Betaproteobacteria;Nitrosomonadales;Thiobacillaceae;Thiobacillus;Thiobacillus thioparus;Thiobacillus thioparus DSM 505 |
| Ga0586324 0000820 8658 10310 | 0.00 | 0.00 | 0.00 | 0.00 | 0.00 | 0.00 | 0.00 | 0.00 | 0.00 | 0.00 | 0.00 | 718038.00 | PAS domain S-box-containing protein | Bacteria;Bacteroidetes;Bacteroidia;Marinilabiliales;Prolixibacteraceae;Sunxiuqinia;Sunxiuqinia dokdonensis;Sunxiuqinia dokdonensis SK |
| Ga0586324 0000830 13202 14503 | 12510000.00 | 25792400.00 | 10758839.92 | 16843500.00 | 41210300.00 | 18976600.00 | 41746800.00 | 16085700.00 | 34036200.00 | 9163000.00 | 24904000.00 | 18345000.00 | hypothetical protein | |
| Ga0586324 0000943 10690 11634 | 0.00 | 0.00 | 0.00 | 0.00 | 0.00 | 0.00 | 1580119.80 | 0.00 | 0.00 | 0.00 | 0.00 | 0.00 | DNA-binding transcriptional LysR family regulator | Bacteria;Proteobacteria;Betaproteobacteria;Nitrosomonadales;Thiobacillaceae;Thiobacillus;Thiobacillus thioparus;Thiobacillus thioparus DSM 505 |
| Ga0586324 0000975 8167 8664 | 0.00 | 2033200.00 | 0.00 | 0.00 | 19364000.00 | 0.00 | 5016000.00 | 29585000.00 | 38556000.00 | 0.00 | 25960000.00 | 0.00 | rubrerythrin | Bacteria;Proteobacteria;Betaproteobacteria;Nitrosomonadales;Thiobacillaceae;Thiobacillus;Thiobacillus thioparus;Thiobacillus thioparus DSM 505 |
| Ga0586324 0001021 11236 11571 | 0.00 | 0.00 | 0.00 | 0.00 | 0.00 | 0.00 | 0.00 | 2909700.00 | 0.00 | 0.00 | 0.00 | 0.00 | TusE/DsrC/DsvC family sulfur relay protein | Bacteria;Proteobacteria;Betaproteobacteria;Nitrosomonadales;Thiobacillaceae;Thiobacillus;Thiobacillus thioparus;Thiobacillus thioparus DSM 505 |
| Ga0586324 0001021 6706 8664 | 0.00 | 1142400.00 | 0.00 | 0.00 | 0.00 | 0.00 | 0.00 | 0.00 | 0.00 | 0.00 | 0.00 | 4807500.00 | NADPH-dependent glutamate synthase beta subunit-like oxidoreductase/Pyruvate/2-oxoacid:fer | Bacteria;Proteobacteria;Betaproteobacteria;Nitrosomonadales;Thiobacillaceae;Thiobacillus;Thiobacillus thioparus;Thiobacillus thioparus DSM 505 |
| Ga0586324 0001096 10846 12051 | 0.00 | 0.00 | 0.00 | 0.00 | 0.00 | 0.00 | 0.00 | 0.00 | 0.00 | 0.00 | 0.00 | 451151.25 | hypothetical protein | Bacteria;Proteobacteria;Deltaproteobacteria;Syntrophobacterales;Syntrophobacteraceae;Syntrophobacter;Syntrophobacter fumaroxidans;Syntrophobacter fumaroxidans MPOB |
| Ga0586324 0001135 4367 6148 | 0.00 | 0.00 | 1634850.66 | 0.00 | 0.00 | 0.00 | 0.00 | 0.00 | 0.00 | 0.00 | 0.00 | 0.00 | alkylation response protein AidB-like acyl-CoA dehydrogenase | Bacteria;Acidobacteria;Acidobacteriia;Bryobacterales;Solibacteraceae;Candidatus Solibacter;Candidatus Solibacter usitatus;Candidatus Solibacter usitatus Ellin6076 |
| Ga0586324 0001224 14088 15227 | 0.00 | 0.00 | 0.00 | 0.00 | 0.00 | 0.00 | 0.00 | 0.00 | 0.00 | 0.00 | 1628000.00 | 0.00 | TRAP-type mannitol/chloroaromatic compound transport system substrate-binding protein | Bacteria;Proteobacteria;Alphaproteobacteria;Hyphomicrobiales;Bradyrhizobiaceae;Bradyrhizobium;Bradyrhizobium sp. Ai1a-2;Bradyrhizobium sp. Ai1a-2 |
| Ga0586324 0001264 5082 6296 | 0.00 | 0.00 | 0.00 | 306400.65 | 0.00 | 0.00 | 0.00 | 0.00 | 0.00 | 0.00 | 0.00 | 0.00 | tetratricopeptide (TPR) repeat protein | Bacteria;Bacteroidetes;Saprospiria;Saprospirales;Saprospiraceae;Aureispira;Aureispira sp. CCB-QB1;Aureispira sp. CCB-QB1 |
| Ga0586324 0001289 8270 12913 | 0.00 | 3529200.00 | 0.00 | 0.00 | 0.00 | 0.00 | 0.00 | 0.00 | 0.00 | 0.00 | 0.00 | 0.00 | hypothetical protein | Bacteria;Firmicutes;Limnochordia;Limnochordales;Limnochordaceae;Limnochorda;Limnochorda pilosa;Limnochorda pilosa HC45 |
| Ga0586324 0001308 640 3012 | 0.00 | 0.00 | 0.00 | 1520000.00 | 0.00 | 1285561.43 | 0.00 | 0.00 | 1286071.02 | 1963500.00 | 704679.36 | 0.00 | type IV pilus secretin PilQ/predicted competence protein | Bacteria;Candidatus Aminicenantes;unclassified Candidatus Aminicenantes;unclassified Candidatus Aminicenantes;unclassified Candidatus Aminicenantes;unclassified Candidatus Aminicenantes;Aminicenantes bacterium SCGC AAA255-A06;Aminicenantes bacterium SCGC AAA255-A06 (contamination screened) |
| Ga0586324 0001373 9826 10515 | 0.00 | 0.00 | 0.00 | 0.00 | 0.00 | 0.00 | 1515324.48 | 2635200.00 | 2000700.00 | 0.00 | 0.00 | 1297500.00 | hypothetical protein | Bacteria;Proteobacteria;Betaproteobacteria;Nitrosomonadales;Thiobacillaceae;Thiobacillus;Thiobacillus thioparus;Thiobacillus thioparus DSM 505 |
| Ga0586324 0001379 20967 22925 | 0.00 | 0.00 | 0.00 | 0.00 | 0.00 | 0.00 | 20907600.00 | 7869000.00 | 6625800.00 | 0.00 | 10190400.00 | 3705000.00 | NADPH-dependent glutamate synthase beta subunit-like oxidoreductase/Pyruvate/2-oxoacid:fer | Bacteria;Proteobacteria;Betaproteobacteria;Nitrosomonadales;Thiobacillaceae;Thiobacillus;Thiobacillus thioparus;Thiobacillus thioparus DSM 505 |
| Ga0586324 0001505 3 3302 | 17410000.00 | 12947200.00 | 7262700.00 | 19218500.00 | 24987800.00 | 15087103.66 | 10465200.00 | 11980400.00 | 10278900.00 | 12469938.25 | 11888800.00 | 13455000.00 | TonB-linked SusC/RagA family outer membrane protein | Bacteria;Bacteroidetes;Flavobacteriia;Flavobacteriales;Flavobacteriaceae;Gaetbulibacter;Gaetbulibacter saemankumensis;Gaetbulibacter saemankumensis DSM 17032 |
| Ga0586324 0001544 9382 9975 | 0.00 | 0.00 | 0.00 | 0.00 | 0.00 | 0.00 | 0.00 | 0.00 | 1142100.00 | 0.00 | 0.00 | 0.00 | CBS domain-containing protein | Bacteria;Proteobacteria;Betaproteobacteria;Nitrosomonadales;Thiobacillaceae;Thiobacillus;Thiobacillus thioparus;Thiobacillus thioparus DSM 505 |
| Ga0586324 0001554 11968 13557 | 0.00 | 0.00 | 0.00 | 0.00 | 0.00 | 0.00 | 0.00 | 0.00 | 0.00 | 1832600.00 | 0.00 | 0.00 | glutamate synthase domain-containing protein 2 | Archaea;Euryarchaeota;Methanomicrobia;Methanomicrobiales;Methanomicrobiaceae;Methanolacinia;Methanolacinia petrolearia;Methanolacinia petrolearia DSM 11571 |
| Ga0586324 0001628 11598 13982 | 0.00 | 0.00 | 0.00 | 0.00 | 0.00 | 0.00 | 0.00 | 0.00 | 0.00 | 0.00 | 0.00 | 319044.75 | hypothetical protein | Bacteria;Proteobacteria;Betaproteobacteria;Nitrosomonadales;Thiobacillaceae;Thiobacillus;Thiobacillus thioparus;Thiobacillus thioparus DSM 505 |
| Ga0586324 0001715 4625 8344 | 0.00 | 999181.12 | 0.00 | 1558000.00 | 3697700.00 | 0.00 | 0.00 | 0.00 | 10335600.00 | 3210901.54 | 19527200.00 | 7005000.00 | 2',3'-cyclic-nucleotide 2'-phosphodiesterase (5'-nucleotidase family) | Bacteria;Actinobacteria;Actinomycetia;Propionibacteriales;Nocardioidaceae;Nocardioides;Nocardioides sp. Root122;Nocardioides sp. Root122 |
| Ga0586324 0001815 16787 17242 | 0.00 | 0.00 | 0.00 | 0.00 | 0.00 | 0.00 | 6748800.00 | 10187000.00 | 8262000.00 | 2949100.00 | 13904000.00 | 10650000.00 | lipid-binding SYLF domain-containing protein | Bacteria;Proteobacteria;Alphaproteobacteria;Hyphomicrobiales;Alsobacteraceae;Alsobacter;Alsobacter soli;Alsobacter soli SH9 |
| Ga0586324 0001823 15552 15887 | 0.00 | 0.00 | 0.00 | 0.00 | 0.00 | 0.00 | 10921200.00 | 5703500.00 | 12393000.00 | 0.00 | 0.00 | 0.00 | ElaB/YqjD/DUF883 family membrane-anchored ribosome-binding protein | Bacteria;Proteobacteria;Betaproteobacteria;Nitrosomonadales;Thiobacillaceae;Thiobacillus;Thiobacillus thioparus;Thiobacillus thioparus DSM 505 |
| Ga0586324 0001878 15789 17378 | 0.00 | 8976000.00 | 0.00 | 0.00 | 0.00 | 0.00 | 0.00 | 0.00 | 8343000.00 | 0.00 | 3872000.00 | 0.00 | glutamate synthase domain-containing protein 2 | Archaea;Euryarchaeota;Methanomicrobia;Methanosarcinales;Methanotrichaceae;Methanothrix;Methanosaeta harundinacea;Methanothrix harundinacea 6Ac |
| Ga0586324 0001918 1909 4815 | 6580000.00 | 2067200.00 | 0.00 | 8711500.00 | 8858000.00 | 8495800.00 | 0.00 | 0.00 | 8505000.00 | 1647800.00 | 22264000.00 | 13072500.00 | hypothetical protein | Bacteria;Acidobacteria;Thermoanaerobaculia;Thermoanaerobaculales;Thermoanaerobaculaceae;Thermoanaerobaculum;Thermoanaerobaculum aquaticum;Thermoanaerobaculum aquaticum MP-01 |
| Ga0586324 0002013 11662 12585 | 0.00 | 0.00 | 990778.66 | 0.00 | 0.00 | 597921.70 | 3237600.00 | 1573800.00 | 0.00 | 0.00 | 0.00 | 1815000.00 | outer membrane protein OmpA-like peptidoglycan-associated protein | Bacteria;Proteobacteria;Betaproteobacteria;Nitrosomonadales;Thiobacillaceae;Annwoodia;Annwoodia aquaesulis;Annwoodia aquaesulis DSM 4255 |
| Ga0586324 0002022 40 4395 | 0.00 | 0.00 | 3971953.74 | 5310500.00 | 0.00 | 928404.35 | 5882400.00 | 2732800.00 | 6342300.00 | 0.00 | 0.00 | 1695000.00 | hypothetical protein | Bacteria;Bacteroidetes;unclassified Bacteroidetes;Bacteroidetes Order II. Incertae sedis;Rhodothermaceae;unclassified Rhodothermaceae;Rhodothermaceae bacterium RA;Halothermobacillus malaysiensis RA |
| Ga0586324 0002091 8926 12357 | 9180000.00 | 5419600.00 | 4199304.40 | 0.00 | 16480000.00 | 892342.46 | 0.00 | 3824700.00 | 5208300.00 | 5289900.00 | 3317600.00 | 3975000.00 | hypothetical protein | Bacteria;Acidobacteria;Acidobacteriia;Acidobacteriales;Acidobacteriaceae;Acidobacterium;Acidobacterium ailaaui;Acidobacterium ailaaui PMMR2 |
| Ga0586324 0002096 10428 11738 | 0.00 | 0.00 | 0.00 | 0.00 | 0.00 | 0.00 | 0.00 | 0.00 | 0.00 | 6546.26 | 0.00 | 0.00 | predicted dehydrogenase | Bacteria;Planctomycetes;Phycisphaerae;Sedimentisphaerales;Anaerohalosphaeraceae;Anaerohalosphaera;Anaerohalosphaera lusitana;Phycisphaerae bacterium ST-NAGAB-D1 |
| Ga0586324 0002126 3622 6714 | 0.00 | 0.00 | 12611200.00 | 0.00 | 0.00 | 0.00 | 0.00 | 0.00 | 0.00 | 0.00 | 7717600.00 | 0.00 | outer membrane receptor protein involved in Fe transport | Bacteria;Proteobacteria;Gammaproteobacteria;Cellvibrionales;Spongiibacteraceae;unclassified Spongiibacteraceae;marine gamma proteobacterium HTCC2143;Marine gamma proteobacterium sp. HTCC2143 |
| Ga0586324 0002126 9140 12235 | 4130000.00 | 1638800.00 | 2301482.07 | 2327500.00 | 1163900.00 | 2964113.16 | 0.00 | 0.00 | 0.00 | 2156000.00 | 492025.60 | 155394.75 | outer membrane receptor protein involved in Fe transport | Bacteria;Proteobacteria;Gammaproteobacteria;Cellvibrionales;Spongiibacteraceae;unclassified Spongiibacteraceae;marine gamma proteobacterium HTCC2143;Marine gamma proteobacterium sp. HTCC2143 |
| Ga0586324 0002173 3821 5230 | 4330000.00 | 0.00 | 0.00 | 0.00 | 0.00 | 0.00 | 8846400.00 | 0.00 | 0.00 | 0.00 | 7541600.00 | 0.00 | glutamine synthetase type I | Bacteria;Proteobacteria;Betaproteobacteria;Nitrosomonadales;Thiobacillaceae;Thiobacillus;Thiobacillus thioparus;Thiobacillus thioparus DSM 505 |
| Ga0586324 0002189 4626 6137 | 3540000.00 | 3366000.00 | 2392834.45 | 2973500.00 | 4181800.00 | 0.00 | 0.00 | 6905200.00 | 8108100.00 | 8824200.00 | 8729600.00 | 6120000.00 | hypothetical protein | Bacteria;Acidobacteria;Acidobacteriia;Acidobacteriales;Acidobacteriaceae;Candidatus Koribacter;Candidatus Koribacter versatilis;Candidatus Koribacter versatilis Ellin345 |
| Ga0586324 0002270 3 18908 | 0.00 | 3806432.60 | 1692299.18 | 1358500.00 | 804797.71 | 272884.70 | 0.00 | 504557.84 | 352980.99 | 0.00 | 1056000.00 | 3105681.00 | hypothetical protein | Bacteria;Cyanobacteria;unclassified Cyanobacteria;Nostocales;Nostocaceae;Nostoc;Nostoc sp. PCC 7524;Nostoc sp. PCC 7524 |
| Ga0586324 0002278 4027 5478 | 3650000.00 | 10492400.00 | 25560200.00 | 48618232.65 | 2904337.35 | 20523411.25 | 0.00 | 0.00 | 0.00 | 5428325.98 | 0.00 | 17864800.50 | hypothetical protein | Bacteria;Chloroflexi;Ardenticatenia;Ardenticatenales;Ardenticatenaceae;Candidatus Promineofilum;Candidatus Promineofilum breve;Candidatus Promineofilum breve Cfx-K |
| Ga0586324 0002303 218 1558 | 5260000.00 | 1672800.00 | 856700.21 | 3534000.00 | 320307.34 | 1173841.66 | 2736000.00 | 2177700.00 | 2551500.00 | 2109800.00 | 1636800.00 | 1650000.00 | crotonobetainyl-CoA:carnitine CoA-transferase CaiB-like acyl-CoA transferase | Bacteria;Proteobacteria;Deltaproteobacteria;Syntrophobacterales;Syntrophobacteraceae;Desulfacinum;Desulfacinum infernum;Desulfacinum infernum DSM 9756 |
| Ga0586324 0002309 206 4396 | 0.00 | 0.00 | 0.00 | 0.00 | 0.00 | 0.00 | 3328800.00 | 0.00 | 0.00 | 0.00 | 0.00 | 0.00 | hypothetical protein | Bacteria;Actinobacteria;Actinomycetia;Propionibacteriales;Kribbellaceae;Kribbella;Kribbella monticola;Kribbella monticola NEAU-SW521 |
| Ga0586324 0002364 17741 18613 | 1100000.00 | 0.00 | 0.00 | 0.00 | 0.00 | 0.00 | 0.00 | 0.00 | 0.00 | 0.00 | 0.00 | 0.00 | hypothetical protein | Bacteria;Bacteroidetes;Flavobacteriia;Flavobacteriales;Flavobacteriaceae;Flavobacterium;Flavobacterium sp. A45;Flavobacterium sp. A45 |
| Ga0586324 0002415 3 482 | 109000000.00 | 0.00 | 0.00 | 0.00 | 0.00 | 0.00 | 0.00 | 0.00 | 0.00 | 0.00 | 0.00 | 0.00 | formyltetrahydrofolate synthetase | Bacteria;Proteobacteria;Deltaproteobacteria;Desulfobacterales;Desulfobacteraceae;Desulfatiglans;Desulfatiglans anilini;Desulfatiglans anilini DSM 4660 |
| Ga0586324 0002443 5377 18369 | 1200000.00 | 0.00 | 0.00 | 0.00 | 631159.28 | 0.00 | 0.00 | 0.00 | 0.00 | 0.00 | 0.00 | 0.00 | Ca2+-binding RTX toxin-like protein | Bacteria;Firmicutes;Clostridia;Eubacteriales;Clostridiaceae;Youngiibacter;Youngiibacter fragilis;Youngiibacter fragilis 232.1 |
| Ga0586324 0002444 4757 7189 | 0.00 | 1394000.00 | 0.00 | 0.00 | 0.00 | 3709655.34 | 0.00 | 0.00 | 0.00 | 0.00 | 6635200.00 | 0.00 | type IV pilus secretin PilQ/predicted competence protein | Bacteria;Candidatus Aminicenantes;unclassified Candidatus Aminicenantes;unclassified Candidatus Aminicenantes;unclassified Candidatus Aminicenantes;unclassified Candidatus Aminicenantes;Aminicenantes bacterium SCGC AAA252-K07;Aminicenantes bacterium SCGC AAA252-K07 (contamination screened) |
| Ga0586324 0002517 8902 12627 | 0.00 | 508987.48 | 0.00 | 1463000.00 | 26151700.00 | 21914400.00 | 0.00 | 14823000.00 | 0.00 | 0.00 | 0.00 | 256328.25 | 2',3'-cyclic-nucleotide 2'-phosphodiesterase (5'-nucleotidase family) | Bacteria;Actinobacteria;Actinomycetia;Propionibacteriales;Nocardioidaceae;Nocardioides;Nocardioides sp. Root122;Nocardioides sp. Root122 |
| Ga0586324 0002541 12112 15198 | 28650000.00 | 24244785.28 | 3411932.01 | 14592000.00 | 16459400.00 | 13425503.83 | 11272484.16 | 12297600.00 | 11080800.00 | 15631000.00 | 15655200.00 | 10462500.00 | hypothetical protein | Bacteria;Acidobacteria;Vicinamibacteria;unclassified Vicinamibacteria;Vicinamibacteraceae;Luteitalea;Luteitalea pratensis;Luteitalea pratensis DSM 100886 |
| Ga0586324 0002544 578 3943 | 7956177.00 | 4086800.00 | 6699700.00 | 0.00 | 567813.25 | 13427441.19 | 8846400.00 | 8228900.00 | 1020535.20 | 0.00 | 4848800.00 | 7650000.00 | hypothetical protein | Bacteria;Acidobacteria;Vicinamibacteria;unclassified Vicinamibacteria;Vicinamibacteraceae;Luteitalea;Luteitalea pratensis;Luteitalea pratensis DSM 100886 |
| Ga0586324 0002581 17259 17771 | 8420000.00 | 0.00 | 0.00 | 0.00 | 0.00 | 0.00 | 0.00 | 2763300.00 | 3110400.00 | 0.00 | 0.00 | 0.00 | tetratricopeptide (TPR) repeat protein | Bacteria;Proteobacteria;Deltaproteobacteria;Syntrophobacterales;Syntrophaceae;Desulfobacca;Desulfobacca acetoxidans;Desulfobacca acetoxidans ASRB2, DSM 11109 |
| Ga0586324 0002626 202 1494 | 0.00 | 0.00 | 0.00 | 0.00 | 0.00 | 0.00 | 0.00 | 0.00 | 1903500.00 | 10626000.00 | 3528800.00 | 1312500.00 | hypothetical protein | Bacteria;Nitrospirae;Nitrospira;Nitrospirales;Nitrospiraceae;Candidatus Magnetoovum;Candidatus Magnetoovum chiemensis;Candidatus Magnetoovum chiemensis CS-04 |
| Ga0586324 0002685 1413 4940 | 3546370.00 | 5453600.00 | 0.00 | 5966000.00 | 5520800.00 | 5161000.00 | 0.00 | 0.00 | 3183300.00 | 1824900.00 | 3335200.00 | 2197500.00 | 2',3'-cyclic-nucleotide 2'-phosphodiesterase (5'-nucleotidase family) | Bacteria;Firmicutes;Clostridia;Eubacteriales;Thermohalobacteraceae;Caloranaerobacter;Caloranaerobacter azorensis;Caloranaerobacter azorensis H53214 |
| Ga0586324 0002696 2864 3589 | 0.00 | 0.00 | 0.00 | 0.00 | 0.00 | 0.00 | 0.00 | 4666500.00 | 4957200.00 | 0.00 | 0.00 | 1575000.00 | flavorubredoxin | Bacteria;Proteobacteria;Betaproteobacteria;Nitrosomonadales;Thiobacillaceae;Thiobacillus;Thiobacillus denitrificans;Thiobacillus denitrificans DSM 12475 |
| Ga0586324 0002712 183 3170 | 5730000.00 | 16061600.00 | 1139776.61 | 5880500.00 | 10227900.00 | 11855178.27 | 0.00 | 1348100.00 | 4416417.27 | 3873100.00 | 4030400.00 | 6854229.75 | hypothetical protein | Bacteria;Candidatus Aminicenantes;unclassified Candidatus Aminicenantes;unclassified Candidatus Aminicenantes;unclassified Candidatus Aminicenantes;unclassified Candidatus Aminicenantes;Aminicenantes bacterium SCGC AAA252-N07;Aminicenantes bacterium SCGC AAA252-N07 (contamination screened) |
| Ga0586324 0002748 1510 3384 | 0.00 | 4311200.00 | 0.00 | 0.00 | 0.00 | 0.00 | 0.00 | 2915800.00 | 5994000.00 | 8693300.00 | 5288800.00 | 0.00 | hypothetical protein | Bacteria;Proteobacteria;Betaproteobacteria;Nitrosomonadales;Sulfuricellaceae;Sulfurirhabdus;Sulfurirhabdus autotrophica;Sulfurirhabdus autotrophica DSM 100309 |
| Ga0586324 0002755 756 2033 | 10000000.00 | 8500000.00 | 11428900.00 | 4664500.00 | 4974900.00 | 11711500.00 | 8618400.00 | 9089000.00 | 9315000.00 | 3072300.00 | 2974400.00 | 6705000.00 | hypothetical protein | Bacteria;Proteobacteria;Deltaproteobacteria;Desulfuromonadales;Geobacteraceae;Geobacter;Geobacter anodireducens;Geobacter anodireducens SD-1 |
| Ga0586324 0002758 2997 3617 | 0.00 | 0.00 | 0.00 | 0.00 | 0.00 | 0.00 | 5084400.00 | 1592100.00 | 5313600.00 | 0.00 | 0.00 | 4275000.00 | hypothetical protein | Bacteria;Bacteroidetes;Bacteroidia;Bacteroidales;Rikenellaceae;Acetobacteroides;Acetobacteroides hydrogenigenes;Acetobacteroides hydrogenigenes DSM 24657 |
| Ga0586324 0002766 11209 14610 | 0.00 | 0.00 | 0.00 | 0.00 | 0.00 | 0.00 | 0.00 | 0.00 | 0.00 | 0.00 | 0.00 | 1845000.00 | hypothetical protein | Bacteria;Acidobacteria;Vicinamibacteria;unclassified Vicinamibacteria;Vicinamibacteraceae;Luteitalea;Luteitalea pratensis;Luteitalea pratensis DSM 100886 |
| Ga0586324 0002793 14337 15797 | 0.00 | 0.00 | 12892700.00 | 0.00 | 0.00 | 0.00 | 0.00 | 0.00 | 0.00 | 0.00 | 0.00 | 0.00 | hypothetical protein | Bacteria;Chloroflexi;Ardenticatenia;Ardenticatenales;Ardenticatenaceae;Candidatus Promineofilum;Candidatus Promineofilum breve;Candidatus Promineofilum breve Cfx-K |
| Ga0586324 0002943 6433 6660 | 0.00 | 0.00 | 0.00 | 3505500.00 | 2235100.00 | 6431400.00 | 0.00 | 1884900.00 | 0.00 | 0.00 | 0.00 | 0.00 | hypothetical protein | |
| Ga0586324 0002994 8995 10023 | 0.00 | 0.00 | 0.00 | 0.00 | 0.00 | 0.00 | 0.00 | 0.00 | 996300.00 | 0.00 | 2631200.00 | 0.00 | TRAP-type C4-dicarboxylate transport system substrate-binding protein | Bacteria;Proteobacteria;Alphaproteobacteria;Hyphomicrobiales;Xanthobacteraceae;Pseudolabrys;Pseudolabrys taiwanensis;Pseudolabrys taiwanensis CC-BB4 |
| Ga0586324 0003034 1566 5366 | 0.00 | 1849600.00 | 1276484.27 | 116542.20 | 0.00 | 0.00 | 0.00 | 811300.00 | 297669.33 | 671640.97 | 0.00 | 1215000.00 | hypothetical protein | Bacteria;Actinobacteria;Actinomycetia;Micrococcales;Microbacteriaceae;Leifsonia;Leifsonia sp. Root60;Leifsonia sp. Root60 |
| Ga0586324 0003138 16106 16723 | 0.00 | 700400.00 | 0.00 | 1729000.00 | 0.00 | 0.00 | 0.00 | 0.00 | 190088.37 | 1593900.00 | 1276000.00 | 0.00 | hypothetical protein | |
| Ga0586324 0003202 120 4136 | 13480000.00 | 18876800.00 | 3549771.30 | 0.00 | 927936.27 | 3166007.51 | 2622000.00 | 19239400.00 | 10756800.00 | 3334100.00 | 1346400.00 | 6217500.00 | hypothetical protein | Bacteria;Rhodothermaeota;Rhodothermia;Rhodothermales;Rubricoccaceae;Rubrivirga;Rubrivirga sp. SAORIC476;Rubrivirga sp. SAORIC476 |
| Ga0586324 0003202 4227 7559 | 60760000.00 | 67292800.00 | 51149366.35 | 39710000.00 | 49306100.00 | 38688400.33 | 37072800.00 | 76158500.00 | 72033300.00 | 49611100.00 | 38447200.00 | 36502500.00 | outer membrane receptor protein involved in Fe transport | Bacteria;Bacteroidetes;unclassified Bacteroidetes;Bacteroidetes Order II. Incertae sedis;Rhodothermaceae;unclassified Rhodothermaceae;Rhodothermaceae bacterium RA;Halothermobacillus malaysiensis RA |
| Ga0586324 0003268 697 1020 | 6490000.00 | 5106800.00 | 11597800.00 | 5833000.00 | 7343900.00 | 9805900.00 | 6361200.00 | 3775900.00 | 4762800.00 | 3180100.00 | 2754400.00 | 1612500.00 | alkylhydroperoxidase/carboxymuconolactone decarboxylase family protein YurZ | Archaea;Euryarchaeota;Methanomicrobia;Methanosarcinales;Methanotrichaceae;Methanothrix;Methanothrix soehngenii;Methanothrix soehngenii GP-6 |
| Ga0586324 0003274 3382 5700 | 0.00 | 0.00 | 0.00 | 0.00 | 0.00 | 0.00 | 0.00 | 1683600.00 | 3304800.00 | 0.00 | 0.00 | 0.00 | arylsulfatase A-like enzyme/Flp pilus assembly protein TadD | Bacteria;Candidatus Aminicenantes;unclassified Candidatus Aminicenantes;unclassified Candidatus Aminicenantes;unclassified Candidatus Aminicenantes;unclassified Candidatus Aminicenantes;Aminicenantes bacterium SCGC AAA252-O03;Aminicenantes bacterium SCGC AAA252-O03 (contamination screened) |
| Ga0586324 0003286 13015 15417 | 0.00 | 0.00 | 0.00 | 0.00 | 0.00 | 0.00 | 0.00 | 3495300.00 | 0.00 | 0.00 | 0.00 | 0.00 | PAS domain S-box-containing protein | Bacteria;Proteobacteria;Alphaproteobacteria;Hyphomicrobiales;Bradyrhizobiaceae;Bradyrhizobium;Bradyrhizobium erythrophlei;Bradyrhizobium erythrophlei GAS242 |
| Ga0586324 0003300 10910 12259 | 0.00 | 0.00 | 0.00 | 0.00 | 5809200.00 | 0.00 | 0.00 | 0.00 | 0.00 | 0.00 | 0.00 | 0.00 | hypothetical protein | Bacteria;Proteobacteria;Gammaproteobacteria;Cellvibrionales;Cellvibrionaceae;Teredinibacter;Teredinibacter turnerae;Teredinibacter turnerae T0609 |
| Ga0586324 0003306 287 4213 | 0.00 | 0.00 | 6643400.00 | 0.00 | 0.00 | 0.00 | 0.00 | 0.00 | 0.00 | 0.00 | 0.00 | 0.00 | 2',3'-cyclic-nucleotide 2'-phosphodiesterase (5'-nucleotidase family) | Bacteria;Firmicutes;Bacilli;Bacillales;Bacillaceae;Bacillus;Bacillus sp. OK048;Bacillus sp. OK048 |
| Ga0586324 0003306 4529 8263 | 8300000.00 | 135027.60 | 0.00 | 178655.10 | 9558400.00 | 11711500.00 | 0.00 | 5221600.00 | 0.00 | 418589.71 | 0.00 | 0.00 | 2',3'-cyclic-nucleotide 2'-phosphodiesterase (5'-nucleotidase family) | Bacteria;Actinobacteria;Actinomycetia;Propionibacteriales;Nocardioidaceae;Nocardioides;Nocardioides sp. Root122;Nocardioides sp. Root122 |
| Ga0586324 0003419 8608 10323 | 0.00 | 0.00 | 0.00 | 0.00 | 2430800.00 | 0.00 | 0.00 | 0.00 | 0.00 | 0.00 | 0.00 | 0.00 | arylsulfatase A-like enzyme | Bacteria;Proteobacteria;Alphaproteobacteria;Hyphomicrobiales;Bradyrhizobiaceae;Bradyrhizobium;unclassified Bradyrhizobium;Bradyrhizobium sp. HAMBI 2115 |
| Ga0586324 0003544 5065 7752 | 0.00 | 0.00 | 0.00 | 0.00 | 0.00 | 0.00 | 0.00 | 0.00 | 0.00 | 1670900.00 | 0.00 | 0.00 | predicted extracellular nuclease | Bacteria;Bacteroidetes;Chitinophagia;Chitinophagales;Chitinophagaceae;Flavisolibacter;Flavisolibacter ginsengiterrae;Flavisolibacter ginsengiterrae DSM 18136 |
| Ga0586324 0003695 2429 5389 | 0.00 | 0.00 | 0.00 | 0.00 | 0.00 | 0.00 | 0.00 | 835700.00 | 0.00 | 0.00 | 0.00 | 0.00 | hypothetical protein | Bacteria;Acidobacteria;Vicinamibacteria;unclassified Vicinamibacteria;Vicinamibacteraceae;Luteitalea;Luteitalea pratensis;Luteitalea pratensis DSM 100886 |
| Ga0586324 0003700 14018 15499 | 17100000.00 | 3685600.00 | 4720360.90 | 11875000.00 | 8755000.00 | 0.00 | 0.00 | 4050400.00 | 5062500.00 | 0.00 | 0.00 | 1867500.00 | hypothetical protein | Viruses;dsDNA viruses, no RNA stage;Caudoviricetes;Caudovirales;Myoviridae;unclassified Myoviridae;Rhodothermus phage RM378;Rhodothermus phage RM378 |
| Ga0586324 0003876 1 5658 | 1600000.00 | 0.00 | 629794.32 | 0.00 | 0.00 | 0.00 | 0.00 | 0.00 | 0.00 | 0.00 | 0.00 | 0.00 | hypothetical protein | Bacteria;Calditrichaeota;Calditrichae;Calditrichales;Calditrichaceae;Caldithrix;Caldithrix abyssi;Caldithrix abyssi LF13, DSM 13497 |
| Ga0586324 0004052 10811 13750 | 7430000.00 | 15947984.92 | 15989200.00 | 7087000.00 | 8353300.00 | 8019400.00 | 8094000.00 | 13420000.00 | 13470300.00 | 9394000.00 | 19835200.00 | 12637500.00 | hypothetical protein | Bacteria;Bacteroidetes;unclassified Bacteroidetes;Bacteroidetes Order II. Incertae sedis;Rhodothermaceae;Rhodothermus;Rhodothermus marinus;Rhodothermus marinus R-10, DSM 4252 |
| Ga0586324 0004052 7199 8215 | 14100000.00 | 0.00 | 0.00 | 0.00 | 0.00 | 0.00 | 0.00 | 0.00 | 0.00 | 0.00 | 0.00 | 0.00 | hypothetical protein | Bacteria;Ignavibacteriae;Ignavibacteria;Ignavibacteriales;Ignavibacteriaceae;Ignavibacterium;Ignavibacterium album;Ignavibacterium album Mat9-16, JCM 16511 |
| Ga0586324 0004052 8270 10756 | 13290000.00 | 20345600.00 | 10170713.23 | 7780500.00 | 2544100.00 | 9370748.30 | 0.00 | 6484300.00 | 2130300.00 | 6152300.00 | 17444558.56 | 11205000.00 | hypothetical protein | Bacteria;Bacteroidetes;unclassified Bacteroidetes;Bacteroidetes Order II. Incertae sedis;Rhodothermaceae;unclassified Rhodothermaceae;Rhodothermaceae bacterium RA;Halothermobacillus malaysiensis RA |
| Ga0586324 0004069 12346 13206 | 0.00 | 0.00 | 595958.02 | 0.00 | 0.00 | 0.00 | 602211.84 | 2592500.00 | 0.00 | 0.00 | 0.00 | 0.00 | uncharacterized coiled-coil DUF342 family protein | Archaea;Euryarchaeota;Methanomicrobia;Methanomicrobiales;Methanoregulaceae;Methanoregula;Methanoregula formicica;Methanoregula formicica SMSP, DSM 22288 |
| Ga0586324 0004081 12120 13007 | 0.00 | 0.00 | 0.00 | 262553.40 | 0.00 | 0.00 | 0.00 | 418706.44 | 1312200.00 | 0.00 | 0.00 | 0.00 | CO/xanthine dehydrogenase FAD-binding subunit | Bacteria;Proteobacteria;Deltaproteobacteria;Syntrophorhabdales;Syntrophorhabdaceae;Syntrophorhabdus;Syntrophorhabdus aromaticivorans;Syntrophorhabdus aromaticivorans UI |
| Ga0586324 0004081 9217 11538 | 0.00 | 0.00 | 0.00 | 1235000.00 | 0.00 | 0.00 | 0.00 | 0.00 | 0.00 | 0.00 | 1610400.00 | 2842500.00 | 4-hydroxybenzoyl-CoA reductase alpha subunit | Bacteria;Proteobacteria;Deltaproteobacteria;Syntrophorhabdales;Syntrophorhabdaceae;Syntrophorhabdus;Syntrophorhabdus aromaticivorans;Syntrophorhabdus aromaticivorans UI |
| Ga0586324 0004174 3 3395 | 6917892.00 | 43057600.00 | 15201000.00 | 13689500.00 | 23597300.00 | 9329500.00 | 9211200.00 | 9564800.00 | 6220800.00 | 10626000.00 | 23584000.00 | 13770000.00 | hypothetical protein | Bacteria;Candidatus Aminicenantes;unclassified Candidatus Aminicenantes;unclassified Candidatus Aminicenantes;unclassified Candidatus Aminicenantes;unclassified Candidatus Aminicenantes;Aminicenantes bacterium SCGC AAA255-E05;Aminicenantes bacterium SCGC AAA255-E05 (contamination screened) |
| Ga0586324 0004186 1559 3556 | 0.00 | 0.00 | 0.00 | 0.00 | 0.00 | 0.00 | 0.00 | 0.00 | 0.00 | 2063600.00 | 0.00 | 0.00 | CHASE2 domain-containing sensor protein | Bacteria;Verrucomicrobia;Verrucomicrobiae;Verrucomicrobiales;Verrucomicrobia subdivision 3;Pedosphaera;Pedosphaera parvula;Pedosphaera parvula Ellin514 |
| Ga0586324 0004207 13545 14624 | 636450.00 | 4474400.00 | 0.00 | 439694.20 | 7704400.00 | 0.00 | 0.00 | 5856000.00 | 0.00 | 12705000.00 | 0.00 | 15300000.00 | excisionase family DNA binding protein | Bacteria;Chloroflexi;Anaerolineae;Anaerolineales;Anaerolineaceae;Levilinea;Levilinea saccharolytica;Levilinea saccharolytica DSM 16555 |
| Ga0586324 0004226 1935 5294 | 0.00 | 2618000.00 | 0.00 | 1235000.00 | 1153600.00 | 0.00 | 0.00 | 451082.80 | 1004400.00 | 0.00 | 506186.56 | 8617500.00 | outer membrane receptor protein involved in Fe transport | Bacteria;Acidobacteria;Acidobacteriia;Bryobacterales;Solibacteraceae;Candidatus Solibacter;Candidatus Solibacter usitatus;Candidatus Solibacter usitatus Ellin6076 |
| Ga0586324 0004232 11672 12823 | 0.00 | 1312400.00 | 0.00 | 988000.00 | 1091800.00 | 0.00 | 0.00 | 939400.00 | 0.00 | 0.00 | 0.00 | 607758.75 | hypothetical protein | Archaea;Euryarchaeota;Methanomicrobia;Methanosarcinales;Methanotrichaceae;Methanothrix;Methanosaeta harundinacea;Methanothrix harundinacea 6Ac |
| Ga0586324 0004239 5405 8590 | 15300000.00 | 25041293.76 | 22347682.59 | 20871500.00 | 18406100.00 | 40097000.00 | 48153600.00 | 63220400.00 | 19043100.00 | 17178700.00 | 17538400.00 | 16875000.00 | hypothetical protein | Bacteria;Candidatus Aminicenantes;unclassified Candidatus Aminicenantes;unclassified Candidatus Aminicenantes;unclassified Candidatus Aminicenantes;unclassified Candidatus Aminicenantes;Aminicenantes bacterium SCGC AAA255-E10;Aminicenantes bacterium SCGC AAA255-E10 (contamination screened) |
| Ga0586324 0004310 1641 2840 | 0.00 | 0.00 | 0.00 | 0.00 | 2914900.00 | 0.00 | 0.00 | 0.00 | 0.00 | 0.00 | 0.00 | 0.00 | hypothetical protein | Bacteria;Bacteroidetes;Bacteroidia;Bacteroidales;Dysgonomonadaceae;Petrimonas;Petrimonas sulfuriphila;Petrimonas sulfuriphila DSM 16547 |
| Ga0586324 0004413 917 4708 | 0.00 | 0.00 | 0.00 | 0.00 | 0.00 | 0.00 | 0.00 | 532767.29 | 0.00 | 0.00 | 0.00 | 0.00 | tetratricopeptide (TPR) repeat protein | Bacteria;Proteobacteria;Alphaproteobacteria;Rhodospirillales;Azospirillaceae;Azospirillum;Azospirillum sp. K2W22B-5;Azospirillum sp. K2W22B-5 |
| Ga0586324 0004512 8742 9896 | 0.00 | 0.00 | 0.00 | 0.00 | 0.00 | 0.00 | 0.00 | 346023.11 | 260589.15 | 0.00 | 0.00 | 0.00 | TRAP-type mannitol/chloroaromatic compound transport system substrate-binding protein | Bacteria;Proteobacteria;Betaproteobacteria;Nitrosomonadales;Thiobacillaceae;Thiobacillus;Thiobacillus denitrificans;Thiobacillus denitrificans RG |
| Ga0586324 0004624 5007 6341 | 4720000.00 | 809200.00 | 0.00 | 14250000.00 | 7292400.00 | 0.00 | 0.00 | 24503700.00 | 1854277.11 | 4196500.00 | 0.00 | 3847500.00 | hypothetical protein | |
| Ga0586324 0004827 6270 7133 | 20620000.00 | 8772000.00 | 24940900.00 | 10545000.00 | 13287000.00 | 7503300.00 | 20337600.00 | 4934900.00 | 16864200.00 | 9933000.00 | 13024000.00 | 8100000.00 | nucleotide-binding universal stress UspA family protein | Archaea;Euryarchaeota;Methanomicrobia;Methanosarcinales;Methanotrichaceae;Methanothrix;Methanothrix soehngenii;Methanothrix soehngenii GP-6 |
| Ga0586324 0004862 5664 9053 | 2436466.00 | 8976000.00 | 11654100.00 | 4018500.00 | 4047900.00 | 979248.14 | 14365769.28 | 8570500.00 | 25668900.00 | 3834600.00 | 11792000.00 | 12720000.00 | hypothetical protein | Bacteria;Acidobacteria;Acidobacteriia;Acidobacteriales;Acidobacteriaceae;Acidipila;Acidipila rosea;Acidipila rosea DSM 103428 |
| Ga0586324 0004903 12667 13812 | 0.00 | 25500000.00 | 0.00 | 0.00 | 0.00 | 0.00 | 0.00 | 0.00 | 0.00 | 0.00 | 0.00 | 0.00 | glutamine synthetase type I | Bacteria;Proteobacteria;Betaproteobacteria;Nitrosomonadales;Sterolibacteriaceae;Sulfurisoma;Sulfurisoma sediminicola;Sulfurisoma sediminicola DSM 26916 |
| Ga0586324 0004993 11044 11217 | 0.00 | 0.00 | 0.00 | 0.00 | 0.00 | 0.00 | 0.00 | 0.00 | 0.00 | 0.00 | 0.00 | 388565.25 | hypothetical protein | Bacteria;Proteobacteria;Deltaproteobacteria;Desulfobacterales;Desulfobacteraceae;Desulfospira;Desulfospira joergensenii;Desulfospira joergensenii DSM 10085 |
| Ga0586324 0005005 11575 13677 | 1210000.00 | 1502800.00 | 1898272.73 | 2973500.00 | 2451400.00 | 0.00 | 0.00 | 1043100.00 | 513943.38 | 3049200.00 | 2182400.00 | 2235000.00 | hypothetical protein | Bacteria;Acidobacteria;Vicinamibacteria;unclassified Vicinamibacteria;Vicinamibacteraceae;Luteitalea;Luteitalea pratensis;Luteitalea pratensis DSM 100886 |
| Ga0586324 0005041 6464 6919 | 0.00 | 0.00 | 2404150.75 | 0.00 | 0.00 | 0.00 | 0.00 | 0.00 | 0.00 | 0.00 | 0.00 | 1320000.00 | rubrerythrin | Archaea;Euryarchaeota;Methanomicrobia;Methanomicrobiales;Methanomicrobiaceae;Methanoculleus;Methanoculleus sp. MAB1;Methanoculleus sp. MAB1 |
| Ga0586324 0005067 5451 6158 | 0.00 | 0.00 | 0.00 | 5415000.00 | 1658300.00 | 1740749.72 | 6748800.00 | 5673000.00 | 1749600.00 | 0.00 | 0.00 | 1755000.00 | outer membrane protein OmpA-like peptidoglycan-associated protein | Bacteria;Candidatus Aminicenantes;unclassified Candidatus Aminicenantes;unclassified Candidatus Aminicenantes;unclassified Candidatus Aminicenantes;unclassified Candidatus Aminicenantes;Aminicenantes bacterium SCGC AAA252-D06;Aminicenantes bacterium SCGC AAA252-D06 (contamination screened) |
| Ga0586324 0005091 4727 9214 | 0.00 | 5242800.00 | 851227.85 | 0.00 | 0.00 | 0.00 | 0.00 | 0.00 | 0.00 | 0.00 | 0.00 | 0.00 | FtsP/CotA-like multicopper oxidase with cupredoxin domain | Bacteria;Proteobacteria;Deltaproteobacteria;Desulfuromonadales;Geobacteraceae;Citrifermentans;Citrifermentans pelophilum;Geobacter pelophilus Dfr2 |
| Ga0586324 0005195 340 2202 | 0.00 | 0.00 | 0.00 | 0.00 | 0.00 | 0.00 | 0.00 | 0.00 | 1053000.00 | 0.00 | 0.00 | 0.00 | hypothetical protein | Bacteria;Ignavibacteriae;Ignavibacteria;Ignavibacteriales;Ignavibacteriaceae;Ignavibacterium;Ignavibacterium album;Ignavibacterium album Mat9-16, JCM 16511 |
| Ga0586324 0005239 8665 10479 | 87453.40 | 0.00 | 2892840.38 | 6697500.00 | 0.00 | 5835900.00 | 0.00 | 0.00 | 1611900.00 | 0.00 | 4109600.00 | 0.00 | uncharacterized LabA/DUF88 family protein | Bacteria;Cyanobacteria;unclassified Cyanobacteria;Pleurocapsales;Hyellaceae;Pleurocapsa;Pleurocapsa sp. PCC 7327;Pleurocapsa minor PCC 7327 |
| Ga0586324 0005320 3 4595 | 1990000.00 | 0.00 | 0.00 | 0.00 | 0.00 | 0.00 | 0.00 | 0.00 | 0.00 | 0.00 | 844108.32 | 0.00 | hypothetical protein | Bacteria;Actinobacteria;Actinomycetia;Micrococcales;Cellulomonadaceae;Cellulomonas;Cellulomonas sp. KRMCY2;Cellulomonas sp. KRMCY2 |
| Ga0586324 0005365 5332 6993 | 5100000.00 | 0.00 | 0.00 | 3097000.00 | 0.00 | 0.00 | 0.00 | 0.00 | 0.00 | 0.00 | 0.00 | 0.00 | hypothetical protein | Bacteria;Proteobacteria;Deltaproteobacteria;Desulfuromonadales;Geobacteraceae;Geoalkalibacter;Geoalkalibacter ferrihydriticus;Geoalkalibacter ferrihydriticus DSM 17813 |
| Ga0586324 0005367 529 3387 | 139378.00 | 2253386.72 | 0.00 | 0.00 | 0.00 | 0.00 | 0.00 | 0.00 | 0.00 | 0.00 | 0.00 | 0.00 | Zn-dependent metalloprotease | Bacteria;Proteobacteria;Betaproteobacteria;Burkholderiales;unclassified Burkholderiales;Paucibacter;Paucibacter sp. KCTC 42545;Paucibacter sp. KCTC 42545 |
| Ga0586324 0005444 5998 8826 | 0.00 | 0.00 | 0.00 | 0.00 | 0.00 | 0.00 | 0.00 | 4648200.00 | 0.00 | 0.00 | 0.00 | 0.00 | CHAT domain-containing protein/tetratricopeptide (TPR) repeat protein | Bacteria;Cyanobacteria;unclassified Cyanobacteria;Oscillatoriales;Desertifilaceae;Desertifilum;Desertifilum sp. IPPAS B-1220;Desertifilum sp. IPPAS B-1220 |
| Ga0586324 0005545 10658 12445 | 0.00 | 0.00 | 0.00 | 0.00 | 0.00 | 5359500.00 | 0.00 | 0.00 | 7889400.00 | 0.00 | 0.00 | 0.00 | formyltetrahydrofolate synthetase | Bacteria;Proteobacteria;Deltaproteobacteria;Syntrophobacterales;Syntrophaceae;Desulfobacca;Desulfobacca acetoxidans;Desulfobacca acetoxidans ASRB2, DSM 11109 |
| Ga0586324 0005580 11752 13101 | 71400000.00 | 64817600.00 | 0.00 | 30685000.00 | 14028600.00 | 0.00 | 0.00 | 1512800.00 | 154099.26 | 87164000.00 | 24824800.00 | 9795000.00 | hypothetical protein | Bacteria;Proteobacteria;Gammaproteobacteria;Legionellales;Coxiellaceae;Rickettsiella;Rickettsiella grylli;Rickettsiella grylli |
| Ga0586324 0005658 2976 5114 | 0.00 | 0.00 | 0.00 | 0.00 | 0.00 | 0.00 | 0.00 | 811300.00 | 599958.90 | 0.00 | 0.00 | 0.00 | hypothetical protein | Bacteria;Candidatus Marinimicrobia;unclassified Candidatus Marinimicrobia;unclassified Candidatus Marinimicrobia;unclassified Candidatus Marinimicrobia;unclassified Candidatus Marinimicrobia;Marinimicrobia bacterium JGI 0000059-E23;Marinimicrobia bacterium JGI 0000059-E23 (contamination screened) |
| Ga0586324 0005662 10776 11555 | 0.00 | 0.00 | 0.00 | 0.00 | 0.00 | 0.00 | 0.00 | 0.00 | 0.00 | 0.00 | 1249600.00 | 0.00 | regulator of protease activity HflC (stomatin/prohibitin superfamily) | Bacteria;Proteobacteria;Betaproteobacteria;Rhodocyclales;Zoogloeaceae;Uliginosibacterium;Uliginosibacterium gangwonense;Uliginosibacterium gangwonense DSM 18521 |
| Ga0586324 0005736 5498 6022 | 0.00 | 0.00 | 0.00 | 0.00 | 0.00 | 0.00 | 0.00 | 7356600.00 | 7533000.00 | 3557400.00 | 5332800.00 | 5047500.00 | hypothetical protein | |
| Ga0586324 0005790 8714 9481 | 0.00 | 0.00 | 0.00 | 0.00 | 0.00 | 0.00 | 0.00 | 0.00 | 0.00 | 0.00 | 266057.44 | 0.00 | regulator of protease activity HflC (stomatin/prohibitin superfamily) | Bacteria;Proteobacteria;Betaproteobacteria;Nitrosomonadales;Thiobacillaceae;Thiobacillus;Thiobacillus denitrificans;Thiobacillus denitrificans RG |
| Ga0586324 0005796 9692 10321 | 0.00 | 0.00 | 0.00 | 0.00 | 0.00 | 0.00 | 6634800.00 | 0.00 | 560579.94 | 0.00 | 0.00 | 0.00 | uncharacterized protein YjgD (DUF1641 family) | Bacteria;Proteobacteria;Betaproteobacteria;Ferrovales;Ferrovaceae;Ferrovum;Ferrovum myxofaciens;Ferrovum myxofaciens P3G |
| Ga0586324 0005798 10334 11242 | 0.00 | 1421200.00 | 0.00 | 0.00 | 0.00 | 0.00 | 0.00 | 0.00 | 0.00 | 0.00 | 0.00 | 0.00 | regulator of protease activity HflC (stomatin/prohibitin superfamily) | Bacteria;Chloroflexi;Anaerolineae;Anaerolineales;Anaerolineaceae;Anaerolinea;Anaerolinea thermophila;Anaerolinea thermophila UNI-1 |
| Ga0586324 0005809 4041 7187 | 0.00 | 0.00 | 0.00 | 0.00 | 0.00 | 0.00 | 0.00 | 0.00 | 1012500.00 | 0.00 | 0.00 | 0.00 | hypothetical protein | Bacteria;Acidobacteria;Acidobacteriia;Bryobacterales;Solibacteraceae;Candidatus Solibacter;Candidatus Solibacter usitatus;Candidatus Solibacter usitatus Ellin6076 |
| Ga0586324 0005933 4185 5228 | 0.00 | 3950800.00 | 0.00 | 0.00 | 0.00 | 45615300.00 | 90647004.24 | 125672200.00 | 78667200.00 | 39578000.00 | 29832000.00 | 46890000.00 | predicted porin | Bacteria;Proteobacteria;Betaproteobacteria;Nitrosomonadales;Thiobacillaceae;Thiobacillus;Thiobacillus denitrificans;Thiobacillus denitrificans ATCC 25259 |
| Ga0586324 0006063 1387 2562 | 0.00 | 0.00 | 0.00 | 0.00 | 1091800.00 | 0.00 | 0.00 | 0.00 | 1247400.00 | 0.00 | 0.00 | 0.00 | TRAP-type mannitol/chloroaromatic compound transport system substrate-binding protein | Bacteria;Proteobacteria;Gammaproteobacteria;Acidiferrobacterales;Acidiferrobacteraceae;Sulfurifustis;Sulfurifustis variabilis;Sulfurifustis variabilis skN76 |
| Ga0586324 0006090 3062 4360 | 31410000.00 | 30858400.00 | 36482400.00 | 19323000.00 | 18406100.00 | 19135400.00 | 12722400.00 | 6161000.00 | 9379800.00 | 29429400.00 | 25264800.00 | 20167500.00 | hypothetical protein | Bacteria;Bacteroidetes;Flavobacteriia;Flavobacteriales;Flavobacteriaceae;Aquimarina;Aquimarina sp. Aq78;Aquimarina sp. Aq78 |
| Ga0586324 0006209 2210 4612 | 2430000.00 | 1591200.00 | 2154009.85 | 4009000.00 | 4161200.00 | 2251188.50 | 0.00 | 3580700.00 | 3134700.00 | 0.00 | 1012000.00 | 2197500.00 | hypothetical protein | Bacteria;Bacteroidetes;Flavobacteriia;Flavobacteriales;Flavobacteriaceae;Aequorivita;Aequorivita sp. CIP111184;Aequorivita sp. CIP 111184 |
| Ga0586324 0006255 5484 6290 | 0.00 | 0.00 | 0.00 | 3448500.00 | 0.00 | 0.00 | 0.00 | 0.00 | 0.00 | 0.00 | 0.00 | 0.00 | hypothetical protein | |

| | | | | | | | | | | | | | | |
|---|---|---|---|---|---|---|---|---|---|---|---|---|---|---|
| Ga0586324 0006265 6134 6814 | 8810000.00 | 1992400.00 | 2449770.64 | 0.00 | 0.00 | 10838100.00 | 1610507.64 | 713700.00 | 790541.37 | 0.00 | 0.00 | 1365000.00 | hypothetical protein | Bacteria;Firmicutes;Clostridia;Eubacteriales;Peptostreptococcaceae;Sporacetigenium;Sporacetigenium mesophilum;Sporacetigenium mesophilum DSM 16796 |
| Ga0586324 0006300 1360 4665 | 3960000.00 | 1169600.00 | 0.00 | 0.00 | 0.00 | 7007867.82 | 1021709.04 | 1537200.00 | 0.00 | 0.00 | 0.00 | 0.00 | hypothetical protein | Bacteria;Bacteroidetes;Flavobacteriia;Flavobacteriales;Flavobacteriaceae;Flavobacterium;Flavobacterium notoginsengisoli;Flavobacterium notoginsengisoli S00128 |
| Ga0586324 0006460 10446 11003 | 0.00 | 2794800.00 | 6699700.00 | 0.00 | 0.00 | 0.00 | 0.00 | 0.00 | 0.00 | 0.00 | 0.00 | 0.00 | hypothetical protein | |
| Ga0586324 0006477 9306 9827 | 0.00 | 0.00 | 0.00 | 0.00 | 0.00 | 0.00 | 0.00 | 0.00 | 0.00 | 0.00 | 3537600.00 | 0.00 | uncharacterized protein (DUF302 family) | Bacteria;Proteobacteria;Betaproteobacteria;Nitrosomonadales;Thiobacillaceae;Thiobacillus;Thiobacillus denitrificans;Thiobacillus denitrificans RG |
| Ga0586324 0006581 6861 8783 | 0.00 | 0.00 | 0.00 | 0.00 | 1936400.00 | 0.00 | 0.00 | 0.00 | 0.00 | 0.00 | 0.00 | 0.00 | hypothetical protein | Bacteria;Proteobacteria;Zetaproteobacteria;unclassified Zetaproteobacteria;unclassified Zetaproteobacteria;unclassified Zetaproteobacteria;unclassified Zetaproteobacteria;Unclassified zetaproteobacterium sp. KV |
| Ga0586324 0006694 1 588 | 22500000.00 | 29308000.00 | 70375000.00 | 0.00 | 30797000.00 | 27353300.00 | 17008800.00 | 16714000.00 | 22437000.00 | 39963000.00 | 37136000.00 | 33900000.00 | hypothetical protein | Bacteria;Proteobacteria;Betaproteobacteria;Nitrosomonadales;Sulfuricellaceae;Sulfurirhabdus;Sulfurirhabdus autotrophica;Sulfurirhabdus autotrophica DSM 100309 |
| Ga0586324 0006694 2688 5267 | 0.00 | 3712800.00 | 0.00 | 0.00 | 0.00 | 1681874.62 | 3328800.00 | 0.00 | 0.00 | 0.00 | 0.00 | 0.00 | hypothetical protein | Bacteria;Proteobacteria;Gammaproteobacteria;Chromatiales;Ectothiorhodospiraceae;Thiogranum;Thiogranum longum;Thiogranum longum DSM 19610 |
| Ga0586324 0006769 6305 9643 | 18820000.00 | 17040800.00 | 13910829.20 | 26144000.00 | 24359500.00 | 20742833.15 | 13292400.00 | 11809600.00 | 15398100.00 | 17579100.00 | 17036800.00 | 14235000.00 | hypothetical protein | Bacteria;Candidatus Aminicenantes;unclassified Candidatus Aminicenantes;unclassified Candidatus Aminicenantes;unclassified Candidatus Aminicenantes;unclassified Candidatus Aminicenantes;Aminicenantes bacterium SCGC AAA252-N07;Aminicenantes bacterium SCGC AAA252-N07 (contamination screened) |
| Ga0586324 0006808 3133 6237 | 0.00 | 0.00 | 2715692.43 | 3268000.00 | 0.00 | 0.00 | 0.00 | 0.00 | 0.00 | 0.00 | 0.00 | 0.00 | hypothetical protein | Bacteria;Bacteroidetes;Chitinophagia;Chitinophagales;Chitinophagaceae;unclassified Chitinophagaceae;Chitinophagaceae bacterium;Chitinophagaceae sp. OAS944 |
| Ga0586324 0006808 9560 11080 | 14900000.00 | 13192000.00 | 18973100.00 | 26790000.00 | 25750000.00 | 6749000.00 | 4947600.00 | 62220000.00 | 12936097.71 | 11704000.00 | 7761600.00 | 35475000.00 | hypothetical protein | Archaea;Euryarchaeota;Methanomicrobia;Methanosarcinales;Methanotrichaceae;Methanothrix;Methanosaeta harundinacea;Methanothrix harundinacea 6Ac |
| Ga0586324 0006897 5865 9974 | 1220000.00 | 0.00 | 0.00 | 0.00 | 0.00 | 0.00 | 0.00 | 0.00 | 0.00 | 0.00 | 0.00 | 0.00 | hypothetical protein | Bacteria;Acidobacteria;Acidobacteriia;Bryobacterales;Solibacteraceae;Candidatus Solibacter;Candidatus Solibacter usitatus;Candidatus Solibacter usitatus Ellin6076 |
| Ga0586324 0006955 7049 8248 | 0.00 | 0.00 | 0.00 | 0.00 | 0.00 | 0.00 | 0.00 | 6087800.00 | 3693600.00 | 3668537.18 | 1628000.00 | 0.00 | hypothetical protein | Bacteria;Proteobacteria;Betaproteobacteria;Nitrosomonadales;Thiobacillaceae;Thiobacillus;Thiobacillus thioparus;Thiobacillus thioparus DSM 505 |
| Ga0586324 0007242 541 4620 | 26323029.00 | 16979600.00 | 30394609.50 | 26372000.00 | 24771500.00 | 26202000.00 | 0.00 | 8893800.00 | 8991000.00 | 9963800.00 | 17512000.00 | 12508731.00 | hypothetical protein | Bacteria;Acidobacteria;Blastocatellia;Blastocatellales;Pyrinomonadaceae;Pyrinomonas;Pyrinomonas methylaliphatogenes;Pyrinomonas methylaliphatogenes K22 |
| Ga0586324 0007443 6044 11257 | 20600000.00 | 1060800.00 | 0.00 | 0.00 | 0.00 | 24415500.00 | 0.00 | 5996300.00 | 7452000.00 | 0.00 | 0.00 | 14775000.00 | PKD repeat protein/FtsP/CotA-like multicopper oxidase with cupredoxin domain | Archaea;Euryarchaeota;Methanomicrobia;Methanomicrobiales;Methanoregulaceae;Methanosphaerula;Methanosphaerula palustris;Methanosphaerula palustris E1-9c, DSM 19958 |
| Ga0586324 0007494 7255 8847 | 0.00 | 958800.00 | 0.00 | 0.00 | 0.00 | 0.00 | 0.00 | 0.00 | 0.00 | 0.00 | 0.00 | 0.00 | arylsulfatase A-like enzyme | Bacteria;Proteobacteria;Alphaproteobacteria;Hyphomicrobiales;Bradyrhizobiaceae;Rhodopseudomonas;Rhodopseudomonas palustris;Rhodopseudomonas palustris 7850, DSM 127 |
| Ga0586324 0007536 9285 10085 | 0.00 | 0.00 | 0.00 | 0.00 | 0.00 | 5161000.00 | 0.00 | 4111400.00 | 0.00 | 0.00 | 0.00 | 0.00 | enoyl-CoA hydratase/carnithine racemase | Bacteria;Proteobacteria;Deltaproteobacteria;Syntrophobacterales;Syntrophaceae;Smithella;Smithella sp. F21;Smithella sp. F21 |
| Ga0586324 0007575 1989 9734 | 0.00 | 2461600.00 | 0.00 | 710547.75 | 1380200.00 | 0.00 | 0.00 | 0.00 | 0.00 | 0.00 | 0.00 | 0.00 | hypothetical protein | Bacteria;Balneolaeota;Balneolia;Balneolales;Balneolaceae;Gracilimonas;Gracilimonas mengyeensis;Gracilimonas mengyeensis DSM 21985 |
| Ga0586324 0007617 6088 9705 | 9470000.00 | 5984000.00 | 15898489.44 | 19693500.00 | 0.00 | 12111918.17 | 1757574.48 | 0.00 | 492995.97 | 3749900.00 | 1962400.00 | 8655000.00 | hypothetical protein | Bacteria;Acidobacteria;Vicinamibacteria;unclassified Vicinamibacteria;Vicinamibacteraceae;Luteitalea;Luteitalea pratensis;Luteitalea pratensis DSM 100886 |
| Ga0586324 0007623 8657 10624 | 2610000.00 | 0.00 | 0.00 | 0.00 | 0.00 | 0.00 | 0.00 | 0.00 | 0.00 | 0.00 | 0.00 | 0.00 | NADPH-dependent glutamate synthase beta subunit-like oxidoreductase/Pyruvate/2-oxoacid:fer | Bacteria;Proteobacteria;Betaproteobacteria;Nitrosomonadales;Gallionellaceae;Sideroxydans;Sideroxydans lithotrophicus;Sideroxydans lithotrophicus ES-1 |
| Ga0586324 0007763 9990 11060 | 96410000.00 | 129914000.00 | 1139450.07 | 57669487.80 | 74057000.00 | 3156959.88 | 9439200.00 | 72211800.00 | 15946275.60 | 40053476.54 | 73664800.00 | 8527500.00 | predicted porin | Bacteria;Proteobacteria;Betaproteobacteria;Nitrosomonadales;Sulfuricellaceae;Sulfuricella;Sulfuricella denitrificans;Sulfuricella denitrificans skB26 |
| Ga0586324 0007801 7031 8065 | 6190000.00 | 8908000.00 | 6981200.00 | 5168000.00 | 0.00 | 0.00 | 0.00 | 0.00 | 0.00 | 4173400.00 | 3115200.00 | 5010000.00 | hypothetical protein | Bacteria;Nitrospirae;Nitrospira;Nitrospirales;Nitrospiraceae;Nitrospira;Candidatus Nitrospira inopinata;Candidatus Nitrospira inopinata ENR4 |
| Ga0586324 0007825 9691 10887 | 0.00 | 0.00 | 0.00 | 0.00 | 0.00 | 0.00 | 0.00 | 0.00 | 256275.09 | 0.00 | 0.00 | 1896031.50 | DNA-binding beta-propeller fold protein YncE | Bacteria;Proteobacteria;Gammaproteobacteria;Chromatiales;Ectothiorhodospiraceae;Inmirania;Inmirania thermothiophila;Inmirania thermothiophila DSM 100275 |
| Ga0586324 0007884 8826 9149 | 2970000.00 | 0.00 | 0.00 | 0.00 | 0.00 | 0.00 | 0.00 | 3214700.00 | 0.00 | 0.00 | 1504800.00 | 0.00 | transcriptional regulator with XRE-family HTH domain | Bacteria;Proteobacteria;Betaproteobacteria;unclassified Betaproteobacteria;unclassified Betaproteobacteria;unclassified Betaproteobacteria;beta proteobacterium L13;beta proteobacterium L13 |
| Ga0586324 0007889 7006 9561 | 0.00 | 1162800.00 | 0.00 | 0.00 | 0.00 | 1155647.15 | 0.00 | 0.00 | 0.00 | 0.00 | 0.00 | 0.00 | hypothetical protein | Bacteria;Ignavibacteriae;Ignavibacteria;Ignavibacteriales;Melioribacteraceae;Melioribacter;Melioribacter roseus;Melioribacter roseus P3M |
| Ga0586324 0007921 363 4004 | 0.00 | 629130.56 | 0.00 | 0.00 | 0.00 | 0.00 | 0.00 | 0.00 | 0.00 | 0.00 | 0.00 | 0.00 | hypothetical protein | Bacteria;Balneolaeota;Balneolia;Balneolales;Balneolaceae;Gracilimonas;Gracilimonas tropica;Gracilimonas tropica DSM 19535 |
| Ga0586324 0007959 2226 4229 | 2290000.00 | 0.00 | 0.00 | 0.00 | 0.00 | 0.00 | 0.00 | 1213900.00 | 1263600.00 | 0.00 | 2868800.00 | 715947.75 | ABC-type transport system substrate-binding protein | Bacteria;Chloroflexi;Anaerolineae;Anaerolineales;Anaerolineaceae;Pelolinea;Pelolinea submarina;Pelolinea submarina DSM 23923 |
| Ga0586324 0007994 3903 5003 | 8950000.00 | 11424000.00 | 0.00 | 0.00 | 0.00 | 0.00 | 0.00 | 0.00 | 0.00 | 0.00 | 0.00 | 7650000.00 | hypothetical protein | Bacteria;Proteobacteria;Alphaproteobacteria;Hyphomicrobiales;Bradyrhizobiaceae;Bradyrhizobium;Bradyrhizobium sp. LTSP885;Bradyrhizobium sp. LTSP885 |
| Ga0586324 0008126 5651 8932 | 0.00 | 0.00 | 0.00 | 0.00 | 0.00 | 0.00 | 0.00 | 0.00 | 0.00 | 0.00 | 0.00 | 15997500.00 | hypothetical protein | Bacteria;Acidobacteria;Acidobacteriia;Acidobacteriales;Acidobacteriaceae;unclassified Acidobacteriaceae;Acidobacteriaceae bacterium KBS 96;Acidobacteriaceae bacterium KBS 96, DSM 24297 |
| Ga0586324 0008226 3061 4953 | 2960000.00 | 6140400.00 | 1229687.71 | 3277500.00 | 6262400.00 | 0.00 | 0.00 | 646600.00 | 4471200.00 | 2695000.00 | 0.00 | 990000.00 | hypothetical protein | Bacteria;Firmicutes;Clostridia;Eubacteriales;Eubacteriales Family XII. Incertae Sedis;Acidaminobacter;Acidaminobacter hydrogenoformans;Acidaminobacter hydrogenoformans DSM 2784 |
| Ga0586324 0008231 2552 2866 | 0.00 | 0.00 | 0.00 | 0.00 | 5108800.00 | 0.00 | 0.00 | 0.00 | 0.00 | 0.00 | 0.00 | 0.00 | sugar (pentulose or hexulose) kinase | Bacteria;Proteobacteria;Deltaproteobacteria;Syntrophobacterales;Syntrophobacteraceae;Desulfacinum;Desulfacinum infernum;Desulfacinum infernum DSM 9756 |
| Ga0586324 0008387 6422 8893 | 0.00 | 0.00 | 0.00 | 0.00 | 1596500.00 | 0.00 | 0.00 | 0.00 | 0.00 | 0.00 | 0.00 | 0.00 | type IV pilus secretin PilQ/predicted competence protein | Bacteria;Candidatus Aminicenantes;unclassified Candidatus Aminicenantes;unclassified Candidatus Aminicenantes;unclassified Candidatus Aminicenantes;unclassified Candidatus Aminicenantes;Aminicenantes bacterium SCGC AAA252-K07;Aminicenantes bacterium SCGC AAA252-K07 (contamination screened) |
| Ga0586324 0008773 3 608 | 0.00 | 3488400.00 | 0.00 | 0.00 | 0.00 | 0.00 | 0.00 | 0.00 | 0.00 | 0.00 | 0.00 | 0.00 | regulator of protease activity HflC (stomatin/prohibitin superfamily) | Bacteria;Bacteroidetes;Bacteroidia;Bacteroidales;Porphyromonadaceae;Microbacter;Microbacter margulisiae;Microbacter margulisiae DSM 27471 |
| Ga0586324 0008808 1 10791 | 0.00 | 0.00 | 0.00 | 0.00 | 0.00 | 0.00 | 0.00 | 0.00 | 0.00 | 0.00 | 0.00 | 157896.00 | hypothetical protein | Bacteria;Cyanobacteria;unclassified Cyanobacteria;Nostocales;Nostocaceae;Nostoc;Nostoc sp. PCC 7524;Nostoc sp. PCC 7524 |
| Ga0586324 0009038 3948 4610 | 0.00 | 0.00 | 0.00 | 2014000.00 | 0.00 | 0.00 | 0.00 | 0.00 | 0.00 | 0.00 | 0.00 | 0.00 | hypothetical protein | Bacteria;Proteobacteria;Alphaproteobacteria;Hyphomicrobiales;Afifellaceae;Afifella;Afifella marina;Afifella marina DSM 2698 |
| Ga0586324 0009076 4828 6243 | 0.00 | 0.00 | 0.00 | 0.00 | 0.00 | 0.00 | 2986800.00 | 1055300.00 | 0.00 | 0.00 | 0.00 | 2340000.00 | glutamine synthetase type I | Bacteria;Proteobacteria;Betaproteobacteria;Rhodocyclales;Rhodocyclaceae;Rugosibacter;Rugosibacter aromaticivorans;Rugosibacter aromaticivorans PG1-Ca6 |
| Ga0586324 0009115 3012 4133 | 0.00 | 0.00 | 0.00 | 0.00 | 0.00 | 230260000.00 | 0.00 | 0.00 | 0.00 | 0.00 | 0.00 | 0.00 | hypothetical protein | Bacteria;Ignavibacteriae;Ignavibacteria;Ignavibacteriales;Melioribacteraceae;Melioribacter;Melioribacter roseus;Melioribacter roseus P3M |
| Ga0586324 0009115 4198 8118 | 5140000.00 | 5752800.00 | 6354772.42 | 3448500.00 | 4954300.00 | 5002200.00 | 1344082.80 | 6893000.00 | 5346000.00 | 5698000.00 | 7312800.00 | 5010000.00 | hypothetical protein | Bacteria;Candidatus Marinimicrobia;unclassified Candidatus Marinimicrobia;unclassified Candidatus Marinimicrobia;unclassified Candidatus Marinimicrobia;unclassified Candidatus Marinimicrobia;Marinimicrobia bacterium JGI 0000059-D20;Marinimicrobia bacterium JGI 0000059-D20 (contamination screened) |
| Ga0586324 0009115 8197 10617 | 8280000.00 | 11634800.00 | 0.00 | 11580500.00 | 11556600.00 | 5768799.06 | 1448969.64 | 9747800.00 | 7848900.00 | 22484000.00 | 13754400.00 | 15337500.00 | hypothetical protein | Bacteria;Candidatus Marinimicrobia;unclassified Candidatus Marinimicrobia;unclassified Candidatus Marinimicrobia;unclassified Candidatus Marinimicrobia;unclassified Candidatus Marinimicrobia;Marinimicrobia bacterium SCGC AAA257-N23;Marinimicrobia bacterium SCGC AAA257-N23 (contamination screened) |
| Ga0586324 0009134 7979 10024 | 0.00 | 0.00 | 0.00 | 0.00 | 0.00 | 0.00 | 0.00 | 0.00 | 1417500.00 | 0.00 | 0.00 | 0.00 | alkyl sulfatase BDS1-like metallo-beta-lactamase superfamily hydrolase | Bacteria;Proteobacteria;Betaproteobacteria;Burkholderiales;Burkholderiaceae;Burkholderia;Burkholderia sp. WP9;Burkholderia sp. WP9 |
| Ga0586324 0009137 61 6249 | 6890000.00 | 0.00 | 0.00 | 4911500.00 | 0.00 | 0.00 | 0.00 | 2372900.00 | 0.00 | 0.00 | 5693600.00 | 0.00 | uncharacterized repeat protein (TIGR01451 family) | Bacteria;Actinobacteria;Actinomycetia;Micrococcales;Cellulomonadaceae;Cellulomonas;Cellulomonas sp. KRMCY2;Cellulomonas sp. KRMCY2 |
| Ga0586324 0009386 2 3211 | 0.00 | 656281.60 | 0.00 | 0.00 | 0.00 | 0.00 | 0.00 | 0.00 | 0.00 | 0.00 | 0.00 | 0.00 | DNA-directed RNA polymerase beta' subunit | Bacteria;Nitrospirae;Thermodesulfovibrionia;Thermodesulfovibrionales;Thermodesulfovibrionaceae;Thermodesulfovibrio;Thermodesulfovibrio sp. N1;Thermodesulfovibrio sp. N1 |
| Ga0586324 0009412 4878 6146 | 7630000.00 | 11050000.00 | 0.00 | 0.00 | 0.00 | 14014100.00 | 4058400.00 | 14518000.00 | 18257400.00 | 0.00 | 0.00 | 7500000.00 | predicted porin | Bacteria;Proteobacteria;Betaproteobacteria;Neisseriales;Chromobacteriaceae;Laribacter;Laribacter hongkongensis;Laribacter hongkongensis HLHK9 |
| Ga0586324 0009413 3602 4123 | 4590000.00 | 8160000.00 | 8895400.00 | 18895500.00 | 23102900.00 | 22721092.09 | 13383600.00 | 11400900.00 | 8667000.00 | 0.00 | 0.00 | 0.00 | uncharacterized membrane protein | Archaea;Euryarchaeota;Methanomicrobia;Methanomicrobiales;Methanomicrobiaceae;Methanoculleus;Methanoculleus thermophilus;Methanoculleus thermophilus DSM 2373 |
| Ga0586324 0009414 4066 4740 | 0.00 | 0.00 | 0.00 | 0.00 | 0.00 | 0.00 | 0.00 | 8601000.00 | 0.00 | 0.00 | 0.00 | 0.00 | hypothetical protein | |
| Ga0586324 0009578 1958 3229 | 0.00 | 1496000.00 | 0.00 | 0.00 | 0.00 | 0.00 | 0.00 | 0.00 | 0.00 | 10857000.00 | 0.00 | 0.00 | hypothetical protein | |
| Ga0586324 0009598 6294 7604 | 0.00 | 0.00 | 0.00 | 0.00 | 0.00 | 0.00 | 0.00 | 308439.18 | 0.00 | 0.00 | 0.00 | 0.00 | hypothetical protein | Bacteria;Proteobacteria;Alphaproteobacteria;Hyphomonadales;Hyphomonadaceae;Hyphomonas;Hyphomonas oceanitis;Hyphomonas oceanitis SCH89 |
| Ga0586324 0009663 9145 9996 | 0.00 | 1462000.00 | 0.00 | 0.00 | 0.00 | 0.00 | 0.00 | 0.00 | 0.00 | 0.00 | 0.00 | 0.00 | glyoxylase-like metal-dependent hydrolase (beta-lactamase superfamily II) | Bacteria;Firmicutes;Clostridia;Eubacteriales;Oscillospiraceae;Sporobacter;Sporobacter termitidis;Sporobacter termitidis DSM 10068 |
| Ga0586324 0009727 3523 6897 | 1390000.00 | 1353200.00 | 5555498.21 | 4265500.00 | 6590735.16 | 8535500.00 | 0.00 | 7747000.00 | 2219400.00 | 954331.84 | 3199978.32 | 8820000.00 | outer membrane receptor protein involved in Fe transport | Bacteria;Acidobacteria;Acidobacteriia;Acidobacteriales;Acidobacteriaceae;Acidipila;Acidipila rosea;Acidipila rosea DSM 103428 |
| Ga0586324 0009852 1464 4643 | 39770000.00 | 27251647.36 | 8601919.36 | 2175500.00 | 0.00 | 4287600.00 | 0.00 | 2019100.00 | 8675100.00 | 8404998.91 | 10216800.00 | 10710000.00 | hypothetical protein | Bacteria;Candidatus Aminicenantes;unclassified Candidatus Aminicenantes;unclassified Candidatus Aminicenantes;unclassified Candidatus Aminicenantes;unclassified Candidatus Aminicenantes;Aminicenantes bacterium SCGC AAA252-O03;Aminicenantes bacterium SCGC AAA252-O03 (contamination screened) |
| Ga0586324 0009912 6735 10136 | 156287.00 | 0.00 | 0.00 | 0.00 | 0.00 | 0.00 | 0.00 | 0.00 | 0.00 | 0.00 | 0.00 | 2100000.00 | hypothetical protein | Bacteria;Acidobacteria;Acidobacteriia;Acidobacteriales;Acidobacteriaceae;unclassified Acidobacteriaceae;Acidobacteriaceae bacterium KBS 96;Acidobacteriaceae bacterium KBS 96, DSM 24297 |
| Ga0586324 0009914 8347 10230 | 2260000.00 | 4216000.00 | 0.00 | 556743.70 | 2544100.00 | 25898811.10 | 0.00 | 342320.41 | 1812170.88 | 3257100.00 | 1258400.00 | 420608.25 | hypothetical protein | |
| Ga0586324 0009916 1491 2981 | 4560000.00 | 4175200.00 | 4138950.80 | 3524500.00 | 4017000.00 | 3683691.54 | 0.00 | 2452200.00 | 2025000.00 | 3703700.00 | 3176800.00 | 2482500.00 | hypothetical protein | Bacteria;Chloroflexi;Anaerolineae;Anaerolineales;Anaerolineaceae;Thermanaerothrix;Thermanaerothrix daxensis;Thermanaerothrix daxensis GNS-1 |
| Ga0586324 0010330 4361 5446 | 0.00 | 10744000.00 | 0.00 | 0.00 | 0.00 | 0.00 | 0.00 | 0.00 | 0.00 | 0.00 | 0.00 | 0.00 | glyoxylase-like metal-dependent hydrolase (beta-lactamase superfamily II) | Bacteria;Proteobacteria;Alphaproteobacteria;Rhodospirillales;Azospirillaceae;Skermanella;Skermanella aerolata;Skermanella aerolata KACC 11604 |
| Ga0586324 0010427 4741 7902 | 0.00 | 0.00 | 0.00 | 0.00 | 0.00 | 0.00 | 0.00 | 0.00 | 0.00 | 0.00 | 0.00 | 9600000.00 | hypothetical protein | Bacteria;Candidatus Aminicenantes;unclassified Candidatus Aminicenantes;unclassified Candidatus Aminicenantes;unclassified Candidatus Aminicenantes;unclassified Candidatus Aminicenantes;Aminicenantes bacterium SCGC AAA252-D06;Aminicenantes bacterium SCGC AAA252-D06 (contamination screened) |
| Ga0586324 0010815 3544 6528 | 0.00 | 1706800.00 | 0.00 | 0.00 | 1565600.00 | 0.00 | 0.00 | 0.00 | 0.00 | 6360200.00 | 11176000.00 | 3540000.00 | hypothetical protein | Bacteria;Candidatus Aminicenantes;unclassified Candidatus Aminicenantes;unclassified Candidatus Aminicenantes;unclassified Candidatus Aminicenantes;unclassified Candidatus Aminicenantes;Aminicenantes bacterium SCGC AAA255-E10;Aminicenantes bacterium SCGC AAA255-E10 (contamination screened) |
| Ga0586324 0010840 8516 9841 | 0.00 | 0.00 | 0.00 | 0.00 | 5304500.00 | 0.00 | 0.00 | 0.00 | 0.00 | 0.00 | 0.00 | 0.00 | predicted porin | Bacteria;Proteobacteria;Alphaproteobacteria;Hyphomicrobiales;unclassified Hyphomicrobiales;Methyloligella;Methyloligella halotolerans;Methyloligella halotolerans VKM B-2706 |
| Ga0586324 0010969 4423 5859 | 0.00 | 0.00 | 0.00 | 0.00 | 0.00 | 0.00 | 0.00 | 1183400.00 | 0.00 | 6814500.00 | 5693600.00 | 0.00 | outer membrane protein OmpA-like peptidoglycan-associated protein | Bacteria;Proteobacteria;Betaproteobacteria;Neisseriales;Neisseriaceae;Vitreoscilla;Vitreoscilla massiliensis;Vitreoscilla massiliensis SN6 |
| Ga0586324 0011052 4826 7567 | 0.00 | 0.00 | 0.00 | 0.00 | 0.00 | 0.00 | 0.00 | 0.00 | 0.00 | 0.00 | 1196800.00 | 0.00 | hypothetical protein | Bacteria;Acidobacteria;Vicinamibacteria;unclassified Vicinamibacteria;Vicinamibacteraceae;Luteitalea;Luteitalea pratensis;Luteitalea pratensis DSM 100886 |
| Ga0586324 0011197 2431 3573 | 13440000.00 | 11689200.00 | 0.00 | 9205500.00 | 19570000.00 | 25481278.26 | 482562000.00 | 112911000.00 | 339633000.00 | 52976000.00 | 54621600.00 | 42075000.00 | predicted porin | Bacteria;Proteobacteria;Betaproteobacteria;Nitrosomonadales;Thiobacillaceae;Thiobacillus;Thiobacillus thioparus;Thiobacillus thioparus DSM 505 |
| Ga0586324 0011240 4940 6322 | 0.00 | 0.00 | 0.00 | 0.00 | 12257000.00 | 0.00 | 0.00 | 54567.06 | 99971.82 | 0.00 | 0.00 | 114553.50 | hypothetical protein | Bacteria;Proteobacteria;Deltaproteobacteria;Syntrophobacterales;Syntrophobacteraceae;Desulfosoma;Desulfosoma caldarium;Desulfosoma caldarium DSM 22027 |
| Ga0586324 0011240 6951 8432 | 38420000.00 | 20400000.00 | 0.00 | 5434000.00 | 2657400.00 | 0.00 | 16666800.00 | 46530800.00 | 14312700.00 | 5035800.00 | 3520000.00 | 12150000.00 | hypothetical protein | Bacteria;Proteobacteria;Deltaproteobacteria;Syntrophobacterales;Syntrophobacteraceae;Desulfosoma;Desulfosoma caldarium;Desulfosoma caldarium DSM 22027 |
| Ga0586324 0011286 2842 7263 | 0.00 | 0.00 | 0.00 | 0.00 | 0.00 | 0.00 | 0.00 | 0.00 | 0.00 | 4242700.00 | 0.00 | 0.00 | hypothetical protein | Bacteria;Acidobacteria;Acidobacteriia;Bryobacterales;Solibacteraceae;Candidatus Solibacter;Candidatus Solibacter usitatus;Candidatus Solibacter usitatus Ellin6076 |
| Ga0586324 0011356 4026 5270 | 9450000.00 | 4671600.00 | 0.00 | 1833500.00 | 5005800.00 | 2460224.88 | 8413200.00 | 4971500.00 | 5046300.00 | 3642100.00 | 3484800.00 | 2820000.00 | hypothetical protein | |
| Ga0586324 0011427 2114 5107 | 0.00 | 0.00 | 0.00 | 0.00 | 0.00 | 0.00 | 603636.84 | 0.00 | 1004400.00 | 640060.96 | 776673.92 | 185211.00 | outer membrane receptor protein involved in Fe transport | Bacteria;Ignavibacteriae;Ignavibacteria;Ignavibacteriales;Ignavibacteriaceae;Ignavibacterium;Ignavibacterium album;Ignavibacterium album Mat9-16, JCM 16511 |
| Ga0586324 0011505 7233 8135 | 0.00 | 0.00 | 0.00 | 0.00 | 2636800.00 | 0.00 | 0.00 | 0.00 | 0.00 | 0.00 | 0.00 | 0.00 | hypothetical protein | Bacteria;Firmicutes;Clostridia;Eubacteriales;Syntrophomonadaceae;Dethiobacter;Dethiobacter alkaliphilus;Dethiobacter alkaliphilus AHT1 |
| Ga0586324 0011667 5513 6070 | 0.00 | 0.00 | 0.00 | 7001500.00 | 0.00 | 6868100.00 | 0.00 | 0.00 | 1863000.00 | 0.00 | 0.00 | 0.00 | phasin family protein | Bacteria;Proteobacteria;Betaproteobacteria;Burkholderiales;unclassified Burkholderiales;Paucibacter;Paucibacter sp. KCTC 42545;Paucibacter sp. KCTC 42545 |
| Ga0586324 0011695 4595 7915 | 0.00 | 4195600.00 | 0.00 | 7467000.00 | 0.00 | 0.00 | 0.00 | 0.00 | 0.00 | 0.00 | 4963200.00 | 3637500.00 | uncharacterized repeat protein (TIGR01451 family) | Bacteria;Actinobacteria;Actinomycetia;Micrococcales;Intrasporangiaceae;Humibacillus;Humibacillus xanthopallidus;Humibacillus xanthopallidus SLBN-155 |
| Ga0586324 0011731 13 438 | 0.00 | 1870000.00 | 0.00 | 5082500.00 | 6046100.00 | 0.00 | 2302800.00 | 4751900.00 | 3977100.00 | 0.00 | 0.00 | 0.00 | rubrerythrin | Bacteria;Nitrospirae;Nitrospira;Nitrospirales;Nitrospiraceae;Nitrospira;Candidatus Nitrospira nitrificans;Candidatus Nitrospira nitrificans COMA2 |
| Ga0586324 0012010 6951 8129 | 0.00 | 897600.00 | 0.00 | 0.00 | 6921600.00 | 0.00 | 0.00 | 0.00 | 1652400.00 | 0.00 | 0.00 | 2025000.00 | hypothetical protein | Bacteria;Proteobacteria;Betaproteobacteria;Burkholderiales;Comamonadaceae;Variovorax;Variovorax sp. YR216;Variovorax sp. YR216 |
| Ga0586324 0012254 789 5090 | 43260000.00 | 58350800.00 | 27530700.00 | 32167000.00 | 36441400.00 | 40347173.52 | 20013267.04 | 21441500.00 | 17876700.00 | 33949300.00 | 34971200.00 | 31297500.00 | hypothetical protein | Bacteria;Acidobacteria;Acidobacteriia;Bryobacterales;Solibacteraceae;Candidatus Solibacter;Candidatus Solibacter usitatus;Candidatus Solibacter usitatus Ellin6076 |
| Ga0586324 0012295 3349 7344 | 0.00 | 0.00 | 0.00 | 0.00 | 893024.42 | 0.00 | 0.00 | 0.00 | 603912.51 | 0.00 | 0.00 | 0.00 | hypothetical protein | Bacteria;Cyanobacteria;unclassified Cyanobacteria;Oscillatoriales;Microcoleaceae;Microcoleus;Microcoleus vaginatus;Microcoleus vaginatus FGP-2 |
| Ga0586324 0012331 395 1750 | 10390000.00 | 7956000.00 | 7713100.00 | 8113000.00 | 7292400.00 | 7756379.56 | 13087200.00 | 6161000.00 | 10538100.00 | 12304600.00 | 13807200.00 | 15405000.00 | hypothetical protein | Bacteria;Proteobacteria;Betaproteobacteria;Nitrosomonadales;Gallionellaceae;Sideroxydans;Sideroxydans lithotrophicus;Sideroxydans lithotrophicus ES-1 |
| Ga0586324 0012870 1488 8417 | 0.00 | 2318800.00 | 1788037.33 | 0.00 | 0.00 | 730261.65 | 3784800.00 | 683200.00 | 1166400.00 | 0.00 | 0.00 | 1417500.00 | hypothetical protein | Bacteria;Ignavibacteriae;Ignavibacteria;Ignavibacteriales;Ignavibacteriaceae;Ignavibacterium;Ignavibacterium album;Ignavibacterium album Mat9-16, JCM 16511 |
| Ga0586324 0012926 7744 9015 | 3830000.00 | 0.00 | 0.00 | 0.00 | 0.00 | 0.00 | 0.00 | 0.00 | 0.00 | 0.00 | 0.00 | 0.00 | hypothetical protein | Bacteria;Proteobacteria;Gammaproteobacteria;Methylococcales;Methylococcaceae;Methylobacter;Methylobacter tundripaludum;Methylobacter tundripaludum OWC-G53F |
| Ga0586324 0012993 7805 9064 | 36200000.00 | 5181600.00 | 0.00 | 0.00 | 0.00 | 0.00 | 0.00 | 0.00 | 3126600.00 | 0.00 | 0.00 | 4882500.00 | predicted porin | Bacteria;Proteobacteria;Betaproteobacteria;Burkholderiales;Comamonadaceae;Acidovorax;Acidovorax konjaci;Acidovorax konjaci S00067 |
| Ga0586324 0013020 372 3521 | 2850000.00 | 0.00 | 0.00 | 0.00 | 0.00 | 0.00 | 0.00 | 0.00 | 0.00 | 0.00 | 0.00 | 0.00 | TonB-linked SusC/RagA family outer membrane protein | Bacteria;Bacteroidetes;Cytophagia;Cytophagales;Fulvivirgaceae;Ohtaekwangia;Ohtaekwangia kribbensis;Ohtaekwangia kribbensis DSM 25221 |
| Ga0586324 0013103 1521 9026 | 20950000.00 | 16367600.00 | 12276687.92 | 14582500.00 | 20909000.00 | 12580862.51 | 19020993.48 | 16244300.00 | 16540200.00 | 15569400.00 | 15200248.80 | 13614717.75 | hypothetical protein | Bacteria;Bacteroidetes;Flavobacteriia;Flavobacteriales;Schleiferiaceae;Schleiferia;Schleiferia thermophila;Schleiferia thermophila Yellowstone |
| Ga0586324 0013203 943 3516 | 0.00 | 5521600.00 | 10471800.00 | 4436500.00 | 2049700.00 | 3700333.78 | 11719200.00 | 4148000.00 | 4754700.00 | 7238000.00 | 623284.64 | 5962500.00 | hypothetical protein | Bacteria;Ignavibacteriae;Ignavibacteria;Ignavibacteriales;Ignavibacteriaceae;Ignavibacterium;Ignavibacterium album;Ignavibacterium album Mat9-16, JCM 16511 |
| Ga0586324 0013213 7270 8994 | 0.00 | 1604800.00 | 0.00 | 0.00 | 0.00 | 0.00 | 0.00 | 0.00 | 1198800.00 | 0.00 | 0.00 | 0.00 | hypothetical protein | Bacteria;Proteobacteria;Betaproteobacteria;Burkholderiales;unclassified Burkholderiales;Ideonella;Ideonella sp. A 288;Ideonella sp. A 288 |
| Ga0586324 0013223 5069 6484 | 0.00 | 70040000.00 | 89517000.00 | 75525000.00 | 122261000.00 | 129025000.00 | 179664000.00 | 62281000.00 | 118503000.00 | 115654000.00 | 129976000.00 | 78000000.00 | crotonobetainyl-CoA:carnitine CoA-transferase CaiB-like acyl-CoA transferase | Bacteria;Proteobacteria;Deltaproteobacteria;Syntrophobacterales;Syntrophobacteraceae;Desulfosoma;Desulfosoma caldarium;Desulfosoma caldarium DSM 22027 |
| Ga0586324 0013223 6484 7968 | 611231.00 | 3338800.00 | 0.00 | 0.00 | 0.00 | 0.00 | 0.00 | 512623.26 | 0.00 | 0.00 | 0.00 | 4102500.00 | crotonobetainyl-CoA:carnitine CoA-transferase CaiB-like acyl-CoA transferase | Bacteria;Proteobacteria;Deltaproteobacteria;Syntrophobacterales;Syntrophobacteraceae;Thermodesulforhabdus;Thermodesulforhabdus norvegica;Thermodesulforhabdus norvegica DSM 9990 |
| Ga0586324 0013301 2913 6005 | 0.00 | 1904000.00 | 0.00 | 0.00 | 0.00 | 0.00 | 0.00 | 0.00 | 0.00 | 0.00 | 0.00 | 0.00 | hypothetical protein | Bacteria;Candidatus Aminicenantes;unclassified Candidatus Aminicenantes;unclassified Candidatus Aminicenantes;unclassified Candidatus Aminicenantes;unclassified Candidatus Aminicenantes;Aminicenantes bacterium SCGC AAA252-D06;Aminicenantes bacterium SCGC AAA252-D06 (contamination screened) |
| Ga0586324 0013419 6211 6753 | 2720000.00 | 0.00 | 0.00 | 4693000.00 | 0.00 | 10679300.00 | 0.00 | 0.00 | 2073600.00 | 10010000.00 | 20944000.00 | 6015000.00 | phasin family protein | Bacteria;Proteobacteria;Betaproteobacteria;Rhodocyclales;Zoogloeaceae;Azoarcus;Azoarcus sp. BH72;Azoarcus sp. BH72 |
| Ga0586324 0013628 3 398 | 0.00 | 0.00 | 0.00 | 0.00 | 0.00 | 0.00 | 0.00 | 3708800.00 | 0.00 | 0.00 | 0.00 | 0.00 | hypothetical protein | Bacteria;Proteobacteria;Deltaproteobacteria;Desulfobacterales;Desulfobacteraceae;Desulfobacula;Desulfobacula toluolica;Desulfobacula toluolica Tol2 |
| Ga0586324 0013705 2693 3889 | 0.00 | 7004000.00 | 0.00 | 21375000.00 | 21238600.00 | 1187748.57 | 0.00 | 878400.00 | 619741.53 | 15122800.00 | 0.00 | 0.00 | hypothetical protein | Bacteria;Proteobacteria;Gammaproteobacteria;Chromatiales;Chromatiaceae;Thiocapsa;Thiocapsa marina;Thiocapsa marina 5811, DSM 5653 |
| Ga0586324 0014020 1068 2210 | 0.00 | 0.00 | 0.00 | 0.00 | 5057300.00 | 0.00 | 0.00 | 0.00 | 0.00 | 0.00 | 0.00 | 0.00 | alkylation response protein AidB-like acyl-CoA dehydrogenase | Bacteria;Proteobacteria;Deltaproteobacteria;Syntrophobacterales;Syntrophobacteraceae;Desulfacinum;Desulfacinum infernum;Desulfacinum infernum DSM 9756 |
| Ga0586324 0014090 6919 7290 | 0.00 | 0.00 | 0.00 | 0.00 | 0.00 | 0.00 | 0.00 | 1714100.00 | 0.00 | 0.00 | 0.00 | 0.00 | TusE/DsrC/DsvC family sulfur relay protein | Bacteria;Proteobacteria;Betaproteobacteria;Nitrosomonadales;Thiobacillaceae;Thiobacillus;Thiobacillus thioparus;Thiobacillus thioparus DSM 505 |
| Ga0586324 0014122 2163 3779 | 0.00 | 0.00 | 2855890.69 | 0.00 | 0.00 | 501180.74 | 2644800.00 | 0.00 | 0.00 | 0.00 | 0.00 | 466001.25 | arylsulfatase A-like enzyme | Bacteria;Proteobacteria;Alphaproteobacteria;Hyphomicrobiales;Bradyrhizobiaceae;Bradyrhizobium;Bradyrhizobium diazoefficiens;Bradyrhizobium japonicum USDA 110 |
| Ga0586324 0014147 5941 7248 | 0.00 | 1135600.00 | 7206400.00 | 0.00 | 0.00 | 0.00 | 0.00 | 0.00 | 0.00 | 0.00 | 0.00 | 0.00 | dienelactone hydrolase | Bacteria;Proteobacteria;Deltaproteobacteria;Myxococcales;Myxococcaceae;Myxococcus;Myxococcus fulvus;Myxococcus fulvus DSM 16525 |
| Ga0586324 0014224 1079 3592 | 0.00 | 0.00 | 0.00 | 0.00 | 0.00 | 0.00 | 0.00 | 0.00 | 0.00 | 588018.20 | 657462.08 | 0.00 | arylsulfatase A-like enzyme | Bacteria;Proteobacteria;Gammaproteobacteria;Methylococcales;Methylococcaceae;Methylomonas;Methylomonas lenta;Methylomonas lenta R-45370 |
| Ga0586324 0014319 700 2226 | 53480000.00 | 107841200.00 | 136844119.94 | 156892500.00 | 98395900.00 | 112891745.76 | 144486226.56 | 29078700.00 | 63269100.00 | 124824700.00 | 140368800.00 | 71940000.00 | hypothetical protein | Bacteria;Proteobacteria;Betaproteobacteria;Nitrosomonadales;Gallionellaceae;Sideroxydans;Sideroxydans lithotrophicus;Sideroxydans lithotrophicus ES-1 |
| Ga0586324 0014477 4097 6829 | 0.00 | 0.00 | 0.00 | 0.00 | 0.00 | 0.00 | 0.00 | 0.00 | 1296000.00 | 0.00 | 0.00 | 0.00 | OmcA/MtrC family decaheme c-type cytochrome | Bacteria;Proteobacteria;Betaproteobacteria;Burkholderiales;Comamonadaceae;Rhodoferax;Rhodoferax ferrireducens;Rhodoferax ferrireducens T118 |
| Ga0586324 0014558 45 2438 | 0.00 | 0.00 | 0.00 | 6298500.00 | 0.00 | 0.00 | 0.00 | 0.00 | 0.00 | 0.00 | 0.00 | 0.00 | type IV pilus secretin PilQ/predicted competence protein | Bacteria;Candidatus Aminicenantes;unclassified Candidatus Aminicenantes;unclassified Candidatus Aminicenantes;unclassified Candidatus Aminicenantes;unclassified Candidatus Aminicenantes;Aminicenantes bacterium SCGC AAA252-K07;Aminicenantes bacterium SCGC AAA252-K07 (contamination screened) |
| Ga0586324 0014663 1826 4933 | 0.00 | 0.00 | 0.00 | 0.00 | 0.00 | 0.00 | 0.00 | 0.00 | 22113000.00 | 32340000.00 | 0.00 | 0.00 | hypothetical protein | Bacteria;Balneolaeota;Balneolia;Balneolales;Balneolaceae;Gracilimonas;Gracilimonas tropica;Gracilimonas tropica DSM 19535 |
| Ga0586324 0014751 1794 5987 | 0.00 | 9438400.00 | 1602607.65 | 6032500.00 | 1668600.00 | 2361872.10 | 0.00 | 0.00 | 689719.05 | 4258100.00 | 7101600.00 | 7862710.50 | glycosidase | Bacteria;Chloroflexi;Chloroflexia;Chloroflexales;Roseiflexaceae;Roseiflexus;Roseiflexus sp. RS-1;Roseiflexus sp. RS-1 |
| Ga0586324 0014792 1554 2573 | 0.00 | 0.00 | 0.00 | 0.00 | 0.00 | 0.00 | 0.00 | 0.00 | 1271700.00 | 0.00 | 0.00 | 0.00 | hypothetical protein | |
| Ga0586324 0015109 771 3761 | 10790000.00 | 20359200.00 | 11316300.00 | 5396000.00 | 5737100.00 | 20078540.99 | 0.00 | 13304100.00 | 10027800.00 | 16339400.00 | 22184800.00 | 21472500.00 | hypothetical protein | Bacteria;Acidobacteria;Acidobacteriia;Acidobacteriales;Acidobacteriaceae;Granulicella;Granulicella sp. GAS466;Granulicella sp. GAS466 |
| Ga0586324 0015869 4394 4819 | 0.00 | 15041600.00 | 0.00 | 3372500.00 | 9970400.00 | 0.00 | 3921600.00 | 2318000.00 | 3426300.00 | 7931000.00 | 14044800.00 | 10170000.00 | hypothetical protein | |
| Ga0586324 0015951 2671 5529 | 3900000.00 | 6855825.28 | 0.00 | 2280000.00 | 8178200.00 | 0.00 | 0.00 | 4288300.00 | 0.00 | 0.00 | 5728800.00 | 614088.00 | hypothetical protein | Bacteria;Acidobacteria;Thermoanaerobaculia;Thermoanaerobaculales;Thermoanaerobaculaceae;Thermoanaerobaculum;Thermoanaerobaculum aquaticum;Thermoanaerobaculum aquaticum MP-01 |
| Ga0586324 0016066 2 2791 | 0.00 | 0.00 | 0.00 | 1349000.00 | 0.00 | 0.00 | 0.00 | 0.00 | 0.00 | 0.00 | 0.00 | 0.00 | hypothetical protein | Bacteria;Acidobacteria;Acidobacteriia;Bryobacterales;Solibacteraceae;Candidatus Solibacter;Candidatus Solibacter usitatus;Candidatus Solibacter usitatus Ellin6076 |
| Ga0586324 0016184 3 389 | 0.00 | 0.00 | 0.00 | 0.00 | 0.00 | 0.00 | 0.00 | 2464400.00 | 0.00 | 0.00 | 0.00 | 0.00 | outer membrane protein OmpA-like peptidoglycan-associated protein | Bacteria;Proteobacteria;Betaproteobacteria;Nitrosomonadales;Thiobacillaceae;Thiobacillus;Thiobacillus thiophilus;Thiobacillus thiophilus DSM 19892 |
| Ga0586324 0016399 3 1931 | 0.00 | 0.00 | 0.00 | 0.00 | 0.00 | 0.00 | 0.00 | 157353.16 | 0.00 | 0.00 | 0.00 | 0.00 | arylsulfatase A-like enzyme | Bacteria;Proteobacteria;Alphaproteobacteria;Hyphomicrobiales;Bradyrhizobiaceae;Bradyrhizobium;Bradyrhizobium elkanii;Bradyrhizobium elkanii USDA 76 |
| Ga0586324 0016455 3 1988 | 0.00 | 2046800.00 | 0.00 | 0.00 | 0.00 | 0.00 | 0.00 | 0.00 | 0.00 | 0.00 | 0.00 | 0.00 | hypothetical protein | Bacteria;Proteobacteria;Deltaproteobacteria;Myxococcales;Anaeromyxobacteraceae;Anaeromyxobacter;Anaeromyxobacter dehalogenans;Anaeromyxobacter dehalogenans 2CP-C |
| Ga0586324 0016587 5364 8102 | 0.00 | 2114800.00 | 0.00 | 0.00 | 0.00 | 0.00 | 0.00 | 0.00 | 0.00 | 0.00 | 0.00 | 0.00 | hypothetical protein | Bacteria;Proteobacteria;Deltaproteobacteria;Syntrophobacterales;Syntrophaceae;Syntrophus;Syntrophus gentianae;Syntrophus gentianae DSM 8423 |
| Ga0586324 0016598 1 3114 | 0.00 | 0.00 | 0.00 | 0.00 | 0.00 | 0.00 | 0.00 | 0.00 | 899100.00 | 0.00 | 0.00 | 892500.00 | tetratricopeptide (TPR) repeat protein | Bacteria;Proteobacteria;Deltaproteobacteria;Myxococcales;Sandaracinaceae;Sandaracinus;Sandaracinus amylolyticus;Sandaracinus amylolyticus DSM 53668 |
| Ga0586324 0016856 4675 5226 | 0.00 | 0.00 | 0.00 | 0.00 | 0.00 | 0.00 | 0.00 | 0.00 | 0.00 | 0.00 | 0.00 | 1867500.00 | hypothetical protein | Bacteria;Proteobacteria;Gammaproteobacteria;Xanthomonadales;Xanthomonadaceae;Stenotrophomonas;Stenotrophomonas rhizophila;Stenotrophomonas rhizophila BIGb0145 |
| Ga0586324 0017085 3285 3830 | 0.00 | 856800.00 | 0.00 | 4123000.00 | 3883100.00 | 3077365.35 | 0.00 | 477825.81 | 0.00 | 0.00 | 3229600.00 | 0.00 | Skp family chaperone for outer membrane proteins | |
| Ga0586324 0017309 3153 4736 | 13100000.00 | 0.00 | 1537035.04 | 0.00 | 0.00 | 15086000.00 | 0.00 | 0.00 | 10449000.00 | 0.00 | 0.00 | 11325000.00 | glutamate synthase domain-containing protein 2 | Bacteria;Candidatus Aminicenantes;unclassified Candidatus Aminicenantes;unclassified Candidatus Aminicenantes;unclassified Candidatus Aminicenantes;unclassified Candidatus Aminicenantes;Aminicenantes bacterium SCGC AAA252-K06;Aminicenantes bacterium SCGC AAA252-K06 (contamination screened) |
| Ga0586324 0017319 7234 7956 | 0.00 | 1693200.00 | 0.00 | 0.00 | 0.00 | 0.00 | 0.00 | 446069.82 | 0.00 | 0.00 | 0.00 | 0.00 | ABC-type transport system substrate-binding protein | Bacteria;Chloroflexi;Thermoflexia;Thermoflexales;Thermoflexaceae;Thermoflexus;Thermoflexus hugenholtzii;Thermoflexus hugenholtzii JAD2 |
| Ga0586324 0017421 2 553 | 0.00 | 0.00 | 0.00 | 0.00 | 0.00 | 0.00 | 0.00 | 0.00 | 0.00 | 0.00 | 0.00 | 420217.50 | hypothetical protein | Bacteria;Acidobacteria;Vicinamibacteria;unclassified Vicinamibacteria;Vicinamibacteraceae;Luteitalea;Luteitalea pratensis;Luteitalea pratensis DSM 100886 |
| Ga0586324 0017641 3804 5144 | 0.00 | 0.00 | 0.00 | 0.00 | 0.00 | 0.00 | 3921600.00 | 0.00 | 0.00 | 0.00 | 0.00 | 0.00 | crotonobetainyl-CoA:carnitine CoA-transferase CaiB-like acyl-CoA transferase | Bacteria;Proteobacteria;Deltaproteobacteria;Syntrophobacterales;Syntrophobacteraceae;Desulfacinum;Desulfacinum infernum;Desulfacinum infernum DSM 9756 |
| Ga0586324 0017867 1019 2167 | 6870000.00 | 4080000.00 | 0.00 | 0.00 | 0.00 | 0.00 | 0.00 | 0.00 | 0.00 | 0.00 | 0.00 | 0.00 | alkylation response protein AidB-like acyl-CoA dehydrogenase | Bacteria;Proteobacteria;Deltaproteobacteria;Syntrophobacterales;Syntrophobacteraceae;Desulfosoma;Desulfosoma caldarium;Desulfosoma caldarium DSM 22027 |
| Ga0586324 0017970 1427 3325 | 1850000.00 | 3678800.00 | 0.00 | 0.00 | 0.00 | 0.00 | 0.00 | 0.00 | 0.00 | 1155000.00 | 1240800.00 | 488720.25 | hypothetical protein | Bacteria;Proteobacteria;Deltaproteobacteria;Desulfuromonadales;Geobacteraceae;Geothermobacter;Geothermobacter sp. HR-1;Geothermobacter sp. HR-1 |
| Ga0586324 0018032 2 2941 | 0.00 | 856800.00 | 0.00 | 0.00 | 0.00 | 0.00 | 0.00 | 0.00 | 0.00 | 0.00 | 0.00 | 0.00 | hypothetical protein | Bacteria;Acidobacteria;Acidobacteriia;Acidobacteriales;Acidobacteriaceae;Terracidiphilus;Terracidiphilus gabretensis;Acidobacteriaceae bacterium S55 |
| Ga0586324 0018180 3636 4640 | 0.00 | 0.00 | 0.00 | 0.00 | 0.00 | 0.00 | 0.00 | 0.00 | 0.00 | 1486100.00 | 0.00 | 0.00 | basic membrane lipoprotein Med (substrate-binding protein (PBP1-ABC) superfamily) | Bacteria;Chloroflexi;Anaerolineae;Anaerolineales;Anaerolineaceae;Pelolinea;Pelolinea submarina;Pelolinea submarina DSM 23923 |
| Ga0586324 0018384 6044 6436 | 0.00 | 0.00 | 0.00 | 0.00 | 2657400.00 | 0.00 | 0.00 | 2507100.00 | 3572100.00 | 0.00 | 0.00 | 0.00 | CBS domain-containing protein | Bacteria;Proteobacteria;Gammaproteobacteria;Methylococcales;Methylococcaceae;Methylovulum;Methylovulum miyakonense;Methylovulum miyakonense HT12 |
| Ga0586324 0018457 6148 7716 | 0.00 | 0.00 | 1581033.49 | 2707500.00 | 0.00 | 1881410.79 | 0.00 | 847900.00 | 0.00 | 0.00 | 0.00 | 0.00 | hypothetical protein | Bacteria;Proteobacteria;Alphaproteobacteria;Hyphomicrobiales;Methylocystaceae;Methylocystis;Methylocystis sp. SC2;Methylocystis sp. SC2 |
| Ga0586324 0018591 535 2022 | 0.00 | 0.00 | 0.00 | 0.00 | 0.00 | 0.00 | 0.00 | 1872700.00 | 0.00 | 0.00 | 0.00 | 0.00 | predicted porin | Bacteria;Proteobacteria;Alphaproteobacteria;Hyphomicrobiales;unclassified Hyphomicrobiales;Methyloligella;Methyloligella halotolerans;Methyloligella halotolerans VKM B-2706 |
| Ga0586324 0018634 2259 6512 | 0.00 | 0.00 | 0.00 | 1035500.00 | 0.00 | 0.00 | 0.00 | 0.00 | 0.00 | 0.00 | 0.00 | 0.00 | hypothetical protein | Bacteria;Acidobacteria;Acidobacteriia;Bryobacterales;Solibacteraceae;Candidatus Solibacter;Candidatus Solibacter usitatus;Candidatus Solibacter usitatus Ellin6076 |
| Ga0586324 0018676 1 4191 | 689554.00 | 0.00 | 0.00 | 0.00 | 0.00 | 0.00 | 0.00 | 0.00 | 0.00 | 0.00 | 0.00 | 0.00 | FtsP/CotA-like multicopper oxidase with cupredoxin domain/fibronectin type 3 domain-containin | Bacteria;Actinobacteria;Actinomycetia;Micrococcales;Cellulomonadaceae;Cellulomonas;Cellulomonas sp. Root137;Cellulomonas sp. Root137 |
| Ga0586324 0018704 2015 3703 | 0.00 | 0.00 | 0.00 | 0.00 | 9702600.00 | 8575200.00 | 0.00 | 0.00 | 0.00 | 5205200.00 | 0.00 | 0.00 | arylsulfatase A-like enzyme | Bacteria;Proteobacteria;Alphaproteobacteria;Hyphomicrobiales;Rhizobiaceae;Rhizobium;Rhizobium sp. Root1220;Rhizobium sp. Root1220 |
| Ga0586324 0018813 3654 4847 | 0.00 | 0.00 | 0.00 | 0.00 | 0.00 | 449816.88 | 0.00 | 0.00 | 0.00 | 0.00 | 0.00 | 0.00 | hypothetical protein | Bacteria;Bacteroidetes;Flavobacteriia;Flavobacteriales;Flavobacteriaceae;Flavobacterium;Flavobacterium aquidurense;Flavobacterium aquidurense DSM 18293 |
| Ga0586324 0018864 6496 6909 | 0.00 | 19856000.00 | 0.00 | 0.00 | 0.00 | 0.00 | 0.00 | 0.00 | 0.00 | 0.00 | 0.00 | 0.00 | hypothetical protein | |
| Ga0586324 0018864 6986 7639 | 17900000.00 | 0.00 | 0.00 | 0.00 | 24514000.00 | 0.00 | 0.00 | 30805000.00 | 14094000.00 | 4219600.00 | 10120000.00 | 12975000.00 | crotonobetainyl-CoA:carnitine CoA-transferase CaiB-like acyl-CoA transferase | Bacteria;Proteobacteria;Deltaproteobacteria;Syntrophobacterales;Syntrophobacteraceae;Thermodesulforhabdus;Thermodesulforhabdus norvegica;Thermodesulforhabdus norvegica DSM 9990 |
| Ga0586324 0019150 5670 5909 | 0.00 | 0.00 | 0.00 | 0.00 | 754945.71 | 0.00 | 0.00 | 0.00 | 0.00 | 0.00 | 0.00 | 0.00 | hypothetical protein | Bacteria;Proteobacteria;Alphaproteobacteria;Rhodospirillales;Acetobacteraceae;Belnapia;Belnapia rosea;Belnapia rosea MJ22 |
| Ga0586324 0019480 4869 6026 | 0.00 | 0.00 | 0.00 | 0.00 | 0.00 | 0.00 | 0.00 | 3111000.00 | 0.00 | 0.00 | 0.00 | 0.00 | alkylation response protein AidB-like acyl-CoA dehydrogenase | Bacteria;Proteobacteria;Deltaproteobacteria;Syntrophobacterales;Syntrophobacteraceae;Thermodesulforhabdus;Thermodesulforhabdus norvegica;Thermodesulforhabdus norvegica DSM 9990 |
| Ga0586324 0019720 2760 5870 | 0.00 | 0.00 | 0.00 | 10317000.00 | 0.00 | 0.00 | 0.00 | 0.00 | 0.00 | 0.00 | 0.00 | 0.00 | hypothetical protein | Bacteria;Candidatus Aminicenantes;unclassified Candidatus Aminicenantes;unclassified Candidatus Aminicenantes;unclassified Candidatus Aminicenantes;unclassified Candidatus Aminicenantes;Aminicenantes bacterium SCGC AAA252-N07;Aminicenantes bacterium SCGC AAA252-N07 (contamination screened) |
| Ga0586324 0019739 2 3793 | 0.00 | 2250800.00 | 0.00 | 0.00 | 0.00 | 0.00 | 0.00 | 0.00 | 0.00 | 299158.09 | 351578.48 | 0.00 | FtsP/CotA-like multicopper oxidase with cupredoxin domain | Bacteria;Actinobacteria;Actinomycetia;Micrococcales;Intrasporangiaceae;Terracoccus;Terracoccus sp. 273MFTsu3.1;Terracoccus sp. 273MFTsu3.1 |
| Ga0586324 0020106 2 5107 | 13080000.00 | 13273600.00 | 25033316.45 | 16862500.00 | 18303100.00 | 13303926.55 | 14012512.92 | 13084500.00 | 13535100.00 | 26272400.00 | 31292800.00 | 13860000.00 | hypothetical protein | Bacteria;Proteobacteria;Alphaproteobacteria;Rhodobacterales;Roseobacteraceae;Sulfitobacter;Sulfitobacter sp. EL44;Sulfitobacter sp. EL44 |
| Ga0586324 0020180 3215 6520 | 46300000.00 | 25955600.00 | 17663854.76 | 10706500.00 | 28613400.00 | 26002892.59 | 32781438.72 | 20727800.00 | 20379600.00 | 0.00 | 6327200.00 | 12577500.00 | hypothetical protein | Bacteria;Proteobacteria;Deltaproteobacteria;Myxococcales;Anaeromyxobacteraceae;Anaeromyxobacter;Anaeromyxobacter sp. Fw109-5;Anaeromyxobacter sp. Fw109-5 |
| Ga0586324 0020336 5824 7050 | 0.00 | 0.00 | 0.00 | 0.00 | 3100300.00 | 0.00 | 0.00 | 780800.00 | 0.00 | 0.00 | 0.00 | 0.00 | hypothetical protein | |
| Ga0586324 0020522 2329 3834 | 0.00 | 843200.00 | 1270026.66 | 0.00 | 1514100.00 | 507417.61 | 0.00 | 0.00 | 732673.35 | 434535.64 | 0.00 | 627588.75 | hypothetical protein | Bacteria;Cyanobacteria;unclassified Cyanobacteria;Chroococcales;Microcystaceae;Microcystis;Microcystis aeruginosa;Microcystis aeruginosa SPC777 |
| Ga0586324 0020924 3 2729 | 0.00 | 0.00 | 0.00 | 0.00 | 0.00 | 1738264.50 | 0.00 | 0.00 | 762486.21 | 1362900.00 | 3344000.00 | 1335000.00 | hypothetical protein | Bacteria;Proteobacteria;Deltaproteobacteria;Myxococcales;Archangiaceae;Archangium;Archangium gephyra;Archangium gephyra DSM 2261 |
| Ga0586324 0020970 4718 5260 | 0.00 | 0.00 | 0.00 | 0.00 | 0.00 | 0.00 | 0.00 | 0.00 | 0.00 | 0.00 | 2534400.00 | 0.00 | phasin family protein | Bacteria;Proteobacteria;Betaproteobacteria;Nitrosomonadales;Sterolibacteriaceae;Sulfurisoma;Sulfurisoma sediminicola;Sulfurisoma sediminicola DSM 26916 |
| Ga0586324 0021181 2 2431 | 0.00 | 0.00 | 0.00 | 0.00 | 0.00 | 0.00 | 0.00 | 198942.96 | 496569.69 | 0.00 | 0.00 | 0.00 | OmcA/MtrC family decaheme c-type cytochrome | Bacteria;Proteobacteria;Betaproteobacteria;Burkholderiales;Comamonadaceae;Rhodoferax;Rhodoferax ferrireducens;Rhodoferax ferrireducens T118 |
| Ga0586324 0021283 306 1268 | 0.00 | 0.00 | 0.00 | 0.00 | 0.00 | 3602278.75 | 0.00 | 0.00 | 0.00 | 0.00 | 0.00 | 0.00 | ABC-type amino acid transport substrate-binding protein | Bacteria;Proteobacteria;Betaproteobacteria;Burkholderiales;Burkholderiaceae;Paraburkholderia;Paraburkholderia aromaticivorans;Paraburkholderia aromaticivorans BN5 |
| Ga0586324 0021304 1455 4256 | 1421689.00 | 276592.72 | 10865900.00 | 4712000.00 | 6097600.00 | 5500252.38 | 1588343.76 | 4282200.00 | 25105336.83 | 0.00 | 6662686.80 | 3967500.00 | hypothetical protein | Bacteria;Acidobacteria;Thermoanaerobaculia;Thermoanaerobaculales;Thermoanaerobaculaceae;Thermoanaerobaculum;Thermoanaerobaculum aquaticum;Thermoanaerobaculum aquaticum MP-01 |
| Ga0586324 0021420 6000 7202 | 19200000.00 | 6745600.00 | 13230500.00 | 3230000.00 | 8600500.00 | 3286461.28 | 0.00 | 6771000.00 | 6237000.00 | 6337100.00 | 7427200.00 | 1770000.00 | ABC-type transport system substrate-binding protein | Bacteria;Proteobacteria;Deltaproteobacteria;Syntrophobacterales;Syntrophobacteraceae;Thermodesulforhabdus;Thermodesulforhabdus norvegica;Thermodesulforhabdus norvegica DSM 9990 |
| Ga0586324 0021458 5210 6415 | 0.00 | 2339200.00 | 0.00 | 0.00 | 0.00 | 0.00 | 0.00 | 0.00 | 0.00 | 0.00 | 0.00 | 0.00 | ABC-type branched-subunit amino acid transport system substrate-binding protein | Bacteria;Proteobacteria;Betaproteobacteria;Burkholderiales;Comamonadaceae;Polaromonas;Polaromonas sp. AET17H-212;Polaromonas sp. AET17H-212 |

| | | | | | | | | | | | | | | |
|---|---|---|---|---|---|---|---|---|---|---|---|---|---|---|
| Ga0586324 0021502 2 1714 | 0.00 | 0.00 | 0.00 | 0.00 | 0.00 | 0.00 | 0.00 | 0.00 | 0.00 | 472449.67 | 0.00 | 1147500.00 | FtsP/CotA-like multicopper oxidase with cupredoxin domain | Bacteria;Proteobacteria;Deltaproteobacteria;Desulfuromonadales;Geobacteraceae;Geobacter;Geobacter metallireducens;Geobacter metallireducens RCH3 |
| Ga0586324 0021508 321 1382 | 2370000.00 | 0.00 | 0.00 | 0.00 | 0.00 | 0.00 | 0.00 | 0.00 | 1344600.00 | 0.00 | 0.00 | 0.00 | hypothetical protein | Archaea;Euryarchaeota;Methanomicrobia;Methanosarcinales;Methanotrichaceae;Methanothrix;Methanothrix soehngenii;Methanothrix soehngenii GP-6 |
| Ga0586324 0021655 2374 3504 | 69200000.00 | 178432000.00 | 55699352.19 | 127175927.15 | 143262700.00 | 190795321.75 | 46192800.00 | 142740000.00 | 134427600.00 | 110464200.00 | 96008000.00 | 145977432.00 | predicted porin | Bacteria;Proteobacteria;Betaproteobacteria;unclassified Betaproteobacteria;unclassified Betaproteobacteria;unclassified Betaproteobacteria;uncultured beta proteobacterium;unclassified betaproteobacterium ENR4 |
| Ga0586324 0021726 2665 4053 | 6880000.00 | 0.00 | 1525865.12 | 0.00 | 0.00 | 0.00 | 0.00 | 0.00 | 0.00 | 0.00 | 0.00 | 1522500.00 | glucose/arabinose dehydrogenase | Bacteria;Proteobacteria;Alphaproteobacteria;Hyphomicrobiales;Bradyrhizobiaceae;Bradyrhizobium;Bradyrhizobium erythrophlei;Bradyrhizobium erythrophlei GAS401 |
| Ga0586324 0021868 5645 7129 | 0.00 | 0.00 | 0.00 | 0.00 | 0.00 | 0.00 | 0.00 | 0.00 | 1169738.01 | 0.00 | 0.00 | 0.00 | hypothetical protein | Bacteria;Acidobacteria;Vicinamibacteria;unclassified Vicinamibacteria;Vicinamibacteraceae;Luteitalea;Luteitalea pratensis;Luteitalea pratensis DSM 100886 |
| Ga0586324 0021876 1316 2569 | 0.00 | 0.00 | 0.00 | 0.00 | 0.00 | 465260.18 | 0.00 | 0.00 | 0.00 | 0.00 | 0.00 | 0.00 | hypothetical protein | Bacteria;Proteobacteria;Deltaproteobacteria;Desulfuromonadales;Geobacteraceae;Geobacter;Geobacter lovleyi;Geobacter lovleyi SZ |
| Ga0586324 0021909 913 5412 | 1130000.00 | 0.00 | 0.00 | 0.00 | 0.00 | 0.00 | 0.00 | 366422.12 | 0.00 | 0.00 | 0.00 | 0.00 | heterodisulfide reductase subunit A-like polyferredoxin | Bacteria;Proteobacteria;Deltaproteobacteria;Desulfobacterales;Desulfobacteraceae;Desulfobacter;Desulfobacter curvatus;Desulfobacter curvatus DSM 3379 |
| Ga0586324 0022034 517 1797 | 0.00 | 0.00 | 0.00 | 0.00 | 0.00 | 0.00 | 0.00 | 0.00 | 291879.45 | 0.00 | 450589.04 | 227520.75 | sugar lactone lactonase YvrE | Bacteria;Proteobacteria;Betaproteobacteria;Burkholderiales;unclassified Burkholderiales;Leptothrix;Leptothrix cholodnii;Leptothrix cholodnii SP-6 |
| Ga0586324 0022044 4845 7106 | 0.00 | 0.00 | 0.00 | 0.00 | 0.00 | 0.00 | 0.00 | 3263500.00 | 0.00 | 1994300.00 | 2288000.00 | 2685000.00 | subtilisin family serine protease | Bacteria;Actinobacteria;Actinomycetia;Kineosporiales;Kineosporiaceae;Kineosporia;Kineosporia sp. A 224;Kineosporia sp. A 224 A 224 |
| Ga0586324 0022155 3546 4643 | 13170000.00 | 5412800.00 | 0.00 | 0.00 | 0.00 | 0.00 | 0.00 | 0.00 | 5459400.00 | 0.00 | 0.00 | 0.00 | kynurenine formamidase | Bacteria;Proteobacteria;Alphaproteobacteria;unclassified Alphaproteobacteria;unclassified Alphaproteobacteria;unclassified Alphaproteobacteria;alpha proteobacterium BAL199;Nisaea sp BAL199 |
| Ga0586324 0022415 1748 5260 | 0.00 | 0.00 | 0.00 | 3087500.00 | 5160300.00 | 0.00 | 0.00 | 0.00 | 0.00 | 2279200.00 | 2684000.00 | 1155000.00 | 2',3'-cyclic-nucleotide 2'-phosphodiesterase (5'-nucleotidase family) | Bacteria;Dictyoglomi;Dictyoglomia;Dictyoglomales;Dictyoglomaceae;Dictyoglomus;Dictyoglomus thermophilum;Dictyoglomus thermophilum H-6-12, ATCC 35947 |
| Ga0586324 0022451 4045 6513 | 0.00 | 0.00 | 796470.47 | 0.00 | 0.00 | 0.00 | 0.00 | 0.00 | 0.00 | 0.00 | 0.00 | 0.00 | ABC-type sugar transport system substrate-binding protein | Bacteria;Chloroflexi;Caldilineae;Caldilineales;Caldilineaceae;Caldilinea;Caldilinea aerophila;Caldilinea aerophila STL-6-O1, DSM 14535 |
| Ga0586324 0022499 1831 5391 | 1940000.00 | 0.00 | 0.00 | 0.00 | 0.00 | 0.00 | 0.00 | 0.00 | 0.00 | 0.00 | 2648800.00 | 2167500.00 | hypothetical protein | Bacteria;Acidobacteria;Vicinamibacteria;unclassified Vicinamibacteria;Vicinamibacteraceae;Luteitalea;Luteitalea pratensis;Luteitalea pratensis DSM 100886 |
| Ga0586324 0022604 5760 6515 | 0.00 | 0.00 | 0.00 | 95902.50 | 0.00 | 0.00 | 0.00 | 0.00 | 0.00 | 0.00 | 0.00 | 0.00 | regulator of protease activity HflC (stomatin/prohibitin superfamily) | Bacteria;Acidobacteria;Vicinamibacteria;unclassified Vicinamibacteria;Vicinamibacteraceae;Luteitalea;Luteitalea pratensis;Luteitalea pratensis DSM 100886 |
| Ga0586324 0022634 6152 6688 | 0.00 | 0.00 | 0.00 | 0.00 | 0.00 | 0.00 | 0.00 | 0.00 | 3547800.00 | 0.00 | 0.00 | 0.00 | invasion protein IalB | Bacteria;Proteobacteria;Alphaproteobacteria;Hyphomicrobiales;Xanthobacteraceae;Pseudolabrys;Pseudolabrys taiwanensis;Pseudolabrys taiwanensis CC-BB4 |
| Ga0586324 0022670 5438 5746 | 0.00 | 0.00 | 0.00 | 0.00 | 0.00 | 4486100.00 | 0.00 | 6527000.00 | 0.00 | 0.00 | 0.00 | 0.00 | chromosome segregation ATPase | Bacteria;Proteobacteria;Gammaproteobacteria;Methylococcales;Methylococcaceae;Methylosarcina;Methylosarcina fibrata;Methylosarcina fibrata AML-C10 |
| Ga0586324 0022884 4352 4969 | 0.00 | 0.00 | 0.00 | 0.00 | 0.00 | 0.00 | 0.00 | 3775900.00 | 0.00 | 0.00 | 0.00 | 0.00 | uncharacterized protein YigD (DUF1641 family) | Bacteria;Proteobacteria;Betaproteobacteria;Ferrovales;Ferrovaceae;Ferrovum;Ferrovum myxofaciens;Ferrovum myxofaciens P3G |
| Ga0586324 0022884 5143 6906 | 14460000.00 | 13906000.00 | 15595100.00 | 0.00 | 0.00 | 4748667.86 | 5784266.52 | 19025900.00 | 8407800.00 | 32540200.00 | 20134400.00 | 14430000.00 | hypothetical protein | Bacteria;Proteobacteria;Alphaproteobacteria;Hyphomicrobiales;Bradyrhizobiaceae;Bradyrhizobium;Bradyrhizobium neotropicale;Bradyrhizobium neotropicale BR 10247 |
| Ga0586324 0022996 3859 5166 | 30620000.00 | 27948000.00 | 43351000.00 | 33373500.00 | 43466000.00 | 38775879.28 | 12882000.00 | 41717900.00 | 24445800.00 | 20713000.00 | 21630400.00 | 14227500.00 | hypothetical protein | |
| Ga0586324 0023006 4207 5454 | 17200000.00 | 13001600.00 | 0.00 | 0.00 | 22866000.00 | 19016300.00 | 0.00 | 9150000.00 | 15309000.00 | 12166000.00 | 0.00 | 0.00 | hypothetical protein | |
| Ga0586324 0023456 3 1310 | 0.00 | 3250400.00 | 0.00 | 0.00 | 146818.26 | 3727675.17 | 2416800.00 | 9333000.00 | 0.00 | 8932000.00 | 12672000.00 | 2272500.00 | hypothetical protein | Bacteria;Spirochaetes;Spirochaetia;Spirochaetales;Spirochaetaceae;Spirochaeta;Spirochaeta cellobiosiphila;Spirochaeta cellobiosiphila DSM 17781 |
| Ga0586324 0023464 4819 6000 | 17400000.00 | 12920000.00 | 35919400.00 | 25032500.00 | 12051000.00 | 0.00 | 18194400.00 | 9028000.00 | 10926900.00 | 12628000.00 | 13288000.00 | 14040000.00 | TRAP-type mannitol/chloroaromatic compound transport system substrate-binding protein | Bacteria;Proteobacteria;Gammaproteobacteria;Acidiferrobacterales;Acidiferrobacteraceae;Sulfuricaulis;Sulfuricaulis limicola;Sulfuricaulis limicola HA5 |
| Ga0586324 0023467 1395 6887 | 18200000.00 | 8989600.00 | 0.00 | 24415000.00 | 25368900.00 | 7588829.68 | 1535564.04 | 8521700.00 | 6763500.00 | 5474700.00 | 4954400.00 | 8857500.00 | uncharacterized repeat protein (TIGR01451 family) | Bacteria;Actinobacteria;Actinomycetia;Propionibacteriales;Kribbellaceae;Kribbella;Kribbella antibiotica;Kribbella antibiotica JCM 13523 |
| Ga0586324 0023837 2941 3822 | 9670000.00 | 7820000.00 | 0.00 | 17527500.00 | 14317000.00 | 18211759.65 | 0.00 | 12444000.00 | 0.00 | 2541000.00 | 11360800.00 | 2587500.00 | hypothetical protein | Bacteria;Proteobacteria;Alphaproteobacteria;Rhodospirillales;Elioraeaceae;Elioraea;Elioraea tepidiphila;Elioraea tepidiphila DSM 17972 |
| Ga0586324 0024049 299 3874 | 0.00 | 0.00 | 0.00 | 0.00 | 0.00 | 0.00 | 0.00 | 0.00 | 0.00 | 0.00 | 0.00 | 2332500.00 | aspartate/methionine/tyrosine aminotransferase/phosphoenolpyruvate carboxylase | Bacteria;Actinobacteria;Actinomycetia;Micrococcales;Micrococcaceae;Micrococcus;Micrococcus luteus;Micrococcus luteus modasa |
| Ga0586324 0024377 1760 5101 | 422665.00 | 754561.32 | 0.00 | 0.00 | 0.00 | 0.00 | 0.00 | 85839.81 | 0.00 | 0.00 | 0.00 | 0.00 | outer membrane receptor protein involved in Fe transport | Bacteria;Acidobacteria;Acidobacteriia;Acidobacteriales;Acidobacteriaceae;Acidipila;Acidipila rosea;Acidipila rosea DSM 103428 |
| Ga0586324 0024787 2734 6072 | 8650000.00 | 0.00 | 0.00 | 0.00 | 0.00 | 0.00 | 0.00 | 0.00 | 0.00 | 0.00 | 0.00 | 0.00 | outer membrane receptor protein involved in Fe transport | Bacteria;Acidobacteria;Vicinamibacteria;unclassified Vicinamibacteria;Vicinamibacteraceae;Luteitalea;Luteitalea pratensis;Luteitalea pratensis DSM 100886 |
| Ga0586324 0024943 2 1927 | 0.00 | 0.00 | 0.00 | 0.00 | 2204200.00 | 2730022.11 | 0.00 | 0.00 | 0.00 | 0.00 | 0.00 | 0.00 | hypothetical protein | |
| Ga0586324 0024975 3742 6693 | 0.00 | 2611200.00 | 0.00 | 62937500.00 | 0.00 | 199404.76 | 0.00 | 0.00 | 1300715.01 | 415621.36 | 0.00 | 499847.25 | hypothetical protein | Bacteria;Acidobacteria;Vicinamibacteria;unclassified Vicinamibacteria;Vicinamibacteraceae;Luteitalea;Luteitalea pratensis;Luteitalea pratensis DSM 100886 |
| Ga0586324 0025111 1244 1675 | 8340000.00 | 10404000.00 | 6981200.00 | 4683500.00 | 0.00 | 29616200.00 | 0.00 | 0.00 | 0.00 | 4111800.00 | 0.00 | 0.00 | nucleotide-binding universal stress UspA family protein | Archaea;Euryarchaeota;Methanomicrobia;Methanosarcinales;Methanotrichaceae;Methanothrix;Methanothrix soehngenii;Methanothrix soehngenii GP-6 |
| Ga0586324 0025540 3648 4586 | 0.00 | 0.00 | 0.00 | 0.00 | 0.00 | 0.00 | 0.00 | 0.00 | 0.00 | 0.00 | 0.00 | 1297500.00 | hypothetical protein | Bacteria;Proteobacteria;Deltaproteobacteria;Syntrophobacterales;Syntrophaceae;Desulfobacca;Desulfobacca acetoxidans;Desulfobacca acetoxidans ASRB2, DSM 11109 |
| Ga0586324 0025573 2834 3958 | 44240000.00 | 26506400.00 | 2784265.83 | 0.00 | 0.00 | 3888908.78 | 0.00 | 8289900.00 | 11097000.00 | 17671500.00 | 68886400.00 | 29670000.00 | predicted porin | Bacteria;Proteobacteria;Betaproteobacteria;Nitrosomonadales;Gallionellaceae;Gallionella;Gallionella capsiferriformans;Gallionella capsiferriformans ES-2 |
| Ga0586324 0025726 331 4308 | 10069770.00 | 15184400.00 | 7769400.00 | 0.00 | 1545000.00 | 7047341.53 | 9154569.36 | 7100400.00 | 7111800.00 | 7584500.00 | 4470400.00 | 2047500.00 | 2',3'-cyclic-nucleotide 2'-phosphodiesterase (5'-nucleotidase family) | Bacteria;Firmicutes;Bacilli;Bacillales;Bacillaceae;Evansella;Evansella cellulosilytica;Bacillus cellulosilyticus N-4, DSM 2522 |
| Ga0586324 0025747 4437 6593 | 0.00 | 1054000.00 | 0.00 | 0.00 | 0.00 | 0.00 | 0.00 | 0.00 | 0.00 | 0.00 | 0.00 | 0.00 | hypothetical protein | Bacteria;Candidatus Aminicenantes;unclassified Candidatus Aminicenantes;unclassified Candidatus Aminicenantes;unclassified Candidatus Aminicenantes;unclassified Candidatus Aminicenantes;Aminicenantes bacterium SCGC AAA255-E05;Aminicenantes bacterium SCGC AAA255-E05 (contamination screened) |
| Ga0586324 0025821 2611 3909 | 0.00 | 2936467.12 | 2812111.81 | 969000.00 | 3460800.00 | 0.00 | 0.00 | 1303305.87 | 1896520.23 | 2094400.00 | 4871942.24 | 1380000.00 | hypothetical protein | Bacteria;Nitrospirae;unclassified Nitrospirae;unclassified Nitrospirae;unclassified Nitrospirae;unclassified Nitrospirae;Nitrospirae bacterium HCH-1;Nitrospirae bacterium HCH-1 |
| Ga0586324 0026258 4188 5327 | 0.00 | 0.00 | 0.00 | 1567500.00 | 0.00 | 0.00 | 0.00 | 0.00 | 0.00 | 0.00 | 0.00 | 0.00 | TRAP-type mannitol/chloroaromatic compound transport system substrate-binding protein | Bacteria;Proteobacteria;Gammaproteobacteria;Acidiferrobacterales;Acidiferrobacteraceae;Sulfuricaulis;Sulfuricaulis limicola;Sulfuricaulis limicola HA5 |
| Ga0586324 0026342 2961 4055 | 0.00 | 0.00 | 0.00 | 0.00 | 0.00 | 0.00 | 0.00 | 0.00 | 0.00 | 1108800.00 | 0.00 | 0.00 | regulator of protease activity HflC (stomatin/prohibitin superfamily) | Bacteria;Chloroflexi;Anaerolineae;Anaerolineales;Anaerolineaceae;Anaerolinea;Anaerolinea thermophila;Anaerolinea thermophila UNI-1 |
| Ga0586324 0026398 2 553 | 0.00 | 5222400.00 | 0.00 | 0.00 | 6509600.00 | 7582700.00 | 4696800.00 | 0.00 | 0.00 | 0.00 | 0.00 | 0.00 | hypothetical protein | Bacteria;Proteobacteria;Deltaproteobacteria;Syntrophobacterales;Syntrophaceae;Desulfobacca;Desulfobacca acetoxidans;Desulfobacca acetoxidans ASRB2, DSM 11109 |
| Ga0586324 0026424 36 2960 | 0.00 | 0.00 | 0.00 | 0.00 | 0.00 | 0.00 | 0.00 | 0.00 | 0.00 | 0.00 | 3150400.00 | 0.00 | hypothetical protein | Bacteria;Ignavibacteriae;Ignavibacteria;Ignavibacteriales;Ignavibacteriaceae;Ignavibacterium;Ignavibacterium album;Ignavibacterium album Mat9-16, JCM 16511 |
| Ga0586324 0026483 1174 3912 | 0.00 | 2679200.00 | 0.00 | 0.00 | 0.00 | 0.00 | 0.00 | 0.00 | 0.00 | 0.00 | 0.00 | 0.00 | hypothetical protein | Bacteria;Proteobacteria;Deltaproteobacteria;Desulfuromonadales;Geobacteraceae;Geobacter;Geobacter uraniireducens;Geobacter uraniireducens Rf4 |
| Ga0586324 0027370 4062 6404 | 0.00 | 0.00 | 0.00 | 0.00 | 0.00 | 0.00 | 0.00 | 0.00 | 0.00 | 0.00 | 0.00 | 1590000.00 | OmcA/MtrC family decaheme c-type cytochrome | Bacteria;Proteobacteria;Deltaproteobacteria;Desulfuromonadales;Geobacteraceae;Geobacter;Geobacter lovleyi;Geobacter lovleyi SZ |
| Ga0586324 0027546 1431 3806 | 0.00 | 1285200.00 | 0.00 | 3990000.00 | 1493500.00 | 0.00 | 0.00 | 1451800.00 | 1142100.00 | 3449600.00 | 1566400.00 | 6075000.00 | type IV pilus secretin PilQ/predicted competence protein | Bacteria;Candidatus Aminicenantes;unclassified Candidatus Aminicenantes;unclassified Candidatus Aminicenantes;unclassified Candidatus Aminicenantes;unclassified Candidatus Aminicenantes;Aminicenantes bacterium SCGC AAA252-K07;Aminicenantes bacterium SCGC AAA252-K07 (contamination screened) |
| Ga0586324 0027645 1 1182 | 0.00 | 0.00 | 0.00 | 0.00 | 0.00 | 0.00 | 0.00 | 0.00 | 0.00 | 0.00 | 2939200.00 | 0.00 | hypothetical protein | Bacteria;Acidobacteria;Vicinamibacteria;unclassified Vicinamibacteria;Vicinamibacteraceae;Luteitalea;Luteitalea pratensis;Luteitalea pratensis DSM 100886 |
| Ga0586324 0027689 169 6369 | 1550000.00 | 0.00 | 0.00 | 0.00 | 0.00 | 0.00 | 0.00 | 0.00 | 0.00 | 0.00 | 0.00 | 1762500.00 | hypothetical protein | |
| Ga0586324 0027862 3590 6352 | 0.00 | 0.00 | 0.00 | 15580000.00 | 16140100.00 | 9091300.00 | 8527200.00 | 12261000.00 | 14158800.00 | 8008000.00 | 11176000.00 | 8265000.00 | outer membrane receptor protein involved in Fe transport | Bacteria;Acidobacteria;Vicinamibacteria;unclassified Vicinamibacteria;Vicinamibacteraceae;Luteitalea;Luteitalea pratensis;Luteitalea pratensis DSM 100886 |
| Ga0586324 0027963 4329 6044 | 1250000.00 | 6936000.00 | 413648.49 | 0.00 | 0.00 | 0.00 | 0.00 | 0.00 | 0.00 | 1401400.00 | 0.00 | 975000.00 | tetratricopeptide (TPR) repeat protein | Bacteria;Bacteroidetes;Bacteroidia;Marinilabiliales;Marinilabiliaceae;Saccharicrinis;Saccharicrinis fermentans;Saccharicrinis fermentans DSM 9555 |
| Ga0586324 0028166 352 3459 | 0.00 | 0.00 | 0.00 | 0.00 | 0.00 | 0.00 | 0.00 | 1213900.00 | 0.00 | 0.00 | 0.00 | 0.00 | hypothetical protein | Bacteria;Candidatus Aminicenantes;unclassified Candidatus Aminicenantes;unclassified Candidatus Aminicenantes;unclassified Candidatus Aminicenantes;unclassified Candidatus Aminicenantes;Aminicenantes bacterium SCGC AAA255-A06;Aminicenantes bacterium SCGC AAA255-A06 (contamination screened) |
| Ga0586324 0028653 4108 5142 | 0.00 | 0.00 | 0.00 | 690254.80 | 0.00 | 0.00 | 0.00 | 0.00 | 0.00 | 2009700.00 | 2340800.00 | 1665000.00 | pyruvate/2-oxoacid:ferredoxin oxidoreductase beta subunit | Bacteria;Firmicutes;Clostridia;Thermoanaerobacterales;Thermoanaerobacteraceae;Moorella;Moorella thermoautotrophica;Moorella thermoautotrophica JW701/3 |
| Ga0586324 0028896 36 2387 | 0.00 | 0.00 | 0.00 | 1149500.00 | 0.00 | 0.00 | 0.00 | 0.00 | 0.00 | 0.00 | 0.00 | 0.00 | hypothetical protein | |
| Ga0586324 0029060 3771 6221 | 2660000.00 | 2040000.00 | 0.00 | 0.00 | 0.00 | 0.00 | 0.00 | 0.00 | 0.00 | 0.00 | 0.00 | 0.00 | uncharacterized repeat protein (TIGR01451 family) | Bacteria;Firmicutes;Clostridia;Halanaerobiales;unclassified Halanaerobiales;Anoxybacter;Anoxybacter fermentans;Anoxybacter fermentans DY22613 |
| Ga0586324 0029102 5068 6219 | 0.00 | 0.00 | 0.00 | 0.00 | 0.00 | 0.00 | 4058400.00 | 0.00 | 2000700.00 | 0.00 | 0.00 | 0.00 | crotonobetainyl-CoA:carnitine CoA-transferase CaiB-like acyl-CoA transferase | Bacteria;Proteobacteria;Deltaproteobacteria;Syntrophobacterales;Syntrophobacteraceae;Desulfacinum;Desulfacinum hydrothermale;Desulfacinum hydrothermale DSM 13146 |
| Ga0586324 0029235 5976 6206 | 39600000.00 | 34748000.00 | 70375000.00 | 23940000.00 | 31003000.00 | 58756000.00 | 0.00 | 0.00 | 15147000.00 | 15323000.00 | 13992000.00 | 19050000.00 | hypothetical protein | Bacteria;Proteobacteria;Deltaproteobacteria;Syntrophobacterales;Syntrophaceae;Desulfobacca;Desulfobacca acetoxidans;Desulfobacca acetoxidans ASRB2, DSM 11109 |
| Ga0586324 0029392 2662 5253 | 0.00 | 1108400.00 | 0.00 | 9139000.00 | 0.00 | 0.00 | 0.00 | 0.00 | 0.00 | 0.00 | 0.00 | 11512500.00 | hypothetical protein | Bacteria;Proteobacteria;Gammaproteobacteria;Chromatiales;Ectothiorhodospiraceae;Thiogranum;Thiogranum longum;Thiogranum longum DSM 19610 |
| Ga0586324 0029427 2888 4132 | 84800000.00 | 63172000.00 | 24428558.74 | 28215000.00 | 51088000.00 | 19858547.41 | 52440000.00 | 46360000.00 | 36053100.00 | 326795700.00 | 381700000.00 | 319095000.00 | hypothetical protein | Bacteria;Proteobacteria;Gammaproteobacteria;Legionellales;Legionellaceae;Legionella;Legionella cherrii;Legionella cherrii ORW |
| Ga0586324 0029649 5408 6166 | 364719.00 | 0.00 | 0.00 | 0.00 | 0.00 | 0.00 | 0.00 | 0.00 | 0.00 | 0.00 | 0.00 | 0.00 | alpha-tubulin suppressor-like RCC1 family protein | Bacteria;Proteobacteria;Deltaproteobacteria;Desulfobacterales;Desulfobacteraceae;Desulfococcus;Desulfococcus multivorans;Desulfococcus multivorans DSM 2059 |
| Ga0586324 0029905 1990 2730 | 0.00 | 0.00 | 0.00 | 0.00 | 0.00 | 0.00 | 0.00 | 0.00 | 2664900.00 | 3041500.00 | 2340800.00 | 0.00 | biopolymer transport protein ExbB/TolQ | Bacteria;Acidobacteria;Acidobacteriia;Acidobacteriales;Acidobacteriaceae;unclassified Acidobacteriaceae;Acidobacteriaceae bacterium URHE0068;Acidobacteriaceae bacterium URHE0068 |
| Ga0586324 0029992 447 2147 | 1490000.00 | 0.00 | 0.00 | 457811.65 | 0.00 | 0.00 | 0.00 | 0.00 | 0.00 | 9394000.00 | 6397600.00 | 5835000.00 | hypothetical protein | Bacteria;Cyanobacteria;unclassified Cyanobacteria;Chroococcales;Microcystaceae;Microcystis;Microcystis aeruginosa;Microcystis aeruginosa CHAOHU 1326 |
| Ga0586324 0030041 3167 5146 | 144732.00 | 0.00 | 0.00 | 2489000.00 | 782917.42 | 0.00 | 1611177.96 | 2305800.00 | 1271700.00 | 0.00 | 0.00 | 0.00 | ABC-type transport system substrate-binding protein | Bacteria;Chloroflexi;Caldilineae;Caldilineales;Caldilineaceae;Caldilinea;Caldilinea aerophila;Caldilinea aerophila STL-6-O1, DSM 14535 |
| Ga0586324 0030050 2968 6126 | 22232140.00 | 2475200.00 | 3184271.70 | 1225500.00 | 988032.65 | 5900900.81 | 513389.88 | 598823.58 | 656737.47 | 3072300.00 | 1504800.00 | 2700000.00 | hypothetical protein | Bacteria;Acidobacteria;Acidobacteriia;Acidobacteriales;Acidobacteriaceae;Acidisarcina;Acidisarcina polymorpha;Acidisarcina polymorpha SBC82 |
| Ga0586324 0030083 422 4954 | 1290000.00 | 3542800.00 | 0.00 | 0.00 | 0.00 | 0.00 | 0.00 | 0.00 | 0.00 | 0.00 | 0.00 | 1065000.00 | FtsP/CotA-like multicopper oxidase with cupredoxin domain | Bacteria;Firmicutes;Bacilli;Bacillales;Paenibacillaceae;Paenibacillus;Paenibacillus sophorae;Paenibacillus sophorae S27 |
| Ga0586324 0030236 3032 6109 | 315653.00 | 0.00 | 0.00 | 0.00 | 0.00 | 0.00 | 0.00 | 0.00 | 0.00 | 0.00 | 0.00 | 0.00 | hypothetical protein | Bacteria;Acidobacteria;Vicinamibacteria;unclassified Vicinamibacteria;Vicinamibacteraceae;Luteitalea;Luteitalea pratensis;Luteitalea pratensis DSM 100886 |
| Ga0586324 0030270 4092 5900 | 0.00 | 3284400.00 | 0.00 | 0.00 | 10815000.00 | 0.00 | 12198000.00 | 8296000.00 | 0.00 | 0.00 | 0.00 | 2407500.00 | hypothetical protein | Bacteria;Ignavibacteriae;Ignavibacteria;Ignavibacteriales;Ignavibacteriaceae;Ignavibacterium;Ignavibacterium album;Ignavibacterium album Mat9-16, JCM 16511 |
| Ga0586324 0030410 1003 5457 | 184534.00 | 6954296.76 | 6193000.00 | 488195.50 | 543390.92 | 18301700.00 | 0.00 | 0.00 | 3888000.00 | 0.00 | 11113615.92 | 0.00 | FtsP/CotA-like multicopper oxidase with cupredoxin domain | Bacteria;Firmicutes;Bacilli;Bacillales;Paenibacillaceae;Paenibacillus;Paenibacillus sophorae;Paenibacillus sophorae S27 |
| Ga0586324 0030502 5331 6083 | 7850000.00 | 3957600.00 | 0.00 | 0.00 | 0.00 | 9647100.00 | 8914800.00 | 1714100.00 | 1215000.00 | 13706000.00 | 4241600.00 | 15450000.00 | hypothetical protein | |
| Ga0586324 0030703 1937 3079 | 0.00 | 0.00 | 0.00 | 0.00 | 0.00 | 0.00 | 0.00 | 0.00 | 0.00 | 3950100.00 | 0.00 | 0.00 | hypothetical protein | Bacteria;Proteobacteria;Deltaproteobacteria;Desulfuromonadales;Geobacteraceae;Geobacter;Geobacter uraniireducens;Geobacter uraniireducens Rf4 |
| Ga0586324 0030709 3 3290 | 14150000.00 | 1638800.00 | 0.00 | 0.00 | 0.00 | 2343963.43 | 2019733.44 | 2116700.00 | 0.00 | 2787400.00 | 1786400.00 | 0.00 | uncharacterized repeat protein (TIGR01451 family) | Bacteria;Proteobacteria;Deltaproteobacteria;Myxococcales;unclassified Myxococcales;Minicystis;Minicystis rosea;Minicystis rosea DSM 24000 |
| Ga0586324 0031100 3 1964 | 1200000.00 | 0.00 | 0.00 | 0.00 | 0.00 | 0.00 | 0.00 | 0.00 | 0.00 | 0.00 | 0.00 | 0.00 | outer membrane receptor protein involved in Fe transport | Bacteria;Acidobacteria;Vicinamibacteria;unclassified Vicinamibacteria;Vicinamibacteraceae;Luteitalea;Luteitalea pratensis;Luteitalea pratensis DSM 100886 |
| Ga0586324 0031105 3660 5375 | 0.00 | 1101600.00 | 0.00 | 0.00 | 10269100.00 | 0.00 | 1293498.72 | 5935300.00 | 0.00 | 2209900.00 | 1936000.00 | 2505000.00 | tetratricopeptide (TPR) repeat protein | Bacteria;Bacteroidetes;Bacteroidia;Marinilabiliales;Marinilabiliaceae;Saccharicrinis;Saccharicrinis fermentans;Saccharicrinis fermentans DSM 9555 |
| Ga0586324 0031316 1685 3109 | 5180000.00 | 8663200.00 | 14703189.77 | 14627994.55 | 27459800.00 | 17297182.81 | 29457600.00 | 14670500.00 | 20177100.00 | 16901500.00 | 5940000.00 | 15532500.00 | hypothetical protein | Bacteria;Proteobacteria;Deltaproteobacteria;Desulfuromonadales;Geobacteraceae;Geobacter;Geobacter metallireducens;Geobacter metallireducens GS-15 |
| Ga0586324 0031445 3 5294 | 0.00 | 0.00 | 0.00 | 0.00 | 0.00 | 0.00 | 0.00 | 0.00 | 0.00 | 6699000.00 | 703532.72 | 4275000.00 | hypothetical protein | Bacteria;Actinobacteria;Actinomycetia;Micrococcales;Cellulomonadaceae;Cellulomonas;Cellulomonas sp. KRMCY2;Cellulomonas sp. KRMCY2 |
| Ga0586324 0031497 4593 5990 | 85912819.00 | 67347200.00 | 178994381.69 | 60941491.10 | 112290600.00 | 172337700.00 | 64638000.00 | 61344958.05 | 65415251.70 | 113028300.00 | 38605600.00 | 253027500.00 | hypothetical protein | Bacteria;Proteobacteria;Deltaproteobacteria;Desulfovibrionales;Desulfovibrionaceae;Halodesulfovibrio;Halodesulfovibrio aestuarii;Halodesulfovibrio aestuarii DSM 10141 |
| Ga0586324 0031701 3 5972 | 0.00 | 0.00 | 0.00 | 0.00 | 0.00 | 0.00 | 0.00 | 0.00 | 0.00 | 0.00 | 496785.52 | 0.00 | GAF domain-containing protein | Bacteria;Proteobacteria;Alphaproteobacteria;Hyphomicrobiales;Bradyrhizobiaceae;Bradyrhizobium;Bradyrhizobium sp. OAE829;Bradyrhizobium sp. OAE829 (Illumina draft) |
| Ga0586324 0031775 3 3590 | 0.00 | 0.00 | 0.00 | 0.00 | 0.00 | 0.00 | 0.00 | 3172000.00 | 0.00 | 0.00 | 0.00 | 0.00 | glutamate synthase domain-containing protein 2/glutamate synthase domain-containing protein | Bacteria;Cyanobacteria;unclassified Cyanobacteria;Chroococcales;Geminocystaceae;Cyanobacterium;Cyanobacterium aponinum;Cyanobacterium aponinum PCC 10605 |
| Ga0586324 0031929 1954 5085 | 0.00 | 0.00 | 1214706.28 | 0.00 | 0.00 | 0.00 | 0.00 | 0.00 | 0.00 | 0.00 | 0.00 | 0.00 | photosystem II stability/assembly factor-like uncharacterized protein | Bacteria;Acidobacteria;Acidobacteriia;Bryobacterales;Solibacteraceae;Candidatus Solibacter;Candidatus Solibacter usitatus;Candidatus Solibacter usitatus Ellin6076 |
| Ga0586324 0032060 4631 5938 | 0.00 | 0.00 | 0.00 | 0.00 | 0.00 | 0.00 | 0.00 | 1500600.00 | 0.00 | 2610300.00 | 583113.52 | 4492500.00 | hypothetical protein | Bacteria;Proteobacteria;Betaproteobacteria;Nitrosomonadales;Thiobacillaceae;Thiobacillus;Thiobacillus thioparus;Thiobacillus thioparus DSM 505 |
| Ga0586324 0032066 4435 4671 | 0.00 | 8840000.00 | 9627300.00 | 0.00 | 0.00 | 15363900.00 | 0.00 | 0.00 | 0.00 | 7338100.00 | 8157600.00 | 5947500.00 | hypothetical protein | Archaea;Euryarchaeota;Methanomicrobia;Methanosarcinales;Methanotrichaceae;Methanothrix;Methanothrix soehngenii;Methanothrix soehngenii GP-6 |
| Ga0586324 0032077 3693 5240 | 0.00 | 0.00 | 0.00 | 0.00 | 0.00 | 0.00 | 2044505.64 | 0.00 | 0.00 | 0.00 | 0.00 | 0.00 | hypothetical protein | Bacteria;Bacteroidetes;Cytophagia;Cytophagales;Fulvivirgaceae;Ohtaekwangia;Ohtaekwangia koreensis;Ohtaekwangia koreensis DSM 25262 |
| Ga0586324 0032077 500 3670 | 2140000.00 | 3597200.00 | 9598553.22 | 4503000.00 | 12751400.00 | 26151600.85 | 11468400.00 | 9760000.00 | 4301100.00 | 2664200.00 | 7339200.00 | 3772500.00 | TonB-linked SusC/RagA family outer membrane protein | Bacteria;Bacteroidetes;Cytophagia;Cytophagales;Fulvivirgaceae;Chryseolinea;Chryseolinea flava;Chryseolinea flava SDU1-6 |
| Ga0586324 0032510 4768 5898 | 0.00 | 0.00 | 0.00 | 0.00 | 0.00 | 0.00 | 0.00 | 0.00 | 0.00 | 0.00 | 0.00 | 488976.75 | hypothetical protein | Bacteria;Bacteroidetes;unclassified Bacteroidetes;Bacteroidetes Order II. Incertae sedis;Rhodothermaceae;unclassified Rhodothermaceae;Rhodothermaceae bacterium RA;Halothermobacillus malaysiensis RA |
| Ga0586324 0032548 5302 5739 | 0.00 | 4059600.00 | 2946803.93 | 4911500.00 | 0.00 | 14371400.00 | 8960400.00 | 7381000.00 | 4082400.00 | 6483400.00 | 24200000.00 | 5752500.00 | phasin family protein | Bacteria;Proteobacteria;Alphaproteobacteria;Rhodospirillales;Acetobacteraceae;Rhodopila;unclassified Rhodopila;Rhodopila sp. LVNP |
| Ga0586324 0032810 3514 4581 | 0.00 | 0.00 | 0.00 | 0.00 | 0.00 | 0.00 | 0.00 | 0.00 | 0.00 | 0.00 | 2455200.00 | 0.00 | signal transduction protein with GAF and PtsI domain | Bacteria;Proteobacteria;Deltaproteobacteria;Syntrophobacterales;Syntrophaceae;Desulfobacca;Desulfobacca acetoxidans;Desulfobacca acetoxidans ASRB2, DSM 11109 |
| Ga0586324 0032864 2738 5872 | 0.00 | 822800.00 | 0.00 | 1995000.00 | 0.00 | 0.00 | 0.00 | 0.00 | 0.00 | 0.00 | 1628000.00 | 1380000.00 | hypothetical protein | Bacteria;Acidobacteria;Acidobacteriia;Acidobacteriales;Acidobacteriaceae;unclassified Acidobacteriaceae;Acidobacteriaceae bacterium KBS 96;Acidobacteriaceae bacterium KBS 96, DSM 24297 |
| Ga0586324 0032979 3 3683 | 1960000.00 | 0.00 | 0.00 | 0.00 | 0.00 | 0.00 | 0.00 | 0.00 | 0.00 | 0.00 | 0.00 | 7800000.00 | hypothetical protein | Bacteria;Acidobacteria;Acidobacteriia;Bryobacterales;Solibacteraceae;Candidatus Solibacter;Candidatus Solibacter usitatus;Candidatus Solibacter usitatus Ellin6076 |
| Ga0586324 0033100 3191 5851 | 12100000.00 | 0.00 | 20774700.00 | 0.00 | 0.00 | 17110700.00 | 0.00 | 0.00 | 0.00 | 0.00 | 0.00 | 0.00 | TonB-linked SusC/RagA family outer membrane protein | Bacteria;Bacteroidetes;Sphingobacteriia;Sphingobacteriales;Sphingobacteriaceae;Mucilaginibacter;Mucilaginibacter sp. OV119;Mucilaginibacter sp. OV119 |
| Ga0586324 0033893 2623 4518 | 9150000.00 | 21338400.00 | 8146542.44 | 0.00 | 0.00 | 8257600.00 | 0.00 | 1323700.00 | 0.00 | 0.00 | 4400000.00 | 12352500.00 | hypothetical protein | Bacteria;Chlorobi;Chlorobia;Chlorobiales;Chlorobiaceae;Chlorobaculum;Chlorobaculum limnaeum;Chlorobaculum limnaeum DSM 1677 |
| Ga0586324 0034103 4942 5703 | 0.00 | 0.00 | 0.00 | 0.00 | 0.00 | 0.00 | 1424078.88 | 0.00 | 0.00 | 0.00 | 0.00 | 0.00 | regulator of protease activity HflC (stomatin/prohibitin superfamily) | Bacteria;Proteobacteria;Deltaproteobacteria;Syntrophorhabdales;Syntrophorhabdaceae;Syntrophorhabdus;Syntrophorhabdus aromaticivorans;Syntrophorhabdus aromaticivorans UI |
| Ga0586324 0034148 1933 3330 | 0.00 | 0.00 | 0.00 | 0.00 | 0.00 | 0.00 | 0.00 | 5721800.00 | 0.00 | 0.00 | 0.00 | 0.00 | HEAT repeat protein | Bacteria;Proteobacteria;Betaproteobacteria;Burkholderiales;Burkholderiaceae;Paraburkholderia;Paraburkholderia sp. WP4 3 2;Paraburkholderia tropici WP4 3 2 |
| Ga0586324 0034223 4211 5683 | 0.00 | 0.00 | 0.00 | 0.00 | 0.00 | 0.00 | 0.00 | 0.00 | 0.00 | 1355200.00 | 0.00 | 0.00 | HEAT repeat protein | Bacteria;Cyanobacteria;unclassified Cyanobacteria;Oscillatoriales;Coleofasciculaceae;Coleofasciculus;Coleofasciculus chthonoplastes;Coleofasciculus chthonoplastes PCC 7420 |
| Ga0586324 0034390 3638 5740 | 6400000.00 | 0.00 | 0.00 | 0.00 | 0.00 | 7741500.00 | 0.00 | 0.00 | 0.00 | 0.00 | 0.00 | 0.00 | OmcA/MtrC family decaheme c-type cytochrome | Bacteria;Proteobacteria;Deltaproteobacteria;Desulfuromonadales;Geobacteraceae;Geobacter;Geobacter lovleyi;Geobacter lovleyi SZ |
| Ga0586324 0034647 3570 4976 | 0.00 | 0.00 | 0.00 | 0.00 | 0.00 | 0.00 | 0.00 | 0.00 | 931500.00 | 0.00 | 0.00 | 0.00 | cytochrome c peroxidase | Bacteria;Proteobacteria;Alphaproteobacteria;Hyphomicrobiales;Beijerinckiaceae;Methylocella;Methylocella silvestris;Methylocella silvestris TVC |
| Ga0586324 0034690 2100 2990 | 0.00 | 0.00 | 4527122.41 | 0.00 | 0.00 | 0.00 | 0.00 | 0.00 | 0.00 | 0.00 | 0.00 | 0.00 | regulator of protease activity HflC (stomatin/prohibitin superfamily) | Bacteria;Bacteroidetes;Sphingobacteriia;Sphingobacteriales;Sphingobacteriaceae;Anseongella;Anseongella ginsenosidimutans;Anseongella ginsenosidimutans DSM 21100 |
| Ga0586324 0034809 3299 5629 | 0.00 | 1917600.00 | 0.00 | 4104000.00 | 0.00 | 11393900.00 | 0.00 | 0.00 | 0.00 | 0.00 | 0.00 | 0.00 | hypothetical protein | Bacteria;Proteobacteria;Betaproteobacteria;unclassified Betaproteobacteria;unclassified Betaproteobacteria;unclassified Betaproteobacteria;Betaproteobacteria bacterium GR16-43;Betaproteobacteria bacterium GR16-43 |
| Ga0586324 0034828 179 1477 | 30700000.00 | 47511600.00 | 66377700.00 | 32034000.00 | 57731500.00 | 32990700.00 | 56316000.00 | 40809000.00 | 49734000.00 | 41218100.00 | 72784800.00 | 26182500.00 | hypothetical protein | Bacteria;Proteobacteria;Alphaproteobacteria;Sphingomonadales;Erythrobacteraceae;Erythrobacter;Erythrobacter neustonensis;Erythrobacter neustonensis DSM 9434 |
| Ga0586324 0034856 3 2381 | 374589.00 | 3304800.00 | 1092985.68 | 1482000.00 | 2327800.00 | 954197.44 | 1155697.80 | 3172000.00 | 1190700.00 | 3149300.00 | 1821600.00 | 2520000.00 | hypothetical protein | Bacteria;Acidobacteria;Thermoanaerobaculia;Thermoanaerobaculales;Thermoanaerobaculaceae;Thermoanaerobaculum;Thermoanaerobaculum aquaticum;Thermoanaerobaculum aquaticum MP-01 |
| Ga0586324 0035021 4349 5689 | 0.00 | 1550400.00 | 0.00 | 0.00 | 0.00 | 0.00 | 0.00 | 0.00 | 0.00 | 0.00 | 0.00 | 0.00 | hypothetical protein | Bacteria;Acidobacteria;Vicinamibacteria;unclassified Vicinamibacteria;Vicinamibacteraceae;Luteitalea;Luteitalea pratensis;Luteitalea pratensis DSM 100886 |
| Ga0586324 0035107 1012 2808 | 0.00 | 0.00 | 0.00 | 0.00 | 0.00 | 0.00 | 0.00 | 165884.62 | 0.00 | 0.00 | 0.00 | 0.00 | methyl-accepting chemotaxis protein | Bacteria;Proteobacteria;Betaproteobacteria;Burkholderiales;Comamonadaceae;Ramlibacter;Ramlibacter lithotrophicus;Ramlibacter lithotrophicus RBP-1 |
| Ga0586324 0035333 2 1945 | 33300000.00 | 0.00 | 26742500.00 | 23275000.00 | 18231000.00 | 16832800.00 | 12859200.00 | 11468000.00 | 12393000.00 | 23100000.00 | 0.00 | 20700000.00 | hypothetical protein | Bacteria;Acidobacteria;Acidobacteriia;Bryobacterales;Solibacteraceae;Candidatus Solibacter;Candidatus Solibacter usitatus;Candidatus Solibacter usitatus Ellin6076 |
| Ga0586324 0035356 400 5352 | 0.00 | 0.00 | 0.00 | 0.00 | 0.00 | 0.00 | 0.00 | 0.00 | 0.00 | 504105.14 | 0.00 | 0.00 | glycosidase/fibronectin type 3 domain-containing protein | Bacteria;Chloroflexi;Chloroflexia;Chloroflexales;Roseiflexaceae;Roseiflexus;Roseiflexus sp. RS-1;Roseiflexus sp. RS-1 |
| Ga0586324 0035758 2858 3379 | 0.00 | 0.00 | 0.00 | 0.00 | 0.00 | 0.00 | 0.00 | 0.00 | 10773000.00 | 0.00 | 0.00 | 0.00 | phage tail-like protein | Bacteria;Bacteroidetes;Chitinophagia;Chitinophagales;Chitinophagaceae;Deminuibacter;Deminuibacter soli;Deminuibacter soli K23C18032701 |
| Ga0586324 0035845 2812 5628 | 0.00 | 520939.84 | 14469100.00 | 698257.60 | 686194.24 | 10719000.00 | 4947600.00 | 3048356.66 | 0.00 | 777700.00 | 0.00 | 1386090.75 | hypothetical protein | Bacteria;Acidobacteria;Vicinamibacteria;unclassified Vicinamibacteria;Vicinamibacteraceae;Luteitalea;Luteitalea pratensis;Luteitalea pratensis DSM 100886 |
| Ga0586324 0036251 323 5596 | 0.00 | 0.00 | 0.00 | 3999500.00 | 0.00 | 0.00 | 0.00 | 841800.00 | 0.00 | 1093400.00 | 0.00 | 0.00 | hypothetical protein | Bacteria;Actinobacteria;Actinomycetia;Propionibacteriales;Kribbellaceae;Kribbella;Kribbella sp. YM55;Kribbella sp. YM55 |
| Ga0586324 0036437 1823 2890 | 0.00 | 0.00 | 0.00 | 0.00 | 0.00 | 0.00 | 0.00 | 0.00 | 0.00 | 0.00 | 0.00 | 7447500.00 | spermidine/putrescine ABC transporter ATP-binding subunit | Bacteria;Proteobacteria;Alphaproteobacteria;Hyphomicrobiales;unclassified Hyphomicrobiales;unclassified Hyphomicrobiales;Rhizobiales bacterium GAS188;Rhizobiales sp. GAS188 |
| Ga0586324 0036550 4744 5574 | 0.00 | 231328.52 | 0.00 | 0.00 | 0.00 | 0.00 | 0.00 | 0.00 | 0.00 | 0.00 | 4435200.00 | 4297500.00 | TRAP-type mannitol/chloroaromatic compound transport system substrate-binding protein | Bacteria;Proteobacteria;Alphaproteobacteria;Rhodospirillales;Azospirillaceae;Azospirillum;Azospirillum sp. OGB3;Azospirillum sp. OGB3 |
| Ga0586324 0036622 618 3944 | 0.00 | 0.00 | 0.00 | 0.00 | 0.00 | 482331.18 | 0.00 | 0.00 | 0.00 | 0.00 | 994400.00 | 1072500.00 | TonB-linked SusC/RagA family outer membrane protein | Bacteria;Bacteroidetes;Flavobacteriia;Flavobacteriales;Flavobacteriaceae;Gaetbulibacter;Gaetbulibacter saemankumensis;Gaetbulibacter saemankumensis DSM 17032 |
| Ga0586324 0036764 3 3644 | 1730000.00 | 0.00 | 0.00 | 0.00 | 0.00 | 0.00 | 0.00 | 0.00 | 0.00 | 0.00 | 0.00 | 0.00 | hypothetical protein | Bacteria;Actinobacteria;Actinomycetia;Micrococcales;Micrococcaceae;Arthrobacter;Arthrobacter sp. PGP41;Arthrobacter sp. PGP41 |
| Ga0586324 0036855 3899 5554 | 0.00 | 992800.00 | 0.00 | 0.00 | 0.00 | 0.00 | 0.00 | 0.00 | 0.00 | 0.00 | 0.00 | 0.00 | formyltetrahydrofolate synthetase | Bacteria;Proteobacteria;Deltaproteobacteria;Syntrophobacterales;Syntrophaceae;Desulfobacca;Desulfobacca acetoxidans;Desulfobacca acetoxidans ASRB2, DSM 11109 |
| Ga0586324 0037002 3 404 | 0.00 | 0.00 | 21450300.00 | 10735000.00 | 0.00 | 0.00 | 0.00 | 0.00 | 0.00 | 0.00 | 0.00 | 0.00 | hypothetical protein | Bacteria;Proteobacteria;Alphaproteobacteria;Hyphomicrobiales;Xanthobacteraceae;Pseudolabrys;Pseudolabrys taiwanensis;Pseudolabrys taiwanensis CC-BB4 |
| Ga0586324 0037909 1 1029 | 0.00 | 992800.00 | 725369.20 | 0.00 | 0.00 | 0.00 | 0.00 | 0.00 | 0.00 | 0.00 | 0.00 | 600969.00 | hypothetical protein | |
| Ga0586324 0038220 1367 2968 | 0.00 | 0.00 | 0.00 | 0.00 | 0.00 | 0.00 | 0.00 | 0.00 | 0.00 | 0.00 | 4593600.00 | 0.00 | arylsulfatase A-like enzyme | Bacteria;Proteobacteria;Alphaproteobacteria;Hyphomicrobiales;Bradyrhizobiaceae;Bradyrhizobium;Bradyrhizobium erythrophlei;Bradyrhizobium erythrophlei GAS478 |
| Ga0586324 0038770 2822 4267 | 0.00 | 0.00 | 0.00 | 0.00 | 0.00 | 0.00 | 0.00 | 548981.70 | 0.00 | 3187800.00 | 2419809.04 | 2155428.00 | hypothetical protein | Bacteria;Chloroflexi;Caldilineae;Caldilineales;Caldilineaceae;Caldilinea;Caldilinea aerophila;Caldilinea aerophila STL-6-O1, DSM 14535 |
| Ga0586324 0039147 2864 4852 | 8870000.00 | 1387200.00 | 8782800.00 | 0.00 | 0.00 | 0.00 | 0.00 | 0.00 | 0.00 | 19357800.00 | 3282400.00 | 0.00 | hypothetical protein | |
| Ga0586324 0039761 979 2583 | 0.00 | 0.00 | 0.00 | 0.00 | 0.00 | 0.00 | 3921600.00 | 0.00 | 0.00 | 0.00 | 0.00 | 0.00 | arylsulfatase A-like enzyme | Bacteria;Proteobacteria;Alphaproteobacteria;Hyphomicrobiales;Bradyrhizobiaceae;Rhodopseudomonas;Rhodopseudomonas palustris;Rhodopseudomonas palustris 7850, DSM 127 |
| Ga0586324 0040066 991 2409 | 0.00 | 0.00 | 0.00 | 0.00 | 1998200.00 | 3295008.69 | 0.00 | 0.00 | 0.00 | 0.00 | 0.00 | 0.00 | hypothetical protein | Bacteria;Proteobacteria;Betaproteobacteria;Nitrosomonadales;Thiobacillaceae;Thiobacillus;Thiobacillus thiophilus;Thiobacillus thiophilus DSM 19892 |
| Ga0586324 0040259 3 1988 | 434000000.00 | 435812000.00 | 627150449.48 | 293208000.00 | 288194000.00 | 344397500.00 | 371594400.00 | 265167000.00 | 339633000.00 | 87202500.00 | 96712000.00 | 117300000.00 | S-layer protein (TIGR01567 family) | Archaea;Euryarchaeota;Methanomicrobia;Methanosarcinales;Methanotrichaceae;Methanothrix;Methanothrix soehngenii;Methanothrix soehngenii GP-6 |
| Ga0586324 0040272 1917 2399 | 4480000.00 | 0.00 | 10134000.00 | 1216000.00 | 0.00 | 0.00 | 0.00 | 0.00 | 1555200.00 | 0.00 | 0.00 | 0.00 | hypothetical protein | Bacteria;Proteobacteria;Alphaproteobacteria;Hyphomicrobiales;unclassified Hyphomicrobiales;Methyloligella;Methyloligella halotolerans;Methyloligella halotolerans VKM B-2706 |
| Ga0586324 0040431 2201 3847 | 0.00 | 0.00 | 0.00 | 0.00 | 0.00 | 0.00 | 0.00 | 6710000.00 | 0.00 | 0.00 | 0.00 | 0.00 | arylsulfatase A-like enzyme | Bacteria;Proteobacteria;Alphaproteobacteria;Hyphomicrobiales;Bradyrhizobiaceae;Bradyrhizobium;Bradyrhizobium pachyrhizi;Bradyrhizobium pachyrhizi BR3262 |
| Ga0586324 0040522 1 2985 | 1260000.00 | 4161600.00 | 0.00 | 0.00 | 0.00 | 0.00 | 1011471.84 | 0.00 | 0.00 | 0.00 | 0.00 | 0.00 | outer membrane receptor protein involved in Fe transport | Bacteria;Ignavibacteriae;Ignavibacteria;Ignavibacteriales;Ignavibacteriaceae;Ignavibacterium;Ignavibacterium album;Ignavibacterium album Mat9-16, JCM 16511 |
| Ga0586324 0041355 3549 4028 | 0.00 | 0.00 | 31077600.00 | 0.00 | 0.00 | 0.00 | 0.00 | 0.00 | 0.00 | 0.00 | 0.00 | 0.00 | predicted Zn-dependent protease | |
| Ga0586324 0041611 2220 3815 | 944278.00 | 0.00 | 0.00 | 0.00 | 0.00 | 1192548.30 | 0.00 | 0.00 | 0.00 | 0.00 | 0.00 | 0.00 | hypothetical protein | Bacteria;Actinobacteria;Actinomycetia;Propionibacteriales;Nocardioidaceae;Nocardioides;Nocardioides sp. Soil805;Nocardioides sp. Soil805 |
| Ga0586324 0041657 3293 4804 | 4680000.00 | 13688400.00 | 29782700.00 | 40517500.00 | 39830100.00 | 0.00 | 7911600.00 | 5227700.00 | 15795000.00 | 13151600.00 | 36291200.00 | 35167500.00 | hypothetical protein | |
| Ga0586324 0041843 2922 4511 | 0.00 | 0.00 | 0.00 | 0.00 | 0.00 | 0.00 | 0.00 | 0.00 | 0.00 | 5682600.00 | 0.00 | 0.00 | glutamate synthase domain-containing protein 2 | Archaea;Euryarchaeota;Methanomicrobia;Methanomicrobiales;Methanospirillaceae;Methanospirillum;Methanospirillum hungatei;Methanospirillum hungatei JF-1 |
| Ga0586324 0042064 267 710 | 0.00 | 0.00 | 0.00 | 0.00 | 0.00 | 0.00 | 0.00 | 646600.00 | 0.00 | 0.00 | 0.00 | 0.00 | phasin | Bacteria;Proteobacteria;Alphaproteobacteria;Hyphomicrobiales;Bradyrhizobiaceae;Bradyrhizobium;Bradyrhizobium erythrophlei;Bradyrhizobium erythrophlei GAS138 |
| Ga0586324 0042322 3214 4620 | 0.00 | 0.00 | 0.00 | 0.00 | 0.00 | 0.00 | 0.00 | 0.00 | 0.00 | 0.00 | 2384800.00 | 0.00 | hypothetical protein | Bacteria;Proteobacteria;Betaproteobacteria;Rhodocyclales;Rhodocyclaceae;Dechlorobacter;Dechlorobacter hydrogenophilus;Dechlorobacter hydrogenophilus LT-1 |
| Ga0586324 0042324 2487 5189 | 9030000.00 | 0.00 | 0.00 | 0.00 | 0.00 | 0.00 | 0.00 | 0.00 | 0.00 | 0.00 | 0.00 | 0.00 | FtsP/CotA-like multicopper oxidase with cupredoxin domain | Bacteria;Proteobacteria;Gammaproteobacteria;Methylococcales;Methylococcaceae;Methyloterricola;Methyloterricola oryzae;Methyloterricola oryzae 73a |
| Ga0586324 0042337 2390 3316 | 0.00 | 1387200.00 | 0.00 | 1320500.00 | 1957000.00 | 0.00 | 0.00 | 976000.00 | 0.00 | 0.00 | 0.00 | 0.00 | hypothetical protein | Bacteria;Proteobacteria;Alphaproteobacteria;Hyphomicrobiales;Bradyrhizobiaceae;Afipia;Afipia broomeae;Afipia broomeae GAS525 |
| Ga0586324 0042364 296 3163 | 5031644.00 | 6772800.00 | 697129.12 | 4018500.00 | 3419600.00 | 767520.10 | 1293642.36 | 8857200.00 | 9558000.00 | 4235000.00 | 5825600.00 | 2107500.00 | hypothetical protein | Bacteria;Acidobacteria;Thermoanaerobaculia;Thermoanaerobaculales;Thermoanaerobaculaceae;Thermoanaerobaculum;Thermoanaerobaculum aquaticum;Thermoanaerobaculum aquaticum MP-01 |
| Ga0586324 0042559 1396 5136 | 14020000.00 | 35360000.00 | 25447600.00 | 60040000.00 | 21733000.00 | 33387700.00 | 18331200.00 | 10919000.00 | 11097000.00 | 35319900.00 | 2376000.00 | 19042500.00 | 2',3'-cyclic-nucleotide 2'-phosphodiesterase (5'-nucleotidase family) | Bacteria;Actinobacteria;Actinomycetia;Propionibacteriales;Nocardioidaceae;Nocardioides;Nocardioides sp. Root122;Nocardioides sp. Root122 |
| Ga0586324 0042577 3527 4549 | 0.00 | 0.00 | 0.00 | 0.00 | 0.00 | 0.00 | 16142400.00 | 0.00 | 8343000.00 | 0.00 | 0.00 | 0.00 | tetratricopeptide (TPR) repeat protein | Bacteria;Candidatus Aminicenantes;unclassified Candidatus Aminicenantes;unclassified Candidatus Aminicenantes;unclassified Candidatus Aminicenantes;unclassified Candidatus Aminicenantes;Aminicenantes bacterium SCGC AAA252-K07;Aminicenantes bacterium SCGC AAA252-K07 (contamination screened) |
| Ga0586324 0042851 4532 5158 | 0.00 | 0.00 | 0.00 | 0.00 | 0.00 | 0.00 | 2485200.00 | 0.00 | 0.00 | 0.00 | 0.00 | 0.00 | regulator of protease activity HflC (stomatin/prohibitin superfamily) | Archaea;Euryarchaeota;Methanomicrobia;Methanosarcinales;Methanotrichaceae;Methanothrix;Methanothrix soehngenii;Methanothrix soehngenii GP-6 |
| Ga0586324 0042853 1863 3236 | 0.00 | 0.00 | 1970719.57 | 1567500.00 | 3326900.00 | 2002547.40 | 2200086.00 | 1494500.00 | 1725300.00 | 1386000.00 | 241618.96 | 511157.25 | hypothetical protein | Bacteria;Proteobacteria;Deltaproteobacteria;Desulfobacterales;Desulfobacteraceae;Desulfatirhabdium;Desulfatirhabdium butyrativorans;Desulfatirhabdium butyrativorans DSM 18734 |
| Ga0586324 0043229 3489 4571 | 0.00 | 0.00 | 0.00 | 0.00 | 0.00 | 0.00 | 519976.80 | 0.00 | 0.00 | 0.00 | 0.00 | 1050000.00 | TRAP-type mannitol/chloroaromatic compound transport system substrate-binding protein | Bacteria;Proteobacteria;Betaproteobacteria;Nitrosomonadales;Sterolibacteriaceae;Sulfurisoma;Sulfurisoma sediminicola;Sulfurisoma sediminicola DSM 26916 |
| Ga0586324 0044164 1122 3857 | 3850000.00 | 0.00 | 0.00 | 0.00 | 0.00 | 0.00 | 0.00 | 0.00 | 0.00 | 0.00 | 0.00 | 0.00 | hypothetical protein | Bacteria;Proteobacteria;Deltaproteobacteria;Syntrophobacterales;Syntrophaceae;Syntrophus;Syntrophus gentianae;Syntrophus gentianae DSM 8423 |
| Ga0586324 0044459 1 861 | 0.00 | 0.00 | 0.00 | 754634.40 | 0.00 | 0.00 | 0.00 | 0.00 | 0.00 | 0.00 | 0.00 | 0.00 | glyoxylase-like metal-dependent hydrolase (beta-lactamase superfamily II) | Bacteria;Proteobacteria;Alphaproteobacteria;Rhodospirillales;Azospirillaceae;Skermanella;Skermanella aerolata;Skermanella aerolata KACC 11604 |
| Ga0586324 0044470 96 3806 | 0.00 | 0.00 | 0.00 | 0.00 | 0.00 | 0.00 | 0.00 | 0.00 | 0.00 | 0.00 | 1513600.00 | 0.00 | 2',3'-cyclic-nucleotide 2'-phosphodiesterase (5'-nucleotidase family) | Bacteria;Actinobacteria;Actinomycetia;Propionibacteriales;Nocardioidaceae;Nocardioides;Nocardioides sp. Root122;Nocardioides sp. Root122 |
| Ga0586324 0044528 3 4436 | 0.00 | 0.00 | 2137665.96 | 0.00 | 0.00 | 0.00 | 0.00 | 0.00 | 0.00 | 0.00 | 0.00 | 0.00 | FtsP/CotA-like multicopper oxidase with cupredoxin domain | Bacteria;Actinobacteria;Actinomycetia;Propionibacteriales;Kribbellaceae;Kribbella;Kribbella sp. VKM Ac-2575;Kribbella voronezhensis VKM Ac-2575 |
| Ga0586324 0044821 853 1986 | 0.00 | 2726800.00 | 0.00 | 0.00 | 0.00 | 0.00 | 0.00 | 0.00 | 0.00 | 0.00 | 0.00 | 0.00 | predicted CXXCH cytochrome family protein | Bacteria;Proteobacteria;Deltaproteobacteria;Desulfuromonadales;Geobacteraceae;Geobacter;Geobacter uraniireducens;Geobacter uraniireducens Rf4 |
| Ga0586324 0045048 2 1849 | 0.00 | 2686000.00 | 0.00 | 0.00 | 0.00 | 0.00 | 0.00 | 2812100.00 | 0.00 | 0.00 | 0.00 | 0.00 | hypothetical protein | Bacteria;Candidatus Aminicenantes;unclassified Candidatus Aminicenantes;unclassified Candidatus Aminicenantes;unclassified Candidatus Aminicenantes;unclassified Candidatus Aminicenantes;Aminicenantes bacterium SCGC AAA252-O03;Aminicenantes bacterium SCGC AAA252-O03 (contamination screened) |
| Ga0586324 0045054 3258 4424 | 6550000.00 | 0.00 | 0.00 | 0.00 | 4377500.00 | 0.00 | 0.00 | 2092300.00 | 0.00 | 0.00 | 0.00 | 0.00 | TRAP-type mannitol/chloroaromatic compound transport system substrate-binding protein | Bacteria;Proteobacteria;Betaproteobacteria;Burkholderiales;Comamonadaceae;Curvibacter;Curvibacter sp. PAE-UM;Curvibacter sp. PAE-UM |
| Ga0586324 0045090 3047 3433 | 0.00 | 0.00 | 0.00 | 0.00 | 0.00 | 0.00 | 0.00 | 0.00 | 1968300.00 | 4712400.00 | 0.00 | 0.00 | nitrogen regulatory protein PII | Bacteria;Proteobacteria;Gammaproteobacteria;Methylococcales;Methylococcaceae;Methylosarcina;Methylosarcina fibrata;Methylosarcina fibrata AML-C10 |

| | | | | | | | | | | | | | | | |
|---|---|---|---|---|---|---|---|---|---|---|---|---|---|---|---|
| Ga0586324 0045173 1262 4975 | 49100000.00 | 9404400.00 | 2205499.58 | 1083000.00 | 1411100.00 | 0.00 | 0.00 | 383369.75 | 5645700.00 | 22530200.00 | 2552000.00 | 27912854.25 | | 2',3'-cyclic-nucleotide 2'-phosphodiesterase (5'-nucleotidase family) | Bacteria;Actinobacteria;Actinomycetia;Micrococcales;Intrasporangiaceae;Intrasporangium;Intrasporangium oryzae;Intrasporangium oryzae NRRL B-24470 |
| Ga0586324 0045345 3419 5014 | 0.00 | 10200000.00 | 0.00 | 9443000.00 | 0.00 | 0.00 | 0.00 | 0.00 | 0.00 | 0.00 | 8738400.00 | 0.00 | | hypothetical protein | Bacteria;Acidobacteria;Acidobacteriia;Acidobacteriales;Acidobacteriaceae;Acidisarcina;Acidisarcina polymorpha;Acidisarcina polymorpha SBC82 |
| Ga0586324 0045456 283 2805 | 0.00 | 788800.00 | 690609.58 | 0.00 | 907655.57 | 0.00 | 0.00 | 0.00 | 0.00 | 0.00 | 0.00 | 1297500.00 | | hypothetical protein | Bacteria;Proteobacteria;Oligoflexia;Bacteriovoracales;Bacteriovoracaceae;Bacteriovorax;Bacteriovorax sp. DB6 IX;Bacteriovorax sp. DB6 IX |
| Ga0586324 0046036 1485 4556 | 6143511.00 | 17217600.00 | 10266896.15 | 8768500.00 | 10176400.00 | 6669600.00 | 3556800.00 | 3239100.00 | 4552200.00 | 5197500.00 | 7233600.00 | 16132500.00 | | outer membrane receptor protein involved in Fe transport | Bacteria;Ignavibacteriae;Ignavibacteria;Ignavibacteriales;Ignavibacteriaceae;Ignavibacterium;Ignavibacterium album;Ignavibacterium album Mat9-16, JCM 16511 |
| Ga0586324 0046036 2 1420 | 0.00 | 0.00 | 0.00 | 0.00 | 0.00 | 0.00 | 0.00 | 322578.37 | 0.00 | 0.00 | 0.00 | 0.00 | | hypothetical protein | Bacteria;Ignavibacteriae;Ignavibacteria;Ignavibacteriales;Melioribacteraceae;Melioribacter;Melioribacter roseus;Melioribacter roseus P3M |
| Ga0586324 0046204 1707 4967 | 0.00 | 0.00 | 0.00 | 0.00 | 5634100.00 | 0.00 | 0.00 | 0.00 | 0.00 | 0.00 | 0.00 | 0.00 | | hypothetical protein | Bacteria;Acidobacteria;Vicinamibacteria;unclassified Vicinamibacteria;Vicinamibacteraceae;Luteitalea;Luteitalea pratensis;Luteitalea pratensis DSM 100886 |
| Ga0586324 0046671 1877 4678 | 0.00 | 3094000.00 | 6981200.00 | 4085000.00 | 6973100.00 | 5200700.00 | 9758400.00 | 5368000.00 | 4932900.00 | 6652800.00 | 6397600.00 | 5182500.00 | | hypothetical protein | Bacteria;Acidobacteria;Vicinamibacteria;unclassified Vicinamibacteria;Vicinamibacteraceae;Luteitalea;Luteitalea pratensis;Luteitalea pratensis DSM 100886 |
| Ga0586324 0047029 3972 4511 | 0.00 | 0.00 | 0.00 | 1358500.00 | 0.00 | 0.00 | 0.00 | 493050.80 | 1093500.00 | 1686300.00 | 0.00 | 729176.25 | | hypothetical protein | Bacteria;Chloroflexi;Anaerolineae;Anaerolineales;Anaerolineaceae;Levilinea;Levilinea saccharolytica;Levilinea saccharolytica DSM 16555 |
| Ga0586324 0047121 4197 4919 | 1190000.00 | 0.00 | 0.00 | 0.00 | 0.00 | 0.00 | 0.00 | 0.00 | 0.00 | 0.00 | 0.00 | 0.00 | | nucleoid-associated protein YgaU | Bacteria;Ignavibacteriae;Ignavibacteria;Ignavibacteriales;Melioribacteraceae;Melioribacter;Melioribacter roseus;Melioribacter roseus P3M |
| Ga0586324 0047138 387 2354 | 0.00 | 0.00 | 0.00 | 0.00 | 0.00 | 0.00 | 0.00 | 0.00 | 0.00 | 4797100.00 | 0.00 | 18675000.00 | | NADPH-dependent glutamate synthase beta subunit-like oxidoreductase/Pyruvate/2-oxoacid:fer | Bacteria;Proteobacteria;Betaproteobacteria;Nitrosomonadales;Gallionellaceae;Sideroxydans;Sideroxydans lithotrophicus;Sideroxydans lithotrophicus ES-1 |
| Ga0586324 0047545 3 2342 | 450465.00 | 357969.00 | 971721.11 | 5861500.00 | 6344800.00 | 870267.67 | 1538605.56 | 2982900.00 | 1692900.00 | 1932700.00 | 3379200.00 | 3637500.00 | | 2',3'-cyclic-nucleotide 2'-phosphodiesterase (5'-nucleotidase family) | Bacteria;Actinobacteria;Actinomycetia;Propionibacteriales;Nocardioidaceae;Nocardioides;Nocardioides soli;Nocardioides soli DSM 105498 |
| Ga0586324 0047604 3 2105 | 530030.00 | 0.00 | 0.00 | 0.00 | 0.00 | 0.00 | 0.00 | 0.00 | 0.00 | 0.00 | 0.00 | 0.00 | | hypothetical protein | Bacteria;Acidobacteria;Acidobacteriia;Bryobacterales;Solibacteraceae;Candidatus Solibacter;Candidatus Solibacter usitatus;Candidatus Solibacter usitatus Ellin6076 |
| Ga0586324 0047748 1104 2624 | 0.00 | 0.00 | 0.00 | 0.00 | 4233300.00 | 0.00 | 0.00 | 2482700.00 | 3029400.00 | 0.00 | 0.00 | 1402500.00 | | acyl-CoA hydrolase | Bacteria;Proteobacteria;Deltaproteobacteria;Syntrophobacterales;Syntrophaceae;Desulfobacca;Desulfobacca acetoxidans;Desulfobacca acetoxidans ASRB2, DSM 11109 |
| Ga0586324 0047828 2044 3390 | 0.00 | 0.00 | 0.00 | 0.00 | 0.00 | 0.00 | 0.00 | 0.00 | 0.00 | 0.00 | 0.00 | 379071.75 | | hypothetical protein | Bacteria;Actinobacteria;Actinomycetia;Micromonosporales;Micromonosporaceae;Micromonospora;Micromonospora echinospora;Micromonospora echinospora ATCC 15837 |
| Ga0586324 0047906 1 1938 | 0.00 | 8092000.00 | 0.00 | 0.00 | 0.00 | 0.00 | 0.00 | 359402.24 | 2988900.00 | 0.00 | 0.00 | 2497500.00 | | hypothetical protein | |
| Ga0586324 0047997 2836 4176 | 1550000.00 | 7330400.00 | 3552051.45 | 14231000.00 | 4356900.00 | 4406700.00 | 5677200.00 | 6002400.00 | 12798000.00 | 2294600.00 | 3150400.00 | 8190000.00 | | predicted porin | Bacteria;Proteobacteria;Betaproteobacteria;Burkholderiales;Alcaligenaceae;Pigmentiphaga;Pigmentiphaga kullae;Pigmentiphaga kullae DSM 13608 |
| Ga0586324 0048155 134 3583 | 7910000.00 | 8044400.00 | 1417977.43 | 1987457.00 | 2791300.00 | 10079733.53 | 5090834.16 | 4965400.00 | 4673700.00 | 3434200.00 | 0.00 | 3697500.00 | | hypothetical protein | Bacteria;Acidobacteria;Acidobacteriia;Acidobacteriales;Acidobacteriaceae;Acidobacterium;Acidobacterium ailaaui;Acidobacterium ailaaui PMMR2 |
| Ga0586324 0048211 82 1383 | 0.00 | 6086000.00 | 0.00 | 0.00 | 0.00 | 0.00 | 0.00 | 0.00 | 0.00 | 0.00 | 0.00 | 0.00 | | YVTN family beta-propeller protein | Bacteria;Planctomycetes;Candidatus Brocadiae;Candidatus Brocadiales;Candidatus Brocadiaceae;Candidatus Brocadia;Candidatus Brocadia sapporoensis;Candidatus Brocadia sapporoensis 40 |
| Ga0586324 0048534 1079 4405 | 0.00 | 1237600.00 | 0.00 | 5871000.00 | 0.00 | 0.00 | 0.00 | 0.00 | 0.00 | 0.00 | 836498.96 | 765000.00 | | hypothetical protein | Bacteria;Acidobacteria;Blastocatellia;Blastocatellales;Pyrinomonadaceae;Pyrinomonas;Pyrinomonas methylaliphatogenes;Pyrinomonas methylaliphatogenes K22 |
| Ga0586324 0048561 1380 4832 | 12570000.00 | 7820000.00 | 0.00 | 14839000.00 | 11433000.00 | 9105341.89 | 0.00 | 5825500.00 | 5880600.00 | 8393000.00 | 10120000.00 | 6660000.00 | | hypothetical protein | Bacteria;Acidobacteria;Acidobacteriia;Acidobacteriales;Acidobacteriaceae;Acidobacterium;unclassified Acidobacterium;Acidobacterium strain C40 |
| Ga0586324 0048631 3432 4844 | 16700000.00 | 0.00 | 5586012.81 | 1985500.00 | 2379300.00 | 0.00 | 0.00 | 0.00 | 0.00 | 0.00 | 0.00 | 0.00 | | tetratricopeptide (TPR) repeat protein | Bacteria;Bacteroidetes;Bacteroidia;Marinilabiliales;Marinilabiliaceae;Saccharicrinis;Saccharicrinis fermentans;Saccharicrinis fermentans DSM 9555 |
| Ga0586324 0048799 479 3190 | 0.00 | 1475600.00 | 0.00 | 0.00 | 0.00 | 0.00 | 0.00 | 0.00 | 1814400.00 | 0.00 | 0.00 | 0.00 | | hypothetical protein | Bacteria;Acidobacteria;Acidobacteriia;Bryobacterales;Solibacteraceae;Candidatus Solibacter;Candidatus Solibacter usitatus;Candidatus Solibacter usitatus Ellin6076 |
| Ga0586324 0048918 3566 4828 | 0.00 | 0.00 | 0.00 | 0.00 | 0.00 | 0.00 | 0.00 | 0.00 | 0.00 | 0.00 | 0.00 | 2302500.00 | | predicted ribosome quality control (RQC) complex YloA/Tae2 family protein | Archaea;Candidatus Thermoplasmatota;Thermoplasmata;Methanomassiliicoccales;Methanomassiliicoccaceae;Methanomassiliicoccus;Methanomassiliicoccus luminyensis;Methanomassiliicoccus luminyensis B10 |
| Ga0586324 0048934 209 1399 | 0.00 | 3706000.00 | 0.00 | 0.00 | 0.00 | 0.00 | 4628400.00 | 3830800.00 | 923400.00 | 8932000.00 | 0.00 | 1387500.00 | | hypothetical protein | Bacteria;Proteobacteria;Deltaproteobacteria;Desulfobacterales;Desulfobacteraceae;Desulfobacter;Desulfobacter postgatei;Desulfobacter postgatei 2ac9 |
| Ga0586324 0049121 1294 2622 | 2370000.00 | 0.00 | 3879024.96 | 2593500.00 | 0.00 | 1235229.77 | 0.00 | 5734000.00 | 1526364.00 | 0.00 | 0.00 | 0.00 | | hypothetical protein | |
| Ga0586324 0049539 2199 3209 | 0.00 | 7820000.00 | 0.00 | 0.00 | 0.00 | 0.00 | 0.00 | 0.00 | 0.00 | 0.00 | 0.00 | 0.00 | | hypothetical protein | Bacteria;Spirochaetes;Spirochaetia;Spirochaetales;Spirochaetaceae;Spirochaeta;Spirochaeta lutea;Spirochaeta lutea JC230 |
| Ga0586324 0049606 3264 4799 | 0.00 | 1856400.00 | 0.00 | 0.00 | 0.00 | 0.00 | 0.00 | 0.00 | 0.00 | 0.00 | 0.00 | 0.00 | | hypothetical protein | Bacteria;Verrucomicrobia;Verrucomicrobiae;Verrucomicrobiales;Verrucomicrobia subdivision 3;Pedosphaera;Pedosphaera parvula;Pedosphaera parvula Ellin514 |
| Ga0586324 0049708 651 3590 | 0.00 | 1496000.00 | 0.00 | 0.00 | 0.00 | 3510944.93 | 0.00 | 0.00 | 0.00 | 0.00 | 0.00 | 0.00 | | outer membrane receptor for ferrienterochelin and colicin | Bacteria;Acidobacteria;Holophagae;Holophagales;Holophagaceae;Geothrix;Geothrix fermentans;Geothrix fermentans DSM 14018 |
| Ga0586324 0050390 3 2897 | 0.00 | 0.00 | 0.00 | 1833500.00 | 0.00 | 0.00 | 0.00 | 0.00 | 0.00 | 0.00 | 1196800.00 | 0.00 | | hypothetical protein | Bacteria;Candidatus Aminicenantes;unclassified Candidatus Aminicenantes;unclassified Candidatus Aminicenantes;unclassified Candidatus Aminicenantes;unclassified Candidatus Aminicenantes;Aminicenantes bacterium SCGC AAA255-E05;Aminicenantes bacterium SCGC AAA255-E05 (contamination screened) |
| Ga0586324 0050395 3990 4763 | 0.00 | 1088000.00 | 0.00 | 0.00 | 0.00 | 0.00 | 0.00 | 0.00 | 0.00 | 0.00 | 0.00 | 0.00 | | uncharacterized coiled-coil DUF342 family protein | Archaea;Euryarchaeota;Methanomicrobia;Methanomicrobiales;Methanoregulaceae;Methanoregula;Methanoregula formicica;Methanoregula formicica SMSP, DSM 22288 |
| Ga0586324 0050501 1042 2631 | 0.00 | 8636000.00 | 0.00 | 10260000.00 | 11021000.00 | 7423900.00 | 5745600.00 | 4642100.00 | 0.00 | 0.00 | 4752000.00 | 4852500.00 | | glutamate synthase domain-containing protein 2 | Bacteria;Firmicutes;Clostridia;Thermoanaerobacterales;Thermoanaerobacterales Family III. Incertae Sedis;Caldicellulosiruptor;Caldicellulosiruptor saccharolyticus;Caldicellulosiruptor saccharolyticus DSM 8903 |
| Ga0586324 0050544 2812 4344 | 2190000.00 | 0.00 | 0.00 | 0.00 | 0.00 | 0.00 | 0.00 | 0.00 | 0.00 | 0.00 | 0.00 | 0.00 | | ABC-type transport system substrate-binding protein | Bacteria;Proteobacteria;Betaproteobacteria;Burkholderiales;Burkholderiaceae;Burkholderia;Burkholderia sp. YI23;Burkholderia sp. YI23 |
| Ga0586324 0050751 2887 4539 | 7770000.00 | 9894000.00 | 19142000.00 | 14725000.00 | 17716000.00 | 19413300.00 | 7683600.00 | 10553000.00 | 11988000.00 | 28721000.00 | 20706400.00 | 27660703.50 | | ABC-type oligopeptide transport system substrate-binding subunit | Bacteria;Actinobacteria;Rubrobacteria;Gaiellales;Gaiellaceae;Gaiella;Gaiella occulta;Gaiella occulta F2-233 |
| Ga0586324 0051039 630 3815 | 856932.00 | 1808800.00 | 3787841.48 | 0.00 | 0.00 | 0.00 | 0.00 | 0.00 | 0.00 | 1601600.00 | 897600.00 | 802500.00 | | hypothetical protein | Bacteria;Acidobacteria;Vicinamibacteria;unclassified Vicinamibacteria;Vicinamibacteraceae;Luteitalea;Luteitalea pratensis;Luteitalea pratensis DSM 100886 |
| Ga0586324 0051059 3 1988 | 0.00 | 796806.32 | 0.00 | 0.00 | 6206911.84 | 0.00 | 0.00 | 0.00 | 172403.64 | 0.00 | 0.00 | 0.00 | | outer membrane receptor protein involved in Fe transport | Bacteria;Acidobacteria;Vicinamibacteria;unclassified Vicinamibacteria;Vicinamibacteraceae;Luteitalea;Luteitalea pratensis;Luteitalea pratensis DSM 100886 |
| Ga0586324 0051133 3 3140 | 0.00 | 0.00 | 0.00 | 0.00 | 0.00 | 0.00 | 0.00 | 2165500.00 | 0.00 | 0.00 | 0.00 | 0.00 | | PAS domain S-box-containing protein | Bacteria;Proteobacteria;Deltaproteobacteria;Desulfuromonadales;Geobacteraceae;Geobacter;Geobacter sp. DSM 2909;Geobacter sp. DSM 2909 |
| Ga0586324 0051458 1610 3097 | 1520000.00 | 10132000.00 | 0.00 | 1805000.00 | 0.00 | 0.00 | 0.00 | 2098400.00 | 22194000.00 | 15092000.00 | 0.00 | 0.00 | | predicted porin | Bacteria;Proteobacteria;Gammaproteobacteria;Chromatiales;Chromatiaceae;Thiobaca;Thiobaca trueperi;Thiobaca trueperi DSM 13587 |
| Ga0586324 0052077 2670 4688 | 0.00 | 0.00 | 6136700.00 | 0.00 | 0.00 | 0.00 | 0.00 | 0.00 | 0.00 | 0.00 | 0.00 | 0.00 | | dienelactone hydrolase | Bacteria;Acidobacteria;Holophagae;Holophagales;Holophagaceae;Geothrix;Geothrix fermentans;Geothrix fermentans DSM 14018 |
| Ga0586324 0052435 2475 4670 | 0.00 | 1156000.00 | 0.00 | 0.00 | 0.00 | 0.00 | 0.00 | 0.00 | 0.00 | 0.00 | 0.00 | 0.00 | | M6 family metalloprotease-like protein | Bacteria;Actinobacteria;Actinomycetia;Propionibacteriales;Nocardioidaceae;Nocardioides;Nocardioides sp. URHA0032;Nocardioides sp. URHA0032 |
| Ga0586324 0052727 1802 2806 | 0.00 | 0.00 | 0.00 | 0.00 | 0.00 | 0.00 | 0.00 | 0.00 | 475570.44 | 0.00 | 0.00 | 0.00 | | basic membrane lipoprotein Med (substrate-binding protein (PBP1-ABC) superfamily) | Bacteria;Chloroflexi;Anaerolineae;Anaerolineales;Anaerolineaceae;Pelolinea;Pelolinea submarina;Pelolinea submarina DSM 23923 |
| Ga0586324 0052923 3 4442 | 8540000.00 | 1808800.00 | 0.00 | 0.00 | 0.00 | 0.00 | 0.00 | 0.00 | 0.00 | 0.00 | 0.00 | 0.00 | | PKD repeat protein/FtsP/CotA-like multicopper oxidase with cupredoxin domain | Archaea;Euryarchaeota;Methanomicrobia;Methanomicrobiales;Methanoregulaceae;Methanosphaerula;Methanosphaerula palustris;Methanosphaerula palustris E1-9c, DSM 19958 |
| Ga0586324 0053119 1941 4280 | 0.00 | 0.00 | 0.00 | 0.00 | 0.00 | 0.00 | 0.00 | 0.00 | 0.00 | 0.00 | 1654400.00 | 0.00 | | OmcA/MtrC family decaheme c-type cytochrome | Bacteria;Proteobacteria;Deltaproteobacteria;Desulfuromonadales;Desulfuromonadaceae;Desulfuromonas;Desulfuromonas soudanensis;Candidatus Desulfuromonas soudanensis WTL |
| Ga0586324 0053273 386 2056 | 0.00 | 0.00 | 0.00 | 0.00 | 0.00 | 0.00 | 2986800.00 | 0.00 | 0.00 | 2063600.00 | 0.00 | 2242500.00 | | hypothetical protein | Bacteria;Proteobacteria;Betaproteobacteria;Burkholderiales;unclassified Burkholderiales;Ideonella;Ideonella sp. A 288;Ideonella sp. A 288 |
| Ga0586324 0053815 1075 2190 | 53090000.00 | 37542800.00 | 54676195.40 | 2023500.00 | 42693500.00 | 235862404.45 | 34004262.00 | 8716900.00 | 29970000.00 | 64298538.15 | 46184025.36 | 75810000.00 | | predicted porin | Bacteria;Proteobacteria;Betaproteobacteria;Burkholderiales;Comamonadaceae;Curvibacter;Curvibacter delicatus;Curvibacter delicatus NBRC 14919 |
| Ga0586324 0054028 362 2959 | 3720000.00 | 4012000.00 | 6136700.00 | 4427000.00 | 0.00 | 0.00 | 0.00 | 2110600.00 | 1611900.00 | 3349500.00 | 0.00 | 4290000.00 | | hypothetical protein | |
| Ga0586324 0054082 1129 2277 | 0.00 | 0.00 | 1903953.40 | 0.00 | 0.00 | 0.00 | 16621200.00 | 0.00 | 0.00 | 3080000.00 | 8870400.00 | 3150000.00 | | TRAP-type mannitol/chloroaromatic compound transport system substrate-binding protein | Bacteria;Proteobacteria;Betaproteobacteria;Burkholderiales;unclassified Burkholderiales;Leptothrix;Leptothrix cholodnii;Leptothrix cholodnii SP-6 |
| Ga0586324 0054502 1195 1527 | 1150000.00 | 0.00 | 0.00 | 0.00 | 0.00 | 0.00 | 0.00 | 0.00 | 0.00 | 0.00 | 0.00 | 0.00 | | hypothetical protein | Bacteria;Proteobacteria;Alphaproteobacteria;Hyphomicrobiales;unclassified Hyphomicrobiales;Methyloceanibacter;Methyloceanibacter superfactus;Methyloceanibacter superfactus R-67175 |
| Ga0586324 0054896 303 3815 | 20870000.00 | 7677200.00 | 7814366.81 | 0.00 | 0.00 | 0.00 | 0.00 | 0.00 | 0.00 | 8439748.24 | 0.00 | 9450000.00 | | hypothetical protein | Bacteria;Candidatus Aminicenantes;unclassified Candidatus Aminicenantes;unclassified Candidatus Aminicenantes;unclassified Candidatus Aminicenantes;unclassified Candidatus Aminicenantes;Aminicenantes bacterium SCGC AAA255-E05;Aminicenantes bacterium SCGC AAA255-E05 (contamination screened) |
| Ga0586324 0054918 1307 3148 | 944794.00 | 0.00 | 0.00 | 0.00 | 0.00 | 0.00 | 0.00 | 0.00 | 0.00 | 0.00 | 0.00 | 0.00 | | hypothetical protein | Bacteria;Proteobacteria;Deltaproteobacteria;Desulfuromonadales;Geobacteraceae;Geobacter;Geobacter argillaceus;Geobacter argillaceus ATCC BAA-1139 |
| Ga0586324 0055162 2084 2800 | 2560000.00 | 2101200.00 | 1881478.44 | 0.00 | 0.00 | 1580032.21 | 2277991.32 | 1262700.00 | 3280500.00 | 1578500.00 | 1460800.00 | 2265000.00 | | hypothetical protein | Bacteria;Spirochaetes;Spirochaetia;Spirochaetales;Spirochaetaceae;Alkalispirochaeta;Alkalispirochaeta odontotermitis;Alkalispirochaeta odontotermitis JC202 |
| Ga0586324 0055598 3 704 | 0.00 | 1672800.00 | 0.00 | 0.00 | 0.00 | 0.00 | 0.00 | 511891.26 | 0.00 | 0.00 | 0.00 | 1132500.00 | | hypothetical protein | Bacteria;Acidobacteria;Acidobacteriia;Bryobacterales;Solibacteraceae;Candidatus Solibacter;Candidatus Solibacter usitatus;Candidatus Solibacter usitatus Ellin6076 |
| Ga0586324 0055922 3 2489 | 0.00 | 1210400.00 | 6756000.00 | 14345000.00 | 0.00 | 0.00 | 0.00 | 0.00 | 0.00 | 5744200.00 | 7374400.00 | 4672500.00 | | hypothetical protein | Bacteria;Acidobacteria;Vicinamibacteria;unclassified Vicinamibacteria;Vicinamibacteraceae;Luteitalea;Luteitalea pratensis;Luteitalea pratensis DSM 100886 |
| Ga0586324 0056218 429 914 | 0.00 | 11152000.00 | 0.00 | 0.00 | 0.00 | 0.00 | 0.00 | 4532300.00 | 0.00 | 0.00 | 3766400.00 | 0.00 | | rubrerythrin | Archaea;Euryarchaeota;Methanomicrobia;Methanomicrobiales;Methanoregulaceae;Methanoregula;Methanoregula formicica;Methanoregula formicica SMSP, DSM 22288 |
| Ga0586324 0056675 3025 4134 | 0.00 | 0.00 | 0.00 | 0.00 | 0.00 | 0.00 | 0.00 | 402286.46 | 0.00 | 0.00 | 0.00 | 0.00 | | TRAP-type mannitol/chloroaromatic compound transport system substrate-binding protein | Bacteria;Proteobacteria;Alphaproteobacteria;Rhodospirillales;Azospirillaceae;Azospirillum;Azospirillum brasilense;Azospirillum brasilense MTCC 4039 |
| Ga0586324 0057259 3 764 | 0.00 | 59675.37 | 0.00 | 0.00 | 0.00 | 0.00 | 0.00 | 0.00 | 0.00 | 0.00 | 0.00 | 0.00 | | regulator of protease activity HflC (stomatin/prohibitin superfamily) | Bacteria;Chloroflexi;Dehalococcoidia;unclassified Dehalococcoidia;unclassified Dehalococcoidia;Dehalogenimonas;Dehalogenimonas formicexedens;Dehalogenimonas formicexedens NSZ-14 |
| Ga0586324 0057287 1654 4476 | 10660000.00 | 6961926.36 | 0.00 | 15618000.00 | 12174110.75 | 5240400.00 | 3283200.00 | 13755500.00 | 14434967.07 | 3780700.00 | 9301600.00 | 4230324.00 | | hypothetical protein | Bacteria;Ignavibacteriae;Ignavibacteria;Ignavibacteriales;Melioribacteraceae;Melioribacter;Melioribacter roseus;Melioribacter roseus P3M |
| Ga0586324 0057358 1151 3748 | 0.00 | 1482400.00 | 2574677.82 | 4807000.00 | 2976700.00 | 2841043.16 | 0.00 | 0.00 | 2721600.00 | 0.00 | 0.00 | 0.00 | | glycolate oxidase subunit GlcD | Bacteria;Proteobacteria;Deltaproteobacteria;Desulfobacterales;Desulfobacteraceae;Desulfatiglans;Desulfatiglans anilini;Desulfatiglans anilini DSM 4660 |
| Ga0586324 0058286 2720 3268 | 0.00 | 0.00 | 0.00 | 0.00 | 0.00 | 0.00 | 0.00 | 0.00 | 0.00 | 0.00 | 1276000.00 | 3487500.00 | | ribosomal subunit interface protein | Bacteria;Proteobacteria;Betaproteobacteria;Nitrosomonadales;Thiobacillaceae;Thiobacillus;Thiobacillus thioparus;Thiobacillus thioparus DSM 505 |
| Ga0586324 0058706 1 2337 | 936552.00 | 6487200.00 | 0.00 | 1197000.00 | 2842800.00 | 0.00 | 0.00 | 0.00 | 3869213.67 | 1663200.00 | 0.00 | 3030000.00 | | hypothetical protein | Bacteria;Calditrichaeota;Calditrichae;Calditrichales;Calditrichaceae;Caldithrix;Caldithrix abyssi;Caldithrix abyssi LF13, DSM 13497 |
| Ga0586324 0058706 2392 4422 | 0.00 | 5188400.00 | 0.00 | 0.00 | 8332700.00 | 0.00 | 5973600.00 | 0.00 | 0.00 | 0.00 | 6089600.00 | 7185000.00 | | hypothetical protein | |
| Ga0586324 0058739 1 1710 | 14963159.00 | 8241600.00 | 8290647.92 | 10222000.00 | 11371200.00 | 24572259.42 | 33538800.00 | 29408100.00 | 5921100.00 | 4173400.00 | 4963200.00 | 6052500.00 | | hypothetical protein | Bacteria;Bacteroidetes;Cytophagia;Cytophagales;Flammeovirgaceae;Flexithrix;Flexithrix dorotheae;Flexithrix dorotheae DSM 6795 |
| Ga0586324 0058739 1740 3296 | 3600000.00 | 4432448.08 | 0.00 | 0.00 | 2080600.00 | 2053732.61 | 4468800.00 | 3782000.00 | 3118500.00 | 624127.35 | 0.00 | 0.00 | | hypothetical protein | Bacteria;Bacteroidetes;Cytophagia;Cytophagales;Cytophagaceae;Dyadobacter;Dyadobacter fermentans;Dyadobacter fermentans NS114, DSM 18053 |
| Ga0586324 0058864 3687 4418 | 0.00 | 37672000.00 | 83324000.00 | 0.00 | 73542000.00 | 0.00 | 4081200.00 | 30134000.00 | 26487000.00 | 53669000.00 | 10208000.00 | 46275000.00 | | ubiquitin | Archaea;Euryarchaeota;Methanomicrobia;Methanosarcinales;Methanotrichaceae;Methanothrix;Methanothrix soehngenii;Methanothrix soehngenii GP-6 |
| Ga0586324 0058874 589 1935 | 0.00 | 0.00 | 0.00 | 0.00 | 0.00 | 0.00 | 0.00 | 0.00 | 5370300.00 | 0.00 | 0.00 | 0.00 | | ABC-type glycerol-3-phosphate transport system substrate-binding protein | Bacteria;Proteobacteria;Alphaproteobacteria;Rhodospirillales;Stellaceae;Stella;Stella humosa;Stella humosa DSM 5900 |
| Ga0586324 0059356 1446 2591 | 0.00 | 0.00 | 0.00 | 0.00 | 0.00 | 16515200.00 | 0.00 | 0.00 | 9801000.00 | 0.00 | 0.00 | 0.00 | | predicted porin | Bacteria;Proteobacteria;Gammaproteobacteria;Acidiferrobacterales;Acidiferrobacteraceae;Sulfuricaulis;Sulfuricaulis limicola;Sulfuricaulis limicola HA5 |
| Ga0586324 0060200 1866 2405 | 0.00 | 0.00 | 3805452.12 | 0.00 | 0.00 | 0.00 | 2713200.00 | 0.00 | 0.00 | 0.00 | 0.00 | 0.00 | | phasin family protein | Bacteria;Proteobacteria;Betaproteobacteria;Burkholderiales;unclassified Burkholderiales;Methylibium;Methylibium sp. CF059;Methylibium sp. CF059 |
| Ga0586324 0060369 1798 2841 | 0.00 | 0.00 | 0.00 | 0.00 | 0.00 | 0.00 | 0.00 | 0.00 | 0.00 | 0.00 | 3687200.00 | 0.00 | | tetratricopeptide (TPR) repeat protein | Bacteria;Proteobacteria;Betaproteobacteria;Nitrosomonadales;Nitrosomonadaceae;Nitrosospira;Nitrosospira sp. Nsp13;Nitrosospira sp. Nsp13 |
| Ga0586324 0060550 1629 3026 | 1320000.00 | 6623200.00 | 0.00 | 0.00 | 1060900.00 | 0.00 | 0.00 | 0.00 | 0.00 | 0.00 | 0.00 | 0.00 | | S-layer protein (TIGR01567 family) | Archaea;Euryarchaeota;Methanomicrobia;Methanosarcinales;Methanotrichaceae;Methanothrix;Methanothrix thermoacetophila;Methanothrix thermoacetophila PT |
| Ga0586324 0060583 3763 4356 | 0.00 | 4902800.00 | 0.00 | 6593000.00 | 3296000.00 | 0.00 | 0.00 | 0.00 | 0.00 | 0.00 | 0.00 | 0.00 | | glutamate synthase domain-containing protein 2 | Bacteria;Proteobacteria;Deltaproteobacteria;Syntrophobacterales;Syntrophaceae;Desulfobacca;Desulfobacca acetoxidans;Desulfobacca acetoxidans ASRB2, DSM 11109 |
| Ga0586324 0060696 656 2350 | 0.00 | 0.00 | 0.00 | 0.00 | 10124900.00 | 0.00 | 0.00 | 0.00 | 0.00 | 0.00 | 0.00 | 0.00 | | outer membrane protein OmpA-like peptidoglycan-associated protein | Bacteria;Acidobacteria;Vicinamibacteria;unclassified Vicinamibacteria;Vicinamibacteraceae;Luteitalea;Luteitalea pratensis;Luteitalea pratensis DSM 100886 |
| Ga0586324 0060810 204 779 | 747973.00 | 4386000.00 | 0.00 | 0.00 | 4511400.00 | 0.00 | 0.00 | 1409100.00 | 0.00 | 1493800.00 | 3634400.00 | 0.00 | | rubrerythrin | Archaea;Euryarchaeota;Methanomicrobia;Methanosarcinales;Methanotrichaceae;Methanothrix;Methanosaeta harundinacea;Methanothrix harundinacea 6Ac |
| Ga0586324 0061010 1 3486 | 2460000.00 | 10125200.00 | 4836451.50 | 0.00 | 3061957.22 | 519152.93 | 0.00 | 0.00 | 0.00 | 720824.72 | 0.00 | 982500.00 | | FtsP/CotA-like multicopper oxidase with cupredoxin domain | Bacteria;Firmicutes;Bacilli;Bacillales;Paenibacillaceae;Paenibacillus;Paenibacillus durus;Paenibacillus durus ATCC 35681 |
| Ga0586324 0061682 285 3326 | 0.00 | 2040000.00 | 0.00 | 563464.95 | 5459000.00 | 4287600.00 | 6740165.64 | 3202500.00 | 2689200.00 | 3164700.00 | 0.00 | 6405000.00 | | hypothetical protein | Bacteria;Acidobacteria;Holophagae;Holophagales;Holophagaceae;Geothrix;Geothrix fermentans;Geothrix fermentans DSM 14018 |
| Ga0586324 0061949 1 1542 | 0.00 | 0.00 | 0.00 | 3277500.00 | 0.00 | 0.00 | 1893275.52 | 671000.00 | 0.00 | 0.00 | 0.00 | 0.00 | | hypothetical protein | Bacteria;Proteobacteria;Betaproteobacteria;Burkholderiales;Burkholderiaceae;Ralstonia;Ralstonia solanacearum;Ralstonia solanacearum PSI07 |
| Ga0586324 0062088 1447 4308 | 0.00 | 0.00 | 0.00 | 0.00 | 0.00 | 0.00 | 0.00 | 0.00 | 5718600.00 | 0.00 | 0.00 | 0.00 | | diguanylate cyclase (GGDEF)-like protein/PAS domain S-box-containing protein | Bacteria;Proteobacteria;Alphaproteobacteria;Hyphomicrobiales;Xanthobacteraceae;Pseudolabrys;Pseudolabrys taiwanensis;Pseudolabrys taiwanensis CC-BB4 |
| Ga0586324 0063155 3069 4271 | 0.00 | 0.00 | 0.00 | 0.00 | 0.00 | 0.00 | 0.00 | 1482300.00 | 0.00 | 0.00 | 0.00 | 0.00 | | cytochrome c peroxidase | Bacteria;Proteobacteria;Deltaproteobacteria;Desulfovibrionales;Desulfovibrionaceae;Desulfohalovibrio;Desulfohalovibrio alkalitolerans;Desulfovibrio alkalitolerans DSM 16529 |
| Ga0586324 0063376 2042 3037 | 0.00 | 0.00 | 0.00 | 0.00 | 0.00 | 0.00 | 3853200.00 | 0.00 | 0.00 | 2864400.00 | 3634400.00 | 3172500.00 | | tripartite ATP-independent transporter DctP family solute receptor | Bacteria;Firmicutes;Negativicutes;Selenomonadales;Sporomusaceae;Anaerosporomusa;Anaerosporomusa subterranea;Anaerosporomusa subterranea RU4 |
| Ga0586324 0063616 1578 2948 | 0.00 | 0.00 | 0.00 | 0.00 | 0.00 | 0.00 | 0.00 | 0.00 | 0.00 | 1208900.00 | 1267200.00 | 907500.00 | | hypothetical protein | |
| Ga0586324 0064760 3 3122 | 0.00 | 0.00 | 4610300.03 | 0.00 | 0.00 | 0.00 | 0.00 | 0.00 | 0.00 | 0.00 | 0.00 | 0.00 | | hypothetical protein | Bacteria;Acidobacteria;Vicinamibacteria;unclassified Vicinamibacteria;Vicinamibacteraceae;Luteitalea;Luteitalea pratensis;Luteitalea pratensis DSM 100886 |
| Ga0586324 0065262 1613 3040 | 0.00 | 13804000.00 | 0.00 | 5415000.00 | 0.00 | 0.00 | 0.00 | 22814000.00 | 907200.00 | 0.00 | 0.00 | 0.00 | | crotonobetainyl-CoA:carnitine CoA-transferase CaiB-like acyl-CoA transferase | Bacteria;Proteobacteria;Deltaproteobacteria;Syntrophobacterales;Syntrophaceae;Desulfobacca;Desulfobacca acetoxidans;Desulfobacca acetoxidans ASRB2, DSM 11109 |
| Ga0586324 0065262 3040 4209 | 0.00 | 13532000.00 | 27755900.00 | 0.00 | 0.00 | 27512100.00 | 0.00 | 13542000.00 | 0.00 | 0.00 | 0.00 | 6502500.00 | | crotonobetainyl-CoA:carnitine CoA-transferase CaiB-like acyl-CoA transferase | Bacteria;Proteobacteria;Deltaproteobacteria;Syntrophobacterales;Syntrophobacteraceae;Desulfosoma;Desulfosoma caldarium;Desulfosoma caldarium DSM 22027 |
| Ga0586324 0065262 76 504 | 0.00 | 0.00 | 0.00 | 0.00 | 0.00 | 0.00 | 0.00 | 4867800.00 | 0.00 | 0.00 | 0.00 | 0.00 | | hypothetical protein | Bacteria;Proteobacteria;Deltaproteobacteria;Syntrophobacterales;Syntrophobacteraceae;Desulfacinum;Desulfacinum hydrothermale;Desulfacinum hydrothermale DSM 13146 |
| Ga0586324 0065522 4 2763 | 0.00 | 0.00 | 1431157.26 | 0.00 | 0.00 | 0.00 | 0.00 | 0.00 | 0.00 | 0.00 | 0.00 | 0.00 | | hypothetical protein | Bacteria;Acidobacteria;Acidobacteriia;Acidobacteriales;Acidobacteriaceae;Granulicella;Granulicella mallensis;Granulicella mallensis MP5ACTX8 |
| Ga0586324 0066633 2325 4163 | 0.00 | 0.00 | 0.00 | 0.00 | 0.00 | 0.00 | 0.00 | 0.00 | 653807.70 | 1686300.00 | 0.00 | 0.00 | | hypothetical protein | Bacteria;Planctomycetes;Planctomycetia;Gemmatales;Gemmataceae;Fimbriiglobus;Fimbriiglobus ruber;Fimbriiglobus ruber SP5 |
| Ga0586324 0066728 376 1353 | 0.00 | 0.00 | 0.00 | 3325000.00 | 2482300.00 | 0.00 | 0.00 | 0.00 | 0.00 | 452434.29 | 0.00 | 0.00 | | tripartite-type tricarboxylate transporter receptor subunit TctC | Bacteria;Proteobacteria;Betaproteobacteria;Burkholderiales;Comamonadaceae;Variovorax;Variovorax sp. Sphag1AA;Variovorax sp. Sphag1AA |
| Ga0586324 0066903 171 4157 | 0.00 | 0.00 | 1438290.47 | 0.00 | 0.00 | 0.00 | 0.00 | 0.00 | 0.00 | 0.00 | 0.00 | 892500.00 | | hypothetical protein | Bacteria;Acidobacteria;Blastocatellia;Blastocatellales;Pyrinomonadaceae;Pyrinomonas;Pyrinomonas methylaliphatogenes;Pyrinomonas methylaliphatogenes K22 |
| Ga0586324 0067674 1 681 | 0.00 | 0.00 | 0.00 | 0.00 | 0.00 | 0.00 | 0.00 | 0.00 | 0.00 | 2079000.00 | 0.00 | 0.00 | | hypothetical protein | |
| Ga0586324 0067734 1597 2466 | 0.00 | 1482400.00 | 0.00 | 0.00 | 0.00 | 0.00 | 0.00 | 0.00 | 0.00 | 0.00 | 0.00 | 0.00 | | hypothetical protein | Archaea;Euryarchaeota;Methanomicrobia;Methanosarcinales;Methanotrichaceae;Methanothrix;Methanothrix soehngenii;Methanothrix soehngenii GP-6 |
| Ga0586324 0068144 1152 1412 | 0.00 | 1026800.00 | 0.00 | 0.00 | 0.00 | 0.00 | 0.00 | 0.00 | 0.00 | 0.00 | 0.00 | 0.00 | | uncharacterized protein with GYD domain | Archaea;Euryarchaeota;Methanomicrobia;Methanomicrobiales;Methanomicrobiaceae;Methanofollis;Methanofollis liminatans;Methanofollis liminatans GKZPZ, DSM 4140 |
| Ga0586324 0068243 263 1990 | 0.00 | 0.00 | 0.00 | 0.00 | 0.00 | 0.00 | 1963271.52 | 0.00 | 0.00 | 0.00 | 0.00 | 0.00 | | PAS domain S-box-containing protein | Bacteria;Proteobacteria;Deltaproteobacteria;Myxococcales;Archangiaceae;Archangium;Archangium sp. Cb G35;Archangium sp. Cb G35 |
| Ga0586324 0068720 2804 3073 | 0.00 | 1094800.00 | 0.00 | 0.00 | 0.00 | 0.00 | 0.00 | 0.00 | 0.00 | 0.00 | 0.00 | 0.00 | | polyhydroxyalkanoate synthesis regulator phasin | Bacteria;Proteobacteria;Deltaproteobacteria;unclassified Deltaproteobacteria;unclassified Deltaproteobacteria;unclassified Deltaproteobacteria;delta proteobacterium NaphS2;delta proteobacterium NaphS2 |
| Ga0586324 0069125 82 1257 | 4780000.00 | 2176000.00 | 5195144.43 | 0.00 | 0.00 | 0.00 | 0.00 | 0.00 | 1571400.00 | 0.00 | 0.00 | 877500.00 | | hypothetical protein | Bacteria;Proteobacteria;Gammaproteobacteria;Cellvibrionales;Halieaceae;Pseudohaliea;Pseudohaliea rubra;Pseudohaliea rubra DSM 19751 |
| Ga0586324 0070012 1261 1935 | 0.00 | 0.00 | 0.00 | 0.00 | 0.00 | 0.00 | 0.00 | 0.00 | 0.00 | 1224300.00 | 0.00 | 0.00 | | hypothetical protein | Bacteria;Proteobacteria;Gammaproteobacteria;Chromatiales;Chromatiaceae;Thioflavicoccus;Thioflavicoccus mobilis;Thioflavicoccus mobilis 8321 |
| Ga0586324 0070106 2335 3486 | 0.00 | 0.00 | 0.00 | 0.00 | 0.00 | 3557378.05 | 0.00 | 4532300.00 | 5459400.00 | 7530600.00 | 0.00 | 0.00 | | predicted porin | Bacteria;Proteobacteria;Betaproteobacteria;Burkholderiales;Burkholderiaceae;Paraburkholderia;Paraburkholderia caffeinilytica;Paraburkholderia caffeinilytica CF1 |
| Ga0586324 0070191 1426 4062 | 0.00 | 594844.28 | 0.00 | 0.00 | 0.00 | 0.00 | 0.00 | 0.00 | 0.00 | 0.00 | 0.00 | 840000.00 | | hypothetical protein | Bacteria;Acidobacteria;Vicinamibacteria;unclassified Vicinamibacteria;Vicinamibacteraceae;Luteitalea;Luteitalea pratensis;Luteitalea pratensis DSM 100886 |
| Ga0586324 0070291 3345 3989 | 0.00 | 0.00 | 0.00 | 0.00 | 0.00 | 0.00 | 0.00 | 0.00 | 0.00 | 0.00 | 0.00 | 2737500.00 | | hypothetical protein | |
| Ga0586324 0071115 3565 4035 | 11836955.00 | 42675182.28 | 38565500.00 | 42275000.00 | 54334665.06 | 36484300.00 | 35568000.00 | 48249695.82 | 40922023.77 | 32863600.00 | 36132800.00 | 32949953.25 | | predicted porin | Bacteria;Proteobacteria;Betaproteobacteria;Burkholderiales;Oxalobacteraceae;Herbaspirillum;Herbaspirillum sp. K1R23-30;Herbaspirillum sp. K1R23-30 |
| Ga0586324 0071290 1 2271 | 0.00 | 0.00 | 0.00 | 0.00 | 2492600.00 | 0.00 | 0.00 | 0.00 | 0.00 | 0.00 | 0.00 | 0.00 | | hypothetical protein | Bacteria;Ignavibacteriae;Ignavibacteria;Ignavibacteriales;Ignavibacteriaceae;Ignavibacterium;Ignavibacterium album;Ignavibacterium album Mat9-16, JCM 16511 |
| Ga0586324 0071466 94 561 | 883715.00 | 0.00 | 0.00 | 0.00 | 0.00 | 0.00 | 0.00 | 0.00 | 0.00 | 0.00 | 0.00 | 0.00 | | hypothetical protein | Bacteria;Proteobacteria;Deltaproteobacteria;Syntrophobacterales;Syntrophobacteraceae;Desulfacinum;Desulfacinum hydrothermale;Desulfacinum hydrothermale DSM 13146 |
| Ga0586324 0071579 2495 3508 | 0.00 | 0.00 | 0.00 | 0.00 | 0.00 | 0.00 | 0.00 | 0.00 | 0.00 | 0.00 | 0.00 | 915000.00 | | TRAP-type C4-dicarboxylate transport system substrate-binding protein | Bacteria;Proteobacteria;Alphaproteobacteria;Rhodospirillales;Acetobacteraceae;Crenalkalicoccus;Crenalkalicoccus roseus;Crenalkalicoccus roseus YIM 78023 |
| Ga0586324 0072105 1 492 | 0.00 | 0.00 | 0.00 | 0.00 | 7168800.00 | 0.00 | 0.00 | 0.00 | 0.00 | 0.00 | 0.00 | 0.00 | | IS30 family transposase | Archaea;Euryarchaeota;Methanomicrobia;Methanosarcinales;Methanotrichaceae;Methanothrix;Methanothrix soehngenii;Methanothrix soehngenii GP-6 |
| Ga0586324 0072641 2 3823 | 0.00 | 0.00 | 0.00 | 0.00 | 0.00 | 0.00 | 0.00 | 0.00 | 0.00 | 5621000.00 | 0.00 | 0.00 | | hypothetical protein | Bacteria;Actinobacteria;Actinomycetia;Micrococcales;Cellulomonadaceae;Cellulomonas;Cellulomonas sp. KRMCY2;Cellulomonas sp. KRMCY2 |
| Ga0586324 0072836 1972 3990 | 0.00 | 0.00 | 0.00 | 0.00 | 0.00 | 0.00 | 3465600.00 | 0.00 | 0.00 | 0.00 | 0.00 | 0.00 | | hypothetical protein | Bacteria;Bacteroidetes;Bacteroidia;Marinilabiliales;Prolixibacteraceae;Mariniphaga;Mariniphaga anaerophila;Mariniphaga anaerophila DSM 26910 |
| Ga0586324 0072935 457 3540 | 5700000.00 | 16136400.00 | 0.00 | 15950500.00 | 8229700.00 | 0.00 | 1433632.08 | 8174000.00 | 7524900.00 | 8393000.00 | 8976000.00 | 3382500.00 | | hypothetical protein | Bacteria;Candidatus Cloacimonetes;unclassified Candidatus Cloacimonetes;unclassified Candidatus Cloacimonetes;unclassified Candidatus Cloacimonetes;unclassified Candidatus Cloacimonetes;Cloacimonetes bacterium JGI 0000039-P09;Cloacimonetes bacterium JGI 0000039-P09 (contamination screened) |
| Ga0586324 0073687 3 902 | 4620000.00 | 8554400.00 | 0.00 | 16245000.00 | 6313900.00 | 0.00 | 0.00 | 8515600.00 | 6852600.00 | 0.00 | 2648800.00 | 1080000.00 | | crotonobetainyl-CoA:carnitine CoA-transferase CaiB-like acyl-CoA transferase | Bacteria;Firmicutes;Clostridia;Thermoanaerobacterales;Thermoanaerobacteraceae;Desulfovirgula;Desulfovirgula thermocuniculi;Desulfovirgula thermocuniculi DSM 16036 |
| Ga0586324 0073896 701 3736 | 0.00 | 0.00 | 0.00 | 0.00 | 0.00 | 0.00 | 0.00 | 0.00 | 0.00 | 0.00 | 0.00 | 1267500.00 | | hypothetical protein | Bacteria;Acidobacteria;Acidobacteriia;Bryobacterales;Solibacteraceae;Candidatus Solibacter;Candidatus Solibacter usitatus;Candidatus Solibacter usitatus Ellin6076 |
| Ga0586324 0074116 1521 3956 | 0.00 | 0.00 | 0.00 | 0.00 | 8559300.00 | 0.00 | 0.00 | 0.00 | 0.00 | 0.00 | 0.00 | 0.00 | | outer membrane receptor protein involved in Fe transport | Bacteria;Acidobacteria;Thermoanaerobaculia;Thermoanaerobaculales;Thermoanaerobaculaceae;Thermoanaerobaculum;Thermoanaerobaculum aquaticum;Thermoanaerobaculum aquaticum MP-01 |
| Ga0586324 0074178 2866 3195 | 1110000.00 | 0.00 | 0.00 | 0.00 | 0.00 | 0.00 | 0.00 | 0.00 | 0.00 | 0.00 | 0.00 | 430826.25 | | 2-oxoacid:acceptor oxidoreductase delta subunit (pyruvate/2-ketoisovalerate family) | Bacteria;Firmicutes;Clostridia;Thermoanaerobacterales;Thermoanaerobacteraceae;Thermanaeromonas;Thermanaeromonas toyohensis;Thermanaeromonas toyohensis ToBE, DSM 14490 |
| Ga0586324 0074902 218 1459 | 0.00 | 0.00 | 0.00 | 0.00 | 0.00 | 0.00 | 0.00 | 0.00 | 0.00 | 0.00 | 0.00 | 1605000.00 | | alkanesulfonate monooxygenase SsuD/methylene tetrahydromethanopterin reductase-like flavin | Bacteria;Proteobacteria;Alphaproteobacteria;Rhodospirillales;Acetobacteraceae;Rhodopila;unclassified Rhodopila;Rhodopila sp. LVNP |
| Ga0586324 0075116 195 3254 | 0.00 | 9928000.00 | 0.00 | 3866500.00 | 11855300.00 | 4367000.00 | 0.00 | 9760000.00 | 0.00 | 0.00 | 11528000.00 | 9000000.00 | | hypothetical protein | Bacteria;Ignavibacteriae;Ignavibacteria;Ignavibacteriales;Melioribacteraceae;Melioribacter;Melioribacter roseus;Melioribacter roseus P3M |
| Ga0586324 0075116 3293 3931 | 0.00 | 479060.68 | 0.00 | 0.00 | 0.00 | 0.00 | 0.00 | 0.00 | 0.00 | 0.00 | 0.00 | 0.00 | | hypothetical protein | Bacteria;Ignavibacteriae;Ignavibacteria;Ignavibacteriales;Melioribacteraceae;Melioribacter;Melioribacter roseus;Melioribacter roseus P3M |
| Ga0586324 0075131 3208 3786 | 0.00 | 0.00 | 8332400.00 | 0.00 | 0.00 | 0.00 | 0.00 | 0.00 | 0.00 | 3395700.00 | 6116000.00 | 2130000.00 | | hypothetical protein | Archaea;Euryarchaeota;Methanomicrobia;Methanosarcinales;Methanotrichaceae;Methanothrix;Methanothrix soehngenii;Methanothrix soehngenii GP-6 |
| Ga0586324 0076212 1 2124 | 0.00 | 0.00 | 0.00 | 0.00 | 1987900.00 | 0.00 | 0.00 | 0.00 | 0.00 | 0.00 | 0.00 | 0.00 | | TonB-linked SusC/RagA family outer membrane protein | Bacteria;Bacteroidetes;Sphingobacteriia;Sphingobacteriales;Sphingobacteriaceae;Daejeonella;Daejeonella rubra;Pedobacter ruber DSM 24536 |
| Ga0586324 0076212 2152 3780 | 0.00 | 0.00 | 0.00 | 3664169.95 | 1874600.00 | 0.00 | 1701623.28 | 1244400.00 | 1109700.00 | 0.00 | 0.00 | 0.00 | | hypothetical protein | Bacteria;Bacteroidetes;Flavobacteriia;Flavobacteriales;Flavobacteriaceae;Muricauda;Muricauda flava;Flagellimonas flava DSM 22638 |
| Ga0586324 0076331 807 1076 | 0.00 | 0.00 | 0.00 | 0.00 | 0.00 | 0.00 | 0.00 | 7564000.00 | 0.00 | 0.00 | 0.00 | 0.00 | | uncharacterized integral membrane protein | |
| Ga0586324 0076404 3430 3897 | 0.00 | 419611.00 | 0.00 | 0.00 | 0.00 | 0.00 | 0.00 | 0.00 | 0.00 | 4034800.00 | 0.00 | 464953.50 | | hypothetical protein | Bacteria;Proteobacteria;Gammaproteobacteria;Cellvibrionales;Halieaceae;Pseudohaliea;Pseudohaliea rubra;Pseudohaliea rubra DSM 19751 |
| Ga0586324 0076466 1688 3229 | 0.00 | 7752000.00 | 0.00 | 0.00 | 0.00 | 0.00 | 0.00 | 0.00 | 0.00 | 15015000.00 | 10648000.00 | 0.00 | | hypothetical protein | |
| Ga0586324 0076692 2179 3483 | 0.00 | 0.00 | 0.00 | 0.00 | 0.00 | 0.00 | 1321761.60 | 0.00 | 0.00 | 0.00 | 0.00 | 0.00 | | hypothetical protein | Bacteria;Proteobacteria;Deltaproteobacteria;Syntrophobacterales;Syntrophaceae;Smithella;Smithella sp. SCADC;Smithella sp. SCADC |
| Ga0586324 0076851 648 3887 | 0.00 | 375380.40 | 842771.59 | 1491500.00 | 6478700.00 | 848309.60 | 0.00 | 0.00 | 0.00 | 426548.43 | 0.00 | 0.00 | | hypothetical protein | Bacteria;Acidobacteria;Vicinamibacteria;unclassified Vicinamibacteria;Vicinamibacteraceae;Luteitalea;Luteitalea pratensis;Luteitalea pratensis DSM 100886 |
| Ga0586324 0076880 3 3026 | 0.00 | 8432000.00 | 1479046.04 | 0.00 | 1215400.00 | 2344419.98 | 0.00 | 0.00 | 633684.87 | 1062600.00 | 1469600.00 | 7665000.00 | | predicted AlkP superfamily phosphohydrolase/phosphomutase | Bacteria;Actinobacteria;Actinomycetia;Propionibacteriales;Kribbellaceae;Kribbella;Kribbella sp. VKM Ac-2527;Kribbella caucasensis VKM Ac-2527 |
| Ga0586324 0077043 1 1059 | 0.00 | 0.00 | 0.00 | 0.00 | 0.00 | 0.00 | 1271483.04 | 0.00 | 0.00 | 0.00 | 0.00 | 0.00 | | arylsulfatase A-like enzyme | Bacteria;Proteobacteria;Alphaproteobacteria;Hyphomicrobiales;Bradyrhizobiaceae;Bradyrhizobium;unclassified Bradyrhizobium;Bradyrhizobium sp. HAMBI 2115 |
| Ga0586324 0077135 3476 3880 | 0.00 | 0.00 | 0.00 | 0.00 | 3399000.00 | 0.00 | 0.00 | 0.00 | 0.00 | 0.00 | 0.00 | 0.00 | | PAS domain-containing protein | Bacteria;Cyanobacteria;unclassified Cyanobacteria;Nostocales;Nostocaceae;Nostoc;Nostoc sp. KVJ20;Nostoc sp. KVJ20 |
| Ga0586324 0077678 1215 3866 | 0.00 | 958800.00 | 0.00 | 0.00 | 0.00 | 0.00 | 0.00 | 916668.96 | 0.00 | 1255100.00 | 2975049.44 | 1020000.00 | | hypothetical protein | Bacteria;Candidatus Aminicenantes;unclassified Candidatus Aminicenantes;unclassified Candidatus Aminicenantes;unclassified Candidatus Aminicenantes;unclassified Candidatus Aminicenantes;Aminicenantes bacterium SCGC AAA252-O03;Aminicenantes bacterium SCGC AAA252-O03 (contamination screened) |
| Ga0586324 0077826 2571 3833 | 5270000.00 | 14534042.56 | 0.00 | 4522000.00 | 2647100.00 | 0.00 | 7090800.00 | 6283000.00 | 4754700.00 | 10626000.00 | 7647200.00 | 930000.00 | | ABC-type sugar transport system substrate-binding protein | Bacteria;Actinobacteria;Actinomycetia;Propionibacteriales;Nocardioidaceae;Nocardioides;Nocardioides immobilis;Nocardioides immobilis CCTCC AB 2017083 |
| Ga0586324 0077958 2395 3630 | 0.00 | 0.00 | 0.00 | 0.00 | 0.00 | 0.00 | 0.00 | 1592100.00 | 0.00 | 0.00 | 0.00 | 0.00 | | uncharacterized protein (TIGR02996 family) | |
| Ga0586324 0077983 2465 3859 | 2460000.00 | 0.00 | 0.00 | 0.00 | 0.00 | 0.00 | 0.00 | 0.00 | 0.00 | 0.00 | 2138400.00 | 2512500.00 | | tetratricopeptide (TPR) repeat protein | Bacteria;Bacteroidetes;Bacteroidia;Marinilabiliales;Marinilabiliaceae;Saccharicrinis;Saccharicrinis fermentans;Saccharicrinis fermentans DSM 9555 |
| Ga0586324 0078057 2917 3660 | 0.00 | 0.00 | 928972.52 | 0.00 | 0.00 | 0.00 | 1039488.48 | 0.00 | 0.00 | 0.00 | 0.00 | 0.00 | | hypothetical protein | |
| Ga0586324 0078062 1 1113 | 61160000.00 | 49483600.00 | 96947879.36 | 104471500.00 | 32506154.19 | 468359392.26 | 175218000.00 | 60072934.20 | 106004700.00 | 114360400.00 | 63069600.00 | 79875000.00 | | predicted porin | Bacteria;Proteobacteria;Gammaproteobacteria;Chromatiales;Halothiobacillaceae;Halothiobacillus;Halothiobacillus sp. LS2;Halothiobacillus sp. LS2 |
| Ga0586324 0078338 3050 3658 | 0.00 | 0.00 | 0.00 | 0.00 | 0.00 | 0.00 | 0.00 | 0.00 | 0.00 | 0.00 | 0.00 | 8250000.00 | | Pyruvate/2-oxoacid:ferredoxin oxidoreductase gamma subunit | Bacteria;Firmicutes;Clostridia;Thermosediminibacterales;Thermosediminibacteraceae;Thermosediminibacter;Thermosediminibacter litoriperuensis;Thermosediminibacter litoriperuensis DSM 16647 |
| Ga0586324 0078482 479 1630 | 0.00 | 0.00 | 0.00 | 0.00 | 0.00 | 0.00 | 0.00 | 1445700.00 | 0.00 | 0.00 | 0.00 | 0.00 | | hypothetical protein | Bacteria;Cyanobacteria;unclassified Cyanobacteria;Nostocales;Hapalosiphonaceae;Fischerella;Fischerella thermalis;Fischerella thermalis CCMEE 5282 |
| Ga0586324 0079091 274 1692 | 12840000.00 | 3964400.00 | 5005452.84 | 2555500.00 | 2183600.00 | 1597921.03 | 6133200.00 | 3208600.00 | 2227500.00 | 1978900.00 | 2692800.00 | 4110000.00 | | hypothetical protein | |
| Ga0586324 0079830 1477 3102 | 0.00 | 508380.24 | 0.00 | 0.00 | 0.00 | 0.00 | 0.00 | 2787700.00 | 0.00 | 0.00 | 0.00 | 0.00 | | hypothetical protein | Bacteria;Bacteroidetes;Cytophagia;Cytophagales;Hymenobacteraceae;Adhaeribacter;Adhaeribacter pallidiroseus;Adhaeribacter pallidiroseus HMF7616 |
| Ga0586324 0079830 3122 3817 | 0.00 | 4216000.00 | 6812300.00 | 5776000.00 | 10547200.00 | 5319800.00 | 5631600.00 | 4105300.00 | 4268700.00 | 4997300.00 | 4725600.00 | 3990000.00 | | hypothetical protein | Bacteria;Bacteroidetes;Cytophagia;Cytophagales;Hymenobacteraceae;Adhaeribacter;Adhaeribacter pallidiroseus;Adhaeribacter pallidiroseus HMF7616 |
| Ga0586324 0079856 48 3344 | 0.00 | 0.00 | 0.00 | 7999000.00 | 0.00 | 0.00 | 0.00 | 0.00 | 0.00 | 0.00 | 0.00 | 0.00 | | hypothetical protein | Bacteria;Proteobacteria;Gammaproteobacteria;unclassified Gammaproteobacteria;unclassified Gammaproteobacteria;unclassified Gammaproteobacteria;Codakia orbicularis gill symbiont;Codakia orbicularis gill symbiont |
| Ga0586324 0080015 3 974 | 0.00 | 0.00 | 2006616.45 | 0.00 | 0.00 | 0.00 | 0.00 | 0.00 | 0.00 | 0.00 | 0.00 | 0.00 | | S1-C subfamily serine protease | Bacteria;Proteobacteria;Deltaproteobacteria;unclassified Deltaproteobacteria;unclassified Deltaproteobacteria;Dissulfuribacter;Dissulfuribacter thermophilus;Dissulfuribacter thermophilus S69 |
| Ga0586324 0080540 699 1871 | 3440000.00 | 0.00 | 0.00 | 3904500.00 | 0.00 | 0.00 | 0.00 | 0.00 | 0.00 | 0.00 | 0.00 | 0.00 | | N-acetylneuraminic acid mutarotase | Bacteria;Proteobacteria;Betaproteobacteria;Burkholderiales;unclassified Burkholderiales;Rubrivivax;Rubrivivax gelatinosus;Rubrivivax gelatinosus CBS |
| Ga0586324 0080768 1 2793 | 0.00 | 1176400.00 | 0.00 | 0.00 | 0.00 | 215755.61 | 0.00 | 0.00 | 0.00 | 0.00 | 0.00 | 0.00 | | hypothetical protein | Bacteria;Acidobacteria;Vicinamibacteria;unclassified Vicinamibacteria;Vicinamibacteraceae;Luteitalea;Luteitalea pratensis;Luteitalea pratensis DSM 100886 |
| Ga0586324 0081101 626 1978 | 0.00 | 11199600.00 | 0.00 | 0.00 | 0.00 | 0.00 | 0.00 | 2397300.00 | 1263600.00 | 0.00 | 3458400.00 | 0.00 | | hypothetical protein | Bacteria;Nitrospirae;Nitrospira;Nitrospirales;Nitrospiraceae;Nitrospira;Nitrospira lenta;Nitrospira lenta N. lenta BS10 |
| Ga0586324 0081562 1649 3781 | 0.00 | 1536800.00 | 0.00 | 0.00 | 0.00 | 0.00 | 0.00 | 0.00 | 0.00 | 0.00 | 0.00 | 0.00 | | hypothetical protein | Bacteria;Acidobacteria;Acidobacteriia;Acidobacteriales;Acidobacteriaceae;unclassified Acidobacteriaceae;Acidobacteriaceae bacterium TAA 166;Acidobacteriaceae bacterium TAA 166 |

| | | | | | | | | | | | | | | | |
|---|---|---|---|---|---|---|---|---|---|---|---|---|---|---|---|
| Ga0586324 0081967 2945 3706 | 0.00 | 0.00 | 0.00 | 0.00 | 0.00 | 3730843.23 | 0.00 | 0.00 | 0.00 | 0.00 | 0.00 | 0.00 | | hypothetical protein | Bacteria;Planctomycetes;Candidatus Brocadiae;Candidatus Brocadiales;Candidatus Brocadiaceae;Candidatus Brocadia;Candidatus Brocadia sinica;Candidatus Brocadia sinica JPN1 |
| Ga0586324 0082082 387 3716 | 0.00 | 0.00 | 0.00 | 0.00 | 2276300.00 | 0.00 | 0.00 | 1415200.00 | 0.00 | 0.00 | 3704800.00 | 3427500.00 | | hypothetical protein | Bacteria;Acidobacteria;Vicinamibacteria;unclassified Vicinamibacteria;Vicinamibacteraceae;Luteitalea;Luteitalea pratensis;Luteitalea pratensis DSM 100886 |
| Ga0586324 0082340 525 3626 | 0.00 | 0.00 | 0.00 | 221714.80 | 0.00 | 0.00 | 0.00 | 0.00 | 0.00 | 0.00 | 0.00 | 0.00 | | hypothetical protein | Bacteria;Acidobacteria;Thermoanaerobaculia;Thermoanaerobaculales;Thermoanaerobaculaceae;Thermoanaerobaculum;Thermoanaerobaculum aquaticum;Thermoanaerobaculum aquaticum MP-01 |
| Ga0586324 0082472 1 3267 | 5710000.00 | 0.00 | 0.00 | 5690500.00 | 5860700.00 | 0.00 | 0.00 | 0.00 | 0.00 | 2402400.00 | 5702400.00 | 5790000.00 | | hypothetical protein | Bacteria;Planctomycetes;Planctomycetia;Planctomycetales;Planctomycetaceae;Planctomyces;Planctomyces sp. SH-PL62;Planctomyces sp. SH-PL62 |
| Ga0586324 0082667 1234 3045 | 0.00 | 0.00 | 0.00 | 0.00 | 1987900.00 | 0.00 | 1735716.12 | 0.00 | 117450000.00 | 0.00 | 0.00 | 0.00 | | formyltetrahydrofolate synthetase | Bacteria;Firmicutes;Clostridia;Eubacteriales;Peptococcaceae;Desulfallas;Desulfallas thermosapovorans;Desulfallas thermosapovorans DSM 6562 |
| Ga0586324 0082969 2490 3680 | 0.00 | 0.00 | 0.00 | 0.00 | 12566000.00 | 0.00 | 0.00 | 0.00 | 0.00 | 0.00 | 0.00 | 0.00 | | hypothetical protein | |
| Ga0586324 0083766 1602 2849 | 0.00 | 0.00 | 0.00 | 0.00 | 0.00 | 0.00 | 69631200.00 | 22838400.00 | 18249300.00 | 20104700.00 | 11193600.00 | 20505000.00 | | predicted porin | Bacteria;Proteobacteria;Betaproteobacteria;Rhodocyclales;Rhodocyclaceae;Aromatoleum;Aromatoleum toluclasticum;Aromatoleum toluclasticum ATCC 700605 |
| Ga0586324 0083793 1401 3050 | 0.00 | 0.00 | 0.00 | 5377000.00 | 0.00 | 0.00 | 0.00 | 0.00 | 0.00 | 0.00 | 0.00 | 0.00 | | thiamine pyrophosphate-dependent acetolactate synthase large subunit-like protein | Bacteria;Proteobacteria;Alphaproteobacteria;Hyphomicrobiales;Hyphomicrobiaceae;Aquabacter;Aquabacter spiritensis;Aquabacter spiritensis DSM 9035 |
| Ga0586324 0084272 1 345 | 0.00 | 0.00 | 0.00 | 0.00 | 0.00 | 0.00 | 0.00 | 0.00 | 0.00 | 7854000.00 | 0.00 | 0.00 | | ABC-type sugar transport system substrate-binding protein | Bacteria;Actinobacteria;Actinomycetia;Propionibacteriales;Nocardioidaceae;Nocardioides;Nocardioides ginsengisegetis;Nocardioides ginsengisegetis DSM 21349 |
| Ga0586324 0084349 1751 2110 | 35500000.00 | 38012000.00 | 0.00 | 22135000.00 | 12051000.00 | 5478600.00 | 8960400.00 | 16043000.00 | 0.00 | 48125000.00 | 53592000.00 | 16575000.00 | | hypothetical protein | |
| Ga0586324 0084810 386 3649 | 0.00 | 526184.00 | 0.00 | 1529500.00 | 8198800.00 | 0.00 | 0.00 | 0.00 | 0.00 | 831600.00 | 497042.48 | 0.00 | | outer membrane receptor protein involved in Fe transport | Bacteria;Acidobacteria;Vicinamibacteria;unclassified Vicinamibacteria;Vicinamibacteraceae;Luteitalea;Luteitalea pratensis;Luteitalea pratensis DSM 100886 |
| Ga0586324 0085723 2432 3691 | 0.00 | 0.00 | 0.00 | 0.00 | 0.00 | 0.00 | 0.00 | 429992.05 | 0.00 | 0.00 | 0.00 | 0.00 | | hypothetical protein | Bacteria;Candidatus Aminicenantes;unclassified Candidatus Aminicenantes;unclassified Candidatus Aminicenantes;unclassified Candidatus Aminicenantes;unclassified Candidatus Aminicenantes;Aminicenantes bacterium SCGC AAA252-D06;Aminicenantes bacterium SCGC AAA252-D06 (contamination screened) |
| Ga0586324 0086302 1929 3386 | 0.00 | 0.00 | 0.00 | 0.00 | 0.00 | 0.00 | 0.00 | 0.00 | 0.00 | 777700.00 | 0.00 | 0.00 | | FAD/FMN-containing dehydrogenase | Bacteria;Proteobacteria;Deltaproteobacteria;unclassified Deltaproteobacteria;unclassified Deltaproteobacteria;unclassified Deltaproteobacteria;delta proteobacterium NaphS2;delta proteobacterium NaphS2 |
| Ga0586324 0086838 1825 3360 | 417720.00 | 0.00 | 0.00 | 0.00 | 0.00 | 0.00 | 0.00 | 0.00 | 0.00 | 0.00 | 0.00 | 0.00 | | glucose/arabinose dehydrogenase | Bacteria;Bacteroidetes;Sphingobacteriia;Sphingobacteriales;Sphingobacteriaceae;Solitalea;Solitalea koreensis;Solitalea koreensis DSM 21342 |
| Ga0586324 0087128 1042 2967 | 0.00 | 0.00 | 3504967.76 | 0.00 | 0.00 | 0.00 | 0.00 | 0.00 | 0.00 | 0.00 | 0.00 | 0.00 | | hypothetical protein | Bacteria;Proteobacteria;Alphaproteobacteria;Rhodospirillales;Rhodospirillaceae;Magnetospirillum;Magnetospirillum magneticum;Magnetospirillum magneticum AMB-1 |
| Ga0586324 0087478 271 3189 | 0.00 | 0.00 | 0.00 | 0.00 | 0.00 | 0.00 | 0.00 | 0.00 | 0.00 | 1886500.00 | 0.00 | 0.00 | | hypothetical protein | Bacteria;Acidobacteria;Vicinamibacteria;unclassified Vicinamibacteria;Vicinamibacteraceae;Luteitalea;Luteitalea pratensis;Luteitalea pratensis DSM 100886 |
| Ga0586324 0088093 2281 3645 | 0.00 | 0.00 | 0.00 | 0.00 | 0.00 | 0.00 | 0.00 | 0.00 | 0.00 | 0.00 | 0.00 | 1260000.00 | | predicted deacylase | Bacteria;Proteobacteria;Gammaproteobacteria;Alteromonadales;Alteromonadaceae;Marinobacter;Marinobacter lipolyticus;Marinobacter lipolyticus SM19 |
| Ga0586324 0088241 701 2422 | 0.00 | 0.00 | 11766700.00 | 0.00 | 0.00 | 0.00 | 0.00 | 0.00 | 0.00 | 0.00 | 0.00 | 0.00 | | hypothetical protein | Bacteria;Proteobacteria;Gammaproteobacteria;Chromatiales;Chromatiaceae;Thiocapsa;Thiocapsa roseopersicina;Thiocapsa roseopersicina DSM 217 |
| Ga0586324 0088823 2 2737 | 4350000.00 | 4406400.00 | 8670200.00 | 0.00 | 0.00 | 0.00 | 0.00 | 0.00 | 0.00 | 0.00 | 0.00 | 2310000.00 | | hypothetical protein | Bacteria;Actinobacteria;Actinomycetia;Micrococcales;Micrococcaceae;Arthrobacter;Arthrobacter psychrochitiniphilus;Arthrobacter psychrochitiniphilus DSM 23143 |
| Ga0586324 0089791 2 2473 | 0.00 | 10628400.00 | 9289500.00 | 3714500.00 | 13245800.00 | 3699825.62 | 5426400.00 | 0.00 | 834300.00 | 0.00 | 2992000.00 | 3277500.00 | | TonB-linked SusC/RagA family outer membrane protein | Bacteria;Bacteroidetes;Cytophagia;Cytophagales;Flammeovirgaceae;Flexithrix;Flexithrix dorotheae;Flexithrix dorotheae DSM 6795 |
| Ga0586324 0089812 1864 3444 | 1150000.00 | 0.00 | 0.00 | 0.00 | 0.00 | 0.00 | 0.00 | 0.00 | 0.00 | 0.00 | 0.00 | 0.00 | | formylglycine-generating enzyme required for sulfatase activity | Bacteria;Proteobacteria;Deltaproteobacteria;Myxococcales;Polyangiaceae;Sorangium;Sorangium cellulosum;Sorangium cellulosum So ceGT47 |
| Ga0586324 0090033 2100 2579 | 58100000.00 | 0.00 | 54106371.84 | 81481500.00 | 9661400.00 | 11347534.37 | 13338000.00 | 2110600.00 | 0.00 | 0.00 | 0.00 | 0.00 | | tetratricopeptide (TPR) repeat protein | Bacteria;Proteobacteria;Deltaproteobacteria;Syntrophobacterales;Syntrophaceae;Desulfobacca;Desulfobacca acetoxidans;Desulfobacca acetoxidans ASRB2, DSM 11109 |
| Ga0586324 0090146 2510 3604 | 0.00 | 0.00 | 0.00 | 0.00 | 0.00 | 0.00 | 0.00 | 0.00 | 0.00 | 0.00 | 4875200.00 | 0.00 | | predicted CXXCH cytochrome family protein | Bacteria;Actinobacteria;Actinomycetia;Micrococcales;Intrasporangiaceae;Intrasporangium;Intrasporangium calvum;Intrasporangium calvum 7KIP, DSM 43043 |
| Ga0586324 0090756 2405 3592 | 0.00 | 26180000.00 | 0.00 | 0.00 | 0.00 | 0.00 | 0.00 | 0.00 | 0.00 | 25756500.00 | 19008000.00 | 0.00 | | formyltetrahydrofolate synthetase | Bacteria;Proteobacteria;Deltaproteobacteria;Syntrophobacterales;Syntrophaceae;Desulfobacca;Desulfobacca acetoxidans;Desulfobacca acetoxidans ASRB2, DSM 11109 |
| Ga0586324 0090920 1742 2719 | 0.00 | 0.00 | 0.00 | 0.00 | 0.00 | 0.00 | 0.00 | 0.00 | 923400.00 | 0.00 | 0.00 | 1312500.00 | | glyoxylase-like metal-dependent hydrolase (beta-lactamase superfamily II) | Bacteria;Proteobacteria;Alphaproteobacteria;Rhodospirillales;Azospirillaceae;Skermanella;Skermanella aerolata;Skermanella aerolata KACC 11604 |
| Ga0586324 0091051 3 1430 | 0.00 | 14892000.00 | 0.00 | 0.00 | 10609000.00 | 5796200.00 | 0.00 | 12566000.00 | 14256000.00 | 0.00 | 0.00 | 0.00 | | hypothetical protein | Bacteria;Proteobacteria;Deltaproteobacteria;Myxococcales;Anaeromyxobacteraceae;Anaeromyxobacter;Anaeromyxobacter sp. Fw109-5;Anaeromyxobacter sp. Fw109-5 |
| Ga0586324 0091085 718 1854 | 0.00 | 0.00 | 0.00 | 0.00 | 0.00 | 0.00 | 0.00 | 219371.25 | 173786.31 | 0.00 | 0.00 | 0.00 | | hypothetical protein | Bacteria;Proteobacteria;Deltaproteobacteria;Desulfobacterales;Desulfobacteraceae;Desulfotignum;Desulfotignum balticum;Desulfotignum balticum DSM 7044 |
| Ga0586324 0091828 2 3433 | 27990000.00 | 10404000.00 | 0.00 | 7296000.00 | 17242200.00 | 0.00 | 0.00 | 3153700.00 | 6042600.00 | 3826900.00 | 7788000.00 | 5842500.00 | | FtsP/CotA-like multicopper oxidase with cupredoxin domain | Bacteria;Proteobacteria;Gammaproteobacteria;Thiotrichales;Thiotrichaceae;Thioploca;Thioploca ingrica;Thioploca ingrica Lake Okotanpe |
| Ga0586324 0091861 2534 2917 | 5020000.00 | 0.00 | 0.00 | 0.00 | 0.00 | 0.00 | 2918400.00 | 0.00 | 0.00 | 0.00 | 0.00 | 0.00 | | hypothetical protein | Bacteria;Proteobacteria;Alphaproteobacteria;Hyphomicrobiales;Methylocystaceae;Methylocystis;Methylocystis parvus;Methylocystis parvus OBBP |
| Ga0586324 0091992 2884 3570 | 0.00 | 0.00 | 0.00 | 0.00 | 0.00 | 0.00 | 0.00 | 0.00 | 0.00 | 8085000.00 | 0.00 | 0.00 | | ABC-type sugar transport system substrate-binding protein | Bacteria;Actinobacteria;Actinomycetia;Propionibacteriales;Nocardioidaceae;Nocardioides;Nocardioides ginsengisegetis;Nocardioides ginsengisegetis DSM 21349 |
| Ga0586324 0092138 3 2045 | 0.00 | 0.00 | 0.00 | 4370000.00 | 1184500.00 | 0.00 | 0.00 | 0.00 | 536104.98 | 0.00 | 0.00 | 0.00 | | beta-glucosidase-like glycosyl hydrolase | Bacteria;Proteobacteria;Betaproteobacteria;Burkholderiales;Burkholderiaceae;Burkholderia;Burkholderia sp. Leaf177;Burkholderia sp. Leaf177 |
| Ga0586324 0092762 2504 3556 | 0.00 | 1679600.00 | 0.00 | 4579000.00 | 0.00 | 0.00 | 0.00 | 4007700.00 | 0.00 | 0.00 | 0.00 | 4042500.00 | | hypothetical protein | |
| Ga0586324 0092816 2093 3229 | 7990000.00 | 0.00 | 0.00 | 0.00 | 66744000.00 | 0.00 | 0.00 | 0.00 | 93410.82 | 0.00 | 0.00 | 0.00 | | hypothetical protein | Bacteria;Proteobacteria;Deltaproteobacteria;Desulfobacterales;Desulfobacteraceae;Desulfotignum;Desulfotignum balticum;Desulfotignum balticum DSM 7044 |
| Ga0586324 0092818 1340 3556 | 0.00 | 0.00 | 0.00 | 0.00 | 0.00 | 0.00 | 0.00 | 0.00 | 0.00 | 2602600.00 | 0.00 | 0.00 | | hypothetical protein | Bacteria;Acidobacteria;Vicinamibacteria;unclassified Vicinamibacteria;Vicinamibacteraceae;Luteitalea;Luteitalea pratensis;Luteitalea pratensis DSM 100886 |
| Ga0586324 0093298 2296 2808 | 0.00 | 0.00 | 0.00 | 0.00 | 2626500.00 | 0.00 | 0.00 | 0.00 | 0.00 | 0.00 | 0.00 | 0.00 | | uncharacterized membrane protein | Bacteria;Proteobacteria;Gammaproteobacteria;Methylococcales;Methylococcaceae;Methylococcus;Methylococcus capsulatus;Methylococcus capsulatus Bath |
| Ga0586324 0094423 1 2133 | 0.00 | 0.00 | 0.00 | 0.00 | 0.00 | 0.00 | 0.00 | 0.00 | 0.00 | 387095.94 | 0.00 | 0.00 | | DNA-directed RNA polymerase beta subunit | Bacteria;Nitrospirae;unclassified Nitrospirae;unclassified Nitrospirae;unclassified Nitrospirae;unclassified Nitrospirae;Nitrospirae bacterium HCH-1;Nitrospirae bacterium HCH-1 |
| Ga0586324 0095103 931 2139 | 0.00 | 0.00 | 0.00 | 0.00 | 0.00 | 0.00 | 2140598.52 | 1988600.00 | 0.00 | 0.00 | 0.00 | 0.00 | | hypothetical protein | Bacteria;Proteobacteria;Deltaproteobacteria;Desulfuromonadales;Geobacteraceae;Geobacter;Geobacter sp. M18;Geobacter sp. M18 |
| Ga0586324 0095116 763 1785 | 0.00 | 0.00 | 0.00 | 0.00 | 0.00 | 0.00 | 0.00 | 0.00 | 435370.95 | 0.00 | 0.00 | 0.00 | | TRAP-type C4-dicarboxylate transport system substrate-binding protein | Bacteria;Proteobacteria;Alphaproteobacteria;Hyphomicrobiales;Bradyrhizobiaceae;Bradyrhizobium;Bradyrhizobium sp. OAE819;Bradyrhizobium sp. OAE819 |
| Ga0586324 0095441 2 436 | 0.00 | 0.00 | 0.00 | 0.00 | 0.00 | 0.00 | 0.00 | 0.00 | 40257000.00 | 0.00 | 0.00 | 0.00 | | predicted porin | |
| Ga0586324 0095441 834 2075 | 25460000.00 | 2441200.00 | 0.00 | 0.00 | 0.00 | 0.00 | 29412000.00 | 0.00 | 15835500.00 | 4920300.00 | 20988000.00 | 11925000.00 | | predicted porin | Bacteria;Proteobacteria;Gammaproteobacteria;Chromatiales;Ectothiorhodospiraceae;Ectothiorhodospira;Ectothiorhodospira mobilis;Ectothiorhodospira marismortui DSM 4180 |
| Ga0586324 0095579 2345 2614 | 0.00 | 0.00 | 4039136.53 | 0.00 | 0.00 | 0.00 | 0.00 | 0.00 | 0.00 | 0.00 | 0.00 | 0.00 | | nucleoid DNA-binding protein | Bacteria;Proteobacteria;Deltaproteobacteria;Desulfobacterales;Desulfobacteraceae;Desulforegula;Desulforegula conservatrix;Desulforegula conservatrix Mb1Pa |
| Ga0586324 0095595 600 1925 | 0.00 | 0.00 | 0.00 | 0.00 | 0.00 | 0.00 | 0.00 | 0.00 | 0.00 | 0.00 | 0.00 | 36825000.00 | | hypothetical protein | Bacteria;Acidobacteria;Acidobacteriia;Acidobacteriales;Acidobacteriaceae;unclassified Acidobacteriaceae;Acidobacteriaceae bacterium URHE0068;Acidobacteriaceae bacterium URHE0068 |
| Ga0586324 0095730 2941 3501 | 0.00 | 1951600.00 | 0.00 | 0.00 | 0.00 | 0.00 | 0.00 | 0.00 | 0.00 | 0.00 | 0.00 | 0.00 | | hypothetical protein | Bacteria;Spirochaetes;Spirochaetia;Spirochaetales;Spirochaetaceae;Spirochaeta;Spirochaeta cellobiosiphila;Spirochaeta cellobiosiphila DSM 17781 |
| Ga0586324 0095754 2843 3502 | 0.00 | 3202800.00 | 0.00 | 0.00 | 0.00 | 0.00 | 0.00 | 0.00 | 0.00 | 0.00 | 0.00 | 0.00 | | acyl-CoA synthetase (AMP-forming)/AMP-acid ligase II | Bacteria;Proteobacteria;Deltaproteobacteria;unclassified Deltaproteobacteria;unclassified Deltaproteobacteria;unclassified Deltaproteobacteria;delta proteobacterium NaphS2;delta proteobacterium NaphS2 |
| Ga0586324 0095806 328 3069 | 0.00 | 0.00 | 3938156.85 | 0.00 | 0.00 | 0.00 | 0.00 | 0.00 | 0.00 | 0.00 | 0.00 | 0.00 | | hypothetical protein | Bacteria;Acidobacteria;Vicinamibacteria;unclassified Vicinamibacteria;Vicinamibacteraceae;Luteitalea;Luteitalea pratensis;Luteitalea pratensis DSM 100886 |
| Ga0586324 0095876 178 1542 | 0.00 | 0.00 | 0.00 | 0.00 | 739287.65 | 0.00 | 0.00 | 378144.49 | 0.00 | 0.00 | 0.00 | 0.00 | | hypothetical protein | Bacteria;Proteobacteria;Deltaproteobacteria;Desulfuromonadales;Geobacteraceae;Geobacter;Geobacter uraniireducens;Geobacter uraniireducens Rf4 |
| Ga0586324 0097990 2 1045 | 0.00 | 0.00 | 0.00 | 0.00 | 0.00 | 1375069.05 | 0.00 | 0.00 | 2138400.00 | 0.00 | 0.00 | 0.00 | | glutamine synthetase type I | Bacteria;Proteobacteria;Betaproteobacteria;Burkholderiales;unclassified Burkholderiales;Methylibium;Methylibium sp. CF059;Methylibium sp. CF059 |
| Ga0586324 0098011 106 1020 | 0.00 | 0.00 | 0.00 | 0.00 | 0.00 | 0.00 | 0.00 | 0.00 | 0.00 | 18287500.00 | 21568800.00 | 10200000.00 | | hypothetical protein | Bacteria;Proteobacteria;Betaproteobacteria;Nitrosomonadales;Sulfuricellaceae;Sulfurirhabdus;Sulfurirhabdus autotrophica;Sulfurirhabdus autotrophica DSM 100309 |
| Ga0586324 0098343 1480 3456 | 2130000.00 | 8629200.00 | 15144700.00 | 0.00 | 2472000.00 | 4883100.00 | 0.00 | 8192300.00 | 2632500.00 | 3580500.00 | 4083200.00 | 0.00 | | MtrB/PioB family decaheme-associated outer membrane protein | Bacteria;Proteobacteria;Betaproteobacteria;Nitrosomonadales;Sulfuricellaceae;Sulfuricella;Sulfuricella sp. T08;Sulfuricella sp. T08 |
| Ga0586324 0098864 1266 1685 | 0.00 | 0.00 | 0.00 | 0.00 | 1524400.00 | 0.00 | 0.00 | 10553000.00 | 0.00 | 0.00 | 0.00 | 0.00 | | rubrerythrin | Bacteria;Acidobacteria;Holophagae;Holophagales;Holophagaceae;Geothrix;Geothrix fermentans;Geothrix fermentans DSM 14018 |
| Ga0586324 0099651 1 2661 | 4070000.00 | 12811200.00 | 0.00 | 0.00 | 14214000.00 | 3325720.61 | 0.00 | 3879600.00 | 646158.87 | 8547000.00 | 0.00 | 10672500.00 | | outer membrane receptor protein involved in Fe transport | Bacteria;Acidobacteria;Acidobacteriia;Acidobacteriales;Acidobacteriaceae;Acidipila;Acidipila rosea;Acidipila rosea DSM 103428 |
| Ga0586324 0099691 1379 2815 | 0.00 | 0.00 | 0.00 | 0.00 | 0.00 | 0.00 | 2804400.00 | 0.00 | 0.00 | 0.00 | 0.00 | 0.00 | | DNA modification methylase | Bacteria;Firmicutes;Bacilli;Lactobacillales;Lactobacillaceae;Apilactobacillus;Apilactobacillus kunkeei;Apilactobacillus kunkeei YH-15 |
| Ga0586324 0099724 336 1196 | 0.00 | 1067600.00 | 0.00 | 0.00 | 0.00 | 0.00 | 0.00 | 0.00 | 0.00 | 0.00 | 0.00 | 0.00 | | uncharacterized coiled-coil DUF342 family protein | Archaea;Euryarchaeota;Methanomicrobia;Methanomicrobiales;Methanoregulaceae;Methanoregula;Methanoregula formicica;Methanoregula formicica SMSP, DSM 22288 |
| Ga0586324 0100181 112 1083 | 0.00 | 0.00 | 0.00 | 0.00 | 0.00 | 0.00 | 0.00 | 0.00 | 0.00 | 11704000.00 | 17749600.00 | 17887500.00 | | molybdenum ABC transporter molybdate-binding protein | Bacteria;Proteobacteria;Betaproteobacteria;Ferrovales;Ferrovaceae;Ferrovum;unclassified Ferrovum;Ferrovum sp. Zulauf-31 |
| Ga0586324 0100543 2 1615 | 2380000.00 | 0.00 | 0.00 | 0.00 | 0.00 | 0.00 | 0.00 | 0.00 | 0.00 | 0.00 | 0.00 | 0.00 | | hypothetical protein | Bacteria;Acidobacteria;Acidobacteriia;Acidobacteriales;Acidobacteriaceae;Candidatus Koribacter;Candidatus Koribacter versatilis;Candidatus Koribacter versatilis Ellin345 |
| Ga0586324 0100718 603 1058 | 8470000.00 | 0.00 | 0.00 | 0.00 | 0.00 | 0.00 | 0.00 | 0.00 | 0.00 | 0.00 | 0.00 | 0.00 | | rubrerythrin | Archaea;Euryarchaeota;Methanomicrobia;Methanomicrobiales;Methanomicrobiaceae;Methanoculleus;Methanoculleus thermophilus;Methanoculleus thermophilus DSM 2373 |
| Ga0586324 0100814 1755 3416 | 0.00 | 0.00 | 0.00 | 0.00 | 0.00 | 0.00 | 0.00 | 0.00 | 0.00 | 0.00 | 0.00 | 4618239.00 | | hypothetical protein | Bacteria;Actinobacteria;Actinomycetia;Propionibacteriales;Nocardioidaceae;Nocardioides;Nocardioides sp. Root122;Nocardioides sp. Root122 |
| Ga0586324 0101138 303 3410 | 13540000.00 | 19230400.00 | 12199478.10 | 5101500.00 | 4492693.14 | 392743.76 | 0.00 | 3031700.00 | 0.00 | 5774421.73 | 6476800.00 | 6060000.00 | | hypothetical protein | Bacteria;Acidobacteria;Vicinamibacteria;unclassified Vicinamibacteria;Vicinamibacteraceae;Luteitalea;Luteitalea pratensis;Luteitalea pratensis DSM 100886 |
| Ga0586324 0101160 1065 3410 | 3180000.00 | 0.00 | 0.00 | 0.00 | 0.00 | 0.00 | 0.00 | 0.00 | 0.00 | 0.00 | 0.00 | 0.00 | | hypothetical protein | Bacteria;Acidobacteria;Acidobacteriia;Acidobacteriales;Acidobacteriaceae;Edaphobacter;Edaphobacter lichenicola;Edaphobacter lichenicola M8UP22 |
| Ga0586324 0101251 374 1027 | 0.00 | 0.00 | 0.00 | 0.00 | 0.00 | 0.00 | 0.00 | 3788100.00 | 0.00 | 0.00 | 0.00 | 0.00 | | curli biogenesis system outer membrane secretion channel CsgG | Bacteria;Acidobacteria;Vicinamibacteria;unclassified Vicinamibacteria;Vicinamibacteraceae;Luteitalea;Luteitalea pratensis;Luteitalea pratensis DSM 100886 |
| Ga0586324 0101533 1489 2151 | 0.00 | 2271200.00 | 0.00 | 0.00 | 5572300.00 | 0.00 | 0.00 | 0.00 | 18549000.00 | 12936000.00 | 0.00 | 0.00 | | biopolymer transport protein ExbB/TolQ | Bacteria;Acidobacteria;Vicinamibacteria;unclassified Vicinamibacteria;Vicinamibacteraceae;Luteitalea;Luteitalea pratensis;Luteitalea pratensis DSM 100886 |
| Ga0586324 0102127 201 3395 | 0.00 | 4610400.00 | 0.00 | 0.00 | 0.00 | 0.00 | 0.00 | 0.00 | 0.00 | 0.00 | 0.00 | 0.00 | | beta-glucosidase-like glycosyl hydrolase | Bacteria;Actinobacteria;Actinomycetia;Motilibacterales;Motilibacteraceae;Motilibacter;Motilibacter rhizosphaerae;Motilibacter rhizosphaerae DSM 45622 |
| Ga0586324 0102186 1786 2646 | 0.00 | 0.00 | 0.00 | 0.00 | 0.00 | 0.00 | 0.00 | 0.00 | 0.00 | 885500.00 | 0.00 | 0.00 | | hypothetical protein | Bacteria;Proteobacteria;Deltaproteobacteria;Desulfovibrionales;Desulfovibrionaceae;Desulfovibrio;Desulfovibrio sp. DMSS-1;Desulfovibrio sp. DMSS-1 |
| Ga0586324 0102227 1245 2870 | 160870000.00 | 46066211.72 | 119243400.00 | 170191016.10 | 70318100.00 | 61523292.47 | 619543912.08 | 131522100.00 | 123306300.00 | 56933800.00 | 114848800.00 | 133965000.00 | | hypothetical protein | Bacteria;Proteobacteria;Alphaproteobacteria;Hyphomicrobiales;Bradyrhizobiaceae;Bradyrhizobium;Bradyrhizobium elkanii;Bradyrhizobium elkanii CCBAU 43297 |
| Ga0586324 0102520 1 861 | 0.00 | 0.00 | 0.00 | 0.00 | 0.00 | 4565500.00 | 0.00 | 0.00 | 0.00 | 0.00 | 0.00 | 0.00 | | hypothetical protein | |
| Ga0586324 0102557 3 3275 | 0.00 | 0.00 | 0.00 | 1425000.00 | 0.00 | 0.00 | 0.00 | 0.00 | 0.00 | 0.00 | 0.00 | 0.00 | | 2',3'-cyclic-nucleotide 2'-phosphodiesterase (5'-nucleotidase family) | Bacteria;Actinobacteria;Actinomycetia;Propionibacteriales;Kribbellaceae;Kribbella;Kribbella catacumbae;Kribbella catacumbae DSM 19601 |
| Ga0586324 0103547 1548 1940 | 0.00 | 0.00 | 42619100.00 | 47120000.00 | 38419000.00 | 40097000.00 | 0.00 | 0.00 | 0.00 | 0.00 | 0.00 | 20925000.00 | | hypothetical protein | Bacteria;Proteobacteria;Gammaproteobacteria;unclassified Gammaproteobacteria;unclassified Gammaproteobacteria;Sedimenticola;unclassified Sedimenticola;Sedimenticola thiotaurini SIP-G1 |
| Ga0586324 0103547 240 1460 | 7590000.00 | 8928400.00 | 0.00 | 9291000.00 | 20826600.00 | 0.00 | 0.00 | 0.00 | 11005681.41 | 13297900.00 | 0.00 | 0.00 | | hypothetical protein | |
| Ga0586324 0103787 165 1697 | 47100000.00 | 18428000.00 | 0.00 | 30590000.00 | 3532900.00 | 0.00 | 0.00 | 6405000.00 | 6723000.00 | 7546000.00 | 10357600.00 | 11475000.00 | | hypothetical protein | |
| Ga0586324 0104162 2749 3264 | 0.00 | 52972000.00 | 0.00 | 0.00 | 164800000.00 | 0.00 | 0.00 | 0.00 | 42687000.00 | 0.00 | 0.00 | 0.00 | | tetratricopeptide (TPR) repeat protein | Bacteria;Proteobacteria;Deltaproteobacteria;Syntrophobacterales;Syntrophaceae;Desulfobacca;Desulfobacca acetoxidans;Desulfobacca acetoxidans ASRB2, DSM 11109 |
| Ga0586324 0104518 2393 3358 | 0.00 | 2040000.00 | 0.00 | 0.00 | 0.00 | 0.00 | 0.00 | 0.00 | 0.00 | 0.00 | 0.00 | 0.00 | | FtsP/CotA-like multicopper oxidase with cupredoxin domain | Bacteria;Proteobacteria;Betaproteobacteria;Burkholderiales;Comamonadaceae;Hydrogenophaga;Hydrogenophaga sp. PAMC20947;Hydrogenophaga sp. PAMC20947 |
| Ga0586324 0104609 268 999 | 1180000.00 | 0.00 | 0.00 | 0.00 | 0.00 | 0.00 | 0.00 | 0.00 | 0.00 | 0.00 | 0.00 | 0.00 | | TRAP-type mannitol/chloroaromatic compound transport system substrate-binding protein | Bacteria;Proteobacteria;Betaproteobacteria;Nitrosomonadales;Thiobacillaceae;Sulfuritortus;Sulfuritortus calidifontis;Sulfuritortus calidifontis DSM 103923 |
| Ga0586324 0105269 713 1291 | 0.00 | 0.00 | 0.00 | 0.00 | 0.00 | 0.00 | 7934400.00 | 4276100.00 | 3150900.00 | 2887500.00 | 4188800.00 | 3765000.00 | | lipid-binding SYLF domain-containing protein | Bacteria;Bacteroidetes;Flavobacteriia;Flavobacteriales;Flavobacteriaceae;Leeuwenhoekiella;Leeuwenhoekiella aequorea;Leeuwenhoekiella aequorea LMG 22550 |
| Ga0586324 0105777 14 1525 | 0.00 | 0.00 | 0.00 | 0.00 | 1112400.00 | 0.00 | 600757.20 | 0.00 | 0.00 | 0.00 | 0.00 | 0.00 | | crotonobetainyl-CoA:carnitine CoA-transferase CaiB-like acyl-CoA transferase | Bacteria;Proteobacteria;Deltaproteobacteria;Syntrophobacterales;Syntrophobacteraceae;Desulfosoma;Desulfosoma caldarium;Desulfosoma caldarium DSM 22027 |
| Ga0586324 0106431 2 1381 | 0.00 | 418193.88 | 0.00 | 0.00 | 0.00 | 0.00 | 0.00 | 646600.00 | 0.00 | 0.00 | 0.00 | 0.00 | | acyl-CoA synthetase (AMP-forming)/AMP-acid ligase II | Bacteria;Proteobacteria;Deltaproteobacteria;Syntrophorhabdales;Syntrophorhabdaceae;Syntrophorhabdus;Syntrophorhabdus aromaticivorans;Syntrophorhabdus aromaticivorans UI |
| Ga0586324 0106547 1143 2231 | 0.00 | 6534800.00 | 563084.45 | 104779.30 | 1091800.00 | 758138.99 | 2074660.92 | 732000.00 | 899100.00 | 893200.00 | 430646.48 | 430861.50 | | TRAP-type mannitol/chloroaromatic compound transport system substrate-binding protein | Bacteria;Proteobacteria;Alphaproteobacteria;Hyphomicrobiales;Bradyrhizobiaceae;Bradyrhizobium;Bradyrhizobium icense;Bradyrhizobium icense LMTR 13 |
| Ga0586324 0106557 2 2101 | 0.00 | 112612.08 | 240134.70 | 0.00 | 0.00 | 718629.55 | 0.00 | 0.00 | 0.00 | 2025100.00 | 0.00 | 0.00 | | hypothetical protein | Bacteria;Acidobacteria;Blastocatellia;Blastocatellales;Pyrinomonadaceae;Pyrinomonas;Pyrinomonas methylaliphatogenes;Pyrinomonas methylaliphatogenes K22 |
| Ga0586324 0106683 291 866 | 0.00 | 0.00 | 0.00 | 0.00 | 0.00 | 0.00 | 0.00 | 0.00 | 112385.88 | 0.00 | 0.00 | 0.00 | | multimeric flavodoxin WrbA | Archaea;Euryarchaeota;Methanomicrobia;Methanomicrobiales;Methanoregulaceae;Methanoregula;Methanoregula formicica;Methanoregula formicica SMSP, DSM 22288 |
| Ga0586324 0107353 2730 3266 | 0.00 | 0.00 | 0.00 | 0.00 | 851977.89 | 0.00 | 0.00 | 0.00 | 0.00 | 0.00 | 0.00 | 0.00 | | uncharacterized membrane protein | Archaea;Euryarchaeota;Methanomicrobia;Methanomicrobiales;Methanomicrobiaceae;Methanofollis;Methanofollis ethanolicus;Methanofollis ethanolicus JCM 15103 |
| Ga0586324 0107408 1898 2449 | 0.00 | 0.00 | 0.00 | 16720000.00 | 0.00 | 0.00 | 0.00 | 0.00 | 0.00 | 0.00 | 0.00 | 0.00 | | CBS domain-containing protein | Bacteria;Proteobacteria;Deltaproteobacteria;Syntrophobacterales;Syntrophaceae;Desulfobacca;Desulfobacca acetoxidans;Desulfobacca acetoxidans ASRB2, DSM 11109 |
| Ga0586324 0108091 2 3304 | 0.00 | 0.00 | 0.00 | 0.00 | 0.00 | 0.00 | 0.00 | 0.00 | 2405700.00 | 0.00 | 0.00 | 0.00 | | uncharacterized protein (TIGR03437 family) | Bacteria;Bacteroidetes;Cytophagia;Cytophagales;Cytophagaceae;Spirosoma;Spirosoma spitsbergense;Spirosoma spitsbergense DSM 19989 |
| Ga0586324 0108141 1425 2837 | 0.00 | 0.00 | 0.00 | 0.00 | 0.00 | 0.00 | 0.00 | 0.00 | 0.00 | 7176400.00 | 3977600.00 | 0.00 | | cytochrome c peroxidase | Bacteria;Proteobacteria;Deltaproteobacteria;Syntrophobacterales;Syntrophaceae;Desulfobacca;Desulfobacca acetoxidans;Desulfobacca acetoxidans ASRB2, DSM 11109 |
| Ga0586324 0108504 1133 3298 | 1050000.00 | 0.00 | 0.00 | 959500.00 | 0.00 | 984941.12 | 0.00 | 1122400.00 | 0.00 | 0.00 | 0.00 | 1455000.00 | | hypothetical protein | Bacteria;Acidobacteria;Vicinamibacteria;unclassified Vicinamibacteria;Vicinamibacteraceae;Luteitalea;Luteitalea pratensis;Luteitalea pratensis DSM 100886 |
| Ga0586324 0108616 3 1712 | 0.00 | 0.00 | 0.00 | 0.00 | 24823000.00 | 0.00 | 0.00 | 0.00 | 0.00 | 0.00 | 0.00 | 0.00 | | hypothetical protein | Bacteria;Bacteroidetes;Cytophagia;Cytophagales;Hymenobacteraceae;Adhaeribacter;Adhaeribacter arboris;Adhaeribacter arboris HMF7605 |
| Ga0586324 0108760 404 3295 | 53950000.00 | 11777600.00 | 58552000.00 | 79904500.00 | 74932500.00 | 24455200.00 | 20791274.40 | 45579200.00 | 38628900.00 | 26565000.00 | 61221600.00 | 51352500.00 | | hypothetical protein | Bacteria;Actinobacteria;Actinomycetia;Micrococcales;Microbacteriaceae;Cnuibacter;Cnuibacter physcomitrellae;Cnuibacter physcomitrellae XA |
| Ga0586324 0109680 2 1786 | 0.00 | 0.00 | 2289433.87 | 0.00 | 0.00 | 0.00 | 0.00 | 0.00 | 0.00 | 0.00 | 0.00 | 0.00 | | outer membrane receptor protein involved in Fe transport | Bacteria;Acidobacteria;Thermoanaerobaculia;Thermoanaerobaculales;Thermoanaerobaculaceae;Thermoanaerobaculum;Thermoanaerobaculum aquaticum;Thermoanaerobaculum aquaticum MP-01 |
| Ga0586324 0109853 1763 3163 | 0.00 | 3808000.00 | 0.00 | 2156500.00 | 0.00 | 0.00 | 0.00 | 0.00 | 0.00 | 0.00 | 0.00 | 6367500.00 | | hypothetical protein | Bacteria;Proteobacteria;Betaproteobacteria;Nitrosomonadales;Gallionellaceae;Sideroxydans;Sideroxydans lithotrophicus;Sideroxydans lithotrophicus ES-1 |
| Ga0586324 0109879 3 2441 | 0.00 | 0.00 | 0.00 | 0.00 | 0.00 | 805953.67 | 0.00 | 0.00 | 0.00 | 0.00 | 0.00 | 0.00 | | hypothetical protein | Bacteria;Acidobacteria;Blastocatellia;Blastocatellales;Pyrinomonadaceae;Pyrinomonas;Pyrinomonas methylaliphatogenes;Pyrinomonas methylaliphatogenes K22 |
| Ga0586324 0109947 1812 3275 | 4800000.00 | 2801600.00 | 6249300.00 | 2394000.00 | 12102500.00 | 8297300.00 | 0.00 | 2842600.00 | 2478600.00 | 0.00 | 3801600.00 | 6247500.00 | | hypothetical protein | Bacteria;Proteobacteria;Gammaproteobacteria;Methylococcales;Methylococcaceae;Methylosarcina;Methylosarcina fibrata;Methylosarcina fibrata AML-C10 |
| Ga0586324 0110009 207 1907 | 851358.00 | 0.00 | 0.00 | 0.00 | 0.00 | 0.00 | 0.00 | 0.00 | 0.00 | 0.00 | 0.00 | 0.00 | | hypothetical protein | |
| Ga0586324 0110046 1 1818 | 0.00 | 0.00 | 0.00 | 0.00 | 0.00 | 0.00 | 0.00 | 13359000.00 | 0.00 | 0.00 | 0.00 | 0.00 | | 2',3'-cyclic-nucleotide 2'-phosphodiesterase (5'-nucleotidase family) | Bacteria;Actinobacteria;Actinomycetia;Propionibacteriales;Nocardioidaceae;Nocardioides;Nocardioides soli;Nocardioides soli DSM 105498 |
| Ga0586324 0111076 1385 3259 | 919256.00 | 0.00 | 0.00 | 0.00 | 0.00 | 0.00 | 0.00 | 0.00 | 0.00 | 0.00 | 0.00 | 0.00 | | hypothetical protein | Bacteria;Acidobacteria;Vicinamibacteria;unclassified Vicinamibacteria;Vicinamibacteraceae;Luteitalea;Luteitalea pratensis;Luteitalea pratensis DSM 100886 |
| Ga0586324 0112141 2606 3247 | 0.00 | 0.00 | 0.00 | 2650500.00 | 0.00 | 0.00 | 0.00 | 0.00 | 0.00 | 0.00 | 0.00 | 0.00 | | glutaredoxin | Bacteria;Actinobacteria;Actinomycetia;Corynebacteriales;Nocardiaceae;Smaragdicoccus;Smaragdicoccus niigatensis;Smaragdicoccus niigatensis DSM 44881 |
| Ga0586324 0112208 1 726 | 0.00 | 754800.00 | 0.00 | 0.00 | 0.00 | 0.00 | 0.00 | 0.00 | 0.00 | 0.00 | 0.00 | 0.00 | | regulator of protease activity HflC (stomatin/prohibitin superfamily) | Bacteria;Bacteroidetes;Chitinophagia;Chitinophagales;Chitinophagaceae;Chitinophaga;Chitinophaga sp. OK723;Chitinophaga sp. OK723 |
| Ga0586324 0112294 1 834 | 0.00 | 0.00 | 0.00 | 0.00 | 0.00 | 0.00 | 1983417.60 | 957700.00 | 0.00 | 3680600.00 | 0.00 | 0.00 | | hypothetical protein | Bacteria;Firmicutes;Bacilli;Bacillales;unclassified Bacillales;Exiguobacterium;Exiguobacterium chiriqhucha;Exiguobacterium chiriqhucha GIC31 |
| Ga0586324 0113197 370 1881 | 1590000.00 | 0.00 | 0.00 | 0.00 | 0.00 | 0.00 | 0.00 | 0.00 | 0.00 | 0.00 | 0.00 | 0.00 | | hypothetical protein | Bacteria;Acidobacteria;Blastocatellia;unclassified Blastocatellia;unclassified Blastocatellia;Chloracidobacterium;Chloracidobacterium thermophilum;Chloracidobacterium thermophilum OC1 |
| Ga0586324 0113587 492 3227 | 0.00 | 0.00 | 0.00 | 0.00 | 0.00 | 0.00 | 2850000.00 | 0.00 | 0.00 | 2625700.00 | 0.00 | 4642500.00 | | TonB-linked SusC/RagA family outer membrane protein | Bacteria;Bacteroidetes;Bacteroidia;Marinilabiliales;Prolixibacteraceae;Prolixibacter;Prolixibacter denitrificans;Prolixibacter denitrificans DSM 27267 |
| Ga0586324 0113844 1396 2484 | 0.00 | 0.00 | 0.00 | 0.00 | 0.00 | 829205.96 | 683008.20 | 574192.39 | 826200.00 | 675485.58 | 0.00 | 0.00 | | TRAP-type mannitol/chloroaromatic compound transport system substrate-binding protein | Bacteria;Proteobacteria;Alphaproteobacteria;Hyphomicrobiales;Bradyrhizobiaceae;Bradyrhizobium;Bradyrhizobium sp. Y36;Bradyrhizobium sp. Y36 |
| Ga0586324 0114433 2619 2975 | 0.00 | 1224000.00 | 4444738.62 | 0.00 | 0.00 | 0.00 | 0.00 | 0.00 | 0.00 | 0.00 | 0.00 | 0.00 | | hypothetical protein | Archaea;Euryarchaeota;Methanobacteria;Methanobacteriales;Methanobacteriaceae;Methanobacterium;Methanobacterium bryantii;Methanobacterium bryantii M.o.H. |
| Ga0586324 0114920 69 2660 | 0.00 | 0.00 | 7713100.00 | 3790500.00 | 2987000.00 | 0.00 | 0.00 | 0.00 | 0.00 | 0.00 | 0.00 | 0.00 | | hypothetical protein | Bacteria;Proteobacteria;Deltaproteobacteria;Desulfobacterales;Desulfobacteraceae;Desulfospira;Desulfospira joergensenii;Desulfospira joergensenii DSM 10085 |
| Ga0586324 0115147 1390 2115 | 0.00 | 0.00 | 0.00 | 4759500.00 | 0.00 | 0.00 | 0.00 | 0.00 | 0.00 | 0.00 | 0.00 | 0.00 | | hypothetical protein | |
| Ga0586324 0115535 2081 3175 | 4980000.00 | 2828800.00 | 0.00 | 4294000.00 | 0.00 | 10877800.00 | 0.00 | 0.00 | 2097900.00 | 0.00 | 0.00 | 6472500.00 | | kynurenine formamidase | Bacteria;Proteobacteria;Alphaproteobacteria;unclassified Alphaproteobacteria;unclassified Alphaproteobacteria;unclassified Alphaproteobacteria;alpha proteobacterium BAL199;Nisaea sp BAL199 |
| Ga0586324 0115624 318 1517 | 0.00 | 0.00 | 0.00 | 0.00 | 0.00 | 0.00 | 0.00 | 0.00 | 0.00 | 0.00 | 0.00 | 4012500.00 | | imidazolonepropionase-like amidohydrolase | Bacteria;Proteobacteria;Deltaproteobacteria;Syntrophobacterales;Syntrophobacteraceae;Syntrophobacter;Syntrophobacter fumaroxidans;Syntrophobacter fumaroxidans MPOB |
| Ga0586324 0115633 2078 3082 | 0.00 | 0.00 | 0.00 | 0.00 | 0.00 | 0.00 | 0.00 | 0.00 | 0.00 | 0.00 | 0.00 | 840000.00 | | basic membrane lipoprotein Med (substrate-binding protein (PBP1-ABC) superfamily) | Bacteria;Chloroflexi;Anaerolineae;Anaerolineales;Anaerolineaceae;Pelolinea;Pelolinea submarina;Pelolinea submarina DSM 23923 |
| Ga0586324 0115966 3 704 | 0.00 | 0.00 | 0.00 | 0.00 | 0.00 | 163564000.00 | 0.00 | 0.00 | 0.00 | 0.00 | 0.00 | 0.00 | | hypothetical protein | Bacteria;Proteobacteria;Alphaproteobacteria;Hyphomicrobiales;Xanthobacteraceae;Pseudolabrys;Pseudolabrys taiwanensis;Pseudolabrys taiwanensis CC-BB4 |
| Ga0586324 0117137 808 1974 | 118360000.00 | 77948400.00 | 14345042.95 | 21660000.00 | 50294900.00 | 97503200.00 | 700302000.00 | 620107700.00 | 601797600.00 | 916253800.00 | ############ | 996832500.00 | | predicted porin | Bacteria;Proteobacteria;Betaproteobacteria;Nitrosomonadales;Thiobacillaceae;Thiobacillus;Thiobacillus denitrificans;Thiobacillus denitrificans RG |
| Ga0586324 0117610 1807 3174 | 0.00 | 0.00 | 0.00 | 0.00 | 0.00 | 0.00 | 0.00 | 0.00 | 0.00 | 1624700.00 | 0.00 | 0.00 | | hypothetical protein | |
| Ga0586324 0117653 775 3156 | 0.00 | 0.00 | 0.00 | 0.00 | 0.00 | 8297300.00 | 0.00 | 0.00 | 0.00 | 0.00 | 0.00 | 0.00 | | subtilisin family serine protease | Bacteria;Acidobacteria;Vicinamibacteria;unclassified Vicinamibacteria;Vicinamibacteraceae;Luteitalea;Luteitalea pratensis;Luteitalea pratensis DSM 100886 |
| Ga0586324 0118476 511 1788 | 8320000.00 | 62254000.00 | 0.00 | 0.00 | 0.00 | 102029000.00 | 95760000.00 | 56730000.00 | 52650000.00 | 38346000.00 | 44000000.00 | 59700000.00 | | hypothetical protein | Bacteria;Verrucomicrobia;Verrucomicrobiae;Verrucomicrobiales;Verrucomicrobia subdivision 3;Pedosphaera;Pedosphaera parvula;Pedosphaera parvula Ellin514 |
| Ga0586324 0119321 2495 3154 | 0.00 | 0.00 | 0.00 | 0.00 | 0.00 | 0.00 | 0.00 | 0.00 | 0.00 | 0.00 | 0.00 | 413322.00 | | TRAP-type mannitol/chloroaromatic compound transport system substrate-binding protein | Bacteria;Proteobacteria;Alphaproteobacteria;Hyphomicrobiales;unclassified Hyphomicrobiales;unclassified Hyphomicrobiales;Rhizobiales bacterium YIM 77505;Rhizobiales bacterium YIM 77505 |
| Ga0586324 0120282 1 723 | 0.00 | 4596800.00 | 0.00 | 0.00 | 3337200.00 | 0.00 | 0.00 | 3068300.00 | 4414500.00 | 0.00 | 0.00 | 454475.25 | | ABC-type sugar transport system substrate-binding protein | |
| Ga0586324 0121655 2239 3123 | 6850000.00 | 13056000.00 | 0.00 | 12825000.00 | 10289700.00 | 4287600.00 | 2019779.04 | 10858000.00 | 0.00 | 16786000.00 | 15664000.00 | 12975000.00 | | hypothetical protein | Bacteria;Proteobacteria;Alphaproteobacteria;Hyphomicrobiales;Bradyrhizobiaceae;Bradyrhizobium;Bradyrhizobium oligotrophicum;Bradyrhizobium oligotrophicum S58 |
| Ga0586324 0122593 2164 2850 | 0.00 | 0.00 | 0.00 | 8873000.00 | 8631400.00 | 1549979.31 | 0.00 | 0.00 | 3272400.00 | 4288900.00 | 0.00 | 0.00 | | hypothetical protein | Bacteria;Actinobacteria;Actinomycetia;Propionibacteriales;Nocardioidaceae;Nocardioides;Nocardioides sp. Soil777;Nocardioides sp. Soil777 |
| Ga0586324 0122809 3 3110 | 0.00 | 3338800.00 | 8163500.00 | 7742500.00 | 6736200.00 | 4644900.00 | 4491600.00 | 5483900.00 | 7419600.00 | 8393000.00 | 0.00 | 7500000.00 | | uncharacterized repeat protein (TIGR01451 family) | Bacteria;Actinobacteria;Actinomycetia;Propionibacteriales;Nocardioidaceae;Nocardioides;Nocardioides sp. YIM ART13;Nocardioides sp. YIM ART13 |
| Ga0586324 0123322 1128 2672 | 2780000.00 | 4284000.00 | 3416593.65 | 0.00 | 0.00 | 0.00 | 4332000.00 | 0.00 | 2357100.00 | 0.00 | 0.00 | 4192500.00 | | hypothetical protein | |
| Ga0586324 0123526 3 3101 | 0.00 | 0.00 | 0.00 | 0.00 | 502825.40 | 0.00 | 0.00 | 695400.00 | 0.00 | 0.00 | 0.00 | 0.00 | | hypothetical protein | Bacteria;Candidatus Cloacimonetes;unclassified Candidatus Cloacimonetes;unclassified Candidatus Cloacimonetes;unclassified Candidatus Cloacimonetes;unclassified Candidatus Cloacimonetes;Cloacimonetes bacterium SCGC AAA252-K16;Cloacimonetes bacterium SCGC AAA252-K16 (contamination screened) |
| Ga0586324 0123817 3 2597 | 0.00 | 2325600.00 | 0.00 | 0.00 | 481115.06 | 0.00 | 0.00 | 0.00 | 0.00 | 0.00 | 0.00 | 0.00 | | beta-glucosidase-like glycosyl hydrolase | Bacteria;Actinobacteria;Thermoleophilia;Solirubrobacterales;Solirubrobacteraceae;Solirubrobacter;Solirubrobacter soli;Solirubrobacter soli DSM 22325 |
| Ga0586324 0123819 1307 1927 | 0.00 | 0.00 | 0.00 | 0.00 | 9455400.00 | 4168500.00 | 5996400.00 | 4465200.00 | 4965300.00 | 0.00 | 0.00 | 0.00 | | hypothetical protein | Bacteria;Acidobacteria;Thermoanaerobaculia;Thermoanaerobaculales;Thermoanaerobaculaceae;Thermoanaerobaculum;Thermoanaerobaculum aquaticum;Thermoanaerobaculum aquaticum MP-01 |
| Ga0586324 0124939 2628 2954 | 0.00 | 0.00 | 0.00 | 0.00 | 0.00 | 0.00 | 0.00 | 0.00 | 0.00 | 1817200.00 | 0.00 | 0.00 | | nucleoid DNA-binding protein | Bacteria;Proteobacteria;Gammaproteobacteria;Chromatiales;Ectothiorhodospiraceae;Thioalkalivibrio;Thioalkalivibrio thiocyanodenitrificans;Thioalkalivibrio thiocyanodenitrificans ARhD 1 |
| Ga0586324 0125077 290 1354 | 0.00 | 5344800.00 | 0.00 | 0.00 | 0.00 | 0.00 | 0.00 | 5953600.00 | 4641300.00 | 9763600.00 | 1988800.00 | 1050000.00 | | hypothetical protein | Bacteria;Proteobacteria;Gammaproteobacteria;Acidiferrobacterales;Acidiferrobacteraceae;Sulfuricaulis;Sulfuricaulis limicola;Sulfuricaulis limicola HA5 |
| Ga0586324 0126038 1201 1878 | 0.00 | 0.00 | 0.00 | 0.00 | 0.00 | 0.00 | 0.00 | 0.00 | 9882000.00 | 0.00 | 0.00 | 0.00 | | hypothetical protein | |
| Ga0586324 0126106 2260 2670 | 0.00 | 0.00 | 0.00 | 2033000.00 | 0.00 | 0.00 | 2644800.00 | 0.00 | 0.00 | 0.00 | 0.00 | 0.00 | | nucleotide-binding universal stress UspA family protein | Bacteria;Proteobacteria;Deltaproteobacteria;Desulfobacterales;Desulfobacteraceae;Desulfatirhabdium;Desulfatirhabdium butyrativorans;Desulfatirhabdium butyrativorans DSM 18734 |
| Ga0586324 0126107 2639 2950 | 0.00 | 0.00 | 0.00 | 0.00 | 0.00 | 2581885.53 | 0.00 | 0.00 | 0.00 | 0.00 | 0.00 | 0.00 | | polyhydroxyalkanoate synthesis regulator phasin | Bacteria;Proteobacteria;Deltaproteobacteria;Syntrophobacterales;Syntrophaceae;Desulfobacca;Desulfobacca acetoxidans;Desulfobacca acetoxidans ASRB2, DSM 11109 |
| Ga0586324 0127233 2228 2755 | 0.00 | 1230800.00 | 4136175.21 | 0.00 | 2193900.00 | 0.00 | 0.00 | 0.00 | 0.00 | 0.00 | 0.00 | 1027500.00 | | cytoskeletal protein CcmA (bactofilin family) | Bacteria;Proteobacteria;Gammaproteobacteria;Xanthomonadales;Xanthomonadaceae;Pseudoxanthomonas;Pseudoxanthomonas sp. OG2;Pseudoxanthomonas sp. OG2 |
| Ga0586324 0127307 704 3058 | 72800000.00 | 14178000.00 | 19085700.00 | 24605000.00 | 2863400.00 | 504388.50 | 0.00 | 11510700.00 | 17382600.00 | 0.00 | 1751200.00 | 3975000.00 | | outer membrane receptor protein involved in Fe transport | Bacteria;Candidatus Aminicenantes;unclassified Candidatus Aminicenantes;unclassified Candidatus Aminicenantes;unclassified Candidatus Aminicenantes;unclassified Candidatus Aminicenantes;Aminicenantes bacterium SCGC AAA252-D06;Aminicenantes bacterium SCGC AAA252-D06 (contamination screened) |
| Ga0586324 0127388 223 1377 | 0.00 | 0.00 | 0.00 | 620042.20 | 0.00 | 0.00 | 0.00 | 0.00 | 0.00 | 0.00 | 0.00 | 0.00 | | alkylation response protein AidB-like acyl-CoA dehydrogenase | Bacteria;Proteobacteria;Deltaproteobacteria;Desulfobacterales;Desulfobacteraceae;Desulfatirhabdium;Desulfatirhabdium butyrativorans;Desulfatirhabdium butyrativorans DSM 18734 |
| Ga0586324 0127514 1512 1997 | 0.00 | 0.00 | 0.00 | 0.00 | 0.00 | 0.00 | 0.00 | 17568000.00 | 22923000.00 | 0.00 | 0.00 | 0.00 | | hypothetical protein | Bacteria;Firmicutes;Clostridia;Thermoanaerobacterales;Thermoanaerobacteraceae;Desulfovirgula;Desulfovirgula thermocuniculi;Desulfovirgula thermocuniculi DSM 16036 |
| Ga0586324 0127514 27 1499 | 71050000.00 | 26764800.00 | 0.00 | 73530000.00 | 58710000.00 | 0.00 | 0.00 | 25986000.00 | 6698700.00 | 0.00 | 0.00 | 8325000.00 | | crotonobetainyl-CoA:carnitine CoA-transferase CaiB-like acyl-CoA transferase | Bacteria;Proteobacteria;Deltaproteobacteria;Syntrophobacterales;Syntrophobacteraceae;Desulfacinum;Desulfacinum infernum;Desulfacinum infernum DSM 9756 |
| Ga0586324 0128825 2 1003 | 0.00 | 0.00 | 0.00 | 0.00 | 0.00 | 0.00 | 0.00 | 0.00 | 440331.39 | 0.00 | 0.00 | 0.00 | | tetratricopeptide (TPR) repeat protein | Bacteria;Proteobacteria;Betaproteobacteria;Nitrosomonadales;Gallionellaceae;Gallionella;Gallionella capsiferriformans;Gallionella capsiferriformans ES-2 |
| Ga0586324 0128983 612 2426 | 0.00 | 0.00 | 0.00 | 0.00 | 0.00 | 3296251.30 | 4126800.00 | 0.00 | 1846800.00 | 0.00 | 0.00 | 0.00 | | hypothetical protein | Bacteria;Proteobacteria;Deltaproteobacteria;Desulfobacterales;Desulfobacteraceae;Desulfococcus;Desulfococcus multivorans;Desulfococcus multivorans DSM 2059 |
| Ga0586324 0129104 1249 3039 | 0.00 | 1482400.00 | 1994697.74 | 1615000.00 | 1792200.00 | 1185096.61 | 0.00 | 2665700.00 | 3555900.00 | 1463000.00 | 3555200.00 | 0.00 | | hypothetical protein | Bacteria;Ignavibacteriae;Ignavibacteria;Ignavibacteriales;Ignavibacteriaceae;Ignavibacterium;Ignavibacterium album;Ignavibacterium album Mat9-16, JCM 16511 |
| Ga0586324 0129350 1 987 | 0.00 | 0.00 | 0.00 | 0.00 | 0.00 | 0.00 | 0.00 | 2604700.00 | 7160400.00 | 12512500.00 | 13261600.00 | 11512500.00 | | hypothetical protein | Bacteria;Proteobacteria;Betaproteobacteria;Nitrosomonadales;Sulfuricellaceae;Sulfuriferula;Sulfuriferula multivorans;Sulfuriferula multivorans TTN |
| Ga0586324 0129453 624 1991 | 8580000.00 | 14960000.00 | 0.00 | 27455000.00 | 19879000.00 | 23343600.00 | 13246800.00 | 17751000.00 | 8991000.00 | 14938000.00 | 14872000.00 | 0.00 | | hypothetical protein | Bacteria;Nitrospirae;Nitrospira;Nitrospirales;Nitrospiraceae;Nitrospira;Nitrospira lenta;Nitrospira lenta N. lenta BS10 |
| Ga0586324 0129526 1792 2112 | 0.00 | 1958400.00 | 44308100.00 | 0.00 | 0.00 | 0.00 | 0.00 | 0.00 | 6966000.00 | 0.00 | 0.00 | 0.00 | | RNA recognition motif-containing protein | Bacteria;Bacteroidetes;Bacteroidia;Marinilabiliales;Marinilabiliaceae;Breznakibacter;Breznakibacter xylanolyticus;Cytophaga xylanolytica DSM 6779 |
| Ga0586324 0129530 81 3032 | 0.00 | 0.00 | 0.00 | 3230000.00 | 5994600.00 | 3269227.51 | 0.00 | 5471700.00 | 7217100.00 | 0.00 | 2807200.00 | 2160000.00 | | hypothetical protein | Bacteria;Acidobacteria;Vicinamibacteria;unclassified Vicinamibacteria;Vicinamibacteraceae;Luteitalea;Luteitalea pratensis;Luteitalea pratensis DSM 100886 |
| Ga0586324 0130896 1412 2155 | 0.00 | 877200.00 | 0.00 | 3914000.00 | 0.00 | 0.00 | 0.00 | 0.00 | 0.00 | 0.00 | 0.00 | 0.00 | | regulator of protease activity HflC (stomatin/prohibitin superfamily) | Bacteria;Proteobacteria;Deltaproteobacteria;Desulfobacterales;Desulfobacteraceae;Desulfatiglans;Desulfatiglans anilini;Desulfatiglans anilini DSM 4660 |
| Ga0586324 0131508 1758 2870 | 5690000.00 | 11009200.00 | 16613139.12 | 488300000.00 | 33001200.00 | 39105595.72 | 527797200.00 | 44889900.00 | 34983900.00 | 15246000.00 | 13279200.00 | 17685000.00 | | predicted porin | Bacteria;Proteobacteria;Betaproteobacteria;Burkholderiales;Comamonadaceae;Caenimonas;Caenimonas koreensis;Caenimonas koreensis DSM 17982 |
| Ga0586324 0132084 2122 3003 | 0.00 | 0.00 | 0.00 | 0.00 | 0.00 | 0.00 | 0.00 | 0.00 | 0.00 | 3680600.00 | 0.00 | 0.00 | | hypothetical protein | Bacteria;Deinococcus-Thermus;Deinococci;Thermales;Thermaceae;Calidithermus;Calidithermus chliarophilus;Meiothermus chliarophilus ALT-8, DSM 9957 |
| Ga0586324 0132358 3 2120 | 1640000.00 | 0.00 | 0.00 | 0.00 | 0.00 | 0.00 | 0.00 | 0.00 | 0.00 | 0.00 | 0.00 | 0.00 | | hypothetical protein | Bacteria;Proteobacteria;Betaproteobacteria;Burkholderiales;Burkholderiaceae;Paraburkholderia;Paraburkholderia sp. OV555;Paraburkholderia sp. OV555 |

| | | | | | | | | | | | | | | | |
|---|---|---|---|---|---|---|---|---|---|---|---|---|---|---|---|
| Ga0586324 0132617 546 2759 | 0.00 | 0.00 | 0.00 | 0.00 | 0.00 | 0.00 | 0.00 | 0.00 | 0.00 | 0.00 | 11176000.00 | 6120000.00 | | anaerobic selenocysteine-containing dehydrogenase | Bacteria;Proteobacteria;Betaproteobacteria;Nitrosomonadales;Gallionellaceae;Gallionella;Gallionella capsiferriformans;Gallionella capsiferriformans ES-2 |
| Ga0586324 0133258 1496 1834 | 0.00 | 0.00 | 0.00 | 0.00 | 0.00 | 12068800.00 | 0.00 | 0.00 | 0.00 | 0.00 | 0.00 | 0.00 | | hypothetical protein | |
| Ga0586324 0133518 1170 2306 | 0.00 | 0.00 | 0.00 | 0.00 | 0.00 | 0.00 | 0.00 | 0.00 | 0.00 | 0.00 | 0.00 | 744393.75 | | TRAP-type mannitol/chloroaromatic compound transport system substrate-binding protein | Bacteria;Proteobacteria;Betaproteobacteria;Rhodocyclales;Zoogloeaceae;Azoarcus;Azoarcus sp. KH32C;Azoarcus sp. KH32C |
| Ga0586324 0133960 802 2985 | 0.00 | 0.00 | 1934552.45 | 0.00 | 0.00 | 0.00 | 0.00 | 0.00 | 0.00 | 0.00 | 0.00 | 0.00 | | hypothetical protein | Bacteria;Acidobacteria;Vicinamibacteria;unclassified Vicinamibacteria;Vicinamibacteraceae;Luteitalea;Luteitalea pratensis;Luteitalea pratensis DSM 100886 |
| Ga0586324 0134179 3 2642 | 27770000.00 | 18768000.00 | 4404754.36 | 8027500.00 | 15862000.00 | 6913766.91 | 5130000.00 | 11126400.00 | 6836400.00 | 14375900.00 | 4232800.00 | 12315000.00 | | hypothetical protein | Bacteria;Acidobacteria;Acidobacteriia;Acidobacteriales;Acidobacteriaceae;Candidatus Koribacter;Candidatus Koribacter versatilis;Candidatus Koribacter versatilis Ellin345 |
| Ga0586324 0134209 549 2159 | 0.00 | 0.00 | 0.00 | 0.00 | 0.00 | 0.00 | 0.00 | 4520100.00 | 0.00 | 0.00 | 0.00 | 0.00 | | enterochelin esterase-like enzyme | Bacteria;Actinobacteria;Thermoleophilia;Solirubrobacterales;Solirubrobacteraceae;Solirubrobacter;Solirubrobacter soli;Solirubrobacter soli DSM 22325 |
| Ga0586324 0134644 752 1162 | 0.00 | 560509.04 | 0.00 | 0.00 | 0.00 | 0.00 | 0.00 | 0.00 | 641879.64 | 0.00 | 0.00 | 0.00 | | nucleotide-binding universal stress UspA family protein | Bacteria;Proteobacteria;Deltaproteobacteria;Syntrophobacterales;Syntrophobacteraceae;Syntrophobacter;Syntrophobacter fumaroxidans;Syntrophobacter fumaroxidans MPOB |
| Ga0586324 0134891 1 1491 | 38700000.00 | 35462000.00 | 34286700.00 | 29022500.00 | 26831500.00 | 25288900.00 | 11992800.00 | 11834000.00 | 9639000.00 | 26873000.00 | 0.00 | 15450000.00 | | hypothetical protein | Bacteria;Thermodesulfobacteria;Thermodesulfobacteria;Thermodesulfobacteriales;Thermodesulfobacteriaceae;Thermosulfurimonas;Thermosulfurimonas dismutans;Thermosulfurimonas dismutans S95 |
| Ga0586324 0136033 1 2961 | 0.00 | 0.00 | 4121300.75 | 0.00 | 0.00 | 0.00 | 0.00 | 0.00 | 0.00 | 2926000.00 | 0.00 | 0.00 | | hypothetical protein | Bacteria;Ignavibacteriae;Ignavibacteria;Ignavibacteriales;Ignavibacteriaceae;Ignavibacterium;Ignavibacterium album;Ignavibacterium album Mat9-16, JCM 16511 |
| Ga0586324 0136111 845 2962 | 2690000.00 | 1339600.00 | 0.00 | 1016500.00 | 1349300.00 | 0.00 | 0.00 | 3867400.00 | 2470500.00 | 0.00 | 0.00 | 3450000.00 | | hypothetical protein | Bacteria;Ignavibacteriae;Ignavibacteria;Ignavibacteriales;Melioribacteraceae;Melioribacter;Melioribacter roseus;Melioribacter roseus P3M |
| Ga0586324 0136349 1313 2959 | 0.00 | 0.00 | 774671.11 | 0.00 | 648582.76 | 0.00 | 0.00 | 0.00 | 0.00 | 0.00 | 0.00 | 0.00 | | ELWxxDGT repeat protein | Bacteria;Bacteroidetes;Chitinophagia;Chitinophagales;Chitinophagaceae;Flavisolibacter;Flavisolibacter ginsengiterrae;Flavisolibacter ginsengiterrae DSM 18136 |
| Ga0586324 0136600 2 2956 | 0.00 | 0.00 | 0.00 | 0.00 | 0.00 | 0.00 | 0.00 | 0.00 | 1611900.00 | 0.00 | 4127200.00 | 0.00 | | outer membrane receptor protein involved in Fe transport | Bacteria;Acidobacteria;Acidobacteriia;Acidobacteriales;Acidobacteriaceae;Acidipila;Acidipila rosea;Acidipila rosea DSM 103428 |
| Ga0586324 0136951 1 1869 | 1310000.00 | 0.00 | 0.00 | 0.00 | 0.00 | 0.00 | 0.00 | 0.00 | 0.00 | 0.00 | 9416000.00 | 0.00 | | hypothetical protein | Bacteria;Proteobacteria;Betaproteobacteria;Nitrosomonadales;Gallionellaceae;Sideroxydans;Sideroxydans lithotrophicus;Sideroxydans lithotrophicus ES-1 |
| Ga0586324 0137335 1806 2948 | 5020000.00 | 6358000.00 | 3361363.35 | 6954000.00 | 0.00 | 6709300.00 | 6931200.00 | 3611200.00 | 2689200.00 | 9240000.00 | 9944000.00 | 9150000.00 | | hypothetical protein | Bacteria;Acidobacteria;Vicinamibacteria;unclassified Vicinamibacteria;Vicinamibacteraceae;Luteitalea;Luteitalea pratensis;Luteitalea pratensis DSM 100886 |
| Ga0586324 0137750 1706 2548 | 0.00 | 0.00 | 0.00 | 0.00 | 0.00 | 0.00 | 0.00 | 0.00 | 0.00 | 5544000.00 | 5702400.00 | 0.00 | | hypothetical protein | Bacteria;Proteobacteria;Betaproteobacteria;Burkholderiales;Burkholderiaceae;Pandoraea;Pandoraea sp. B-6;Pandoraea sp. B-6 |
| Ga0586324 0138105 884 2932 | 0.00 | 0.00 | 0.00 | 0.00 | 0.00 | 0.00 | 0.00 | 0.00 | 0.00 | 0.00 | 2824800.00 | 0.00 | | PAS domain S-box-containing protein | Bacteria;Proteobacteria;Deltaproteobacteria;Desulfuromonadales;Geobacteraceae;Geothermobacter;Geothermobacter sp. HR-1;Geothermobacter sp. HR-1 |
| Ga0586324 0138379 3 1247 | 0.00 | 3182400.00 | 0.00 | 5880500.00 | 0.00 | 0.00 | 0.00 | 0.00 | 0.00 | 3873100.00 | 0.00 | 0.00 | | hypothetical protein | Bacteria;Proteobacteria;Deltaproteobacteria;Syntrophobacterales;Syntrophaceae;Smithella;Smithella sp. F21;Smithella sp. F21 |
| Ga0586324 0138992 499 1512 | 0.00 | 0.00 | 0.00 | 0.00 | 0.00 | 0.00 | 0.00 | 0.00 | 0.00 | 25718000.00 | 0.00 | 0.00 | | ABC-type sugar transport system substrate-binding protein | Bacteria;Actinobacteria;Actinomycetia;Propionibacteriales;Nocardioidaceae;Nocardioides;Nocardioides ginsengisegetis;Nocardioides ginsengisegetis DSM 21349 |
| Ga0586324 0140040 3 2021 | 2430000.00 | 2169200.00 | 3342930.73 | 2460500.00 | 2595600.00 | 0.00 | 0.00 | 0.00 | 0.00 | 0.00 | 0.00 | 0.00 | | hypothetical protein | Bacteria;Acidobacteria;Vicinamibacteria;unclassified Vicinamibacteria;Vicinamibacteraceae;Luteitalea;Luteitalea pratensis;Luteitalea pratensis DSM 100886 |
| Ga0586324 0140839 2133 2915 | 86800000.00 | 0.00 | 0.00 | 0.00 | 0.00 | 0.00 | 0.00 | 0.00 | 0.00 | 0.00 | 0.00 | 104250000.00 | | hypothetical protein | Bacteria;Proteobacteria;Alphaproteobacteria;Rhodobacterales;Rhodobacteraceae;Rhodovulum;Rhodovulum sp. PH10;Rhodovulum sp. PH10 |
| Ga0586324 0141116 1747 2223 | 7410000.00 | 3155200.00 | 7262700.00 | 2099500.00 | 2894300.00 | 16968581.94 | 6384000.00 | 3971100.00 | 4487400.00 | 0.00 | 2340800.00 | 4552500.00 | | predicted Zn-dependent protease | |
| Ga0586324 0141433 151 1275 | 0.00 | 0.00 | 0.00 | 0.00 | 0.00 | 0.00 | 1294739.04 | 0.00 | 290243.25 | 2648800.00 | 0.00 | 0.00 | | TRAP-type mannitol/chloroaromatic compound transport system substrate-binding protein | Bacteria;Proteobacteria;Betaproteobacteria;Burkholderiales;Alcaligenaceae;Bordetella;Bordetella flabilis;Bordetella flabilis AU10664 |
| Ga0586324 0141847 2 2905 | 0.00 | 0.00 | 0.00 | 0.00 | 0.00 | 0.00 | 0.00 | 0.00 | 0.00 | 2102100.00 | 0.00 | 0.00 | | hypothetical protein | Bacteria;Actinobacteria;Actinomycetia;Pseudonocardiales;Pseudonocardiaceae;Amycolatopsis;Amycolatopsis sp. CA-126428;Amycolatopsis sp. CA-126428 |
| Ga0586324 0142327 2 2899 | 0.00 | 0.00 | 0.00 | 0.00 | 0.00 | 0.00 | 0.00 | 0.00 | 0.00 | 1085700.00 | 0.00 | 0.00 | | hypothetical protein | Bacteria;Actinobacteria;Actinomycetia;Micrococcales;Cellulomonadaceae;Cellulomonas;Cellulomonas sp. KRMCY2;Cellulomonas sp. KRMCY2 |
| Ga0586324 0142372 645 2828 | 0.00 | 0.00 | 1336297.39 | 0.00 | 0.00 | 0.00 | 0.00 | 0.00 | 0.00 | 3819200.00 | 0.00 | 2475000.00 | | hypothetical protein | Bacteria;Proteobacteria;Alphaproteobacteria;Hyphomicrobiales;Bradyrhizobiaceae;Bradyrhizobium;Bradyrhizobium erythrophlei;Bradyrhizobium erythrophlei GAS401 |
| Ga0586324 0142456 3 1004 | 0.00 | 5426400.00 | 18691600.00 | 5871000.00 | 9352400.00 | 14530200.00 | 14227200.00 | 7686000.00 | 8586000.00 | 6036800.00 | 11176000.00 | 6105000.00 | | alkylation response protein AidB-like acyl-CoA dehydrogenase | Bacteria;Proteobacteria;Deltaproteobacteria;Desulfarculales;Desulfarculaceae;Desulfocarbo;Desulfocarbo indianensis;Desulfocarbo indianensis SCBM |
| Ga0586324 0143291 1458 2597 | 0.00 | 0.00 | 0.00 | 0.00 | 0.00 | 536196.14 | 0.00 | 0.00 | 0.00 | 0.00 | 0.00 | 0.00 | | TRAP-type mannitol/chloroaromatic compound transport system substrate-binding protein | Bacteria;Proteobacteria;Alphaproteobacteria;Hyphomicrobiales;Hyphomicrobiaceae;Rhodoplanes;Rhodoplanes sp. Z2-YC6860;Rhodoplanes sp. Z2-YC6860 |
| Ga0586324 0144152 1 2100 | 22700000.00 | 166598.64 | 3899270.44 | 0.00 | 4727700.00 | 1561845.64 | 0.00 | 210129.14 | 0.00 | 0.00 | 692845.12 | 1608152.25 | | FtsP/CotA-like multicopper oxidase with cupredoxin domain | Bacteria;Firmicutes;Bacilli;Bacillales;Paenibacillaceae;Paenibacillus;Paenibacillus sophorae;Paenibacillus sophorae S27 |
| Ga0586324 0144219 1 2184 | 0.00 | 2672400.00 | 0.00 | 0.00 | 0.00 | 0.00 | 6201600.00 | 0.00 | 6083100.00 | 0.00 | 5913600.00 | 0.00 | | hypothetical protein | Bacteria;Spirochaetes;Spirochaetia;Spirochaetales;Spirochaetaceae;Spirochaeta;Spirochaeta africana;Spirochaeta africana Z-7692, DSM 8902 |
| Ga0586324 0144368 863 2380 | 9670000.00 | 8092000.00 | 11364250.71 | 5035000.00 | 10289700.00 | 12711411.99 | 4696800.00 | 7460300.00 | 5872500.00 | 3318700.00 | 6564800.00 | 4575000.00 | | hypothetical protein | Bacteria;Proteobacteria;Deltaproteobacteria;Desulfobacterales;Desulfobulbaceae;Desulfoprunum;Desulfoprunum benzoelyticum;Desulfoprunum benzoelyticum DSM 28570 |
| Ga0586324 0145683 1 2580 | 4960000.00 | 4270400.00 | 5315052.17 | 7619000.00 | 5077900.00 | 5161000.00 | 3328800.00 | 2238700.00 | 3102300.00 | 0.00 | 3960000.00 | 3727500.00 | | subtilisin family serine protease | Bacteria;Proteobacteria;Gammaproteobacteria;Nevskiales;Steroidobacteraceae;Povalibacter;Povalibacter uvarum;Povalibacter uvarum DSM 26723 |
| Ga0586324 0145691 558 2867 | 0.00 | 1740800.00 | 2828816.02 | 0.00 | 0.00 | 3468767.65 | 0.00 | 0.00 | 0.00 | 0.00 | 0.00 | 0.00 | | hypothetical protein | Bacteria;Actinobacteria;Actinomycetia;Streptosporangiales;Streptosporangiaceae;Nonomuraea;Nonomuraea longispora;Nonomuraea longispora KC201 |
| Ga0586324 0145835 3 1346 | 23400000.00 | 5875200.00 | 0.00 | 490687.35 | 2739800.00 | 0.00 | 0.00 | 5459500.00 | 2251800.00 | 0.00 | 0.00 | 0.00 | | hypothetical protein | Bacteria;Candidatus Aminicenantes;unclassified Candidatus Aminicenantes;unclassified Candidatus Aminicenantes;unclassified Candidatus Aminicenantes;unclassified Candidatus Aminicenantes;Aminicenantes bacterium SCGC AAA252-D06;Aminicenantes bacterium SCGC AAA252-D06 (contamination screened) |
| Ga0586324 0145836 1525 1863 | 0.00 | 0.00 | 3458897.47 | 0.00 | 0.00 | 4843400.00 | 0.00 | 0.00 | 2057400.00 | 0.00 | 0.00 | 0.00 | | TusE/DsrC/DsvC family sulfur relay protein | Bacteria;Proteobacteria;Gammaproteobacteria;Chromatiales;Chromatiaceae;Thioflavicoccus;Thioflavicoccus mobilis;Thioflavicoccus mobilis 8321 |
| Ga0586324 0146181 1 507 | 0.00 | 0.00 | 0.00 | 0.00 | 0.00 | 0.00 | 0.00 | 0.00 | 0.00 | 0.00 | 0.00 | 14775000.00 | | rubrerythrin | Bacteria;Deferribacteres;Deferribacteres;Deferribacterales;Deferribacteraceae;Calditerrivibrio;Calditerrivibrio nitroreducens;Calditerrivibrio nitroreducens Yu37-1, DSM 19672 |
| Ga0586324 0146489 561 2861 | 0.00 | 1156000.00 | 0.00 | 0.00 | 0.00 | 0.00 | 0.00 | 0.00 | 531191.52 | 0.00 | 0.00 | 0.00 | | OmcA/MtrC family decaheme c-type cytochrome | Bacteria;Acidobacteria;Holophagae;Holophagales;Holophagaceae;Geothrix;Geothrix fermentans;Geothrix fermentans DSM 14018 |
| Ga0586324 0146720 2039 2860 | 0.00 | 0.00 | 0.00 | 0.00 | 0.00 | 0.00 | 4788000.00 | 0.00 | 0.00 | 0.00 | 0.00 | 327981.75 | | hypothetical protein | Bacteria;Ignavibacteriae;Ignavibacteria;Ignavibacteriales;Melioribacteraceae;Melioribacter;Melioribacter roseus;Melioribacter roseus P3M |
| Ga0586324 0146720 3 1991 | 0.00 | 0.00 | 0.00 | 0.00 | 0.00 | 0.00 | 935727.96 | 0.00 | 0.00 | 0.00 | 0.00 | 0.00 | | hypothetical protein | Bacteria;Ignavibacteriae;Ignavibacteria;Ignavibacteriales;Melioribacteraceae;Melioribacter;Melioribacter roseus;Melioribacter roseus P3M |
| Ga0586324 0146806 1 2568 | 0.00 | 0.00 | 4384328.72 | 0.00 | 0.00 | 0.00 | 0.00 | 0.00 | 0.00 | 0.00 | 0.00 | 0.00 | | hypothetical protein | Bacteria;Acidobacteria;Vicinamibacteria;unclassified Vicinamibacteria;Vicinamibacteraceae;Luteitalea;Luteitalea pratensis;Luteitalea pratensis DSM 100886 |
| Ga0586324 0146915 2421 2780 | 4010000.00 | 0.00 | 0.00 | 0.00 | 0.00 | 0.00 | 0.00 | 0.00 | 0.00 | 0.00 | 0.00 | 0.00 | | quercetin dioxygenase-like cupin family protein | Bacteria;Proteobacteria;Alphaproteobacteria;Rhodobacterales;Rhodobacteraceae;Shimia;Shimia sp. SK013;Shimia sp. SK013 |
| Ga0586324 0147449 1295 2710 | 0.00 | 14416000.00 | 0.00 | 0.00 | 0.00 | 0.00 | 0.00 | 0.00 | 0.00 | 0.00 | 0.00 | 0.00 | | glutamine synthetase type I | Bacteria;Proteobacteria;Betaproteobacteria;Burkholderiales;unclassified Burkholderiales;unclassified Burkholderiales;Burkholderiales bacterium GJ-E10;Burkholderiales bacterium GJ-E10 |
| Ga0586324 0148124 554 2521 | 869278.00 | 0.00 | 0.00 | 0.00 | 0.00 | 0.00 | 0.00 | 0.00 | 0.00 | 0.00 | 0.00 | 0.00 | | uncharacterized membrane protein YqiK | Bacteria;Proteobacteria;Deltaproteobacteria;Myxococcales;Labilitrichaceae;Labilithrix;Labilithrix luteola;Labilithrix luteola DSM 27648 |
| Ga0586324 0148280 2 2668 | 3400000.00 | 2856000.00 | 0.00 | 3828500.00 | 4120000.00 | 6537633.23 | 4400400.00 | 6228100.00 | 3337200.00 | 2464000.00 | 6362400.00 | 7654036.50 | | TonB-linked SusC/RagA family outer membrane protein | Bacteria;Bacteroidetes;Cytophagia;Cytophagales;Hymenobacteraceae;Adhaeribacter;Adhaeribacter pallidiroseus;Adhaeribacter pallidiroseus HMF7616 |
| Ga0586324 0148299 3 2012 | 0.00 | 0.00 | 0.00 | 0.00 | 0.00 | 0.00 | 1450887.12 | 0.00 | 0.00 | 0.00 | 1179200.00 | 2415000.00 | | MtrB/PioB family decaheme-associated outer membrane protein | Bacteria;Proteobacteria;Betaproteobacteria;Rhodocyclales;Rhodocyclaceae;Niveibacterium;Niveibacterium umoris;Niveibacterium umoris DSM 106739 |
| Ga0586324 0148552 2 2842 | 0.00 | 0.00 | 0.00 | 829693.90 | 0.00 | 0.00 | 0.00 | 0.00 | 0.00 | 0.00 | 0.00 | 0.00 | | FtsP/CotA-like multicopper oxidase with cupredoxin domain | Bacteria;Firmicutes;Bacilli;Bacillales;Paenibacillaceae;Paenibacillus;Paenibacillus sophorae;Paenibacillus sophorae S27 |
| Ga0586324 0148899 173 2839 | 0.00 | 0.00 | 0.00 | 0.00 | 0.00 | 0.00 | 0.00 | 0.00 | 0.00 | 0.00 | 200869.68 | 0.00 | | hypothetical protein | Bacteria;Proteobacteria;Betaproteobacteria;Nitrosomonadales;Thiobacillaceae;Thiobacillus;Thiobacillus thiophilus;Thiobacillus thiophilus DSM 19892 |
| Ga0586324 0149277 478 2835 | 0.00 | 0.00 | 0.00 | 0.00 | 0.00 | 0.00 | 0.00 | 0.00 | 0.00 | 0.00 | 0.00 | 1987500.00 | | hypothetical protein | Bacteria;Acidobacteria;Acidobacteriia;Acidobacteriales;Acidobacteriaceae;unclassified Acidobacteriaceae;Acidobacteriaceae bacterium KBS 146;Acidobacteriaceae sp. KBS 146 |
| Ga0586324 0149500 2 2833 | 0.00 | 306382.16 | 0.00 | 0.00 | 0.00 | 0.00 | 0.00 | 0.00 | 0.00 | 0.00 | 0.00 | 0.00 | | FtsP/CotA-like multicopper oxidase with cupredoxin domain | Bacteria;Actinobacteria;Actinomycetia;Micrococcales;Intrasporangiaceae;Terrabacter;Terrabacter sp. 28;Terrabacter sp. 28 |
| Ga0586324 0149742 503 796 | 0.00 | 0.00 | 0.00 | 2935500.00 | 0.00 | 0.00 | 0.00 | 0.00 | 0.00 | 0.00 | 0.00 | 0.00 | | gas vesicle protein | Bacteria;Firmicutes;Bacilli;Bacillales;Bacillaceae;Lederbergia;Lederbergia lentus;Bacillus lentus NBRC 16444 |
| Ga0586324 0149799 998 2830 | 642826.00 | 0.00 | 0.00 | 919591.45 | 0.00 | 0.00 | 575973.60 | 538664.77 | 0.00 | 0.00 | 0.00 | 862500.00 | | hypothetical protein | Bacteria;Acidobacteria;Vicinamibacteria;unclassified Vicinamibacteria;Vicinamibacteraceae;Luteitalea;Luteitalea pratensis;Luteitalea pratensis DSM 100886 |
| Ga0586324 0150197 423 2828 | 0.00 | 6800000.00 | 0.00 | 0.00 | 0.00 | 0.00 | 0.00 | 0.00 | 0.00 | 1663200.00 | 0.00 | 0.00 | | 2',3'-cyclic-nucleotide 2'-phosphodiesterase (5'-nucleotidase family) | Bacteria;Actinobacteria;Actinomycetia;Propionibacteriales;Nocardioidaceae;Nocardioides;Nocardioides soli;Nocardioides soli DSM 105498 |
| Ga0586324 0151145 492 989 | 0.00 | 0.00 | 0.00 | 5291500.00 | 5943100.00 | 0.00 | 0.00 | 0.00 | 0.00 | 0.00 | 0.00 | 0.00 | | rubrerythrin | Bacteria;Planctomycetes;Candidatus Brocadiae;Candidatus Brocadiales;Candidatus Brocadiaceae;Candidatus Jettenia;Candidatus Jettenia caeni;Planctomycetaceae bacterium KSU-1 |
| Ga0586324 0151594 515 1522 | 0.00 | 0.00 | 4331812.08 | 0.00 | 0.00 | 0.00 | 0.00 | 0.00 | 0.00 | 0.00 | 0.00 | 11250000.00 | | ABC-type sugar transport system substrate-binding protein | Bacteria;Actinobacteria;Actinomycetia;Propionibacteriales;Nocardioidaceae;Nocardioides;Nocardioides ginsengisegetis;Nocardioides ginsengisegetis DSM 21349 |
| Ga0586324 0151875 3 2273 | 9630000.00 | 4970800.00 | 0.00 | 36575000.00 | 7848600.00 | 1155285.88 | 0.00 | 5093500.00 | 7630200.00 | 15554000.00 | 12645600.00 | 3225000.00 | | hypothetical protein | Bacteria;Ignavibacteriae;Ignavibacteria;Ignavibacteriales;Ignavibacteriaceae;Ignavibacterium;Ignavibacterium album;Ignavibacterium album Mat9-16, JCM 16511 |
| Ga0586324 0151943 1 360 | 0.00 | 0.00 | 0.00 | 0.00 | 0.00 | 0.00 | 0.00 | 0.00 | 11178000.00 | 0.00 | 18832000.00 | 12375000.00 | | TRAP-type mannitol/chloroaromatic compound transport system substrate-binding protein | Bacteria;Proteobacteria;Alphaproteobacteria;Rhodospirillales;Azospirillaceae;Azospirillum;Azospirillum sp. B506;Azospirillum sp. B506 |
| Ga0586324 0152667 1 2580 | 1996861.00 | 4970800.00 | 0.00 | 4778500.00 | 2472000.00 | 4724300.00 | 0.00 | 771437.11 | 378661.23 | 0.00 | 0.00 | 0.00 | | hypothetical protein | Bacteria;Candidatus Aminicenantes;unclassified Candidatus Aminicenantes;unclassified Candidatus Aminicenantes;unclassified Candidatus Aminicenantes;unclassified Candidatus Aminicenantes;Aminicenantes bacterium SCGC AAA252-D06;Aminicenantes bacterium SCGC AAA252-D06 (contamination screened) |
| Ga0586324 0153306 452 2800 | 737820.00 | 748000.00 | 0.00 | 0.00 | 0.00 | 0.00 | 0.00 | 0.00 | 0.00 | 0.00 | 0.00 | 0.00 | | hypothetical protein | Bacteria;Proteobacteria;Betaproteobacteria;Burkholderiales;Comamonadaceae;Variovorax;Variovorax paradoxus;Variovorax paradoxus H084 |
| Ga0586324 0153762 721 2796 | 0.00 | 0.00 | 0.00 | 0.00 | 0.00 | 0.00 | 0.00 | 0.00 | 16605000.00 | 1270500.00 | 0.00 | 3120000.00 | | MtrB/PioB family decaheme-associated outer membrane protein | Bacteria;Proteobacteria;Betaproteobacteria;Nitrosomonadales;Sulfuricellaceae;Sulfuricella;Sulfuricella sp. T08;Sulfuricella sp. T08 |
| Ga0586324 0154561 381 2534 | 95430000.00 | 99511200.00 | 63229637.08 | 29516500.00 | 38728000.00 | 17841529.36 | 16324800.00 | 118479762.59 | 70583400.00 | 20393195.13 | 9829600.00 | 22267500.00 | | S-layer protein (TIGR01567 family) | Archaea;Euryarchaeota;Methanomicrobia;Methanosarcinales;Methanotrichaceae;Methanothrix;Methanothrix soehngenii;Methanothrix soehngenii GP-6 |
| Ga0586324 0154948 1787 2215 | 0.00 | 0.00 | 0.00 | 0.00 | 0.00 | 0.00 | 0.00 | 1348100.00 | 0.00 | 0.00 | 0.00 | 637732.50 | | hypothetical protein | Bacteria;Cyanobacteria;unclassified Cyanobacteria;Synechococcales;Merismopediaceae;Aphanocapsa;Aphanocapsa montana;Aphanocapsa montana BDHKU210001 |
| Ga0586324 0155123 3 1877 | 6120000.00 | 0.00 | 6530800.00 | 5348500.00 | 8590200.00 | 6510800.00 | 0.00 | 0.00 | 0.00 | 0.00 | 5561600.00 | 8925000.00 | | TonB-linked SusC/RagA family outer membrane protein | Bacteria;Bacteroidetes;Cytophagia;Cytophagales;Fulvivirgaceae;Ohtaekwangia;Ohtaekwangia koreensis;Ohtaekwangia koreensis DSM 25262 |
| Ga0586324 0155568 1120 2781 | 0.00 | 0.00 | 0.00 | 0.00 | 0.00 | 29576500.00 | 0.00 | 0.00 | 0.00 | 0.00 | 0.00 | 0.00 | | ELWxxDGT repeat protein | Bacteria;Cyanobacteria;unclassified Cyanobacteria;Chroococcidiopsidales;Chroococcidiopsidaceae;Chroococcidiopsis;Chroococcidiopsis sp. PCC 6712;Chroococcidiopsis sp. PCC 6712 |
| Ga0586324 0155642 637 1203 | 2040000.00 | 0.00 | 0.00 | 0.00 | 0.00 | 0.00 | 0.00 | 0.00 | 0.00 | 0.00 | 0.00 | 0.00 | | hypothetical protein | |
| Ga0586324 0155787 211 720 | 5440000.00 | 1768000.00 | 0.00 | 0.00 | 11319700.00 | 24941834.66 | 6999600.00 | 7246800.00 | 10189800.00 | 3118500.00 | 2147200.00 | 0.00 | | tetratricopeptide (TPR) repeat protein | Bacteria;Proteobacteria;Deltaproteobacteria;Syntrophobacterales;Syntrophaceae;Desulfobacca;Desulfobacca acetoxidans;Desulfobacca acetoxidans ASRB2, DSM 11109 |
| Ga0586324 0155862 1241 2644 | 0.00 | 0.00 | 0.00 | 0.00 | 0.00 | 0.00 | 0.00 | 0.00 | 0.00 | 0.00 | 7884800.00 | 0.00 | | cytochrome c peroxidase | Bacteria;Proteobacteria;Deltaproteobacteria;Syntrophobacterales;Syntrophaceae;Desulfomonile;Desulfomonile tiedjei;Desulfomonile tiedjei DCB-1, DSM 6799 |
| Ga0586324 0155936 1341 2624 | 0.00 | 0.00 | 0.00 | 0.00 | 0.00 | 0.00 | 0.00 | 0.00 | 0.00 | 0.00 | 1610400.00 | 0.00 | | hypothetical protein | Bacteria;Firmicutes;Bacilli;Bacillales;Bacillaceae;Halalkalibacillus;Halalkalibacillus halophilus;Halalkalibacillus halophilus DSM 18494 |
| Ga0586324 0156239 1 528 | 0.00 | 0.00 | 0.00 | 0.00 | 0.00 | 0.00 | 0.00 | 0.00 | 0.00 | 1101100.00 | 0.00 | 0.00 | | hypothetical protein | Bacteria;Proteobacteria;Gammaproteobacteria;Chromatiales;Ectothiorhodospiraceae;Thioalkalivibrio;Thioalkalivibrio thiocyanodenitrificans;Thioalkalivibrio thiocyanodenitrificans ARhD 1 |
| Ga0586324 0159027 1 2754 | 3650000.00 | 1761200.00 | 0.00 | 0.00 | 1369900.00 | 2856228.41 | 2713200.00 | 0.00 | 0.00 | 1524600.00 | 848004.08 | 0.00 | | hypothetical protein | Bacteria;Bacteroidetes;Flavobacteriia;Flavobacteriales;Weeksellaceae;Chryseobacterium;Chryseobacterium koreense;Chryseobacterium koreense CCUG 49689 |
| Ga0586324 0159904 722 2137 | 3700000.00 | 0.00 | 0.00 | 0.00 | 889344.23 | 6384573.85 | 4005002.40 | 3135400.00 | 6115500.00 | 0.00 | 0.00 | 1230000.00 | | hypothetical protein | Bacteria;Nitrospirae;Thermodesulfovibrionia;Thermodesulfovibrionales;Thermodesulfovibrionaceae;Thermodesulfovibrio;Thermodesulfovibrio islandicus;Thermodesulfovibrio islandicus DSM 12570 |
| Ga0586324 0160572 1771 2742 | 697023.00 | 1217200.00 | 0.00 | 0.00 | 1266900.00 | 4168500.00 | 3784800.00 | 0.00 | 392769.81 | 1824900.00 | 870611.28 | 485268.00 | | hypothetical protein | Bacteria;Proteobacteria;Gammaproteobacteria;Legionellales;Coxiellaceae;Rickettsiella;Rickettsiella grylli;Rickettsiella grylli |
| Ga0586324 0163316 368 1645 | 0.00 | 0.00 | 0.00 | 0.00 | 18128000.00 | 20445500.00 | 0.00 | 0.00 | 0.00 | 120689800.00 | 159491200.00 | 5632500.00 | | predicted porin | Bacteria;Proteobacteria;Betaproteobacteria;Ferrovales;Ferrovaceae;Ferrovum;Ferrovum myxofaciens;Ferrovum myxofaciens JA12 |
| Ga0586324 0163521 1 873 | 0.00 | 0.00 | 0.00 | 0.00 | 0.00 | 0.00 | 0.00 | 0.00 | 0.00 | 38577000.00 | 41888000.00 | 0.00 | | hypothetical protein | |
| Ga0586324 0163640 542 988 | 0.00 | 0.00 | 0.00 | 0.00 | 0.00 | 0.00 | 0.00 | 0.00 | 0.00 | 0.00 | 1126400.00 | 0.00 | | nucleotide-binding universal stress UspA family protein | Bacteria;Proteobacteria;Deltaproteobacteria;Desulfuromonadales;Geobacteraceae;Geobacter;Geobacter argillaceus;Geobacter argillaceus ATCC BAA-1139 |
| Ga0586324 0163738 117 1454 | 0.00 | 0.00 | 0.00 | 0.00 | 0.00 | 0.00 | 0.00 | 3556300.00 | 0.00 | 0.00 | 0.00 | 0.00 | | ABC-type glycerol-3-phosphate transport system substrate-binding protein | Bacteria;Proteobacteria;Alphaproteobacteria;unclassified Alphaproteobacteria;unclassified Alphaproteobacteria;Phreatobacter;Phreatobacter stygius;Phreatobacter stygius KCTC 52518 |
| Ga0586324 0164308 2 2710 | 0.00 | 0.00 | 0.00 | 0.00 | 0.00 | 0.00 | 0.00 | 1549400.00 | 0.00 | 0.00 | 0.00 | 2002500.00 | | hypothetical protein | Bacteria;Acidobacteria;Acidobacteriia;Bryobacterales;Solibacteraceae;Candidatus Solibacter;Candidatus Solibacter usitatus;Candidatus Solibacter usitatus Ellin6076 |
| Ga0586324 0164788 1 2709 | 0.00 | 0.00 | 0.00 | 0.00 | 0.00 | 0.00 | 2039106.60 | 0.00 | 0.00 | 0.00 | 0.00 | 0.00 | | hypothetical protein | Bacteria;Bacteroidetes;Cytophagia;Cytophagales;Hymenobacteraceae;Hymenobacter;Hymenobacter sp. 1B;Hymenobacter artiguensis 1B |
| Ga0586324 0165161 1907 2704 | 10410000.00 | 7548000.00 | 8951700.00 | 0.00 | 0.00 | 7781200.00 | 0.00 | 5563200.00 | 5086800.00 | 5174400.00 | 0.00 | 5280000.00 | | hypothetical protein | Archaea;Euryarchaeota;Methanomicrobia;Methanomicrobiales;Methanoregulaceae;Methanoregula;Methanoregula formicica;Methanoregula formicica SMSP, DSM 22288 |
| Ga0586324 0166102 1621 2541 | 0.00 | 1958400.00 | 0.00 | 0.00 | 0.00 | 0.00 | 0.00 | 0.00 | 0.00 | 0.00 | 0.00 | 0.00 | | hypothetical protein | Bacteria;Proteobacteria;Gammaproteobacteria;Chromatiales;unclassified Chromatiales;Thiohalobacter;Thiohalobacter thiocyanaticus;Thiohalobacter thiocyanaticus FOKN1 |
| Ga0586324 0166726 1 855 | 5610000.00 | 0.00 | 0.00 | 0.00 | 6344800.00 | 0.00 | 0.00 | 3165900.00 | 0.00 | 0.00 | 0.00 | 0.00 | | uncharacterized surface protein with fasciclin (FAS1) repeats | Archaea;Euryarchaeota;Methanomicrobia;Methanosarcinales;Methanosarcinaceae;Methanosarcina;Methanosarcina lacustris;Methanosarcina lacustris Z-7289 |
| Ga0586324 0166767 1 1269 | 6110000.00 | 3440800.00 | 0.00 | 1349000.00 | 785806.57 | 0.00 | 5517600.00 | 3824700.00 | 5540400.00 | 5667200.00 | 6265600.00 | 10725000.00 | | hypothetical protein | Bacteria;Proteobacteria;Betaproteobacteria;Nitrosomonadales;Gallionellaceae;Sideroxydans;Sideroxydans lithotrophicus;Sideroxydans lithotrophicus ES-1 |
| Ga0586324 0169075 1350 2675 | 0.00 | 1475600.00 | 0.00 | 0.00 | 0.00 | 0.00 | 0.00 | 0.00 | 0.00 | 0.00 | 0.00 | 0.00 | | regulator of protease activity HflC (stomatin/prohibitin superfamily) | Bacteria;Proteobacteria;Deltaproteobacteria;Desulfuromonadales;Geobacteraceae;Geobacter;Geobacter thiogenes;Geobacter thiogenes ATCC BAA-34 |
| Ga0586324 0169741 90 821 | 0.00 | 0.00 | 18635300.00 | 0.00 | 0.00 | 0.00 | 0.00 | 15738000.00 | 0.00 | 0.00 | 13376000.00 | 0.00 | | opacity protein-like surface antigen | |
| Ga0586324 0170692 3 1151 | 0.00 | 0.00 | 0.00 | 0.00 | 0.00 | 0.00 | 0.00 | 976000.00 | 0.00 | 0.00 | 0.00 | 0.00 | | hypothetical protein | |
| Ga0586324 0170820 326 1624 | 1130000.00 | 0.00 | 0.00 | 0.00 | 5489900.00 | 0.00 | 0.00 | 0.00 | 0.00 | 0.00 | 0.00 | 0.00 | | hypothetical protein | Bacteria;Proteobacteria;Betaproteobacteria;Burkholderiales;unclassified Burkholderiales;Paucibacter;Paucibacter toxinivorans;Paucibacter toxinivorans DSM 16998 |
| Ga0586324 0172373 3 2651 | 0.00 | 0.00 | 0.00 | 0.00 | 0.00 | 0.00 | 0.00 | 0.00 | 0.00 | 0.00 | 8342400.00 | 0.00 | | hypothetical protein | Bacteria;Acidobacteria;Vicinamibacteria;unclassified Vicinamibacteria;Vicinamibacteraceae;Luteitalea;Luteitalea pratensis;Luteitalea pratensis DSM 100886 |
| Ga0586324 0172546 1063 2040 | 0.00 | 0.00 | 0.00 | 0.00 | 0.00 | 0.00 | 0.00 | 0.00 | 0.00 | 540424.50 | 552533.52 | 0.00 | | tripartite-type tricarboxylate transporter receptor subunit TctC | Bacteria;Proteobacteria;Betaproteobacteria;Burkholderiales;Oxalobacteraceae;Herbaspirillum;Herbaspirillum sp. K1R23-30;Herbaspirillum sp. K1R23-30 |
| Ga0586324 0176240 3 467 | 0.00 | 0.00 | 0.00 | 0.00 | 0.00 | 0.00 | 0.00 | 0.00 | 0.00 | 0.00 | 0.00 | 6952500.00 | | predicted GTPase | Archaea;Euryarchaeota;Thermococci;Thermococcales;Thermococcaceae;Thermococcus;Thermococcus sibiricus;Thermococcus sibiricus MM 739 |
| Ga0586324 0178111 2 2611 | 0.00 | 3094000.00 | 0.00 | 4246500.00 | 0.00 | 0.00 | 0.00 | 0.00 | 2049300.00 | 0.00 | 0.00 | 0.00 | | TonB-linked SusC/RagA family outer membrane protein | Bacteria;Bacteroidetes;Chitinophagia;Chitinophagales;Chitinophagaceae;Terrimonas;Terrimonas ferruginea;Terrimonas ferruginea DSM 30193 |
| Ga0586324 0178114 574 1980 | 0.00 | 1217200.00 | 0.00 | 0.00 | 0.00 | 0.00 | 0.00 | 0.00 | 0.00 | 475669.04 | 0.00 | 0.00 | | PAS domain S-box-containing protein | Bacteria;Proteobacteria;Deltaproteobacteria;Desulfobacterales;Desulfobacteraceae;Desulfosarcina;Desulfosarcina cetonica;Desulfosarcina cetonica JCM 12296 |
| Ga0586324 0178531 517 1173 | 0.00 | 5820800.00 | 0.00 | 0.00 | 0.00 | 0.00 | 0.00 | 2128900.00 | 1595700.00 | 0.00 | 0.00 | 0.00 | | hypothetical protein | Bacteria;Proteobacteria;Alphaproteobacteria;Rhodospirillales;Acetobacteraceae;Rhodopila;unclassified Rhodopila;Rhodopila sp. LVNP |
| Ga0586324 0178761 1263 2606 | 0.00 | 0.00 | 0.00 | 0.00 | 0.00 | 0.00 | 0.00 | 0.00 | 1952100.00 | 0.00 | 11704000.00 | 3022500.00 | | CO/xanthine dehydrogenase Mo-binding subunit | Bacteria;Proteobacteria;Alphaproteobacteria;Rhodospirillales;Acetobacteraceae;Rhodopila;unclassified Rhodopila;Rhodopila sp. LVNP |
| Ga0586324 0178906 2196 2606 | 0.00 | 788800.00 | 0.00 | 0.00 | 0.00 | 0.00 | 0.00 | 0.00 | 0.00 | 0.00 | 0.00 | 0.00 | | hypothetical protein | |
| Ga0586324 0180960 2 1957 | 0.00 | 15164000.00 | 36088300.00 | 0.00 | 0.00 | 0.00 | 0.00 | 0.00 | 1871100.00 | 12551000.00 | 3300000.00 | 12675000.00 | | nitrate/TMAO reductase-like tetraheme cytochrome c subunit | |
| Ga0586324 0182056 214 1623 | 0.00 | 0.00 | 0.00 | 0.00 | 0.00 | 0.00 | 0.00 | 0.00 | 0.00 | 0.00 | 374136.40 | 0.00 | | glutamine synthetase type I | Bacteria;Proteobacteria;Betaproteobacteria;unclassified Betaproteobacteria;unclassified Betaproteobacteria;unclassified Betaproteobacteria;Betaproteobacteria bacterium GR16-43;Betaproteobacteria bacterium GR16-43 |
| Ga0586324 0182213 1498 2583 | 3240000.00 | 2522800.00 | 0.00 | 10162755.15 | 1012894.79 | 5399200.00 | 0.00 | 2616900.00 | 0.00 | 1509200.00 | 1047200.00 | 0.00 | | predicted porin | Bacteria;Proteobacteria;Betaproteobacteria;Burkholderiales;unclassified Burkholderiales;Methylibium;Methylibium sp. CF059;Methylibium sp. CF059 |
| Ga0586324 0182717 1689 1961 | 0.00 | 0.00 | 0.00 | 0.00 | 0.00 | 0.00 | 0.00 | 0.00 | 1644300.00 | 0.00 | 0.00 | 0.00 | | nucleoid DNA-binding protein | Bacteria;Proteobacteria;Betaproteobacteria;unclassified Betaproteobacteria;unclassified Betaproteobacteria;unclassified Betaproteobacteria;unclassified Betaproteobacteria;beta proteobacterium NB0016 |
| Ga0586324 0183334 1 2511 | 0.00 | 0.00 | 0.00 | 3657500.00 | 0.00 | 0.00 | 0.00 | 0.00 | 1352700.00 | 0.00 | 0.00 | 0.00 | | hypothetical protein | |
| Ga0586324 0183648 802 2040 | 0.00 | 0.00 | 0.00 | 0.00 | 0.00 | 0.00 | 0.00 | 0.00 | 0.00 | 0.00 | 1971200.00 | 13275000.00 | | uncharacterized membrane protein | Bacteria;Proteobacteria;Deltaproteobacteria;Desulfobacterales;Desulfobacteraceae;Desulfatiglans;Desulfatiglans anilini;Desulfatiglans anilini DSM 4660 |
| Ga0586324 0185105 367 2082 | 2590000.00 | 0.00 | 0.00 | 0.00 | 0.00 | 0.00 | 0.00 | 0.00 | 0.00 | 0.00 | 0.00 | 0.00 | | tetratricopeptide (TPR) repeat protein | Bacteria;Bacteroidetes;Bacteroidia;Marinilabiliales;Marinilabiliaceae;Saccharicrinis;Saccharicrinis fermentans;Saccharicrinis fermentans DSM 9555 |
| Ga0586324 0185168 3 1811 | 0.00 | 1421200.00 | 0.00 | 0.00 | 0.00 | 0.00 | 0.00 | 0.00 | 0.00 | 0.00 | 0.00 | 1515000.00 | | hypothetical protein | Bacteria;Acidobacteria;Vicinamibacteria;unclassified Vicinamibacteria;Vicinamibacteraceae;Luteitalea;Luteitalea pratensis;Luteitalea pratensis DSM 100886 |
| Ga0586324 0185715 211 1221 | 0.00 | 0.00 | 0.00 | 0.00 | 0.00 | 0.00 | 0.00 | 0.00 | 2462400.00 | 0.00 | 0.00 | 0.00 | | hypothetical protein | Bacteria;Bacteroidetes;Bacteroidia;Bacteroidales;Lentimicrobiaceae;Lentimicrobium;Lentimicrobium saccharophilum;Bacteroidales bacterium TBC1 |
| Ga0586324 0186329 1660 2016 | 0.00 | 0.00 | 0.00 | 0.00 | 2379300.00 | 0.00 | 0.00 | 0.00 | 0.00 | 0.00 | 0.00 | 0.00 | | hypothetical protein | Bacteria;Actinobacteria;Actinomycetia;Pseudonocardiales;Pseudonocardiaceae;Amycolatopsis;Amycolatopsis taiwanensis;Amycolatopsis taiwanensis DSM 45107 |
| Ga0586324 0186400 3 1169 | 0.00 | 2488800.00 | 0.00 | 0.00 | 0.00 | 0.00 | 0.00 | 0.00 | 0.00 | 0.00 | 0.00 | 0.00 | | ABC-type sugar transport system substrate-binding protein | Bacteria;Proteobacteria;Alphaproteobacteria;Hyphomicrobiales;Devosiaceae;Maritalea;Maritalea myrionectae;Maritalea myrionectae DSM 19524 |
| Ga0586324 0186738 479 2554 | 11370000.00 | 24364400.00 | 18942005.51 | 15988500.00 | 13245800.00 | 4780003.07 | 12262407.72 | 33958700.00 | 5880600.00 | 17694600.00 | 44352000.00 | 18292500.00 | | hypothetical protein | Bacteria;Candidatus Aminicenantes;unclassified Candidatus Aminicenantes;unclassified Candidatus Aminicenantes;unclassified Candidatus Aminicenantes;unclassified Candidatus Aminicenantes;Aminicenantes bacterium SCGC AAA255-E10;Aminicenantes bacterium SCGC AAA255-E10 (contamination screened) |
| Ga0586324 0189433 33 2537 | 37730000.00 | 19516000.00 | 3127555.08 | 10640000.00 | 2781000.00 | 14014100.00 | 0.00 | 0.00 | 0.00 | 22168300.00 | 14168000.00 | 0.00 | | hypothetical protein | Bacteria;Actinobacteria;Actinomycetia;Kineosporiales;Kineosporiaceae;Kineococcus;Kineococcus xinjiangensis;Kineococcus xinjiangensis DSM 22857 |
| Ga0586324 0190262 871 2373 | 0.00 | 0.00 | 0.00 | 0.00 | 1781900.00 | 6431400.00 | 0.00 | 6100000.00 | 0.00 | 6183100.00 | 0.00 | 0.00 | | hypothetical protein | Bacteria;Proteobacteria;Gammaproteobacteria;Chromatiales;Chromatiaceae;Nitrosococcus;Nitrosococcus oceani;Nitrosococcus oceani C-27 |
| Ga0586324 0190791 968 2374 | 81762463.00 | 81940000.00 | 18973100.00 | 21375000.00 | 21290100.00 | 76859200.00 | 10237200.00 | 19574900.00 | 8497396.53 | 35982100.00 | 8157600.00 | 37349590.50 | | hypothetical protein | Bacteria;Proteobacteria;Deltaproteobacteria;Syntrophorhabdales;Syntrophorhabdaceae;Syntrophorhabdus;Syntrophorhabdus aromaticivorans;Syntrophorhabdus aromaticivorans UI |
| Ga0586324 0191079 1007 2527 | 0.00 | 0.00 | 0.00 | 0.00 | 0.00 | 3953060.01 | 2302800.00 | 2250900.00 | 0.00 | 0.00 | 0.00 | 1882500.00 | | type II secretory pathway component GspD/PulD (secretin) | Bacteria;Verrucomicrobia;Verrucomicrobiae;Verrucomicrobiales;Verrucomicrobia subdivision 3;Pedosphaera;Pedosphaera parvula;Pedosphaera parvula Ellin514 |
| Ga0586324 0192507 3 2519 | 1250000.00 | 3087200.00 | 0.00 | 2736000.00 | 5129400.00 | 0.00 | 0.00 | 3885700.00 | 3037500.00 | 5343800.00 | 0.00 | 2085000.00 | | hypothetical protein | |
| Ga0586324 0192931 951 2120 | 0.00 | 0.00 | 0.00 | 0.00 | 0.00 | 0.00 | 0.00 | 0.00 | 0.00 | 1355200.00 | 0.00 | 0.00 | | TRAP-type mannitol/chloroaromatic compound transport system substrate-binding protein | Bacteria;Proteobacteria;Gammaproteobacteria;Acidiferrobacterales;Acidiferrobacteraceae;Sulfuricaulis;Sulfuricaulis limicola;Sulfuricaulis limicola HA5 |
| Ga0586324 0193166 1194 2516 | 0.00 | 2189600.00 | 0.00 | 0.00 | 724358.83 | 0.00 | 0.00 | 0.00 | 0.00 | 1031800.00 | 1056000.00 | 0.00 | | hypothetical protein | Bacteria;Proteobacteria;Deltaproteobacteria;Syntrophobacterales;Syntrophaceae;Desulfobacca;Desulfobacca acetoxidans;Desulfobacca acetoxidans ASRB2, DSM 11109 |
| Ga0586324 0194166 3 1217 | 0.00 | 2536400.00 | 0.00 | 2650500.00 | 2688300.00 | 0.00 | 0.00 | 2501000.00 | 1741500.00 | 1848000.00 | 1170400.00 | 0.00 | | hypothetical protein | Bacteria;Ignavibacteriae;Ignavibacteria;Ignavibacteriales;Melioribacteraceae;Melioribacter;Melioribacter roseus;Melioribacter roseus P3M |
| Ga0586324 0194384 1318 2334 | 0.00 | 0.00 | 0.00 | 0.00 | 0.00 | 0.00 | 0.00 | 0.00 | 0.00 | 1093400.00 | 0.00 | 0.00 | | hypothetical protein | |
| Ga0586324 0194467 3 1619 | 888834.00 | 1674848.84 | 11091100.00 | 5586000.00 | 1472900.00 | 488929.32 | 0.00 | 124435.73 | 681528.33 | 810358.01 | 840668.40 | 1809078.75 | | hypothetical protein | Bacteria;Bacteroidetes;Cytophagia;Cytophagales;Cyclobacteriaceae;Cecembia;Cecembia rubra;Cecembia rubra DSM 28057 |
| Ga0586324 0194633 1 2505 | 990897.00 | 1006400.00 | 1627649.89 | 3885500.00 | 5500200.00 | 0.00 | 4572068.04 | 3599000.00 | 1620000.00 | 4211900.00 | 1892000.00 | 2869262.25 | | hypothetical protein | |
| Ga0586324 0194657 395 2161 | 0.00 | 0.00 | 0.00 | 0.00 | 0.00 | 0.00 | 0.00 | 0.00 | 5799600.00 | 0.00 | 0.00 | 0.00 | | choline dehydrogenase-like flavoprotein | Bacteria;Bacteroidetes;Chitinophagia;Chitinophagales;Chitinophagaceae;Chitinophaga;Chitinophaga sp. OAE865;Chitinophaga sp. OAE865 (Illumina draft) |
| Ga0586324 0195056 285 1397 | 1130000.00 | 0.00 | 0.00 | 0.00 | 0.00 | 0.00 | 0.00 | 0.00 | 0.00 | 0.00 | 2578400.00 | 0.00 | | TRAP-type mannitol/chloroaromatic compound transport system substrate-binding protein | Bacteria;Proteobacteria;Alphaproteobacteria;Rhodospirillales;Azospirillaceae;Azospirillum;Azospirillum oryzae;Azospirillum oryzae A2P |
| Ga0586324 0195207 3 2504 | 13500000.00 | 16796000.00 | 0.00 | 0.00 | 25956000.00 | 0.00 | 29412000.00 | 21655000.00 | 21951000.00 | 18788000.00 | 0.00 | 13350000.00 | | hypothetical protein | Bacteria;Bacteroidetes;Bacteroidia;Bacteroidales;Lentimicrobiaceae;Lentimicrobium;Lentimicrobium saccharophilum;Bacteroidales bacterium TBC1 |
| Ga0586324 0195320 163 1803 | 0.00 | 3733200.00 | 2023191.17 | 5652500.00 | 5366300.00 | 0.00 | 0.00 | 2360700.00 | 0.00 | 7777000.00 | 0.00 | 3112500.00 | | hypothetical protein | Bacteria;Chloroflexi;Ardenticatenia;Ardenticatenales;Ardenticatenaceae;Candidatus Promineofilum;Candidatus Promineofilum breve;Candidatus Promineofilum breve Cfx-K |
| Ga0586324 0195553 1618 2082 | 0.00 | 0.00 | 0.00 | 0.00 | 0.00 | 1320957.95 | 0.00 | 0.00 | 0.00 | 0.00 | 0.00 | 0.00 | | nucleotide-binding universal stress UspA family protein | Bacteria;Proteobacteria;Deltaproteobacteria;Syntrophobacterales;Syntrophobacteraceae;Syntrophobacter;Syntrophobacter fumaroxidans;Syntrophobacter fumaroxidans MPOB |
| Ga0586324 0196852 1414 2493 | 0.00 | 0.00 | 0.00 | 0.00 | 1709800.00 | 0.00 | 0.00 | 1438182.97 | 1668600.00 | 3380300.00 | 4435200.00 | 1170000.00 | | thiamine pyrophosphate-dependent acetolactate synthase large subunit-like protein | Bacteria;Proteobacteria;Alphaproteobacteria;Rhodospirillales;Acetobacteraceae;Rhodopila;Rhodopila globiformis;Rhodopila globiformis DSM 161 |
| Ga0586324 0196878 3 2063 | 0.00 | 0.00 | 0.00 | 0.00 | 0.00 | 0.00 | 0.00 | 0.00 | 94711.68 | 0.00 | 0.00 | 0.00 | | hypothetical protein | Bacteria;Acidobacteria;Blastocatellia;Blastocatellales;Pyrinomonadaceae;Pyrinomonas;Pyrinomonas methylaliphatogenes;Pyrinomonas methylaliphatogenes K22 |
| Ga0586324 0199441 1 342 | 13000000.00 | 10948000.00 | 3807794.20 | 0.00 | 0.00 | 4128800.00 | 0.00 | 5227700.00 | 5208300.00 | 0.00 | 0.00 | 4567500.00 | | hypothetical protein | Bacteria;Proteobacteria;Gammaproteobacteria;unclassified Gammaproteobacteria;Candidatus Competibacteraceae;Candidatus Contendobacter;Candidatus Contendobacter odensis;Candidatus Contendobacter odensis Run B J11 |
| Ga0586324 0199851 2 1345 | 41800000.00 | 0.00 | 5855200.00 | 0.00 | 0.00 | 0.00 | 10032000.00 | 0.00 | 0.00 | 0.00 | 0.00 | 0.00 | | hypothetical protein | Bacteria;Proteobacteria;Alphaproteobacteria;Hyphomicrobiales;Bradyrhizobiaceae;Bradyrhizobium;Bradyrhizobium lablabi;Bradyrhizobium lablabi CCBAU 23086 |
| Ga0586324 0200373 378 2474 | 0.00 | 11723200.00 | 13737200.00 | 8331500.00 | 3532900.00 | 3316970.73 | 798636.12 | 2013000.00 | 791098.65 | 2810500.00 | 2420000.00 | 1252500.00 | | hypothetical protein | Bacteria;Acidobacteria;Vicinamibacteria;unclassified Vicinamibacteria;Vicinamibacteraceae;Luteitalea;Luteitalea pratensis;Luteitalea pratensis DSM 100886 |
| Ga0586324 0201477 869 1999 | 0.00 | 0.00 | 0.00 | 0.00 | 0.00 | 69872000.00 | 101688000.00 | 45628000.00 | 65367000.00 | 1863400.00 | 0.00 | 0.00 | | hypothetical protein | Bacteria;Proteobacteria;Deltaproteobacteria;Desulfobacterales;Desulfobacteraceae;Desulfotignum;Desulfotignum balticum;Desulfotignum balticum DSM 7044 |
| Ga0586324 0202875 65 2458 | 0.00 | 0.00 | 0.00 | 0.00 | 0.00 | 448832.32 | 0.00 | 0.00 | 0.00 | 0.00 | 0.00 | 0.00 | | DNA polymerase III epsilon subunit family exonuclease | Bacteria;Firmicutes;Clostridia;Thermoanaerobacterales;Thermoanaerobacterales Family IV. Incertae Sedis;Mahella;Mahella australiensis;Mahella australiensis 50-1 BON, DSM 15567 |
| Ga0586324 0204000 1 942 | 0.00 | 0.00 | 0.00 | 0.00 | 0.00 | 0.00 | 0.00 | 0.00 | 0.00 | 0.00 | 0.00 | 559623.75 | | tripartite-type tricarboxylate transporter receptor subunit TctC | Bacteria;Proteobacteria;Betaproteobacteria;Burkholderiales;Alcaligenaceae;Bordetella;Bordetella genomosp. 9;Bordetella genomosp. 9 AU21707 |
| Ga0586324 0204441 1552 2451 | 0.00 | 4590000.00 | 0.00 | 0.00 | 0.00 | 0.00 | 10921200.00 | 3397700.00 | 0.00 | 0.00 | 0.00 | 1192500.00 | | hypothetical protein | |
| Ga0586324 0205320 892 2445 | 0.00 | 0.00 | 0.00 | 0.00 | 3996400.00 | 0.00 | 0.00 | 0.00 | 0.00 | 0.00 | 0.00 | 9900000.00 | | hypothetical protein | Bacteria;Acidobacteria;Acidobacteriia;Bryobacterales;Solibacteraceae;Candidatus Solibacter;Candidatus Solibacter usitatus;Candidatus Solibacter usitatus Ellin6076 |
| Ga0586324 0205926 1247 2128 | 0.00 | 11832000.00 | 0.00 | 1263500.00 | 0.00 | 0.00 | 0.00 | 0.00 | 0.00 | 0.00 | 3106400.00 | 0.00 | | hypothetical protein | |
| Ga0586324 0207203 2175 2435 | 0.00 | 0.00 | 0.00 | 0.00 | 2266000.00 | 0.00 | 0.00 | 0.00 | 0.00 | 0.00 | 0.00 | 0.00 | | hypothetical protein | Bacteria;Actinobacteria;Actinomycetia;Micrococcales;Micrococcaceae;Arthrobacter;Arthrobacter sulfonylureivorans;Arthrobacter sulfonylureivorans LAM7117 |
| Ga0586324 0207759 768 2153 | 0.00 | 0.00 | 0.00 | 0.00 | 0.00 | 0.00 | 1233628.20 | 0.00 | 0.00 | 0.00 | 0.00 | 0.00 | | hypothetical protein | Bacteria;Proteobacteria;Deltaproteobacteria;Syntrophobacterales;Syntrophobacteraceae;Desulfosoma;Desulfosoma caldarium;Desulfosoma caldarium DSM 22027 |
| Ga0586324 0207897 1250 2431 | 0.00 | 822800.00 | 0.00 | 0.00 | 0.00 | 0.00 | 0.00 | 0.00 | 0.00 | 0.00 | 0.00 | 0.00 | | hypothetical protein | Bacteria;Acidobacteria;Acidobacteriia;Bryobacterales;Bryobacteraceae;Bryobacter;Bryobacter aggregatus;Bryobacter aggregatus DSM 18758 |
| Ga0586324 0209481 51 1343 | 0.00 | 0.00 | 0.00 | 0.00 | 0.00 | 0.00 | 0.00 | 2025200.00 | 0.00 | 12089000.00 | 0.00 | 10080000.00 | | TRAP-type mannitol/chloroaromatic compound transport system substrate-binding protein | Bacteria;Proteobacteria;Alphaproteobacteria;Hyphomicrobiales;Bradyrhizobiaceae;Tardiphaga;Tardiphaga sp. OV697;Tardiphaga sp. OV697 |
| Ga0586324 0211638 3 2411 | 0.00 | 0.00 | 0.00 | 0.00 | 0.00 | 0.00 | 0.00 | 3782000.00 | 0.00 | 0.00 | 0.00 | 0.00 | | FtsP/CotA-like multicopper oxidase with cupredoxin domain | Bacteria;Firmicutes;Bacilli;Bacillales;Paenibacillaceae;Paenibacillus;Paenibacillus sophorae;Paenibacillus sophorae S27 |
| Ga0586324 0212515 1 1833 | 0.00 | 0.00 | 0.00 | 0.00 | 0.00 | 0.00 | 0.00 | 0.00 | 931500.00 | 0.00 | 0.00 | 542205.00 | | hypothetical protein | Bacteria;Proteobacteria;Betaproteobacteria;Burkholderiales;Comamonadaceae;Variovorax;Variovorax paradoxus;Variovorax paradoxus MEDvA23 |
| Ga0586324 0212549 514 1593 | 0.00 | 0.00 | 0.00 | 0.00 | 0.00 | 0.00 | 0.00 | 774700.00 | 0.00 | 0.00 | 0.00 | 0.00 | | TRAP-type C4-dicarboxylate transport system substrate-binding protein | Bacteria;Proteobacteria;Deltaproteobacteria;unclassified Deltaproteobacteria;unclassified Deltaproteobacteria;unclassified Deltaproteobacteria;delta proteobacterium NaphS2;delta proteobacterium NaphS2 |
| Ga0586324 0212671 248 1621 | 0.00 | 389118.44 | 0.00 | 8641325.40 | 0.00 | 0.00 | 0.00 | 0.00 | 0.00 | 0.00 | 0.00 | 0.00 | | cytochrome c peroxidase | Bacteria;Proteobacteria;Deltaproteobacteria;Syntrophobacterales;Syntrophaceae;Desulfomonile;Desulfomonile tiedjei;Desulfomonile tiedjei DCB-1, DSM 6799 |

| | | | | | | | | | | | | | | | |
|---|---|---|---|---|---|---|---|---|---|---|---|---|---|---|---|
| Ga0586324 0213623 3 1358 | 0.00 | 0.00 | 19479800.00 | 15580000.00 | 0.00 | 40896692.98 | 52234800.00 | 5514400.00 | 0.00 | 5828900.00 | 5209600.00 | 0.00 | | hypothetical protein | Bacteria;Proteobacteria;Alphaproteobacteria;Hyphomicrobiales;Bradyrhizobiaceae;Bradyrhizobium;Bradyrhizobium elkanii;Bradyrhizobium elkanii WSM2783 |
| Ga0586324 0214395 563 1408 | 0.00 | 0.00 | 0.00 | 0.00 | 0.00 | 0.00 | 674699.88 | 1244400.00 | 0.00 | 0.00 | 0.00 | 0.00 | | chromosome segregation ATPase | Bacteria;Chloroflexi;Caldilineae;Caldilineales;Caldilineaceae;Caldilinea;Caldilinea aerophila;Caldilinea aerophila STL-6-O1, DSM 14535 |
| Ga0586324 0216499 2 1066 | 0.00 | 7344000.00 | 0.00 | 0.00 | 0.00 | 0.00 | 0.00 | 0.00 | 0.00 | 1917300.00 | 0.00 | 0.00 | | hypothetical protein | Bacteria;Proteobacteria;Deltaproteobacteria;Desulfobacterales;Desulfobacteraceae;Desulfobacter;Desulfobacter curvatus;Desulfobacter curvatus DSM 3379 |
| Ga0586324 0216595 876 2216 | 3590000.00 | 6847600.00 | 0.00 | 2584000.00 | 0.00 | 0.00 | 1480235.28 | 0.00 | 1757700.00 | 3372600.00 | 2226400.00 | 2077500.00 | | ABC-type glycerol-3-phosphate transport system substrate-binding protein | Bacteria;Proteobacteria;Alphaproteobacteria;Rhodospirillales;Acetobacteraceae;Rhodopila;unclassified Rhodopila;Rhodopila sp. LVNP |
| Ga0586324 0217059 935 2311 | 0.00 | 0.00 | 0.00 | 0.00 | 1308100.00 | 0.00 | 0.00 | 0.00 | 633892.23 | 0.00 | 0.00 | 0.00 | | hypothetical protein | Bacteria;Nitrospirae;Thermodesulfovibrionia;Thermodesulfovibrionales;Thermodesulfovibrionaceae;Thermodesulfovibrio;Thermodesulfovibrio islandicus;Thermodesulfovibrio islandicus DSM 12570 |
| Ga0586324 0217126 598 1059 | 229952.00 | 0.00 | 0.00 | 0.00 | 1452300.00 | 0.00 | 0.00 | 0.00 | 2122200.00 | 0.00 | 0.00 | 0.00 | | rubrerythrin | Bacteria;Proteobacteria;Deltaproteobacteria;Syntrophobacterales;Syntrophobacteraceae;Desulfacinum;Desulfacinum infernum;Desulfacinum infernum DSM 9756 |
| Ga0586324 0217619 698 2380 | 0.00 | 6235600.00 | 19648700.00 | 7999000.00 | 11330000.00 | 17190100.00 | 4879200.00 | 5758400.00 | 8343000.00 | 9009000.00 | 8685600.00 | 7875000.00 | | outer membrane receptor protein involved in Fe transport | Bacteria;Bacteroidetes;Sphingobacteriia;Sphingobacteriales;Sphingobacteriaceae;Pedobacter;Pedobacter psychrotolerans;Pedobacter psychrotolerans DSM 103236 |
| Ga0586324 0218114 3 2339 | 0.00 | 2529600.00 | 0.00 | 0.00 | 0.00 | 0.00 | 2209586.76 | 2159400.00 | 3563842.86 | 0.00 | 4012800.00 | 1912500.00 | | hypothetical protein | Bacteria;Acidobacteria;Acidobacteriia;Acidobacteriales;Acidobacteriaceae;Acidobacterium;unclassified Acidobacterium;Acidobacterium strain C40 |
| Ga0586324 0218207 118 1449 | 0.00 | 0.00 | 0.00 | 0.00 | 0.00 | 0.00 | 0.00 | 0.00 | 0.00 | 0.00 | 0.00 | 1035000.00 | | FtsP/CotA-like multicopper oxidase with cupredoxin domain | Bacteria;Actinobacteria;Actinomycetia;Micrococcales;Intrasporangiaceae;Humibacillus;Humibacillus xanthopallidus;Humibacillus xanthopallidus DSM 21776 |
| Ga0586324 0219075 1368 2033 | 0.00 | 0.00 | 0.00 | 1016500.00 | 0.00 | 0.00 | 0.00 | 0.00 | 0.00 | 0.00 | 0.00 | 0.00 | | hypothetical protein | |
| Ga0586324 0219647 1 558 | 0.00 | 17952000.00 | 0.00 | 0.00 | 15347000.00 | 0.00 | 0.00 | 16897000.00 | 10611000.00 | 29621900.00 | 27711200.00 | 29175000.00 | | hypothetical protein | Bacteria;Proteobacteria;Betaproteobacteria;Burkholderiales;Burkholderiaceae;Cupriavidus;Cupriavidus necator;Cupriavidus necator H16 |
| Ga0586324 0220031 1176 2264 | 0.00 | 0.00 | 0.00 | 0.00 | 0.00 | 0.00 | 0.00 | 0.00 | 425706.84 | 0.00 | 0.00 | 0.00 | | TRAP-type mannitol/chloroaromatic compound transport system substrate-binding protein | Bacteria;Proteobacteria;Betaproteobacteria;unclassified Betaproteobacteria;unclassified Betaproteobacteria;unclassified Betaproteobacteria;Betaproteobacteria bacterium UKL13-2;Betaproteobacteria bacterium UKL13-2 |
| Ga0586324 0220603 3 1037 | 2340000.00 | 0.00 | 0.00 | 0.00 | 0.00 | 0.00 | 2736000.00 | 0.00 | 0.00 | 1663200.00 | 0.00 | 0.00 | | TRAP-type mannitol/chloroaromatic compound transport system substrate-binding protein | Bacteria;Proteobacteria;Alphaproteobacteria;Hyphomicrobiales;Bradyrhizobiaceae;Bradyrhizobium;Bradyrhizobium sp. Ai1a-2;Bradyrhizobium sp. Ai1a-2 |
| Ga0586324 0220656 946 2364 | 0.00 | 0.00 | 0.00 | 988000.00 | 0.00 | 0.00 | 0.00 | 0.00 | 0.00 | 0.00 | 0.00 | 0.00 | | type IV pilus secretin PilQ/predicted competence protein | Bacteria;Proteobacteria;Deltaproteobacteria;Syntrophobacterales;Syntrophaceae;Desulfomonile;Desulfomonile tiedjei;Desulfomonile tiedjei DCB-1, DSM 6799 |
| Ga0586324 0220679 685 2184 | 0.00 | 0.00 | 0.00 | 0.00 | 0.00 | 0.00 | 0.00 | 193047.31 | 0.00 | 0.00 | 0.00 | 0.00 | | hypothetical protein | Bacteria;Proteobacteria;Deltaproteobacteria;Syntrophobacterales;Syntrophobacteraceae;Syntrophobacter;Syntrophobacter fumaroxidans;Syntrophobacter fumaroxidans MPOB |
| Ga0586324 0221336 1 1044 | 0.00 | 0.00 | 0.00 | 0.00 | 956552.76 | 0.00 | 0.00 | 503395.18 | 0.00 | 0.00 | 0.00 | 0.00 | | hypothetical protein | Bacteria;Proteobacteria;Gammaproteobacteria;Chromatiales;Chromatiaceae;Lamprocystis;Lamprocystis purpurea;Lamprocystis purpurea DSM 4197 |
| Ga0586324 0221460 3 2360 | 1970000.00 | 0.00 | 0.00 | 3420000.00 | 2276300.00 | 0.00 | 0.00 | 0.00 | 0.00 | 0.00 | 0.00 | 0.00 | | Ca2+-binding RTX toxin-like protein | Bacteria;Planctomycetes;Planctomycetia;Pirellulales;Pirellulaceae;Rhodopirellula;Rhodopirellula baltica;Rhodopirellula baltica SWK14 |
| Ga0586324 0221543 85 2031 | 15540000.00 | 11206400.00 | 14525400.00 | 4645500.00 | 4655600.00 | 3813181.03 | 18308400.00 | 3300100.00 | 5372287.74 | 0.00 | 19351200.00 | 18480000.00 | | hypothetical protein | Bacteria;Spirochaetes;Spirochaetia;Leptospirales;Leptospiraceae;Leptonema;Leptonema illini;Leptonema illini 3055, DSM 21528 |
| Ga0586324 0221833 3 1247 | 883710000.00 | 46464400.00 | 19879670.75 | 851314.00 | 37986400.00 | 27439774.54 | 191034733.92 | 17482600.00 | 2167357.50 | 50935500.00 | 1786400.00 | 3735000.00 | | hypothetical protein | Bacteria;Proteobacteria;Alphaproteobacteria;Hyphomicrobiales;Bradyrhizobiaceae;Bradyrhizobium;Bradyrhizobium erythrophlei;Bradyrhizobium erythrophlei GAS138 |
| Ga0586324 0221858 916 1887 | 0.00 | 0.00 | 0.00 | 0.00 | 0.00 | 0.00 | 0.00 | 0.00 | 4941000.00 | 0.00 | 0.00 | 0.00 | | hypothetical protein | Bacteria;Proteobacteria;Betaproteobacteria;Nitrosomonadales;Thiobacillaceae;Thiobacillus;Thiobacillus thiophilus;Thiobacillus thiophilus DSM 19892 |
| Ga0586324 0222323 1 2358 | 6380000.00 | 6528000.00 | 10359200.00 | 0.00 | 0.00 | 16991600.00 | 7615200.00 | 9455000.00 | 5418900.00 | 0.00 | 0.00 | 4200000.00 | | outer membrane protein OmpA-like peptidoglycan-associated protein | Bacteria;Proteobacteria;Betaproteobacteria;unclassified Betaproteobacteria;unclassified Betaproteobacteria;unclassified Betaproteobacteria;Betaproteobacteria bacterium GR16-43;Betaproteobacteria bacterium GR16-43 |
| Ga0586324 0222340 321 2357 | 1370000.00 | 1638800.00 | 0.00 | 1653000.00 | 0.00 | 0.00 | 0.00 | 0.00 | 0.00 | 0.00 | 0.00 | 0.00 | | hypothetical protein | Bacteria;Acidobacteria;Thermoanaerobaculia;Thermoanaerobaculales;Thermoanaerobaculaceae;Thermoanaerobaculum;Thermoanaerobaculum aquaticum;Thermoanaerobaculum aquaticum MP-01 |
| Ga0586324 0224289 571 2349 | 0.00 | 0.00 | 0.00 | 0.00 | 0.00 | 0.00 | 0.00 | 0.00 | 0.00 | 17933300.00 | 22704000.00 | 0.00 | | hypothetical protein | Bacteria;Acidobacteria;Acidobacteriia;Bryobacterales;Solibacteraceae;Candidatus Solibacter;Candidatus Solibacter usitatus;Candidatus Solibacter usitatus Ellin6076 |
| Ga0586324 0225538 2 1603 | 99100000.00 | 11832000.00 | 0.00 | 17480000.00 | 38213000.00 | 27265090.57 | 5745600.00 | 14762000.00 | 10044000.00 | 3264800.00 | 0.00 | 13950000.00 | | TonB-linked SusC/RagA family outer membrane protein | Bacteria;Bacteroidetes;Bacteroidia;Marinilabiliales;Prolixibacteraceae;Sunxiuqinia;Sunxiuqinia elliptica;Sunxiuqinia elliptica 114D |
| Ga0586324 0225626 2 1693 | 0.00 | 0.00 | 0.00 | 0.00 | 0.00 | 0.00 | 0.00 | 0.00 | 0.00 | 0.00 | 0.00 | 1245000.00 | | predicted porin | Bacteria;Proteobacteria;Alphaproteobacteria;Hyphomicrobiales;Bradyrhizobiaceae;Bradyrhizobium;Bradyrhizobium erythrophlei;Bradyrhizobium erythrophlei GAS401 |
| Ga0586324 0226452 1328 1825 | 0.00 | 3087200.00 | 0.00 | 0.00 | 0.00 | 0.00 | 0.00 | 0.00 | 0.00 | 0.00 | 0.00 | 0.00 | | rubrerythrin | Bacteria;Proteobacteria;Deltaproteobacteria;Desulfovibrionales;Desulfovibrionaceae;Desulfovibrio;Desulfovibrio inopinatus;Desulfovibrio inopinatus DSM 10711 |
| Ga0586324 0227644 3 2330 | 0.00 | 1645600.00 | 0.00 | 1871500.00 | 0.00 | 0.00 | 0.00 | 0.00 | 0.00 | 3110800.00 | 0.00 | 0.00 | | outer membrane receptor protein involved in Fe transport | Bacteria;Acidobacteria;Thermoanaerobaculia;Thermoanaerobaculales;Thermoanaerobaculaceae;Thermoanaerobaculum;Thermoanaerobaculum aquaticum;Thermoanaerobaculum aquaticum MP-01 |
| Ga0586324 0228501 3 2327 | 3550000.00 | 3298000.00 | 8445000.00 | 4075500.00 | 0.00 | 3172816.06 | 3762000.00 | 2128900.00 | 0.00 | 4265800.00 | 3863200.00 | 1620000.00 | | hypothetical protein | Bacteria;Acidobacteria;Vicinamibacteria;unclassified Vicinamibacteria;Vicinamibacteraceae;Luteitalea;Luteitalea pratensis;Luteitalea pratensis DSM 100886 |
| Ga0586324 0228527 1384 2328 | 0.00 | 12172000.00 | 0.00 | 15485000.00 | 11845000.00 | 0.00 | 0.00 | 11651000.00 | 0.00 | 6029100.00 | 4479200.00 | 0.00 | | hypothetical protein | Bacteria;Proteobacteria;Deltaproteobacteria;Syntrophobacterales;Syntrophobacteraceae;Desulfosoma;Desulfosoma caldarium;Desulfosoma caldarium DSM 22027 |
| Ga0586324 0228612 1275 1952 | 0.00 | 1217200.00 | 0.00 | 5823500.00 | 0.00 | 0.00 | 5084400.00 | 3397700.00 | 2883600.00 | 2887500.00 | 0.00 | 0.00 | | biopolymer transport protein ExbB/TolQ | Bacteria;Candidatus Aminicenantes;unclassified Candidatus Aminicenantes;unclassified Candidatus Aminicenantes;unclassified Candidatus Aminicenantes;unclassified Candidatus Aminicenantes;Aminicenantes bacterium SCGC AAA252-P13;Aminicenantes bacterium SCGC AAA252-P13 (contamination screened) |
| Ga0586324 0228619 1565 2044 | 11000000.00 | 7888000.00 | 16158100.00 | 12929310.00 | 16377000.00 | 19330708.52 | 6156000.00 | 6161000.00 | 4625100.00 | 10395000.00 | 4576000.00 | 3442500.00 | | hypothetical protein | Bacteria;Proteobacteria;Deltaproteobacteria;Syntrophobacterales;Syntrophaceae;Desulfobacca;Desulfobacca acetoxidans;Desulfobacca acetoxidans ASRB2, DSM 11109 |
| Ga0586324 0229010 1042 2100 | 0.00 | 0.00 | 0.00 | 1558000.00 | 0.00 | 0.00 | 0.00 | 0.00 | 0.00 | 0.00 | 0.00 | 0.00 | | transcriptional regulator with GAF, ATPase, and Fis domain | Bacteria;Proteobacteria;Deltaproteobacteria;Desulfobacterales;Desulfobacteraceae;Desulfatirhabdium;Desulfatirhabdium butyrativorans;Desulfatirhabdium butyrativorans DSM 18734 |
| Ga0586324 0229504 1 360 | 0.00 | 0.00 | 0.00 | 0.00 | 0.00 | 0.00 | 0.00 | 0.00 | 636950.79 | 0.00 | 0.00 | 0.00 | | hypothetical protein | |
| Ga0586324 0232875 198 1973 | 22600000.00 | 17884000.00 | 17959700.00 | 37430000.00 | 32754000.00 | 30172670.93 | 19790400.00 | 18605000.00 | 28026000.00 | 7007000.00 | 22932800.00 | 15975000.00 | | formyltetrahydrofolate synthetase | Bacteria;Chloroflexi;Dehalococcoidia;unclassified Dehalococcoidia;unclassified Dehalococcoidia;Dehalogenimonas;Dehalogenimonas sp. GP;Dehalogenimonas sp. GP |
| Ga0586324 0233789 511 2088 | 0.00 | 0.00 | 0.00 | 0.00 | 0.00 | 0.00 | 0.00 | 5471700.00 | 0.00 | 0.00 | 0.00 | 0.00 | | glutamate synthase domain-containing protein 2 | Bacteria;Proteobacteria;Deltaproteobacteria;Syntrophobacterales;Syntrophaceae;Desulfobacca;Desulfobacca acetoxidans;Desulfobacca acetoxidans ASRB2, DSM 11109 |
| Ga0586324 0234781 1199 2086 | 0.00 | 285056.68 | 0.00 | 0.00 | 0.00 | 0.00 | 0.00 | 0.00 | 0.00 | 0.00 | 0.00 | 0.00 | | regulator of protease activity HflC (stomatin/prohibitin superfamily) | Bacteria;Acidobacteria;Thermoanaerobaculia;Thermoanaerobaculales;Thermoanaerobaculaceae;Thermoanaerobaculum;Thermoanaerobaculum aquaticum;Thermoanaerobaculum aquaticum MP-01 |
| Ga0586324 0235137 1143 2132 | 0.00 | 0.00 | 176434.07 | 0.00 | 266678.33 | 0.00 | 0.00 | 0.00 | 0.00 | 0.00 | 0.00 | 1215000.00 | | RND family efflux transporter MFP subunit | Bacteria;Proteobacteria;Alphaproteobacteria;Hyphomicrobiales;Bradyrhizobiaceae;Afipia;Afipia massiliensis;Afipia massiliensis DSM 17498 |
| Ga0586324 0236504 1 2292 | 0.00 | 3808000.00 | 1275673.55 | 1121000.00 | 707093.97 | 768842.11 | 0.00 | 382911.03 | 2438729.37 | 4435200.00 | 0.00 | 3045000.00 | | hypothetical protein | Bacteria;Candidatus Aminicenantes;unclassified Candidatus Aminicenantes;unclassified Candidatus Aminicenantes;unclassified Candidatus Aminicenantes;unclassified Candidatus Aminicenantes;Aminicenantes bacterium SCGC AAA252-O03;Aminicenantes bacterium SCGC AAA252-O03 (contamination screened) |
| Ga0586324 0237303 942 1562 | 0.00 | 0.00 | 0.00 | 0.00 | 0.00 | 0.00 | 0.00 | 646600.00 | 842400.00 | 0.00 | 0.00 | 2055000.00 | | hypothetical protein | Bacteria;Proteobacteria;Alphaproteobacteria;Hyphomicrobiales;unclassified Hyphomicrobiales;unclassified Hyphomicrobiales;Rhizobiales bacterium GAS191;Rhizobiales sp. GAS191 |
| Ga0586324 0238168 997 1752 | 0.00 | 0.00 | 0.00 | 687267.05 | 792977.43 | 0.00 | 0.00 | 0.00 | 1069200.00 | 1031800.00 | 1478400.00 | 0.00 | | regulator of protease activity HflC (stomatin/prohibitin superfamily) | Bacteria;Proteobacteria;Deltaproteobacteria;Desulfuromonadales;Geobacteraceae;Geobacter;Geobacter sulfurreducens;Geobacter sulfurreducens PCA |
| Ga0586324 0238224 2 2284 | 0.00 | 0.00 | 0.00 | 0.00 | 0.00 | 0.00 | 0.00 | 0.00 | 0.00 | 631058.12 | 0.00 | 0.00 | | 2',3'-cyclic-nucleotide 2'-phosphodiesterase (5'-nucleotidase family) | Bacteria;Firmicutes;Clostridia;Halanaerobiales;unclassified Halanaerobiales;Anoxybacter;Anoxybacter fermentans;Anoxybacter fermentans DY22613 |
| Ga0586324 0238419 290 2281 | 0.00 | 5582800.00 | 0.00 | 0.00 | 2513200.00 | 0.00 | 0.00 | 0.00 | 0.00 | 0.00 | 357240.40 | 1732500.00 | | hypothetical protein | Bacteria;Acidobacteria;Acidobacteriia;Acidobacteriales;Acidobacteriaceae;Terracidiphilus;Terracidiphilus gabretensis;Acidobacteriaceae bacterium S55 |
| Ga0586324 0238739 1450 1866 | 0.00 | 0.00 | 32259900.00 | 0.00 | 0.00 | 0.00 | 0.00 | 0.00 | 0.00 | 0.00 | 0.00 | 0.00 | | rubrerythrin | Bacteria;Proteobacteria;Gammaproteobacteria;Chromatiales;Chromatiaceae;Lamprocystis;Lamprocystis purpurea;Lamprocystis purpurea DSM 4197 |
| Ga0586324 0239771 3 560 | 0.00 | 0.00 | 0.00 | 0.00 | 0.00 | 0.00 | 0.00 | 12749000.00 | 0.00 | 0.00 | 0.00 | 0.00 | | formyltetrahydrofolate synthetase | Bacteria;Proteobacteria;Deltaproteobacteria;Syntrophobacterales;Syntrophaceae;Desulfobacca;Desulfobacca acetoxidans;Desulfobacca acetoxidans ASRB2, DSM 11109 |
| Ga0586324 0239839 95 1990 | 2760000.00 | 2495600.00 | 0.00 | 0.00 | 0.00 | 0.00 | 0.00 | 0.00 | 0.00 | 534627.17 | 0.00 | 0.00 | | hypothetical protein | Bacteria;Proteobacteria;Deltaproteobacteria;Syntrophobacterales;Syntrophobacteraceae;Syntrophobacter;Syntrophobacter fumaroxidans;Syntrophobacter fumaroxidans MPOB |
| Ga0586324 0240123 3 1118 | 0.00 | 0.00 | 0.00 | 0.00 | 0.00 | 1851234.82 | 0.00 | 0.00 | 0.00 | 0.00 | 0.00 | 0.00 | | TRAP-type mannitol/chloroaromatic compound transport system substrate-binding protein | Bacteria;Proteobacteria;Betaproteobacteria;Burkholderiales;unclassified Burkholderiales;Rivibacter;Rivibacter subsaxonicus;Rivibacter subsaxonicus DSM 19570 |
| Ga0586324 0240626 3 2273 | 1910000.00 | 1264800.00 | 0.00 | 0.00 | 0.00 | 0.00 | 0.00 | 0.00 | 0.00 | 0.00 | 0.00 | 468711.75 | | hypothetical protein | Bacteria;Candidatus Aminicenantes;unclassified Candidatus Aminicenantes;unclassified Candidatus Aminicenantes;unclassified Candidatus Aminicenantes;unclassified Candidatus Aminicenantes;Aminicenantes bacterium SCGC AAA255-E05;Aminicenantes bacterium SCGC AAA255-E05 (contamination screened) |
| Ga0586324 0241003 1664 2170 | 39200000.00 | 25432000.00 | 0.00 | 67260000.00 | 0.00 | 63123000.00 | 0.00 | 26535000.00 | 31347000.00 | 0.00 | 0.00 | 15825000.00 | | tetratricopeptide (TPR) repeat protein | Bacteria;Proteobacteria;Deltaproteobacteria;Syntrophobacterales;Syntrophaceae;Desulfobacca;Desulfobacca acetoxidans;Desulfobacca acetoxidans ASRB2, DSM 11109 |
| Ga0586324 0242092 358 2202 | 678118.00 | 0.00 | 0.00 | 0.00 | 0.00 | 0.00 | 0.00 | 0.00 | 0.00 | 0.00 | 0.00 | 0.00 | | hypothetical protein | Bacteria;Proteobacteria;Deltaproteobacteria;Syntrophobacterales;Syntrophaceae;Desulfomonile;Desulfomonile tiedjei;Desulfomonile tiedjei DCB-1, DSM 6799 |
| Ga0586324 0243056 968 1390 | 0.00 | 0.00 | 0.00 | 0.00 | 0.00 | 0.00 | 782236.08 | 0.00 | 0.00 | 0.00 | 0.00 | 0.00 | | rhodanese-related sulfurtransferase | Bacteria;Proteobacteria;Betaproteobacteria;Nitrosomonadales;Gallionellaceae;Sideroxydans;Sideroxydans lithotrophicus;Sideroxydans lithotrophicus ES-1 |
| Ga0586324 0244248 2 2257 | 4620000.00 | 1530000.00 | 6339943.00 | 3695500.00 | 0.00 | 0.00 | 3146400.00 | 0.00 | 0.00 | 2017400.00 | 1663200.00 | 1282500.00 | | hypothetical protein | Bacteria;Proteobacteria;Betaproteobacteria;unclassified Betaproteobacteria;unclassified Betaproteobacteria;unclassified Betaproteobacteria;Betaproteobacteria bacterium GR16-43;Betaproteobacteria bacterium GR16-43 |
| Ga0586324 0244519 276 827 | 0.00 | 1271600.00 | 0.00 | 0.00 | 0.00 | 0.00 | 0.00 | 0.00 | 0.00 | 0.00 | 0.00 | 0.00 | | hypothetical protein | Archaea;Euryarchaeota;Methanomicrobia;Methanosarcinales;Methanotrichaceae;Methanothrix;Methanothrix soehngenii;Methanothrix soehngenii GP-6 |
| Ga0586324 0245479 3 1154 | 11400000.00 | 4732800.00 | 13680900.00 | 0.00 | 15553000.00 | 12068800.00 | 0.00 | 8540000.00 | 10287000.00 | 11627000.00 | 12056000.00 | 11550000.00 | | hypothetical protein | Bacteria;Candidatus Aminicenantes;unclassified Candidatus Aminicenantes;unclassified Candidatus Aminicenantes;unclassified Candidatus Aminicenantes;unclassified Candidatus Aminicenantes;Aminicenantes bacterium SCGC AAA255-E05;Aminicenantes bacterium SCGC AAA255-E05 (contamination screened) |
| Ga0586324 0245549 1 2253 | 0.00 | 0.00 | 0.00 | 0.00 | 7632300.00 | 0.00 | 0.00 | 0.00 | 2551500.00 | 0.00 | 0.00 | 0.00 | | hypothetical protein | Bacteria;Chloroflexi;Chloroflexia;Chloroflexales;Roseiflexaceae;Roseiflexus;Roseiflexus castenholzii;Roseiflexus castenholzii HLO8, DSM 13941 |
| Ga0586324 0245575 1 2250 | 0.00 | 0.00 | 1039129.10 | 0.00 | 0.00 | 0.00 | 0.00 | 0.00 | 1320300.00 | 0.00 | 0.00 | 0.00 | | outer membrane receptor protein involved in Fe transport | Bacteria;Proteobacteria;Gammaproteobacteria;Cellvibrionales;Spongiibacteraceae;unclassified Spongiibacteraceae;marine gamma proteobacterium HTCC2143;Marine gamma proteobacterium sp. HTCC2143 |
| Ga0586324 0246032 1055 2092 | 9390000.00 | 46332611.24 | 0.00 | 111150000.00 | 149375033.12 | 17547400.00 | 107388000.00 | 134119292.73 | 154629000.00 | 30122400.00 | 65876800.00 | 37067826.75 | | predicted porin | Bacteria;Proteobacteria;Betaproteobacteria;Nitrosomonadales;Thiobacillaceae;Sulfuritortus;Sulfuritortus calidifontis;Sulfuritortus calidifontis DSM 103923 |
| Ga0586324 0246476 928 2247 | 0.00 | 0.00 | 0.00 | 0.00 | 0.00 | 1651516.03 | 0.00 | 0.00 | 0.00 | 0.00 | 0.00 | 523455.75 | | hypothetical protein | Bacteria;Acidobacteria;Vicinamibacteria;unclassified Vicinamibacteria;Vicinamibacteraceae;Luteitalea;Luteitalea pratensis;Luteitalea pratensis DSM 100886 |
| Ga0586324 0246766 3 1172 | 0.00 | 0.00 | 0.00 | 0.00 | 0.00 | 6788700.00 | 0.00 | 4684800.00 | 1093500.00 | 0.00 | 3414400.00 | 626761.50 | | hypothetical protein | Bacteria;Proteobacteria;Gammaproteobacteria;Cellvibrionales;Halieaceae;Pseudohaliea;Pseudohaliea rubra;Pseudohaliea rubra DSM 19751 |
| Ga0586324 0247189 209 994 | 0.00 | 0.00 | 0.00 | 0.00 | 0.00 | 0.00 | 0.00 | 0.00 | 0.00 | 1624700.00 | 0.00 | 2145000.00 | | uncharacterized protein (TIGR02001 family) | Bacteria;Proteobacteria;Gammaproteobacteria;Chromatiales;Halothiobacillaceae;Halothiobacillus;Halothiobacillus neapolitanus;Halothiobacillus neapolitanus c2, ATCC 23641 |
| Ga0586324 0247490 1260 2243 | 0.00 | 0.00 | 0.00 | 0.00 | 0.00 | 0.00 | 0.00 | 0.00 | 0.00 | 1136177.35 | 0.00 | 0.00 | | TRAP-type mannitol/chloroaromatic compound transport system substrate-binding protein | Bacteria;Proteobacteria;Alphaproteobacteria;Hyphomicrobiales;Xanthobacteraceae;Pseudolabrys;Pseudolabrys taiwanensis;Pseudolabrys taiwanensis CC-BB4 |
| Ga0586324 0248711 187 1533 | 0.00 | 0.00 | 0.00 | 0.00 | 0.00 | 0.00 | 6247200.00 | 0.00 | 0.00 | 0.00 | 0.00 | 0.00 | | ABC-type glycerol-3-phosphate transport system substrate-binding protein | Bacteria;Proteobacteria;Alphaproteobacteria;Rhodospirillales;unclassified Rhodospirillales;unclassified Rhodospirillales;Rhodospirillales bacterium URHD0088;Rhodospirillales bacterium URHD0088 |
| Ga0586324 0250766 1238 2230 | 36300000.00 | 104584000.00 | 226100800.00 | 174980500.00 | 199614000.00 | 94089000.00 | 39900000.00 | 194407000.00 | 130653000.00 | 113729000.00 | 82280000.00 | 186300000.00 | | predicted porin | Bacteria;Proteobacteria;Betaproteobacteria;Burkholderiales;Burkholderiaceae;Quisquiliibacterium;Quisquiliibacterium transsilvanicum;Quisquiliibacterium transsilvanicum DSM 29781 |
| Ga0586324 0252062 2 2179 | 0.00 | 0.00 | 0.00 | 2242000.00 | 0.00 | 0.00 | 0.00 | 0.00 | 0.00 | 0.00 | 0.00 | 0.00 | | hypothetical protein | Bacteria;Acidobacteria;Blastocatellia;Blastocatellales;Pyrinomonadaceae;Pyrinomonas;Pyrinomonas methylaliphatogenes;Pyrinomonas methylaliphatogenes K22 |
| Ga0586324 0252154 207 791 | 1380000.00 | 0.00 | 0.00 | 12445000.00 | 0.00 | 0.00 | 0.00 | 7808000.00 | 8262000.00 | 0.00 | 0.00 | 0.00 | | opacity protein-like surface antigen | |
| Ga0586324 0252590 94 609 | 1650000.00 | 0.00 | 0.00 | 0.00 | 0.00 | 0.00 | 0.00 | 0.00 | 0.00 | 0.00 | 0.00 | 0.00 | | tetratricopeptide (TPR) repeat protein | Bacteria;Proteobacteria;Deltaproteobacteria;Syntrophobacterales;Syntrophaceae;Desulfobacca;Desulfobacca acetoxidans;Desulfobacca acetoxidans ASRB2, DSM 11109 |
| Ga0586324 0252895 667 2220 | 0.00 | 0.00 | 0.00 | 0.00 | 0.00 | 0.00 | 0.00 | 0.00 | 4374000.00 | 0.00 | 0.00 | 0.00 | | hypothetical protein | Bacteria;Bacteroidetes;Cytophagia;Cytophagales;Cyclobacteriaceae;Algoriphagus;Algoriphagus mannitolivorans;Algoriphagus mannitolivorans DSM 15301 |
| Ga0586324 0254111 586 2217 | 8190000.00 | 4807600.00 | 0.00 | 12920000.00 | 6779519.74 | 0.00 | 0.00 | 3745400.00 | 10206000.00 | 0.00 | 0.00 | 0.00 | | hypothetical protein | Bacteria;Candidatus Aminicenantes;unclassified Candidatus Aminicenantes;unclassified Candidatus Aminicenantes;unclassified Candidatus Aminicenantes;unclassified Candidatus Aminicenantes;Aminicenantes bacterium SCGC AAA252-D06;Aminicenantes bacterium SCGC AAA252-D06 (contamination screened) |
| Ga0586324 0254255 1148 2215 | 72900000.00 | 56372000.00 | 0.00 | 20900000.00 | 0.00 | 57962000.00 | 72276000.00 | 44408000.00 | 73629000.00 | 24178000.00 | 0.00 | 4582500.00 | | crotonobetainyl-CoA:carnitine CoA-transferase CaiB-like acyl-CoA transferase | Archaea;Euryarchaeota;Archaeoglobi;Archaeoglobales;Archaeoglobaceae;Ferroglobus;Ferroglobus placidus;Ferroglobus placidus AEDII12DO, DSM 10642 |
| Ga0586324 0255123 663 2213 | 0.00 | 0.00 | 1062116.39 | 0.00 | 0.00 | 1332137.47 | 0.00 | 707600.00 | 668546.46 | 0.00 | 0.00 | 0.00 | | NADPH-dependent 2,4-dienoyl-CoA reductase/sulfur reductase-like enzyme | Bacteria;Cyanobacteria;unclassified Cyanobacteria;Oscillatoriales;Microcoleaceae;Planktothrix;Planktothrix serta;Planktothrix serta PCC 8927 |
| Ga0586324 0255425 91 2211 | 0.00 | 4406400.00 | 0.00 | 0.00 | 0.00 | 0.00 | 0.00 | 0.00 | 0.00 | 0.00 | 0.00 | 0.00 | | hypothetical protein | Bacteria;Acidobacteria;Holophagae;Holophagales;Holophagaceae;Geothrix;Geothrix fermentans;Geothrix fermentans DSM 14018 |
| Ga0586324 0255914 1 1443 | 0.00 | 1115200.00 | 960348.51 | 1672000.00 | 0.00 | 0.00 | 542621.76 | 689300.00 | 842400.00 | 893200.00 | 1337600.00 | 855000.00 | | hypothetical protein | Bacteria;Actinobacteria;Actinomycetia;Micrococcales;Micrococcaceae;Sinomonas;Sinomonas mesophila;Sinomonas mesophila MPKL 26 |
| Ga0586324 0256352 1 1119 | 0.00 | 1203600.00 | 0.00 | 0.00 | 0.00 | 0.00 | 0.00 | 1433500.00 | 1247400.00 | 2748900.00 | 2842400.00 | 2242500.00 | | predicted porin | Bacteria;Proteobacteria;Betaproteobacteria;Burkholderiales;Alcaligenaceae;Candidimonas;Candidimonas bauzanensis;Candidimonas bauzanensis CGMCC 1.10190 |
| Ga0586324 0259919 2 2194 | 0.00 | 0.00 | 3806138.98 | 0.00 | 0.00 | 0.00 | 0.00 | 0.00 | 0.00 | 0.00 | 0.00 | 0.00 | | hypothetical protein | Bacteria;Acidobacteria;Blastocatellia;Blastocatellales;Pyrinomonadaceae;Pyrinomonas;Pyrinomonas methylaliphatogenes;Pyrinomonas methylaliphatogenes K22 |
| Ga0586324 0260591 985 2190 | 0.00 | 0.00 | 0.00 | 0.00 | 0.00 | 1161721.25 | 0.00 | 0.00 | 0.00 | 0.00 | 0.00 | 0.00 | | ABC-type sugar transport system substrate-binding protein | Bacteria;Chloroflexi;Caldilineae;Caldilineales;Caldilineaceae;Caldilinea;Caldilinea aerophila;Caldilinea aerophila STL-6-O1, DSM 14535 |
| Ga0586324 0261629 936 2114 | 38130000.00 | 15572000.00 | 44308100.00 | 12331000.00 | 161699700.00 | 138751500.00 | 156567600.00 | 98881000.00 | 115028100.00 | 20790000.00 | 12883200.00 | 57405000.00 | | hypothetical protein | Bacteria;Proteobacteria;Deltaproteobacteria;Desulfobacterales;Desulfobacteraceae;Desulfobacter;Desulfobacter postgatei;Desulfobacter postgatei 2ac9 |
| Ga0586324 0263436 3 1979 | 0.00 | 0.00 | 0.00 | 0.00 | 0.00 | 0.00 | 0.00 | 0.00 | 4568400.00 | 0.00 | 0.00 | 0.00 | | NADPH-dependent glutamate synthase beta subunit-like oxidoreductase/Pyruvate/2-oxoacid:fer | Bacteria;Proteobacteria;Betaproteobacteria;Nitrosomonadales;Thiobacillaceae;Thiobacillus;Thiobacillus denitrificans;Thiobacillus denitrificans DSM 12475 |
| Ga0586324 0263697 2 1951 | 0.00 | 2699600.00 | 0.00 | 3439000.00 | 3944900.00 | 2565826.88 | 0.00 | 3074400.00 | 2041200.00 | 0.00 | 0.00 | 0.00 | | hypothetical protein | Bacteria;Firmicutes;Bacilli;Bacillales;Paenibacillaceae;Paenibacillus;Paenibacillus sophorae;Paenibacillus sophorae S27 |
| Ga0586324 0263886 3 2177 | 18400000.00 | 3264000.00 | 0.00 | 11875000.00 | 7343900.00 | 0.00 | 0.00 | 4941000.00 | 2330570.88 | 12551000.00 | 3520000.00 | 2752500.00 | | hypothetical protein | Bacteria;Ignavibacteriae;Ignavibacteria;Ignavibacteriales;Ignavibacteriaceae;Ignavibacterium;Ignavibacterium album;Ignavibacterium album Mat9-16, JCM 16511 |
| Ga0586324 0264545 1 1479 | 0.00 | 0.00 | 0.00 | 0.00 | 0.00 | 0.00 | 1125207.36 | 0.00 | 3126600.00 | 1370600.00 | 0.00 | 0.00 | | hypothetical protein | Bacteria;Bacteroidetes;Flavobacteriia;Flavobacteriales;Flavobacteriaceae;Xanthomarina;Xanthomarina gelatinilytica;Xanthomarina gelatinilytica AK20 |
| Ga0586324 0265229 1 1824 | 0.00 | 0.00 | 0.00 | 0.00 | 0.00 | 0.00 | 0.00 | 0.00 | 0.00 | 0.00 | 0.00 | 1957500.00 | | hypothetical protein | Bacteria;Proteobacteria;Betaproteobacteria;Nitrosomonadales;Thiobacillaceae;Thiobacillus;Thiobacillus thioparus;Thiobacillus thioparus DSM 505 |
| Ga0586324 0265334 349 1950 | 0.00 | 0.00 | 0.00 | 0.00 | 10083700.00 | 0.00 | 0.00 | 0.00 | 0.00 | 0.00 | 4884000.00 | 0.00 | | arylsulfatase A-like enzyme | Bacteria;Proteobacteria;Alphaproteobacteria;Hyphomicrobiales;Bradyrhizobiaceae;Rhodopseudomonas;Rhodopseudomonas palustris;Rhodopseudomonas palustris 7850, DSM 127 |
| Ga0586324 0265803 3 1568 | 0.00 | 2733600.00 | 0.00 | 0.00 | 0.00 | 0.00 | 0.00 | 0.00 | 0.00 | 0.00 | 0.00 | 0.00 | | hypothetical protein | Bacteria;Actinobacteria;Actinomycetia;Kineosporiales;Kineosporiaceae;Kineosporia;Kineosporia sp. A 224;Kineosporia sp. A 224 A 224 |
| Ga0586324 0266855 601 1275 | 0.00 | 0.00 | 0.00 | 3638500.00 | 0.00 | 1496590.75 | 0.00 | 0.00 | 1846800.00 | 0.00 | 0.00 | 0.00 | | DNA-binding NarL/FixJ family response regulator | Bacteria;Proteobacteria;Deltaproteobacteria;Syntrophobacterales;Syntrophaceae;Desulfobacca;Desulfobacca acetoxidans;Desulfobacca acetoxidans ASRB2, DSM 11109 |
| Ga0586324 0269553 2 2158 | 0.00 | 0.00 | 2399494.74 | 0.00 | 0.00 | 0.00 | 0.00 | 0.00 | 0.00 | 4281200.00 | 3396800.00 | 4267500.00 | | hypothetical protein | Bacteria;Acidobacteria;Acidobacteriia;Acidobacteriales;Acidobacteriaceae;unclassified Acidobacteriaceae;Acidobacteriaceae bacterium KBS 96;Acidobacteriaceae bacterium KBS 96, DSM 24297 |
| Ga0586324 0269715 1 1215 | 0.00 | 0.00 | 0.00 | 0.00 | 0.00 | 0.00 | 0.00 | 0.00 | 0.00 | 1963500.00 | 2138400.00 | 1507500.00 | | predicted porin | Bacteria;Proteobacteria;Betaproteobacteria;Burkholderiales;Burkholderiaceae;Paraburkholderia;Paraburkholderia caffeinilytica;Paraburkholderia caffeinilytica CF1 |
| Ga0586324 0270730 1 1836 | 2700000.00 | 4930000.00 | 0.00 | 0.00 | 0.00 | 0.00 | 0.00 | 0.00 | 2292300.00 | 0.00 | 0.00 | 0.00 | | hypothetical protein | Bacteria;Proteobacteria;Deltaproteobacteria;Myxococcales;Anaeromyxobacteraceae;Anaeromyxobacter;Anaeromyxobacter sp. Fw109-5;Anaeromyxobacter sp. Fw109-5 |
| Ga0586324 0270887 1060 1839 | 0.00 | 0.00 | 8163500.00 | 0.00 | 0.00 | 0.00 | 0.00 | 0.00 | 0.00 | 0.00 | 0.00 | 22500000.00 | | hypothetical protein | Bacteria;Proteobacteria;Betaproteobacteria;Nitrosomonadales;Gallionellaceae;Sideroxydans;Sideroxydans lithotrophicus;Sideroxydans lithotrophicus ES-1 |
| Ga0586324 0271438 1000 1161 | 0.00 | 0.00 | 0.00 | 0.00 | 0.00 | 0.00 | 537792.72 | 0.00 | 0.00 | 0.00 | 0.00 | 0.00 | | hypothetical protein | |
| Ga0586324 0271611 2 1672 | 0.00 | 0.00 | 0.00 | 0.00 | 2647100.00 | 0.00 | 0.00 | 0.00 | 0.00 | 0.00 | 0.00 | 0.00 | | NADH:ubiquinone oxidoreductase subunit F (NADH-binding) | Bacteria;Firmicutes;Clostridia;Thermoanaerobacterales;Thermoanaerobacteraceae;Desulfovirgula;Desulfovirgula thermocuniculi;Desulfovirgula thermocuniculi DSM 16036 |
| Ga0586324 0272017 782 1009 | 0.00 | 0.00 | 0.00 | 0.00 | 0.00 | 0.00 | 5380800.00 | 0.00 | 3094200.00 | 0.00 | 0.00 | 0.00 | | hypothetical protein | |
| Ga0586324 0272349 322 2148 | 4120000.00 | 0.00 | 0.00 | 0.00 | 2425122.64 | 0.00 | 0.00 | 0.00 | 0.00 | 0.00 | 0.00 | 2632500.00 | | hypothetical protein | Bacteria;Acidobacteria;Vicinamibacteria;unclassified Vicinamibacteria;Vicinamibacteraceae;Luteitalea;Luteitalea pratensis;Luteitalea pratensis DSM 100886 |
| Ga0586324 0272541 3 2147 | 2030000.00 | 6555200.00 | 0.00 | 0.00 | 0.00 | 0.00 | 0.00 | 2958500.00 | 2778300.00 | 3811500.00 | 6960800.00 | 847500.00 | | hypothetical protein | |
| Ga0586324 0273092 2 1762 | 0.00 | 0.00 | 0.00 | 0.00 | 0.00 | 0.00 | 0.00 | 0.00 | 0.00 | 0.00 | 2798400.00 | 0.00 | | 2',3'-cyclic-nucleotide 2'-phosphodiesterase (5'-nucleotidase family) | Bacteria;Actinobacteria;Actinomycetia;Propionibacteriales;Nocardioidaceae;Nocardioides;Nocardioides soli;Nocardioides soli DSM 105498 |
| Ga0586324 0273198 2 964 | 0.00 | 0.00 | 0.00 | 0.00 | 0.00 | 0.00 | 0.00 | 0.00 | 0.00 | 0.00 | 4012800.00 | 2160000.00 | | tripartite-type tricarboxylate transporter receptor subunit TctC | Bacteria;Proteobacteria;Betaproteobacteria;unclassified Betaproteobacteria;unclassified Betaproteobacteria;unclassified Betaproteobacteria;Betaproteobacteria bacterium GR16-43;Betaproteobacteria bacterium GR16-43 |
| Ga0586324 0273244 21 2144 | 0.00 | 0.00 | 0.00 | 0.00 | 0.00 | 1066528.59 | 0.00 | 0.00 | 0.00 | 0.00 | 0.00 | 0.00 | | hypothetical protein | Bacteria;Proteobacteria;Gammaproteobacteria;unclassified Gammaproteobacteria;unclassified Gammaproteobacteria;Gallaecimonas;Gallaecimonas mangrovi;Gallaecimonas mangrovi HK-28 |
| Ga0586324 0274672 2 2140 | 0.00 | 0.00 | 0.00 | 0.00 | 0.00 | 0.00 | 416683.68 | 0.00 | 0.00 | 0.00 | 0.00 | 0.00 | | heterodisulfide reductase subunit A-like polyferredoxin | Bacteria;Firmicutes;Clostridia;Thermoanaerobacterales;Thermoanaerobacteraceae;Calderihabitans;Calderihabitans maritimus;Calderihabitans maritimus KKC1 |
| Ga0586324 0275160 487 1377 | 0.00 | 0.00 | 0.00 | 0.00 | 0.00 | 0.00 | 0.00 | 0.00 | 0.00 | 0.00 | 9680000.00 | 11400000.00 | | hypothetical protein | Bacteria;Proteobacteria;Alphaproteobacteria;Hyphomicrobiales;Methylobacteriaceae;Methylorubrum;Methylorubrum thiocyanatum;Methylobacterium thiocyanatum DSM 11490 |
| Ga0586324 0275461 1397 1837 | 1260000.00 | 0.00 | 0.00 | 0.00 | 0.00 | 0.00 | 0.00 | 0.00 | 0.00 | 0.00 | 0.00 | 0.00 | | hypothetical protein | |
| Ga0586324 0276372 423 1199 | 0.00 | 0.00 | 0.00 | 0.00 | 1998200.00 | 0.00 | 0.00 | 0.00 | 0.00 | 0.00 | 0.00 | 0.00 | | CBS domain-containing protein | Archaea;Euryarchaeota;Methanomicrobia;Methanosarcinales;Methanotrichaceae;Methanothrix;Methanothrix soehngenii;Methanothrix soehngenii GP-6 |
| Ga0586324 0277199 3 1697 | 0.00 | 0.00 | 0.00 | 0.00 | 0.00 | 0.00 | 0.00 | 0.00 | 0.00 | 1347500.00 | 0.00 | 2250000.00 | | hypothetical protein | Bacteria;Acidobacteria;Acidobacteriia;Acidobacteriales;Acidobacteriaceae;Candidatus Koribacter;Candidatus Koribacter versatilis;Candidatus Koribacter versatilis Ellin345 |
| Ga0586324 0277538 911 2128 | 9480000.00 | 0.00 | 1872346.58 | 3059000.00 | 0.00 | 0.00 | 0.00 | 0.00 | 0.00 | 1193500.00 | 0.00 | 0.00 | | hypothetical protein | Bacteria;Bacteroidetes;Flavobacteriia;Flavobacteriales;Flavobacteriaceae;Formosa;Formosa sp. Hel1 33 131;Formosa sp. Hel1 33 131 |
| Ga0586324 0278677 1 2124 | 0.00 | 0.00 | 0.00 | 0.00 | 0.00 | 0.00 | 2918400.00 | 0.00 | 0.00 | 2025100.00 | 0.00 | 3030000.00 | | hypothetical protein | Bacteria;Acidobacteria;Blastocatellia;Blastocatellales;Pyrinomonadaceae;Pyrinomonas;Pyrinomonas methylaliphatogenes;Pyrinomonas methylaliphatogenes K22 |
| Ga0586324 0279008 3 725 | 0.00 | 0.00 | 0.00 | 0.00 | 0.00 | 0.00 | 0.00 | 1756800.00 | 0.00 | 0.00 | 0.00 | 0.00 | | hypothetical protein | Bacteria;Acidobacteria;Acidobacteriia;Acidobacteriales;Acidobacteriaceae;Candidatus Koribacter;Candidatus Koribacter versatilis;Candidatus Koribacter versatilis Ellin345 |
| Ga0586324 0280017 2 1816 | 0.00 | 0.00 | 0.00 | 0.00 | 0.00 | 0.00 | 0.00 | 0.00 | 0.00 | 0.00 | 0.00 | 3120000.00 | | hypothetical protein | Bacteria;Acidobacteria;Acidobacteriia;Acidobacteriales;Acidobacteriaceae;unclassified Acidobacteriaceae;Acidobacteriaceae bacterium KBS 89;Acidobacteriaceae bacterium KBS 89, DSM 24296 |
| Ga0586324 0280050 404 2119 | 6050000.00 | 3760400.00 | 483063.01 | 0.00 | 0.00 | 0.00 | 0.00 | 0.00 | 0.00 | 0.00 | 0.00 | 0.00 | | hypothetical protein | Bacteria;Proteobacteria;Deltaproteobacteria;Myxococcales;Polyangiaceae;Sorangium;Sorangium cellulosum;Sorangium cellulosum So0008-312 |
| Ga0586324 0280279 47 1531 | 12400000.00 | 18428000.00 | 0.00 | 6954000.00 | 0.00 | 0.00 | 0.00 | 0.00 | 6844500.00 | 0.00 | 0.00 | 0.00 | | crotonobetainyl-CoA:carnitine CoA-transferase CaiB-like acyl-CoA transferase | Bacteria;Proteobacteria;Deltaproteobacteria;Syntrophobacterales;Syntrophobacteraceae;Desulfacinum;Desulfacinum hydrothermale;Desulfacinum hydrothermale DSM 13146 |
| Ga0586324 0281102 88 930 | 0.00 | 0.00 | 0.00 | 0.00 | 0.00 | 0.00 | 3192000.00 | 0.00 | 0.00 | 0.00 | 0.00 | 0.00 | | MoxR-like ATPase | Bacteria;Proteobacteria;Gammaproteobacteria;unclassified Gammaproteobacteria;unclassified Gammaproteobacteria;Dechloromarinus;Dechloromarinus chlorophilus;Dechloromarinus chlorophilus NSS |
| Ga0586324 0283670 1083 1835 | 0.00 | 5752800.00 | 0.00 | 0.00 | 0.00 | 0.00 | 0.00 | 0.00 | 0.00 | 3919300.00 | 0.00 | 0.00 | | hypothetical protein | Bacteria;Proteobacteria;Deltaproteobacteria;Syntrophobacterales;Syntrophaceae;Desulfobacca;Desulfobacca acetoxidans;Desulfobacca acetoxidans ASRB2, DSM 11109 |
| Ga0586324 0283842 734 1786 | 3780000.00 | 13056000.00 | 0.00 | 1672000.00 | 0.00 | 0.00 | 0.00 | 0.00 | 0.00 | 8701000.00 | 8791200.00 | 0.00 | | predicted porin | Bacteria;Proteobacteria;Gammaproteobacteria;Acidiferrobacterales;Acidiferrobacteraceae;Sulfuricaulis;Sulfuricaulis limicola;Sulfuricaulis limicola HA5 |
| Ga0586324 0286080 2 2101 | 5600000.00 | 5929600.00 | 965944.73 | 0.00 | 0.00 | 1858877.07 | 0.00 | 49660749.65 | 0.00 | 0.00 | 0.00 | 0.00 | | S-layer protein (TIGR01567 family) | Archaea;Euryarchaeota;Methanomicrobia;Methanosarcinales;Methanotrichaceae;Methanothrix;Methanothrix soehngenii;Methanothrix soehngenii GP-6 |
| Ga0586324 0286442 2 847 | 31400000.00 | 353908.04 | 1113281.83 | 1377500.00 | 497184.09 | 592732.91 | 1219442.04 | 360292.23 | 1142100.00 | 1463000.00 | 2024000.00 | 532873.50 | | cytochrome c peroxidase | Bacteria;Proteobacteria;Gammaproteobacteria;Methylococcales;Methylococcaceae;Methylosarcina;Methylosarcina fibrata;Methylosarcina fibrata AML-C10 |
| Ga0586324 0286877 3 2096 | 1740000.00 | 0.00 | 0.00 | 0.00 | 0.00 | 0.00 | 0.00 | 0.00 | 0.00 | 0.00 | 0.00 | 0.00 | | glycosidase | Bacteria;Chloroflexi;Chloroflexia;Chloroflexales;Chloroflexaceae;Chloroflexus;Chloroflexus aurantiacus;Chloroflexus aurantiacus Y-400-fl |
| Ga0586324 0288278 3 2093 | 18900000.00 | 0.00 | 0.00 | 0.00 | 0.00 | 0.00 | 0.00 | 0.00 | 0.00 | 0.00 | 0.00 | 0.00 | | hypothetical protein | Bacteria;Acidobacteria;Acidobacteriia;Acidobacteriales;Acidobacteriaceae;Terracidiphilus;Terracidiphilus gabretensis;Acidobacteriaceae bacterium S55 |
| Ga0586324 0289068 1 1422 | 0.00 | 0.00 | 0.00 | 0.00 | 2163000.00 | 0.00 | 0.00 | 0.00 | 0.00 | 0.00 | 0.00 | 0.00 | | hypothetical protein | Bacteria;Proteobacteria;Gammaproteobacteria;Xanthomonadales;Rhodanobacteraceae;Rhodanobacter;Rhodanobacter sp. TND4EH1;Rhodanobacter sp. TND4EH1 |
| Ga0586324 0289519 2 2089 | 1110000.00 | 1475600.00 | 0.00 | 432482.75 | 0.00 | 0.00 | 0.00 | 0.00 | 0.00 | 0.00 | 1487200.00 | 0.00 | | hypothetical protein | Bacteria;Acidobacteria;Blastocatellia;Blastocatellales;Pyrinomonadaceae;Pyrinomonas;Pyrinomonas methylaliphatogenes;Pyrinomonas methylaliphatogenes K22 |
| Ga0586324 0289759 3 2087 | 0.00 | 0.00 | 0.00 | 20520000.00 | 0.00 | 0.00 | 0.00 | 0.00 | 0.00 | 0.00 | 0.00 | 0.00 | | type II secretory pathway component GspD/PulD (secretin) | Bacteria;Verrucomicrobia;Verrucomicrobiae;Verrucomicrobiales;Verrucomicrobia subdivision 3;Pedosphaera;Pedosphaera parvula;Pedosphaera parvula Ellin514 |
| Ga0586324 0289961 297 1487 | 0.00 | 2658800.00 | 0.00 | 6992000.00 | 0.00 | 0.00 | 0.00 | 0.00 | 0.00 | 0.00 | 0.00 | 1950000.00 | | predicted porin | Bacteria;Proteobacteria;Betaproteobacteria;unclassified Betaproteobacteria;unclassified Betaproteobacteria;unclassified Betaproteobacteria;uncultured beta proteobacterium;unclassified betaproteobacterium ENR4 |
| Ga0586324 0290093 1 1902 | 0.00 | 0.00 | 0.00 | 0.00 | 0.00 | 0.00 | 0.00 | 0.00 | 556180.02 | 0.00 | 0.00 | 0.00 | | alkylation response protein AidB-like acyl-CoA dehydrogenase | Bacteria;Proteobacteria;Deltaproteobacteria;Syntrophobacterales;Syntrophaceae;Smithella;Smithella sp. F21;Smithella sp. F21 |
| Ga0586324 0290120 921 2087 | 0.00 | 0.00 | 0.00 | 0.00 | 0.00 | 0.00 | 0.00 | 0.00 | 0.00 | 0.00 | 0.00 | 1470000.00 | | hypothetical protein | Bacteria;Firmicutes;Bacilli;Bacillales;Paenibacillaceae;Paenibacillus;Paenibacillus sophorae;Paenibacillus sophorae S27 |
| Ga0586324 0290331 1216 1815 | 0.00 | 3910000.00 | 4146427.44 | 2916500.00 | 1730400.00 | 3437761.95 | 1756648.80 | 1342000.00 | 0.00 | 1178100.00 | 976800.00 | 703482.00 | | hypothetical protein | Bacteria;Proteobacteria;Alphaproteobacteria;Rhodospirillales;Azospirillaceae;Skermanella;Skermanella aerolata;Skermanella aerolata KACC 11604 |
| Ga0586324 0292310 3 731 | 0.00 | 0.00 | 10021400.00 | 17565500.00 | 9146400.00 | 7820900.00 | 6452400.00 | 6081700.00 | 11186100.00 | 6583500.00 | 0.00 | 0.00 | | hypothetical protein | Bacteria;Proteobacteria;Betaproteobacteria;Burkholderiales;unclassified Burkholderiales;Paucibacter;Paucibacter toxinivorans;Paucibacter toxinivorans DSM 16998 |
| Ga0586324 0293523 3 2075 | 2670000.00 | 979200.00 | 0.00 | 0.00 | 0.00 | 0.00 | 1767745.56 | 1665300.00 | 1409400.00 | 0.00 | 0.00 | 0.00 | | hypothetical protein | Bacteria;Bacteroidetes;unclassified Bacteroidetes;Bacteroidetes Order II. Incertae sedis;Rhodothermaceae;Rhodothermus;Rhodothermus marinus;Rhodothermus marinus SG0.5JP17-172 |
| Ga0586324 0294505 3 1253 | 1940000.00 | 1781600.00 | 0.00 | 0.00 | 0.00 | 1526377.66 | 0.00 | 0.00 | 0.00 | 0.00 | 0.00 | 1230000.00 | | hypothetical protein | Bacteria;Verrucomicrobia;Verrucomicrobiae;Verrucomicrobiales;Verrucomicrobiaceae;Haloferula;Haloferula sp. BvORR071;Haloferula sp. BvORR071 |
| Ga0586324 0295046 782 2071 | 0.00 | 0.00 | 0.00 | 7362500.00 | 0.00 | 0.00 | 0.00 | 0.00 | 0.00 | 0.00 | 0.00 | 0.00 | | arylsulfatase A-like enzyme | Bacteria;Proteobacteria;Alphaproteobacteria;Hyphomicrobiales;Hyphomicrobiaceae;Hyphomicrobium;Hyphomicrobium sp. 99;Hyphomicrobium sp. 99 |
| Ga0586324 0295157 352 1635 | 0.00 | 0.00 | 0.00 | 0.00 | 0.00 | 0.00 | 0.00 | 5404600.00 | 4868100.00 | 0.00 | 0.00 | 0.00 | | hypothetical protein | Bacteria;Nitrospirae;Nitrospira;Nitrospirales;Nitrospiraceae;Nitrospira;Candidatus Nitrospira nitrificans;Candidatus Nitrospira nitrificans COMA2 |
| Ga0586324 0296156 152 1705 | 0.00 | 9764800.00 | 0.00 | 0.00 | 0.00 | 0.00 | 0.00 | 0.00 | 5985900.00 | 0.00 | 0.00 | 0.00 | | hypothetical protein | Bacteria;Proteobacteria;Betaproteobacteria;Nitrosomonadales;Sulfuricellaceae;Sulfurirhabdus;Sulfurirhabdus autotrophica;Sulfurirhabdus autotrophica DSM 100309 |
| Ga0586324 0296195 1 2067 | 1750000.00 | 1312400.00 | 0.00 | 0.00 | 0.00 | 0.00 | 0.00 | 0.00 | 0.00 | 0.00 | 5385600.00 | 0.00 | | FtsP/CotA-like multicopper oxidase with cupredoxin domain | Bacteria;Firmicutes;Bacilli;Bacillales;Paenibacillaceae;Paenibacillus;Paenibacillus sophorae;Paenibacillus sophorae S27 |
| Ga0586324 0297002 212 1420 | 929886.00 | 0.00 | 0.00 | 0.00 | 0.00 | 0.00 | 0.00 | 0.00 | 0.00 | 5005000.00 | 0.00 | 4275000.00 | | predicted porin | Bacteria;Proteobacteria;Betaproteobacteria;Burkholderiales;Oxalobacteraceae;Janthinobacterium;Janthinobacterium sp. CG3;Janthinobacterium sp. CG3 |
| Ga0586324 0297087 632 1051 | 0.00 | 0.00 | 0.00 | 0.00 | 0.00 | 0.00 | 6543600.00 | 0.00 | 0.00 | 0.00 | 0.00 | 0.00 | | hypothetical protein | Bacteria;Proteobacteria;Gammaproteobacteria;Methylococcales;Methylococcaceae;Methylobacter;Methylobacter tundripaludum;Methylobacter tundripaludum SV96 |
| Ga0586324 0297127 282 1757 | 0.00 | 0.00 | 0.00 | 0.00 | 0.00 | 0.00 | 1775326.56 | 841800.00 | 0.00 | 0.00 | 0.00 | 0.00 | | hypothetical protein | Bacteria;Bacteroidetes;Flavobacteriia;Flavobacteriales;Flavobacteriaceae;Flavobacterium;Flavobacterium sp. Leaf82;Flavobacterium sp. Leaf82 |
| Ga0586324 0298249 3 761 | 14100000.00 | 0.00 | 14356500.00 | 18905000.00 | 18643000.00 | 10004400.00 | 6019200.00 | 6466000.00 | 9882000.00 | 18179700.00 | 8448000.00 | 8925000.00 | | hypothetical protein | |
| Ga0586324 0298397 568 1263 | 0.00 | 0.00 | 0.00 | 0.00 | 0.00 | 0.00 | 0.00 | 0.00 | 2972700.00 | 0.00 | 0.00 | 0.00 | | ferredoxin | Bacteria;Proteobacteria;Deltaproteobacteria;Syntrophobacterales;Syntrophaceae;Desulfobacca;Desulfobacca acetoxidans;Desulfobacca acetoxidans ASRB2, DSM 11109 |
| Ga0586324 0298565 3 893 | 0.00 | 0.00 | 0.00 | 0.00 | 0.00 | 1187681.08 | 0.00 | 0.00 | 0.00 | 0.00 | 0.00 | 0.00 | | hypothetical protein | Archaea;Euryarchaeota;Methanomicrobia;Methanosarcinales;Methanotrichaceae;Methanothrix;Methanothrix soehngenii;Methanothrix soehngenii GP-6 |
| Ga0586324 0299573 1 1443 | 4520000.00 | 5678000.00 | 15074713.47 | 4246500.00 | 3409300.00 | 13259800.00 | 0.00 | 2147200.00 | 10035900.00 | 5682600.00 | 4488000.00 | 3337500.00 | | hypothetical protein | Bacteria;Proteobacteria;Deltaproteobacteria;Syntrophobacterales;Syntrophaceae;Desulfobacca;Desulfobacca acetoxidans;Desulfobacca acetoxidans ASRB2, DSM 11109 |
| Ga0586324 0299629 333 869 | 3780000.00 | 0.00 | 0.00 | 0.00 | 0.00 | 2154757.20 | 0.00 | 4587200.00 | 9720000.00 | 0.00 | 0.00 | 2610000.00 | | uncharacterized membrane protein | Archaea;Euryarchaeota;Methanomicrobia;Methanosarcinales;Methanosarcinaceae;Methanosarcina;Methanosarcina barkeri;Methanosarcina barkeri Fusaro, DSM 804 |
| Ga0586324 0300546 2 532 | 0.00 | 0.00 | 0.00 | 14630000.00 | 0.00 | 13696500.00 | 0.00 | 0.00 | 4050000.00 | 0.00 | 0.00 | 0.00 | | hypothetical protein | Archaea;Euryarchaeota;Archaeoglobi;Archaeoglobales;Archaeoglobaceae;Geoglobus;Geoglobus ahangari;Geoglobus ahangari 234 |
| Ga0586324 0300789 696 2054 | 0.00 | 20277600.00 | 35994881.41 | 0.00 | 0.00 | 7265100.00 | 0.00 | 0.00 | 3426300.00 | 7792400.00 | 6626400.00 | 7102500.00 | | hypothetical protein | Bacteria;Proteobacteria;Betaproteobacteria;Nitrosomonadales;Sulfuricellaceae;Sulfurirhabdus;Sulfurirhabdus autotrophica;Sulfurirhabdus autotrophica DSM 100309 |
| Ga0586324 0301278 3 1217 | 0.00 | 0.00 | 0.00 | 0.00 | 0.00 | 3699639.03 | 0.00 | 0.00 | 0.00 | 0.00 | 0.00 | 0.00 | | hypothetical protein | |
| Ga0586324 0301844 2 697 | 2170000.00 | 0.00 | 0.00 | 0.00 | 0.00 | 0.00 | 0.00 | 0.00 | 0.00 | 0.00 | 0.00 | 0.00 | | hypothetical protein | Bacteria;Proteobacteria;Deltaproteobacteria;Desulfobacterales;Desulfobacteraceae;Candidatus Magnetomorum;Candidatus Magnetomorum sp. HK-1;Candidatus Magnetomorum sp. HK-1 |

| | | | | | | | | | | | | | | | |
|---|---|---|---|---|---|---|---|---|---|---|---|---|---|---|---|
| Ga0586324 0302558 1 2049 | 4080000.00 | 0.00 | 0.00 | 0.00 | 0.00 | 0.00 | 0.00 | 0.00 | 0.00 | 0.00 | 0.00 | 0.00 | | hypothetical protein | Bacteria;Acidobacteria;Acidobacteriia;Bryobacterales;Solibacteraceae;Candidatus Solibacter;Candidatus Solibacter usitatus;Candidatus Solibacter usitatus Ellin6076 |
| Ga0586324 0302583 1061 2047 | 1810000.00 | 5222400.00 | 4437881.28 | 2726500.00 | 4552600.00 | 4066431.30 | 6498000.00 | 10656700.00 | 8326800.00 | 2537164.63 | 3467200.00 | 1972500.00 | | hypothetical protein | |
| Ga0586324 0304858 1468 2040 | 0.00 | 0.00 | 0.00 | 0.00 | 0.00 | 0.00 | 0.00 | 5996300.00 | 6407100.00 | 11319000.00 | 10560000.00 | 7132500.00 | | glutamine synthetase | Bacteria;Proteobacteria;Betaproteobacteria;Nitrosomonadales;Thiobacillaceae;Sulfuritortus;Sulfuritortus calidifontis;Sulfuritortus calidifontis DSM 103923 |
| Ga0586324 0305298 2 808 | 0.00 | 0.00 | 0.00 | 0.00 | 0.00 | 0.00 | 0.00 | 0.00 | 0.00 | 9779000.00 | 0.00 | 0.00 | | hypothetical protein | Bacteria;Actinobacteria;Actinomycetia;Jiangellales;Jiangellaceae;Jiangella;Jiangella aurantiaca;Jiangella aurantiaca 8K307 |
| Ga0586324 0307745 581 1195 | 0.00 | 11893200.00 | 11991900.00 | 3059000.00 | 18776900.00 | 5280100.00 | 11673600.00 | 11919400.00 | 0.00 | 23223200.00 | 20988000.00 | 11212500.00 | | hypothetical protein | |
| Ga0586324 0308321 676 1446 | 0.00 | 270478.84 | 0.00 | 0.00 | 0.00 | 361924.65 | 0.00 | 0.00 | 0.00 | 0.00 | 0.00 | 0.00 | | regulator of protease activity HflC (stomatin/prohibitin superfamily) | Bacteria;Acidobacteria;Thermoanaerobaculia;Thermoanaerobaculales;Thermoanaerobaculaceae;Thermoanaerobaculum;Thermoanaerobaculum aquaticum;Thermoanaerobaculum aquaticum MP-01 |
| Ga0586324 0311341 71 2020 | 0.00 | 897600.00 | 0.00 | 0.00 | 0.00 | 0.00 | 0.00 | 0.00 | 0.00 | 816200.00 | 0.00 | 0.00 | | hypothetical protein | Bacteria;Proteobacteria;Betaproteobacteria;Nitrosomonadales;Nitrosomonadaceae;Nitrosomonas;Nitrosomonas sp. Nm141;Nitrosomonas sp. Nm141 |
| Ga0586324 0312272 2 2017 | 3740000.00 | 2573432.80 | 3352569.29 | 0.00 | 1957000.00 | 5838933.08 | 1075327.80 | 269895.72 | 477456.12 | 2202200.00 | 3634400.00 | 322188.00 | | hypothetical protein | Bacteria;Acidobacteria;Vicinamibacteria;unclassified Vicinamibacteria;Vicinamibacteraceae;Luteitalea;Luteitalea pratensis;Luteitalea pratensis DSM 100886 |
| Ga0586324 0312585 962 1717 | 0.00 | 0.00 | 0.00 | 0.00 | 0.00 | 0.00 | 0.00 | 0.00 | 3385800.00 | 0.00 | 0.00 | 0.00 | | regulator of protease activity HflC (stomatin/prohibitin superfamily) | Bacteria;Bacteroidetes;Chitinophagia;Chitinophagales;Chitinophagaceae;Niastella;Niastella sp. CF465;Niastella sp. CF465 |
| Ga0586324 0312836 1648 2016 | 0.00 | 0.00 | 0.00 | 0.00 | 0.00 | 5756500.00 | 7888800.00 | 0.00 | 0.00 | 0.00 | 0.00 | 0.00 | | methanogenic corrinoid protein MtbC1 | Bacteria;Firmicutes;Clostridia;Halanaerobiales;Halobacteroidaceae;Selenihalanaerobacter;Selenihalanaerobacter shriftii;Selenihalanaerobacter shriftii ATCC BAA-73 |
| Ga0586324 0313955 195 821 | 0.00 | 0.00 | 0.00 | 0.00 | 0.00 | 0.00 | 5130000.00 | 0.00 | 0.00 | 0.00 | 0.00 | 0.00 | | methanogenic corrinoid protein MtbC1 | Bacteria;Firmicutes;Clostridia;Halanaerobiales;Halobacteroidaceae;Selenihalanaerobacter;Selenihalanaerobacter shriftii;Selenihalanaerobacter shriftii ATCC BAA-73 |
| Ga0586324 0317618 490 2004 | 0.00 | 0.00 | 0.00 | 0.00 | 0.00 | 0.00 | 0.00 | 2336300.00 | 0.00 | 0.00 | 0.00 | 0.00 | | predicted porin | Bacteria;Proteobacteria;Alphaproteobacteria;Hyphomicrobiales;unclassified Hyphomicrobiales;Methyloceanibacter;Methyloceanibacter methanicus;Methyloceanibacter methanicus R-67174 |
| Ga0586324 0318678 185 1201 | 0.00 | 0.00 | 0.00 | 0.00 | 0.00 | 0.00 | 0.00 | 0.00 | 0.00 | 703810.03 | 0.00 | 0.00 | | hypothetical protein | Bacteria;Proteobacteria;Gammaproteobacteria;Chromatiales;Thioalkalispiraceae;Thiohalophilus;Thiohalophilus thiocyanatoxydans;Thiohalophilus thiocyanatoxydans DSM 16326 |
| Ga0586324 0318780 1515 2000 | 22430000.00 | 26316000.00 | 0.00 | 17290000.00 | 0.00 | 0.00 | 0.00 | 17873000.00 | 4050000.00 | 0.00 | 22176000.00 | 16800000.00 | | hypothetical protein | |
| Ga0586324 0318996 3 2000 | 16620000.00 | 1156000.00 | 0.00 | 0.00 | 0.00 | 3403893.88 | 0.00 | 2812100.00 | 0.00 | 5751900.00 | 0.00 | 729431.25 | | hypothetical protein | Bacteria;Proteobacteria;Betaproteobacteria;unclassified Betaproteobacteria;unclassified Betaproteobacteria;unclassified Betaproteobacteria;Betaproteobacteria bacterium GR16-43;Betaproteobacteria bacterium GR16-43 |
| Ga0586324 0319524 3 578 | 0.00 | 0.00 | 0.00 | 0.00 | 5489900.00 | 0.00 | 0.00 | 0.00 | 0.00 | 0.00 | 0.00 | 0.00 | | polyhydroxyalkanoate synthesis repressor PhaR | Bacteria;Proteobacteria;Alphaproteobacteria;Hyphomicrobiales;Bradyrhizobiaceae;Bradyrhizobium;Bradyrhizobium sp. URHD0069;Bradyrhizobium sp. URHD0069 |
| Ga0586324 0319665 483 1574 | 0.00 | 0.00 | 0.00 | 4487693.60 | 0.00 | 0.00 | 0.00 | 9333000.00 | 10854000.00 | 0.00 | 0.00 | 0.00 | | predicted porin | Bacteria;Proteobacteria;Betaproteobacteria;Nitrosomonadales;Thiobacillaceae;Thiobacillus;Thiobacillus denitrificans;Thiobacillus denitrificans ATCC 25259 |
| Ga0586324 0320264 1024 1995 | 0.00 | 5453600.00 | 0.00 | 3353500.00 | 0.00 | 12545200.00 | 0.00 | 4782400.00 | 8982900.00 | 0.00 | 0.00 | 0.00 | | predicted porin | Bacteria;Proteobacteria;Betaproteobacteria;Nitrosomonadales;Sulfuricellaceae;Sulfuricella;Sulfuricella sp. T08;Sulfuricella sp. T08 |
| Ga0586324 0321156 684 1994 | 62000000.00 | 63920000.00 | 124986000.00 | 75810000.00 | 81370000.00 | 84561000.00 | 77064000.00 | 48495000.00 | 41310000.00 | 71764000.00 | 75407200.00 | 84660000.00 | | hypothetical protein | Bacteria;Proteobacteria;Deltaproteobacteria;Desulfobacterales;Desulfobacteraceae;Desulfofaba;Desulfofaba hansenii;Desulfofaba hansenii DSM 12642 |
| Ga0586324 0321852 2 601 | 0.00 | 0.00 | 0.00 | 0.00 | 0.00 | 0.00 | 0.00 | 0.00 | 0.00 | 2541000.00 | 3036000.00 | 0.00 | | hypothetical protein | |
| Ga0586324 0322396 633 1133 | 0.00 | 11492000.00 | 0.00 | 0.00 | 13390000.00 | 1426615.53 | 0.00 | 0.00 | 3377700.00 | 0.00 | 0.00 | 0.00 | | rubrerythrin | Bacteria;Chlorobi;Chlorobia;Chlorobiales;Chlorobiaceae;Chlorobium;Chlorobium phaeobacteroides;Chlorobium phaeobacteroides BS1 |
| Ga0586324 0322845 57 1139 | 625750.00 | 0.00 | 0.00 | 0.00 | 0.00 | 0.00 | 1027919.76 | 0.00 | 0.00 | 0.00 | 0.00 | 0.00 | | TRAP-type mannitol/chloroaromatic compound transport system substrate-binding protein | Bacteria;Proteobacteria;Alphaproteobacteria;Hyphomicrobiales;Xanthobacteraceae;Pseudolabrys;Pseudolabrys taiwanensis;Pseudolabrys taiwanensis CC-BB4 |
| Ga0586324 0323183 1091 1987 | 0.00 | 720800.00 | 243658.52 | 0.00 | 0.00 | 1412795.96 | 1466382.00 | 1140700.00 | 1790100.00 | 1470700.00 | 1144000.00 | 628708.50 | | TRAP-type mannitol/chloroaromatic compound transport system substrate-binding protein | Bacteria;Proteobacteria;Alphaproteobacteria;Hyphomicrobiales;Bradyrhizobiaceae;Bradyrhizobium;Bradyrhizobium lablabi;Bradyrhizobium lablabi GAS499 |
| Ga0586324 0323387 256 1719 | 0.00 | 1142400.00 | 0.00 | 0.00 | 0.00 | 0.00 | 0.00 | 0.00 | 0.00 | 0.00 | 1522400.00 | 0.00 | | hypothetical protein | Bacteria;Proteobacteria;Gammaproteobacteria;Chromatiales;Chromatiaceae;Nitrosococcus;Nitrosococcus halophilus;Nitrosococcus halophilus Nc4 |
| Ga0586324 0325293 1252 1875 | 0.00 | 0.00 | 0.00 | 0.00 | 0.00 | 0.00 | 0.00 | 0.00 | 0.00 | 2979900.00 | 3713600.00 | 0.00 | | opacity protein-like surface antigen | Bacteria;Proteobacteria;Betaproteobacteria;Rhodocyclales;Rhodocyclaceae;Rhodocyclus;Rhodocyclus tenuis;Rhodocyclus tenuis DSM 109 |
| Ga0586324 0326165 3 1979 | 0.00 | 8726537.92 | 0.00 | 15960000.00 | 0.00 | 8932500.00 | 0.00 | 0.00 | 0.00 | 0.00 | 0.00 | 6600000.00 | | hypothetical protein | |
| Ga0586324 0328677 267 1973 | 0.00 | 0.00 | 0.00 | 0.00 | 0.00 | 7066600.00 | 0.00 | 0.00 | 0.00 | 0.00 | 0.00 | 0.00 | | Zn-dependent protease with chaperone function | Bacteria;Planctomycetes;Planctomycetia;Gemmatales;Gemmataceae;Gemmata;Gemmata massiliana;Gemmata massiliana IIL30 |
| Ga0586324 0328691 1214 1972 | 0.00 | 0.00 | 0.00 | 0.00 | 0.00 | 0.00 | 0.00 | 0.00 | 0.00 | 1370600.00 | 0.00 | 0.00 | | MFS family permease | Bacteria;Proteobacteria;Deltaproteobacteria;Myxococcales;Anaeromyxobacteraceae;Anaeromyxobacter;Anaeromyxobacter sp. Fw109-5;Anaeromyxobacter sp. Fw109-5 |
| Ga0586324 0329116 907 1200 | 0.00 | 0.00 | 0.00 | 0.00 | 3357800.00 | 0.00 | 0.00 | 0.00 | 0.00 | 0.00 | 0.00 | 0.00 | | hypothetical protein | Bacteria;Proteobacteria;Deltaproteobacteria;Syntrophobacterales;Syntrophaceae;Desulfobacca;Desulfobacca acetoxidans;Desulfobacca acetoxidans ASRB2, DSM 11109 |
| Ga0586324 0329536 2 754 | 35000000.00 | 0.00 | 0.00 | 0.00 | 0.00 | 0.00 | 0.00 | 0.00 | 0.00 | 0.00 | 0.00 | 0.00 | | hypothetical protein | Bacteria;Acidobacteria;Acidobacteriia;Acidobacteriales;Acidobacteriaceae;unclassified Acidobacteriaceae;Acidobacteriaceae bacterium KBS 96;Acidobacteriaceae bacterium KBS 96, DSM 24297 |
| Ga0586324 0329564 2 1567 | 0.00 | 0.00 | 0.00 | 0.00 | 0.00 | 2525035.13 | 2850000.00 | 0.00 | 0.00 | 1755600.00 | 0.00 | 3697500.00 | | hypothetical protein | Bacteria;Bacteroidetes;Cytophagia;Cytophagales;Cytophagaceae;Dyadobacter;Dyadobacter psychrophilus;Dyadobacter psychrophilus DSM 22270 |
| Ga0586324 0331667 1 1890 | 36260000.00 | 53168520.00 | 0.00 | 28595000.00 | 26347400.00 | 0.00 | 0.00 | 24314600.00 | 32173200.00 | 44128700.00 | 21692000.00 | 2490000.00 | | transketolase | Bacteria;Proteobacteria;Gammaproteobacteria;Methylococcales;Methylococcaceae;Methylomonas;Methylomonas lenta;Methylomonas lenta R-45370 |
| Ga0586324 0331992 1 936 | 0.00 | 0.00 | 0.00 | 0.00 | 0.00 | 0.00 | 0.00 | 1427400.00 | 1806300.00 | 0.00 | 0.00 | 0.00 | | TRAP-type mannitol/chloroaromatic compound transport system substrate-binding protein | Bacteria;Proteobacteria;Alphaproteobacteria;Hyphomicrobiales;Bradyrhizobiaceae;Bradyrhizobium;Bradyrhizobium sp. Ai1a-2;Bradyrhizobium sp. Ai1a-2 |
| Ga0586324 0332560 1173 1877 | 0.00 | 0.00 | 9289500.00 | 0.00 | 0.00 | 0.00 | 0.00 | 0.00 | 0.00 | 0.00 | 0.00 | 0.00 | | predicted membrane protein | Bacteria;Firmicutes;Clostridia;Eubacteriales;unclassified Eubacteriales;Natranaerovirga;Natranaerovirga pectinivora;Natranaerovirga pectinivora DSM 24629 |
| Ga0586324 0333072 750 1961 | 0.00 | 2250800.00 | 0.00 | 3334500.00 | 9651100.00 | 0.00 | 3716400.00 | 2031300.00 | 2478600.00 | 3757600.00 | 3528800.00 | 2557500.00 | | hypothetical protein | Bacteria;Firmicutes;Clostridia;Thermoanaerobacterales;Thermoanaerobacteraceae;Thermodesulfitimonas;Thermodesulfitimonas autotrophica;Thermodesulfitimonas autotrophica DSM 102936 |
| Ga0586324 0333352 3 1082 | 0.00 | 0.00 | 0.00 | 0.00 | 0.00 | 0.00 | 1074007.68 | 0.00 | 0.00 | 0.00 | 0.00 | 0.00 | | hypothetical protein | Bacteria;Verrucomicrobia;Verrucomicrobiae;Verrucomicrobiales;Verrucomicrobia subdivision 3;Pedosphaera;Pedosphaera parvula;Pedosphaera parvula Ellin514 |
| Ga0586324 0333962 672 1958 | 0.00 | 0.00 | 0.00 | 0.00 | 0.00 | 0.00 | 4833600.00 | 9625800.00 | 7565400.00 | 36652000.00 | 33492800.00 | 23887500.00 | | NADPH-dependent glutamate synthase beta subunit-like oxidoreductase | Bacteria;Proteobacteria;Betaproteobacteria;Nitrosomonadales;Sulfuricellaceae;Sulfurirhabdus;Sulfurirhabdus autotrophica;Sulfurirhabdus autotrophica DSM 100309 |
| Ga0586324 0334128 641 1912 | 2601453.00 | 85775795.00 | 28994500.00 | 18430000.00 | 143695300.00 | 41288000.00 | 22070400.00 | 102785000.00 | 89262000.00 | 88396000.00 | 36080000.00 | 72375000.00 | | hypothetical protein | Bacteria;Proteobacteria;Alphaproteobacteria;Sphingomonadales;Erythrobacteraceae;Erythrobacter;Erythrobacter neustonensis;Erythrobacter neustonensis DSM 9434 |
| Ga0586324 0334753 1302 1958 | 106000000.00 | 85408000.00 | 0.00 | 0.00 | 0.00 | 0.00 | 0.00 | 0.00 | 0.00 | 10857000.00 | 0.00 | 0.00 | | hypothetical protein | Bacteria;Proteobacteria;Betaproteobacteria;Burkholderiales;Burkholderiaceae;Quisquiliibacterium;Quisquiliibacterium transsilvanicum;Quisquiliibacterium transsilvanicum DSM 29781 |
| Ga0586324 0335176 173 1957 | 0.00 | 0.00 | 0.00 | 0.00 | 0.00 | 0.00 | 3625200.00 | 0.00 | 0.00 | 0.00 | 0.00 | 0.00 | | outer membrane receptor protein involved in Fe transport | Bacteria;Acidobacteria;Thermoanaerobaculia;Thermoanaerobaculales;Thermoanaerobaculaceae;Thermoanaerobaculum;Thermoanaerobaculum aquaticum;Thermoanaerobaculum aquaticum MP-01 |
| Ga0586324 0335724 1 954 | 0.00 | 0.00 | 0.00 | 0.00 | 0.00 | 0.00 | 0.00 | 0.00 | 0.00 | 0.00 | 1654400.00 | 0.00 | | hypothetical protein | |
| Ga0586324 0335741 77 979 | 0.00 | 0.00 | 0.00 | 0.00 | 0.00 | 0.00 | 0.00 | 0.00 | 0.00 | 0.00 | 1909600.00 | 0.00 | | gliding motility-associated protein GldL | Bacteria;Bacteroidetes;Flavobacteriia;Flavobacteriales;Crocinitomicaceae;Brumimicrobium;Brumimicrobium sp. C305;Brumimicrobium sp. C305 |
| Ga0586324 0335965 745 1953 | 0.00 | 343537.36 | 0.00 | 0.00 | 0.00 | 0.00 | 0.00 | 0.00 | 0.00 | 0.00 | 0.00 | 0.00 | | hypothetical protein | Bacteria;Chloroflexi;Anaerolineae;Anaerolineales;Anaerolineaceae;Thermanaerothrix;Thermanaerothrix daxensis;Thermanaerothrix daxensis GNS-1 |
| Ga0586324 0336812 222 1952 | 0.00 | 1196800.00 | 0.00 | 0.00 | 2204200.00 | 0.00 | 0.00 | 0.00 | 0.00 | 0.00 | 0.00 | 0.00 | | hypothetical protein | Bacteria;Actinobacteria;Actinomycetia;Streptomycetales;Streptomycetaceae;Kitasatospora;Kitasatospora cineracea;Kitasatospora cineracea DSM 44780 |
| Ga0586324 0337200 3 1952 | 0.00 | 0.00 | 0.00 | 0.00 | 1874600.00 | 0.00 | 0.00 | 0.00 | 0.00 | 0.00 | 0.00 | 0.00 | | hypothetical protein | Archaea;Euryarchaeota;Halobacteria;Halobacteriales;Haloarculaceae;Natronomonas;Natronomonas moolapensis;Natronomonas moolapensis 8.8.11, DSM 18674 |
| Ga0586324 0337362 545 1744 | 0.00 | 0.00 | 0.00 | 0.00 | 0.00 | 0.00 | 12973200.00 | 0.00 | 0.00 | 0.00 | 0.00 | 0.00 | | predicted porin | Bacteria;Proteobacteria;Gammaproteobacteria;Chromatiales;Chromatiaceae;Thioflavicoccus;Thioflavicoccus mobilis;Thioflavicoccus mobilis 8321 |
| Ga0586324 0338714 797 1948 | 0.00 | 0.00 | 0.00 | 0.00 | 0.00 | 0.00 | 1797932.76 | 0.00 | 0.00 | 0.00 | 0.00 | 0.00 | | hypothetical protein | Bacteria;Proteobacteria;Alphaproteobacteria;Hyphomicrobiales;Bradyrhizobiaceae;Bradyrhizobium;Bradyrhizobium sp. URHD0069;Bradyrhizobium sp. URHD0069 |
| Ga0586324 0339013 188 1945 | 26360000.00 | 45478400.00 | 2729880.03 | 36537000.00 | 63551000.00 | 2754786.97 | 20428800.00 | 57279000.00 | 38750400.00 | 32147500.00 | 27588000.00 | 37899185.25 | | hypothetical protein | Bacteria;Proteobacteria;Deltaproteobacteria;Syntrophobacterales;Syntrophobacteraceae;Desulfosoma;Desulfosoma caldarium;Desulfosoma caldarium DSM 22027 |
| Ga0586324 0339423 206 1237 | 3850000.00 | 21467600.00 | 18128600.00 | 7609500.00 | 14821700.00 | 25765300.00 | 22321200.00 | 18702600.00 | 17544600.00 | 23315600.00 | 17661600.00 | 12997500.00 | | predicted porin | Bacteria;Proteobacteria;Betaproteobacteria;Burkholderiales;unclassified Burkholderiales;Methylibium;Methylibium sp. CF059;Methylibium sp. CF059 |
| Ga0586324 0339689 114 1454 | 0.00 | 2135200.00 | 0.00 | 6327000.00 | 2719200.00 | 0.00 | 0.00 | 0.00 | 3361500.00 | 0.00 | 0.00 | 0.00 | | crotonobetainyl-CoA:carnitine CoA-transferase CaiB-like acyl-CoA transferase | Bacteria;Proteobacteria;Deltaproteobacteria;Syntrophobacterales;Syntrophobacteraceae;Desulfacinum;Desulfacinum infernum;Desulfacinum infernum DSM 9756 |
| Ga0586324 0340171 3 401 | 0.00 | 0.00 | 0.00 | 0.00 | 2956100.00 | 0.00 | 0.00 | 0.00 | 0.00 | 0.00 | 0.00 | 0.00 | | hypothetical protein | |
| Ga0586324 0340732 1631 1942 | 0.00 | 0.00 | 0.00 | 0.00 | 0.00 | 0.00 | 0.00 | 0.00 | 2478600.00 | 0.00 | 1830400.00 | 0.00 | | phasin family protein | Bacteria;Proteobacteria;Betaproteobacteria;Rhodocyclales;Rhodocyclaceae;Aromatoleum;Aromatoleum aromaticum;Aromatoleum aromaticum EbN1 |
| Ga0586324 0341052 739 1926 | 0.00 | 0.00 | 0.00 | 0.00 | 0.00 | 0.00 | 0.00 | 0.00 | 0.00 | 0.00 | 0.00 | 606827.25 | | predicted porin | Bacteria;Proteobacteria;Betaproteobacteria;Burkholderiales;Burkholderiaceae;Cupriavidus;Cupriavidus basilensis;Cupriavidus basilensis 4G11 |
| Ga0586324 0341413 1313 1939 | 0.00 | 0.00 | 0.00 | 0.00 | 0.00 | 9051600.00 | 0.00 | 0.00 | 0.00 | 0.00 | 0.00 | 0.00 | | predicted porin | Bacteria;Proteobacteria;Betaproteobacteria;Burkholderiales;Oxalobacteraceae;Janthinobacterium;Janthinobacterium sp. CG3;Janthinobacterium sp. CG3 |
| Ga0586324 0341514 63 1940 | 0.00 | 992800.00 | 0.00 | 0.00 | 0.00 | 0.00 | 0.00 | 0.00 | 0.00 | 0.00 | 0.00 | 0.00 | | hypothetical protein | Bacteria;Bacteroidetes;Chitinophagia;Chitinophagales;Chitinophagaceae;Chitinophaga;Chitinophaga terrae (ex Kim and Jung 2007);Chitinophaga terrae DSM 23920 |
| Ga0586324 0343559 1 1935 | 558482.00 | 2774400.00 | 0.00 | 0.00 | 0.00 | 0.00 | 0.00 | 0.00 | 0.00 | 0.00 | 0.00 | 0.00 | | hypothetical protein | Bacteria;Proteobacteria;Deltaproteobacteria;Myxococcales;Anaeromyxobacteraceae;Anaeromyxobacter;Anaeromyxobacter sp. PSR-1;Anaeromyxobacter sp. PSR-1 |
| Ga0586324 0343707 1 1935 | 0.00 | 4005200.00 | 0.00 | 0.00 | 5283900.00 | 0.00 | 0.00 | 0.00 | 4511700.00 | 5921300.00 | 0.00 | 0.00 | | CO/xanthine dehydrogenase Mo-binding subunit | Bacteria;Proteobacteria;Deltaproteobacteria;Syntrophorhabdales;Syntrophorhabdaceae;Syntrophorhabdus;Syntrophorhabdus aromaticivorans;Syntrophorhabdus aromaticivorans UI |
| Ga0586324 0344736 49 1011 | 0.00 | 0.00 | 0.00 | 0.00 | 0.00 | 0.00 | 0.00 | 263920.77 | 0.00 | 0.00 | 0.00 | 0.00 | | ABC-type sugar transport system substrate-binding protein | Bacteria;Chloroflexi;Chloroflexia;Chloroflexales;Oscillochloridaceae;Oscillochloris;Oscillochloris trichoides;Oscillochloris trichoides DG6 |
| Ga0586324 0345379 368 1930 | 0.00 | 0.00 | 0.00 | 0.00 | 3326900.00 | 0.00 | 0.00 | 0.00 | 0.00 | 0.00 | 0.00 | 0.00 | | hypothetical protein | Bacteria;Acidobacteria;Acidobacteriia;Acidobacteriales;Acidobacteriaceae;unclassified Acidobacteriaceae;Acidobacteriaceae bacterium KBS 96;Acidobacteriaceae bacterium KBS 96, DSM 24297 |
| Ga0586324 0346591 69 1928 | 0.00 | 0.00 | 0.00 | 0.00 | 0.00 | 0.00 | 0.00 | 1634800.00 | 0.00 | 0.00 | 0.00 | 0.00 | | multidrug efflux pump subunit AcrB | Bacteria;Thermodesulfobacteria;Thermodesulfobacteria;Thermodesulfobacteriales;Thermodesulfobacteriaceae;Thermosulfuriphilus;Thermosulfuriphilus ammonigenes;Thermosulfuriphilus ammonigenes DSM 102941 |
| Ga0586324 0346999 1 1926 | 0.00 | 0.00 | 0.00 | 0.00 | 0.00 | 0.00 | 0.00 | 0.00 | 230896.17 | 0.00 | 0.00 | 0.00 | | NADPH-dependent glutamate synthase beta subunit-like oxidoreductase | Bacteria;Proteobacteria;Deltaproteobacteria;Syntrophobacterales;Syntrophaceae;Desulfomonile;Desulfomonile tiedjei;Desulfomonile tiedjei DCB-1, DSM 6799 |
| Ga0586324 0347370 3 1250 | 0.00 | 0.00 | 68123000.00 | 0.00 | 0.00 | 81782000.00 | 30552000.00 | 0.00 | 42525000.00 | 0.00 | 0.00 | 19200000.00 | | predicted porin | Bacteria;Proteobacteria;Deltaproteobacteria;Desulfuromonadales;Geobacteraceae;Geobacter;Geobacter argillaceus;Geobacter argillaceus ATCC BAA-1139 |
| Ga0586324 0347786 605 1924 | 5370000.00 | 5875200.00 | 0.00 | 0.00 | 0.00 | 0.00 | 0.00 | 0.00 | 0.00 | 0.00 | 0.00 | 0.00 | | putative cell wall-binding protein | Bacteria;Actinobacteria;Actinomycetia;Acidothermales;Acidothermaceae;Acidothermus;Acidothermus cellulolyticus;Acidothermus cellulolyticus 11B |
| Ga0586324 0350138 1 1917 | 0.00 | 0.00 | 2924824.41 | 0.00 | 0.00 | 0.00 | 2553600.00 | 0.00 | 0.00 | 0.00 | 0.00 | 2085000.00 | | alkyl sulfatase BDS1-like metallo-beta-lactamase superfamily hydrolase | Bacteria;Proteobacteria;Alphaproteobacteria;Hyphomicrobiales;Bradyrhizobiaceae;Rhodopseudomonas;Rhodopseudomonas palustris;Rhodopseudomonas palustris 7850, DSM 127 |
| Ga0586324 0350256 3 983 | 39100000.00 | 31397211.60 | 75723500.00 | 63460000.00 | 18952000.00 | 37159200.00 | 52827600.00 | 64782000.00 | 46008000.00 | 4835600.00 | 0.00 | 11100000.00 | | hypothetical protein | Bacteria;Proteobacteria;Alphaproteobacteria;Sphingomonadales;Erythrobacteraceae;Erythrobacter;Erythrobacter neustonensis;Erythrobacter neustonensis DSM 9434 |
| Ga0586324 0350405 557 1918 | 2080000.00 | 1842800.00 | 0.00 | 959500.00 | 0.00 | 0.00 | 3556800.00 | 1665300.00 | 1522800.00 | 0.00 | 0.00 | 2062500.00 | | hypothetical protein | Bacteria;Nitrospirae;Thermodesulfovibrionia;Thermodesulfovibrionales;Thermodesulfovibrionaceae;Thermodesulfovibrio;Thermodesulfovibrio islandicus;Thermodesulfovibrio islandicus DSM 12570 |
| Ga0586324 0351704 356 1915 | 5050000.00 | 0.00 | 0.00 | 0.00 | 0.00 | 0.00 | 0.00 | 0.00 | 0.00 | 916300.00 | 464229.92 | 885000.00 | | hypothetical protein | Bacteria;Proteobacteria;Gammaproteobacteria;Methylococcales;Methylococcaceae;Methylomonas;Methylomonas koyamae;Methylomonas koyamae R-45383 |
| Ga0586324 0352375 892 1914 | 0.00 | 0.00 | 0.00 | 0.00 | 0.00 | 16237300.00 | 0.00 | 10492000.00 | 0.00 | 0.00 | 0.00 | 0.00 | | hypothetical protein | Bacteria;Acidobacteria;Thermoanaerobaculia;Thermoanaerobaculales;Thermoanaerobaculaceae;Thermoanaerobaculum;Thermoanaerobaculum aquaticum;Thermoanaerobaculum aquaticum MP-01 |
| Ga0586324 0352663 1193 1912 | 0.00 | 1285200.00 | 0.00 | 0.00 | 0.00 | 0.00 | 0.00 | 0.00 | 0.00 | 0.00 | 0.00 | 0.00 | | enoyl-CoA hydratase/carnithine racemase | Bacteria;Proteobacteria;Deltaproteobacteria;Desulfarculales;Desulfarculaceae;Desulfarculus;Desulfarculus baarsii;Desulfarculus baarsii 2st14, DSM 2075 |
| Ga0586324 0353095 186 1910 | 0.00 | 0.00 | 0.00 | 0.00 | 0.00 | 2536464.76 | 0.00 | 0.00 | 0.00 | 0.00 | 0.00 | 0.00 | | hypothetical protein | Bacteria;Proteobacteria;Betaproteobacteria;Burkholderiales;Oxalobacteraceae;Noviherbaspirillum;Noviherbaspirillum autotrophicum;Noviherbaspirillum autotrophicum TSA66 |
| Ga0586324 0353681 166 1425 | 0.00 | 5610000.00 | 0.00 | 3838000.00 | 11124000.00 | 0.00 | 0.00 | 3794200.00 | 0.00 | 1278200.00 | 1707200.00 | 2965164.00 | | hypothetical protein | Bacteria;Nitrospirae;Nitrospira;Nitrospirales;Nitrospiraceae;Candidatus Magnetoovum;Candidatus Magnetoovum chiemensis;Candidatus Magnetoovum chiemensis CS-04 |
| Ga0586324 0353806 1 324 | 0.00 | 0.00 | 0.00 | 0.00 | 0.00 | 0.00 | 0.00 | 0.00 | 2016900.00 | 0.00 | 0.00 | 0.00 | | dipeptidyl aminopeptidase/acylaminoacyl peptidase | Bacteria;Bacteroidetes;Chitinophagia;Chitinophagales;Chitinophagaceae;Flavisolibacter;Flavisolibacter ginsengiterrae;Flavisolibacter ginsengiterrae DSM 18136 |
| Ga0586324 0353885 2 691 | 0.00 | 0.00 | 0.00 | 9880000.00 | 589626.59 | 0.00 | 6224400.00 | 0.00 | 6220800.00 | 0.00 | 0.00 | 0.00 | | alkylation response protein AidB-like acyl-CoA dehydrogenase | Bacteria;Proteobacteria;Deltaproteobacteria;Desulfobacterales;Desulfobacteraceae;Desulfatiglans;Desulfatiglans anilini;Desulfatiglans anilini DSM 4660 |
| Ga0586324 0355536 2 1906 | 0.00 | 0.00 | 0.00 | 0.00 | 0.00 | 0.00 | 0.00 | 3599000.00 | 0.00 | 0.00 | 0.00 | 0.00 | | hypothetical protein | |
| Ga0586324 0356765 36 1532 | 4330000.00 | 6738800.00 | 0.00 | 0.00 | 0.00 | 0.00 | 0.00 | 3001200.00 | 1757700.00 | 0.00 | 2684000.00 | 1245000.00 | | hypothetical protein | Bacteria;Nitrospirae;Nitrospira;Nitrospirales;Nitrospiraceae;Nitrospira;Nitrospira marina;Nitrospira marina Nb-295 |
| Ga0586324 0357651 1 1233 | 0.00 | 0.00 | 0.00 | 0.00 | 45011000.00 | 39183900.00 | 28500000.00 | 28609000.00 | 0.00 | 0.00 | 2587200.00 | 32250000.00 | | hypothetical protein | Bacteria;Proteobacteria;Gammaproteobacteria;Legionellales;Legionellaceae;Legionella;Legionella drancourtii;Legionella drancourtii LLAP12 |
| Ga0586324 0358338 261 1532 | 7970000.00 | 4188800.00 | 47292000.00 | 34675000.00 | 74160000.00 | 81742300.00 | 37392000.00 | 38606900.00 | 71093700.00 | 50427300.00 | 83248000.00 | 33225000.00 | | hypothetical protein | Bacteria;Proteobacteria;Deltaproteobacteria;Myxococcales;Anaeromyxobacteraceae;Anaeromyxobacter;Anaeromyxobacter dehalogenans;Anaeromyxobacter dehalogenans 2CP-C |
| Ga0586324 0358511 3 1748 | 0.00 | 4052800.00 | 0.00 | 0.00 | 0.00 | 0.00 | 0.00 | 0.00 | 0.00 | 432580.61 | 0.00 | 2625000.00 | | outer membrane receptor protein involved in Fe transport | Bacteria;Bacteroidetes;Chitinophagia;Chitinophagales;Chitinophagaceae;Hydrobacter;Hydrobacter penzbergensis;Hydrobacter penzbergensis DSM 25353 |
| Ga0586324 0363619 1168 1659 | 0.00 | 2495600.00 | 0.00 | 6289000.00 | 4717400.00 | 0.00 | 0.00 | 3983300.00 | 0.00 | 0.00 | 0.00 | 0.00 | | uncharacterized surface protein with fasciclin (FAS1) repeats | Bacteria;Bacteroidetes;Cytophagia;Cytophagales;Cyclobacteriaceae;Algoriphagus;Algoriphagus mannitolivorans;Algoriphagus mannitolivorans DSM 15301 |
| Ga0586324 0363986 2 862 | 27300000.00 | 13124000.00 | 0.00 | 46455000.00 | 53354000.00 | 42479000.00 | 58368000.00 | 44286000.00 | 37746000.00 | 0.00 | 0.00 | 24525000.00 | | predicted porin | Bacteria;Proteobacteria;Betaproteobacteria;Burkholderiales;unclassified Burkholderiales;Rubrivivax;Rubrivivax benzoatilyticus;Rubrivivax benzoatilyticus JA2 |
| Ga0586324 0364021 3 1886 | 0.00 | 0.00 | 0.00 | 0.00 | 8549000.00 | 0.00 | 0.00 | 0.00 | 0.00 | 0.00 | 0.00 | 0.00 | | hypothetical protein | Bacteria;Proteobacteria;Betaproteobacteria;unclassified Betaproteobacteria;unclassified Betaproteobacteria;unclassified Betaproteobacteria;Betaproteobacteria bacterium GR16-43;Betaproteobacteria bacterium GR16-43 |
| Ga0586324 0365186 244 1884 | 0.00 | 0.00 | 0.00 | 0.00 | 0.00 | 0.00 | 0.00 | 0.00 | 0.00 | 0.00 | 1214400.00 | 0.00 | | putative PEP-CTERM system TPR-repeat lipoprotein | Bacteria;Proteobacteria;Betaproteobacteria;Nitrosomonadales;Sulfuricellaceae;Sulfurirhabdus;Sulfurirhabdus autotrophica;Sulfurirhabdus autotrophica DSM 100309 |
| Ga0586324 0366095 1 1329 | 0.00 | 0.00 | 0.00 | 452078.40 | 0.00 | 0.00 | 0.00 | 0.00 | 0.00 | 0.00 | 0.00 | 0.00 | | hypothetical protein | |
| Ga0586324 0367678 578 1078 | 0.00 | 0.00 | 0.00 | 0.00 | 0.00 | 0.00 | 0.00 | 0.00 | 0.00 | 546847.84 | 0.00 | 1860000.00 | | 2-oxoacid:acceptor oxidoreductase delta subunit (pyruvate/2-ketoisovalerate family) | Bacteria;Firmicutes;Clostridia;Thermoanaerobacterales;Thermoanaerobacteraceae;Thermanaeromonas;Thermanaeromonas toyohensis;Thermanaeromonas toyohensis ToBE, DSM 14490 |
| Ga0586324 0367877 3 977 | 0.00 | 1475600.00 | 8107200.00 | 0.00 | 2121800.00 | 3663202.37 | 4537200.00 | 1116300.00 | 3349597.86 | 8470000.00 | 3590400.00 | 3720000.00 | | hypothetical protein | Bacteria;Proteobacteria;Gammaproteobacteria;Chromatiales;Chromatiaceae;Lamprocystis;Lamprocystis purpurea;Lamprocystis purpurea DSM 4197 |
| Ga0586324 0368378 3 1874 | 16400000.00 | 22236000.00 | 10190300.00 | 16340000.00 | 16068000.00 | 11354200.00 | 68400000.00 | 8601000.00 | 6674400.00 | 7638400.00 | 6177600.00 | 4845000.00 | | FtsP/CotA-like multicopper oxidase with cupredoxin domain | Bacteria;Actinobacteria;Actinomycetia;Micromonosporales;Micromonosporaceae;Micromonospora;Micromonospora humi;Micromonospora humi DSM 45647 |
| Ga0586324 0368767 801 1451 | 0.00 | 0.00 | 0.00 | 5434000.00 | 0.00 | 0.00 | 0.00 | 0.00 | 0.00 | 0.00 | 0.00 | 0.00 | | hypothetical protein | Bacteria;Acidobacteria;Acidobacteriia;Acidobacteriales;Acidobacteriaceae;Granulicella;Granulicella sp. GAS466;Granulicella sp. GAS466 |
| Ga0586324 0369096 349 1875 | 0.00 | 4454000.00 | 0.00 | 0.00 | 0.00 | 0.00 | 0.00 | 0.00 | 0.00 | 0.00 | 0.00 | 0.00 | | hypothetical protein | Bacteria;Proteobacteria;Betaproteobacteria;Burkholderiales;Comamonadaceae;Delftia;Delftia sp. HK171;Delftia sp. HK171 (v2) |
| Ga0586324 0369100 1 300 | 0.00 | 0.00 | 533003.36 | 0.00 | 0.00 | 0.00 | 0.00 | 0.00 | 0.00 | 0.00 | 0.00 | 0.00 | | hypothetical protein | Bacteria;Proteobacteria;Gammaproteobacteria;Methylococcales;Methylococcaceae;Methylobacter;Methylobacter tundripaludum;Methylobacter tundripaludum SV96 |
| Ga0586324 0369460 952 1413 | 0.00 | 0.00 | 0.00 | 19855000.00 | 0.00 | 0.00 | 0.00 | 0.00 | 0.00 | 0.00 | 0.00 | 0.00 | | nucleotide-binding universal stress UspA family protein | Archaea;Euryarchaeota;Methanomicrobia;Methanosarcinales;Methanotrichaceae;Methanothrix;Methanothrix thermoacetophila;Methanothrix thermoacetophila PT |
| Ga0586324 0369526 960 1520 | 0.00 | 0.00 | 0.00 | 0.00 | 0.00 | 0.00 | 0.00 | 0.00 | 0.00 | 0.00 | 2728000.00 | 0.00 | | phasin family protein | Bacteria;Proteobacteria;Betaproteobacteria;unclassified Betaproteobacteria;unclassified Betaproteobacteria;unclassified Betaproteobacteria;Betaproteobacteria bacterium GR16-43;Betaproteobacteria bacterium GR16-43 |
| Ga0586324 0369753 1033 1872 | 0.00 | 0.00 | 0.00 | 467750.55 | 0.00 | 0.00 | 0.00 | 0.00 | 882900.00 | 0.00 | 0.00 | 0.00 | | OmcA/MtrC family decaheme c-type cytochrome | Bacteria;Proteobacteria;Betaproteobacteria;Burkholderiales;Comamonadaceae;Rhodoferax;Rhodoferax ferrireducens;Rhodoferax ferrireducens T118 |
| Ga0586324 0370213 1046 1870 | 5820000.00 | 0.00 | 9739900.00 | 9785000.00 | 8188500.00 | 12902500.00 | 6292800.00 | 0.00 | 4997700.00 | 6953100.00 | 8280800.00 | 0.00 | | hypothetical protein | Bacteria;Ignavibacteriae;Ignavibacteria;Ignavibacteriales;Ignavibacteriaceae;Ignavibacterium;Ignavibacterium album;Ignavibacterium album Mat9-16, JCM 16511 |
| Ga0586324 0370479 2 1870 | 0.00 | 2862800.00 | 3538038.38 | 0.00 | 0.00 | 0.00 | 0.00 | 0.00 | 0.00 | 0.00 | 1117600.00 | 0.00 | | hypothetical protein | Bacteria;Verrucomicrobia;Opitutae;Opitutales;Opitutaceae;Lacunisphaera;Lacunisphaera limnophila;Opitutaceae bacterium IG16b |
| Ga0586324 0371984 200 1588 | 0.00 | 0.00 | 0.00 | 4626500.00 | 1771600.00 | 1067401.99 | 0.00 | 0.00 | 0.00 | 0.00 | 0.00 | 0.00 | | hypothetical protein | Bacteria;Proteobacteria;Deltaproteobacteria;Syntrophobacterales;Syntrophobacteraceae;Desulfacinum;Desulfacinum infernum;Desulfacinum infernum DSM 9756 |
| Ga0586324 0373321 192 869 | 0.00 | 0.00 | 0.00 | 0.00 | 0.00 | 0.00 | 0.00 | 1372500.00 | 1053000.00 | 0.00 | 0.00 | 0.00 | | biopolymer transport protein ExbB/TolQ | Bacteria;Candidatus Aminicenantes;unclassified Candidatus Aminicenantes;unclassified Candidatus Aminicenantes;unclassified Candidatus Aminicenantes;unclassified Candidatus Aminicenantes;Aminicenantes bacterium SCGC AAA252-P13;Aminicenantes bacterium SCGC AAA252-P13 (contamination screened) |
| Ga0586324 0375376 3 677 | 0.00 | 0.00 | 0.00 | 1539000.00 | 0.00 | 0.00 | 0.00 | 0.00 | 0.00 | 0.00 | 0.00 | 0.00 | | 3-hydroxyisobutyrate dehydrogenase-like beta-hydroxyacid dehydrogenase | Bacteria;Proteobacteria;Alphaproteobacteria;Hyphomicrobiales;Hyphomicrobiaceae;Rhodoplanes;Rhodoplanes sp. Z2-YC6860;Rhodoplanes sp. Z2-YC6860 |
| Ga0586324 0375864 205 546 | 0.00 | 1761200.00 | 0.00 | 0.00 | 0.00 | 0.00 | 0.00 | 0.00 | 0.00 | 0.00 | 0.00 | 0.00 | | RNA recognition motif-containing protein | Bacteria;Bacteroidetes;Bacteroidia;Marinilabiliales;Prolixibacteraceae;Sunxiuqinia;Sunxiuqinia dokdonensis;Sunxiuqinia dokdonensis SK |
| Ga0586324 0377614 110 1855 | 3700000.00 | 0.00 | 0.00 | 2945000.00 | 0.00 | 0.00 | 0.00 | 2183800.00 | 2122200.00 | 4643100.00 | 0.00 | 0.00 | | hypothetical protein | Bacteria;Balneolaeota;Balneolia;Balneolales;Balneolaceae;Gracilimonas;Gracilimonas mengyeensis;Gracilimonas mengyeensis DSM 21985 |
| Ga0586324 0377691 2 1855 | 0.00 | 4644400.00 | 4826610.26 | 2337000.00 | 2626500.00 | 623671.12 | 3739200.00 | 3184200.00 | 3280500.00 | 4812500.00 | 3572800.00 | 4087500.00 | | hypothetical protein | Archaea;Euryarchaeota;Methanomicrobia;Methanomicrobiales;Methanoregulaceae;Methanoregula;Methanoregula formicica;Methanoregula formicica SMSP, DSM 22288 |
| Ga0586324 0378165 3 1853 | 1430000.00 | 279949.88 | 0.00 | 0.00 | 0.00 | 0.00 | 797842.68 | 0.00 | 2850636.24 | 0.00 | 493402.80 | 832500.00 | | hypothetical protein | Bacteria;Acidobacteria;Vicinamibacteria;unclassified Vicinamibacteria;Vicinamibacteraceae;Luteitalea;Luteitalea pratensis;Luteitalea pratensis DSM 100886 |
| Ga0586324 0378185 3 1853 | 0.00 | 2556971.36 | 0.00 | 0.00 | 2080600.00 | 0.00 | 3169200.00 | 1939800.00 | 0.00 | 1678600.00 | 1320000.00 | 3420000.00 | | hypothetical protein | Bacteria;Candidatus Aminicenantes;unclassified Candidatus Aminicenantes;unclassified Candidatus Aminicenantes;unclassified Candidatus Aminicenantes;unclassified Candidatus Aminicenantes;Aminicenantes bacterium SCGC AAA252-O03;Aminicenantes bacterium SCGC AAA252-O03 (contamination screened) |
| Ga0586324 0378948 1 1722 | 0.00 | 0.00 | 0.00 | 0.00 | 0.00 | 0.00 | 0.00 | 0.00 | 0.00 | 0.00 | 6899200.00 | 1732500.00 | | hypothetical protein | Bacteria;Acidobacteria;Acidobacteriia;Acidobacteriales;Acidobacteriaceae;unclassified Acidobacteriaceae;Acidobacteriaceae bacterium URHE0068;Acidobacteriaceae bacterium URHE0068 |
| Ga0586324 0381737 368 1612 | 0.00 | 5800400.00 | 0.00 | 0.00 | 8888900.00 | 0.00 | 0.00 | 0.00 | 10871981.19 | 0.00 | 11352000.00 | 1507500.00 | | predicted porin | Bacteria;Proteobacteria;Betaproteobacteria;Burkholderiales;Burkholderiaceae;Quisquiliibacterium;Quisquiliibacterium transsilvanicum;Quisquiliibacterium transsilvanicum DSM 29781 |
| Ga0586324 0382262 135 1370 | 0.00 | 0.00 | 0.00 | 0.00 | 0.00 | 0.00 | 0.00 | 860100.00 | 0.00 | 0.00 | 0.00 | 817500.00 | | TRAP-type mannitol/chloroaromatic compound transport system substrate-binding protein | Bacteria;Proteobacteria;Alphaproteobacteria;Hyphomicrobiales;Bradyrhizobiaceae;Bradyrhizobium;Bradyrhizobium lablabi;Bradyrhizobium lablabi GAS165 |
| Ga0586324 0382325 249 1844 | 0.00 | 4909600.00 | 0.00 | 0.00 | 0.00 | 0.00 | 0.00 | 0.00 | 13608000.00 | 0.00 | 15136000.00 | 0.00 | | ABC-type transport system substrate-binding protein | Bacteria;Chloroflexi;Chloroflexia;Chloroflexales;Oscillochloridaceae;Oscillochloris;Oscillochloris trichoides;Oscillochloris trichoides DG6 |
| Ga0586324 0383543 28 495 | 3770000.00 | 6358000.00 | 0.00 | 0.00 | 0.00 | 0.00 | 3739200.00 | 3251300.00 | 2940300.00 | 8547000.00 | 6916800.00 | 10125000.00 | | hypothetical protein | |
| Ga0586324 0384837 490 1839 | 5210000.00 | 0.00 | 0.00 | 0.00 | 0.00 | 0.00 | 0.00 | 0.00 | 0.00 | 0.00 | 0.00 | 0.00 | | hypothetical protein | Bacteria;Actinobacteria;Actinomycetia;Micrococcales;Intrasporangiaceae;Terrabacter;Terrabacter sp. 28;Terrabacter sp. 28 |
| Ga0586324 0389241 1 525 | 5520000.00 | 0.00 | 0.00 | 0.00 | 0.00 | 0.00 | 0.00 | 0.00 | 0.00 | 0.00 | 0.00 | 0.00 | | uncharacterized protein (DUF1015 family) | Bacteria;Acidobacteria;Blastocatellia;Blastocatellales;Pyrinomonadaceae;Pyrinomonas;Pyrinomonas methylaliphatogenes;Pyrinomonas methylaliphatogenes K22 |
| Ga0586324 0392444 318 1670 | 0.00 | 0.00 | 0.00 | 4740500.00 | 0.00 | 0.00 | 0.00 | 2226500.00 | 0.00 | 5043500.00 | 1786400.00 | 2197500.00 | | predicted CXXCH cytochrome family protein | Bacteria;Actinobacteria;Actinomycetia;Micrococcales;Intrasporangiaceae;Intrasporangium;Intrasporangium calvum;Intrasporangium calvum 7KIP, DSM 43043 |
| Ga0586324 0394323 195 1061 | 0.00 | 5807200.00 | 0.00 | 0.00 | 0.00 | 0.00 | 7136400.00 | 0.00 | 0.00 | 0.00 | 0.00 | 0.00 | | uncharacterized surface protein with fasciclin (FAS1) repeats/plastocyanin | Bacteria;Deinococcus-Thermus;Deinococci;Deinococcales;Deinococcaceae;Deinococcus;Deinococcus puniceus;Deinococcus puniceus DY1 |
| Ga0586324 0395179 264 1334 | 298668.00 | 0.00 | 0.00 | 0.00 | 0.00 | 0.00 | 0.00 | 0.00 | 0.00 | 0.00 | 0.00 | 0.00 | | excisionase family DNA binding protein | Bacteria;Chloroflexi;Caldilineae;Caldilineales;Caldilineaceae;Caldilinea;Caldilinea aerophila;Caldilinea aerophila STL-6-O1, DSM 14535 |
| Ga0586324 0397200 3 458 | 2850000.00 | 0.00 | 0.00 | 0.00 | 0.00 | 0.00 | 0.00 | 0.00 | 0.00 | 0.00 | 0.00 | 0.00 | | hypothetical protein | Bacteria;Proteobacteria;Deltaproteobacteria;Desulfobacterales;Desulfobulbaceae;Desulfofustis;Desulfofustis glycolicus;Desulfofustis glycolicus DSM 9705 |
| Ga0586324 0399994 3 638 | 2100000.00 | 0.00 | 0.00 | 0.00 | 0.00 | 0.00 | 0.00 | 0.00 | 0.00 | 0.00 | 0.00 | 1777500.00 | | hypothetical protein | Bacteria;Planctomycetes;Candidatus Brocadiae;Candidatus Brocadiales;Candidatus Brocadiaceae;Candidatus Brocadia;Candidatus Brocadia sinica;Candidatus Brocadia sinica JPN1 |
| Ga0586324 0401694 158 1804 | 0.00 | 0.00 | 0.00 | 0.00 | 1431700.00 | 1233431.36 | 0.00 | 866200.00 | 1204408.44 | 0.00 | 0.00 | 0.00 | | hypothetical protein | Bacteria;Firmicutes;Bacilli;Bacillales;Paenibacillaceae;Paenibacillus;Paenibacillus sp. 453mf;Paenibacillus sp. 453MF |
| Ga0586324 0402153 1 696 | 0.00 | 0.00 | 0.00 | 0.00 | 0.00 | 0.00 | 2024272.92 | 0.00 | 0.00 | 2086700.00 | 0.00 | 0.00 | | hypothetical protein | Bacteria;Proteobacteria;Deltaproteobacteria;Syntrophobacterales;Syntrophobacteraceae;Desulfoglaeba;Desulfoglaeba alkanexedens;Desulfoglaeba alkanexedens ALDC |
| Ga0586324 0403311 183 1802 | 0.00 | 7004000.00 | 7882000.00 | 0.00 | 5397200.00 | 0.00 | 0.00 | 16775000.00 | 17496000.00 | 17787000.00 | 14960000.00 | 517476.00 | | hypothetical protein | Bacteria;Acidobacteria;Acidobacteriia;Acidobacteriales;Acidobacteriaceae;Terriglobus;Terriglobus saanensis;Terriglobus saanensis SP1PR4, DSM 23119 |
| Ga0586324 0406334 720 1796 | 8840000.00 | 20264000.00 | 5014365.13 | 38836000.00 | 7343900.00 | 8995067.20 | 8436000.00 | 2513200.00 | 3134700.00 | 4643100.00 | 3150400.00 | 29550000.00 | | hypothetical protein | |
| Ga0586324 0408798 91 1449 | 0.00 | 0.00 | 0.00 | 0.00 | 2173300.00 | 0.00 | 0.00 | 0.00 | 0.00 | 0.00 | 0.00 | 0.00 | | crotonobetainyl-CoA:carnitine CoA-transferase CaiB-like acyl-CoA transferase | Archaea;Euryarchaeota;Archaeoglobi;Archaeoglobales;Archaeoglobaceae;Ferroglobus;Ferroglobus placidus;Ferroglobus placidus AEDII12DO, DSM 10642 |
| Ga0586324 0409773 3 1286 | 0.00 | 0.00 | 25144272.49 | 0.00 | 21012000.00 | 25805000.00 | 0.00 | 2269200.00 | 0.00 | 2741200.00 | 9328000.00 | 3592500.00 | | hypothetical protein | Bacteria;Proteobacteria;Deltaproteobacteria;Desulfovibrionales;Desulfovibrionaceae;Halodesulfovibrio;Halodesulfovibrio aestuarii;Halodesulfovibrio aestuarii aestuarii ATCC 29578 |
| Ga0586324 0410126 3 1790 | 2620000.00 | 5956800.00 | 7431600.00 | 0.00 | 0.00 | 12704000.00 | 0.00 | 0.00 | 0.00 | 0.00 | 0.00 | 0.00 | | hypothetical protein | Bacteria;Acidobacteria;Vicinamibacteria;unclassified Vicinamibacteria;Vicinamibacteraceae;Luteitalea;Luteitalea pratensis;Luteitalea pratensis DSM 100886 |
| Ga0586324 0410548 3 542 | 0.00 | 0.00 | 0.00 | 0.00 | 0.00 | 0.00 | 1875081.12 | 0.00 | 1733400.00 | 571811.24 | 0.00 | 922500.00 | | hypothetical protein | Bacteria;Proteobacteria;Gammaproteobacteria;Methylococcales;Methylococcaceae;Methylosarcina;Methylosarcina fibrata;Methylosarcina fibrata AML-C10 |
| Ga0586324 0410550 1 1095 | 0.00 | 0.00 | 0.00 | 0.00 | 0.00 | 0.00 | 0.00 | 0.00 | 654957.90 | 0.00 | 0.00 | 0.00 | | hypothetical protein | Bacteria;Balneolaeota;Balneolia;Balneolales;Balneolaceae;Aliifodinibius;Aliifodinibius sediminis;Aliifodinibius sediminis DSM 21194 |
| Ga0586324 0412931 452 1492 | 0.00 | 5644000.00 | 0.00 | 8170000.00 | 0.00 | 0.00 | 8892000.00 | 5886500.00 | 5386500.00 | 0.00 | 0.00 | 4417500.00 | | predicted porin | Bacteria;Proteobacteria;Betaproteobacteria;Burkholderiales;unclassified Burkholderiales;unclassified Burkholderiales;Burkholderiales bacterium JOSHI 001;Burkholderiales sp. JOSHI 001 |
| Ga0586324 0413577 1140 1760 | 0.00 | 0.00 | 0.00 | 0.00 | 0.00 | 0.00 | 0.00 | 0.00 | 0.00 | 1749600.00 | 0.00 | 0.00 | | hypothetical protein | Bacteria;Acidobacteria;Thermoanaerobaculia;Thermoanaerobaculales;Thermoanaerobaculaceae;Thermoanaerobaculum;Thermoanaerobaculum aquaticum;Thermoanaerobaculum aquaticum MP-01 |
| Ga0586324 0415975 379 1776 | 7340000.00 | 4488000.00 | 0.00 | 0.00 | 0.00 | 11314500.00 | 0.00 | 9516000.00 | 0.00 | 0.00 | 0.00 | 6465000.00 | | hypothetical protein | Bacteria;Verrucomicrobia;unclassified Verrucomicrobia;unclassified Verrucomicrobia;unclassified Verrucomicrobia;unclassified Verrucomicrobia;Verrucomicrobia bacterium LW23;Verrucomicrobia bacterium LW23 |
| Ga0586324 0416886 3 1052 | 0.00 | 0.00 | 0.00 | 0.00 | 6313900.00 | 0.00 | 0.00 | 1634800.00 | 0.00 | 0.00 | 2666400.00 | 2115000.00 | | hypothetical protein | Bacteria;Acidobacteria;Thermoanaerobaculia;Thermoanaerobaculales;Thermoanaerobaculaceae;Thermoanaerobaculum;Thermoanaerobaculum aquaticum;Thermoanaerobaculum aquaticum MP-01 |
| Ga0586324 0417258 220 1140 | 76600000.00 | 40800000.00 | 15201000.00 | 10355000.00 | 10207300.00 | 1224983.20 | 0.00 | 2427800.00 | 8829000.00 | 0.00 | 3414400.00 | 1965000.00 | | hypothetical protein | Bacteria;Proteobacteria;Gammaproteobacteria;Methylococcales;Methylococcaceae;Methylovulum;Methylovulum miyakonense;Methylovulum miyakonense HT12 |
| Ga0586324 0417845 889 1773 | 0.00 | 0.00 | 0.00 | 0.00 | 0.00 | 0.00 | 1914570.72 | 0.00 | 1814400.00 | 0.00 | 0.00 | 0.00 | | crotonobetainyl-CoA:carnitine CoA-transferase CaiB-like acyl-CoA transferase | Bacteria;Proteobacteria;Deltaproteobacteria;Syntrophobacterales;Syntrophobacteraceae;Thermodesulforhabdus;Thermodesulforhabdus norvegica;Thermodesulforhabdus norvegica DSM 9990 |
| Ga0586324 0418092 3 1772 | 0.00 | 890800.00 | 0.00 | 0.00 | 0.00 | 1055075.14 | 0.00 | 0.00 | 0.00 | 0.00 | 0.00 | 0.00 | | hypothetical protein | Bacteria;Acidobacteria;Vicinamibacteria;unclassified Vicinamibacteria;Vicinamibacteraceae;Luteitalea;Luteitalea pratensis;Luteitalea pratensis DSM 100886 |
| Ga0586324 0418393 3 1013 | 0.00 | 1190000.00 | 0.00 | 761882.90 | 1874600.00 | 1629073.62 | 1131249.36 | 1177300.00 | 1644300.00 | 3542000.00 | 0.00 | 1905000.00 | | hypothetical protein | Bacteria;Proteobacteria;Deltaproteobacteria;Syntrophobacterales;Syntrophaceae;Desulfomonile;Desulfomonile tiedjei;Desulfomonile tiedjei DCB-1, DSM 6799 |
| Ga0586324 0419448 3 1772 | 0.00 | 0.00 | 0.00 | 0.00 | 0.00 | 0.00 | 0.00 | 0.00 | 0.00 | 0.00 | 0.00 | 501364.50 | | hypothetical protein | Bacteria;Ignavibacteriae;Ignavibacteria;Ignavibacteriales;Melioribacteraceae;Melioribacter;Melioribacter roseus;Melioribacter roseus P3M |
| Ga0586324 0420644 1 1770 | 1860000.00 | 1101600.00 | 0.00 | 0.00 | 0.00 | 0.00 | 0.00 | 695400.00 | 0.00 | 0.00 | 0.00 | 0.00 | | hypothetical protein | Bacteria;Proteobacteria;Alphaproteobacteria;Sphingomonadales;Sphingomonadaceae;Sphingomonas;Sphingomonas sp. R1S6C;Sphingomonas sp. R1S6C |
| Ga0586324 0421182 1361 1768 | 0.00 | 4998000.00 | 0.00 | 0.00 | 0.00 | 0.00 | 0.00 | 0.00 | 0.00 | 0.00 | 0.00 | 0.00 | | hypothetical protein | Bacteria;Proteobacteria;Alphaproteobacteria;Rhodospirillales;Acetobacteraceae;Rhodopila;unclassified Rhodopila;Rhodopila sp. LVNP |
| Ga0586324 0421320 1242 1514 | 0.00 | 0.00 | 0.00 | 0.00 | 3718300.00 | 0.00 | 0.00 | 0.00 | 0.00 | 0.00 | 0.00 | 0.00 | | hypothetical protein | Bacteria;Proteobacteria;Deltaproteobacteria;Desulfobacterales;Desulfobacteraceae;Desulfatiglans;Desulfatiglans anilini;Desulfatiglans anilini DSM 4660 |
| Ga0586324 0430070 183 1751 | 0.00 | 0.00 | 0.00 | 0.00 | 0.00 | 0.00 | 0.00 | 0.00 | 0.00 | 0.00 | 0.00 | 3697500.00 | | hypothetical protein | Bacteria;Acidobacteria;Acidobacteriia;Acidobacteriales;Acidobacteriaceae;unclassified Acidobacteriaceae;Acidobacteriaceae bacterium KBS 89;Acidobacteriaceae bacterium KBS 89, DSM 24296 |
| Ga0586324 0433357 1 1746 | 0.00 | 0.00 | 0.00 | 0.00 | 0.00 | 0.00 | 0.00 | 0.00 | 29484000.00 | 0.00 | 0.00 | 0.00 | | pyruvate/2-oxoacid:ferredoxin oxidoreductase alpha subunit/Pyruvate/2-oxoacid:ferredoxin oxic | Bacteria;Proteobacteria;Deltaproteobacteria;Syntrophobacterales;Syntrophaceae;Desulfobacca;Desulfobacca acetoxidans;Desulfobacca acetoxidans ASRB2, DSM 11109 |
| Ga0586324 0435825 122 1087 | 0.00 | 0.00 | 0.00 | 0.00 | 0.00 | 0.00 | 6315600.00 | 0.00 | 0.00 | 0.00 | 0.00 | 0.00 | | VWFA-related protein | Bacteria;Acidobacteria;Acidobacteriia;Acidobacteriales;Acidobacteriaceae;Acidobacterium;Acidobacterium ailaaui;Acidobacterium ailaaui PMMR2 |
| Ga0586324 0436050 1 1515 | 3600000.00 | 9785200.00 | 5855200.00 | 7410000.00 | 7642600.00 | 4843400.00 | 2827200.00 | 9656300.00 | 9963000.00 | 6175400.00 | 7761600.00 | 3255000.00 | | hypothetical protein | Bacteria;Acidobacteria;Acidobacteriia;Bryobacterales;Bryobacteraceae;Bryobacter;Bryobacter aggregatus;Bryobacter aggregatus DSM 18758 |

| | | | | | | | | | | | | | | | |
|---|---|---|---|---|---|---|---|---|---|---|---|---|---|---|---|
| Ga0586324 0436056 152 961 | 0.00 | 0.00 | 0.00 | 0.00 | 0.00 | 0.00 | 0.00 | 0.00 | 0.00 | 48587000.00 | 25608000.00 | 24900000.00 | | cytochrome c553 | Bacteria;Proteobacteria;Betaproteobacteria;Nitrosomonadales;Gallionellaceae;Sideroxydans;Sideroxydans lithotrophicus;Sideroxydans lithotrophicus ES-1 |
| Ga0586324 0436530 2 847 | 0.00 | 0.00 | 0.00 | 0.00 | 0.00 | 0.00 | 0.00 | 0.00 | 0.00 | 156459.38 | 0.00 | 0.00 | | hypothetical protein | Bacteria;Actinobacteria;Actinomycetia;Propionibacteriales;Nocardioidaceae;Nocardioides;Nocardioides sp. Root122;Nocardioides sp. Root122 |
| Ga0586324 0436831 479 1465 | 0.00 | 1319200.00 | 0.00 | 0.00 | 0.00 | 0.00 | 0.00 | 0.00 | 0.00 | 0.00 | 0.00 | 0.00 | | ABC-type glycerol-3-phosphate transport system substrate-binding protein | Bacteria;Proteobacteria;Alphaproteobacteria;Hyphomicrobiales;Reyranellaceae;Reyranella;Reyranella sp. OAS946;Reyranella sp. OAS946 |
| Ga0586324 0438706 502 1641 | 1520000.00 | 6154000.00 | 0.00 | 0.00 | 0.00 | 0.00 | 0.00 | 0.00 | 0.00 | 0.00 | 0.00 | 0.00 | | TRAP-type mannitol/chloroaromatic compound transport system substrate-binding protein | Bacteria;Proteobacteria;Alphaproteobacteria;Hyphomicrobiales;Bradyrhizobiaceae;Bradyrhizobium;Bradyrhizobium sp. OAE819;Bradyrhizobium sp. OAE819 |
| Ga0586324 0440108 423 914 | 0.00 | 0.00 | 0.00 | 0.00 | 0.00 | 0.00 | 0.00 | 0.00 | 0.00 | 2117500.00 | 2534400.00 | 0.00 | | nucleotide-binding universal stress UspA family protein | Bacteria;Proteobacteria;Deltaproteobacteria;Syntrophobacterales;Syntrophaceae;Desulfobacca;Desulfobacca acetoxidans;Desulfobacca acetoxidans ASRB2, DSM 11109 |
| Ga0586324 0443053 1 1728 | 1420000.00 | 2203200.00 | 0.00 | 1320500.00 | 3986100.00 | 0.00 | 0.00 | 2604700.00 | 0.00 | 0.00 | 0.00 | 1395000.00 | | hypothetical protein | Bacteria;Verrucomicrobia;Opitutae;Opitutales;Opitutaceae;Opitutus;Opitutus terrae;Opitutus terrae PB90-1 |
| Ga0586324 0443863 1 1728 | 0.00 | 3386400.00 | 0.00 | 0.00 | 0.00 | 0.00 | 5928000.00 | 1464000.00 | 4147200.00 | 0.00 | 0.00 | 0.00 | | hypothetical protein | |
| Ga0586324 0445288 2 1186 | 4560000.00 | 4386000.00 | 0.00 | 3458000.00 | 2914900.00 | 0.00 | 0.00 | 0.00 | 1701000.00 | 3542000.00 | 2930400.00 | 0.00 | | predicted peptidase | Bacteria;Actinobacteria;Actinomycetia;Streptomycetales;Streptomycetaceae;Streptomyces;Streptomyces sp. PRh5;Streptomyces sp. PRh5 |
| Ga0586324 0446604 1 576 | 0.00 | 0.00 | 0.00 | 0.00 | 0.00 | 0.00 | 0.00 | 0.00 | 0.00 | 0.00 | 0.00 | 5235000.00 | | radical SAM superfamily enzyme YgiQ (UPF0313 family) | Bacteria;Proteobacteria;Deltaproteobacteria;Desulfarculales;Desulfarculaceae;Dethiosulfatarculus;Dethiosulfatarculus sandiegensis;Dethiosulfatarculus sandiegensis SPR |
| Ga0586324 0446753 1028 1723 | 0.00 | 0.00 | 0.00 | 0.00 | 1091800.00 | 0.00 | 0.00 | 0.00 | 0.00 | 0.00 | 0.00 | 0.00 | | hypothetical protein | |
| Ga0586324 0446899 2 1723 | 0.00 | 2216800.00 | 0.00 | 3040000.00 | 3450500.00 | 0.00 | 4468800.00 | 628300.00 | 2956500.00 | 2310000.00 | 0.00 | 2482500.00 | | PKD repeat protein | Bacteria;Actinobacteria;Actinomycetia;Micrococcales;Microbacteriaceae;Leifsonia;Leifsonia sp. Leaf325;Leifsonia sp. Leaf325 |
| Ga0586324 0447788 1193 1648 | 0.00 | 924800.00 | 505531.21 | 1149500.00 | 2774701.55 | 0.00 | 226247.14 | 0.00 | 0.00 | 0.00 | 0.00 | 0.00 | | hypothetical protein | |
| Ga0586324 0450067 3 1667 | 837188.00 | 0.00 | 0.00 | 0.00 | 0.00 | 0.00 | 0.00 | 0.00 | 0.00 | 0.00 | 0.00 | 1860000.00 | | hypothetical protein | Bacteria;Acidobacteria;Vicinamibacteria;unclassified Vicinamibacteria;Vicinamibacteraceae;Luteitalea;Luteitalea pratensis;Luteitalea pratensis DSM 100886 |
| Ga0586324 0451313 578 1714 | 52500000.00 | 12920000.00 | 47348300.00 | 66690000.00 | 74160000.00 | 10957200.00 | 20064000.00 | 29585000.00 | 25920000.00 | 36290100.00 | 136840000.00 | 28500000.00 | | hypothetical protein | Bacteria;Proteobacteria;Alphaproteobacteria;Hyphomicrobiales;Xanthobacteraceae;Pseudolabrys;Pseudolabrys taiwanensis;Pseudolabrys taiwanensis CC-BB4 |
| Ga0586324 0455379 3 1709 | 0.00 | 0.00 | 0.00 | 0.00 | 0.00 | 1938646.28 | 0.00 | 2714500.00 | 0.00 | 1332100.00 | 3009600.00 | 2265000.00 | | outer membrane protein OmpA-like peptidoglycan-associated protein | Bacteria;Bacteroidetes;Bacteroidia;Bacteroidales;Lentimicrobiaceae;Lentimicrobium;Lentimicrobium saccharophilum;Bacteroidales bacterium TBC1 |
| Ga0586324 0456990 1 1704 | 0.00 | 0.00 | 0.00 | 0.00 | 0.00 | 4525800.00 | 0.00 | 0.00 | 0.00 | 0.00 | 0.00 | 0.00 | | hypothetical protein | Bacteria;Proteobacteria;Betaproteobacteria;unclassified Betaproteobacteria;unclassified Betaproteobacteria;unclassified Betaproteobacteria;Betaproteobacteria bacterium GR16-43;Betaproteobacteria bacterium GR16-43 |
| Ga0586324 0457635 1 1530 | 0.00 | 0.00 | 0.00 | 10260000.00 | 0.00 | 0.00 | 0.00 | 0.00 | 0.00 | 0.00 | 0.00 | 0.00 | | hypothetical protein | Bacteria;Proteobacteria;Alphaproteobacteria;Hyphomicrobiales;Bradyrhizobiaceae;Bradyrhizobium;Bradyrhizobium elkanii;Bradyrhizobium elkanii USDA 76 |
| Ga0586324 0459983 895 1701 | 0.00 | 0.00 | 0.00 | 0.00 | 0.00 | 0.00 | 0.00 | 0.00 | 291331.08 | 0.00 | 0.00 | 0.00 | | NAD(P)-dependent dehydrogenase (short-subunit alcohol dehydrogenase family) | Bacteria;Proteobacteria;Alphaproteobacteria;Hyphomicrobiales;unclassified Hyphomicrobiales;Pseudorhodoplanes;Pseudorhodoplanes sinuspersici;Pseudorhodoplanes sinuspersici RIPI110 |
| Ga0586324 0460731 3 773 | 0.00 | 0.00 | 4964916.84 | 8046500.00 | 0.00 | 5716800.00 | 0.00 | 4166300.00 | 0.00 | 0.00 | 0.00 | 0.00 | | hypothetical protein | |
| Ga0586324 0461948 127 1698 | 0.00 | 625912.12 | 0.00 | 0.00 | 0.00 | 0.00 | 0.00 | 0.00 | 0.00 | 0.00 | 0.00 | 0.00 | | hypothetical protein | Bacteria;Proteobacteria;Betaproteobacteria;unclassified Betaproteobacteria;unclassified Betaproteobacteria;unclassified Betaproteobacteria;Betaproteobacteria bacterium GR16-43;Betaproteobacteria bacterium GR16-43 |
| Ga0586324 0462240 3 1697 | 0.00 | 0.00 | 2436979.28 | 0.00 | 0.00 | 0.00 | 0.00 | 0.00 | 0.00 | 962500.00 | 0.00 | 0.00 | | uncharacterized repeat protein (TIGR01451 family) | Bacteria;Actinobacteria;Actinomycetia;Micrococcales;Intrasporangiaceae;Humibacillus;Humibacillus xanthopallidus;Humibacillus xanthopallidus SLBN-155 |
| Ga0586324 0462454 842 1696 | 5220000.00 | 2189600.00 | 2611593.73 | 23180000.00 | 16583000.00 | 1743663.70 | 0.00 | 17202000.00 | 1377000.00 | 592525.01 | 18128000.00 | 12675000.00 | | hypothetical protein | Bacteria;Verrucomicrobia;Verrucomicrobiae;Verrucomicrobiales;Verrucomicrobia subdivision 3;Pedosphaera;Pedosphaera parvula;Pedosphaera parvula Ellin514 |
| Ga0586324 0462920 3 1208 | 41010000.00 | 46022400.00 | 11203700.00 | 35519611.75 | 23576700.00 | 0.00 | 8846400.00 | 38341194.98 | 35302231.62 | 56125300.00 | 59118400.00 | 100809164.25 | | hypothetical protein | Bacteria;Proteobacteria;Betaproteobacteria;Nitrosomonadales;Gallionellaceae;Gallionella;Gallionella capsiferriformans;Gallionella capsiferriformans ES-2 |
| Ga0586324 0463858 985 1695 | 0.00 | 0.00 | 0.00 | 0.00 | 0.00 | 0.00 | 0.00 | 2562000.00 | 0.00 | 0.00 | 0.00 | 0.00 | | uncharacterized protein YgfA (UPF0365 family) | Bacteria;Spirochaetes;Spirochaetia;Spirochaetales;Spirochaetaceae;Spirochaeta;Spirochaeta isovalerica;Spirochaeta isovalerica DSM 2461 |
| Ga0586324 0465592 481 912 | 29500000.00 | 1421200.00 | 0.00 | 0.00 | 0.00 | 0.00 | 0.00 | 0.00 | 0.00 | 0.00 | 0.00 | 0.00 | | nucleotide-binding universal stress UspA family protein | Archaea;Euryarchaeota;Methanomicrobia;Methanosarcinales;Methanotrichaceae;Methanothrix;Methanothrix soehngenii;Methanothrix soehngenii GP-6 |
| Ga0586324 0466835 2 1690 | 0.00 | 0.00 | 893233.28 | 0.00 | 0.00 | 0.00 | 0.00 | 0.00 | 0.00 | 0.00 | 0.00 | 0.00 | | hypothetical protein | Bacteria;Bacteroidetes;unclassified Bacteroidetes;Bacteroidetes Order II. Incertae sedis;Rhodothermaceae;unclassified Rhodothermaceae;Rhodothermaceae bacterium RA;Halothermobacillus malaysiensis RA |
| Ga0586324 0467224 1 1392 | 1490000.00 | 0.00 | 4770270.85 | 0.00 | 0.00 | 0.00 | 0.00 | 0.00 | 0.00 | 0.00 | 0.00 | 0.00 | | predicted ATP-dependent protease | Bacteria;Proteobacteria;Deltaproteobacteria;Syntrophobacterales;Syntrophobacteraceae;Syntrophobacter;Syntrophobacter fumaroxidans;Syntrophobacter fumaroxidans MPOB |
| Ga0586324 0468321 1 1686 | 0.00 | 1156000.00 | 984478.69 | 0.00 | 1452300.00 | 1135717.75 | 238939.44 | 945500.00 | 931500.00 | 993300.00 | 932800.00 | 483494.25 | | hypothetical protein | Bacteria;Rhodothermaeota;Rhodothermia;Rhodothermales;Rubricoccaceae;Rubricoccus;Rubricoccus marinus;Rubricoccus marinus SG-29 |
| Ga0586324 0468560 492 1541 | 0.00 | 0.00 | 0.00 | 0.00 | 0.00 | 0.00 | 0.00 | 0.00 | 0.00 | 0.00 | 0.00 | 325862.25 | | LDH2 family malate/lactate/ureidoglycolate dehydrogenase | Bacteria;Proteobacteria;Alphaproteobacteria;Rhodospirillales;Acetobacteraceae;Rhodopila;Rhodopila globiformis;Rhodopila globiformis DSM 161 |
| Ga0586324 0470825 441 1682 | 0.00 | 0.00 | 0.00 | 0.00 | 0.00 | 0.00 | 0.00 | 0.00 | 0.00 | 0.00 | 3599200.00 | 0.00 | | hypothetical protein | Bacteria;Proteobacteria;Betaproteobacteria;Nitrosomonadales;Sulfuricellaceae;Sulfurirhabdus;Sulfurirhabdus autotrophica;Sulfurirhabdus autotrophica DSM 100309 |
| Ga0586324 0475905 3 1676 | 0.00 | 0.00 | 0.00 | 0.00 | 0.00 | 0.00 | 0.00 | 0.00 | 891000.00 | 0.00 | 0.00 | 0.00 | | ABC-type bacteriocin/lantibiotic exporter with double-glycine peptidase domain | Bacteria;Actinobacteria;Actinomycetia;Streptomycetales;Streptomycetaceae;Kitasatospora;Kitasatospora sp. MBT63;Kitasatospora sp. MBT63 |
| Ga0586324 0476544 236 1675 | 0.00 | 1822400.00 | 0.00 | 0.00 | 1606800.00 | 905755.50 | 0.00 | 0.00 | 1012500.00 | 4211900.00 | 3801600.00 | 2932500.00 | | hypothetical protein | Bacteria;Chloroflexi;Anaerolineae;Anaerolineales;Anaerolineaceae;Anaerolinea;Anaerolinea thermophila;Anaerolinea thermophila UNI-1 |
| Ga0586324 0477008 720 1673 | 0.00 | 0.00 | 0.00 | 5263000.00 | 0.00 | 0.00 | 0.00 | 10675000.00 | 0.00 | 0.00 | 0.00 | 0.00 | | uncharacterized 2Fe-2S/4Fe-4S cluster protein (DUF4445 family) | Bacteria;Proteobacteria;Deltaproteobacteria;Syntrophobacterales;Syntrophaceae;Desulfomonile;Desulfomonile tiedjei;Desulfomonile tiedjei DCB-1, DSM 6799 |
| Ga0586324 0477957 17 859 | 0.00 | 3651600.00 | 0.00 | 4113500.00 | 10403000.00 | 0.00 | 10123200.00 | 0.00 | 16848000.00 | 0.00 | 0.00 | 16200000.00 | | cytochrome c peroxidase | Bacteria;Proteobacteria;Deltaproteobacteria;Syntrophobacterales;Syntrophaceae;Desulfomonile;Desulfomonile tiedjei;Desulfomonile tiedjei DCB-1, DSM 6799 |
| Ga0586324 0478838 2 1669 | 600331.00 | 0.00 | 0.00 | 0.00 | 0.00 | 0.00 | 0.00 | 0.00 | 0.00 | 0.00 | 0.00 | 0.00 | | hypothetical protein | Bacteria;Acidobacteria;Acidobacteriia;Acidobacteriales;Acidobacteriaceae;Candidatus Koribacter;Candidatus Koribacter versatilis;Candidatus Koribacter versatilis Ellin345 |
| Ga0586324 0479015 524 1669 | 0.00 | 3617600.00 | 8332400.00 | 1577000.00 | 2348400.00 | 3216132.73 | 5768400.00 | 3812500.00 | 4252500.00 | 3080000.00 | 2191200.00 | 5190000.00 | | ABC-type transport system substrate-binding protein | Bacteria;Proteobacteria;Deltaproteobacteria;Syntrophobacterales;Syntrophobacteraceae;Desulfacinum;Desulfacinum infernum;Desulfacinum infernum DSM 9756 |
| Ga0586324 0480922 200 622 | 0.00 | 26044000.00 | 0.00 | 0.00 | 0.00 | 0.00 | 0.00 | 0.00 | 0.00 | 0.00 | 0.00 | 0.00 | | hypothetical protein | |
| Ga0586324 0481767 121 1665 | 0.00 | 0.00 | 0.00 | 0.00 | 769793.16 | 0.00 | 0.00 | 0.00 | 0.00 | 0.00 | 0.00 | 0.00 | | methyl-accepting chemotaxis protein | Bacteria;Caldiserica;unclassified Caldiserica;unclassified Caldiserica;unclassified Caldiserica;unclassified Caldiserica;Caldiserica bacterium JGI 0000059-M03;Caldiserica bacterium JGI 0000059-M03 (contamination screened) |
| Ga0586324 0482689 776 1663 | 0.00 | 2176000.00 | 0.00 | 500688.00 | 0.00 | 1561575.68 | 0.00 | 207031.56 | 1150200.00 | 0.00 | 0.00 | 0.00 | | TRAP-type mannitol/chloroaromatic compound transport system substrate-binding protein | Bacteria;Proteobacteria;Alphaproteobacteria;Hyphomicrobiales;Xanthobacteraceae;Pseudolabrys;Pseudolabrys sp. Root1462;Pseudolabrys sp. Root1462 |
| Ga0586324 0483823 3 929 | 0.00 | 0.00 | 1158811.64 | 0.00 | 0.00 | 0.00 | 0.00 | 0.00 | 0.00 | 0.00 | 0.00 | 0.00 | | hypothetical protein | Bacteria;Proteobacteria;Gammaproteobacteria;unclassified Gammaproteobacteria;Candidatus Competibacteraceae;Candidatus Competibacter;Candidatus Competibacter denitrificans;Candidatus Competibacter denitrificans Run A D11 |
| Ga0586324 0484262 538 1092 | 0.00 | 0.00 | 0.00 | 3040000.00 | 0.00 | 0.00 | 0.00 | 0.00 | 0.00 | 0.00 | 0.00 | 0.00 | | L-amino acid N-acyltransferase YncA | Bacteria;Candidatus Calescamantes;unclassified Candidatus Calescamantes;unclassified Candidatus Calescamantes;unclassified Candidatus Calescamantes;unclassified Candidatus Calescamantes;Calescamantes bacterium SCGC AAA471-M6;Calescamantes bacterium SCGC AAA471-M6 (contamination screened) |
| Ga0586324 0484747 644 1660 | 0.00 | 0.00 | 0.00 | 0.00 | 0.00 | 1566776.38 | 0.00 | 0.00 | 0.00 | 0.00 | 0.00 | 0.00 | | FtsP/CotA-like multicopper oxidase with cupredoxin domain | Bacteria;Actinobacteria;Actinomycetia;Micrococcales;Intrasporangiaceae;Humibacillus;Humibacillus xanthopallidus;Humibacillus xanthopallidus DSM 21776 |
| Ga0586324 0485533 3 1661 | 0.00 | 0.00 | 0.00 | 0.00 | 0.00 | 0.00 | 0.00 | 580476.00 | 0.00 | 0.00 | 0.00 | 0.00 | | N-methylhydantoinase A/oxoprolinase/acetone carboxylase beta subunit | Bacteria;Proteobacteria;Deltaproteobacteria;Desulfobacterales;Desulfobacteraceae;Desulfatiglans;Desulfatiglans anilini;Desulfatiglans anilini DSM 4660 |
| Ga0586324 0487306 1 1656 | 0.00 | 0.00 | 1419413.08 | 0.00 | 6386000.00 | 3503703.65 | 3100800.00 | 3800300.00 | 3183300.00 | 1593900.00 | 3273600.00 | 0.00 | | hypothetical protein | Bacteria;Acidobacteria;Vicinamibacteria;unclassified Vicinamibacteria;Vicinamibacteraceae;Luteitalea;Luteitalea pratensis;Luteitalea pratensis DSM 100886 |
| Ga0586324 0488071 776 1657 | 0.00 | 434576.44 | 0.00 | 0.00 | 0.00 | 0.00 | 0.00 | 0.00 | 0.00 | 0.00 | 0.00 | 0.00 | | hypothetical protein | Bacteria;Bacteroidetes;Bacteroidia;Bacteroidales;Bacteroidaceae;Bacteroides;Bacteroides ovatus;Bacteroides ovatus CL02T12C04 |
| Ga0586324 0489048 91 1656 | 0.00 | 0.00 | 0.00 | 0.00 | 0.00 | 0.00 | 0.00 | 0.00 | 0.00 | 0.00 | 0.00 | 22725000.00 | | cytochrome c peroxidase | Bacteria;Proteobacteria;Gammaproteobacteria;Methylococcales;Methylococcaceae;Methylobacter;Methylobacter tundripaludum;Methylobacter tundripaludum SV96 |
| Ga0586324 0489420 1 1653 | 11939339.00 | 0.00 | 0.00 | 4189500.00 | 0.00 | 0.00 | 0.00 | 0.00 | 0.00 | 0.00 | 0.00 | 0.00 | | TonB-linked SusC/RagA family outer membrane protein | Bacteria;Bacteroidetes;Flavobacteriia;Flavobacteriales;Flavobacteriaceae;Flavobacterium;Flavobacterium weaverense;Flavobacterium weaverense DSM 19727 |
| Ga0586324 0490050 300 1652 | 728800.00 | 6976800.00 | 3038370.25 | 12065000.00 | 12916200.00 | 35293300.00 | 0.00 | 23411800.00 | 21287599.47 | 0.00 | 650900.80 | 183702.00 | | Flp pilus assembly protein TadD | Bacteria;Bacteroidetes;Bacteroidia;Marinilabiliales;Marinifilaceae;Ancylomarina;Ancylomarina subtilis;Ancylomarina subtilis DSM 28825 |
| Ga0586324 0492625 213 1649 | 1150000.00 | 0.00 | 0.00 | 0.00 | 0.00 | 0.00 | 0.00 | 0.00 | 0.00 | 0.00 | 0.00 | 0.00 | | outer membrane protein assembly factor BamB | Bacteria;Planctomycetes;Planctomycetia;Gemmatales;Gemmataceae;Gemmata;Gemmata obscuriglobus;Gemmata obscuriglobus UQM 2246 |
| Ga0586324 0493847 2 1648 | 0.00 | 0.00 | 0.00 | 0.00 | 722470.84 | 0.00 | 0.00 | 0.00 | 0.00 | 0.00 | 0.00 | 0.00 | | hypothetical protein | Bacteria;Acidobacteria;Acidobacteriia;Bryobacterales;Solibacteraceae;Candidatus Solibacter;Candidatus Solibacter usitatus;Candidatus Solibacter usitatus Ellin6076 |
| Ga0586324 0496316 1 1644 | 0.00 | 653694.20 | 0.00 | 0.00 | 648396.33 | 0.00 | 0.00 | 1049200.00 | 818100.00 | 0.00 | 0.00 | 0.00 | | hypothetical protein | Bacteria;Candidatus Aminicenantes;unclassified Candidatus Aminicenantes;unclassified Candidatus Aminicenantes;unclassified Candidatus Aminicenantes;unclassified Candidatus Aminicenantes;Aminicenantes bacterium SCGC AAA255-E05;Aminicenantes bacterium SCGC AAA255-E05 (contamination screened) |
| Ga0586324 0496888 2 1642 | 0.00 | 6310400.00 | 12160800.00 | 6621500.00 | 9723200.00 | 0.00 | 6247200.00 | 4892200.00 | 4382100.00 | 7700000.00 | 8976000.00 | 0.00 | | hypothetical protein | Bacteria;Acidobacteria;Acidobacteriia;Acidobacteriales;Acidobacteriaceae;unclassified Acidobacteriaceae;Acidobacteriaceae bacterium KBS 96;Acidobacteriaceae bacterium KBS 96, DSM 24297 |
| Ga0586324 0500324 1 1638 | 0.00 | 0.00 | 0.00 | 0.00 | 0.00 | 0.00 | 0.00 | 0.00 | 0.00 | 3041500.00 | 1416800.00 | 0.00 | | hypothetical protein | Bacteria;Proteobacteria;Gammaproteobacteria;Methylococcales;Methylococcaceae;Methyloterricola;Methyloterricola oryzae;Methyloterricola oryzae 73a |
| Ga0586324 0501285 2 1198 | 0.00 | 0.00 | 0.00 | 0.00 | 0.00 | 0.00 | 0.00 | 0.00 | 1992600.00 | 0.00 | 0.00 | 0.00 | | outer membrane protein OmpA-like peptidoglycan-associated protein | Bacteria;Bacteroidetes;Bacteroidia;Bacteroidales;Paludibacteraceae;Paludibacter;Paludibacter propionicigenes;Paludibacter propionicigenes WB4, DSM 17365 |
| Ga0586324 0501574 177 1637 | 0.00 | 0.00 | 0.00 | 0.00 | 0.00 | 945947.78 | 0.00 | 0.00 | 0.00 | 0.00 | 0.00 | 0.00 | | heterodisulfide reductase subunit A-like polyferredoxin | Bacteria;Proteobacteria;Deltaproteobacteria;Desulfobacterales;Desulfobacteraceae;Desulfatirhabdium;Desulfatirhabdium butyrativorans;Desulfatirhabdium butyrativorans DSM 18734 |
| Ga0586324 0501577 832 1611 | 0.00 | 884000.00 | 0.00 | 0.00 | 0.00 | 7940000.00 | 0.00 | 1543300.00 | 1101600.00 | 2525600.00 | 0.00 | 0.00 | | CBS domain-containing protein | Archaea;Euryarchaeota;Methanomicrobia;Methanosarcinales;Methanotrichaceae;Methanothrix;Methanothrix soehngenii;Methanothrix soehngenii GP-6 |
| Ga0586324 0501784 3 1637 | 0.00 | 952000.00 | 0.00 | 1729000.00 | 0.00 | 0.00 | 0.00 | 0.00 | 0.00 | 0.00 | 0.00 | 20025000.00 | | outer membrane receptor protein involved in Fe transport | Bacteria;Acidobacteria;Blastocatellia;Blastocatellales;Pyrinomonadaceae;Pyrinomonas;Pyrinomonas methylaliphatogenes;Pyrinomonas methylaliphatogenes K22 |
| Ga0586324 0501861 2 1636 | 0.00 | 754800.00 | 0.00 | 1909500.00 | 0.00 | 0.00 | 0.00 | 0.00 | 0.00 | 0.00 | 0.00 | 0.00 | | FtsP/CotA-like multicopper oxidase with cupredoxin domain | Bacteria;Proteobacteria;Deltaproteobacteria;Desulfuromonadales;Geobacteraceae;Geobacter;Geobacter metallireducens;Geobacter metallireducens RCH3 |
| Ga0586324 0502058 1 1635 | 0.00 | 7820000.00 | 0.00 | 0.00 | 0.00 | 0.00 | 0.00 | 0.00 | 0.00 | 0.00 | 0.00 | 0.00 | | hypothetical protein | Bacteria;Acidobacteria;Acidobacteriia;Acidobacteriales;Acidobacteriaceae;Acidobacterium;Acidobacterium capsulatum;Acidobacterium capsulatum ATCC 51196 |
| Ga0586324 0502402 1 252 | 0.00 | 1863200.00 | 2919470.28 | 0.00 | 1699500.00 | 1583736.22 | 1676249.16 | 1598200.00 | 1871100.00 | 3688300.00 | 4030400.00 | 5422500.00 | | hypothetical protein | Bacteria;Proteobacteria;Alphaproteobacteria;Hyphomicrobiales;Hyphomicrobiaceae;Hyphomicrobium;unclassified Hyphomicrobium;Hyphomicrobium sp. VS |
| Ga0586324 0502436 2 1297 | 0.00 | 0.00 | 0.00 | 0.00 | 0.00 | 0.00 | 0.00 | 0.00 | 939600.00 | 0.00 | 0.00 | 0.00 | | ABC-type transport system substrate-binding protein | Bacteria;Proteobacteria;Betaproteobacteria;Burkholderiales;Oxalobacteraceae;Oxalobacter;Oxalobacter formigenes;Oxalobacter formigenes OXCC13 |
| Ga0586324 0507971 1 1578 | 0.00 | 0.00 | 0.00 | 0.00 | 0.00 | 0.00 | 0.00 | 0.00 | 0.00 | 0.00 | 0.00 | 4852500.00 | | hypothetical protein | Bacteria;Proteobacteria;Alphaproteobacteria;Hyphomicrobiales;Bradyrhizobiaceae;Bradyrhizobium;Bradyrhizobium valentinum;Bradyrhizobium valentinum LmjM6 |
| Ga0586324 0510685 3 1625 | 521674.00 | 0.00 | 0.00 | 0.00 | 0.00 | 0.00 | 0.00 | 0.00 | 0.00 | 0.00 | 0.00 | 201213.75 | | hypothetical protein | Bacteria;Chloroflexi;Ardenticatenia;Ardenticatenales;Ardenticatenaceae;Candidatus Promineofilum;Candidatus Promineofilum breve;Candidatus Promineofilum breve Cfx-K |
| Ga0586324 0511939 311 1621 | 0.00 | 1319200.00 | 0.00 | 0.00 | 0.00 | 0.00 | 0.00 | 0.00 | 0.00 | 0.00 | 0.00 | 0.00 | | hypothetical protein | Bacteria;Acidobacteria;Acidobacteriia;Acidobacteriales;Acidobacteriaceae;Edaphobacter;Edaphobacter modestus;Edaphobacter modestus DSM 18101 |
| Ga0586324 0513090 2 409 | 9240000.00 | 10744000.00 | 10415500.00 | 11020000.00 | 22866000.00 | 5081600.00 | 12357600.00 | 17366700.00 | 11340000.00 | 27412000.00 | 27632000.00 | 23872500.00 | | hypothetical protein | Bacteria;Proteobacteria;Deltaproteobacteria;Desulfobacterales;Desulfobacteraceae;Desulfococcus;Desulfococcus oleovorans;Desulfococcus oleovorans Hxd3 |
| Ga0586324 0513275 1201 1539 | 0.00 | 0.00 | 0.00 | 0.00 | 0.00 | 8654600.00 | 0.00 | 0.00 | 0.00 | 0.00 | 0.00 | 0.00 | | nitrogen regulatory protein PII | Bacteria;Proteobacteria;Alphaproteobacteria;Rhodospirillales;unclassified Rhodospirillales;unclassified Rhodospirillales;Rhodospirillales bacterium URHD0017;Rhodospirillales bacterium URHD0017 |
| Ga0586324 0514206 746 1618 | 0.00 | 0.00 | 0.00 | 0.00 | 0.00 | 0.00 | 0.00 | 0.00 | 2875500.00 | 0.00 | 0.00 | 0.00 | | hypothetical protein | Bacteria;Proteobacteria;Betaproteobacteria;Rhodocyclales;Zoogloeaceae;Thauera;Thauera chlorobenzoica;Thauera chlorobenzoica 3CB-1 |
| Ga0586324 0517271 688 1452 | 0.00 | 0.00 | 0.00 | 0.00 | 0.00 | 0.00 | 0.00 | 0.00 | 0.00 | 0.00 | 0.00 | 182001.00 | | hypothetical protein | Bacteria;Proteobacteria;Deltaproteobacteria;Desulfovibrionales;Desulfovibrionaceae;Desulfocurvibacter;Desulfocurvibacter africanus;Desulfovibrio africanus DSM 2603 |
| Ga0586324 0517395 347 967 | 0.00 | 0.00 | 0.00 | 0.00 | 0.00 | 0.00 | 0.00 | 0.00 | 1765800.00 | 0.00 | 0.00 | 0.00 | | hypothetical protein | Bacteria;Acidobacteria;Thermoanaerobaculia;Thermoanaerobaculales;Thermoanaerobaculaceae;Thermoanaerobaculum;Thermoanaerobaculum aquaticum;Thermoanaerobaculum aquaticum MP-01 |
| Ga0586324 0518064 3 1613 | 0.00 | 0.00 | 0.00 | 0.00 | 1771600.00 | 0.00 | 0.00 | 599196.90 | 374117.13 | 1370600.00 | 1311200.00 | 587360.25 | | hypothetical protein | Bacteria;Acidobacteria;Thermoanaerobaculia;Thermoanaerobaculales;Thermoanaerobaculaceae;Thermoanaerobaculum;Thermoanaerobaculum aquaticum;Thermoanaerobaculum aquaticum MP-01 |
| Ga0586324 0521023 2 1609 | 0.00 | 0.00 | 0.00 | 0.00 | 0.00 | 0.00 | 0.00 | 0.00 | 0.00 | 1894200.00 | 0.00 | 0.00 | | hypothetical protein | Bacteria;Firmicutes;Bacilli;Bacillales;Paenibacillaceae;Paenibacillus;Paenibacillus sophorae;Paenibacillus sophorae S27 |
| Ga0586324 0521281 3 1484 | 0.00 | 0.00 | 0.00 | 0.00 | 0.00 | 877687.60 | 0.00 | 0.00 | 0.00 | 0.00 | 0.00 | 0.00 | | hypothetical protein | Bacteria;Actinobacteria;Actinomycetia;Jiangellales;Jiangellaceae;Jiangella;Jiangella rhizosphaerae;Jiangella rhizosphaerae NEAU-YY265 |
| Ga0586324 0525804 3 1055 | 0.00 | 0.00 | 0.00 | 3743000.00 | 0.00 | 0.00 | 0.00 | 0.00 | 0.00 | 0.00 | 0.00 | 0.00 | | crotonobetainyl-CoA:carnitine CoA-transferase CaiB-like acyl-CoA transferase | Bacteria;Proteobacteria;Deltaproteobacteria;Syntrophobacterales;Syntrophaceae;Desulfobacca;Desulfobacca acetoxidans;Desulfobacca acetoxidans ASRB2, DSM 11109 |
| Ga0586324 0526838 1 1062 | 0.00 | 0.00 | 0.00 | 1244500.00 | 0.00 | 0.00 | 0.00 | 0.00 | 0.00 | 0.00 | 0.00 | 0.00 | | ABC-type transport system substrate-binding protein | Bacteria;Firmicutes;Bacilli;Bacillales;Paenibacillaceae;Paenibacillus;Paenibacillus fonticola;Paenibacillus fonticola DSM 21315 |
| Ga0586324 0527464 3 1601 | 0.00 | 1217200.00 | 0.00 | 0.00 | 0.00 | 0.00 | 0.00 | 0.00 | 0.00 | 0.00 | 0.00 | 0.00 | | hypothetical protein | Bacteria;Bacteroidetes;Flavobacteriia;Flavobacteriales;Flavobacteriaceae;Flavobacterium;Flavobacterium fluvii;Flavobacterium fluvii DSM 19978 |
| Ga0586324 0528358 1339 1599 | 0.00 | 0.00 | 0.00 | 0.00 | 0.00 | 0.00 | 0.00 | 0.00 | 0.00 | 0.00 | 289497.12 | 0.00 | | osmotically-inducible protein OsmY | Bacteria;Proteobacteria;Betaproteobacteria;Nitrosomonadales;Sterolibacteriaceae;Sterolibacterium;Sterolibacterium denitrificans;Sterolibacterium denitrificans Chol |
| Ga0586324 0528865 1 822 | 0.00 | 0.00 | 0.00 | 0.00 | 0.00 | 0.00 | 0.00 | 0.00 | 0.00 | 625263.10 | 0.00 | 0.00 | | hypothetical protein | Bacteria;Proteobacteria;Betaproteobacteria;Burkholderiales;Oxalobacteraceae;Collimonas;Collimonas sp. OK607;Collimonas sp. OK607 |
| Ga0586324 0530916 853 1596 | 2180000.00 | 0.00 | 0.00 | 0.00 | 0.00 | 0.00 | 0.00 | 0.00 | 0.00 | 0.00 | 0.00 | 0.00 | | signal transduction protein with GAF and PtsI domain | Bacteria;Proteobacteria;Deltaproteobacteria;Syntrophobacterales;Syntrophaceae;Desulfobacca;Desulfobacca acetoxidans;Desulfobacca acetoxidans ASRB2, DSM 11109 |
| Ga0586324 0531432 263 1414 | 0.00 | 0.00 | 0.00 | 0.00 | 0.00 | 0.00 | 0.00 | 0.00 | 3231900.00 | 0.00 | 0.00 | 0.00 | | TRAP-type mannitol/chloroaromatic compound transport system substrate-binding protein | Bacteria;Proteobacteria;Betaproteobacteria;Burkholderiales;Comamonadaceae;Rhodoferax;Rhodoferax antarcticus;Rhodoferax antarcticus DSM 24876 |
| Ga0586324 0533600 43 1593 | 0.00 | 0.00 | 0.00 | 1320500.00 | 0.00 | 0.00 | 0.00 | 0.00 | 0.00 | 0.00 | 0.00 | 0.00 | | TonB-dependent SusC/RagA subfamily outer membrane receptor | Bacteria;Bacteroidetes;Bacteroidia;Bacteroidales;Paludibacteraceae;Paludibacter;Paludibacter propionicigenes;Paludibacter propionicigenes WB4, DSM 17365 |
| Ga0586324 0534969 2 1084 | 0.00 | 0.00 | 28206300.00 | 22990000.00 | 0.00 | 0.00 | 22572000.00 | 0.00 | 16038000.00 | 23793000.00 | 18832000.00 | 17775000.00 | | hypothetical protein | Bacteria;Proteobacteria;Deltaproteobacteria;Syntrophobacterales;Syntrophaceae;Desulfobacca;Desulfobacca acetoxidans;Desulfobacca acetoxidans ASRB2, DSM 11109 |
| Ga0586324 0536776 1 831 | 0.00 | 3080400.00 | 10021400.00 | 4759500.00 | 6581700.00 | 6788700.00 | 4149600.00 | 3965000.00 | 4017600.00 | 4981900.00 | 4796000.00 | 4717500.00 | | predicted porin | Bacteria;Proteobacteria;Betaproteobacteria;unclassified Betaproteobacteria;unclassified Betaproteobacteria;unclassified Betaproteobacteria;uncultured beta proteobacterium;unclassified betaproteobacterium ENR4 |
| Ga0586324 0537210 1 1419 | 9140000.00 | 0.00 | 0.00 | 0.00 | 0.00 | 0.00 | 0.00 | 0.00 | 0.00 | 0.00 | 0.00 | 5887500.00 | | hypothetical protein | Bacteria;Nitrospirae;Thermodesulfovibrionia;Thermodesulfovibrionales;Thermodesulfovibrionaceae;Thermodesulfovibrio;Thermodesulfovibrio islandicus;Thermodesulfovibrio islandicus DSM 12570 |
| Ga0586324 0538537 2 922 | 22000000.00 | 25833200.00 | 25841700.00 | 64505000.00 | 36565000.00 | 58994200.00 | 41541600.00 | 119932100.00 | 27102600.00 | 39039000.00 | 39688000.00 | 37665000.00 | | predicted porin | Bacteria;Proteobacteria;Gammaproteobacteria;Chromatiales;Ectothiorhodospiraceae;Ectothiorhodosinus;Ectothiorhodosinus mongolicus;Ectothiorhodosinus mongolicus M9 |
| Ga0586324 0539449 242 1585 | 0.00 | 5154400.00 | 0.00 | 0.00 | 0.00 | 0.00 | 0.00 | 2989000.00 | 0.00 | 5212900.00 | 0.00 | 0.00 | | hypothetical protein | Bacteria;Ignavibacteriae;Ignavibacteria;Ignavibacteriales;Melioribacteraceae;Melioribacter;Melioribacter roseus;Melioribacter roseus P3M |
| Ga0586324 0541299 1 1578 | 24770000.00 | 18713600.00 | 15032100.00 | 0.00 | 0.00 | 0.00 | 0.00 | 3013400.00 | 0.00 | 0.00 | 0.00 | 13147500.00 | | hypothetical protein | Bacteria;Chloroflexi;Dehalococcoidia;Dehalococcoidales;Dehalococcoidaceae;Dehalococcoides;Dehalococcoides mccartyi;Dehalococcoides mccartyi 11a |
| Ga0586324 0544921 114 1577 | 0.00 | 593683.52 | 0.00 | 0.00 | 0.00 | 0.00 | 0.00 | 0.00 | 0.00 | 0.00 | 0.00 | 0.00 | | FtsP/CotA-like multicopper oxidase with cupredoxin domain | Bacteria;Firmicutes;Bacilli;Bacillales;Paenibacillaceae;Paenibacillus;Paenibacillus sophorae;Paenibacillus sophorae S27 |
| Ga0586324 0545814 386 1576 | 2650000.00 | 2890000.00 | 4292002.35 | 0.00 | 2358700.00 | 1954117.37 | 2394000.00 | 2299700.00 | 1895400.00 | 2279200.00 | 2296800.00 | 2557500.00 | | hypothetical protein | Bacteria;Candidatus Aminicenantes;unclassified Candidatus Aminicenantes;unclassified Candidatus Aminicenantes;unclassified Candidatus Aminicenantes;unclassified Candidatus Aminicenantes;Aminicenantes bacterium SCGC AAA255-E05;Aminicenantes bacterium SCGC AAA255-E05 (contamination screened) |
| Ga0586324 0547836 3 1574 | 0.00 | 622320.36 | 0.00 | 0.00 | 0.00 | 0.00 | 0.00 | 0.00 | 0.00 | 0.00 | 0.00 | 0.00 | | FtsP/CotA-like multicopper oxidase with cupredoxin domain | Bacteria;Proteobacteria;Deltaproteobacteria;Desulfuromonadales;Geobacteraceae;Geobacter;Geobacter metallireducens;Geobacter metallireducens RCH3 |
| Ga0586324 0548085 296 1459 | 0.00 | 0.00 | 0.00 | 0.00 | 4387800.00 | 0.00 | 0.00 | 0.00 | 0.00 | 0.00 | 0.00 | 0.00 | | Fe-S oxidoreductase | Bacteria;Proteobacteria;Deltaproteobacteria;Syntrophobacterales;Syntrophobacteraceae;Desulfacinum;Desulfacinum infernum;Desulfacinum infernum DSM 9756 |
| Ga0586324 0550414 2 1570 | 4320000.00 | 0.00 | 0.00 | 0.00 | 3697700.00 | 0.00 | 0.00 | 1836100.00 | 0.00 | 0.00 | 3493600.00 | 5040000.00 | | hypothetical protein | Bacteria;Acidobacteria;Acidobacteriia;Bryobacterales;Solibacteraceae;Candidatus Solibacter;Candidatus Solibacter usitatus;Candidatus Solibacter usitatus Ellin6076 |
| Ga0586324 0551318 3 1571 | 0.00 | 2488800.00 | 0.00 | 0.00 | 0.00 | 1355080.10 | 0.00 | 0.00 | 0.00 | 0.00 | 0.00 | 0.00 | | hypothetical protein | Bacteria;Proteobacteria;Gammaproteobacteria;Chromatiales;Chromatiaceae;Lamprocystis;Lamprocystis purpurea;Lamprocystis purpurea DSM 4197 |
| Ga0586324 0554550 1 1566 | 60550000.00 | 22208800.00 | 47460900.00 | 22420000.00 | 67950750.06 | 2875125.61 | 38258400.00 | 13450500.00 | 23182200.00 | 18902342.69 | 41624000.00 | 42667500.00 | | hypothetical protein | |
| Ga0586324 0555214 3 1004 | 0.00 | 0.00 | 0.00 | 0.00 | 3378400.00 | 0.00 | 0.00 | 0.00 | 0.00 | 0.00 | 0.00 | 0.00 | | lysophospholipase L1-like esterase | Bacteria;Proteobacteria;Deltaproteobacteria;Myxococcales;Polyangiaceae;Chondromyces;Chondromyces apiculatus;Chondromyces apiculatus DSM 436 |
| Ga0586324 0555686 367 906 | 0.00 | 0.00 | 0.00 | 0.00 | 0.00 | 0.00 | 0.00 | 0.00 | 0.00 | 6421800.00 | 19219200.00 | 0.00 | | phasin family protein | Bacteria;Proteobacteria;Betaproteobacteria;Nitrosomonadales;Sterolibacteriaceae;Sulfurisoma;Sulfurisoma sediminicola;Sulfurisoma sediminicola DSM 26916 |
| Ga0586324 0557008 982 1563 | 0.00 | 0.00 | 0.00 | 6773500.00 | 6581700.00 | 4803700.00 | 0.00 | 2787700.00 | 2656800.00 | 0.00 | 0.00 | 0.00 | | S-layer protein (TIGR01567 family) | Archaea;Euryarchaeota;Methanomicrobia;Methanosarcinales;Methanosarcinaceae;Methanosarcina;Methanosarcina barkeri;Methanosarcina barkeri 227 |
| Ga0586324 0558881 3 1562 | 0.00 | 0.00 | 2125358.78 | 0.00 | 0.00 | 0.00 | 0.00 | 0.00 | 0.00 | 0.00 | 0.00 | 0.00 | | hypothetical protein | Bacteria;Candidatus Aminicenantes;unclassified Candidatus Aminicenantes;unclassified Candidatus Aminicenantes;unclassified Candidatus Aminicenantes;unclassified Candidatus Aminicenantes;Aminicenantes bacterium SCGC AAA255-A06;Aminicenantes bacterium SCGC AAA255-A06 (contamination screened) |
| Ga0586324 0561677 699 1559 | 0.00 | 0.00 | 0.00 | 0.00 | 0.00 | 0.00 | 0.00 | 0.00 | 1425600.00 | 0.00 | 0.00 | 0.00 | | uncharacterized protein (TIGR02001 family) | Bacteria;Proteobacteria;Gammaproteobacteria;Chromatiales;Chromatiaceae;Candidatus Tenderia;Candidatus Tenderia electrophaga;Candidatus Tenderia electrophaga NRL1 |
| Ga0586324 0562398 62 1132 | 0.00 | 0.00 | 0.00 | 0.00 | 0.00 | 0.00 | 0.00 | 0.00 | 0.00 | 4966500.00 | 0.00 | 0.00 | | sialic acid synthase SpsE | Bacteria;Proteobacteria;Alphaproteobacteria;Rhodospirillales;Rhodospirillaceae;Aestuariispira;Aestuariispira insulae;Aestuariispira insulae CECT 8488 |
| Ga0586324 0568518 414 1517 | 0.00 | 356474.36 | 0.00 | 0.00 | 8167900.00 | 15307331.47 | 37118400.00 | 13072300.00 | 17884800.00 | 6406400.00 | 4276800.00 | 711347.25 | | predicted porin | Bacteria;Proteobacteria;Gammaproteobacteria;Chromatiales;unclassified Chromatiales;Thiohalobacter;Thiohalobacter thiocyanaticus;Thiohalobacter thiocyanaticus FOKN1 |
| Ga0586324 0572317 1 1545 | 0.00 | 0.00 | 0.00 | 0.00 | 0.00 | 1083516.22 | 0.00 | 0.00 | 4811400.00 | 1586200.00 | 2798400.00 | 1642500.00 | | TonB-dependent SusC/RagA subfamily outer membrane receptor | Bacteria;Bacteroidetes;Sphingobacteriia;Sphingobacteriales;Sphingobacteriaceae;Mucilaginibacter;unclassified Mucilaginibacter;Mucilaginibacter sp. YR332 |
| Ga0586324 0573950 1 1542 | 0.00 | 2536400.00 | 0.00 | 0.00 | 0.00 | 0.00 | 0.00 | 0.00 | 0.00 | 0.00 | 0.00 | 0.00 | | outer membrane receptor protein involved in Fe transport | Bacteria;Bacteroidetes;Bacteroidia;Marinilabiliales;Prolixibacteraceae;Prolixibacter;Prolixibacter denitrificans;Prolixibacter denitrificans DSM 27267 |
| Ga0586324 0575048 2 1069 | 0.00 | 0.00 | 0.00 | 0.00 | 3223900.00 | 0.00 | 0.00 | 0.00 | 0.00 | 0.00 | 0.00 | 0.00 | | TonB-dependent SusC/RagA subfamily outer membrane receptor | Bacteria;Bacteroidetes;Bacteroidia;Marinilabiliales;Prolixibacteraceae;Sunxiuqinia;Sunxiuqinia elliptica;Sunxiuqinia elliptica CGMCC 1.9156 |
| Ga0586324 0575401 201 1541 | 0.00 | 0.00 | 0.00 | 0.00 | 0.00 | 0.00 | 0.00 | 547424.98 | 0.00 | 0.00 | 0.00 | 0.00 | | hypothetical protein | Bacteria;Proteobacteria;Deltaproteobacteria;Desulfobacterales;Desulfobacteraceae;Desulfococcus;Desulfococcus multivorans;Desulfococcus multivorans DSM 2059 |
| Ga0586324 0575872 1 1542 | 0.00 | 0.00 | 0.00 | 0.00 | 0.00 | 0.00 | 0.00 | 0.00 | 0.00 | 0.00 | 0.00 | 4087500.00 | | FtsP/CotA-like multicopper oxidase with cupredoxin domain | Bacteria;Proteobacteria;Deltaproteobacteria;Desulfuromonadales;Geobacteraceae;Citrifermentans;Citrifermentans bremense;Geobacter bremensis R1 |
| Ga0586324 0576310 1 1029 | 0.00 | 0.00 | 0.00 | 1738500.00 | 2369000.00 | 2056209.89 | 4590023.04 | 12993000.00 | 3329100.00 | 1093400.00 | 2525600.00 | 2115000.00 | | predicted porin | Bacteria;Proteobacteria;Gammaproteobacteria;Chromatiales;unclassified Chromatiales;Thiohalobacter;Thiohalobacter thiocyanaticus;Thiohalobacter thiocyanaticus FOKN1 |
| Ga0586324 0577967 685 1539 | 0.00 | 0.00 | 0.00 | 0.00 | 0.00 | 0.00 | 0.00 | 4629900.00 | 0.00 | 3626700.00 | 6318400.00 | 9150000.00 | | predicted porin | Bacteria;Proteobacteria;Gammaproteobacteria;Chromatiales;Halothiobacillaceae;Halothiobacillus;Halothiobacillus sp. WRN-7;Halothiobacillus sp. WRN-7 |
| Ga0586324 0578507 362 1537 | 0.00 | 3610800.00 | 6587100.00 | 8531000.00 | 4851300.00 | 16225374.12 | 6156000.00 | 2787700.00 | 5109769.98 | 3649800.00 | 1645600.00 | 2047500.00 | | hypothetical protein | Bacteria;Proteobacteria;Betaproteobacteria;Rhodocyclales;Zoogloeaceae;Thauera;Thauera chlorobenzoica;Thauera chlorobenzoica 3CB-1 |
| Ga0586324 0578674 1 1539 | 7150000.00 | 2223600.00 | 0.00 | 0.00 | 0.00 | 0.00 | 0.00 | 0.00 | 0.00 | 0.00 | 0.00 | 4636314.00 | | hypothetical protein | Bacteria;Proteobacteria;Deltaproteobacteria;Desulfobacterales;Desulfobulbaceae;Desulfofustis;Desulfofustis glycolicus;Desulfofustis glycolicus DSM 9705 |
| Ga0586324 0580255 1197 1535 | 35700000.00 | 11369600.00 | 0.00 | 0.00 | 0.00 | 0.00 | 0.00 | 0.00 | 0.00 | 0.00 | 0.00 | 0.00 | | hypothetical protein | |
| Ga0586324 0581205 81 1184 | 77739672.00 | 59159557.32 | 38058800.00 | 40506339.40 | 2894300.00 | 109770500.00 | 92887200.00 | 81325200.00 | 83129525.64 | 39650745.75 | 35463725.44 | 56257500.00 | | predicted porin | Bacteria;Proteobacteria;Betaproteobacteria;Burkholderiales;unclassified Burkholderiales;Rhizobacter;Rhizobacter sp. SG703;Rhizobacter sp. SG703 |
| Ga0586324 0583424 742 1530 | 0.00 | 0.00 | 0.00 | 0.00 | 2348400.00 | 0.00 | 0.00 | 0.00 | 0.00 | 0.00 | 0.00 | 0.00 | | hypothetical protein | Bacteria;Nitrospirae;Nitrospira;Nitrospirales;Nitrospiraceae;Leptospirillum;Leptospirillum ferriphilum;Leptospirillum ferriphilum DSM 14647 |
| Ga0586324 0584782 28 1530 | 0.00 | 0.00 | 0.00 | 0.00 | 0.00 | 0.00 | 0.00 | 0.00 | 0.00 | 2233000.00 | 0.00 | 0.00 | | hypothetical protein | Bacteria;Proteobacteria;Alphaproteobacteria;Hyphomicrobiales;Bradyrhizobiaceae;Afipia;Afipia sp. 1NLS2;Afipia sp. 1NLS2 |
| Ga0586324 0586174 1 678 | 0.00 | 0.00 | 0.00 | 0.00 | 0.00 | 0.00 | 0.00 | 0.00 | 0.00 | 0.00 | 0.00 | 2257500.00 | | hypothetical protein | Bacteria;Proteobacteria;Deltaproteobacteria;Desulfuromonadales;Geobacteraceae;Citrifermentans;Citrifermentans pelophilum;Geobacter pelophilus Dfr2 |
| Ga0586324 0586484 579 1340 | 0.00 | 0.00 | 0.00 | 1672000.00 | 0.00 | 0.00 | 0.00 | 0.00 | 0.00 | 0.00 | 0.00 | 0.00 | | opacity protein-like surface antigen | |
| Ga0586324 0586858 288 1529 | 933638.00 | 0.00 | 0.00 | 0.00 | 0.00 | 0.00 | 0.00 | 0.00 | 0.00 | 2548700.00 | 0.00 | 0.00 | | crotonobetainyl-CoA:carnitine CoA-transferase CaiB-like acyl-CoA transferase | Bacteria;Proteobacteria;Deltaproteobacteria;Syntrophobacterales;Syntrophobacteraceae;Desulfacinum;Desulfacinum hydrothermale;Desulfacinum hydrothermale DSM 13146 |
| Ga0586324 0587530 79 1182 | 0.00 | 26588000.00 | 72064000.00 | 0.00 | 0.00 | 91707000.00 | 49020000.00 | 0.00 | 1660500.00 | 35343000.00 | 36344000.00 | 0.00 | | predicted porin | Bacteria;Proteobacteria;Betaproteobacteria;Burkholderiales;unclassified Burkholderiales;Rhizobacter;Rhizobacter sp. SG703;Rhizobacter sp. SG703 |
| Ga0586324 0588133 187 681 | 0.00 | 0.00 | 0.00 | 0.00 | 6046100.00 | 0.00 | 0.00 | 0.00 | 0.00 | 0.00 | 0.00 | 0.00 | | rubrerythrin | Bacteria;Proteobacteria;Deltaproteobacteria;Syntrophobacterales;Syntrophaceae;Desulfobacca;Desulfobacca acetoxidans;Desulfobacca acetoxidans ASRB2, DSM 11109 |
| Ga0586324 0588618 601 1527 | 0.00 | 2318800.00 | 0.00 | 0.00 | 0.00 | 0.00 | 0.00 | 4166300.00 | 0.00 | 0.00 | 0.00 | 0.00 | | uncharacterized protein YgfA (UPF0365 family) | Bacteria;Verrucomicrobia;unclassified Verrucomicrobia;unclassified Verrucomicrobia;unclassified Verrucomicrobia;unclassified Verrucomicrobia;Verrucomicrobia bacterium LW23;Verrucomicrobia bacterium LW23 |
| Ga0586324 0590036 3 1526 | 0.00 | 0.00 | 0.00 | 0.00 | 0.00 | 1425341.16 | 0.00 | 0.00 | 0.00 | 0.00 | 0.00 | 0.00 | | uncharacterized protein YfaS (alpha-2-macroglobulin family) | Bacteria;Planctomycetes;Planctomycetia;Gemmatales;Gemmataceae;Zavarzinella;Zavarzinella formosa;Zavarzinella formosa DSM 19928 |
| Ga0586324 0591079 1 1395 | 0.00 | 2856000.00 | 0.00 | 3467500.00 | 4841000.00 | 0.00 | 0.00 | 0.00 | 0.00 | 0.00 | 0.00 | 0.00 | | hypothetical protein | Bacteria;Proteobacteria;Betaproteobacteria;Burkholderiales;Oxalobacteraceae;Massilia;Massilia umbonata;Massilia umbonata CECT 7753 |
| Ga0586324 0591800 58 1524 | 0.00 | 1842800.00 | 1422244.97 | 1520000.00 | 3326900.00 | 4890861.35 | 0.00 | 300062.66 | 436111.29 | 566383.51 | 950400.00 | 997500.00 | | hypothetical protein | Bacteria;Actinobacteria;Actinomycetia;Propionibacteriales;Nocardioidaceae;Nocardioides;Nocardioides oleivorans;Nocardioides oleivorans CGMCC 4.6882 |
| Ga0586324 0593482 3 1520 | 0.00 | 0.00 | 0.00 | 0.00 | 0.00 | 0.00 | 0.00 | 2659600.00 | 1279800.00 | 0.00 | 0.00 | 0.00 | | hypothetical protein | Archaea;Euryarchaeota;Methanomicrobia;Methanosarcinales;Methanotrichaceae;Methanothrix;Methanothrix soehngenii;Methanothrix soehngenii GP-6 |
| Ga0586324 0594674 958 1521 | 7190000.00 | 5861600.00 | 11823000.00 | 0.00 | 7601400.00 | 0.00 | 0.00 | 0.00 | 2405700.00 | 1255100.00 | 0.00 | 0.00 | | hypothetical protein | Bacteria;Proteobacteria;Deltaproteobacteria;Desulfuromonadales;Geobacteraceae;Geobacter;Geobacter argillaceus;Geobacter argillaceus ATCC BAA-1139 |
| Ga0586324 0595475 118 603 | 0.00 | 0.00 | 0.00 | 0.00 | 0.00 | 0.00 | 19220400.00 | 1860500.00 | 3207600.00 | 0.00 | 0.00 | 3225000.00 | | hypothetical protein | Bacteria;Proteobacteria;Deltaproteobacteria;Syntrophobacterales;Syntrophaceae;Desulfobacca;Desulfobacca acetoxidans;Desulfobacca acetoxidans ASRB2, DSM 11109 |
| Ga0586324 0596894 561 1517 | 2950000.00 | 0.00 | 0.00 | 0.00 | 0.00 | 0.00 | 0.00 | 0.00 | 0.00 | 0.00 | 0.00 | 0.00 | | ABC-type sugar transport system substrate-binding protein | Bacteria;Proteobacteria;Alphaproteobacteria;Hyphomicrobiales;Stappiaceae;Pseudovibrio;Pseudovibrio sp. Tun.PSC04-5.I4;Pseudovibrio sp. Tun.PSC04-5.I4 |
| Ga0586324 0597332 703 1518 | 0.00 | 0.00 | 0.00 | 0.00 | 0.00 | 0.00 | 0.00 | 8113000.00 | 0.00 | 0.00 | 0.00 | 0.00 | | hypothetical protein | Bacteria;Synergistetes;Synergistia;Synergistales;Synergistaceae;Acetomicrobium;Acetomicrobium thermoterrenum;Acetomicrobium thermoterrenum DSM 13490 |
| Ga0586324 0598746 353 1516 | 0.00 | 0.00 | 0.00 | 0.00 | 0.00 | 0.00 | 0.00 | 0.00 | 0.00 | 4889159.66 | 1988800.00 | 0.00 | | TonB-dependent SusC/RagA subfamily outer membrane receptor | Bacteria;Gemmatimonadetes;Gemmatimonadetes;Gemmatimonadales;Gemmatimonadaceae;Gemmatimonas;Gemmatimonas phototrophica;Gemmatimonas phototrophica AP64 |
| Ga0586324 0598959 77 1516 | 66830000.00 | 128853200.00 | 43895111.35 | 42436500.00 | 57989000.00 | 74242227.61 | 29146623.96 | 29761900.00 | 27888300.00 | 63020071.73 | 41060800.00 | 55204856.25 | | hypothetical protein | Bacteria;Proteobacteria;Gammaproteobacteria;Alteromonadales;Alteromonadaceae;Marinobacter;Marinobacter sp.;Marinobacter sp. MIT1353 |
| Ga0586324 0599385 407 781 | 0.00 | 0.00 | 0.00 | 2223000.00 | 3357800.00 | 0.00 | 0.00 | 0.00 | 0.00 | 0.00 | 0.00 | 0.00 | | predicted Fe-Mo cluster-binding NifX family protein | Bacteria;Proteobacteria;Deltaproteobacteria;Syntrophobacterales;Syntrophaceae;Syntrophus;Syntrophus gentianae;Syntrophus gentianae DSM 8423 |
| Ga0586324 0599822 974 1513 | 0.00 | 0.00 | 0.00 | 0.00 | 0.00 | 0.00 | 0.00 | 0.00 | 0.00 | 3010700.00 | 0.00 | 0.00 | | hypothetical protein | Bacteria;Acidobacteria;Vicinamibacteria;unclassified Vicinamibacteria;Vicinamibacteraceae;Luteitalea;Luteitalea pratensis;Luteitalea pratensis DSM 100886 |
| Ga0586324 0599871 3 1286 | 0.00 | 0.00 | 0.00 | 0.00 | 0.00 | 3304052.35 | 0.00 | 0.00 | 0.00 | 0.00 | 0.00 | 0.00 | | TonB-dependent SusC/RagA subfamily outer membrane receptor | Bacteria;Bacteroidetes;Bacteroidia;Marinilabiliales;Prolixibacteraceae;Prolixibacter;Prolixibacter denitrificans;Prolixibacter denitrificans DSM 27267 |
| Ga0586324 0600172 2 1513 | 10200000.00 | 12546000.00 | 18579000.00 | 26077500.00 | 14832000.00 | 14093500.00 | 21409200.00 | 13291900.00 | 0.00 | 9317000.00 | 14405600.00 | 23625000.00 | | hypothetical protein | Bacteria;Ignavibacteriae;Ignavibacteria;Ignavibacteriales;Ignavibacteriaceae;Ignavibacterium;Ignavibacterium album;Ignavibacterium album Mat9-16, JCM 16511 |
| Ga0586324 0608649 3 1505 | 0.00 | 0.00 | 0.00 | 0.00 | 0.00 | 0.00 | 6042000.00 | 0.00 | 0.00 | 8239000.00 | 0.00 | 8400000.00 | | hypothetical protein | Bacteria;Acidobacteria;Acidobacteriia;Acidobacteriales;Acidobacteriaceae;unclassified Acidobacteriaceae;Acidobacteriaceae bacterium KBS 96;Acidobacteriaceae bacterium KBS 96, DSM 24297 |
| Ga0586324 0609038 3 977 | 0.00 | 3046400.00 | 0.00 | 0.00 | 0.00 | 0.00 | 0.00 | 0.00 | 1458000.00 | 0.00 | 2050400.00 | 0.00 | | hypothetical protein | Bacteria;Proteobacteria;Gammaproteobacteria;Chromatiales;Chromatiaceae;Thiodictyon;Candidatus Thiodictyon syntrophicum;Candidatus Thiodictyon syntrophicum Cad16 |
| Ga0586324 0610059 119 1432 | 0.00 | 0.00 | 0.00 | 0.00 | 19085900.00 | 0.00 | 0.00 | 0.00 | 0.00 | 4165700.00 | 0.00 | 0.00 | | hypothetical protein | Bacteria;Verrucomicrobia;Verrucomicrobiae;Verrucomicrobiales;Verrucomicrobia subdivision 3;Pedosphaera;Pedosphaera parvula;Pedosphaera parvula Ellin514 |
| Ga0586324 0614866 1 999 | 0.00 | 0.00 | 0.00 | 0.00 | 0.00 | 0.00 | 0.00 | 0.00 | 2770200.00 | 0.00 | 0.00 | 0.00 | | hypothetical protein | Bacteria;Proteobacteria;Gammaproteobacteria;Acidiferrobacterales;Acidiferrobacteraceae;Sulfuricaulis;Sulfuricaulis limicola;Sulfuricaulis limicola HA5 |
| Ga0586324 0616806 291 1268 | 2790000.00 | 1523200.00 | 0.00 | 0.00 | 0.00 | 6709300.00 | 0.00 | 0.00 | 0.00 | 2502500.00 | 3933600.00 | 780000.00 | | glyoxylase-like metal-dependent hydrolase (beta-lactamase superfamily II) | Bacteria;Proteobacteria;Alphaproteobacteria;Rhodospirillales;Azospirillaceae;Skermanella;Skermanella aerolata;Skermanella aerolata KACC 11604 |
| Ga0586324 0617049 1 1074 | 0.00 | 0.00 | 0.00 | 0.00 | 0.00 | 0.00 | 0.00 | 0.00 | 0.00 | 349720.91 | 0.00 | 0.00 | | prepilin-type N-terminal cleavage/methylation domain-containing protein | |

| | | | | | | | | | | | | | | | |
|---|---|---|---|---|---|---|---|---|---|---|---|---|---|---|---|
| Ga0586324 0623202 291 1031 | 7170000.00 | 0.00 | 0.00 | 0.00 | 0.00 | 0.00 | 0.00 | 0.00 | 0.00 | 0.00 | 0.00 | 0.00 | | hypothetical protein | |
| Ga0586324 0623578 1 1170 | 0.00 | 12512000.00 | 0.00 | 0.00 | 0.00 | 0.00 | 0.00 | 0.00 | 0.00 | 0.00 | 0.00 | 0.00 | | arylsulfatase A-like enzyme | Bacteria;Acidobacteria;Acidobacteriia;Acidobacteriales;Acidobacteriaceae;Edaphobacter;Edaphobacter modestus;Edaphobacter modestus DSM 18101 |
| Ga0586324 0624186 2 1489 | 0.00 | 0.00 | 0.00 | 0.00 | 0.00 | 0.00 | 0.00 | 0.00 | 0.00 | 854700.00 | 0.00 | 0.00 | | hypothetical protein | Bacteria;Firmicutes;Bacilli;Bacillales;Paenibacillaceae;Paenibacillus;Paenibacillus sophorae;Paenibacillus sophorae S27 |
| Ga0586324 0625040 3 1487 | 0.00 | 2876400.00 | 0.00 | 3885500.00 | 3399000.00 | 0.00 | 0.00 | 2232600.00 | 0.00 | 9463300.00 | 0.00 | 10620000.00 | | Ca2+-binding RTX toxin-like protein | Bacteria;Proteobacteria;Betaproteobacteria;Neisseriales;Chromobacteriaceae;Aquaspirillum;Aquaspirillum serpens;Aquaspirillum serpens DSM 68 |
| Ga0586324 0626027 780 1448 | 0.00 | 0.00 | 0.00 | 2954500.00 | 0.00 | 0.00 | 0.00 | 0.00 | 0.00 | 1540000.00 | 0.00 | 2010000.00 | | corrinoid protein of di/trimethylamine methyltransferase | Bacteria;Spirochaetes;Spirochaetia;Spirochaetales;Spirochaetaceae;Alkalispirochaeta;Alkalispirochaeta odontotermitis;Alkalispirochaeta odontotermitis JC202 |
| Ga0586324 0627586 9 1352 | 0.00 | 0.00 | 0.00 | 0.00 | 0.00 | 2170537.95 | 0.00 | 0.00 | 0.00 | 0.00 | 0.00 | 0.00 | | long-subunit fatty acid transport protein | Bacteria;Proteobacteria;Deltaproteobacteria;Desulfobacterales;Desulfobacteraceae;Desulfotignum;Desulfotignum phosphitoxidans;Desulfotignum phosphitoxidans FiPS-3, DSM 13687 |
| Ga0586324 0628742 32 1483 | 0.00 | 0.00 | 0.00 | 0.00 | 0.00 | 0.00 | 0.00 | 0.00 | 0.00 | 0.00 | 0.00 | 1207500.00 | | hypothetical protein | Bacteria;Bacteroidetes;Cytophagia;Cytophagales;Hymenobacteraceae;Hymenobacter;Hymenobacter sp. YR204;Hymenobacter sp. YR204 |
| Ga0586324 0629515 2 1483 | 0.00 | 359179.40 | 0.00 | 0.00 | 1843700.00 | 0.00 | 0.00 | 1561600.00 | 899100.00 | 2733500.00 | 0.00 | 1830000.00 | | hypothetical protein | Bacteria;Acidobacteria;Acidobacteriia;Acidobacteriales;Acidobacteriaceae;Granulicella;Granulicella aggregans;Granulicella aggregans M8UP14 |
| Ga0586324 0630029 3 1481 | 0.00 | 0.00 | 2875910.97 | 2033000.00 | 10238200.00 | 0.00 | 3602400.00 | 0.00 | 5467500.00 | 0.00 | 2261600.00 | 2850000.00 | | hypothetical protein | Bacteria;Actinobacteria;Actinomycetia;Micrococcales;Intrasporangiaceae;Humibacillus;Humibacillus xanthopallidus;Humibacillus xanthopallidus DSM 21776 |
| Ga0586324 0631260 36 1481 | 0.00 | 0.00 | 0.00 | 0.00 | 0.00 | 4525800.00 | 0.00 | 0.00 | 0.00 | 0.00 | 0.00 | 0.00 | | predicted molibdopterin-dependent oxidoreductase YjgC | Bacteria;Proteobacteria;Deltaproteobacteria;Syntrophobacterales;Syntrophaceae;Desulfobacca;Desulfobacca acetoxidans;Desulfobacca acetoxidans ASRB2, DSM 11109 |
| Ga0586324 0631499 1 99 | 1700000.00 | 897600.00 | 24659400.00 | 0.00 | 0.00 | 0.00 | 0.00 | 921100.00 | 14985000.00 | 0.00 | 5112800.00 | 0.00 | | translation elongation factor EF-Tu-like GTPase | |
| Ga0586324 0631655 1 1482 | 7220000.00 | 0.00 | 0.00 | 0.00 | 0.00 | 0.00 | 0.00 | 0.00 | 0.00 | 0.00 | 0.00 | 0.00 | | glucose/arabinose dehydrogenase | Bacteria;Proteobacteria;Alphaproteobacteria;Hyphomicrobiales;Bradyrhizobiaceae;Bradyrhizobium;Bradyrhizobium sp. ARR65;Bradyrhizobium sp. ARR65 |
| Ga0586324 0631789 1 702 | 0.00 | 0.00 | 0.00 | 0.00 | 0.00 | 0.00 | 0.00 | 0.00 | 0.00 | 0.00 | 0.00 | 7875000.00 | | hypothetical protein | Bacteria;Proteobacteria;Alphaproteobacteria;Hyphomicrobiales;Methylocystaceae;Methylosinus;Methylosinus trichosporium;Methylosinus trichosporium OB3b |
| Ga0586324 0634904 2 1288 | 6050000.00 | 0.00 | 0.00 | 9880000.00 | 0.00 | 0.00 | 0.00 | 0.00 | 0.00 | 21868000.00 | 11704000.00 | 17250000.00 | | predicted porin | Bacteria;Proteobacteria;Betaproteobacteria;Burkholderiales;Burkholderiaceae;Paraburkholderia;Paraburkholderia caffeinilytica;Paraburkholderia caffeinilytica CF1 |
| Ga0586324 0637655 1 1278 | 0.00 | 1747600.00 | 0.00 | 3486500.00 | 2739800.00 | 0.00 | 1307874.12 | 2324100.00 | 1782000.00 | 0.00 | 0.00 | 0.00 | | NodT family efflux transporter outer membrane factor (OMF) lipoprotein | Bacteria;Proteobacteria;Alphaproteobacteria;Hyphomicrobiales;Methylocystaceae;Methylocystis;Methylocystis parvus;Methylocystis parvus OBBP |
| Ga0586324 0638644 251 1474 | 0.00 | 0.00 | 0.00 | 0.00 | 0.00 | 0.00 | 0.00 | 0.00 | 0.00 | 1701700.00 | 0.00 | 0.00 | | hypothetical protein | |
| Ga0586324 0640389 3 1472 | 0.00 | 586417.72 | 1036865.84 | 0.00 | 2904600.00 | 0.00 | 0.00 | 2858971.79 | 2389500.00 | 4150300.00 | 831154.72 | 0.00 | | hypothetical protein | Bacteria;Calditrichaeota;Calditrichae;Calditrichales;Calditrichaceae;Caldithrix;Caldithrix abyssi;Caldithrix abyssi LF13, DSM 13497 |
| Ga0586324 0640602 841 1275 | 0.00 | 0.00 | 0.00 | 0.00 | 0.00 | 0.00 | 0.00 | 0.00 | 0.00 | 0.00 | 816324.08 | 0.00 | | cytoskeletal protein CcmA (bactofilin family) | Bacteria;Acidobacteria;Vicinamibacteria;unclassified Vicinamibacteria;Vicinamibacteraceae;Luteitalea;Luteitalea pratensis;Luteitalea pratensis DSM 100886 |
| Ga0586324 0640650 3 1472 | 0.00 | 0.00 | 0.00 | 2945000.00 | 0.00 | 0.00 | 0.00 | 0.00 | 1631870.55 | 0.00 | 0.00 | 0.00 | | hypothetical protein | Bacteria;Nitrospirae;Nitrospira;Nitrospirales;Nitrospiraceae;Nitrospira;Nitrospira lenta;Nitrospira lenta N. lenta BS10 |
| Ga0586324 0641639 508 1470 | 0.00 | 0.00 | 0.00 | 0.00 | 0.00 | 8416400.00 | 0.00 | 0.00 | 0.00 | 0.00 | 10824000.00 | 0.00 | | alkyl sulfatase BDS1-like metallo-beta-lactamase superfamily hydrolase | Archaea;Euryarchaeota;Methanomicrobia;Methanosarcinales;Methanosarcinaceae;Methanosarcina;Methanosarcina mazei;Methanosarcina mazei S-6 |
| Ga0586324 0641859 287 1414 | 20300000.00 | 8173600.00 | 6530800.00 | 0.00 | 2482300.00 | 5637400.00 | 51938400.00 | 50782500.00 | 7558217.73 | 35512400.00 | 29190601.44 | 1845000.00 | | hypothetical protein | Bacteria;Proteobacteria;Deltaproteobacteria;Desulfobacterales;Desulfobacteraceae;Desulfotignum;Desulfotignum balticum;Desulfotignum balticum DSM 7044 |
| Ga0586324 0644740 374 931 | 0.00 | 3454400.00 | 0.00 | 0.00 | 0.00 | 0.00 | 0.00 | 0.00 | 0.00 | 0.00 | 0.00 | 3405000.00 | | lipid-binding SYLF domain-containing protein | Bacteria;Proteobacteria;Gammaproteobacteria;Methylococcales;Methylococcaceae;Methylobacter;Methylobacter luteus;Methylobacter luteus IMV-B-3098 |
| Ga0586324 0645937 823 1467 | 0.00 | 0.00 | 0.00 | 0.00 | 0.00 | 4962500.00 | 0.00 | 0.00 | 0.00 | 4265800.00 | 0.00 | 2767500.00 | | hypothetical protein | Bacteria;Chloroflexi;Anaerolineae;Anaerolineales;Anaerolineaceae;Anaerolinea;Anaerolinea thermophila;Anaerolinea thermophila UNI-1 |
| Ga0586324 0647176 397 1299 | 843312.00 | 0.00 | 0.00 | 0.00 | 0.00 | 0.00 | 0.00 | 0.00 | 0.00 | 0.00 | 0.00 | 2137500.00 | | hypothetical protein | Bacteria;Firmicutes;Clostridia;Eubacteriales;Syntrophomonadaceae;Dethiobacter;Dethiobacter alkaliphilus;Dethiobacter alkaliphilus AHT1 |
| Ga0586324 0647673 1 1464 | 0.00 | 0.00 | 0.00 | 0.00 | 0.00 | 0.00 | 0.00 | 0.00 | 0.00 | 0.00 | 0.00 | 990000.00 | | TonB-linked SusC/RagA family outer membrane protein | Bacteria;Bacteroidetes;Cytophagia;Cytophagales;Cyclobacteriaceae;Lunatimonas;Lunatimonas lonarensis;Lunatimonas lonarensis AK24 |
| Ga0586324 0648793 854 1462 | 0.00 | 7140000.00 | 0.00 | 0.00 | 0.00 | 0.00 | 0.00 | 1104100.00 | 0.00 | 0.00 | 0.00 | 0.00 | | transketolase N-terminal domain/subunit | Bacteria;Proteobacteria;Gammaproteobacteria;Methylococcales;Methylococcaceae;Methylomonas;Methylomonas methanica;Methylomonas methanica MC09 |
| Ga0586324 0649312 3 1013 | 0.00 | 391350.20 | 0.00 | 0.00 | 3677100.00 | 0.00 | 0.00 | 0.00 | 0.00 | 0.00 | 0.00 | 0.00 | | type II secretory pathway component GspD/PulD (secretin) | Bacteria;Verrucomicrobia;Verrucomicrobiae;Verrucomicrobiales;Verrucomicrobia subdivision 3;Pedosphaera;Pedosphaera parvula;Pedosphaera parvula Ellin514 |
| Ga0586324 0652207 294 1460 | 0.00 | 384393.12 | 0.00 | 0.00 | 0.00 | 0.00 | 0.00 | 0.00 | 0.00 | 0.00 | 0.00 | 0.00 | | ABC-type multidrug transport system fused ATPase/permease subunit | Bacteria;Bacteroidetes;Chitinophagia;Chitinophagales;Chitinophagaceae;Chitinophaga;Chitinophaga niabensis;Chitinophaga niabensis DSM 24787 |
| Ga0586324 0654594 73 1428 | 21000000.00 | 16592000.00 | 40254500.00 | 0.00 | 0.00 | 40216100.00 | 12631200.00 | 0.00 | 0.00 | 0.00 | 0.00 | 11250000.00 | | hypothetical protein | Bacteria;Proteobacteria;Deltaproteobacteria;Syntrophorhabdales;Syntrophorhabdaceae;Syntrophorhabdus;Syntrophorhabdus aromaticivorans;Syntrophorhabdus aromaticivorans UI |
| Ga0586324 0654702 3 1457 | 0.00 | 8568000.00 | 0.00 | 0.00 | 16150400.00 | 0.00 | 10237200.00 | 11584878.44 | 20355300.00 | 11195800.00 | 3212000.00 | 7192500.00 | | outer membrane receptor for ferrienterochelin and colicin | Bacteria;Acidobacteria;Thermoanaerobaculia;Thermoanaerobaculales;Thermoanaerobaculaceae;Thermoanaerobaculum;Thermoanaerobaculum aquaticum;Thermoanaerobaculum aquaticum MP-01 |
| Ga0586324 0654815 217 1326 | 0.00 | 0.00 | 0.00 | 0.00 | 0.00 | 0.00 | 0.00 | 0.00 | 0.00 | 0.00 | 265678.16 | 1147500.00 | | TRAP-type mannitol/chloroaromatic compound transport system substrate-binding protein | Bacteria;Proteobacteria;Alphaproteobacteria;Rhodospirillales;Azospirillaceae;Azospirillum;Azospirillum brasilense;Azospirillum brasilense MTCC 4039 |
| Ga0586324 0656114 1 1350 | 0.00 | 0.00 | 0.00 | 0.00 | 0.00 | 0.00 | 0.00 | 0.00 | 0.00 | 823900.00 | 0.00 | 0.00 | | FtsP/CotA-like multicopper oxidase with cupredoxin domain | Bacteria;Proteobacteria;Gammaproteobacteria;Methylococcales;Methylococcaceae;Methylosarcina;Methylosarcina fibrata;Methylosarcina fibrata AML-C10 |
| Ga0586324 0662689 248 1450 | 699082.00 | 0.00 | 0.00 | 0.00 | 0.00 | 0.00 | 0.00 | 0.00 | 0.00 | 0.00 | 0.00 | 0.00 | | hypothetical protein | Bacteria;Acidobacteria;Acidobacteriia;Acidobacteriales;Acidobacteriaceae;Terriglobus;Terriglobus roseus;Terriglobus roseus AB35.6 |
| Ga0586324 0664301 1 480 | 510850.00 | 461017.56 | 0.00 | 0.00 | 0.00 | 0.00 | 0.00 | 0.00 | 0.00 | 156670.36 | 0.00 | 0.00 | | hypothetical protein | Bacteria;Bacteroidetes;Cytophagia;Cytophagales;Cytophagaceae;Spirosoma;Spirosoma linguale;Spirosoma linguale DSM 74 |
| Ga0586324 0666656 1 1446 | 0.00 | 0.00 | 0.00 | 0.00 | 2090900.00 | 0.00 | 0.00 | 1073600.00 | 0.00 | 1216600.00 | 1408000.00 | 1417500.00 | | hypothetical protein | Bacteria;Acidobacteria;Acidobacteriia;Bryobacterales;Bryobacteraceae;Bryobacter;Bryobacter aggregatus;Bryobacter aggregatus DSM 18758 |
| Ga0586324 0667440 1 1446 | 0.00 | 1326000.00 | 0.00 | 0.00 | 0.00 | 0.00 | 0.00 | 0.00 | 0.00 | 0.00 | 0.00 | 0.00 | | hypothetical protein | Bacteria;Acidobacteria;Vicinamibacteria;unclassified Vicinamibacteria;Vicinamibacteraceae;Luteitalea;Luteitalea pratensis;Luteitalea pratensis DSM 100886 |
| Ga0586324 0667647 118 990 | 0.00 | 53788000.00 | 0.00 | 0.00 | 0.00 | 0.00 | 0.00 | 0.00 | 0.00 | 0.00 | 1469600.00 | 0.00 | | HK97 family phage major capsid protein | |
| Ga0586324 0667693 1121 1444 | 0.00 | 7956000.00 | 0.00 | 0.00 | 11433000.00 | 0.00 | 0.00 | 12200000.00 | 26406000.00 | 0.00 | 11616000.00 | 4597500.00 | | predicted porin | Bacteria;Proteobacteria;Betaproteobacteria;unclassified Betaproteobacteria;unclassified Betaproteobacteria;unclassified Betaproteobacteria;uncultured beta proteobacterium;unclassified betaproteobacterium ENR4 |
| Ga0586324 0667693 3 596 | 0.00 | 5293953.68 | 0.00 | 0.00 | 872088.64 | 0.00 | 0.00 | 2952400.00 | 0.00 | 327614.98 | 6899200.00 | 5644442.25 | | predicted porin | Bacteria;Proteobacteria;Betaproteobacteria;Burkholderiales;unclassified Burkholderiales;Methylibium;Methylibium petroleiphilum;Methylibium petroleiphilum PM1 |
| Ga0586324 0668343 966 1445 | 0.00 | 0.00 | 0.00 | 0.00 | 2822200.00 | 0.00 | 0.00 | 0.00 | 0.00 | 0.00 | 0.00 | 0.00 | | regulator of protease activity HflC (stomatin/prohibitin superfamily) | Bacteria;Acidobacteria;Thermoanaerobaculia;Thermoanaerobaculales;Thermoanaerobaculaceae;Thermoanaerobaculum;Thermoanaerobaculum aquaticum;Thermoanaerobaculum aquaticum MP-01 |
| Ga0586324 0671861 794 1441 | 0.00 | 6004400.00 | 44195500.00 | 45030000.00 | 50058000.00 | 22390800.00 | 69768000.00 | 15677000.00 | 20817000.00 | 11396000.00 | 5984000.00 | 34500000.00 | | hypothetical protein | Bacteria;Deferribacteres;Deferribacteres;Deferribacterales;Deferribacteraceae;Deferribacter;Deferribacter desulfuricans;Deferribacter desulfuricans SSM1 |
| Ga0586324 0672599 3 1442 | 24200000.00 | 9656000.00 | 0.00 | 0.00 | 13699000.00 | 0.00 | 0.00 | 0.00 | 208980000.00 | 0.00 | 0.00 | 0.00 | | hypothetical protein | Bacteria;Proteobacteria;Alphaproteobacteria;Hyphomicrobiales;unclassified Hyphomicrobiales;Methyloceanibacter;Methyloceanibacter superfactus;Methyloceanibacter superfactus R-67175 |
| Ga0586324 0672829 1 1116 | 0.00 | 2822000.00 | 0.00 | 0.00 | 0.00 | 0.00 | 0.00 | 0.00 | 0.00 | 1801800.00 | 1865600.00 | 0.00 | | hypothetical protein | |
| Ga0586324 0676300 2 1438 | 0.00 | 0.00 | 0.00 | 0.00 | 0.00 | 0.00 | 0.00 | 0.00 | 2519100.00 | 1001000.00 | 4655200.00 | 0.00 | | FtsP/CotA-like multicopper oxidase with cupredoxin domain | Bacteria;Proteobacteria;Gammaproteobacteria;Methylococcales;Methylococcaceae;Methyloterricola;Methyloterricola oryzae;Methyloterricola oryzae 73a |
| Ga0586324 0677737 239 787 | 0.00 | 0.00 | 0.00 | 0.00 | 1442000.00 | 0.00 | 0.00 | 0.00 | 0.00 | 0.00 | 0.00 | 0.00 | | hypothetical protein | Bacteria;Proteobacteria;Deltaproteobacteria;Desulfobacterales;Desulfobacteraceae;Desulfatiglans;Desulfatiglans anilini;Desulfatiglans anilini DSM 4660 |
| Ga0586324 0679426 461 1435 | 0.00 | 0.00 | 0.00 | 0.00 | 0.00 | 0.00 | 0.00 | 0.00 | 0.00 | 0.00 | 0.00 | 113567.25 | | cytochrome c peroxidase | Bacteria;Proteobacteria;Deltaproteobacteria;Desulfovibrionales;Desulfovibrionaceae;Desulfocurvibacter;Desulfocurvibacter africanus;Desulfocurvibacter africanus Walvis Bay |
| Ga0586324 0679987 1 726 | 0.00 | 0.00 | 0.00 | 2413000.00 | 0.00 | 0.00 | 0.00 | 0.00 | 0.00 | 0.00 | 0.00 | 0.00 | | hypothetical protein | Bacteria;Proteobacteria;Alphaproteobacteria;Hyphomicrobiales;Bradyrhizobiaceae;Bradyrhizobium;unclassified Bradyrhizobium;Bradyrhizobium sp. HAMBI 2115 |
| Ga0586324 0680719 2 1432 | 3700000.00 | 7330400.00 | 0.00 | 0.00 | 0.00 | 0.00 | 1772189.28 | 1091900.00 | 1790100.00 | 5443900.00 | 4004000.00 | 7560000.00 | | subtilisin family serine protease | Bacteria;Chloroflexi;Anaerolineae;Anaerolineales;Anaerolineaceae;Levilinea;Levilinea saccharolytica;Levilinea saccharolytica DSM 16555 |
| Ga0586324 0682989 3 1430 | 1850000.00 | 0.00 | 0.00 | 0.00 | 0.00 | 0.00 | 0.00 | 0.00 | 0.00 | 0.00 | 0.00 | 0.00 | | hypothetical protein | Bacteria;Firmicutes;Bacilli;Bacillales;Paenibacillaceae;Paenibacillus;Paenibacillus beijingensis;Paenibacillus beijingensis DSM 24997 |
| Ga0586324 0685542 262 1380 | 6220000.00 | 6840800.00 | 14207198.03 | 12179000.00 | 4429000.00 | 0.00 | 20141985.12 | 42456000.00 | 24543000.00 | 14306600.00 | 8641600.00 | 26047500.00 | | hypothetical protein | Bacteria;Proteobacteria;Betaproteobacteria;Burkholderiales;unclassified Burkholderiales;Thiomonas;Thiomonas sp. FB-Cd;Thiomonas sp. FB-Cd, DSM 25617 |
| Ga0586324 0686148 620 1429 | 0.00 | 0.00 | 47629800.00 | 0.00 | 0.00 | 0.00 | 0.00 | 0.00 | 0.00 | 0.00 | 0.00 | 0.00 | | DNA-binding GntR family transcriptional regulator | Bacteria;Proteobacteria;Deltaproteobacteria;Syntrophorhabdales;Syntrophorhabdaceae;Syntrophorhabdus;Syntrophorhabdus aromaticivorans;Syntrophorhabdus aromaticivorans UI |
| Ga0586324 0686314 2 1429 | 0.00 | 0.00 | 0.00 | 0.00 | 0.00 | 0.00 | 0.00 | 0.00 | 0.00 | 14168000.00 | 0.00 | 0.00 | | ABC-type transport system substrate-binding protein | Bacteria;Chloroflexi;Anaerolineae;Anaerolineales;Anaerolineaceae;Anaerolinea;Anaerolinea thermophila;Anaerolinea thermophila UNI-1 |
| Ga0586324 0686655 214 1428 | 2120000.00 | 2026400.00 | 2402304.11 | 0.00 | 0.00 | 1923500.73 | 0.00 | 1988600.00 | 1790100.00 | 3595900.00 | 4347200.00 | 2557500.00 | | hypothetical protein | Bacteria;Acidobacteria;Acidobacteriia;Acidobacteriales;Acidobacteriaceae;unclassified Acidobacteriaceae;Acidobacteriaceae bacterium KBS 96;Acidobacteriaceae bacterium KBS 96, DSM 24297 |
| Ga0586324 0688744 1 477 | 0.00 | 0.00 | 0.00 | 0.00 | 3326900.00 | 468372.66 | 0.00 | 707600.00 | 751275.00 | 0.00 | 0.00 | 0.00 | | peroxiredoxin | Bacteria;Acidobacteria;Acidobacteriia;Bryobacterales;Solibacteraceae;Candidatus Solibacter;Candidatus Solibacter usitatus;Candidatus Solibacter usitatus Ellin6076 |
| Ga0586324 0689337 238 1242 | 8710000.00 | 2754000.00 | 3117792.66 | 5253500.00 | 5253000.00 | 5041900.00 | 3488400.00 | 4379800.00 | 7500600.00 | 1771000.00 | 3062400.00 | 0.00 | | predicted porin | Bacteria;Proteobacteria;Betaproteobacteria;Burkholderiales;unclassified Burkholderiales;Leptothrix;Leptothrix cholodnii;Leptothrix cholodnii SP-6 |
| Ga0586324 0690399 2 883 | 0.00 | 1822400.00 | 0.00 | 0.00 | 0.00 | 0.00 | 0.00 | 0.00 | 0.00 | 0.00 | 215801.52 | 0.00 | | outer membrane receptor protein involved in Fe transport | Bacteria;Bacteroidetes;unclassified Bacteroidetes;Bacteroidetes Order II. Incertae sedis;Rhodothermaceae;unclassified Rhodothermaceae;Rhodothermaceae bacterium RA;Halothermobacillus malaysiensis RA |
| Ga0586324 0692256 402 1424 | 39050000.00 | 25160000.00 | 30514600.00 | 3363000.00 | 0.00 | 50498400.00 | 59721127.56 | 27267000.00 | 31266000.00 | 17332700.00 | 27174400.00 | 19950000.00 | | predicted porin | Bacteria;Proteobacteria;Betaproteobacteria;Burkholderiales;Burkholderiaceae;Quisquiliibacterium;Quisquiliibacterium transsilvanicum;Quisquiliibacterium transsilvanicum DSM 29781 |
| Ga0586324 0693875 748 1152 | 0.00 | 0.00 | 0.00 | 0.00 | 0.00 | 0.00 | 0.00 | 0.00 | 0.00 | 1955800.00 | 0.00 | 0.00 | | predicted flap endonuclease-1-like 5' DNA nuclease | Bacteria;Proteobacteria;Alphaproteobacteria;Rhodospirillales;Rhodospirillaceae;Roseospirillum;Roseospirillum parvum;Roseospirillum parvum 930I |
| Ga0586324 0694573 2 631 | 0.00 | 0.00 | 0.00 | 0.00 | 0.00 | 0.00 | 0.00 | 0.00 | 0.00 | 3819200.00 | 0.00 | 0.00 | | hypothetical protein | |
| Ga0586324 0695138 1 1419 | 0.00 | 0.00 | 0.00 | 0.00 | 4398100.00 | 0.00 | 0.00 | 0.00 | 0.00 | 0.00 | 0.00 | 0.00 | | hypothetical protein | Bacteria;Proteobacteria;Alphaproteobacteria;Hyphomicrobiales;Bradyrhizobiaceae;Bradyrhizobium;Bradyrhizobium erythrophlei;Bradyrhizobium erythrophlei GAS242 |
| Ga0586324 0696161 151 1146 | 0.00 | 0.00 | 0.00 | 0.00 | 0.00 | 0.00 | 0.00 | 16531000.00 | 0.00 | 0.00 | 0.00 | 0.00 | | tripartite ATP-independent transporter DctP family solute receptor | Bacteria;Firmicutes;Negativicutes;Selenomonadales;Sporomusaceae;Anaerospora;Anaerospora hongkongensis;Anaerospora hongkongensis DSM 15969 |
| Ga0586324 0696509 1 1224 | 10400000.00 | 9044000.00 | 0.00 | 0.00 | 19570000.00 | 18500200.00 | 11787600.00 | 13603000.00 | 10773000.00 | 0.00 | 3696000.00 | 0.00 | | hypothetical protein | Bacteria;Verrucomicrobia;Verrucomicrobiae;Verrucomicrobiales;Verrucomicrobia subdivision 3;Pedosphaera;Pedosphaera parvula;Pedosphaera parvula Ellin514 |
| Ga0586324 0701924 2 1096 | 0.00 | 0.00 | 0.00 | 0.00 | 0.00 | 934811.93 | 0.00 | 0.00 | 0.00 | 2725800.00 | 0.00 | 0.00 | | predicted porin | Bacteria;Proteobacteria;Betaproteobacteria;Burkholderiales;Oxalobacteraceae;Janthinobacterium;Janthinobacterium sp. CG3;Janthinobacterium sp. CG3 |
| Ga0586324 0702425 268 1413 | 0.00 | 9384000.00 | 0.00 | 0.00 | 0.00 | 0.00 | 33880800.00 | 13298000.00 | 0.00 | 0.00 | 0.00 | 0.00 | | hypothetical protein | Bacteria;Proteobacteria;Alphaproteobacteria;Hyphomicrobiales;unclassified Hyphomicrobiales;Methyloceanibacter;Methyloceanibacter superfactus;Methyloceanibacter superfactus R-67175 |
| Ga0586324 0705170 291 1412 | 28050000.00 | 25071600.00 | 10373128.62 | 25678500.00 | 33538498.47 | 10083800.00 | 19243200.00 | 31219800.00 | 32634900.00 | 17748500.00 | 12320000.00 | 15787500.00 | | hypothetical protein | Bacteria;Proteobacteria;Gammaproteobacteria;Thiotrichales;unclassified Thiotrichales;Thioflexothrix;Thioflexothrix psekupsii;Thioflexothrix psekupsii D-3 |
| Ga0586324 0705441 354 1034 | 0.00 | 0.00 | 0.00 | 0.00 | 0.00 | 0.00 | 0.00 | 171426.47 | 0.00 | 0.00 | 0.00 | 0.00 | | putative intracellular protease/amidase | Bacteria;Proteobacteria;Betaproteobacteria;Nitrosomonadales;Nitrosomonadaceae;Nitrosospira;Nitrosospira sp. NpAV;Nitrosospira sp. NpAV |
| Ga0586324 0706703 711 1409 | 1190000.00 | 3134800.00 | 0.00 | 0.00 | 0.00 | 0.00 | 0.00 | 0.00 | 0.00 | 0.00 | 0.00 | 0.00 | | predicted porin | Bacteria;Proteobacteria;Betaproteobacteria;Burkholderiales;Burkholderiaceae;Quisquiliibacterium;Quisquiliibacterium transsilvanicum;Quisquiliibacterium transsilvanicum DSM 29781 |
| Ga0586324 0710811 544 1407 | 0.00 | 523323.24 | 0.00 | 0.00 | 0.00 | 0.00 | 0.00 | 0.00 | 2316600.00 | 0.00 | 0.00 | 0.00 | | hypothetical protein | Bacteria;Spirochaetes;Spirochaetia;Spirochaetales;Spirochaetaceae;Spirochaeta;Spirochaeta isovalerica;Spirochaeta isovalerica DSM 2461 |
| Ga0586324 0711829 115 996 | 0.00 | 0.00 | 0.00 | 0.00 | 0.00 | 0.00 | 0.00 | 0.00 | 0.00 | 0.00 | 0.00 | 9450000.00 | | outer membrane protein OmpA-like peptidoglycan-associated protein | Bacteria;Proteobacteria;Gammaproteobacteria;Xanthomonadales;Xanthomonadaceae;Pseudoxanthomonas;Pseudoxanthomonas spadix;Pseudoxanthomonas spadix BD-a59 |
| Ga0586324 0712841 1 1404 | 7640000.00 | 4549200.00 | 1946758.29 | 2251500.00 | 2997300.00 | 8972200.00 | 7774800.00 | 2934100.00 | 2583900.00 | 0.00 | 0.00 | 0.00 | | hypothetical protein | Bacteria;Acidobacteria;Vicinamibacteria;unclassified Vicinamibacteria;Vicinamibacteraceae;Luteitalea;Luteitalea pratensis;Luteitalea pratensis DSM 100886 |
| Ga0586324 0713593 419 1306 | 0.00 | 0.00 | 0.00 | 0.00 | 0.00 | 564133.03 | 0.00 | 0.00 | 0.00 | 0.00 | 0.00 | 0.00 | | hypothetical protein | |
| Ga0586324 0714763 496 1341 | 0.00 | 0.00 | 0.00 | 0.00 | 0.00 | 0.00 | 0.00 | 0.00 | 0.00 | 2802800.00 | 0.00 | 0.00 | | hypothetical protein | Bacteria;Proteobacteria;Gammaproteobacteria;Pseudomonadales;Pseudomonadaceae;Pseudomonas;Pseudomonas abietaniphila;Pseudomonas abietaniphila LMG 20220 |
| Ga0586324 0716829 3 1400 | 0.00 | 2264400.00 | 0.00 | 0.00 | 0.00 | 0.00 | 0.00 | 0.00 | 0.00 | 0.00 | 0.00 | 0.00 | | hypothetical protein | Bacteria;Acidobacteria;Vicinamibacteria;unclassified Vicinamibacteria;Vicinamibacteraceae;Luteitalea;Luteitalea pratensis;Luteitalea pratensis DSM 100886 |
| Ga0586324 0718031 3 701 | 1160000.00 | 0.00 | 0.00 | 0.00 | 0.00 | 0.00 | 0.00 | 0.00 | 850500.00 | 0.00 | 0.00 | 0.00 | | hypothetical protein | Bacteria;Gemmatimonadetes;Gemmatimonadetes;Gemmatimonadales;Gemmatimonadaceae;Gemmatimonas;Gemmatimonas phototrophica;Gemmatimonas phototrophica AP64 |
| Ga0586324 0719362 580 852 | 0.00 | 0.00 | 0.00 | 0.00 | 0.00 | 0.00 | 0.00 | 0.00 | 1223100.00 | 0.00 | 0.00 | 0.00 | | hypothetical protein | Bacteria;Proteobacteria;Alphaproteobacteria;Caulobacterales;Caulobacteraceae;Phenylobacterium;Phenylobacterium sp. Root77;Phenylobacterium sp. Root77 |
| Ga0586324 0720194 313 1044 | 0.00 | 0.00 | 0.00 | 0.00 | 0.00 | 0.00 | 0.00 | 0.00 | 0.00 | 2825900.00 | 2622400.00 | 1845000.00 | | hypothetical protein | Bacteria;Proteobacteria;Betaproteobacteria;Nitrosomonadales;Sulfuricellaceae;Sulfurirhabdus;Sulfurirhabdus autotrophica;Sulfurirhabdus autotrophica DSM 100309 |
| Ga0586324 0721328 666 1397 | 0.00 | 0.00 | 0.00 | 0.00 | 0.00 | 6828400.00 | 0.00 | 0.00 | 0.00 | 0.00 | 0.00 | 0.00 | | RND family efflux transporter MFP subunit | Bacteria;Proteobacteria;Deltaproteobacteria;Desulfobacterales;Desulfobulbaceae;Desulfoprunum;Desulfoprunum benzoelyticum;Desulfoprunum benzoelyticum DSM 28570 |
| Ga0586324 0724213 688 1395 | 0.00 | 0.00 | 0.00 | 0.00 | 0.00 | 1692732.57 | 0.00 | 0.00 | 0.00 | 0.00 | 0.00 | 0.00 | | hypothetical protein | Bacteria;Proteobacteria;Betaproteobacteria;Burkholderiales;Burkholderiaceae;Ralstonia;Ralstonia solanacearum;Ralstonia solanacearum PSI07 |
| Ga0586324 0726582 1 1392 | 0.00 | 0.00 | 0.00 | 0.00 | 0.00 | 0.00 | 0.00 | 0.00 | 0.00 | 2109800.00 | 0.00 | 0.00 | | 2',3'-cyclic-nucleotide 2'-phosphodiesterase (5'-nucleotidase family) | Bacteria;Firmicutes;Clostridia;Halanaerobiales;unclassified Halanaerobiales;Anoxybacter;Anoxybacter fermentans;Anoxybacter fermentans DY22613 |
| Ga0586324 0728827 3 857 | 3450000.00 | 0.00 | 0.00 | 0.00 | 0.00 | 0.00 | 0.00 | 0.00 | 0.00 | 0.00 | 0.00 | 0.00 | | outer membrane receptor protein involved in Fe transport | Bacteria;Candidatus Aminicenantes;unclassified Candidatus Aminicenantes;unclassified Candidatus Aminicenantes;unclassified Candidatus Aminicenantes;unclassified Candidatus Aminicenantes;Aminicenantes bacterium SCGC AAA252-N07;Aminicenantes bacterium SCGC AAA252-N07 (contamination screened) |
| Ga0586324 0731099 286 771 | 0.00 | 0.00 | 0.00 | 0.00 | 0.00 | 0.00 | 0.00 | 0.00 | 1944000.00 | 0.00 | 0.00 | 0.00 | | hypothetical protein | Bacteria;Nitrospirae;Nitrospira;Nitrospirales;Nitrospiraceae;Nitrospira;Nitrospira sp. ND1;Nitrospira sp. ND1 |
| Ga0586324 0731822 1 1389 | 0.00 | 0.00 | 0.00 | 0.00 | 0.00 | 0.00 | 0.00 | 695400.00 | 0.00 | 0.00 | 0.00 | 0.00 | | heterodisulfide reductase subunit A-like polyferredoxin | Bacteria;Proteobacteria;Deltaproteobacteria;Syntrophobacterales;Syntrophaceae;Desulfobacca;Desulfobacca acetoxidans;Desulfobacca acetoxidans ASRB2, DSM 11109 |
| Ga0586324 0732096 268 1389 | 115260000.00 | 145574400.00 | 45833216.33 | 25270000.00 | 104349300.00 | 71460000.00 | 100502400.00 | 205698100.00 | 50827500.00 | 125710200.00 | 79299528.00 | 36750000.00 | | predicted porin | Bacteria;Proteobacteria;Betaproteobacteria;Neisseriales;Chromobacteriaceae;Aquaspirillum;Aquaspirillum sp. LM1;Aquaspirillum sp. LM1 |
| Ga0586324 0732414 3 1388 | 0.00 | 0.00 | 0.00 | 0.00 | 0.00 | 0.00 | 0.00 | 0.00 | 11664000.00 | 0.00 | 0.00 | 0.00 | | alkylation response protein AidB-like acyl-CoA dehydrogenase | Bacteria;Firmicutes;Clostridia;Eubacteriales;Peptococcaceae;Desulfitispora;Desulfitispora alkaliphila;Desulfitispora alkaliphila DSM 22410 |
| Ga0586324 0733230 497 1096 | 0.00 | 0.00 | 4553352.58 | 3401000.00 | 0.00 | 0.00 | 0.00 | 0.00 | 0.00 | 0.00 | 0.00 | 0.00 | | phasin family protein | Bacteria;Proteobacteria;Betaproteobacteria;Burkholderiales;unclassified Burkholderiales;Paucibacter;Paucibacter oligotrophus;Paucibacter oligotrophus S00239 |
| Ga0586324 0734409 2 1228 | 0.00 | 9248000.00 | 17058900.00 | 11115000.00 | 11433000.00 | 13339200.00 | 13885200.00 | 8967000.00 | 8667000.00 | 28721000.00 | 20064000.00 | 21150000.00 | | hypothetical protein | |
| Ga0586324 0734491 3 1388 | 0.00 | 9180000.00 | 0.00 | 11495000.00 | 7477800.00 | 0.00 | 0.00 | 0.00 | 0.00 | 0.00 | 0.00 | 0.00 | | maltose-binding protein MalE | Bacteria;Chloroflexi;Ardenticatenia;Ardenticatenales;Ardenticatenaceae;Candidatus Promineofilum;Candidatus Promineofilum breve;Candidatus Promineofilum breve Cfx-K |
| Ga0586324 0736485 189 1385 | 0.00 | 0.00 | 0.00 | 0.00 | 0.00 | 2510056.32 | 0.00 | 0.00 | 0.00 | 0.00 | 0.00 | 0.00 | | hypothetical protein | Bacteria;Bacteroidetes;Bacteroidia;Marinilabiliales;Marinilabiliaceae;Saccharicrinis;Saccharicrinis fermentans;Saccharicrinis fermentans DSM 9555 |
| Ga0586324 0740185 1057 1383 | 0.00 | 2074000.00 | 0.00 | 0.00 | 0.00 | 0.00 | 0.00 | 0.00 | 0.00 | 0.00 | 0.00 | 0.00 | | outer membrane protein OmpA-like peptidoglycan-associated protein | Bacteria;Proteobacteria;Deltaproteobacteria;Desulfobacterales;Desulfobulbaceae;Desulfobulbus;Desulfobulbus mediterraneus;Desulfobulbus mediterraneus DSM 13871 |
| Ga0586324 0741515 1 1380 | 897348.00 | 0.00 | 0.00 | 0.00 | 0.00 | 0.00 | 0.00 | 0.00 | 0.00 | 0.00 | 0.00 | 0.00 | | hypothetical protein | Bacteria;Bacteroidetes;Cytophagia;Cytophagales;Hymenobacteraceae;Adhaeribacter;Adhaeribacter aquaticus;Adhaeribacter aquaticus DSM 16391 |
| Ga0586324 0742451 2 1252 | 0.00 | 4141200.00 | 7431600.00 | 0.00 | 0.00 | 0.00 | 0.00 | 0.00 | 2616300.00 | 0.00 | 0.00 | 0.00 | | hypothetical protein | Bacteria;Actinobacteria;Actinomycetia;Jiangellales;Jiangellaceae;Jiangella;Jiangella aurantiaca;Jiangella aurantiaca 8K307 |
| Ga0586324 0742541 526 1380 | 0.00 | 0.00 | 0.00 | 0.00 | 0.00 | 567146.26 | 6452400.00 | 504592.00 | 0.00 | 2833600.00 | 3484800.00 | 2625000.00 | | TRAP-type mannitol/chloroaromatic compound transport system substrate-binding protein | Bacteria;Proteobacteria;Gammaproteobacteria;Acidiferrobacterales;Acidiferrobacteraceae;Sulfuricaulis;Sulfuricaulis limicola;Sulfuricaulis limicola HA5 |
| Ga0586324 0742542 3 1379 | 0.00 | 0.00 | 0.00 | 0.00 | 946915.05 | 0.00 | 0.00 | 0.00 | 0.00 | 0.00 | 0.00 | 0.00 | | hypothetical protein | Bacteria;Actinobacteria;Actinomycetia;Jiangellales;Jiangellaceae;Jiangella;Jiangella aurantiaca;Jiangella aurantiaca 8K307 |
| Ga0586324 0744853 494 1378 | 7620000.00 | 7412000.00 | 16383300.00 | 9880000.00 | 0.00 | 16634300.00 | 12768000.00 | 0.00 | 0.00 | 0.00 | 0.00 | 9600000.00 | | hypothetical protein | |
| Ga0586324 0747529 612 1079 | 0.00 | 0.00 | 6474500.00 | 0.00 | 8868300.00 | 0.00 | 4240800.00 | 0.00 | 0.00 | 0.00 | 0.00 | 2722500.00 | | hypothetical protein | Bacteria;Proteobacteria;Deltaproteobacteria;Syntrophobacterales;Syntrophobacteraceae;Desulfacinum;Desulfacinum hydrothermale;Desulfacinum hydrothermale DSM 13146 |
| Ga0586324 0748246 2 1303 | 0.00 | 1224000.00 | 0.00 | 0.00 | 0.00 | 0.00 | 0.00 | 0.00 | 0.00 | 0.00 | 0.00 | 0.00 | | predicted porin | Bacteria;Nitrospirae;Thermodesulfovibrionia;Thermodesulfovibrionales;Thermodesulfovibrionaceae;Thermodesulfovibrio;Thermodesulfovibrio islandicus;Thermodesulfovibrio islandicus DSM 12570 |
| Ga0586324 0750196 2 1375 | 0.00 | 4318000.00 | 0.00 | 0.00 | 0.00 | 0.00 | 0.00 | 0.00 | 0.00 | 0.00 | 0.00 | 0.00 | | hypothetical protein | Bacteria;Proteobacteria;Deltaproteobacteria;Desulfuromonadales;Geobacteraceae;Geobacter;Geobacter thiogenes;Geobacter thiogenes ATCC BAA-34 |
| Ga0586324 0750827 3 1373 | 0.00 | 0.00 | 0.00 | 0.00 | 0.00 | 0.00 | 0.00 | 0.00 | 0.00 | 0.00 | 1786400.00 | 0.00 | | hypothetical protein | Bacteria;Proteobacteria;Deltaproteobacteria;Myxococcales;Archangiaceae;Cystobacter;Cystobacter ferrugineus;Cystobacter ferrugineus Cbfe23 |
| Ga0586324 0751243 308 1372 | 0.00 | 42683600.00 | 37211078.51 | 88497.63 | 159021700.00 | 0.00 | 358776.24 | 36256.45 | 50527800.00 | 65057300.00 | 1654400.00 | 53188.35 | | hypothetical protein | Bacteria;Proteobacteria;Deltaproteobacteria;Syntrophobacterales;Syntrophaceae;Syntrophus;Syntrophus aciditrophicus;Syntrophus aciditrophicus SB |
| Ga0586324 0754673 1 1326 | 26890000.00 | 8282400.00 | 8282810.96 | 0.00 | 0.00 | 3125831.11 | 0.00 | 0.00 | 3612600.00 | 7492100.00 | 9240000.00 | 14535000.00 | | hypothetical protein | Bacteria;Nitrospirae;Thermodesulfovibrionia;Thermodesulfovibrionales;Thermodesulfovibrionaceae;Thermodesulfovibrio;Thermodesulfovibrio hydrogeniphilus;Thermodesulfovibrio hydrogeniphilus DSM 18151 |
| Ga0586324 0757298 3 926 | 0.00 | 0.00 | 0.00 | 2536500.00 | 0.00 | 0.00 | 0.00 | 0.00 | 0.00 | 0.00 | 0.00 | 0.00 | | predicted porin | Bacteria;Proteobacteria;Betaproteobacteria;Rhodocyclales;Zoogloeaceae;Azoarcus;Azoarcus sp. CIB;Azoarcus sp. CIB |
| Ga0586324 0758255 3 1367 | 0.00 | 0.00 | 16439600.00 | 0.00 | 0.00 | 14133200.00 | 0.00 | 0.00 | 5119200.00 | 0.00 | 0.00 | 0.00 | | 2',3'-cyclic-nucleotide 2'-phosphodiesterase (5'-nucleotidase family) | Bacteria;Proteobacteria;Deltaproteobacteria;Myxococcales;Myxococcaceae;Myxococcus;Myxococcus hansupus;Myxococcus hansupus DSM 436 |
| Ga0586324 0758537 1 1368 | 252991.00 | 0.00 | 0.00 | 0.00 | 0.00 | 0.00 | 0.00 | 0.00 | 0.00 | 0.00 | 0.00 | 0.00 | | hypothetical protein | Bacteria;Proteobacteria;Betaproteobacteria;Burkholderiales;unclassified Burkholderiales;Rhizobacter;Rhizobacter sp. Root404;Rhizobacter sp. Root404 |
| Ga0586324 0758947 2 1366 | 5570000.00 | 1271600.00 | 0.00 | 0.00 | 0.00 | 0.00 | 0.00 | 0.00 | 0.00 | 0.00 | 0.00 | 0.00 | | hypothetical protein | Bacteria;Candidatus Aminicenantes;unclassified Candidatus Aminicenantes;unclassified Candidatus Aminicenantes;unclassified Candidatus Aminicenantes;unclassified Candidatus Aminicenantes;Aminicenantes bacterium SCGC AAA255-A06;Aminicenantes bacterium SCGC AAA255-A06 (contamination screened) |
| Ga0586324 0770902 1 1359 | 0.00 | 0.00 | 0.00 | 0.00 | 0.00 | 0.00 | 0.00 | 780800.00 | 233868.87 | 0.00 | 0.00 | 0.00 | | hypothetical protein | Bacteria;Bacteroidetes;unclassified Bacteroidetes;Bacteroidetes Order II. Incertae sedis;Rhodothermaceae;unclassified Rhodothermaceae;Rhodothermaceae bacterium RA;Halothermobacillus malaysiensis RA |
| Ga0586324 0772207 2 1357 | 0.00 | 0.00 | 0.00 | 0.00 | 0.00 | 0.00 | 0.00 | 0.00 | 4706100.00 | 0.00 | 0.00 | 0.00 | | acyl-CoA reductase-like NAD-dependent aldehyde dehydrogenase | Bacteria;Chloroflexi;Caldilineae;Caldilineales;Caldilineaceae;Caldilinea;Caldilinea aerophila;Caldilinea aerophila STL-6-O1, DSM 14535 |
| Ga0586324 0772399 68 1102 | 3310000.00 | 5773200.00 | 0.00 | 0.00 | 0.00 | 0.00 | 0.00 | 0.00 | 0.00 | 5621000.00 | 2384800.00 | 0.00 | | hypothetical protein | |
| Ga0586324 0781862 2 1351 | 0.00 | 6419200.00 | 13624600.00 | 5681000.00 | 6777400.00 | 0.00 | 0.00 | 0.00 | 2802600.00 | 15507800.00 | 8923200.00 | 7305000.00 | | hypothetical protein | Bacteria;Proteobacteria;Betaproteobacteria;Burkholderiales;Comamonadaceae;Acidovorax;Acidovorax sp. JS42;Acidovorax sp. JS42 |
| Ga0586324 0782166 1 1350 | 0.00 | 0.00 | 0.00 | 0.00 | 0.00 | 0.00 | 5928000.00 | 0.00 | 0.00 | 0.00 | 1707200.00 | 0.00 | | hypothetical protein | Bacteria;Actinobacteria;Actinomycetia;Propionibacteriales;Nocardioidaceae;Nocardioides;Nocardioides sp. Root122;Nocardioides sp. Root122 |
| Ga0586324 0782853 3 1349 | 0.00 | 0.00 | 0.00 | 677279.70 | 0.00 | 0.00 | 0.00 | 0.00 | 565806.87 | 0.00 | 0.00 | 0.00 | | hypothetical protein | Bacteria;Chloroflexi;Chloroflexia;Chloroflexales;Chloroflexaceae;Chloroflexus;Chloroflexus islandicus;Chloroflexus islandicus isl-2 |
| Ga0586324 0782865 556 1350 | 837128.00 | 1428000.00 | 0.00 | 659334.20 | 0.00 | 0.00 | 0.00 | 0.00 | 270872.91 | 0.00 | 0.00 | 0.00 | | DNA-binding MurR/RpiR family transcriptional regulator | Bacteria;Chloroflexi;Caldilineae;Caldilineales;Caldilineaceae;Caldilinea;Caldilinea aerophila;Caldilinea aerophila STL-6-O1, DSM 14535 |
| Ga0586324 0783364 2 1348 | 0.00 | 0.00 | 185555.23 | 0.00 | 1864300.00 | 1238056.41 | 4856400.00 | 0.00 | 0.00 | 0.00 | 0.00 | 1845000.00 | | hypothetical protein | |
| Ga0586324 0786081 3 638 | 11600000.00 | 12797600.00 | 29557500.00 | 17046966.25 | 29252000.00 | 29254505.21 | 23836433.28 | 25955500.00 | 30770160.93 | 16508800.00 | 12408000.00 | 8850000.00 | | predicted porin | Bacteria;Proteobacteria;Betaproteobacteria;Burkholderiales;unclassified Burkholderiales;Aquabacterium;Aquabacterium tepidiphilum;Aquabacterium tepidiphilum YIM 730274 |
| Ga0586324 0788462 1 1293 | 914521.00 | 1366800.00 | 1388448.08 | 7001500.00 | 0.00 | 0.00 | 0.00 | 0.00 | 2081700.00 | 0.00 | 0.00 | 0.00 | | hypothetical protein | Bacteria;Candidatus Aminicenantes;unclassified Candidatus Aminicenantes;unclassified Candidatus Aminicenantes;unclassified Candidatus Aminicenantes;unclassified Candidatus Aminicenantes;Aminicenantes bacterium SCGC AAA252-O03;Aminicenantes bacterium SCGC AAA252-O03 (contamination screened) |
| Ga0586324 0789034 507 1004 | 797712.00 | 0.00 | 0.00 | 0.00 | 0.00 | 0.00 | 180775.73 | 0.00 | 432663.12 | 0.00 | 0.00 | 0.00 | | hypothetical protein | Bacteria;Proteobacteria;Deltaproteobacteria;Syntrophobacterales;Syntrophaceae;Desulfomonile;Desulfomonile tiedjei;Desulfomonile tiedjei DCB-1, DSM 6799 |
| Ga0586324 0793451 343 1341 | 0.00 | 0.00 | 0.00 | 0.00 | 0.00 | 0.00 | 0.00 | 13603000.00 | 0.00 | 0.00 | 0.00 | 0.00 | | arylsulfatase A-like enzyme | Bacteria;Proteobacteria;Alphaproteobacteria;Hyphomicrobiales;Xanthobacteraceae;Pseudolabrys;Pseudolabrys taiwanensis;Pseudolabrys taiwanensis CC-BB4 |
| Ga0586324 0795231 336 1340 | 0.00 | 0.00 | 0.00 | 0.00 | 0.00 | 0.00 | 0.00 | 5191100.00 | 0.00 | 6083000.00 | 6626400.00 | 6457500.00 | | hypothetical protein | Bacteria;Proteobacteria;Betaproteobacteria;Nitrosomonadales;Sulfuricellaceae;Sulfurirhabdus;Sulfurirhabdus autotrophica;Sulfurirhabdus autotrophica DSM 100309 |
| Ga0586324 0800591 1 1128 | 0.00 | 0.00 | 21281400.00 | 0.00 | 0.00 | 0.00 | 0.00 | 0.00 | 0.00 | 0.00 | 0.00 | 0.00 | | uncharacterized protein (DUF2252 family) | Bacteria;Actinobacteria;Actinomycetia;Micrococcales;Intrasporangiaceae;Humibacillus;Humibacillus xanthopallidus;Humibacillus xanthopallidus DSM 21776 |
| Ga0586324 0800743 3 323 | 0.00 | 1468800.00 | 0.00 | 0.00 | 0.00 | 0.00 | 0.00 | 0.00 | 0.00 | 0.00 | 0.00 | 0.00 | | outer membrane protein OmpA-like peptidoglycan-associated protein | Bacteria;Bacteroidetes;Sphingobacteriia;Sphingobacteriales;Sphingobacteriaceae;Solitalea;Solitalea koreensis;Solitalea koreensis DSM 21342 |
| Ga0586324 0801608 220 717 | 0.00 | 0.00 | 0.00 | 708199.35 | 0.00 | 0.00 | 0.00 | 0.00 | 0.00 | 0.00 | 0.00 | 0.00 | | rubrerythrin | Bacteria;Proteobacteria;Deltaproteobacteria;Syntrophobacterales;Syntrophaceae;Smithella;Smithella sp. F21;Smithella sp. F21 |
| Ga0586324 0802380 3 917 | 0.00 | 0.00 | 0.00 | 0.00 | 0.00 | 0.00 | 0.00 | 0.00 | 286336.62 | 0.00 | 0.00 | 0.00 | | hypothetical protein | Bacteria;Acidobacteria;Vicinamibacteria;unclassified Vicinamibacteria;Vicinamibacteraceae;Luteitalea;Luteitalea pratensis;Luteitalea pratensis DSM 100886 |
| Ga0586324 0805710 367 1332 | 0.00 | 0.00 | 0.00 | 438432.60 | 437694.38 | 131992.18 | 451330.56 | 272815.18 | 412458.48 | 0.00 | 414036.48 | 234277.50 | | crotonobetainyl-CoA:carnitine CoA-transferase CaiB-like acyl-CoA transferase | Bacteria;Proteobacteria;Deltaproteobacteria;Syntrophobacterales;Syntrophobacteraceae;Desulfacinum;Desulfacinum infernum;Desulfacinum infernum DSM 9756 |
| Ga0586324 0805749 331 1221 | 0.00 | 0.00 | 0.00 | 0.00 | 0.00 | 0.00 | 0.00 | 0.00 | 0.00 | 2209900.00 | 5095200.00 | 0.00 | | hypothetical protein | |
| Ga0586324 0806340 1125 1331 | 2650000.00 | 0.00 | 0.00 | 0.00 | 0.00 | 0.00 | 0.00 | 0.00 | 0.00 | 2025100.00 | 0.00 | 0.00 | | hypothetical protein | Bacteria;Proteobacteria;Gammaproteobacteria;Methylococcales;Methylococcaceae;Methyloglobulus;Methyloglobulus morosus;Methyloglobulus morosus KoM1 |
| Ga0586324 0807951 20 469 | 0.00 | 0.00 | 0.00 | 0.00 | 0.00 | 0.00 | 0.00 | 0.00 | 0.00 | 1470700.00 | 0.00 | 0.00 | | hypothetical protein | |
| Ga0586324 0808344 1 1332 | 1270000.00 | 0.00 | 0.00 | 0.00 | 0.00 | 0.00 | 0.00 | 0.00 | 0.00 | 0.00 | 0.00 | 3592500.00 | | hypothetical protein | Bacteria;Acidobacteria;Vicinamibacteria;unclassified Vicinamibacteria;Vicinamibacteraceae;Luteitalea;Luteitalea pratensis;Luteitalea pratensis DSM 100886 |
| Ga0586324 0811485 1 1329 | 52690000.00 | 25309600.00 | 2569475.70 | 41255912.20 | 47936200.00 | 71586535.81 | 28591200.00 | 22661500.00 | 5394600.00 | 25548600.00 | 20143200.00 | 3097689.75 | | hypothetical protein | Bacteria;Proteobacteria;Gammaproteobacteria;Alteromonadales;Alteromonadaceae;Marinobacter;Marinobacter sp.;Marinobacter sp. MIT1353 |
| Ga0586324 0815256 1 765 | 0.00 | 0.00 | 0.00 | 0.00 | 0.00 | 0.00 | 0.00 | 286886.66 | 0.00 | 1370600.00 | 0.00 | 0.00 | | hypothetical protein | Bacteria;Proteobacteria;Deltaproteobacteria;Desulfuromonadales;Geobacteraceae;Geobacter;Geobacter lovleyi;Geobacter lovleyi SZ |
| Ga0586324 0817451 1 1008 | 0.00 | 0.00 | 0.00 | 0.00 | 0.00 | 0.00 | 6976800.00 | 0.00 | 4600800.00 | 0.00 | 0.00 | 4552500.00 | | tripartite-type tricarboxylate transporter receptor subunit TctC | Bacteria;Proteobacteria;Alphaproteobacteria;Hyphomicrobiales;Xanthobacteraceae;Pseudolabrys;Pseudolabrys taiwanensis;Pseudolabrys taiwanensis CC-BB4 |
| Ga0586324 0823408 163 1185 | 9570000.00 | 9044000.00 | 13906100.00 | 13680000.00 | 0.00 | 25368300.00 | 0.00 | 0.00 | 0.00 | 0.00 | 0.00 | 0.00 | | hypothetical protein | Bacteria;Proteobacteria;Alphaproteobacteria;Hyphomicrobiales;Bradyrhizobiaceae;Bradyrhizobium;Bradyrhizobium erythrophlei;Bradyrhizobium erythrophlei GAS138 |
| Ga0586324 0823749 3 1319 | 0.00 | 0.00 | 0.00 | 0.00 | 0.00 | 0.00 | 0.00 | 646600.00 | 3807000.00 | 0.00 | 0.00 | 0.00 | | outer membrane receptor for ferrienterochelin and colicin | Bacteria;Acidobacteria;Thermoanaerobaculia;Thermoanaerobaculales;Thermoanaerobaculaceae;Thermoanaerobaculum;Thermoanaerobaculum aquaticum;Thermoanaerobaculum aquaticum MP-01 |
| Ga0586324 0823999 1 1254 | 0.00 | 1251200.00 | 17030068.77 | 7429000.00 | 0.00 | 0.00 | 0.00 | 0.00 | 0.00 | 0.00 | 0.00 | 1502136.75 | | TonB-dependent SusC/RagA subfamily outer membrane receptor | Bacteria;Bacteroidetes;Flavobacteriia;Flavobacteriales;Flavobacteriaceae;Arenibacter;Arenibacter nanhaiticus;Arenibacter nanhaiticus CGMCC 1.8863 |
| Ga0586324 0825165 2 349 | 0.00 | 0.00 | 0.00 | 1054500.00 | 0.00 | 0.00 | 0.00 | 0.00 | 0.00 | 0.00 | 0.00 | 0.00 | | rubrerythrin | Bacteria;Thermotogae;Thermotogae;Thermotogales;Fervidobacteriaceae;Fervidobacterium;Fervidobacterium nodosum;Fervidobacterium nodosum Rt17-B1 |
| Ga0586324 0827412 3 1175 | 0.00 | 924800.00 | 0.00 | 0.00 | 0.00 | 0.00 | 0.00 | 0.00 | 0.00 | 0.00 | 0.00 | 0.00 | | hypothetical protein | Bacteria;Acidobacteria;Acidobacteriia;Bryobacterales;Solibacteraceae;Candidatus Solibacter;Candidatus Solibacter usitatus;Candidatus Solibacter usitatus Ellin6076 |
| Ga0586324 0827902 2 1210 | 0.00 | 21148000.00 | 0.00 | 0.00 | 41612000.00 | 42082000.00 | 0.00 | 19825000.00 | 0.00 | 0.00 | 0.00 | 0.00 | | predicted deacylase | Bacteria;Proteobacteria;Gammaproteobacteria;Alteromonadales;Alteromonadaceae;Marinobacter;Marinobacter lipolyticus;Marinobacter lipolyticus SM19 |
| Ga0586324 0828132 986 1318 | 0.00 | 0.00 | 0.00 | 0.00 | 0.00 | 0.00 | 0.00 | 0.00 | 0.00 | 0.00 | 0.00 | 41625000.00 | | hypothetical protein | Bacteria;Firmicutes;Bacilli;Bacillales;Paenibacillaceae;Paenibacillus;Paenibacillus sp. FJAT-26967;Paenibacillus sp. FJAT-26967 |
| Ga0586324 0828249 300 1316 | 615706.00 | 0.00 | 0.00 | 0.00 | 0.00 | 0.00 | 0.00 | 0.00 | 0.00 | 0.00 | 0.00 | 0.00 | | predicted dehydrogenase | Bacteria;Proteobacteria;Betaproteobacteria;Burkholderiales;unclassified Burkholderiales;Rhizobacter;Rhizobacter sp. SG490;Rhizobacter sp. SG490 |
| Ga0586324 0831115 3 1316 | 0.00 | 0.00 | 0.00 | 0.00 | 0.00 | 0.00 | 0.00 | 0.00 | 0.00 | 0.00 | 12540793.76 | 0.00 | | hypothetical protein | Bacteria;Actinobacteria;Actinomycetia;Propionibacteriales;Kribbellaceae;Kribbella;Kribbella catacumbae;Kribbella catacumbae DSM 19601 |
| Ga0586324 0831257 2 1315 | 0.00 | 0.00 | 0.00 | 1111500.00 | 0.00 | 0.00 | 0.00 | 0.00 | 0.00 | 0.00 | 0.00 | 0.00 | | hypothetical protein | Bacteria;Actinobacteria;Actinomycetia;Propionibacteriales;Nocardioidaceae;Nocardioides;Nocardioides sp. Root122;Nocardioides sp. Root122 |

| | | | | | | | | | | | | | | | |
|---|---|---|---|---|---|---|---|---|---|---|---|---|---|---|---|
| Ga0586324_0832278_182_1264 | 24220000.00 | 0.00 | 0.00 | 5681000.00 | 33568892.74 | 12428589.19 | 27382800.00 | 22185700.00 | 22477500.00 | 15700300.00 | 596264.24 | 4177500.00 | | predicted porin | Bacteria;Proteobacteria;Betaproteobacteria;Burkholderiales;unclassified Burkholderiales;Aquincola;Aquincola tertiaricarbonis;Aquincola tertiaricarbonis MIMtkpLc11 |
| Ga0586324_0837258_3_1310 | 6230000.00 | 897600.00 | 0.00 | 0.00 | 0.00 | 0.00 | 6641530.56 | 0.00 | 12789900.00 | 2186800.00 | 5517600.00 | 1650000.00 | | hypothetical protein | Bacteria;Atribacterota;unclassified Atribacterota;unclassified Atribacterota;unclassified Atribacterota;unclassified Atribacterota;Atribacteria bacterium SCGC AB-164-P05;Atribacteria bacterium SCGC AB-164-P05 (contamination screened) |
| Ga0586324_0838761_95_1177 | 0.00 | 0.00 | 0.00 | 0.00 | 0.00 | 1411898.74 | 0.00 | 0.00 | 0.00 | 0.00 | 0.00 | 0.00 | | TRAP-type mannitol/chloroaromatic compound transport system substrate-binding protein | Bacteria;Proteobacteria;Betaproteobacteria;Burkholderiales;unclassified Burkholderiales;Burkholderiales bacterium JOSHI_001;Burkholderiales sp. JOSHI_001 |
| Ga0586324_0842214_2_952 | 7520000.00 | 6772800.00 | 22871734.25 | 7742500.00 | 11999500.00 | 0.00 | 0.00 | 6380600.00 | 5386500.00 | 0.00 | 0.00 | 2632500.00 | | hypothetical protein | Bacteria;Proteobacteria;Alphaproteobacteria;Sphingomonadales;Erythrobacteraceae;Erythrobacter;Erythrobacter neustonensis;Erythrobacter neustonensis DSM 9434 |
| Ga0586324_0843122_1_636 | 0.00 | 0.00 | 0.00 | 0.00 | 0.00 | 0.00 | 0.00 | 0.00 | 0.00 | 0.00 | 0.00 | 1410000.00 | | hypothetical protein | Archaea;Euryarchaeota;Methanomicrobia;Methanosarcinales;Methanotrichaceae;Methanothrix;Methanothrix soehngenii;Methanothrix soehngenii GP-6 |
| Ga0586324_0844806_1_1284 | 0.00 | 0.00 | 0.00 | 0.00 | 0.00 | 0.00 | 0.00 | 0.00 | 0.00 | 0.00 | 0.00 | 3015000.00 | | Ni,Fe-hydrogenase I large subunit | Bacteria;Firmicutes;Clostridia;Eubacteriales;Peptococcaceae;Desulfosporosinus;Desulfosporosinus sp. HMP52;Desulfosporosinus sp. HMP52 |
| Ga0586324_0845207_38_1306 | 0.00 | 0.00 | 0.00 | 0.00 | 0.00 | 0.00 | 0.00 | 0.00 | 0.00 | 0.00 | 0.00 | 2955000.00 | | TRAP-type mannitol/chloroaromatic compound transport system substrate-binding protein | Bacteria;Proteobacteria;Alphaproteobacteria;Hyphomicrobiales;Bradyrhizobiaceae;Bradyrhizobium;Bradyrhizobium lablabi;Bradyrhizobium lablabi GAS499 |
| Ga0586324_0856097_3_875 | 24730000.00 | 33857200.00 | 0.00 | 35340000.00 | 8807205.55 | 0.00 | 6452400.00 | 61830758.39 | 8829000.00 | 69270000.80 | 68393600.00 | 54082500.00 | | predicted porin | Bacteria;Proteobacteria;Betaproteobacteria;Burkholderiales;Oxalobacteraceae;Duganella;Duganella sp. Leaf61;Duganella sp. Leaf61 |
| Ga0586324_0856904_633_1211 | 0.00 | 0.00 | 0.00 | 0.00 | 0.00 | 0.00 | 28728000.00 | 0.00 | 0.00 | 0.00 | 0.00 | 0.00 | | rubrerythrin | Bacteria;Proteobacteria;Deltaproteobacteria;Syntrophorhabdales;Syntrophorhabdaceae;Syntrophorhabdus;Syntrophorhabdus aromaticivorans;Syntrophorhabdus aromaticivorans UI |
| Ga0586324_0859217_289_1296 | 0.00 | 0.00 | 0.00 | 0.00 | 811896.47 | 0.00 | 0.00 | 0.00 | 0.00 | 0.00 | 0.00 | 0.00 | | hypothetical protein | Bacteria;Proteobacteria;Deltaproteobacteria;Desulfuromonadales;Geobacteraceae;Geobacter;Geobacter lovleyi;Geobacter lovleyi SZ |
| Ga0586324_0861778_3_1133 | 0.00 | 0.00 | 0.00 | 27692500.00 | 13184000.00 | 0.00 | 0.00 | 0.00 | 0.00 | 3226300.00 | 0.00 | 0.00 | | hypothetical protein | Bacteria;Acidobacteria;Vicinamibacteria;unclassified Vicinamibacteria;Vicinamibacteraceae;Luteitalea;Luteitalea pratensis;Luteitalea pratensis DSM 100886 |
| Ga0586324_0864959_495_1292 | 0.00 | 0.00 | 0.00 | 1149500.00 | 0.00 | 0.00 | 0.00 | 0.00 | 0.00 | 0.00 | 0.00 | 0.00 | | TRAP-type mannitol/chloroaromatic compound transport system substrate-binding protein | Bacteria;Proteobacteria;Alphaproteobacteria;Rhodospirillales;Azospirillaceae;Azospirillum;Azospirillum sp. TSA2s;Azospirillum sp. TSA2S |
| Ga0586324_0865959_336_1292 | 0.00 | 0.00 | 0.00 | 0.00 | 0.00 | 687099.81 | 0.00 | 1000400.00 | 0.00 | 0.00 | 0.00 | 0.00 | | hypothetical protein | Bacteria;Candidatus Aminicenantes;unclassified Candidatus Aminicenantes;unclassified Candidatus Aminicenantes;unclassified Candidatus Aminicenantes;unclassified Candidatus Aminicenantes;Aminicenantes bacterium SCGC AAA252-D06;Aminicenantes bacterium SCGC AAA252-D06 (contamination screened) |
| Ga0586324_0867371_244_1290 | 11100000.00 | 2801600.00 | 0.00 | 13575500.00 | 3790400.00 | 0.00 | 0.00 | 0.00 | 0.00 | 12766600.00 | 4382400.00 | 3885000.00 | | hypothetical protein | |
| Ga0586324_0870941_126_695 | 0.00 | 0.00 | 0.00 | 0.00 | 0.00 | 0.00 | 0.00 | 0.00 | 2656800.00 | 2094400.00 | 3581600.00 | 2932500.00 | | osmotically-inducible protein OsmY | Bacteria;Proteobacteria;Betaproteobacteria;Nitrosomonadales;Sulfuricellaceae;Sulfuricella;Sulfuricella sp. T08;Sulfuricella sp. T08 |
| Ga0586324_0871187_531_1289 | 0.00 | 3039600.00 | 4716121.51 | 11685000.00 | 8899200.00 | 11195400.00 | 5266800.00 | 2141100.00 | 4179600.00 | 1955800.00 | 4153600.00 | 2812500.00 | | hypothetical protein | Bacteria;Proteobacteria;Deltaproteobacteria;Desulfobacterales;Desulfobacteraceae;Desulfococcus;Desulfococcus oleovorans;Desulfococcus oleovorans Hxd3 |
| Ga0586324_0875604_1_360 | 0.00 | 3236800.00 | 0.00 | 0.00 | 0.00 | 0.00 | 0.00 | 0.00 | 0.00 | 0.00 | 0.00 | 0.00 | | predicted porin | Bacteria;Proteobacteria;Betaproteobacteria;Rhodocyclales;Azonexaceae;Dechloromonas;Dechloromonas denitrificans;Dechloromonas denitrificans ATCC BAA-841 |
| Ga0586324_0875963_129_1286 | 3350000.00 | 7208000.00 | 0.00 | 5728500.00 | 6725900.00 | 0.00 | 4582800.00 | 6063400.00 | 6147900.00 | 3241700.00 | 3766400.00 | 3607500.00 | | subtilisin family serine protease | Bacteria;Proteobacteria;Gammaproteobacteria;Chromatiales;Woeseiaceae;unclassified Woeseiaceae;unclassified Woeseiaceae;uncultured Woeseiaceae SAG 1868_B |
| Ga0586324_0880334_3_1232 | 0.00 | 1489200.00 | 0.00 | 1919000.00 | 0.00 | 0.00 | 0.00 | 0.00 | 2907900.00 | 0.00 | 1126400.00 | 0.00 | | hypothetical protein | Bacteria;Acidobacteria;Vicinamibacteria;unclassified Vicinamibacteria;Vicinamibacteraceae;Luteitalea;Luteitalea pratensis;Luteitalea pratensis DSM 100886 |
| Ga0586324_0881612_326_1282 | 0.00 | 0.00 | 0.00 | 0.00 | 0.00 | 0.00 | 0.00 | 3483100.00 | 0.00 | 4727800.00 | 6740800.00 | 4425000.00 | | hypothetical protein | Bacteria;Proteobacteria;Betaproteobacteria;Nitrosomonadales;Thiobacillaceae;Thiobacillus;Thiobacillus thiophilus;Thiobacillus thiophilus DSM 19892 |
| Ga0586324_0882066_850_1281 | 0.00 | 0.00 | 0.00 | 0.00 | 0.00 | 6590200.00 | 0.00 | 0.00 | 0.00 | 0.00 | 0.00 | 0.00 | | membrane protease subunit (stomatin/prohibitin family) | Bacteria;Chloroflexi;Anaerolineae;Anaerolineales;Anaerolineaceae;Pelolinea;Pelolinea submarina;Pelolinea submarina DSM 23923 |
| Ga0586324_0882864_722_1174 | 3050000.00 | 0.00 | 0.00 | 0.00 | 0.00 | 0.00 | 0.00 | 0.00 | 0.00 | 0.00 | 0.00 | 0.00 | | hypothetical protein | |
| Ga0586324_0883910_2_1279 | 0.00 | 5671200.00 | 0.00 | 0.00 | 0.00 | 0.00 | 3214800.00 | 0.00 | 0.00 | 0.00 | 2455200.00 | 3330000.00 | | hypothetical protein | |
| Ga0586324_0884729_208_1281 | 1920000.00 | 1611600.00 | 0.00 | 0.00 | 0.00 | 0.00 | 0.00 | 0.00 | 0.00 | 513137.24 | 0.00 | 0.00 | | arylsulfatase A-like enzyme | Bacteria;Proteobacteria;Alphaproteobacteria;Hyphomicrobiales;Bradyrhizobiaceae;Bradyrhizobium;Bradyrhizobium sp. URHA0002;Bradyrhizobium sp. URHA0002 |
| Ga0586324_0886746_1_1083 | 0.00 | 0.00 | 0.00 | 0.00 | 0.00 | 0.00 | 0.00 | 0.00 | 7476300.00 | 0.00 | 0.00 | 0.00 | | hypothetical protein | Bacteria;Planctomycetes;Planctomycetia;Pirellulales;Thermoguttaceae;Thermogutta;Thermogutta terrifontis;Thermogutta terrifontis R1 |
| Ga0586324_0890948_1_1092 | 6330000.00 | 0.00 | 0.00 | 5491000.00 | 5623800.00 | 0.00 | 0.00 | 0.00 | 6561000.00 | 0.00 | 6520800.00 | 0.00 | | predicted porin | Bacteria;Proteobacteria;Betaproteobacteria;Rhodocyclales;Rhodocyclaceae;Rugosibacter;Rugosibacter aromaticivorans;Rugosibacter aromaticivorans PG1-Ca6 |
| Ga0586324_0893399_907_1275 | 0.00 | 3719600.00 | 0.00 | 0.00 | 0.00 | 0.00 | 0.00 | 0.00 | 0.00 | 0.00 | 0.00 | 0.00 | | hypothetical protein | |
| Ga0586324_0893500_2_1273 | 0.00 | 5440000.00 | 0.00 | 0.00 | 0.00 | 0.00 | 0.00 | 0.00 | 0.00 | 0.00 | 0.00 | 0.00 | | hypothetical protein | Bacteria;Proteobacteria;Deltaproteobacteria;Desulfobacterales;Desulfobulbaceae;Desulfofustis;Desulfofustis glycolicus;Desulfofustis glycolicus DSM 9705 |
| Ga0586324_0894461_375_1274 | 0.00 | 0.00 | 2548571.51 | 0.00 | 4243600.00 | 0.00 | 0.00 | 3806400.00 | 0.00 | 0.00 | 2833600.00 | 0.00 | | membrane protease subunit (stomatin/prohibitin family) | Archaea;Thaumarchaeota;unclassified Thaumarchaeota;unclassified Thaumarchaeota;unclassified Thaumarchaeota;unclassified Thaumarchaeota;Aigarchaeota archaeon JGI 0000106-J15;Aigarchaeota archaeon JGI 0000106-J15 (contamination screened) |
| Ga0586324_0896139_479_982 | 0.00 | 0.00 | 0.00 | 0.00 | 0.00 | 0.00 | 7793525.64 | 42944000.00 | 0.00 | 0.00 | 0.00 | 0.00 | | ferritin-like metal-binding protein YciE | Bacteria;Proteobacteria;Alphaproteobacteria;Hyphomicrobiales;Rhizobiaceae;Rhizobium;Rhizobium leguminosarum;Rhizobium leguminosarum ATCC 14479 |
| Ga0586324_0896683_3_875 | 1970000.00 | 0.00 | 0.00 | 0.00 | 0.00 | 0.00 | 0.00 | 0.00 | 0.00 | 0.00 | 0.00 | 0.00 | | hypothetical protein | Bacteria;Acidobacteria;Acidobacteriia;Acidobacteriales;Acidobacteriaceae;Edaphobacter;Edaphobacter modestus;Edaphobacter modestus DSM 18101 |
| Ga0586324_0899242_2_889 | 372166.00 | 986000.00 | 189514.81 | 763190.10 | 739030.15 | 142106.55 | 488895.84 | 713700.00 | 601591.05 | 793100.00 | 775539.60 | 1080000.00 | | TRAP-type mannitol/chloroaromatic compound transport system substrate-binding protein | Bacteria;Proteobacteria;Betaproteobacteria;unclassified Betaproteobacteria;unclassified Betaproteobacteria;unclassified Betaproteobacteria;Betaproteobacteria bacterium GR16-43;Betaproteobacteria bacterium GR16-43 |
| Ga0586324_0900074_665_1270 | 0.00 | 1774800.00 | 0.00 | 0.00 | 0.00 | 0.00 | 0.00 | 0.00 | 0.00 | 0.00 | 0.00 | 0.00 | | hypothetical protein | Bacteria;Bacteroidetes;Cytophagia;Cytophagales;Cytophagaceae;Cytophaga;Cytophaga aurantiaca;Cytophaga aurantiaca DSM 3654 |
| Ga0586324_0901930_48_1004 | 0.00 | 0.00 | 4430900.08 | 0.00 | 0.00 | 0.00 | 0.00 | 0.00 | 2891700.00 | 0.00 | 0.00 | 0.00 | | predicted flap endonuclease-1-like 5' DNA nuclease | Bacteria;Aquificae;Aquificae;Desulfurobacteriales;Desulfurobacteriaceae;Thermovibrio;Thermovibrio ammonificans;Thermovibrio ammonificans HB-1 |
| Ga0586324_0903654_1_1269 | 0.00 | 2420800.00 | 0.00 | 0.00 | 0.00 | 0.00 | 0.00 | 0.00 | 0.00 | 629949.32 | 0.00 | 0.00 | | D-xylose ABC transporter substrate-binding protein | Bacteria;Chloroflexi;Anaerolineae;Anaerolineales;Anaerolineaceae;Levilinea;Levilinea saccharolytica;Levilinea saccharolytica DSM 16555 |
| Ga0586324_0910832_959_1264 | 27800000.00 | 27540000.00 | 39353700.00 | 32870000.00 | 53560000.00 | 42876000.00 | 69084000.00 | 70760000.00 | 81000000.00 | 65758000.00 | 78584000.00 | 69300000.00 | | predicted porin | |
| Ga0586324_0912481_2_1264 | 0.00 | 0.00 | 0.00 | 2270500.00 | 1023153.59 | 0.00 | 0.00 | 0.00 | 1223100.00 | 650183.38 | 0.00 | 0.00 | | hypothetical protein | |
| Ga0586324_0913927_3_722 | 4170000.00 | 3481600.00 | 0.00 | 2175500.00 | 3285700.00 | 0.00 | 0.00 | 3702700.00 | 0.00 | 0.00 | 0.00 | 0.00 | | predicted porin | Bacteria;Proteobacteria;Betaproteobacteria;unclassified Betaproteobacteria;unclassified Betaproteobacteria;unclassified Betaproteobacteria;Betaproteobacteria bacterium UKL13-2;Betaproteobacteria bacterium UKL13-2 |
| Ga0586324_0914692_133_1260 | 0.00 | 0.00 | 0.00 | 0.00 | 0.00 | 0.00 | 0.00 | 0.00 | 0.00 | 0.00 | 1672000.00 | 0.00 | | hypothetical protein | Bacteria;Rhodothermaeota;Rhodothermia;Rhodothermales;Rubricoccaceae;Rubrivirga;Rubrivirga marina;Rubrivirga marina SAORIC-28 |
| Ga0586324_0915873_2_937 | 0.00 | 1203600.00 | 0.00 | 0.00 | 0.00 | 0.00 | 0.00 | 0.00 | 0.00 | 0.00 | 0.00 | 0.00 | | crotonobetainyl-CoA:carnitine CoA-transferase CaiB-like acyl-CoA transferase | Bacteria;Proteobacteria;Deltaproteobacteria;Syntrophobacterales;Syntrophaceae;Desulfobacca;Desulfobacca acetoxidans;Desulfobacca acetoxidans ASRB2, DSM 11109 |
| Ga0586324_0916226_1_351 | 0.00 | 583516.16 | 0.00 | 0.00 | 0.00 | 0.00 | 0.00 | 0.00 | 996300.00 | 0.00 | 0.00 | 0.00 | | hypothetical protein | |
| Ga0586324_0919283_2_526 | 0.00 | 0.00 | 0.00 | 0.00 | 0.00 | 0.00 | 0.00 | 0.00 | 7241400.00 | 0.00 | 0.00 | 0.00 | | hypothetical protein | Bacteria;Verrucomicrobia;Verrucomicrobiae;Verrucomicrobiales;Verrucomicrobia subdivision 3;Pedosphaera;Pedosphaera parvula;Pedosphaera parvula Ellin514 |
| Ga0586324_0926379_3_1256 | 0.00 | 2794800.00 | 0.00 | 0.00 | 0.00 | 0.00 | 0.00 | 0.00 | 0.00 | 0.00 | 0.00 | 0.00 | | hypothetical protein | Bacteria;Bacteroidetes;Flavobacteriia;Flavobacteriales;Flavobacteriaceae;Flavobacterium;Flavobacterium circumlabens;Flavobacterium circumlabens P5626 |
| Ga0586324_0927304_1_1254 | 1470000.00 | 441278.52 | 287220.08 | 0.00 | 0.00 | 0.00 | 0.00 | 0.00 | 0.00 | 3013927.84 | 1848000.00 | 0.00 | | ABC-type xylose transport system substrate-binding protein | Bacteria;Chloroflexi;Ardenticatenia;Ardenticatenales;Ardenticatenaceae;Candidatus Promineofilum;Candidatus Promineofilum breve;Candidatus Promineofilum breve Cfx-K |
| Ga0586324_0929048_1_1254 | 0.00 | 0.00 | 4813126.41 | 0.00 | 0.00 | 0.00 | 0.00 | 0.00 | 0.00 | 0.00 | 0.00 | 0.00 | | hypothetical protein | Bacteria;Proteobacteria;Deltaproteobacteria;Desulfobacterales;Desulfobulbaceae;Desulfobulbus;Desulfobulbus japonicus;Desulfobulbus japonicus DSM 18378 |
| Ga0586324_0931941_4_708 | 0.00 | 0.00 | 11034800.00 | 23465000.00 | 40994000.00 | 42876000.00 | 32604000.00 | 24766000.00 | 30537000.00 | 12936000.00 | 0.00 | 8100000.00 | | peptidoglycan hydrolase CwlO-like protein | Bacteria;Ignavibacteriae;Ignavibacteria;Ignavibacteriales;Ignavibacteriaceae;Ignavibacterium;Ignavibacterium album;Ignavibacterium album Mat9-16, JCM 16511 |
| Ga0586324_0932511_285_1250 | 0.00 | 0.00 | 0.00 | 3809500.00 | 1606800.00 | 2533141.87 | 0.00 | 0.00 | 0.00 | 0.00 | 0.00 | 0.00 | | hypothetical protein | |
| Ga0586324_0933126_3_1250 | 0.00 | 0.00 | 7600500.00 | 14725000.00 | 13802000.00 | 4605200.00 | 0.00 | 0.00 | 13527000.00 | 0.00 | 5376800.00 | 0.00 | | formyltetrahydrofolate synthetase | Bacteria;Proteobacteria;Deltaproteobacteria;Desulfobacterales;Desulfobacteraceae;Desulfatirhabdium;Desulfatirhabdium butyrativorans;Desulfatirhabdium butyrativorans DSM 18734 |
| Ga0586324_0933217_328_1251 | 41970000.00 | 30008400.00 | 37419068.73 | 55670000.00 | 70307800.00 | 34578700.00 | 24076800.00 | 53497000.00 | 25531200.00 | 16793700.00 | 30272000.00 | 14932500.00 | | predicted porin | Bacteria;Proteobacteria;Betaproteobacteria;Rhodocyclales;Rhodocyclaceae;Azospira;unclassified Azospira;Azospira sp. ZAP |
| Ga0586324_0934620_2_1249 | 0.00 | 0.00 | 0.00 | 0.00 | 1236000.00 | 0.00 | 0.00 | 388020.39 | 0.00 | 2594900.00 | 1346400.00 | 0.00 | | hypothetical protein | Bacteria;Acidobacteria;Acidobacteriia;Bryobacterales;Solibacteraceae;Candidatus Solibacter;Candidatus Solibacter usitatus;Candidatus Solibacter usitatus Ellin6076 |
| Ga0586324_0935747_3_515 | 0.00 | 4508400.00 | 20155400.00 | 0.00 | 0.00 | 9686800.00 | 17578800.00 | 7991000.00 | 7476300.00 | 0.00 | 8272000.00 | 6952500.00 | | hypothetical protein | Bacteria;Proteobacteria;Alphaproteobacteria;Hyphomicrobiales;Bradyrhizobiaceae;Bradyrhizobium;Bradyrhizobium symbiodeficiens;Bradyrhizobium symbiodeficiens 85S1MB |
| Ga0586324_0938334_3_887 | 0.00 | 0.00 | 0.00 | 0.00 | 0.00 | 0.00 | 1117464.48 | 0.00 | 0.00 | 0.00 | 0.00 | 0.00 | | hypothetical protein | |
| Ga0586324_0939811_1_1248 | 0.00 | 3298000.00 | 0.00 | 0.00 | 0.00 | 0.00 | 0.00 | 0.00 | 0.00 | 0.00 | 0.00 | 0.00 | | hypothetical protein | Bacteria;Firmicutes;Bacilli;Bacillales;Paenibacillaceae;Paenibacillus;Paenibacillus durus;Paenibacillus durus VPI 6563-1 |
| Ga0586324_0940624_1_1245 | 0.00 | 1346400.00 | 0.00 | 0.00 | 0.00 | 328303.12 | 4058400.00 | 2470500.00 | 2114100.00 | 3826900.00 | 1645600.00 | 3000000.00 | | hypothetical protein | Bacteria;Proteobacteria;Gammaproteobacteria;Chromatiales;Ectothiorhodospiraceae;Thiogranum;Thiogranum longum;Thiogranum longum DSM 19610 |
| Ga0586324_0942165_2_973 | 4790000.00 | 2182800.00 | 2705243.15 | 4398500.00 | 3790400.00 | 0.00 | 9120000.00 | 6420603.80 | 7970400.00 | 4412100.00 | 3819200.00 | 7050000.00 | | hypothetical protein | Bacteria;Proteobacteria;Gammaproteobacteria;Pseudomonadales;Moraxellaceae;Fluviicoccus;Fluviicoccus keumensis;Fluviicoccus keumensis DSM 105135 |
| Ga0586324_0942710_3_467 | 0.00 | 0.00 | 0.00 | 0.00 | 500987.88 | 0.00 | 0.00 | 0.00 | 0.00 | 0.00 | 0.00 | 0.00 | | hypothetical protein | Bacteria;Bacteroidetes;Cytophagia;Cytophagales;Cytophagaceae;Siphonobacter;Siphonobacter sp. BAB-5405;Siphonobacter sp. BAB-5405 |
| Ga0586324_0943400_3_1244 | 5710000.00 | 6052000.00 | 0.00 | 1330000.00 | 3285700.00 | 2363940.47 | 0.00 | 1384700.00 | 1838700.00 | 5728800.00 | 2244000.00 | 1672500.00 | | hypothetical protein | Bacteria;Acidobacteria;Vicinamibacteria;unclassified Vicinamibacteria;Vicinamibacteraceae;Luteitalea;Luteitalea pratensis;Luteitalea pratensis DSM 100886 |
| Ga0586324_0945992_264_1244 | 776679.00 | 1774800.00 | 0.00 | 0.00 | 1761300.00 | 0.00 | 0.00 | 1213900.00 | 691265.34 | 1247400.00 | 994400.00 | 0.00 | | hypothetical protein | Bacteria;Proteobacteria;Betaproteobacteria;Rhodocyclales;Rhodocyclaceae;Rhodocyclus;Rhodocyclus tenuis;Rhodocyclus tenuis DSM 109 |
| Ga0586324_0948965_219_791 | 0.00 | 2033200.00 | 0.00 | 3743000.00 | 0.00 | 0.00 | 0.00 | 0.00 | 5678100.00 | 0.00 | 0.00 | 0.00 | | lipid-binding SYLF domain-containing protein | Bacteria;Proteobacteria;Alphaproteobacteria;Rhodobacterales;Roseobacteraceae;Ruegeria;Ruegeria atlantica;Ruegeria atlantica CECT 4293 |
| Ga0586324_0952320_966_1241 | 0.00 | 0.00 | 0.00 | 0.00 | 0.00 | 0.00 | 0.00 | 0.00 | 0.00 | 0.00 | 0.00 | 1987500.00 | | hypothetical protein | |
| Ga0586324_0954743_291_623 | 5290000.00 | 0.00 | 0.00 | 0.00 | 0.00 | 12783400.00 | 9188400.00 | 0.00 | 0.00 | 0.00 | 0.00 | 0.00 | | hypothetical protein | Bacteria;Proteobacteria;Deltaproteobacteria;Syntrophobacterales;Syntrophaceae;Desulfobacca;Desulfobacca acetoxidans;Desulfobacca acetoxidans ASRB2, DSM 11109 |
| Ga0586324_0956657_669_1238 | 0.00 | 0.00 | 4257360.96 | 0.00 | 0.00 | 0.00 | 0.00 | 0.00 | 0.00 | 0.00 | 0.00 | 0.00 | | poly(hydroxyalkanoate) granule-associated protein | Bacteria;Proteobacteria;Betaproteobacteria;unclassified Betaproteobacteria;unclassified Betaproteobacteria;unclassified Betaproteobacteria;beta proteobacterium AAP65;Rubrivivax sp. AAP65 |
| Ga0586324_0957269_3_884 | 184078.00 | 0.00 | 0.00 | 0.00 | 0.00 | 0.00 | 0.00 | 0.00 | 0.00 | 0.00 | 0.00 | 0.00 | | hypothetical protein | |
| Ga0586324_0957735_1_1236 | 0.00 | 870400.00 | 0.00 | 0.00 | 0.00 | 0.00 | 0.00 | 0.00 | 0.00 | 885500.00 | 0.00 | 0.00 | | hypothetical protein | |
| Ga0586324_0960131_1_1194 | 0.00 | 809200.00 | 0.00 | 0.00 | 0.00 | 0.00 | 0.00 | 0.00 | 0.00 | 0.00 | 0.00 | 0.00 | | hypothetical protein | |
| Ga0586324_0966067_3_980 | 0.00 | 562771.40 | 0.00 | 0.00 | 0.00 | 0.00 | 0.00 | 0.00 | 0.00 | 0.00 | 0.00 | 1695000.00 | | uncharacterized protein (DUF2252 family) | Bacteria;Actinobacteria;Actinomycetia;Streptomycetales;Streptomycetaceae;Streptomyces;Streptomyces sp. TLI_053;Streptomyces sp. TLI_053 |
| Ga0586324_0969107_1054_1230 | 3710000.00 | 19176000.00 | 0.00 | 81510000.00 | 49543000.00 | 0.00 | 0.00 | 17080000.00 | 42201000.00 | 37576000.00 | 0.00 | 0.00 | | predicted porin | |
| Ga0586324_0969709_126_1229 | 0.00 | 4848400.00 | 0.00 | 0.00 | 2461700.00 | 0.00 | 0.00 | 0.00 | 0.00 | 0.00 | 0.00 | 1500000.00 | | hypothetical protein | Bacteria;Proteobacteria;Alphaproteobacteria;Hyphomicrobiales;Bradyrhizobiaceae;Bradyrhizobium;Bradyrhizobium zhanjiangense;Bradyrhizobium zhanjiangense CCBAU 51778 |
| Ga0586324_0970199_2_1117 | 0.00 | 0.00 | 0.00 | 0.00 | 0.00 | 0.00 | 0.00 | 3294000.00 | 0.00 | 0.00 | 0.00 | 0.00 | | hypothetical protein | Bacteria;Candidatus Marinimicrobia;unclassified Candidatus Marinimicrobia;unclassified Candidatus Marinimicrobia;unclassified Candidatus Marinimicrobia;unclassified Candidatus Marinimicrobia;Marinimicrobia bacterium SCGC AAA257-N23;Marinimicrobia bacterium SCGC AAA257-N23 (contamination screened) |
| Ga0586324_0971183_272_1228 | 0.00 | 0.00 | 0.00 | 0.00 | 0.00 | 0.00 | 1152261.84 | 1335900.00 | 1628100.00 | 3072300.00 | 0.00 | 3022500.00 | | TRAP-type mannitol/chloroaromatic compound transport system substrate-binding protein | Bacteria;Proteobacteria;Betaproteobacteria;Rhodocyclales;Zoogloeaceae;Azoarcus;Azoarcus sp. KH32C;Azoarcus sp. KH32C |
| Ga0586324_0977936_3_371 | 0.00 | 0.00 | 0.00 | 0.00 | 0.00 | 34697800.00 | 0.00 | 0.00 | 0.00 | 0.00 | 0.00 | 4290000.00 | | predicted Zn-dependent protease | |
| Ga0586324_0979697_3_1103 | 0.00 | 0.00 | 0.00 | 0.00 | 0.00 | 0.00 | 5152800.00 | 0.00 | 0.00 | 0.00 | 0.00 | 0.00 | | hypothetical protein | Bacteria;Proteobacteria;Deltaproteobacteria;Desulfuromonadales;Geobacteraceae;Geobacter;Geobacter soli;Geobacter soli GSS01 |
| Ga0586324_0985984_3_1220 | 0.00 | 0.00 | 0.00 | 5434000.00 | 2987000.00 | 1854633.14 | 0.00 | 768600.00 | 332759.34 | 0.00 | 0.00 | 0.00 | | hypothetical protein | Bacteria;Bacteroidetes;Cytophagia;Cytophagales;Hymenobacteraceae;Pontibacter;Pontibacter sp. S10-8;Pontibacter sp. S10-8 |
| Ga0586324_0987266_309_1220 | 0.00 | 0.00 | 0.00 | 0.00 | 0.00 | 0.00 | 0.00 | 0.00 | 0.00 | 0.00 | 2068000.00 | 0.00 | | hypothetical protein | Bacteria;Nitrospirae;Nitrospira;Nitrospirales;Nitrospiraceae;Nitrospira;Nitrospira japonica;Nitrospira japonica NJ11 |
| Ga0586324_0990923_3_995 | 0.00 | 0.00 | 0.00 | 0.00 | 0.00 | 0.00 | 0.00 | 0.00 | 0.00 | 0.00 | 0.00 | 6150000.00 | | Fe-S oxidoreductase | Bacteria;Proteobacteria;Betaproteobacteria;Nitrosomonadales;Gallionellaceae;Sideroxydans;Sideroxydans lithotrophicus;Sideroxydans lithotrophicus ES-1 |
| Ga0586324_0991056_1_771 | 9630000.00 | 0.00 | 0.00 | 0.00 | 0.00 | 0.00 | 0.00 | 0.00 | 0.00 | 0.00 | 0.00 | 0.00 | | hypothetical protein | |
| Ga0586324_0994704_156_1217 | 12900000.00 | 4399600.00 | 13737200.00 | 0.00 | 0.00 | 10679300.00 | 5198400.00 | 4367600.00 | 3539700.00 | 7777000.00 | 9064000.00 | 6270000.00 | | hypothetical protein | |
| Ga0586324_0995456_178_735 | 0.00 | 2461600.00 | 0.00 | 1824000.00 | 5386900.00 | 0.00 | 0.00 | 0.00 | 0.00 | 0.00 | 2543200.00 | 2422500.00 | | phasin family protein | Bacteria;Proteobacteria;Betaproteobacteria;Neisseriales;Chromobacteriaceae;Andreprevotia;Andreprevotia chitinilytica;Andreprevotia chitinilytica DSM 18519 |
| Ga0586324_0995897_1_279 | 0.00 | 0.00 | 0.00 | 7381500.00 | 3131200.00 | 0.00 | 0.00 | 0.00 | 0.00 | 0.00 | 0.00 | 0.00 | | hypothetical protein | |
| Ga0586324_0997304_137_1063 | 0.00 | 0.00 | 0.00 | 0.00 | 0.00 | 0.00 | 0.00 | 0.00 | 0.00 | 11473000.00 | 12777600.00 | 3592500.00 | | hypothetical protein | Bacteria;Proteobacteria;Betaproteobacteria;Burkholderiales;Comamonadaceae;Rhodoferax;Rhodoferax sp. TH121;Rhodoferax sp. TH121 |
| Ga0586324_1004304_684_1211 | 0.00 | 4569600.00 | 0.00 | 0.00 | 0.00 | 0.00 | 0.00 | 0.00 | 0.00 | 0.00 | 0.00 | 0.00 | | CBS domain-containing protein | Archaea;Euryarchaeota;Methanomicrobia;Methanosarcinales;Methanotrichaceae;Methanothrix;Methanothrix soehngenii;Methanothrix soehngenii GP-6 |
| Ga0586324_1004564_625_1212 | 0.00 | 0.00 | 0.00 | 0.00 | 343602.85 | 0.00 | 0.00 | 19818.05 | 0.00 | 0.00 | 0.00 | 33838.28 | | TRAP-type mannitol/chloroaromatic compound transport system substrate-binding protein | Bacteria;Proteobacteria;Gammaproteobacteria;Acidiferrobacterales;Acidiferrobacteraceae;Sulfuricaulis;Sulfuricaulis limicola;Sulfuricaulis limicola HA5 |
| Ga0586324_1005885_25_1140 | 56850000.00 | 207230000.00 | 34969607.74 | 94751286.20 | 141681181.35 | 83647900.00 | 172026000.00 | 71026255.24 | 91794852.18 | 33067599.18 | 121501600.00 | 84810000.00 | | predicted porin | Bacteria;Proteobacteria;Betaproteobacteria;Burkholderiales;Comamonadaceae;Curvibacter;Curvibacter delicatus;Curvibacter delicatus NBRC 14919 |
| Ga0586324_1006231_414_1211 | 0.00 | 0.00 | 0.00 | 0.00 | 0.00 | 0.00 | 0.00 | 0.00 | 0.00 | 0.00 | 5728800.00 | 0.00 | | predicted porin | Bacteria;Proteobacteria;Betaproteobacteria;unclassified Betaproteobacteria;unclassified Betaproteobacteria;unclassified Betaproteobacteria;beta proteobacterium AAP65;Rubrivivax sp. AAP65 |
| Ga0586324_1008183_79_1146 | 0.00 | 0.00 | 0.00 | 4047000.00 | 3965500.00 | 2558712.64 | 1763716.80 | 2000800.00 | 2316600.00 | 0.00 | 0.00 | 0.00 | | signal transduction protein with GAF and PtsI domain | Bacteria;Proteobacteria;Deltaproteobacteria;Syntrophobacterales;Syntrophaceae;Desulfobacca;Desulfobacca acetoxidans;Desulfobacca acetoxidans ASRB2, DSM 11109 |
| Ga0586324_1008977_395_1210 | 19000000.00 | 18482400.00 | 9514700.00 | 15361500.00 | 16304900.00 | 12505500.00 | 14037383.16 | 18885600.00 | 53411400.00 | 15723400.00 | 13886400.00 | 10320000.00 | | predicted porin | Bacteria;Proteobacteria;Betaproteobacteria;Burkholderiales;Comamonadaceae;Polaromonas;Polaromonas sp. C04;Polaromonas sp. C04 |
| Ga0586324_1009347_352_858 | 0.00 | 0.00 | 0.00 | 14440000.00 | 0.00 | 12386400.00 | 0.00 | 5490000.00 | 0.00 | 0.00 | 0.00 | 0.00 | | tetratricopeptide (TPR) repeat protein | Bacteria;Proteobacteria;Deltaproteobacteria;Syntrophobacterales;Syntrophaceae;Desulfobacca;Desulfobacca acetoxidans;Desulfobacca acetoxidans ASRB2, DSM 11109 |
| Ga0586324_1009697_343_1185 | 43300000.00 | 31892000.00 | 85688600.00 | 46569000.00 | 68495000.00 | 0.00 | 0.00 | 0.00 | 0.00 | 0.00 | 0.00 | 33900000.00 | | hypothetical protein | |
| Ga0586324_1011537_53_1207 | 0.00 | 0.00 | 1095513.55 | 0.00 | 0.00 | 0.00 | 0.00 | 544171.85 | 1717200.00 | 3003000.00 | 3097600.00 | 1357500.00 | | hypothetical protein | |
| Ga0586324_1012643_2_1207 | 0.00 | 0.00 | 0.00 | 2137500.00 | 0.00 | 0.00 | 0.00 | 0.00 | 0.00 | 0.00 | 0.00 | 0.00 | | tetratricopeptide (TPR) repeat protein | Bacteria;Bacteroidetes;Bacteroidia;Marinilabiliales;Marinifilaceae;Marinifilum;Marinifilum fragile;Marinifilum fragile JCM 15579 |
| Ga0586324_1013260_1_1206 | 0.00 | 0.00 | 0.00 | 0.00 | 0.00 | 0.00 | 0.00 | 0.00 | 0.00 | 0.00 | 1038400.00 | 0.00 | | hypothetical protein | Bacteria;Firmicutes;Bacilli;Bacillales;unclassified Bacillales;Gemella;Gemella sanguinis;Gemella sanguinis UMB0186 |
| Ga0586324_1013411_1_1044 | 0.00 | 1897200.00 | 0.00 | 153672000.00 | 3244500.00 | 0.00 | 0.00 | 9150000.00 | 0.00 | 885500.00 | 0.00 | 0.00 | | hypothetical protein | Bacteria;Proteobacteria;Deltaproteobacteria;Desulfobacterales;Desulfobacteraceae;Desulfotignum;Desulfotignum balticum;Desulfotignum balticum DSM 7044 |
| Ga0586324_1014882_171_812 | 1590000.00 | 0.00 | 0.00 | 0.00 | 0.00 | 0.00 | 0.00 | 0.00 | 0.00 | 0.00 | 0.00 | 0.00 | | polyhydroxyalkanoate synthesis repressor PhaR | Bacteria;Proteobacteria;Alphaproteobacteria;Hyphomicrobiales;Bradyrhizobiaceae;Bradyrhizobium;Bradyrhizobium valentinum;Bradyrhizobium valentinum LmjM3 |
| Ga0586324_1017938_3_1205 | 0.00 | 0.00 | 0.00 | 1339500.00 | 0.00 | 0.00 | 0.00 | 603773.12 | 0.00 | 0.00 | 0.00 | 1777500.00 | | hypothetical protein | Bacteria;Acidobacteria;Vicinamibacteria;unclassified Vicinamibacteria;Vicinamibacteraceae;Luteitalea;Luteitalea pratensis;Luteitalea pratensis DSM 100886 |
| Ga0586324_1019909_450_1205 | 0.00 | 0.00 | 0.00 | 0.00 | 0.00 | 0.00 | 0.00 | 0.00 | 0.00 | 0.00 | 0.00 | 1897500.00 | | glyoxylase-like metal-dependent hydrolase (beta-lactamase superfamily II) | Bacteria;Proteobacteria;Alphaproteobacteria;Rhodospirillales;Azospirillaceae;Skermanella;Skermanella aerolata;Skermanella aerolata KACC 11604 |
| Ga0586324_1028844_1_1200 | 14400000.00 | 19448000.00 | 14975800.00 | 34485000.00 | 34608000.00 | 17229800.00 | 2026432.08 | 31476000.00 | 38070000.00 | 19635000.00 | 0.00 | 17325000.00 | | crotonobetainyl-CoA:carnitine CoA-transferase CaiB-like acyl-CoA transferase | Bacteria;Proteobacteria;Deltaproteobacteria;Syntrophobacterales;Syntrophobacteraceae;Thermodesulforhabdus;Thermodesulforhabdus norvegica;Thermodesulforhabdus norvegica DSM 9990 |
| Ga0586324_1030611_317_1198 | 0.00 | 0.00 | 0.00 | 0.00 | 0.00 | 0.00 | 0.00 | 2281400.00 | 0.00 | 0.00 | 0.00 | 0.00 | | tetratricopeptide (TPR) repeat protein | Bacteria;Bacteroidetes;Bacteroidia;Marinilabiliales;Marinilabiliaceae;Thermophagus;Thermophagus xiamenensis;Thermophagus xiamenensis HS1 |
| Ga0586324_1032536_1_303 | 0.00 | 4814400.00 | 0.00 | 0.00 | 0.00 | 0.00 | 0.00 | 0.00 | 0.00 | 2440900.00 | 0.00 | 0.00 | | hypothetical protein | |
| Ga0586324_1034205_2_1117 | 0.00 | 0.00 | 0.00 | 0.00 | 0.00 | 3445364.50 | 0.00 | 0.00 | 0.00 | 0.00 | 0.00 | 0.00 | | hypothetical protein | Bacteria;Proteobacteria;Deltaproteobacteria;Desulfobacterales;Desulfobulbaceae;Desulfopila;Desulfopila aestuarii;Desulfopila aestuarii DSM 18488 |
| Ga0586324_1035859_206_586 | 0.00 | 0.00 | 0.00 | 0.00 | 0.00 | 0.00 | 0.00 | 0.00 | 2025000.00 | 0.00 | 0.00 | 0.00 | | DNA-binding transcriptional MerR regulator | Bacteria;Proteobacteria;Betaproteobacteria;Nitrosomonadales;Sulfuricellaceae;Sulfuricella;Sulfuricella sp. T08;Sulfuricella sp. T08 |
| Ga0586324_1041972_123_1193 | 0.00 | 0.00 | 0.00 | 15390000.00 | 0.00 | 17348900.00 | 0.00 | 4605500.00 | 3474900.00 | 0.00 | 0.00 | 0.00 | | hypothetical protein | Bacteria;Bacteroidetes;Chitinophagia;Chitinophagales;Chitinophagaceae;Filimonas;Filimonas endophytica;Filimonas endophytica 3197 |
| Ga0586324_1042889_1_1191 | 0.00 | 615091.96 | 0.00 | 0.00 | 0.00 | 0.00 | 0.00 | 0.00 | 0.00 | 0.00 | 0.00 | 0.00 | | uncharacterized protein (DUF2252 family) | Bacteria;Actinobacteria;Actinomycetia;Micrococcales;Micrococcaceae;Arthrobacter;Arthrobacter sp. 49Tsu3.1M3;Arthrobacter sp. 49Tsu3.1M3 |
| Ga0586324_1044065_1_1191 | 0.00 | 0.00 | 0.00 | 1681500.00 | 2575000.00 | 0.00 | 255882.12 | 1653100.00 | 793704.42 | 0.00 | 0.00 | 0.00 | | hypothetical protein | Bacteria;Bacteroidetes;Bacteroidia;Marinilabiliales;Prolixibacteraceae;Mariniphaga;Mariniphaga anaerophila;Mariniphaga anaerophila DSM 26910 |
| Ga0586324_1046399_2_826 | 0.00 | 1414400.00 | 0.00 | 0.00 | 5963700.00 | 0.00 | 0.00 | 0.00 | 247372.38 | 0.00 | 0.00 | 0.00 | | hypothetical protein | Bacteria;Proteobacteria;Deltaproteobacteria;Myxococcales;Myxococcaceae;Corallococcus;Corallococcus sp. CA031C;Corallococcus sp. CA031C |
| Ga0586324_1048020_1_921 | 0.00 | 863600.00 | 2848982.68 | 2736000.00 | 0.00 | 5200700.00 | 4514400.00 | 6710000.00 | 5062500.00 | 0.00 | 6309600.00 | 5902500.00 | | long-subunit fatty acid transport protein | |
| Ga0586324_1049001_3_326 | 0.00 | 3515600.00 | 0.00 | 0.00 | 0.00 | 0.00 | 0.00 | 0.00 | 0.00 | 1601600.00 | 2640000.00 | 0.00 | | HSP20 family molecular chaperone IbpA | Bacteria;Planctomycetes;Planctomycetia;Gemmatales;Gemmataceae;Fimbriiglobus;Fimbriiglobus ruber;Fimbriiglobus ruber SP5 |
| Ga0586324_1050748_1_849 | 0.00 | 0.00 | 0.00 | 0.00 | 0.00 | 0.00 | 0.00 | 0.00 | 0.00 | 4981900.00 | 3757600.00 | 3915000.00 | | hypothetical protein | |
| Ga0586324_1051125_336_1187 | 0.00 | 0.00 | 0.00 | 791571.35 | 6077000.00 | 4446400.00 | 0.00 | 0.00 | 0.00 | 0.00 | 0.00 | 0.00 | | uncharacterized protein YqfA (UPF0365 family) | Bacteria;Bacteroidetes;Bacteroidia;Bacteroidales;Lentimicrobiaceae;Lentimicrobium;Lentimicrobium saccharophilum;Bacteroidales bacterium TBC1 |
| Ga0586324_1051175_148_504 | 0.00 | 0.00 | 0.00 | 2479500.00 | 0.00 | 0.00 | 0.00 | 0.00 | 0.00 | 0.00 | 0.00 | 0.00 | | putative exosortase-associated protein (TIGR04073 family) | |
| Ga0586324_1054392_287_1186 | 0.00 | 0.00 | 0.00 | 0.00 | 0.00 | 0.00 | 0.00 | 0.00 | 0.00 | 1547700.00 | 0.00 | 0.00 | | hypothetical protein | Bacteria;Proteobacteria;Betaproteobacteria;Rhodocyclales;Zoogloeaceae;Thauera;Thauera aminoaromatica;Thauera aminoaromatica S2 |
| Ga0586324_1056957_1_1185 | 0.00 | 134340.12 | 0.00 | 0.00 | 6087300.00 | 7900300.00 | 0.00 | 0.00 | 0.00 | 0.00 | 0.00 | 0.00 | | hypothetical protein | Bacteria;Proteobacteria;Deltaproteobacteria;Desulfuromonadales;Geobacteraceae;Geobacter;Geobacter metallireducens;Geobacter metallireducens RCH3 |
| Ga0586324_1058318_3_944 | 4202142.00 | 0.00 | 0.00 | 0.00 | 13168980.54 | 0.00 | 11491200.00 | 0.00 | 1579500.00 | 4242700.00 | 4980800.00 | 4942500.00 | | predicted porin | Bacteria;Proteobacteria;Betaproteobacteria;Neisseriales;Chromobacteriaceae;Pseudogulbenkiania;Pseudogulbenkiania sp. MAI-1;Pseudogulbenkiania sp. MAI-1 |
| Ga0586324_1061525_3_602 | 0.00 | 0.00 | 0.00 | 0.00 | 0.00 | 0.00 | 0.00 | 0.00 | 5038200.00 | 0.00 | 2648800.00 | 2430000.00 | | TRAP-type mannitol/chloroaromatic compound transport system substrate-binding protein | Bacteria;Proteobacteria;Betaproteobacteria;unclassified Betaproteobacteria;unclassified Betaproteobacteria;unclassified Betaproteobacteria;Betaproteobacteria bacterium UKL13-2;Betaproteobacteria bacterium UKL13-2 |
| Ga0586324_1065628_233_628 | 0.00 | 0.00 | 0.00 | 0.00 | 0.00 | 1891339.76 | 0.00 | 0.00 | 0.00 | 0.00 | 0.00 | 0.00 | | CheY-like chemotaxis protein | Bacteria;Proteobacteria;Deltaproteobacteria;unclassified Deltaproteobacteria;unclassified Deltaproteobacteria;unclassified Deltaproteobacteria;delta proteobacterium NaphS2;delta proteobacterium NaphS2 |
| Ga0586324_1066592_160_960 | 0.00 | 0.00 | 0.00 | 1216000.00 | 0.00 | 0.00 | 0.00 | 1573800.00 | 0.00 | 1732500.00 | 0.00 | 0.00 | | NAD(P)-dependent dehydrogenase (short-subunit alcohol dehydrogenase family) | Bacteria;Proteobacteria;Alphaproteobacteria;Rhodospirillales;Acetobacteraceae;Rhodopila;unclassified Rhodopila;Rhodopila sp. LVNP |
| Ga0586324_1066672_110_1180 | 0.00 | 0.00 | 5431576.28 | 0.00 | 0.00 | 0.00 | 0.00 | 0.00 | 0.00 | 0.00 | 0.00 | 0.00 | | TRAP-type mannitol/chloroaromatic compound transport system substrate-binding protein | Bacteria;Proteobacteria;Alphaproteobacteria;Rhodospirillales;Azospirillaceae;Azospirillum;Azospirillum sp. B506;Azospirillum sp. B506 |
| Ga0586324_1067530_1_1182 | 0.00 | 0.00 | 0.00 | 0.00 | 0.00 | 0.00 | 0.00 | 374014.18 | 0.00 | 0.00 | 0.00 | 0.00 | | hypothetical protein | Bacteria;Chloroflexi;Thermoflexia;Thermoflexales;Thermoflexaceae;Thermoflexus;Thermoflexus hugenholtzii;Thermoflexus hugenholtzii JAD2 |
| Ga0586324_1067592_300_1181 | 0.00 | 0.00 | 0.00 | 0.00 | 0.00 | 0.00 | 0.00 | 0.00 | 0.00 | 5513200.00 | 0.00 | 0.00 | | hypothetical protein | Bacteria;Proteobacteria;Gammaproteobacteria;Xanthomonadales;Xanthomonadaceae;Lysobacter;Lysobacter sp. cf310;Lysobacter sp. CF310 |
| Ga0586324_1068789_3_1181 | 0.00 | 3182400.00 | 0.00 | 0.00 | 0.00 | 0.00 | 0.00 | 0.00 | 0.00 | 0.00 | 0.00 | 0.00 | | hypothetical protein | Bacteria;Bacteroidetes;Cytophagia;Cytophagales;Fulvivirgaceae;Ohtaekwangia;Ohtaekwangia kribbensis;Ohtaekwangia kribbensis DSM 25221 |
| Ga0586324_1069875_2_1180 | 93200000.00 | 44470040.92 | 27589685.51 | 103331500.00 | 82709000.00 | 81504100.00 | 27633600.00 | 67261547.52 | 26487000.00 | 34765500.00 | 9143200.00 | 50550000.00 | | predicted porin | Bacteria;Proteobacteria;Betaproteobacteria;Burkholderiales;Comamonadaceae;Rhodoferax;Rhodoferax sp. YR267;Rhodoferax sp. YR267 |
| Ga0586324_1072117_2_1177 | 0.00 | 0.00 | 0.00 | 0.00 | 0.00 | 0.00 | 69194.58 | 0.00 | 0.00 | 0.00 | 0.00 | 0.00 | | 2',3'-cyclic-nucleotide 2'-phosphodiesterase (5'-nucleotidase family) | Bacteria;Actinobacteria;Actinomycetia;Pseudonocardiales;Pseudonocardiaceae;Lentzea;Lentzea aerocolonigenes;Lentzea aerocolonigenes DSM 40034 |
| Ga0586324_1072507_2_172 | 0.00 | 0.00 | 0.00 | 0.00 | 3223900.00 | 0.00 | 0.00 | 0.00 | 0.00 | 0.00 | 0.00 | 0.00 | | predicted porin | Bacteria;Proteobacteria;Betaproteobacteria;Burkholderiales;unclassified Burkholderiales;Rubrivivax;Rubrivivax gelatinosus;Rubrivivax gelatinosus DSM 1709 |
| Ga0586324_1074242_3_827 | 0.00 | 0.00 | 0.00 | 0.00 | 0.00 | 0.00 | 0.00 | 817400.00 | 0.00 | 0.00 | 0.00 | 0.00 | | co-chaperonin GroES (HSP10) | Bacteria;Chloroflexi;Anaerolineae;Anaerolineales;Anaerolineaceae;Anaerolinea;Anaerolinea thermophila;Anaerolinea thermophila UNI-1 |
| Ga0586324_1077457_1_1176 | 0.00 | 0.00 | 0.00 | 0.00 | 0.00 | 0.00 | 21204000.00 | 0.00 | 0.00 | 0.00 | 0.00 | 0.00 | | TRAP-type mannitol/chloroaromatic compound transport system substrate-binding protein | Bacteria;Proteobacteria;Betaproteobacteria;Burkholderiales;unclassified Burkholderiales;Leptothrix;Leptothrix cholodnii;Leptothrix cholodnii SP-6 |
| Ga0586324_1082574_590_1120 | 0.00 | 0.00 | 0.00 | 0.00 | 0.00 | 3125529.39 | 0.00 | 0.00 | 0.00 | 0.00 | 0.00 | 0.00 | | hypothetical protein | Bacteria;Proteobacteria;Gammaproteobacteria;Methylococcales;Methylococcaceae;Methylomonas;Methylomonas sp. 11b;Methylomonas sp. 11b |
| Ga0586324_1082587_96_1172 | 7970000.00 | 1353200.00 | 0.00 | 0.00 | 0.00 | 0.00 | 0.00 | 0.00 | 0.00 | 0.00 | 0.00 | 0.00 | | hypothetical protein | |
| Ga0586324_1082700_1_831 | 0.00 | 0.00 | 0.00 | 0.00 | 890082.74 | 0.00 | 0.00 | 0.00 | 0.00 | 0.00 | 0.00 | 0.00 | | alkylation response protein AidB-like acyl-CoA dehydrogenase | Bacteria;Firmicutes;Clostridia;Thermoanaerobacterales;Thermoanaerobacteraceae;Carboxydothermus;Carboxydothermus islandicus;Carboxydothermus islandicus SET IS-9 |
| Ga0586324_1089945_9_1169 | 0.00 | 7004000.00 | 0.00 | 0.00 | 0.00 | 0.00 | 0.00 | 0.00 | 0.00 | 0.00 | 0.00 | 0.00 | | amidohydrolase | Bacteria;Actinobacteria;Actinomycetia;Micrococcales;Micrococcaceae;Arthrobacter;Arthrobacter sp. CAL618;Arthrobacter sp. CAL618 |
| Ga0586324_1091495_3_425 | 0.00 | 0.00 | 0.00 | 0.00 | 0.00 | 0.00 | 0.00 | 0.00 | 5159700.00 | 0.00 | 0.00 | 0.00 | | hypothetical protein | Bacteria;Candidatus Poribacteria;unclassified Candidatus Poribacteria;unclassified Candidatus Poribacteria;unclassified Candidatus Poribacteria;unclassified Candidatus Poribacteria;Candidatus Poribacteria bacterium WGA-4C;Candidatus Poribacteria sp. WGA-4C (contamination screened) |
| Ga0586324_1096083_3_986 | 0.00 | 0.00 | 2464639.47 | 0.00 | 0.00 | 0.00 | 0.00 | 0.00 | 0.00 | 0.00 | 0.00 | 0.00 | | hypothetical protein | Bacteria;Proteobacteria;Betaproteobacteria;Burkholderiales;Comamonadaceae;Polaromonas;Polaromonas sp. EUR3 1.2.1;Polaromonas sp. EUR3 1.2.1 |
| Ga0586324_1096257_30_1166 | 0.00 | 1910800.00 | 3552912.84 | 0.00 | 0.00 | 0.00 | 0.00 | 1665300.00 | 0.00 | 4458300.00 | 2428800.00 | 1470000.00 | | hypothetical protein | Bacteria;Acidobacteria;Vicinamibacteria;unclassified Vicinamibacteria;Vicinamibacteraceae;Luteitalea;Luteitalea pratensis;Luteitalea pratensis DSM 100886 |
| Ga0586324_1098093_1_1167 | 5870000.00 | 5093200.00 | 0.00 | 0.00 | 0.00 | 7066600.00 | 3670800.00 | 0.00 | 0.00 | 0.00 | 0.00 | 4020000.00 | | hypothetical protein | Bacteria;Acidobacteria;Vicinamibacteria;unclassified Vicinamibacteria;Vicinamibacteraceae;Luteitalea;Luteitalea pratensis;Luteitalea pratensis DSM 100886 |
| Ga0586324_1098429_1_1047 | 0.00 | 15844000.00 | 5545195.31 | 0.00 | 16480000.00 | 0.00 | 0.00 | 0.00 | 12636000.00 | 35420000.00 | 26400000.00 | 0.00 | | kynurenine formamidase | Bacteria;Proteobacteria;Alphaproteobacteria;unclassified Alphaproteobacteria;unclassified Alphaproteobacteria;unclassified Alphaproteobacteria;alpha proteobacterium BAL199;Nisaea sp BAL199 |
| Ga0586324_1102824_36_668 | 0.00 | 0.00 | 0.00 | 0.00 | 3656500.00 | 0.00 | 0.00 | 0.00 | 0.00 | 0.00 | 0.00 | 0.00 | | corrinoid protein of di/trimethylamine methyltransferase | Archaea;Candidatus Thermoplasmatota;Thermoplasmata;Methanomassiliicoccales;Methanomassiliicoccaceae;Methanomassiliicoccus;Methanomassiliicoccus luminyensis;Methanomassiliicoccus luminyensis B10 |
| Ga0586324_1103380_229_1164 | 32100000.00 | 0.00 | 0.00 | 0.00 | 26368000.00 | 0.00 | 0.00 | 0.00 | 0.00 | 0.00 | 0.00 | 0.00 | | hypothetical protein | |
| Ga0586324_1107050_1_1161 | 0.00 | 0.00 | 0.00 | 1273000.00 | 0.00 | 0.00 | 0.00 | 0.00 | 0.00 | 0.00 | 0.00 | 0.00 | | hypothetical protein | Bacteria;Proteobacteria;Betaproteobacteria;Nitrosomonadales;Nitrosomonadaceae;Nitrosospira;Nitrosospira multiformis;Nitrosospira multiformis NI1 |
| Ga0586324_1107320_537_1073 | 0.00 | 0.00 | 0.00 | 0.00 | 0.00 | 0.00 | 0.00 | 0.00 | 0.00 | 0.00 | 3951200.00 | 0.00 | | hypothetical protein | |
| Ga0586324_1110628_227_1162 | 0.00 | 0.00 | 0.00 | 0.00 | 0.00 | 0.00 | 0.00 | 289760.98 | 0.00 | 0.00 | 0.00 | 0.00 | | sugar phosphate isomerase/epimerase | Bacteria;Bacteroidetes;Bacteroidia;Bacteroidales;Bacteroidaceae;Bacteroides;Bacteroides sp. 2_1_33B;Bacteroides sp. 2_1_33B |
| Ga0586324_1111253_2_421 | 0.00 | 0.00 | 0.00 | 0.00 | 0.00 | 0.00 | 377207.76 | 0.00 | 0.00 | 0.00 | 0.00 | 0.00 | | crotonobetainyl-CoA:carnitine CoA-transferase CaiB-like acyl-CoA transferase | Bacteria;Proteobacteria;Deltaproteobacteria;Syntrophobacterales;Syntrophaceae;Desulfobacca;Desulfobacca acetoxidans;Desulfobacca acetoxidans ASRB2, DSM 11109 |

| | | | | | | | | | | | | | | | |
|---|---|---|---|---|---|---|---|---|---|---|---|---|---|---|---|
| Ga0586324 1111253 421 1161 | 10100000.00 | 3284400.00 | 311833.88 | 3952000.00 | 6674400.00 | 2162232.71 | 0.00 | 0.00 | 38151000.00 | 0.00 | 0.00 | 2925000.00 | | crotonobetainyl-CoA:carnitine CoA-transferase CaiB-like acyl-CoA transferase | Bacteria;Proteobacteria;Deltaproteobacteria;Syntrophobacterales;Syntrophobacteraceae;Desulfosoma;Desulfosoma caldarium;Desulfosoma caldarium DSM 22027 |
| Ga0586324 1112219 1 1161 | 0.00 | 1373600.00 | 0.00 | 1282500.00 | 1606800.00 | 0.00 | 848574.96 | 483997.79 | 718596.36 | 0.00 | 0.00 | 1057500.00 | | hypothetical protein | Bacteria;Acidobacteria;Vicinamibacteria;unclassified Vicinamibacteria;Vicinamibacteraceae;Luteitalea;Luteitalea pratensis;Luteitalea pratensis DSM 100886 |
| Ga0586324 1113574 302 1159 | 0.00 | 0.00 | 0.00 | 0.00 | 0.00 | 0.00 | 0.00 | 0.00 | 0.00 | 0.00 | 0.00 | 772500.00 | | hypothetical protein | Bacteria;Actinobacteria;Actinomycetia;Micromonosporales;Micromonosporaceae;Micromonospora;Micromonospora rhizosphaerae;Micromonospora rhizosphaerae DSM 45431 |
| Ga0586324 1113723 1 1158 | 3030000.00 | 0.00 | 0.00 | 0.00 | 0.00 | 0.00 | 0.00 | 0.00 | 0.00 | 0.00 | 0.00 | 0.00 | | hypothetical protein | Bacteria;Proteobacteria;Betaproteobacteria;Burkholderiales;Burkholderiaceae;Burkholderia;Burkholderia sp. H160;Burkholderia sp. H160 |
| Ga0586324 1113878 381 1160 | 0.00 | 0.00 | 0.00 | 515045.35 | 5819500.00 | 0.00 | 0.00 | 0.00 | 0.00 | 0.00 | 0.00 | 0.00 | | uncharacterized protein YqfA (UPF0365 family) | Bacteria;Verrucomicrobia;unclassified Verrucomicrobia;unclassified Verrucomicrobia;unclassified Verrucomicrobia;unclassified Verrucomicrobia;Verrucomicrobia bacterium LW23;Verrucomicrobia bacterium LW23 |
| Ga0586324 1114174 3 1160 | 0.00 | 707200.00 | 0.00 | 764819.35 | 843057.06 | 0.00 | 0.00 | 273291.59 | 0.00 | 0.00 | 0.00 | 0.00 | | TRAP-type mannitol/chloroaromatic compound transport system substrate-binding protein | Bacteria;Proteobacteria;Betaproteobacteria;Burkholderiales;Comamonadaceae;Polaromonas;Polaromonas sp. EUR3 1.2.1;Polaromonas sp. EUR3 1.2.1 |
| Ga0586324 1114201 2 646 | 4260000.00 | 0.00 | 0.00 | 0.00 | 0.00 | 0.00 | 0.00 | 2531500.00 | 0.00 | 0.00 | 0.00 | 0.00 | | hypothetical protein | Bacteria;Proteobacteria;Alphaproteobacteria;Rhodobacterales;Rhodobacteraceae;Yoonia;Yoonia vestfoldensis;Loktanella vestfoldensis DSM 16212 |
| Ga0586324 1114764 3 881 | 0.00 | 0.00 | 0.00 | 600179.60 | 0.00 | 0.00 | 0.00 | 0.00 | 0.00 | 0.00 | 0.00 | 0.00 | | hypothetical protein | Bacteria;Acidobacteria;Acidobacteriia;Bryobacterales;Bryobacteraceae;Bryobacter;Bryobacter aggregatus;Bryobacter aggregatus DSM 18758 |
| Ga0586324 1115828 1 315 | 0.00 | 0.00 | 0.00 | 0.00 | 6736200.00 | 0.00 | 0.00 | 3574600.00 | 3750300.00 | 3773000.00 | 0.00 | 2670000.00 | | hypothetical protein | |
| Ga0586324 1115917 364 1158 | 0.00 | 0.00 | 0.00 | 0.00 | 0.00 | 0.00 | 0.00 | 0.00 | 0.00 | 0.00 | 0.00 | 2917500.00 | | hypothetical protein | Bacteria;Proteobacteria;Alphaproteobacteria;Hyphomicrobiales;Bradyrhizobiaceae;Bradyrhizobium;Bradyrhizobium elkanii;Bradyrhizobium elkanii USDA 76 |
| Ga0586324 1117630 172 1158 | 6910000.00 | 7684000.00 | 0.00 | 8673500.00 | 9517200.00 | 7543000.00 | 13292400.00 | 7564000.00 | 5086800.00 | 6899200.00 | 6476800.00 | 0.00 | | uncharacterized protein (TIGR02001 family) | Bacteria;Proteobacteria;Alphaproteobacteria;Hyphomicrobiales;Bradyrhizobiaceae;Bradyrhizobium;Bradyrhizobium sp. YR681;Bradyrhizobium sp. YR681 |
| Ga0586324 1119900 145 969 | 332519.00 | 1285200.00 | 12723800.00 | 227576.30 | 0.00 | 0.00 | 1527611.40 | 0.00 | 0.00 | 0.00 | 0.00 | 0.00 | | DNA-binding MurR/RpiR family transcriptional regulator | Bacteria;Chloroflexi;Caldilineae;Caldilineales;Caldilineaceae;Caldilinea;Caldilinea aerophila;Caldilinea aerophila STL-6-O1, DSM 14535 |
| Ga0586324 1121337 3 1157 | 0.00 | 0.00 | 0.00 | 0.00 | 0.00 | 4525800.00 | 5084400.00 | 0.00 | 0.00 | 0.00 | 0.00 | 3900000.00 | | hypothetical protein | Bacteria;Proteobacteria;Gammaproteobacteria;Legionellales;Legionellaceae;Legionella;Legionella drancourtii;Legionella drancourtii LLAP12 |
| Ga0586324 1124176 232 552 | 0.00 | 0.00 | 3583652.64 | 0.00 | 0.00 | 0.00 | 0.00 | 0.00 | 0.00 | 0.00 | 0.00 | 0.00 | | hypothetical protein | Bacteria;Proteobacteria;Alphaproteobacteria;Hyphomicrobiales;Methylobacteriaceae;Microvirga;Microvirga flocculans;Microvirga flocculans ATCC BAA-817 |
| Ga0586324 1124915 2 703 | 1360000.00 | 0.00 | 0.00 | 323584.25 | 155723.64 | 0.00 | 0.00 | 584893.01 | 0.00 | 0.00 | 0.00 | 511647.00 | | hypothetical protein | Bacteria;Proteobacteria;Gammaproteobacteria;Methylococcales;Methylococcaceae;Methylovulum;Methylovulum miyakonense;Methylovulum miyakonense HT12 |
| Ga0586324 1125509 5 1153 | 0.00 | 0.00 | 0.00 | 0.00 | 0.00 | 0.00 | 6224400.00 | 0.00 | 0.00 | 2079000.00 | 0.00 | 0.00 | | predicted porin | Bacteria;Proteobacteria;Betaproteobacteria;Burkholderiales;Alcaligenaceae;Pigmentiphaga;Pigmentiphaga kullae;Pigmentiphaga kullae DSM 13608 |
| Ga0586324 1125515 3 1154 | 0.00 | 0.00 | 0.00 | 0.00 | 3388700.00 | 0.00 | 0.00 | 0.00 | 0.00 | 0.00 | 0.00 | 0.00 | | hypothetical protein | Bacteria;Actinobacteria;Actinomycetia;Propionibacteriales;Kribbellaceae;Kribbella;Kribbella catacumbae;Kribbella catacumbae DSM 19601 |
| Ga0586324 1127032 3 1154 | 1400000.00 | 0.00 | 0.00 | 9595000.00 | 8301800.00 | 0.00 | 0.00 | 1439600.00 | 0.00 | 2510200.00 | 0.00 | 0.00 | | hypothetical protein | Bacteria;Proteobacteria;Betaproteobacteria;Burkholderiales;unclassified Burkholderiales;Paucibacter;Paucibacter toxinivorans;Paucibacter toxinivorans DSM 16998 |
| Ga0586324 1130629 391 738 | 5850000.00 | 0.00 | 0.00 | 0.00 | 17819000.00 | 0.00 | 10488000.00 | 9943000.00 | 9234000.00 | 0.00 | 0.00 | 0.00 | | hypothetical protein | Archaea;Euryarchaeota;Methanomicrobia;Methanosarcinales;Methanotrichaceae;Methanothrix;Methanothrix soehngenii;Methanothrix soehngenii GP-6 |
| Ga0586324 1131084 218 1153 | 1560000.00 | 20536000.00 | 17453000.00 | 24510000.00 | 1050600.00 | 16197600.00 | 23712000.00 | 0.00 | 0.00 | 1108800.00 | 0.00 | 0.00 | | cytochrome c peroxidase | Bacteria;Proteobacteria;Deltaproteobacteria;Desulfuromonadales;Geobacteraceae;Geobacter;Geobacter soli;Geobacter soli GSS01 |
| Ga0586324 1134909 2 751 | 37110000.00 | 78349600.00 | 77074700.00 | 112005000.00 | 96717000.00 | 102426000.00 | 64820400.00 | 65379800.00 | 35073000.00 | 86193800.00 | 71720000.00 | 48300000.00 | | hypothetical protein | |
| Ga0586324 1135954 92 1150 | 0.00 | 0.00 | 0.00 | 0.00 | 2492600.00 | 0.00 | 0.00 | 0.00 | 0.00 | 0.00 | 0.00 | 0.00 | | hypothetical protein | Bacteria;Proteobacteria;Gammaproteobacteria;Oceanospirillales;Oleiphilaceae;Oleiphilus;Oleiphilus messinensis;Oleiphilus messinensis ME102 |
| Ga0586324 1136113 1 945 | 208980000.00 | 170578000.00 | 282435604.66 | 137237000.00 | 162626700.00 | 136758401.20 | 78454800.00 | 104767500.00 | 88160400.00 | 226537234.00 | 180400000.00 | 280005000.00 | | predicted porin | Bacteria;Proteobacteria;Alphaproteobacteria;Rhodospirillales;Acetobacteraceae;Rhodopila;unclassified Rhodopila;Rhodopila sp. LVNP |
| Ga0586324 1137507 2 673 | 8780000.00 | 7276000.00 | 0.00 | 12635000.00 | 0.00 | 11790900.00 | 22344000.00 | 20618000.00 | 17415000.00 | 27181000.00 | 24464000.00 | 15600000.00 | | TRAP-type mannitol/chloroaromatic compound transport system substrate-binding protein | Bacteria;Proteobacteria;Betaproteobacteria;Burkholderiales;unclassified Burkholderiales;Thiomonas;Thiomonas sp. X19;Thiomonas sp. X19 |
| Ga0586324 1137683 1 1149 | 0.00 | 0.00 | 0.00 | 0.00 | 0.00 | 0.00 | 0.00 | 0.00 | 0.00 | 3264800.00 | 0.00 | 0.00 | | hypothetical protein | Bacteria;Proteobacteria;Gammaproteobacteria;Methylococcales;Methylococcaceae;Methylobacter;Methylobacter tundripaludum;Methylobacter tundripaludum SV96 |
| Ga0586324 1138232 144 1070 | 7330000.00 | 2971600.00 | 0.00 | 0.00 | 0.00 | 0.00 | 0.00 | 2641300.00 | 2535300.00 | 0.00 | 0.00 | 1237500.00 | | C1A family cysteine protease | Archaea;Euryarchaeota;Methanomicrobia;Methanosarcinales;Methanotrichaceae;Methanothrix;Methanothrix soehngenii;Methanothrix soehngenii GP-6 |
| Ga0586324 1139648 2 1147 | 0.00 | 0.00 | 0.00 | 0.00 | 0.00 | 1961195.88 | 0.00 | 0.00 | 3855600.00 | 0.00 | 0.00 | 0.00 | | ABC-type branched-subunit amino acid transport system substrate-binding protein | Bacteria;Chloroflexi;Anaerolineae;Anaerolineales;Anaerolineaceae;Flexilinea;Flexilinea flocculi;Flexilinea flocculi TC1 |
| Ga0586324 1139912 673 1149 | 536293.00 | 0.00 | 0.00 | 0.00 | 0.00 | 0.00 | 0.00 | 0.00 | 0.00 | 0.00 | 0.00 | 0.00 | | Spy/CpxP family protein refolding chaperone | Bacteria;Proteobacteria;Deltaproteobacteria;Syntrophobacterales;Syntrophaceae;Desulfobacca;Desulfobacca acetoxidans;Desulfobacca acetoxidans ASRB2, DSM 11109 |
| Ga0586324 1140000 1 876 | 0.00 | 1115200.00 | 1678348.04 | 900740.60 | 0.00 | 0.00 | 0.00 | 0.00 | 776699.28 | 0.00 | 0.00 | 745059.00 | | hypothetical protein | |
| Ga0586324 1140521 3 1148 | 0.00 | 0.00 | 0.00 | 0.00 | 0.00 | 0.00 | 0.00 | 0.00 | 0.00 | 0.00 | 0.00 | 1785000.00 | | hypothetical protein | Bacteria;Proteobacteria;Alphaproteobacteria;Hyphomicrobiales;Bradyrhizobiaceae;Bradyrhizobium;Bradyrhizobium erythrophlei;Bradyrhizobium erythrophlei GAS138 |
| Ga0586324 1143221 134 631 | 0.00 | 16592000.00 | 0.00 | 0.00 | 0.00 | 0.00 | 0.00 | 0.00 | 0.00 | 0.00 | 0.00 | 0.00 | | rubrerythrin | Archaea;Euryarchaeota;Methanomicrobia;Methanosarcinales;Methanotrichaceae;Methanothrix;Methanothrix soehngenii;Methanothrix soehngenii GP-6 |
| Ga0586324 1144702 389 1144 | 0.00 | 8160000.00 | 0.00 | 0.00 | 0.00 | 0.00 | 0.00 | 0.00 | 0.00 | 0.00 | 0.00 | 0.00 | | hypothetical protein | Bacteria;Proteobacteria;Betaproteobacteria;Burkholderiales;Comamonadaceae;Curvibacter;Curvibacter gracilis;Curvibacter gracilis ATCC BAA-807 |
| Ga0586324 1145133 2 1144 | 0.00 | 0.00 | 0.00 | 0.00 | 3193000.00 | 0.00 | 0.00 | 0.00 | 0.00 | 0.00 | 0.00 | 0.00 | | hypothetical protein | Bacteria;Candidatus Aminicenantes;unclassified Candidatus Aminicenantes;unclassified Candidatus Aminicenantes;unclassified Candidatus Aminicenantes;unclassified Candidatus Aminicenantes;Aminicenantes bacterium SCGC AAA252-O03;Aminicenantes bacterium SCGC AAA252-O03 (contamination screened) |
| Ga0586324 1145994 2 1144 | 7370000.00 | 0.00 | 0.00 | 0.00 | 0.00 | 0.00 | 0.00 | 0.00 | 0.00 | 0.00 | 0.00 | 0.00 | | hypothetical protein | Bacteria;Acidobacteria;Acidobacteriia;Bryobacterales;Bryobacteraceae;Bryobacter;Bryobacter aggregatus;Bryobacter aggregatus DSM 18758 |
| Ga0586324 1154480 363 1142 | 7480000.00 | 0.00 | 0.00 | 0.00 | 0.00 | 0.00 | 0.00 | 0.00 | 0.00 | 0.00 | 0.00 | 0.00 | | predicted porin | |
| Ga0586324 1158173 379 1065 | 0.00 | 0.00 | 0.00 | 0.00 | 0.00 | 6034400.00 | 0.00 | 0.00 | 0.00 | 0.00 | 0.00 | 0.00 | | multimeric flavodoxin WrbA | Bacteria;Proteobacteria;Deltaproteobacteria;Syntrophobacterales;Syntrophobacteraceae;Desulfacinum;Desulfacinum hydrothermale;Desulfacinum hydrothermale DSM 13146 |
| Ga0586324 1163174 105 1001 | 0.00 | 0.00 | 0.00 | 911688.40 | 1514100.00 | 0.00 | 0.00 | 0.00 | 0.00 | 0.00 | 0.00 | 0.00 | | nucleotide-binding universal stress UspA family protein | Bacteria;Proteobacteria;Alphaproteobacteria;Rhodospirillales;Acetobacteraceae;Rhodopila;unclassified Rhodopila;Rhodopila sp. LVNP |
| Ga0586324 1165225 2 1138 | 2420000.00 | 8384400.00 | 0.00 | 0.00 | 0.00 | 0.00 | 0.00 | 0.00 | 0.00 | 4920300.00 | 0.00 | 4755000.00 | | hypothetical protein | Bacteria;Proteobacteria;Gammaproteobacteria;Chromatiales;Chromatiaceae;Thiorhodococcus;Thiorhodococcus drewsii;Thiorhodococcus drewsii AZ1 |
| Ga0586324 1165554 2 841 | 0.00 | 0.00 | 0.00 | 0.00 | 0.00 | 0.00 | 0.00 | 1927600.00 | 931500.00 | 0.00 | 0.00 | 0.00 | | UbiD family decarboxylase | Bacteria;Proteobacteria;Deltaproteobacteria;Syntrophorhabdales;Syntrophorhabdaceae;Syntrophorhabdus;Syntrophorhabdus aromaticivorans;Syntrophorhabdus aromaticivorans UI |
| Ga0586324 1166656 419 1135 | 0.00 | 0.00 | 0.00 | 0.00 | 0.00 | 0.00 | 0.00 | 13969000.00 | 0.00 | 0.00 | 0.00 | 0.00 | | TRAP-type mannitol/chloroaromatic compound transport system substrate-binding protein | Bacteria;Proteobacteria;Betaproteobacteria;Nitrosomonadales;Sterolibacteriaceae;Sulfurisoma;Sulfurisoma sediminicola;Sulfurisoma sediminicola DSM 26916 |
| Ga0586324 1176824 1 513 | 0.00 | 0.00 | 0.00 | 0.00 | 0.00 | 0.00 | 0.00 | 0.00 | 0.00 | 0.00 | 0.00 | 1087500.00 | | curli biogenesis system outer membrane secretion channel CsgG | Bacteria;Acidobacteria;Vicinamibacteria;unclassified Vicinamibacteria;Vicinamibacteraceae;Luteitalea;Luteitalea pratensis;Luteitalea pratensis DSM 100886 |
| Ga0586324 1177332 3 1001 | 0.00 | 4596800.00 | 0.00 | 0.00 | 0.00 | 0.00 | 0.00 | 0.00 | 0.00 | 4966500.00 | 0.00 | 3247500.00 | | hypothetical protein | Bacteria;Proteobacteria;Deltaproteobacteria;Syntrophorhabdales;Syntrophorhabdaceae;Syntrophorhabdus;Syntrophorhabdus aromaticivorans;Syntrophorhabdus aromaticivorans UI |
| Ga0586324 1177365 1 1131 | 0.00 | 0.00 | 0.00 | 0.00 | 0.00 | 0.00 | 0.00 | 0.00 | 1636200.00 | 0.00 | 0.00 | 0.00 | | hypothetical protein | Bacteria;Calditrichaeota;Calditrichae;Calditrichales;Calditrichaceae;Caldithrix;Caldithrix abyssi;Caldithrix abyssi LF13, DSM 13497 |
| Ga0586324 1183064 2 1129 | 0.00 | 0.00 | 0.00 | 0.00 | 2523500.00 | 0.00 | 0.00 | 1830000.00 | 6212700.00 | 0.00 | 4778400.00 | 595422.75 | | hypothetical protein | Bacteria;Acidobacteria;Acidobacteriia;Acidobacteriales;Acidobacteriaceae;unclassified Acidobacteriaceae;Acidobacteriaceae bacterium KBS 83;Acidobacteriaceae bacterium KBS 83 |
| Ga0586324 1186629 219 1127 | 0.00 | 0.00 | 0.00 | 0.00 | 0.00 | 0.00 | 0.00 | 0.00 | 866700.00 | 728704.90 | 1135200.00 | 1417500.00 | | hypothetical protein | Bacteria;Proteobacteria;Gammaproteobacteria;Salinisphaerales;Salinisphaeraceae;Abyssibacter;Abyssibacter profundi;Abyssibacter profundi OUC007 |
| Ga0586324 1190357 3 1127 | 0.00 | 3354538.60 | 0.00 | 3087500.00 | 3821300.00 | 10807491.30 | 8983200.00 | 1110200.00 | 0.00 | 0.00 | 0.00 | 5805000.00 | | hypothetical protein | Bacteria;Candidatus Aminicenantes;unclassified Candidatus Aminicenantes;unclassified Candidatus Aminicenantes;unclassified Candidatus Aminicenantes;unclassified Candidatus Aminicenantes;Aminicenantes bacterium SCGC AAA252-D06;Aminicenantes bacterium SCGC AAA252-D06 (contamination screened) |
| Ga0586324 1191181 1 1125 | 0.00 | 0.00 | 0.00 | 0.00 | 0.00 | 0.00 | 0.00 | 0.00 | 0.00 | 924000.00 | 0.00 | 877500.00 | | hypothetical protein | Bacteria;Candidatus Aminicenantes;unclassified Candidatus Aminicenantes;unclassified Candidatus Aminicenantes;unclassified Candidatus Aminicenantes;unclassified Candidatus Aminicenantes;Aminicenantes bacterium SCGC AAA255-F06;Aminicenantes bacterium SCGC AAA255-F06 (contamination screened) |
| Ga0586324 1191601 1 870 | 0.00 | 0.00 | 0.00 | 0.00 | 0.00 | 0.00 | 0.00 | 0.00 | 0.00 | 6367900.00 | 0.00 | 0.00 | | hypothetical protein | Bacteria;Acidobacteria;Vicinamibacteria;unclassified Vicinamibacteria;Vicinamibacteraceae;Luteitalea;Luteitalea pratensis;Luteitalea pratensis DSM 100886 |
| Ga0586324 1193179 299 1126 | 0.00 | 0.00 | 0.00 | 0.00 | 251401.37 | 0.00 | 0.00 | 0.00 | 0.00 | 0.00 | 0.00 | 0.00 | | alkylation response protein AidB-like acyl-CoA dehydrogenase | Bacteria;Proteobacteria;Deltaproteobacteria;Desulfobacterales;Desulfobacteraceae;Desulfatiglans;Desulfatiglans anilini;Desulfatiglans anilini DSM 4660 |
| Ga0586324 1195797 284 760 | 2320000.00 | 4372400.00 | 3714178.56 | 5728500.00 | 17201000.00 | 17626800.00 | 0.00 | 22936000.00 | 972000.00 | 23716000.00 | 0.00 | 1305000.00 | | predicted Zn-dependent protease | |
| Ga0586324 1200127 1 1122 | 8580000.00 | 0.00 | 0.00 | 0.00 | 0.00 | 0.00 | 0.00 | 0.00 | 0.00 | 0.00 | 5931200.00 | 0.00 | | ABC-type xylose transport system substrate-binding protein | Bacteria;Chloroflexi;Ardenticatenia;Ardenticatenales;Ardenticatenaceae;Candidatus Promineofilum;Candidatus Promineofilum breve;Candidatus Promineofilum breve Cfx-K |
| Ga0586324 1200675 228 686 | 0.00 | 0.00 | 0.00 | 0.00 | 0.00 | 0.00 | 0.00 | 0.00 | 0.00 | 0.00 | 3634400.00 | 2887500.00 | | hypothetical protein | Bacteria;Proteobacteria;Alphaproteobacteria;Hyphomicrobiales;Alsobacteraceae;Alsobacter;Alsobacter soli;Alsobacter soli SH9 |
| Ga0586324 1200882 2 376 | 0.00 | 0.00 | 0.00 | 0.00 | 0.00 | 0.00 | 0.00 | 0.00 | 1944000.00 | 0.00 | 0.00 | 0.00 | | hypothetical protein | Bacteria;Bacteroidetes;Bacteroidia;Marinilabiliales;Prolixibacteraceae;Draconibacterium;Draconibacterium sediminis;Draconibacterium sediminis JN14CK-3 |
| Ga0586324 1201805 1 657 | 0.00 | 2244000.00 | 0.00 | 0.00 | 0.00 | 0.00 | 0.00 | 0.00 | 0.00 | 0.00 | 0.00 | 0.00 | | regulator of protease activity HflC (stomatin/prohibitin superfamily) | Bacteria;Firmicutes;Clostridia;Eubacteriales;Peptococcaceae;Desulfitobacterium;Desulfitobacterium chlororespirans;Desulfitobacterium chlororespirans DSM 11544 |
| Ga0586324 1202791 2 946 | 10660000.00 | 33816400.00 | 84675200.00 | 44830500.00 | 0.00 | 4883100.00 | 70064400.00 | 49428300.00 | 52593300.00 | 69122900.00 | 64944000.00 | 27795000.00 | | hypothetical protein | Bacteria;Proteobacteria;Gammaproteobacteria;Chromatiales;Ectothiorhodospiraceae;Thiorhodospira;Thiorhodospira sibirica;Thiorhodospira sibirica A12, ATCC 700588 |
| Ga0586324 1204498 2 1120 | 0.00 | 0.00 | 1737716.39 | 3971000.00 | 3635900.00 | 0.00 | 0.00 | 0.00 | 0.00 | 0.00 | 950400.00 | 0.00 | | 2',3'-cyclic-nucleotide 2'-phosphodiesterase (5'-nucleotidase family) | Bacteria;Firmicutes;Clostridia;Eubacteriales;Thermohalobacteraceae;Caloranaerobacter;Caloranaerobacter sp. TR13;Caloranaerobacter sp. TR13 |
| Ga0586324 1205440 469 1119 | 0.00 | 0.00 | 0.00 | 0.00 | 0.00 | 0.00 | 0.00 | 0.00 | 0.00 | 0.00 | 0.00 | 2040000.00 | | predicted porin | Bacteria;Proteobacteria;Betaproteobacteria;Rhodocyclales;Zoogloeaceae;Azoarcus;Azoarcus sp. CIB;Azoarcus sp. CIB |
| Ga0586324 1206772 1 1119 | 604654.00 | 2271200.00 | 0.00 | 0.00 | 0.00 | 0.00 | 0.00 | 0.00 | 0.00 | 0.00 | 0.00 | 0.00 | | hypothetical protein | Bacteria;Candidatus Aminicenantes;unclassified Candidatus Aminicenantes;unclassified Candidatus Aminicenantes;unclassified Candidatus Aminicenantes;unclassified Candidatus Aminicenantes;Aminicenantes bacterium SCGC AAA252-D06;Aminicenantes bacterium SCGC AAA252-D06 (contamination screened) |
| Ga0586324 1210763 532 1119 | 14500000.00 | 22644000.00 | 21731800.00 | 4769000.00 | 5459000.00 | 11711500.00 | 131556000.00 | 128905200.00 | 98658000.00 | 91476000.00 | 163856000.00 | 135180000.00 | | predicted porin | |
| Ga0586324 1212660 481 1116 | 0.00 | 0.00 | 0.00 | 0.00 | 0.00 | 0.00 | 0.00 | 0.00 | 0.00 | 0.00 | 0.00 | 2407500.00 | | glutamate synthase domain-containing protein 2 | Bacteria;Proteobacteria;Deltaproteobacteria;Syntrophobacterales;Syntrophaceae;Desulfobacca;Desulfobacca acetoxidans;Desulfobacca acetoxidans ASRB2, DSM 11109 |
| Ga0586324 1213847 3 491 | 3570000.00 | 2869600.00 | 0.00 | 0.00 | 5273600.00 | 5677100.00 | 0.00 | 2799900.00 | 2154600.00 | 0.00 | 0.00 | 0.00 | | phasin family protein | Bacteria;Proteobacteria;Alphaproteobacteria;Rhodospirillales;Rhodospirillaceae;Dongia;Dongia sp. URHE0060;Dongia sp. URHE0060 |
| Ga0586324 1215850 2 1117 | 0.00 | 809200.00 | 0.00 | 0.00 | 0.00 | 0.00 | 0.00 | 0.00 | 0.00 | 0.00 | 0.00 | 0.00 | | hypothetical protein | Bacteria;Proteobacteria;Gammaproteobacteria;Xanthomonadales;Rhodanobacteraceae;Frateuria;Frateuria sp. Soil773;Frateuria sp. Soil773 |
| Ga0586324 1217132 2 1117 | 128860000.00 | 169694000.00 | 190121153.37 | 172710000.00 | 149586900.00 | 102981800.00 | 56886000.00 | 74395600.00 | 104166000.00 | 66628100.00 | 61679200.00 | 51105000.00 | | S-layer protein (TIGR01567 family) | Archaea;Euryarchaeota;Methanomicrobia;Methanosarcinales;Methanotrichaceae;Methanothrix;Methanothrix thermoacetophila;Methanothrix thermoacetophila PT |
| Ga0586324 1218125 2 748 | 600990.00 | 0.00 | 4738635.88 | 0.00 | 6756800.00 | 7041477.84 | 3055200.00 | 0.00 | 0.00 | 0.00 | 0.00 | 0.00 | | PGF-CTERM protein | Archaea;Euryarchaeota;Methanomicrobia;Methanosarcinales;Methanosarcinaceae;Methanosarcina;Methanosarcina vacuolata;Methanosarcina vacuolata Z-761 |
| Ga0586324 1218556 2 757 | 0.00 | 0.00 | 0.00 | 0.00 | 0.00 | 0.00 | 0.00 | 0.00 | 0.00 | 0.00 | 0.00 | 693217.50 | | crotonobetainyl-CoA:carnitine CoA-transferase CaiB-like acyl-CoA transferase | Archaea;Euryarchaeota;Archaeoglobi;Archaeoglobales;Archaeoglobaceae;Ferroglobus;Ferroglobus placidus;Ferroglobus placidus AEDII12DO, DSM 10642 |
| Ga0586324 1218692 3 1115 | 0.00 | 0.00 | 0.00 | 1358500.00 | 0.00 | 0.00 | 0.00 | 0.00 | 0.00 | 0.00 | 0.00 | 0.00 | | hypothetical protein | Bacteria;Ignavibacteriae;Ignavibacteria;Ignavibacteriales;Ignavibacteriaceae;Ignavibacterium;Ignavibacterium album;Ignavibacterium album Mat9-16, JCM 16511 |
| Ga0586324 1222452 3 353 | 0.00 | 0.00 | 0.00 | 0.00 | 0.00 | 0.00 | 0.00 | 0.00 | 0.00 | 23100000.00 | 0.00 | 0.00 | | hypothetical protein | Bacteria;Proteobacteria;Betaproteobacteria;Nitrosomonadales;Methylophilaceae;Methylobacillus;Methylobacillus glycogenes;Methylobacillus glycogenes JCM 2850 |
| Ga0586324 1224909 21 1112 | 5400000.00 | 5154400.00 | 0.00 | 12445000.00 | 11845000.00 | 0.00 | 0.00 | 6344000.00 | 6666300.00 | 7630700.00 | 0.00 | 5175000.00 | | hypothetical protein | Bacteria;Acidobacteria;Acidobacteriia;Bryobacterales;Bryobacteraceae;Bryobacter;Bryobacter aggregatus;Bryobacter aggregatus DSM 18758 |
| Ga0586324 1227182 633 1112 | 0.00 | 0.00 | 0.00 | 0.00 | 0.00 | 0.00 | 0.00 | 13054000.00 | 0.00 | 8393000.00 | 11528000.00 | 0.00 | | hypothetical protein | |
| Ga0586324 1228529 1 1092 | 0.00 | 0.00 | 0.00 | 0.00 | 0.00 | 0.00 | 0.00 | 0.00 | 0.00 | 0.00 | 0.00 | 624875.25 | | ABC-type transport system substrate-binding protein | Bacteria;Proteobacteria;Deltaproteobacteria;Syntrophobacterales;Syntrophobacteraceae;Desulfosoma;Desulfosoma caldarium;Desulfosoma caldarium DSM 22027 |
| Ga0586324 1229838 1 1110 | 0.00 | 0.00 | 0.00 | 0.00 | 0.00 | 6709300.00 | 0.00 | 0.00 | 0.00 | 0.00 | 0.00 | 0.00 | | formyltetrahydrofolate synthetase | Bacteria;Firmicutes;Clostridia;Eubacteriales;Peptococcaceae;Desulfurispora;Desulfurispora thermophila;Desulfurispora thermophila DSM 16022 |
| Ga0586324 1231789 1 1104 | 0.00 | 0.00 | 0.00 | 0.00 | 1266900.00 | 0.00 | 0.00 | 0.00 | 0.00 | 0.00 | 0.00 | 0.00 | | methylmalonyl-CoA mutase cobalamin-binding domain/chain | Bacteria;Bacteroidetes;Sphingobacteriia;Sphingobacteriales;Sphingobacteriaceae;Pedobacter;Pedobacter indicus;Pedobacter indicus SM1810 |
| Ga0586324 1234041 3 605 | 0.00 | 1802000.00 | 0.00 | 0.00 | 0.00 | 0.00 | 0.00 | 0.00 | 0.00 | 0.00 | 0.00 | 0.00 | | hypothetical protein | |
| Ga0586324 1237833 3 1109 | 1320000.00 | 0.00 | 0.00 | 0.00 | 0.00 | 0.00 | 0.00 | 0.00 | 0.00 | 0.00 | 0.00 | 2077500.00 | | hypothetical protein | Bacteria;Proteobacteria;Gammaproteobacteria;Alteromonadales;Pseudoalteromonadaceae;Algicola;Algicola sagamiensis;Algicola sagamiensis DSM 14643 |
| Ga0586324 1238086 3 1109 | 0.00 | 0.00 | 0.00 | 0.00 | 26162000.00 | 22152600.00 | 31692000.00 | 3690500.00 | 20655000.00 | 17248000.00 | 15928000.00 | 7800000.00 | | TRAP-type mannitol/chloroaromatic compound transport system substrate-binding protein | Bacteria;Proteobacteria;Alphaproteobacteria;Rhodospirillales;Rhodospirillaceae;Indioceanicola;Indioceanicola profundi;Indioceanicola profundi SCSIO 08040 |
| Ga0586324 1238281 3 836 | 0.00 | 1074400.00 | 0.00 | 0.00 | 0.00 | 0.00 | 0.00 | 276729.55 | 359456.13 | 0.00 | 1795200.00 | 1080000.00 | | hypothetical protein | Bacteria;Proteobacteria;Betaproteobacteria;Burkholderiales;Burkholderiaceae;Pandoraea;Pandoraea sp. B-6;Pandoraea sp. B-6 |
| Ga0586324 1239554 2 1108 | 0.00 | 0.00 | 0.00 | 6222500.00 | 6540500.00 | 4962500.00 | 0.00 | 3007300.00 | 3045600.00 | 2895200.00 | 1381600.00 | 0.00 | | hypothetical protein | Bacteria;Acidobacteria;Acidobacteriia;Bryobacterales;Bryobacteraceae;Bryobacter;Bryobacter aggregatus;Bryobacter aggregatus DSM 18758 |
| Ga0586324 1243763 315 1028 | 0.00 | 0.00 | 0.00 | 0.00 | 0.00 | 0.00 | 0.00 | 0.00 | 0.00 | 0.00 | 281033.28 | 0.00 | | hypothetical protein | |
| Ga0586324 1248437 2 1003 | 0.00 | 0.00 | 0.00 | 0.00 | 0.00 | 0.00 | 0.00 | 0.00 | 0.00 | 5012700.00 | 0.00 | 0.00 | | hypothetical protein | Bacteria;Proteobacteria;Gammaproteobacteria;Chromatiales;unclassified Chromatiales;unclassified Chromatiales;unclassified Chromatiales;Chromatiales bacterium |
| Ga0586324 1254642 3 1100 | 0.00 | 0.00 | 0.00 | 0.00 | 0.00 | 0.00 | 0.00 | 0.00 | 0.00 | 2648800.00 | 0.00 | 0.00 | | hypothetical protein | Bacteria;Gemmatimonadetes;Gemmatimonadetes;Gemmatimonadales;Gemmatimonadaceae;Gemmatimonas;Gemmatimonas phototrophica;Gemmatimonas phototrophica AP64 |
| Ga0586324 1255047 208 798 | 4610000.00 | 0.00 | 4124425.40 | 4750000.00 | 5150000.00 | 4049400.00 | 4924800.00 | 5471700.00 | 0.00 | 4335100.00 | 5473600.00 | 5017500.00 | | hypothetical protein | Bacteria;Balneolaeota;Balneolia;Balneolales;Balneolaceae;Gracilimonas;Gracilimonas amylolytica;Gracilimonas amylolytica LA399 |
| Ga0586324 1255245 97 1101 | 0.00 | 0.00 | 0.00 | 0.00 | 0.00 | 0.00 | 0.00 | 0.00 | 0.00 | 13937000.00 | 0.00 | 0.00 | | formyltetrahydrofolate synthetase | Bacteria;Chloroflexi;Caldilineae;Caldilineales;Caldilineaceae;Caldilinea;Caldilinea aerophila;Caldilinea aerophila STL-6-O1, DSM 14535 |
| Ga0586324 1256193 3 1070 | 22700000.00 | 4644400.00 | 9176900.00 | 22952000.00 | 1514100.00 | 0.00 | 0.00 | 2013000.00 | 1830600.00 | 4535300.00 | 2376000.00 | 1762500.00 | | hypothetical protein | Bacteria;Spirochaetes;Spirochaetia;Spirochaetales;Spirochaetaceae;Spirochaeta;Spirochaeta cellobiosiphila;Spirochaeta cellobiosiphila DSM 17781 |
| Ga0586324 1256217 3 1100 | 0.00 | 0.00 | 0.00 | 2802500.00 | 0.00 | 0.00 | 0.00 | 0.00 | 0.00 | 0.00 | 0.00 | 0.00 | | hypothetical protein | |
| Ga0586324 1258207 1 960 | 0.00 | 0.00 | 2333556.18 | 0.00 | 2276300.00 | 0.00 | 0.00 | 0.00 | 1328400.00 | 0.00 | 0.00 | 0.00 | | ABC-type sugar transport system substrate-binding protein | Bacteria;Chloroflexi;Caldilineae;Caldilineales;Caldilineaceae;Caldilinea;Caldilinea aerophila;Caldilinea aerophila STL-6-O1, DSM 14535 |
| Ga0586324 1259791 3 1100 | 7400000.00 | 7276000.00 | 16527484.30 | 11115000.00 | 14121300.00 | 13995814.18 | 16210800.00 | 9406200.00 | 4762800.00 | 5859700.00 | 7304000.00 | 9375000.00 | | hypothetical protein | Bacteria;Candidatus Aminicenantes;unclassified Candidatus Aminicenantes;unclassified Candidatus Aminicenantes;unclassified Candidatus Aminicenantes;unclassified Candidatus Aminicenantes;Aminicenantes bacterium SCGC AAA252-D06;Aminicenantes bacterium SCGC AAA252-D06 (contamination screened) |
| Ga0586324 1260694 426 1100 | 0.00 | 4535600.00 | 7713100.00 | 969000.00 | 1215400.00 | 8416400.00 | 707424.72 | 1134600.00 | 548735.31 | 5320700.00 | 5051200.00 | 3045000.00 | | hypothetical protein | Bacteria;Proteobacteria;Deltaproteobacteria;Desulfuromonadales;Desulfuromonadaceae;Pelobacter;Pelobacter propionicus;Pelobacter propionicus DSM 2379 |
| Ga0586324 1261009 1 1098 | 0.00 | 0.00 | 0.00 | 0.00 | 0.00 | 0.00 | 544108.32 | 1366400.00 | 0.00 | 0.00 | 0.00 | 0.00 | | outer membrane receptor protein involved in Fe transport | Bacteria;Bacteroidetes;Cytophagia;Cytophagales;Cytophagaceae;Dyadobacter;Dyadobacter crusticola;Dyadobacter crusticola DSM 16708 |
| Ga0586324 1261837 60 1100 | 0.00 | 0.00 | 0.00 | 0.00 | 0.00 | 0.00 | 0.00 | 0.00 | 1741500.00 | 0.00 | 0.00 | 0.00 | | hypothetical protein | Bacteria;Calditrichaeota;Calditrichae;Calditrichales;Calditrichaceae;Caldithrix;Caldithrix abyssi;Caldithrix abyssi LF13, DSM 13497 |
| Ga0586324 1261967 595 1098 | 0.00 | 0.00 | 0.00 | 0.00 | 0.00 | 2286068.92 | 0.00 | 0.00 | 2891700.00 | 839300.00 | 0.00 | 0.00 | | hypothetical protein | Bacteria;Proteobacteria;Alphaproteobacteria;Hyphomicrobiales;Bradyrhizobiaceae;Bradyrhizobium;Bradyrhizobium sp. ARR65;Bradyrhizobium sp. ARR65 |
| Ga0586324 1264346 2 958 | 0.00 | 2767600.00 | 0.00 | 0.00 | 2235100.00 | 0.00 | 0.00 | 0.00 | 0.00 | 0.00 | 0.00 | 5190000.00 | | hypothetical protein | Bacteria;Proteobacteria;Deltaproteobacteria;Desulfuromonadales;Geobacteraceae;Geobacter;Geobacter argillaceus;Geobacter argillaceus ATCC BAA-1139 |
| Ga0586324 1267515 2 808 | 48500000.00 | 3155200.00 | 0.00 | 34865000.00 | 0.00 | 0.00 | 0.00 | 0.00 | 0.00 | 18326000.00 | 0.00 | 0.00 | | hypothetical protein | Bacteria;Proteobacteria;Deltaproteobacteria;Syntrophobacterales;Syntrophaceae;Syntrophus;Syntrophus aciditrophicus;Syntrophus aciditrophicus SB |
| Ga0586324 1270481 1 417 | 18900000.00 | 0.00 | 0.00 | 0.00 | 0.00 | 14490500.00 | 0.00 | 6222000.00 | 5880600.00 | 8470000.00 | 9504000.00 | 8850000.00 | | hypothetical protein | |
| Ga0586324 1270499 3 761 | 32149600.00 | 27268000.00 | 771338.15 | 29260000.00 | 31827000.00 | 33387700.00 | 29640000.00 | 36752500.00 | 31047300.00 | 28028000.00 | 41800000.00 | 22275000.00 | | predicted porin | Bacteria;Proteobacteria;Betaproteobacteria;Burkholderiales;unclassified Burkholderiales;Rhizobacter;Rhizobacter sp. SG703;Rhizobacter sp. SG703 |
| Ga0586324 1271210 2 1096 | 0.00 | 336039.00 | 0.00 | 0.00 | 0.00 | 0.00 | 0.00 | 0.00 | 0.00 | 0.00 | 0.00 | 0.00 | | type II secretory pathway component GspD/PulD (secretin) | Bacteria;Proteobacteria;Deltaproteobacteria;Desulfobacterales;Desulfobulbaceae;Desulfocapsa;Desulfocapsa sulfexigens;Desulfocapsa sulfexigens DSM 10523 |
| Ga0586324 1272446 3 725 | 0.00 | 1502800.00 | 0.00 | 930026.25 | 1277200.00 | 0.00 | 0.00 | 282110.97 | 0.00 | 0.00 | 0.00 | 0.00 | | crotonobetainyl-CoA:carnitine CoA-transferase CaiB-like acyl-CoA transferase | Bacteria;Proteobacteria;Deltaproteobacteria;Syntrophobacterales;Syntrophobacteraceae;Desulfacinum;Desulfacinum infernum;Desulfacinum infernum DSM 9756 |
| Ga0586324 1273218 2 784 | 73600000.00 | 54060000.00 | 57989000.00 | 34105000.00 | 41200000.00 | 57803200.00 | 25992000.00 | 37393000.00 | 46008000.00 | 94710000.00 | 95304000.00 | 91425000.00 | | predicted porin | Bacteria;Proteobacteria;Betaproteobacteria;Burkholderiales;Burkholderiaceae;Cupriavidus;Cupriavidus sp. GA3-3;Cupriavidus sp. GA3-3 |
| Ga0586324 1275577 2 505 | 6090000.00 | 0.00 | 0.00 | 0.00 | 0.00 | 0.00 | 0.00 | 0.00 | 0.00 | 0.00 | 0.00 | 1770000.00 | | 2,4-dienoyl-CoA reductase-like NADH-dependent reductase (Old Yellow Enzyme family) | Bacteria;Firmicutes;Clostridia;Eubacteriales;Lachnospiraceae;Lacrimispora;Lacrimispora aerotolerans;Lachnoclostridium aerotolerans DSM 5434 |
| Ga0586324 1278279 22 1065 | 0.00 | 0.00 | 0.00 | 16055000.00 | 0.00 | 0.00 | 0.00 | 0.00 | 0.00 | 0.00 | 0.00 | 0.00 | | excisionase family DNA binding protein | Bacteria;Chloroflexi;Anaerolineae;Anaerolineales;Anaerolineaceae;Pelolinea;Pelolinea submarina;Pelolinea submarina DSM 23923 |
| Ga0586324 1282377 660 1091 | 0.00 | 0.00 | 0.00 | 0.00 | 0.00 | 0.00 | 1585983.96 | 0.00 | 0.00 | 0.00 | 0.00 | 0.00 | | hypothetical protein | Bacteria;Spirochaetes;Spirochaetia;Spirochaetales;Spirochaetaceae;Spirochaeta;Spirochaeta thermophila;Spirochaeta thermophila DSM 6192 |
| Ga0586324 1284207 1 558 | 0.00 | 0.00 | 77131000.00 | 0.00 | 0.00 | 0.00 | 0.00 | 0.00 | 0.00 | 0.00 | 0.00 | 0.00 | | hypothetical protein | Bacteria;Proteobacteria;Alphaproteobacteria;Hyphomicrobiales;Xanthobacteraceae;Pseudolabrys;Pseudolabrys taiwanensis;Pseudolabrys taiwanensis CC-BB4 |
| Ga0586324 1284923 3 1091 | 0.00 | 0.00 | 0.00 | 0.00 | 0.00 | 0.00 | 1019440.44 | 0.00 | 0.00 | 0.00 | 0.00 | 0.00 | | hypothetical protein | Bacteria;Proteobacteria;Betaproteobacteria;unclassified Betaproteobacteria;unclassified Betaproteobacteria;unclassified Betaproteobacteria;Betaproteobacteria bacterium GR16-43;Betaproteobacteria bacterium GR16-43 |
| Ga0586324 1289132 1 1089 | 0.00 | 0.00 | 0.00 | 0.00 | 0.00 | 0.00 | 0.00 | 0.00 | 0.00 | 0.00 | 0.00 | 12300000.00 | | arylsulfatase A-like enzyme | Bacteria;Proteobacteria;Alphaproteobacteria;Hyphomicrobiales;Bradyrhizobiaceae;Bradyrhizobium;Bradyrhizobium erythrophlei;Bradyrhizobium erythrophlei GAS478 |
| Ga0586324 1293439 81 1088 | 724463.00 | 0.00 | 0.00 | 0.00 | 0.00 | 0.00 | 0.00 | 0.00 | 0.00 | 0.00 | 0.00 | 0.00 | | hypothetical protein | Bacteria;Actinobacteria;Actinomycetia;Propionibacteriales;Nocardioidaceae;Nocardioides;Nocardioides sp.;Nocardioides sp. LS1 |
| Ga0586324 1298771 2 733 | 0.00 | 0.00 | 0.00 | 0.00 | 0.00 | 0.00 | 4879200.00 | 0.00 | 0.00 | 0.00 | 0.00 | 0.00 | | hypothetical protein | |
| Ga0586324 1300353 3 1085 | 0.00 | 0.00 | 3085650.99 | 0.00 | 0.00 | 1810073.86 | 0.00 | 695400.00 | 0.00 | 1285900.00 | 0.00 | 0.00 | | hypothetical protein | Bacteria;Acidobacteria;Vicinamibacteria;unclassified Vicinamibacteria;Vicinamibacteraceae;Luteitalea;Luteitalea pratensis;Luteitalea pratensis DSM 100886 |
| Ga0586324 1303656 190 1083 | 4050000.00 | 2529600.00 | 0.00 | 0.00 | 0.00 | 0.00 | 0.00 | 0.00 | 0.00 | 0.00 | 0.00 | 0.00 | | hypothetical protein | Bacteria;Acidobacteria;Acidobacteriia;Acidobacteriales;Acidobacteriaceae;Granulicella;Granulicella rosea;Granulicella rosea DSM 18704 |
| Ga0586324 1304203 1 1083 | 0.00 | 0.00 | 0.00 | 0.00 | 1833400.00 | 0.00 | 0.00 | 0.00 | 0.00 | 0.00 | 0.00 | 0.00 | | hypothetical protein | Bacteria;Acidobacteria;Vicinamibacteria;unclassified Vicinamibacteria;Vicinamibacteraceae;Luteitalea;Luteitalea pratensis;Luteitalea pratensis DSM 100886 |
| Ga0586324 1312918 2 1081 | 0.00 | 0.00 | 0.00 | 0.00 | 0.00 | 0.00 | 0.00 | 4044300.00 | 0.00 | 0.00 | 0.00 | 3150000.00 | | TonB-dependent SusC/RagA subfamily outer membrane receptor | Bacteria;Bacteroidetes;Cytophagia;Cytophagales;Cyclobacteriaceae;Cyclobacterium;unclassified Cyclobacterium;Cyclobacterium marinum Atlantic-IS |
| Ga0586324 1318078 3 977 | 0.00 | 945200.00 | 0.00 | 0.00 | 582515.47 | 0.00 | 0.00 | 0.00 | 0.00 | 0.00 | 0.00 | 0.00 | | hypothetical protein | |
| Ga0586324 1318478 1 1077 | 0.00 | 0.00 | 0.00 | 0.00 | 0.00 | 0.00 | 0.00 | 0.00 | 0.00 | 36252.22 | 0.00 | 0.00 | | hypothetical protein | Bacteria;Acidobacteria;Vicinamibacteria;unclassified Vicinamibacteria;Vicinamibacteraceae;Luteitalea;Luteitalea pratensis;Luteitalea pratensis DSM 100886 |
| Ga0586324 1319350 2 916 | 0.00 | 0.00 | 0.00 | 0.00 | 1946700.00 | 0.00 | 0.00 | 0.00 | 0.00 | 0.00 | 0.00 | 0.00 | | FAD/FMN-containing dehydrogenase | Bacteria;Proteobacteria;Alphaproteobacteria;Hyphomicrobiales;unclassified Hyphomicrobiales;Nordella;Nordella sp. OAE833;Nordella sp. OAE833 |
| Ga0586324 1321289 3 1076 | 0.00 | 0.00 | 0.00 | 0.00 | 0.00 | 0.00 | 0.00 | 0.00 | 0.00 | 0.00 | 1434400.00 | 0.00 | | hypothetical protein | Bacteria;Proteobacteria;Betaproteobacteria;Nitrosomonadales;Nitrosomonadaceae;Nitrosospira;Nitrosospira multiformis;Nitrosospira multiformis NI1 |
| Ga0586324 1323342 3 326 | 0.00 | 1251200.00 | 0.00 | 0.00 | 1658300.00 | 0.00 | 0.00 | 0.00 | 0.00 | 0.00 | 0.00 | 0.00 | | uncharacterized membrane protein | Bacteria;Proteobacteria;Gammaproteobacteria;Methylococcales;Methylococcaceae;Methylomonas;Methylomonas lenta;Methylomonas lenta R-45370 |
| Ga0586324 1323559 2 787 | 0.00 | 0.00 | 0.00 | 7315000.00 | 0.00 | 0.00 | 0.00 | 0.00 | 0.00 | 0.00 | 0.00 | 0.00 | | alkylation response protein AidB-like acyl-CoA dehydrogenase | Bacteria;Proteobacteria;Deltaproteobacteria;Syntrophorhabdales;Syntrophorhabdaceae;Syntrophorhabdus;Syntrophorhabdus aromaticivorans;Syntrophorhabdus aromaticivorans UI |
| Ga0586324 1324352 3 1076 | 0.00 | 0.00 | 0.00 | 0.00 | 0.00 | 0.00 | 0.00 | 0.00 | 0.00 | 2594900.00 | 2138400.00 | 0.00 | | hypothetical protein | Bacteria;Actinobacteria;Actinomycetia;Micrococcales;Cellulomonadaceae;Cellulomonas;Cellulomonas sp. KRMCY2;Cellulomonas sp. KRMCY2 |
| Ga0586324 1327325 2 1075 | 20200000.00 | 19040000.00 | 0.00 | 30115000.00 | 0.00 | 0.00 | 0.00 | 8235000.00 | 0.00 | 9394000.00 | 8263200.00 | 17250000.00 | | hypothetical protein | Bacteria;Actinobacteria;Thermoleophilia;Solirubrobacterales;unclassified Solirubrobacterales;unclassified Solirubrobacterales;Solirubrobacterales bacterium URHD0059;Solirubrobacterales bacterium URHD0059 |
| Ga0586324 1328209 2 514 | 1450000.00 | 0.00 | 0.00 | 0.00 | 0.00 | 0.00 | 0.00 | 0.00 | 0.00 | 0.00 | 0.00 | 0.00 | | hypothetical protein | |
| Ga0586324 1329925 1 1074 | 0.00 | 3427200.00 | 0.00 | 0.00 | 6272700.00 | 0.00 | 0.00 | 0.00 | 0.00 | 0.00 | 0.00 | 0.00 | | arylsulfatase A-like enzyme | Bacteria;Proteobacteria;Alphaproteobacteria;Hyphomicrobiales;Bradyrhizobiaceae;Bradyrhizobium;Bradyrhizobium elkanii;Bradyrhizobium elkanii USDA 76 |
| Ga0586324 1331311 446 1015 | 0.00 | 0.00 | 0.00 | 0.00 | 1091800.00 | 0.00 | 0.00 | 0.00 | 0.00 | 0.00 | 0.00 | 0.00 | | hypothetical protein | |
| Ga0586324 1332325 2 976 | 4280000.00 | 2400400.00 | 0.00 | 0.00 | 0.00 | 4962500.00 | 0.00 | 0.00 | 0.00 | 0.00 | 3599200.00 | 3330000.00 | | hypothetical protein | |
| Ga0586324 1334619 2 271 | 0.00 | 0.00 | 0.00 | 0.00 | 13905000.00 | 0.00 | 0.00 | 0.00 | 0.00 | 0.00 | 0.00 | 0.00 | | tripartite-type tricarboxylate transporter receptor subunit TctC | Bacteria;Proteobacteria;Deltaproteobacteria;Desulfuromonadales;Desulfuromonadaceae;Pelobacter;Pelobacter seleniigenes;Pelobacter seleniigenes DSM 18267 |
| Ga0586324 1337318 3 449 | 0.00 | 2590800.00 | 0.00 | 2090000.00 | 2399900.00 | 0.00 | 1758598.20 | 0.00 | 1684800.00 | 0.00 | 0.00 | 1410000.00 | | hypothetical protein | |
| Ga0586324 1339002 3 1070 | 0.00 | 626685.28 | 0.00 | 0.00 | 0.00 | 0.00 | 0.00 | 0.00 | 0.00 | 655763.57 | 0.00 | 747242.25 | | hypothetical protein | Bacteria;Chloroflexi;Anaerolineae;Anaerolineales;Anaerolineaceae;Thermanaerothrix;Thermanaerothrix daxensis;Thermanaerothrix daxensis GNS-1 |
| Ga0586324 1339902 1 549 | 997005.00 | 856800.00 | 0.00 | 1168500.00 | 0.00 | 0.00 | 0.00 | 0.00 | 972000.00 | 0.00 | 0.00 | 0.00 | | hypothetical protein | Bacteria;Proteobacteria;Betaproteobacteria;Burkholderiales;Comamonadaceae;Variovorax;Variovorax soli;Variovorax soli NBRC 106424 |
| Ga0586324 1341408 970 1071 | 0.00 | 0.00 | 0.00 | 0.00 | 0.00 | 13180400.00 | 0.00 | 0.00 | 0.00 | 0.00 | 0.00 | 0.00 | | anaerobic selenocysteine-containing dehydrogenase | Bacteria;Proteobacteria;Gammaproteobacteria;Enterobacterales;Pectobacteriaceae;Brenneria;Brenneria salicis;Brenneria salicis ATCC 15712 |
| Ga0586324 1347867 2 1069 | 1910000.00 | 0.00 | 0.00 | 0.00 | 3048800.00 | 0.00 | 0.00 | 774700.00 | 0.00 | 847000.00 | 0.00 | 0.00 | | hypothetical protein | Bacteria;Acidobacteria;Vicinamibacteria;unclassified Vicinamibacteria;Vicinamibacteraceae;Luteitalea;Luteitalea pratensis;Luteitalea pratensis DSM 100886 |
| Ga0586324 1349688 2 865 | 0.00 | 0.00 | 0.00 | 1482000.00 | 0.00 | 0.00 | 0.00 | 0.00 | 0.00 | 0.00 | 0.00 | 0.00 | | hypothetical protein | Bacteria;Proteobacteria;Alphaproteobacteria;Hyphomicrobiales;Parvibaculaceae;Tepidicaulis;Tepidicaulis sp. EA10;Tepidicaulis sp. EA10 |
| Ga0586324 1349883 437 1066 | 0.00 | 0.00 | 0.00 | 0.00 | 1503800.00 | 0.00 | 0.00 | 0.00 | 0.00 | 0.00 | 0.00 | 0.00 | | hypothetical protein | Bacteria;Proteobacteria;Alphaproteobacteria;Hyphomicrobiales;Devosiaceae;Devosia;Devosia sp. Root635;Devosia sp. Root635 |
| Ga0586324 1350515 3 1067 | 1780000.00 | 7480000.00 | 0.00 | 0.00 | 0.00 | 0.00 | 0.00 | 0.00 | 0.00 | 0.00 | 0.00 | 0.00 | | hypothetical protein | Bacteria;Acidobacteria;Acidobacteriia;Acidobacteriales;Acidobacteriaceae;unclassified Acidobacteriaceae;Acidobacteriaceae bacterium KBS 96;Acidobacteriaceae bacterium KBS 96, DSM 24297 |
| Ga0586324 1353152 166 1065 | 0.00 | 0.00 | 0.00 | 0.00 | 0.00 | 0.00 | 0.00 | 0.00 | 0.00 | 0.00 | 0.00 | 125366.25 | | hypothetical protein | Bacteria;Proteobacteria;Deltaproteobacteria;Syntrophobacterales;Syntrophobacteraceae;Syntrophobacter;Syntrophobacter fumaroxidans;Syntrophobacter fumaroxidans MPOB |
| Ga0586324 1354401 319 1065 | 0.00 | 0.00 | 0.00 | 0.00 | 0.00 | 0.00 | 0.00 | 0.00 | 0.00 | 1747900.00 | 0.00 | 0.00 | | hypothetical protein | Bacteria;Nitrospirae;Nitrospira;Nitrospirales;Nitrospiraceae;Nitrospira;Candidatus Nitrospira nitrificans;Candidatus Nitrospira nitrificans COMA2 |
| Ga0586324 1356066 612 1064 | 0.00 | 0.00 | 0.00 | 0.00 | 0.00 | 0.00 | 0.00 | 2507100.00 | 0.00 | 0.00 | 2226400.00 | 2467500.00 | | hypothetical protein | Bacteria;Proteobacteria;Betaproteobacteria;Burkholderiales;Burkholderiaceae;Cupriavidus;Cupriavidus necator;Cupriavidus necator PHE3-6 |
| Ga0586324 1357445 652 1065 | 0.00 | 0.00 | 0.00 | 0.00 | 0.00 | 0.00 | 0.00 | 0.00 | 0.00 | 885500.00 | 0.00 | 0.00 | | hypothetical protein | Archaea;Candidatus Diapherotrites;unclassified Candidatus Diapherotrites;unclassified Candidatus Diapherotrites;unclassified Candidatus Diapherotrites;Candidatus Iainarchaeum;Candidatus Iainarchaeum andersonii;Candidatus Iainarchaeum andersonii SCGC AAA011-E11 (contamination screened) |
| Ga0586324 1358866 3 1064 | 0.00 | 0.00 | 0.00 | 0.00 | 1968973.75 | 0.00 | 0.00 | 0.00 | 0.00 | 0.00 | 0.00 | 0.00 | | cytochrome c peroxidase | Bacteria;Planctomycetes;Planctomycetia;Pirellulales;Pirellulaceae;Blastopirellula;Blastopirellula cremea;Blastopirellula cremea HEX-2 MGV |
| Ga0586324 1359902 3 245 | 21000000.00 | 0.00 | 24828300.00 | 0.00 | 21733000.00 | 15880000.00 | 0.00 | 0.00 | 0.00 | 0.00 | 6617600.00 | 0.00 | | hypothetical protein | Bacteria;Proteobacteria;Deltaproteobacteria;Syntrophobacterales;Syntrophaceae;Desulfobacca;Desulfobacca acetoxidans;Desulfobacca acetoxidans ASRB2, DSM 11109 |
| Ga0586324 1363071 1 1062 | 0.00 | 0.00 | 0.00 | 0.00 | 1967300.00 | 0.00 | 0.00 | 0.00 | 0.00 | 0.00 | 0.00 | 0.00 | | hypothetical protein | Bacteria;Acidobacteria;Vicinamibacteria;unclassified Vicinamibacteria;Vicinamibacteraceae;Luteitalea;Luteitalea pratensis;Luteitalea pratensis DSM 100886 |
| Ga0586324 1363408 1 414 | 0.00 | 0.00 | 0.00 | 0.00 | 0.00 | 0.00 | 0.00 | 0.00 | 0.00 | 0.00 | 398251.92 | 0.00 | | hypothetical protein | Bacteria;Proteobacteria;Betaproteobacteria;Burkholderiales;Burkholderiaceae;Burkholderia;Burkholderia mallei;Burkholderia mallei 102 |
| Ga0586324 1364007 1 432 | 0.00 | 0.00 | 0.00 | 0.00 | 0.00 | 0.00 | 0.00 | 0.00 | 0.00 | 0.00 | 0.00 | 212742.75 | | hypothetical protein | Bacteria;Spirochaetes;Spirochaetia;Spirochaetales;Spirochaetaceae;Spirochaeta;Spirochaeta cellobiosiphila;Spirochaeta cellobiosiphila DSM 17781 |

| | | | | | | | | | | | | | | | |
|---|---|---|---|---|---|---|---|---|---|---|---|---|---|---|---|
| Ga0586324 1365278 2 1063 | 0.00 | 1672800.00 | 0.00 | 0.00 | 0.00 | 0.00 | 0.00 | 0.00 | 0.00 | 0.00 | 0.00 | 0.00 | | hypothetical protein | Bacteria;Proteobacteria;Gammaproteobacteria;Methylococcales;Methylococcaceae;Methylosarcina;Methylosarcina fibrata;Methylosarcina fibrata AML-C10 |
| Ga0586324 1368067 1 1062 | 0.00 | 0.00 | 0.00 | 0.00 | 0.00 | 0.00 | 0.00 | 0.00 | 786929.58 | 0.00 | 0.00 | 0.00 | | hypothetical protein | Bacteria;Acidobacteria;Acidobacteriia;Bryobacterales;Solibacteraceae;Candidatus Solibacter;Candidatus Solibacter usitatus;Candidatus Solibacter usitatus Ellin6076 |
| Ga0586324 1369658 526 1062 | 9240000.00 | 0.00 | 0.00 | 4769000.00 | 5654700.00 | 0.00 | 0.00 | 0.00 | 0.00 | 0.00 | 17512000.00 | 9000000.00 | | predicted porin | Bacteria;Proteobacteria;Betaproteobacteria;Burkholderiales;Burkholderiaceae;Cupriavidus;Cupriavidus sp. GA3-3;Cupriavidus sp. GA3-3 |
| Ga0586324 1370041 1 720 | 0.00 | 0.00 | 0.00 | 0.00 | 0.00 | 0.00 | 0.00 | 215913.16 | 0.00 | 0.00 | 385344.08 | 298866.75 | | TRAP-type mannitol/chloroaromatic compound transport system substrate-binding protein | Bacteria;Proteobacteria;Betaproteobacteria;Burkholderiales;Comamonadaceae;Polaromonas;Polaromonas naphthalenivorans;Polaromonas naphthalenivorans CJ2 |
| Ga0586324 1371469 473 1060 | 0.00 | 0.00 | 0.00 | 1054500.00 | 0.00 | 0.00 | 0.00 | 0.00 | 0.00 | 0.00 | 0.00 | 0.00 | | hypothetical protein | Bacteria;Bacteroidetes;Bacteroidia;Marinilabiliales;Marinifilaceae;Labilibaculum;Labilibaculum manganireducens;Labilibaculum manganireducens 59.10-2M |
| Ga0586324 1372194 171 905 | 0.00 | 0.00 | 0.00 | 7961000.00 | 0.00 | 0.00 | 4126800.00 | 0.00 | 0.00 | 0.00 | 0.00 | 1830000.00 | | outer membrane protein OmpA-like peptidoglycan-associated protein | Bacteria;Proteobacteria;Alphaproteobacteria;Rhodospirillales;Rhodospirillaceae;Dongia;Dongia sp. URHE0060;Dongia sp. URHE0060 |
| Ga0586324 1379355 3 569 | 4540000.00 | 0.00 | 0.00 | 0.00 | 0.00 | 0.00 | 0.00 | 0.00 | 0.00 | 0.00 | 3467200.00 | 0.00 | | hypothetical protein | Bacteria;Proteobacteria;Betaproteobacteria;unclassified Betaproteobacteria;unclassified Betaproteobacteria;unclassified Betaproteobacteria;Betaproteobacteria bacterium GR16-43;Betaproteobacteria bacterium GR16-43 |
| Ga0586324 1381886 598 1056 | 0.00 | 9112000.00 | 0.00 | 0.00 | 0.00 | 0.00 | 0.00 | 0.00 | 0.00 | 0.00 | 0.00 | 0.00 | | hypothetical protein | |
| Ga0586324 1383702 48 1055 | 0.00 | 3202800.00 | 0.00 | 0.00 | 0.00 | 0.00 | 0.00 | 0.00 | 0.00 | 0.00 | 0.00 | 0.00 | | hypothetical protein | Bacteria;Proteobacteria;Alphaproteobacteria;Hyphomicrobiales;Bradyrhizobiaceae;Bradyrhizobium;Bradyrhizobium japonicum;Bradyrhizobium japonicum 22 |
| Ga0586324 1386765 3 1055 | 0.00 | 0.00 | 0.00 | 0.00 | 0.00 | 0.00 | 0.00 | 0.00 | 1182600.00 | 0.00 | 0.00 | 0.00 | | NADPH-dependent glutamate synthase beta subunit-like oxidoreductase | Bacteria;Proteobacteria;Deltaproteobacteria;Syntrophobacterales;Syntrophaceae;Desulfobacca;Desulfobacca acetoxidans;Desulfobacca acetoxidans ASRB2, DSM 11109 |
| Ga0586324 1394906 314 952 | 1600000.00 | 0.00 | 0.00 | 0.00 | 0.00 | 0.00 | 0.00 | 0.00 | 0.00 | 0.00 | 0.00 | 0.00 | | Pyruvate/2-oxoacid:ferredoxin oxidoreductase gamma subunit | Bacteria;Firmicutes;Clostridia;Thermosediminibacterales;Thermosediminibacteraceae;Thermosediminibacter;Thermosediminibacter litoriperuensis;Thermosediminibacter litoriperuensis DSM 16647 |
| Ga0586324 1396216 12 479 | 0.00 | 7004000.00 | 8613900.00 | 3382000.00 | 3357800.00 | 0.00 | 3876000.00 | 0.00 | 0.00 | 4627700.00 | 0.00 | 3307500.00 | | hypothetical protein | Bacteria;Proteobacteria;Deltaproteobacteria;Syntrophobacterales;Syntrophobacteraceae;Desulfacinum;Desulfacinum infernum;Desulfacinum infernum DSM 9756 |
| Ga0586324 1397920 3 1037 | 0.00 | 0.00 | 0.00 | 0.00 | 0.00 | 0.00 | 3921600.00 | 0.00 | 0.00 | 7854000.00 | 0.00 | 0.00 | | hypothetical protein | Bacteria;Proteobacteria;Deltaproteobacteria;Syntrophobacterales;Syntrophaceae;Smithella;Smithella sp. F21;Smithella sp. F21 |
| Ga0586324 1408358 3 425 | 1870000.00 | 0.00 | 0.00 | 0.00 | 0.00 | 0.00 | 0.00 | 144659.06 | 0.00 | 0.00 | 597605.36 | 0.00 | | TRAP-type mannitol/chloroaromatic compound transport system substrate-binding protein | Bacteria;Proteobacteria;Gammaproteobacteria;Acidiferrobacterales;Acidiferrobacteraceae;Sulfuricaulis;Sulfuricaulis limicola;Sulfuricaulis limicola HA5 |
| Ga0586324 1412303 1 1047 | 0.00 | 0.00 | 0.00 | 0.00 | 0.00 | 0.00 | 0.00 | 0.00 | 0.00 | 0.00 | 2640000.00 | 0.00 | | outer membrane protein OmpA-like peptidoglycan-associated protein | Bacteria;Acidobacteria;Acidobacteriia;Acidobacteriales;Acidobacteriaceae;Terriglobus;Terriglobus roseus;Terriglobus roseus AB35.6 |
| Ga0586324 1412895 509 1048 | 0.00 | 2944400.00 | 0.00 | 0.00 | 4315700.00 | 0.00 | 0.00 | 3275700.00 | 3442500.00 | 0.00 | 0.00 | 0.00 | | CBS domain-containing protein | Archaea;Euryarchaeota;Methanomicrobia;Methanosarcinales;Methanotrichaceae;Methanothrix;Methanothrix soehngenii;Methanothrix soehngenii GP-6 |
| Ga0586324 1415173 3 1046 | 0.00 | 0.00 | 0.00 | 0.00 | 4521700.00 | 0.00 | 0.00 | 0.00 | 0.00 | 0.00 | 0.00 | 0.00 | | predicted ATP-dependent protease | Bacteria;Proteobacteria;Deltaproteobacteria;Syntrophobacterales;Syntrophobacteraceae;Syntrophobacter;Syntrophobacter fumaroxidans;Syntrophobacter fumaroxidans MPOB |
| Ga0586324 1417493 601 1044 | 18200000.00 | 0.00 | 66997000.00 | 0.00 | 0.00 | 0.00 | 0.00 | 3190300.00 | 3167100.00 | 0.00 | 0.00 | 0.00 | | hypothetical protein | Bacteria;Proteobacteria;Deltaproteobacteria;Desulfobacterales;Desulfobacteraceae;Desulfotignum;Desulfotignum balticum;Desulfotignum balticum DSM 7044 |
| Ga0586324 1417565 670 1044 | 3190000.00 | 0.00 | 0.00 | 0.00 | 0.00 | 0.00 | 0.00 | 0.00 | 0.00 | 0.00 | 0.00 | 0.00 | | hypothetical protein | |
| Ga0586324 1419469 1 1014 | 13360000.00 | 27446115.80 | 0.00 | 3885500.00 | 18910800.00 | 37079800.00 | 8415208.68 | 32696000.00 | 58142131.29 | 403816.49 | 577353.92 | 23610000.00 | | S-layer protein (TIGR01567 family) | Archaea;Euryarchaeota;Methanomicrobia;Methanosarcinales;Methanotrichaceae;Methanothrix;Methanothrix soehngenii;Methanothrix soehngenii GP-6 |
| Ga0586324 1420025 199 585 | 0.00 | 0.00 | 216340.63 | 0.00 | 0.00 | 0.00 | 0.00 | 0.00 | 0.00 | 0.00 | 0.00 | 0.00 | | hypothetical protein | Bacteria;Proteobacteria;Gammaproteobacteria;Methylococcales;Methylococcaceae;Methylobacter;Methylobacter tundripaludum;Methylobacter tundripaludum SV96 |
| Ga0586324 1420540 3 1043 | 2310000.00 | 0.00 | 0.00 | 0.00 | 0.00 | 0.00 | 0.00 | 0.00 | 0.00 | 0.00 | 0.00 | 0.00 | | hypothetical protein | |
| Ga0586324 1422659 2 940 | 0.00 | 0.00 | 0.00 | 0.00 | 0.00 | 0.00 | 0.00 | 0.00 | 0.00 | 0.00 | 0.00 | 490085.25 | | TRAP-type mannitol/chloroaromatic compound transport system substrate-binding protein | Bacteria;Proteobacteria;Betaproteobacteria;unclassified Betaproteobacteria;unclassified Betaproteobacteria;unclassified Betaproteobacteria;uncultured beta proteobacterium;unclassified betaproteobacterium ENR4 |
| Ga0586324 1429645 92 1042 | 1760000.00 | 3094000.00 | 7600500.00 | 0.00 | 0.00 | 0.00 | 0.00 | 0.00 | 0.00 | 0.00 | 0.00 | 0.00 | | TRAP-type C4-dicarboxylate transport system substrate-binding protein | Bacteria;Proteobacteria;Deltaproteobacteria;Desulfobacterales;Desulfobacteraceae;Desulfatirhabdium;Desulfatirhabdium butyrativorans;Desulfatirhabdium butyrativorans DSM 18734 |
| Ga0586324 1437845 3 1040 | 0.00 | 0.00 | 0.00 | 0.00 | 0.00 | 0.00 | 0.00 | 388894.52 | 0.00 | 0.00 | 0.00 | 0.00 | | peroxiredoxin | Bacteria;Armatimonadetes;Armatimonadia;Armatimonadales;Armatimonadaceae;Armatimonas;Armatimonas rosea;Armatimonas rosea DSM 23562 |
| Ga0586324 1438947 45 1040 | 852205.00 | 0.00 | 0.00 | 0.00 | 0.00 | 0.00 | 0.00 | 0.00 | 0.00 | 0.00 | 0.00 | 0.00 | | glucose/arabinose dehydrogenase | Bacteria;Proteobacteria;Alphaproteobacteria;Hyphomicrobiales;Bradyrhizobiaceae;Bradyrhizobium;Bradyrhizobium elkanii;Bradyrhizobium elkanii WSM1741 |
| Ga0586324 1439672 1 1038 | 5710000.00 | 0.00 | 0.00 | 0.00 | 0.00 | 0.00 | 0.00 | 0.00 | 0.00 | 5674900.00 | 0.00 | 4822500.00 | | hypothetical protein | |
| Ga0586324 1442646 1 810 | 0.00 | 0.00 | 0.00 | 0.00 | 0.00 | 0.00 | 0.00 | 0.00 | 0.00 | 0.00 | 0.00 | 2347500.00 | | hypothetical protein | Bacteria;Proteobacteria;Gammaproteobacteria;Pseudomonadales;Pseudomonadaceae;Pseudomonas;Pseudomonas benzenivorans;Pseudomonas benzenivorans DSM 8628 |
| Ga0586324 1443345 3 572 | 0.00 | 0.00 | 0.00 | 0.00 | 5768000.00 | 0.00 | 0.00 | 0.00 | 0.00 | 0.00 | 0.00 | 0.00 | | uncharacterized membrane protein | Archaea;Euryarchaeota;Methanomicrobia;Methanomicrobiales;Methanomicrobiaceae;Methanofollis;Methanofollis ethanolicus;Methanofollis ethanolicus JCM 15103 |
| Ga0586324 1443955 371 913 | 2090000.00 | 0.00 | 0.00 | 0.00 | 0.00 | 0.00 | 0.00 | 0.00 | 0.00 | 0.00 | 0.00 | 0.00 | | phasin family protein | Bacteria;Proteobacteria;Betaproteobacteria;Neisseriales;Chromobacteriaceae;Andreprevotia;Andreprevotia lacus;Andreprevotia lacus DSM 23236 |
| Ga0586324 1444259 1 1038 | 0.00 | 0.00 | 0.00 | 0.00 | 0.00 | 0.00 | 0.00 | 0.00 | 0.00 | 0.00 | 10648000.00 | 0.00 | | LysM repeat protein | Bacteria;Bacteroidetes;Bacteroidia;Bacteroidales;Odoribacteraceae;Butyricimonas;Butyricimonas synergistica;Butyricimonas synergistica DSM 23225 |
| Ga0586324 1446617 95 1036 | 0.00 | 0.00 | 0.00 | 0.00 | 0.00 | 0.00 | 0.00 | 1933700.00 | 963900.00 | 0.00 | 0.00 | 915000.00 | | TRAP-type mannitol/chloroaromatic compound transport system substrate-binding protein | Bacteria;Proteobacteria;Alphaproteobacteria;Hyphomicrobiales;Methylobacteriaceae;Microvirga;Microvirga ossetica;Microvirga ossetica V5/3m |
| Ga0586324 1446774 79 411 | 0.00 | 0.00 | 0.00 | 0.00 | 0.00 | 0.00 | 0.00 | 0.00 | 0.00 | 1832600.00 | 0.00 | 0.00 | | phasin family protein | Bacteria;Proteobacteria;Alphaproteobacteria;Hyphomicrobiales;unclassified Hyphomicrobiales;Methyloceanibacter;Methyloceanibacter superfactus;Methyloceanibacter superfactus R-67175 |
| Ga0586324 1447800 1 1035 | 0.00 | 0.00 | 3527532.80 | 0.00 | 0.00 | 1562556.27 | 0.00 | 0.00 | 0.00 | 0.00 | 0.00 | 0.00 | | outer membrane receptor for ferrienterochelin and colicin | Bacteria;Proteobacteria;Gammaproteobacteria;unclassified Gammaproteobacteria;unclassified Gammaproteobacteria;Gallaecimonas;Gallaecimonas mangrovi;Gallaecimonas mangrovi HK-28 |
| Ga0586324 1448126 3 1037 | 0.00 | 0.00 | 0.00 | 2147000.00 | 2080600.00 | 0.00 | 0.00 | 0.00 | 0.00 | 0.00 | 0.00 | 0.00 | | hypothetical protein | Bacteria;Bacteroidetes;Chitinophagia;Chitinophagales;Chitinophagaceae;Cnuella;Cnuella takakiae;Cnuella takakiae DSM 26897 |
| Ga0586324 1451166 1 1035 | 0.00 | 0.00 | 0.00 | 3638500.00 | 0.00 | 2740756.99 | 0.00 | 0.00 | 0.00 | 3003000.00 | 1874400.00 | 1695000.00 | | hypothetical protein | Bacteria;Actinobacteria;Actinomycetia;Streptosporangiales;Streptosporangiaceae;Thermoactinospora;Thermoactinospora rubra;Thermoactinospora rubra YIM 77501 |
| Ga0586324 1452752 2 304 | 2690000.00 | 2366400.00 | 3687554.29 | 2394000.00 | 0.00 | 0.00 | 0.00 | 0.00 | 1182600.00 | 0.00 | 0.00 | 1702500.00 | | NAD(P)-dependent dehydrogenase (short-subunit alcohol dehydrogenase family) | Bacteria;Proteobacteria;Alphaproteobacteria;Rhodospirillales;Acetobacteraceae;Rhodopila;unclassified Rhodopila;Rhodopila sp. LVNP |
| Ga0586324 1458344 3 581 | 0.00 | 2019600.00 | 0.00 | 6555000.00 | 5541400.00 | 26360800.00 | 0.00 | 0.00 | 4098600.00 | 0.00 | 0.00 | 0.00 | | crotonobetainyl-CoA:carnitine CoA-transferase CaiB-like acyl-CoA transferase | Archaea;Euryarchaeota;Archaeoglobi;Archaeoglobales;Archaeoglobaceae;Ferroglobus;Ferroglobus placidus;Ferroglobus placidus AEDII12DO, DSM 10642 |
| Ga0586324 1458344 600 1034 | 0.00 | 0.00 | 0.00 | 0.00 | 0.00 | 0.00 | 0.00 | 0.00 | 0.00 | 1755600.00 | 0.00 | 0.00 | | crotonobetainyl-CoA:carnitine CoA-transferase CaiB-like acyl-CoA transferase | Bacteria;Firmicutes;Clostridia;Thermoanaerobacterales;Thermoanaerobacteraceae;Desulfovirgula;Desulfovirgula thermocuniculi;Desulfovirgula thermocuniculi DSM 16036 |
| Ga0586324 1458585 67 1032 | 0.00 | 0.00 | 0.00 | 0.00 | 0.00 | 0.00 | 0.00 | 1171200.00 | 0.00 | 0.00 | 0.00 | 0.00 | | PAS domain S-box-containing protein | Archaea;Euryarchaeota;Methanomicrobia;Methanomicrobiales;Methanospirillaceae;Methanospirillum;Methanospirillum hungatei;Methanospirillum hungatei JF-1 |
| Ga0586324 1465229 2 1030 | 0.00 | 0.00 | 0.00 | 0.00 | 0.00 | 1892066.27 | 0.00 | 0.00 | 0.00 | 0.00 | 0.00 | 0.00 | | hypothetical protein | |
| Ga0586324 1465319 425 1030 | 0.00 | 5984000.00 | 0.00 | 2973500.00 | 2502900.00 | 0.00 | 0.00 | 0.00 | 1887300.00 | 0.00 | 0.00 | 1672500.00 | | hypothetical protein | Bacteria;Proteobacteria;Gammaproteobacteria;Alteromonadales;Alteromonadaceae;Marinobacter;Marinobacter sp. Arc7-DN-1;Marinobacter sp. Arc7-DN-1 |
| Ga0586324 1466044 1 1029 | 0.00 | 4270400.00 | 5362158.38 | 0.00 | 0.00 | 3191510.79 | 8299200.00 | 3367200.00 | 1798200.00 | 0.00 | 0.00 | 7755000.00 | | hypothetical protein | Bacteria;Proteobacteria;Deltaproteobacteria;Myxococcales;Anaeromyxobacteraceae;Anaeromyxobacter;Anaeromyxobacter sp. Fw109-5;Anaeromyxobacter sp. Fw109-5 |
| Ga0586324 1467393 602 1030 | 0.00 | 0.00 | 0.00 | 0.00 | 0.00 | 0.00 | 0.00 | 0.00 | 0.00 | 0.00 | 0.00 | 8250000.00 | | phasin family protein | Bacteria;Proteobacteria;Betaproteobacteria;Rhodocyclales;Azonexaceae;Dechloromonas;Dechloromonas agitata;Dechloromonas agitata CKB |
| Ga0586324 1470069 1 1029 | 1410000.00 | 0.00 | 0.00 | 0.00 | 0.00 | 0.00 | 0.00 | 0.00 | 0.00 | 0.00 | 0.00 | 1372500.00 | | hypothetical protein | Bacteria;Actinobacteria;Actinomycetia;Micrococcales;Intrasporangiaceae;Intrasporangium;Intrasporangium flavum;Intrasporangium flavum MUSC 78 |
| Ga0586324 1470453 2 1030 | 1390000.00 | 1652400.00 | 0.00 | 0.00 | 0.00 | 0.00 | 0.00 | 0.00 | 0.00 | 0.00 | 0.00 | 0.00 | | hypothetical protein | Bacteria;Acidobacteria;Vicinamibacteria;unclassified Vicinamibacteria;Vicinamibacteraceae;Luteitalea;Luteitalea pratensis;Luteitalea pratensis DSM 100886 |
| Ga0586324 1472446 235 1029 | 50700000.00 | 40936000.00 | 0.00 | 0.00 | 17098000.00 | 0.00 | 0.00 | 0.00 | 0.00 | 7569100.00 | 0.00 | 0.00 | | predicted porin | Bacteria;Proteobacteria;Alphaproteobacteria;Hyphomicrobiales;Bradyrhizobiaceae;Bradyrhizobium;Bradyrhizobium erythrophlei;Bradyrhizobium erythrophlei GAS401 |
| Ga0586324 1486583 294 851 | 1620000.00 | 2298400.00 | 2127779.68 | 0.00 | 0.00 | 2470872.42 | 0.00 | 0.00 | 0.00 | 0.00 | 0.00 | 0.00 | | lipid-binding SYLF domain-containing protein | Bacteria;Proteobacteria;Alphaproteobacteria;Rhodospirillales;Acetobacteraceae;Rhodopila;Rhodopila globiformis;Rhodopila globiformis DSM 161 |
| Ga0586324 1489418 754 1023 | 2410000.00 | 0.00 | 0.00 | 0.00 | 0.00 | 0.00 | 0.00 | 0.00 | 794824.65 | 0.00 | 0.00 | 0.00 | | hypothetical protein | |
| Ga0586324 1491450 2 1024 | 0.00 | 0.00 | 0.00 | 11875000.00 | 15965000.00 | 0.00 | 0.00 | 0.00 | 0.00 | 0.00 | 0.00 | 0.00 | | formyltetrahydrofolate synthetase | Bacteria;Proteobacteria;Deltaproteobacteria;Desulfobacterales;Desulfobacteraceae;Desulfatirhabdium;Desulfatirhabdium butyrativorans;Desulfatirhabdium butyrativorans DSM 18734 |
| Ga0586324 1491555 172 1023 | 0.00 | 3026000.00 | 12104500.00 | 0.00 | 5912200.00 | 12267300.00 | 7318800.00 | 6161000.00 | 11866500.00 | 7045500.00 | 6177600.00 | 6900000.00 | | hypothetical protein | Bacteria;Acidobacteria;Blastocatellia;Blastocatellales;Pyrinomonadaceae;Pyrinomonas;Pyrinomonas methylaliphatogenes;Pyrinomonas methylaliphatogenes K22 |
| Ga0586324 1492076 2 1024 | 0.00 | 0.00 | 0.00 | 0.00 | 1936400.00 | 0.00 | 0.00 | 1213900.00 | 0.00 | 1309000.00 | 269163.84 | 473940.75 | | hypothetical protein | Bacteria;Acidobacteria;Vicinamibacteria;unclassified Vicinamibacteria;Vicinamibacteraceae;Luteitalea;Luteitalea pratensis;Luteitalea pratensis DSM 100886 |
| Ga0586324 1494830 160 639 | 1540000.00 | 1468800.00 | 0.00 | 3002000.00 | 0.00 | 0.00 | 0.00 | 0.00 | 0.00 | 0.00 | 0.00 | 0.00 | | tetratricopeptide (TPR) repeat protein | |
| Ga0586324 1497193 2 1021 | 0.00 | 2284800.00 | 0.00 | 0.00 | 0.00 | 0.00 | 0.00 | 0.00 | 0.00 | 0.00 | 0.00 | 0.00 | | hypothetical protein | Bacteria;Bacteroidetes;Bacteroidia;Bacteroidales;Dysgonomonadaceae;Petrimonas;Petrimonas sulfuriphila;Petrimonas sulfuriphila DSM 16547 |
| Ga0586324 1500156 72 1019 | 0.00 | 0.00 | 0.00 | 0.00 | 0.00 | 1719010.00 | 0.00 | 0.00 | 0.00 | 0.00 | 0.00 | 3285000.00 | | hypothetical protein | |
| Ga0586324 1503550 152 859 | 0.00 | 0.00 | 0.00 | 4370000.00 | 4418700.00 | 0.00 | 0.00 | 0.00 | 2705400.00 | 0.00 | 0.00 | 0.00 | | nucleoid-associated protein YgaU | Bacteria;Ignavibacteriae;Ignavibacteria;Ignavibacteriales;Ignavibacteriaceae;Ignavibacterium;Ignavibacterium album;Ignavibacterium album Mat9-16, JCM 16511 |
| Ga0586324 1505883 2 619 | 10400000.00 | 7888000.00 | 0.00 | 0.00 | 0.00 | 4168500.00 | 0.00 | 9150000.00 | 11421000.00 | 22561000.00 | 10824000.00 | 17025000.00 | | predicted porin | Bacteria;Proteobacteria;Betaproteobacteria;unclassified Betaproteobacteria;unclassified Betaproteobacteria;unclassified Betaproteobacteria;Betaproteobacteria bacterium GR16-43;Betaproteobacteria bacterium GR16-43 |
| Ga0586324 1506729 1 840 | 0.00 | 0.00 | 0.00 | 0.00 | 0.00 | 0.00 | 3328800.00 | 7625000.00 | 0.00 | 0.00 | 0.00 | 0.00 | | transketolase C-terminal domain/subunit | Bacteria;Proteobacteria;Gammaproteobacteria;Methylococcales;Methylococcaceae;Methylobacter;Methylobacter tundripaludum;Methylobacter tundripaludum SV96 |
| Ga0586324 1514654 1 1017 | 2090000.00 | 1904000.00 | 2199325.72 | 3952000.00 | 3841900.00 | 3368644.25 | 2713200.00 | 3416000.00 | 3450600.00 | 0.00 | 2508000.00 | 0.00 | | hypothetical protein | Bacteria;Acidobacteria;Holophagae;Holophagales;Holophagaceae;Geothrix;Geothrix fermentans;Geothrix fermentans DSM 14018 |
| Ga0586324 1514912 47 1015 | 0.00 | 0.00 | 0.00 | 4009000.00 | 0.00 | 0.00 | 0.00 | 605408.53 | 0.00 | 0.00 | 0.00 | 0.00 | | hypothetical protein | Bacteria;Proteobacteria;Gammaproteobacteria;Chromatiales;Chromatiaceae;Thiodictyon;Candidatus Thiodictyon syntrophicum;Candidatus Thiodictyon syntrophicum Cad16 |
| Ga0586324 1517033 652 1014 | 2780000.00 | 0.00 | 0.00 | 0.00 | 4676200.00 | 0.00 | 3602400.00 | 4257800.00 | 2932200.00 | 3411100.00 | 2191200.00 | 0.00 | | predicted porin | Bacteria;Proteobacteria;Betaproteobacteria;Burkholderiales;unclassified Burkholderiales;Rhizobacter;Rhizobacter sp. Root404;Rhizobacter sp. Root404 |
| Ga0586324 1518474 3 1016 | 0.00 | 5596400.00 | 0.00 | 3258500.00 | 3296000.00 | 0.00 | 0.00 | 0.00 | 0.00 | 9702000.00 | 2252800.00 | 0.00 | | hypothetical protein | Bacteria;Actinobacteria;Actinomycetia;Propionibacteriales;Nocardioidaceae;Nocardioides;Nocardioides sp. Root122;Nocardioides sp. Root122 |
| Ga0586324 1518624 3 632 | 39700000.00 | 75888000.00 | 35637900.00 | 11789500.00 | 10712000.00 | 22311400.00 | 0.00 | 27999000.00 | 27054000.00 | 127327200.00 | 110767514.00 | 91950000.00 | | predicted porin | Bacteria;Proteobacteria;Betaproteobacteria;Burkholderiales;Burkholderiaceae;Cupriavidus;Cupriavidus sp. GA3-3;Cupriavidus sp. GA3-3 |
| Ga0586324 1523736 2 1015 | 0.00 | 0.00 | 0.00 | 0.00 | 0.00 | 0.00 | 0.00 | 817400.00 | 0.00 | 0.00 | 0.00 | 0.00 | | hypothetical protein | Bacteria;Candidatus Aminicenantes;unclassified Candidatus Aminicenantes;unclassified Candidatus Aminicenantes;unclassified Candidatus Aminicenantes;unclassified Candidatus Aminicenantes;Aminicenantes bacterium SCGC AAA255-A06;Aminicenantes bacterium SCGC AAA255-A06 (contamination screened) |
| Ga0586324 1525566 1 813 | 2710000.00 | 4195600.00 | 0.00 | 0.00 | 0.00 | 0.00 | 0.00 | 0.00 | 0.00 | 0.00 | 0.00 | 2497500.00 | | hypothetical protein | Bacteria;Bacteroidetes;Bacteroidia;Marinilabiliales;Prolixibacteraceae;Mariniphaga;Mariniphaga anaerophila;Mariniphaga anaerophila DSM 26910 |
| Ga0586324 1528392 3 1013 | 2470000.00 | 2740400.00 | 3151899.20 | 3420000.00 | 0.00 | 0.00 | 0.00 | 1439600.00 | 143040.33 | 2833600.00 | 0.00 | 2002500.00 | | hypothetical protein | |
| Ga0586324 1531845 441 1010 | 0.00 | 0.00 | 0.00 | 0.00 | 0.00 | 0.00 | 0.00 | 2708400.00 | 0.00 | 0.00 | 0.00 | 0.00 | | signal transduction histidine kinase | Bacteria;Proteobacteria;Alphaproteobacteria;Rhodospirillales;Azospirillaceae;Azospirillum;Azospirillum palustre;Azospirillum palustre B2 |
| Ga0586324 1533858 63 1010 | 0.00 | 0.00 | 0.00 | 0.00 | 0.00 | 0.00 | 0.00 | 0.00 | 980100.00 | 0.00 | 0.00 | 318744.75 | | TonB-dependent SusC/RagA subfamily outer membrane receptor | Bacteria;Bacteroidetes;Flavobacteriia;Flavobacteriales;Flavobacteriaceae;Arenibacter;Arenibacter nanhaiticus;Arenibacter nanhaiticus CGMCC 1.8863 |
| Ga0586324 1534989 1 330 | 0.00 | 12512000.00 | 0.00 | 0.00 | 1545000.00 | 1435428.93 | 0.00 | 0.00 | 0.00 | 0.00 | 0.00 | 0.00 | | predicted porin | Bacteria;Proteobacteria;Betaproteobacteria;Burkholderiales;unclassified Burkholderiales;Leptothrix;Leptothrix cholodnii;Leptothrix cholodnii SP-6 |
| Ga0586324 1542277 3 1007 | 0.00 | 0.00 | 0.00 | 0.00 | 0.00 | 0.00 | 0.00 | 0.00 | 0.00 | 0.00 | 2358400.00 | 2985000.00 | | hypothetical protein | Bacteria;Proteobacteria;Deltaproteobacteria;Myxococcales;Myxococcaceae;Myxococcus;Myxococcus stipitatus;Myxococcus stipitatus DSM 14675 |
| Ga0586324 1544993 103 1008 | 0.00 | 0.00 | 0.00 | 0.00 | 0.00 | 0.00 | 0.00 | 0.00 | 0.00 | 1378300.00 | 0.00 | 0.00 | | hypothetical protein | Bacteria;Proteobacteria;Deltaproteobacteria;Syntrophobacterales;Syntrophaceae;Desulfobacca;Desulfobacca acetoxidans;Desulfobacca acetoxidans ASRB2, DSM 11109 |
| Ga0586324 1552963 1 966 | 0.00 | 3597200.00 | 2522121.77 | 0.00 | 9373000.00 | 6312300.00 | 10191600.00 | 5361900.00 | 6860700.00 | 4296600.00 | 4144800.00 | 3637500.00 | | hypothetical protein | Bacteria;Nitrospirae;Thermodesulfovibrionia;Thermodesulfovibrionales;Thermodesulfovibrionaceae;Thermodesulfovibrio;Thermodesulfovibrio islandicus;Thermodesulfovibrio islandicus DSM 12570 |
| Ga0586324 1553958 3 1004 | 0.00 | 0.00 | 1406993.30 | 0.00 | 0.00 | 0.00 | 0.00 | 0.00 | 0.00 | 0.00 | 0.00 | 0.00 | | hypothetical protein | Bacteria;Spirochaetes;Spirochaetia;Leptospirales;Leptospiraceae;Turneriella;Turneriella parva;Turneriella parva H, DSM 21527 |
| Ga0586324 1556371 366 1004 | 0.00 | 1509600.00 | 0.00 | 0.00 | 0.00 | 0.00 | 0.00 | 0.00 | 0.00 | 0.00 | 0.00 | 0.00 | | hypothetical protein | Bacteria;Ignavibacteriae;Ignavibacteria;Ignavibacteriales;Melioribacteraceae;Melioribacter;Melioribacter roseus;Melioribacter roseus P3M |
| Ga0586324 1561089 1 1002 | 0.00 | 0.00 | 0.00 | 0.00 | 481119.18 | 0.00 | 0.00 | 0.00 | 0.00 | 0.00 | 0.00 | 0.00 | | TonB-dependent SusC/RagA subfamily outer membrane receptor | Bacteria;Bacteroidetes;Cytophagia;Cytophagales;Fulvivirgaceae;Ohtaekwangia;Ohtaekwangia koreensis;Ohtaekwangia koreensis DSM 25262 |
| Ga0586324 1566317 3 1001 | 0.00 | 0.00 | 0.00 | 4522000.00 | 543583.53 | 0.00 | 0.00 | 0.00 | 0.00 | 0.00 | 1724800.00 | 0.00 | | hypothetical protein | Bacteria;Bacteroidetes;Saprospiria;Saprospirales;Lewinellaceae;Lewinella;Lewinella cohaerens;Lewinella cohaerens DSM 23179 |
| Ga0586324 1568044 3 662 | 0.00 | 0.00 | 0.00 | 0.00 | 0.00 | 0.00 | 0.00 | 0.00 | 0.00 | 502160.12 | 0.00 | 0.00 | | basic membrane lipoprotein Med (substrate-binding protein (PBP1-ABC) superfamily) | Bacteria;Chloroflexi;Anaerolineae;Anaerolineales;Anaerolineaceae;Thermanaerothrix;Thermanaerothrix daxensis;Thermanaerothrix daxensis GNS-1 |
| Ga0586324 1571972 295 738 | 2240000.00 | 0.00 | 0.00 | 3068500.00 | 1617100.00 | 0.00 | 0.00 | 829600.00 | 0.00 | 0.00 | 0.00 | 0.00 | | nucleotide-binding universal stress UspA family protein | Bacteria;Proteobacteria;Gammaproteobacteria;Xanthomonadales;Rhodanobacteraceae;Rudaea;Rudaea cellulosilytica;Rudaea cellulosilytica DSM 22992 |
| Ga0586324 1575508 3 998 | 0.00 | 0.00 | 0.00 | 0.00 | 0.00 | 0.00 | 0.00 | 0.00 | 0.00 | 575077.58 | 0.00 | 571025.25 | | ABC-type xylose transport system substrate-binding protein | Bacteria;Chloroflexi;Ardenticatenia;Ardenticatenales;Ardenticatenaceae;Candidatus Promineofilum;Candidatus Promineofilum breve;Candidatus Promineofilum breve Cfx-K |
| Ga0586324 1576282 1 999 | 0.00 | 0.00 | 27024000.00 | 0.00 | 0.00 | 0.00 | 0.00 | 0.00 | 0.00 | 0.00 | 0.00 | 0.00 | | hypothetical protein | |
| Ga0586324 1579037 1 999 | 40800000.00 | 6800000.00 | 25053500.00 | 4417500.00 | 0.00 | 14530200.00 | 0.00 | 0.00 | 1344600.00 | 8778000.00 | 12038400.00 | 5527500.00 | | hypothetical protein | Bacteria;Proteobacteria;Deltaproteobacteria;Syntrophobacterales;Syntrophaceae;Smithella;Smithella sp. SCADC;Smithella sp. SCADC |
| Ga0586324 1583275 268 996 | 0.00 | 0.00 | 0.00 | 0.00 | 0.00 | 0.00 | 3990000.00 | 0.00 | 0.00 | 0.00 | 0.00 | 0.00 | | TRAP-type mannitol/chloroaromatic compound transport system substrate-binding protein | Bacteria;Proteobacteria;Deltaproteobacteria;Syntrophobacterales;Syntrophaceae;Desulfomonile;Desulfomonile tiedjei;Desulfomonile tiedjei DCB-1, DSM 6799 |
| Ga0586324 1583351 175 996 | 0.00 | 0.00 | 0.00 | 0.00 | 0.00 | 0.00 | 0.00 | 0.00 | 497692.35 | 0.00 | 0.00 | 0.00 | | TRAP-type mannitol/chloroaromatic compound transport system substrate-binding protein | Bacteria;Proteobacteria;Alphaproteobacteria;Rhodospirillales;Rhodospirillaceae;Limibacillus;Limibacillus halophilus;Limibacillus halophilus CECT 8803 |
| Ga0586324 1584565 1 996 | 0.00 | 0.00 | 0.00 | 0.00 | 0.00 | 0.00 | 0.00 | 153706.20 | 0.00 | 0.00 | 0.00 | 0.00 | | hypothetical protein | |
| Ga0586324 1586041 2 997 | 746948.00 | 0.00 | 0.00 | 0.00 | 0.00 | 0.00 | 0.00 | 0.00 | 0.00 | 0.00 | 0.00 | 0.00 | | FtsP/CotA-like multicopper oxidase with cupredoxin domain | Bacteria;Chloroflexi;Ktedonobacteria;Ktedonobacterales;Dictyobacteraceae;Dictyobacter;Dictyobacter aurantiacus;Dictyobacter aurantiacus S27 |
| Ga0586324 1586130 667 996 | 0.00 | 0.00 | 0.00 | 1757500.00 | 0.00 | 0.00 | 0.00 | 0.00 | 0.00 | 0.00 | 0.00 | 0.00 | | hydroxymethylpyrimidine/phosphomethylpyrimidine kinase | Archaea;Euryarchaeota;Halobacteria;Haloferacales;Halorubraceae;Halorubrum;Halorubrum coriense;Halorubrum coriense ACM 3911, Ch2 |
| Ga0586324 1586837 248 997 | 3730000.00 | 4630800.00 | 3053013.88 | 5044500.00 | 5665000.00 | 1774276.37 | 6224400.00 | 5331400.00 | 4422600.00 | 7430500.00 | 7339200.00 | 6007500.00 | | TRAP-type mannitol/chloroaromatic compound transport system substrate-binding protein | Bacteria;Proteobacteria;Alphaproteobacteria;Rhodospirillales;Rhodospirillaceae;Dongia;Dongia mobilis;Dongia mobilis CGMCC 1.7660 |
| Ga0586324 1589288 72 995 | 0.00 | 0.00 | 0.00 | 0.00 | 12360000.00 | 0.00 | 0.00 | 1701900.00 | 0.00 | 0.00 | 0.00 | 0.00 | | hypothetical protein | Bacteria;Proteobacteria;Deltaproteobacteria;Desulfuromonadales;Geobacteraceae;Geobacter;Geobacter anodireducens;Geobacter anodireducens SD-1 |
| Ga0586324 1590391 3 995 | 0.00 | 0.00 | 0.00 | 0.00 | 0.00 | 0.00 | 0.00 | 0.00 | 0.00 | 14399000.00 | 0.00 | 0.00 | | DNA-directed RNA polymerase subunit A'' | Archaea;Euryarchaeota;Thermococci;Thermococcales;Thermococcaceae;Palaeococcus;Palaeococcus pacificus;Palaeococcus pacificus DY20341 |
| Ga0586324 1592327 2 994 | 0.00 | 0.00 | 0.00 | 0.00 | 0.00 | 0.00 | 0.00 | 0.00 | 0.00 | 0.00 | 4065600.00 | 3202500.00 | | hypothetical protein | Bacteria;Acidobacteria;Acidobacteriia;Acidobacteriales;Acidobacteriaceae;Acidobacterium;unclassified Acidobacterium;Acidobacterium strain C40 |
| Ga0586324 1593547 431 994 | 0.00 | 0.00 | 0.00 | 0.00 | 0.00 | 0.00 | 0.00 | 0.00 | 0.00 | 1701700.00 | 0.00 | 0.00 | | TRAP-type mannitol/chloroaromatic compound transport system substrate-binding protein | Bacteria;Proteobacteria;Alphaproteobacteria;Hyphomicrobiales;Bradyrhizobiaceae;Afipia;Afipia clevelandensis;Afipia clevelandensis ATCC 49720 |
| Ga0586324 1595330 1 993 | 293000000.00 | 0.00 | 0.00 | 0.00 | 0.00 | 0.00 | 0.00 | 0.00 | 0.00 | 0.00 | 0.00 | 0.00 | | ABC-type xylose transport system substrate-binding protein | Bacteria;Chloroflexi;Ardenticatenia;Ardenticatenales;Ardenticatenaceae;Candidatus Promineofilum;Candidatus Promineofilum breve;Candidatus Promineofilum breve Cfx-K |
| Ga0586324 1601028 470 994 | 0.00 | 0.00 | 2417274.28 | 7115500.00 | 12463000.00 | 6193200.00 | 775747.20 | 719800.00 | 2754000.00 | 0.00 | 0.00 | 5520000.00 | | uncharacterized surface protein with fascilin (FAS1) repeats | Bacteria;Cyanobacteria;unclassified Cyanobacteria;Nostocales;Calotrichaceae;Calothrix;Calothrix brevissima;Calothrix brevissima NIES-22 |
| Ga0586324 1605670 268 990 | 0.00 | 12716000.00 | 0.00 | 13015000.00 | 13802000.00 | 0.00 | 0.00 | 8906000.00 | 0.00 | 0.00 | 0.00 | 0.00 | | crotonobetainyl-CoA:carnitine CoA-transferase CaiB-like acyl-CoA transferase | Bacteria;Proteobacteria;Deltaproteobacteria;Syntrophobacterales;Syntrophobacteraceae;Desulfacinum;Desulfacinum infernum;Desulfacinum infernum DSM 9756 |
| Ga0586324 1608853 2 991 | 0.00 | 3495200.00 | 0.00 | 3002000.00 | 4707100.00 | 0.00 | 0.00 | 3855200.00 | 0.00 | 0.00 | 4215200.00 | 3495000.00 | | hypothetical protein | Bacteria;Acidobacteria;Vicinamibacteria;unclassified Vicinamibacteria;Vicinamibacteraceae;Luteitalea;Luteitalea pratensis;Luteitalea pratensis DSM 100886 |
| Ga0586324 1610701 1 888 | 10600000.00 | 2998800.00 | 0.00 | 0.00 | 0.00 | 0.00 | 0.00 | 0.00 | 0.00 | 2933700.00 | 0.00 | 1485000.00 | | hypothetical protein | Bacteria;Candidatus Aminicenantes;unclassified Candidatus Aminicenantes;unclassified Candidatus Aminicenantes;unclassified Candidatus Aminicenantes;unclassified Candidatus Aminicenantes;Aminicenantes bacterium SCGC AAA252-O03;Aminicenantes bacterium SCGC AAA252-O03 (contamination screened) |
| Ga0586324 1611608 2 298 | 13800000.00 | 3835200.00 | 0.00 | 0.00 | 0.00 | 7185700.00 | 0.00 | 0.00 | 3175200.00 | 5097400.00 | 0.00 | 0.00 | | hypothetical protein | Bacteria;Proteobacteria;Deltaproteobacteria;Syntrophorhabdales;Syntrophorhabdaceae;Syntrophorhabdus;Syntrophorhabdus aromaticivorans;Syntrophorhabdus aromaticivorans UI |
| Ga0586324 1618358 3 632 | 0.00 | 9248000.00 | 0.00 | 0.00 | 5726800.00 | 0.00 | 966106.68 | 0.00 | 0.00 | 0.00 | 0.00 | 1282500.00 | | ABC-type glycerol-3-phosphate transport system substrate-binding protein | Bacteria;Firmicutes;Bacilli;Bacillales;Paenibacillaceae;Paenibacillus;Paenibacillus sp. 453mf;Paenibacillus sp. 453MF |
| Ga0586324 1621654 199 987 | 309588.00 | 12199200.00 | 0.00 | 2033000.00 | 8126700.00 | 0.00 | 0.00 | 3009011.66 | 0.00 | 2625700.00 | 6952000.00 | 1620000.00 | | predicted porin | Bacteria;Proteobacteria;Betaproteobacteria;Burkholderiales;Oxalobacteraceae;Duganella;Duganella sp. Leaf61;Duganella sp. Leaf61 |
| Ga0586324 1622117 3 986 | 0.00 | 22535200.00 | 0.00 | 0.00 | 17613000.00 | 0.00 | 0.00 | 0.00 | 0.00 | 9933000.00 | 13974400.00 | 15757500.00 | | hypothetical protein | Bacteria;Proteobacteria;Betaproteobacteria;Burkholderiales;Oxalobacteraceae;Massilia;Massilia sp. Root351;Massilia sp. Root351 |
| Ga0586324 1624342 1 987 | 5450000.00 | 2590800.00 | 0.00 | 2118500.00 | 0.00 | 1838900.03 | 0.00 | 1396900.00 | 0.00 | 576341.92 | 0.00 | 900000.00 | | S-layer protein (TIGR01567 family) | Archaea;Euryarchaeota;Methanomicrobia;Methanosarcinales;Methanotrichaceae;Methanothrix;Methanothrix soehngenii;Methanothrix soehngenii GP-6 |
| Ga0586324 1626743 3 908 | 0.00 | 0.00 | 0.00 | 3714500.00 | 0.00 | 0.00 | 0.00 | 0.00 | 0.00 | 0.00 | 5042400.00 | 0.00 | | hypothetical protein | Bacteria;Acidobacteria;Acidobacteriia;Acidobacteriales;Acidobacteriaceae;unclassified Acidobacteriaceae;Acidobacteriaceae bacterium URHE0068;Acidobacteriaceae bacterium URHE0068 |
| Ga0586324 1629279 2 985 | 4200000.00 | 0.00 | 0.00 | 0.00 | 0.00 | 0.00 | 0.00 | 0.00 | 0.00 | 0.00 | 0.00 | 0.00 | | ABC-type sugar transport system substrate-binding protein | Bacteria;Proteobacteria;Alphaproteobacteria;Hyphomicrobiales;Devosiaceae;Maritalea;Maritalea myrionectae;Maritalea myrionectae DSM 19524 |
| Ga0586324 1630704 3 716 | 0.00 | 0.00 | 0.00 | 0.00 | 0.00 | 0.00 | 0.00 | 0.00 | 0.00 | 0.00 | 0.00 | 832500.00 | | NAD(P)-dependent dehydrogenase (short-subunit alcohol dehydrogenase family) | Bacteria;Firmicutes;Bacilli;Bacillales;Bacillaceae;Peribacillus;Peribacillus huizhouensis;Bacillus huizhouensis DSM 105481 |
| Ga0586324 1631268 1 957 | 0.00 | 610679.44 | 0.00 | 0.00 | 0.00 | 0.00 | 0.00 | 0.00 | 0.00 | 0.00 | 0.00 | 0.00 | | regulator of protease activity HflC (stomatin/prohibitin superfamily) | Bacteria;Chloroflexi;Anaerolineae;Anaerolineales;Anaerolineaceae;Thermanaerothrix;Thermanaerothrix daxensis;Thermanaerothrix daxensis GNS-1 |
| Ga0586324 1631684 1 672 | 0.00 | 0.00 | 0.00 | 0.00 | 0.00 | 0.00 | 0.00 | 0.00 | 1174500.00 | 0.00 | 0.00 | 0.00 | | predicted porin | Bacteria;Proteobacteria;Betaproteobacteria;Burkholderiales;Burkholderiaceae;Cupriavidus;Cupriavidus gilardii;Cupriavidus gilardii J4 |
| Ga0586324 1634862 1 984 | 0.00 | 0.00 | 0.00 | 1729000.00 | 0.00 | 0.00 | 0.00 | 0.00 | 0.00 | 0.00 | 0.00 | 0.00 | | hypothetical protein | Bacteria;Actinobacteria;Actinomycetia;Streptomycetales;Streptomycetaceae;Streptomyces;Streptomyces sp. LAM7114;Streptomyces sp. LAM7114 |
| Ga0586324 1635764 1 699 | 0.00 | 0.00 | 0.00 | 0.00 | 0.00 | 3648164.01 | 0.00 | 0.00 | 0.00 | 0.00 | 0.00 | 0.00 | | aryl-alcohol dehydrogenase-like predicted oxidoreductase | Bacteria;Proteobacteria;Alphaproteobacteria;Hyphomicrobiales;Hyphomicrobiaceae;Rhodoplanes;Rhodoplanes sp. Z2-YC6860;Rhodoplanes sp. Z2-YC6860 |
| Ga0586324 1639016 3 983 | 180717843.00 | 4943600.00 | 129193259.59 | 15903000.00 | 13276700.00 | 134980000.00 | 50091600.00 | 46116000.00 | 11218500.00 | 19481000.00 | 39776000.00 | 3397500.00 | | S-layer protein (TIGR01567 family) | Archaea;Euryarchaeota;Methanomicrobia;Methanosarcinales;Methanotrichaceae;Methanothrix;Methanothrix soehngenii;Methanothrix soehngenii GP-6 |
| Ga0586324 1639143 3 872 | 0.00 | 0.00 | 0.00 | 0.00 | 0.00 | 0.00 | 0.00 | 4825100.00 | 4527900.00 | 0.00 | 0.00 | 0.00 | | hypothetical protein | |
| Ga0586324 1639961 152 982 | 0.00 | 0.00 | 0.00 | 0.00 | 0.00 | 0.00 | 0.00 | 0.00 | 619898.67 | 0.00 | 0.00 | 0.00 | | TRAP-type mannitol/chloroaromatic compound transport system substrate-binding protein | Bacteria;Proteobacteria;Alphaproteobacteria;Hyphomicrobiales;Bradyrhizobiaceae;Bradyrhizobium;Bradyrhizobium lablabi;Bradyrhizobium lablabi GAS499 |
| Ga0586324 1644650 247 843 | 0.00 | 0.00 | 5054754.75 | 0.00 | 0.00 | 0.00 | 0.00 | 0.00 | 3231900.00 | 0.00 | 0.00 | 0.00 | | polyhydroxyalkanoate synthesis repressor PhaR | Bacteria;Proteobacteria;Alphaproteobacteria;Hyphomicrobiales;Bradyrhizobiaceae;Bradyrhizobium;Bradyrhizobium erythrophlei;Bradyrhizobium erythrophlei GAS242 |
| Ga0586324 1660606 2 976 | 3100000.00 | 0.00 | 0.00 | 0.00 | 0.00 | 0.00 | 0.00 | 0.00 | 0.00 | 0.00 | 0.00 | 0.00 | | predicted permease | Bacteria;Acidobacteria;Acidobacteriia;Bryobacterales;Solibacteraceae;Candidatus Solibacter;Candidatus Solibacter usitatus;Candidatus Solibacter usitatus Ellin6076 |
| Ga0586324 1667001 1 135 | 6410000.00 | 9588000.00 | 10077700.00 | 4341500.00 | 5273600.00 | 3070128.04 | 0.00 | 0.00 | 0.00 | 8316000.00 | 9328000.00 | 7237500.00 | | hypothetical protein | |
| Ga0586324 1668152 8 976 | 0.00 | 952000.00 | 4763965.25 | 6051500.00 | 3914000.00 | 5597700.00 | 3237600.00 | 1592100.00 | 1798200.00 | 0.00 | 2420000.00 | 1372500.00 | | hypothetical protein | Viruses;dsDNA viruses, no RNA stage;Caudoviricetes;Caudovirales;Myoviridae;Alcyoneusvirus;Klebsiella virus RaK2;Klebsiella phage vB_KleM_RaK2 |
| Ga0586324 1671142 2 895 | 1320000.00 | 0.00 | 0.00 | 0.00 | 0.00 | 0.00 | 0.00 | 0.00 | 1020600.00 | 1386000.00 | 0.00 | 1080000.00 | | TonB-dependent SusC/RagA subfamily outer membrane receptor | Bacteria;Bacteroidetes;Cytophagia;Cytophagales;Fulvivirgaceae;Ohtaekwangia;Ohtaekwangia kribbensis;Ohtaekwangia kribbensis DSM 25221 |
| Ga0586324 1675685 2 973 | 0.00 | 0.00 | 0.00 | 0.00 | 0.00 | 0.00 | 0.00 | 0.00 | 0.00 | 1008700.00 | 0.00 | 0.00 | | hypothetical protein | Bacteria;Proteobacteria;Gammaproteobacteria;Methylococcales;Methylococcaceae;Methylocaldum;Methylocaldum sp. 0917;Methylocaldum sp. 0917 |
| Ga0586324 1678408 1 972 | 0.00 | 0.00 | 0.00 | 0.00 | 1050600.00 | 0.00 | 0.00 | 0.00 | 0.00 | 0.00 | 0.00 | 0.00 | | hypothetical protein | |
| Ga0586324 1682828 3 971 | 2200000.00 | 3338800.00 | 0.00 | 0.00 | 0.00 | 0.00 | 0.00 | 0.00 | 0.00 | 0.00 | 0.00 | 0.00 | | hypothetical protein | Bacteria;Acidobacteria;Acidobacteriia;Bryobacterales;Bryobacteraceae;Bryobacter;Bryobacter aggregatus;Bryobacter aggregatus DSM 18758 |
| Ga0586324 1684652 2 937 | 0.00 | 0.00 | 0.00 | 0.00 | 3553500.00 | 1367208.45 | 0.00 | 0.00 | 0.00 | 0.00 | 0.00 | 0.00 | | TonB-dependent SusC/RagA subfamily outer membrane receptor | Bacteria;Bacteroidetes;Cytophagia;Cytophagales;Fulvivirgaceae;Ohtaekwangia;Ohtaekwangia koreensis;Ohtaekwangia koreensis DSM 25262 |
| Ga0586324 1687319 1 972 | 0.00 | 0.00 | 0.00 | 1510500.00 | 0.00 | 2174611.17 | 3853200.00 | 0.00 | 0.00 | 0.00 | 0.00 | 0.00 | | tetratricopeptide (TPR) repeat protein | Bacteria;Bacteroidetes;Bacteroidia;Bacteroidales;Porphyromonadaceae;Microbacter;Microbacter margulisiae;Microbacter margulisiae DSM 27471 |
| Ga0586324 1693477 1 969 | 0.00 | 0.00 | 0.00 | 0.00 | 0.00 | 0.00 | 0.00 | 0.00 | 0.00 | 9504000.00 | 0.00 | 0.00 | | hypothetical protein | Bacteria;Actinobacteria;Actinomycetia;Propionibacteriales;Nocardioidaceae;Nocardioides;Nocardioides sp. Root122;Nocardioides sp. Root122 |
| Ga0586324 1697359 2 967 | 401676.00 | 0.00 | 0.00 | 0.00 | 0.00 | 4446400.00 | 0.00 | 0.00 | 0.00 | 0.00 | 0.00 | 0.00 | | S-layer protein (TIGR01567 family) | Archaea;Euryarchaeota;Methanomicrobia;Methanosarcinales;Methanosarcinaceae;Methanosarcina;Methanosarcina barkeri;Methanosarcina barkeri 227 |
| Ga0586324 1697946 2 967 | 0.00 | 0.00 | 0.00 | 0.00 | 0.00 | 2816238.60 | 0.00 | 0.00 | 0.00 | 0.00 | 0.00 | 0.00 | | hypothetical protein | Bacteria;Ignavibacteriae;Ignavibacteria;Ignavibacteriales;Melioribacteraceae;Melioribacter;Melioribacter roseus;Melioribacter roseus P3M |
| Ga0586324 1700146 1 969 | 0.00 | 0.00 | 0.00 | 8588000.00 | 0.00 | 0.00 | 9804000.00 | 0.00 | 0.00 | 0.00 | 0.00 | 0.00 | | hypothetical protein | Bacteria;Proteobacteria;Gammaproteobacteria;Xanthomonadales;Rhodanobacteraceae;Rhodanobacter;Rhodanobacter sp. Soil772;Rhodanobacter sp. Soil772 |
| Ga0586324 1706433 1 966 | 0.00 | 0.00 | 0.00 | 0.00 | 0.00 | 0.00 | 0.00 | 0.00 | 0.00 | 0.00 | 941600.00 | 0.00 | | hypothetical protein | Bacteria;Acidobacteria;Acidobacteriia;Acidobacteriales;Acidobacteriaceae;Granulicella;Granulicella rosea;Granulicella rosea DSM 18704 |
| Ga0586324 1713737 2 964 | 4650000.00 | 0.00 | 0.00 | 0.00 | 0.00 | 0.00 | 0.00 | 0.00 | 0.00 | 0.00 | 0.00 | 0.00 | | hypothetical protein | Bacteria;Firmicutes;Clostridia;Thermoanaerobacterales;Thermoanaerobacterales Family III. Incertae Sedis;Caldicellulosiruptor;Caldicellulosiruptor kronotskyensis;Caldicellulosiruptor kronotskyensis 2002 |
| Ga0586324 1715395 3 965 | 0.00 | 0.00 | 0.00 | 0.00 | 1184500.00 | 0.00 | 0.00 | 0.00 | 0.00 | 0.00 | 0.00 | 0.00 | | hypothetical protein | Bacteria;Acidobacteria;Vicinamibacteria;unclassified Vicinamibacteria;Vicinamibacteraceae;Luteitalea;Luteitalea pratensis;Luteitalea pratensis DSM 100886 |
| Ga0586324 1715748 1 963 | 16100000.00 | 15028000.00 | 11823000.00 | 18620000.00 | 14008000.00 | 16515200.00 | 10738800.00 | 6100000.00 | 6698700.00 | 0.00 | 0.00 | 9525000.00 | | hypothetical protein | Bacteria;Candidatus Aminicenantes;unclassified Candidatus Aminicenantes;unclassified Candidatus Aminicenantes;unclassified Candidatus Aminicenantes;unclassified Candidatus Aminicenantes;Aminicenantes bacterium SCGC AAA252-O03;Aminicenantes bacterium SCGC AAA252-O03 (contamination screened) |
| Ga0586324 1717968 565 963 | 0.00 | 8364000.00 | 0.00 | 0.00 | 0.00 | 0.00 | 0.00 | 0.00 | 0.00 | 0.00 | 0.00 | 0.00 | | hypothetical protein | |
| Ga0586324 1718386 1 705 | 16800000.00 | 11696000.00 | 0.00 | 5282000.00 | 0.00 | 0.00 | 0.00 | 8181000.00 | 0.00 | 0.00 | 0.00 | 0.00 | | hypothetical protein | Bacteria;Proteobacteria;Gammaproteobacteria;Cellvibrionales;Spongiibacteraceae;Oceanicoccus;Oceanicoccus sagamiensis;Oceanicoccus sagamiensis NBRC 107125 |
| Ga0586324 1718757 3 962 | 0.00 | 0.00 | 0.00 | 0.00 | 0.00 | 0.00 | 0.00 | 0.00 | 0.00 | 0.00 | 0.00 | 318786.00 | | hypothetical protein | Bacteria;Acidobacteria;Acidobacteriia;Acidobacteriales;Acidobacteriaceae;unclassified Acidobacteriaceae;Acidobacteriaceae bacterium KBS 96;Acidobacteriaceae bacterium KBS 96, DSM 24297 |
| Ga0586324 1723146 134 961 | 0.00 | 0.00 | 1005957.14 | 0.00 | 0.00 | 0.00 | 0.00 | 0.00 | 0.00 | 0.00 | 0.00 | 0.00 | | ABC-type transport system substrate-binding protein | Bacteria;Proteobacteria;Deltaproteobacteria;unclassified Deltaproteobacteria;unclassified Deltaproteobacteria;unclassified Deltaproteobacteria;SAR324 cluster bacterium JCVI-SC AAA005;SAR324 cluster bacterium JCVI-SC AAA005 |
| Ga0586324 1723243 1 564 | 0.00 | 0.00 | 0.00 | 0.00 | 0.00 | 0.00 | 0.00 | 0.00 | 0.00 | 2571800.00 | 0.00 | 0.00 | | hypothetical protein | Bacteria;Acidobacteria;Acidobacteriia;Acidobacteriales;Acidobacteriaceae;Acidobacterium;Acidobacterium ailaaui;Acidobacterium ailaaui PMMR2 |
| Ga0586324 1725205 1 708 | 6660000.00 | 0.00 | 0.00 | 0.00 | 0.00 | 0.00 | 0.00 | 0.00 | 0.00 | 0.00 | 0.00 | 0.00 | | uncharacterized linocin/CFP29 family protein | Archaea;Euryarchaeota;Methanomicrobia;Methanomicrobiales;Methanoregulaceae;Methanosphaerula;Methanosphaerula palustris;Methanosphaerula palustris E1-9c, DSM 19958 |
| Ga0586324 1727314 38 517 | 0.00 | 0.00 | 0.00 | 0.00 | 11433000.00 | 0.00 | 0.00 | 0.00 | 0.00 | 0.00 | 0.00 | 0.00 | | hypothetical protein | Bacteria;Deinococcus-Thermus;Deinococci;Thermales;Thermaceae;Thermus;Thermus islandicus;Thermus islandicus DSM 21543 |
| Ga0586324 1730756 2 781 | 988059.00 | 0.00 | 0.00 | 0.00 | 0.00 | 0.00 | 0.00 | 0.00 | 0.00 | 0.00 | 0.00 | 0.00 | | DNA-binding winged helix-turn-helix (wHTH) protein | Bacteria;Proteobacteria;Betaproteobacteria;Burkholderiales;Comamonadaceae;Pelomonas;Pelomonas sp. BT06;Pelomonas sp. BT06 |
| Ga0586324 1733437 1 960 | 0.00 | 0.00 | 0.00 | 12065000.00 | 0.00 | 0.00 | 0.00 | 0.00 | 0.00 | 0.00 | 0.00 | 0.00 | | predicted porin | Bacteria;Proteobacteria;Gammaproteobacteria;Chromatiales;Chromatiaceae;Thiorhodococcus;Thiorhodococcus drewsii;Thiorhodococcus drewsii AZ1 |
| Ga0586324 1736223 2 958 | 0.00 | 0.00 | 0.00 | 0.00 | 0.00 | 0.00 | 0.00 | 0.00 | 0.00 | 0.00 | 0.00 | 952500.00 | | hypothetical protein | Bacteria;Proteobacteria;Alphaproteobacteria;Hyphomicrobiales;Rhizobiaceae;Rhizobium;Rhizobium leguminosarum;Rhizobium leguminosarum bv. viciae Vc2 |

| | | | | | | | | | | | | | | |
|---|---|---|---|---|---|---|---|---|---|---|---|---|---|---|
| Ga0586324 1740362 338 958 | 0.00 | 1768000.00 | 0.00 | 0.00 | 0.00 | 0.00 | 0.00 | 113043.98 | 0.00 | 0.00 | 0.00 | 183094.50 | hypothetical protein | |
| Ga0586324 1740907 3 749 | 0.00 | 6364800.00 | 0.00 | 0.00 | 14832000.00 | 13974400.00 | 56316000.00 | 8845000.00 | 12798000.00 | 7700000.00 | 8888000.00 | 12000000.00 | predicted porin | Bacteria;Proteobacteria;Betaproteobacteria;Burkholderiales;Burkholderiaceae;Quisquiliibacterium;Quisquiliibacterium transsilvanicum;Quisquiliibacterium transsilvanicum DSM 29781 |
| Ga0586324 1744020 1 657 | 0.00 | 0.00 | 0.00 | 1463000.00 | 0.00 | 0.00 | 0.00 | 0.00 | 0.00 | 0.00 | 0.00 | 0.00 | CBS domain-containing protein | Bacteria;Proteobacteria;Alphaproteobacteria;Hyphomicrobiales;Bradyrhizobiaceae;Bradyrhizobium;Bradyrhizobium erythrophlei;Bradyrhizobium erythrophlei GAS242 |
| Ga0586324 1744428 3 956 | 0.00 | 0.00 | 0.00 | 0.00 | 0.00 | 1533170.33 | 0.00 | 0.00 | 0.00 | 0.00 | 0.00 | 0.00 | hypothetical protein | Bacteria;Bacteroidetes;Bacteroidia;Marinilabiliales;Prolixibacteraceae;Mariniphaga;Mariniphaga anaerophila;Mariniphaga anaerophila DSM 26910 |
| Ga0586324 1757155 121 954 | 0.00 | 469425.76 | 0.00 | 0.00 | 0.00 | 0.00 | 0.00 | 0.00 | 0.00 | 0.00 | 0.00 | 0.00 | hypothetical protein | Bacteria;Proteobacteria;Alphaproteobacteria;Hyphomicrobiales;Bradyrhizobiaceae;Rhodopseudomonas;Rhodopseudomonas palustris;Rhodopseudomonas palustris BAL398 |
| Ga0586324 1762535 2 358 | 0.00 | 17612000.00 | 23983800.00 | 6479000.00 | 16480000.00 | 23621500.00 | 0.00 | 0.00 | 10773000.00 | 4758600.00 | 0.00 | 4477500.00 | hypothetical protein | |
| Ga0586324 1763957 315 782 | 0.00 | 0.00 | 0.00 | 4750000.00 | 3965500.00 | 0.00 | 0.00 | 0.00 | 0.00 | 0.00 | 0.00 | 2092500.00 | hypothetical protein | Bacteria;Bacteroidetes;Cytophagia;Cytophagales;Fulvivirgaceae;Chryseolinea;Chryseolinea soli;Chryseolinea soli KIS68-18 |
| Ga0586324 1772297 2 805 | 0.00 | 0.00 | 0.00 | 0.00 | 0.00 | 0.00 | 0.00 | 0.00 | 0.00 | 2656500.00 | 0.00 | 0.00 | TonB-dependent SusC/RagA subfamily outer membrane receptor | Bacteria;Bacteroidetes;Bacteroidia;Bacteroidales;Lentimicrobiaceae;Lentimicrobium;Lentimicrobium saccharophilum;Bacteroidales bacterium TBC1 |
| Ga0586324 1774388 3 950 | 3870000.00 | 0.00 | 0.00 | 0.00 | 0.00 | 0.00 | 0.00 | 0.00 | 0.00 | 0.00 | 0.00 | 0.00 | hypothetical protein | Archaea;Euryarchaeota;Methanomicrobia;Methanosarcinales;Methanotrichaceae;Methanothrix;Methanothrix soehngenii;Methanothrix soehngenii GP-6 |
| Ga0586324 1775024 114 950 | 224612.00 | 0.00 | 0.00 | 0.00 | 0.00 | 0.00 | 0.00 | 0.00 | 0.00 | 0.00 | 0.00 | 0.00 | hypothetical protein | Bacteria;Acidobacteria;Acidobacteriia;Acidobacteriales;Acidobacteriaceae;unclassified Acidobacteriaceae;Acidobacteriaceae bacterium URHE0068;Acidobacteriaceae bacterium URHE0068 |
| Ga0586324 1776467 2 949 | 1520000.00 | 0.00 | 0.00 | 0.00 | 0.00 | 0.00 | 0.00 | 0.00 | 0.00 | 0.00 | 0.00 | 0.00 | hypothetical protein | Bacteria;Acidobacteria;Vicinamibacteria;unclassified Vicinamibacteria;Vicinamibacteraceae;Luteitalea;Luteitalea pratensis;Luteitalea pratensis DSM 100886 |
| Ga0586324 1776896 83 949 | 0.00 | 0.00 | 0.00 | 0.00 | 0.00 | 3521874.34 | 0.00 | 0.00 | 0.00 | 0.00 | 0.00 | 0.00 | hypothetical protein | Bacteria;Bacteroidetes;Cytophagia;Cytophagales;Cyclobacteriaceae;Cecembia;Cecembia calidifontis;Cecembia calidifontis DSM 21411 |
| Ga0586324 1777428 52 948 | 0.00 | 0.00 | 48305400.00 | 0.00 | 0.00 | 27432700.00 | 12608400.00 | 0.00 | 0.00 | 25872000.00 | 0.00 | 21375000.00 | hypothetical protein | Bacteria;Firmicutes;Bacilli;Bacillales;Alicyclobacillaceae;Tumebacillus;Tumebacillus permanentifrigoris;Tumebacillus permanentifrigoris DSM 18773 |
| Ga0586324 1782301 1 810 | 0.00 | 0.00 | 0.00 | 0.00 | 0.00 | 0.00 | 0.00 | 0.00 | 0.00 | 0.00 | 0.00 | 21750000.00 | arylsulfatase A-like enzyme | Bacteria;Proteobacteria;Alphaproteobacteria;Hyphomicrobiales;Methylocystaceae;Methylocystis;Methylocystis sp. ATCC 49242;Methylocystis sp. Rockwell, ATCC 49242 |
| Ga0586324 1784481 2 949 | 0.00 | 0.00 | 0.00 | 0.00 | 0.00 | 0.00 | 0.00 | 0.00 | 0.00 | 0.00 | 1064800.00 | 0.00 | hypothetical protein | Bacteria;Acidobacteria;Acidobacteriia;Acidobacteriales;Acidobacteriaceae;Edaphobacter;Edaphobacter aggregans;Edaphobacter aggregans EB153 |
| Ga0586324 1785065 1 948 | 16600000.00 | 0.00 | 0.00 | 42845000.00 | 0.00 | 0.00 | 0.00 | 26779000.00 | 0.00 | 36883000.00 | 29216000.00 | 20850000.00 | hypothetical protein | |
| Ga0586324 1787738 332 946 | 0.00 | 2781200.00 | 0.00 | 0.00 | 0.00 | 0.00 | 0.00 | 0.00 | 0.00 | 0.00 | 0.00 | 0.00 | hypothetical protein | |
| Ga0586324 1789881 252 638 | 0.00 | 0.00 | 0.00 | 0.00 | 0.00 | 0.00 | 0.00 | 3226900.00 | 0.00 | 0.00 | 0.00 | 0.00 | uncharacterized spore protein YtfJ | Bacteria;Proteobacteria;Betaproteobacteria;Burkholderiales;Comamonadaceae;Rhodoferax;Rhodoferax ferrireducens;Rhodoferax ferrireducens T118 |
| Ga0586324 1790359 2 904 | 0.00 | 0.00 | 0.00 | 0.00 | 0.00 | 0.00 | 0.00 | 0.00 | 0.00 | 3126200.00 | 2983200.00 | 0.00 | hypothetical protein | Bacteria;Chloroflexi;Caldilineae;Caldilineales;Caldilineaceae;Caldilinea;Caldilinea aerophila;Caldilinea aerophila STL-6-O1, DSM 14535 |
| Ga0586324 1794208 2 208 | 0.00 | 0.00 | 0.00 | 0.00 | 0.00 | 0.00 | 0.00 | 0.00 | 748941.39 | 0.00 | 0.00 | 31875000.00 | RNA recognition motif-containing protein | Bacteria;Planctomycetes;Candidatus Brocadiae;Candidatus Brocadiales;Candidatus Brocadiaceae;Candidatus Jettenia;Candidatus Jettenia caeni;Planctomycetaceae bacterium KSU-1 |
| Ga0586324 1794688 1 945 | 0.00 | 0.00 | 0.00 | 0.00 | 0.00 | 0.00 | 0.00 | 0.00 | 0.00 | 0.00 | 0.00 | 84750000.00 | hypothetical protein | Bacteria;Proteobacteria;Alphaproteobacteria;Hyphomicrobiales;Xanthobacteraceae;Pseudolabrys;Pseudolabrys taiwanensis;Pseudolabrys taiwanensis CC-BB4 |
| Ga0586324 1795535 3 944 | 1160000.00 | 0.00 | 0.00 | 0.00 | 0.00 | 0.00 | 0.00 | 0.00 | 0.00 | 0.00 | 0.00 | 0.00 | hypothetical protein | Bacteria;Proteobacteria;Gammaproteobacteria;Xanthomonadales;Rhodanobacteraceae;Oleiagrimonas;Oleiagrimonas sp. MCCC 1A03011;Oleiagrimonas sp. MCCC 1A03011 |
| Ga0586324 1795759 552 923 | 0.00 | 0.00 | 684365.91 | 0.00 | 0.00 | 0.00 | 0.00 | 0.00 | 0.00 | 0.00 | 0.00 | 0.00 | transposase | Bacteria;Proteobacteria;Alphaproteobacteria;Hyphomicrobiales;Bradyrhizobiaceae;Nitrobacter;Nitrobacter sp. Nb-311A;Nitrobacter sp. Nb-311A |
| Ga0586324 1796410 3 944 | 0.00 | 0.00 | 0.00 | 4028000.00 | 0.00 | 0.00 | 0.00 | 0.00 | 0.00 | 0.00 | 0.00 | 0.00 | ABC-type glutathione transport system ATPase component | Bacteria;Proteobacteria;Epsilonproteobacteria;Campylobacterales;Campylobacteraceae;Campylobacter;Campylobacter concisus;Campylobacter concisus UNSW2 |
| Ga0586324 1800902 650 943 | 0.00 | 0.00 | 0.00 | 0.00 | 0.00 | 0.00 | 0.00 | 3788100.00 | 0.00 | 0.00 | 0.00 | 0.00 | TusE/DsrC/DsvC family sulfur relay protein | Bacteria;Proteobacteria;Betaproteobacteria;Nitrosomonadales;Thiobacillaceae;Thiobacillus;Thiobacillus thioparus;Thiobacillus thioparus DSM 505 |
| Ga0586324 1802464 653 943 | 0.00 | 0.00 | 0.00 | 0.00 | 0.00 | 0.00 | 0.00 | 0.00 | 0.00 | 1871100.00 | 1425600.00 | 1230000.00 | FAD/FMN-containing dehydrogenase | Bacteria;Proteobacteria;Betaproteobacteria;Burkholderiales;Comamonadaceae;Polaromonas;Polaromonas jejuensis;Polaromonas jejuensis NBRC 106434 |
| Ga0586324 1807967 2 943 | 0.00 | 0.00 | 0.00 | 0.00 | 18952000.00 | 0.00 | 0.00 | 0.00 | 0.00 | 0.00 | 0.00 | 0.00 | methyl-accepting chemotaxis protein | Bacteria;Actinobacteria;Thermoleophilia;Solirubrobacterales;Solirubrobacteraceae;Solirubrobacter;Solirubrobacter pauli;Solirubrobacter pauli DSM 14954 |
| Ga0586324 1808817 527 943 | 0.00 | 0.00 | 0.00 | 0.00 | 0.00 | 4962500.00 | 0.00 | 0.00 | 0.00 | 0.00 | 4743200.00 | 3735000.00 | polyhydroxyalkanoate synthesis repressor PhaR | Bacteria;Proteobacteria;Alphaproteobacteria;Hyphomicrobiales;Bradyrhizobiaceae;Bradyrhizobium;Bradyrhizobium erythrophlei;Bradyrhizobium erythrophlei GAS242 |
| Ga0586324 1809896 2 943 | 4050000.00 | 0.00 | 0.00 | 0.00 | 4140600.00 | 0.00 | 0.00 | 0.00 | 0.00 | 0.00 | 3986400.00 | 0.00 | hypothetical protein | Bacteria;Actinobacteria;Thermoleophilia;Solirubrobacterales;Solirubrobacteraceae;Solirubrobacter;Solirubrobacter soli;Solirubrobacter soli DSM 22325 |
| Ga0586324 1814081 2 940 | 0.00 | 0.00 | 17903400.00 | 15485000.00 | 0.00 | 60224900.00 | 17898000.00 | 14152000.00 | 18711000.00 | 0.00 | 0.00 | 16575000.00 | crotonobetainyl-CoA:carnitine CoA-transferase CaiB-like acyl-CoA transferase | Bacteria;Proteobacteria;Deltaproteobacteria;Syntrophobacterales;Syntrophobacteraceae;Desulfosoma;Desulfosoma caldarium;Desulfosoma caldarium DSM 22027 |
| Ga0586324 1816385 1 924 | 3300000.00 | 7208000.00 | 0.00 | 0.00 | 7014300.00 | 0.00 | 10921200.00 | 4764100.00 | 7776000.00 | 8085000.00 | 11176000.00 | 4905000.00 | hypothetical protein | Bacteria;Actinobacteria;Actinomycetia;Propionibacteriales;Nocardioidaceae;Nocardioides;Nocardioides sp. Root122;Nocardioides sp. Root122 |
| Ga0586324 1817786 134 940 | 22300000.00 | 32096000.00 | 31528000.00 | 41990000.00 | 75396000.00 | 47640000.00 | 24396000.00 | 34587000.00 | 19602000.00 | 0.00 | 16544000.00 | 8475000.00 | predicted porin | Bacteria;Proteobacteria;Betaproteobacteria;Nitrosomonadales;Gallionellaceae;Sideroxydans;Sideroxydans lithotrophicus;Sideroxydans lithotrophicus ES-1 |
| Ga0586324 1820745 144 941 | 556927.00 | 8636000.00 | 0.00 | 0.00 | 0.00 | 0.00 | 16963200.00 | 15372000.00 | 14499000.00 | 13475000.00 | 17072000.00 | 20527075.50 | predicted porin | Bacteria;Proteobacteria;Betaproteobacteria;Nitrosomonadales;Sulfuricellaceae;Sulfuricella;Sulfuricella denitrificans;Sulfuricella denitrificans skB26 |
| Ga0586324 1836335 1 936 | 0.00 | 0.00 | 0.00 | 0.00 | 0.00 | 1175894.15 | 0.00 | 0.00 | 0.00 | 0.00 | 0.00 | 0.00 | hypothetical protein | Bacteria;Proteobacteria;Gammaproteobacteria;Oceanospirillales;Alcanivoracaceae;Alcanivorax;Alcanivorax sp. MD8A;Alcanivorax sp. MD8A |
| Ga0586324 1838477 2 937 | 0.00 | 0.00 | 0.00 | 0.00 | 0.00 | 0.00 | 0.00 | 0.00 | 0.00 | 15015000.00 | 0.00 | 0.00 | hypothetical protein | Bacteria;Proteobacteria;Betaproteobacteria;Burkholderiales;Comamonadaceae;Acidovorax;Acidovorax sp. JS42;Acidovorax sp. JS42 |
| Ga0586324 1847052 1 933 | 620818.00 | 1584400.00 | 0.00 | 0.00 | 0.00 | 0.00 | 0.00 | 390296.91 | 1887300.00 | 1355200.00 | 447156.16 | 0.00 | hypothetical protein | Bacteria;Proteobacteria;Alphaproteobacteria;Hyphomicrobiales;Bradyrhizobiaceae;Rhodopseudomonas;Rhodopseudomonas sp. AAP120;Rhodopseudomonas sp. AAP120 |
| Ga0586324 1849890 181 933 | 0.00 | 0.00 | 0.00 | 0.00 | 0.00 | 0.00 | 2986800.00 | 0.00 | 0.00 | 0.00 | 0.00 | 0.00 | hypothetical protein | Bacteria;Proteobacteria;Betaproteobacteria;Nitrosomonadales;Gallionellaceae;Sideroxydans;Sideroxydans lithotrophicus;Sideroxydans lithotrophicus ES-1 |
| Ga0586324 1852152 124 690 | 11900000.00 | 14592800.00 | 0.00 | 10450000.00 | 7529300.00 | 0.00 | 0.00 | 0.00 | 0.00 | 0.00 | 0.00 | 0.00 | hypothetical protein | Bacteria;Proteobacteria;Deltaproteobacteria;Syntrophobacterales;Syntrophaceae;Desulfobacca;Desulfobacca acetoxidans;Desulfobacca acetoxidans ASRB2, DSM 11109 |
| Ga0586324 1857431 344 931 | 0.00 | 0.00 | 0.00 | 0.00 | 0.00 | 0.00 | 0.00 | 1732400.00 | 0.00 | 0.00 | 0.00 | 0.00 | phenylphosphate carboxylase alpha subunit | Bacteria;Proteobacteria;Deltaproteobacteria;Syntrophorhabdales;Syntrophorhabdaceae;Syntrophorhabdus;Syntrophorhabdus aromaticivorans;Syntrophorhabdus aromaticivorans UI |
| Ga0586324 1858939 72 932 | 0.00 | 0.00 | 0.00 | 0.00 | 15759000.00 | 0.00 | 0.00 | 0.00 | 0.00 | 0.00 | 0.00 | 0.00 | probable F420-dependent oxidoreductase | Bacteria;Proteobacteria;Alphaproteobacteria;Rhodospirillales;Acetobacteraceae;Rhodopila;Rhodopila globiformis;Rhodopila globiformis DSM 161 |
| Ga0586324 1871440 184 930 | 1620000.00 | 0.00 | 0.00 | 0.00 | 0.00 | 0.00 | 0.00 | 0.00 | 0.00 | 0.00 | 0.00 | 0.00 | hypothetical protein | Bacteria;Proteobacteria;Deltaproteobacteria;Syntrophobacterales;Syntrophaceae;Syntrophus;Syntrophus gentianae;Syntrophus gentianae DSM 8423 |
| Ga0586324 1872419 3 917 | 0.00 | 594038.48 | 0.00 | 0.00 | 0.00 | 0.00 | 0.00 | 0.00 | 0.00 | 0.00 | 0.00 | 0.00 | nitrous oxide reductase accessory protein NosL | Bacteria;Proteobacteria;Gammaproteobacteria;Methylococcales;Methylococcaceae;Methylobacter;Methylobacter tundripaludum;Methylobacter tundripaludum OWC-G53F |
| Ga0586324 1874174 1 927 | 6990000.00 | 5100000.00 | 0.00 | 0.00 | 38110000.00 | 6272600.00 | 0.00 | 0.00 | 0.00 | 0.00 | 0.00 | 0.00 | hypothetical protein | Bacteria;Spirochaetes;Spirochaetia;Spirochaetales;Spirochaetaceae;Spirochaeta;Spirochaeta cellobiosiphila;Spirochaeta cellobiosiphila DSM 17781 |
| Ga0586324 1874235 3 929 | 0.00 | 0.00 | 0.00 | 0.00 | 0.00 | 0.00 | 0.00 | 0.00 | 923400.00 | 0.00 | 0.00 | 0.00 | DNA-directed RNA polymerase beta' subunit | Bacteria;Actinobacteria;Rubrobacteria;Gaiellales;Gaiellaceae;Gaiella;Gaiella occulta;Gaiella occulta F2-233 |
| Ga0586324 1874661 289 849 | 0.00 | 0.00 | 0.00 | 1805000.00 | 0.00 | 0.00 | 0.00 | 0.00 | 0.00 | 0.00 | 0.00 | 0.00 | hypothetical protein | Bacteria;Proteobacteria;Deltaproteobacteria;Syntrophobacterales;Syntrophaceae;Desulfobacca;Desulfobacca acetoxidans;Desulfobacca acetoxidans ASRB2, DSM 11109 |
| Ga0586324 1875223 3 929 | 2650000.00 | 0.00 | 0.00 | 0.00 | 0.00 | 0.00 | 0.00 | 0.00 | 0.00 | 0.00 | 0.00 | 0.00 | hypothetical protein | Bacteria;Nitrospirae;Thermodesulfovibrionia;Thermodesulfovibrionales;Thermodesulfovibrionaceae;Thermodesulfovibrio;Thermodesulfovibrio islandicus;Thermodesulfovibrio islandicus DSM 12570 |
| Ga0586324 1877802 234 929 | 0.00 | 0.00 | 0.00 | 0.00 | 1895200.00 | 0.00 | 0.00 | 0.00 | 0.00 | 0.00 | 0.00 | 0.00 | TRAP-type mannitol/chloroaromatic compound transport system substrate-binding protein | Bacteria;Proteobacteria;Deltaproteobacteria;unclassified Deltaproteobacteria;unclassified Deltaproteobacteria;unclassified Deltaproteobacteria;delta proteobacterium NaphS2;delta proteobacterium NaphS2 |
| Ga0586324 1880991 503 883 | 0.00 | 6052000.00 | 0.00 | 0.00 | 0.00 | 0.00 | 0.00 | 0.00 | 0.00 | 0.00 | 0.00 | 0.00 | hypothetical protein | Archaea;Euryarchaeota;Methanomicrobia;Methanosarcinales;Methanotrichaceae;Methanothrix;Methanothrix soehngenii;Methanothrix soehngenii GP-6 |
| Ga0586324 1885560 3 926 | 422873.00 | 0.00 | 0.00 | 0.00 | 0.00 | 0.00 | 0.00 | 0.00 | 0.00 | 0.00 | 0.00 | 0.00 | hypothetical protein | Bacteria;Proteobacteria;Betaproteobacteria;Burkholderiales;unclassified Burkholderiales;Paucibacter;Paucibacter toxinivorans;Paucibacter toxinivorans DSM 16998 |
| Ga0586324 1886540 134 925 | 0.00 | 1931200.00 | 0.00 | 1520000.00 | 1864300.00 | 0.00 | 0.00 | 0.00 | 1036800.00 | 3526600.00 | 0.00 | 0.00 | hypothetical protein | Bacteria;Actinobacteria;Actinomycetia;Micrococcales;Intrasporangiaceae;Intrasporangium;Intrasporangium oryzae;Intrasporangium oryzae NRRL B-24470 |
| Ga0586324 1895824 500 925 | 1440000.00 | 720800.00 | 0.00 | 0.00 | 0.00 | 0.00 | 0.00 | 0.00 | 1952100.00 | 0.00 | 0.00 | 0.00 | hypothetical protein | Bacteria;Acidobacteria;Thermoanaerobaculia;Thermoanaerobaculales;Thermoanaerobaculaceae;Thermoanaerobaculum;Thermoanaerobaculum aquaticum;Thermoanaerobaculum aquaticum MP-01 |
| Ga0586324 1896196 1 567 | 0.00 | 0.00 | 0.00 | 0.00 | 1400800.00 | 0.00 | 2016206.28 | 2135000.00 | 2219400.00 | 1563100.00 | 0.00 | 0.00 | hypothetical protein | Bacteria;Proteobacteria;Deltaproteobacteria;Desulfovibrionales;Desulfovibrionaceae;Desulfocurvibacter;Desulfocurvibacter africanus;Desulfovibrio africanus DSM 2603 |
| Ga0586324 1897111 1 924 | 0.00 | 0.00 | 0.00 | 0.00 | 0.00 | 0.00 | 2576400.00 | 1323700.00 | 0.00 | 2071300.00 | 1381600.00 | 3292500.00 | hypothetical protein | Bacteria;Proteobacteria;Gammaproteobacteria;Acidiferrobacterales;Acidiferrobacteraceae;Sulfuricaulis;Sulfuricaulis limicola;Sulfuricaulis limicola HA5 |
| Ga0586324 1898378 3 923 | 7670000.00 | 8024000.00 | 14919500.00 | 9091500.00 | 13081000.00 | 11155700.00 | 0.00 | 4367600.00 | 2972700.00 | 5351500.00 | 3924800.00 | 3360000.00 | hypothetical protein | Bacteria;Actinobacteria;Actinomycetia;Micrococcales;Microbacteriaceae;Cryobacterium;Cryobacterium sp. MDB2-B;Cryobacterium sp. MDB2-B |
| Ga0586324 1910812 227 922 | 4330000.00 | 0.00 | 0.00 | 0.00 | 0.00 | 0.00 | 0.00 | 0.00 | 0.00 | 0.00 | 0.00 | 0.00 | uncharacterized protein (TIGR02001 family) | Bacteria;Proteobacteria;Betaproteobacteria;Nitrosomonadales;Thiobacillaceae;Thiobacillus;Thiobacillus thioparus;Thiobacillus thioparus DSM 505 |
| Ga0586324 1911824 3 152 | 3610000.00 | 0.00 | 0.00 | 0.00 | 0.00 | 0.00 | 0.00 | 0.00 | 0.00 | 0.00 | 0.00 | 0.00 | hypothetical protein | |
| Ga0586324 1913768 316 921 | 0.00 | 0.00 | 0.00 | 0.00 | 0.00 | 0.00 | 0.00 | 0.00 | 0.00 | 0.00 | 816966.48 | 0.00 | hypothetical protein | Bacteria;Acidobacteria;Acidobacteriia;Acidobacteriales;Acidobacteriaceae;unclassified Acidobacteriaceae;Acidobacteriaceae bacterium KBS 96;Acidobacteriaceae bacterium KBS 96, DSM 24297 |
| Ga0586324 1916254 2 898 | 0.00 | 924800.00 | 0.00 | 0.00 | 0.00 | 0.00 | 0.00 | 0.00 | 0.00 | 0.00 | 0.00 | 0.00 | hypothetical protein | Bacteria;Actinobacteria;Actinomycetia;Streptosporangiales;Streptosporangiaceae;Thermobispora;Thermobispora bispora;Thermobispora bispora R51, DSM 43833 |
| Ga0586324 1922734 3 917 | 0.00 | 843200.00 | 1618810.79 | 0.00 | 0.00 | 0.00 | 0.00 | 695400.00 | 0.00 | 0.00 | 0.00 | 679850.25 | hypothetical protein | |
| Ga0586324 1928159 1 918 | 0.00 | 2495600.00 | 4195093.16 | 0.00 | 0.00 | 0.00 | 0.00 | 1659200.00 | 0.00 | 0.00 | 0.00 | 0.00 | hypothetical protein | Bacteria;Bacteroidetes;Bacteroidia;Marinilabiliales;Marinilabiliaceae;Breznakibacter;Breznakibacter xylanolyticus;Cytophaga xylanolytica DSM 6779 |
| Ga0586324 1930196 2 916 | 0.00 | 0.00 | 25166100.00 | 0.00 | 0.00 | 0.00 | 0.00 | 0.00 | 0.00 | 0.00 | 0.00 | 0.00 | hypothetical protein | |
| Ga0586324 1932545 2 748 | 0.00 | 5093200.00 | 0.00 | 0.00 | 0.00 | 0.00 | 0.00 | 0.00 | 0.00 | 0.00 | 0.00 | 6555000.00 | hypothetical protein | Bacteria;Spirochaetes;Spirochaetia;Leptospirales;Leptospiraceae;Leptonema;Leptonema illini;Leptonema illini 3055, DSM 21528 |
| Ga0586324 1938506 239 631 | 4880000.00 | 2006000.00 | 0.00 | 0.00 | 6725900.00 | 0.00 | 0.00 | 2427800.00 | 2130300.00 | 0.00 | 0.00 | 1282500.00 | hypothetical protein | Bacteria;Proteobacteria;Gammaproteobacteria;unclassified Gammaproteobacteria;unclassified Gammaproteobacteria;Sedimenticola;unclassified Sedimenticola;Sedimenticola thiotaurini SIP-G1 |
| Ga0586324 1938506 721 915 | 0.00 | 9112000.00 | 0.00 | 21375000.00 | 22454000.00 | 0.00 | 0.00 | 0.00 | 0.00 | 19789000.00 | 14960000.00 | 11625000.00 | hypothetical protein | |
| Ga0586324 1939160 193 915 | 0.00 | 0.00 | 0.00 | 0.00 | 0.00 | 0.00 | 0.00 | 0.00 | 0.00 | 0.00 | 1812800.00 | 0.00 | NAD(P)-dependent dehydrogenase (short-subunit alcohol dehydrogenase family) | Bacteria;Proteobacteria;Alphaproteobacteria;Rhodospirillales;Rhodospirillaceae;Dongia;Dongia mobilis;Dongia mobilis CGMCC 1.7660 |
| Ga0586324 1942008 288 731 | 1870000.00 | 0.00 | 0.00 | 0.00 | 0.00 | 0.00 | 2037143.52 | 0.00 | 0.00 | 3896200.00 | 0.00 | 0.00 | uncharacterized membrane protein | Bacteria;Atribacterota;unclassified Atribacterota;unclassified Atribacterota;unclassified Atribacterota;unclassified Atribacterota;Atribacteria bacterium SCGC AB-164-P05;Atribacteria bacterium SCGC AB-164-P05 (contamination screened) |
| Ga0586324 1943226 3 914 | 0.00 | 0.00 | 0.00 | 0.00 | 0.00 | 0.00 | 0.00 | 0.00 | 0.00 | 0.00 | 0.00 | 510390.75 | succinate dehydrogenase/fumarate reductase flavoprotein subunit | Bacteria;Proteobacteria;Gammaproteobacteria;Chromatiales;Chromatiaceae;Thioflavicoccus;Thioflavicoccus mobilis;Thioflavicoccus mobilis 8321 |
| Ga0586324 1948765 1 912 | 258564.00 | 0.00 | 1815686.26 | 2356000.00 | 0.00 | 2483159.57 | 0.00 | 0.00 | 0.00 | 0.00 | 0.00 | 1890000.00 | hypothetical protein | Bacteria;Acidobacteria;Acidobacteriia;Acidobacteriales;Acidobacteriaceae;unclassified Acidobacteriaceae;Acidobacteriaceae bacterium KBS 96;Acidobacteriaceae bacterium KBS 96, DSM 24297 |
| Ga0586324 1953432 3 911 | 0.00 | 0.00 | 0.00 | 1672000.00 | 2111500.00 | 0.00 | 0.00 | 0.00 | 0.00 | 0.00 | 0.00 | 0.00 | hypothetical protein | Bacteria;Acidobacteria;Acidobacteriia;Bryobacterales;Solibacteraceae;Candidatus Solibacter;Candidatus Solibacter usitatus;Candidatus Solibacter usitatus Ellin6076 |
| Ga0586324 1968500 1 840 | 0.00 | 0.00 | 5798900.00 | 0.00 | 0.00 | 0.00 | 0.00 | 0.00 | 0.00 | 0.00 | 0.00 | 0.00 | arylsulfatase A-like enzyme | Bacteria;Proteobacteria;Betaproteobacteria;Nitrosomonadales;Sterolibacteriaceae;Sulfurisoma;Sulfurisoma sediminicola;Sulfurisoma sediminicola DSM 26916 |
| Ga0586324 1973313 2 907 | 0.00 | 0.00 | 0.00 | 0.00 | 0.00 | 0.00 | 0.00 | 0.00 | 0.00 | 0.00 | 0.00 | 667755.75 | NADPH-dependent glutamate synthase beta subunit-like oxidoreductase | Bacteria;Proteobacteria;Betaproteobacteria;Burkholderiales;unclassified Burkholderiales;Ideonella;Ideonella sp. A 288;Ideonella sp. A 288 |
| Ga0586324 1978044 1 906 | 6070000.00 | 0.00 | 0.00 | 0.00 | 0.00 | 0.00 | 0.00 | 0.00 | 0.00 | 0.00 | 0.00 | 0.00 | hypothetical protein | Bacteria;Nitrospirae;Nitrospira;Nitrospirales;Nitrospiraceae;Candidatus Magnetoovum;Candidatus Magnetoovum chiemensis;Candidatus Magnetoovum chiemensis CS-04 |
| Ga0586324 1979020 1 906 | 0.00 | 0.00 | 0.00 | 0.00 | 0.00 | 0.00 | 0.00 | 1799500.00 | 0.00 | 0.00 | 0.00 | 0.00 | hypothetical protein | Bacteria;Proteobacteria;Betaproteobacteria;Nitrosomonadales;Sterolibacteriaceae;Sulfurisoma;Sulfurisoma sediminicola;Sulfurisoma sediminicola DSM 26916 |
| Ga0586324 1979588 117 908 | 0.00 | 0.00 | 0.00 | 9281500.00 | 8260600.00 | 0.00 | 0.00 | 0.00 | 0.00 | 0.00 | 0.00 | 0.00 | cytochrome c peroxidase | Bacteria;Proteobacteria;Deltaproteobacteria;Myxococcales;Polyangiaceae;Sorangium;Sorangium cellulosum;Sorangium cellulosum So ce 56 |
| Ga0586324 1984204 3 542 | 0.00 | 0.00 | 0.00 | 0.00 | 0.00 | 0.00 | 0.00 | 0.00 | 8910000.00 | 0.00 | 0.00 | 0.00 | hypothetical protein | |
| Ga0586324 1992160 2 904 | 0.00 | 0.00 | 0.00 | 1900000.00 | 0.00 | 0.00 | 0.00 | 0.00 | 1555200.00 | 0.00 | 0.00 | 0.00 | hypothetical protein | Bacteria;Acidobacteria;Thermoanaerobaculia;Thermoanaerobaculales;Thermoanaerobaculaceae;Thermoanaerobaculum;Thermoanaerobaculum aquaticum;Thermoanaerobaculum aquaticum MP-01 |
| Ga0586324 1992449 1 279 | 0.00 | 218491.48 | 0.00 | 0.00 | 1102100.00 | 0.00 | 0.00 | 0.00 | 0.00 | 0.00 | 0.00 | 0.00 | hypothetical protein | Bacteria;Bacteroidetes;Cytophagia;Cytophagales;Cytophagaceae;Dyadobacter;Dyadobacter arcticus;Dyadobacter arcticus DSM 102865 |
| Ga0586324 1995406 1 891 | 0.00 | 0.00 | 0.00 | 0.00 | 2914900.00 | 0.00 | 0.00 | 0.00 | 0.00 | 0.00 | 0.00 | 0.00 | crotonobetainyl-CoA:carnitine CoA-transferase CaiB-like acyl-CoA transferase | Bacteria;Proteobacteria;Deltaproteobacteria;Syntrophobacterales;Syntrophaceae;Desulfobacca;Desulfobacca acetoxidans;Desulfobacca acetoxidans ASRB2, DSM 11109 |
| Ga0586324 2002716 2 523 | 0.00 | 0.00 | 0.00 | 0.00 | 0.00 | 0.00 | 12266400.00 | 0.00 | 7905600.00 | 0.00 | 0.00 | 0.00 | DNA-binding NarL/FixJ family response regulator | Bacteria;Planctomycetes;Planctomycetia;Pirellulales;Pirellulaceae;Roseimaritima;Roseimaritima ulvae;Roseimaritima ulvae DSM 25454 |
| Ga0586324 2007165 2 901 | 31000000.00 | 7820000.00 | 0.00 | 4389000.00 | 0.00 | 0.00 | 0.00 | 0.00 | 0.00 | 0.00 | 0.00 | 0.00 | hypothetical protein | Bacteria;Candidatus Aminicenantes;unclassified Candidatus Aminicenantes;unclassified Candidatus Aminicenantes;unclassified Candidatus Aminicenantes;unclassified Candidatus Aminicenantes;Aminicenantes bacterium SCGC AAA252-N07;Aminicenantes bacterium SCGC AAA252-N07 (contamination screened) |
| Ga0586324 2008660 2 901 | 0.00 | 0.00 | 8050900.00 | 7999000.00 | 0.00 | 7860600.00 | 4947600.00 | 0.00 | 0.00 | 0.00 | 0.00 | 0.00 | hypothetical protein | Bacteria;Actinobacteria;Actinomycetia;Micrococcales;Intrasporangiaceae;Humibacillus;Humibacillus xanthopallidus;Humibacillus xanthopallidus DSM 21776 |
| Ga0586324 2011212 1 900 | 0.00 | 0.00 | 0.00 | 0.00 | 0.00 | 0.00 | 0.00 | 0.00 | 5337900.00 | 0.00 | 0.00 | 0.00 | hypothetical protein | Bacteria;Acidobacteria;Acidobacteriia;Acidobacteriales;Acidobacteriaceae;unclassified Acidobacteriaceae;Acidobacteriaceae bacterium KBS 96;Acidobacteriaceae bacterium KBS 96, DSM 24297 |
| Ga0586324 2012904 1 417 | 0.00 | 3576800.00 | 0.00 | 0.00 | 0.00 | 0.00 | 0.00 | 0.00 | 0.00 | 0.00 | 0.00 | 0.00 | ABC-type sugar transport system substrate-binding protein | Bacteria;Firmicutes;Clostridia;Eubacteriales;Eubacteriales Family XII. Incertae Sedis;Fusibacter;Fusibacter sp. 3D3;Fusibacter sp. 3D3 |
| Ga0586324 2014401 3 899 | 0.00 | 0.00 | 0.00 | 0.00 | 0.00 | 0.00 | 0.00 | 0.00 | 0.00 | 1162700.00 | 0.00 | 0.00 | hypothetical protein | Bacteria;Ignavibacteriae;Ignavibacteria;Ignavibacteriales;Melioribacteraceae;Melioribacter;Melioribacter roseus;Melioribacter roseus P3M |
| Ga0586324 2014498 1 900 | 576116.00 | 0.00 | 0.00 | 0.00 | 0.00 | 0.00 | 0.00 | 0.00 | 0.00 | 0.00 | 0.00 | 0.00 | hypothetical protein | Bacteria;Bacteroidetes;Bacteroidia;Marinilabiliales;Prolixibacteraceae;Prolixibacter;Prolixibacter bellariivorans;Prolixibacter bellariivorans ATCC BAA-1284 |
| Ga0586324 2017405 494 901 | 0.00 | 2352800.00 | 0.00 | 0.00 | 0.00 | 0.00 | 0.00 | 0.00 | 0.00 | 12166000.00 | 13904000.00 | 0.00 | hypothetical protein | Bacteria;Firmicutes;Clostridia;Thermosediminibacterales;Thermosediminibacteraceae;Thermosediminibacter;Thermosediminibacter oceani;Thermosediminibacter oceani JW/IW-1228P, DSM 16646 |
| Ga0586324 2023075 1 348 | 0.00 | 0.00 | 0.00 | 0.00 | 0.00 | 0.00 | 0.00 | 0.00 | 0.00 | 0.00 | 26752000.00 | 0.00 | rubrerythrin | Bacteria;Proteobacteria;Deltaproteobacteria;Desulfarculales;Desulfarculaceae;Desulfocarbo;Desulfocarbo indianensis;Desulfocarbo indianensis SCBM |
| Ga0586324 2024497 1 900 | 0.00 | 628829.32 | 0.00 | 0.00 | 0.00 | 0.00 | 0.00 | 0.00 | 6593400.00 | 0.00 | 0.00 | 0.00 | crotonobetainyl-CoA:carnitine CoA-transferase CaiB-like acyl-CoA transferase | Bacteria;Firmicutes;Clostridia;Thermoanaerobacterales;Thermoanaerobacteraceae;Desulfovirgula;Desulfovirgula thermocuniculi;Desulfovirgula thermocuniculi DSM 16036 |
| Ga0586324 2031541 329 898 | 0.00 | 0.00 | 0.00 | 0.00 | 2842800.00 | 0.00 | 0.00 | 0.00 | 0.00 | 3033800.00 | 0.00 | 0.00 | hypothetical protein | Bacteria;Proteobacteria;Gammaproteobacteria;Nevskiales;Steroidobacteraceae;Povalibacter;Povalibacter uvarum;Povalibacter uvarum DSM 26723 |
| Ga0586324 2035550 2 763 | 0.00 | 0.00 | 0.00 | 0.00 | 0.00 | 0.00 | 0.00 | 0.00 | 0.00 | 0.00 | 0.00 | 4470000.00 | hypothetical protein | Bacteria;Cyanobacteria;unclassified Cyanobacteria;Nostocales;Nostocaceae;Anabaena;Anabaena cylindrica;Anabaena cylindrica PCC 7122 |
| Ga0586324 2037903 2 895 | 15456106.00 | 10206800.00 | 0.00 | 43158500.00 | 14626000.00 | 16852380.04 | 0.00 | 2165500.00 | 866700.00 | 658461.65 | 0.00 | 0.00 | hypothetical protein | Bacteria;Proteobacteria;Deltaproteobacteria;Syntrophobacterales;Syntrophaceae;Syntrophus;Syntrophus aciditrophicus;Syntrophus aciditrophicus SB |
| Ga0586324 2038310 1 897 | 0.00 | 0.00 | 0.00 | 0.00 | 0.00 | 0.00 | 8299200.00 | 12566000.00 | 0.00 | 17232600.00 | 23056000.00 | 3300000.00 | NADPH-dependent glutamate synthase beta subunit-like oxidoreductase | Bacteria;Proteobacteria;Betaproteobacteria;Nitrosomonadales;Thiobacillaceae;Thiobacillus;Thiobacillus denitrificans;Thiobacillus denitrificans ATCC 25259 |
| Ga0586324 2039068 1 753 | 0.00 | 0.00 | 0.00 | 0.00 | 0.00 | 0.00 | 0.00 | 0.00 | 0.00 | 406926.52 | 0.00 | 0.00 | predicted dehydrogenase | Bacteria;Acidobacteria;Blastocatellia;Blastocatellales;Pyrinomonadaceae;Pyrinomonas;Pyrinomonas methylaliphatogenes;Pyrinomonas methylaliphatogenes K22 |
| Ga0586324 2044649 584 895 | 8430000.00 | 0.00 | 0.00 | 0.00 | 0.00 | 0.00 | 0.00 | 0.00 | 0.00 | 0.00 | 0.00 | 0.00 | predicted porin | Bacteria;Proteobacteria;Betaproteobacteria;Burkholderiales;Comamonadaceae;Acidovorax;Acidovorax sp. 62;Acidovorax sp. 62 |
| Ga0586324 2054240 3 821 | 953475.00 | 0.00 | 0.00 | 0.00 | 0.00 | 0.00 | 0.00 | 0.00 | 0.00 | 0.00 | 0.00 | 0.00 | hypothetical protein | Bacteria;Bacteroidetes;Cytophagia;Cytophagales;Cyclobacteriaceae;Cecembia;Cecembia calidifontis;Cecembia calidifontis DSM 21411 |
| Ga0586324 2054627 1 894 | 0.00 | 1203600.00 | 0.00 | 824532.55 | 4851300.00 | 0.00 | 0.00 | 0.00 | 0.00 | 0.00 | 0.00 | 0.00 | hypothetical protein | Bacteria;Spirochaetes;Spirochaetia;Leptospirales;Leptospiraceae;Leptonema;Leptonema illini;Leptonema illini 3055, DSM 21528 |
| Ga0586324 2054845 412 681 | 0.00 | 1815600.00 | 0.00 | 0.00 | 0.00 | 6987200.00 | 0.00 | 0.00 | 1976400.00 | 0.00 | 0.00 | 945000.00 | hypothetical protein | Bacteria;Proteobacteria;Deltaproteobacteria;unclassified Deltaproteobacteria;unclassified Deltaproteobacteria;unclassified Deltaproteobacteria;delta proteobacterium NaphS2;delta proteobacterium NaphS2 |
| Ga0586324 2057925 3 893 | 0.00 | 0.00 | 0.00 | 0.00 | 0.00 | 0.00 | 0.00 | 0.00 | 0.00 | 154000000.00 | 0.00 | 0.00 | predicted porin | Bacteria;Proteobacteria;Gammaproteobacteria;Chromatiales;Chromatiaceae;Thiorhodococcus;Thiorhodococcus drewsii;Thiorhodococcus drewsii AZ1 |
| Ga0586324 2059257 2 748 | 0.00 | 0.00 | 0.00 | 0.00 | 0.00 | 0.00 | 0.00 | 2989000.00 | 3248100.00 | 0.00 | 0.00 | 0.00 | opacity protein-like surface antigen | Bacteria;Verrucomicrobia;Verrucomicrobiae;Verrucomicrobiales;Verrucomicrobia subdivision 3;Pedosphaera;Pedosphaera parvula;Pedosphaera parvula Ellin514 |
| Ga0586324 2061808 2 892 | 0.00 | 0.00 | 0.00 | 0.00 | 0.00 | 0.00 | 0.00 | 0.00 | 0.00 | 722735.86 | 2411200.00 | 0.00 | hypothetical protein | |
| Ga0586324 2071498 1 402 | 0.00 | 0.00 | 0.00 | 950000.00 | 0.00 | 0.00 | 0.00 | 0.00 | 0.00 | 0.00 | 950400.00 | 0.00 | hypothetical protein | Bacteria;Acidobacteria;Acidobacteriia;Acidobacteriales;Acidobacteriaceae;unclassified Acidobacteriaceae;Acidobacteriaceae bacterium KBS 96;Acidobacteriaceae bacterium KBS 96, DSM 24297 |
| Ga0586324 2073414 1 888 | 0.00 | 0.00 | 0.00 | 0.00 | 0.00 | 0.00 | 0.00 | 7381000.00 | 0.00 | 0.00 | 0.00 | 1125000.00 | DNA-directed RNA polymerase beta subunit | Bacteria;Nitrospirae;unclassified Nitrospirae;unclassified Nitrospirae;unclassified Nitrospirae;unclassified Nitrospirae;Nitrospirae bacterium HCH-1;Nitrospirae bacterium HCH-1 |
| Ga0586324 2074905 322 888 | 6790000.00 | 6670800.00 | 0.00 | 10355000.00 | 10815000.00 | 1478638.41 | 0.00 | 5148400.00 | 0.00 | 1247400.00 | 1064800.00 | 1612500.00 | hypothetical protein | Bacteria;Nitrospirae;Nitrospira;Nitrospirales;Nitrospiraceae;Candidatus Magnetoovum;Candidatus Magnetoovum chiemensis;Candidatus Magnetoovum chiemensis CS-04 |
| Ga0586324 2080404 605 889 | 0.00 | 0.00 | 0.00 | 0.00 | 0.00 | 1496400.19 | 0.00 | 7564000.00 | 0.00 | 2879800.00 | 0.00 | 0.00 | predicted porin | Bacteria;Proteobacteria;Gammaproteobacteria;unclassified Gammaproteobacteria;Candidatus Competibacteraceae;Candidatus Contendobacter;Candidatus Contendobacter odensis;Candidatus Contendobacter odensis Run B J11 |
| Ga0586324 2081701 3 623 | 0.00 | 1727200.00 | 0.00 | 0.00 | 0.00 | 0.00 | 0.00 | 0.00 | 0.00 | 0.00 | 0.00 | 2062500.00 | hypothetical protein | Bacteria;Firmicutes;Bacilli;Bacillales;Paenibacillaceae;Paenibacillus;Paenibacillus donghaensis;Paenibacillus donghaensis KCTC 13049 |
| Ga0586324 2081730 2 889 | 0.00 | 0.00 | 0.00 | 0.00 | 0.00 | 15086000.00 | 0.00 | 0.00 | 0.00 | 0.00 | 0.00 | 0.00 | arylsulfatase A-like enzyme | Bacteria;Proteobacteria;Alphaproteobacteria;Hyphomicrobiales;Bradyrhizobiaceae;Bradyrhizobium;Bradyrhizobium sp. th.b2;Bradyrhizobium sp. Th.b2 |
| Ga0586324 2086814 1 765 | 0.00 | 0.00 | 0.00 | 3999500.00 | 0.00 | 0.00 | 0.00 | 1165100.00 | 0.00 | 0.00 | 0.00 | 0.00 | GAF domain-containing protein | Bacteria;Proteobacteria;Deltaproteobacteria;Syntrophobacterales;Syntrophaceae;Desulfobacca;Desulfobacca acetoxidans;Desulfobacca acetoxidans ASRB2, DSM 11109 |
| Ga0586324 2088181 1 888 | 0.00 | 0.00 | 0.00 | 0.00 | 0.00 | 0.00 | 1077977.16 | 391812.15 | 0.00 | 1062600.00 | 1610400.00 | 1380000.00 | hypothetical protein | Bacteria;Proteobacteria;Betaproteobacteria;Burkholderiales;Burkholderiaceae;Limnobacter;Limnobacter thiooxidans;Limnobacter thiooxidans DSM 13612 |
| Ga0586324 2089928 139 885 | 1140000.00 | 0.00 | 0.00 | 0.00 | 0.00 | 0.00 | 0.00 | 0.00 | 0.00 | 0.00 | 0.00 | 0.00 | predicted porin | Bacteria;Proteobacteria;Gammaproteobacteria;Cellvibrionales;Cellvibrionaceae;Cellvibrio;Cellvibrio sp. PSBB023;Cellvibrio sp. PSBB023 |
| Ga0586324 2090822 2 886 | 0.00 | 1482400.00 | 0.00 | 0.00 | 0.00 | 332343.39 | 0.00 | 0.00 | 0.00 | 0.00 | 0.00 | 0.00 | outer membrane receptor protein involved in Fe transport | Bacteria;Verrucomicrobia;Opitutae;Opitutales;Opitutaceae;Opitutus;Opitutus terrae;Opitutus terrae PB90-1 |
| Ga0586324 2094769 229 816 | 0.00 | 0.00 | 0.00 | 0.00 | 0.00 | 0.00 | 0.00 | 1226100.00 | 0.00 | 0.00 | 0.00 | 0.00 | hypothetical protein | Bacteria;Bacteroidetes;Flavobacteriia;Flavobacteriales;Weeksellaceae;Chryseobacterium;Chryseobacterium piperi;Chryseobacterium piperi CTM |
| Ga0586324 2098706 3 884 | 0.00 | 2305200.00 | 0.00 | 3705000.00 | 0.00 | 0.00 | 4924800.00 | 0.00 | 0.00 | 0.00 | 0.00 | 1410000.00 | ABC-type transport system substrate-binding protein | Bacteria;Chloroflexi;Caldilineae;Caldilineales;Caldilineaceae;Caldilinea;Caldilinea aerophila;Caldilinea aerophila STL-6-O1, DSM 14535 |
| Ga0586324 2100579 1 885 | 0.00 | 0.00 | 0.00 | 0.00 | 0.00 | 4089100.00 | 0.00 | 0.00 | 0.00 | 0.00 | 0.00 | 0.00 | ABC-type transport system substrate-binding protein | Bacteria;Proteobacteria;Deltaproteobacteria;Syntrophobacterales;Syntrophobacteraceae;Desulfacinum;Desulfacinum hydrothermale;Desulfacinum hydrothermale DSM 13146 |
| Ga0586324 2101278 340 885 | 0.00 | 0.00 | 0.00 | 0.00 | 0.00 | 0.00 | 0.00 | 0.00 | 0.00 | 0.00 | 0.00 | 930000.00 | TRAP-type mannitol/chloroaromatic compound transport system substrate-binding protein | Bacteria;Proteobacteria;Betaproteobacteria;Nitrosomonadales;Sulfuricellaceae;Sulfurirhabdus;Sulfurirhabdus autotrophica;Sulfurirhabdus autotrophica DSM 100309 |
| Ga0586324 2101863 3 884 | 0.00 | 3168800.00 | 0.00 | 7258000.00 | 5325100.00 | 0.00 | 0.00 | 3196400.00 | 2033100.00 | 0.00 | 0.00 | 0.00 | hypothetical protein | Bacteria;Acidobacteria;Vicinamibacteria;unclassified Vicinamibacteria;Vicinamibacteraceae;Luteitalea;Luteitalea pratensis;Luteitalea pratensis DSM 100886 |
| Ga0586324 2101954 384 884 | 0.00 | 0.00 | 0.00 | 0.00 | 0.00 | 0.00 | 1687022.16 | 0.00 | 611504.64 | 0.00 | 0.00 | 0.00 | methanogenic corrinoid protein MtbC1 | Bacteria;Proteobacteria;Deltaproteobacteria;Syntrophobacterales;Syntrophaceae;Desulfomonile;Desulfomonile tiedjei;Desulfomonile tiedjei DCB-1, DSM 6799 |
| Ga0586324 2102491 1 693 | 0.00 | 0.00 | 0.00 | 0.00 | 0.00 | 0.00 | 0.00 | 0.00 | 1101600.00 | 0.00 | 0.00 | 0.00 | uncharacterized protein (TIGR02001 family) | Bacteria;Proteobacteria;Betaproteobacteria;Nitrosomonadales;Gallionellaceae;Ferriphaselus;Ferriphaselus amnicola;Ferriphaselus amnicola OYT1 |
| Ga0586324 2104721 35 883 | 0.00 | 2488800.00 | 0.00 | 0.00 | 0.00 | 0.00 | 0.00 | 0.00 | 0.00 | 0.00 | 0.00 | 0.00 | hypothetical protein | Bacteria;Cyanobacteria;unclassified Cyanobacteria;Nostocales;Calotrichaceae;Calothrix;Calothrix sp. NIES-2100;Calothrix sp. NIES-2100 |
| Ga0586324 2106348 2 883 | 43800000.00 | 57395891.64 | 4842306.70 | 71673996.40 | 82220827.38 | 0.00 | 10123200.00 | 41785000.00 | 18111600.00 | 48664000.00 | 54594664.96 | 33959038.50 | TRAP-type mannitol/chloroaromatic compound transport system substrate-binding protein | Bacteria;Proteobacteria;Alphaproteobacteria;Hyphomicrobiales;Bradyrhizobiaceae;Bradyrhizobium;Bradyrhizobium elkanii;Bradyrhizobium elkanii WSM2783 |
| Ga0586324 2107450 3 836 | 0.00 | 0.00 | 0.00 | 0.00 | 0.00 | 0.00 | 6589200.00 | 1037000.00 | 0.00 | 0.00 | 1654400.00 | 0.00 | hypothetical protein | Bacteria;Proteobacteria;Betaproteobacteria;unclassified Betaproteobacteria;unclassified Betaproteobacteria;unclassified Betaproteobacteria;Betaproteobacteria bacterium UKL13-2;Betaproteobacteria bacterium UKL13-2 |
| Ga0586324 2107649 1 882 | 7770000.00 | 4195600.00 | 5126745.56 | 0.00 | 0.00 | 4883100.00 | 0.00 | 15982000.00 | 0.00 | 6699000.00 | 5341600.00 | 5707500.00 | hypothetical protein | Bacteria;Acidobacteria;Vicinamibacteria;unclassified Vicinamibacteria;Vicinamibacteraceae;Luteitalea;Luteitalea pratensis;Luteitalea pratensis DSM 100886 |
| Ga0586324 2108185 2 775 | 0.00 | 0.00 | 0.00 | 0.00 | 0.00 | 0.00 | 0.00 | 593093.85 | 0.00 | 0.00 | 0.00 | 0.00 | transcriptional regulator with PAS, ATPase and Fis domain | Bacteria;Verrucomicrobia;Verrucomicrobiae;Verrucomicrobiales;Verrucomicrobia subdivision 3;Pedosphaera;Pedosphaera parvula;Pedosphaera parvula Ellin514 |
| Ga0586324 2113673 246 881 | 0.00 | 1598000.00 | 0.00 | 0.00 | 0.00 | 0.00 | 0.00 | 0.00 | 0.00 | 0.00 | 0.00 | 0.00 | hypothetical protein | Bacteria;Proteobacteria;Deltaproteobacteria;Myxococcales;Anaeromyxobacteraceae;Anaeromyxobacter;Anaeromyxobacter sp. Fw109-5;Anaeromyxobacter sp. Fw109-5 |
| Ga0586324 2115497 3 881 | 0.00 | 0.00 | 0.00 | 0.00 | 0.00 | 0.00 | 0.00 | 1220000.00 | 0.00 | 0.00 | 0.00 | 0.00 | hypothetical protein | Bacteria;Acidobacteria;Holophagae;Holophagales;Holophagaceae;Geothrix;Geothrix fermentans;Geothrix fermentans DSM 14018 |
| Ga0586324 2116229 3 743 | 0.00 | 0.00 | 0.00 | 2546000.00 | 2235100.00 | 0.00 | 0.00 | 0.00 | 0.00 | 0.00 | 0.00 | 0.00 | uncharacterized membrane protein | Bacteria;Actinobacteria;Actinomycetia;Streptomycetales;Streptomycetaceae;Streptomyces;Streptomyces sp. NRRL S-149;Streptomyces sp. NRRL S-149 |
| Ga0586324 2117842 2 883 | 0.00 | 0.00 | 0.00 | 0.00 | 0.00 | 0.00 | 0.00 | 3263500.00 | 0.00 | 0.00 | 0.00 | 0.00 | hypothetical protein | Bacteria;Proteobacteria;Alphaproteobacteria;Rhodospirillales;Rhodospirillaceae;Dongia;Dongia sp. URHE0060;Dongia sp. URHE0060 |
| Ga0586324 2118604 2 880 | 0.00 | 635808.16 | 0.00 | 0.00 | 0.00 | 0.00 | 0.00 | 0.00 | 0.00 | 1108800.00 | 0.00 | 518082.00 | hypothetical protein | |
| Ga0586324 2121436 439 882 | 2100000.00 | 73607.28 | 480791.87 | 14630000.00 | 69319000.00 | 17190100.00 | 0.00 | 18910000.00 | 46575000.00 | 689717.49 | 7594400.00 | 6855000.00 | hypothetical protein | |
| Ga0586324 2128572 3 461 | 0.00 | 0.00 | 0.00 | 0.00 | 0.00 | 0.00 | 0.00 | 0.00 | 915300.00 | 0.00 | 0.00 | 0.00 | nucleotide-binding universal stress UspA family protein | Bacteria;Proteobacteria;Betaproteobacteria;Rhodocyclales;Zoogloeaceae;unclassified Zoogloeaceae;Zoogloeaceae bacterium Par-f-2;Zoogloeaceae bacterium Par-f-2 |
| Ga0586324 2136697 3 767 | 0.00 | 0.00 | 0.00 | 2793000.00 | 0.00 | 0.00 | 0.00 | 0.00 | 0.00 | 0.00 | 0.00 | 0.00 | MoxR-like ATPase | Bacteria;Spirochaetes;Spirochaetia;Spirochaetales;Spirochaetaceae;Alkalispirochaeta;Alkalispirochaeta odontotermitis;Alkalispirochaeta odontotermitis JC202 |
| Ga0586324 2140075 2 814 | 0.00 | 0.00 | 0.00 | 0.00 | 0.00 | 0.00 | 0.00 | 0.00 | 0.00 | 7184100.00 | 0.00 | 0.00 | NADPH-dependent glutamate synthase beta subunit-like oxidoreductase | Bacteria;Proteobacteria;Betaproteobacteria;Burkholderiales;unclassified Burkholderiales;Ideonella;Ideonella sp. A 288;Ideonella sp. A 288 |
| Ga0586324 2141407 3 575 | 0.00 | 0.00 | 0.00 | 0.00 | 0.00 | 0.00 | 0.00 | 878400.00 | 0.00 | 0.00 | 0.00 | 0.00 | hypothetical protein | Bacteria;Proteobacteria;Gammaproteobacteria;Methylococcales;Methylococcaceae;Methylobacter;Methylobacter luteus;Methylobacter luteus IMV-B-3098 |
| Ga0586324 2145006 300 692 | 7430000.00 | 0.00 | 0.00 | 0.00 | 0.00 | 0.00 | 0.00 | 0.00 | 0.00 | 0.00 | 0.00 | 0.00 | CBS domain-containing protein | Bacteria;Proteobacteria;Gammaproteobacteria;Methylococcales;Methylococcaceae;Methylovulum;Methylovulum miyakonense;Methylovulum miyakonense HT12 |
| Ga0586324 2145052 3 878 | 942276.00 | 1319200.00 | 0.00 | 2888000.00 | 3213600.00 | 0.00 | 0.00 | 701500.00 | 0.00 | 0.00 | 0.00 | 1462500.00 | hypothetical protein | Bacteria;Acidobacteria;Vicinamibacteria;unclassified Vicinamibacteria;Vicinamibacteraceae;Luteitalea;Luteitalea pratensis;Luteitalea pratensis DSM 100886 |
| Ga0586324 2145423 1 696 | 0.00 | 0.00 | 0.00 | 0.00 | 0.00 | 0.00 | 0.00 | 0.00 | 0.00 | 0.00 | 4549600.00 | 0.00 | Flp pilus assembly protein TadB | |
| Ga0586324 2146840 2 877 | 4890000.00 | 0.00 | 0.00 | 0.00 | 0.00 | 0.00 | 0.00 | 0.00 | 0.00 | 0.00 | 0.00 | 3472500.00 | hypothetical protein | Bacteria;Cyanobacteria;unclassified Cyanobacteria;Nostocales;Nostocaceae;Nostoc;Nostoc piscinale;Nostoc piscinale CENA21 |
| Ga0586324 2149807 110 877 | 0.00 | 44268000.00 | 2230324.50 | 3980500.00 | 24853900.00 | 0.00 | 1291145.76 | 878400.00 | 0.00 | 0.00 | 0.00 | 0.00 | hypothetical protein | Bacteria;Proteobacteria;Gammaproteobacteria;Methylococcales;Methylococcaceae;Methylomicrobium;Methylomicrobium agile;Methylomicrobium agile ATCC 35068 |
| Ga0586324 2149954 3 875 | 0.00 | 2278000.00 | 0.00 | 0.00 | 0.00 | 0.00 | 0.00 | 0.00 | 0.00 | 0.00 | 0.00 | 0.00 | ABC-type sugar transport system substrate-binding protein | Bacteria;Chloroflexi;Caldilineae;Caldilineales;Caldilineaceae;Caldilinea;Caldilinea aerophila;Caldilinea aerophila STL-6-O1, DSM 14535 |
| Ga0586324 2154412 572 874 | 0.00 | 0.00 | 0.00 | 0.00 | 0.00 | 6629900.00 | 0.00 | 2952400.00 | 0.00 | 0.00 | 0.00 | 0.00 | acyl-coenzyme A synthetase/AMP-(fatty) acid ligase | Bacteria;Proteobacteria;Deltaproteobacteria;Syntrophobacterales;Syntrophaceae;Desulfobacca;Desulfobacca acetoxidans;Desulfobacca acetoxidans ASRB2, DSM 11109 |

| | | | | | | | | | | | | | | | |
|---|---|---|---|---|---|---|---|---|---|---|---|---|---|---|---|
| Ga0586324 2156704 3 875 | 0.00 | 0.00 | 0.00 | 3885500.00 | 0.00 | 0.00 | 0.00 | 2793800.00 | 2494800.00 | 0.00 | 0.00 | 0.00 | | cytochrome c peroxidase | Bacteria;Proteobacteria;Deltaproteobacteria;Desulfovibrionales;Desulfovibrionaceae;Desulfohalovibrio;Desulfohalovibrio alkalitolerans;Desulfovibrio alkalitolerans DSM 16529 |
| Ga0586324 2165615 2 874 | 0.00 | 0.00 | 0.00 | 0.00 | 0.00 | 0.00 | 0.00 | 0.00 | 0.00 | 1963500.00 | 0.00 | 0.00 | | signal transduction histidine kinase | Bacteria;Proteobacteria;Alphaproteobacteria;Maricaulales;Maricaulaceae;Euryhalocaulis;Euryhalocaulis caribicus;Euryhalocaulis caribicus JL2009 |
| Ga0586324 2168727 3 848 | 6510000.00 | 7792800.00 | 10134000.00 | 4389000.00 | 0.00 | 3807305.43 | 14614800.00 | 18544000.00 | 13883400.00 | 15453900.00 | 19096000.00 | 15412500.00 | | predicted porin | Bacteria;Proteobacteria;Betaproteobacteria;Nitrosomonadales;Thiobacillaceae;Thiobacillus;Thiobacillus denitrificans;Thiobacillus denitrificans ATCC 25259 |
| Ga0586324 2171270 3 872 | 18590000.00 | 12938827.84 | 17589285.41 | 15409000.00 | 17705700.00 | 12376244.74 | 7785292.56 | 3074400.00 | 7549200.00 | 19457900.00 | 14546400.00 | 17167500.00 | | hypothetical protein | Bacteria;Proteobacteria;Betaproteobacteria;Burkholderiales;Comamonadaceae;Acidovorax;Acidovorax sp. JS42;Acidovorax sp. JS42 |
| Ga0586324 2175162 3 872 | 1380000.00 | 0.00 | 0.00 | 0.00 | 0.00 | 0.00 | 0.00 | 0.00 | 0.00 | 0.00 | 0.00 | 0.00 | | hypothetical protein | |
| Ga0586324 2178557 76 864 | 3530000.00 | 0.00 | 0.00 | 0.00 | 0.00 | 0.00 | 0.00 | 0.00 | 0.00 | 0.00 | 0.00 | 0.00 | | uncharacterized linocin/CFP29 family protein | Archaea;Euryarchaeota;Methanomicrobia;Methanomicrobiales;Methanoregulaceae;Methanosphaerula;Methanosphaerula palustris;Methanosphaerula palustris E1-9c, DSM 19958 |
| Ga0586324 2179249 1 870 | 0.00 | 449834.28 | 0.00 | 0.00 | 0.00 | 0.00 | 0.00 | 0.00 | 0.00 | 0.00 | 731728.80 | 253566.00 | | hypothetical protein | Bacteria;Ignavibacteriae;Ignavibacteria;Ignavibacteriales;Ignavibacteriaceae;Ignavibacterium;Ignavibacterium album;Ignavibacterium album Mat9-16, JCM 16511 |
| Ga0586324 2183609 1 870 | 0.00 | 348339.52 | 0.00 | 0.00 | 0.00 | 0.00 | 0.00 | 0.00 | 0.00 | 0.00 | 0.00 | 0.00 | | TRAP-type mannitol/chloroaromatic compound transport system substrate-binding protein | Bacteria;Proteobacteria;Alphaproteobacteria;Hyphomicrobiales;Hyphomicrobiaceae;Hyphomicrobium;Hyphomicrobium nitrativorans;Hyphomicrobium nitrativorans NL23 |
| Ga0586324 2187337 37 870 | 0.00 | 0.00 | 0.00 | 0.00 | 0.00 | 0.00 | 0.00 | 0.00 | 0.00 | 0.00 | 0.00 | 1402500.00 | | hypothetical protein | |
| Ga0586324 2191410 1 825 | 0.00 | 2536400.00 | 0.00 | 0.00 | 0.00 | 0.00 | 0.00 | 0.00 | 0.00 | 0.00 | 0.00 | 0.00 | | hypothetical protein | Bacteria;Proteobacteria;Deltaproteobacteria;Syntrophobacterales;Syntrophaceae;Desulfobacca;Desulfobacca acetoxidans;Desulfobacca acetoxidans ASRB2, DSM 11109 |
| Ga0586324 2191575 1 870 | 0.00 | 1754400.00 | 99096.45 | 3173000.00 | 3615300.00 | 0.00 | 911049.24 | 1634800.00 | 1571400.00 | 2440900.00 | 1856800.00 | 1215000.00 | | hypothetical protein | |
| Ga0586324 2197211 1 867 | 0.00 | 0.00 | 0.00 | 0.00 | 0.00 | 7741500.00 | 0.00 | 0.00 | 0.00 | 0.00 | 0.00 | 0.00 | | arylsulfatase A-like enzyme | Bacteria;Proteobacteria;Alphaproteobacteria;Hyphomicrobiales;Bradyrhizobiaceae;Bradyrhizobium;Bradyrhizobium neotropicale;Bradyrhizobium neotropicale BR 10247 |
| Ga0586324 2201727 2 868 | 0.00 | 1305600.00 | 0.00 | 0.00 | 0.00 | 0.00 | 0.00 | 551978.02 | 0.00 | 2479400.00 | 0.00 | 1305000.00 | | hypothetical protein | |
| Ga0586324 2203265 416 868 | 0.00 | 0.00 | 5080528.89 | 0.00 | 0.00 | 0.00 | 0.00 | 0.00 | 0.00 | 0.00 | 700305.76 | 3622500.00 | | predicted AlkP superfamily pyrophosphatase or phosphodiesterase | Bacteria;Actinobacteria;Actinomycetia;Micrococcales;Intrasporangiaceae;Intrasporangium;Intrasporangium oryzae;Intrasporangium oryzae NRRL B-24470 |
| Ga0586324 2203764 3 866 | 1980000.00 | 0.00 | 0.00 | 4294000.00 | 0.00 | 0.00 | 4058400.00 | 3641700.00 | 0.00 | 0.00 | 0.00 | 0.00 | | hypothetical protein | |
| Ga0586324 2207454 3 779 | 0.00 | 16592000.00 | 0.00 | 25745000.00 | 30900000.00 | 0.00 | 0.00 | 23119000.00 | 31185000.00 | 26180000.00 | 22000000.00 | 7725000.00 | | hypothetical protein | Bacteria;Proteobacteria;Alphaproteobacteria;Hyphomicrobiales;unclassified Hyphomicrobiales;Nordella;Nordella sp. OAE833;Nordella sp. OAE833 |
| Ga0586324 2209801 1 774 | 0.00 | 2332400.00 | 3805283.22 | 0.00 | 0.00 | 0.00 | 0.00 | 0.00 | 0.00 | 0.00 | 0.00 | 0.00 | | ABC-type sugar transport system substrate-binding protein | Bacteria;Chloroflexi;Caldilineae;Caldilineales;Caldilineaceae;Caldilinea;Caldilinea aerophila;Caldilinea aerophila STL-6-O1, DSM 14535 |
| Ga0586324 2216085 3 866 | 0.00 | 0.00 | 0.00 | 0.00 | 0.00 | 0.00 | 11628000.00 | 0.00 | 0.00 | 0.00 | 0.00 | 0.00 | | ATPase subunit of ABC transporter with duplicated ATPase domains | Bacteria;Proteobacteria;Alphaproteobacteria;Hyphomicrobiales;Xanthobacteraceae;Pseudolabrys;Pseudolabrys sp. Root1462;Pseudolabrys sp. Root1462 |
| Ga0586324 2222635 250 864 | 3120000.00 | 570391.48 | 0.00 | 463998.05 | 4851300.00 | 0.00 | 0.00 | 5673000.00 | 0.00 | 5705700.00 | 391903.60 | 305453.25 | | hypothetical protein | Bacteria;Verrucomicrobia;Verrucomicrobiae;Verrucomicrobiales;Verrucomicrobia subdivision 3;Pedosphaera;Pedosphaera parvula;Pedosphaera parvula Ellin514 |
| Ga0586324 2222960 3 827 | 0.00 | 986000.00 | 0.00 | 0.00 | 0.00 | 0.00 | 0.00 | 0.00 | 0.00 | 0.00 | 0.00 | 1005000.00 | | hypothetical protein | Bacteria;Acidobacteria;Acidobacteriia;Acidobacteriales;Acidobacteriaceae;Acidobacterium;Acidobacterium ailaaui;Acidobacterium ailaaui PMMR2 |
| Ga0586324 2224652 1 864 | 0.00 | 0.00 | 0.00 | 0.00 | 0.00 | 0.00 | 0.00 | 0.00 | 0.00 | 6098400.00 | 0.00 | 0.00 | | hypothetical protein | Bacteria;Proteobacteria;Alphaproteobacteria;Rhodospirillales;Azospirillaceae;Azospirillum;Azospirillum baldaniorum;Azospirillum baldaniorum Sp245 |
| Ga0586324 2225761 497 862 | 4950000.00 | 3767200.00 | 4177094.05 | 5339000.00 | 5047000.00 | 0.00 | 4833600.00 | 3489200.00 | 2956500.00 | 2533300.00 | 0.00 | 3307500.00 | | predicted porin | Bacteria;Proteobacteria;Betaproteobacteria;Burkholderiales;Comamonadaceae;Caldimonas;Caldimonas taiwanensis;Caldimonas taiwanensis NBRC 104434 |
| Ga0586324 2232517 1 861 | 685004.00 | 0.00 | 0.00 | 3458000.00 | 0.00 | 0.00 | 0.00 | 0.00 | 0.00 | 0.00 | 0.00 | 0.00 | | hypothetical protein | Bacteria;Proteobacteria;Gammaproteobacteria;Cellvibrionales;Halieaceae;Pseudohaliea;Pseudohaliea rubra;Pseudohaliea rubra DSM 19751 |
| Ga0586324 2234628 469 861 | 0.00 | 9112000.00 | 10302900.00 | 11305000.00 | 0.00 | 11711500.00 | 0.00 | 0.00 | 0.00 | 0.00 | 0.00 | 7650000.00 | | hypothetical protein | Bacteria;Acidobacteria;Blastocatellia;unclassified Blastocatellia;unclassified Blastocatellia;Chloracidobacterium;Chloracidobacterium thermophilum;Chloracidobacterium thermophilum B |
| Ga0586324 2245556 287 715 | 12000000.00 | 0.00 | 3277999.94 | 7058500.00 | 36771000.00 | 42479000.00 | 7569600.00 | 6893000.00 | 6172200.00 | 0.00 | 0.00 | 6922500.00 | | hypothetical protein | Bacteria;Proteobacteria;Deltaproteobacteria;Syntrophobacterales;Syntrophobacteraceae;Desulfacinum;Desulfacinum hydrothermale;Desulfacinum hydrothermale DSM 13146 |
| Ga0586324 2247443 326 859 | 0.00 | 0.00 | 0.00 | 11685000.00 | 13905000.00 | 10322000.00 | 8208000.00 | 7503000.00 | 6123600.00 | 0.00 | 0.00 | 0.00 | | hypothetical protein | Bacteria;Firmicutes;Clostridia;Eubacteriales;unclassified Eubacteriales;unclassified Eubacteriales;Clostridiales bacterium PH28  bin88;Clostridiales bacterium PH28  bin88 |
| Ga0586324 2250130 363 860 | 0.00 | 0.00 | 0.00 | 0.00 | 0.00 | 8456100.00 | 0.00 | 0.00 | 0.00 | 0.00 | 0.00 | 0.00 | | Pyruvate/2-oxoacid:ferredoxin oxidoreductase gamma subunit | Bacteria;Firmicutes;Clostridia;Thermoanaerobacterales;Thermoanaerobacteraceae;Thermanaeromonas;Thermanaeromonas toyohensis;Thermanaeromonas toyohensis ToBE, DSM 14490 |
| Ga0586324 2252457 324 860 | 0.00 | 0.00 | 0.00 | 0.00 | 0.00 | 0.00 | 0.00 | 7991000.00 | 0.00 | 0.00 | 0.00 | 0.00 | | predicted porin | Bacteria;Proteobacteria;Gammaproteobacteria;unclassified Gammaproteobacteria;unclassified Gammaproteobacteria;unclassified Gammaproteobacteria;Codakia orbicularis gill symbiont;Codakia orbicularis gill symbiont |
| Ga0586324 2253379 1 786 | 0.00 | 0.00 | 0.00 | 0.00 | 0.00 | 0.00 | 0.00 | 0.00 | 0.00 | 513641.59 | 0.00 | 0.00 | | tetratricopeptide (TPR) repeat protein | Bacteria;Bacteroidetes;Bacteroidia;Marinilabiliales;Marinilabiliaceae;Saccharicrinis;Saccharicrinis fermentans;Saccharicrinis fermentans DSM 9555 |
| Ga0586324 2254475 398 859 | 2290000.00 | 795600.00 | 0.00 | 1311000.00 | 1339000.00 | 0.00 | 0.00 | 383833.35 | 121648.23 | 0.00 | 0.00 | 197721.00 | | TRAP-type mannitol/chloroaromatic compound transport system substrate-binding protein | Bacteria;Proteobacteria;Alphaproteobacteria;Hyphomicrobiales;Hyphomicrobiaceae;Rhodomicrobium;Rhodomicrobium vannielii;Rhodomicrobium vannielii R  RK  2 |
| Ga0586324 2254945 1 858 | 703655.00 | 3291200.00 | 0.00 | 4085000.00 | 0.00 | 7143411.56 | 10351200.00 | 4019900.00 | 3240000.00 | 2517900.00 | 2085600.00 | 1942500.00 | | hypothetical protein | Bacteria;Proteobacteria;Deltaproteobacteria;Syntrophobacterales;Syntrophobacteraceae;Desulfosoma;Desulfosoma caldarium;Desulfosoma caldarium DSM 22027 |
| Ga0586324 2271121 3 857 | 3480000.00 | 8228000.00 | 14591192.18 | 4161000.00 | 5582600.00 | 11116000.00 | 0.00 | 0.00 | 0.00 | 0.00 | 0.00 | 2842500.00 | | FtsP/CotA-like multicopper oxidase with cupredoxin domain | Bacteria;Proteobacteria;Gammaproteobacteria;Methylococcales;Methylococcaceae;Methylosarcina;Methylosarcina fibrata;Methylosarcina fibrata AML-C10 |
| Ga0586324 2272996 338 775 | 0.00 | 0.00 | 0.00 | 0.00 | 0.00 | 0.00 | 0.00 | 0.00 | 0.00 | 0.00 | 4769600.00 | 3135000.00 | | flavorubredoxin | Bacteria;Proteobacteria;Deltaproteobacteria;Syntrophobacterales;Syntrophobacteraceae;Desulfacinum;Desulfacinum hydrothermale;Desulfacinum hydrothermale DSM 13146 |
| Ga0586324 2300210 3 851 | 0.00 | 4617200.00 | 0.00 | 0.00 | 4490800.00 | 0.00 | 8025600.00 | 0.00 | 0.00 | 0.00 | 0.00 | 0.00 | | hypothetical protein | |
| Ga0586324 2309659 1 849 | 12000000.00 | 3855600.00 | 0.00 | 6479000.00 | 0.00 | 0.00 | 6338400.00 | 0.00 | 0.00 | 0.00 | 0.00 | 0.00 | | ABC-type xylose transport system substrate-binding protein | Bacteria;Chloroflexi;Anaerolineae;Anaerolineales;Anaerolineaceae;Pelolinea;Pelolinea submarina;Pelolinea submarina DSM 23923 |
| Ga0586324 2312748 2 718 | 0.00 | 6759200.00 | 0.00 | 8550000.00 | 0.00 | 0.00 | 0.00 | 6588000.00 | 6423300.00 | 0.00 | 6292000.00 | 6337500.00 | | opacity protein-like surface antigen | Bacteria;Proteobacteria;Deltaproteobacteria;Syntrophobacterales;Syntrophaceae;Desulfobacca;Desulfobacca acetoxidans;Desulfobacca acetoxidans ASRB2, DSM 11109 |
| Ga0586324 2314049 529 849 | 0.00 | 0.00 | 0.00 | 0.00 | 4006700.00 | 0.00 | 1709961.24 | 4422500.00 | 2057400.00 | 5266800.00 | 0.00 | 4597500.00 | | predicted porin | Bacteria;Proteobacteria;Betaproteobacteria;unclassified Betaproteobacteria;unclassified Betaproteobacteria;unclassified Betaproteobacteria;uncultured beta proteobacterium;unclassified betaproteobacterium ENR4 |
| Ga0586324 2319744 2 847 | 3040000.00 | 0.00 | 0.00 | 0.00 | 0.00 | 4565500.00 | 0.00 | 8302100.00 | 4422600.00 | 1524600.00 | 0.00 | 0.00 | | hypothetical protein | Bacteria;Proteobacteria;Deltaproteobacteria;Desulfuromonadales;Geobacteraceae;Geobacter;Geobacter uraniireducens;Geobacter uraniireducens Rf4 |
| Ga0586324 2326094 1 762 | 0.00 | 0.00 | 0.00 | 0.00 | 0.00 | 2974355.76 | 0.00 | 0.00 | 0.00 | 0.00 | 0.00 | 0.00 | | uncharacterized protein YbaA (DUF1428 family) | Bacteria;Proteobacteria;Alphaproteobacteria;Hyphomicrobiales;Reyranellaceae;Reyranella;Reyranella massiliensis;Reyranella massiliensis 521, DSM 23428 |
| Ga0586324 2326338 2 847 | 0.00 | 1489200.00 | 0.00 | 0.00 | 0.00 | 0.00 | 0.00 | 0.00 | 0.00 | 0.00 | 0.00 | 1147500.00 | | hypothetical protein | |
| Ga0586324 2329767 144 848 | 0.00 | 0.00 | 5203741.44 | 3553000.00 | 4933700.00 | 0.00 | 6589200.00 | 0.00 | 3701700.00 | 0.00 | 3423200.00 | 2797500.00 | | NAD(P)-dependent dehydrogenase (short-subunit alcohol dehydrogenase family) | Bacteria;Proteobacteria;Alphaproteobacteria;Rhodospirillales;Acetobacteraceae;Rhodopila;unclassified Rhodopila;Rhodopila sp. LVNP |
| Ga0586324 2342382 183 455 | 0.00 | 0.00 | 20324300.00 | 0.00 | 0.00 | 0.00 | 0.00 | 0.00 | 0.00 | 0.00 | 0.00 | 0.00 | | hypothetical protein | Archaea;Euryarchaeota;Methanomicrobia;Methanosarcinales;Methanotrichaceae;Methanothrix;Methanothrix soehngenii;Methanothrix soehngenii GP-6 |
| Ga0586324 2343064 1 846 | 18230000.00 | 26125600.00 | 54069540.38 | 0.00 | 3574100.00 | 14411100.00 | 10374000.00 | 0.00 | 13381200.00 | 7561400.00 | 0.00 | 9345000.00 | | S-layer protein (TIGR01567 family) | Archaea;Euryarchaeota;Methanomicrobia;Methanosarcinales;Methanotrichaceae;Methanothrix;Methanosaeta harundinacea;Methanothrix harundinacea 6Ac |
| Ga0586324 2344751 240 845 | 0.00 | 0.00 | 0.00 | 0.00 | 1122700.00 | 0.00 | 0.00 | 0.00 | 0.00 | 0.00 | 0.00 | 0.00 | | Ca2+-binding RTX toxin-like protein | Bacteria;Cyanobacteria;unclassified Cyanobacteria;Chroococcales;Microcystaceae;Microcystis;Microcystis aeruginosa;Microcystis aeruginosa NIES-87 |
| Ga0586324 2346331 118 843 | 0.00 | 0.00 | 0.00 | 3743000.00 | 0.00 | 0.00 | 0.00 | 0.00 | 0.00 | 0.00 | 0.00 | 0.00 | | glutamate synthase domain-containing protein 2 | Bacteria;Proteobacteria;Deltaproteobacteria;Desulfarculales;Desulfarculaceae;Desulfocarbo;Desulfocarbo indianensis;Desulfocarbo indianensis SCBM |
| Ga0586324 2366441 1 840 | 0.00 | 0.00 | 0.00 | 150005.95 | 0.00 | 0.00 | 0.00 | 0.00 | 0.00 | 0.00 | 0.00 | 0.00 | | hypothetical protein | Bacteria;Actinobacteria;Actinomycetia;Streptomycetales;Streptomycetaceae;Streptomyces;Streptomyces odonnellii;Streptomyces odonnellii NRRL B-24891 |
| Ga0586324 2366627 321 842 | 11200000.00 | 0.00 | 0.00 | 0.00 | 0.00 | 23859700.00 | 0.00 | 0.00 | 0.00 | 0.00 | 0.00 | 0.00 | | adenylate kinase family enzyme | Archaea;Euryarchaeota;Methanomicrobia;Methanosarcinales;Methanotrichaceae;Methanothrix;Methanothrix soehngenii;Methanothrix soehngenii GP-6 |
| Ga0586324 2367798 3 842 | 2930000.00 | 5752800.00 | 3431479.37 | 0.00 | 0.00 | 1534385.15 | 0.00 | 2720600.00 | 0.00 | 0.00 | 3511200.00 | 3105000.00 | | hypothetical protein | Bacteria;Nitrospirae;Thermodesulfovibrionia;Thermodesulfovibrionales;Thermodesulfovibrionaceae;Thermodesulfovibrio;Thermodesulfovibrio hydrogeniphilus;Thermodesulfovibrio hydrogeniphilus DSM 18151 |
| Ga0586324 2369402 186 548 | 0.00 | 0.00 | 0.00 | 0.00 | 0.00 | 0.00 | 0.00 | 0.00 | 0.00 | 0.00 | 0.00 | 2407500.00 | | RNA recognition motif-containing protein | Bacteria;Proteobacteria;Deltaproteobacteria;Syntrophobacterales;Syntrophaceae;Desulfobacca;Desulfobacca acetoxidans;Desulfobacca acetoxidans ASRB2, DSM 11109 |
| Ga0586324 2379708 1 840 | 0.00 | 22304000.00 | 0.00 | 0.00 | 0.00 | 0.00 | 0.00 | 0.00 | 0.00 | 0.00 | 0.00 | 0.00 | | hypothetical protein | Bacteria;Proteobacteria;Gammaproteobacteria;Chromatiales;Chromatiaceae;Thiorhodococcus;Thiorhodococcus drewsii;Thiorhodococcus drewsii AZ1 |
| Ga0586324 2379962 59 838 | 0.00 | 0.00 | 0.00 | 1368000.00 | 0.00 | 0.00 | 0.00 | 0.00 | 0.00 | 0.00 | 0.00 | 0.00 | | hypothetical protein | Archaea;Euryarchaeota;Methanomicrobia;Methanosarcinales;Methanotrichaceae;Methanothrix;Methanothrix soehngenii;Methanothrix soehngenii GP-6 |
| Ga0586324 2388315 3 683 | 0.00 | 0.00 | 0.00 | 693932.25 | 0.00 | 0.00 | 0.00 | 0.00 | 0.00 | 415647.54 | 0.00 | 0.00 | | nucleoid-associated protein YgaU | Bacteria;Candidatus Latescibacteria;unclassified Candidatus Latescibacteria;unclassified Candidatus Latescibacteria;unclassified Candidatus Latescibacteria;unclassified Candidatus Latescibacteria;Latescibacteria bacterium SCGC AAA257-K07;Latescibacteria bacterium SCGC AAA257-K07 (contamination screened) |
| Ga0586324 2388983 3 668 | 0.00 | 0.00 | 0.00 | 0.00 | 0.00 | 0.00 | 49020000.00 | 0.00 | 0.00 | 0.00 | 0.00 | 0.00 | | opacity protein-like surface antigen | Bacteria;Proteobacteria;Deltaproteobacteria;Syntrophobacterales;Syntrophaceae;Desulfobacca;Desulfobacca acetoxidans;Desulfobacca acetoxidans ASRB2, DSM 11109 |
| Ga0586324 2392586 495 836 | 83500000.00 | 32300000.00 | 68123000.00 | 47690000.00 | 51500000.00 | 56374000.00 | 25080000.00 | 25559000.00 | 22356000.00 | 30492000.00 | 30184000.00 | 32850000.00 | | phasin family protein | Bacteria;Proteobacteria;Gammaproteobacteria;unclassified Gammaproteobacteria;Candidatus Competibacteraceae;Candidatus Contendobacter;Candidatus Contendobacter odensis;Candidatus Contendobacter odensis Run  B  J11 |
| Ga0586324 2393178 2 838 | 0.00 | 0.00 | 0.00 | 0.00 | 0.00 | 0.00 | 0.00 | 0.00 | 0.00 | 0.00 | 0.00 | 1387500.00 | | hypothetical protein | Bacteria;Proteobacteria;Deltaproteobacteria;Syntrophobacterales;Syntrophaceae;Desulfomonile;Desulfomonile tiedjei;Desulfomonile tiedjei DCB-1, DSM 6799 |
| Ga0586324 2399817 3 680 | 0.00 | 0.00 | 2807371.35 | 0.00 | 0.00 | 0.00 | 0.00 | 0.00 | 1490400.00 | 0.00 | 1953600.00 | 0.00 | | predicted porin | Bacteria;Proteobacteria;Betaproteobacteria;Burkholderiales;unclassified Burkholderiales;Leptothrix;Leptothrix cholodnii;Leptothrix cholodnii SP-6 |
| Ga0586324 2401007 3 836 | 0.00 | 938400.00 | 0.00 | 0.00 | 3687400.00 | 0.00 | 0.00 | 0.00 | 1725300.00 | 0.00 | 0.00 | 0.00 | | nucleotide-binding universal stress UspA family protein | Archaea;Euryarchaeota;Methanomicrobia;Methanosarcinales;Methanotrichaceae;Methanothrix;Methanothrix soehngenii;Methanothrix soehngenii GP-6 |
| Ga0586324 2402590 2 835 | 0.00 | 0.00 | 0.00 | 99750000.00 | 0.00 | 0.00 | 0.00 | 0.00 | 0.00 | 0.00 | 0.00 | 0.00 | | TonB-dependent SusC/RagA subfamily outer membrane receptor | Bacteria;Bacteroidetes;Cytophagia;Cytophagales;Cytophagaceae;Spirosoma;Spirosoma radiotolerans;Spirosoma radiotolerans DG5A |
| Ga0586324 2408819 1 834 | 0.00 | 0.00 | 0.00 | 0.00 | 0.00 | 0.00 | 0.00 | 0.00 | 0.00 | 0.00 | 0.00 | 1372500.00 | | hypothetical protein | Bacteria;Acidobacteria;Acidobacteriia;Acidobacteriales;Acidobacteriaceae;unclassified Acidobacteriaceae;Acidobacteriaceae bacterium TAA 166;Acidobacteriaceae bacterium TAA 166 |
| Ga0586324 2410994 289 834 | 4240000.00 | 7888000.00 | 5630000.00 | 5443500.00 | 5592900.00 | 0.00 | 0.00 | 6466000.00 | 0.00 | 0.00 | 0.00 | 0.00 | | predicted porin | Bacteria;Proteobacteria;Betaproteobacteria;Burkholderiales;Comamonadaceae;Ramlibacter;Ramlibacter sp. Leaf400;Ramlibacter sp. Leaf400 |
| Ga0586324 2413928 3 833 | 0.00 | 0.00 | 0.00 | 852988.85 | 0.00 | 4089100.00 | 0.00 | 0.00 | 0.00 | 0.00 | 0.00 | 0.00 | | crotonobetainyl-CoA:carnitine CoA-transferase CaiB-like acyl-CoA transferase | Bacteria;Proteobacteria;Deltaproteobacteria;Syntrophobacterales;Syntrophaceae;Desulfobacca;Desulfobacca acetoxidans;Desulfobacca acetoxidans ASRB2, DSM 11109 |
| Ga0586324 2418108 64 276 | 3620000.00 | 0.00 | 0.00 | 0.00 | 0.00 | 0.00 | 0.00 | 0.00 | 0.00 | 0.00 | 0.00 | 0.00 | | predicted RNA-binding protein with TRAM domain | Archaea;Euryarchaeota;Methanomicrobia;Methanosarcinales;Candidatus Methanoperedenaceae;Candidatus Methanoperedens;Candidatus Methanoperedens nitroreducens;Candidatus Methanoperedens nitroreducens ANME-2d |
| Ga0586324 2420610 2 832 | 0.00 | 0.00 | 0.00 | 0.00 | 0.00 | 0.00 | 0.00 | 0.00 | 783915.57 | 0.00 | 0.00 | 0.00 | | hypothetical protein | Bacteria;Actinobacteria;Actinomycetia;Micrococcales;Intrasporangiaceae;Humibacillus;Humibacillus xanthopallidus;Humibacillus xanthopallidus DSM 21776 |
| Ga0586324 2421346 2 607 | 1050000.00 | 4637600.00 | 0.00 | 7296000.00 | 0.00 | 0.00 | 7204800.00 | 5050800.00 | 4398300.00 | 13552000.00 | 0.00 | 4387500.00 | | DNA-binding NarL/FixJ family response regulator | Bacteria;Planctomycetes;Planctomycetia;Pirellulales;Pirellulaceae;Blastopirellula;Blastopirellula marina;Blastopirellula marina DSM 3645 |
| Ga0586324 2422625 276 833 | 0.00 | 0.00 | 0.00 | 0.00 | 0.00 | 0.00 | 0.00 | 0.00 | 0.00 | 0.00 | 0.00 | 1935000.00 | | hypothetical protein | Bacteria;Proteobacteria;Deltaproteobacteria;Desulfobacterales;Desulfobulbaceae;Desulfoprunum;Desulfoprunum benzoelyticum;Desulfoprunum benzoelyticum DSM 28570 |
| Ga0586324 2425334 2 832 | 0.00 | 0.00 | 0.00 | 0.00 | 0.00 | 0.00 | 0.00 | 2397300.00 | 0.00 | 0.00 | 0.00 | 0.00 | | opacity protein-like surface antigen | Bacteria;Proteobacteria;Deltaproteobacteria;Syntrophobacterales;Syntrophaceae;Desulfobacca;Desulfobacca acetoxidans;Desulfobacca acetoxidans ASRB2, DSM 11109 |
| Ga0586324 2425397 3 833 | 1140000.00 | 1142400.00 | 0.00 | 0.00 | 0.00 | 0.00 | 0.00 | 0.00 | 0.00 | 15169000.00 | 0.00 | 2647500.00 | | hypothetical protein | Bacteria;Acidobacteria;Acidobacteriia;Acidobacteriales;Acidobacteriaceae;unclassified Acidobacteriaceae;Acidobacteriaceae bacterium KBS 96;Acidobacteriaceae bacterium KBS 96, DSM 24297 |
| Ga0586324 2432542 2 412 | 0.00 | 0.00 | 0.00 | 0.00 | 0.00 | 0.00 | 0.00 | 1945900.00 | 0.00 | 0.00 | 0.00 | 0.00 | | 3-polyprenyl-4-hydroxybenzoate decarboxylase | Bacteria;Proteobacteria;Deltaproteobacteria;Desulfobacterales;Desulfobacteraceae;Desulfosarcina;Desulfosarcina variabilis;Desulfosarcina variabilis Montpellier |
| Ga0586324 2438590 221 718 | 0.00 | 0.00 | 0.00 | 2555500.00 | 0.00 | 0.00 | 0.00 | 0.00 | 0.00 | 0.00 | 0.00 | 0.00 | | rubrerythrin | Archaea;Euryarchaeota;Methanomicrobia;Methanosarcinales;Methanotrichaceae;Methanothrix;Methanothrix soehngenii;Methanothrix soehngenii GP-6 |
| Ga0586324 2447211 64 828 | 2360000.00 | 9180000.00 | 0.00 | 0.00 | 0.00 | 0.00 | 0.00 | 774700.00 | 0.00 | 0.00 | 0.00 | 627231.00 | | predicted porin | Bacteria;Proteobacteria;Gammaproteobacteria;Chromatiales;Ectothiorhodospiraceae;Ectothiorhodosinus;Ectothiorhodosinus mongolicus;Ectothiorhodosinus mongolicus M9 |
| Ga0586324 2451556 2 829 | 0.00 | 0.00 | 0.00 | 0.00 | 0.00 | 0.00 | 0.00 | 0.00 | 0.00 | 0.00 | 10560000.00 | 0.00 | | hypothetical protein | |
| Ga0586324 2452343 1 828 | 0.00 | 0.00 | 0.00 | 0.00 | 0.00 | 0.00 | 8367600.00 | 0.00 | 2737800.00 | 0.00 | 6098400.00 | 0.00 | | predicted porin | Bacteria;Proteobacteria;Alphaproteobacteria;Hyphomicrobiales;Bradyrhizobiaceae;Bradyrhizobium;Bradyrhizobium lablabi;Bradyrhizobium lablabi GAS165 |
| Ga0586324 2464892 188 574 | 0.00 | 0.00 | 0.00 | 0.00 | 0.00 | 0.00 | 0.00 | 7747000.00 | 0.00 | 0.00 | 0.00 | 0.00 | | hypothetical protein | Bacteria;Proteobacteria;Gammaproteobacteria;Methylococcales;Methylococcaceae;Methylobacter;Methylobacter tundripaludum;Methylobacter tundripaludum 31/32 |
| Ga0586324 2466642 2 826 | 1430000.00 | 0.00 | 0.00 | 0.00 | 0.00 | 0.00 | 0.00 | 0.00 | 0.00 | 0.00 | 0.00 | 0.00 | | predicted porin | Bacteria;Proteobacteria;Gammaproteobacteria;Chromatiales;Chromatiaceae;Thiobaca;Thiobaca trueperi;Thiobaca trueperi DSM 13587 |
| Ga0586324 2468616 3 827 | 0.00 | 454616.72 | 821749.17 | 0.00 | 0.00 | 1058540.95 | 1557700.56 | 1049200.00 | 0.00 | 2795100.00 | 3625600.00 | 1717500.00 | | hypothetical protein | Bacteria;Candidatus Aminicenantes;unclassified Candidatus Aminicenantes;unclassified Candidatus Aminicenantes;unclassified Candidatus Aminicenantes;unclassified Candidatus Aminicenantes;Aminicenantes bacterium JGI 0000014-B08;Aminicenantes bacterium JGI 0000014-B08 (contamination screened) |
| Ga0586324 2471117 1 825 | 4460000.00 | 2080800.00 | 3815935.18 | 5757000.00 | 3708000.00 | 0.00 | 0.00 | 0.00 | 0.00 | 0.00 | 0.00 | 0.00 | | hypothetical protein | Bacteria;Proteobacteria;Gammaproteobacteria;Alteromonadales;Pseudoalteromonadaceae;Pseudoalteromonas;Pseudoalteromonas sp. BSi20495;Pseudoalteromonas sp. BSi20495 |
| Ga0586324 2474729 265 825 | 0.00 | 3610800.00 | 0.00 | 0.00 | 1102100.00 | 0.00 | 1930284.48 | 2037400.00 | 2308500.00 | 0.00 | 2103200.00 | 2722500.00 | | predicted AlkP superfamily pyrophosphatase or phosphodiesterase | Bacteria;Chloroflexi;Thermoflexia;Thermoflexales;Thermoflexaceae;Thermoflexus;Thermoflexus hugenholtzii;Thermoflexus hugenholtzii JAD2 |
| Ga0586324 2474981 2 826 | 0.00 | 0.00 | 0.00 | 0.00 | 2791300.00 | 0.00 | 0.00 | 1122400.00 | 0.00 | 0.00 | 0.00 | 0.00 | | hypothetical protein | Bacteria;Acidobacteria;Acidobacteriia;Bryobacterales;Solibacteraceae;Candidatus Solibacter;Candidatus Solibacter usitatus;Candidatus Solibacter usitatus Ellin6076 |
| Ga0586324 2485340 3 824 | 0.00 | 4195600.00 | 0.00 | 5054000.00 | 0.00 | 0.00 | 0.00 | 0.00 | 0.00 | 0.00 | 0.00 | 0.00 | | FtsP/CotA-like multicopper oxidase with cupredoxin domain | Bacteria;Proteobacteria;Deltaproteobacteria;Desulfuromonadales;Geobacteraceae;Geobacter;Geobacter metallireducens;Geobacter metallireducens RCH3 |
| Ga0586324 2486252 2 823 | 0.00 | 0.00 | 0.00 | 0.00 | 0.00 | 0.00 | 0.00 | 0.00 | 0.00 | 0.00 | 0.00 | 1740000.00 | | hypothetical protein | Bacteria;Acidobacteria;Acidobacteriia;Acidobacteriales;Acidobacteriaceae;unclassified Acidobacteriaceae;Acidobacteriaceae bacterium KBS 146;Acidobacteriaceae sp. KBS 146 |
| Ga0586324 2487540 1 825 | 2910000.00 | 2108000.00 | 0.00 | 4522000.00 | 8806500.00 | 5558000.00 | 5791200.00 | 6405000.00 | 11599200.00 | 4027100.00 | 6626400.00 | 1905000.00 | | predicted porin | Bacteria;Proteobacteria;Betaproteobacteria;Burkholderiales;unclassified Burkholderiales;Rhizobacter;Rhizobacter sp. Root404;Rhizobacter sp. Root404 |
| Ga0586324 2491040 3 683 | 25530000.00 | 0.00 | 0.00 | 0.00 | 0.00 | 0.00 | 0.00 | 0.00 | 0.00 | 0.00 | 0.00 | 0.00 | | hypothetical protein | Archaea;Euryarchaeota;Methanomicrobia;Methanomicrobiales;Methanomicrobiaceae;Methanofollis;Methanofollis ethanolicus;Methanofollis ethanolicus JCM 15103 |
| Ga0586324 2494074 1 822 | 0.00 | 0.00 | 0.00 | 0.00 | 0.00 | 0.00 | 5472000.00 | 0.00 | 0.00 | 0.00 | 5772800.00 | 0.00 | | hypothetical protein | Bacteria;Proteobacteria;Alphaproteobacteria;Hyphomicrobiales;Xanthobacteraceae;Pseudolabrys;Pseudolabrys taiwanensis;Pseudolabrys taiwanensis CC-BB4 |
| Ga0586324 2502886 2 823 | 2990000.00 | 2665175.00 | 4909748.47 | 0.00 | 3265100.00 | 3380097.70 | 0.00 | 1506700.00 | 0.00 | 9994600.00 | 0.00 | 2840364.75 | | hypothetical protein | Bacteria;Proteobacteria;Deltaproteobacteria;Desulfuromonadales;Geobacteraceae;Geobacter;Geobacter argillaceus;Geobacter argillaceus ATCC BAA-1139 |
| Ga0586324 2509994 160 726 | 0.00 | 0.00 | 0.00 | 0.00 | 0.00 | 0.00 | 0.00 | 0.00 | 23490000.00 | 0.00 | 0.00 | 0.00 | | hypothetical protein | |
| Ga0586324 2512394 134 820 | 0.00 | 0.00 | 0.00 | 0.00 | 0.00 | 0.00 | 0.00 | 0.00 | 0.00 | 0.00 | 0.00 | 2482500.00 | | membrane protease subunit (stomatin/prohibitin family) | Bacteria;Chloroflexi;Anaerolineae;Anaerolineales;Anaerolineaceae;Levilinea;Levilinea saccharolytica;Levilinea saccharolytica DSM 16555 |
| Ga0586324 2514214 2 739 | 0.00 | 4263600.00 | 2498532.07 | 2774000.00 | 0.00 | 0.00 | 0.00 | 0.00 | 0.00 | 0.00 | 0.00 | 0.00 | | hypothetical protein | |
| Ga0586324 2520056 2 820 | 6000000.00 | 0.00 | 0.00 | 0.00 | 0.00 | 11274800.00 | 0.00 | 0.00 | 0.00 | 0.00 | 0.00 | 0.00 | | membrane protease subunit (stomatin/prohibitin family) | Bacteria;Chloroflexi;Anaerolineae;Anaerolineales;Anaerolineaceae;Pelolinea;Pelolinea submarina;Pelolinea submarina DSM 23923 |
| Ga0586324 2523018 2 820 | 0.00 | 3971200.00 | 1171349.65 | 0.00 | 0.00 | 1319663.73 | 7774800.00 | 3513600.00 | 2608200.00 | 2633400.00 | 0.00 | 2370000.00 | | hypothetical protein | |
| Ga0586324 2529203 1 819 | 0.00 | 0.00 | 0.00 | 0.00 | 0.00 | 0.00 | 0.00 | 0.00 | 2559600.00 | 0.00 | 0.00 | 0.00 | | hypothetical protein | Bacteria;Acidobacteria;Blastocatellia;Blastocatellales;Pyrinomonadaceae;Pyrinomonas;Pyrinomonas methylaliphatogenes;Pyrinomonas methylaliphatogenes K22 |
| Ga0586324 2532916 3 410 | 0.00 | 2305200.00 | 0.00 | 5073000.00 | 0.00 | 0.00 | 0.00 | 0.00 | 0.00 | 0.00 | 0.00 | 0.00 | | crotonobetainyl-CoA:carnitine CoA-transferase CaiB-like acyl-CoA transferase | Archaea;Euryarchaeota;Archaeoglobi;Archaeoglobales;Archaeoglobaceae;Ferroglobus;Ferroglobus placidus;Ferroglobus placidus AEDII12DO, DSM 10642 |
| Ga0586324 2539751 167 817 | 0.00 | 1482400.00 | 0.00 | 0.00 | 0.00 | 0.00 | 0.00 | 0.00 | 0.00 | 0.00 | 0.00 | 0.00 | | hypothetical protein | |
| Ga0586324 2544508 1 789 | 0.00 | 0.00 | 0.00 | 251366.20 | 0.00 | 0.00 | 0.00 | 0.00 | 0.00 | 0.00 | 0.00 | 0.00 | | uncharacterized membrane protein YebE (DUF533 family) | Bacteria;Proteobacteria;Deltaproteobacteria;Desulfuromonadales;Desulfuromonadaceae;Desulfuromonas;Desulfuromonas acetexigens;Desulfuromonas acetexigens DSM 1397 |
| Ga0586324 2544626 1 816 | 2740000.00 | 0.00 | 4144637.10 | 0.00 | 5180900.00 | 9060453.10 | 0.00 | 2006900.00 | 0.00 | 2148300.00 | 795534.96 | 2910000.00 | | hypothetical protein | Bacteria;Proteobacteria;Deltaproteobacteria;Desulfobacterales;Desulfobulbaceae;Desulfobulbus;Desulfobulbus propionicus;Desulfobulbus propionicus 1pr3, DSM 2032 |
| Ga0586324 2547054 66 815 | 0.00 | 816000.00 | 0.00 | 0.00 | 0.00 | 0.00 | 0.00 | 0.00 | 0.00 | 0.00 | 0.00 | 0.00 | | hypothetical protein | Bacteria;Proteobacteria;Deltaproteobacteria;Syntrophobacterales;Syntrophobacteraceae;Syntrophobacter;Syntrophobacter fumaroxidans;Syntrophobacter fumaroxidans MPOB |
| Ga0586324 2557152 1 813 | 0.00 | 3821600.00 | 0.00 | 0.00 | 0.00 | 0.00 | 0.00 | 0.00 | 0.00 | 0.00 | 0.00 | 0.00 | | ABC-type branched-subunit amino acid transport system substrate-binding protein | Bacteria;Chloroflexi;Thermoflexia;Thermoflexales;Thermoflexaceae;Thermoflexus;Thermoflexus hugenholtzii;Thermoflexus hugenholtzii JAD2 |
| Ga0586324 2573791 2 502 | 5775224.00 | 3638000.00 | 3417787.21 | 0.00 | 0.00 | 2936946.45 | 4446000.00 | 19337000.00 | 3693600.00 | 4019400.00 | 5024800.00 | 5190000.00 | | hypothetical protein | |
| Ga0586324 2577396 1 315 | 0.00 | 0.00 | 0.00 | 0.00 | 0.00 | 0.00 | 0.00 | 0.00 | 0.00 | 1216600.00 | 0.00 | 354850.50 | | hypothetical protein | Bacteria;Proteobacteria;Gammaproteobacteria;unclassified Gammaproteobacteria;unclassified Gammaproteobacteria;unclassified Gammaproteobacteria;Solemya velum gill symbiont;Solemya velum gill symbiont WH |
| Ga0586324 2579085 2 811 | 0.00 | 0.00 | 0.00 | 0.00 | 0.00 | 3072422.70 | 2736000.00 | 0.00 | 0.00 | 0.00 | 0.00 | 0.00 | | hypothetical protein | |
| Ga0586324 2584493 2 511 | 0.00 | 0.00 | 0.00 | 0.00 | 0.00 | 0.00 | 0.00 | 0.00 | 0.00 | 1031800.00 | 6740800.00 | 4545000.00 | | hypothetical protein | |
| Ga0586324 2600651 231 671 | 0.00 | 0.00 | 0.00 | 0.00 | 0.00 | 0.00 | 0.00 | 0.00 | 0.00 | 0.00 | 0.00 | 1890000.00 | | hypothetical protein | Bacteria;Proteobacteria;Gammaproteobacteria;Methylococcales;Methylococcaceae;Methylobacter;Methylobacter luteus;Methylobacter luteus IMV-B-3098 |
| Ga0586324 2605084 158 808 | 0.00 | 0.00 | 0.00 | 3230000.00 | 0.00 | 0.00 | 0.00 | 0.00 | 0.00 | 0.00 | 1408000.00 | 0.00 | | hypothetical protein | Bacteria;Acidobacteria;Vicinamibacteria;unclassified Vicinamibacteria;Vicinamibacteraceae;Luteitalea;Luteitalea pratensis;Luteitalea pratensis DSM 100886 |
| Ga0586324 2606230 3 809 | 0.00 | 0.00 | 0.00 | 0.00 | 0.00 | 0.00 | 0.00 | 0.00 | 0.00 | 3141600.00 | 0.00 | 0.00 | | hypothetical protein | Bacteria;Proteobacteria;Gammaproteobacteria;Chromatiales;Ectothiorhodospiraceae;Thioalkalivibrio;Thioalkalivibrio thiocyanodenitrificans;Thioalkalivibrio thiocyanodenitrificans ARhD 1 |
| Ga0586324 2609516 1 807 | 1940000.00 | 0.00 | 2386782.20 | 1577000.00 | 2533800.00 | 0.00 | 2228428.68 | 0.00 | 0.00 | 1794100.00 | 1716000.00 | 0.00 | | outer membrane receptor protein involved in Fe transport | Bacteria;Acidobacteria;Acidobacteriia;Acidobacteriales;Acidobacteriaceae;Granulicella;Granulicella aggregans;Granulicella aggregans M8UP14 |
| Ga0586324 2614721 1 807 | 0.00 | 0.00 | 0.00 | 0.00 | 0.00 | 0.00 | 0.00 | 2702300.00 | 4608900.00 | 0.00 | 0.00 | 0.00 | | methionine synthase II (cobalamin-independent) | Bacteria;Proteobacteria;Gammaproteobacteria;Methylococcales;Methylococcaceae;Methyloglobulus;Methyloglobulus morosus;Methyloglobulus morosus KoM1 |
| Ga0586324 2624640 1 804 | 13300000.00 | 0.00 | 14525400.00 | 0.00 | 0.00 | 0.00 | 0.00 | 0.00 | 0.00 | 0.00 | 0.00 | 0.00 | | hypothetical protein | Bacteria;Acidobacteria;Acidobacteriia;Bryobacterales;Solibacteraceae;Candidatus Solibacter;Candidatus Solibacter usitatus;Candidatus Solibacter usitatus Ellin6076 |
| Ga0586324 2635386 2 718 | 0.00 | 0.00 | 0.00 | 0.00 | 0.00 | 0.00 | 0.00 | 2580300.00 | 0.00 | 0.00 | 0.00 | 0.00 | | hypothetical protein | |
| Ga0586324 2635491 3 86 | 0.00 | 0.00 | 2125809.18 | 0.00 | 0.00 | 0.00 | 0.00 | 0.00 | 0.00 | 0.00 | 0.00 | 0.00 | | hypothetical protein | |
| Ga0586324 2635516 1 804 | 0.00 | 0.00 | 0.00 | 0.00 | 0.00 | 0.00 | 0.00 | 0.00 | 0.00 | 3072300.00 | 0.00 | 0.00 | | alkylation response protein AidB-like acyl-CoA dehydrogenase | Bacteria;Proteobacteria;Deltaproteobacteria;Syntrophobacterales;Syntrophobacteraceae;Desulfacinum;Desulfacinum infernum;Desulfacinum infernum DSM 9756 |
| Ga0586324 2640591 33 710 | 0.00 | 2944400.00 | 0.00 | 19665000.00 | 0.00 | 0.00 | 0.00 | 0.00 | 0.00 | 0.00 | 0.00 | 0.00 | | corrinoid protein of di/trimethylamine methyltransferase | Bacteria;Chloroflexi;Caldilineae;Caldilineales;Caldilineaceae;Caldilinea;Caldilinea aerophila;Caldilinea aerophila STL-6-O1, DSM 14535 |
| Ga0586324 2648129 2 802 | 0.00 | 0.00 | 0.00 | 0.00 | 1606800.00 | 0.00 | 0.00 | 0.00 | 0.00 | 0.00 | 0.00 | 0.00 | | hypothetical protein | |
| Ga0586324 2648421 2 487 | 0.00 | 0.00 | 0.00 | 758701.35 | 10227900.00 | 0.00 | 0.00 | 1628700.00 | 0.00 | 0.00 | 0.00 | 0.00 | | regulator of protease activity HflC (stomatin/prohibitin superfamily) | Bacteria;Verrucomicrobia;Spartobacteria;Chthoniobacterales;Chthoniobacteraceae;Chthoniobacter;Chthoniobacter flavus;Chthoniobacter flavus Ellin428 |
| Ga0586324 2651712 1 801 | 20800000.00 | 11832000.00 | 5686300.00 | 6669000.00 | 10712000.00 | 0.00 | 6270000.00 | 0.00 | 0.00 | 11935000.00 | 9416000.00 | 8775000.00 | | hypothetical protein | Bacteria;Acidobacteria;Blastocatellia;Blastocatellales;Pyrinomonadaceae;Pyrinomonas;Pyrinomonas methylaliphatogenes;Pyrinomonas methylaliphatogenes K22 |
| Ga0586324 2657171 3 800 | 0.00 | 0.00 | 0.00 | 0.00 | 0.00 | 0.00 | 0.00 | 0.00 | 251616.78 | 0.00 | 0.00 | 0.00 | | hypothetical protein | Bacteria;Actinobacteria;Actinomycetia;Propionibacteriales;Nocardioidaceae;Nocardioides;Nocardioides guangzhouensis;Nocardioides guangzhouensis 130 |
| Ga0586324 2657697 1 801 | 0.00 | 0.00 | 0.00 | 0.00 | 0.00 | 0.00 | 0.00 | 0.00 | 0.00 | 0.00 | 0.00 | 77380.50 | | hypothetical protein | |
| Ga0586324 2660418 210 521 | 0.00 | 0.00 | 0.00 | 0.00 | 3481400.00 | 0.00 | 0.00 | 0.00 | 0.00 | 0.00 | 0.00 | 0.00 | | ElaB/YqiD/DUF883 family membrane-anchored ribosome-binding protein | Bacteria;Proteobacteria;Betaproteobacteria;Nitrosomonadales;Sterolibacteriaceae;Sulfurisoma;Sulfurisoma sediminicola;Sulfurisoma sediminicola DSM 26916 |
| Ga0586324 2683611 1 798 | 0.00 | 0.00 | 0.00 | 0.00 | 0.00 | 0.00 | 0.00 | 0.00 | 0.00 | 0.00 | 0.00 | 482370.75 | | hypothetical protein | |
| Ga0586324 2684694 2 799 | 8170000.00 | 0.00 | 0.00 | 0.00 | 0.00 | 0.00 | 49476000.00 | 0.00 | 0.00 | 0.00 | 0.00 | 0.00 | | hypothetical protein | Bacteria;Proteobacteria;Alphaproteobacteria;Hyphomicrobiales;Bradyrhizobiaceae;Bradyrhizobium;Bradyrhizobium elkanii;Bradyrhizobium elkanii WSM2783 |
| Ga0586324 2685023 41 799 | 0.00 | 5052400.00 | 0.00 | 7381500.00 | 9888000.00 | 0.00 | 0.00 | 3739300.00 | 3661200.00 | 4142600.00 | 2164800.00 | 2040000.00 | | hypothetical protein | Bacteria;Proteobacteria;Deltaproteobacteria;Syntrophobacterales;Syntrophobacteraceae;Syntrophobacter;Syntrophobacter fumaroxidans;Syntrophobacter fumaroxidans MPOB |
| Ga0586324 2694240 2 511 | 9740000.00 | 0.00 | 0.00 | 0.00 | 0.00 | 0.00 | 0.00 | 0.00 | 0.00 | 0.00 | 0.00 | 0.00 | | predicted porin | Bacteria;Proteobacteria;Betaproteobacteria;Rhodocyclales;Azonexaceae;Azonexus;Azonexus hydrophilus;Azonexus hydrophilus DSM 23864 |
| Ga0586324 2697845 3 368 | 0.00 | 6303600.00 | 0.00 | 0.00 | 4738000.00 | 0.00 | 0.00 | 0.00 | 0.00 | 0.00 | 0.00 | 6555000.00 | | predicted CXXCH cytochrome family protein | Bacteria;Proteobacteria;Deltaproteobacteria;Desulfuromonadales;Geobacteraceae;Citrifermentans;Citrifermentans bremense;Geobacter bremensis R1 |
| Ga0586324 2700724 3 797 | 0.00 | 2427600.00 | 0.00 | 0.00 | 0.00 | 0.00 | 0.00 | 0.00 | 3450600.00 | 0.00 | 0.00 | 0.00 | | hypothetical protein | Bacteria;Proteobacteria;Alphaproteobacteria;Hyphomicrobiales;unclassified Hyphomicrobiales;Methyloceanibacter;Methyloceanibacter methanicus;Methyloceanibacter methanicus R-67174 |
| Ga0586324 2701238 1 348 | 3390000.00 | 0.00 | 0.00 | 0.00 | 0.00 | 0.00 | 0.00 | 0.00 | 0.00 | 0.00 | 0.00 | 0.00 | | catechol 2,3-dioxygenase-like lactoylglutathione lyase family enzyme | |
| Ga0586324 2701576 278 796 | 0.00 | 0.00 | 0.00 | 4056500.00 | 6241800.00 | 0.00 | 0.00 | 9211000.00 | 0.00 | 0.00 | 0.00 | 5430000.00 | | hypothetical protein | Bacteria;Proteobacteria;Deltaproteobacteria;Syntrophobacterales;Syntrophobacteraceae;Desulfacinum;Desulfacinum hydrothermale;Desulfacinum hydrothermale DSM 13146 |
| Ga0586324 2712955 2 793 | 0.00 | 0.00 | 0.00 | 0.00 | 1596500.00 | 1784912.00 | 1269843.72 | 939400.00 | 0.00 | 2517900.00 | 0.00 | 0.00 | | regulator of protease activity HflC (stomatin/prohibitin superfamily) | Bacteria;Candidatus Sumerlaeota;unclassified Candidatus Sumerlaeota;unclassified Candidatus Sumerlaeota;unclassified Candidatus Sumerlaeota;unclassified Candidatus Sumerlaeota;BRC1 bacterium SCGC AAA257-C11;BRC1 bacterium SCGC AAA257-C11 (contamination screened) |
| Ga0586324 2712983 2 793 | 0.00 | 911200.00 | 0.00 | 0.00 | 0.00 | 0.00 | 0.00 | 0.00 | 0.00 | 0.00 | 730446.64 | 0.00 | | outer membrane receptor for ferrienterochelin and colicin | Bacteria;Acidobacteria;Holophagae;Holophagales;Holophagaceae;Geothrix;Geothrix fermentans;Geothrix fermentans DSM 14018 |
| Ga0586324 2715973 1 795 | 0.00 | 1326000.00 | 0.00 | 0.00 | 0.00 | 0.00 | 0.00 | 0.00 | 0.00 | 0.00 | 0.00 | 0.00 | | hypothetical protein | |
| Ga0586324 2718002 3 794 | 0.00 | 0.00 | 3118800.43 | 0.00 | 0.00 | 0.00 | 0.00 | 0.00 | 2025000.00 | 0.00 | 2244000.00 | 0.00 | | hypothetical protein | |
| Ga0586324 2723279 3 575 | 4200000.00 | 3461200.00 | 0.00 | 3885500.00 | 5726800.00 | 0.00 | 0.00 | 0.00 | 0.00 | 4758600.00 | 0.00 | 0.00 | | regulator of protease activity HflC (stomatin/prohibitin superfamily) | Bacteria;Chloroflexi;Dehalococcoidia;unclassified Dehalococcoidia;unclassified Dehalococcoidia;Dehalogenimonas;Dehalogenimonas formicexedens;Dehalogenimonas formicexedens NSZ-14 |
| Ga0586324 2724147 100 507 | 0.00 | 816000.00 | 0.00 | 0.00 | 0.00 | 0.00 | 0.00 | 0.00 | 0.00 | 0.00 | 0.00 | 0.00 | | rubrerythrin | Bacteria;Proteobacteria;Deltaproteobacteria;Myxococcales;Anaeromyxobacteraceae;Anaeromyxobacter;Anaeromyxobacter sp. PSR-1;Anaeromyxobacter sp. PSR-1 |
| Ga0586324 2725508 2 793 | 0.00 | 0.00 | 0.00 | 2555500.00 | 0.00 | 0.00 | 0.00 | 1439600.00 | 0.00 | 0.00 | 0.00 | 488319.75 | | hypothetical protein | Bacteria;Acidobacteria;Acidobacteriia;Bryobacterales;Bryobacteraceae;Bryobacter;Bryobacter aggregatus;Bryobacter aggregatus DSM 18758 |
| Ga0586324 2729160 1 792 | 4960000.00 | 0.00 | 14506927.97 | 10545000.00 | 7508700.00 | 0.00 | 3192000.00 | 2940200.00 | 3815100.00 | 5567100.00 | 5764000.00 | 4087500.00 | | predicted porin | Bacteria;Proteobacteria;Betaproteobacteria;Rhodocyclales;Rhodocyclaceae;Aromatoleum;Aromatoleum toluclasticum;Aromatoleum toluclasticum ATCC 700605 |
| Ga0586324 2735052 1 792 | 8240000.00 | 1564000.00 | 0.00 | 0.00 | 0.00 | 15205100.00 | 0.00 | 8845000.00 | 7557300.00 | 10010000.00 | 3326400.00 | 7350000.00 | | hypothetical protein | Archaea;Euryarchaeota;Methanomicrobia;Methanomicrobiales;Methanoregulaceae;Methanoregula;Methanoregula formicica;Methanoregula formicica SMSP, DSM 22288 |
| Ga0586324 2747094 2 661 | 562317.00 | 0.00 | 0.00 | 0.00 | 0.00 | 0.00 | 0.00 | 0.00 | 0.00 | 0.00 | 0.00 | 0.00 | | hypothetical protein | |
| Ga0586324 2767218 142 789 | 0.00 | 1380400.00 | 1663625.59 | 2318000.00 | 0.00 | 0.00 | 1783256.40 | 0.00 | 0.00 | 1794100.00 | 1971200.00 | 1500000.00 | | hypothetical protein | |
| Ga0586324 2768523 3 788 | 0.00 | 138269.16 | 0.00 | 0.00 | 0.00 | 847384.59 | 0.00 | 0.00 | 0.00 | 0.00 | 0.00 | 0.00 | | hypothetical protein | |
| Ga0586324 2769818 2 787 | 0.00 | 0.00 | 0.00 | 0.00 | 0.00 | 0.00 | 0.00 | 1817800.00 | 0.00 | 0.00 | 0.00 | 0.00 | | CO/xanthine dehydrogenase Mo-binding subunit | Bacteria;Proteobacteria;Alphaproteobacteria;Rhodospirillales;Acetobacteraceae;Rhodopila;Rhodopila globiformis;Rhodopila globiformis DSM 161 |
| Ga0586324 2775043 3 788 | 0.00 | 0.00 | 0.00 | 0.00 | 42642000.00 | 0.00 | 0.00 | 0.00 | 0.00 | 0.00 | 0.00 | 0.00 | | formyltetrahydrofolate synthetase | Bacteria;Proteobacteria;Deltaproteobacteria;Syntrophobacterales;Syntrophobacteraceae;Syntrophobacter;Syntrophobacter fumaroxidans;Syntrophobacter fumaroxidans MPOB |
| Ga0586324 2783753 3 785 | 29100000.00 | 34340000.00 | 29444900.00 | 25669000.00 | 23072000.00 | 29894100.00 | 0.00 | 10736000.00 | 0.00 | 11750200.00 | 12144000.00 | 14040000.00 | | hypothetical protein | |

| | | | | | | | | | | | | | | |
|---|---|---|---|---|---|---|---|---|---|---|---|---|---|---|
| Ga0586324 2783756 1 453 | 0.00 | 3889600.00 | 0.00 | 0.00 | 0.00 | 0.00 | 0.00 | 0.00 | 0.00 | 0.00 | 0.00 | 0.00 | hypothetical protein | |
| Ga0586324 2788353 230 784 | 0.00 | 0.00 | 0.00 | 0.00 | 0.00 | 0.00 | 0.00 | 0.00 | 2891700.00 | 0.00 | 0.00 | 0.00 | predicted porin | Bacteria;Proteobacteria;Betaproteobacteria;Burkholderiales;Comamonadaceae;Variovorax;Variovorax sp. BK119;Variovorax sp. BK119 |
| Ga0586324 2792658 3 326 | 0.00 | 0.00 | 8163500.00 | 0.00 | 0.00 | 0.00 | 0.00 | 0.00 | 0.00 | 0.00 | 0.00 | 0.00 | methanogenic corrinoid protein MtbC1 | Bacteria;Bacteroidetes;Bacteroidia;Bacteroidales;unclassified Bacteroidales;unclassified Bacteroidales;Bacteroidales bacterium KHT7;Bacteroidales bacterium KHT7 |
| Ga0586324 2798287 31 363 | 0.00 | 0.00 | 0.00 | 4674000.00 | 10186700.00 | 0.00 | 0.00 | 0.00 | 0.00 | 0.00 | 0.00 | 0.00 | hypothetical protein | Bacteria;Proteobacteria;Deltaproteobacteria;Syntrophobacterales;Syntrophaceae;Desulfobacca;Desulfobacca acetoxidans;Desulfobacca acetoxidans ASRB2, DSM 11109 |
| Ga0586324 2805763 3 782 | 0.00 | 0.00 | 0.00 | 0.00 | 0.00 | 0.00 | 0.00 | 0.00 | 1053000.00 | 0.00 | 0.00 | 0.00 | NADPH-dependent glutamate synthase beta subunit-like oxidoreductase | Bacteria;Proteobacteria;Betaproteobacteria;Nitrosomonadales;Thiobacillaceae;Thiobacillus;Thiobacillus thioparus;Thiobacillus thioparus DSM 505 |
| Ga0586324 2806687 108 782 | 0.00 | 0.00 | 0.00 | 0.00 | 0.00 | 5200700.00 | 0.00 | 0.00 | 0.00 | 0.00 | 0.00 | 0.00 | formylglycine-generating enzyme required for sulfatase activity | Bacteria;Proteobacteria;Gammaproteobacteria;Oceanospirillales;Oceanospirillaceae;Marinobacterium;Marinobacterium stanieri;Marinobacterium stanieri S30 |
| Ga0586324 2808636 3 443 | 0.00 | 972400.00 | 0.00 | 1168500.00 | 801109.28 | 0.00 | 0.00 | 744200.00 | 0.00 | 900900.00 | 0.00 | 385626.00 | hypothetical protein | Bacteria;Proteobacteria;Deltaproteobacteria;Desulfuromonadales;Geobacteraceae;Geobacter;Geobacter sp. DSM 2909;Geobacter sp. DSM 2909 |
| Ga0586324 2811270 54 614 | 0.00 | 0.00 | 0.00 | 0.00 | 0.00 | 0.00 | 0.00 | 0.00 | 0.00 | 2533300.00 | 0.00 | 0.00 | uncharacterized membrane protein | Archaea;Euryarchaeota;Methanomicrobia;Methanosarcinales;Methanosarcinaceae;Methanosarcina;Methanosarcina barkeri;Methanosarcina barkeri Fusaro, DSM 804 |
| Ga0586324 2819406 3 782 | 0.00 | 0.00 | 0.00 | 0.00 | 0.00 | 0.00 | 0.00 | 0.00 | 0.00 | 0.00 | 0.00 | 3862500.00 | hypothetical protein | Bacteria;Proteobacteria;Alphaproteobacteria;Hyphomicrobiales;Bradyrhizobiaceae;Bradyrhizobium;Bradyrhizobium neotropicale;Bradyrhizobium neotropicale BR 10247 |
| Ga0586324 2834180 2 781 | 0.00 | 195330.00 | 0.00 | 0.00 | 0.00 | 0.00 | 0.00 | 0.00 | 0.00 | 0.00 | 0.00 | 0.00 | regulator of protease activity HflC (stomatin/prohibitin superfamily) | Bacteria;Chloroflexi;Anaerolineae;Anaerolineales;Anaerolineaceae;Anaerolinea;Anaerolinea thermophila;Anaerolinea thermophila UNI-1 |
| Ga0586324 2843680 2 475 | 0.00 | 4467600.00 | 0.00 | 0.00 | 0.00 | 0.00 | 0.00 | 0.00 | 0.00 | 0.00 | 0.00 | 0.00 | rubrerythrin | Bacteria;Proteobacteria;Deltaproteobacteria;Syntrophobacterales;Syntrophaceae;Smithella;Smithella sp. SCADC;Smithella sp. SCADC |
| Ga0586324 2845761 1 777 | 0.00 | 0.00 | 0.00 | 2451000.00 | 0.00 | 0.00 | 0.00 | 0.00 | 0.00 | 0.00 | 0.00 | 0.00 | L-serine deaminase | Bacteria;Acidobacteria;Vicinamibacteria;unclassified Vicinamibacteria;Vicinamibacteraceae;Luteitalea;Luteitalea pratensis;Luteitalea pratensis DSM 100886 |
| Ga0586324 2848270 2 778 | 3660000.00 | 0.00 | 2279750.27 | 5500500.00 | 5840100.00 | 4803700.00 | 3237600.00 | 3092700.00 | 3523500.00 | 0.00 | 0.00 | 0.00 | predicted molibdopterin-dependent oxidoreductase YjgC | Bacteria;Proteobacteria;Deltaproteobacteria;Syntrophobacterales;Syntrophobacteraceae;Desulfosoma;Desulfosoma caldarium;Desulfosoma caldarium DSM 22027 |
| Ga0586324 2860823 3 776 | 0.00 | 2849200.00 | 0.00 | 0.00 | 0.00 | 0.00 | 0.00 | 0.00 | 0.00 | 0.00 | 0.00 | 0.00 | hypothetical protein | Bacteria;Proteobacteria;Deltaproteobacteria;Desulfuromonadales;Geobacteraceae;Geobacter;Geobacter uraniireducens;Geobacter uraniireducens Rf4 |
| Ga0586324 2866194 1 777 | 0.00 | 80277.40 | 388231.29 | 0.00 | 0.00 | 0.00 | 0.00 | 1988600.00 | 0.00 | 0.00 | 2569600.00 | 1409894.25 | hypothetical protein | |
| Ga0586324 2867014 3 692 | 0.00 | 0.00 | 0.00 | 0.00 | 0.00 | 0.00 | 0.00 | 0.00 | 5281200.00 | 9086000.00 | 8703200.00 | 0.00 | hypothetical protein | |
| Ga0586324 2868405 1 777 | 0.00 | 0.00 | 0.00 | 0.00 | 0.00 | 0.00 | 2063691.84 | 0.00 | 0.00 | 0.00 | 0.00 | 0.00 | hypothetical protein | |
| Ga0586324 2869390 486 776 | 0.00 | 1550400.00 | 0.00 | 0.00 | 0.00 | 0.00 | 0.00 | 0.00 | 0.00 | 0.00 | 0.00 | 0.00 | hypothetical protein | Bacteria;Acidobacteria;Blastocatellia;Blastocatellales;Pyrinomonadaceae;Pyrinomonas;Pyrinomonas methylaliphatogenes;Pyrinomonas methylaliphatogenes K22 |
| Ga0586324 2881119 1 774 | 0.00 | 0.00 | 0.00 | 0.00 | 0.00 | 0.00 | 0.00 | 0.00 | 0.00 | 0.00 | 0.00 | 217923.00 | arylsulfatase A-like enzyme | Bacteria;Proteobacteria;Deltaproteobacteria;Desulfovibrionales;Desulfovibrionaceae;Desulfovibrio;Desulfovibrio magneticus;Desulfovibrio cf. magneticus IFRC170 |
| Ga0586324 2900031 1 771 | 0.00 | 0.00 | 0.00 | 0.00 | 0.00 | 0.00 | 0.00 | 0.00 | 0.00 | 0.00 | 654592.40 | 0.00 | hypothetical protein | Bacteria;Proteobacteria;Gammaproteobacteria;Chromatiales;Woeseiaceae;unclassified Woeseiaceae;unclassified Woeseiaceae;uncultured Woeseiaceae SAG 1868 B |
| Ga0586324 2905725 1 528 | 13400000.00 | 24388293.16 | 42956900.00 | 0.00 | 1266900.00 | 0.00 | 3556800.00 | 0.00 | 0.00 | 12628000.00 | 0.00 | 13875000.00 | hypothetical protein | Bacteria;Proteobacteria;Deltaproteobacteria;Syntrophorhabdales;Syntrophorhabdaceae;Syntrophorhabdus;Syntrophorhabdus aromaticivorans;Syntrophorhabdus aromaticivorans UI |
| Ga0586324 2916236 130 609 | 10830000.00 | 7187600.00 | 25616500.00 | 11010500.00 | 35442300.00 | 11433600.00 | 55404000.00 | 0.00 | 2114100.00 | 4612300.00 | 0.00 | 2955000.00 | hypothetical protein | Bacteria;Proteobacteria;Deltaproteobacteria;Syntrophobacterales;Syntrophaceae;Desulfobacca;Desulfobacca acetoxidans;Desulfobacca acetoxidans ASRB2, DSM 11109 |
| Ga0586324 2931864 2 760 | 0.00 | 0.00 | 0.00 | 0.00 | 0.00 | 0.00 | 0.00 | 0.00 | 0.00 | 0.00 | 0.00 | 1710000.00 | uncharacterized protein (DUF362 family) | Bacteria;Proteobacteria;Deltaproteobacteria;Desulfuromonadales;Geobacteraceae;Geobacter;Geobacter lovleyi;Geobacter lovleyi SZ |
| Ga0586324 2935187 2 769 | 0.00 | 754800.00 | 0.00 | 0.00 | 0.00 | 0.00 | 0.00 | 896700.00 | 0.00 | 0.00 | 0.00 | 0.00 | hypothetical protein | Bacteria;Acidobacteria;Acidobacteriia;Acidobacteriales;Acidobacteriaceae;Acidobacterium;Acidobacterium ailaaui;Acidobacterium ailaaui PMMR2 |
| Ga0586324 2936548 412 768 | 0.00 | 0.00 | 0.00 | 0.00 | 0.00 | 0.00 | 0.00 | 0.00 | 0.00 | 931700.00 | 0.00 | 0.00 | hypothetical protein | Bacteria;Acidobacteria;Acidobacteriia;Acidobacteriales;Acidobacteriaceae;Edaphobacter;Edaphobacter lichenicola;Edaphobacter lichenicola M8UP22 |
| Ga0586324 2942204 1 768 | 15400000.00 | 38624000.00 | 62211500.00 | 0.00 | 14172800.00 | 23661200.00 | 42864000.00 | 19886000.00 | 53784000.00 | 14784000.00 | 33096800.00 | 34425000.00 | predicted porin | Bacteria;Proteobacteria;Betaproteobacteria;Nitrosomonadales;Sulfuricellaceae;Sulfuricella;Sulfuricella denitrificans;Sulfuricella denitrificans skB26 |
| Ga0586324 2942625 3 767 | 0.00 | 0.00 | 0.00 | 2185000.00 | 0.00 | 0.00 | 0.00 | 0.00 | 0.00 | 0.00 | 0.00 | 0.00 | Pyruvate/2-oxoacid:ferredoxin oxidoreductase gamma subunit | Bacteria;Proteobacteria;Deltaproteobacteria;Syntrophobacterales;Syntrophaceae;Desulfobacca;Desulfobacca acetoxidans;Desulfobacca acetoxidans ASRB2, DSM 11109 |
| Ga0586324 2949218 1 768 | 0.00 | 0.00 | 0.00 | 0.00 | 0.00 | 0.00 | 1582176.36 | 0.00 | 0.00 | 0.00 | 0.00 | 0.00 | hypothetical protein | Bacteria;Spirochaetes;Spirochaetia;Leptospirales;Leptospiraceae;Leptonema;Leptonema illini;Leptonema illini 3055, DSM 21528 |
| Ga0586324 2962898 2 640 | 0.00 | 0.00 | 0.00 | 0.00 | 0.00 | 0.00 | 0.00 | 4788500.00 | 0.00 | 0.00 | 0.00 | 0.00 | hypothetical protein | Bacteria;Proteobacteria;Alphaproteobacteria;Hyphomicrobiales;Hyphomicrobiaceae;Hyphomicrobium;Hyphomicrobium facile;Hyphomicrobium facile facile DSM 1565 |
| Ga0586324 2964674 163 765 | 0.00 | 0.00 | 0.00 | 3353500.00 | 3203300.00 | 0.00 | 0.00 | 0.00 | 0.00 | 3842300.00 | 1848000.00 | 3187500.00 | predicted porin | Bacteria;Proteobacteria;Betaproteobacteria;Burkholderiales;Burkholderiaceae;Cupriavidus;Cupriavidus basilensis;Cupriavidus basilensis OR16 |
| Ga0586324 2969469 290 766 | 0.00 | 0.00 | 0.00 | 0.00 | 0.00 | 0.00 | 0.00 | 1311500.00 | 0.00 | 0.00 | 0.00 | 0.00 | hypothetical protein | Bacteria;Proteobacteria;Alphaproteobacteria;Sphingomonadales;Sphingomonadaceae;Sphingobium;Sphingobium xanthum;Sphingobium xanthum DSM 100901 |
| Ga0586324 2974987 1 369 | 0.00 | 0.00 | 0.00 | 0.00 | 0.00 | 9925000.00 | 0.00 | 0.00 | 0.00 | 0.00 | 0.00 | 2340000.00 | Flp pilus assembly protein TadD | Bacteria;Proteobacteria;Deltaproteobacteria;Syntrophobacterales;Syntrophaceae;Desulfobacca;Desulfobacca acetoxidans;Desulfobacca acetoxidans ASRB2, DSM 11109 |
| Ga0586324 2977018 2 763 | 0.00 | 4046000.00 | 0.00 | 5538500.00 | 0.00 | 2692315.05 | 3055200.00 | 14078800.00 | 6658200.00 | 5443900.00 | 5121600.00 | 4380000.00 | predicted porin | Bacteria;Proteobacteria;Betaproteobacteria;unclassified Betaproteobacteria;unclassified Betaproteobacteria;unclassified Betaproteobacteria;Betaproteobacteria bacterium GR16-43;Betaproteobacteria bacterium GR16-43 |
| Ga0586324 2979412 1 765 | 0.00 | 0.00 | 0.00 | 2147000.00 | 0.00 | 0.00 | 0.00 | 0.00 | 0.00 | 0.00 | 0.00 | 0.00 | hypothetical protein | Bacteria;Planctomycetes;Planctomycetia;Gemmatales;Gemmataceae;Gemmata;Gemmata massiliana;Gemmata massiliana IIL30 |
| Ga0586324 2995727 81 761 | 3580000.00 | 0.00 | 7544200.00 | 6232000.00 | 8147300.00 | 7940000.00 | 7455600.00 | 7137000.00 | 6269400.00 | 2633400.00 | 3520000.00 | 2287500.00 | predicted porin | |
| Ga0586324 2999479 3 761 | 0.00 | 1094800.00 | 0.00 | 0.00 | 0.00 | 0.00 | 0.00 | 0.00 | 0.00 | 0.00 | 0.00 | 0.00 | hypothetical protein | Bacteria;Candidatus Aminicenantes;unclassified Candidatus Aminicenantes;unclassified Candidatus Aminicenantes;unclassified Candidatus Aminicenantes;unclassified Candidatus Aminicenantes;Aminicenantes bacterium SCGC AAA255-E10;Aminicenantes bacterium SCGC AAA255-E10 (contamination screened) |
| Ga0586324 3003954 3 761 | 0.00 | 3536000.00 | 0.00 | 0.00 | 3337200.00 | 0.00 | 2269254.36 | 2183800.00 | 874800.00 | 2202200.00 | 3511200.00 | 780000.00 | MFS family permease | Bacteria;Proteobacteria;Deltaproteobacteria;Myxococcales;Anaeromyxobacteraceae;Anaeromyxobacter;Anaeromyxobacter sp. Fw109-5;Anaeromyxobacter sp. Fw109-5 |
| Ga0586324 3004846 1 762 | 0.00 | 0.00 | 0.00 | 0.00 | 0.00 | 0.00 | 0.00 | 0.00 | 0.00 | 0.00 | 22264000.00 | 0.00 | crotonobetainyl-CoA:carnitine CoA-transferase CaiB-like acyl-CoA transferase | Bacteria;Firmicutes;Clostridia;Thermoanaerobacterales;Thermoanaerobacteraceae;Desulfovirgula;Desulfovirgula thermocuniculi;Desulfovirgula thermocuniculi DSM 16036 |
| Ga0586324 3013475 2 760 | 0.00 | 0.00 | 0.00 | 0.00 | 1823100.00 | 0.00 | 0.00 | 0.00 | 0.00 | 0.00 | 0.00 | 0.00 | 2',3'-cyclic-nucleotide 2'-phosphodiesterase (5'-nucleotidase family) | Bacteria;Thermotogae;Thermotogae;Petrotogales;Petrotogaceae;Marinitoga;Marinitoga piezophila;Marinitoga piezophila KA3 |
| Ga0586324 3016226 2 760 | 0.00 | 0.00 | 0.00 | 0.00 | 0.00 | 0.00 | 0.00 | 707600.00 | 573166.53 | 0.00 | 0.00 | 0.00 | hypothetical protein | Bacteria;Proteobacteria;Betaproteobacteria;Burkholderiales;Burkholderiaceae;Pandoraea;Pandoraea thiooxydans;Pandoraea thiooxydans DSM 25325 |
| Ga0586324 3016679 1 759 | 0.00 | 0.00 | 0.00 | 0.00 | 0.00 | 0.00 | 0.00 | 0.00 | 0.00 | 0.00 | 1707200.00 | 0.00 | iron-only hydrogenase group A | Bacteria;Ignavibacteriae;Ignavibacteria;Ignavibacteriales;Melioribacteraceae;Melioribacter;Melioribacter roseus;Melioribacter roseus P3M |
| Ga0586324 3017385 1 759 | 0.00 | 0.00 | 0.00 | 0.00 | 0.00 | 0.00 | 1391933.16 | 0.00 | 0.00 | 0.00 | 0.00 | 0.00 | ABC-type sugar transport system substrate-binding protein | |
| Ga0586324 3030626 2 757 | 3010000.00 | 0.00 | 0.00 | 0.00 | 0.00 | 0.00 | 0.00 | 0.00 | 0.00 | 11473000.00 | 2816000.00 | 9975000.00 | hypothetical protein | Bacteria;Proteobacteria;Alphaproteobacteria;Hyphomicrobiales;Bradyrhizobiaceae;Bradyrhizobium;Bradyrhizobium elkanii;Bradyrhizobium elkanii USDA 76 |
| Ga0586324 3031177 1 759 | 0.00 | 0.00 | 0.00 | 2223000.00 | 0.00 | 0.00 | 0.00 | 0.00 | 0.00 | 0.00 | 0.00 | 0.00 | hypothetical protein | |
| Ga0586324 3033053 1 759 | 427944.00 | 0.00 | 0.00 | 0.00 | 0.00 | 0.00 | 0.00 | 0.00 | 0.00 | 0.00 | 0.00 | 0.00 | serine phosphatase RsbU (regulator of sigma subunit) | Bacteria;Nitrospirae;Nitrospira;Nitrospirales;Nitrospiraceae;Candidatus Magnetobacterium;Candidatus Magnetobacterium casensis;Candidatus Magnetobacterium casensis MYR-1 |
| Ga0586324 3033599 3 758 | 2550000.00 | 0.00 | 0.00 | 0.00 | 0.00 | 0.00 | 0.00 | 0.00 | 0.00 | 4527600.00 | 4822400.00 | 0.00 | hypothetical protein | |
| Ga0586324 3034432 2 757 | 0.00 | 0.00 | 0.00 | 0.00 | 0.00 | 0.00 | 0.00 | 0.00 | 802537.47 | 0.00 | 0.00 | 0.00 | NADH:ubiquinone oxidoreductase subunit F (NADH-binding) | Bacteria;Firmicutes;Clostridia;Eubacteriales;Peptococcaceae;Desulfofundulus;Desulfofundulus australicus;Desulfofundulus australicus DSM 11792 |
| Ga0586324 3036154 38 757 | 6980000.00 | 0.00 | 12386000.00 | 0.00 | 0.00 | 0.00 | 0.00 | 0.00 | 0.00 | 0.00 | 0.00 | 0.00 | hypothetical protein | |
| Ga0586324 3041236 66 758 | 0.00 | 68680000.00 | 0.00 | 0.00 | 0.00 | 0.00 | 0.00 | 0.00 | 0.00 | 0.00 | 0.00 | 0.00 | hypothetical protein | |
| Ga0586324 3046798 326 676 | 0.00 | 1625200.00 | 0.00 | 0.00 | 0.00 | 0.00 | 0.00 | 0.00 | 0.00 | 0.00 | 0.00 | 0.00 | RNA recognition motif-containing protein | Bacteria;Nitrospirae;Nitrospira;Nitrospirales;Nitrospiraceae;Nitrospira;Nitrospira lenta;Nitrospira lenta N. lenta BS10 |
| Ga0586324 3055055 2 628 | 0.00 | 204540.60 | 421598.61 | 0.00 | 0.00 | 0.00 | 0.00 | 0.00 | 0.00 | 3441900.00 | 0.00 | 0.00 | long-subunit fatty acid transport protein | |
| Ga0586324 3059346 3 719 | 0.00 | 11471600.00 | 0.00 | 7533500.00 | 0.00 | 0.00 | 0.00 | 1226100.00 | 0.00 | 7145600.00 | 6292000.00 | 0.00 | hypothetical protein | Bacteria;Verrucomicrobia;Verrucomicrobiae;Verrucomicrobiales;Verrucomicrobia subdivision 3;Pedosphaera;Pedosphaera parvula;Pedosphaera parvula Ellin514 |
| Ga0586324 3060087 2 754 | 0.00 | 0.00 | 0.00 | 0.00 | 0.00 | 0.00 | 0.00 | 0.00 | 0.00 | 17787000.00 | 420803.68 | 0.00 | hypothetical protein | Bacteria;Deferribacteres;Deferribacteres;Deferribacterales;Deferribacteraceae;Deferribacter;Deferribacter desulfuricans;Deferribacter desulfuricans SSM1 |
| Ga0586324 3063347 1 756 | 0.00 | 0.00 | 0.00 | 0.00 | 0.00 | 0.00 | 0.00 | 0.00 | 0.00 | 0.00 | 2094400.00 | 0.00 | TRAP-type mannitol/chloroaromatic compound transport system substrate-binding protein | Bacteria;Proteobacteria;Betaproteobacteria;Nitrosomonadales;Thiobacillaceae;Sulfuritortus;Sulfuritortus calidifontis;Sulfuritortus calidifontis DSM 103923 |
| Ga0586324 3066749 1 756 | 0.00 | 0.00 | 0.00 | 0.00 | 0.00 | 0.00 | 0.00 | 0.00 | 0.00 | 0.00 | 8800000.00 | 0.00 | hypothetical protein | Bacteria;Proteobacteria;Betaproteobacteria;Nitrosomonadales;Nitrosomonadaceae;Nitrosomonas;Nitrosomonas sp. Nm166;Nitrosomonas sp. Nm166 |
| Ga0586324 3076159 2 316 | 4230000.00 | 0.00 | 0.00 | 0.00 | 0.00 | 0.00 | 0.00 | 0.00 | 0.00 | 0.00 | 0.00 | 0.00 | hypothetical protein | |
| Ga0586324 3077858 1 606 | 0.00 | 0.00 | 0.00 | 0.00 | 0.00 | 0.00 | 924191.16 | 1738500.00 | 0.00 | 0.00 | 0.00 | 0.00 | hypothetical protein | Bacteria;Proteobacteria;Gammaproteobacteria;Enterobacterales;Bruguierivoracaceae;Sodalis;Sodalis sp.;Sodalis lignotolerans 159R |
| Ga0586324 3078369 3 710 | 0.00 | 0.00 | 0.00 | 0.00 | 20291000.00 | 0.00 | 0.00 | 0.00 | 0.00 | 0.00 | 0.00 | 0.00 | hypothetical protein | |
| Ga0586324 3083489 3 752 | 0.00 | 0.00 | 0.00 | 0.00 | 0.00 | 0.00 | 673347.84 | 0.00 | 0.00 | 0.00 | 0.00 | 0.00 | predicted porin | Bacteria;Proteobacteria;Gammaproteobacteria;Chromatiales;unclassified Chromatiales;Thiohalobacter;Thiohalobacter thiocyanaticus;Thiohalobacter thiocyanaticus FOKN1 |
| Ga0586324 3084917 435 752 | 0.00 | 0.00 | 0.00 | 213180000.00 | 0.00 | 0.00 | 14751600.00 | 5020300.00 | 0.00 | 8316000.00 | 0.00 | 8625000.00 | hypothetical protein | Bacteria;Proteobacteria;Alphaproteobacteria;Hyphomicrobiales;Bradyrhizobiaceae;Bradyrhizobium;Bradyrhizobium erythrophlei;Bradyrhizobium erythrophlei GAS478 |
| Ga0586324 3086107 1 753 | 0.00 | 0.00 | 0.00 | 0.00 | 0.00 | 0.00 | 0.00 | 0.00 | 0.00 | 0.00 | 1056000.00 | 0.00 | hypothetical protein | Bacteria;Acidobacteria;Vicinamibacteria;unclassified Vicinamibacteria;Vicinamibacteraceae;Luteitalea;Luteitalea pratensis;Luteitalea pratensis DSM 100886 |
| Ga0586324 3096435 532 753 | 0.00 | 0.00 | 0.00 | 0.00 | 0.00 | 0.00 | 11878800.00 | 0.00 | 0.00 | 0.00 | 0.00 | 0.00 | hypothetical protein | Bacteria;Proteobacteria;Alphaproteobacteria;Hyphomicrobiales;Bradyrhizobiaceae;Bradyrhizobium;Bradyrhizobium erythrophlei;Bradyrhizobium erythrophlei GAS242 |
| Ga0586324 3110341 120 749 | 4680000.00 | 8228000.00 | 5855200.00 | 1064000.00 | 3223900.00 | 0.00 | 4446000.00 | 2006900.00 | 5799600.00 | 7176400.00 | 7374400.00 | 2610000.00 | hypothetical protein | |
| Ga0586324 3113641 3 749 | 0.00 | 0.00 | 0.00 | 0.00 | 0.00 | 2698401.06 | 0.00 | 2763300.00 | 0.00 | 0.00 | 0.00 | 0.00 | hypothetical protein | |
| Ga0586324 3117983 2 748 | 0.00 | 0.00 | 0.00 | 0.00 | 0.00 | 0.00 | 0.00 | 10187000.00 | 0.00 | 0.00 | 0.00 | 0.00 | ABC-type nitrate/sulfonate/bicarbonate transport system substrate-binding protein | Bacteria;Actinobacteria;Actinomycetia;Corynebacteriales;Nocardiaceae;Rhodococcus;Rhodococcus rhodochrous;Rhodococcus rhodochrous 11Y |
| Ga0586324 3129392 3 527 | 0.00 | 0.00 | 0.00 | 0.00 | 9218500.00 | 0.00 | 37255200.00 | 5258200.00 | 0.00 | 10857000.00 | 0.00 | 0.00 | crotonobetainyl-CoA:carnitine CoA-transferase CaiB-like acyl-CoA transferase | Bacteria;Proteobacteria;Deltaproteobacteria;Syntrophobacterales;Syntrophaceae;Desulfobacca;Desulfobacca acetoxidans;Desulfobacca acetoxidans ASRB2, DSM 11109 |
| Ga0586324 3130442 3 749 | 0.00 | 0.00 | 0.00 | 0.00 | 2945800.00 | 0.00 | 0.00 | 0.00 | 0.00 | 0.00 | 0.00 | 0.00 | S-layer protein (TIGR01567 family) | Archaea;Euryarchaeota;Methanomicrobia;Methanosarcinales;Methanotrichaceae;Methanothrix;Methanothrix soehngenii;Methanothrix soehngenii GP-6 |
| Ga0586324 3140304 3 662 | 0.00 | 0.00 | 9008000.00 | 4227500.00 | 3749200.00 | 5637400.00 | 0.00 | 0.00 | 0.00 | 0.00 | 0.00 | 0.00 | hypothetical protein | Bacteria;Firmicutes;Bacilli;Bacillales;Bacillaceae;Bacillus;Bacillus sp. AFS006103;Bacillus sp. AFS006103 |
| Ga0586324 3142728 1 747 | 0.00 | 0.00 | 0.00 | 0.00 | 3182700.00 | 0.00 | 0.00 | 0.00 | 0.00 | 0.00 | 0.00 | 0.00 | hypothetical protein | Bacteria;Acidobacteria;Thermoanaerobaculia;Thermoanaerobaculales;Thermoanaerobaculaceae;Thermoanaerobaculum;Thermoanaerobaculum aquaticum;Thermoanaerobaculum aquaticum MP-01 |
| Ga0586324 3143676 1 747 | 0.00 | 0.00 | 0.00 | 0.00 | 0.00 | 2477625.39 | 0.00 | 0.00 | 0.00 | 0.00 | 0.00 | 0.00 | hypothetical protein | Bacteria;Proteobacteria;Deltaproteobacteria;Desulfuromonadales;Geobacteraceae;Geobacter;Geobacter argillaceus;Geobacter argillaceus ATCC BAA-1139 |
| Ga0586324 3143681 1 567 | 27800000.00 | 26384000.00 | 52809400.00 | 36195000.00 | 61903000.00 | 12584900.00 | 32832000.00 | 41663000.00 | 18549000.00 | 0.00 | 42064000.00 | 35325000.00 | hypothetical protein | Bacteria;Proteobacteria;Alphaproteobacteria;Rhodospirillales;Azospirillaceae;Skermanella;Skermanella aerolata;Skermanella aerolata KACC 11604 |
| Ga0586324 3144979 3 746 | 0.00 | 0.00 | 7600500.00 | 0.00 | 0.00 | 0.00 | 0.00 | 0.00 | 0.00 | 0.00 | 0.00 | 1582500.00 | arylsulfatase A-like enzyme | Bacteria;Planctomycetes;Planctomycetia;Pirellulales;Pirellulaceae;Rhodopirellula;Rhodopirellula bahusiensis;Rhodopirellula bahusiensis SWK21 |
| Ga0586324 3151576 110 745 | 3220000.00 | 0.00 | 0.00 | 0.00 | 0.00 | 0.00 | 0.00 | 0.00 | 0.00 | 0.00 | 0.00 | 0.00 | opacity protein-like surface antigen | Bacteria;Proteobacteria;Deltaproteobacteria;Syntrophobacterales;Syntrophaceae;Desulfobacca;Desulfobacca acetoxidans;Desulfobacca acetoxidans ASRB2, DSM 11109 |
| Ga0586324 3168225 3 419 | 0.00 | 7391600.00 | 8107200.00 | 6887500.00 | 7261500.00 | 0.00 | 5061600.00 | 7076000.00 | 7411500.00 | 6575800.00 | 7321600.00 | 0.00 | predicted porin | Bacteria;Proteobacteria;Betaproteobacteria;Nitrosomonadales;Sulfuricellaceae;Sulfuricella;Sulfuricella denitrificans;Sulfuricella denitrificans skB26 |
| Ga0586324 3168636 2 745 | 3540000.00 | 0.00 | 0.00 | 0.00 | 0.00 | 0.00 | 0.00 | 0.00 | 0.00 | 0.00 | 0.00 | 0.00 | ABC-type uncharacterized transport system YnjBCD substrate-binding protein | Bacteria;Firmicutes;Bacilli;Bacillales;Paenibacillaceae;Paenibacillus;Paenibacillus naphthalenovorans;Paenibacillus naphthalenovorans PR-N1 |
| Ga0586324 3177177 1 621 | 0.00 | 0.00 | 0.00 | 0.00 | 2296900.00 | 0.00 | 0.00 | 0.00 | 0.00 | 0.00 | 0.00 | 0.00 | tetratricopeptide (TPR) repeat protein | Bacteria;Bacteroidetes;Sphingobacteriia;Sphingobacteriales;Sphingobacteriaceae;Pedobacter;Pedobacter ginsengisoli;Pedobacter ginsengisoli T01R-27 |
| Ga0586324 3179110 58 744 | 6620000.00 | 0.00 | 0.00 | 0.00 | 0.00 | 0.00 | 0.00 | 0.00 | 0.00 | 0.00 | 0.00 | 0.00 | cytochrome c peroxidase | Bacteria;Proteobacteria;Deltaproteobacteria;Syntrophobacterales;Syntrophaceae;Desulfomonile;Desulfomonile tiedjei;Desulfomonile tiedjei DCB-1, DSM 6799 |
| Ga0586324 3180045 1 579 | 0.00 | 11560000.00 | 0.00 | 0.00 | 0.00 | 0.00 | 0.00 | 0.00 | 0.00 | 0.00 | 0.00 | 0.00 | hypothetical protein | |
| Ga0586324 3180872 2 742 | 4160000.00 | 0.00 | 0.00 | 0.00 | 12669000.00 | 0.00 | 9188400.00 | 8967000.00 | 0.00 | 0.00 | 7488800.00 | 2092500.00 | hypothetical protein | Bacteria;Proteobacteria;Deltaproteobacteria;Syntrophorhabdales;Syntrophorhabdaceae;Syntrophorhabdus;Syntrophorhabdus aromaticivorans;Syntrophorhabdus aromaticivorans UI |
| Ga0586324 3184817 1 744 | 0.00 | 573178.80 | 0.00 | 0.00 | 0.00 | 0.00 | 0.00 | 0.00 | 0.00 | 0.00 | 0.00 | 0.00 | crotonobetainyl-CoA:carnitine CoA-transferase CaiB-like acyl-CoA transferase | Bacteria;Firmicutes;Clostridia;Thermoanaerobacterales;Thermoanaerobacteraceae;Desulfovirgula;Desulfovirgula thermocuniculi;Desulfovirgula thermocuniculi DSM 16036 |
| Ga0586324 3187790 319 741 | 5490000.00 | 0.00 | 0.00 | 0.00 | 0.00 | 0.00 | 0.00 | 0.00 | 0.00 | 0.00 | 0.00 | 0.00 | hypothetical protein | Bacteria;Firmicutes;Clostridia;Thermosediminibacterales;Thermosediminibacteraceae;Caldanaerovirga;Caldanaerovirga acetigignens;Caldanaerovirga acetigignens DSM 18802 |
| Ga0586324 3190524 1 741 | 0.00 | 0.00 | 0.00 | 0.00 | 0.00 | 0.00 | 0.00 | 725900.00 | 0.00 | 0.00 | 0.00 | 0.00 | hypothetical protein | Bacteria;Proteobacteria;Deltaproteobacteria;Myxococcales;Kofleriaceae;Haliangium;Haliangium sp. UPWRP 2;Haliangium sp. TSM HC |
| Ga0586324 3194319 2 742 | 0.00 | 0.00 | 0.00 | 0.00 | 0.00 | 0.00 | 0.00 | 0.00 | 0.00 | 3511200.00 | 0.00 | 0.00 | hypothetical protein | |
| Ga0586324 3195143 2 742 | 0.00 | 610016.44 | 1196977.41 | 0.00 | 0.00 | 0.00 | 0.00 | 0.00 | 0.00 | 0.00 | 0.00 | 0.00 | hypothetical protein | Bacteria;Proteobacteria;Deltaproteobacteria;Desulfuromonadales;Geobacteraceae;Geobacter;Geobacter argillaceus;Geobacter argillaceus ATCC BAA-1139 |
| Ga0586324 3197384 3 743 | 2870000.00 | 0.00 | 0.00 | 4370000.00 | 0.00 | 0.00 | 0.00 | 0.00 | 0.00 | 0.00 | 0.00 | 0.00 | aldehyde:ferredoxin oxidoreductase | |
| Ga0586324 3202881 1 456 | 0.00 | 0.00 | 0.00 | 0.00 | 0.00 | 10917500.00 | 0.00 | 0.00 | 0.00 | 0.00 | 0.00 | 0.00 | hypothetical protein | Bacteria;Bacteroidetes;Flavobacteriia;Flavobacteriales;Flavobacteriaceae;Bizionia;Bizionia paragorgiae;Bizionia paragorgiae DSM 23842 |
| Ga0586324 3212627 1 741 | 0.00 | 0.00 | 4441298.69 | 0.00 | 0.00 | 0.00 | 0.00 | 0.00 | 0.00 | 0.00 | 0.00 | 0.00 | hypothetical protein | |
| Ga0586324 3219854 149 739 | 2150000.00 | 2475200.00 | 11541500.00 | 2850000.00 | 2502900.00 | 2647886.78 | 1966862.52 | 2061800.00 | 5823900.00 | 0.00 | 1830400.00 | 2497500.00 | NAD(P)-dependent dehydrogenase (short-subunit alcohol dehydrogenase family) | Bacteria;Proteobacteria;Alphaproteobacteria;Rhodospirillales;Acetobacteraceae;Rhodopila;Rhodopila globiformis;Rhodopila globiformis DSM 161 |
| Ga0586324 3222710 2 547 | 0.00 | 4331600.00 | 0.00 | 0.00 | 0.00 | 0.00 | 0.00 | 0.00 | 0.00 | 0.00 | 0.00 | 0.00 | hypothetical protein | Bacteria;Bacteroidetes;Bacteroidia;Marinilabiliales;Prolixibacteraceae;Tangfeifania;Tangfeifania diversioriginum;Tangfeifania diversioriginum DSM 27063 |
| Ga0586324 3223203 1 738 | 0.00 | 4596800.00 | 7319000.00 | 0.00 | 0.00 | 0.00 | 3100800.00 | 0.00 | 4382100.00 | 0.00 | 8791200.00 | 4207500.00 | hypothetical protein | Bacteria;Proteobacteria;Deltaproteobacteria;Desulfuromonadales;Geobacteraceae;Geobacter;Geobacter lovleyi;Geobacter lovleyi SZ |
| Ga0586324 3225345 3 572 | 0.00 | 1421200.00 | 0.00 | 0.00 | 0.00 | 0.00 | 0.00 | 0.00 | 0.00 | 1424500.00 | 2103200.00 | 0.00 | PAS domain-containing protein | Bacteria;Proteobacteria;Deltaproteobacteria;Syntrophobacterales;Syntrophaceae;Syntrophus;Syntrophus gentianae;Syntrophus gentianae DSM 8423 |
| Ga0586324 3226712 2 739 | 0.00 | 0.00 | 0.00 | 0.00 | 0.00 | 0.00 | 0.00 | 244950.38 | 1555200.00 | 0.00 | 1786400.00 | 0.00 | TRAP-type mannitol/chloroaromatic compound transport system substrate-binding protein | Bacteria;Proteobacteria;Betaproteobacteria;Rhodocyclales;Zoogloeaceae;Azoarcus;Azoarcus sp. KH32C;Azoarcus sp. KH32C |
| Ga0586324 3228666 1 225 | 0.00 | 0.00 | 0.00 | 0.00 | 0.00 | 0.00 | 0.00 | 0.00 | 0.00 | 0.00 | 2842400.00 | 0.00 | hypothetical protein | Eukaryota;Streptophyta;Lycopodiopsida;Selaginellales;Selaginellaceae;Selaginella;Selaginella moellendorffii;Selaginella moellendorffii |
| Ga0586324 3238389 2 739 | 0.00 | 0.00 | 0.00 | 0.00 | 2739800.00 | 3284682.72 | 0.00 | 0.00 | 1166400.00 | 0.00 | 2032800.00 | 1275000.00 | gliding motility-associated protein GldL | Bacteria;Bacteroidetes;Flavobacteriia;Flavobacteriales;Crocinitomicaceae;Crocinitomix;Crocinitomix algicola;Crocinitomix algicola 0182 |
| Ga0586324 3244907 3 737 | 7990000.00 | 0.00 | 0.00 | 0.00 | 0.00 | 0.00 | 0.00 | 0.00 | 0.00 | 0.00 | 0.00 | 0.00 | hypothetical protein | Bacteria;Acidobacteria;Blastocatellia;Blastocatellales;Pyrinomonadaceae;Pyrinomonas;Pyrinomonas methylaliphatogenes;Pyrinomonas methylaliphatogenes K22 |
| Ga0586324 3245545 1 738 | 0.00 | 0.00 | 0.00 | 0.00 | 4583500.00 | 0.00 | 0.00 | 0.00 | 2187000.00 | 0.00 | 0.00 | 0.00 | hypothetical protein | |
| Ga0586324 3247217 3 287 | 0.00 | 0.00 | 5607074.64 | 6051500.00 | 0.00 | 0.00 | 27588000.00 | 0.00 | 0.00 | 0.00 | 0.00 | 8775000.00 | hypothetical protein | Bacteria;Actinobacteria;Actinomycetia;Corynebacteriales;Mycobacteriaceae;Mycobacterium;Mycobacterium sp. 3519A;Mycobacterium sp. 3519A |
| Ga0586324 3249732 1 738 | 5140000.00 | 0.00 | 0.00 | 4360500.00 | 0.00 | 0.00 | 0.00 | 0.00 | 0.00 | 0.00 | 0.00 | 0.00 | hypothetical protein | Bacteria;Chloroflexi;Caldilineae;Caldilineales;Caldilineaceae;Caldilinea;Caldilinea aerophila;Caldilinea aerophila STL-6-O1, DSM 14535 |
| Ga0586324 3250028 2 736 | 0.00 | 0.00 | 0.00 | 0.00 | 0.00 | 0.00 | 0.00 | 0.00 | 0.00 | 0.00 | 4716800.00 | 0.00 | hypothetical protein | Bacteria;Acidobacteria;Holophagae;Holophagales;Holophagaceae;Geothrix;Geothrix fermentans;Geothrix fermentans DSM 14018 |
| Ga0586324 3263202 3 734 | 0.00 | 748000.00 | 0.00 | 0.00 | 0.00 | 0.00 | 0.00 | 0.00 | 0.00 | 0.00 | 0.00 | 0.00 | Neuraminidase (sialidase) | |
| Ga0586324 3273291 2 733 | 0.00 | 0.00 | 0.00 | 0.00 | 0.00 | 0.00 | 0.00 | 359613.30 | 0.00 | 0.00 | 0.00 | 0.00 | autotransporter-associated beta strand protein | Bacteria;Proteobacteria;Betaproteobacteria;Burkholderiales;Comamonadaceae;Pelomonas;Pelomonas saccharophila;Pelomonas saccharophila DSM 654 |
| Ga0586324 3279176 232 735 | 0.00 | 0.00 | 0.00 | 0.00 | 0.00 | 0.00 | 0.00 | 288364.08 | 0.00 | 0.00 | 0.00 | 0.00 | phasin | Bacteria;Proteobacteria;Alphaproteobacteria;Hyphomicrobiales;Xanthobacteraceae;Pseudolabrys;Pseudolabrys taiwanensis;Pseudolabrys taiwanensis CC-BB4 |
| Ga0586324 3286310 3 734 | 0.00 | 0.00 | 0.00 | 0.00 | 536363.23 | 0.00 | 0.00 | 0.00 | 0.00 | 900900.00 | 0.00 | 0.00 | hypothetical protein | Bacteria;Spirochaetes;Spirochaetia;Spirochaetales;Spirochaetaceae;Sediminispirochaeta;Sediminispirochaeta smaragdinae;Sediminispirochaeta smaragdinae DSM 11293 |
| Ga0586324 3287422 2 733 | 0.00 | 0.00 | 0.00 | 0.00 | 0.00 | 0.00 | 0.00 | 0.00 | 2073600.00 | 0.00 | 0.00 | 0.00 | Ca2+-binding RTX toxin-like protein | Bacteria;Proteobacteria;Alphaproteobacteria;Hyphomicrobiales;Cohaesibacteraceae;Cohaesibacter;Cohaesibacter gelatinilyticus;Cohaesibacter gelatinilyticus DSM 18289 |
| Ga0586324 3294900 1 462 | 0.00 | 0.00 | 0.00 | 0.00 | 0.00 | 0.00 | 0.00 | 0.00 | 0.00 | 4127200.00 | 0.00 | 0.00 | lipid-binding SYLF domain-containing protein | Bacteria;Proteobacteria;Betaproteobacteria;Nitrosomonadales;Sterolibacteriaceae;Sulfurisoma;Sulfurisoma sediminicola;Sulfurisoma sediminicola DSM 26916 |
| Ga0586324 3295986 165 731 | 0.00 | 539609.92 | 0.00 | 0.00 | 0.00 | 0.00 | 0.00 | 0.00 | 0.00 | 0.00 | 0.00 | 0.00 | glutamate synthase domain-containing protein 2 | Archaea;Euryarchaeota;Methanomicrobia;Methanomicrobiales;Methanomicrobiaceae;Methanofollis;Methanofollis liminatans;Methanofollis liminatans GKZPZ, DSM 4140 |
| Ga0586324 3300968 3 731 | 0.00 | 4535600.00 | 0.00 | 12825000.00 | 0.00 | 0.00 | 0.00 | 0.00 | 0.00 | 0.00 | 17336000.00 | 0.00 | crotonobetainyl-CoA:carnitine CoA-transferase CaiB-like acyl-CoA transferase | Bacteria;Proteobacteria;Deltaproteobacteria;Syntrophobacterales;Syntrophaceae;Desulfobacca;Desulfobacca acetoxidans;Desulfobacca acetoxidans ASRB2, DSM 11109 |
| Ga0586324 3308670 1 732 | 0.00 | 0.00 | 1561711.33 | 455508.85 | 0.00 | 0.00 | 0.00 | 547998.99 | 0.00 | 0.00 | 0.00 | 0.00 | outer membrane receptor protein involved in Fe transport | Bacteria;Proteobacteria;Betaproteobacteria;Burkholderiales;Burkholderiaceae;Burkholderia;Burkholderia lata;Burkholderia cepacia 383 |
| Ga0586324 3311842 1 732 | 0.00 | 1999200.00 | 0.00 | 0.00 | 0.00 | 0.00 | 0.00 | 0.00 | 0.00 | 0.00 | 0.00 | 0.00 | S-layer protein (TIGR01567 family) | Archaea;Euryarchaeota;Methanomicrobia;Methanosarcinales;Methanotrichaceae;Methanothrix;Methanothrix soehngenii;Methanothrix soehngenii GP-6 |
| Ga0586324 3313172 192 731 | 0.00 | 2543200.00 | 0.00 | 2812000.00 | 0.00 | 0.00 | 2644800.00 | 0.00 | 0.00 | 1555400.00 | 3229600.00 | 2377500.00 | predicted porin | Bacteria;Proteobacteria;Betaproteobacteria;Neisseriales;Chromobacteriaceae;Aquaspirillum;Aquaspirillum sp. LM1;Aquaspirillum sp. LM1 |
| Ga0586324 3315992 1 729 | 0.00 | 0.00 | 0.00 | 0.00 | 0.00 | 0.00 | 0.00 | 0.00 | 0.00 | 862400.00 | 0.00 | 0.00 | ABC-type oligopeptide transport system substrate-binding subunit | Bacteria;Actinobacteria;Rubrobacteria;Gaiellales;Gaiellaceae;Gaiella;Gaiella occulta;Gaiella occulta F2-233 |
| Ga0586324 3316062 1 477 | 0.00 | 0.00 | 0.00 | 0.00 | 0.00 | 0.00 | 2416800.00 | 0.00 | 0.00 | 0.00 | 1654400.00 | 588264.75 | membrane protease subunit (stomatin/prohibitin family) | Bacteria;Chloroflexi;Anaerolineae;Anaerolineales;Anaerolineaceae;Thermanaerothrix;Thermanaerothrix daxensis;Thermanaerothrix daxensis GNS-1 |
| Ga0586324 3316590 2 250 | 0.00 | 999600.00 | 0.00 | 0.00 | 0.00 | 0.00 | 9918000.00 | 529334.21 | 866700.00 | 19250000.00 | 200109.36 | 351805.50 | predicted porin | |
| Ga0586324 3317128 449 730 | 6820000.00 | 0.00 | 0.00 | 0.00 | 0.00 | 0.00 | 0.00 | 0.00 | 0.00 | 0.00 | 0.00 | 5302500.00 | hypothetical protein | Bacteria;Spirochaetes;Spirochaetia;Spirochaetales;Spirochaetaceae;Spirochaeta;Spirochaeta cellobiosiphila;Spirochaeta cellobiosiphila DSM 17781 |
| Ga0586324 3318552 1 729 | 7950000.00 | 4970800.00 | 0.00 | 0.00 | 0.00 | 0.00 | 0.00 | 0.00 | 0.00 | 0.00 | 0.00 | 0.00 | hypothetical protein | Bacteria;Spirochaetes;Spirochaetia;Spirochaetales;Spirochaetaceae;Sediminispirochaeta;Sediminispirochaeta smaragdinae;Sediminispirochaeta smaragdinae DSM 11293 |
| Ga0586324 3328433 1 594 | 1140000.00 | 1108400.00 | 0.00 | 0.00 | 0.00 | 0.00 | 0.00 | 0.00 | 327312.90 | 993300.00 | 2094400.00 | 0.00 | hypothetical protein | Bacteria;Spirochaetes;Spirochaetia;Spirochaetales;Spirochaetaceae;Spirochaeta;Spirochaeta thermophila;Spirochaeta thermophila DSM 6192 |
| Ga0586324 3337742 2 727 | 2810000.00 | 0.00 | 2181715.08 | 3239500.00 | 0.00 | 3744023.63 | 4367725.32 | 0.00 | 0.00 | 0.00 | 0.00 | 1980000.00 | predicted porin | Bacteria;Proteobacteria;Betaproteobacteria;Burkholderiales;unclassified Burkholderiales;Aquincola;Aquincola tertiaricarbonis;Aquincola tertiaricarbonis MIMtkpLc11 |
| Ga0586324 3341574 3 728 | 327054.00 | 4093600.00 | 9796200.00 | 5662000.00 | 0.00 | 3518519.69 | 0.00 | 7564000.00 | 7273800.00 | 21467600.00 | 0.00 | 12150000.00 | predicted porin | Bacteria;Proteobacteria;Betaproteobacteria;unclassified Betaproteobacteria;unclassified Betaproteobacteria;unclassified Betaproteobacteria;Betaproteobacteria bacterium GR16-43;Betaproteobacteria bacterium GR16-43 |
| Ga0586324 3343666 2 727 | 0.00 | 393162.40 | 0.00 | 1387000.00 | 0.00 | 0.00 | 0.00 | 0.00 | 0.00 | 0.00 | 0.00 | 0.00 | hypothetical protein | Bacteria;Proteobacteria;Gammaproteobacteria;Methylococcales;Methylococcaceae;Methylovulum;Methylovulum miyakonense;Methylovulum miyakonense HT12 |
| Ga0586324 3347838 1 729 | 5550000.00 | 1645600.00 | 0.00 | 0.00 | 3903700.00 | 0.00 | 0.00 | 0.00 | 1790100.00 | 0.00 | 749365.76 | 1777500.00 | hypothetical protein | Bacteria;Proteobacteria;Alphaproteobacteria;Hyphomicrobiales;Rhizobiaceae;Rhizobium;Rhizobium mesoamericanum;Rhizobium mesoamericanum STM3625 |
| Ga0586324 3358820 190 636 | 3260000.00 | 0.00 | 0.00 | 0.00 | 0.00 | 0.00 | 0.00 | 0.00 | 0.00 | 0.00 | 0.00 | 0.00 | hypothetical protein | Bacteria;Nitrospirae;Nitrospira;Nitrospirales;Nitrospiraceae;Nitrospira;Nitrospira moscoviensis;Nitrospira moscoviensis NSP M-1 |
| Ga0586324 3371845 24 725 | 2860000.00 | 3189200.00 | 5387223.14 | 0.00 | 0.00 | 3554745.94 | 0.00 | 0.00 | 1595700.00 | 0.00 | 0.00 | 1455000.00 | hypothetical protein | Bacteria;Cyanobacteria;unclassified Cyanobacteria;Oscillatoriales;Microcoleaceae;Microcoleus;Microcoleus vaginatus;Microcoleus vaginatus PCC 9802 |
| Ga0586324 3405089 3 722 | 0.00 | 29606258.88 | 30007900.00 | 5785500.00 | 11010700.00 | 8654600.00 | 20953200.00 | 5703500.00 | 10044000.00 | 0.00 | 5781600.00 | 3622500.00 | hypothetical protein | Archaea;Euryarchaeota;Methanomicrobia;Methanosarcinales;Methanotrichaceae;Methanothrix;Methanothrix thermoacetophila;Methanothrix thermoacetophila PT |
| Ga0586324 3405998 3 464 | 0.00 | 822800.00 | 0.00 | 0.00 | 0.00 | 0.00 | 0.00 | 0.00 | 0.00 | 0.00 | 0.00 | 685509.00 | hypothetical protein | |
| Ga0586324 3413602 44 721 | 0.00 | 1400800.00 | 0.00 | 0.00 | 0.00 | 0.00 | 0.00 | 0.00 | 0.00 | 0.00 | 0.00 | 0.00 | rubrerythrin | Bacteria;Proteobacteria;Deltaproteobacteria;Syntrophobacterales;Syntrophaceae;Smithella;Smithella sp. SCADC;Smithella sp. SCADC |
| Ga0586324 3430809 3 719 | 0.00 | 802400.00 | 0.00 | 0.00 | 0.00 | 0.00 | 0.00 | 0.00 | 0.00 | 0.00 | 0.00 | 0.00 | arylsulfatase A-like enzyme | Bacteria;Planctomycetes;Planctomycetia;Planctomycetales;Planctomycetaceae;Planctopirus;Planctopirus limnophila;Planctopirus limnophila DSM 3776 |
| Ga0586324 3432092 3 413 | 0.00 | 0.00 | 0.00 | 0.00 | 0.00 | 0.00 | 0.00 | 0.00 | 0.00 | 0.00 | 0.00 | 10050000.00 | hypothetical protein | Bacteria;Ignavibacteriae;Ignavibacteria;Ignavibacteriales;Melioribacteraceae;Melioribacter;Melioribacter roseus;Melioribacter roseus P3M |
| Ga0586324 3434163 45 719 | 0.00 | 0.00 | 0.00 | 0.00 | 0.00 | 333347.80 | 0.00 | 0.00 | 0.00 | 0.00 | 0.00 | 177591.00 | TRAP-type mannitol/chloroaromatic compound transport system substrate-binding protein | Bacteria;Proteobacteria;Alphaproteobacteria;Hyphomicrobiales;Bradyrhizobiaceae;Bradyrhizobium;Bradyrhizobium jicamae;Bradyrhizobium jicamae PAC68 |
| Ga0586324 3438917 1 720 | 0.00 | 0.00 | 0.00 | 0.00 | 0.00 | 0.00 | 0.00 | 0.00 | 0.00 | 0.00 | 1188000.00 | 0.00 | hypothetical protein | Bacteria;Acidobacteria;Vicinamibacteria;unclassified Vicinamibacteria;Vicinamibacteraceae;Luteitalea;Luteitalea pratensis;Luteitalea pratensis DSM 100886 |
| Ga0586324 3441505 1 720 | 3500000.00 | 4501365.40 | 6211899.91 | 2441500.00 | 3254800.00 | 1721939.86 | 5138189.76 | 44285989.63 | 1443838.77 | 0.00 | 1126400.00 | 1170000.00 | hypothetical protein | Archaea;Euryarchaeota;Methanomicrobia;Methanosarcinales;Methanotrichaceae;Methanothrix;Methanothrix thermoacetophila;Methanothrix thermoacetophila PT |
| Ga0586324 3454655 2 634 | 12900000.00 | 12512000.00 | 0.00 | 21185000.00 | 21012000.00 | 8456100.00 | 14341200.00 | 12078000.00 | 13608000.00 | 14630000.00 | 13640000.00 | 10575000.00 | hypothetical protein | |
| Ga0586324 3477544 386 691 | 0.00 | 0.00 | 0.00 | 0.00 | 10506000.00 | 13855300.00 | 0.00 | 0.00 | 0.00 | 0.00 | 0.00 | 0.00 | RNA recognition motif-containing protein | Bacteria;Bacteroidetes;Bacteroidia;Marinilabiliales;Marinilabiliaceae;Anaerophaga;Anaerophaga thermohalophila;Anaerophaga thermohalophila Fru22, DSM 12881 |
| Ga0586324 3478395 2 715 | 0.00 | 0.00 | 0.00 | 0.00 | 0.00 | 0.00 | 0.00 | 0.00 | 675970.11 | 0.00 | 0.00 | 0.00 | Ca2+-binding RTX toxin-like protein | Bacteria;Planctomycetes;Planctomycetia;Pirellulales;Pirellulaceae;Roseimaritima;Roseimaritima ulvae;Roseimaritima ulvae DSM 25454 |
| Ga0586324 3479584 272 715 | 17300000.00 | 12988000.00 | 7882000.00 | 24225000.00 | 38934000.00 | 5875600.00 | 9028800.00 | 9882000.00 | 5370300.00 | 15246000.00 | 4004000.00 | 10650000.00 | tetratricopeptide (TPR) repeat protein | Bacteria;Proteobacteria;Deltaproteobacteria;Syntrophobacterales;Syntrophaceae;Desulfobacca;Desulfobacca acetoxidans;Desulfobacca acetoxidans ASRB2, DSM 11109 |
| Ga0586324 3480561 3 431 | 0.00 | 0.00 | 0.00 | 0.00 | 0.00 | 0.00 | 0.00 | 0.00 | 1312200.00 | 0.00 | 0.00 | 0.00 | hypothetical protein | Bacteria;Proteobacteria;Deltaproteobacteria;Desulfobacterales;Desulfobacteraceae;Desulfonema;Desulfonema limicola;Desulfonema limicola Jadebusen, DSM 2076 |
| Ga0586324 3485832 3 716 | 0.00 | 0.00 | 0.00 | 0.00 | 0.00 | 0.00 | 0.00 | 0.00 | 0.00 | 0.00 | 0.00 | 5685000.00 | hypothetical protein | Bacteria;Candidatus Aminicenantes;unclassified Candidatus Aminicenantes;unclassified Candidatus Aminicenantes;unclassified Candidatus Aminicenantes;unclassified Candidatus Aminicenantes;Aminicenantes bacterium SCGC AAA255-E05;Aminicenantes bacterium SCGC AAA255-E05 (contamination screened) |
| Ga0586324 3493594 2 613 | 2420000.00 | 4800800.00 | 7769400.00 | 6963500.00 | 0.00 | 3729723.69 | 5266800.00 | 0.00 | 4827600.00 | 11896500.00 | 7163200.00 | 5407500.00 | hypothetical protein | Bacteria;Acidobacteria;Acidobacteriia;Acidobacteriales;Acidobacteriaceae;unclassified Acidobacteriaceae;Acidobacteriaceae bacterium KBS 96;Acidobacteriaceae bacterium KBS 96, DSM 24297 |
| Ga0586324 3499211 330 713 | 0.00 | 0.00 | 0.00 | 0.00 | 0.00 | 0.00 | 0.00 | 0.00 | 0.00 | 0.00 | 0.00 | 12900000.00 | hypothetical protein | |
| Ga0586324 3516154 3 713 | 0.00 | 0.00 | 0.00 | 0.00 | 0.00 | 0.00 | 0.00 | 0.00 | 0.00 | 0.00 | 0.00 | 795000.00 | methyl-accepting chemotaxis protein | Bacteria;Proteobacteria;Betaproteobacteria;Nitrosomonadales;Thiobacillaceae;Sulfuritortus;Sulfuritortus calidifontis;Sulfuritortus calidifontis DSM 103923 |

| | | | | | | | | | | | | | | |
|---|---|---|---|---|---|---|---|---|---|---|---|---|---|---|
| Ga0586324_3519527_2_712 | 0.00 | 0.00 | 0.00 | 0.00 | 0.00 | 0.00 | 0.00 | 0.00 | 0.00 | 0.00 | 0.00 | 11250000.00 | hypothetical protein | Bacteria;Actinobacteria;Thermoleophilia;Solirubrobacterales;Solirubrobacteraceae;Solirubrobacter;Solirubrobacter soli;Solirubrobacter soli DSM 22325 |
| Ga0586324_3520287_2_712 | 0.00 | 0.00 | 0.00 | 0.00 | 0.00 | 0.00 | 0.00 | 0.00 | 0.00 | 0.00 | 7532800.00 | 0.00 | hypothetical protein | Bacteria;Proteobacteria;Alphaproteobacteria;Rhodobacterales;Rhodobacteraceae;Rhodovulum;Rhodovulum sp. PH10;Rhodovulum sp. PH10 |
| Ga0586324_3524173_3_371 | 0.00 | 5650800.00 | 0.00 | 0.00 | 0.00 | 0.00 | 0.00 | 0.00 | 0.00 | 0.00 | 0.00 | 0.00 | crotonobetainyl-CoA:carnitine CoA-transferase CaiB-like acyl-CoA transferase | Archaea;Euryarchaeota;Archaeoglobi;Archaeoglobales;Archaeoglobaceae;Ferroglobus;Ferroglobus placidus;Ferroglobus placidus AEDII12DO, DSM 10642 |
| Ga0586324_3528080_70_711 | 0.00 | 17000000.00 | 0.00 | 12635000.00 | 62521000.00 | 0.00 | 47424000.00 | 13481000.00 | 31023000.00 | 0.00 | 0.00 | 11700000.00 | hypothetical protein | Bacteria;Proteobacteria;Alphaproteobacteria;Hyphomicrobiales;Bradyrhizobiaceae;Bradyrhizobium;Bradyrhizobium macuxiense;Bradyrhizobium macuxiense BR 10355 |
| Ga0586324_3530595_100_711 | 0.00 | 0.00 | 5518723.05 | 0.00 | 0.00 | 0.00 | 0.00 | 0.00 | 0.00 | 0.00 | 0.00 | 0.00 | regulator of protease activity HflC (stomatin/prohibitin superfamily) | Bacteria;Proteobacteria;Deltaproteobacteria;Desulfuromonadales;Geobacteraceae;Geobacter;Geobacter sp. DSM 2909;Geobacter sp. DSM 2909 |
| Ga0586324_3533941_3_710 | 0.00 | 0.00 | 0.00 | 0.00 | 0.00 | 0.00 | 1568291.16 | 1152900.00 | 0.00 | 0.00 | 0.00 | 1102500.00 | ABC-type branched-subunit amino acid transport system substrate-binding protein | Bacteria;Actinobacteria;Actinomycetia;Streptosporangiales;Streptosporangiaceae;Nonomuraea;Nonomuraea coxensis;Nonomuraea coxensis DSM 45129 |
| Ga0586324_3546496_64_711 | 0.00 | 0.00 | 0.00 | 0.00 | 11536000.00 | 0.00 | 0.00 | 6527000.00 | 6982200.00 | 0.00 | 0.00 | 0.00 | glutamate synthase domain-containing protein 2 | Archaea;Euryarchaeota;Methanomicrobia;Methanosarcinales;Methanotrichaceae;Methanothrix;Methanothrix soehngenii;Methanothrix soehngenii GP-6 |
| Ga0586324_3553212_1_711 | 0.00 | 22440000.00 | 0.00 | 25080000.00 | 36256000.00 | 0.00 | 0.00 | 14884000.00 | 24462000.00 | 20944000.00 | 16280000.00 | 0.00 | hypothetical protein | Bacteria;Proteobacteria;Deltaproteobacteria;Desulfobacterales;Desulfobacteraceae;Desulfobacter;Desulfobacter curvatus;Desulfobacter curvatus DSM 3379 |
| Ga0586324_3553310_3_242 | 2620000.00 | 2216800.00 | 4610896.81 | 3144500.00 | 3326900.00 | 3278977.83 | 0.00 | 0.00 | 1522800.00 | 3765300.00 | 3264800.00 | 2242500.00 | predicted porin | Bacteria;Proteobacteria;Betaproteobacteria;unclassified Betaproteobacteria;unclassified Betaproteobacteria;unclassified Betaproteobacteria;uncultured beta proteobacterium;unclassified betaproteobacterium ENR4 |
| Ga0586324_3554025_1_666 | 0.00 | 0.00 | 0.00 | 3410500.00 | 3306300.00 | 1482147.89 | 0.00 | 2513200.00 | 16524000.00 | 4750900.00 | 0.00 | 1492500.00 | predicted porin | Bacteria;Proteobacteria;Betaproteobacteria;Burkholderiales;Oxalobacteraceae;Massilia;Massilia niastensis;Massilia niastensis DSM 21313 |
| Ga0586324_3554292_1_705 | 0.00 | 0.00 | 0.00 | 0.00 | 0.00 | 0.00 | 0.00 | 0.00 | 0.00 | 0.00 | 0.00 | 12090000.00 | kynurenine formamidase | Bacteria;Proteobacteria;Alphaproteobacteria;Rhodobacterales;Acuticoccaceae;Acuticoccus;Acuticoccus kandeliae;Acuticoccus kandeliae J103 |
| Ga0586324_3560638_296_709 | 0.00 | 0.00 | 0.00 | 0.00 | 0.00 | 0.00 | 0.00 | 0.00 | 0.00 | 2086700.00 | 4919200.00 | 4275000.00 | hypothetical protein | |
| Ga0586324_3564620_1_426 | 7070000.00 | 17000000.00 | 15144700.00 | 5386500.00 | 0.00 | 83767000.00 | 0.00 | 22570000.00 | 47547000.00 | 64834000.00 | 23496000.00 | 68175000.00 | hypothetical protein | |
| Ga0586324_3567605_1_708 | 111170000.00 | 76309600.00 | 25111973.18 | 36860000.00 | 61495858.51 | 123744900.00 | 23871600.00 | 21594000.00 | 17455500.00 | 68737900.00 | 39582400.00 | 15120000.00 | hypothetical protein | Bacteria;Proteobacteria;Deltaproteobacteria;Desulfobacterales;Desulfobacteraceae;Desulfatirhabdium;Desulfatirhabdium butyrativorans;Desulfatirhabdium butyrativorans DSM 18734 |
| Ga0586324_3568790_1_708 | 2200000.00 | 0.00 | 8050900.00 | 0.00 | 0.00 | 0.00 | 0.00 | 0.00 | 0.00 | 0.00 | 0.00 | 0.00 | TRAP-type mannitol/chloroaromatic compound transport system substrate-binding protein | Bacteria;Proteobacteria;Alphaproteobacteria;Hyphomicrobiales;Bradyrhizobiaceae;Bradyrhizobium;Bradyrhizobium valentinum;Bradyrhizobium valentinum LmjM3 |
| Ga0586324_3569559_1_708 | 0.00 | 5161200.00 | 7150100.00 | 8075000.00 | 8868300.00 | 4089100.00 | 8527200.00 | 15067000.00 | 13122000.00 | 11988900.00 | 11440000.00 | 10875000.00 | hypothetical protein | Bacteria;Proteobacteria;Alphaproteobacteria;Sphingomonadales;Erythrobacteraceae;Altererythrobacter;Altererythrobacter epoxidivorans;Altererythrobacter epoxidivorans CGMCC 1.7731 |
| Ga0586324_3600209_2_706 | 0.00 | 0.00 | 0.00 | 0.00 | 0.00 | 0.00 | 0.00 | 0.00 | 0.00 | 850500.00 | 0.00 | 0.00 | hypothetical protein | |
| Ga0586324_3614667_2_706 | 0.00 | 3359200.00 | 3833022.23 | 5377000.00 | 7446900.00 | 5399200.00 | 0.00 | 5368000.00 | 4179600.00 | 6183100.00 | 6538400.00 | 6142500.00 | hypothetical protein | Bacteria;Acidobacteria;Acidobacteriia;Acidobacteriales;Acidobacteriaceae;Edaphobacter;Edaphobacter dinghuensis;Edaphobacter dinghuensis EB95 |
| Ga0586324_3622761_3_704 | 24900000.00 | 0.00 | 0.00 | 0.00 | 0.00 | 0.00 | 0.00 | 0.00 | 0.00 | 0.00 | 0.00 | 0.00 | predicted porin | |
| Ga0586324_3638456_1_474 | 0.00 | 0.00 | 0.00 | 0.00 | 0.00 | 0.00 | 0.00 | 0.00 | 0.00 | 0.00 | 846961.28 | 0.00 | EpsD family peptidyl-prolyl cis-trans isomerase | Bacteria;Proteobacteria;Betaproteobacteria;Nitrosomonadales;Thiobacillaceae;Thiobacillus;Thiobacillus denitrificans;Thiobacillus denitrificans DSM 12475 |
| Ga0586324_3638910_3_704 | 0.00 | 0.00 | 0.00 | 0.00 | 0.00 | 0.00 | 0.00 | 3617300.00 | 0.00 | 0.00 | 0.00 | 0.00 | Tfp pilus assembly protein PilF | |
| Ga0586324_3642467_3_704 | 5300000.00 | 0.00 | 0.00 | 0.00 | 0.00 | 0.00 | 0.00 | 0.00 | 0.00 | 0.00 | 0.00 | 4957500.00 | hypothetical protein | |
| Ga0586324_3650511_2_703 | 0.00 | 3940534.72 | 0.00 | 0.00 | 0.00 | 2754497.16 | 0.00 | 0.00 | 0.00 | 0.00 | 0.00 | 681587.25 | hypothetical protein | Bacteria;Proteobacteria;Deltaproteobacteria;Desulfobacterales;Desulfobacteraceae;Desulfobacterium;Desulfobacterium autotrophicum;Desulfobacterium autotrophicum HRM2, DSM 3382 |
| Ga0586324_3652739_1_702 | 0.00 | 0.00 | 0.00 | 0.00 | 0.00 | 0.00 | 0.00 | 0.00 | 899100.00 | 0.00 | 0.00 | 0.00 | hypothetical protein | Bacteria;Verrucomicrobia;Opitutae;Opitutales;Opitutaceae;Opitutus;Opitutus sp. GAS368;Opitutus sp. GAS368 |
| Ga0586324_3656447_3_653 | 20600000.00 | 0.00 | 5798900.00 | 10165000.00 | 0.00 | 0.00 | 0.00 | 0.00 | 0.00 | 0.00 | 0.00 | 6712500.00 | predicted porin | Bacteria;Proteobacteria;Gammaproteobacteria;Pseudomonadales;Pseudomonadaceae;Pseudomonas;Pseudomonas gingeri;Pseudomonas gingeri NCPPB 3146 |
| Ga0586324_3657468_3_497 | 38400000.00 | 0.00 | 0.00 | 0.00 | 0.00 | 0.00 | 0.00 | 6093900.00 | 0.00 | 0.00 | 0.00 | 0.00 | rubrerythrin | Bacteria;Planctomycetes;Candidatus Brocadiae;Candidatus Brocadiales;Candidatus Brocadiaceae;Candidatus Kuenenia;Candidatus Kuenenia stuttgartiensis;Candidatus Kuenenia stuttgartiensis MBR1 |
| Ga0586324_3659984_52_702 | 173000000.00 | 0.00 | 0.00 | 0.00 | 0.00 | 0.00 | 90060000.00 | 0.00 | 59454000.00 | 0.00 | 0.00 | 30600000.00 | hypothetical protein | Bacteria;Proteobacteria;Alphaproteobacteria;Rhodobacterales;Rhodobacteraceae;Rhodovulum;Rhodovulum sp. PH10;Rhodovulum sp. PH10 |
| Ga0586324_3674147_1_699 | 0.00 | 0.00 | 0.00 | 0.00 | 0.00 | 0.00 | 0.00 | 0.00 | 1563300.00 | 0.00 | 0.00 | 0.00 | hypothetical protein | Bacteria;Proteobacteria;Alphaproteobacteria;Sphingomonadales;Erythrobacteraceae;Altererythrobacter;Altererythrobacter xiamenensis;Altererythrobacter xiamenensis CGMCC 1.12494 |
| Ga0586324_3674156_1_699 | 0.00 | 1292000.00 | 0.00 | 0.00 | 0.00 | 0.00 | 0.00 | 0.00 | 0.00 | 0.00 | 0.00 | 0.00 | formyltetrahydrofolate synthetase | Bacteria;Firmicutes;Clostridia;Eubacteriales;unclassified Eubacteriales;unclassified Eubacteriales;Clostridiales bacterium PH28_bin88;Clostridiales bacterium PH28_bin88 |
| Ga0586324_3690547_3_224 | 0.00 | 9044000.00 | 0.00 | 32870000.00 | 20909000.00 | 0.00 | 0.00 | 11407000.00 | 17091000.00 | 0.00 | 0.00 | 0.00 | predicted porin | Bacteria;Proteobacteria;Betaproteobacteria;Rhodocyclales;Zoogloeaceae;Thauera;Thauera sp. 28;Thauera sp. 28 |
| Ga0586324_3696922_1_699 | 0.00 | 0.00 | 0.00 | 0.00 | 1833400.00 | 0.00 | 0.00 | 1793400.00 | 0.00 | 924000.00 | 0.00 | 0.00 | hypothetical protein | Bacteria;Proteobacteria;Deltaproteobacteria;Syntrophobacterales;Syntrophobacteraceae;Thermodesulforhabdus;Thermodesulforhabdus norvegica;Thermodesulforhabdus norvegica DSM 9990 |
| Ga0586324_3709196_168_590 | 0.00 | 0.00 | 0.00 | 0.00 | 0.00 | 0.00 | 0.00 | 0.00 | 0.00 | 0.00 | 0.00 | 2992500.00 | uncharacterized surface protein with fasciclin (FAS1) repeats | Bacteria;Cyanobacteria;unclassified Cyanobacteria;Synechococcales;Merismopediaceae;Merismopedia;Merismopedia glauca;Merismopedia glauca CCAP 1448/3 |
| Ga0586324_3722431_3_695 | 0.00 | 0.00 | 0.00 | 0.00 | 0.00 | 225879.11 | 0.00 | 0.00 | 0.00 | 0.00 | 0.00 | 0.00 | TonB-dependent SusC/RagA subfamily outer membrane receptor | Bacteria;Bacteroidetes;Cytophagia;Cytophagales;Flammeovirgaceae;Flexithrix;Flexithrix dorotheae;Flexithrix dorotheae DSM 6795 |
| Ga0586324_3730128_488_697 | 0.00 | 4025600.00 | 0.00 | 0.00 | 0.00 | 0.00 | 0.00 | 0.00 | 0.00 | 0.00 | 0.00 | 0.00 | hypothetical protein | |
| Ga0586324_3731788_3_695 | 0.00 | 169371.00 | 0.00 | 0.00 | 0.00 | 0.00 | 0.00 | 0.00 | 0.00 | 660649.99 | 0.00 | 0.00 | hypothetical protein | Bacteria;Acidobacteria;Thermoanaerobaculia;Thermoanaerobaculales;Thermoanaerobaculaceae;Thermoanaerobaculum;Thermoanaerobaculum aquaticum;Thermoanaerobaculum aquaticum MP-01 |
| Ga0586324_3743110_1_696 | 0.00 | 0.00 | 0.00 | 0.00 | 14523000.00 | 17348900.00 | 0.00 | 0.00 | 0.00 | 0.00 | 0.00 | 0.00 | hypothetical protein | Bacteria;Proteobacteria;Deltaproteobacteria;Syntrophobacterales;Syntrophobacteraceae;Syntrophobacter;Syntrophobacter fumaroxidans;Syntrophobacter fumaroxidans MPOB |
| Ga0586324_3745399_2_694 | 0.00 | 0.00 | 0.00 | 0.00 | 0.00 | 0.00 | 0.00 | 0.00 | 0.00 | 0.00 | 4268000.00 | 0.00 | hypothetical protein | Bacteria;Acidobacteria;Acidobacteriia;Acidobacteriales;Acidobacteriaceae;Candidatus Koribacter;Candidatus Koribacter versatilis;Candidatus Koribacter versatilis Ellin345 |
| Ga0586324_3758571_2_694 | 0.00 | 1101600.00 | 0.00 | 0.00 | 0.00 | 0.00 | 0.00 | 0.00 | 655274.61 | 176837.43 | 0.00 | 0.00 | predicted CXXCH cytochrome family protein | Bacteria;Firmicutes;Clostridia;Eubacteriales;Peptococcaceae;Thermincola;Thermincola ferriacetica;Thermincola ferriacetica Z-0001 |
| Ga0586324_3763019_3_692 | 0.00 | 1074400.00 | 1585419.26 | 0.00 | 0.00 | 1745497.84 | 0.00 | 0.00 | 1344600.00 | 0.00 | 0.00 | 0.00 | hypothetical protein | Bacteria;Rhodothermaeota;Rhodothermia;Rhodothermales;Rubricoccaceae;Rubricoccus;Rubricoccus marinus;Rubricoccus marinus SG-29 |
| Ga0586324_3796379_146_619 | 0.00 | 0.00 | 0.00 | 0.00 | 0.00 | 0.00 | 0.00 | 11956000.00 | 0.00 | 0.00 | 0.00 | 0.00 | predicted Zn-dependent protease | |
| Ga0586324_3798063_3_692 | 0.00 | 0.00 | 3857889.94 | 0.00 | 0.00 | 0.00 | 0.00 | 0.00 | 0.00 | 0.00 | 3036000.00 | 3382500.00 | parallel beta-helix repeat protein | Archaea;Euryarchaeota;Methanomicrobia;Methanosarcinales;Methanosarcinaceae;Methanohalophilus;Methanohalophilus mahii;Methanohalophilus mahii SLP, DSM 5219 |
| Ga0586324_3800472_1_690 | 22400000.00 | 37400000.00 | 25109800.00 | 19665000.00 | 47380000.00 | 11195400.00 | 27998400.00 | 28853000.00 | 28188000.00 | 22022000.00 | 39424000.00 | 44895000.00 | hypothetical protein | |
| Ga0586324_3802614_1_690 | 0.00 | 7276000.00 | 0.00 | 0.00 | 0.00 | 0.00 | 0.00 | 0.00 | 0.00 | 0.00 | 0.00 | 0.00 | ATP-dependent Clp protease ATP-binding subunit ClpA | Bacteria;Verrucomicrobia;Verrucomicrobiae;Verrucomicrobiales;Verrucomicrobia subdivision 3;Pedosphaera;Pedosphaera parvula;Pedosphaera parvula Ellin514 |
| Ga0586324_3804726_2_691 | 0.00 | 1667731.28 | 0.00 | 1368000.00 | 1194800.00 | 0.00 | 0.00 | 1049200.00 | 0.00 | 2510200.00 | 0.00 | 1545000.00 | hypothetical protein | |
| Ga0586324_3807355_2_691 | 0.00 | 0.00 | 0.00 | 1491500.00 | 0.00 | 0.00 | 0.00 | 0.00 | 0.00 | 0.00 | 0.00 | 0.00 | glyoxylase-like metal-dependent hydrolase (beta-lactamase superfamily II) | Bacteria;Proteobacteria;Alphaproteobacteria;Hyphomicrobiales;Bradyrhizobiaceae;Bradyrhizobium;Bradyrhizobium sp. Tv2a-2;Bradyrhizobium sp. TV2a.2 |
| Ga0586324_3808139_222_689 | 8090000.00 | 7888000.00 | 11091100.00 | 14725000.00 | 0.00 | 12412030.32 | 18285600.00 | 8906000.00 | 3588300.00 | 8316000.00 | 7198400.00 | 2257500.00 | hypothetical protein | |
| Ga0586324_3814894_1_690 | 0.00 | 1666000.00 | 0.00 | 0.00 | 0.00 | 4764000.00 | 0.00 | 0.00 | 0.00 | 0.00 | 0.00 | 0.00 | hypothetical protein | Bacteria;Proteobacteria;Gammaproteobacteria;Xanthomonadales;Rhodanobacteraceae;Ahniella;Ahniella affigens;Ahniella affigens D13 |
| Ga0586324_3815812_2_379 | 0.00 | 0.00 | 0.00 | 0.00 | 0.00 | 0.00 | 0.00 | 0.00 | 0.00 | 0.00 | 3599200.00 | 0.00 | hypothetical protein | Bacteria;Actinobacteria;Actinomycetia;Streptosporangiales;Streptosporangiaceae;Sphaerisporangium;Sphaerisporangium album;Sphaerisporangium album CCTCC AA 208026 |
| Ga0586324_3831585_2_688 | 0.00 | 0.00 | 0.00 | 0.00 | 0.00 | 0.00 | 0.00 | 1750700.00 | 0.00 | 0.00 | 0.00 | 0.00 | hypothetical protein | Bacteria;Nitrospirae;Thermodesulfovibrionia;Thermodesulfovibrionales;Thermodesulfovibrionaceae;Thermodesulfovibrio;Thermodesulfovibrio islandicus;Thermodesulfovibrio islandicus DSM 12570 |
| Ga0586324_3842194_1_687 | 0.00 | 0.00 | 0.00 | 0.00 | 0.00 | 0.00 | 0.00 | 0.00 | 0.00 | 0.00 | 0.00 | 2902500.00 | hypothetical protein | Bacteria;Actinobacteria;Actinomycetia;Micrococcales;Intrasporangiaceae;Intrasporangium;Intrasporangium chromatireducens;Intrasporangium chromatireducens Q5-1 |
| Ga0586324_3843662_3_686 | 0.00 | 1203600.00 | 0.00 | 0.00 | 0.00 | 0.00 | 0.00 | 4855600.00 | 0.00 | 461099.10 | 0.00 | 0.00 | hypothetical protein | |
| Ga0586324_3855478_170_685 | 0.00 | 542359.16 | 0.00 | 0.00 | 0.00 | 0.00 | 0.00 | 0.00 | 0.00 | 0.00 | 0.00 | 0.00 | outer membrane receptor protein involved in Fe transport | |
| Ga0586324_3870956_147_416 | 0.00 | 0.00 | 0.00 | 0.00 | 1081500.00 | 0.00 | 0.00 | 0.00 | 0.00 | 0.00 | 0.00 | 0.00 | nucleoid DNA-binding protein | Bacteria;Proteobacteria;Deltaproteobacteria;Desulfobacterales;Desulfobacteraceae;Desulforegula;Desulforegula conservatrix;Desulforegula conservatrix Mb1Pa |
| Ga0586324_3876937_1_684 | 0.00 | 2250800.00 | 0.00 | 0.00 | 0.00 | 0.00 | 0.00 | 0.00 | 0.00 | 0.00 | 2684000.00 | 0.00 | hypothetical protein | Bacteria;Bacteroidetes;Cytophagia;Cytophagales;Fulvivirgaceae;Ohtaekwangia;Ohtaekwangia koreensis;Ohtaekwangia koreensis DSM 25262 |
| Ga0586324_3886312_3_683 | 8170000.00 | 4154800.00 | 0.00 | 13338000.00 | 0.00 | 5835900.00 | 10305600.00 | 4703100.00 | 4811400.00 | 0.00 | 3511200.00 | 3517500.00 | hypothetical protein | Bacteria;Nitrospirae;unclassified Nitrospirae;unclassified Nitrospirae;unclassified Nitrospirae;unclassified Nitrospirae;Nitrospirae bacterium HCH-1;Nitrospirae bacterium HCH-1 |
| Ga0586324_3886919_232_684 | 2850000.00 | 1822400.00 | 2922127.64 | 6555000.00 | 0.00 | 0.00 | 0.00 | 1390800.00 | 1547100.00 | 0.00 | 0.00 | 0.00 | hypothetical protein | Bacteria;Verrucomicrobia;Verrucomicrobiae;Verrucomicrobiales;Verrucomicrobia subdivision 3;Pedosphaera;Pedosphaera parvula;Pedosphaera parvula Ellin514 |
| Ga0586324_3887468_3_683 | 0.00 | 0.00 | 0.00 | 0.00 | 0.00 | 0.00 | 0.00 | 2867000.00 | 0.00 | 0.00 | 0.00 | 0.00 | subtilisin family serine protease | Bacteria;Firmicutes;Limnochordia;Limnochordales;Limnochordaceae;Limnochorda;Limnochorda pilosa;Limnochorda pilosa HC45 |
| Ga0586324_3891068_327_683 | 0.00 | 0.00 | 0.00 | 2394000.00 | 0.00 | 0.00 | 0.00 | 0.00 | 0.00 | 0.00 | 0.00 | 0.00 | hypothetical protein | Bacteria;Actinobacteria;Actinomycetia;Propionibacteriales;Kribbellaceae;Kribbella;Kribbella sp. VKM Ac-2568;Kribbella pushchinensis VKM Ac-2568 |
| Ga0586324_3904508_225_683 | 0.00 | 0.00 | 0.00 | 0.00 | 0.00 | 0.00 | 0.00 | 0.00 | 0.00 | 19712000.00 | 0.00 | 0.00 | hypothetical protein | |
| Ga0586324_3909454_258_683 | 44900000.00 | 6460000.00 | 29895300.00 | 41705000.00 | 7941300.00 | 37437100.00 | 36571200.00 | 7503000.00 | 7395300.00 | 22792000.00 | 26065600.00 | 9030000.00 | predicted porin | Bacteria;Proteobacteria;Betaproteobacteria;Burkholderiales;Oxalobacteraceae;Herbaspirillum;Herbaspirillum autotrophicum;Herbaspirillum autotrophicum IAM 14942 |
| Ga0586324_3918447_3_683 | 6840000.00 | 3032800.00 | 0.00 | 0.00 | 0.00 | 0.00 | 0.00 | 1665300.00 | 1296000.00 | 1809500.00 | 2358400.00 | 2025000.00 | hypothetical protein | Bacteria;Proteobacteria;Gammaproteobacteria;Methylococcales;Methylococcaceae;Methylosarcina;Methylosarcina fibrata;Methylosarcina fibrata AML-C10 |
| Ga0586324_3919987_3_683 | 0.00 | 0.00 | 0.00 | 0.00 | 0.00 | 33030400.00 | 0.00 | 0.00 | 26892000.00 | 0.00 | 0.00 | 0.00 | S-layer protein (TIGR01567 family) | Archaea;Euryarchaeota;Methanomicrobia;Methanosarcinales;Methanotrichaceae;Methanothrix;Methanothrix thermoacetophila;Methanothrix thermoacetophila PT |
| Ga0586324_3927666_1_588 | 0.00 | 0.00 | 0.00 | 0.00 | 0.00 | 0.00 | 0.00 | 0.00 | 18873000.00 | 0.00 | 0.00 | 0.00 | hypothetical protein | Bacteria;Acidobacteria;Acidobacteriia;Acidobacteriales;Acidobacteriaceae;unclassified Acidobacteriaceae;Acidobacteriaceae bacterium KBS 146;Acidobacteriaceae sp. KBS 146 |
| Ga0586324_3930851_176_682 | 544576.00 | 493633.08 | 0.00 | 870946.70 | 13657800.00 | 7618207.68 | 722864.88 | 732000.00 | 980100.00 | 3542000.00 | 1416800.00 | 0.00 | predicted porin | Bacteria;Proteobacteria;Betaproteobacteria;Burkholderiales;unclassified Burkholderiales;Aquabacterium;Aquabacterium tepidiphilum;Aquabacterium tepidiphilum YIM 730274 |
| Ga0586324_3931990_1_681 | 4550000.00 | 2978400.00 | 7319000.00 | 0.00 | 0.00 | 0.00 | 0.00 | 0.00 | 0.00 | 0.00 | 0.00 | 0.00 | S-layer protein (TIGR01567 family) | Archaea;Euryarchaeota;Methanomicrobia;Methanosarcinales;Methanotrichaceae;Methanothrix;Methanothrix thermoacetophila;Methanothrix thermoacetophila PT |
| Ga0586324_3935348_1_681 | 0.00 | 11696000.00 | 0.00 | 4227500.00 | 23896000.00 | 0.00 | 11126400.00 | 4471300.00 | 11826000.00 | 8778000.00 | 7295200.00 | 10125000.00 | predicted porin | Bacteria;Proteobacteria;Gammaproteobacteria;Chromatiales;Chromatiaceae;Thiobaca;Thiobaca trueperi;Thiobaca trueperi DSM 13587 |
| Ga0586324_3957913_3_680 | 0.00 | 0.00 | 0.00 | 0.00 | 0.00 | 0.00 | 310998.84 | 0.00 | 0.00 | 0.00 | 0.00 | 0.00 | predicted porin | Bacteria;Proteobacteria;Gammaproteobacteria;Chromatiales;Ectothiorhodospiraceae;Thiogranum;Thiogranum longum;Thiogranum longum DSM 19610 |
| Ga0586324_3959299_1_678 | 0.00 | 4637600.00 | 0.00 | 0.00 | 0.00 | 0.00 | 0.00 | 0.00 | 1336500.00 | 0.00 | 0.00 | 0.00 | hypothetical protein | Bacteria;Proteobacteria;Deltaproteobacteria;Syntrophorhabdales;Syntrophorhabdaceae;Syntrophorhabdus;Syntrophorhabdus aromaticivorans;Syntrophorhabdus aromaticivorans UI |
| Ga0586324_3970132_3_677 | 0.00 | 5249600.00 | 0.00 | 0.00 | 0.00 | 0.00 | 0.00 | 0.00 | 0.00 | 0.00 | 0.00 | 0.00 | hypothetical protein | Bacteria;Spirochaetes;Spirochaetia;Spirochaetales;Spirochaetaceae;Spirochaeta;Spirochaeta africana;Spirochaeta africana Z-7692, DSM 8902 |
| Ga0586324_3972977_2_679 | 0.00 | 0.00 | 0.00 | 0.00 | 0.00 | 0.00 | 1822301.40 | 0.00 | 1984500.00 | 2356200.00 | 0.00 | 0.00 | hypothetical protein | |
| Ga0586324_3977899_276_677 | 0.00 | 0.00 | 0.00 | 0.00 | 0.00 | 0.00 | 0.00 | 0.00 | 0.00 | 0.00 | 3036000.00 | 0.00 | hypothetical protein | Bacteria;Proteobacteria;Gammaproteobacteria;Methylococcales;Methylococcaceae;Methylomonas;Methylomonas sp. 11b;Methylomonas sp. 11b |
| Ga0586324_3982290_513_677 | 0.00 | 0.00 | 0.00 | 0.00 | 0.00 | 0.00 | 0.00 | 0.00 | 0.00 | 1478400.00 | 0.00 | 0.00 | hypothetical protein | |
| Ga0586324_3983887_227_676 | 0.00 | 12784000.00 | 3877431.67 | 0.00 | 47792000.00 | 11830600.00 | 39900000.00 | 0.00 | 10935000.00 | 0.00 | 0.00 | 0.00 | TRAP-type mannitol/chloroaromatic compound transport system substrate-binding protein | Bacteria;Proteobacteria;Betaproteobacteria;Burkholderiales;Oxalobacteraceae;Noviherbaspirillum;Noviherbaspirillum massiliense;Noviherbaspirillum massiliense JC206 |
| Ga0586324_3983937_209_676 | 0.00 | 0.00 | 0.00 | 0.00 | 0.00 | 0.00 | 0.00 | 1793400.00 | 0.00 | 0.00 | 0.00 | 0.00 | hypothetical protein | Bacteria;Proteobacteria;Gammaproteobacteria;Methylococcales;Methylococcaceae;Methylomicrobium;Methylomicrobium lacus;Methylosarcina lacus LW14 |
| Ga0586324_3998242_358_675 | 0.00 | 4916400.00 | 0.00 | 0.00 | 0.00 | 0.00 | 0.00 | 2202100.00 | 0.00 | 3534300.00 | 0.00 | 2385000.00 | hypothetical protein | Bacteria;Proteobacteria;Deltaproteobacteria;Syntrophobacterales;Syntrophaceae;Smithella;Smithella sp. SCADC;Smithella sp. SCADC |
| Ga0586324_3998485_3_452 | 0.00 | 632578.16 | 0.00 | 0.00 | 0.00 | 0.00 | 0.00 | 0.00 | 0.00 | 0.00 | 0.00 | 0.00 | membrane protease subunit (stomatin/prohibitin family) | Bacteria;Chloroflexi;Chloroflexia;Kallotenuales;Kallotenuaceae;Kallotenue;Kallotenue papyrolyticum;Kallotenue papyrolyticum JKG1 |
| Ga0586324_4003741_1_675 | 0.00 | 0.00 | 0.00 | 0.00 | 0.00 | 1538041.52 | 0.00 | 0.00 | 0.00 | 0.00 | 0.00 | 0.00 | hypothetical protein | Bacteria;Candidatus Poribacteria;unclassified Candidatus Poribacteria;unclassified Candidatus Poribacteria;unclassified Candidatus Poribacteria;unclassified Candidatus Poribacteria;uncultured Poribacteria bacterium;Uncultured Poribacteria bacterium AGPOR5 |
| Ga0586324_4004618_3_677 | 3920000.00 | 0.00 | 0.00 | 0.00 | 0.00 | 0.00 | 0.00 | 0.00 | 0.00 | 0.00 | 0.00 | 0.00 | hypothetical protein | Bacteria;Chlorobi;Chlorobia;Chlorobiales;Chlorobiaceae;Chlorobaculum;Chlorobaculum limnaeum;Chlorobaculum limnaeum DSM 1677 |
| Ga0586324_4008061_2_565 | 0.00 | 0.00 | 0.00 | 0.00 | 7240900.00 | 0.00 | 0.00 | 0.00 | 0.00 | 0.00 | 0.00 | 0.00 | hypothetical protein | Bacteria;Spirochaetes;Spirochaetia;Spirochaetales;Spirochaetaceae;Spirochaeta;Spirochaeta lutea;Spirochaeta lutea JC230 |
| Ga0586324_4011692_2_553 | 0.00 | 0.00 | 0.00 | 0.00 | 0.00 | 0.00 | 0.00 | 0.00 | 0.00 | 346502.31 | 0.00 | 0.00 | NAD(P)-dependent dehydrogenase (short-subunit alcohol dehydrogenase family) | Bacteria;Proteobacteria;Alphaproteobacteria;Hyphomicrobiales;Rhizobiaceae;Rhizobium;Rhizobium gallicum;Rhizobium gallicum IE4872 |
| Ga0586324_4012610_2_676 | 1720000.00 | 2271200.00 | 0.00 | 2356000.00 | 2049700.00 | 0.00 | 1148730.12 | 1561600.00 | 882900.00 | 2802800.00 | 3291200.00 | 1042500.00 | hypothetical protein | |
| Ga0586324_4016646_404_676 | 5600000.00 | 2516000.00 | 0.00 | 0.00 | 0.00 | 0.00 | 0.00 | 0.00 | 0.00 | 0.00 | 0.00 | 0.00 | hypothetical protein | |
| Ga0586324_4022590_66_674 | 0.00 | 0.00 | 0.00 | 0.00 | 0.00 | 0.00 | 0.00 | 0.00 | 0.00 | 0.00 | 305875.68 | 0.00 | predicted AlkP superfamily pyrophosphatase or phosphodiesterase | Bacteria;Actinobacteria;Actinomycetia;Propionibacteriales;Nocardioidaceae;Nocardioides;Nocardioides sp. OAS665;Nocardioides sp. OAS665 |
| Ga0586324_4025802_1_675 | 0.00 | 0.00 | 0.00 | 0.00 | 0.00 | 0.00 | 0.00 | 0.00 | 0.00 | 2156000.00 | 2323200.00 | 2025000.00 | hypothetical protein | Bacteria;Acidobacteria;Acidobacteriia;Bryobacterales;Solibacteraceae;Candidatus Solibacter;Candidatus Solibacter usitatus;Candidatus Solibacter usitatus Ellin6076 |
| Ga0586324_4030034_1_675 | 0.00 | 0.00 | 0.00 | 0.00 | 15656000.00 | 0.00 | 0.00 | 0.00 | 0.00 | 0.00 | 0.00 | 0.00 | hypothetical protein | |
| Ga0586324_4032538_174_674 | 0.00 | 0.00 | 0.00 | 2232500.00 | 0.00 | 0.00 | 3511200.00 | 1610400.00 | 3410100.00 | 0.00 | 0.00 | 0.00 | uncharacterized membrane protein | Bacteria;Proteobacteria;Gammaproteobacteria;unclassified Gammaproteobacteria;Candidatus Competibacteraceae;Candidatus Contendobacter;Candidatus Contendobacter odensis;Candidatus Contendobacter odensis Run_B_J11 |
| Ga0586324_4032539_1_675 | 2360000.00 | 8024000.00 | 0.00 | 0.00 | 0.00 | 0.00 | 0.00 | 0.00 | 0.00 | 109370800.00 | 8439024.00 | 0.00 | predicted porin | Bacteria;Proteobacteria;Betaproteobacteria;Rhodocyclales;Azonexaceae;Dechloromonas;Dechloromonas denitrificans;Dechloromonas denitrificans ATCC BAA-841 |
| Ga0586324_4051335_1_672 | 5660000.00 | 0.00 | 0.00 | 4256000.00 | 0.00 | 0.00 | 0.00 | 0.00 | 0.00 | 0.00 | 3828000.00 | 0.00 | hypothetical protein | Bacteria;Acidobacteria;Vicinamibacteria;unclassified Vicinamibacteria;Vicinamibacteraceae;Luteitalea;Luteitalea pratensis;Luteitalea pratensis DSM 100886 |
| Ga0586324_4052799_3_671 | 0.00 | 0.00 | 0.00 | 0.00 | 0.00 | 0.00 | 0.00 | 0.00 | 0.00 | 0.00 | 0.00 | 1327500.00 | hypothetical protein | Bacteria;Verrucomicrobia;Verrucomicrobiae;Verrucomicrobiales;Verrucomicrobia subdivision 3;Pedosphaera;Pedosphaera parvula;Pedosphaera parvula Ellin514 |
| Ga0586324_4054075_3_671 | 0.00 | 0.00 | 0.00 | 0.00 | 0.00 | 0.00 | 0.00 | 0.00 | 0.00 | 0.00 | 0.00 | 1530000.00 | DNA-binding transcriptional LysR family regulator | Bacteria;Proteobacteria;Alphaproteobacteria;Hyphomicrobiales;Phyllobacteriaceae;Mesorhizobium;Mesorhizobium sp. M6A.T.Cr.TU.016.01.1.1;Mesorhizobium sp. M6A.T.Cr.TU.016.01.1.1 |
| Ga0586324_4064928_3_383 | 1630000.00 | 0.00 | 0.00 | 0.00 | 0.00 | 0.00 | 0.00 | 0.00 | 0.00 | 0.00 | 0.00 | 0.00 | Tfp pilus assembly protein PilF | Bacteria;Proteobacteria;Deltaproteobacteria;Syntrophobacterales;Syntrophaceae;Desulfobacca;Desulfobacca acetoxidans;Desulfobacca acetoxidans ASRB2, DSM 11109 |
| Ga0586324_4068171_3_671 | 0.00 | 2114800.00 | 0.00 | 0.00 | 0.00 | 0.00 | 0.00 | 0.00 | 0.00 | 0.00 | 0.00 | 0.00 | TRAP-type mannitol/chloroaromatic compound transport system substrate-binding protein | Bacteria;Proteobacteria;Betaproteobacteria;Burkholderiales;Oxalobacteraceae;unclassified Oxalobacteraceae;Oxalobacteraceae bacterium;Oxalobacteraceae sp. OAS666 |
| Ga0586324_4071999_3_671 | 0.00 | 0.00 | 0.00 | 0.00 | 0.00 | 5002200.00 | 0.00 | 0.00 | 0.00 | 0.00 | 0.00 | 0.00 | predicted porin | Bacteria;Proteobacteria;Betaproteobacteria;unclassified Betaproteobacteria;unclassified Betaproteobacteria;unclassified Betaproteobacteria;Betaproteobacteria bacterium GR16-43;Betaproteobacteria bacterium GR16-43 |
| Ga0586324_4082241_3_476 | 239277.00 | 0.00 | 0.00 | 0.00 | 0.00 | 0.00 | 0.00 | 0.00 | 0.00 | 0.00 | 0.00 | 0.00 | rubrerythrin | Bacteria;Proteobacteria;Deltaproteobacteria;Syntrophobacterales;Syntrophobacteraceae;Desulfacinum;Desulfacinum infernum;Desulfacinum infernum DSM 9756 |
| Ga0586324_4084643_237_671 | 0.00 | 1258000.00 | 0.00 | 2546000.00 | 0.00 | 3461379.48 | 0.00 | 1482300.00 | 2656800.00 | 1794100.00 | 1962400.00 | 0.00 | hypothetical protein | Bacteria;Cyanobacteria;unclassified Cyanobacteria;Chroococcales;Microcystaceae;Microcystis;Microcystis aeruginosa;Microcystis aeruginosa FACHB-524 |
| Ga0586324_4086762_3_671 | 0.00 | 0.00 | 0.00 | 0.00 | 0.00 | 0.00 | 0.00 | 0.00 | 0.00 | 0.00 | 9944000.00 | 0.00 | hypothetical protein | Bacteria;Acidobacteria;Acidobacteriia;Acidobacteriales;Acidobacteriaceae;Terriglobus;Terriglobus roseus;Terriglobus roseus AB35.6 |
| Ga0586324_4088508_3_671 | 0.00 | 0.00 | 0.00 | 48383500.00 | 43507200.00 | 47441500.00 | 8960400.00 | 10797000.00 | 9234000.00 | 0.00 | 0.00 | 5392500.00 | hypothetical protein | Bacteria;Proteobacteria;Deltaproteobacteria;Desulfobacterales;Desulfobacteraceae;Desulfatiglans;Desulfatiglans anilini;Desulfatiglans anilini DSM 4660 |
| Ga0586324_4089212_2_670 | 0.00 | 0.00 | 2937536.95 | 0.00 | 0.00 | 0.00 | 0.00 | 0.00 | 0.00 | 0.00 | 0.00 | 0.00 | glutamate synthase domain-containing protein 2 | Bacteria;Firmicutes;Clostridia;Thermoanaerobacterales;Thermoanaerobacterales Family III. Incertae Sedis;Caldicellulosiruptor;Caldicellulosiruptor bescii;Caldicellulosiruptor bescii DSM 6725 |
| Ga0586324_4091162_1_498 | 0.00 | 0.00 | 0.00 | 0.00 | 0.00 | 0.00 | 0.00 | 0.00 | 0.00 | 4481400.00 | 0.00 | 0.00 | hypothetical protein | Bacteria;Proteobacteria;Deltaproteobacteria;Syntrophobacterales;Syntrophaceae;Desulfobacca;Desulfobacca acetoxidans;Desulfobacca acetoxidans ASRB2, DSM 11109 |
| Ga0586324_4095173_113_487 | 0.00 | 0.00 | 0.00 | 0.00 | 0.00 | 0.00 | 5928000.00 | 0.00 | 0.00 | 0.00 | 0.00 | 0.00 | hypothetical protein | Bacteria;Proteobacteria;Gammaproteobacteria;Methylococcales;Methylococcaceae;Methyloglobulus;Methyloglobulus morosus;Methyloglobulus morosus KoM1 |
| Ga0586324_4095214_425_670 | 0.00 | 0.00 | 0.00 | 0.00 | 0.00 | 0.00 | 0.00 | 0.00 | 0.00 | 0.00 | 450369.04 | 0.00 | hypothetical protein | Bacteria;Proteobacteria;Deltaproteobacteria;Myxococcales;unclassified Myxococcales;Minicystis;Minicystis rosea;Minicystis rosea DSM 24000 |
| Ga0586324_4098118_2_412 | 0.00 | 2108000.00 | 0.00 | 8607000.00 | 5819500.00 | 0.00 | 0.00 | 4575000.00 | 4179600.00 | 2664200.00 | 870279.52 | 0.00 | hypothetical protein | |
| Ga0586324_4105491_3_668 | 0.00 | 0.00 | 0.00 | 0.00 | 0.00 | 0.00 | 0.00 | 0.00 | 0.00 | 6267800.00 | 0.00 | 0.00 | hypothetical protein | Bacteria;Proteobacteria;Gammaproteobacteria;Thiotrichales;Piscirickettsiaceae;Cycloclasticus;Cycloclasticus sp. P1;Cycloclasticus sp. P1 |
| Ga0586324_4111442_36_347 | 0.00 | 0.00 | 0.00 | 0.00 | 0.00 | 0.00 | 0.00 | 2238700.00 | 0.00 | 0.00 | 0.00 | 0.00 | hypothetical protein | Bacteria;Actinobacteria;Acidimicrobiia;Acidimicrobiales;Ilumatobacteraceae;Ilumatobacter;Ilumatobacter nonamiensis;Ilumatobacter nonamiensis YM16-303 |
| Ga0586324_4115170_3_668 | 24920000.00 | 0.00 | 0.00 | 16216500.00 | 23793000.00 | 27710600.00 | 31486800.00 | 22997000.00 | 7119900.00 | 0.00 | 6881600.00 | 0.00 | hypothetical protein | |
| Ga0586324_4127448_3_668 | 0.00 | 0.00 | 0.00 | 0.00 | 0.00 | 0.00 | 204372.59 | 0.00 | 0.00 | 0.00 | 0.00 | 787500.00 | hypothetical protein | Bacteria;Nitrospirae;Thermodesulfovibrionia;Thermodesulfovibrionales;Thermodesulfovibrionaceae;Thermodesulfovibrio;Thermodesulfovibrio yellowstonii;Thermodesulfovibrio yellowstonii DSM 11347 |
| Ga0586324_4131977_2_448 | 0.00 | 0.00 | 0.00 | 5158500.00 | 0.00 | 0.00 | 0.00 | 0.00 | 0.00 | 0.00 | 0.00 | 0.00 | hypothetical protein | |
| Ga0586324_4132496_1_666 | 0.00 | 1925618.56 | 0.00 | 0.00 | 291777.37 | 0.00 | 0.00 | 0.00 | 0.00 | 0.00 | 0.00 | 0.00 | transketolase C-terminal domain/subunit | Bacteria;Proteobacteria;Gammaproteobacteria;Methylococcales;Methylococcaceae;Methylobacter;Methylobacter tundripaludum;Methylobacter tundripaludum SV96 |
| Ga0586324_4138233_1_666 | 0.00 | 2679200.00 | 0.00 | 0.00 | 0.00 | 0.00 | 0.00 | 0.00 | 0.00 | 0.00 | 0.00 | 0.00 | hypothetical protein | Bacteria;Actinobacteria;Actinomycetia;Micromonosporales;Micromonosporaceae;Micromonospora;Micromonospora humi;Micromonospora humi DSM 45647 |
| Ga0586324_4138309_1_666 | 0.00 | 0.00 | 218925.93 | 0.00 | 786970.47 | 684983.80 | 804796.68 | 608870.89 | 674840.97 | 484407.00 | 491656.00 | 191352.00 | TRAP-type mannitol/chloroaromatic compound transport system substrate-binding protein | Bacteria;Proteobacteria;Alphaproteobacteria;Hyphomicrobiales;Bradyrhizobiaceae;Bradyrhizobium;Bradyrhizobium erythrophlei;Bradyrhizobium erythrophlei GAS242 |
| Ga0586324_4149026_2_667 | 0.00 | 0.00 | 11710400.00 | 0.00 | 0.00 | 0.00 | 0.00 | 0.00 | 6390900.00 | 0.00 | 0.00 | 0.00 | PAS domain S-box-containing protein | Bacteria;Proteobacteria;Deltaproteobacteria;Desulfuromonadales;Desulfuromonadaceae;Pelobacter;Pelobacter propionicus;Pelobacter propionicus DSM 2379 |
| Ga0586324_4151850_3_665 | 0.00 | 0.00 | 0.00 | 0.00 | 0.00 | 0.00 | 0.00 | 0.00 | 0.00 | 377288.45 | 0.00 | 0.00 | hypothetical protein | Bacteria;Actinobacteria;Actinomycetia;Micromonosporales;Micromonosporaceae;Asanoa;Asanoa ferruginea;Asanoa ferruginea DSM 44099 |
| Ga0586324_4152111_3_665 | 0.00 | 5528400.00 | 0.00 | 0.00 | 0.00 | 0.00 | 0.00 | 0.00 | 0.00 | 0.00 | 0.00 | 0.00 | glutamate synthase domain-containing protein 2 | Bacteria;Firmicutes;Clostridia;Eubacteriales;Oscillospiraceae;Papillibacter;Papillibacter cinnamivorans;Papillibacter cinnamivorans DSM 12816 |
| Ga0586324_4160434_329_664 | 0.00 | 0.00 | 0.00 | 0.00 | 0.00 | 3735067.31 | 0.00 | 0.00 | 0.00 | 0.00 | 0.00 | 0.00 | hypothetical protein | Bacteria;Proteobacteria;Deltaproteobacteria;Desulfobacterales;Desulfobacteraceae;Desulfococcus;Desulfococcus multivorans;Desulfococcus multivorans DSM 2059 |
| Ga0586324_4169612_1_489 | 0.00 | 2108000.00 | 0.00 | 0.00 | 1545000.00 | 0.00 | 0.00 | 847900.00 | 1142100.00 | 0.00 | 0.00 | 0.00 | hypothetical protein | Bacteria;Proteobacteria;Deltaproteobacteria;Desulfuromonadales;Geobacteraceae;Geobacter;Geobacter pickeringii;Geobacter pickeringii G13 |
| Ga0586324_4172811_2_334 | 0.00 | 0.00 | 0.00 | 2992500.00 | 0.00 | 0.00 | 0.00 | 0.00 | 0.00 | 0.00 | 0.00 | 0.00 | hypothetical protein | Bacteria;Proteobacteria;Deltaproteobacteria;Syntrophobacterales;Syntrophobacteraceae;Desulfacinum;Desulfacinum infernum;Desulfacinum infernum DSM 9756 |
| Ga0586324_4174321_1_426 | 2730000.00 | 3862400.00 | 0.00 | 5491000.00 | 0.00 | 0.00 | 0.00 | 0.00 | 0.00 | 1509200.00 | 1504800.00 | 1155000.00 | hypothetical protein | |
| Ga0586324_4174504_3_665 | 1330000.00 | 0.00 | 0.00 | 2660000.00 | 2801600.00 | 0.00 | 0.00 | 0.00 | 1717200.00 | 0.00 | 0.00 | 0.00 | hypothetical protein | |
| Ga0586324_4178147_3_665 | 0.00 | 0.00 | 0.00 | 0.00 | 0.00 | 0.00 | 0.00 | 0.00 | 2737800.00 | 0.00 | 0.00 | 0.00 | predicted porin | Bacteria;Proteobacteria;Betaproteobacteria;Burkholderiales;Burkholderiaceae;Caballeronia;Caballeronia pedi;Caballeronia pedi LMG 29323 |
| Ga0586324_4200877_1_663 | 1690000.00 | 0.00 | 0.00 | 0.00 | 0.00 | 0.00 | 0.00 | 0.00 | 0.00 | 0.00 | 0.00 | 0.00 | hypothetical protein | Bacteria;Acidobacteria;Vicinamibacteria;unclassified Vicinamibacteria;Vicinamibacteraceae;Luteitalea;Luteitalea pratensis;Luteitalea pratensis DSM 100886 |
| Ga0586324_4202606_3_662 | 0.00 | 2155600.00 | 9064300.00 | 10545000.00 | 13802000.00 | 15205100.00 | 7660800.00 | 0.00 | 8343000.00 | 2625700.00 | 0.00 | 0.00 | hypothetical protein | Archaea;Euryarchaeota;Methanomicrobia;Methanosarcinales;Methanosarcinaceae;Methanosarcina;Methanosarcina vacuolata;Methanosarcina vacuolata Z-761 |
| Ga0586324_4211374_3_662 | 0.00 | 918000.00 | 0.00 | 0.00 | 0.00 | 0.00 | 0.00 | 0.00 | 534918.33 | 0.00 | 0.00 | 2212500.00 | hypothetical protein | |
| Ga0586324_4212198_3_305 | 0.00 | 0.00 | 0.00 | 0.00 | 0.00 | 0.00 | 0.00 | 939400.00 | 0.00 | 0.00 | 0.00 | 0.00 | osmotically-inducible protein OsmY | Bacteria;Proteobacteria;Betaproteobacteria;Nitrosomonadales;Nitrosomonadaceae;Nitrosomonas;Nitrosomonas eutropha;Nitrosomonas eutropha C91 |
| Ga0586324_4214625_1_630 | 0.00 | 0.00 | 0.00 | 0.00 | 0.00 | 0.00 | 0.00 | 0.00 | 0.00 | 1763300.00 | 0.00 | 2227500.00 | opacity protein-like surface antigen | Bacteria;Proteobacteria;Betaproteobacteria;Burkholderiales;Oxalobacteraceae;Herbaspirillum;Herbaspirillum hiltneri;Herbaspirillum hiltneri N3 |
| Ga0586324_4222624_75_662 | 4050000.00 | 0.00 | 0.00 | 0.00 | 0.00 | 0.00 | 0.00 | 0.00 | 0.00 | 0.00 | 0.00 | 0.00 | hypothetical protein | |
| Ga0586324_4224435_2_661 | 0.00 | 0.00 | 0.00 | 0.00 | 0.00 | 0.00 | 0.00 | 0.00 | 0.00 | 22792000.00 | 1135200.00 | 0.00 | hypothetical protein | |
| Ga0586324_4226202_3_662 | 1110000.00 | 0.00 | 0.00 | 0.00 | 0.00 | 22112900.00 | 0.00 | 0.00 | 0.00 | 416076.43 | 0.00 | 6210000.00 | predicted porin | Bacteria;Proteobacteria;Betaproteobacteria;Burkholderiales;Oxalobacteraceae;Herbaspirillum;Herbaspirillum rhizosphaerae;Herbaspirillum rhizosphaerae UMS-37 |
| Ga0586324_4227637_1_660 | 0.00 | 3420400.00 | 0.00 | 0.00 | 0.00 | 0.00 | 0.00 | 0.00 | 0.00 | 0.00 | 0.00 | 1687500.00 | hypothetical protein | |
| Ga0586324_4235832_281_661 | 0.00 | 1645600.00 | 0.00 | 0.00 | 0.00 | 0.00 | 0.00 | 0.00 | 0.00 | 0.00 | 0.00 | 0.00 | phasin family protein | Bacteria;Proteobacteria;Betaproteobacteria;Rhodocyclales;Azonexaceae;Dechloromonas;Dechloromonas sp. SG708;Dechloromonas sp. SG708 |
| Ga0586324_4235857_157_660 | 8830000.00 | 0.00 | 0.00 | 0.00 | 0.00 | 0.00 | 0.00 | 0.00 | 0.00 | 0.00 | 0.00 | 0.00 | regulator of protease activity HflC (stomatin/prohibitin superfamily) | Bacteria;Proteobacteria;Deltaproteobacteria;Desulfuromonadales;Geobacteraceae;Geobacter;Geobacter sp. DSM 2909;Geobacter sp. DSM 2909 |
| Ga0586324_4241758_152_661 | 9303505.80 | 6324000.00 | 5366836.91 | 5899500.00 | 0.00 | 5677100.00 | 6292800.00 | 4941000.00 | 4714200.00 | 9471000.00 | 7972800.00 | 10425000.00 | predicted porin | Bacteria;Proteobacteria;Betaproteobacteria;Burkholderiales;unclassified Burkholderiales;Rubrivivax;Rubrivivax benzoatilyticus;Rubrivivax benzoatilyticus JA2 |
| Ga0586324_4243396_133_660 | 0.00 | 190189200.00 | 243779000.00 | 194750000.00 | 218360000.00 | 214380000.00 | 282720000.00 | 126880000.00 | 105300000.00 | 174020000.00 | 191840000.00 | 169500000.00 | hypothetical protein | Bacteria;Proteobacteria;Alphaproteobacteria;Hyphomicrobiales;Bradyrhizobiaceae;Bradyrhizobium;Bradyrhizobium sp. OAE819;Bradyrhizobium sp. OAE819 |
| Ga0586324_4247355_269_658 | 5490000.00 | 0.00 | 0.00 | 0.00 | 0.00 | 0.00 | 0.00 | 0.00 | 16362000.00 | 0.00 | 0.00 | 0.00 | phasin family protein | Bacteria;Proteobacteria;Gammaproteobacteria;unclassified Gammaproteobacteria;Candidatus Competibacteraceae;Candidatus Contendobacter;Candidatus Contendobacter odensis;Candidatus Contendobacter odensis Run_B_J11 |
| Ga0586324_4251737_396_659 | 0.00 | 0.00 | 0.00 | 0.00 | 0.00 | 0.00 | 0.00 | 0.00 | 0.00 | 403527.74 | 0.00 | 0.00 | hypothetical protein | |
| Ga0586324_4254669_3_659 | 2410000.00 | 0.00 | 1849775.91 | 0.00 | 1864300.00 | 1405832.58 | 3328800.00 | 0.00 | 0.00 | 2456300.00 | 2798400.00 | 2287500.00 | TRAP-type mannitol/chloroaromatic compound transport system substrate-binding protein | Bacteria;Proteobacteria;Alphaproteobacteria;Hyphomicrobiales;Bradyrhizobiaceae;Afipia;Afipia sp. P52-10;Afipia sp. P52-10 |
| Ga0586324_4262153_1_660 | 3140000.00 | 0.00 | 0.00 | 0.00 | 0.00 | 0.00 | 0.00 | 0.00 | 0.00 | 0.00 | 0.00 | 0.00 | predicted porin | |
| Ga0586324_4264875_3_659 | 0.00 | 0.00 | 2095480.37 | 0.00 | 0.00 | 0.00 | 0.00 | 628300.00 | 0.00 | 0.00 | 0.00 | 736863.00 | acetyl-CoA acetyltransferase | Bacteria;Proteobacteria;Deltaproteobacteria;unclassified Deltaproteobacteria;unclassified Deltaproteobacteria;unclassified Deltaproteobacteria;delta proteobacterium NaphS2;delta proteobacterium NaphS2 |
| Ga0586324_4268610_3_659 | 2700000.00 | 0.00 | 0.00 | 0.00 | 0.00 | 0.00 | 0.00 | 0.00 | 0.00 | 0.00 | 0.00 | 0.00 | hypothetical protein | Archaea;Euryarchaeota;Methanomicrobia;Methanomicrobiales;Methanoregulaceae;Methanoregula;Methanoregula formicica;Methanoregula formicica SMSP, DSM 22288 |
| Ga0586324_4284506_56_562 | 0.00 | 0.00 | 0.00 | 0.00 | 0.00 | 0.00 | 136800000.00 | 0.00 | 0.00 | 0.00 | 0.00 | 0.00 | tetratricopeptide (TPR) repeat protein | Bacteria;Proteobacteria;Deltaproteobacteria;Syntrophobacterales;Syntrophaceae;Desulfobacca;Desulfobacca acetoxidans;Desulfobacca acetoxidans ASRB2, DSM 11109 |

| | | | | | | | | | | | | | | | |
|---|---|---|---|---|---|---|---|---|---|---|---|---|---|---|---|
| Ga0586324 4284924 71 658 | 7180000.00 | 0.00 | 9627300.00 | 0.00 | 0.00 | 6312300.00 | 0.00 | 0.00 | 4260600.00 | 0.00 | 0.00 | 0.00 | | hypothetical protein | |
| Ga0586324 4288109 3 656 | 3800000.00 | 6446400.00 | 2831022.98 | 1862000.00 | 3213600.00 | 2405403.15 | 3625200.00 | 5752300.00 | 3564000.00 | 1324400.00 | 206976.88 | 1455000.00 | | S-layer protein (TIGR01567 family) | Archaea;Euryarchaeota;Methanomicrobia;Methanosarcinales;Methanotrichaceae;Methanothrix;Methanothrix soehngenii;Methanothrix soehngenii GP-6 |
| Ga0586324 4292377 1 657 | 0.00 | 0.00 | 1896375.42 | 0.00 | 0.00 | 0.00 | 0.00 | 0.00 | 0.00 | 0.00 | 0.00 | 0.00 | | hypothetical protein | Bacteria;Proteobacteria;Alphaproteobacteria;Hyphomicrobiales;Xanthobacteraceae;Pseudolabrys;Pseudolabrys taiwanensis;Pseudolabrys taiwanensis CC-BB4 |
| Ga0586324 4319551 1 654 | 0.00 | 362927.56 | 373712.64 | 502355.25 | 398880.89 | 236574.68 | 234687.24 | 0.00 | 212261.31 | 0.00 | 358285.84 | 0.00 | | cytochrome c peroxidase | Bacteria;Proteobacteria;Deltaproteobacteria;Syntrophobacterales;Syntrophobacteraceae;Desulfoglaeba;Desulfoglaeba alkanexedens;Desulfoglaeba alkanexedens ALDC |
| Ga0586324 4328507 2 655 | 0.00 | 0.00 | 1816829.15 | 0.00 | 5294200.00 | 0.00 | 0.00 | 0.00 | 2081700.00 | 1963500.00 | 0.00 | 0.00 | | hypothetical protein | Bacteria;Actinobacteria;Actinomycetia;Micrococcales;Intrasporangiaceae;Intrasporangium;Intrasporangium oryzae;Intrasporangium oryzae NRRL B-24470 |
| Ga0586324 4333406 1 654 | 1950000.00 | 4698800.00 | 0.00 | 0.00 | 0.00 | 0.00 | 0.00 | 0.00 | 0.00 | 0.00 | 0.00 | 0.00 | | bacillopeptidase F (M6 metalloprotease family) | Bacteria;Candidatus Cloacimonetes;unclassified Candidatus Cloacimonetes;unclassified Candidatus Cloacimonetes;unclassified Candidatus Cloacimonetes;unclassified Candidatus Cloacimonetes;Cloacimonetes bacterium SCGC AAA252-K16;Cloacimonetes bacterium SCGC AAA252-K16 (contamination screened) |
| Ga0586324 4336290 3 653 | 17500000.00 | 4943600.00 | 15932900.00 | 13048458.05 | 0.00 | 7979700.00 | 7090800.00 | 0.00 | 1449900.00 | 7184100.00 | 0.00 | 19867500.00 | | predicted porin | Bacteria;Proteobacteria;Betaproteobacteria;Rhodocyclales;Azonexaceae;Azonexus;Azonexus hydrophilus;Azonexus hydrophilus DSM 23864 |
| Ga0586324 4338054 3 653 | 0.00 | 5854800.00 | 0.00 | 997500.00 | 11330000.00 | 0.00 | 0.00 | 0.00 | 0.00 | 0.00 | 0.00 | 0.00 | | hypothetical protein | Bacteria;Proteobacteria;Gammaproteobacteria;Alteromonadales;Alteromonadaceae;Marinobacter;Marinobacter sp.;Marinobacter sp. MIT1353 |
| Ga0586324 4343361 2 652 | 0.00 | 0.00 | 0.00 | 1548500.00 | 0.00 | 0.00 | 0.00 | 0.00 | 0.00 | 0.00 | 6943200.00 | 0.00 | | hypothetical protein | Bacteria;Proteobacteria;Alphaproteobacteria;Hyphomicrobiales;Xanthobacteraceae;Pseudolabrys;Pseudolabrys taiwanensis;Pseudolabrys taiwanensis CC-BB4 |
| Ga0586324 4352577 2 652 | 90200000.00 | 62016000.00 | 197387800.00 | 34390000.00 | 16583000.00 | 125849000.00 | 0.00 | 0.00 | 10530000.00 | 3803800.00 | 3088800.00 | 14625000.00 | | hypothetical protein | Archaea;Euryarchaeota;Methanomicrobia;Methanosarcinales;Methanotrichaceae;Methanothrix;Methanothrix soehngenii;Methanothrix soehngenii GP-6 |
| Ga0586324 4357828 3 653 | 0.00 | 0.00 | 0.00 | 0.00 | 10300000.00 | 0.00 | 0.00 | 0.00 | 0.00 | 0.00 | 0.00 | 0.00 | | outer membrane receptor for ferrienterochelin and colicin | Bacteria;Acidobacteria;Blastocatellia;Blastocatellales;Pyrinomonadaceae;Pyrinomonas;Pyrinomonas methylaliphatogenes;Pyrinomonas methylaliphatogenes K22 |
| Ga0586324 4361707 14 652 | 5570000.00 | 2223600.00 | 5234092.77 | 0.00 | 0.00 | 0.00 | 3146400.00 | 0.00 | 0.00 | 0.00 | 0.00 | 0.00 | | hypothetical protein | |
| Ga0586324 4361715 3 653 | 3850000.00 | 2309794.76 | 0.00 | 4332000.00 | 4130300.00 | 0.00 | 0.00 | 0.00 | 0.00 | 0.00 | 0.00 | 2617500.00 | | hypothetical protein | Bacteria;Acidobacteria;Acidobacteriia;Bryobacterales;Bryobacteraceae;Bryobacter;Bryobacter aggregatus;Bryobacter aggregatus DSM 18758 |
| Ga0586324 4364312 3 587 | 0.00 | 0.00 | 0.00 | 0.00 | 0.00 | 0.00 | 0.00 | 0.00 | 0.00 | 0.00 | 5218400.00 | 0.00 | | hypothetical protein | |
| Ga0586324 4381176 2 652 | 0.00 | 0.00 | 0.00 | 0.00 | 0.00 | 0.00 | 0.00 | 0.00 | 0.00 | 3157000.00 | 0.00 | 0.00 | | hypothetical protein | Bacteria;Proteobacteria;Deltaproteobacteria;Desulfuromonadales;Geobacteraceae;Geobacter;Geobacter metallireducens;Geobacter metallireducens GS-15 |
| Ga0586324 4383295 1 282 | 0.00 | 13872000.00 | 0.00 | 0.00 | 0.00 | 0.00 | 0.00 | 0.00 | 0.00 | 0.00 | 0.00 | 0.00 | | RNA recognition motif-containing protein | Bacteria;Bacteroidetes;Bacteroidia;Marinilabiliales;Prolixibacteraceae;Draconibacterium;Draconibacterium orientale;Draconibacterium orientale FH5 |
| Ga0586324 4384000 8 652 | 0.00 | 0.00 | 637237.18 | 0.00 | 0.00 | 0.00 | 0.00 | 0.00 | 0.00 | 0.00 | 0.00 | 0.00 | | TRAP-type mannitol/chloroaromatic compound transport system substrate-binding protein | Bacteria;Proteobacteria;Alphaproteobacteria;Hyphomicrobiales;Bradyrhizobiaceae;Bradyrhizobium;Bradyrhizobium sp. Ai1a-2;Bradyrhizobium sp. Ai1a-2 |
| Ga0586324 4388607 178 468 | 1630000.00 | 0.00 | 0.00 | 0.00 | 0.00 | 0.00 | 0.00 | 0.00 | 0.00 | 0.00 | 0.00 | 0.00 | | uncharacterized protein with GYD domain | Bacteria;Nitrospirae;Nitrospira;Nitrospirales;Nitrospiraceae;Nitrospira;Nitrospira marina;Nitrospira marina Nb-295 |
| Ga0586324 4392760 1 213 | 0.00 | 171126.08 | 0.00 | 0.00 | 0.00 | 0.00 | 0.00 | 0.00 | 826200.00 | 0.00 | 0.00 | 0.00 | | cystathionine beta-lyase/cystathionine gamma-synthase | |
| Ga0586324 4402229 1 651 | 0.00 | 1346400.00 | 0.00 | 0.00 | 1431700.00 | 0.00 | 0.00 | 994300.00 | 0.00 | 0.00 | 0.00 | 0.00 | | hypothetical protein | |
| Ga0586324 4412595 2 649 | 0.00 | 0.00 | 0.00 | 0.00 | 0.00 | 0.00 | 0.00 | 0.00 | 0.00 | 0.00 | 0.00 | 52375.05 | | hypothetical protein | |
| Ga0586324 4412632 92 649 | 2640000.00 | 0.00 | 0.00 | 0.00 | 0.00 | 0.00 | 0.00 | 0.00 | 0.00 | 0.00 | 0.00 | 0.00 | | hypothetical protein | |
| Ga0586324 4414353 1 531 | 4750000.00 | 0.00 | 0.00 | 0.00 | 0.00 | 0.00 | 0.00 | 0.00 | 0.00 | 0.00 | 0.00 | 0.00 | | hypothetical protein | Bacteria;Proteobacteria;Deltaproteobacteria;Desulfobacterales;Desulfobacteraceae;Desulfotignum;Desulfotignum balticum;Desulfotignum balticum DSM 7044 |
| Ga0586324 4414407 2 649 | 1294096.00 | 0.00 | 0.00 | 794442.25 | 114466.99 | 0.00 | 0.00 | 0.00 | 0.00 | 0.00 | 0.00 | 3772500.00 | | hypothetical protein | Bacteria;Acidobacteria;Acidobacteriia;Acidobacteriales;Acidobacteriaceae;Granulicella;Granulicella sp. GAS466;Granulicella sp. GAS466 |
| Ga0586324 4429402 2 442 | 0.00 | 12172000.00 | 0.00 | 0.00 | 0.00 | 0.00 | 11559600.00 | 0.00 | 0.00 | 0.00 | 0.00 | 0.00 | | hypothetical protein | Bacteria;Proteobacteria;Deltaproteobacteria;Syntrophobacterales;Syntrophobacteraceae;Desulfacinum;Desulfacinum infernum;Desulfacinum infernum DSM 9756 |
| Ga0586324 4430588 3 647 | 0.00 | 1074400.00 | 0.00 | 0.00 | 0.00 | 1616290.22 | 4248807.36 | 0.00 | 0.00 | 0.00 | 1029600.00 | 0.00 | | hypothetical protein | Bacteria;Bacteroidetes;Bacteroidia;Marinilabiliales;Prolixibacteraceae;Prolixibacter;Prolixibacter denitrificans;Prolixibacter denitrificans DSM 27267 |
| Ga0586324 4437708 416 649 | 0.00 | 3631200.00 | 0.00 | 1415500.00 | 0.00 | 0.00 | 0.00 | 0.00 | 3458700.00 | 0.00 | 0.00 | 0.00 | | NAD(P)H-nitrite reductase large subunit | Bacteria;Proteobacteria;Deltaproteobacteria;Syntrophobacterales;Syntrophaceae;Syntrophus;Syntrophus aciditrophicus;Syntrophus aciditrophicus SB |
| Ga0586324 4440395 2 646 | 0.00 | 0.00 | 0.00 | 0.00 | 0.00 | 0.00 | 0.00 | 0.00 | 2049300.00 | 0.00 | 0.00 | 0.00 | | arylsulfatase A-like enzyme | Bacteria;Proteobacteria;Alphaproteobacteria;Hyphomicrobiales;Bradyrhizobiaceae;Bradyrhizobium;Bradyrhizobium erythrophlei;Bradyrhizobium erythrophlei GAS138 |
| Ga0586324 4448312 3 647 | 0.00 | 0.00 | 0.00 | 0.00 | 5603200.00 | 4128800.00 | 0.00 | 0.00 | 0.00 | 0.00 | 0.00 | 0.00 | | hypothetical protein | Bacteria;Proteobacteria;Deltaproteobacteria;Desulfuromonadales;Geobacteraceae;Geobacter;Geobacter argillaceus;Geobacter argillaceus ATCC BAA-1139 |
| Ga0586324 4451036 2 646 | 0.00 | 0.00 | 0.00 | 8350500.00 | 9043400.00 | 0.00 | 0.00 | 10553000.00 | 0.00 | 10857000.00 | 10032000.00 | 9975000.00 | | predicted porin | Bacteria;Proteobacteria;Betaproteobacteria;Nitrosomonadales;Sterolibacteriaceae;Sulfurisoma;Sulfurisoma sediminicola;Sulfurisoma sediminicola DSM 26916 |
| Ga0586324 4459780 217 645 | 0.00 | 0.00 | 0.00 | 0.00 | 0.00 | 0.00 | 0.00 | 0.00 | 5111100.00 | 0.00 | 0.00 | 0.00 | | hypothetical protein | |
| Ga0586324 4473295 1 282 | 0.00 | 2944400.00 | 0.00 | 0.00 | 0.00 | 0.00 | 0.00 | 0.00 | 0.00 | 0.00 | 0.00 | 0.00 | | crotonobetainyl-CoA:carnitine CoA-transferase CaiB-like acyl-CoA transferase | |
| Ga0586324 4477176 1 645 | 0.00 | 0.00 | 0.00 | 3667000.00 | 3975800.00 | 0.00 | 0.00 | 1366400.00 | 0.00 | 0.00 | 0.00 | 0.00 | | hypothetical protein | Bacteria;Proteobacteria;Deltaproteobacteria;Syntrophobacterales;Syntrophaceae;Desulfobacca;Desulfobacca acetoxidans;Desulfobacca acetoxidans ASRB2, DSM 11109 |
| Ga0586324 4482022 1 645 | 14200000.00 | 8704000.00 | 13455700.00 | 15105000.00 | 15965000.00 | 13498000.00 | 5608800.00 | 8235000.00 | 6763500.00 | 11473000.00 | 10208000.00 | 11925000.00 | | predicted porin | Bacteria;Proteobacteria;Betaproteobacteria;Neisseriales;Chromobacteriaceae;Pseudogulbenkiania;Pseudogulbenkiania ferrooxidans;Pseudogulbenkiania ferrooxidans 2002 |
| Ga0586324 4483073 3 644 | 0.00 | 0.00 | 0.00 | 0.00 | 2018800.00 | 0.00 | 0.00 | 0.00 | 0.00 | 0.00 | 0.00 | 0.00 | | hypothetical protein | Bacteria;Acidobacteria;Vicinamibacteria;unclassified Vicinamibacteria;Vicinamibacteraceae;Luteitalea;Luteitalea pratensis;Luteitalea pratensis DSM 100886 |
| Ga0586324 4500463 2 643 | 13200000.00 | 12770400.00 | 5407479.88 | 17160243.30 | 14317000.00 | 8568288.23 | 3214800.00 | 7015000.00 | 6204600.00 | 7007000.00 | 7128000.00 | 5760000.00 | | hypothetical protein | |
| Ga0586324 4511420 3 644 | 0.00 | 0.00 | 0.00 | 0.00 | 0.00 | 0.00 | 0.00 | 0.00 | 0.00 | 0.00 | 649553.52 | 0.00 | | EpsD family peptidyl-prolyl cis-trans isomerase | Bacteria;Proteobacteria;Betaproteobacteria;Nitrosomonadales;Thiobacillaceae;Thiobacillus;Thiobacillus denitrificans;Thiobacillus denitrificans RG |
| Ga0586324 4527108 3 641 | 0.00 | 0.00 | 0.00 | 0.00 | 0.00 | 0.00 | 0.00 | 0.00 | 0.00 | 0.00 | 2780800.00 | 0.00 | | CheY-like chemotaxis protein | Bacteria;Proteobacteria;Betaproteobacteria;Burkholderiales;Comamonadaceae;Variovorax;Variovorax sp. OV329;Variovorax sp. OV329 |
| Ga0586324 4530052 3 641 | 0.00 | 0.00 | 0.00 | 0.00 | 0.00 | 0.00 | 0.00 | 0.00 | 0.00 | 5105100.00 | 0.00 | 0.00 | | ABC-type oligopeptide transport system substrate-binding subunit | Bacteria;Proteobacteria;Alphaproteobacteria;Hyphomicrobiales;Rhizobiaceae;Rhizobium;Rhizobium flavum;Rhizobium flavum CCTCC AB 2013042 |
| Ga0586324 4534538 3 641 | 0.00 | 7140000.00 | 0.00 | 0.00 | 0.00 | 30846900.00 | 0.00 | 0.00 | 0.00 | 0.00 | 0.00 | 0.00 | | S-layer protein (TIGR01567 family) | Archaea;Euryarchaeota;Methanomicrobia;Methanosarcinales;Methanotrichaceae;Methanothrix;Methanothrix thermoacetophila;Methanothrix thermoacetophila PT |
| Ga0586324 4557333 1 642 | 0.00 | 0.00 | 0.00 | 2831000.00 | 4830700.00 | 0.00 | 0.00 | 0.00 | 0.00 | 0.00 | 0.00 | 0.00 | | hypothetical protein | |
| Ga0586324 4559319 20 499 | 0.00 | 0.00 | 0.00 | 0.00 | 0.00 | 4327300.00 | 0.00 | 0.00 | 0.00 | 0.00 | 0.00 | 0.00 | | tetratricopeptide (TPR) repeat protein | |
| Ga0586324 4565370 1 639 | 0.00 | 3434000.00 | 0.00 | 0.00 | 0.00 | 0.00 | 0.00 | 0.00 | 0.00 | 0.00 | 0.00 | 0.00 | | hypothetical protein | Bacteria;Acidobacteria;Acidobacteriia;Acidobacteriales;Acidobacteriaceae;Edaphobacter;Edaphobacter modestus;Edaphobacter modestus DSM 18101 |
| Ga0586324 4566916 3 641 | 0.00 | 0.00 | 10640700.00 | 0.00 | 0.00 | 0.00 | 33060000.00 | 0.00 | 11907000.00 | 0.00 | 0.00 | 0.00 | | hypothetical protein | Bacteria;Verrucomicrobia;Verrucomicrobiae;Verrucomicrobiales;Verrucomicrobia subdivision 3;Pedosphaera;Pedosphaera parvula;Pedosphaera parvula Ellin514 |
| Ga0586324 4570116 1 468 | 0.00 | 0.00 | 0.00 | 0.00 | 0.00 | 0.00 | 0.00 | 0.00 | 0.00 | 0.00 | 1188000.00 | 0.00 | | predicted porin | Bacteria;Proteobacteria;Betaproteobacteria;unclassified Betaproteobacteria;unclassified Betaproteobacteria;unclassified Betaproteobacteria;uncultured beta proteobacterium;unclassified betaproteobacterium ENR4 |
| Ga0586324 4581734 3 638 | 0.00 | 0.00 | 0.00 | 0.00 | 0.00 | 0.00 | 0.00 | 0.00 | 0.00 | 0.00 | 20064000.00 | 0.00 | | arylsulfatase A-like enzyme | Bacteria;Proteobacteria;Alphaproteobacteria;Hyphomicrobiales;Bradyrhizobiaceae;Bradyrhizobium;Bradyrhizobium japonicum;Bradyrhizobium japonicum SEMIA 5079 |
| Ga0586324 4586705 109 639 | 0.00 | 0.00 | 3798696.12 | 0.00 | 0.00 | 0.00 | 0.00 | 0.00 | 0.00 | 0.00 | 0.00 | 0.00 | | hypothetical protein | Bacteria;Verrucomicrobia;Verrucomicrobiae;Verrucomicrobiales;Verrucomicrobia subdivision 3;Pedosphaera;Pedosphaera parvula;Pedosphaera parvula Ellin514 |
| Ga0586324 4605855 174 638 | 0.00 | 1332800.00 | 0.00 | 0.00 | 0.00 | 0.00 | 0.00 | 0.00 | 0.00 | 2687300.00 | 0.00 | 0.00 | | hypothetical protein | Bacteria;Proteobacteria;Gammaproteobacteria;Thiotrichales;Thiotrichaceae;Achromatium;uncultured Achromatium sp.;Achromatium sp. Cell8 |
| Ga0586324 4612819 1 636 | 0.00 | 2631600.00 | 0.00 | 6479000.00 | 0.00 | 0.00 | 0.00 | 0.00 | 0.00 | 0.00 | 3440800.00 | 0.00 | | hypothetical protein | Bacteria;Proteobacteria;Gammaproteobacteria;Chromatiales;Ectothiorhodospiraceae;Thiorhodospira;Thiorhodospira sibirica;Thiorhodospira sibirica A12, ATCC 700588 |
| Ga0586324 4618810 352 636 | 0.00 | 20060000.00 | 0.00 | 0.00 | 0.00 | 0.00 | 0.00 | 0.00 | 0.00 | 0.00 | 0.00 | 0.00 | | predicted porin | Bacteria;Proteobacteria;Betaproteobacteria;Burkholderiales;unclassified Burkholderiales;Rhizobacter;Rhizobacter sp. Root404;Rhizobacter sp. Root404 |
| Ga0586324 4622718 122 637 | 0.00 | 0.00 | 0.00 | 0.00 | 0.00 | 0.00 | 0.00 | 0.00 | 760693.68 | 0.00 | 0.00 | 0.00 | | hypothetical protein | |
| Ga0586324 4626290 1 636 | 0.00 | 0.00 | 2003199.04 | 0.00 | 0.00 | 0.00 | 0.00 | 0.00 | 0.00 | 0.00 | 0.00 | 0.00 | | TonB-dependent receptor | Bacteria;Ignavibacteriae;Ignavibacteria;Ignavibacteriales;Melioribacteraceae;Melioribacter;Melioribacter roseus;Melioribacter roseus P3M |
| Ga0586324 4636875 1 624 | 0.00 | 497101.08 | 0.00 | 0.00 | 0.00 | 0.00 | 0.00 | 0.00 | 0.00 | 0.00 | 0.00 | 0.00 | | hypothetical protein | |
| Ga0586324 4645488 2 637 | 0.00 | 0.00 | 0.00 | 0.00 | 0.00 | 0.00 | 0.00 | 0.00 | 1790100.00 | 0.00 | 0.00 | 0.00 | | hypothetical protein | Bacteria;Firmicutes;Bacilli;Bacillales;Thermoactinomycetaceae;Shimazuella;Shimazuella kribbensis;Shimazuella kribbensis DSM 45090 |
| Ga0586324 4654546 3 635 | 0.00 | 1319200.00 | 0.00 | 0.00 | 2688300.00 | 740305.75 | 0.00 | 0.00 | 0.00 | 0.00 | 0.00 | 1567500.00 | | hypothetical protein | Bacteria;Bacteroidetes;Cytophagia;Cytophagales;Flammeovirgaceae;Flexithrix;Flexithrix dorotheae;Flexithrix dorotheae DSM 6795 |
| Ga0586324 4669613 3 296 | 4050000.00 | 2536400.00 | 2399680.53 | 2498500.00 | 2111500.00 | 1732623.13 | 2251821.48 | 1695800.00 | 1814400.00 | 0.00 | 0.00 | 596761.50 | | TRAP-type mannitol/chloroaromatic compound transport system substrate-binding protein | Bacteria;Proteobacteria;Alphaproteobacteria;Hyphomicrobiales;Bradyrhizobiaceae;Afipia;Afipia sp. P52-10;Afipia sp. P52-10 |
| Ga0586324 4678068 2 634 | 1890000.00 | 1332800.00 | 0.00 | 0.00 | 1030000.00 | 0.00 | 0.00 | 2232600.00 | 1757700.00 | 0.00 | 1029600.00 | 0.00 | | predicted porin | Bacteria;Proteobacteria;Betaproteobacteria;Nitrosomonadales;Sulfuricellaceae;Sulfuriferula;Sulfuriferula thiophila;Sulfuriferula thiophila mst6 |
| Ga0586324 4679736 1 216 | 0.00 | 0.00 | 0.00 | 0.00 | 0.00 | 0.00 | 0.00 | 0.00 | 0.00 | 0.00 | 324309.04 | 0.00 | | uncharacterized membrane protein YqjE | |
| Ga0586324 4680016 3 635 | 1490000.00 | 0.00 | 0.00 | 0.00 | 0.00 | 0.00 | 0.00 | 0.00 | 0.00 | 0.00 | 0.00 | 0.00 | | ABC-type oligopeptide transport system ATPase subunit | Bacteria;Chloroflexi;Anaerolineae;Anaerolineales;Anaerolineaceae;Anaerolinea;Anaerolinea thermophila;Anaerolinea thermophila UNI-1 |
| Ga0586324 4694232 1 633 | 0.00 | 2815200.00 | 0.00 | 0.00 | 0.00 | 0.00 | 0.00 | 0.00 | 0.00 | 0.00 | 0.00 | 0.00 | | hypothetical protein | Bacteria;Proteobacteria;Betaproteobacteria;Nitrosomonadales;Nitrosomonadaceae;Nitrosospira;Nitrosospira multiformis;Nitrosospira multiformis Nl1 |
| Ga0586324 4705894 2 631 | 0.00 | 538337.64 | 0.00 | 0.00 | 0.00 | 0.00 | 0.00 | 0.00 | 0.00 | 0.00 | 0.00 | 0.00 | | hypothetical protein | Bacteria;Proteobacteria;Deltaproteobacteria;Myxococcales;Polyangiaceae;Sorangium;Sorangium cellulosum;Sorangium cellulosum So0157-25 |
| Ga0586324 4710843 74 595 | 0.00 | 2828800.00 | 0.00 | 0.00 | 6365400.00 | 7384200.00 | 0.00 | 0.00 | 0.00 | 0.00 | 0.00 | 0.00 | | Flp pilus assembly protein TadD | |
| Ga0586324 4712126 2 631 | 0.00 | 1536800.00 | 0.00 | 0.00 | 0.00 | 0.00 | 0.00 | 0.00 | 0.00 | 0.00 | 0.00 | 0.00 | | predicted porin | Bacteria;Proteobacteria;Betaproteobacteria;Burkholderiales;Alcaligenaceae;Candidimonas;Candidimonas bauzanensis;Candidimonas bauzanensis CGMCC 1.10190 |
| Ga0586324 4716483 2 631 | 0.00 | 0.00 | 0.00 | 0.00 | 0.00 | 0.00 | 0.00 | 0.00 | 0.00 | 0.00 | 0.00 | 549291.75 | | hypothetical protein | |
| Ga0586324 4717159 3 632 | 0.00 | 0.00 | 0.00 | 0.00 | 0.00 | 0.00 | 0.00 | 0.00 | 0.00 | 0.00 | 0.00 | 5467500.00 | | hypothetical protein | Bacteria;Chloroflexi;Caldilineae;Caldilineales;Caldilineaceae;Caldilinea;Caldilinea aerophila;Caldilinea aerophila STL-6-O1, DSM 14535 |
| Ga0586324 4720353 3 632 | 352235.00 | 0.00 | 0.00 | 0.00 | 0.00 | 0.00 | 0.00 | 0.00 | 0.00 | 0.00 | 0.00 | 0.00 | | hypothetical protein | |
| Ga0586324 4723372 330 632 | 0.00 | 657572.24 | 0.00 | 0.00 | 0.00 | 2012464.46 | 0.00 | 6893000.00 | 5459400.00 | 0.00 | 0.00 | 0.00 | | predicted porin | Bacteria;Proteobacteria;Betaproteobacteria;Burkholderiales;Oxalobacteraceae;unclassified Oxalobacteraceae;Oxalobacteraceae bacterium IMCC9480;beta proteobacterium sp. IMCC9480 |
| Ga0586324 4732531 1 630 | 0.00 | 212087.24 | 0.00 | 0.00 | 0.00 | 0.00 | 0.00 | 0.00 | 0.00 | 0.00 | 0.00 | 0.00 | | hypothetical protein | Bacteria;Proteobacteria;Betaproteobacteria;Nitrosomonadales;Sulfuricellaceae;Sulfurirhabdus;Sulfurirhabdus autotrophica;Sulfurirhabdus autotrophica DSM 100309 |
| Ga0586324 4744838 3 629 | 0.00 | 0.00 | 0.00 | 0.00 | 0.00 | 0.00 | 0.00 | 0.00 | 0.00 | 0.00 | 0.00 | 2625000.00 | | UbiD family decarboxylase | Bacteria;Proteobacteria;Deltaproteobacteria;Syntrophorhabdales;Syntrophorhabdaceae;Syntrophorhabdus;Syntrophorhabdus aromaticivorans;Syntrophorhabdus aromaticivorans UI |
| Ga0586324 4751609 2 631 | 0.00 | 4297600.00 | 12667500.00 | 0.00 | 0.00 | 8575200.00 | 8071200.00 | 3996753.55 | 0.00 | 124609100.00 | 112112000.00 | 46545000.00 | | predicted porin | Bacteria;Proteobacteria;Betaproteobacteria;Burkholderiales;unclassified Burkholderiales;unclassified Burkholderiales;Burkholderiales bacterium GJ-E10;Burkholderiales bacterium GJ-E10 |
| Ga0586324 4763592 31 630 | 0.00 | 0.00 | 0.00 | 0.00 | 0.00 | 0.00 | 0.00 | 3080500.00 | 3969000.00 | 0.00 | 0.00 | 2310000.00 | | hypothetical protein | Bacteria;Actinobacteria;Actinomycetia;Streptomycetales;Streptomycetaceae;Kitasatospora;Kitasatospora atroaurantiaca;Kitasatospora atroaurantiaca DSM 41649 |
| Ga0586324 4796343 3 626 | 0.00 | 0.00 | 0.00 | 0.00 | 0.00 | 0.00 | 0.00 | 0.00 | 3458700.00 | 0.00 | 3106400.00 | 0.00 | | hypothetical protein | |
| Ga0586324 4797735 3 626 | 91816306.00 | 126242000.00 | 0.00 | 125732500.00 | 262165900.00 | 6946809.22 | 9507600.00 | 171397800.00 | 110459700.00 | 633428.95 | 1879254.96 | 57225000.00 | | predicted porin | Bacteria;Proteobacteria;Betaproteobacteria;Rhodocyclales;Azonexaceae;Dechloromonas;Dechloromonas denitrificans;Dechloromonas denitrificans ATCC BAA-841 |
| Ga0586324 4810397 2 625 | 0.00 | 1081200.00 | 0.00 | 3182500.00 | 2832500.00 | 0.00 | 0.00 | 70760000.00 | 1360800.00 | 0.00 | 0.00 | 0.00 | | hypothetical protein | Bacteria;Bacteroidetes;Bacteroidia;Marinilabiliales;Marinilabiliaceae;Saccharicrinis;Saccharicrinis fermentans;Saccharicrinis fermentans DSM 9555 |
| Ga0586324 4812608 2 625 | 0.00 | 0.00 | 0.00 | 0.00 | 2214500.00 | 0.00 | 0.00 | 0.00 | 0.00 | 0.00 | 0.00 | 0.00 | | crotonobetainyl-CoA:carnitine CoA-transferase CaiB-like acyl-CoA transferase | Archaea;Euryarchaeota;Archaeoglobi;Archaeoglobales;Archaeoglobaceae;Geoglobus;Geoglobus ahangari;Geoglobus ahangari 234 |
| Ga0586324 4825068 2 625 | 0.00 | 0.00 | 0.00 | 1102000.00 | 0.00 | 0.00 | 0.00 | 0.00 | 0.00 | 0.00 | 0.00 | 0.00 | | tetratricopeptide (TPR) repeat protein | Bacteria;Proteobacteria;Betaproteobacteria;Burkholderiales;unclassified Burkholderiales;Ideonella;Ideonella sp. A 288;Ideonella sp. A 288 |
| Ga0586324 4825453 1 627 | 0.00 | 0.00 | 0.00 | 0.00 | 0.00 | 0.00 | 0.00 | 19459000.00 | 0.00 | 17633000.00 | 0.00 | 0.00 | | hypothetical protein | |
| Ga0586324 4837861 2 625 | 0.00 | 0.00 | 0.00 | 0.00 | 0.00 | 0.00 | 0.00 | 707600.00 | 0.00 | 4373600.00 | 3203200.00 | 2092500.00 | | hypothetical protein | Bacteria;Proteobacteria;Betaproteobacteria;Nitrosomonadales;Thiobacillaceae;Thiobacillus;Thiobacillus thiophilus;Thiobacillus thiophilus DSM 19892 |
| Ga0586324 4839887 3 626 | 0.00 | 0.00 | 0.00 | 0.00 | 2688300.00 | 0.00 | 0.00 | 0.00 | 0.00 | 0.00 | 0.00 | 0.00 | | ABC-type sugar transport system substrate-binding protein | Bacteria;Chloroflexi;Caldilineae;Caldilineales;Caldilineaceae;Caldilinea;Caldilinea aerophila;Caldilinea aerophila STL-6-O1, DSM 14535 |
| Ga0586324 4845237 1 624 | 0.00 | 0.00 | 0.00 | 1206500.00 | 863427.37 | 0.00 | 747308.76 | 1598200.00 | 0.00 | 0.00 | 0.00 | 0.00 | | hypothetical protein | Bacteria;Proteobacteria;Deltaproteobacteria;Syntrophobacterales;Syntrophobacteraceae;Desulfosoma;Desulfosoma caldarium;Desulfosoma caldarium DSM 22027 |
| Ga0586324 4850573 2 625 | 0.00 | 0.00 | 0.00 | 0.00 | 0.00 | 0.00 | 0.00 | 0.00 | 0.00 | 0.00 | 7673600.00 | 0.00 | | hypothetical protein | Bacteria;Atribacterota;unclassified Atribacterota;unclassified Atribacterota;unclassified Atribacterota;unclassified Atribacterota;Atribacteria bacterium JGI 0000059-I14;Atribacteria bacterium JGI 0000059-I14 (contamination screened) |
| Ga0586324 4850698 3 623 | 0.00 | 0.00 | 0.00 | 0.00 | 8260600.00 | 0.00 | 0.00 | 0.00 | 0.00 | 0.00 | 0.00 | 0.00 | | hypothetical protein | Bacteria;Proteobacteria;Gammaproteobacteria;unclassified Gammaproteobacteria;Candidatus Competibacteraceae;Candidatus Competibacter;Candidatus Competibacter denitrificans;Candidatus Competibacter denitrificans Run A D11 |
| Ga0586324 4851065 121 624 | 0.00 | 0.00 | 0.00 | 0.00 | 0.00 | 0.00 | 0.00 | 0.00 | 4228200.00 | 0.00 | 0.00 | 0.00 | | hypothetical protein | Bacteria;Bacteroidetes;Bacteroidia;Marinilabiliales;Prolixibacteraceae;Sunxiuqinia;Sunxiuqinia dokdonensis;Sunxiuqinia dokdonensis SK |
| Ga0586324 4855055 125 625 | 0.00 | 1060800.00 | 0.00 | 0.00 | 1998200.00 | 0.00 | 0.00 | 0.00 | 730240.92 | 0.00 | 275113.52 | 0.00 | | hypothetical protein | |
| Ga0586324 4859779 1 624 | 1050000.00 | 2950921.20 | 1247557.33 | 0.00 | 675187.66 | 2355218.38 | 2652166.68 | 927200.00 | 2535300.00 | 0.00 | 0.00 | 1170000.00 | | S-layer protein (TIGR01567 family) | Archaea;Euryarchaeota;Methanomicrobia;Methanosarcinales;Methanotrichaceae;Methanothrix;Methanothrix soehngenii;Methanothrix soehngenii GP-6 |
| Ga0586324 4864616 116 625 | 0.00 | 0.00 | 0.00 | 0.00 | 0.00 | 0.00 | 0.00 | 0.00 | 0.00 | 347023.60 | 0.00 | 0.00 | | hypothetical protein | |
| Ga0586324 4867658 146 622 | 0.00 | 0.00 | 0.00 | 0.00 | 0.00 | 0.00 | 0.00 | 0.00 | 0.00 | 0.00 | 0.00 | 7327500.00 | | alkylation response protein AidB-like acyl-CoA dehydrogenase | Bacteria;Nitrospirae;Thermodesulfovibrionia;Thermodesulfovibrionales;Thermodesulfovibrionaceae;Thermodesulfovibrio;Thermodesulfovibrio hydrogeniphilus;Thermodesulfovibrio hydrogeniphilus DSM 18151 |
| Ga0586324 4879808 164 622 | 2990000.00 | 0.00 | 0.00 | 790573.85 | 0.00 | 0.00 | 0.00 | 0.00 | 0.00 | 0.00 | 0.00 | 0.00 | | rubrerythrin | Bacteria;Planctomycetes;Candidatus Brocadiae;Candidatus Brocadiales;Candidatus Brocadiaceae;Candidatus Jettenia;Candidatus Jettenia caeni;Planctomycetaceae bacterium KSU-1 |
| Ga0586324 4883289 135 623 | 1040000.00 | 0.00 | 0.00 | 0.00 | 0.00 | 0.00 | 0.00 | 701500.00 | 0.00 | 0.00 | 0.00 | 0.00 | | predicted porin | Bacteria;Proteobacteria;Gammaproteobacteria;Chromatiales;Ectothiorhodospiraceae;Thioalkalivibrio;Thioalkalivibrio sp. ALJ24;Thioalkalivibrio sp. ALJ24 |
| Ga0586324 4891251 17 622 | 0.00 | 263038.28 | 0.00 | 0.00 | 0.00 | 0.00 | 0.00 | 0.00 | 0.00 | 0.00 | 0.00 | 0.00 | | transketolase N-terminal domain/subunit | Bacteria;Proteobacteria;Gammaproteobacteria;Methylococcales;Methylococcaceae;Methylomonas;Methylomonas methanica;Methylomonas methanica MC09 |
| Ga0586324 4905657 2 622 | 0.00 | 0.00 | 0.00 | 0.00 | 0.00 | 0.00 | 0.00 | 0.00 | 1458000.00 | 0.00 | 0.00 | 0.00 | | hypothetical protein | |
| Ga0586324 4907945 1 621 | 13120000.00 | 1373600.00 | 0.00 | 0.00 | 0.00 | 0.00 | 0.00 | 0.00 | 0.00 | 0.00 | 0.00 | 0.00 | | hypothetical protein | Bacteria;Spirochaetes;Spirochaetia;Spirochaetales;Treponemataceae;Treponema;Treponema caldarium;Treponema caldarium DSM 7334 |
| Ga0586324 4930896 3 620 | 0.00 | 0.00 | 0.00 | 0.00 | 0.00 | 0.00 | 0.00 | 0.00 | 0.00 | 0.00 | 144136.96 | 227985.75 | | hypothetical protein | Bacteria;Actinobacteria;Thermoleophilia;Solirubrobacterales;Solirubrobacteraceae;Solirubrobacter;Solirubrobacter pauli;Solirubrobacter pauli DSM 14954 |
| Ga0586324 4938958 3 620 | 0.00 | 0.00 | 0.00 | 0.00 | 0.00 | 0.00 | 0.00 | 0.00 | 0.00 | 0.00 | 1689600.00 | 0.00 | | hypothetical protein | Bacteria;Bacteroidetes;Bacteroidia;Marinilabiliales;Prolixibacteraceae;Sunxiuqinia;Sunxiuqinia elliptica;Sunxiuqinia elliptica CGMCC 1.9156 |
| Ga0586324 4951546 3 620 | 0.00 | 0.00 | 0.00 | 0.00 | 0.00 | 0.00 | 20793600.00 | 0.00 | 0.00 | 0.00 | 0.00 | 0.00 | | protein-export membrane protein SecD | Bacteria;Calditrichaeota;Calditrichae;Calditrichales;Calditrichaceae;Caldithrix;Caldithrix abyssi;Caldithrix abyssi LF13, DSM 13497 |
| Ga0586324 4955744 3 620 | 2180000.00 | 822800.00 | 2358474.56 | 0.00 | 0.00 | 2022746.76 | 1748541.12 | 0.00 | 955800.00 | 893200.00 | 0.00 | 473068.50 | | predicted porin | Bacteria;Proteobacteria;Betaproteobacteria;Burkholderiales;Comamonadaceae;Limnohabitans;Limnohabitans sp. 63ED37-2;Limnohabitans sp. 63ED37-2 |
| Ga0586324 4955800 407 619 | 0.00 | 0.00 | 0.00 | 0.00 | 0.00 | 0.00 | 0.00 | 0.00 | 3183300.00 | 0.00 | 0.00 | 0.00 | | hypothetical protein | |
| Ga0586324 4957523 1 381 | 0.00 | 0.00 | 0.00 | 0.00 | 8435700.00 | 0.00 | 0.00 | 3879600.00 | 0.00 | 0.00 | 0.00 | 0.00 | | Tfp pilus assembly protein PilF | Bacteria;Proteobacteria;Deltaproteobacteria;Syntrophobacterales;Syntrophaceae;Desulfobacca;Desulfobacca acetoxidans;Desulfobacca acetoxidans ASRB2, DSM 11109 |
| Ga0586324 4961278 3 545 | 0.00 | 3685600.00 | 0.00 | 0.00 | 0.00 | 0.00 | 0.00 | 0.00 | 0.00 | 0.00 | 0.00 | 0.00 | | hypothetical protein | Bacteria;Acidobacteria;Vicinamibacteria;unclassified Vicinamibacteria;Vicinamibacteraceae;Luteitalea;Luteitalea pratensis;Luteitalea pratensis DSM 100886 |
| Ga0586324 4967588 126 617 | 0.00 | 0.00 | 0.00 | 0.00 | 0.00 | 0.00 | 0.00 | 12993000.00 | 14742000.00 | 0.00 | 0.00 | 0.00 | | hypothetical protein | Bacteria;Proteobacteria;Deltaproteobacteria;Desulfuromonadales;Geobacteraceae;Geobacter;Geobacter sp. M18;Geobacter sp. M18 |
| Ga0586324 4968752 12 617 | 0.00 | 0.00 | 0.00 | 0.00 | 0.00 | 0.00 | 0.00 | 0.00 | 0.00 | 0.00 | 1496000.00 | 0.00 | | hypothetical protein | |
| Ga0586324 4986499 3 581 | 0.00 | 18020000.00 | 1365708.51 | 14155000.00 | 12679300.00 | 0.00 | 0.00 | 21708875.20 | 35633555.64 | 0.00 | 0.00 | 2663930.25 | | predicted porin | Bacteria;Proteobacteria;Betaproteobacteria;Burkholderiales;Oxalobacteraceae;Herbaspirillum;Herbaspirillum sp. meg3;Herbaspirillum sp. meg3 |
| Ga0586324 5001939 1 618 | 0.00 | 0.00 | 0.00 | 0.00 | 0.00 | 0.00 | 0.00 | 293998.65 | 0.00 | 0.00 | 0.00 | 0.00 | | hypothetical protein | Bacteria;Proteobacteria;Deltaproteobacteria;Myxococcales;unclassified Myxococcales;Enhygromyxa;Enhygromyxa salina;Enhygromyxa salina SWB007 |
| Ga0586324 5005209 31 615 | 0.00 | 0.00 | 0.00 | 3648000.00 | 0.00 | 0.00 | 0.00 | 0.00 | 0.00 | 0.00 | 0.00 | 0.00 | | membrane protease subunit (stomatin/prohibitin family) | Archaea;Thaumarchaeota;unclassified Thaumarchaeota;unclassified Thaumarchaeota;unclassified Thaumarchaeota;unclassified Thaumarchaeota;Aigarchaeota archaeon JGI 0000106-J15;Aigarchaeota archaeon JGI 0000106-J15 (contamination screened) |
| Ga0586324 5029923 2 616 | 78700000.00 | 91868000.00 | 44653888.97 | 119225000.00 | 137597700.00 | 29131026.30 | 199446757.44 | 315919000.00 | 261006300.00 | 138677000.00 | 320434400.00 | 105150000.00 | | predicted porin | Bacteria;Proteobacteria;Gammaproteobacteria;Chromatiales;Halothiobacillaceae;Halothiobacillus;Halothiobacillus sp. LS2;Halothiobacillus sp. LS2 |
| Ga0586324 5032467 3 425 | 0.00 | 0.00 | 12926316.73 | 0.00 | 1122700.00 | 0.00 | 0.00 | 0.00 | 0.00 | 0.00 | 3071200.00 | 2010000.00 | | hypothetical protein | |
| Ga0586324 5049212 1 570 | 0.00 | 0.00 | 0.00 | 0.00 | 0.00 | 0.00 | 0.00 | 0.00 | 0.00 | 0.00 | 10648000.00 | 7387500.00 | | hypothetical protein | |
| Ga0586324 5053283 3 614 | 0.00 | 0.00 | 0.00 | 0.00 | 3718300.00 | 0.00 | 0.00 | 5142300.00 | 0.00 | 0.00 | 0.00 | 0.00 | | hypothetical protein | Bacteria;Proteobacteria;Deltaproteobacteria;Desulfuromonadales;Geobacteraceae;Geobacter;Geobacter thiogenes;Geobacter thiogenes ATCC BAA-34 |
| Ga0586324 5074524 3 614 | 1940000.00 | 0.00 | 0.00 | 0.00 | 0.00 | 0.00 | 3306000.00 | 0.00 | 0.00 | 0.00 | 0.00 | 0.00 | | hypothetical protein | Bacteria;Actinobacteria;Actinomycetia;Micromonosporales;Micromonosporaceae;Plantactinospora;Plantactinospora sp. CNZ320;Plantactinospora sp. CNZ320 |
| Ga0586324 5077614 2 541 | 0.00 | 0.00 | 21731800.00 | 24320000.00 | 0.00 | 0.00 | 12608400.00 | 10614000.00 | 13608000.00 | 0.00 | 0.00 | 0.00 | | hypothetical protein | |
| Ga0586324 5077777 2 613 | 0.00 | 0.00 | 0.00 | 0.00 | 0.00 | 0.00 | 0.00 | 0.00 | 0.00 | 0.00 | 0.00 | 1155000.00 | | hypothetical protein | Bacteria;Proteobacteria;Deltaproteobacteria;Desulfuromonadales;Geobacteraceae;Geobacter;Geobacter thiogenes;Geobacter thiogenes ATCC BAA-34 |
| Ga0586324 5079091 3 614 | 0.00 | 0.00 | 0.00 | 0.00 | 0.00 | 392521.44 | 0.00 | 0.00 | 0.00 | 0.00 | 0.00 | 0.00 | | uncharacterized caspase-like protein | Bacteria;Proteobacteria;Betaproteobacteria;Burkholderiales;Comamonadaceae;Curvibacter;Curvibacter lanceolatus;Curvibacter lanceolatus ATCC 14669 |
| Ga0586324 5087987 105 611 | 0.00 | 0.00 | 0.00 | 3296500.00 | 3903700.00 | 0.00 | 0.00 | 0.00 | 0.00 | 0.00 | 0.00 | 0.00 | | Fe-S oxidoreductase | Bacteria;Proteobacteria;Deltaproteobacteria;Syntrophobacterales;Syntrophobacteraceae;Desulfosoma;Desulfosoma caldarium;Desulfosoma caldarium DSM 22027 |
| Ga0586324 5090206 3 611 | 0.00 | 1060800.00 | 0.00 | 0.00 | 0.00 | 9686800.00 | 0.00 | 0.00 | 558148.32 | 0.00 | 594894.08 | 0.00 | | TRAP-type mannitol/chloroaromatic compound transport system substrate-binding protein | Bacteria;Proteobacteria;Alphaproteobacteria;Rhodospirillales;Azospirillaceae;Azospirillum;Azospirillum doebereinerae;Azospirillum doebereinerae DSM 13131 |
| Ga0586324 5092157 2 208 | 0.00 | 843200.00 | 0.00 | 0.00 | 0.00 | 0.00 | 0.00 | 0.00 | 0.00 | 0.00 | 0.00 | 0.00 | | arylsulfatase A-like enzyme | Bacteria;Proteobacteria;Betaproteobacteria;Nitrosomonadales;Nitrosomonadaceae;Nitrosospira;Nitrosospira sp. Nsp2;Nitrosospira sp. Nsp2 |
| Ga0586324 5106902 2 610 | 0.00 | 0.00 | 0.00 | 0.00 | 0.00 | 0.00 | 0.00 | 0.00 | 0.00 | 0.00 | 3546400.00 | 0.00 | | hypothetical protein | Bacteria;Proteobacteria;Deltaproteobacteria;Syntrophobacterales;Syntrophaceae;Smithella;Smithella sp. SCADC;Smithella sp. SCADC |
| Ga0586324 5117163 1 612 | 0.00 | 3685600.00 | 0.00 | 1111500.00 | 0.00 | 0.00 | 0.00 | 1945900.00 | 0.00 | 0.00 | 0.00 | 2610000.00 | | methionine synthase II (cobalamin-independent) | Bacteria;Proteobacteria;Gammaproteobacteria;Methylococcales;Methylococcaceae;Methyloglobulus;Methyloglobulus morosus;Methyloglobulus morosus KoM1 |
| Ga0586324 5127387 2 385 | 0.00 | 0.00 | 0.00 | 0.00 | 0.00 | 0.00 | 0.00 | 0.00 | 2114100.00 | 3518900.00 | 0.00 | 0.00 | | hypothetical protein | Bacteria;Proteobacteria;Deltaproteobacteria;Syntrophorhabdales;Syntrophorhabdaceae;Syntrophorhabdus;Syntrophorhabdus aromaticivorans;Syntrophorhabdus aromaticivorans UI |
| Ga0586324 5127473 91 609 | 291986.00 | 437214.16 | 0.00 | 0.00 | 270765.37 | 0.00 | 0.00 | 0.00 | 0.00 | 1301300.00 | 664975.52 | 728991.00 | | predicted AlkP superfamily pyrophosphatase or phosphodiesterase | Bacteria;Actinobacteria;Actinomycetia;Propionibacteriales;Nocardioidaceae;Nocardioides;Nocardioides sp. OAS665;Nocardioides sp. OAS665 |
| Ga0586324 5168570 2 412 | 4960000.00 | 0.00 | 26235800.00 | 9785000.00 | 6952500.00 | 27750300.00 | 21454800.00 | 12810000.00 | 0.00 | 17787000.00 | 14344000.00 | 10200000.00 | | predicted porin | Bacteria;Proteobacteria;Betaproteobacteria;unclassified Betaproteobacteria;unclassified Betaproteobacteria;unclassified Betaproteobacteria;uncultured beta proteobacterium;unclassified betaproteobacterium ENR4 |
| Ga0586324 5179700 3 608 | 25200000.00 | 16320000.00 | 23308200.00 | 21755000.00 | 23587000.00 | 22827500.00 | 1012844.40 | 16531000.00 | 16200000.00 | 0.00 | 11176000.00 | 12000000.00 | | hypothetical protein | Bacteria;Proteobacteria;Alphaproteobacteria;Rhodobacterales;Rhodobacteraceae;Rhodovulum;Rhodovulum sp. PH10;Rhodovulum sp. PH10 |
| Ga0586324 5188501 1 606 | 0.00 | 3862400.00 | 0.00 | 0.00 | 3512300.00 | 0.00 | 0.00 | 0.00 | 0.00 | 4750900.00 | 5104000.00 | 0.00 | | hypothetical protein | |
| Ga0586324 5197326 202 606 | 0.00 | 0.00 | 0.00 | 0.00 | 0.00 | 1014120.62 | 1251546.72 | 933300.00 | 0.00 | 0.00 | 0.00 | 975000.00 | | TRAP-type mannitol/chloroaromatic compound transport system substrate-binding protein | Bacteria;Proteobacteria;Betaproteobacteria;Nitrosomonadales;Sterolibacteriaceae;Sulfurisoma;Sulfurisoma sediminicola;Sulfurisoma sediminicola DSM 26916 |
| Ga0586324 5199782 3 608 | 0.00 | 0.00 | 0.00 | 0.00 | 0.00 | 0.00 | 0.00 | 0.00 | 0.00 | 0.00 | 3300000.00 | 0.00 | | predicted double-glycine peptidase | Bacteria;Firmicutes;Negativicutes;Selenomonadales;Sporomusaceae;Anaeroarcus;Anaeroarcus burkinensis;Anaeroarcus burkinensis DSM 6283 |
| Ga0586324 5200960 1 606 | 2510000.00 | 5984000.00 | 2827217.10 | 4170500.00 | 6359941.00 | 4287600.00 | 3534000.00 | 4495700.00 | 7452000.00 | 0.00 | 0.00 | 0.00 | | crotonobetainyl-CoA:carnitine CoA-transferase CaiB-like acyl-CoA transferase | Bacteria;Proteobacteria;Deltaproteobacteria;Syntrophobacterales;Syntrophaceae;Desulfobacca;Desulfobacca acetoxidans;Desulfobacca acetoxidans ASRB2, DSM 11109 |
| Ga0586324 5213357 2 607 | 2340000.00 | 0.00 | 0.00 | 0.00 | 0.00 | 0.00 | 0.00 | 0.00 | 0.00 | 0.00 | 0.00 | 0.00 | | hypothetical protein | Bacteria;Spirochaetes;Spirochaetia;Spirochaetales;Spirochaetaceae;Spirochaeta;Spirochaeta lutea;Spirochaeta lutea JC230 |
| Ga0586324 5223885 104 607 | 0.00 | 2210000.00 | 0.00 | 0.00 | 0.00 | 0.00 | 0.00 | 0.00 | 0.00 | 1478400.00 | 0.00 | 0.00 | | septal ring factor EnvC (AmiA/AmiB activator) | |
| Ga0586324 5225718 1 369 | 1600000.00 | 1434800.00 | 0.00 | 0.00 | 0.00 | 0.00 | 0.00 | 0.00 | 0.00 | 0.00 | 0.00 | 0.00 | | acyl-coenzyme A synthetase/AMP-(fatty) acid ligase | Bacteria;Proteobacteria;Deltaproteobacteria;Syntrophobacterales;Syntrophaceae;Desulfobacca;Desulfobacca acetoxidans;Desulfobacca acetoxidans ASRB2, DSM 11109 |
| Ga0586324 5229989 2 604 | 0.00 | 0.00 | 0.00 | 0.00 | 0.00 | 0.00 | 0.00 | 0.00 | 0.00 | 1717100.00 | 0.00 | 0.00 | | DmsE family decaheme c-type cytochrome | Bacteria;Proteobacteria;Gammaproteobacteria;Chromatiales;Chromatiaceae;Imhoffiella;Imhoffiella purpurea;Imhoffiella purpurea AK35 |
| Ga0586324 5236916 2 604 | 0.00 | 0.00 | 4980264.22 | 2593500.00 | 3811000.00 | 4009700.00 | 0.00 | 0.00 | 0.00 | 0.00 | 0.00 | 3022500.00 | | hypothetical protein | Bacteria;Actinobacteria;Actinomycetia;Micrococcales;Intrasporangiaceae;Intrasporangium;Intrasporangium oryzae;Intrasporangium oryzae NRRL B-24470 |
| Ga0586324 5297773 2 601 | 964019.00 | 0.00 | 0.00 | 0.00 | 0.00 | 491184.28 | 467484.36 | 756400.00 | 0.00 | 1301300.00 | 0.00 | 0.00 | | hypothetical protein | |
| Ga0586324 5299391 2 601 | 4430000.00 | 0.00 | 0.00 | 0.00 | 0.00 | 0.00 | 0.00 | 0.00 | 0.00 | 0.00 | 0.00 | 0.00 | | hypothetical protein | Bacteria;Spirochaetes;Spirochaetia;Spirochaetales;Spirochaetaceae;Spirochaeta;Spirochaeta cellobiosiphila;Spirochaeta cellobiosiphila DSM 17781 |
| Ga0586324 5302596 3 602 | 5680000.00 | 0.00 | 0.00 | 10640000.00 | 0.00 | 0.00 | 0.00 | 1555500.00 | 0.00 | 6714400.00 | 5086400.00 | 0.00 | | hypothetical protein | Bacteria;Candidatus Aminicenantes;unclassified Candidatus Aminicenantes;unclassified Candidatus Aminicenantes;unclassified Candidatus Aminicenantes;unclassified Candidatus Aminicenantes;Aminicenantes bacterium SCGC AAA252-N07;Aminicenantes bacterium SCGC AAA252-N07 (contamination screened) |
| Ga0586324 5303111 3 602 | 0.00 | 0.00 | 0.00 | 0.00 | 3986100.00 | 40891000.00 | 5130000.00 | 0.00 | 0.00 | 0.00 | 0.00 | 0.00 | | crotonobetainyl-CoA:carnitine CoA-transferase CaiB-like acyl-CoA transferase | Bacteria;Proteobacteria;Deltaproteobacteria;Syntrophobacterales;Syntrophobacteraceae;Desulfacinum;Desulfacinum infernum;Desulfacinum infernum DSM 9756 |
| Ga0586324 5303965 1 297 | 0.00 | 7276000.00 | 0.00 | 0.00 | 0.00 | 0.00 | 0.00 | 0.00 | 0.00 | 0.00 | 0.00 | 0.00 | | predicted porin | Bacteria;Proteobacteria;Betaproteobacteria;Burkholderiales;Comamonadaceae;Curvibacter;Curvibacter delicatus;Curvibacter delicatus NBRC 14919 |
| Ga0586324 5313817 3 602 | 0.00 | 4290800.00 | 9796200.00 | 0.00 | 0.00 | 0.00 | 0.00 | 0.00 | 0.00 | 0.00 | 0.00 | 0.00 | | S-layer protein (TIGR01567 family) | Archaea;Euryarchaeota;Methanomicrobia;Methanosarcinales;Methanotrichaceae;Methanothrix;Methanothrix soehngenii;Methanothrix soehngenii GP-6 |
| Ga0586324 5318697 3 380 | 0.00 | 0.00 | 0.00 | 2460500.00 | 3759500.00 | 0.00 | 0.00 | 2366800.00 | 3402000.00 | 0.00 | 0.00 | 0.00 | | hypothetical protein | Bacteria;Chloroflexi;Anaerolineae;Anaerolineales;Anaerolineaceae;Levilinea;Levilinea saccharolytica;Levilinea saccharolytica DSM 16555 |
| Ga0586324 5323120 3 602 | 2110000.00 | 1020000.00 | 0.00 | 0.00 | 0.00 | 0.00 | 0.00 | 0.00 | 0.00 | 0.00 | 0.00 | 0.00 | | hypothetical protein | Bacteria;Acidobacteria;Vicinamibacteria;unclassified Vicinamibacteria;Vicinamibacteraceae;Luteitalea;Luteitalea pratensis;Luteitalea pratensis DSM 100886 |
| Ga0586324 5336629 1 600 | 712183.00 | 0.00 | 0.00 | 0.00 | 0.00 | 0.00 | 0.00 | 0.00 | 0.00 | 0.00 | 0.00 | 0.00 | | crotonobetainyl-CoA:carnitine CoA-transferase CaiB-like acyl-CoA transferase | Bacteria;Proteobacteria;Deltaproteobacteria;Syntrophobacterales;Syntrophobacteraceae;Desulfacinum;Desulfacinum infernum;Desulfacinum infernum DSM 9756 |

| | | | | | | | | | | | | | | | |
|---|---|---|---|---|---|---|---|---|---|---|---|---|---|---|---|
| Ga0586324 5346249 78 578 | 0.00 | 0.00 | 0.00 | 0.00 | 0.00 | 16515200.00 | 0.00 | 0.00 | 0.00 | 0.00 | 0.00 | 0.00 | | hypothetical protein | Bacteria;Proteobacteria;Alphaproteobacteria;Hyphomicrobiales;Bradyrhizobiaceae;Bradyrhizobium;Bradyrhizobium tropiciagri;Bradyrhizobium tropiciagri SEMIA 6148 |
| Ga0586324 5359152 3 599 | 0.00 | 0.00 | 0.00 | 9975000.00 | 11433000.00 | 1961993.85 | 1580895.00 | 3056100.00 | 3790800.00 | 0.00 | 0.00 | 0.00 | | hypothetical protein | Bacteria;Bacteroidetes;Bacteroidia;Bacteroidales;Rikenellaceae;Acetobacteroides;Acetobacteroides hydrogenigenes;Acetobacteroides hydrogenigenes DSM 24657 |
| Ga0586324 5361378 2 598 | 0.00 | 7888000.00 | 0.00 | 0.00 | 0.00 | 0.00 | 0.00 | 0.00 | 0.00 | 34596100.00 | 18779200.00 | 0.00 | | hypothetical protein | Bacteria;Deferribacteres;Deferribacteres;Deferribacterales;Deferribacteraceae;Deferribacter;Deferribacter desulfuricans;Deferribacter desulfuricans SSM1 |
| Ga0586324 5366788 1 492 | 0.00 | 0.00 | 4802344.96 | 3230000.00 | 1843700.00 | 0.00 | 0.00 | 0.00 | 0.00 | 0.00 | 0.00 | 0.00 | | hypothetical protein | Bacteria;Bacteroidetes;Cytophagia;Cytophagales;Hymenobacteraceae;Adhaeribacter;Adhaeribacter pallidiroseus;Adhaeribacter pallidiroseus HMF7616 |
| Ga0586324 5375570 41 598 | 0.00 | 0.00 | 805360.24 | 0.00 | 0.00 | 1883649.87 | 0.00 | 0.00 | 0.00 | 0.00 | 0.00 | 7267500.00 | | hypothetical protein | Bacteria;Chloroflexi;Caldilineae;Caldilineales;Caldilineaceae;Caldilinea;Caldilinea aerophila;Caldilinea aerophila STL-6-O1, DSM 14535 |
| Ga0586324 5380116 166 597 | 0.00 | 2094400.00 | 0.00 | 1425000.00 | 0.00 | 0.00 | 0.00 | 0.00 | 0.00 | 0.00 | 0.00 | 0.00 | | hypothetical protein | |
| Ga0586324 5380774 1 213 | 0.00 | 0.00 | 0.00 | 0.00 | 0.00 | 0.00 | 0.00 | 0.00 | 0.00 | 0.00 | 1179200.00 | 0.00 | | hypothetical protein | Bacteria;Bacteroidetes;unclassified Bacteroidetes;Bacteroidetes Order II. Incertae sedis;Rhodothermaceae;Rhodothermus;Rhodothermus marinus;Rhodothermus marinus SG0.5JP17-172 |
| Ga0586324 5393624 1 597 | 0.00 | 3774000.00 | 0.00 | 1016500.00 | 852749.36 | 0.00 | 0.00 | 0.00 | 0.00 | 3542000.00 | 0.00 | 0.00 | | formyltetrahydrofolate synthetase | Bacteria;Proteobacteria;Deltaproteobacteria;Desulfobacterales;Desulfobacteraceae;Desulfatirhabdium;Desulfatirhabdium butyrativorans;Desulfatirhabdium butyrativorans DSM 18734 |
| Ga0586324 5396188 2 550 | 0.00 | 0.00 | 0.00 | 7049000.00 | 4624700.00 | 0.00 | 0.00 | 0.00 | 0.00 | 0.00 | 0.00 | 0.00 | | hypothetical protein | |
| Ga0586324 5423981 258 596 | 0.00 | 0.00 | 0.00 | 0.00 | 0.00 | 0.00 | 908666.64 | 0.00 | 0.00 | 0.00 | 0.00 | 0.00 | | rubrerythrin | Bacteria;Proteobacteria;Deltaproteobacteria;Myxococcales;unclassified Myxococcales;Enhygromyxa;Enhygromyxa salina;Enhygromyxa salina SWB007 |
| Ga0586324 5424176 3 404 | 9930000.00 | 8500000.00 | 58214200.00 | 0.00 | 0.00 | 48592800.00 | 0.00 | 2757200.00 | 0.00 | 36575000.00 | 0.00 | 0.00 | | hypothetical protein | Bacteria;Proteobacteria;Alphaproteobacteria;Hyphomicrobiales;Bradyrhizobiaceae;Bradyrhizobium;Bradyrhizobium sp. OAE819;Bradyrhizobium sp. OAE819 |
| Ga0586324 5436841 3 575 | 2170000.00 | 0.00 | 0.00 | 0.00 | 0.00 | 0.00 | 0.00 | 0.00 | 0.00 | 0.00 | 0.00 | 0.00 | | hypothetical protein | |
| Ga0586324 5437854 1 597 | 0.00 | 6147200.00 | 0.00 | 0.00 | 0.00 | 15046300.00 | 23484000.00 | 6649000.00 | 12150000.00 | 0.00 | 0.00 | 13575000.00 | | hypothetical protein | Bacteria;Proteobacteria;Deltaproteobacteria;Syntrophobacterales;Syntrophaceae;Syntrophus;Syntrophus aciditrophicus;Syntrophus aciditrophicus SB |
| Ga0586324 5445327 1 594 | 37660000.00 | 7110069.80 | 0.00 | 15580000.00 | 16171000.00 | 0.00 | 0.00 | 1055300.00 | 18966453.75 | 8162000.00 | 33088000.00 | 8490000.00 | | hypothetical protein | Bacteria;Proteobacteria;Deltaproteobacteria;Syntrophorhabdales;Syntrophorhabdaceae;Syntrophorhabdus;Syntrophorhabdus aromaticivorans;Syntrophorhabdus aromaticivorans UI |
| Ga0586324 5452194 1 594 | 0.00 | 5168000.00 | 0.00 | 0.00 | 8167900.00 | 0.00 | 8937600.00 | 8845000.00 | 5807700.00 | 8008000.00 | 6151200.00 | 0.00 | | basic membrane lipoprotein Med (substrate-binding protein (PBP1-ABC) superfamily) | Bacteria;Chloroflexi;Anaerolineae;Anaerolineales;Anaerolineaceae;Levilinea;Levilinea saccharolytica;Levilinea saccharolytica DSM 16555 |
| Ga0586324 5463285 1 594 | 0.00 | 0.00 | 0.00 | 0.00 | 0.00 | 0.00 | 2485200.00 | 0.00 | 0.00 | 0.00 | 0.00 | 0.00 | | hypothetical protein | Bacteria;Firmicutes;Clostridia;Thermoanaerobacterales;Thermoanaerobacteraceae;Thermacetogenium;Thermacetogenium phaeum;Thermacetogenium phaeum PB |
| Ga0586324 5467169 2 337 | 0.00 | 0.00 | 0.00 | 0.00 | 2090900.00 | 0.00 | 0.00 | 1043100.00 | 0.00 | 0.00 | 29145600.00 | 2670000.00 | | hypothetical protein | |
| Ga0586324 5472342 1 489 | 7190000.00 | 7820000.00 | 0.00 | 8588000.00 | 5376600.00 | 0.00 | 3328800.00 | 3660000.00 | 3183300.00 | 0.00 | 6899200.00 | 3795000.00 | | opacity protein-like surface antigen | Bacteria;Acidobacteria;Blastocatellia;unclassified Blastocatellia;unclassified Blastocatellia;Chloracidobacterium;Chloracidobacterium thermophilum;Chloracidobacterium thermophilum B |
| Ga0586324 5472643 2 595 | 0.00 | 0.00 | 0.00 | 0.00 | 0.00 | 0.00 | 8276400.00 | 4135800.00 | 0.00 | 0.00 | 0.00 | 0.00 | | hypothetical protein | Bacteria;Proteobacteria;Betaproteobacteria;Burkholderiales;Burkholderiaceae;Ralstonia;blood disease bacterium A2-HR MARDI;blood disease bacterium A2-HR MARDI |
| Ga0586324 5489267 2 592 | 0.00 | 0.00 | 0.00 | 0.00 | 0.00 | 0.00 | 0.00 | 0.00 | 539810.73 | 0.00 | 0.00 | 0.00 | | hypothetical protein | |
| Ga0586324 5495458 79 594 | 0.00 | 0.00 | 2043858.90 | 0.00 | 0.00 | 0.00 | 0.00 | 0.00 | 0.00 | 0.00 | 0.00 | 0.00 | | hypothetical protein | Bacteria;Proteobacteria;Betaproteobacteria;Burkholderiales;Burkholderiaceae;Cupriavidus;Cupriavidus sp. YR651;Cupriavidus sp. YR651 |
| Ga0586324 5500600 1 474 | 3720000.00 | 0.00 | 0.00 | 0.00 | 0.00 | 0.00 | 0.00 | 0.00 | 0.00 | 0.00 | 0.00 | 0.00 | | hypothetical protein | Bacteria;Proteobacteria;Deltaproteobacteria;Syntrophobacterales;Syntrophaceae;Desulfobacca;Desulfobacca acetoxidans;Desulfobacca acetoxidans ASRB2, DSM 11109 |
| Ga0586324 5514866 2 592 | 0.00 | 1842800.00 | 0.00 | 0.00 | 0.00 | 0.00 | 0.00 | 0.00 | 0.00 | 0.00 | 0.00 | 0.00 | | hypothetical protein | |
| Ga0586324 5515547 3 593 | 22000000.00 | 0.00 | 38565500.00 | 16737941.70 | 16603664.89 | 0.00 | 0.00 | 707600.00 | 0.00 | 18326000.00 | 0.00 | 687770.25 | | hypothetical protein | Bacteria;Proteobacteria;Gammaproteobacteria;Methylococcales;Methylococcaceae;Methylomicrobium;Methylomicrobium lacus;Methylosarcina lacus LW14 |
| Ga0586324 5526562 3 593 | 6300000.00 | 4584135.00 | 3232909.27 | 2859500.00 | 2430800.00 | 4247900.00 | 2207963.40 | 1305400.00 | 602865.99 | 3395700.00 | 1742400.00 | 2070000.00 | | hypothetical protein | |
| Ga0586324 5526862 1 591 | 1580000.00 | 1584400.00 | 0.00 | 0.00 | 0.00 | 0.00 | 0.00 | 0.00 | 0.00 | 0.00 | 0.00 | 2587500.00 | | FtsP/CotA-like multicopper oxidase with cupredoxin domain | Bacteria;Proteobacteria;Betaproteobacteria;Burkholderiales;Oxalobacteraceae;Massilia;Massilia sp. WF1;Massilia sp. WF1 |
| Ga0586324 5540973 2 592 | 0.00 | 0.00 | 0.00 | 0.00 | 0.00 | 0.00 | 0.00 | 0.00 | 468382.50 | 0.00 | 0.00 | 0.00 | | hypothetical protein | Bacteria;Acidobacteria;Acidobacteriia;Acidobacteriales;Acidobacteriaceae;Acidipila;Acidipila sp.;Acidipila sp. EB88 |
| Ga0586324 5546971 1 591 | 0.00 | 10880000.00 | 0.00 | 11115000.00 | 0.00 | 0.00 | 0.00 | 5471700.00 | 0.00 | 5621000.00 | 9680000.00 | 5205000.00 | | hypothetical protein | Bacteria;Verrucomicrobia;Verrucomicrobiae;Verrucomicrobiales;Verrucomicrobia subdivision 3;Pedosphaera;Pedosphaera parvula;Pedosphaera parvula Ellin514 |
| Ga0586324 5559611 2 535 | 0.00 | 0.00 | 51007800.00 | 29450000.00 | 1493500.00 | 91707000.00 | 65892000.00 | 25498000.00 | 35883000.00 | 0.00 | 0.00 | 0.00 | | hypothetical protein | |
| Ga0586324 5564881 1 591 | 0.00 | 0.00 | 0.00 | 716892.80 | 0.00 | 0.00 | 0.00 | 0.00 | 0.00 | 0.00 | 0.00 | 0.00 | | predicted Zn-dependent protease | Bacteria;Proteobacteria;Deltaproteobacteria;Syntrophobacterales;Syntrophaceae;Desulfobacca;Desulfobacca acetoxidans;Desulfobacca acetoxidans ASRB2, DSM 11109 |
| Ga0586324 5576234 136 591 | 0.00 | 2638400.00 | 0.00 | 0.00 | 0.00 | 0.00 | 0.00 | 0.00 | 0.00 | 0.00 | 0.00 | 0.00 | | ABC-type branched-subunit amino acid transport system substrate-binding protein | Bacteria;Spirochaetes;Spirochaetia;Spirochaetales;Spirochaetaceae;Spirochaeta;Spirochaeta cellobiosiphila;Spirochaeta cellobiosiphila DSM 17781 |
| Ga0586324 5577485 2 589 | 0.00 | 4365600.00 | 5558099.27 | 13680000.00 | 2585300.00 | 11314500.00 | 8344800.00 | 7320000.00 | 0.00 | 7469000.00 | 7532800.00 | 6030000.00 | | alkylation response protein AidB-like acyl-CoA dehydrogenase | Bacteria;Proteobacteria;Deltaproteobacteria;Syntrophobacterales;Syntrophobacteraceae;Desulfacinum;Desulfacinum infernum;Desulfacinum infernum DSM 9756 |
| Ga0586324 5579163 3 590 | 0.00 | 0.00 | 0.00 | 890028.40 | 0.00 | 4525800.00 | 1557390.48 | 0.00 | 2551500.00 | 0.00 | 0.00 | 2667562.50 | | outer membrane receptor protein involved in Fe transport | Bacteria;Acidobacteria;Acidobacteriia;Bryobacterales;Bryobacteraceae;Bryobacter;Bryobacter aggregatus;Bryobacter aggregatus DSM 18758 |
| Ga0586324 5594715 3 395 | 15900000.00 | 0.00 | 0.00 | 0.00 | 0.00 | 0.00 | 0.00 | 0.00 | 0.00 | 0.00 | 0.00 | 0.00 | | predicted porin | Bacteria;Proteobacteria;Betaproteobacteria;Nitrosomonadales;Sulfuricellaceae;Sulfuricella;Sulfuricella denitrificans;Sulfuricella denitrificans skB26 |
| Ga0586324 5602001 2 589 | 0.00 | 1523200.00 | 2961937.37 | 0.00 | 0.00 | 0.00 | 0.00 | 0.00 | 0.00 | 0.00 | 0.00 | 2250000.00 | | hypothetical protein | Bacteria;Actinobacteria;Actinomycetia;Motilibacterales;Motilibacteraceae;Motilibacter;Motilibacter peucedani;Motilibacter peucedani DSM 45328 |
| Ga0586324 5602447 128 589 | 0.00 | 0.00 | 0.00 | 0.00 | 0.00 | 6669600.00 | 0.00 | 0.00 | 0.00 | 0.00 | 0.00 | 0.00 | | TRAP-type mannitol/chloroaromatic compound transport system substrate-binding protein | Bacteria;Proteobacteria;Betaproteobacteria;Nitrosomonadales;Sterolibacteriaceae;Sulfurisoma;Sulfurisoma sediminicola;Sulfurisoma sediminicola DSM 26916 |
| Ga0586324 5610397 92 589 | 0.00 | 0.00 | 0.00 | 0.00 | 0.00 | 0.00 | 0.00 | 0.00 | 0.00 | 222284.37 | 0.00 | 0.00 | | signal transduction histidine kinase | Bacteria;Planctomycetes;Candidatus Brocadiae;Candidatus Brocadiales;Candidatus Brocadiaceae;Candidatus Brocadia;Candidatus Brocadia fulgida;Candidatus Brocadia fulgida Brocadia fulgida enrichment bioreactor |
| Ga0586324 5614727 276 587 | 0.00 | 91120000.00 | 255039000.00 | 0.00 | 0.00 | 0.00 | 0.00 | 66490000.00 | 0.00 | 0.00 | 0.00 | 138000000.00 | | hypothetical protein | Bacteria;Proteobacteria;Alphaproteobacteria;Hyphomicrobiales;Xanthobacteraceae;Pseudolabrys;Pseudolabrys taiwanensis;Pseudolabrys taiwanensis CC-BB4 |
| Ga0586324 5616021 2 589 | 0.00 | 10608000.00 | 1397061.98 | 2413000.00 | 2616200.00 | 0.00 | 3990000.00 | 1769000.00 | 2219400.00 | 20066200.00 | 809863.12 | 945000.00 | | hypothetical protein | |
| Ga0586324 5643928 1 588 | 0.00 | 14892000.00 | 0.00 | 6773500.00 | 9774700.00 | 0.00 | 0.00 | 0.00 | 0.00 | 0.00 | 0.00 | 0.00 | | S-layer protein (TIGR01567 family) | Archaea;Euryarchaeota;Methanomicrobia;Methanosarcinales;Methanosarcinaceae;Methanosarcina;Methanosarcina sp. Kolksee;Methanosarcina sp. Kolksee |
| Ga0586324 5674008 2 586 | 37600000.00 | 1502800.00 | 34511900.00 | 16435000.00 | 32826100.00 | 29576500.00 | 13725600.00 | 0.00 | 0.00 | 14168000.00 | 98560000.00 | 13492500.00 | | hypothetical protein | Bacteria;Proteobacteria;Alphaproteobacteria;Hyphomicrobiales;Bradyrhizobiaceae;Bradyrhizobium;Bradyrhizobium sp. Y-H1;Bradyrhizobium sp. Y-H1 |
| Ga0586324 5682518 248 586 | 0.00 | 0.00 | 0.00 | 0.00 | 2605900.00 | 0.00 | 0.00 | 0.00 | 0.00 | 0.00 | 0.00 | 0.00 | | hypothetical protein | Bacteria;Proteobacteria;Deltaproteobacteria;Syntrophorhabdales;Syntrophorhabdaceae;Syntrophorhabdus;Syntrophorhabdus aromaticivorans;Syntrophorhabdus aromaticivorans UI |
| Ga0586324 5684259 3 584 | 553648.00 | 0.00 | 0.00 | 0.00 | 0.00 | 0.00 | 0.00 | 0.00 | 0.00 | 0.00 | 0.00 | 0.00 | | ABC-type sugar transport system substrate-binding protein | Bacteria;Chloroflexi;Chloroflexia;Chloroflexales;Oscillochloridaceae;Oscillochloris;Oscillochloris trichoides;Oscillochloris trichoides DG6 |
| Ga0586324 5685683 7 585 | 0.00 | 0.00 | 0.00 | 3771500.00 | 0.00 | 0.00 | 0.00 | 0.00 | 0.00 | 0.00 | 0.00 | 0.00 | | hypothetical protein | Bacteria;Proteobacteria;Deltaproteobacteria;Syntrophobacterales;Syntrophaceae;Desulfobacca;Desulfobacca acetoxidans;Desulfobacca acetoxidans ASRB2, DSM 11109 |
| Ga0586324 5689281 3 584 | 92890000.00 | 113288000.00 | 90643000.00 | 87875000.00 | 86211000.00 | 100441000.00 | 98268000.00 | 77634700.00 | 18225000.00 | 74074000.00 | 69062400.00 | 68880000.00 | | predicted porin | Bacteria;Proteobacteria;Betaproteobacteria;Rhodocyclales;Zoogloeaceae;Zoogloea;Zoogloea sp. LCSB751;Zoogloea schifflangensis LCSB751 |
| Ga0586324 5700756 2 406 | 19200000.00 | 4270400.00 | 3343274.16 | 5823500.00 | 5500200.00 | 3558620.66 | 15344400.00 | 2092300.00 | 3458700.00 | 5505500.00 | 9072800.00 | 12832500.00 | | 2',3'-cyclic-nucleotide 2'-phosphodiesterase (5'-nucleotidase family) | Bacteria;Actinobacteria;Actinomycetia;Propionibacteriales;Nocardioidaceae;Nocardioides;Nocardioides sp. Root122;Nocardioides sp. Root122 |
| Ga0586324 5706123 1 585 | 0.00 | 0.00 | 0.00 | 0.00 | 0.00 | 0.00 | 0.00 | 0.00 | 4147200.00 | 0.00 | 0.00 | 0.00 | | glutamate synthase domain-containing protein 2 | Bacteria;Firmicutes;Clostridia;Thermoanaerobacterales;Thermoanaerobacterales Family III. Incertae Sedis;Caldicellulosiruptor;Caldicellulosiruptor bescii;Caldicellulosiruptor bescii DSM 6725 |
| Ga0586324 5718944 3 305 | 0.00 | 0.00 | 0.00 | 0.00 | 3378400.00 | 0.00 | 0.00 | 0.00 | 0.00 | 0.00 | 0.00 | 0.00 | | NAD(P)-dependent dehydrogenase (short-subunit alcohol dehydrogenase family) | Bacteria;Acidobacteria;Acidobacteriia;Acidobacteriales;Acidobacteriaceae;Acidobacterium;unclassified Acidobacterium;Acidobacterium strain C40 |
| Ga0586324 5722911 1 582 | 22100000.00 | 24548000.00 | 30176800.00 | 28880000.00 | 3223900.00 | 0.00 | 0.00 | 1262700.00 | 14256000.00 | 0.00 | 0.00 | 0.00 | | crotonobetainyl-CoA:carnitine CoA-transferase CaiB-like acyl-CoA transferase | Archaea;Euryarchaeota;Archaeoglobi;Archaeoglobales;Archaeoglobaceae;Geoglobus;Geoglobus ahangari;Geoglobus ahangari 234 |
| Ga0586324 5743807 3 581 | 10900000.00 | 16524000.00 | 9458400.00 | 22610000.00 | 18643000.00 | 7265100.00 | 6840000.00 | 8479000.00 | 12798000.00 | 20790000.00 | 21648000.00 | 13875000.00 | | hypothetical protein | |
| Ga0586324 5754796 2 583 | 0.00 | 2087600.00 | 0.00 | 0.00 | 0.00 | 0.00 | 0.00 | 0.00 | 0.00 | 0.00 | 1654400.00 | 0.00 | | predicted porin | Bacteria;Proteobacteria;Gammaproteobacteria;Chromatiales;Chromatiaceae;Thiocystis;Thiocystis violascens;Thiocystis violascens 611, DSM 198 |
| Ga0586324 5756862 1 183 | 0.00 | 0.00 | 0.00 | 0.00 | 0.00 | 0.00 | 0.00 | 0.00 | 0.00 | 0.00 | 3124000.00 | 0.00 | | DNA-directed RNA polymerase beta' subunit | |
| Ga0586324 5759810 3 581 | 0.00 | 0.00 | 0.00 | 0.00 | 0.00 | 0.00 | 0.00 | 0.00 | 0.00 | 0.00 | 0.00 | 570251.25 | | lactate dehydrogenase-like 2-hydroxyacid dehydrogenase | Bacteria;Proteobacteria;Alphaproteobacteria;Rhodobacterales;Rhodobacteraceae;Thioclava;unclassified Thioclava;Thioclava electrotrophica elox9 |
| Ga0586324 5774728 2 580 | 3420000.00 | 0.00 | 0.00 | 0.00 | 0.00 | 0.00 | 0.00 | 6405000.00 | 7411500.00 | 0.00 | 0.00 | 0.00 | | predicted porin | Bacteria;Proteobacteria;Betaproteobacteria;Burkholderiales;Comamonadaceae;Ramlibacter;Ramlibacter tataouinensis;Ramlibacter tataouinensis 5-10 |
| Ga0586324 5784555 201 527 | 0.00 | 0.00 | 3069763.13 | 0.00 | 0.00 | 6907800.00 | 4742400.00 | 0.00 | 3029400.00 | 0.00 | 0.00 | 0.00 | | RNA recognition motif-containing protein | Bacteria;Bacteroidetes;Cytophagia;Cytophagales;Cytophagaceae;Cytophaga;Cytophaga aurantiaca;Cytophaga aurantiaca DSM 3654 |
| Ga0586324 5787153 3 281 | 0.00 | 0.00 | 0.00 | 0.00 | 0.00 | 0.00 | 0.00 | 0.00 | 0.00 | 0.00 | 0.00 | 2077500.00 | | hypothetical protein | Bacteria;Proteobacteria;Betaproteobacteria;Nitrosomonadales;Sterolibacteriaceae;Sulfurisoma;Sulfurisoma sediminicola;Sulfurisoma sediminicola DSM 26916 |
| Ga0586324 5808256 52 579 | 311000000.00 | 0.00 | 136809000.00 | 282150000.00 | 0.00 | 17904700.00 | 61788000.00 | 0.00 | 0.00 | 0.00 | 11616000.00 | 46200000.00 | | predicted porin | Bacteria;Proteobacteria;Gammaproteobacteria;Chromatiales;Chromatiaceae;Thiobaca;Thiobaca trueperi;Thiobaca trueperi DSM 13587 |
| Ga0586324 5816093 3 578 | 4180000.00 | 7969600.00 | 8490056.89 | 0.00 | 7508700.00 | 3625280.93 | 1214551.44 | 1878800.00 | 2592000.00 | 3765300.00 | 0.00 | 6037500.00 | | hypothetical protein | |
| Ga0586324 5827156 2 580 | 12300000.00 | 10132000.00 | 12836400.00 | 17100000.00 | 0.00 | 15284500.00 | 0.00 | 10126000.00 | 10530000.00 | 0.00 | 0.00 | 0.00 | | hypothetical protein | Bacteria;Bacteroidetes;Saprospiria;Saprospirales;Lewinellaceae;Lewinella;Lewinella antarctica;Lewinella antarctica DSM 105096 |
| Ga0586324 5827524 3 578 | 0.00 | 0.00 | 0.00 | 0.00 | 0.00 | 0.00 | 0.00 | 0.00 | 0.00 | 985600.00 | 0.00 | 0.00 | | hypothetical protein | |
| Ga0586324 5839191 2 577 | 0.00 | 0.00 | 0.00 | 0.00 | 0.00 | 0.00 | 4195200.00 | 0.00 | 0.00 | 0.00 | 0.00 | 0.00 | | PAS domain S-box-containing protein | Bacteria;Proteobacteria;Alphaproteobacteria;Hyphomicrobiales;Methylobacteriaceae;Microvirga;Microvirga massiliensis;Microvirga massiliensis JC119 |
| Ga0586324 5841570 3 578 | 0.00 | 0.00 | 0.00 | 0.00 | 0.00 | 0.00 | 0.00 | 0.00 | 356536.89 | 0.00 | 0.00 | 0.00 | | acetyltransferase-like isoleucine patch superfamily enzyme | |
| Ga0586324 5850781 1 579 | 0.00 | 0.00 | 0.00 | 0.00 | 4429000.00 | 0.00 | 0.00 | 0.00 | 0.00 | 0.00 | 0.00 | 0.00 | | ABC-type transport system substrate-binding protein | Bacteria;Chloroflexi;Anaerolineae;Anaerolineales;Anaerolineaceae;Levilinea;Levilinea saccharolytica;Levilinea saccharolytica DSM 16555 |
| Ga0586324 5863745 1 576 | 0.00 | 0.00 | 0.00 | 0.00 | 1957000.00 | 9607400.00 | 0.00 | 1695800.00 | 0.00 | 0.00 | 2974400.00 | 0.00 | | alkylation response protein AidB-like acyl-CoA dehydrogenase | Bacteria;Proteobacteria;Deltaproteobacteria;Syntrophobacterales;Syntrophobacteraceae;Desulfosoma;Desulfosoma caldarium;Desulfosoma caldarium DSM 22027 |
| Ga0586324 5869787 1 576 | 0.00 | 651203.36 | 0.00 | 0.00 | 0.00 | 0.00 | 0.00 | 0.00 | 366785.82 | 0.00 | 0.00 | 445089.00 | | hypothetical protein | |
| Ga0586324 5872343 2 463 | 0.00 | 1054000.00 | 1102123.17 | 1092500.00 | 1122700.00 | 0.00 | 0.00 | 0.00 | 381630.69 | 1386000.00 | 1152800.00 | 1537500.00 | | hypothetical protein | Bacteria;Chloroflexi;Anaerolineae;Anaerolineales;Anaerolineaceae;Anaerolinea;Anaerolinea thermophila;Anaerolinea thermophila UNI-1 |
| Ga0586324 5884550 1 576 | 4400000.00 | 0.00 | 0.00 | 0.00 | 2018800.00 | 0.00 | 0.00 | 0.00 | 0.00 | 0.00 | 0.00 | 3750000.00 | | hypothetical protein | Bacteria;Acidobacteria;Acidobacteriia;Acidobacteriales;Acidobacteriaceae;Silvibacterium;Silvibacterium bohemicum;Acidobacteriaceae bacterium S15 |
| Ga0586324 5897807 3 332 | 3550000.00 | 1802000.00 | 0.00 | 2413000.00 | 2255700.00 | 0.00 | 0.00 | 1549400.00 | 1474200.00 | 1463000.00 | 2094400.00 | 1110000.00 | | hypothetical protein | |
| Ga0586324 5902702 3 575 | 0.00 | 0.00 | 636612.25 | 2394000.00 | 0.00 | 0.00 | 0.00 | 0.00 | 0.00 | 1848000.00 | 1839200.00 | 0.00 | | hypothetical protein | |
| Ga0586324 5910313 1 297 | 0.00 | 0.00 | 4318756.11 | 0.00 | 0.00 | 0.00 | 0.00 | 0.00 | 0.00 | 0.00 | 0.00 | 0.00 | | TusE/DsrC/DsvC family sulfur relay protein | Bacteria;Proteobacteria;Gammaproteobacteria;Chromatiales;Chromatiaceae;Thioflavicoccus;Thioflavicoccus mobilis;Thioflavicoccus mobilis 8321 |
| Ga0586324 5931602 2 574 | 0.00 | 0.00 | 0.00 | 0.00 | 1194800.00 | 0.00 | 0.00 | 0.00 | 0.00 | 0.00 | 0.00 | 0.00 | | tripartite-type tricarboxylate transporter receptor subunit TctC | Bacteria;Proteobacteria;Betaproteobacteria;Burkholderiales;Comamonadaceae;Curvibacter;Curvibacter sp. PAE-UM;Curvibacter sp. PAE-UM |
| Ga0586324 5939419 1 573 | 0.00 | 2373200.00 | 0.00 | 0.00 | 0.00 | 0.00 | 0.00 | 0.00 | 0.00 | 0.00 | 0.00 | 0.00 | | L-ascorbate metabolism protein UlaG (beta-lactamase superfamily) | Bacteria;Candidatus Latescibacteria;unclassified Candidatus Latescibacteria;unclassified Candidatus Latescibacteria;unclassified Candidatus Latescibacteria;unclassified Candidatus Latescibacteria;Latescibacteria bacterium SCGC AAA257-K07;Latescibacteria bacterium SCGC AAA257-K07 (contamination screened) |
| Ga0586324 5944697 3 575 | 0.00 | 0.00 | 5414872.07 | 0.00 | 0.00 | 4883100.00 | 0.00 | 0.00 | 0.00 | 0.00 | 0.00 | 0.00 | | hypothetical protein | Bacteria;Acidobacteria;Acidobacteriia;Acidobacteriales;Acidobacteriaceae;Candidatus Koribacter;Candidatus Koribacter versatilis;Candidatus Koribacter versatilis Ellin345 |
| Ga0586324 5945592 3 575 | 0.00 | 0.00 | 0.00 | 0.00 | 0.00 | 3045109.10 | 0.00 | 0.00 | 0.00 | 0.00 | 0.00 | 0.00 | | outer membrane receptor for Fe3+-dicitrate | Bacteria;Bacteroidetes;Bacteroidia;Marinilabiliales;Prolixibacteraceae;Draconibacterium;Draconibacterium sediminis;Draconibacterium sediminis JN14CK-3 |
| Ga0586324 5965526 142 573 | 0.00 | 0.00 | 0.00 | 3809500.00 | 0.00 | 31998200.00 | 19699200.00 | 0.00 | 11016000.00 | 5898200.00 | 0.00 | 0.00 | | hypothetical protein | |
| Ga0586324 5967043 3 362 | 0.00 | 0.00 | 0.00 | 13517057.90 | 0.00 | 0.00 | 0.00 | 0.00 | 2802600.00 | 0.00 | 0.00 | 0.00 | | hypothetical protein | Bacteria;Proteobacteria;Deltaproteobacteria;Syntrophobacterales;Syntrophaceae;Desulfobacca;Desulfobacca acetoxidans;Desulfobacca acetoxidans ASRB2, DSM 11109 |
| Ga0586324 5968970 3 572 | 0.00 | 0.00 | 0.00 | 2137500.00 | 0.00 | 0.00 | 0.00 | 0.00 | 0.00 | 0.00 | 0.00 | 0.00 | | heme/copper-type cytochrome/quinol oxidase subunit 2 | Bacteria;Proteobacteria;Deltaproteobacteria;Desulfobacterales;Desulfobulbaceae;Desulfobulbus;Desulfobulbus propionicus;Desulfobulbus propionicus 1pr3, DSM 2032 |
| Ga0586324 5976279 3 572 | 6800000.00 | 0.00 | 616276.69 | 0.00 | 7910400.00 | 0.00 | 0.00 | 0.00 | 0.00 | 0.00 | 4056800.00 | 3915000.00 | | hypothetical protein | Bacteria;Bacteroidetes;Cytophagia;Cytophagales;Cytophagaceae;Dyadobacter;Dyadobacter sp. SG02;Dyadobacter sp. SG02 |
| Ga0586324 5983049 3 572 | 15300000.00 | 9860000.00 | 0.00 | 0.00 | 0.00 | 0.00 | 0.00 | 0.00 | 7144200.00 | 0.00 | 0.00 | 0.00 | | hypothetical protein | |
| Ga0586324 5984516 3 572 | 0.00 | 0.00 | 0.00 | 0.00 | 0.00 | 0.00 | 0.00 | 0.00 | 0.00 | 0.00 | 0.00 | 257979.75 | | hypothetical protein | |
| Ga0586324 5988059 1 573 | 0.00 | 884000.00 | 4346956.78 | 0.00 | 0.00 | 1359208.90 | 0.00 | 0.00 | 0.00 | 0.00 | 0.00 | 0.00 | | hypothetical protein | Bacteria;Candidatus Aminicenantes;unclassified Candidatus Aminicenantes;unclassified Candidatus Aminicenantes;unclassified Candidatus Aminicenantes;unclassified Candidatus Aminicenantes;Aminicenantes bacterium SCGC AAA252-D06;Aminicenantes bacterium SCGC AAA252-D06 (contamination screened) |
| Ga0586324 6009366 1 396 | 0.00 | 0.00 | 0.00 | 0.00 | 0.00 | 0.00 | 0.00 | 0.00 | 0.00 | 0.00 | 0.00 | 2602500.00 | | hypothetical protein | Bacteria;Actinobacteria;Actinomycetia;Nakamurellales;Nakamurellaceae;Nakamurella;Nakamurella multipartita;Nakamurella multipartita Y-104, DSM 44233 |
| Ga0586324 6016906 1 570 | 0.00 | 1523200.00 | 0.00 | 0.00 | 0.00 | 0.00 | 0.00 | 0.00 | 0.00 | 0.00 | 0.00 | 0.00 | | hypothetical protein | Bacteria;Acidobacteria;Blastocatellia;Blastocatellales;Pyrinomonadaceae;Pyrinomonas;Pyrinomonas methylaliphatogenes;Pyrinomonas methylaliphatogenes K22 |
| Ga0586324 6018169 137 571 | 5260000.00 | 0.00 | 0.00 | 14060000.00 | 12772000.00 | 5240400.00 | 260934.60 | 0.00 | 4203900.00 | 1647800.00 | 0.00 | 6375000.00 | | methanogenic corrinoid protein MtbC1 | Bacteria;Proteobacteria;Deltaproteobacteria;Syntrophobacterales;Syntrophaceae;Desulfomonile;Desulfomonile tiedjei;Desulfomonile tiedjei DCB-1, DSM 6799 |
| Ga0586324 6024456 178 570 | 0.00 | 5256400.00 | 0.00 | 0.00 | 0.00 | 0.00 | 0.00 | 0.00 | 0.00 | 0.00 | 0.00 | 0.00 | | uncharacterized protein YqfA (UPF0365 family) | Bacteria;Planctomycetes;Planctomycetia;Pirellulales;Pirellulaceae;Pirellula;Pirellula staleyi;Pirellula staleyi DSM 6068 |
| Ga0586324 6039124 3 569 | 0.00 | 0.00 | 0.00 | 0.00 | 0.00 | 0.00 | 0.00 | 713700.00 | 0.00 | 0.00 | 0.00 | 0.00 | | DNA-directed RNA polymerase beta' subunit | Bacteria;Nitrospirae;Nitrospira;Nitrospirales;Nitrospiraceae;Candidatus Magnetobacterium;Candidatus Magnetobacterium casensis;Candidatus Magnetobacterium casensis MYR-1 |
| Ga0586324 6040357 2 571 | 26780000.00 | 27125200.00 | 30852400.00 | 15798500.00 | 41962200.00 | 61693800.00 | 35248800.00 | 34227100.00 | 33890400.00 | 35258300.00 | 28168800.00 | 27502500.00 | | predicted porin | |
| Ga0586324 6041793 36 569 | 0.00 | 0.00 | 0.00 | 0.00 | 0.00 | 0.00 | 21705600.00 | 0.00 | 0.00 | 7553700.00 | 25696000.00 | 0.00 | | TRAP-type mannitol/chloroaromatic compound transport system substrate-binding protein | Bacteria;Proteobacteria;Betaproteobacteria;Burkholderiales;unclassified Burkholderiales;Paucibacter;Paucibacter toxinivorans;Paucibacter toxinivorans DSM 16998 |
| Ga0586324 6055520 99 569 | 3390000.00 | 0.00 | 0.00 | 0.00 | 0.00 | 0.00 | 0.00 | 0.00 | 0.00 | 0.00 | 0.00 | 0.00 | | regulator of protease activity HflC (stomatin/prohibitin superfamily) | Bacteria;Firmicutes;Bacilli;Bacillales;Paenibacillaceae;Aneurinibacillus;Aneurinibacillus thermoaerophilus;Aneurinibacillus thermoaerophilus DSM 10154 |
| Ga0586324 6056732 1 492 | 9180000.00 | 0.00 | 0.00 | 0.00 | 0.00 | 0.00 | 0.00 | 0.00 | 0.00 | 0.00 | 0.00 | 0.00 | | rubrerythrin | Bacteria;Proteobacteria;Deltaproteobacteria;Myxococcales;unclassified Myxococcales;Enhygromyxa;Enhygromyxa salina;Enhygromyxa salina SWB005 |
| Ga0586324 6076268 59 397 | 0.00 | 0.00 | 800023.00 | 0.00 | 0.00 | 0.00 | 0.00 | 0.00 | 0.00 | 0.00 | 0.00 | 0.00 | | RNA recognition motif-containing protein | Bacteria;Verrucomicrobia;Verrucomicrobiae;Verrucomicrobiales;Verrucomicrobia subdivision 3;Pedosphaera;Pedosphaera parvula;Pedosphaera parvula Ellin514 |
| Ga0586324 6079364 16 570 | 0.00 | 0.00 | 33780000.00 | 43320000.00 | 330483.74 | 0.00 | 0.00 | 0.00 | 0.00 | 0.00 | 0.00 | 0.00 | | crotonobetainyl-CoA:carnitine CoA-transferase CaiB-like acyl-CoA transferase | Bacteria;Proteobacteria;Deltaproteobacteria;Syntrophobacterales;Syntrophaceae;Desulfobacca;Desulfobacca acetoxidans;Desulfobacca acetoxidans ASRB2, DSM 11109 |
| Ga0586324 6085872 203 568 | 856623.00 | 5508000.00 | 0.00 | 0.00 | 0.00 | 0.00 | 0.00 | 1262700.00 | 1198800.00 | 0.00 | 0.00 | 0.00 | | rubrerythrin | Bacteria;Proteobacteria;Gammaproteobacteria;Alteromonadales;Ferrimonadaceae;Ferrimonas;Ferrimonas futtsuensis;Ferrimonas futtsuensis DSM 18154 |
| Ga0586324 6105840 2 568 | 0.00 | 3447600.00 | 0.00 | 0.00 | 0.00 | 7066600.00 | 0.00 | 0.00 | 545068.44 | 0.00 | 22792000.00 | 8550000.00 | | hypothetical protein | Bacteria;Spirochaetes;Spirochaetia;Spirochaetales;Spirochaetaceae;Spirochaeta;Spirochaeta cellobiosiphila;Spirochaeta cellobiosiphila DSM 17781 |
| Ga0586324 6111432 3 461 | 364505.00 | 0.00 | 0.00 | 0.00 | 3069400.00 | 4367000.00 | 2941200.00 | 0.00 | 0.00 | 0.00 | 0.00 | 0.00 | | DNA-binding NarL/FixJ family response regulator | Bacteria;Planctomycetes;Planctomycetia;Pirellulales;Pirellulaceae;Blastopirellula;Blastopirellula marina;Blastopirellula marina DSM 3645 |
| Ga0586324 6114427 2 538 | 0.00 | 6031600.00 | 0.00 | 12540000.00 | 0.00 | 0.00 | 0.00 | 0.00 | 9882000.00 | 8932000.00 | 9680000.00 | 9300000.00 | | hypothetical protein | Bacteria;Proteobacteria;Alphaproteobacteria;Hyphomicrobiales;Bradyrhizobiaceae;Bradyrhizobium;Bradyrhizobium lablabi;Bradyrhizobium lablabi CCBAU 23086 |
| Ga0586324 6126801 1 567 | 0.00 | 3950800.00 | 0.00 | 0.00 | 0.00 | 0.00 | 0.00 | 0.00 | 0.00 | 1955800.00 | 0.00 | 930000.00 | | hypothetical protein | Bacteria;Proteobacteria;Alphaproteobacteria;Hyphomicrobiales;Xanthobacteraceae;Pseudolabrys;Pseudolabrys taiwanensis;Pseudolabrys taiwanensis CC-BB4 |
| Ga0586324 6127443 3 566 | 6580000.00 | 7480000.00 | 30514600.00 | 7286500.00 | 9723200.00 | 18936900.00 | 0.00 | 5490000.00 | 6852600.00 | 9471000.00 | 9240000.00 | 11257500.00 | | hypothetical protein | Bacteria;Proteobacteria;Deltaproteobacteria;Desulfuromonadales;Geobacteraceae;Geobacter;Geobacter sulfurreducens;Geobacter sulfurreducens PCA |
| Ga0586324 6135958 249 566 | 2780000.00 | 0.00 | 0.00 | 0.00 | 0.00 | 0.00 | 0.00 | 0.00 | 0.00 | 0.00 | 0.00 | 0.00 | | hypothetical protein | Bacteria;Proteobacteria;Gammaproteobacteria;Oceanospirillales;Alcanivoracaceae;Alcanivorax;Alcanivorax nanhaiticus;Alcanivorax nanhaiticus 19-m-6 |
| Ga0586324 6138590 104 568 | 0.00 | 0.00 | 0.00 | 1301500.00 | 1905500.00 | 908808.43 | 1218856.08 | 408966.57 | 164772.63 | 0.00 | 0.00 | 0.00 | | hypothetical protein | Bacteria;Bacteroidetes;Bacteroidia;Bacteroidales;Odoribacteraceae;Culturomica;Culturomica massiliensis;Culturomica massiliensis Marseille-P2698 |
| Ga0586324 6139812 2 568 | 0.00 | 0.00 | 0.00 | 0.00 | 0.00 | 0.00 | 0.00 | 0.00 | 1611900.00 | 0.00 | 0.00 | 2107500.00 | | hypothetical protein | |
| Ga0586324 6146789 2 565 | 0.00 | 0.00 | 0.00 | 0.00 | 0.00 | 4525800.00 | 0.00 | 0.00 | 0.00 | 0.00 | 0.00 | 0.00 | | FtsP/CotA-like multicopper oxidase with cupredoxin domain | Bacteria;Actinobacteria;Actinomycetia;Streptomycetales;Streptomycetaceae;Streptomyces;Streptomyces odonnellii;Streptomyces odonnellii NRRL B-24891 |
| Ga0586324 6149843 3 329 | 0.00 | 0.00 | 0.00 | 0.00 | 0.00 | 0.00 | 9165600.00 | 0.00 | 0.00 | 0.00 | 0.00 | 0.00 | | DNA-directed RNA polymerase specialized sigma24 family protein | |
| Ga0586324 6170759 273 566 | 0.00 | 5596400.00 | 0.00 | 0.00 | 0.00 | 0.00 | 0.00 | 0.00 | 0.00 | 11704000.00 | 8800000.00 | 0.00 | | hypothetical protein | |
| Ga0586324 6206415 1 564 | 3200000.00 | 0.00 | 0.00 | 0.00 | 0.00 | 0.00 | 0.00 | 0.00 | 0.00 | 0.00 | 0.00 | 0.00 | | hypothetical protein | |
| Ga0586324 6221783 416 562 | 0.00 | 0.00 | 0.00 | 0.00 | 0.00 | 0.00 | 0.00 | 0.00 | 0.00 | 0.00 | 692033.76 | 0.00 | | hypothetical protein | |
| Ga0586324 6222485 3 533 | 0.00 | 0.00 | 0.00 | 0.00 | 0.00 | 1612348.01 | 0.00 | 0.00 | 0.00 | 0.00 | 0.00 | 0.00 | | hypothetical protein | Bacteria;Proteobacteria;Deltaproteobacteria;Syntrophorhabdales;Syntrophorhabdaceae;Syntrophorhabdus;Syntrophorhabdus aromaticivorans;Syntrophorhabdus aromaticivorans UI |
| Ga0586324 6223498 65 562 | 0.00 | 0.00 | 0.00 | 0.00 | 0.00 | 0.00 | 0.00 | 0.00 | 0.00 | 1493800.00 | 0.00 | 0.00 | | hypothetical protein | Bacteria;Proteobacteria;Betaproteobacteria;Burkholderiales;Comamonadaceae;Diaphorobacter;Diaphorobacter polyhydroxybutyrativorans;Diaphorobacter polyhydroxybutyrativorans SL-205 |
| Ga0586324 6227742 1 564 | 0.00 | 0.00 | 0.00 | 0.00 | 0.00 | 0.00 | 0.00 | 799100.00 | 0.00 | 0.00 | 0.00 | 0.00 | | outer membrane receptor protein involved in Fe transport | Bacteria;Verrucomicrobia;Opitutae;Opitutales;Opitutaceae;Lacunisphaera;Lacunisphaera limnophila;Opitutaceae bacterium IG16b |
| Ga0586324 6233368 1 564 | 2160000.00 | 0.00 | 0.00 | 0.00 | 0.00 | 0.00 | 0.00 | 21106000.00 | 18063000.00 | 0.00 | 0.00 | 23625000.00 | | predicted porin | Bacteria;Proteobacteria;Gammaproteobacteria;Chromatiales;Ectothiorhodospiraceae;Thiorhodospira;Thiorhodospira sibirica;Thiorhodospira sibirica A12, ATCC 700588 |
| Ga0586324 6265534 3 563 | 157410000.00 | 13411701.20 | 33208825.24 | 13110000.00 | 81277300.00 | 24176315.44 | 6270000.00 | 8723000.00 | 16248600.00 | 4858700.00 | 12672000.00 | 5130000.00 | | S-layer protein (TIGR01567 family) | Archaea;Euryarchaeota;Methanomicrobia;Methanosarcinales;Methanotrichaceae;Methanothrix;Methanothrix soehngenii;Methanothrix soehngenii GP-6 |
| Ga0586324 6278472 1 405 | 0.00 | 1054000.00 | 0.00 | 0.00 | 0.00 | 0.00 | 0.00 | 0.00 | 0.00 | 0.00 | 0.00 | 0.00 | | uncharacterized repeat protein (TIGR01451 family) | Bacteria;Proteobacteria;Gammaproteobacteria;Thiotrichales;Thiotrichaceae;Achromatium;Achromatium sp. WMS3;Achromatium sp. WMS3 |
| Ga0586324 6278695 249 560 | 0.00 | 0.00 | 6474500.00 | 0.00 | 0.00 | 0.00 | 0.00 | 0.00 | 0.00 | 0.00 | 0.00 | 0.00 | | hypothetical protein | Bacteria;Spirochaetes;Spirochaetia;Spirochaetales;Spirochaetaceae;Spirochaeta;Spirochaeta cellobiosiphila;Spirochaeta cellobiosiphila DSM 17781 |
| Ga0586324 6278976 3 560 | 0.00 | 0.00 | 0.00 | 23275000.00 | 0.00 | 0.00 | 0.00 | 0.00 | 0.00 | 0.00 | 0.00 | 0.00 | | formyltetrahydrofolate synthetase | Bacteria;Proteobacteria;Deltaproteobacteria;Syntrophobacterales;Syntrophaceae;Desulfobacca;Desulfobacca acetoxidans;Desulfobacca acetoxidans ASRB2, DSM 11109 |
| Ga0586324 6298694 3 560 | 0.00 | 0.00 | 0.00 | 0.00 | 0.00 | 0.00 | 13611600.00 | 0.00 | 710016.03 | 3873100.00 | 0.00 | 8025000.00 | | predicted porin | Bacteria;Proteobacteria;Betaproteobacteria;Nitrosomonadales;Thiobacillaceae;Thiobacillus;Thiobacillus denitrificans;Thiobacillus denitrificans ATCC 25259 |
| Ga0586324 6301605 40 561 | 0.00 | 3270800.00 | 0.00 | 0.00 | 0.00 | 0.00 | 0.00 | 0.00 | 2494800.00 | 0.00 | 0.00 | 0.00 | | crotonobetainyl-CoA:carnitine CoA-transferase CaiB-like acyl-CoA transferase | Bacteria;Proteobacteria;Deltaproteobacteria;Syntrophobacterales;Syntrophaceae;Desulfobacca;Desulfobacca acetoxidans;Desulfobacca acetoxidans ASRB2, DSM 11109 |
| Ga0586324 6302518 402 560 | 4730000.00 | 0.00 | 0.00 | 0.00 | 0.00 | 13537700.00 | 4902000.00 | 1769000.00 | 0.00 | 2040500.00 | 583970.64 | 5632500.00 | | hypothetical protein | |
| Ga0586324 6305856 3 560 | 0.00 | 0.00 | 0.00 | 0.00 | 4923400.00 | 0.00 | 0.00 | 0.00 | 0.00 | 0.00 | 0.00 | 0.00 | | hypothetical protein | |
| Ga0586324 6317369 106 561 | 0.00 | 0.00 | 0.00 | 0.00 | 0.00 | 0.00 | 0.00 | 0.00 | 850500.00 | 0.00 | 0.00 | 0.00 | | phasin family protein | Bacteria;Proteobacteria;Betaproteobacteria;Rhodocyclales;Azonexaceae;Dechloromonas;Dechloromonas sp. JJ;Dechloromonas sp. JJ |
| Ga0586324 6321954 2 559 | 0.00 | 0.00 | 0.00 | 0.00 | 0.00 | 0.00 | 0.00 | 0.00 | 0.00 | 0.00 | 1760000.00 | 0.00 | | hypothetical protein | |
| Ga0586324 6328206 2 559 | 0.00 | 0.00 | 0.00 | 0.00 | 0.00 | 0.00 | 0.00 | 0.00 | 0.00 | 0.00 | 0.00 | 9600000.00 | | hypothetical protein | |
| Ga0586324 6328377 2 559 | 0.00 | 0.00 | 0.00 | 0.00 | 0.00 | 0.00 | 0.00 | 0.00 | 0.00 | 0.00 | 0.00 | 840000.00 | | Mg-chelatase subunit ChlI | Bacteria;Chloroflexi;Caldilineae;Caldilineales;Caldilineaceae;Caldilinea;Caldilinea aerophila;Caldilinea aerophila STL-6-O1, DSM 14535 |
| Ga0586324 6342592 1 399 | 0.00 | 0.00 | 0.00 | 1254000.00 | 0.00 | 0.00 | 0.00 | 0.00 | 0.00 | 1054900.00 | 0.00 | 0.00 | | uncharacterized membrane protein | Archaea;Euryarchaeota;Methanomicrobia;Methanomicrobiales;Methanomicrobiaceae;Methanofollis;Methanofollis ethanolicus;Methanofollis ethanolicus JCM 15103 |
| Ga0586324 6352747 256 558 | 34600000.00 | 0.00 | 1242940.73 | 0.00 | 0.00 | 183017000.00 | 620999.04 | 0.00 | 0.00 | 0.00 | 87208000.00 | 68250000.00 | | hypothetical protein | Bacteria;Proteobacteria;Gammaproteobacteria;Methylococcales;Crenotrichaceae;Crenothrix;Crenothrix polyspora;Crenothrix polyspora ERS1545888 |
| Ga0586324 6356043 164 559 | 0.00 | 299363.88 | 0.00 | 0.00 | 0.00 | 0.00 | 0.00 | 0.00 | 0.00 | 0.00 | 0.00 | 0.00 | | regulator of protease activity HflC (stomatin/prohibitin superfamily) | Bacteria;Chloroflexi;Anaerolineae;Anaerolineales;Anaerolineaceae;Levilinea;Levilinea saccharolytica;Levilinea saccharolytica DSM 16555 |
| Ga0586324 6387807 2 472 | 0.00 | 23596000.00 | 0.00 | 2926000.00 | 0.00 | 29735300.00 | 24624000.00 | 12322000.00 | 17577000.00 | 0.00 | 0.00 | 0.00 | | hypothetical protein | |
| Ga0586324 6392377 3 557 | 0.00 | 0.00 | 33892600.00 | 0.00 | 0.00 | 2106323.20 | 0.00 | 0.00 | 0.00 | 0.00 | 0.00 | 1132500.00 | | crotonobetainyl-CoA:carnitine CoA-transferase CaiB-like acyl-CoA transferase | Bacteria;Proteobacteria;Deltaproteobacteria;Syntrophobacterales;Syntrophobacteraceae;Thermodesulforhabdus;Thermodesulforhabdus norvegica;Thermodesulforhabdus norvegica DSM 9990 |
| Ga0586324 6417014 2 490 | 0.00 | 0.00 | 0.00 | 0.00 | 0.00 | 0.00 | 0.00 | 0.00 | 0.00 | 0.00 | 490934.40 | 94574.25 | | predicted AlkP superfamily pyrophosphatase or phosphodiesterase | Bacteria;Actinobacteria;Actinomycetia;Propionibacteriales;Nocardioidaceae;Nocardioides;Nocardioides sp. Root122;Nocardioides sp. Root122 |
| Ga0586324 6428208 1 555 | 3810000.00 | 3128000.00 | 0.00 | 2973500.00 | 0.00 | 0.00 | 0.00 | 0.00 | 0.00 | 0.00 | 0.00 | 0.00 | | hypothetical protein | Bacteria;Proteobacteria;Deltaproteobacteria;Desulfuromonadales;Geobacteraceae;Geobacter;Geobacter metallireducens;Geobacter metallireducens RCH3 |
| Ga0586324 6441731 234 554 | 0.00 | 0.00 | 0.00 | 0.00 | 1658300.00 | 0.00 | 0.00 | 0.00 | 0.00 | 0.00 | 0.00 | 0.00 | | hypothetical protein | |
| Ga0586324 6441858 132 554 | 0.00 | 0.00 | 0.00 | 0.00 | 0.00 | 0.00 | 0.00 | 0.00 | 0.00 | 0.00 | 2833600.00 | 0.00 | | MFS family permease | Bacteria;Proteobacteria;Betaproteobacteria;Nitrosomonadales;Sterolibacteriaceae;Sulfurisoma;Sulfurisoma sediminicola;Sulfurisoma sediminicola BSN1 |
| Ga0586324 6443864 1 408 | 0.00 | 0.00 | 0.00 | 0.00 | 2173300.00 | 0.00 | 0.00 | 0.00 | 0.00 | 0.00 | 0.00 | 0.00 | | hypothetical protein | |
| Ga0586324 6456173 1 108 | 2160000.00 | 6079200.00 | 0.00 | 0.00 | 0.00 | 7106300.00 | 0.00 | 0.00 | 0.00 | 0.00 | 0.00 | 3607500.00 | | hypothetical protein | |
| Ga0586324 6467580 2 553 | 0.00 | 1054000.00 | 0.00 | 0.00 | 0.00 | 0.00 | 0.00 | 0.00 | 0.00 | 0.00 | 0.00 | 0.00 | | uncharacterized protein YqfA (UPF0365 family) | Bacteria;Planctomycetes;Planctomycetia;Pirellulales;Thermoguttaceae;Thermogutta;Thermogutta terrifontis;Thermogutta terrifontis R1 |
| Ga0586324 6477021 41 553 | 0.00 | 0.00 | 5911500.00 | 5196500.00 | 0.00 | 0.00 | 0.00 | 0.00 | 0.00 | 0.00 | 0.00 | 2685000.00 | | hypothetical protein | |
| Ga0586324 6479445 3 554 | 0.00 | 0.00 | 2574953.69 | 0.00 | 0.00 | 6550500.00 | 0.00 | 2086200.00 | 2033100.00 | 0.00 | 0.00 | 0.00 | | membrane protease subunit (stomatin/prohibitin family) | Bacteria;Chloroflexi;Anaerolineae;Anaerolineales;Anaerolineaceae;Levilinea;Levilinea saccharolytica;Levilinea saccharolytica DSM 16555 |
| Ga0586324 6481401 1 555 | 0.00 | 0.00 | 0.00 | 14915000.00 | 0.00 | 0.00 | 0.00 | 0.00 | 0.00 | 0.00 | 0.00 | 0.00 | | TonB-dependent SusC/RagA subfamily outer membrane receptor | Bacteria;Bacteroidetes;Cytophagia;Cytophagales;Cytophagaceae;Arsenicibacter;Arsenicibacter rosenii;Arsenicibacter rosenii SM-1 |
| Ga0586324 6483354 1 555 | 0.00 | 0.00 | 0.00 | 0.00 | 0.00 | 0.00 | 0.00 | 0.00 | 0.00 | 0.00 | 0.00 | 900000.00 | | hypothetical protein | |
| Ga0586324 6497966 408 554 | 0.00 | 0.00 | 45208190.62 | 0.00 | 0.00 | 0.00 | 0.00 | 0.00 | 1976400.00 | 0.00 | 0.00 | 0.00 | | rubrerythrin | Bacteria;Proteobacteria;Deltaproteobacteria;Syntrophobacterales;Syntrophaceae;Smithella;Smithella sp. F21;Smithella sp. F21 |

| | | | | | | | | | | | | | | | |
|---|---|---|---|---|---|---|---|---|---|---|---|---|---|---|---|
| Ga0586324 6500964 200 553 | 0.00 | 0.00 | 0.00 | 0.00 | 0.00 | 0.00 | 0.00 | 0.00 | 1490400.00 | 0.00 | 0.00 | 0.00 | | hypothetical protein | Bacteria;Proteobacteria;Betaproteobacteria;Nitrosomonadales;Thiobacillaceae;Thiobacillus;Thiobacillus denitrificans;Thiobacillus denitrificans DSM 12475 |
| Ga0586324 6518123 115 552 | 0.00 | 2427497.32 | 45715600.00 | 21394000.00 | 29344700.00 | 0.00 | 66348000.00 | 37393000.00 | 38370056.40 | 0.00 | 24904000.00 | 6067500.00 | | predicted porin | |
| Ga0586324 6534138 3 551 | 0.00 | 0.00 | 0.00 | 3315500.00 | 2441100.00 | 0.00 | 0.00 | 0.00 | 0.00 | 0.00 | 0.00 | 0.00 | | alkyl sulfatase BDS1-like metallo-beta-lactamase superfamily hydrolase | Bacteria;Proteobacteria;Gammaproteobacteria;Chromatiales;Chromatiaceae;Thiodictyon;Candidatus Thiodictyon syntrophicum;Candidatus Thiodictyon syntrophicum Cad16 |
| Ga0586324 6555273 3 551 | 0.00 | 0.00 | 0.00 | 0.00 | 0.00 | 0.00 | 0.00 | 0.00 | 0.00 | 1632400.00 | 1478400.00 | 0.00 | | hypothetical protein | Bacteria;Proteobacteria;Betaproteobacteria;Nitrosomonadales;Gallionellaceae;Sideroxydans;Sideroxydans lithotrophicus;Sideroxydans lithotrophicus ES-1 |
| Ga0586324 6564511 2 550 | 0.00 | 3230000.00 | 1883657.25 | 0.00 | 0.00 | 2183313.41 | 0.00 | 0.00 | 0.00 | 0.00 | 0.00 | 0.00 | | hypothetical protein | |
| Ga0586324 6594640 2 550 | 0.00 | 0.00 | 0.00 | 0.00 | 0.00 | 12108500.00 | 0.00 | 0.00 | 0.00 | 0.00 | 5491200.00 | 2737500.00 | | hypothetical protein | Bacteria;Acidobacteria;Acidobacteriia;Bryobacterales;Solibacteraceae;Candidatus Solibacter;Candidatus Solibacter usitatus;Candidatus Solibacter usitatus Ellin6076 |
| Ga0586324 6600004 3 551 | 24457442.00 | 29700761.20 | 36738519.96 | 4379500.00 | 18677463.80 | 21618055.38 | 4661421.24 | 16555400.00 | 8950500.00 | 20254081.54 | 20319200.00 | 22702500.00 | | hypothetical protein | Bacteria;Proteobacteria;Gammaproteobacteria;Alteromonadales;Alteromonadaceae;Marinobacter;Marinobacter manganoxydans;Marinobacter manganoxydans MnI7-9 |
| Ga0586324 6614850 3 548 | 0.00 | 1475600.00 | 0.00 | 0.00 | 0.00 | 0.00 | 0.00 | 0.00 | 0.00 | 0.00 | 0.00 | 0.00 | | arylsulfatase A-like enzyme | Bacteria;Proteobacteria;Gammaproteobacteria;Methylococcales;Methylococcaceae;Methylomonas;Methylomonas lenta;Methylomonas lenta R-45370 |
| Ga0586324 6642143 2 532 | 0.00 | 0.00 | 0.00 | 0.00 | 0.00 | 0.00 | 0.00 | 0.00 | 0.00 | 0.00 | 1443200.00 | 0.00 | | hypothetical protein | |
| Ga0586324 6645759 69 482 | 817096.00 | 3821600.00 | 0.00 | 0.00 | 0.00 | 4367000.00 | 0.00 | 2360700.00 | 0.00 | 0.00 | 0.00 | 0.00 | | nucleotide-binding universal stress UspA family protein | Bacteria;Proteobacteria;Deltaproteobacteria;Syntrophobacterales;Syntrophobacteraceae;Syntrophobacter;Syntrophobacter fumaroxidans;Syntrophobacter fumaroxidans MPOB |
| Ga0586324 6648023 1 549 | 0.00 | 0.00 | 0.00 | 0.00 | 0.00 | 0.00 | 0.00 | 0.00 | 0.00 | 0.00 | 0.00 | 475910.25 | | hypothetical protein | Bacteria;Planctomycetes;Phycisphaerae;Sedimentisphaerales;Anaerohalosphaeraceae;Anaerohalosphaera;Anaerohalosphaera lusitana;Phycisphaerae bacterium ST-NAGAB-D1 |
| Ga0586324 6650458 1 549 | 0.00 | 14348000.00 | 18916800.00 | 0.00 | 0.00 | 0.00 | 0.00 | 0.00 | 0.00 | 14553000.00 | 0.00 | 12150000.00 | | hypothetical protein | Bacteria;Proteobacteria;Deltaproteobacteria;Syntrophobacterales;Syntrophaceae;Desulfobacca;Desulfobacca acetoxidans;Desulfobacca acetoxidans ASRB2, DSM 11109 |
| Ga0586324 6652733 2 547 | 0.00 | 0.00 | 0.00 | 0.00 | 0.00 | 0.00 | 0.00 | 2513200.00 | 0.00 | 0.00 | 0.00 | 0.00 | | hypothetical protein | Archaea;Euryarchaeota;Methanomicrobia;Methanomicrobiales;Methanoregulaceae;Methanoregula;Methanoregula formicica;Methanoregula formicica SMSP, DSM 22288 |
| Ga0586324 6665884 3 548 | 0.00 | 0.00 | 0.00 | 0.00 | 0.00 | 0.00 | 0.00 | 0.00 | 0.00 | 847000.00 | 0.00 | 0.00 | | hypothetical protein | |
| Ga0586324 6666956 105 548 | 0.00 | 4467600.00 | 5798900.00 | 0.00 | 0.00 | 0.00 | 0.00 | 0.00 | 0.00 | 0.00 | 4611200.00 | 0.00 | | hypothetical protein | |
| Ga0586324 6678079 116 547 | 618337.00 | 0.00 | 0.00 | 0.00 | 0.00 | 0.00 | 0.00 | 0.00 | 0.00 | 0.00 | 0.00 | 0.00 | | hypothetical protein | Bacteria;Proteobacteria;Gammaproteobacteria;Methylococcales;Methylococcaceae;Methylosarcina;Methylosarcina fibrata;Methylosarcina fibrata AML-C10 |
| Ga0586324 6683257 193 546 | 0.00 | 0.00 | 0.00 | 6317500.00 | 8054600.00 | 0.00 | 0.00 | 3751500.00 | 5054400.00 | 4504500.00 | 3792800.00 | 3052500.00 | | Tfp pilus assembly protein PilF | Bacteria;Proteobacteria;Deltaproteobacteria;Syntrophobacterales;Syntrophaceae;Desulfobacca;Desulfobacca acetoxidans;Desulfobacca acetoxidans ASRB2, DSM 11109 |
| Ga0586324 6684052 397 546 | 0.00 | 0.00 | 0.00 | 291439.10 | 0.00 | 0.00 | 0.00 | 0.00 | 0.00 | 0.00 | 0.00 | 0.00 | | hypothetical protein | |
| Ga0586324 6685601 3 548 | 0.00 | 23460000.00 | 0.00 | 0.00 | 0.00 | 0.00 | 0.00 | 0.00 | 0.00 | 0.00 | 0.00 | 0.00 | | hypothetical protein | Bacteria;Spirochaetes;Spirochaetia;Spirochaetales;Spirochaetaceae;Spirochaeta;Spirochaeta cellobiosiphila;Spirochaeta cellobiosiphila DSM 17781 |
| Ga0586324 6702973 3 545 | 0.00 | 0.00 | 0.00 | 0.00 | 0.00 | 0.00 | 0.00 | 4196800.00 | 0.00 | 3896200.00 | 0.00 | 0.00 | | predicted porin | |
| Ga0586324 6704802 3 545 | 10600000.00 | 15640000.00 | 14187600.00 | 12540000.00 | 7529300.00 | 33983200.00 | 6201600.00 | 4953200.00 | 5580900.00 | 4874100.00 | 5368000.00 | 19950000.00 | | crotonobetainyl-CoA:carnitine CoA-transferase CaiB-like acyl-CoA transferase | Bacteria;Proteobacteria;Deltaproteobacteria;Syntrophobacterales;Syntrophaceae;Desulfobacca;Desulfobacca acetoxidans;Desulfobacca acetoxidans ASRB2, DSM 11109 |
| Ga0586324 6705497 73 546 | 0.00 | 0.00 | 0.00 | 0.00 | 0.00 | 0.00 | 0.00 | 0.00 | 406060.29 | 0.00 | 0.00 | 0.00 | | predicted porin | Bacteria;Proteobacteria;Betaproteobacteria;Rhodocyclales;Azonexaceae;Dechloromonas;Dechloromonas denitrificans;Dechloromonas denitrificans ATCC BAA-841 |
| Ga0586324 6716505 1 546 | 0.00 | 0.00 | 0.00 | 0.00 | 0.00 | 0.00 | 0.00 | 0.00 | 0.00 | 5921300.00 | 0.00 | 0.00 | | hypothetical protein | |
| Ga0586324 6727311 3 446 | 0.00 | 16292800.00 | 5425287.57 | 6536000.00 | 30024500.00 | 5319800.00 | 6384000.00 | 15262200.00 | 21011400.00 | 20335700.00 | 19324800.00 | 0.00 | | predicted porin | Bacteria;Proteobacteria;Betaproteobacteria;Burkholderiales;Comamonadaceae;Acidovorax;Acidovorax temperans;Acidovorax temperans CB2 |
| Ga0586324 6759811 1 396 | 0.00 | 1122000.00 | 0.00 | 0.00 | 0.00 | 0.00 | 0.00 | 0.00 | 0.00 | 0.00 | 0.00 | 0.00 | | hypothetical protein | |
| Ga0586324 6765189 1 543 | 0.00 | 0.00 | 0.00 | 5567000.00 | 0.00 | 0.00 | 0.00 | 0.00 | 0.00 | 0.00 | 0.00 | 0.00 | | arylsulfatase A-like enzyme | Bacteria;Proteobacteria;Alphaproteobacteria;Hyphomicrobiales;Methylobacteriaceae;Methylobacterium;Methylobacterium pseudosasicola;Methylobacterium pseudosasicola BL36 |
| Ga0586324 6782896 73 543 | 0.00 | 0.00 | 0.00 | 0.00 | 370050.16 | 0.00 | 1236717.60 | 1055300.00 | 0.00 | 0.00 | 0.00 | 601796.25 | | predicted porin | Bacteria;Proteobacteria;Betaproteobacteria;Burkholderiales;unclassified Burkholderiales;Rhizobacter;Rhizobacter sp. SG703;Rhizobacter sp. SG703 |
| Ga0586324 6785386 3 545 | 4700000.00 | 0.00 | 0.00 | 10640000.00 | 0.00 | 0.00 | 0.00 | 0.00 | 0.00 | 0.00 | 0.00 | 1740000.00 | | hypothetical protein | Bacteria;Spirochaetes;Spirochaetia;Spirochaetales;Spirochaetaceae;Sediminispirochaeta;Sediminispirochaeta smaragdinae;Sediminispirochaeta smaragdinae DSM 11293 |
| Ga0586324 6806423 119 544 | 0.00 | 0.00 | 26010600.00 | 0.00 | 20703000.00 | 25606500.00 | 0.00 | 0.00 | 0.00 | 0.00 | 0.00 | 0.00 | | hypothetical protein | Bacteria;Proteobacteria;Deltaproteobacteria;Desulfuromonadales;Geobacteraceae;Geobacter;Geobacter sulfurreducens;Geobacter sulfurreducens AM-1 |
| Ga0586324 6806427 3 542 | 0.00 | 2080800.00 | 0.00 | 0.00 | 0.00 | 0.00 | 0.00 | 0.00 | 0.00 | 0.00 | 0.00 | 0.00 | | hypothetical protein | Bacteria;Acidobacteria;Acidobacteriia;Acidobacteriales;Acidobacteriaceae;Edaphobacter;Edaphobacter modestus;Edaphobacter modestus DSM 18101 |
| Ga0586324 6808879 134 544 | 0.00 | 0.00 | 0.00 | 0.00 | 0.00 | 0.00 | 0.00 | 0.00 | 0.00 | 0.00 | 0.00 | 3862500.00 | | hypothetical protein | Bacteria;Proteobacteria;Deltaproteobacteria;Myxococcales;Polyangiaceae;Sorangium;Sorangium cellulosum;Sorangium cellulosum So ceGT47 |
| Ga0586324 6814055 72 542 | 56300000.00 | 42160000.00 | 94021000.00 | 59185000.00 | 71585000.00 | 84958000.00 | 4035600.00 | 58499000.00 | 52326000.00 | 53053000.00 | 57816000.00 | 49500000.00 | | hypothetical protein | Bacteria;Verrucomicrobia;Verrucomicrobiae;Verrucomicrobiales;Verrucomicrobia subdivision 3;Pedosphaera;Pedosphaera parvula;Pedosphaera parvula Ellin514 |
| Ga0586324 6818435 2 541 | 0.00 | 0.00 | 0.00 | 0.00 | 0.00 | 0.00 | 0.00 | 1640900.00 | 2276100.00 | 0.00 | 0.00 | 0.00 | | predicted porin | |
| Ga0586324 6821322 1 519 | 0.00 | 0.00 | 0.00 | 4351000.00 | 0.00 | 0.00 | 0.00 | 0.00 | 0.00 | 0.00 | 0.00 | 0.00 | | anaerobic selenocysteine-containing dehydrogenase | Bacteria;Proteobacteria;Deltaproteobacteria;Syntrophobacterales;Syntrophaceae;Desulfobacca;Desulfobacca acetoxidans;Desulfobacca acetoxidans ASRB2, DSM 11109 |
| Ga0586324 6825143 2 541 | 0.00 | 0.00 | 0.00 | 0.00 | 0.00 | 0.00 | 0.00 | 0.00 | 1765800.00 | 0.00 | 768916.72 | 1462500.00 | | hypothetical protein | |
| Ga0586324 6831133 2 439 | 0.00 | 782000.00 | 0.00 | 0.00 | 0.00 | 0.00 | 0.00 | 0.00 | 0.00 | 0.00 | 0.00 | 0.00 | | hypothetical protein | |
| Ga0586324 6837861 2 541 | 0.00 | 0.00 | 0.00 | 0.00 | 0.00 | 0.00 | 0.00 | 0.00 | 0.00 | 0.00 | 880000.00 | 273993.75 | | hypothetical protein | |
| Ga0586324 6842115 3 275 | 0.00 | 0.00 | 0.00 | 0.00 | 0.00 | 0.00 | 0.00 | 0.00 | 0.00 | 1293600.00 | 0.00 | 0.00 | | hypothetical protein | Bacteria;Proteobacteria;Deltaproteobacteria;Desulfuromonadales;Geobacteraceae;Geobacter;Geobacter sp. DSM 9736;Geobacter sp. DSM 9736 |
| Ga0586324 6845717 1 543 | 0.00 | 0.00 | 0.00 | 0.00 | 0.00 | 0.00 | 0.00 | 0.00 | 0.00 | 0.00 | 0.00 | 1777500.00 | | hypothetical protein | Bacteria;Proteobacteria;Betaproteobacteria;Burkholderiales;Comamonadaceae;Acidovorax;Acidovorax sp. JS42;Acidovorax sp. JS42 |
| Ga0586324 6848699 2 310 | 0.00 | 0.00 | 1276664.43 | 0.00 | 0.00 | 0.00 | 0.00 | 0.00 | 0.00 | 0.00 | 0.00 | 0.00 | | ABC-type branched-subunit amino acid transport system substrate-binding protein | |
| Ga0586324 6849609 3 542 | 0.00 | 1720400.00 | 0.00 | 0.00 | 0.00 | 0.00 | 0.00 | 0.00 | 0.00 | 0.00 | 0.00 | 0.00 | | hypothetical protein | Bacteria;Verrucomicrobia;Verrucomicrobiae;Verrucomicrobiales;Verrucomicrobia subdivision 3;Pedosphaera;Pedosphaera parvula;Pedosphaera parvula Ellin514 |
| Ga0586324 6850044 2 541 | 0.00 | 0.00 | 0.00 | 56430000.00 | 0.00 | 0.00 | 0.00 | 0.00 | 0.00 | 0.00 | 0.00 | 0.00 | | RNA polymerase sigma factor (sigma-70 family) | Bacteria;Bacteroidetes;Sphingobacteriia;Sphingobacteriales;Sphingobacteriaceae;Mucilaginibacter;Mucilaginibacter sp. OK268;Mucilaginibacter sp. OK268 |
| Ga0586324 6871856 2 541 | 0.00 | 0.00 | 0.00 | 0.00 | 0.00 | 5280100.00 | 0.00 | 0.00 | 0.00 | 0.00 | 0.00 | 0.00 | | hypothetical protein | |
| Ga0586324 6881028 2 541 | 0.00 | 5644000.00 | 0.00 | 8065500.00 | 6499300.00 | 0.00 | 5266800.00 | 510524.25 | 5540400.00 | 3634400.00 | 0.00 | 3285000.00 | | hypothetical protein | Bacteria;Acidobacteria;Vicinamibacteria;unclassified Vicinamibacteria;Vicinamibacteraceae;Luteitalea;Luteitalea pratensis;Luteitalea pratensis DSM 100886 |
| Ga0586324 6891663 2 541 | 0.00 | 0.00 | 593036.05 | 0.00 | 0.00 | 0.00 | 0.00 | 0.00 | 0.00 | 0.00 | 0.00 | 0.00 | | hypothetical protein | Bacteria;Spirochaetes;Spirochaetia;Spirochaetales;Spirochaetaceae;Spirochaeta;Spirochaeta cellobiosiphila;Spirochaeta cellobiosiphila DSM 17781 |
| Ga0586324 6935371 1 540 | 0.00 | 0.00 | 946870.29 | 0.00 | 0.00 | 0.00 | 0.00 | 0.00 | 0.00 | 0.00 | 0.00 | 0.00 | | hypothetical protein | Bacteria;Proteobacteria;Deltaproteobacteria;Myxococcales;Anaeromyxobacteraceae;Anaeromyxobacter;Anaeromyxobacter sp. K;Anaeromyxobacter sp. K |
| Ga0586324 6965096 3 539 | 0.00 | 0.00 | 0.00 | 0.00 | 0.00 | 0.00 | 0.00 | 0.00 | 1960200.00 | 0.00 | 0.00 | 0.00 | | hypothetical protein | Bacteria;Acidobacteria;Acidobacteriia;Acidobacteriales;Acidobacteriaceae;unclassified Acidobacteriaceae;Acidobacteriaceae bacterium KBS 96;Acidobacteriaceae bacterium KBS 96, DSM 24297 |
| Ga0586324 6970513 1 537 | 1340000.00 | 0.00 | 0.00 | 0.00 | 0.00 | 0.00 | 0.00 | 0.00 | 0.00 | 0.00 | 0.00 | 0.00 | | 2-keto-4-pentenoate hydratase/2-oxohepta-3-ene-1,7-dioic acid hydratase in catechol pathway | Bacteria;Verrucomicrobia;Verrucomicrobiae;Verrucomicrobiales;Verrucomicrobia subdivision 3;Pedosphaera;Pedosphaera parvula;Pedosphaera parvula Ellin514 |
| Ga0586324 6976588 1 537 | 0.00 | 0.00 | 0.00 | 0.00 | 0.00 | 0.00 | 0.00 | 616100.00 | 0.00 | 0.00 | 0.00 | 0.00 | | TRAP-type mannitol/chloroaromatic compound transport system substrate-binding protein | Bacteria;Proteobacteria;Alphaproteobacteria;Hyphomicrobiales;Bradyrhizobiaceae;Bradyrhizobium;Bradyrhizobium erythrophlei;Bradyrhizobium erythrophlei GAS242 |
| Ga0586324 6992251 3 536 | 0.00 | 0.00 | 0.00 | 0.00 | 0.00 | 0.00 | 0.00 | 589328.93 | 0.00 | 0.00 | 0.00 | 0.00 | | hypothetical protein | Bacteria;Acidobacteria;Vicinamibacteria;unclassified Vicinamibacteria;Vicinamibacteraceae;Luteitalea;Luteitalea pratensis;Luteitalea pratensis DSM 100886 |
| Ga0586324 7001686 1 537 | 4740000.00 | 10852800.00 | 15538800.00 | 66500000.00 | 0.00 | 0.00 | 0.00 | 10431000.00 | 0.00 | 0.00 | 0.00 | 0.00 | | S-layer protein (TIGR01567 family) | Archaea;Euryarchaeota;Methanomicrobia;Methanosarcinales;Methanotrichaceae;Methanothrix;Methanothrix soehngenii;Methanothrix soehngenii GP-6 |
| Ga0586324 7006101 3 536 | 6420000.00 | 0.00 | 0.00 | 0.00 | 0.00 | 0.00 | 0.00 | 0.00 | 0.00 | 0.00 | 0.00 | 0.00 | | hypothetical protein | Bacteria;Bacteroidetes;Flavobacteriia;Flavobacteriales;Flavobacteriaceae;Aquimarina;Aquimarina sp. Aq107;Aquimarina sp. Aq107 |
| Ga0586324 7021604 2 535 | 4130000.00 | 0.00 | 0.00 | 0.00 | 0.00 | 0.00 | 0.00 | 0.00 | 0.00 | 0.00 | 0.00 | 0.00 | | hypothetical protein | Bacteria;Bacteroidetes;Cytophagia;Cytophagales;Fulvivirgaceae;Chryseolinea;Chryseolinea flava;Chryseolinea flava SDU1-6 |
| Ga0586324 7030339 3 536 | 0.00 | 0.00 | 0.00 | 0.00 | 0.00 | 0.00 | 0.00 | 0.00 | 0.00 | 2787400.00 | 0.00 | 0.00 | | homoaconitase/3-isopropylmalate dehydratase large subunit | Bacteria;Firmicutes;Negativicutes;Acidaminococcales;Acidaminococcaceae;Acidaminococcus;Acidaminococcus massiliensis;Acidaminococcus massiliensis Marseille-P2828 |
| Ga0586324 7030911 1 378 | 0.00 | 0.00 | 0.00 | 0.00 | 2101200.00 | 0.00 | 0.00 | 0.00 | 0.00 | 0.00 | 0.00 | 0.00 | | hypothetical protein | |
| Ga0586324 7040853 2 535 | 0.00 | 3468000.00 | 7093800.00 | 7248500.00 | 0.00 | 0.00 | 0.00 | 0.00 | 6188400.00 | 0.00 | 0.00 | 0.00 | | crotonobetainyl-CoA:carnitine CoA-transferase CaiB-like acyl-CoA transferase | Bacteria;Proteobacteria;Deltaproteobacteria;Syntrophobacterales;Syntrophaceae;Desulfobacca;Desulfobacca acetoxidans;Desulfobacca acetoxidans ASRB2, DSM 11109 |
| Ga0586324 7052797 3 536 | 0.00 | 2937600.00 | 0.00 | 0.00 | 0.00 | 0.00 | 0.00 | 0.00 | 0.00 | 0.00 | 0.00 | 0.00 | | HEAT repeat protein | Bacteria;Cyanobacteria;unclassified Cyanobacteria;Nostocales;Calotrichaceae;Calothrix;Calothrix sp. NIES-2098;Calothrix sp. NIES-2098 |
| Ga0586324 7073425 1 534 | 0.00 | 0.00 | 0.00 | 0.00 | 0.00 | 0.00 | 0.00 | 0.00 | 1960200.00 | 0.00 | 0.00 | 0.00 | | glutamate synthase domain-containing protein 2 | Bacteria;Proteobacteria;Deltaproteobacteria;Syntrophobacterales;Syntrophobacteraceae;Syntrophobacter;Syntrophobacter fumaroxidans;Syntrophobacter fumaroxidans MPOB |
| Ga0586324 7105759 1 534 | 0.00 | 0.00 | 0.00 | 0.00 | 0.00 | 0.00 | 0.00 | 58438000.00 | 0.00 | 0.00 | 0.00 | 0.00 | | predicted porin | Bacteria;Proteobacteria;Betaproteobacteria;Burkholderiales;Burkholderiaceae;Paraburkholderia;Paraburkholderia ginsengiterrae;Paraburkholderia ginsengiterrae DCY85-1 |
| Ga0586324 7124842 1 258 | 49330000.00 | 13334800.00 | 110348000.00 | 22505500.00 | 37347800.00 | 13418600.00 | 17282400.00 | 13725000.00 | 22275000.00 | 15708000.00 | 0.00 | 7800000.00 | | hypothetical protein | Bacteria;Proteobacteria;Alphaproteobacteria;Hyphomicrobiales;Bradyrhizobiaceae;Bradyrhizobium;Bradyrhizobium macuxiense;Bradyrhizobium macuxiense BR 10355 |
| Ga0586324 7138375 2 532 | 0.00 | 0.00 | 0.00 | 3106500.00 | 0.00 | 0.00 | 0.00 | 1433500.00 | 0.00 | 0.00 | 2692800.00 | 3772500.00 | | hypothetical protein | Bacteria;Proteobacteria;Alphaproteobacteria;Hyphomicrobiales;Bradyrhizobiaceae;Bradyrhizobium;Bradyrhizobium sp. R5;Bradyrhizobium sp. R5 |
| Ga0586324 7157085 2 532 | 0.00 | 566338.00 | 0.00 | 0.00 | 0.00 | 0.00 | 0.00 | 0.00 | 0.00 | 0.00 | 0.00 | 0.00 | | TRAP-type mannitol/chloroaromatic compound transport system substrate-binding protein | Bacteria;Proteobacteria;Alphaproteobacteria;Hyphomicrobiales;Bradyrhizobiaceae;Bradyrhizobium;Bradyrhizobium lablabi;Bradyrhizobium lablabi GAS499 |
| Ga0586324 7184268 2 532 | 0.00 | 0.00 | 0.00 | 0.00 | 0.00 | 0.00 | 0.00 | 1384700.00 | 0.00 | 0.00 | 0.00 | 0.00 | | hypothetical protein | Bacteria;Proteobacteria;Deltaproteobacteria;Syntrophobacterales;Syntrophobacteraceae;Desulfacinum;Desulfacinum infernum;Desulfacinum infernum DSM 9756 |
| Ga0586324 7192212 1 531 | 0.00 | 0.00 | 0.00 | 0.00 | 0.00 | 0.00 | 0.00 | 0.00 | 0.00 | 2525600.00 | 3115200.00 | 0.00 | | pimeloyl-ACP methyl ester carboxylesterase | Bacteria;Calditrichaeota;Calditrichae;Calditrichales;Calditrichaceae;Caldithrix;Caldithrix abyssi;Caldithrix abyssi LF13, DSM 13497 |
| Ga0586324 7195914 2 529 | 0.00 | 2108000.00 | 0.00 | 0.00 | 0.00 | 0.00 | 0.00 | 0.00 | 0.00 | 0.00 | 0.00 | 0.00 | | hypothetical protein | Bacteria;Acidobacteria;Acidobacteriia;Acidobacteriales;Acidobacteriaceae;unclassified Acidobacteriaceae;Acidobacteriaceae bacterium KBS 96;Acidobacteriaceae bacterium KBS 96, DSM 24297 |
| Ga0586324 7213556 2 529 | 0.00 | 0.00 | 7825700.00 | 0.00 | 0.00 | 0.00 | 0.00 | 0.00 | 0.00 | 0.00 | 0.00 | 0.00 | | hypothetical protein | Bacteria;Acidobacteria;Acidobacteriia;Acidobacteriales;Acidobacteriaceae;unclassified Acidobacteriaceae;Acidobacteriaceae bacterium KBS 96;Acidobacteriaceae bacterium KBS 96, DSM 24297 |
| Ga0586324 7224035 2 529 | 0.00 | 0.00 | 0.00 | 0.00 | 0.00 | 0.00 | 0.00 | 0.00 | 711458.64 | 0.00 | 0.00 | 0.00 | | aspartyl aminopeptidase | Bacteria;Proteobacteria;Deltaproteobacteria;Syntrophobacterales;Syntrophaceae;Desulfobacca;Desulfobacca acetoxidans;Desulfobacca acetoxidans ASRB2, DSM 11109 |
| Ga0586324 7243124 1 528 | 5840000.00 | 9662800.00 | 0.00 | 12723832.60 | 8631400.00 | 863336.05 | 2864459.76 | 10900700.00 | 8286300.00 | 4768244.25 | 14643200.00 | 10027500.00 | | hypothetical protein | |
| Ga0586324 7262128 3 452 | 0.00 | 0.00 | 842416.90 | 0.00 | 0.00 | 571092.44 | 0.00 | 0.00 | 0.00 | 0.00 | 0.00 | 712578.75 | | rubrerythrin | Archaea;Euryarchaeota;Methanomicrobia;Methanomicrobiales;Methanoregulaceae;Methanoregula;Methanoregula formicica;Methanoregula formicica SMSP, DSM 22288 |
| Ga0586324 7278967 1 441 | 0.00 | 0.00 | 0.00 | 0.00 | 0.00 | 0.00 | 0.00 | 0.00 | 0.00 | 0.00 | 8316000.00 | 0.00 | | phasin family protein | Bacteria;Proteobacteria;Betaproteobacteria;Rhodocyclales;Azonexaceae;Dechloromonas;Dechloromonas agitata;Dechloromonas agitata CKB |
| Ga0586324 7284450 1 528 | 0.00 | 0.00 | 0.00 | 0.00 | 0.00 | 0.00 | 0.00 | 0.00 | 642671.82 | 0.00 | 0.00 | 0.00 | | hypothetical protein | |
| Ga0586324 7296191 1 510 | 0.00 | 0.00 | 0.00 | 10165000.00 | 0.00 | 0.00 | 0.00 | 0.00 | 0.00 | 0.00 | 0.00 | 0.00 | | polyhydroxyalkanoate synthesis repressor PhaR | Bacteria;Proteobacteria;Betaproteobacteria;unclassified Betaproteobacteria;unclassified Betaproteobacteria;unclassified Betaproteobacteria;uncultured beta proteobacterium;unclassified betaproteobacterium ENR4 |
| Ga0586324 7307291 1 435 | 0.00 | 0.00 | 0.00 | 0.00 | 0.00 | 1109678.52 | 0.00 | 0.00 | 0.00 | 0.00 | 0.00 | 0.00 | | uncharacterized membrane protein YphA (DoxX/SURF4 family) | Bacteria;Verrucomicrobia;Opitutae;Opitutales;Opitutaceae;Opitutus;Opitutus sp. ER46;Opitutus sp. ER46 |
| Ga0586324 7319541 2 526 | 0.00 | 0.00 | 0.00 | 0.00 | 0.00 | 0.00 | 0.00 | 0.00 | 0.00 | 0.00 | 0.00 | 3615000.00 | | methyl-accepting chemotaxis protein | Bacteria;Acidobacteria;Vicinamibacteria;unclassified Vicinamibacteria;Vicinamibacteraceae;Luteitalea;Luteitalea pratensis;Luteitalea pratensis DSM 100886 |
| Ga0586324 7324349 184 528 | 0.00 | 0.00 | 0.00 | 0.00 | 0.00 | 0.00 | 0.00 | 0.00 | 0.00 | 0.00 | 1425600.00 | 0.00 | | RNA recognition motif-containing protein | Bacteria;Proteobacteria;Deltaproteobacteria;Syntrophobacterales;Syntrophaceae;Desulfobacca;Desulfobacca acetoxidans;Desulfobacca acetoxidans ASRB2, DSM 11109 |
| Ga0586324 7329544 2 526 | 0.00 | 0.00 | 0.00 | 0.00 | 8425400.00 | 5280100.00 | 0.00 | 0.00 | 0.00 | 0.00 | 0.00 | 0.00 | | hypothetical protein | Bacteria;Bacteroidetes;Bacteroidia;Marinilabiliales;Marinilabiliaceae;Saccharicrinis;Saccharicrinis fermentans;Saccharicrinis fermentans DSM 9555 |
| Ga0586324 7356142 3 491 | 0.00 | 0.00 | 0.00 | 0.00 | 470035.35 | 0.00 | 0.00 | 1622600.00 | 1044900.00 | 2371600.00 | 1214400.00 | 900000.00 | | hypothetical protein | Bacteria;Proteobacteria;Alphaproteobacteria;Rhodobacterales;Rhodobacteraceae;Rhodovulum;Rhodovulum sp. PH10;Rhodovulum sp. PH10 |
| Ga0586324 7356590 1 525 | 0.00 | 7398400.00 | 1396617.21 | 11685000.00 | 15038000.00 | 0.00 | 0.00 | 5154500.00 | 1798200.00 | 8331400.00 | 0.00 | 2032500.00 | | hypothetical protein | |
| Ga0586324 7363109 3 524 | 0.00 | 0.00 | 0.00 | 0.00 | 0.00 | 0.00 | 0.00 | 2116700.00 | 0.00 | 0.00 | 0.00 | 0.00 | | hypothetical protein | Bacteria;Verrucomicrobia;Verrucomicrobiae;Verrucomicrobiales;Verrucomicrobia subdivision 3;Pedosphaera;Pedosphaera parvula;Pedosphaera parvula Ellin514 |
| Ga0586324 7364953 1 219 | 0.00 | 0.00 | 0.00 | 0.00 | 0.00 | 0.00 | 3283200.00 | 0.00 | 0.00 | 0.00 | 3317600.00 | 0.00 | | nucleotide-binding universal stress UspA family protein | Archaea;Euryarchaeota;Methanomicrobia;Methanosarcinales;Methanotrichaceae;Methanothrix;Methanothrix soehngenii;Methanothrix soehngenii GP-6 |
| Ga0586324 7373361 225 524 | 0.00 | 0.00 | 0.00 | 1159000.00 | 0.00 | 0.00 | 0.00 | 0.00 | 0.00 | 0.00 | 0.00 | 0.00 | | predicted porin | Bacteria;Proteobacteria;Betaproteobacteria;Burkholderiales;Burkholderiaceae;Cupriavidus;Cupriavidus necator;Cupriavidus necator NH9 |
| Ga0586324 7378358 2 526 | 0.00 | 0.00 | 0.00 | 0.00 | 0.00 | 0.00 | 16917600.00 | 0.00 | 0.00 | 0.00 | 0.00 | 0.00 | | crotonobetainyl-CoA:carnitine CoA-transferase CaiB-like acyl-CoA transferase | Bacteria;Proteobacteria;Deltaproteobacteria;Syntrophobacterales;Syntrophaceae;Desulfobacca;Desulfobacca acetoxidans;Desulfobacca acetoxidans ASRB2, DSM 11109 |
| Ga0586324 7423889 1 525 | 0.00 | 6392000.00 | 0.00 | 0.00 | 0.00 | 0.00 | 0.00 | 0.00 | 0.00 | 0.00 | 0.00 | 0.00 | | hypothetical protein | Bacteria;Acidobacteria;Acidobacteriia;Acidobacteriales;Acidobacteriaceae;unclassified Acidobacteriaceae;Acidobacteriaceae bacterium KBS 96;Acidobacteriaceae bacterium KBS 96, DSM 24297 |
| Ga0586324 7430453 3 524 | 0.00 | 0.00 | 0.00 | 0.00 | 1534700.00 | 984691.01 | 0.00 | 0.00 | 1004400.00 | 0.00 | 0.00 | 0.00 | | hypothetical protein | Bacteria;Proteobacteria;Gammaproteobacteria;Chromatiales;Chromatiaceae;Thiocapsa;Thiocapsa roseopersicina;Thiocapsa roseopersicina DSM 217 |
| Ga0586324 7454960 153 524 | 0.00 | 0.00 | 0.00 | 13110000.00 | 15450000.00 | 0.00 | 0.00 | 0.00 | 10773000.00 | 0.00 | 0.00 | 0.00 | | hypothetical protein | Bacteria;Proteobacteria;Deltaproteobacteria;Syntrophobacterales;Syntrophobacteraceae;Desulfacinum;Desulfacinum hydrothermale;Desulfacinum hydrothermale DSM 13146 |
| Ga0586324 7456080 76 522 | 0.00 | 0.00 | 0.00 | 11495000.00 | 0.00 | 0.00 | 0.00 | 0.00 | 0.00 | 0.00 | 0.00 | 0.00 | | hypothetical protein | Bacteria;Proteobacteria;Deltaproteobacteria;Desulfovibrionales;Desulfovibrionaceae;Desulfovibrio;unclassified Desulfovibrio;Desulfovibrio sp. DF1 |
| Ga0586324 7458741 2 475 | 0.00 | 0.00 | 0.00 | 0.00 | 4871900.00 | 0.00 | 0.00 | 0.00 | 0.00 | 0.00 | 0.00 | 0.00 | | hypothetical protein | Bacteria;Proteobacteria;Gammaproteobacteria;Methylococcales;Methylococcaceae;Methylomicrobium;Methylomicrobium lacus;Methylosarcina lacus LW14 |
| Ga0586324 7480506 1 522 | 1170000.00 | 6228800.00 | 1676518.29 | 0.00 | 0.00 | 0.00 | 0.00 | 0.00 | 2227500.00 | 0.00 | 0.00 | 0.00 | | hypothetical protein | |
| Ga0586324 7480949 51 482 | 0.00 | 0.00 | 0.00 | 5871000.00 | 9084600.00 | 8059100.00 | 0.00 | 0.00 | 0.00 | 0.00 | 0.00 | 0.00 | | nucleoid-associated protein YgaU | Bacteria;Proteobacteria;Deltaproteobacteria;Desulfobacterales;Desulfobacteraceae;Desulfatiglans;Desulfatiglans anilini;Desulfatiglans anilini DSM 4660 |
| Ga0586324 7494817 51 521 | 0.00 | 0.00 | 0.00 | 0.00 | 0.00 | 0.00 | 0.00 | 0.00 | 0.00 | 0.00 | 1443200.00 | 0.00 | | hypothetical protein | Bacteria;Planctomycetes;Planctomycetia;Pirellulales;Pirellulaceae;Roseimaritima;Roseimaritima ulvae;Roseimaritima ulvae DSM 25454 |
| Ga0586324 7500570 236 520 | 0.00 | 0.00 | 0.00 | 0.00 | 0.00 | 1514757.47 | 0.00 | 0.00 | 0.00 | 0.00 | 0.00 | 0.00 | | F0F1-type ATP synthase membrane subunit b/b' | |
| Ga0586324 7505343 3 521 | 0.00 | 1509600.00 | 0.00 | 0.00 | 0.00 | 0.00 | 0.00 | 0.00 | 0.00 | 0.00 | 0.00 | 0.00 | | hypothetical protein | Bacteria;Proteobacteria;Deltaproteobacteria;Desulfuromonadales;Geobacteraceae;Geobacter;Geobacter lovleyi;Geobacter lovleyi SZ |
| Ga0586324 7509093 3 521 | 7290000.00 | 3502243.44 | 0.00 | 15458869.30 | 11927060.10 | 0.00 | 0.00 | 106872.00 | 800779.77 | 277925.34 | 3528800.00 | 2204051.18 | | hypothetical protein | Bacteria;Proteobacteria;Deltaproteobacteria;Syntrophorhabdales;Syntrophorhabdaceae;Syntrophorhabdus;Syntrophorhabdus aromaticivorans;Syntrophorhabdus aromaticivorans UI |
| Ga0586324 7515800 1 522 | 5160000.00 | 5684800.00 | 0.00 | 0.00 | 0.00 | 0.00 | 0.00 | 0.00 | 0.00 | 0.00 | 0.00 | 0.00 | | formyltetrahydrofolate synthetase | Bacteria;Proteobacteria;Deltaproteobacteria;Syntrophobacterales;Syntrophaceae;Desulfobacca;Desulfobacca acetoxidans;Desulfobacca acetoxidans ASRB2, DSM 11109 |
| Ga0586324 7522681 2 358 | 0.00 | 0.00 | 0.00 | 5396000.00 | 0.00 | 0.00 | 0.00 | 0.00 | 0.00 | 0.00 | 0.00 | 0.00 | | phasin family protein | Bacteria;Proteobacteria;Betaproteobacteria;Neisseriales;Chromobacteriaceae;Andreprevotia;Andreprevotia lacus;Andreprevotia lacus DSM 23236 |
| Ga0586324 7525373 1 522 | 11100000.00 | 20672000.00 | 7037500.00 | 7856500.00 | 6561100.00 | 17388600.00 | 12790800.00 | 5843800.00 | 5734800.00 | 5259100.00 | 5984000.00 | 0.00 | | hypothetical protein | Bacteria;Spirochaetes;Spirochaetia;Spirochaetales;Spirochaetaceae;Spirochaeta;Spirochaeta cellobiosiphila;Spirochaeta cellobiosiphila DSM 17781 |
| Ga0586324 7528696 3 521 | 21700000.00 | 16449200.00 | 0.00 | 18715000.00 | 0.00 | 0.00 | 0.00 | 0.00 | 14175000.00 | 0.00 | 0.00 | 0.00 | | predicted porin | Bacteria;Proteobacteria;Betaproteobacteria;Burkholderiales;Burkholderiaceae;Cupriavidus;Cupriavidus basilensis;Cupriavidus basilensis 4G11 |
| Ga0586324 7531963 3 521 | 0.00 | 0.00 | 0.00 | 0.00 | 0.00 | 0.00 | 0.00 | 0.00 | 0.00 | 0.00 | 153715.76 | 0.00 | | hypothetical protein | |
| Ga0586324 7539507 1 519 | 6210000.00 | 0.00 | 0.00 | 0.00 | 0.00 | 0.00 | 0.00 | 0.00 | 0.00 | 0.00 | 0.00 | 0.00 | | hypothetical protein | Bacteria;Spirochaetes;Spirochaetia;Spirochaetales;Spirochaetaceae;Spirochaeta;Spirochaeta cellobiosiphila;Spirochaeta cellobiosiphila DSM 17781 |
| Ga0586324 7547629 3 521 | 0.00 | 0.00 | 10134000.00 | 0.00 | 0.00 | 0.00 | 0.00 | 0.00 | 0.00 | 0.00 | 0.00 | 0.00 | | uncharacterized delta-60 repeat protein | Bacteria;Cyanobacteria;unclassified Cyanobacteria;Nostocales;Nostocaceae;Cylindrospermum;Cylindrospermum stagnale;Cylindrospermum stagnale PCC 7417 |
| Ga0586324 7549341 28 519 | 0.00 | 0.00 | 0.00 | 368679.80 | 838021.39 | 0.00 | 0.00 | 0.00 | 0.00 | 0.00 | 0.00 | 0.00 | | uncharacterized membrane protein | Archaea;Euryarchaeota;Methanomicrobia;Methanomicrobiales;Methanomicrobiaceae;Methanofollis;Methanofollis ethanolicus;Methanofollis ethanolicus JCM 15103 |
| Ga0586324 7549856 1 519 | 0.00 | 10404000.00 | 0.00 | 0.00 | 0.00 | 0.00 | 0.00 | 0.00 | 0.00 | 2302300.00 | 0.00 | 0.00 | | predicted porin | Bacteria;Proteobacteria;Betaproteobacteria;Burkholderiales;Burkholderiaceae;Quisquiliibacterium;Quisquiliibacterium transsilvanicum;Quisquiliibacterium transsilvanicum DSM 29781 |
| Ga0586324 7562769 1 519 | 0.00 | 4256800.00 | 0.00 | 0.00 | 0.00 | 0.00 | 0.00 | 0.00 | 321677.73 | 0.00 | 0.00 | 0.00 | | crotonobetainyl-CoA:carnitine CoA-transferase CaiB-like acyl-CoA transferase | Bacteria;Proteobacteria;Deltaproteobacteria;Syntrophobacterales;Syntrophobacteraceae;Desulfosoma;Desulfosoma caldarium;Desulfosoma caldarium DSM 22027 |
| Ga0586324 7563730 3 518 | 770065.00 | 585495.64 | 549772.32 | 0.00 | 0.00 | 310284.88 | 0.00 | 744200.00 | 487538.19 | 398398.77 | 0.00 | 567709.50 | | outer membrane receptor protein involved in Fe transport | Bacteria;Acidobacteria;Vicinamibacteria;unclassified Vicinamibacteria;Vicinamibacteraceae;Luteitalea;Luteitalea pratensis;Luteitalea pratensis DSM 100886 |
| Ga0586324 7566828 2 520 | 0.00 | 0.00 | 0.00 | 0.00 | 0.00 | 2042029.05 | 0.00 | 0.00 | 0.00 | 0.00 | 0.00 | 0.00 | | regulator of protease activity HflC (stomatin/prohibitin superfamily) | Bacteria;Acidobacteria;Acidobacteriia;Acidobacteriales;Acidobacteriaceae;unclassified Acidobacteriaceae;Acidobacteriaceae bacterium URHE0068;Acidobacteriaceae bacterium URHE0068 |
| Ga0586324 7575483 3 518 | 0.00 | 0.00 | 0.00 | 0.00 | 0.00 | 0.00 | 0.00 | 0.00 | 4673700.00 | 0.00 | 0.00 | 0.00 | | hypothetical protein | Bacteria;Proteobacteria;Deltaproteobacteria;Desulfovibrionales;Desulfovibrionaceae;Desulfovibrio;Desulfovibrio ferrophilus;Desulfovibrio ferrophilus IS5 |
| Ga0586324 7579407 80 520 | 0.00 | 904400.00 | 0.00 | 0.00 | 0.00 | 0.00 | 0.00 | 0.00 | 0.00 | 0.00 | 0.00 | 0.00 | | regulator of protease activity HflC (stomatin/prohibitin superfamily) | Bacteria;Chloroflexi;Dehalococcoidia;Dehalococcoidales;Dehalococcoidaceae;Dehalococcoides;Dehalococcoides mccartyi;Dehalococcoides mccartyi CBDB1 |
| Ga0586324 7627822 3 416 | 0.00 | 0.00 | 0.00 | 0.00 | 0.00 | 0.00 | 0.00 | 577122.22 | 0.00 | 0.00 | 0.00 | 0.00 | | uncharacterized protein (DUF169 family) | Archaea;Euryarchaeota;Methanomicrobia;Methanosarcinales;Methanotrichaceae;Methanothrix;Methanothrix soehngenii;Methanothrix soehngenii GP-6 |
| Ga0586324 7637327 2 517 | 0.00 | 0.00 | 0.00 | 3277500.00 | 0.00 | 0.00 | 0.00 | 0.00 | 0.00 | 0.00 | 0.00 | 0.00 | | hypothetical protein | Bacteria;Firmicutes;Clostridia;Thermosediminibacterales;Thermosediminibacteraceae;Caldanaerovirga;Caldanaerovirga acetigignens;Caldanaerovirga acetigignens DSM 18802 |
| Ga0586324 7649411 1 516 | 2670000.00 | 0.00 | 0.00 | 13775000.00 | 0.00 | 0.00 | 0.00 | 0.00 | 0.00 | 0.00 | 0.00 | 0.00 | | hypothetical protein | |
| Ga0586324 7661154 2 469 | 935400.00 | 0.00 | 0.00 | 0.00 | 0.00 | 0.00 | 0.00 | 0.00 | 0.00 | 0.00 | 0.00 | 0.00 | | hypothetical protein | Bacteria;Spirochaetes;Spirochaetia;Spirochaetales;Spirochaetaceae;Spirochaeta;Spirochaeta cellobiosiphila;Spirochaeta cellobiosiphila DSM 17781 |
| Ga0586324 7662106 19 516 | 0.00 | 0.00 | 0.00 | 0.00 | 0.00 | 0.00 | 0.00 | 4684800.00 | 0.00 | 0.00 | 0.00 | 0.00 | | ABC-type nitrate/sulfonate/bicarbonate transport system substrate-binding protein | Bacteria;Actinobacteria;Actinomycetia;Corynebacteriales;Mycobacteriaceae;Mycobacterium;Mycobacterium sp. AZCC_0083;Mycobacterium sp. AZCC_0083 |
| Ga0586324 7686371 172 357 | 0.00 | 0.00 | 0.00 | 0.00 | 0.00 | 3600023.79 | 0.00 | 0.00 | 0.00 | 8239000.00 | 7374400.00 | 0.00 | | hypothetical protein | |
| Ga0586324 7688082 2 517 | 9050000.00 | 8908000.00 | 11485200.00 | 6099000.00 | 4521700.00 | 0.00 | 0.00 | 2092300.00 | 2713500.00 | 4419800.00 | 4549600.00 | 4222500.00 | | hypothetical protein | Bacteria;Proteobacteria;Alphaproteobacteria;Hyphomicrobiales;Bradyrhizobiaceae;Bradyrhizobium;Bradyrhizobium sp. UASWS1016;Bradyrhizobium sp. UASWS1016 |
| Ga0586324 7696292 2 517 | 9270000.00 | 166402800.00 | 1825054.58 | 165889000.00 | 11309400.00 | 120365215.18 | 16146932.64 | 70150000.00 | 40362300.00 | 50881776.33 | 747818.72 | 2107500.00 | | S-layer protein (TIGR01567 family) | Archaea;Euryarchaeota;Methanomicrobia;Methanosarcinales;Methanotrichaceae;Methanothrix;Methanothrix soehngenii;Methanothrix soehngenii GP-6 |
| Ga0586324 7704192 3 515 | 0.00 | 0.00 | 0.00 | 0.00 | 6180000.00 | 0.00 | 0.00 | 0.00 | 0.00 | 0.00 | 0.00 | 0.00 | | tetratricopeptide (TPR) repeat protein | Bacteria;Proteobacteria;Deltaproteobacteria;Syntrophobacterales;Syntrophaceae;Desulfomonile;Desulfomonile tiedjei;Desulfomonile tiedjei DCB-1, DSM 6799 |
| Ga0586324 7710929 2 514 | 0.00 | 0.00 | 0.00 | 0.00 | 1266900.00 | 0.00 | 0.00 | 0.00 | 0.00 | 1617000.00 | 1672000.00 | 1395000.00 | | hypothetical protein | Bacteria;Proteobacteria;Deltaproteobacteria;Desulfobacterales;Desulfobacteraceae;Desulfobacter;Desulfobacter curvatus;Desulfobacter curvatus DSM 3379 |
| Ga0586324 7740484 2 514 | 46000000.00 | 33612400.00 | 37101700.00 | 28500000.00 | 25287284.86 | 0.00 | 0.00 | 0.00 | 18792000.00 | 23947000.00 | 0.00 | 0.00 | | S-layer protein (TIGR01567 family) | Archaea;Euryarchaeota;Methanomicrobia;Methanosarcinales;Methanotrichaceae;Methanothrix;Methanothrix soehngenii;Methanothrix soehngenii GP-6 |
| Ga0586324 7742448 2 484 | 0.00 | 1904000.00 | 0.00 | 0.00 | 0.00 | 0.00 | 0.00 | 0.00 | 0.00 | 1609300.00 | 0.00 | 0.00 | | hypothetical protein | Bacteria;Proteobacteria;Gammaproteobacteria;Methylococcales;Methylococcaceae;Methylobacter;Methylobacter tundripaludum;Methylobacter tundripaludum SV96 |
| Ga0586324 7750554 1 414 | 0.00 | 2828800.00 | 0.00 | 0.00 | 8909500.00 | 0.00 | 0.00 | 0.00 | 0.00 | 0.00 | 0.00 | 0.00 | | Tfp pilus assembly protein PilF | Bacteria;Proteobacteria;Deltaproteobacteria;Syntrophobacterales;Syntrophaceae;Desulfobacca;Desulfobacca acetoxidans;Desulfobacca acetoxidans ASRB2, DSM 11109 |
| Ga0586324 7752769 1 513 | 0.00 | 0.00 | 0.00 | 0.00 | 112185.54 | 0.00 | 0.00 | 0.00 | 0.00 | 0.00 | 0.00 | 0.00 | | hypothetical protein | Bacteria;Actinobacteria;Thermoleophilia;Solirubrobacterales;Solirubrobacteraceae;Solirubrobacter;Solirubrobacter pauli;Solirubrobacter pauli DSM 14954 |
| Ga0586324 7755703 3 431 | 0.00 | 0.00 | 0.00 | 0.00 | 0.00 | 0.00 | 0.00 | 0.00 | 0.00 | 0.00 | 1540000.00 | 0.00 | | 3-hydroxyisobutyrate dehydrogenase-like beta-hydroxyacid dehydrogenase | Bacteria;Proteobacteria;Alphaproteobacteria;Rhodospirillales;Acetobacteraceae;Rhodopila;Rhodopila globiformis;Rhodopila globiformis DSM 161 |
| Ga0586324 7778023 1 513 | 0.00 | 0.00 | 0.00 | 0.00 | 0.00 | 0.00 | 0.00 | 0.00 | 4941000.00 | 0.00 | 0.00 | 0.00 | | hypothetical protein | Bacteria;Bacteroidetes;Bacteroidia;Bacteroidales;Bacteroidaceae;Bacteroides;Bacteroides fragilis;Bacteroides fragilis KLE1257 |
| Ga0586324 7779405 2 454 | 0.00 | 0.00 | 7656800.00 | 0.00 | 0.00 | 25884400.00 | 5973600.00 | 8662000.00 | 0.00 | 0.00 | 0.00 | 0.00 | | tetratricopeptide (TPR) repeat protein | Bacteria;Proteobacteria;Deltaproteobacteria;Syntrophobacterales;Syntrophaceae;Desulfobacca;Desulfobacca acetoxidans;Desulfobacca acetoxidans ASRB2, DSM 11109 |
| Ga0586324 7780851 1 426 | 0.00 | 0.00 | 17847100.00 | 0.00 | 0.00 | 0.00 | 0.00 | 0.00 | 0.00 | 0.00 | 0.00 | 0.00 | | nucleotide-binding universal stress UspA family protein | Archaea;Euryarchaeota;Methanomicrobia;Methanosarcinales;Methanotrichaceae;Methanothrix;Methanothrix thermoacetophila;Methanothrix thermoacetophila PT |
| Ga0586324 7784478 3 512 | 0.00 | 0.00 | 0.00 | 0.00 | 0.00 | 0.00 | 0.00 | 0.00 | 0.00 | 3349500.00 | 0.00 | 0.00 | | ornithine carbamoyltransferase | Bacteria;Chloroflexi;Anaerolineae;Anaerolineales;Anaerolineaceae;Levilinea;Levilinea saccharolytica;Levilinea saccharolytica DSM 16555 |
| Ga0586324 7787473 194 460 | 0.00 | 0.00 | 0.00 | 0.00 | 1163900.00 | 0.00 | 0.00 | 0.00 | 0.00 | 0.00 | 0.00 | 0.00 | | hypothetical protein | Bacteria;Proteobacteria;Alphaproteobacteria;Hyphomicrobiales;Bradyrhizobiaceae;Bradyrhizobium;Bradyrhizobium lablabi;Bradyrhizobium lablabi CCBAU 23086 |
| Ga0586324 7790135 3 386 | 5440000.00 | 0.00 | 0.00 | 0.00 | 0.00 | 0.00 | 0.00 | 0.00 | 0.00 | 0.00 | 0.00 | 0.00 | | hypothetical protein | |
| Ga0586324 7824212 1 513 | 0.00 | 353117.88 | 0.00 | 0.00 | 0.00 | 0.00 | 0.00 | 0.00 | 0.00 | 0.00 | 0.00 | 0.00 | | hypothetical protein | Bacteria;Acidobacteria;Acidobacteriia;Bryobacterales;Solibacteraceae;Candidatus Solibacter;Candidatus Solibacter usitatus;Candidatus Solibacter usitatus Ellin6076 |
| Ga0586324 7835427 197 511 | 0.00 | 0.00 | 0.00 | 874993.70 | 648579.67 | 11671800.00 | 0.00 | 0.00 | 0.00 | 0.00 | 0.00 | 0.00 | | hypothetical protein | Bacteria;Proteobacteria;Deltaproteobacteria;Syntrophobacterales;Syntrophobacteraceae;Desulfacinum;Desulfacinum infernum;Desulfacinum infernum DSM 9756 |
| Ga0586324 7840144 1 513 | 0.00 | 748000.00 | 0.00 | 0.00 | 0.00 | 0.00 | 0.00 | 0.00 | 0.00 | 0.00 | 0.00 | 0.00 | | regulator of protease activity HflC (stomatin/prohibitin superfamily) | Bacteria;Chloroflexi;Dehalococcoidia;unclassified Dehalococcoidia;unclassified Dehalococcoidia;Dehalogenimonas;Dehalogenimonas formicexedens;Dehalogenimonas formicexedens NSZ-14 |
| Ga0586324 7866964 3 512 | 0.00 | 0.00 | 0.00 | 0.00 | 148320000.00 | 0.00 | 0.00 | 0.00 | 0.00 | 0.00 | 0.00 | 0.00 | | formyltetrahydrofolate synthetase | Bacteria;Proteobacteria;Deltaproteobacteria;Syntrophorhabdales;Syntrophorhabdaceae;Syntrophorhabdus;Syntrophorhabdus aromaticivorans;Syntrophorhabdus aromaticivorans UI |
| Ga0586324 7880037 1 510 | 0.00 | 3332000.00 | 0.00 | 0.00 | 0.00 | 0.00 | 0.00 | 0.00 | 0.00 | 0.00 | 0.00 | 0.00 | | hypothetical protein | Bacteria;Proteobacteria;Betaproteobacteria;Nitrosomonadales;Nitrosomonadaceae;Nitrosospira;Nitrosospira multiformis;Nitrosospira multiformis Nl1 |
| Ga0586324 7912113 190 507 | 15100000.00 | 0.00 | 24152700.00 | 17765000.00 | 0.00 | 0.00 | 6931200.00 | 18056000.00 | 16200000.00 | 0.00 | 0.00 | 0.00 | | transposase | Bacteria;Actinobacteria;Actinomycetia;Corynebacteriales;Mycobacteriaceae;Mycobacterium;Mycobacterium sp. ACS4331;Mycobacterium sp. ACS4331 |
| Ga0586324 7953831 2 508 | 1090000.00 | 979200.00 | 0.00 | 1862000.00 | 1699500.00 | 301519.91 | 0.00 | 713700.00 | 891000.00 | 116891.39 | 0.00 | 840000.00 | | crotonobetainyl-CoA:carnitine CoA-transferase CaiB-like acyl-CoA transferase | Bacteria;Proteobacteria;Deltaproteobacteria;Syntrophobacterales;Syntrophobacteraceae;Desulfacinum;Desulfacinum infernum;Desulfacinum infernum DSM 9756 |
| Ga0586324 7960554 3 287 | 0.00 | 0.00 | 887023.39 | 2527000.00 | 0.00 | 0.00 | 2302800.00 | 1543300.00 | 1733400.00 | 0.00 | 0.00 | 0.00 | | hypothetical protein | Bacteria;Proteobacteria;Deltaproteobacteria;Syntrophobacterales;Syntrophobacteraceae;Desulfacinum;Desulfacinum hydrothermale;Desulfacinum hydrothermale DSM 13146 |
| Ga0586324 7967655 3 509 | 40170000.00 | 40256000.00 | 0.00 | 0.00 | 2492600.00 | 0.00 | 0.00 | 3373300.00 | 4090500.00 | 16170000.00 | 6916800.00 | 7185000.00 | | predicted porin | Bacteria;Proteobacteria;Betaproteobacteria;Burkholderiales;Oxalobacteraceae;Janthinobacterium;Janthinobacterium sp. CG3;Janthinobacterium sp. CG3 |
| Ga0586324 7973264 2 508 | 12330000.00 | 11437600.00 | 2865326.57 | 0.00 | 0.00 | 3064665.32 | 0.00 | 866200.00 | 1830600.00 | 0.00 | 0.00 | 0.00 | | hypothetical protein | Bacteria;Proteobacteria;Betaproteobacteria;Nitrosomonadales;Methylophilaceae;unclassified Methylophilaceae;unclassified Methylophilaceae;Betaproteobacterium LSUCC0135 |
| Ga0586324 7978770 1 507 | 2200000.00 | 0.00 | 0.00 | 0.00 | 0.00 | 0.00 | 0.00 | 0.00 | 0.00 | 0.00 | 0.00 | 0.00 | | Xaa-Pro aminopeptidase | Bacteria;Proteobacteria;Betaproteobacteria;Burkholderiales;Burkholderiaceae;Paraburkholderia;Paraburkholderia nodosa;Paraburkholderia nodosa DSM 21604 |

| | | | | | | | | | | | | | | | |
|---|---|---|---|---|---|---|---|---|---|---|---|---|---|---|---|
| Ga0586324 7991814 3 509 | 0.00 | 0.00 | 0.00 | 7115500.00 | 8559300.00 | 0.00 | 0.00 | 3684400.00 | 2713500.00 | 0.00 | 0.00 | 0.00 | | hypothetical protein | |
| Ga0586324 8001321 2 481 | 2160000.00 | 3257200.00 | 9739900.00 | 0.00 | 0.00 | 0.00 | 0.00 | 0.00 | 1587600.00 | 7168700.00 | 0.00 | 9075000.00 | | hypothetical protein | |
| Ga0586324 8002305 3 506 | 0.00 | 10281600.00 | 6812300.00 | 8132000.00 | 21630000.00 | 7463600.00 | 10960094.52 | 7902889.77 | 13632300.00 | 9548000.00 | 20215605.52 | 8325000.00 | | predicted porin | |
| Ga0586324 8005696 31 507 | 0.00 | 0.00 | 0.00 | 0.00 | 9836500.00 | 0.00 | 0.00 | 0.00 | 0.00 | 0.00 | 0.00 | 0.00 | | hypothetical protein | |
| Ga0586324 8006368 1 411 | 2270000.00 | 4658000.00 | 0.00 | 0.00 | 0.00 | 6788700.00 | 0.00 | 0.00 | 0.00 | 0.00 | 5728800.00 | 0.00 | | hypothetical protein | Bacteria;Proteobacteria;Alphaproteobacteria;Rhodospirillales;Azospirillaceae;Skermanella;Skermanella aerolata;Skermanella aerolata KACC 11604 |
| Ga0586324 8014833 3 506 | 0.00 | 1196800.00 | 0.00 | 0.00 | 0.00 | 0.00 | 0.00 | 0.00 | 0.00 | 0.00 | 0.00 | 1665000.00 | | hypothetical protein | Bacteria;Bacteroidetes;Sphingobacteriia;Sphingobacteriales;Sphingobacteriaceae;Mucilaginibacter;Mucilaginibacter sp. OV119;Mucilaginibacter sp. OV119 |
| Ga0586324 8039342 1 507 | 1860000.00 | 13736000.00 | 27418100.00 | 25460000.00 | 15553000.00 | 18976600.00 | 0.00 | 6100000.00 | 6868800.00 | 18480000.00 | 24472800.00 | 23835000.00 | | Ca2+-binding RTX toxin-like protein | Bacteria;Proteobacteria;Betaproteobacteria;Burkholderiales;Comamonadaceae;Acidovorax;Acidovorax sp. JS42;Acidovorax sp. JS42 |
| Ga0586324 8040324 3 506 | 0.00 | 0.00 | 0.00 | 0.00 | 0.00 | 0.00 | 0.00 | 0.00 | 0.00 | 0.00 | 3854400.00 | 0.00 | | TonB-dependent SusC/RagA subfamily outer membrane receptor | Bacteria;Bacteroidetes;Bacteroidia;Marinilabiliales;Prolixibacteraceae;Draconibacterium;Draconibacterium sediminis;Draconibacterium sediminis JN14CK-3 |
| Ga0586324 8053228 1 507 | 7060000.00 | 258414.28 | 1404397.87 | 0.00 | 0.00 | 0.00 | 9895200.00 | 366074.42 | 24440598.18 | 12940696.23 | 2883726.56 | 0.00 | | predicted porin | |
| Ga0586324 8066310 3 506 | 0.00 | 0.00 | 0.00 | 0.00 | 0.00 | 0.00 | 0.00 | 0.00 | 0.00 | 0.00 | 0.00 | 2475000.00 | | hypothetical protein | |
| Ga0586324 8066638 3 506 | 2060000.00 | 2427600.00 | 5547008.17 | 6403000.00 | 3378400.00 | 6431400.00 | 0.00 | 0.00 | 3029400.00 | 5621000.00 | 6837600.00 | 3517500.00 | | hypothetical protein | Bacteria;Proteobacteria;Alphaproteobacteria;Hyphomicrobiales;Roseiarcaceae;Roseiarcus;Roseiarcus fermentans;Roseiarcus fermentans DSM 24875 |
| Ga0586324 8074151 2 166 | 0.00 | 0.00 | 0.00 | 0.00 | 0.00 | 0.00 | 0.00 | 0.00 | 0.00 | 258130.18 | 0.00 | 0.00 | | hypothetical protein | |
| Ga0586324 8076464 182 505 | 0.00 | 0.00 | 0.00 | 0.00 | 0.00 | 0.00 | 0.00 | 0.00 | 1466100.00 | 0.00 | 0.00 | 0.00 | | plastocyanin | Archaea;Euryarchaeota;Methanomicrobia;Methanosarcinales;Methanosarcinaceae;Methanosarcina;Methanosarcina sp. DSM 11855;Methanosarcina sp. DSM 11855 |
| Ga0586324 8091791 262 504 | 1730000.00 | 1169600.00 | 0.00 | 0.00 | 0.00 | 0.00 | 0.00 | 0.00 | 0.00 | 0.00 | 0.00 | 0.00 | | hypothetical protein | |
| Ga0586324 8107284 3 506 | 0.00 | 2305200.00 | 0.00 | 0.00 | 0.00 | 0.00 | 0.00 | 0.00 | 0.00 | 0.00 | 0.00 | 0.00 | | hypothetical protein | Bacteria;Bacteroidetes;Chitinophagia;Chitinophagales;Chitinophagaceae;Sediminibacterium;Sediminibacterium magnilacihabitans;Vibrionimonas magnilacihabitans DSM 22423 |
| Ga0586324 8134037 1 504 | 0.00 | 0.00 | 0.00 | 0.00 | 0.00 | 2196886.84 | 0.00 | 0.00 | 0.00 | 0.00 | 0.00 | 0.00 | | uncharacterized protein (DUF2252 family) | Bacteria;Actinobacteria;Actinomycetia;Micrococcales;Cellulomonadaceae;Cellulomonas;Cellulomonas sp. HLT2-17;Cellulomonas sp. HLT2-17 |
| Ga0586324 8168256 2 502 | 0.00 | 863600.00 | 0.00 | 0.00 | 0.00 | 0.00 | 0.00 | 0.00 | 0.00 | 0.00 | 0.00 | 0.00 | | hypothetical protein | |
| Ga0586324 8173697 1 504 | 0.00 | 0.00 | 0.00 | 0.00 | 0.00 | 0.00 | 0.00 | 0.00 | 0.00 | 0.00 | 6415200.00 | 0.00 | | predicted porin | Bacteria;Proteobacteria;Betaproteobacteria;Burkholderiales;Comamonadaceae;Polaromonas;Polaromonas jejuensis;Polaromonas jejuensis NBRC 106434 |
| Ga0586324 8180559 2 502 | 874759.00 | 1700000.00 | 1990908.75 | 0.00 | 0.00 | 0.00 | 0.00 | 0.00 | 0.00 | 0.00 | 0.00 | 0.00 | | hypothetical protein | Bacteria;Bacteroidetes;Chitinophagia;Chitinophagales;Chitinophagaceae;Sediminibacterium;Sediminibacterium magnilacihabitans;Vibrionimonas magnilacihabitans DSM 22423 |
| Ga0586324 8188900 3 503 | 0.00 | 1258000.00 | 0.00 | 0.00 | 0.00 | 0.00 | 0.00 | 0.00 | 0.00 | 0.00 | 0.00 | 0.00 | | hypothetical protein | Bacteria;Proteobacteria;Betaproteobacteria;Nitrosomonadales;Sulfuricellaceae;Sulfurirhabdus;Sulfurirhabdus autotrophica;Sulfurirhabdus autotrophica DSM 100309 |
| Ga0586324 8191844 3 503 | 0.00 | 0.00 | 0.00 | 0.00 | 0.00 | 0.00 | 1723011.96 | 0.00 | 0.00 | 0.00 | 0.00 | 0.00 | | predicted porin | |
| Ga0586324 8192136 3 503 | 0.00 | 0.00 | 10865900.00 | 6251000.00 | 1390500.00 | 6550500.00 | 0.00 | 0.00 | 3037500.00 | 750977.15 | 0.00 | 0.00 | | outer membrane protein OmpA-like peptidoglycan-associated protein | Bacteria;Proteobacteria;Gammaproteobacteria;Alteromonadales;Alteromonadaceae;unclassified Alteromonadaceae;Alteromonadaceae bacterium 2719K.STB50.0a.01;Alteromonadaceae bacterium 2719K.STB50.0a.01 |
| Ga0586324 8209022 3 431 | 0.00 | 0.00 | 0.00 | 0.00 | 0.00 | 0.00 | 0.00 | 719800.00 | 0.00 | 0.00 | 0.00 | 0.00 | | regulator of protease activity HflC (stomatin/prohibitin superfamily) | Bacteria;Proteobacteria;Deltaproteobacteria;Desulfuromonadales;Geobacteraceae;Geobacter;Geobacter metallireducens;Geobacter metallireducens GS-15 |
| Ga0586324 8223907 73 501 | 0.00 | 0.00 | 0.00 | 0.00 | 2678000.00 | 0.00 | 0.00 | 3104900.00 | 3790800.00 | 3957800.00 | 0.00 | 3097500.00 | | hypothetical protein | Bacteria;Proteobacteria;Gammaproteobacteria;Methylococcales;Methylococcaceae;Methylosarcina;Methylosarcina fibrata;Methylosarcina fibrata AML-C10 |
| Ga0586324 8226485 3 503 | 0.00 | 0.00 | 0.00 | 3562500.00 | 4233300.00 | 0.00 | 8527200.00 | 4995900.00 | 5864400.00 | 4897200.00 | 5068800.00 | 0.00 | | hypothetical protein | |
| Ga0586324 8237060 247 501 | 0.00 | 0.00 | 0.00 | 0.00 | 0.00 | 0.00 | 0.00 | 0.00 | 2235600.00 | 4211900.00 | 4699200.00 | 2707500.00 | | hypothetical protein | Bacteria;Planctomycetes;Planctomycetia;Pirellulales;Pirellulaceae;Rhodopirellula;Rhodopirellula sp. SWK7;Rhodopirellula sp. SWK7 |
| Ga0586324 8262125 1 501 | 6710000.00 | 4515200.00 | 0.00 | 0.00 | 566619.48 | 2285012.90 | 0.00 | 2531500.00 | 2405700.00 | 31493000.00 | 353019.04 | 0.00 | | hypothetical protein | Bacteria;Verrucomicrobia;Verrucomicrobiae;Verrucomicrobiales;Verrucomicrobia subdivision 3;Pedosphaera;Pedosphaera parvula;Pedosphaera parvula Ellin514 |
| Ga0586324 8276734 48 500 | 2470000.00 | 0.00 | 0.00 | 0.00 | 0.00 | 0.00 | 0.00 | 0.00 | 0.00 | 0.00 | 0.00 | 0.00 | | Spy/CpxP family protein refolding chaperone | Bacteria;Planctomycetes;Planctomycetia;Gemmatales;Gemmataceae;Fimbriiglobus;Fimbriiglobus ruber;Fimbriiglobus ruber SP5 |
| Ga0586324 8278434 1 501 | 0.00 | 0.00 | 0.00 | 0.00 | 0.00 | 0.00 | 1581068.28 | 0.00 | 0.00 | 0.00 | 0.00 | 0.00 | | cytochrome c-type biogenesis protein CcmH/NrfG | Bacteria;Cyanobacteria;unclassified Cyanobacteria;Nostocales;Fortieaceae;Aulosira;Aulosira laxa;Aulosira laxa NIES-50 |
| Ga0586324 8302785 1 501 | 20540000.00 | 24364400.00 | 6024100.00 | 14630000.00 | 16191600.00 | 3822050.01 | 2325600.00 | 20636300.00 | 8861400.00 | 24817100.00 | 9398400.00 | 15202500.00 | | hypothetical protein | Bacteria;Proteobacteria;Gammaproteobacteria;Alteromonadales;Alteromonadaceae;Marinobacter;Marinobacter sp.;Marinobacter sp. MIT1353 |
| Ga0586324 8309713 403 498 | 0.00 | 0.00 | 0.00 | 0.00 | 0.00 | 0.00 | 0.00 | 0.00 | 0.00 | 0.00 | 0.00 | 4320000.00 | | hypothetical protein | |
| Ga0586324 8315782 2 499 | 0.00 | 0.00 | 787484.99 | 0.00 | 0.00 | 0.00 | 4751923.56 | 319702.83 | 0.00 | 0.00 | 608108.16 | 0.00 | | hypothetical protein | Bacteria;Proteobacteria;Alphaproteobacteria;Hyphomicrobiales;Bradyrhizobiaceae;Bradyrhizobium;Bradyrhizobium sp. Ai1a-2;Bradyrhizobium sp. Ai1a-2 |
| Ga0586324 8321491 2 499 | 0.00 | 0.00 | 0.00 | 0.00 | 0.00 | 0.00 | 0.00 | 0.00 | 0.00 | 5544000.00 | 0.00 | 0.00 | | predicted porin | Bacteria;Proteobacteria;Gammaproteobacteria;Acidiferrobacterales;Acidiferrobacteraceae;Sulfuricaulis;Sulfuricaulis limicola;Sulfuricaulis limicola HA5 |
| Ga0586324 8334022 10 498 | 0.00 | 0.00 | 0.00 | 0.00 | 0.00 | 0.00 | 0.00 | 0.00 | 0.00 | 0.00 | 0.00 | 817500.00 | | rhodanese-related sulfurtransferase | Bacteria;Proteobacteria;Betaproteobacteria;Nitrosomonadales;Sulfuricellaceae;Sulfuricella;Sulfuricella sp. T08;Sulfuricella sp. T08 |
| Ga0586324 8336218 2 499 | 0.00 | 0.00 | 0.00 | 0.00 | 0.00 | 0.00 | 0.00 | 0.00 | 0.00 | 2063600.00 | 141033.20 | 0.00 | | AcrR family transcriptional regulator | Bacteria;Actinobacteria;Nitriliruptoria;Egibacterales;Egibacteraceae;Egibacter;Egibacter rhizosphaerae;Egibacter rhizosphaerae EGI 80759 |
| Ga0586324 8336674 136 498 | 0.00 | 0.00 | 0.00 | 0.00 | 0.00 | 10401400.00 | 0.00 | 0.00 | 0.00 | 0.00 | 0.00 | 0.00 | | hypothetical protein | Bacteria;Proteobacteria;Deltaproteobacteria;Desulfuromonadales;Geobacteraceae;Geobacter;Geobacter lovleyi;Geobacter lovleyi SZ |

Supplementary Table 3. Pairwise comparison of key functional proteins identified by metaproteomics.

| KO number | Description | Comparison | | | | | | | | | |
|---|---|---|---|---|---|---|---|---|---|---|---|
| | | S-O- vs. S+O- | | S-O+vs. S+O+ | | S-O- vs. S-O+ | | S+O- vs. S+O+ | | S-O- vs. S+O+ | |
| | | log2FoldChange | *padj* | log2FoldChange | *padj* | log2FoldChange | *padj* | log2FoldChange | *padj* | log2FoldChange | *padj* |
| K00437 | [NiFe] hydrogenase large subunit | 1.67 | 0.79 | 68633.75 | 0.86 | 0.15 | 0.98 | -1.00 | 0.78 | 0.71 | 0.85 |
| K00674 | 2,3,4,5-tetrahydropyridine-2-carboxylate N-succinyltransferase | -17.58 | 0.00 | 23009.43 | 0.58 | -0.16 | 0.15 | 29.44 | 0.00 | -1.25 | 0.69 |
| K01834 | 2,3-bisphosphoglycerate-dependent phosphoglycerate mutase | -19.25 | 0.00 | 20300.91 | 0.00 | -1.43 | 0.67 | NA | NA | -19.16 | 0.00 |
| K15633 | 2,3-bisphosphoglycerate-independent phosphoglycerate mutase | NA | NA | 46407.24 | 0.00 | 17.31 | 0.00 | 18.56 | 0.00 | 18.48 | 0.00 |
| K15635 | 2,3-bisphosphoglycerate-independent phosphoglycerate mutase | -0.45 | 0.00 | 2885.15 | 0.00 | -15.96 | 0.00 | -0.34 | 0.00 | -0.83 | 0.00 |
| K06178 | 23S rRNA pseudouridine2605 synthase | -16.38 | 0.00 | 29163.57 | 0.00 | 1.89 | 0.00 | NA | NA | -16.29 | 0.00 |
| K00065 | 2-deoxy-D-gluconate 3-dehydrogenase | -18.40 | 0.00 | 0.00 | NA | -18.42 | 0.00 | NA | NA | -18.31 | 0.00 |
| K00042 | 2-hydroxy-3-oxopropionate reductase | -0.39 | 0.36 | 58576.80 | 0.21 | 0.09 | 0.95 | -0.65 | 0.51 | -1.10 | 0.26 |
| K12261 | 2-hydroxyacyl-CoA lyase 1 | 16.14 | 0.00 | 0.00 | NA | NA | NA | -16.07 | 0.00 | NA | NA |
| K01649 | 2-isopropylmalate synthase | 15.79 | 0.00 | 42747.62 | 0.53 | 17.60 | 0.00 | 2.40 | 0.00 | 18.14 | 0.00 |
| K00658 | 2-oxoglutarate dehydrogenase E2 component (dihydrolipoamide succinyltransferase) | 1.19 | 0.76 | 9580.01 | 0.00 | -30.00 | 0.00 | -2.06 | 0.00 | -0.85 | 0.80 |
| K00174 | 2-oxoglutarate ferredoxin oxidoreductase subunit alpha | NA | NA | 10633.54 | 0.00 | NA | NA | 17.02 | 0.00 | 16.94 | 0.00 |
| K11381 | 2-oxoisovalerate dehydrogenase E1 component | -16.05 | 0.00 | 0.00 | NA | -16.07 | 0.00 | NA | NA | -15.96 | 0.00 |
| K14652 | 3,4-dihydroxy 2-butanone 4-phosphate synthase/GTP cyclohydrolase II | NA | NA | 17881.60 | 0.00 | 29.70 | 0.00 | NA | NA | NA | NA |
| K03786 | 3-dehydroquinate dehydratase-2 | NA | NA | 7952.36 | 0.00 | NA | NA | 29.63 | 0.00 | 29.55 | 0.00 |
| K03856 | 3-deoxy-7-phosphoheptulonate synthase | 18.56 | 0.00 | 10601.55 | 0.00 | 15.51 | 0.00 | -2.38 | 0.00 | 16.15 | 0.00 |
| K08093 | 3-hexulose-6-phosphate synthase | -2.17 | 0.48 | 367544.63 | 0.47 | -0.31 | 0.95 | 4.03 | 0.15 | 1.86 | 0.07 |
| K00074 | 3-hydroxybutyryl-CoA dehydrogenase | -0.23 | 0.96 | 41052.80 | 0.84 | -0.85 | 0.82 | 0.24 | 0.97 | -0.03 | 1.00 |
| K00052 | 3-isopropylmalate dehydrogenase | 16.53 | 0.00 | 12634.16 | 0.00 | 17.01 | 0.00 | -16.46 | 0.00 | NA | NA |
| K00606 | 3-methyl-2-oxobutanoate hydroxymethyltransferase | NA | NA | 14254.83 | 0.00 | 17.19 | 0.00 | NA | NA | NA | NA |
| K01969 | 3-methylcrotonyl-CoA carboxylase beta subunit | 0.90 | 0.00 | 16300.01 | 0.93 | 1.13 | 0.00 | 0.63 | 0.87 | 1.54 | 0.62 |
| K00059 | 3-oxoacyl-[acyl-carrier protein] reductase | 0.41 | 0.93 | 59235.58 | 0.95 | 0.08 | 0.97 | 0.08 | 0.98 | 0.44 | 0.79 |
| K01607 | 4-carboxymuconolactone decarboxylase | -1.39 | 0.72 | 24044.72 | NA | -2.76 | 0.39 | -0.80 | 0.83 | -2.26 | 0.00 |
| K00205 | 4Fe-4S ferredoxin | NA | NA | 57219.30 | 0.00 | 19.19 | 0.00 | NA | NA | NA | NA |
| K14534 | 4-hydroxybutyryl-CoA dehydratase/vinylacetyl-CoA-Delta-isomerase | 0.12 | 0.98 | 147455.78 | NA | 1.60 | 0.67 | -19.08 | 0.00 | -30.00 | 0.00 |
| K00215 | 4-hydroxy-tetrahydrodipicolinate reductase | 1.36 | 0.00 | 9182.22 | 0.00 | -16.53 | 0.00 | -1.06 | 0.00 | 0.27 | 0.06 |
| K01714 | 4-hydroxy-tetrahydrodipicolinate synthase | -0.15 | 0.97 | 102190.62 | 0.18 | 1.15 | 0.12 | 0.61 | 0.88 | 0.39 | 0.74 |
| K01588 | 5-(carboxyamino)imidazole ribonucleotide mutase | NA | NA | 9649.46 | 0.00 | NA | NA | 16.88 | 0.00 | 16.80 | 0.00 |
| K00320 | 5,10-methylenetetrahydromethanopterin reductase | 0.03 | 0.97 | 4523272.16 | 0.00 | -0.42 | 0.35 | -1.29 | 0.00 | -1.29 | 0.01 |
| K10713 | 5,6,7,8-tetrahydromethanopterin hydro-lyase | -0.34 | 0.70 | 909390.17 | 0.91 | -0.69 | 0.38 | -0.05 | 0.87 | -0.46 | 0.54 |
| K15023 | 5-methyltetrahydrofolate corrinoid/iron sulfur protein methyltransferase | 1.55 | 0.00 | 84015.35 | 0.82 | -0.09 | 0.89 | -1.92 | 0.53 | -0.45 | 0.91 |
| K00548 | 5-methyltetrahydrofolate--homocysteine methyltransferase | -16.26 | 0.00 | 69140.96 | 0.67 | 2.73 | 0.40 | 30.00 | 0.00 | 1.44 | 0.67 |
| K07538 | 6-hydroxycyclohex-1-ene-1-carbonyl-CoA dehydrogenase | -0.12 | 0.98 | 56005.69 | NA | 2.41 | NA | -3.41 | 0.00 | -3.52 | 0.16 |
| K07539 | 6-oxo-cyclohex-1-ene-carbonyl-CoA hydrolase | -1.77 | 0.02 | 117952.20 | 0.59 | -1.54 | 0.15 | 0.88 | 0.00 | -0.93 | 0.26 |
| K00033 | 6-phosphogluconate dehydrogenase | -30.00 | 0.00 | 40453.04 | 0.82 | 0.91 | 0.88 | 18.19 | 0.00 | 1.51 | 0.63 |
| K01990 | ABC-2 type transport system ATP-binding protein | NA | NA | 31428.41 | 0.59 | 17.82 | 0.00 | 29.37 | 0.00 | 29.81 | 0.00 |
| K00925 | acetate kinase | 19.07 | 0.00 | 1887.48 | 0.00 | 14.27 | 0.00 | -19.00 | 0.00 | NA | NA |
| K00023 | acetoacetyl-CoA reductase | 0.77 | 0.91 | 26741.58 | 0.01 | -1.78 | 0.58 | -0.44 | 0.91 | 0.26 | 0.95 |
| K01907 | acetoacetyl-CoA synthetase | 18.37 | 0.00 | 28185.40 | 0.00 | NA | NA | 0.01 | 0.92 | 18.35 | 0.00 |
| K04767 | acetoin utilization protein AcuB | 19.76 | 0.00 | 0.00 | NA | NA | NA | -19.69 | 0.00 | NA | NA |
| K01652 | acetolactate synthase-1/2/3 large subunit | 1.10 | 0.86 | 87888.38 | 0.71 | 1.96 | 0.54 | 1.42 | 0.68 | 2.46 | 0.42 |
| K01653 | acetolactate synthase-1/3 small subunit | 1.30 | 0.67 | 0.00 | NA | -28.52 | 0.00 | -16.17 | 0.00 | -28.58 | 0.00 |
| K00632 | acetyl-CoA acyltransferase | NA | NA | 19334.13 | 0.00 | 17.63 | 0.00 | NA | NA | NA | NA |
| K00626 | acetyl-CoA C-acetyltransferase | 0.38 | 0.76 | 273841.16 | 0.01 | 1.77 | 0.03 | -0.68 | 0.56 | -0.32 | 0.78 |
| K00192 | acetyl-CoA decarbonylase/synthase complex subunit alpha | -0.53 | 0.74 | 448709.56 | 0.01 | -0.55 | 0.11 | -1.75 | 0.17 | -2.24 | 0.00 |
| K00193 | acetyl-CoA decarbonylase/synthase complex subunit beta | -0.27 | 0.63 | 2206607.44 | 0.00 | -0.71 | 0.11 | -1.07 | 0.00 | -1.36 | 0.00 |
| K00194 | acetyl-CoA decarbonylase/synthase complex subunit delta | 0.03 | 0.96 | 805307.40 | 0.00 | -0.25 | 0.59 | -1.08 | 0.00 | -1.05 | 0.00 |
| K00195 | acetyl-CoA decarbonylase/synthase complex subunit epsilon | 1.05 | 0.01 | 14231.50 | 0.00 | -0.41 | 0.12 | -18.57 | 0.00 | -17.55 | 0.00 |
| K00197 | acetyl-CoA decarbonylase/synthase complex subunit gamma | 1.22 | 0.00 | 1365269.44 | 0.00 | 0.56 | 0.13 | -1.40 | 0.00 | -0.18 | 0.68 |
| K14138 | acetyl-CoA synthase | 1.53 | 0.00 | 1343606.58 | 0.04 | 0.26 | 0.30 | -1.90 | 0.00 | -0.41 | 0.33 |
| K01895 | acetyl-CoA synthetase | -0.48 | 0.07 | 5519292.35 | 0.00 | -0.66 | 0.01 | -0.75 | 0.00 | -1.28 | 0.00 |
| K00930 | acetylglutamate kinase | -15.97 | 0.00 | 14762.30 | 0.48 | 0.14 | 0.57 | 16.66 | 0.00 | 0.69 | 0.28 |
| K00821 | acetylornithine/N-succinyldiaminopimelate aminotransferase | NA | NA | 2592.72 | 0.00 | NA | NA | 14.99 | 0.00 | 14.91 | 0.00 |
| K01681 | aconitate hydratase | -15.67 | 0.00 | 57195.49 | 0.01 | 3.50 | 0.02 | 16.01 | 0.00 | 0.31 | 0.03 |
| K01682 | aconitate hydratase 2/2-methylisocitrate dehydratase | 1.57 | 0.05 | 43651.23 | 0.88 | -0.25 | 0.96 | -2.03 | 0.00 | -0.51 | 0.60 |
| K01251 | adenosylhomocysteinase | -1.82 | 0.01 | 266423.68 | 0.21 | -0.32 | 0.66 | 1.17 | 0.07 | -0.69 | 0.10 |
| K00939 | adenylate kinase | 1.02 | 0.12 | 1790168.21 | 0.00 | 1.80 | 0.08 | -1.91 | 0.00 | -0.96 | 0.21 |
| K01756 | adenylosuccinate lyase | -17.52 | 0.00 | 25839.07 | 0.00 | 0.58 | 0.00 | NA | NA | -17.43 | 0.00 |
| K01939 | adenylosuccinate synthase | -1.78 | 0.57 | 46535.58 | 0.81 | 0.05 | 0.94 | 1.41 | NA | -0.41 | 0.92 |
| K00394 | adenylylsulfate reductase subunit A | 1.18 | 0.00 | 11718565.35 | 0.20 | 0.52 | 0.00 | -0.89 | 0.01 | 0.26 | 0.43 |
| K00395 | adenylylsulfate reductase subunit B | -0.22 | 0.68 | 773444.02 | 0.79 | -0.37 | 0.61 | 0.13 | 0.49 | -0.13 | 0.78 |
| K12543 | adhesin transport system outer membrane protein | 0.57 | 0.89 | 324668.98 | 0.02 | 2.49 | 0.43 | 4.32 | 0.00 | 4.80 | 0.06 |
| K03274 | ADP-L-glycero-D-manno-heptose 6-epimerase | NA | NA | 5959.28 | 0.00 | NA | NA | 16.19 | 0.00 | 16.11 | 0.00 |
| K01872 | alanyl-tRNA synthetase | -1.10 | 0.00 | 69121.25 | 0.62 | -0.93 | 0.80 | 1.62 | 0.07 | 0.47 | 0.68 |
| K00114 | alcohol dehydrogenase (cytochrome c) | -1.16 | 0.76 | 45572.82 | 0.50 | -1.23 | 0.72 | 1.77 | 0.57 | 0.66 | 0.92 |
| K00138 | aldehyde dehydrogenase | -0.41 | 0.92 | 14858.45 | 0.00 | -0.89 | 0.00 | -30.00 | 0.00 | -18.11 | 0.00 |
| K00128 | aldehyde dehydrogenase (NAD+) | 17.44 | 0.00 | 3253.71 | 0.00 | 15.06 | 0.00 | -17.37 | 0.00 | NA | NA |
| K03738 | aldehyde:ferredoxin oxidoreductase | -1.08 | 0.02 | 431771.64 | 0.13 | -1.50 | 0.00 | -0.52 | 0.24 | -1.63 | 0.00 |
| K10232 | alpha-glucoside transport system substrate-binding protein | -0.61 | 0.60 | 342959.91 | 0.03 | 0.15 | 0.91 | -0.26 | 0.80 | -0.89 | 0.32 |
| K01426 | amidase | -1.72 | 0.00 | 0.00 | NA | -17.95 | 0.00 | -16.08 | 0.00 | -17.84 | 0.00 |
| K00764 | amidophosphoribosyltransferase | NA | NA | 5004.02 | 0.00 | NA | NA | 15.93 | 0.00 | 15.86 | 0.00 |
| K03392 | aminocarboxymuconate-semialdehyde decarboxylase | 16.30 | 0.00 | 82962.21 | 0.00 | NA | NA | 3.63 | 0.00 | 19.91 | 0.00 |
| K00605 | aminomethyltransferase | -18.39 | 0.00 | 0.00 | NA | -18.41 | 0.00 | NA | NA | -18.30 | 0.00 |
| K22010 | AmiR/NasT family two-component response regulator | 0.30 | 0.01 | 44500.70 | 0.33 | 1.05 | 0.00 | -1.26 | 0.68 | -1.04 | 0.75 |
| K03320 | Amt family ammonium transporter | NA | NA | 53053.07 | 0.46 | 18.68 | 0.00 | 30.00 | 0.00 | 29.18 | 0.00 |
| K00766 | anthranilate phosphoribosyltransferase | NA | NA | 20838.11 | 0.00 | NA | NA | 17.99 | 0.00 | 17.91 | 0.00 |
| K04749 | anti-sigma B factor antagonist | -1.65 | 0.00 | 68161.33 | 0.00 | -19.60 | 0.00 | 1.78 | 0.00 | 0.09 | 0.61 |
| K01584 | arginine decarboxylase | NA | NA | 6366.49 | 0.00 | NA | NA | 29.32 | 0.00 | 29.23 | 0.00 |
| K01478 | arginine deiminase | 0.19 | 0.78 | 73415.94 | 0.00 | 0.58 | 0.00 | 1.23 | 0.01 | 1.39 | 0.00 |
| K01940 | argininosuccinate synthase | -16.68 | 0.00 | 51519.80 | 0.23 | 1.06 | 0.02 | 18.56 | 0.00 | 1.85 | 0.00 |
| K08356 | arsenite oxidase large subunit | NA | NA | 6560.22 | 0.00 | NA | NA | 16.33 | 0.00 | 16.25 | 0.00 |
| K01551 | arsenite-transporting ATPase | -17.66 | 0.00 | 0.00 | NA | -17.68 | 0.00 | NA | NA | -17.57 | 0.00 |
| K07289 | AsmA protein | NA | NA | 51741.31 | 0.00 | 17.83 | 0.00 | 18.50 | 0.00 | 18.42 | 0.00 |
| K09758 | aspartate 4-decarboxylase | NA | NA | 2647.87 | 0.00 | 27.93 | 0.00 | NA | NA | NA | NA |
| K00812 | aspartate aminotransferase | NA | NA | 4167.81 | 0.00 | NA | NA | 15.67 | 0.00 | 15.59 | 0.00 |
| K00609 | aspartate carbamoyltransferase catalytic subunit | -1.30 | 0.00 | 16564.48 | 0.80 | -1.06 | 0.75 | 1.32 | 0.68 | -0.08 | 0.98 |
| K00133 | aspartate-semialdehyde dehydrogenase | -15.17 | 0.00 | 18112.45 | 0.92 | 1.35 | 0.00 | 16.81 | 0.00 | 1.61 | 0.00 |
| K01876 | aspartyl-tRNA synthetase | -1.16 | 0.75 | 114823.83 | 0.35 | 1.10 | 0.27 | 3.09 | 0.25 | 1.89 | 0.02 |
| K02434 | aspartyl-tRNA(Asn)/glutamyl-tRNA(Gln) amidotransferase subunit B | -15.28 | 0.00 | 22631.47 | 0.00 | -15.30 | 0.00 | 18.11 | 0.00 | 2.80 | 0.00 |
| K03593 | ATP-binding protein involved in chromosome partitioning | 19.42 | 0.00 | 133753.19 | 0.82 | 30.00 | 0.00 | 0.53 | 0.90 | 30.00 | 0.00 |
| K03694 | ATP-dependent Clp protease ATP-binding subunit ClpA | 2.32 | 0.00 | 32193.63 | 0.31 | 0.77 | 0.43 | -0.41 | 0.04 | 1.88 | 0.00 |
| K03695 | ATP-dependent Clp protease ATP-binding subunit ClpB | 0.29 | 0.79 | 294904.69 | 0.74 | 0.59 | 0.54 | 0.63 | 0.21 | 0.90 | 0.09 |
| K03696 | ATP-dependent Clp protease ATP-binding subunit ClpC | -0.21 | NA | 50063.35 | 0.76 | -0.39 | 0.95 | 1.02 | 0.79 | 0.85 | 0.88 |
| K03544 | ATP-dependent Clp protease ATP-binding subunit ClpX | 30.00 | 0.00 | 108836.37 | 0.18 | 19.57 | 0.00 | -0.56 | 0.90 | 18.71 | 0.00 |
| K01358 | ATP-dependent Clp protease protease subunit | NA | NA | 11750.44 | 0.38 | 14.62 | 0.00 | 29.05 | 0.00 | 29.77 | 0.00 |
| K03667 | ATP-dependent HslUV protease ATP-binding subunit HslU | 20.63 | 0.00 | 183062.42 | 0.00 | NA | NA | 0.45 | 0.00 | 21.05 | 0.00 |
| K01338 | ATP-dependent Lon protease | -2.38 | 0.41 | 160979.77 | 0.54 | 1.72 | 0.76 | 5.76 | 0.00 | 3.26 | 0.26 |
| K21449 | autotransporter adhesin | 17.46 | 0.00 | 24964.60 | 0.13 | 17.47 | 0.00 | -1.01 | 0.23 | 16.43 | 0.00 |
| K03594 | bacterioferritin | 0.33 | 0.96 | 62446.80 | 0.63 | 0.78 | 0.84 | -0.49 | 0.93 | -0.21 | 0.97 |
| K07335 | basic membrane protein A | -0.36 | 0.53 | 585200.82 | 0.00 | -0.37 | 0.12 | -0.52 | 0.29 | -0.89 | 0.00 |
| K05349 | beta-glucosidase | -29.61 | 0.00 | 7025.19 | 0.00 | 0.18 | 0.96 | NA | NA | -29.66 | 0.00 |
| K03561 | biopolymer transport protein ExbB | 0.62 | 0.19 | 145016.19 | 0.82 | 0.50 | 0.51 | -0.15 | 0.91 | 0.42 | 0.69 |
| K03559 | biopolymer transport protein ExbD | -18.67 | 0.00 | 0.00 | NA | -18.69 | 0.00 | NA | NA | -18.59 | 0.00 |
| K00826 | branched-chain amino acid aminotransferase | 0.06 | 0.72 | 0.00 | NA | -17.57 | 0.00 | -17.49 | 0.00 | -17.46 | 0.00 |
| K01999 | branched-chain amino acid transport system substrate-binding protein | -0.59 | 0.05 | 4047015.01 | 0.87 | -0.54 | 0.05 | 0.22 | 0.27 | -0.38 | 0.18 |
| K00248 | butyryl-CoA dehydrogenase | 0.31 | 0.95 | 37262.59 | 0.94 | -0.26 | 0.95 | -0.56 | 0.92 | -0.23 | 0.95 |
| K11688 | C4-dicarboxylate-binding protein DctP | -18.29 | 0.00 | 20157.81 | 0.00 | -2.77 | 0.00 | 17.60 | 0.00 | -0.72 | 0.00 |
| K21449 | Ca2+-binding RTX toxin-like protein | 17.46 | 0.00 | 24964.60 | 0.13 | 17.47 | 0.00 | -1.01 | 0.23 | 16.43 | 0.00 |
| K01955 | carbamoyl-phosphate synthase large subunit | -15.37 | 0.00 | 48515.64 | 0.28 | 0.85 | 0.80 | 19.04 | 0.00 | 3.59 | 0.00 |
| K01673 | carbonic anhydrase | 2.08 | 0.02 | 552246.75 | NA | 2.93 | 0.00 | -0.56 | NA | 1.35 | NA |
| K00198 | carbon-monoxide dehydrogenase catalytic subunit | 1.09 | 0.02 | 1069467.30 | 0.00 | 0.19 | 0.82 | -2.24 | 0.00 | -1.20 | 0.01 |
| K03520 | carbon-monoxide dehydrogenase large subunit | 30.00 | 0.00 | 63196.23 | 0.08 | 17.39 | 0.00 | 2.06 | 0.49 | 19.07 | 0.00 |
| K03519 | carbon-monoxide dehydrogenase medium subunit | 29.85 | 0.00 | 43666.24 | 0.43 | 29.75 | 0.00 | 1.84 | 0.53 | 18.61 | 0.00 |
| K03797 | carboxyl-terminal processing protease | -30.00 | 0.00 | 83398.12 | 0.09 | -0.39 | 0.94 | 19.54 | 0.00 | 1.21 | 0.72 |
| K01061 | carboxymethylenebutenolidase | NA | NA | 21367.65 | 0.00 | NA | NA | 18.04 | 0.00 | 17.95 | 0.00 |
| K03782 | catalase-peroxidase | -0.71 | 0.62 | 252715.64 | 0.99 | -0.32 | 0.64 | 0.72 | NA | -0.09 | NA |
| K14393 | cation/acetate symporter | NA | NA | 226209.75 | 0.52 | 20.40 | 0.00 | 20.10 | 0.00 | 20.06 | 0.00 |
| K03798 | cell division protease FtsH | -3.74 | 0.18 | 71900.41 | 0.11 | -0.74 | NA | -2.50 | 0.57 | -6.28 | 0.00 |
| K03531 | cell division protein FtsZ | -0.28 | 0.80 | 40560.85 | 0.90 | -1.32 | 0.71 | -1.04 | 0.11 | -1.36 | 0.00 |
| K04077 | chaperonin GroEL | 0.43 | 0.08 | 64384886.01 | 0.99 | 0.41 | 0.14 | 0.18 | 0.14 | 0.57 | 0.05 |
| K22447 | chaperonin GroEL (HSP60 family) | 0.13 | 0.89 | 1232062.84 | 0.03 | -0.02 | 0.98 | -0.65 | 0.14 | -0.51 | 0.36 |
| K04078 | chaperonin GroES | -0.30 | 0.89 | 188508.31 | 0.09 | -3.93 | 0.00 | -2.33 | 0.00 | -2.71 | 0.02 |
| K06596 | chemosensory pili system protein ChpA (sensor histidine kinase/response regulator) | NA | NA | 27747.04 | 0.04 | 13.25 | 0.00 | 30.00 | 0.00 | 30.00 | 0.00 |
| K02557 | chemotaxis protein MotB | 3.28 | 0.16 | 11345.38 | 0.00 | -28.70 | 0.00 | -1.16 | 0.72 | 2.19 | 0.64 |
| K03529 | chromosome segregation protein | NA | NA | 18616.18 | 0.93 | 16.67 | 0.00 | 29.72 | 0.00 | 28.69 | 0.00 |
| K01644 | citrate lyase subunit beta/citryl-CoA lyase | -0.90 | 0.83 | 19368.02 | 0.69 | -2.51 | 0.38 | -0.56 | 0.89 | -1.48 | 0.12 |
| K01647 | citrate synthase | 1.63 | 0.80 | 250683.15 | 0.02 | 2.39 | 0.49 | -1.29 | 0.71 | 0.31 | 0.94 |
| K11645 | class I fructose-bisphosphate aldolase | -0.21 | 0.98 | 128957.75 | NA | 0.70 | 0.87 | -0.16 | 0.98 | -0.35 | 0.95 |
| K00440 | coenzyme F420 hydrogenase subunit alpha | 1.62 | 0.19 | 662512.69 | 0.95 | 0.43 | 0.80 | -1.06 | 0.12 | 0.58 | 0.70 |
| K00441 | coenzyme F420 hydrogenase subunit beta | -19.34 | 0.00 | 0.00 | NA | -19.36 | 0.00 | NA | NA | -19.25 | 0.00 |
| K00443 | coenzyme F420 hydrogenase subunit gamma | NA | NA | 11766.91 | 0.00 | NA | NA | 17.17 | 0.00 | 17.09 | 0.00 |
| K13195 | cold-inducible RNA-binding protein | -0.22 | 0.97 | 59620.21 | 0.06 | 0.64 | 0.87 | 1.36 | 0.67 | 1.05 | 0.76 |
| K03704 | CspA family cold shock protein | 2.58 | 0.00 | 24341.35 | 0.99 | 2.39 | 0.41 | -0.09 | 0.98 | 2.42 | 0.38 |
| K01937 | CTP synthase | NA | NA | 6637.27 | 0.00 | NA | NA | 29.35 | 0.00 | 29.31 | 0.00 |
| K07537 | cyclohexa-1,5-dienecarbonyl-CoA hydratase | 27.28 | 0.00 | 1152.14 | 0.00 | 13.49 | 0.00 | -28.52 | 0.00 | NA | NA |
| K04487 | cysteine desulfurase | NA | NA | 61592.04 | 0.84 | 30.00 | 0.00 | 18.30 | 0.00 | 18.24 | 0.00 |
| K01738 | cysteine synthase A | 18.73 | 0.00 | 10232.15 | 0.00 | 15.45 | 0.00 | -2.59 | 0.00 | 16.11 | 0.00 |
| K00405 | cytochrome c oxidase cbb3-type subunit 2 | NA | NA | 49636.26 | 0.00 | NA | NA | 19.25 | 0.00 | 19.17 | 0.00 |

| | | | | | | | | | | | |
|---|---|---|---|---|---|---|---|---|---|---|---|
| K00406 | cytochrome c oxidase cbb3-type subunit 3 | NA | NA | 6475.65 | 0.00 | NA | NA | 16.31 | 0.00 | 16.23 | 0.00 |
| K02275 | cytochrome c oxidase subunit 2 | 18.89 | 0.00 | 24583.71 | 0.74 | 29.22 | NA | -1.25 | 0.70 | 30.00 | 0.00 |
| K00428 | cytochrome c peroxidase | 0.02 | 0.97 | 340352.05 | 0.39 | -0.90 | 0.14 | -1.18 | 0.00 | -1.20 | 0.00 |
| K00058 | D-3-phosphoglycerate dehydrogenase | 1.29 | 0.75 | 81522.07 | 0.00 | 0.37 | 0.94 | -2.67 | 0.00 | -1.47 | 0.67 |
| K00809 | deoxyhypusine synthase | -16.50 | 0.00 | 0.00 | NA | -16.52 | 0.00 | NA | NA | -16.41 | 0.00 |
| K03273 | D-glycero-D-manno-heptose 1,7-bisphosphate phosphatase | NA | NA | 4452.22 | 0.00 | NA | NA | 15.77 | 0.00 | 15.69 | 0.00 |
| K00382 | dihydrolipoamide dehydrogenase | -14.87 | 0.00 | 69561.26 | 0.00 | 3.30 | 0.00 | 19.01 | 0.00 | 4.11 | 0.00 |
| K05878 | dihydroxyacetone kinase-like protein | 18.16 | 0.00 | 0.00 | NA | NA | NA | -18.09 | 0.00 | NA | NA |
| K01687 | dihydroxy-acid dehydratase | 19.30 | 0.00 | 22629.88 | 0.47 | 28.26 | 0.00 | -1.50 | 0.64 | 29.84 | 0.00 |
| K02469 | DNA gyrase subunit A | NA | NA | 13288.53 | 0.00 | NA | NA | 30.00 | 0.00 | 29.43 | 0.00 |
| K02470 | DNA gyrase subunit B | NA | NA | 6384.65 | 0.00 | 16.03 | 0.00 | NA | NA | NA | NA |
| K06915 | DNA helicase HerA-like ATPase | -3.21 | 0.22 | 45197.34 | 0.00 | 1.57 | 0.69 | -13.91 | 0.00 | -30.00 | 0.00 |
| K02342 | DNA polymerase III subunit epsilon | -18.59 | 0.00 | 2217.60 | 0.00 | -18.61 | 0.00 | 14.76 | 0.00 | -3.86 | 0.00 |
| K02338 | DNA polymerase-3 subunit beta | 0.91 | 0.89 | 110002.23 | 0.04 | 2.44 | 0.43 | 0.38 | 0.92 | 1.30 | 0.69 |
| K03168 | DNA topoisomerase-1 | NA | NA | 27706.35 | 0.00 | NA | NA | 18.40 | 0.00 | 18.33 | 0.00 |
| K03622 | DNA-binding protein | -30.00 | 0.00 | 98609.69 | 0.00 | -30.00 | 0.00 | 20.24 | 0.00 | 1.02 | 0.78 |
| K03746 | DNA-binding protein H-NS | 0.24 | 0.91 | 37522.96 | 0.00 | -19.67 | 0.00 | -1.06 | 0.77 | -0.90 | 0.81 |
| K03530 | DNA-binding protein HU-beta | 0.37 | 0.94 | 217314.38 | 0.07 | 0.91 | NA | -0.15 | 0.91 | 0.10 | 0.98 |
| K21900 | DNA-binding transcriptional LysR family regulator | NA | NA | 26015.61 | 0.00 | 16.10 | 0.00 | 17.88 | 0.00 | 17.80 | 0.00 |
| K03041 | DNA-directed RNA polymerase subunit A' | 1.24 | NA | 32466.74 | 0.00 | 0.90 | NA | -2.48 | 0.00 | -1.27 | 0.71 |
| K03042 | DNA-directed RNA polymerase subunit A" | 0.17 | 0.17 | 232214.33 | 0.00 | -0.41 | 0.00 | 3.81 | 0.00 | 3.95 | 0.00 |
| K03040 | DNA-directed RNA polymerase subunit alpha | 1.10 | 0.79 | 598083.81 | 0.22 | 1.45 | 0.69 | 0.95 | 0.00 | 2.07 | 0.51 |
| K03045 | DNA-directed RNA polymerase subunit B" | -19.24 | 0.00 | 0.00 | NA | -19.26 | 0.00 | NA | NA | -19.16 | 0.00 |
| K03043 | DNA-directed RNA polymerase subunit beta | 0.31 | 0.37 | 2290147.78 | 0.00 | 0.43 | 0.10 | 1.04 | 0.00 | 1.33 | 0.00 |
| K03046 | DNA-directed RNA polymerase subunit beta' | -0.50 | 0.37 | 2087422.55 | 0.16 | -0.31 | 0.62 | 0.83 | 0.00 | 0.33 | 0.50 |
| K03047 | DNA-directed RNA polymerase subunit D | -17.21 | 0.00 | 14842.50 | 0.80 | -1.15 | 0.72 | 16.79 | 0.00 | -0.48 | 0.56 |
| K10543 | D-xylose transport system substrate-binding protein | -2.02 | 0.00 | 1220505.49 | 0.00 | -1.71 | 0.00 | 1.31 | 0.00 | -0.76 | 0.00 |
| K03522 | electron transfer flavoprotein alpha subunit | 1.58 | 0.00 | 124881.64 | 0.24 | 0.79 | 0.23 | -3.34 | 0.21 | -1.83 | 0.56 |
| K03521 | electron transfer flavoprotein beta subunit | 2.24 | 0.00 | 21174.61 | 0.84 | 0.44 | 0.00 | -2.09 | 0.46 | 0.10 | 0.98 |
| K03231 | elongation factor 1-alpha | -0.74 | 0.02 | 439797.30 | 0.76 | -1.36 | 0.00 | -0.49 | 0.07 | -1.25 | 0.00 |
| K02355 | elongation factor G | -1.00 | 0.25 | 842099.09 | 0.12 | 1.60 | 0.04 | 3.13 | 0.00 | 2.16 | 0.00 |
| K02357 | elongation factor Ts | -30.00 | 0.00 | 46466.66 | 0.39 | -0.60 | 0.90 | 18.64 | 0.00 | 0.88 | 0.81 |
| K02358 | elongation factor Tu | 0.03 | 0.90 | 10971717.26 | 0.11 | 0.59 | 0.00 | 0.96 | 0.00 | 0.96 | 0.00 |
| K21449 | ELWxxDGT repeat protein | 17.46 | 0.00 | 24964.60 | 0.13 | 17.47 | 0.00 | -1.01 | 0.23 | 16.43 | 0.00 |
| K01689 | enolase | -0.68 | 0.46 | 421869.25 | 0.00 | -0.93 | 0.02 | 1.26 | 0.09 | 0.55 | 0.00 |
| K00208 | enoyl-[acyl-carrier protein] reductase I | -0.22 | 0.81 | 67836.30 | 0.89 | -0.01 | 0.98 | 0.02 | 1.00 | -0.24 | 0.95 |
| K04027 | ethanolamine utilization protein EutM | 0.34 | 0.85 | 89997.89 | 0.28 | -1.34 | 0.18 | -0.67 | 0.58 | -0.38 | 0.78 |
| K03701 | excinuclease ABC subunit A | 0.39 | 0.96 | 82729.60 | 0.28 | 1.59 | 0.64 | 2.41 | 0.42 | 2.83 | 0.33 |
| K14127 | F420-non-reducing hydrogenase iron-sulfur subunit | 15.50 | 0.00 | 948.94 | 0.00 | 13.28 | 0.00 | -15.43 | 0.00 | NA | NA |
| K14126 | F420-non-reducing hydrogenase large subunit | NA | NA | 89700.92 | 0.00 | 19.84 | 0.00 | NA | NA | NA | NA |
| K04564 | Fe-Mn family superoxide dismutase | -0.32 | 0.83 | 219392.75 | 0.44 | -0.22 | 0.74 | -0.01 | 0.99 | -0.39 | 0.63 |
| K00528 | ferredoxin--NADP+ reductase | NA | NA | 15043.38 | 0.00 | 17.26 | 0.00 | NA | NA | NA | NA |
| K02217 | ferritin | 1.05 | 0.00 | 39943.67 | 0.96 | 1.07 | 0.00 | 0.44 | 0.91 | 1.40 | 0.67 |
| K09013 | Fe-S cluster assembly ATP-binding protein | 29.13 | 0.00 | 23322.93 | 0.00 | 17.90 | 0.00 | -29.68 | 0.00 | NA | NA |
| K03773 | FKBP-type peptidyl-prolyl cis-trans isomerase FklB | NA | NA | 33428.08 | 0.00 | 18.42 | 0.00 | NA | NA | NA | NA |
| K02390 | flagellar hook protein FlgE | 18.50 | 0.00 | 24358.21 | 0.00 | NA | NA | -0.33 | 0.00 | 18.14 | 0.00 |
| K02406 | flagellin | -0.14 | 0.83 | 1508496.94 | 0.96 | 0.05 | 0.98 | 0.57 | 0.44 | 0.36 | 0.68 |
| K09165 | flavin-binding protein dodecin | 1.35 | 0.72 | 94477.65 | 0.95 | 0.90 | 0.82 | -0.35 | 0.92 | 0.92 | 0.88 |
| K00124 | formate dehydrogenase iron-sulfur subunit | 16.95 | 0.00 | 15087.93 | 0.00 | 17.27 | 0.00 | -16.88 | 0.00 | NA | NA |
| K00123 | formate dehydrogenase major subunit | NA | NA | 10047.73 | 0.00 | 16.68 | 0.00 | NA | NA | NA | NA |
| K00125 | formate dehydrogenase subunit beta | -1.88 | 0.62 | 77627.24 | 0.44 | -2.74 | 0.06 | 0.04 | 0.99 | -1.95 | 0.17 |
| K01938 | formate--tetrahydrofolate ligase | 0.77 | 0.24 | 1146473.89 | 0.17 | -0.25 | 0.80 | -0.12 | 0.87 | 0.62 | 0.33 |
| K00200 | formylmethanofuran dehydrogenase subunit A | -16.67 | 0.00 | 0.00 | NA | -16.69 | 0.00 | NA | NA | -16.58 | 0.00 |
| K00201 | formylmethanofuran dehydrogenase subunit B | -17.66 | 0.00 | 14175.68 | 0.40 | -2.51 | 0.35 | 17.07 | 0.00 | -0.62 | 0.00 |
| K00202 | formylmethanofuran dehydrogenase subunit C | -2.19 | 0.00 | 0.00 | NA | -18.62 | 0.00 | -16.29 | 0.00 | -18.51 | 0.00 |
| K10552 | fructose transport system substrate-binding protein | -16.23 | 0.00 | 9815.06 | 0.72 | -0.19 | 0.06 | 28.57 | 0.00 | -0.82 | 0.79 |
| K03841 | fructose-1,6-bisphosphatase I | 15.70 | 0.00 | 0.00 | NA | NA | NA | -15.63 | 0.00 | NA | NA |
| K01623 | fructose-bisphosphate aldolase class I | 16.48 | 0.00 | 8781.99 | 0.00 | 16.49 | 0.00 | -16.41 | 0.00 | NA | NA |
| K01624 | fructose-bisphosphate aldolase class II | -1.54 | 0.69 | 422674.15 | 0.41 | 1.14 | 0.40 | 2.36 | 0.42 | 0.74 | 0.58 |
| K22349 | FtsP/CotA-like multicopper oxidase with cupredoxin domain | NA | NA | 6351.87 | 0.00 | NA | NA | 16.28 | 0.00 | 16.20 | 0.00 |
| K02111 | F-type H+-transporting ATPase subunit alpha | 0.30 | 0.31 | 4601571.02 | 0.71 | -0.34 | 0.38 | -0.57 | 0.02 | -0.29 | 0.31 |
| K02109 | F-type H+-transporting ATPase subunit b | 1.42 | 0.05 | 95604.74 | 0.44 | 0.80 | 0.05 | -0.20 | 0.86 | 1.20 | 0.00 |
| K02112 | F-type H+-transporting ATPase subunit beta | 0.67 | 0.00 | 4612297.97 | 0.01 | 0.60 | 0.00 | -0.39 | 0.09 | 0.27 | 0.37 |
| K02113 | F-type H+-transporting ATPase subunit delta | -1.26 | 0.72 | 16118.48 | 0.79 | -1.77 | 0.56 | 0.19 | 0.96 | -1.08 | 0.00 |
| K02114 | F-type H+-transporting ATPase subunit epsilon | 19.38 | 0.00 | 0.00 | NA | NA | NA | -19.31 | 0.00 | NA | NA |
| K02115 | F-type H+-transporting ATPase subunit gamma | 18.99 | 0.00 | 34313.63 | 0.02 | 18.10 | 0.00 | -2.53 | 0.00 | 16.40 | 0.00 |
| K01676 | fumarate hydratase class I | -15.32 | 0.00 | 0.00 | NA | -15.34 | 0.00 | NA | NA | -15.23 | 0.00 |
| K03711 | Fur family ferric uptake transcriptional regulator | NA | NA | 34596.31 | 0.00 | 18.47 | 0.00 | NA | NA | NA | NA |
| K01711 | GDPmannose 4,6-dehydratase | NA | NA | 18642.81 | 0.00 | 17.11 | 0.00 | 15.96 | 0.00 | 15.88 | 0.00 |
| K09969 | general L-amino acid transport system substrate-binding protein | -1.02 | 0.08 | 319327.75 | 1.00 | 0.17 | 0.81 | 1.45 | 0.02 | 0.37 | 0.38 |
| K11477 | glc operon protein GlcG | NA | NA | 22854.21 | 0.52 | 27.74 | 0.00 | 30.00 | 0.00 | 29.92 | 0.00 |
| K01053 | gluconolactonase | -0.66 | 0.13 | 48488.08 | 0.55 | -2.76 | 0.57 | 0.52 | 0.90 | -0.20 | 0.96 |
| K00820 | glucosamine--fructose-6-phosphate aminotransferase (isomerizing) | 1.91 | 0.50 | 77584.65 | 0.73 | 4.03 | 0.17 | 1.18 | 0.83 | 3.05 | 0.27 |
| K17315 | glucose/mannose transport system substrate-binding protein | -1.17 | 0.32 | 331452.22 | 0.71 | -0.58 | 0.16 | 1.02 | 0.40 | -0.20 | 0.77 |
| K00975 | glucose-1-phosphate adenylyltransferase | NA | NA | 29871.78 | 0.00 | 16.98 | 0.00 | 17.74 | 0.00 | 17.67 | 0.00 |
| K00973 | glucose-1-phosphate thymidylyltransferase | NA | NA | 47553.77 | 0.00 | NA | NA | 19.18 | 0.00 | 19.11 | 0.00 |
| K01039 | glutaconate CoA-transferase subunit A | -0.38 | 0.94 | 85114.83 | 0.93 | -0.98 | 0.80 | -0.21 | 0.95 | -0.56 | 0.93 |
| K01040 | glutaconate CoA-transferase subunit B | -2.38 | 0.43 | 128132.75 | 0.89 | -0.64 | 0.62 | 1.88 | 0.53 | -0.52 | 0.22 |
| K01615 | glutaconyl-CoA decarboxylase | 29.37 | 0.00 | 0.00 | NA | NA | NA | -29.87 | 0.00 | NA | NA |
| K01295 | glutamate carboxypeptidase | NA | NA | 37118.65 | 0.00 | NA | NA | 18.83 | 0.00 | 18.75 | 0.00 |
| K00262 | glutamate dehydrogenase (NADP+) | 0.49 | 0.24 | 567778.63 | 0.28 | -0.69 | 0.17 | -0.44 | 0.21 | 0.01 | 0.99 |
| K00620 | glutamate N-acetyltransferase/amino-acid N-acetyltransferase | 16.74 | 0.00 | 23317.50 | 0.91 | 16.97 | 0.00 | 0.32 | 0.13 | 17.04 | 0.00 |
| K01776 | glutamate racemase | NA | NA | 7517.37 | 0.00 | NA | NA | 16.52 | 0.00 | 16.44 | 0.00 |
| K00265 | glutamate synthase (NADPH/NADH) large chain | -0.52 | 0.90 | 84554.72 | 0.09 | 0.31 | 0.80 | 2.26 | 0.42 | 1.72 | 0.00 |
| K00266 | glutamate synthase (NADPH/NADH) small chain | 0.25 | 0.06 | 52570.81 | 0.85 | -1.57 | 0.64 | -2.00 | 0.00 | -1.78 | 0.00 |
| K10001 | glutamate/aspartate transport system substrate-binding protein | 0.23 | 0.96 | 47998.61 | 0.95 | 0.40 | 0.95 | 0.24 | 0.95 | 0.34 | 0.95 |
| K01845 | glutamate-1-semialdehyde 2,1-aminomutase | -2.36 | 0.00 | 21513.87 | 0.50 | -4.30 | 0.10 | -0.14 | 0.05 | -2.53 | 0.00 |
| K00147 | glutamate-5-semialdehyde dehydrogenase | -18.13 | 0.00 | 257077.62 | NA | 2.94 | NA | 19.53 | 0.00 | 1.39 | 0.01 |
| K01425 | glutaminase | -0.03 | 0.98 | 24981.91 | 0.08 | -0.57 | 0.61 | -1.43 | 0.02 | -1.52 | 0.08 |
| K01915 | glutamine synthetase | -1.49 | 0.00 | 1739451.89 | 0.21 | 0.32 | 0.60 | 2.44 | 0.00 | 0.94 | 0.00 |
| K16173 | glutaryl-CoA dehydrogenase (non-decarboxylating) | 0.79 | 0.88 | 47992.45 | 0.00 | 0.39 | 0.94 | -3.05 | 0.00 | -2.40 | 0.46 |
| K00799 | glutathione S-transferase | NA | NA | 53297.66 | 0.12 | 28.65 | 0.00 | 19.21 | 0.00 | 19.15 | 0.00 |
| K13889 | glutathione transport system substrate-binding protein | -17.06 | 0.00 | 2150.05 | 0.00 | -17.08 | 0.00 | 14.72 | 0.00 | -2.38 | 0.00 |
| K00134 | glyceraldehyde 3-phosphate dehydrogenase | -0.45 | 0.55 | 835229.34 | 0.12 | -0.68 | 0.39 | 0.57 | 0.13 | 0.13 | 0.83 |
| K00018 | glycerate dehydrogenase | NA | NA | 2317.76 | 0.00 | NA | NA | 14.82 | 0.00 | 14.75 | 0.00 |
| K00864 | glycerol kinase | 0.24 | 0.83 | 714308.65 | 0.91 | 0.27 | 0.79 | 0.25 | 0.30 | 0.40 | 0.66 |
| K17321 | glycerol transport system substrate-binding protein | 0.28 | 0.96 | 136349.44 | 0.74 | -0.01 | 1.00 | -0.19 | 0.96 | 0.08 | 0.95 |
| K00113 | glycerol-3-phosphate dehydrogenase subunit C | NA | NA | 45085.31 | 0.47 | 16.71 | 0.00 | 30.00 | 0.00 | 30.00 | 0.00 |
| K02002 | glycine betaine/proline transport system substrate-binding protein | -29.15 | 0.00 | 9272.46 | 0.00 | -29.15 | 0.00 | 16.82 | 0.00 | 1.38 | 0.64 |
| K00283 | glycine dehydrogenase subunit 2 | NA | NA | 34482.25 | 0.96 | 17.40 | 0.00 | 30.00 | 0.00 | 29.77 | 0.00 |
| K00600 | glycine hydroxymethyltransferase | -30.00 | 0.00 | 67785.17 | 0.11 | 0.12 | 0.98 | 18.34 | 0.00 | -0.37 | 0.93 |
| K00104 | glycolate oxidase | 0.69 | 0.86 | 28373.87 | 0.67 | 0.33 | 0.94 | -0.05 | 0.92 | 0.57 | 0.88 |
| K01879 | glycyl-tRNA synthetase beta chain | NA | NA | 5571.35 | 0.00 | 15.83 | 0.00 | NA | NA | NA | NA |
| K01951 | GMP synthase (glutamine-hydrolysing) | 1.16 | 0.00 | 19112.80 | 0.75 | 0.31 | 0.95 | 0.00 | 0.99 | 1.13 | 0.00 |
| K03979 | GTP-binding protein | NA | NA | 12192.78 | 0.75 | 28.67 | 0.00 | 16.52 | 0.00 | 16.44 | 0.00 |
| K06207 | GTP-binding protein | 1.06 | 0.80 | 252328.07 | 0.28 | 3.07 | 0.55 | -1.77 | 0.56 | -0.88 | 0.88 |
| K06942 | GTP-binding protein YchF | 14.45 | 0.00 | 3975.43 | 0.00 | 15.34 | 0.00 | -14.38 | 0.00 | NA | NA |
| K12132 | HAMP domain-containing protein | 14.98 | 0.00 | 28479.21 | 0.87 | 29.83 | 0.00 | 2.68 | 0.32 | 30.00 | 0.00 |
| K03388 | heterodisulfide reductase subunit A | -0.01 | 0.99 | 77970.79 | 0.49 | -0.33 | 0.69 | -1.75 | 0.56 | -1.79 | 0.57 |
| K03389 | heterodisulfide reductase subunit B | 16.48 | 0.00 | 29490.27 | 0.00 | 18.24 | 0.00 | -16.41 | 0.00 | NA | NA |
| K11254 | histone H4 | -1.09 | 0.37 | 481684.87 | 0.93 | -1.11 | 0.35 | 0.13 | 0.94 | -0.96 | 0.54 |
| K00003 | homoserine dehydrogenase | 0.98 | 0.00 | 17337.22 | 0.44 | 1.39 | 0.29 | -0.22 | 0.70 | 0.72 | 0.13 |
| K03666 | host factor-I protein | -0.58 | 0.00 | 67785.09 | 0.89 | -0.44 | 0.72 | 0.66 | 0.87 | 0.00 | 1.00 |
| K13993 | HSP20 family protein | 0.96 | 0.04 | 222513.78 | 0.05 | -0.61 | 0.59 | 0.02 | 0.95 | 0.93 | 0.06 |
| K06281 | hydrogenase large subunit | 0.67 | 0.52 | 464425.59 | 0.37 | 0.31 | 0.75 | -0.78 | 0.41 | -0.12 | 0.91 |
| K10535 | hydroxylamine dehydrogenase | -18.38 | 0.00 | 0.00 | NA | -18.41 | 0.00 | NA | NA | -18.30 | 0.00 |
| K05601 | hydroxylamine reductase | 0.70 | 0.88 | 323543.38 | 0.86 | 1.18 | 0.77 | 0.79 | 0.08 | 1.44 | 0.67 |
| K07045 | hypothetical protein | -16.62 | 0.00 | 0.00 | NA | -16.64 | 0.00 | NA | NA | -16.53 | 0.00 |
| K09924 | hypothetical protein | 0.24 | 0.03 | 659.85 | 0.00 | -2.39 | 0.00 | -15.31 | 0.00 | -15.10 | 0.00 |
| K21449 | hypothetical protein | 17.46 | 0.00 | 24964.60 | 0.13 | 17.47 | 0.00 | -1.01 | 0.23 | 16.43 | 0.00 |
| K12132 | hypothetical protein | 14.98 | 0.00 | 28479.21 | 0.87 | 29.83 | 0.00 | 2.68 | 0.32 | 30.00 | 0.00 |
| K06907 | hypothetical protein | 0.95 | 0.00 | 673157.22 | 0.62 | -0.12 | 0.94 | -0.39 | 0.23 | 0.53 | 0.00 |
| K21572 | hypothetical protein | 1.25 | 0.00 | 199291.38 | 0.27 | 0.34 | 0.15 | -1.49 | 0.08 | -0.27 | 0.81 |
| K21619 | hypothetical protein | 17.80 | 0.00 | 18853.48 | 0.00 | 17.59 | 0.00 | -17.73 | 0.00 | NA | NA |
| K20370 | hypothetical protein | 18.49 | 0.00 | 0.00 | NA | NA | NA | -18.42 | 0.00 | NA | NA |
| K01693 | imidazoleglycerol-phosphate dehydratase | -17.42 | 0.00 | 19829.59 | 0.00 | -17.44 | 0.00 | 30.00 | 0.00 | 0.43 | 0.92 |
| K09607 | immune inhibitor A | -1.45 | 0.06 | 390686.87 | 0.09 | -2.00 | 0.02 | 0.77 | 0.24 | -0.74 | 0.51 |
| K00088 | IMP dehydrogenase | 0.11 | 0.98 | 125338.34 | 0.61 | 0.91 | 0.82 | 0.57 | 0.56 | 0.75 | 0.85 |
| K04764 | integration host factor subunit alpha | 19.04 | 0.00 | 27308.76 | 0.00 | 18.12 | 0.00 | -18.97 | 0.00 | NA | NA |
| K09004 | intracellular sulfur oxidation DsrE/DsrF family protein | 15.68 | 0.00 | 1204481.50 | 0.01 | 22.23 | 0.00 | 7.42 | 0.00 | 23.08 | 0.00 |
| K02014 | iron complex outermembrane receptor protein | 0.34 | 0.13 | 3859883.90 | 0.05 | 0.26 | 0.39 | 0.54 | 0.01 | 0.87 | 0.00 |
| K02012 | iron(III) transport system substrate-binding protein | 1.18 | 0.00 | 4698.15 | 0.00 | -0.11 | 0.34 | -16.80 | 0.00 | -15.66 | 0.00 |
| K00031 | isocitrate dehydrogenase | -1.48 | 0.00 | 7470.58 | 0.00 | -3.78 | 0.17 | -18.38 | 0.00 | -19.89 | 0.00 |
| K01637 | isocitrate lyase | -30.00 | 0.00 | 76279.55 | 0.90 | 0.07 | 0.98 | 30.00 | 0.00 | -0.01 | 1.00 |
| K07303 | isoquinoline 1-oxidoreductase beta subunit | -0.16 | 0.97 | 24292.69 | 0.00 | -30.00 | 0.00 | 0.27 | 0.94 | 0.14 | 0.98 |
| K00253 | isovaleryl-CoA dehydrogenase | NA | NA | 47518.33 | 0.00 | NA | NA | 19.18 | 0.00 | 19.10 | 0.00 |
| K15987 | K(+)-stimulated pyrophosphate-energized sodium pump | 29.86 | 0.00 | 204830.08 | NA | 30.00 | NA | 3.65 | 0.16 | 20.44 | 0.00 |
| K00053 | ketol-acid reductoisomerase | 0.66 | 0.88 | 212228.79 | 0.70 | 2.16 | 0.50 | 1.85 | 0.00 | 2.42 | 0.42 |
| K02863 | large subunit ribosomal protein L1 | -0.63 | 0.89 | 235356.01 | 0.73 | 1.63 | 0.10 | 2.70 | 0.34 | 2.07 | 0.00 |
| K02864 | large subunit ribosomal protein L10 | 2.22 | 0.46 | 5457.87 | 0.00 | -0.36 | 0.94 | -30.00 | 0.00 | -16.21 | 0.00 |
| K02867 | large subunit ribosomal protein L11 | -3.41 | 0.21 | 42299.94 | 0.78 | -1.34 | 0.71 | 2.97 | 0.29 | -0.46 | 0.94 |
| K02874 | large subunit ribosomal protein L14 | 0.69 | NA | 220534.17 | 0.00 | 0.61 | NA | 1.93 | 0.00 | 2.58 | 0.06 |

| | | | | | | | | | | | |
|---|---|---|---|---|---|---|---|---|---|---|---|
| K02879 | large subunit ribosomal protein L17 | 18.56 | 0.00 | 0.00 | NA | NA | NA | -18.49 | 0.00 | NA | NA |
| K02884 | large subunit ribosomal protein L19 | NA | NA | 898286.98 | 0.00 | 23.06 | 0.00 | 19.56 | 0.00 | 19.47 | 0.00 |
| K02886 | large subunit ribosomal protein L2 | -30.00 | 0.00 | 52393.32 | 0.87 | -0.08 | 0.99 | 18.11 | 0.00 | -0.17 | 0.96 |
| K02890 | large subunit ribosomal protein L22 | -16.17 | 0.00 | 8291.43 | 0.00 | 0.29 | 0.00 | NA | NA | -16.08 | 0.00 |
| K02892 | large subunit ribosomal protein L23 | -18.16 | 0.00 | 169040.74 | 0.00 | 1.45 | 0.00 | 20.20 | 0.00 | 2.01 | 0.00 |
| K02897 | large subunit ribosomal protein L25 | NA | NA | 43407.84 | 0.00 | NA | NA | 19.06 | 0.00 | 18.98 | 0.00 |
| K02906 | large subunit ribosomal protein L3 | -17.31 | 0.00 | 33132.29 | 0.00 | -17.33 | 0.00 | 18.66 | 0.00 | 1.31 | 0.00 |
| K02907 | large subunit ribosomal protein L30 | 0.21 | 0.97 | 45507.64 | 0.19 | -0.17 | 0.97 | -0.88 | 0.09 | -0.71 | 0.85 |
| K02910 | large subunit ribosomal protein L31e | 16.17 | 0.00 | 0.00 | NA | NA | NA | -16.10 | 0.00 | NA | NA |
| K02926 | large subunit ribosomal protein L4 | -16.24 | 0.00 | 24311.54 | 0.25 | 1.05 | 0.00 | 16.89 | 0.00 | 0.59 | 0.36 |
| K02931 | large subunit ribosomal protein L5 | -0.03 | 0.99 | 332394.16 | 0.02 | 1.94 | 0.57 | 3.01 | 0.01 | 2.99 | 0.30 |
| K02933 | large subunit ribosomal protein L6 | -1.95 | 0.05 | 184119.82 | 0.02 | 0.78 | 0.54 | 3.92 | 0.00 | 2.00 | 0.05 |
| K02935 | large subunit ribosomal protein L7/L12 | 30.00 | 0.00 | 21402.78 | 0.00 | 17.77 | 0.00 | -30.00 | 0.00 | NA | NA |
| K02939 | large subunit ribosomal protein L9 | -30.00 | 0.00 | 224128.69 | 0.00 | 1.41 | 0.68 | 20.89 | 0.00 | 2.73 | 0.35 |
| K01424 | L-asparaginase | 0.81 | 0.00 | 49857.42 | 0.85 | 0.44 | 0.04 | -0.40 | 0.84 | 0.36 | 0.85 |
| K03744 | LemA protein | -0.24 | 0.85 | 198600.80 | 0.57 | 1.01 | 0.21 | 1.23 | 0.00 | 0.98 | 0.10 |
| K01255 | leucyl aminopeptidase | 15.95 | 0.00 | 27113.96 | 1.00 | 30.00 | 0.00 | 1.34 | 0.67 | 30.00 | 0.00 |
| K20523 | lipid-binding SYLF domain-containing protein | 0.36 | 0.93 | 77627.43 | 0.01 | -0.03 | 0.99 | 2.12 | 0.03 | 2.33 | 0.45 |
| K10206 | LL-diaminopimelate aminotransferase | 17.06 | 0.00 | 0.00 | NA | NA | NA | -16.99 | 0.00 | NA | NA |
| K01897 | long-chain acyl-CoA synthetase | 0.44 | 0.00 | 2602.27 | 0.00 | -0.95 | 0.00 | -16.05 | 0.00 | -15.65 | 0.00 |
| K06076 | long-chain fatty acid transport protein | 0.15 | 0.88 | 6412298.98 | 0.00 | 1.86 | 0.00 | 2.71 | 0.00 | 2.86 | 0.00 |
| K01752 | L-serine dehydratase | -1.44 | NA | 79437.27 | 0.21 | -0.15 | 0.96 | -1.62 | 0.62 | -3.16 | NA |
| K01843 | lysine 2,3-aminomutase | 16.57 | 0.00 | 0.00 | NA | NA | NA | -16.50 | 0.00 | NA | NA |
| K00024 | malate dehydrogenase | -0.53 | 0.85 | 237347.83 | 0.00 | 1.43 | 0.28 | 0.83 | 0.58 | 0.35 | 0.82 |
| K00029 | malate dehydrogenase (oxaloacetate-decarboxylating)(NADP+) | -1.30 | 0.00 | 44218.38 | 0.62 | -0.39 | 0.00 | -0.10 | 0.98 | -1.41 | 0.67 |
| K00140 | malonate-semialdehyde dehydrogenase (acetylating)/methylmalonate-semialdehyde dehydro | -17.69 | 0.00 | 0.00 | NA | -17.71 | 0.00 | NA | NA | -17.60 | 0.00 |
| K15986 | manganese-dependent inorganic pyrophosphatase | -1.70 | 0.02 | 479566.51 | 0.36 | -1.35 | 0.00 | 0.25 | 0.80 | -1.50 | 0.00 |
| K03585 | membrane fusion protein (multidrug efflux system) | NA | NA | 8094.68 | 0.00 | NA | NA | 16.63 | 0.00 | 16.55 | 0.00 |
| K04087 | membrane protease subunit HflC | -1.14 | 0.00 | 64470.36 | NA | 0.35 | NA | 1.56 | 0.00 | 0.38 | 0.01 |
| K04088 | membrane protease subunit HflK | 17.40 | 0.00 | 32095.99 | 0.64 | 16.73 | 0.00 | 0.63 | 0.87 | 30.00 | 0.00 |
| K10944 | methane/ammonia monooxygenase subunit A | -23.41 | 0.00 | 464436.64 | NA | -1.07 | NA | NA | NA | -23.32 | 0.00 |
| K10945 | methane/ammonia monooxygenase subunit B | 1.43 | NA | 236522.12 | 0.13 | -0.63 | 0.41 | -2.79 | NA | -1.41 | 0.00 |
| K14081 | methanol corrinoid protein | -0.95 | 0.00 | 287757.68 | 0.02 | -2.14 | 0.00 | -2.31 | 0.00 | -3.28 | 0.00 |
| K04480 | methanol--5-hydroxybenzimidazolylcobamide Co-methyltransferase | -0.57 | 0.25 | 806715.77 | 0.05 | -1.80 | 0.00 | -1.76 | 0.00 | -2.39 | 0.00 |
| K01874 | methionyl-tRNA synthetase | -17.53 | 0.00 | 6986.58 | 0.00 | -1.33 | 0.00 | NA | NA | -17.45 | 0.00 |
| K00400 | methyl coenzyme M reductase system subunit A2 | -0.04 | 0.99 | 21803.85 | 0.00 | -1.99 | 0.53 | -0.27 | 0.28 | -0.40 | 0.92 |
| K03406 | methyl-accepting chemotaxis protein | -2.33 | 0.66 | 227573.27 | 0.09 | 0.55 | 0.90 | 4.25 | 0.11 | 1.82 | 0.59 |
| K00399 | methyl-coenzyme M reductase alpha subunit | -2.44 | 0.00 | 377608.88 | 0.06 | -1.32 | 0.39 | -0.80 | 0.25 | -3.25 | 0.00 |
| K00401 | methyl-coenzyme M reductase beta subunit | -0.53 | 0.00 | 18320421.28 | 0.35 | -0.96 | 0.00 | -0.38 | 0.02 | -0.93 | 0.00 |
| K00402 | methyl-coenzyme M reductase gamma subunit | -0.76 | 0.02 | 5340193.12 | 0.31 | -1.76 | 0.00 | -1.15 | 0.00 | -1.96 | 0.00 |
| K01491 | methylenetetrahydrofolate dehydrogenase (NADP+)/methenyltetrahydrofolate cyclohydrolase | 2.59 | 0.01 | 78275.78 | 0.74 | 0.28 | 0.85 | -2.92 | 0.30 | -0.39 | 0.93 |
| K00297 | methylenetetrahydrofolate reductase (NADPH) | 30.00 | 0.00 | 21196.96 | 0.00 | 17.25 | 0.00 | -1.63 | 0.60 | 16.26 | 0.00 |
| K00319 | methylenetetrahydromethanopterin dehydrogenase | -0.74 | 0.43 | 473418.28 | 0.97 | -1.21 | 0.15 | -0.29 | 0.51 | -1.00 | 0.23 |
| K10540 | methyl-galactoside transport system substrate-binding protein | 0.19 | 0.09 | 0.00 | NA | -15.19 | 0.00 | -15.24 | 0.00 | -15.09 | 0.00 |
| K01849 | methylmalonyl-CoA mutase | -0.67 | 0.00 | 0.00 | NA | -19.26 | 0.00 | -18.45 | 0.00 | -19.15 | 0.00 |
| K01847 | methylmalonyl-CoA mutase | -16.86 | 0.00 | 0.00 | NA | -16.88 | 0.00 | NA | NA | -16.77 | 0.00 |
| K01848 | methylmalonyl-CoA mutase N-terminal domain/subunit | -0.01 | 0.99 | 27781.44 | 0.14 | -2.02 | 0.17 | -3.12 | 0.00 | -3.23 | 0.00 |
| K07050 | misacylated tRNA(Ala) deacylase | -1.10 | 0.00 | 7740.36 | 0.00 | -18.75 | 0.00 | -1.06 | 0.00 | -2.20 | 0.00 |
| K04043 | molecular chaperone DnaK | -1.05 | 0.32 | 2310208.09 | 0.52 | -0.87 | 0.44 | 0.67 | 0.13 | -0.46 | 0.70 |
| K04079 | molecular chaperone HtpG | -2.61 | 0.00 | 463872.15 | 0.01 | -0.61 | 0.04 | 1.66 | 0.00 | -0.97 | 0.00 |
| K04080 | molecular chaperone IbpA | -0.98 | 0.89 | 92720.54 | 0.35 | -1.21 | 0.77 | 1.18 | 0.74 | 0.23 | 0.95 |
| K02020 | molybdate transport system substrate-binding protein | NA | NA | 70918.99 | 0.00 | NA | NA | 19.77 | 0.00 | 19.69 | 0.00 |
| K03924 | MoxR-like ATPase | -2.61 | 0.00 | 24325.79 | 0.70 | -2.98 | 0.30 | -1.13 | 0.00 | -3.77 | 0.00 |
| K10112 | multiple sugar transport system ATP-binding protein | -3.76 | 0.03 | 55305.55 | 0.90 | -3.34 | 0.32 | 0.26 | 0.90 | -3.50 | 0.02 |
| K10111 | multiple sugar transport system ATP-binding protein | -30.00 | 0.00 | 3705.30 | 0.00 | -30.00 | 0.00 | 15.50 | 0.00 | -1.12 | 0.73 |
| K02026 | multiple sugar transport system permease protein | NA | NA | 8169.16 | 0.00 | 16.38 | 0.00 | NA | NA | NA | NA |
| K02027 | multiple sugar transport system substrate-binding protein | -1.77 | 0.00 | 133804.32 | 0.78 | -2.23 | 0.00 | -0.36 | 0.68 | -2.18 | 0.00 |
| K01858 | myo-inositol-1-phosphate synthase | -1.41 | 0.62 | 4637.70 | 0.00 | -0.27 | 0.95 | -27.93 | 0.00 | -15.73 | 0.00 |
| K02600 | N utilization substance protein A | 0.53 | 0.00 | 88104.32 | 0.99 | 1.51 | 0.67 | 1.09 | 0.19 | 1.56 | 0.05 |
| K00346 | Na+-transporting NADH:ubiquinone oxidoreductase subunit A | -18.88 | 0.00 | 27674.71 | 0.65 | -2.63 | 0.36 | 30.00 | 0.00 | -0.91 | 0.79 |
| K00145 | N-acetyl-gamma-glutamyl-phosphate reductase | NA | NA | 12338.53 | 0.00 | 30.00 | 0.00 | NA | NA | NA | NA |
| K13075 | N-acyl homoserine lactone hydrolase | -0.18 | 0.97 | 18886.68 | 0.94 | -1.88 | 0.57 | -1.27 | 0.69 | -1.57 | 0.77 |
| K00324 | NAD(P) transhydrogenase subunit alpha | -20.02 | 0.00 | 112716.87 | 0.11 | -0.29 | 0.82 | 18.69 | 0.00 | -1.33 | 0.25 |
| K00331 | NADH-quinone oxidoreductase subunit B | 18.75 | 0.00 | 16747.48 | 0.00 | 30.00 | 0.00 | -18.68 | 0.00 | NA | NA |
| K00332 | NADH-quinone oxidoreductase subunit C | NA | NA | 19906.00 | 0.00 | NA | NA | 17.93 | 0.00 | 17.85 | 0.00 |
| K00333 | NADH-quinone oxidoreductase subunit D | 17.83 | 0.00 | 0.00 | NA | NA | NA | -17.76 | 0.00 | NA | NA |
| K00336 | NADH-quinone oxidoreductase subunit G | NA | NA | 7612.64 | 0.00 | NA | NA | 16.53 | 0.00 | 16.46 | 0.00 |
| K00344 | NADPH2:quinone reductase | NA | NA | 11256.21 | 0.00 | NA | NA | 17.10 | 0.00 | 17.03 | 0.00 |
| K00370 | nitrate reductase alpha subunit | -1.25 | 0.75 | 24659.96 | 0.49 | -2.64 | 0.38 | 0.50 | 0.90 | -0.80 | 0.41 |
| K00371 | nitrate reductase beta subunit | 0.71 | 0.68 | 241748.49 | 0.00 | 1.93 | 0.01 | 0.07 | 0.96 | 0.74 | 0.44 |
| K02567 | nitrate reductase NapA | NA | NA | 6441.08 | 0.00 | 28.13 | 0.00 | NA | NA | NA | NA |
| K15576 | nitrate/nitrite transport system substrate-binding protein | NA | NA | 26539.18 | 0.00 | 16.80 | 0.00 | 17.58 | 0.00 | 17.50 | 0.00 |
| K03385 | nitrite reductase (cytochrome c-552) | 0.23 | 0.96 | 78141.01 | 0.01 | 2.24 | 0.04 | -0.53 | 0.89 | -0.31 | 0.22 |
| K15864 | nitrite reductase (NO-forming)/hydroxylamine reductase | -19.39 | 0.00 | 38045.32 | 0.49 | -1.92 | 0.00 | 18.08 | 0.00 | -1.36 | 0.02 |
| K02585 | nitrogen fixation protein NifB | -1.15 | 0.72 | 4618.59 | 0.00 | -1.83 | 0.00 | 0.84 | 0.78 | -0.32 | 0.02 |
| K04751 | nitrogen regulatory protein P-II 1 | 2.06 | 0.00 | 388533.39 | 0.18 | 1.08 | 0.01 | -1.16 | 0.05 | 0.88 | 0.04 |
| K04752 | nitrogen regulatory protein P-II 2 | 2.52 | 0.00 | 310863.58 | 0.15 | 2.00 | 0.00 | -0.84 | 0.09 | 1.66 | 0.00 |
| K00376 | nitrous-oxide reductase | -2.71 | 0.34 | 193538.88 | 0.31 | 0.57 | 0.73 | 2.29 | 0.43 | -0.43 | 0.43 |
| K02051 | NitT/TauT family transport system substrate-binding protein | -29.86 | 0.00 | 58736.49 | 0.96 | 0.37 | 0.94 | 18.51 | 0.00 | 0.42 | 0.92 |
| K09759 | nondiscriminating aspartyl-tRNA synthetase | -15.70 | 0.00 | 0.00 | NA | -15.72 | 0.00 | NA | NA | -15.62 | 0.00 |
| K07001 | NTE family protein | NA | NA | 15838.05 | 0.00 | 14.31 | 0.00 | 17.41 | 0.00 | 17.33 | 0.00 |
| K00940 | nucleoside-diphosphate kinase | -18.26 | 0.00 | 24959.83 | 0.00 | -0.44 | 0.00 | 15.49 | 0.00 | -2.80 | 0.00 |
| K01740 | O-acetylhomoserine (thiol)-lyase | 0.81 | 0.85 | 34974.54 | 0.90 | -0.71 | 0.91 | -0.97 | 0.78 | -0.19 | 0.98 |
| K02523 | octaprenyl-diphosphate synthase | NA | NA | 13474.51 | 0.00 | NA | NA | 30.00 | 0.00 | 30.00 | 0.00 |
| K10823 | oligopeptide transport system ATP-binding protein | -17.11 | 0.00 | 37118.65 | 0.00 | -17.13 | 0.00 | 18.83 | 0.00 | 1.69 | 0.00 |
| K15580 | oligopeptide transport system substrate-binding protein | -0.77 | 0.86 | 41519.88 | 0.00 | 0.32 | 0.95 | -17.65 | 0.00 | -30.00 | 0.00 |
| K03286 | OOP family OmpA-OmpF porin | -0.29 | 0.73 | 3571615.88 | 0.06 | 0.27 | 0.57 | 1.03 | 0.02 | 0.75 | 0.02 |
| K19691 | osomolarity two-component system, sensor histidine kinase NIK1 | NA | NA | 29679.23 | 0.00 | NA | NA | 18.50 | 0.00 | 18.43 | 0.00 |
| K16079 | outer membrane immunogenic protein | 0.87 | 0.84 | 815547.83 | 0.05 | 1.91 | 0.58 | 0.11 | 0.42 | 1.01 | 0.78 |
| K12686 | outer membrane lipase/esterase | -0.37 | 0.96 | 13702.32 | 0.27 | -1.75 | 0.72 | 1.04 | 0.74 | 0.59 | 0.87 |
| K03634 | outer membrane lipoprotein carrier protein | NA | NA | 0.00 | NA | NA | NA | NA | NA | NA | NA |
| K12340 | outer membrane protein | -0.45 | 0.92 | 71522.19 | 0.12 | -1.96 | 0.51 | 2.11 | 0.48 | 1.64 | 0.00 |
| K07274 | outer membrane protein | 1.10 | 0.79 | 89204.88 | 0.00 | 1.67 | 0.00 | -0.37 | 0.92 | 0.66 | 0.16 |
| K06142 | outer membrane protein | 3.55 | 0.00 | 26143.35 | 0.17 | -3.03 | 0.00 | -2.95 | 0.32 | 0.43 | 0.92 |
| K17713 | outer membrane protein assembly factor BamB | NA | NA | 19586.64 | 0.00 | NA | NA | 30.00 | 0.00 | 30.00 | 0.00 |
| K07287 | outer membrane protein assembly factor BamC | NA | NA | 23330.65 | 0.00 | NA | NA | 18.14 | 0.00 | 18.07 | 0.00 |
| K07277 | outer membrane protein insertion porin family | -0.63 | 0.54 | 240262.77 | 0.56 | 0.40 | 0.61 | 1.67 | 0.03 | 0.99 | 0.00 |
| K01577 | oxalyl-CoA decarboxylase | -16.74 | 0.00 | 0.00 | NA | -16.76 | 0.00 | NA | NA | -16.65 | 0.00 |
| K03497 | ParB family chromosome partitioning protein | -7.14 | 0.00 | 16633.06 | 0.00 | -8.00 | 0.00 | 3.56 | 0.00 | -3.64 | 0.00 |
| K01728 | pectate lyase | -0.04 | 0.83 | 3196.49 | 0.00 | -15.81 | 0.00 | -0.46 | 0.00 | -0.53 | 0.00 |
| K02032 | peptide/nickel transport system ATP-binding protein | 0.26 | 0.04 | 41403.15 | 0.00 | -18.01 | 0.00 | 0.74 | 0.00 | 0.96 | 0.00 |
| K02035 | peptide/nickel transport system substrate-binding protein | -0.74 | 0.04 | 1376513.92 | 0.02 | -0.64 | 0.02 | 0.61 | 0.03 | -0.15 | 0.56 |
| K03640 | peptidoglycan-associated lipoprotein | -0.45 | 0.20 | 166532.39 | 0.22 | -2.22 | 0.00 | -0.91 | 0.00 | -1.37 | 0.00 |
| K03769 | peptidyl-prolyl cis-trans isomerase C | 1.47 | 0.81 | 73569.37 | 0.72 | 0.81 | 0.90 | 0.47 | 0.91 | 1.89 | 0.56 |
| K03770 | peptidyl-prolyl cis-trans isomerase D | NA | NA | 40581.94 | 0.50 | 29.84 | 0.00 | 18.55 | 0.00 | 18.45 | 0.00 |
| K03771 | peptidyl-prolyl cis-trans isomerase SurA | NA | NA | 14876.08 | 0.29 | 14.57 | 0.00 | 29.38 | 0.00 | 29.99 | 0.00 |
| K03386 | peroxiredoxin (alkyl hydroperoxide reductase subunit C) | 0.55 | 0.94 | 92974.26 | 0.09 | 0.20 | 0.96 | 0.47 | 0.91 | 1.10 | 0.76 |
| K03564 | peroxiredoxin Q/BCP | 29.51 | 0.00 | 22717.61 | 0.00 | NA | NA | 1.59 | 0.58 | 18.04 | 0.00 |
| K21449 | PGF-CTERM protein | 17.46 | 0.00 | 24964.60 | 0.13 | 17.47 | 0.00 | -1.01 | 0.23 | 16.43 | 0.00 |
| K03969 | phage shock protein A | 0.68 | 0.92 | 9138.99 | NA | 0.21 | 0.96 | -0.95 | NA | -0.30 | 0.96 |
| K06907 | phage tail sheath protein FI | 0.95 | 0.00 | 673157.22 | 0.62 | -0.12 | 0.94 | -0.39 | 0.23 | 0.53 | 0.00 |
| K00625 | phosphate acetyltransferase | 0.70 | 0.88 | 31211.60 | 0.95 | -0.64 | 0.87 | -1.42 | 0.79 | -0.69 | 0.85 |
| K00634 | phosphate butyryltransferase | NA | NA | 26926.37 | 0.00 | 15.68 | 0.00 | 18.06 | 0.00 | 17.99 | 0.00 |
| K06217 | phosphate starvation-inducible PhoH-like protein | NA | NA | 16925.41 | 0.00 | NA | NA | 17.69 | 0.00 | 17.61 | 0.00 |
| K02036 | phosphate transport system ATP-binding protein | NA | NA | 7750.75 | 0.00 | NA | NA | 16.57 | 0.00 | 16.49 | 0.00 |
| K02040 | phosphate transport system substrate-binding protein | -2.33 | 0.00 | 252676.25 | 0.00 | -0.94 | 0.03 | 3.48 | 0.00 | 1.14 | 0.00 |
| K07221 | phosphate-selective porin OprO/OprP | 2.15 | 0.46 | 0.00 | NA | -30.00 | 0.00 | -18.63 | 0.00 | -30.00 | 0.00 |
| K01610 | phosphoenolpyruvate carboxykinase (ATP) | -1.71 | 0.00 | 6208.43 | 0.00 | -0.74 | 0.00 | -14.94 | 0.00 | -16.69 | 0.00 |
| K01596 | phosphoenolpyruvate carboxykinase (GTP) | -30.00 | 0.00 | 17064.30 | 0.36 | -1.47 | 0.64 | 29.54 | 0.00 | 0.82 | 0.88 |
| K01835 | phosphoglucomutase | NA | NA | 17318.45 | 0.00 | 17.47 | 0.00 | NA | NA | NA | NA |
| K00927 | phosphoglycerate kinase | 0.81 | 0.30 | 396500.71 | 0.01 | 2.24 | 0.00 | 0.90 | 0.01 | 1.65 | 0.02 |
| K15778 | phosphomannomutase/phosphoglucomutase | NA | NA | 82656.44 | 0.00 | 19.40 | 0.00 | 17.65 | 0.00 | 17.59 | 0.00 |
| K03147 | phosphomethylpyrimidine synthase | -3.00 | 0.00 | 16872.03 | 0.71 | -0.58 | 0.47 | 1.82 | 0.51 | -1.19 | 0.71 |
| K00602 | phosphoribosylaminoimidazolecarboxamide formyltransferase/IMP cyclohydrolase | -1.49 | 0.00 | 20207.33 | 0.00 | -0.30 | 0.00 | -16.42 | 0.00 | -17.95 | 0.00 |
| K01923 | phosphoribosylaminoimidazole-succinocarboxamide synthase | NA | NA | 30151.36 | NA | 30.00 | NA | 16.72 | 0.00 | 16.65 | 0.00 |
| K01814 | phosphoribosylformimino-5-aminoimidazole carboxamide ribotide isomerase | NA | NA | 14183.62 | 0.00 | 15.38 | 0.00 | 16.95 | 0.00 | 16.87 | 0.00 |
| K00855 | phosphoribulokinase | 18.38 | 0.00 | 299802.24 | 0.78 | 30.00 | 0.00 | 2.69 | 0.00 | 21.08 | 0.00 |
| K00831 | phosphoserine aminotransferase | 30.00 | 0.00 | 20953.81 | 0.00 | 17.74 | 0.00 | -30.00 | 0.00 | NA | NA |
| K02704 | photosystem II CP47 chlorophyll apoprotein | 1.50 | 0.00 | 255411.36 | 0.40 | 4.85 | 0.00 | 1.48 | 0.65 | 2.99 | 0.31 |
| K02703 | photosystem II P680 reaction center D1 protein | 30.00 | 0.00 | 0.00 | NA | NA | NA | -30.00 | 0.00 | NA | NA |
| K02706 | photosystem II P680 reaction center D2 protein | 1.82 | 0.00 | 43430.68 | 0.00 | 0.56 | 0.90 | -19.95 | 0.00 | -18.16 | 0.00 |
| K02280 | pilus assembly protein CpaC | 1.47 | 0.06 | 167360.29 | 0.10 | 1.10 | 0.74 | 3.83 | 0.01 | 5.32 | 0.00 |
| K02283 | pilus assembly protein CpaF | NA | NA | 46370.86 | 0.10 | 18.19 | 0.00 | 17.77 | 0.00 | 17.70 | 0.00 |
| K08086 | pilus assembly protein FimV | NA | NA | 4635.05 | 0.00 | NA | NA | 15.82 | 0.00 | 15.75 | 0.00 |
| K02030 | polar amino acid transport system substrate-binding protein | -1.57 | 0.02 | 387365.48 | 0.61 | -0.68 | 0.46 | 1.40 | 0.00 | -0.25 | 0.77 |
| K00962 | polyribonucleotide nucleotidyltransferase | 0.52 | 0.72 | 704449.98 | 0.72 | 1.47 | 0.18 | 1.27 | 0.00 | 1.83 | 0.06 |
| K01698 | porphobilinogen synthase | NA | NA | 26794.06 | 0.67 | 30.00 | 0.00 | 16.74 | 0.00 | 16.66 | 0.00 |
| K11206 | predicted amidohydrolase | NA | NA | 10091.16 | 0.00 | 12.82 | 0.00 | 16.84 | 0.00 | 16.76 | 0.00 |
| K07004 | predicted extracellular nuclease | 0.09 | 0.98 | 3900.41 | 0.00 | -30.00 | 0.00 | -1.74 | 0.00 | -1.74 | 0.58 |
| K07092 | predicted peroxiredoxin | NA | NA | 21303.65 | 0.80 | 16.78 | 0.00 | 17.01 | 0.00 | 16.93 | 0.00 |
| K07006 | predicted pyridoxine 5'-phosphate oxidase superfamily flavin-nucleotide-binding protein | -17.53 | 0.00 | 9843.79 | 0.00 | -0.83 | 0.00 | NA | NA | -17.44 | 0.00 |
| K07045 | predicted TIM-barrel fold metal-dependent hydrolase | -16.62 | 0.00 | 0.00 | NA | -16.64 | 0.00 | NA | NA | -16.53 | 0.00 |

| | | | | | | | | | | | |
|---|---|---|---|---|---|---|---|---|---|---|---|
| K07744 | predicted transcriptional regulator | 0.03 | 0.99 | 8281.53 | 0.00 | -0.57 | 0.60 | -30.00 | 0.00 | -16.94 | 0.00 |
| K03070 | preprotein translocase subunit SecA | 2.78 | 0.00 | 229719.40 | 0.06 | 1.22 | 0.31 | -0.17 | 0.72 | 2.53 | 0.00 |
| K03072 | preprotein translocase subunit SecD | 17.69 | 0.00 | 19593.67 | 0.96 | 28.84 | 0.00 | -0.96 | 0.77 | 29.77 | 0.00 |
| K04802 | proliferating cell nuclear antigen | -1.42 | 0.00 | 6540.82 | 0.00 | -2.61 | 0.36 | -17.15 | 0.00 | -18.60 | 0.00 |
| K01881 | prolyl-tRNA synthetase | NA | NA | 7750.75 | 0.00 | NA | NA | 16.57 | 0.00 | 16.49 | 0.00 |
| K01966 | propionyl-CoA carboxylase beta chain | 3.37 | 0.00 | 42494.76 | 0.00 | 3.56 | 0.00 | -0.82 | 0.37 | 2.53 | 0.00 |
| K01908 | propionyl-CoA synthetase | -0.44 | 0.51 | 82499.12 | 0.34 | 0.87 | 0.28 | 0.84 | 0.16 | 0.39 | 0.58 |
| K04773 | protease-4 | NA | NA | 12572.94 | 0.55 | 27.25 | 0.00 | 29.94 | 0.00 | 29.84 | 0.00 |
| K03432 | proteasome alpha subunit | 0.14 | 0.81 | 160964.34 | 0.95 | -1.55 | 0.01 | -1.49 | 0.00 | -1.41 | 0.00 |
| K03433 | proteasome beta subunit | -0.21 | 0.07 | 0.00 | NA | -16.65 | 0.00 | -16.29 | 0.00 | -16.54 | 0.00 |
| K0627 | protein phosphatase 1 regulatory subunit | NA | NA | 79140.69 | 0.00 | NA | NA | 19.92 | 0.00 | 19.84 | 0.00 |
| K22169 | proton-translocating NADH-quinone oxidoreductase chain N | NA | NA | 11856.50 | 0.00 | 16.92 | 0.00 | NA | NA | NA | NA |
| K02003 | putative ABC transport system ATP-binding protein | -28.70 | 0.00 | 0.00 | NA | -28.70 | 0.00 | NA | NA | -28.59 | 0.00 |
| K01989 | putative ABC transport system substrate-binding protein | NA | NA | 3284.83 | 0.00 | NA | NA | 15.33 | 0.00 | 15.25 | 0.00 |
| K10546 | putative multiple sugar transport system substrate-binding protein | -0.26 | 0.57 | 1020653.61 | 0.99 | -0.53 | 0.14 | -0.08 | 0.87 | -0.37 | 0.23 |
| K07572 | putative nucleotide binding protein | -16.77 | 0.00 | 0.00 | NA | -16.79 | 0.00 | NA | NA | -16.68 | 0.00 |
| K07283 | putative salt-induced outer membrane protein | NA | NA | 3704.17 | 0.00 | 15.24 | 0.00 | NA | NA | NA | NA |
| K02052 | putative spermidine/putrescine transport system ATP-binding protein | NA | NA | 36537.08 | 0.00 | NA | NA | 18.80 | 0.00 | 18.72 | 0.00 |
| K02055 | putative spermidine/putrescine transport system substrate-binding protein | -16.69 | 0.00 | 0.00 | NA | -16.71 | 0.00 | NA | NA | -16.60 | 0.00 |
| K07795 | putative tricarboxylic transport membrane protein | 0.12 | 0.92 | 516720.04 | 0.00 | 1.29 | 0.01 | 2.55 | 0.00 | 2.62 | 0.00 |
| K11073 | putrescine transport system substrate-binding protein | -18.86 | 0.00 | 0.00 | NA | -18.88 | 0.00 | NA | NA | -18.78 | 0.00 |
| K06215 | pyridoxal 5'-phosphate synthase pdxS subunit | 30.00 | 0.00 | 110316.00 | 0.60 | 19.80 | 0.00 | -0.95 | 0.87 | 30.00 | NA |
| K00756 | pyrimidine-nucleoside phosphorylase | -0.39 | 0.92 | 194380.52 | 0.00 | -30.00 | 0.00 | 4.87 | 0.00 | 4.38 | 0.09 |
| K00895 | pyrophosphate--fructose-6-phosphate 1-phosphotransferase | -1.56 | 0.00 | 77670.11 | 0.99 | -1.58 | 0.65 | 0.14 | 0.29 | -1.47 | 0.00 |
| K00163 | pyruvate dehydrogenase E1 component | 17.72 | 0.00 | 0.00 | NA | NA | NA | -17.65 | 0.00 | NA | NA |
| K00162 | pyruvate dehydrogenase E1 component beta subunit | -16.67 | 0.00 | 6659.90 | 0.00 | -16.69 | 0.00 | 16.35 | 0.00 | -0.35 | 0.01 |
| K00169 | pyruvate ferredoxin oxidoreductase alpha subunit | -0.68 | 0.92 | 100681.84 | 0.80 | 0.01 | 1.00 | 1.67 | 0.75 | 0.88 | 0.88 |
| K00170 | pyruvate ferredoxin oxidoreductase beta subunit | 18.75 | 0.00 | 9229.50 | 0.00 | NA | NA | -1.99 | 0.00 | 16.74 | 0.00 |
| K00172 | pyruvate ferredoxin oxidoreductase gamma subunit | 19.07 | 0.00 | 219748.19 | 0.00 | 21.13 | 0.00 | -19.00 | 0.00 | NA | NA |
| K00873 | pyruvate kinase | -17.55 | 0.00 | 43164.11 | 0.98 | 0.34 | 0.95 | 18.10 | 0.00 | 0.53 | 0.50 |
| K01006 | pyruvate,orthophosphate dikinase | 0.33 | 0.57 | 486184.50 | 0.32 | -0.24 | 0.72 | -0.80 | 0.07 | -0.49 | 0.23 |
| K01007 | pyruvate,water dikinase | 0.14 | 0.90 | 38119.39 | 0.93 | -1.59 | 0.64 | -1.27 | 0.70 | -1.13 | 0.75 |
| K03737 | pyruvate-ferredoxin/flavodoxin oxidoreductase | 0.02 | 0.99 | 171254.12 | 0.88 | -0.22 | 0.87 | 0.00 | 1.00 | 0.00 | 1.00 |
| K16885 | quinone-modifying oxidoreductase subunit QmoA | 2.78 | 0.02 | 328979.93 | 0.66 | 2.51 | 0.03 | 0.21 | 0.88 | 2.90 | 0.00 |
| K16886 | quinone-modifying oxidoreductase subunit QmoB | 1.89 | 0.00 | 653111.61 | 0.18 | 1.26 | 0.10 | 0.16 | 0.54 | 1.99 | 0.00 |
| K10117 | raffinose/stachyose/melibiose transport system substrate-binding protein | -1.70 | 0.01 | 360134.08 | 0.99 | -0.88 | 0.45 | 1.07 | NA | -0.74 | NA |
| K07463 | RecJ-like exonuclease | 30.00 | 0.00 | 0.00 | NA | NA | NA | -30.00 | 0.00 | NA | NA |
| K03554 | recombination associated protein RdgC | 18.58 | 0.00 | 10359.59 | 0.00 | 16.73 | 0.00 | -18.51 | 0.00 | NA | NA |
| K03553 | recombination protein RecA | -0.23 | 0.80 | 175589.79 | 0.01 | 0.56 | 0.40 | 0.54 | 0.02 | 0.29 | 0.67 |
| K10559 | rhamnose transport system substrate-binding protein | -30.00 | 0.00 | 29653.63 | 0.00 | -0.10 | 0.98 | 17.84 | 0.00 | 0.91 | 0.79 |
| K08300 | ribonuclease E | -30.00 | 0.00 | 55817.18 | 0.53 | -1.06 | 0.86 | 18.98 | 0.00 | 0.49 | 0.91 |
| K12574 | ribonuclease J | -1.63 | 0.22 | 2339.92 | 0.00 | -2.26 | 0.09 | -15.15 | 0.00 | -16.74 | 0.00 |
| K00989 | ribonuclease PH | -15.43 | 0.00 | 0.00 | NA | -15.45 | 0.00 | NA | NA | -15.34 | 0.00 |
| K00525 | ribonucleoside-diphosphate reductase alpha chain | -17.62 | 0.00 | 62577.92 | 0.16 | 0.64 | 0.00 | 18.68 | 0.00 | 1.02 | 0.00 |
| K10439 | ribose transport system substrate-binding protein | -1.22 | 0.00 | 542522.63 | 0.31 | -1.31 | 0.00 | 0.32 | 0.20 | -0.91 | 0.00 |
| K00948 | ribose-phosphate pyrophosphokinase | NA | NA | 19358.80 | 0.39 | 17.29 | 0.00 | 27.88 | 0.00 | 27.83 | 0.00 |
| K02838 | ribosome recycling factor | -18.99 | 0.00 | 0.00 | NA | -19.01 | 0.00 | NA | NA | -18.90 | 0.00 |
| K01601 | ribulose-bisphosphate carboxylase large chain | 3.79 | 0.00 | 193450.46 | 0.00 | 3.44 | 0.00 | -0.90 | 0.27 | 2.87 | 0.00 |
| K01783 | ribulose-phosphate 3-epimerase | -17.35 | 0.00 | 195461.62 | 0.49 | 3.52 | 0.28 | 29.96 | 0.00 | 1.07 | 0.76 |
| K03086 | RNA polymerase primary sigma factor | 0.16 | 0.22 | 63581.27 | 0.34 | -0.90 | 0.80 | 1.31 | 0.05 | 1.44 | 0.03 |
| K03599 | RNA polymerase-associated protein | NA | NA | 53297.66 | 0.12 | 28.65 | 0.00 | 19.21 | 0.00 | 19.15 | 0.00 |
| K03569 | rod shape-determining protein MreB | 1.29 | 0.02 | 228760.58 | 0.52 | 1.85 | 0.01 | 1.40 | 0.25 | 2.65 | 0.01 |
| K00789 | S-adenosylmethionine synthetase | -2.79 | 0.32 | 214805.86 | 0.53 | 0.21 | 0.77 | 3.35 | 0.21 | 0.59 | 0.28 |
| K04771 | serine protease Do | -1.39 | 0.28 | 561344.58 | 0.00 | -0.39 | 0.75 | 5.32 | 0.00 | 3.89 | 0.00 |
| K07180 | serine protein kinase | 0.73 | 0.47 | 112515.49 | 0.33 | 1.56 | 0.02 | 0.16 | 0.91 | 0.85 | 0.42 |
| K07258 | serine-type D-Ala-D-Ala carboxypeptidase | -18.48 | 0.00 | 0.00 | NA | -18.50 | 0.00 | NA | NA | -18.40 | 0.00 |
| K03598 | sigma-E factor negative regulatory protein RseB | NA | NA | 17969.43 | 0.00 | NA | NA | 30.00 | 0.00 | 29.79 | 0.00 |
| K03106 | signal recognition particle subunit SRP54 | -1.82 | 0.78 | 34756.01 | 0.00 | -29.94 | 0.00 | 2.81 | 0.28 | 0.89 | 0.80 |
| K02058 | simple sugar transport system substrate-binding protein | -1.65 | 0.18 | 144316.49 | 0.90 | -1.74 | 0.12 | 0.16 | 0.92 | -1.51 | 0.00 |
| K03111 | single-strand DNA-binding protein | 20.43 | 0.00 | 0.00 | NA | NA | NA | -20.36 | 0.00 | NA | NA |
| K02945 | small subunit ribosomal protein S1 | 1.38 | 0.11 | 495333.61 | 0.35 | 3.21 | 0.00 | 2.86 | 0.00 | 4.24 | 0.00 |
| K02946 | small subunit ribosomal protein S10 | -1.27 | 0.84 | 67117.78 | 0.76 | -0.84 | 0.90 | 1.74 | 0.74 | 0.48 | 0.94 |
| K02948 | small subunit ribosomal protein S11 | -0.41 | 0.93 | 39208.43 | 0.82 | -1.30 | 0.82 | -0.21 | 0.00 | -0.67 | 0.86 |
| K02956 | small subunit ribosomal protein S15 | NA | NA | 32533.67 | 0.56 | 17.85 | 0.00 | 29.78 | 0.00 | 29.75 | 0.00 |
| K02967 | small subunit ribosomal protein S2 | 1.29 | NA | 228610.71 | 0.07 | 1.85 | 0.59 | 1.41 | 0.00 | 2.58 | 0.40 |
| K02982 | small subunit ribosomal protein S3 | -3.36 | NA | 325558.31 | 0.24 | -0.11 | 0.96 | 3.73 | 0.00 | 0.33 | NA |
| K02984 | small subunit ribosomal protein S3Ae | -16.60 | 0.00 | 0.00 | NA | -16.61 | 0.00 | NA | NA | -16.50 | 0.00 |
| K02986 | small subunit ribosomal protein S4 | 0.16 | 0.97 | 141744.43 | 0.50 | -0.21 | 0.98 | 1.41 | 0.01 | 1.51 | 0.66 |
| K02988 | small subunit ribosomal protein S5 | -18.27 | 0.00 | 73931.21 | 0.94 | 0.32 | 0.95 | 18.86 | 0.00 | 0.58 | 0.70 |
| K02992 | small subunit ribosomal protein S7 | -1.36 | 0.00 | 403003.91 | 0.88 | 0.49 | 0.42 | 2.01 | 0.00 | 0.63 | 0.19 |
| K02994 | small subunit ribosomal protein S8 | -29.10 | 0.00 | 27572.29 | 0.51 | 2.35 | 0.40 | 29.54 | 0.00 | 1.06 | 0.83 |
| K02996 | small subunit ribosomal protein S9 | NA | NA | 41104.73 | 0.00 | NA | NA | 18.97 | 0.00 | 18.89 | 0.00 |
| K05813 | sn-glycerol 3-phosphate transport system substrate-binding protein | -17.92 | 0.00 | 41971.96 | 0.00 | -17.94 | 0.00 | 19.00 | 0.00 | 1.05 | 0.00 |
| K06324 | spore coat protein A | 0.72 | 0.53 | 210509.95 | 0.32 | -0.22 | 0.87 | -1.38 | 0.05 | -0.68 | 0.58 |
| K21677 | squalene-associated FAD-dependent desaturase | 15.31 | 0.00 | 0.00 | NA | NA | NA | -15.24 | 0.00 | NA | NA |
| K00688 | starch phosphorylase | -18.32 | 0.00 | 5490.05 | 0.00 | -2.50 | 0.38 | NA | NA | -18.23 | 0.00 |
| K04047 | starvation-inducible DNA-binding protein | 1.24 | 0.83 | 48396.51 | 0.93 | 1.42 | 0.80 | 0.22 | 0.95 | 1.37 | 0.67 |
| K11085 | subfamily B ATP-binding cassette protein MsbA | NA | NA | 3162.13 | 0.00 | 15.01 | 0.00 | NA | NA | NA | NA |
| K00239 | succinate dehydrogenase / fumarate reductase flavoprotein subunit | 2.42 | 0.46 | 72068.01 | 0.81 | -0.10 | 0.98 | -3.12 | 0.28 | -0.83 | 0.89 |
| K01902 | succinyl-CoA synthetase alpha subunit | -0.57 | 0.90 | 70657.85 | 0.41 | 0.27 | 0.95 | -0.88 | 0.80 | -1.47 | 0.78 |
| K01903 | succinyl-CoA synthetase beta subunit | -29.98 | 0.00 | 20335.56 | 0.00 | 1.42 | 0.66 | NA | NA | -29.82 | 0.00 |
| K00958 | sulfate adenylyltransferase | 0.11 | 0.89 | 884661.92 | 0.00 | 0.10 | 0.91 | -1.09 | 0.00 | -1.04 | 0.01 |
| K06020 | sulfate-transporting ATPase | NA | NA | 11445.39 | 0.55 | 27.74 | NA | 16.80 | 0.00 | 16.70 | 0.00 |
| K17229 | sulfide dehydrogenase [flavocytochrome c] flavoprotein subunit | NA | NA | 16421.03 | 0.00 | NA | NA | 30.00 | 0.00 | 30.00 | 0.00 |
| K17218 | sulfide:quinone oxidoreductase | -5.42 | 0.02 | 483106.22 | 0.82 | 1.32 | 0.06 | 7.02 | 0.00 | 1.57 | 0.02 |
| K05301 | sulfite dehydrogenase | -18.77 | 0.00 | 741518.01 | 0.00 | 2.13 | 0.01 | 22.75 | 0.00 | 3.95 | 0.00 |
| K11180 | sulfite reductase alpha subunit | 0.42 | 0.44 | 3353409.30 | 0.75 | 1.23 | 0.01 | 1.06 | 0.00 | 1.48 | 0.00 |
| K11181 | sulfite reductase beta subunit | 0.46 | 0.25 | 2707265.26 | 0.00 | 0.51 | 0.11 | -0.06 | 0.85 | 0.36 | 0.27 |
| K15515 | sulfoacetaldehyde dehydrogenase | NA | NA | 7964.72 | 0.00 | 16.35 | 0.00 | NA | NA | NA | NA |
| K17222 | sulfur-oxidizing protein SoxA | -18.73 | 0.00 | 4528310.68 | 0.06 | 5.59 | 0.00 | 24.98 | 0.00 | 6.22 | 0.00 |
| K17224 | sulfur-oxidizing protein SoxB | 0.88 | 0.83 | 323318.82 | 0.44 | 2.73 | 0.59 | 3.85 | 0.00 | 4.76 | 0.06 |
| K17223 | sulfur-oxidizing protein SoxX | NA | NA | 158812.59 | 0.00 | 19.85 | 0.00 | 19.73 | 0.00 | 19.65 | 0.00 |
| K17226 | sulfur-oxidizing protein SoxY | 16.98 | 0.00 | 13224.84 | 0.00 | NA | NA | 0.31 | 0.00 | 17.26 | 0.00 |
| K17227 | sulfur-oxidizing protein SoxZ | NA | NA | 215479.51 | 0.00 | 21.08 | 0.00 | 16.97 | 0.00 | 16.88 | 0.00 |
| K05919 | superoxide reductase | -0.40 | 0.55 | 145398.88 | 0.00 | -0.89 | 0.05 | -1.12 | 0.00 | -1.58 | 0.00 |
| K05795 | tellurium resistance protein TerD | -17.56 | 0.00 | 0.00 | NA | -17.58 | 0.00 | NA | NA | -17.47 | 0.00 |
| K00577 | tetrahydromethanopterin S-methyltransferase subunit A | 1.00 | 0.81 | 284542.10 | 0.00 | 2.25 | 0.51 | -2.73 | 0.00 | -1.72 | 0.60 |
| K00584 | tetrahydromethanopterin S-methyltransferase subunit H | -0.31 | 0.41 | 372286.04 | 0.65 | -0.99 | 0.11 | -0.75 | 0.05 | -1.09 | 0.00 |
| K22447 | thermosome | 0.13 | 0.89 | 1232062.84 | 0.03 | -0.02 | 0.98 | -0.65 | 0.14 | -0.51 | 0.36 |
| K11065 | thiol peroxidase | NA | NA | 5460.38 | 0.00 | 28.81 | 0.00 | NA | NA | NA | NA |
| K03673 | thiol:disulfide interchange protein DsbA | -18.24 | 0.00 | 63800.64 | 0.54 | 0.02 | 0.98 | 18.76 | 0.00 | 0.51 | 0.25 |
| K03671 | thioredoxin 1 | 30.00 | 0.00 | 74819.09 | 0.00 | 19.60 | 0.00 | -30.00 | 0.00 | NA | NA |
| K01754 | threonine dehydratase | NA | NA | 60922.80 | 0.63 | 18.16 | 0.00 | 18.69 | 0.00 | 18.62 | 0.00 |
| K01868 | threonyl-tRNA synthetase | NA | NA | 11957.30 | 0.00 | NA | NA | 17.19 | 0.00 | 17.11 | 0.00 |
| K03568 | TldD protein | NA | NA | 10142.60 | 0.01 | 14.63 | 0.00 | 16.55 | 0.00 | 16.50 | 0.00 |
| K00616 | transaldolase | -0.67 | 0.92 | 281733.04 | 0.20 | 0.33 | 0.95 | 0.39 | 0.92 | -0.22 | 0.96 |
| K03124 | transcription initiation factor TFIIB | -0.86 | 0.00 | 13411.33 | 0.00 | -1.19 | 0.00 | -17.36 | 0.00 | -18.25 | 0.00 |
| K03120 | transcription initiation factor TFIID TATA-box-binding protein | 0.60 | 0.89 | 52615.00 | 0.52 | 1.26 | 0.20 | -0.87 | 0.88 | -0.26 | 0.95 |
| K03628 | transcription termination factor Rho | -0.16 | 0.74 | 1068436.86 | 0.99 | -0.18 | 0.80 | 0.24 | 0.54 | 0.07 | 0.91 |
| K02601 | transcriptional antiterminator NusG | 1.07 | 0.00 | 26176.43 | 0.65 | -0.39 | 0.56 | -1.54 | 0.00 | -0.51 | 0.38 |
| K13525 | transitional endoplasmic reticulum ATPase | -2.17 | 0.53 | 99342.50 | 0.16 | -1.88 | 0.60 | 1.11 | 0.15 | -1.02 | 0.78 |
| K00615 | transketolase | -1.81 | 0.00 | 1586040.58 | 0.00 | -0.72 | 0.25 | 0.10 | 0.03 | -1.77 | 0.00 |
| K02519 | translation initiation factor IF-2 | 0.52 | 0.89 | 41538.16 | 0.89 | 2.20 | 0.00 | 1.55 | 0.76 | 2.03 | 0.50 |
| K02520 | translation initiation factor IF-3 | NA | NA | 52927.78 | 0.89 | 30.00 | 0.00 | 18.16 | 0.00 | 18.08 | 0.00 |
| K09800 | translocation and assembly module TamB | NA | NA | 21069.60 | 0.00 | NA | NA | 18.01 | 0.00 | 17.93 | 0.00 |
| K07080 | TRAP transporter TAXI family solute receptor | -2.42 | 0.00 | 257735.54 | 0.26 | -2.02 | 0.00 | 0.93 | 0.01 | -1.50 | 0.00 |
| K21395 | TRAP-type C4-dicarboxylate transport system substrate-binding protein | 1.16 | 0.87 | 2956.36 | 0.00 | -1.28 | 0.80 | -29.76 | 0.00 | -29.76 | 0.00 |
| K03545 | trigger factor | 2.63 | 0.00 | 23734.51 | 0.52 | 2.21 | 0.43 | 1.19 | 0.00 | 3.77 | 0.00 |
| K01803 | triosephosphate isomerase | -0.26 | 0.96 | 42182.42 | 0.24 | 1.68 | 0.01 | -0.57 | 0.92 | -0.88 | 0.78 |
| K21395 | tripartite ATP-independent transporter DctP family solute receptor | 1.16 | 0.87 | 2956.36 | 0.00 | -1.28 | 0.80 | -29.76 | 0.00 | -29.76 | 0.00 |
| K04085 | tRNA 2-thiouridine synthesizing protein A | NA | NA | 24782.46 | 0.00 | 30.00 | 0.00 | NA | NA | NA | NA |
| K07237 | tRNA 2-thiouridine synthesizing protein B | NA | NA | 2915.89 | 0.00 | NA | NA | 28.21 | 0.00 | 28.14 | 0.00 |
| K07235 | tRNA 2-thiouridine synthesizing protein D | 0.74 | 0.00 | 104081.59 | 0.01 | 2.17 | 0.00 | 2.60 | 0.00 | 3.30 | 0.00 |
| K06001 | tryptophan synthase beta chain | 0.28 | 0.96 | 51652.15 | 0.89 | 0.50 | 0.91 | -0.17 | 0.98 | 0.16 | 0.97 |
| K01867 | tryptophanyl-tRNA synthetase | NA | NA | 4121.36 | 0.00 | NA | NA | 28.76 | 0.00 | 28.68 | 0.00 |
| K23077 | TusE/DsrC/DsvC family sulfur relay protein | 0.31 | 0.88 | 414387.57 | 0.03 | 0.75 | 0.40 | -0.30 | 0.83 | 0.03 | 0.98 |
| K02660 | twitching motility protein PilJ | 16.22 | 0.00 | 121045.34 | 0.06 | 18.66 | 0.00 | 3.72 | 0.00 | 19.88 | 0.00 |
| K02669 | twitching motility protein PilT | -12.57 | 0.00 | 111329.42 | 0.62 | 5.99 | 0.02 | 19.90 | 0.00 | 7.27 | 0.00 |
| K02670 | twitching motility protein PilU | NA | NA | 27551.99 | 0.67 | 17.55 | 0.00 | 28.94 | 0.00 | 29.82 | 0.00 |
| K02658 | twitching motility two-component system response regulator PilH | NA | NA | 2361.22 | 0.00 | NA | NA | 14.85 | 0.00 | 14.77 | 0.00 |
| K13587 | two-component system cell cycle sensor histidine kinase/response regulator CckA | NA | NA | 727.17 | 0.00 | 12.89 | 0.00 | NA | NA | NA | NA |
| K07712 | two-component system nitrogen regulation response regulator GlnG | NA | NA | 13992.16 | 0.55 | 27.94 | 0.00 | 30.00 | 0.00 | 30.00 | 0.00 |
| K02482 | two-component system NtrC family sensor kinase | NA | NA | 3794.62 | 0.00 | 28.43 | 0.00 | NA | NA | NA | NA |
| K14981 | two-component system response regulator ChvI | -18.61 | 0.00 | 29422.95 | 0.00 | -0.33 | 0.00 | NA | NA | -18.52 | 0.00 |
| K02650 | type IV pilus assembly protein PilA | NA | NA | 23159.58 | 0.00 | 17.89 | 0.00 | NA | NA | NA | NA |
| K02652 | type IV pilus assembly protein PilB | -16.66 | 0.00 | 0.00 | NA | -16.69 | 0.00 | NA | NA | -16.58 | 0.00 |
| K02656 | type IV pilus assembly protein PilF | NA | NA | 14874.38 | 0.83 | 16.26 | 0.00 | 16.49 | 0.00 | 16.41 | 0.00 |
| K02662 | type IV pilus assembly protein PilM | 17.55 | 0.00 | 88160.65 | 0.21 | 19.29 | 0.00 | 0.85 | 0.18 | 18.36 | 0.00 |
| K02663 | type IV pilus assembly protein PilN | -16.11 | 0.00 | 133290.18 | 0.13 | 3.87 | 0.00 | 18.89 | 0.00 | 2.71 | 0.00 |
| K02664 | type IV pilus assembly protein PilO | NA | NA | 12565.02 | 0.88 | 28.79 | 0.00 | 29.57 | 0.00 | 29.39 | 0.00 |
| K02666 | type IV pilus assembly protein PilQ | -0.22 | 0.55 | 2563983.19 | 0.00 | 0.50 | 0.27 | 1.63 | 0.00 | 1.37 | 0.00 |
| K11901 | type VI secretion system protein ImpB | -16.63 | 0.00 | 0.00 | NA | -16.65 | 0.00 | NA | NA | -16.54 | 0.00 |

| | | | | | | | | | | | |
|---|---|---|---|---|---|---|---|---|---|---|---|
| K11900 | type VI secretion system protein ImpC | -0.94 | 0.00 | 39796.02 | 0.40 | -2.22 | 0.00 | -0.65 | 0.26 | -1.60 | 0.01 |
| K11907 | type VI secretion system protein VasG | -0.22 | 0.12 | 29554.81 | 0.00 | 0.16 | 0.83 | -1.18 | 0.00 | -1.44 | 0.00 |
| K11903 | type VI secretion system secreted protein Hcp | 18.61 | 0.00 | 1741.86 | 0.00 | NA | NA | -4.25 | 0.00 | 14.33 | 0.00 |
| K01866 | tyrosyl-tRNA synthetase | NA | NA | 1812.82 | 0.00 | NA | NA | 27.27 | 0.00 | 26.97 | 0.00 |
| K00413 | ubiquinol-cytochrome c reductase cytochrome c1 subunit | -0.01 | 1.00 | 32573.81 | 0.73 | 2.08 | 0.51 | 1.04 | 0.85 | 1.06 | 0.75 |
| K00411 | ubiquinol-cytochrome c reductase iron-sulfur subunit | NA | NA | 123917.49 | 0.00 | NA | NA | 20.57 | 0.00 | 20.49 | 0.00 |
| K00012 | UDPglucose 6-dehydrogenase | NA | NA | 20618.92 | 0.88 | 30.00 | 0.00 | 29.70 | 0.00 | 29.68 | 0.00 |
| K08678 | UDP-glucuronate decarboxylase | -18.37 | 0.00 | 0.00 | NA | -18.39 | 0.00 | NA | NA | -18.28 | 0.00 |
| K02473 | UDP-N-acetylglucosamine 4-epimerase | NA | NA | 2699.49 | 0.00 | NA | NA | 15.04 | 0.00 | 14.97 | 0.00 |
| K09702 | uncharacterized protein | -18.08 | 0.00 | 1597.77 | 0.00 | -18.10 | 0.00 | 14.29 | 0.00 | -3.82 | 0.00 |
| K09807 | uncharacterized protein YggE | -1.97 | 0.00 | 0.00 | NA | -17.53 | 0.00 | -15.42 | 0.00 | -17.43 | 0.00 |
| K11959 | urea transport system substrate-binding protein | -0.94 | 0.82 | 48145.89 | 0.94 | -0.44 | 0.60 | 0.57 | 0.92 | -0.41 | 0.92 |
| K02302 | uroporphyrin-III C-methyltransferase/precorrin-2 dehydrogenase/sirohydrochlorin ferrochelata | NA | NA | 10044.38 | 0.00 | NA | NA | 29.12 | 0.00 | 29.10 | 0.00 |
| K00963 | UTP--glucose-1-phosphate uridylyltransferase | NA | NA | 63201.20 | 0.49 | 18.15 | 0.00 | 18.75 | 0.00 | 18.69 | 0.00 |
| K02117 | V/A-type H+-transporting ATPase subunit A | -0.07 | 0.81 | 1502380.58 | 0.00 | -0.56 | 0.00 | -0.95 | 0.00 | -1.04 | 0.00 |
| K02118 | V/A-type H+-transporting ATPase subunit B | -0.11 | 0.85 | 1426580.53 | 0.00 | -0.45 | 0.03 | -0.80 | 0.03 | -0.92 | 0.00 |
| K02119 | V/A-type H+-transporting ATPase subunit C | 1.07 | 0.03 | 216138.22 | 0.18 | 0.96 | 0.03 | -0.38 | 0.32 | 0.70 | 0.17 |
| K02121 | V/A-type H+-transporting ATPase subunit E | -0.91 | 0.01 | 440133.54 | 0.13 | -0.98 | 0.00 | -0.40 | 0.45 | -1.35 | 0.00 |
| K02122 | V/A-type H+-transporting ATPase subunit F | -17.18 | 0.00 | 0.00 | NA | -17.20 | 0.00 | NA | NA | -17.09 | 0.00 |
| K02123 | V/A-type H+-transporting ATPase subunit I | 2.87 | 0.01 | 35195.51 | 0.00 | 1.62 | 0.00 | -19.65 | 0.00 | -16.82 | 0.00 |
| K01805 | xylose isomerase | 16.36 | 0.00 | 2616.81 | 0.00 | NA | NA | -1.41 | 0.00 | 14.92 | 0.00 |
| K01621 | xylulose-5-phosphate/fructose-6-phosphate phosphoketolase | -13.69 | 0.00 | 5025.29 | 0.48 | 0.11 | 0.31 | 28.57 | 0.00 | 1.74 | 0.53 |
| K07263 | zinc protease | NA | NA | 30885.96 | 0.83 | 17.54 | 0.00 | 30.00 | 0.00 | 30.00 | 0.00 |
| K00765 | ATP phosphoribosyltransferase | NA | NA | 17624.61 | 0.00 | NA | NA | 17.75 | 0.00 | 17.67 | 0.00 |
| K23107 | 1-deoxyxylulose-5-phosphate synthase | NA | NA | 17605.24 | 0.00 | NA | NA | 17.75 | 0.00 | 17.67 | 0.00 |
| K03735 | ethanolamine ammonia-lyase large subunit | -17.85 | 0.00 | 11722.85 | 0.00 | -0.89 | 0.00 | NA | NA | -17.76 | 0.00 |
| K01810 | glucose-6-phosphate isomerase | NA | NA | 77196.78 | 0.00 | 19.26 | 0.00 | 17.71 | 0.00 | 17.63 | 0.00 |
| K03149 | thiazole synthase | NA | NA | 16475.66 | 0.00 | NA | NA | 17.65 | 0.00 | 17.58 | 0.00 |
| K02665 | type IV pilus assembly protein PilP | NA | NA | 27296.61 | 0.65 | 29.56 | 0.00 | 17.82 | 0.00 | 17.73 | 0.00 |
| K00177 | 2-oxoglutarate ferredoxin oxidoreductase subunit gamma | -17.56 | 0.00 | 0.00 | NA | -17.58 | 0.00 | NA | NA | -17.48 | 0.00 |
| K22222 | tubulin-like protein CetZ | -17.56 | 0.00 | 0.00 | NA | -17.58 | 0.00 | NA | NA | -17.48 | 0.00 |
| K03071 | preprotein translocase subunit SecB | NA | NA | 13905.14 | 0.00 | NA | NA | 17.41 | 0.00 | 17.33 | 0.00 |
| K16787 | energy-coupling factor transport system ATP-binding protein | NA | NA | 12736.34 | 0.00 | NA | NA | 17.28 | 0.00 | 17.20 | 0.00 |
| K00368 | nitrite reductase (NO-forming) | -17.38 | 0.00 | 0.00 | NA | -17.41 | 0.00 | NA | NA | -17.30 | 0.00 |
| K07041 | uncharacterized protein | NA | NA | 12697.60 | 0.00 | NA | NA | 17.28 | 0.00 | 17.20 | 0.00 |
| K03500 | 16S rRNA (cytosine967-C5)-methyltransferase | NA | NA | 12619.65 | 0.00 | NA | NA | 17.27 | 0.00 | 17.19 | 0.00 |
| K08691 | malyl-CoA/(S)-citramalyl-CoA lyase | -17.36 | 0.00 | 0.00 | NA | -17.38 | 0.00 | NA | NA | -17.28 | 0.00 |
| K03390 | heterodisulfide reductase subunit C2 | -17.30 | 0.00 | 0.00 | NA | -17.33 | 0.00 | NA | NA | -17.22 | 0.00 |
| K06911 | quercetin 2,3-dioxygenase | NA | NA | 11732.42 | 0.00 | NA | NA | 17.16 | 0.00 | 17.09 | 0.00 |
| K03183 | demethylmenaquinone methyltransferase / 2-methoxy-6-polyprenyl-1,4-benzoquinol methylas | -17.26 | 0.00 | 0.00 | NA | -17.28 | 0.00 | NA | NA | -17.17 | 0.00 |
| K02314 | replicative DNA helicase | 29.55 | 0.00 | 11334.16 | 0.00 | NA | NA | 0.54 | 0.88 | 17.04 | 0.00 |
| K13812 | bifunctional enzyme Fae/Hps | NA | NA | 11142.35 | 0.00 | NA | NA | 17.09 | 0.00 | 17.01 | 0.00 |
| K19064 | lysine 6-dehydrogenase | -17.18 | 0.00 | 0.00 | NA | -17.20 | 0.00 | NA | NA | -17.09 | 0.00 |
| K00259 | alanine dehydrogenase | NA | NA | 10817.79 | 0.00 | NA | NA | 17.05 | 0.00 | 16.97 | 0.00 |
| K08301 | ribonuclease G | NA | NA | 9510.56 | 0.00 | NA | NA | 16.86 | 0.00 | 16.78 | 0.00 |
| K02031 | peptide/nickel transport system ATP-binding protein | -16.86 | 0.00 | 0.00 | NA | -16.88 | 0.00 | NA | NA | -16.77 | 0.00 |
| K17725 | sulfur dioxygenase | NA | NA | 16786.99 | 0.96 | 29.50 | 0.00 | 16.74 | 0.00 | 16.66 | 0.00 |
| K19804 | lipopolysaccharide assembly protein B | NA | NA | 8374.36 | 0.00 | NA | NA | 16.68 | 0.00 | 16.60 | 0.00 |
| K02188 | cobalt-precorrin-5B (C1)-methyltransferase | -16.73 | 0.00 | 0.00 | NA | -16.75 | 0.00 | NA | NA | -16.65 | 0.00 |
| K21417 | acetoin:2,6-dichlorophenolindophenol oxidoreductase subunit beta | -16.67 | 0.00 | 0.00 | NA | -16.69 | 0.00 | NA | NA | -16.58 | 0.00 |
| K02454 | general secretion pathway protein E | -16.66 | 0.00 | 0.00 | NA | -16.69 | 0.00 | NA | NA | -16.58 | 0.00 |
| K12339 | S-sulfo-L-cysteine synthase | -16.62 | 0.00 | 0.00 | NA | -16.64 | 0.00 | NA | NA | -16.53 | 0.00 |
| K13628 | iron-sulfur cluster assembly protein | NA | NA | 7439.41 | 0.00 | NA | NA | 16.51 | 0.00 | 16.43 | 0.00 |
| K01258 | tripeptide aminopeptidase | NA | NA | 24537.92 | 0.00 | 17.45 | 0.00 | 16.51 | 0.00 | 16.43 | 0.00 |
| K06044 | (1->4)-alpha-D-glucan 1-alpha-D-glucosylmutase | NA | NA | 7324.14 | 0.00 | NA | NA | 16.48 | 0.00 | 16.41 | 0.00 |
| K00705 | 4-alpha-glucanotransferase | NA | NA | 7324.14 | 0.00 | NA | NA | 16.48 | 0.00 | 16.41 | 0.00 |
| K07275 | outer membrane protein | -16.56 | 0.00 | 15743.82 | 0.00 | 0.82 | 0.00 | NA | NA | -16.47 | 0.00 |
| K12538 | outer membrane protein, type I secretion system | NA | NA | 7027.92 | 0.00 | NA | NA | 16.43 | 0.00 | 16.35 | 0.00 |
| K03590 | cell division protein FtsA | -16.43 | 0.00 | 14745.38 | 0.00 | 0.85 | 0.00 | NA | NA | -16.34 | 0.00 |
| K01995 | branched-chain amino acid transport system ATP-binding protein | NA | NA | 6981.19 | 0.00 | 12.69 | 0.00 | 16.28 | 0.00 | 16.20 | 0.00 |
| K01998 | branched-chain amino acid transport system permease protein | NA | NA | 6348.57 | 0.00 | NA | NA | 16.28 | 0.00 | 16.20 | 0.00 |
| K00681 | gamma-glutamyltranspeptidase / glutathione hydrolase | -16.36 | 0.00 | 0.00 | NA | -16.38 | 0.00 | NA | NA | -16.28 | 0.00 |
| K01608 | tartronate-semialdehyde synthase | NA | NA | 14831.10 | 0.00 | 16.49 | 0.00 | 16.21 | 0.00 | 16.13 | 0.00 |
| K02343 | DNA polymerase III subunit gamma/tau | NA | NA | 5900.70 | 0.00 | NA | NA | 16.17 | 0.00 | 16.09 | 0.00 |
| K09065 | N-acetylornithine carbamoyltransferase | -16.22 | 0.00 | 0.00 | NA | -16.24 | 0.00 | NA | NA | -16.13 | 0.00 |
| K06869 | uncharacterized protein | -0.26 | 0.02 | 0.00 | NA | -16.21 | 0.00 | -15.81 | 0.00 | -16.11 | 0.00 |
| K01782 | 3-hydroxyacyl-CoA dehydrogenase / enoyl-CoA hydratase / 3-hydroxybutyryl-CoA epimerase | NA | NA | 5484.48 | 0.00 | NA | NA | 16.07 | 0.00 | 15.99 | 0.00 |
| K01280 | tripeptidyl-peptidase II | 0.31 | 0.00 | 0.00 | NA | -16.17 | 0.00 | -16.34 | 0.00 | -16.06 | 0.00 |
| K00677 | UDP-N-acetylglucosamine acyltransferase | -16.15 | 0.00 | 0.00 | NA | -16.17 | 0.00 | NA | NA | -16.06 | 0.00 |
| K01945 | phosphoribosylamine---glycine ligase | -16.05 | 0.00 | 0.00 | NA | -16.07 | 0.00 | NA | NA | -15.97 | 0.00 |
| K02674 | type IV pilus assembly protein PilY1 | NA | NA | 5102.28 | 0.00 | NA | NA | 15.96 | 0.00 | 15.88 | 0.00 |
| K00549 | 5-methyltetrahydropteroyltriglutamate--homocysteine methyltransferase | NA | NA | 4868.90 | 0.00 | NA | NA | 15.90 | 0.00 | 15.82 | 0.00 |
| K01126 | glycerophosphoryl diester phosphodiesterase | NA | NA | 7935.58 | 0.81 | 27.60 | 0.00 | 15.83 | 0.00 | 15.75 | 0.00 |
| K04567 | lysyl-tRNA synthetase, class II | NA | NA | 4165.45 | 0.00 | NA | NA | 15.67 | 0.00 | 15.59 | 0.00 |
| K02410 | flagellar motor switch protein FliG | NA | NA | 3716.16 | 0.00 | NA | NA | 15.51 | 0.00 | 15.43 | 0.00 |
| K02990 | small subunit ribosomal protein S6 | NA | NA | 3689.24 | 0.00 | NA | NA | 15.50 | 0.00 | 15.42 | 0.00 |
| K01042 | L-seryl-tRNA(Ser) seleniumtransferase | -15.53 | 0.00 | 0.00 | NA | -15.55 | 0.00 | NA | NA | -15.44 | 0.00 |
| K00872 | homoserine kinase | NA | NA | 3364.67 | 0.00 | NA | NA | 15.36 | 0.00 | 15.28 | 0.00 |
| K09949 | UDP-2,3-diacylglucosamine hydrolase | -15.19 | 0.00 | 9606.68 | 0.00 | 1.48 | 0.00 | NA | NA | -15.10 | 0.00 |
| K04748 | nitric oxide reductase NorQ protein | NA | NA | 33243.22 | 0.00 | NA | NA | 18.66 | 0.00 | 18.57 | 0.00 |
| K16898 | ATP-dependent helicase/nuclease subunit A | -15.01 | 0.00 | 0.00 | NA | -15.03 | 0.00 | NA | NA | -14.92 | 0.00 |
| K07646 | two-component system, OmpR family, sensor histidine kinase KdpD | -14.95 | 0.00 | 0.00 | NA | -14.97 | 0.00 | NA | NA | -14.86 | 0.00 |
| K02340 | DNA polymerase III subunit delta | -14.87 | 0.00 | 0.00 | NA | -14.89 | 0.00 | NA | NA | -14.79 | 0.00 |
| K04065 | hyperosmotically inducible periplasmic protein | NA | NA | 23685.26 | 0.46 | 17.10 | 0.00 | 16.95 | 0.00 | 16.89 | 0.00 |
| K03565 | regulatory protein | -14.83 | 0.00 | 0.00 | NA | -14.85 | 0.00 | NA | NA | -14.74 | 0.00 |
| K03546 | DNA repair protein SbcC/Rad50 | 4.37 | 0.00 | 0.00 | NA | -14.83 | 0.00 | -19.06 | 0.00 | -14.73 | 0.00 |
| K00055 | aryl-alcohol dehydrogenase | NA | NA | 1847.22 | 0.00 | NA | NA | 14.50 | 0.00 | 14.42 | 0.00 |
| K01792 | glucose-6-phosphate 1-epimerase | NA | NA | 10186.99 | 0.00 | 16.44 | 0.00 | 14.35 | 0.00 | 14.27 | 0.00 |
| K02304 | precorrin-2 dehydrogenase | NA | NA | 1452.73 | 0.00 | NA | NA | 14.15 | 0.00 | 14.07 | 0.00 |
| K01736 | chorismate synthase | NA | NA | 1428.17 | 0.00 | NA | NA | 14.13 | 0.00 | 14.05 | 0.00 |
| K00790 | UDP-N-acetylglucosamine 1-carboxyvinyltransferase | NA | NA | 1099.35 | 0.00 | NA | NA | 13.75 | 0.00 | 13.67 | 0.00 |
| K01962 | acetyl-CoA carboxylase carboxyl transferase subunit alpha | NA | NA | 1058.72 | 0.00 | NA | NA | 13.69 | 0.00 | 13.62 | 0.00 |
| K02078 | acyl carrier protein | NA | NA | 898.57 | 0.00 | NA | NA | 13.46 | 0.00 | 13.38 | 0.00 |
| K20920 | polysaccharide biosynthesis protein VpsM | -16.99 | 0.00 | 101265.45 | 0.00 | 1.51 | 0.00 | 19.68 | 0.00 | 2.66 | 0.00 |
| K10822 | nonpolar-amino-acid-transporting ATPase | NA | NA | 22925.84 | 0.53 | 16.50 | 0.00 | 17.47 | 0.00 | 17.34 | 0.00 |
| K08884 | serine/threonine protein kinase, bacterial | 14.98 | 0.00 | 28479.21 | 0.87 | 29.83 | 0.00 | 2.68 | 0.32 | 30.00 | 0.00 |
| K03768 | peptidyl-prolyl cis-trans isomerase B (cyclophilin B) | -0.85 | 0.83 | 0.00 | NA | -30.00 | 0.00 | -16.44 | 0.00 | -30.00 | 0.00 |
| K02487 | type IV pili sensor histidine kinase | NA | NA | 19211.39 | 0.00 | NA | NA | 30.00 | 0.00 | 30.00 | 0.00 |
| K01023 | arylsulfate sulfotransferase | 1.05 | 0.78 | 10211.07 | 0.00 | 0.28 | 0.96 | -17.35 | 0.00 | -29.96 | 0.00 |
| K07337 | penicillin-binding protein activator | NA | NA | 12427.37 | 0.70 | 15.48 | 0.00 | 28.72 | 0.00 | 29.62 | 0.00 |
| K22187 | alpha-ketoglutaric semialdehyde dehydrogenase | NA | NA | 8127.60 | 0.00 | NA | NA | 29.63 | 0.00 | 29.58 | 0.00 |
| K07175 | PhoH-like ATPase | NA | NA | 19669.70 | 0.86 | 29.98 | 0.00 | 29.30 | 0.00 | 29.39 | 0.00 |
| K02303 | uroporphyrin-III C-methyltransferase | NA | NA | 10044.38 | 0.00 | NA | NA | 29.12 | 0.00 | 29.10 | 0.00 |
| K01622 | fructose 1,6-bisphosphate aldolase/phosphatase | -29.14 | 0.00 | 0.00 | NA | -29.14 | 0.00 | NA | NA | -29.00 | 0.00 |
| K08070 | 2-alkenal reductase | NA | NA | 5242.07 | 0.00 | NA | NA | 28.93 | 0.00 | 28.90 | 0.00 |
| K01991 | polysaccharide biosynthesis/export protein | 30.00 | 0.00 | 20858.76 | 0.76 | 29.18 | 0.00 | -0.86 | 0.88 | 28.87 | 0.00 |
| K06346 | spoIIIJ-associated protein | NA | NA | 3706.15 | 0.00 | NA | NA | 28.59 | 0.00 | 28.55 | 0.00 |
| K01586 | diaminopimelate decarboxylase | 19.16 | 0.00 | 18263.66 | 0.36 | 30.00 | 0.00 | -4.70 | 0.04 | 27.58 | 0.00 |
| K03518 | aerobic carbon-monoxide dehydrogenase | NA | NA | 1872.33 | 0.00 | NA | NA | 27.57 | 0.00 | 27.33 | 0.00 |
| K01130 | arylsulfatase | 0.89 | 0.46 | 41346.12 | 0.89 | -1.90 | 0.57 | -2.34 | 0.01 | -1.46 | 0.00 |
| K07192 | flotillin | -1.60 | 0.00 | 9069.14 | 0.00 | -17.60 | 0.00 | 0.81 | 0.00 | -0.83 | 0.00 |
| K03657 | ATP-dependent DNA helicase UvrD/PcrA | -15.01 | 0.00 | 4887.96 | 0.00 | -1.25 | 0.00 | 15.46 | 0.00 | 0.42 | 0.00 |
| K05520 | deglycase | -17.25 | 0.00 | 16484.16 | 0.00 | -17.27 | 0.00 | 17.65 | 0.00 | 0.37 | 0.01 |
| K00020 | 3-hydroxyisobutyrate dehydrogenase | -0.44 | 0.22 | 58576.80 | 0.21 | 0.05 | 0.98 | -0.65 | 0.51 | -1.14 | 0.23 |
| K11179 | tRNA 2-thiouridine synthesizing protein E | 1.46 | 0.00 | 118951.34 | 0.25 | 0.38 | 0.83 | -2.51 | 0.00 | -1.07 | 0.29 |
| K00611 | ornithine carbamoyltransferase | 1.08 | 0.00 | 58814.32 | 0.00 | 1.94 | 0.00 | -0.83 | 0.00 | 0.21 | 0.38 |
| K18584 | actin-related protein 3 | 0.42 | 0.92 | 151012.11 | 0.00 | 3.35 | 0.22 | 1.97 | 0.00 | 2.41 | 0.39 |
| K02120 | V/A-type H+/Na+-transporting ATPase subunit D | 1.05 | 0.81 | 9252.41 | 0.78 | -2.38 | 0.63 | -4.22 | 0.10 | -3.13 | 0.49 |
| K02044 | phosphonate transport system substrate-binding protein | 1.77 | 0.15 | 27492.98 | 0.56 | -0.74 | 0.56 | -3.71 | 0.18 | -1.94 | 0.51 |
| K02878 | large subunit ribosomal protein L16 | -30.00 | 0.00 | 33859.99 | 0.66 | -2.98 | 0.56 | 18.12 | 0.00 | -1.92 | 0.59 |
| K00335 | NADH-quinone oxidoreductase subunit F | -0.58 | 0.89 | 45464.86 | 0.87 | -0.79 | 0.83 | 0.42 | 0.91 | -0.21 | 0.59 |
| K00135 | succinate-semialdehyde dehydrogenase / glutarate-semialdehyde dehydrogenase | -16.94 | 0.00 | 3566.94 | 0.00 | -16.96 | 0.00 | 28.26 | 0.00 | -1.53 | 0.60 |
| K05592 | ATP-dependent RNA helicase DeaD | 0.07 | 0.99 | 17162.37 | 0.26 | 1.75 | 0.73 | -0.90 | 0.00 | -0.81 | 0.79 |
| K03684 | ribonuclease D | 0.70 | 0.54 | 345860.60 | 0.01 | 0.44 | 0.72 | -0.45 | 0.00 | 0.27 | 0.82 |
| K18933 | tyrosine decarboxylase / aspartate 1-decarboxylase | -0.82 | 0.90 | 26015.43 | 0.58 | -2.11 | 0.68 | 0.05 | 0.99 | -0.73 | 0.84 |
| K00027 | malate dehydrogenase (oxaloacetate-decarboxylating) | 2.26 | 0.46 | 45376.21 | 0.60 | 0.50 | 0.91 | -2.85 | 0.30 | -0.56 | 0.93 |
| K11187 | peroxiredoxin 5 | -1.10 | 0.76 | 24813.51 | 0.88 | -0.95 | 0.79 | 0.84 | 0.88 | -0.35 | 0.93 |
| K09860 | outer membrane protein FlgP | -29.68 | 0.00 | 7794.08 | 0.01 | -1.14 | 0.72 | 15.88 | 0.00 | -0.30 | 0.94 |
| K17285 | methanethiol oxidase | -19.95 | 0.00 | 111921.66 | 0.59 | -1.96 | NA | 30.00 | 0.00 | 0.09 | 0.98 |
| K01302 | carboxypeptidase Q | NA | NA | 3072.53 | 0.00 | 14.97 | 0.00 | NA | NA | NA | NA |
| K05962 | two-component system, LuxR family, sensor histidine kinase TtrS | 29.41 | 0.00 | 60213.02 | 0.00 | 30.00 | 0.00 | -29.93 | 0.00 | NA | NA |
| K01028 | 3-oxoacid CoA-transferase subunit A | NA | NA | 7346.74 | 0.00 | 29.36 | 0.00 | NA | NA | NA | NA |
| K03079 | L-ribulose-5-phosphate 3-epimerase | 19.08 | 0.00 | 0.00 | NA | NA | NA | -19.01 | 0.00 | NA | NA |
| K02474 | UDP-N-acetyl-D-glucosamine/UDP-N-acetyl-D-galactosamine dehydrogenase | NA | NA | 10538.29 | 0.00 | 16.75 | 0.00 | NA | NA | NA | NA |
| K09691 | homopolymeric O-antigen transport system ATP-binding protein | NA | NA | 28609.65 | 0.00 | 18.19 | 0.00 | NA | NA | NA | NA |
| K11891 | type VI secretion system protein ImpL | NA | NA | 1680.56 | 0.00 | 14.10 | 0.00 | NA | NA | NA | NA |
| K01497 | GTP cyclohydrolase II | NA | NA | 16595.14 | 0.00 | 30.00 | 0.00 | NA | NA | NA | NA |
| K0701 | putative glutamine amidotransferase | 15.75 | 0.00 | 0.00 | NA | NA | NA | -15.67 | 0.00 | NA | NA |
| K03044 | DNA-directed RNA polymerase subunit B' | 16.16 | 0.00 | 21672.07 | 0.00 | 17.79 | 0.00 | -16.09 | 0.00 | NA | NA |
| K00712 | poly(glycerol-phosphate) alpha-glucosyltransferase | NA | NA | 1325.14 | 0.00 | 13.76 | 0.00 | NA | NA | NA | NA |
| K00184 | dimethyl sulfoxide reductase iron-sulfur subunit | NA | NA | 7009.85 | 0.00 | 16.16 | 0.00 | NA | NA | NA | NA |
| K02851 | UDP-GlcNAc:undecaprenyl-phosphate/decaprenyl-phosphate GlcNAc-1-phosphate transfera | NA | NA | 8682.00 | 0.00 | 16.47 | 0.00 | NA | NA | NA | NA |

| | | | | | | | | | | | |
|---|---|---|---|---|---|---|---|---|---|---|---|
| K01170 | tRNA-intron endonuclease, archaea type | 16.52 | 0.00 | 0.00 | NA | NA | NA | -16.45 | 0.00 | NA | NA |
| K01933 | phosphoribosylformylglycinamidine cyclo-ligase | 17.11 | 0.00 | 4005.13 | 0.00 | 15.35 | 0.00 | -17.04 | 0.00 | NA | NA |
| K05594 | ElaB protein | NA | NA | 57238.03 | 0.00 | 19.20 | 0.00 | NA | NA | NA | NA |
| K01092 | myo-inositol-1(or 4)-monophosphatase | NA | NA | 1593.93 | 0.00 | 14.03 | 0.00 | NA | NA | NA | NA |
| K08264 | heterodisulfide reductase subunit D | 18.00 | 0.00 | 0.00 | NA | NA | NA | -17.93 | 0.00 | NA | NA |
| K13924 | two-component system, chemotaxis family, CheB/CheR fusion protein | 17.78 | 0.00 | 0.00 | NA | NA | NA | -17.71 | 0.00 | NA | NA |
| K03615 | H+/Na+-translocating ferredoxin:NAD+ oxidoreductase subunit C | -0.99 | 0.80 | 16346.23 | 0.51 | -1.01 | 0.10 | -0.48 | 0.90 | -1.43 | NA |
| K02837 | peptide chain release factor 3 | 15.97 | 0.00 | 0.00 | NA | NA | NA | -15.90 | 0.00 | NA | NA |
| K01926 | redox-sensing transcriptional repressor | 30.00 | 0.00 | 25667.15 | 0.00 | 30.00 | 0.00 | -30.00 | 0.00 | NA | NA |
| K01417 | extracellular elastinolytic metalloproteinase | NA | NA | 976.66 | 0.00 | 13.32 | 0.00 | NA | NA | NA | NA |
| K02316 | DNA primase | NA | NA | 11559.49 | 0.00 | 16.88 | 0.00 | NA | NA | NA | NA |
| K13819 | NifU-like protein | NA | NA | 3750.70 | 0.00 | 15.26 | 0.00 | NA | NA | NA | NA |
| K11076 | putrescine transport system ATP-binding protein | NA | NA | 8250.84 | 0.00 | 16.40 | 0.00 | NA | NA | NA | NA |
| K00374 | nitrate reductase gamma subunit | NA | NA | 5179.79 | 0.00 | 15.73 | 0.00 | NA | NA | NA | NA |
| K16887 | quinone-modifying oxidoreductase, subunit QmoC | 16.78 | 0.00 | 0.00 | NA | NA | NA | -16.71 | 0.00 | NA | NA |
| K00015 | glyoxylate reductase | 14.98 | 0.00 | 0.00 | NA | NA | NA | -14.91 | 0.00 | NA | NA |
| K14170 | chorismate mutase / prephenate dehydratase | NA | NA | 3403.20 | 0.00 | 15.12 | 0.00 | NA | NA | NA | NA |
| K04518 | prephenate dehydratase | NA | NA | 3403.20 | 0.00 | 15.12 | 0.00 | NA | NA | NA | NA |
| K00412 | ubiquinol-cytochrome c reductase cytochrome b subunit | NA | NA | 8454.79 | 0.00 | 16.43 | 0.00 | NA | NA | NA | NA |
| K20976 | histidine phosphotransfer protein HptB | NA | NA | 0.00 | NA | NA | NA | NA | NA | NA | NA |
| K06147 | ATP-binding cassette, subfamily B | NA | NA | 3162.13 | 0.00 | 15.01 | 0.00 | NA | NA | NA | NA |
| K17758 | ADP-dependent NAD(P)H-hydrate dehydratase | 18.77 | 0.00 | 0.00 | NA | NA | NA | -18.70 | 0.00 | NA | NA |
| K17759 | NAD(P)H-hydrate epimerase | 18.77 | 0.00 | 0.00 | NA | NA | NA | -18.70 | 0.00 | NA | NA |
| K01733 | threonine synthase | NA | NA | 1529.32 | NA | 27.13 | NA | NA | NA | NA | NA |
| K17836 | beta-lactamase class A | NA | NA | 2477.53 | 0.00 | 14.66 | 0.00 | NA | NA | NA | NA |
| K00566 | tRNA-uridine 2-sulfurtransferase | 11.72 | 0.00 | 0.00 | NA | NA | NA | -11.65 | 0.00 | NA | NA |
| K03821 | poly[(R)-3-hydroxyalkanoate] polymerase subunit PhaC | 17.65 | 0.00 | 0.00 | NA | NA | NA | -17.57 | 0.00 | NA | NA |
| K01638 | malate synthase | 15.58 | 0.00 | 0.00 | NA | NA | NA | -15.51 | 0.00 | NA | NA |
| K03703 | excinuclease ABC subunit C | 17.07 | 0.00 | 0.00 | NA | NA | NA | -17.00 | 0.00 | NA | NA |
| K03641 | TolB protein | NA | NA | 0.00 | NA | NA | NA | NA | NA | NA | NA |
| K06886 | hemoglobin | 14.61 | 0.00 | 0.00 | NA | NA | NA | -14.54 | 0.00 | NA | NA |
| K07250 | 4-aminobutyrate aminotransferase / (S)-3-amino-2-methylpropionate transaminase / 5-aminov | 29.26 | 0.00 | 0.00 | NA | NA | NA | -30.00 | 0.00 | NA | NA |
| K00823 | 4-aminobutyrate aminotransferase | 29.26 | 0.00 | 0.00 | NA | NA | NA | -30.00 | 0.00 | NA | NA |
| K07214 | iron(III)-enterobactin esterase | NA | NA | 22315.58 | 0.00 | 17.83 | 0.00 | NA | NA | NA | NA |
| K01159 | crossover junction endodeoxyribonuclease RuvC | 16.13 | 0.00 | 0.00 | NA | NA | NA | -16.06 | 0.00 | NA | NA |
| K00931 | glutamate 5-kinase | NA | NA | 111451.99 | NA | 30.00 | NA | NA | NA | NA | NA |
| K07638 | two-component system, OmpR family, osmolarity sensor histidine kinase | 17.55 | 0.00 | 0.00 | NA | NA | NA | -17.48 | 0.00 | NA | NA |
| K18682 | ribonucrease Y | NA | NA | 0.00 | NA | NA | NA | NA | NA | NA | NA |
| K16306 | fructose-bisphosphate aldolase / 2-amino-3,7-dideoxy-D-threo-hept-6-ulosonate synthase | 30.00 | 0.00 | 10148.71 | 0.00 | 16.70 | 0.00 | -30.00 | 0.00 | NA | NA |
| K01890 | phenylalanyl-tRNA synthetase beta chain | NA | NA | 10864.99 | 0.00 | 16.79 | 0.00 | NA | NA | NA | NA |
| K01209 | alpha-L-arabinofuranosidase | 14.68 | 0.00 | 0.00 | NA | NA | NA | -14.61 | 0.00 | NA | NA |
| K07536 | 2-ketocyclohexanecarboxyl-CoA hydrolase | NA | NA | 18367.87 | 0.00 | 17.55 | 0.00 | NA | NA | NA | NA |
| K12257 | SecD/SecF fusion protein | 17.69 | 0.00 | 0.00 | NA | NA | NA | -17.62 | 0.00 | NA | NA |
| K04044 | molecular chaperone HscA | NA | NA | 1462.26 | 0.00 | 13.90 | 0.00 | NA | NA | NA | NA |
| K00763 | nicotinate phosphoribosyltransferase | 21.39 | 0.00 | 0.00 | NA | NA | NA | -21.32 | 0.00 | NA | NA |
| K07482 | transposase, IS30 family | 14.35 | 0.00 | 0.00 | NA | NA | NA | -14.28 | 0.00 | NA | NA |
| K06959 | protein Tex | 15.44 | 0.00 | 0.00 | NA | NA | NA | -15.37 | 0.00 | NA | NA |
| K06200 | carbon starvation protein | NA | NA | 15355.24 | 0.00 | 17.29 | 0.00 | NA | NA | NA | NA |
| K15256 | tRNA (cmo5U34)-methyltransferase | NA | NA | 14091.47 | 0.00 | 17.17 | 0.00 | NA | NA | NA | NA |
| K14128 | F420-non-reducing hydrogenase small subunit | NA | NA | 13823.18 | 0.00 | 17.14 | 0.00 | NA | NA | NA | NA |
| K00784 | ribonuclease Z | NA | NA | 7964.72 | 0.00 | 16.35 | 0.00 | NA | NA | NA | NA |
| K06413 | stage V sporulation protein K | NA | NA | 5123.36 | 0.00 | 15.71 | 0.00 | NA | NA | NA | NA |
| K02904 | large subunit ribosomal protein L29 | 18.18 | 0.00 | 0.00 | NA | NA | NA | -18.11 | 0.00 | NA | NA |
| K10701 | acetophenone carboxylase | 16.04 | 0.00 | 0.00 | NA | NA | NA | -15.97 | 0.00 | NA | NA |
| K01473 | N-methylhydantoinase A | 16.04 | 0.00 | 0.00 | NA | NA | NA | -15.97 | 0.00 | NA | NA |
| K00343 | NADH-quinone oxidoreductase subunit N | NA | NA | 11856.50 | 0.00 | 16.92 | 0.00 | NA | NA | NA | NA |
| K01744 | aspartate ammonia-lyase | 17.16 | 0.00 | 0.00 | NA | NA | NA | -17.09 | 0.00 | NA | NA |
| K02659 | twitching motility protein PilI | NA | NA | 9546.78 | 0.00 | 16.61 | 0.00 | NA | NA | NA | NA |
| K05501 | TetR/AcrR family transcriptional regulator | NA | NA | 13371.23 | 0.00 | 17.09 | 0.00 | NA | NA | NA | NA |
| K01692 | enoyl-CoA hydratase | 18.07 | 0.00 | 0.00 | NA | NA | NA | -18.00 | 0.00 | NA | NA |
| K05607 | methylglutaconyl-CoA hydratase | 18.07 | 0.00 | 0.00 | NA | NA | NA | -18.00 | 0.00 | NA | NA |
| K06865 | ATPase | NA | NA | 2369.12 | 0.00 | 14.60 | 0.00 | NA | NA | NA | NA |
| K18332 | NADP-reducing hydrogenase subunit HndD | NA | NA | 5541.15 | 0.00 | 15.82 | 0.00 | NA | NA | NA | NA |
| K07246 | tartrate dehydrogenase/decarboxylase / D-malate dehydrogenase | 18.97 | 0.00 | 0.00 | NA | NA | NA | -18.90 | 0.00 | NA | NA |

NA, Not available

Supplementary Table 4. Comparison of stoichiometric fluxes and marker protein abundances using two-way ANOVA.

| Modeling | | | | Proteomics | | | |
|---|---|---|---|---|---|---|---|
| Reactions | Treatments | | | Maker proteins | Treatments | | |
| | F (1,8) (*p*-value) | | | | F (1,8) (*p*-value) | | |
| | $SO_4^{2-}$ | $O_2$ | Interaction | | $SO_4^{2-}$ | $O_2$ | Interaction |
| Aerobic Heterotroph | 5.1 | 3633 (***) | 5.1 | Cytochrome c oxidase (ccoN) | 72.8 (***) | 141.9 (***) | 72.8 (***) |
| Lactate Fermentation | 113.7 (***) | 34.8 (***) | 44.1 (***) | L-lactate dehydrogenase (Ldh) | 1.7 | 0.02 | 0.1 |
| Hydrogenic acetogenesis | 16.2 (**) | 4545 (***) | 24.5 (**) | Acetate kinase (Ack) | 0.6 | 0.6 | 1.4 |
| Sulfidogenic lactate oxidation | 50.4 (***) | 50.4 (***) | 50.4 (***) | Sulfide reductase (Srd) | 0.7 | 0.2 | 0.08 |
| Sulfidogenic hydrogen oxidation | 14784 (***) | 14784 (***) | 14784 (***) | Dissimilatory sulfite reductase (Dsr) | 10.6 (**) | 0.03 | 0 |
| Hydrogenic Methanogenesis | 1147 (***) | 1064 (***) | 1141 (***) | Ferredoxin oxidoreductase (Frt) | 0.8 | 0.6 | 4.9 |
| Acetoclastic Methanogenesis | 1728 (***) | 19020 (***) | 1744 (***) | Phosphate acetyltransferase (Pta) | 9.1 (*) | 0.01 | 0.7 |
| Methane oxidation | 2.1 | 145.8 (***) | 2.1 | Methane/ammonia monooxygenase (Pmo) | 5.2 | 17.9 (**) | 1.7 |
| Butyrate production | 6.2 | 217.2 (***) | 6.2 | 3-hydroxyisobutyrate dehydrogenase (HIBADH) | 2 | 0 | 0 |

* for *padj* < 0.05, ** for *padj* < 0.01 and *** for *padj* < 0.001.
